# Supplementary material for: Spatial maps of prostate cancer transcriptomes reveal an unexplored landscape of heterogeneity
Source: Nat Commun. 2018 Jun 20;9:2419. doi: 10.1038/s41467-018-04724-5 (PMC6010471; doi:10.1038/s41467-018-04724-5)
Supplement: Supplementary file 1 — Supplementary Information [file 41467_2018_4724_MOESM1_ESM.pdf]

Spatial Maps of Prostate Cancer Transcriptomes Reveal an Unexplored Landscape of Heterogeneity

Berglund *et al.*

Supplementary information

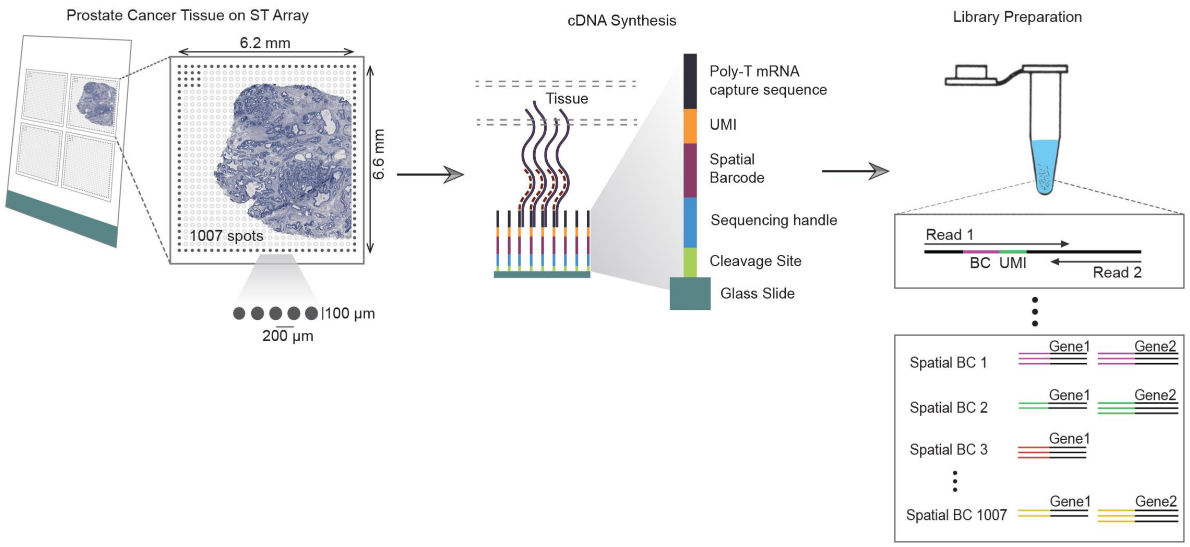

Supplementary Figure 1a. Spatially-resolved transcriptome profiling in prostate cancer. Tissue sections are placed on the array, fixed, H&E stained and imaged. The tissue is permeabilized, transcripts are captured and reverse transcribed. After tissue removal, barcoded cDNA is enzymatically released from the array and used for further library preparation and sequencing. The spatial barcode is used to connect every transcript with the spot it derives from, the UMI to correct for PCR amplification bias.

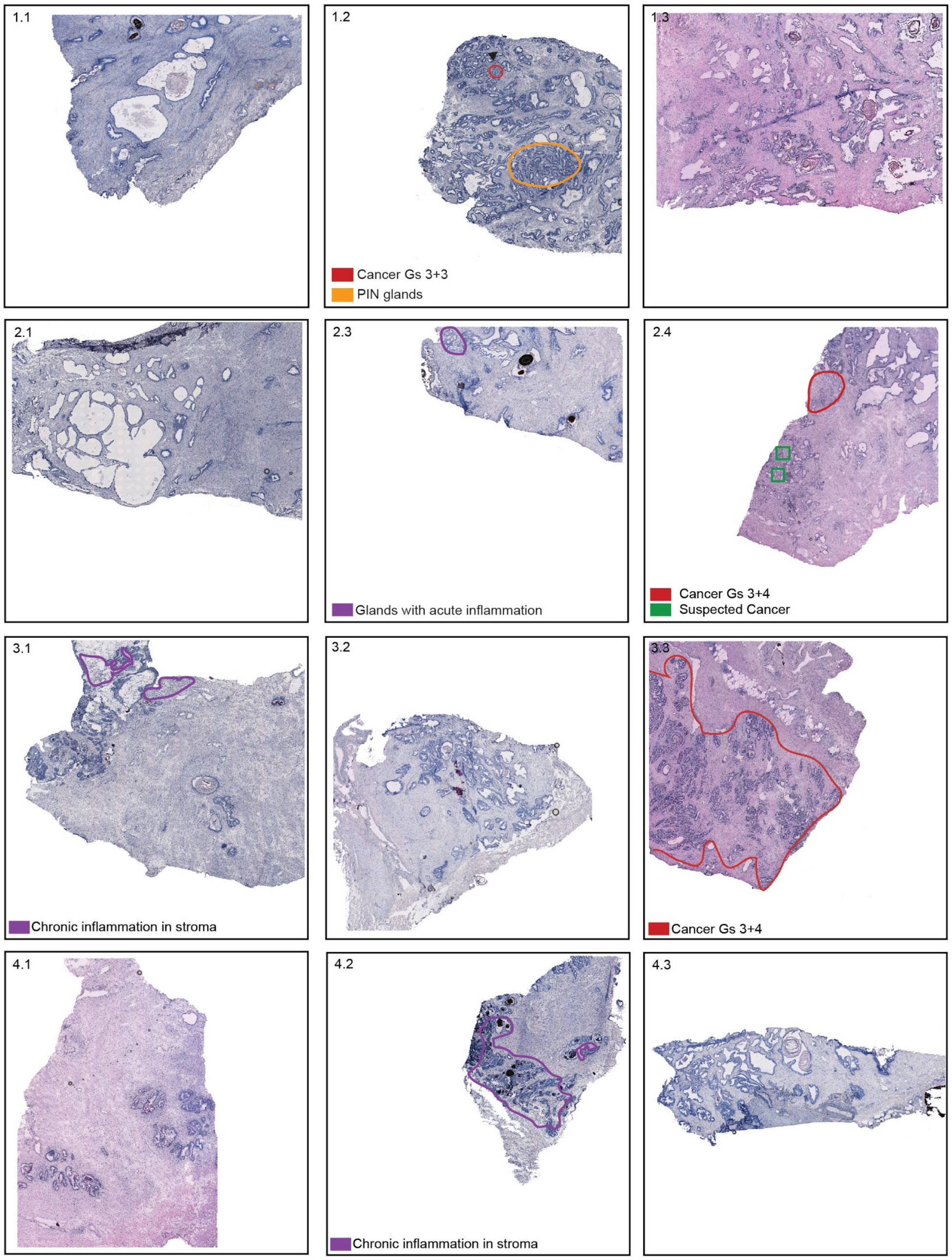

Supplementary Figure 1b. Histological prostate cancer tissue sections annotated by a pathologist are colored; cancer (red), PIN (yellow), suspected cancer (green) and inflammation (purple).

a

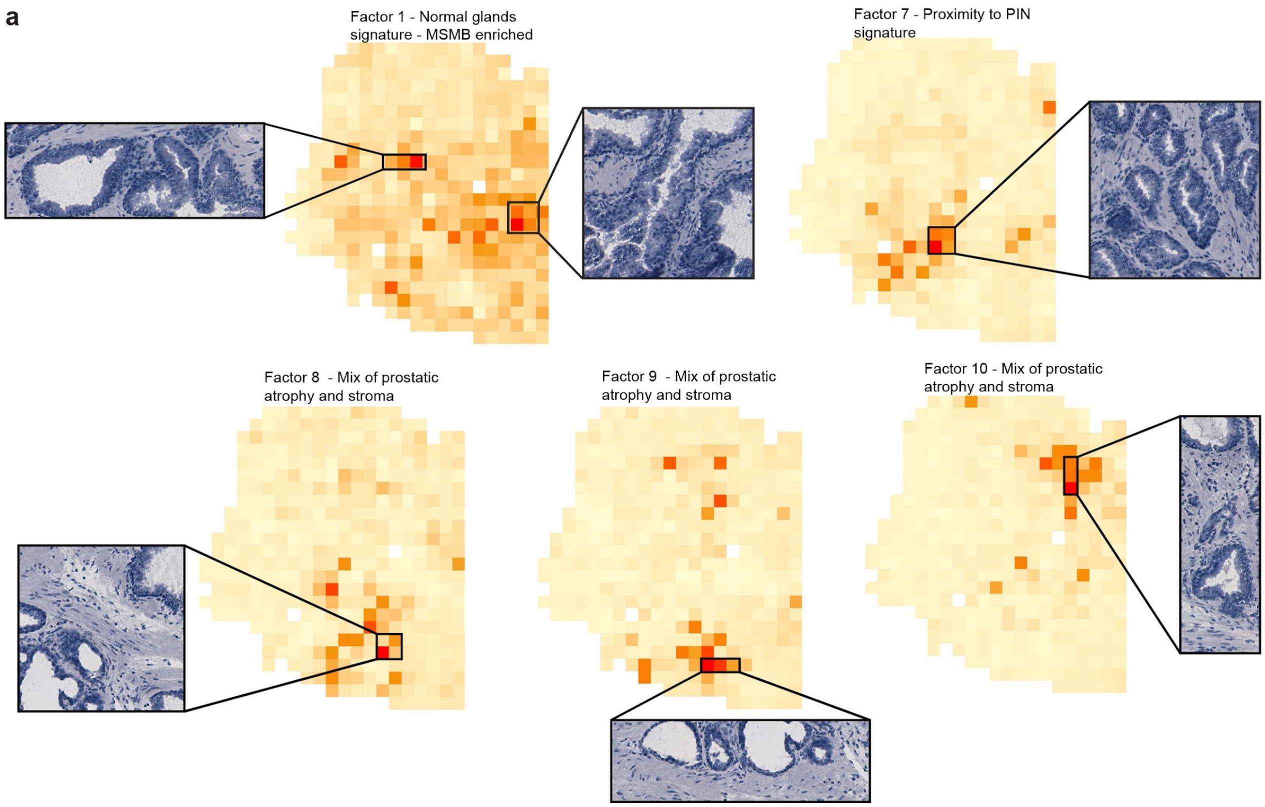

b

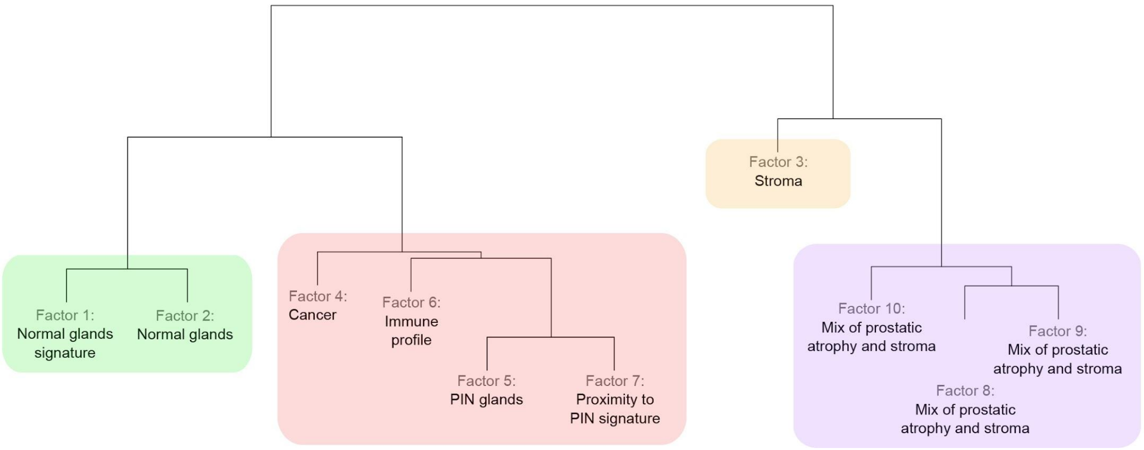

Supplementary Figure 2. Remaining activity maps from factor analysis in Fig. 2. a Factor activity maps of one cancer sample corresponding to normal gland signatures, proximity to PIN signature and mix of stroma and prostatic atrophy. Enlarged boxes show examples of the histology specific for the given factor. **b** Hierarchical clustering of all 10 factors.

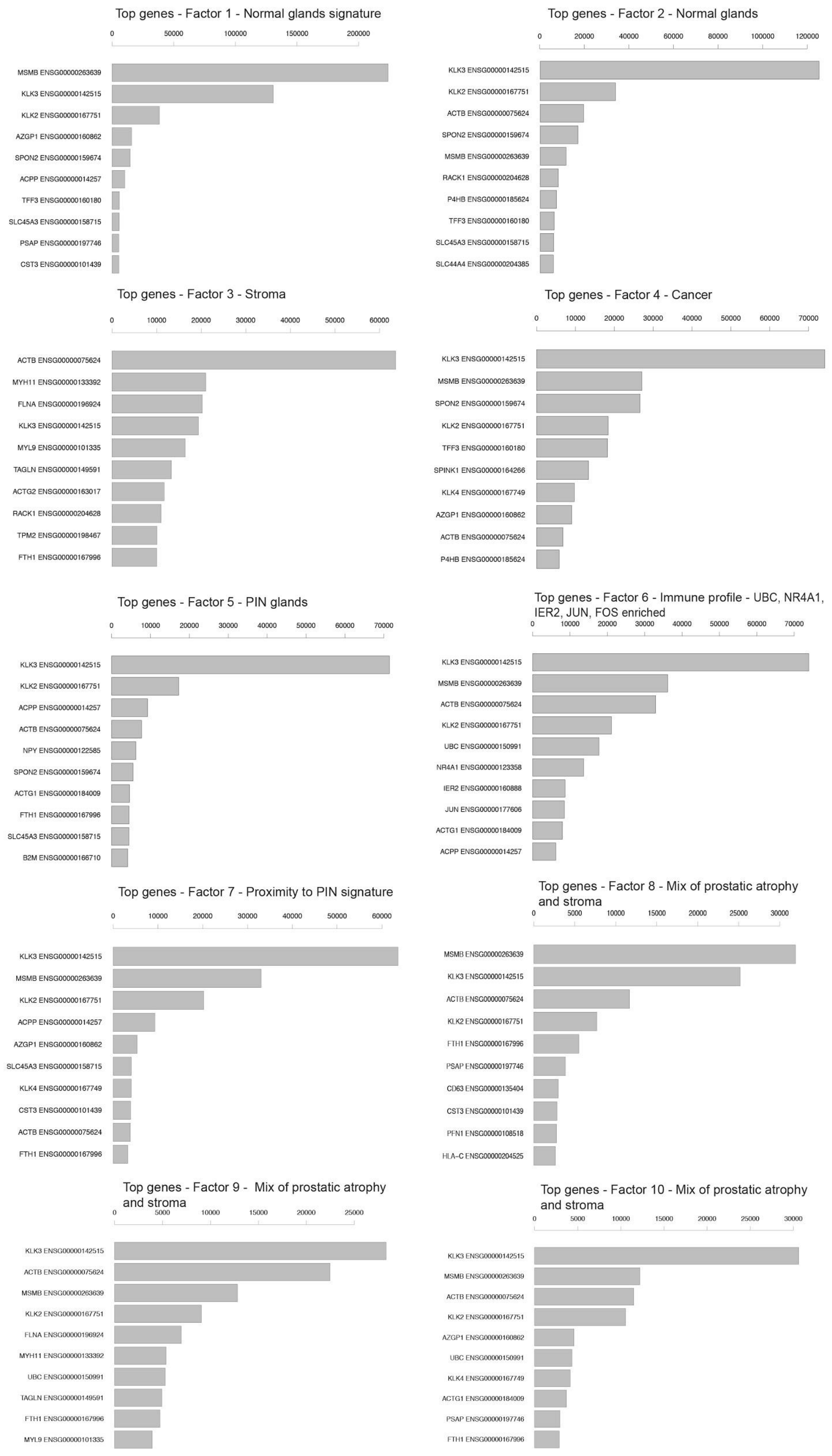

Supplementary Figure 3. Expected number of reads explained by all ten factors of the factor analysis in Fig. 2a. Only the ten highest-expressed genes are shown.

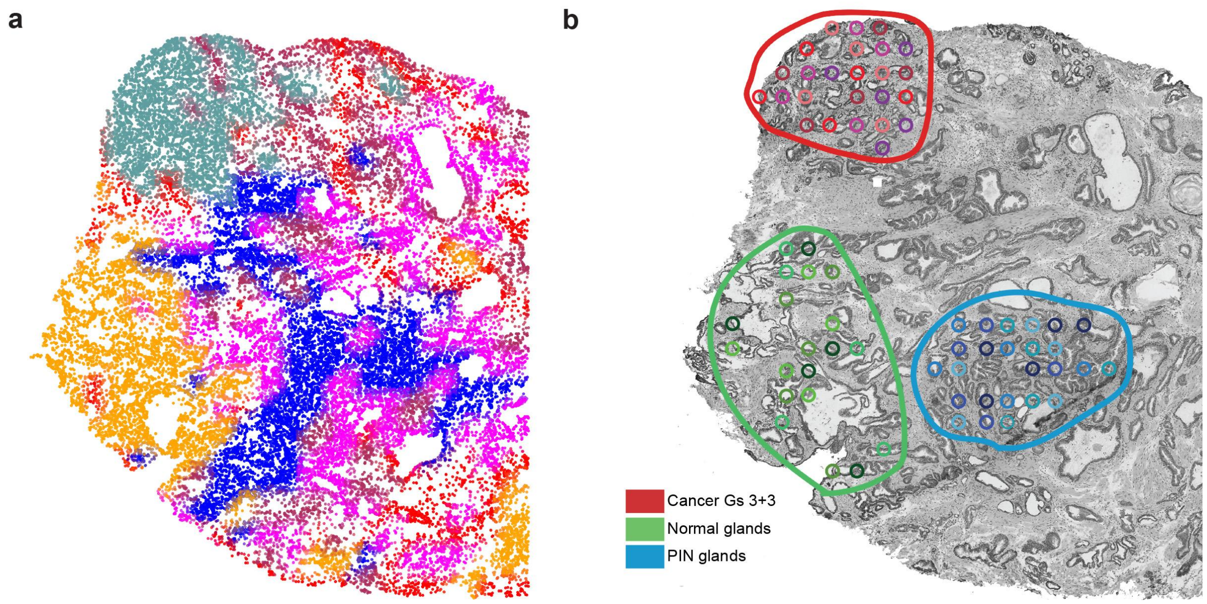

Supplementary Figure 4. a Hierarchical clustering of the spatial features revealed 6 clusters. Each cluster was assigned a color and the cluster identity was interpolated across the tissue structure to visualize major spatial patterns within the sample. This analysis correlates well with our factor based method and the morphology: orange: normal epithelium, green: cancer, blue and pink: PIN, red and purple: stroma. **b** We manually defined three regions based on factor activities and morphological information; Gs 3+3 (red), normal glands (green) and PIN (blue). In addition, spot replicates extracted from three different areas used for downstream analysis are shown. Each replicate (with 4-5 spots) is uniquely colored.

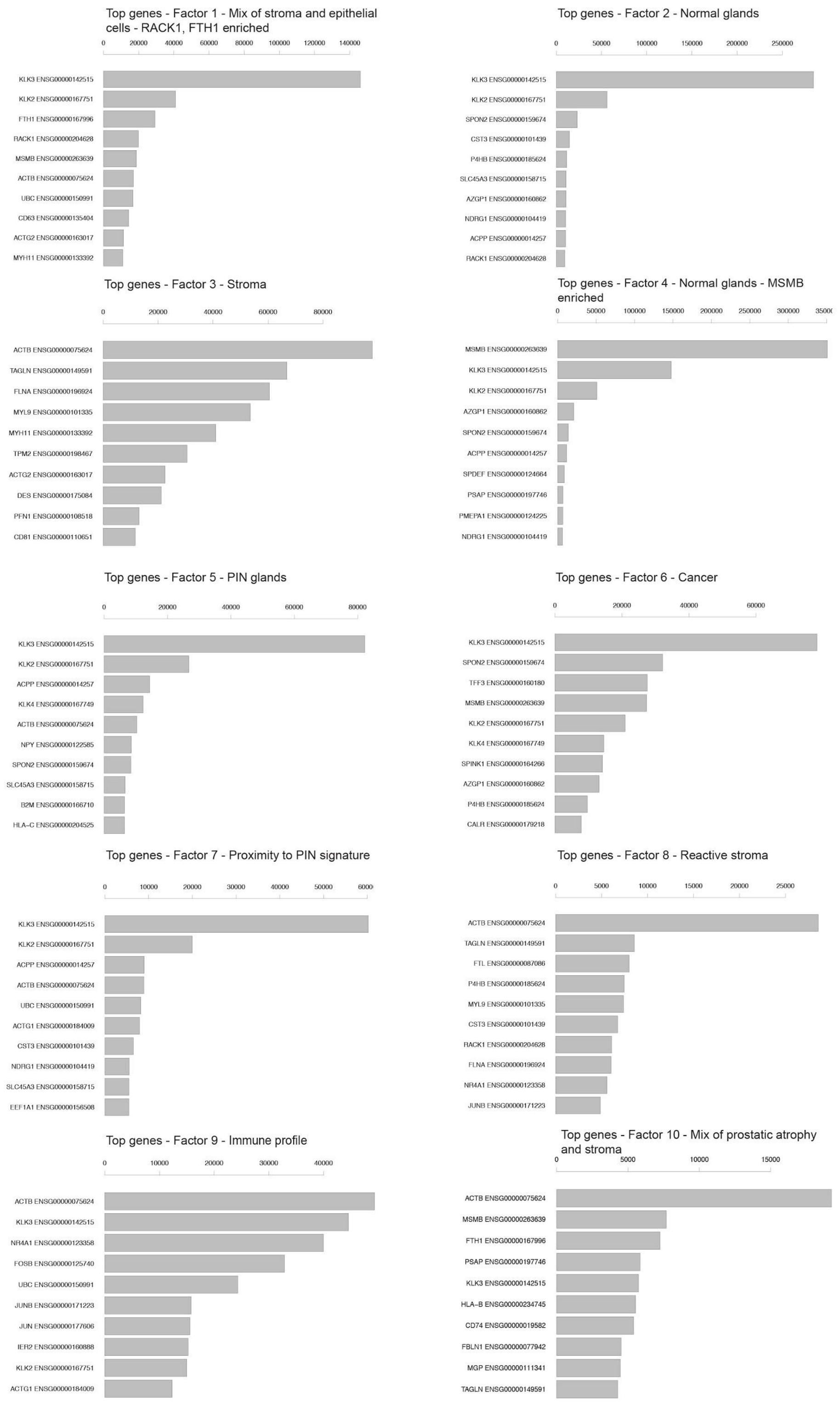

Supplementary Figure 5. Expected number of reads explained by all ten factors from factor analysis in Fig. 3b. Only the ten highest-expressed genes are shown.

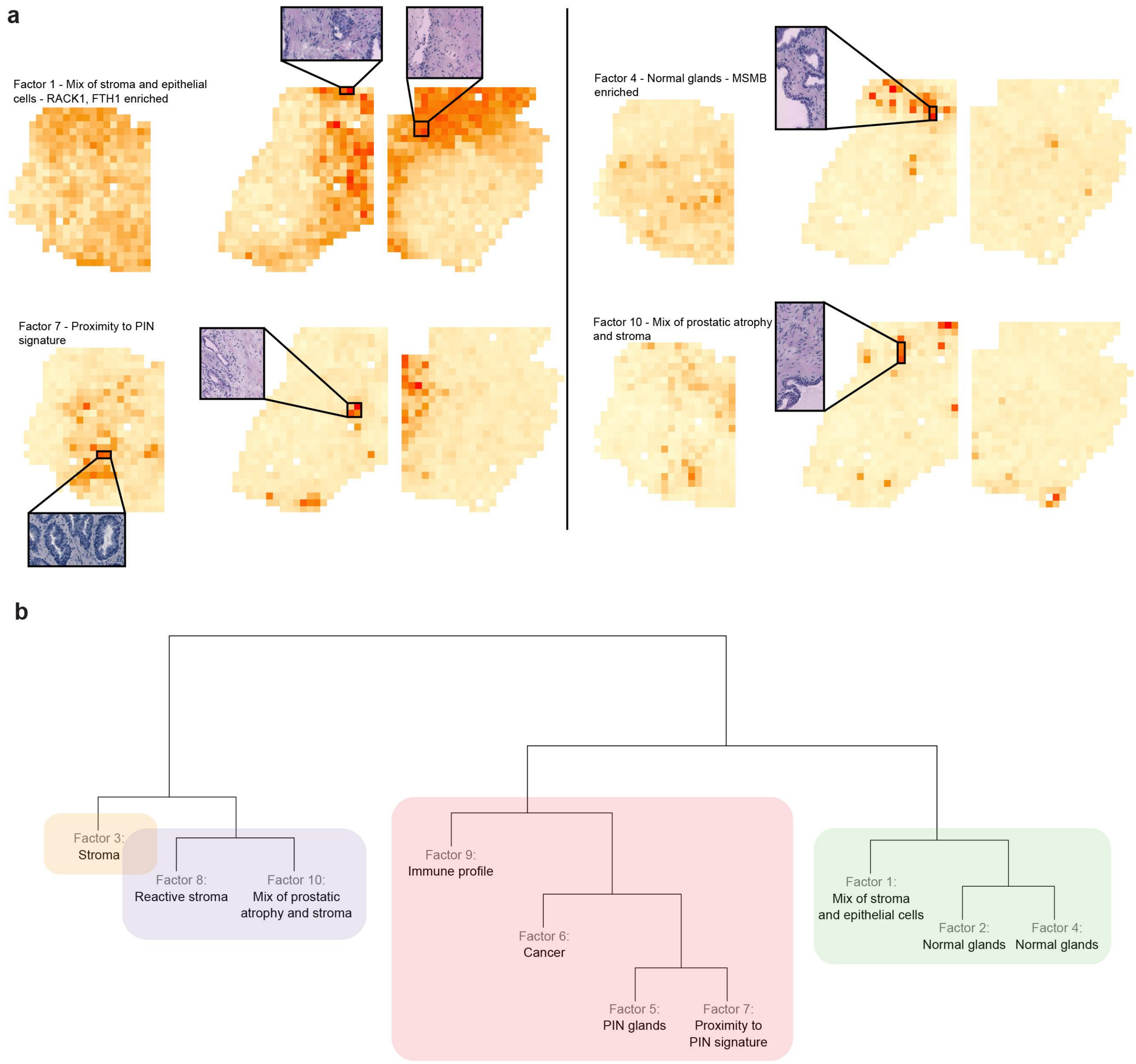

Supplementary Figure 6. Remaining activity maps from factor analysis in Fig. 3b. a Factor activity maps of three cancer containing samples corresponding to either normal glands or mix of stroma and epithelial cells. Enlarged boxes show examples of the histology specific for that factor. **b** Hierarchical clustering suggest that immune reactive stroma and stroma cluster close whereas PIN, cancer and inflammation are observed in another cluster. Normal glands (with and without MSMB) are neither similar to cancer nor to stroma cells.

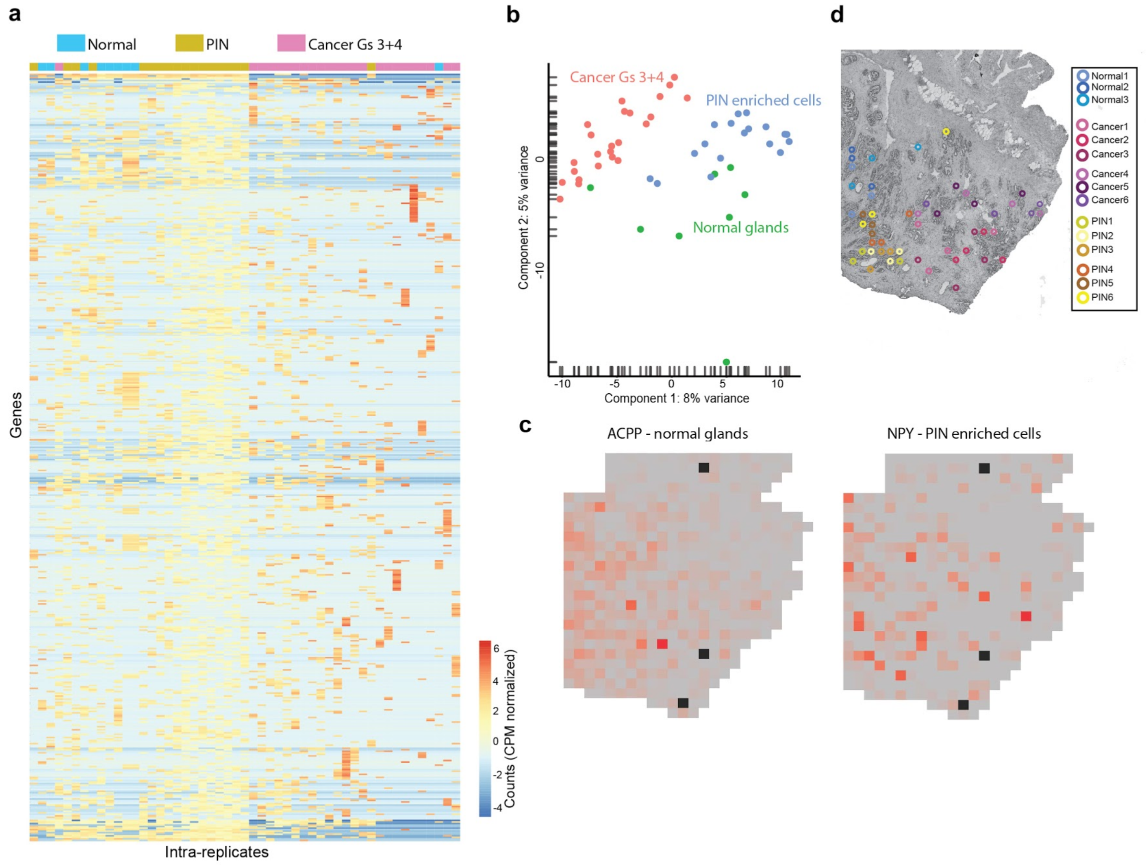

Supplementary Figure 7. Analysis between three factor activity clusters in sample 3.3 (cancer, normal and PIN enriched areas). a Heatmap of the 500 most variable genes between cancer, PIN and normal regions suggest that the normal and PIN regions identified by ST are different from the cancer region. **b** PCA plot separates normal and PIN glands from cancer cells. **c** Heatmaps of ACPP and NPY genes with absolute transcript counts show that the genes are expressed more in the PIN and normal regions compared to the cancer region identified by ST. **d** Spot replicates extracted from three different areas used for analysis in **a** and **b** are shown. Each replicate (with 3-4 spots) is uniquely colored.

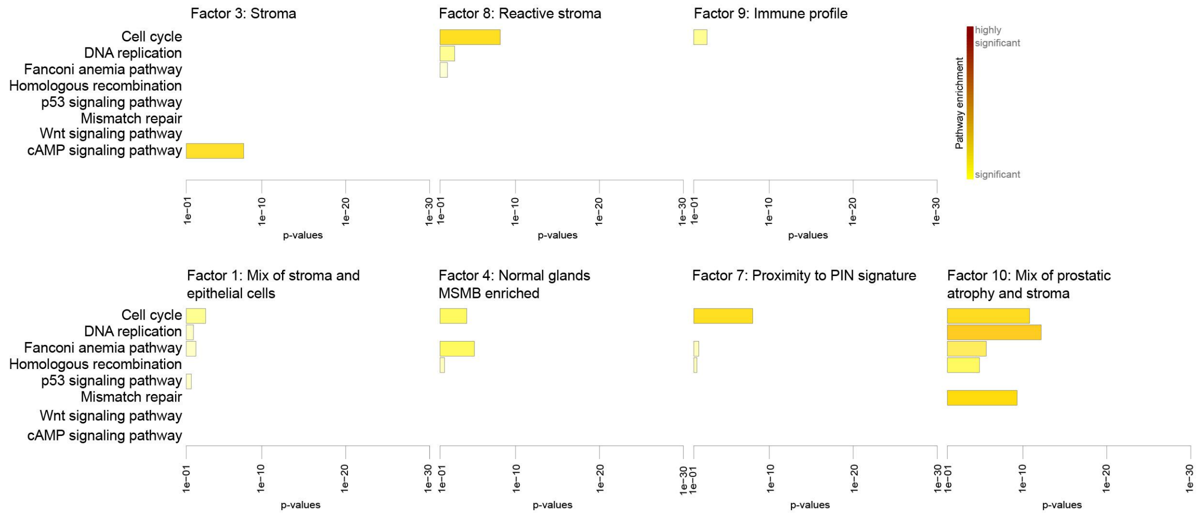

Supplementary Figure 8. Pathway enrichment. Remaining pathways enriched in all cancer samples from analysis seen in Fig. 3c.

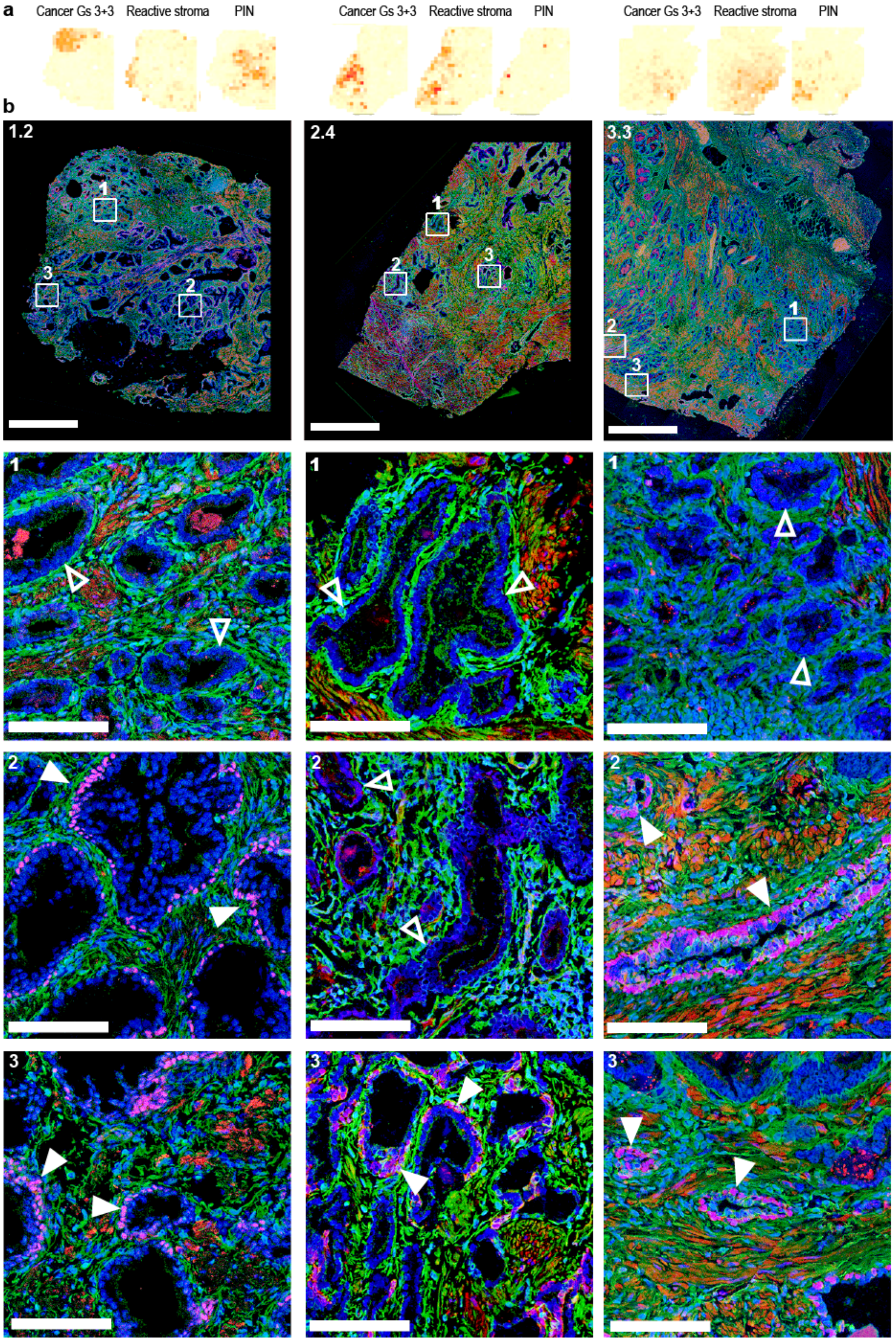
a
Cancer Gs 3+3
Reactive stroma
PIN
b
1.2
2.4
3.3
1
2
3

Supplementary Figure 9. Glands in cancer areas lack P63-staining basal cells. **a** Factor activity maps from the factor analysis of the three cancer samples reproduced from Fig. 3. **b** Fluorescence microscopy images of neighboring tissue sections stained by IHC for P63 (red), vimentin (green) and DNA (blue). Areas marked by white rectangles in the tissue sections are shown as close-ups below each section. Filled and unfilled triangles respectively point at glands with or without P63-stained basal cells. Scale bars in tissue sections and close-ups respectively indicate 1 mm and 100 μm

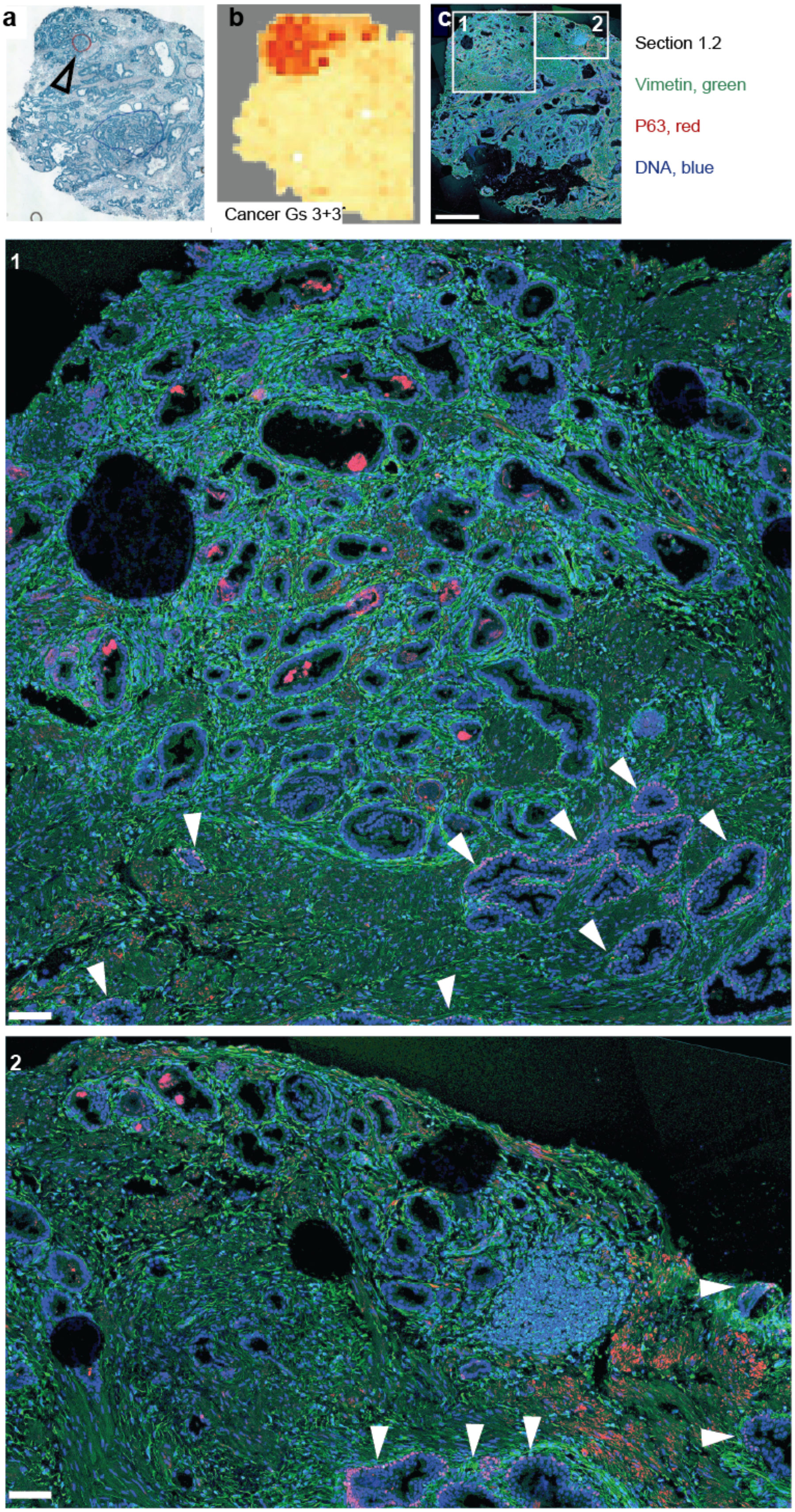
a
b
Cancer Gs 3+3
c
1
2
Section 1.2
Vimetin, green
P63, red
DNA, blue
1
2

Supplementary Figure 10. Glands in the area marked as cancer by the ST "cancer" factor lack P63 staining. **a** H&E staining of section 1.2 with the cancer annotation done by the pathologist. **b** Factor activity maps from the factor analysis of the three cancer samples reproduced from Fig. 3. **c** Fluorescence microscopy images of the tissue sections stained by IHC for vimentin (green), P63 (red) and DNA (blue). Areas marked with white rectangles in the tissue sections corresponds to, and are slightly bigger than the "cancer" factor in **b**. Close up of the white rectangles are shown in the middle and lower image. Filled triangles point at glands with P63-stained basal cells. Notice that glands outside the area of the "cancer" factor have basal cells with P63 staining. Scale bars in tissue sections and close-ups respectively indicate 1 mm and 100 μm.

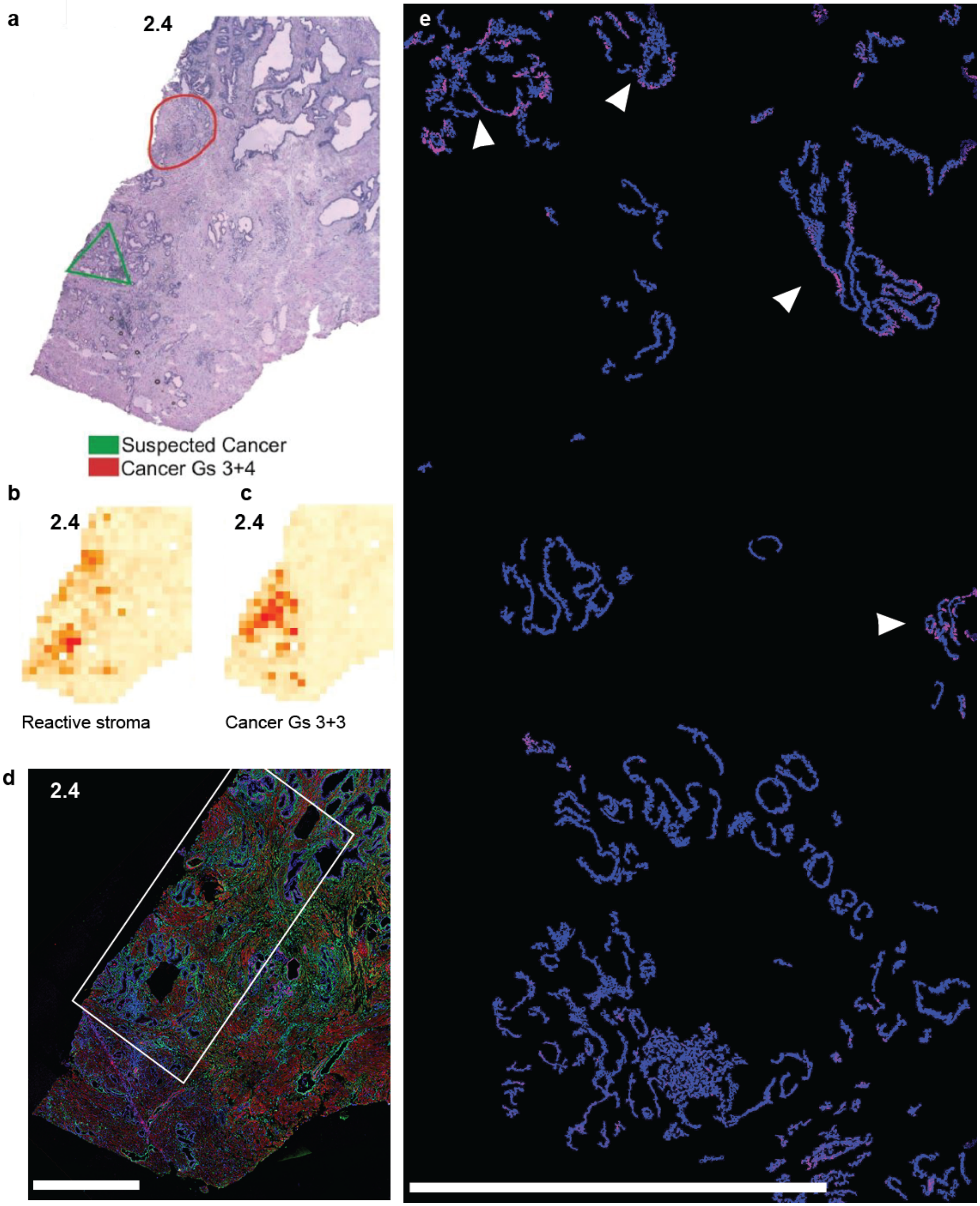
a
2.4
Suspected Cancer
Cancer Gs 3+4
b
2.4
Reactive stroma
c
2.4
Cancer Gs 3+3
d
2.4
e

Supplementary Figure 11. Glands in the area marked as cancer by the ST "cancer" factor lack P63 staining. **a** H&E staining of section 2.4 with the cancer annotation done by the pathologist. **b and c** Factor activity maps from sample 2.4 reproduced from Fig. 3. **d** Fluorescence microscopy images of the tissue sections stained by IHC for vimentin (green), P63 (red) and DNA (blue). Area marked with white rectangle in the tissue section corresponds to and is slightly larger than the "cancer" and "reactive stroma" factors in **b** and **c**. **e** Close up of box in **d**. The P63 signal shown is extracted by using the DNA image as a mask. Only P63 signal from epithelial nuclei is shown. Filled triangles point at glands with P63-stained basal cells. Notice that glands outside the area of the cancer factor in the upper part of the close up have basal cells with P63 staining. Scale bars in tissue sections and close-ups respectively indicate 1 mm.

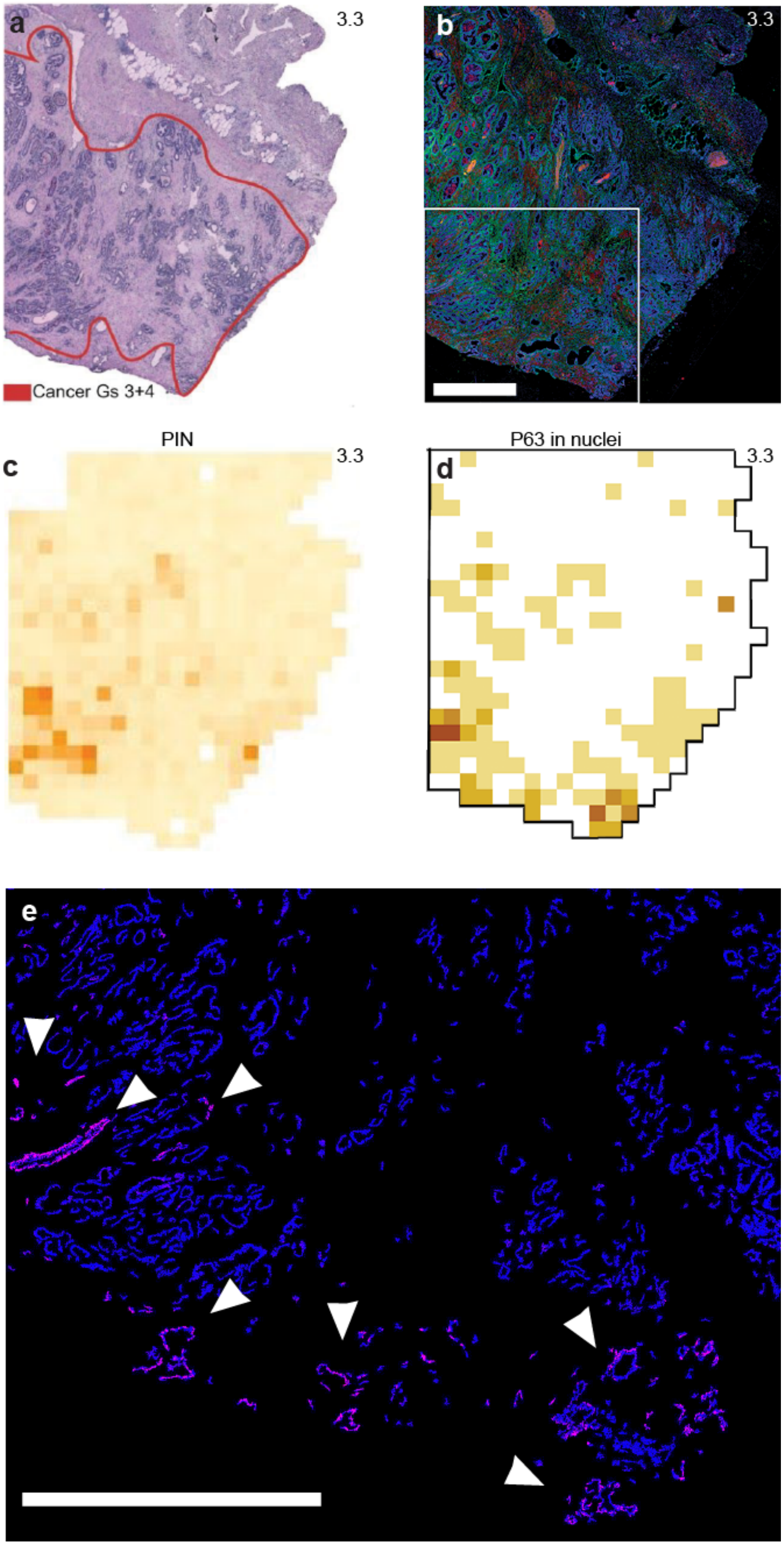
a
3.3
Cancer Gs 3+4
b
3.3
PIN
c
3.3
P63 in nuclei
d
3.3
e

Supplementary Figure 12. Sample 3.3 shows activity of the ST “PIN” factor in a region annotated as cancer. IHC reveals that a fraction of glands in this region are P63 positive. **a** H&E staining of section 3.3 with the cancer annotation done by the pathologist. **b** Factor activity map for “PIN” from cancer sample 3.3 reproduced from Fig. 3. **c** Fluorescence microscopy images of the tissue sections stained by IHC for vimentin (green), P63 (red) and DNA (blue). Area marked with white rectangle in the tissue sections contains the area positive for the “PIN” factor in **b**. **d** Protein distribution map of P63 signal solely from epithelial nuclei. **e** Close up of box in **c**. Only P63 signal from epithelial nuclei is shown. Filled triangles point at glands with P63-stained basal cells. Note that the areas with basal cells correspond to the areas positive for the “PIN” factor and areas annotated as cancerous or occasional PIN glands by the pathologist. Scale bars in tissue sections and close-ups respectively indicate 1 mm**.**

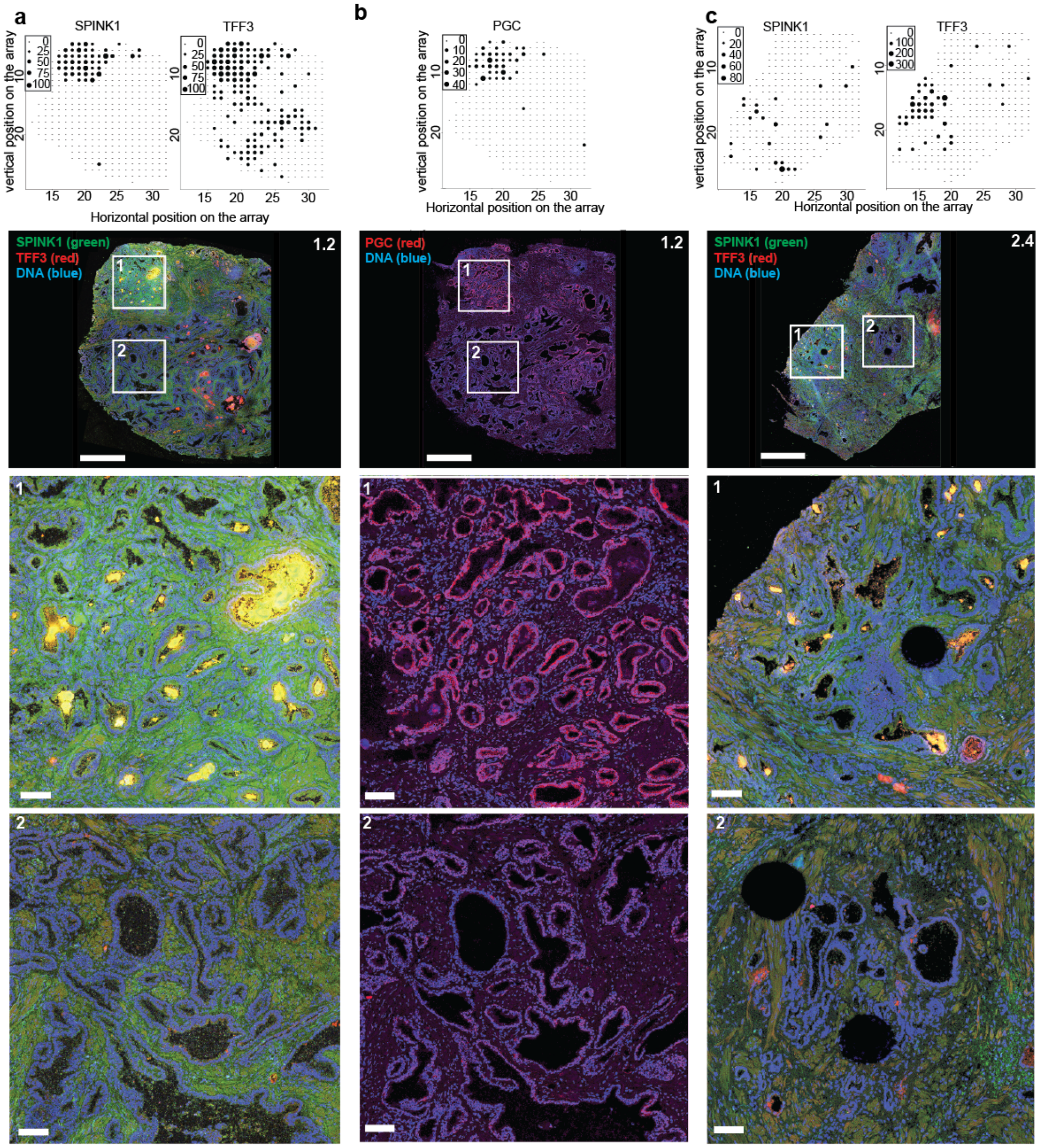

Supplementary Figure 13. Consistent spatial expression patterns of mRNA and protein

measured by ST and IHC. Circle size in array dot plots indicates normalized ST counts. Proteins stained by IHC indicated by colored labels, nuclei stained with DAPI (blue). Areas marked with numbered, white rectangles in the tissue sections contain cancerous (1) and normal regions (2) and are shown as close-ups in the two bottom rows. Scale bars in tissue sections and close-ups indicate 1mm and 100μm, respectively. **a** SPINK1 and TFF3 in section 1.2. **b** PGC in section 1.2. **c** SPINK1 and TFF3 in section 2.4. Note: colocalization of SPINK1 and TFF3 shows as yellow. SPINK1, TFF3, PGC expression levels were zero in the ST data for section 3.3. and hence were excluded from IHC.

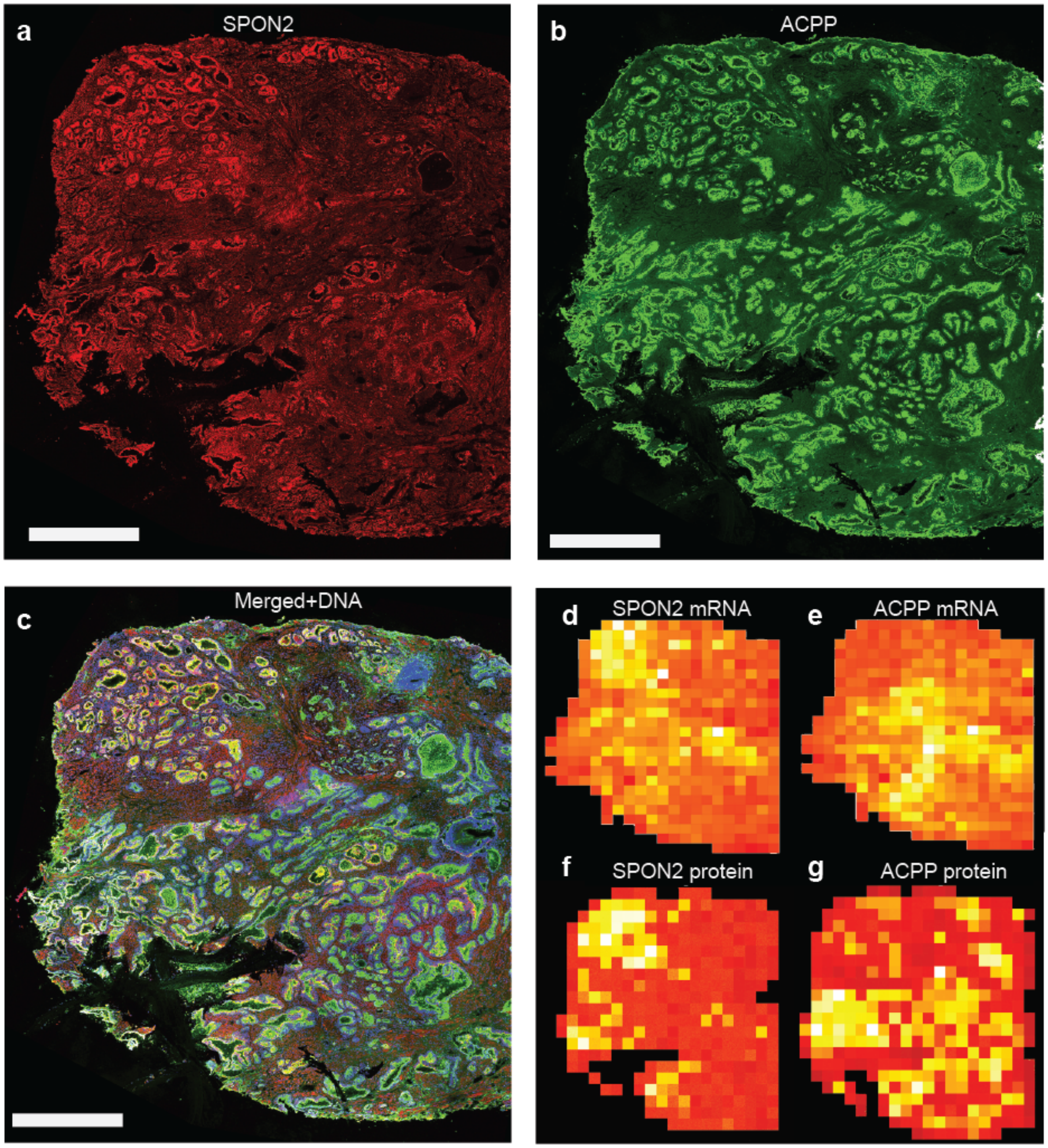

Supplementary Figure 14. Comparison of protein and mRNA localisation in tissue section 1.2. **a** IHC image of SPON2 (red). **b** IHC image of ACPP (green). **c** Merged image of SPON2, ACPP and DNA (blue). **d** Heatmap of ST-mRNA data for SPON2. **e** Heatmap of ST-mRNA data for ACPP. **f** Heatmap of IHC data in **a**. **g** Heatmap of IHC data in **b**. Scale bars in IHC images indicate 1 mm.

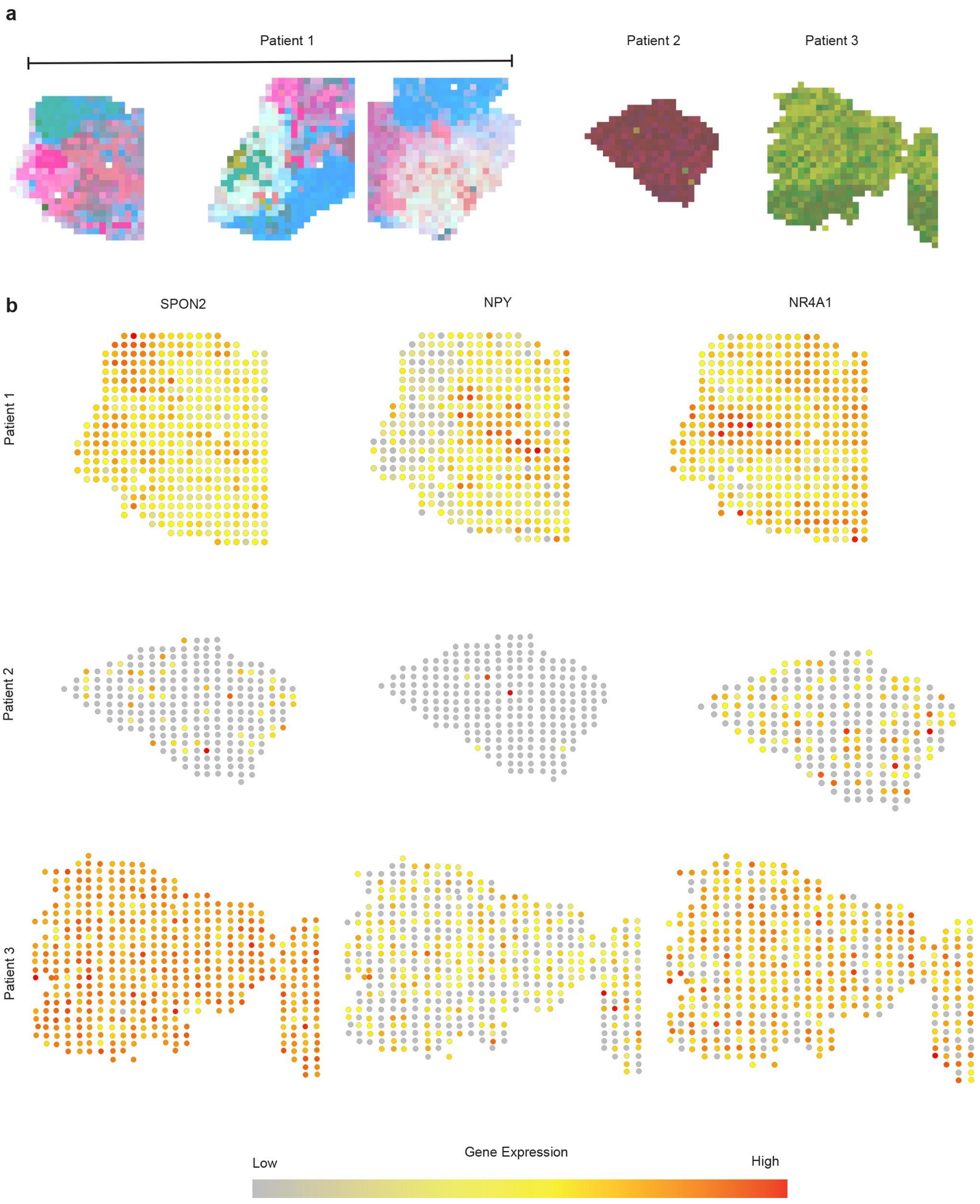

Supplementary Figure 15. **Analysis between 3 patients.** Results demonstrate high inter-tumor heterogeneity, although some genes (e.g. SPON2, NPY and NR4A1) in patient 1 were also shared between patient 2 and 3.

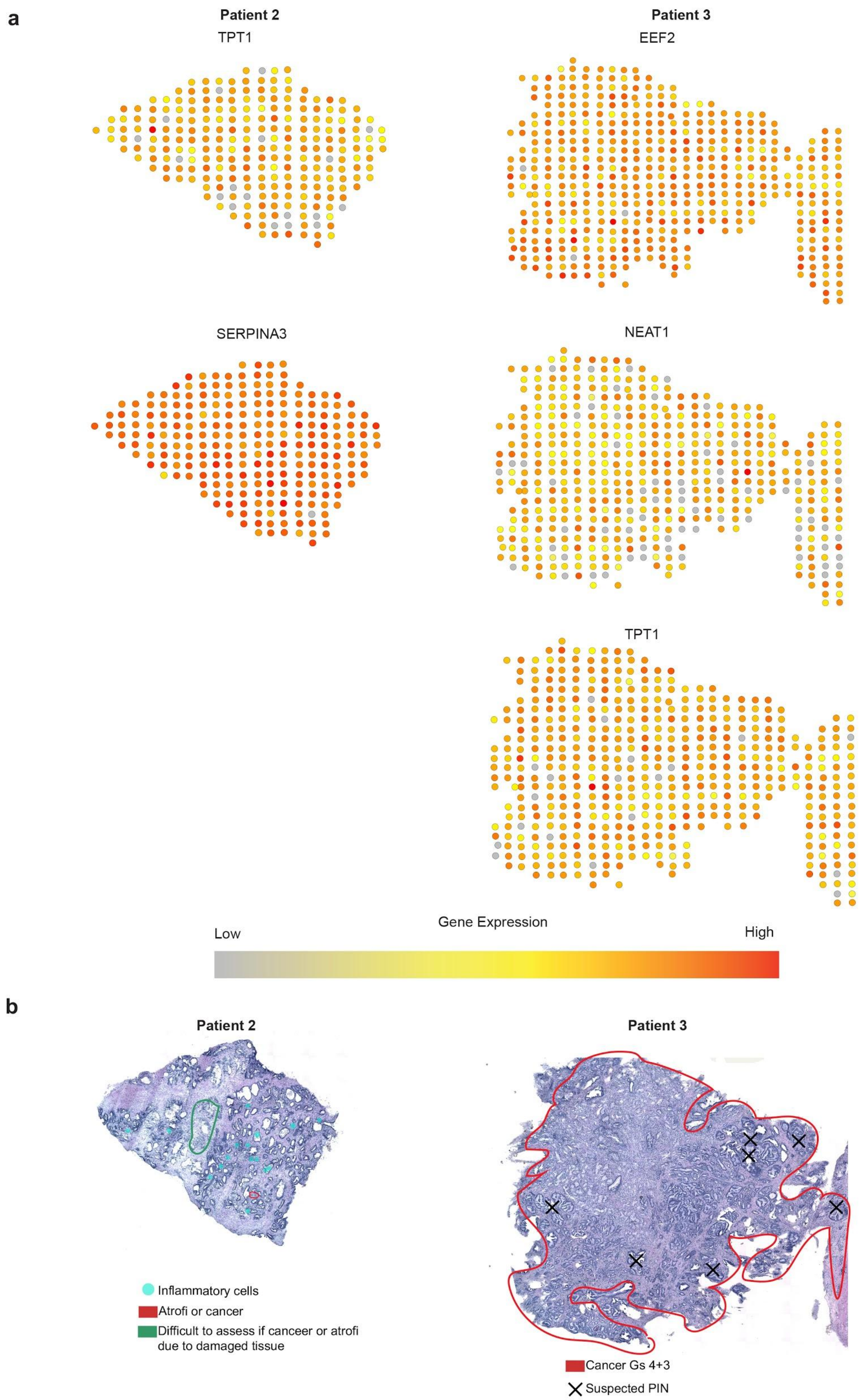
a
Patient 2
TPT1
SERPINA3
Patient 3
EEF2
NEAT1
TPT1
Gene Expression
Low
High
b
Patient 2
Inflammatory cells
Atrofi or cancer
Difficult to assess if canceer or atrofi due to damaged tissue
Patient 3
Cancer Gs 4+3
Suspected PIN

Supplementary Figure 16. Examples of unique sets of transcripts in each tumor. a TPT1 and SERPINA3 was highly expressed in patient 2 and EEF2, NEAT1 and TPT1 in patient 3. **b** Histological prostate cancer tissue sections annotated by a pathologist are colored.

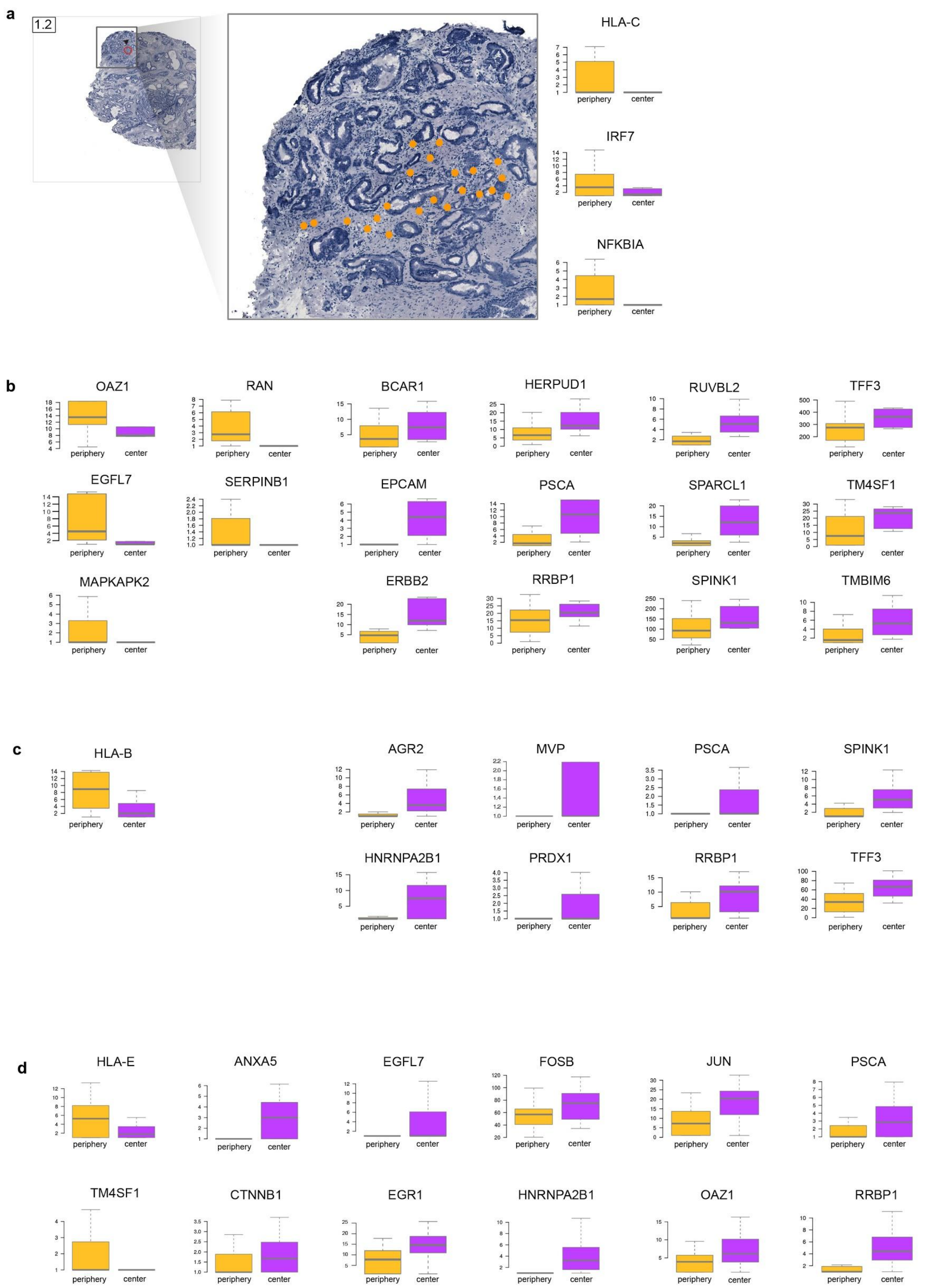

Supplementary Figure 17. a Annotated inflammation (orange color) by a pathologist and

immune-related genes upregulated in the periphery of sample 1.2. **b, c, d** Remaining box plots from analysis in Fig. 4 showing expression levels of noteworthy genes significantly upregulated in either periphery or the cancer center of sample 1.2 (b), sample 2.4 (c) and sample 3.3 (d).

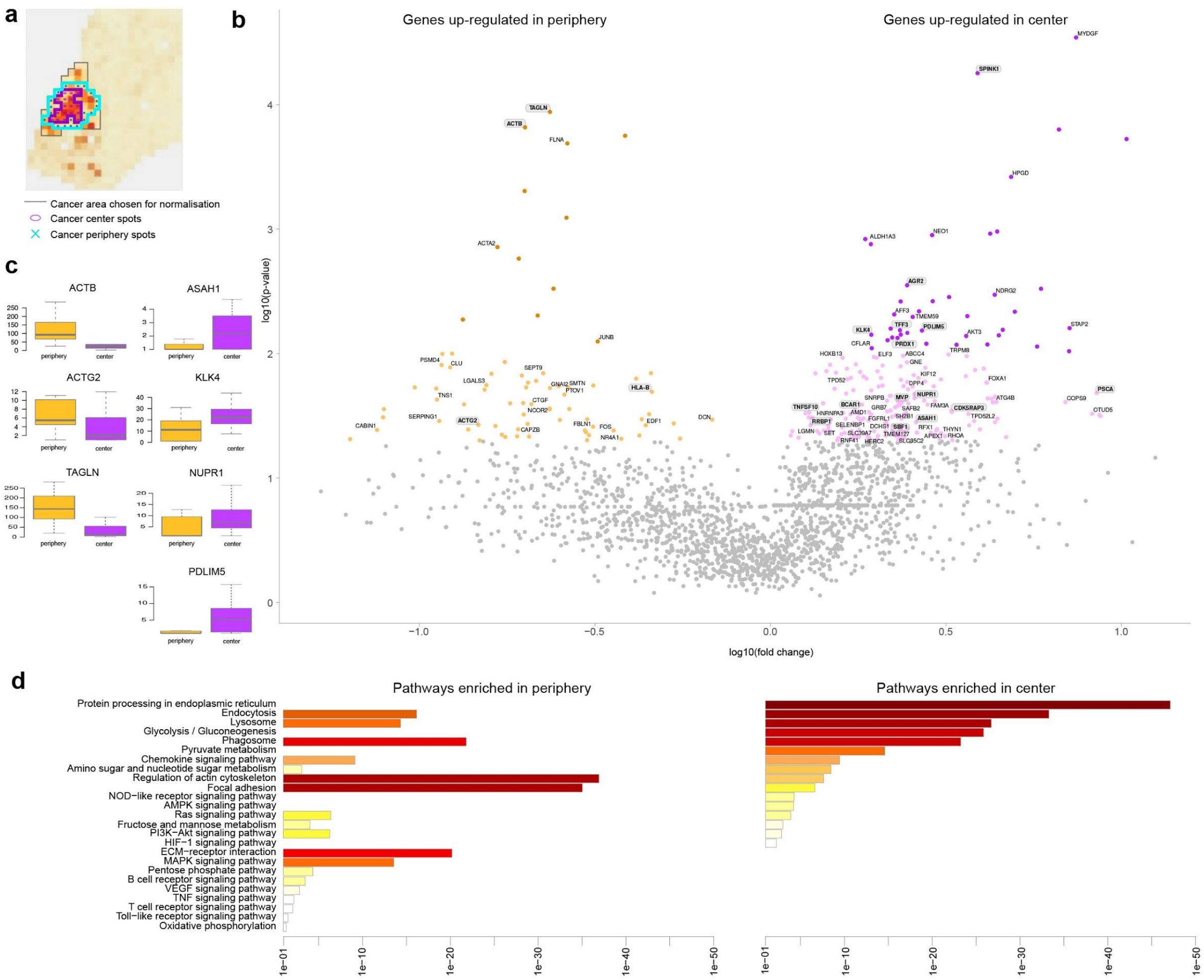

Supplementary Figure 18. Spatial comparison of periphery and center of sample 2.4. a Area comprising spots taken for normalisation of ST counts, within this area spots are chosen as periphery and center. Choice of spots is based on the activity of the factors "cancer" and "reactive stroma". **b** Volcano plot of significantly differentially expressed genes between periphery and center. **c** Box plots showing expression levels of noteworthy genes significantly upregulated in either periphery or the cancer center. **d** Enriched pathways for significantly ($p < 0.05$) differentially expressed genes in center and periphery.

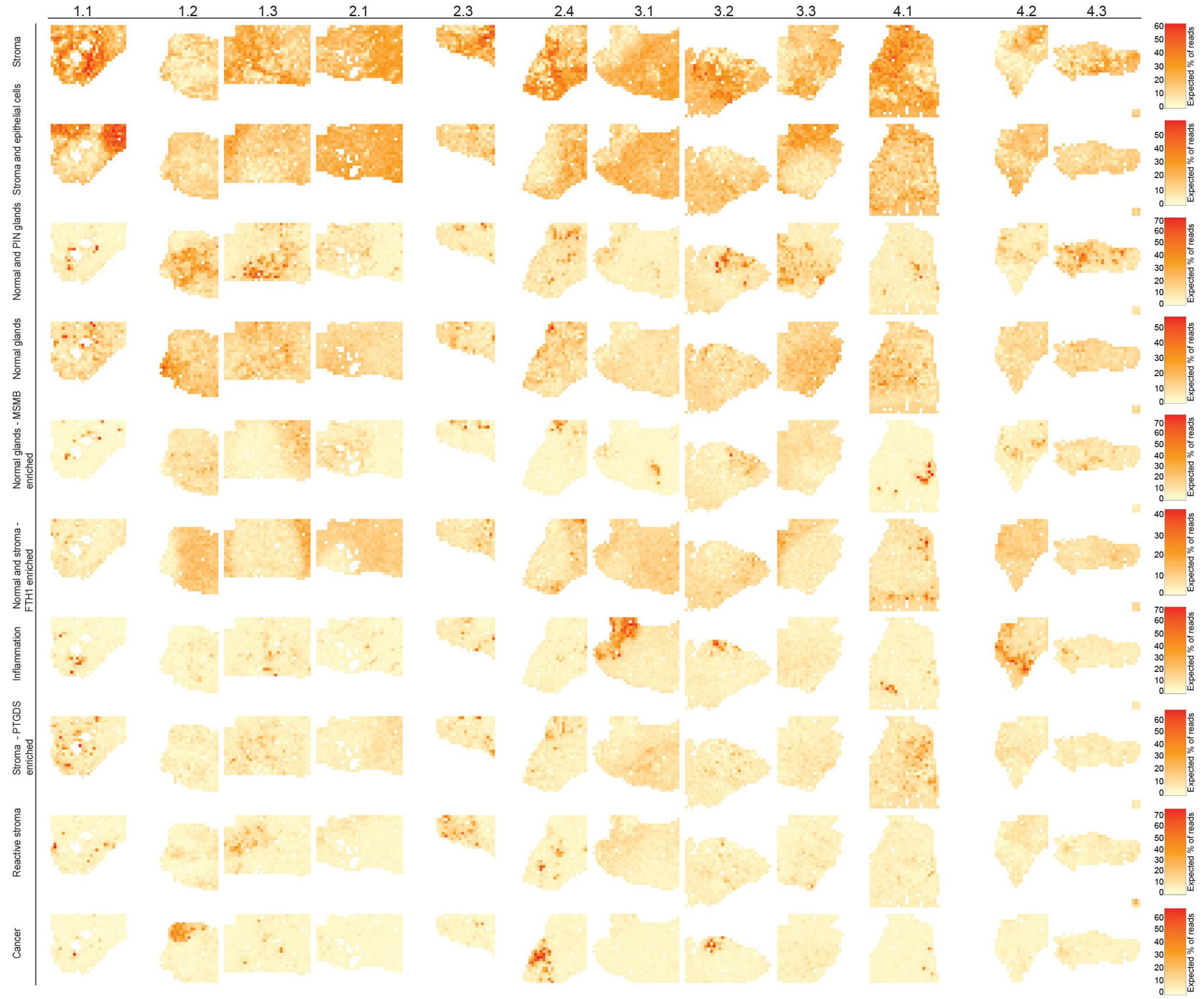

Supplementary Figure 19. Factor activity maps based on an analysis of all 12 samples. Scale bars equal relative frequencies expressed in percentage.

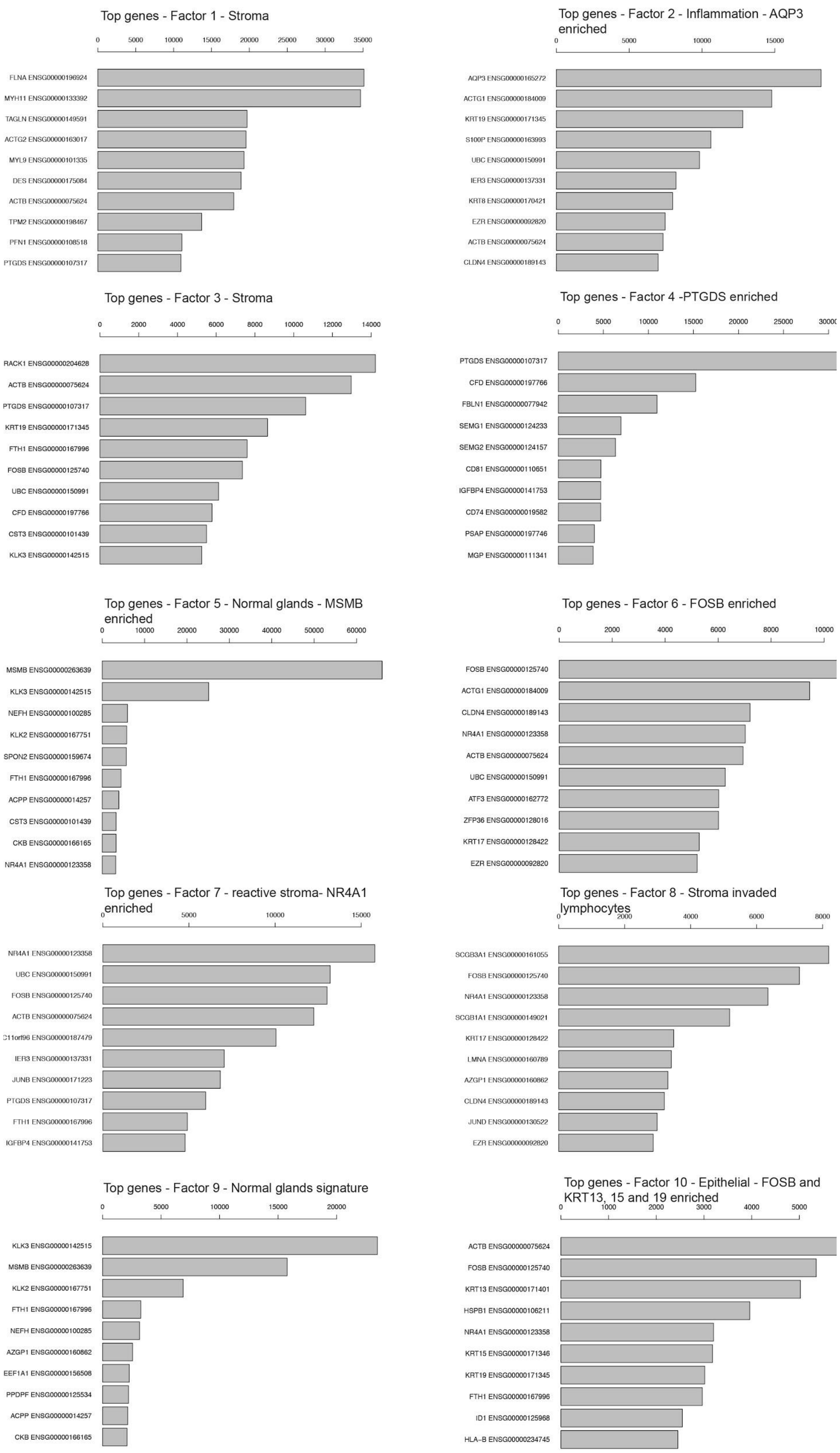

Supplementary Figure 20. Expected number of reads explained by factors from factor analysis in Fig. 5b. Only the ten highest-expressed genes are shown.

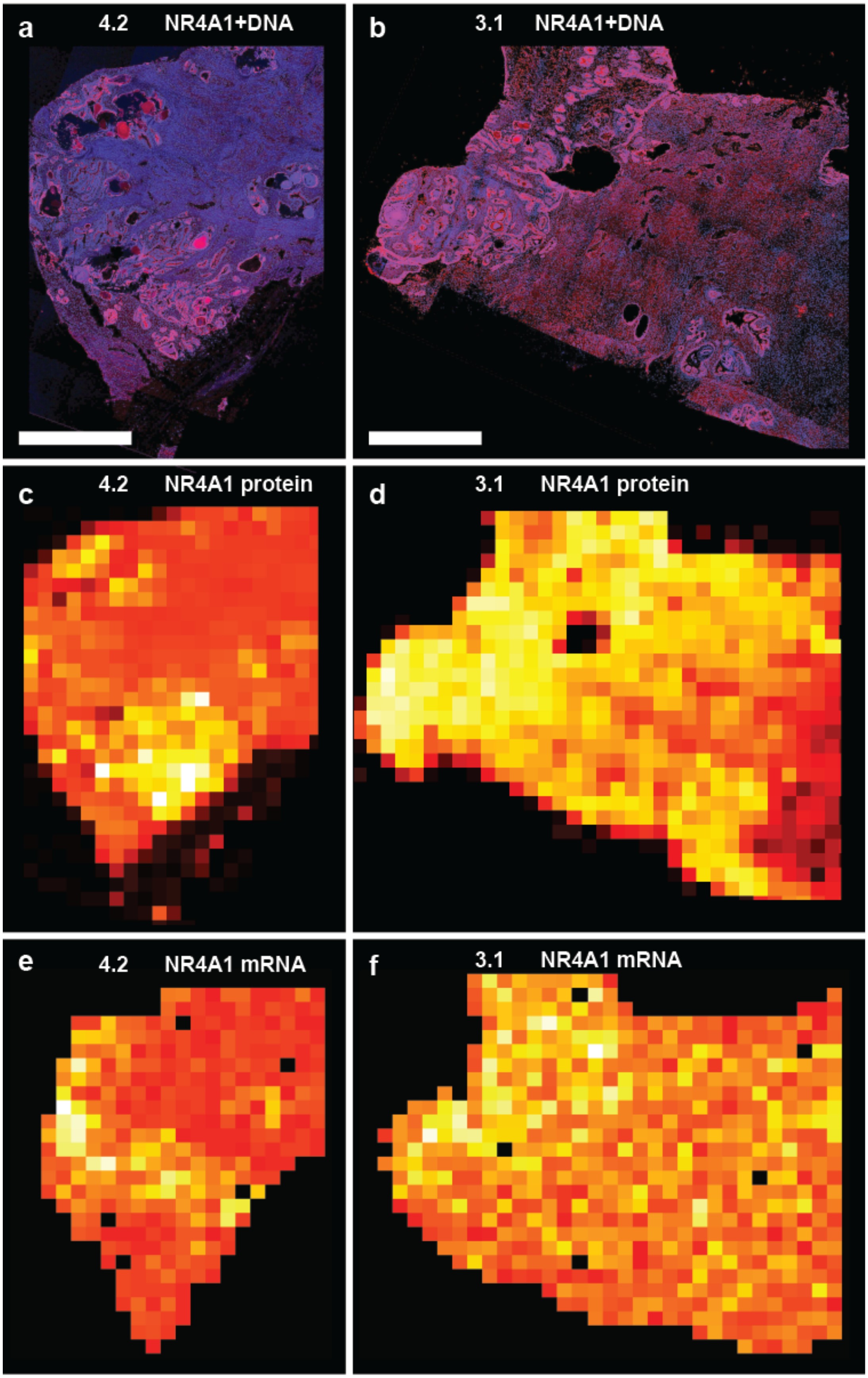
a
4.2
NR4A1+DNA
b
3.1
NR4A1+DNA
c
4.2
NR4A1 protein
d
3.1
NR4A1 protein
e
4.2
NR4A1 mRNA
f
3.1
NR4A1 mRNA

Supplementary Figure 21. Comparison of protein and mRNA localisation in tissue sections 4.2 and 3.1. a IHC image of NR4A1 (red) and DNA (blue) in section 4.2. **b** IHC image of NR4A1 (red) and DNA (blue) in section 3.1. **c** Heatmap of IHC data of NR4A1 shown in **a**. **d** Heatmap of IHC data of NR4A1 shown in **b**. **e** Heatmap of ST-mRNA data of NR4A1 in section 4.2. **f** Heatmap of ST-mRNA data of NR4A1 in section 3.1. Scale bars in IHC images indicate 1 mm.

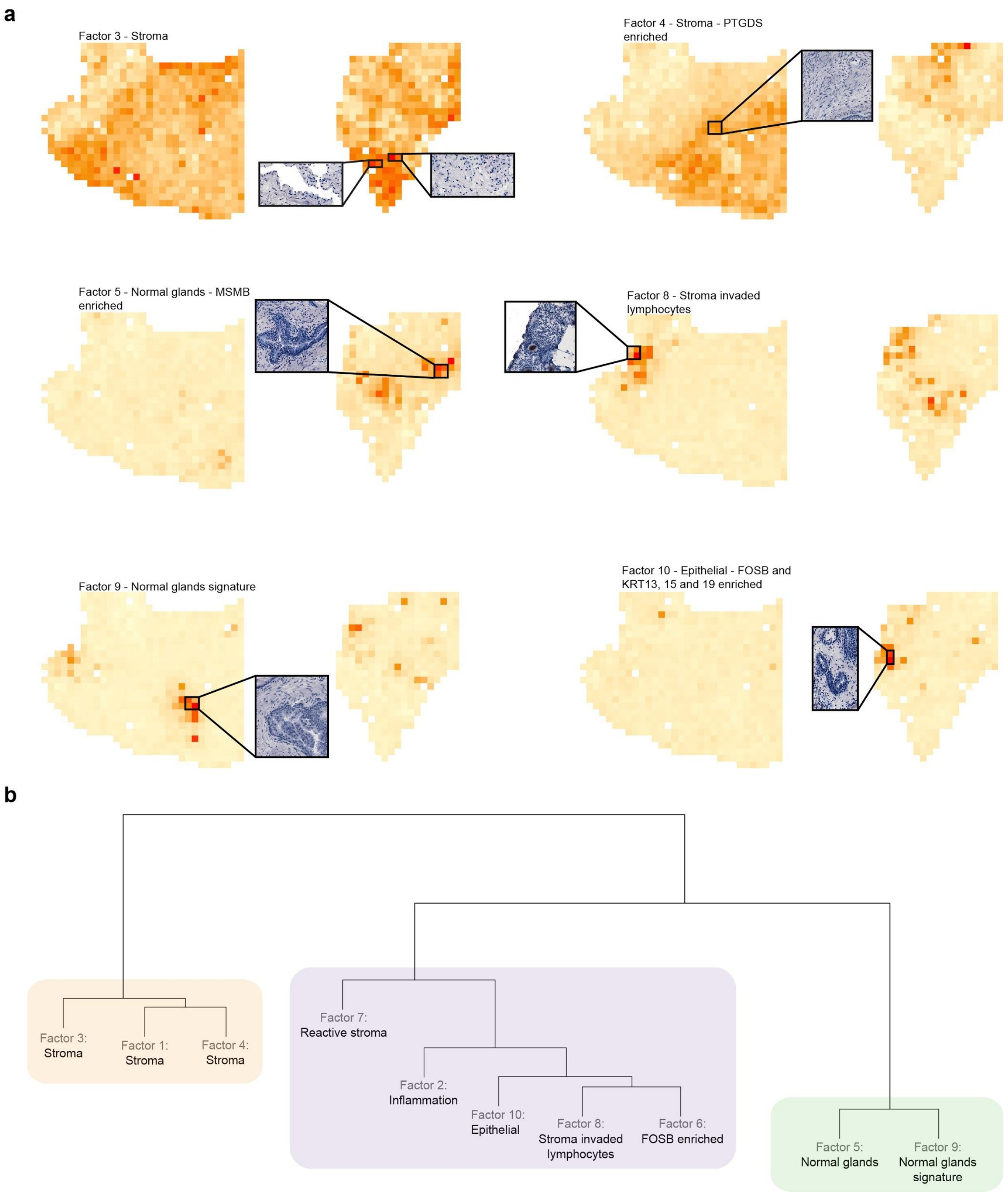
a
Factor 3 - Stroma
Factor 4 - Stroma - PTGDS enriched
Factor 5 - Normal glands - MSMB enriched
Factor 8 - Stroma invaded lymphocytes
Factor 9 - Normal glands signature
Factor 10 - Epithelial - FOSB and KRT13, 15 and 19 enriched
b
Factor 3: Stroma
Factor 1: Stroma
Factor 4: Stroma
Factor 7: Reactive stroma
Factor 2: Inflammation
Factor 10: Epithelial
Factor 8: Stroma invaded lymphocytes
Factor 6: FOSB enriched
Factor 5: Normal glands
Factor 9: Normal glands signature

Supplementary Figure 22. Remaining activity maps from factor analysis in Fig. 5. a Factor activity maps of two samples with inflammation corresponding to different types of stroma or epithelial signatures. Enlarged boxes show examples of the histology specific for that factor. **b** Hierarchical clustering reveal that stroma cluster by their own, separated from normal, inflammation and reactive stroma.

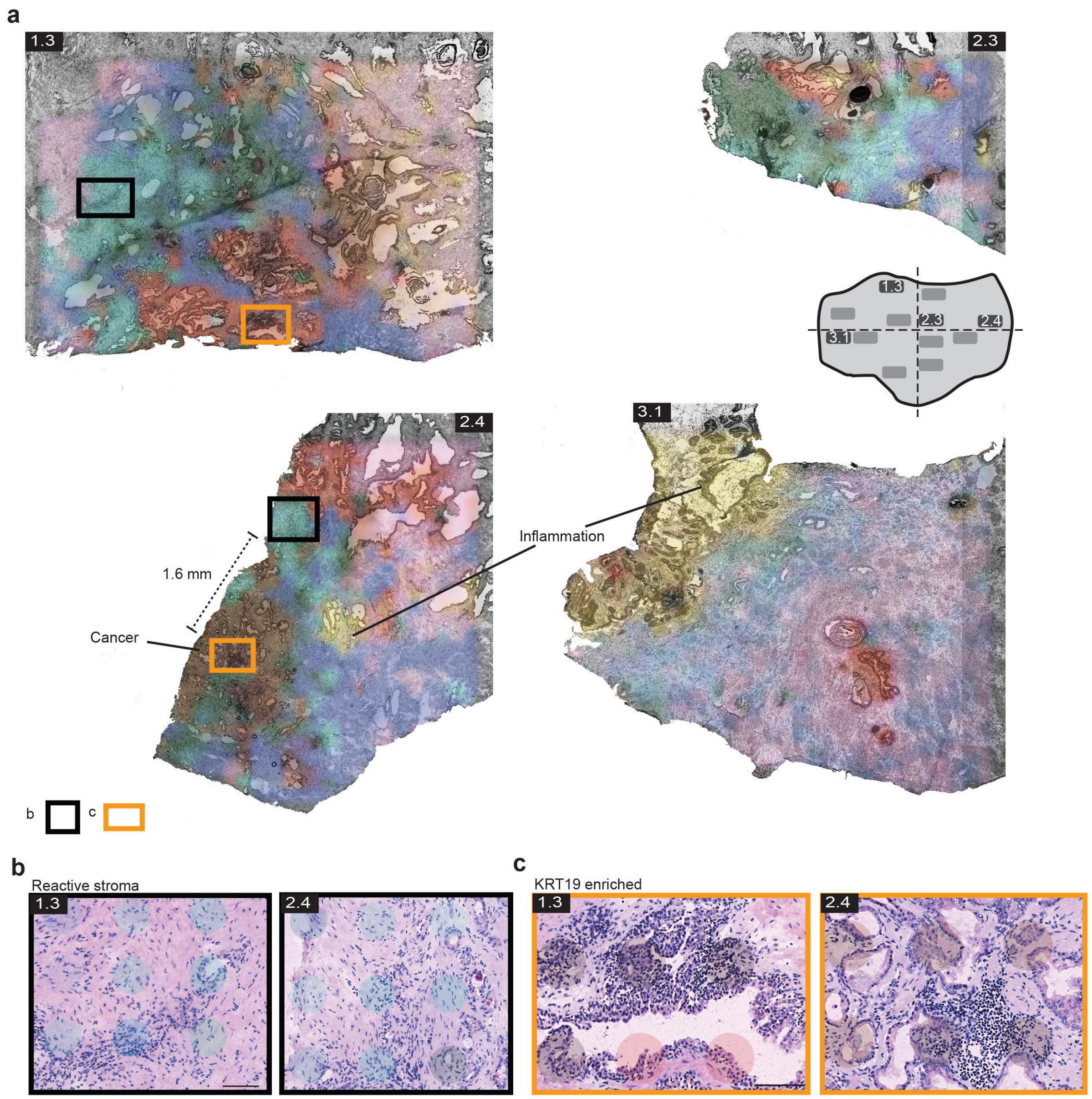

Supplementary Figure 23. Microenvironment vicinal to cancer and inflammation. a t-SNE summary of factor activity maps based on a joint factor analysis of the four samples (Supplementary data 5), linearly interpolated and superimposed on histological images for visibility of morphology. **b and c** Close-ups of reactive stroma and KRT19-enriched regions shown in black and orange rectangles in **a**. Position of array spots are visible behind the tissue section.

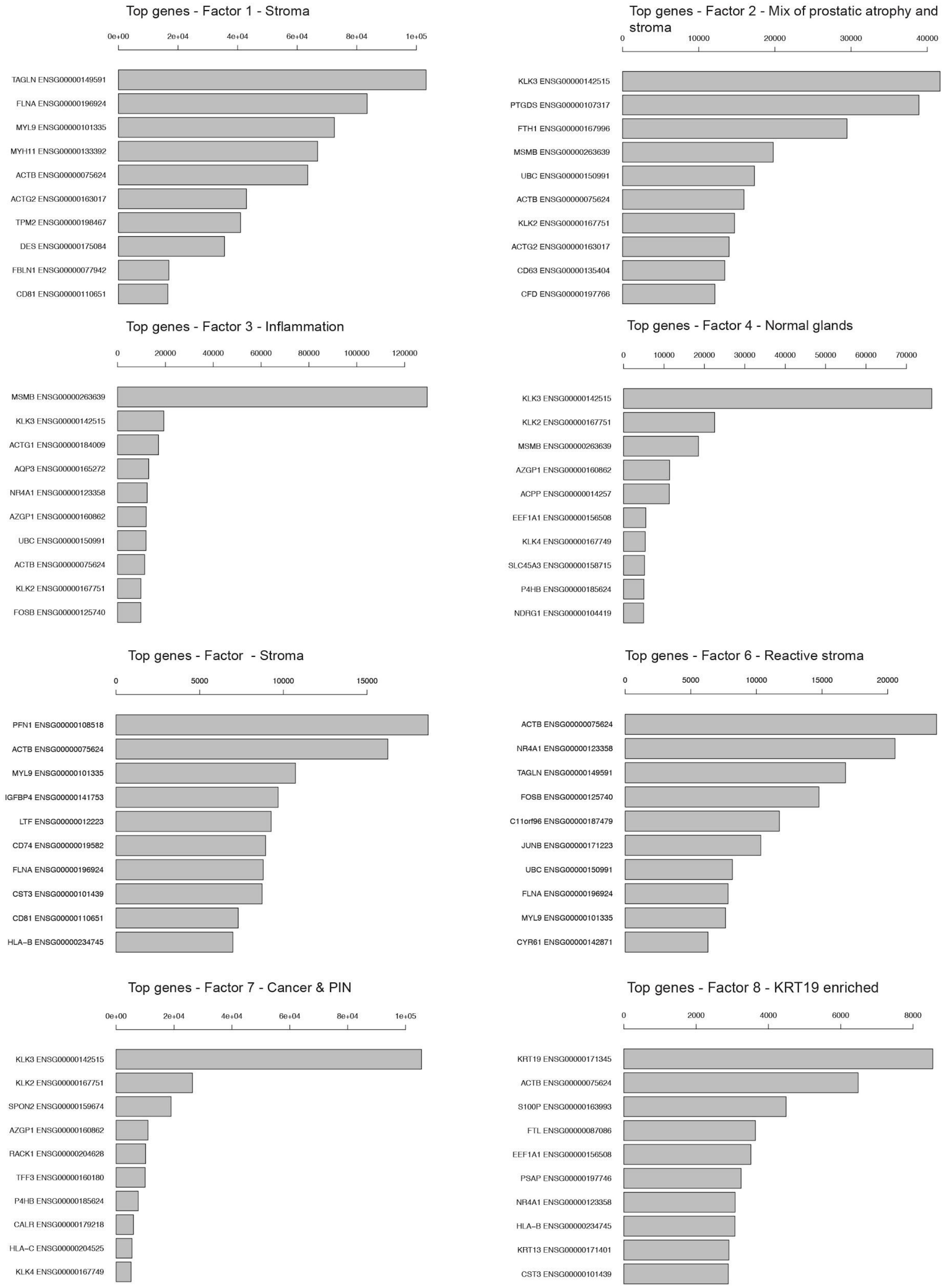

Supplementary Figure 24. Expected number of reads explained by factors from factor analysis in Supplementary Fig. 23. Only the ten highest-expressed genes are shown.

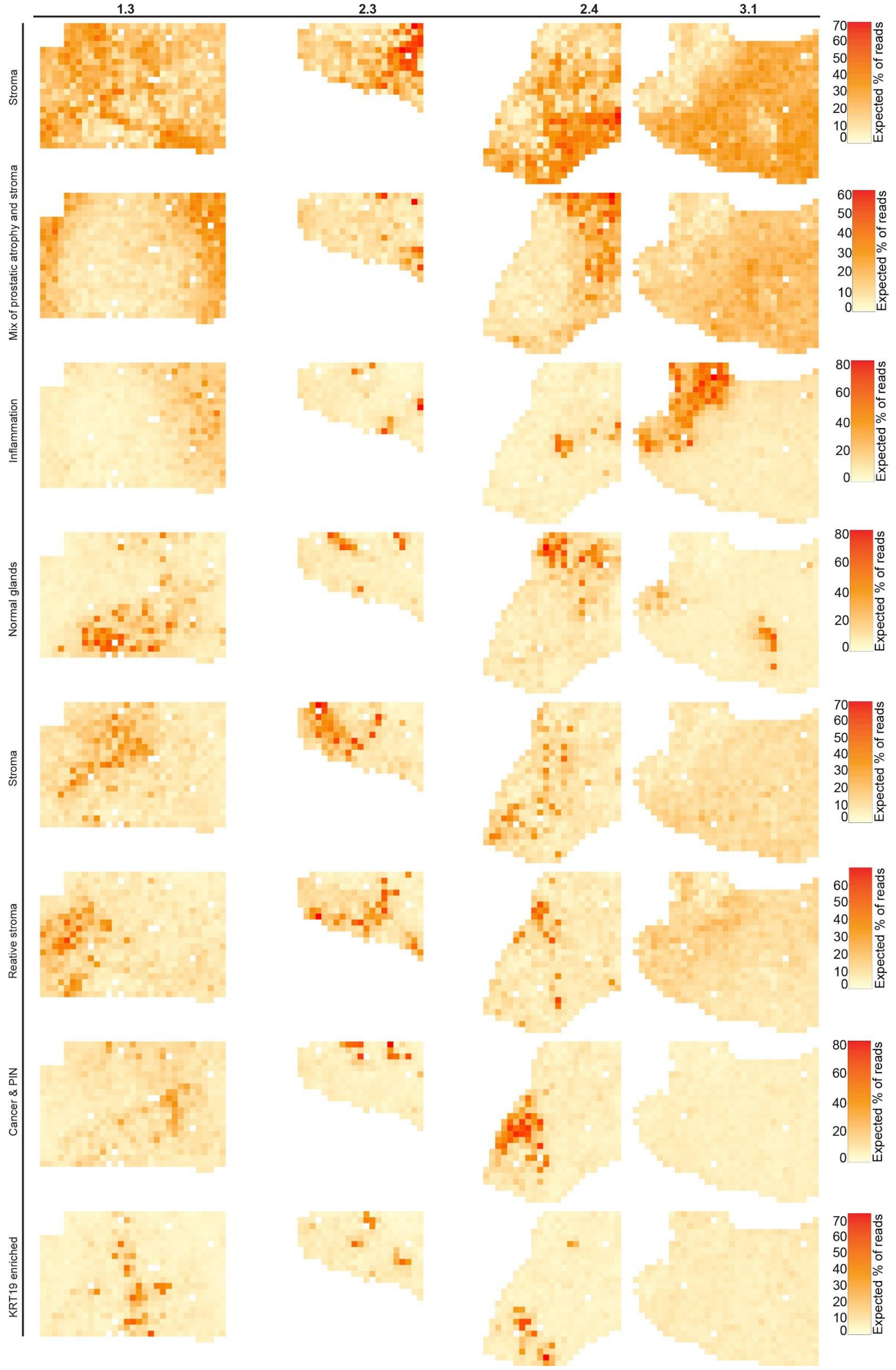

Supplementary Figure 25. Factor activity maps from analysis in Supplementary Fig. 23. Scale bar equals relative frequencies expressed in percentage.

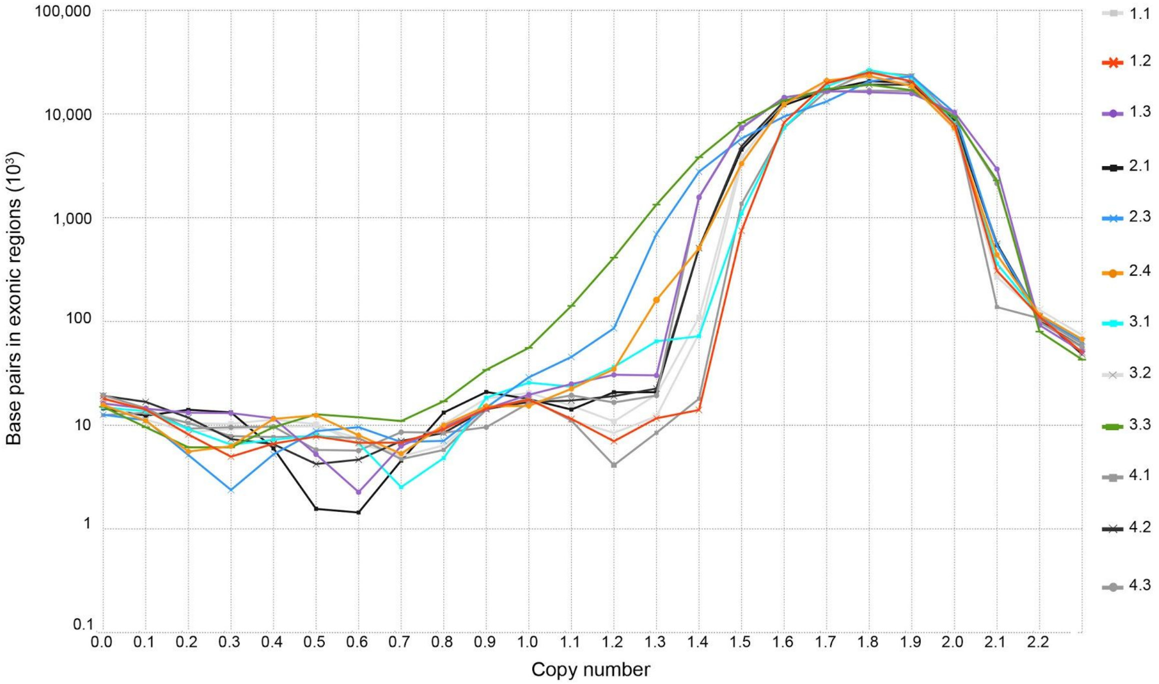

Supplementary Figure 26. Copy number analysis for deleted segments. Affected base pairs in exonic regions per rounded copy number value below 2.2 for each sample are presented. Sample 3.3 with the largest cancerous area shows the most deleted base pairs.

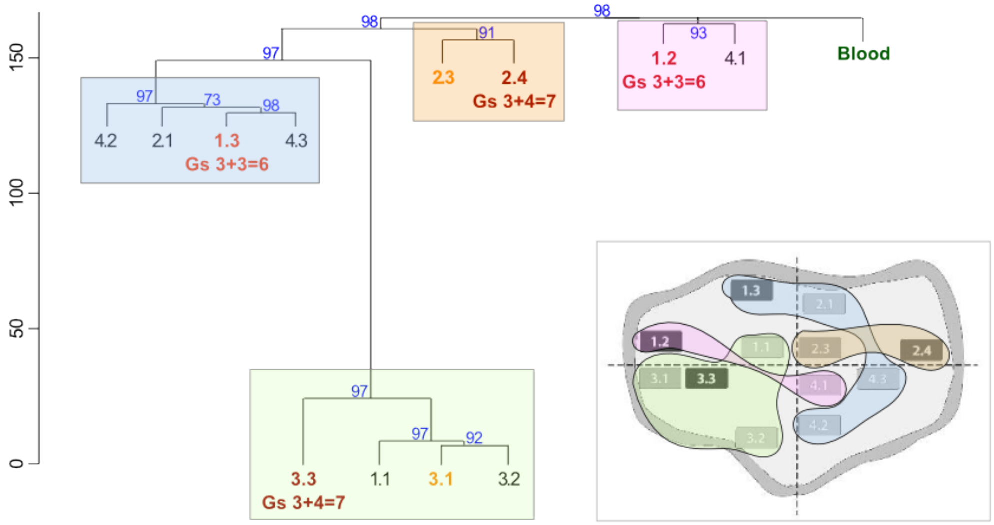

Supplementary Figure 27. **Similarity tree based on Euclidean distance and hierarchical clustering (ward.D2).** Segments of the whole genome with a CNV<1.6 (deletions) and >2.3 (amplifications) were considered. Four clusters were revealed and each cluster contains one cancerous sample. 1.2 was ending up close to blood in the tree, whereas sample 3.3 shows the biggest difference to blood. Further, 3.3 contains the highest number of genetic structural variations of the twelve samples.

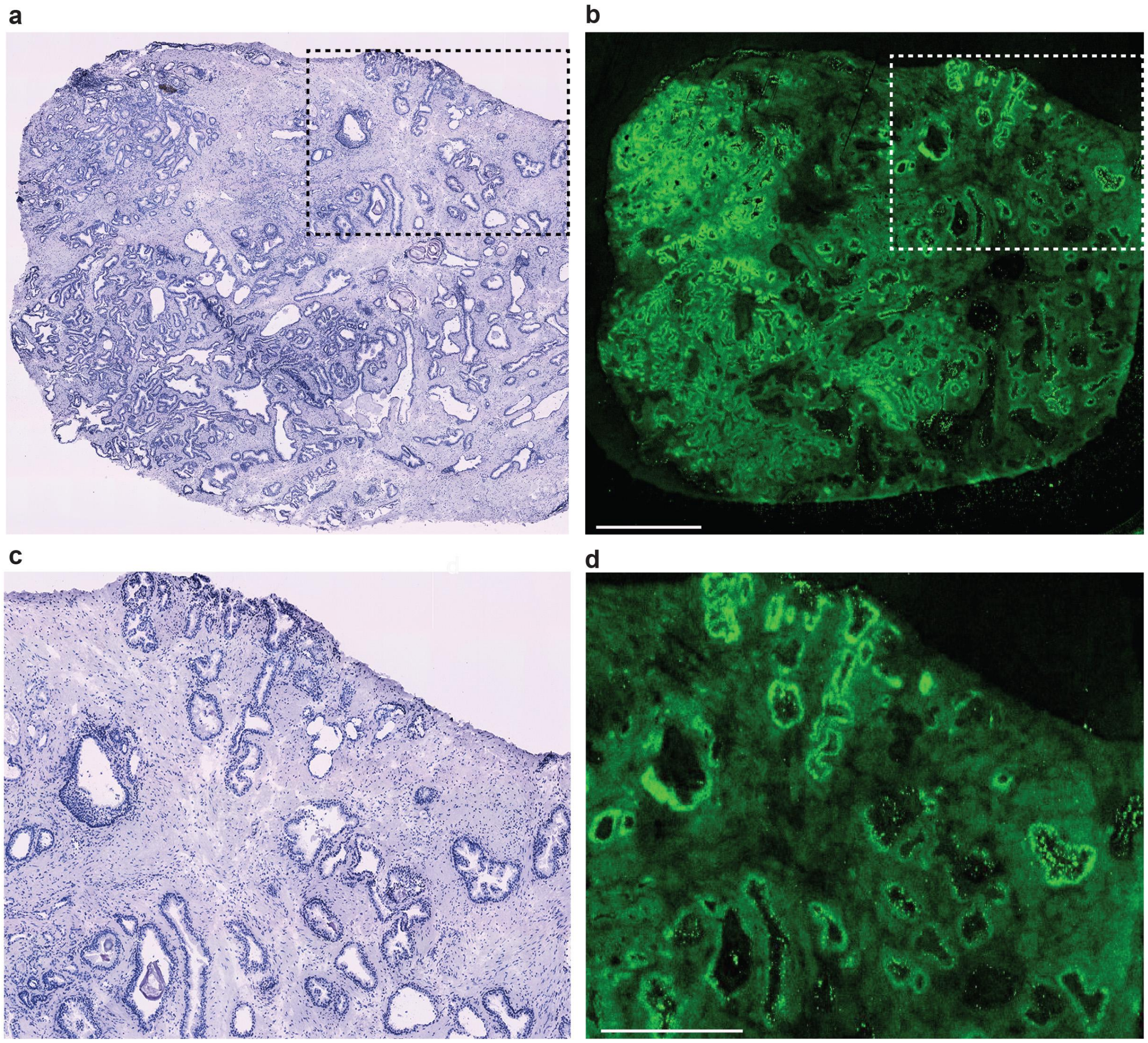

Supplementary Figure 28. Quality control assay of a prostate cancer tissue section. a Hematoxylin and eosin staining of prostate cancer tissue. **b** Fluorescent cDNA signal after tissue removal. Scale bar=1.5mm. **c** and **d** Magnification of box in **a and b**. Scale bar=750µm. The results display that a permeabilization time of 10 min gives maximized signal within tissue and minimized diffusion outside the tissue.

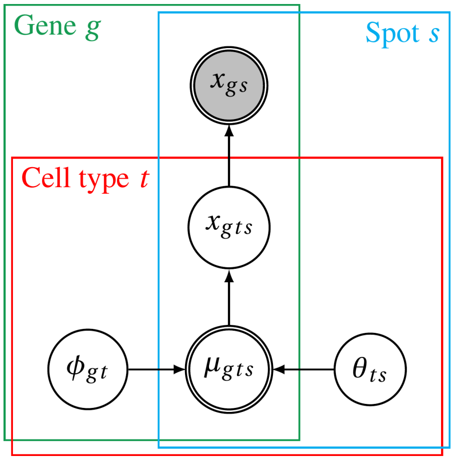

Supplementary Figure 29. Graphical model representation of the core Poisson regression model. Plates for genes, spots, and cell types indicate replication. Circled nodes are random variables. Doubly-circled nodes are deterministic random variables. The shaded node is observed.

Supplementary Table 1. Overview of samples and data evaluation for patient 1. Table includes pathological annotation and sequencing data statistics for all tissue sections, number of spots covered by tissue, genes per spots, unique transcripts covered by tissue and unique transcripts per spot. Also, the (relative) area in % of the given cell type is given. Numbers in parentheses in the three last columns represent proportions annotated as cancer.

Sample	Pathological annotation	Spots covered by tissue	Genes per spots	Unique transcripts covered by tissue (x 10^4)	Unique transcripts per spot	Area [%]		
						Stroma	*Epithelium*	*Lumen*
1.1	No cancer	432	802	123.4	2857	75.7	11.0	13.3
1.2	Gs 3+3 & PIN3	406	2895	366.2	9019	53.6(0.21)	34.5(0.35)	11.9(0.04)
1.3	Gs 3+3 (outside of area covered by array spots)	629	1377	282.0	4483	82.0(0.82)	8.9(0.19)	9.1(0.05)
2.1	No cancer	547	2869	481.9	8810	70.3	8.3	19.6
2.3	Inflammation	483	346	92.0	1904	81.8	10.4	7.8
2.4	Gs 3+4, suspected Gs	448	790	150.3	3356	79.3(4.0)	9.7(0.15)	11.0(0.01)
3.1	Inflammation	627	1374	152.8	2436	85.6	7.3	7.1
3.2	No cancer	523	553	38.0	727	82.5	8.6	8.9
3.3	Gs 3+4, PIN	501	1200	223.2	4457	83.9(48.16)	11.0(10.22)	5.1(2.54)
4.1	No cancer	688	503	150.9	2194	93.0	2.7	4.3
4.2	Inflammation	324	2062	150.0	4629	77.7	17.5	4.8
4.3	No cancer	302	588	23.4	776	67.9	17.3	14.8

Supplementary Table 2. Calculation of epitel, strom and lumen for factors in Fig. 2. The sum of the 10 strongest spots for every factor.

factor	area				%		
	epitel	stroma	lumen	tot	epitel	stroma	lumen
1	294178	45377	68615	408170	72	11	17
8	150981	174211	82978	408170	37	43	20
9	56453	287172	64545	408170	14	70	16
10	184395	150806	72970	408170	45	37	18
7	229570	129345	49255	408170	56	32	12

Supplementary Note 1

Factor Analysis for Spatial Transcriptome Deconvolution

Here we give the mathematical details of the model to perform factor analysis on count matrices such as applicable for spatial gene expression data. First, in section 1 we describe a model to analyze count data of single experiments. Conditional posteriors for this model are given in section 2, and section 3 explains how inference is performed with it. Sections 4 and 5 explain extensions of the model to jointly analyze multiple experiments and to perform spatial smoothing. How parameters are initialized is explained in section 6. Section 7 gives several remarks on practical application of the model, such as on the number of factors to use, on convergence and number of iterations, as well as on how to extract gene expression profiles and spatial activity maps from the inferred parameters. Section 8 concludes the outline of the method's model with a discussion of its limitations. Section 9 explains notation, and probability distributions used in this document are defined in section 10. Proofs for the lemmata are collected in section 12.

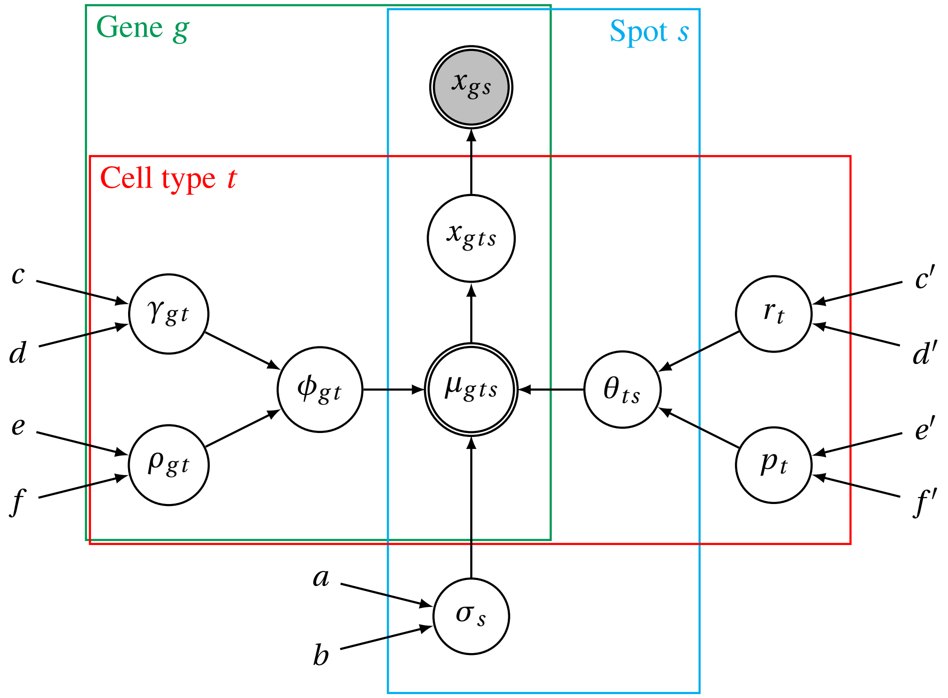

Supplementary Figure 30: Graphical model representation of the Poisson factor analysis single-experiment model. Circled nodes are random variables, those without circles are parameters. Doubly-circled nodes are deterministic random variables. The shaded node is observed.

1 Poisson factor analysis single-experiment model

We propose the following Poisson factor analysis single-experiment model here:

$$x_{gs} = \sum_{t=1}^{T} x_{gts} \tag{1}$$

$$x_{gts} \sim \text{Pois}\left(\mu_{gts}\right) \tag{2}$$

$$\mu_{gts} = \phi_{gt}\theta_{ts}\sigma_s \tag{3}$$

$$\phi_{gt} \sim \text{Gamma}\left(\gamma_{gt}, \frac{1-\rho_{gt}}{\rho_{gt}}\right) \tag{4}$$

$$\theta_{ts} \sim \text{Gamma}\left(r_t, \frac{1-p_t}{p_t}\right) \tag{5}$$

$$\sigma_s \sim \text{Gamma}\left(a, b\right) \tag{6}$$

$$\gamma_{gt} \sim \text{Gamma}\left(c, d\right) \tag{7}$$

$$\rho_{gt} \sim \text{Beta}\left(e, f\right) \tag{8}$$

$$r_t \sim \text{Gamma}\left(c', d'\right) \tag{9}$$

$$p_t \sim \text{Beta}\left(e', f'\right) \tag{10}$$

The corresponding graphical model is depicted in suppl. fig. 30. Default values of the hyper parameters are given in table T1.

2 Conditional posteriors for x_{gts}

Lemma 1 (Conditional posterior of x_{gts} after marginalizing out θ_{ts})**.** *In the Poisson factor analysis model, the conditional posterior of x_{gts} after marginalizing out θ_{ts} is given by*

$$Pr\left(x_{gts} \mid -\theta_{ts}\right) = NB\left(x_{gts}; r_t, \frac{\mu_{gts}}{\mu_{gts} + \theta_{ts}\frac{1-p_t}{p_t}}\right). \tag{11}$$

Lemma 2 (Conditional posterior of x_{gts} after marginalizing out ϕ_{gt})**.** *In the Poisson factor analysis model, the conditional posterior of x_{gts} after marginalizing out ϕ_{gt} is given by*

$$Pr\left(x_{gts} \mid -\phi_{gt}\right) = NB\left(x_{gts}; \gamma_{gt}, \frac{\mu_{gts}}{\mu_{gts} + \phi_{gt}\frac{1-\rho_{gt}}{\rho_{gt}}}\right). \tag{12}$$

Table T1: Default values for hyperparameters in the Poisson factor analysis model.

Parameter	Value
a	10
b	10
c	1
d	0.001
e	2
f	2
c'	1
d'	1
e'	0.05
f'	0.95

Lemma 3 (Mean of x_{gts} after marginalizing out ϕ_{gt})**.** *In the Poisson factor analysis model, the conditional posterior expected value of x_{gts} after marginalizing out ϕ_{gt} is given by*

$$\mathbb{E}\left[x_{gts} \mid -\phi_{gt}\right] = \gamma_{gt} \frac{\mu_{gts}}{\phi_{gt}} \frac{\rho_{gt}}{1-\rho_{gt}}.$$

Lemma 4 (Variance of x_{gts} after marginalizing out ϕ_{gt})**.** *In the Poisson factor analysis model, the conditional posterior variance of x_{gts} after marginalizing out ϕ_{gt} is given by*

$$Var\left[x_{gts} \mid -\phi_{gt}\right] = \gamma_{gt} \frac{\mu_{gts}}{\phi_{gt}} \frac{\rho_{gt}}{1-\rho_{gt}} \left(\frac{\mu_{gts}}{\phi_{gt}} \frac{\rho_{gt}}{1-\rho_{gt}} + 1\right).$$

Lemma 5 (Variance-to-mean ratio of x_{gts} after marginalizing out ϕ_{gt})**.** *In the Poisson factor analysis model, the conditional posterior variance-to-mean ratio of x_{gts} after marginalizing out ϕ_{gt} is given by*

$$\frac{Var\left[x_{gts} \mid -\phi_{gt}\right]}{\mathbb{E}\left[x_{gts} \mid -\phi_{gt}\right]} = \frac{\mu_{gts}}{\phi_{gt}} \frac{\rho_{gt}}{1-\rho_{gt}} + 1.$$

3 Parameter inference

The parameters x_{gts}, ϕ_{gt}, θ_{ts}, and σ_s of the Poisson factor analysis model can be optimized with Gibbs sampling iterations using the full conditional posteriors given below in lemmata 6 to 9. The

parameters γ_{gt}, ρ_{gt}, r_t, and p_t, lacking conjugacy, can be sampled with Metropolis-Hastings steps based on the conditional posteriors given in lemmata 1 and 2.

Lemma 6 (Sampling x_{gts})**.** *In the Poisson factor analysis model, denoting* $y_{gts} = \frac{\mu_{gts}}{\sum_t \mu_{gts}}$*, the full conditional posterior of* $[x_{g1s},\ldots,x_{gTs}]$ *is given by*

$$Pr\left([x_{g1s},\ldots,x_{gTs}] \mid -\right) \sim Mult\left(x_{gs}, [y_{g1s},\ldots,y_{gTs}]\right). \tag{13}$$

Lemma 7 (Sampling ϕ_{gt})**.** *In the Poisson factor analysis model, the full conditional posterior of* ϕ_{gt} *is given by*

$$Pr\left(\phi_{gt} \mid -\right) \sim Gamma\left(\gamma_{gt} + \sum_s x_{gts}, \frac{1-\rho_{gt}}{\rho_{gt}} + \sum_s \frac{\mu_{gts}}{\phi_{gt}}\right). \tag{14}$$

Lemma 8 (Sampling θ_{ts})**.** *In the Poisson factor analysis model, the full conditional posterior of* θ_{ts} *is given by*

$$Pr\left(\theta_{ts} \mid -\right) \sim Gamma\left(r_t + \sum_g x_{gts}, \frac{1-p_t}{p_t} + \sum_g \frac{\mu_{gts}}{\theta_{ts}}\right). \tag{15}$$

Lemma 9 (Sampling σ_s)**.** *In the Poisson factor analysis model, the full conditional posterior of* σ_s *is given by*

$$Pr\left(\sigma_s \mid -\right) \sim Gamma\left(a + \sum_{g,t} x_{gts}, b + \sum_{g,t} \frac{\mu_{gts}}{\sigma_s}\right).$$

4 Extension to multiple experiments

When multiple experiments are jointly analyzed, gene expression profile differences are frequently observed between the experiments. Regardless whether such differences are of biological or technical origin, we can extend the model to capture the expression differences. Between-experiment variability is allowed in our model by replacing eq. (3) with eq. (16) and amending the model with

eqs. (17) to (22), as follows:

$$\mu_{gts} = \phi_{gt}\varphi_{\epsilon(s)gt}\psi_{\epsilon(s)g}\theta_{ts}\sigma_s \tag{16}$$

$$\varphi_{egt} \sim \text{Gamma}\left(\lambda_{egt}, \frac{1-\varrho_{egt}}{\varrho_{egt}}\right) \tag{17}$$

$$\lambda_{egt} \sim \text{Gamma}\left(c', d'\right) \tag{18}$$

$$\varrho_{egt} \sim \text{Beta}\left(e', f'\right) \tag{19}$$

$$\psi_{eg} \sim \text{Gamma}\left(\beta_{eg}, \frac{1-\pi_{eg}}{\pi_{eg}}\right) \tag{20}$$

$$\beta_{eg} \sim \text{Gamma}\left(c', d'\right) \tag{21}$$

$$\pi_{eg} \sim \text{Beta}\left(e', f'\right), \tag{22}$$

where $\epsilon(s)$ gives the experiment that spot s is part of. The default values of the hyper-parameters c', d', e', f' are based on the values given in table T1. Specifically, they are chosen 50 times larger than those for the global random variables in order for the experiment-local random variables to have the same means while allowing for less variance around the means.

When count data of only one experiment is given then the value of the experiment-local variables φ_{egt} and ψ_{eg} is kept fixed at 1. Otherwise, the above described extension is used.

The expressions given for the conditional posteriors of the parameters x_{gts}, ϕ_{gt}, θ_{ts}, and σ_s of the Poisson factor analysis multi-experiment model are identical to those of the Poisson factor analysis single-experiment model given in sections 2 and 3, and their derivation is analogous. Corresponding expressions for the conditional posteriors of the parameters φ_{egt}, ψ_{eg} can also be derived analogously.

5 Extension for spatial smoothing

Spatial activities θ_{ts} in the model described so far are conditionally independent of each other and depend only on read counts in the spot s. In order to discover factors that have locally smoothly varying spatial activities, the modeling framework offers the following experimental feature.

We replace the spatial activities θ_{ts} used throughout the preceding sections by a product $\psi_{ts}\epsilon_{ts}$

of a smooth spatial field ψ_{ts} and a residual spatial activity ϵ_{ts}. In this way, local spot-specific deviations from the smooth field ψ_{ts} are captured by the residual spatial activities ϵ.

Updating the smooth spatial field ψ The smooth spatial field ψ is updated from the following conditional posterior,

$$\Pr\left(\psi_{ts} \mid -\right) \sim \text{Gamma}\left(r_t + \sum_{s'}\sum_{g} w_{s's} x_{gts'}, p_t + \sum_{s' \neq s}\sum_{g} w_{s's}\sigma_{s'}\psi_{s'}\phi_{gt}\right), \tag{23}$$

where $W = \left(w_{ij}\right)_{i=1,\ldots,S;j=1,\ldots,S}$ is a row-stochastic matrix such that $\sum_j w_{ij} = 1$ for all i and such that $w_{ij} \geq 0$ for all i and j. Specifically, we use $w_{ij} = \frac{K(d_{ij})}{\sum_l K(d_{il})}$ where d_{ij} gives the Euclidean distance between spots i and j, and $K(x) = e^{\frac{-x^2}{2\varsigma^2}}$ with a default value for the length scale ς of 1.

Updating the residual spatial activities ϵ The residual spatial field ϵ is updated from the following conditional posterior,

$$\Pr\left(\epsilon_{ts} \mid -\right) \sim \text{Gamma}\left(\alpha + \sum_{g} x_{gts}, \beta + \psi_{ts}\sigma_s \sum_{g} \phi_{gt}\right), \tag{24}$$

where the default values for α and β are both 100.

6 Initialization

In general, prior parameters are initialized before parameters that depend on them. Most parameters are initialized by sampling from their conditional distributions based on their respective priors. But some parameters are initialized to deterministic values. Specifically, the parameters are initialized as follows.

First, the gene expression prior parameters γ_{gt} and ρ_{gt} are sampled from their conditional distributions in eqs. (7) and (8). Then, the gene expression parameters ϕ_{gt} are sampled using eq. (4). Next, the experiment-local, type-dependent gene expression prior parameters λ_{egt} and ϱ_{egt} are sampled using eqs. (18) and (19) before the experiment-local, type-dependent gene expression parameters φ_{egt} are initialized with eq. (17). Similarly, the experiment-local, type-independent gene expression prior parameters β_{et} and π_{et} are sampled with eqs. (21) and (22) before the experiment-local,

type-independent gene expression parameters ψ_{et} are initialized from eq. (20). All of these are sampled from their conditional distributions based on their prior parameters.

The r_t parameters are sampled from their conditional distribution given in eq. (9) and the p_t parameters are initialized to the value $\frac{1}{2}$. Then, the spatial activity variables θ_{ts} are sampled from their conditional distribution using eq. (5).

Finally, the spot scaling variables σ_s are initialized so as to be proportional to the number of reads observed in the spots and so that their mean is equal to one.

The count contributions x_{gts} need not be initialized since they are the first variables to be sampled in every iteration, and so will be implicitly initialized in the first iteration.

7 Usage notes

Convergence and number of iterations With Monte-Carlo Markov chain (MCMC) sampling, convergence of sampling is difficult to assess in general. For this reason it is left to the analyst to check convergence by inspecting the log-likelihood during sampling, and then deciding on a suitable number of MCMC iterations to perform. The default number of 2000 iterations typically appears sufficient in our experience and was used for all experiments described in the manuscript.

Number of factors The number of factors to allow for is determined by the analyst performing factor analysis. A too large number will yield factors that explain detailed, but perhaps overly specific patterns. Contrarily, a too small number of factors may over-compress the data and miss interesting patterns. All factor analyses presented in this manuscript allowed for ten factors.

Factor gene expression profiles Based on the model we can easily calculate the expected number of reads $x_{gt} = \sum_s x_{gts}$ explained by a given factor t for every gene g across some set of spots, $\mathbb{E}[x_{gt}] = \mathbb{E}[\sum_s x_{gts}] = \sum_s \mu_{gts}$. These expected numbers of explained reads are a useful way to represent the gene expression profiles of the factors.

Spatial factor activity maps Similarly to the gene expression profiles, also the factor activity maps presented in the manuscript are based on expected numbers of reads $x_{ts} = \sum_g x_{gts}$ explained by a given factor t in a spot s, $\mathbb{E}[x_{ts}] = \mathbb{E}\left[\sum_g x_{gts}\right] = \sum_g \mu_{gts}$. Specifically, they give relative numbers of expected counts of explained reads, $\frac{\mathbb{E}[x_{ts}]}{\sum_t \mathbb{E}[x_{ts}]}$.

8 Limitations

Gene expression is often modeled with the negative binomial distribution. In contrast, the model presented here essentially relies on the Poisson distribution for this purpose. The reason why the negative binomial distribution is often preferred is that it allows the variance to exceed the mean (i.e. it allows to model over-dispersion), while for the Poisson distribution these are always identical, and, empirically, gene expression data often displays over-dispersion. However, as lemma 2 shows, the conditional posterior probability distribution of x_{gts} after marginalizing out ϕ_{gt} is negative binomially distributed. Based on this, one might be lead to use the conditional mean and variance of x_{gts} after marginalizing out ϕ_{gt} as a basis to perform differential gene expression analysis. In spite of this, we want to warn against over-interpreting the model parameters for the purpose of variance estimation since the variance scaling of the model is not as would be desired. Specifically, this is visible from the variance-to-mean ratio given in lemma 5, which scales linearly in $\frac{\mu_{gts}}{\phi_{gt}}$, and thus also linearly in θ_{ts} and σ_s. In other words, as the mean increases, due to e.g. increases in sequencing depth, so does the over-dispersion, which seems undesired.

We argue however, that despite this short-coming the Poisson factorization approach is useful to decompose convolved gene expression measurements from multiple cell types. The reasoning is two-fold, with a computational and empirical component, as well as a probabilistic one. On the computational and empirical side, we argue that the presented method based on Poisson factorization is computationally practical, and furthermore, biologically useful, as demonstrated here by the application in the main manuscript. Second, and on the probabilistic side, we argue that among mean and variance, the mean is the more important quantity to estimate, since a wrong mean estimate would (most likely) imply a wrong variance estimate, but not necessarily vice-versa. Thus, we hope that a factorization based on the Poisson distribution in order to be useful only needs to

successfully estimate the (deconvolved) mean expression, while a factorization based on a more accurate modeling of the gene expression variance might be expected to reflect reality even better.

9 Notation

The following notational conventions are used in this manuscript.

9.1 Full conditional probability distributions

For a model consisting of random variables $x_1,\dots,x_n$, the full conditional probability distribution of a set of variables, e.g. of x_1 and x_2 given the remaining variables will be denoted by

$$\Pr(x_1,x_2 \mid -) := \Pr(x_1,x_2 \mid x_3,\dots,x_n)\,.$$

9.2 Marginalized conditionals

When we consider a conditional probability distribution of a set of random variables (e.g. x_1 and x_2) after some other set of random variables has been marginalized out (e.g. x_3 and x_4), we will use the following notation:

$$\Pr(x_1,x_2 \mid -x_3,x_4) := \iint \Pr(x_1,x_2,x_3,x_4 \mid x_5,\dots,x_n)\, dx_3 dx_4.$$

10 Relevant probability distributions

Since some probability distributions have more than one commonly used definition, we define here the specific forms that will be used in this document.

10.1 Discrete probability distributions

We will first consider probability distributions for discrete random variables.

10.1.1 Poisson distribution

The probability mass function of a Poisson-distributed non-negative, integer-valued random variable X with positive, real-valued rate parameter $\lambda \in \mathbb{R}$ is given by

$$\Pr(X = k \mid \lambda) = \frac{\lambda^k e^{-\lambda}}{k!}. \tag{25}$$

The mean and variance of this distribution are equal to the rate parameter λ, $\mathbb{E}[X] = \mathrm{Var}[X] = \lambda$.

10.1.2 Binomial distribution

This distribution gives the probability of observing k successes in a series of n i.i.d. Bernoulli trials with probability parameter p,

$$\begin{aligned} \Pr(X = k \mid n, p) &= \binom{n}{k} p^k (1-p)^{n-k} \\ &= \frac{n!}{k!\,(n-k)!} p^k (1-p)^{n-k} \\ &= \frac{\Gamma(n+1)}{\Gamma(k+1)\,\Gamma(n-k+1)} p^k (1-p)^{n-k}. \end{aligned} \tag{26}$$

The mean of this distribution is $\mathbb{E}[X] = np$ and its variance is $\mathrm{Var}[X] = np(1-p)$.

10.1.3 Negative binomial distribution

This distribution gives the probability of observing k successes in i.i.d. Bernoulli trials with probability parameter p before $r > 0$ failures are seen,

$$\Pr(X = k \mid r, p) = \binom{k+r-1}{k} p^k (1-p)^r \tag{27}$$

$$= \frac{(k+r-1)!}{k!\,(r-1)!} p^k (1-p)^r \tag{28}$$

$$= \frac{\Gamma(k+r)}{\Gamma(k+1)\,\Gamma(r)} p^k (1-p)^r. \tag{29}$$

For this parameterization, the mean is given by

$$\mathbb{E}[X] = \frac{rp}{1-p} \tag{30}$$

and the variance by

$$\mathrm{Var}[X] = \frac{rp}{(1-p)^2}. \tag{31}$$

10.1.4 Multinomial distribution

Given a probability distribution $\boldsymbol{p} = [p_1, \ldots, p_k]$,

$$\begin{aligned}\Pr\left(\boldsymbol{X} = [x_1, \ldots, x_k] \mid n, \boldsymbol{p}, \sum_{i=1}^{k} x_i = n\right) &= \frac{n!}{\prod_{i=1}^{k} x_i!} \prod_{i=1}^{k} p_i^{x_i} \\ &= \frac{\Gamma(n+1)}{\prod_{i=1}^{k} \Gamma(x_i+1)} \prod_{i=1}^{k} p_i^{x_i}\end{aligned} \tag{32}$$

10.2 Continuous probability distributions

Next, we present definitions for some probability distributions of continuous random variables.

10.2.1 Beta distribution

A random variable X is beta-distributed with positive, real-valued parameters α and β if its probability density function is

$$\begin{aligned}\Pr(X = x \mid \alpha, \beta) &= \frac{1}{B(\alpha, \beta)} x^{\alpha-1}(1-x)^{\beta-1} \\ &= \frac{\Gamma(\alpha+\beta)}{\Gamma(\alpha)\Gamma(\beta)} x^{\alpha-1}(1-x)^{\beta-1}.\end{aligned} \tag{33}$$

10.2.2 Gamma distribution

The probability density function of a gamma-distributed positive, real-valued random variable λ with positive, real-valued parameters α and β is given by

$$\Pr(\lambda \mid \alpha, \beta) = \frac{\beta^{\alpha}}{\Gamma(\alpha)} \lambda^{\alpha-1} e^{-\beta\lambda}. \tag{34}$$

Note that there exists also another parameterization for Gamma-distributed random variables in which the second parameter is equal to the inverse of β. To avoid confusion, we will consistently only use the above parameterization in this document.

11 Supplementary lemmata

Lemma 10. *Suppose that $x_1, \dots, x_T$ are independent random variables with $x_t \sim Pois(\lambda_t)$ and $x = \sum_t x_t$. Set $\lambda = \sum_t \lambda_t$ and let $y_1, \dots, y_T$ be random variables such that*

$$y \sim Pois(\lambda)$$
$$[y_1, \dots, y_T] \mid y \sim Mult\left(y, \left[\frac{\lambda_1}{\lambda}, \dots, \frac{\lambda_T}{\lambda}\right]\right).$$

Then $[x, x_1, \dots, x_T]$ has the same distribution as $[y, y_1, \dots, y_T]$.

12 Proofs

Proof of Lemma 1. [Conditional posterior of x_{gts} after marginalizing out θ_{ts}] Let us denote $\zeta = \frac{1-p_t}{p_t}$. We can consider how many reads are attributed to a given cell type t in spot s for gene g after marginalizing out θ_{ts},

$$\begin{aligned}
\Pr(x_{gts} \mid -\theta_{ts}) &= \int_0^\infty \text{Pois}(x_{gts}; \mu_{gts})\, \text{Gamma}(\theta_{ts}; r_t, \zeta)\, d\theta_{ts} && (35)\\
&= \int_0^\infty \text{Pois}(x_{gts}; \phi_{gt}\theta_{ts}\sigma_s)\, \text{Gamma}(\theta_{ts}; r_t, \zeta)\, d\theta_{ts} && (36)\\
&= \int_0^\infty \frac{(\phi_{gt}\theta_{ts}\sigma_s)^{x_{gts}}}{x_{gts}!} e^{-\phi_{gt}\theta_{ts}\sigma_s} \frac{\zeta^{r_t}}{\Gamma(r_t)} \theta_{ts}^{r_t-1} e^{-\zeta\theta_{ts}}\, d\theta_{ts} && (37)\\
&= \frac{(\phi_{gt}\sigma_s)^{x_{gts}}}{x_{gts}!} \frac{\zeta^{r_t}}{\Gamma(r_t)} \int_0^\infty \theta_{ts}^{r_t + x_{gts} - 1} e^{-\theta_{ts}(\phi_{gt}\sigma_s + \zeta)}\, d\theta_{ts} && (38)\\
&= \frac{(\phi_{gt}\sigma_s)^{x_{gts}}}{x_{gts}!} \frac{\zeta^{r_t}}{\Gamma(r_t)} \Gamma(r_t + x_{gts}) (\phi_{gt}\sigma_s + \zeta)^{-r_t - x_{gts}} && (39)\\
&= \left(\frac{\phi_{gt}\sigma_s}{\phi_{gt}\sigma_s + \zeta}\right)^{x_{gts}} \frac{\Gamma(r_t + x_{gts})}{x_{gts}!\Gamma(r_t)} \left(\frac{\zeta}{\phi_{gt}\sigma_s + \zeta}\right)^{r_t} && (40)\\
&= \text{NB}\left(x_{gts}; r_t, \frac{\phi_{gt}\sigma_s}{\phi_{gt}\sigma_s + \zeta}\right) && (41)\\
&= \text{NB}\left(x_{gts}; r_t, \frac{\phi_{gt}\sigma_s}{\phi_{gt}\sigma_s + \frac{1-p_t}{p_t}}\right). && (42)
\end{aligned}$$

□

Proof of Lemma 2. [Conditional posterior of x_{gts} after marginalizing out ϕ_{gt}] Let us again denote $\zeta = \frac{1-\rho_{gt}}{\rho_{gt}}$. We can consider how many reads are attributed to a given cell type t in spot s for gene g after marginalizing out ϕ_{gt},

$$\begin{aligned}
\Pr\left(x_{gts} \mid -\phi_{gt}\right) &= \int_0^\infty \text{Pois}\left(x_{gts};\mu_{gts}\right)\text{Gamma}\left(\phi_{gt};\gamma_{gt},\zeta\right)d\phi_{gt} && (43)\\
&= \int_0^\infty \text{Pois}\left(x_{gts};\phi_{gt}\theta_{ts}\sigma_s\right)\text{Gamma}\left(\phi_{gt};\gamma_{gt},\zeta\right)d\phi_{gt} && (44)\\
&= \int_0^\infty \frac{\left(\phi_{gt}\theta_{ts}\sigma_s\right)^{x_{gts}}}{x_{gts}!}e^{-\phi_{gt}\theta_{ts}\sigma_s}\frac{\zeta^{\gamma_{gt}}}{\Gamma\left(\gamma_{gt}\right)}\phi_{gt}^{\gamma_{gt}-1}e^{-\zeta\phi_{gt}}d\phi_{gt} && (45)\\
&= \frac{(\theta_{ts}\sigma_s)^{x_{gts}}}{x_{gts}!}\frac{\zeta^{\gamma_{gt}}}{\Gamma\left(\gamma_{gt}\right)}\int_0^\infty \phi_{gt}^{\gamma_{gt}+x_{gts}-1}e^{-\phi_{gt}(\theta_{ts}\sigma_s+\zeta)}d\phi_{gt} && (46)\\
&= \frac{(\theta_{ts}\sigma_s)^{x_{gts}}}{x_{gts}!}\frac{\zeta^{\gamma_{gt}}}{\Gamma\left(\gamma_{gt}\right)}\Gamma\left(\gamma_{gt}+x_{gts}\right)(\theta_{ts}\sigma_s+\zeta)^{-\gamma_{gt}-x_{gts}} && (47)\\
&= \left(\frac{\theta_{ts}\sigma_s}{\theta_{ts}\sigma_s+\zeta}\right)^{x_{gts}}\frac{\Gamma\left(\gamma_{gt}+x_{gts}\right)}{x_{gts}!\Gamma\left(\gamma_{gt}\right)}\left(\frac{\zeta}{\theta_{ts}\sigma_s+\gamma}\right)^{\gamma_{gt}} && (48)\\
&= \text{NB}\left(x_{gts};\gamma_{gt},\frac{\theta_{ts}\sigma_s}{\theta_{ts}\sigma_s+\zeta}\right) && (49)\\
&= \text{NB}\left(x_{gts};\gamma_{gt},\frac{\theta_{ts}\sigma_s}{\theta_{ts}\sigma_s+\frac{1-\rho_{gt}}{\rho_{gt}}}\right). && (50)
\end{aligned}$$

□

Proof of Lemma 3. This follows from eq. (30) in combination with lemma 2. □

Proof of Lemma 4. This follows from eq. (31) in combination with lemma 2. □

Proof of Lemma 5. This follows from lemmata 3 and 4. □

Proof of Lemma 6. [Poisson factor analysis model: Sampling x_{gts}] Use lemma 10 and eqs. (1) and (2). □

Proof of Lemma 7. [Sampling ϕ_{gt}] Writing $\zeta = \frac{1-\rho_{gt}}{\rho_{gt}}$, the relevant terms for the conditional posterior of ϕ_{gt} are

$$\begin{aligned}
\Pr\left(\phi_{gt} \mid -\right) &\propto \Pr\left(\phi_{gt} \mid \gamma_{gt},\rho_{gt}\right)\prod_s \Pr\left(x_{gts} \mid \mu_{gts}\right)\\
&= \text{Gamma}\left(\phi_{gt};\gamma_{gt},\zeta\right)\prod_s \text{Pois}\left(x_{gts};\mu_{gts}\right)
\end{aligned}$$

$$
\begin{aligned}
&= \frac{\zeta^{\gamma_{gt}}}{\Gamma\left(\gamma_{gt}\right)} \phi_{gt}^{\gamma_{gt}-1} e^{-\zeta\phi_{gt}} \prod_s \frac{\mu_{gts}^{x_{gts}} e^{-\mu_{gts}}}{x_{gts}!} \\
&= \frac{\zeta^{\gamma_{gt}}}{\Gamma\left(\gamma_{gt}\right)} \phi_{gt}^{\gamma_{gt}-1} e^{-\zeta\phi_{gt}} \prod_s \frac{\left(\phi_{gt}\theta_{ts}\sigma_s\right)^{x_{gts}} e^{-\phi_{gt}\theta_{ts}\sigma_s}}{x_{gts}!} \\
&= \frac{\zeta^{\gamma_{gt}}}{\Gamma\left(\gamma_{gt}\right)} \phi_{gt}^{\gamma_{gt}+\sum_s x_{gts}-1} e^{-\phi_{gt}(\zeta+\sum_s \theta_{ts}\sigma_s)} \prod_s \frac{(\theta_{ts}\sigma_s)^{x_{gts}}}{x_{gts}!} \\
&\propto \text{Gamma}\left(\phi_{gt}; \gamma_{gt} + \sum_s x_{gts}, \zeta + \sum_s \theta_{ts}\sigma_s\right) \\
&= \text{Gamma}\left(\phi_{gt}; \gamma_{gt} + \sum_s x_{gts}, \frac{1-\rho_{gt}}{\rho_{gt}} + \sum_s \theta_{ts}\sigma_s\right) \\
&= \text{Gamma}\left(\gamma_{gt} + \sum_s x_{gts}, \frac{1-\rho_{gt}}{\rho_{gt}} + \sum_s \frac{\mu_{gts}}{\phi_{gt}}\right).
\end{aligned}
$$

□

Proof of Lemma 8. [Sampling θ_{ts}] Writing $\zeta = \frac{1-p_t}{p_t}$, the relevant terms for the conditional posterior of θ_{ts} are

$$
\begin{aligned}
\Pr(\theta_{ts} \mid -) &\propto \Pr\left(\theta_{ts} \mid r_t, p_t\right) \prod_g \Pr\left(x_{gts} \mid \mu_{gts}\right) \\
&= \text{Gamma}(\theta_{ts}; r_t, \zeta) \prod_g \text{Pois}\left(x_{gts}; \mu_{gts}\right) \\
&= \frac{\zeta^{r_t}}{\Gamma(r_t)} \theta_{ts}^{r_t-1} e^{-\zeta\theta_{ts}} \prod_g \frac{\mu_{gts}^{x_{gts}} e^{-\mu_{gts}}}{x_{gts}!} \\
&= \frac{\zeta^{r_t}}{\Gamma(r_t)} \theta_{ts}^{r_t-1} e^{-\zeta\theta_{ts}} \prod_g \frac{\left(\phi_{gt}\theta_{ts}\sigma_s\right)^{x_{gts}} e^{-\phi_{gt}\theta_{ts}\sigma_s}}{x_{gts}!} \\
&= \frac{\zeta^{r_t}}{\Gamma(r_t)} \theta_{ts}^{r_t+\sum_g x_{gts}-1} e^{-\theta_{ts}\left(\zeta+\sum_g \phi_{gt}\sigma_s\right)} \prod_g \frac{\left(\phi_{gt}\sigma_s\right)^{x_{gts}}}{x_{gts}!} \\
&\propto \text{Gamma}\left(\theta_{ts}; r_t + \sum_g x_{gts}, \zeta + \sum_g \phi_{gt}\sigma_s\right) \\
&= \text{Gamma}\left(\theta_{ts}; r_t + \sum_g x_{gts}, \frac{1-p_t}{p_t} + \sum_g \phi_{gt}\sigma_s\right) \\
&= \text{Gamma}\left(r_t + \sum_g x_{gts}, \frac{1-p_t}{p_t} + \sum_g \frac{\mu_{gts}}{\theta_{ts}}\right).
\end{aligned}
$$

□

Proof of Lemma 9. [Sampling σ_s]

$$\begin{aligned}
\Pr(\sigma_s \mid -) &\propto \text{Gamma}(\sigma_s; a, b) \prod_{g,t} \text{Pois}\left(x_{gts}; \phi_{gt}\theta_{ts}\sigma_s\right) \\
&= \frac{b^a}{\Gamma(a)} \sigma_s^{a-1} e^{-b\sigma_s} \prod_{g,t} \frac{\left(\phi_{gt}\theta_{ts}\sigma_s\right)^{x_{gts}} e^{-\phi_{gt}\theta_{ts}\sigma_s}}{x_{gts}!} \\
&= \frac{b^a}{\Gamma(a)} \sigma_s^{a+\sum_{g,t} x_{gts}-1} e^{-\sigma_s\left(b+\sum_{g,t}\phi_{gt}\theta_{ts}\right)} \prod_{g,t} \frac{\left(\phi_{gt}\theta_{ts}\right)^{x_{gts}}}{x_{gts}!} \\
&\propto \text{Gamma}\left(\sigma_s; a + \sum_{g,t} x_{gts}, b + \sum_{g,t} \phi_{gt}\theta_{ts}\right) \\
&= \text{Gamma}\left(a + \sum_{g,t} x_{gts}, b + \sum_{g,t} \frac{\mu_{gts}}{\sigma_s}\right).
\end{aligned}$$

□

Proof of Lemma 10.

$$\Pr(x, [x_1, \ldots, x_T]) = \prod_t \text{Pois}(x_t; \lambda_t) = \prod_t \frac{\lambda_t^{x_t} e^{-\lambda_t}}{x_t!} = e^{-\sum_t \lambda_t} \prod_t \frac{\lambda_t^{x_t}}{x_t!} = e^{-\lambda} \prod_t \frac{\lambda_t^{x_t}}{x_t!}$$

$$\begin{aligned}
\Pr\left(y, [y_1, \ldots, y_T]\right) &= \Pr\left(y\right) \Pr\left([y_1, \ldots, y_T] \mid y\right) = \text{Pois}\left(y; \lambda\right) \text{Mult}\left([y_1, \ldots, y_T]; y, \left[\frac{\lambda_1}{\lambda}, \ldots, \frac{\lambda_T}{\lambda}\right]\right) \\
&= \frac{\lambda^y e^{-\lambda}}{y!} \cdot \frac{y!}{\prod_t y_t!} \prod_t \left(\frac{\lambda_t}{\lambda}\right)^{y_t} = \frac{\lambda^y e^{-\lambda}}{\lambda^{\sum_t y_t}} \prod_t \frac{\lambda_t^{y_t}}{y_t!} = \frac{\lambda^y e^{-\lambda}}{\lambda^y} \prod_t \frac{\lambda_t^{y_t}}{y_t!} = e^{-\lambda} \prod_t \frac{\lambda_t^{y_t}}{y_t!}
\end{aligned}$$

□
