# Supplementary material for: Spatial maps of prostate cancer transcriptomes reveal an unexplored landscape of heterogeneity
Source: Nat Commun. 2018 Jun 20;9:2419. doi: 10.1038/s41467-018-04724-5 (PMC6010471; doi:10.1038/s41467-018-04724-5)
Supplement: Supplementary file 3 — Description of Additional Supplementary Files [file 41467_2018_4724_MOESM3_ESM.pdf]

Description of Additional Supplementary Files

File Name: Supplementary Data 1
Description: Data files and PDFs with the results of the factor analysis for sample 1.2.

File Name: Supplementary Data 2
Description: Results from gene expression analysis for sample 1.2

File Name: Supplementary Data 3
Description: Data files and PDFs with the results of the factor analysis for samples 1.2, 2.4 and 3.3.

File Name: Supplementary Data 4
Description: Results from pathway analysis of all ten factors in Fig. 3

File Name: Supplementary Data 5
Description: Data files and PDFs with the results of the factor analysis for Patient 1, 2 and 3.

File Name: Supplementary Data 6
Description: Data files and PDFs with the results of the factor analysis for all 12 samples.

File Name: Supplementary Data 7
Description: Data files and PDFs with the results of the factor analysis for samples 3.1 and 4.2.

File Name: Supplementary Data 8
Description: Results from Pathway analysis with IPA software (normal stroma) in Fig 5.

File Name: Supplementary Data 9
Description: Results from Pathway analysis with IPA software (reactive stroma) in Fig 5.

File Name: Supplementary Data 10
Description: Data files and PDFs with the results of the factor analysis for samples 1.3, 2.3, 2.4, and 3.1.

File Name: Supplementary Data 11
Description: Results from gene expression analysis for sample 3.3

File Name: Supplementary Data 12
Description: Results from pathway analysis of center and periphery of cancer in sample 1.2

File Name: Supplementary Data 13
Description: Results from pathway analysis of center and periphery of cancer in sample 3.3

File Name: Supplementary Data 14
Description: Results from pathway analysis of center and periphery of cancer in sample 2.4
