# Supplementary material for: Spatial maps of prostate cancer transcriptomes reveal an unexplored landscape of heterogeneity
Source: Nat Commun. 2018 Jun 20;9:2419. doi: 10.1038/s41467-018-04724-5 (PMC6010471; doi:10.1038/s41467-018-04724-5)
Supplement: Supplementary file 4 — Supplementary Data 1 [file 41467_2018_4724_MOESM4_ESM.zip › Supplementary Dataset 1/top-genes.pdf]

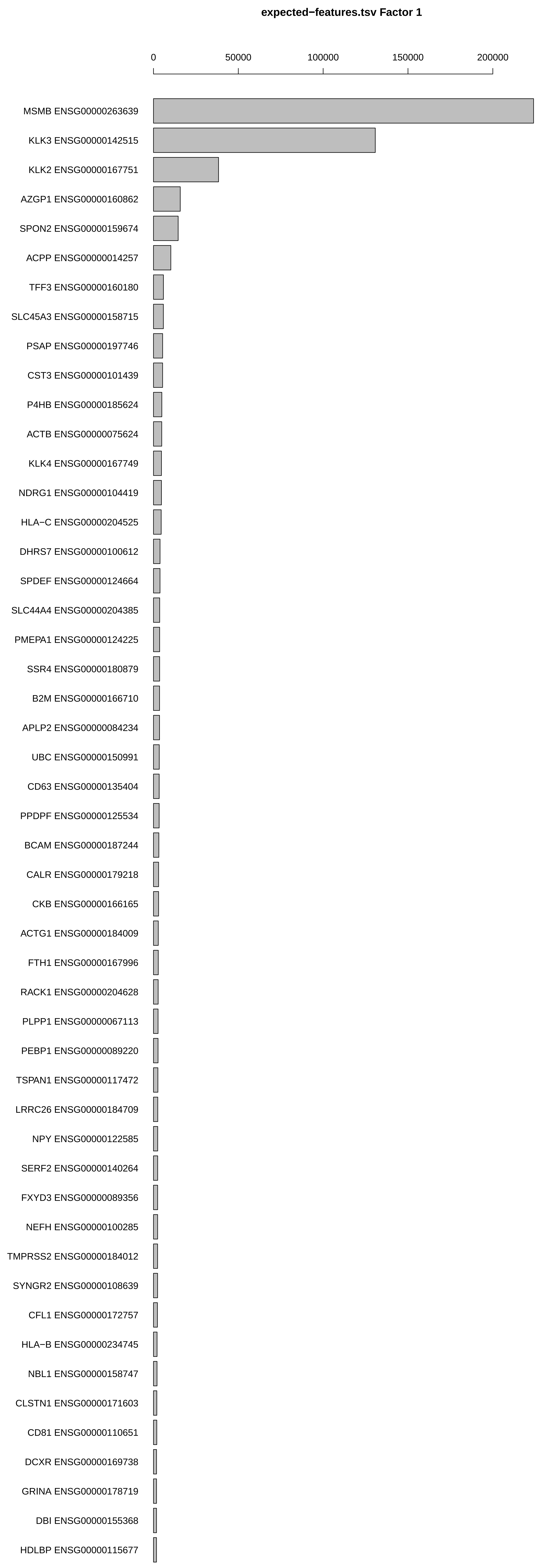
expected−features.tsv Factor 1
0
50000
100000
150000
200000
MSMB ENSG00000263639
KLK3 ENSG00000142515
KLK2 ENSG00000167751
AZGP1 ENSG00000160862
SPON2 ENSG00000159674
ACPP ENSG00000014257
TFF3 ENSG00000160180
SLC45A3 ENSG00000158715
PSAP ENSG00000197746
CST3 ENSG00000101439
P4HB ENSG00000185624
ACTB ENSG00000075624
KLK4 ENSG00000167749
NDRG1 ENSG00000104419
HLA−C ENSG00000204525
DHRS7 ENSG00000100612
SPDEF ENSG00000124664
SLC44A4 ENSG00000204385
PMEPA1 ENSG00000124225
SSR4 ENSG00000180879
B2M ENSG00000166710
APLP2 ENSG00000084234
UBC ENSG00000150991
CD63 ENSG00000135404
PPDPF ENSG00000125534
BCAM ENSG00000187244
CALR ENSG00000179218
CKB ENSG00000166165
ACTG1 ENSG00000184009
FTH1 ENSG00000167996
RACK1 ENSG00000204628
PLPP1 ENSG00000067113
PEBP1 ENSG00000089220
TSPAN1 ENSG00000117472
LRRC26 ENSG00000184709
NPY ENSG00000122585
SERF2 ENSG00000140264
FXYD3 ENSG00000089356
NEFH ENSG00000100285
TMPRSS2 ENSG00000184012
SYNGR2 ENSG00000108639
CFL1 ENSG00000172757
HLA−B ENSG00000234745
NBL1 ENSG00000158747
CLSTN1 ENSG00000171603
CD81 ENSG00000110651
DCXR ENSG00000169738
GRINA ENSG00000178719
DBI ENSG00000155368
HDLBP ENSG00000115677

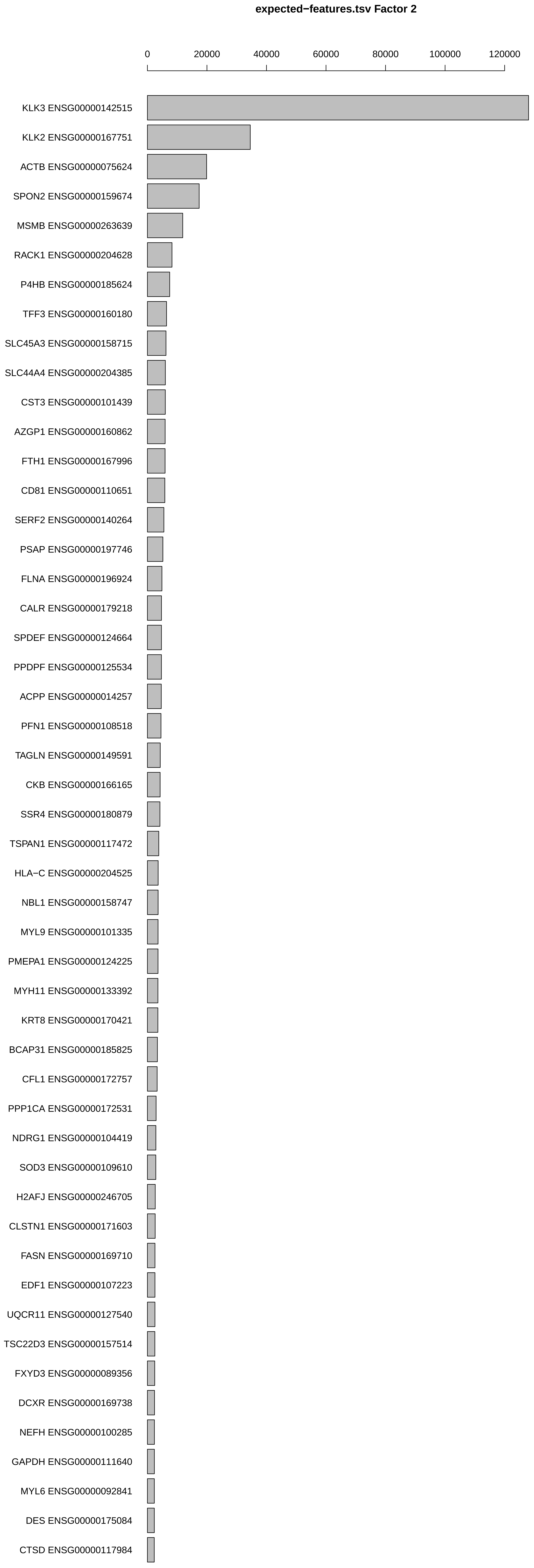
expected−features.tsv Factor 2
0
20000
40000
60000
80000
100000
120000
KLK3 ENSG00000142515
KLK2 ENSG00000167751
ACTB ENSG00000075624
SPON2 ENSG00000159674
MSMB ENSG00000263639
RACK1 ENSG00000204628
P4HB ENSG00000185624
TFF3 ENSG00000160180
SLC45A3 ENSG00000158715
SLC44A4 ENSG00000204385
CST3 ENSG00000101439
AZGP1 ENSG00000160862
FTH1 ENSG00000167996
CD81 ENSG00000110651
SERF2 ENSG00000140264
PSAP ENSG00000197746
FLNA ENSG00000196924
CALR ENSG00000179218
SPDEF ENSG00000124664
PPDPF ENSG00000125534
ACPP ENSG00000014257
PFN1 ENSG00000108518
TAGLN ENSG00000149591
CKB ENSG00000166165
SSR4 ENSG00000180879
TSPAN1 ENSG00000117472
HLA−C ENSG00000204525
NBL1 ENSG00000158747
MYL9 ENSG00000101335
PMEPA1 ENSG00000124225
MYH11 ENSG00000133392
KRT8 ENSG00000170421
BCAP31 ENSG00000185825
CFL1 ENSG00000172757
PPP1CA ENSG00000172531
NDRG1 ENSG00000104419
SOD3 ENSG00000109610
H2AFJ ENSG00000246705
CLSTN1 ENSG00000171603
FASN ENSG00000169710
EDF1 ENSG00000107223
UQCR11 ENSG00000127540
TSC22D3 ENSG00000157514
FXYD3 ENSG00000089356
DCXR ENSG00000169738
NEFH ENSG00000100285
GAPDH ENSG00000111640
MYL6 ENSG00000092841
DES ENSG00000175084
CTSD ENSG00000117984

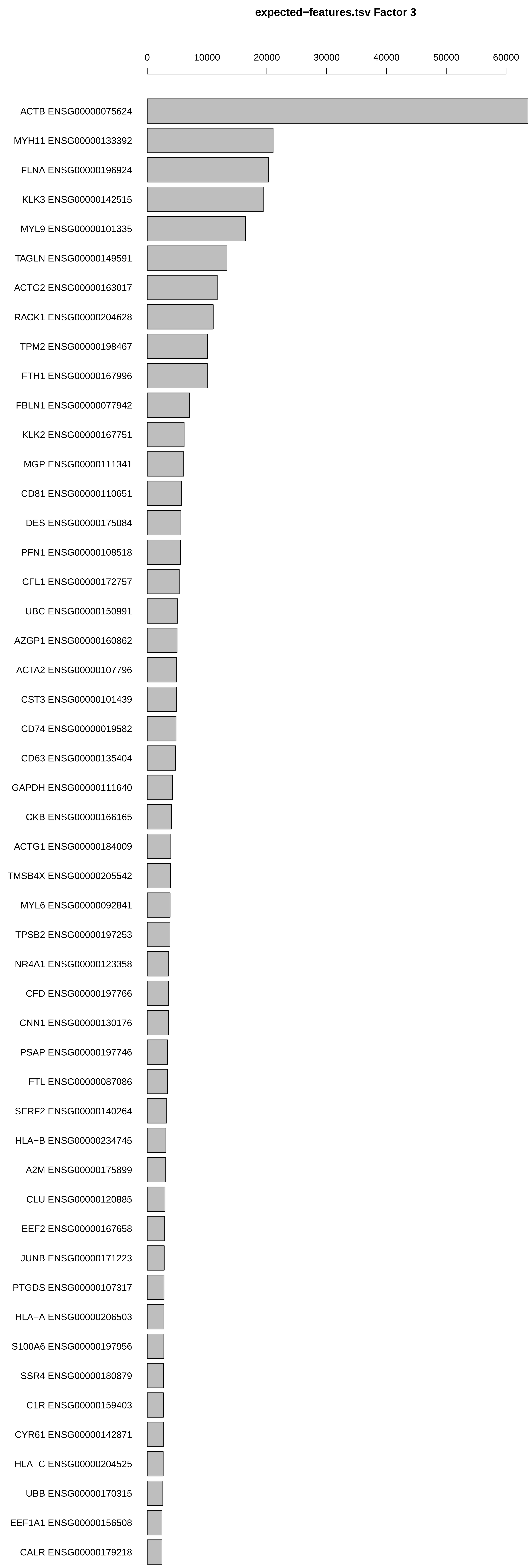

expected−features.tsv Factor 3
0
10000
20000
30000
40000
50000
60000
ACTB ENSG00000075624
MYH11 ENSG00000133392
FLNA ENSG00000196924
KLK3 ENSG00000142515
MYL9 ENSG00000101335
TAGLN ENSG00000149591
ACTG2 ENSG00000163017
RACK1 ENSG00000204628
TPM2 ENSG00000198467
FTH1 ENSG00000167996
FBLN1 ENSG00000077942
KLK2 ENSG00000167751
MGP ENSG00000111341
CD81 ENSG00000110651
DES ENSG00000175084
PFN1 ENSG00000108518
CFL1 ENSG00000172757
UBC ENSG00000150991
AZGP1 ENSG00000160862
ACTA2 ENSG00000107796
CST3 ENSG00000101439
CD74 ENSG00000019582
CD63 ENSG00000135404
GAPDH ENSG00000111640
CKB ENSG00000166165
ACTG1 ENSG00000184009
TMSB4X ENSG00000205542
MYL6 ENSG00000092841
TPSB2 ENSG00000197253
NR4A1 ENSG00000123358
CFD ENSG00000197766
CNN1 ENSG00000130176
PSAP ENSG00000197746
FTL ENSG00000087086
SERF2 ENSG00000140264
HLA−B ENSG00000234745
A2M ENSG00000175899
CLU ENSG00000120885
EEF2 ENSG00000167658
JUNB ENSG00000171223
PTGDS ENSG00000107317
HLA−A ENSG00000206503
S100A6 ENSG00000197956
SSR4 ENSG00000180879
C1R ENSG00000159403
CYR61 ENSG00000142871
HLA−C ENSG00000204525
UBB ENSG00000170315
EEF1A1 ENSG00000156508
CALR ENSG00000179218

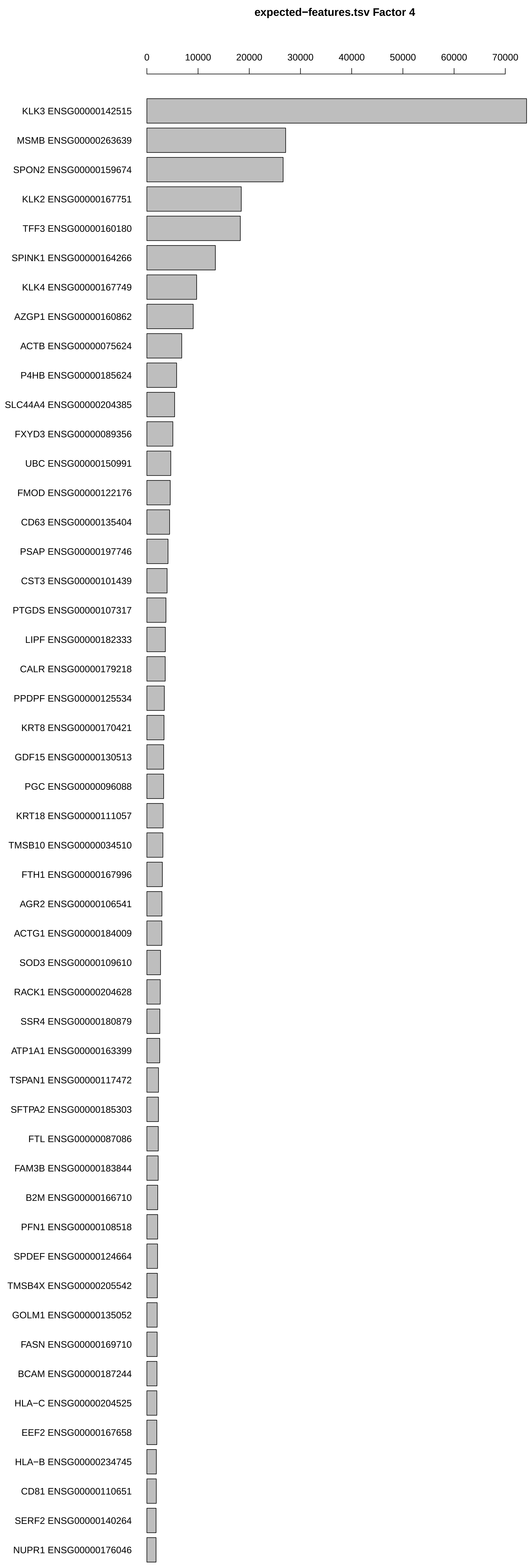
expected−features.tsv Factor 4
0
10000
20000
30000
40000
50000
60000
70000
KLK3 ENSG00000142515
MSMB ENSG00000263639
SPON2 ENSG00000159674
KLK2 ENSG00000167751
TFF3 ENSG00000160180
SPINK1 ENSG00000164266
KLK4 ENSG00000167749
AZGP1 ENSG00000160862
ACTB ENSG00000075624
P4HB ENSG00000185624
SLC44A4 ENSG00000204385
FXYD3 ENSG00000089356
UBC ENSG00000150991
FMOD ENSG00000122176
CD63 ENSG00000135404
PSAP ENSG00000197746
CST3 ENSG00000101439
PTGDS ENSG00000107317
LIPF ENSG00000182333
CALR ENSG00000179218
PPDPF ENSG00000125534
KRT8 ENSG00000170421
GDF15 ENSG00000130513
PGC ENSG00000096088
KRT18 ENSG00000111057
TMSB10 ENSG00000034510
FTH1 ENSG00000167996
AGR2 ENSG00000106541
ACTG1 ENSG00000184009
SOD3 ENSG00000109610
RACK1 ENSG00000204628
SSR4 ENSG00000180879
ATP1A1 ENSG00000163399
TSPAN1 ENSG00000117472
SFTPA2 ENSG00000185303
FTL ENSG00000087086
FAM3B ENSG00000183844
B2M ENSG00000166710
PFN1 ENSG00000108518
SPDEF ENSG00000124664
TMSB4X ENSG00000205542
GOLM1 ENSG00000135052
FASN ENSG00000169710
BCAM ENSG00000187244
HLA−C ENSG00000204525
EEF2 ENSG00000167658
HLA−B ENSG00000234745
CD81 ENSG00000110651
SERF2 ENSG00000140264
NUPR1 ENSG00000176046

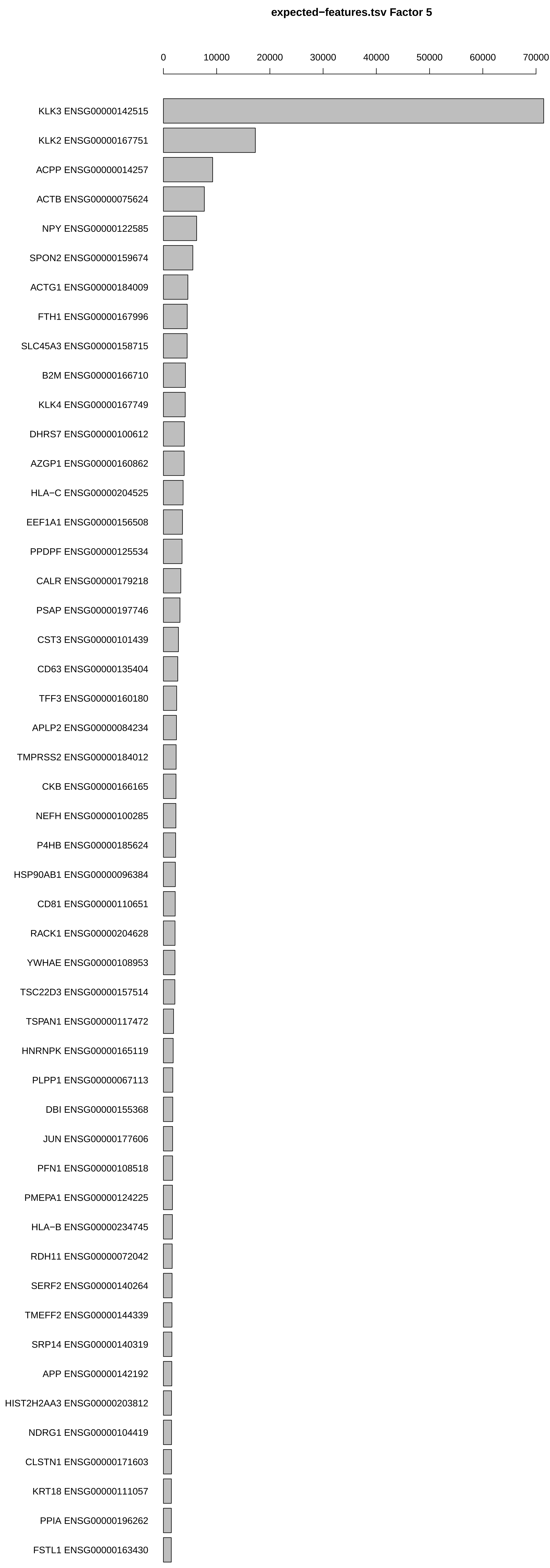

expected−features.tsv Factor 5
0
10000
20000
30000
40000
50000
60000
70000
KLK3 ENSG00000142515
KLK2 ENSG00000167751
ACPP ENSG00000014257
ACTB ENSG00000075624
NPY ENSG00000122585
SPON2 ENSG00000159674
ACTG1 ENSG00000184009
FTH1 ENSG00000167996
SLC45A3 ENSG00000158715
B2M ENSG00000166710
KLK4 ENSG00000167749
DHRS7 ENSG00000100612
AZGP1 ENSG00000160862
HLA−C ENSG00000204525
EEF1A1 ENSG00000156508
PPDPF ENSG00000125534
CALR ENSG00000179218
PSAP ENSG00000197746
CST3 ENSG00000101439
CD63 ENSG00000135404
TFF3 ENSG00000160180
APLP2 ENSG00000084234
TMPRSS2 ENSG00000184012
CKB ENSG00000166165
NEFH ENSG00000100285
P4HB ENSG00000185624
HSP90AB1 ENSG00000096384
CD81 ENSG00000110651
RACK1 ENSG00000204628
YWHAE ENSG00000108953
TSC22D3 ENSG00000157514
TSPAN1 ENSG00000117472
HNRNPK ENSG00000165119
PLPP1 ENSG00000067113
DBI ENSG00000155368
JUN ENSG00000177606
PFN1 ENSG00000108518
PMEPA1 ENSG00000124225
HLA−B ENSG00000234745
RDH11 ENSG00000072042
SERF2 ENSG00000140264
TMEFF2 ENSG00000144339
SRP14 ENSG00000140319
APP ENSG00000142192
HIST2H2AA3 ENSG00000203812
NDRG1 ENSG00000104419
CLSTN1 ENSG00000171603
KRT18 ENSG00000111057
PPIA ENSG00000196262
FSTL1 ENSG00000163430

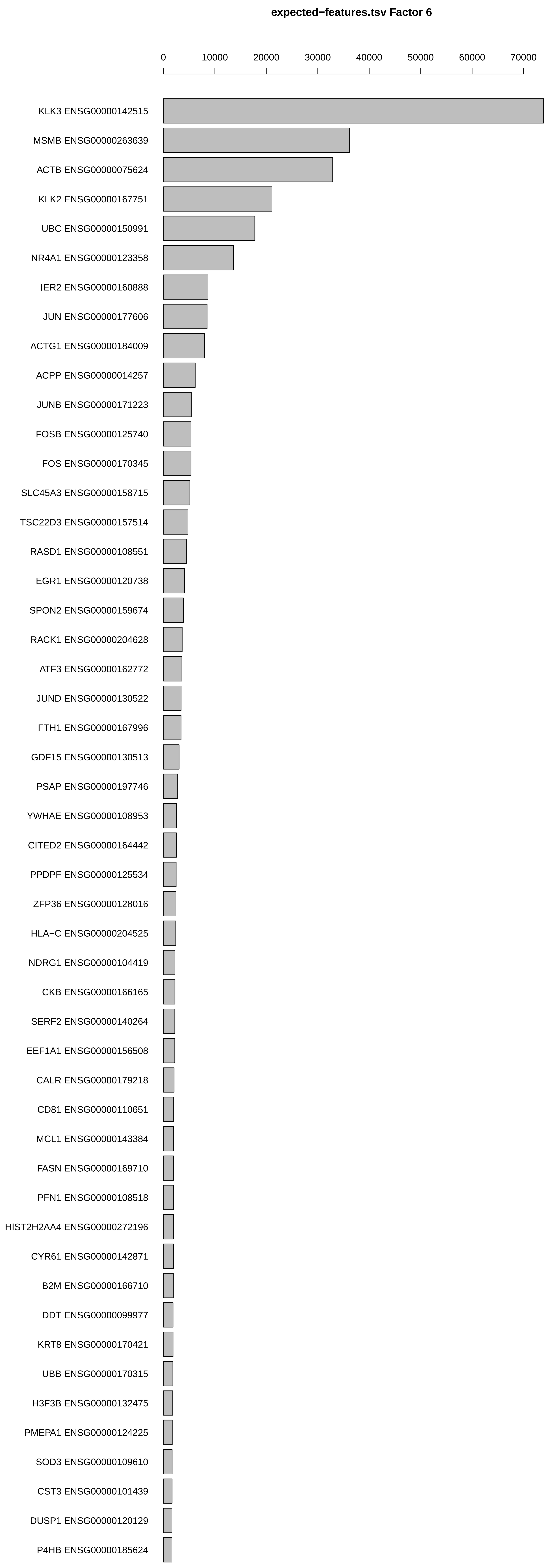
expected−features.tsv Factor 6
0
10000
20000
30000
40000
50000
60000
70000
KLK3 ENSG00000142515
MSMB ENSG00000263639
ACTB ENSG00000075624
KLK2 ENSG00000167751
UBC ENSG00000150991
NR4A1 ENSG00000123358
IER2 ENSG00000160888
JUN ENSG00000177606
ACTG1 ENSG00000184009
ACPP ENSG00000014257
JUNB ENSG00000171223
FOSB ENSG00000125740
FOS ENSG00000170345
SLC45A3 ENSG00000158715
TSC22D3 ENSG00000157514
RASD1 ENSG00000108551
EGR1 ENSG00000120738
SPON2 ENSG00000159674
RACK1 ENSG00000204628
ATF3 ENSG00000162772
JUND ENSG00000130522
FTH1 ENSG00000167996
GDF15 ENSG00000130513
PSAP ENSG00000197746
YWHAE ENSG00000108953
CITED2 ENSG00000164442
PPDPF ENSG00000125534
ZFP36 ENSG00000128016
HLA−C ENSG00000204525
NDRG1 ENSG00000104419
CKB ENSG00000166165
SERF2 ENSG00000140264
EEF1A1 ENSG00000156508
CALR ENSG00000179218
CD81 ENSG00000110651
MCL1 ENSG00000143384
FASN ENSG00000169710
PFN1 ENSG00000108518
HIST2H2AA4 ENSG00000272196
CYR61 ENSG00000142871
B2M ENSG00000166710
DDT ENSG00000099977
KRT8 ENSG00000170421
UBB ENSG00000170315
H3F3B ENSG00000132475
PMEPA1 ENSG00000124225
SOD3 ENSG00000109610
CST3 ENSG00000101439
DUSP1 ENSG00000120129
P4HB ENSG00000185624

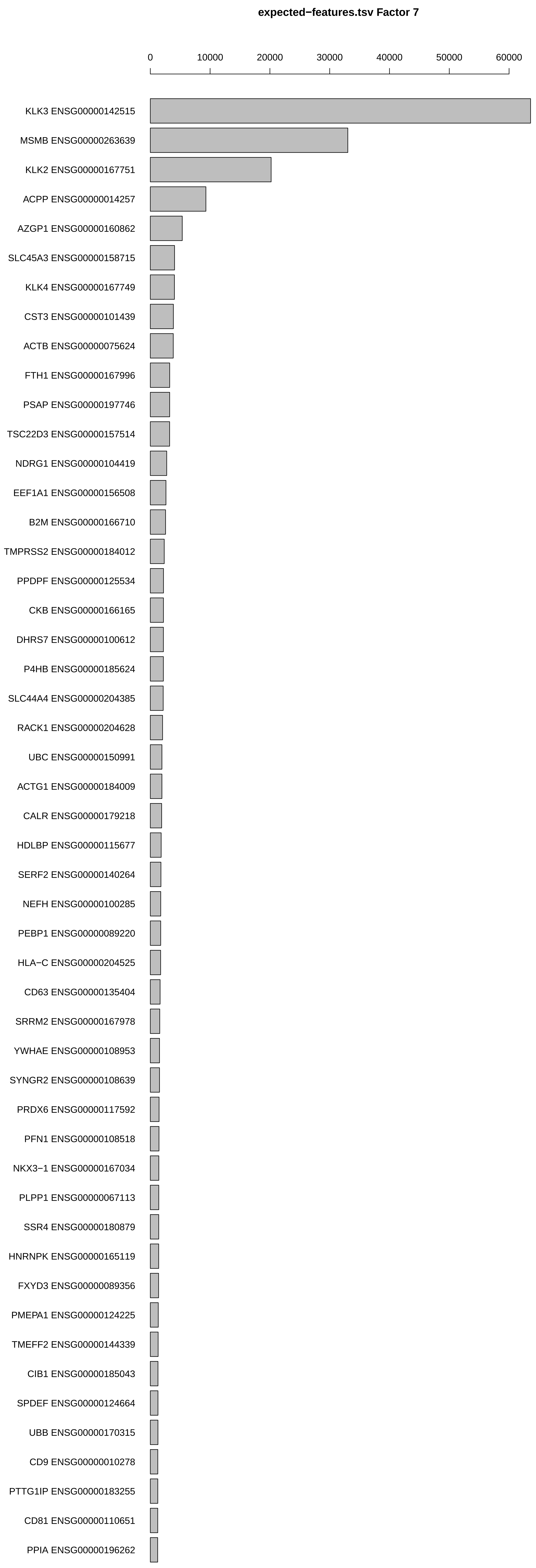
expected−features.tsv Factor 7
0
10000
20000
30000
40000
50000
60000
KLK3 ENSG00000142515
MSMB ENSG00000263639
KLK2 ENSG00000167751
ACPP ENSG00000014257
AZGP1 ENSG00000160862
SLC45A3 ENSG00000158715
KLK4 ENSG00000167749
CST3 ENSG00000101439
ACTB ENSG00000075624
FTH1 ENSG00000167996
PSAP ENSG00000197746
TSC22D3 ENSG00000157514
NDRG1 ENSG00000104419
EEF1A1 ENSG00000156508
B2M ENSG00000166710
TMPRSS2 ENSG00000184012
PPDPF ENSG00000125534
CKB ENSG00000166165
DHRS7 ENSG00000100612
P4HB ENSG00000185624
SLC44A4 ENSG00000204385
RACK1 ENSG00000204628
UBC ENSG00000150991
ACTG1 ENSG00000184009
CALR ENSG00000179218
HDLBP ENSG00000115677
SERF2 ENSG00000140264
NEFH ENSG00000100285
PEBP1 ENSG00000089220
HLA−C ENSG00000204525
CD63 ENSG00000135404
SRRM2 ENSG00000167978
YWHAE ENSG00000108953
SYNGR2 ENSG00000108639
PRDX6 ENSG00000117592
PFN1 ENSG00000108518
NKX3−1 ENSG00000167034
PLPP1 ENSG00000067113
SSR4 ENSG00000180879
HNRNPK ENSG00000165119
FXYD3 ENSG00000089356
PMEPA1 ENSG00000124225
TMEFF2 ENSG00000144339
CIB1 ENSG00000185043
SPDEF ENSG00000124664
UBB ENSG00000170315
CD9 ENSG00000010278
PTTG1IP ENSG00000183255
CD81 ENSG00000110651
PPIA ENSG00000196262

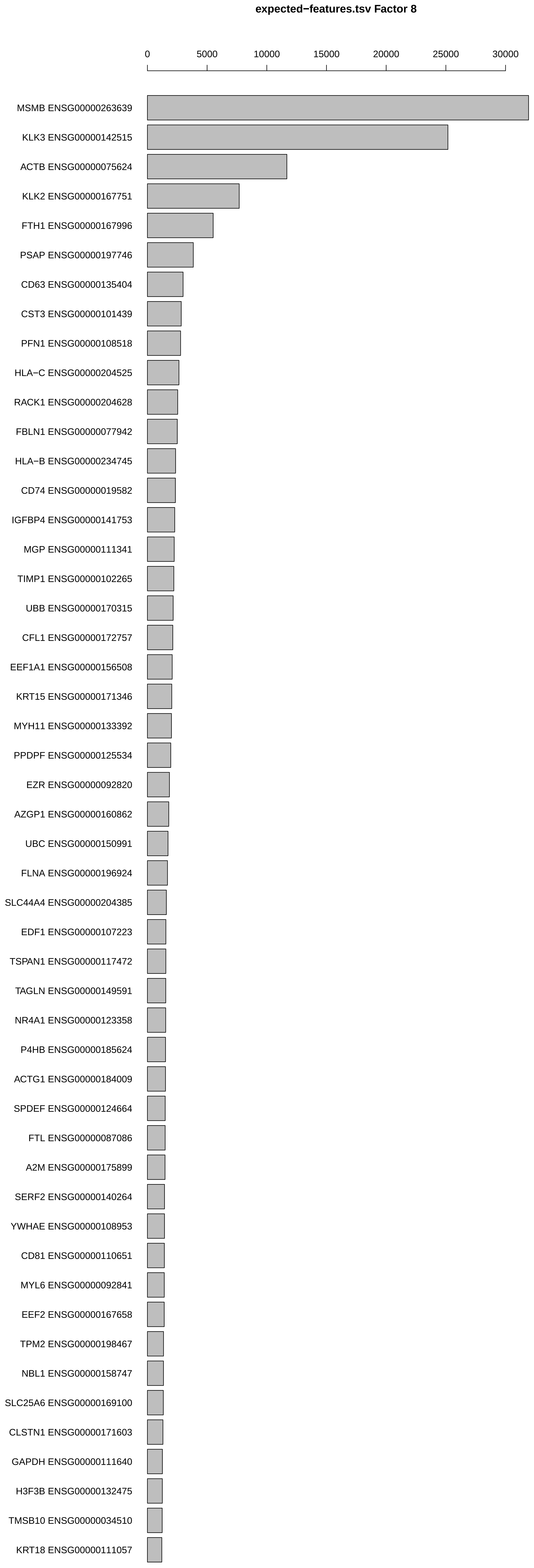
expected−features.tsv Factor 8
0
5000
10000
15000
20000
25000
30000
MSMB ENSG00000263639
KLK3 ENSG00000142515
ACTB ENSG00000075624
KLK2 ENSG00000167751
FTH1 ENSG00000167996
PSAP ENSG00000197746
CD63 ENSG00000135404
CST3 ENSG00000101439
PFN1 ENSG00000108518
HLA−C ENSG00000204525
RACK1 ENSG00000204628
FBLN1 ENSG00000077942
HLA−B ENSG00000234745
CD74 ENSG00000019582
IGFBP4 ENSG00000141753
MGP ENSG00000111341
TIMP1 ENSG00000102265
UBB ENSG00000170315
CFL1 ENSG00000172757
EEF1A1 ENSG00000156508
KRT15 ENSG00000171346
MYH11 ENSG00000133392
PPDPF ENSG00000125534
EZR ENSG00000092820
AZGP1 ENSG00000160862
UBC ENSG00000150991
FLNA ENSG00000196924
SLC44A4 ENSG00000204385
EDF1 ENSG00000107223
TSPAN1 ENSG00000117472
TAGLN ENSG00000149591
NR4A1 ENSG00000123358
P4HB ENSG00000185624
ACTG1 ENSG00000184009
SPDEF ENSG00000124664
FTL ENSG00000087086
A2M ENSG00000175899
SERF2 ENSG00000140264
YWHAE ENSG00000108953
CD81 ENSG00000110651
MYL6 ENSG00000092841
EEF2 ENSG00000167658
TPM2 ENSG00000198467
NBL1 ENSG00000158747
SLC25A6 ENSG00000169100
CLSTN1 ENSG00000171603
GAPDH ENSG00000111640
H3F3B ENSG00000132475
TMSB10 ENSG00000034510
KRT18 ENSG00000111057

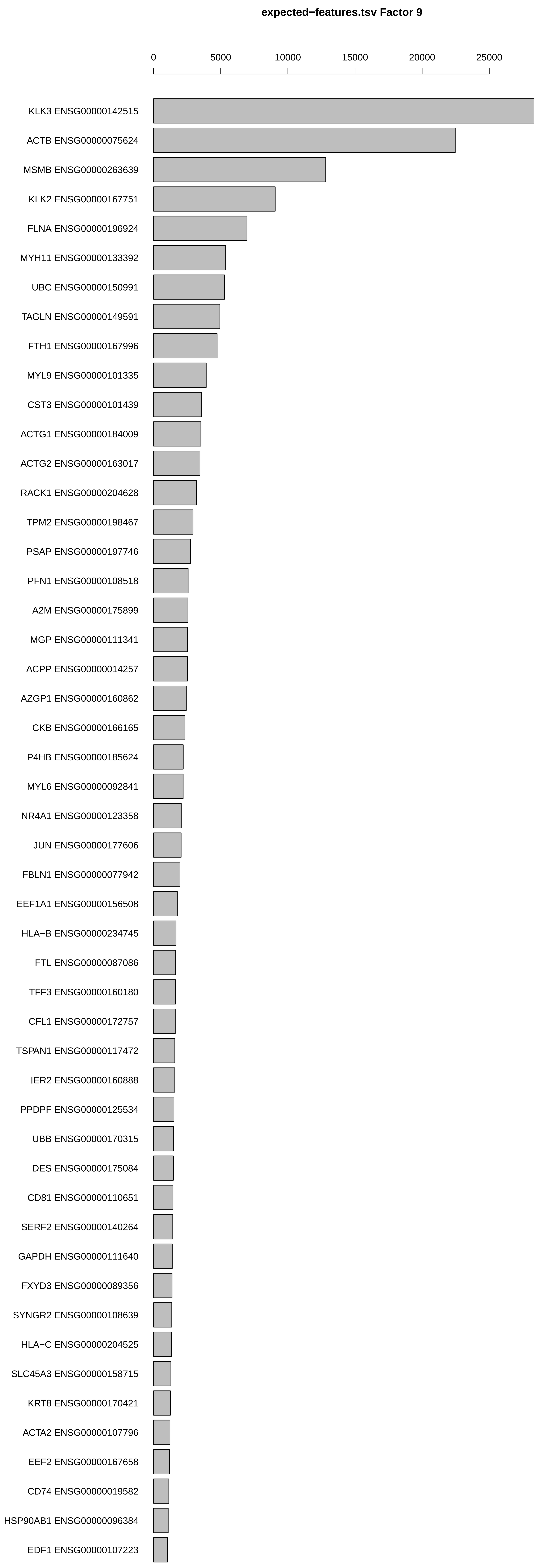
expected−features.tsv Factor 9
0
5000
10000
15000
20000
25000
KLK3 ENSG00000142515
ACTB ENSG00000075624
MSMB ENSG00000263639
KLK2 ENSG00000167751
FLNA ENSG00000196924
MYH11 ENSG00000133392
UBC ENSG00000150991
TAGLN ENSG00000149591
FTH1 ENSG00000167996
MYL9 ENSG00000101335
CST3 ENSG00000101439
ACTG1 ENSG00000184009
ACTG2 ENSG00000163017
RACK1 ENSG00000204628
TPM2 ENSG00000198467
PSAP ENSG00000197746
PFN1 ENSG00000108518
A2M ENSG00000175899
MGP ENSG00000111341
ACPP ENSG00000014257
AZGP1 ENSG00000160862
CKB ENSG00000166165
P4HB ENSG00000185624
MYL6 ENSG00000092841
NR4A1 ENSG00000123358
JUN ENSG00000177606
FBLN1 ENSG00000077942
EEF1A1 ENSG00000156508
HLA−B ENSG00000234745
FTL ENSG00000087086
TFF3 ENSG00000160180
CFL1 ENSG00000172757
TSPAN1 ENSG00000117472
IER2 ENSG00000160888
PPDPF ENSG00000125534
UBB ENSG00000170315
DES ENSG00000175084
CD81 ENSG00000110651
SERF2 ENSG00000140264
GAPDH ENSG00000111640
FXYD3 ENSG00000089356
SYNGR2 ENSG00000108639
HLA−C ENSG00000204525
SLC45A3 ENSG00000158715
KRT8 ENSG00000170421
ACTA2 ENSG00000107796
EEF2 ENSG00000167658
CD74 ENSG00000019582
HSP90AB1 ENSG00000096384
EDF1 ENSG00000107223

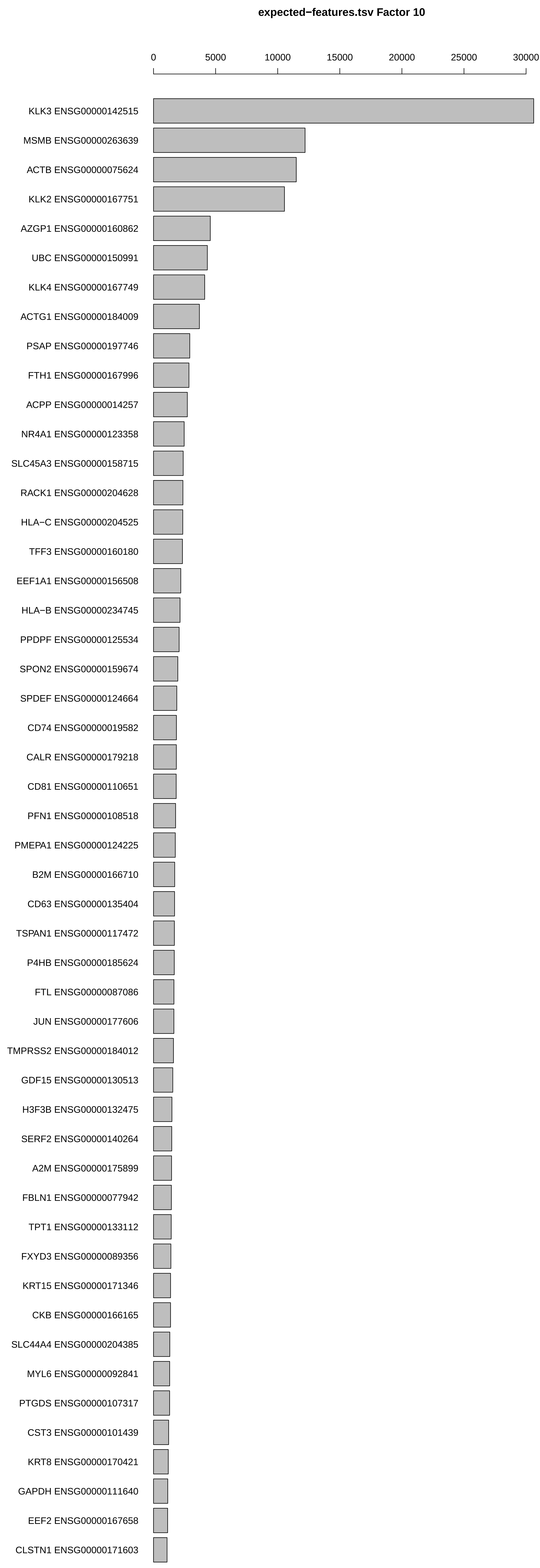
expected−features.tsv Factor 10
0
5000
10000
15000
20000
25000
30000
KLK3 ENSG00000142515
MSMB ENSG00000263639
ACTB ENSG00000075624
KLK2 ENSG00000167751
AZGP1 ENSG00000160862
UBC ENSG00000150991
KLK4 ENSG00000167749
ACTG1 ENSG00000184009
PSAP ENSG00000197746
FTH1 ENSG00000167996
ACPP ENSG00000014257
NR4A1 ENSG00000123358
SLC45A3 ENSG00000158715
RACK1 ENSG00000204628
HLA−C ENSG00000204525
TFF3 ENSG00000160180
EEF1A1 ENSG00000156508
HLA−B ENSG00000234745
PPDPF ENSG00000125534
SPON2 ENSG00000159674
SPDEF ENSG00000124664
CD74 ENSG00000019582
CALR ENSG00000179218
CD81 ENSG00000110651
PFN1 ENSG00000108518
PMEPA1 ENSG00000124225
B2M ENSG00000166710
CD63 ENSG00000135404
TSPAN1 ENSG00000117472
P4HB ENSG00000185624
FTL ENSG00000087086
JUN ENSG00000177606
TMPRSS2 ENSG00000184012
GDF15 ENSG00000130513
H3F3B ENSG00000132475
SERF2 ENSG00000140264
A2M ENSG00000175899
FBLN1 ENSG00000077942
TPT1 ENSG00000133112
FXYD3 ENSG00000089356
KRT15 ENSG00000171346
CKB ENSG00000166165
SLC44A4 ENSG00000204385
MYL6 ENSG00000092841
PTGDS ENSG00000107317
CST3 ENSG00000101439
KRT8 ENSG00000170421
GAPDH ENSG00000111640
EEF2 ENSG00000167658
CLSTN1 ENSG00000171603

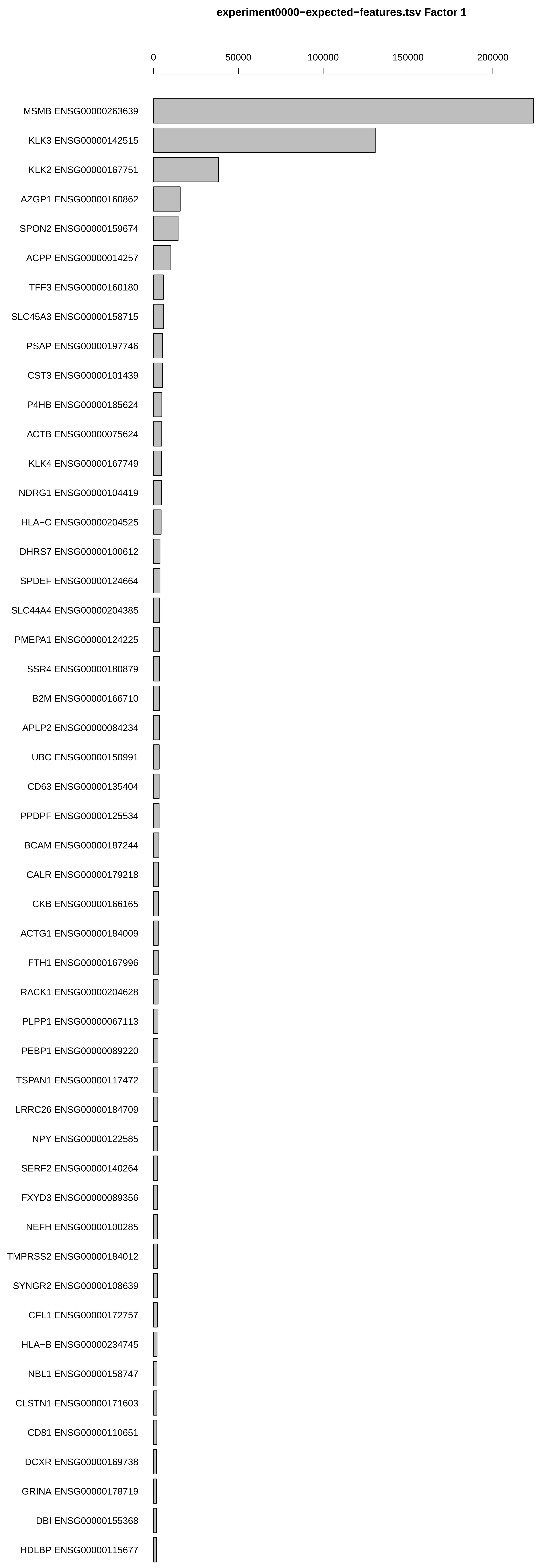

experiment0000−expected−features.tsv Factor 1
0
50000
100000
150000
200000
MSMB ENSG00000263639
KLK3 ENSG00000142515
KLK2 ENSG00000167751
AZGP1 ENSG00000160862
SPON2 ENSG00000159674
ACPP ENSG00000014257
TFF3 ENSG00000160180
SLC45A3 ENSG00000158715
PSAP ENSG00000197746
CST3 ENSG00000101439
P4HB ENSG00000185624
ACTB ENSG00000075624
KLK4 ENSG00000167749
NDRG1 ENSG00000104419
HLA−C ENSG00000204525
DHRS7 ENSG00000100612
SPDEF ENSG00000124664
SLC44A4 ENSG00000204385
PMEPA1 ENSG00000124225
SSR4 ENSG00000180879
B2M ENSG00000166710
APLP2 ENSG00000084234
UBC ENSG00000150991
CD63 ENSG00000135404
PPDPF ENSG00000125534
BCAM ENSG00000187244
CALR ENSG00000179218
CKB ENSG00000166165
ACTG1 ENSG00000184009
FTH1 ENSG00000167996
RACK1 ENSG00000204628
PLPP1 ENSG00000067113
PEBP1 ENSG00000089220
TSPAN1 ENSG00000117472
LRRC26 ENSG00000184709
NPY ENSG00000122585
SERF2 ENSG00000140264
FXYD3 ENSG00000089356
NEFH ENSG00000100285
TMPRSS2 ENSG00000184012
SYNGR2 ENSG00000108639
CFL1 ENSG00000172757
HLA−B ENSG00000234745
NBL1 ENSG00000158747
CLSTN1 ENSG00000171603
CD81 ENSG00000110651
DCXR ENSG00000169738
GRINA ENSG00000178719
DBI ENSG00000155368
HDLBP ENSG00000115677

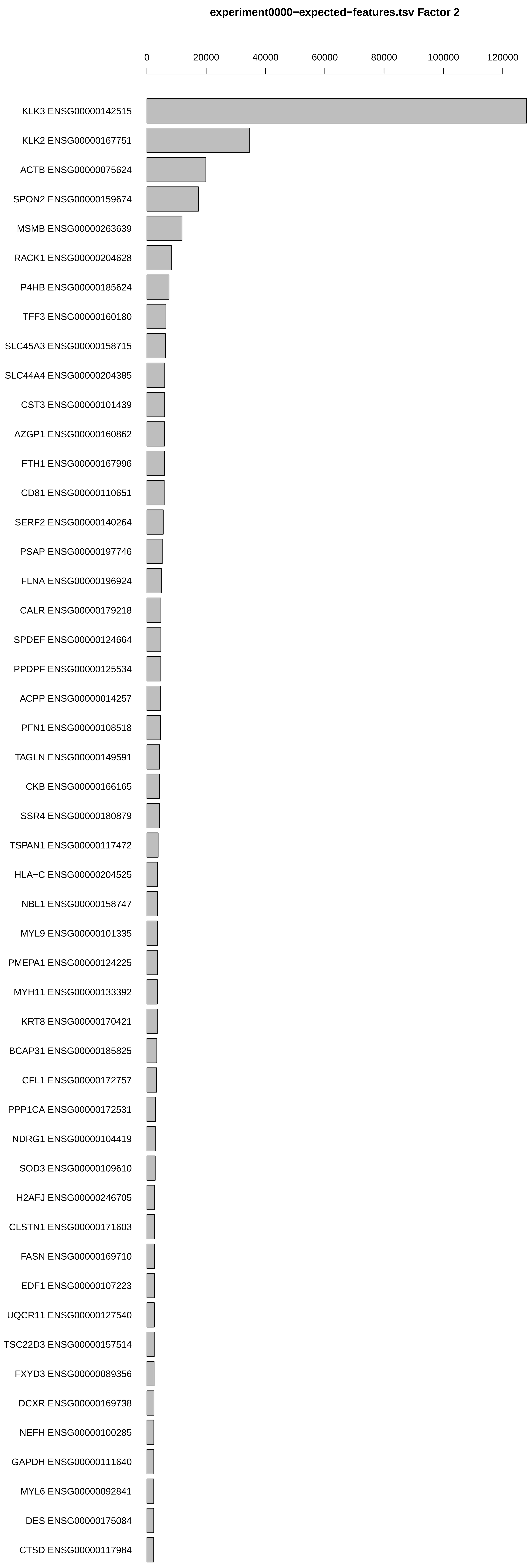
experiment0000−expected−features.tsv Factor 2
0
20000
40000
60000
80000
100000
120000
KLK3 ENSG00000142515
KLK2 ENSG00000167751
ACTB ENSG00000075624
SPON2 ENSG00000159674
MSMB ENSG00000263639
RACK1 ENSG00000204628
P4HB ENSG00000185624
TFF3 ENSG00000160180
SLC45A3 ENSG00000158715
SLC44A4 ENSG00000204385
CST3 ENSG00000101439
AZGP1 ENSG00000160862
FTH1 ENSG00000167996
CD81 ENSG00000110651
SERF2 ENSG00000140264
PSAP ENSG00000197746
FLNA ENSG00000196924
CALR ENSG00000179218
SPDEF ENSG00000124664
PPDPF ENSG00000125534
ACPP ENSG00000014257
PFN1 ENSG00000108518
TAGLN ENSG00000149591
CKB ENSG00000166165
SSR4 ENSG00000180879
TSPAN1 ENSG00000117472
HLA−C ENSG00000204525
NBL1 ENSG00000158747
MYL9 ENSG00000101335
PMEPA1 ENSG00000124225
MYH11 ENSG00000133392
KRT8 ENSG00000170421
BCAP31 ENSG00000185825
CFL1 ENSG00000172757
PPP1CA ENSG00000172531
NDRG1 ENSG00000104419
SOD3 ENSG00000109610
H2AFJ ENSG00000246705
CLSTN1 ENSG00000171603
FASN ENSG00000169710
EDF1 ENSG00000107223
UQCR11 ENSG00000127540
TSC22D3 ENSG00000157514
FXYD3 ENSG00000089356
DCXR ENSG00000169738
NEFH ENSG00000100285
GAPDH ENSG00000111640
MYL6 ENSG00000092841
DES ENSG00000175084
CTSD ENSG00000117984

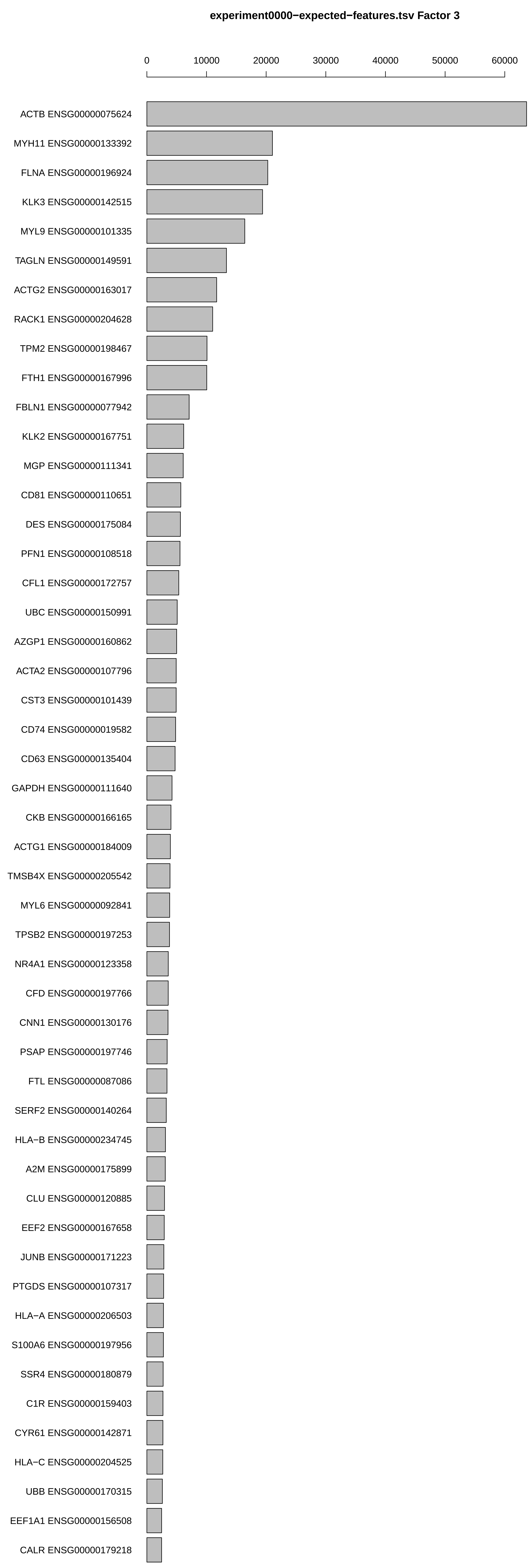
experiment0000−expected−features.tsv Factor 3
0
10000
20000
30000
40000
50000
60000
ACTB ENSG00000075624
MYH11 ENSG00000133392
FLNA ENSG00000196924
KLK3 ENSG00000142515
MYL9 ENSG00000101335
TAGLN ENSG00000149591
ACTG2 ENSG00000163017
RACK1 ENSG00000204628
TPM2 ENSG00000198467
FTH1 ENSG00000167996
FBLN1 ENSG00000077942
KLK2 ENSG00000167751
MGP ENSG00000111341
CD81 ENSG00000110651
DES ENSG00000175084
PFN1 ENSG00000108518
CFL1 ENSG00000172757
UBC ENSG00000150991
AZGP1 ENSG00000160862
ACTA2 ENSG00000107796
CST3 ENSG00000101439
CD74 ENSG00000019582
CD63 ENSG00000135404
GAPDH ENSG00000111640
CKB ENSG00000166165
ACTG1 ENSG00000184009
TMSB4X ENSG00000205542
MYL6 ENSG00000092841
TPSB2 ENSG00000197253
NR4A1 ENSG00000123358
CFD ENSG00000197766
CNN1 ENSG00000130176
PSAP ENSG00000197746
FTL ENSG00000087086
SERF2 ENSG00000140264
HLA−B ENSG00000234745
A2M ENSG00000175899
CLU ENSG00000120885
EEF2 ENSG00000167658
JUNB ENSG00000171223
PTGDS ENSG00000107317
HLA−A ENSG00000206503
S100A6 ENSG00000197956
SSR4 ENSG00000180879
C1R ENSG00000159403
CYR61 ENSG00000142871
HLA−C ENSG00000204525
UBB ENSG00000170315
EEF1A1 ENSG00000156508
CALR ENSG00000179218

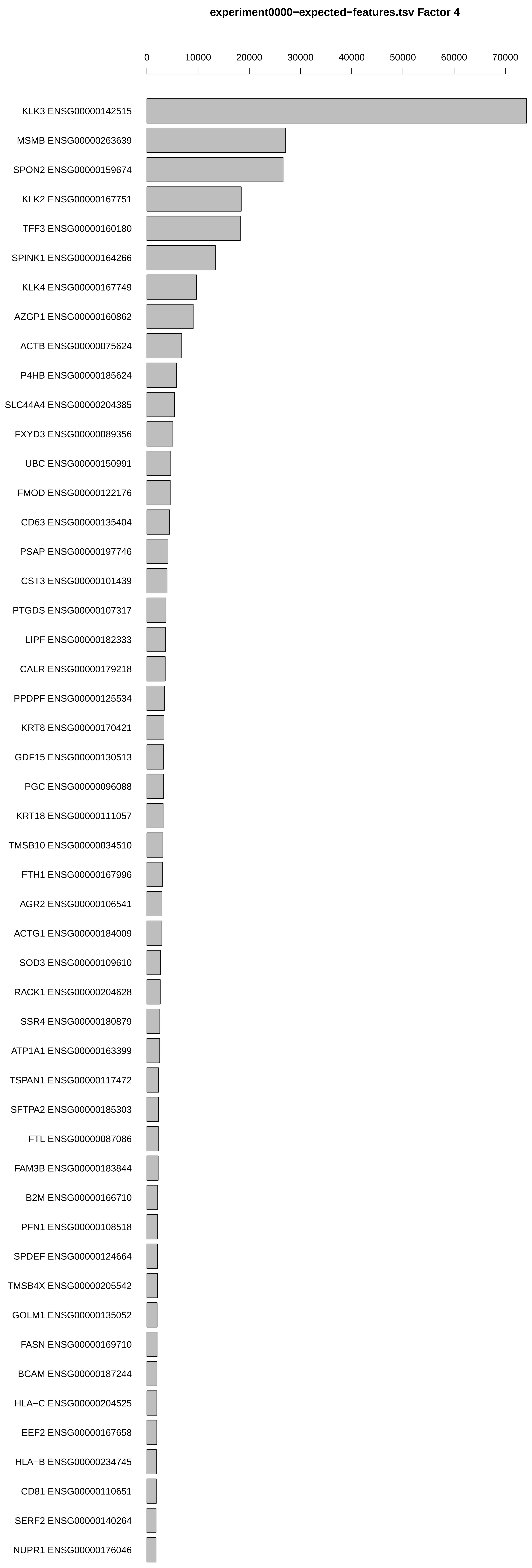
experiment0000−expected−features.tsv Factor 4
0
10000
20000
30000
40000
50000
60000
70000
KLK3 ENSG00000142515
MSMB ENSG00000263639
SPON2 ENSG00000159674
KLK2 ENSG00000167751
TFF3 ENSG00000160180
SPINK1 ENSG00000164266
KLK4 ENSG00000167749
AZGP1 ENSG00000160862
ACTB ENSG00000075624
P4HB ENSG00000185624
SLC44A4 ENSG00000204385
FXYD3 ENSG00000089356
UBC ENSG00000150991
FMOD ENSG00000122176
CD63 ENSG00000135404
PSAP ENSG00000197746
CST3 ENSG00000101439
PTGDS ENSG00000107317
LIPF ENSG00000182333
CALR ENSG00000179218
PPDPF ENSG00000125534
KRT8 ENSG00000170421
GDF15 ENSG00000130513
PGC ENSG00000096088
KRT18 ENSG00000111057
TMSB10 ENSG00000034510
FTH1 ENSG00000167996
AGR2 ENSG00000106541
ACTG1 ENSG00000184009
SOD3 ENSG00000109610
RACK1 ENSG00000204628
SSR4 ENSG00000180879
ATP1A1 ENSG00000163399
TSPAN1 ENSG00000117472
SFTPA2 ENSG00000185303
FTL ENSG00000087086
FAM3B ENSG00000183844
B2M ENSG00000166710
PFN1 ENSG00000108518
SPDEF ENSG00000124664
TMSB4X ENSG00000205542
GOLM1 ENSG00000135052
FASN ENSG00000169710
BCAM ENSG00000187244
HLA−C ENSG00000204525
EEF2 ENSG00000167658
HLA−B ENSG00000234745
CD81 ENSG00000110651
SERF2 ENSG00000140264
NUPR1 ENSG00000176046

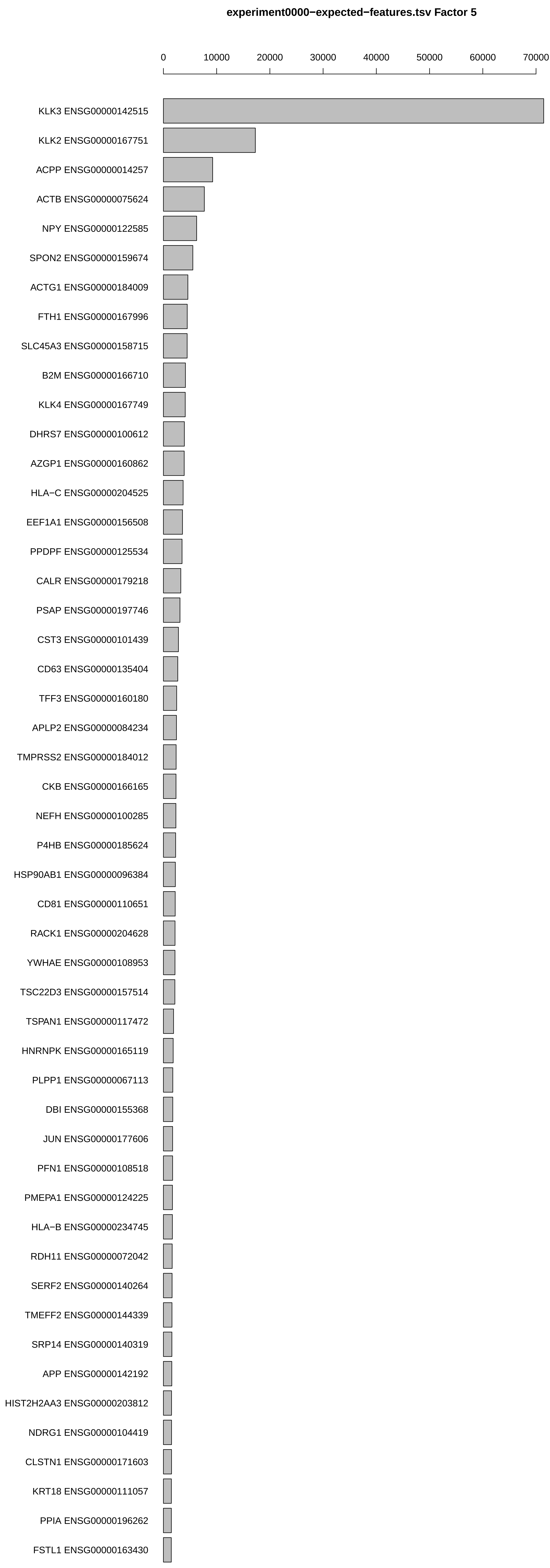
experiment0000−expected−features.tsv Factor 5
0
10000
20000
30000
40000
50000
60000
70000
KLK3 ENSG00000142515
KLK2 ENSG00000167751
ACPP ENSG00000014257
ACTB ENSG00000075624
NPY ENSG00000122585
SPON2 ENSG00000159674
ACTG1 ENSG00000184009
FTH1 ENSG00000167996
SLC45A3 ENSG00000158715
B2M ENSG00000166710
KLK4 ENSG00000167749
DHRS7 ENSG00000100612
AZGP1 ENSG00000160862
HLA−C ENSG00000204525
EEF1A1 ENSG00000156508
PPDPF ENSG00000125534
CALR ENSG00000179218
PSAP ENSG00000197746
CST3 ENSG00000101439
CD63 ENSG00000135404
TFF3 ENSG00000160180
APLP2 ENSG00000084234
TMPRSS2 ENSG00000184012
CKB ENSG00000166165
NEFH ENSG00000100285
P4HB ENSG00000185624
HSP90AB1 ENSG00000096384
CD81 ENSG00000110651
RACK1 ENSG00000204628
YWHAE ENSG00000108953
TSC22D3 ENSG00000157514
TSPAN1 ENSG00000117472
HNRNPK ENSG00000165119
PLPP1 ENSG00000067113
DBI ENSG00000155368
JUN ENSG00000177606
PFN1 ENSG00000108518
PMEPA1 ENSG00000124225
HLA−B ENSG00000234745
RDH11 ENSG00000072042
SERF2 ENSG00000140264
TMEFF2 ENSG00000144339
SRP14 ENSG00000140319
APP ENSG00000142192
HIST2H2AA3 ENSG00000203812
NDRG1 ENSG00000104419
CLSTN1 ENSG00000171603
KRT18 ENSG00000111057
PPIA ENSG00000196262
FSTL1 ENSG00000163430

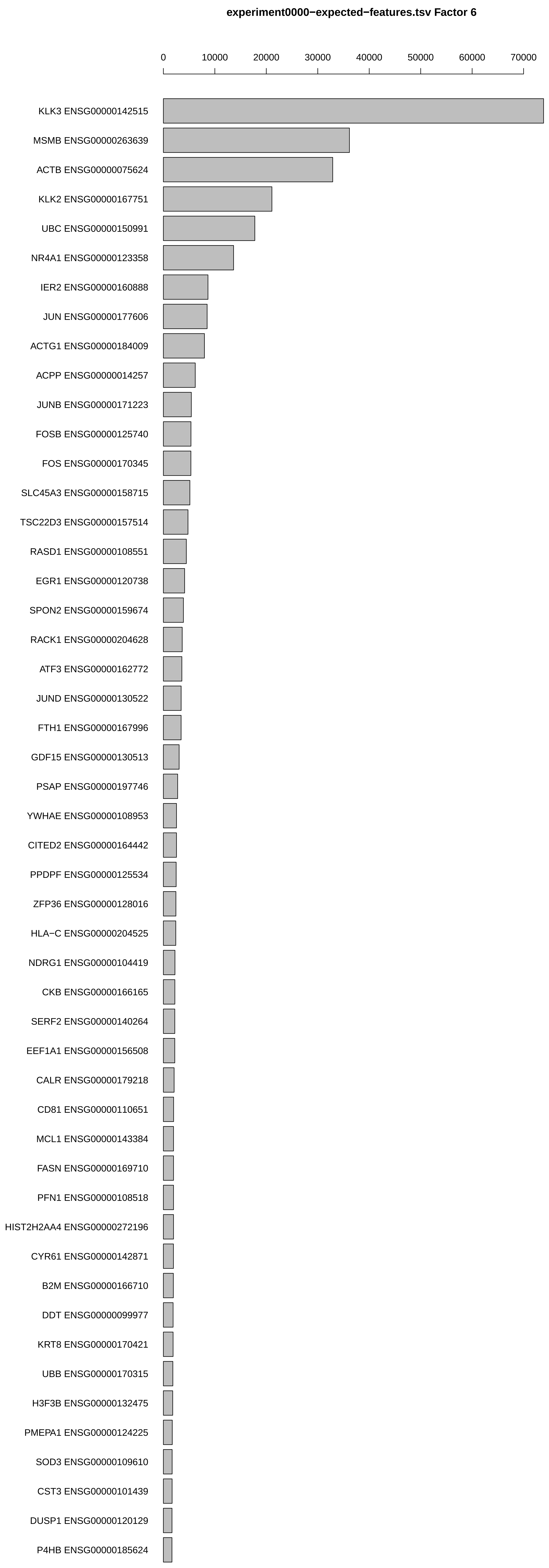
experiment0000−expected−features.tsv Factor 6
0
10000
20000
30000
40000
50000
60000
70000
KLK3 ENSG00000142515
MSMB ENSG00000263639
ACTB ENSG00000075624
KLK2 ENSG00000167751
UBC ENSG00000150991
NR4A1 ENSG00000123358
IER2 ENSG00000160888
JUN ENSG00000177606
ACTG1 ENSG00000184009
ACPP ENSG00000014257
JUNB ENSG00000171223
FOSB ENSG00000125740
FOS ENSG00000170345
SLC45A3 ENSG00000158715
TSC22D3 ENSG00000157514
RASD1 ENSG00000108551
EGR1 ENSG00000120738
SPON2 ENSG00000159674
RACK1 ENSG00000204628
ATF3 ENSG00000162772
JUND ENSG00000130522
FTH1 ENSG00000167996
GDF15 ENSG00000130513
PSAP ENSG00000197746
YWHAE ENSG00000108953
CITED2 ENSG00000164442
PPDPF ENSG00000125534
ZFP36 ENSG00000128016
HLA−C ENSG00000204525
NDRG1 ENSG00000104419
CKB ENSG00000166165
SERF2 ENSG00000140264
EEF1A1 ENSG00000156508
CALR ENSG00000179218
CD81 ENSG00000110651
MCL1 ENSG00000143384
FASN ENSG00000169710
PFN1 ENSG00000108518
HIST2H2AA4 ENSG00000272196
CYR61 ENSG00000142871
B2M ENSG00000166710
DDT ENSG00000099977
KRT8 ENSG00000170421
UBB ENSG00000170315
H3F3B ENSG00000132475
PMEPA1 ENSG00000124225
SOD3 ENSG00000109610
CST3 ENSG00000101439
DUSP1 ENSG00000120129
P4HB ENSG00000185624

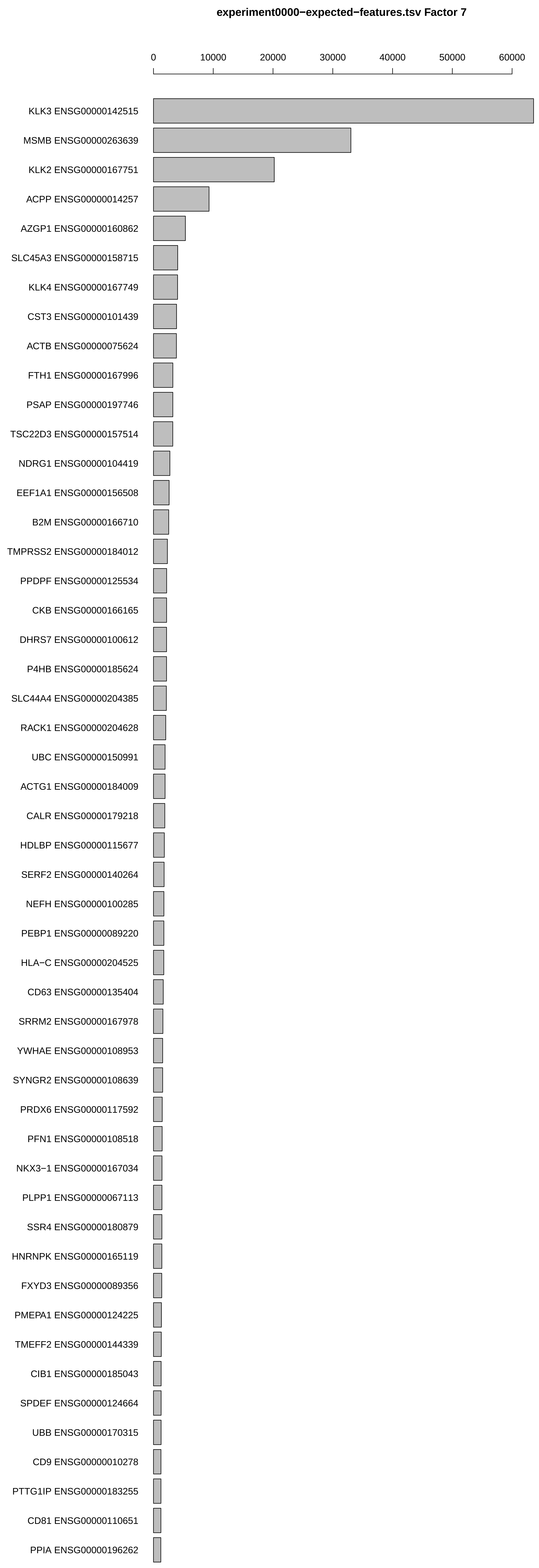
experiment0000−expected−features.tsv Factor 7
0
10000
20000
30000
40000
50000
60000
KLK3 ENSG00000142515
MSMB ENSG00000263639
KLK2 ENSG00000167751
ACPP ENSG00000014257
AZGP1 ENSG00000160862
SLC45A3 ENSG00000158715
KLK4 ENSG00000167749
CST3 ENSG00000101439
ACTB ENSG00000075624
FTH1 ENSG00000167996
PSAP ENSG00000197746
TSC22D3 ENSG00000157514
NDRG1 ENSG00000104419
EEF1A1 ENSG00000156508
B2M ENSG00000166710
TMPRSS2 ENSG00000184012
PPDPF ENSG00000125534
CKB ENSG00000166165
DHRS7 ENSG00000100612
P4HB ENSG00000185624
SLC44A4 ENSG00000204385
RACK1 ENSG00000204628
UBC ENSG00000150991
ACTG1 ENSG00000184009
CALR ENSG00000179218
HDLBP ENSG00000115677
SERF2 ENSG00000140264
NEFH ENSG00000100285
PEBP1 ENSG00000089220
HLA−C ENSG00000204525
CD63 ENSG00000135404
SRRM2 ENSG00000167978
YWHAE ENSG00000108953
SYNGR2 ENSG00000108639
PRDX6 ENSG00000117592
PFN1 ENSG00000108518
NKX3−1 ENSG00000167034
PLPP1 ENSG00000067113
SSR4 ENSG00000180879
HNRNPK ENSG00000165119
FXYD3 ENSG00000089356
PMEPA1 ENSG00000124225
TMEFF2 ENSG00000144339
CIB1 ENSG00000185043
SPDEF ENSG00000124664
UBB ENSG00000170315
CD9 ENSG00000010278
PTTG1IP ENSG00000183255
CD81 ENSG00000110651
PPIA ENSG00000196262

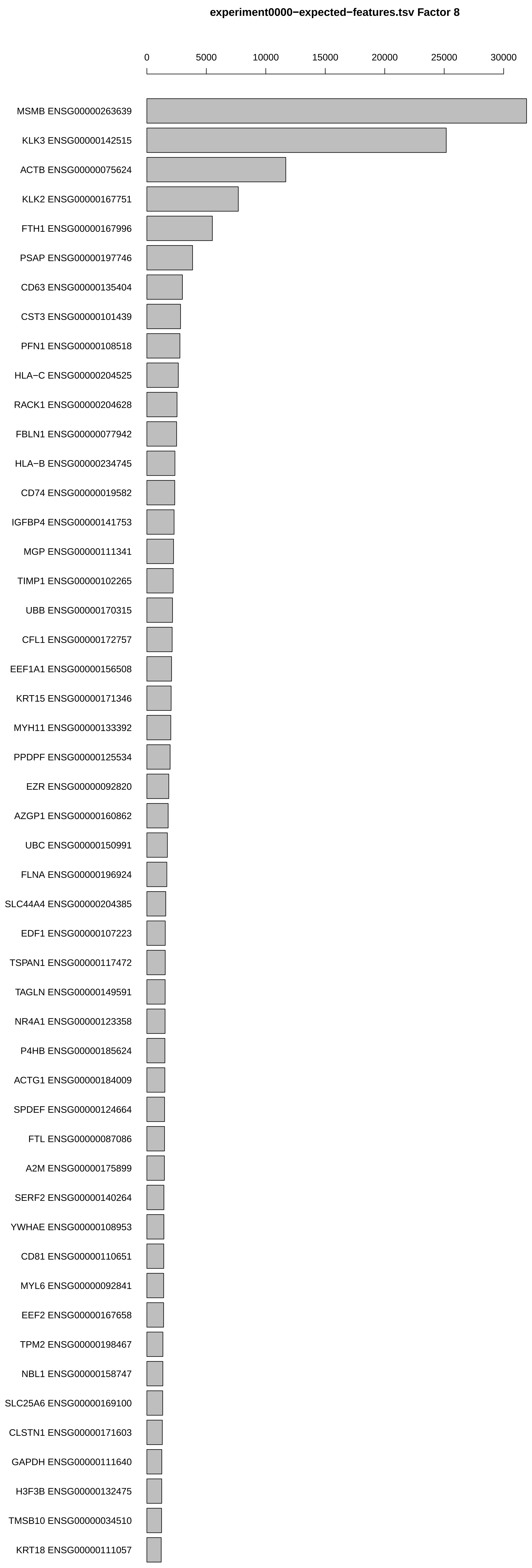
experiment0000−expected−features.tsv Factor 8
0
5000
10000
15000
20000
25000
30000
MSMB ENSG00000263639
KLK3 ENSG00000142515
ACTB ENSG00000075624
KLK2 ENSG00000167751
FTH1 ENSG00000167996
PSAP ENSG00000197746
CD63 ENSG00000135404
CST3 ENSG00000101439
PFN1 ENSG00000108518
HLA−C ENSG00000204525
RACK1 ENSG00000204628
FBLN1 ENSG00000077942
HLA−B ENSG00000234745
CD74 ENSG00000019582
IGFBP4 ENSG00000141753
MGP ENSG00000111341
TIMP1 ENSG00000102265
UBB ENSG00000170315
CFL1 ENSG00000172757
EEF1A1 ENSG00000156508
KRT15 ENSG00000171346
MYH11 ENSG00000133392
PPDPF ENSG00000125534
EZR ENSG00000092820
AZGP1 ENSG00000160862
UBC ENSG00000150991
FLNA ENSG00000196924
SLC44A4 ENSG00000204385
EDF1 ENSG00000107223
TSPAN1 ENSG00000117472
TAGLN ENSG00000149591
NR4A1 ENSG00000123358
P4HB ENSG00000185624
ACTG1 ENSG00000184009
SPDEF ENSG00000124664
FTL ENSG00000087086
A2M ENSG00000175899
SERF2 ENSG00000140264
YWHAE ENSG00000108953
CD81 ENSG00000110651
MYL6 ENSG00000092841
EEF2 ENSG00000167658
TPM2 ENSG00000198467
NBL1 ENSG00000158747
SLC25A6 ENSG00000169100
CLSTN1 ENSG00000171603
GAPDH ENSG00000111640
H3F3B ENSG00000132475
TMSB10 ENSG00000034510
KRT18 ENSG00000111057

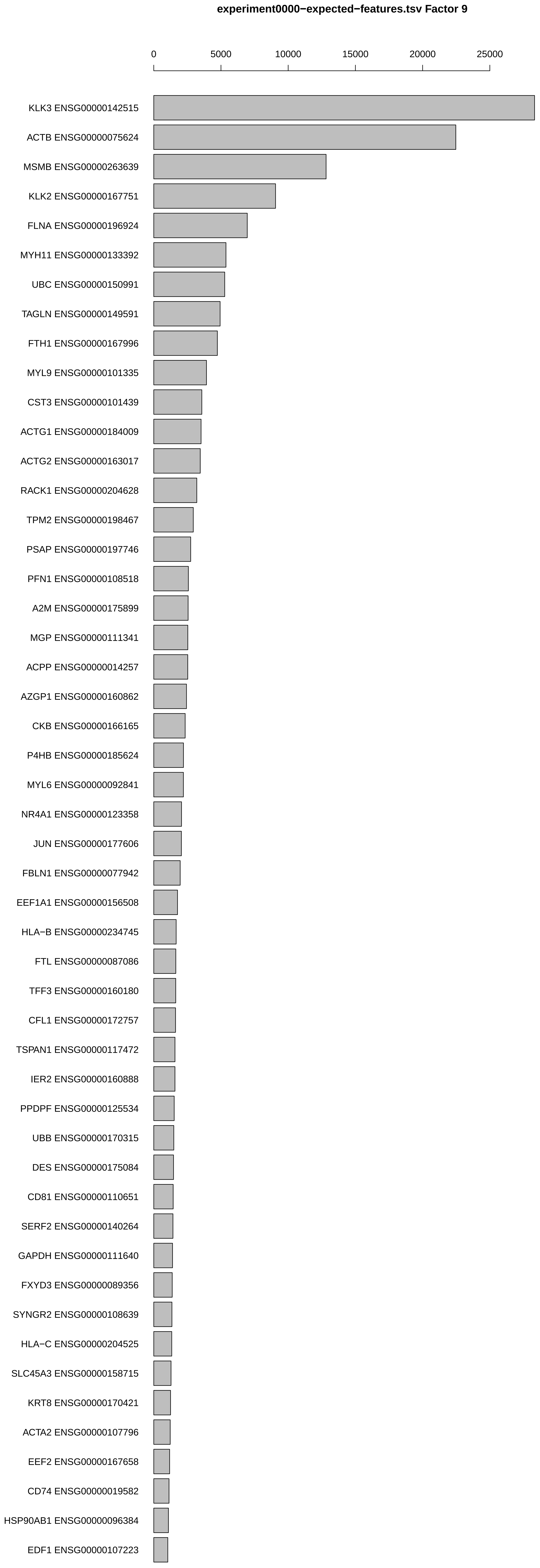
experiment0000−expected−features.tsv Factor 9
0
5000
10000
15000
20000
25000
KLK3 ENSG00000142515
ACTB ENSG00000075624
MSMB ENSG00000263639
KLK2 ENSG00000167751
FLNA ENSG00000196924
MYH11 ENSG00000133392
UBC ENSG00000150991
TAGLN ENSG00000149591
FTH1 ENSG00000167996
MYL9 ENSG00000101335
CST3 ENSG00000101439
ACTG1 ENSG00000184009
ACTG2 ENSG00000163017
RACK1 ENSG00000204628
TPM2 ENSG00000198467
PSAP ENSG00000197746
PFN1 ENSG00000108518
A2M ENSG00000175899
MGP ENSG00000111341
ACPP ENSG00000014257
AZGP1 ENSG00000160862
CKB ENSG00000166165
P4HB ENSG00000185624
MYL6 ENSG00000092841
NR4A1 ENSG00000123358
JUN ENSG00000177606
FBLN1 ENSG00000077942
EEF1A1 ENSG00000156508
HLA−B ENSG00000234745
FTL ENSG00000087086
TFF3 ENSG00000160180
CFL1 ENSG00000172757
TSPAN1 ENSG00000117472
IER2 ENSG00000160888
PPDPF ENSG00000125534
UBB ENSG00000170315
DES ENSG00000175084
CD81 ENSG00000110651
SERF2 ENSG00000140264
GAPDH ENSG00000111640
FXYD3 ENSG00000089356
SYNGR2 ENSG00000108639
HLA−C ENSG00000204525
SLC45A3 ENSG00000158715
KRT8 ENSG00000170421
ACTA2 ENSG00000107796
EEF2 ENSG00000167658
CD74 ENSG00000019582
HSP90AB1 ENSG00000096384
EDF1 ENSG00000107223

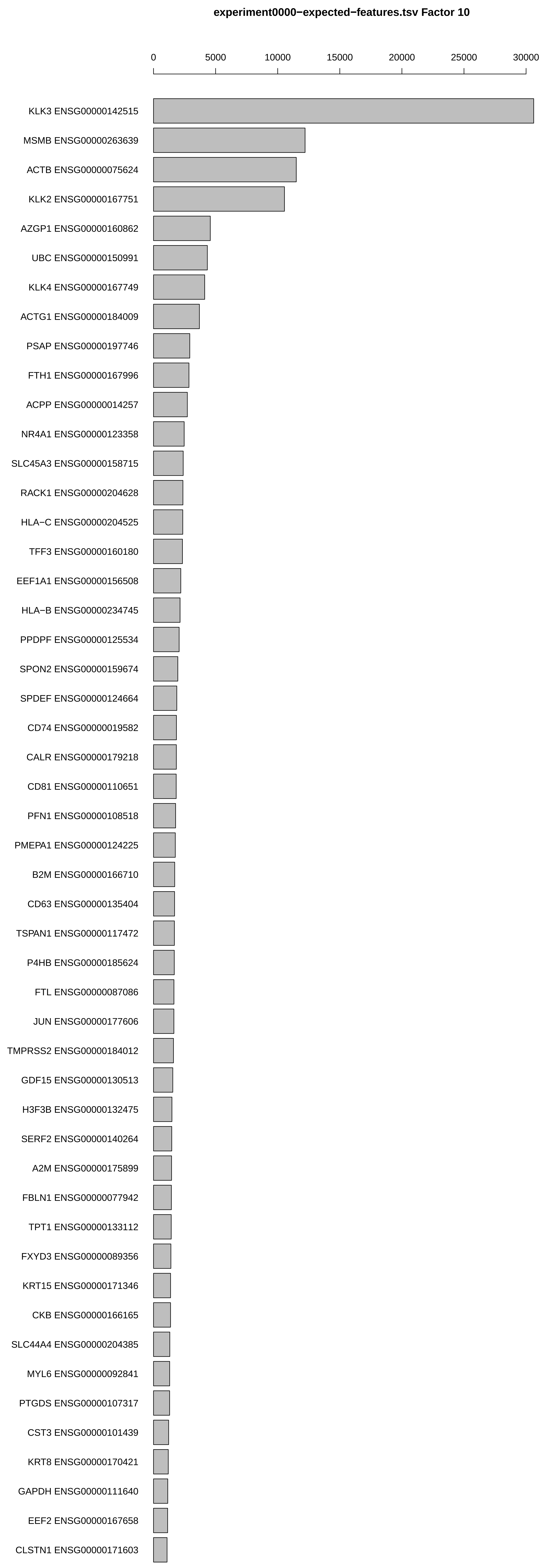
experiment0000−expected−features.tsv Factor 10
0
5000
10000
15000
20000
25000
30000
KLK3 ENSG00000142515
MSMB ENSG00000263639
ACTB ENSG00000075624
KLK2 ENSG00000167751
AZGP1 ENSG00000160862
UBC ENSG00000150991
KLK4 ENSG00000167749
ACTG1 ENSG00000184009
PSAP ENSG00000197746
FTH1 ENSG00000167996
ACPP ENSG00000014257
NR4A1 ENSG00000123358
SLC45A3 ENSG00000158715
RACK1 ENSG00000204628
HLA−C ENSG00000204525
TFF3 ENSG00000160180
EEF1A1 ENSG00000156508
HLA−B ENSG00000234745
PPDPF ENSG00000125534
SPON2 ENSG00000159674
SPDEF ENSG00000124664
CD74 ENSG00000019582
CALR ENSG00000179218
CD81 ENSG00000110651
PFN1 ENSG00000108518
PMEPA1 ENSG00000124225
B2M ENSG00000166710
CD63 ENSG00000135404
TSPAN1 ENSG00000117472
P4HB ENSG00000185624
FTL ENSG00000087086
JUN ENSG00000177606
TMPRSS2 ENSG00000184012
GDF15 ENSG00000130513
H3F3B ENSG00000132475
SERF2 ENSG00000140264
A2M ENSG00000175899
FBLN1 ENSG00000077942
TPT1 ENSG00000133112
FXYD3 ENSG00000089356
KRT15 ENSG00000171346
CKB ENSG00000166165
SLC44A4 ENSG00000204385
MYL6 ENSG00000092841
PTGDS ENSG00000107317
CST3 ENSG00000101439
KRT8 ENSG00000170421
GAPDH ENSG00000111640
EEF2 ENSG00000167658
CLSTN1 ENSG00000171603
