# Supplementary figures and images for: Spatial maps of prostate cancer transcriptomes reveal an unexplored landscape of heterogeneity
Source: Nat Commun. 2018 Jun 20;9:2419. doi: 10.1038/s41467-018-04724-5 (PMC6010471; doi:10.1038/s41467-018-04724-5)

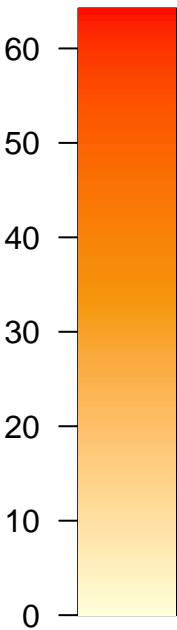

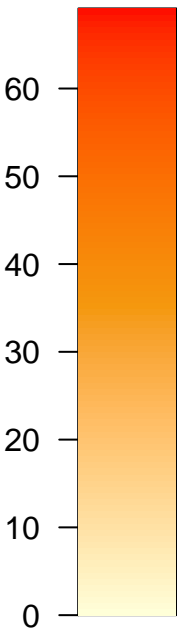

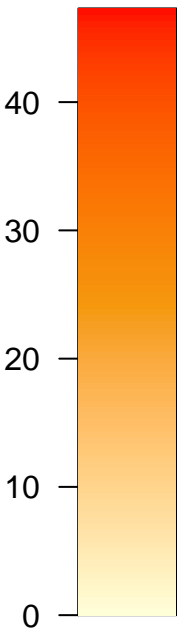

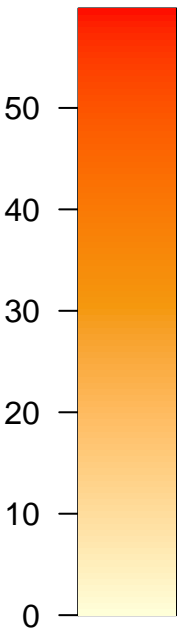

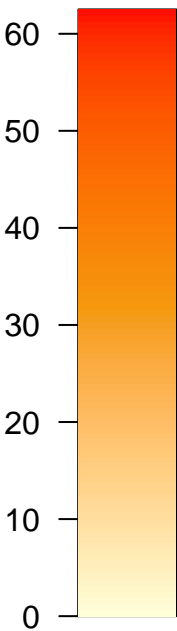

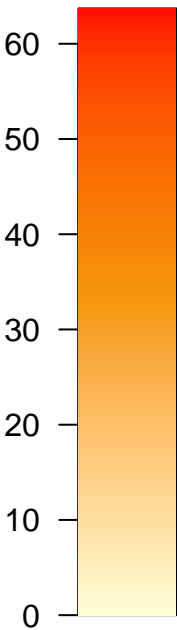

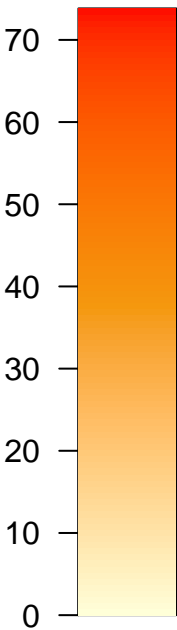

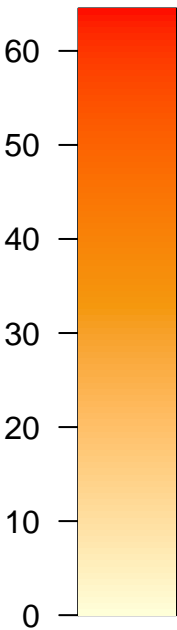

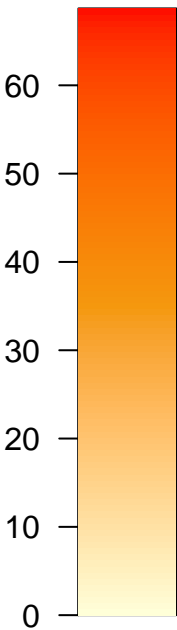

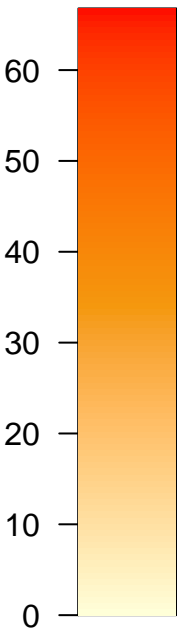

Supplement: Supplementary file 4 — Supplementary Data 1 [file 41467_2018_4724_MOESM4_ESM.zip › Supplementary Dataset 1/joint-mix-profiles-rel-common-scale-matrix-colorbar.pdf]

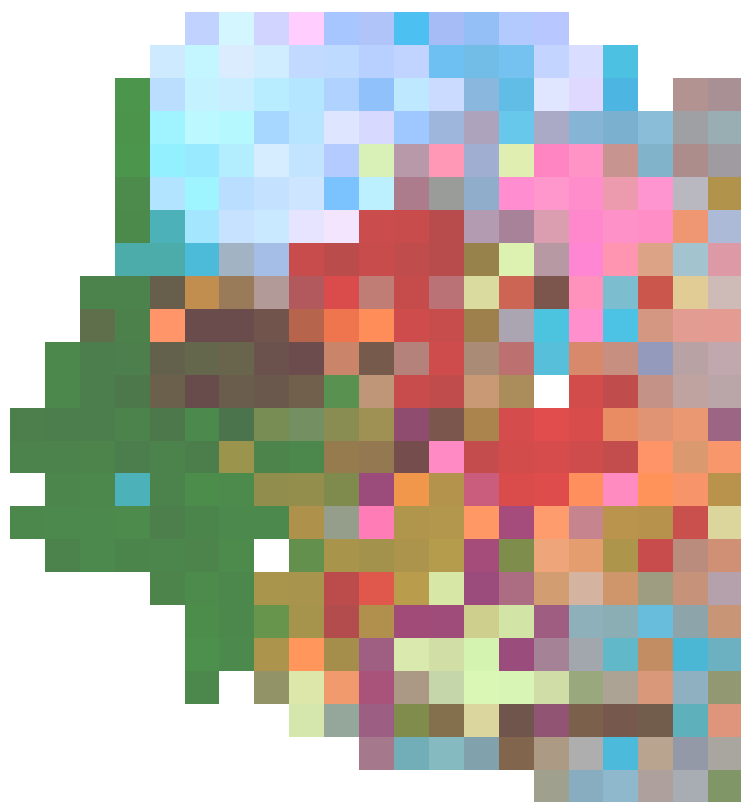

Supplement: Supplementary file 4 — Supplementary Data 1 [file 41467_2018_4724_MOESM4_ESM.zip › Supplementary Dataset 1/joint-mix-dimensionality-reduction-tSNE-matrix.pdf]

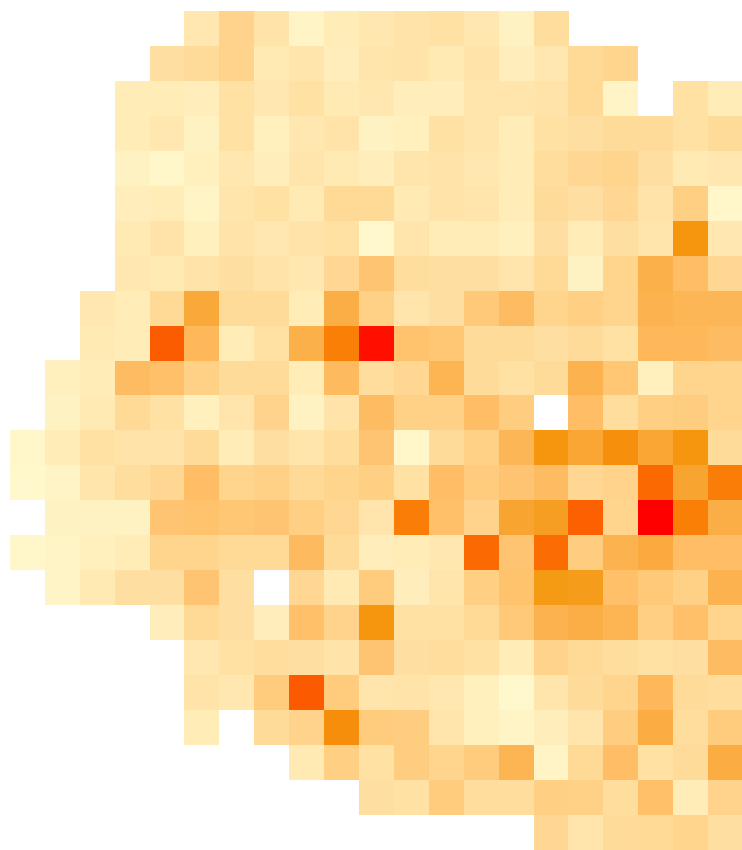

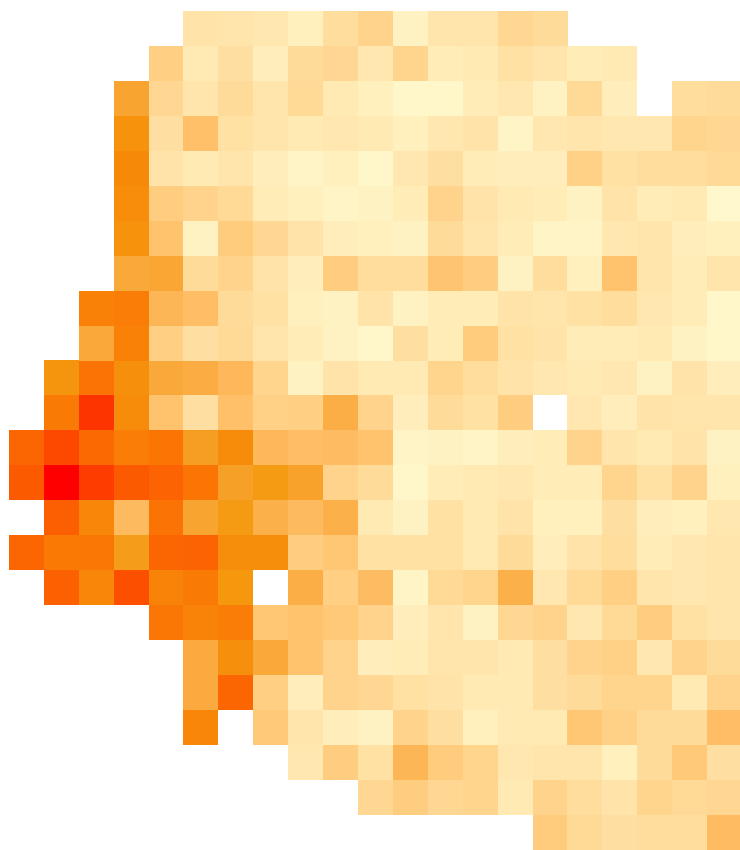

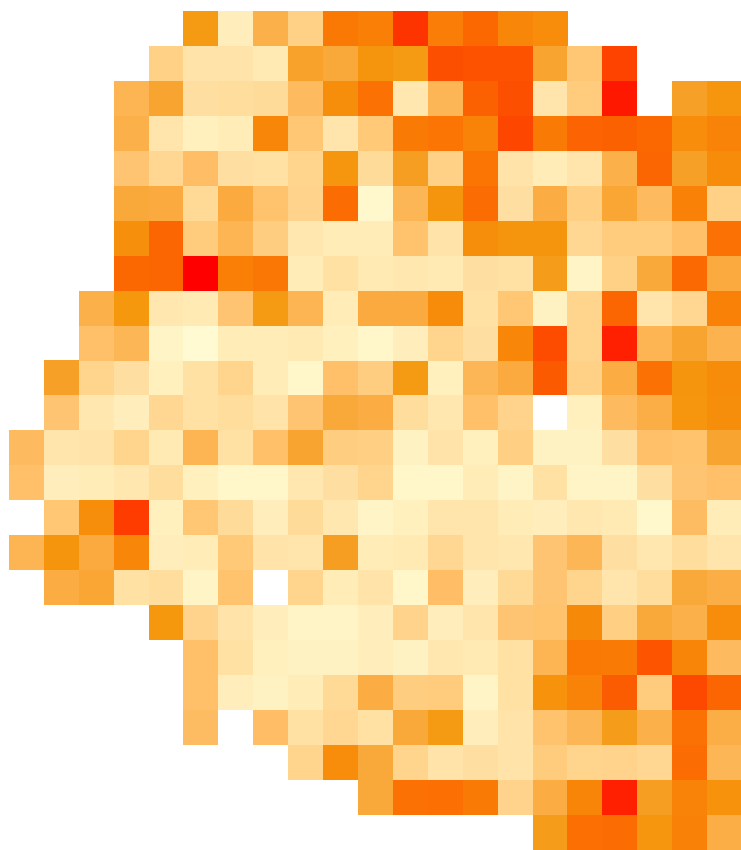

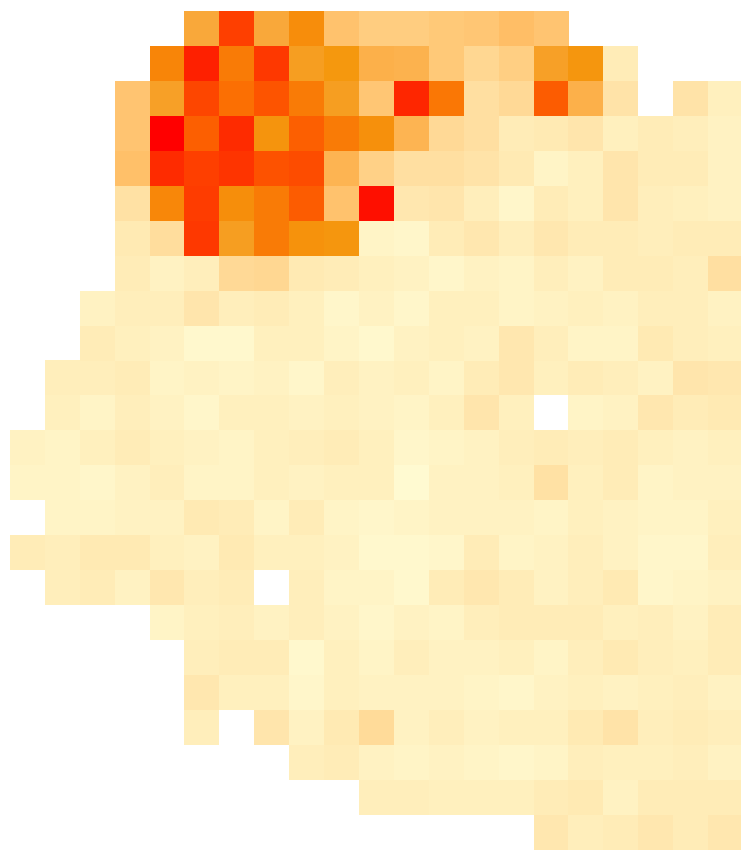

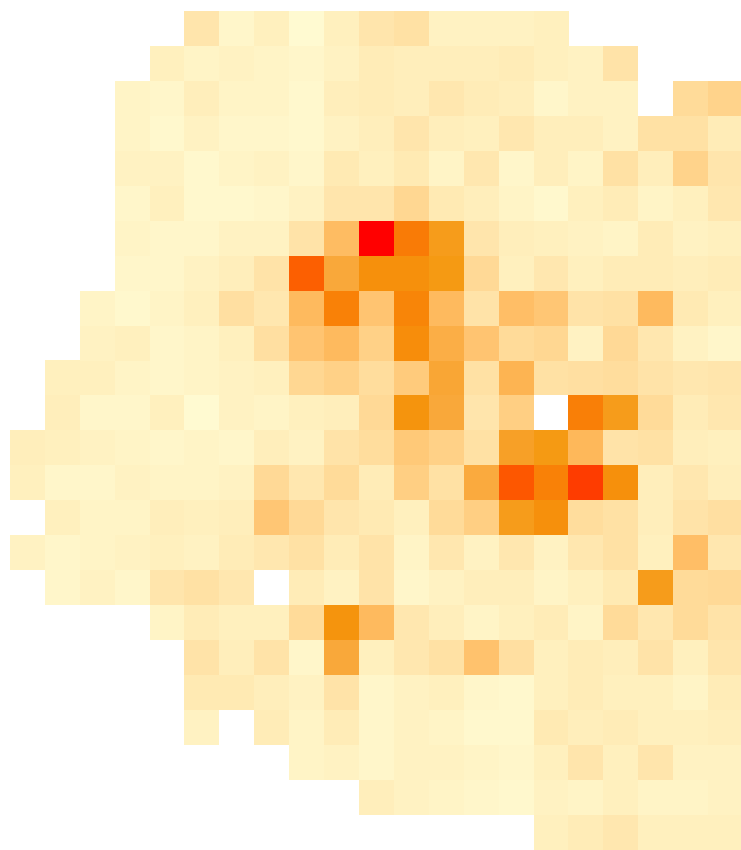

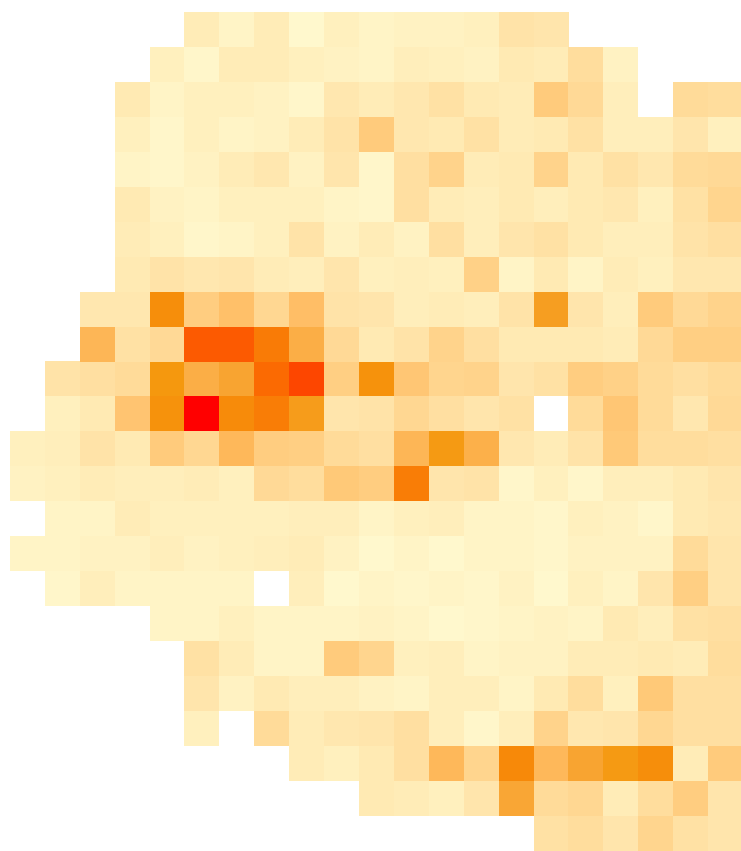

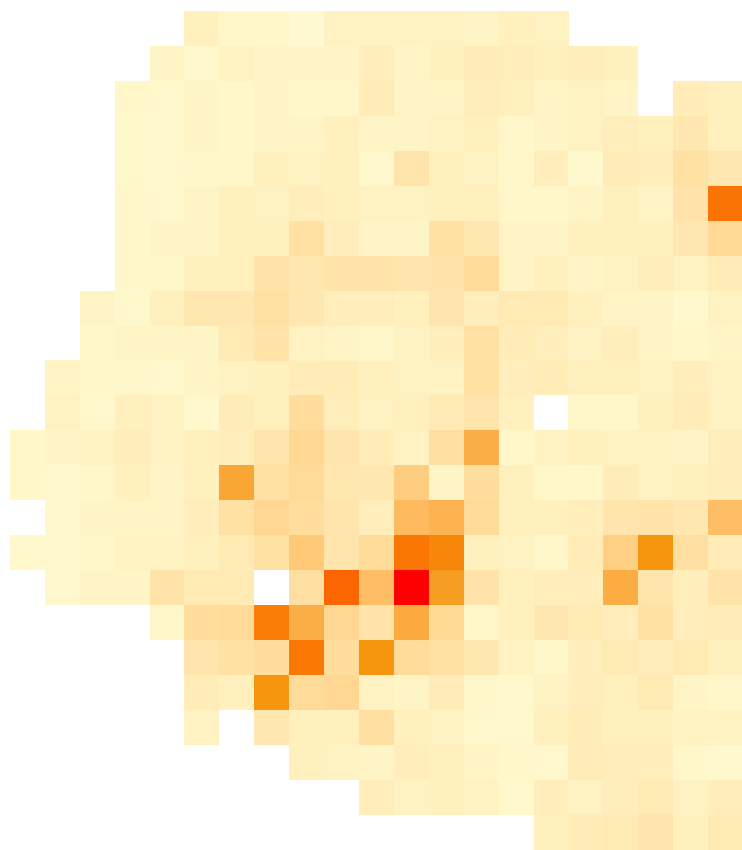

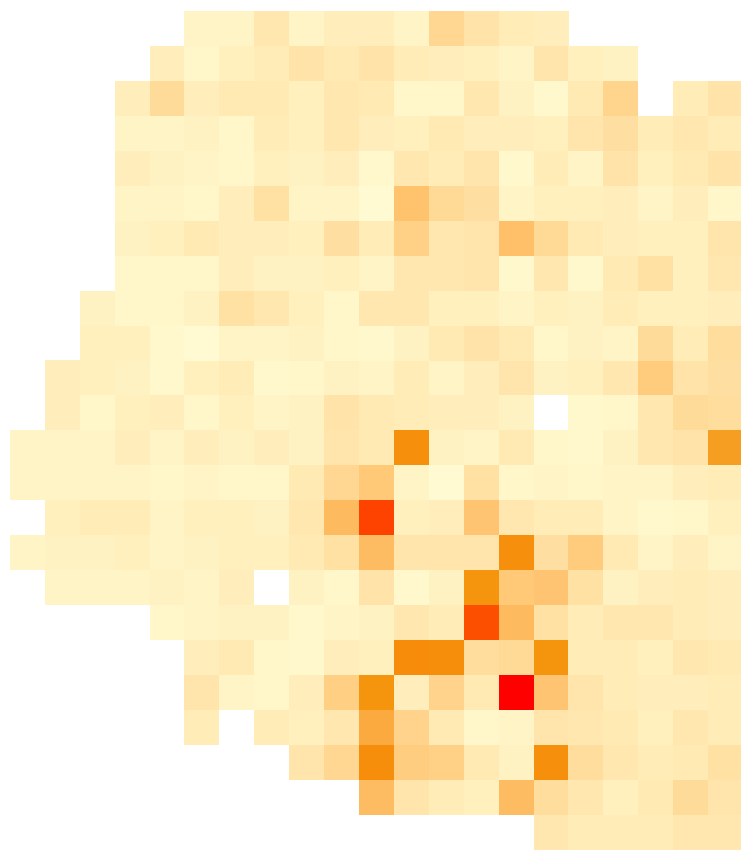

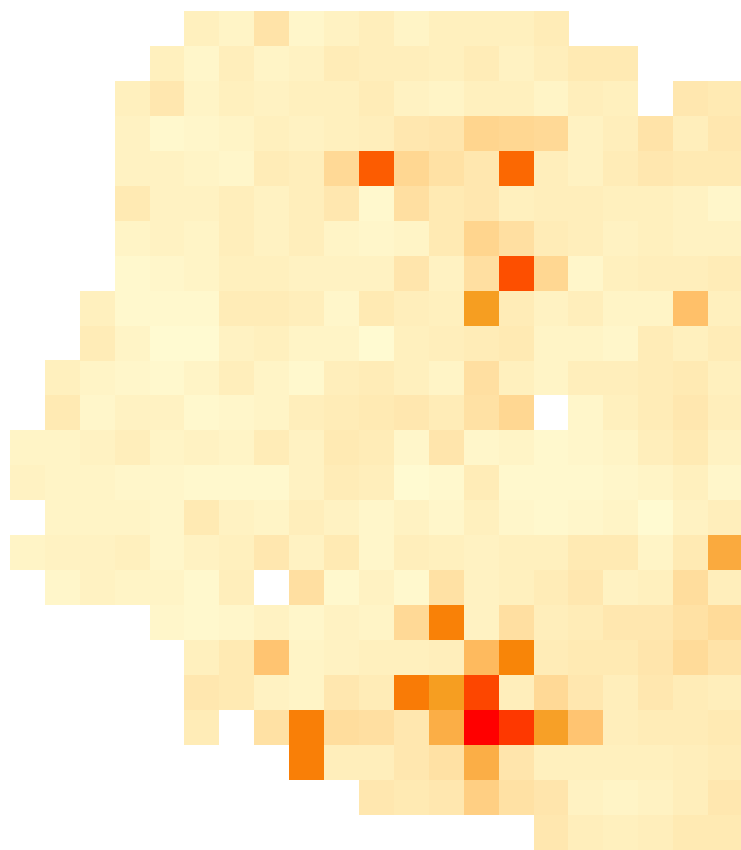

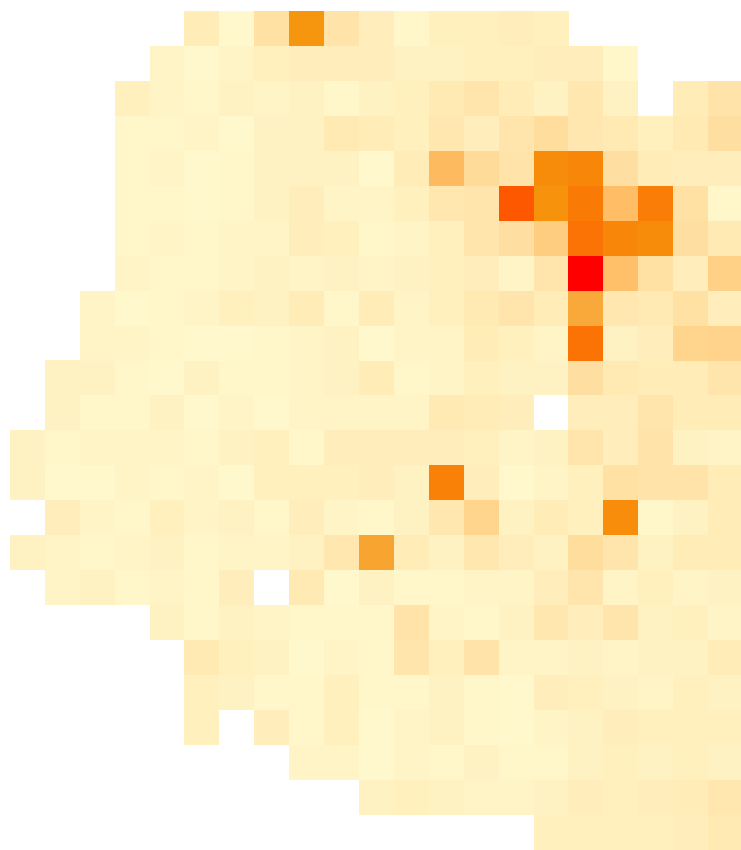

Supplement: Supplementary file 4 — Supplementary Data 1 [file 41467_2018_4724_MOESM4_ESM.zip › Supplementary Dataset 1/joint-mix-profiles-rel-common-scale-matrix.pdf]

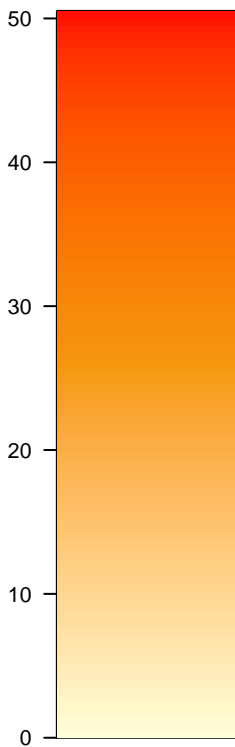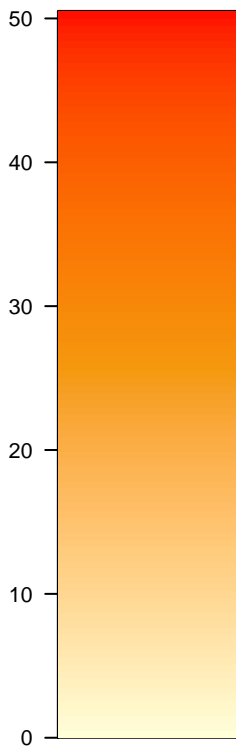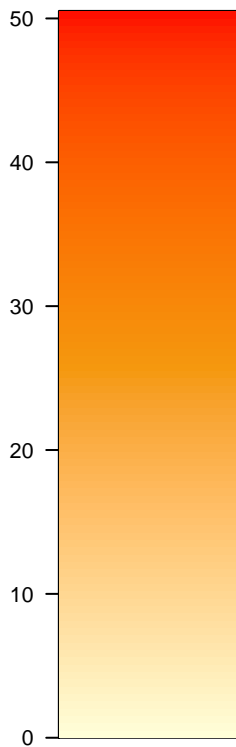

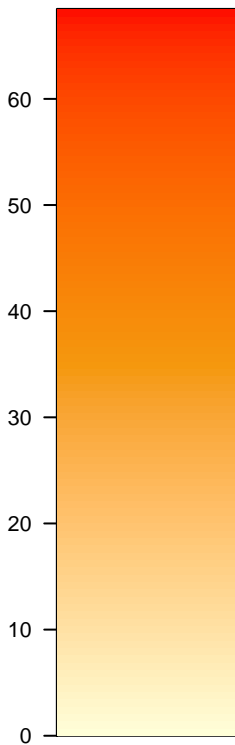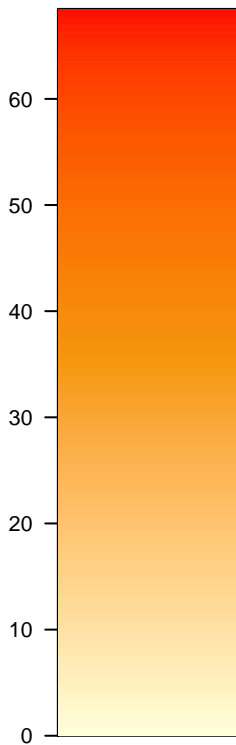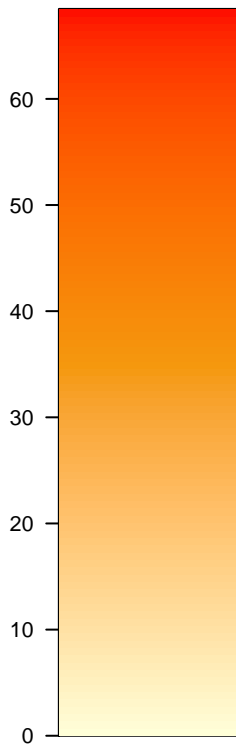

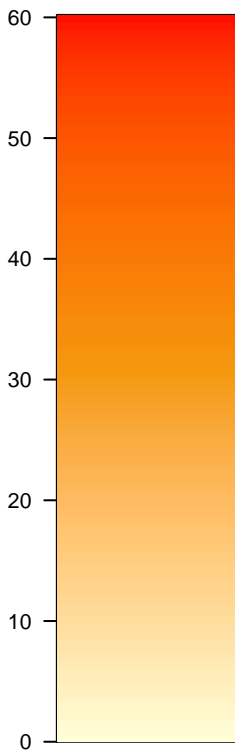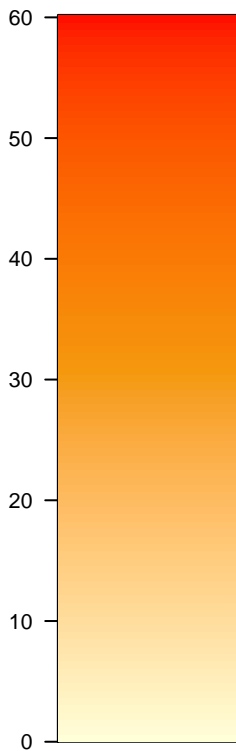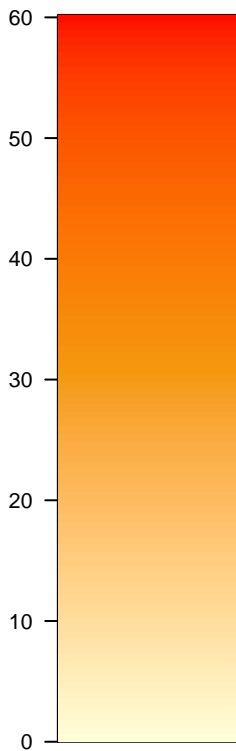

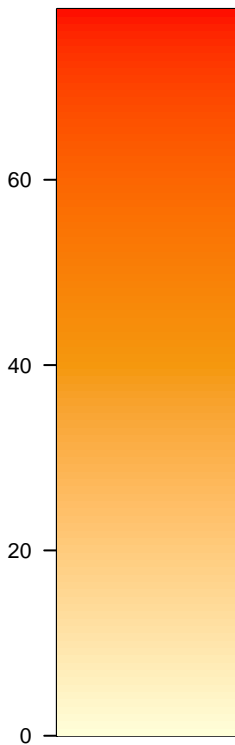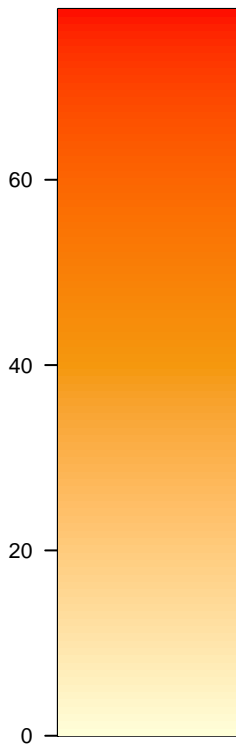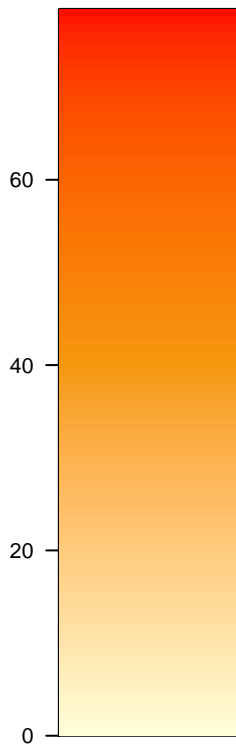

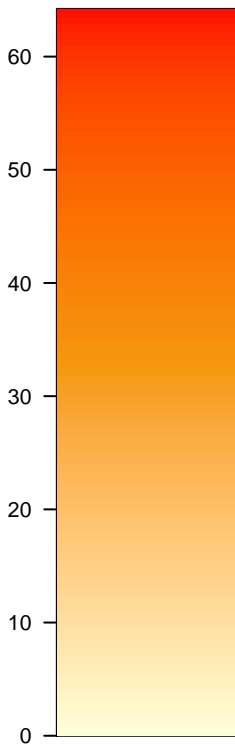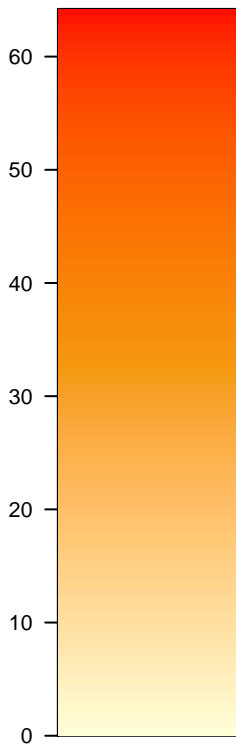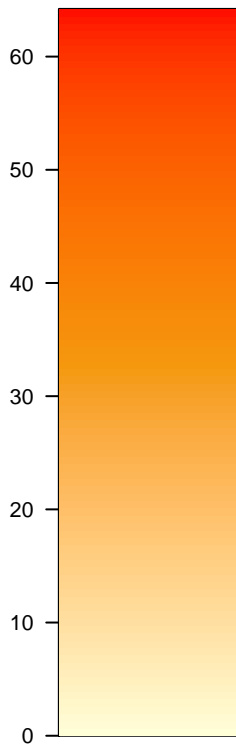

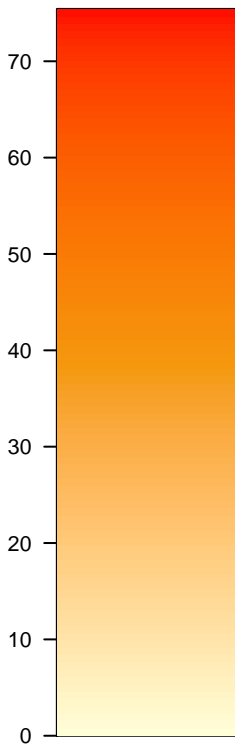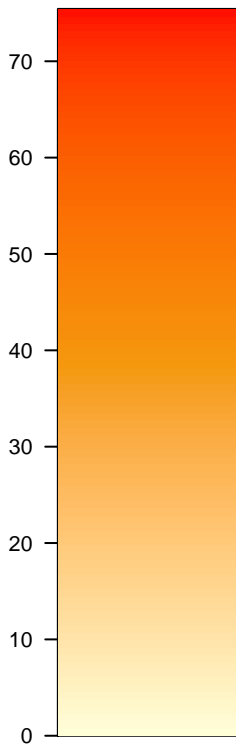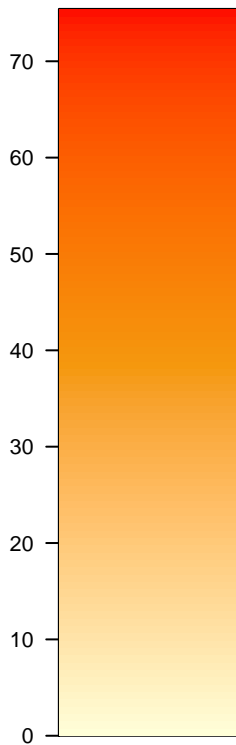

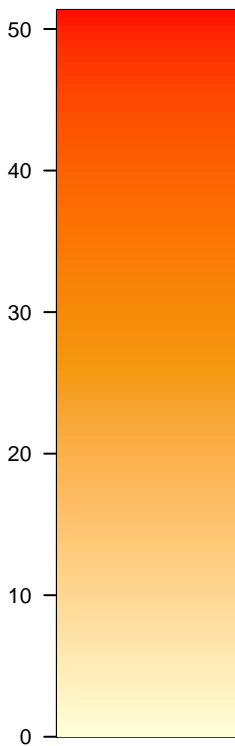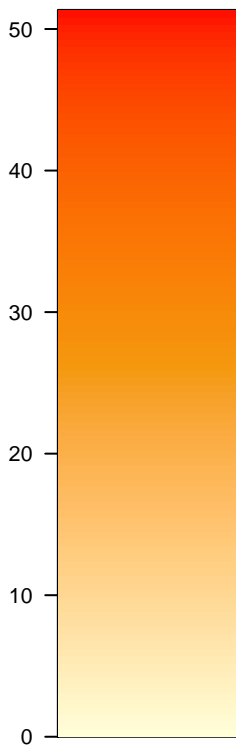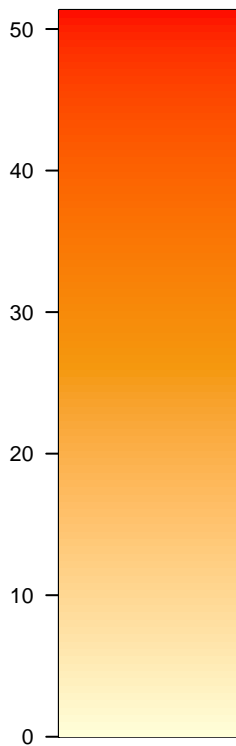

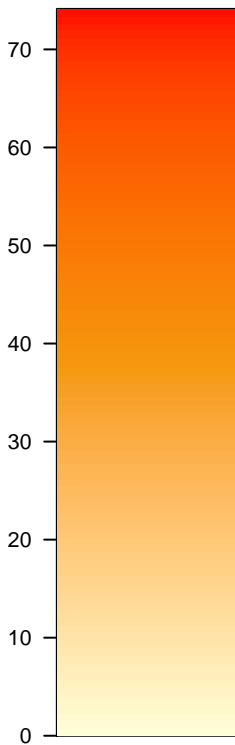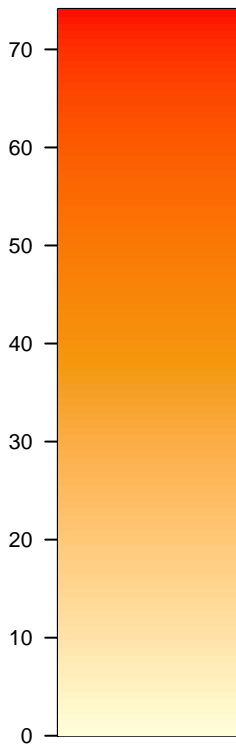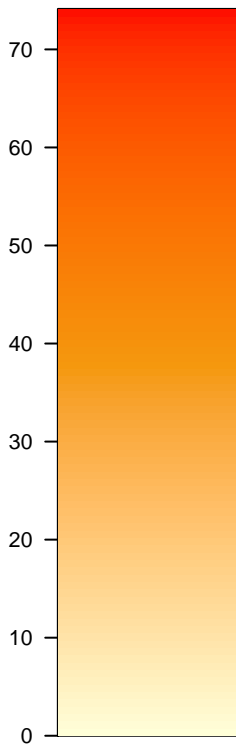

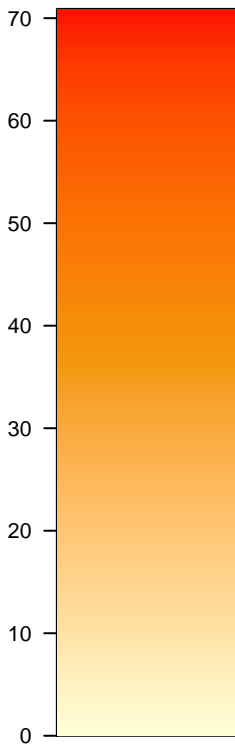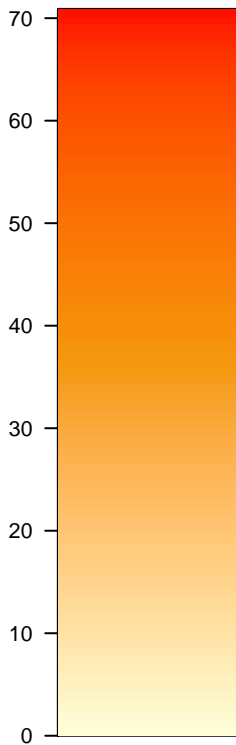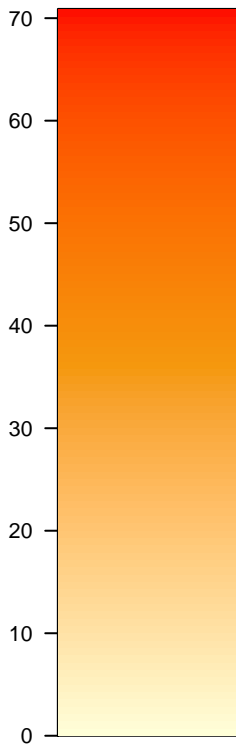

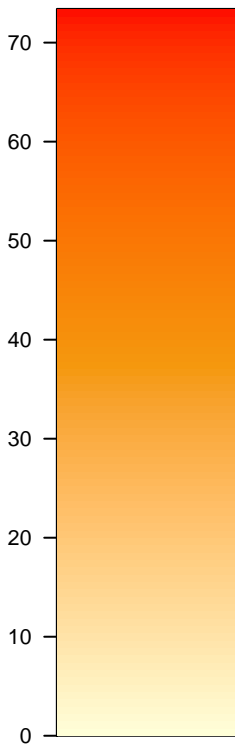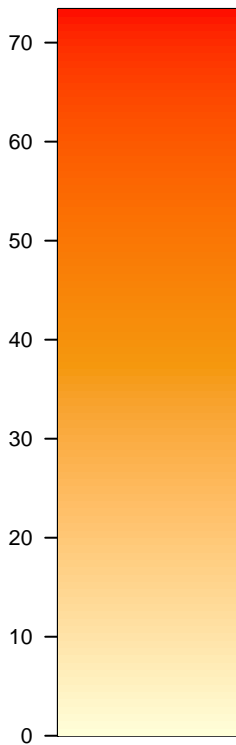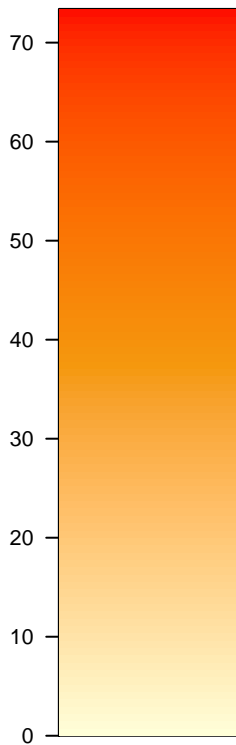

Supplement: Supplementary file 6 — Supplementary Data 3 [file 41467_2018_4724_MOESM6_ESM.zip › Supplementary Dataset 2/joint-mix-profiles-rel-common-scale-matrix-colorbar.pdf]

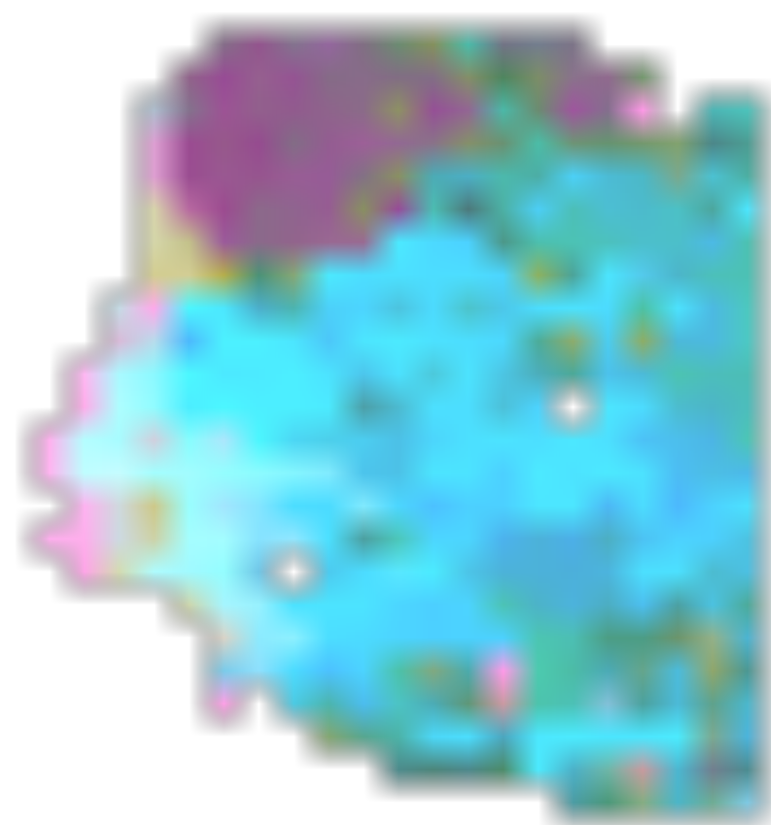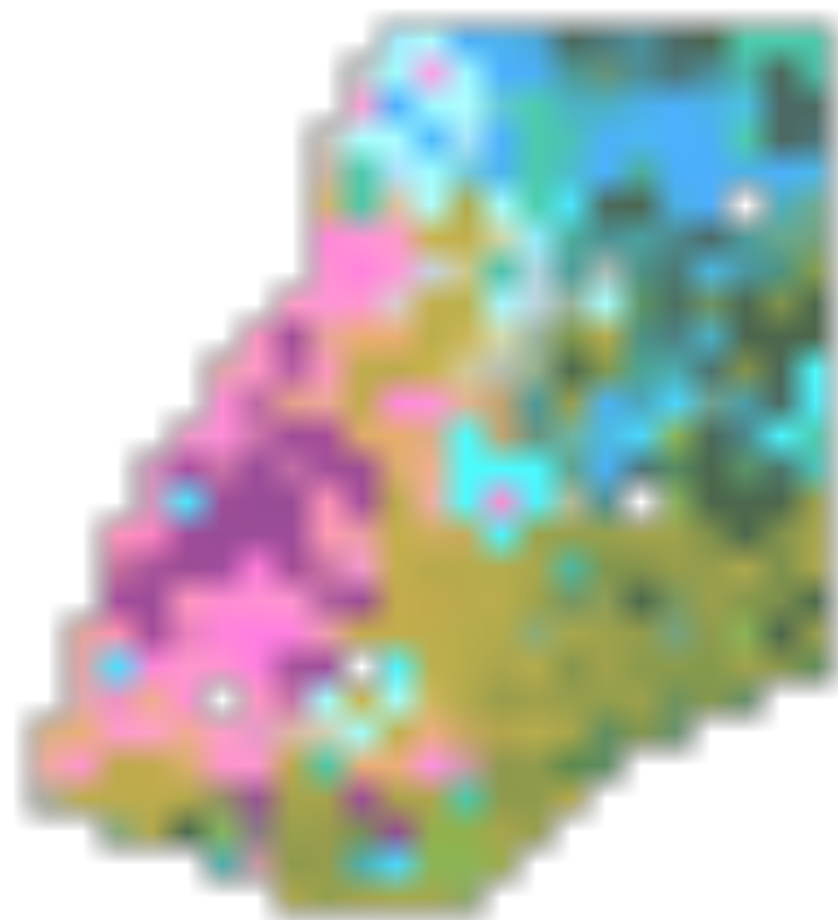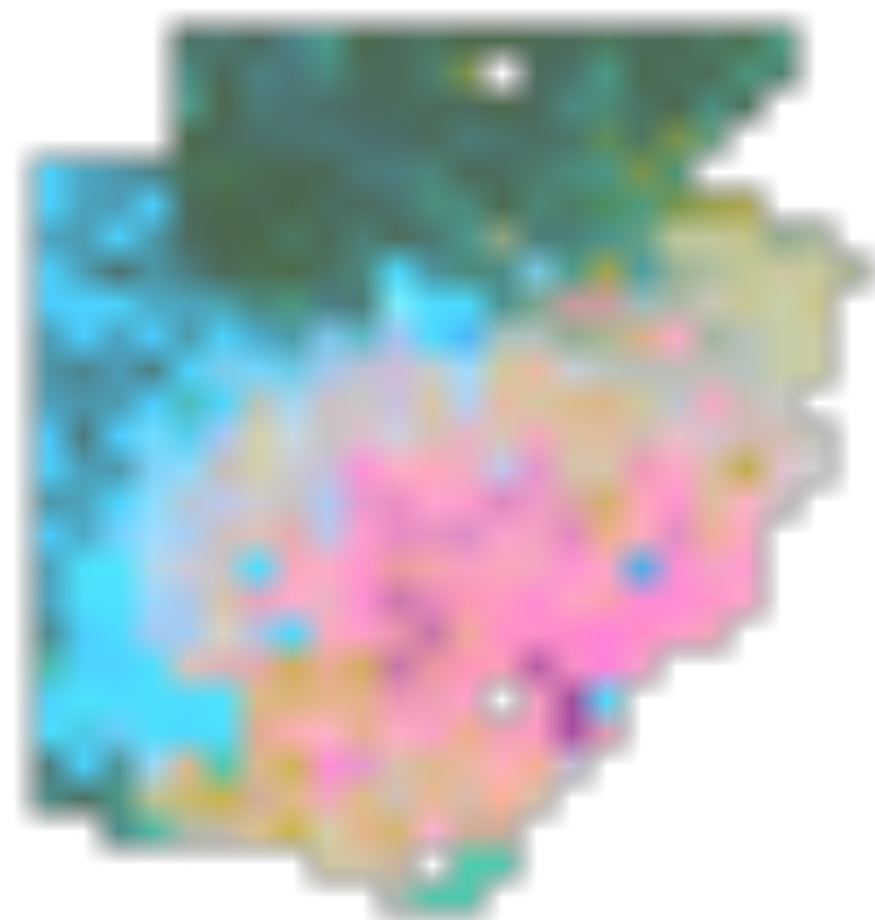

Supplement: Supplementary file 6 — Supplementary Data 3 [file 41467_2018_4724_MOESM6_ESM.zip › Supplementary Dataset 2/joint-mix-dimensionality-reduction-tSNE-matrix.pdf]

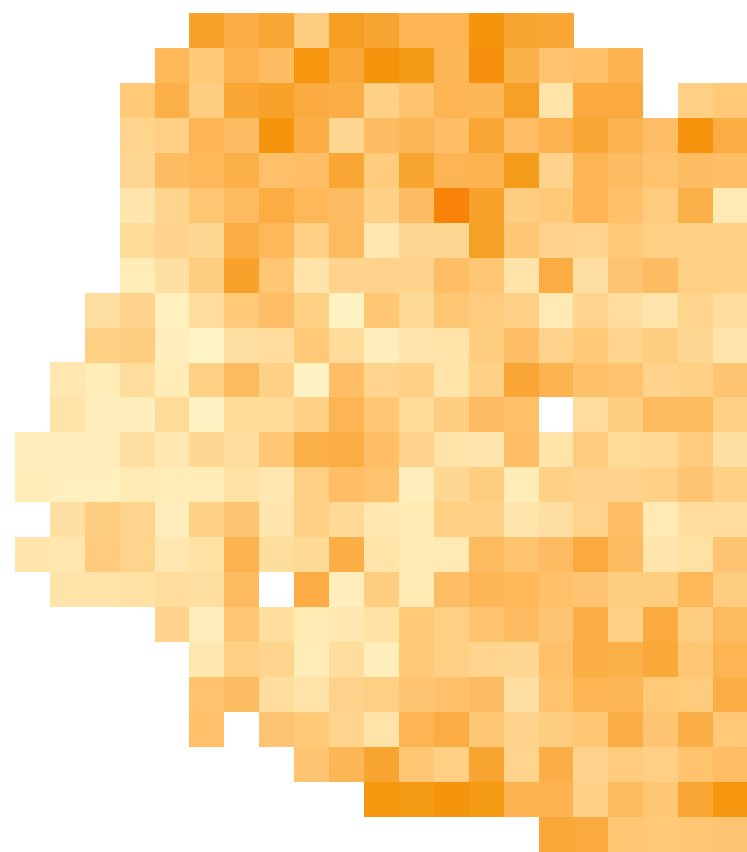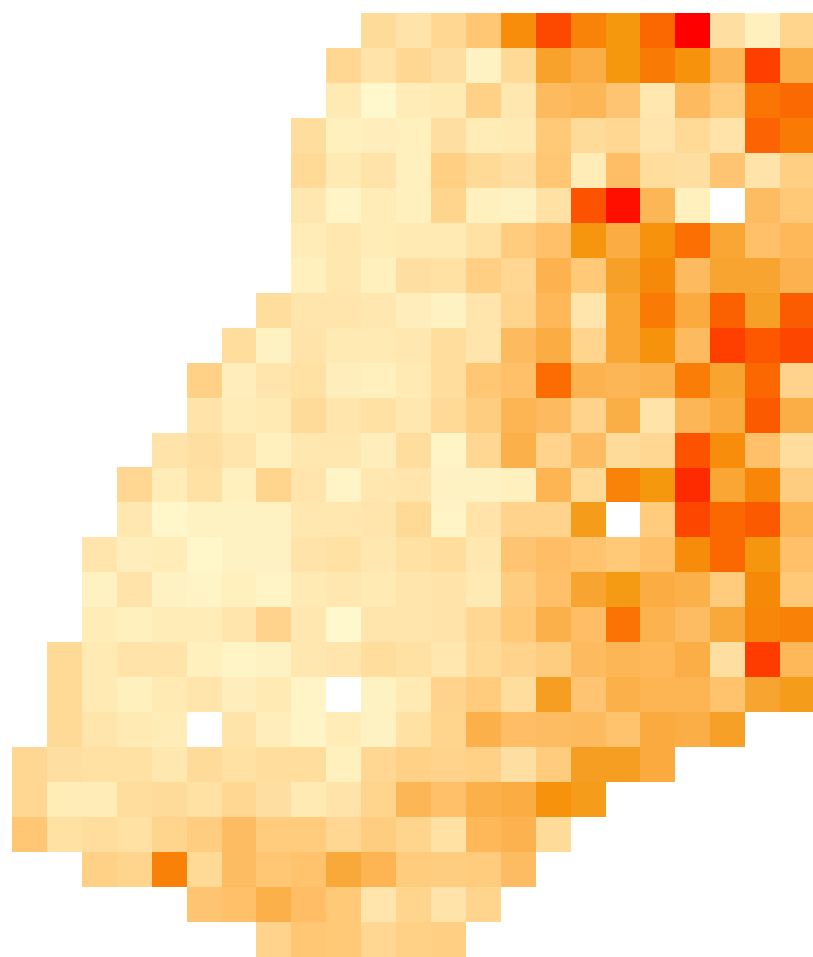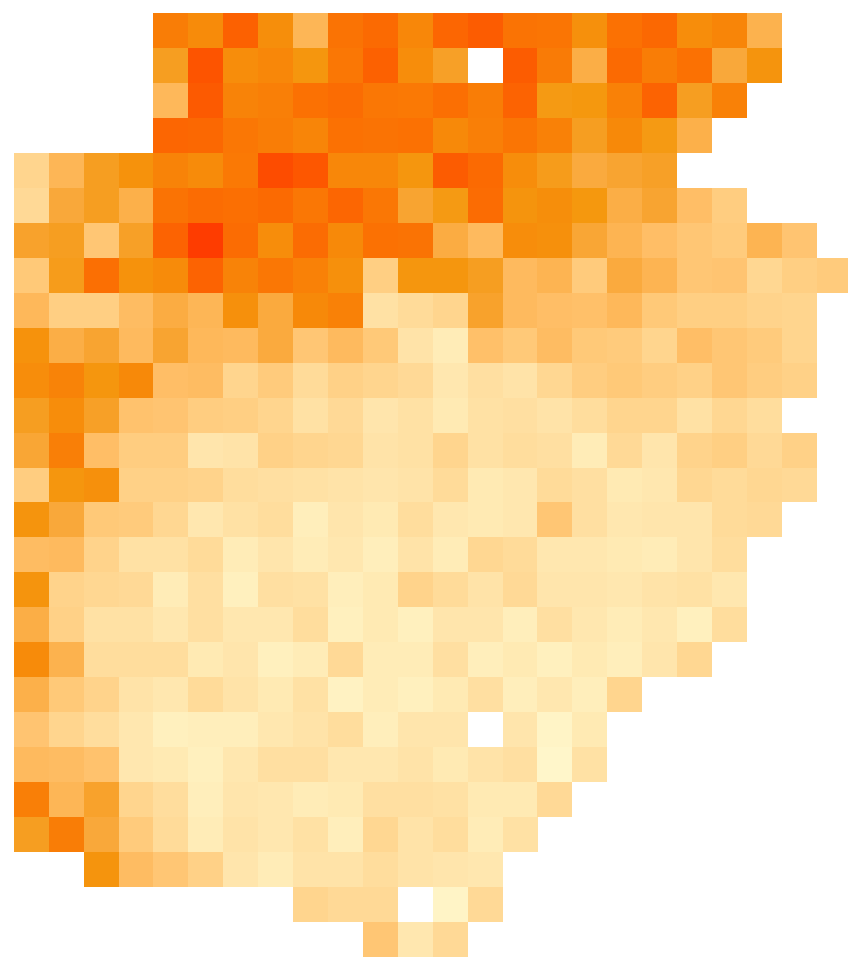

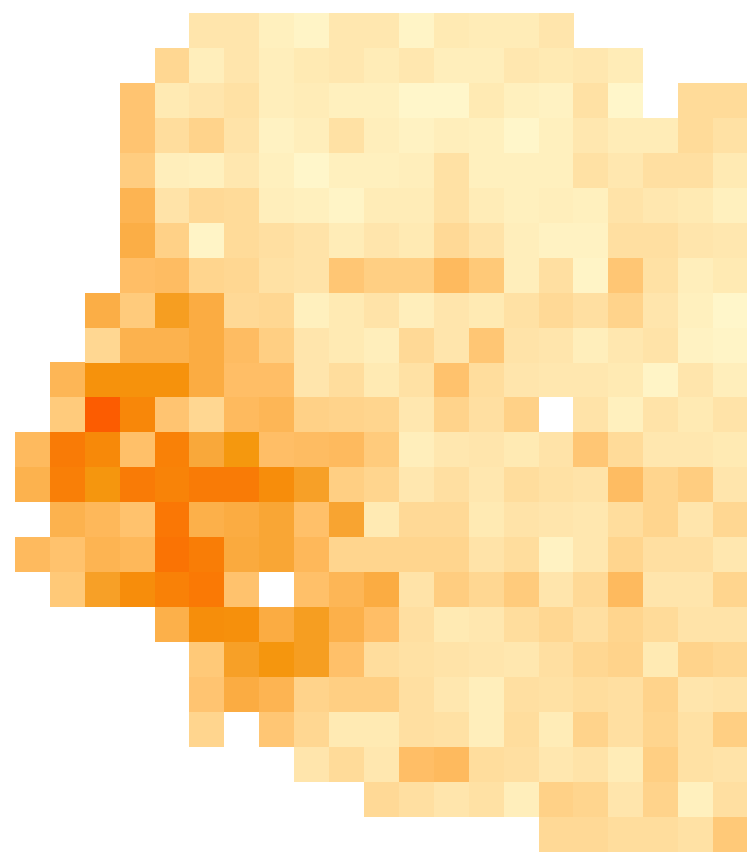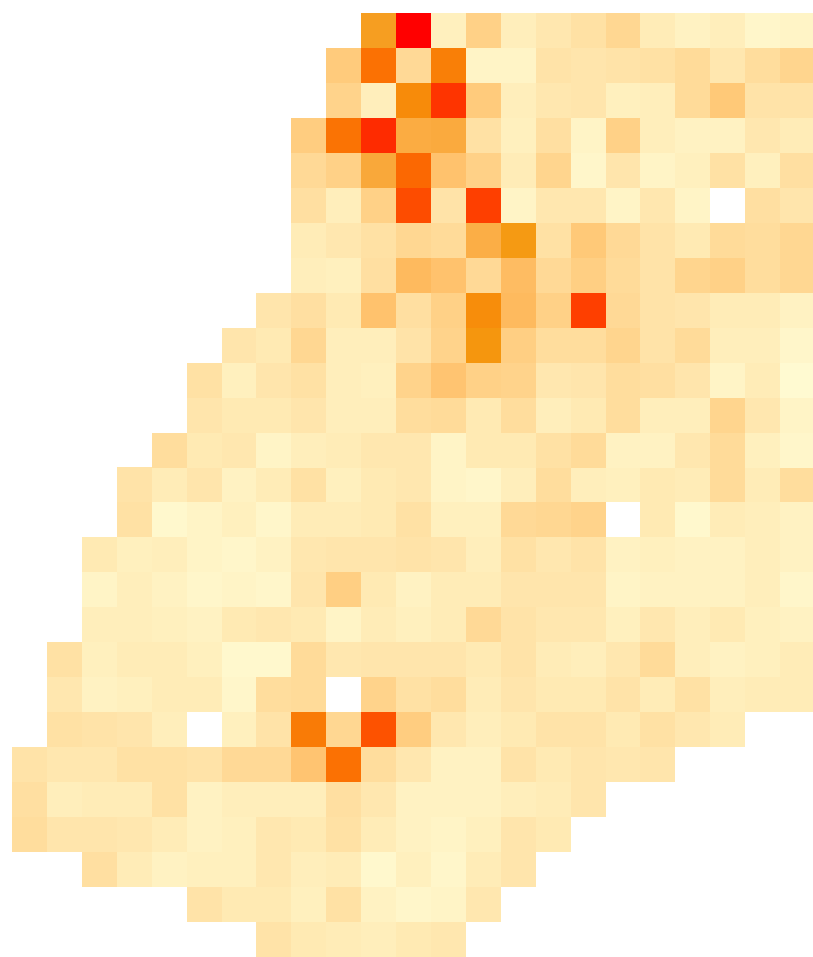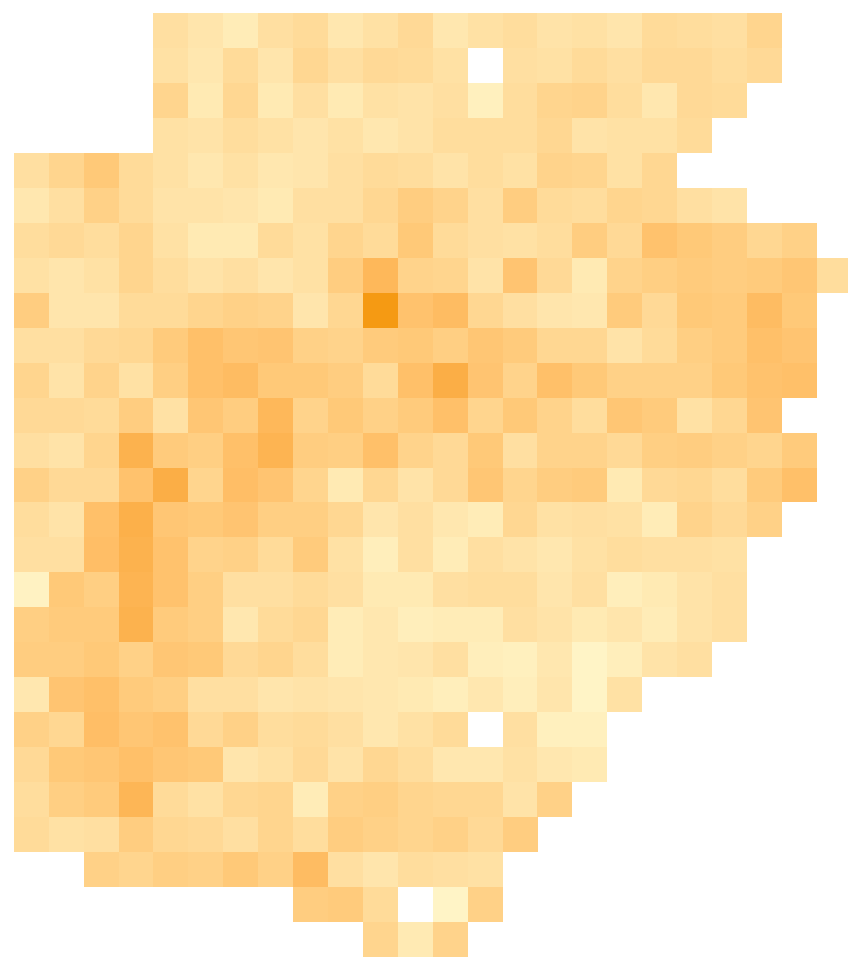

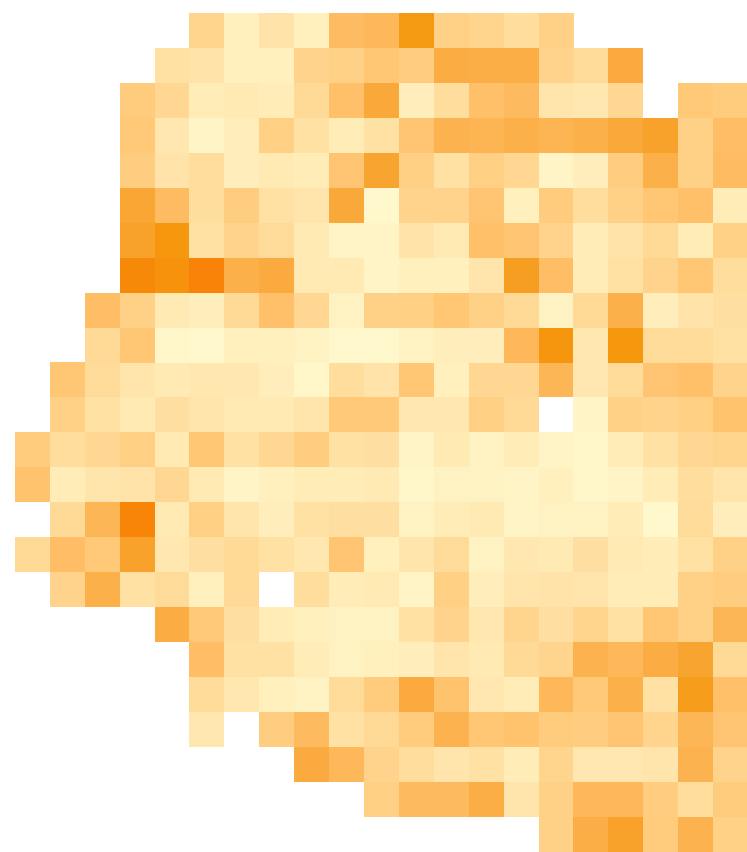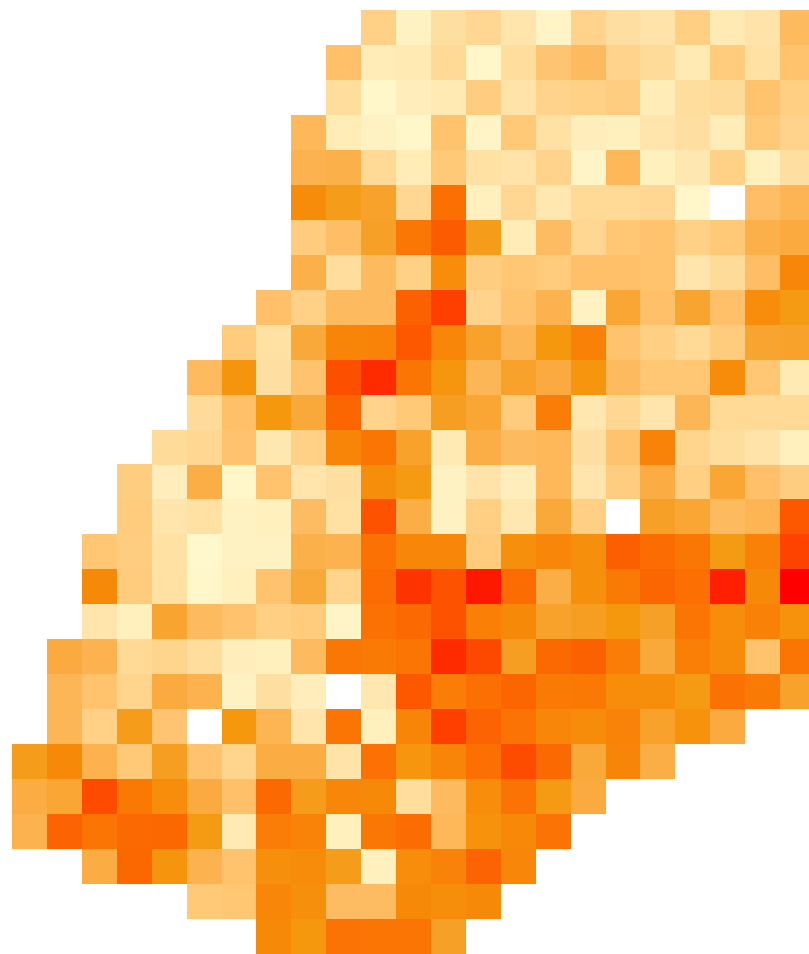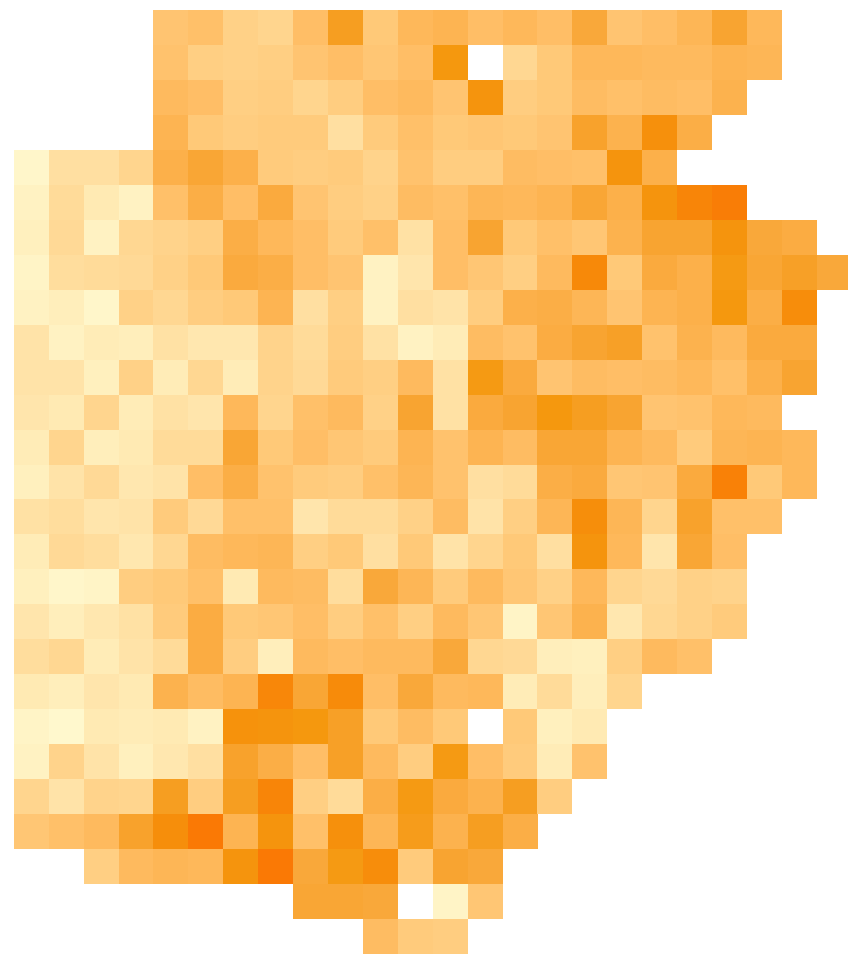

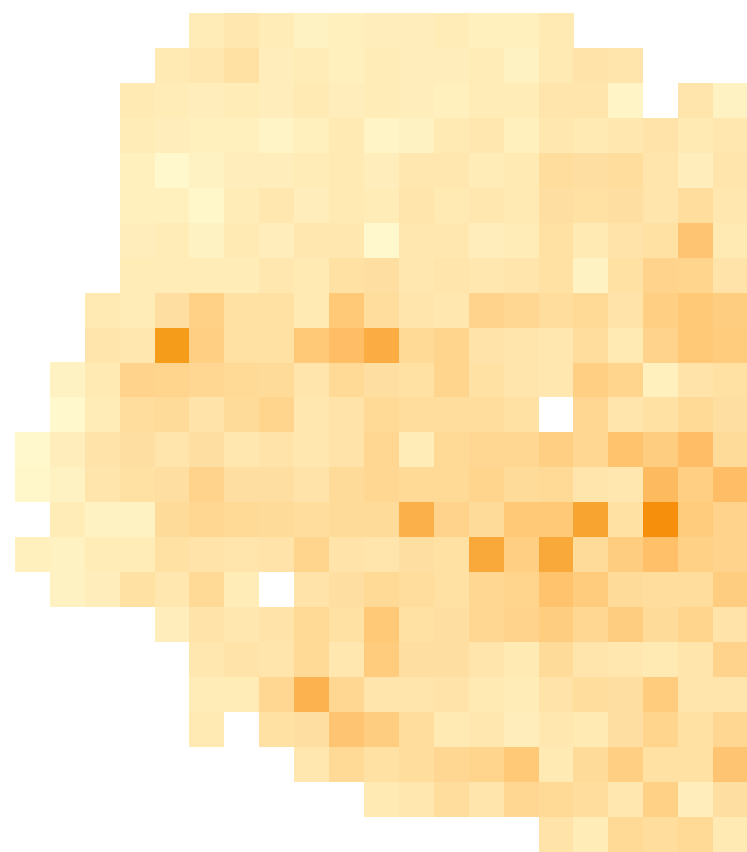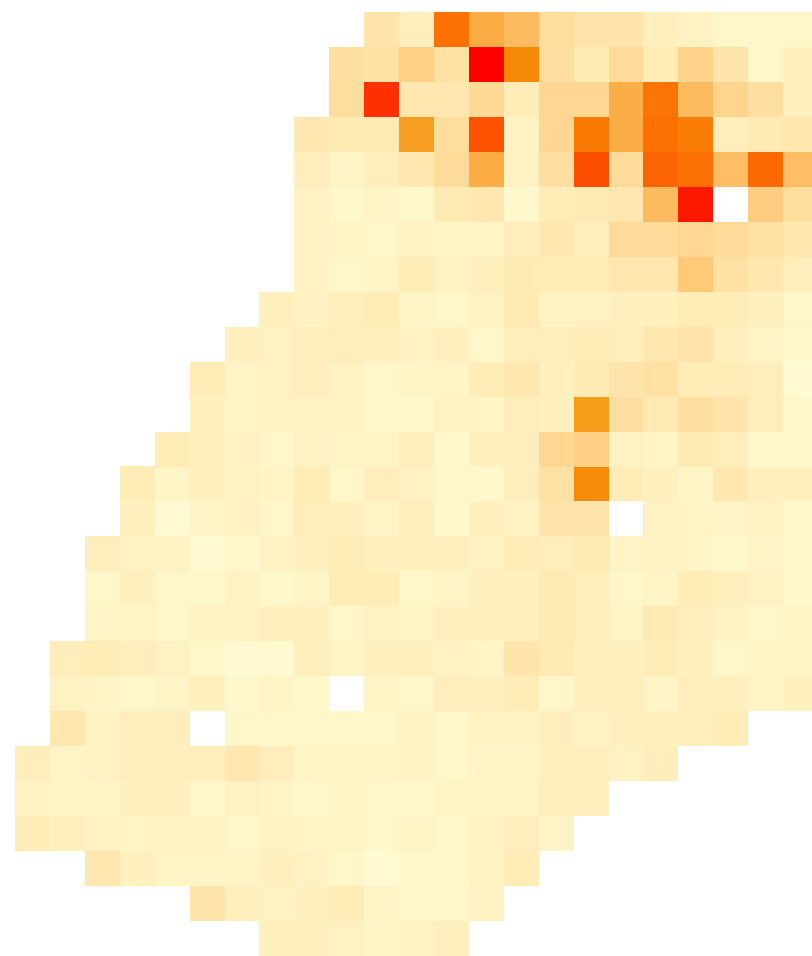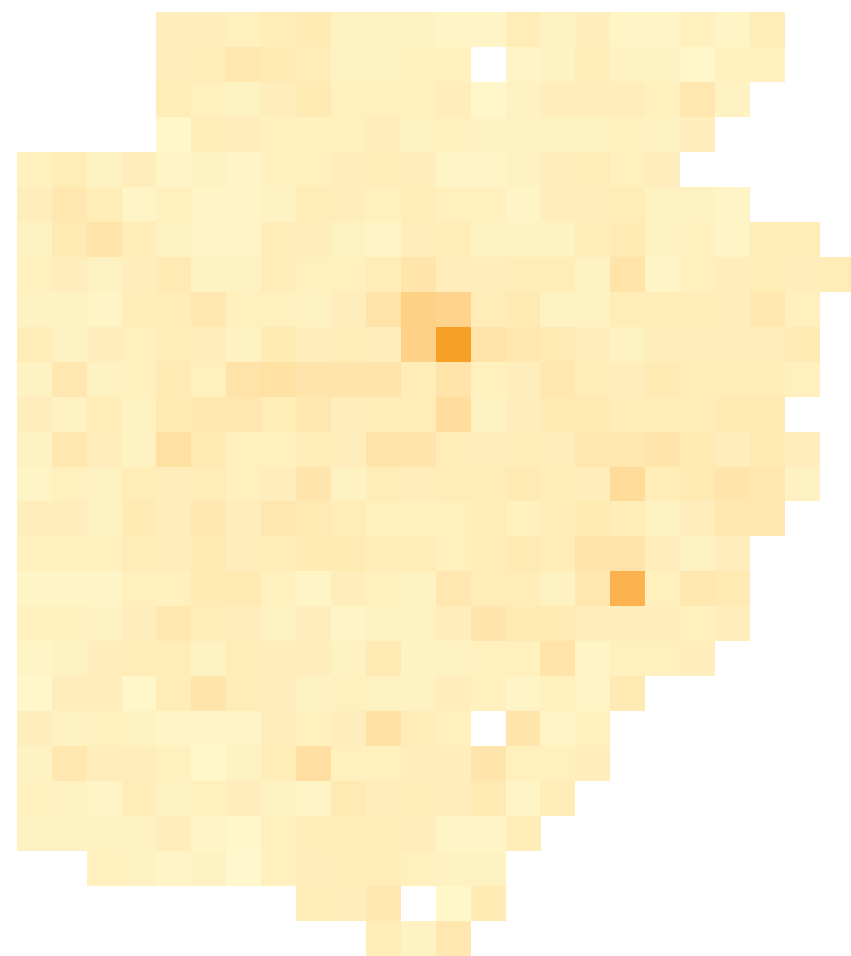

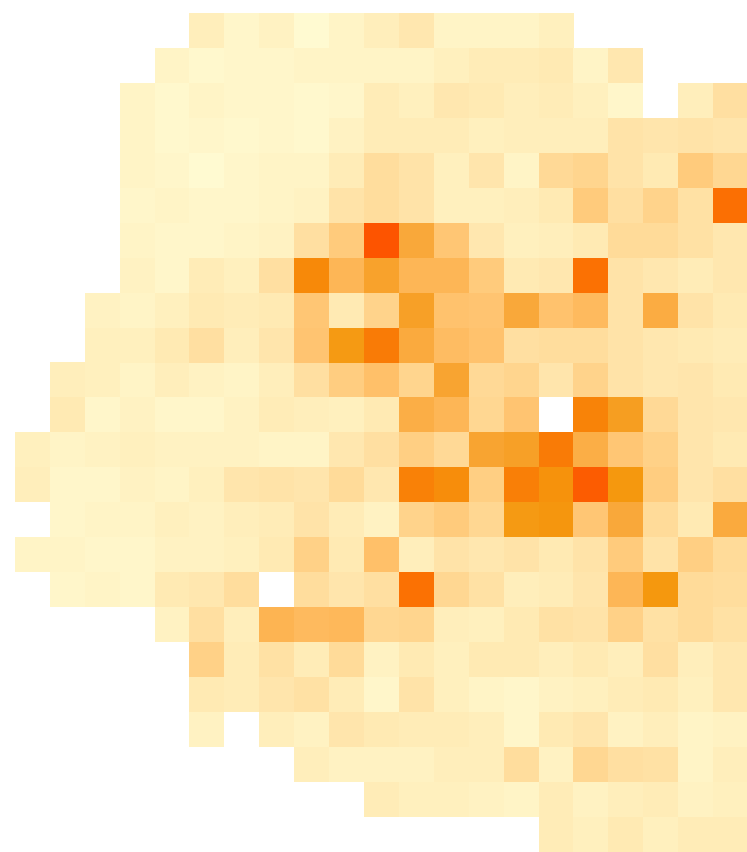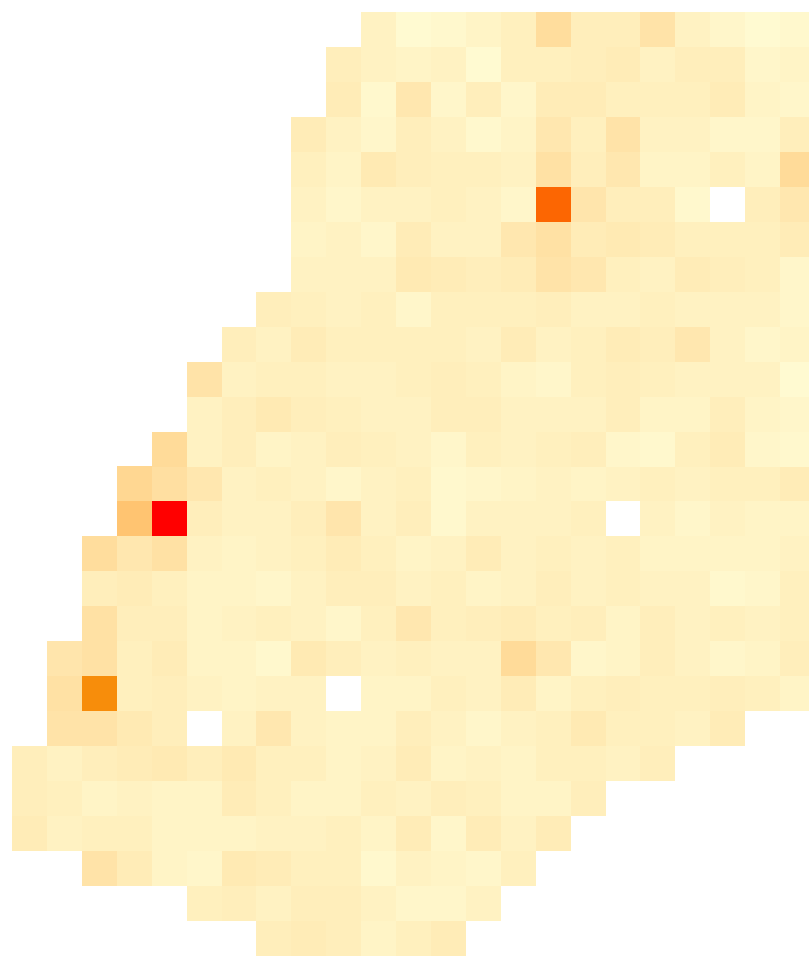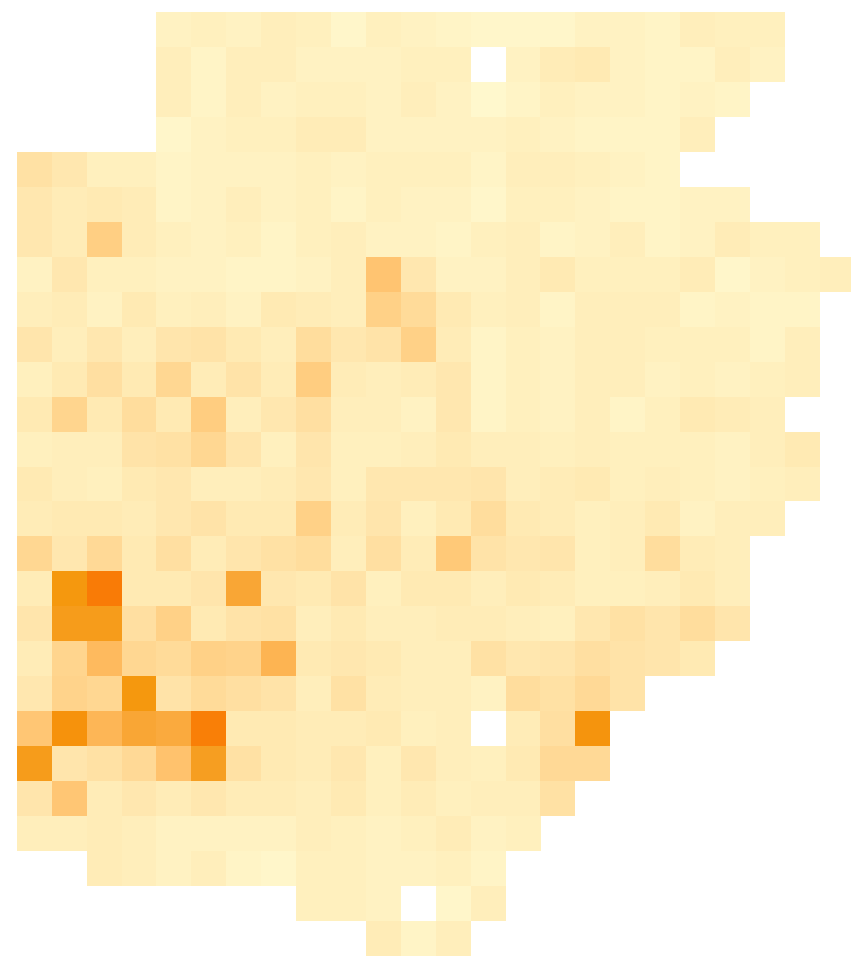

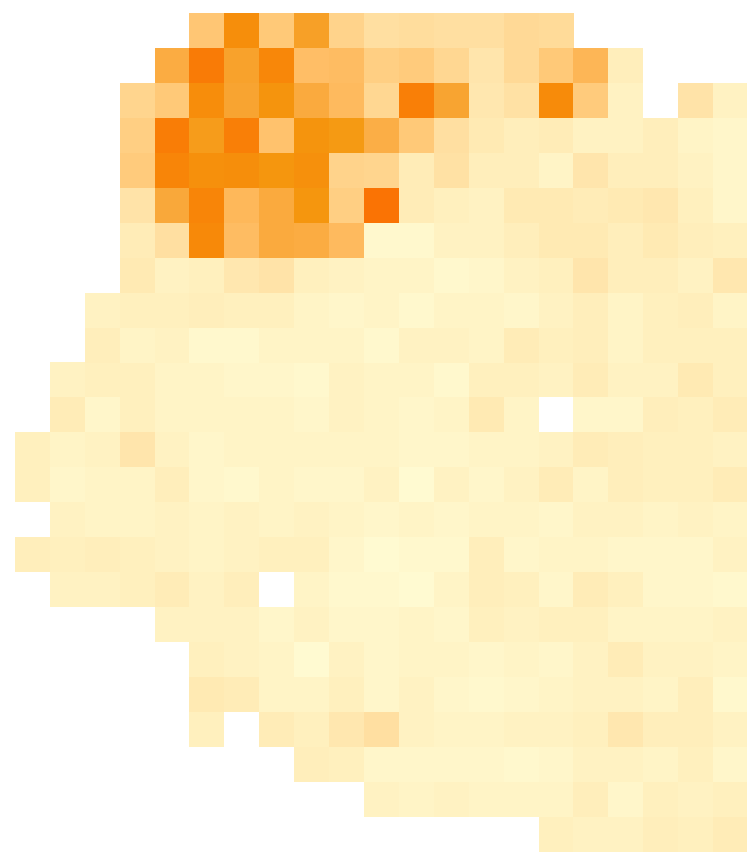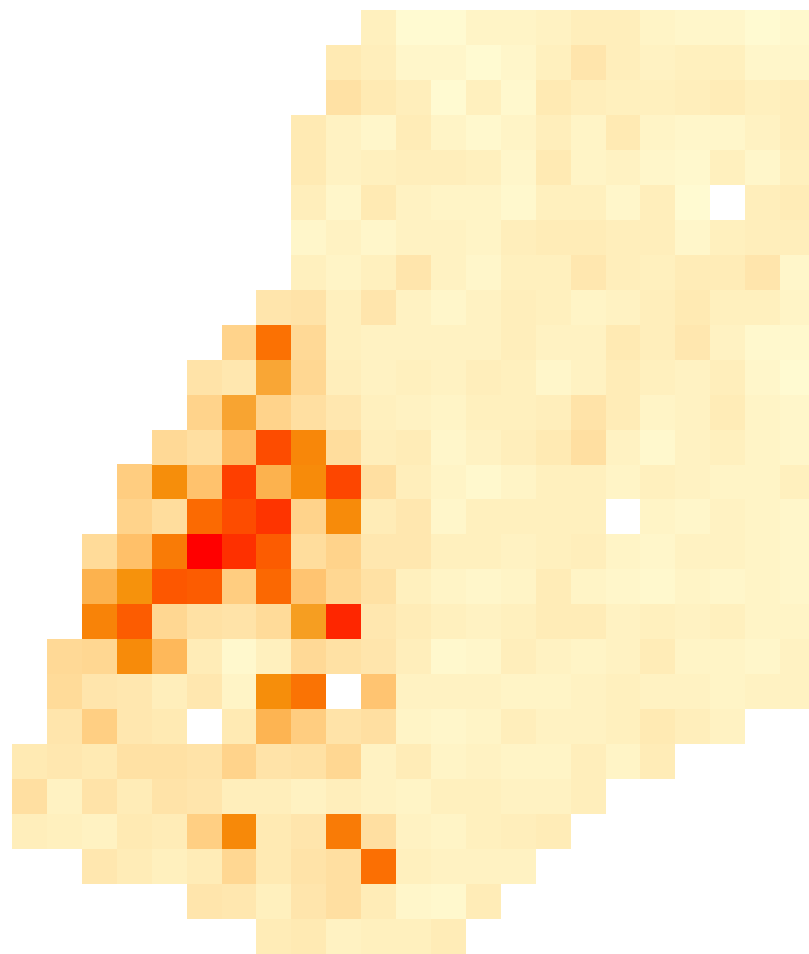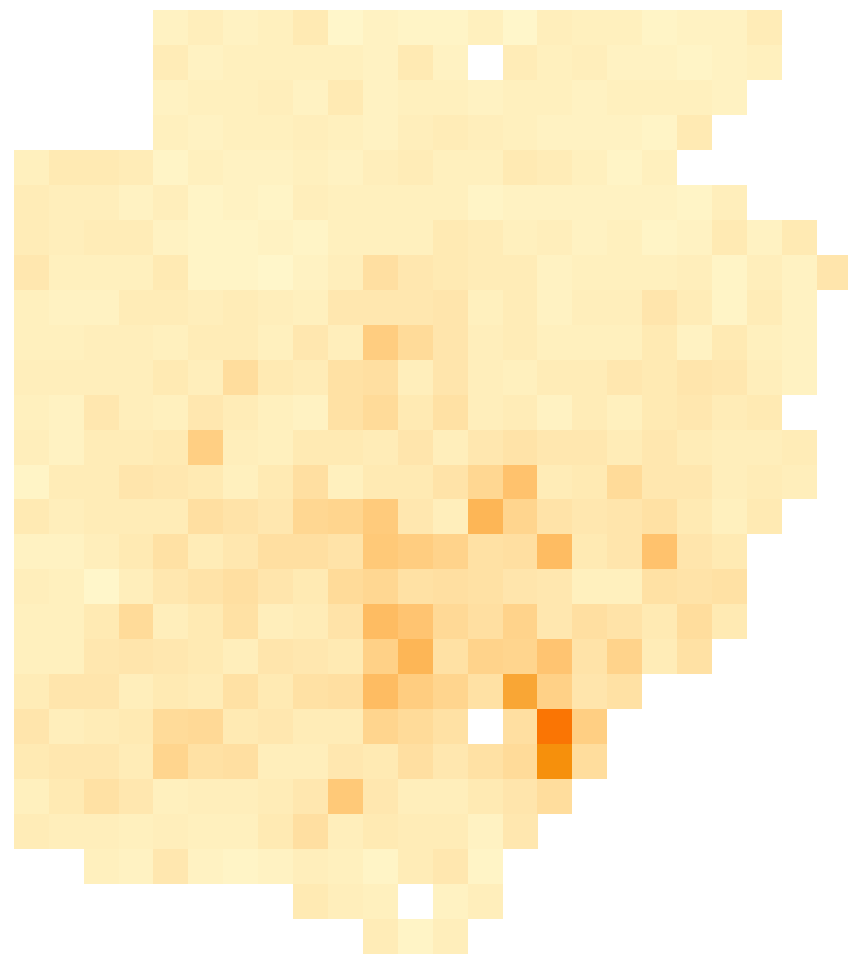

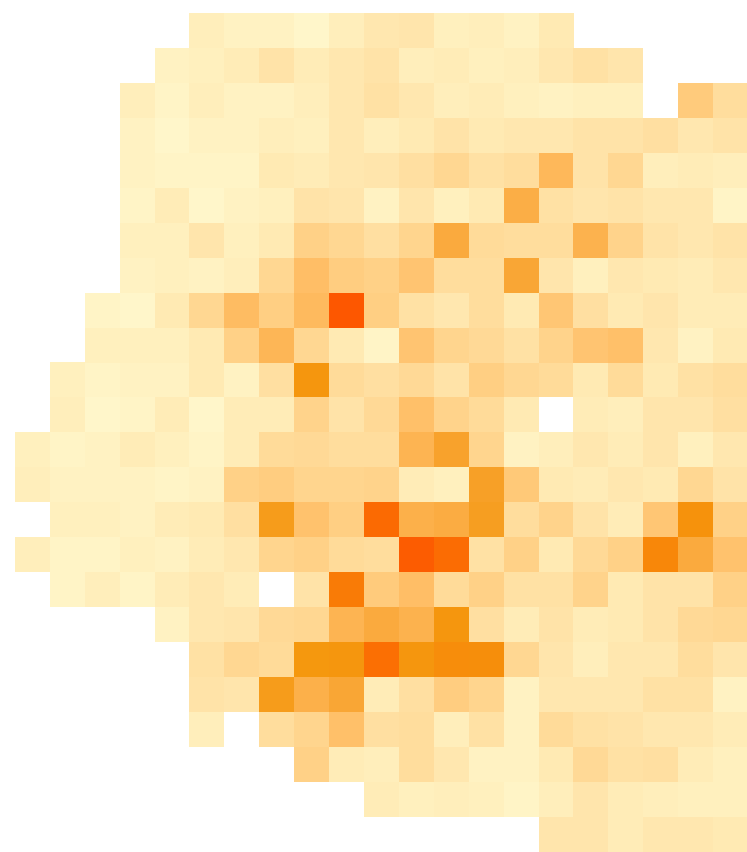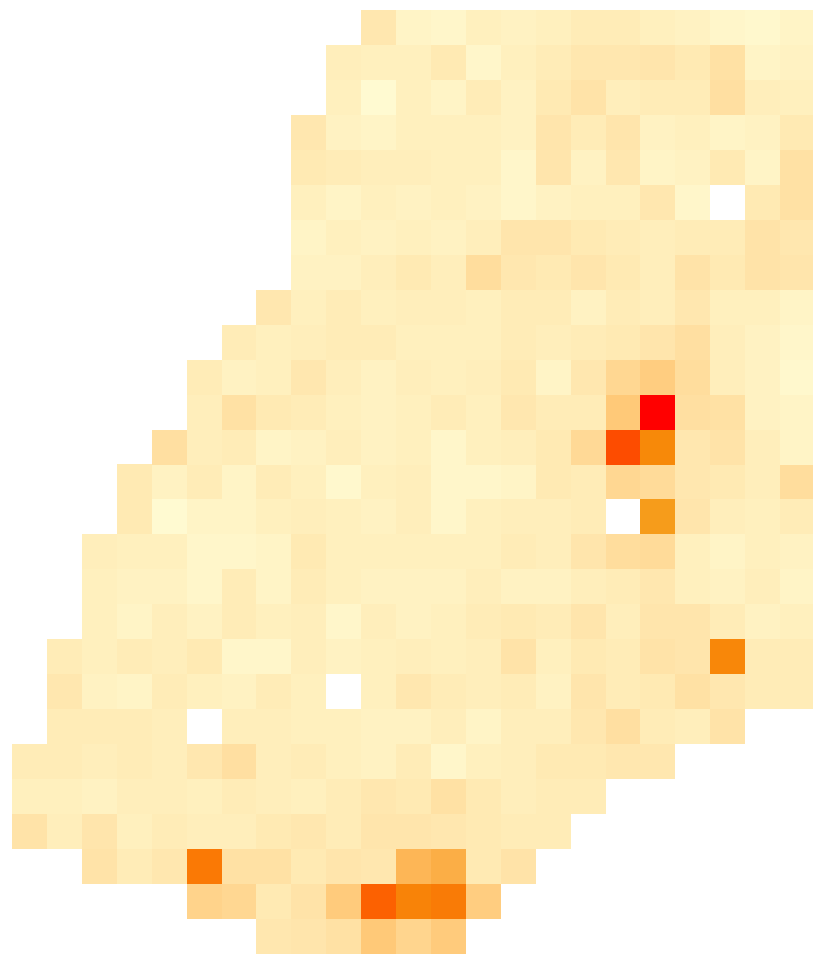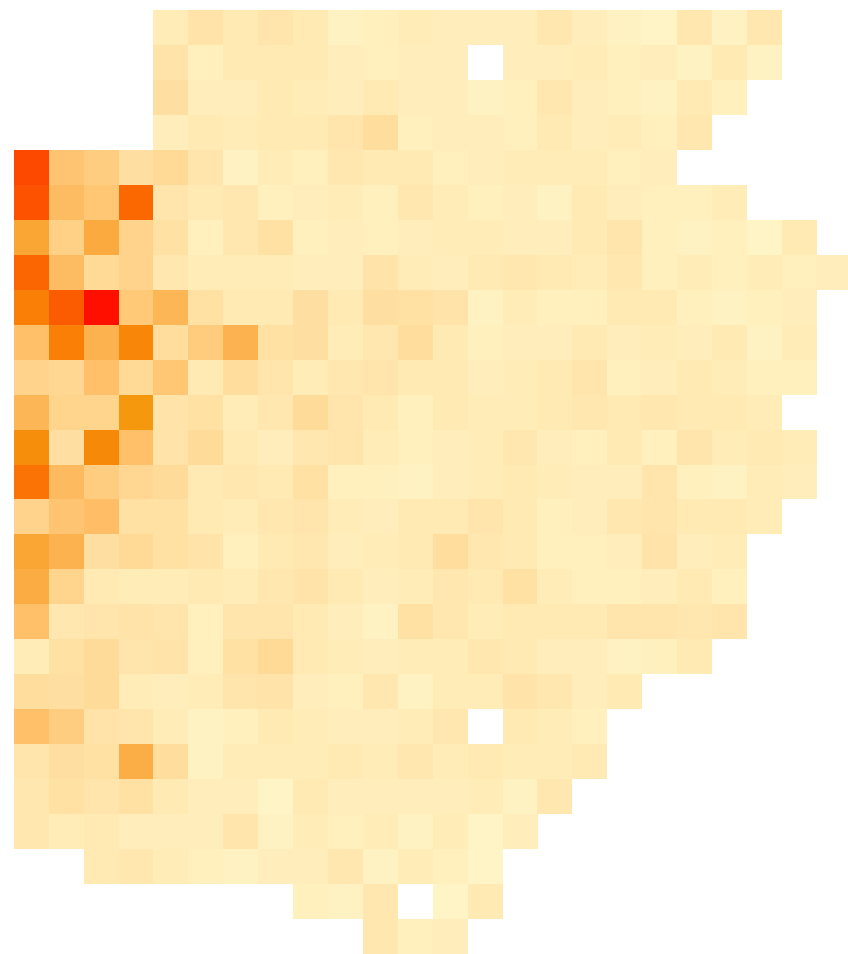

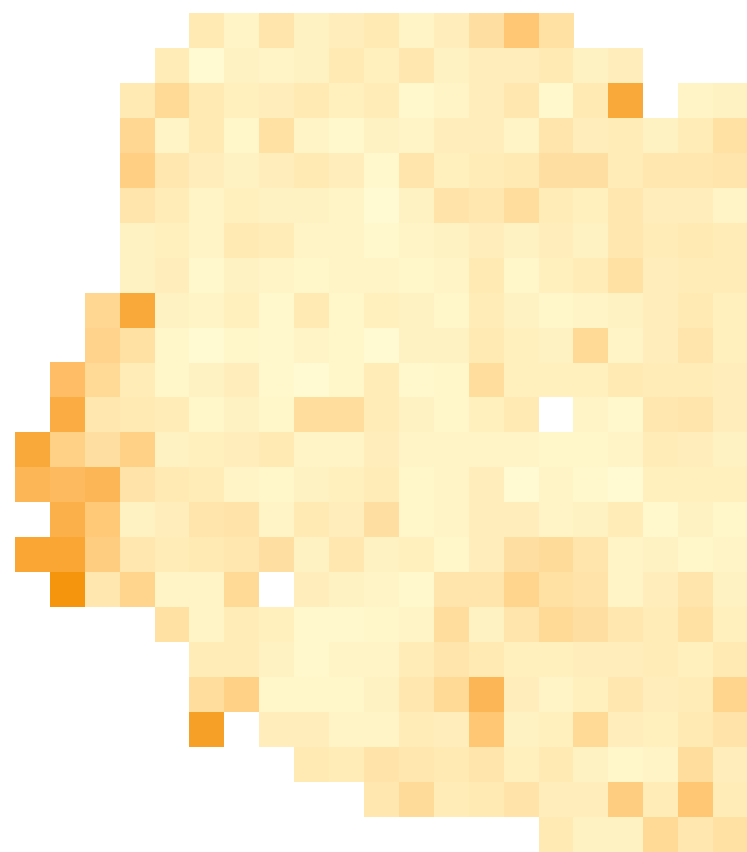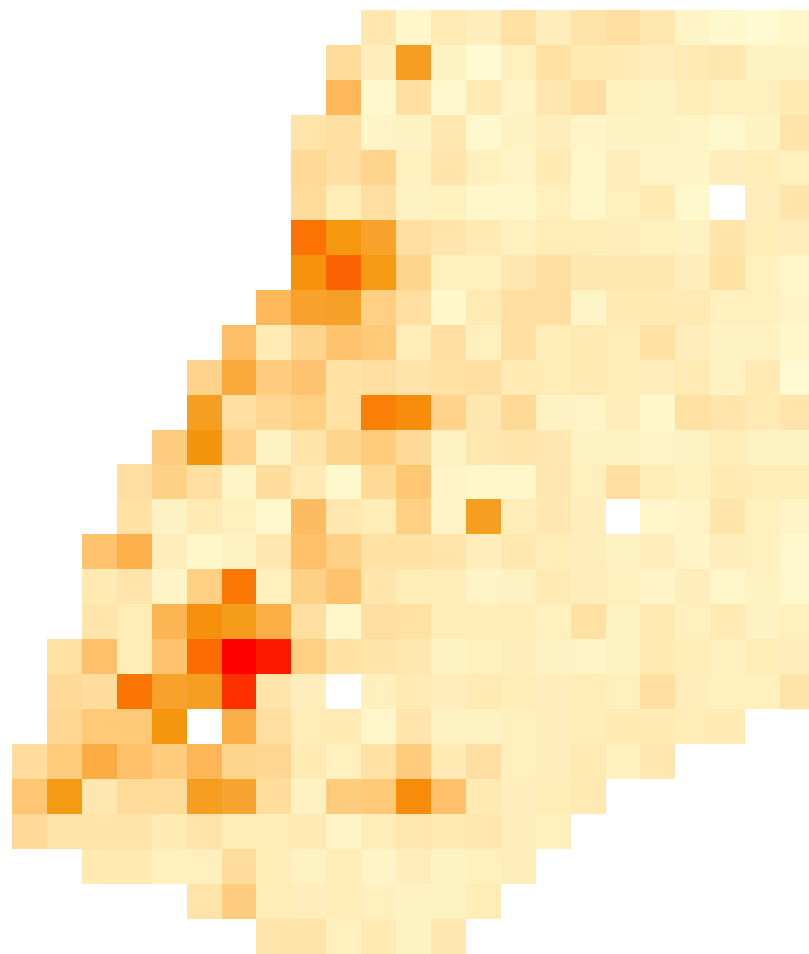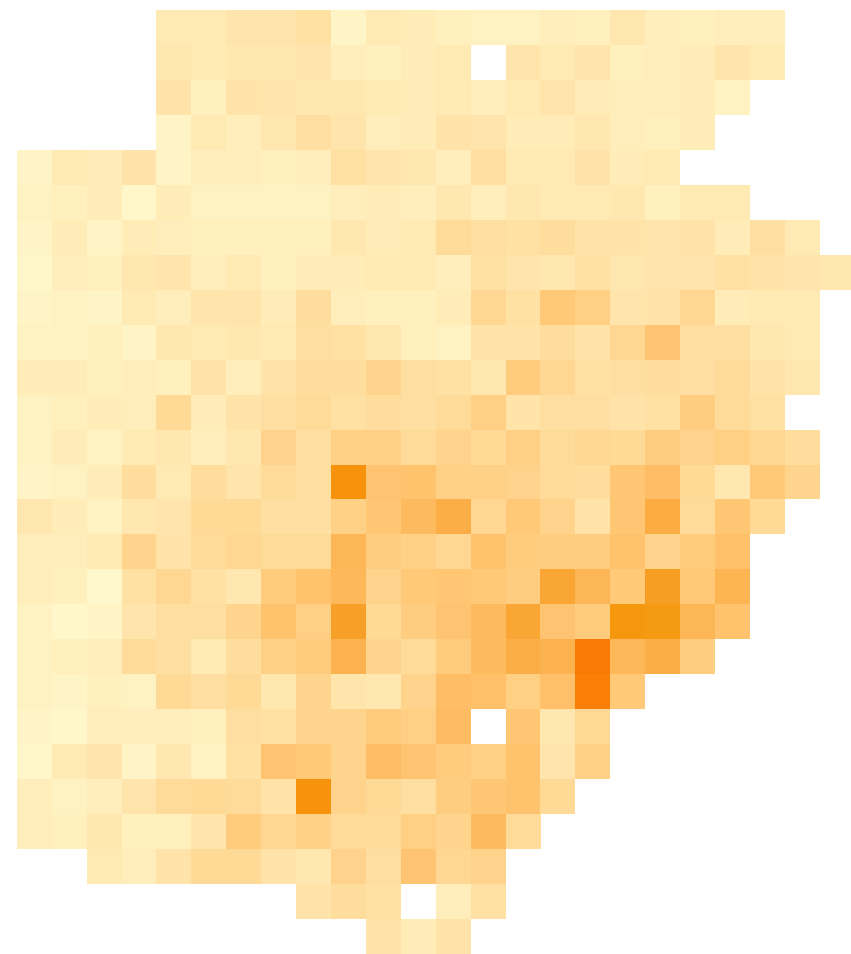

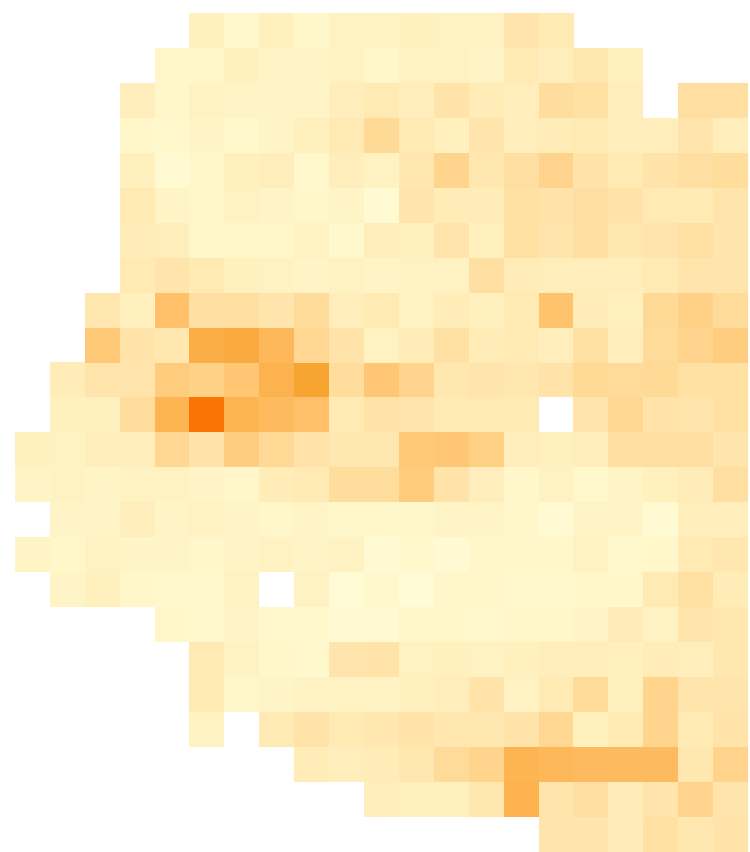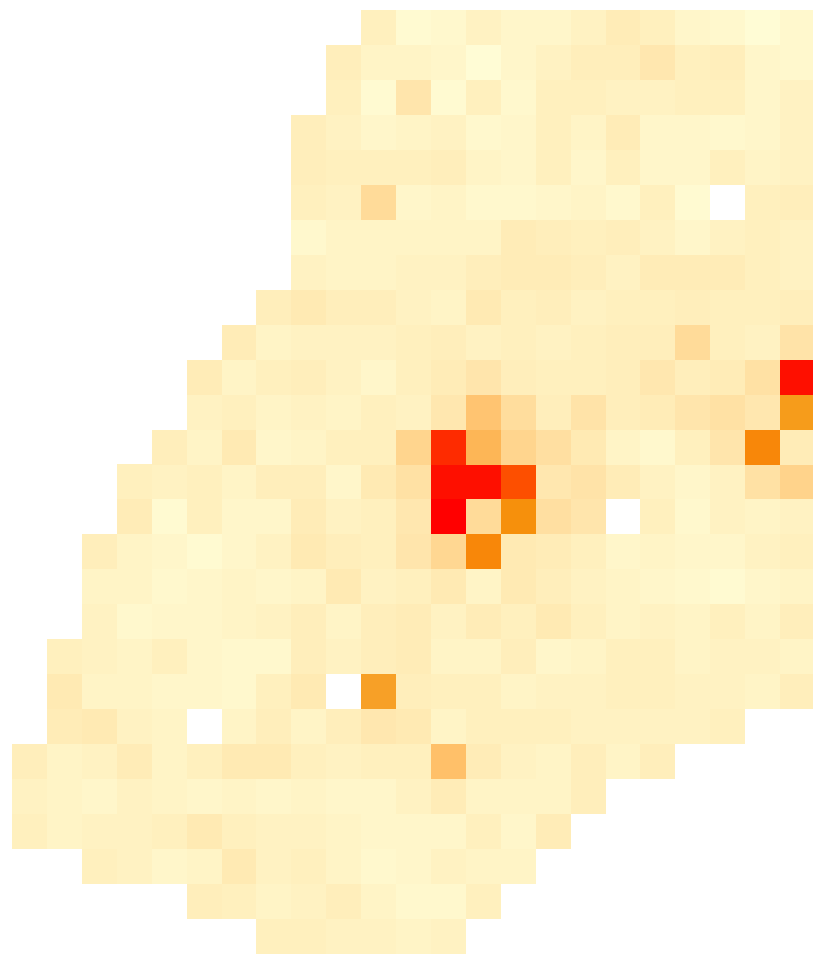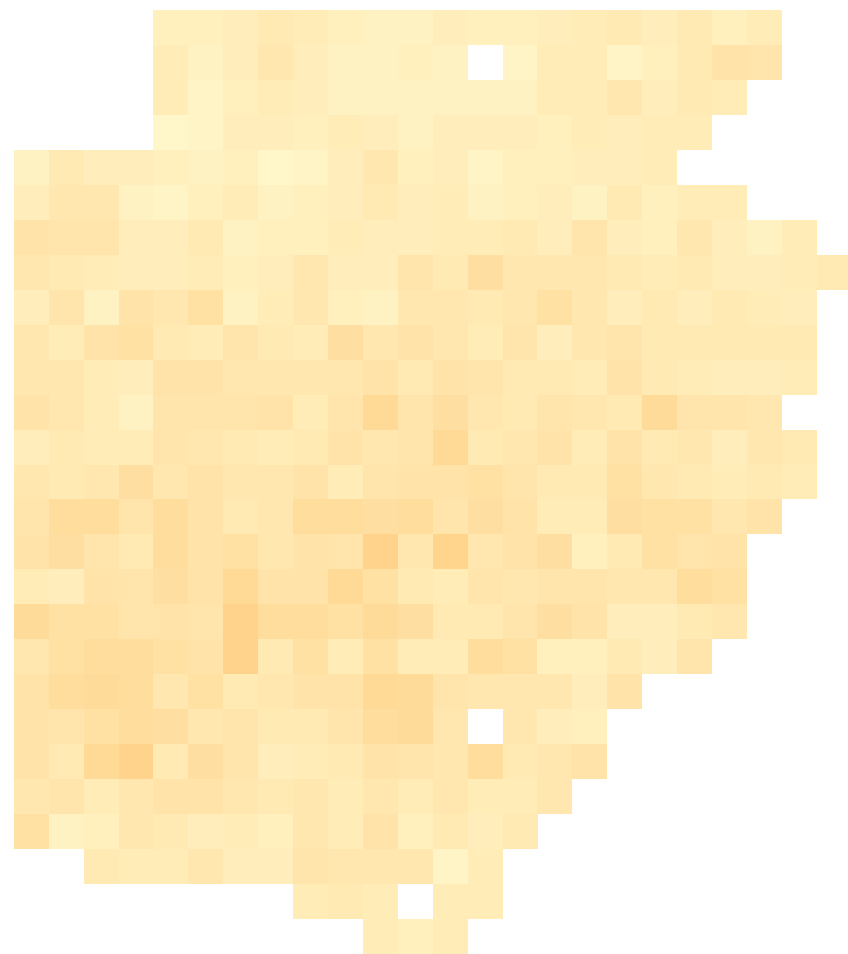

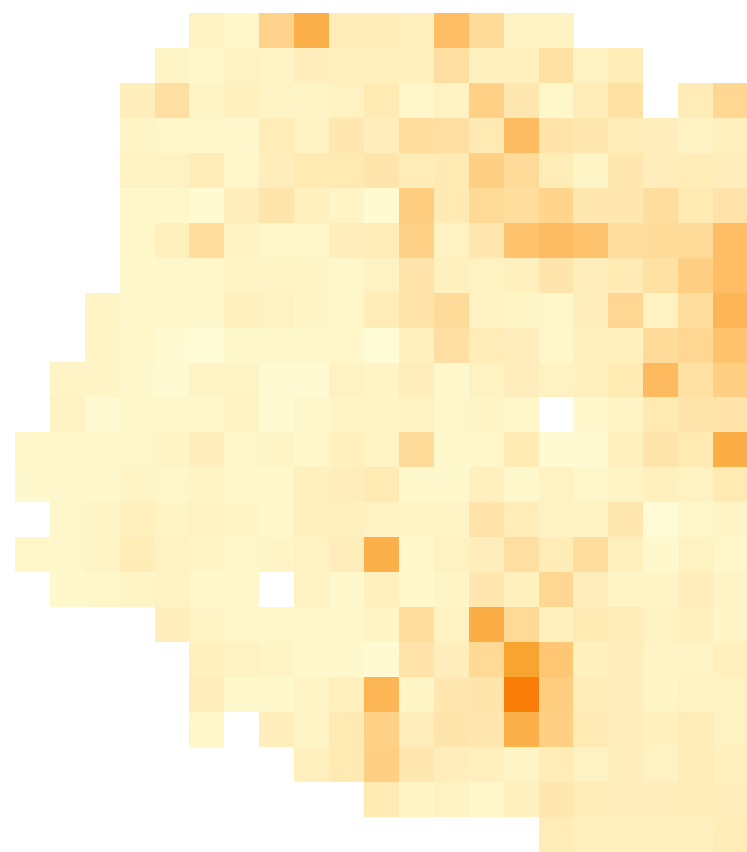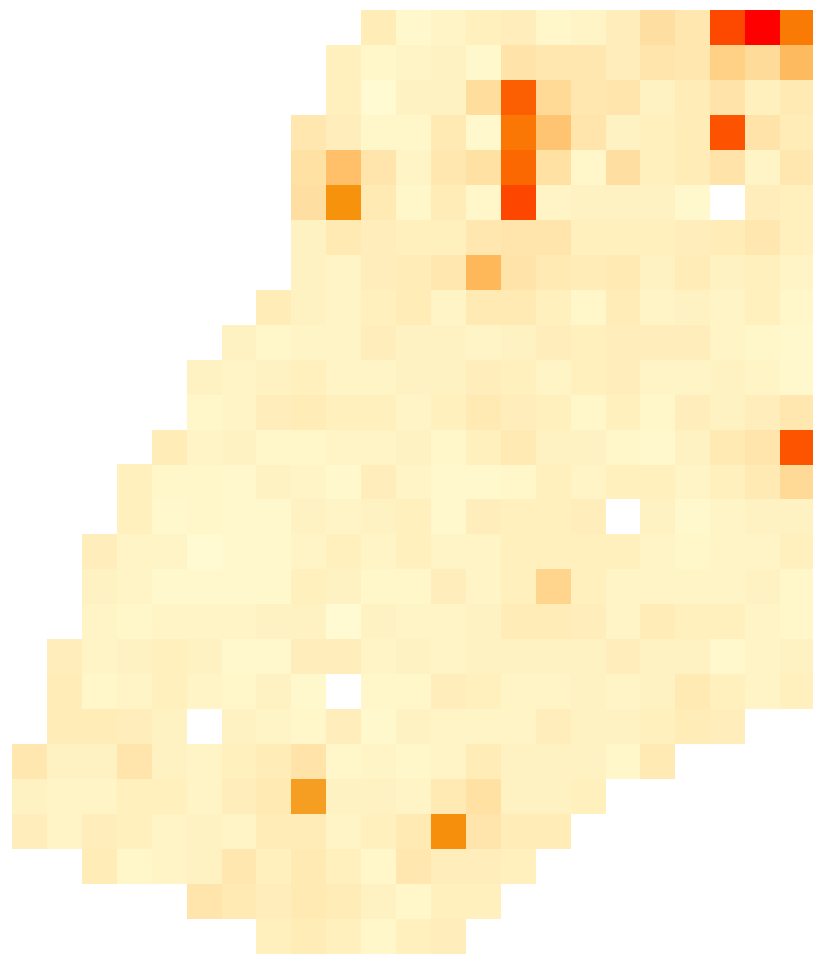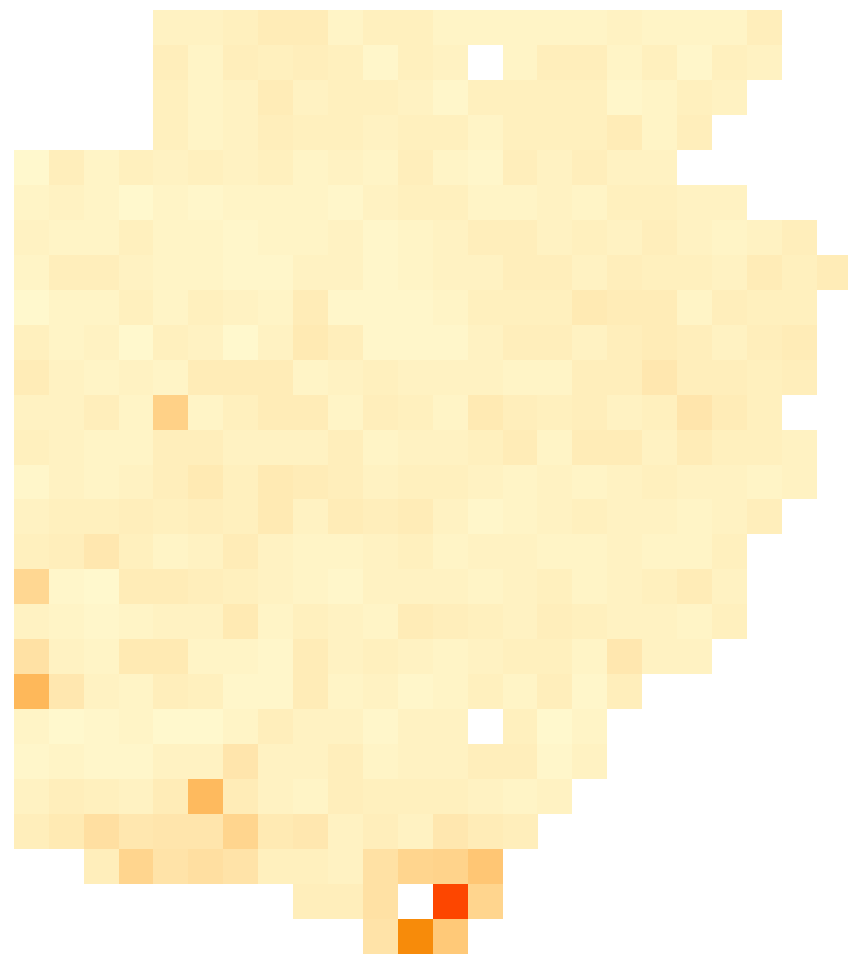

Supplement: Supplementary file 6 — Supplementary Data 3 [file 41467_2018_4724_MOESM6_ESM.zip › Supplementary Dataset 2/joint-mix-profiles-rel-common-scale-matrix.pdf]

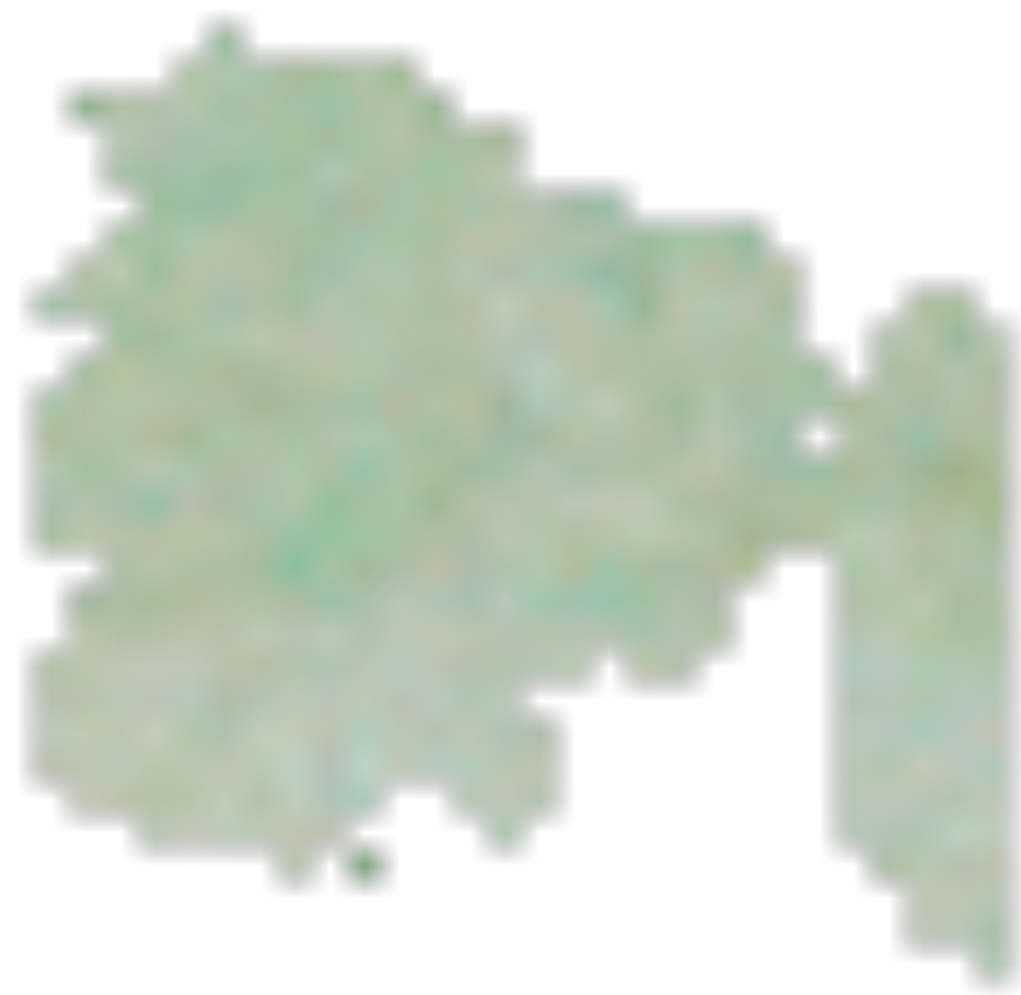

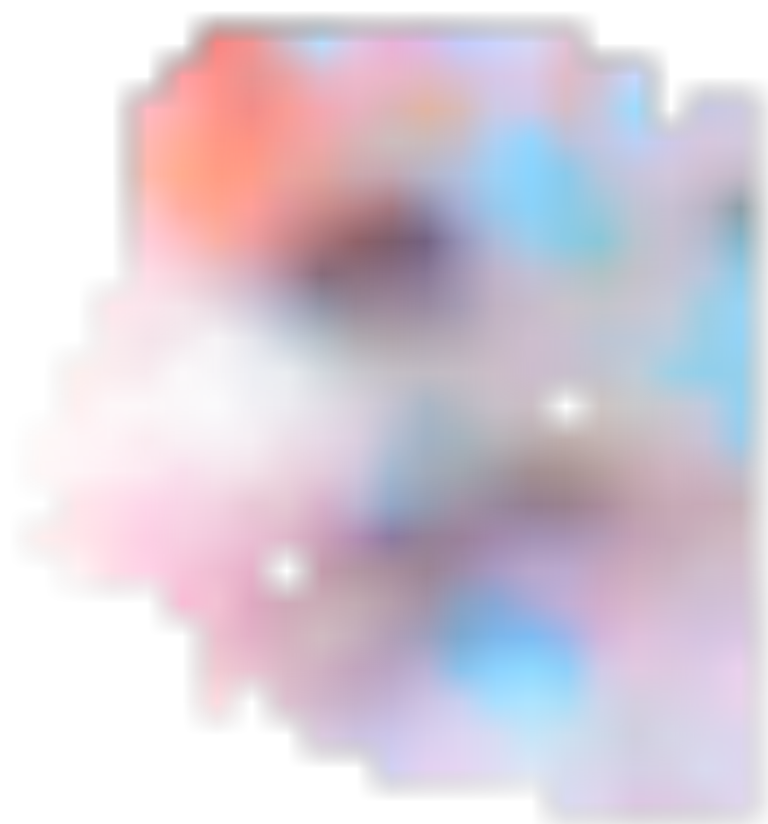

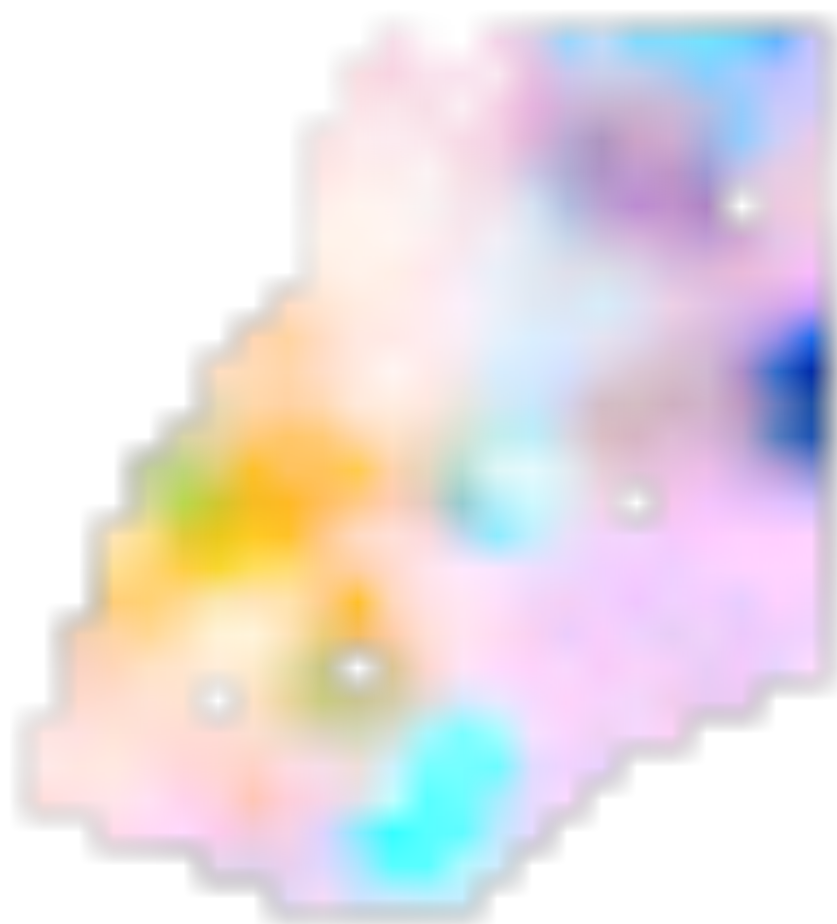

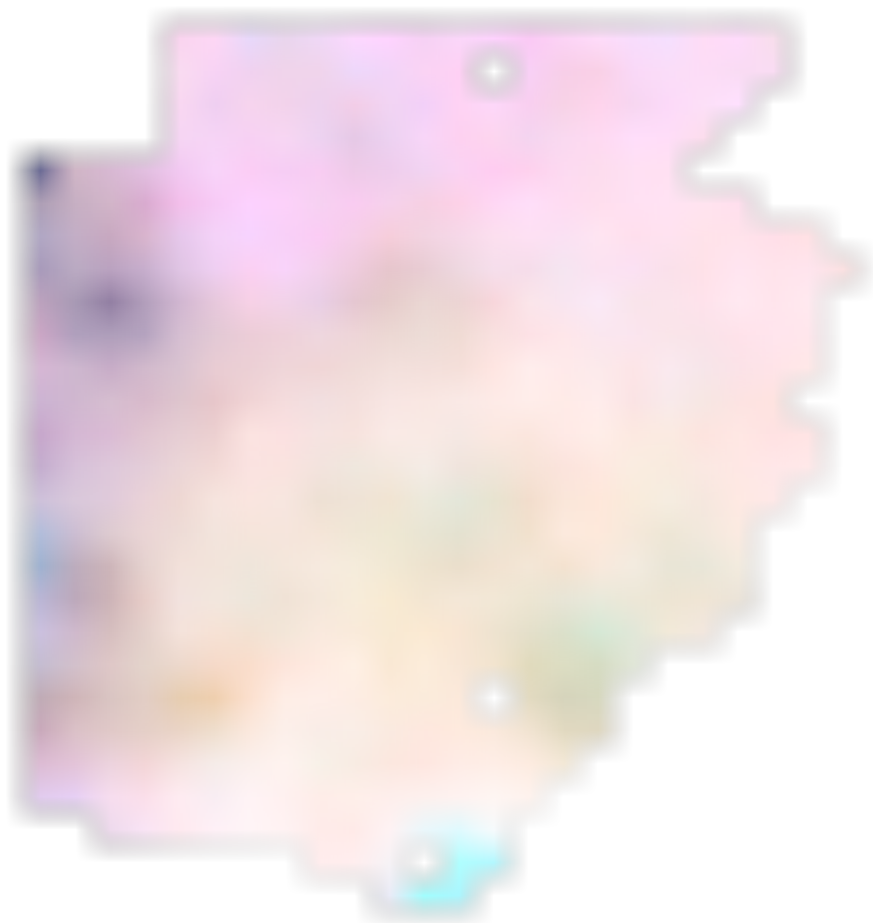

Supplement: Supplementary file 8 — Supplementary Data 5 [file 41467_2018_4724_MOESM8_ESM.zip › Supplementary Dataset 7/joint-field-dimensionality-reduction-PCA-matrix-split.pdf.interpolated.pdf]

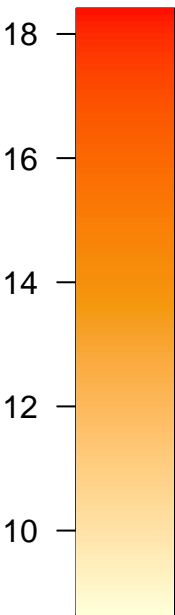

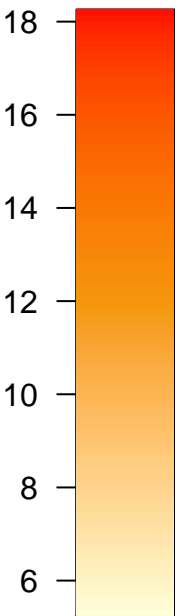

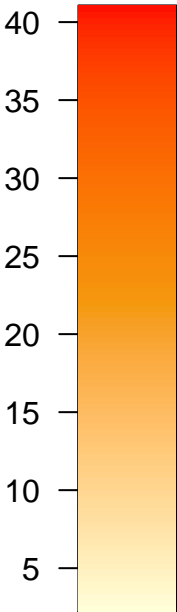

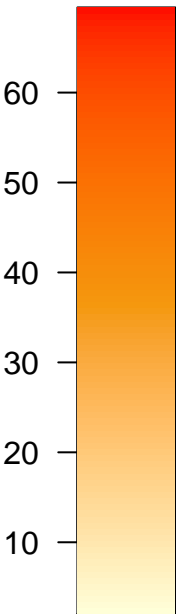

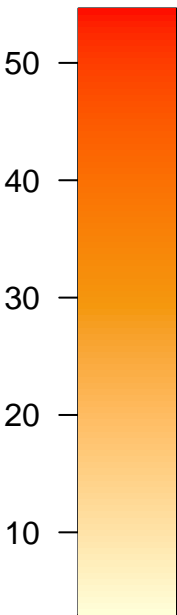

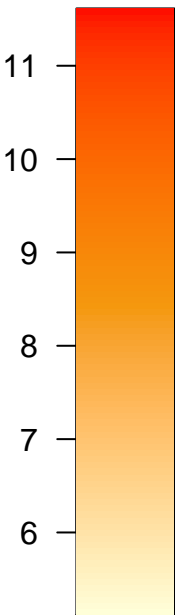

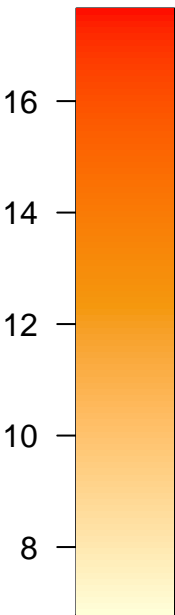

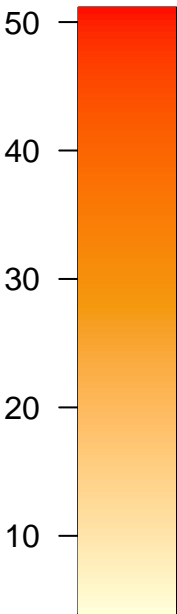

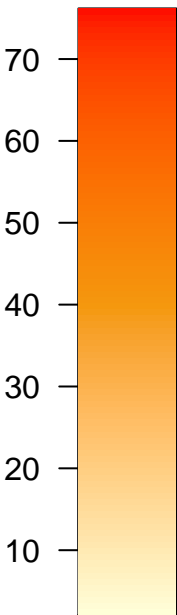

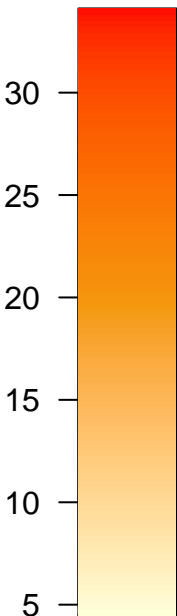

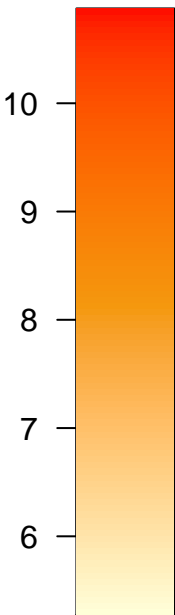

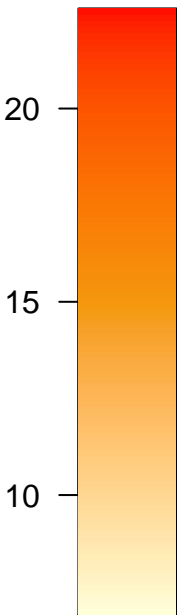

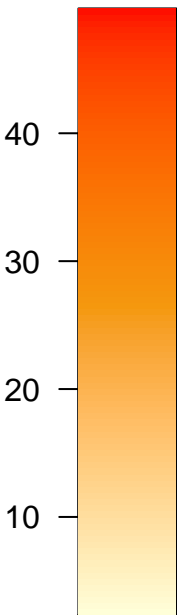

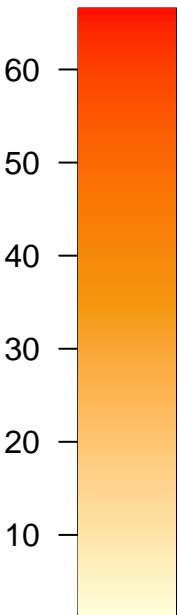

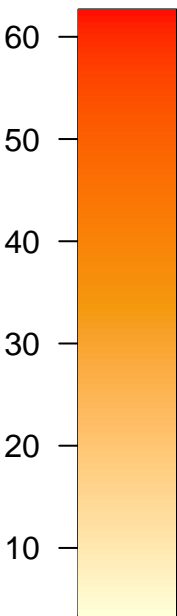

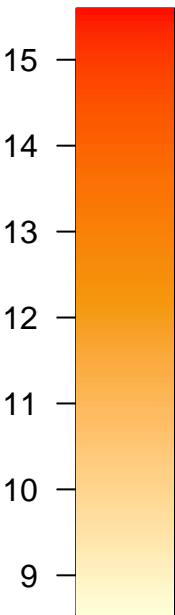

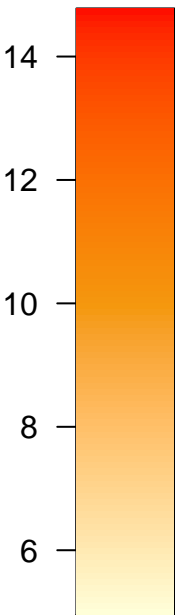

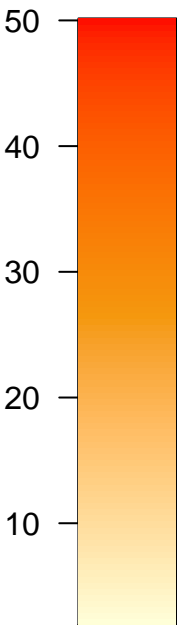

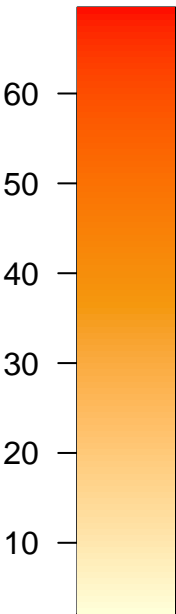

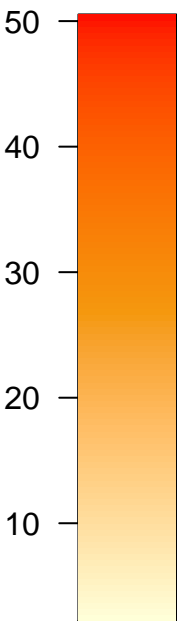

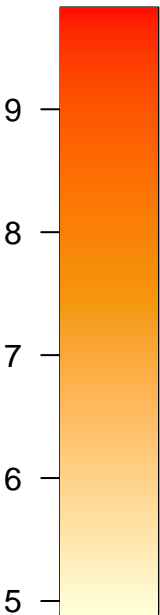

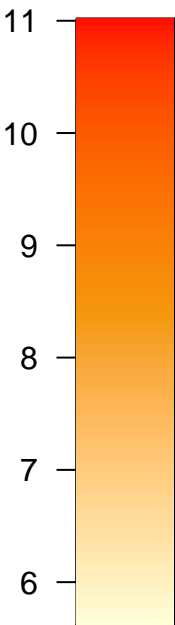

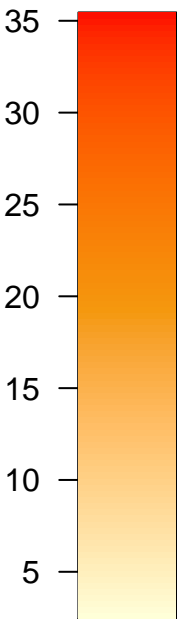

70

60

50

40

30

20

10

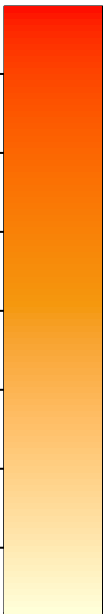

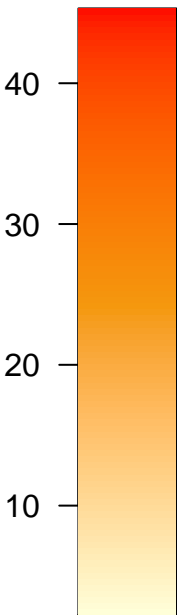

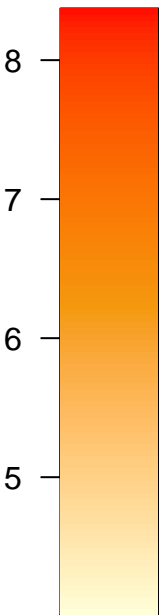

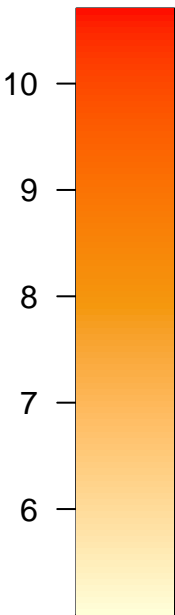

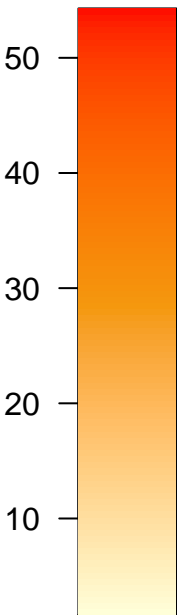

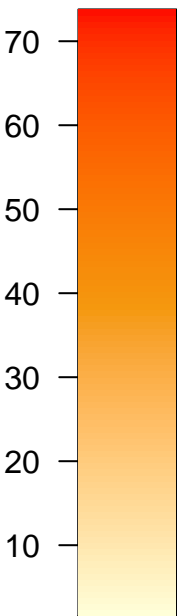

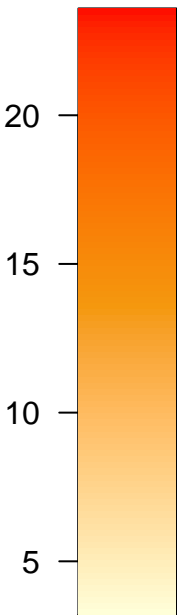

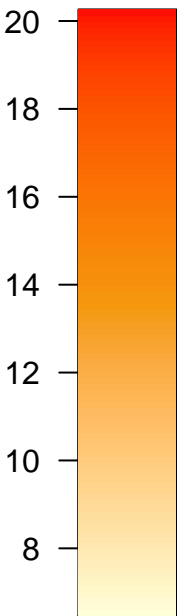

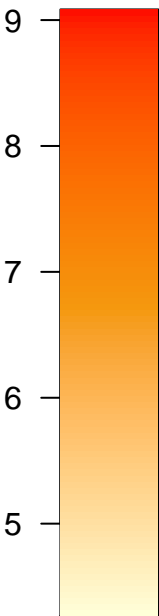

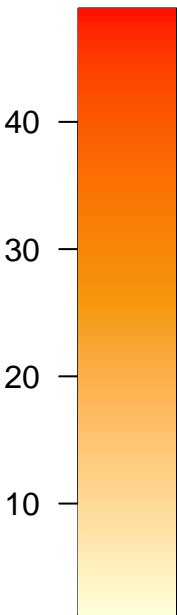

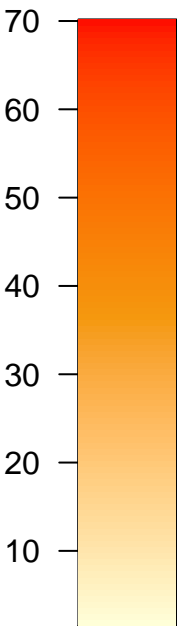

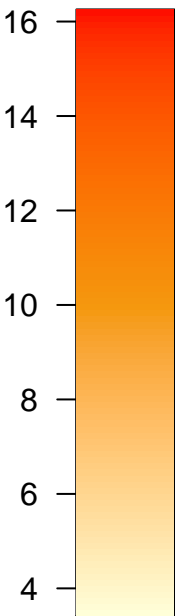

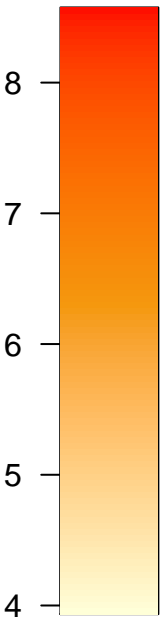

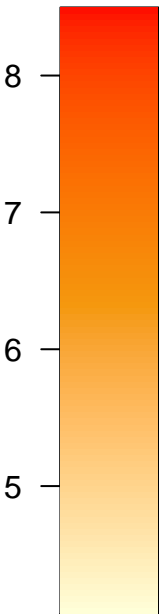

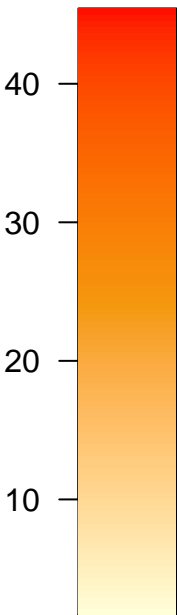

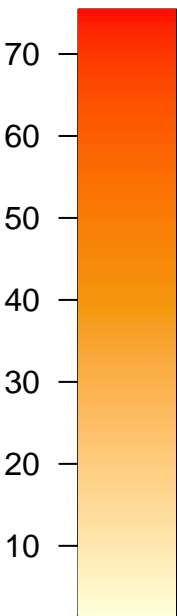

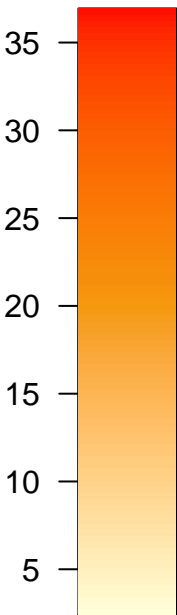

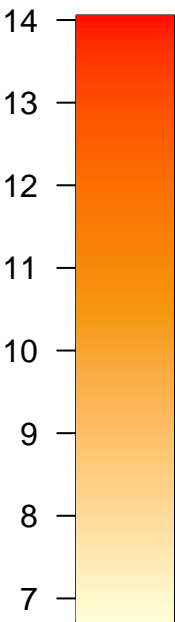

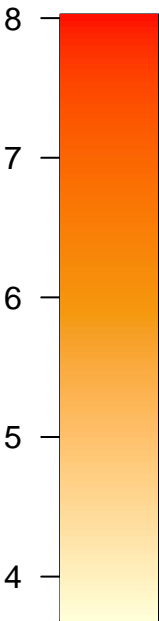

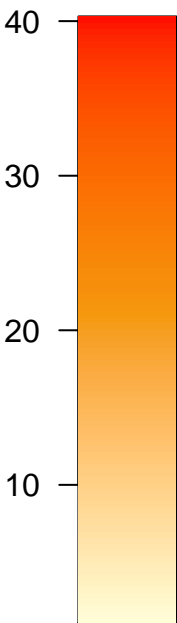

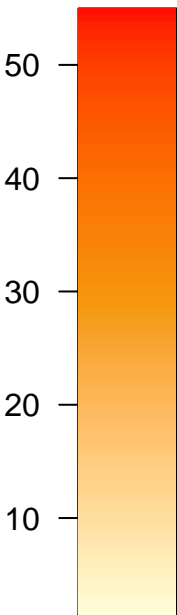

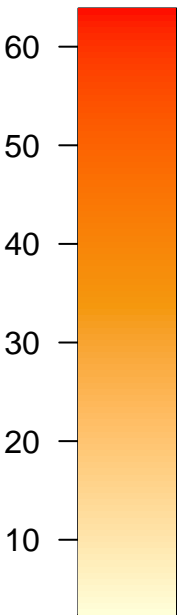

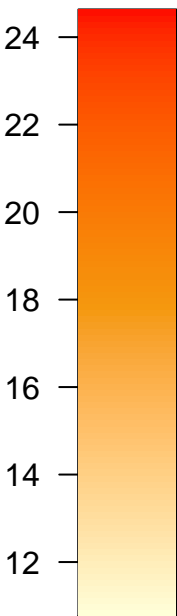

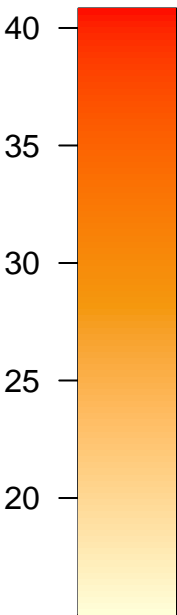

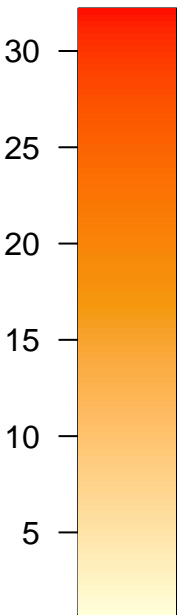

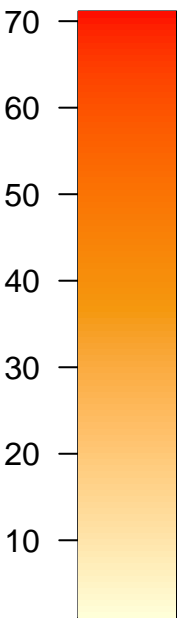

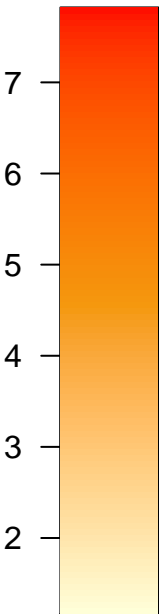

Supplement: Supplementary file 8 — Supplementary Data 5 [file 41467_2018_4724_MOESM8_ESM.zip › Supplementary Dataset 7/joint-mix-profiles-rel-individual-scale-dots-split-colorbar.pdf]

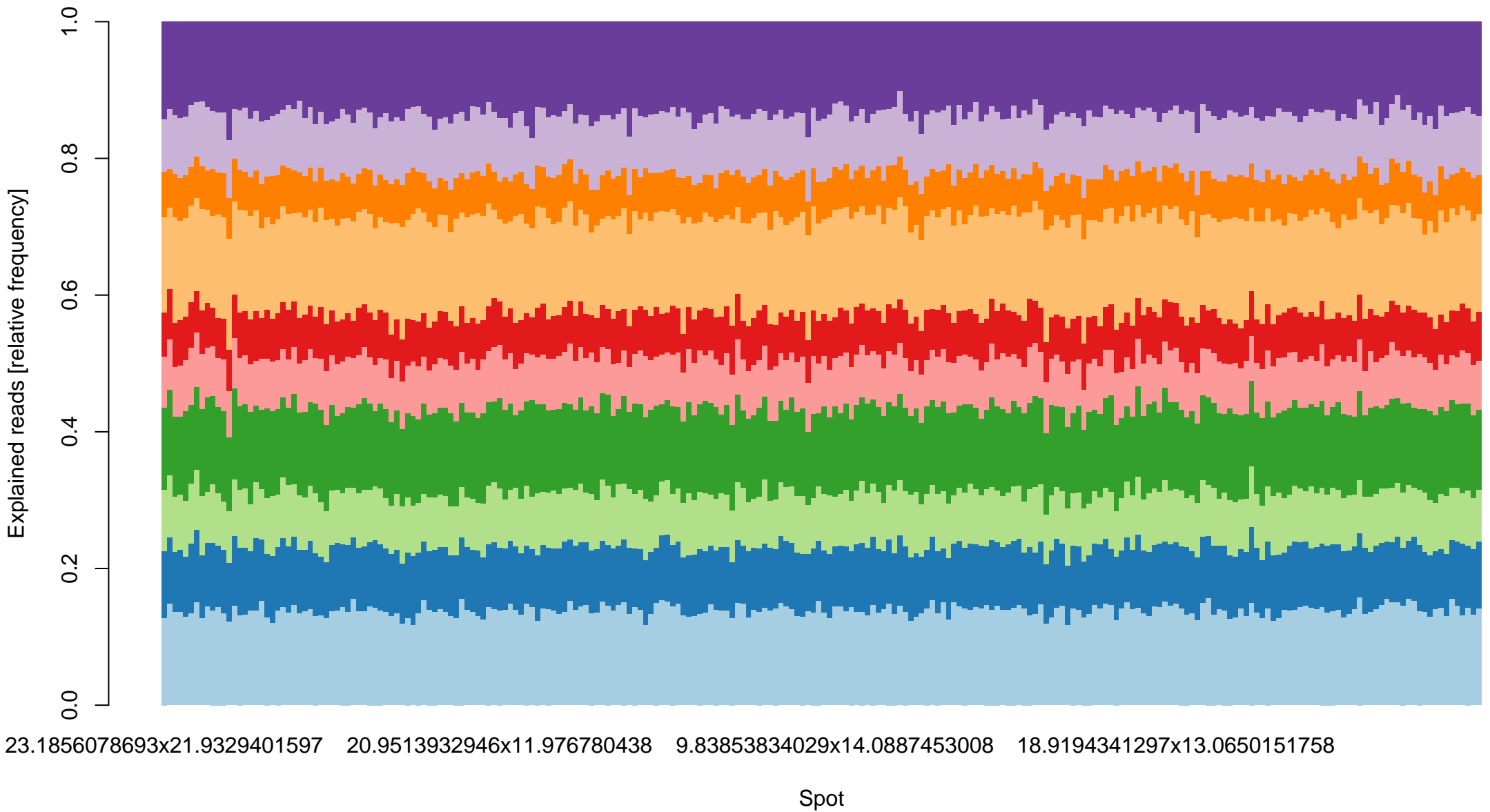

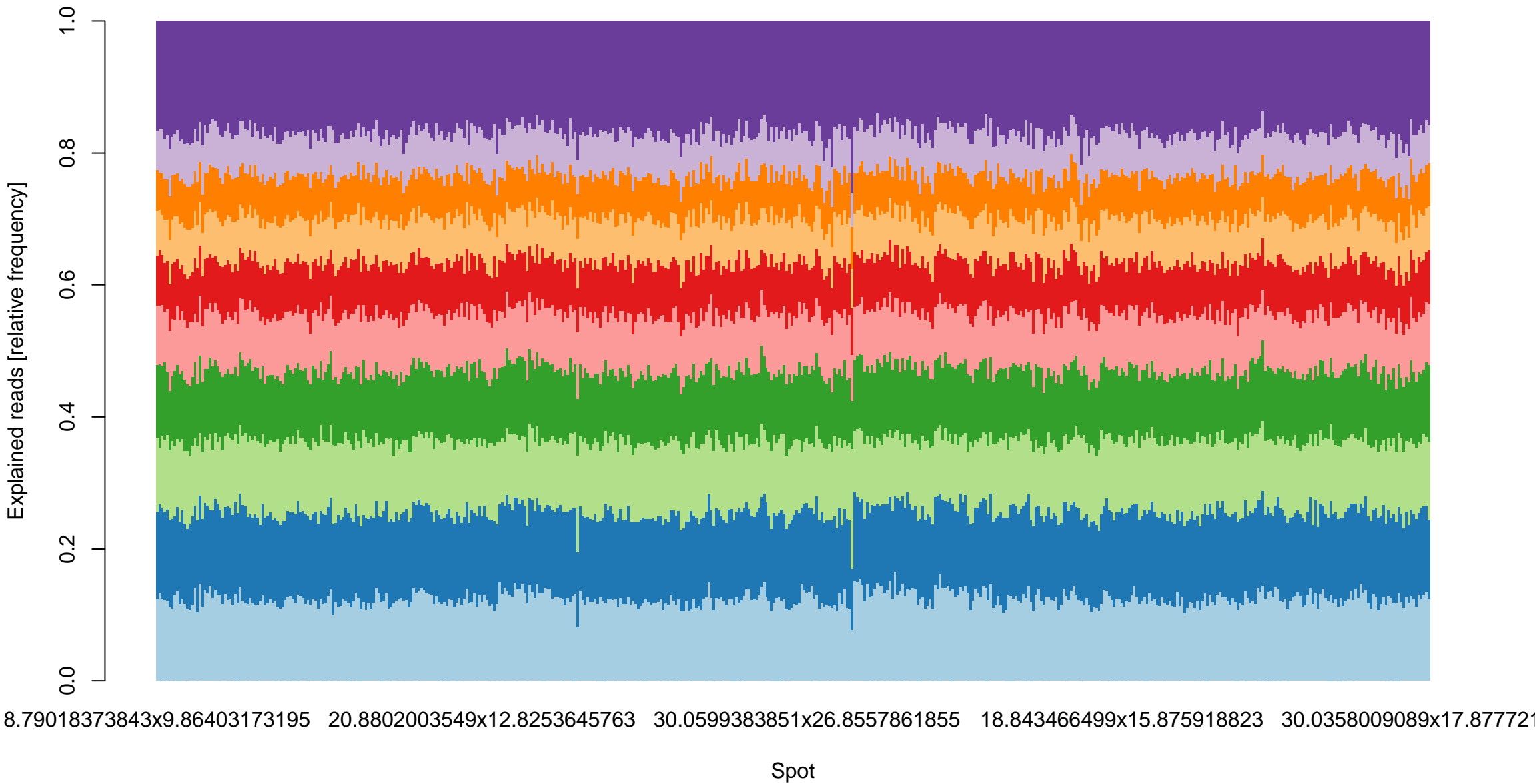

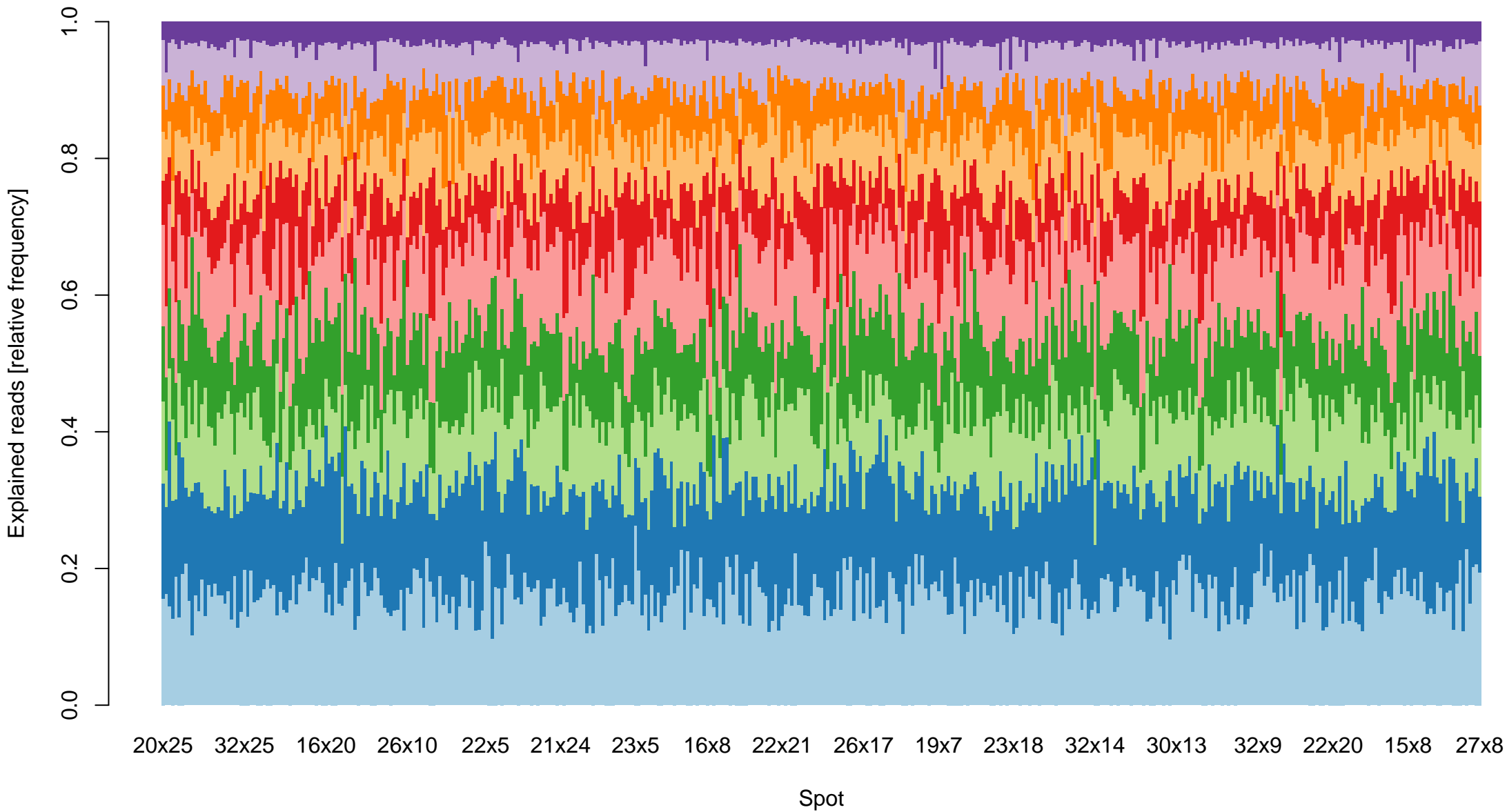

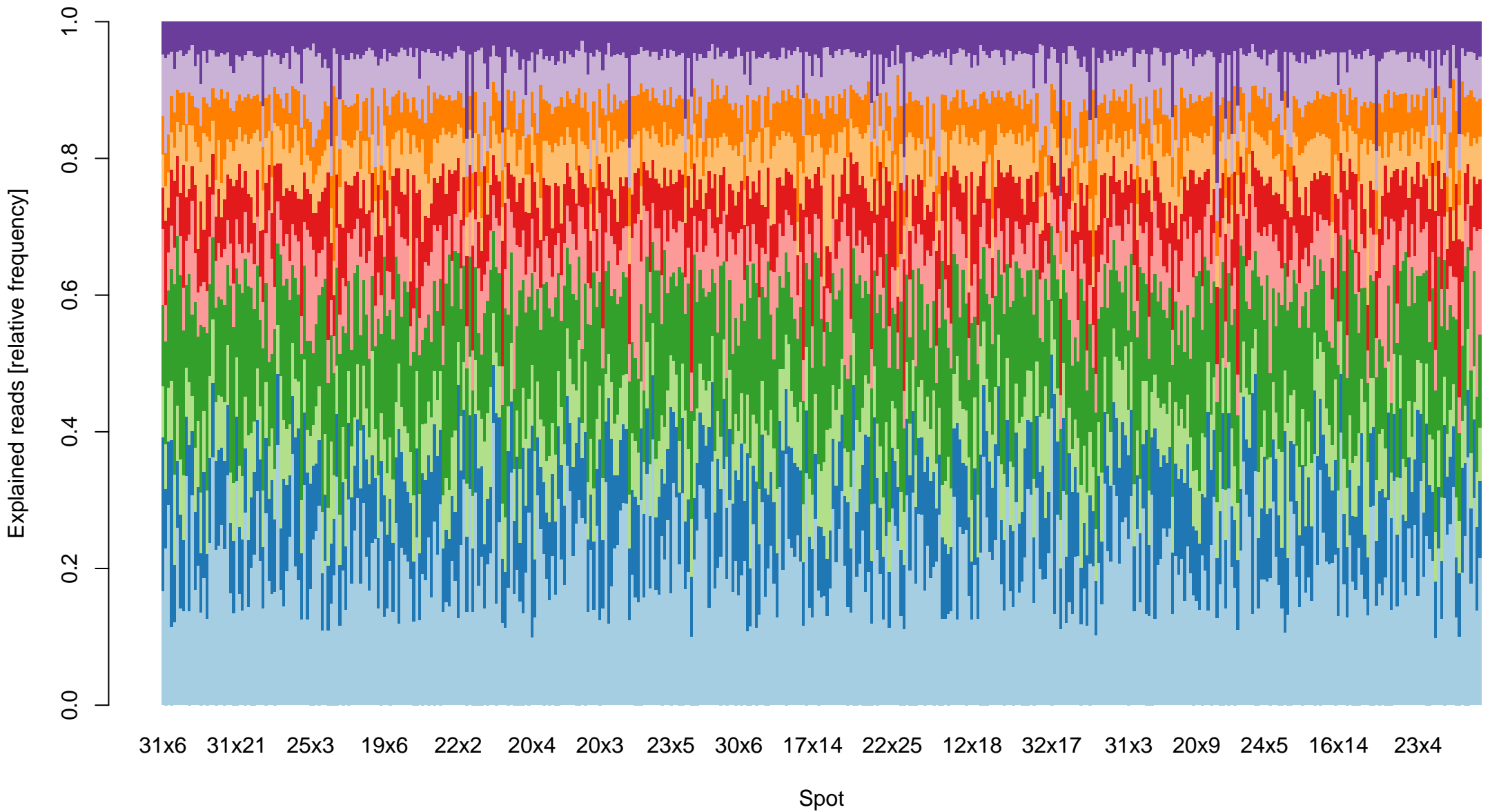

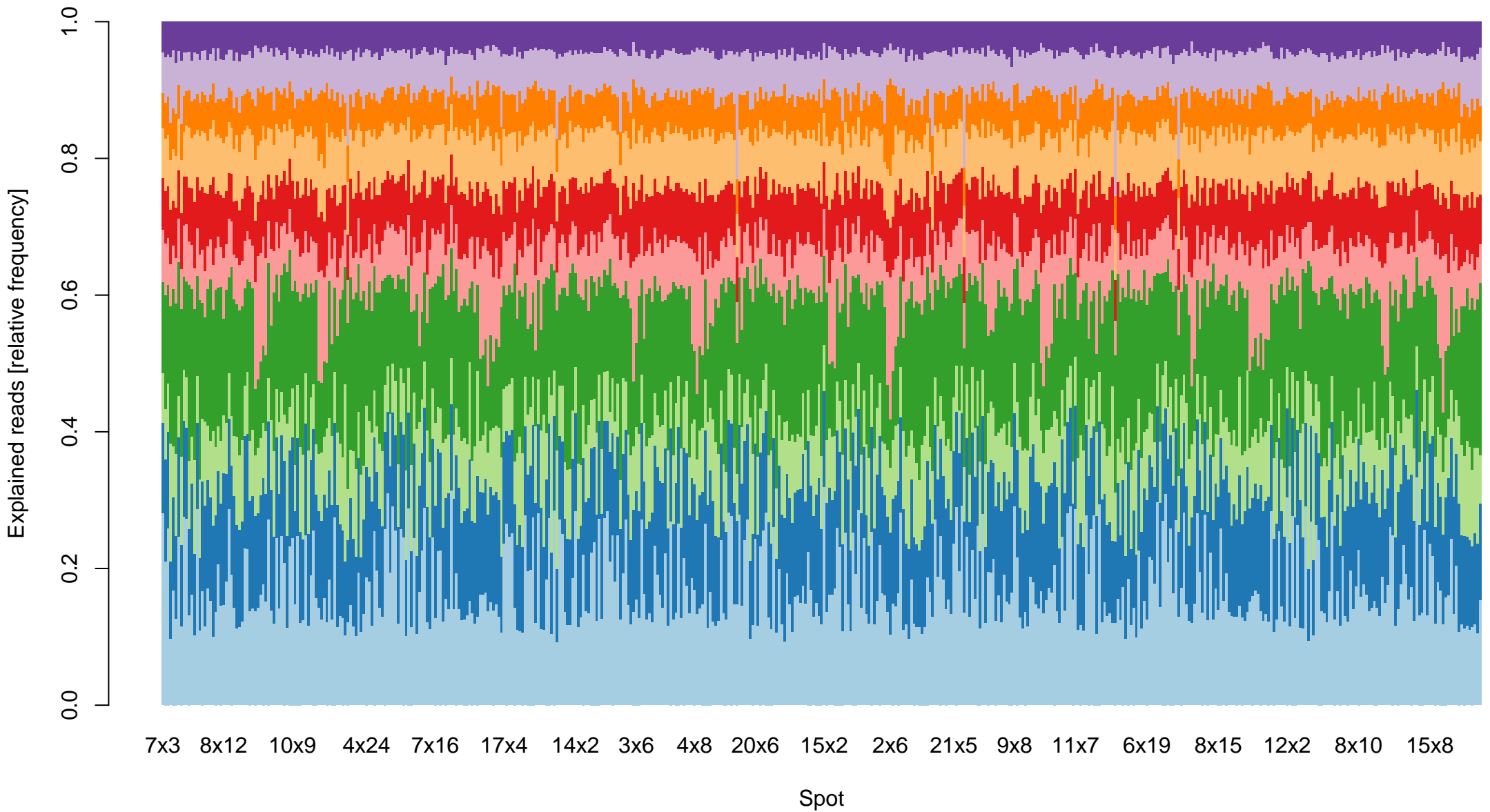

Supplement: Supplementary file 8 — Supplementary Data 5 [file 41467_2018_4724_MOESM8_ESM.zip › Supplementary Dataset 7/joint-field-factor-strength-spotbarplot-rel-freq.pdf]

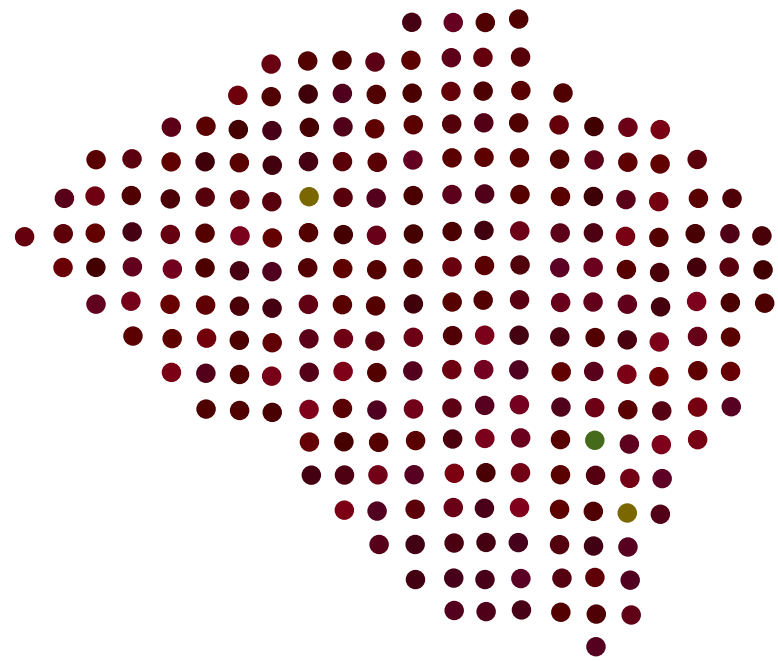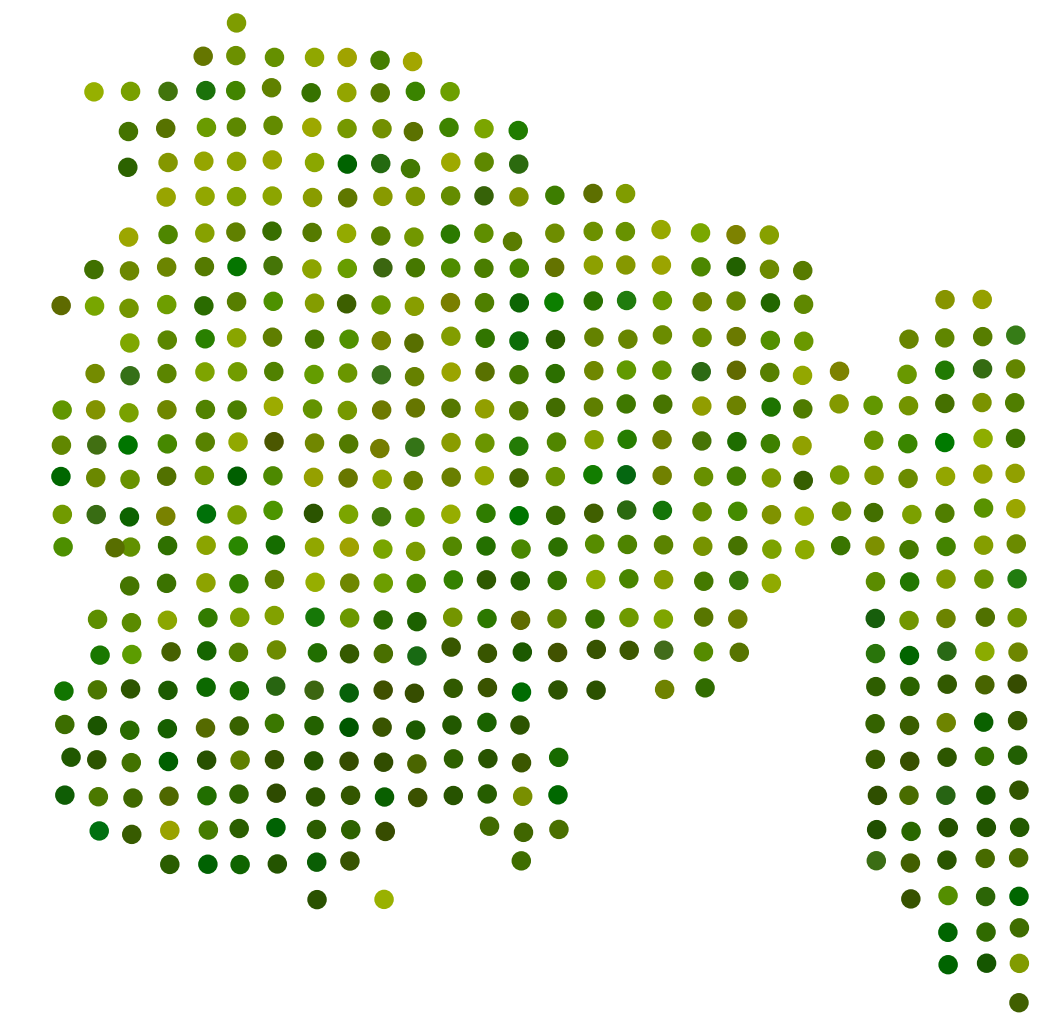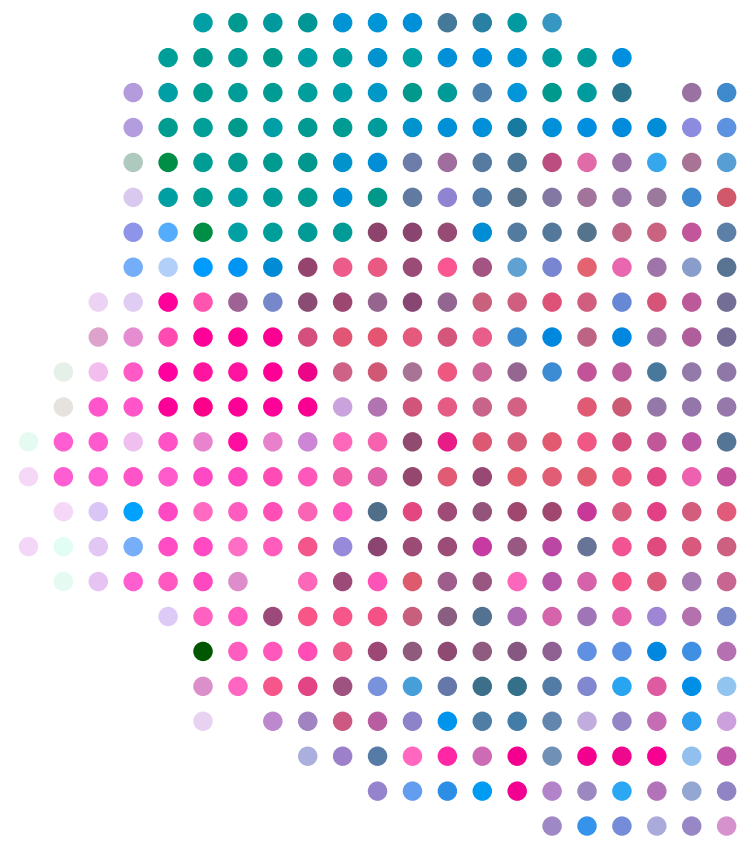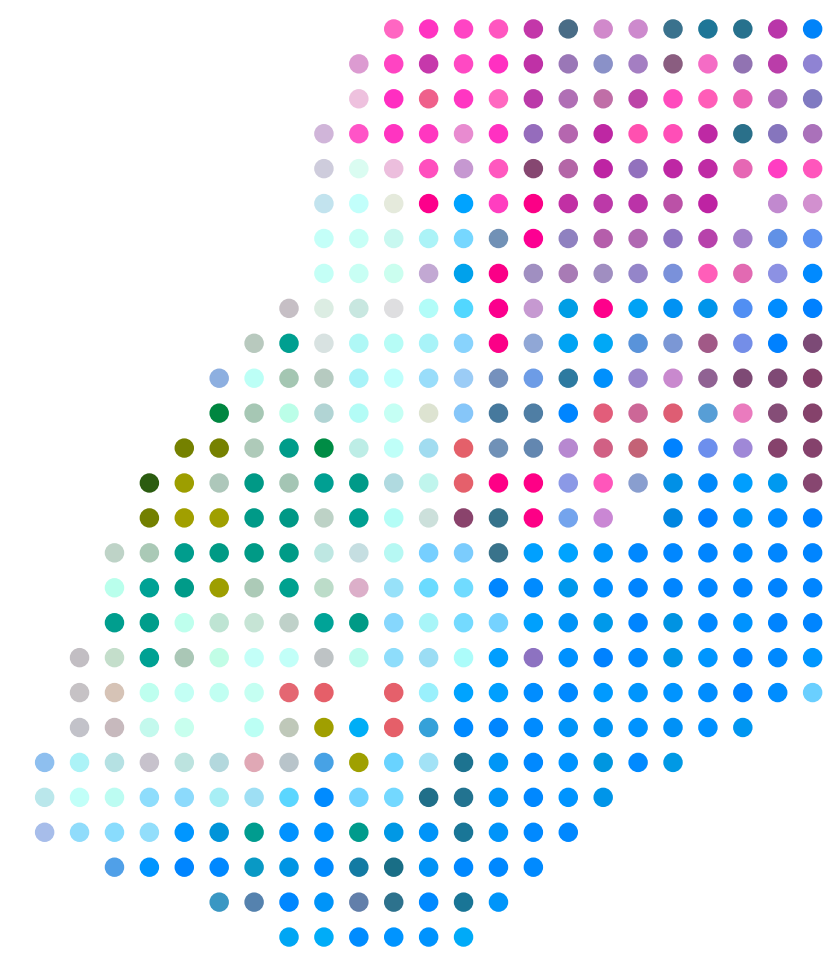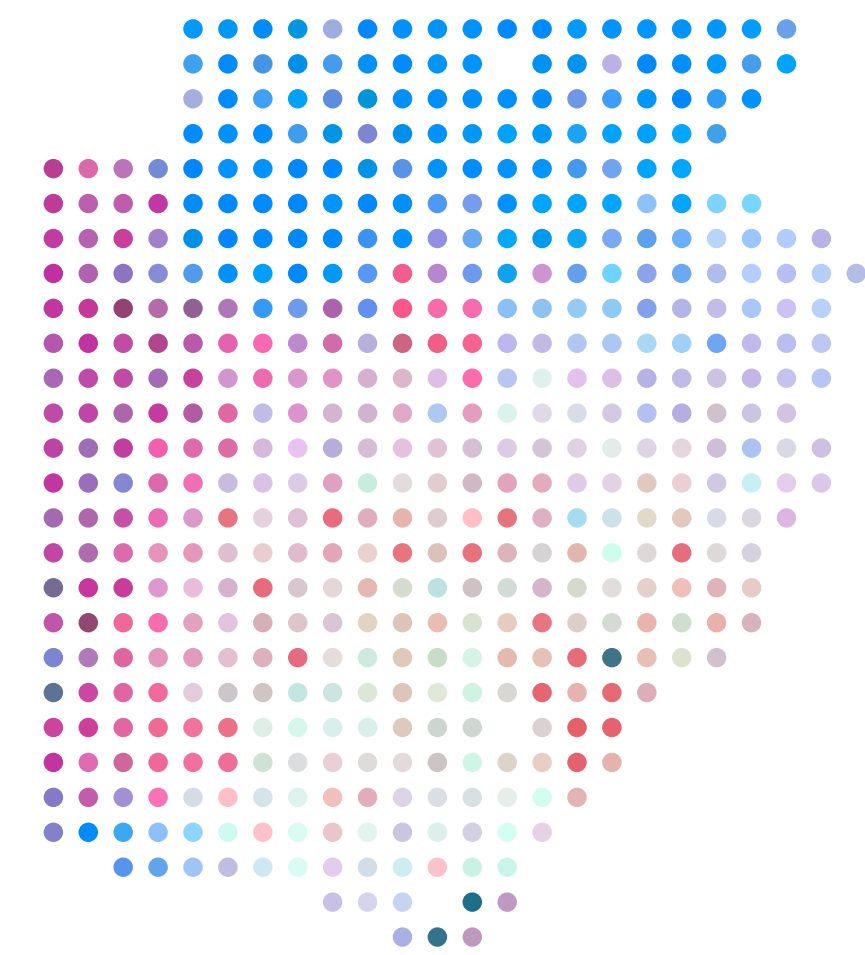

Supplement: Supplementary file 8 — Supplementary Data 5 [file 41467_2018_4724_MOESM8_ESM.zip › Supplementary Dataset 7/joint-mix-dimensionality-reduction-tSNE-dots.pdf]

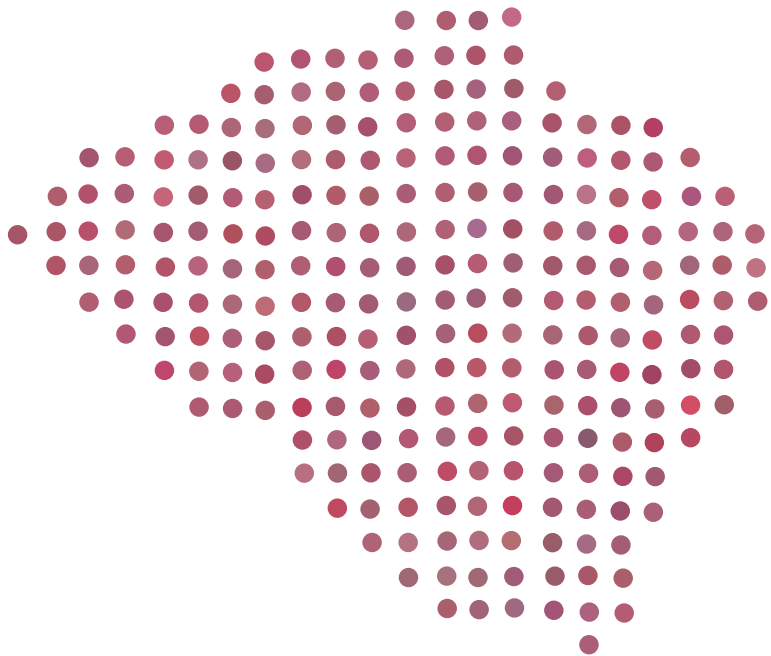

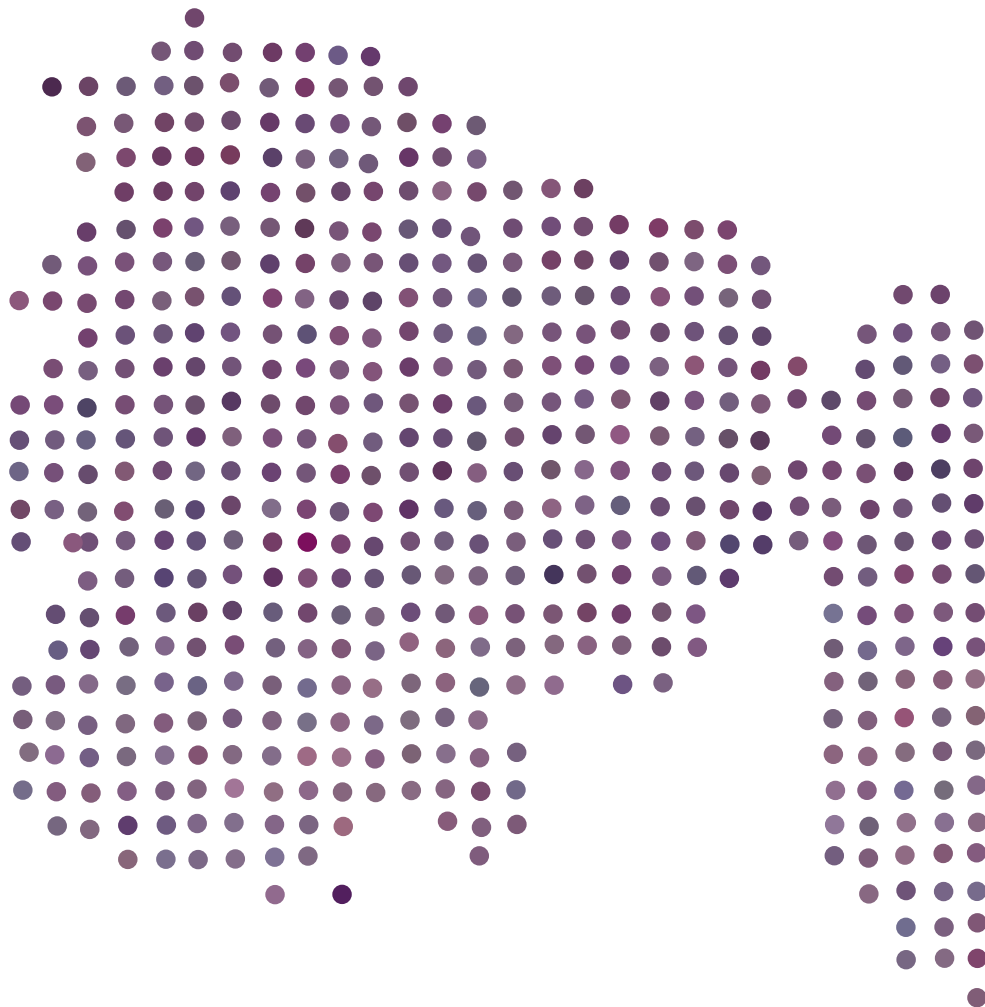

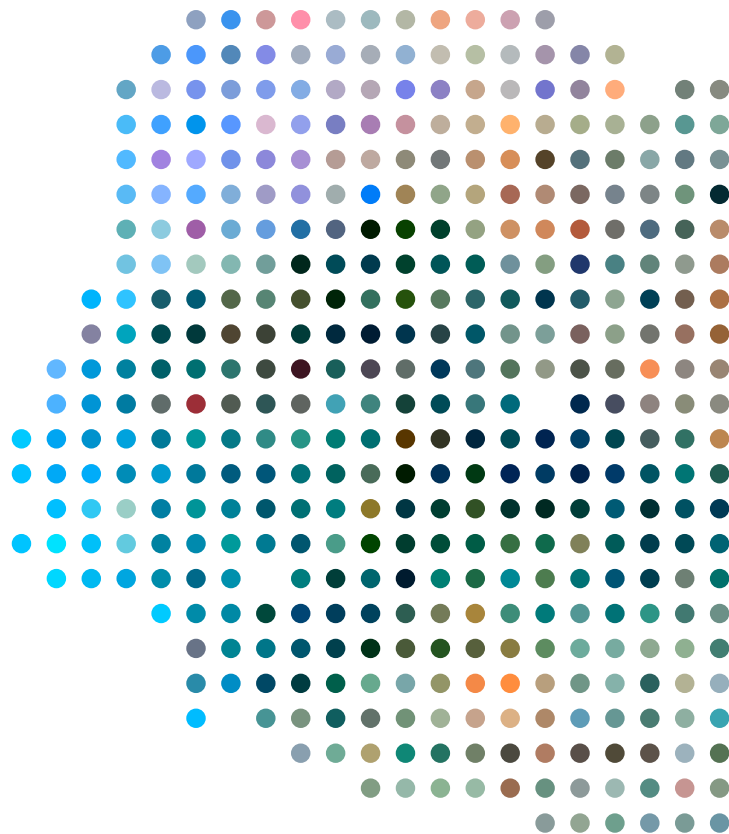

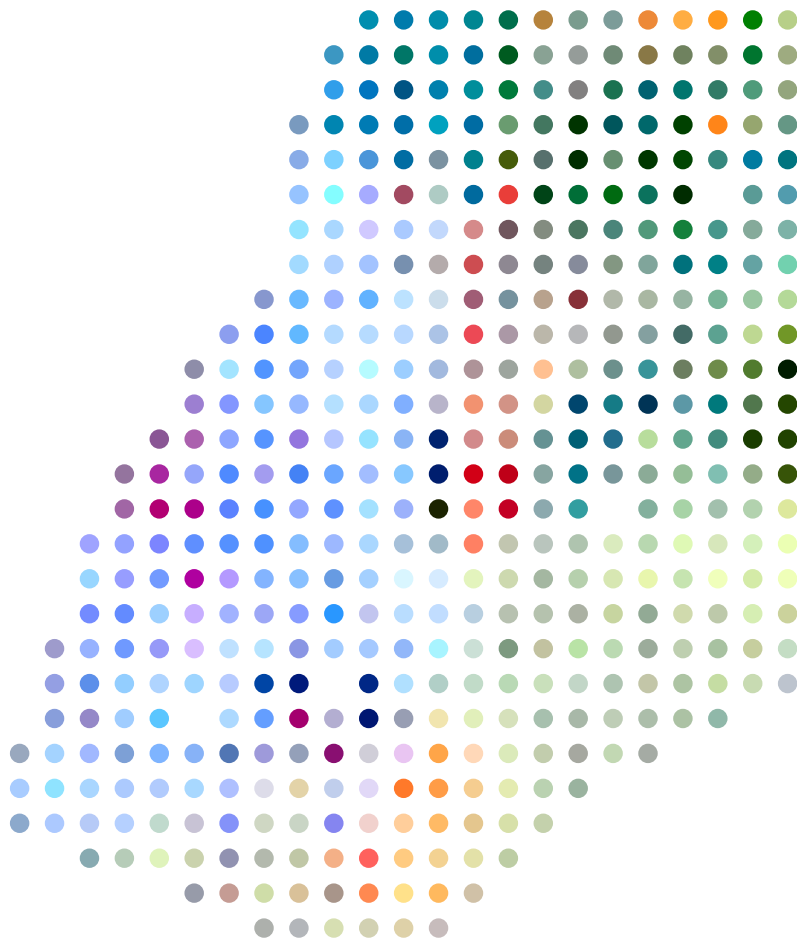

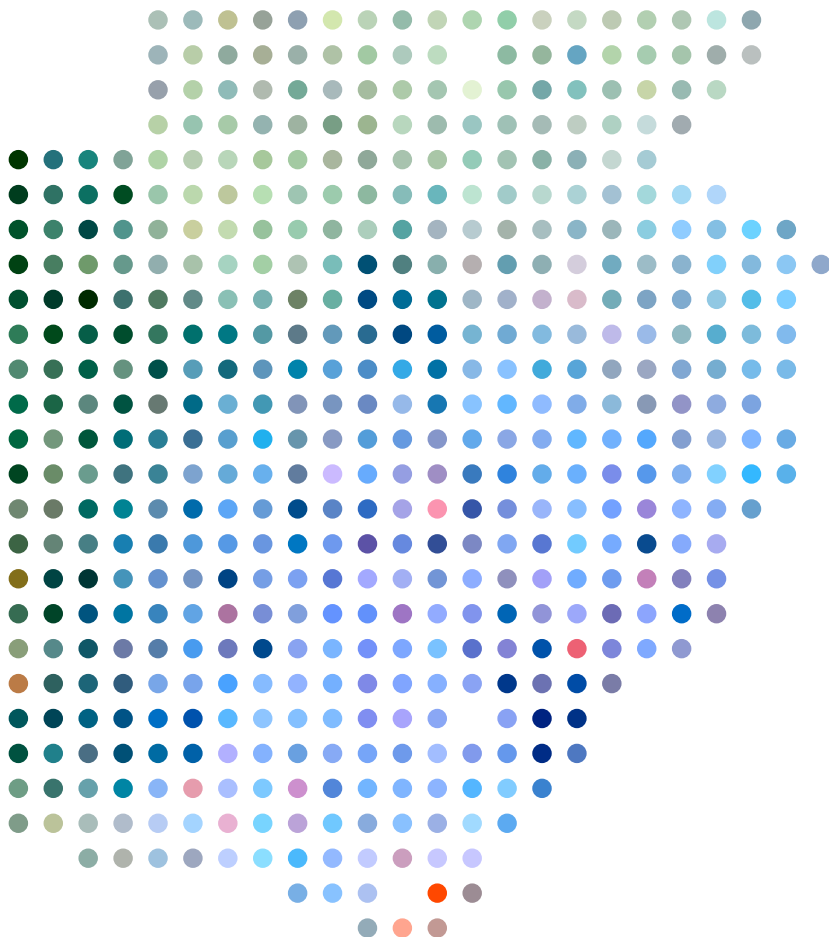

Supplement: Supplementary file 8 — Supplementary Data 5 [file 41467_2018_4724_MOESM8_ESM.zip › Supplementary Dataset 7/joint-mix-dimensionality-reduction-PCA-dots-split.pdf]

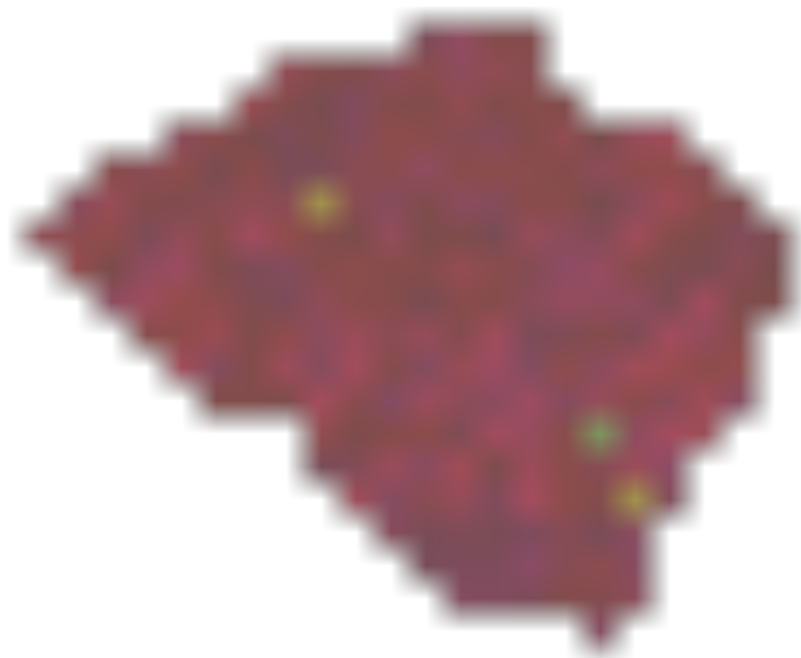

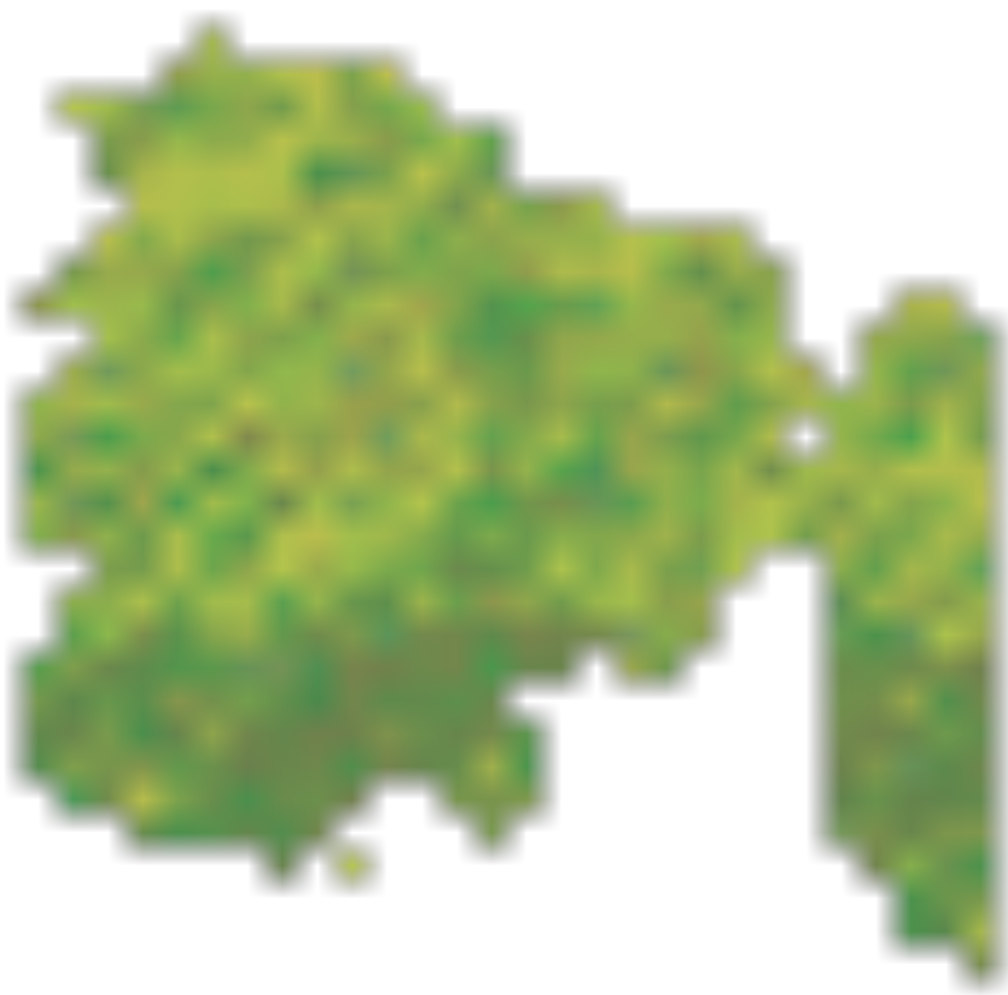

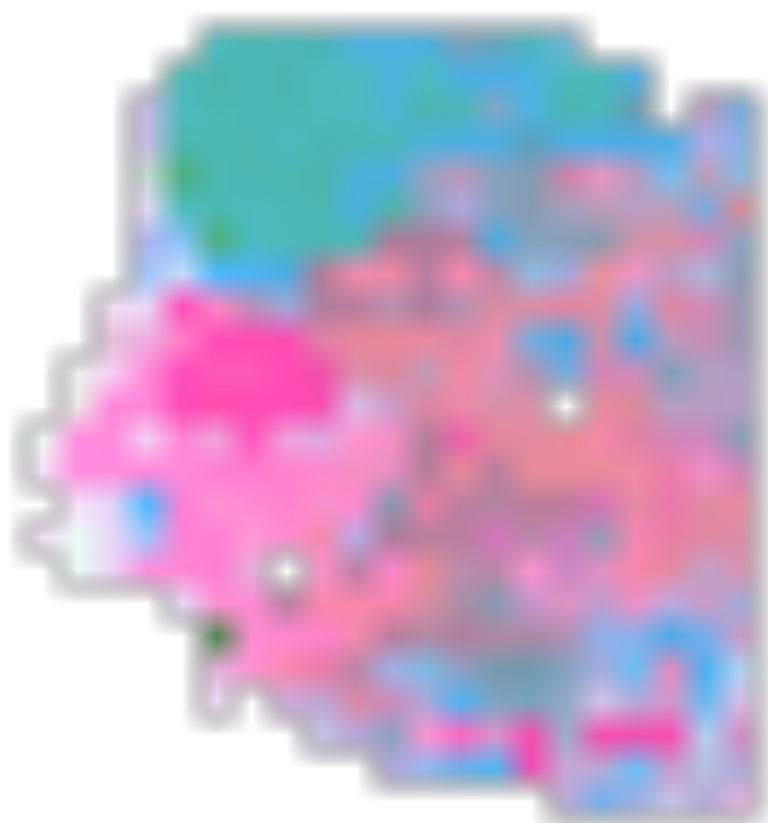

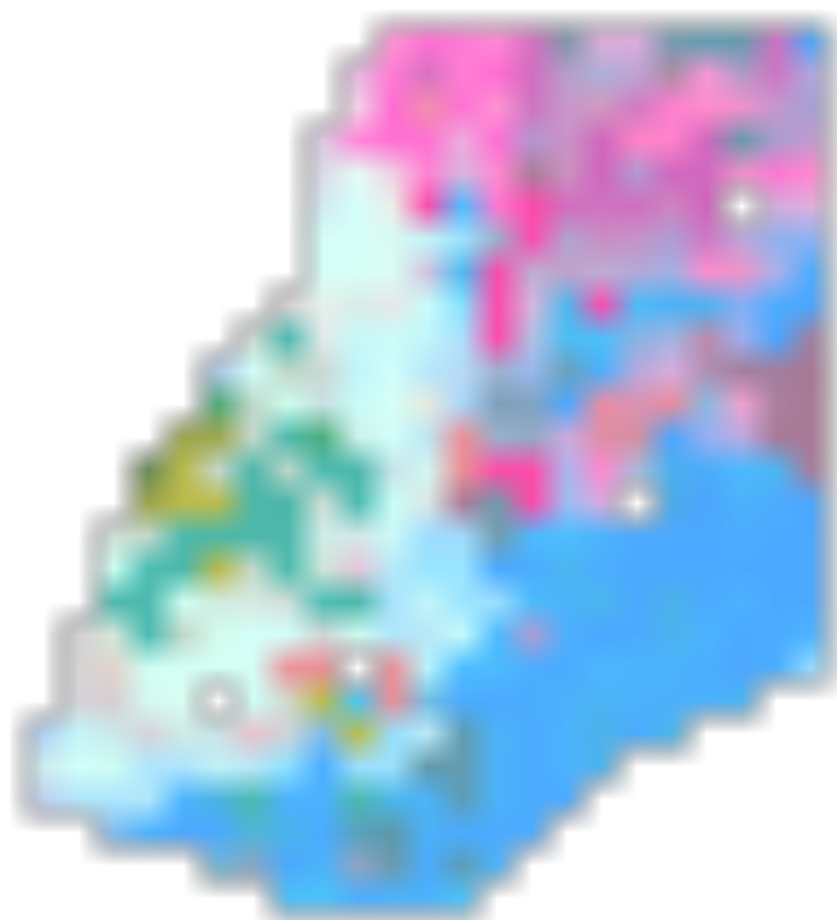

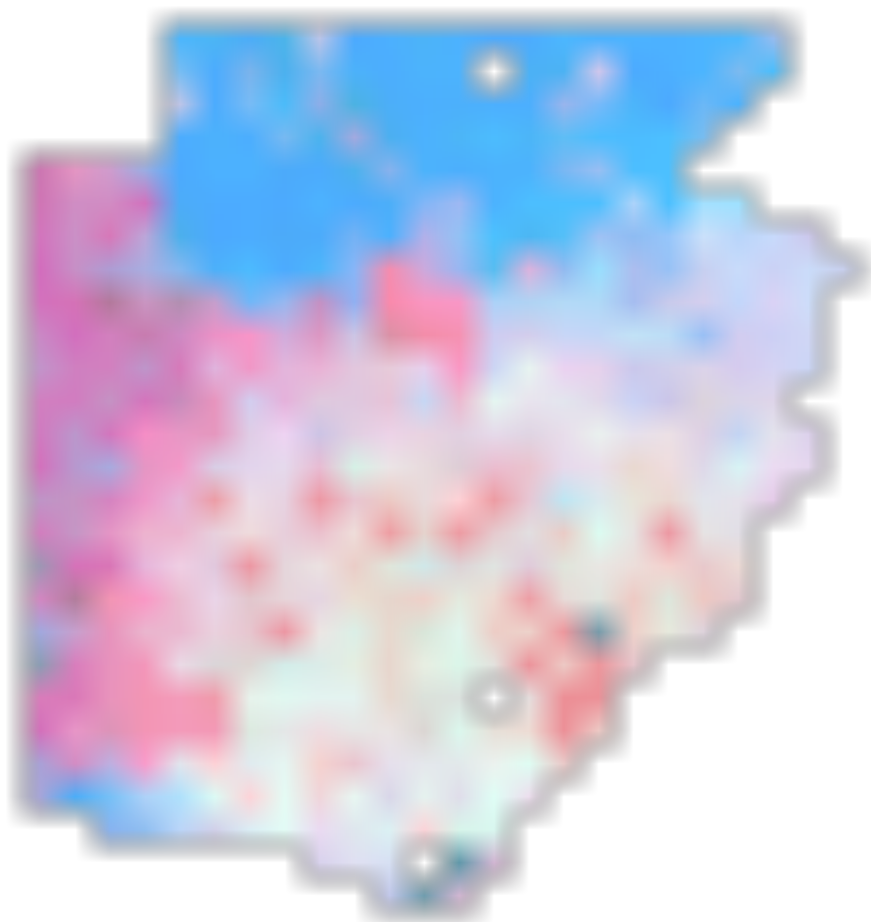

Supplement: Supplementary file 8 — Supplementary Data 5 [file 41467_2018_4724_MOESM8_ESM.zip › Supplementary Dataset 7/joint-mix-dimensionality-reduction-tSNE-matrix-split.pdf.interpolated.pdf]

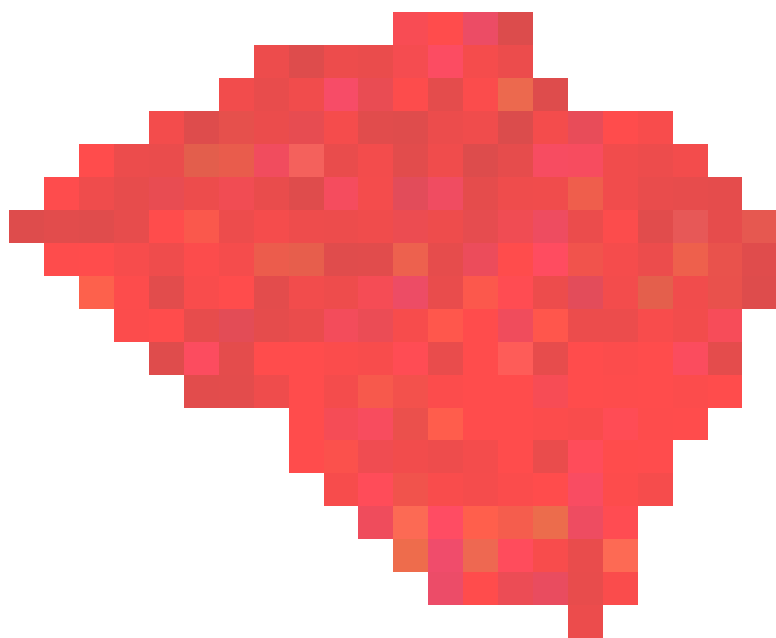

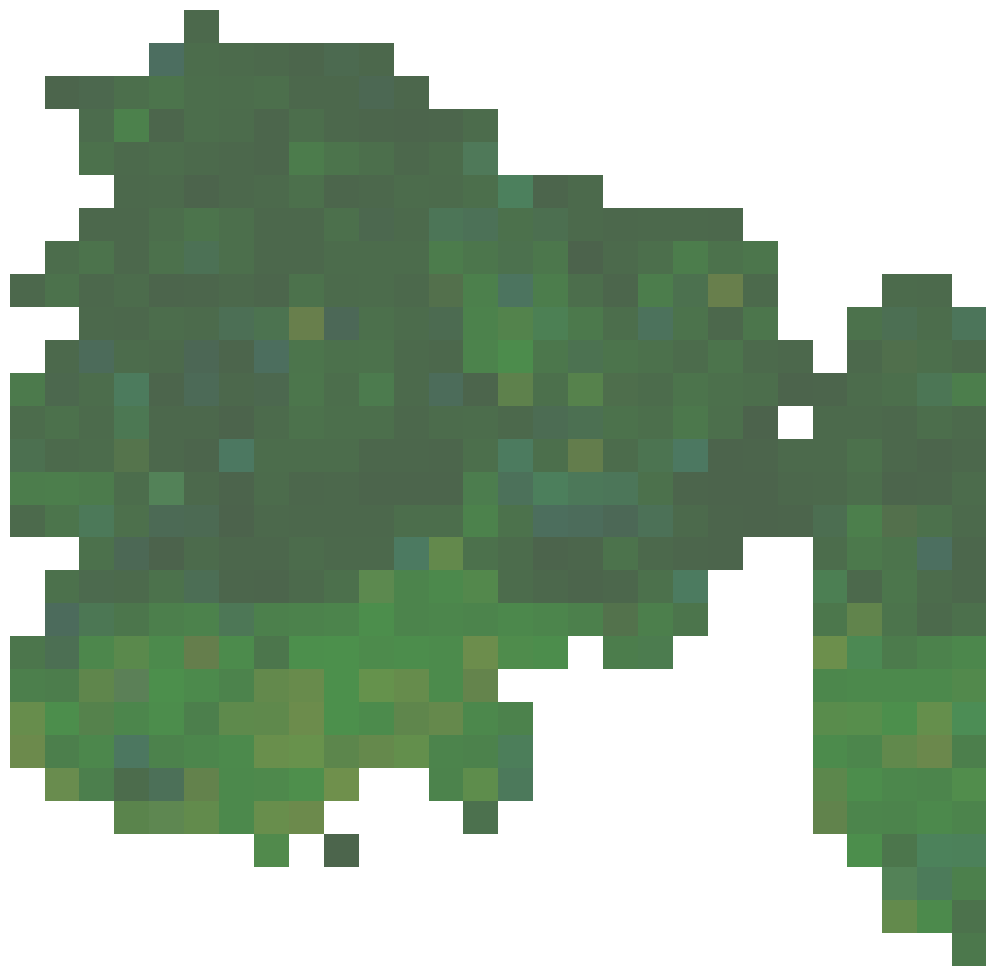

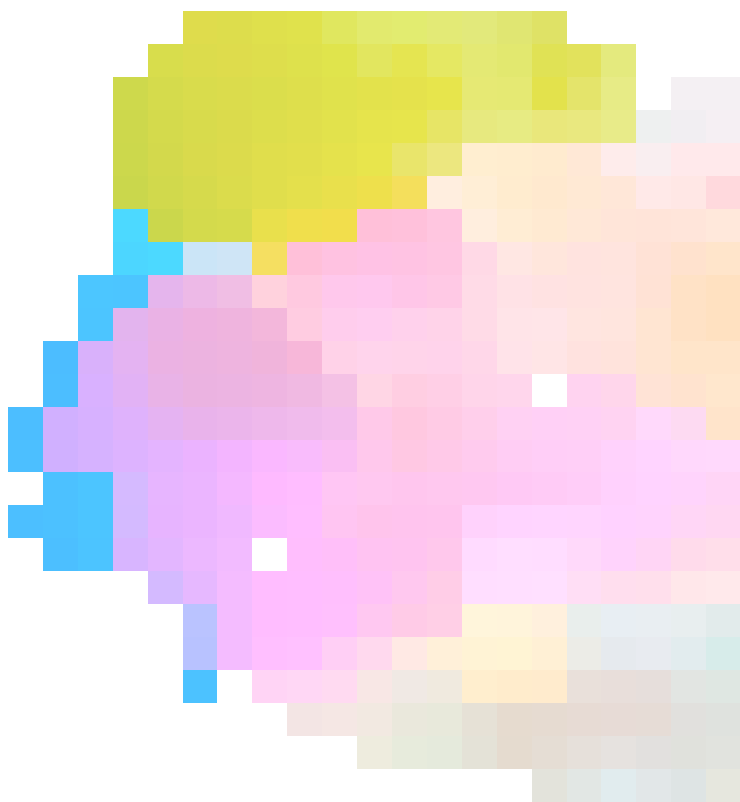

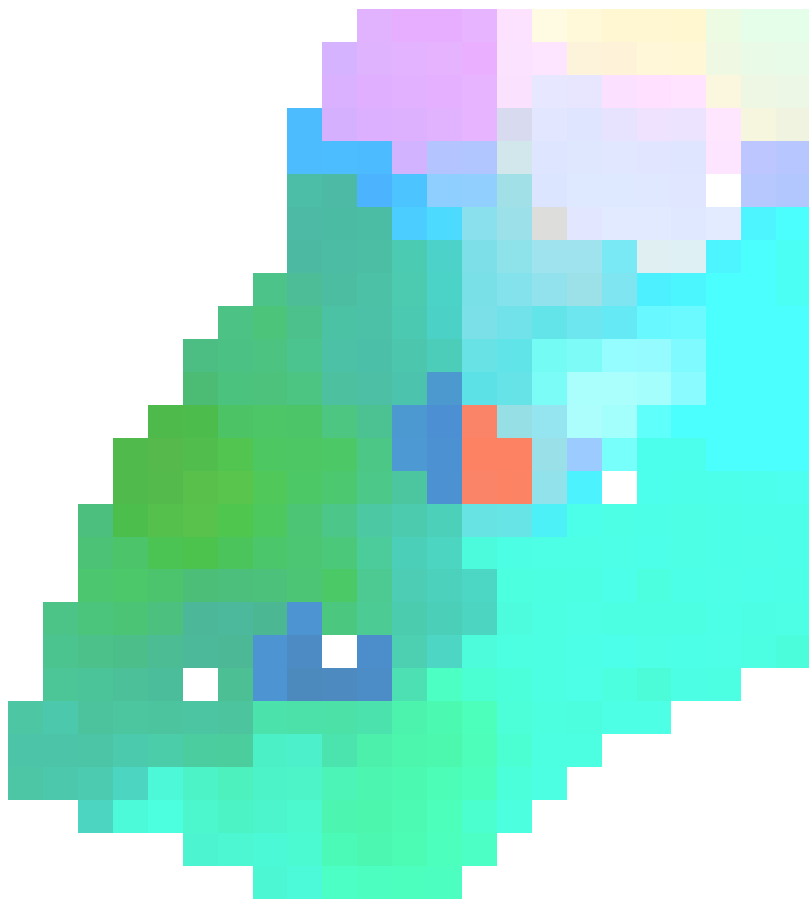

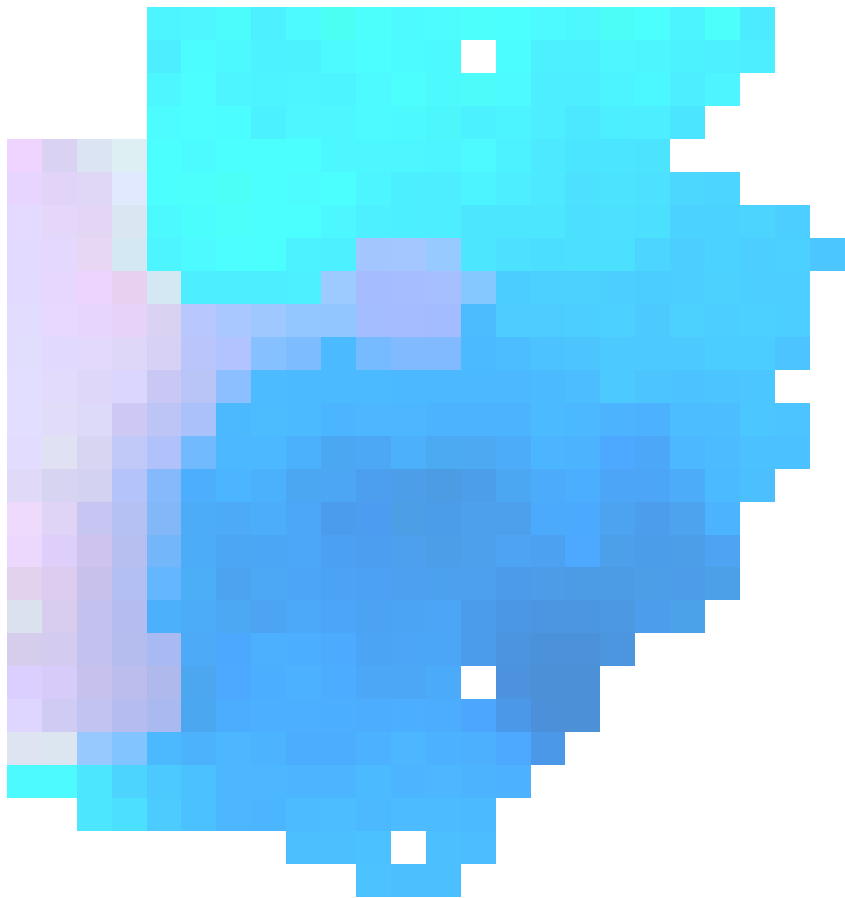

Supplement: Supplementary file 8 — Supplementary Data 5 [file 41467_2018_4724_MOESM8_ESM.zip › Supplementary Dataset 7/joint-field-dimensionality-reduction-tSNE-matrix-split.pdf]

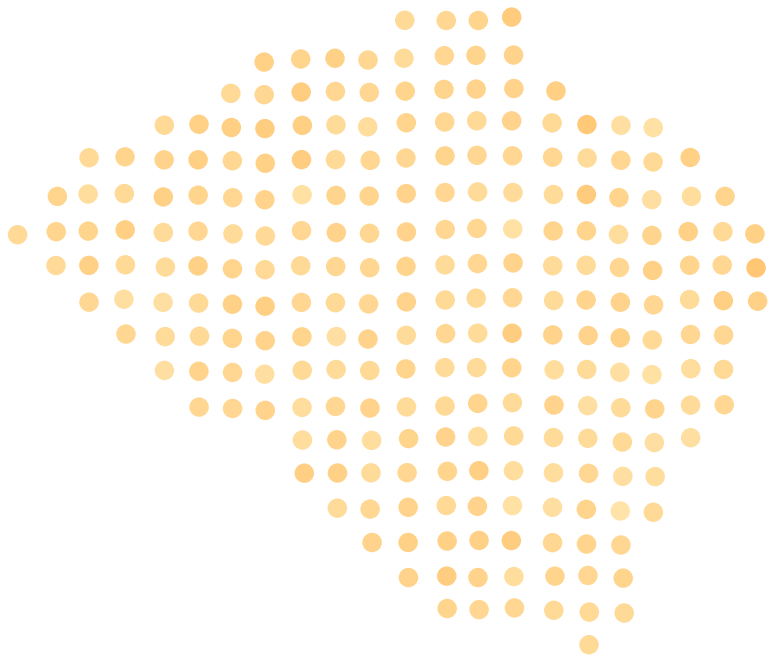

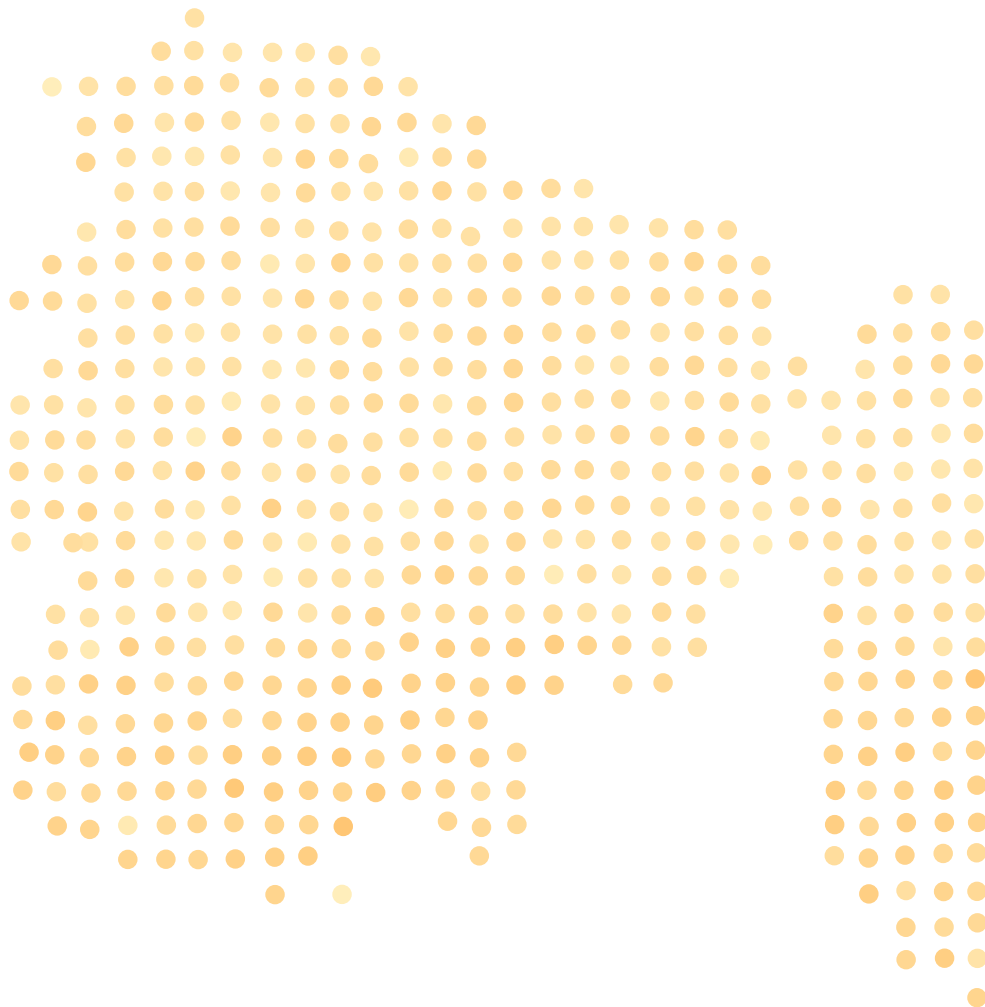

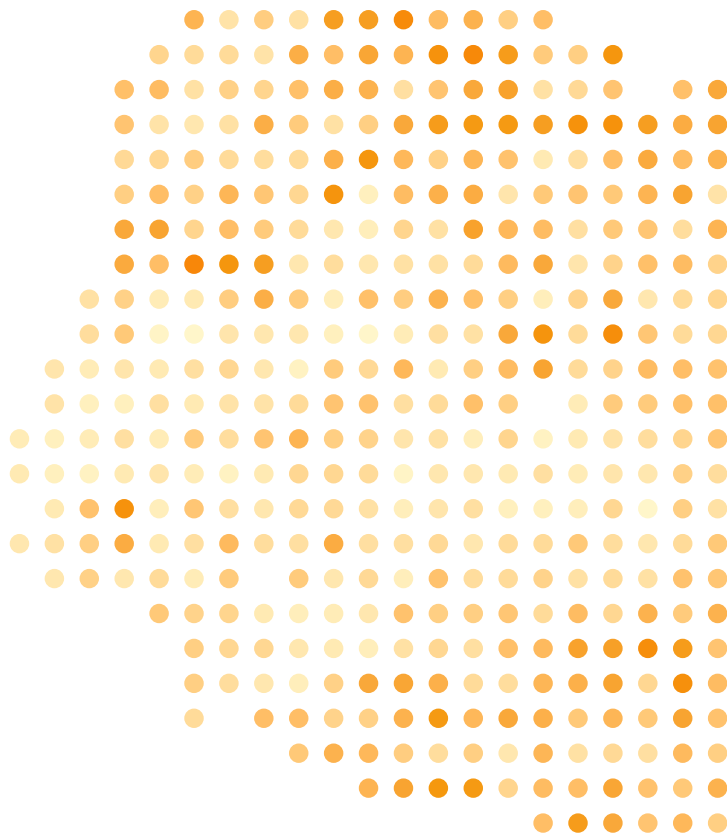

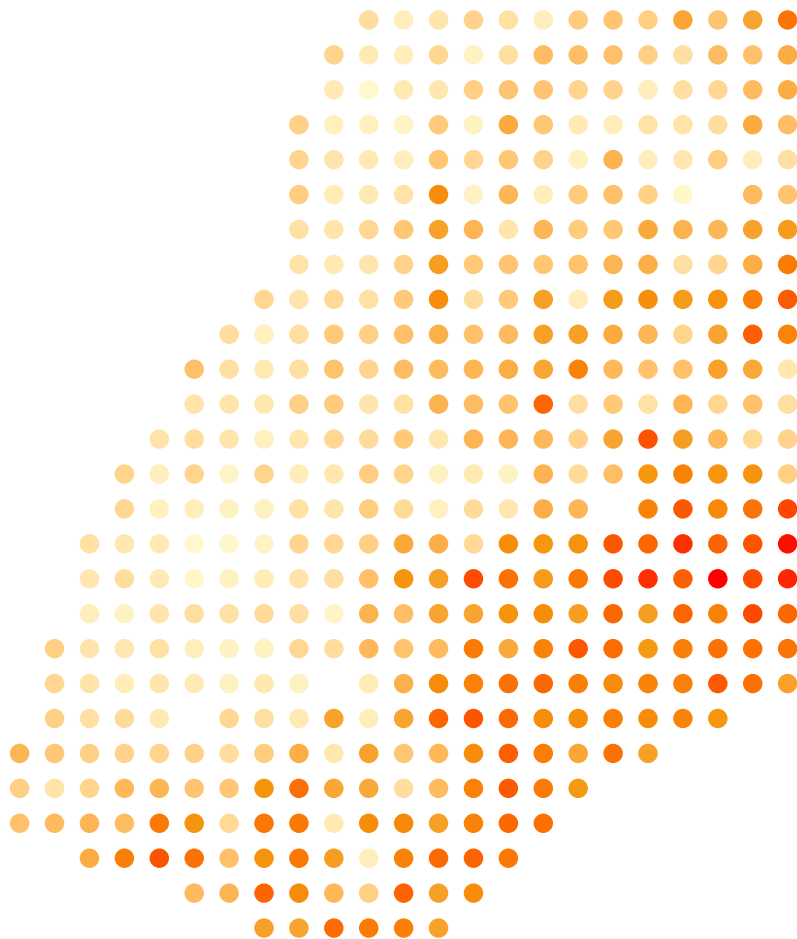

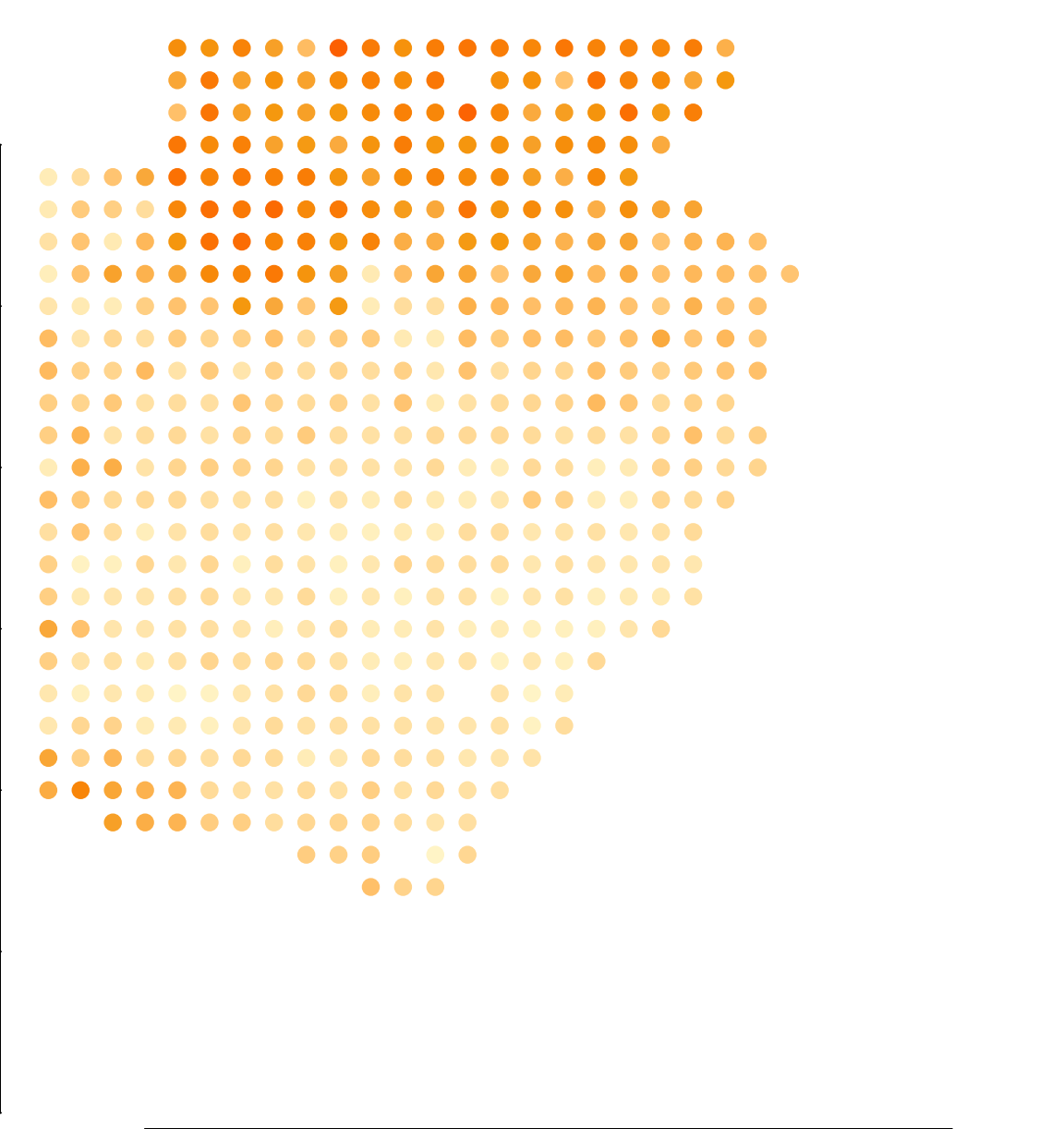

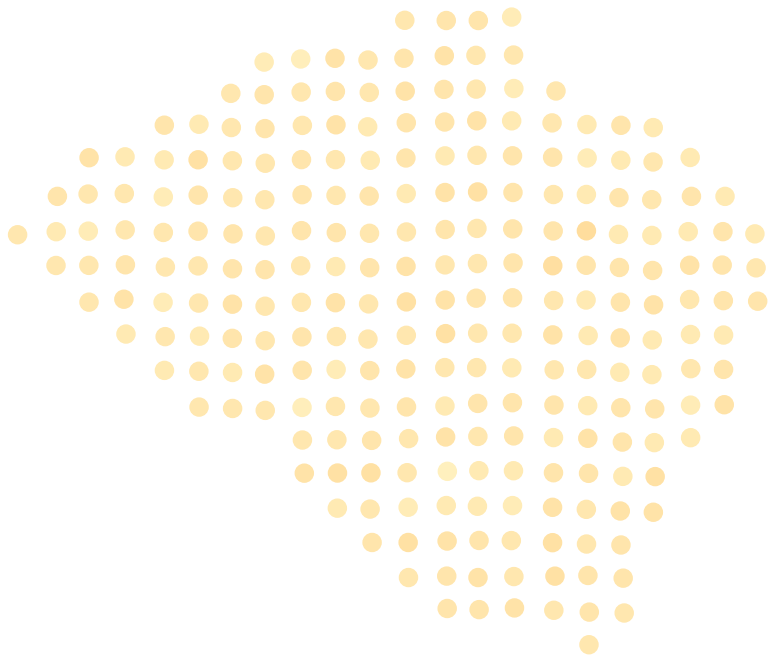

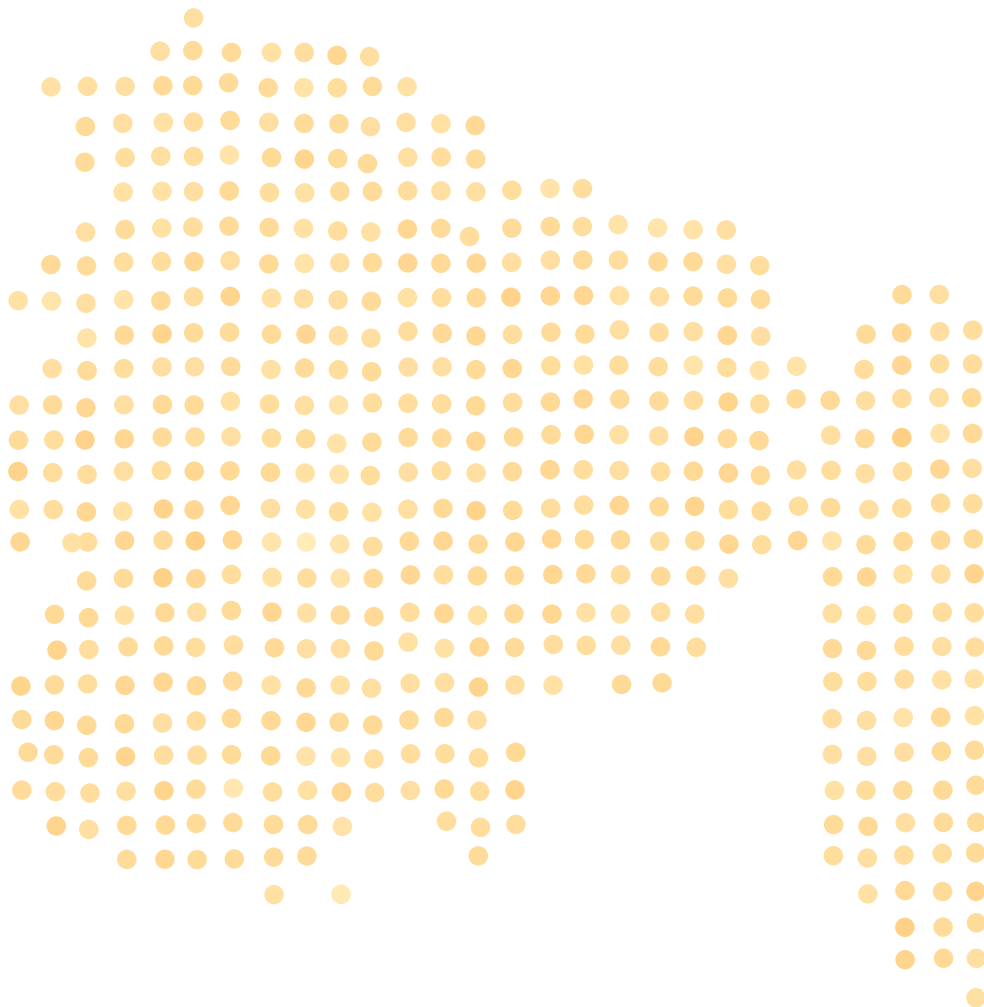

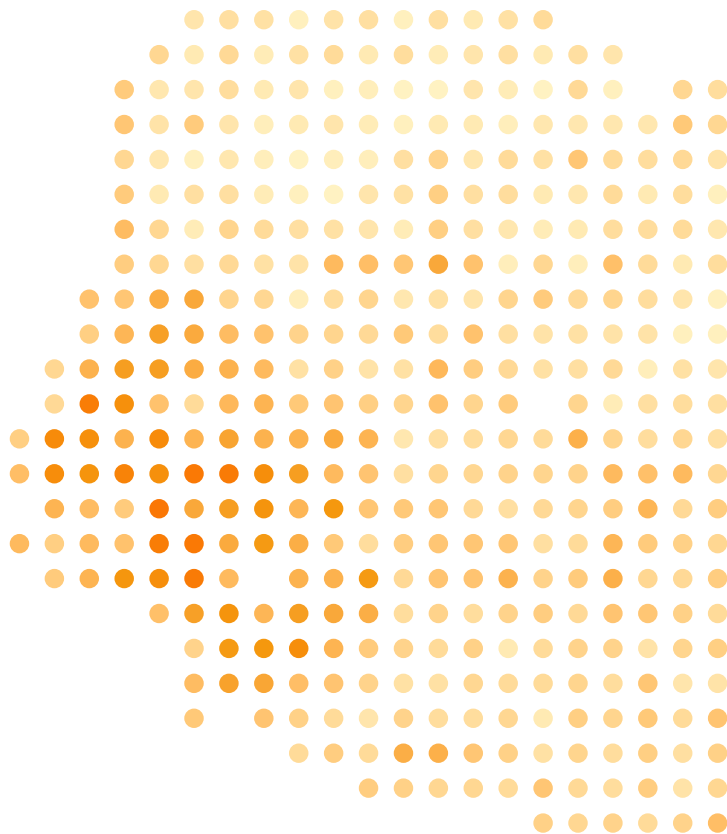

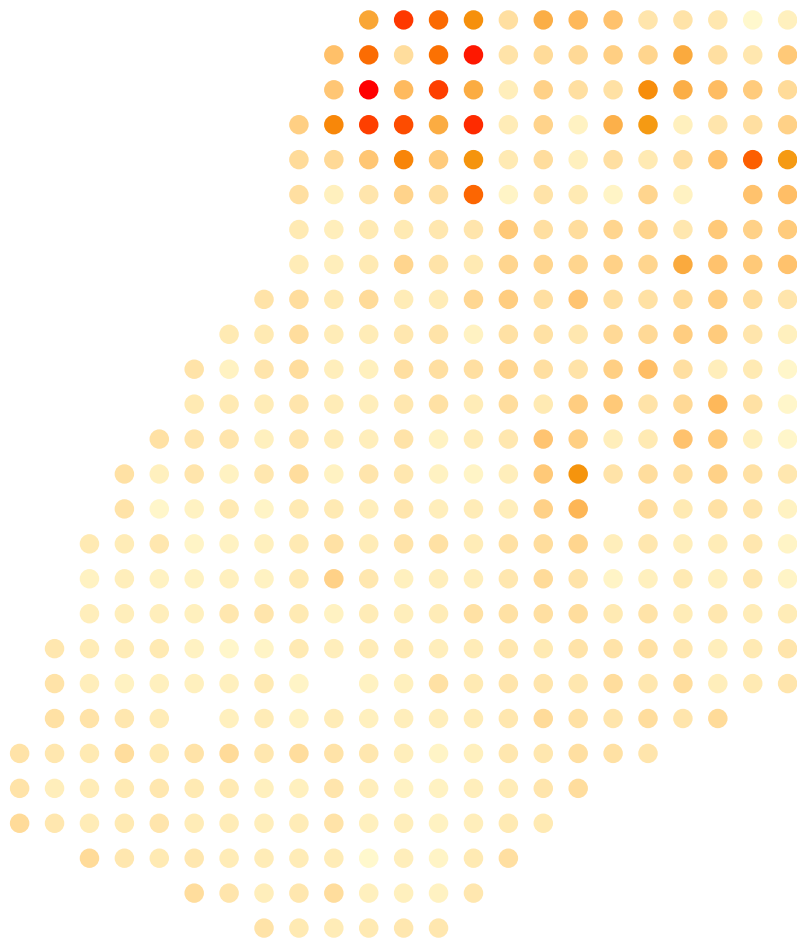

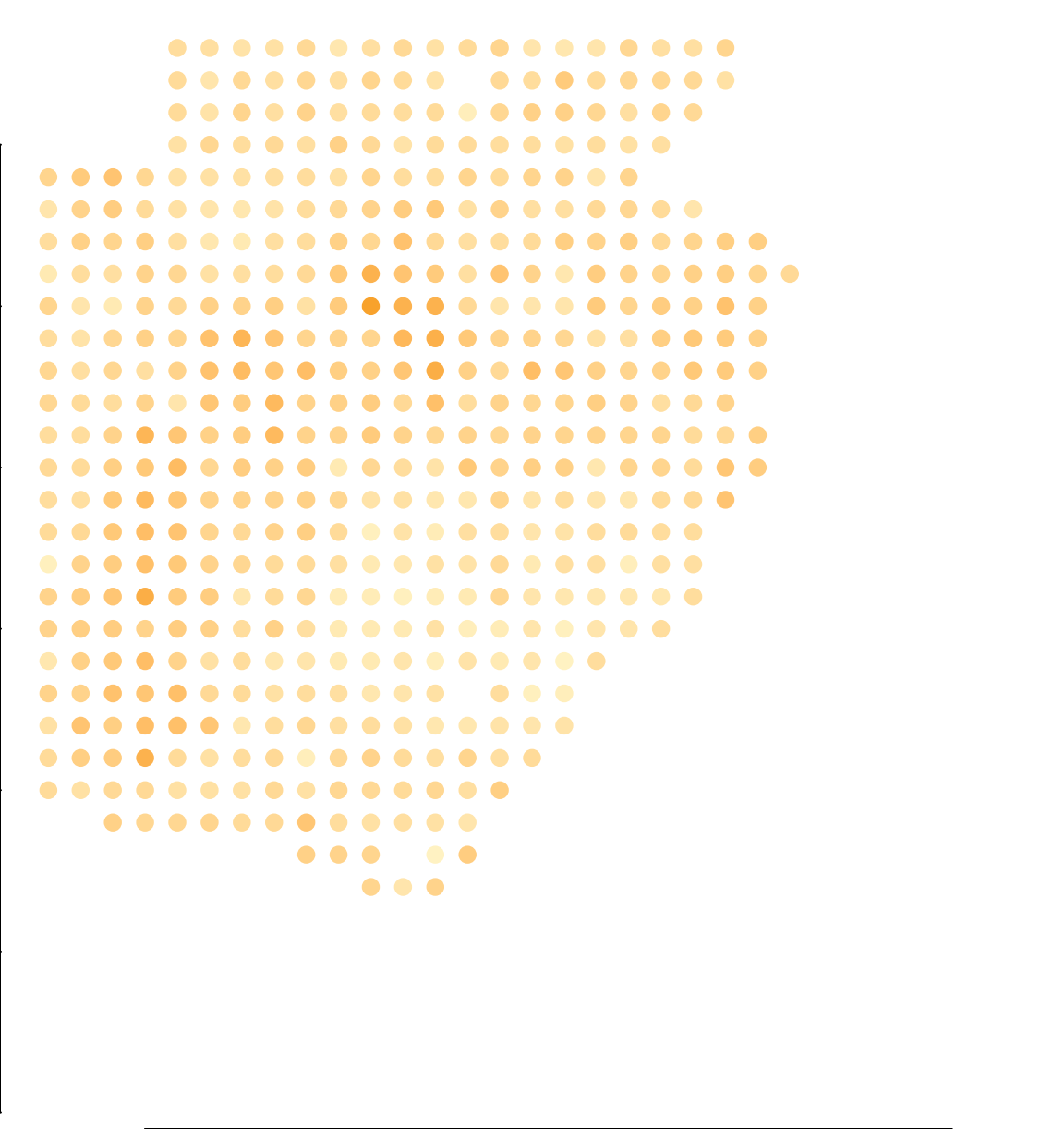

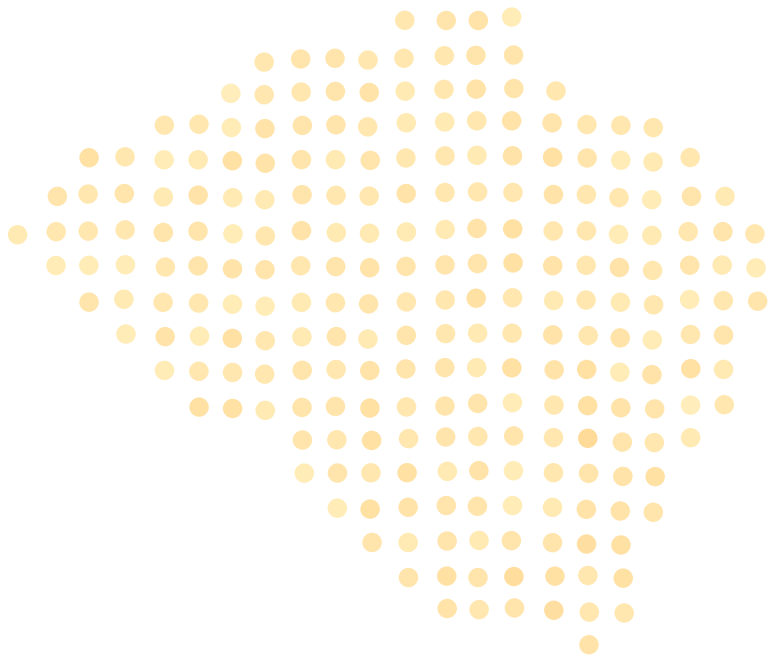

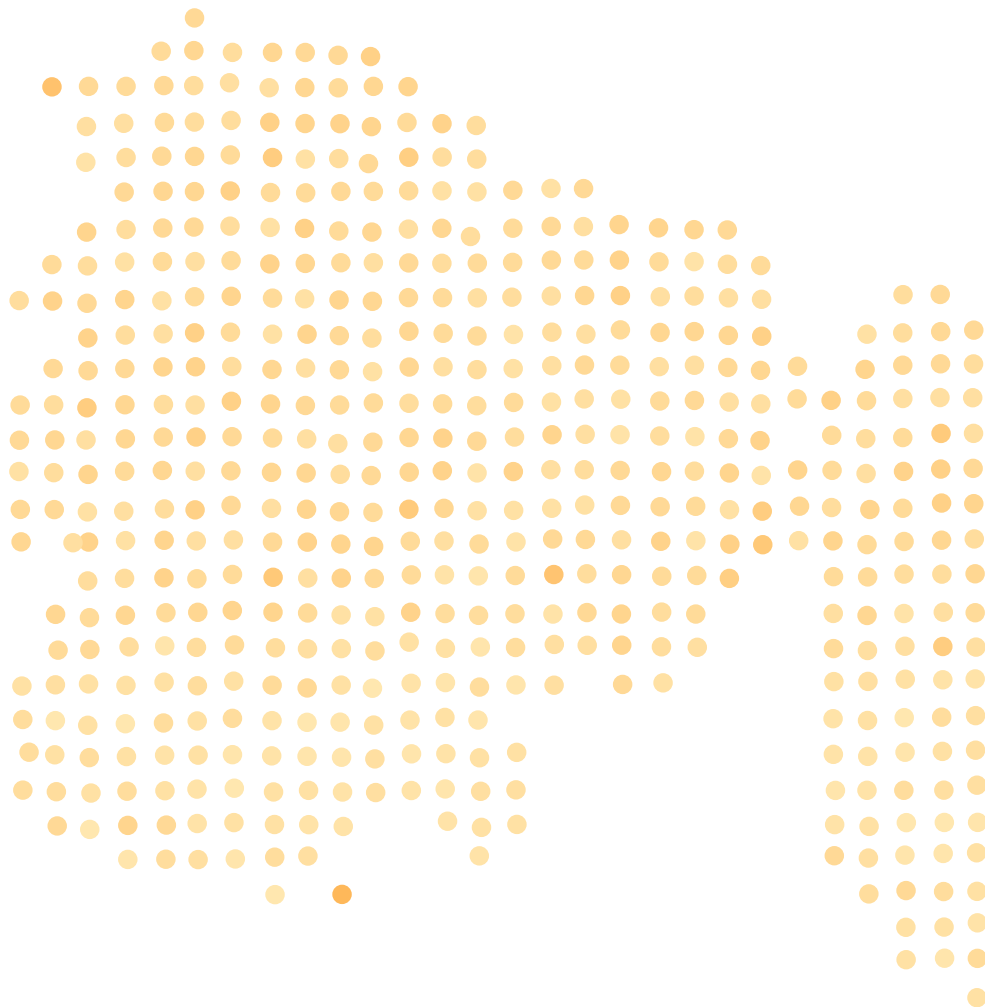

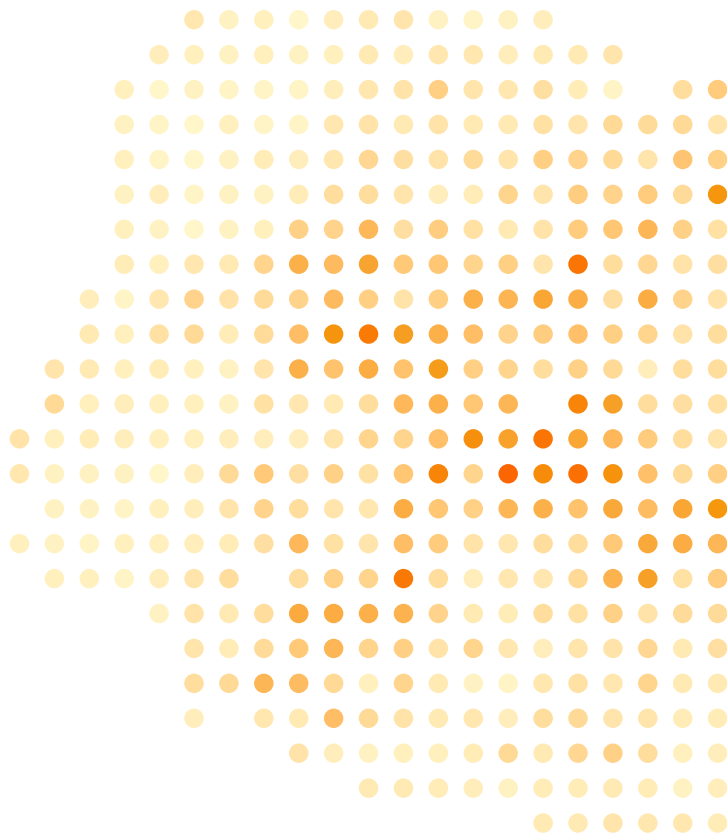

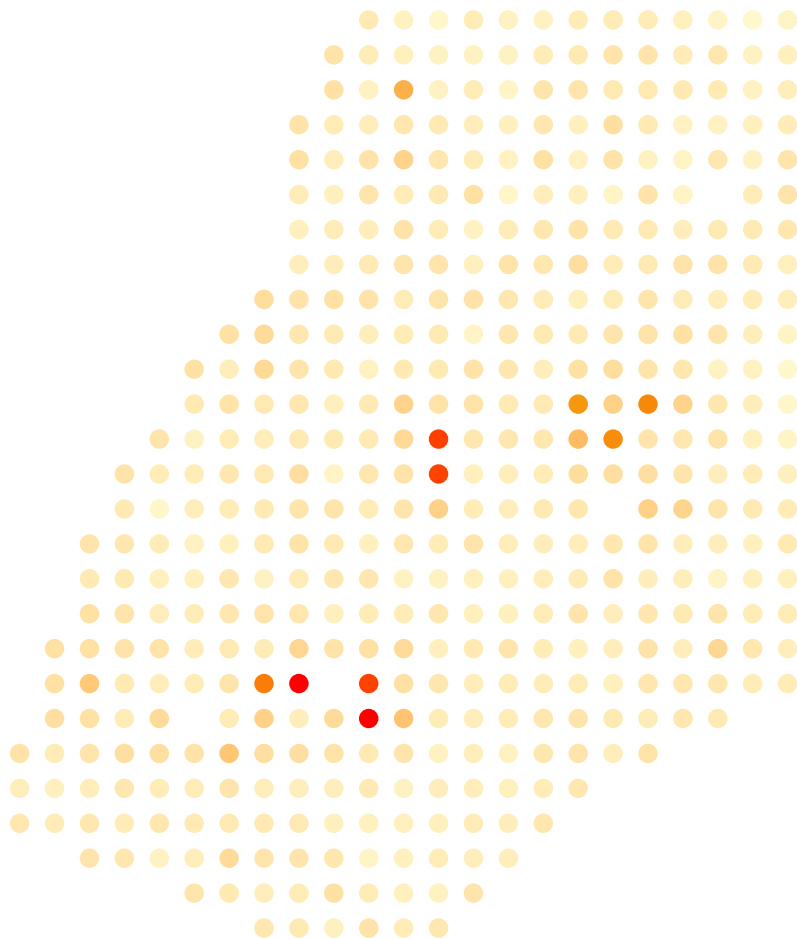

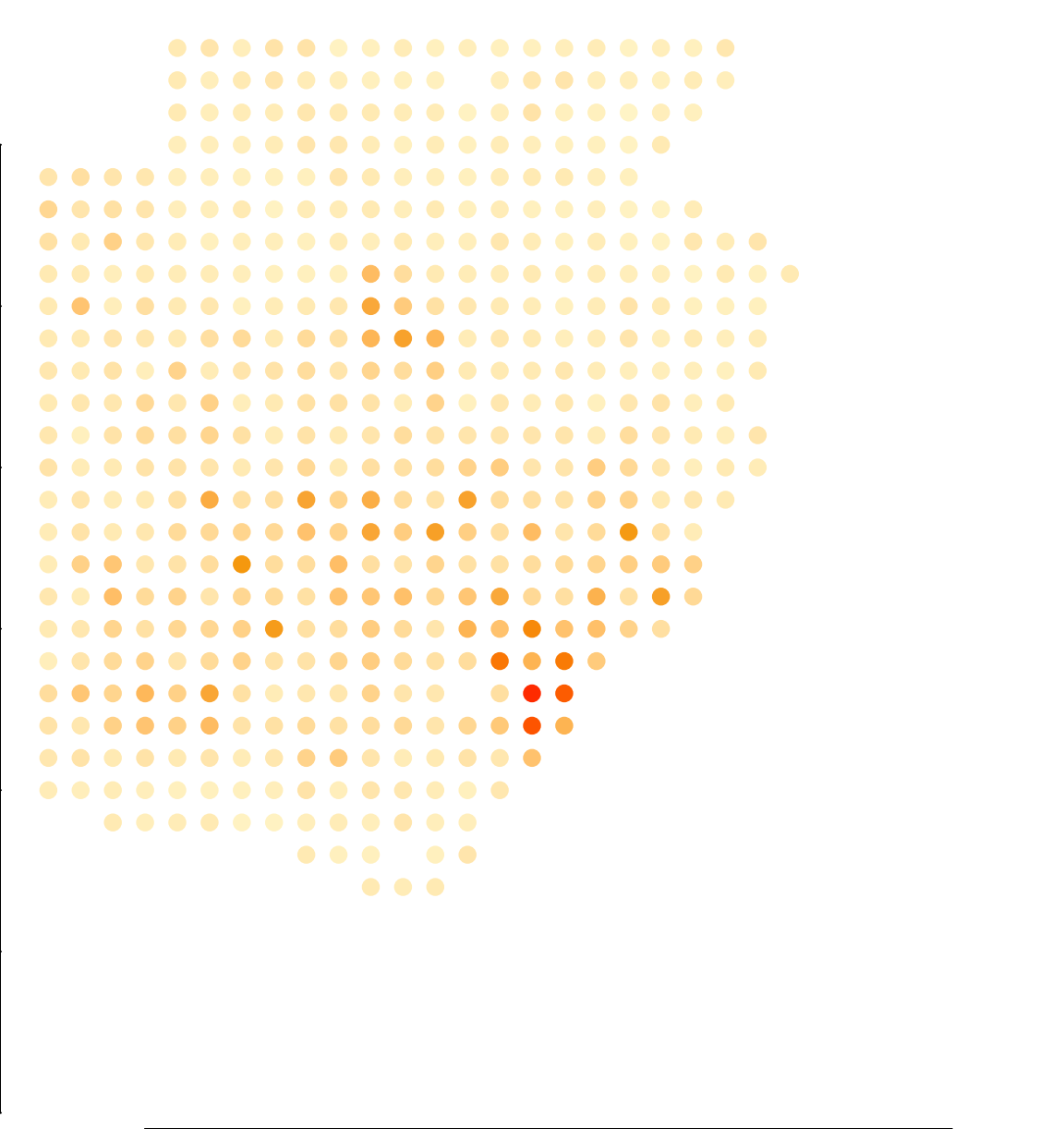

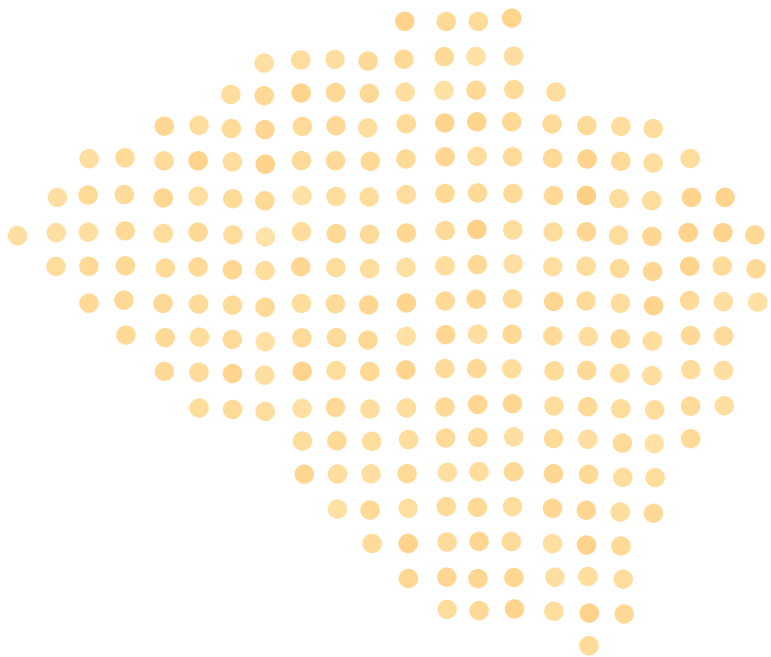

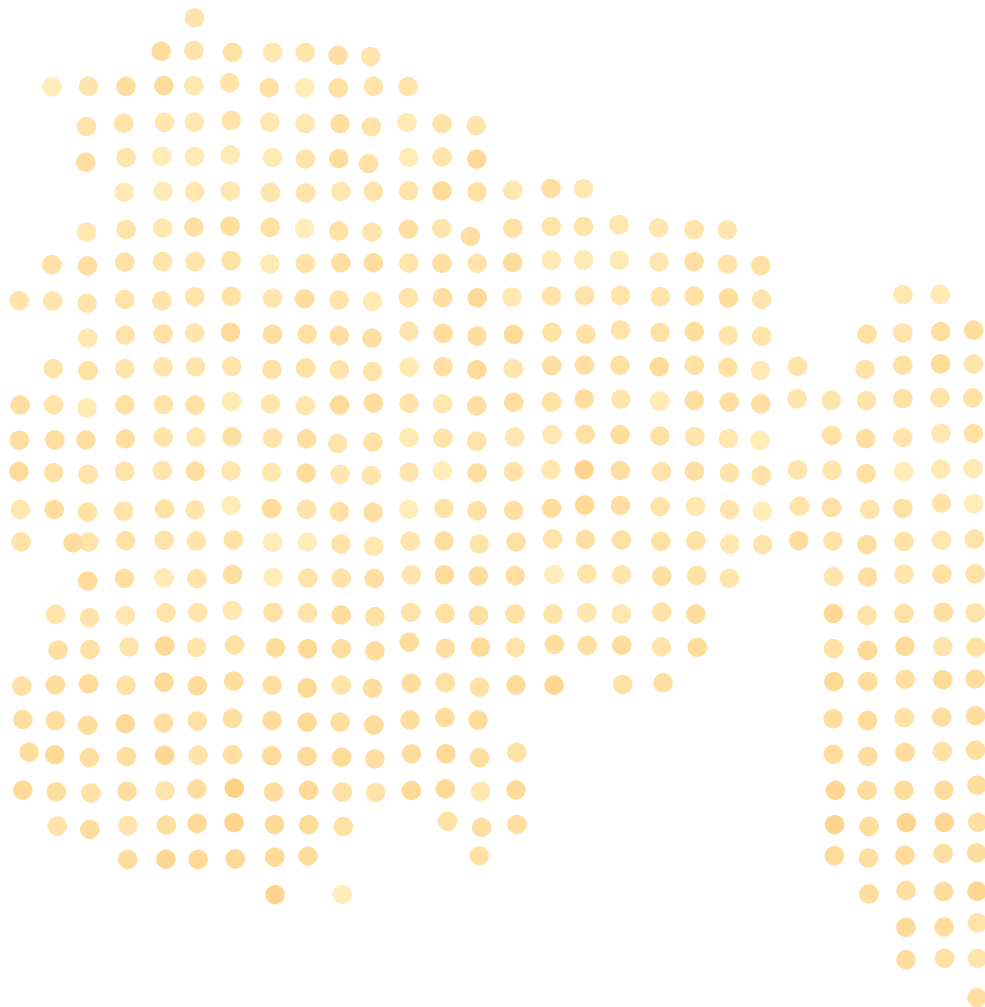

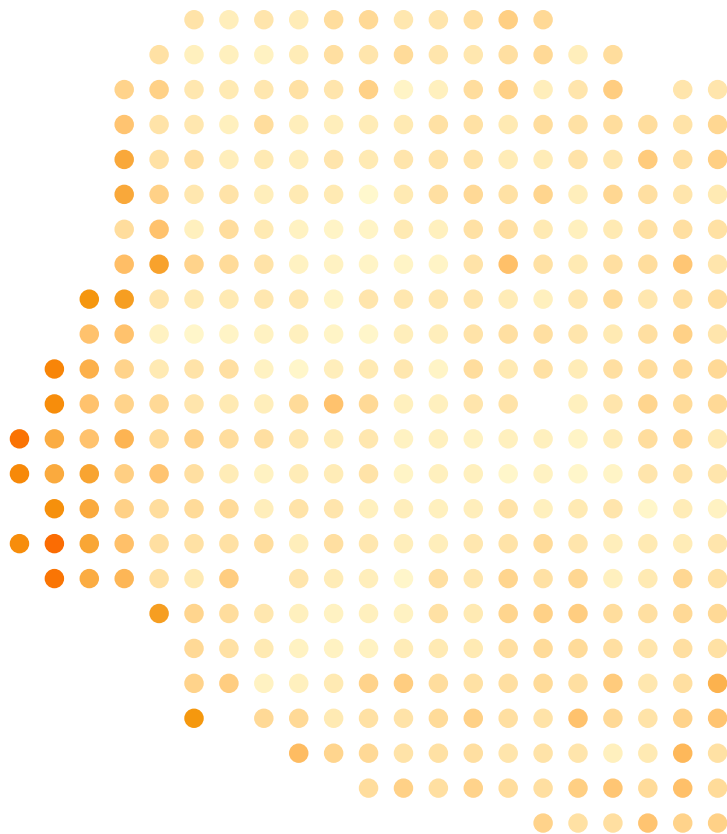

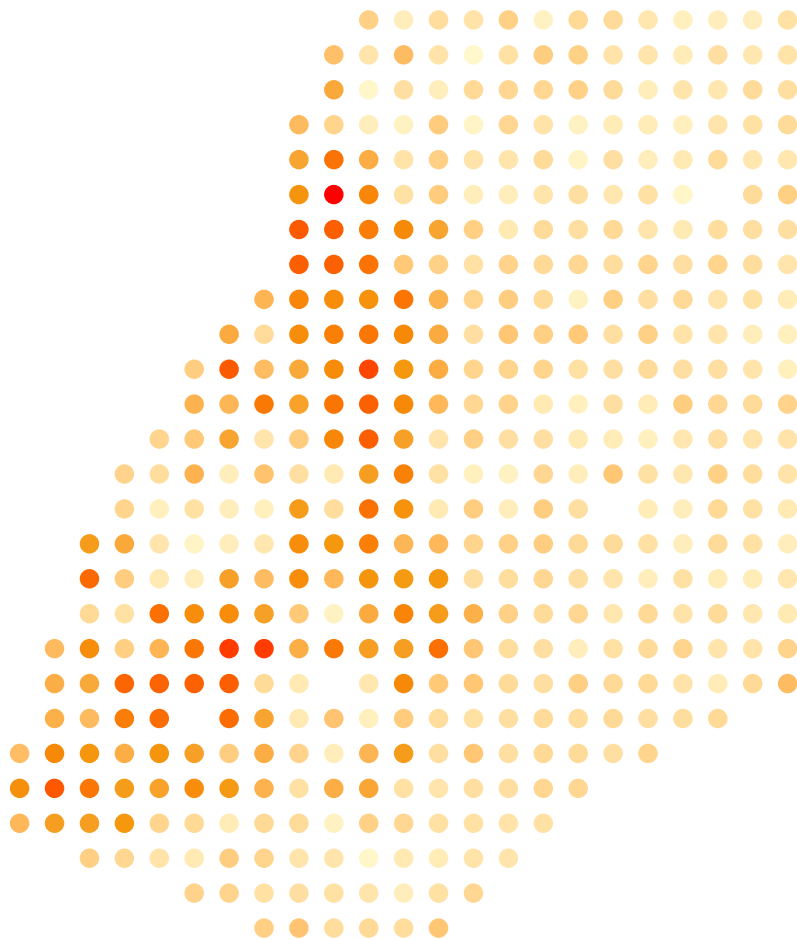

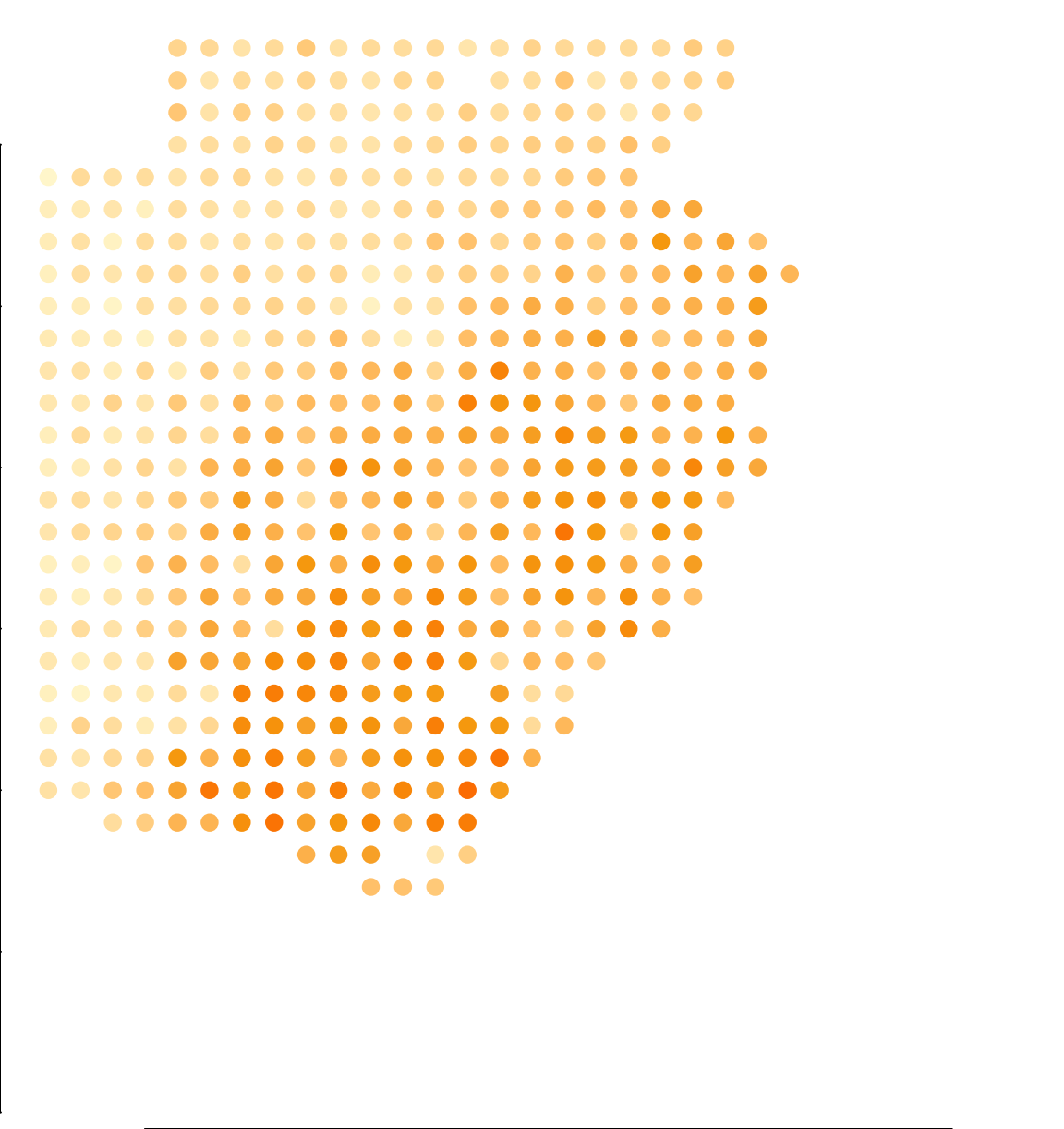

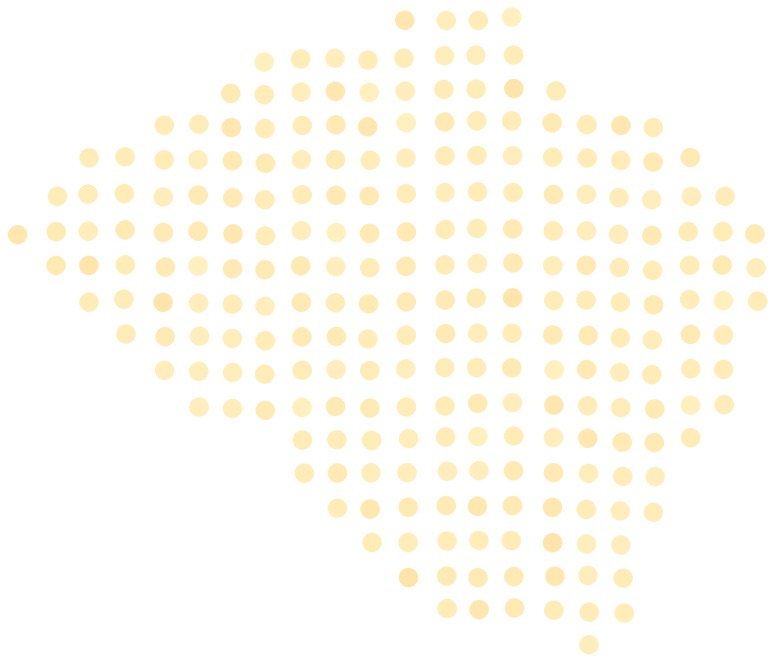

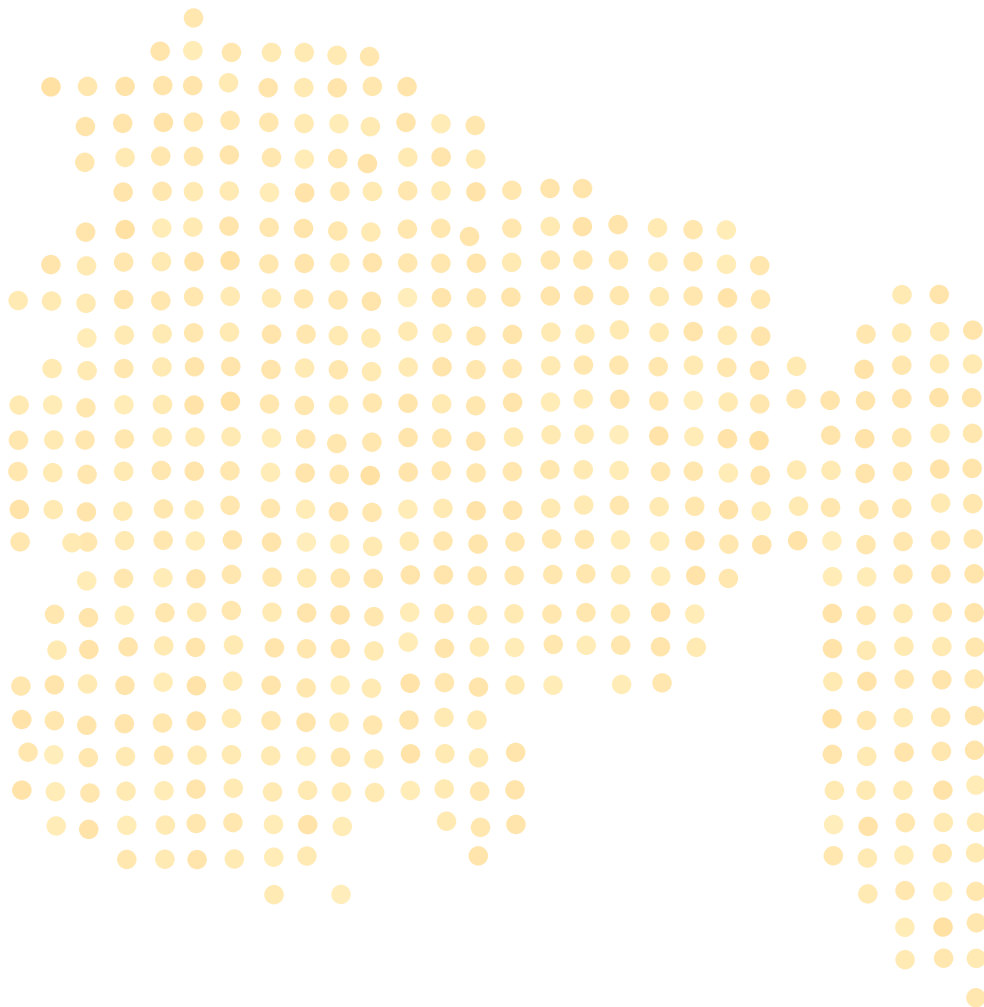

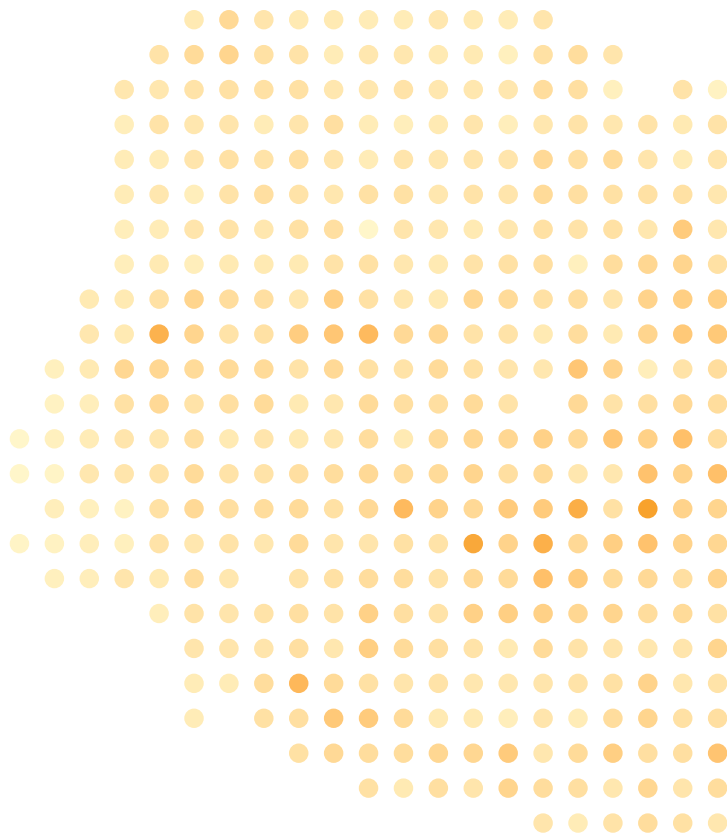

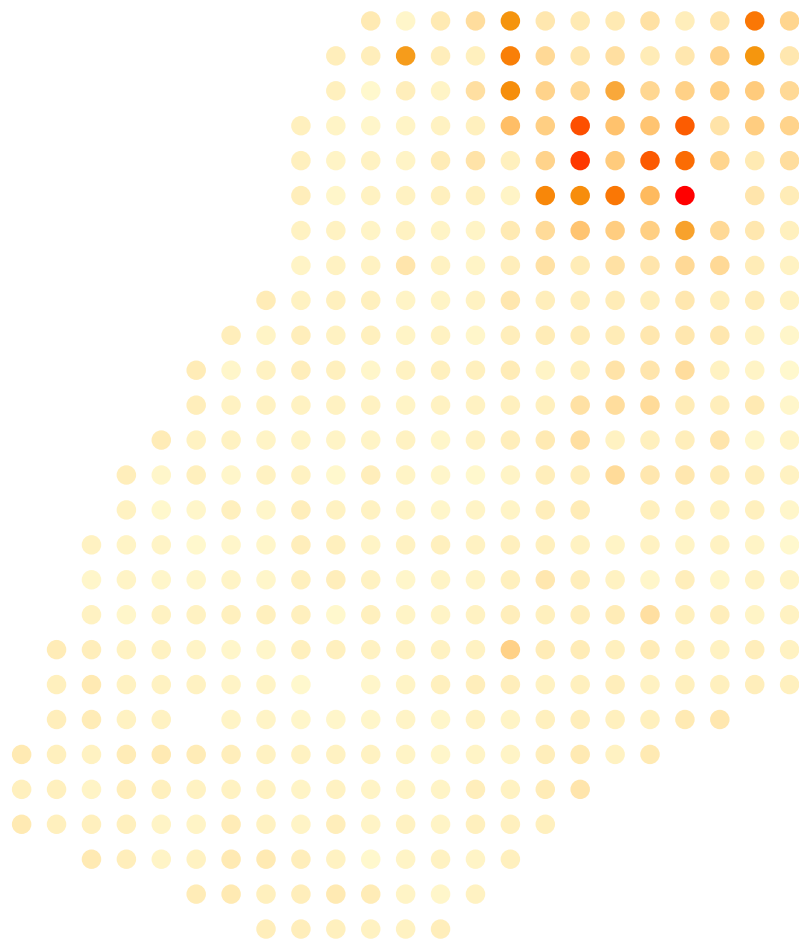

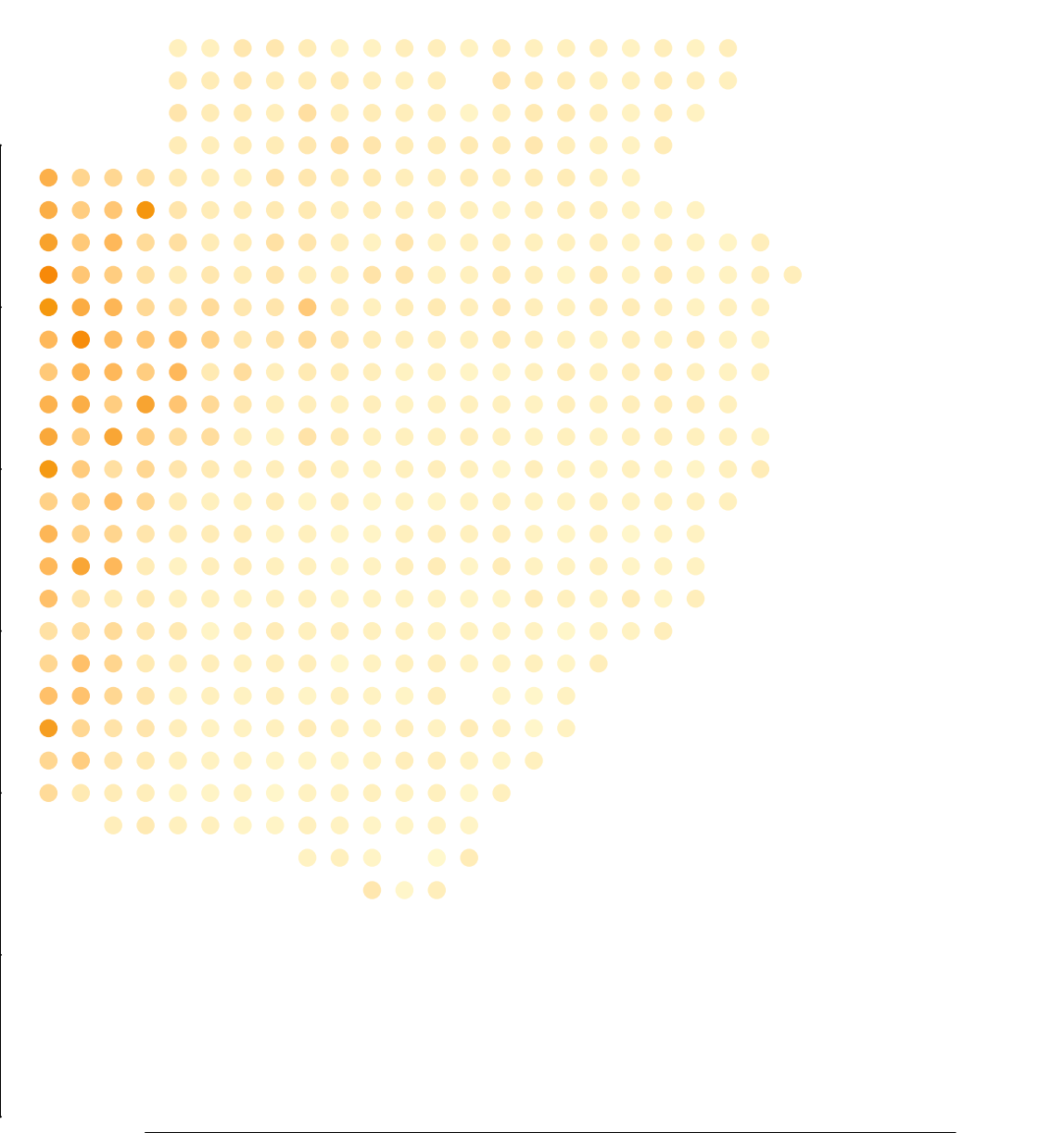

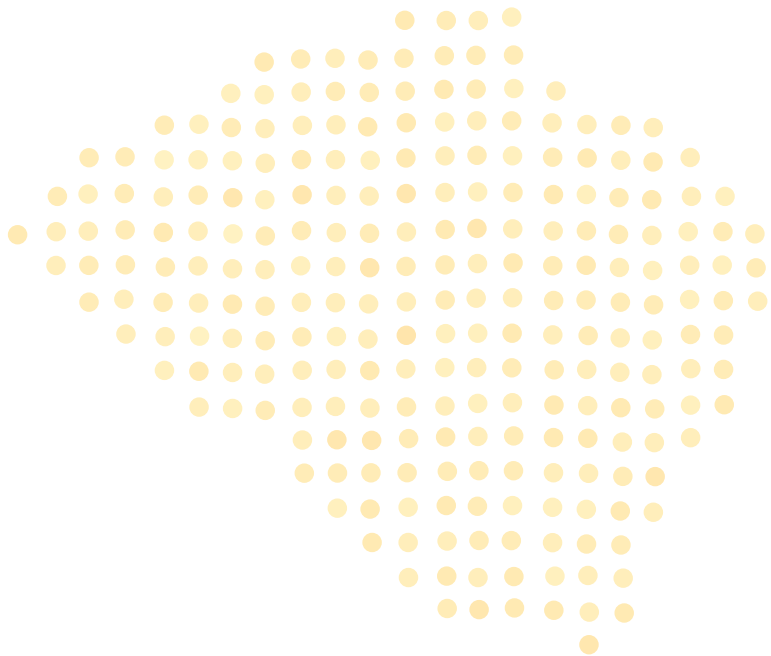

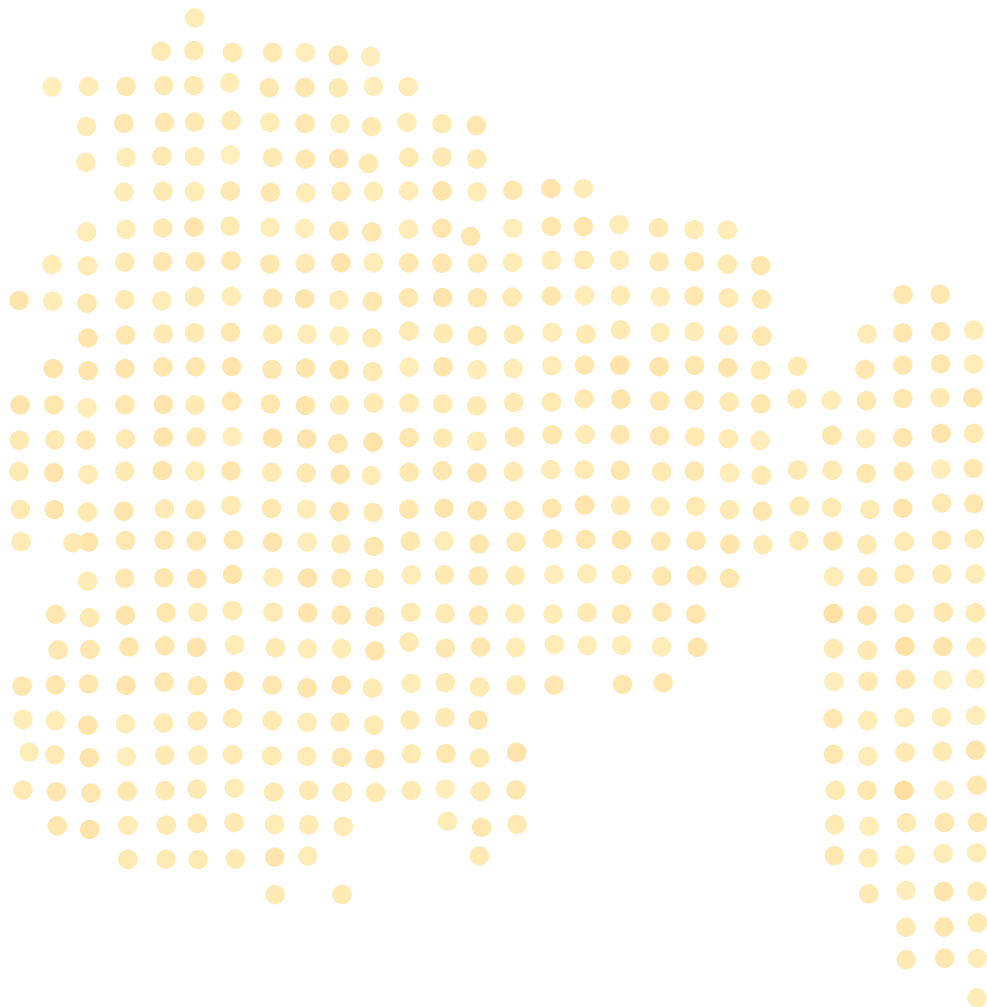

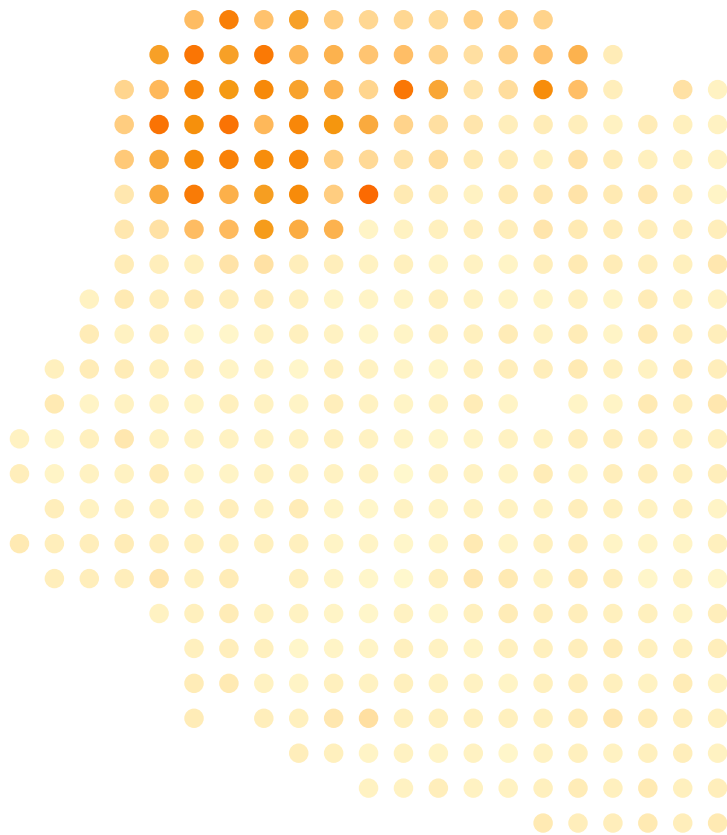

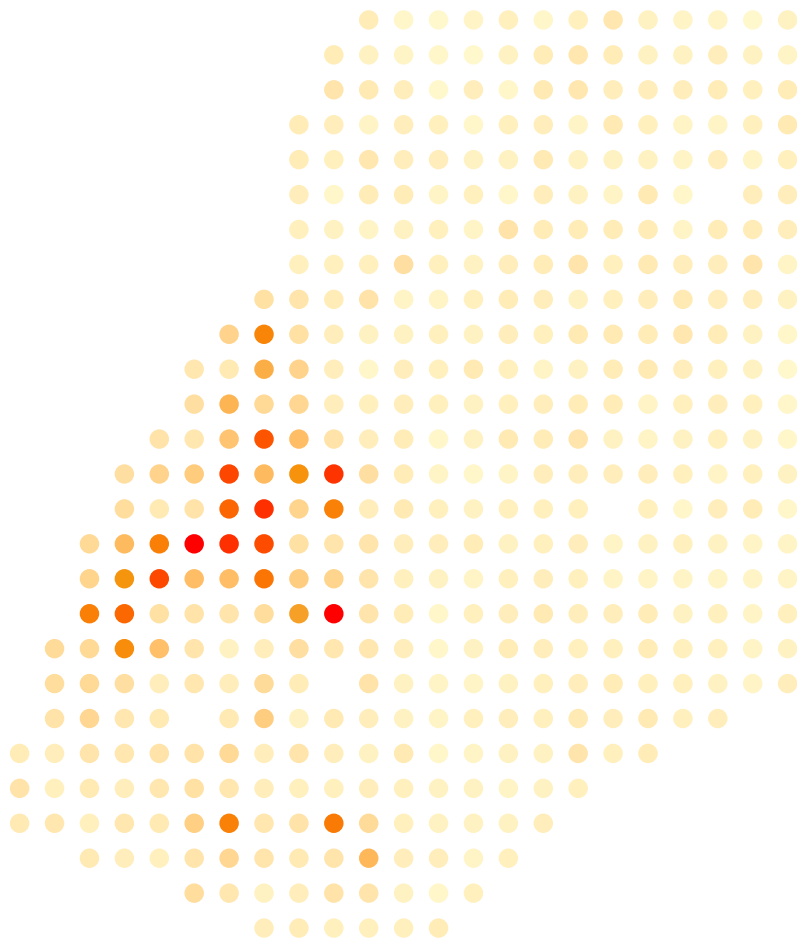

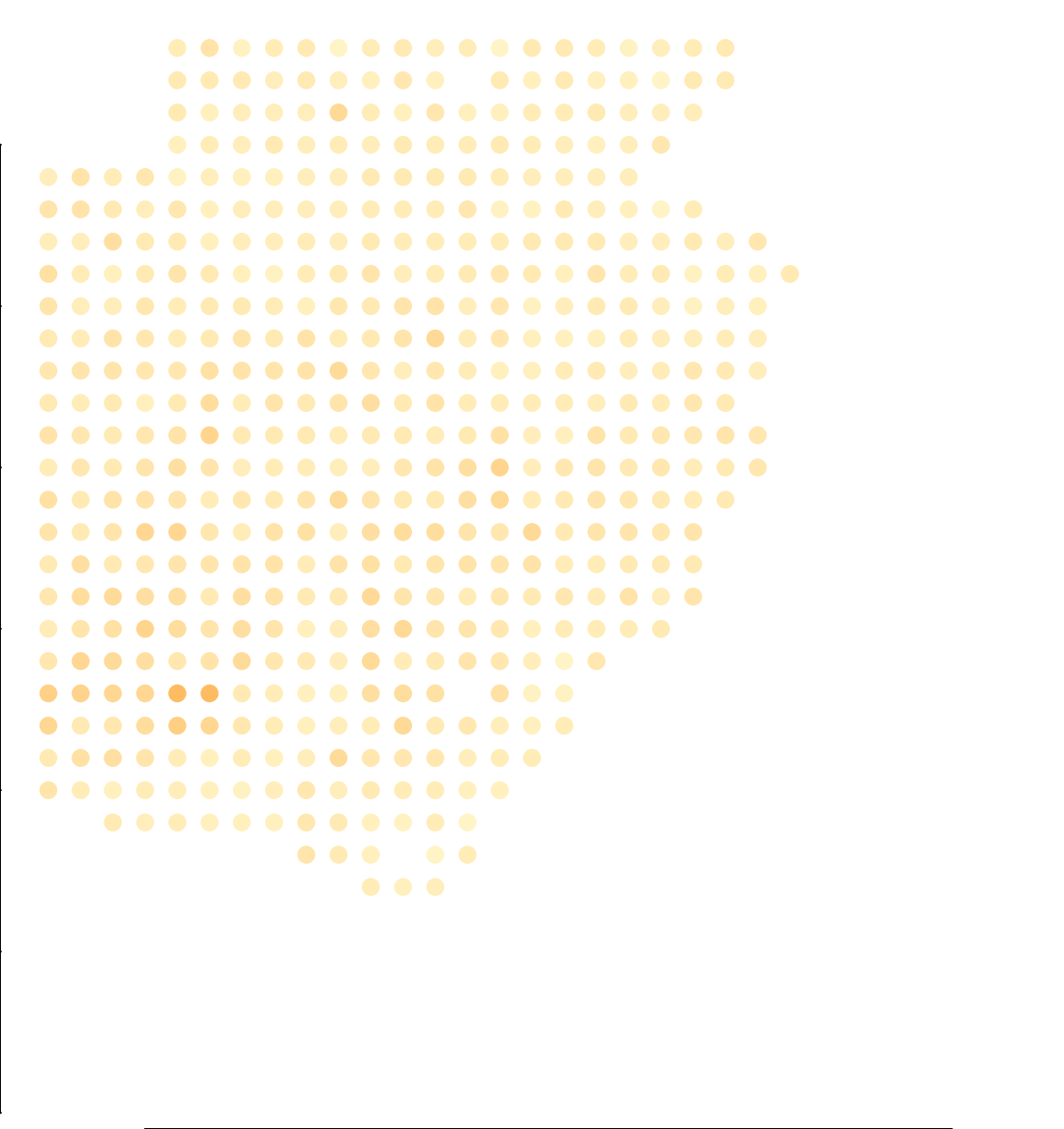

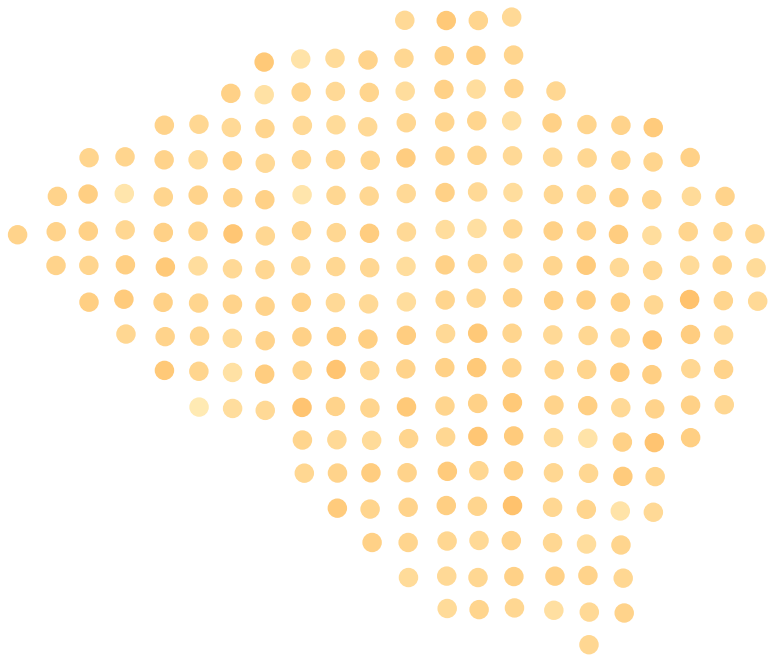

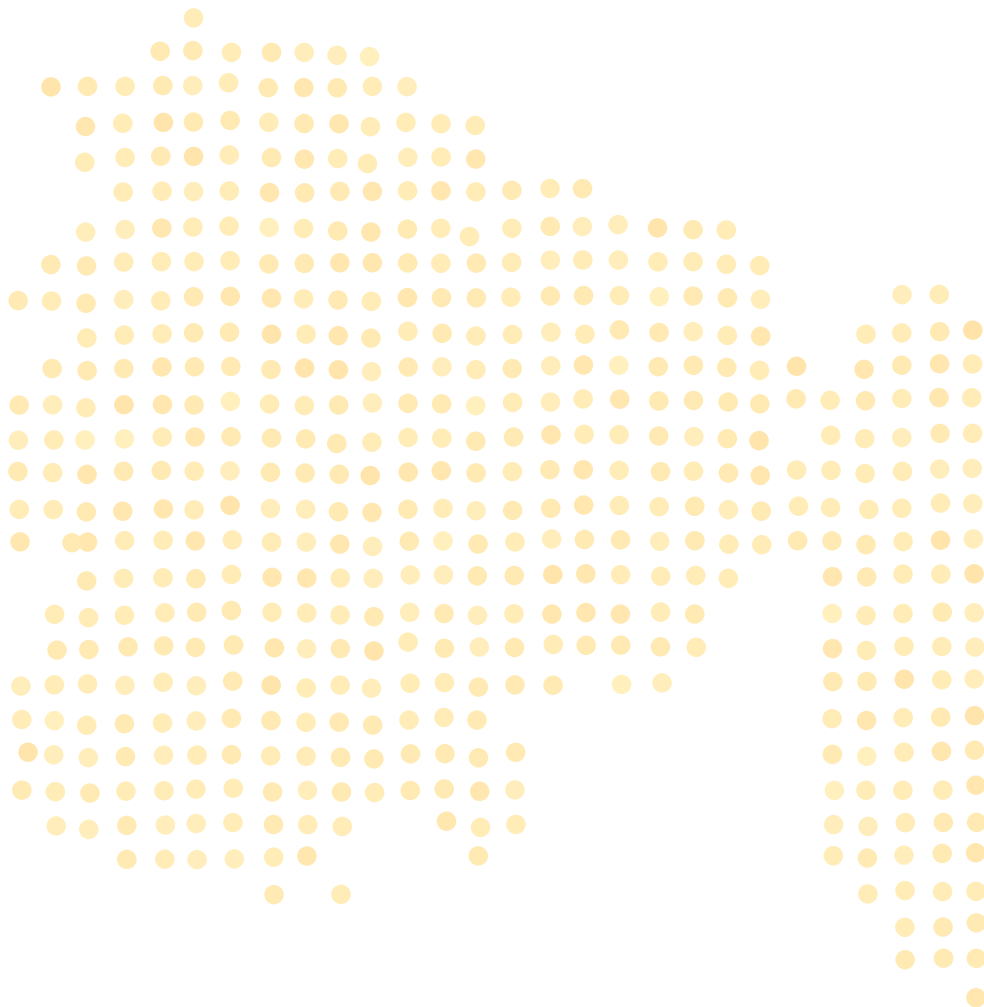

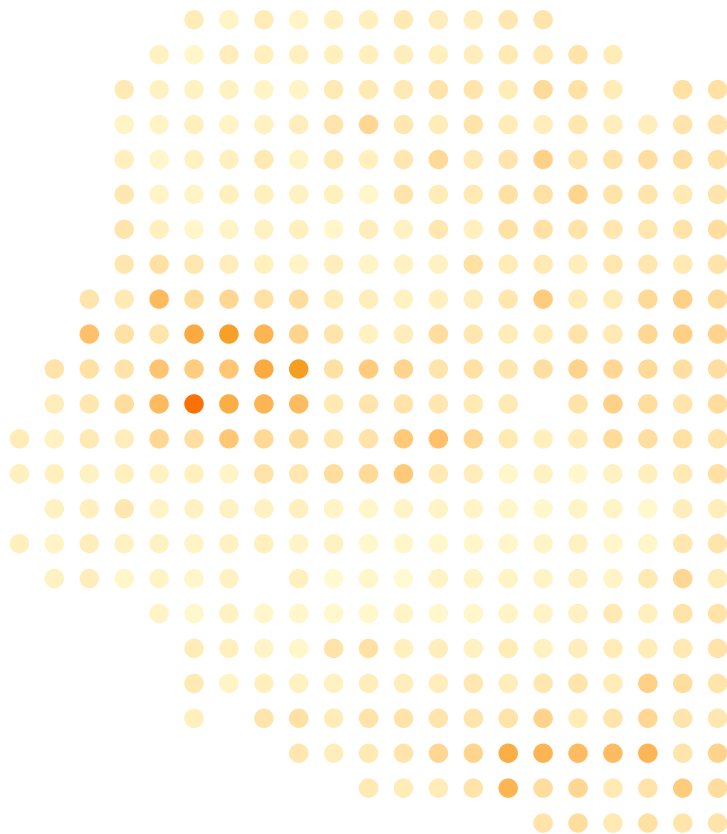

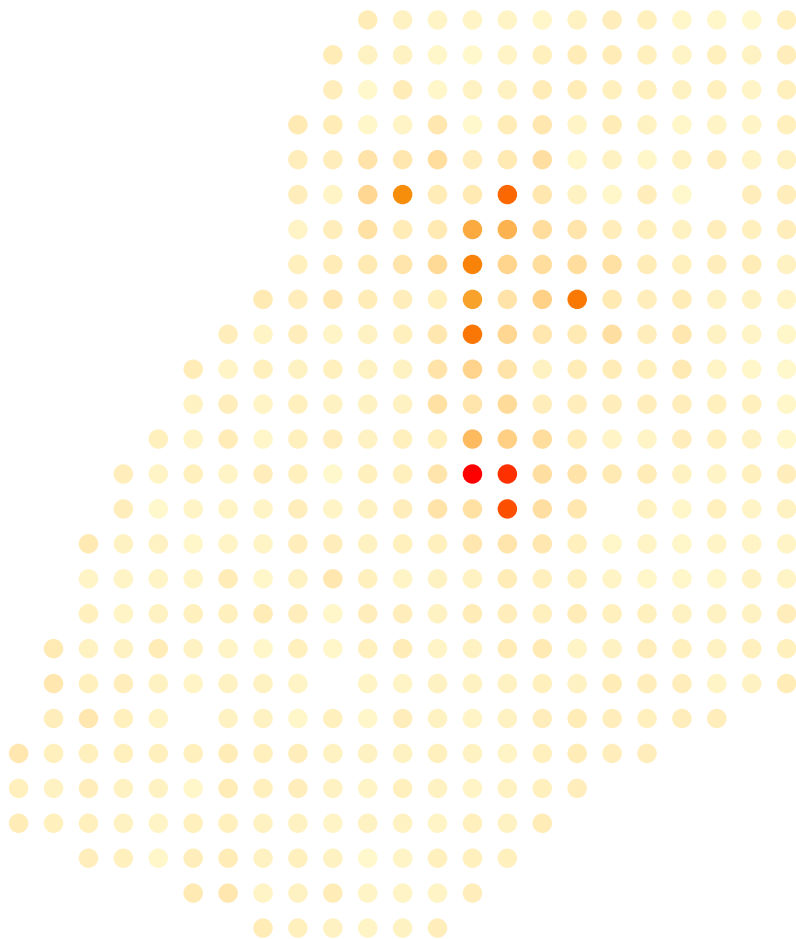

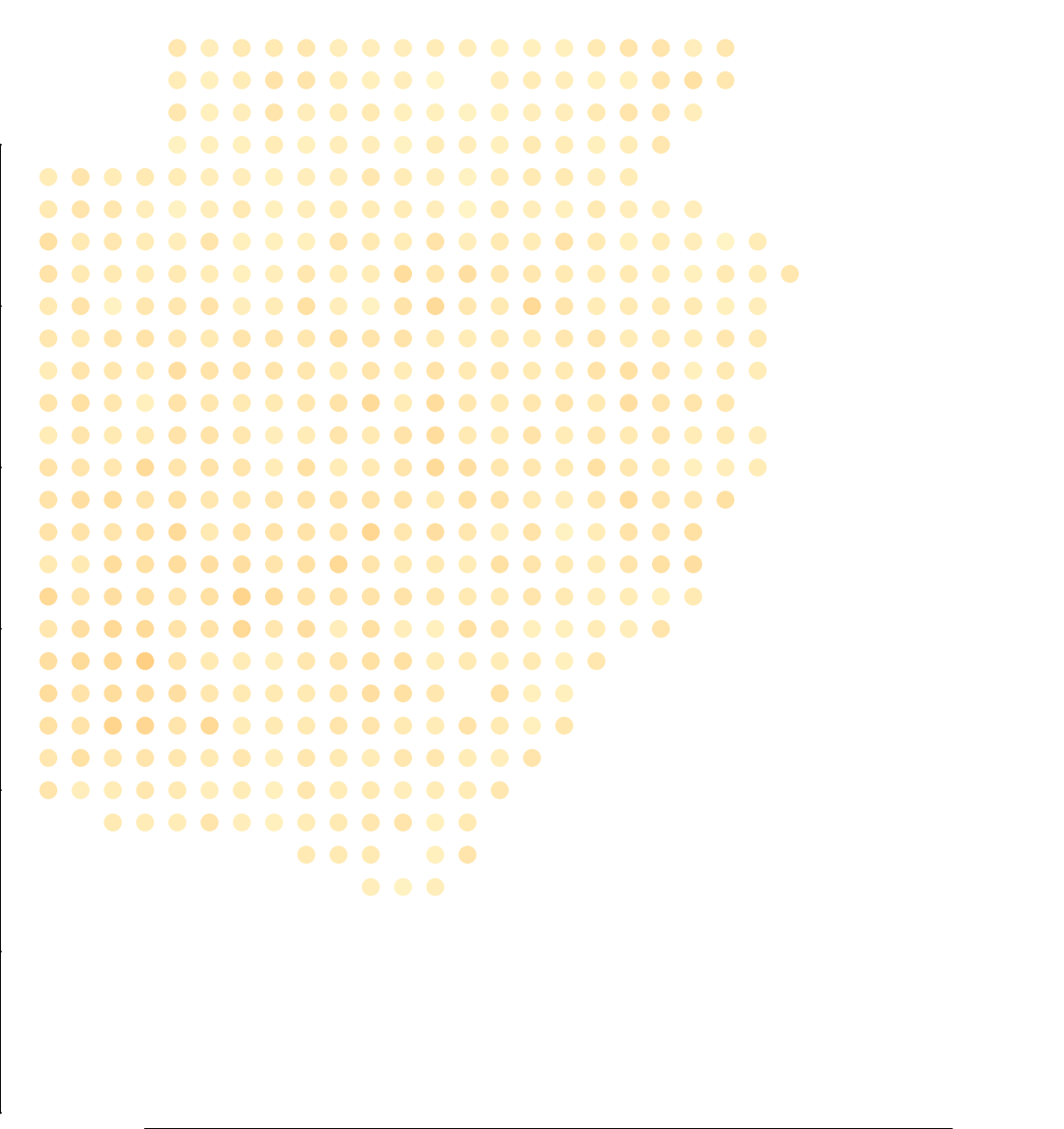

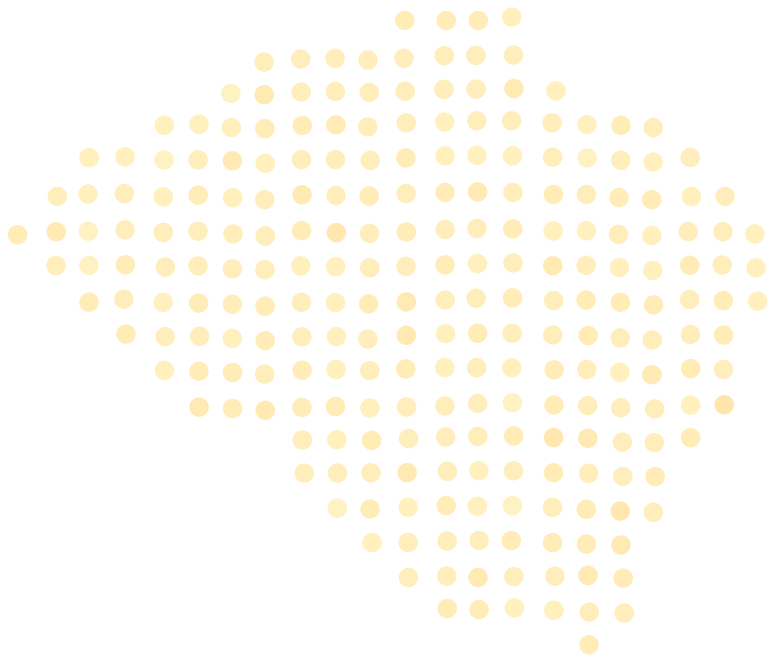

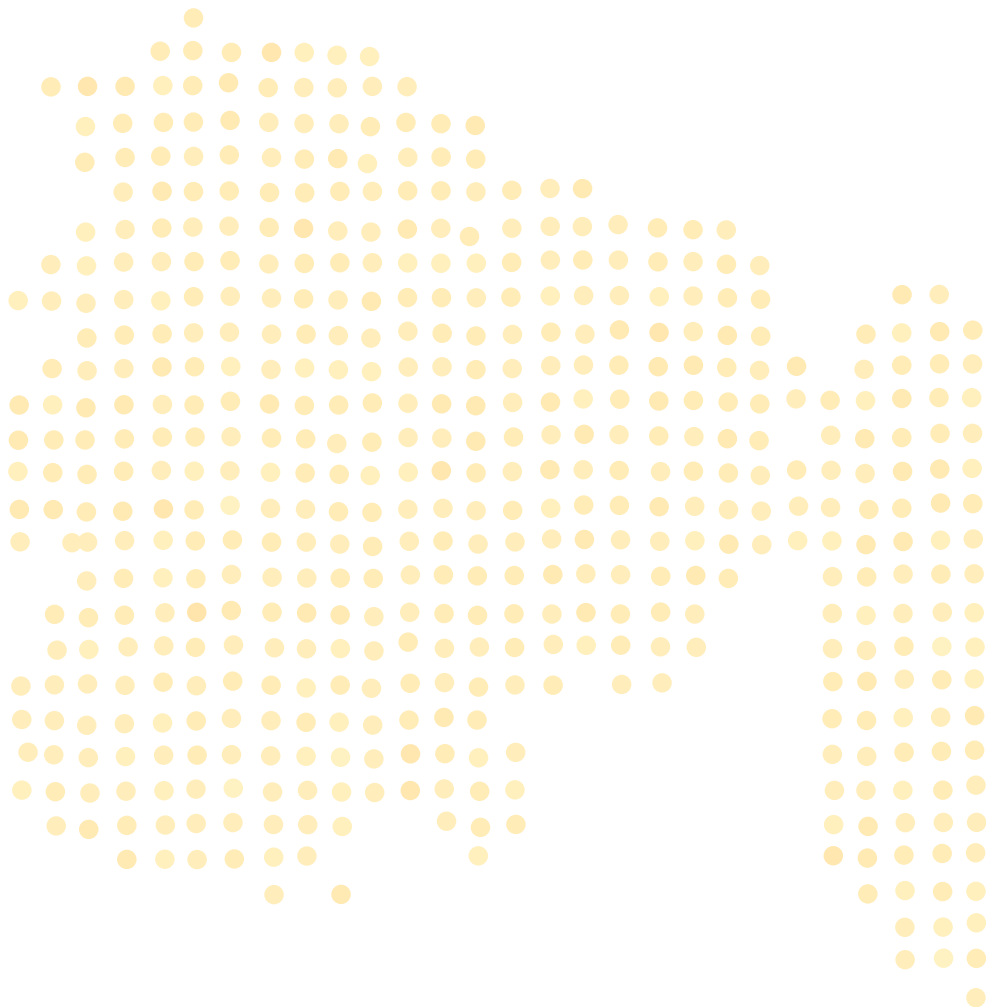

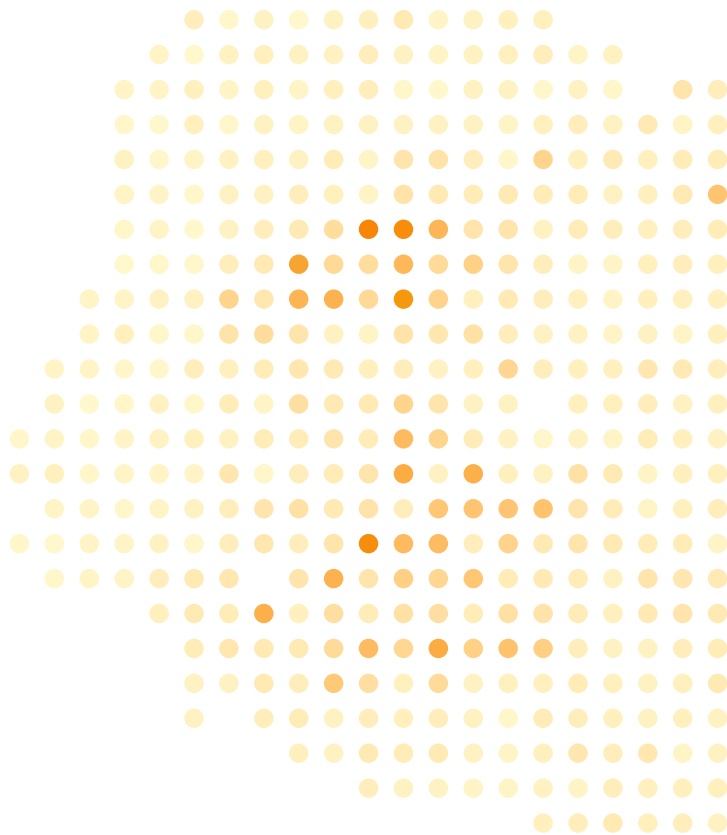





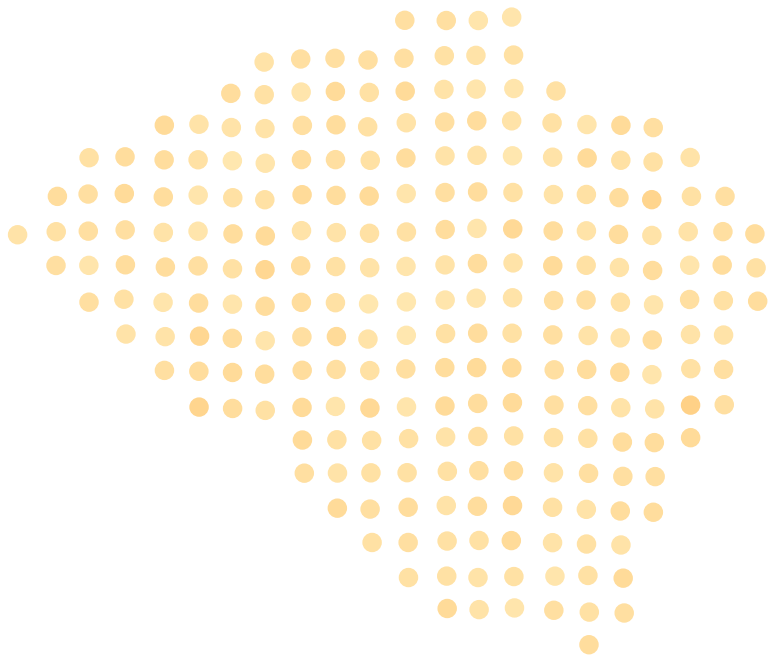

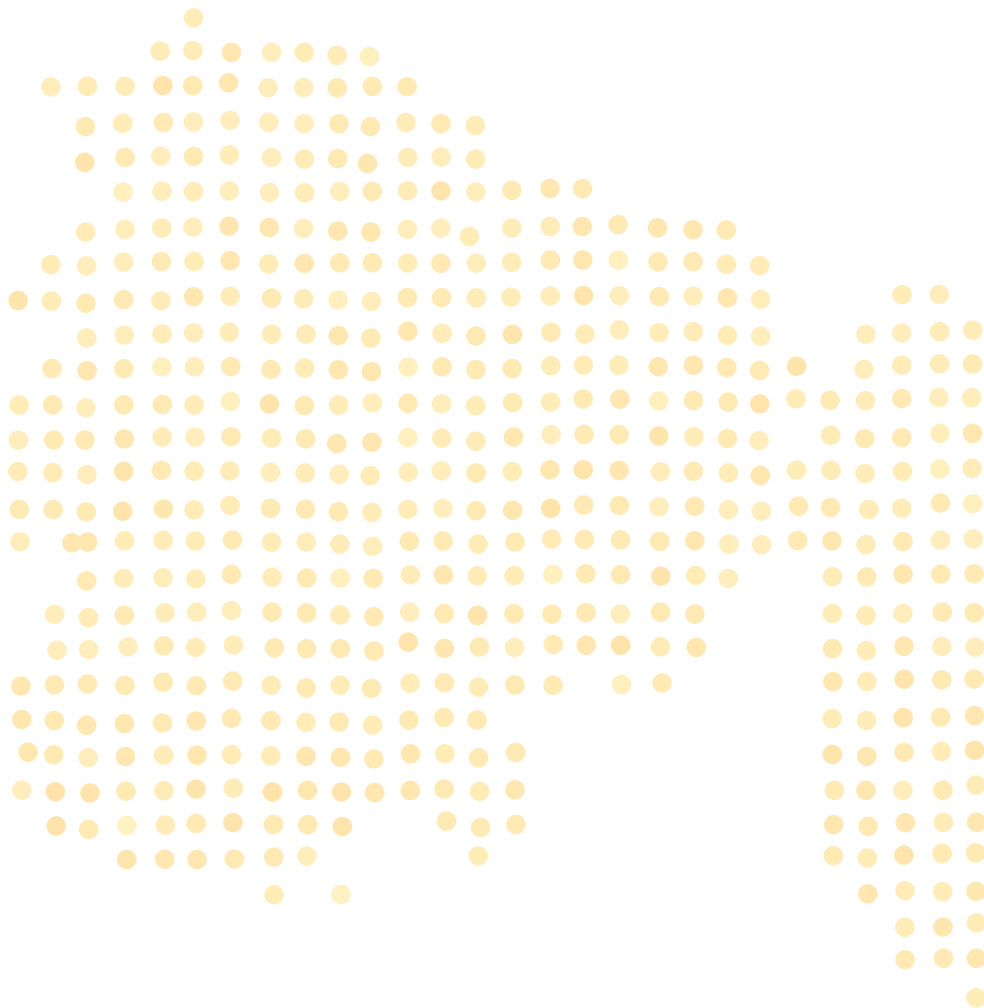

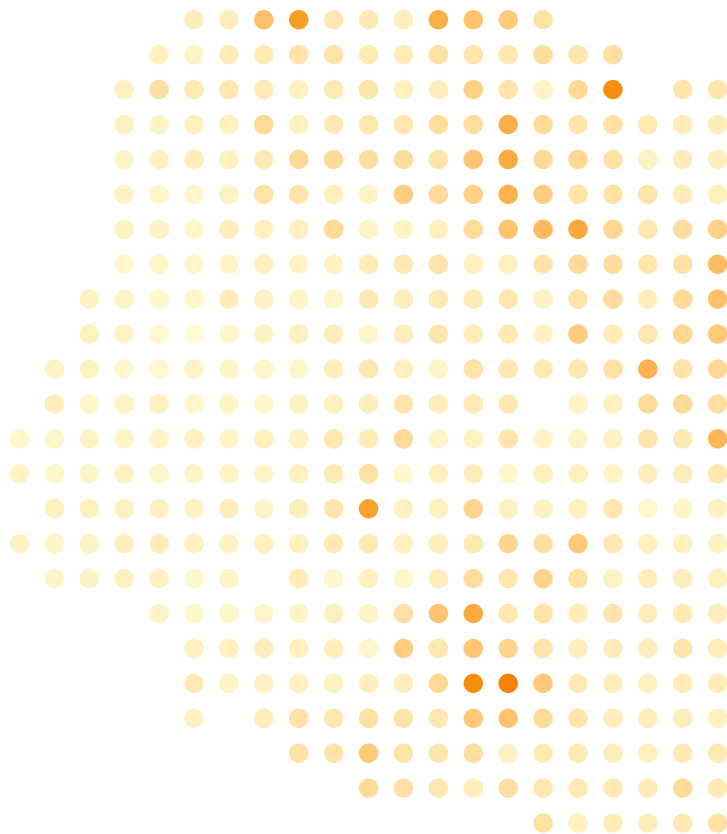

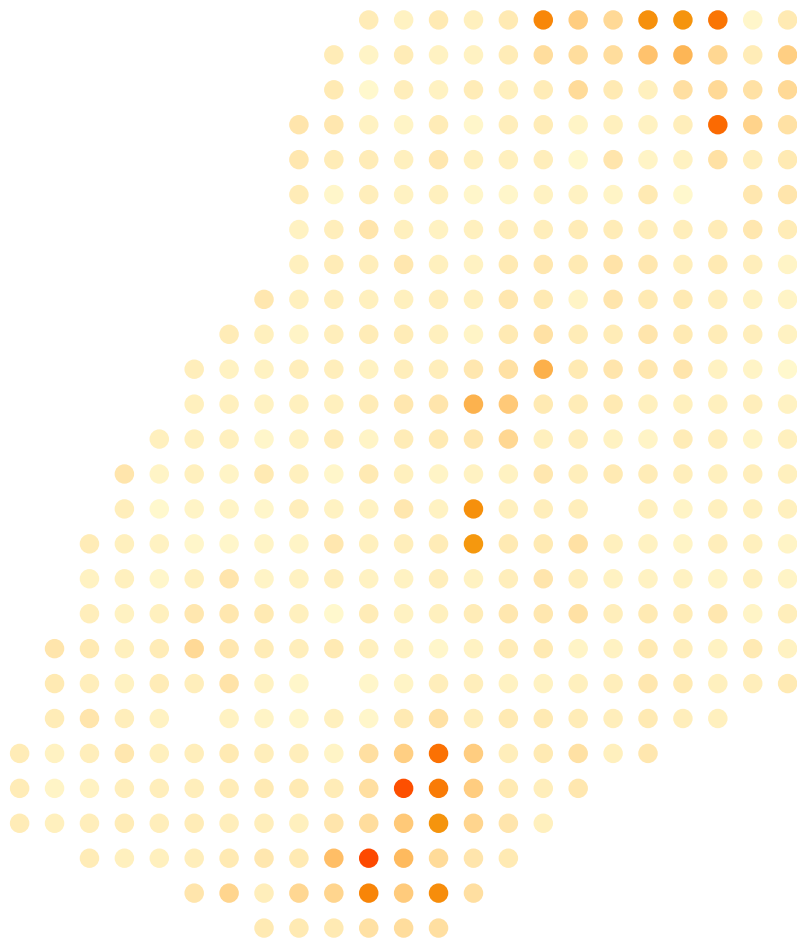

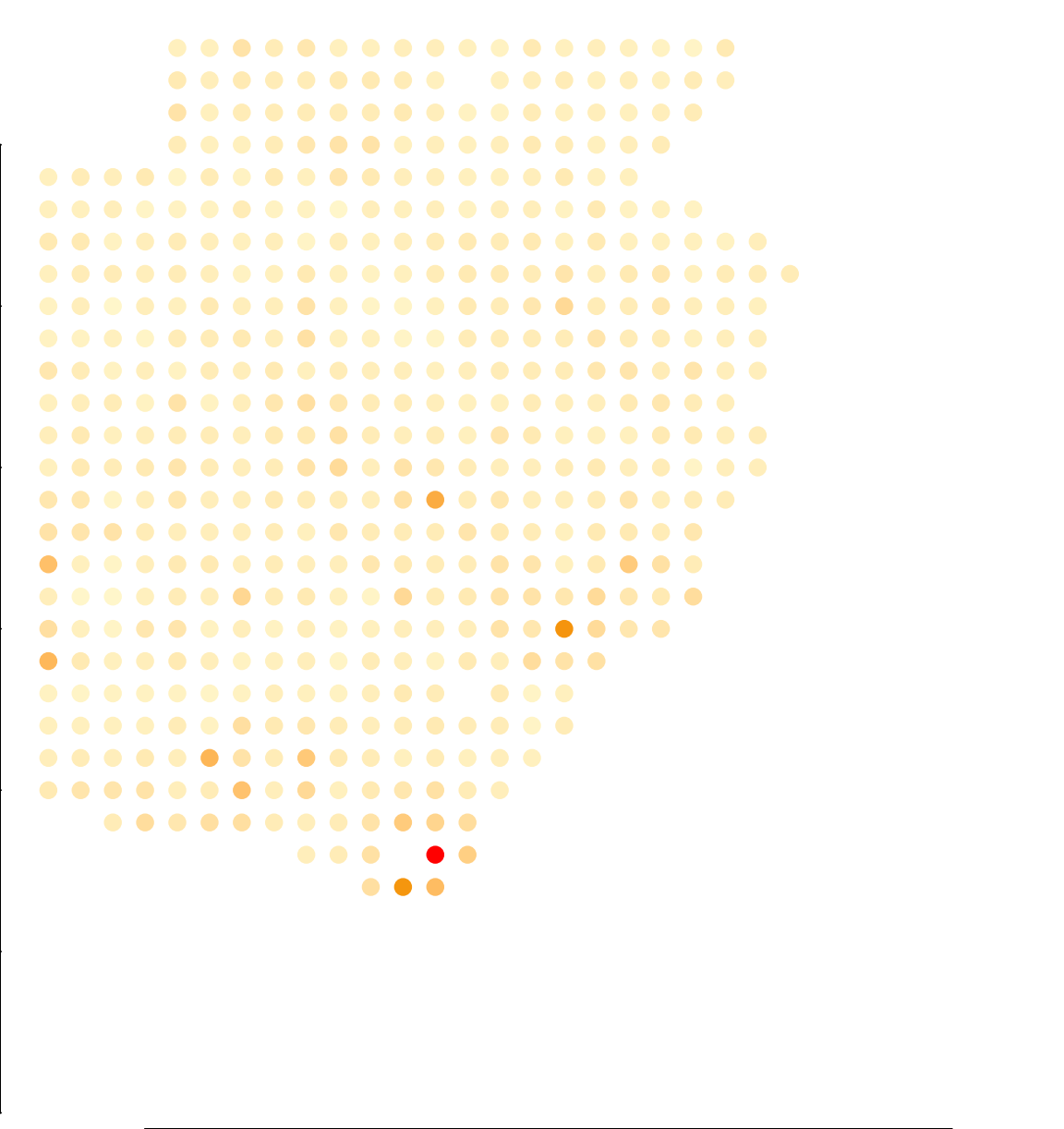

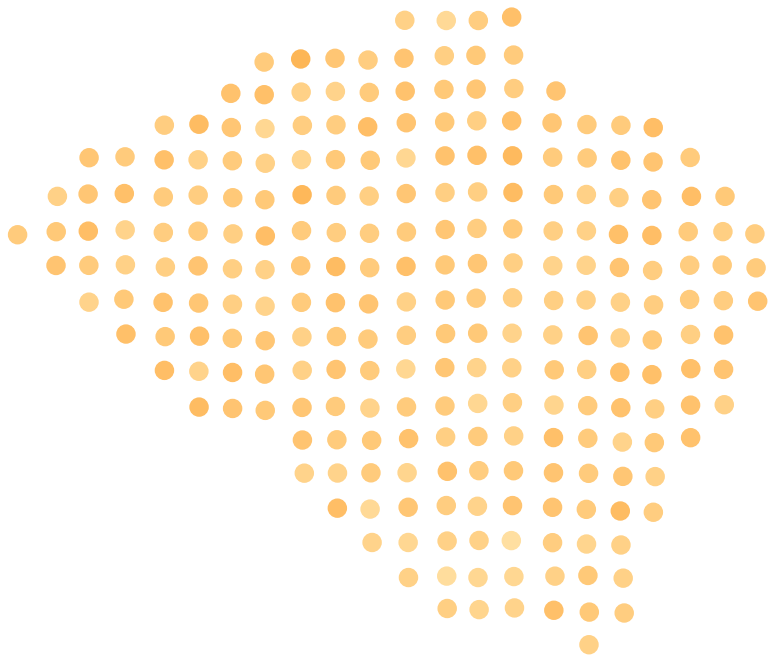

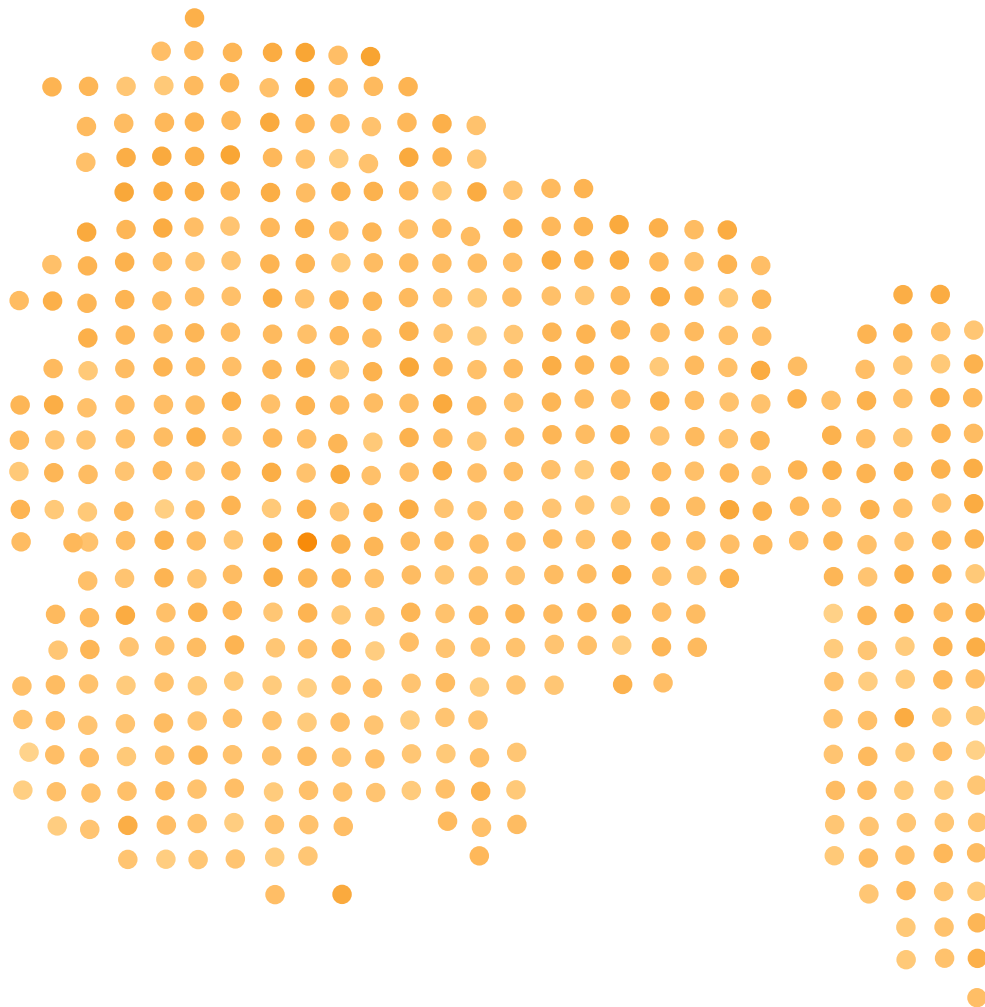

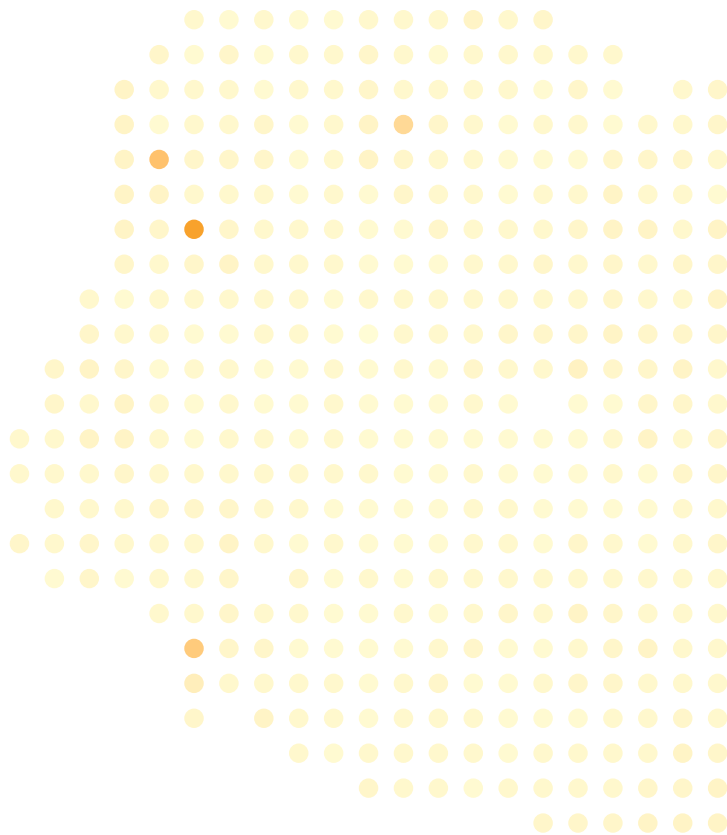

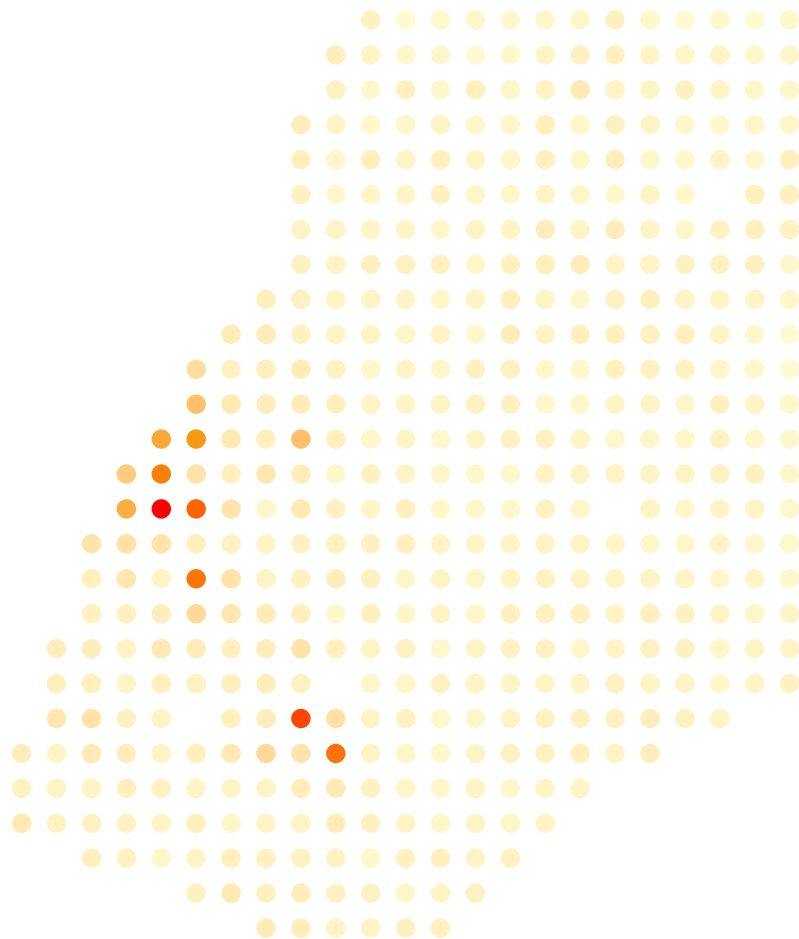

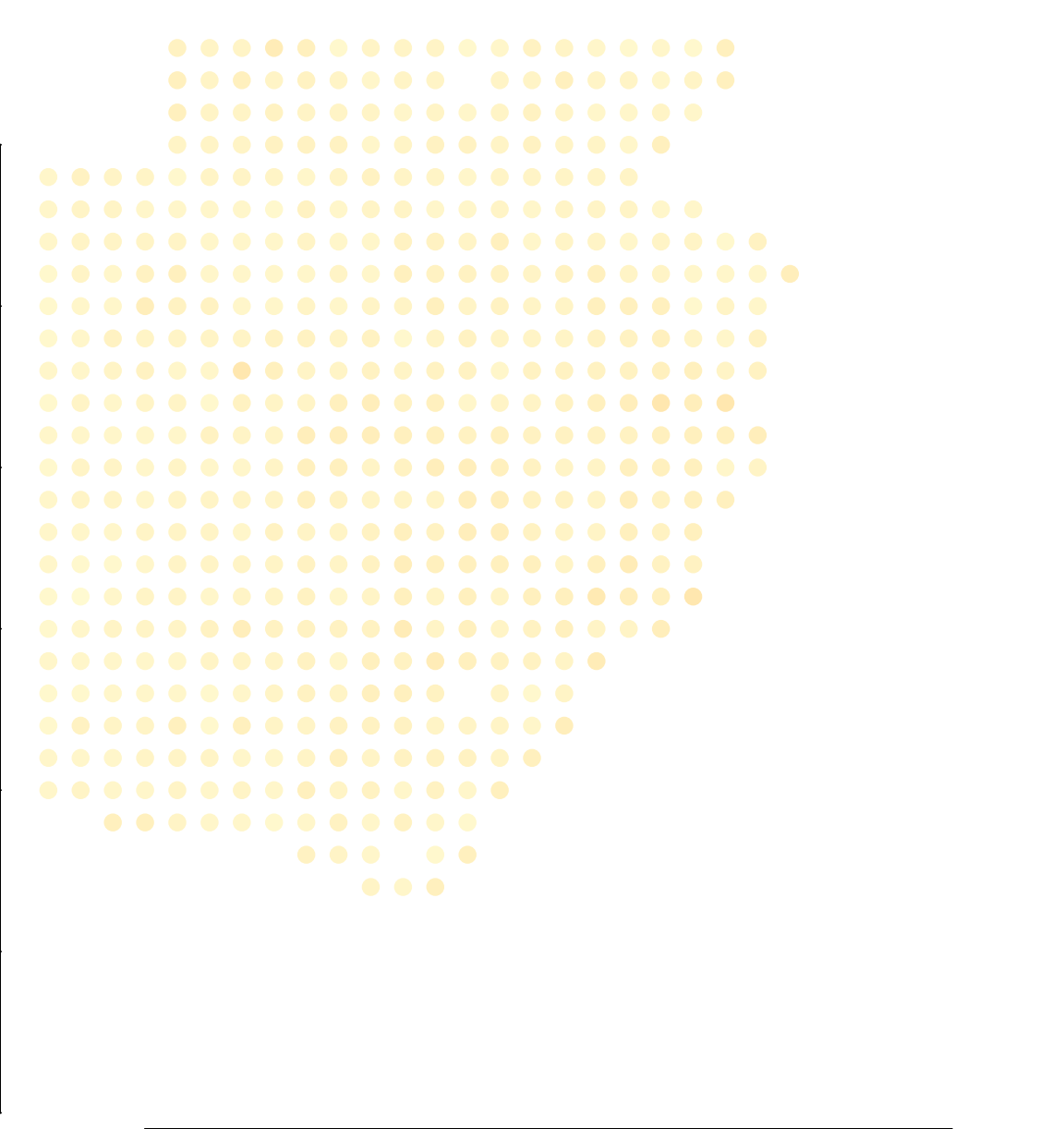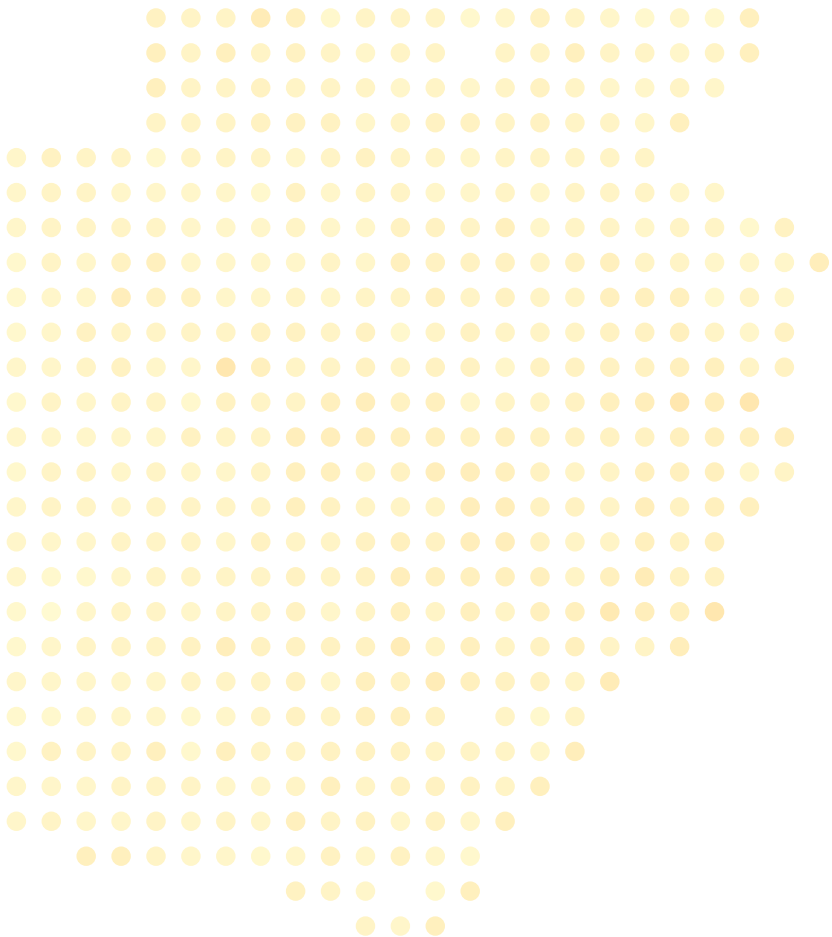

Supplement: Supplementary file 8 — Supplementary Data 5 [file 41467_2018_4724_MOESM8_ESM.zip › Supplementary Dataset 7/joint-mix-profiles-rel-common-scale-dots-split.pdf]

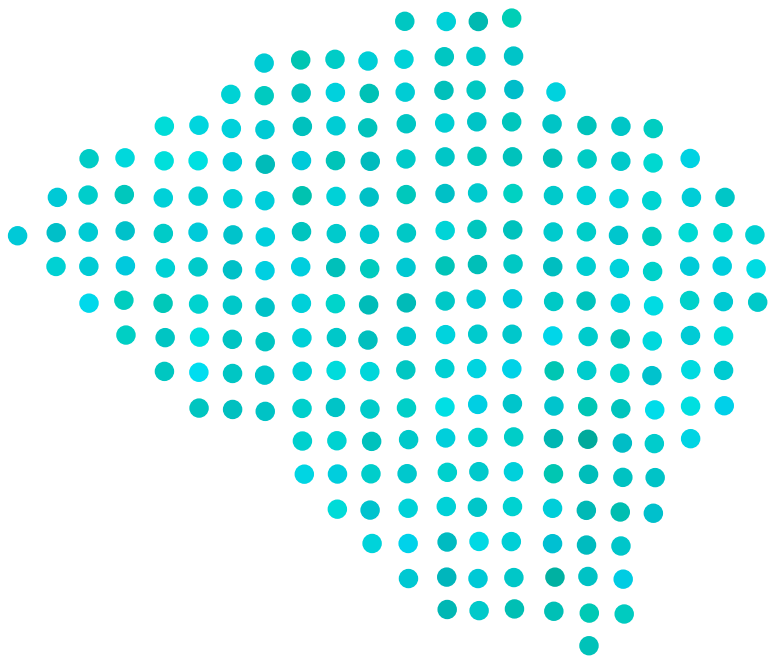

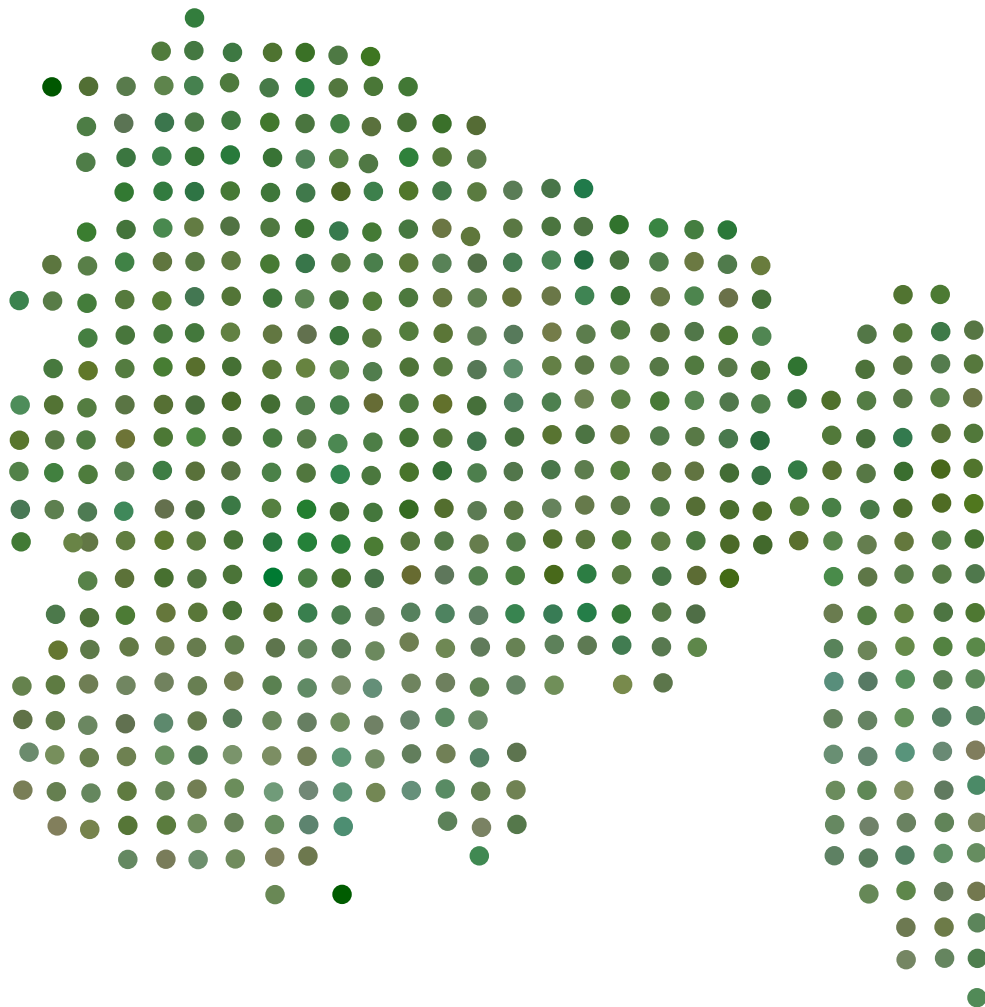

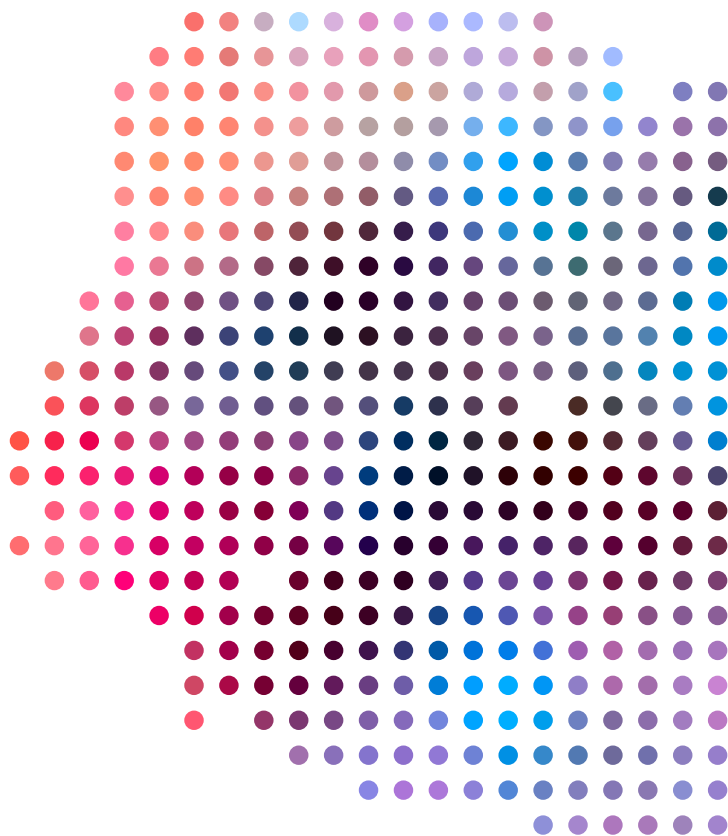

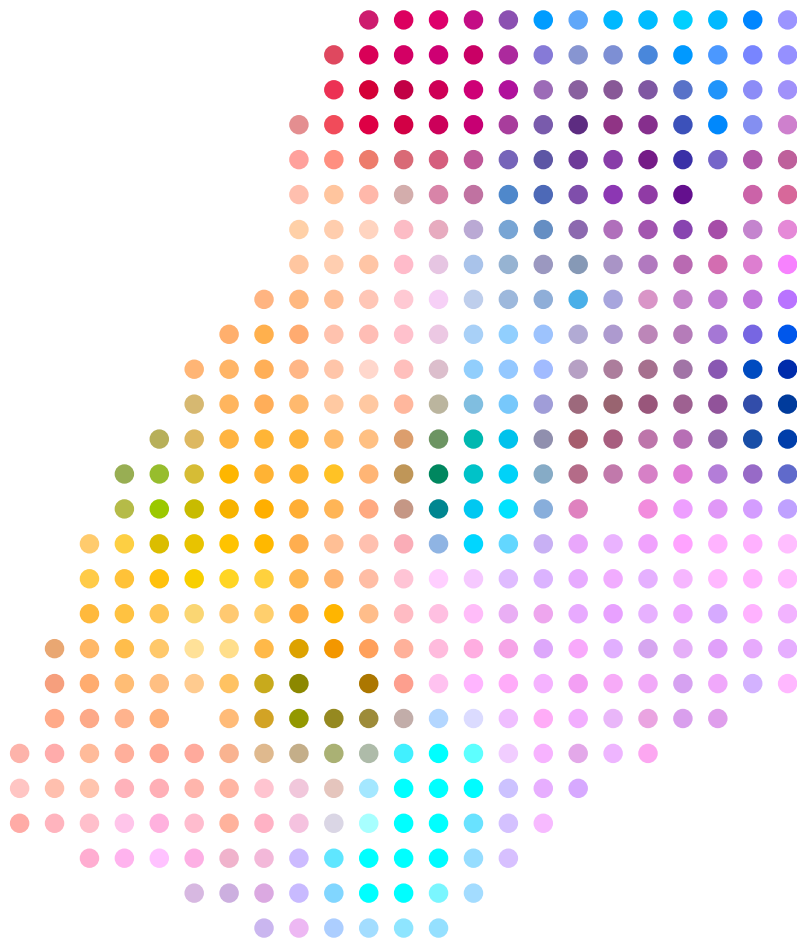

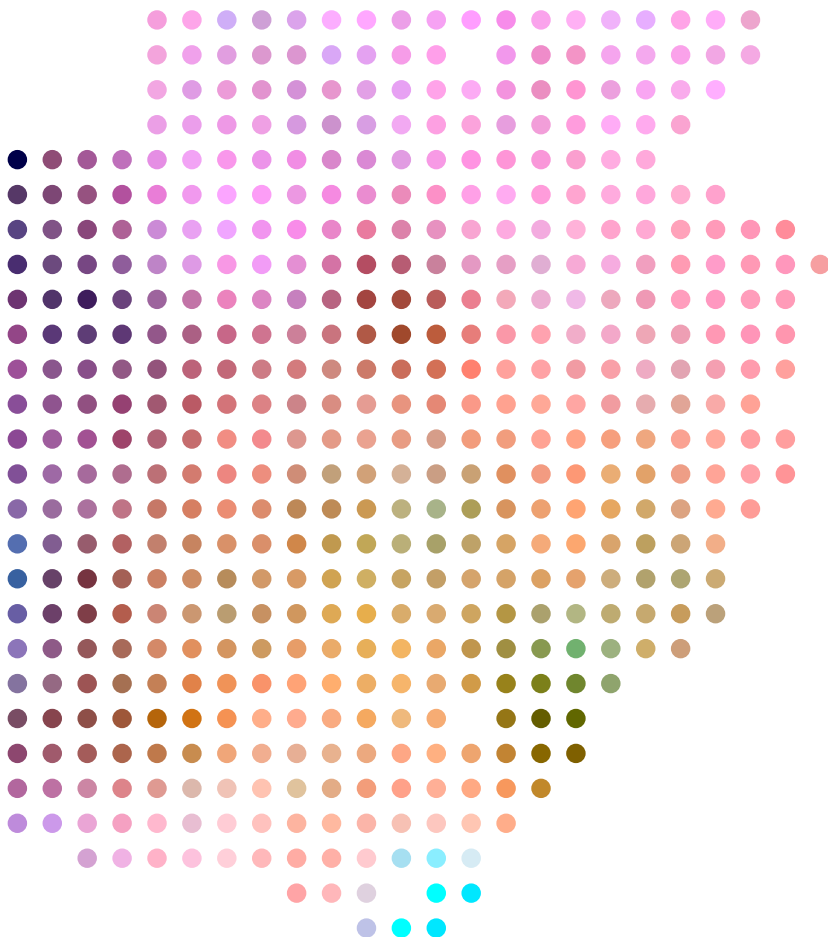

Supplement: Supplementary file 8 — Supplementary Data 5 [file 41467_2018_4724_MOESM8_ESM.zip › Supplementary Dataset 7/joint-field-dimensionality-reduction-PCA-dots-split.pdf]

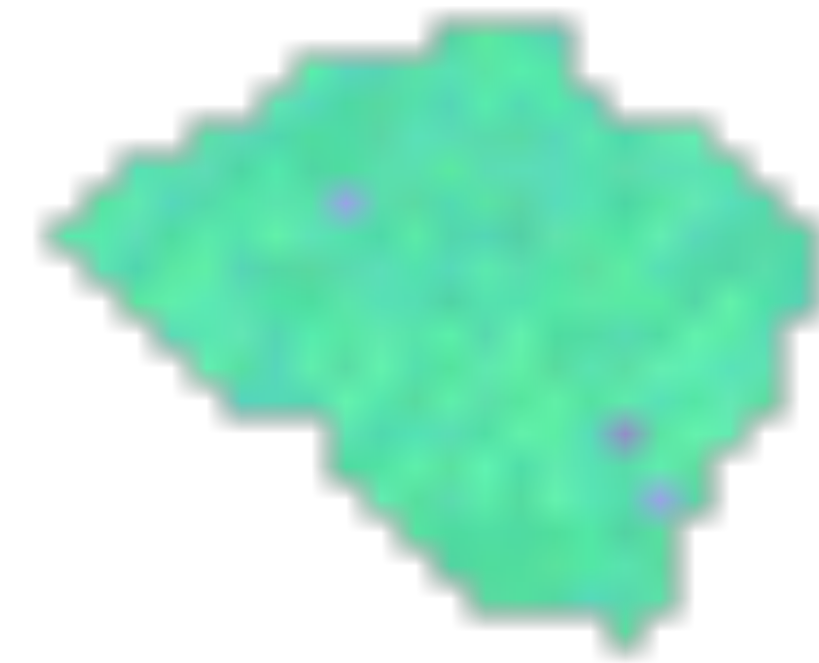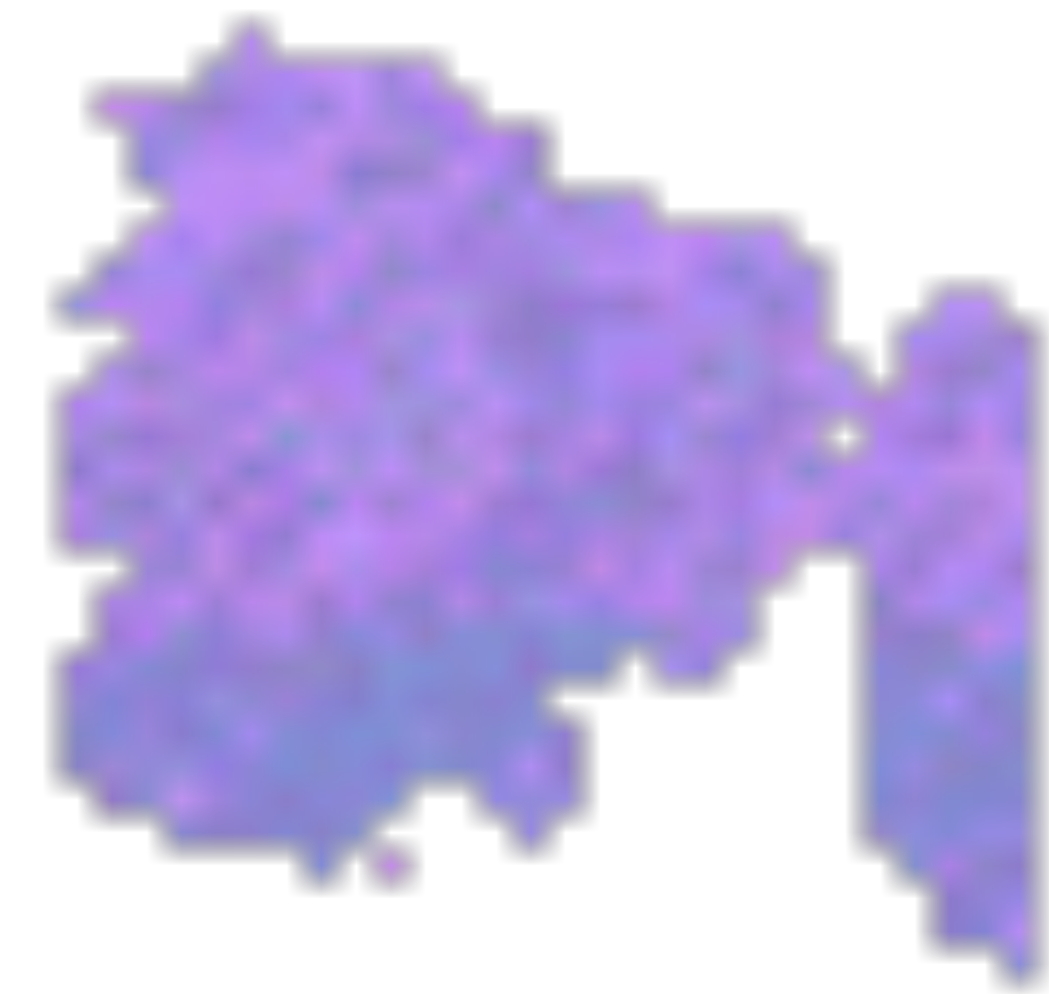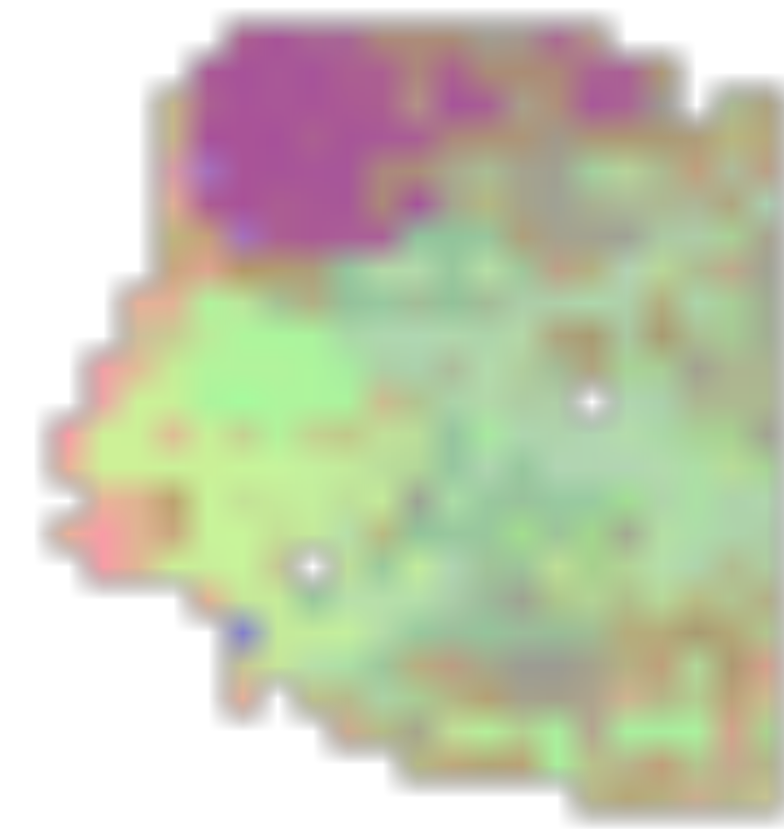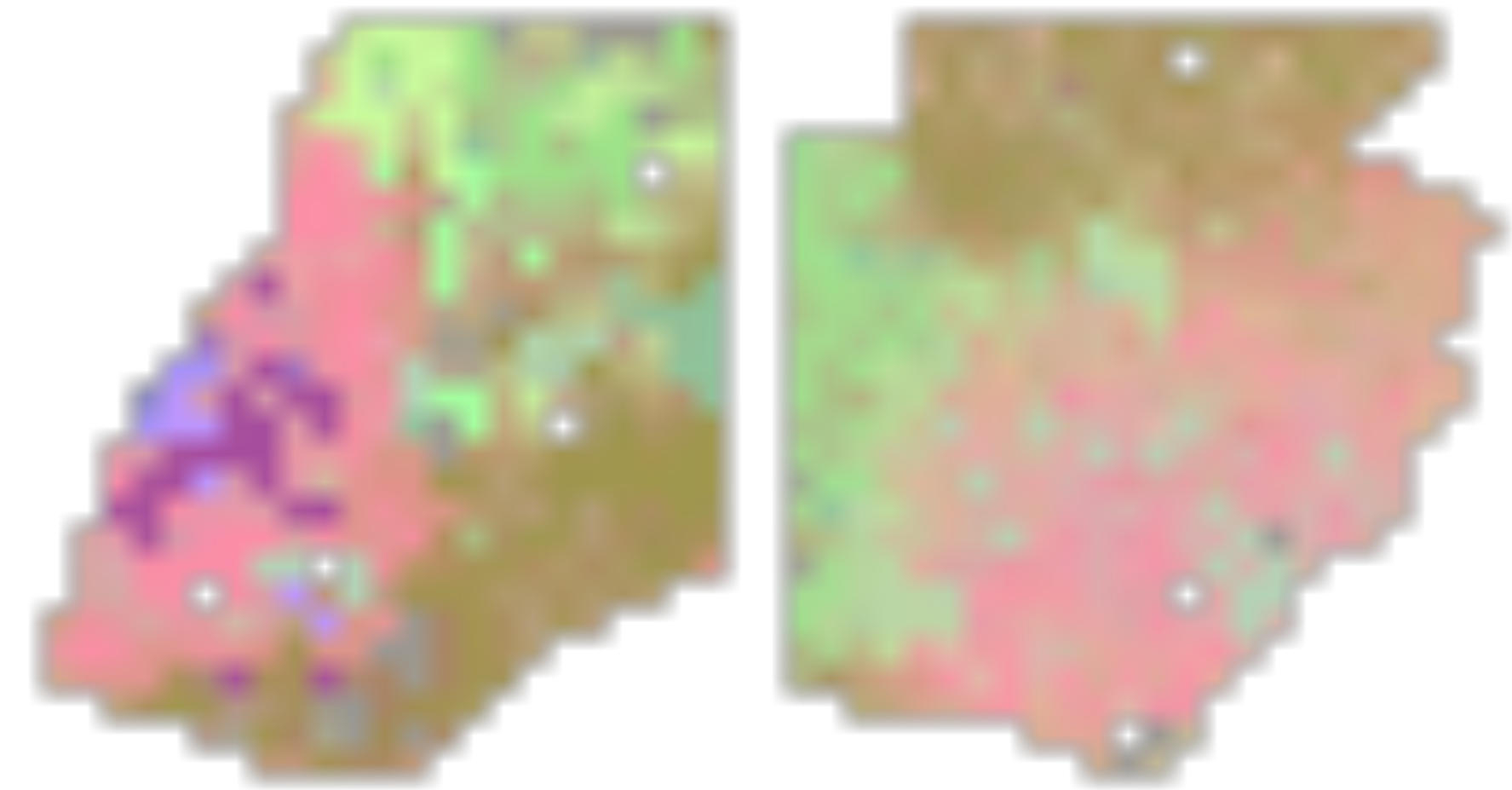

Supplement: Supplementary file 8 — Supplementary Data 5 [file 41467_2018_4724_MOESM8_ESM.zip › Supplementary Dataset 7/joint-mix-dimensionality-reduction-tSNE-matrix-rgb.pdf.interpolated.pdf]

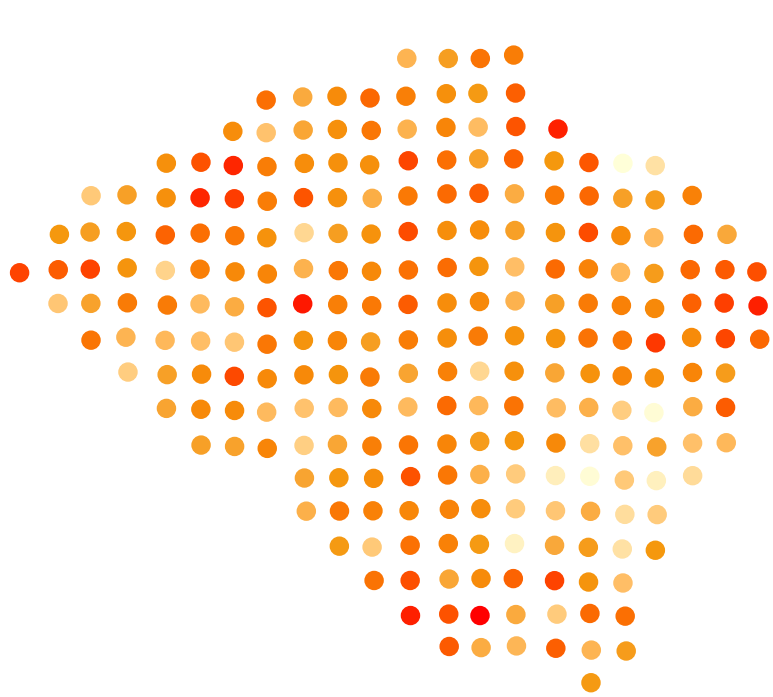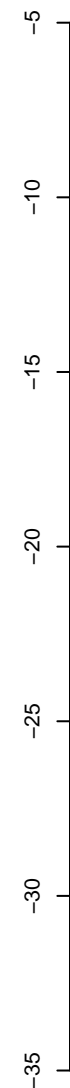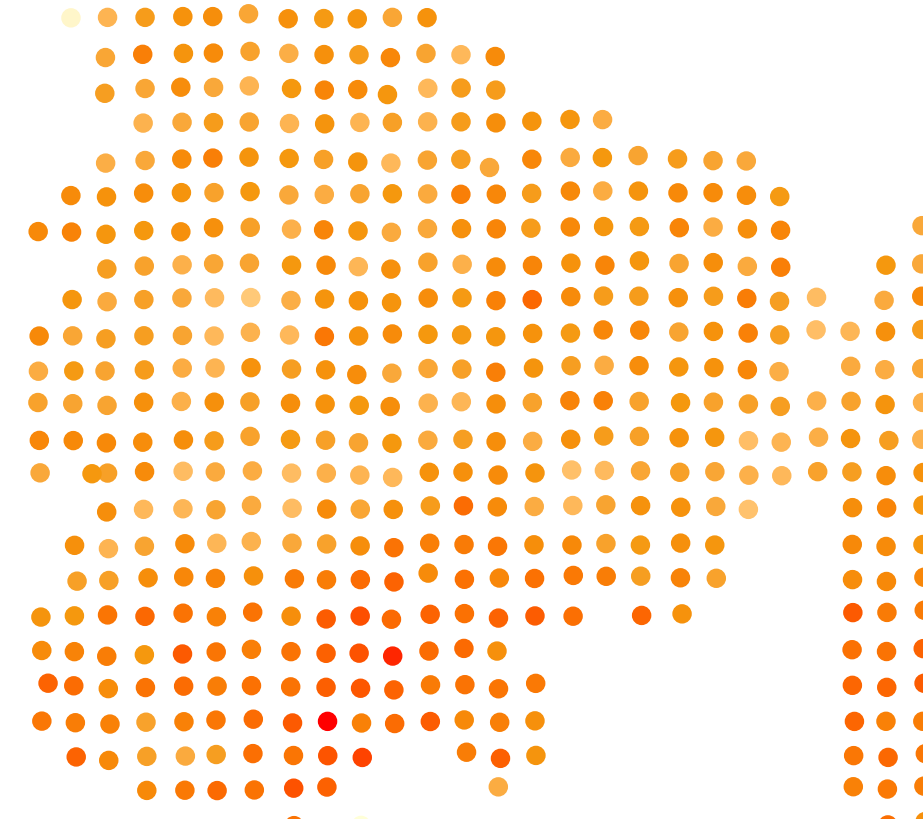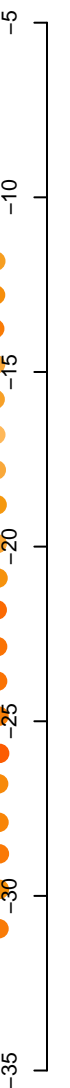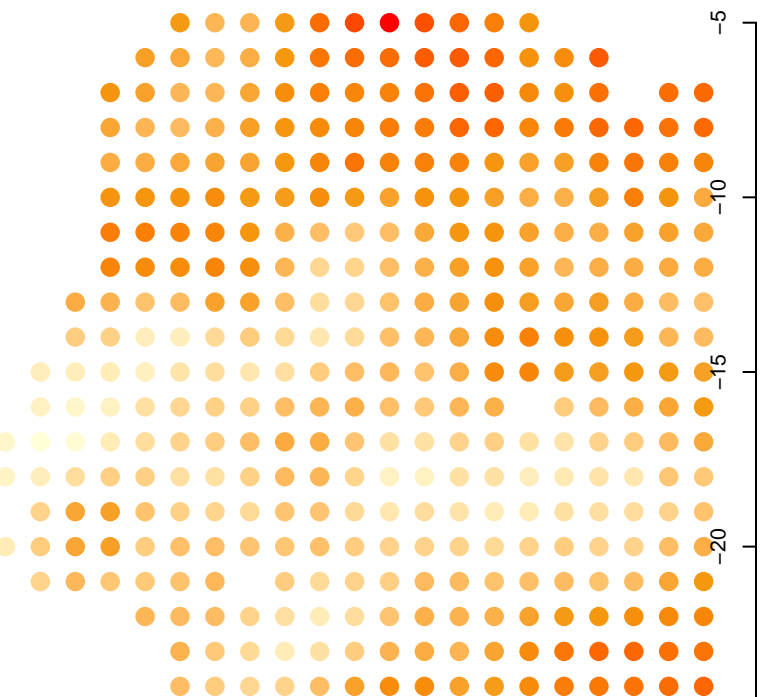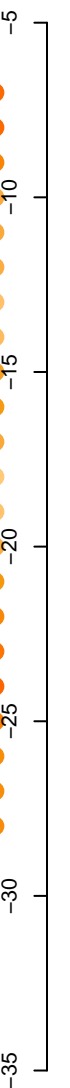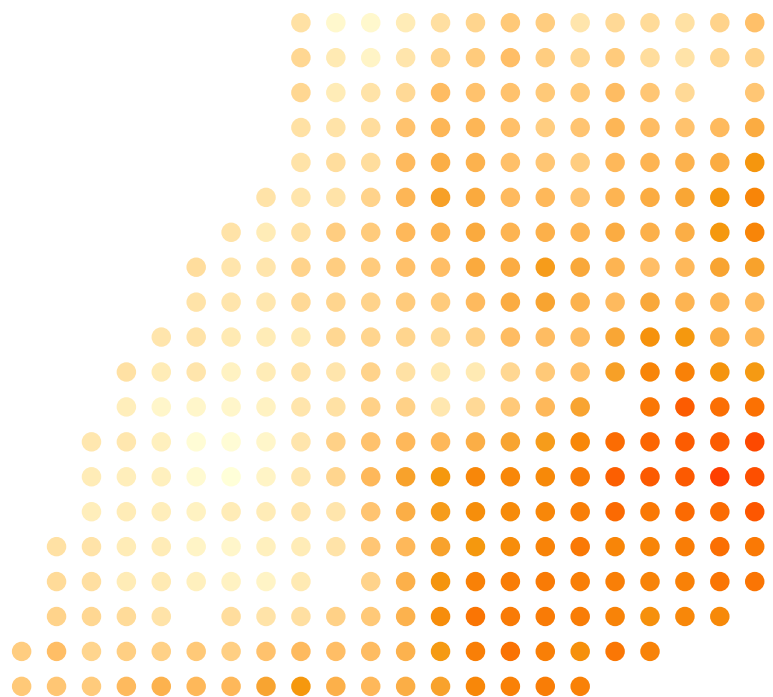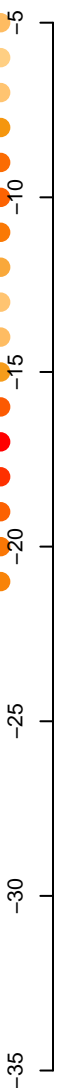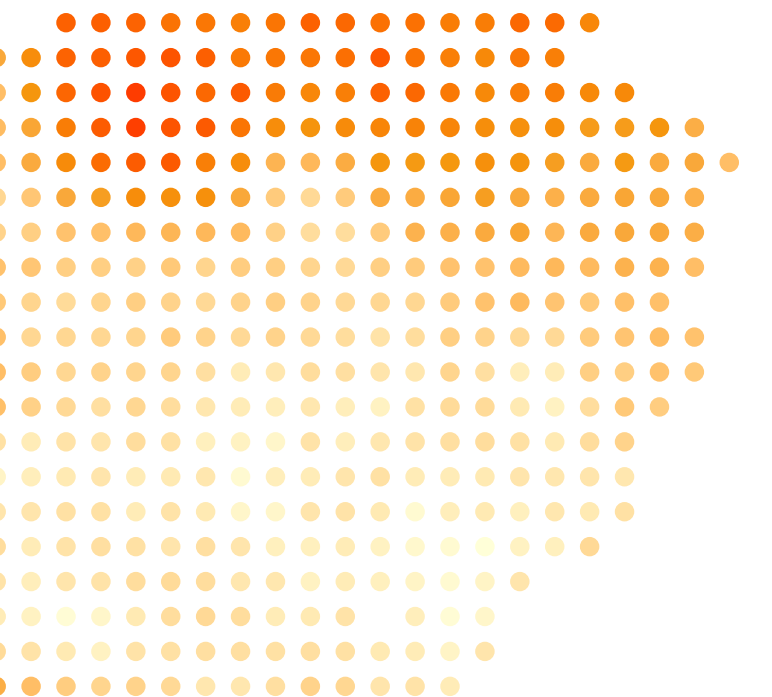

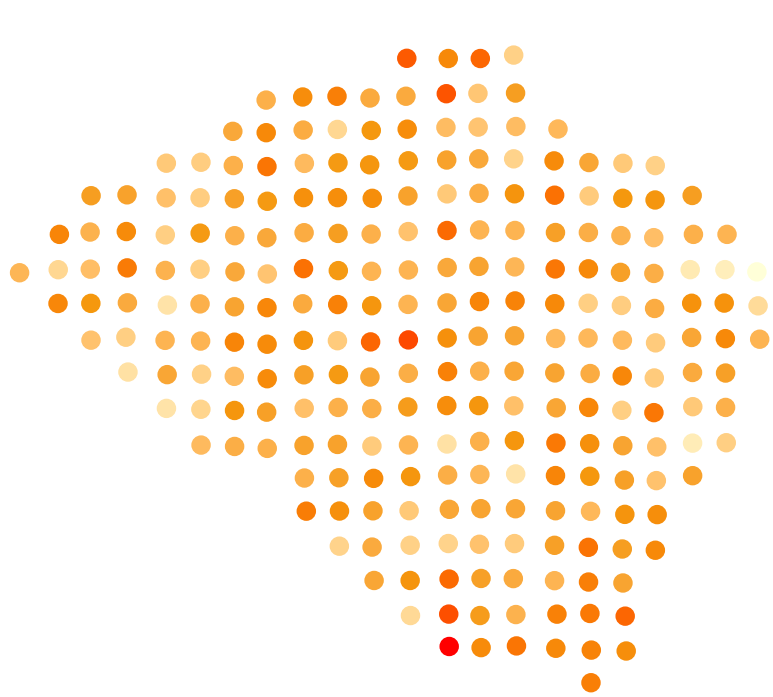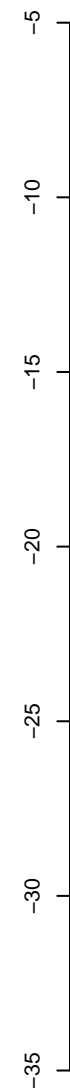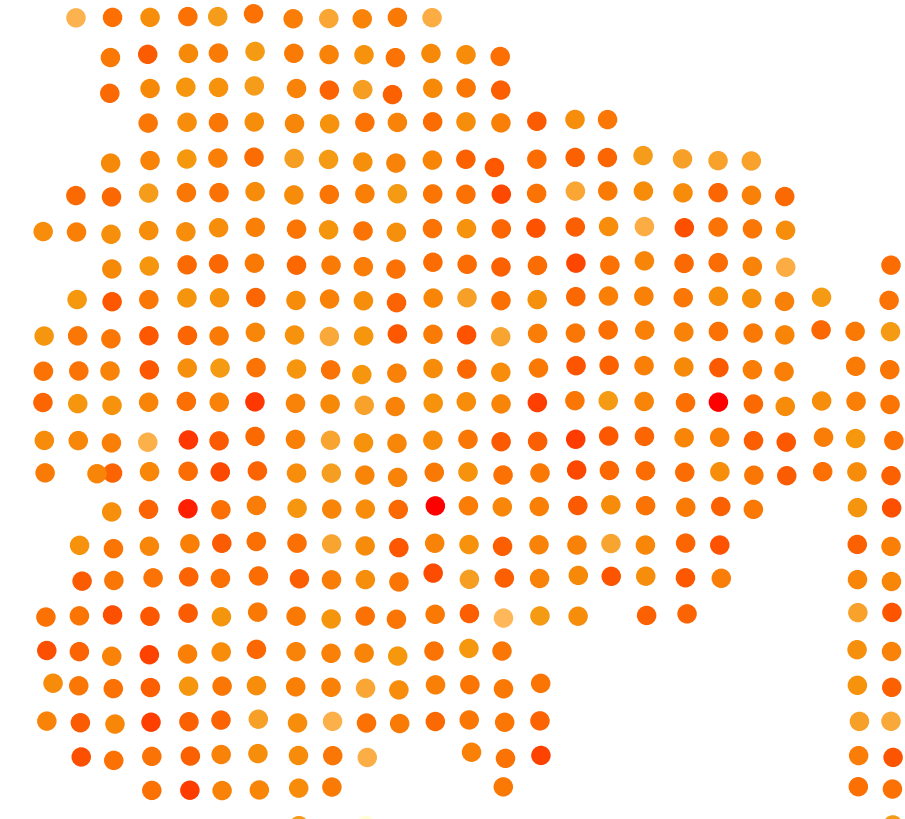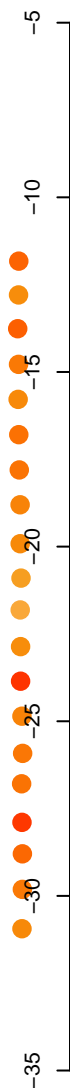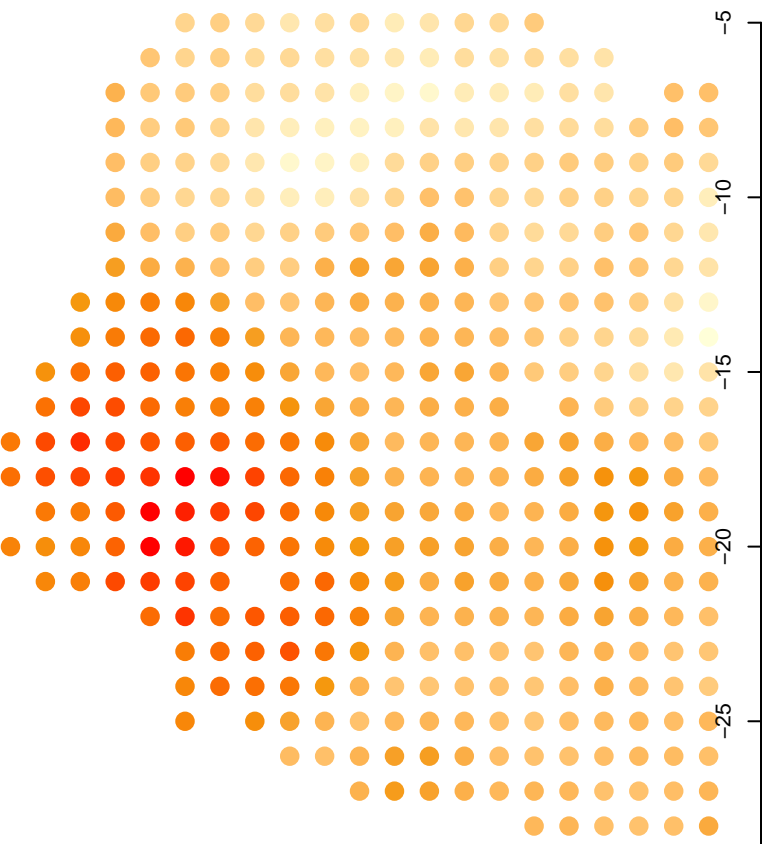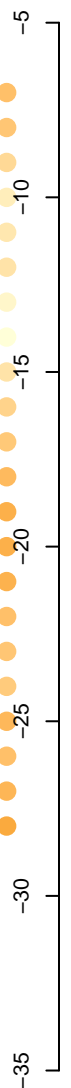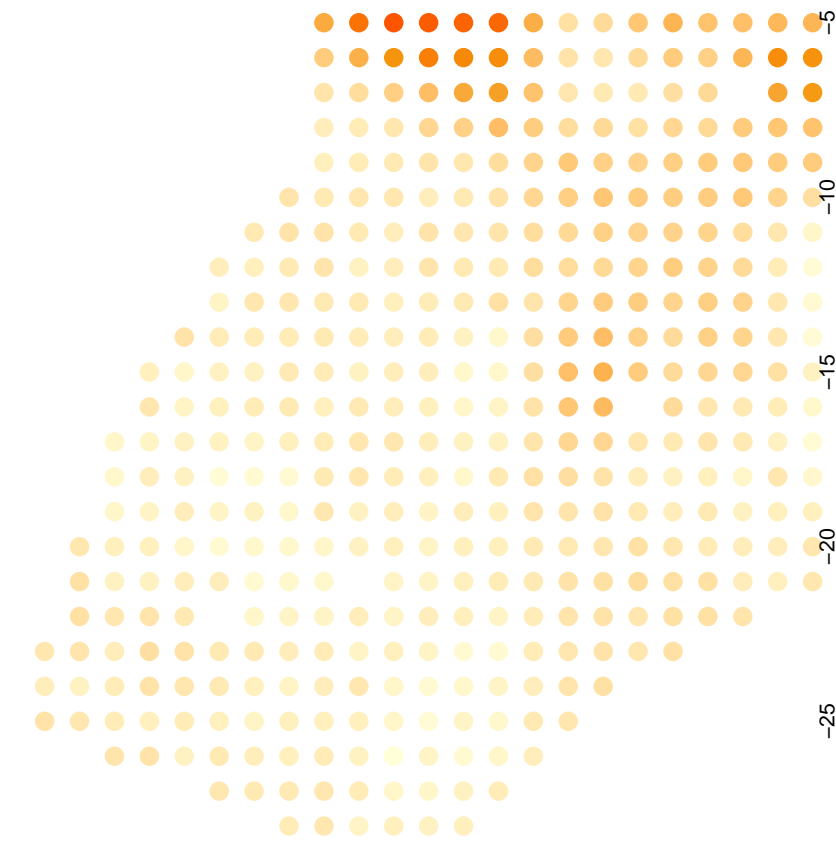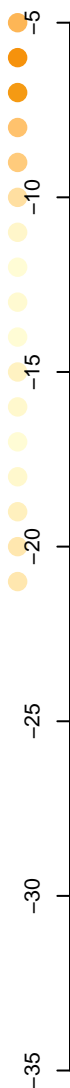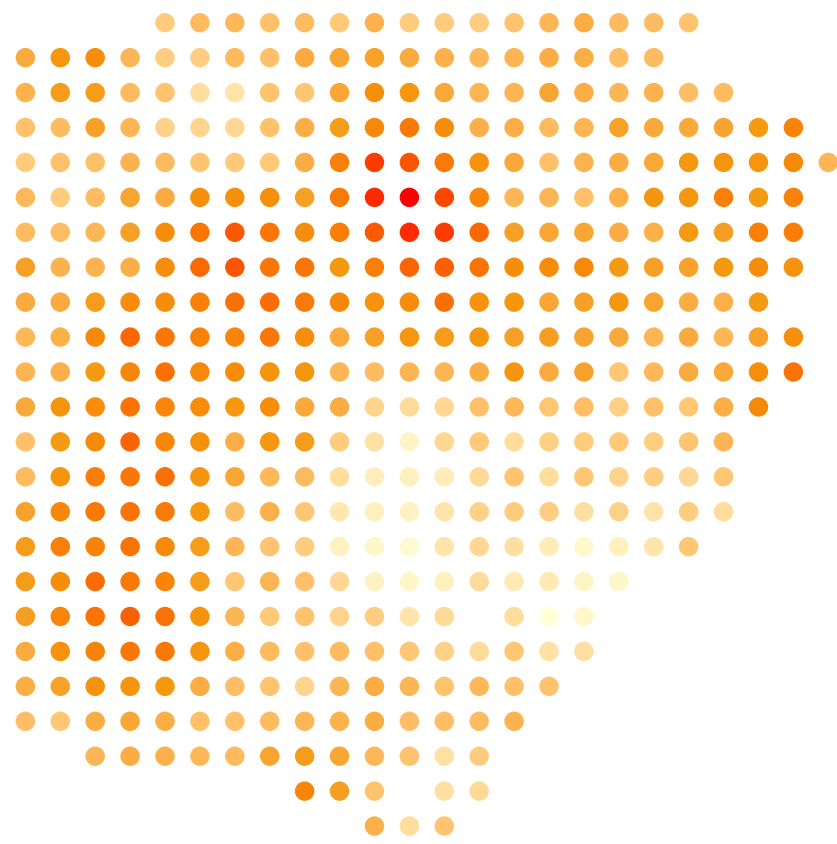

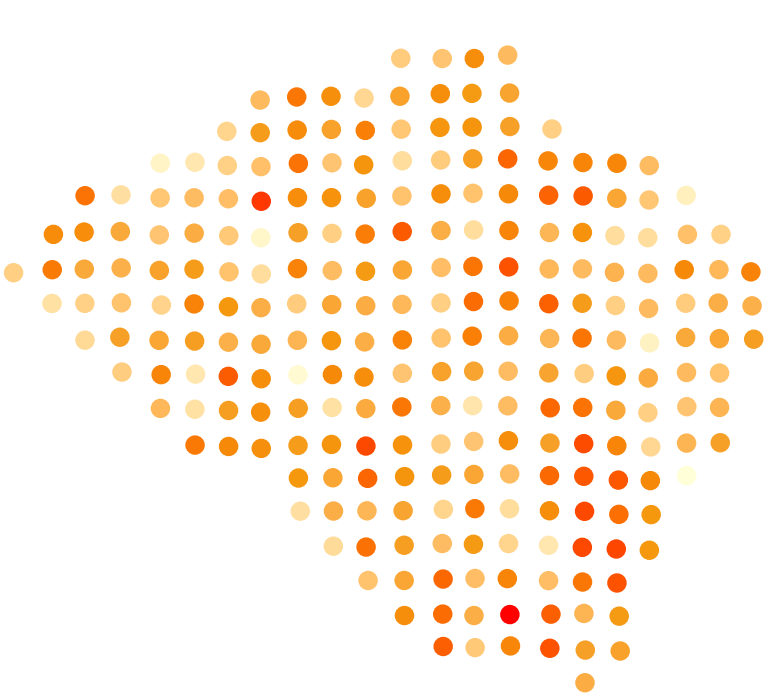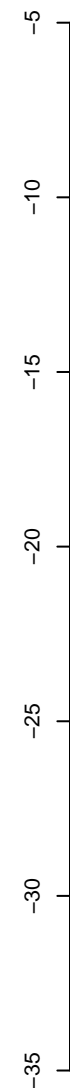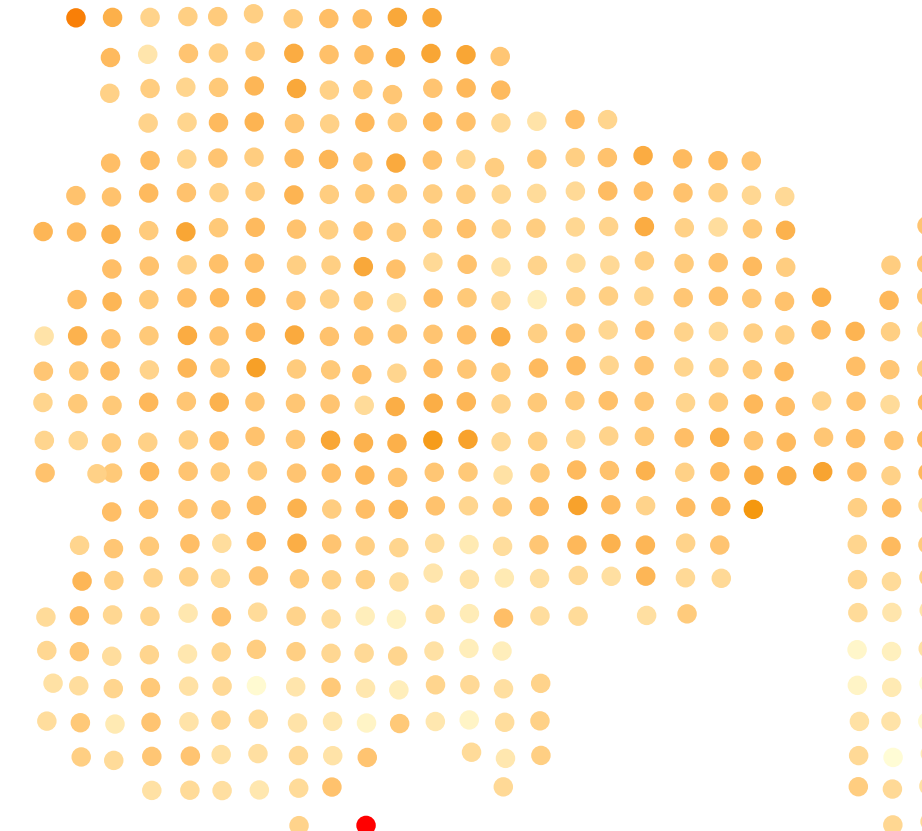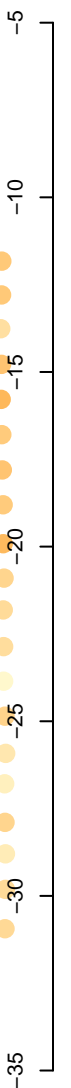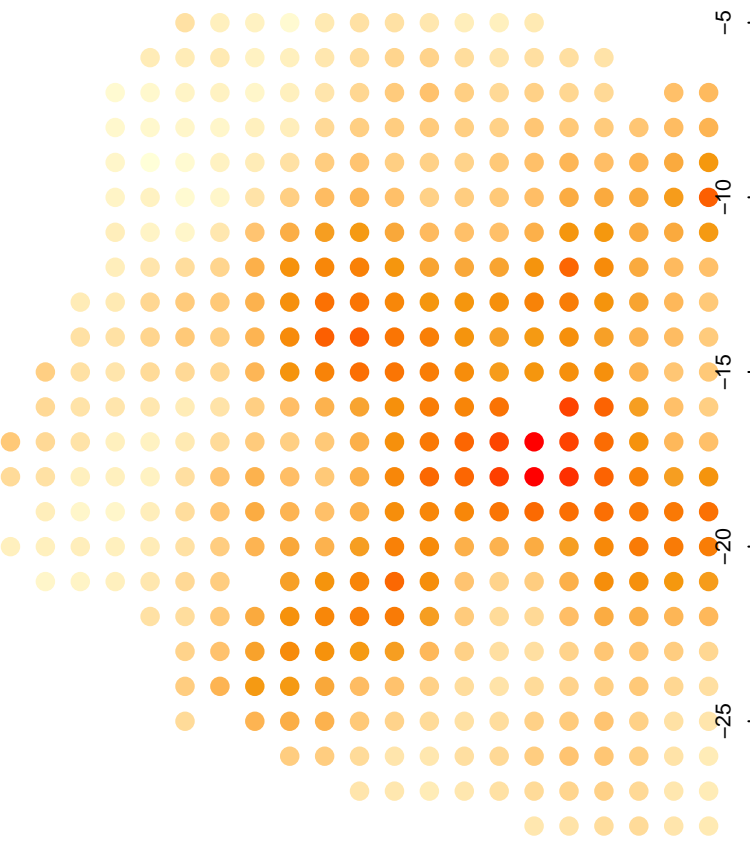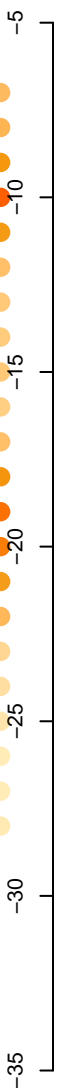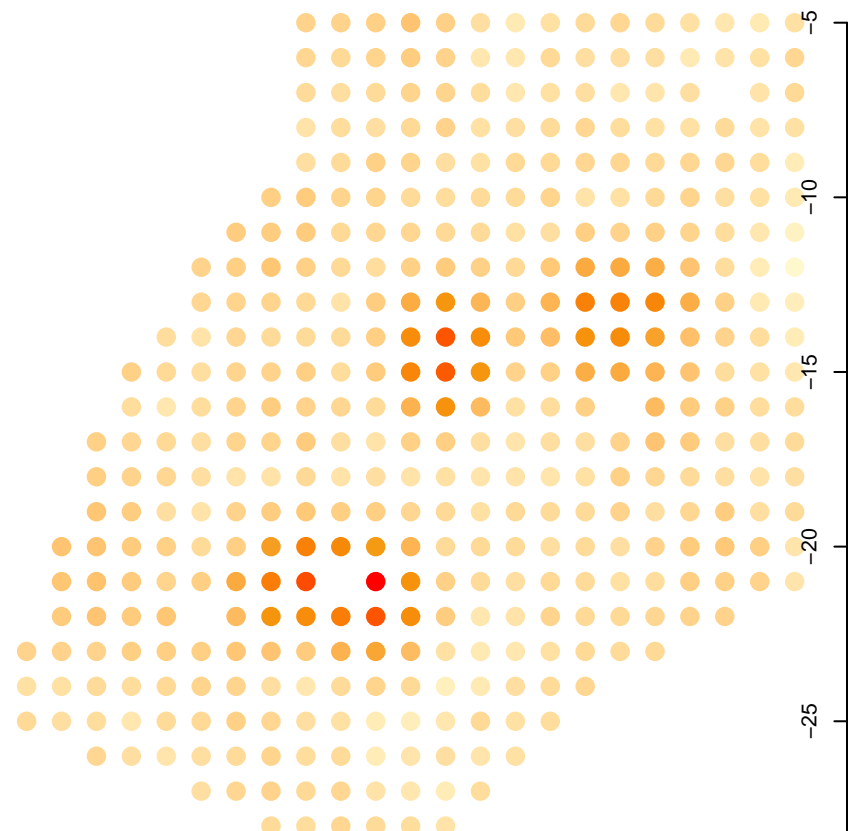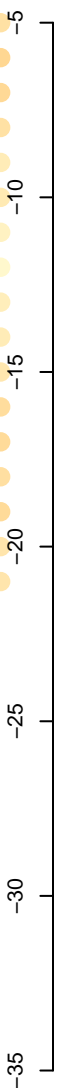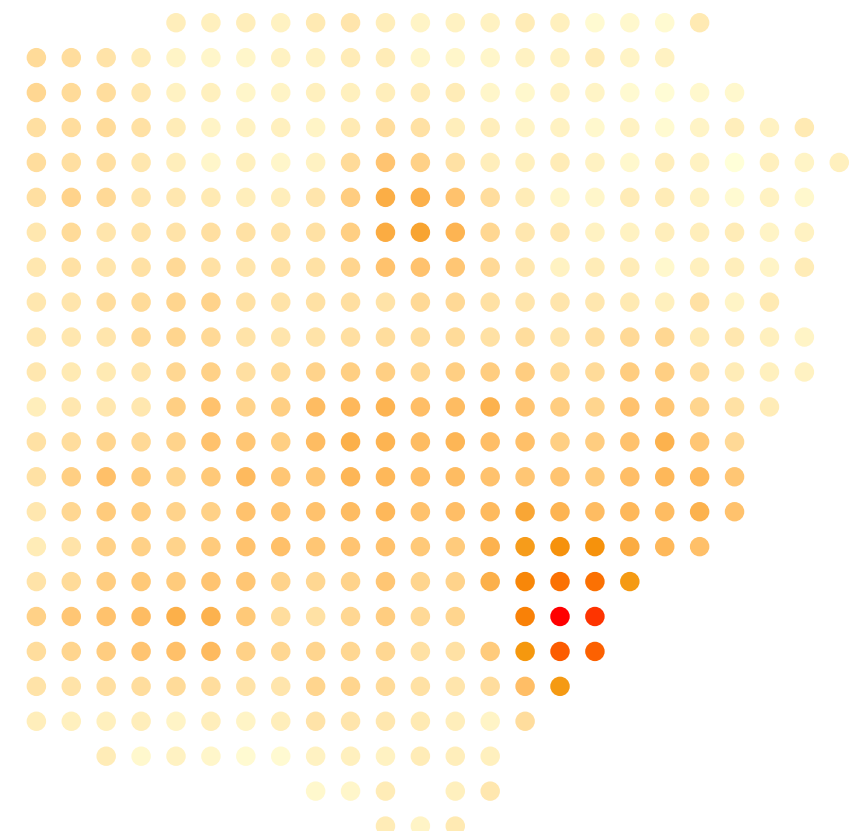

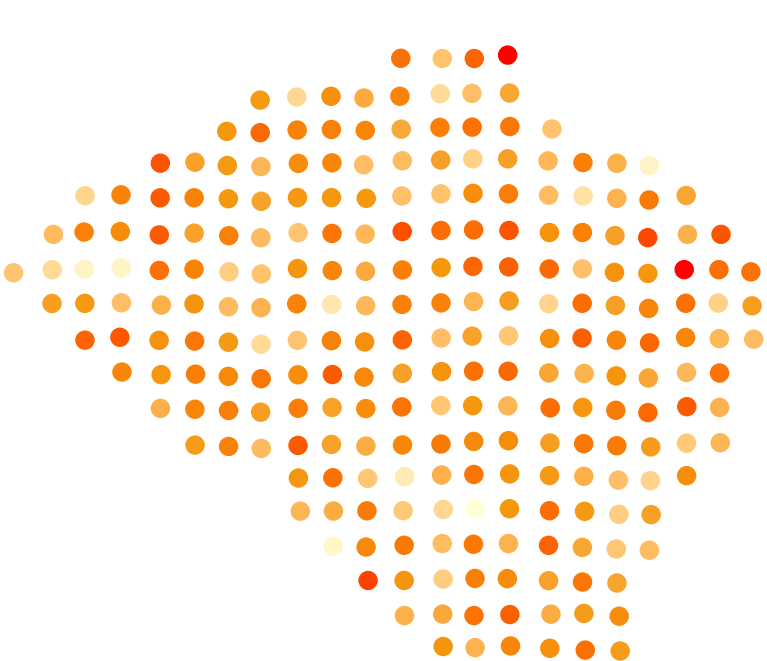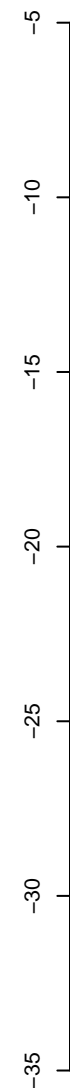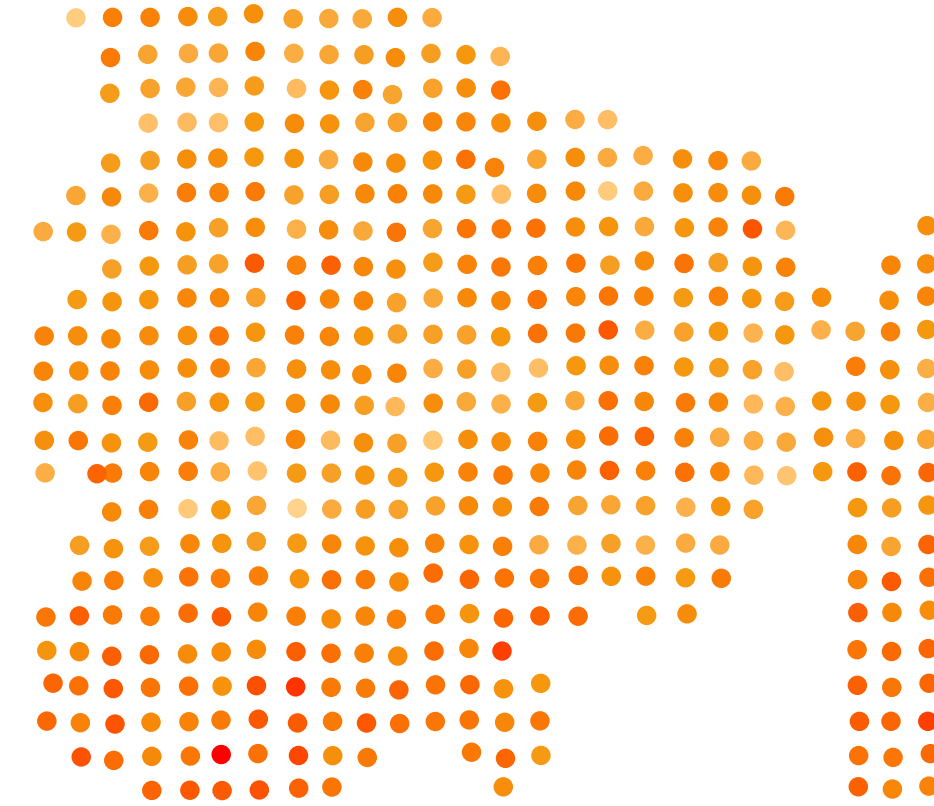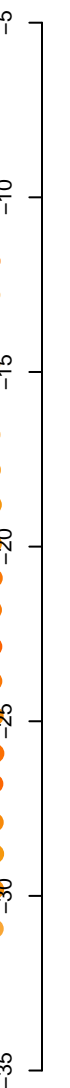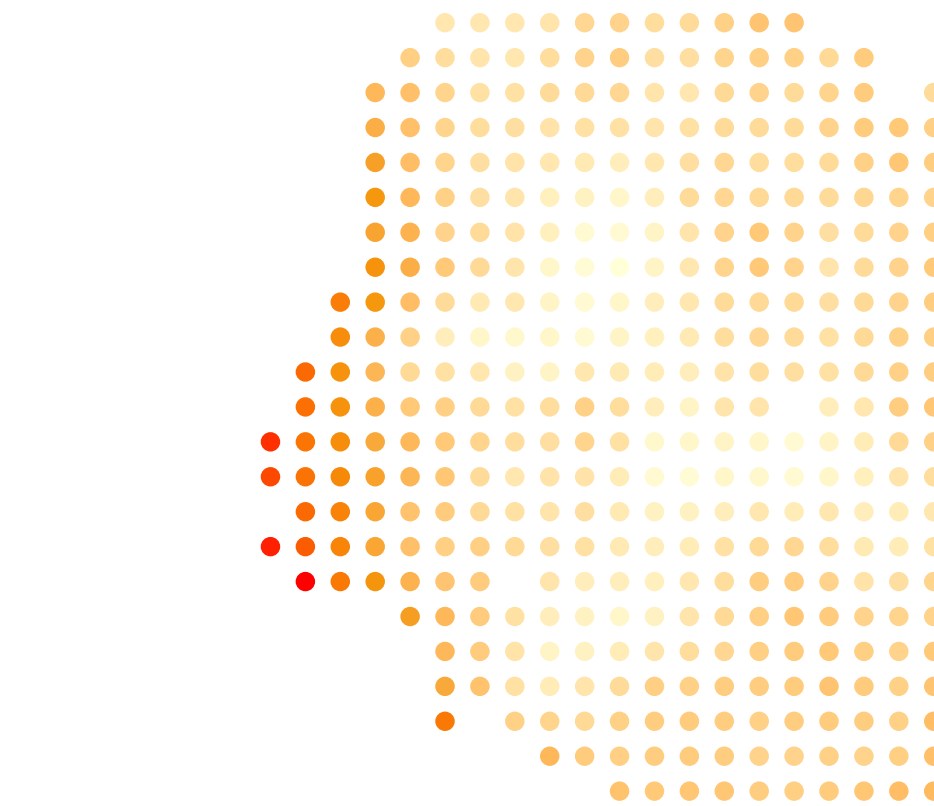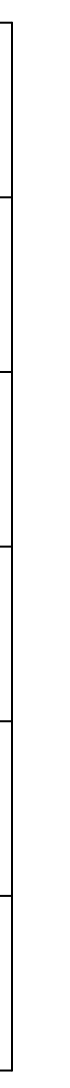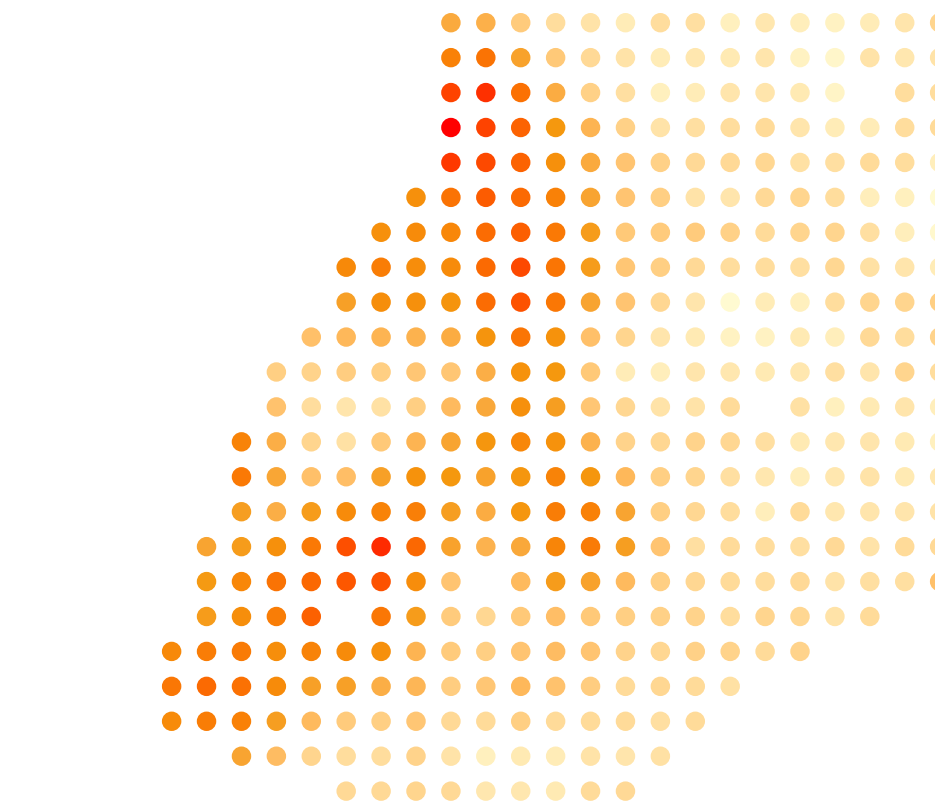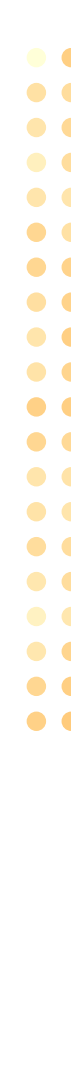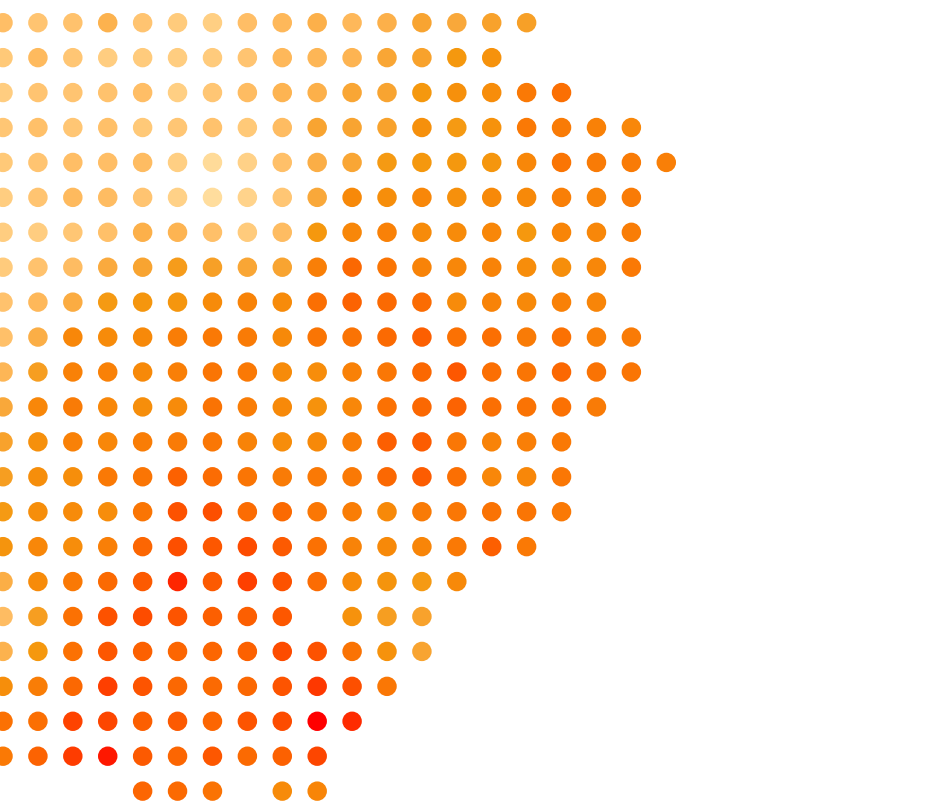

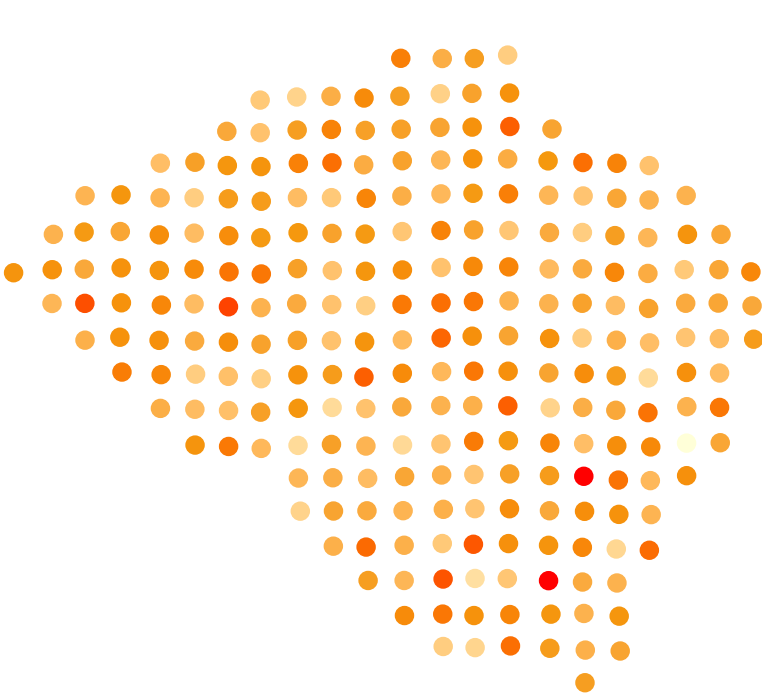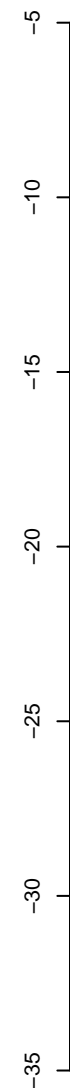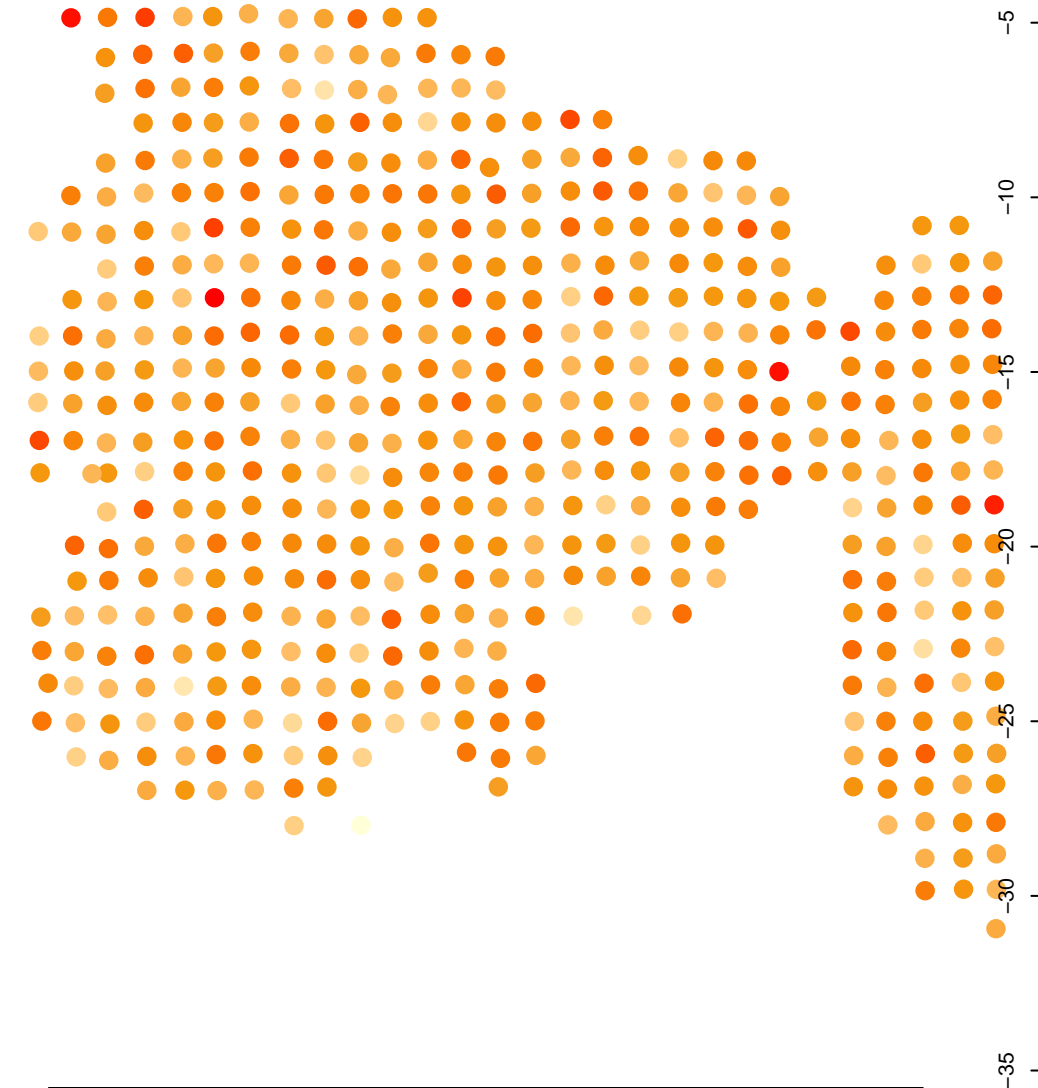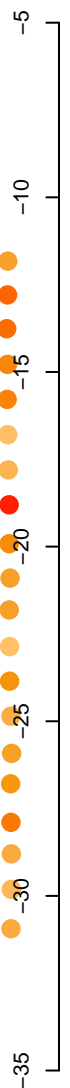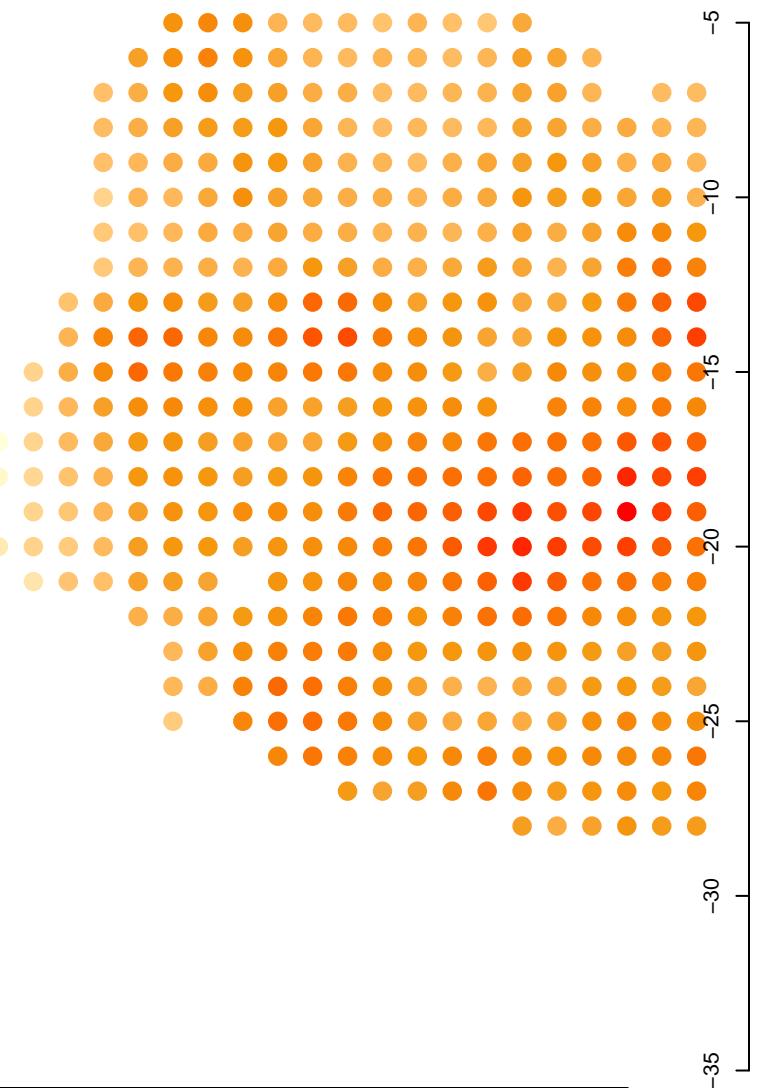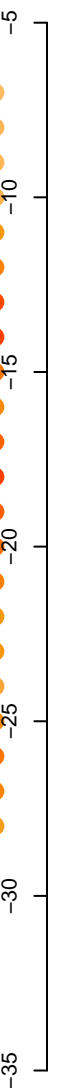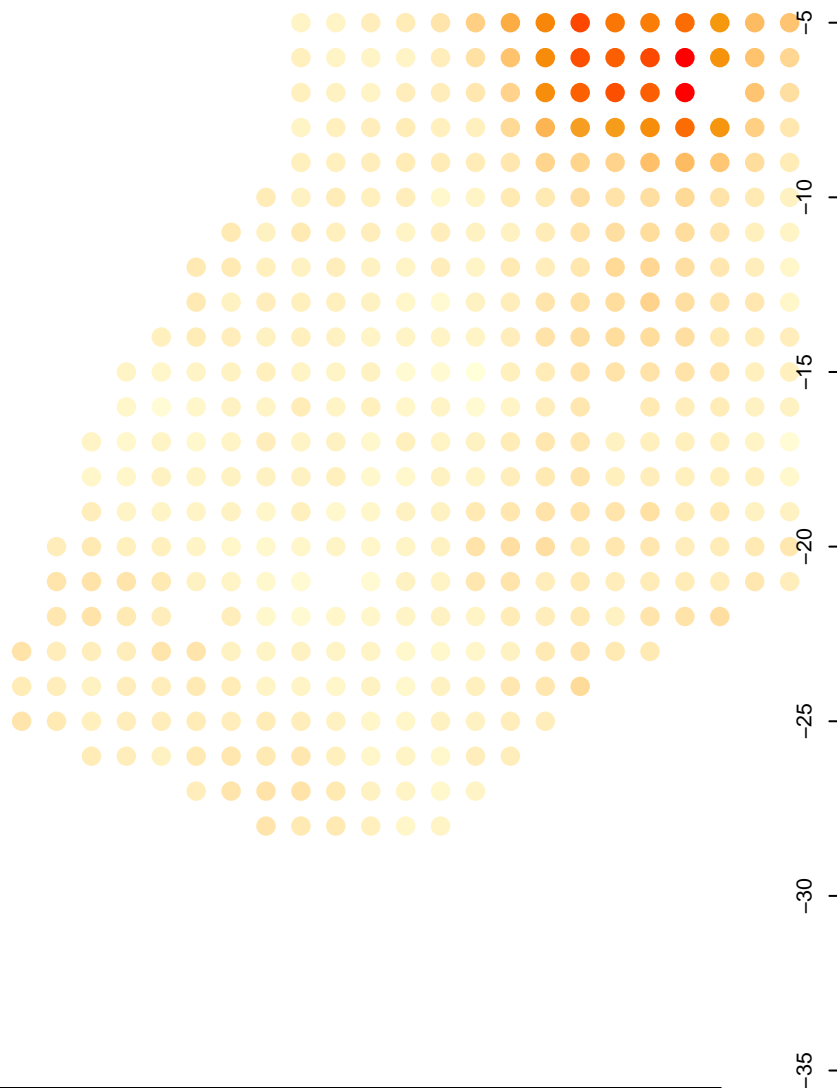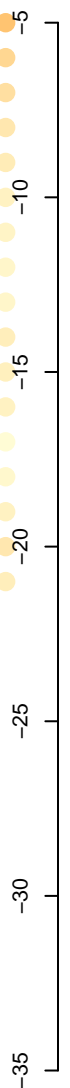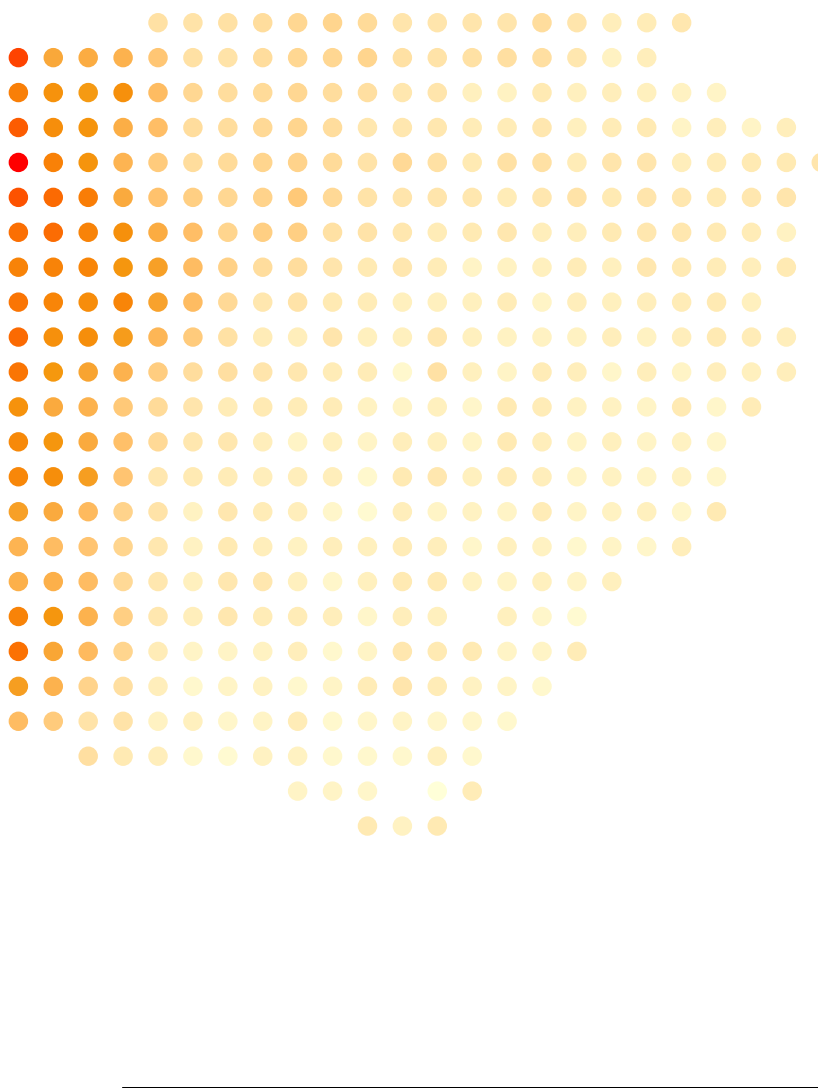

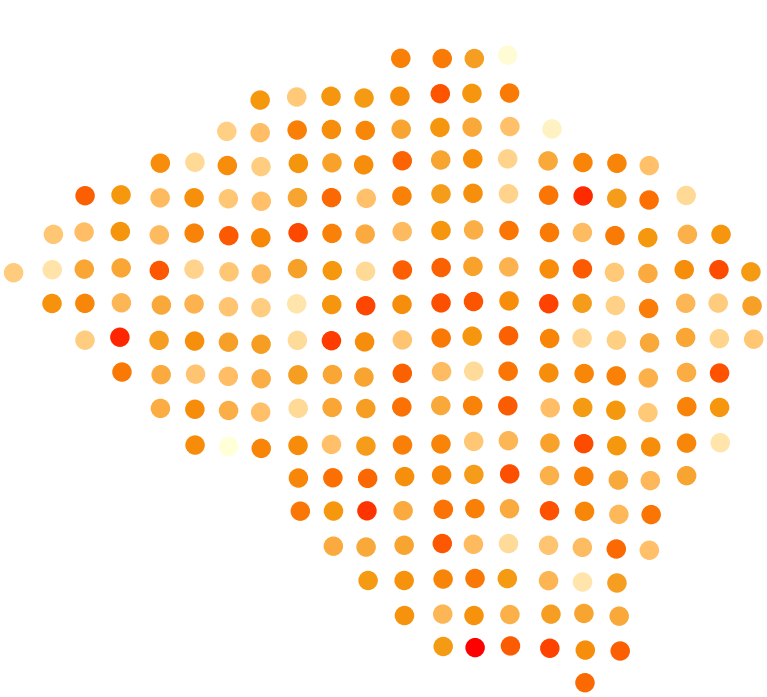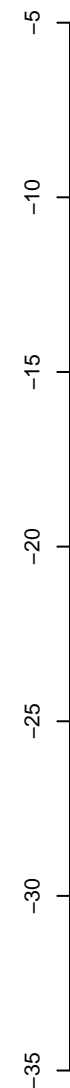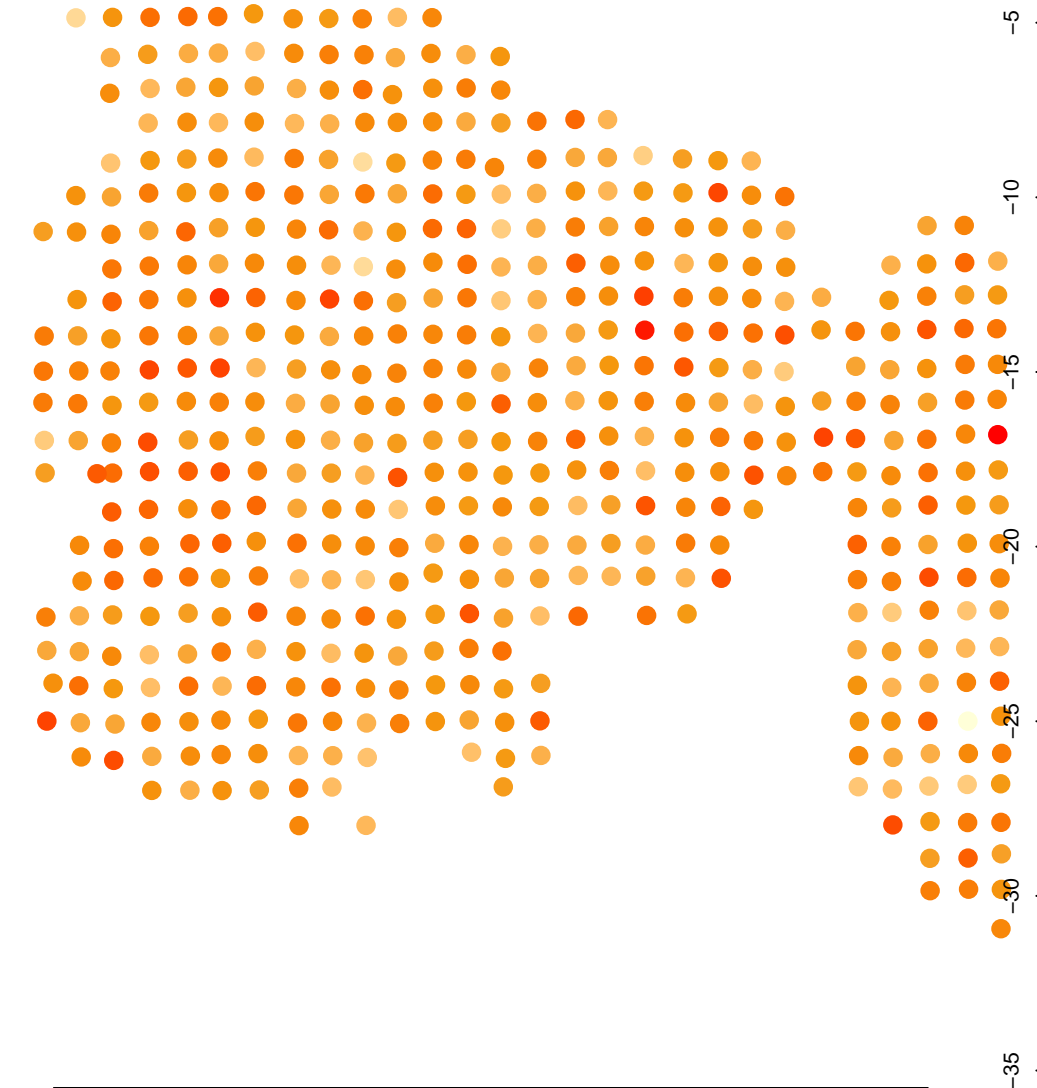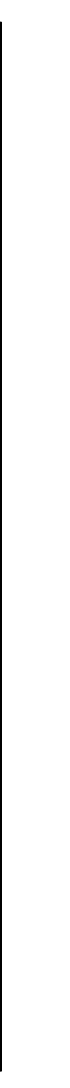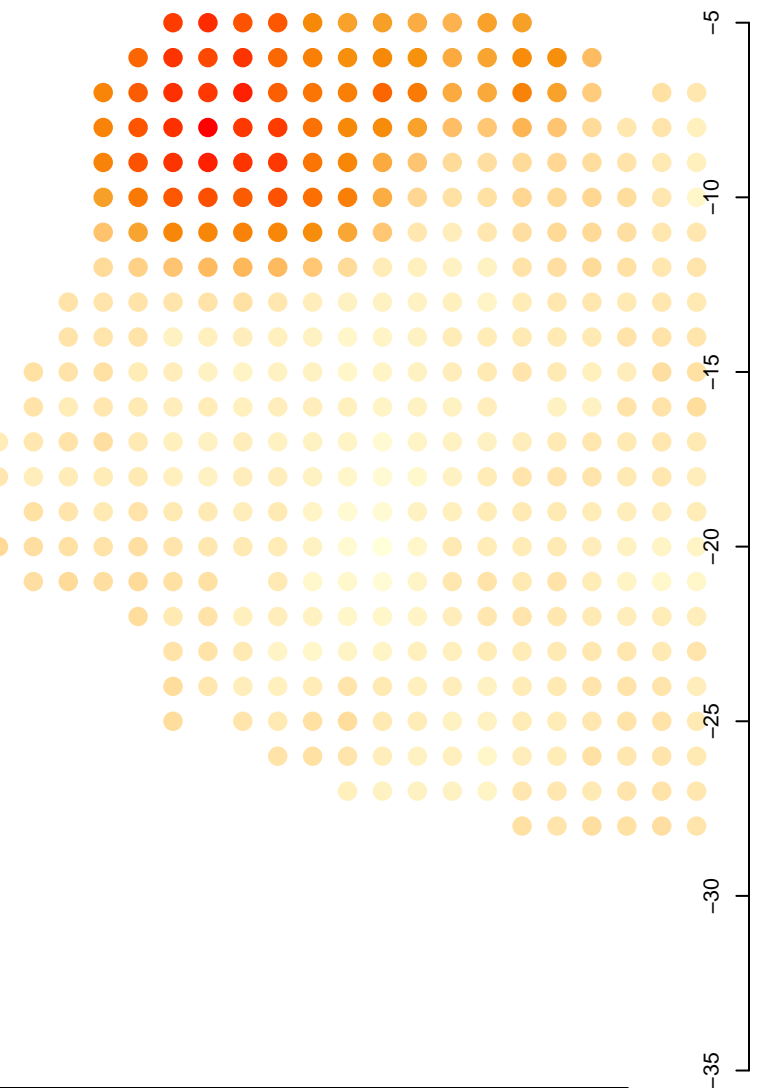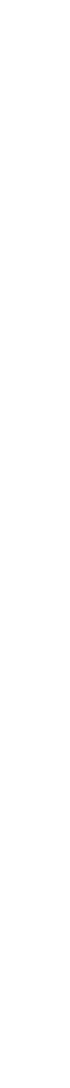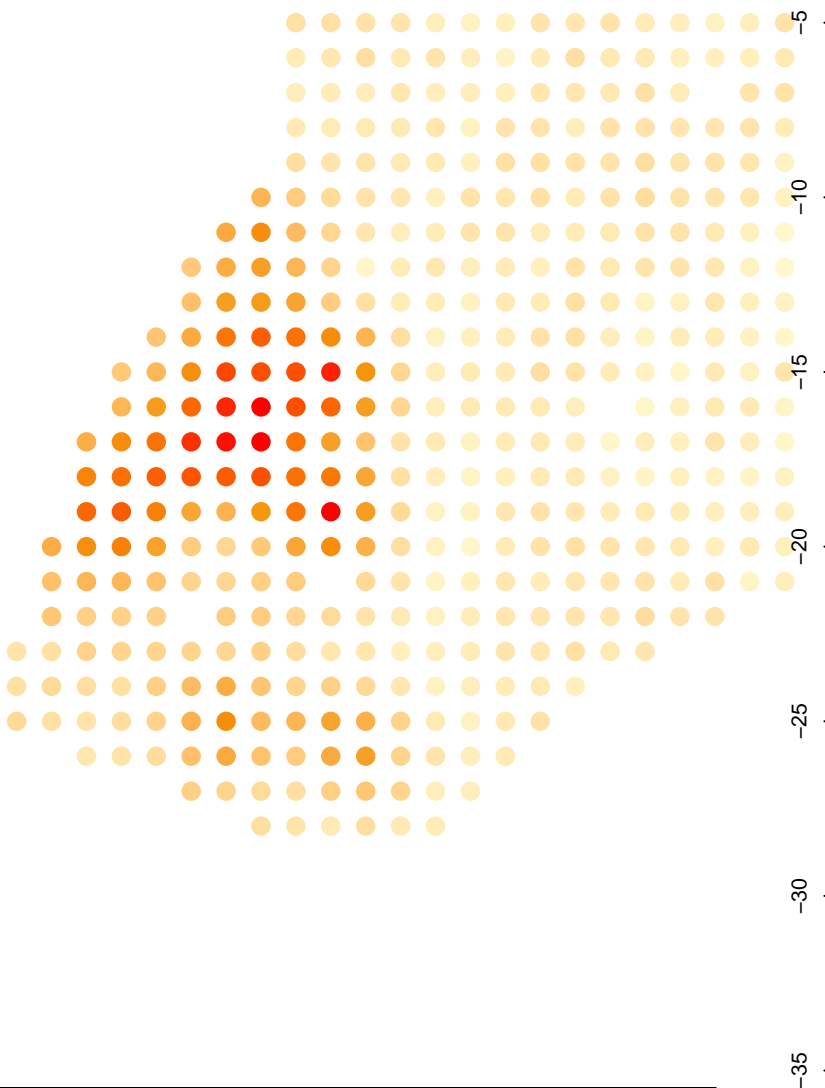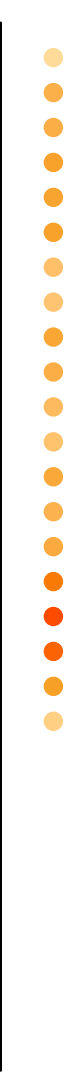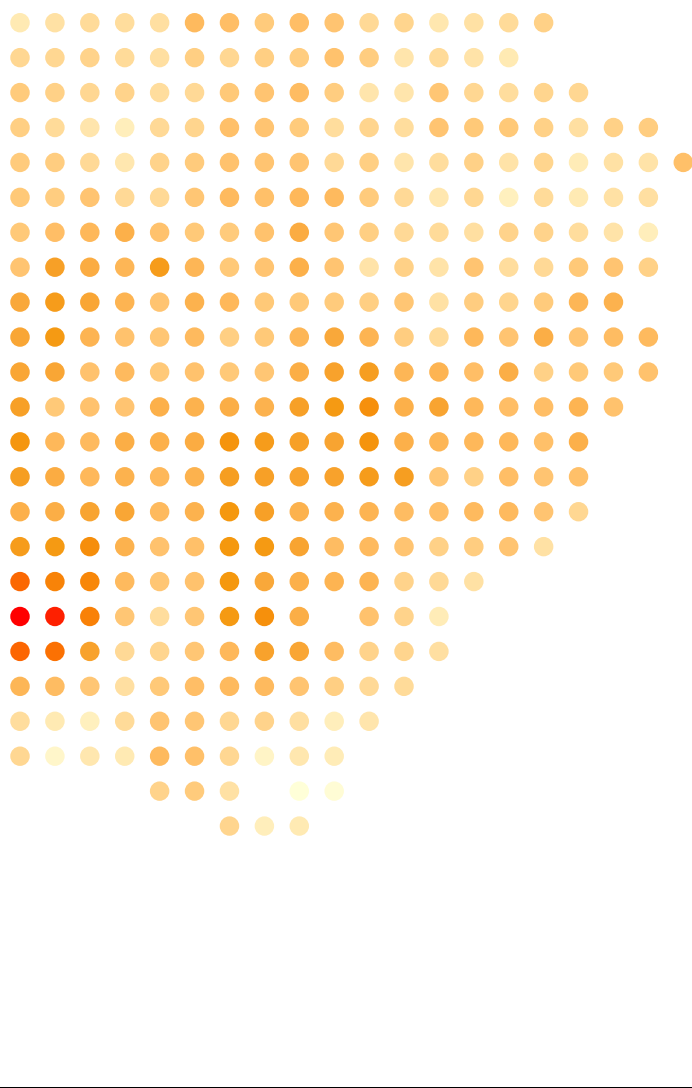

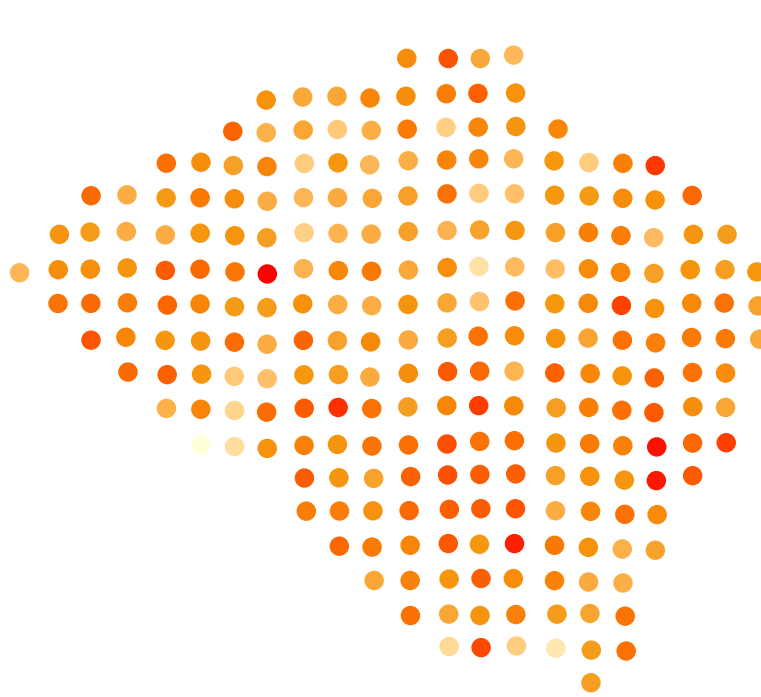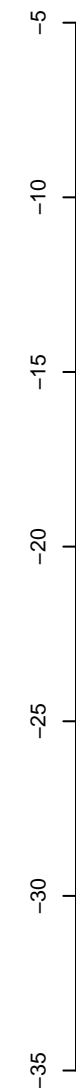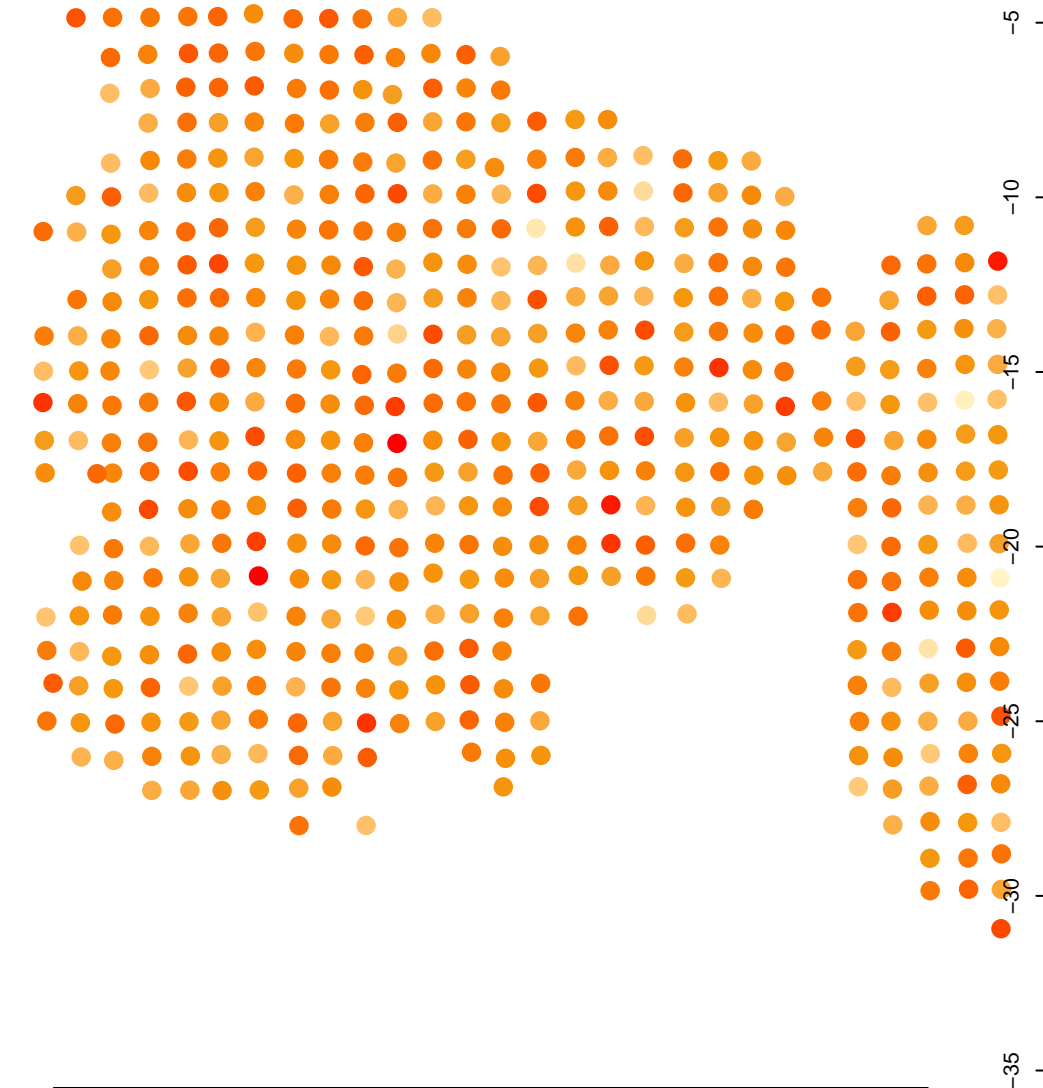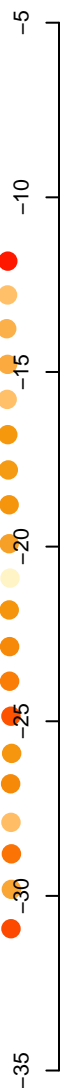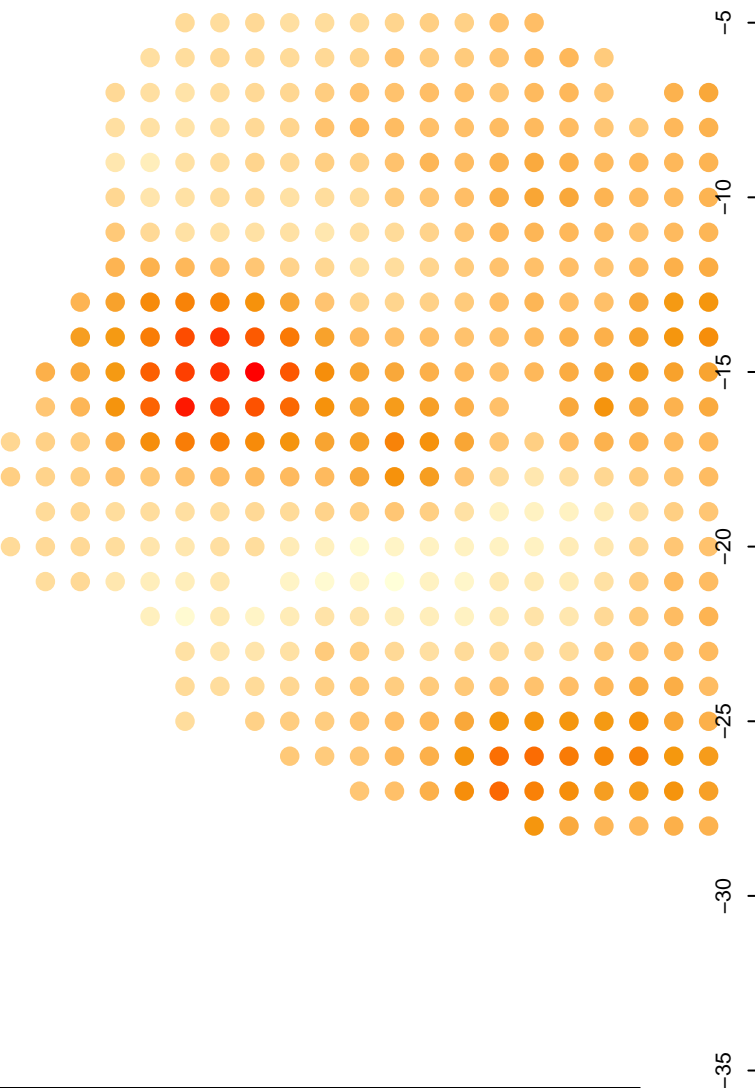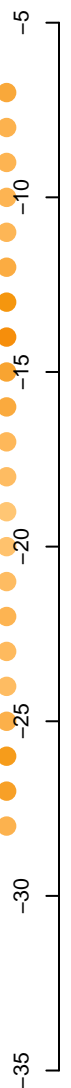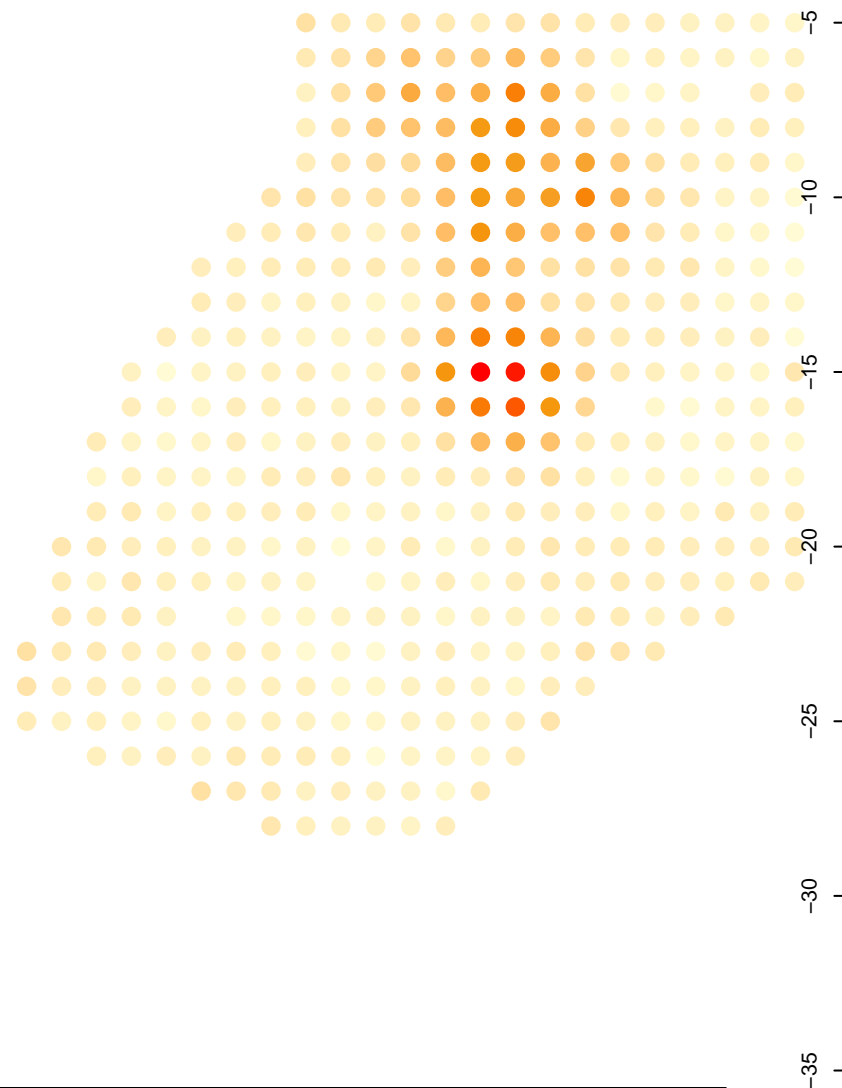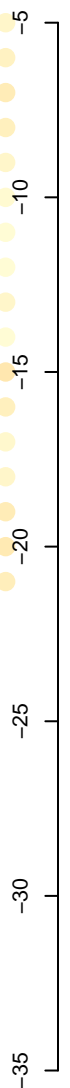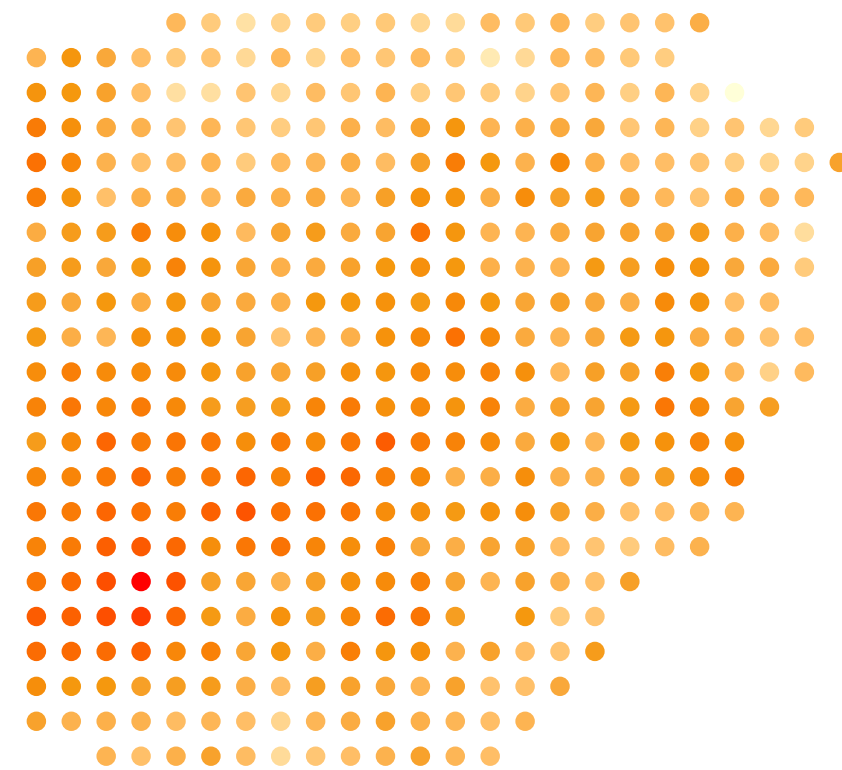

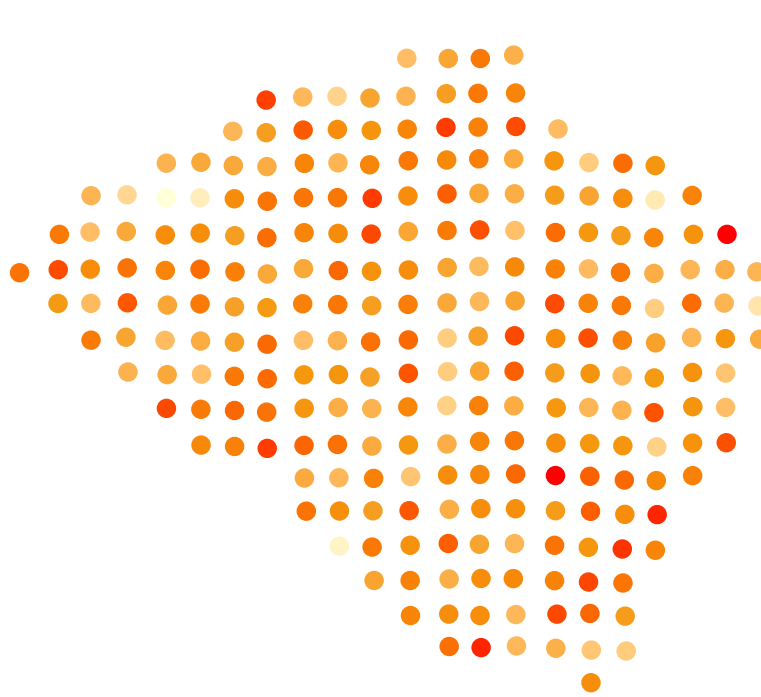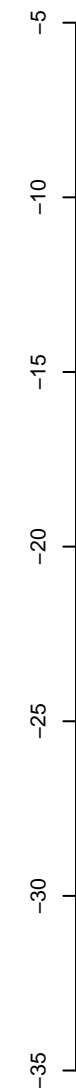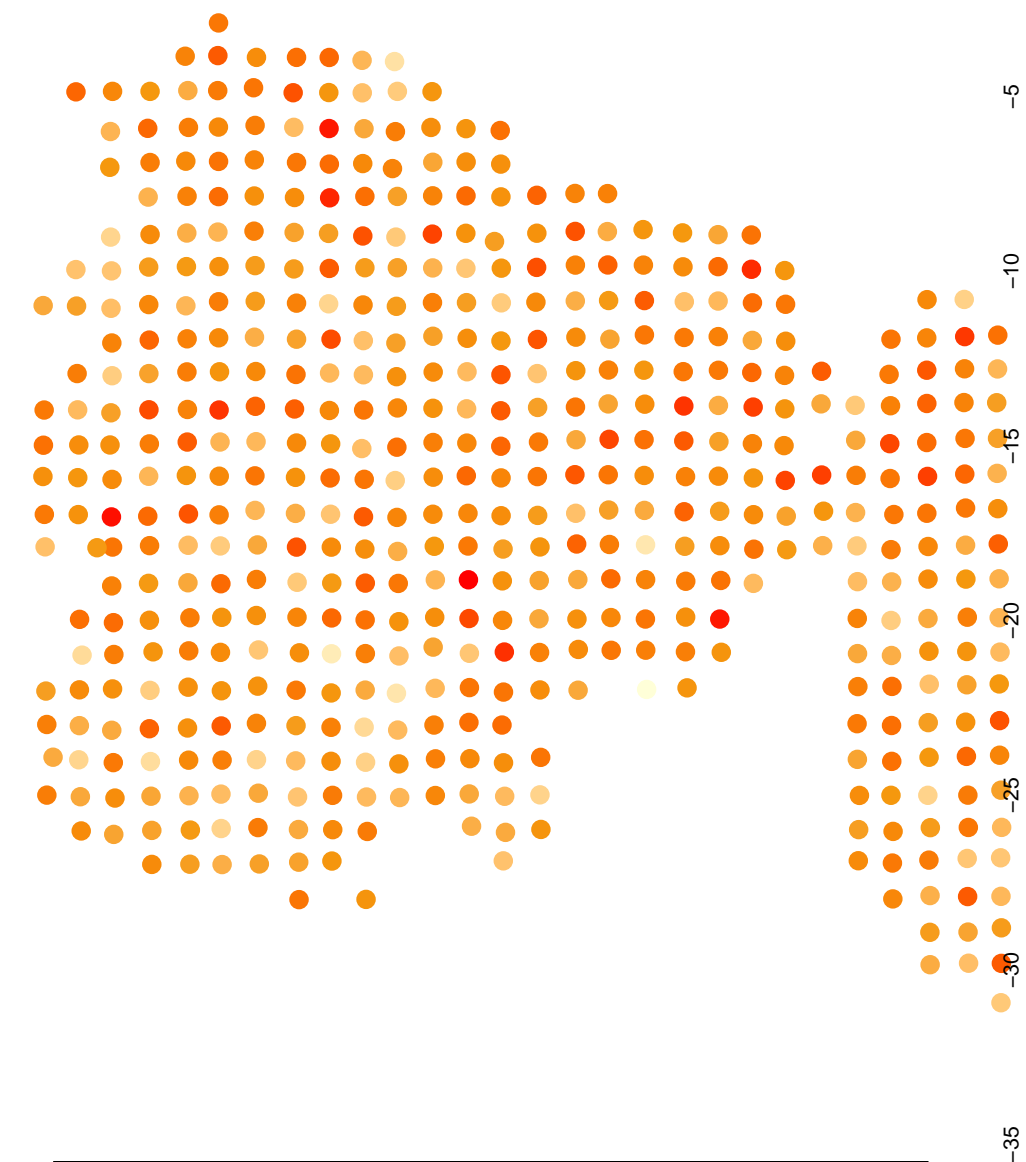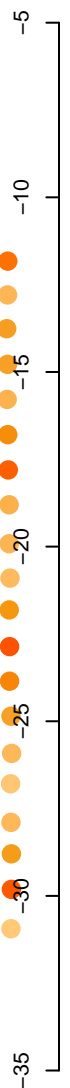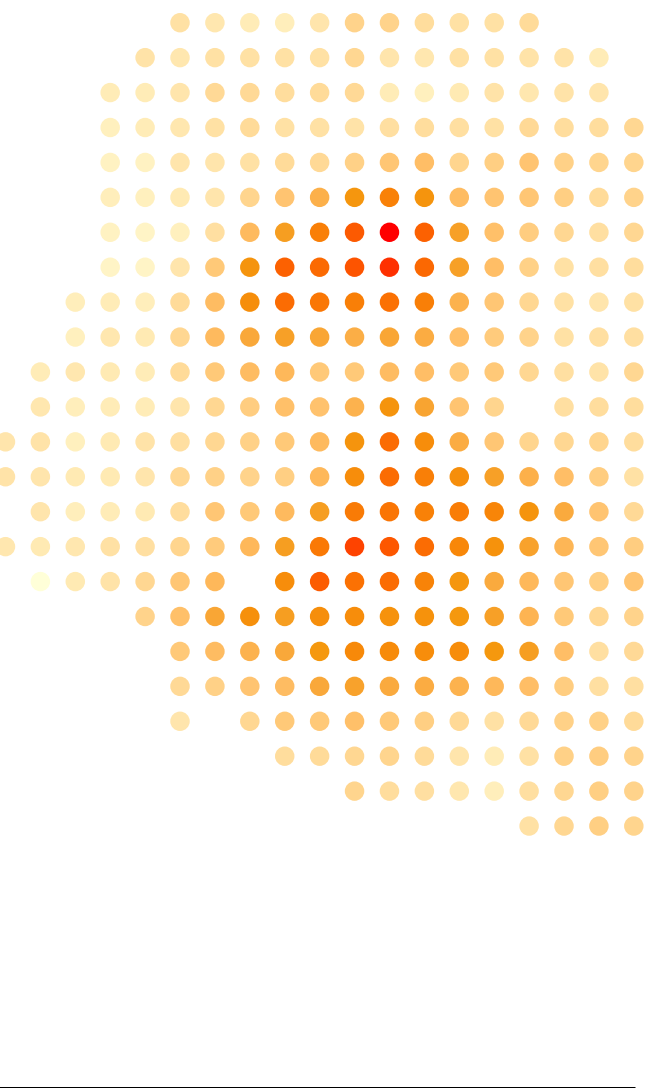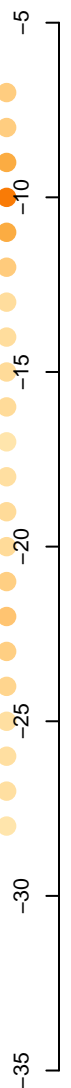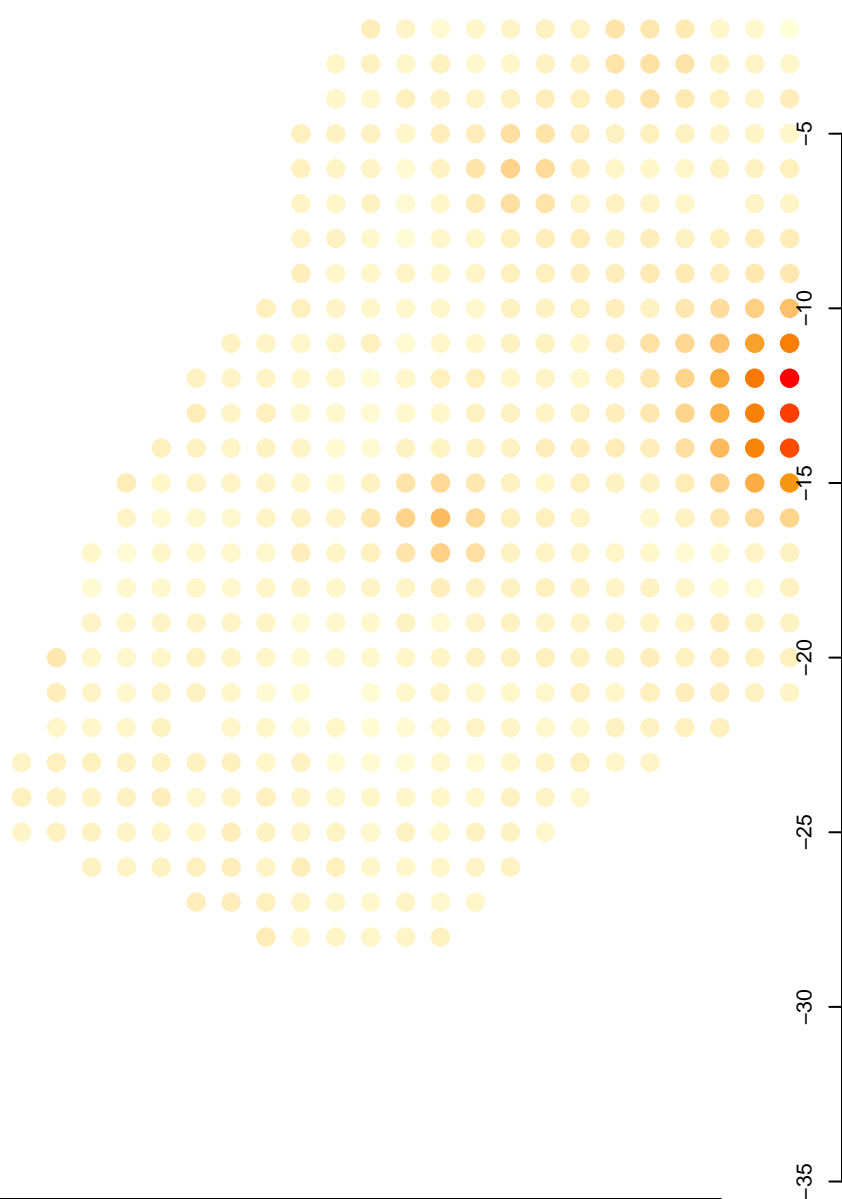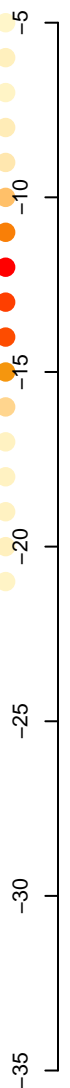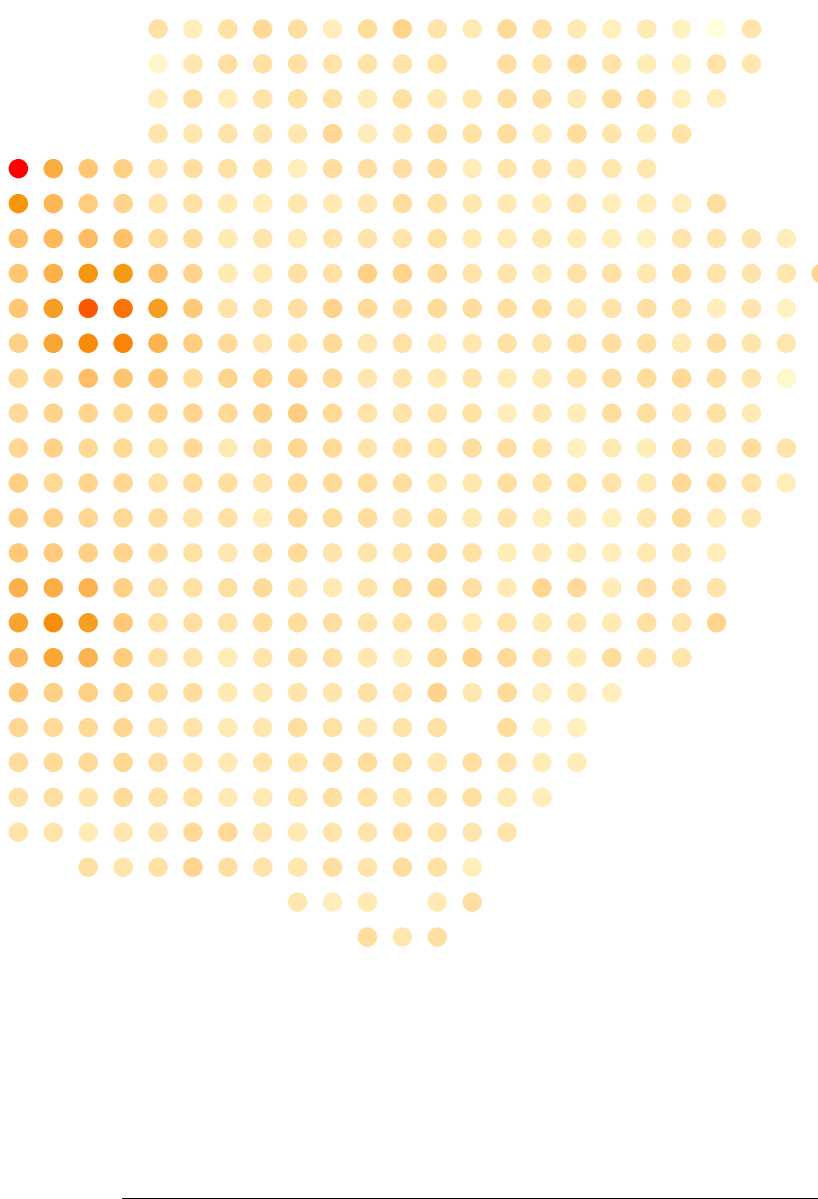

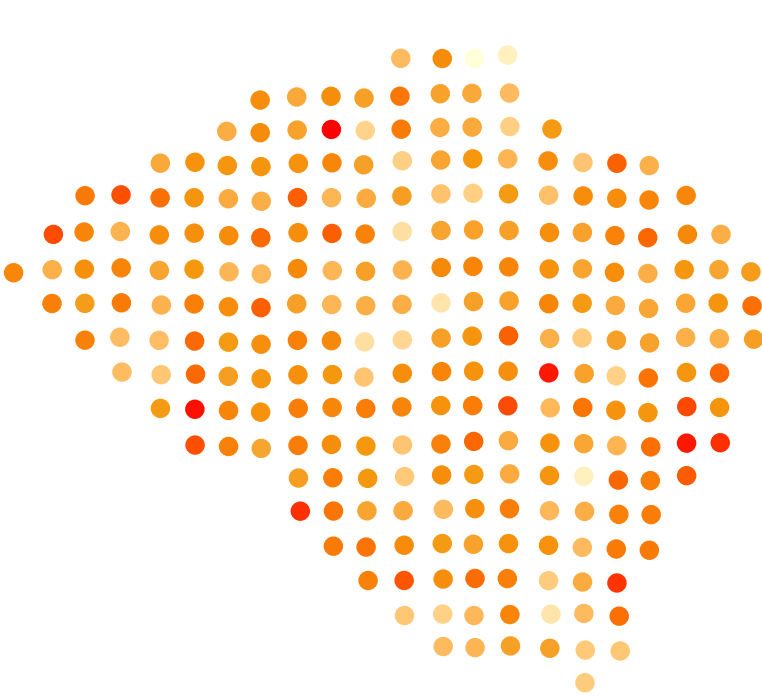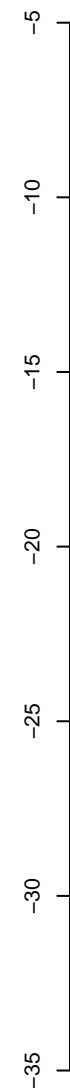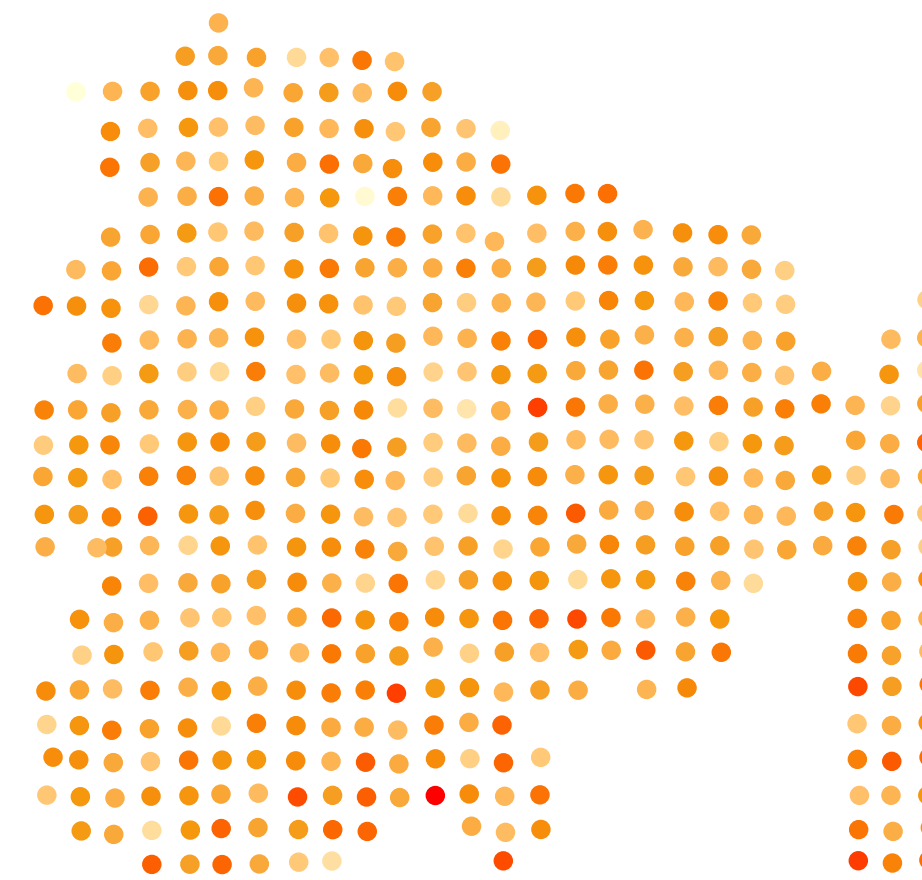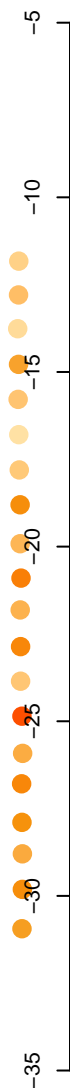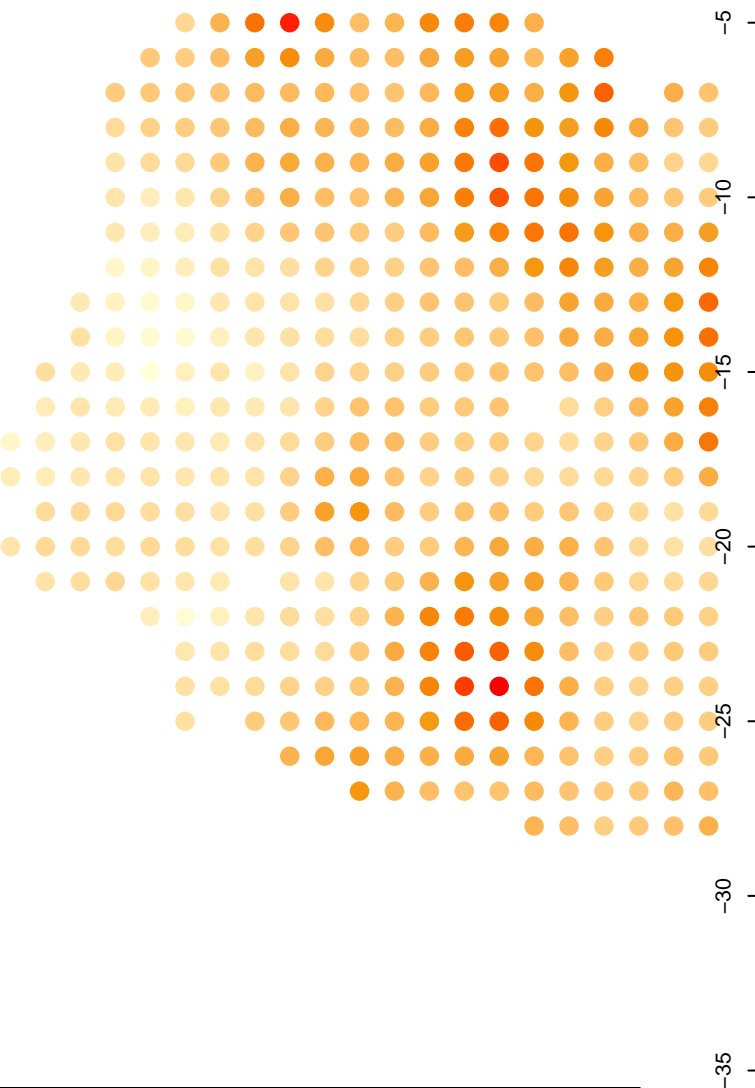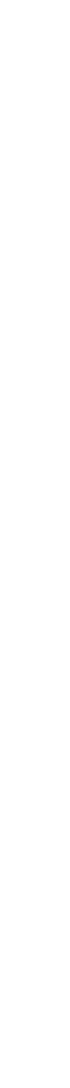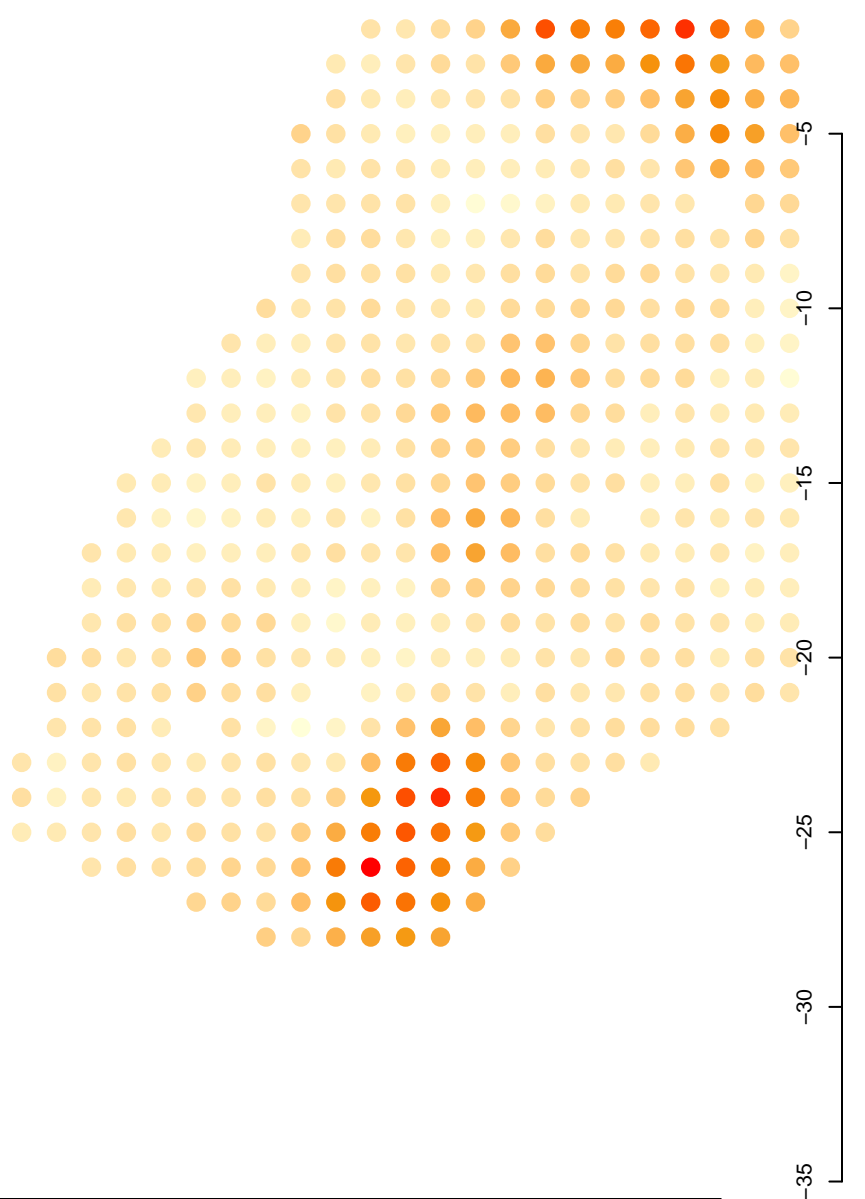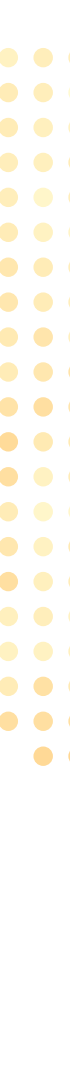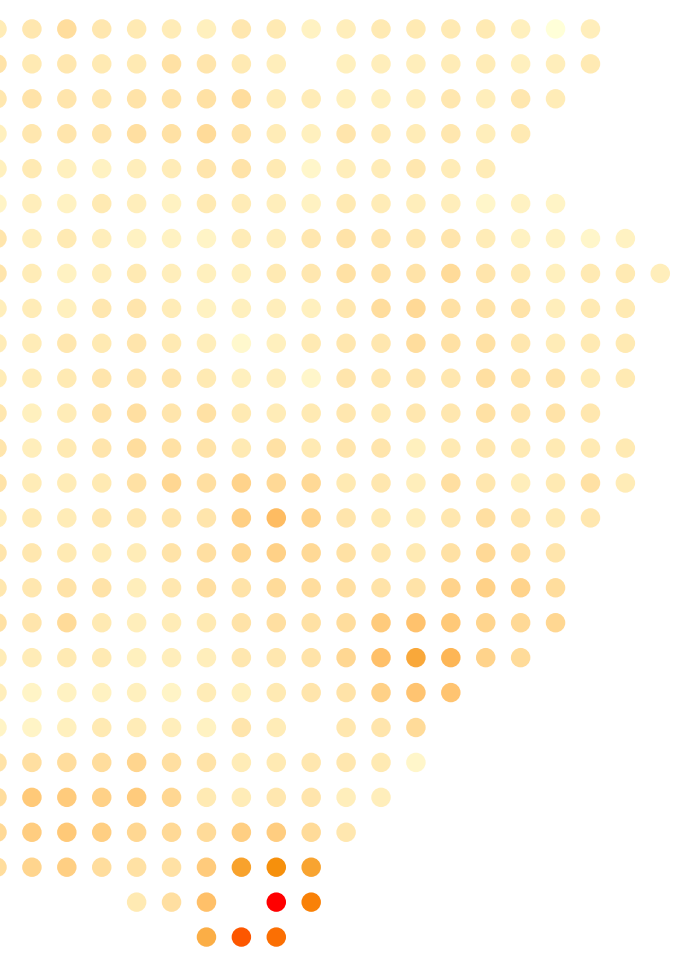

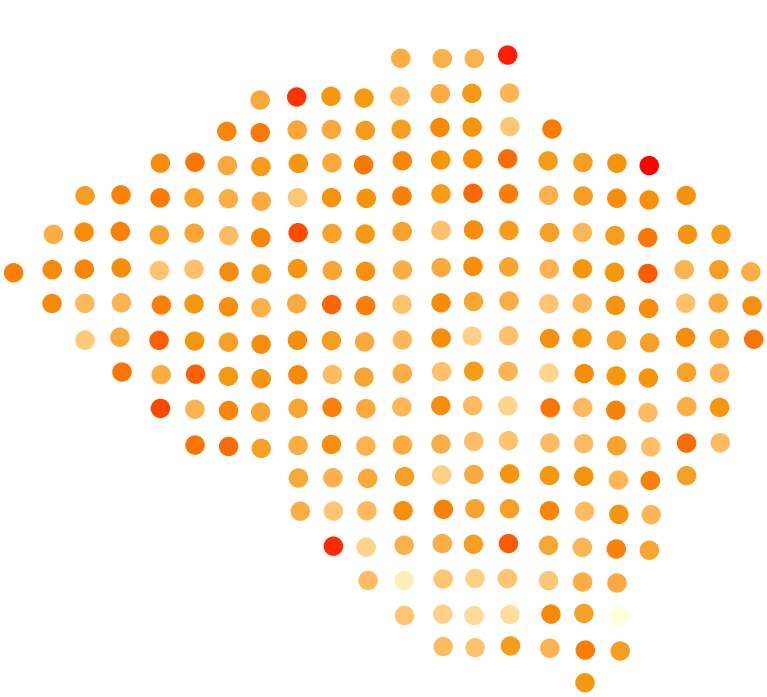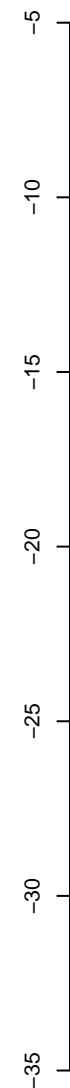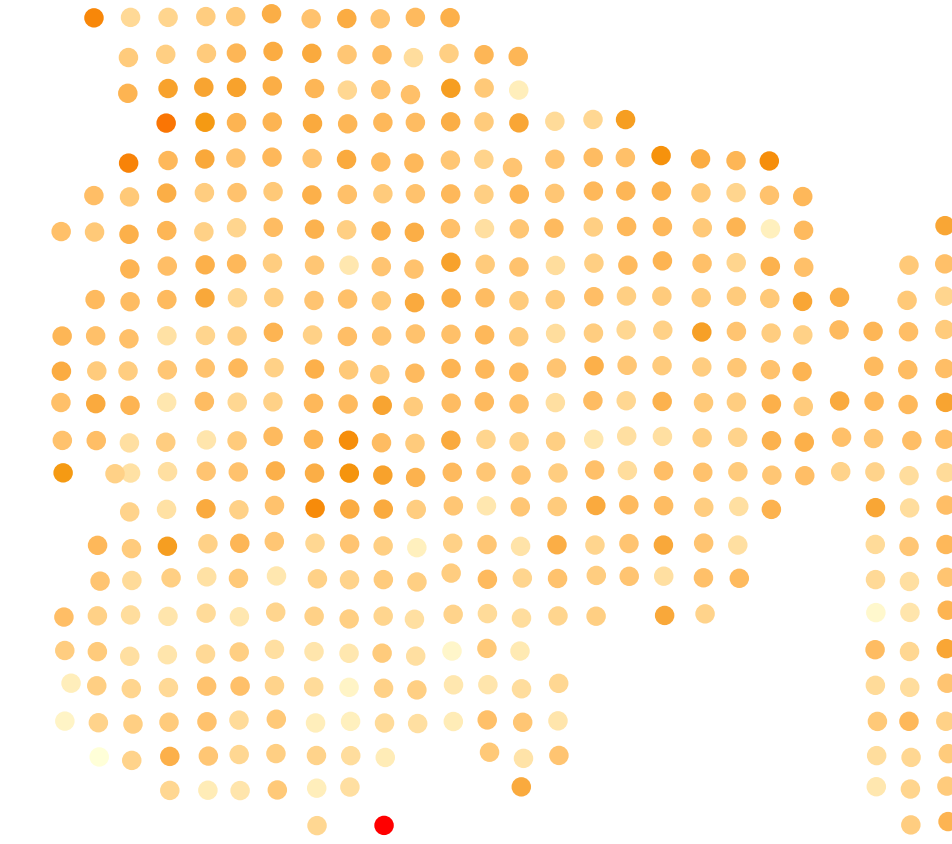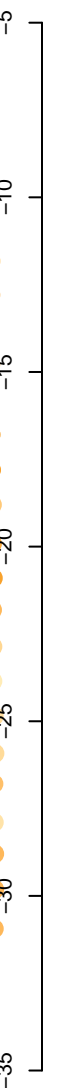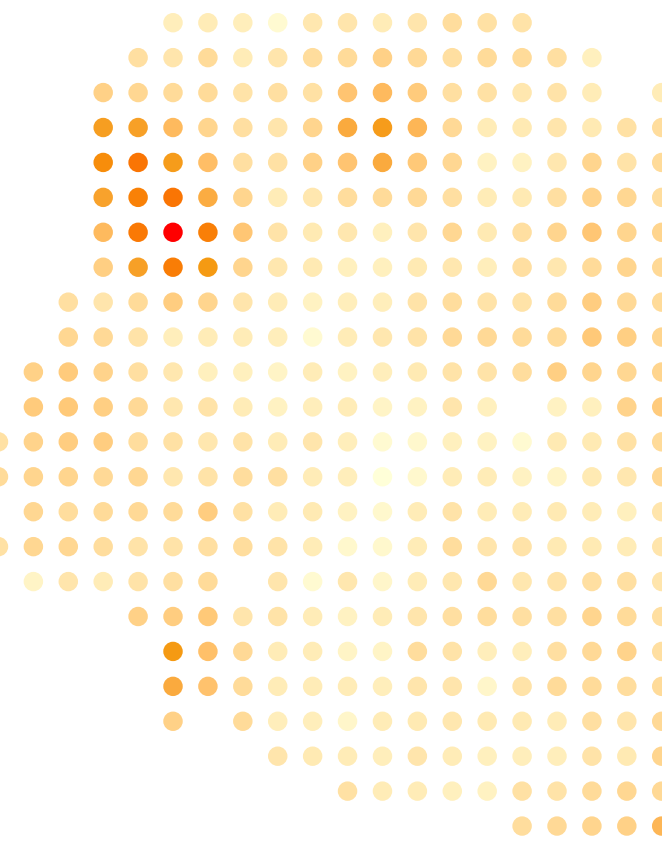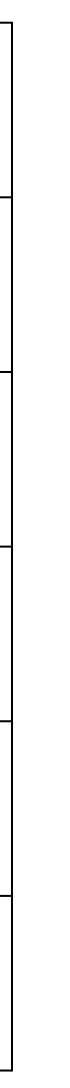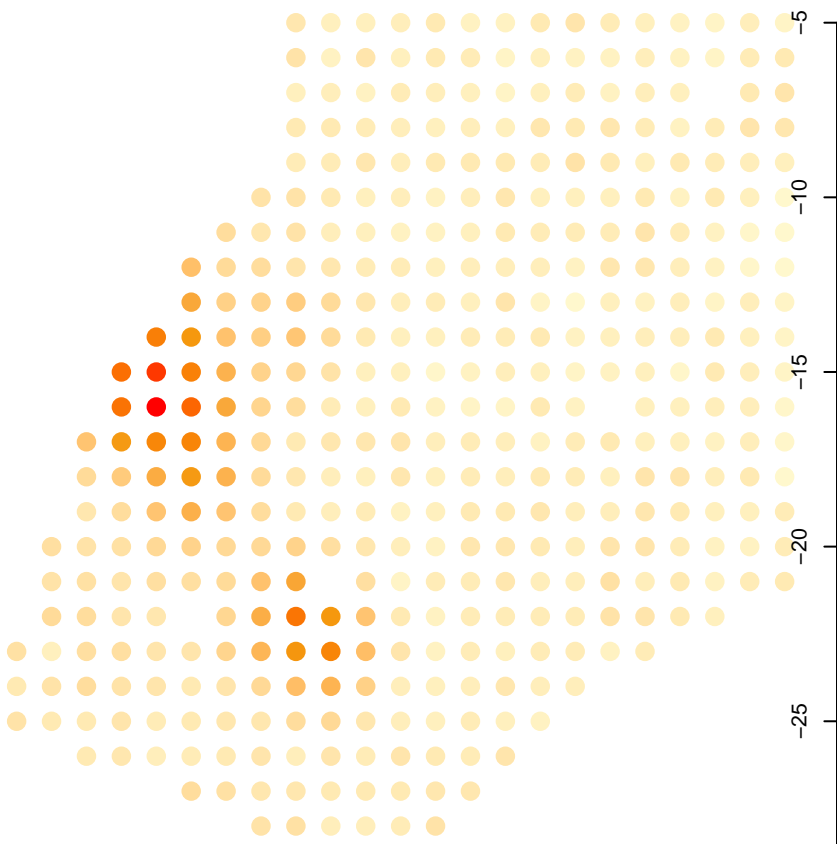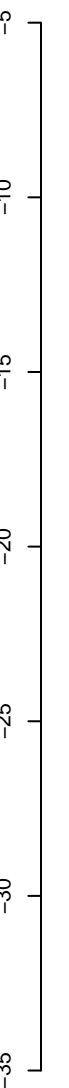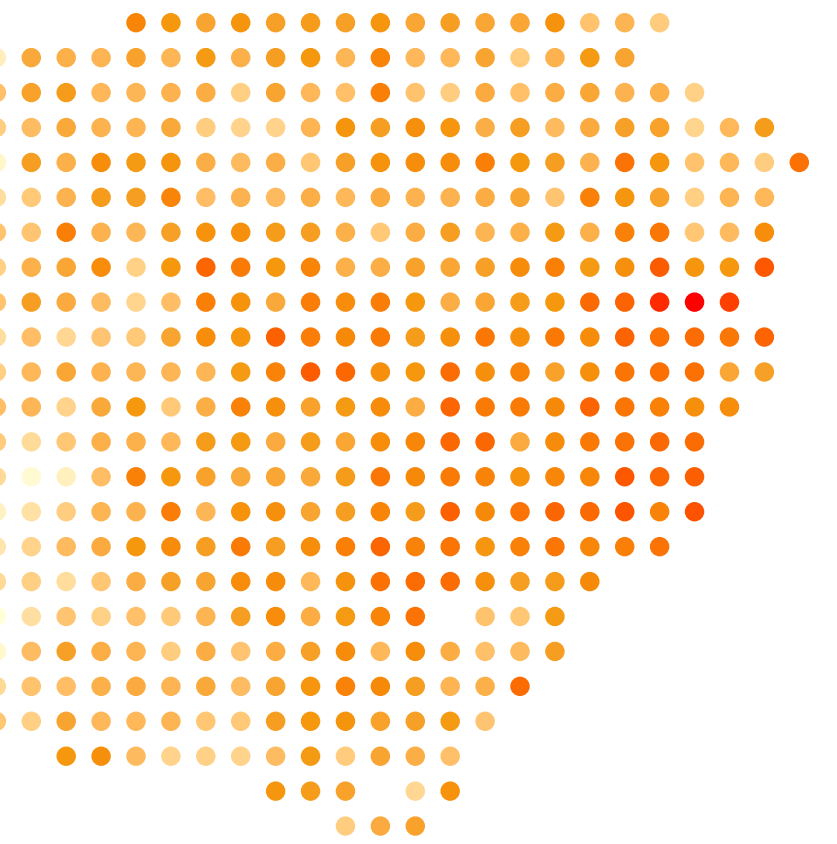

Supplement: Supplementary file 8 — Supplementary Data 5 [file 41467_2018_4724_MOESM8_ESM.zip › Supplementary Dataset 7/joint-field-profiles-rel-individual-scale-dots.pdf]

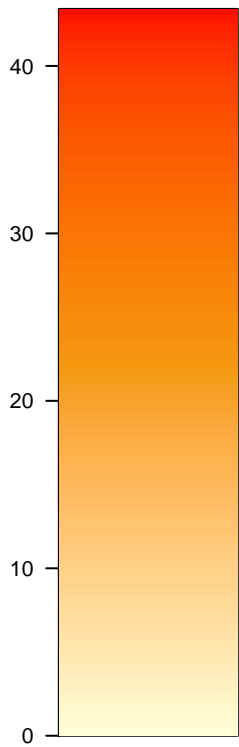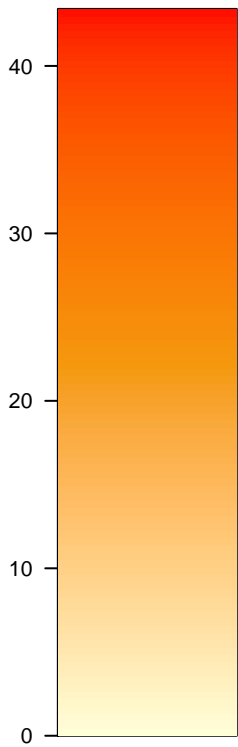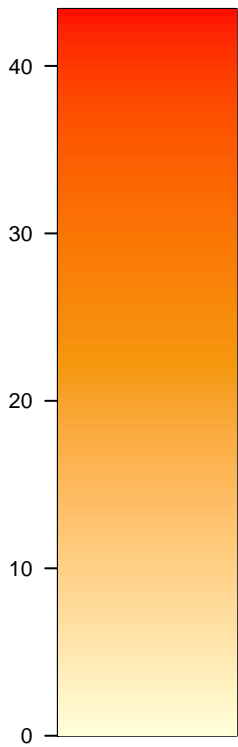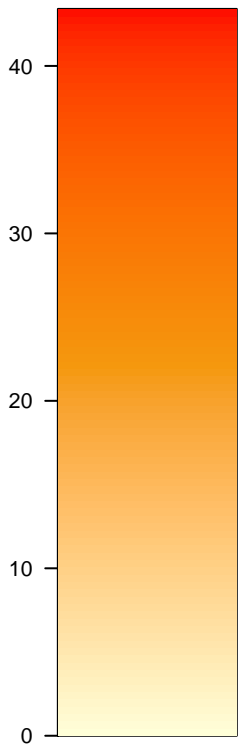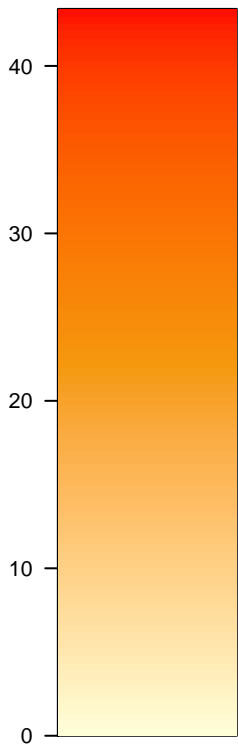

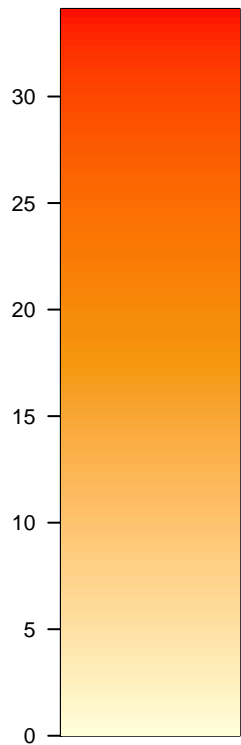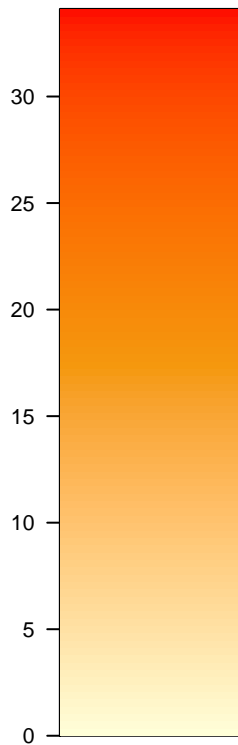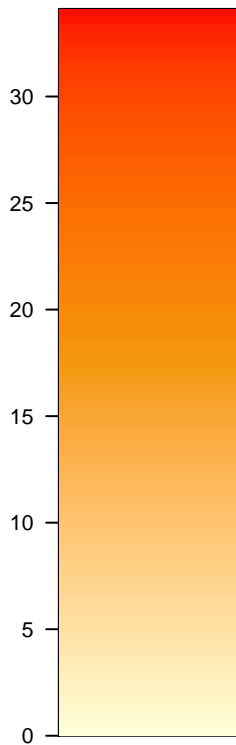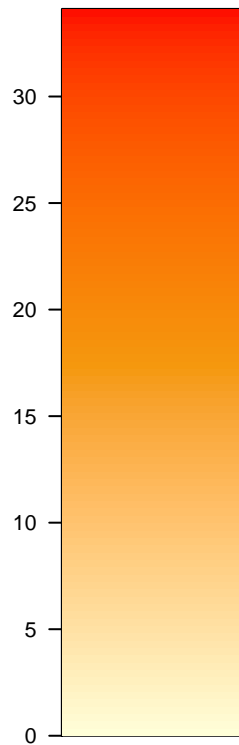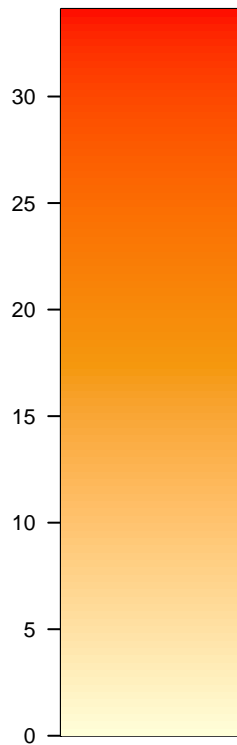

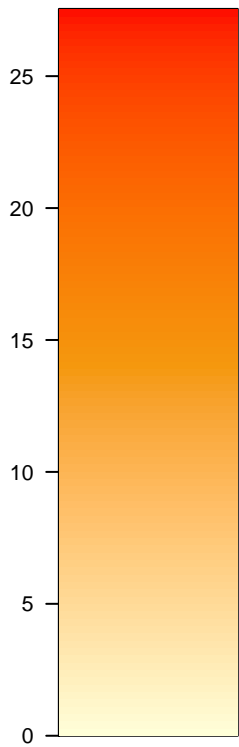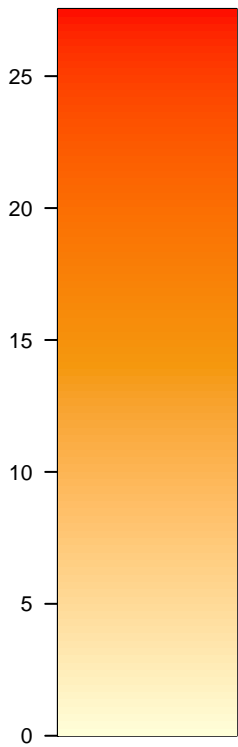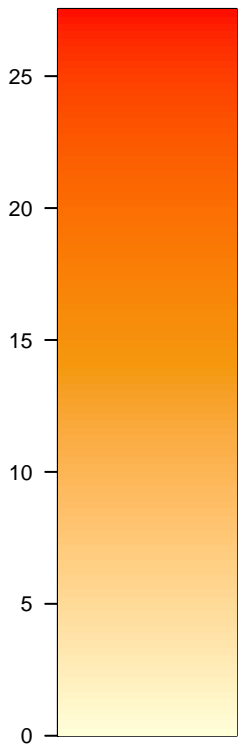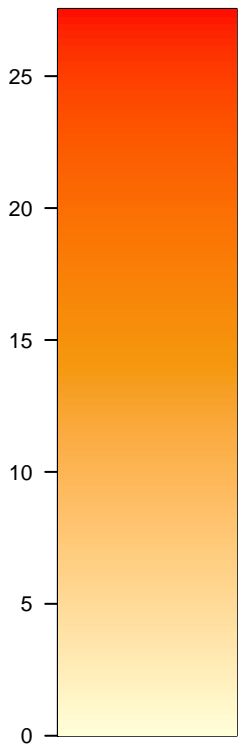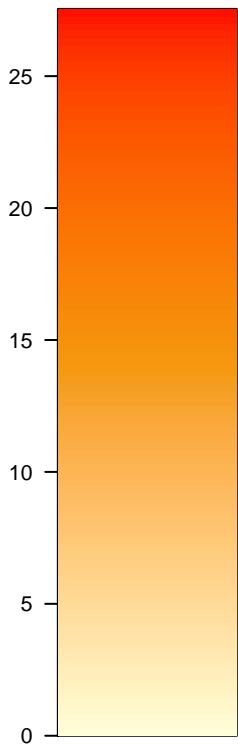

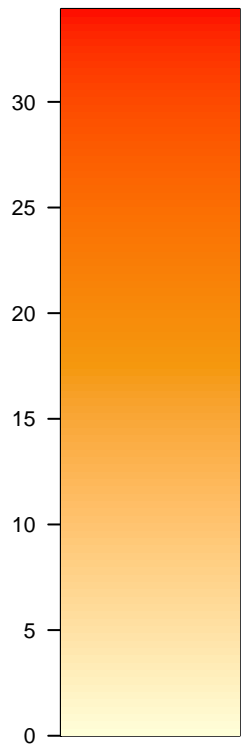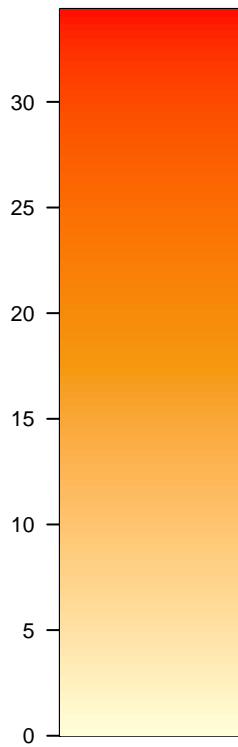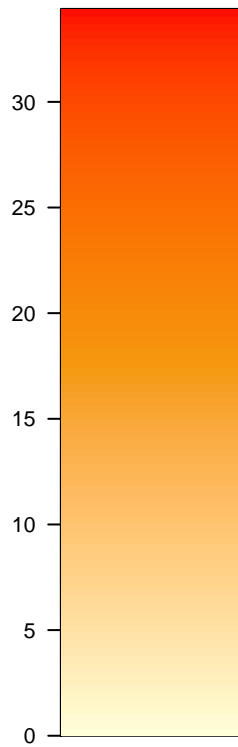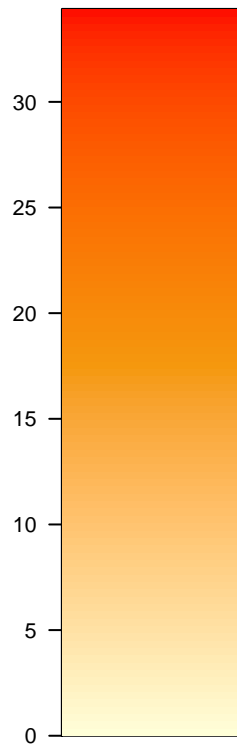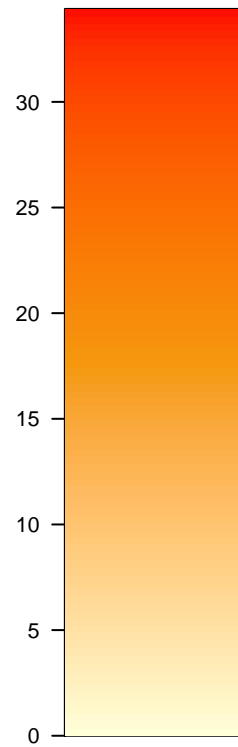

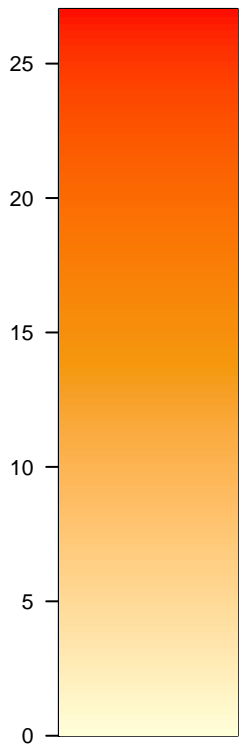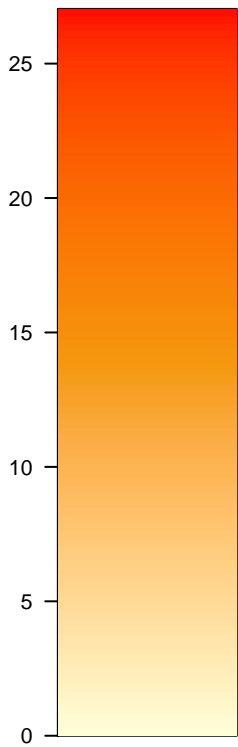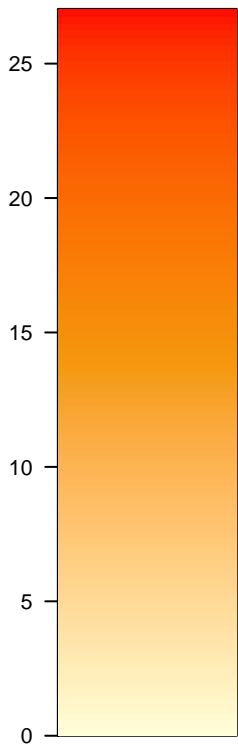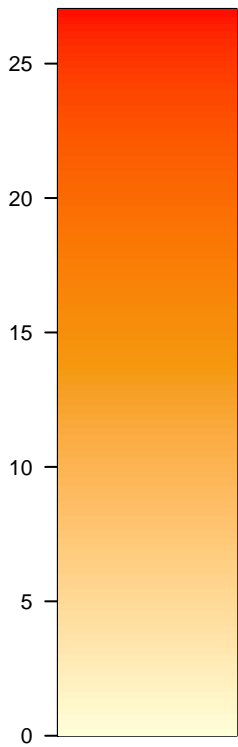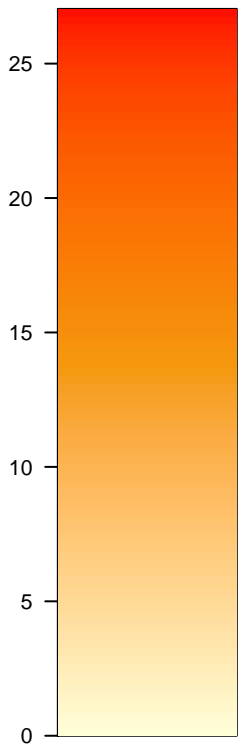

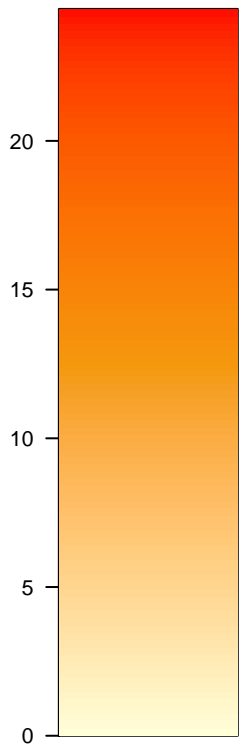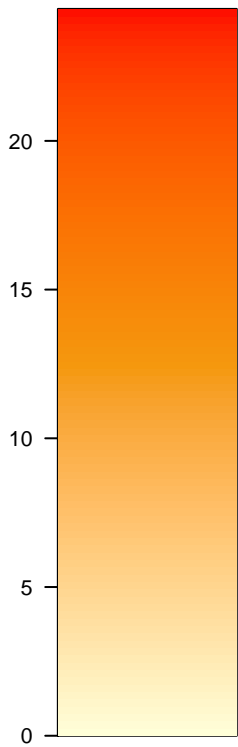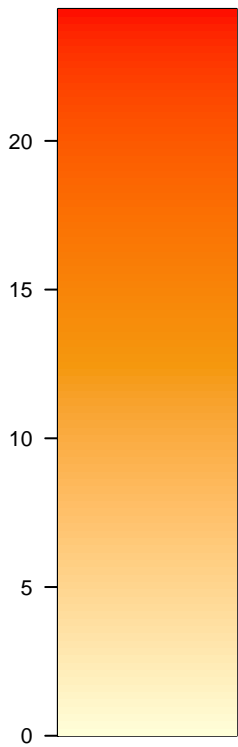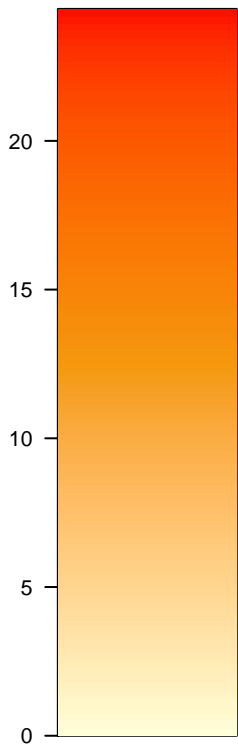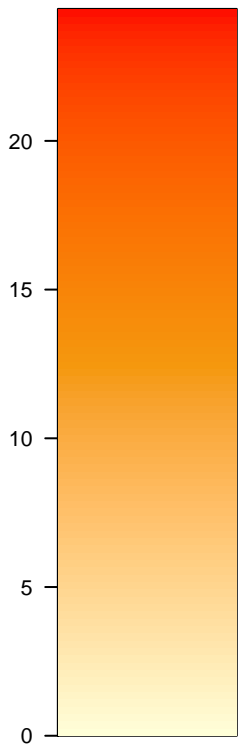

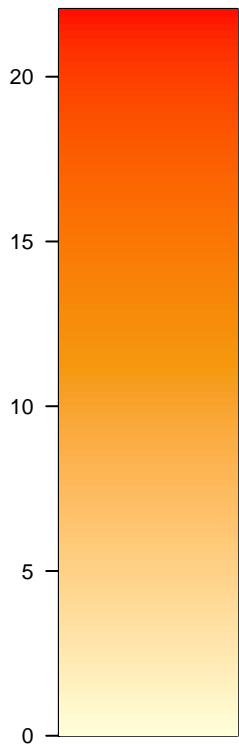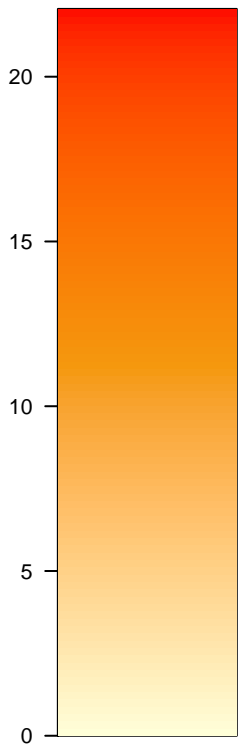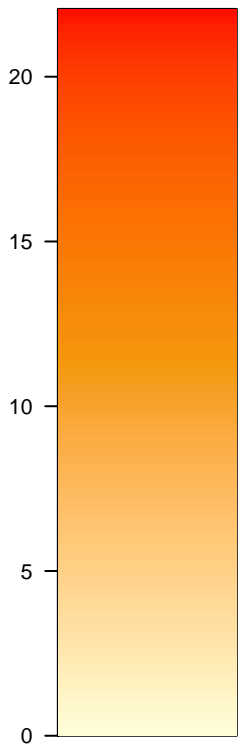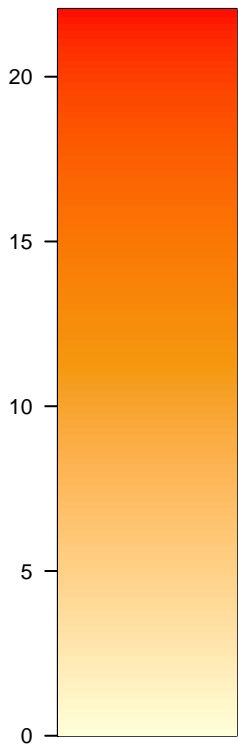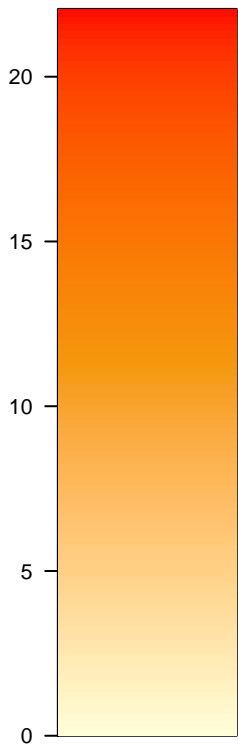

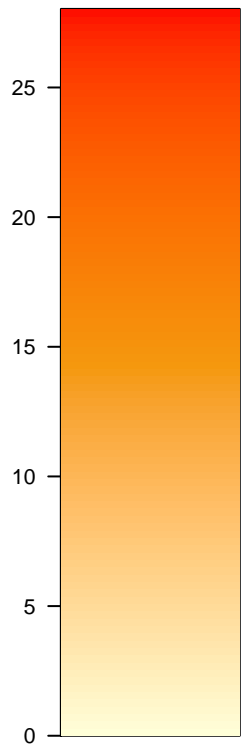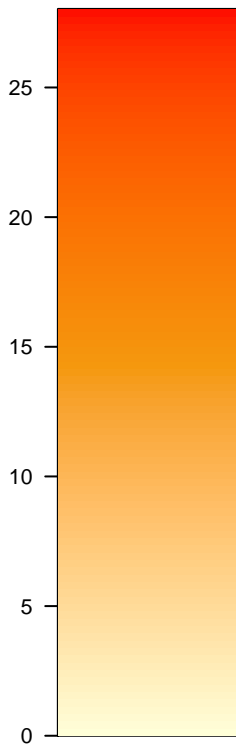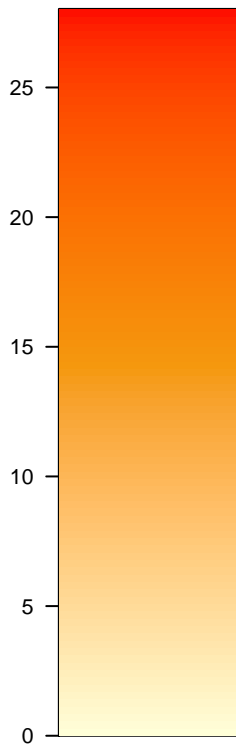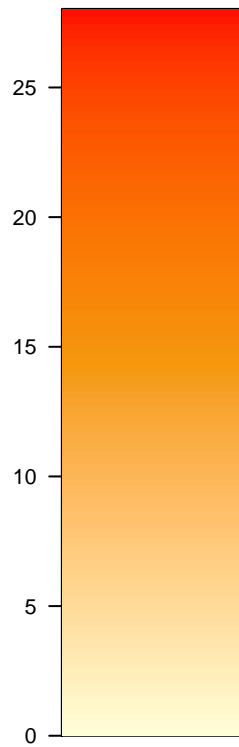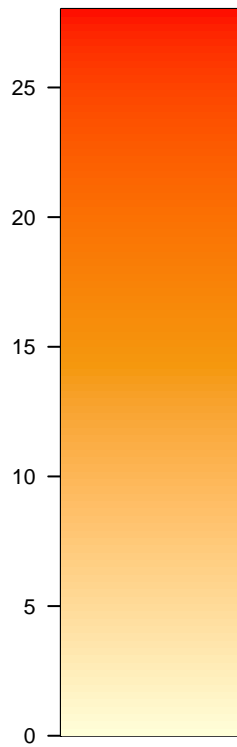

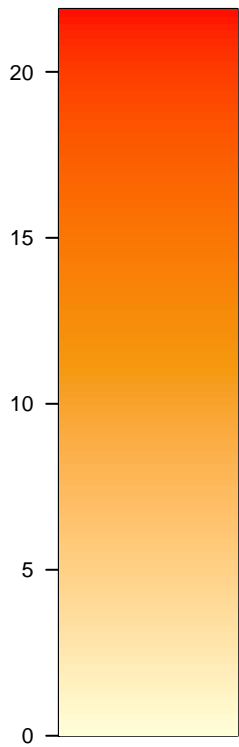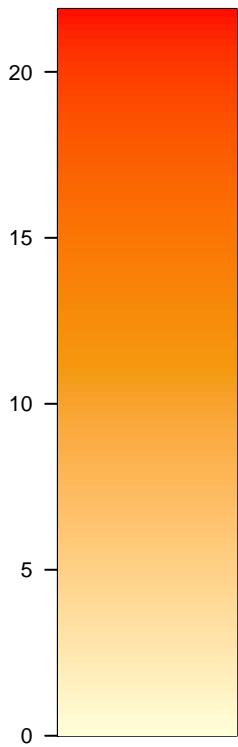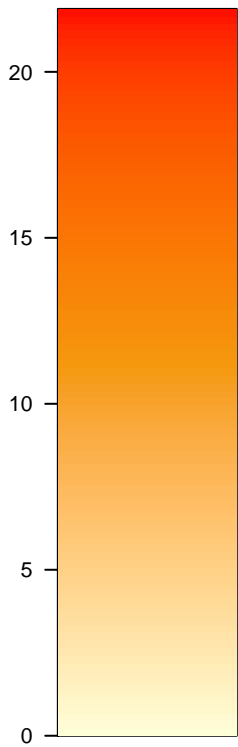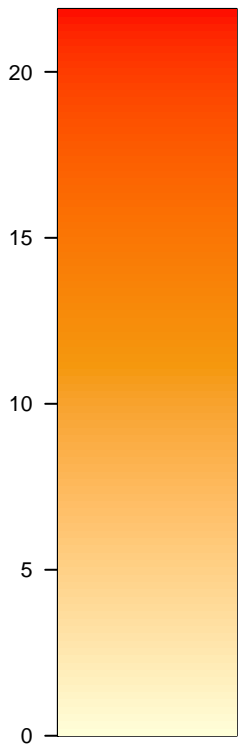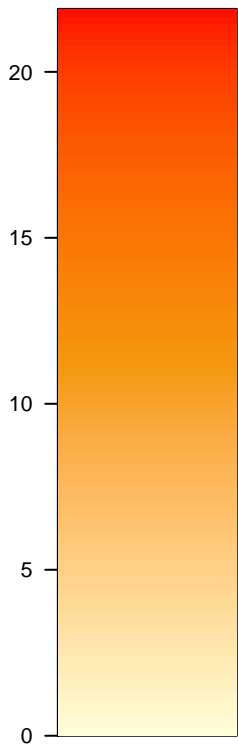

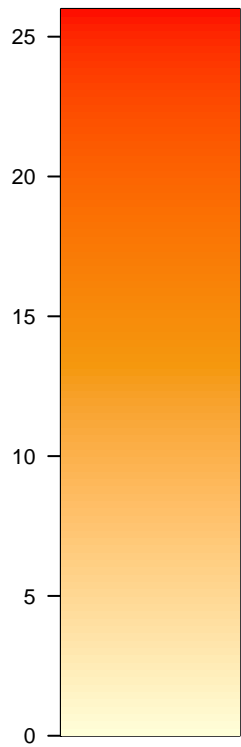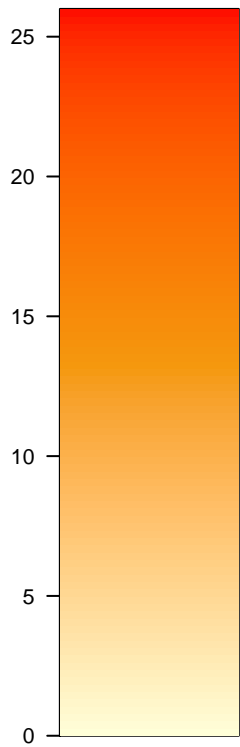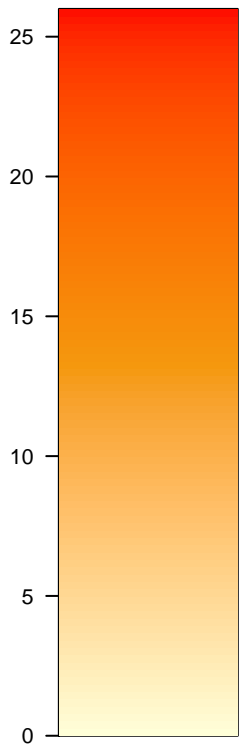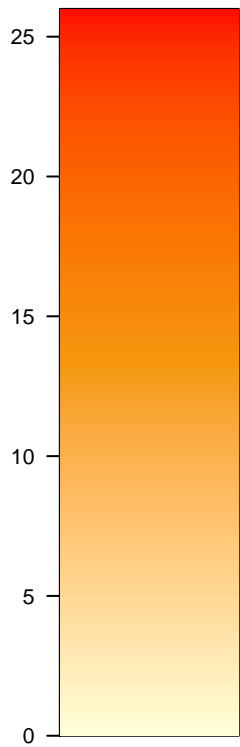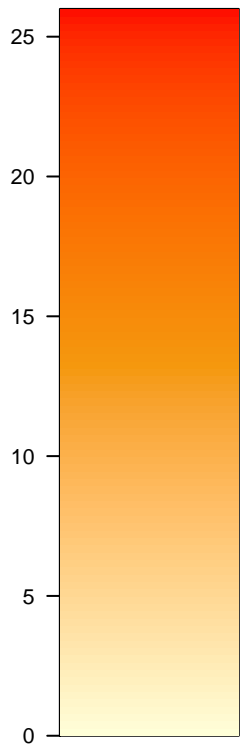

Supplement: Supplementary file 8 — Supplementary Data 5 [file 41467_2018_4724_MOESM8_ESM.zip › Supplementary Dataset 7/joint-field-profiles-rel-common-scale-dots-colorbar.pdf]

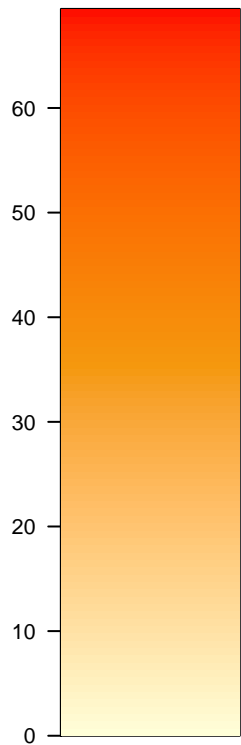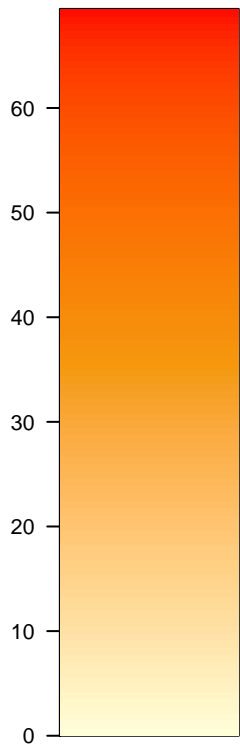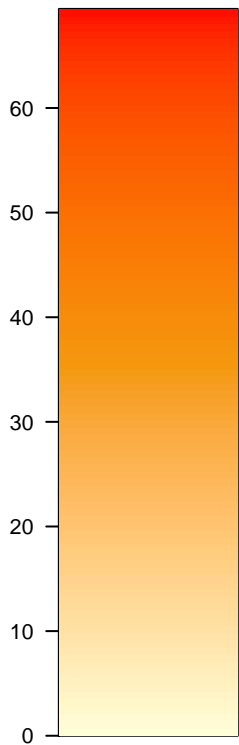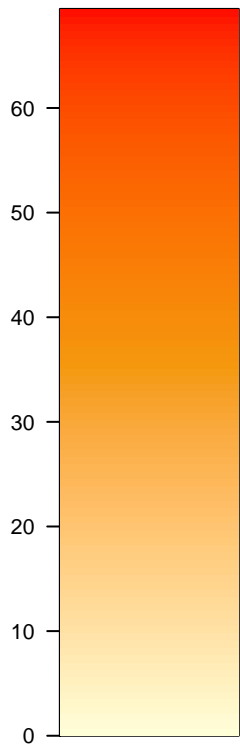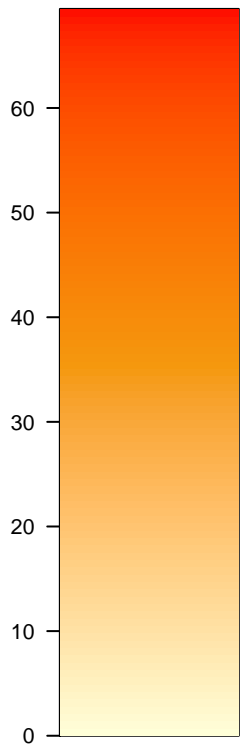

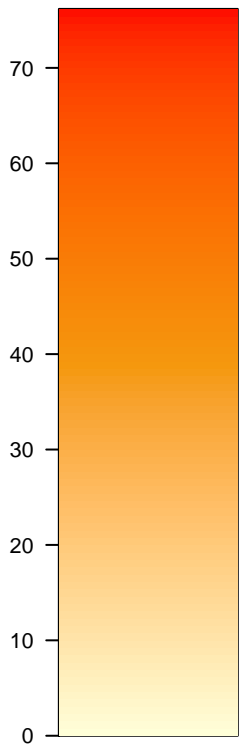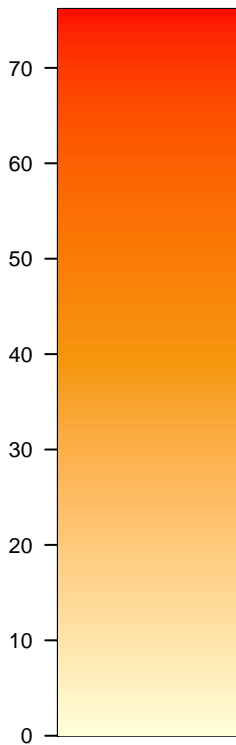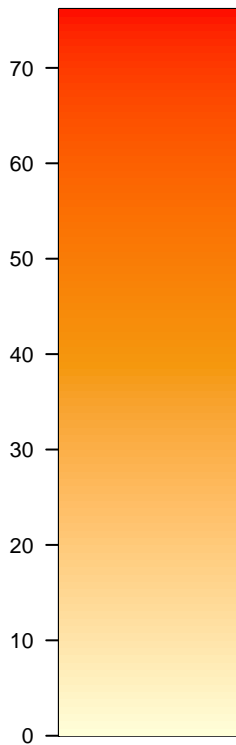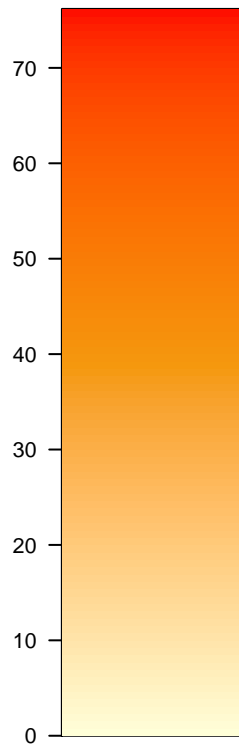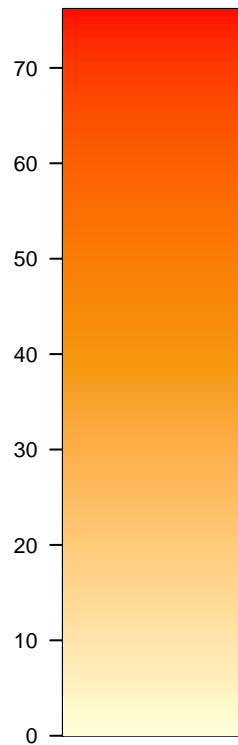

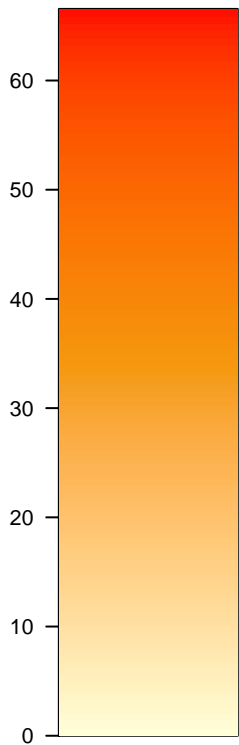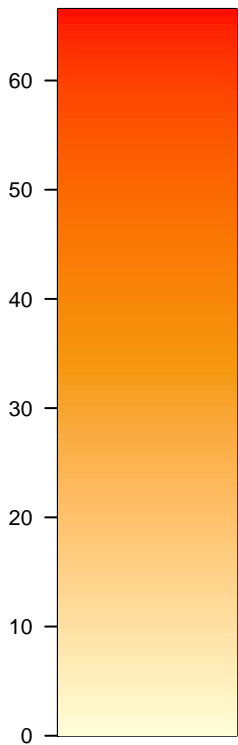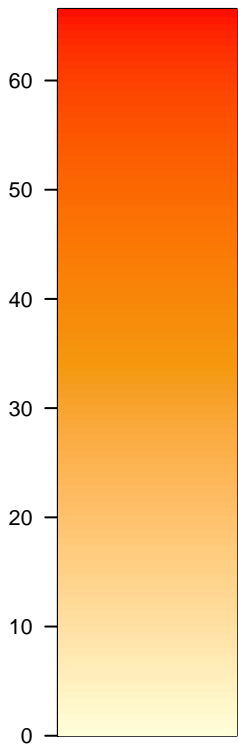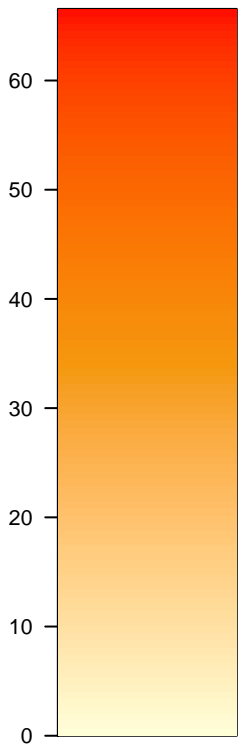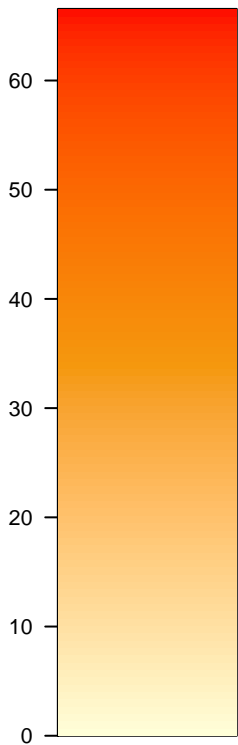

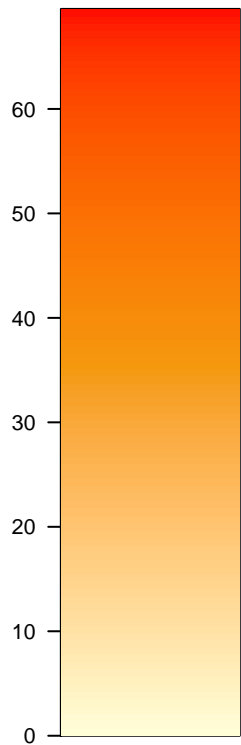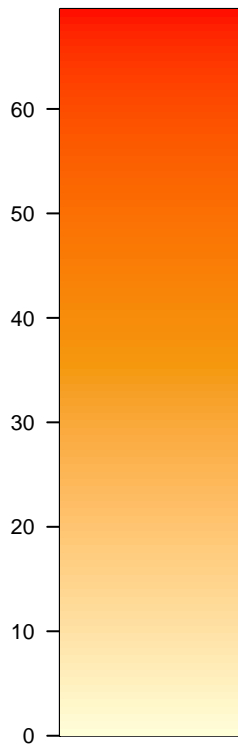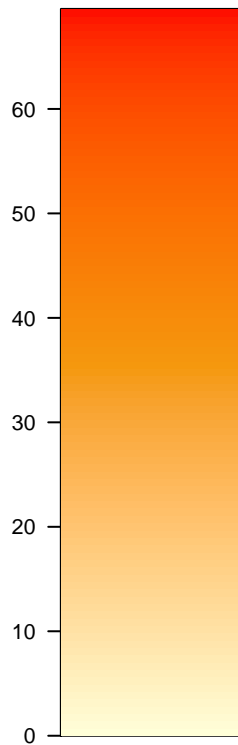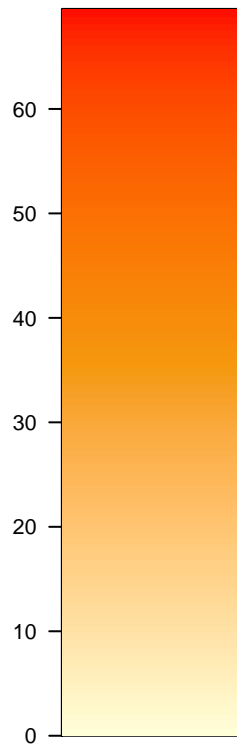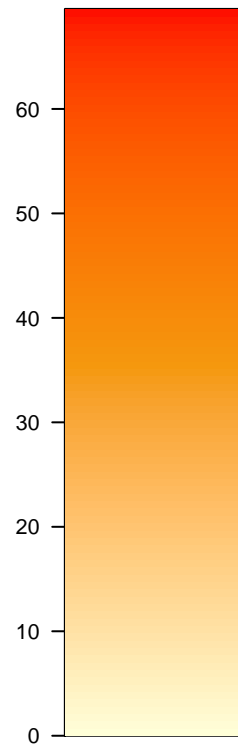

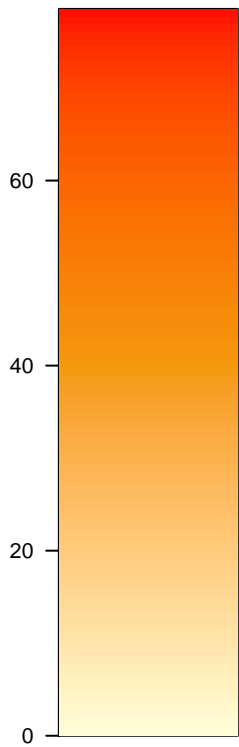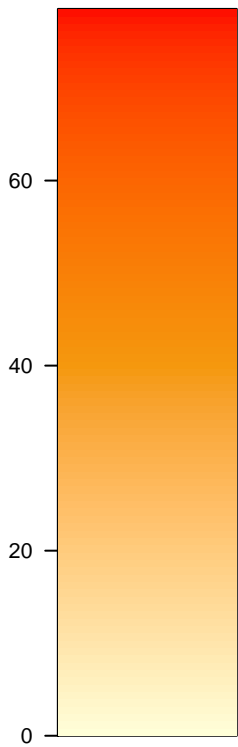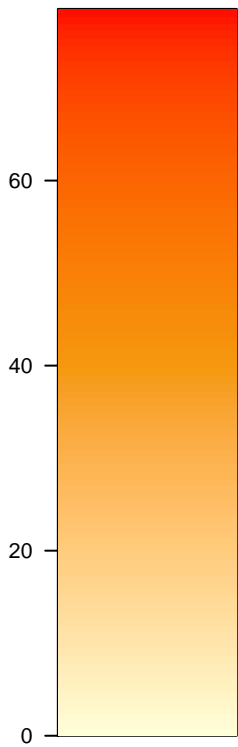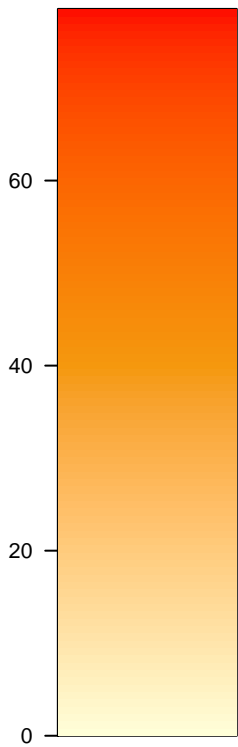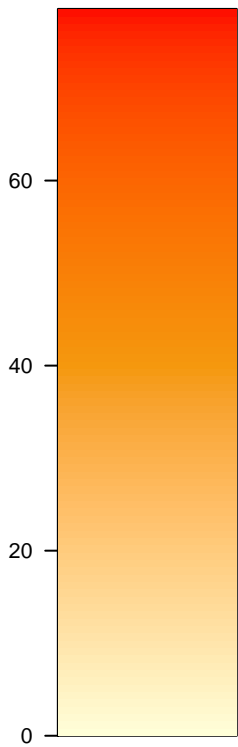

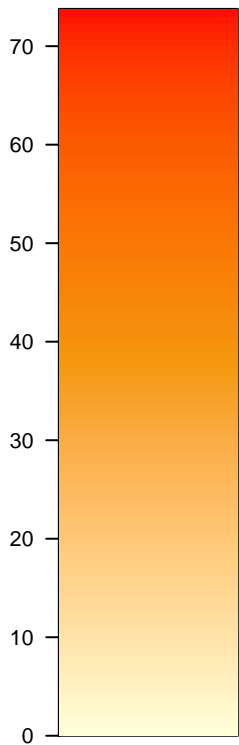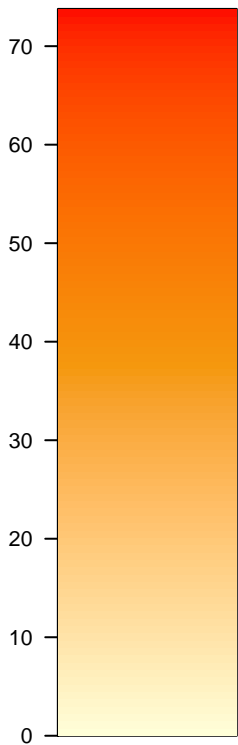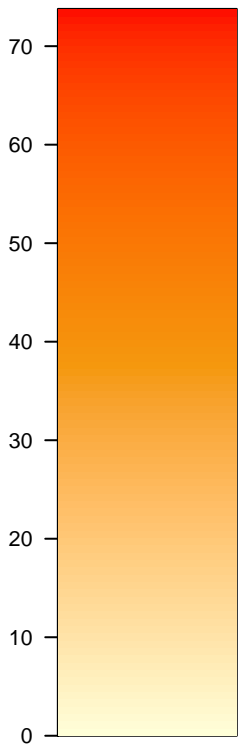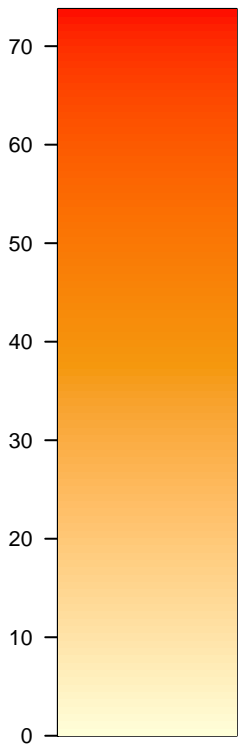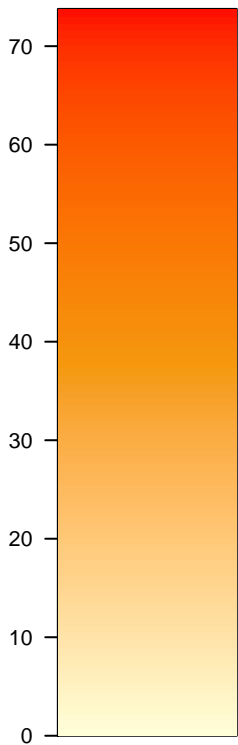

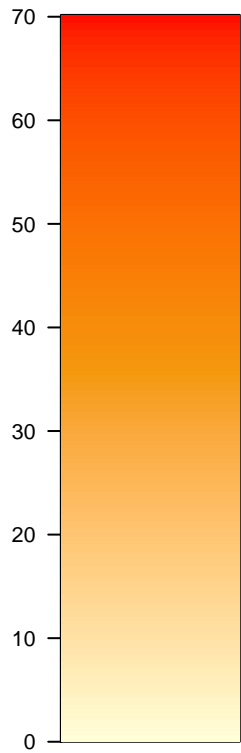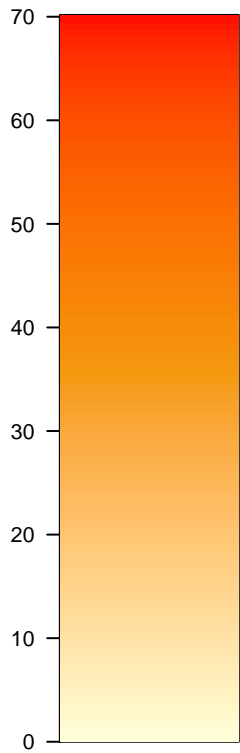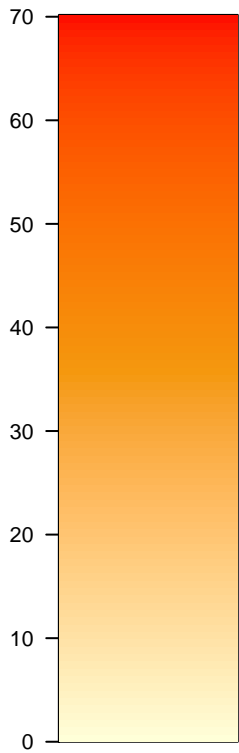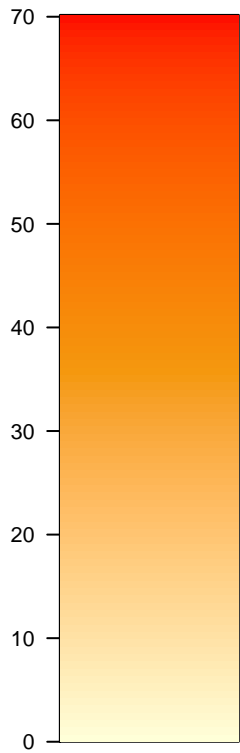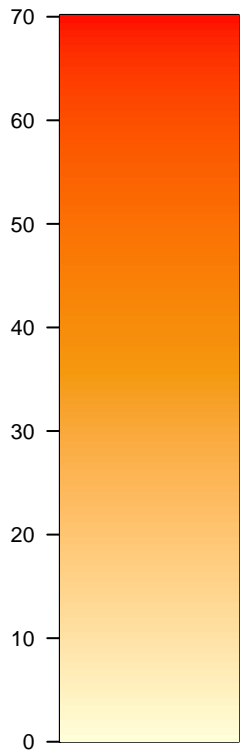

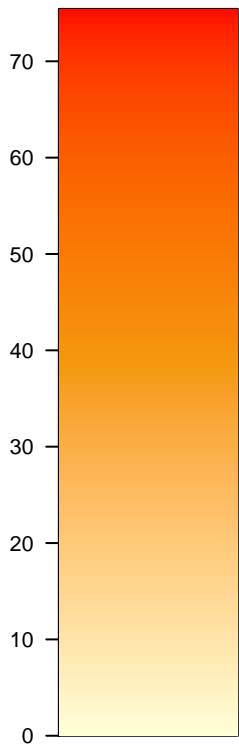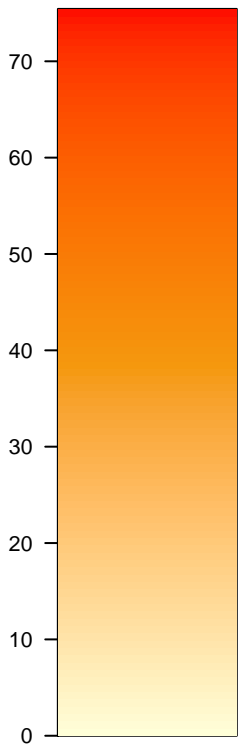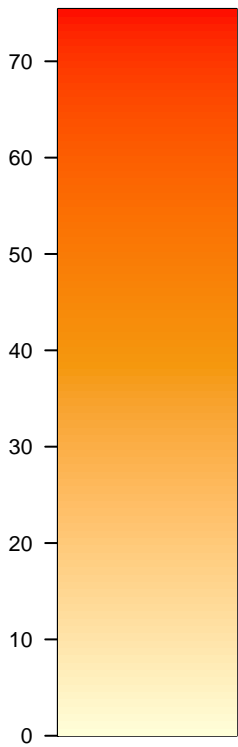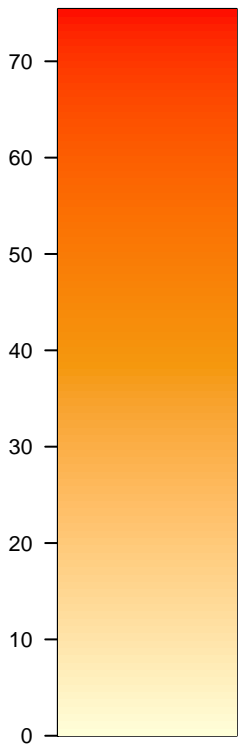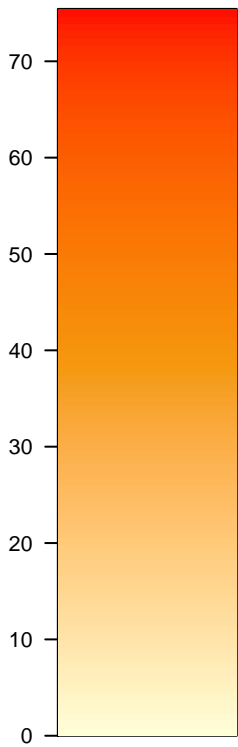

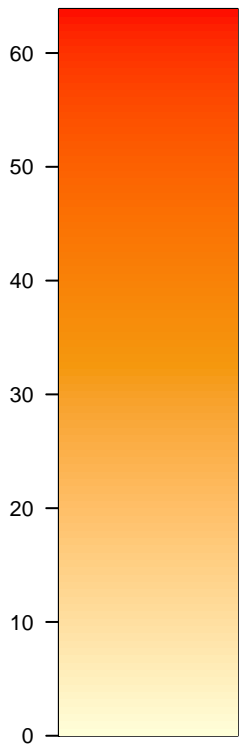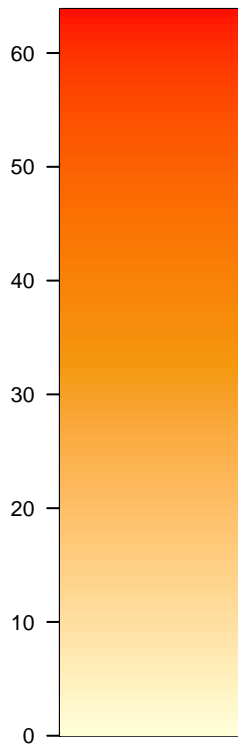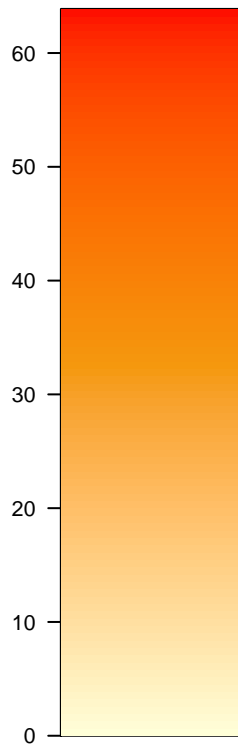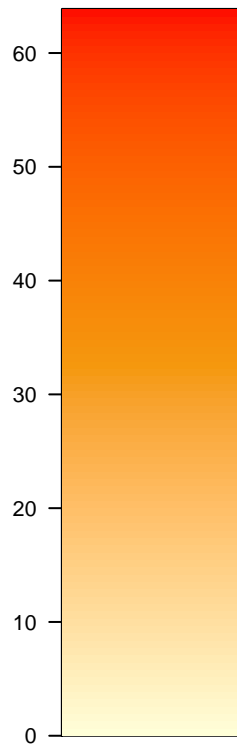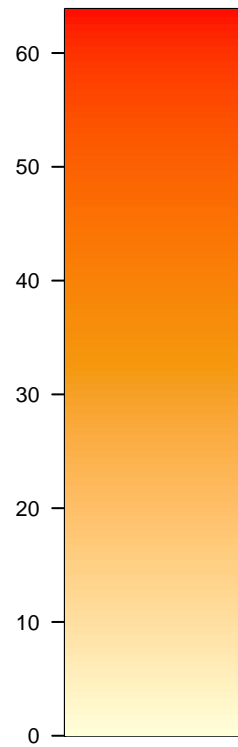

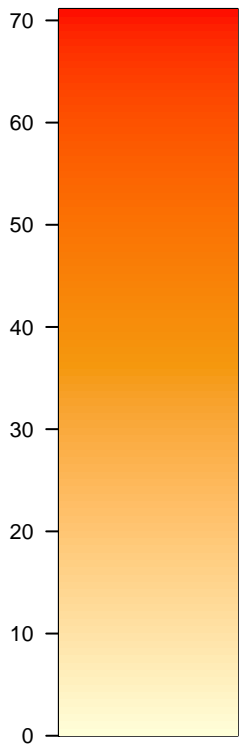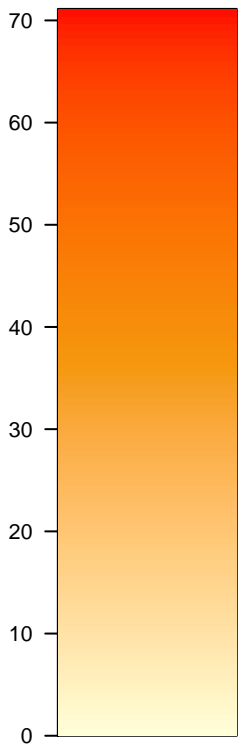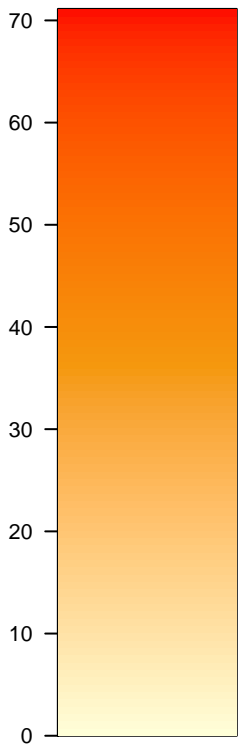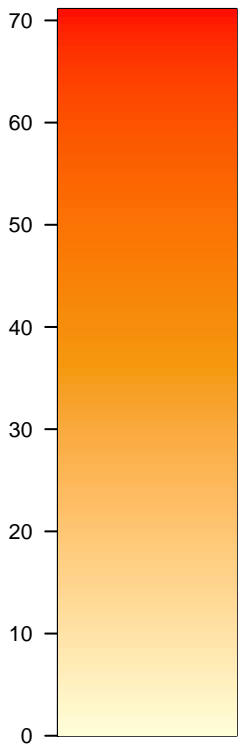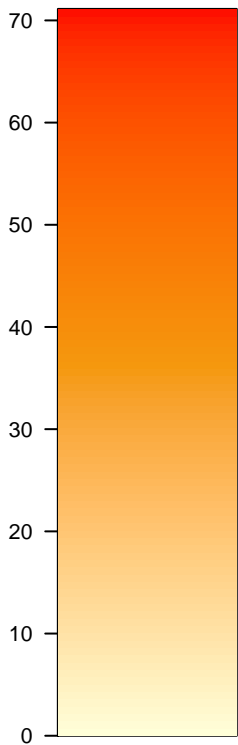

Supplement: Supplementary file 8 — Supplementary Data 5 [file 41467_2018_4724_MOESM8_ESM.zip › Supplementary Dataset 7/joint-mix-profiles-rel-common-scale-dots-colorbar.pdf]

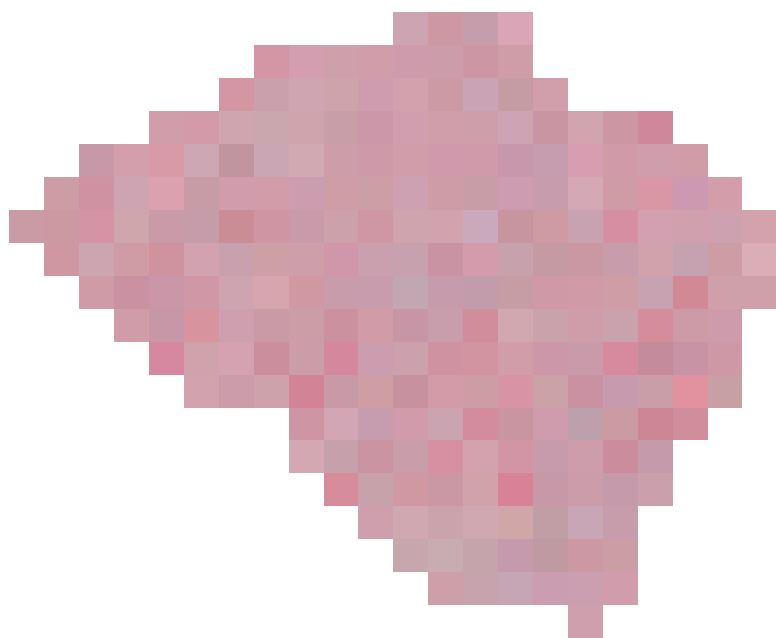

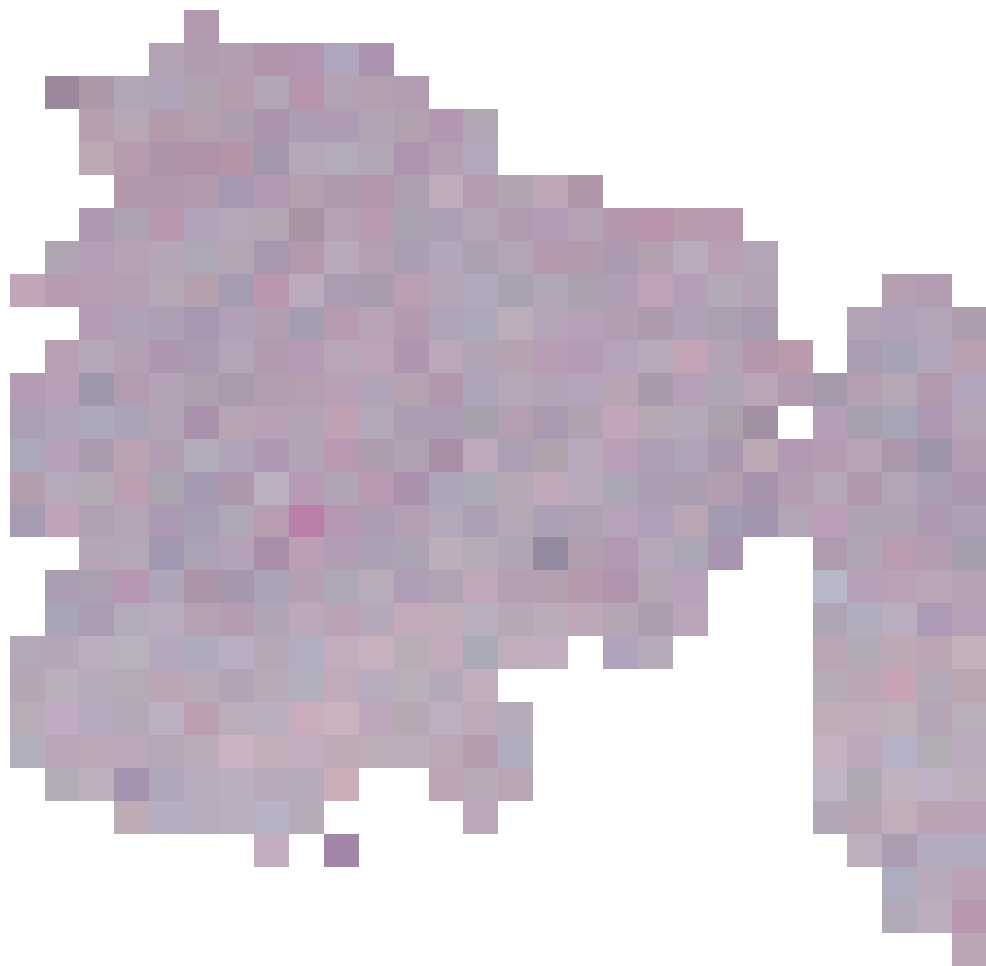

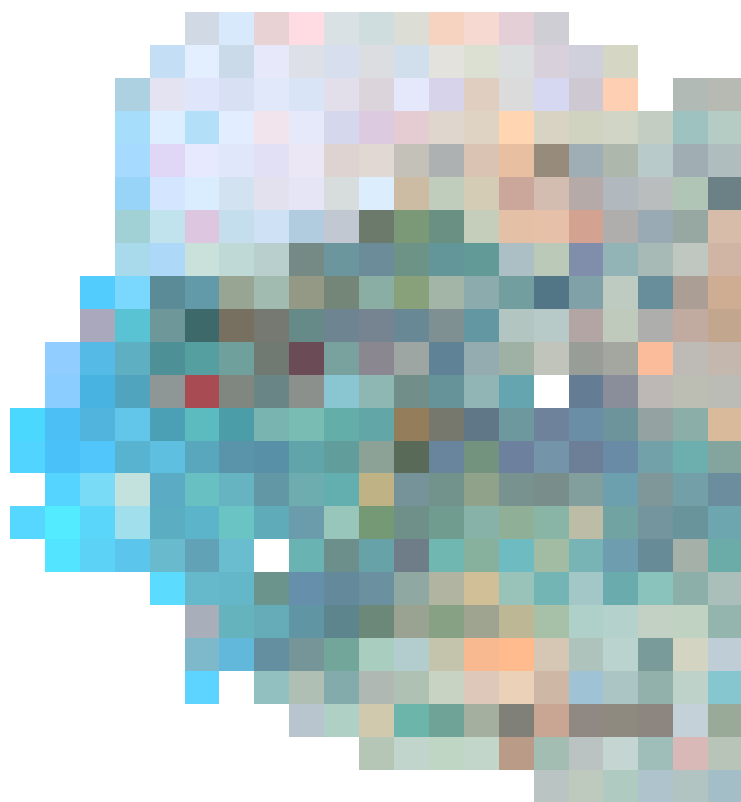

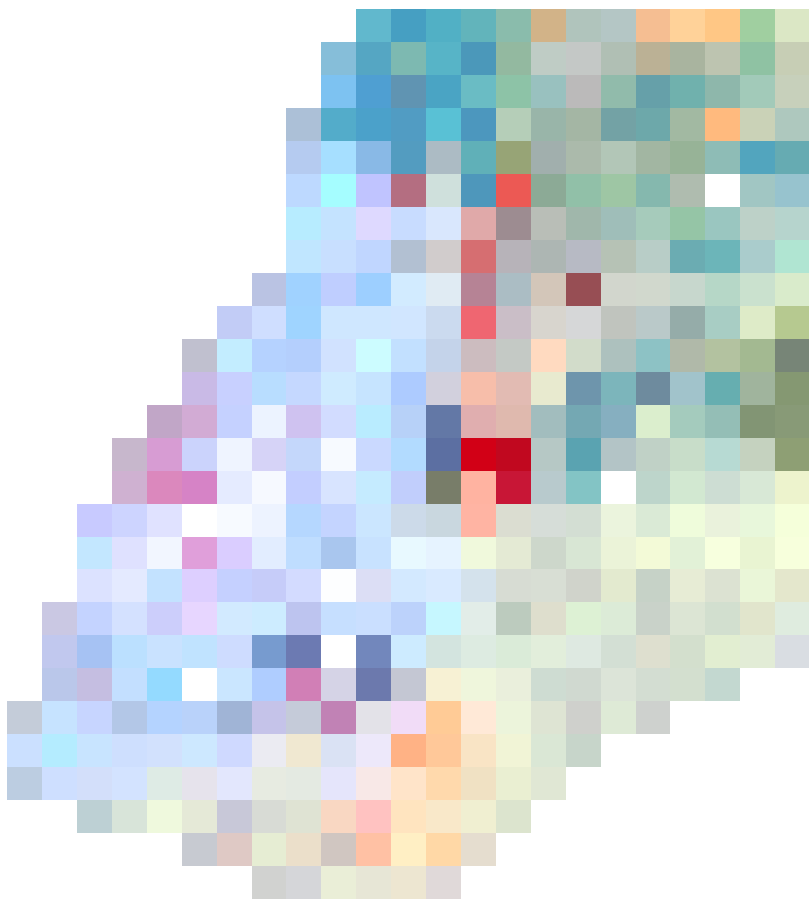

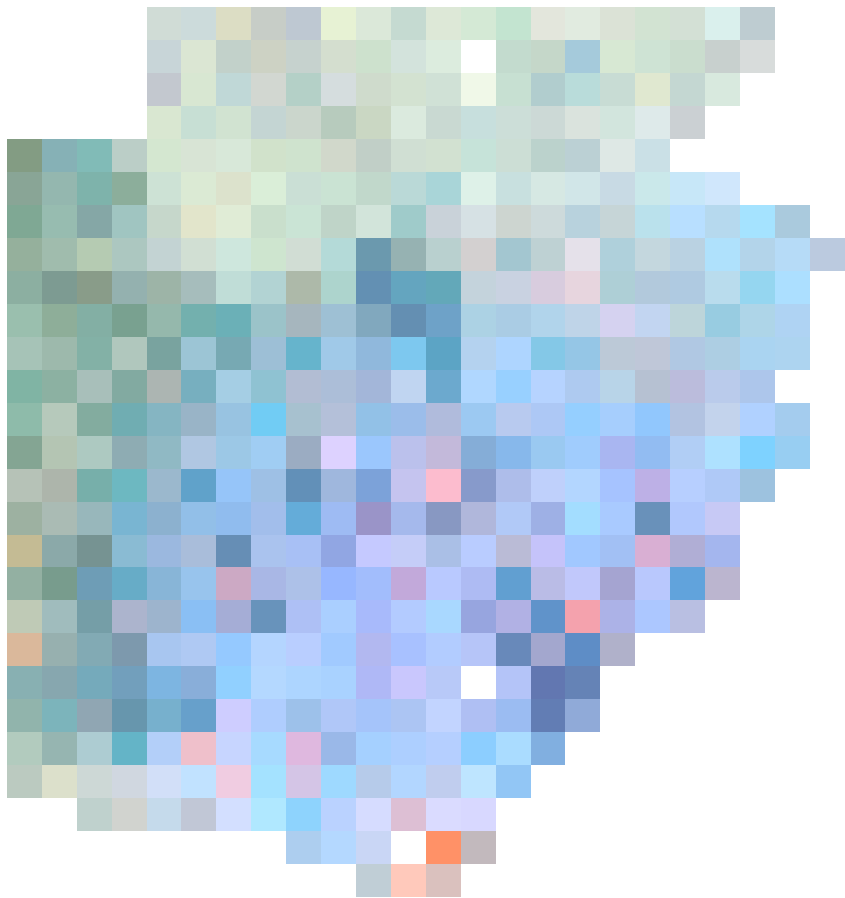

Supplement: Supplementary file 8 — Supplementary Data 5 [file 41467_2018_4724_MOESM8_ESM.zip › Supplementary Dataset 7/joint-mix-dimensionality-reduction-PCA-matrix-split.pdf]

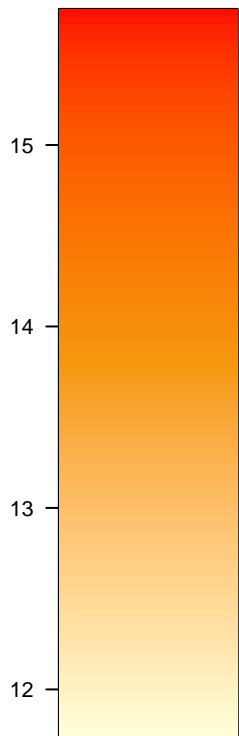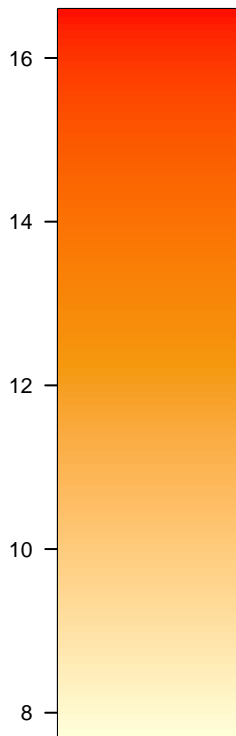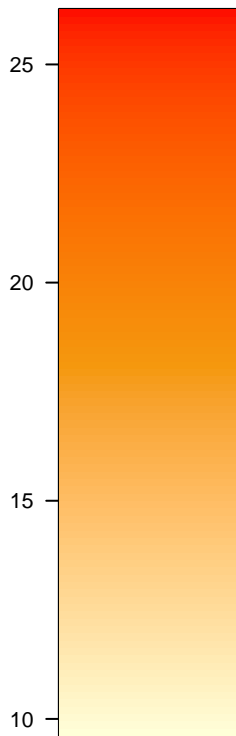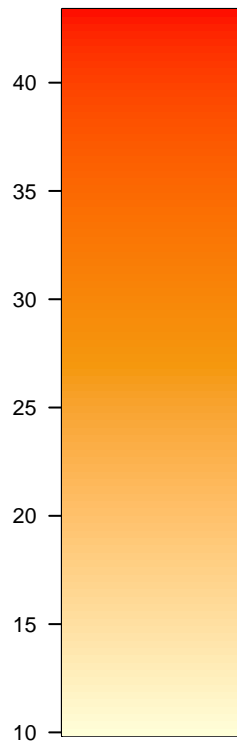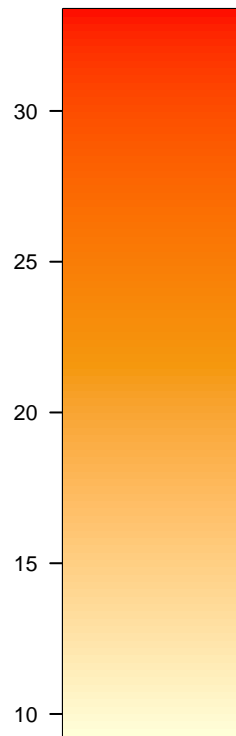

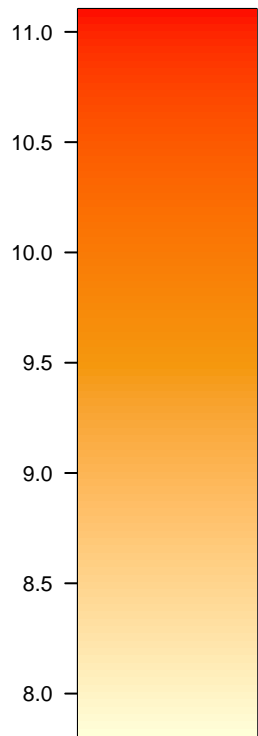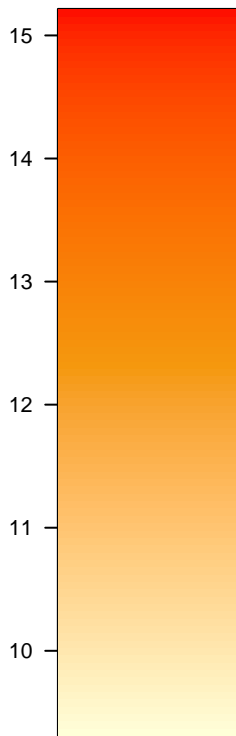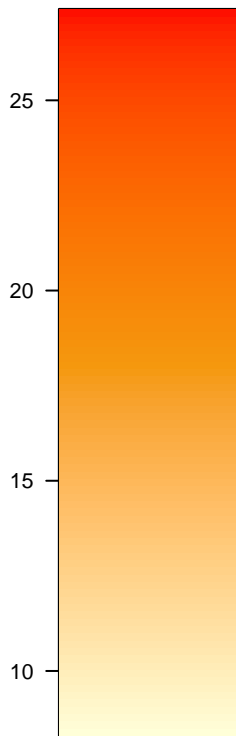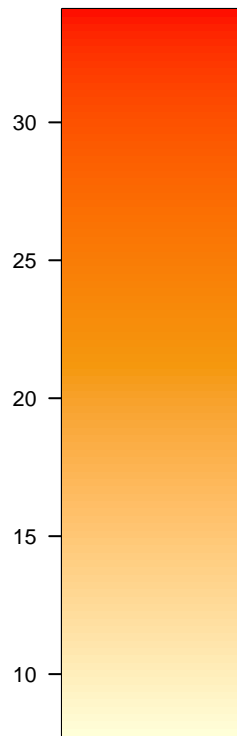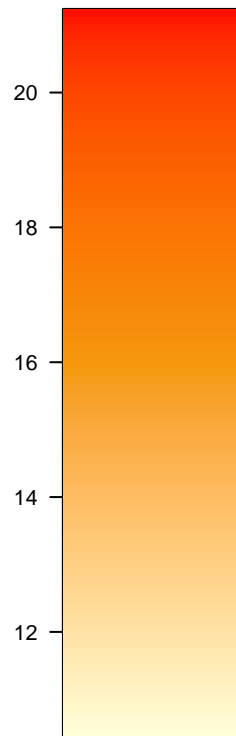

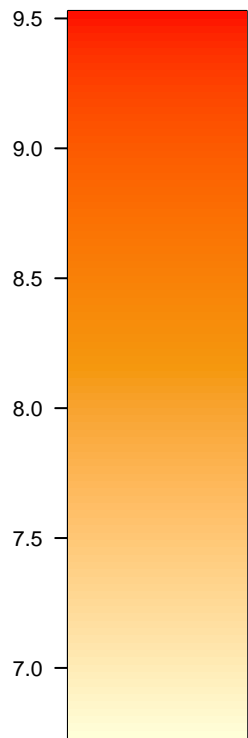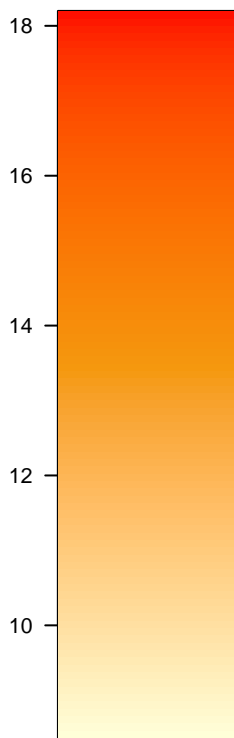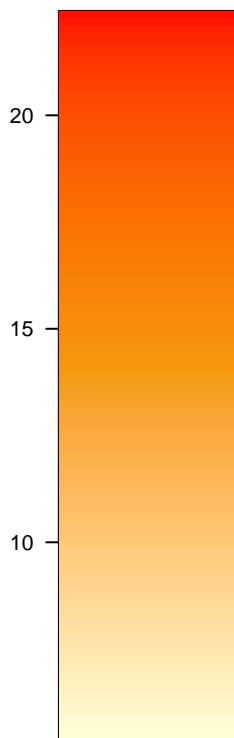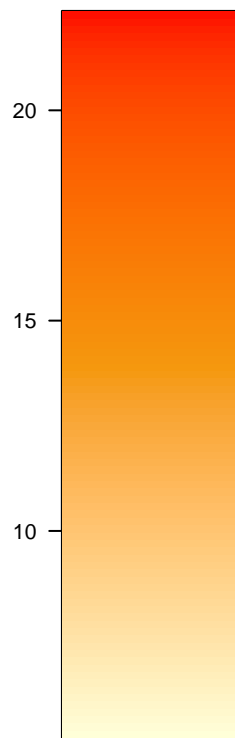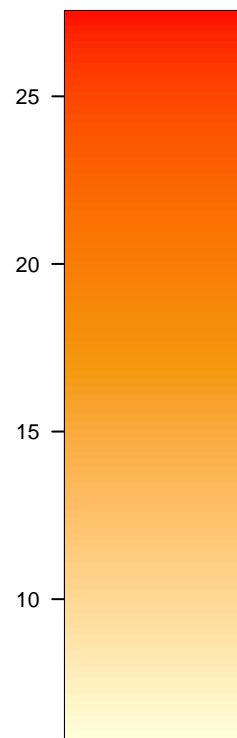

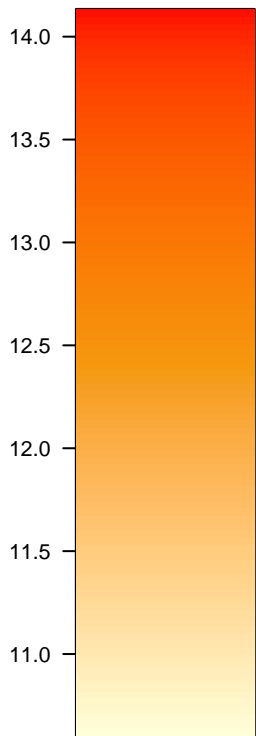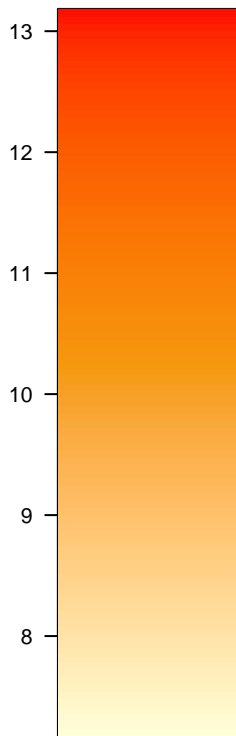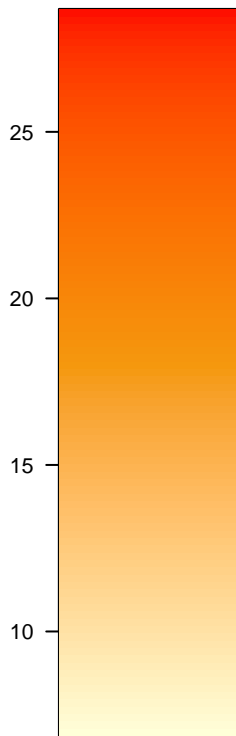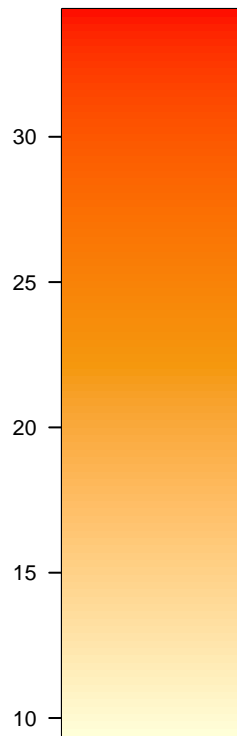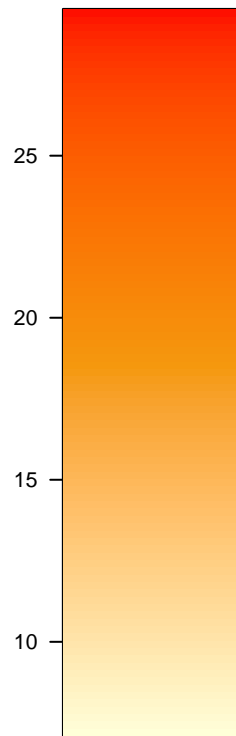

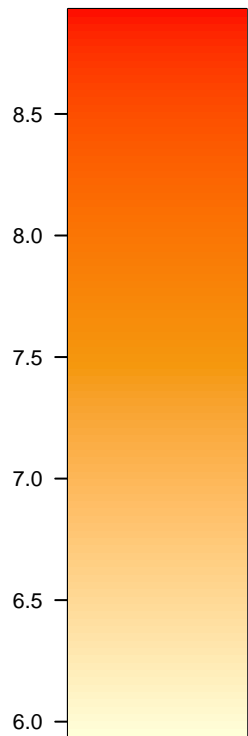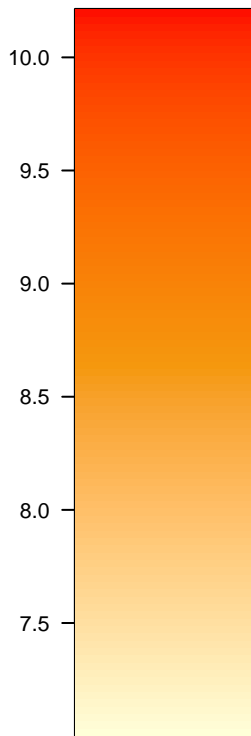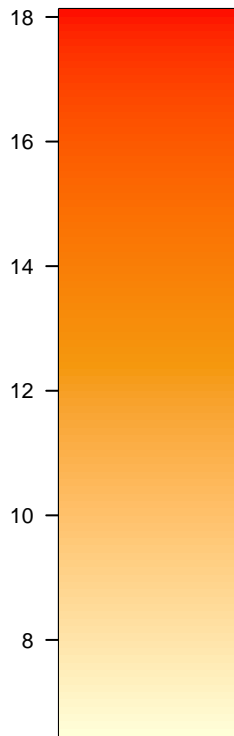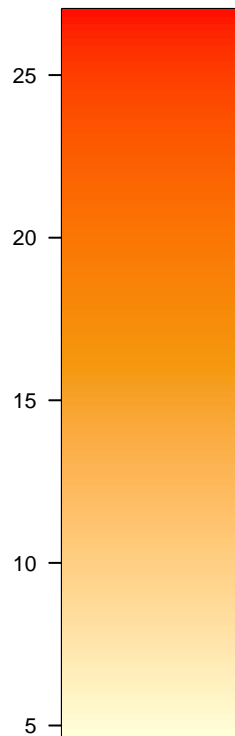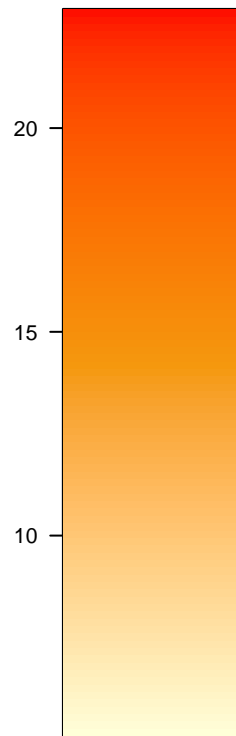

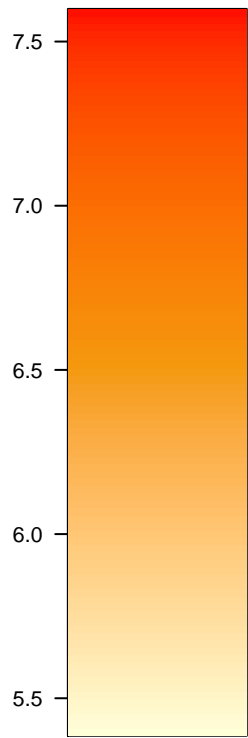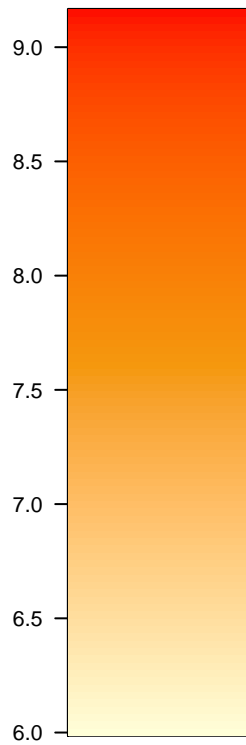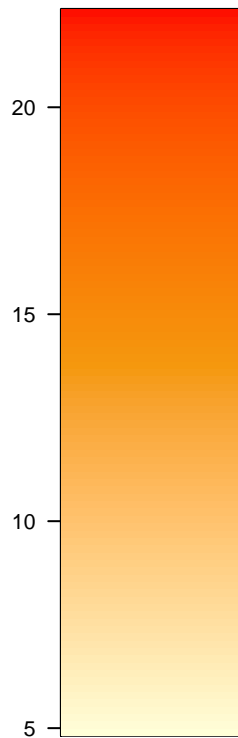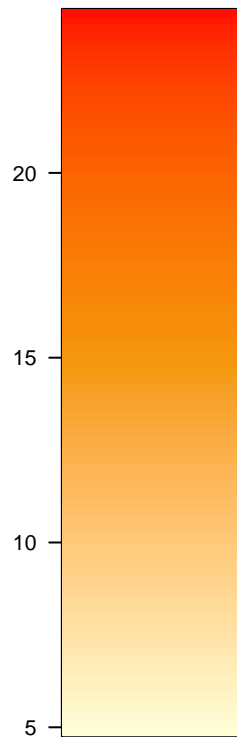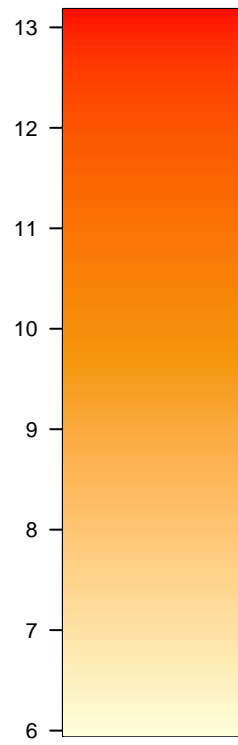

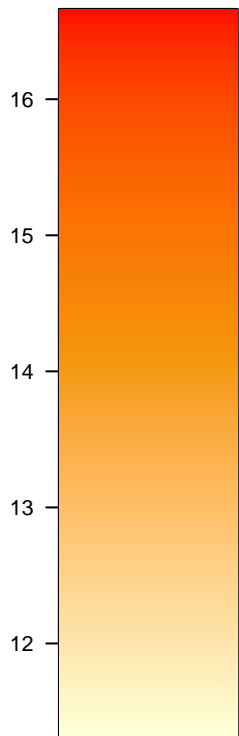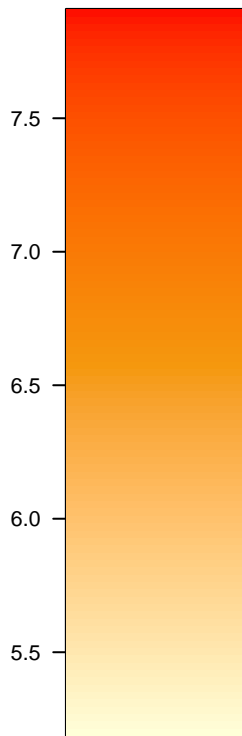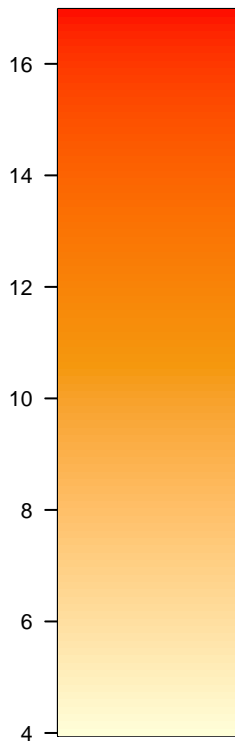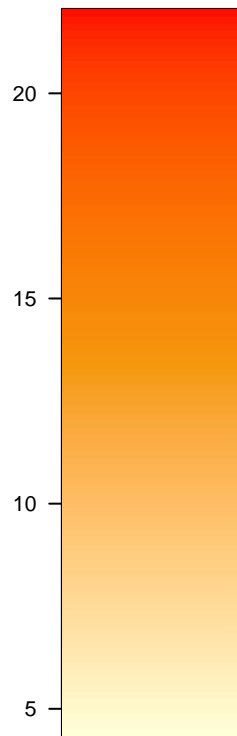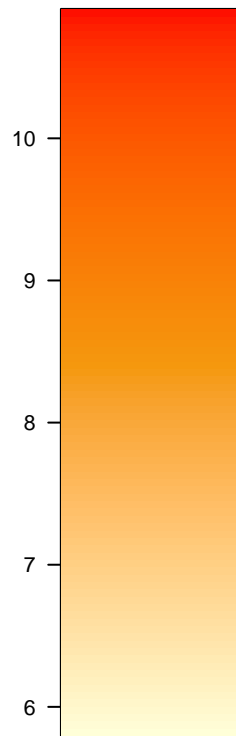

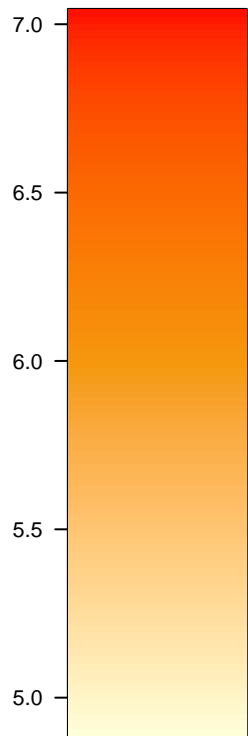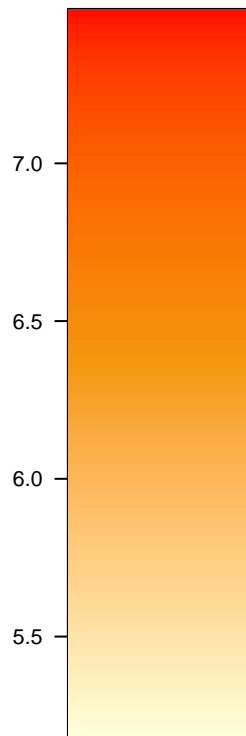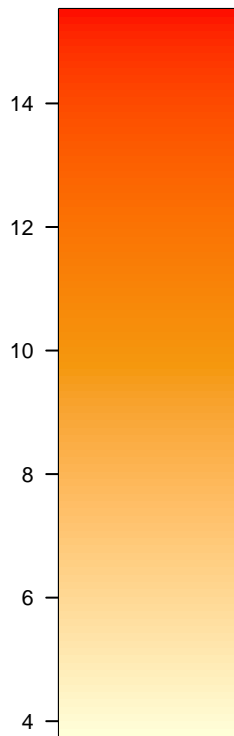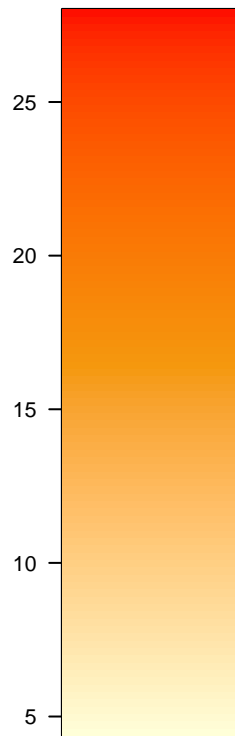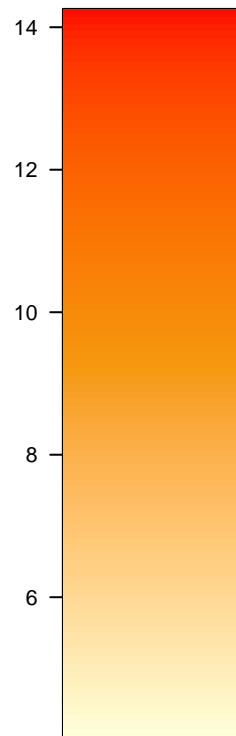

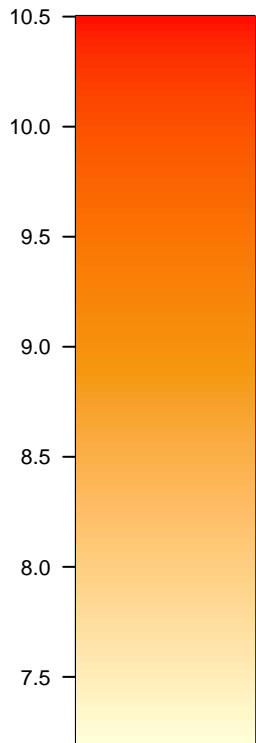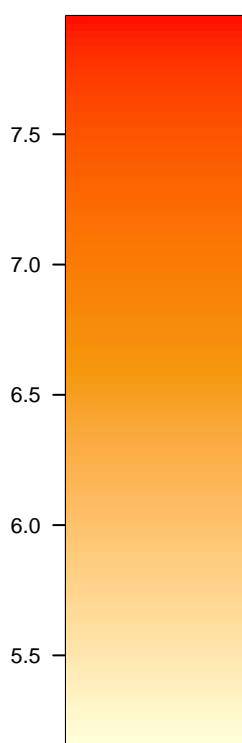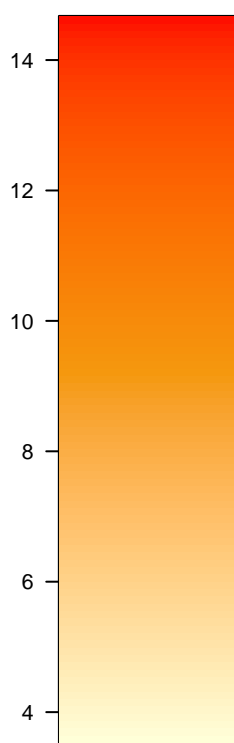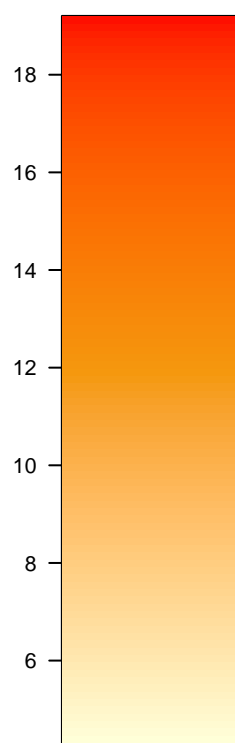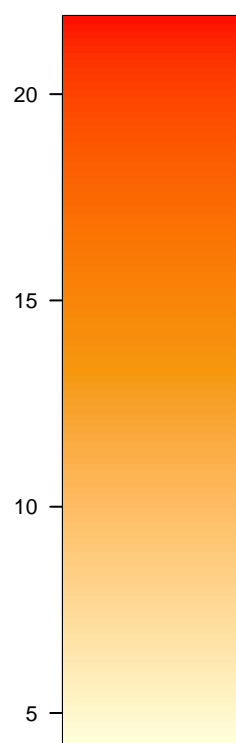

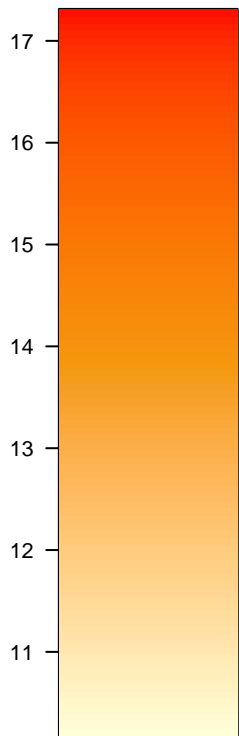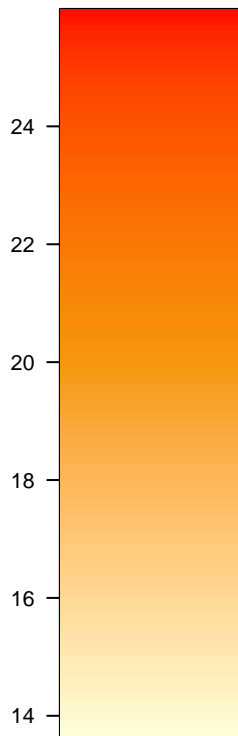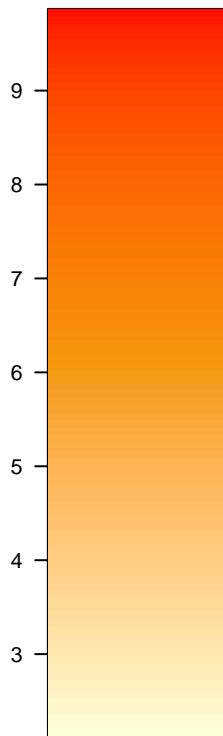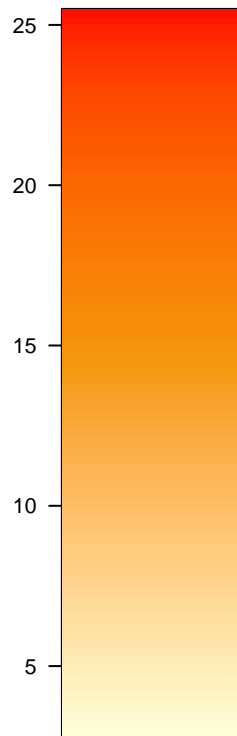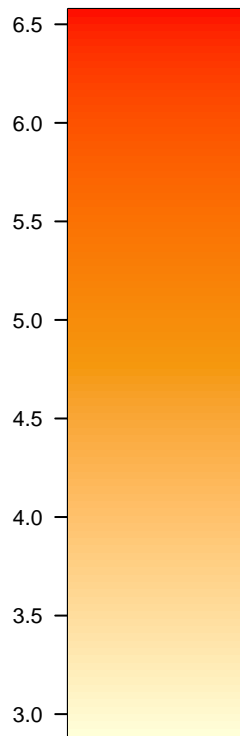

Supplement: Supplementary file 8 — Supplementary Data 5 [file 41467_2018_4724_MOESM8_ESM.zip › Supplementary Dataset 7/joint-field-profiles-rel-individual-scale-dots-colorbar.pdf]

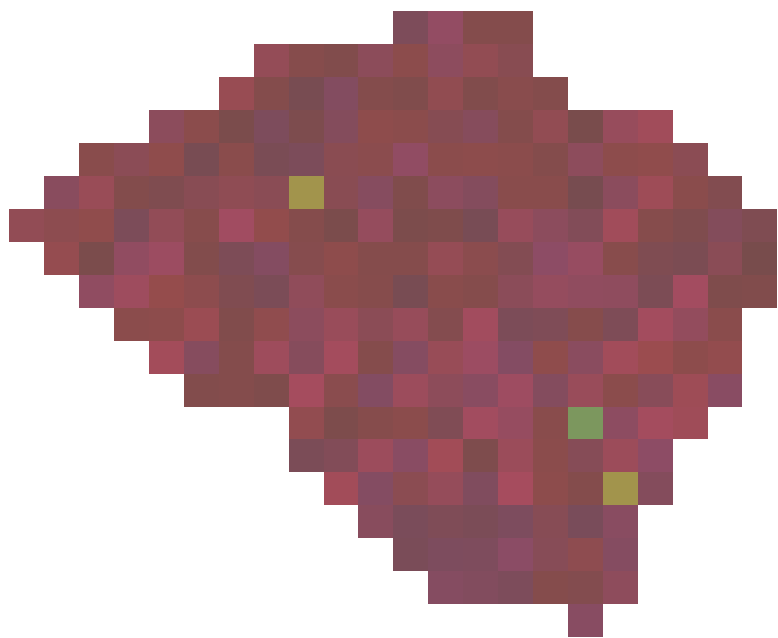

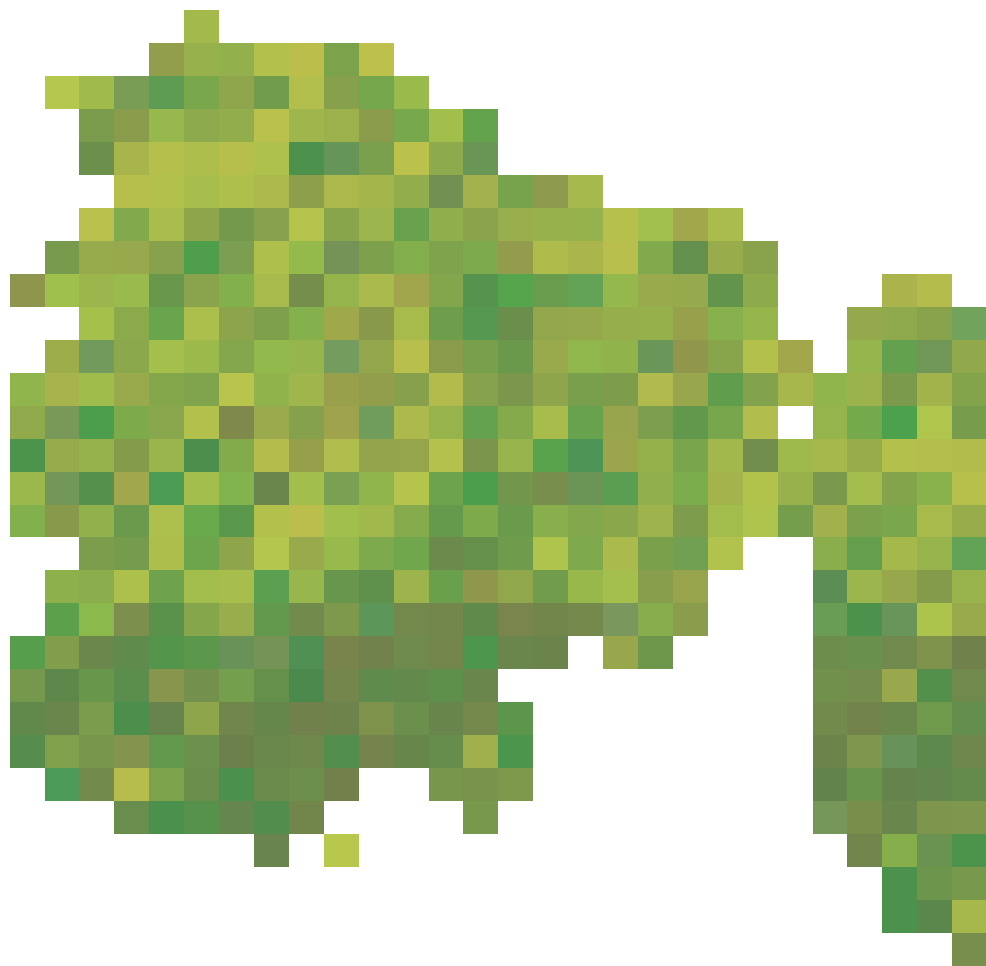

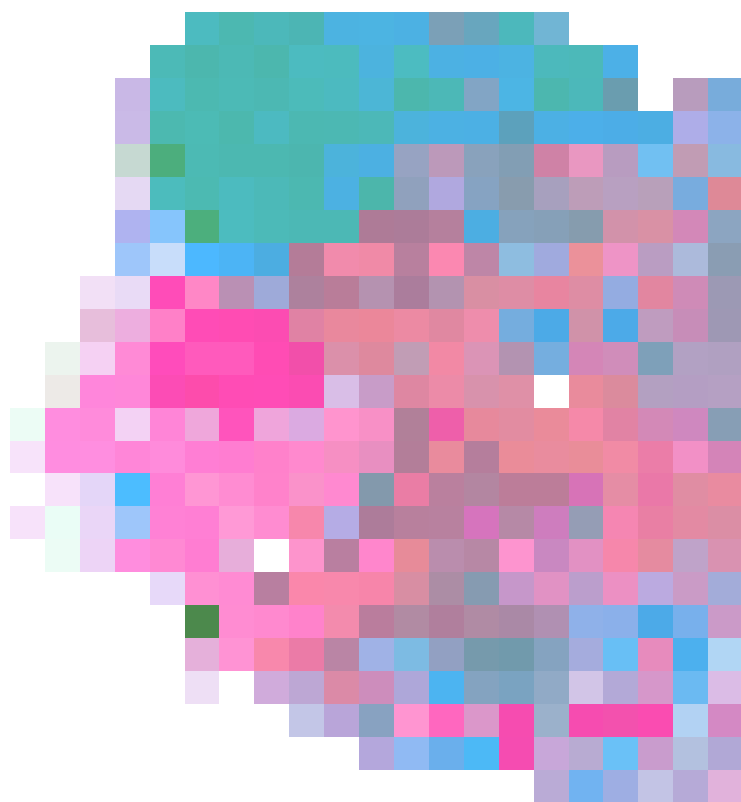

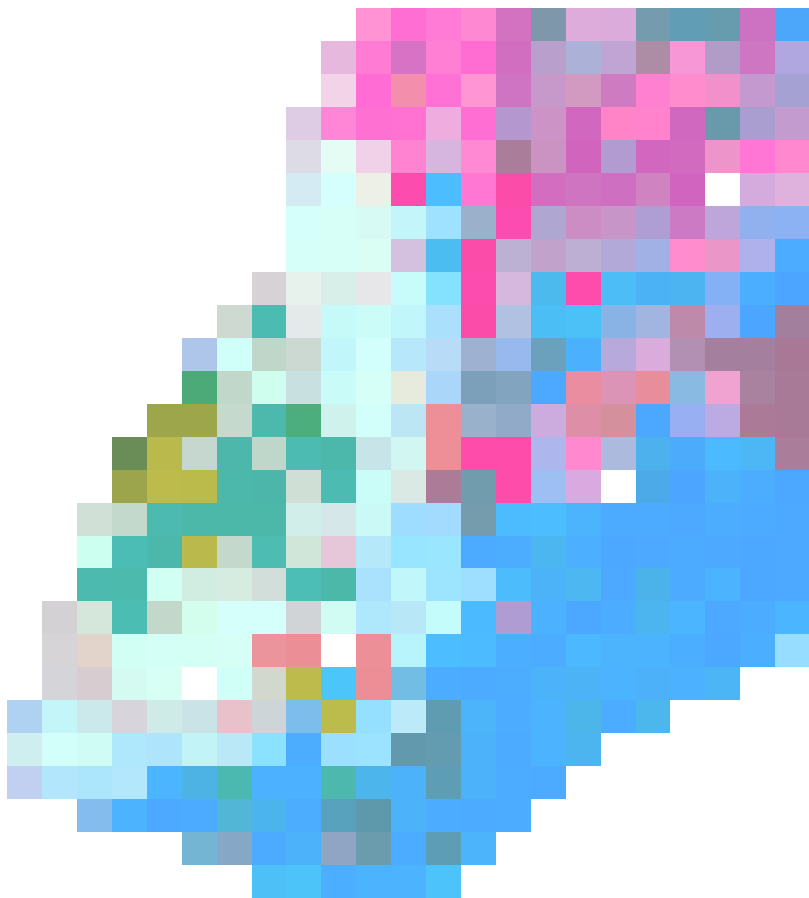

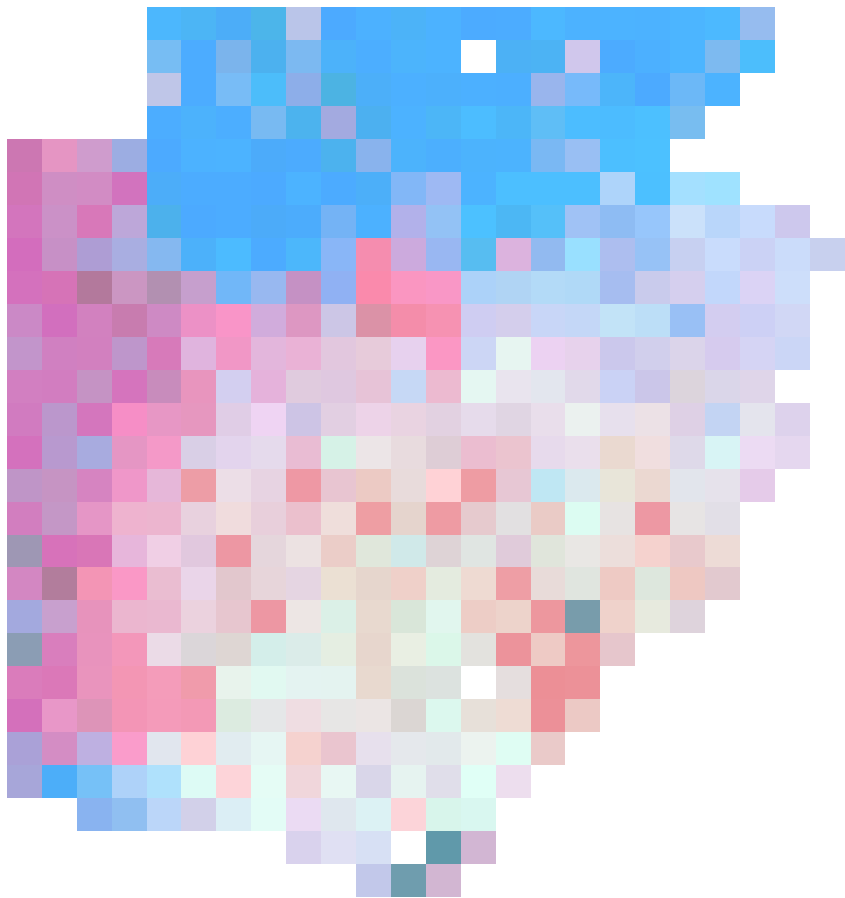

Supplement: Supplementary file 8 — Supplementary Data 5 [file 41467_2018_4724_MOESM8_ESM.zip › Supplementary Dataset 7/joint-mix-dimensionality-reduction-tSNE-matrix-split.pdf]

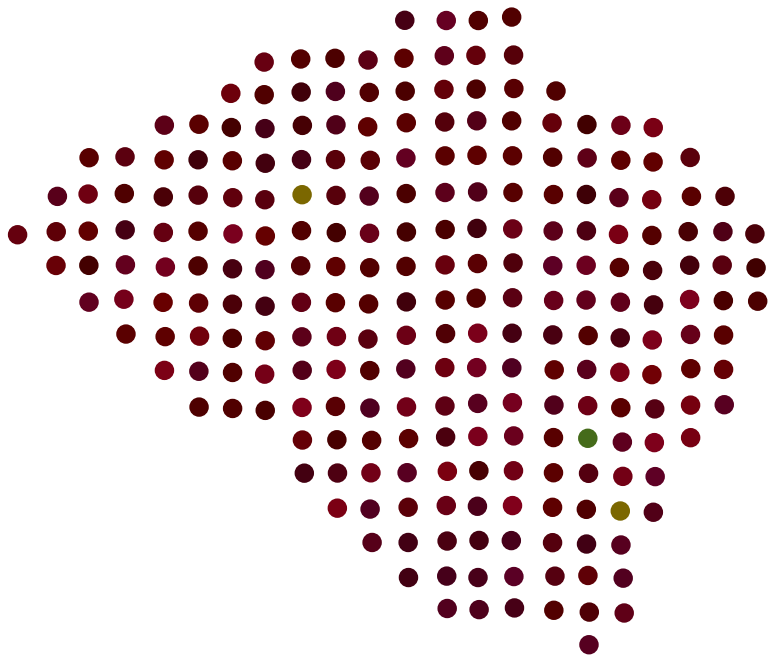

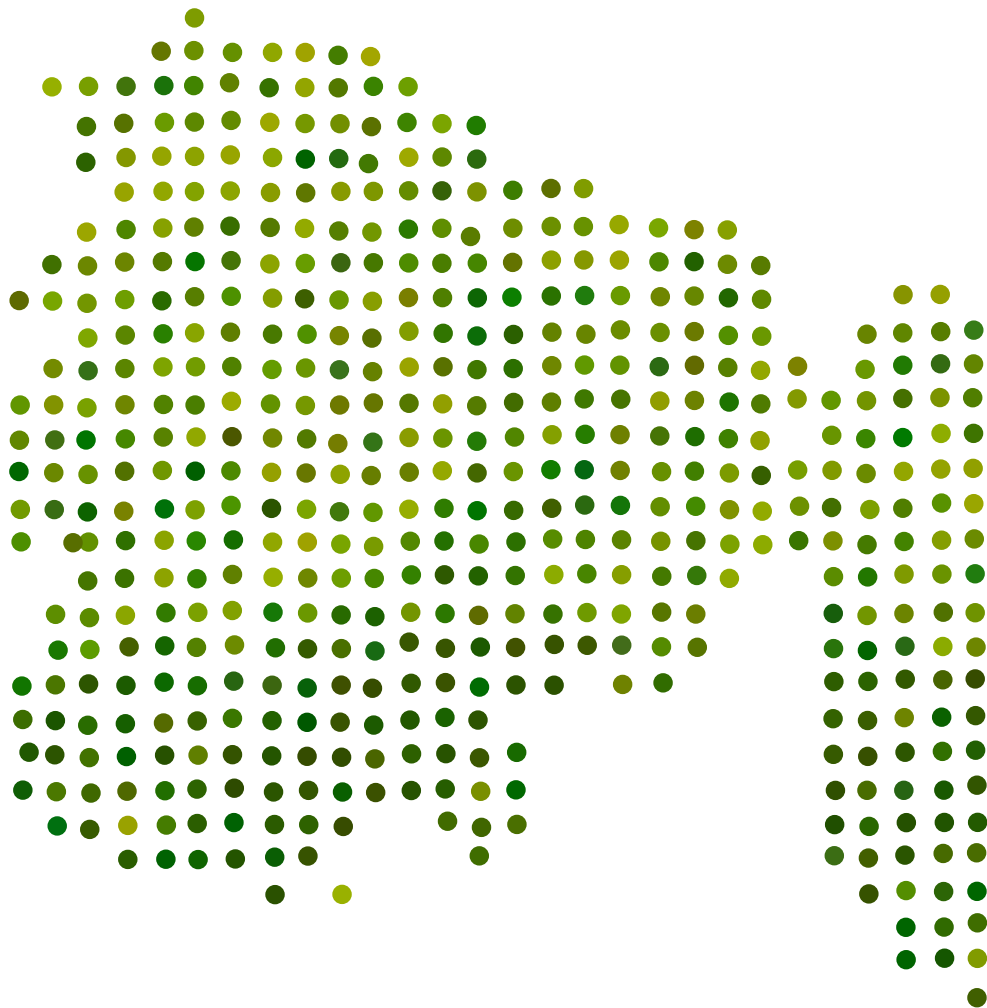

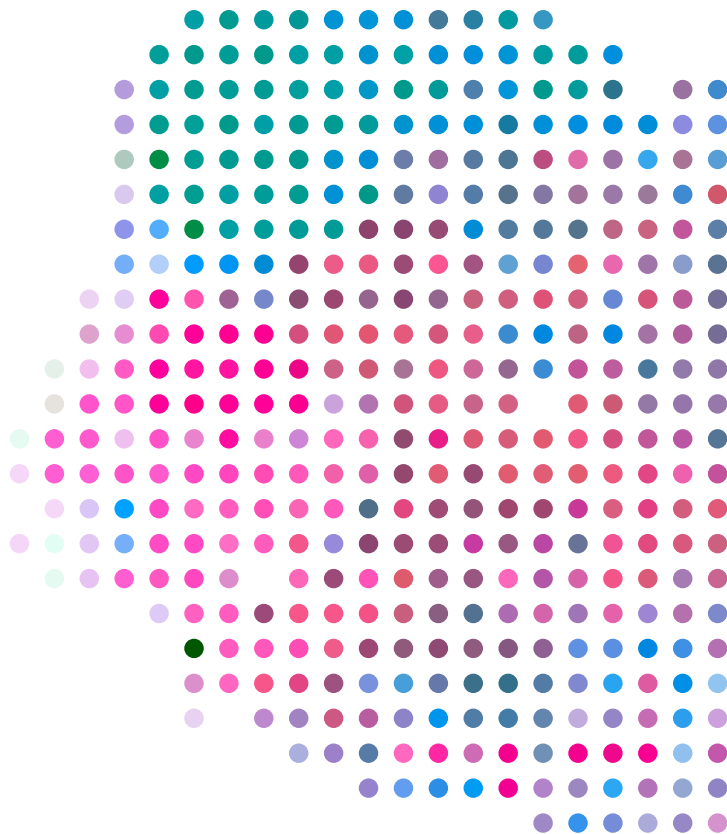

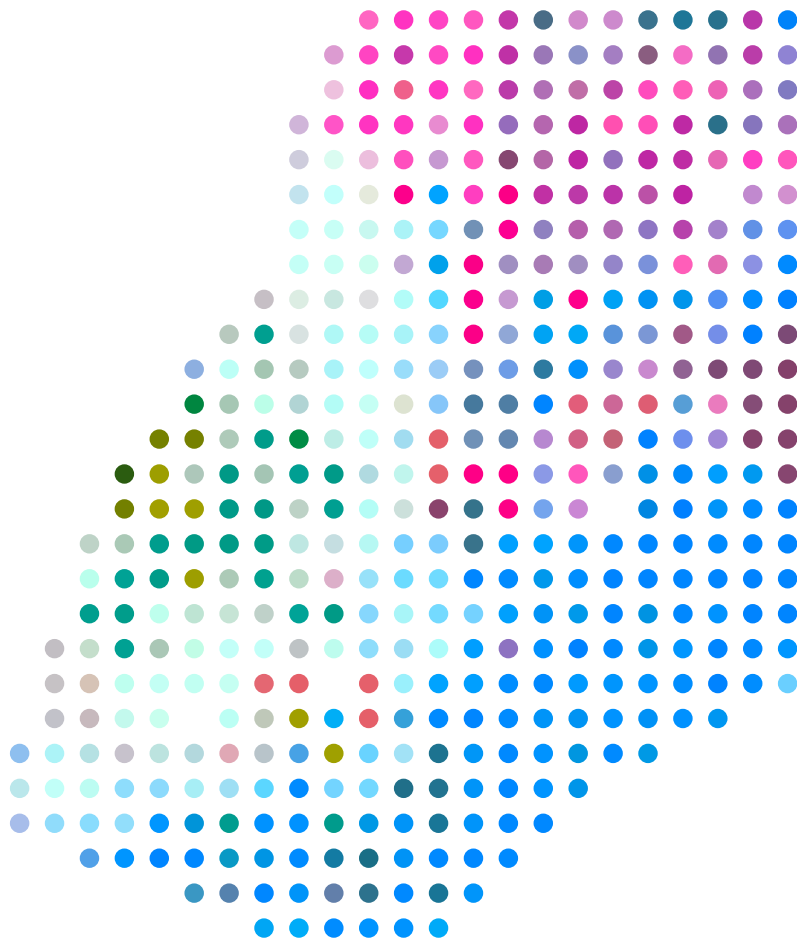

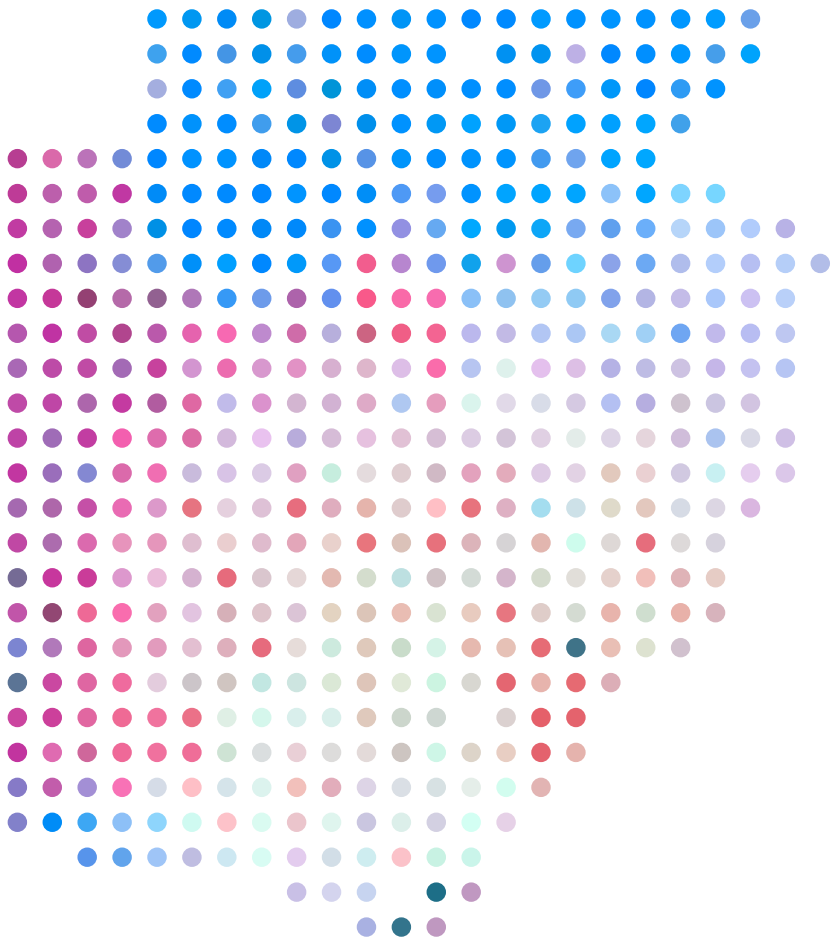

Supplement: Supplementary file 8 — Supplementary Data 5 [file 41467_2018_4724_MOESM8_ESM.zip › Supplementary Dataset 7/joint-mix-dimensionality-reduction-tSNE-dots-split.pdf]

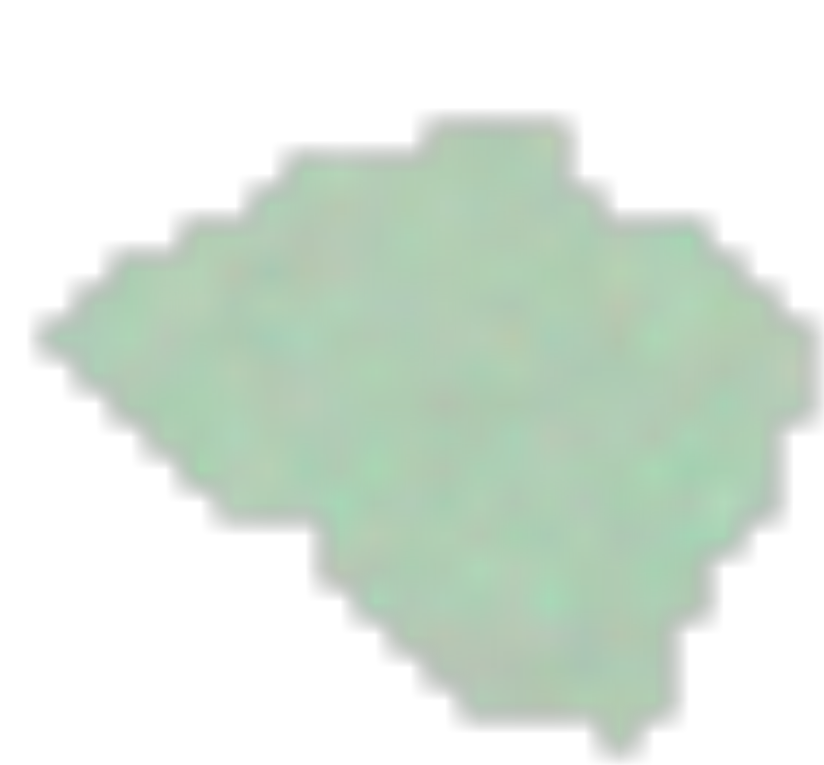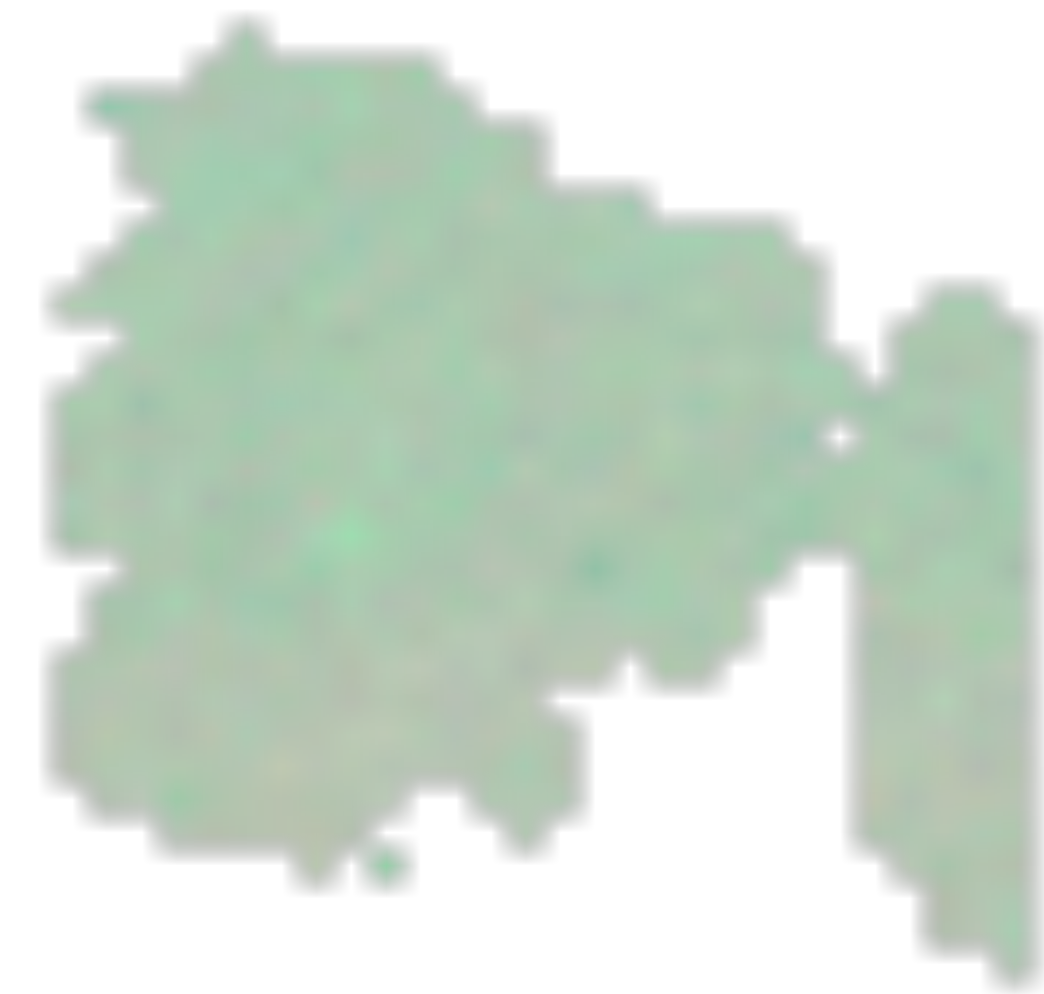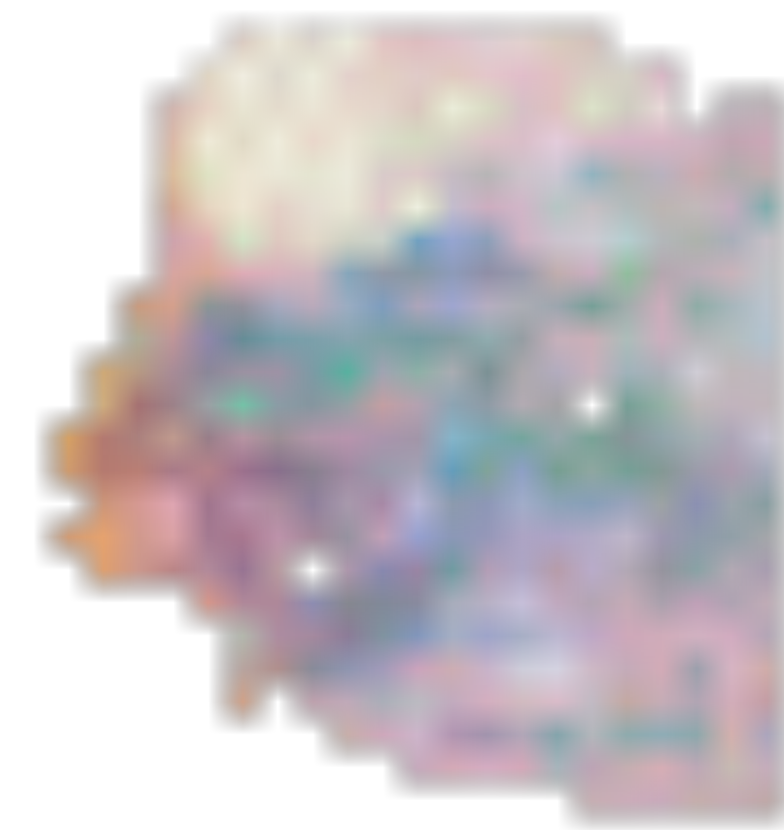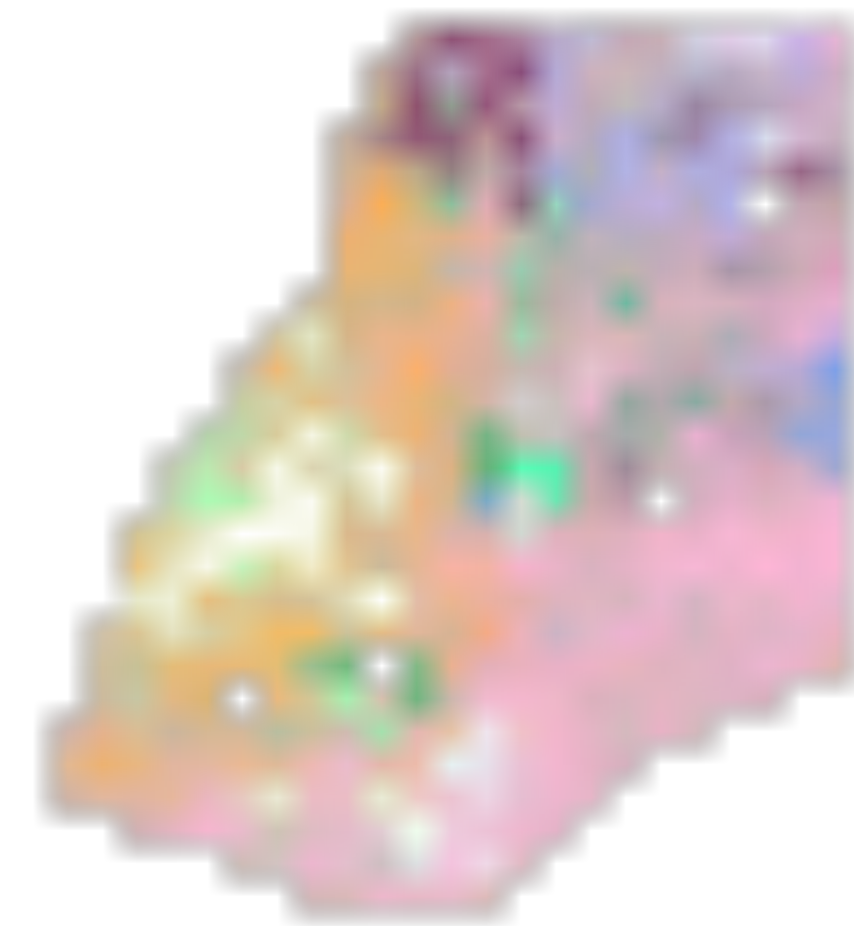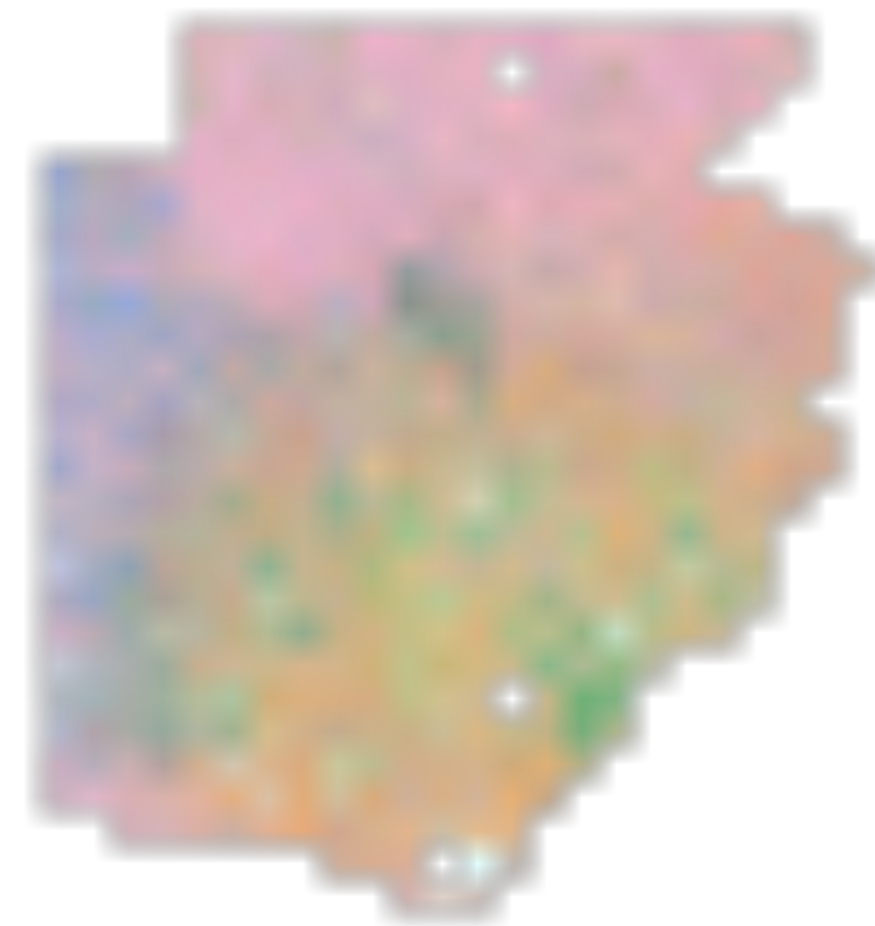

Supplement: Supplementary file 8 — Supplementary Data 5 [file 41467_2018_4724_MOESM8_ESM.zip › Supplementary Dataset 7/joint-mix-dimensionality-reduction-PCA-matrix-rgb.pdf.interpolated.pdf]

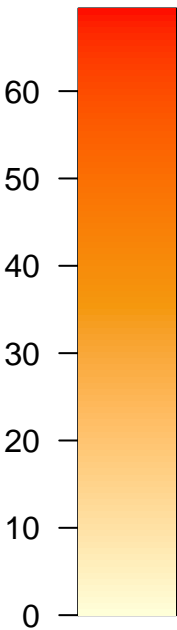

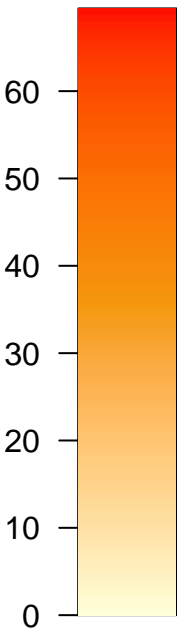

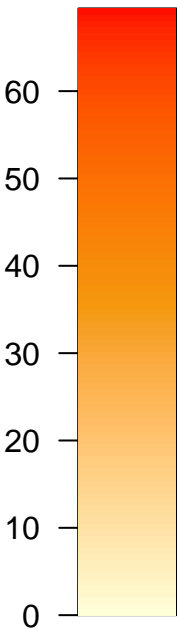

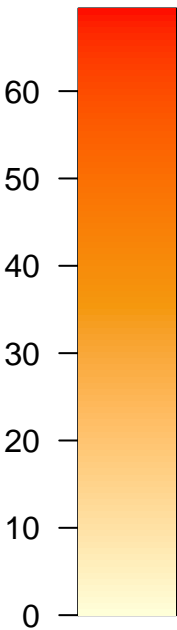

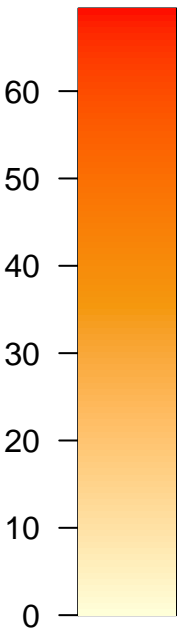

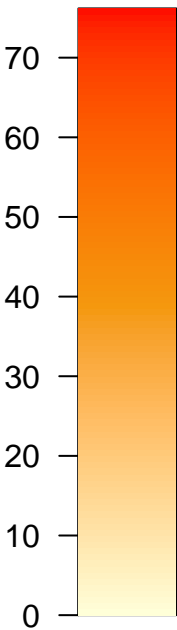

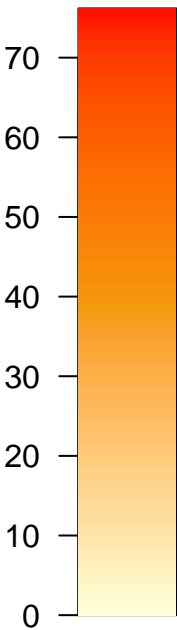

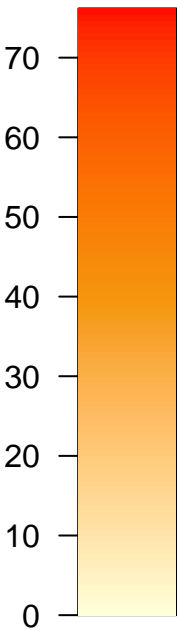

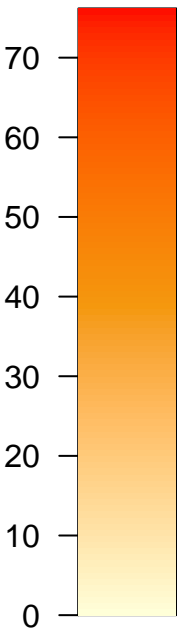

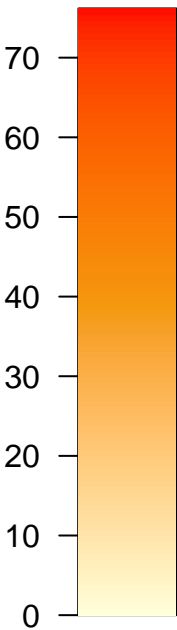

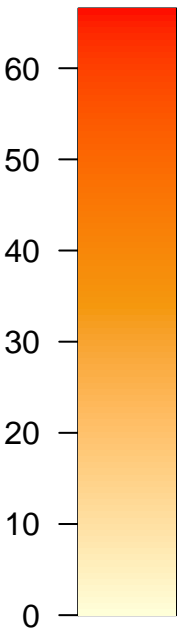

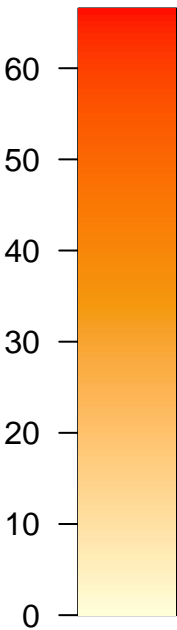

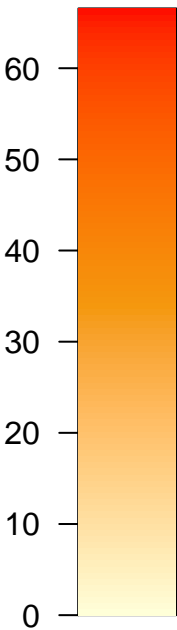

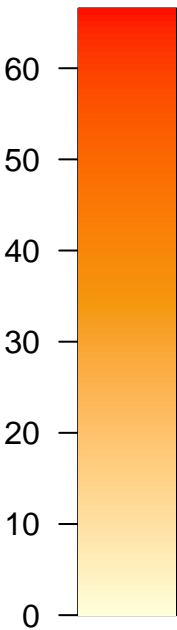

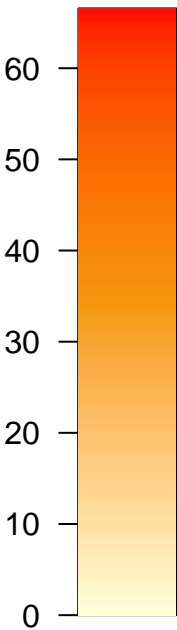

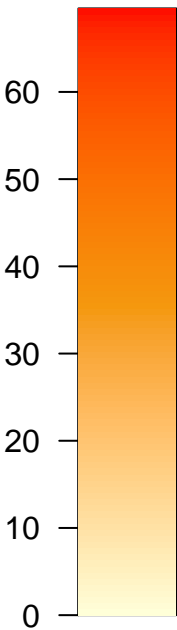

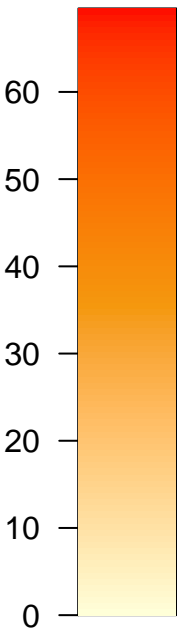

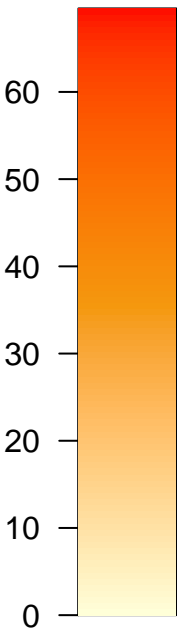

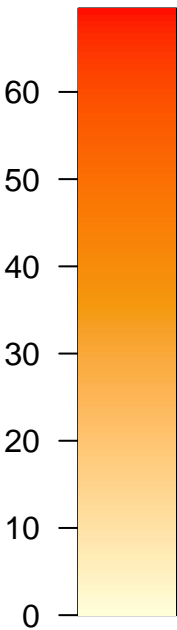

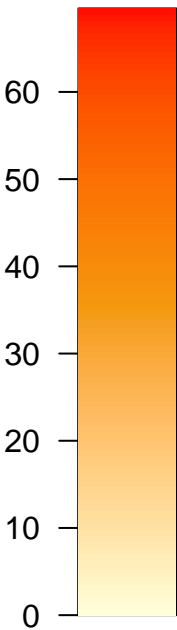

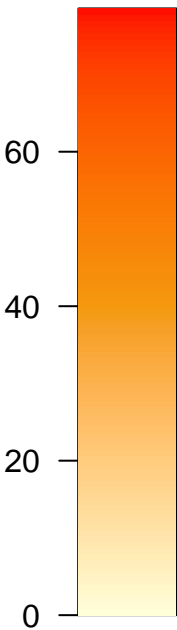

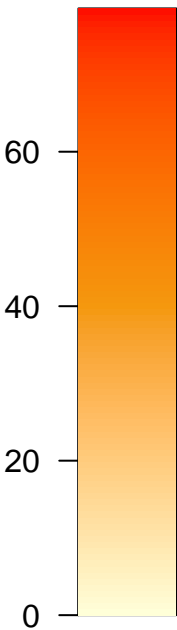

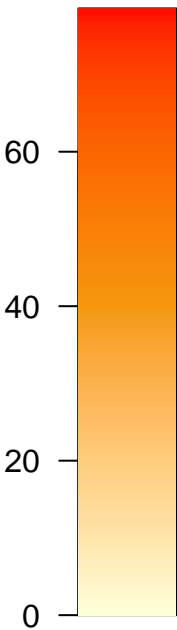

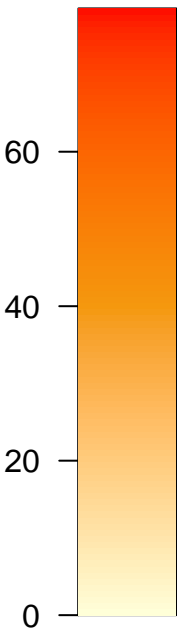

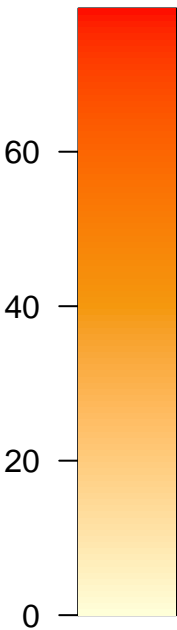

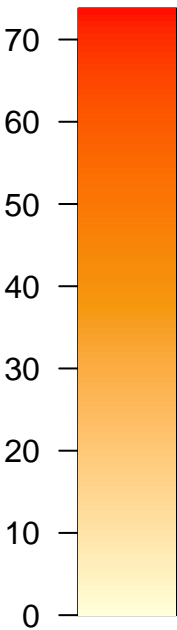

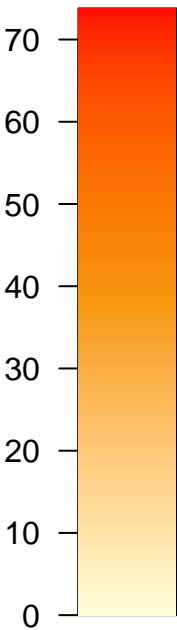

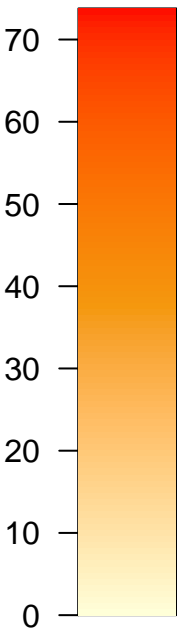

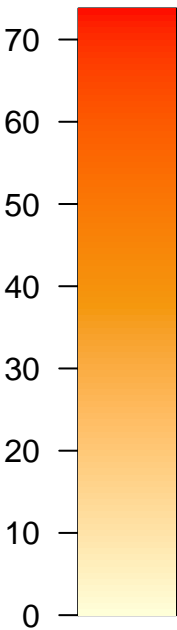

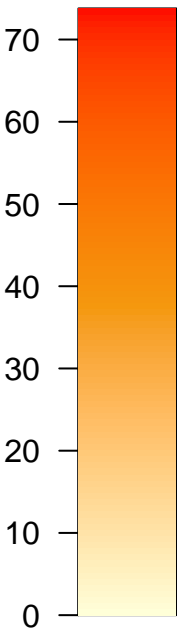

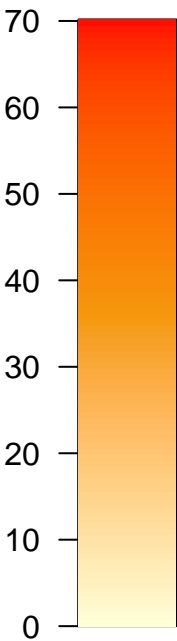

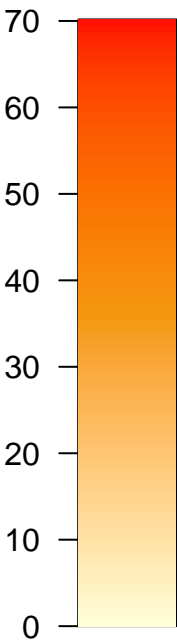

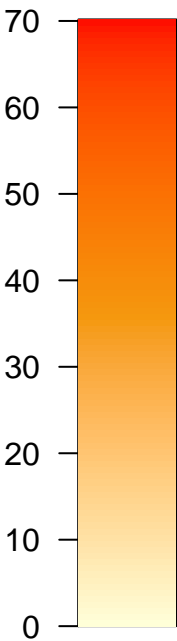

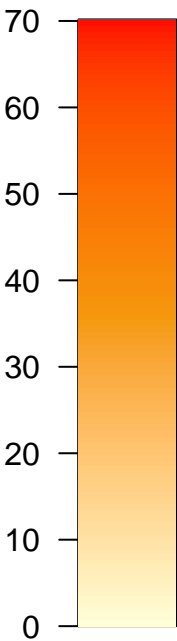

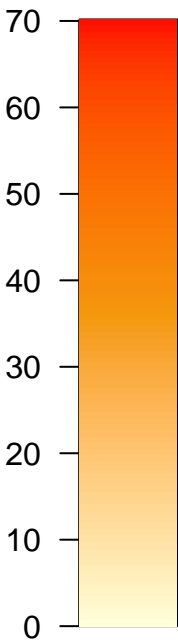

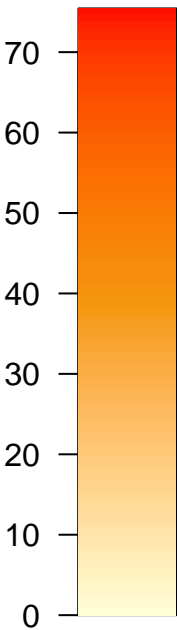

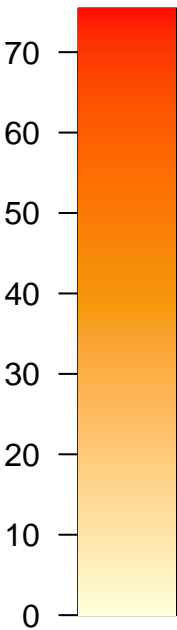

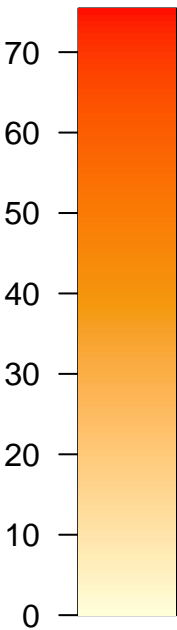

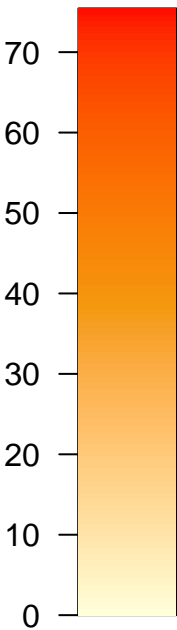

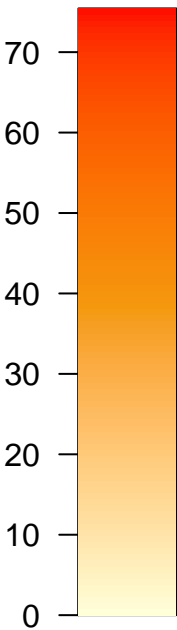

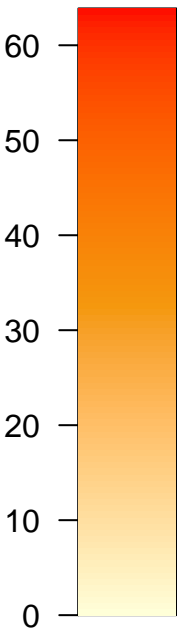

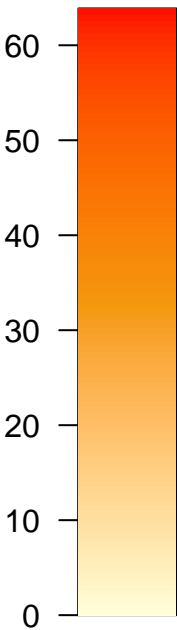

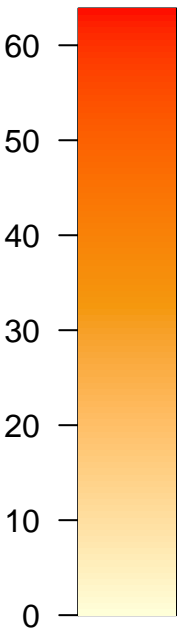

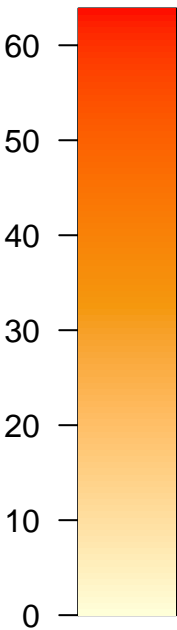

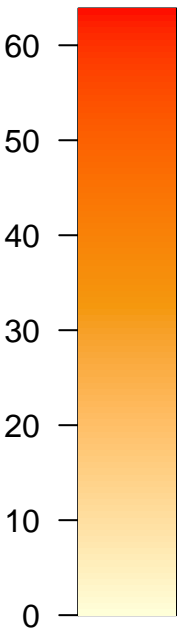

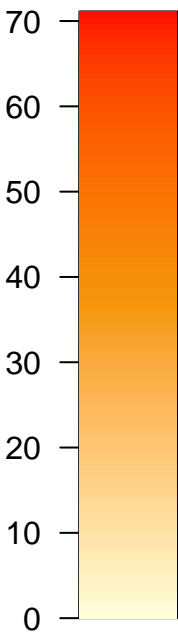

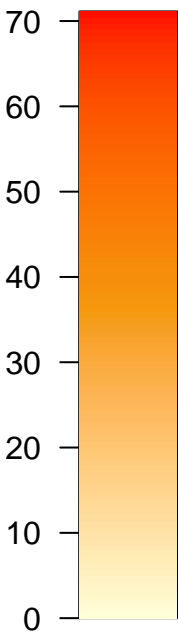

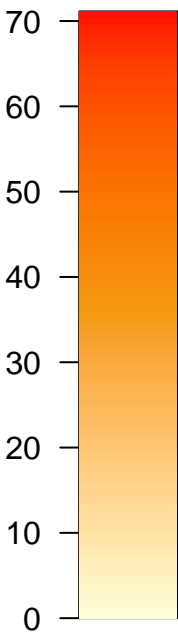

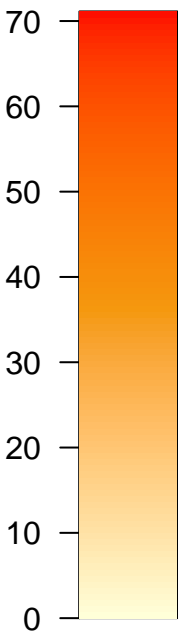

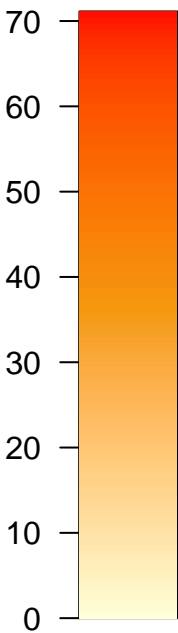

Supplement: Supplementary file 8 — Supplementary Data 5 [file 41467_2018_4724_MOESM8_ESM.zip › Supplementary Dataset 7/joint-mix-profiles-rel-common-scale-dots-split-colorbar.pdf]

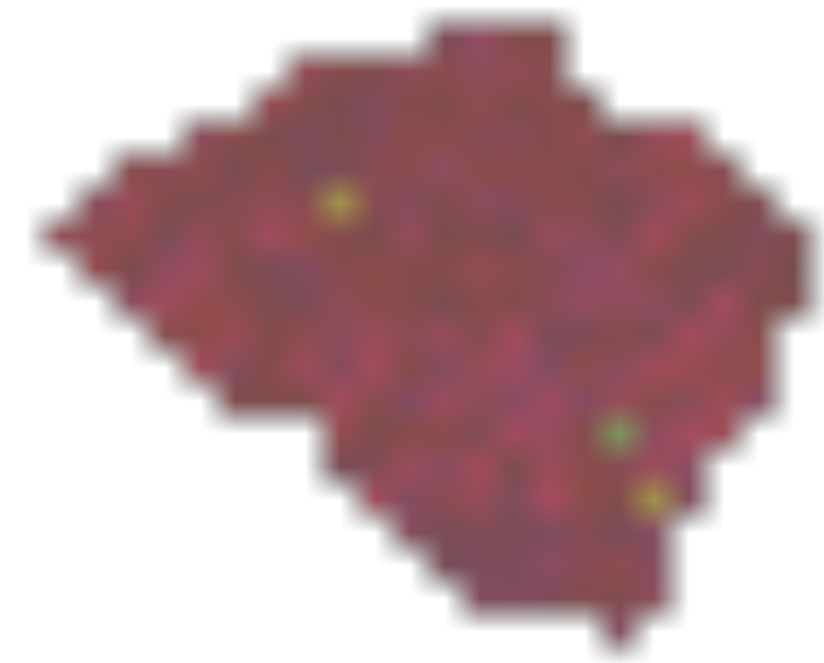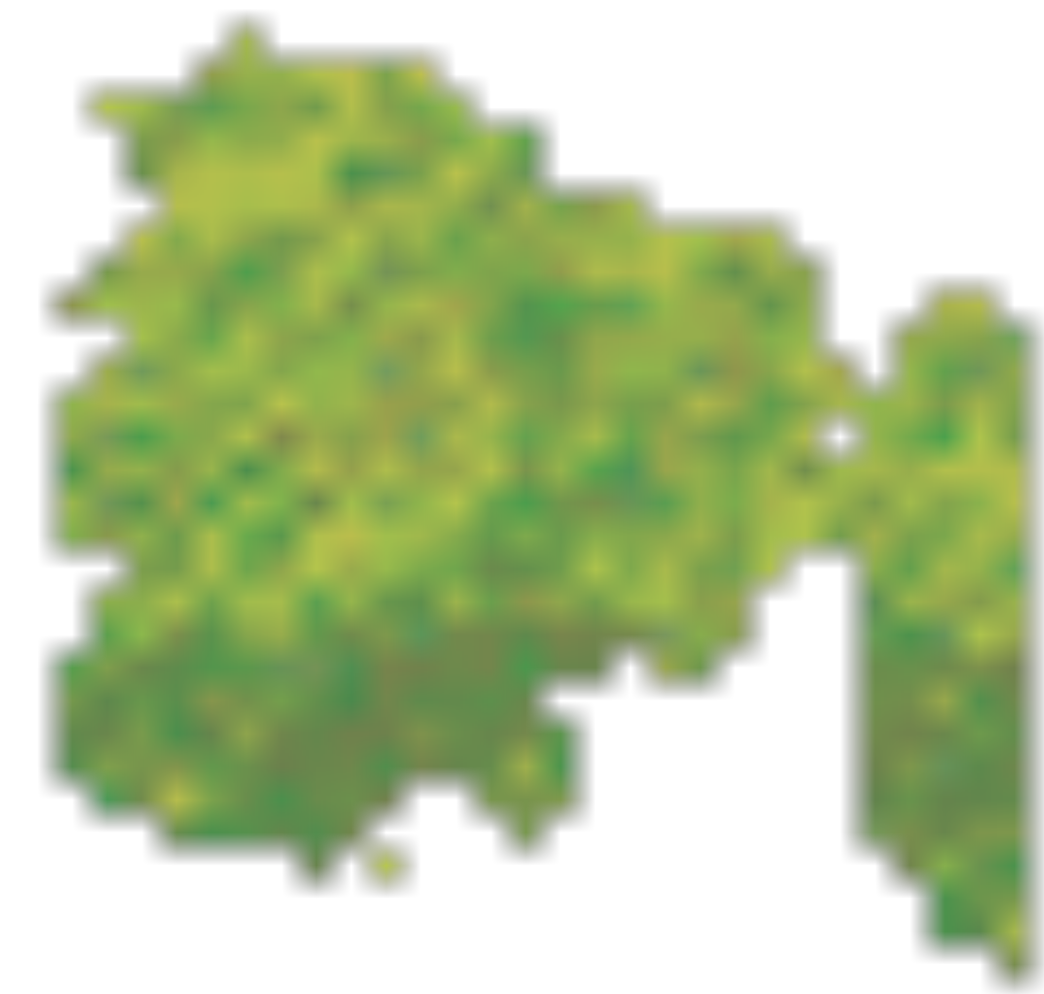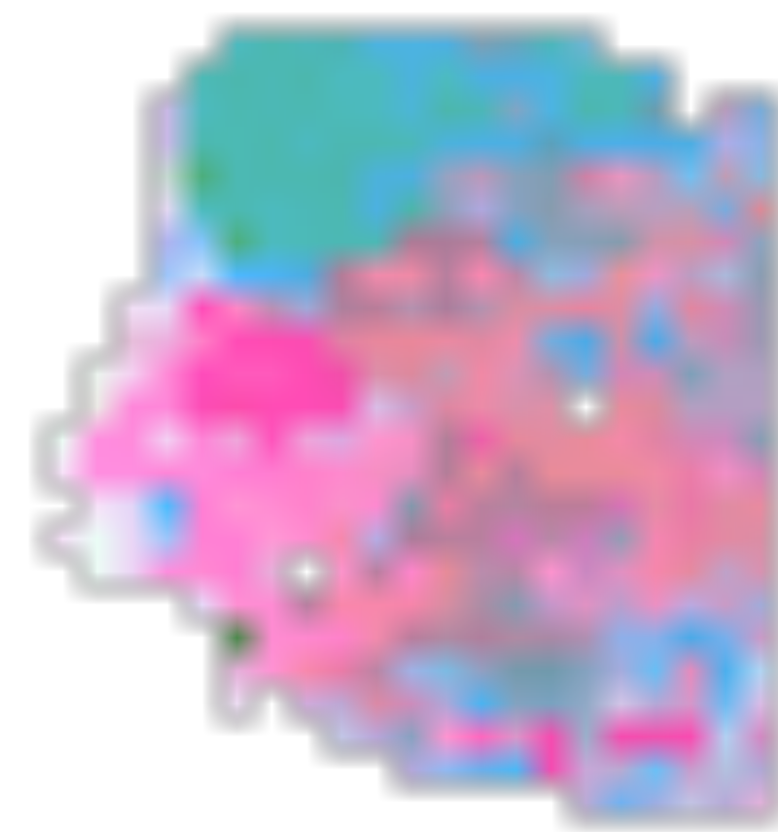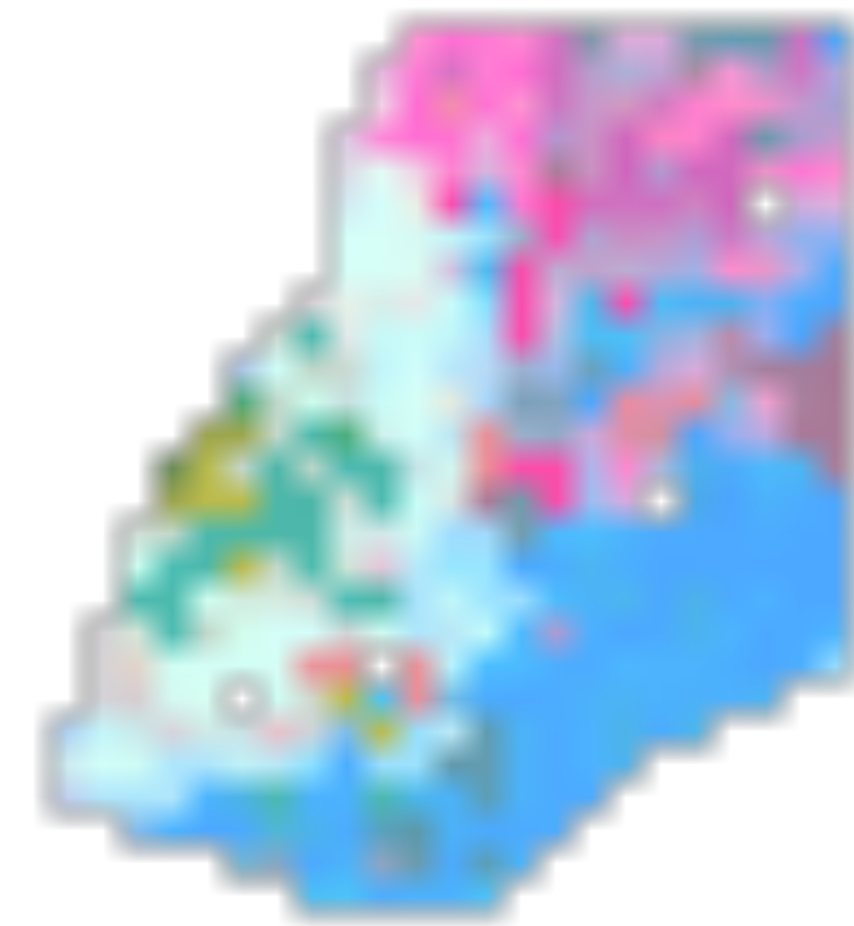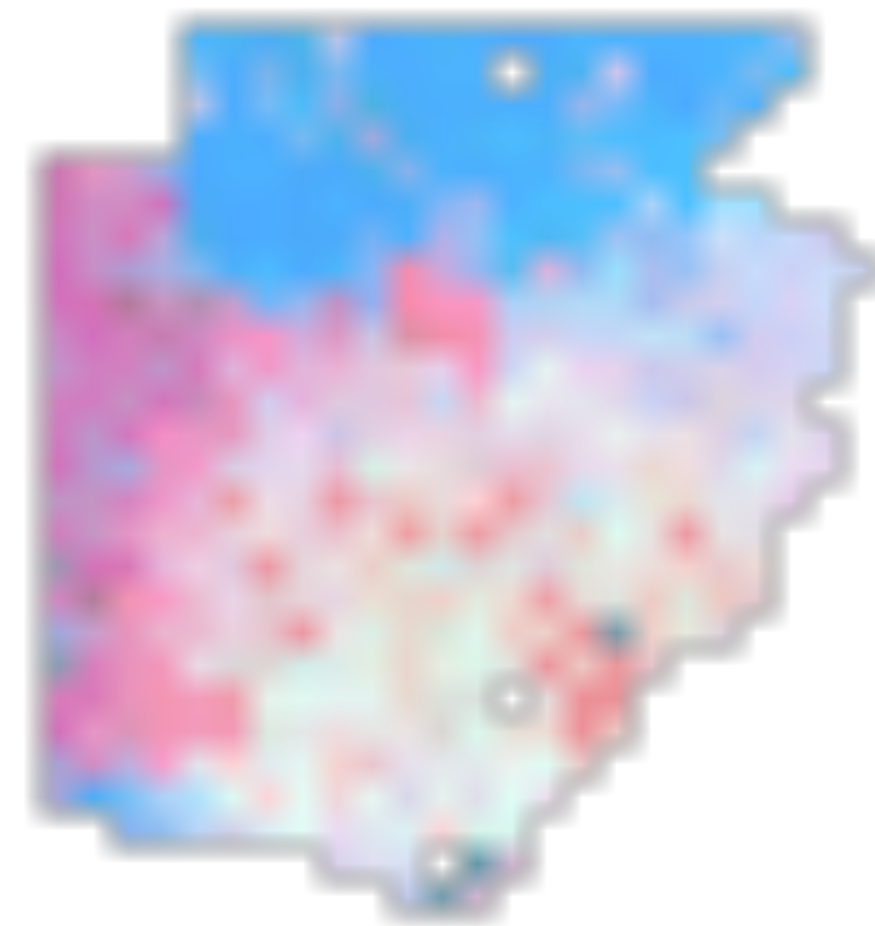

Supplement: Supplementary file 8 — Supplementary Data 5 [file 41467_2018_4724_MOESM8_ESM.zip › Supplementary Dataset 7/joint-mix-dimensionality-reduction-tSNE-matrix.pdf.interpolated.pdf]

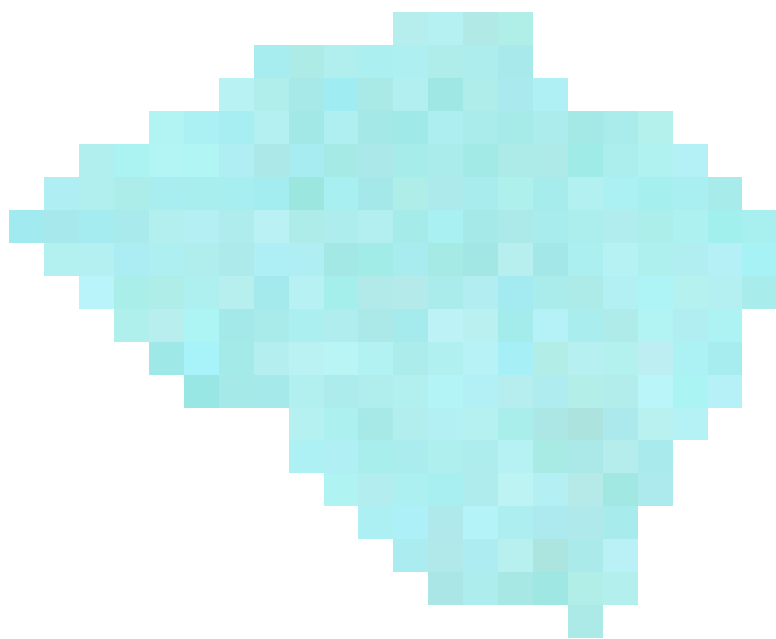

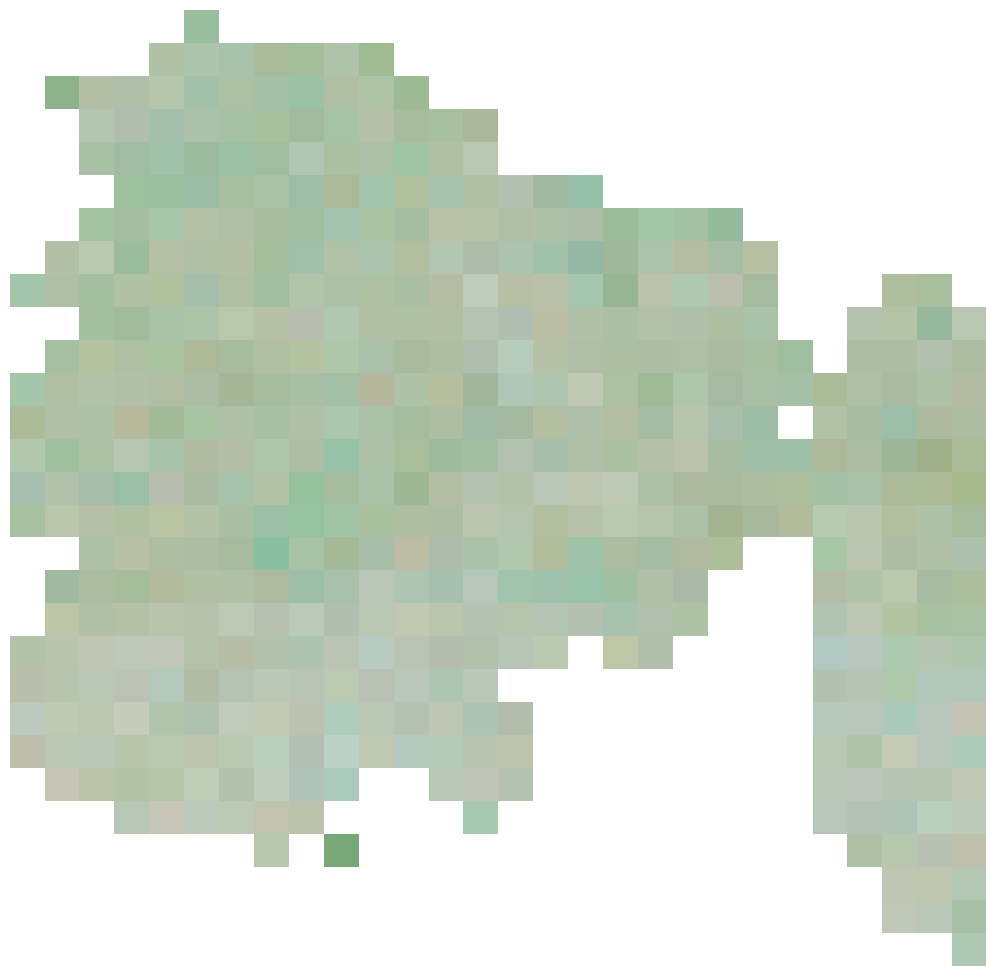

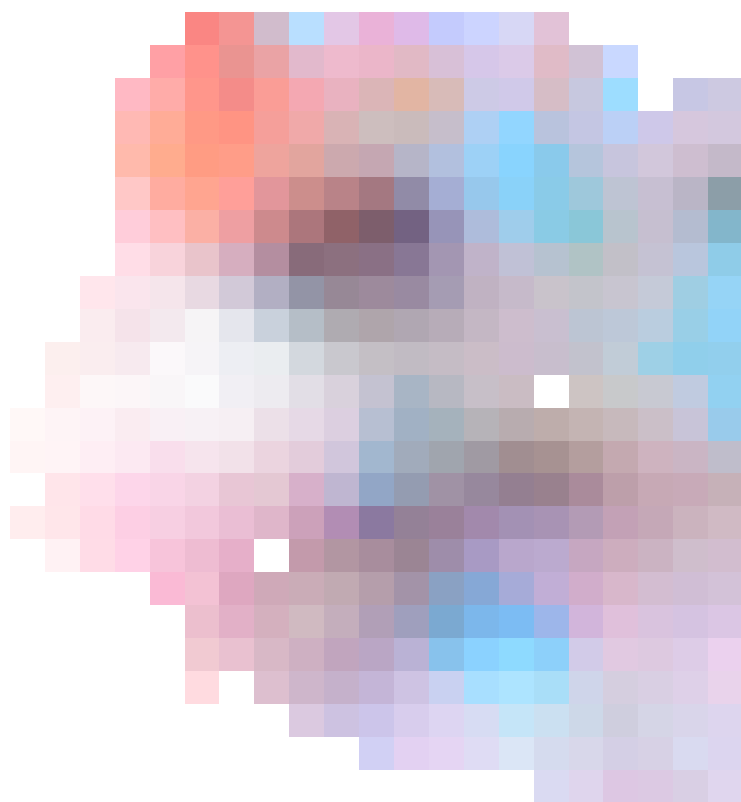

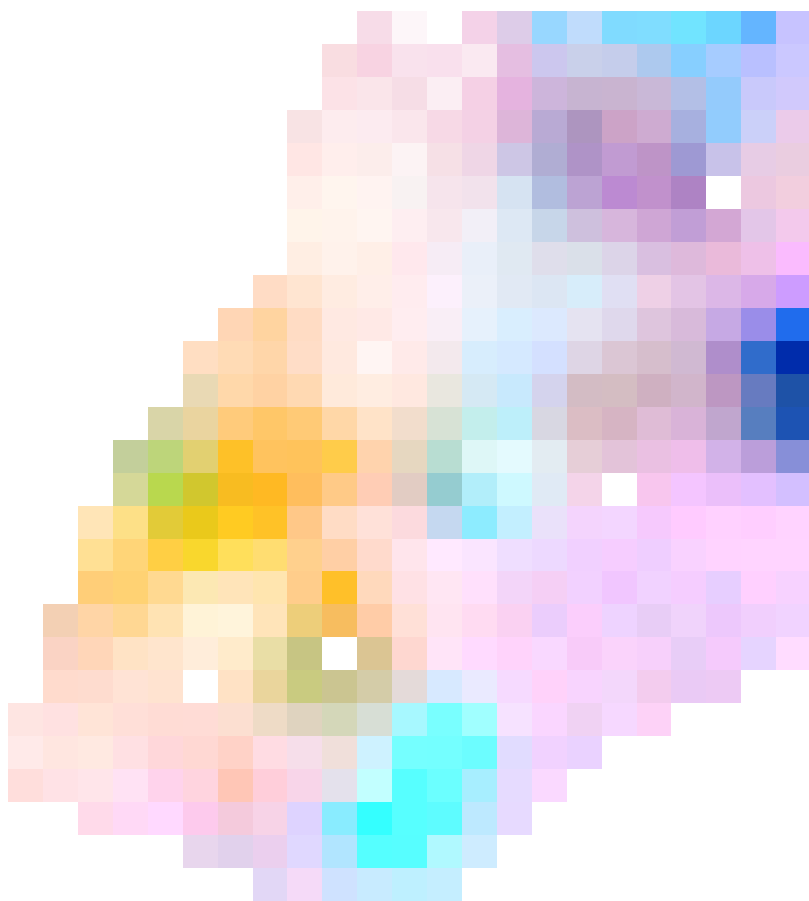

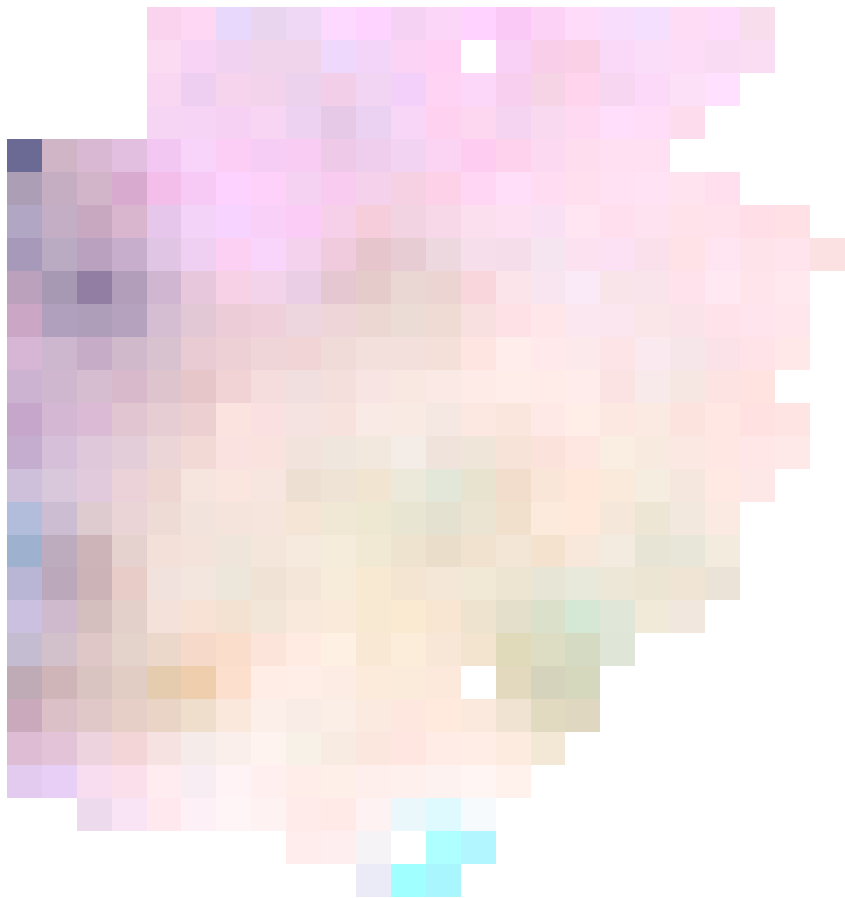

Supplement: Supplementary file 8 — Supplementary Data 5 [file 41467_2018_4724_MOESM8_ESM.zip › Supplementary Dataset 7/joint-field-dimensionality-reduction-PCA-matrix-split.pdf]

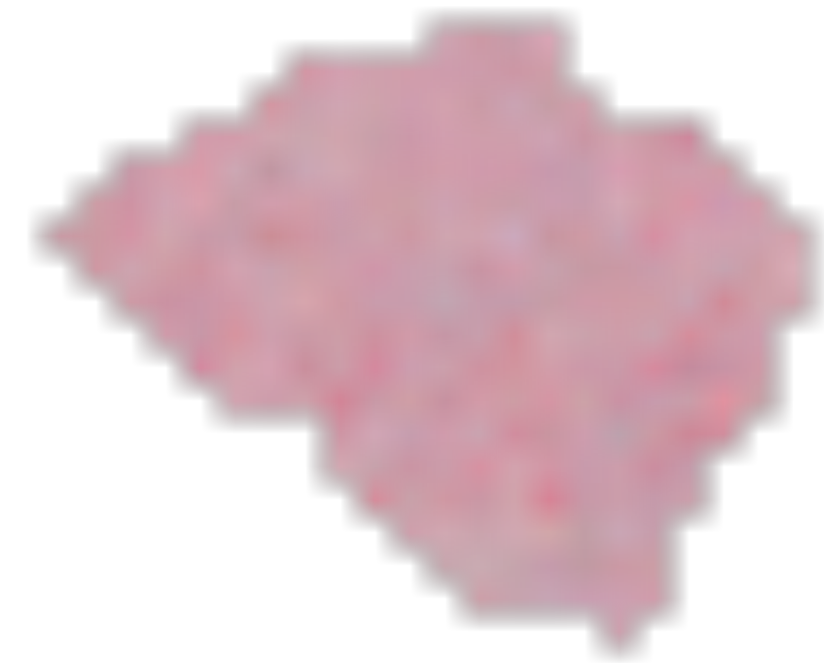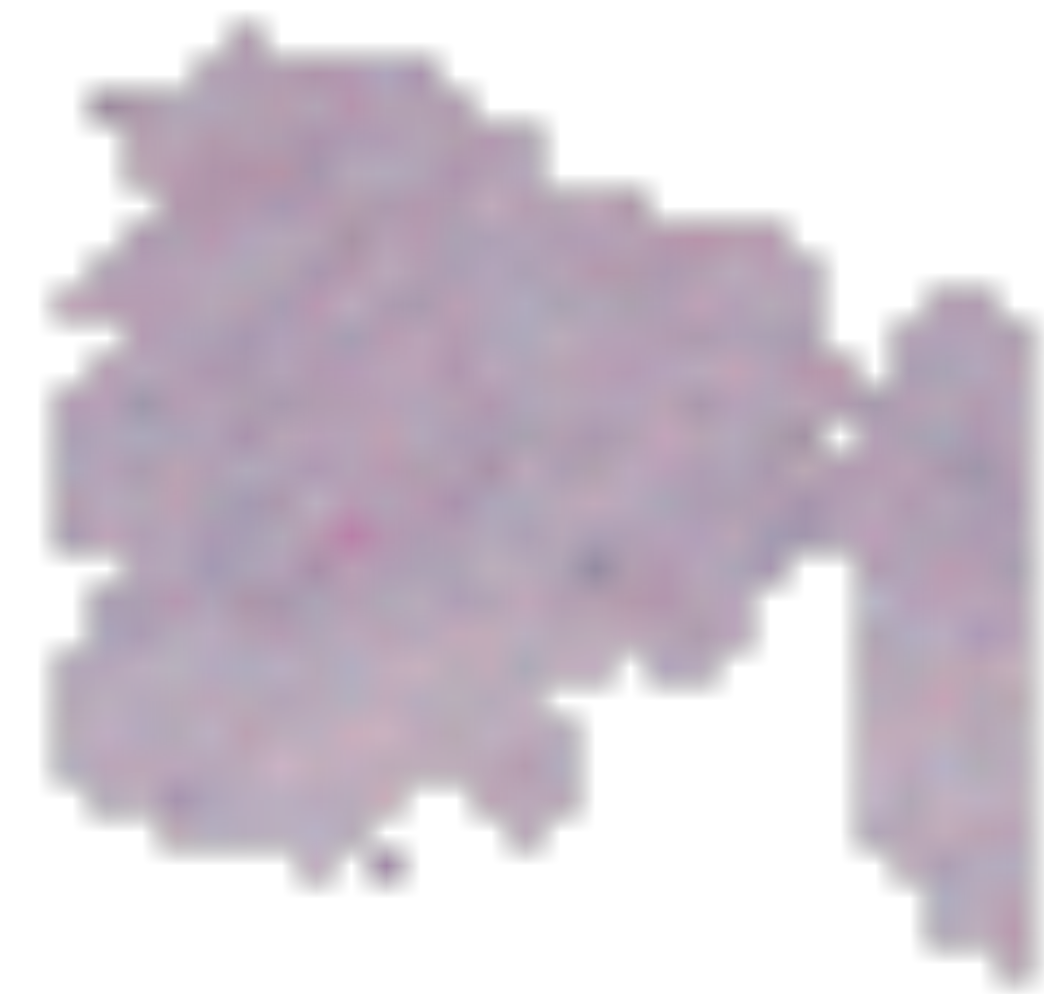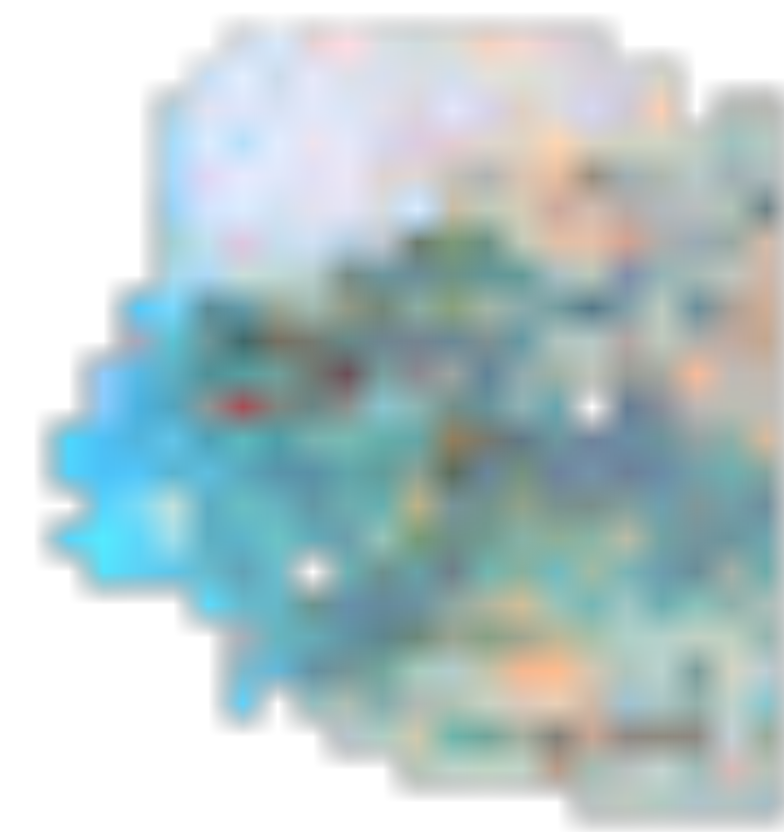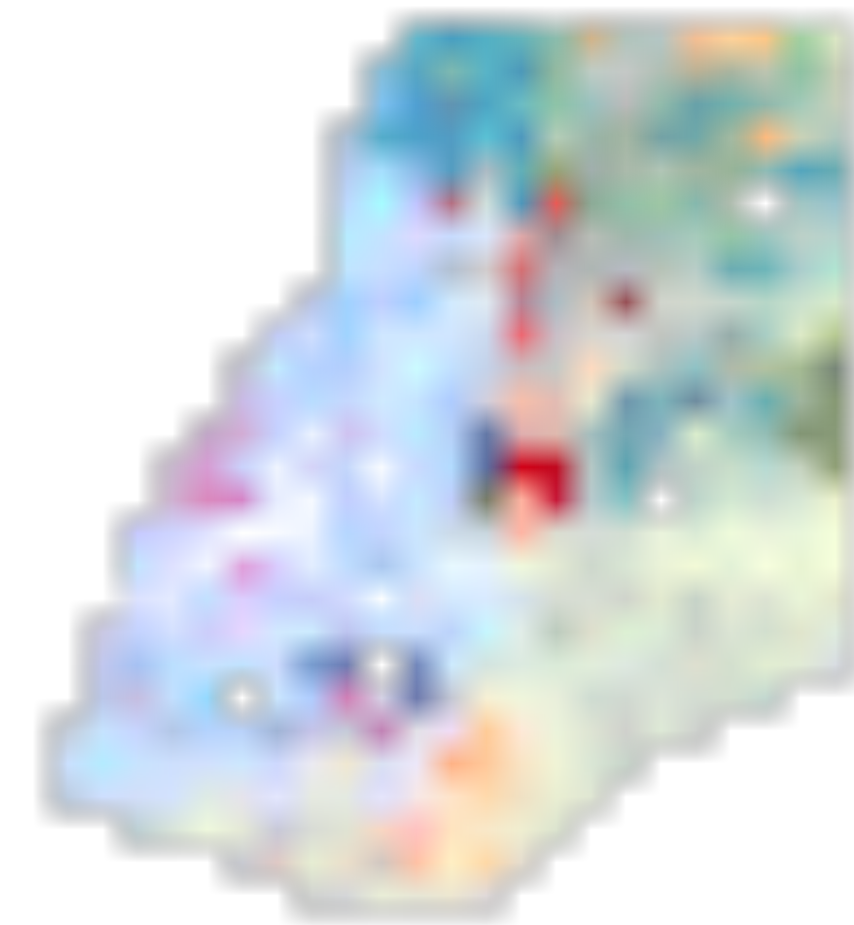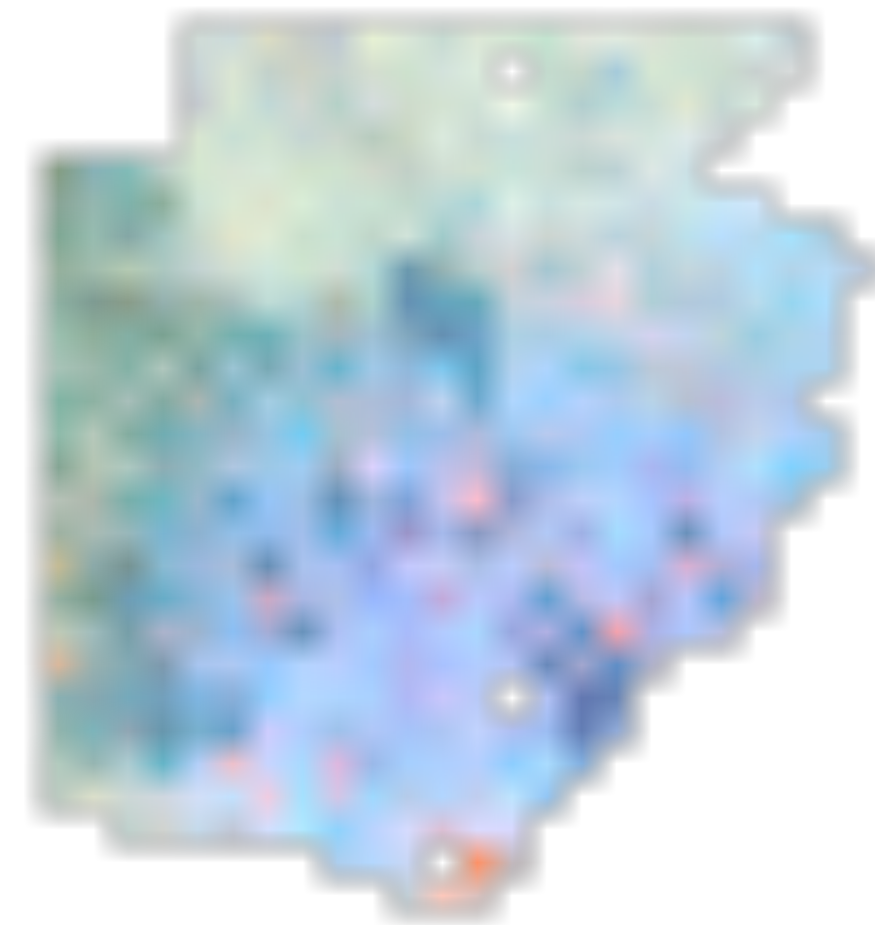

Supplement: Supplementary file 8 — Supplementary Data 5 [file 41467_2018_4724_MOESM8_ESM.zip › Supplementary Dataset 7/joint-mix-dimensionality-reduction-PCA-matrix.pdf.interpolated.pdf]

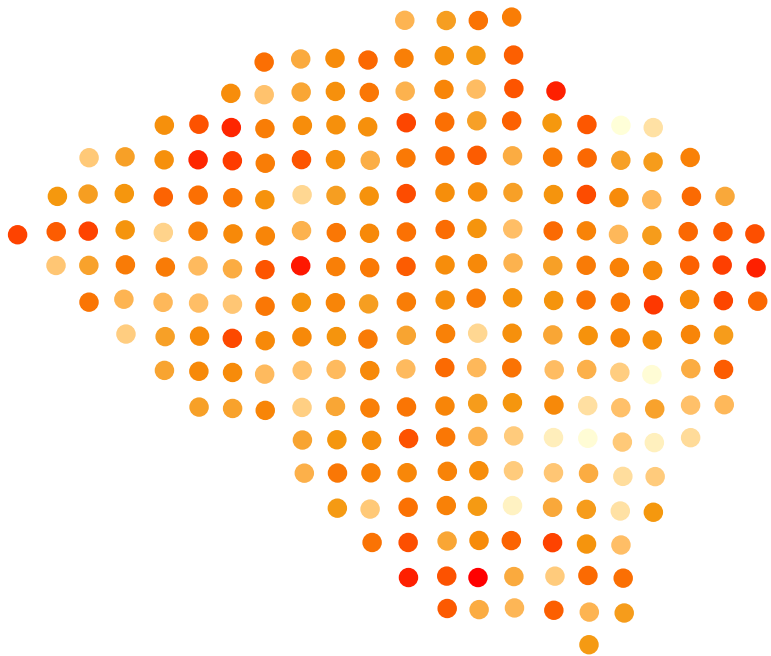

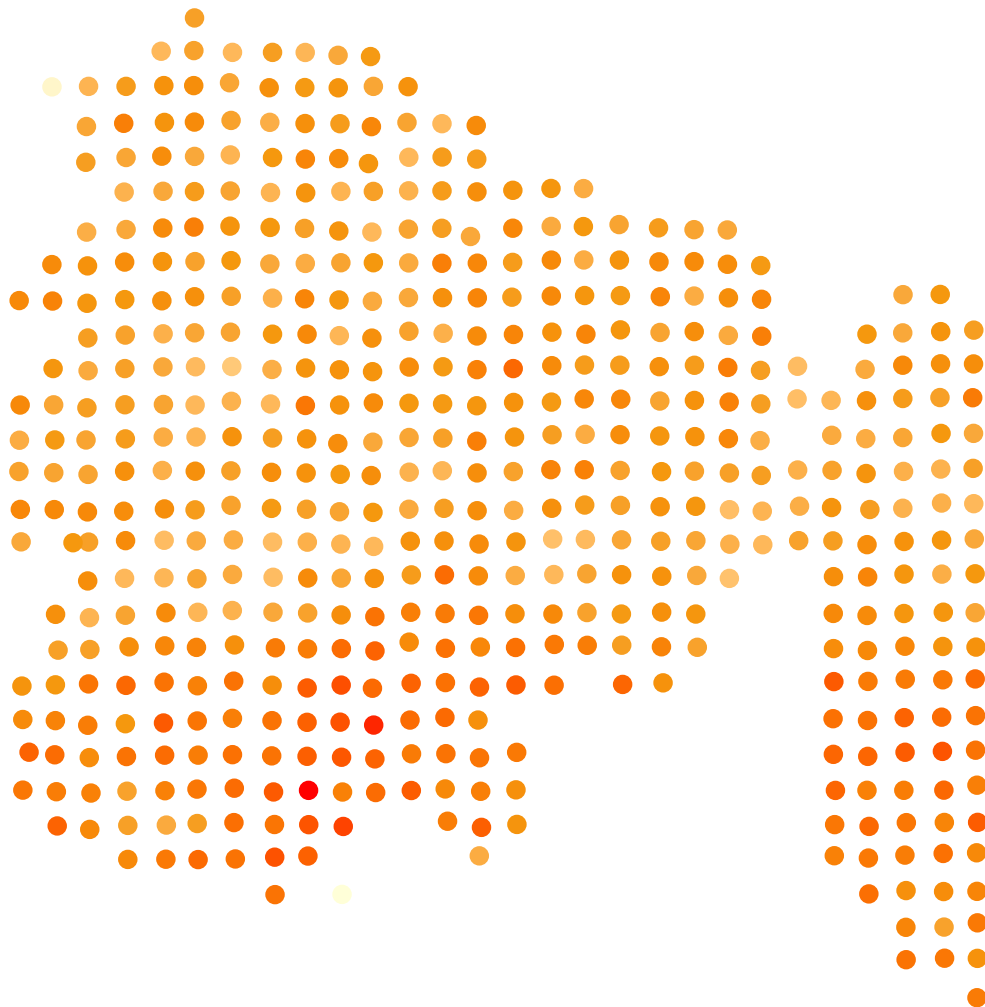

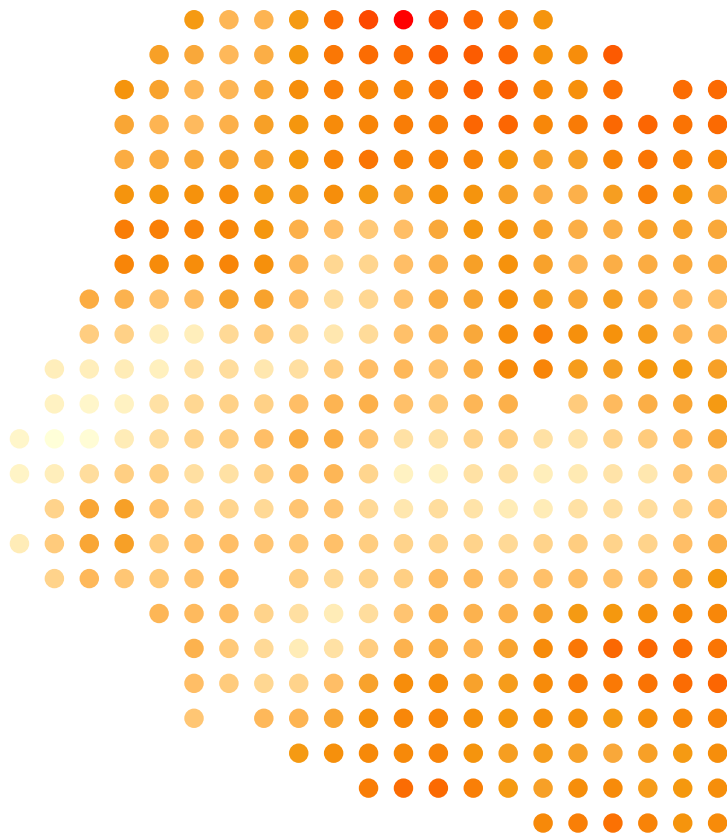

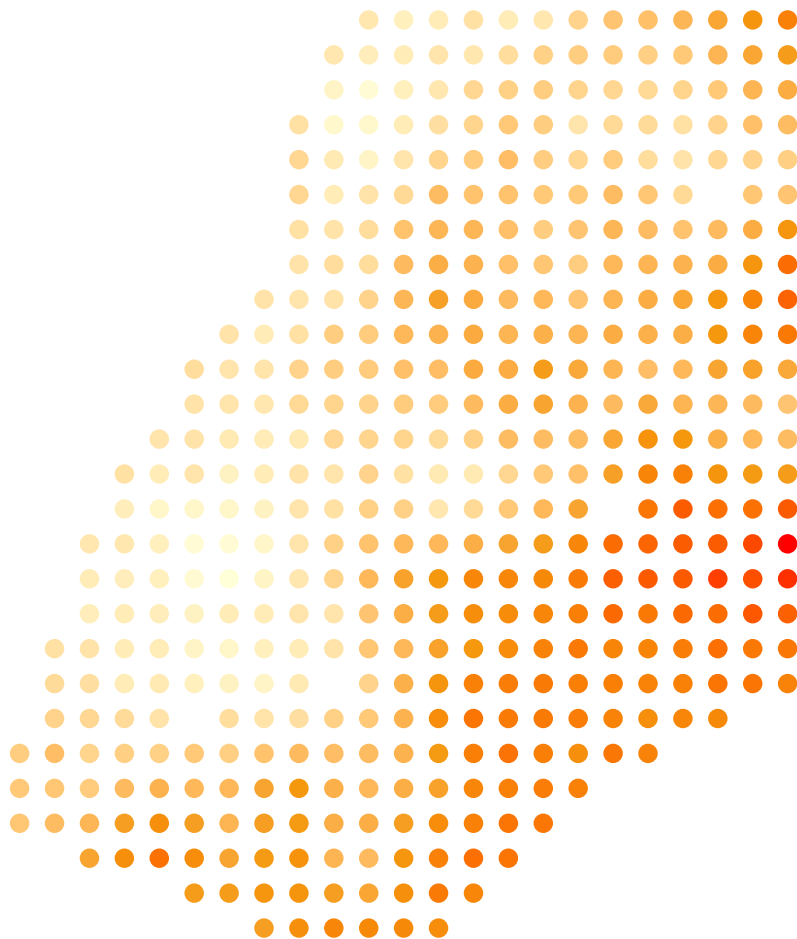

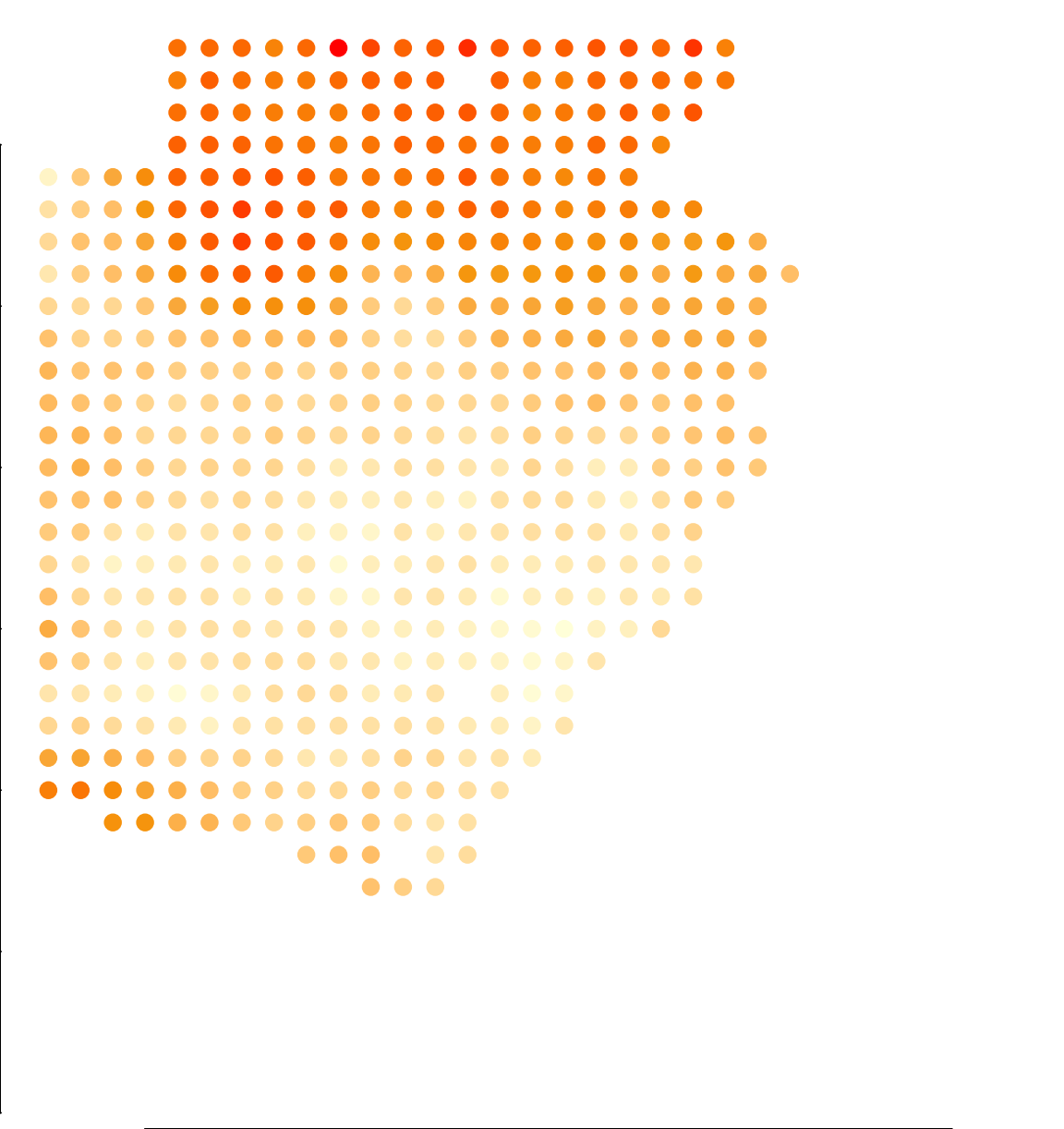

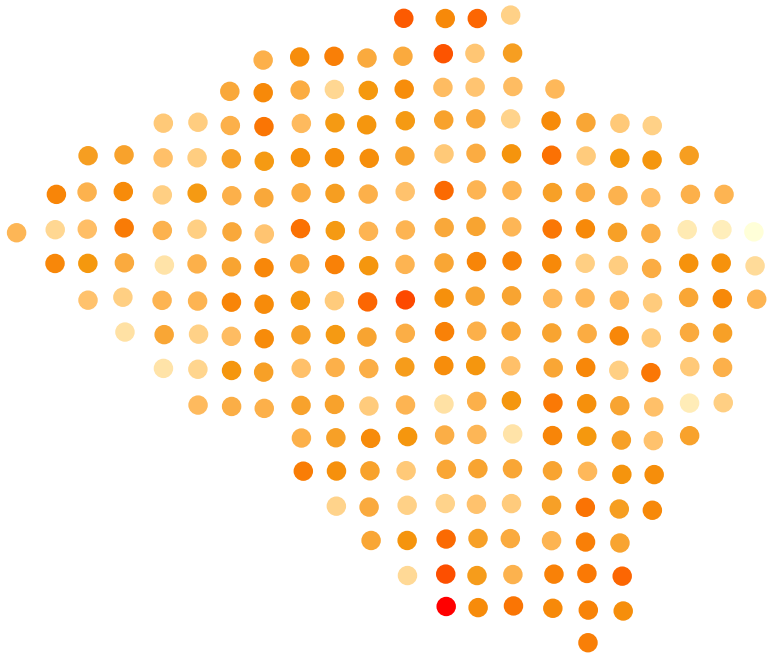

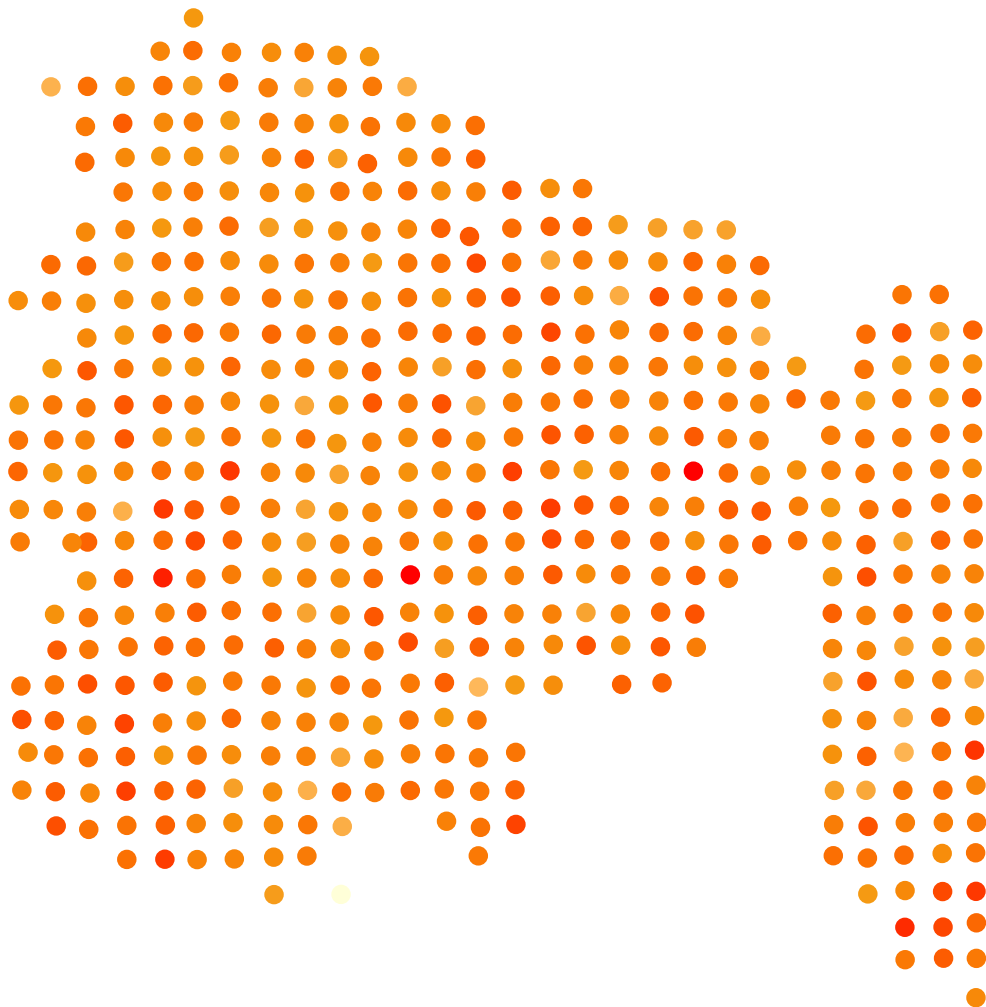

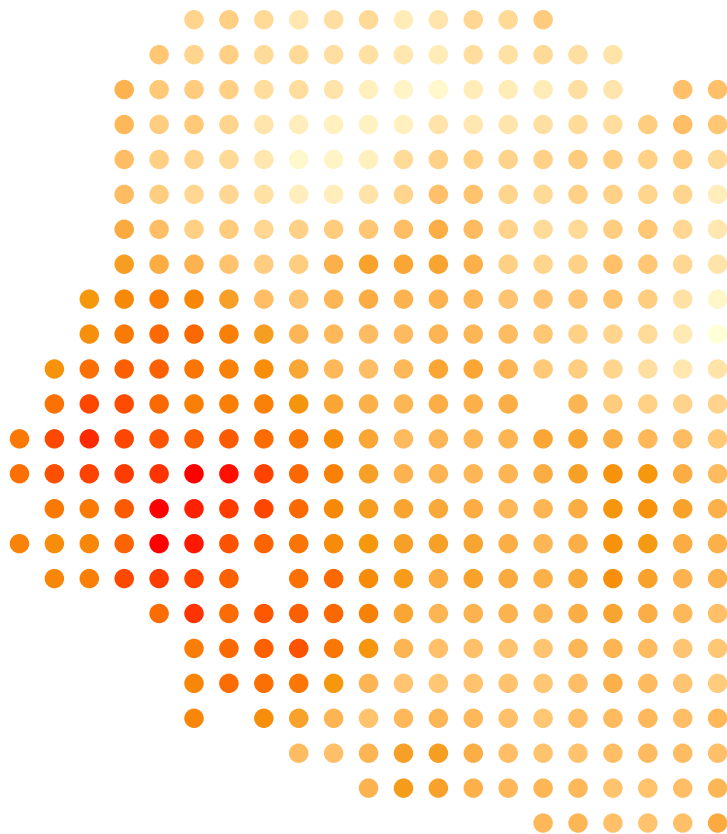

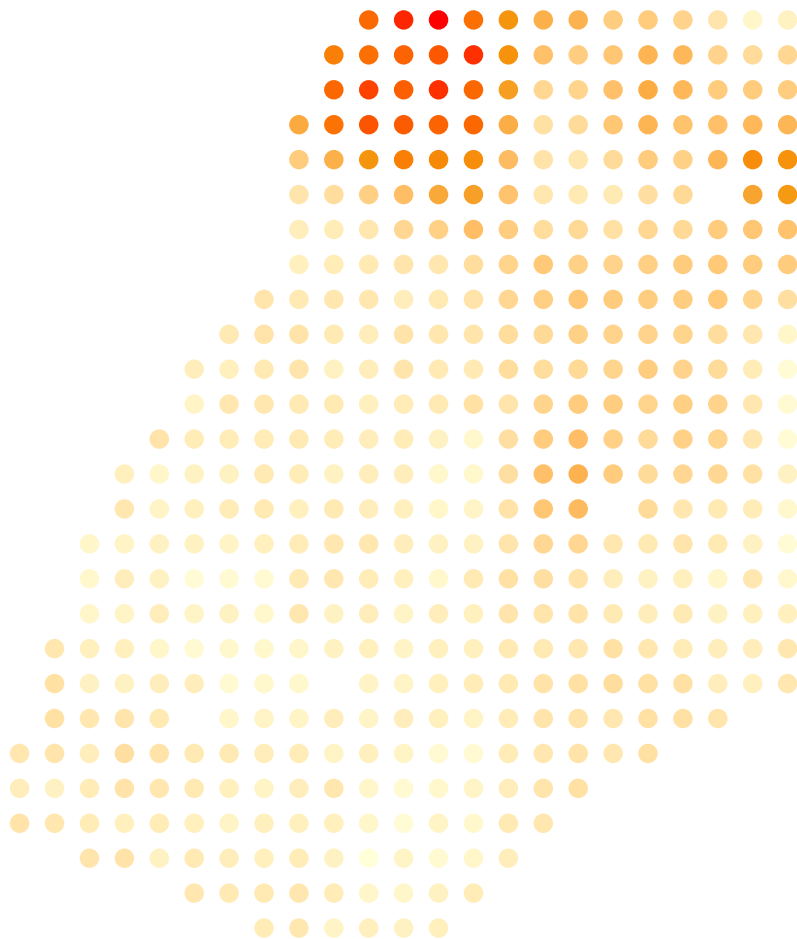

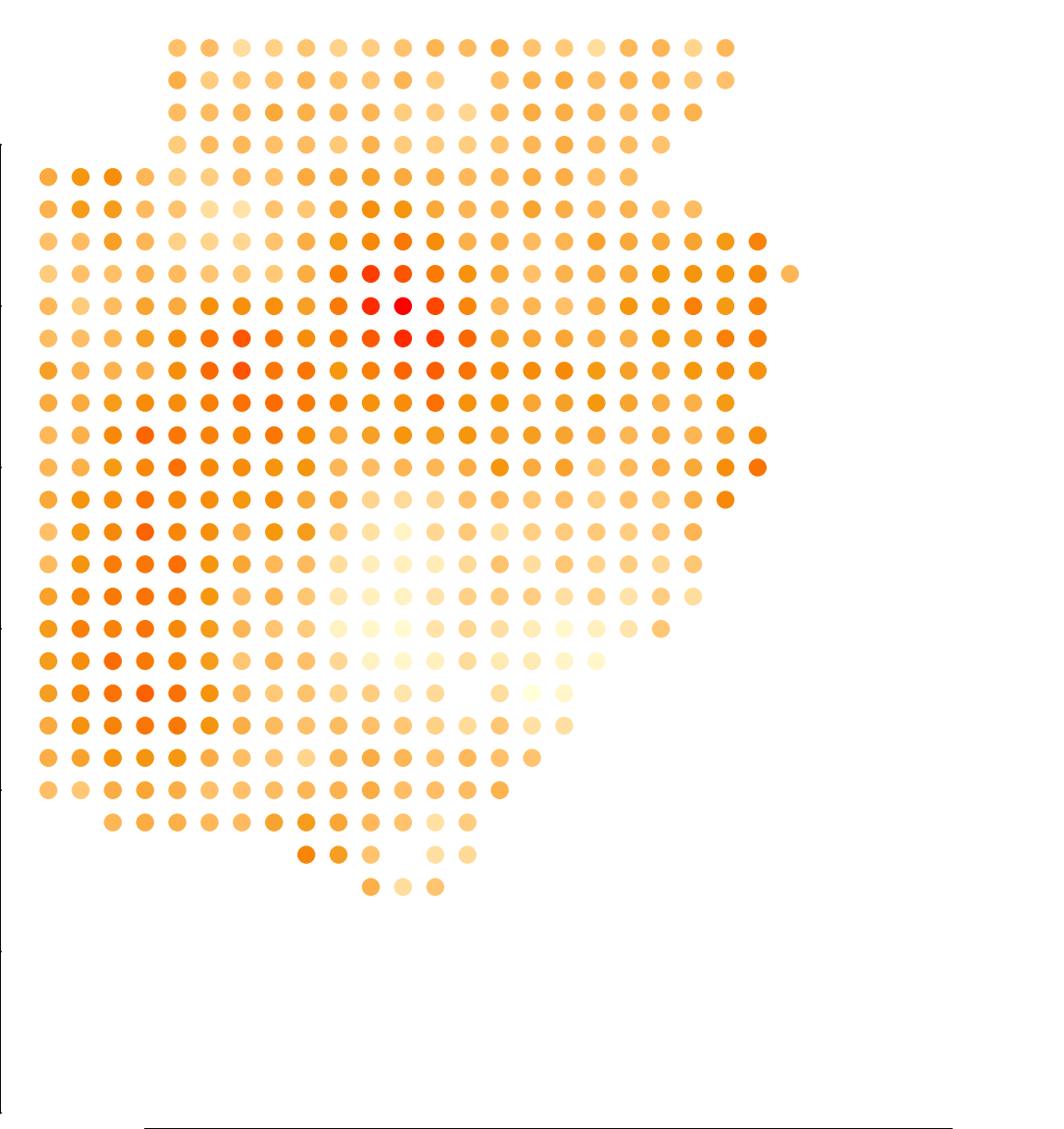

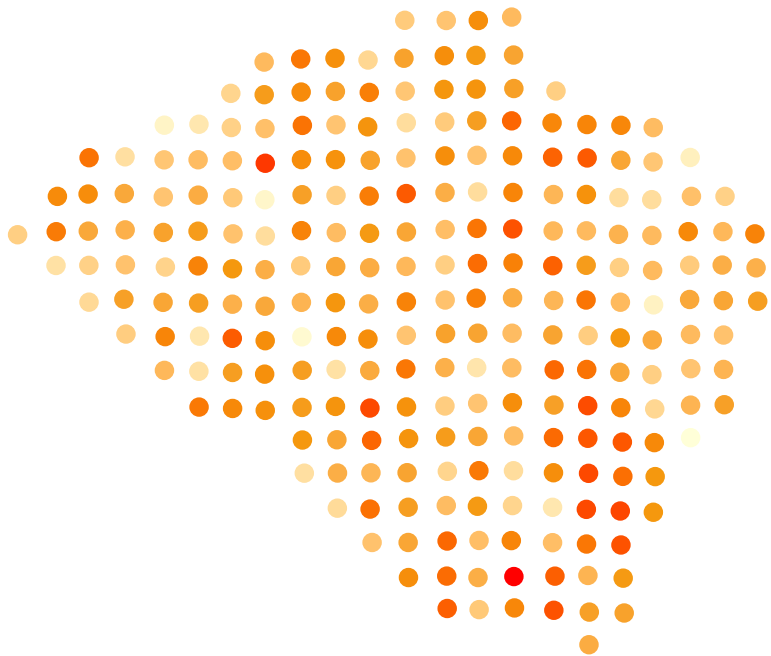

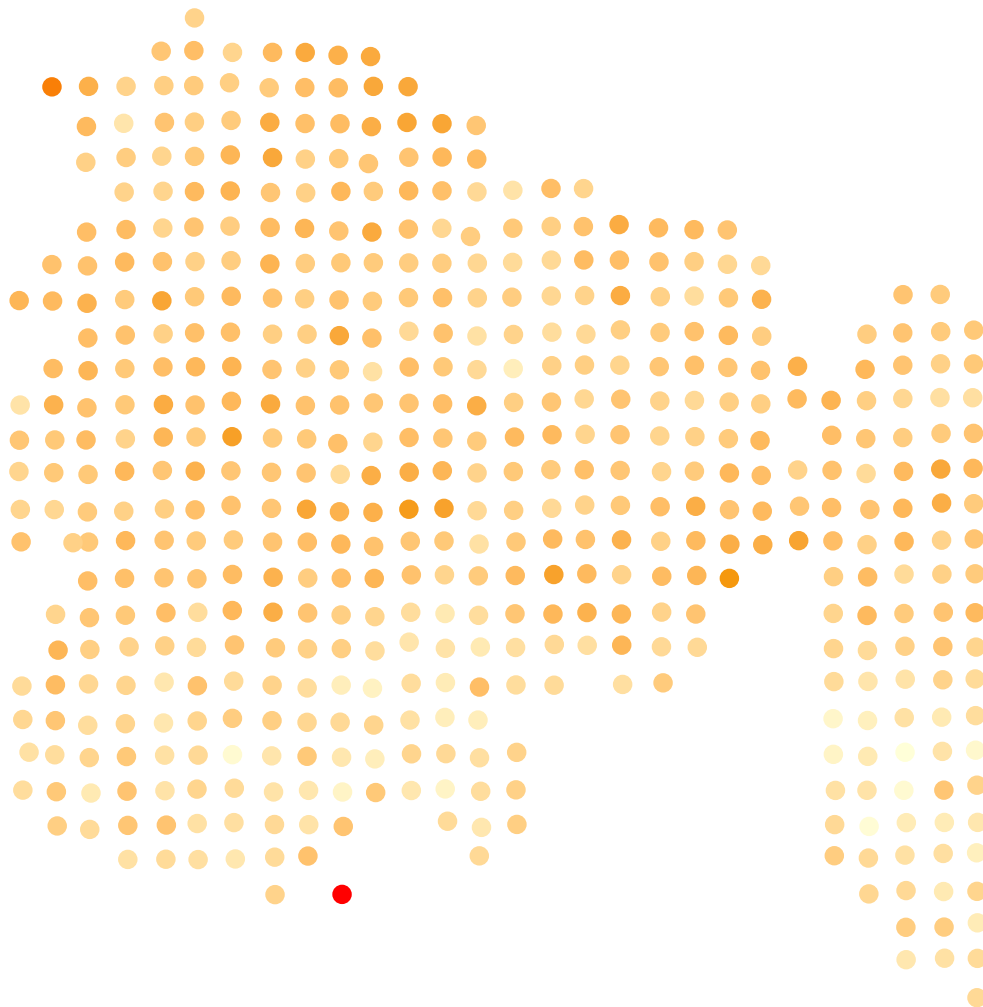

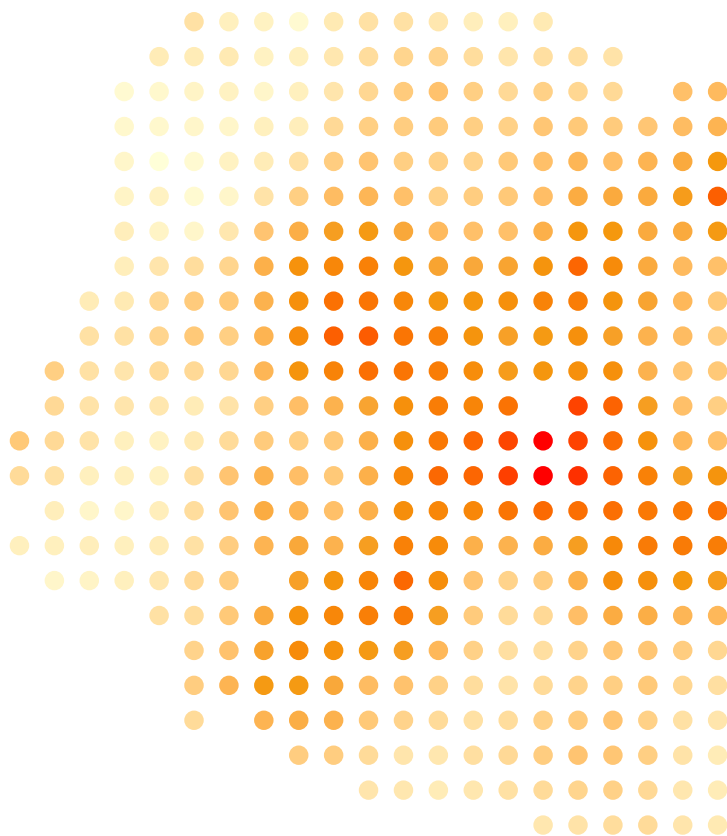

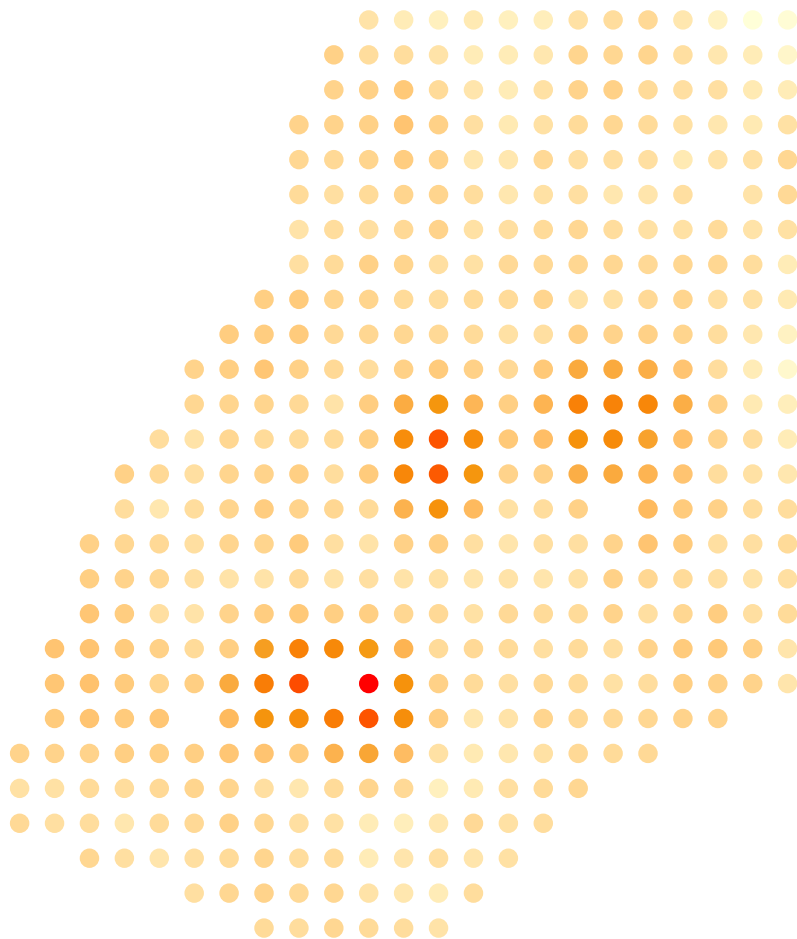

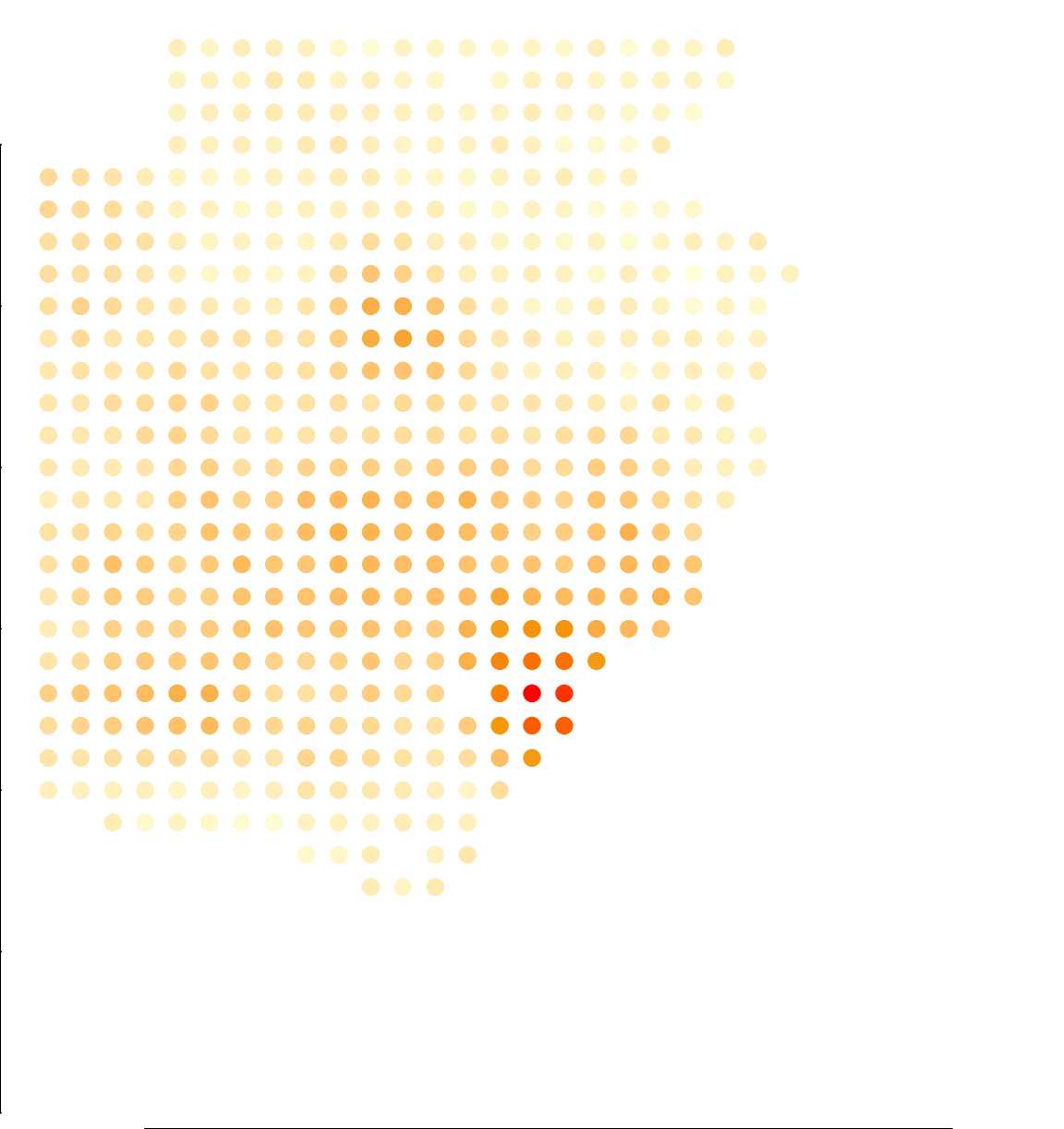

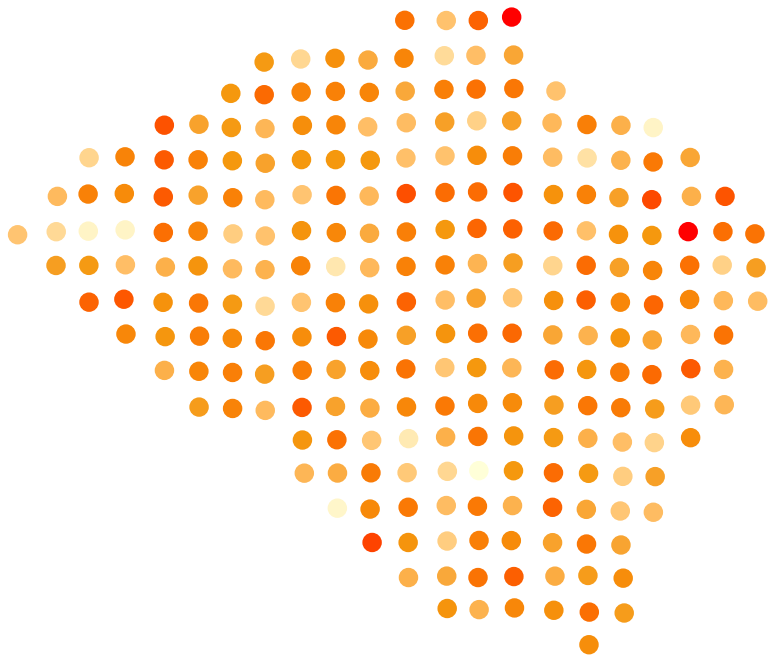

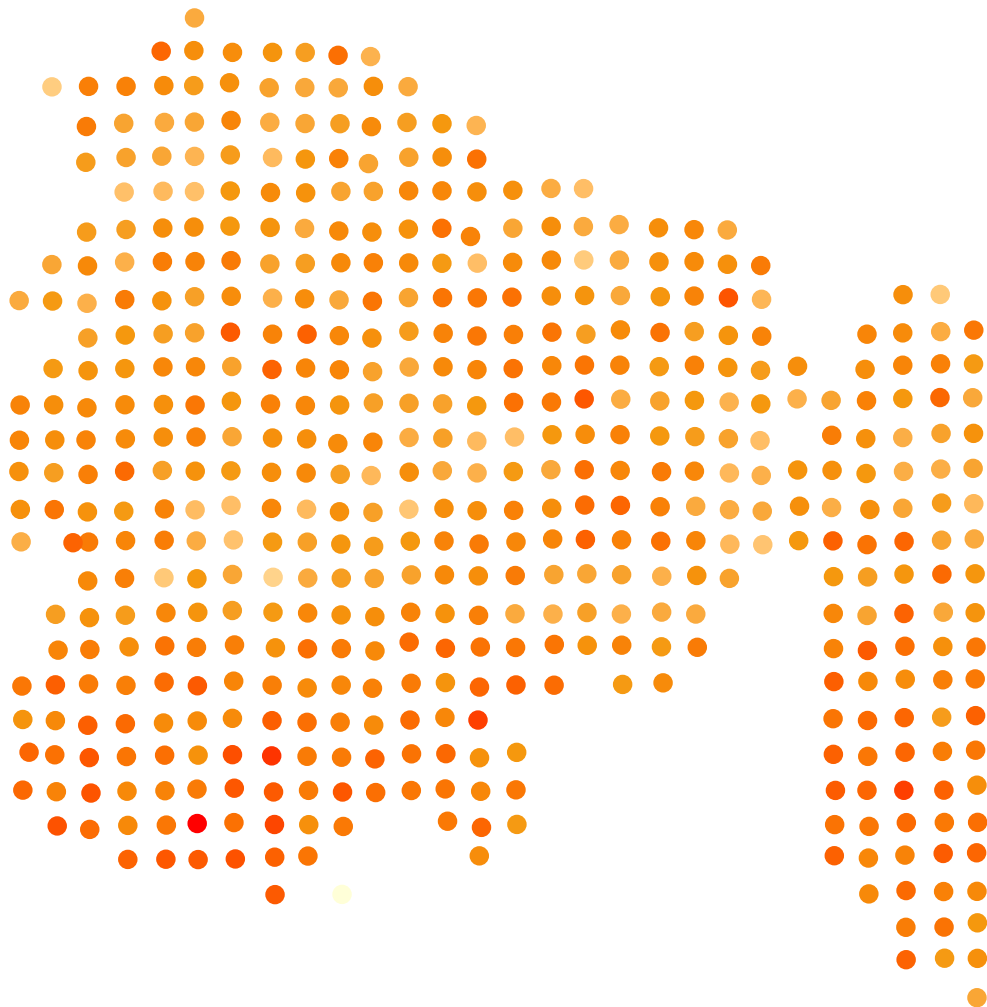

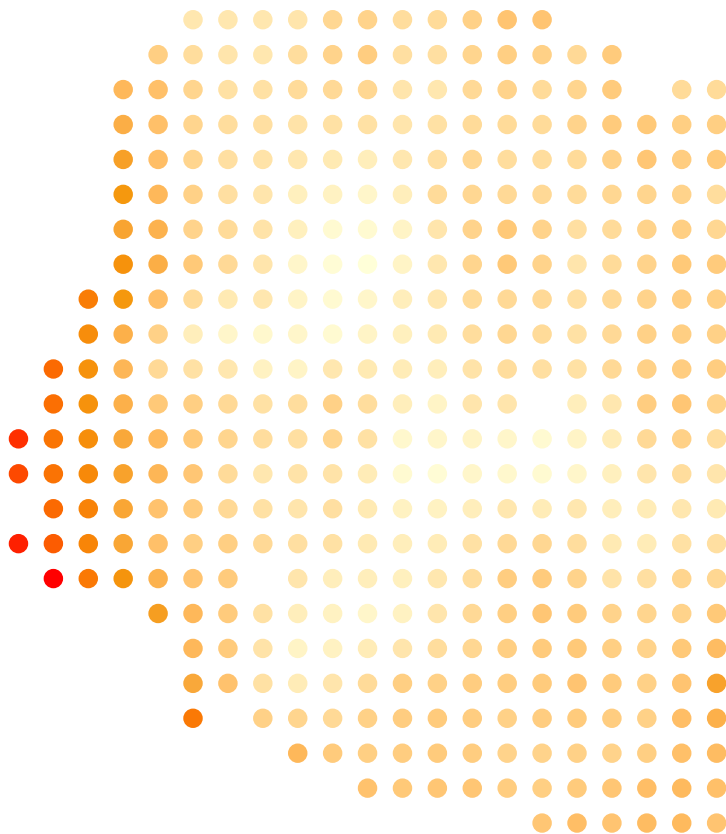

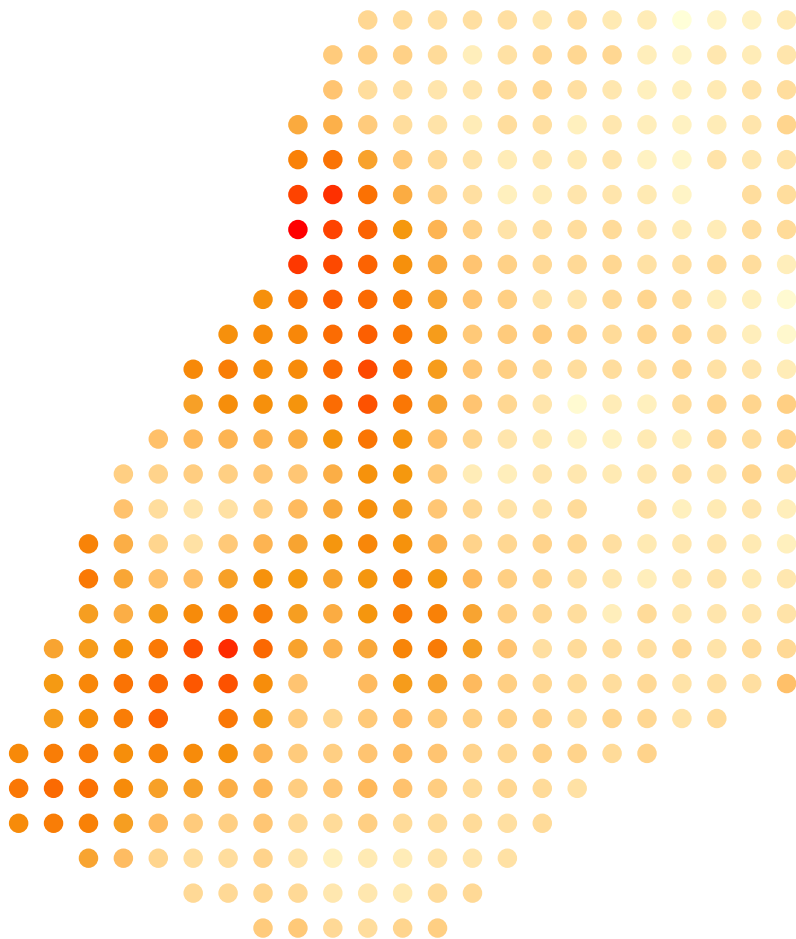

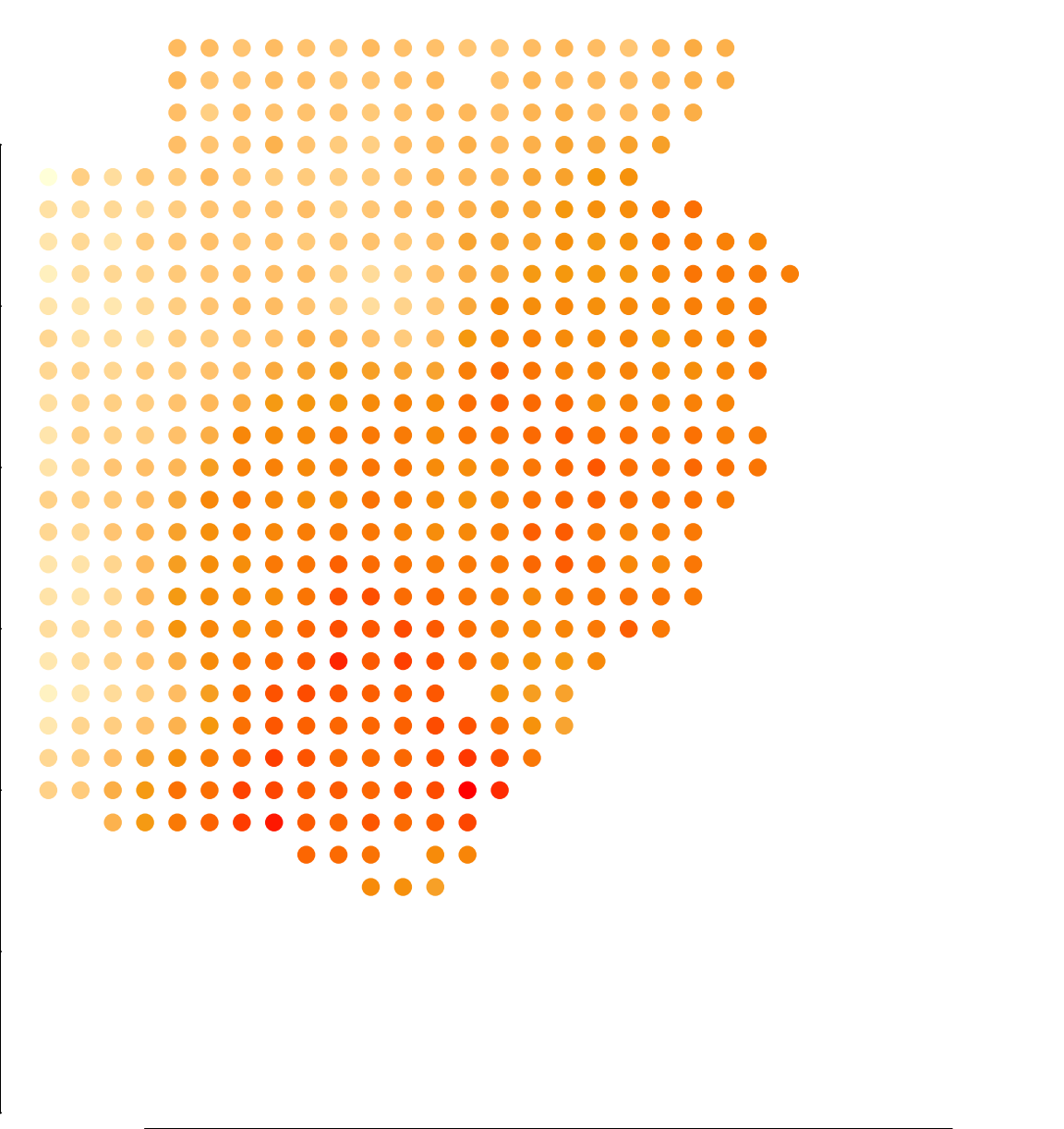

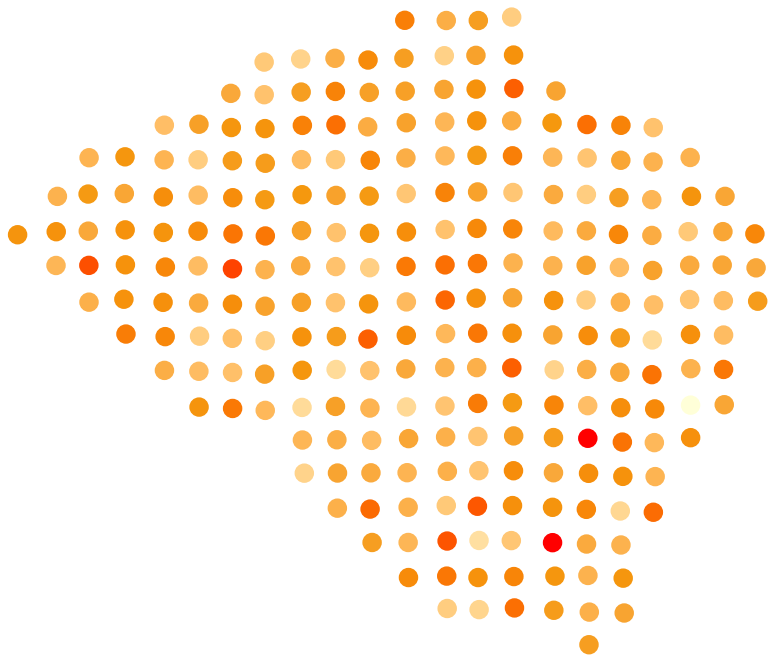

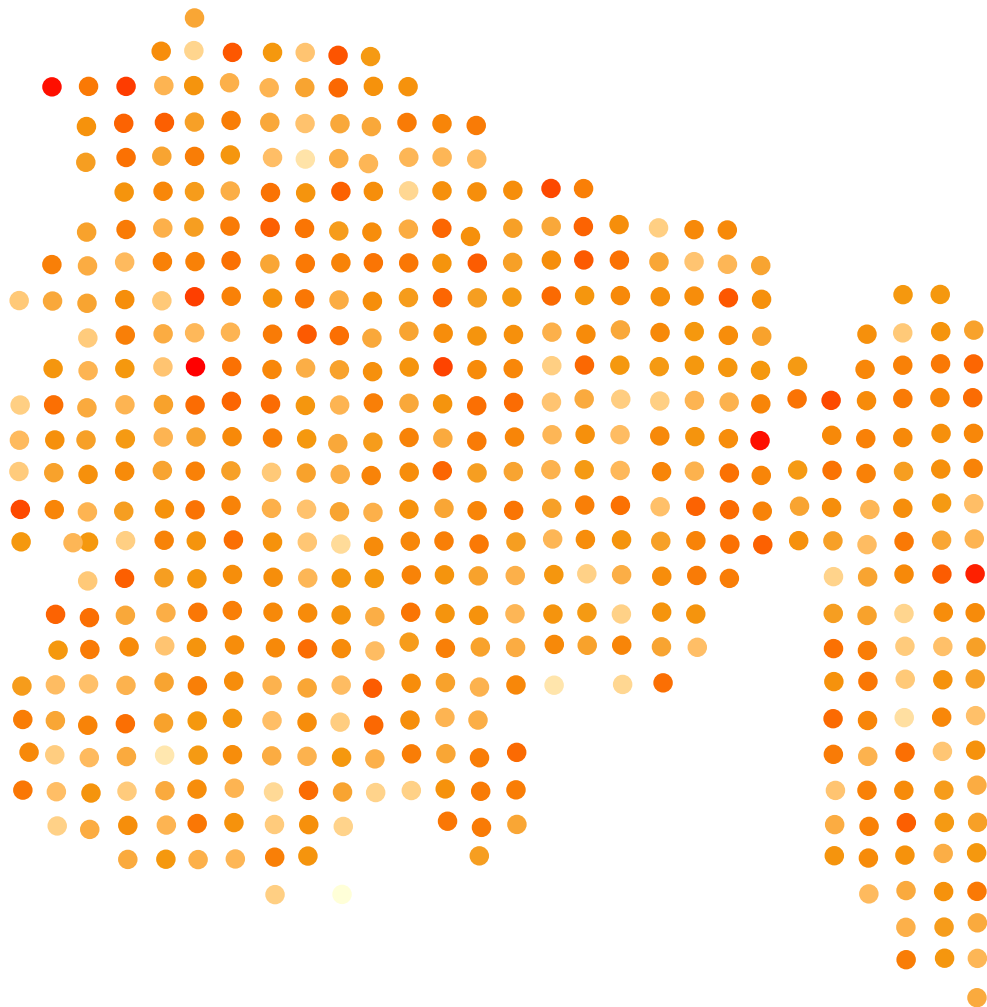

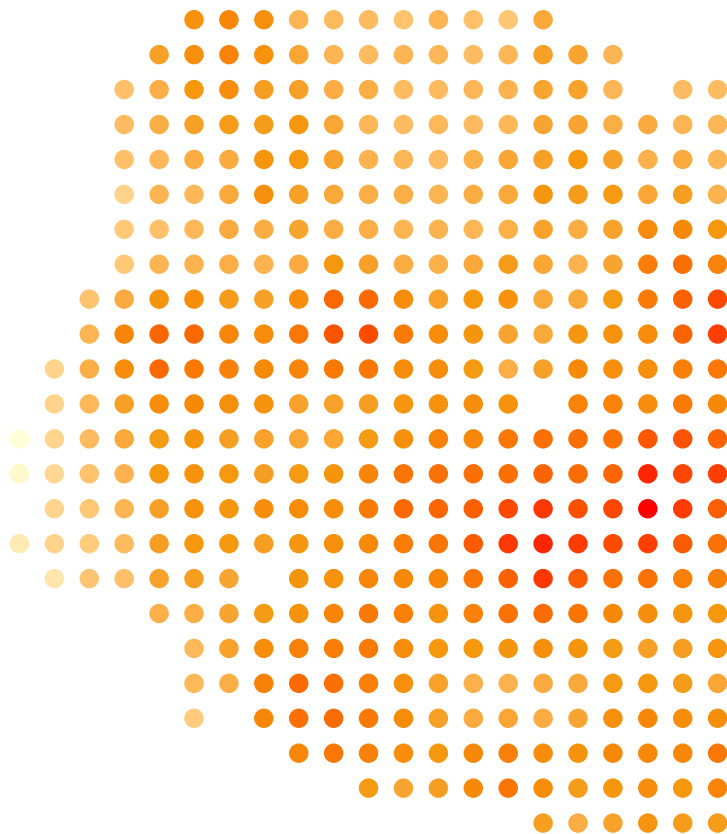

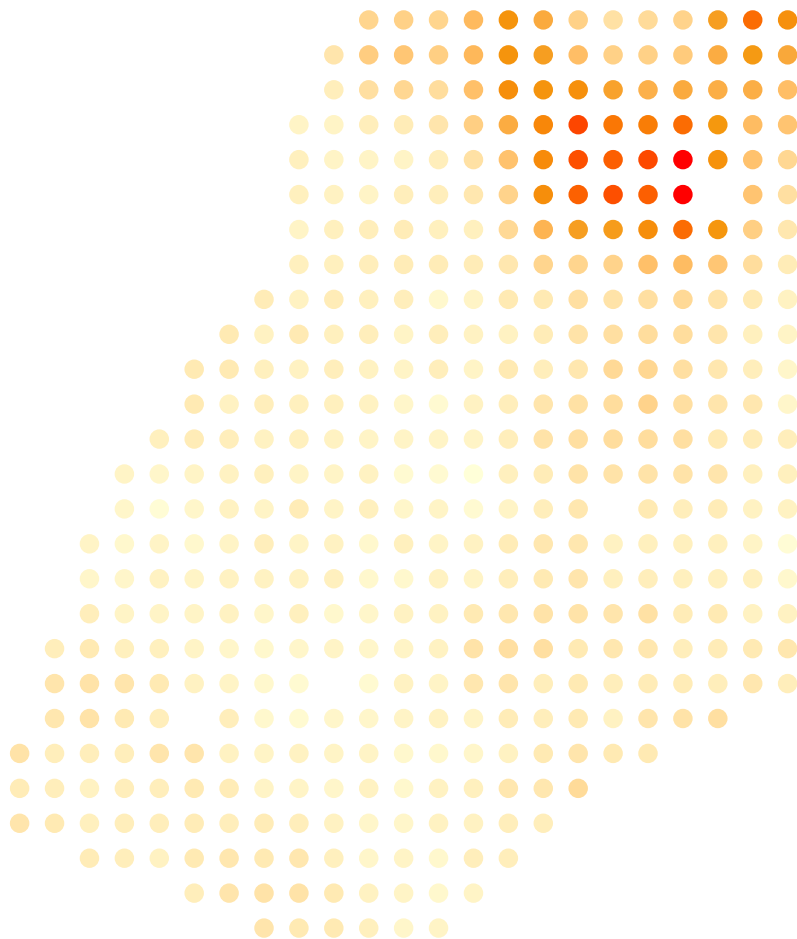

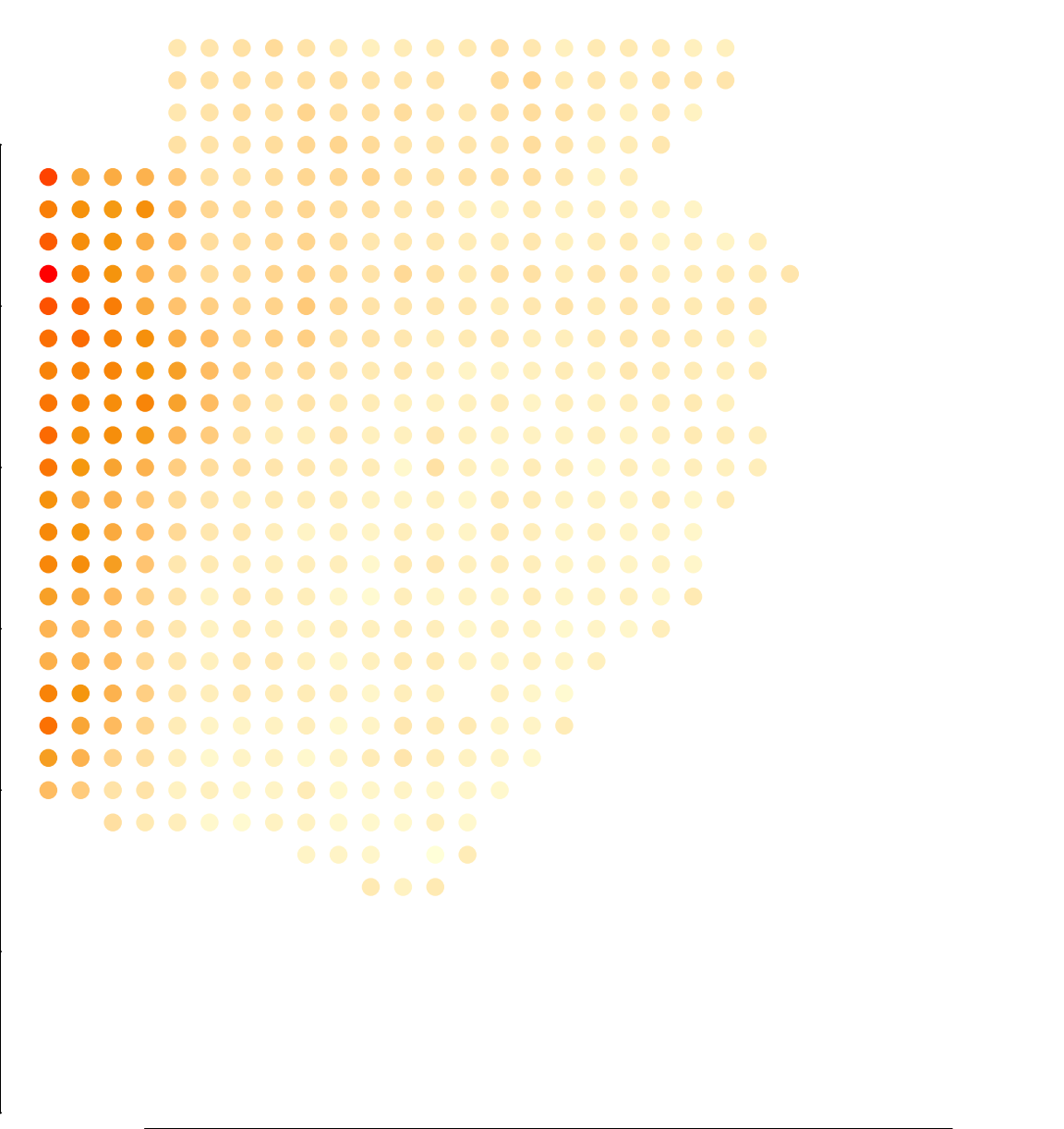

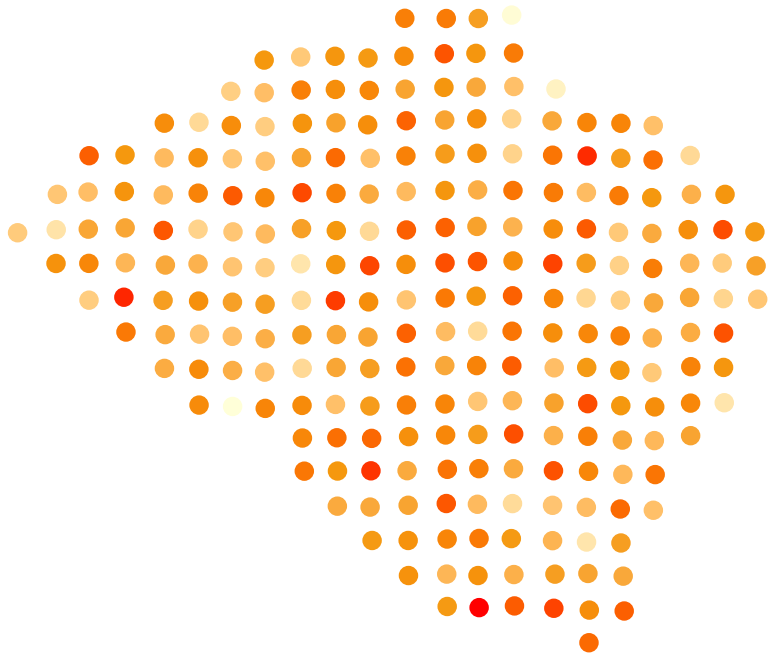

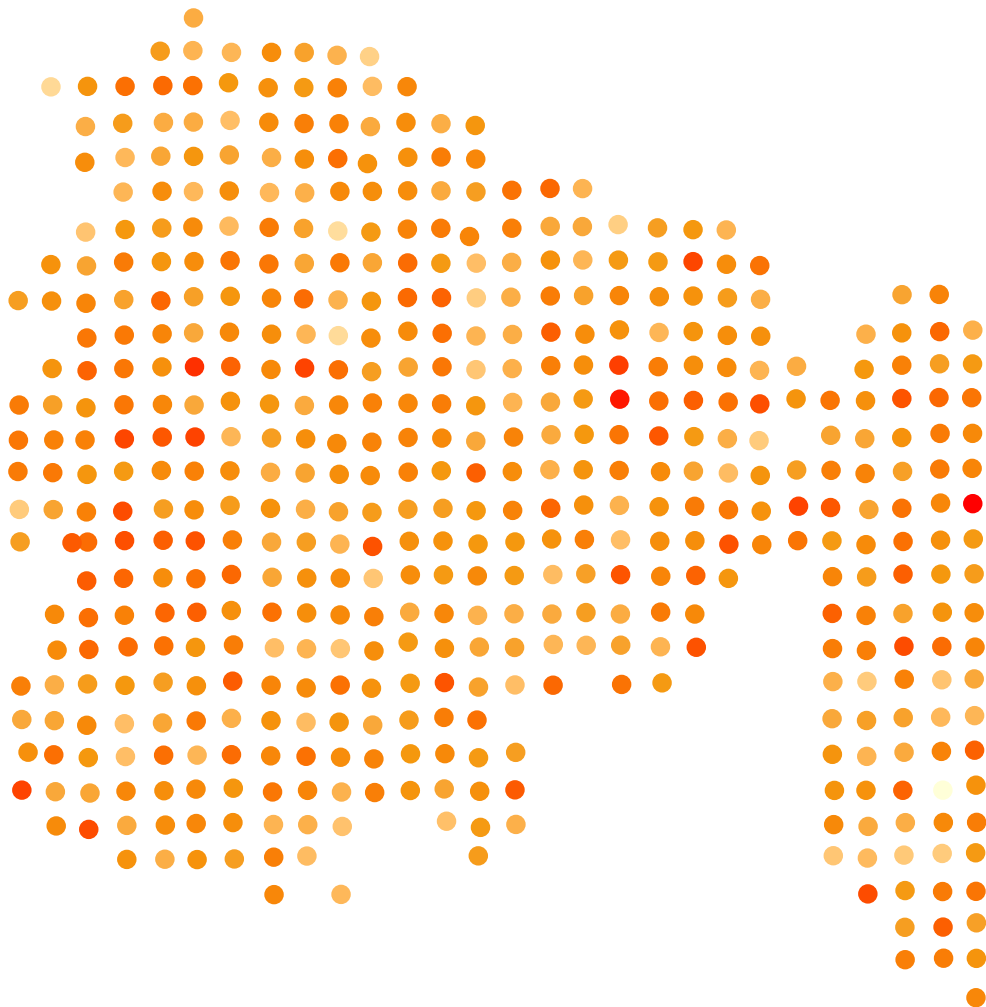

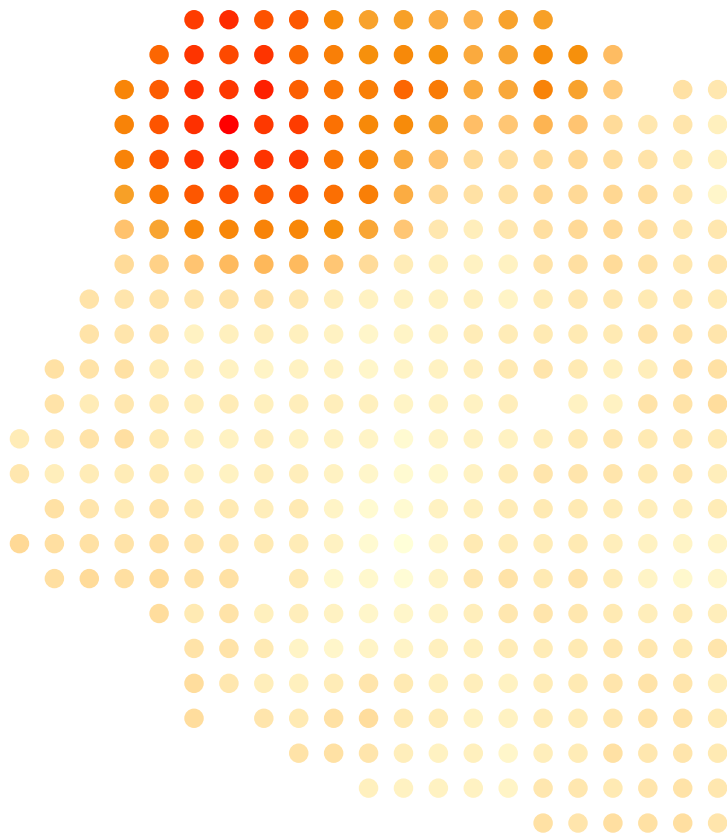

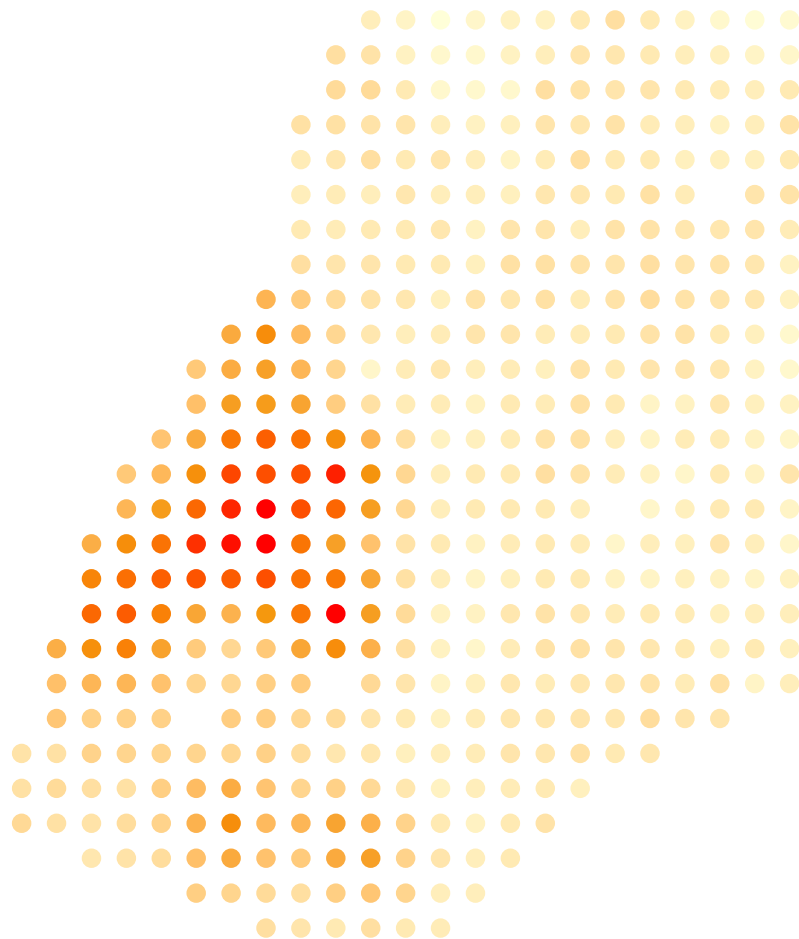

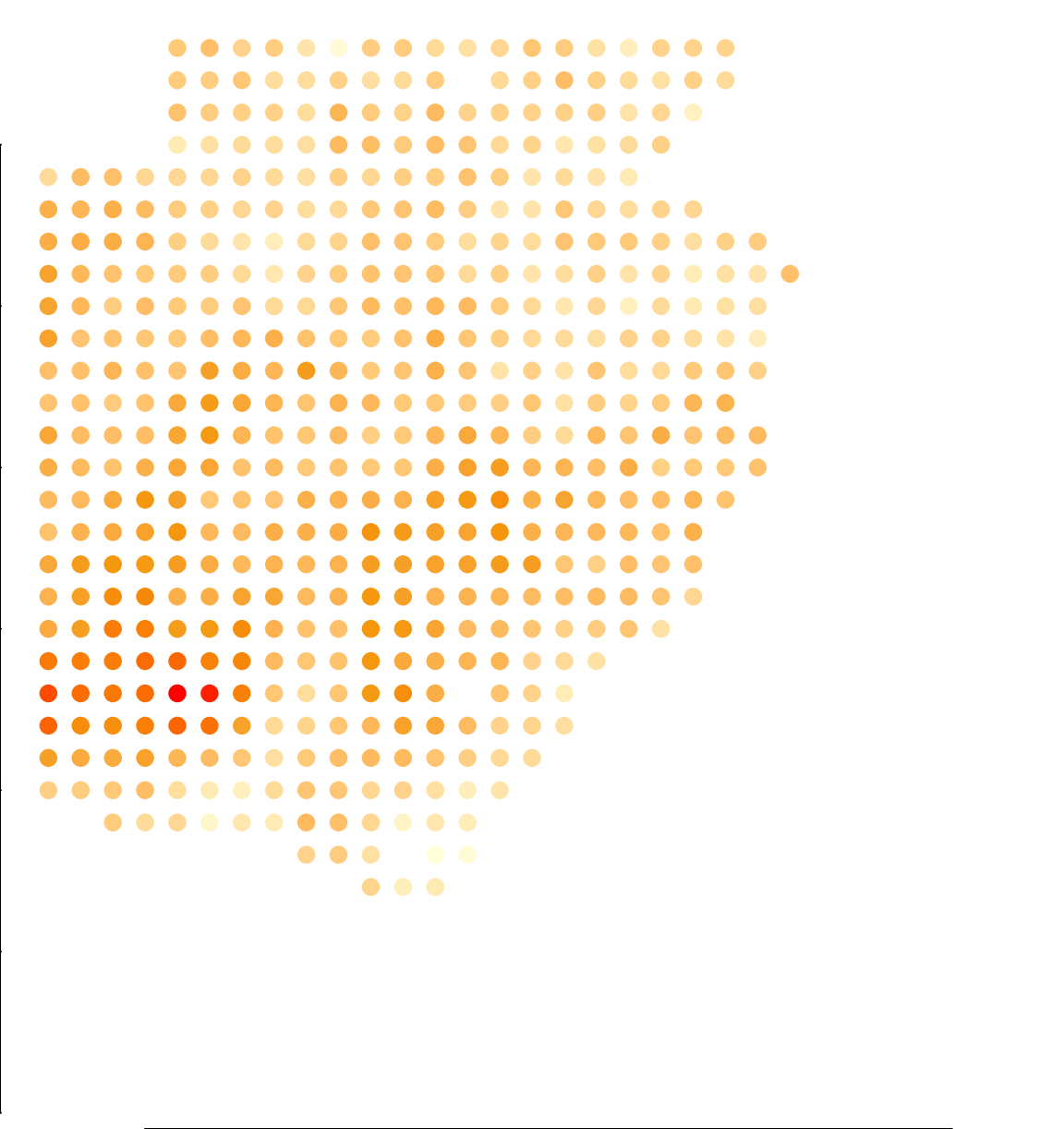

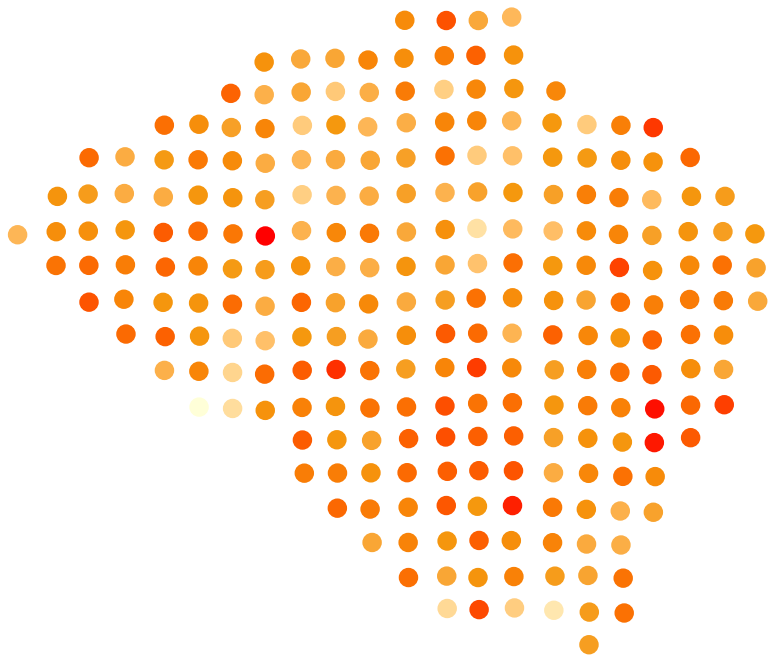

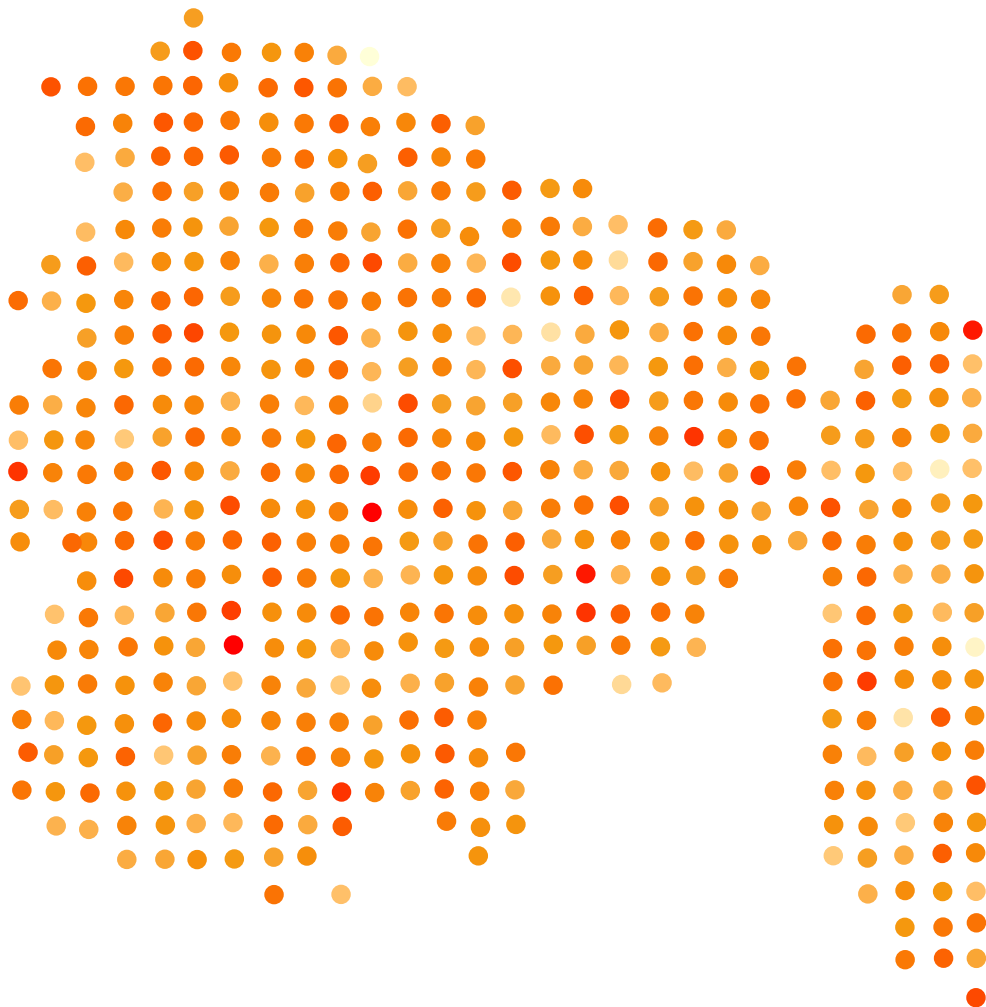

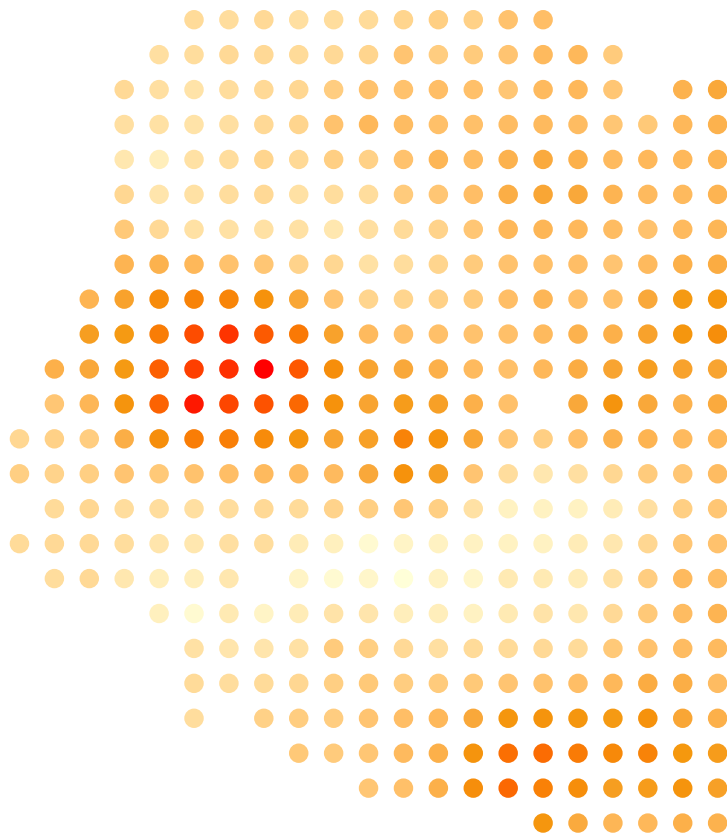

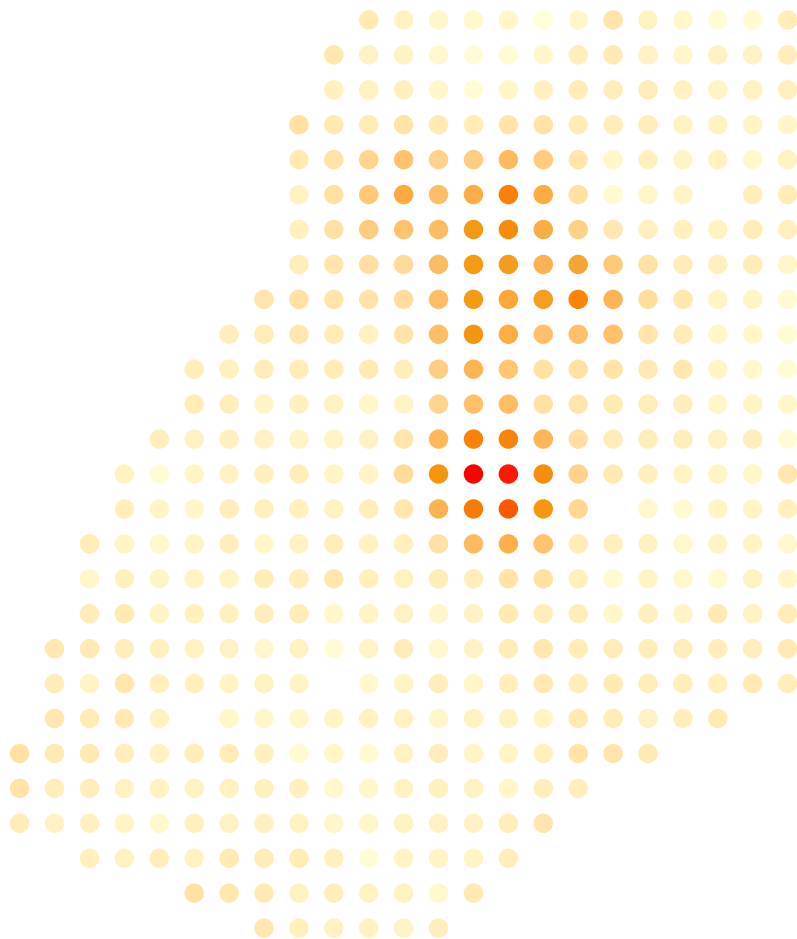

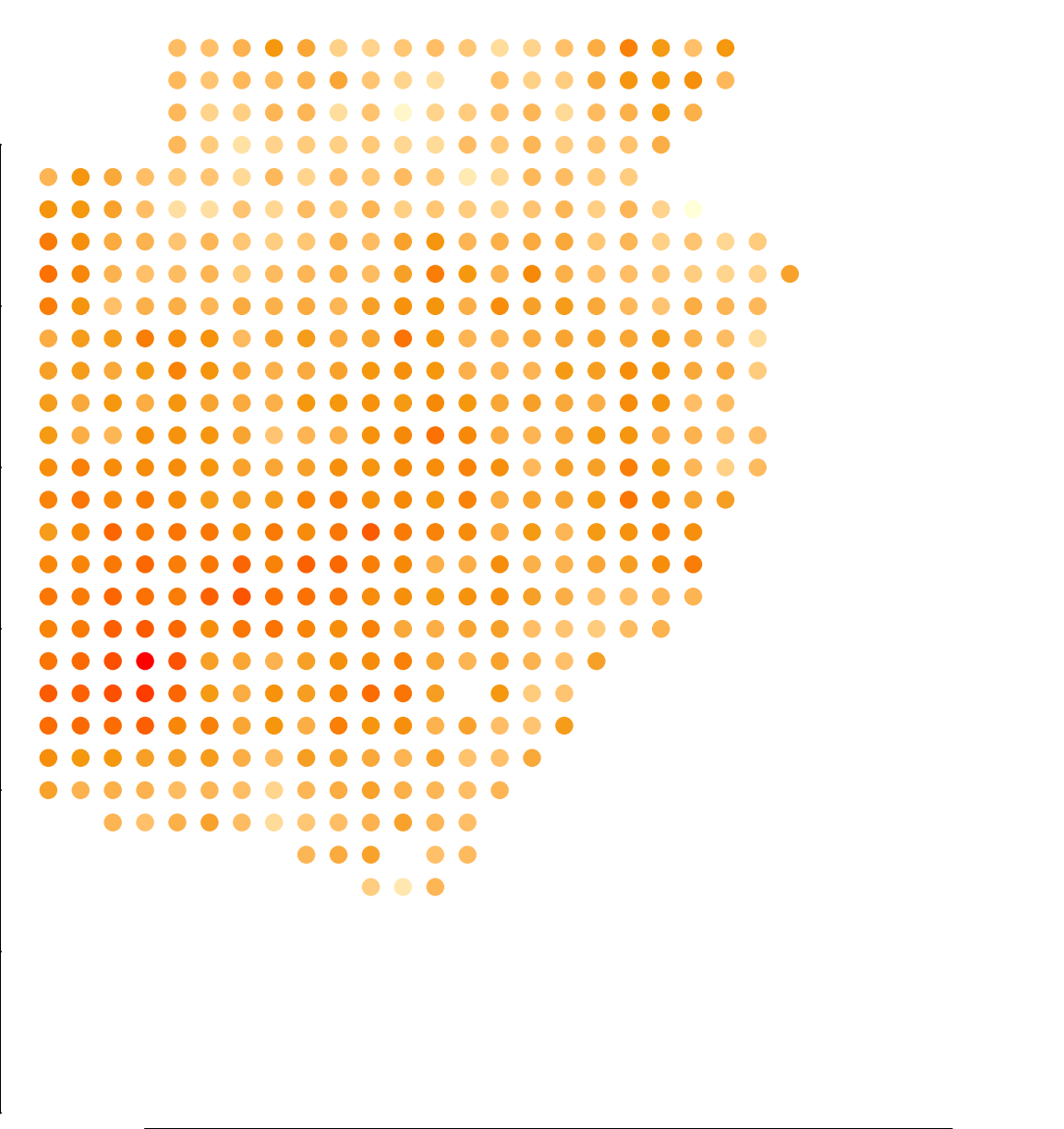

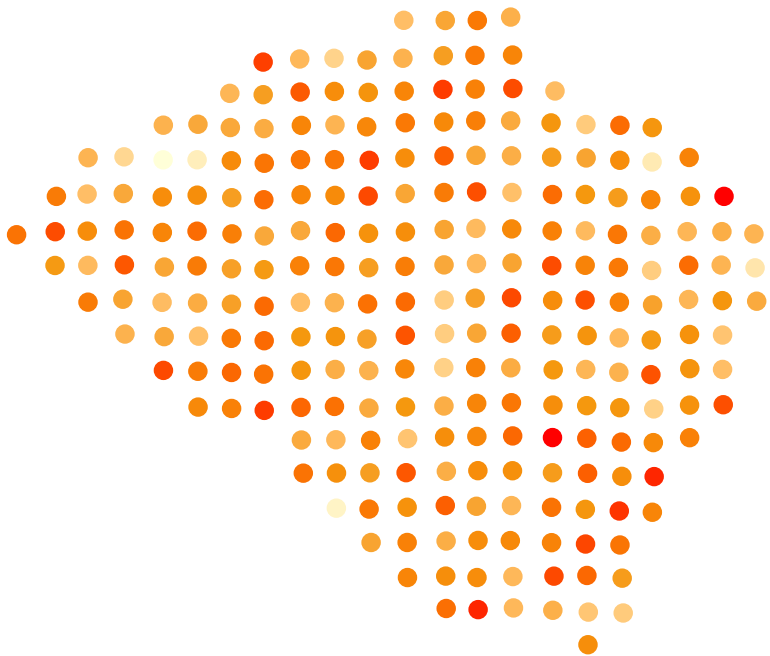

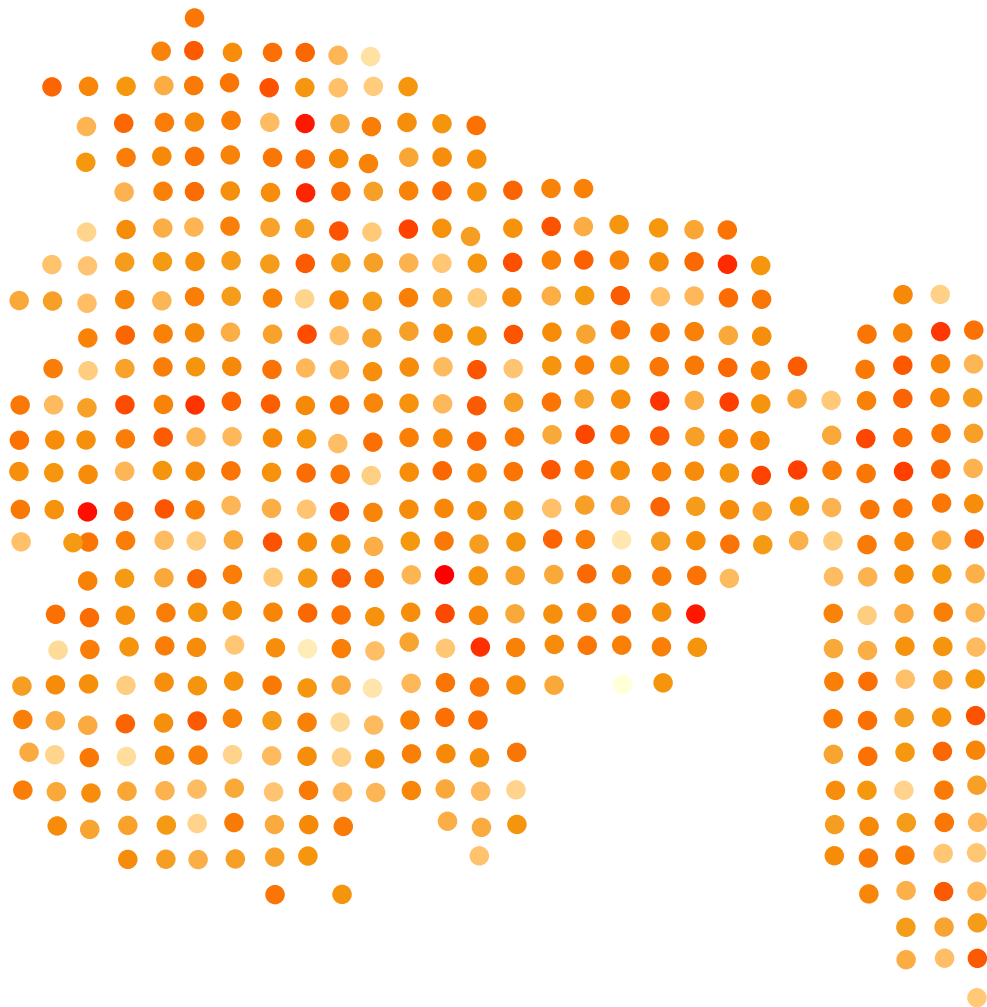

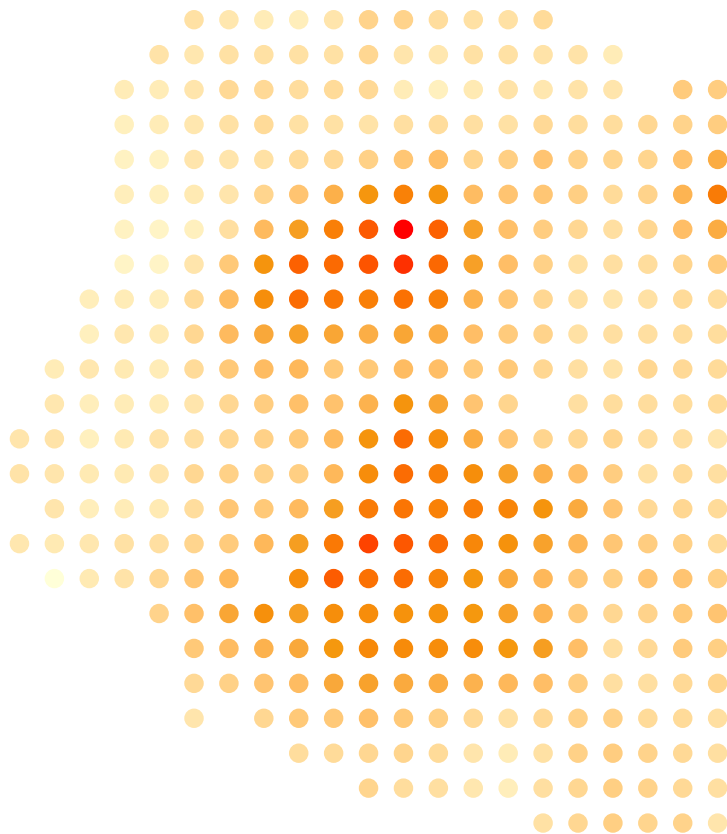

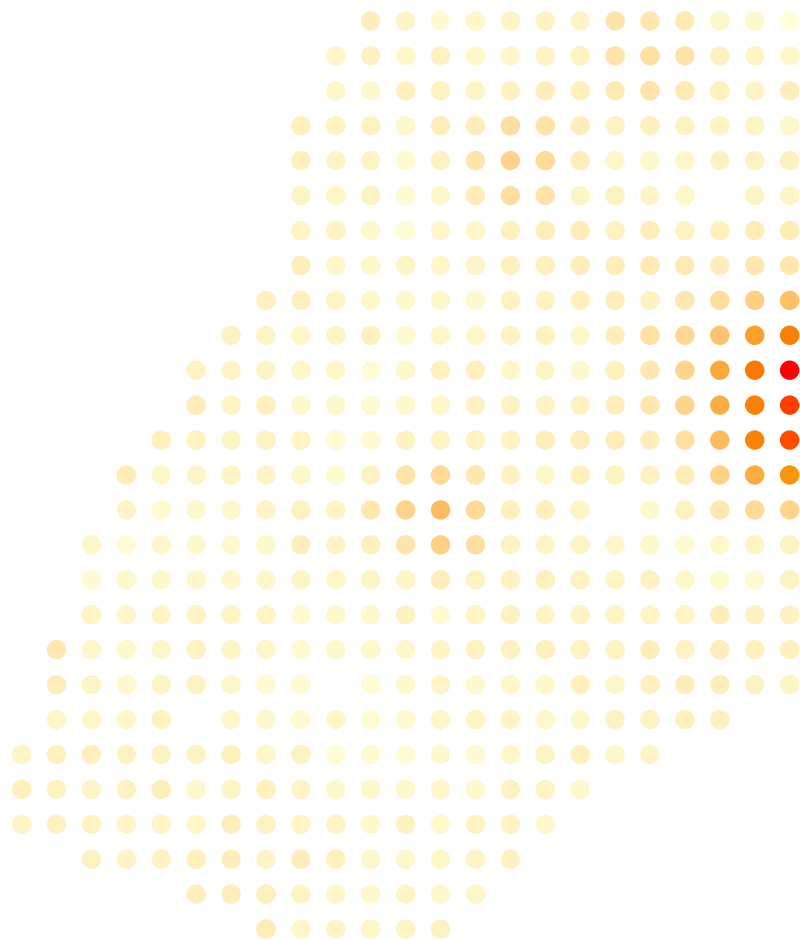

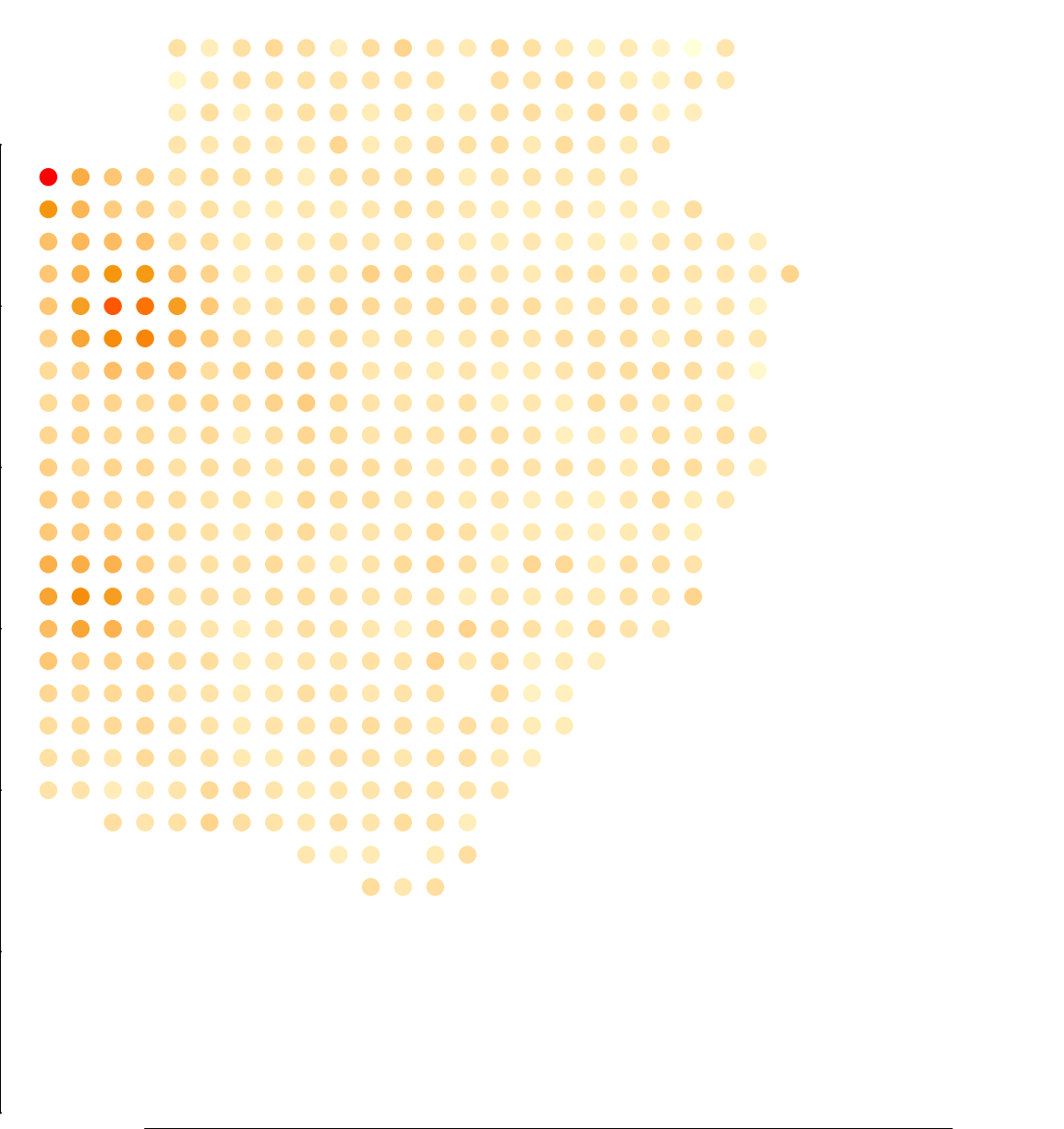

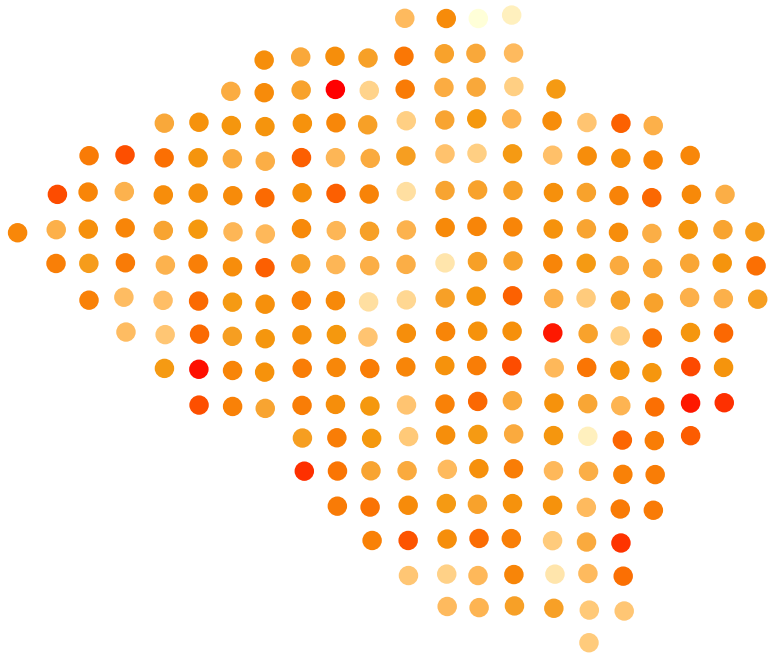

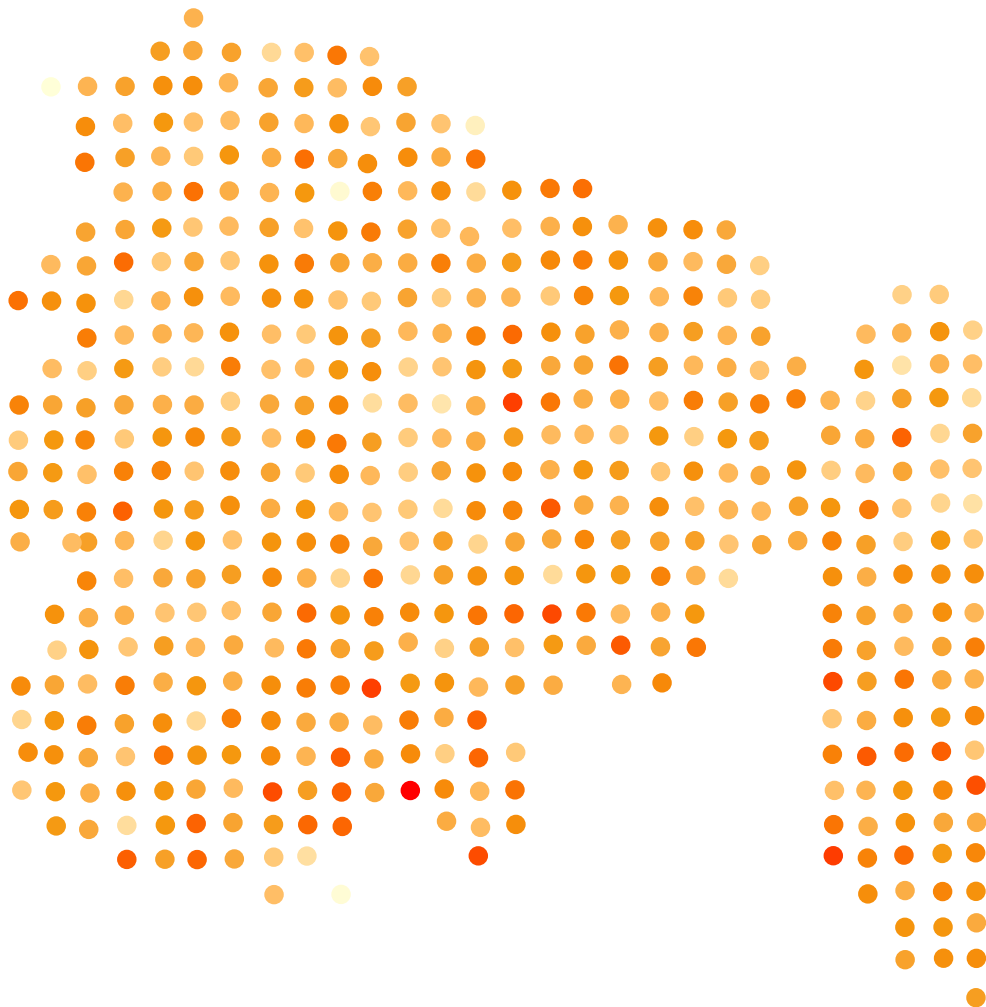

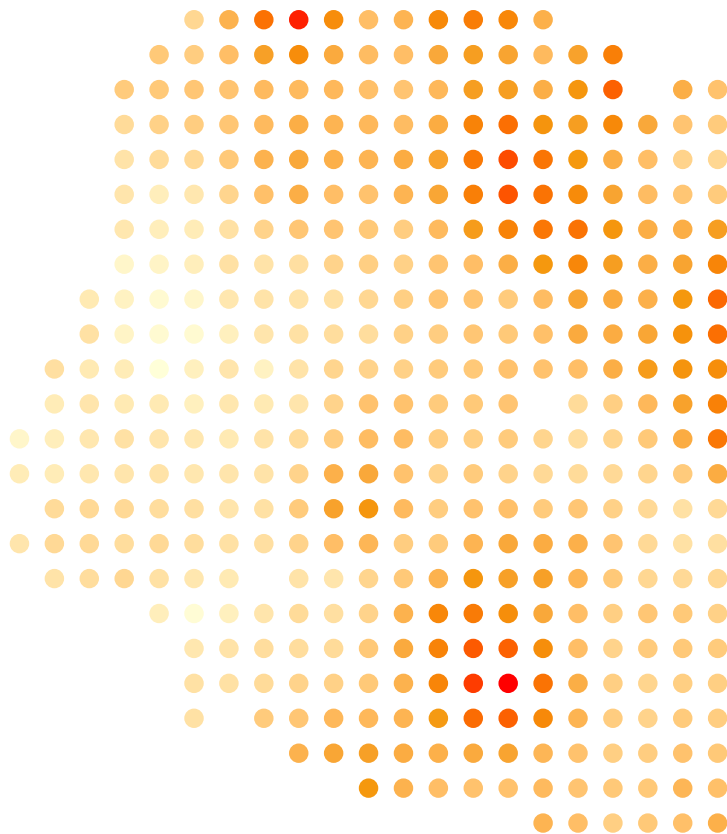

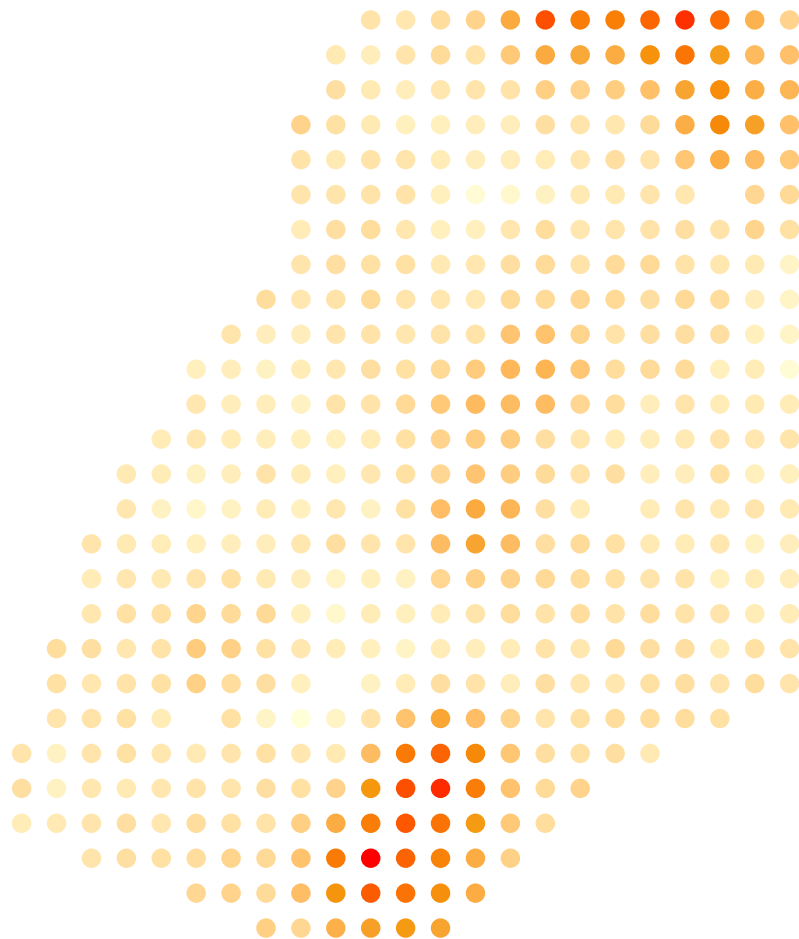

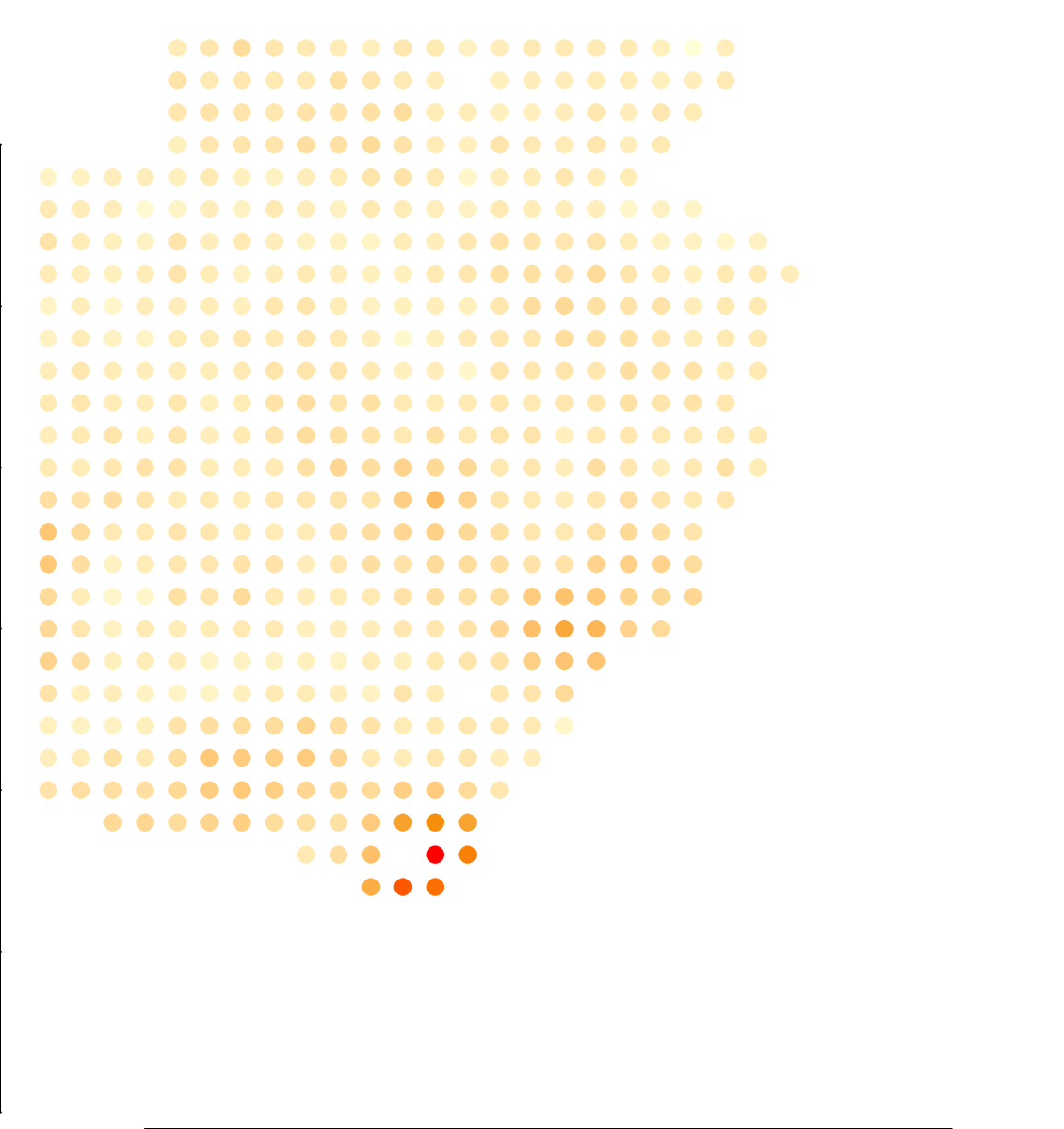

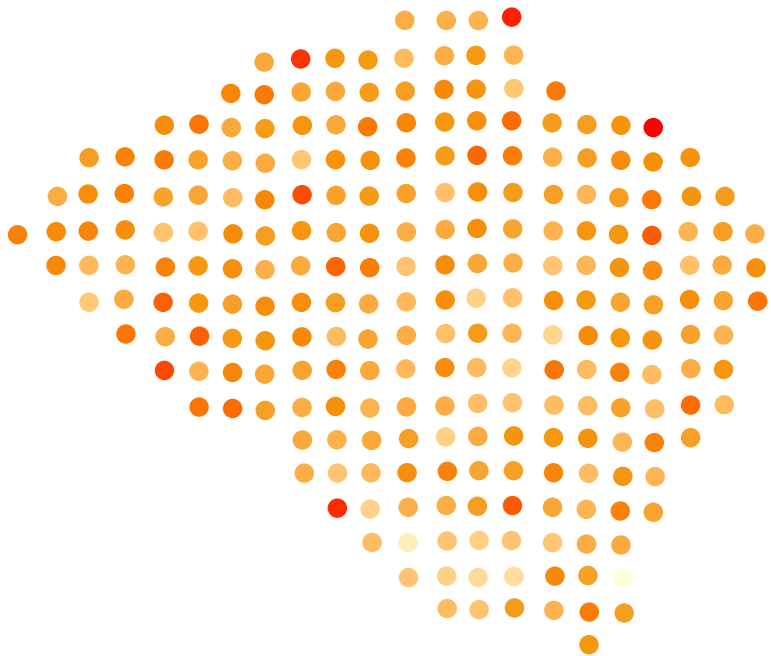

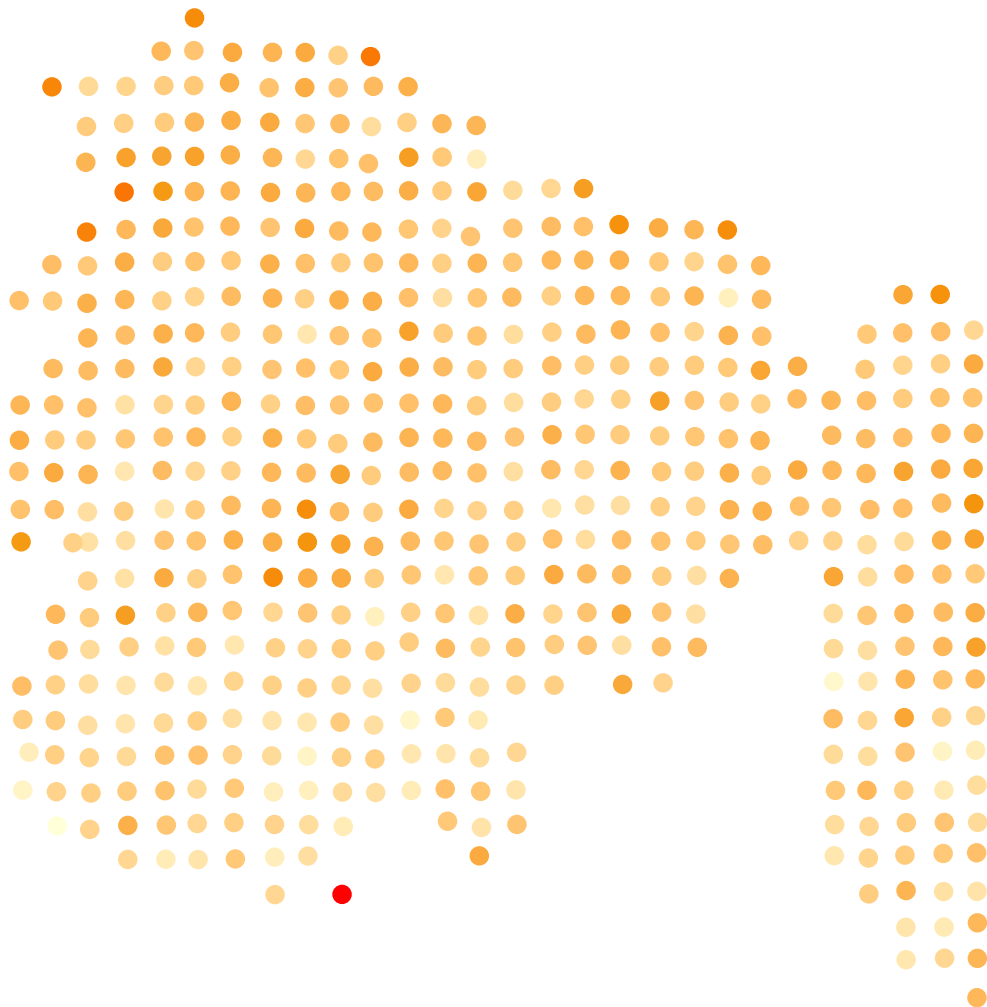

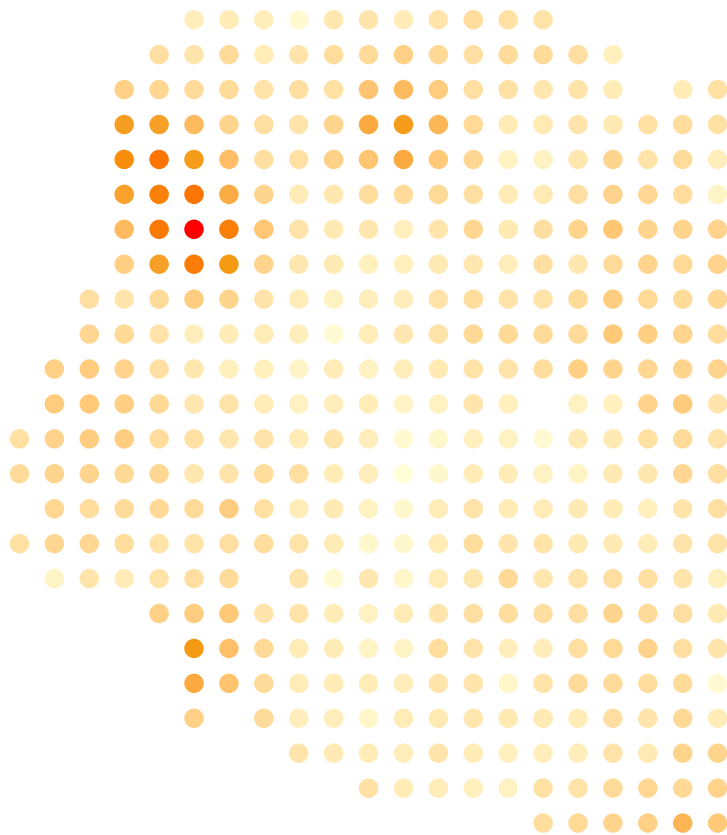

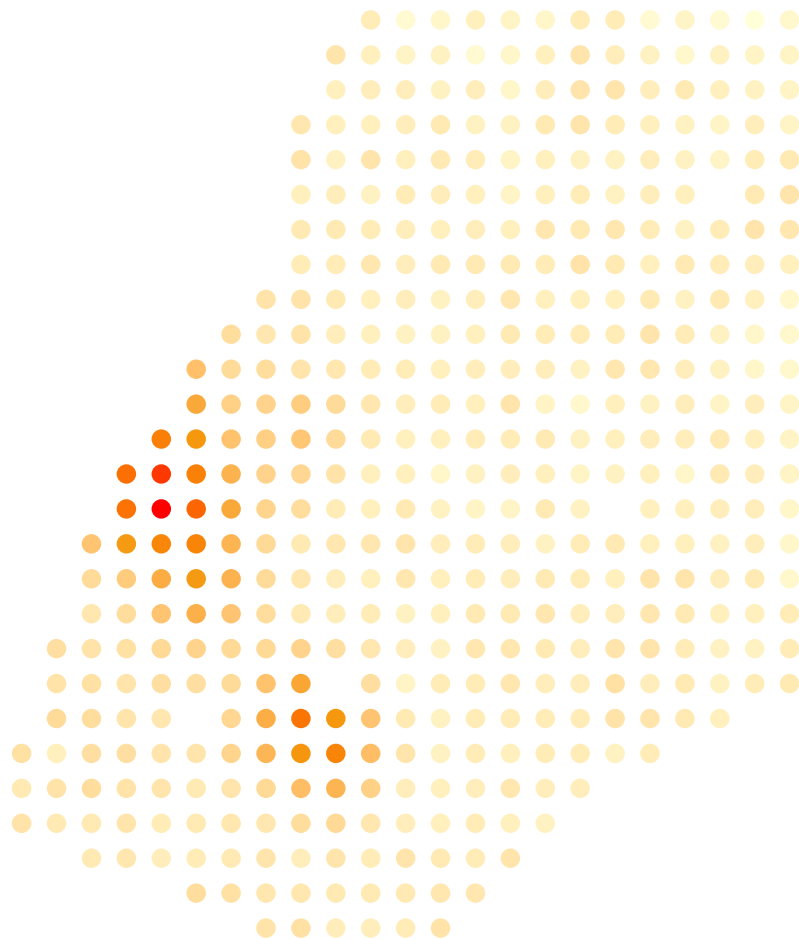

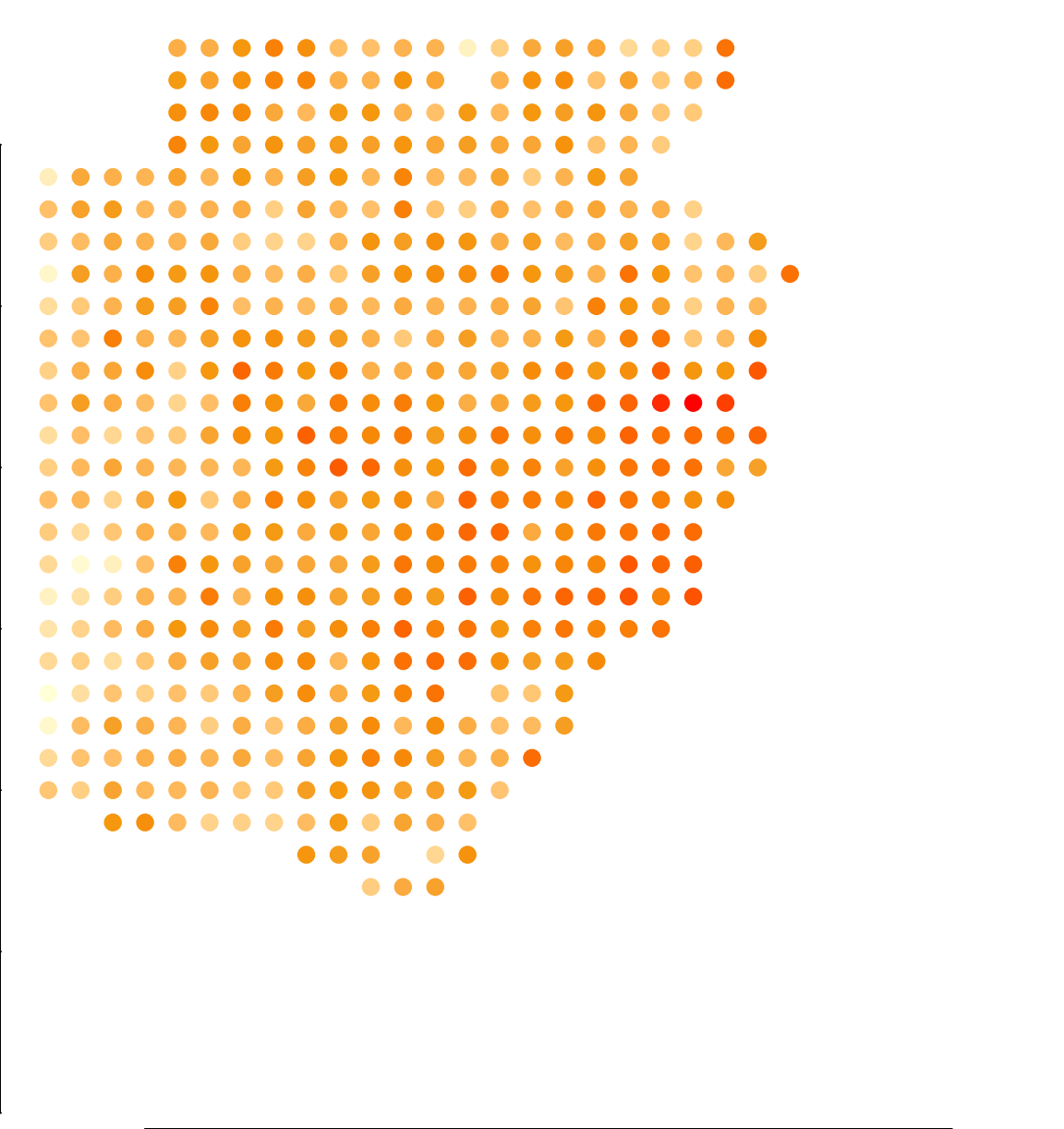

Supplement: Supplementary file 8 — Supplementary Data 5 [file 41467_2018_4724_MOESM8_ESM.zip › Supplementary Dataset 7/joint-field-profiles-rel-individual-scale-dots-split.pdf]

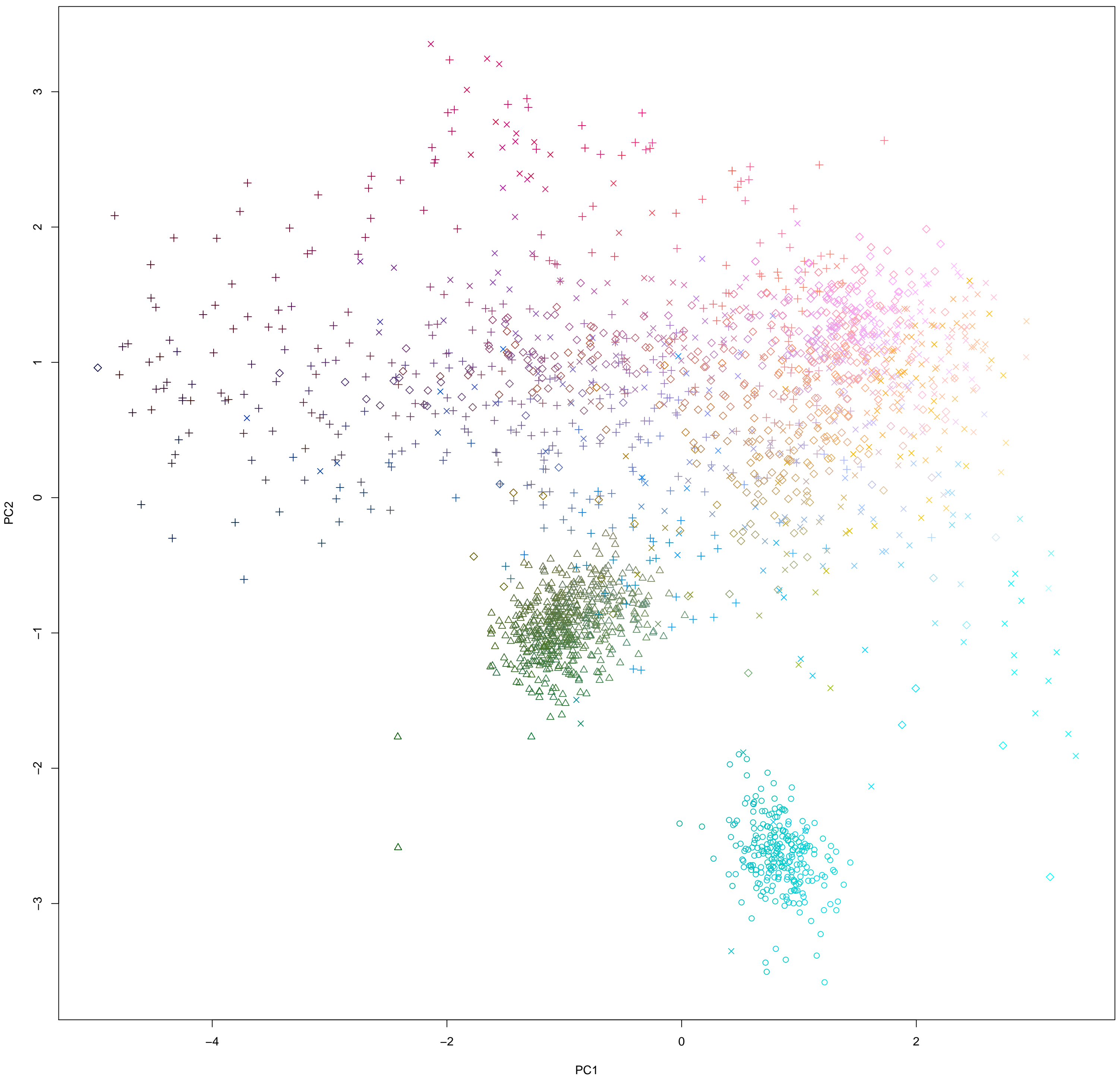

Supplement: Supplementary file 8 — Supplementary Data 5 [file 41467_2018_4724_MOESM8_ESM.zip › Supplementary Dataset 7/joint-field-dimensionality-reduction-PCA-2d.pdf]

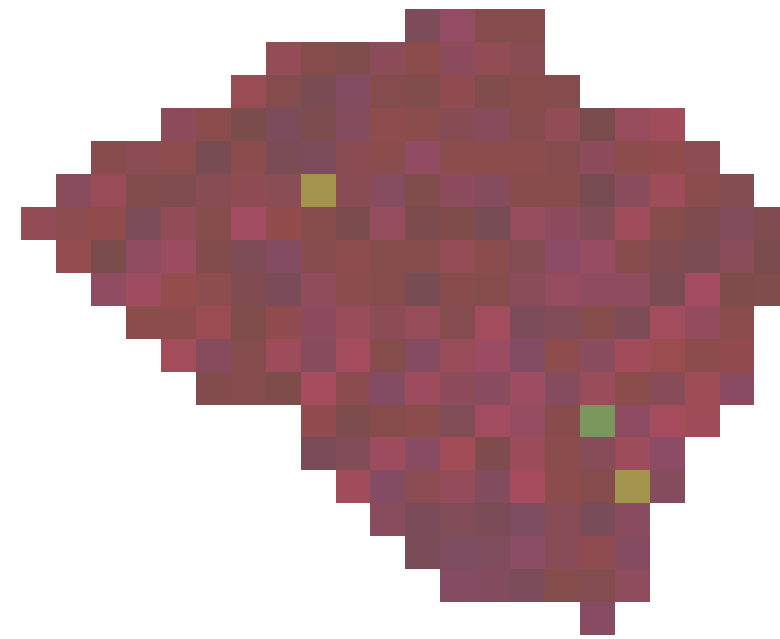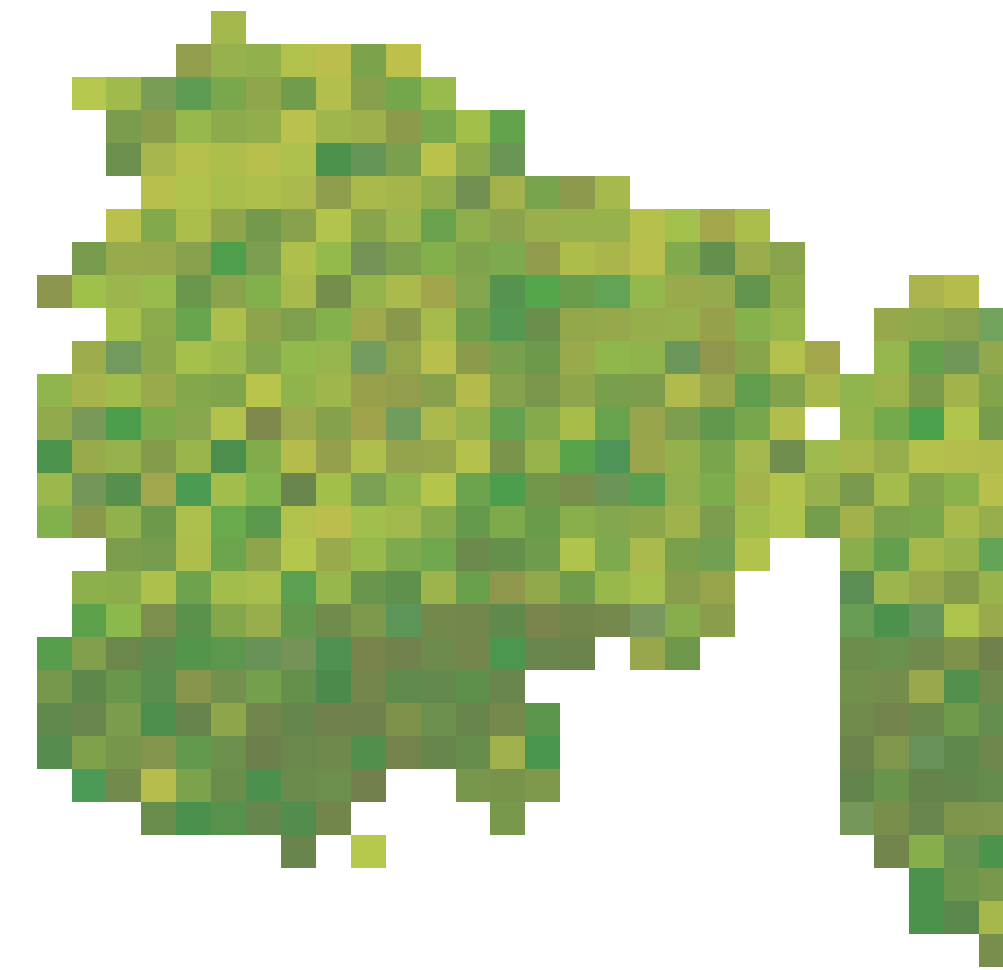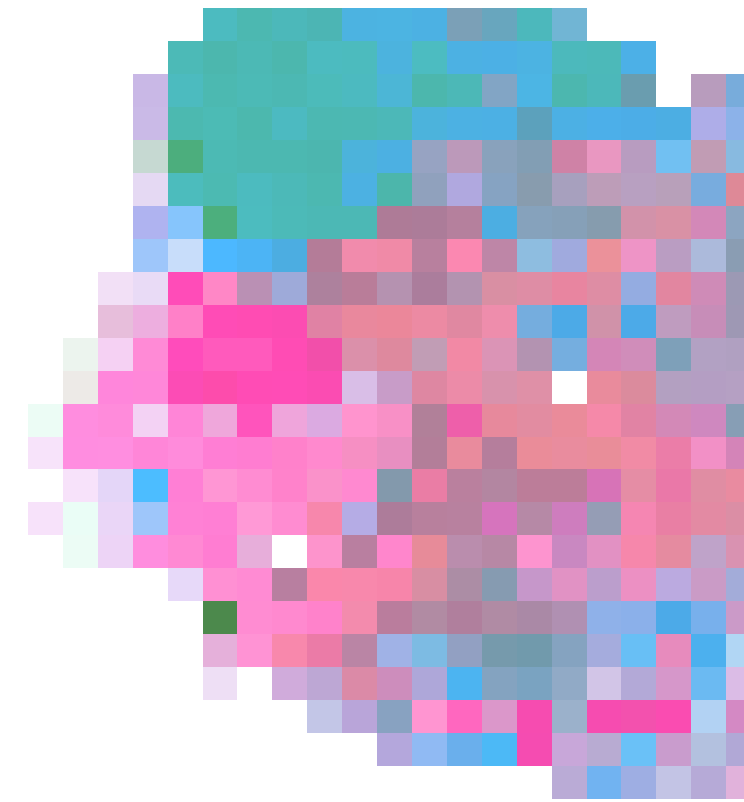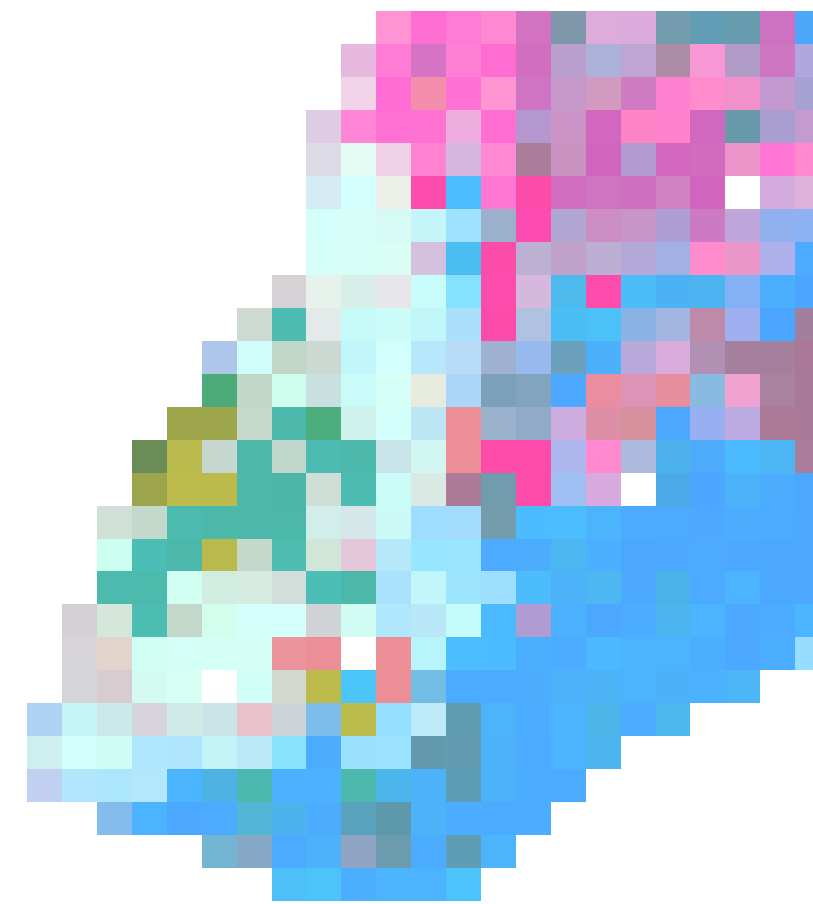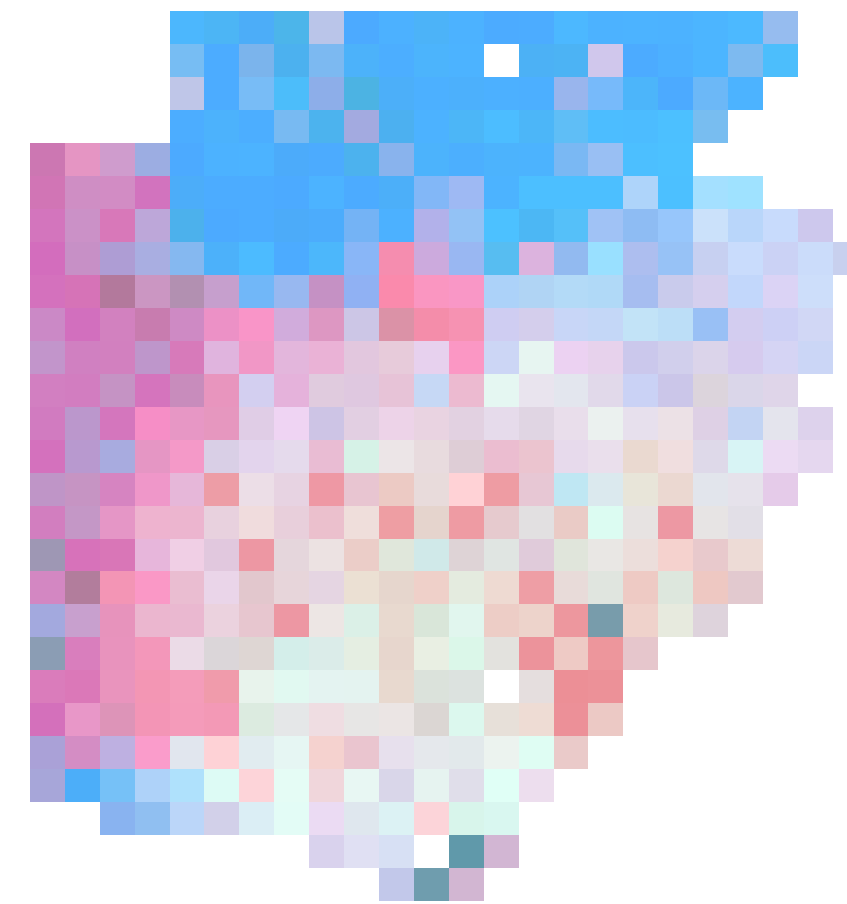

Supplement: Supplementary file 8 — Supplementary Data 5 [file 41467_2018_4724_MOESM8_ESM.zip › Supplementary Dataset 7/joint-mix-dimensionality-reduction-tSNE-matrix.pdf]

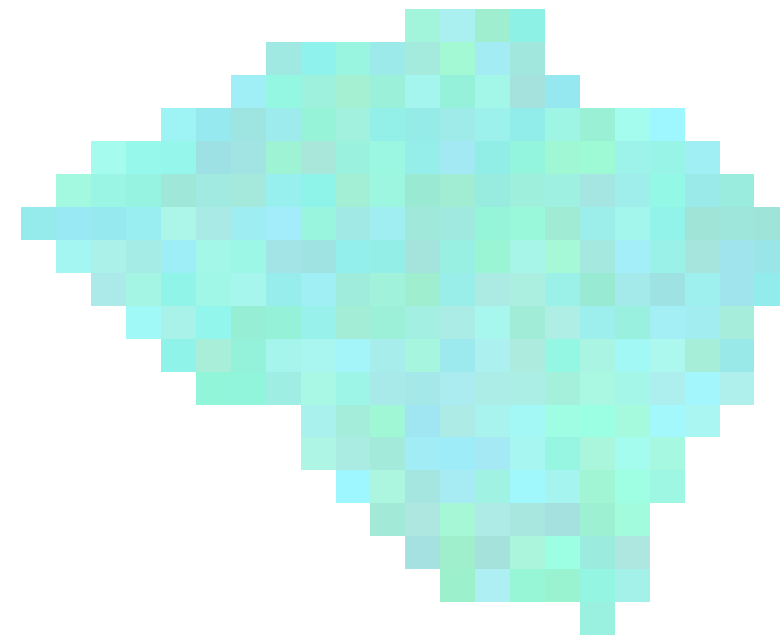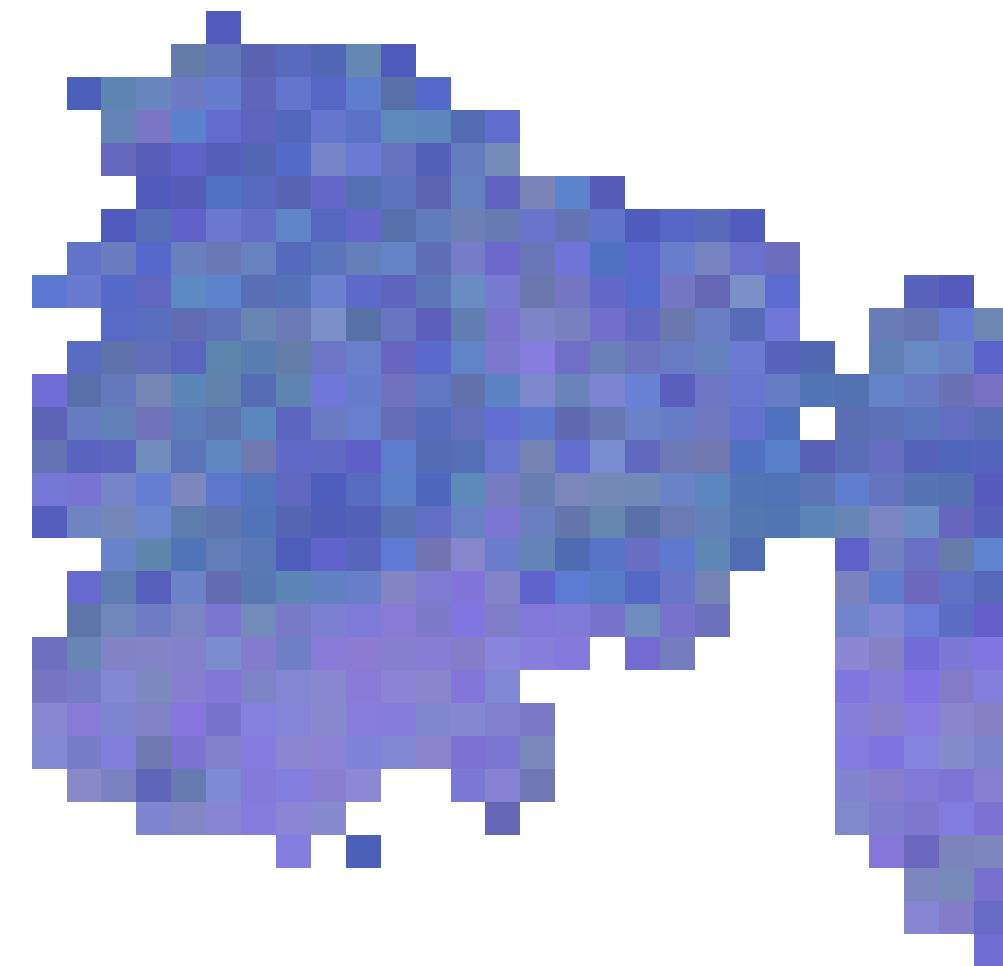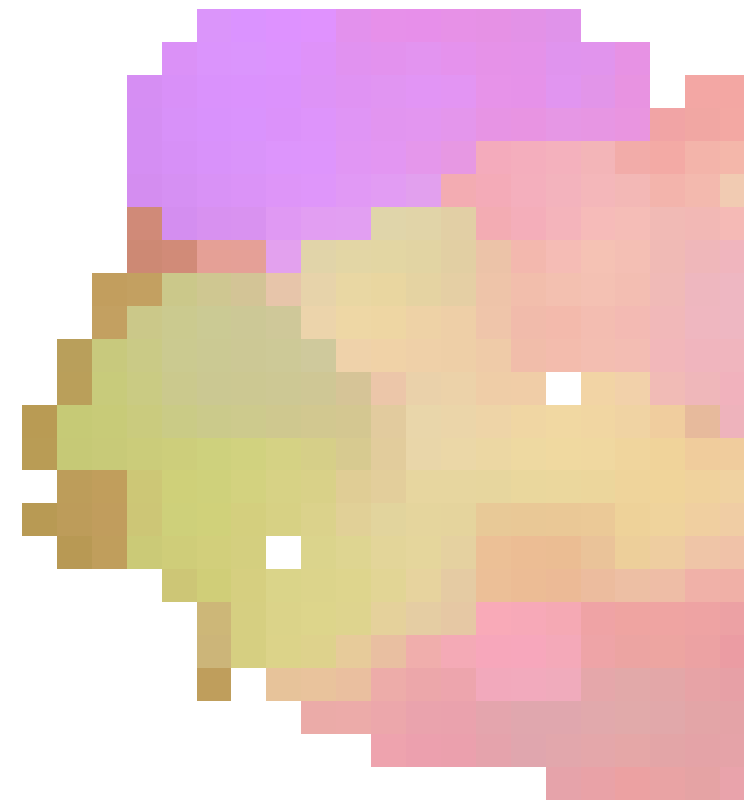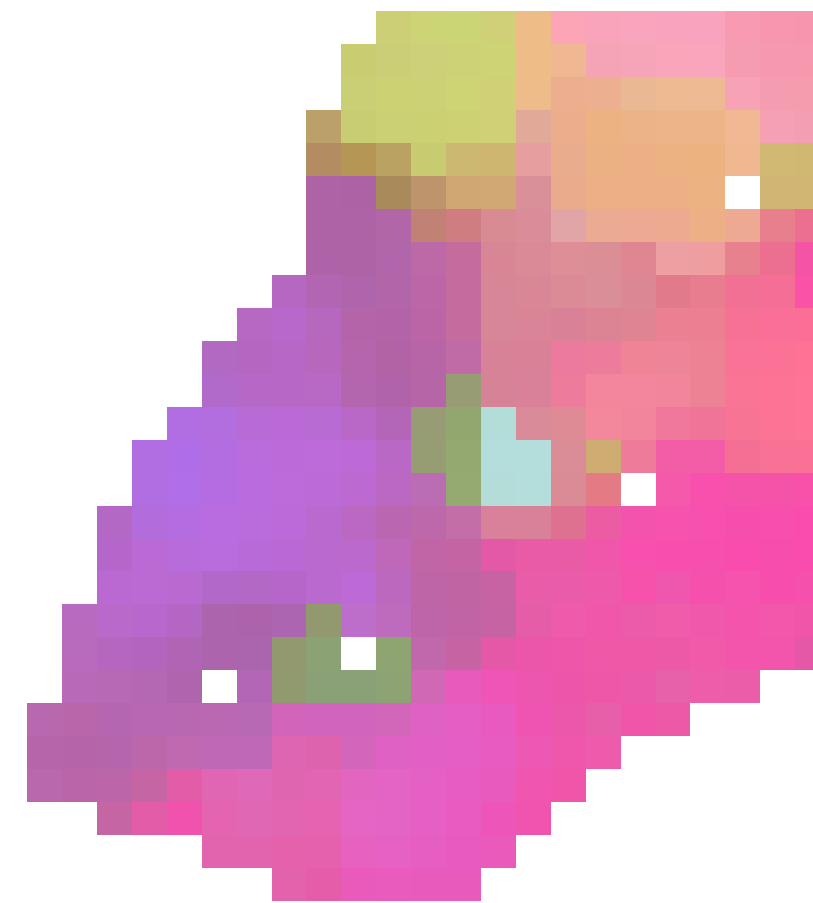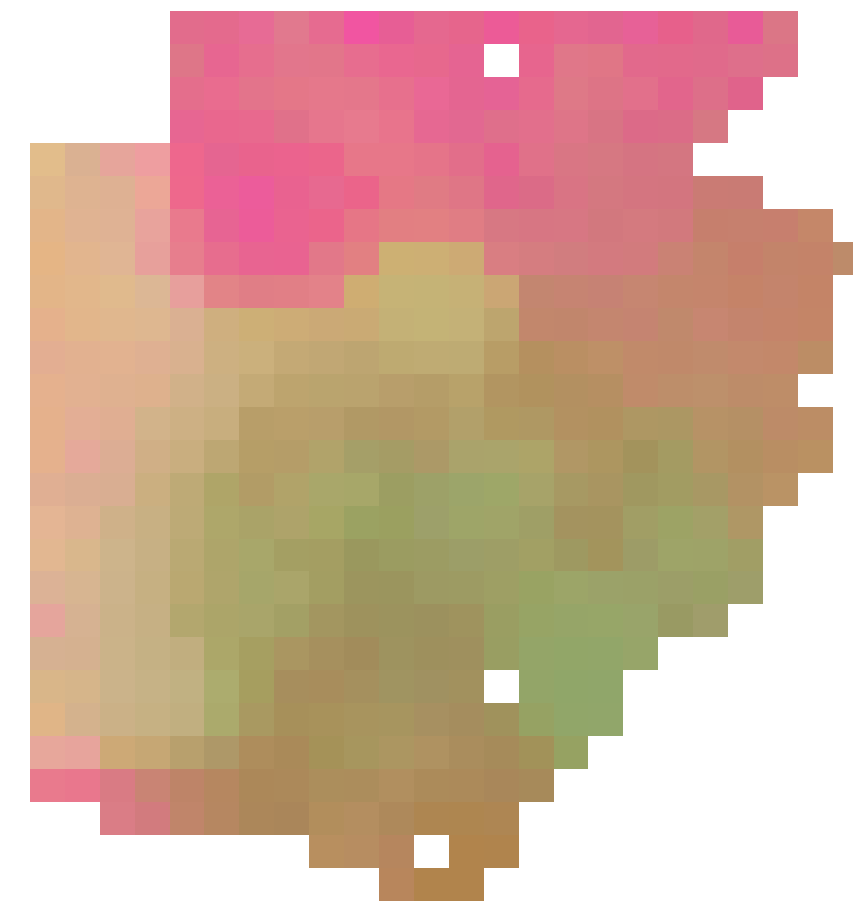

Supplement: Supplementary file 8 — Supplementary Data 5 [file 41467_2018_4724_MOESM8_ESM.zip › Supplementary Dataset 7/joint-field-dimensionality-reduction-tSNE-matrix-rgb.pdf]

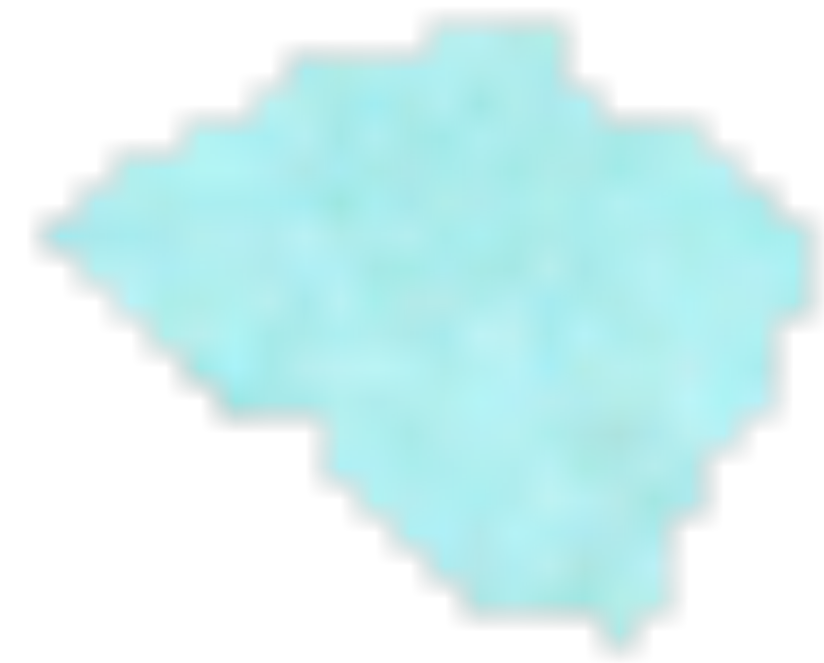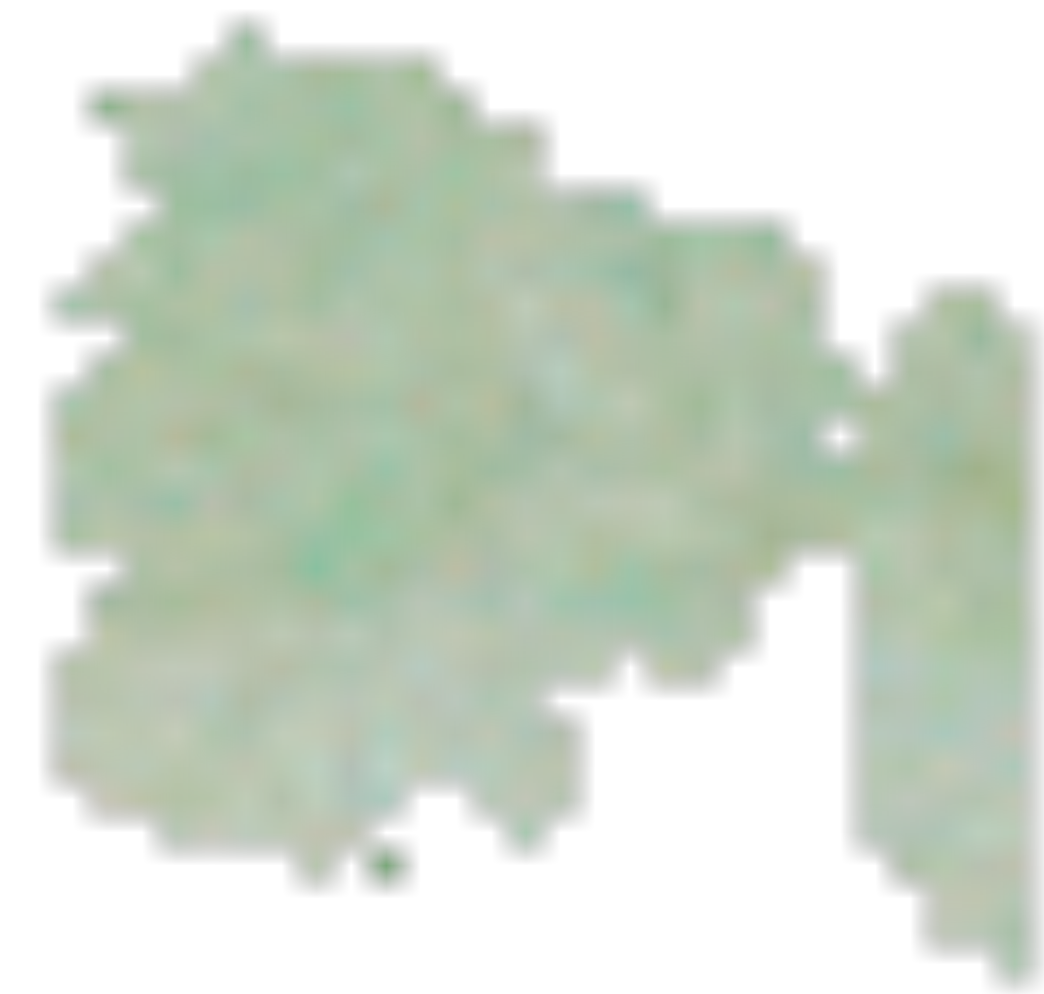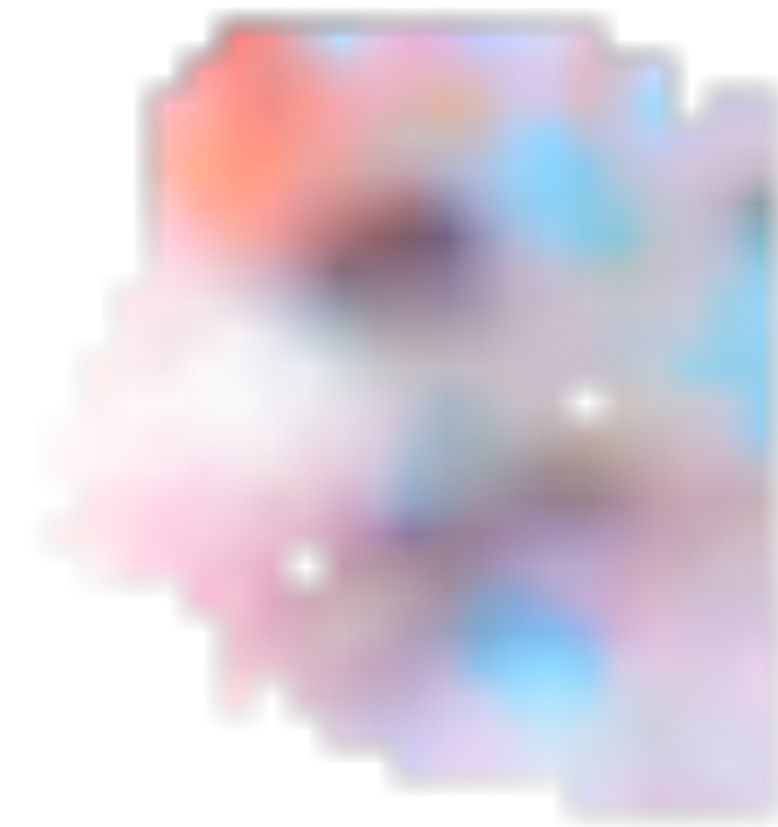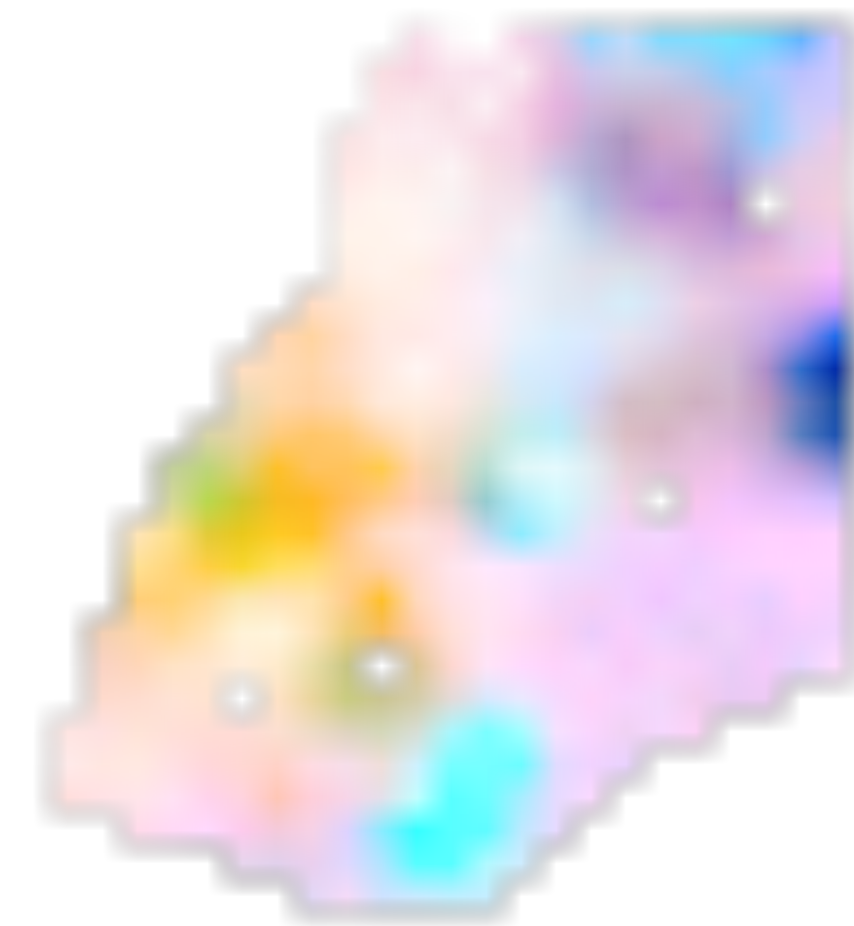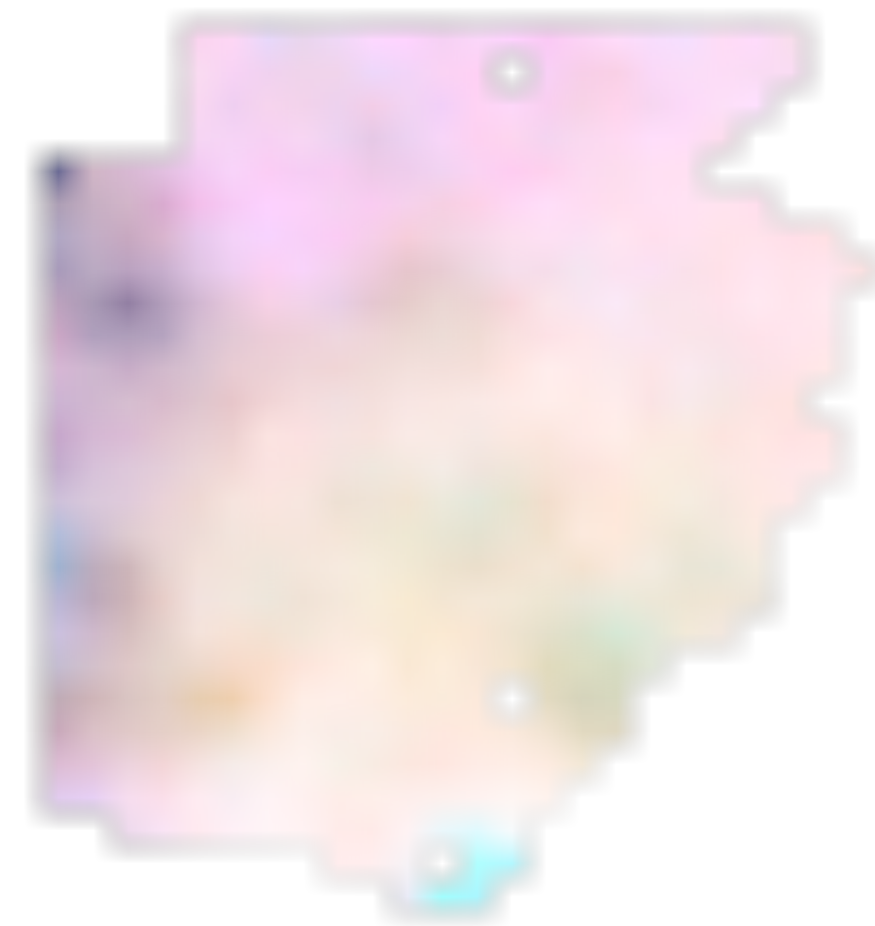

Supplement: Supplementary file 8 — Supplementary Data 5 [file 41467_2018_4724_MOESM8_ESM.zip › Supplementary Dataset 7/joint-field-dimensionality-reduction-PCA-matrix.pdf.interpolated.pdf]

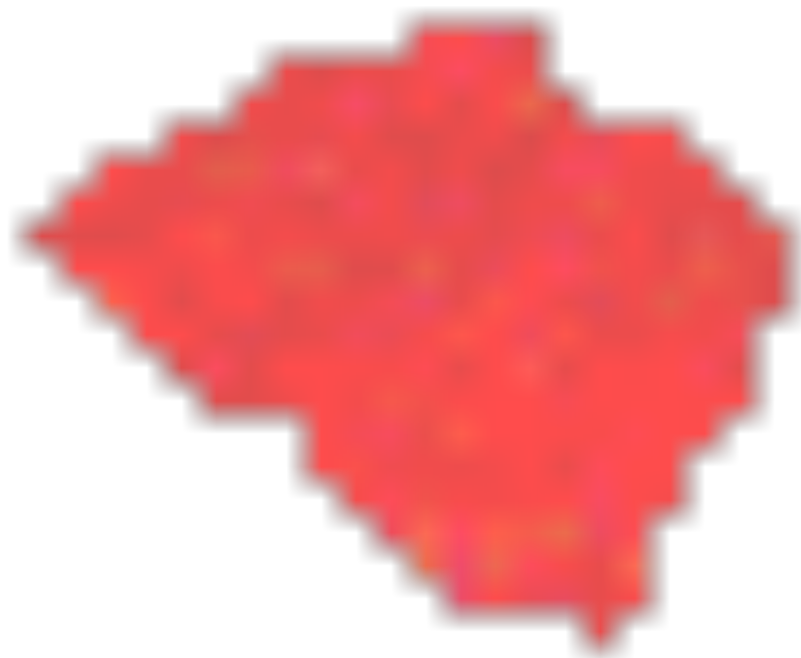

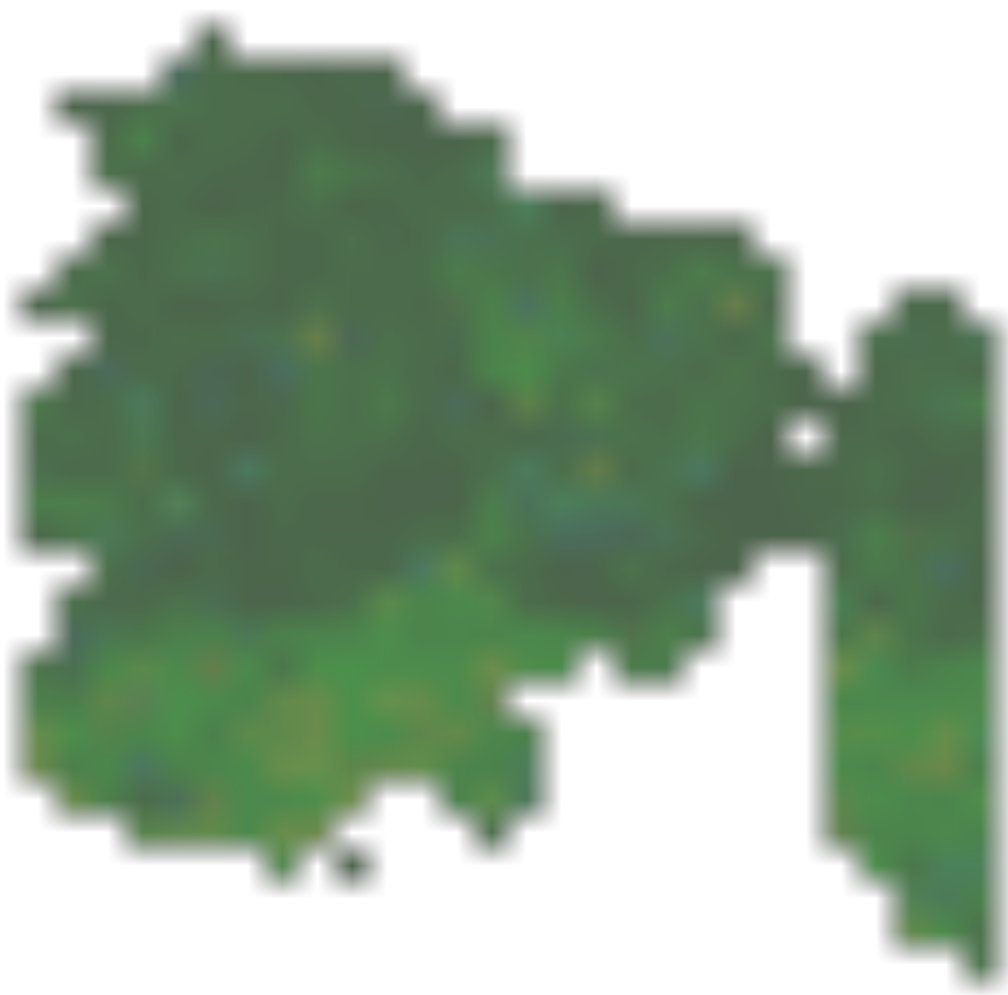

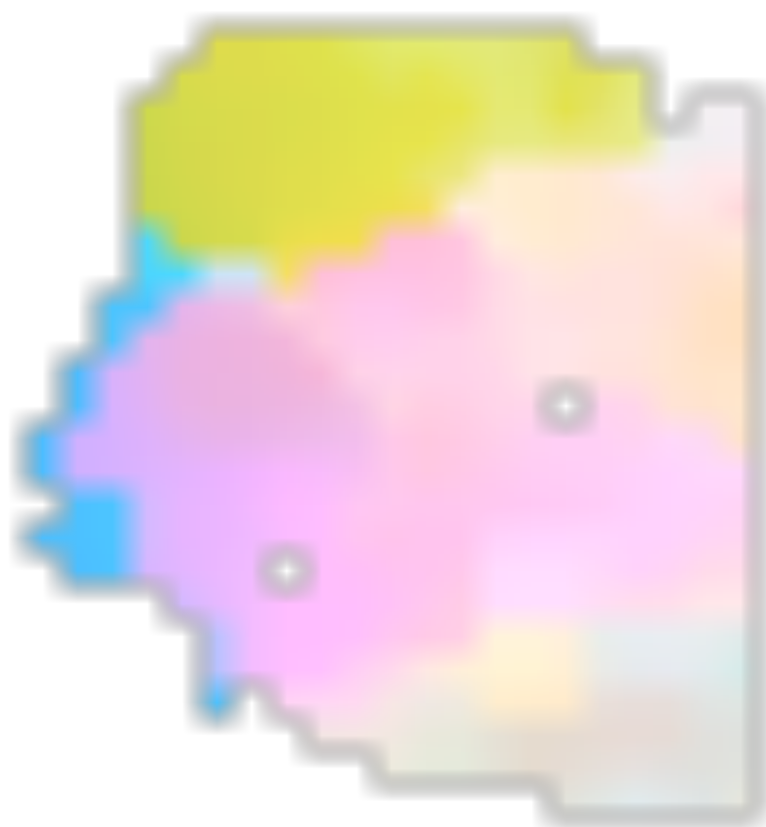

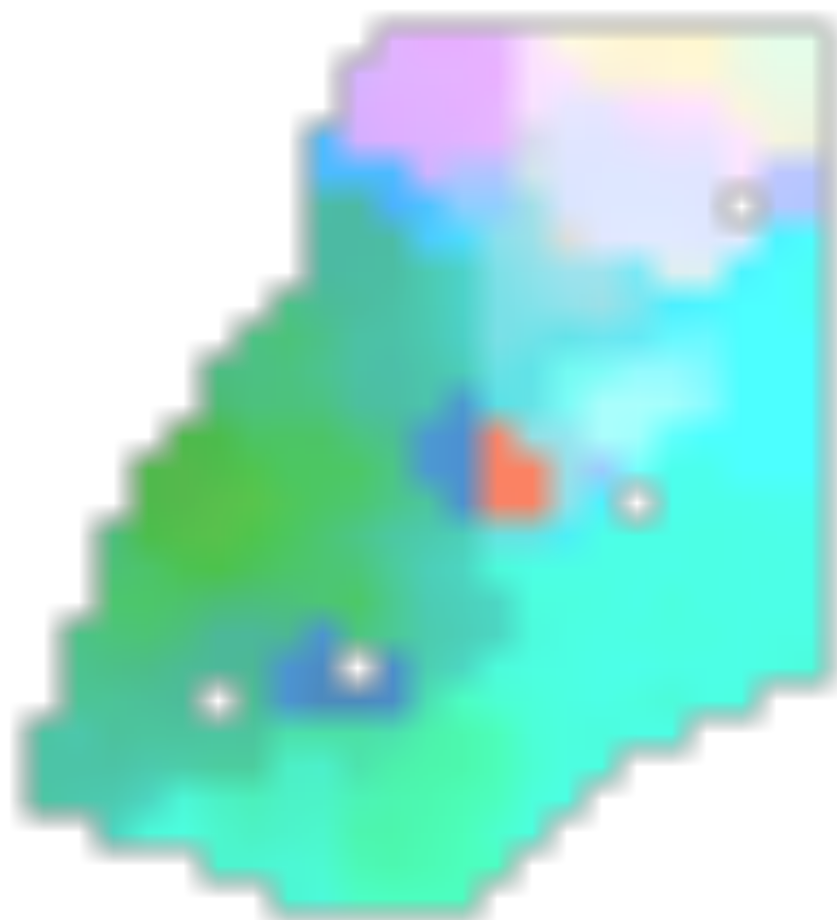

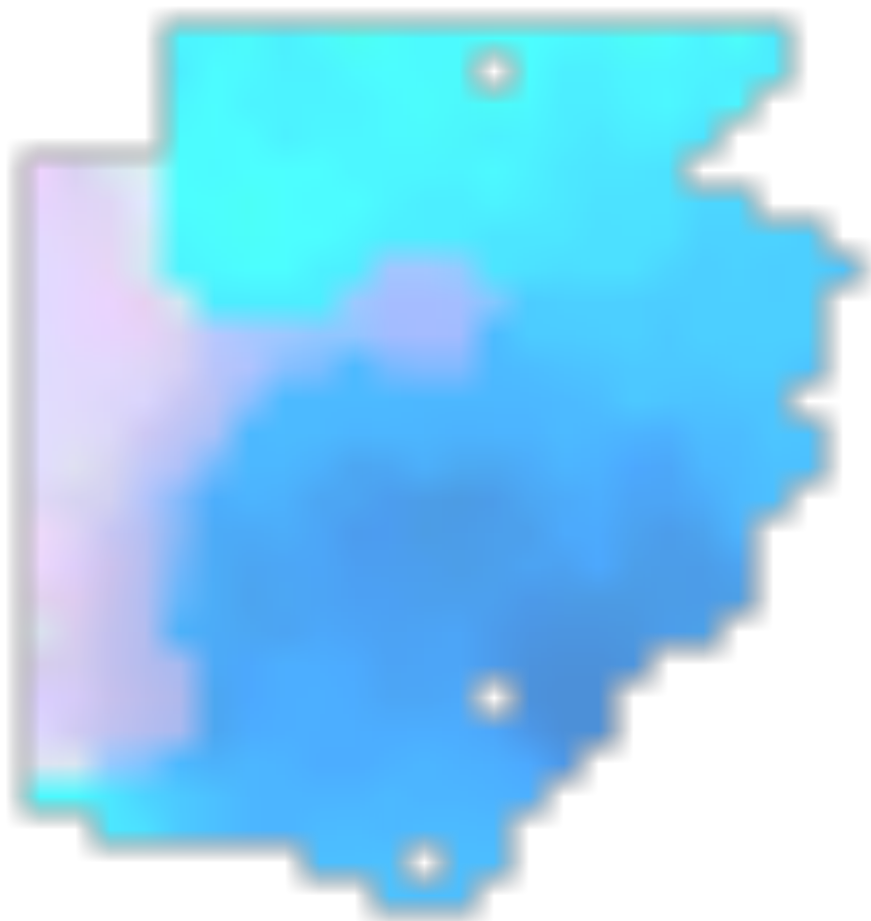

Supplement: Supplementary file 8 — Supplementary Data 5 [file 41467_2018_4724_MOESM8_ESM.zip › Supplementary Dataset 7/joint-field-dimensionality-reduction-tSNE-matrix-split.pdf.interpolated.pdf]

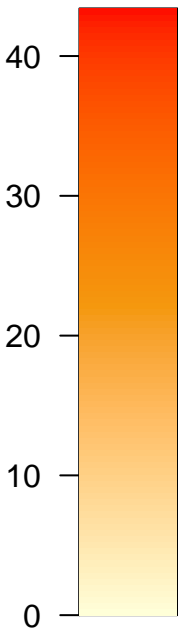

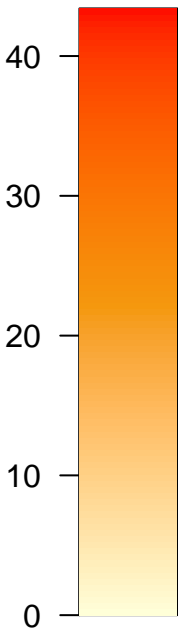

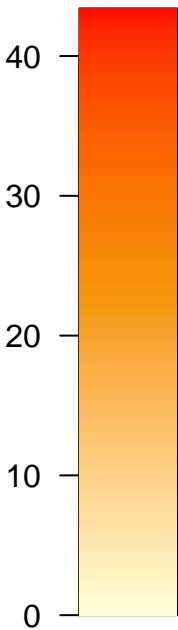

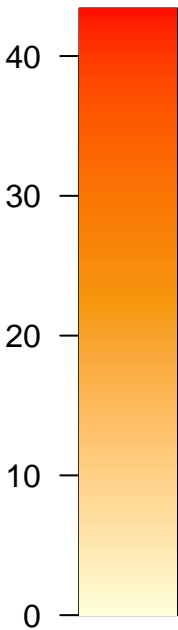

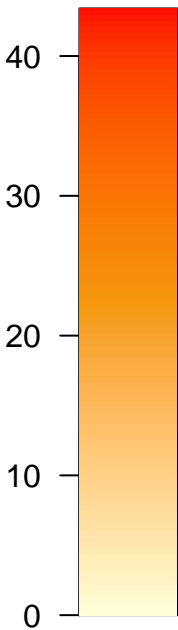

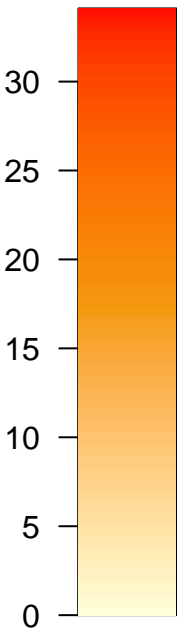

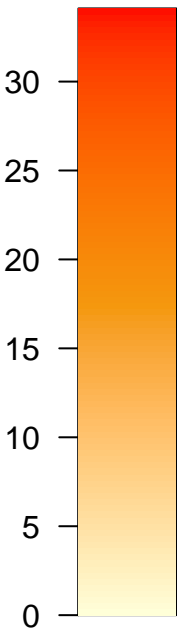

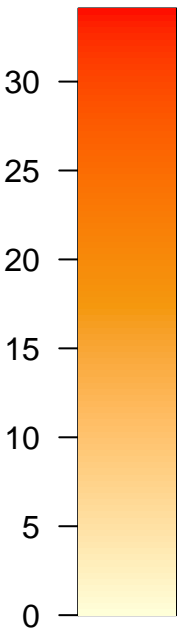

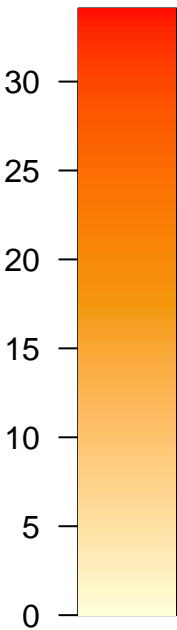

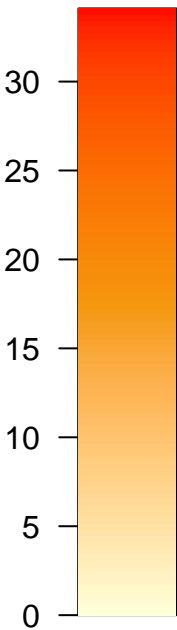

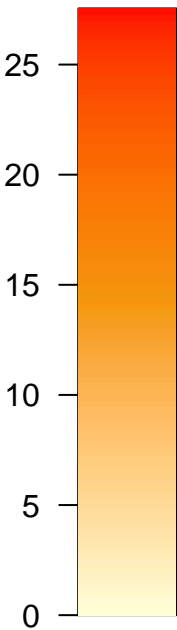

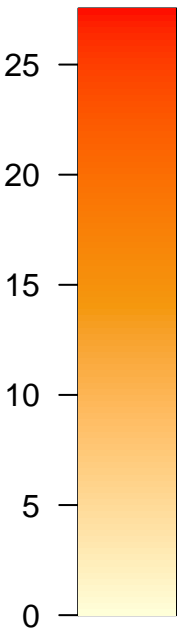

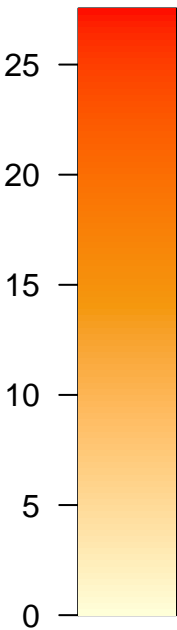

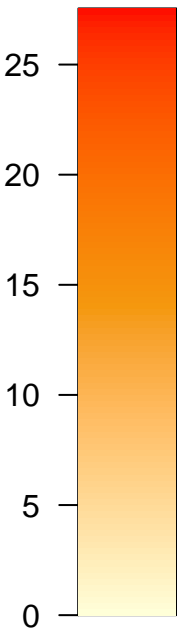

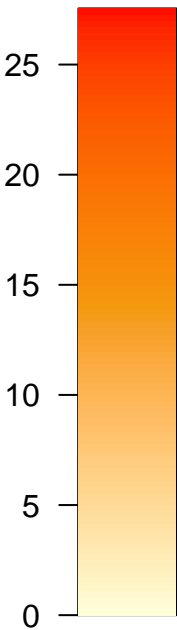

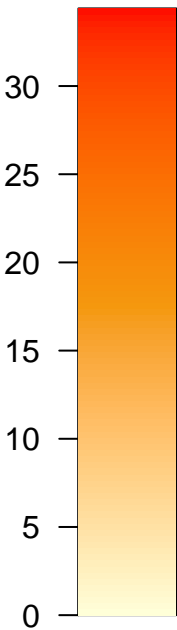

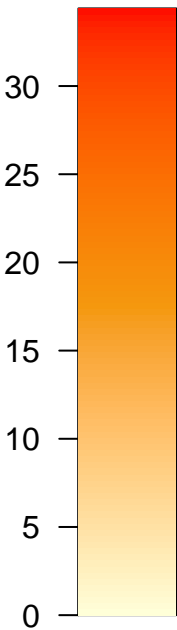

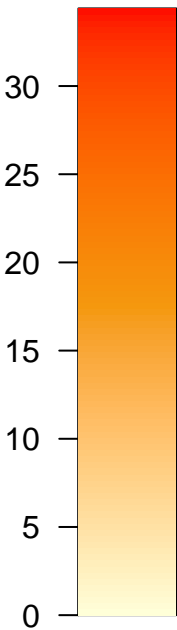

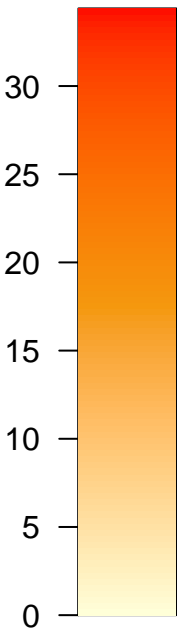

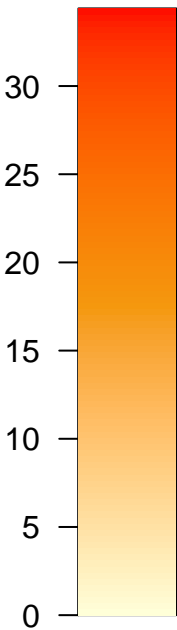

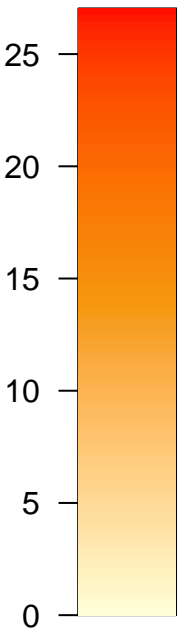

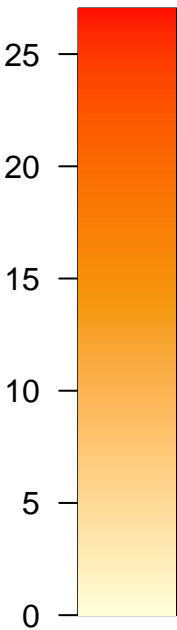

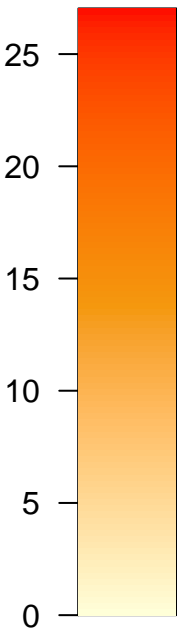

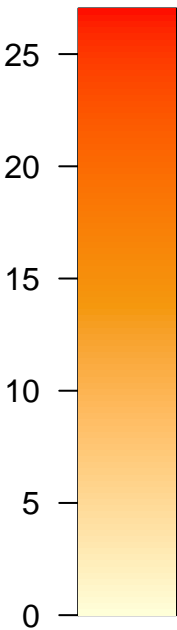

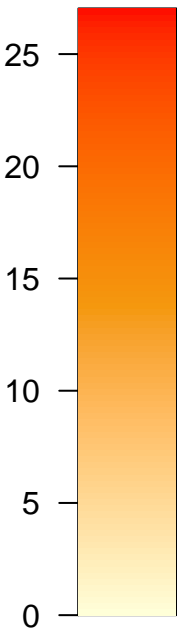

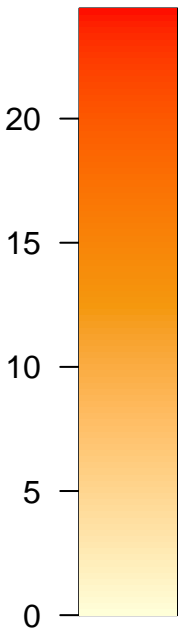

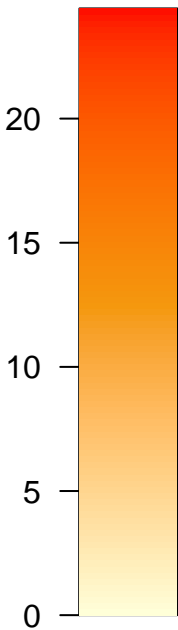

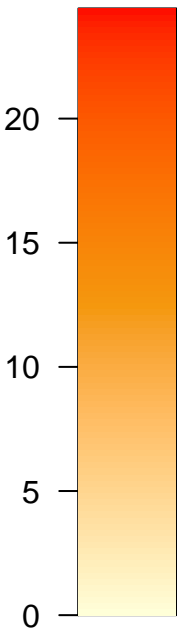

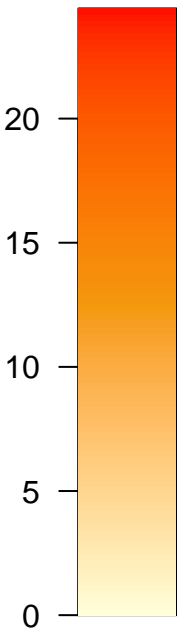

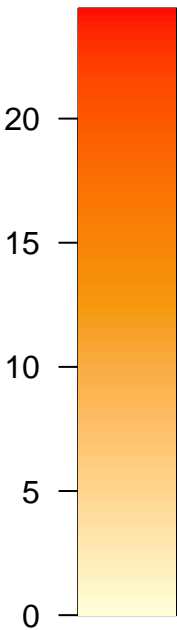

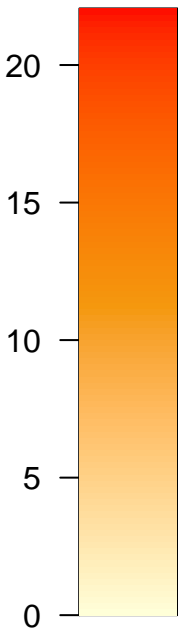

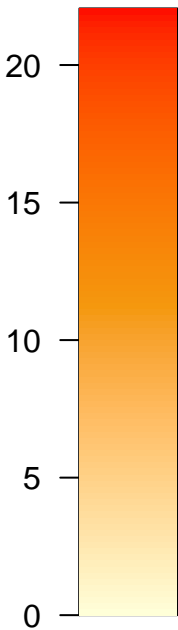

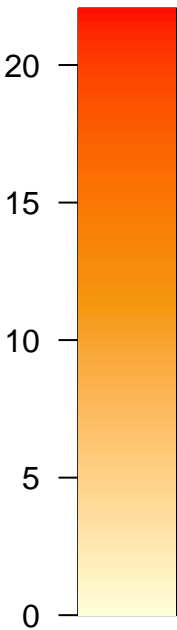

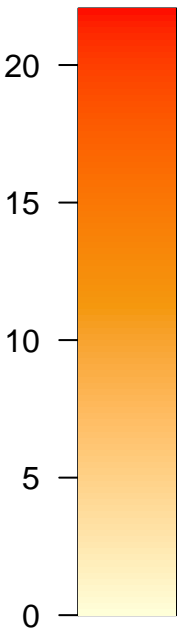

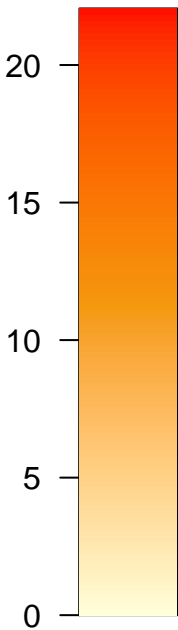

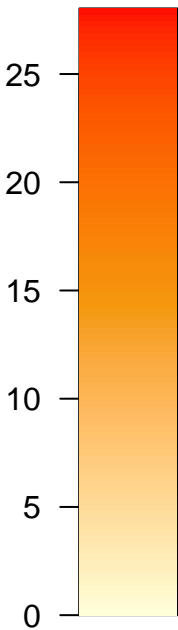

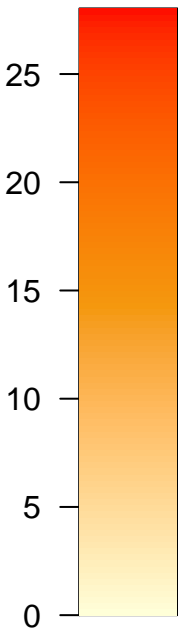

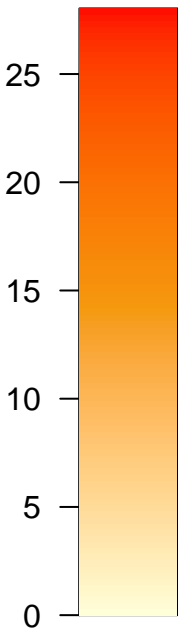

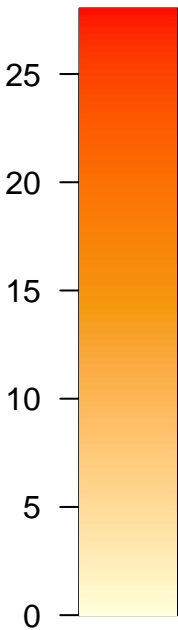

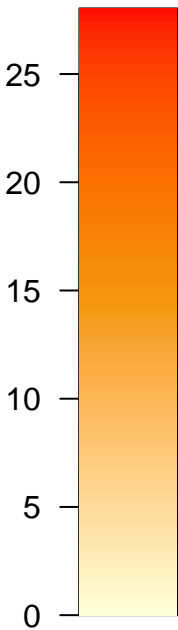

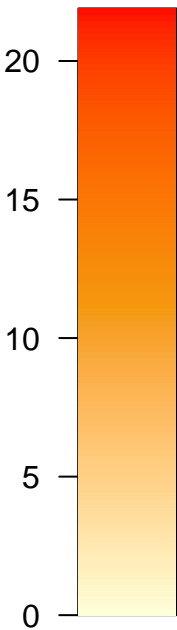

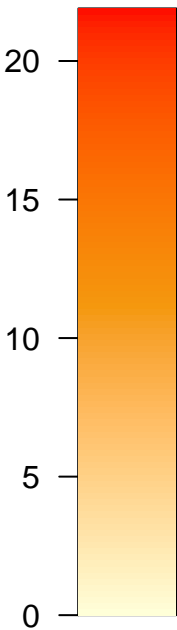

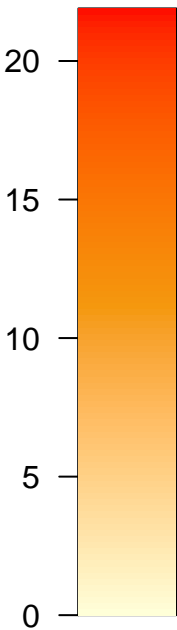

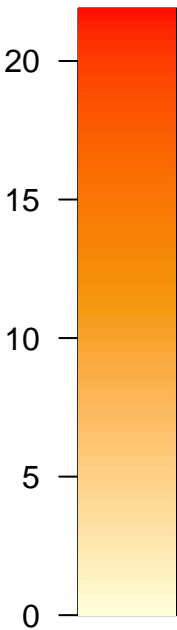

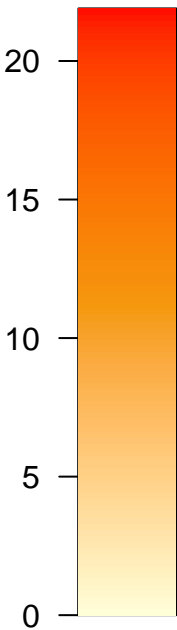

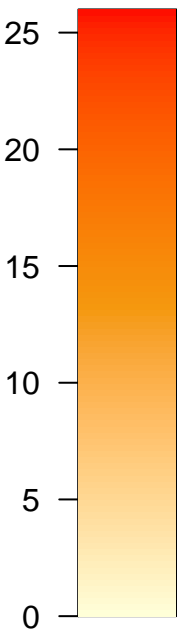

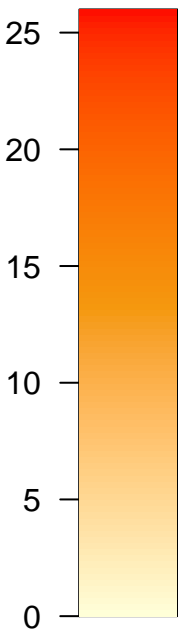

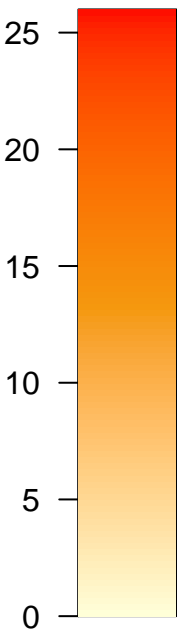

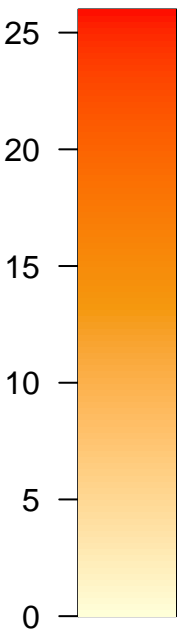

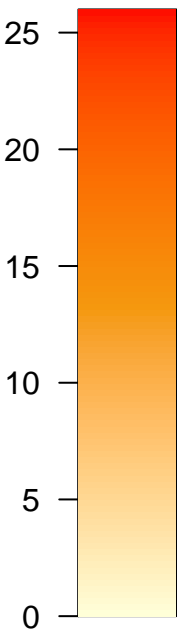

Supplement: Supplementary file 8 — Supplementary Data 5 [file 41467_2018_4724_MOESM8_ESM.zip › Supplementary Dataset 7/joint-field-profiles-rel-common-scale-dots-split-colorbar.pdf]

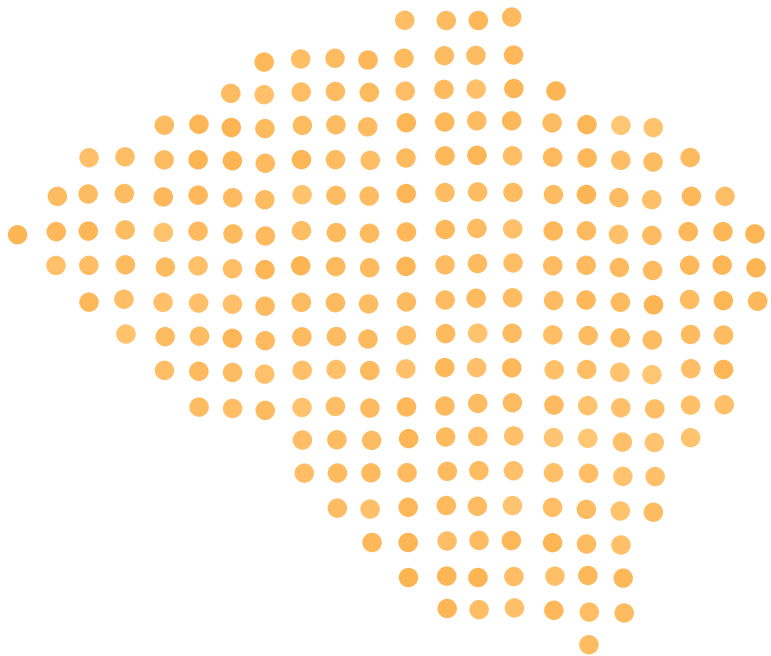

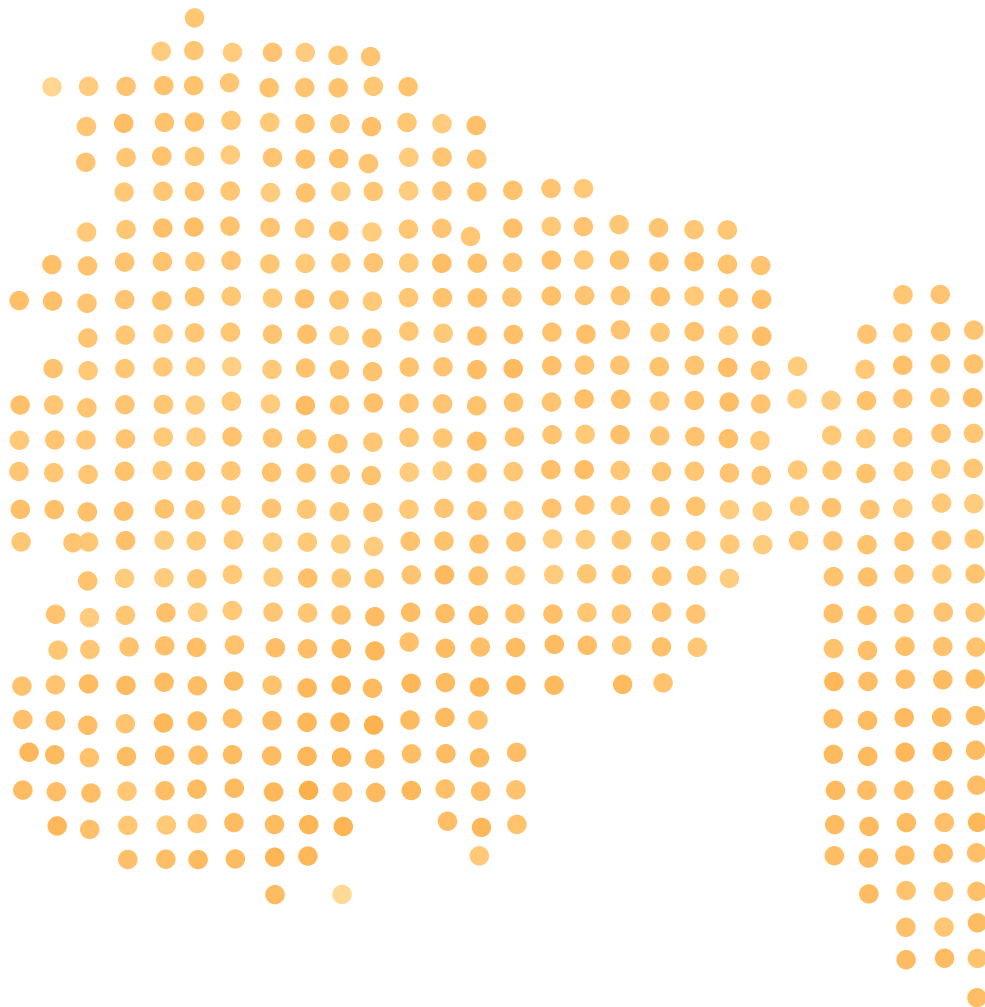

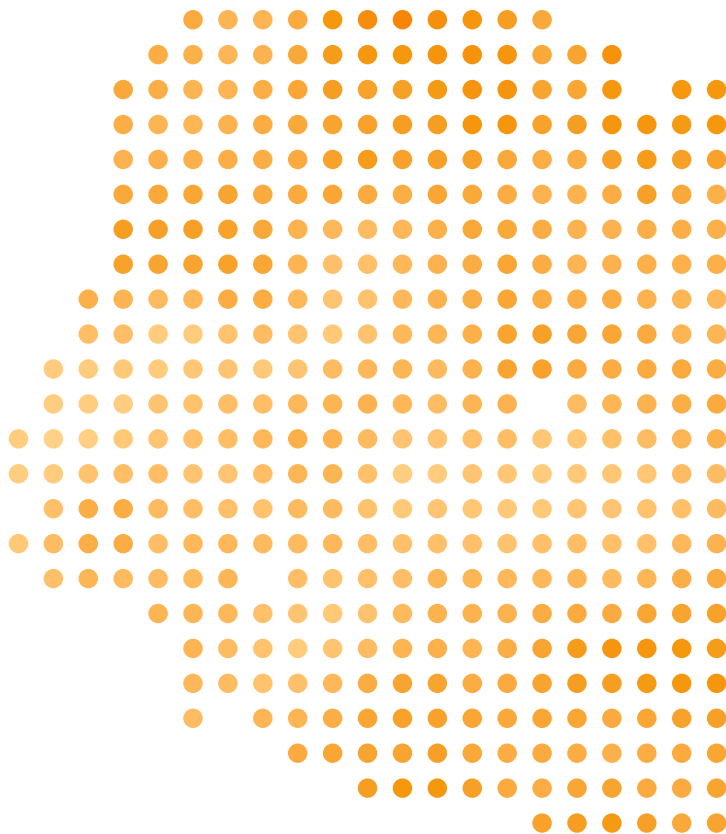

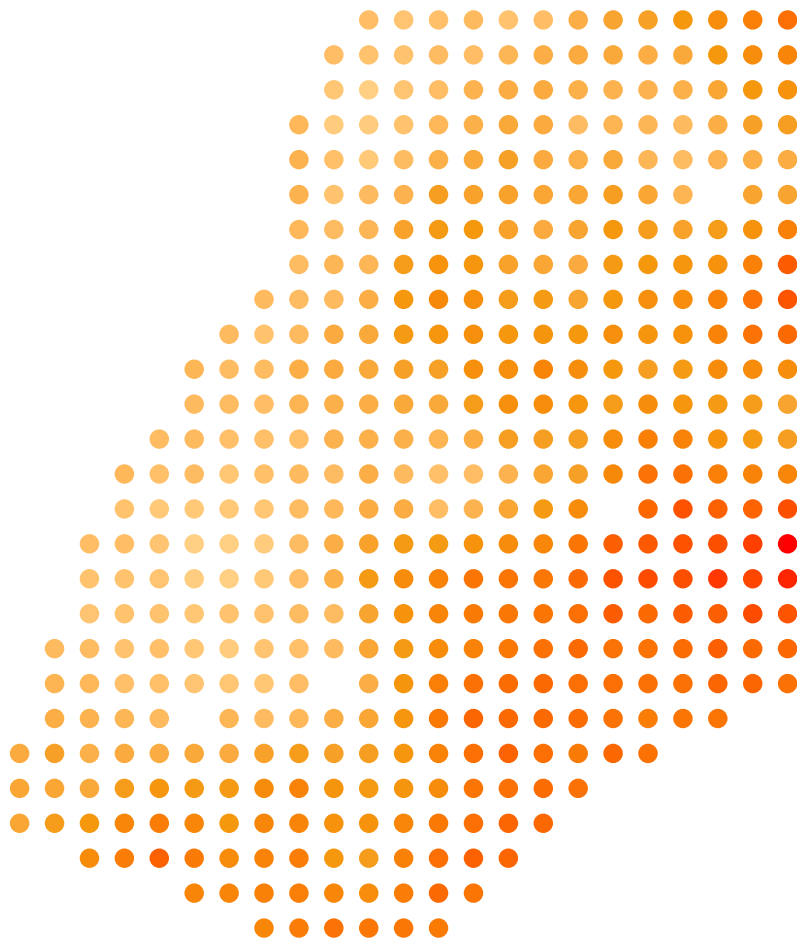

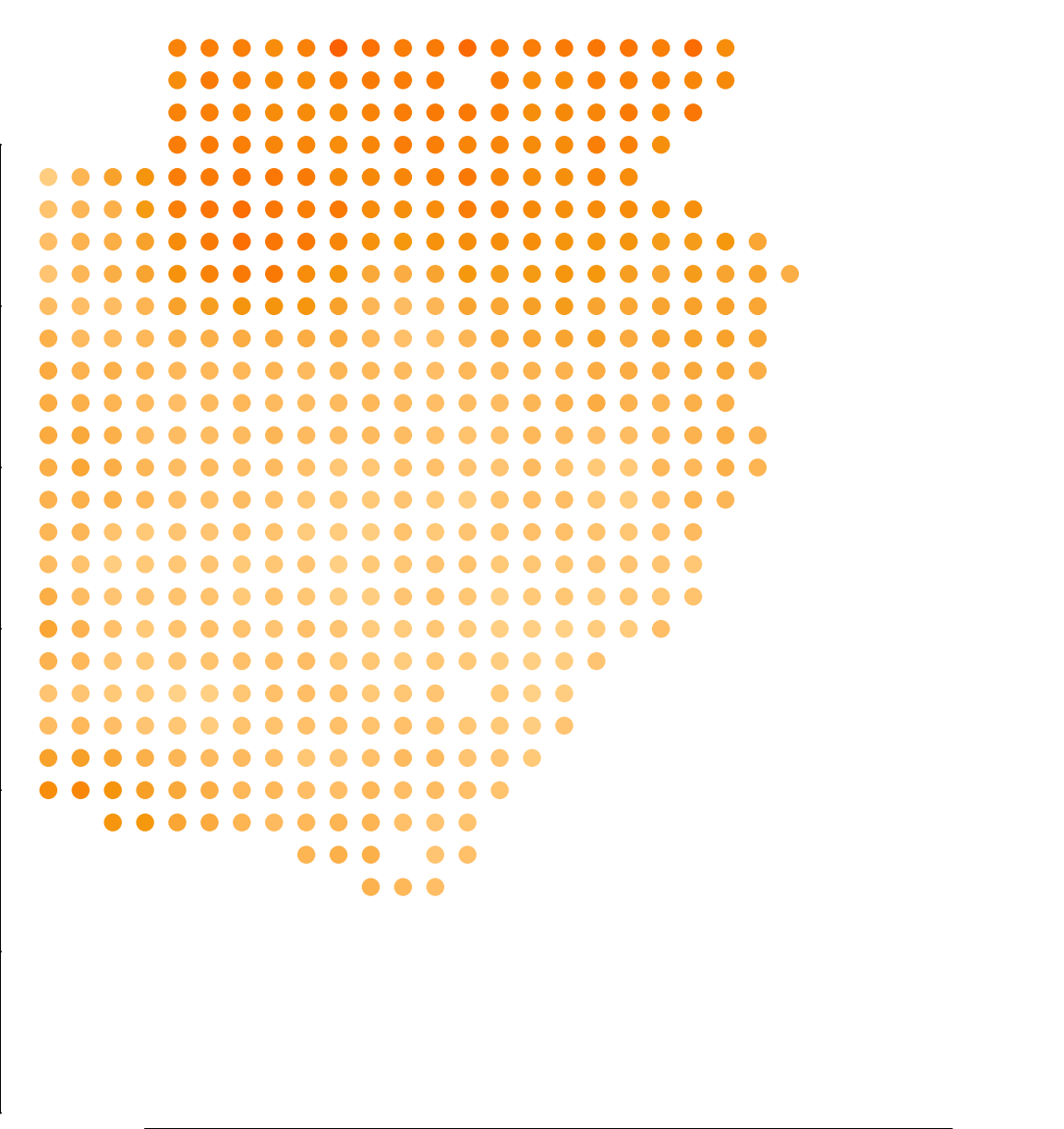

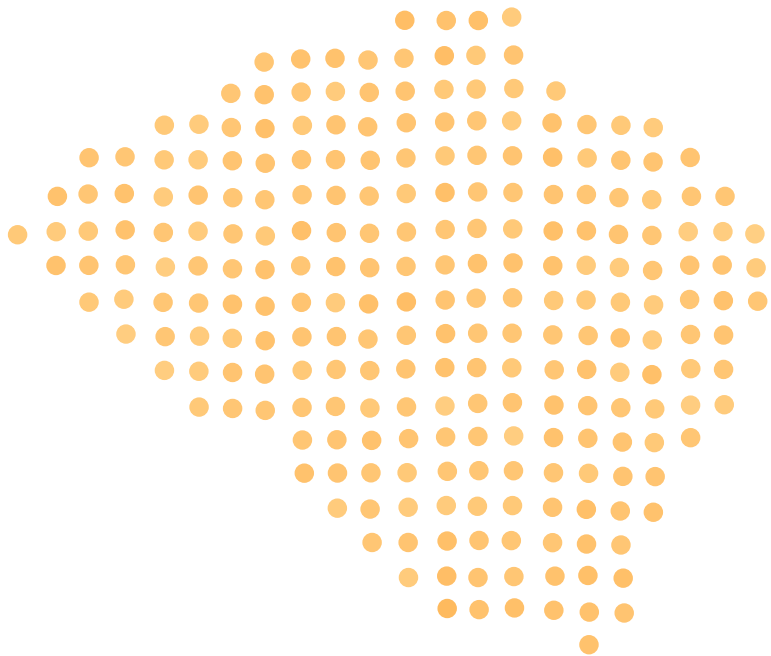

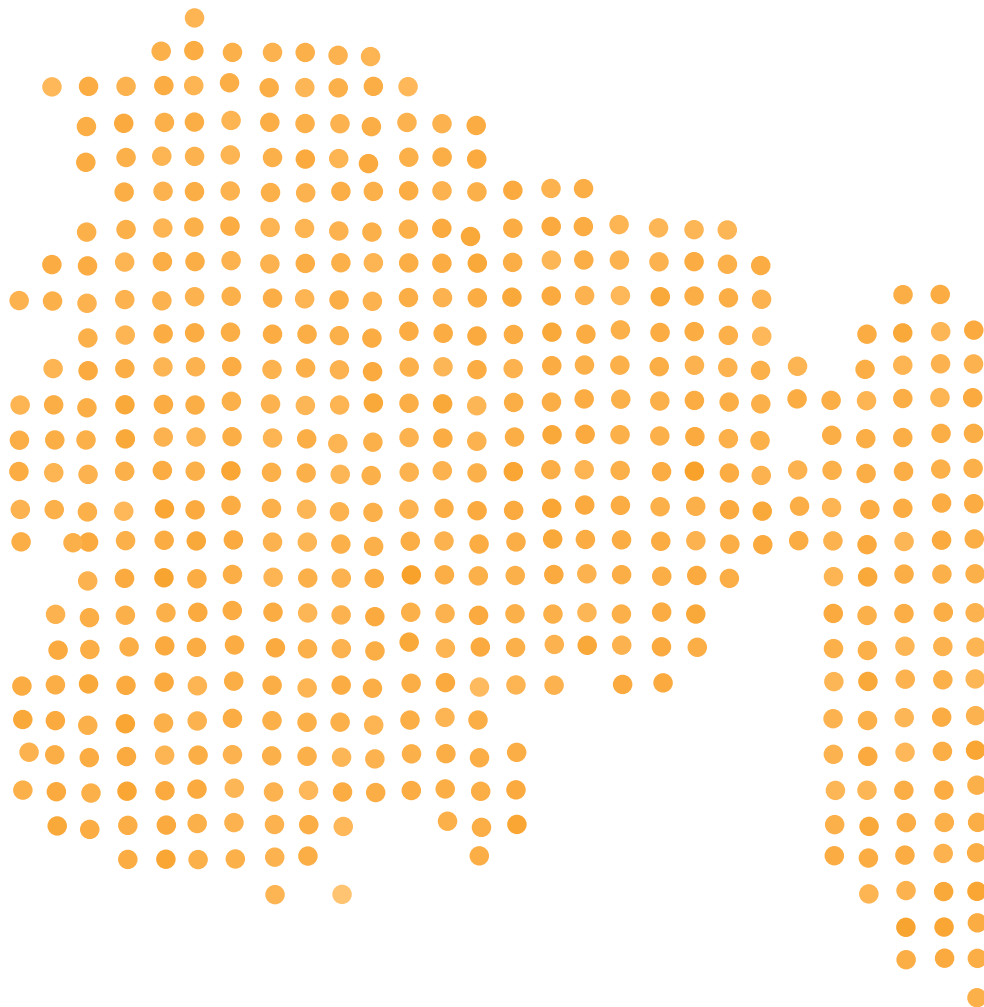

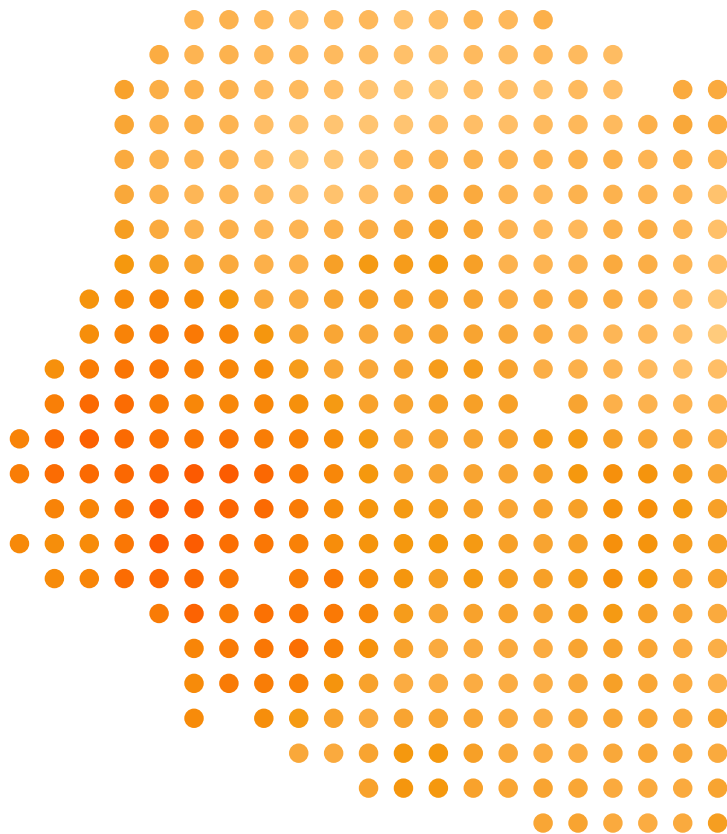

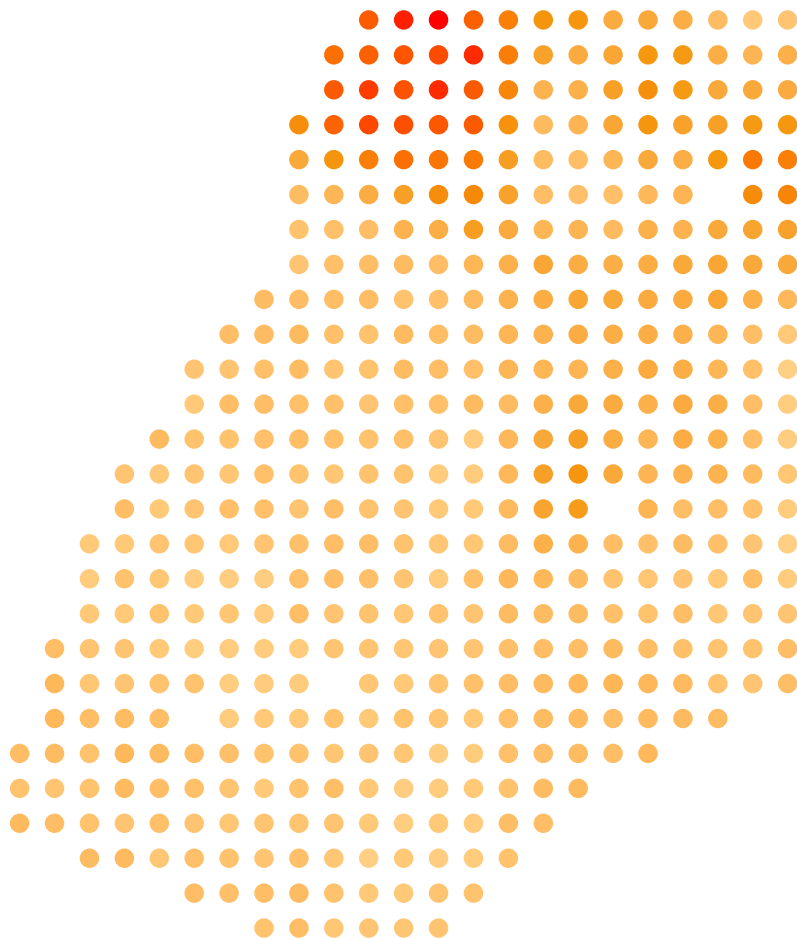

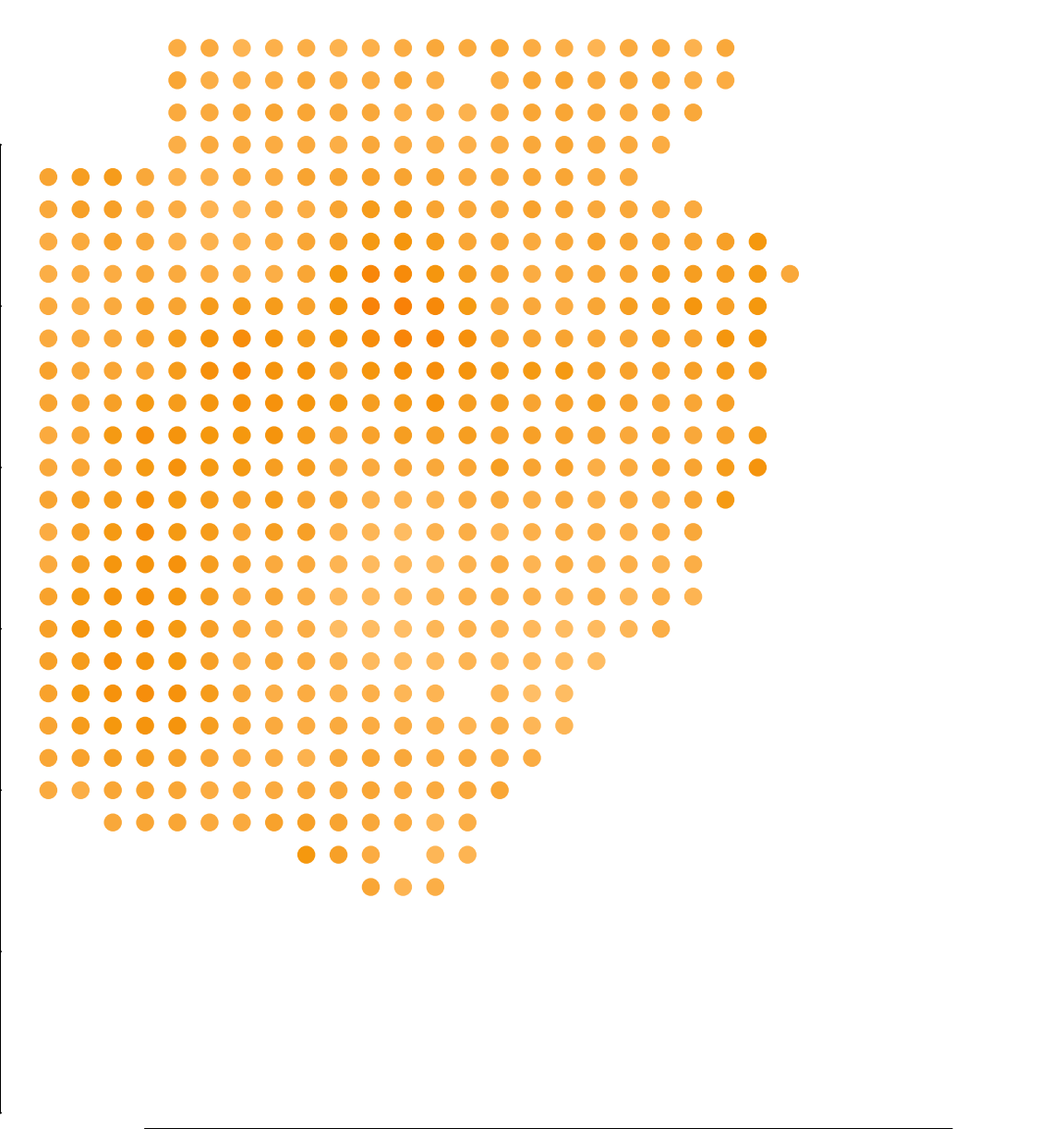

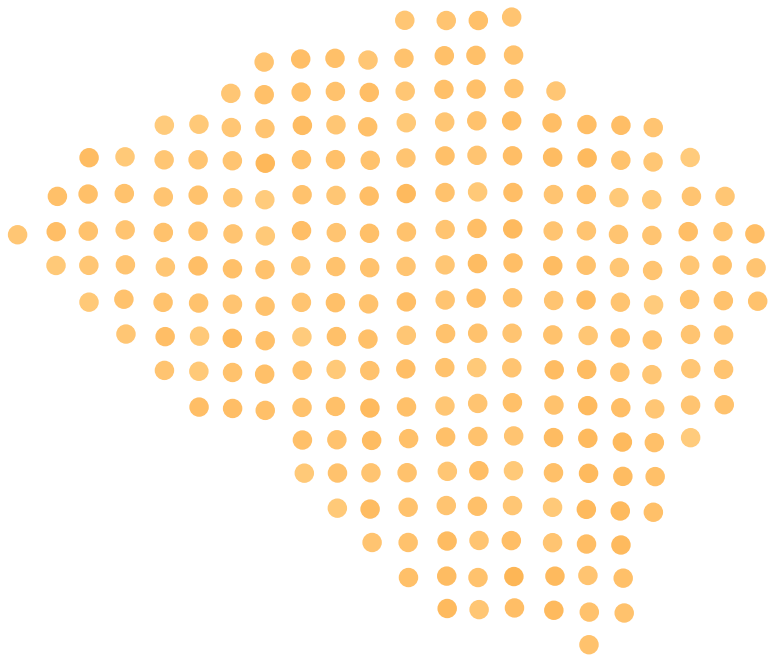

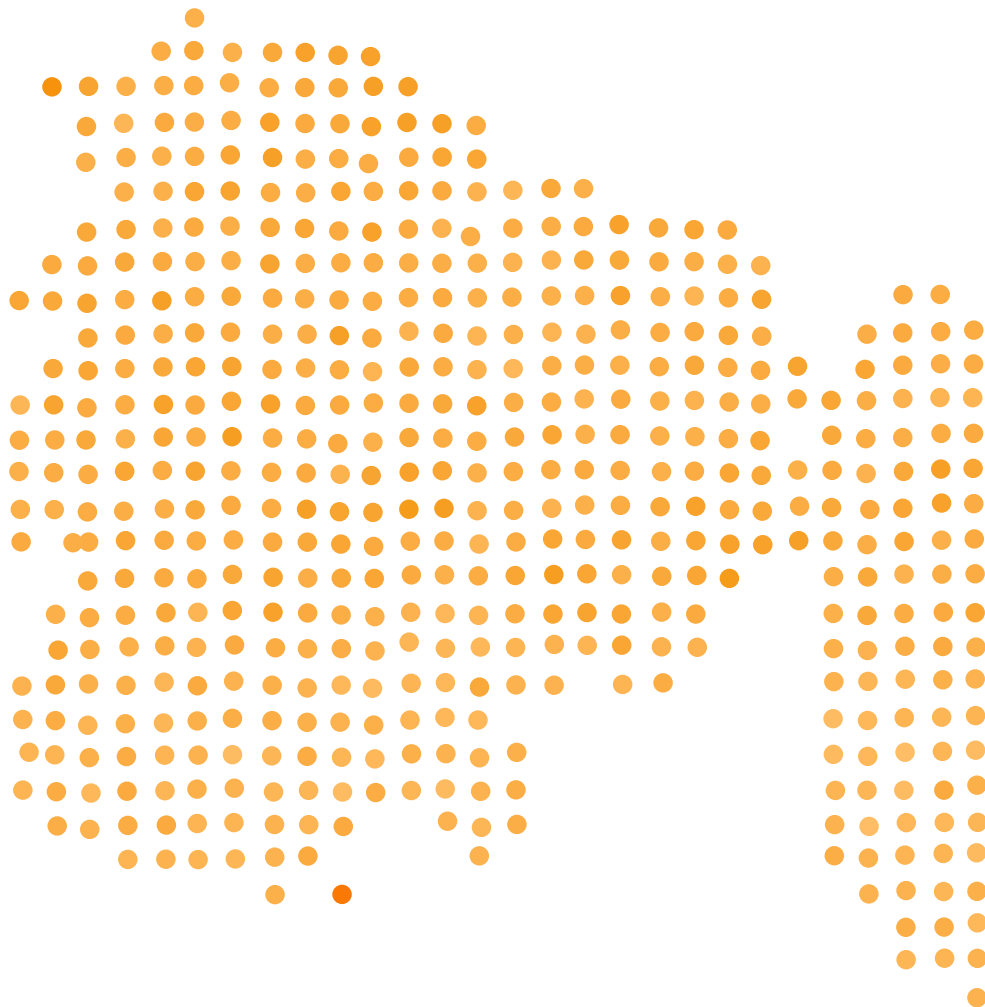

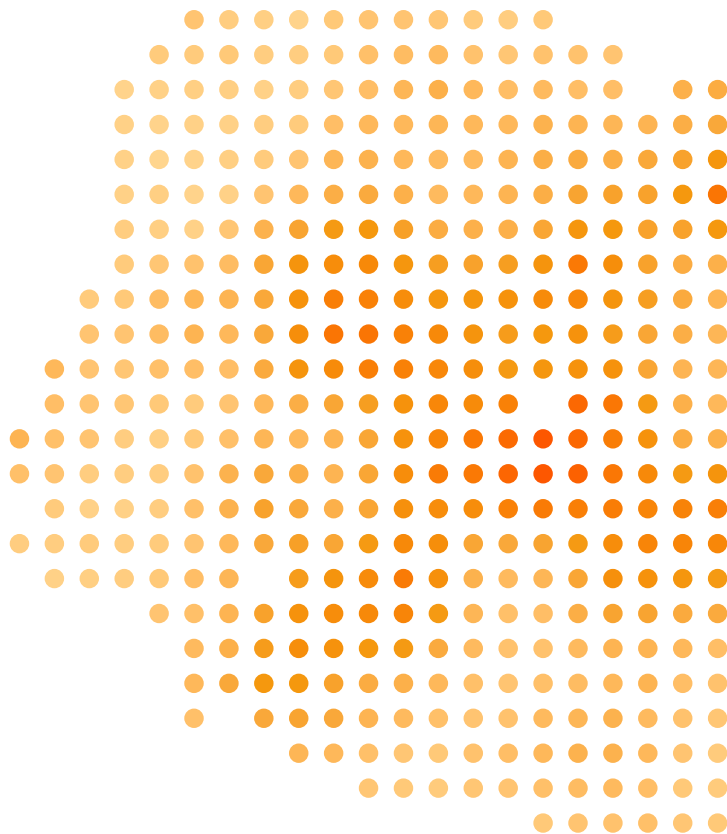

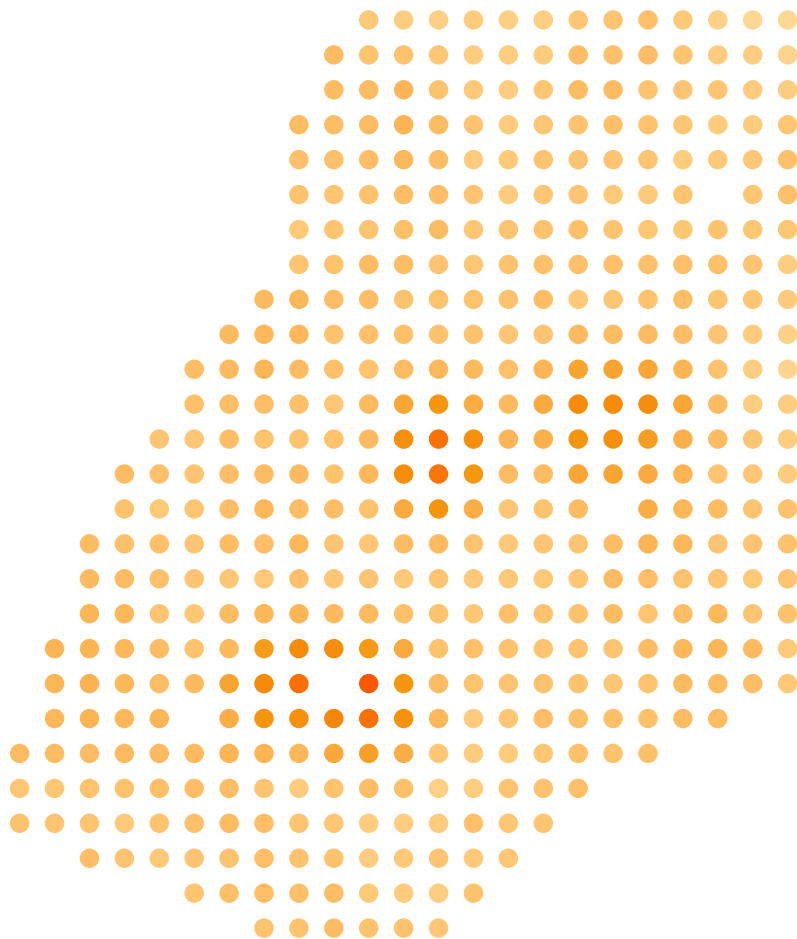

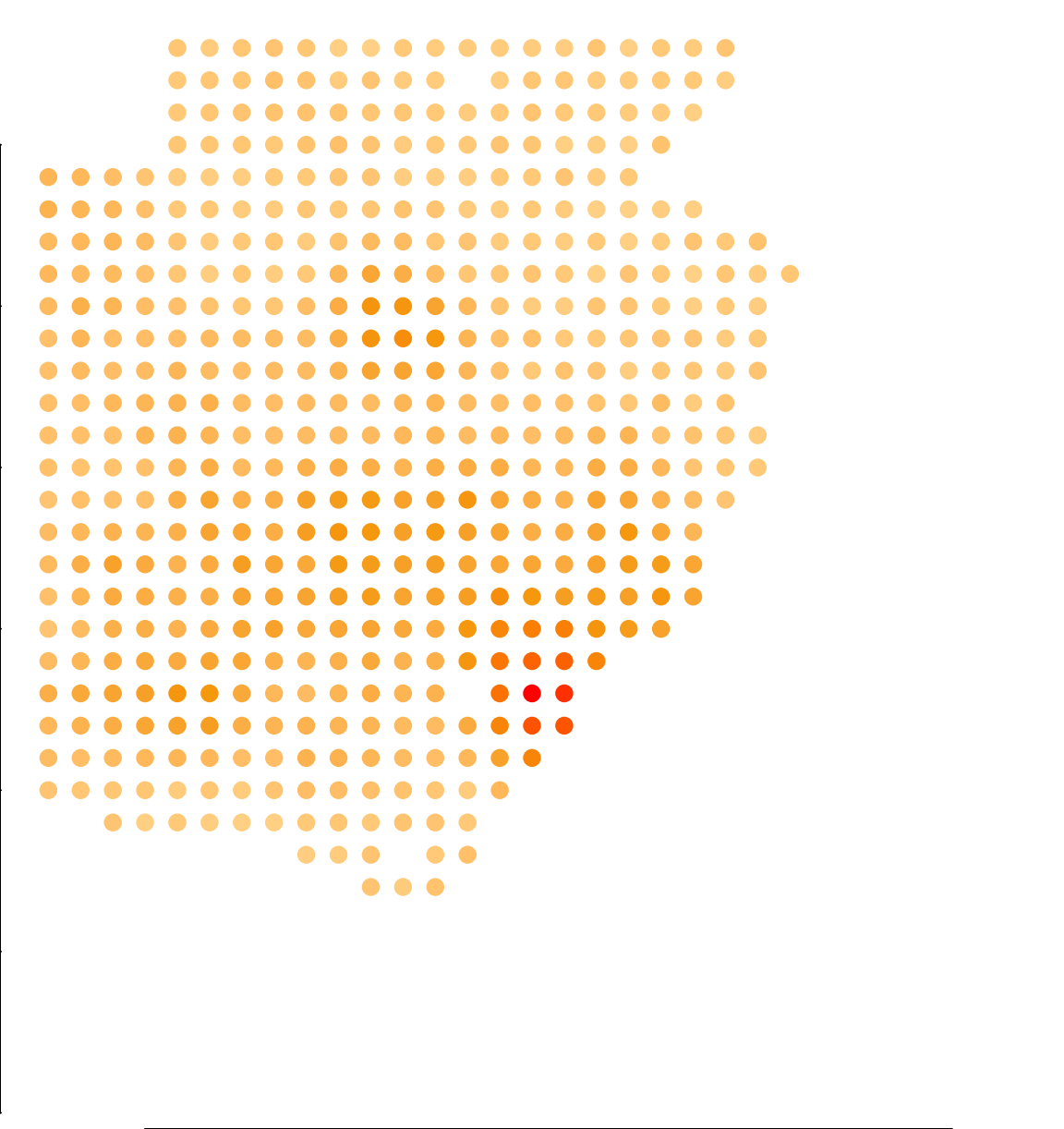

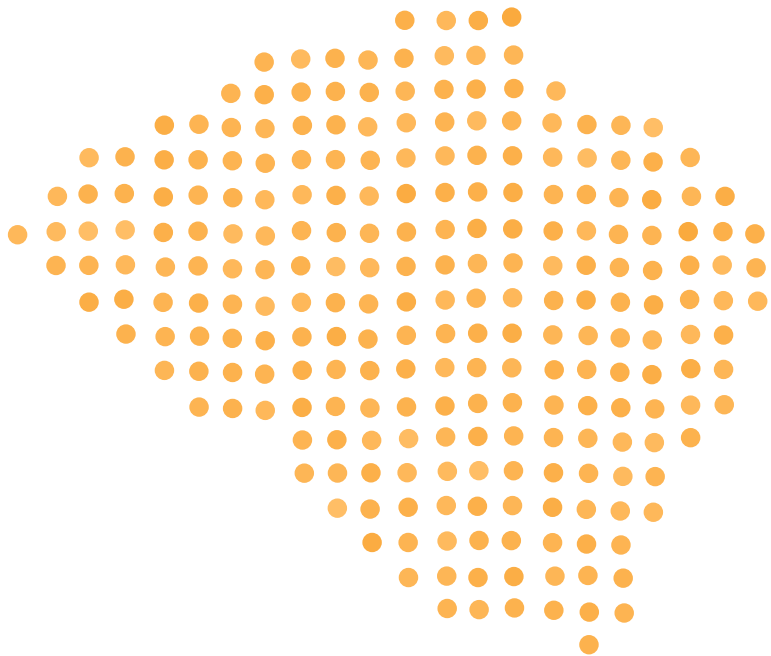

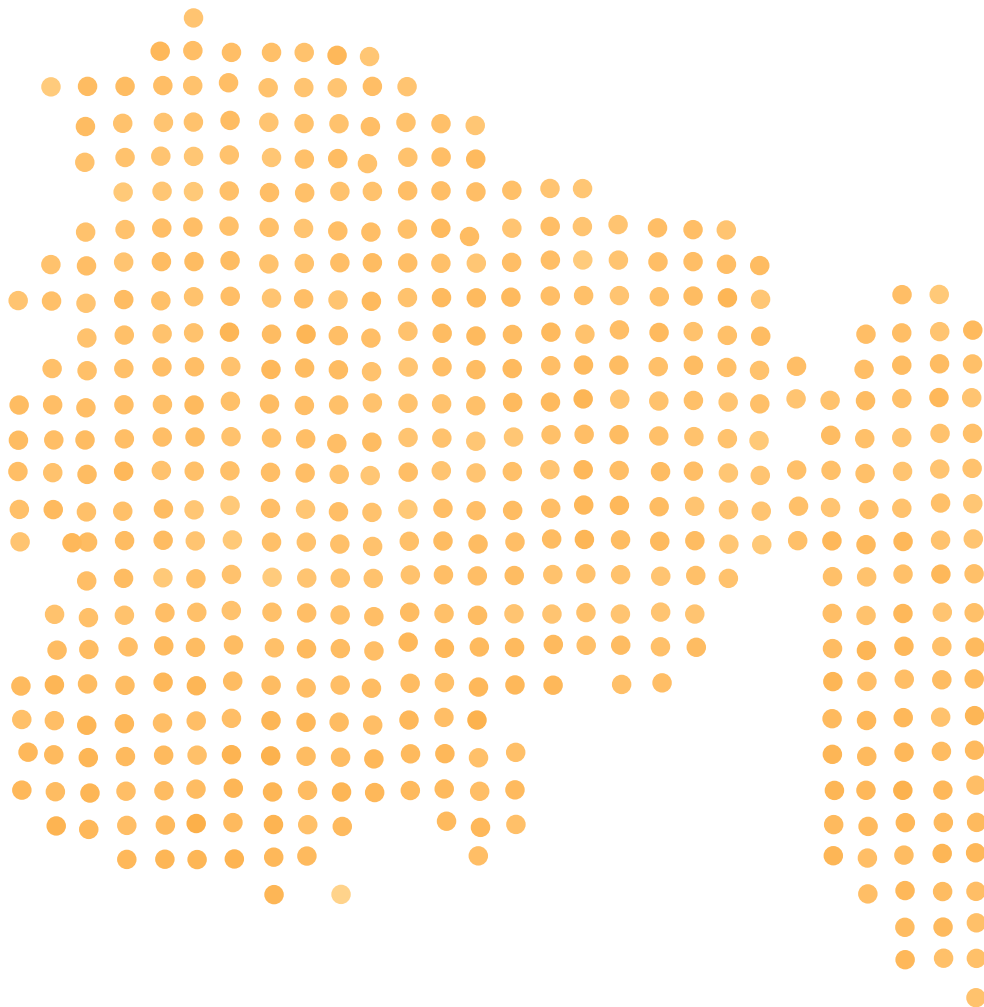

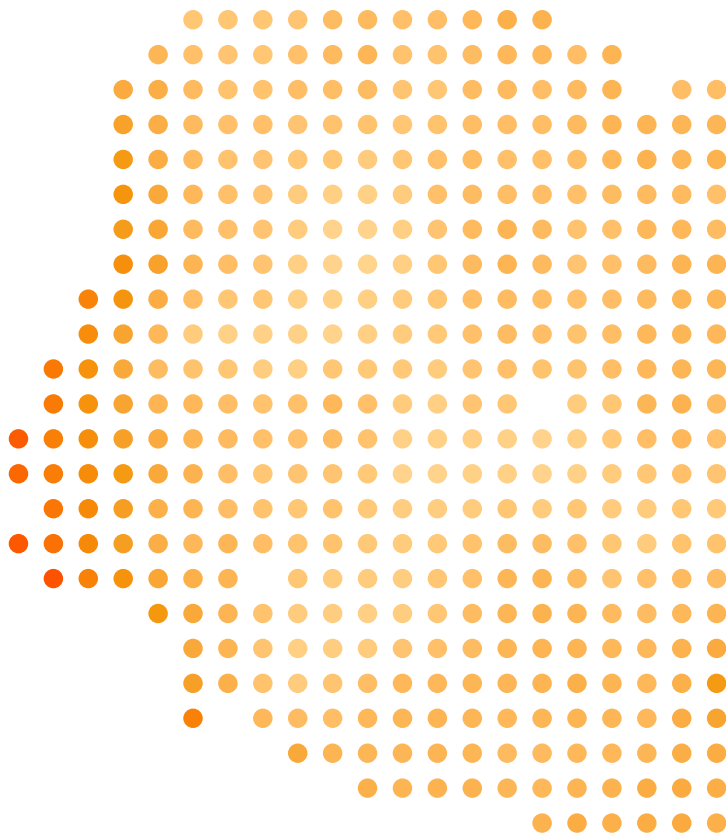

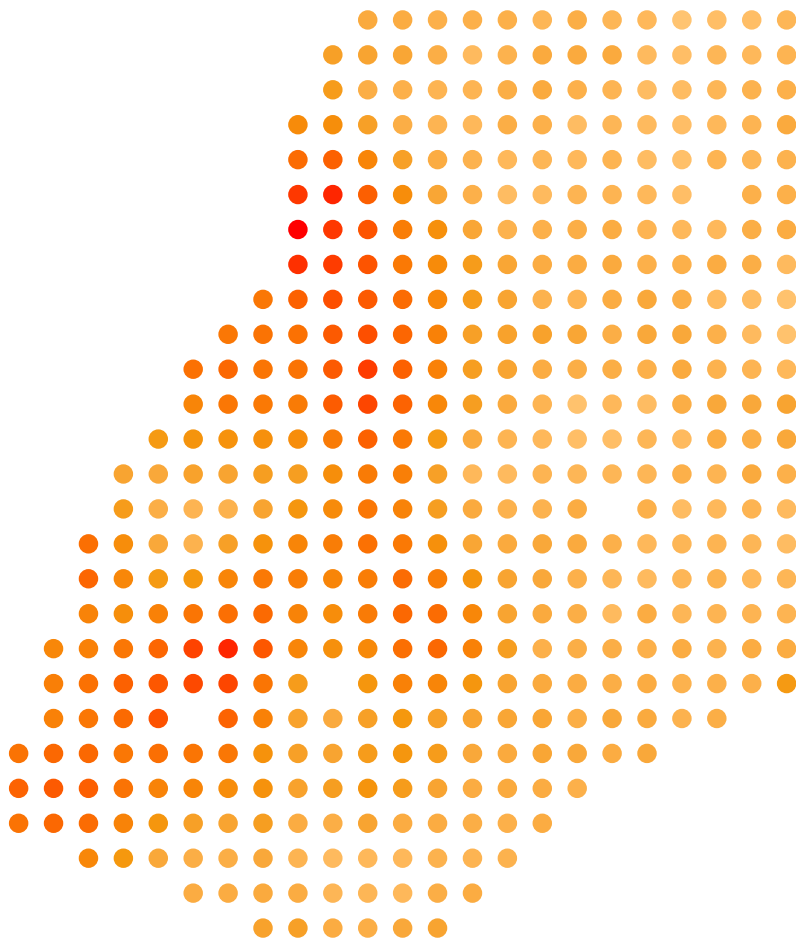

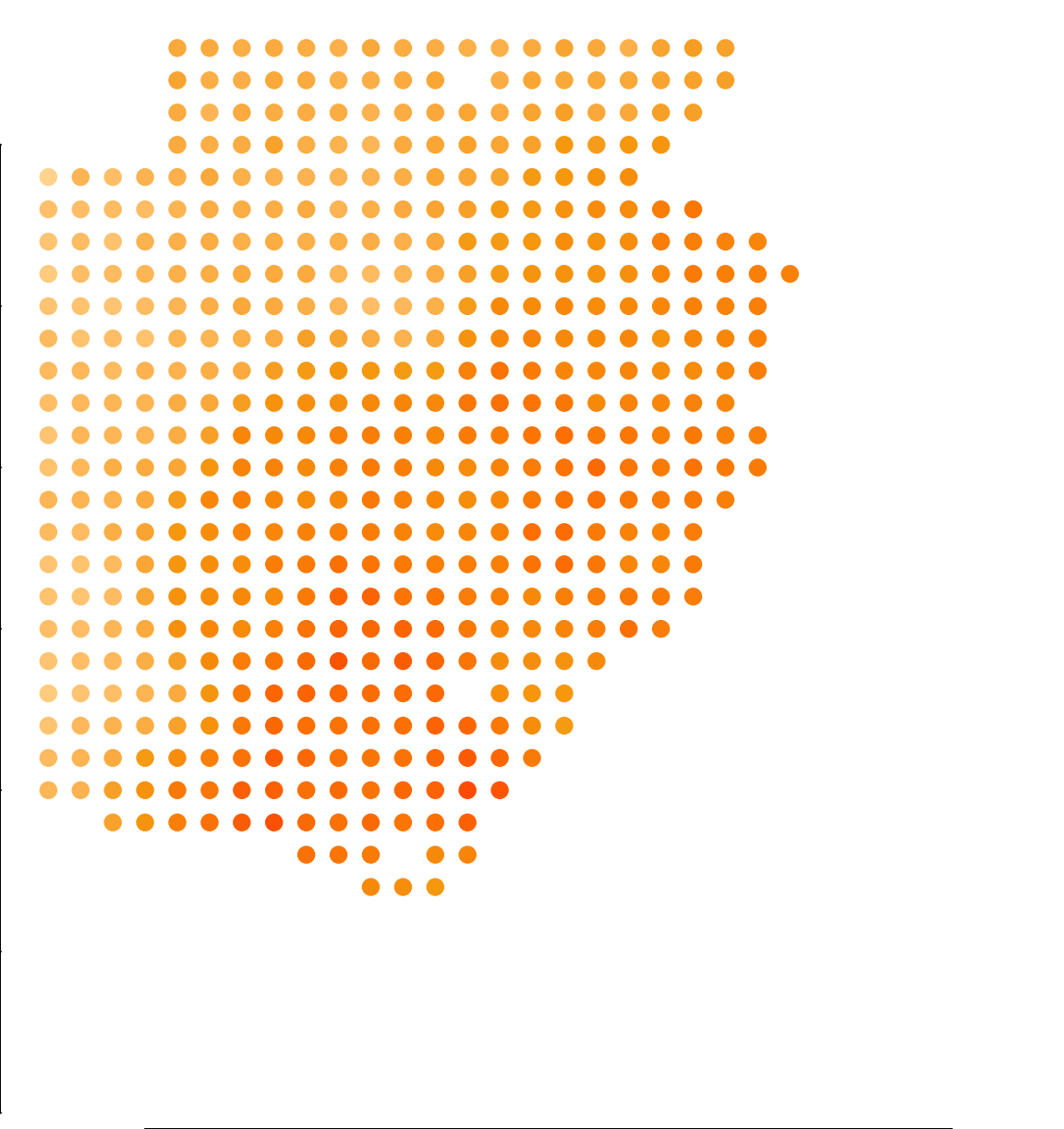

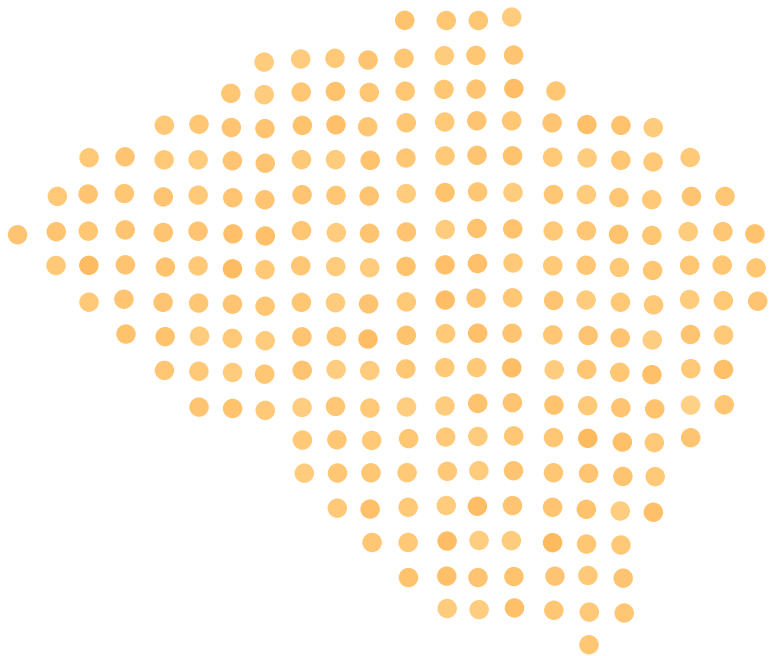

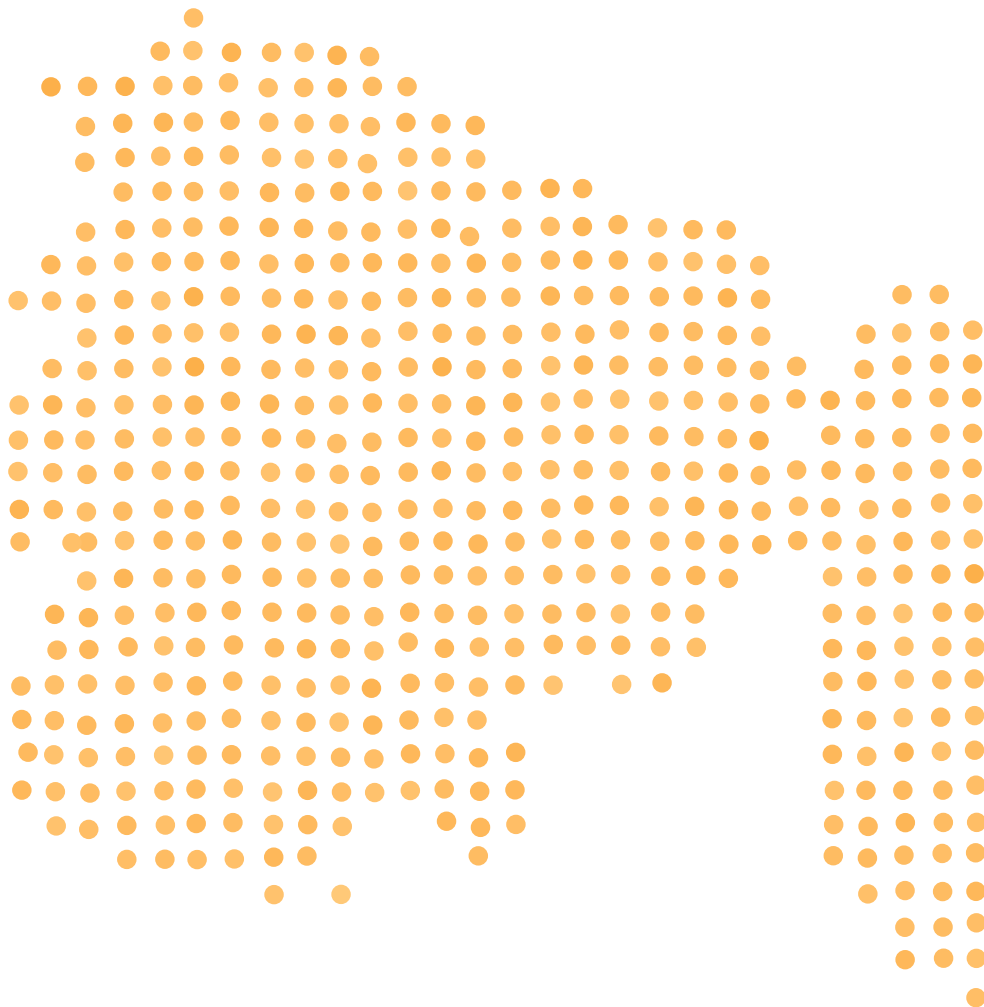

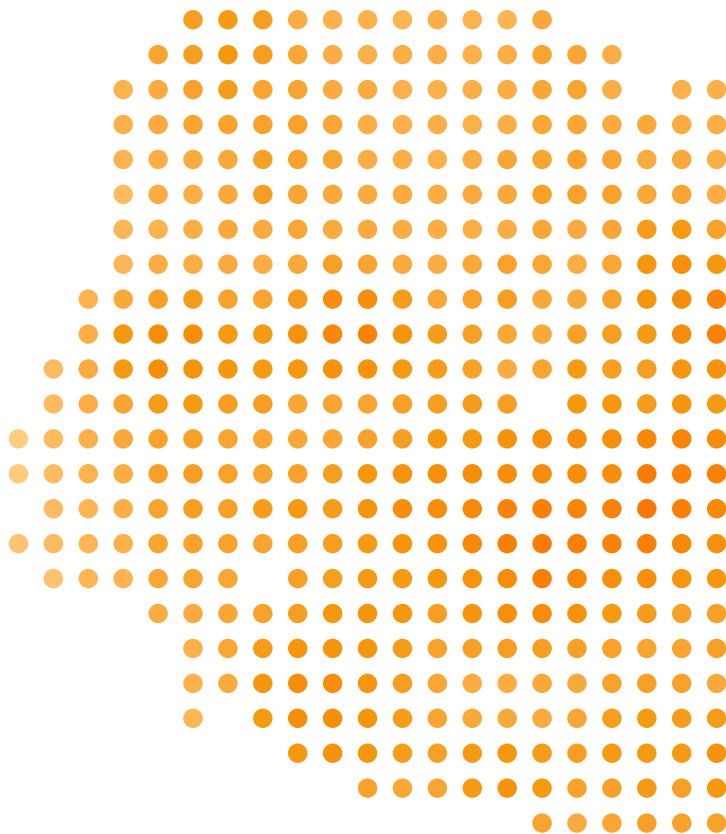

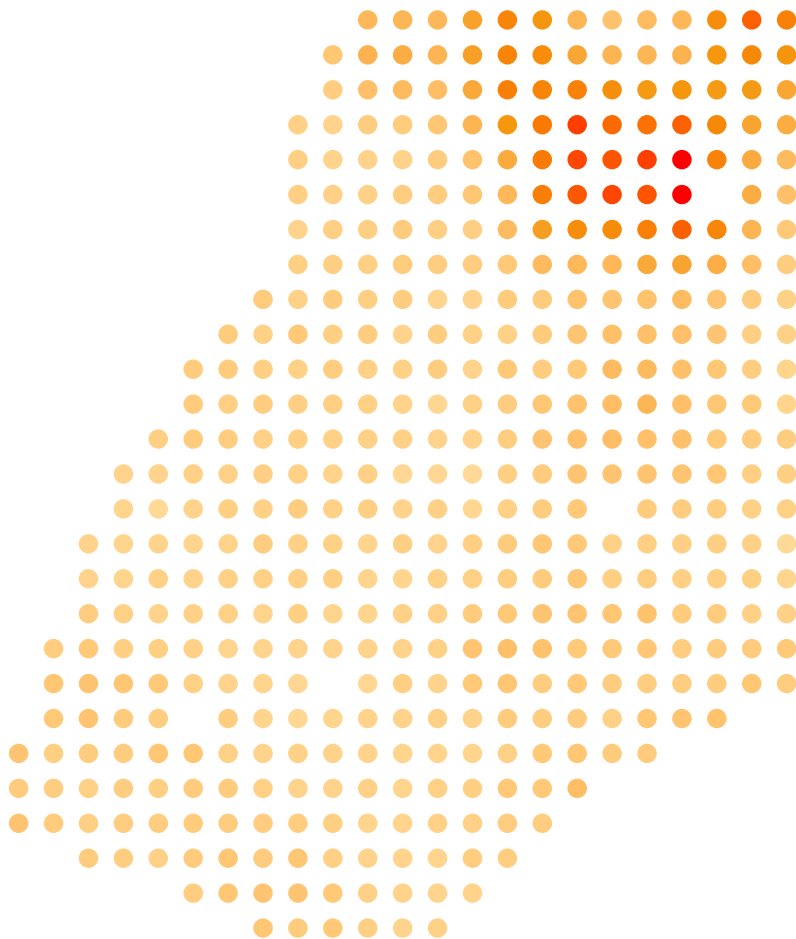

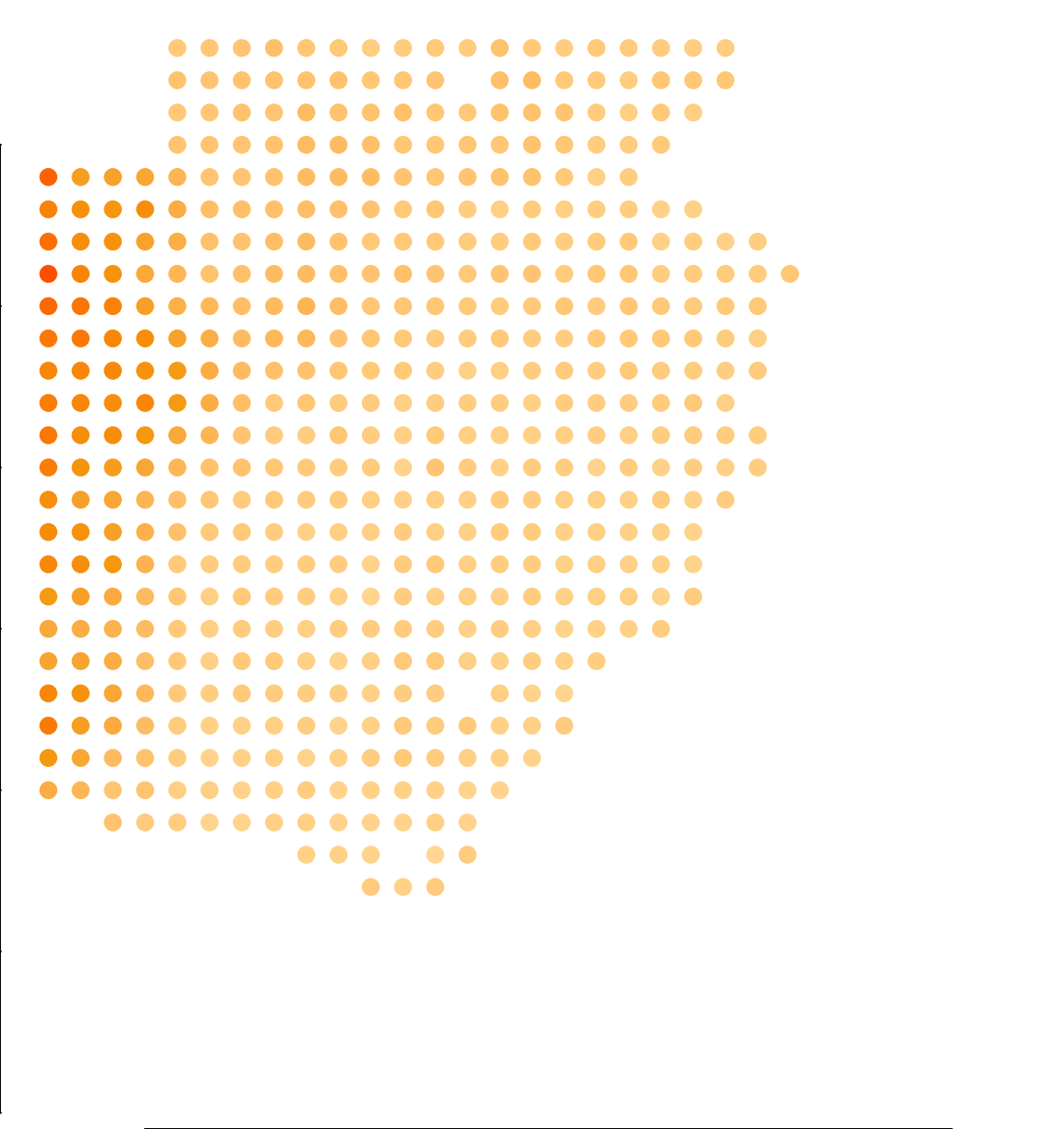

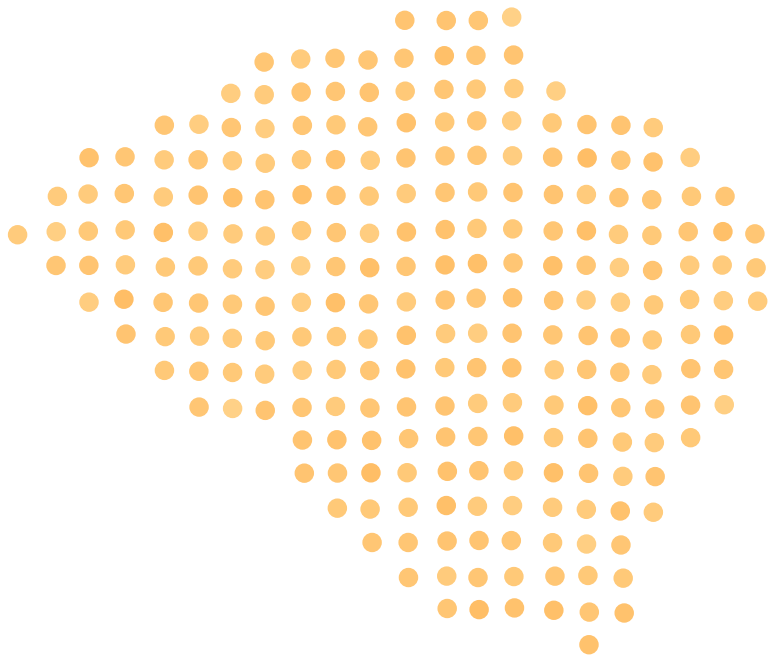

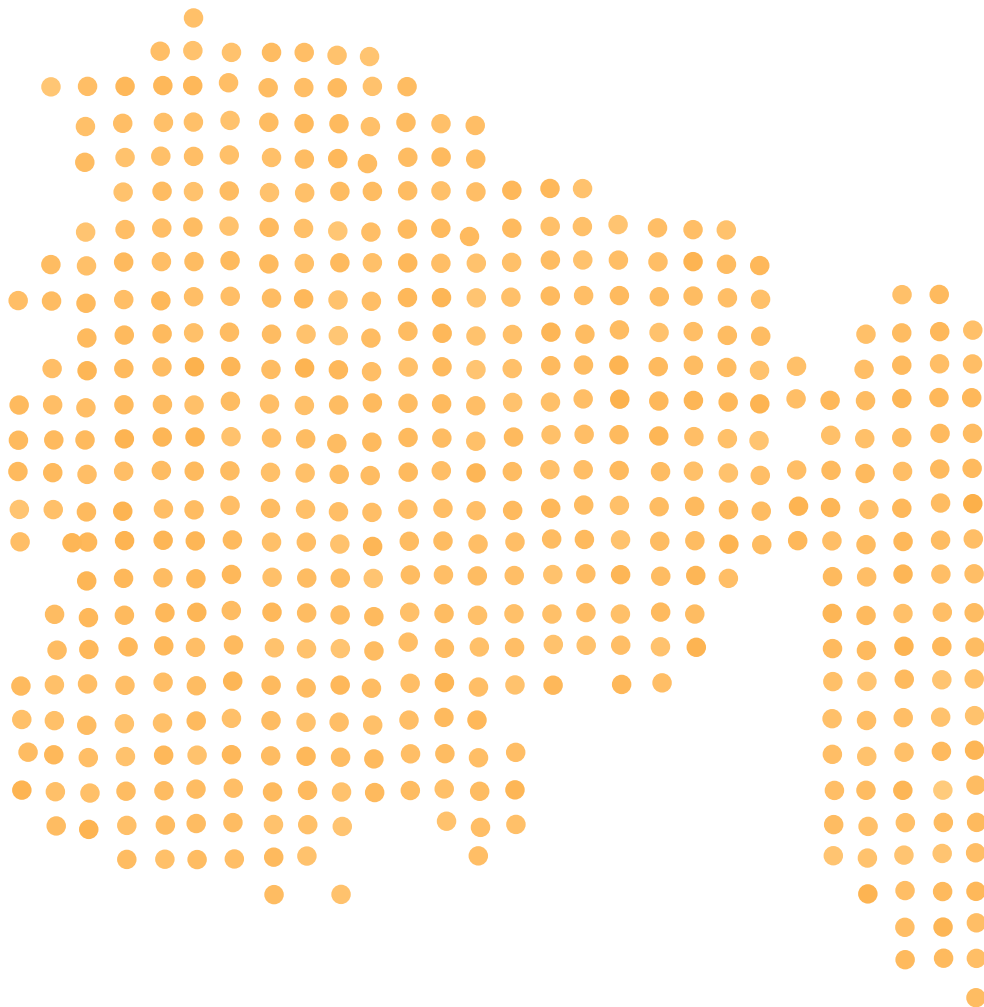

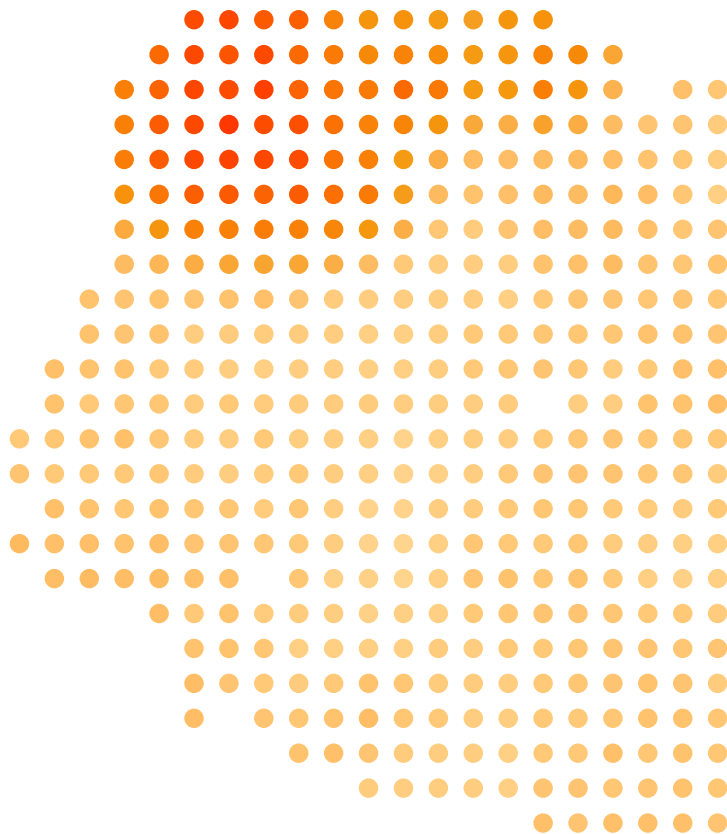

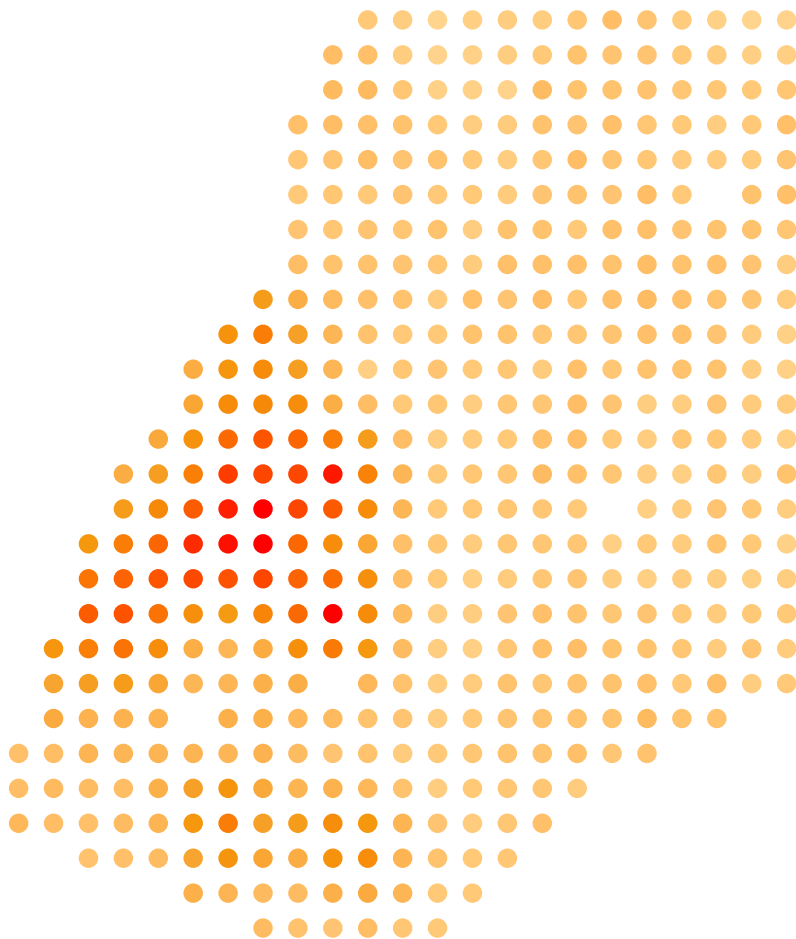

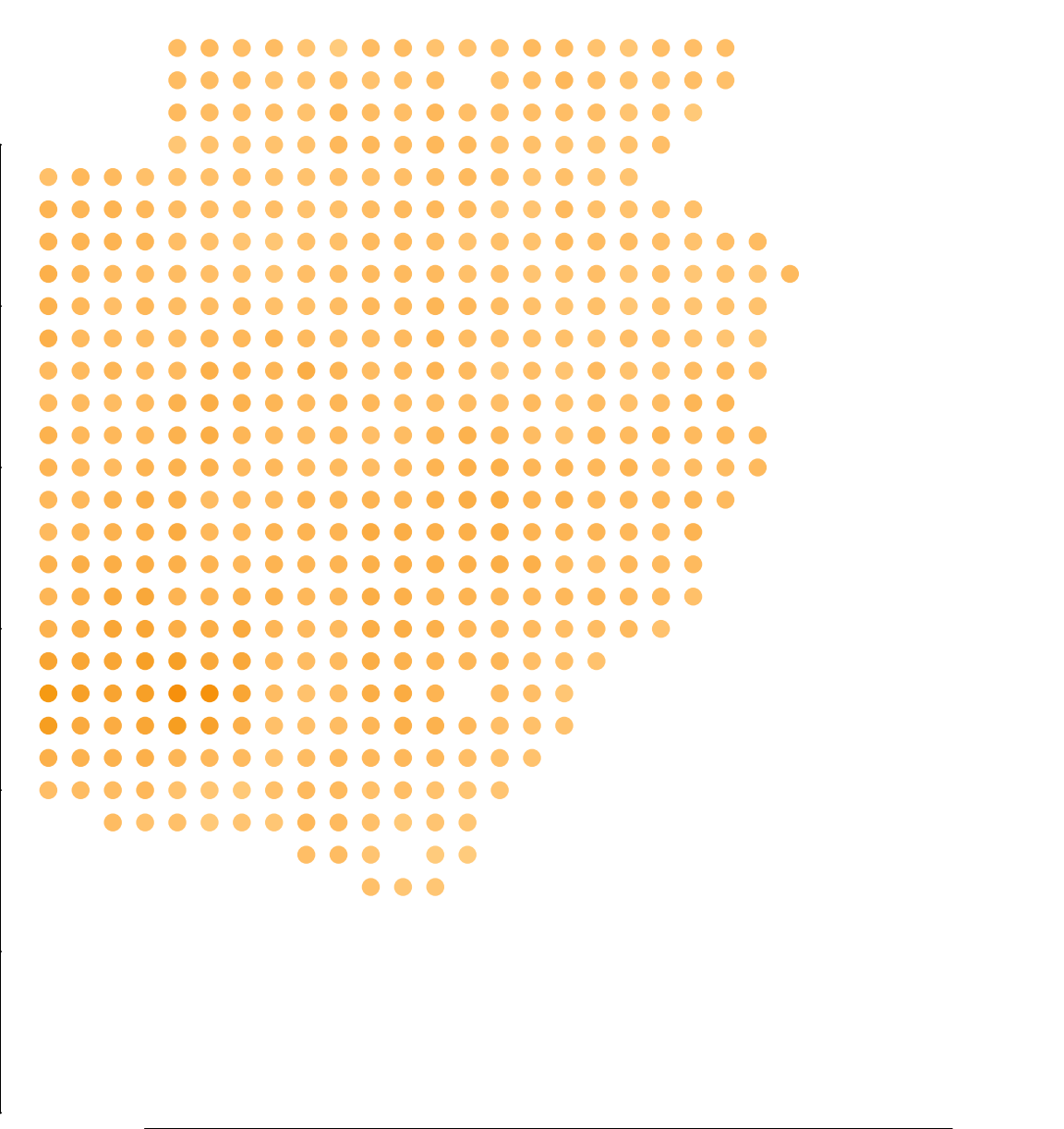

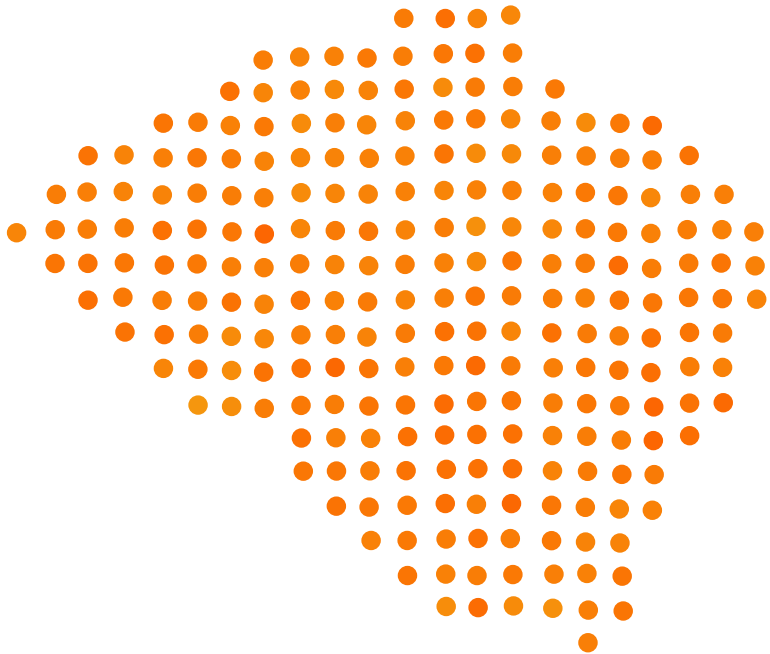

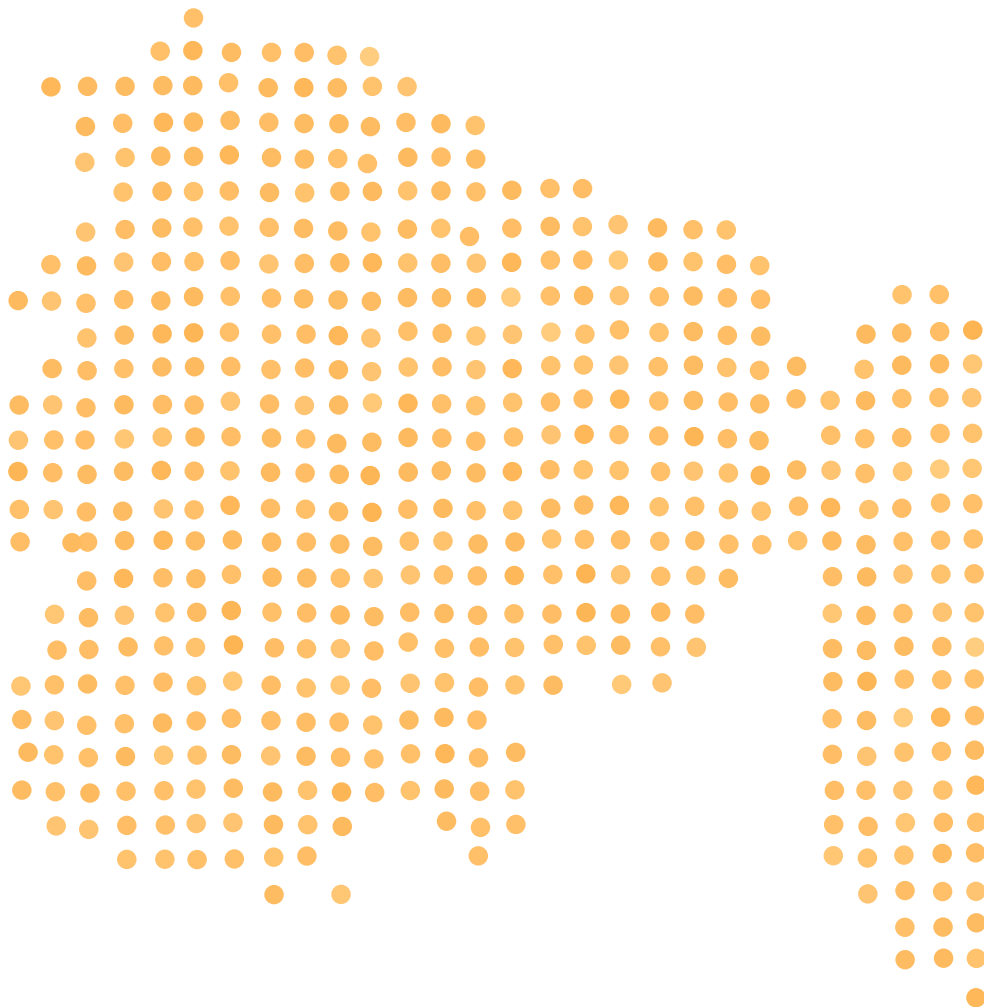

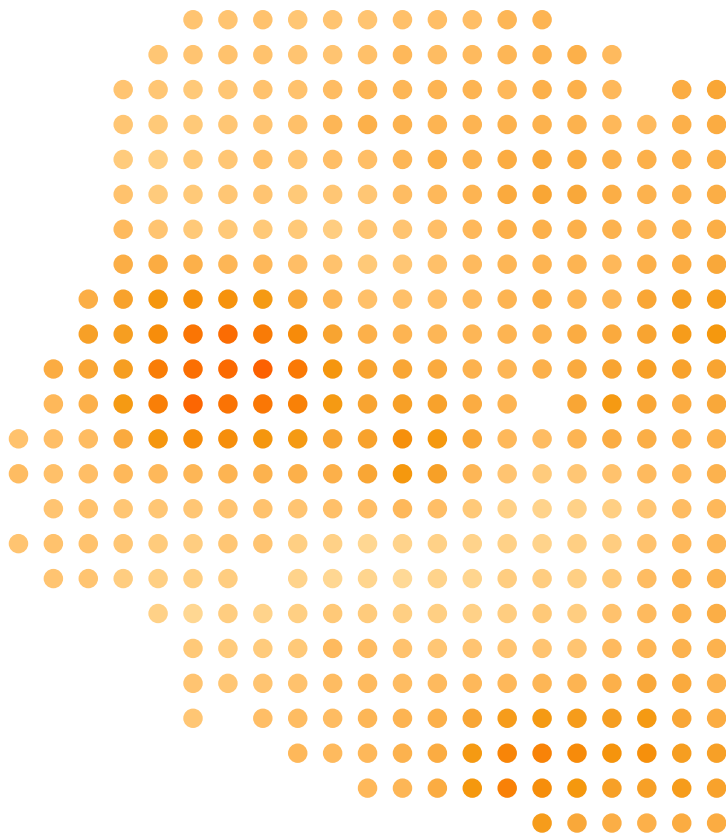

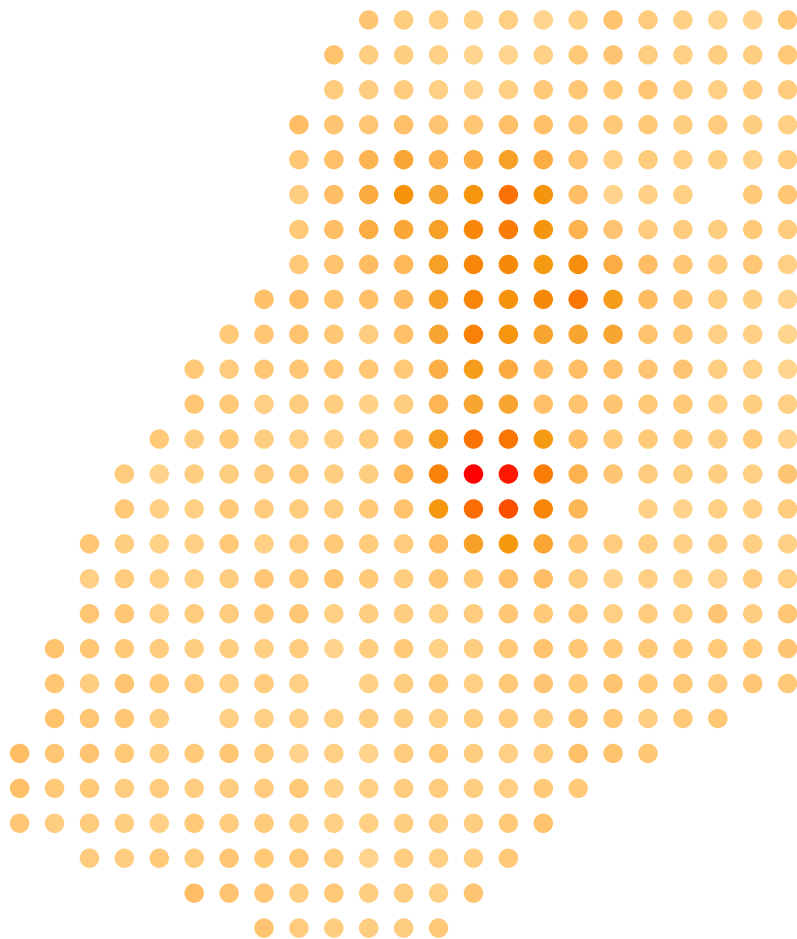

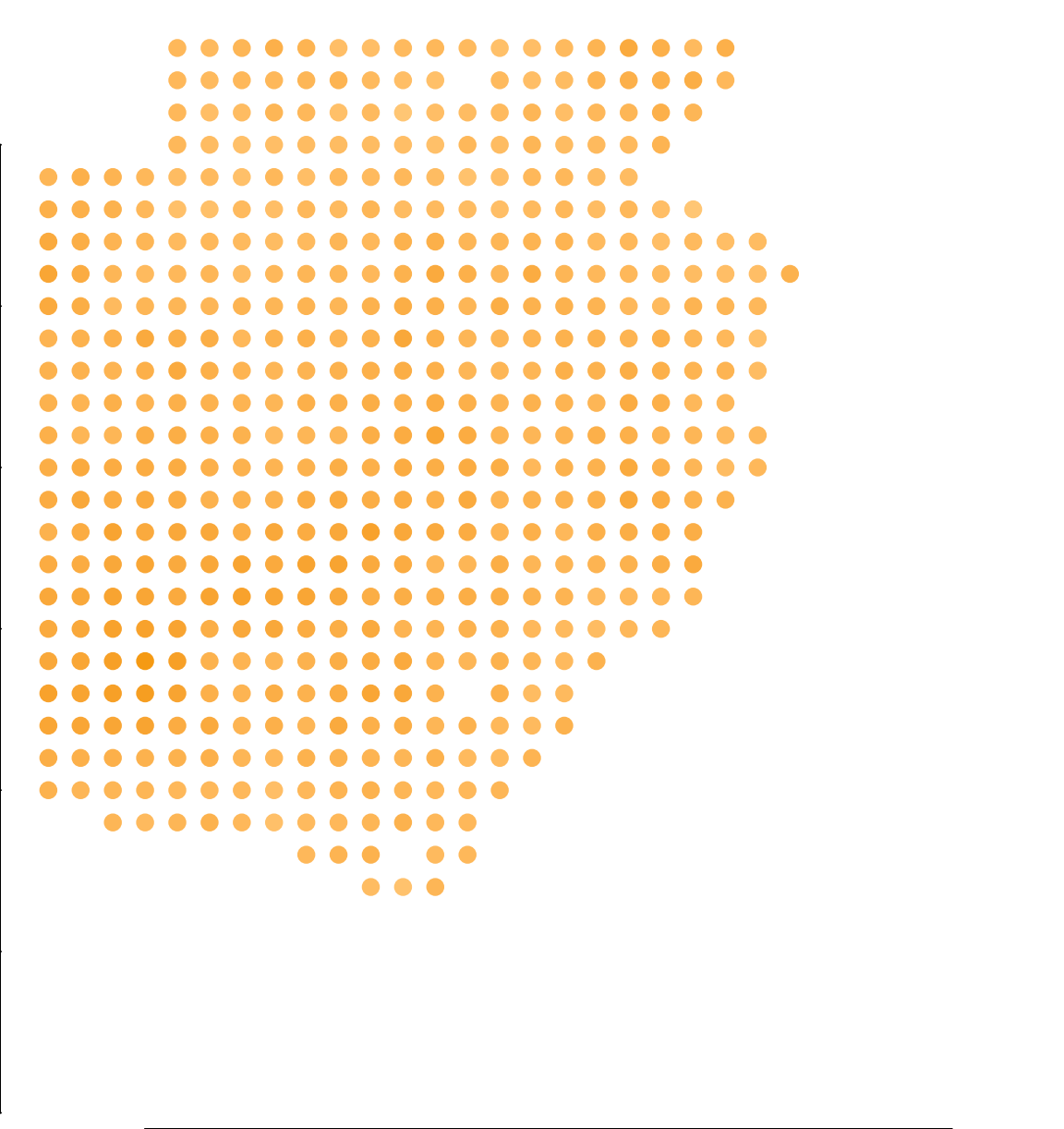

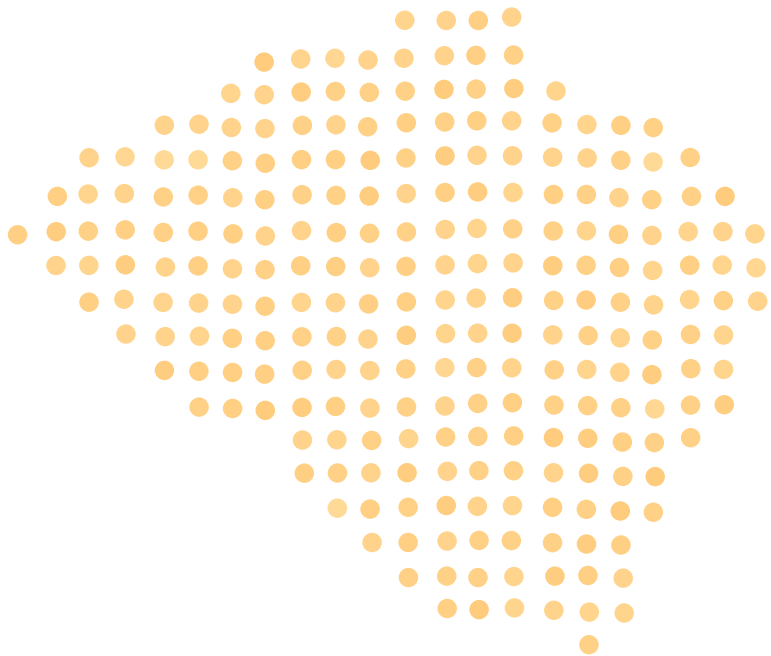

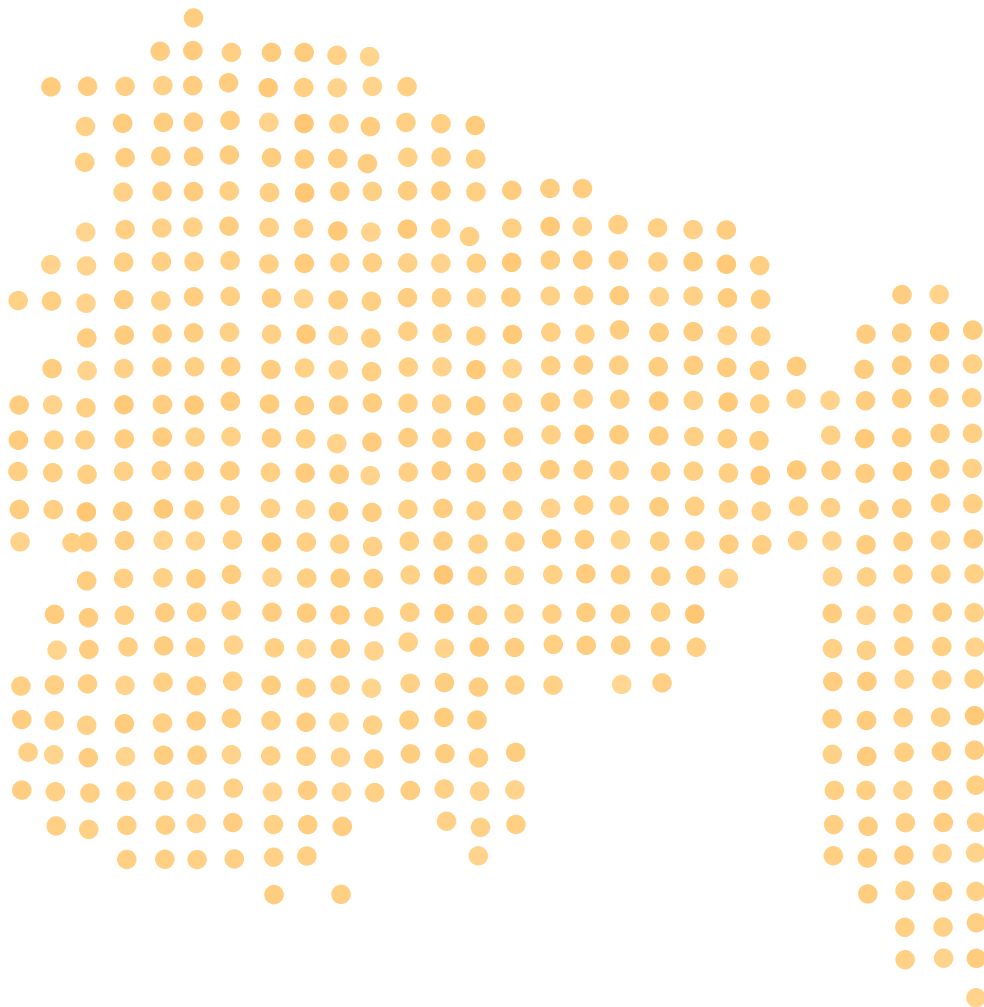

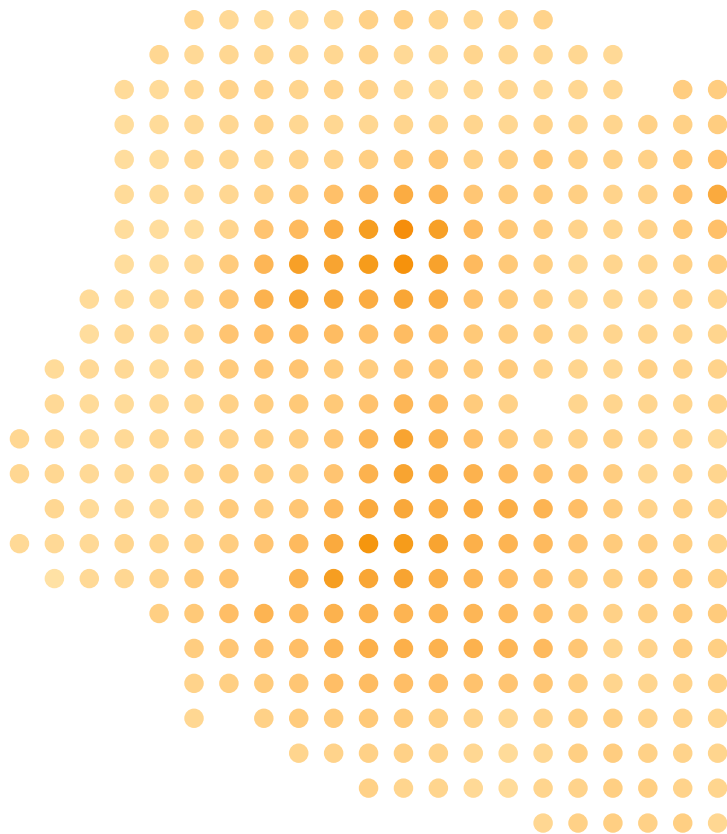

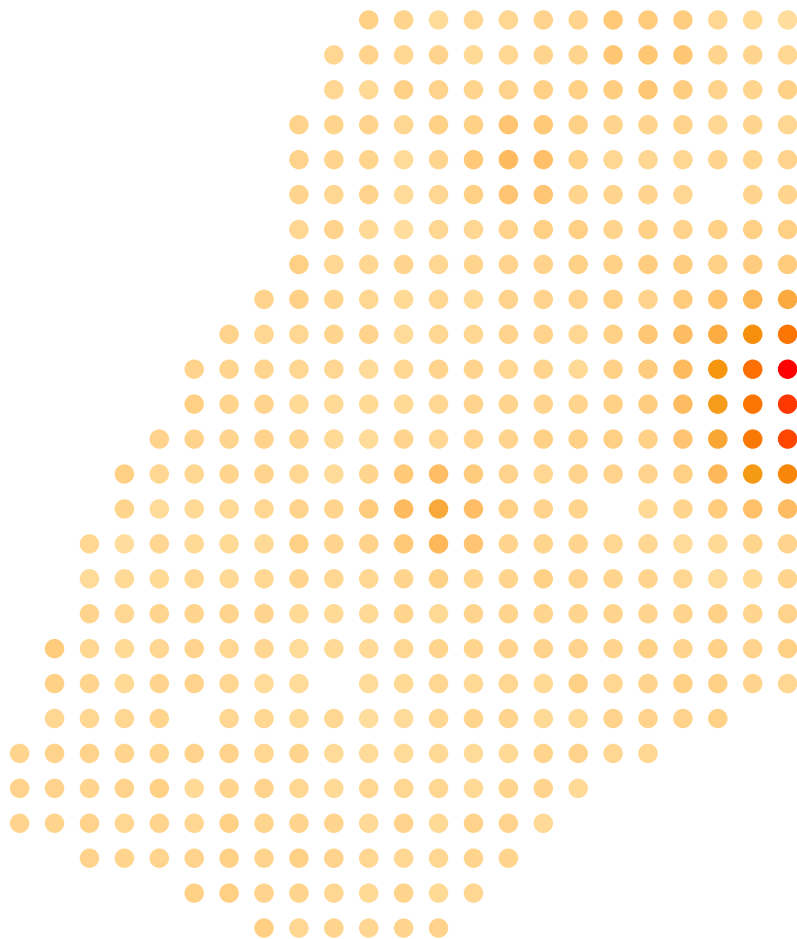

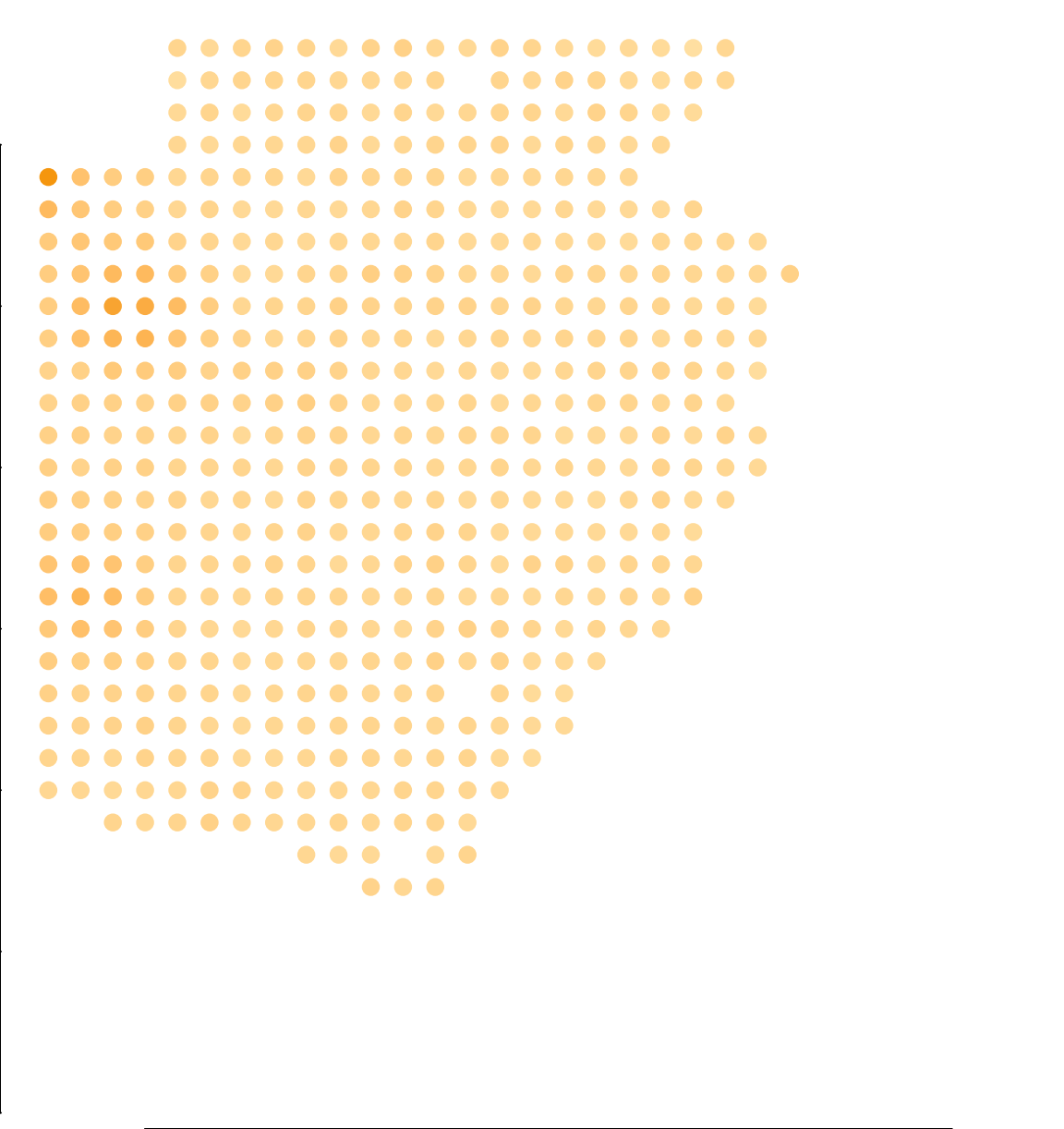

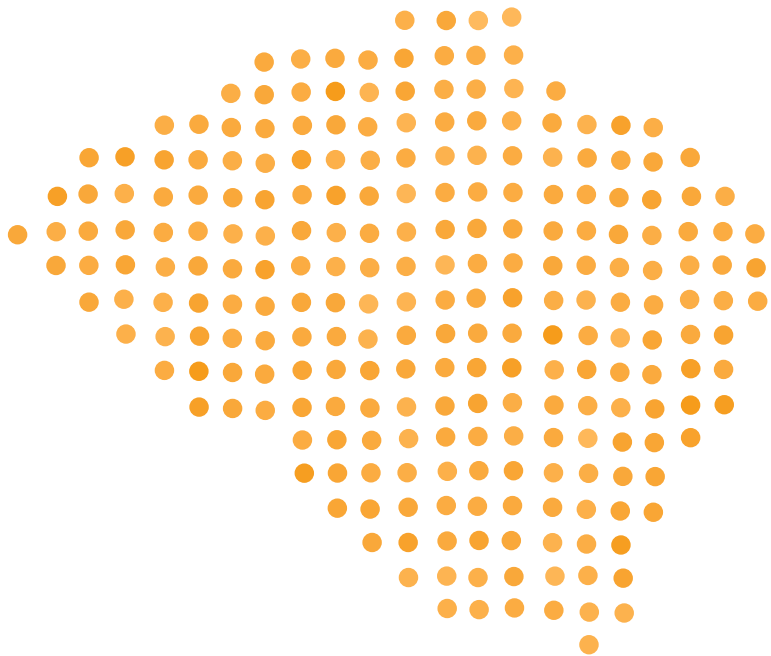

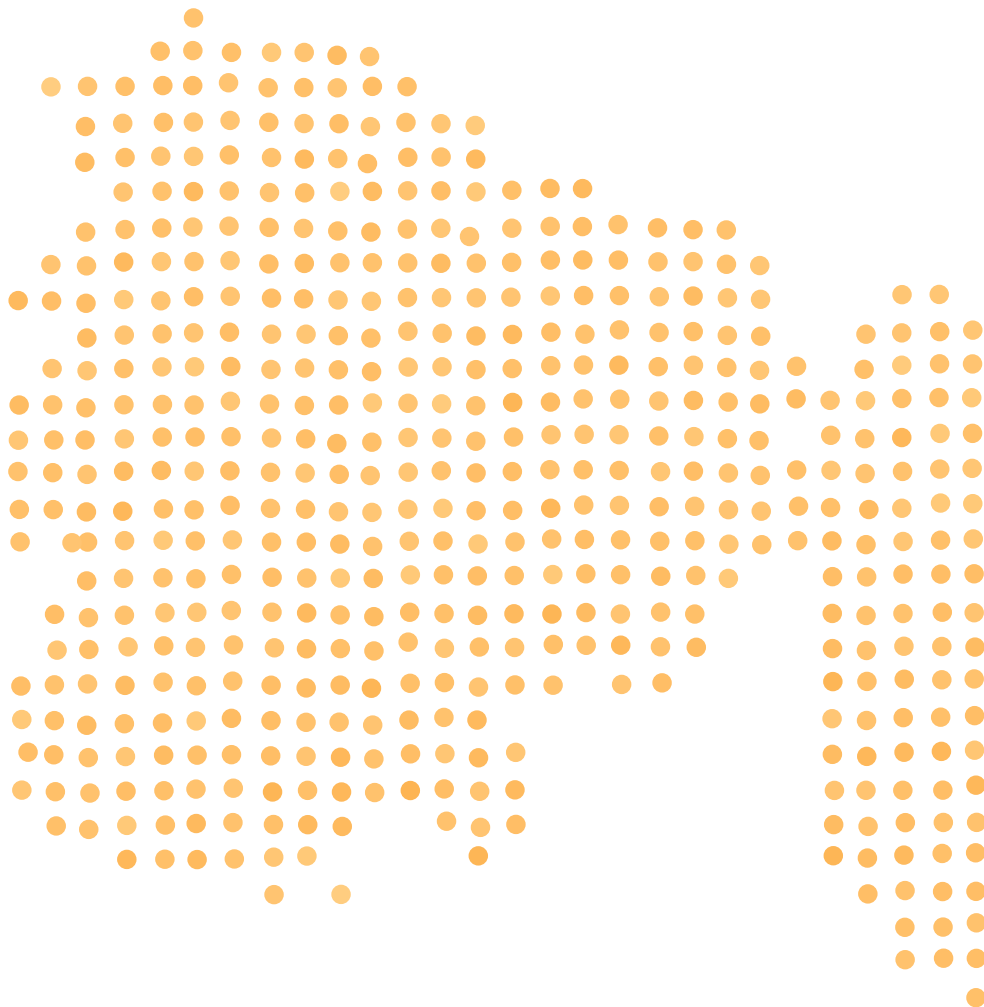

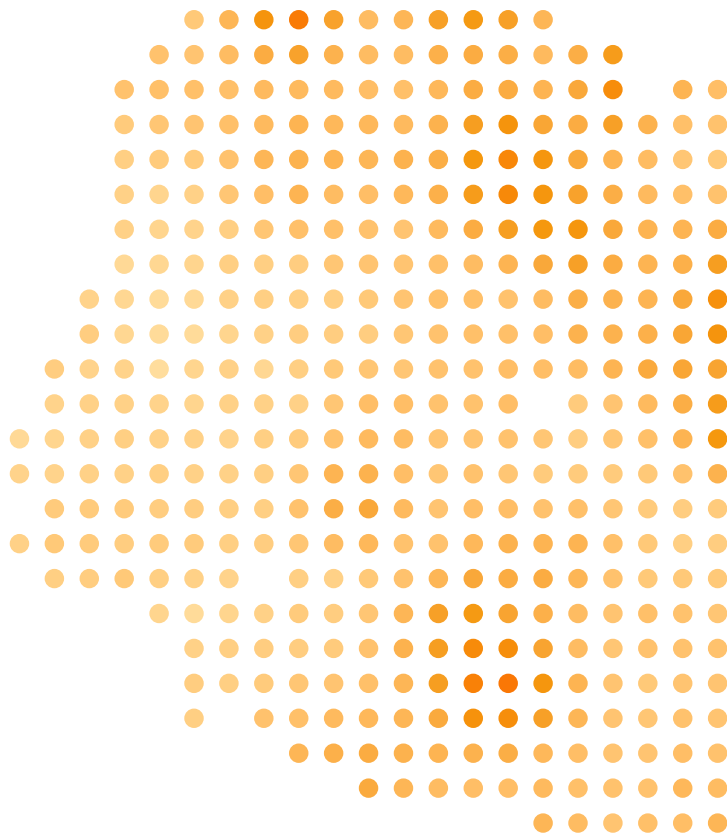

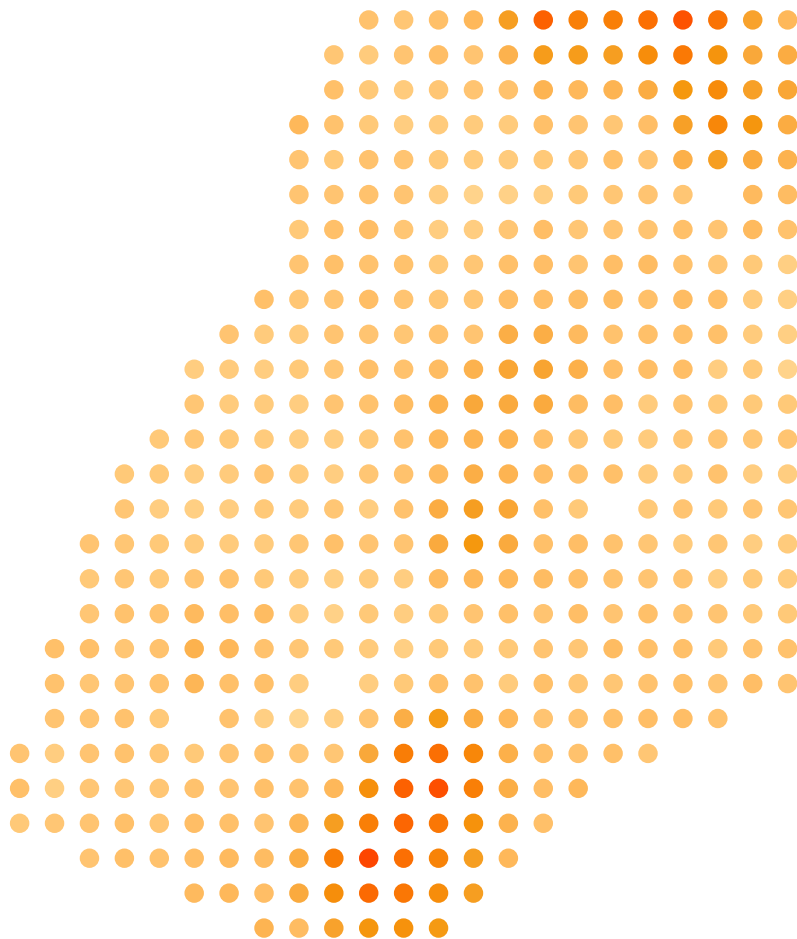

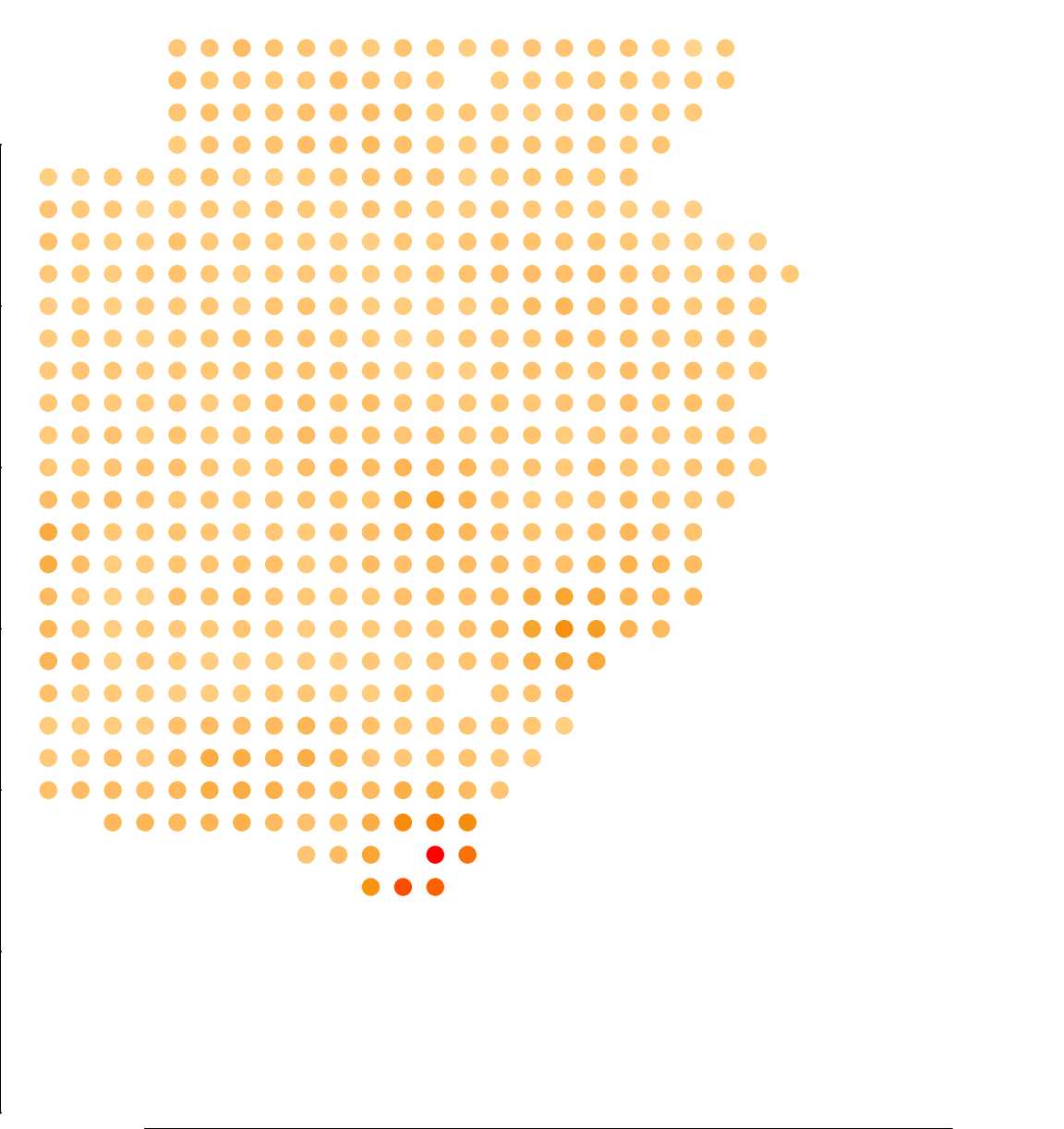

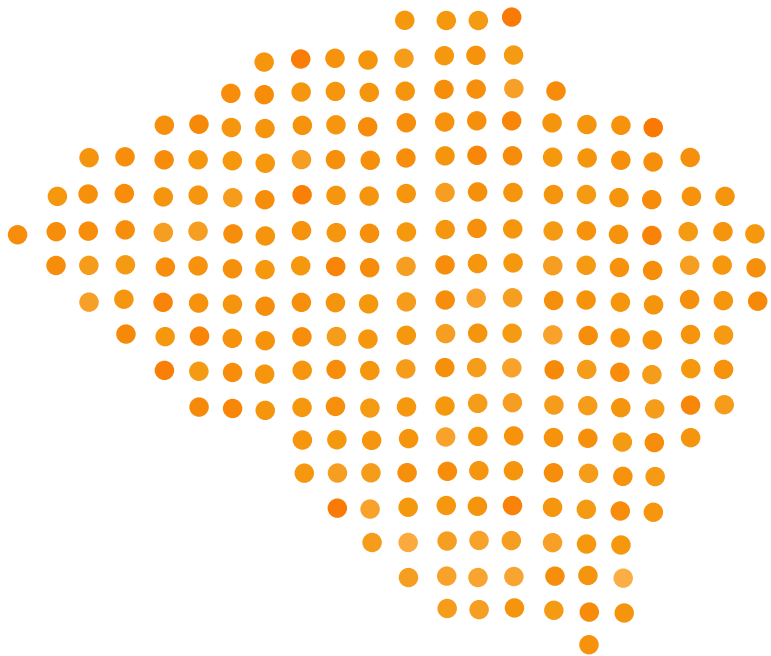

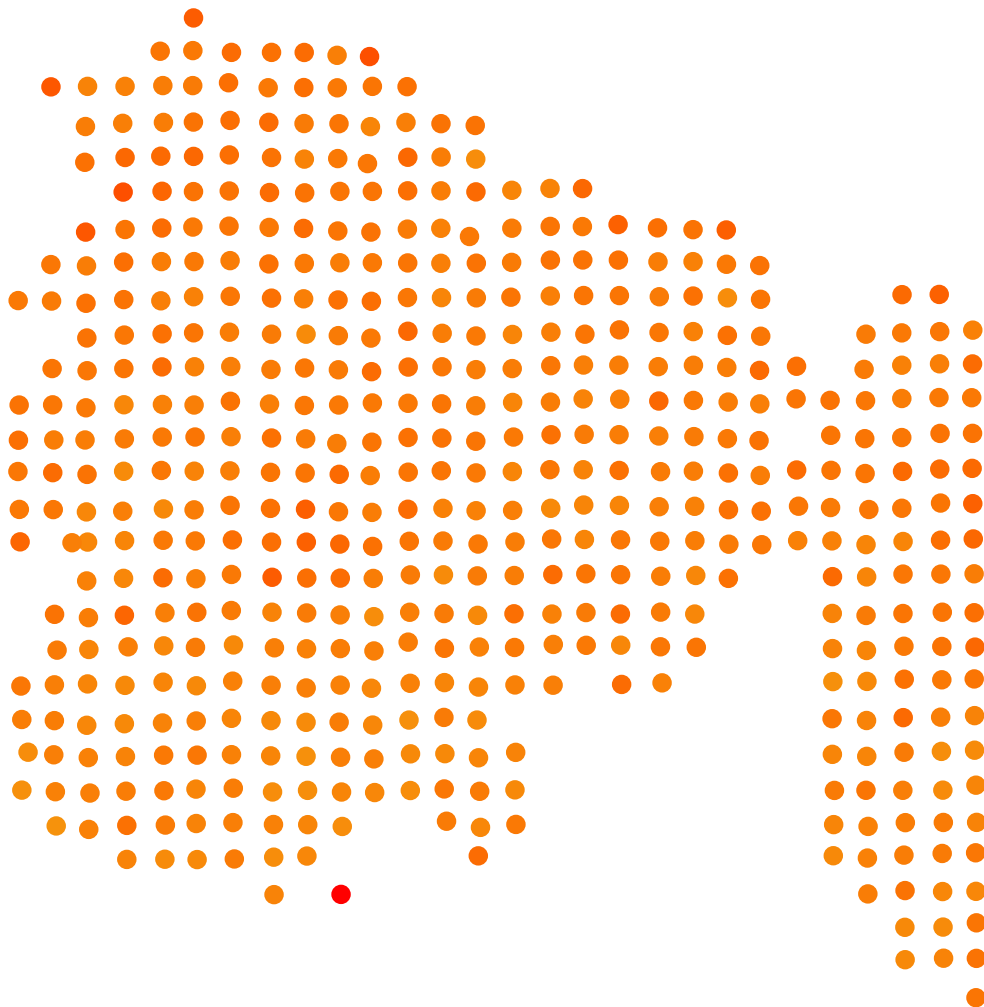

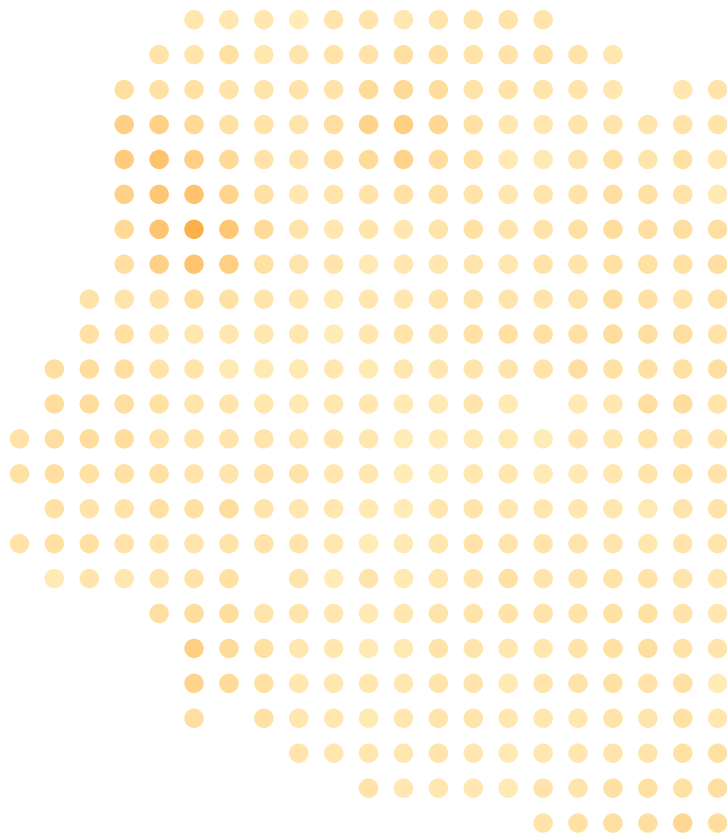

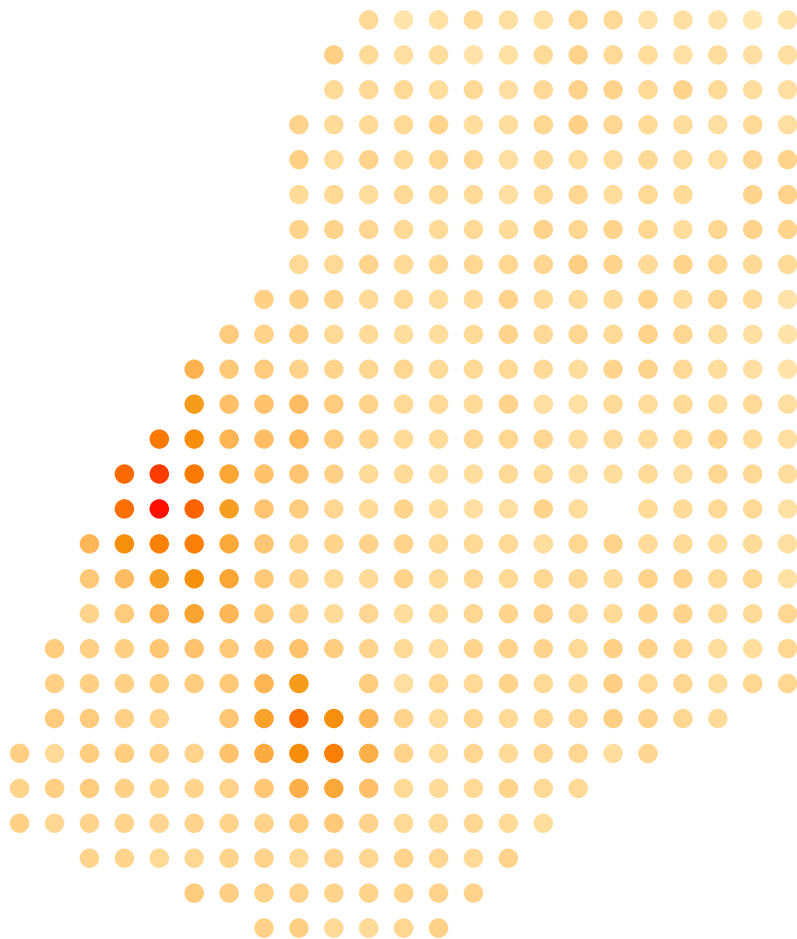

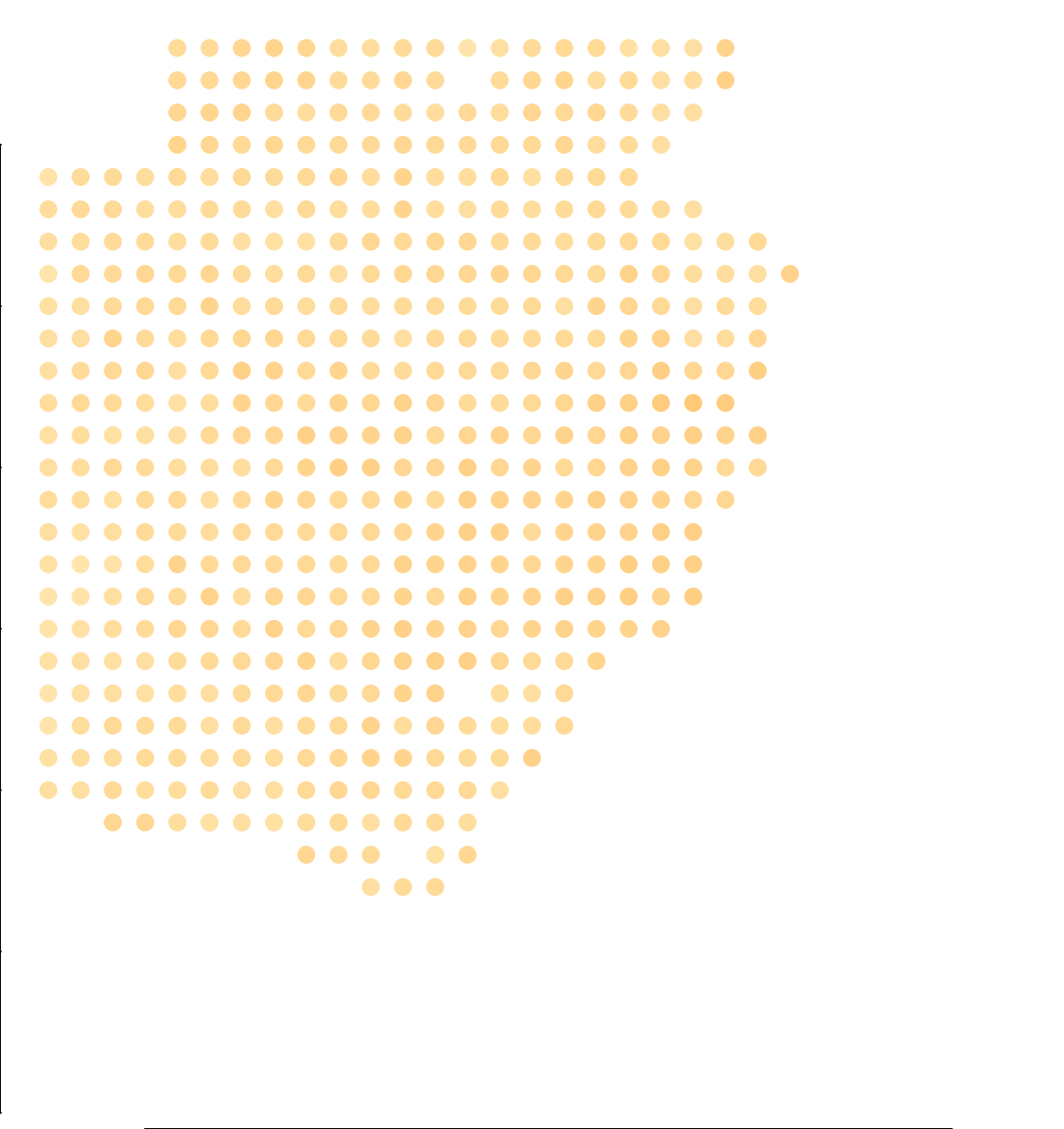

Supplement: Supplementary file 8 — Supplementary Data 5 [file 41467_2018_4724_MOESM8_ESM.zip › Supplementary Dataset 7/joint-field-profiles-rel-common-scale-dots-split.pdf]

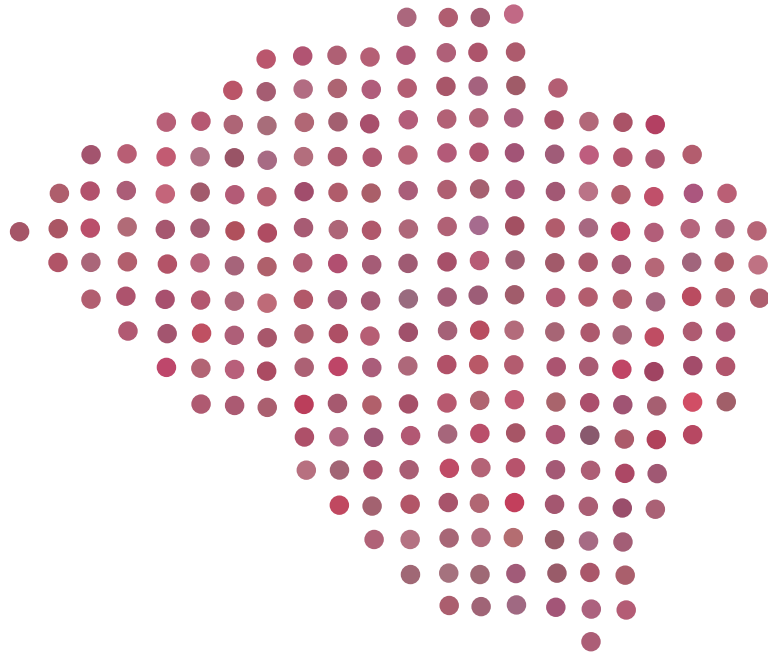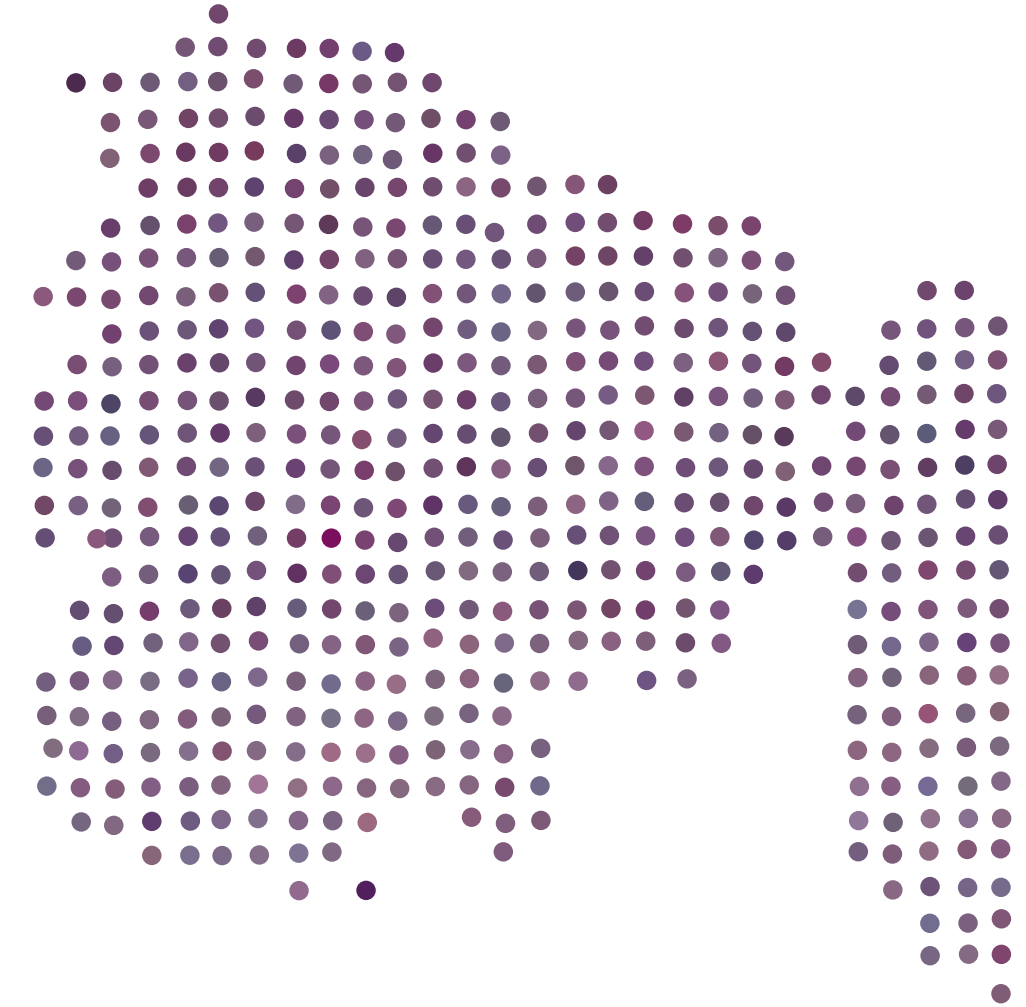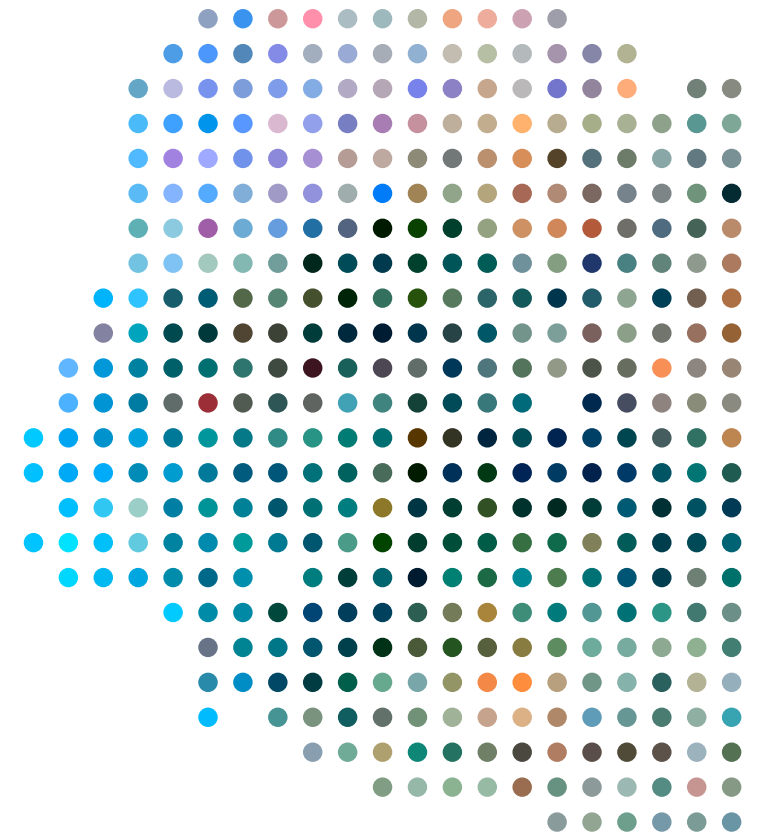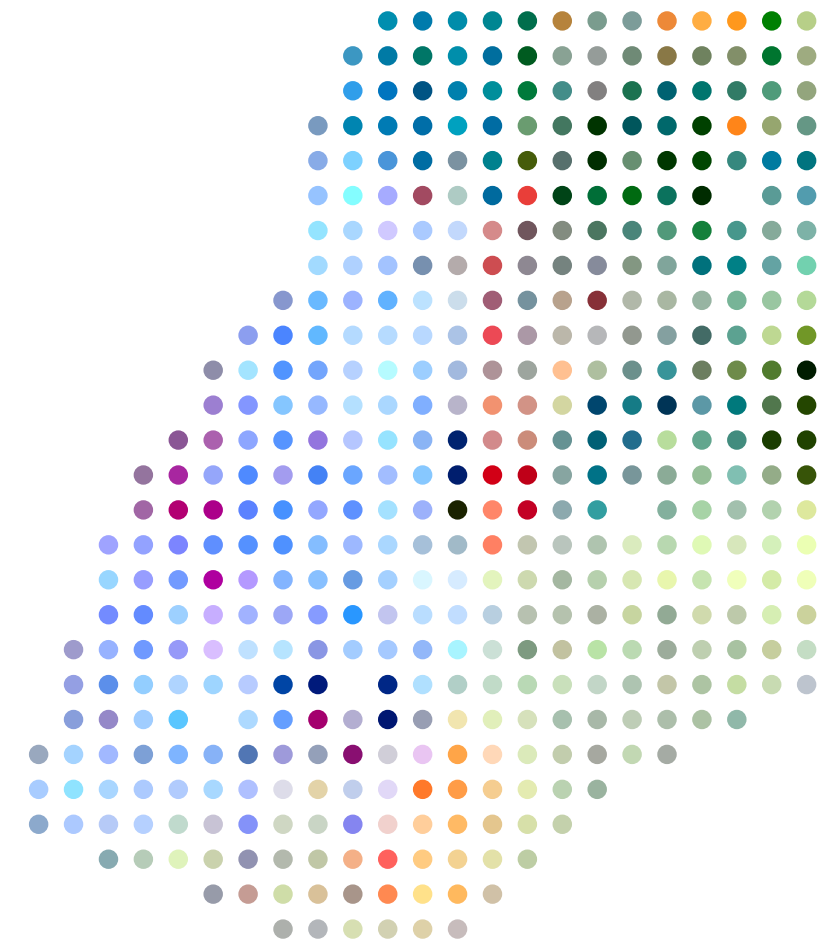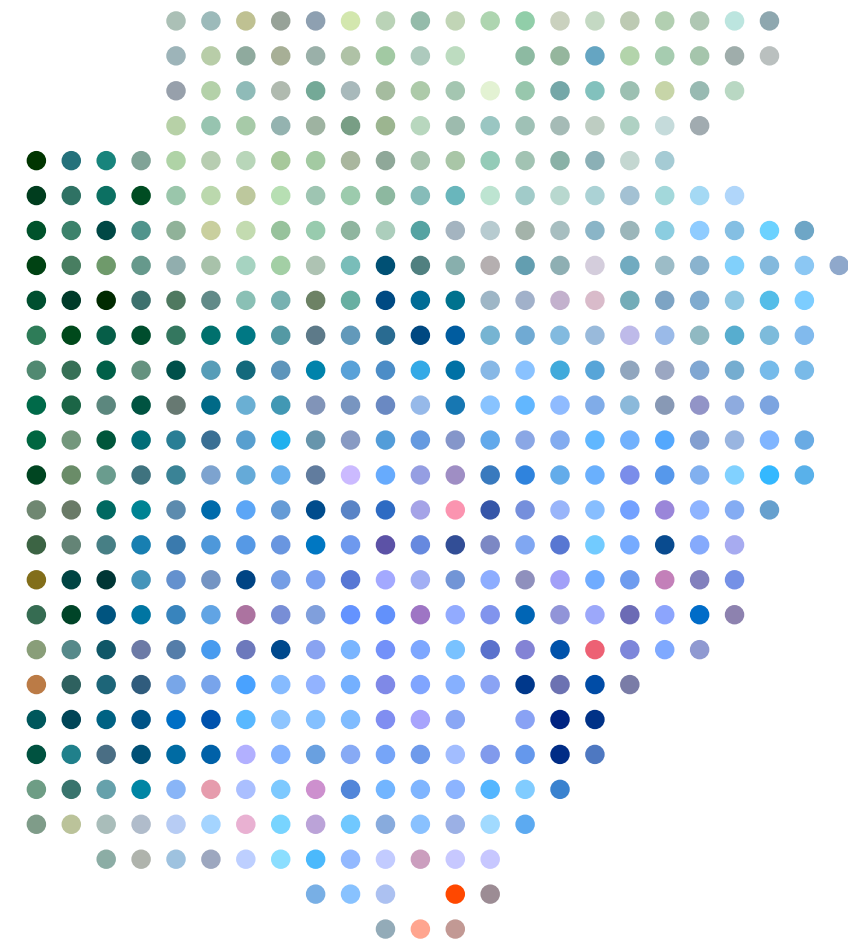

Supplement: Supplementary file 8 — Supplementary Data 5 [file 41467_2018_4724_MOESM8_ESM.zip › Supplementary Dataset 7/joint-mix-dimensionality-reduction-PCA-dots.pdf]

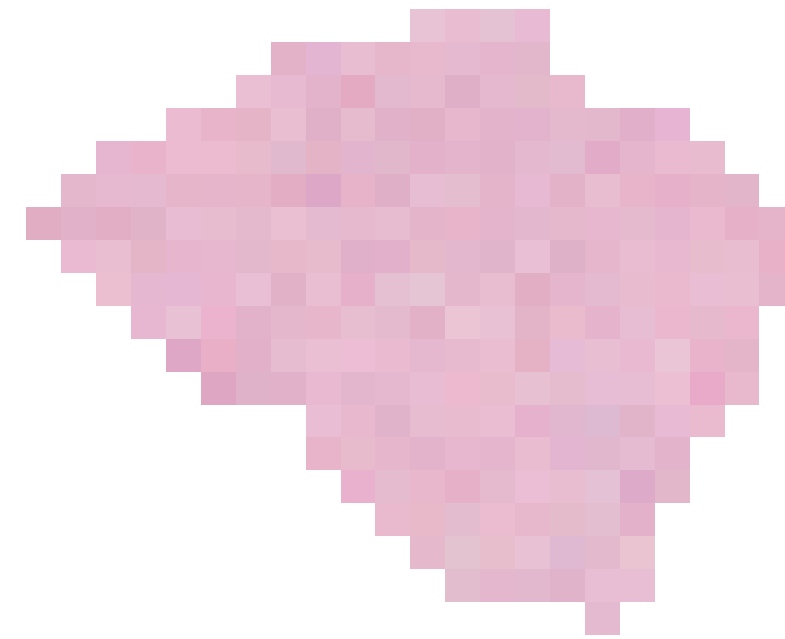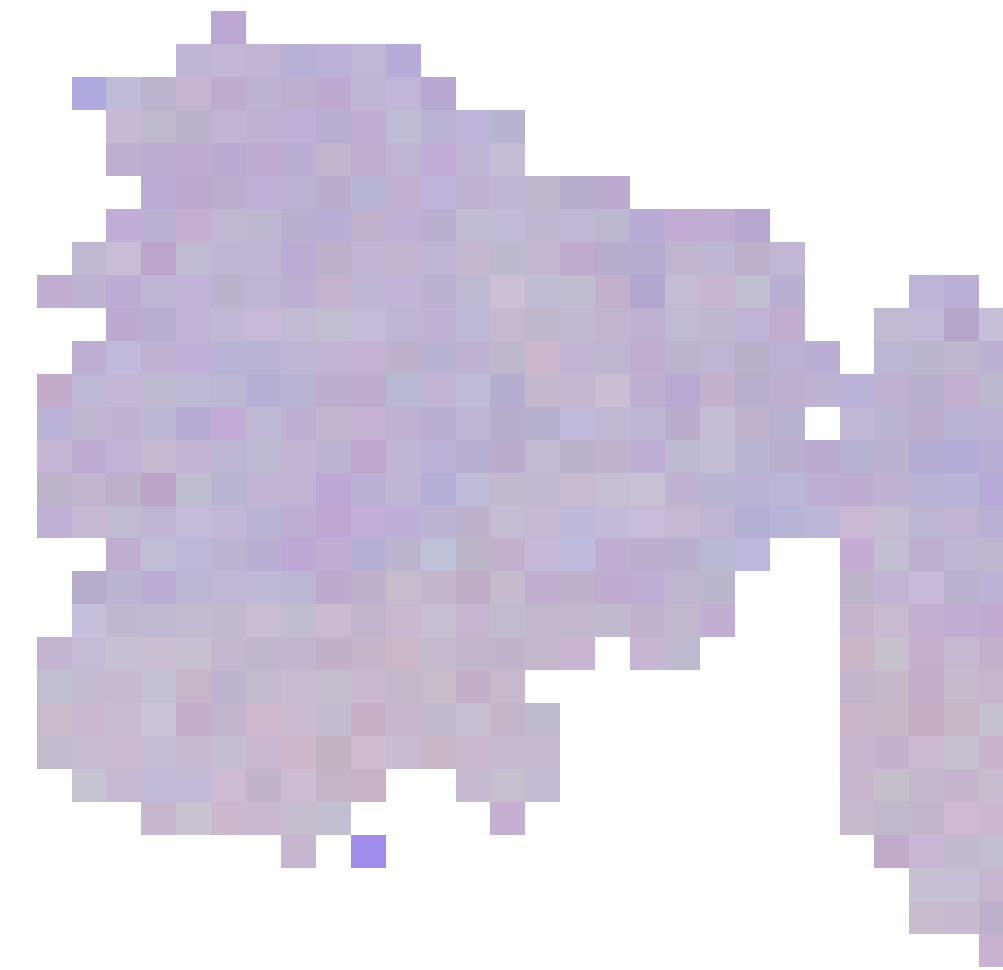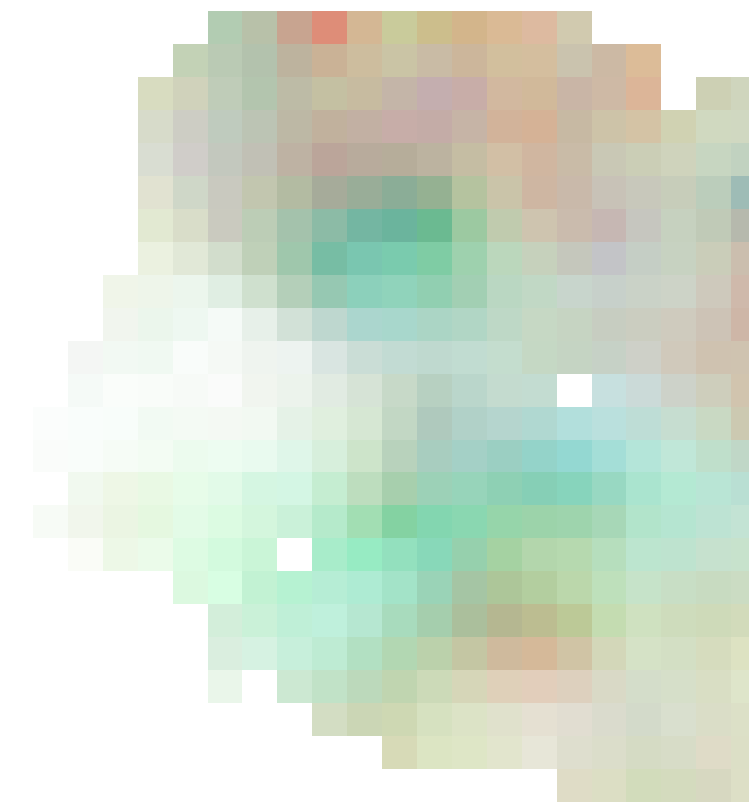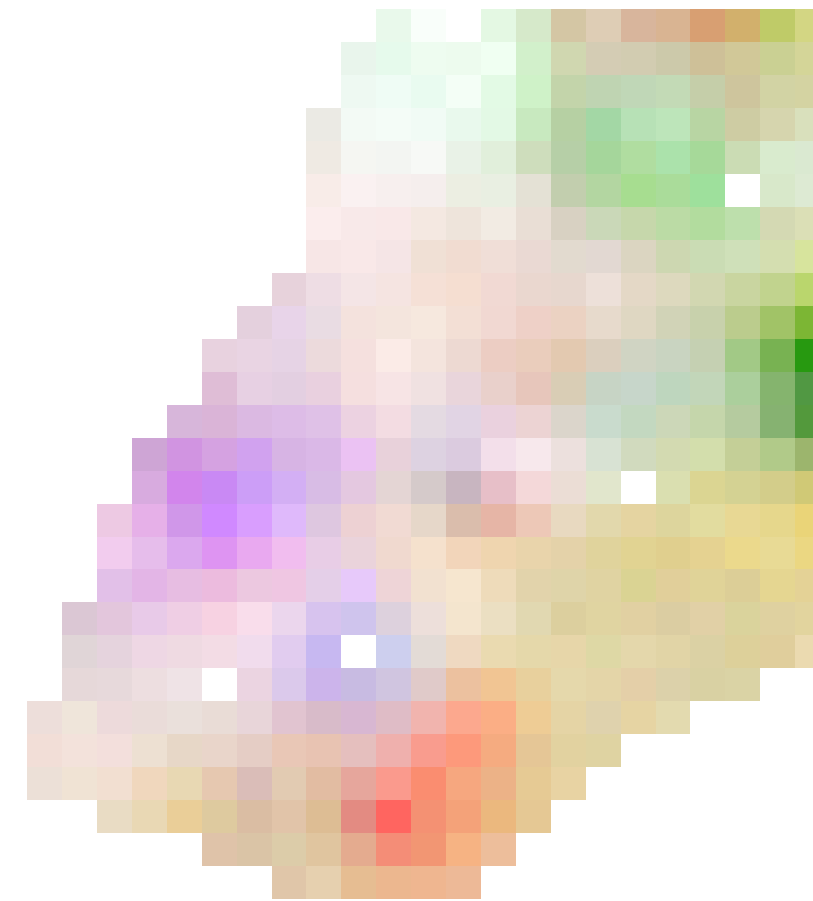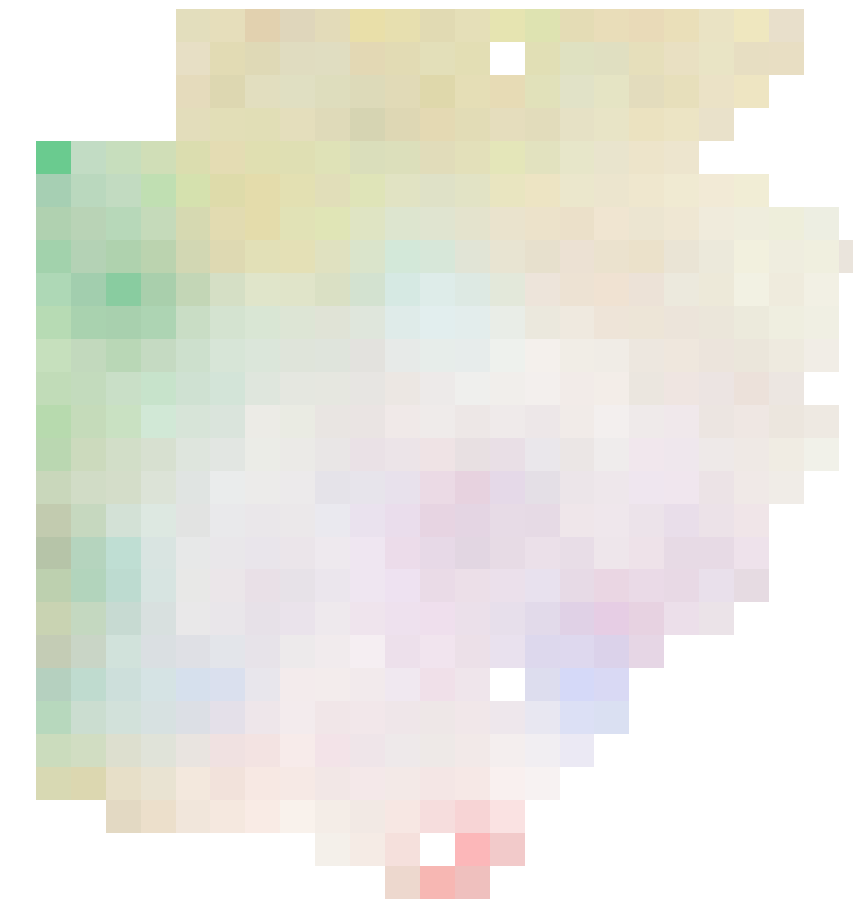

Supplement: Supplementary file 8 — Supplementary Data 5 [file 41467_2018_4724_MOESM8_ESM.zip › Supplementary Dataset 7/joint-field-dimensionality-reduction-PCA-matrix-rgb.pdf]

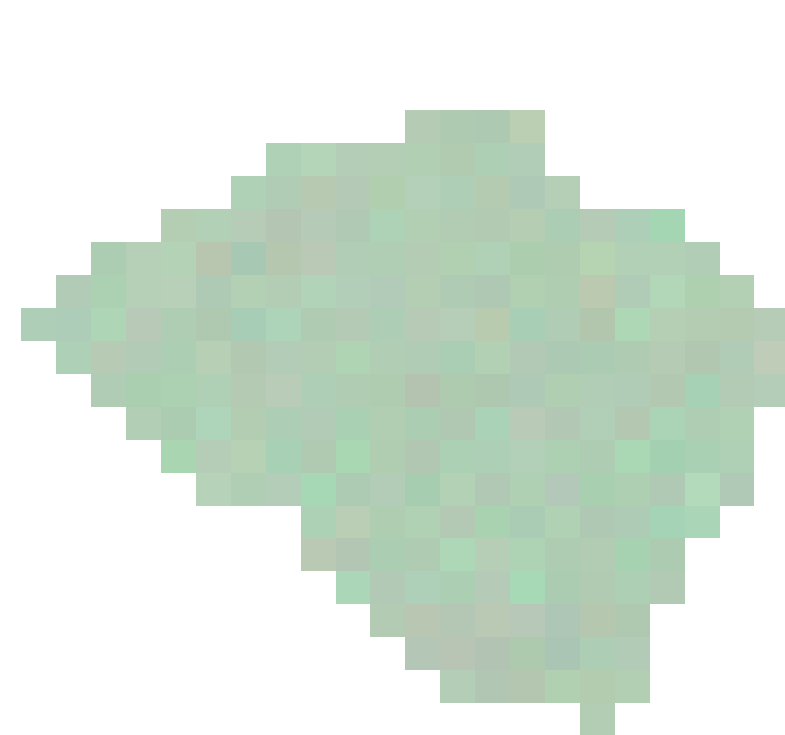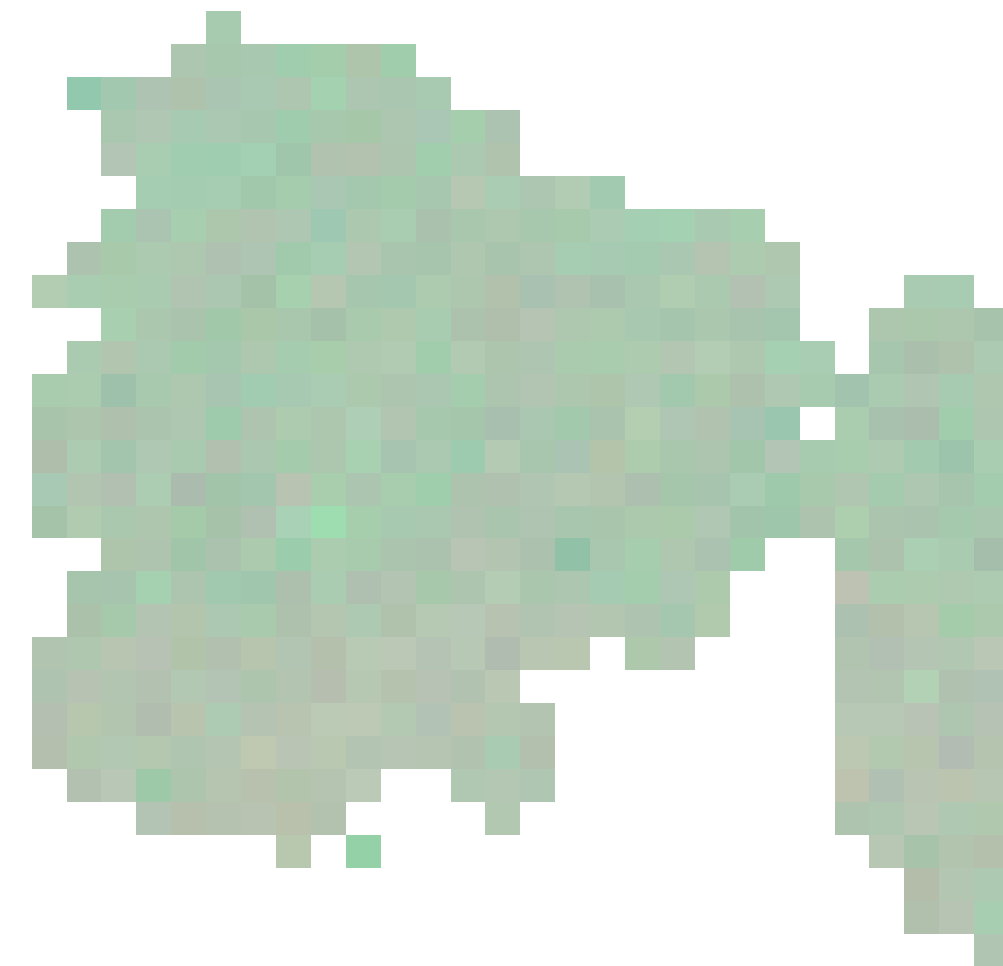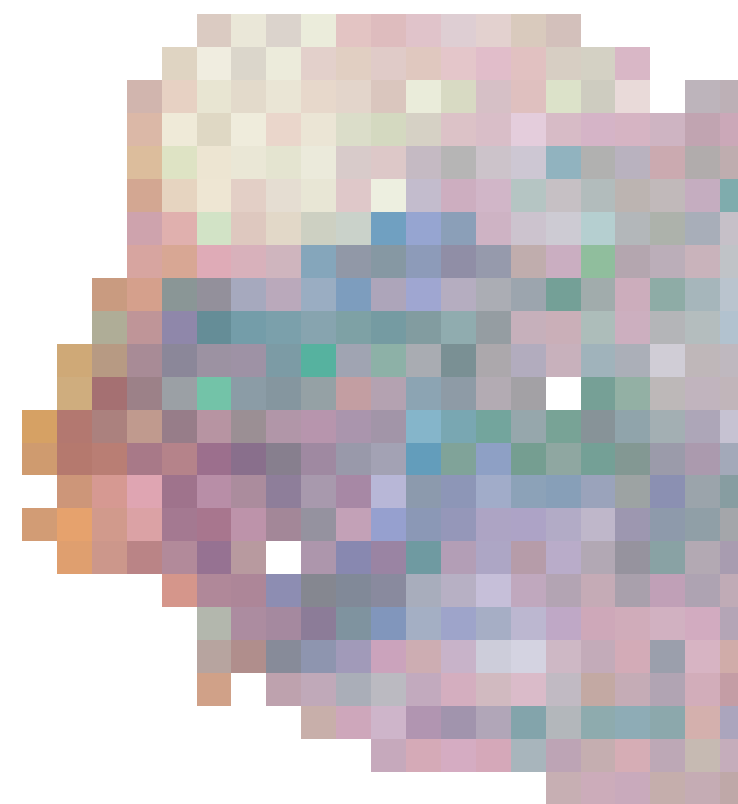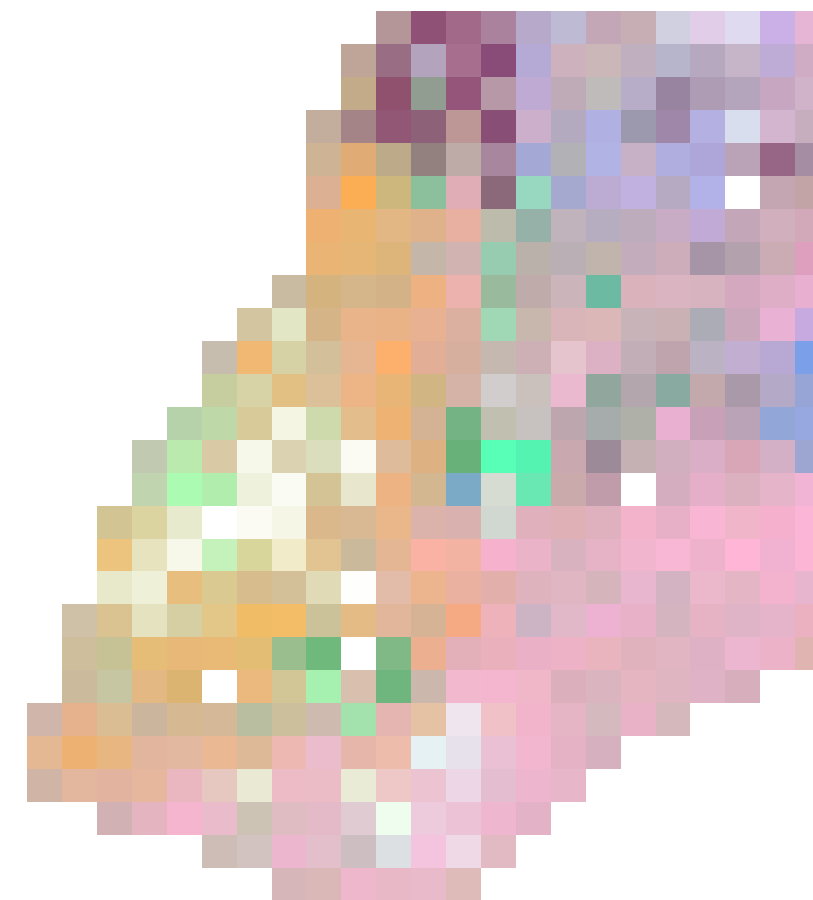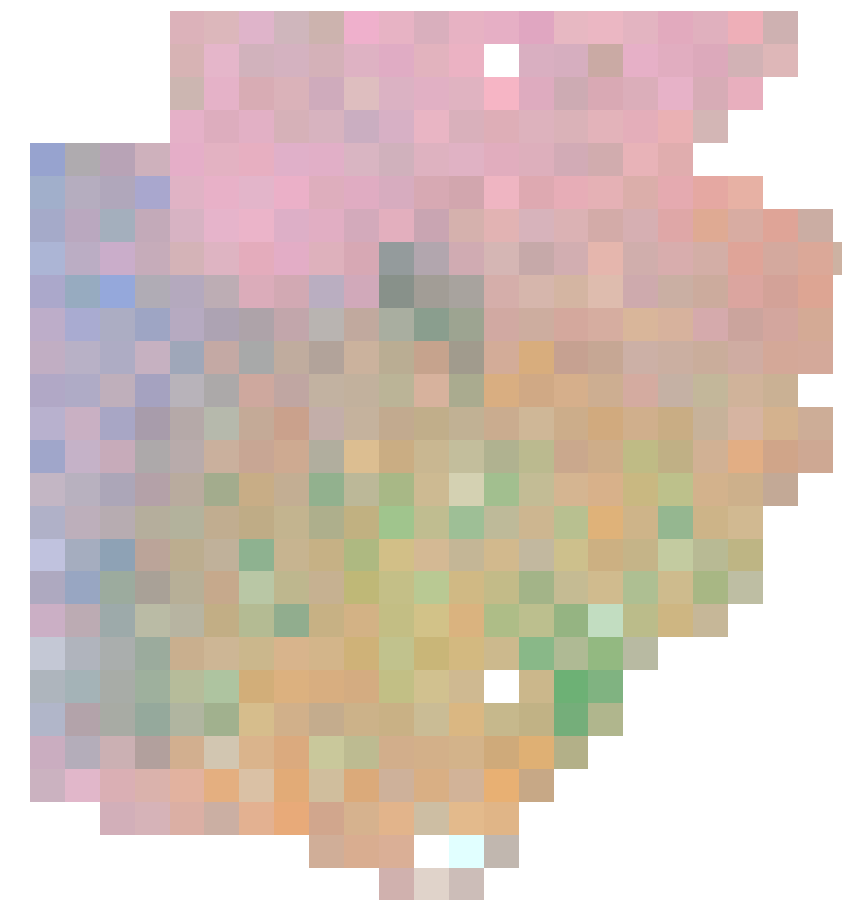

Supplement: Supplementary file 8 — Supplementary Data 5 [file 41467_2018_4724_MOESM8_ESM.zip › Supplementary Dataset 7/joint-mix-dimensionality-reduction-PCA-matrix-rgb.pdf]

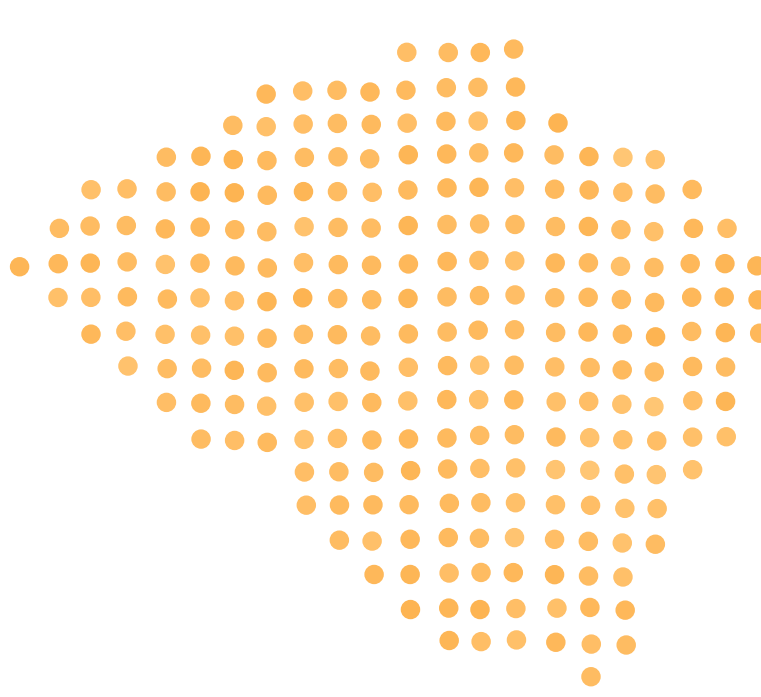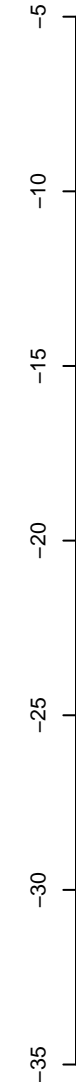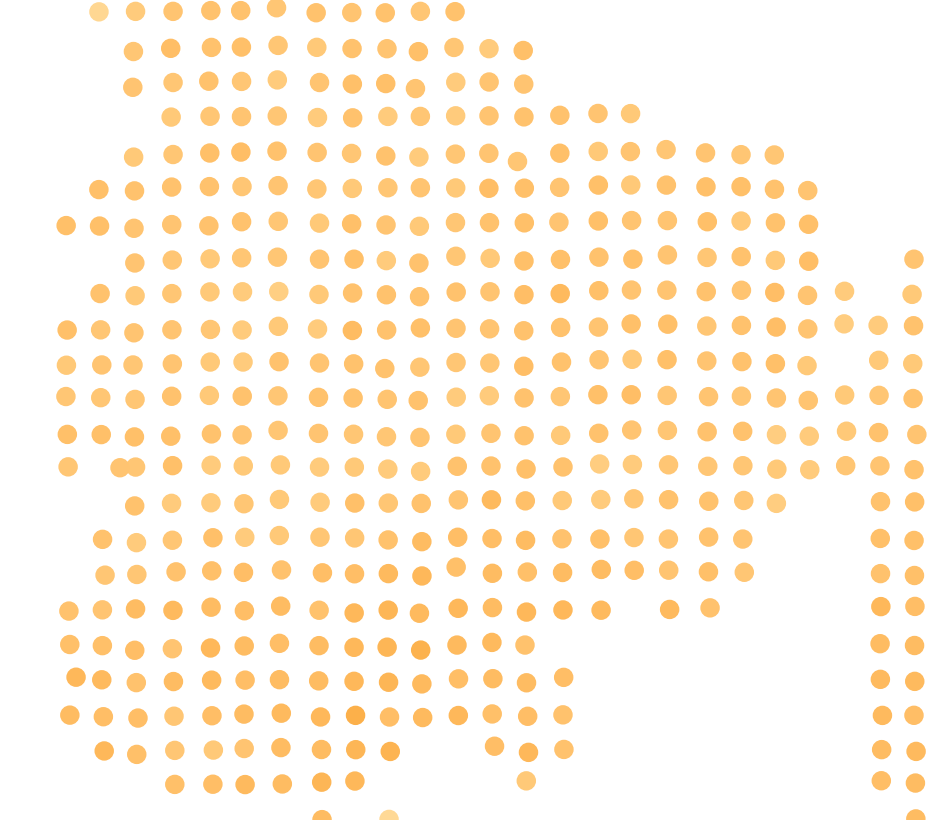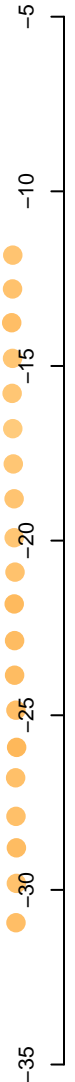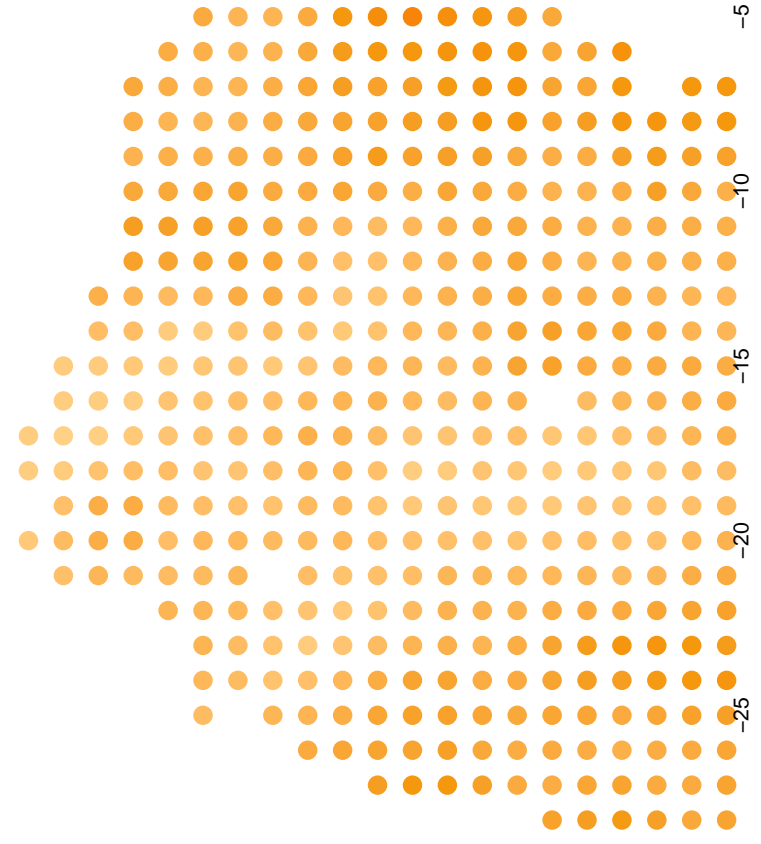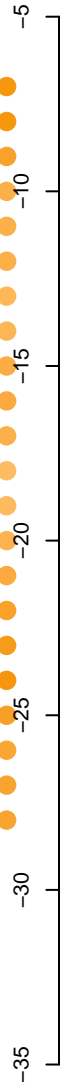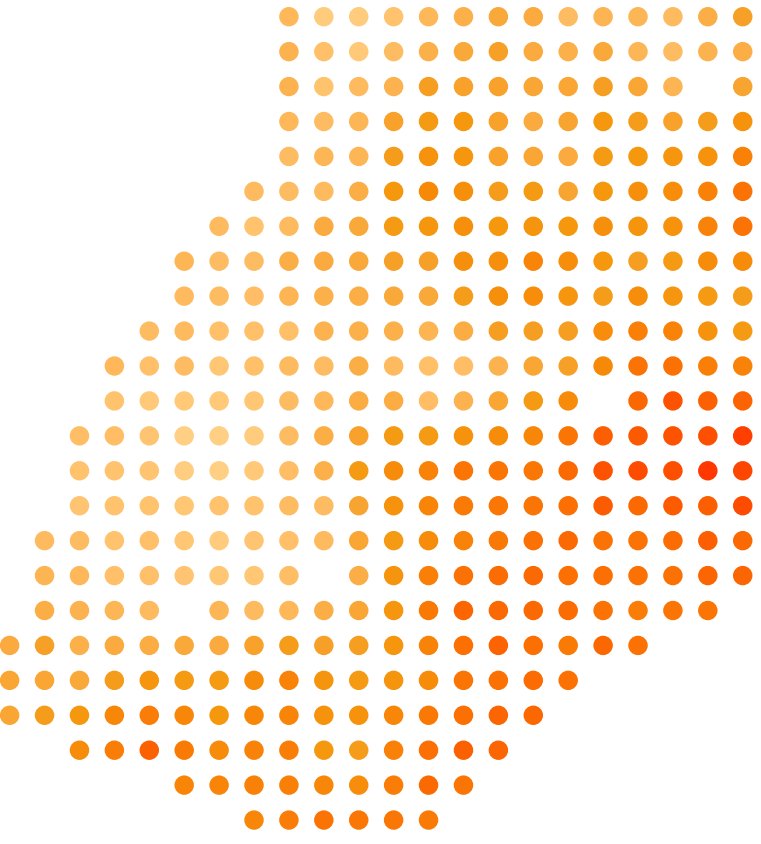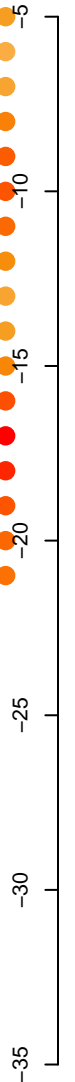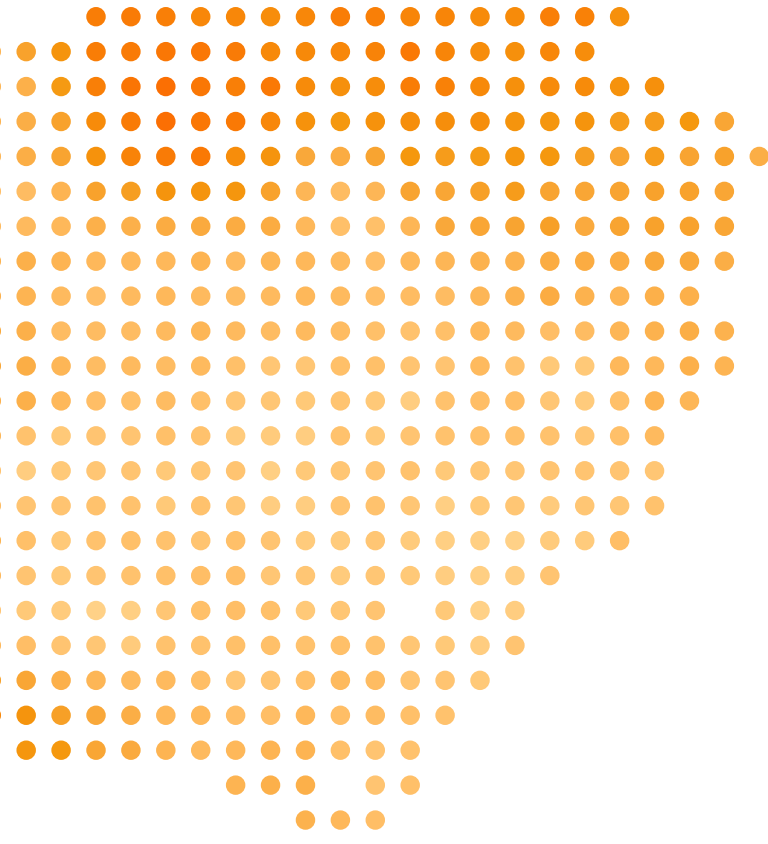

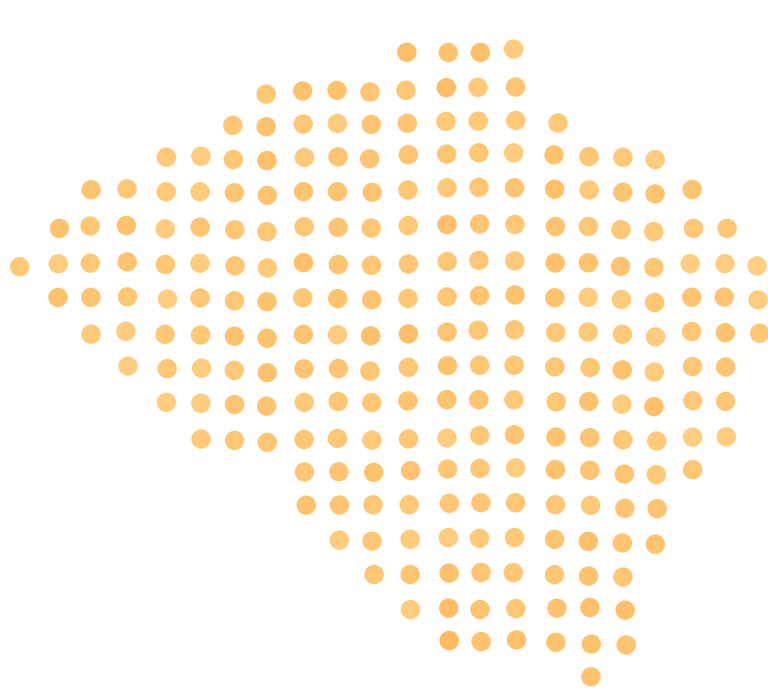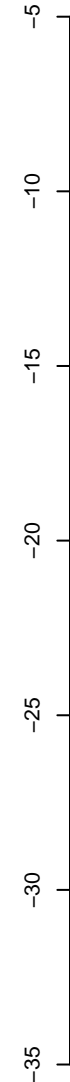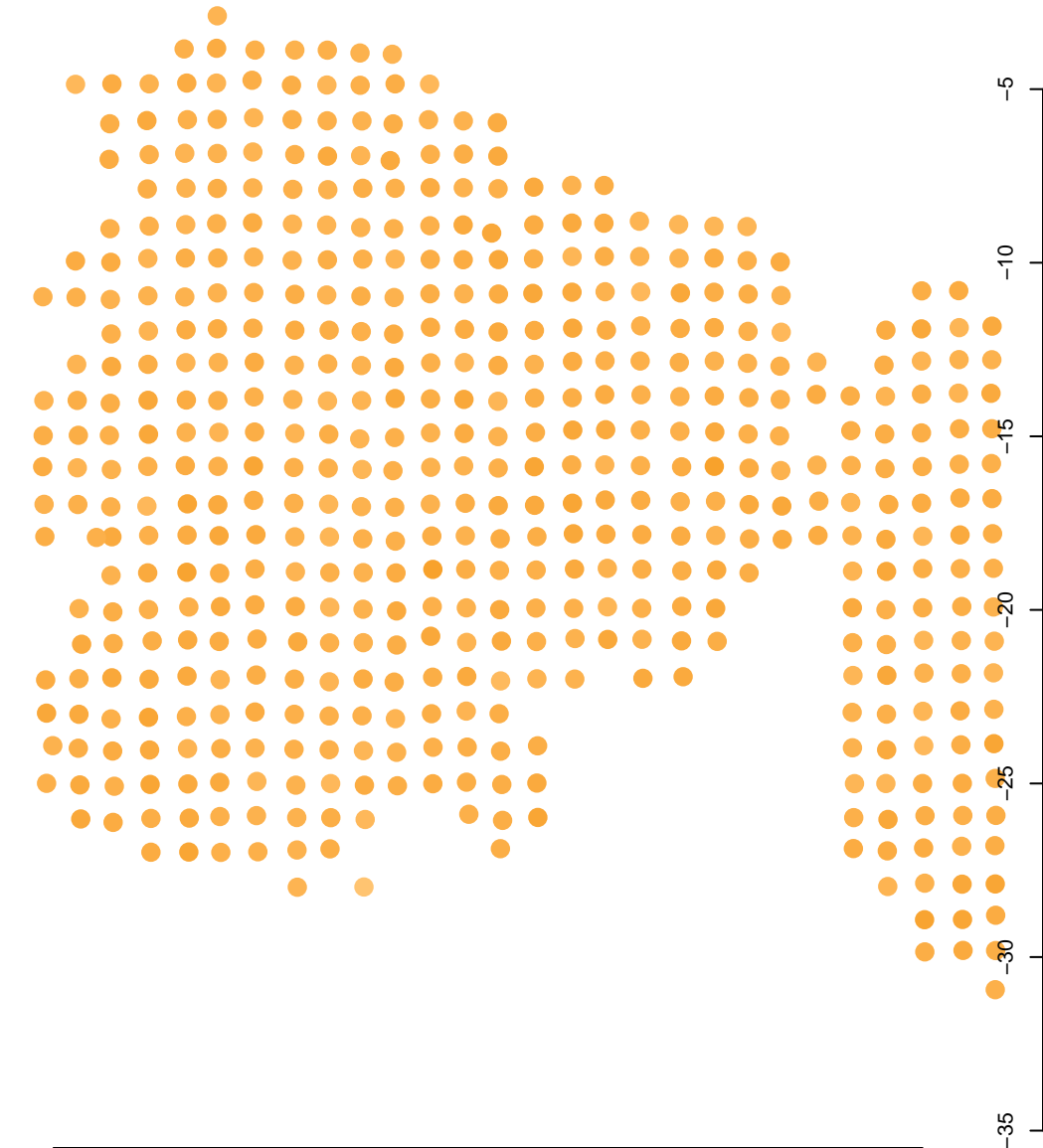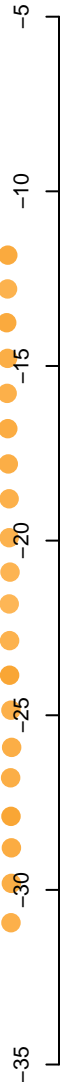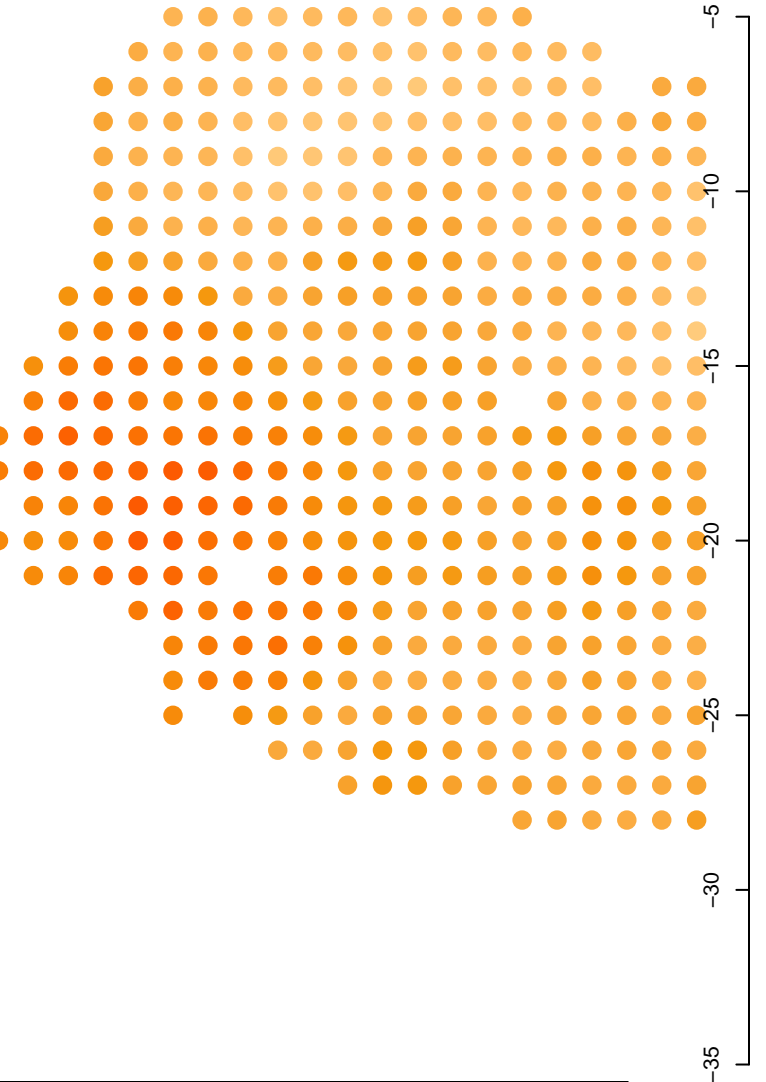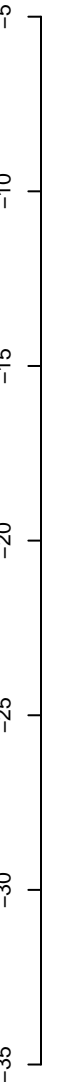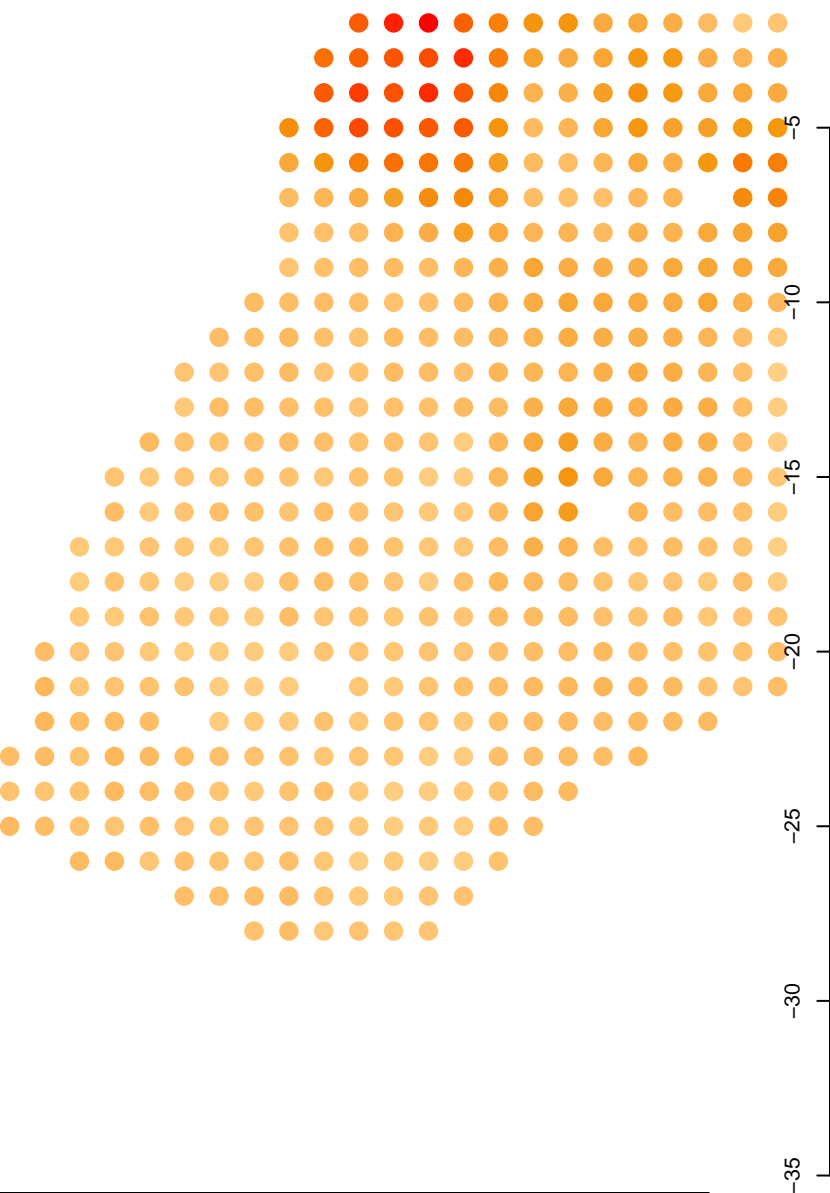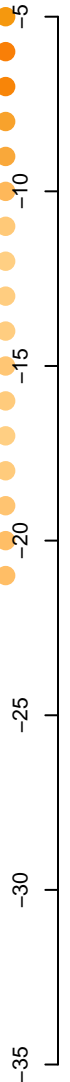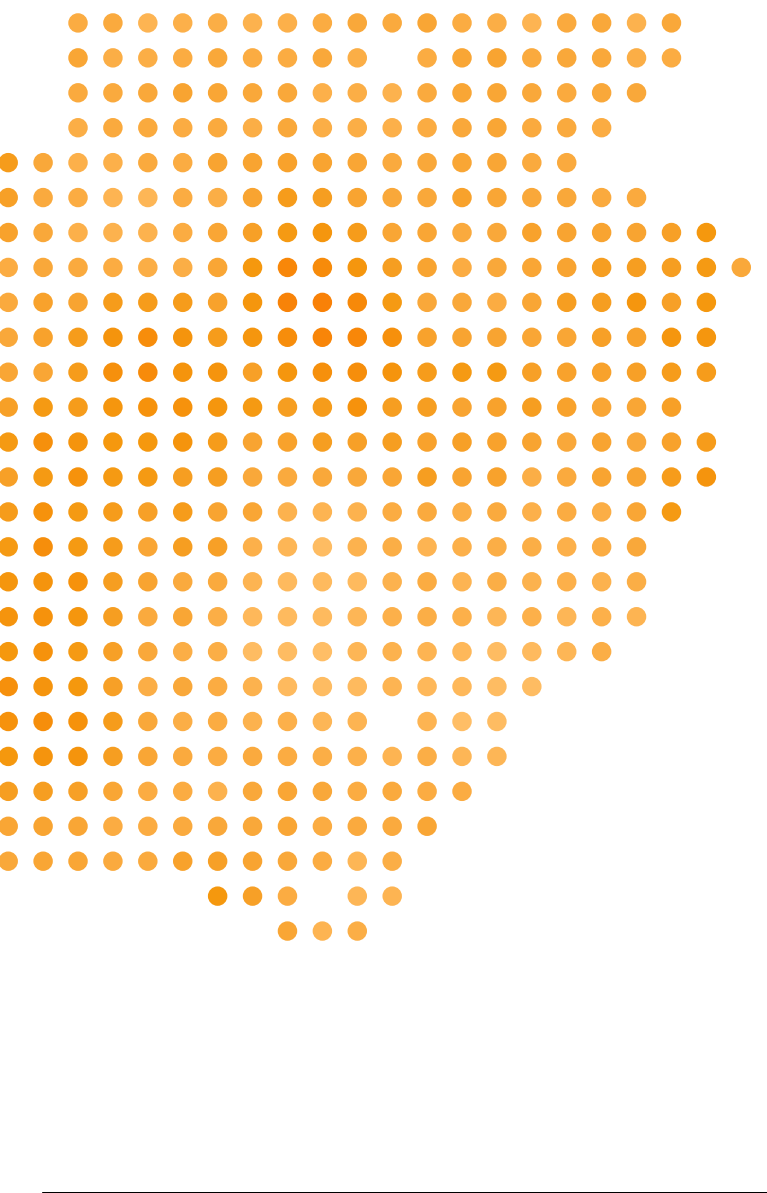

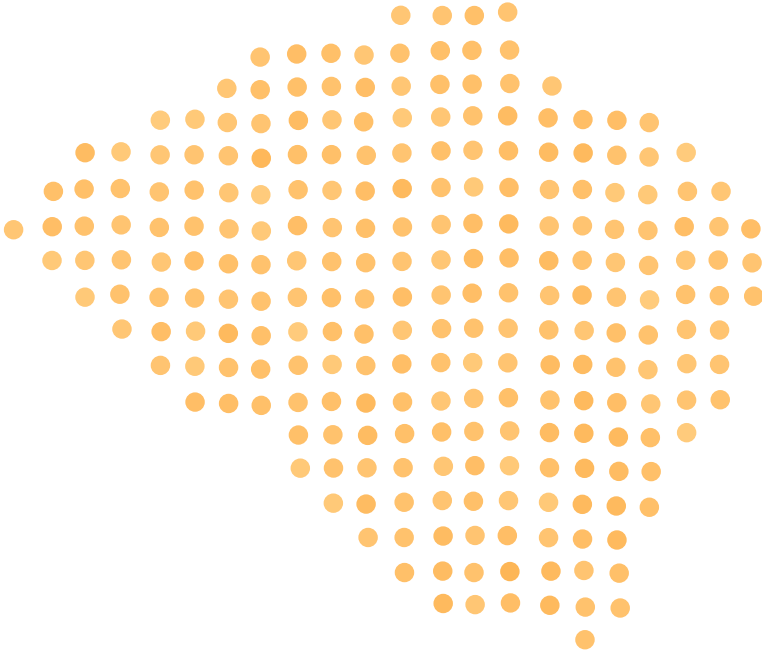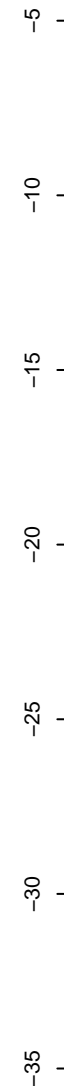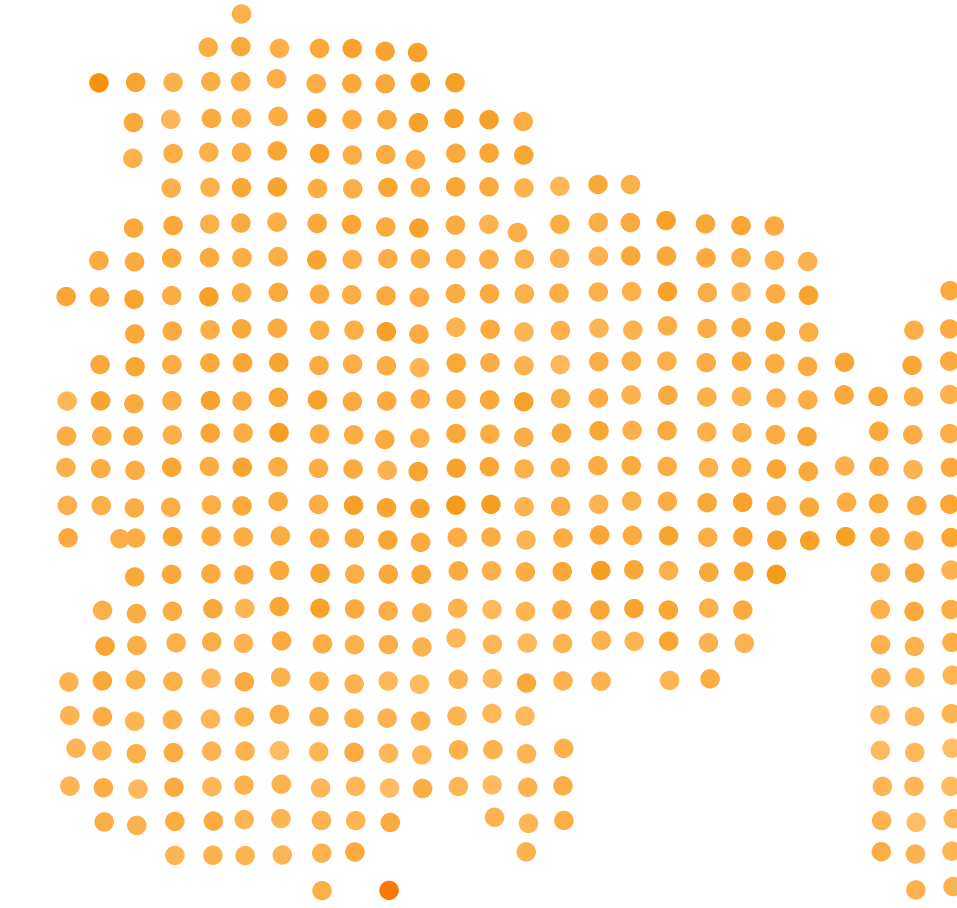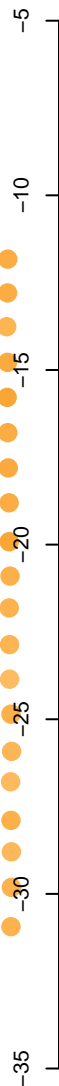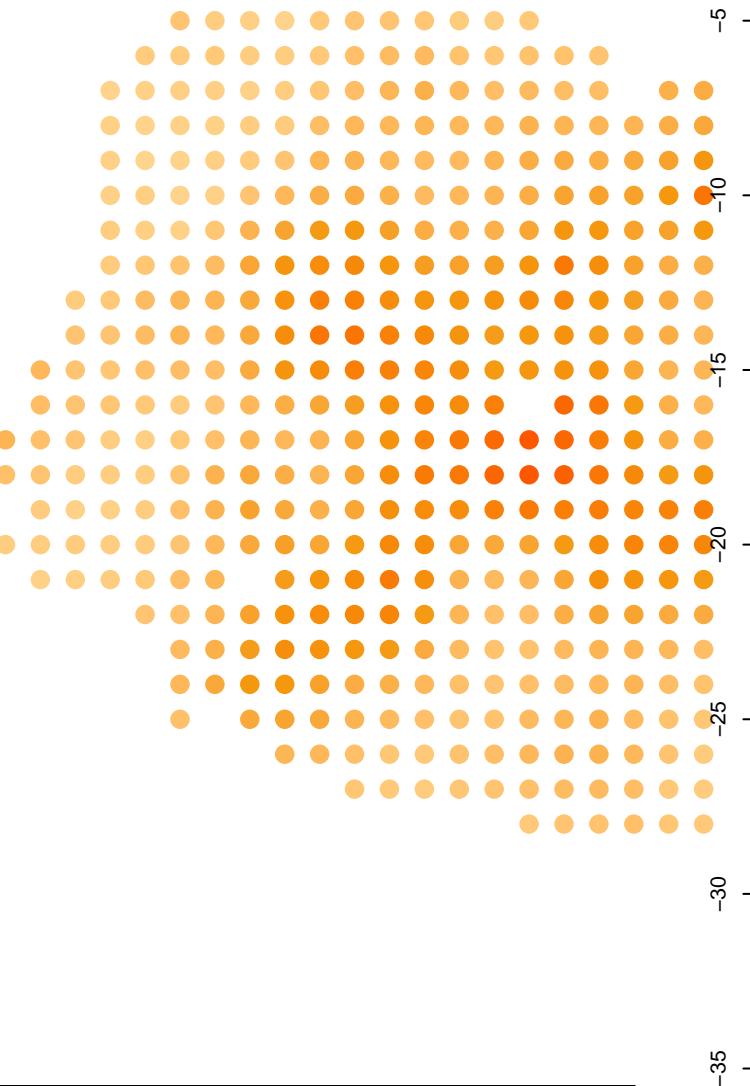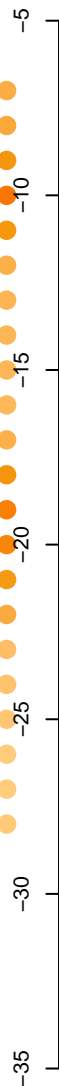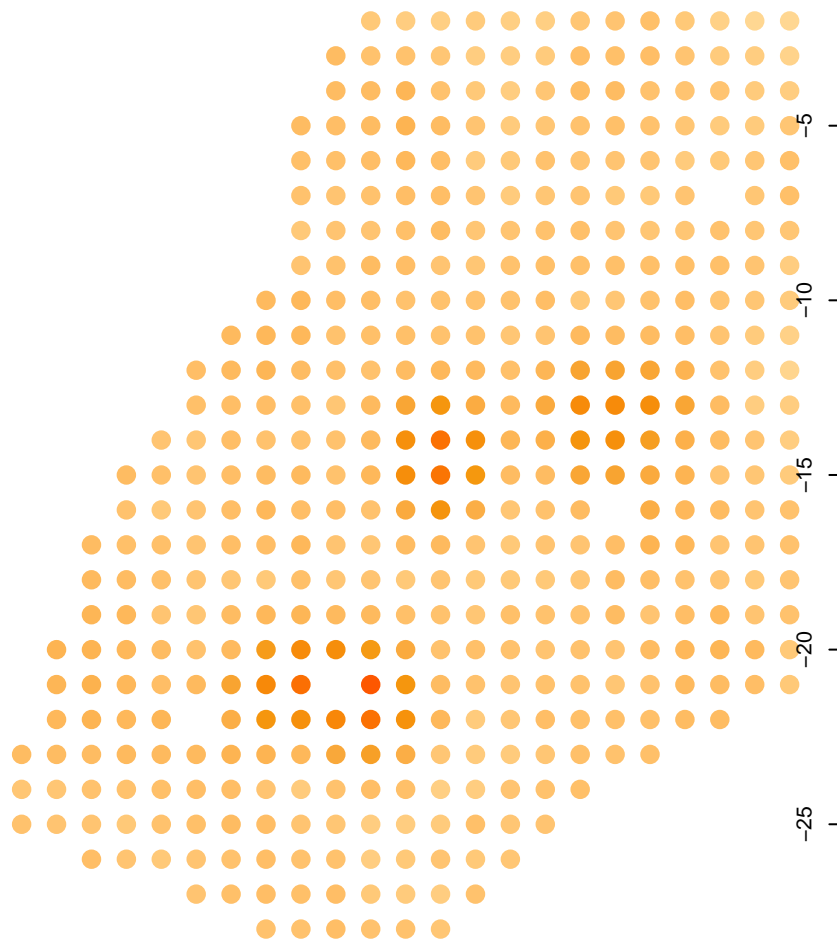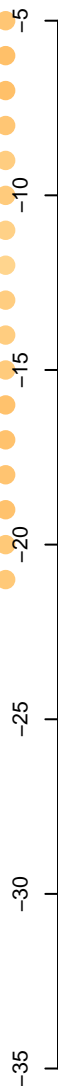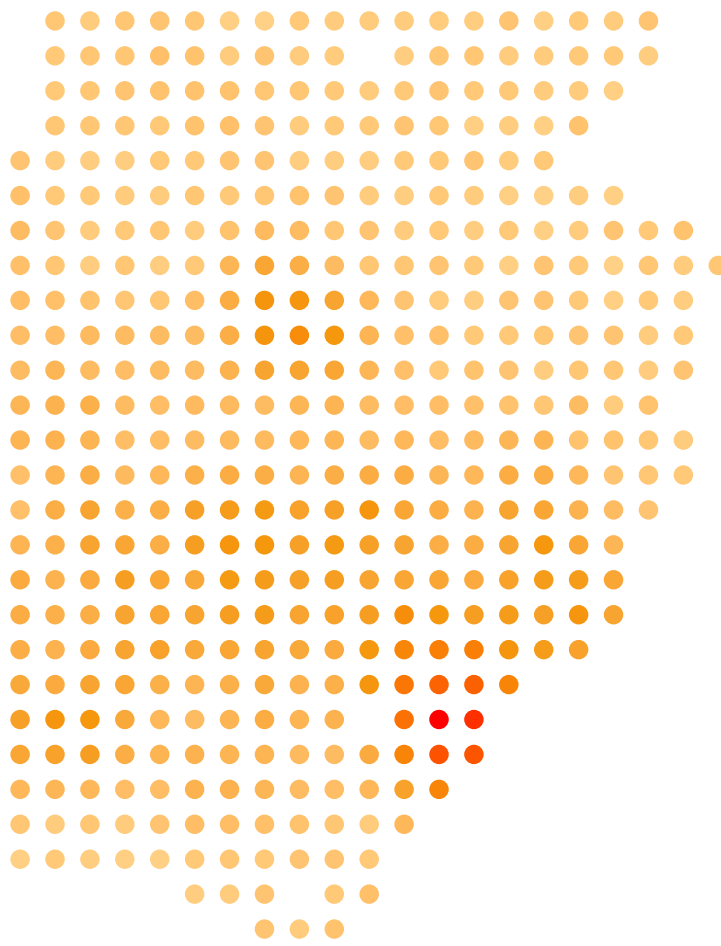

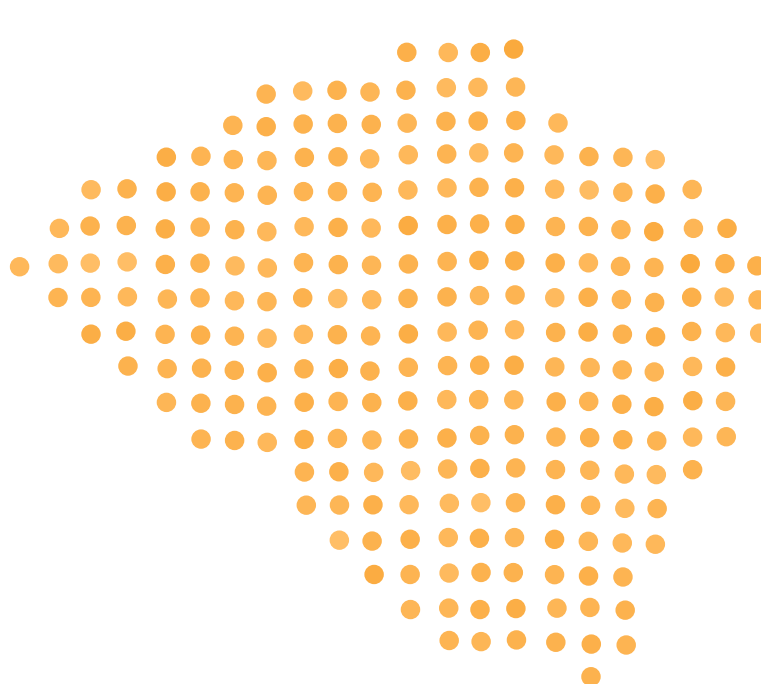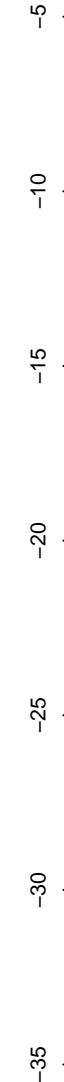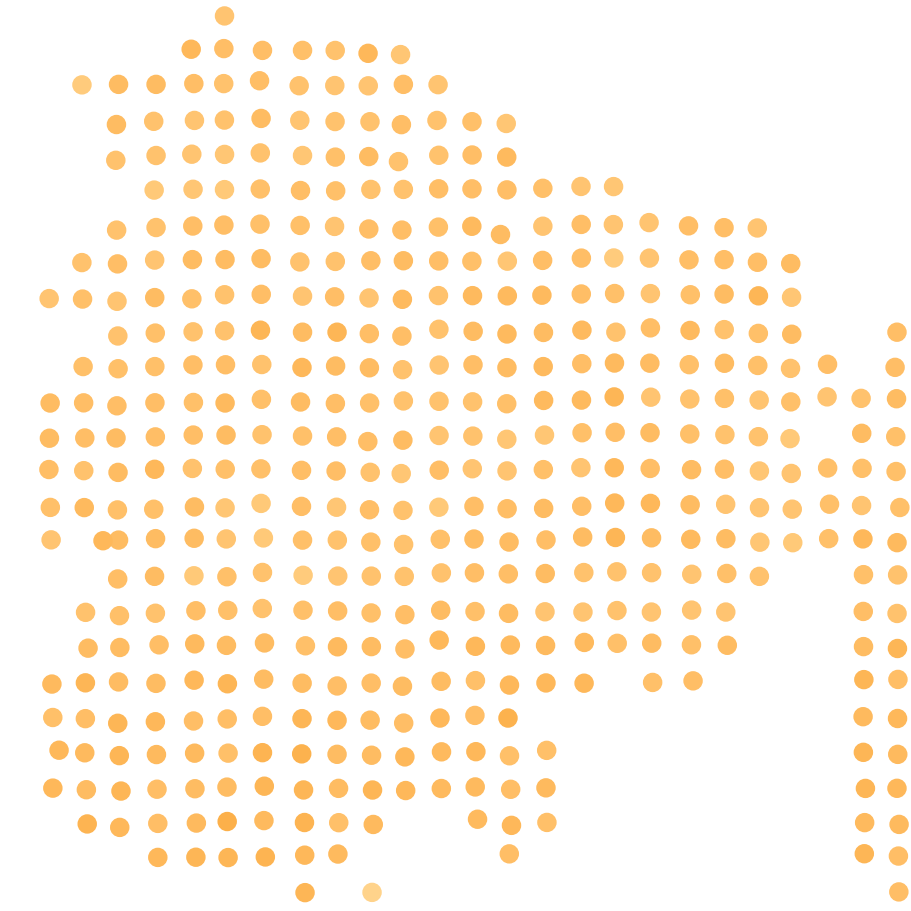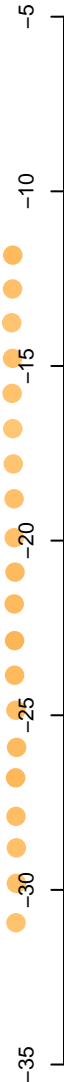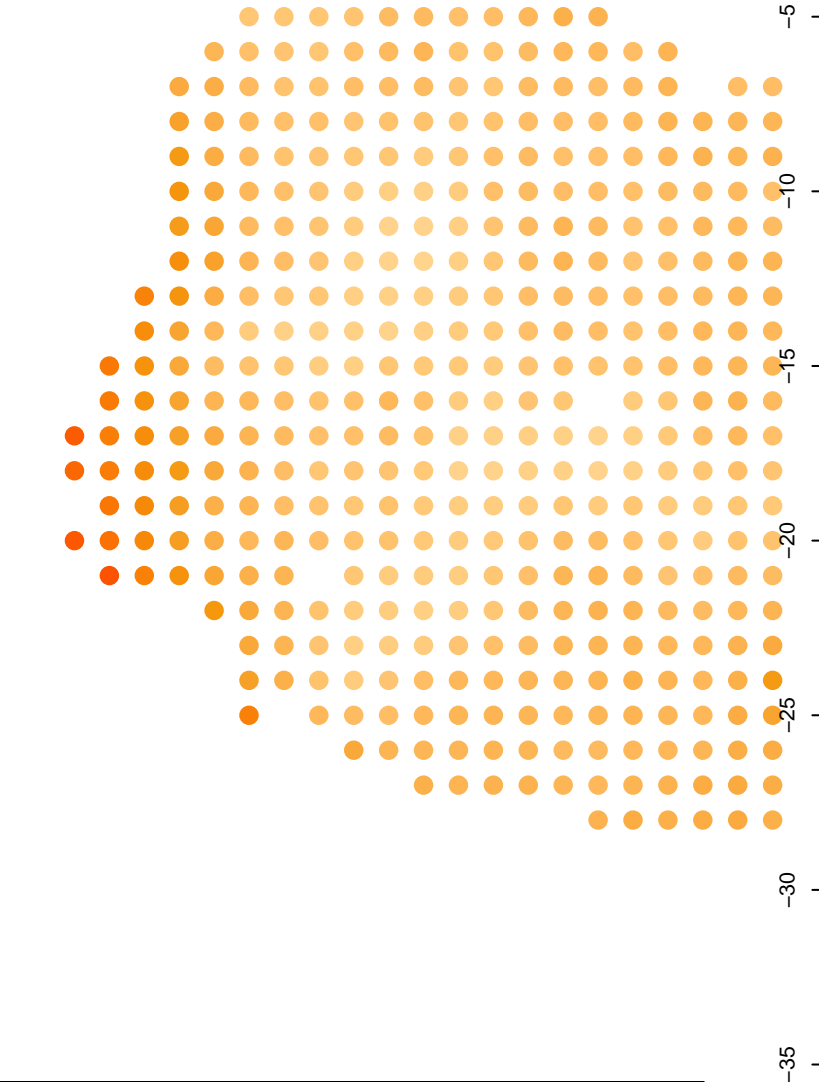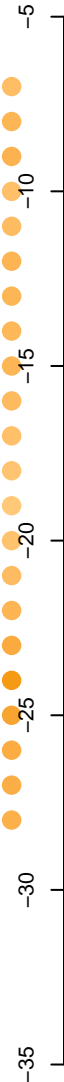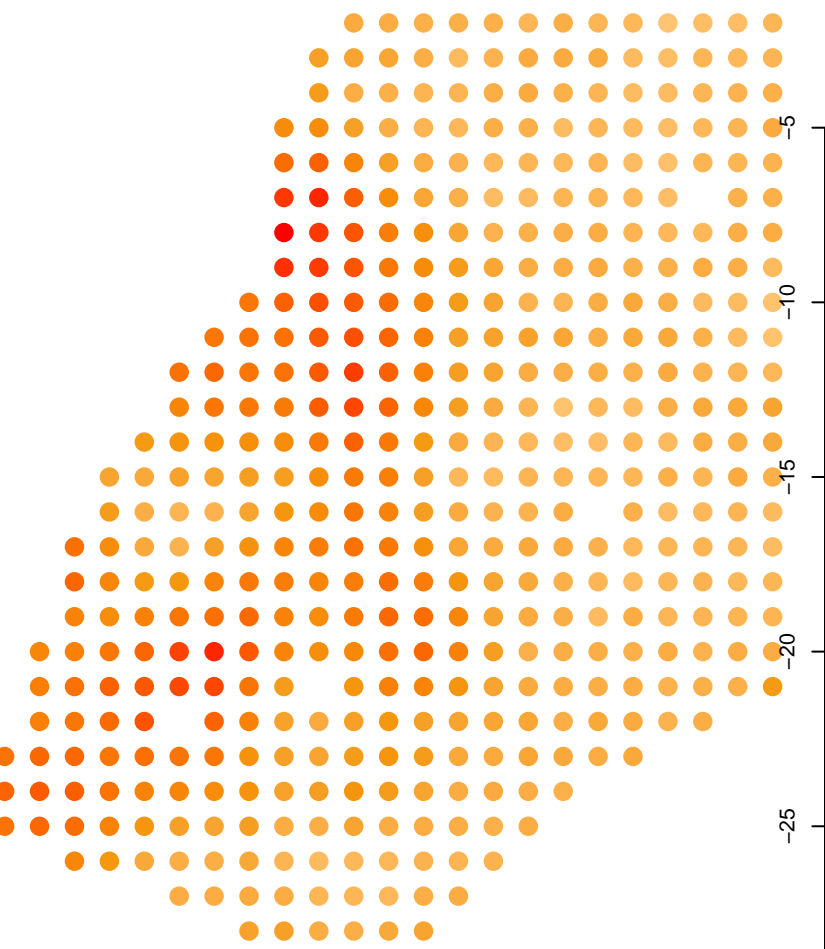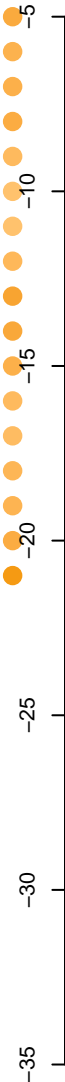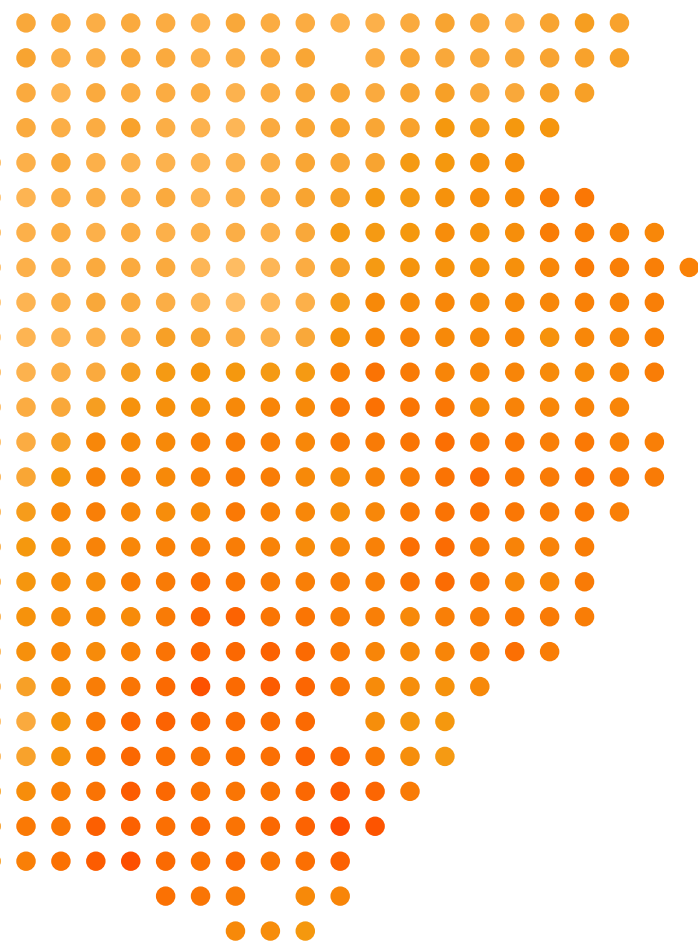

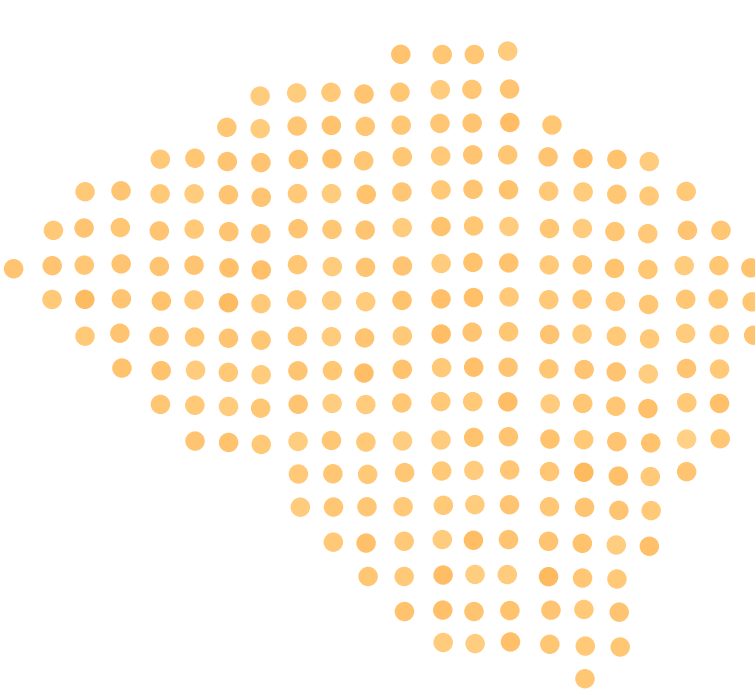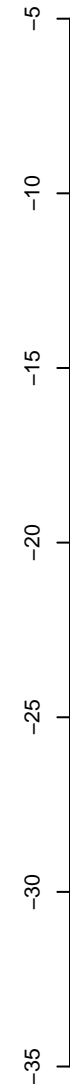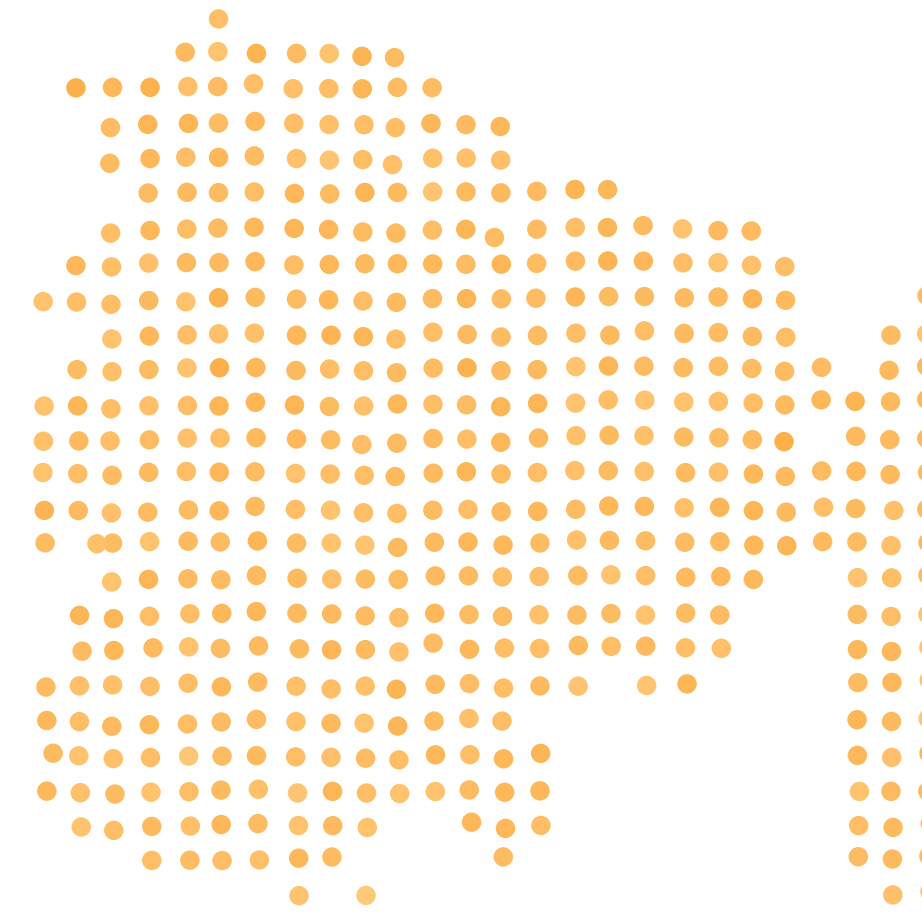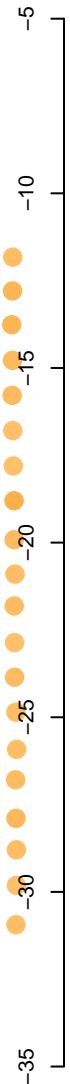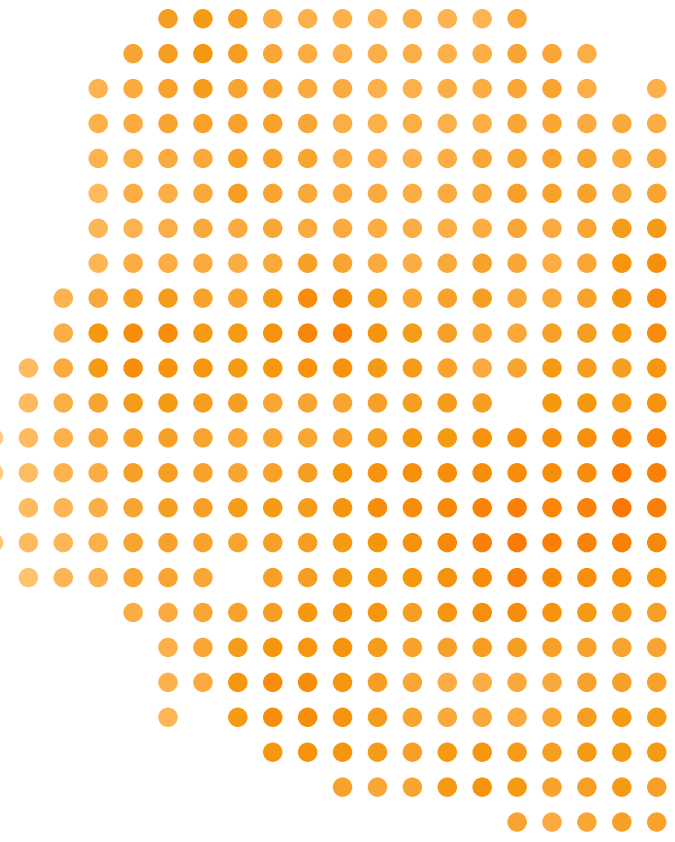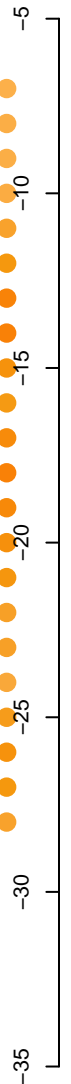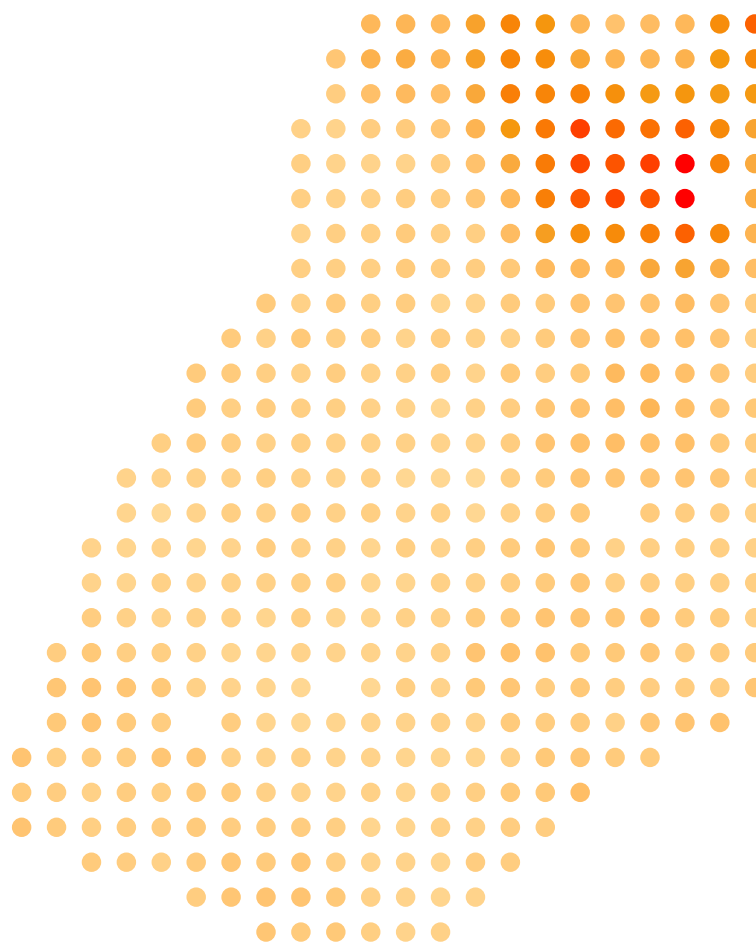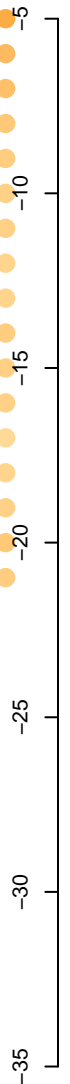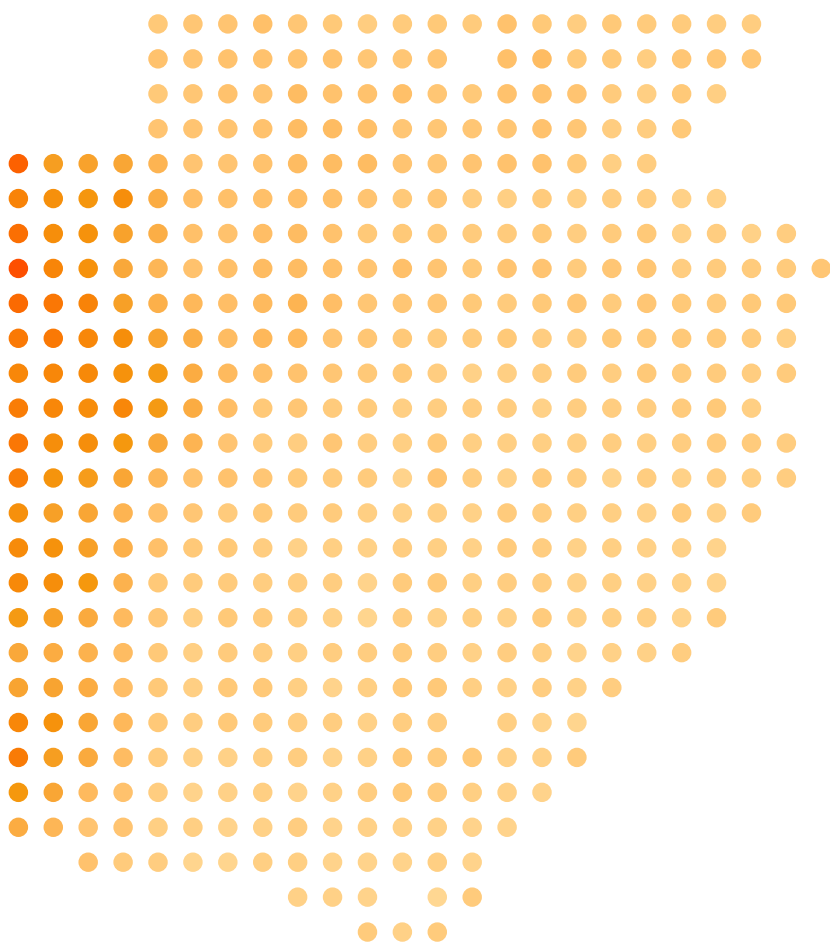

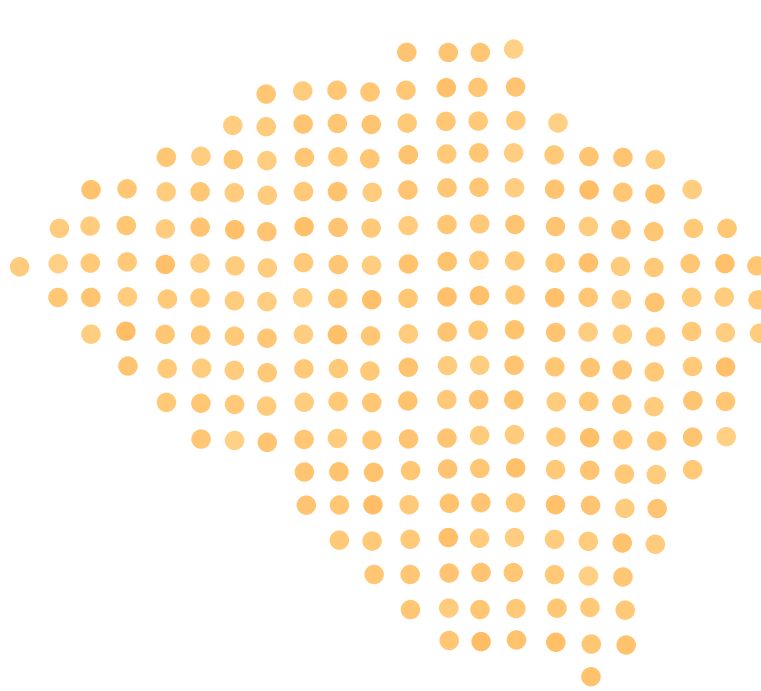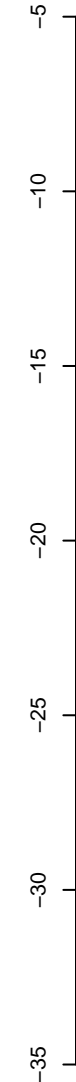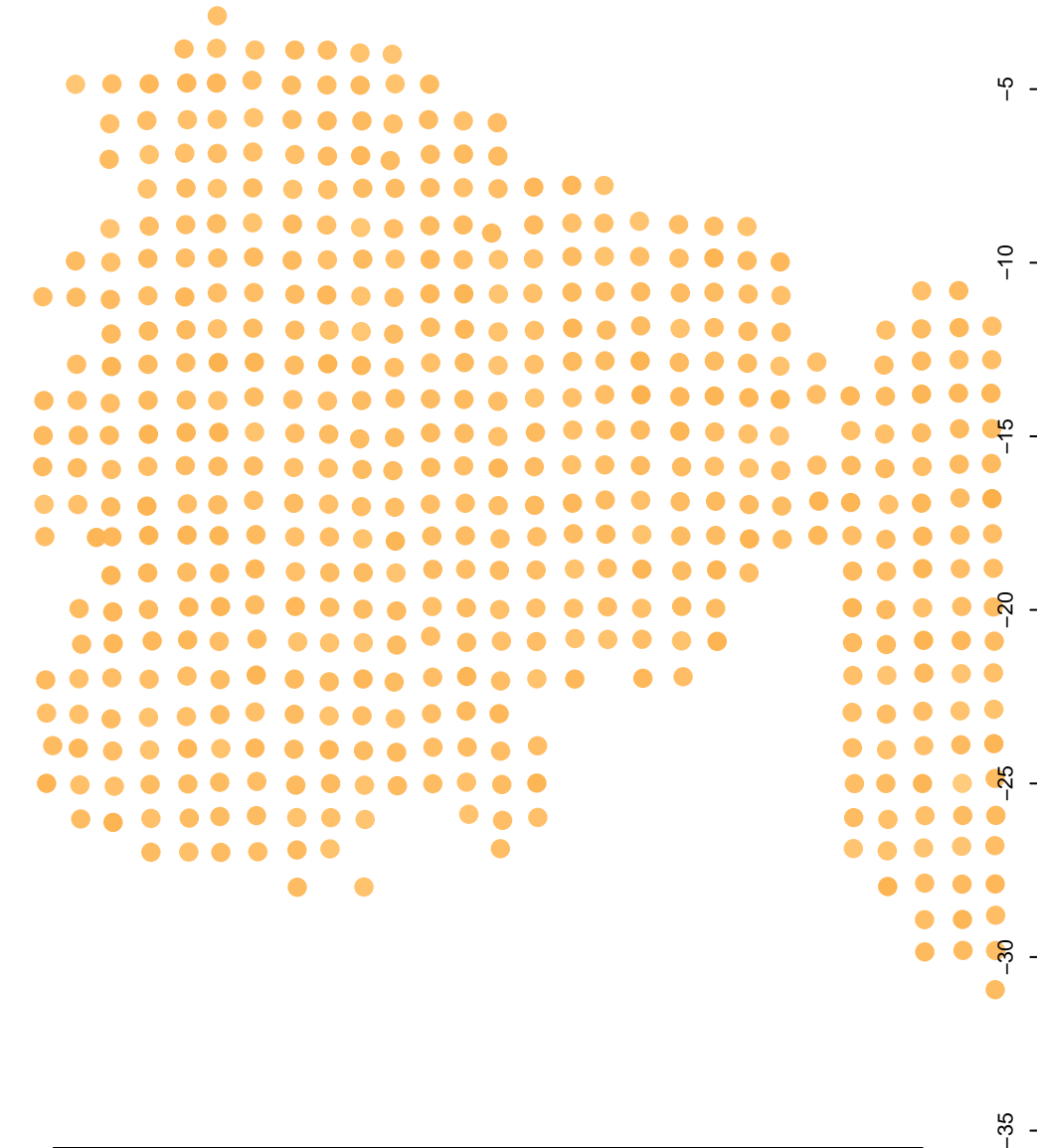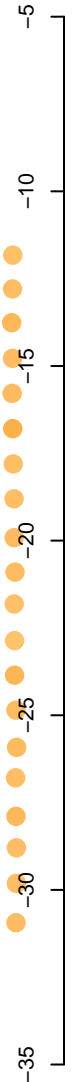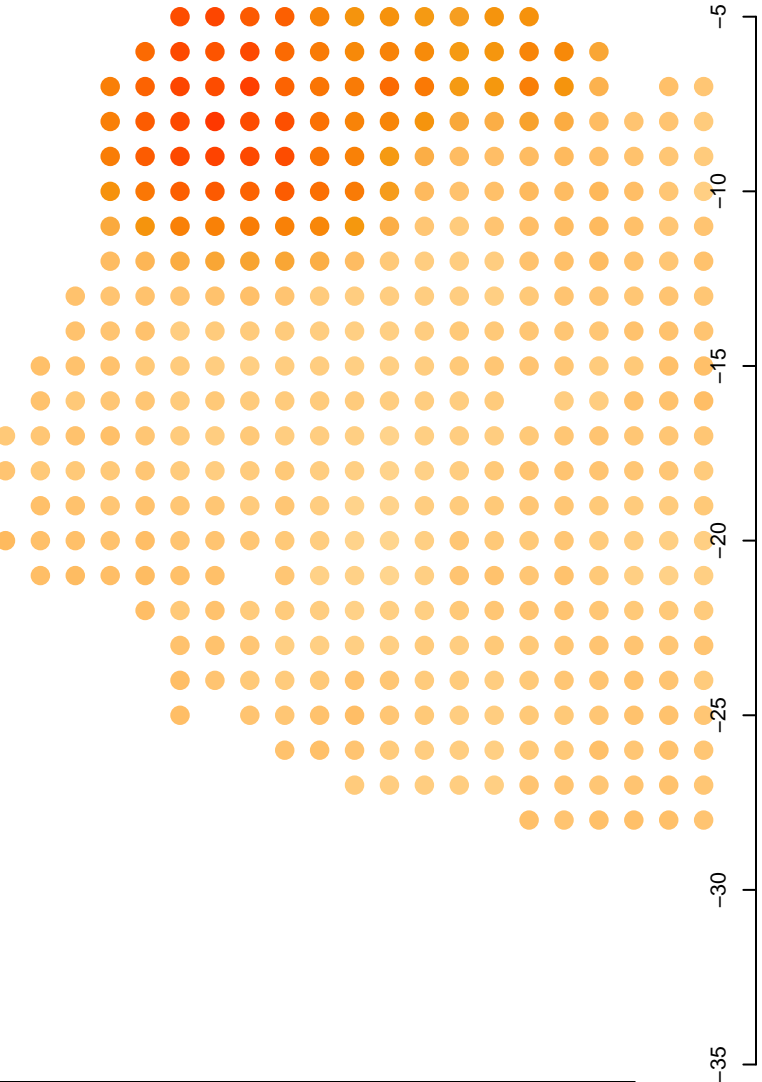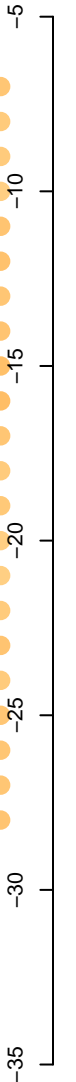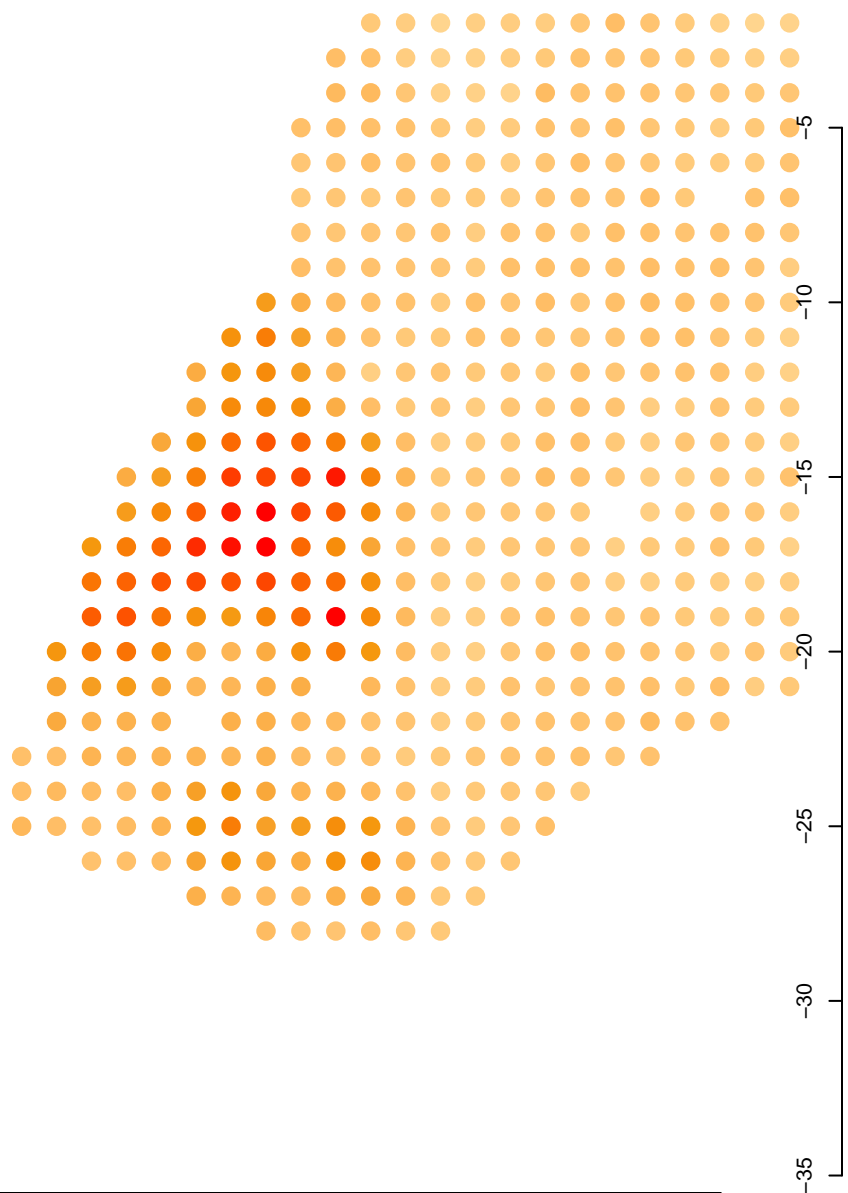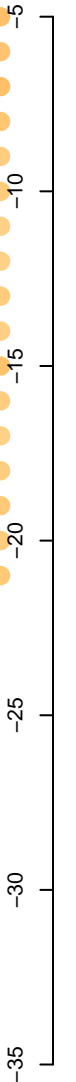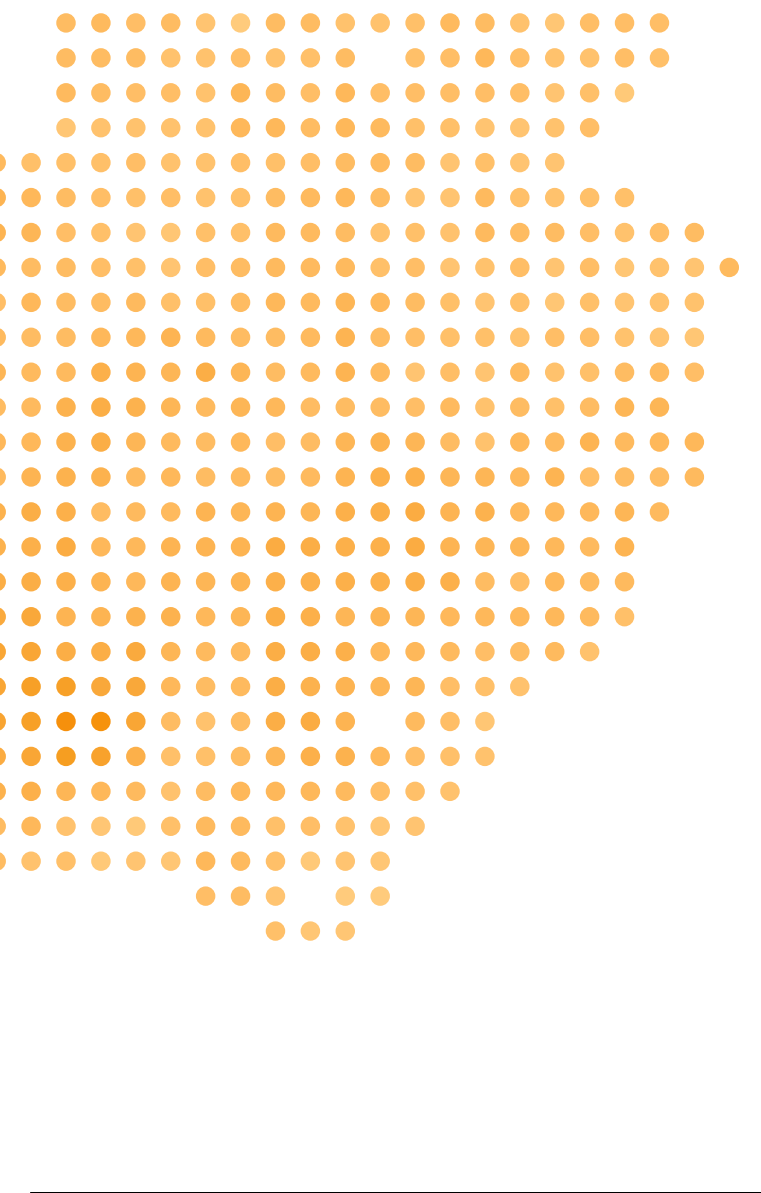

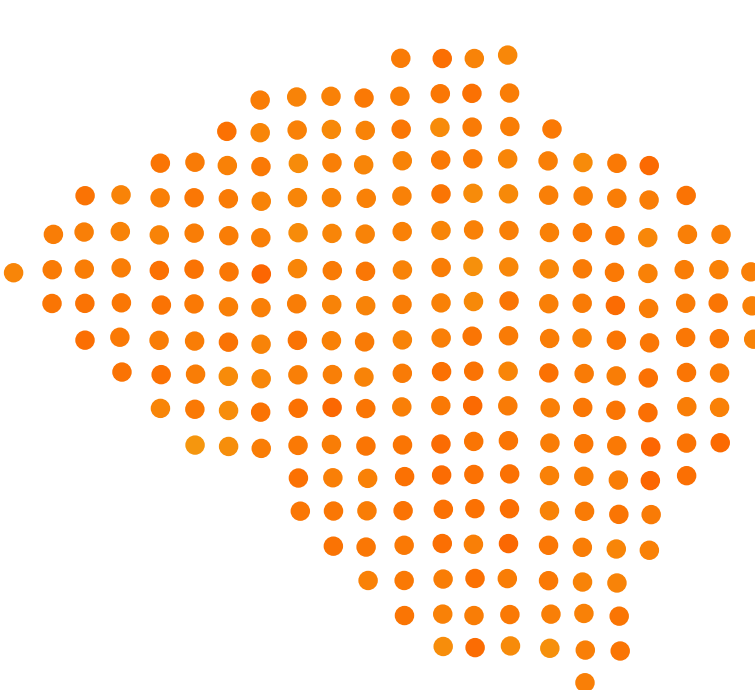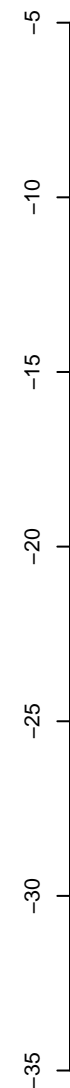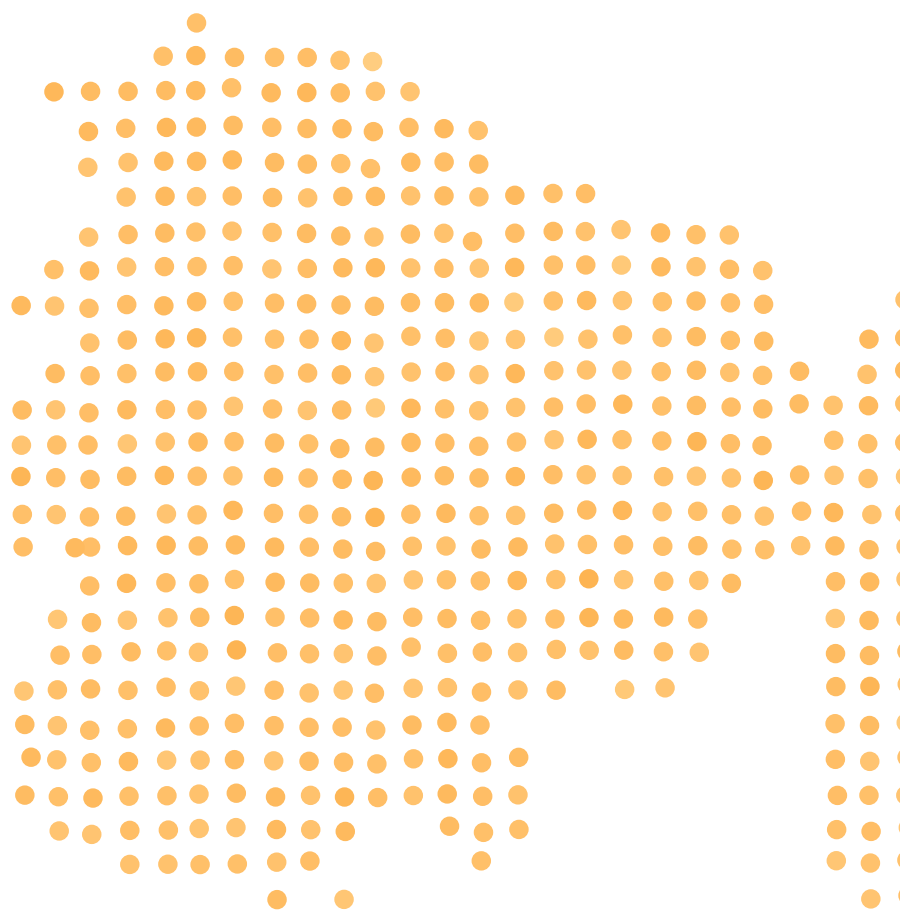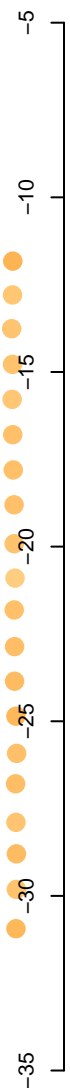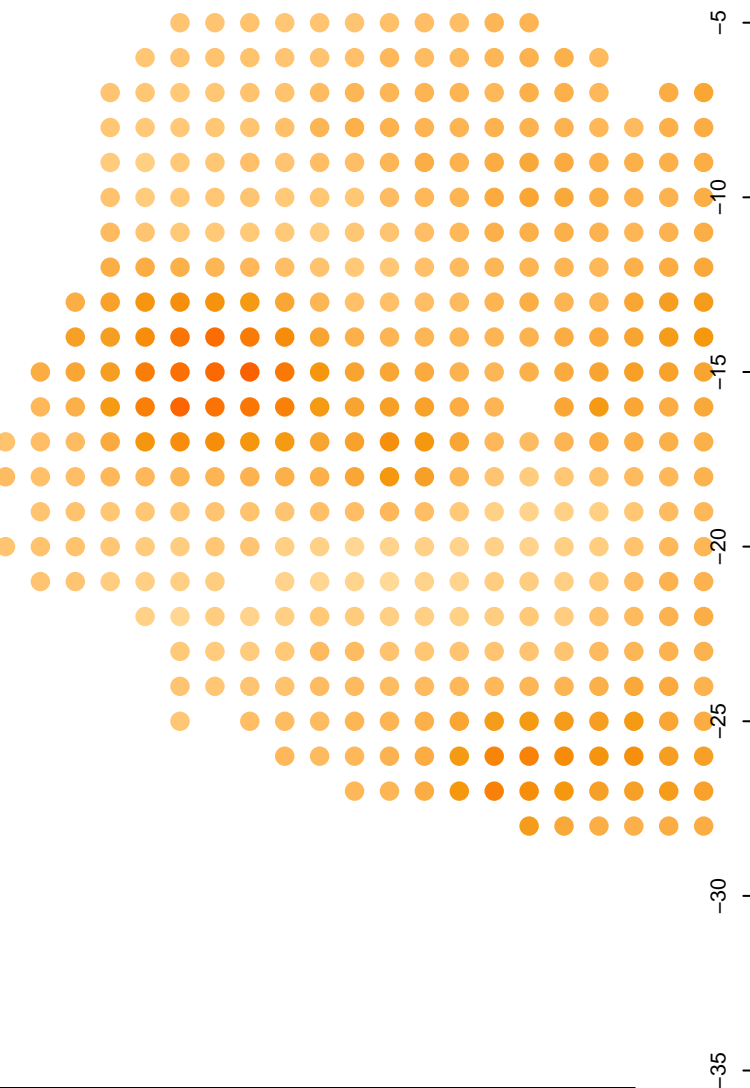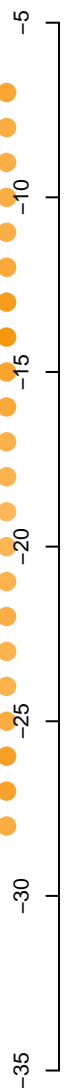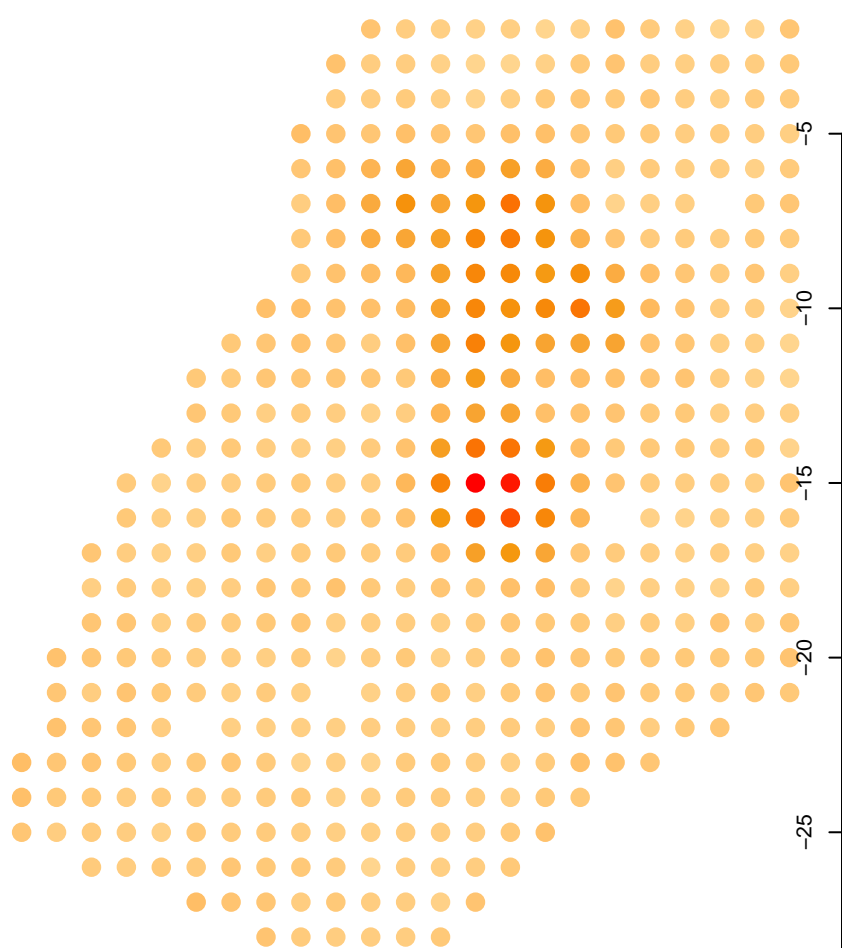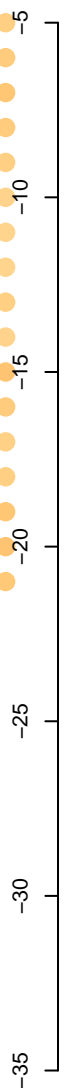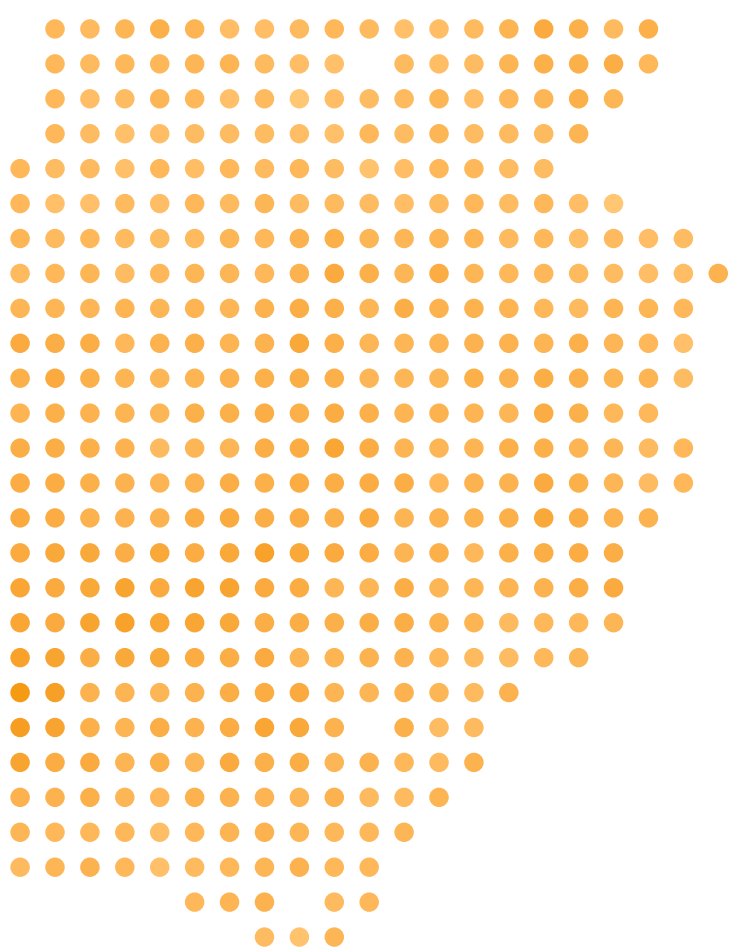

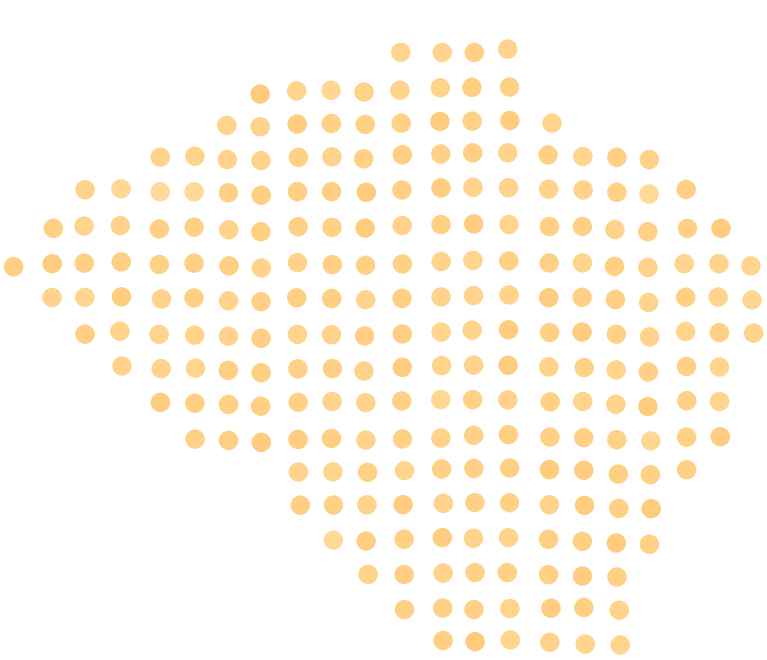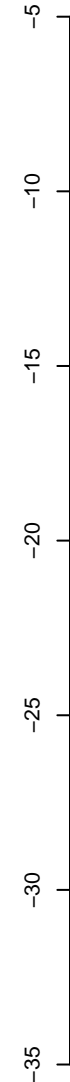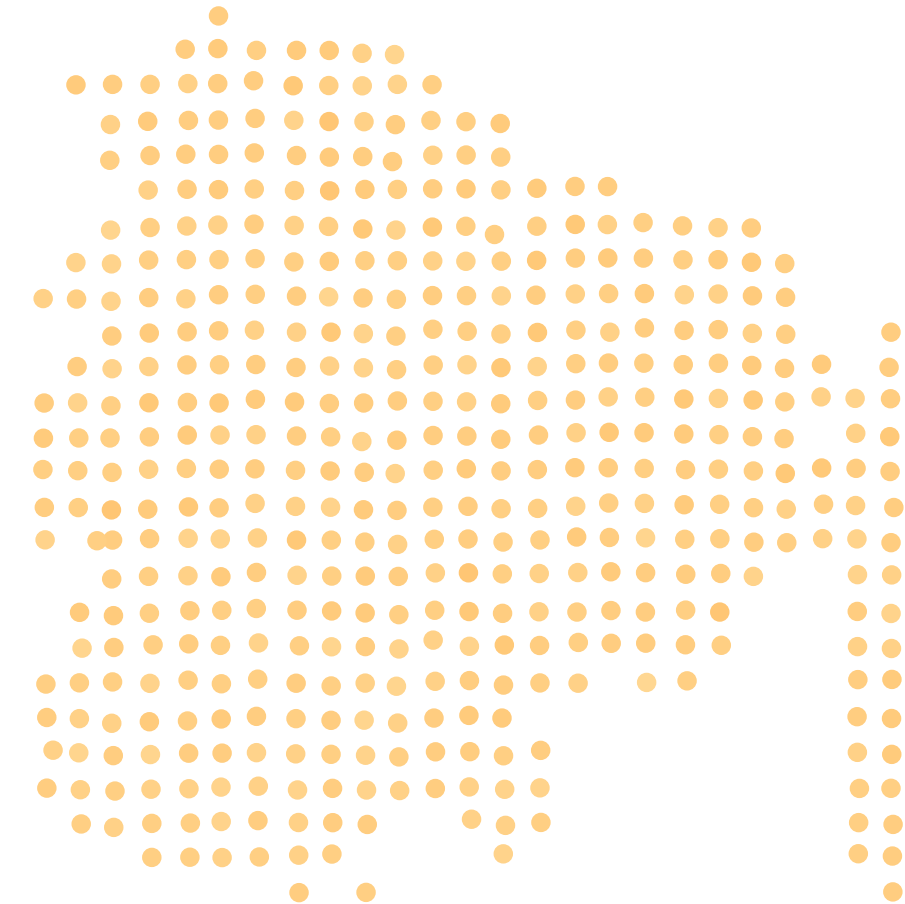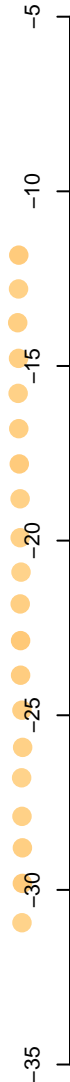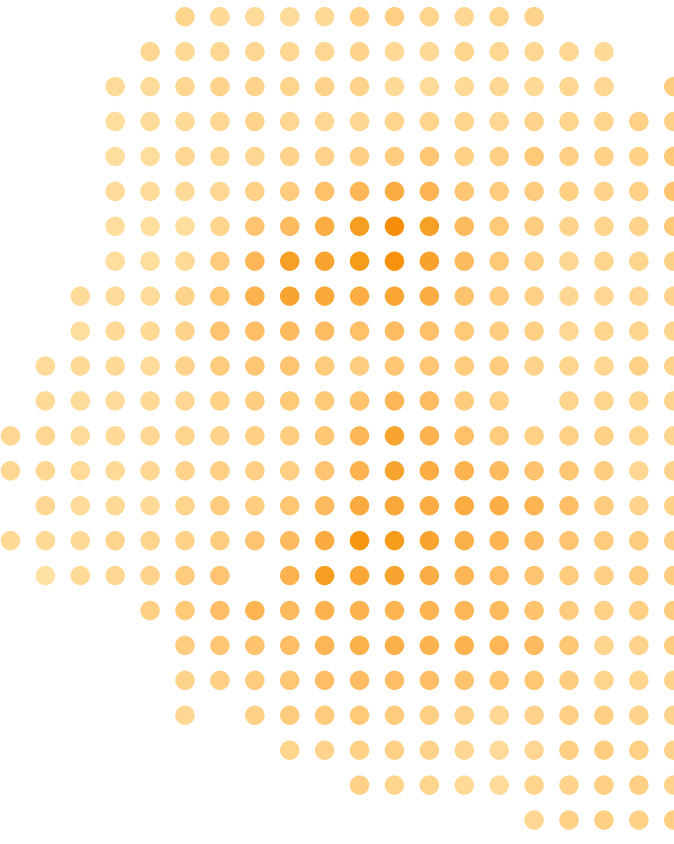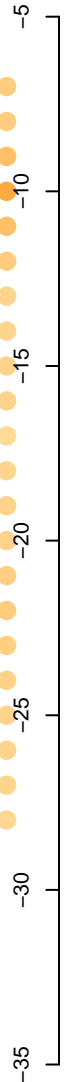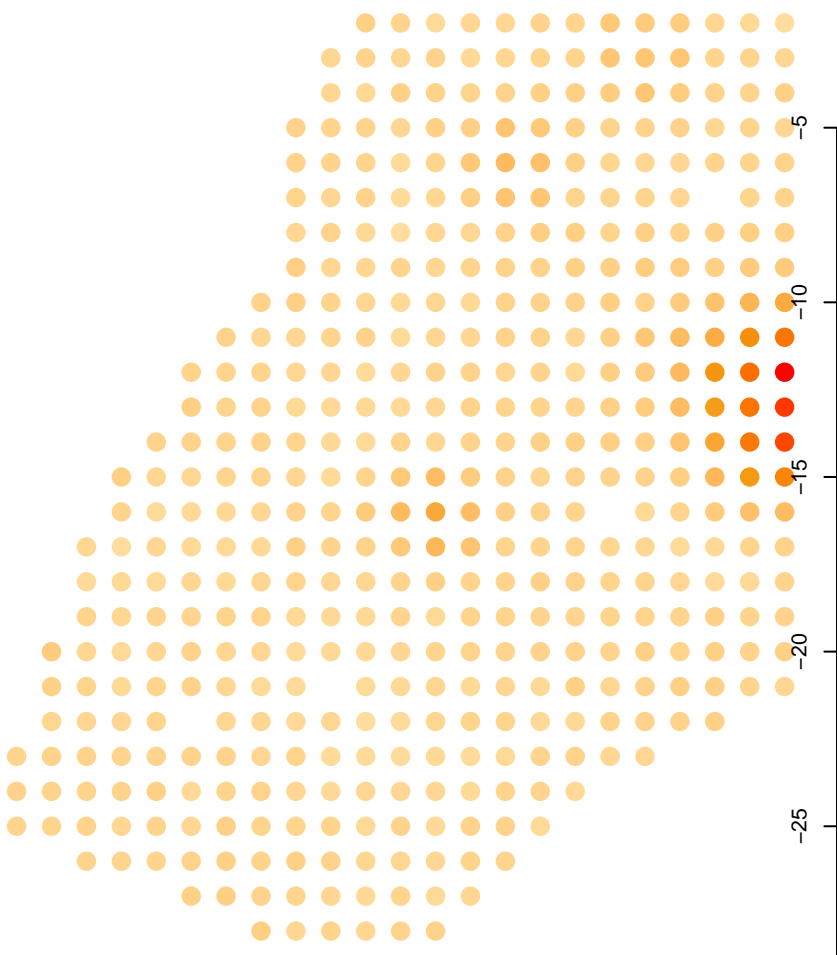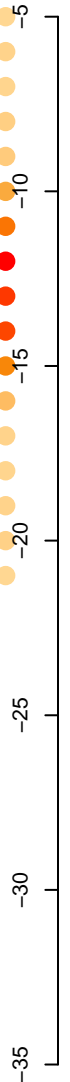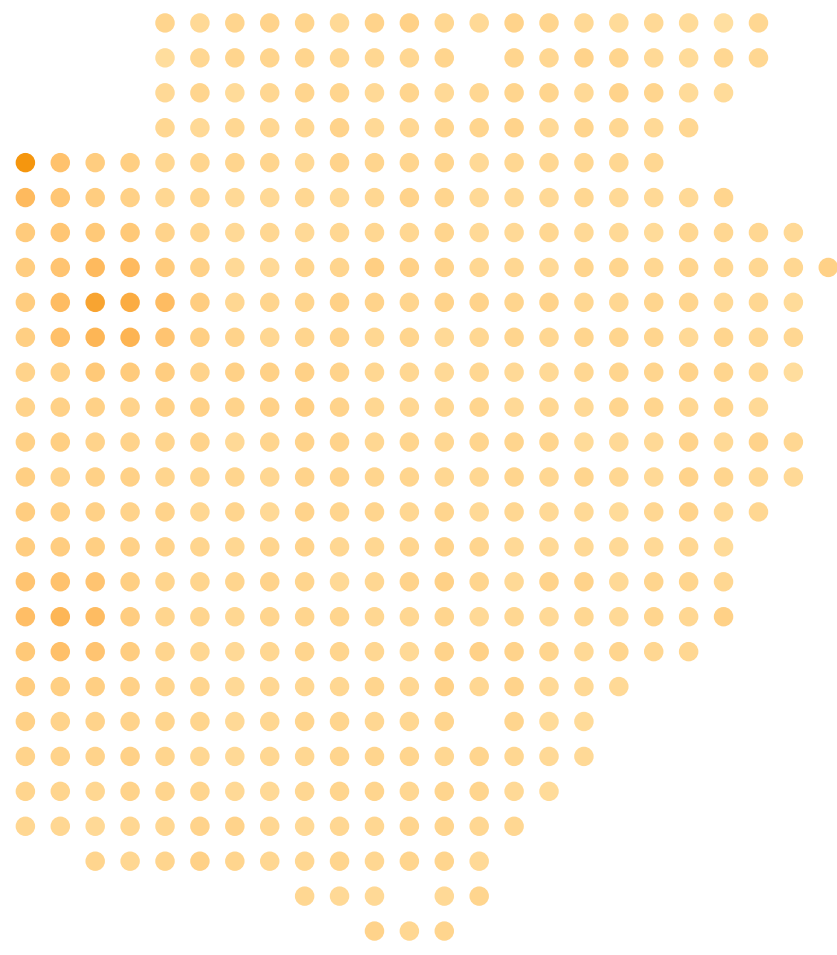

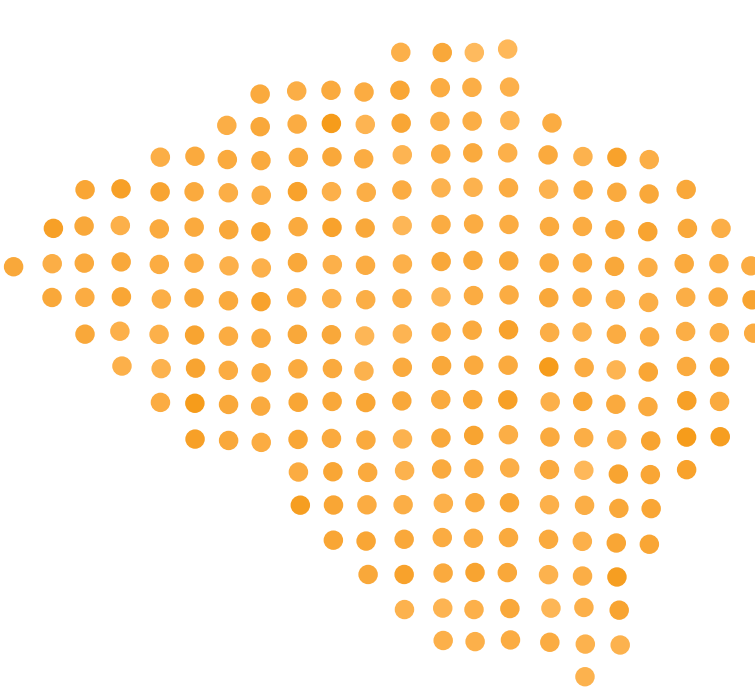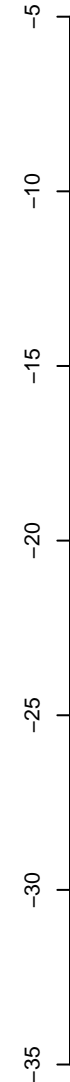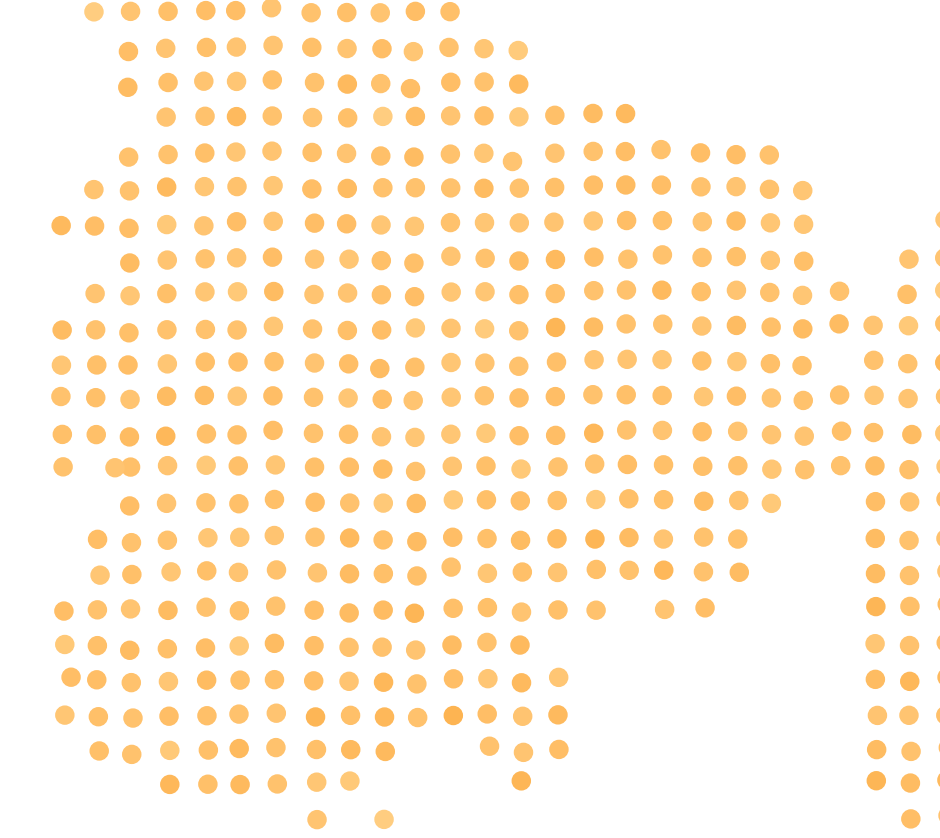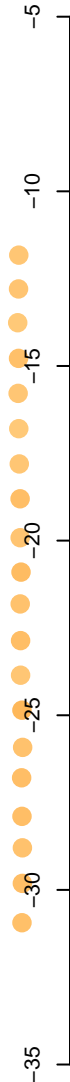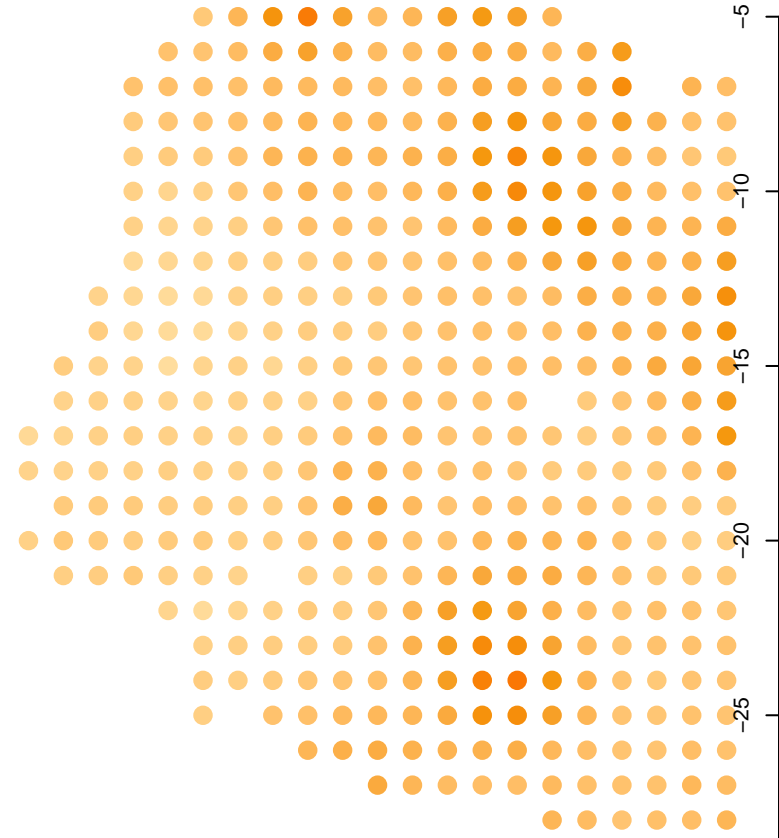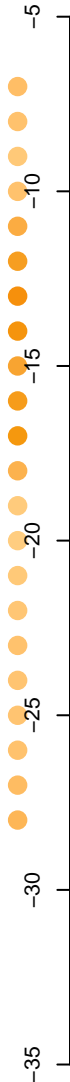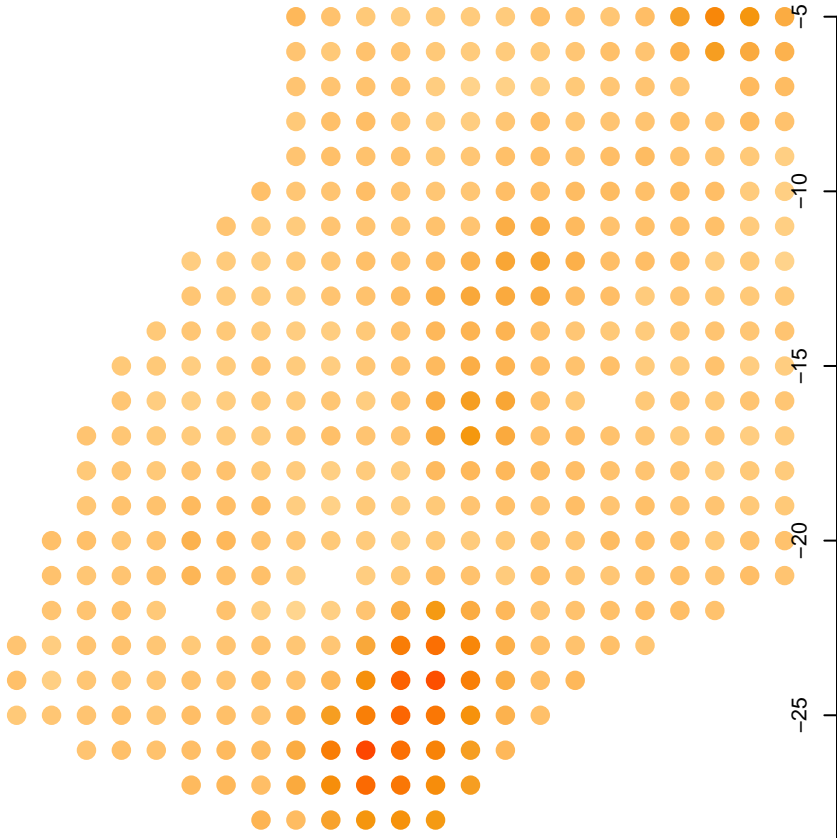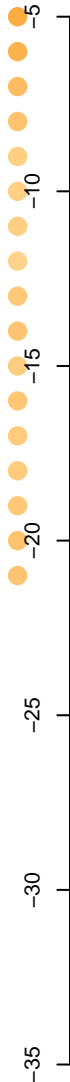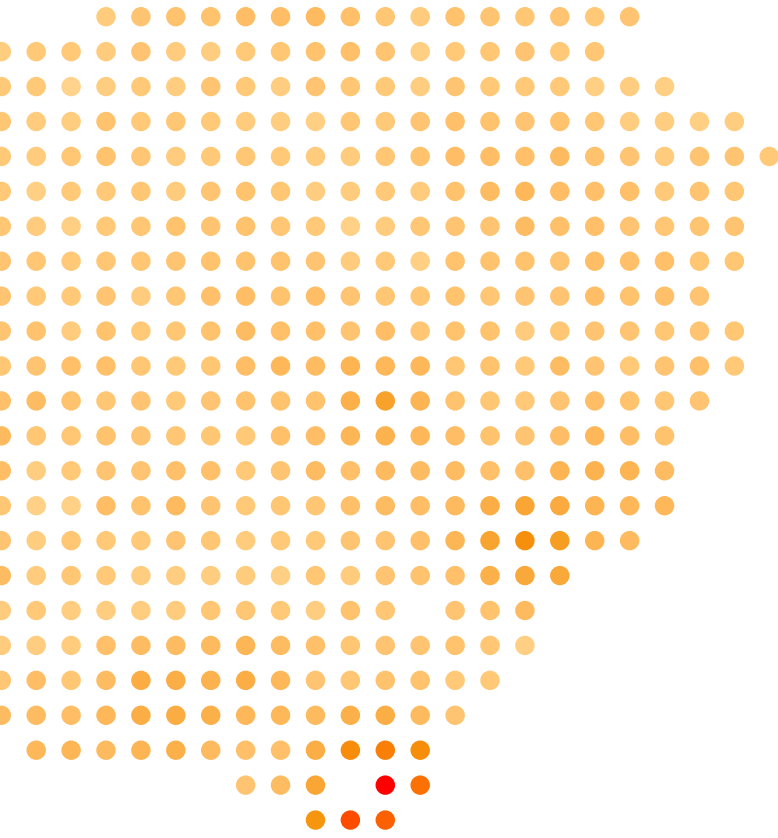

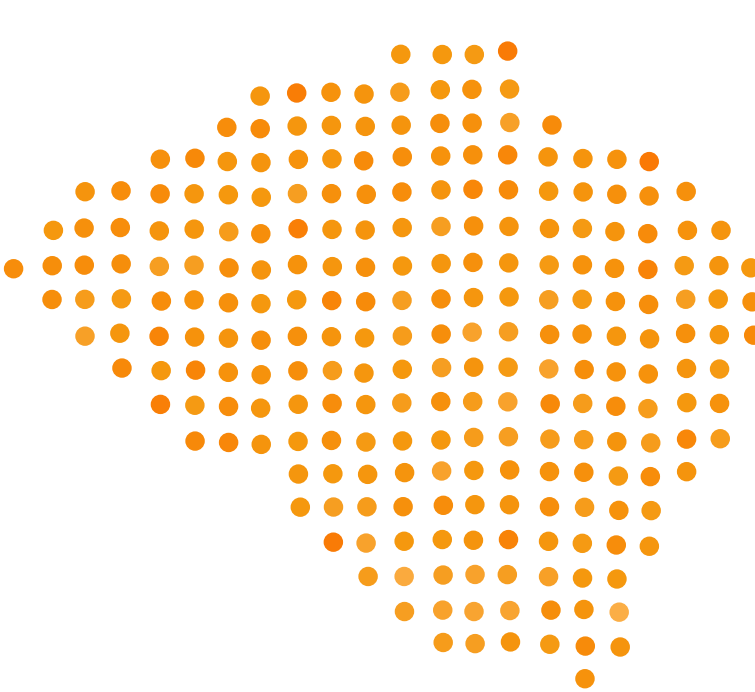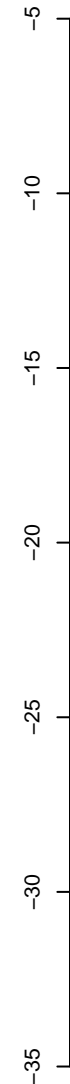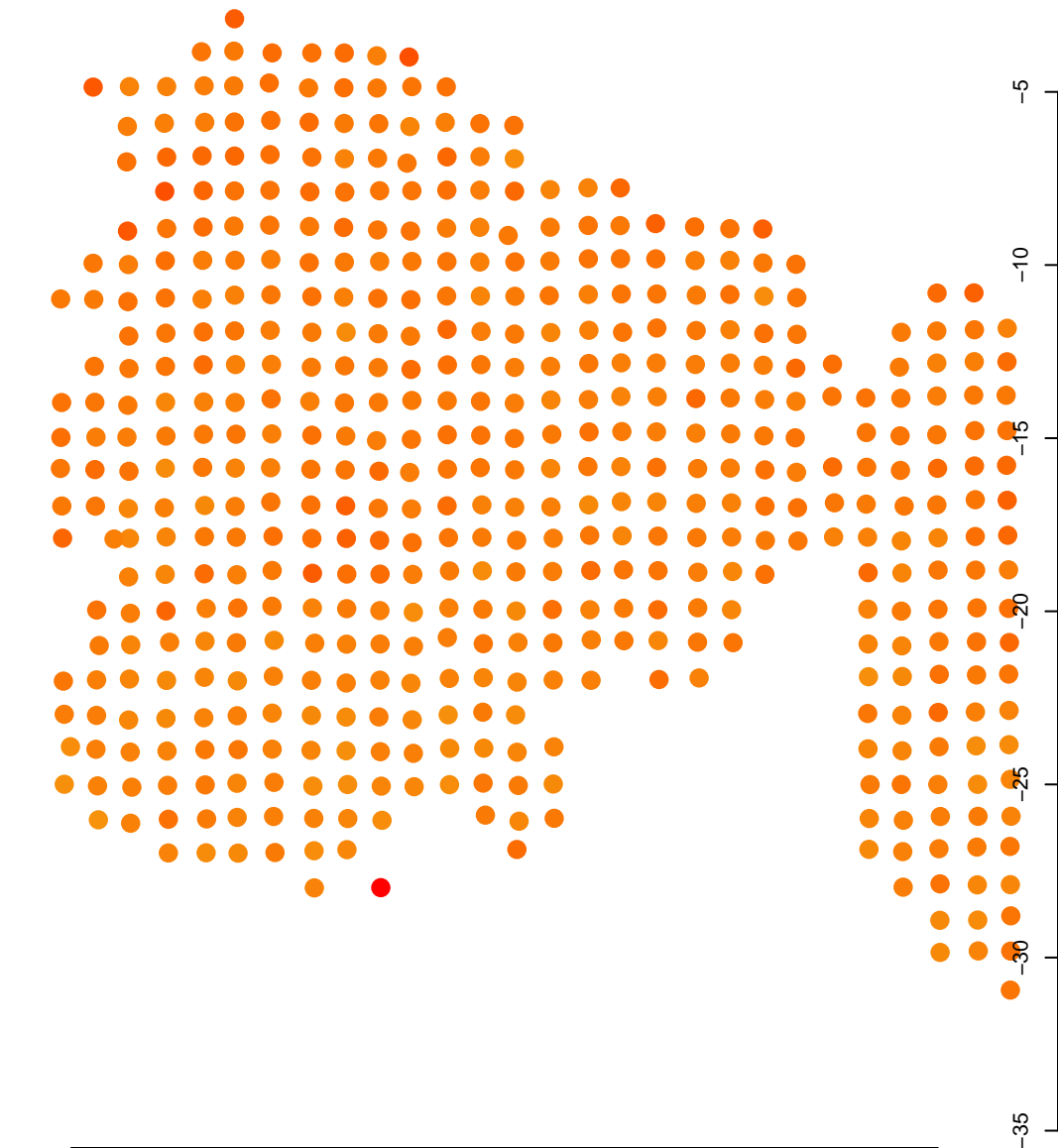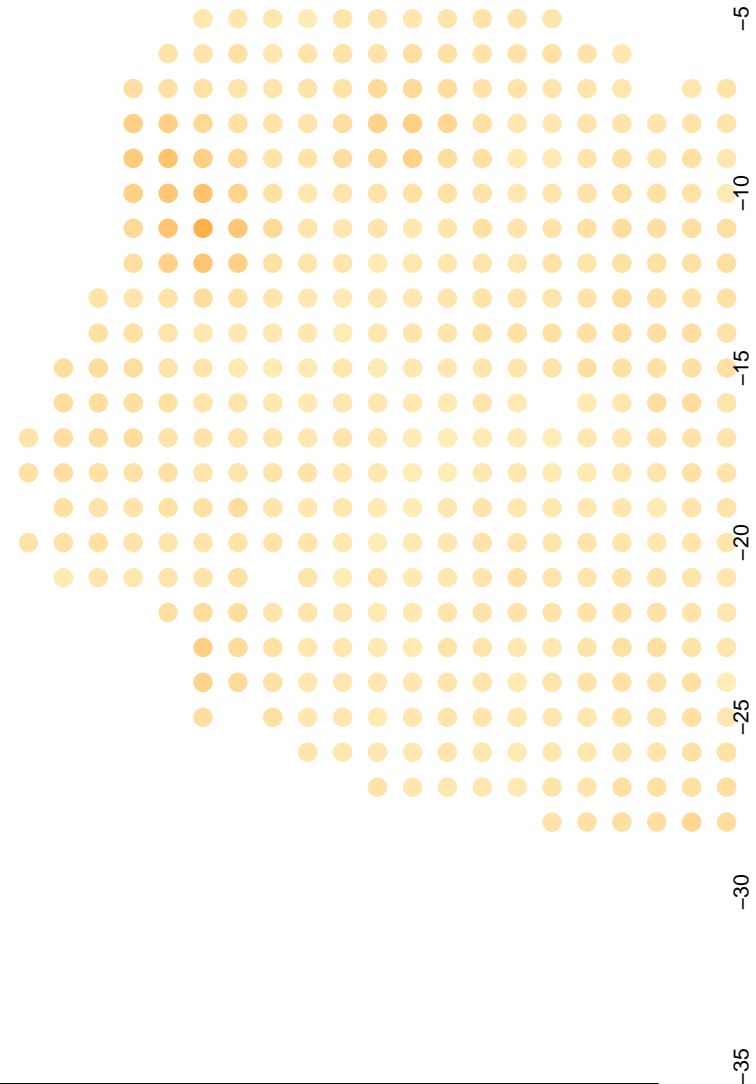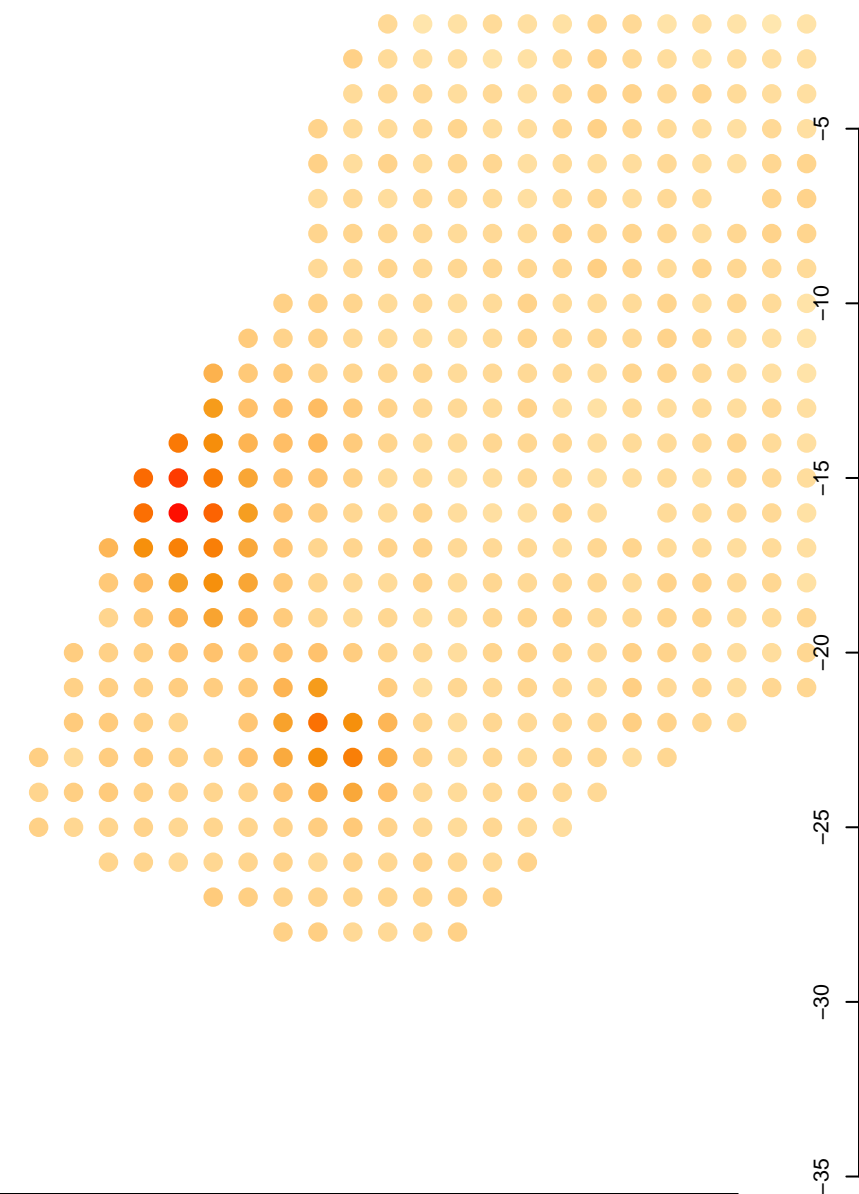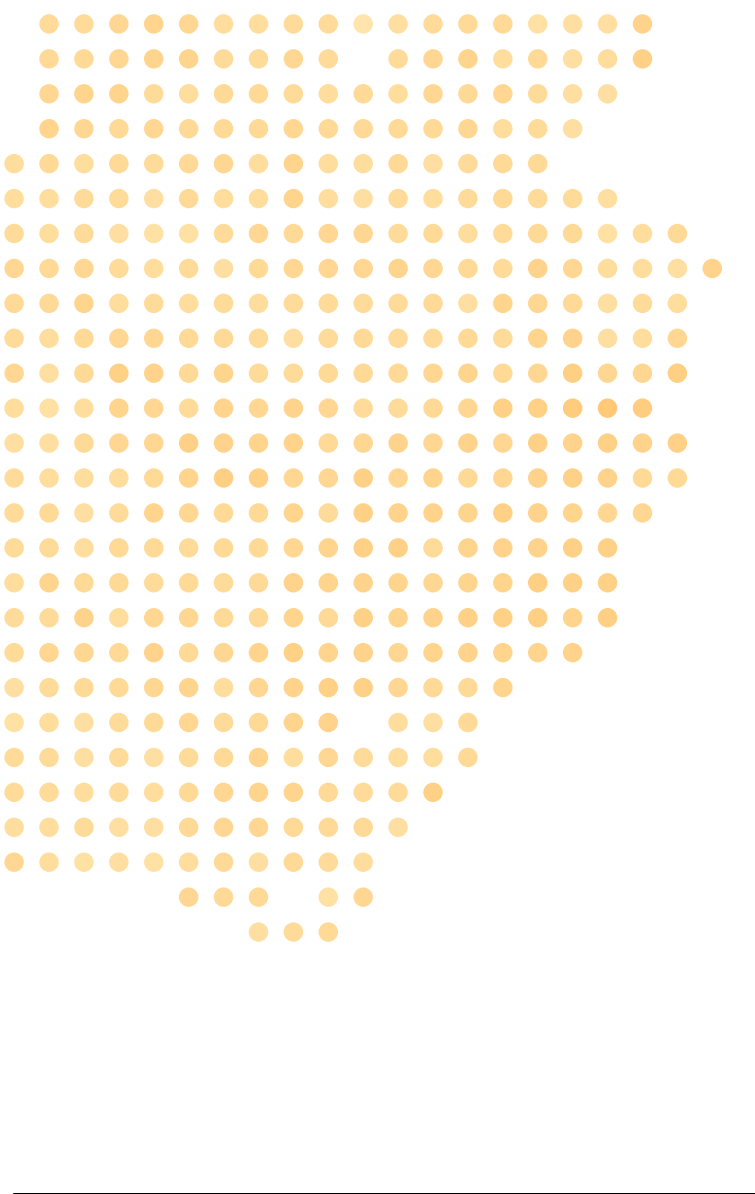

Supplement: Supplementary file 8 — Supplementary Data 5 [file 41467_2018_4724_MOESM8_ESM.zip › Supplementary Dataset 7/joint-field-profiles-rel-common-scale-dots.pdf]

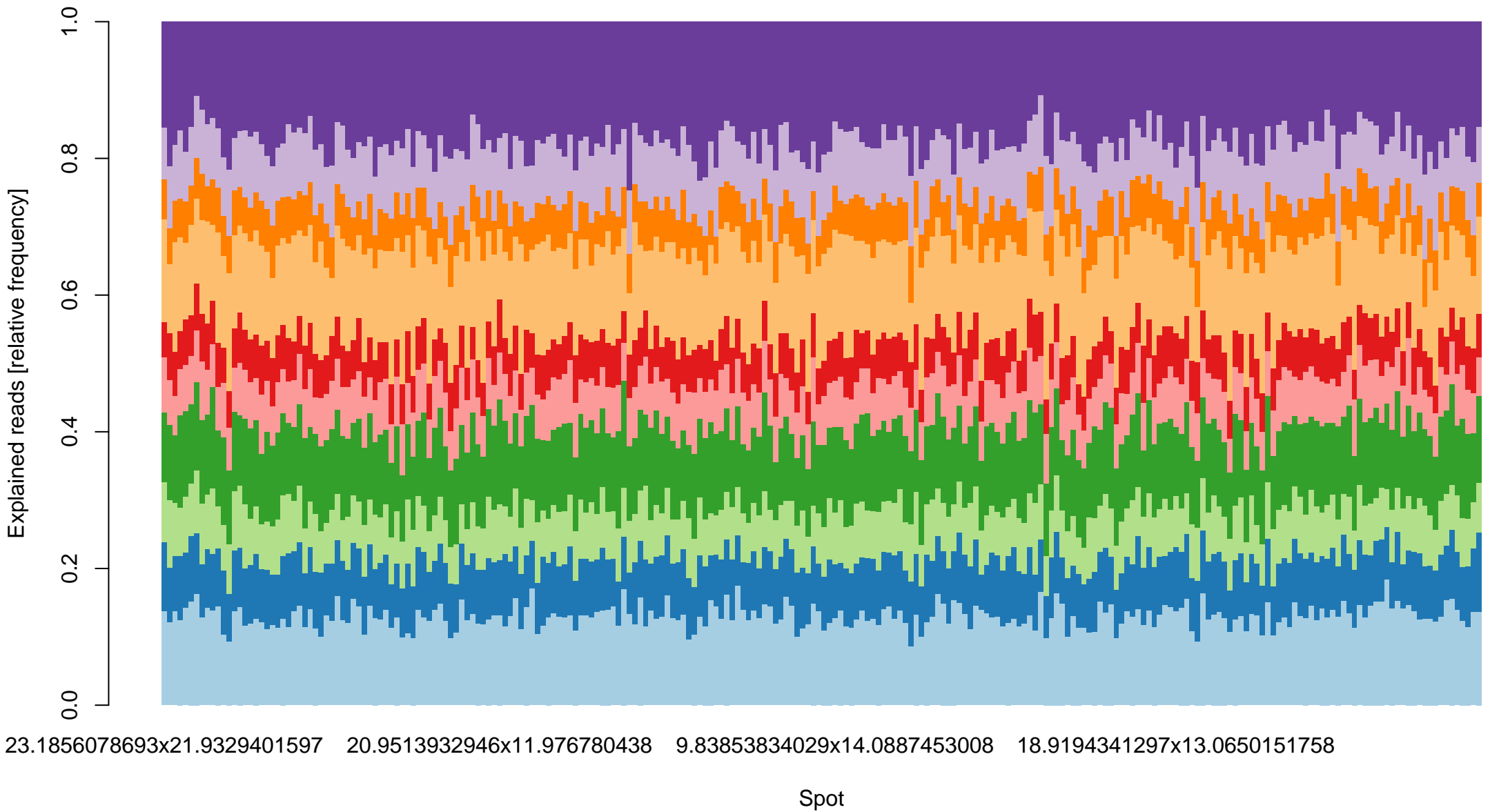

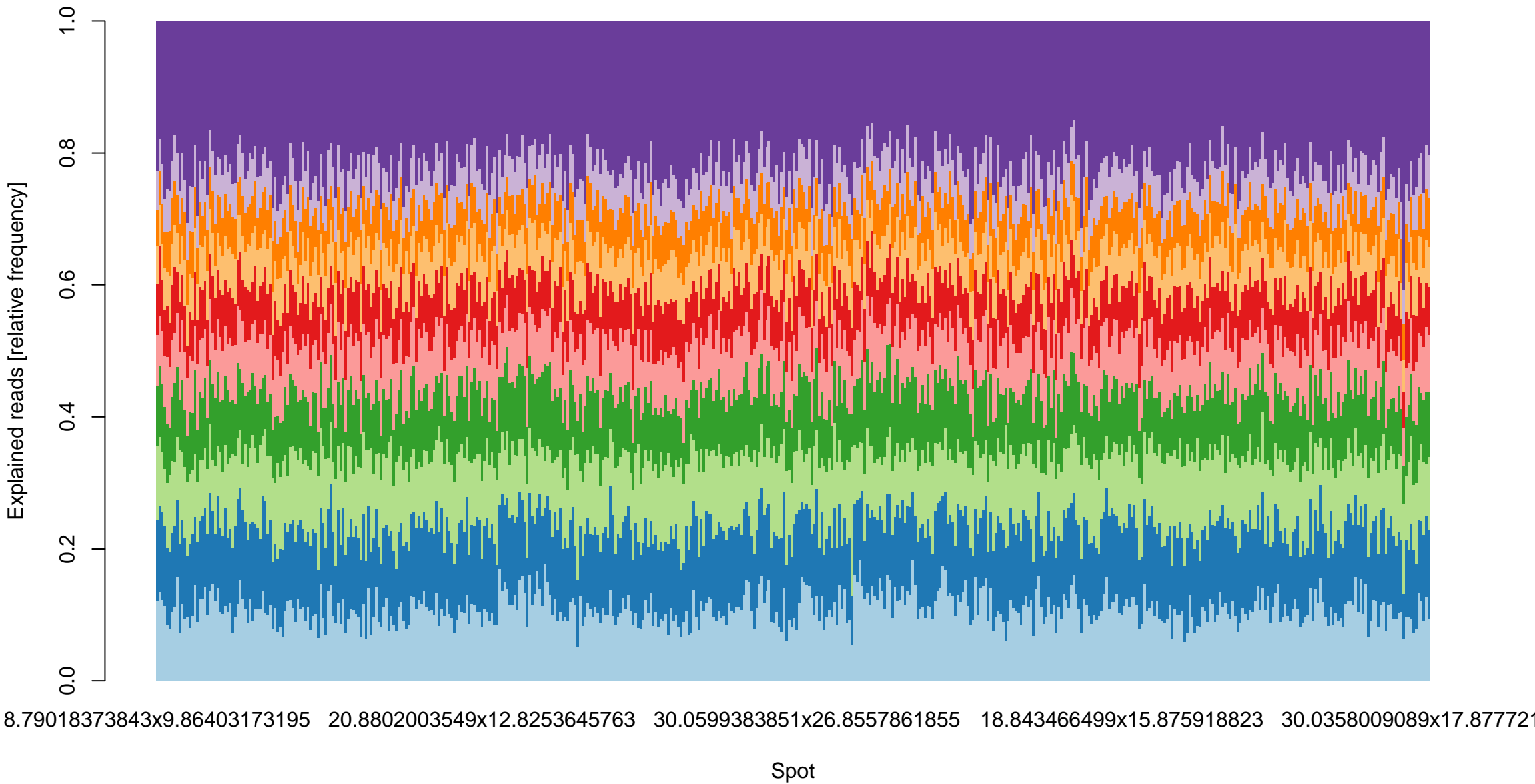

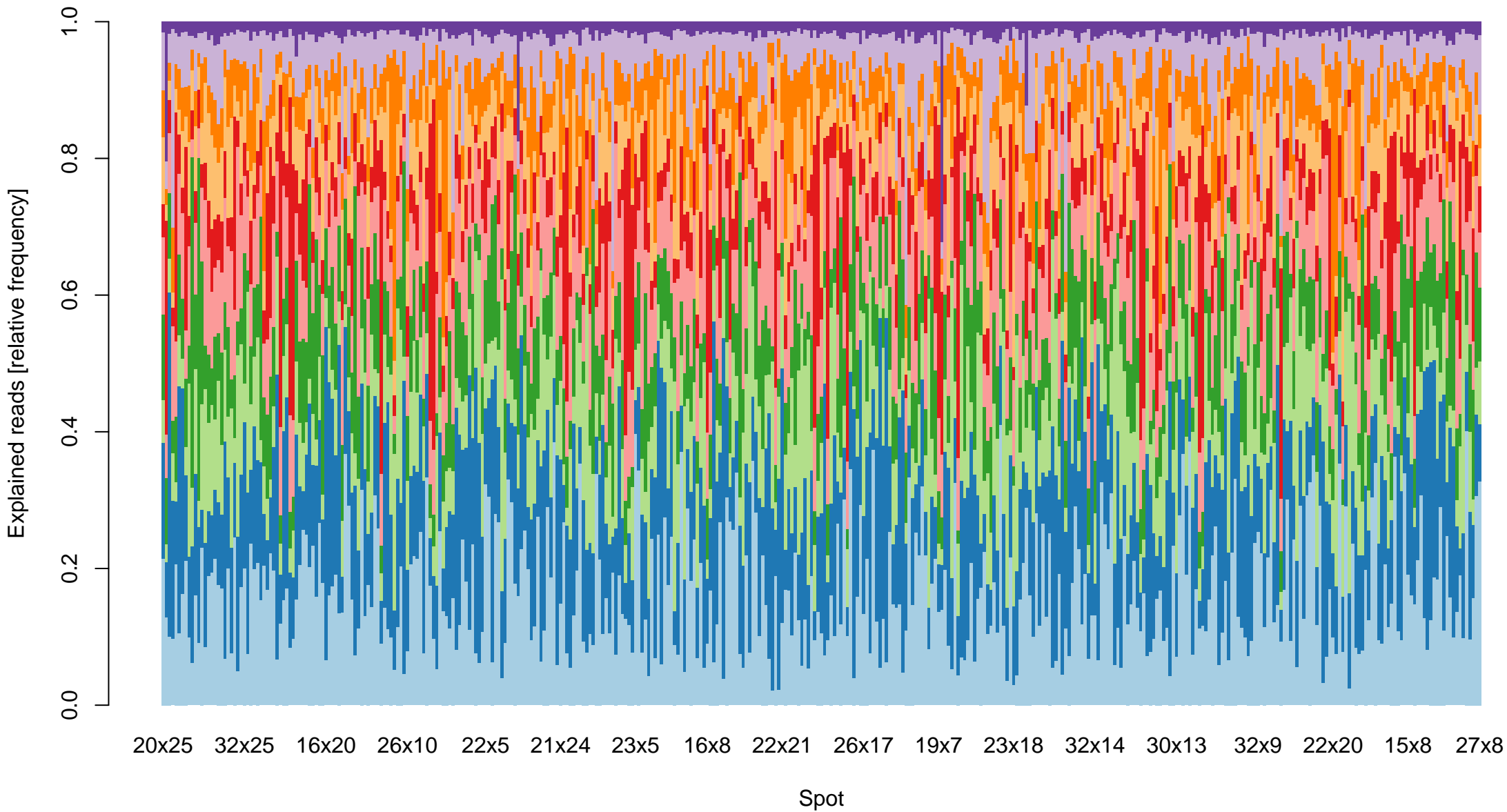

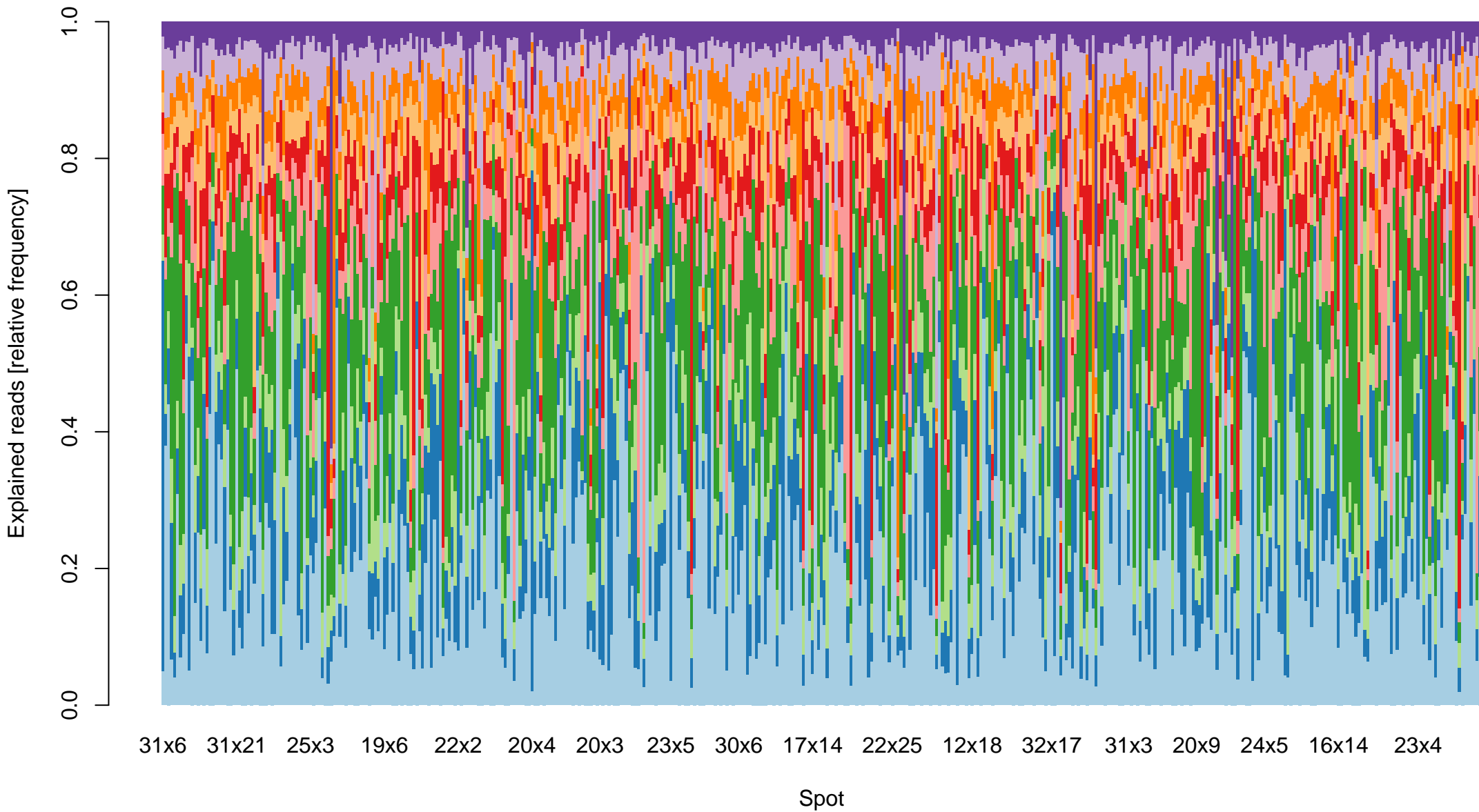

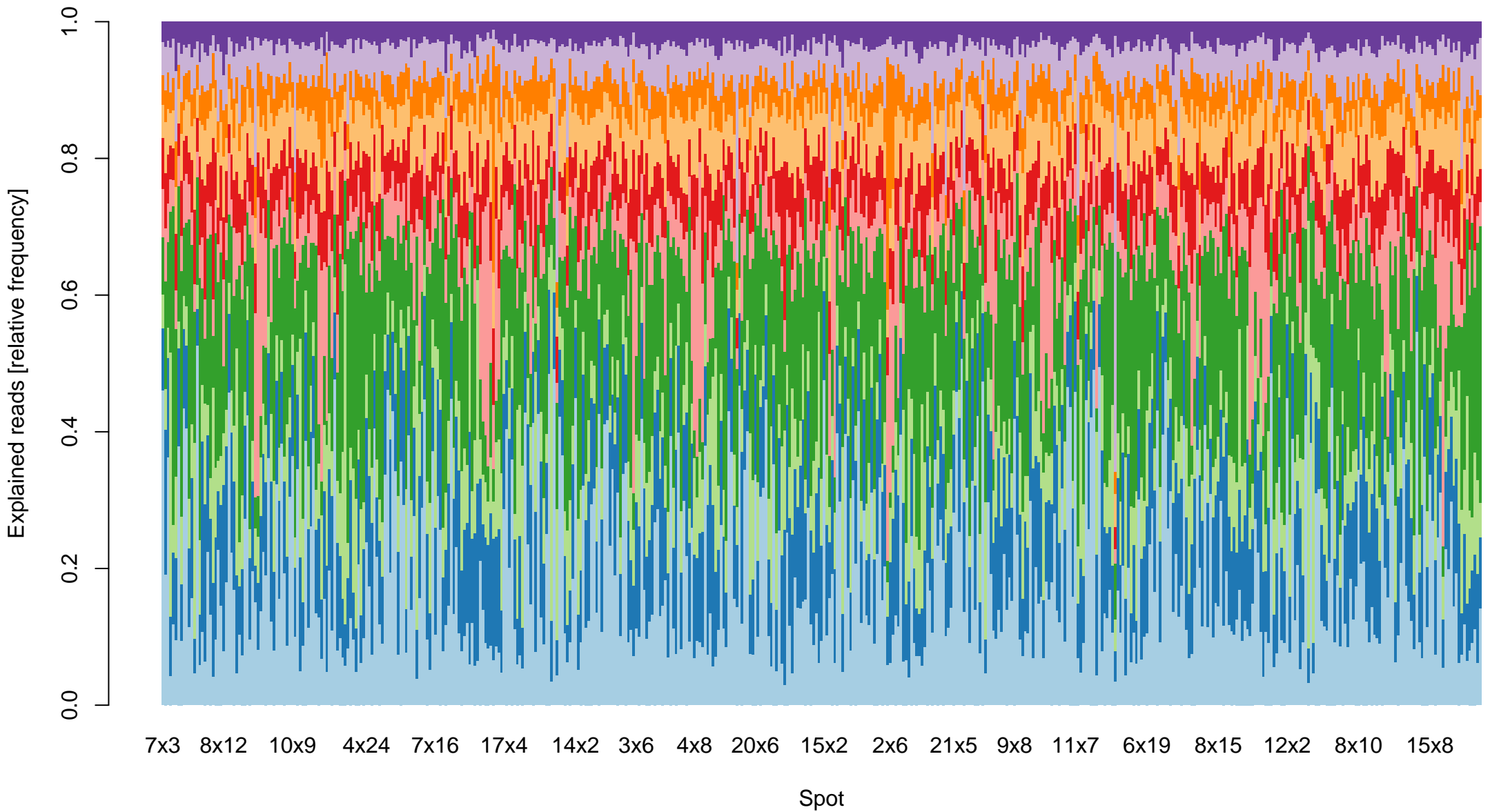

Supplement: Supplementary file 8 — Supplementary Data 5 [file 41467_2018_4724_MOESM8_ESM.zip › Supplementary Dataset 7/joint-mix-factor-strength-spotbarplot-rel-freq.pdf]

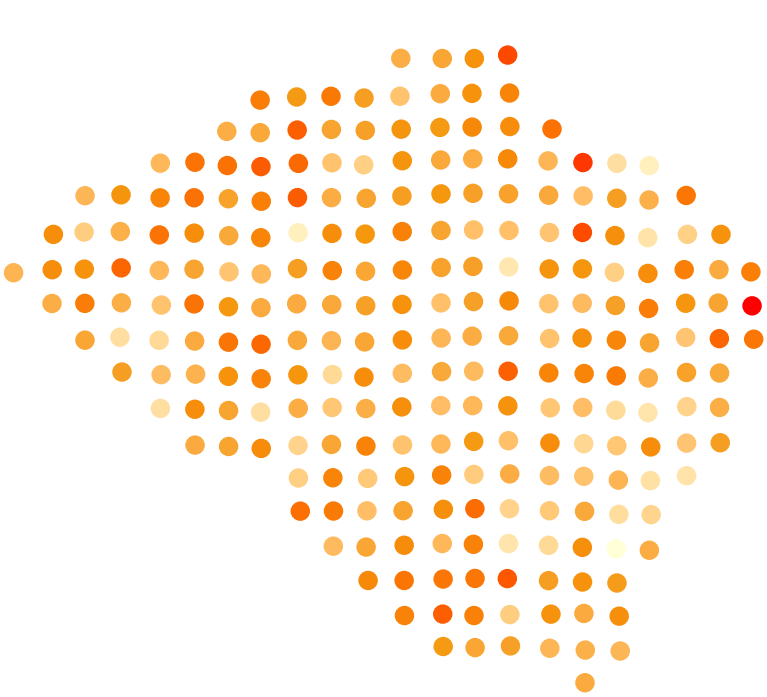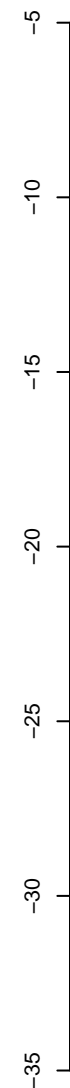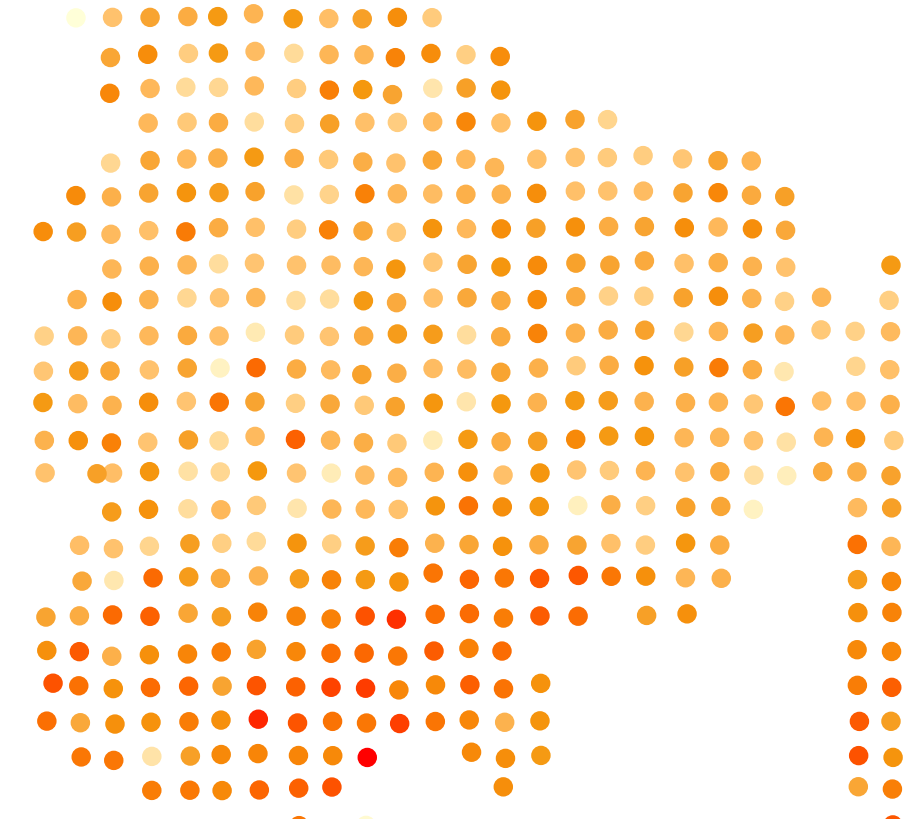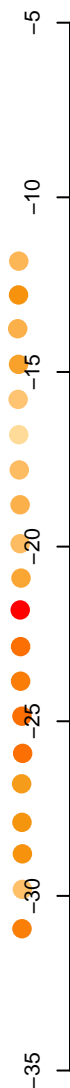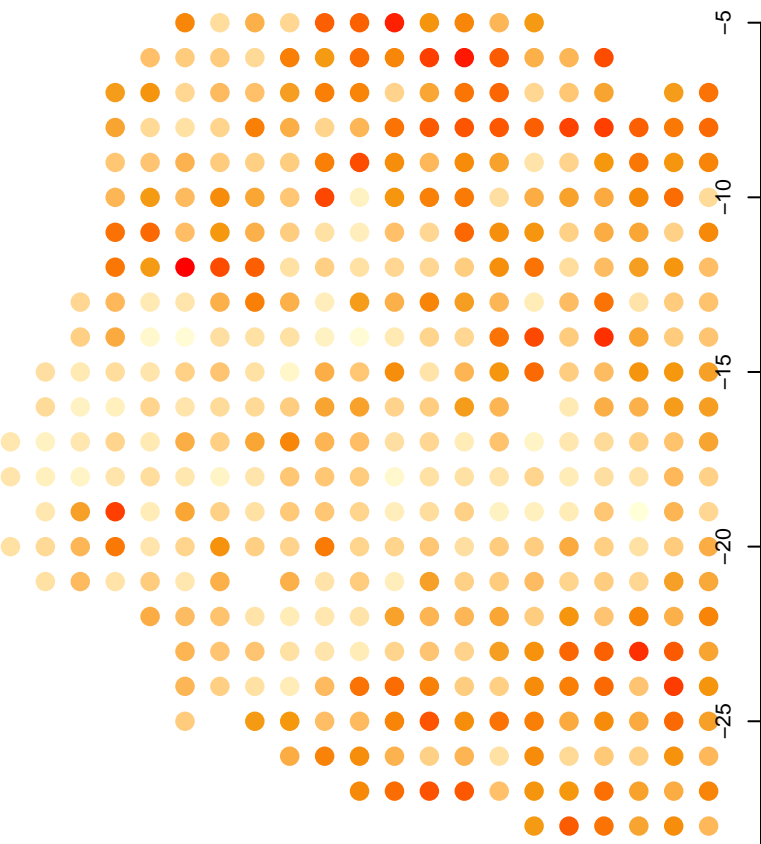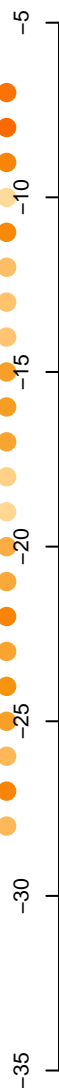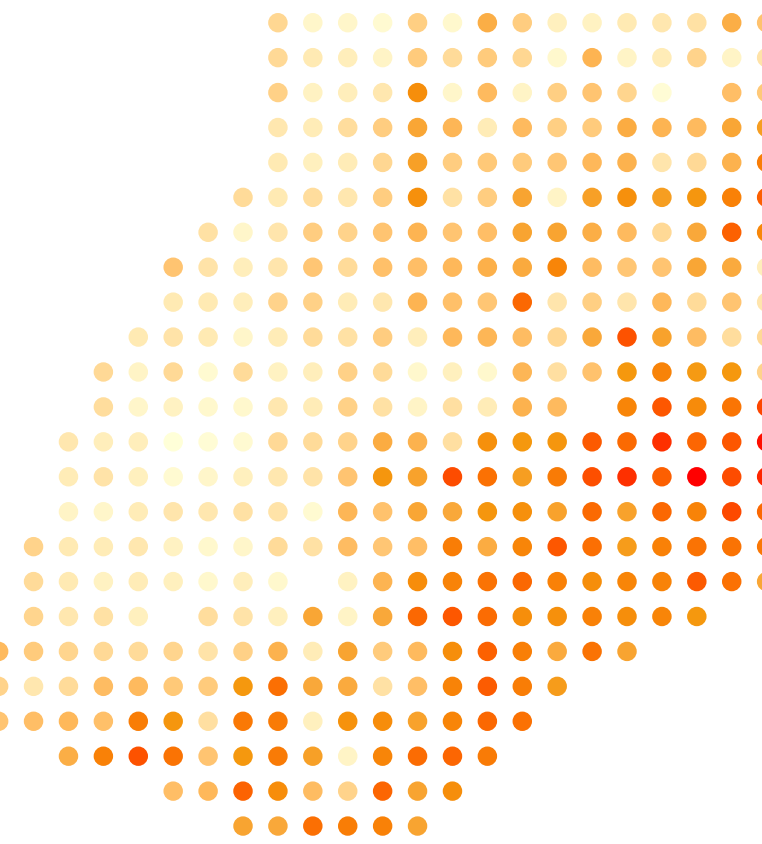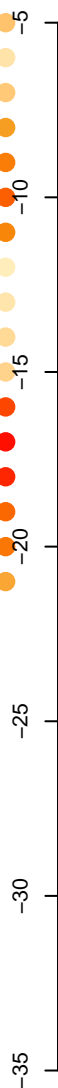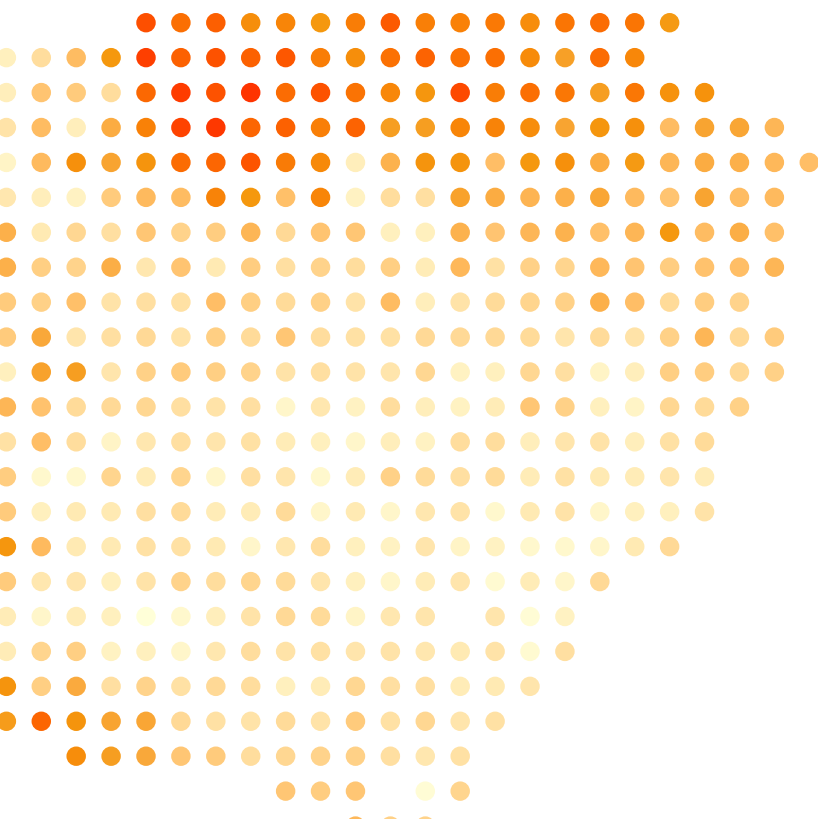

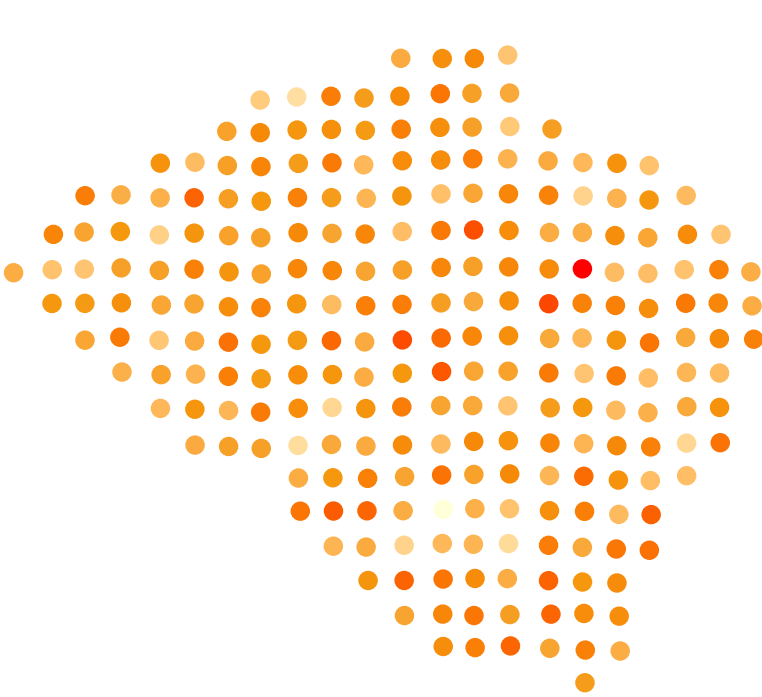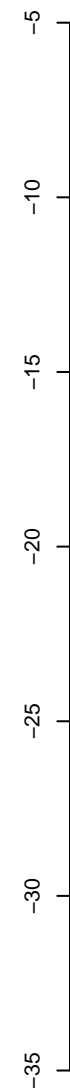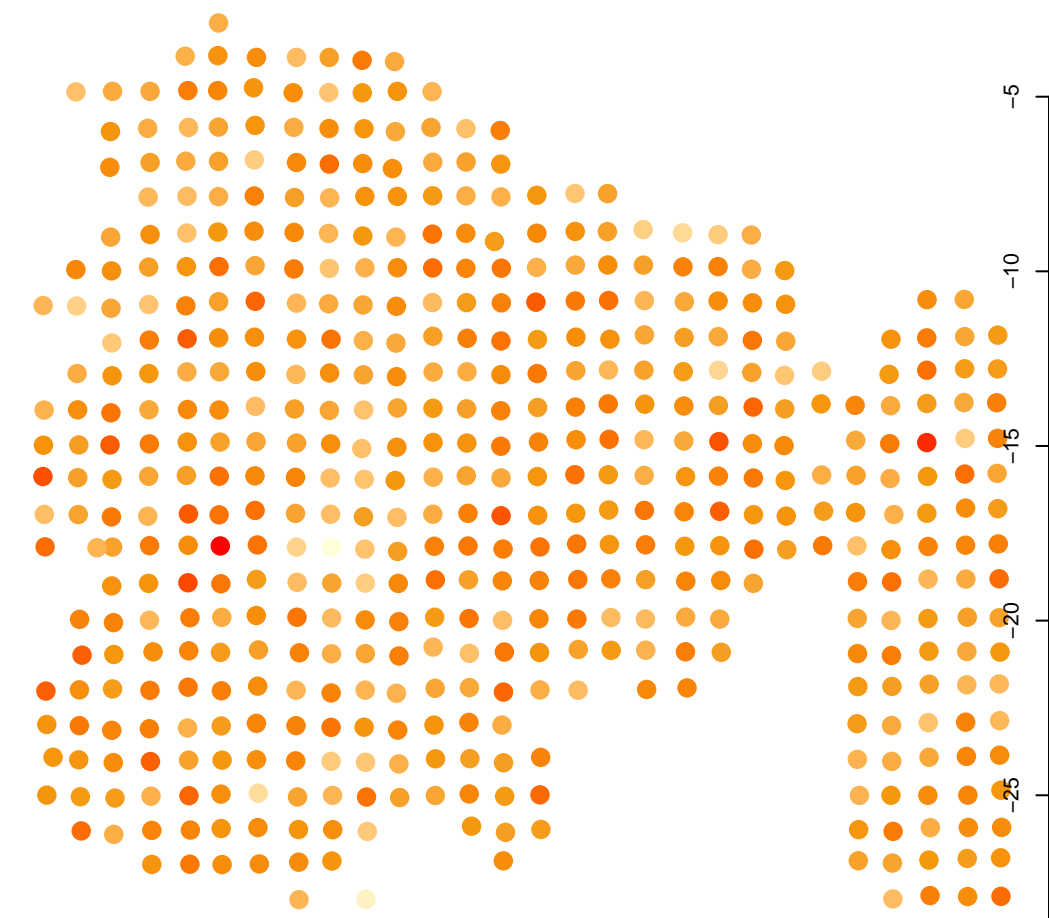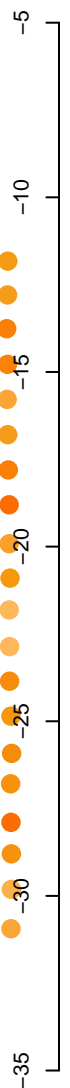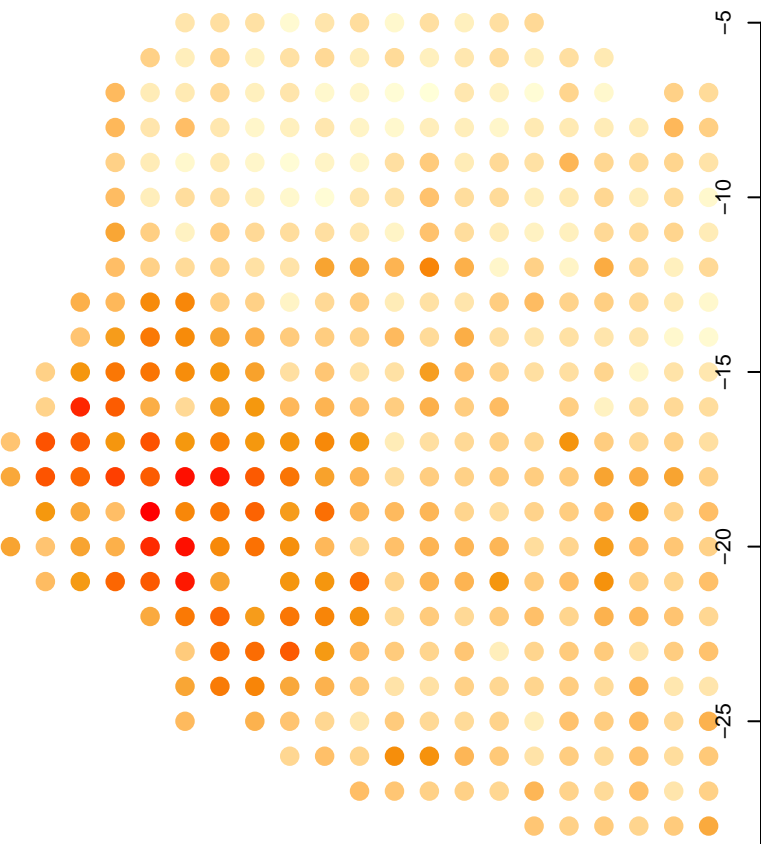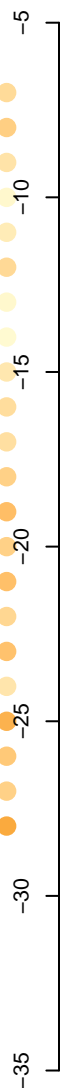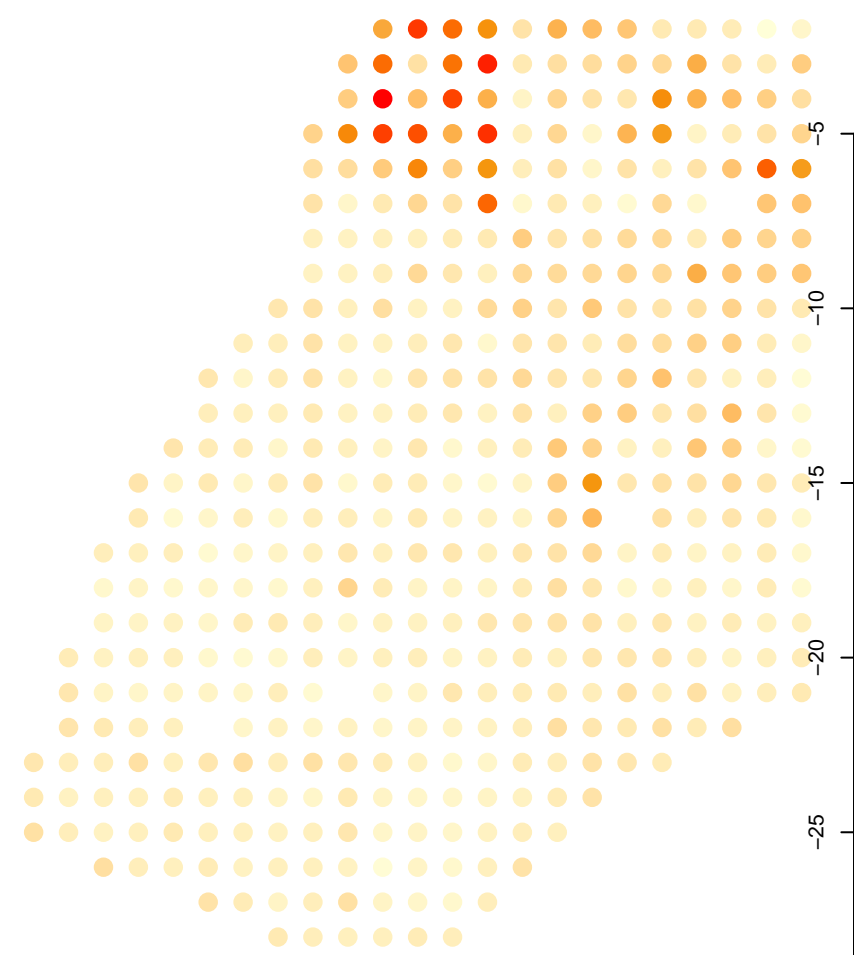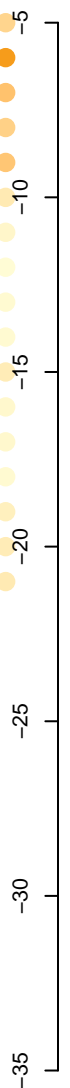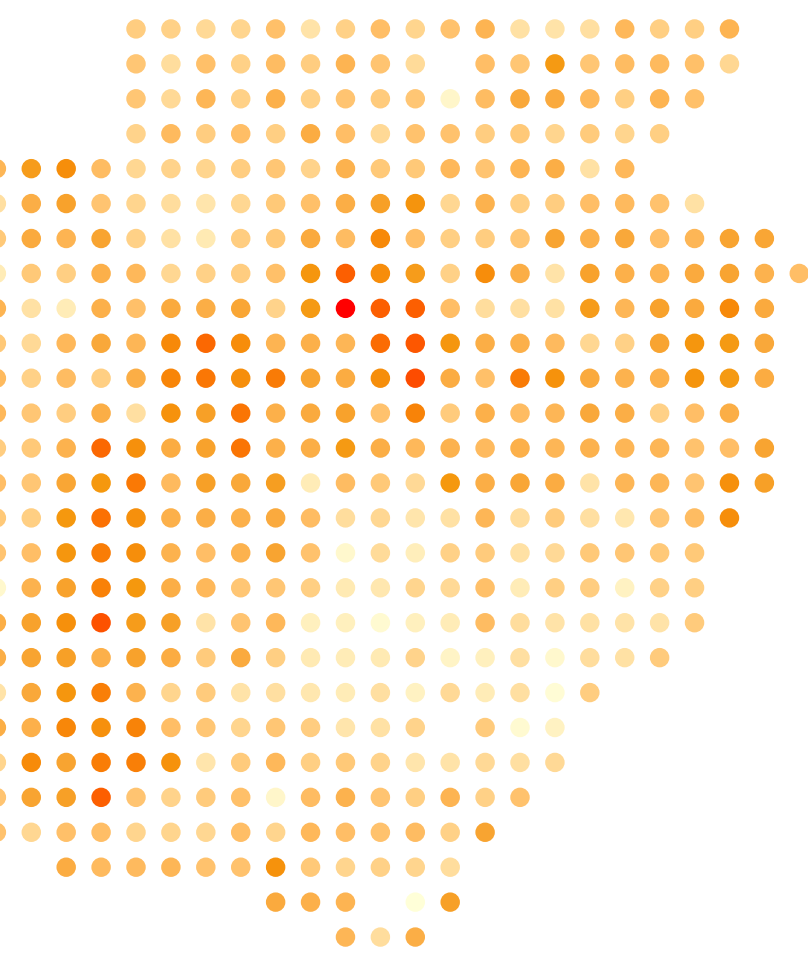

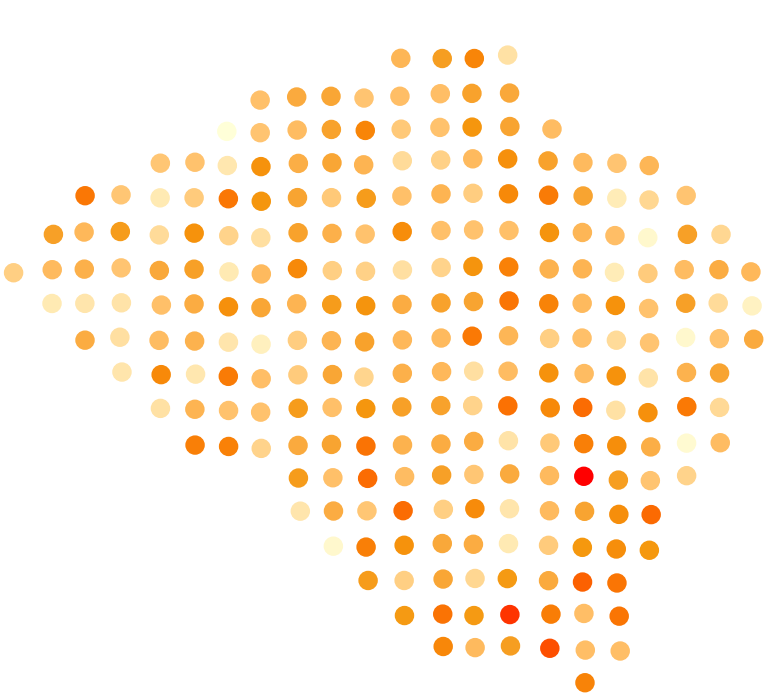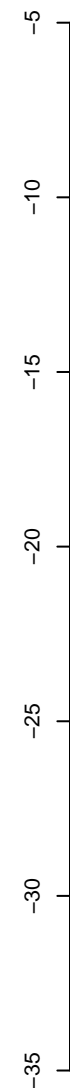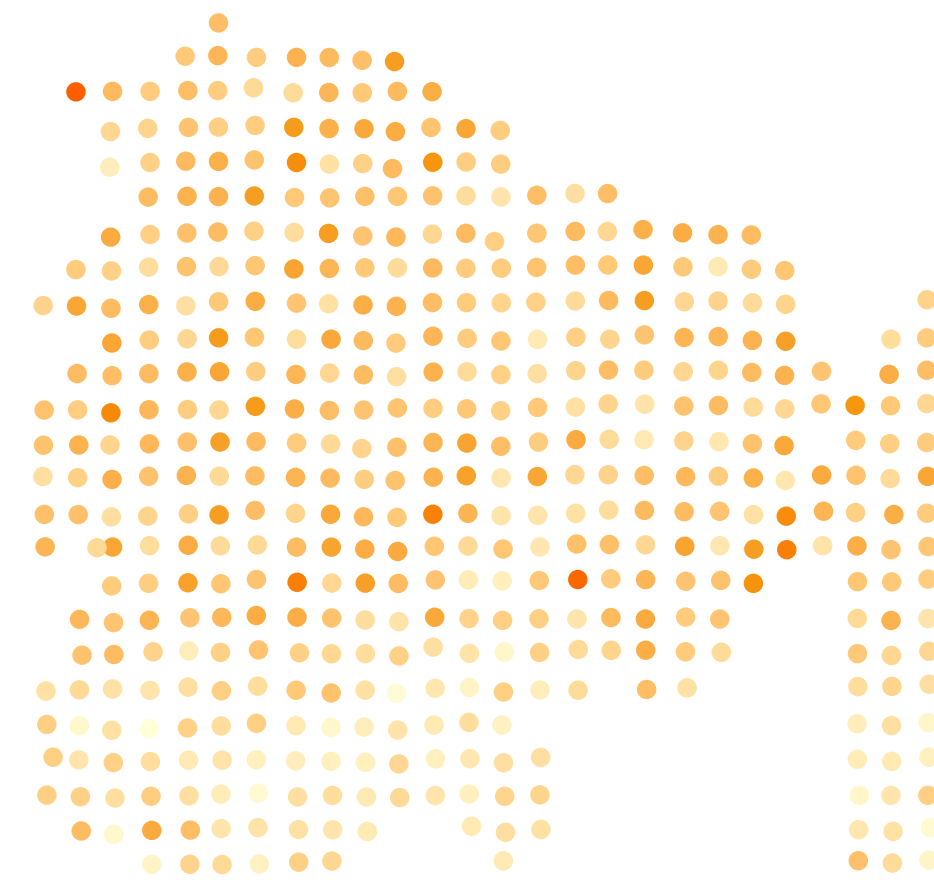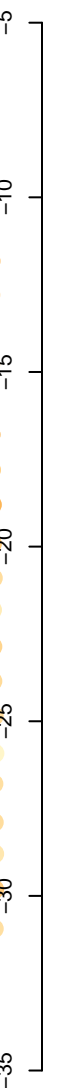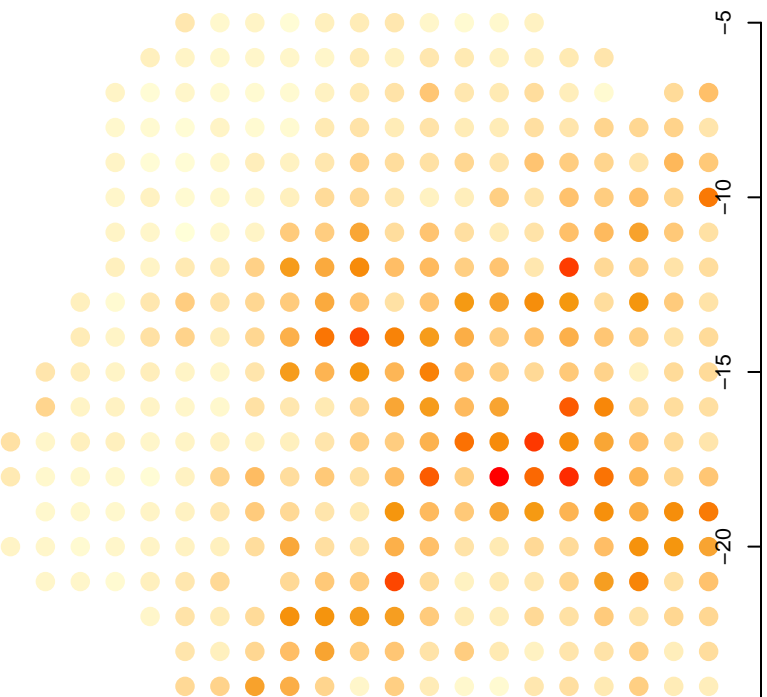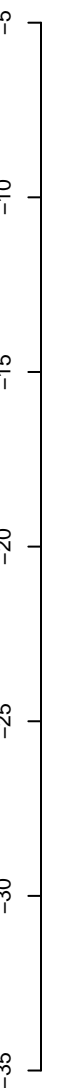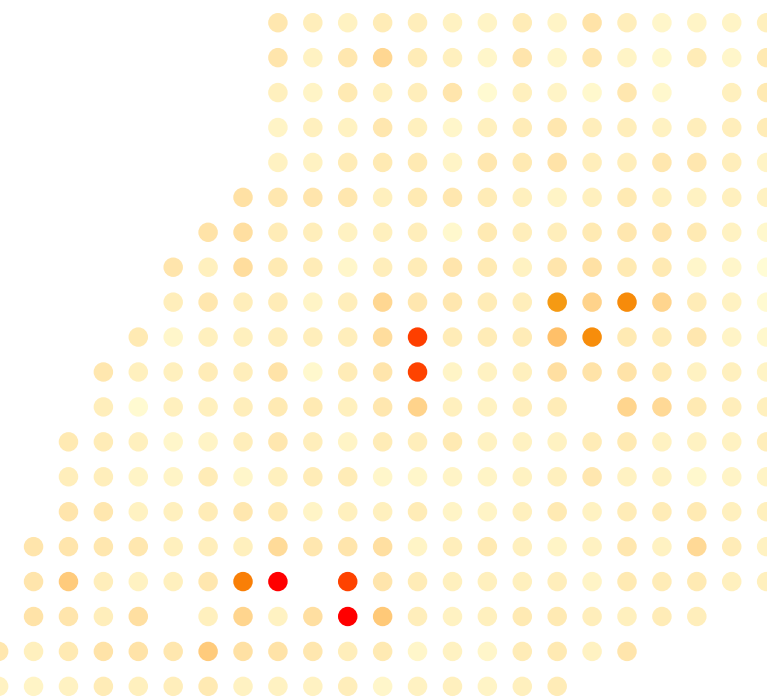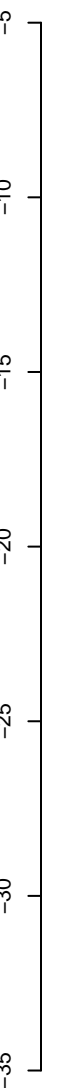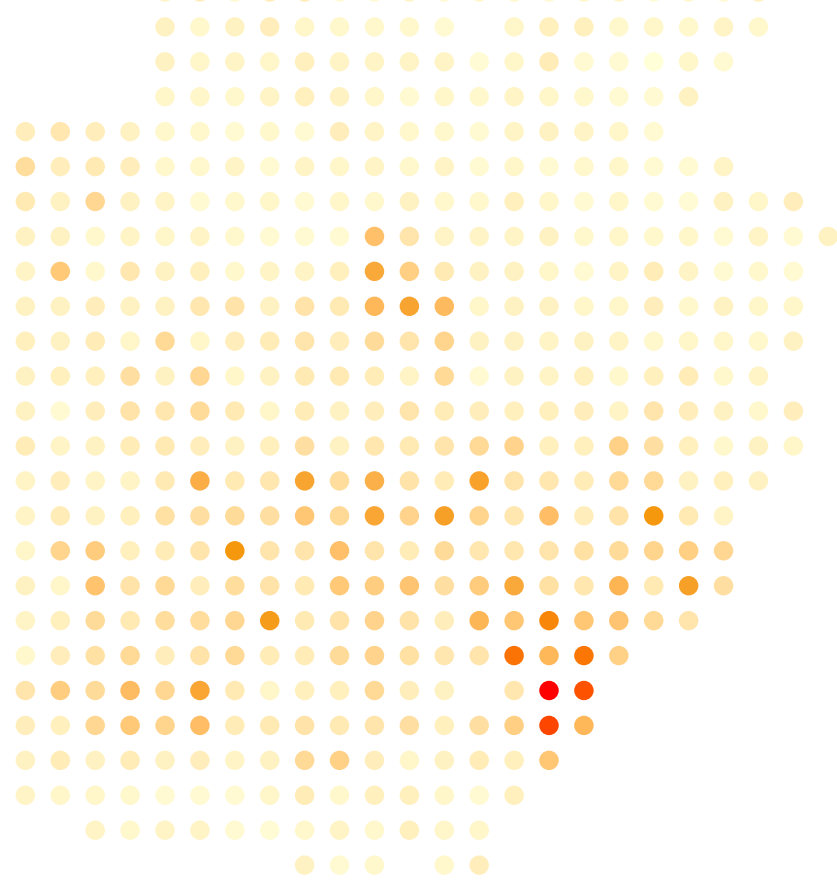

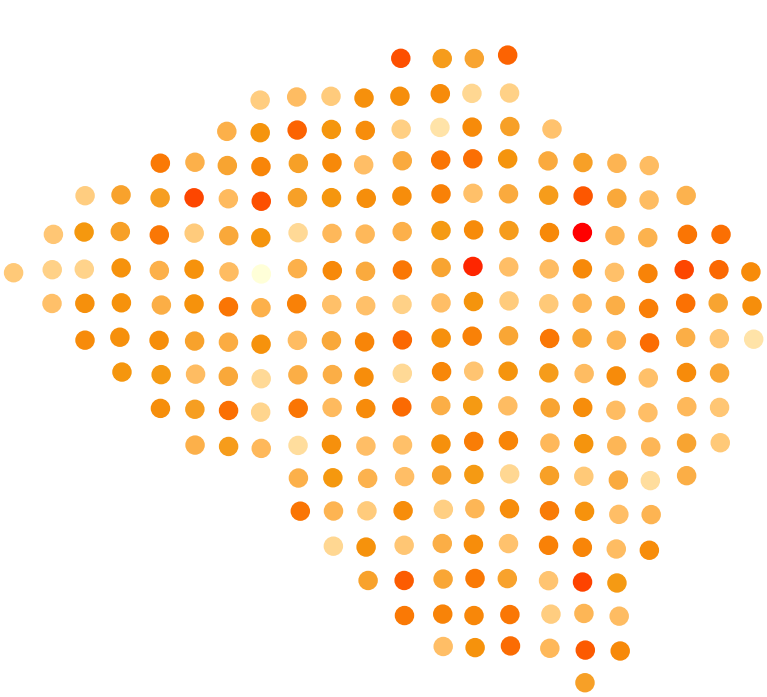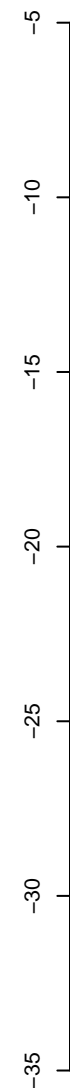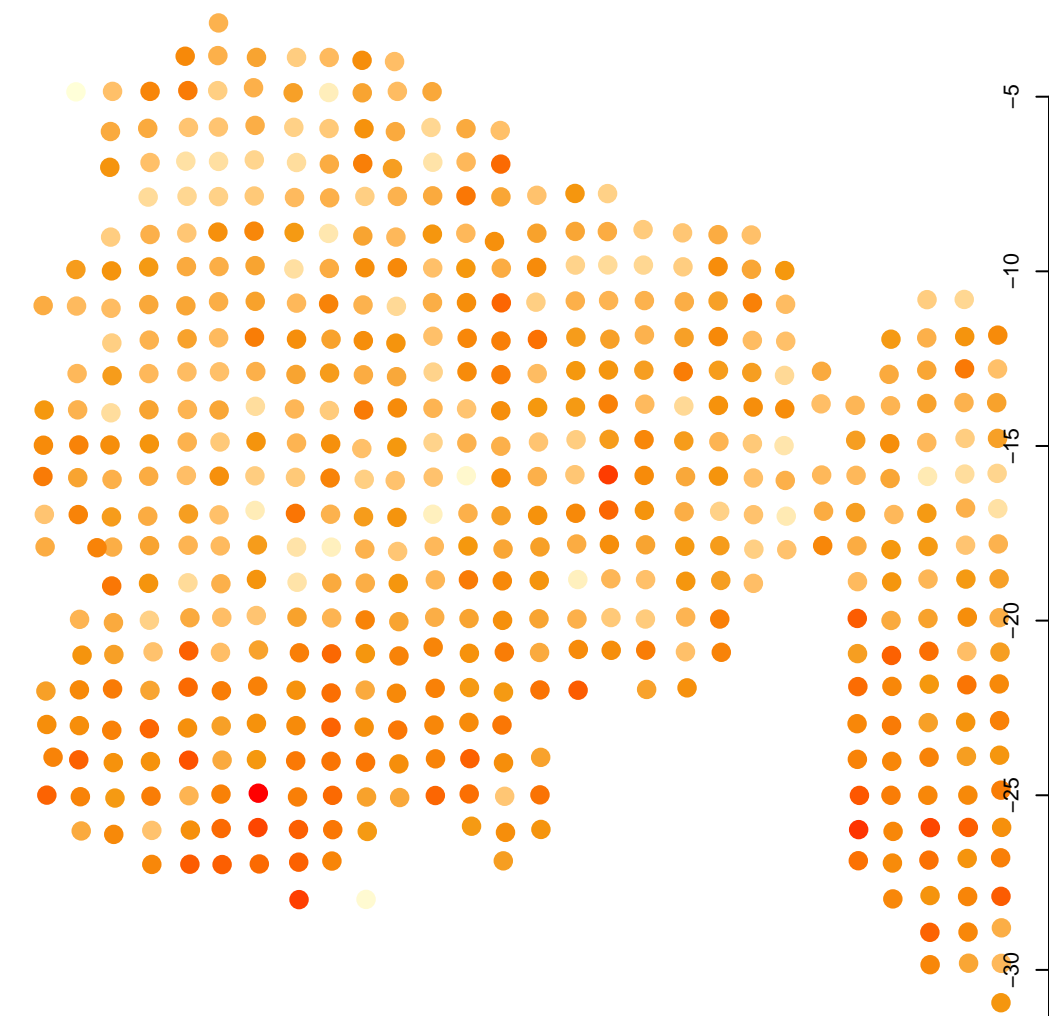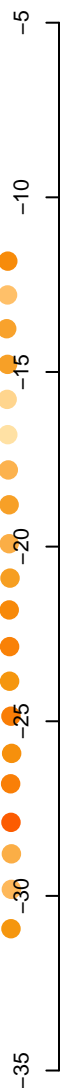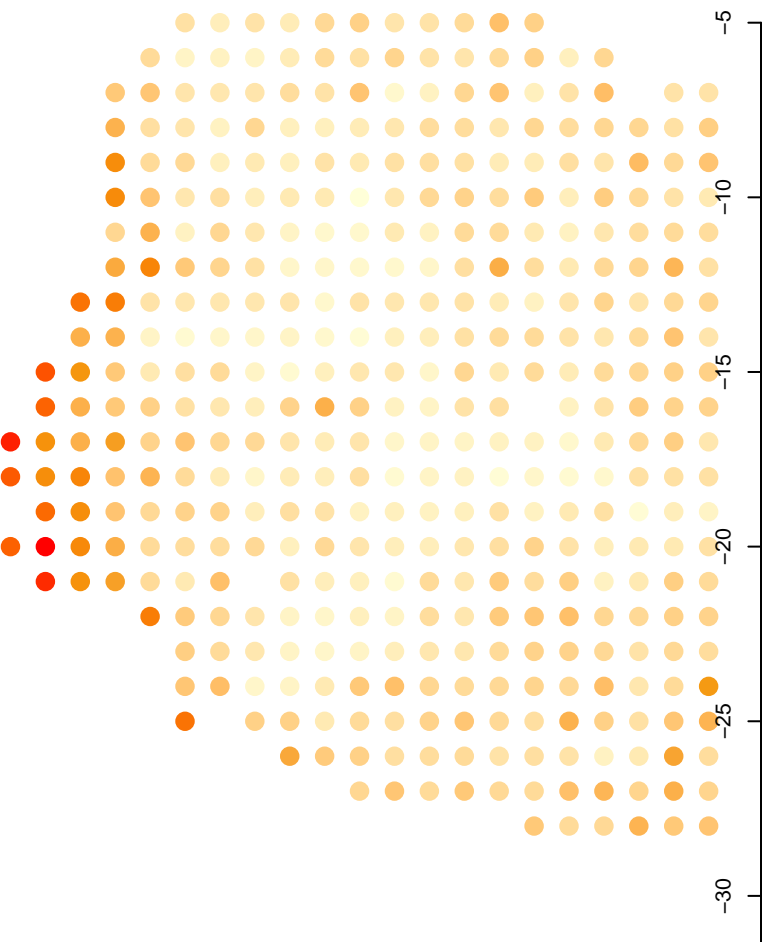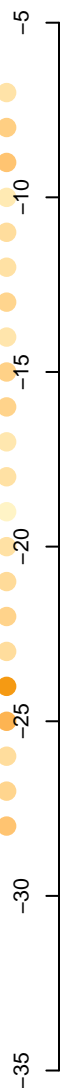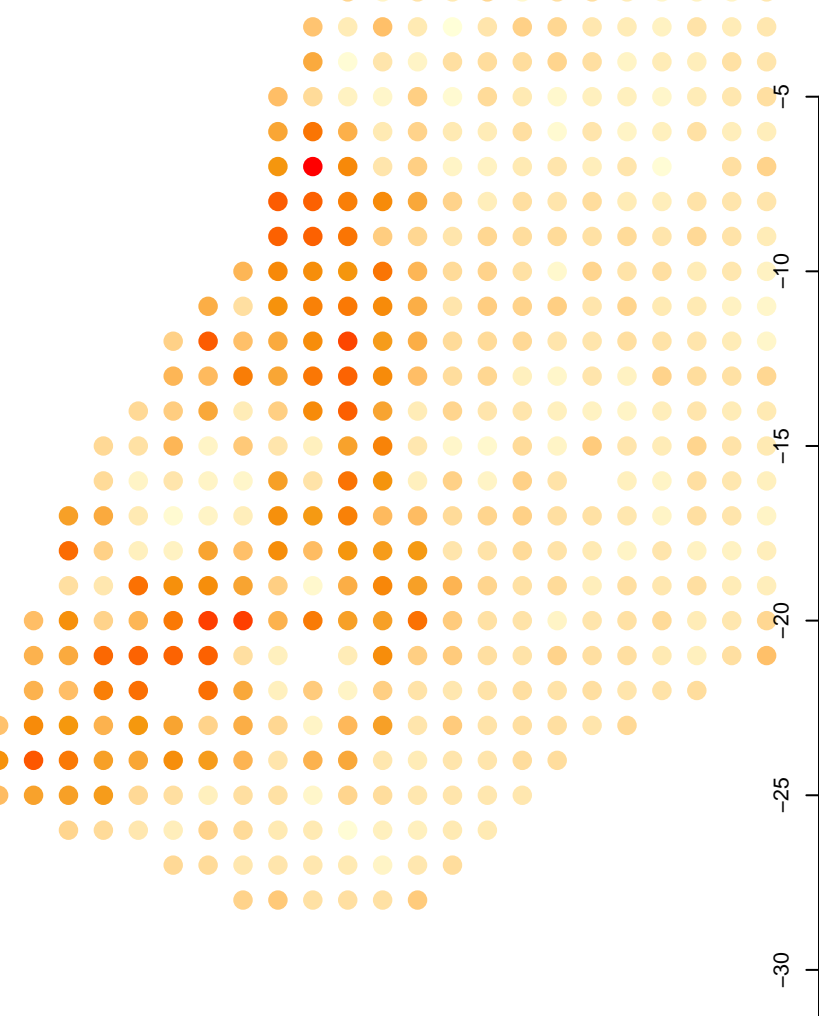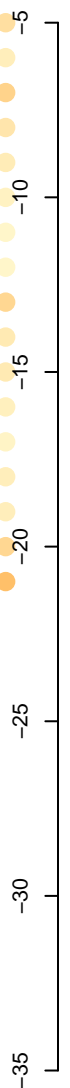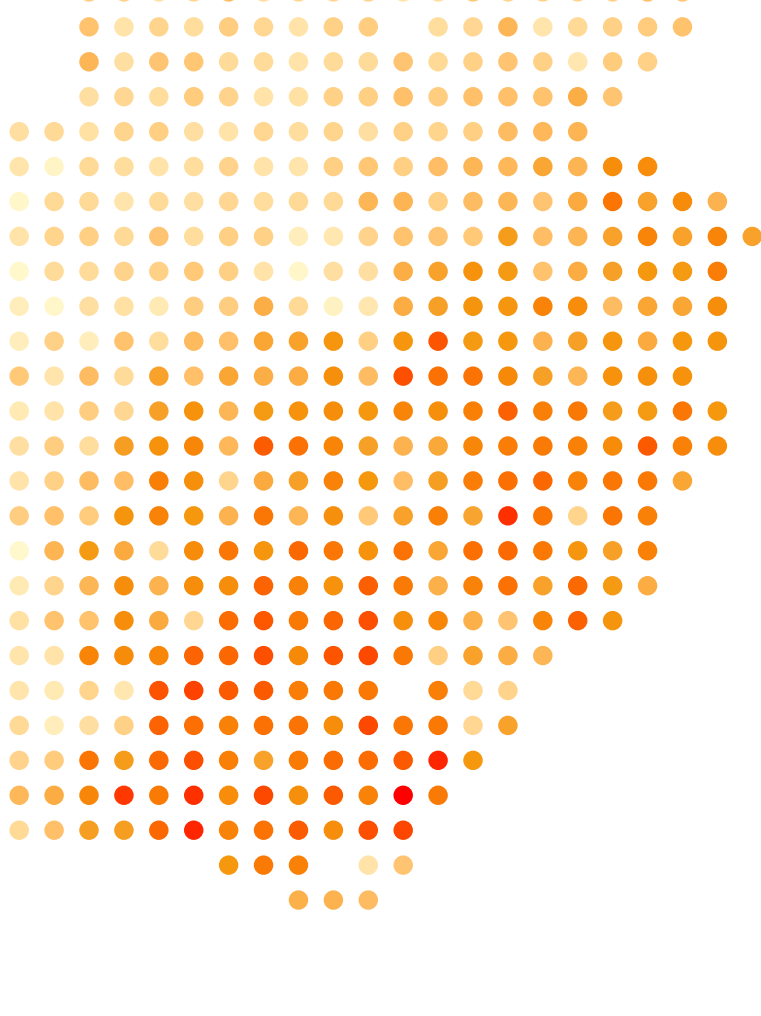

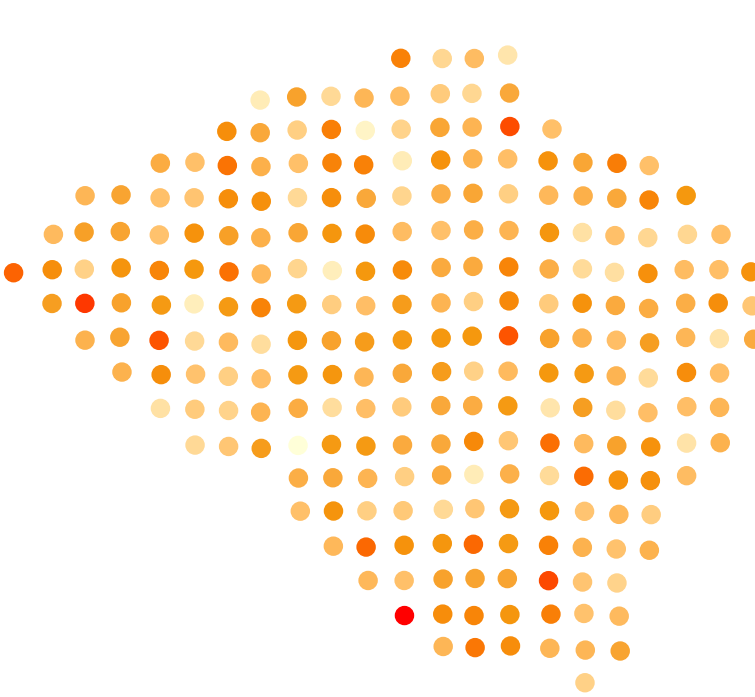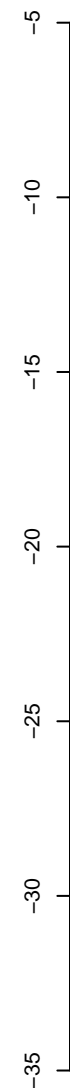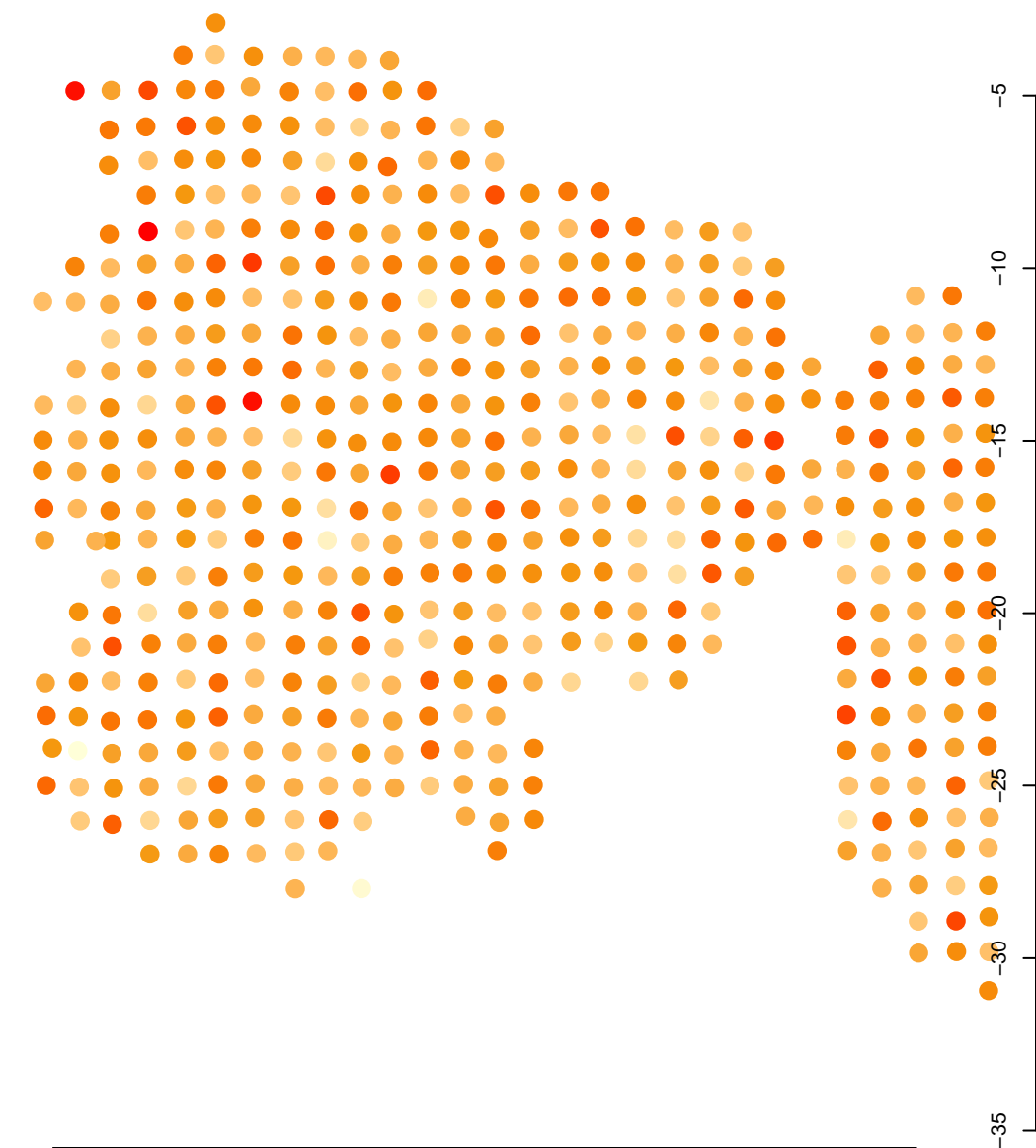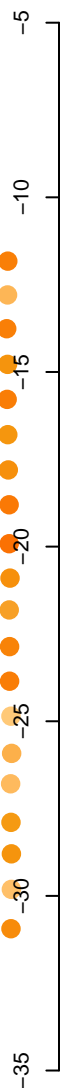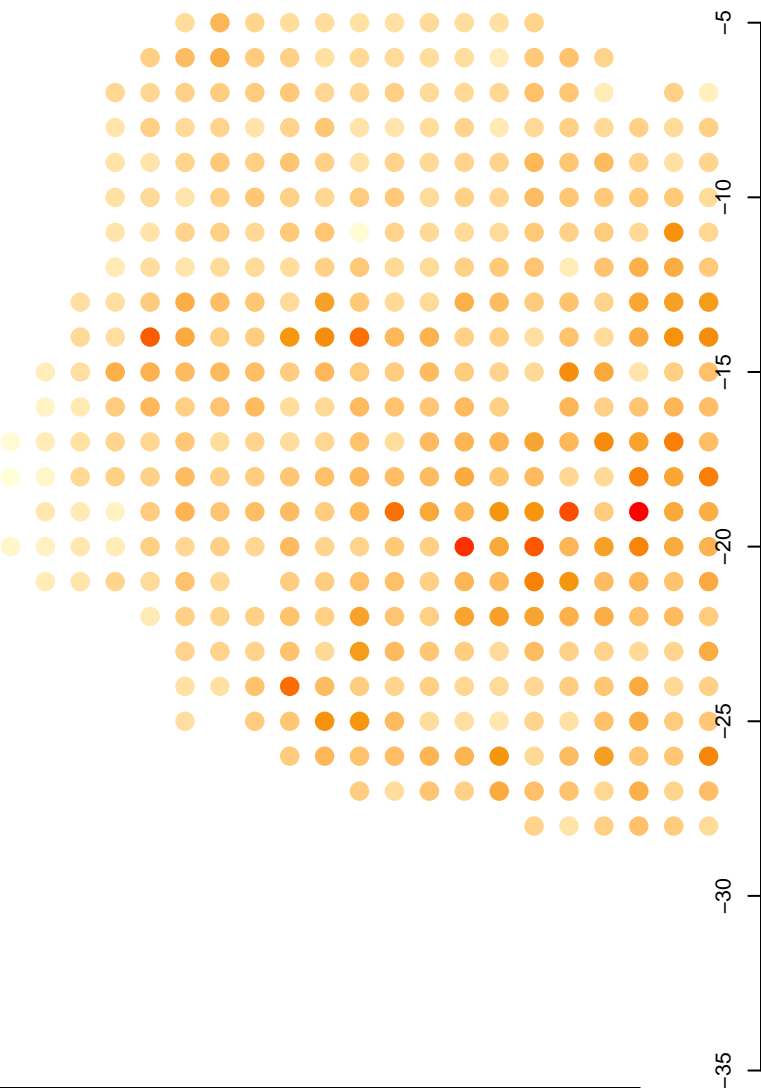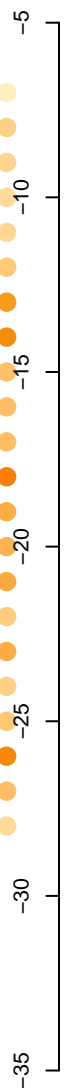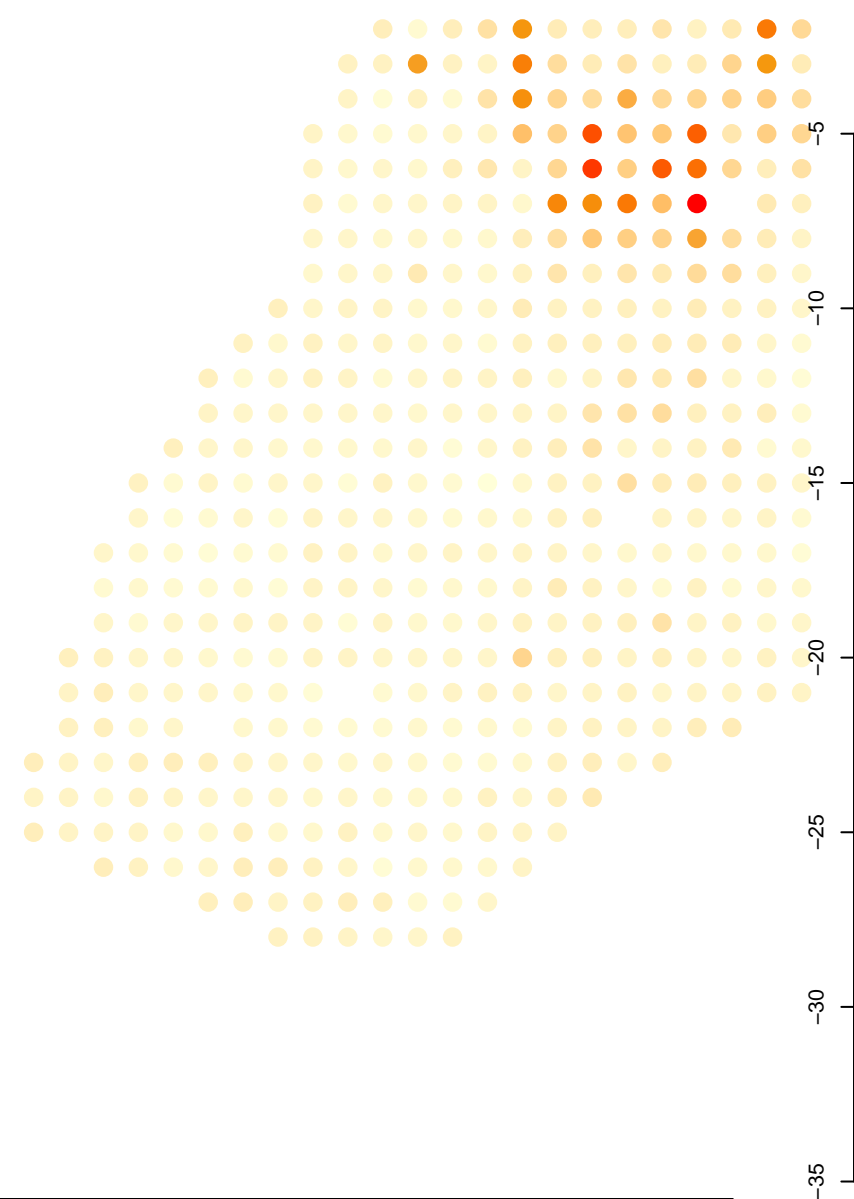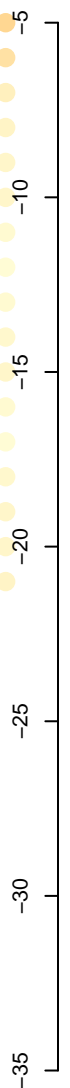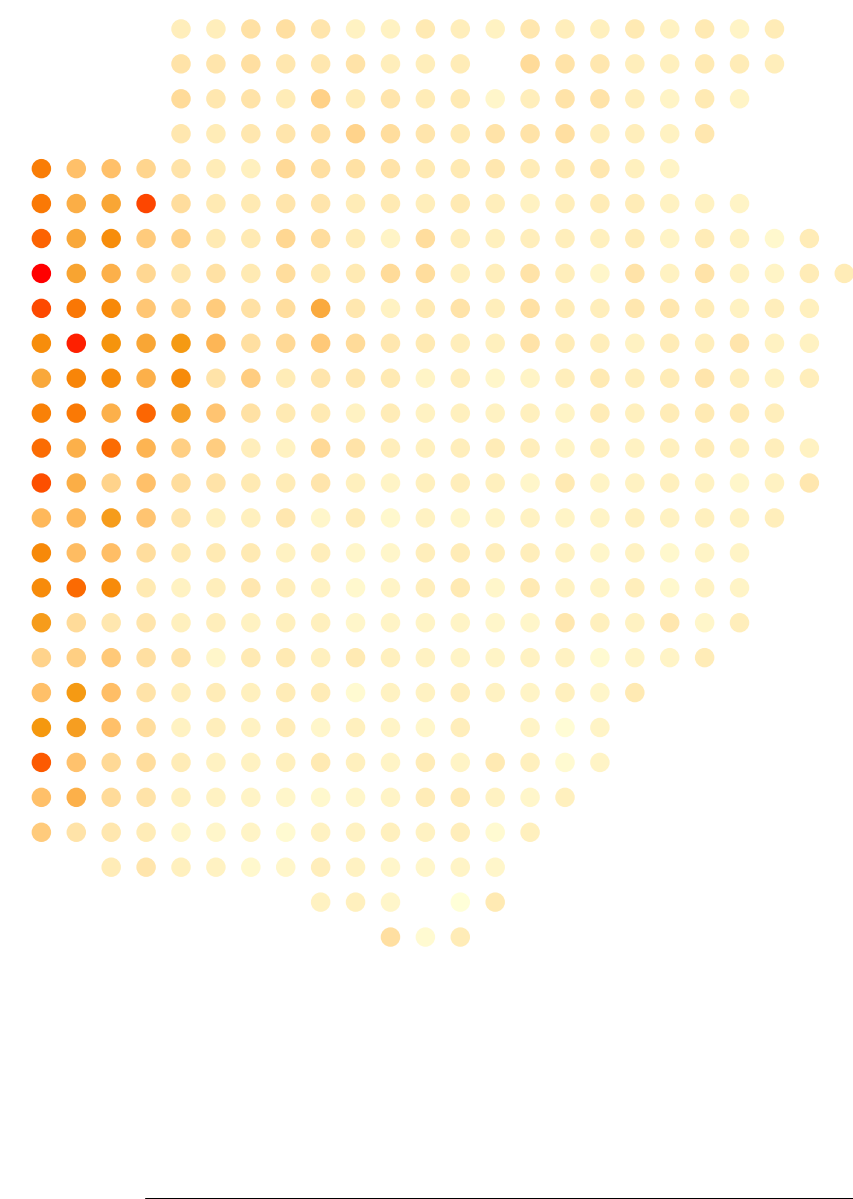

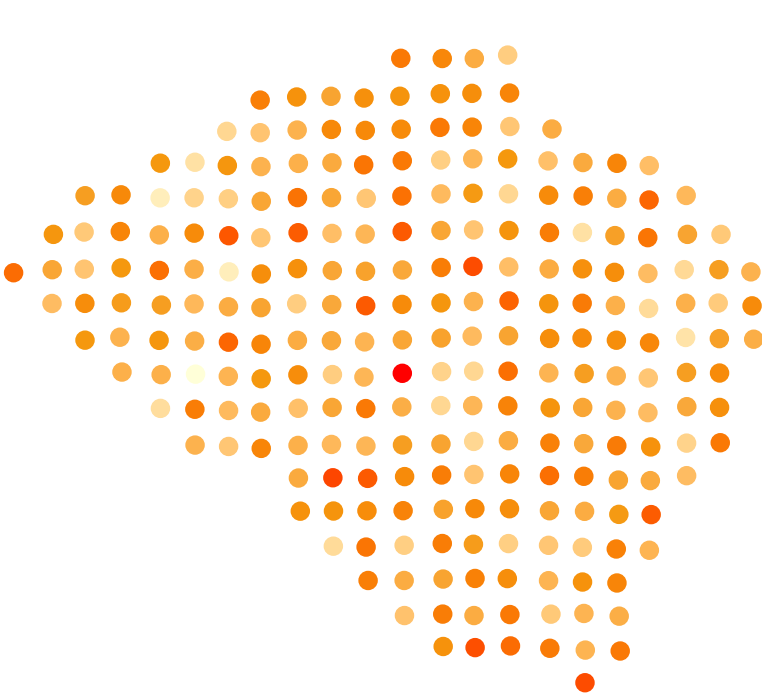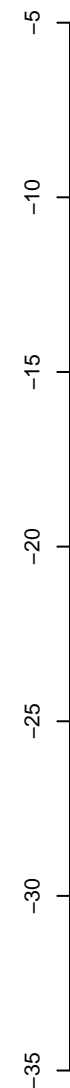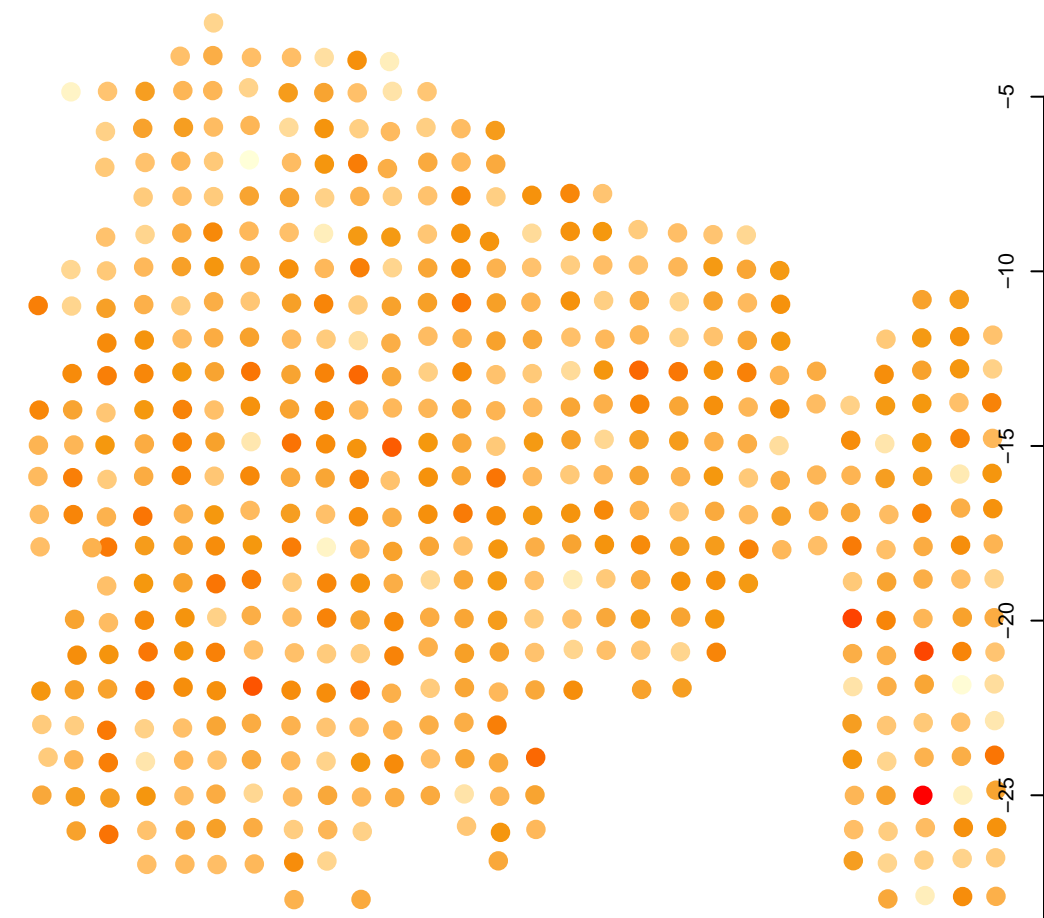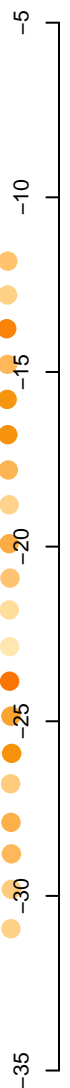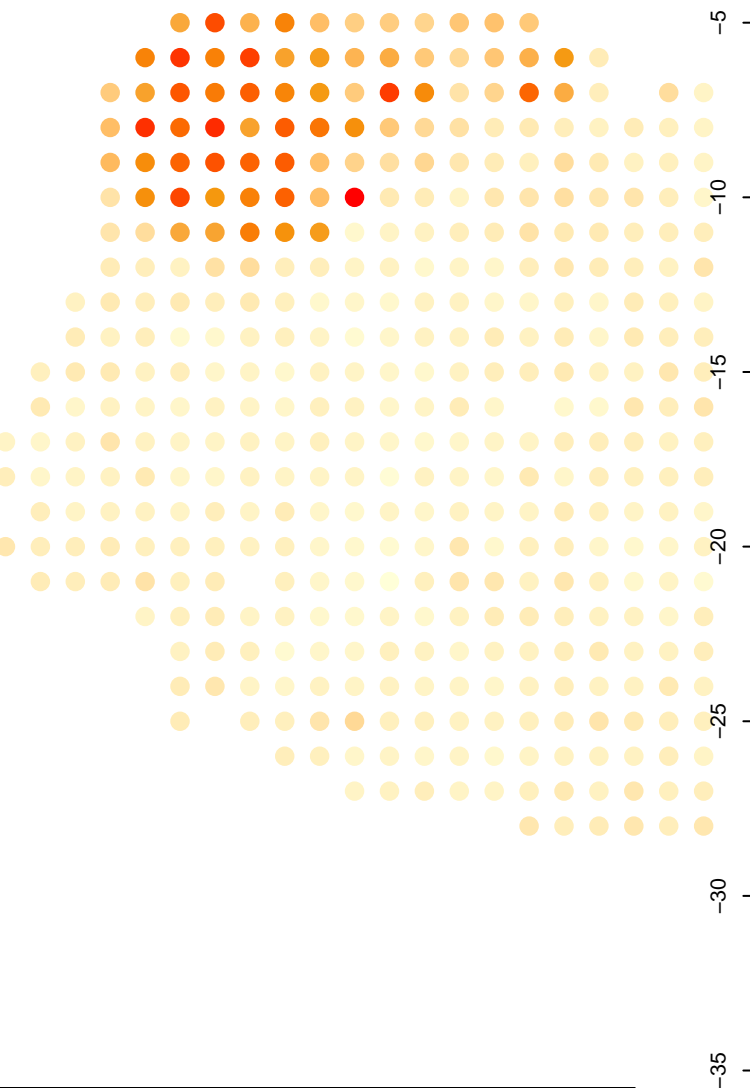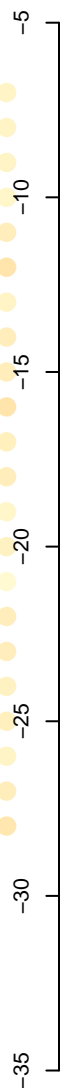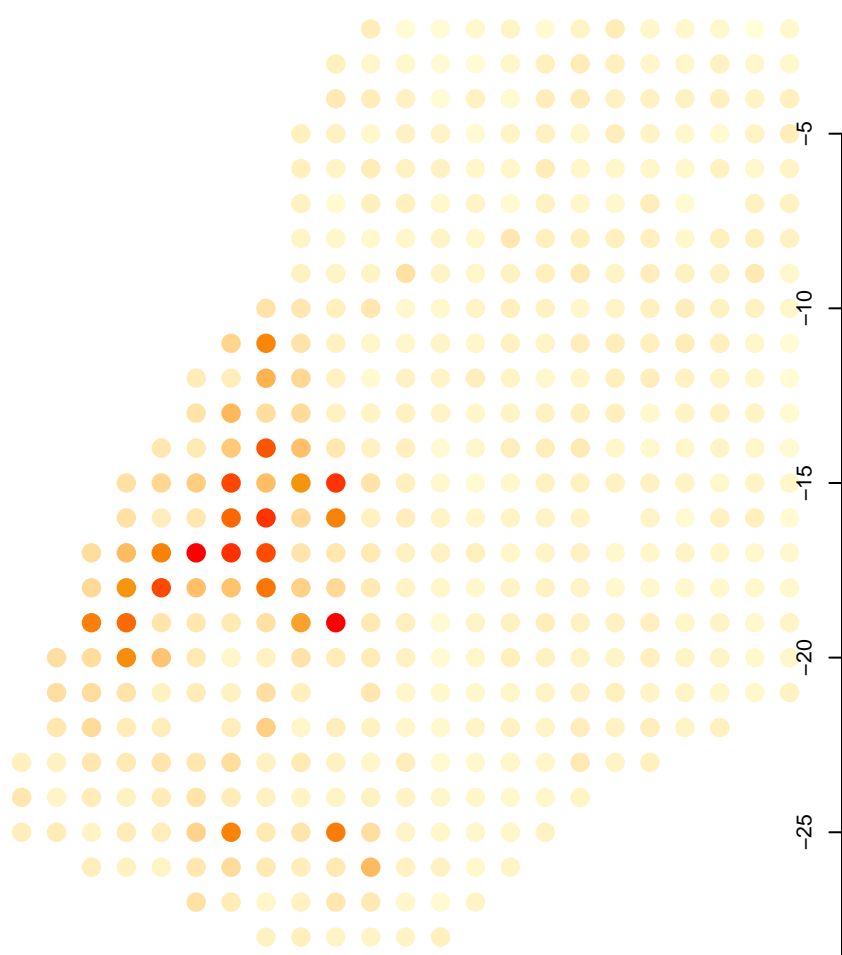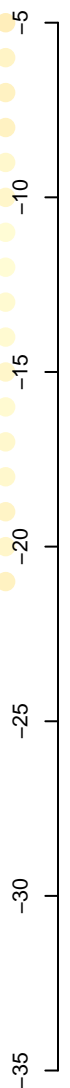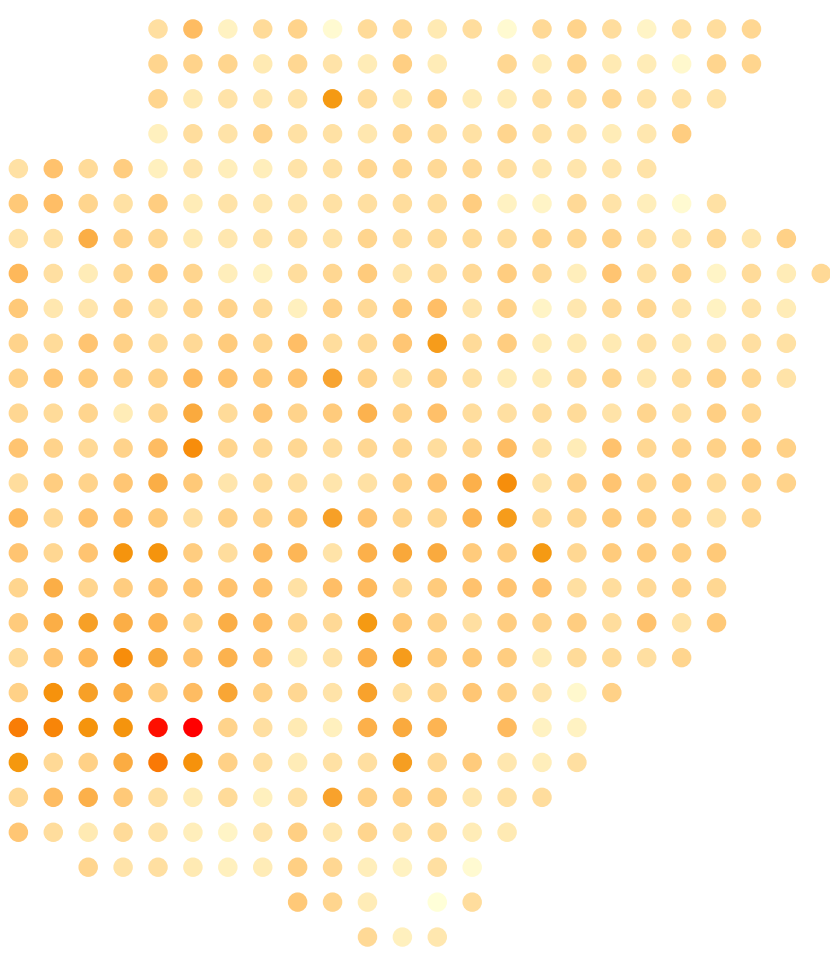

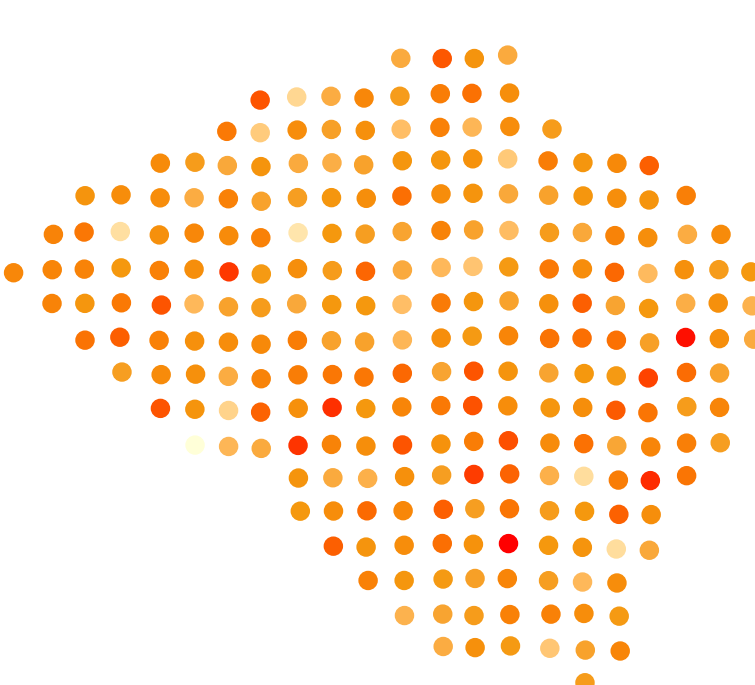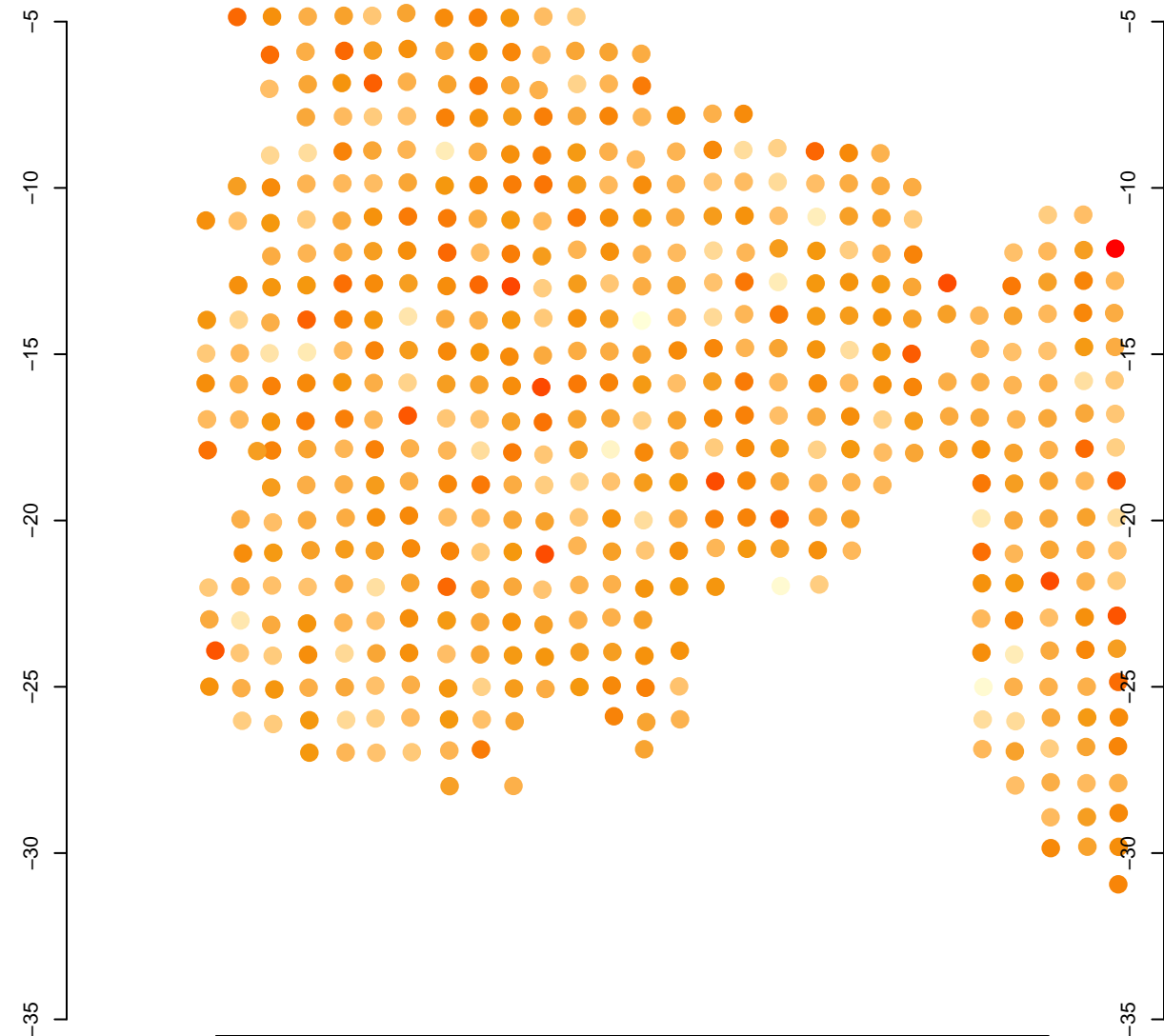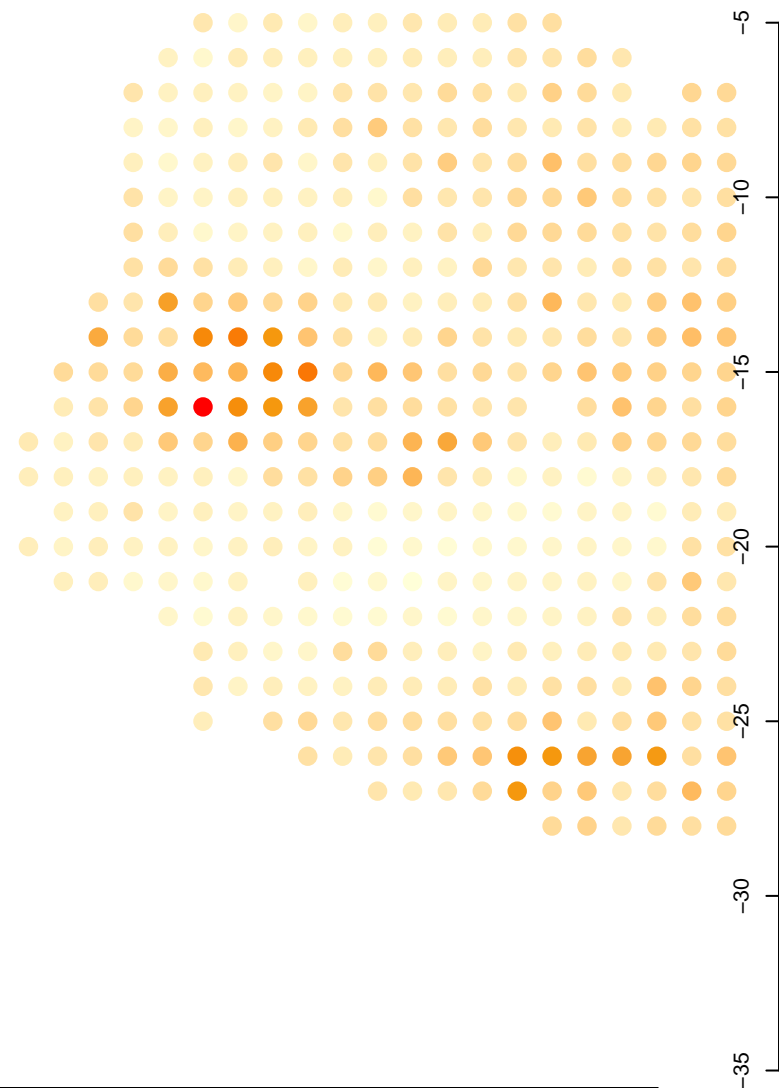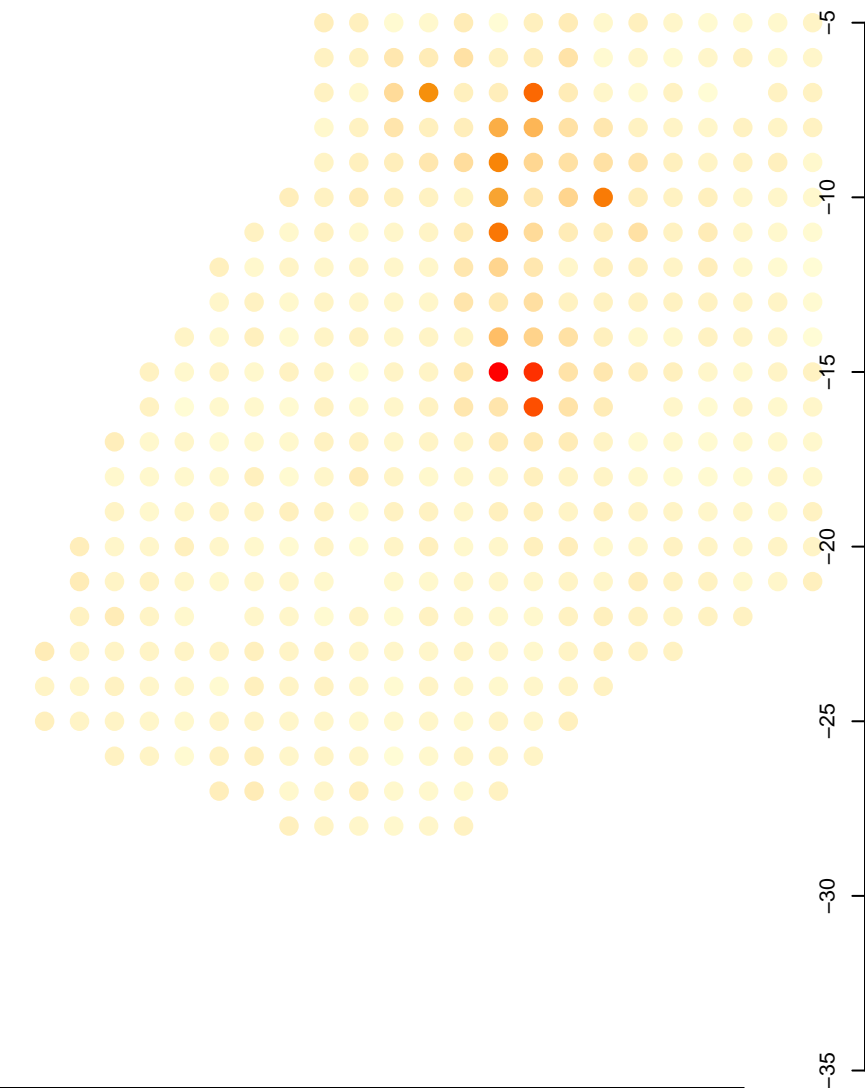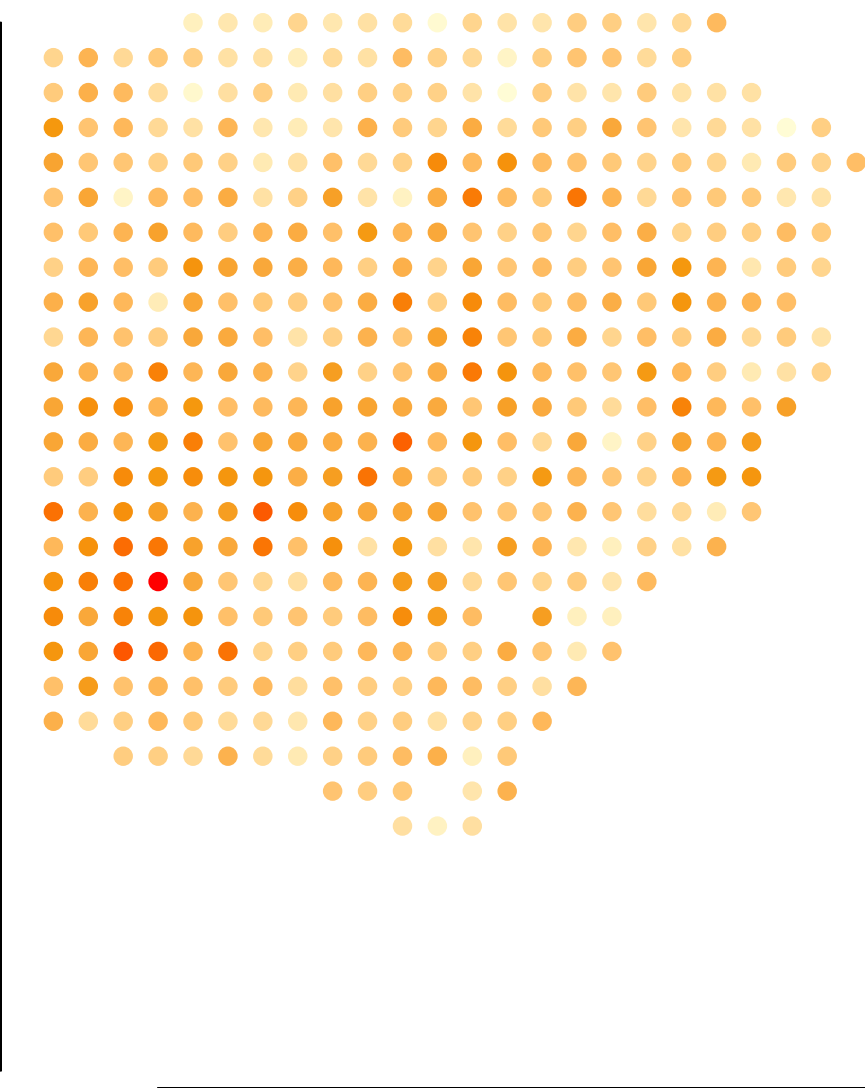

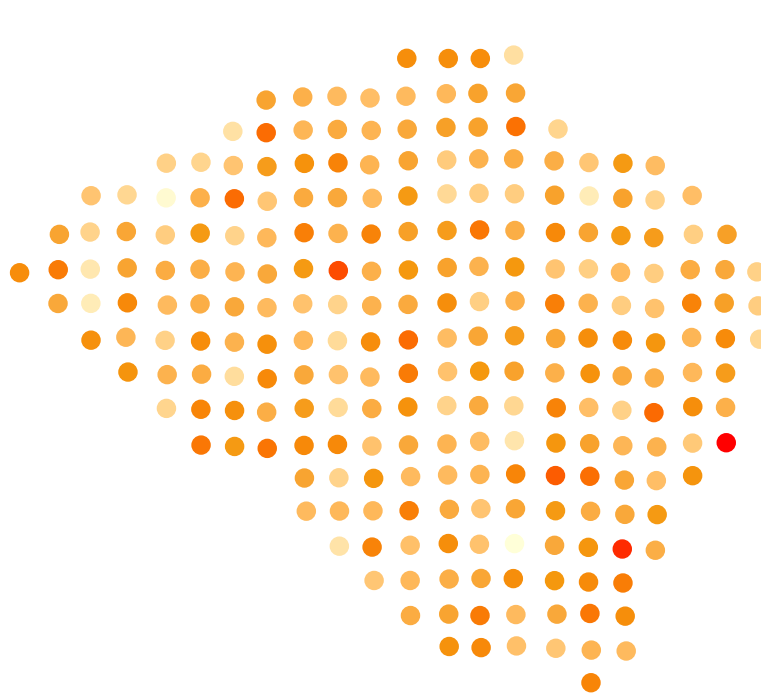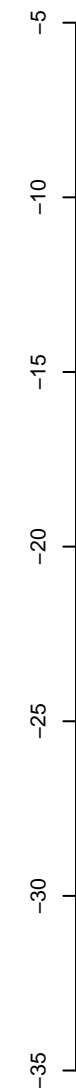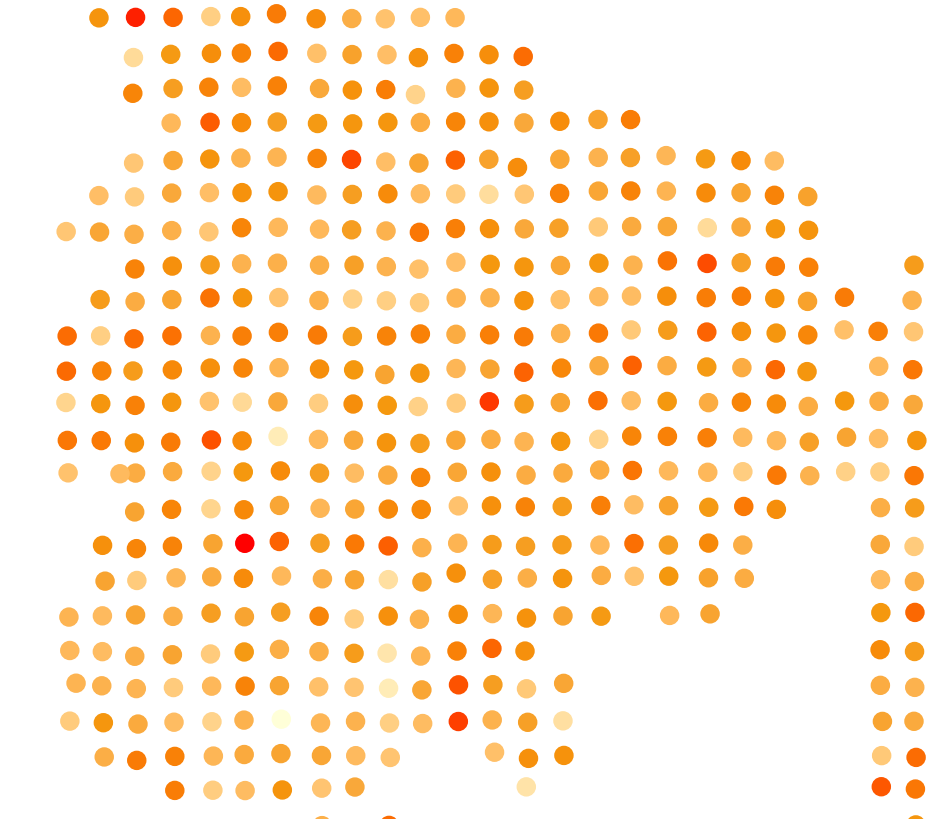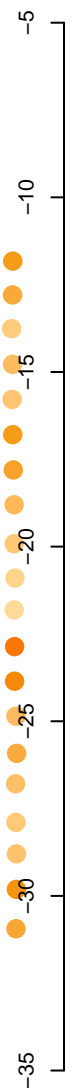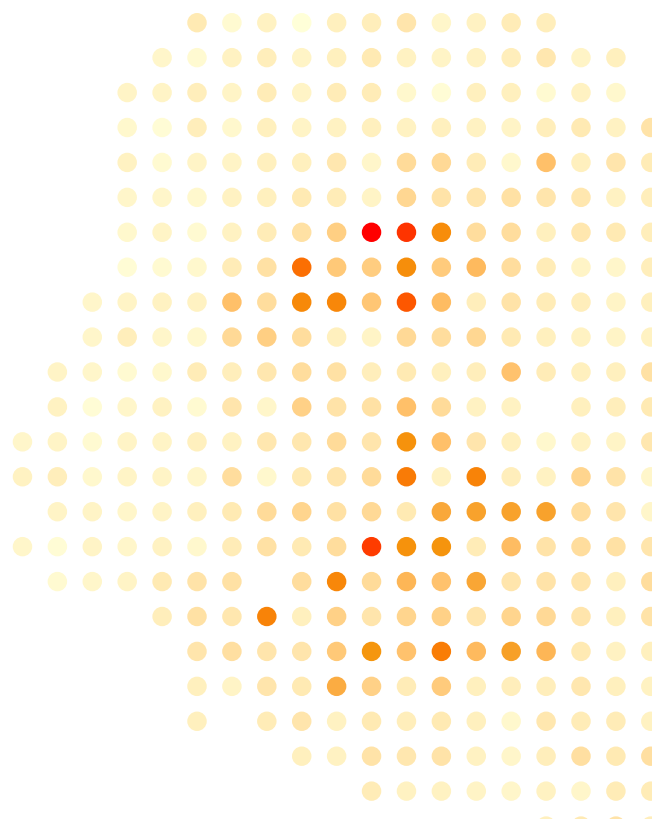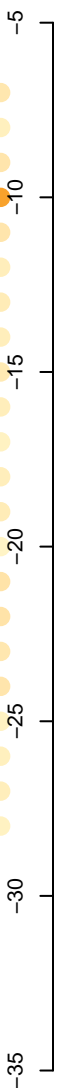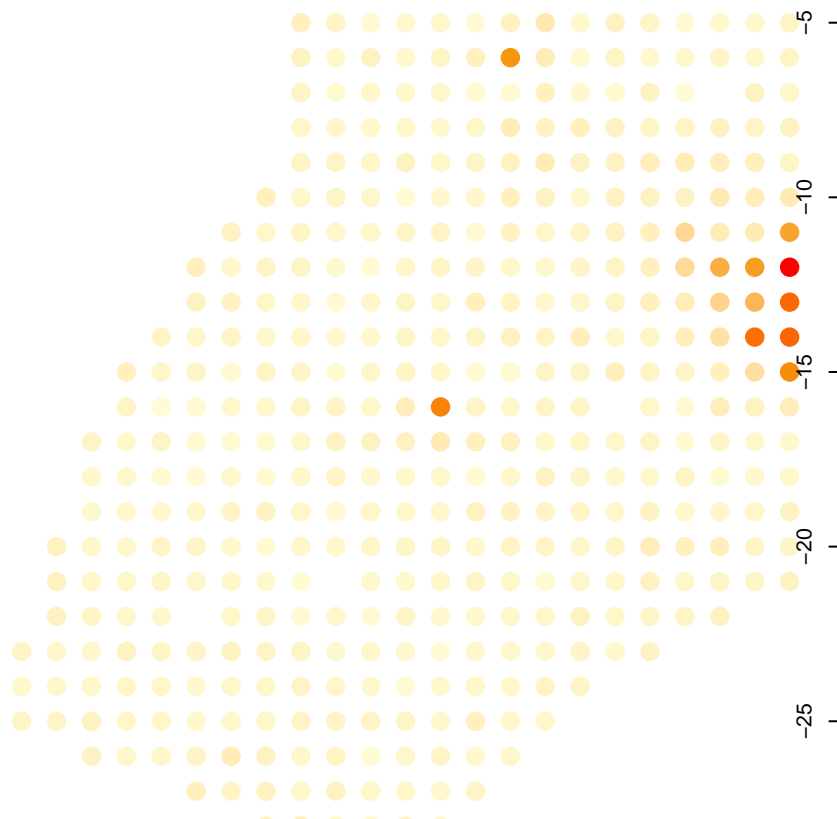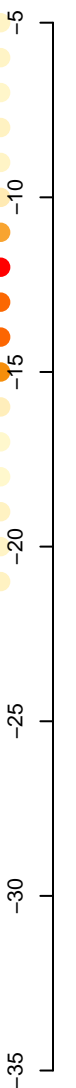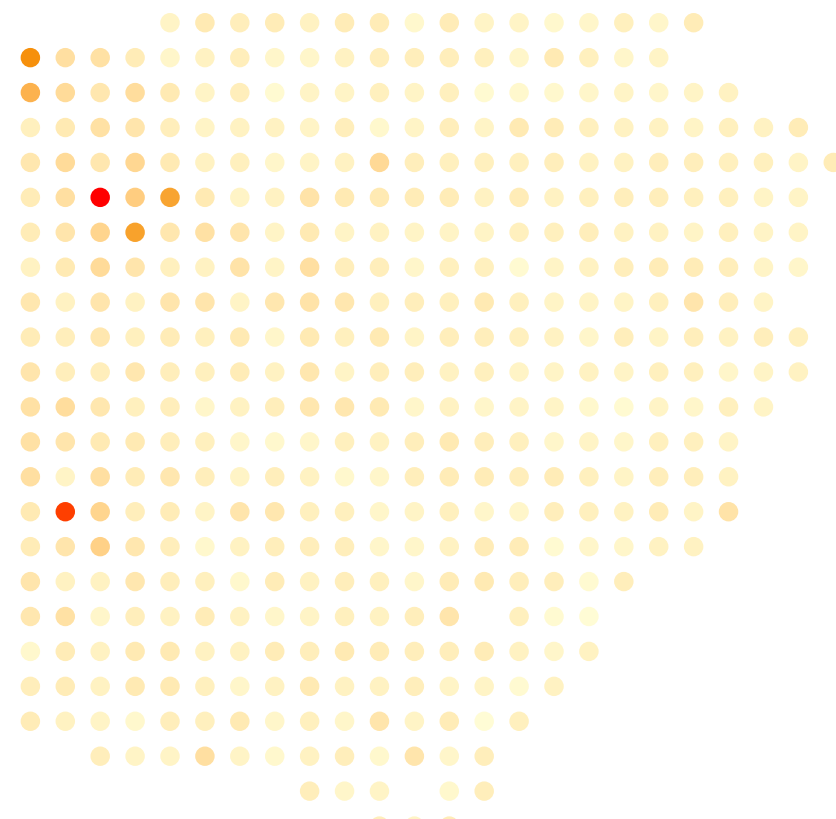

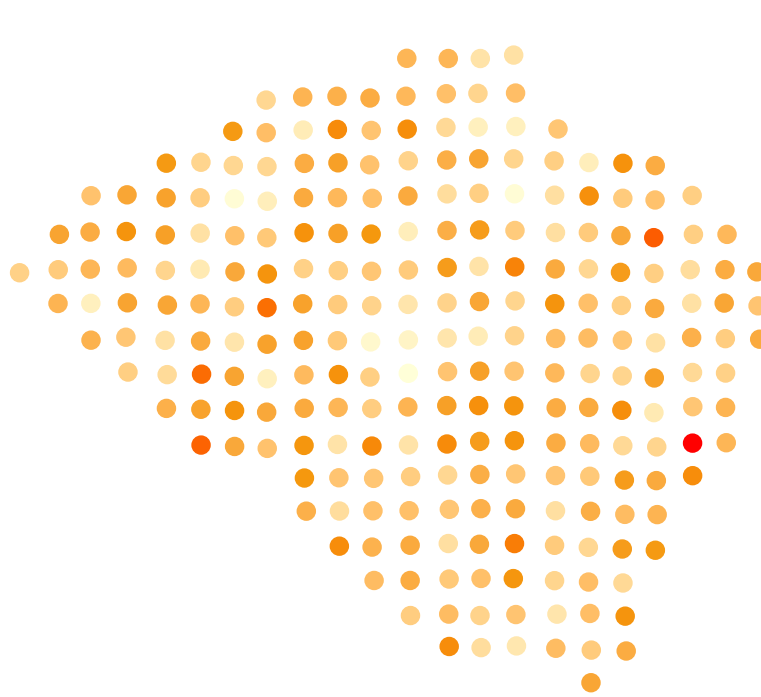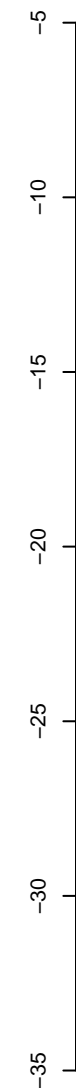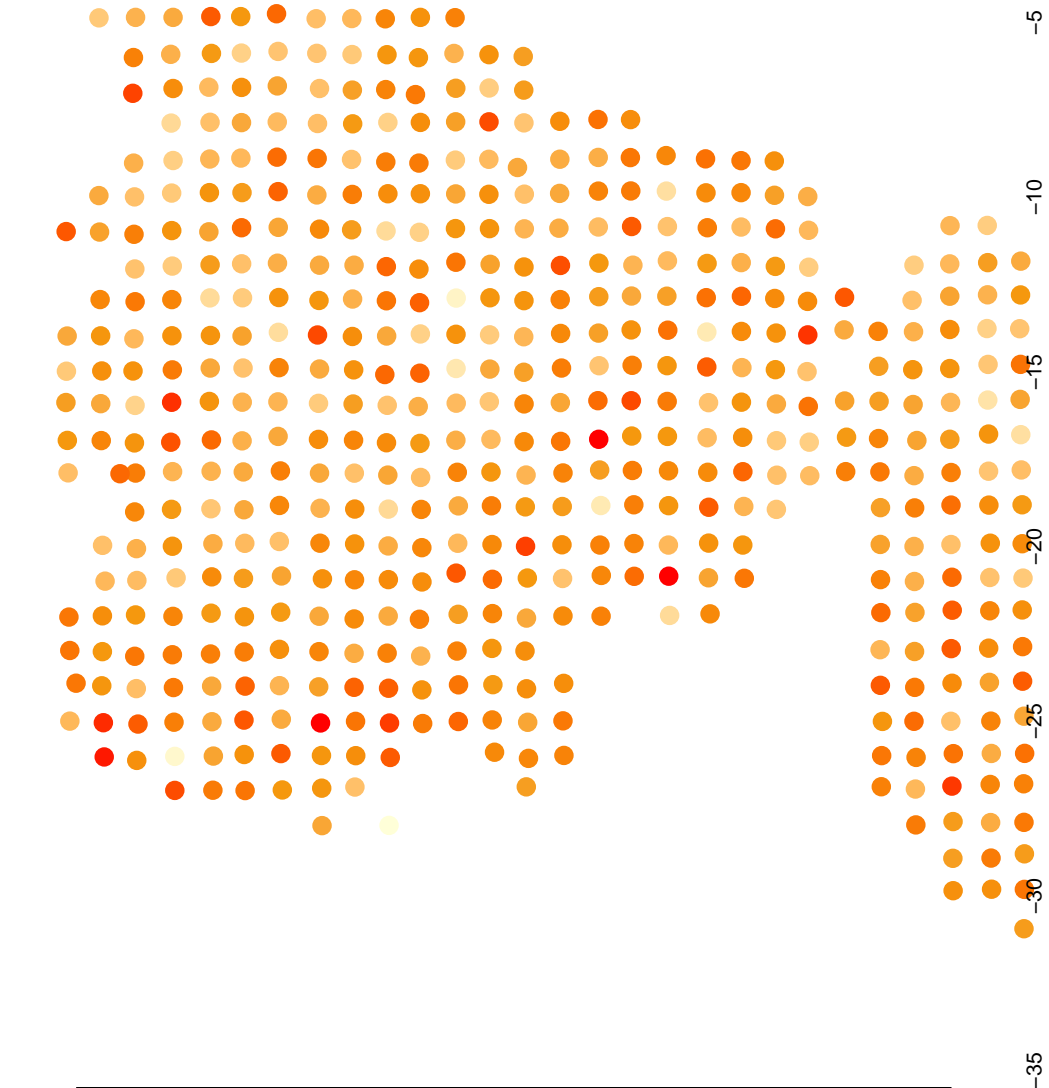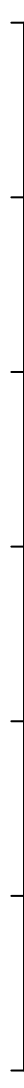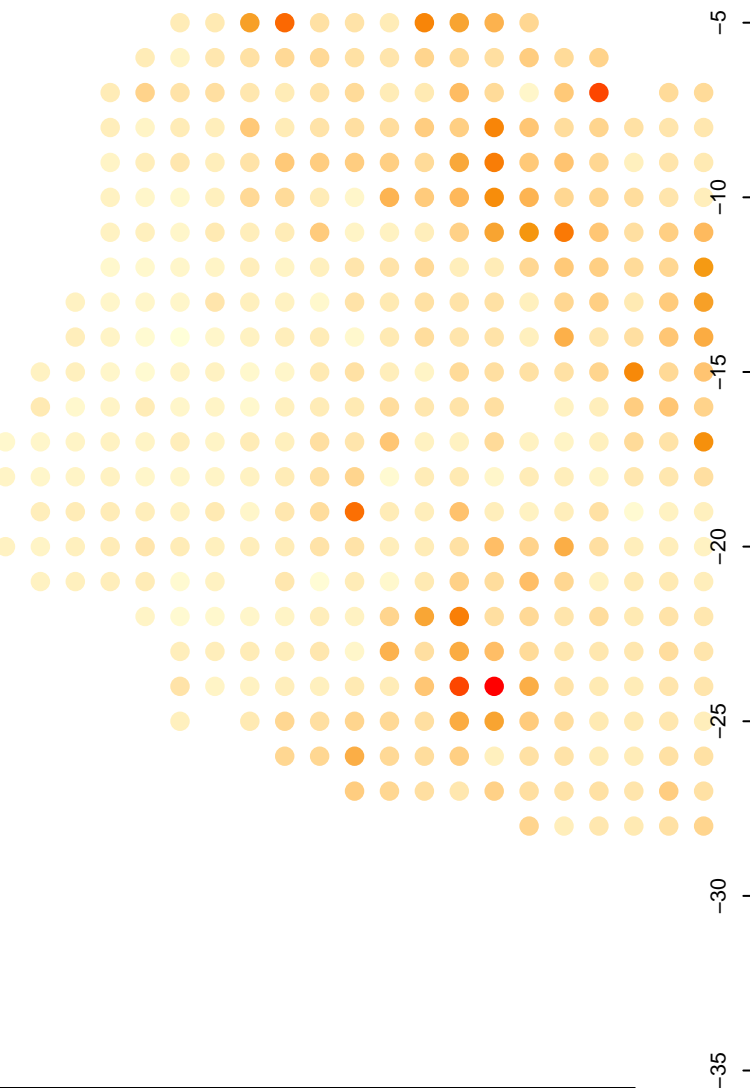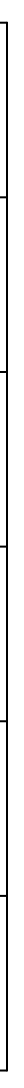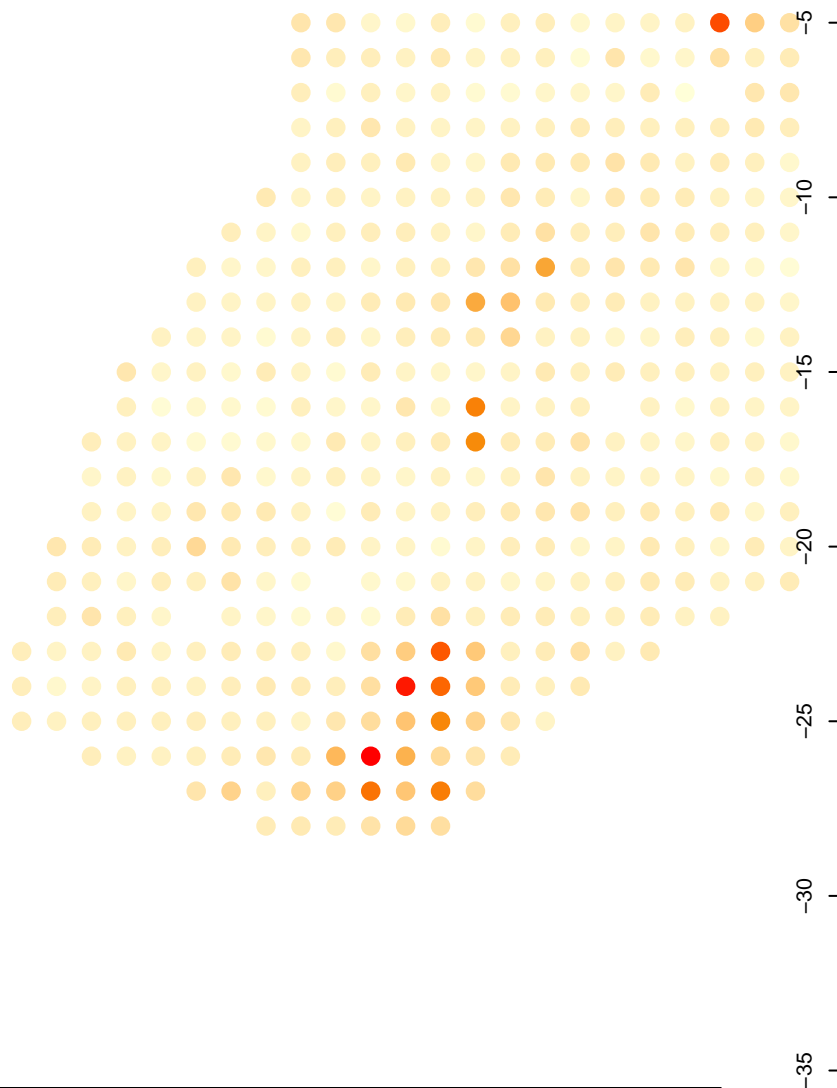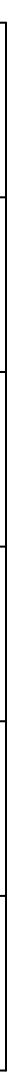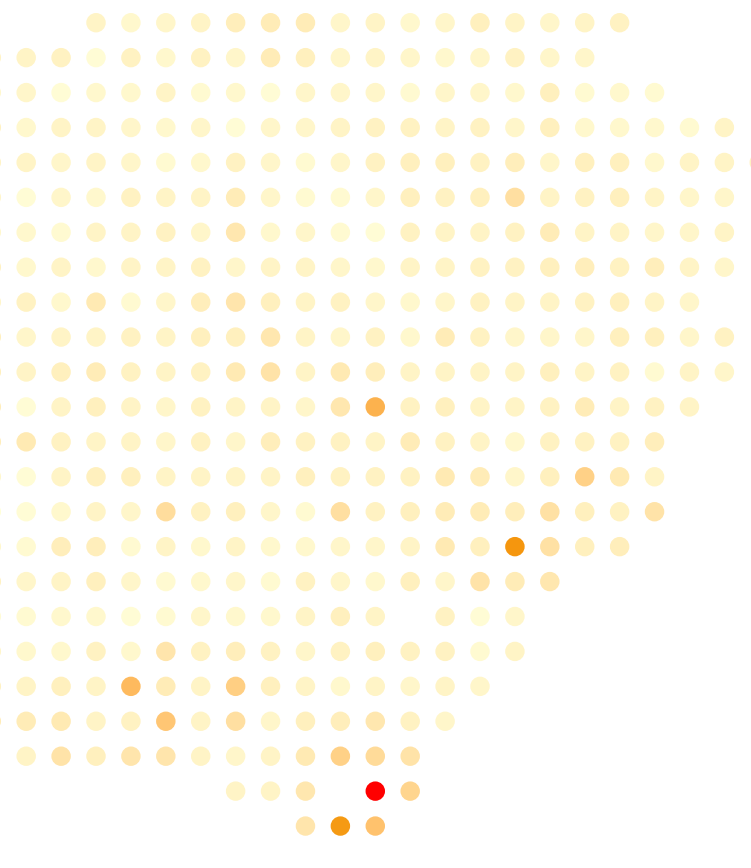

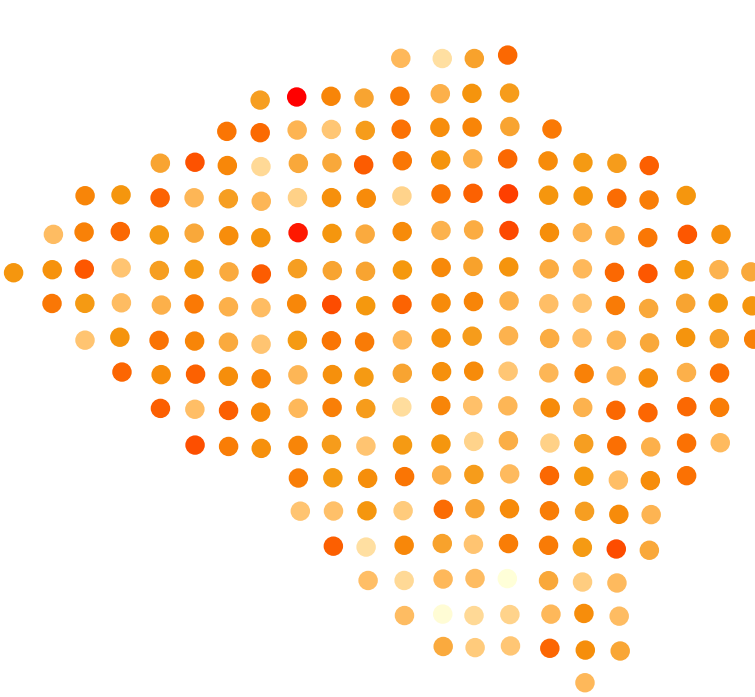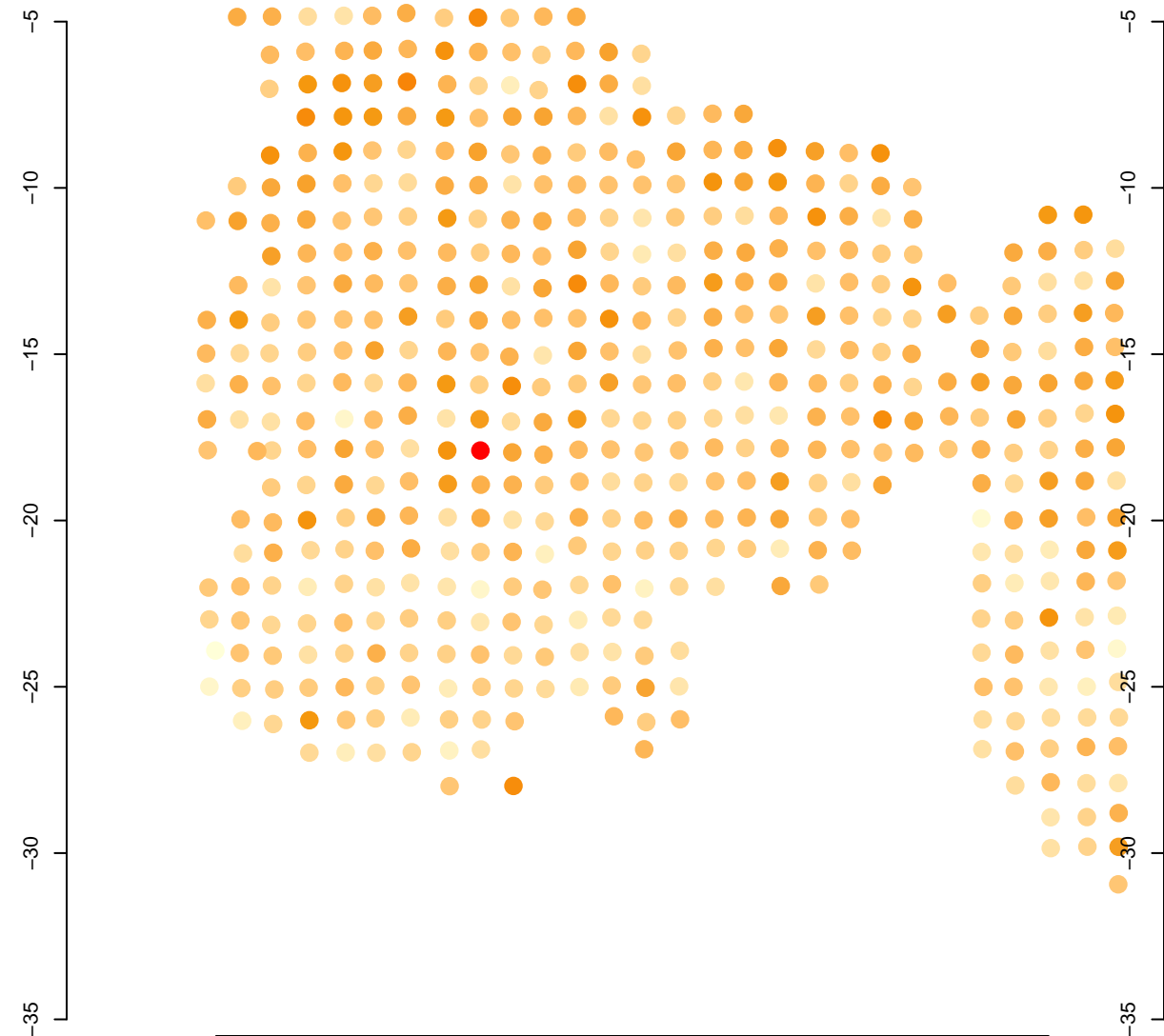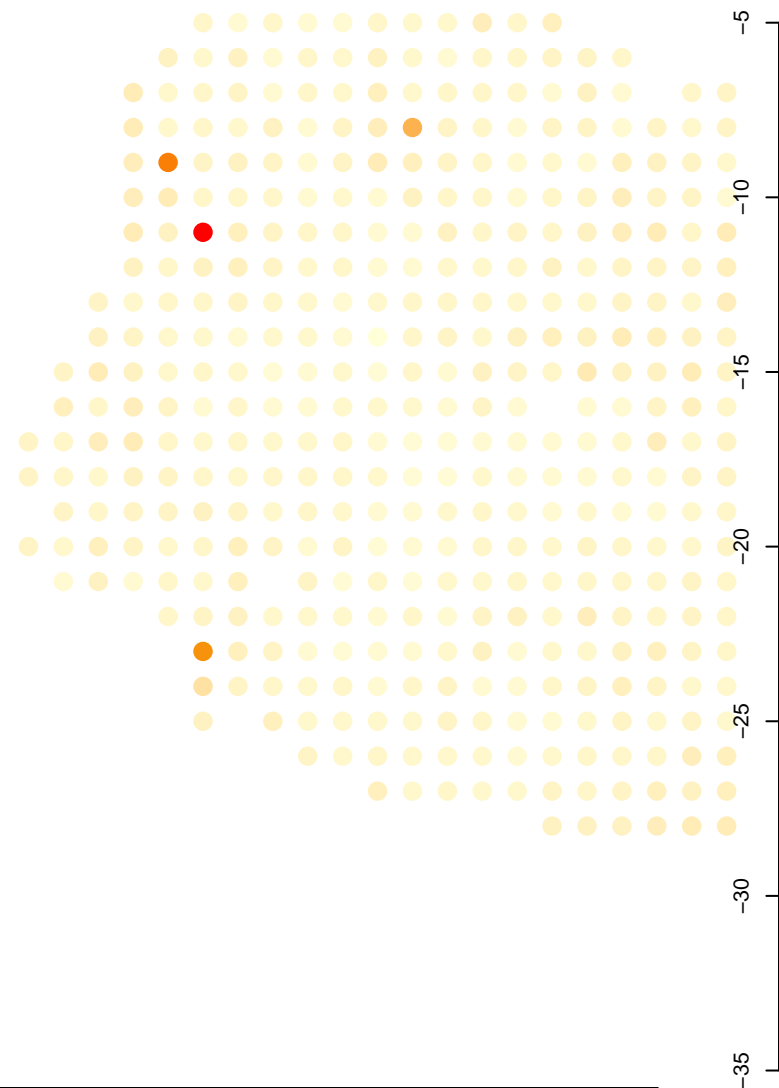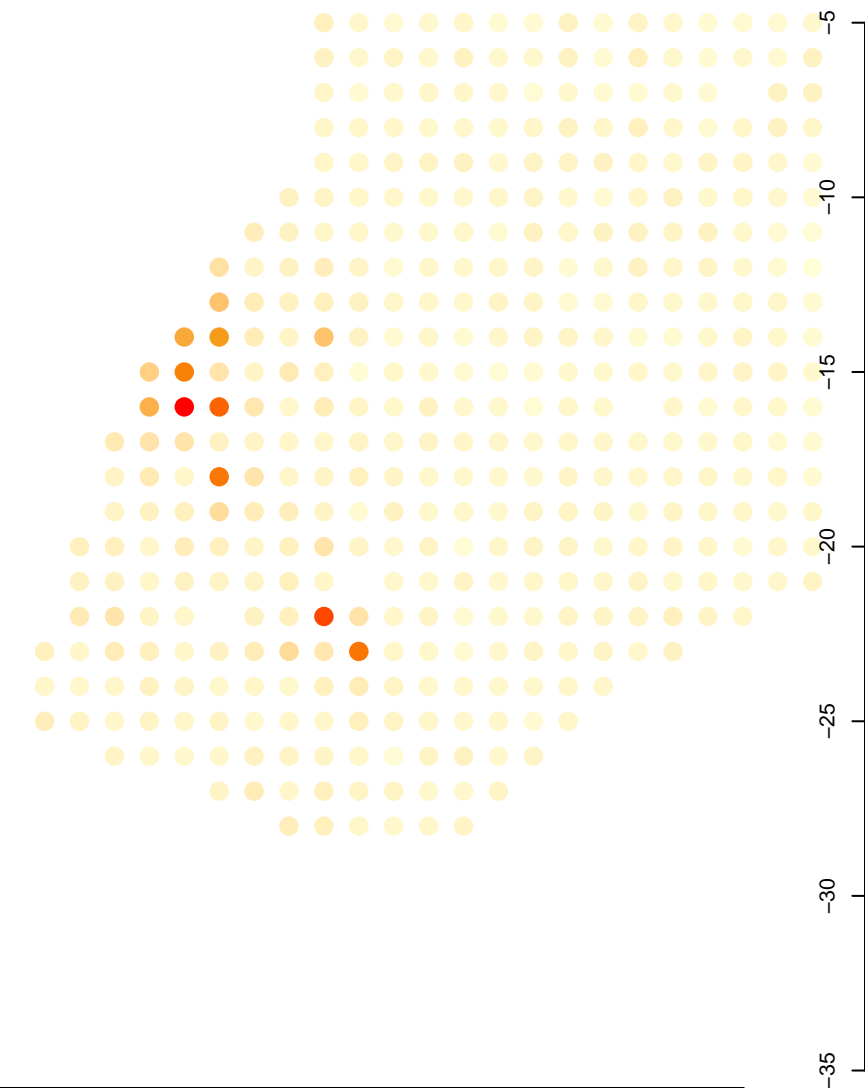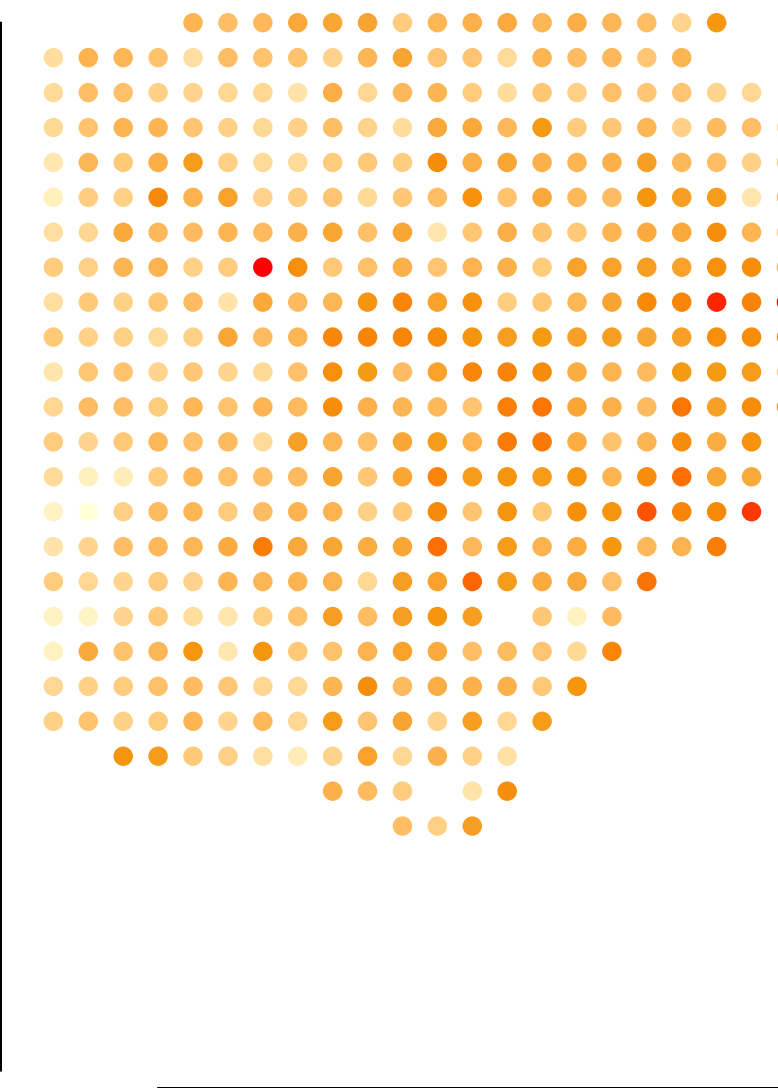

Supplement: Supplementary file 8 — Supplementary Data 5 [file 41467_2018_4724_MOESM8_ESM.zip › Supplementary Dataset 7/joint-mix-profiles-rel-individual-scale-dots.pdf]

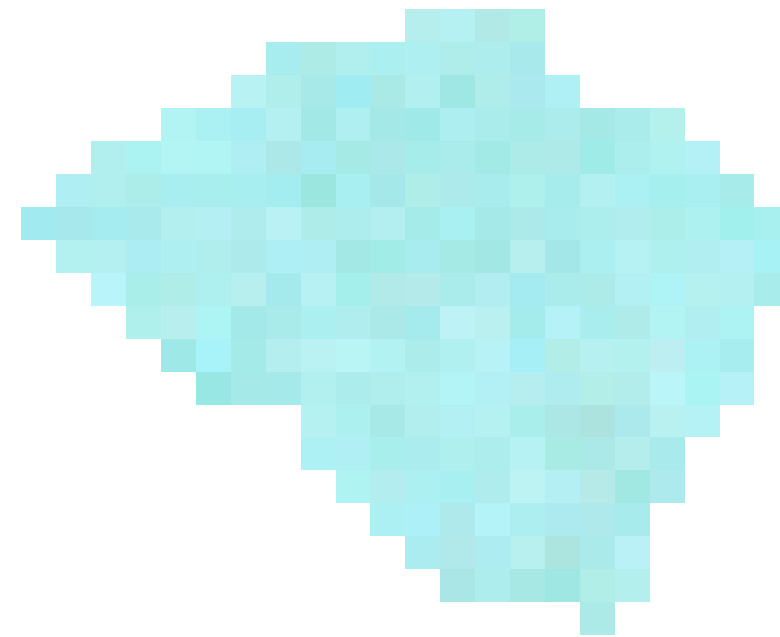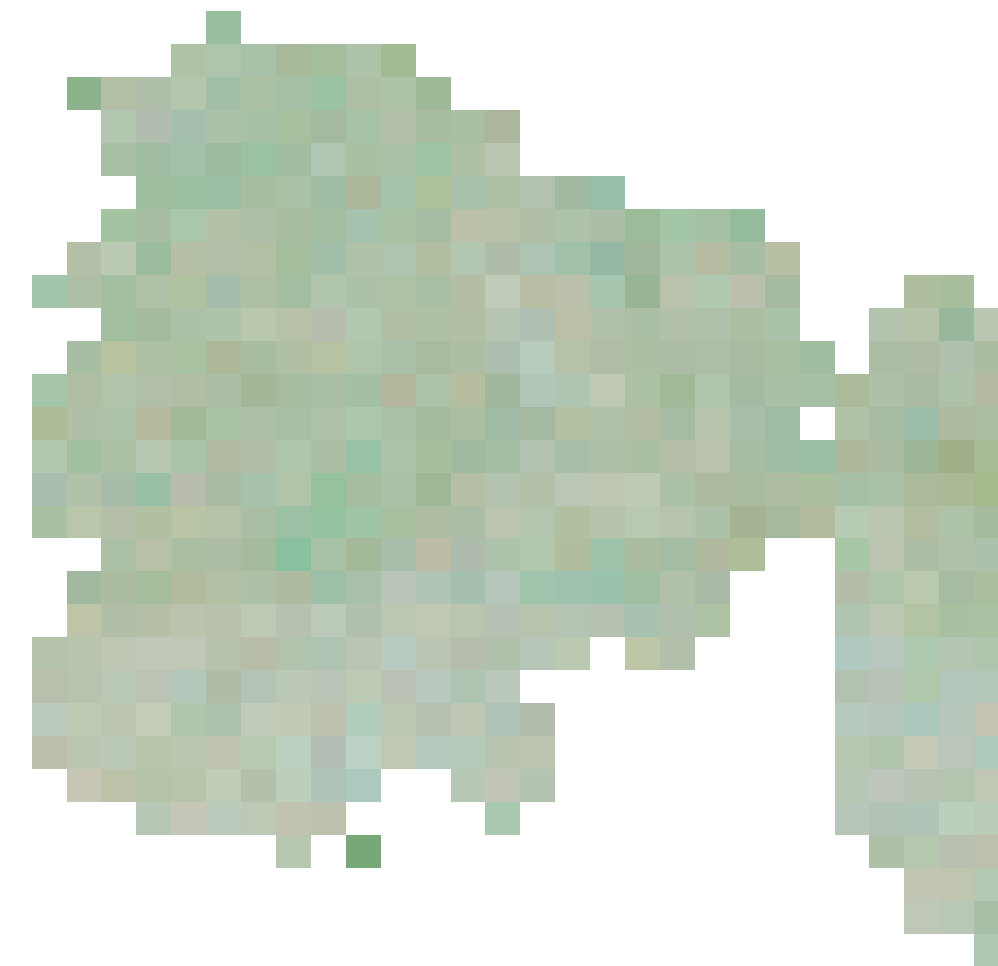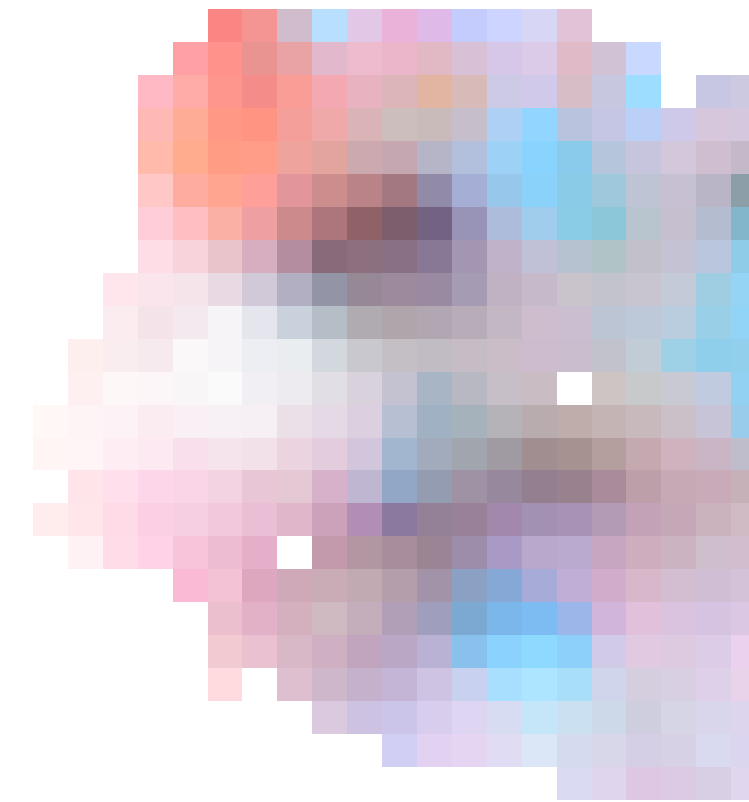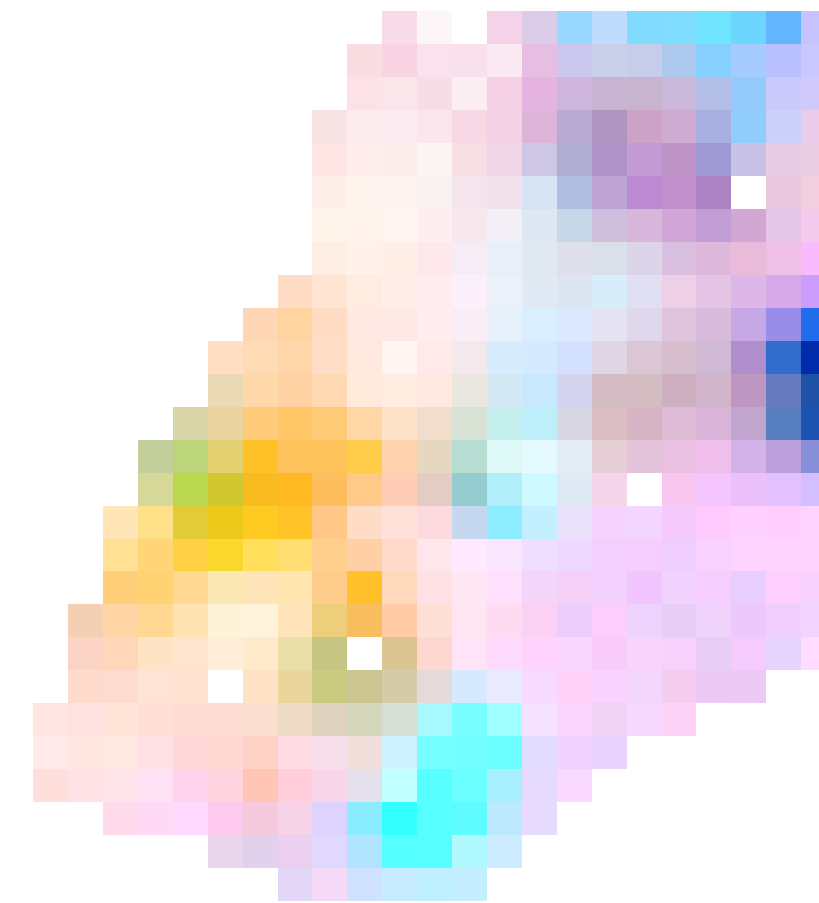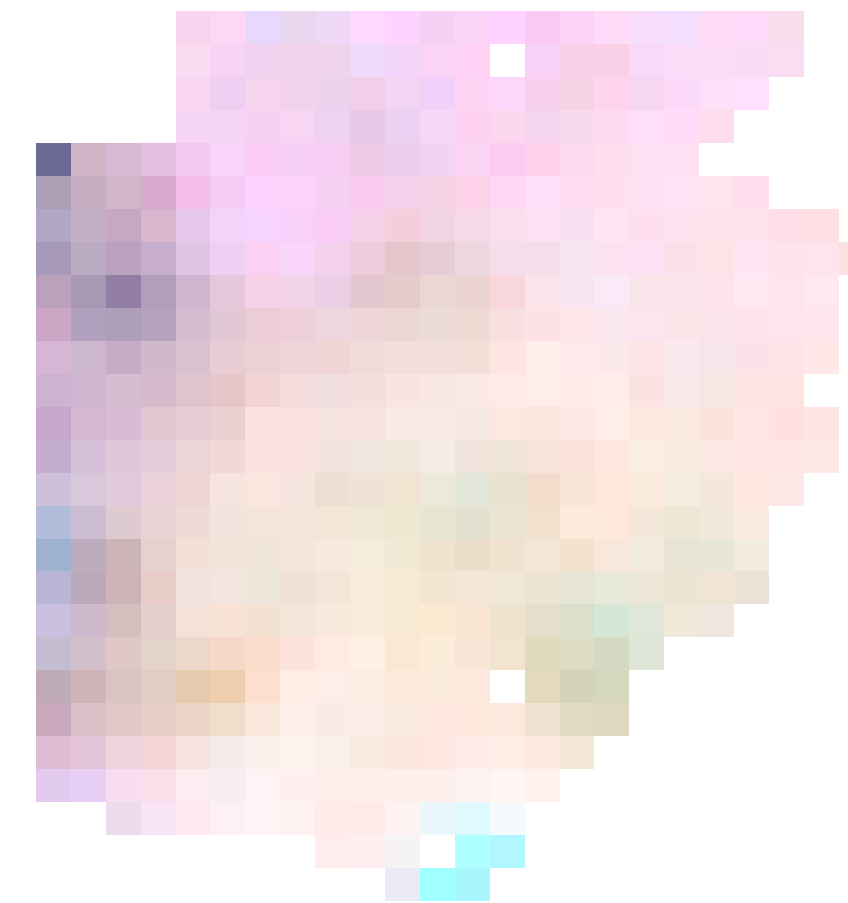

Supplement: Supplementary file 8 — Supplementary Data 5 [file 41467_2018_4724_MOESM8_ESM.zip › Supplementary Dataset 7/joint-field-dimensionality-reduction-PCA-matrix.pdf]

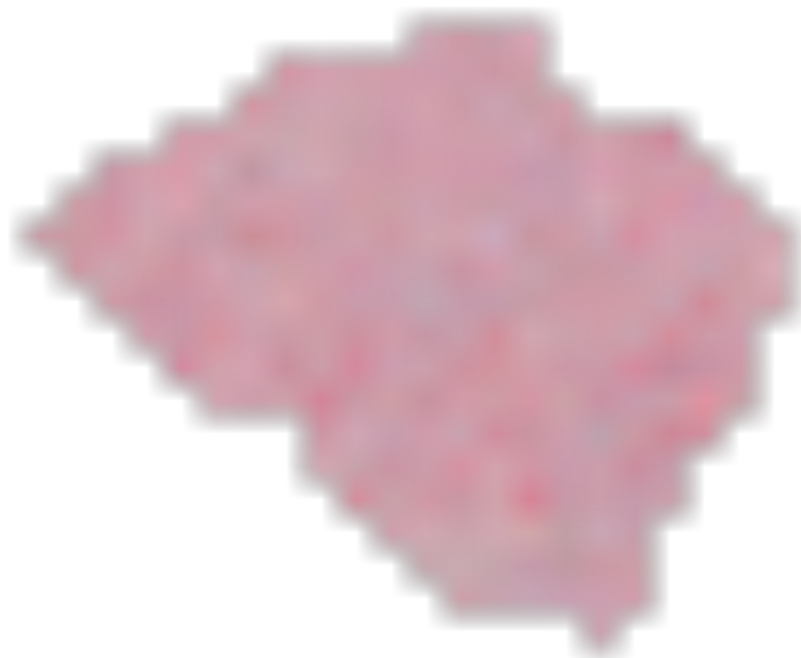

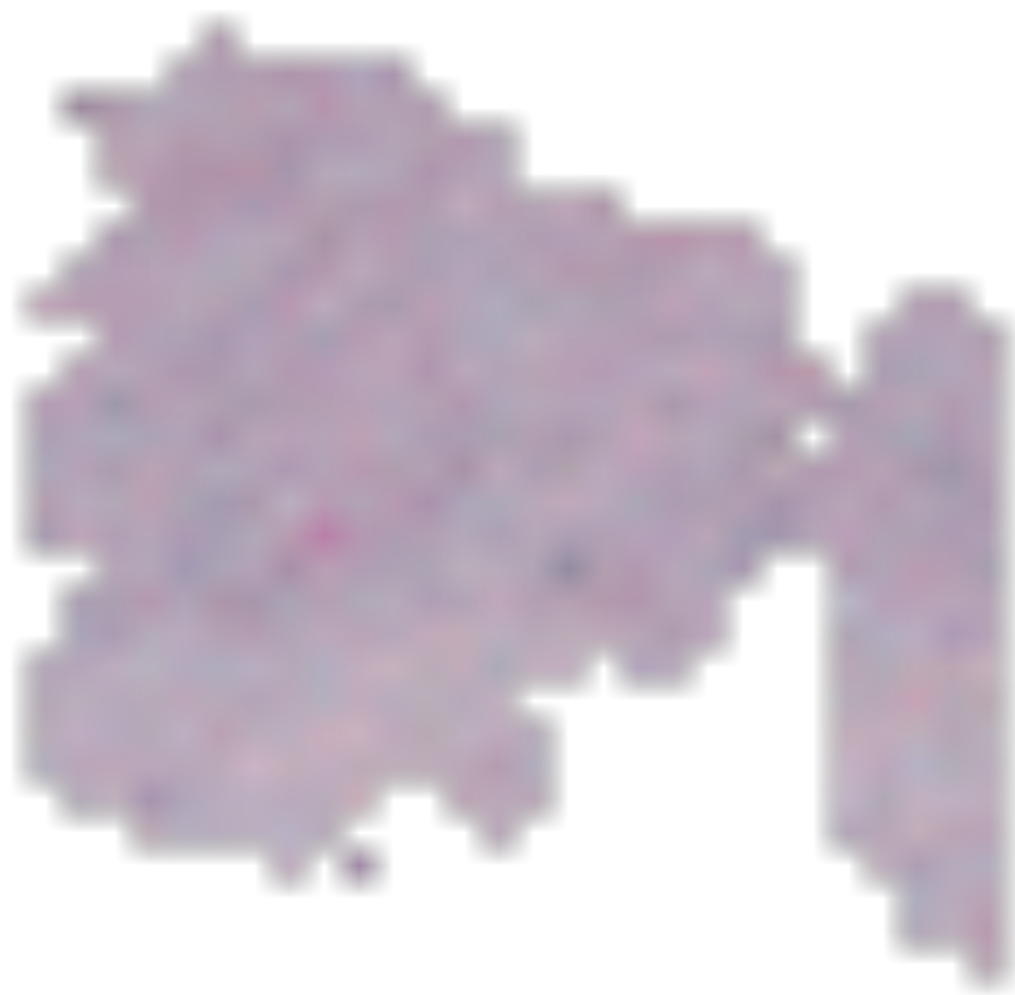

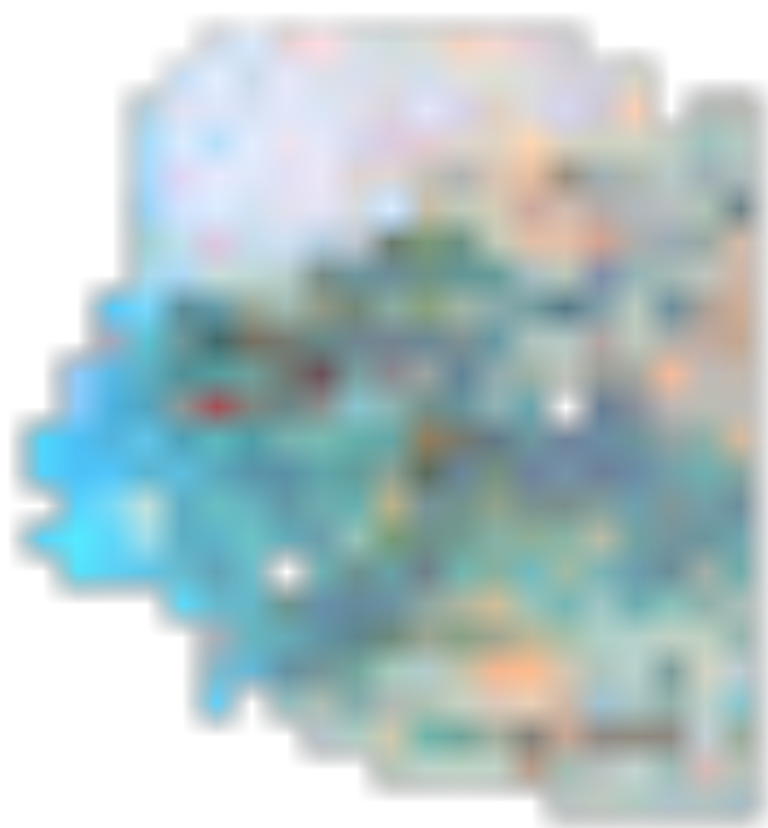

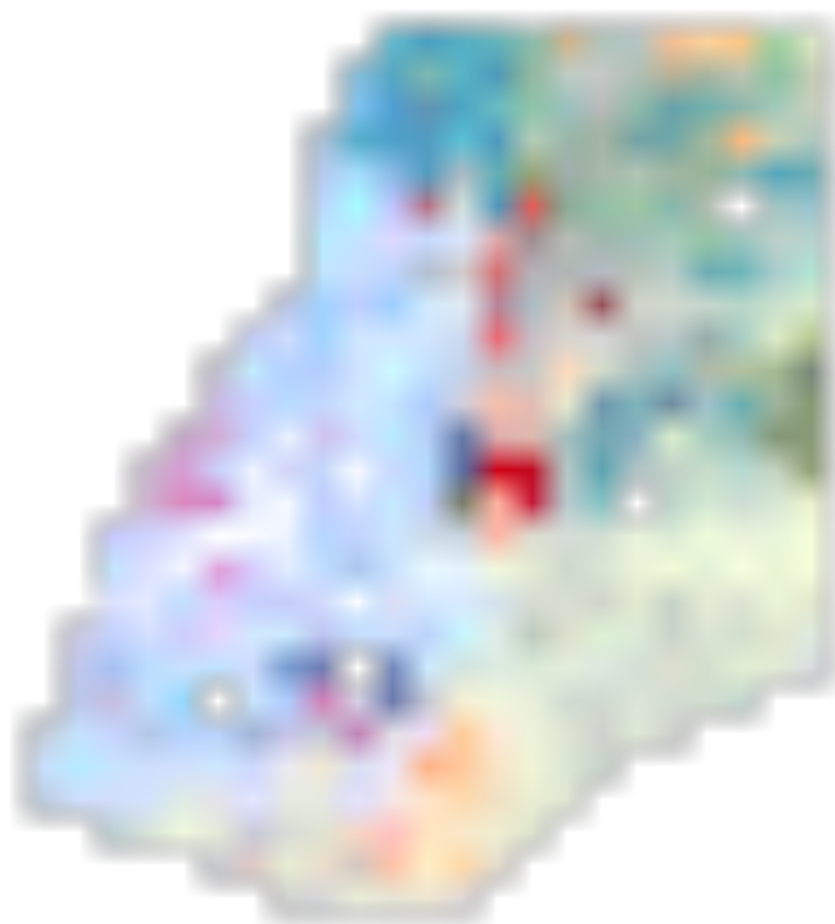

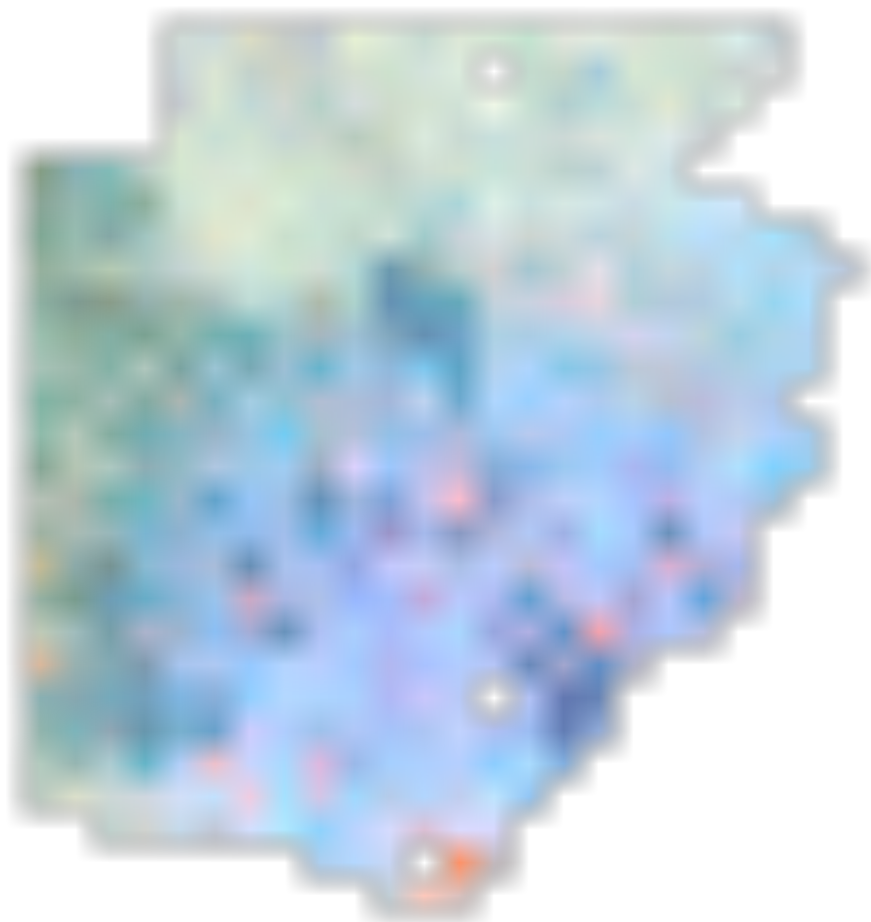

Supplement: Supplementary file 8 — Supplementary Data 5 [file 41467_2018_4724_MOESM8_ESM.zip › Supplementary Dataset 7/joint-mix-dimensionality-reduction-PCA-matrix-split.pdf.interpolated.pdf]

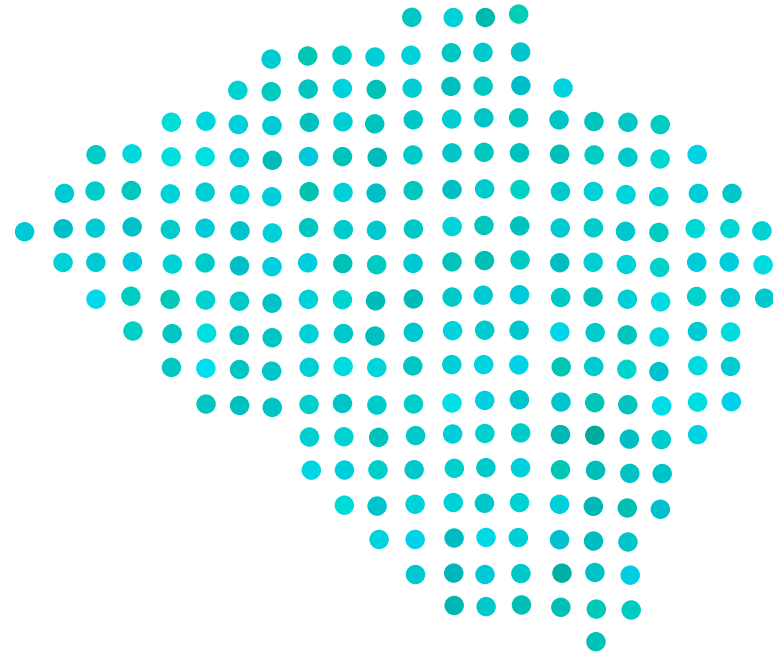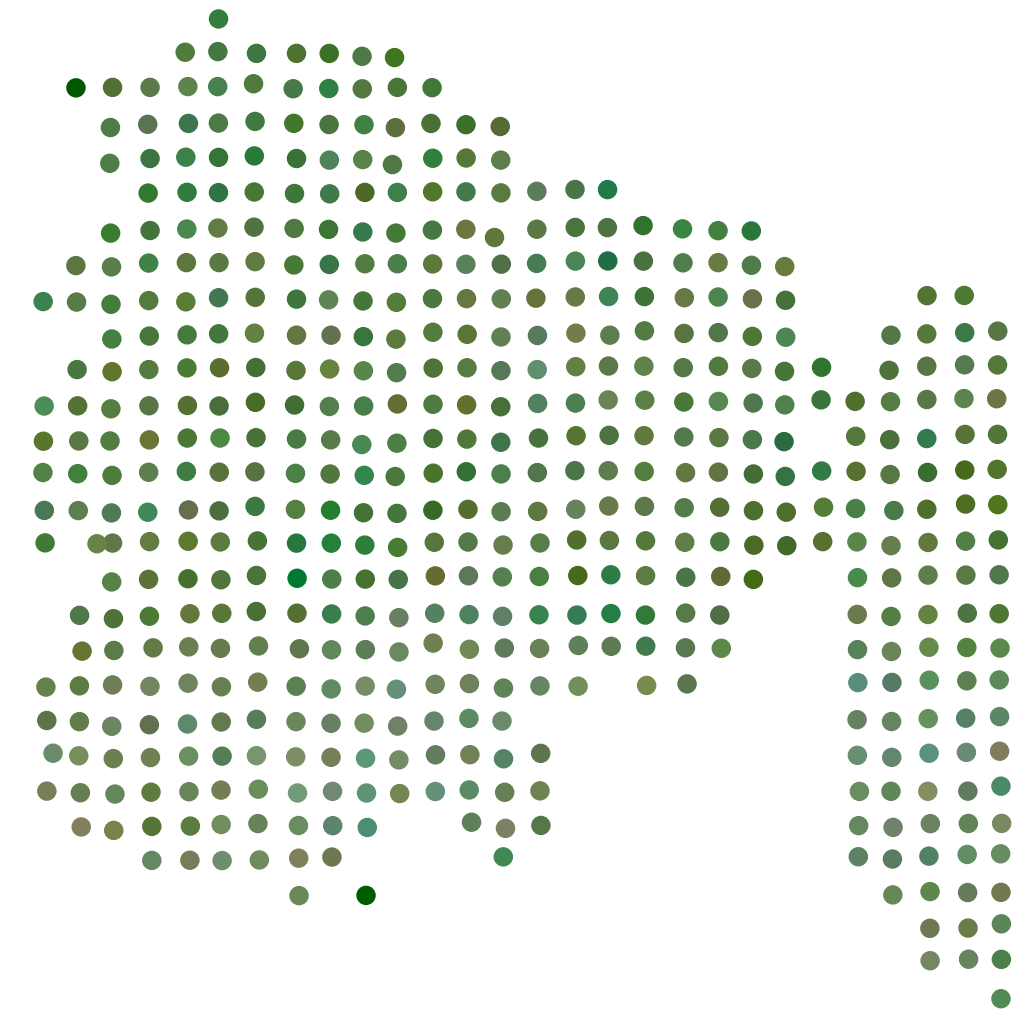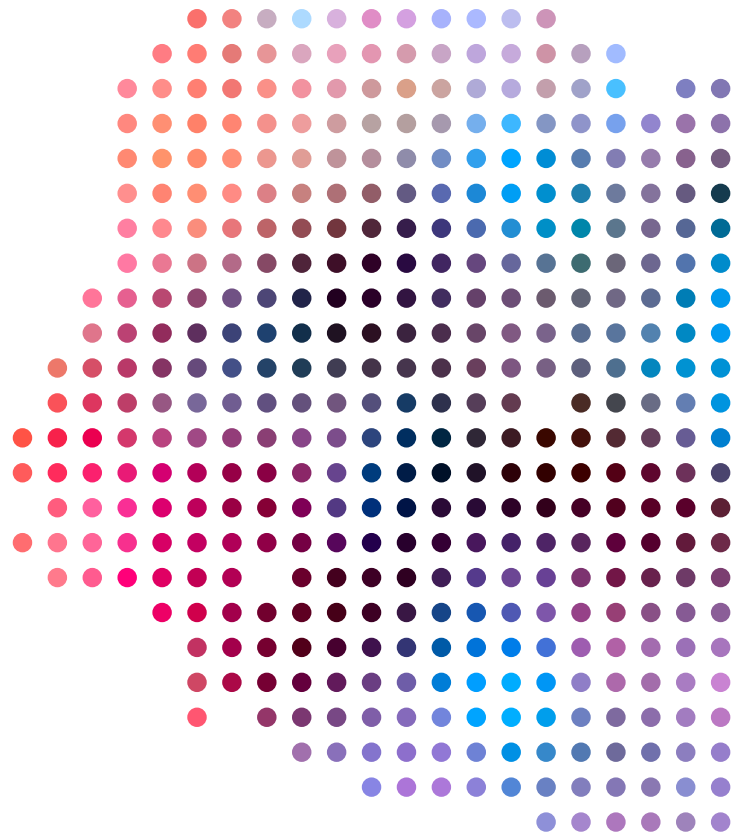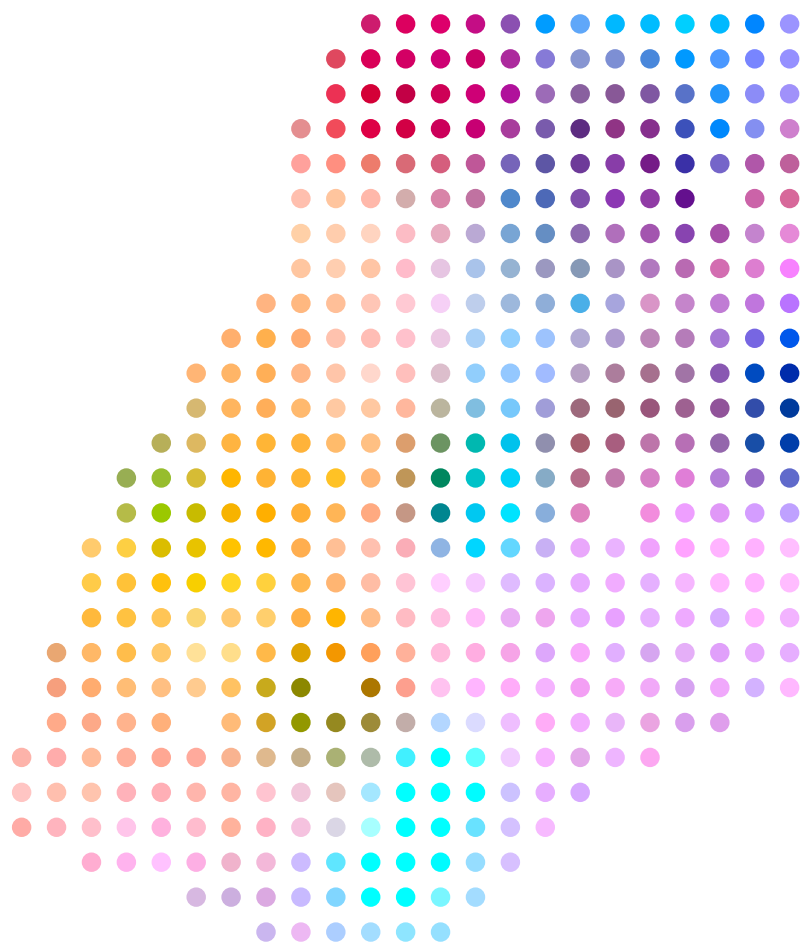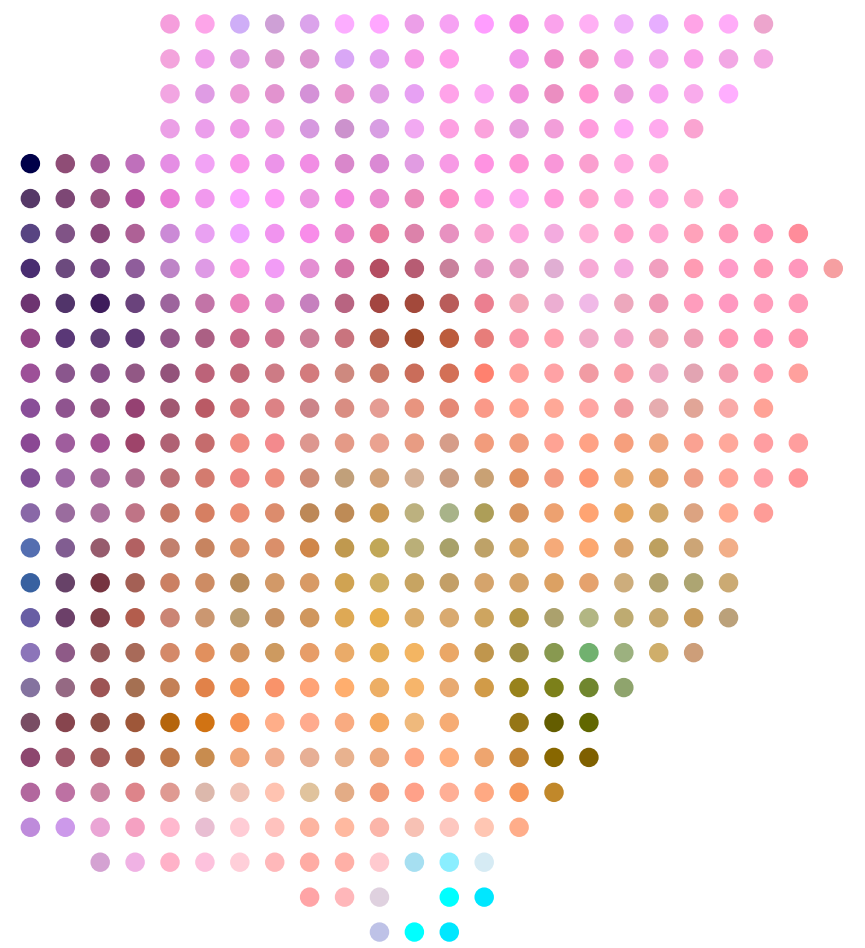

Supplement: Supplementary file 8 — Supplementary Data 5 [file 41467_2018_4724_MOESM8_ESM.zip › Supplementary Dataset 7/joint-field-dimensionality-reduction-PCA-dots.pdf]

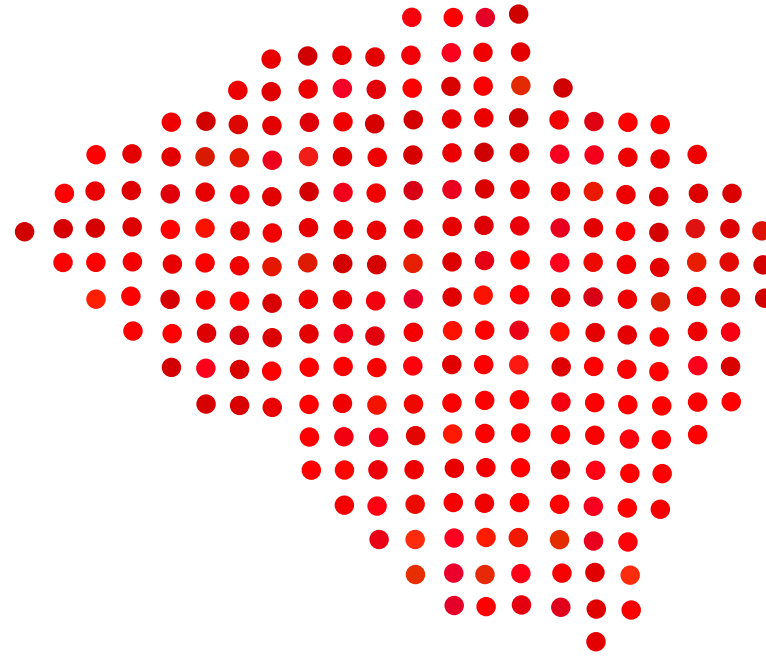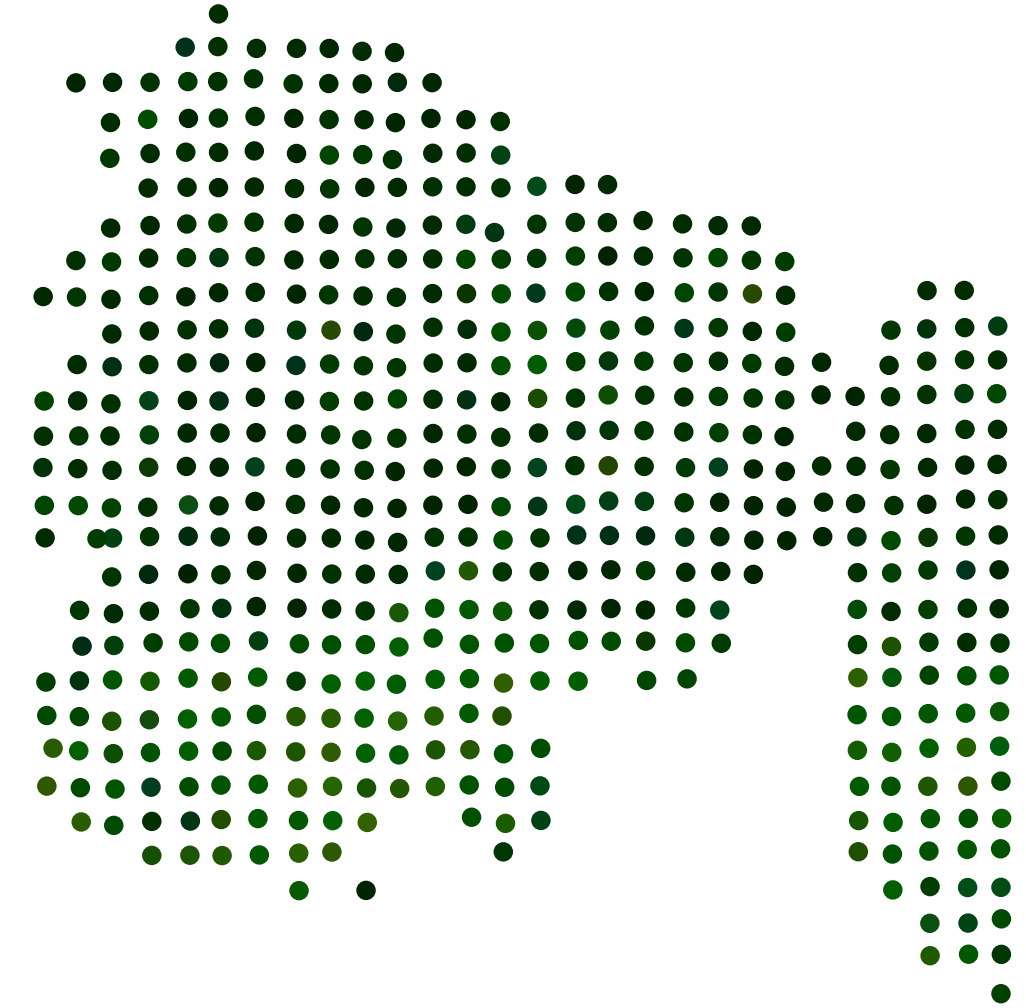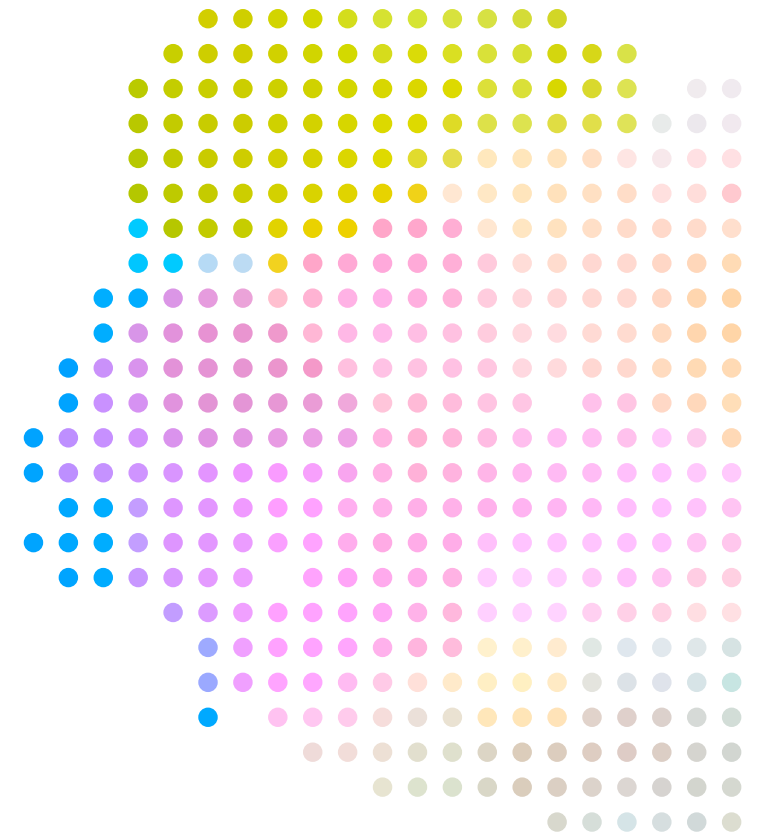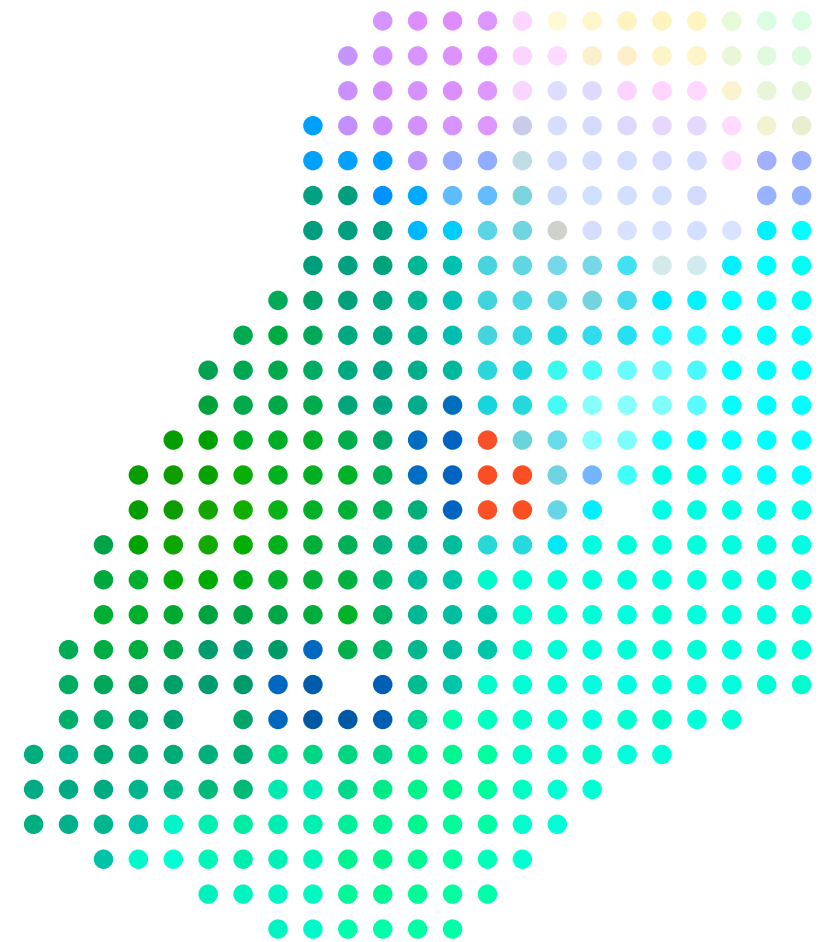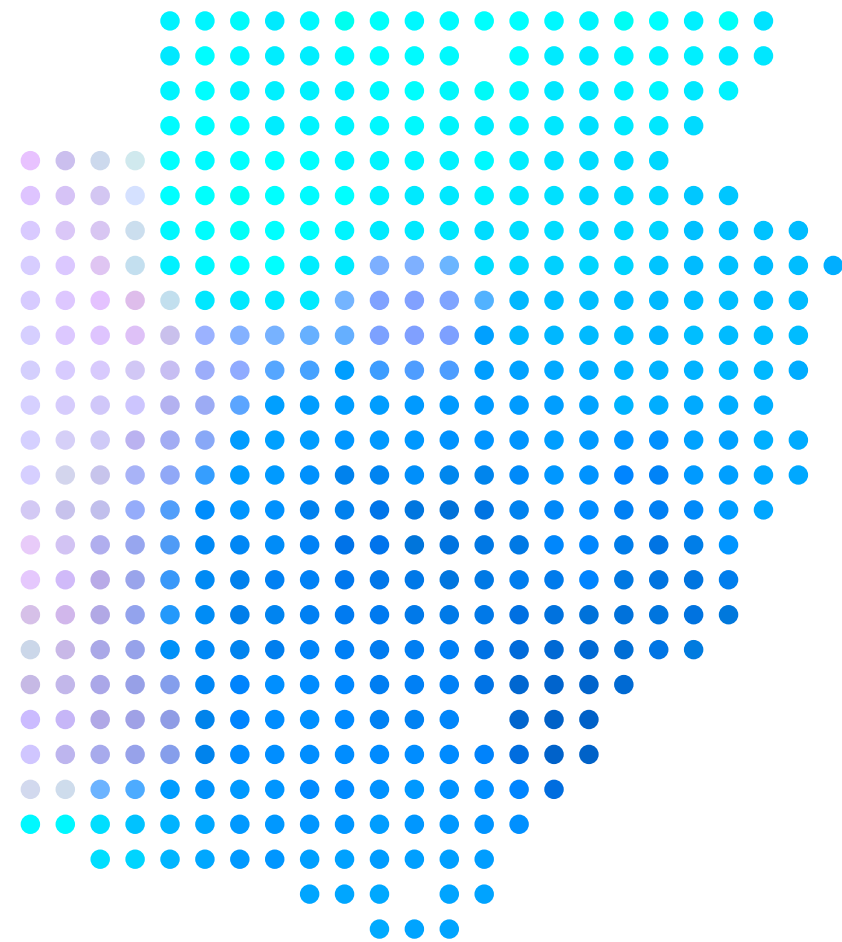

Supplement: Supplementary file 8 — Supplementary Data 5 [file 41467_2018_4724_MOESM8_ESM.zip › Supplementary Dataset 7/joint-field-dimensionality-reduction-tSNE-dots.pdf]

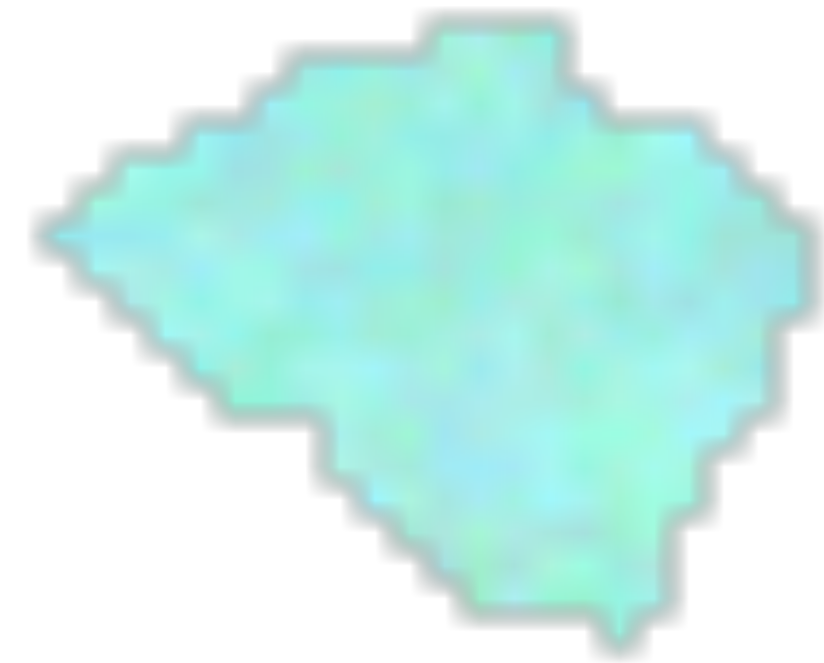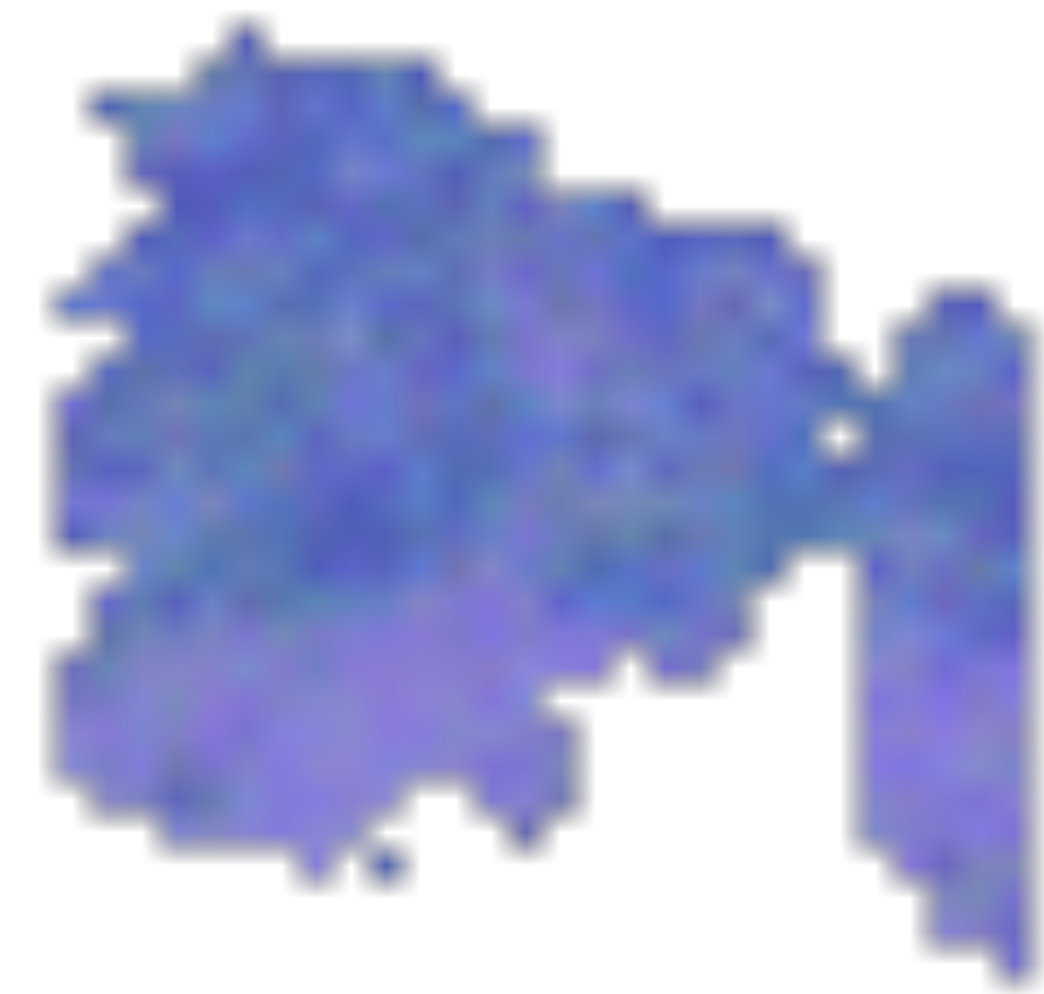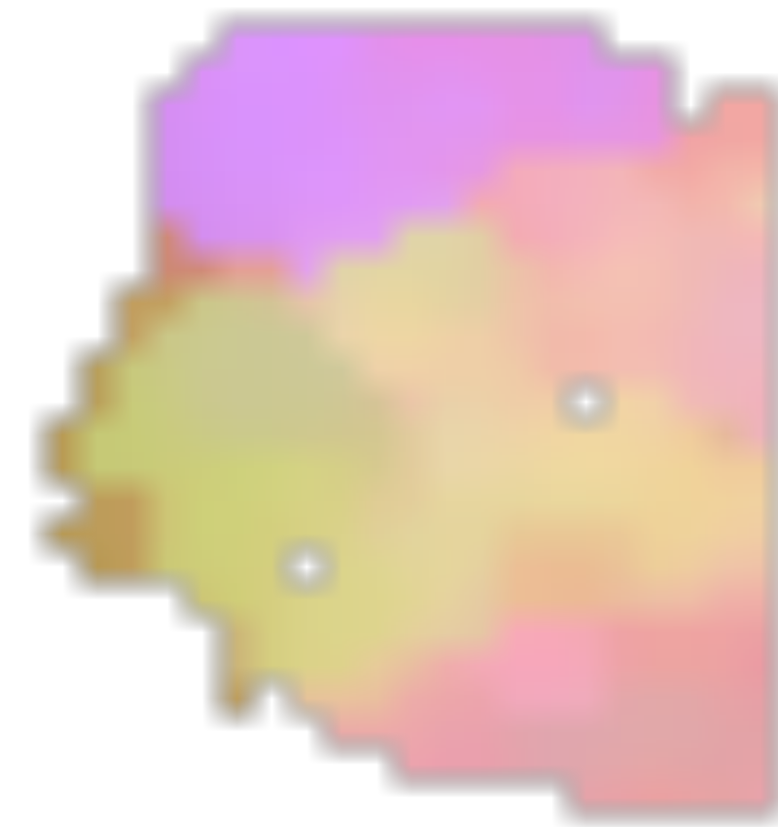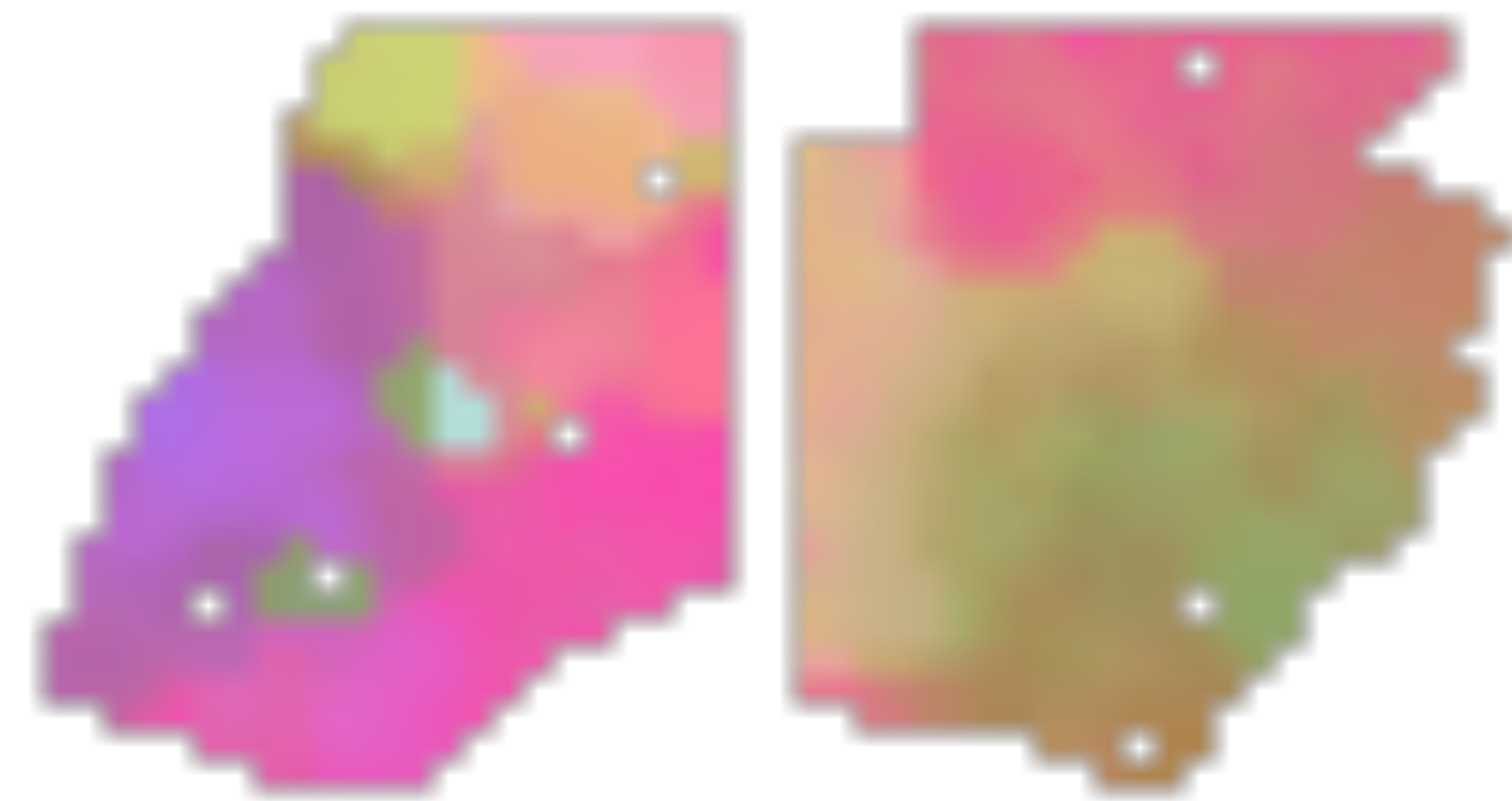

Supplement: Supplementary file 8 — Supplementary Data 5 [file 41467_2018_4724_MOESM8_ESM.zip › Supplementary Dataset 7/joint-field-dimensionality-reduction-tSNE-matrix-rgb.pdf.interpolated.pdf]

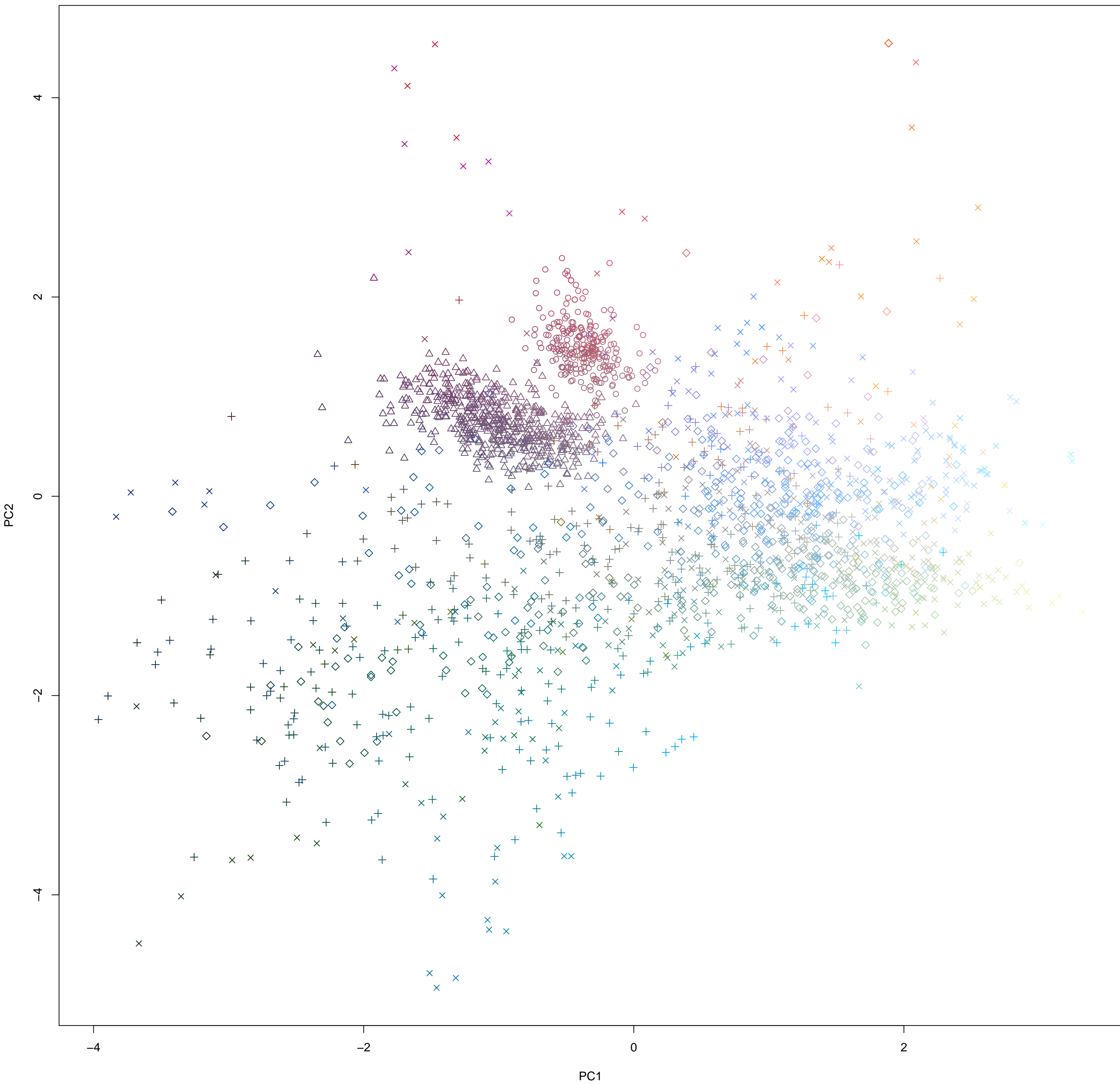

Supplement: Supplementary file 8 — Supplementary Data 5 [file 41467_2018_4724_MOESM8_ESM.zip › Supplementary Dataset 7/joint-mix-dimensionality-reduction-PCA-2d.pdf]

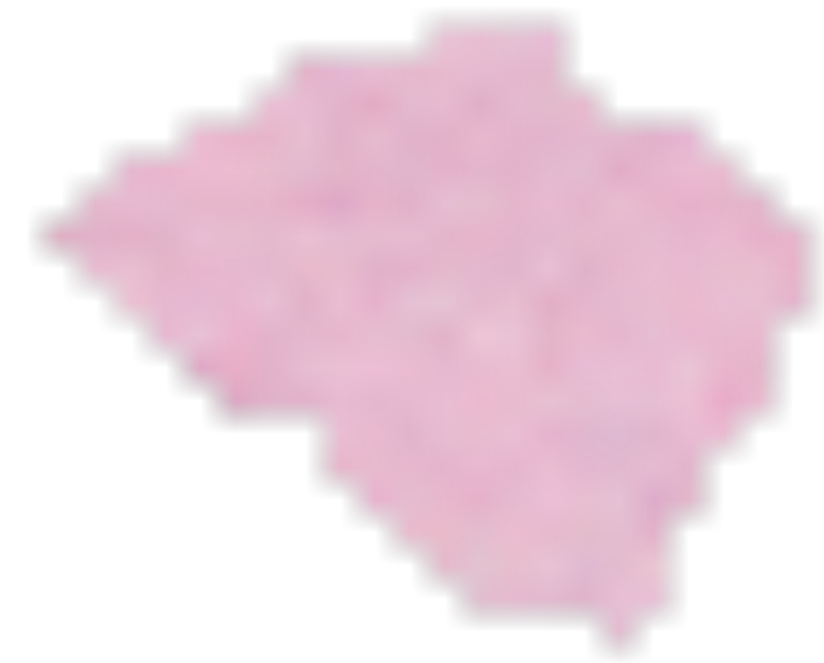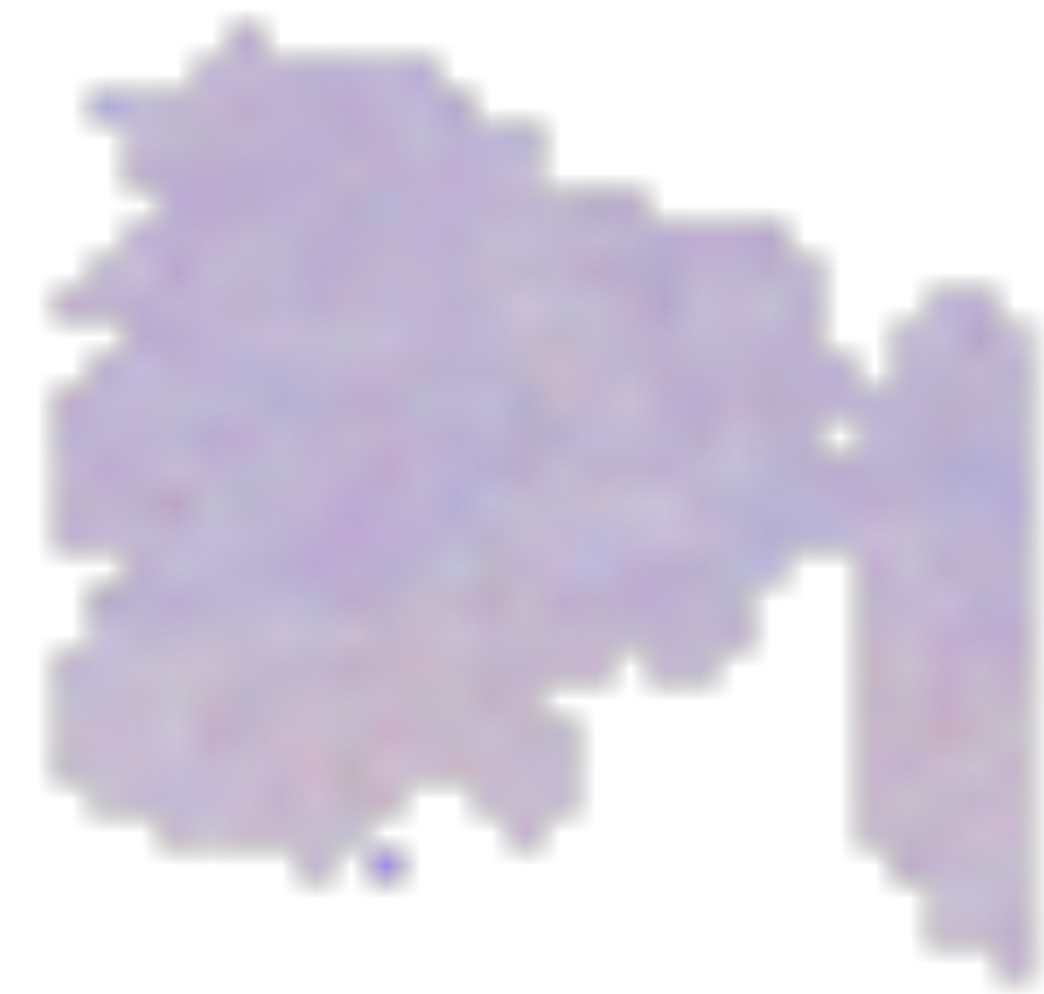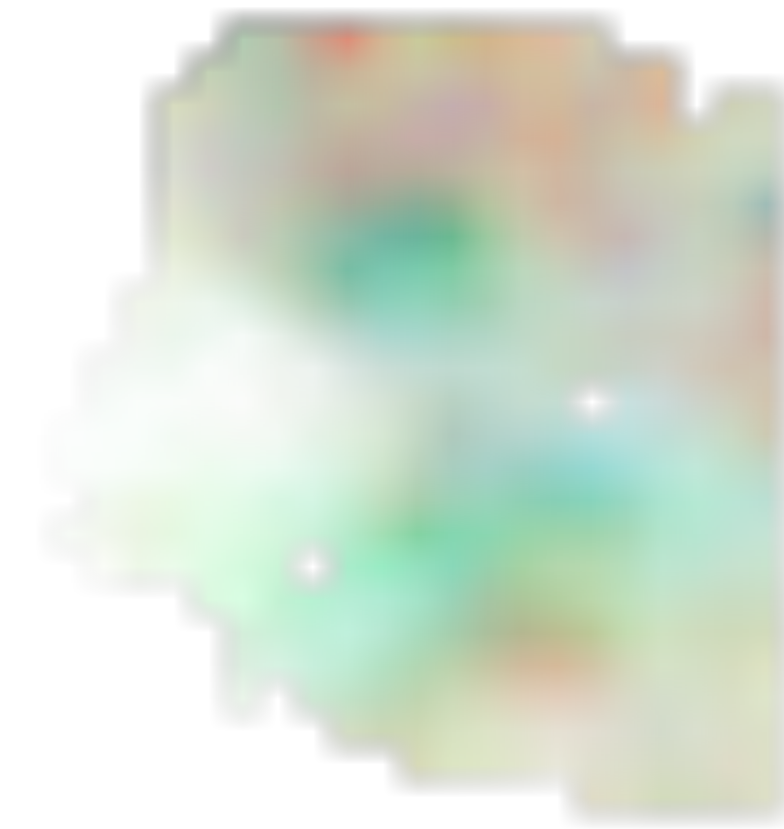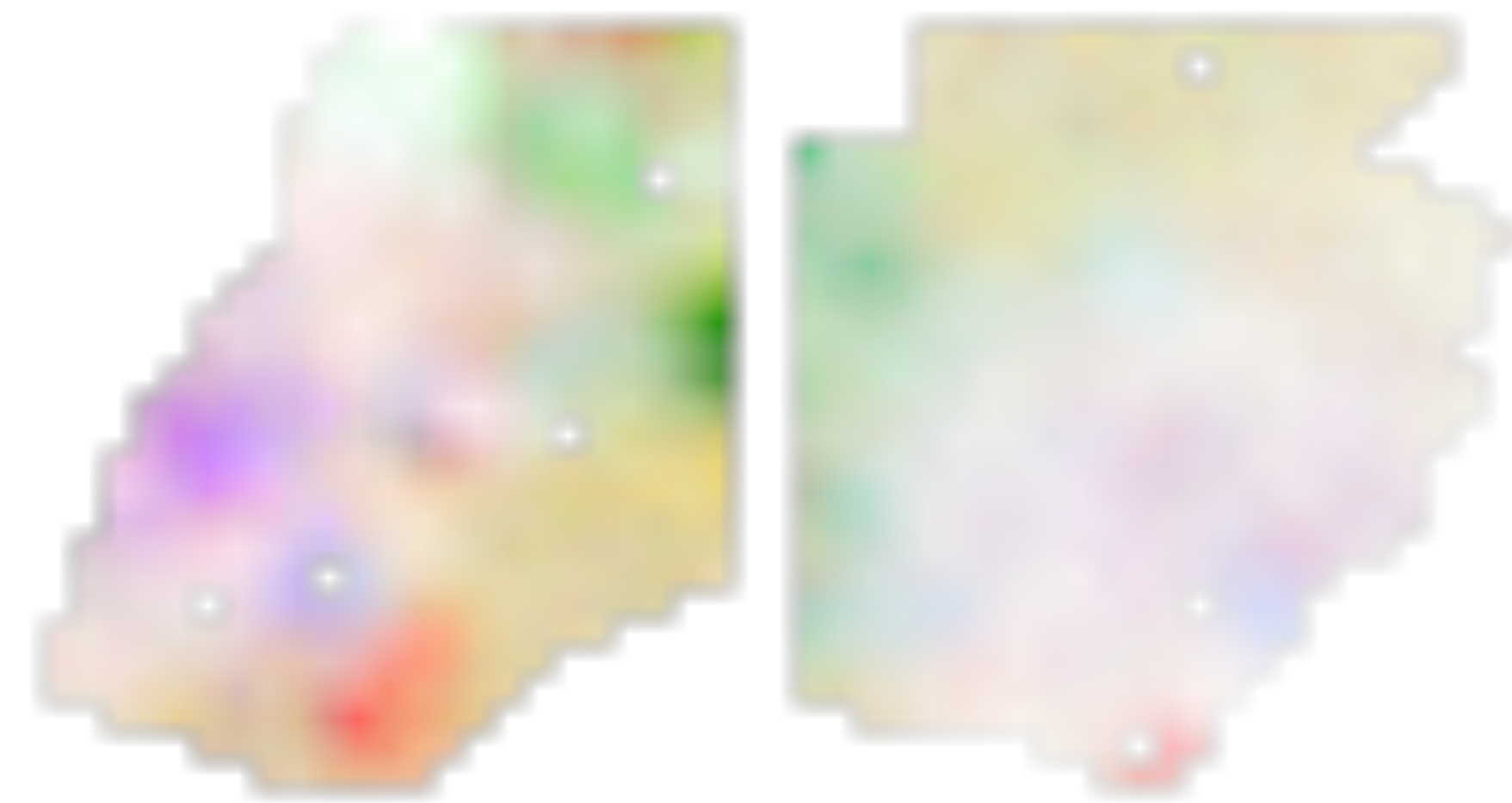

Supplement: Supplementary file 8 — Supplementary Data 5 [file 41467_2018_4724_MOESM8_ESM.zip › Supplementary Dataset 7/joint-field-dimensionality-reduction-PCA-matrix-rgb.pdf.interpolated.pdf]

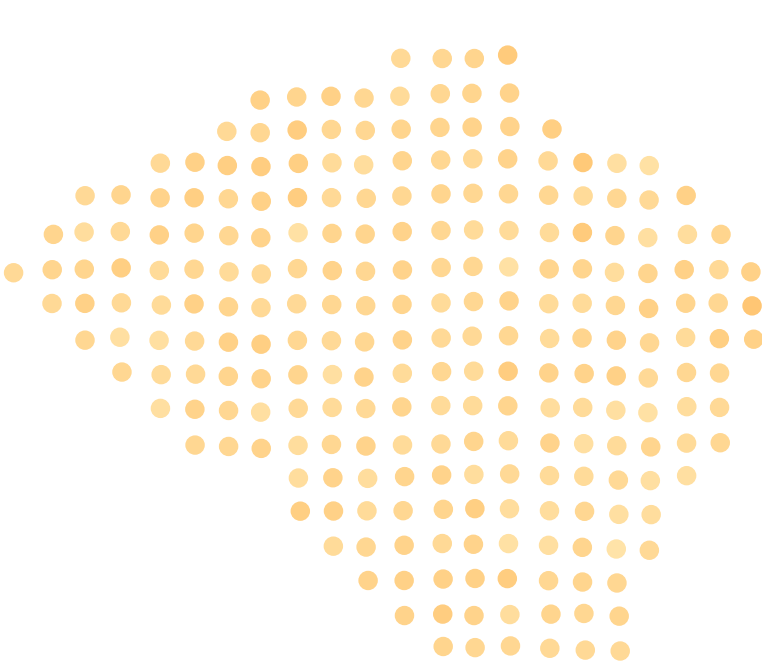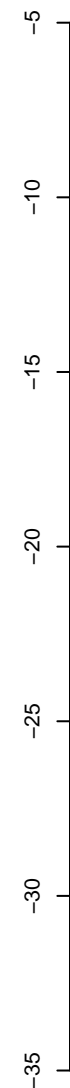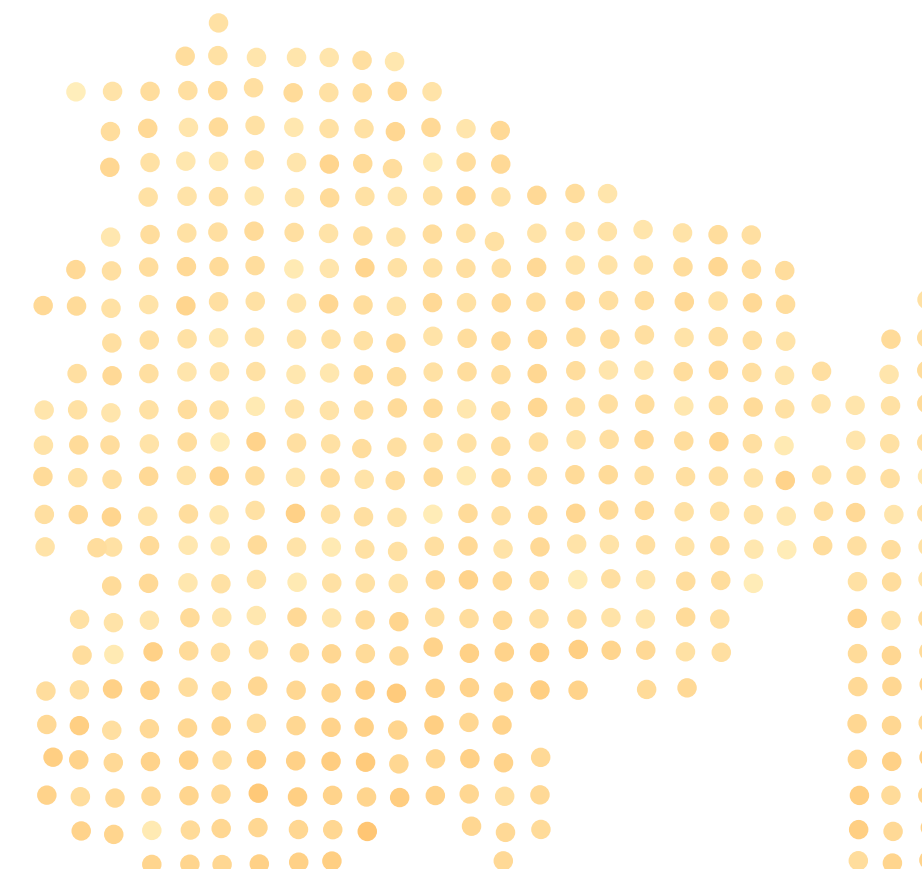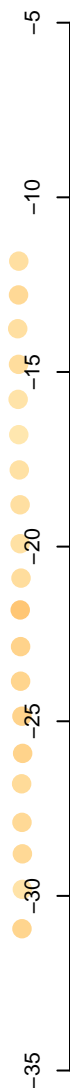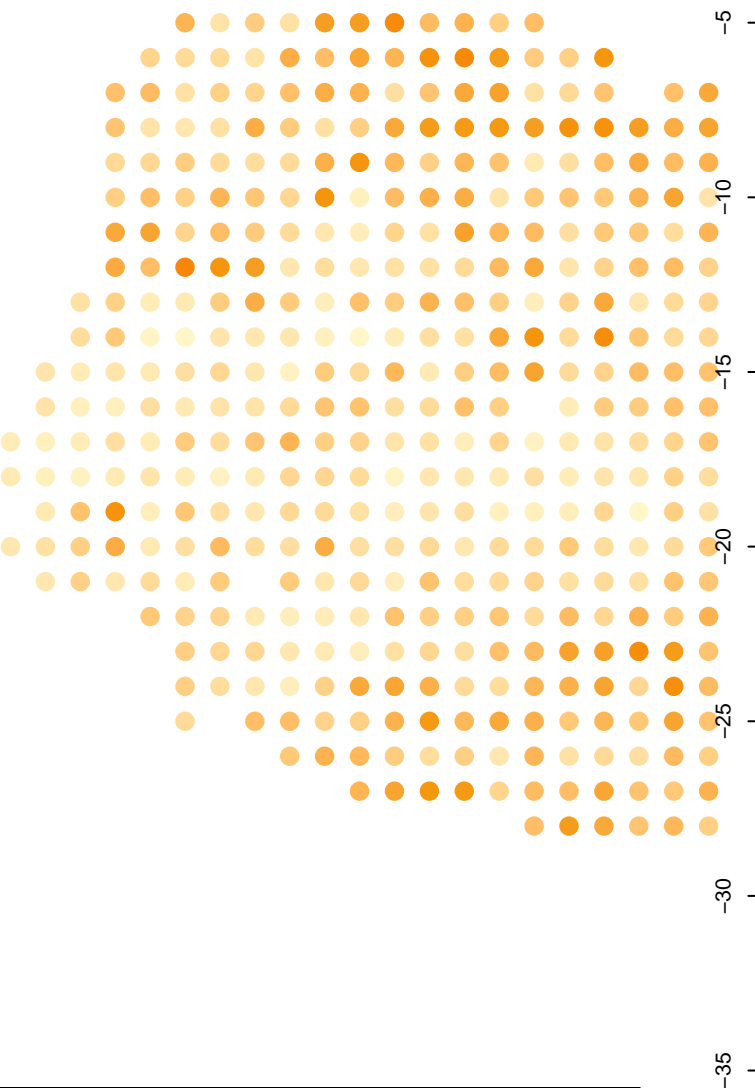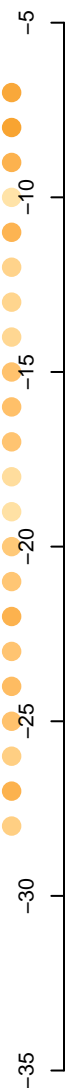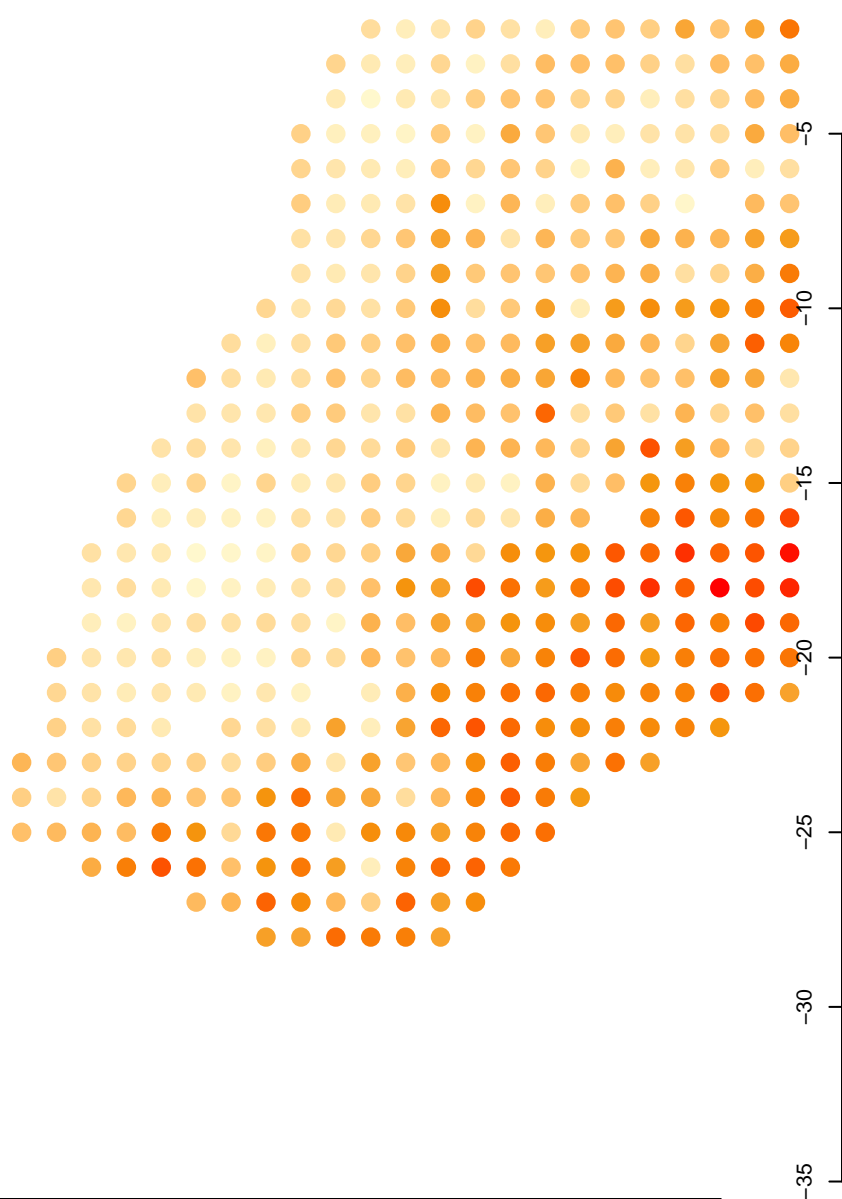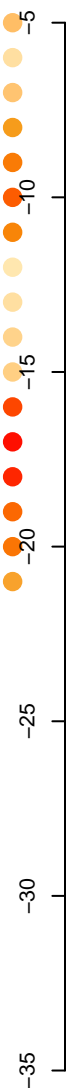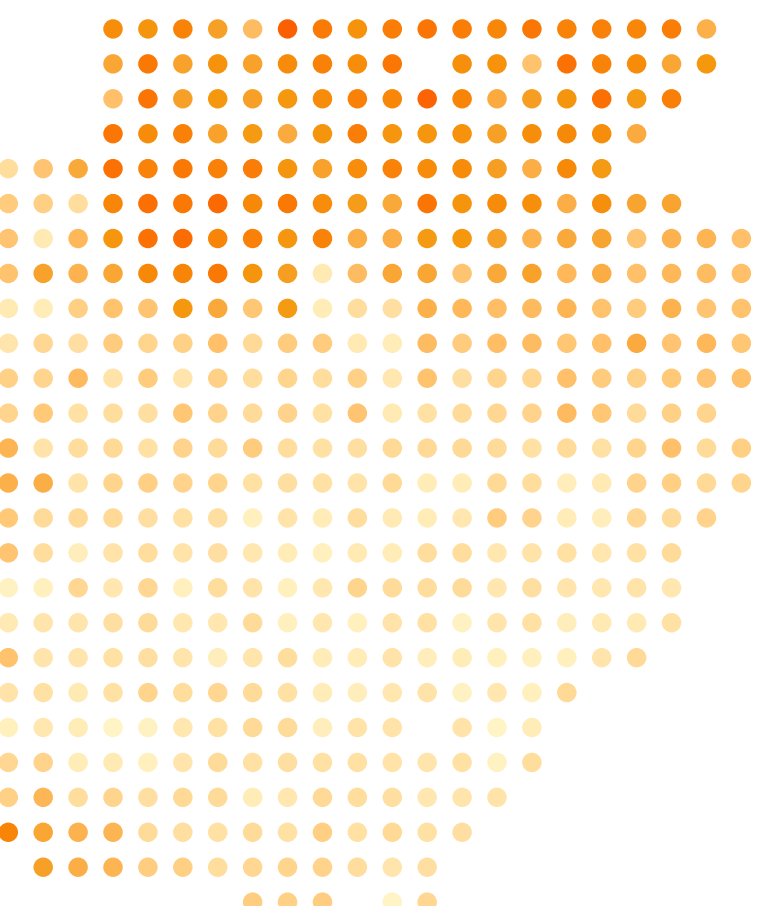

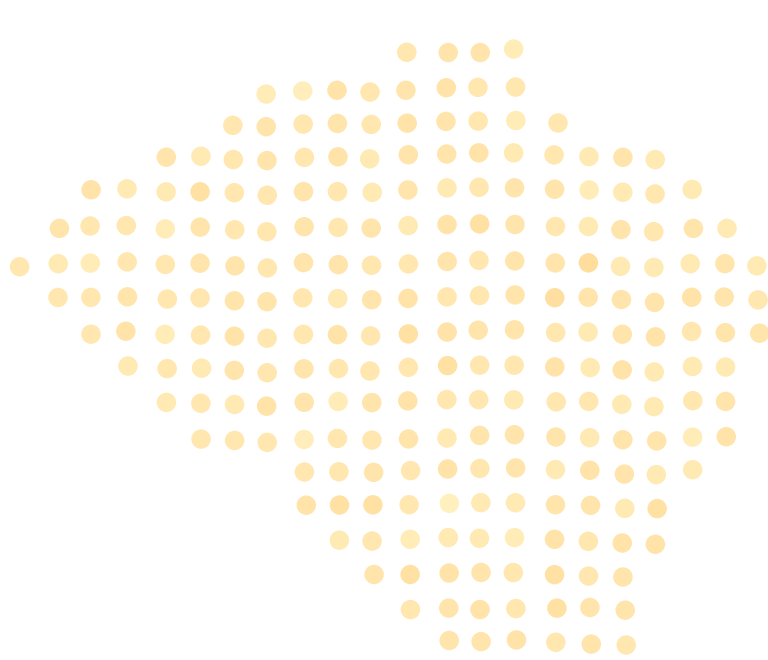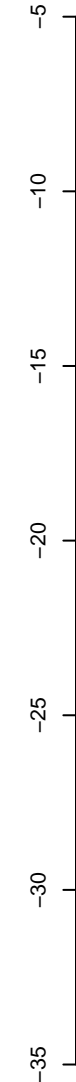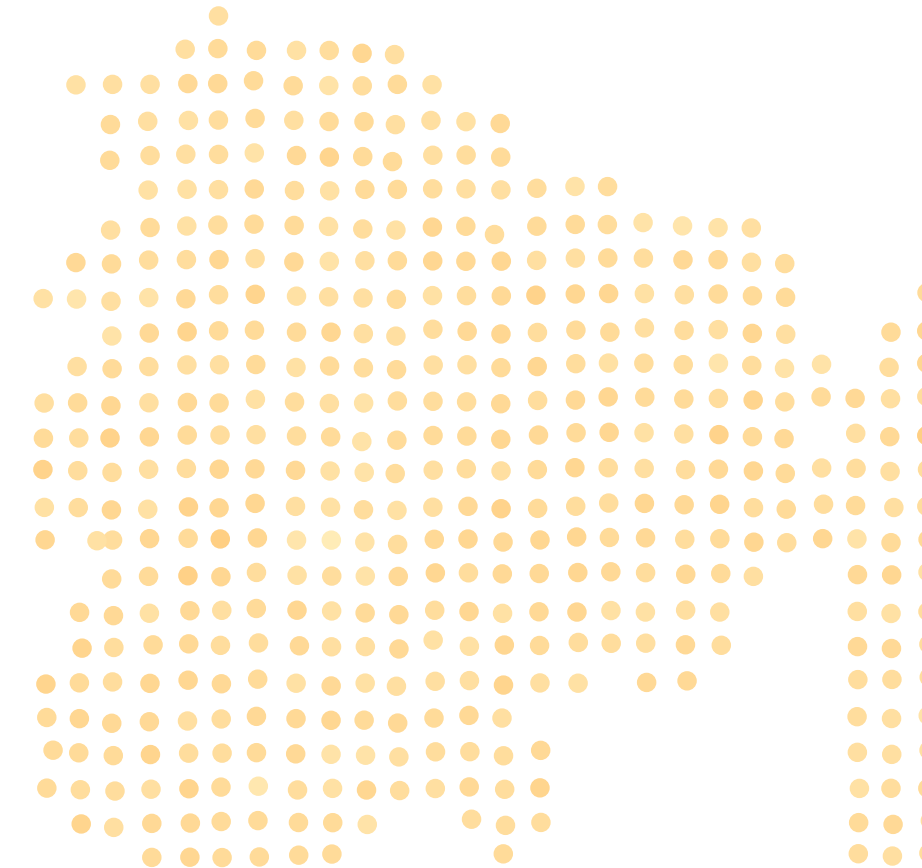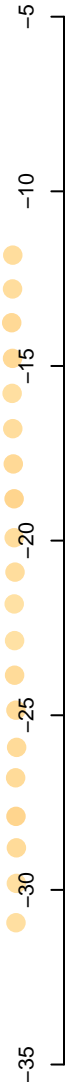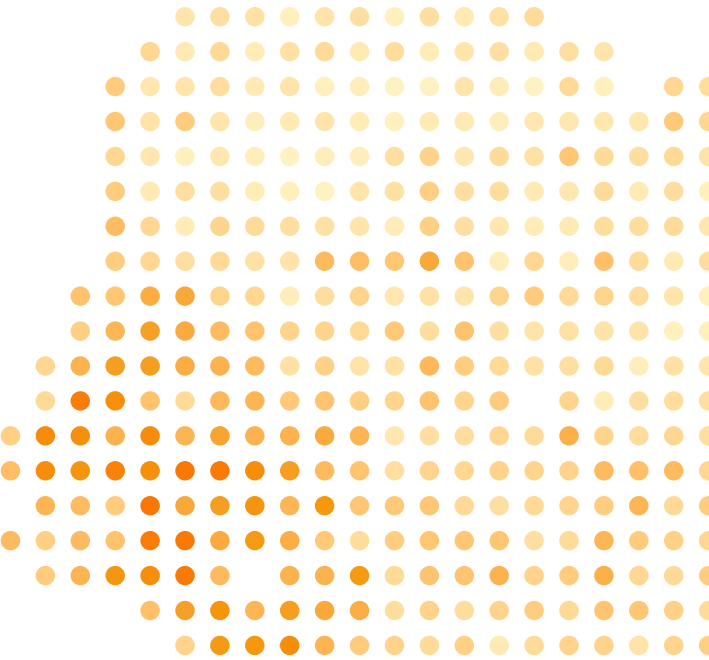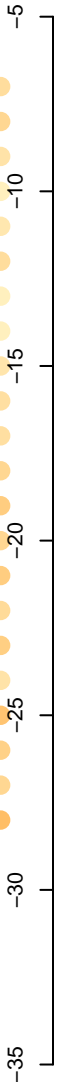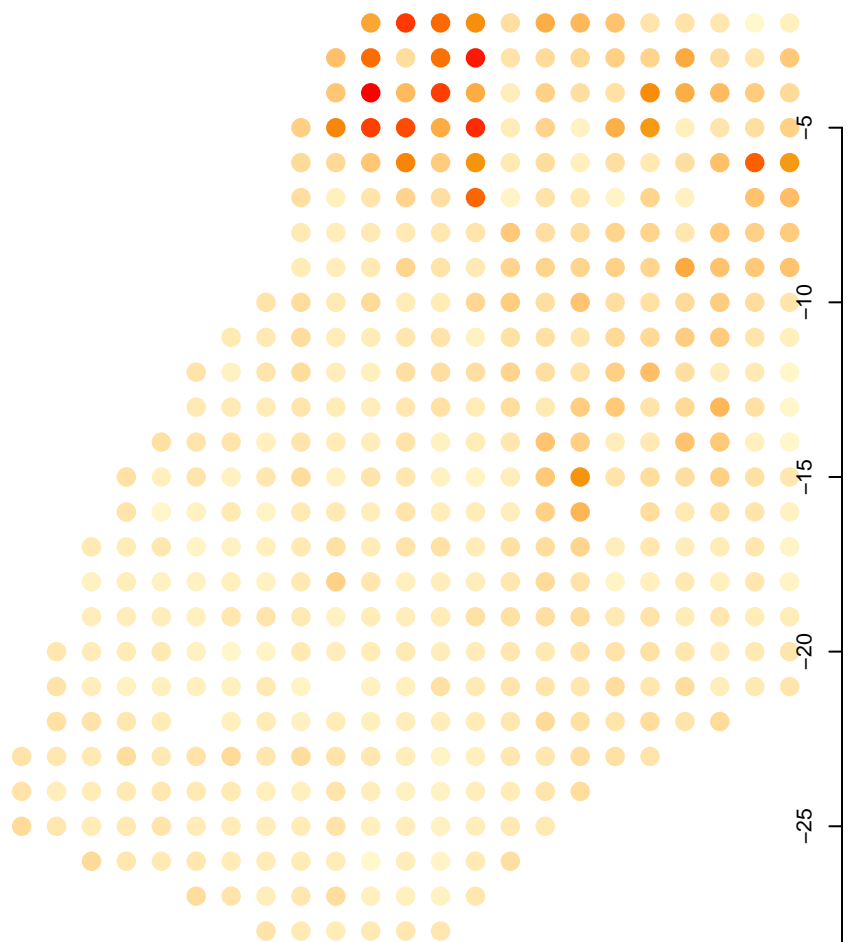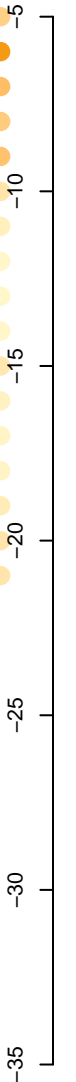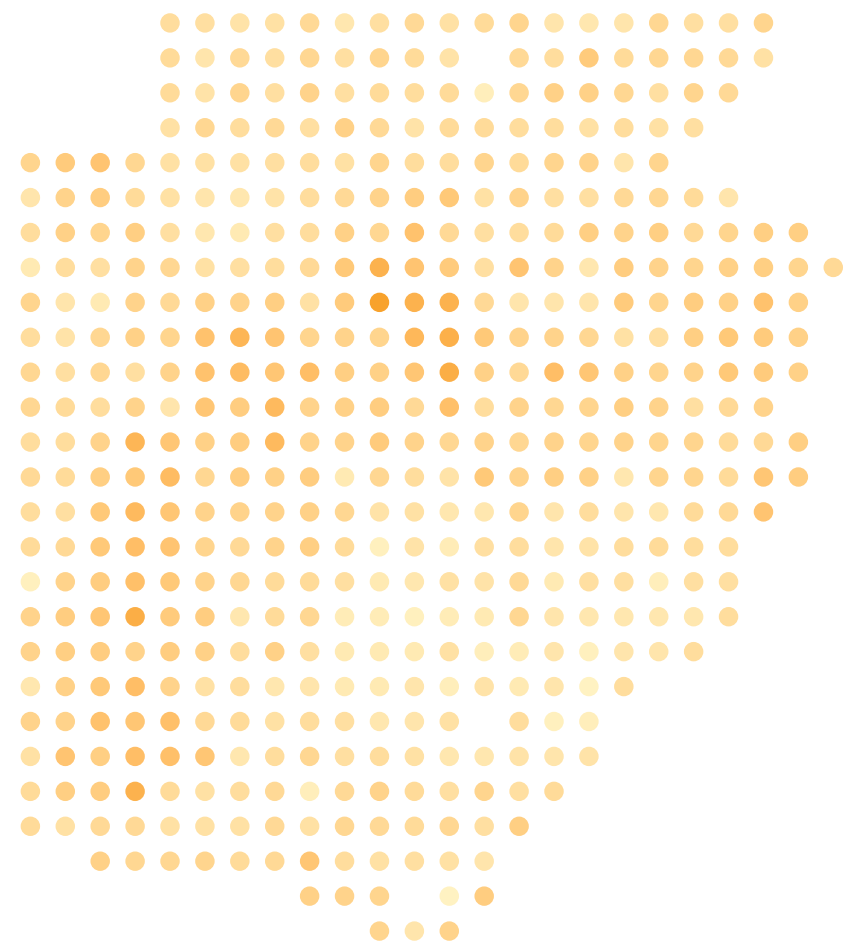

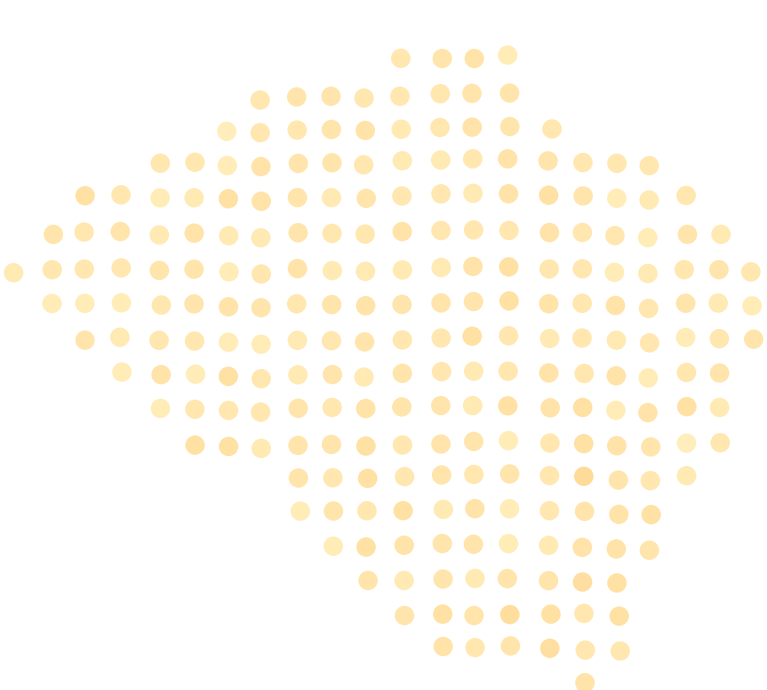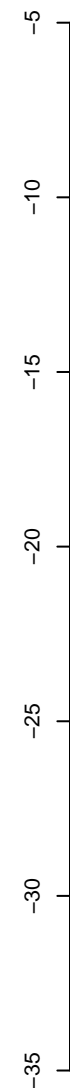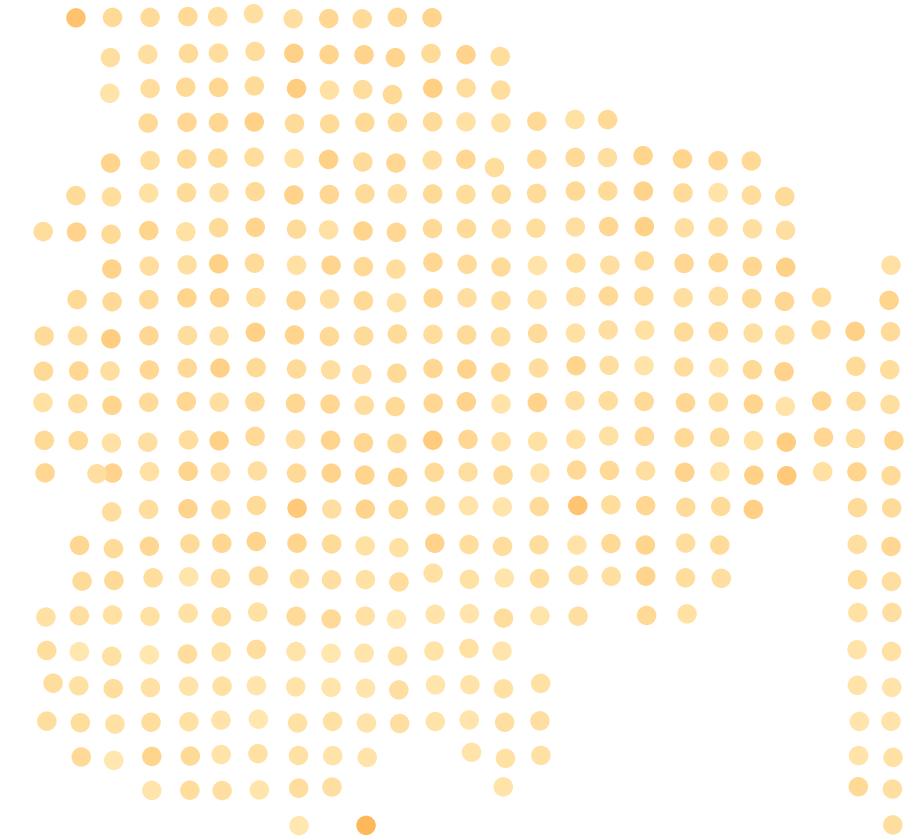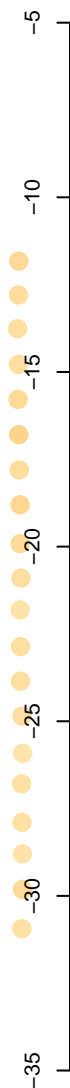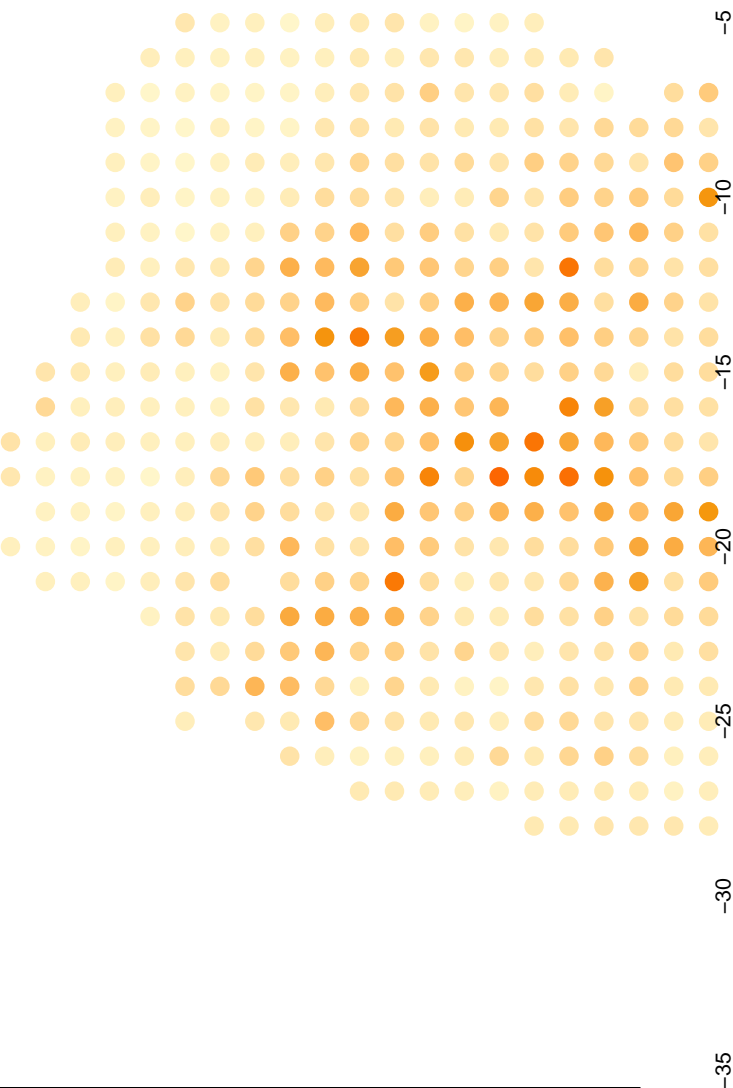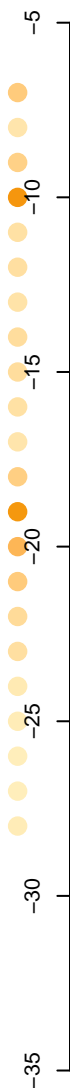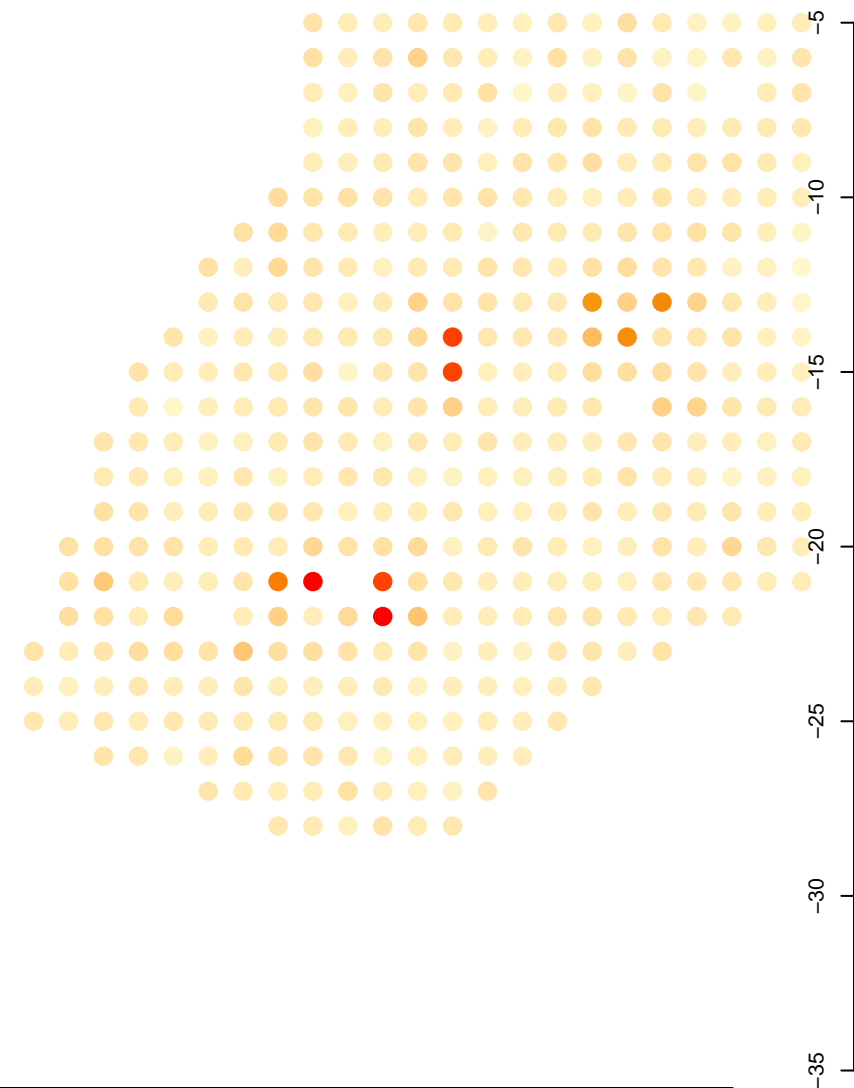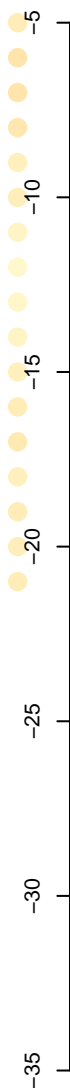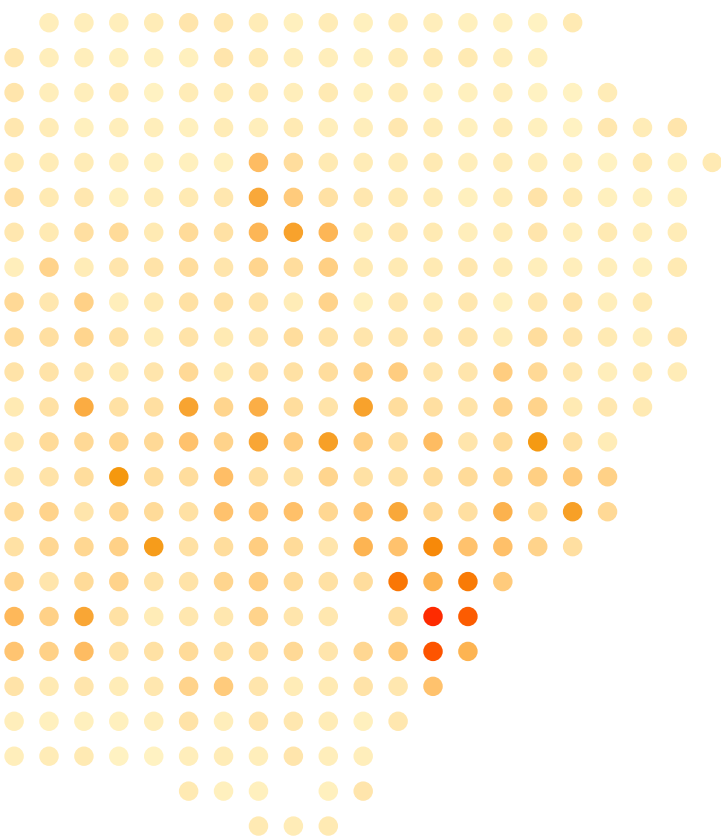

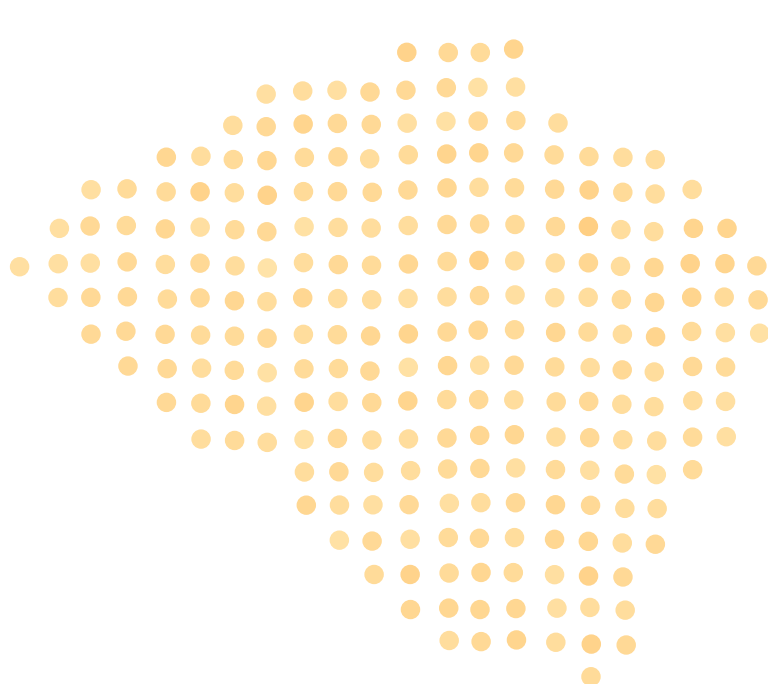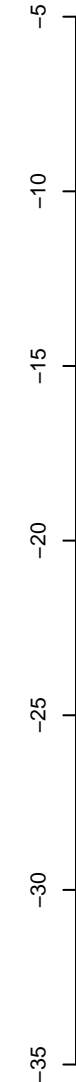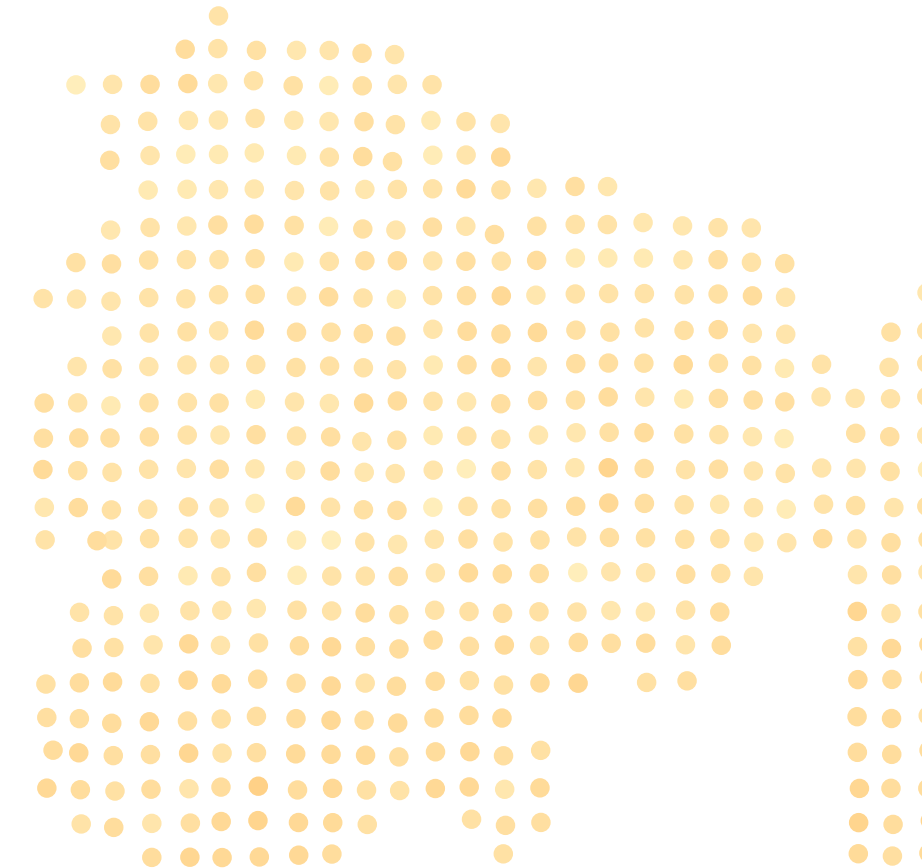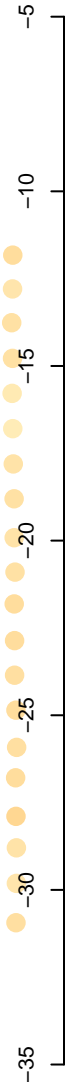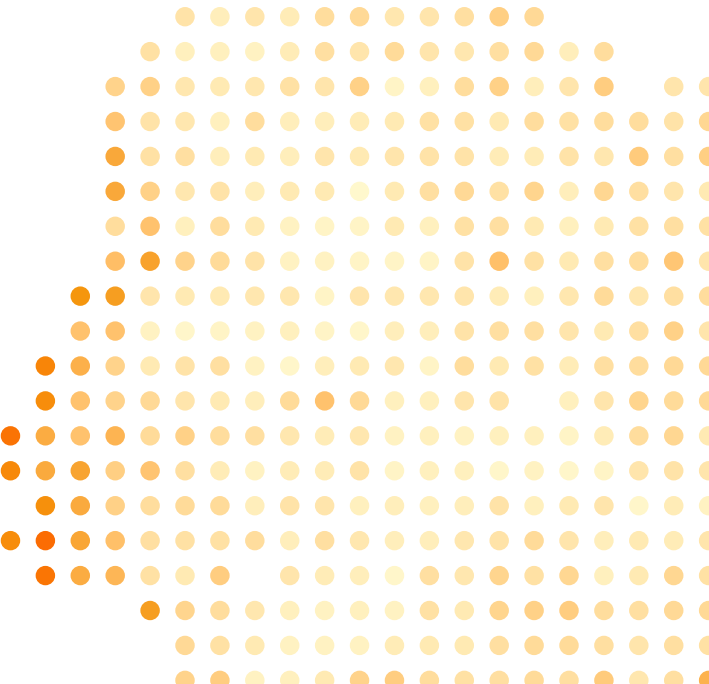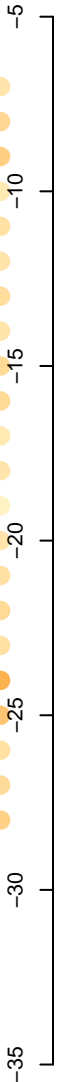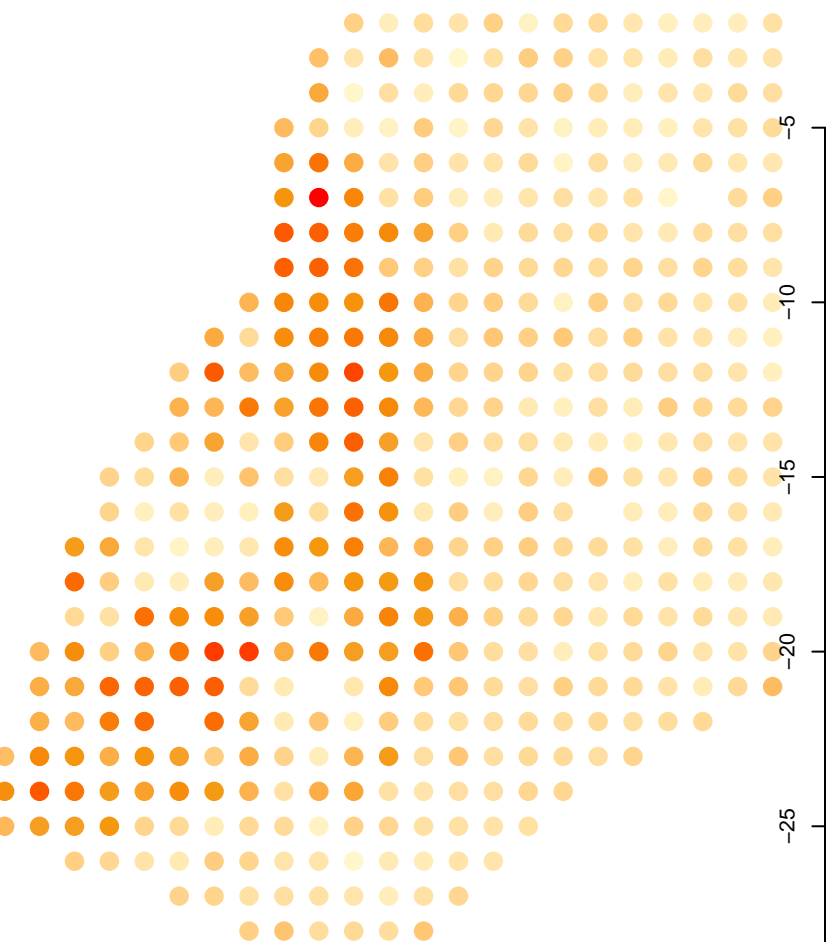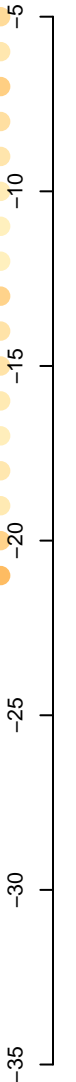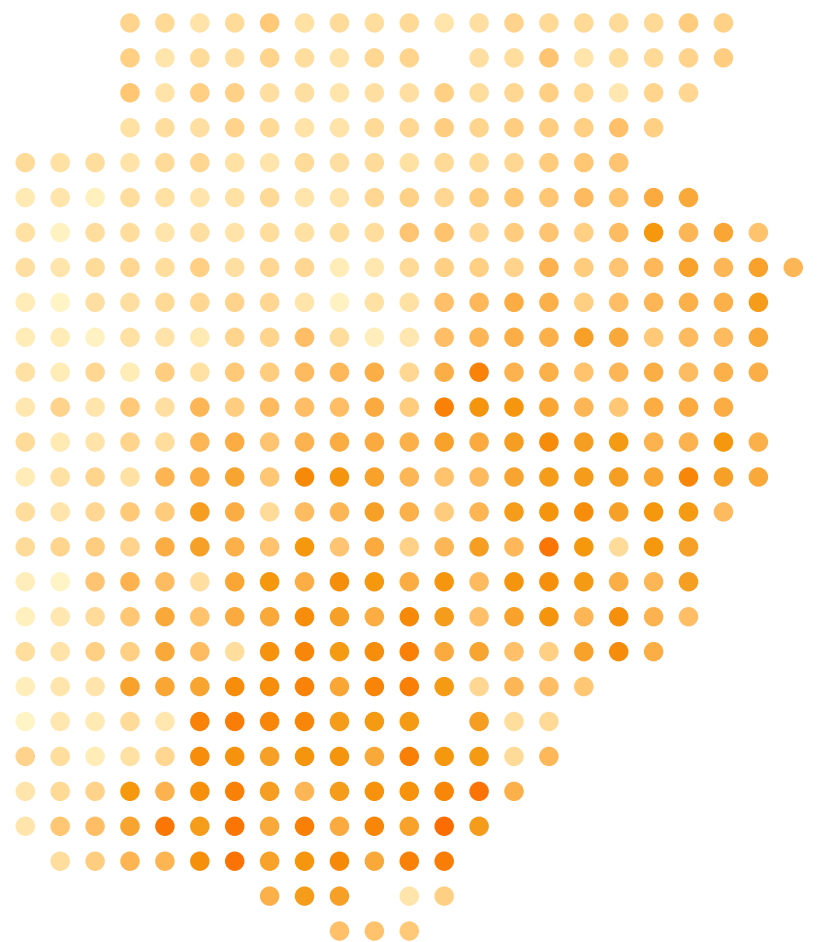

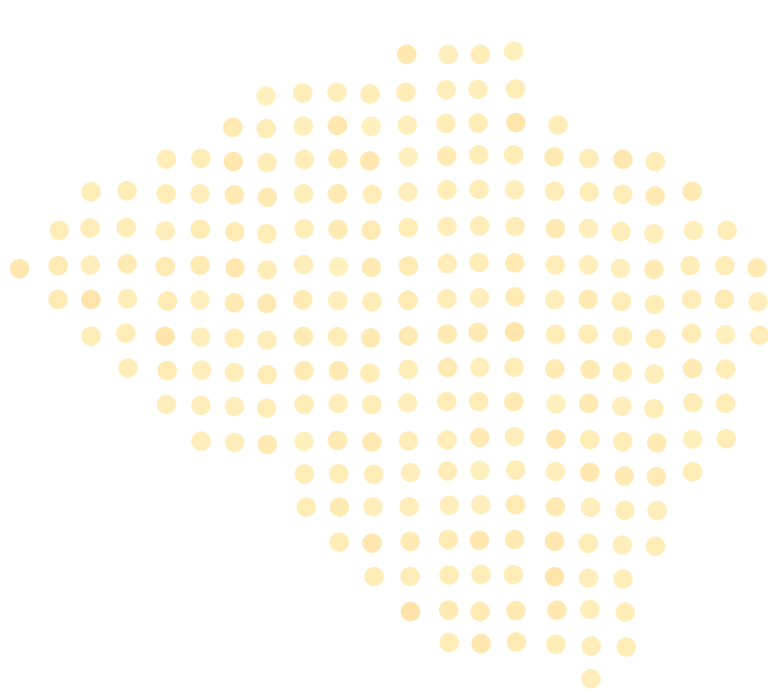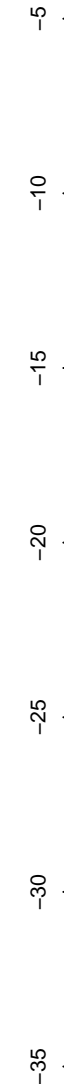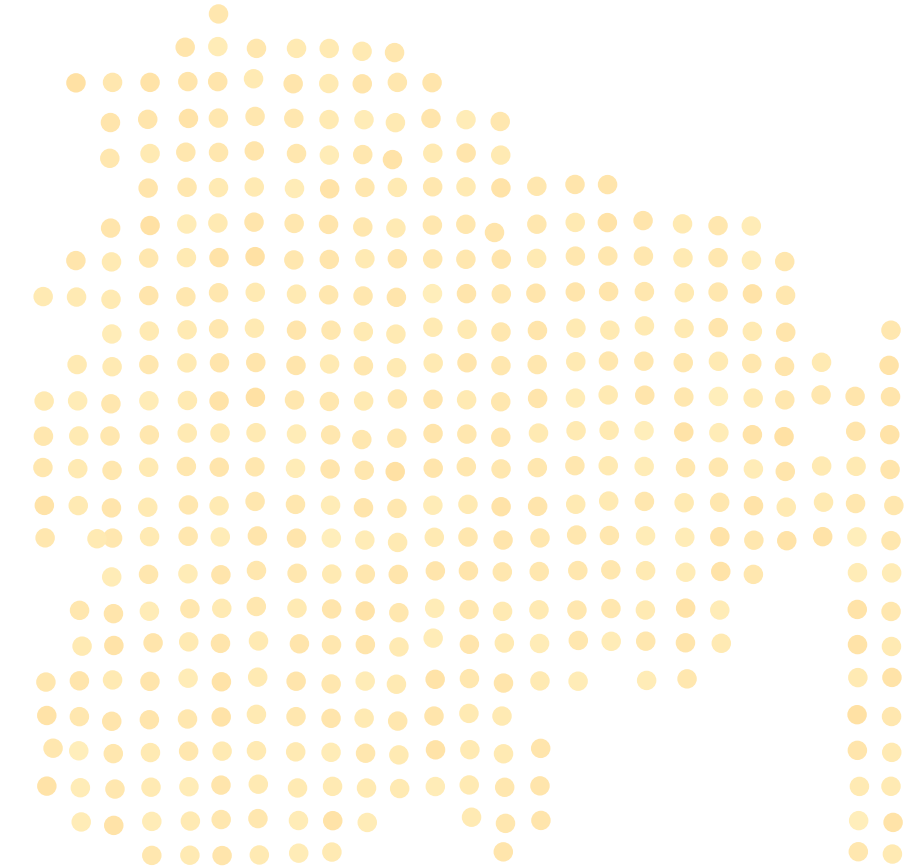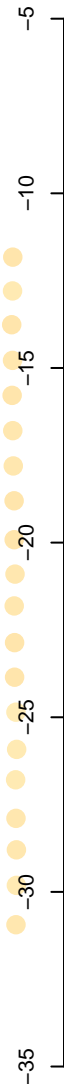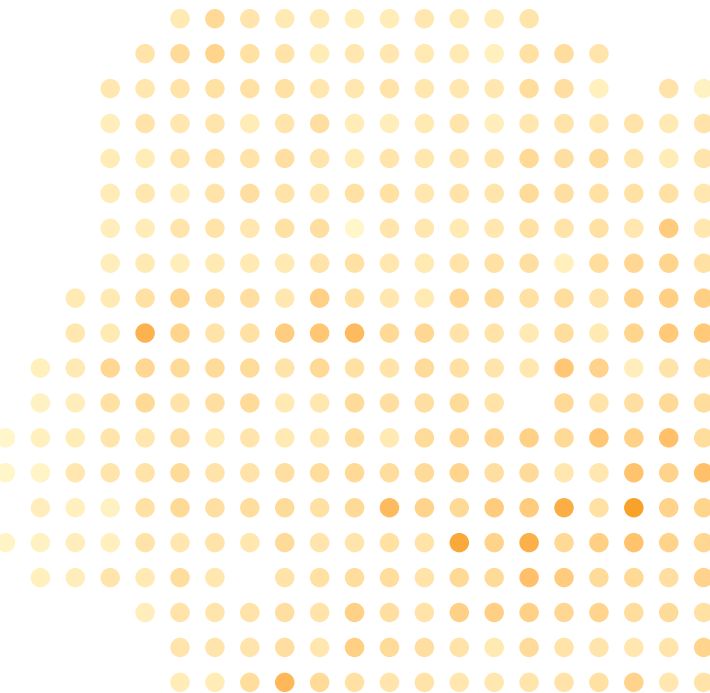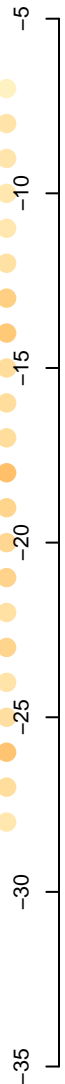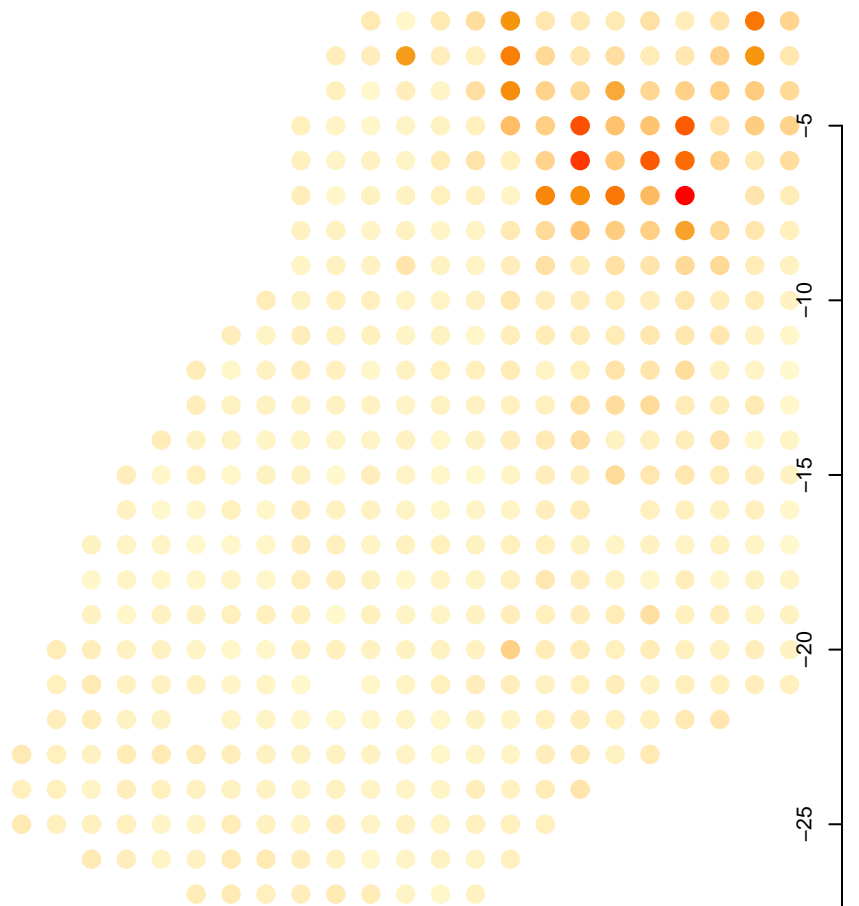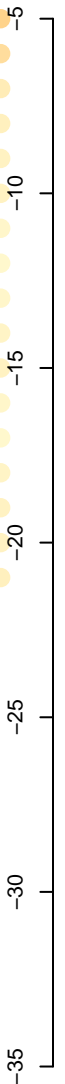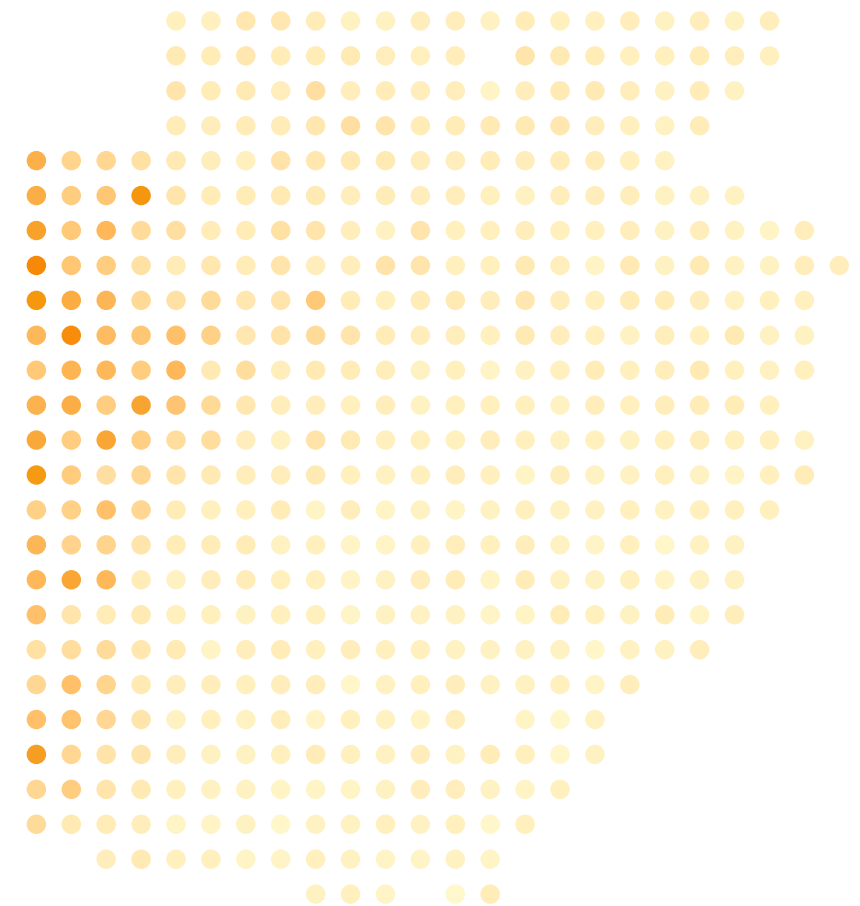

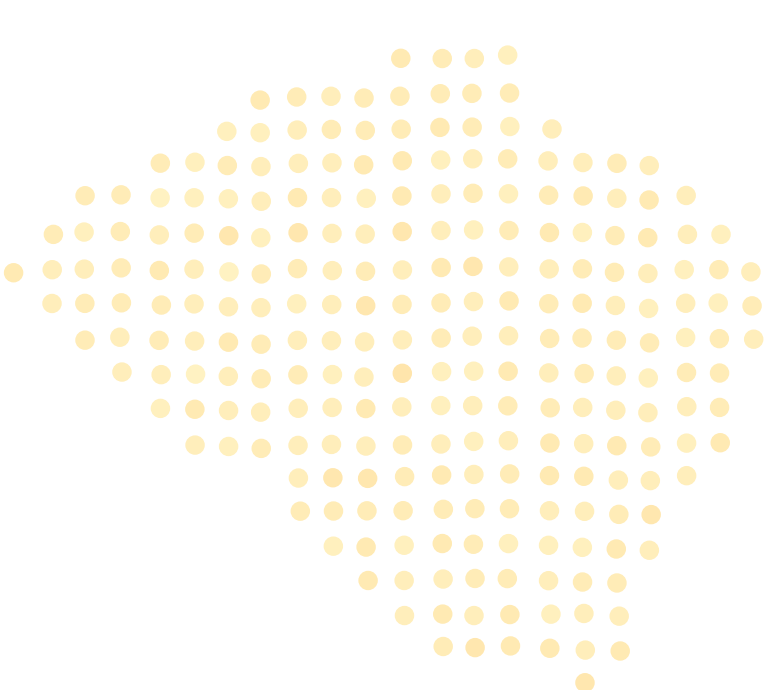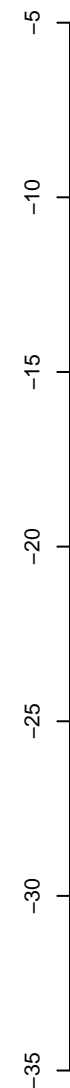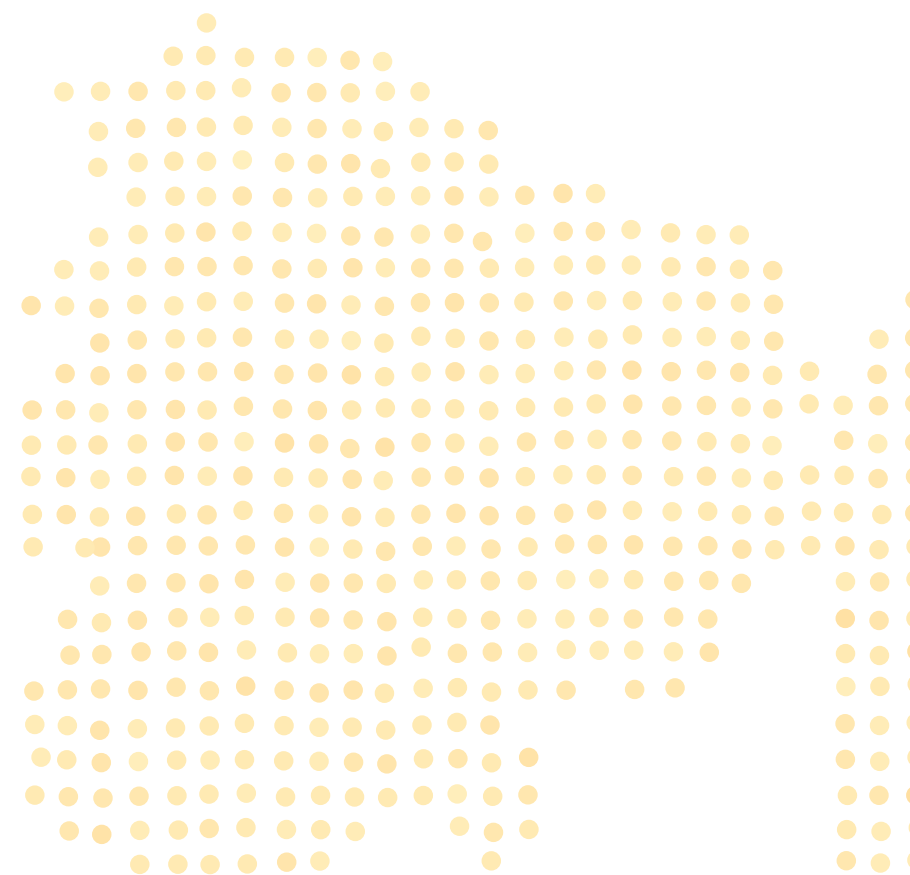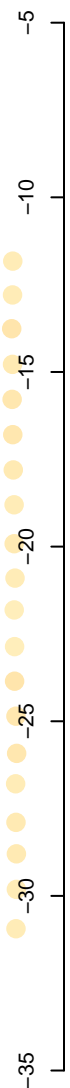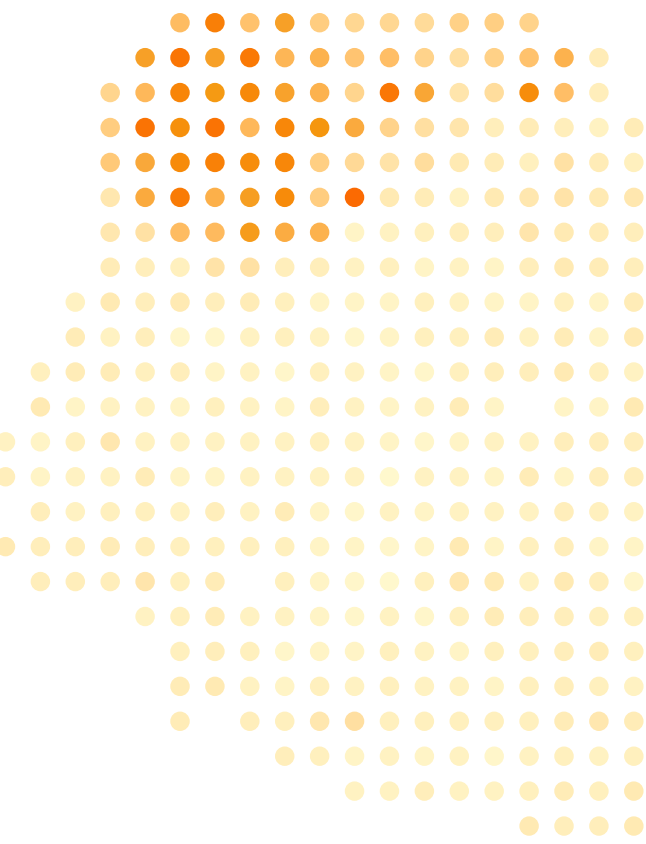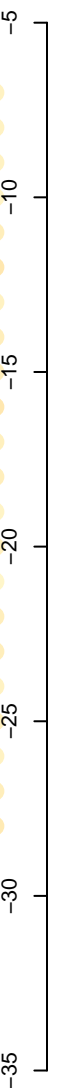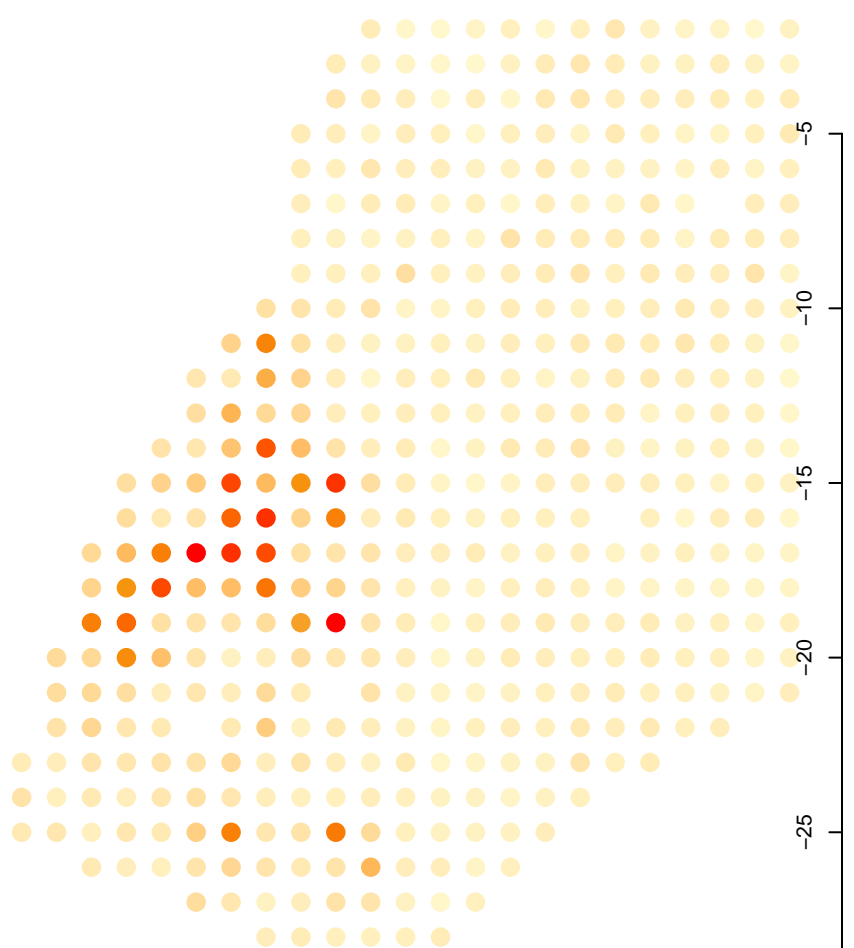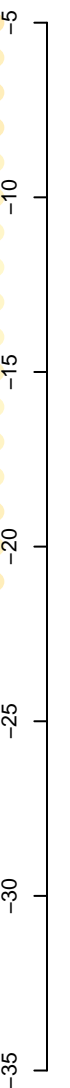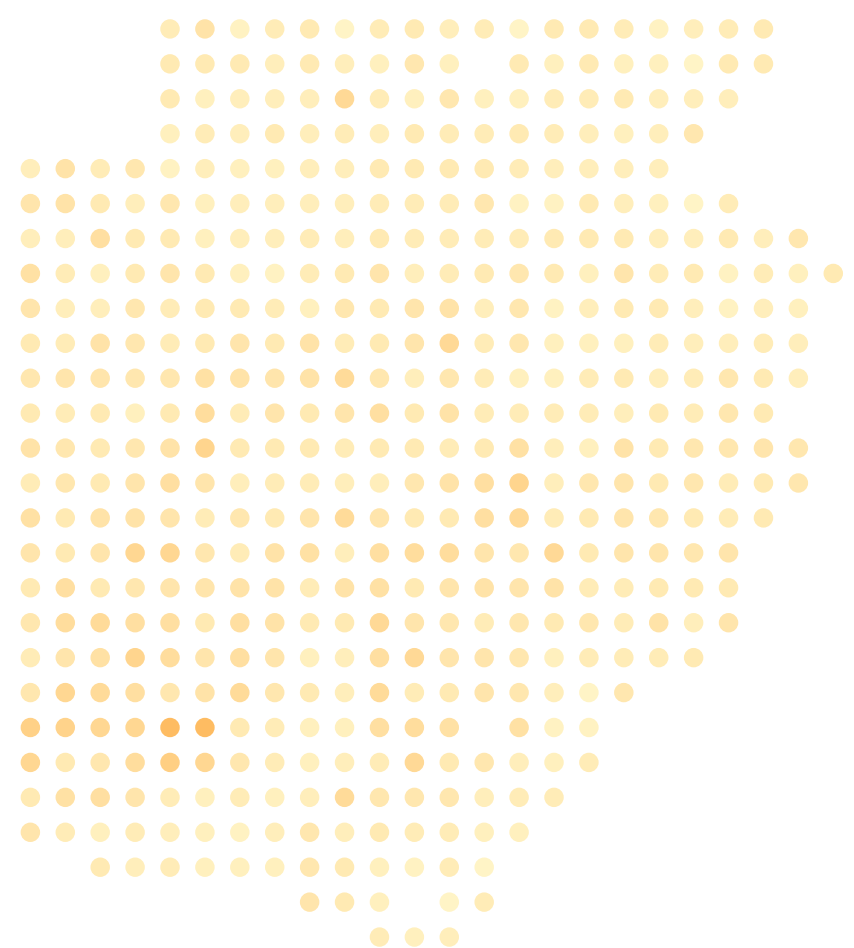

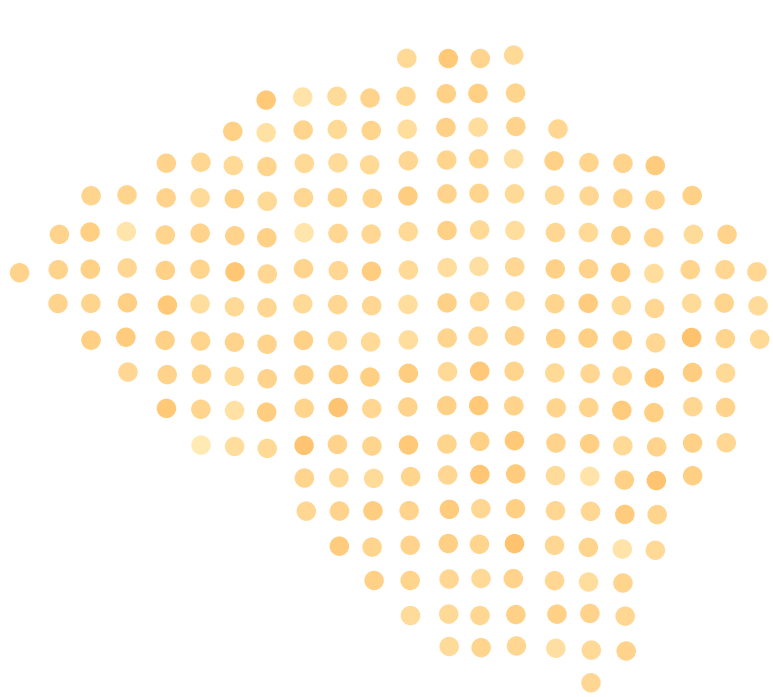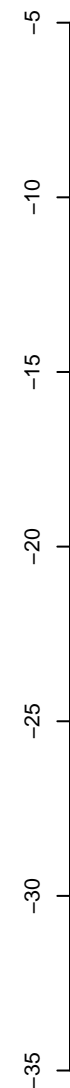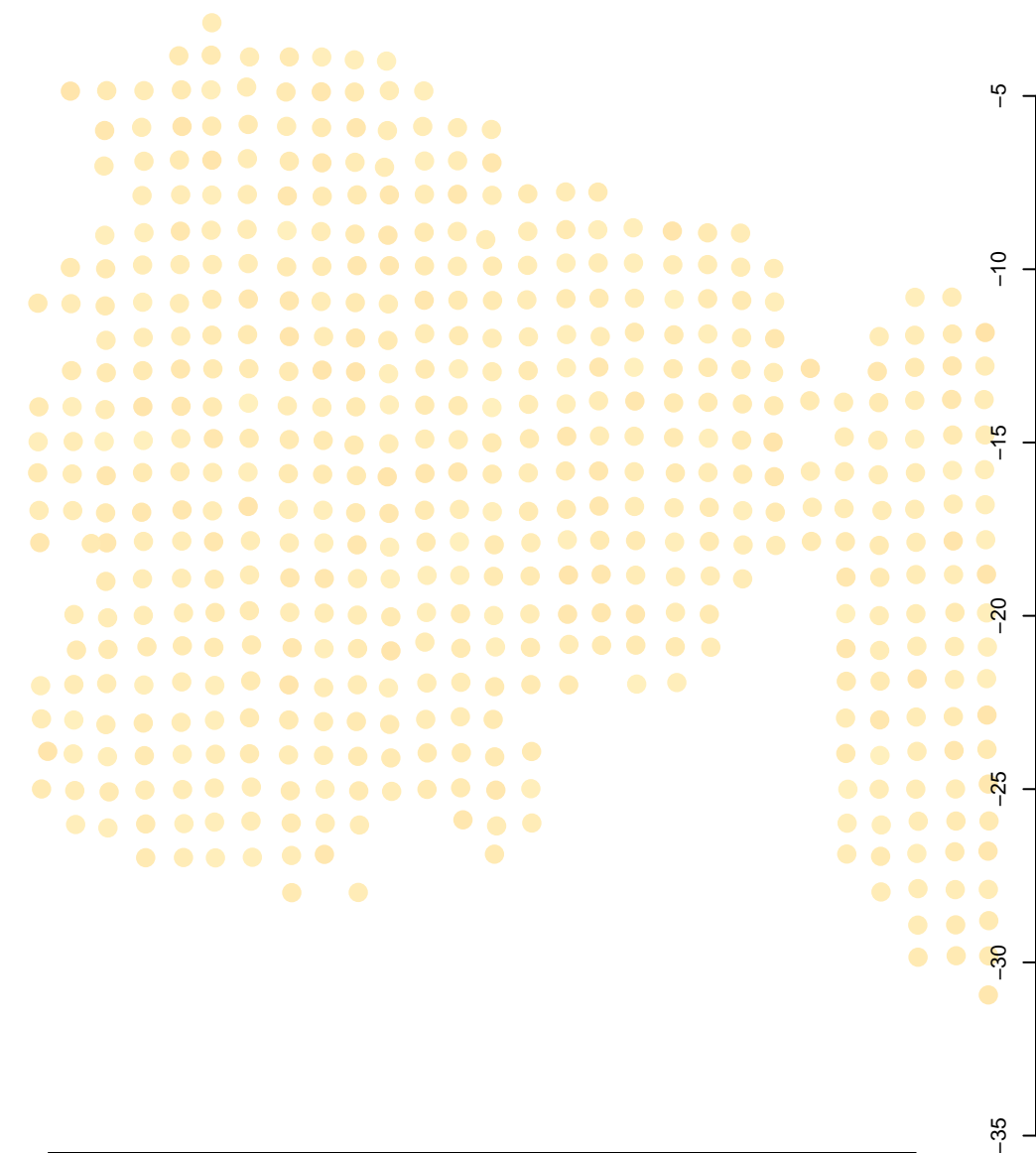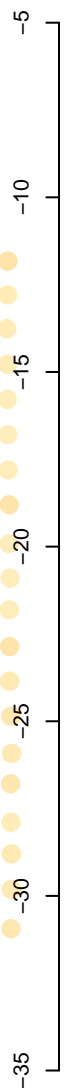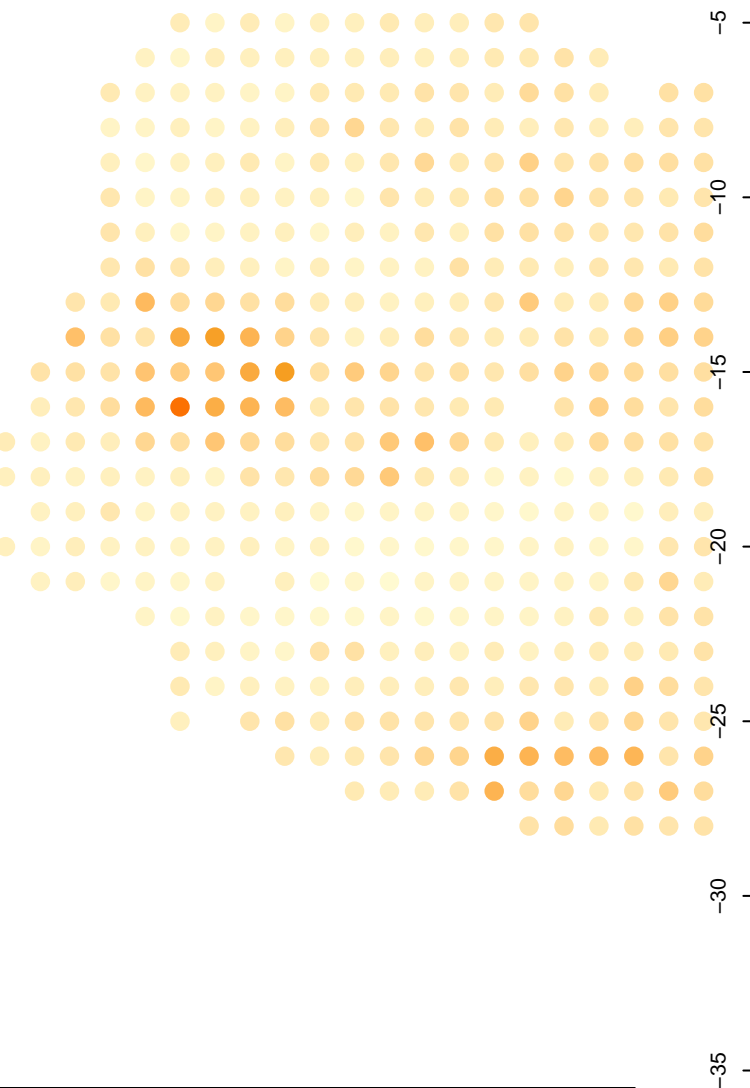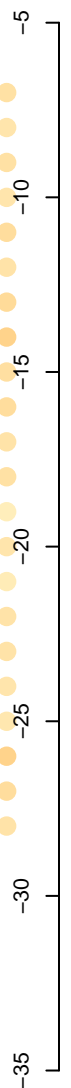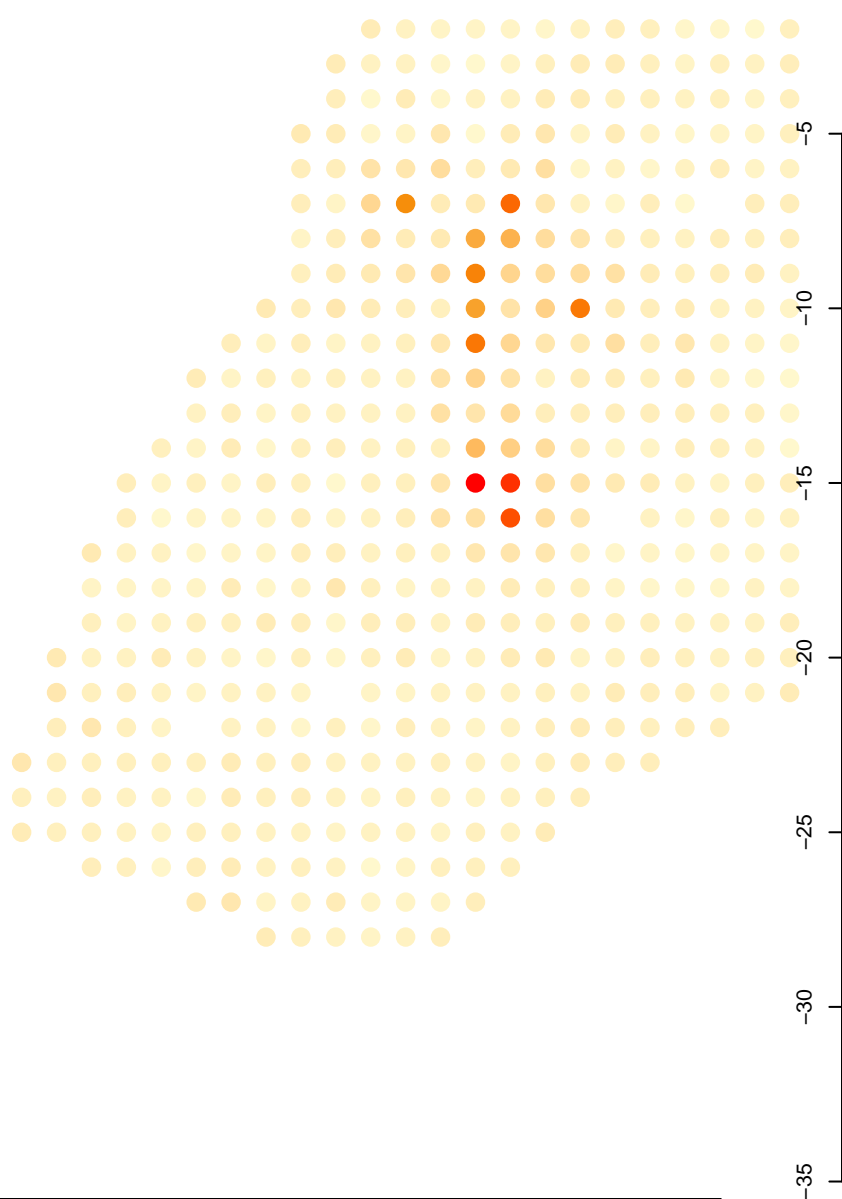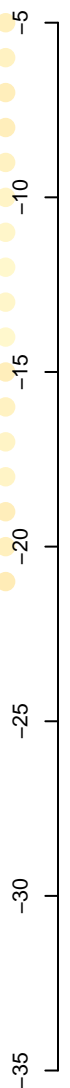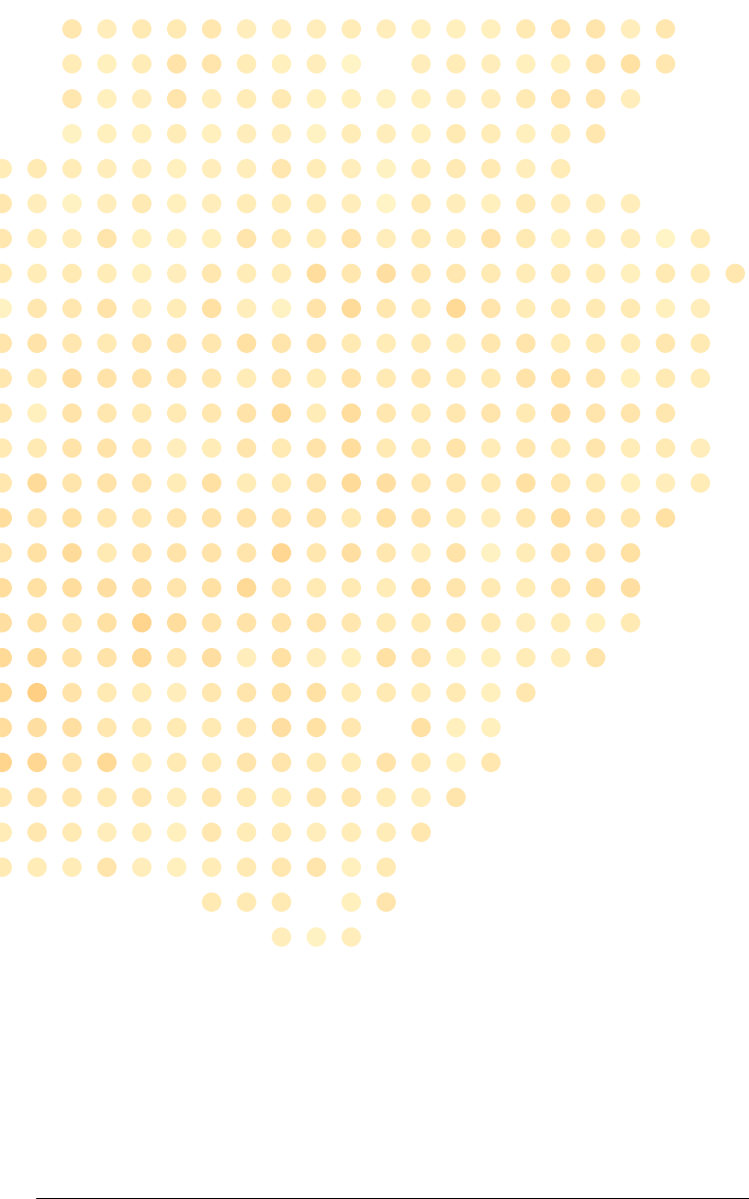

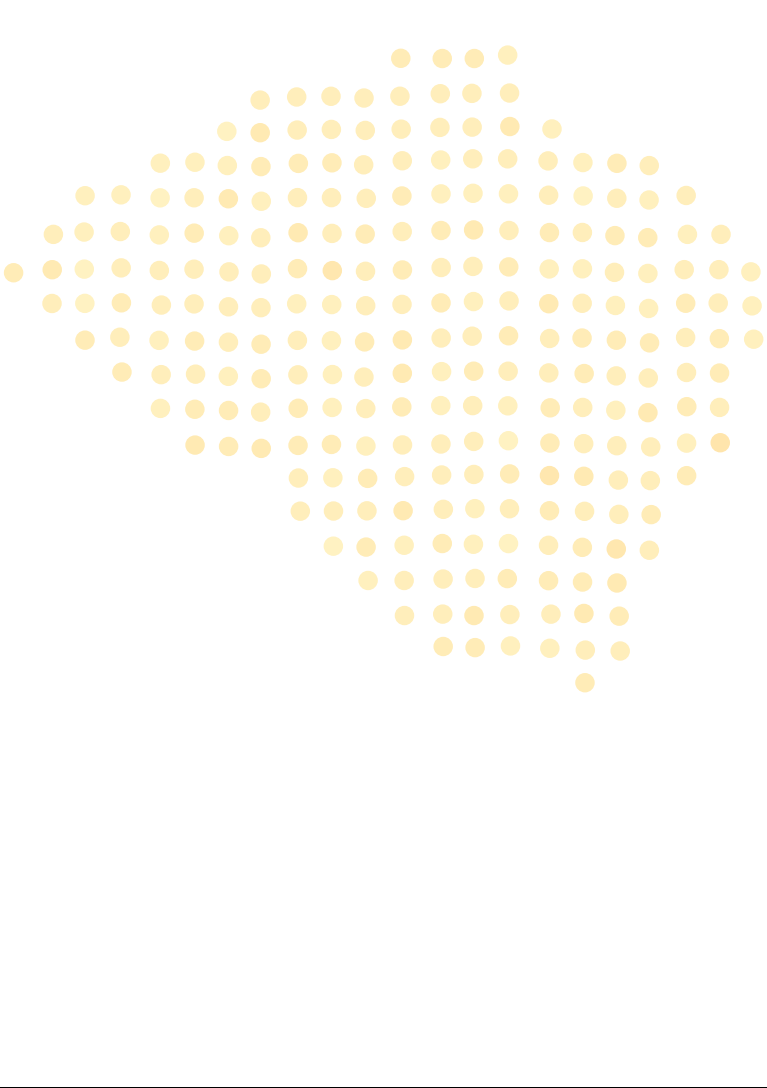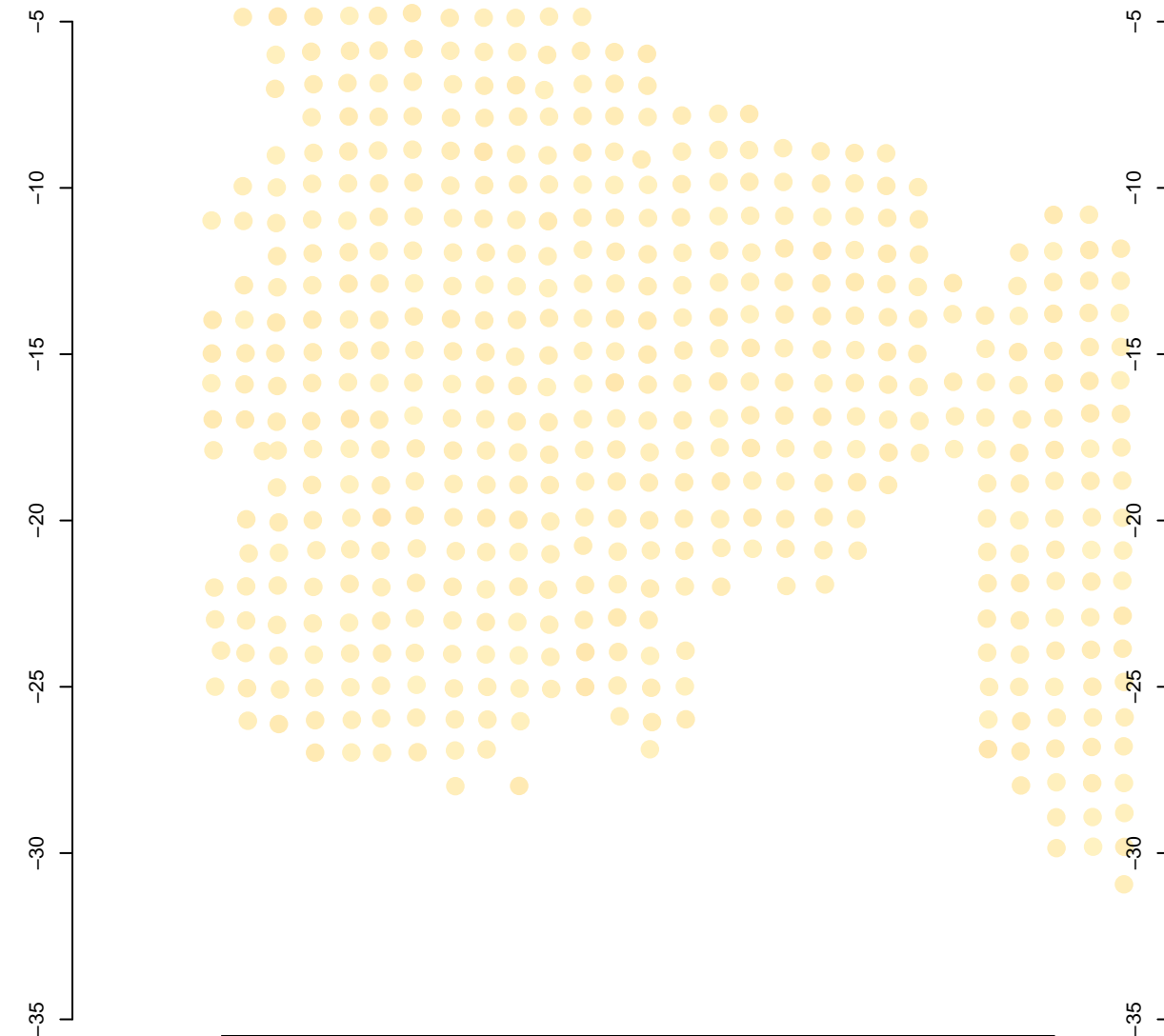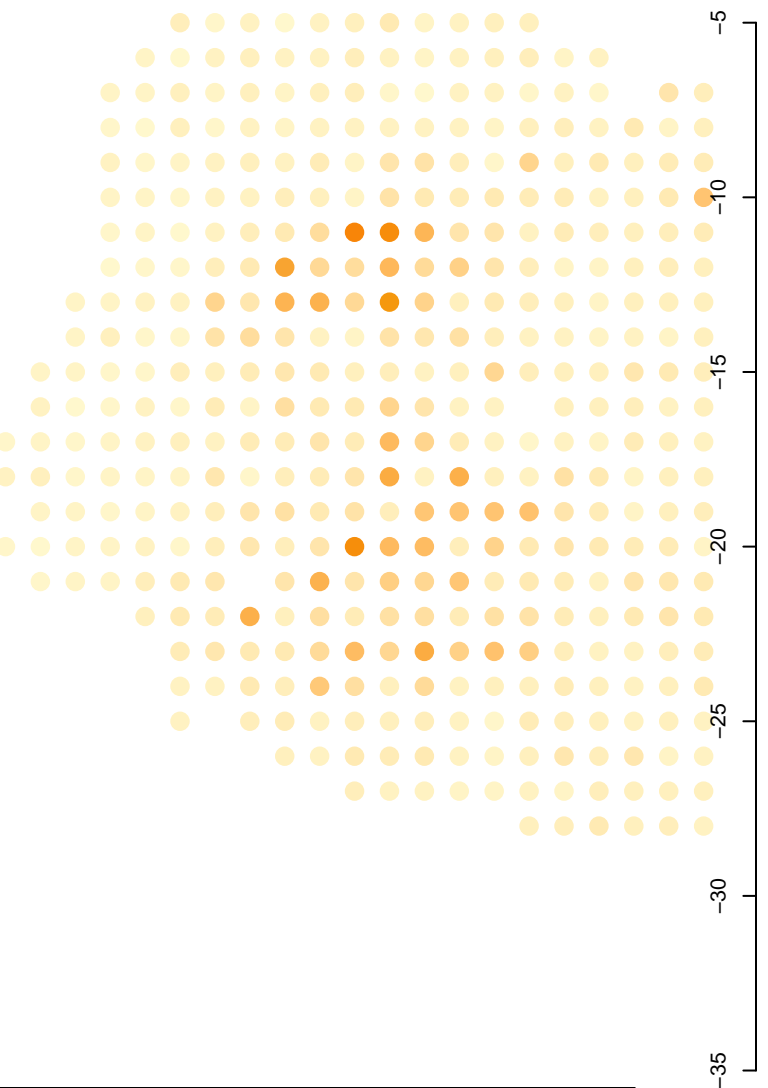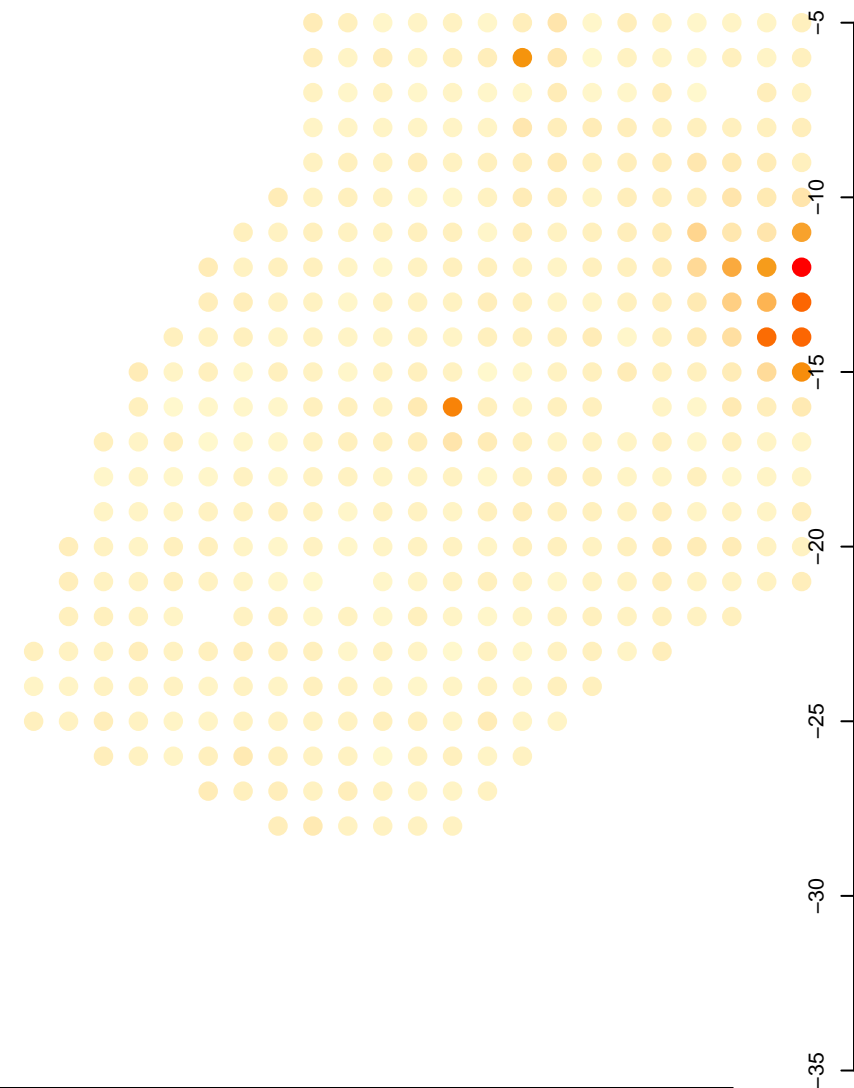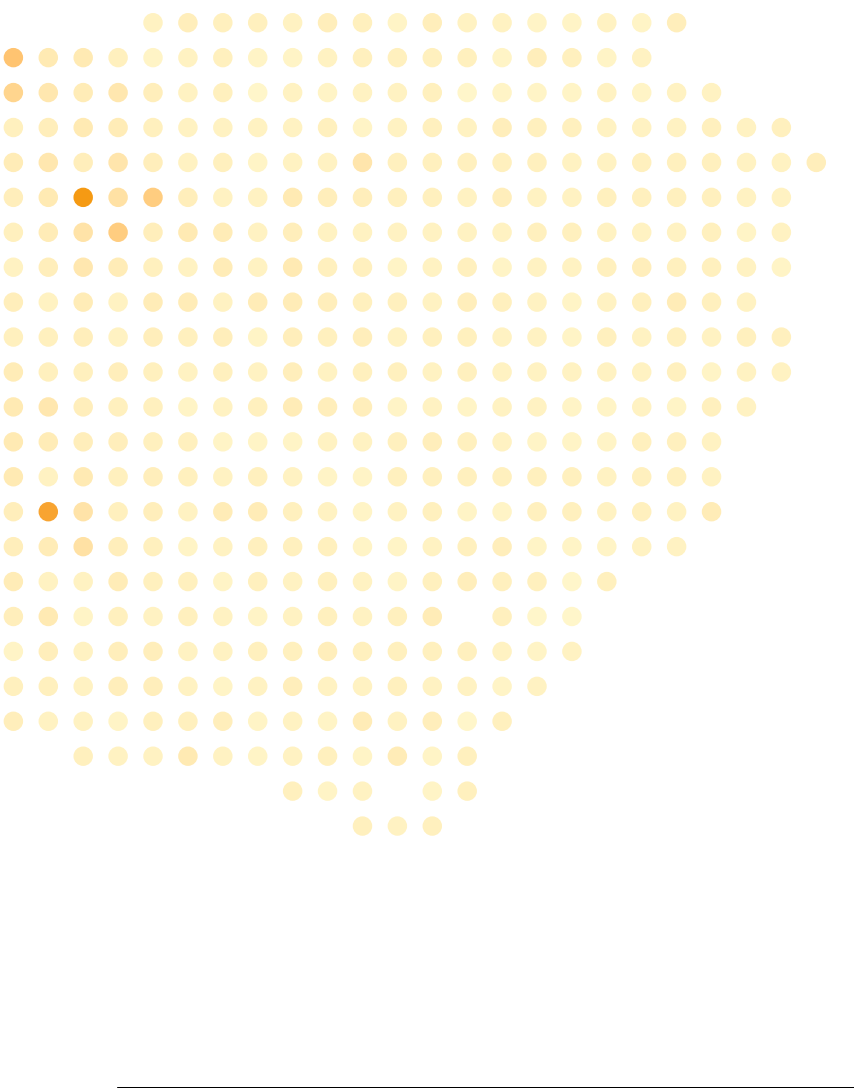

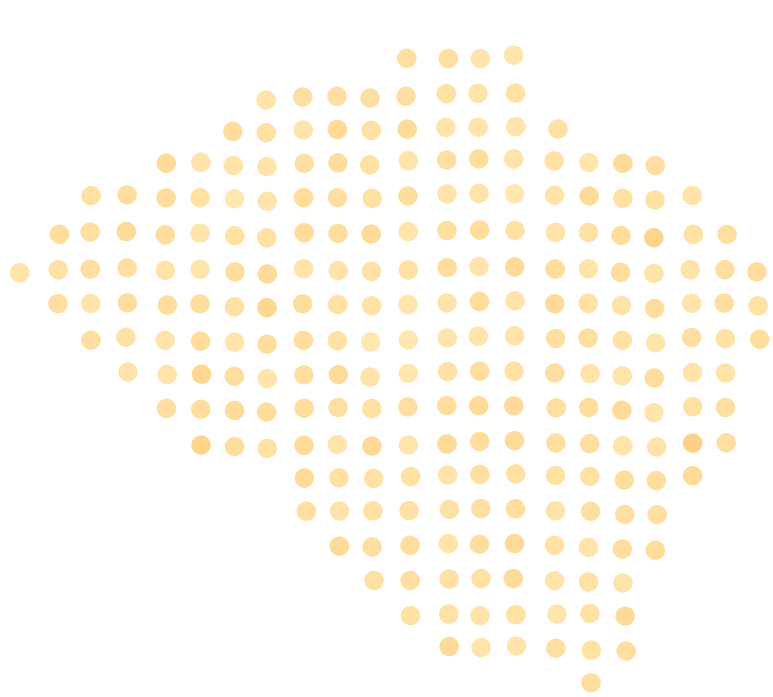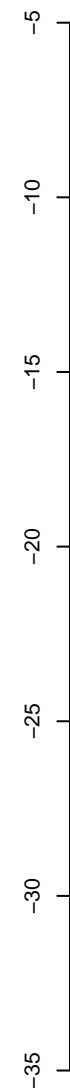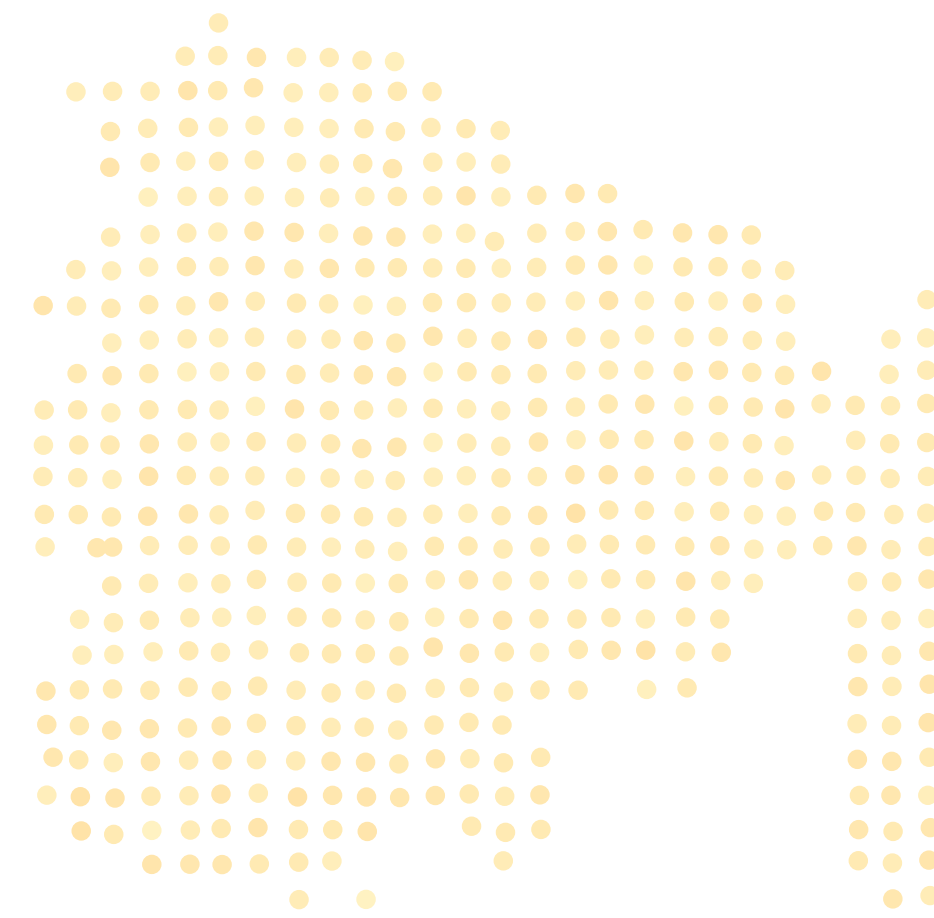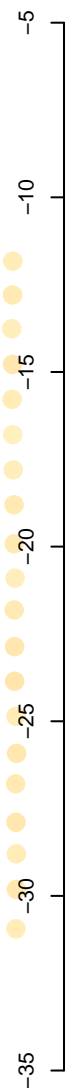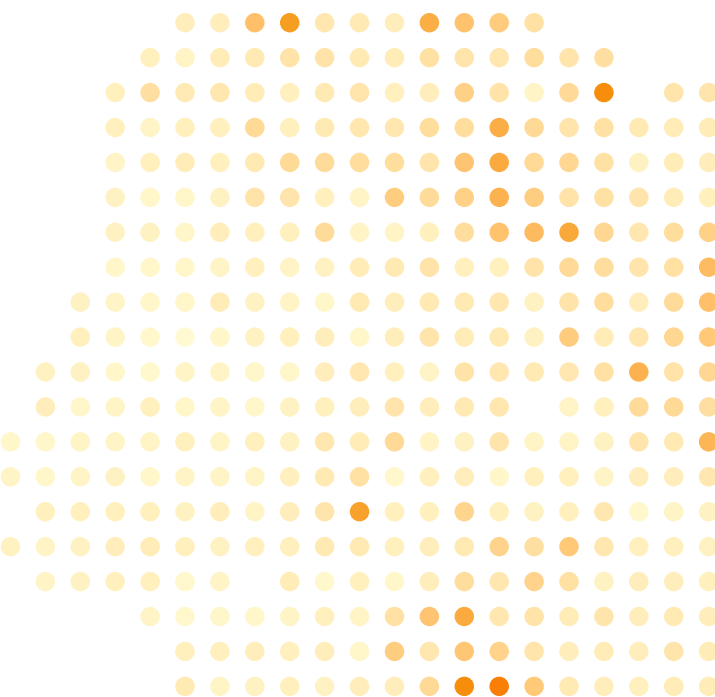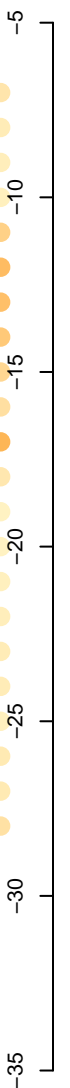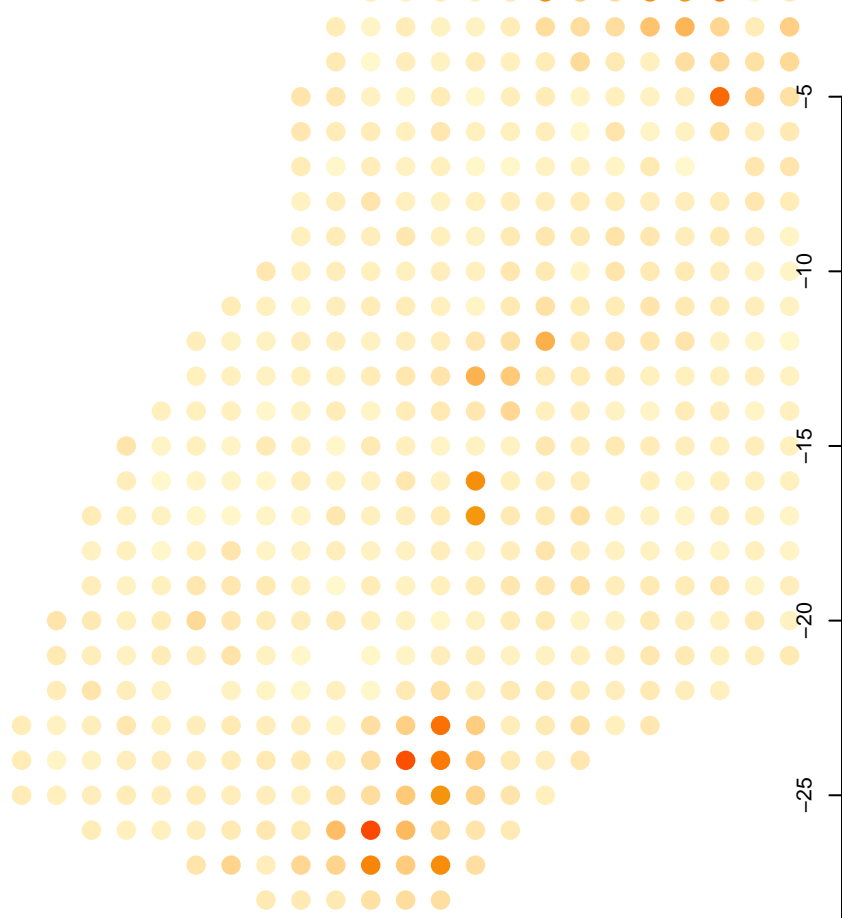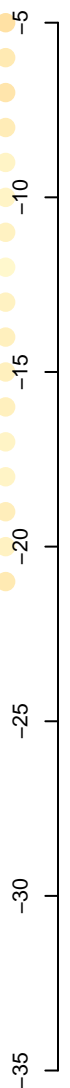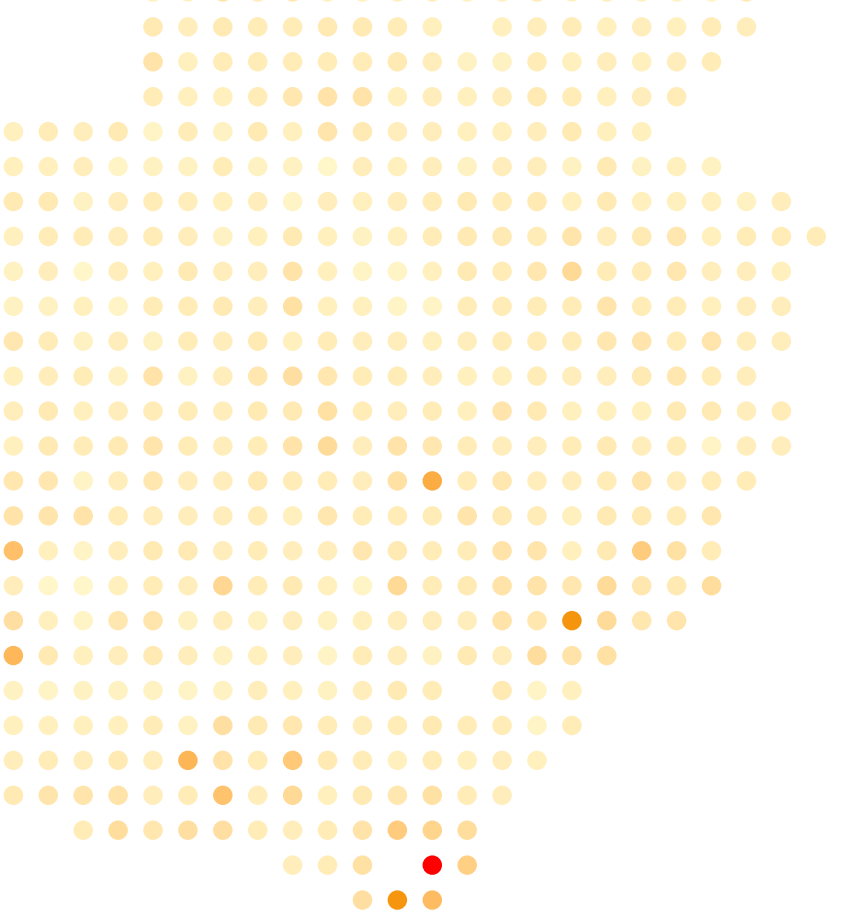

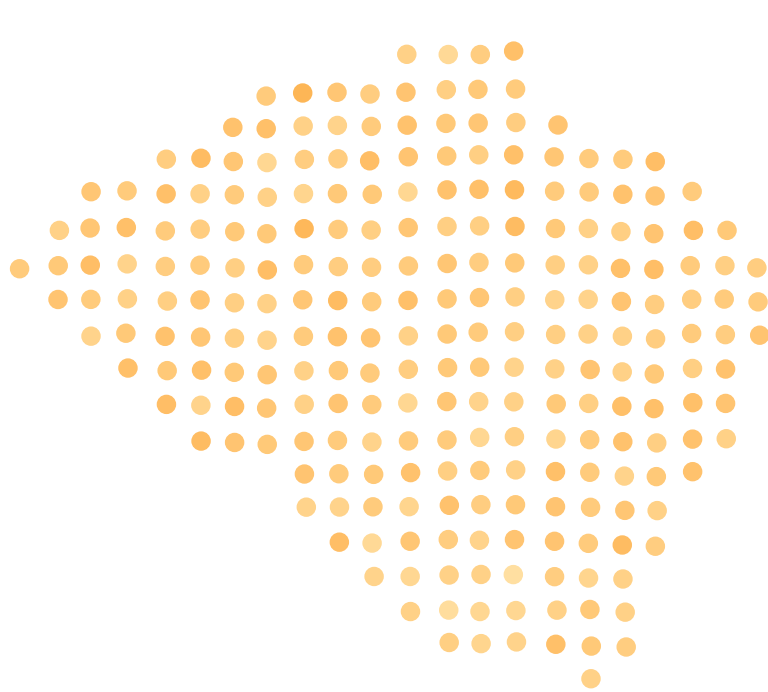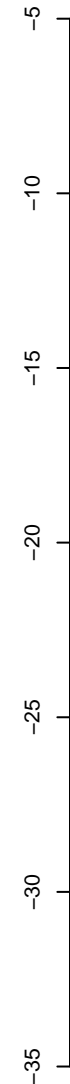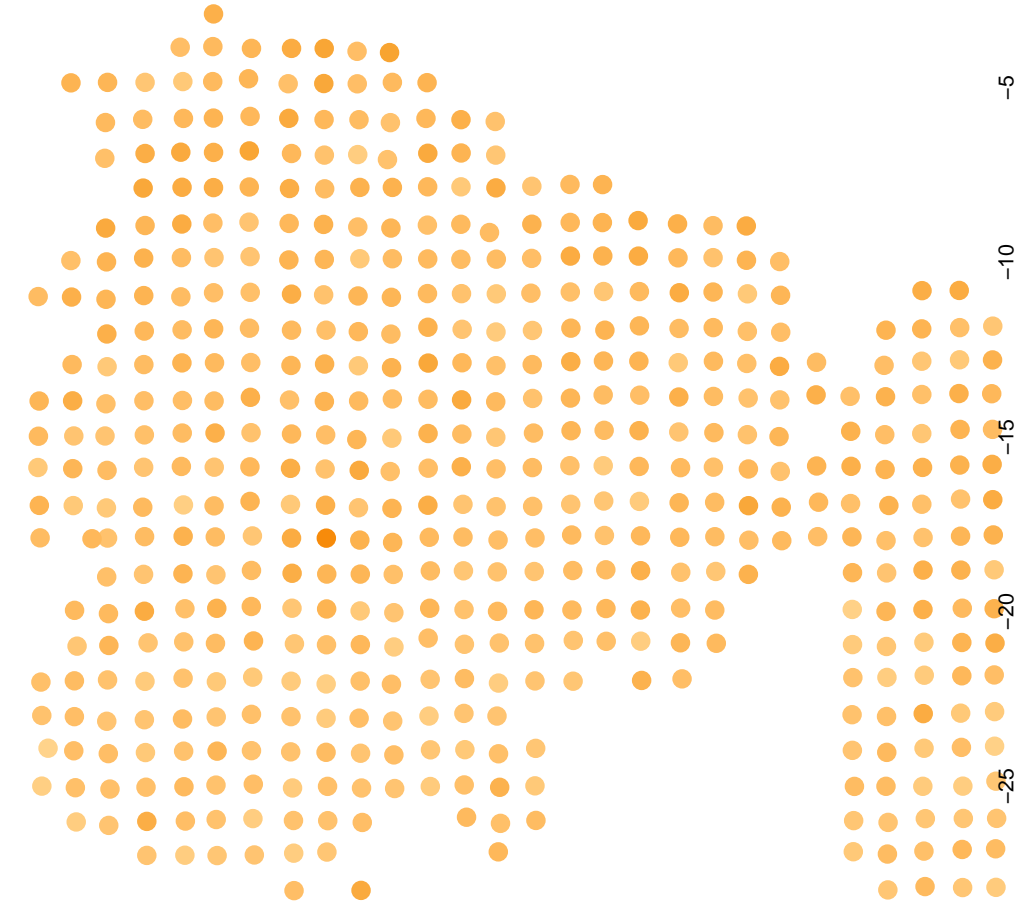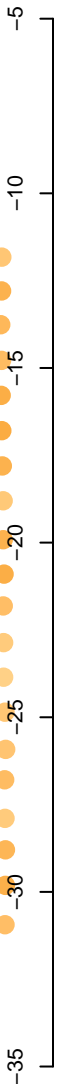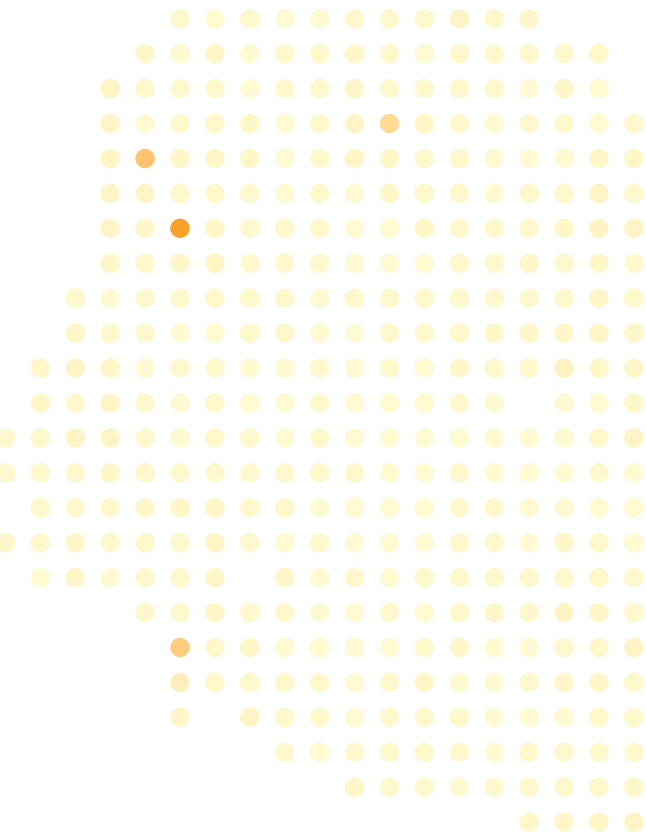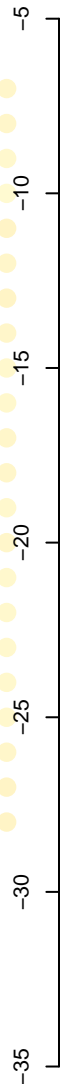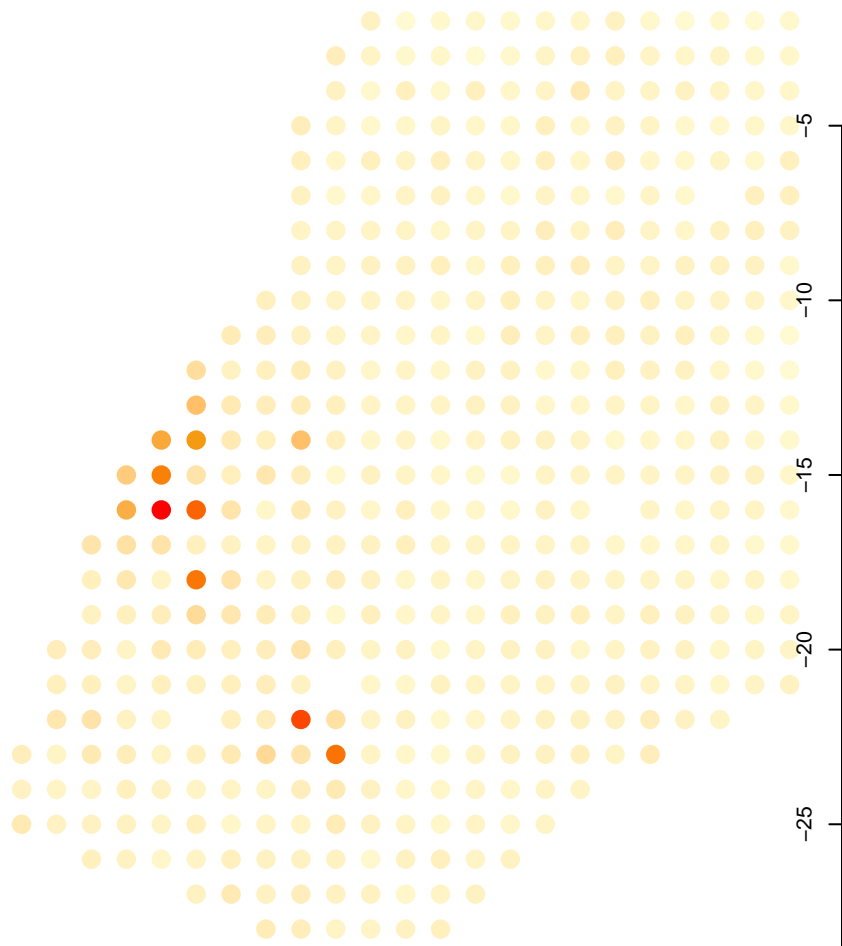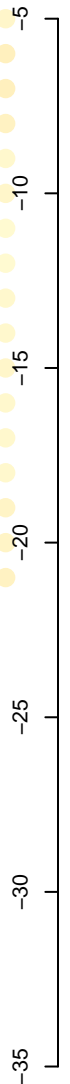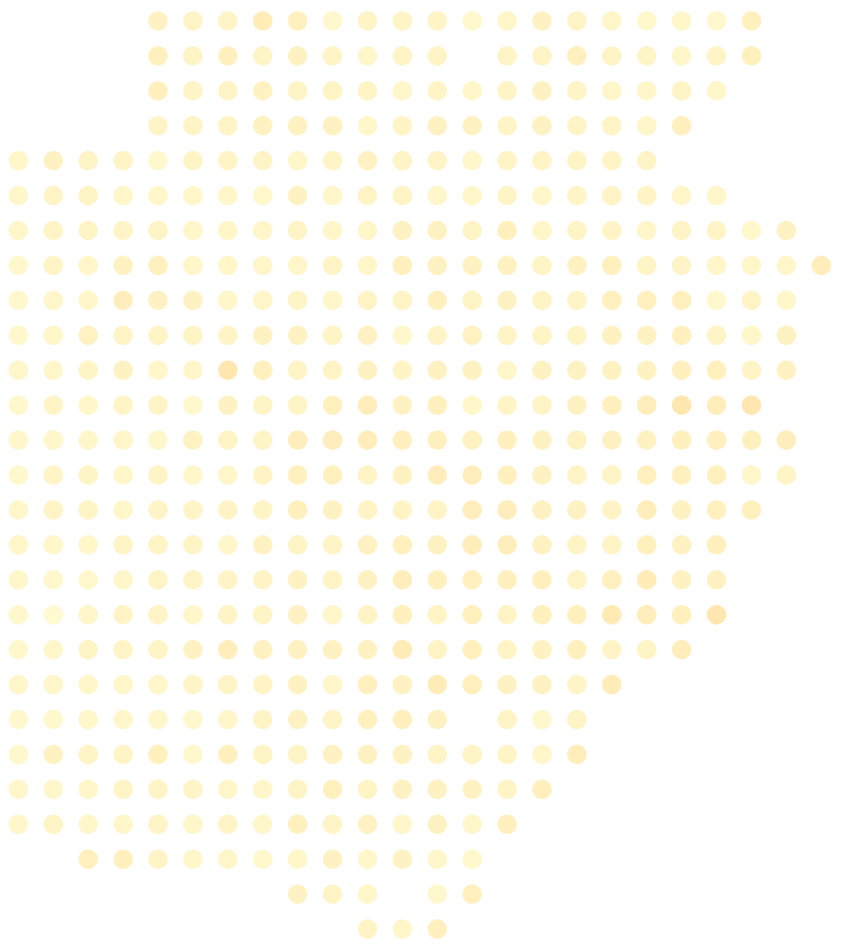

Supplement: Supplementary file 8 — Supplementary Data 5 [file 41467_2018_4724_MOESM8_ESM.zip › Supplementary Dataset 7/joint-mix-profiles-rel-common-scale-dots.pdf]

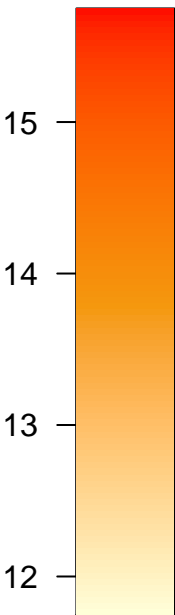

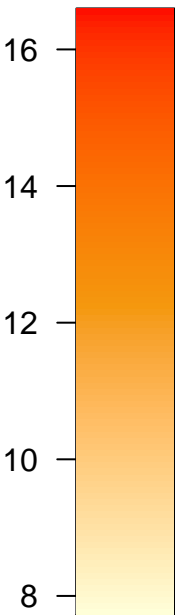

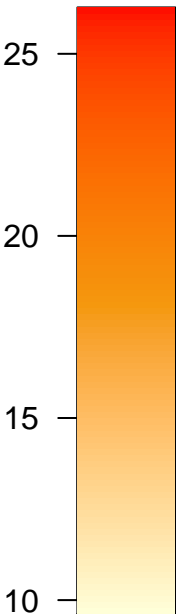

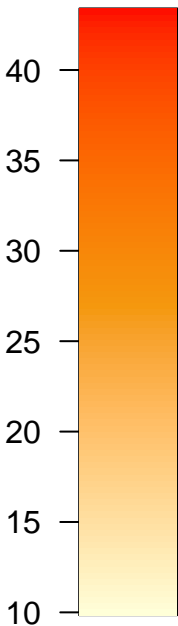

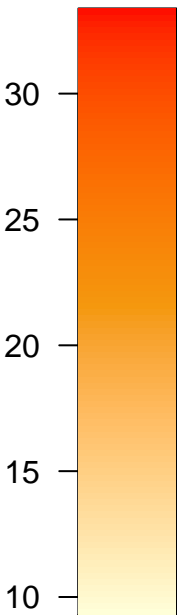

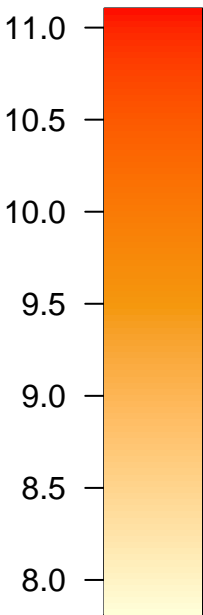

15

14

13

12

11

10

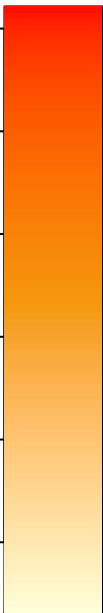

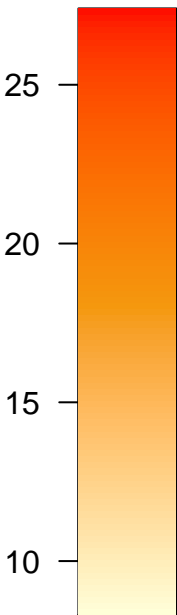

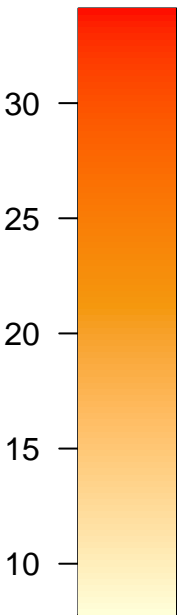

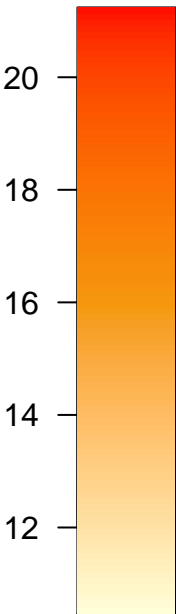

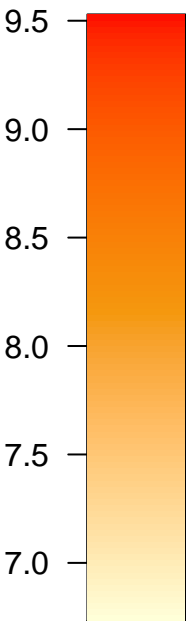

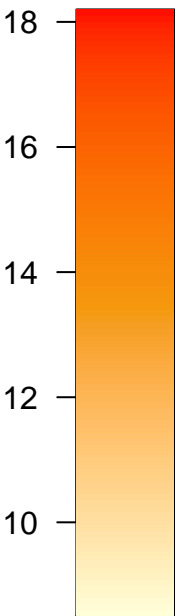

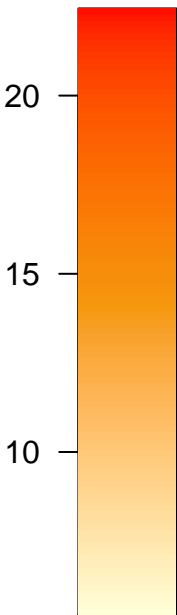

20

15

10

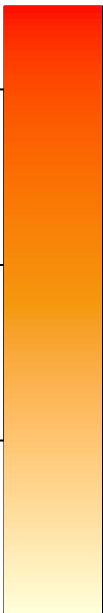

25

20

15

10

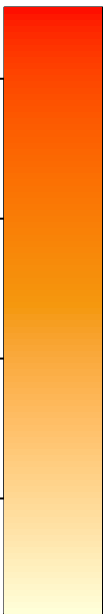

14.0

13.5

13.0

12.5

12.0

11.5

11.0

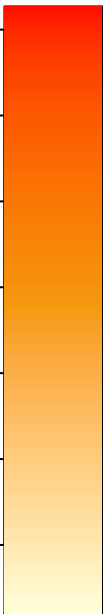

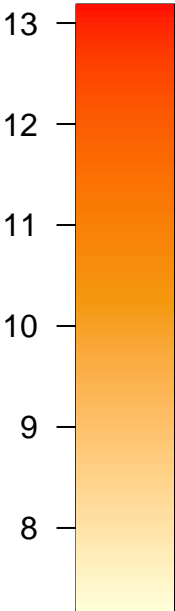

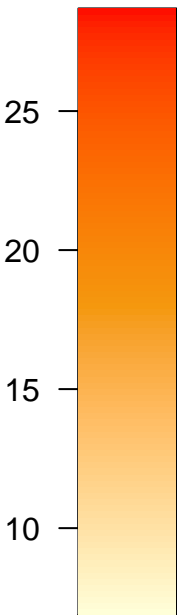

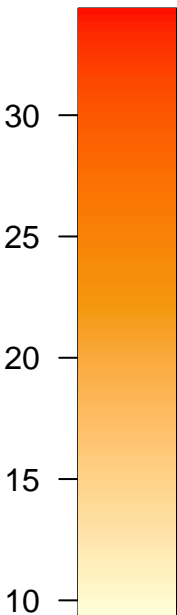

25

20

15

10

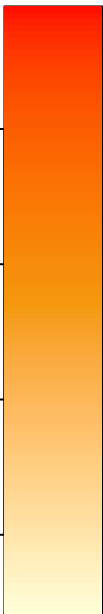

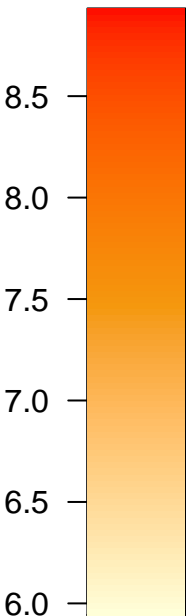

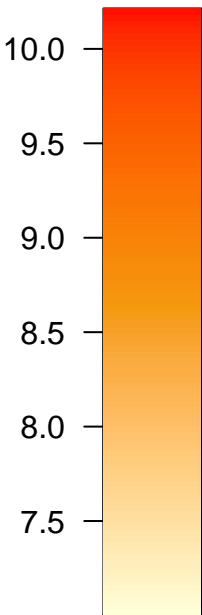

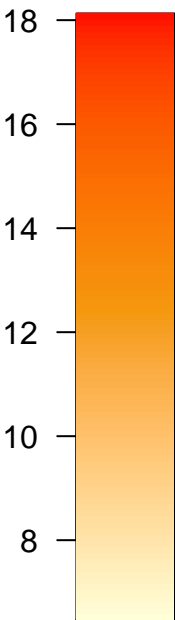

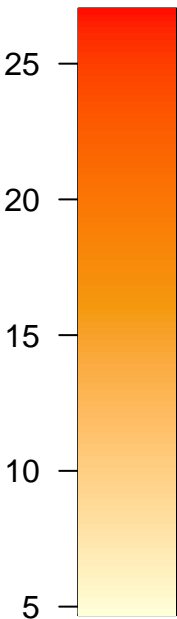

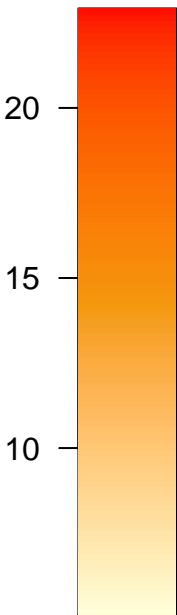

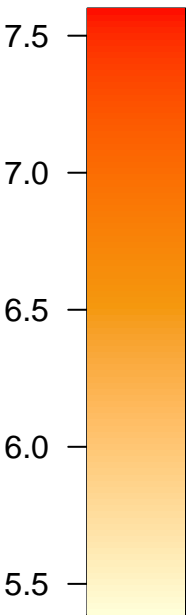

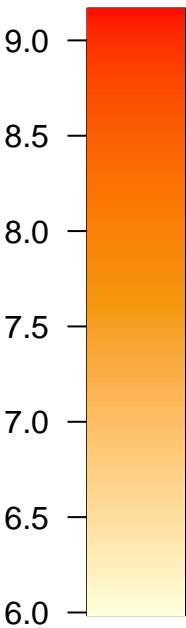

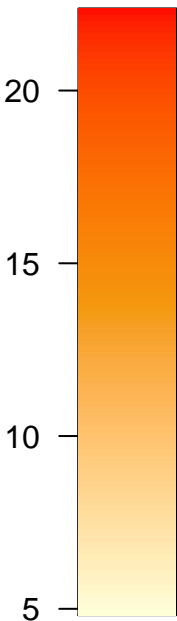

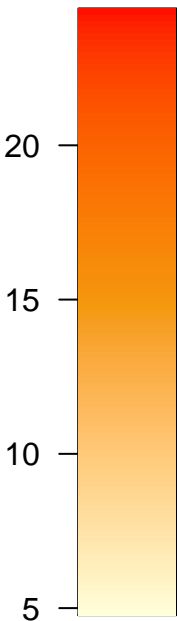

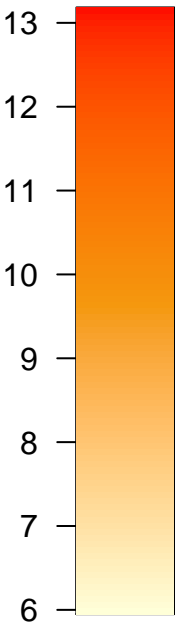

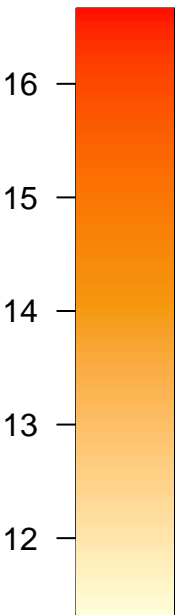

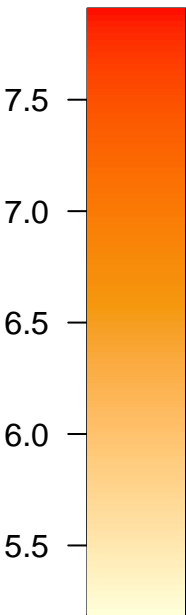

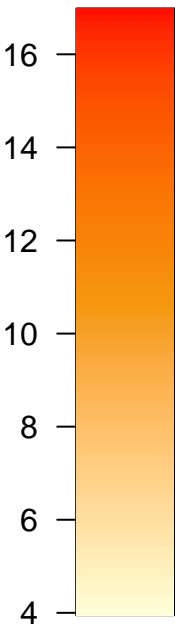

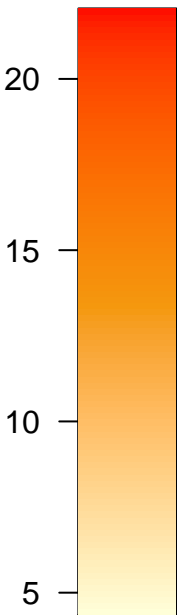

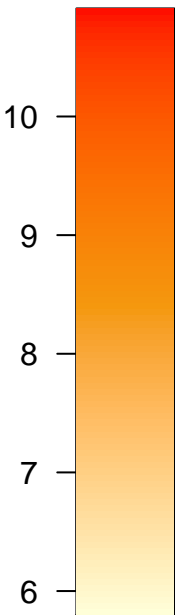

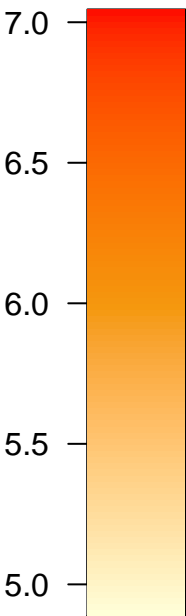

7.0

6.5

6.0

5.5

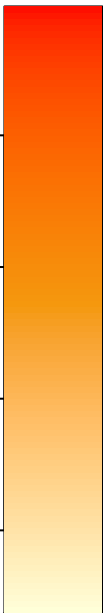

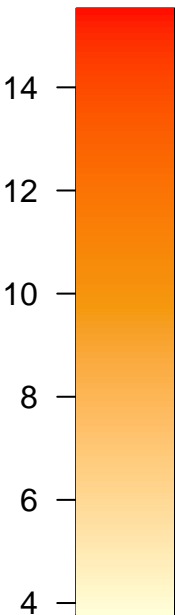

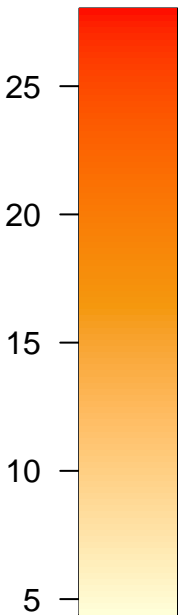

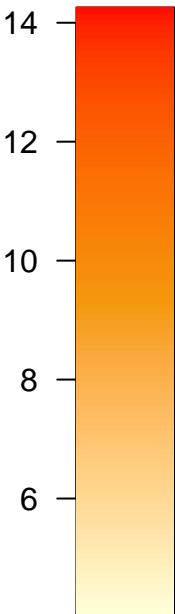

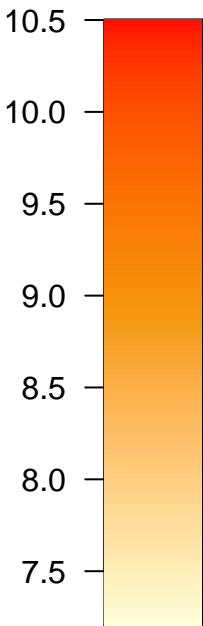

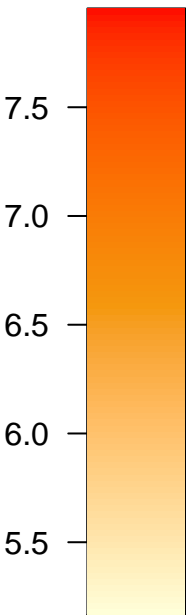

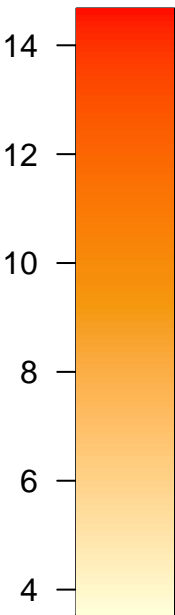

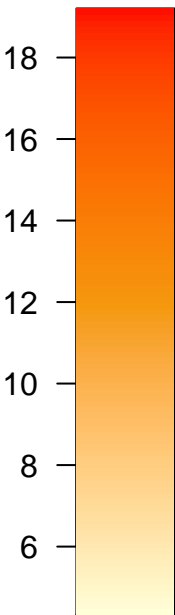

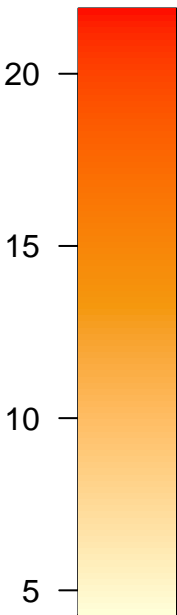

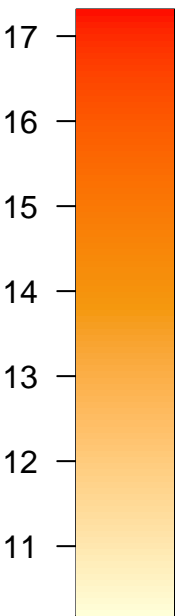

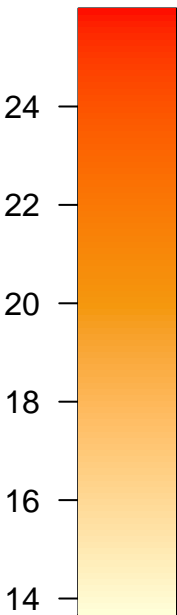

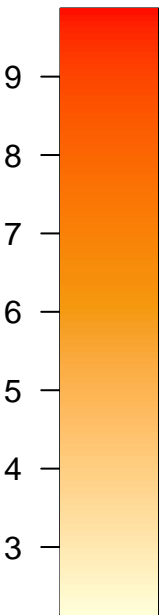

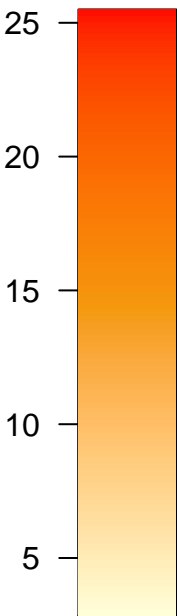

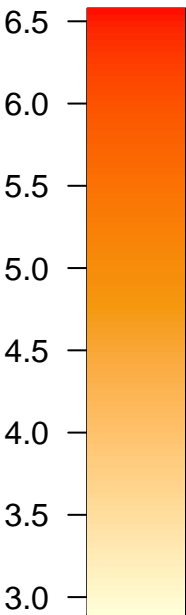

Supplement: Supplementary file 8 — Supplementary Data 5 [file 41467_2018_4724_MOESM8_ESM.zip › Supplementary Dataset 7/joint-field-profiles-rel-individual-scale-dots-split-colorbar.pdf]

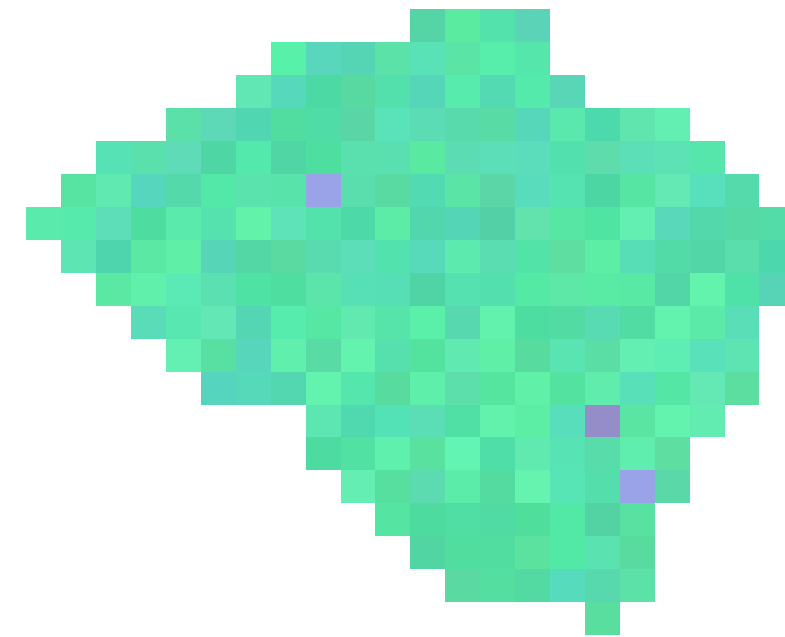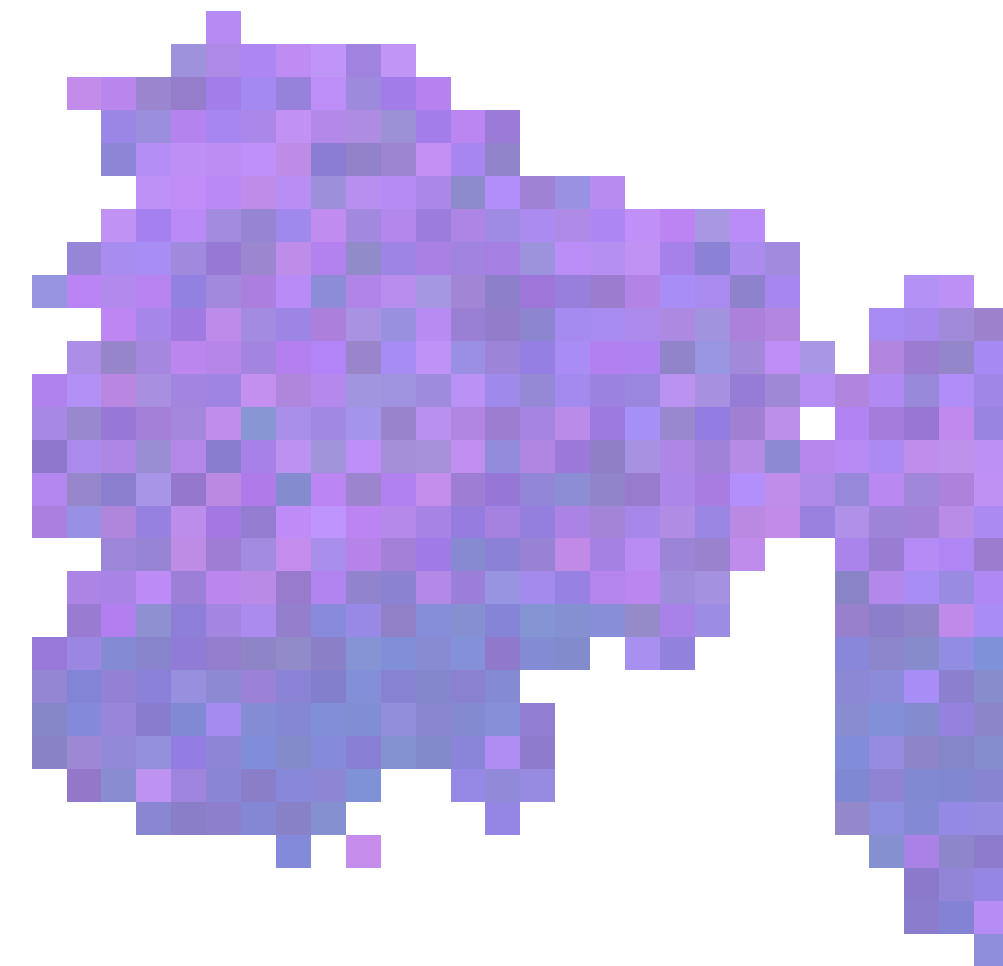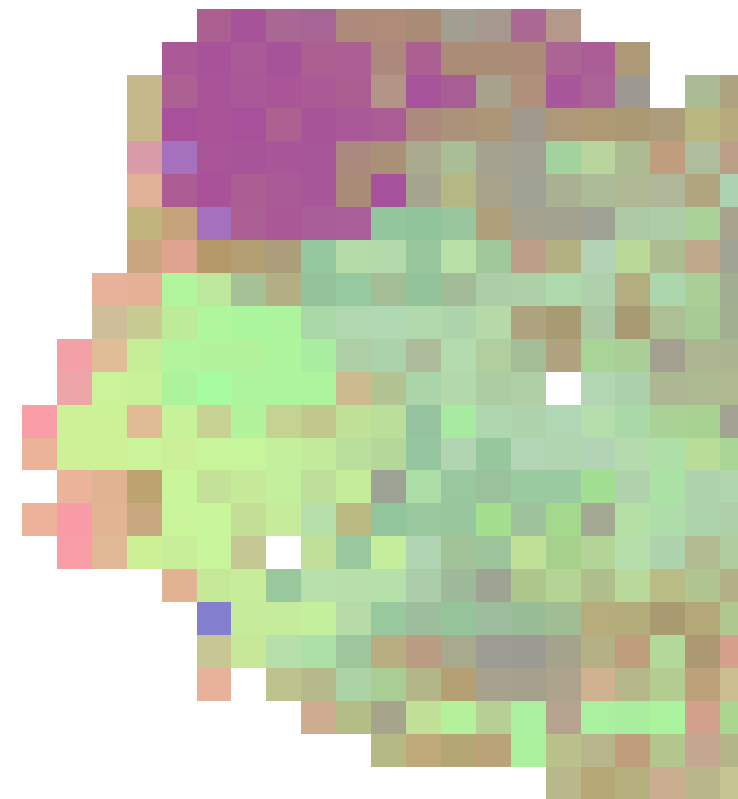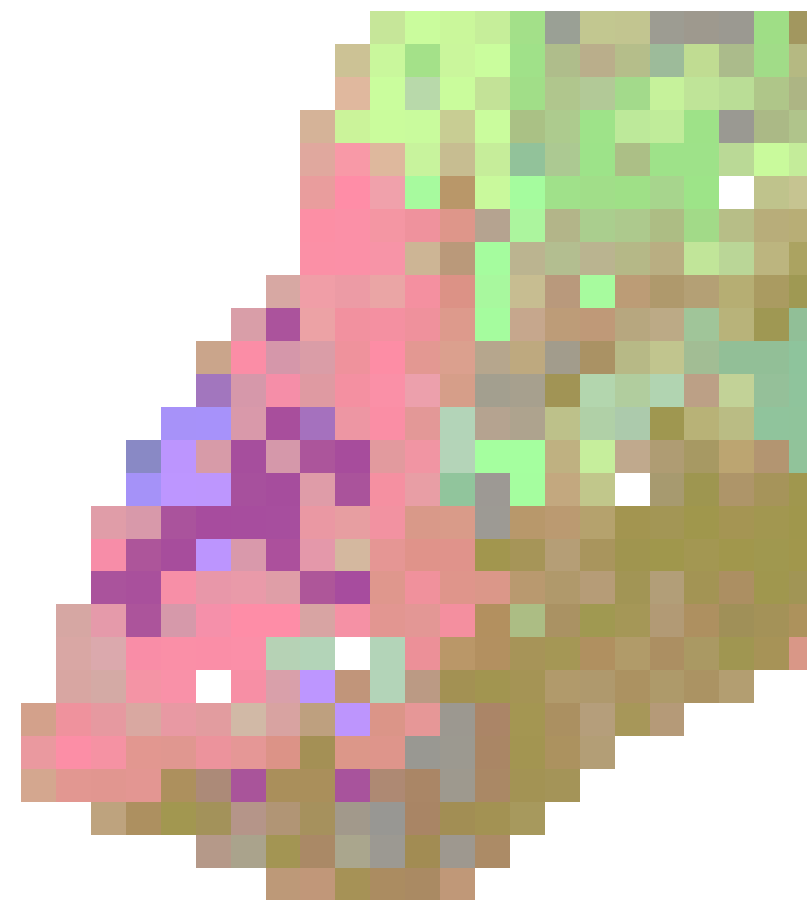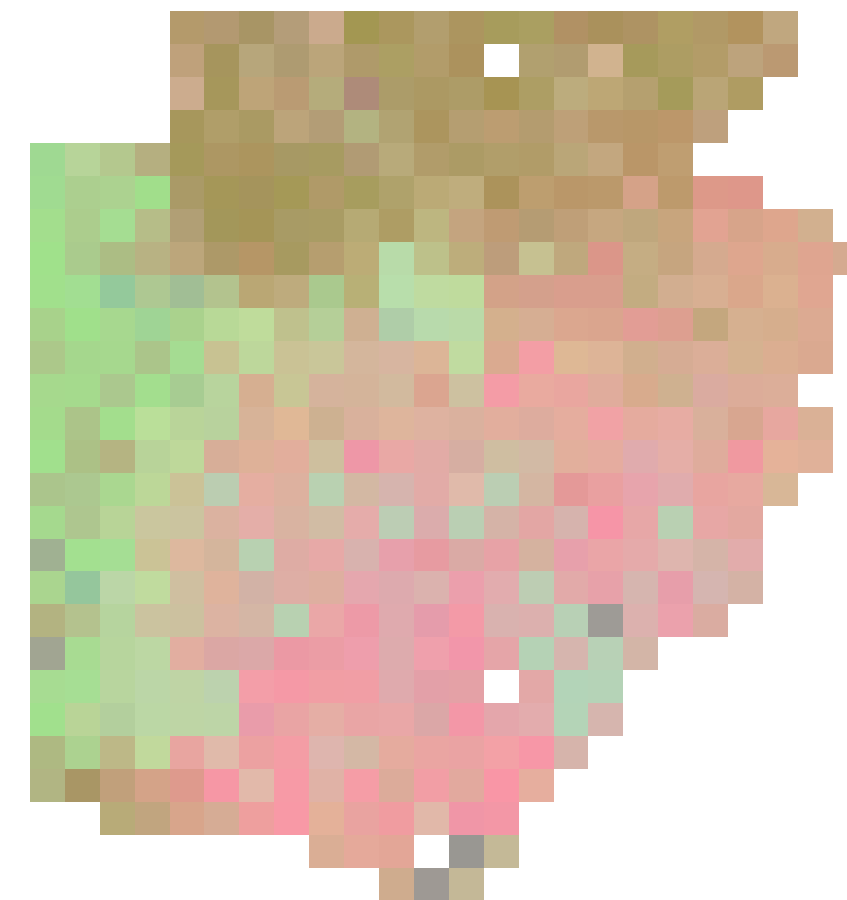

Supplement: Supplementary file 8 — Supplementary Data 5 [file 41467_2018_4724_MOESM8_ESM.zip › Supplementary Dataset 7/joint-mix-dimensionality-reduction-tSNE-matrix-rgb.pdf]

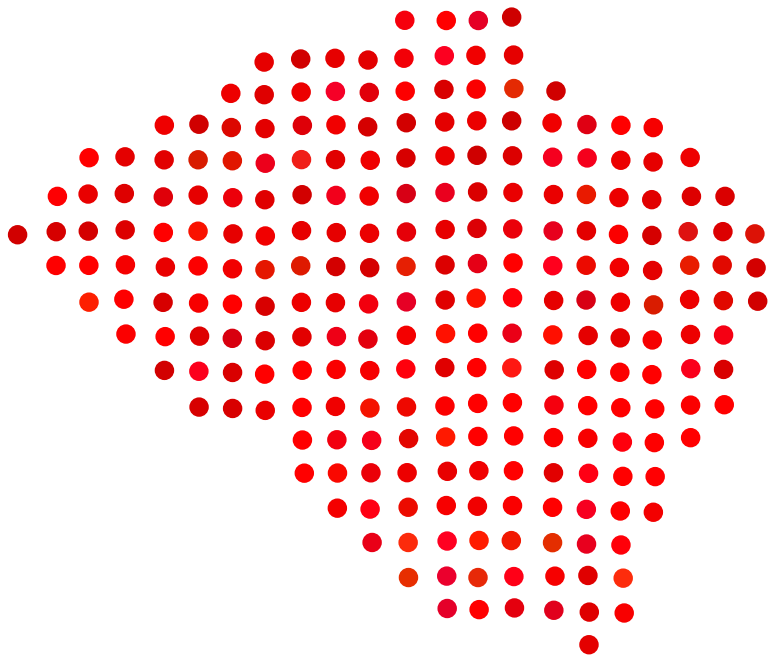

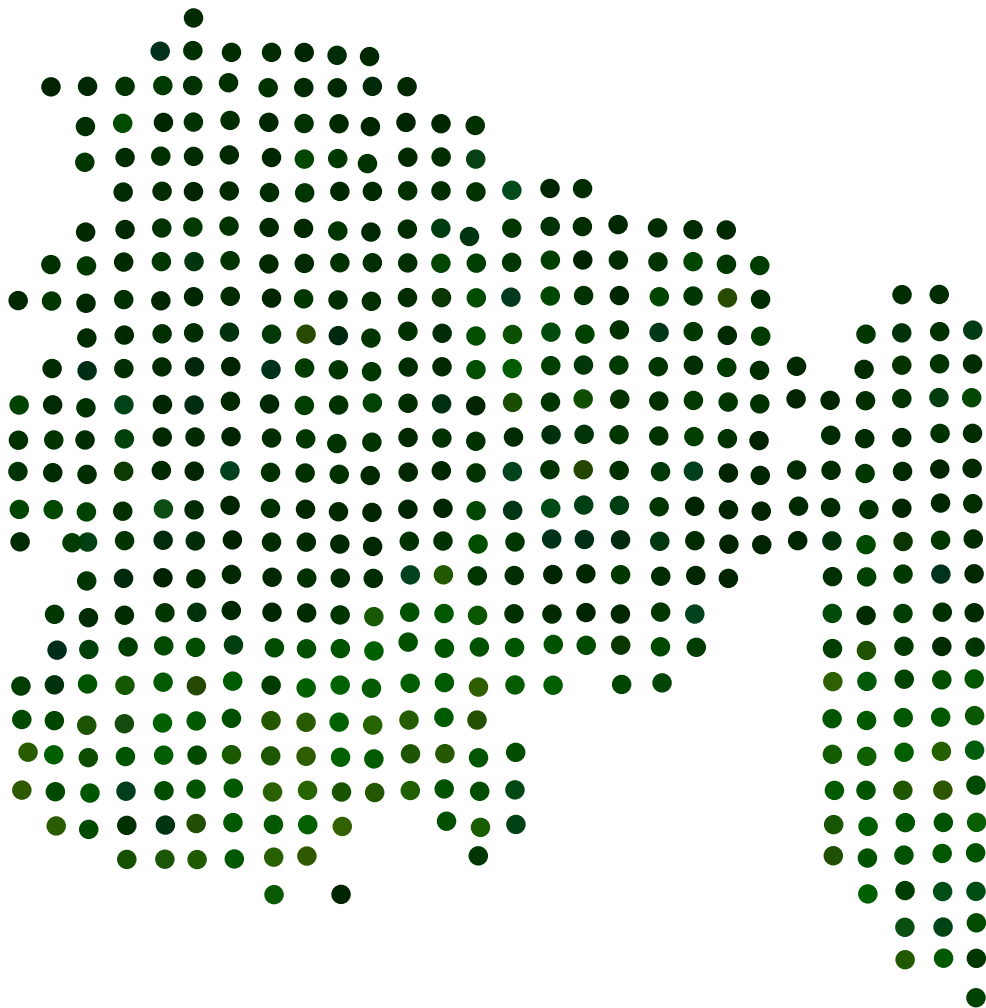

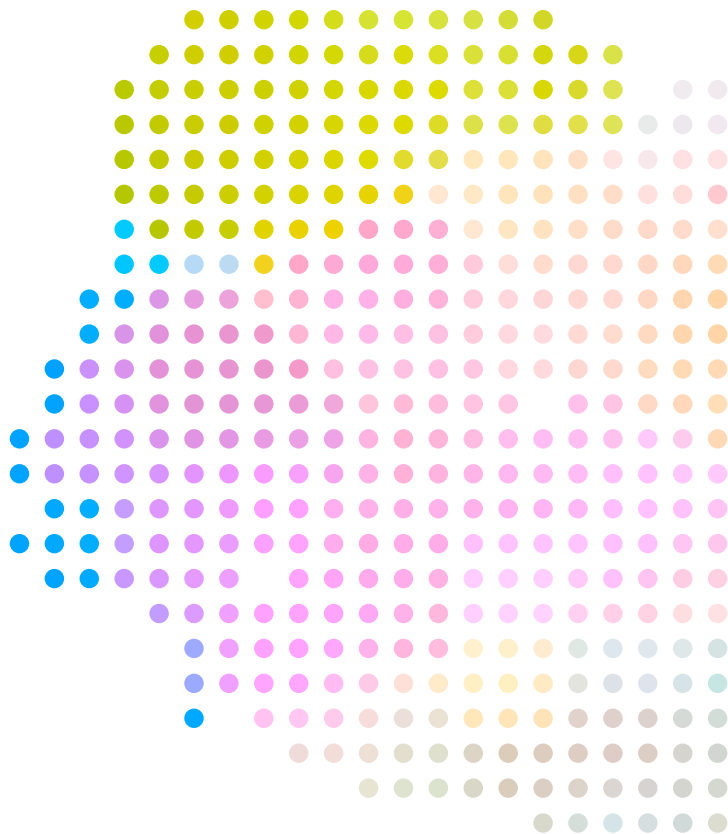

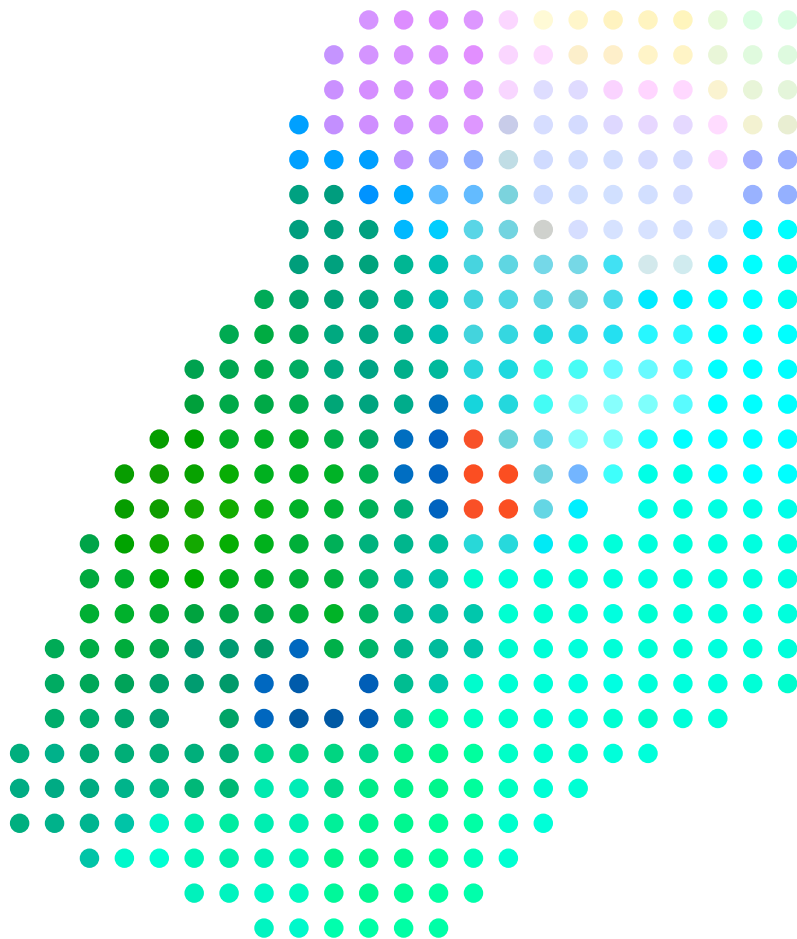

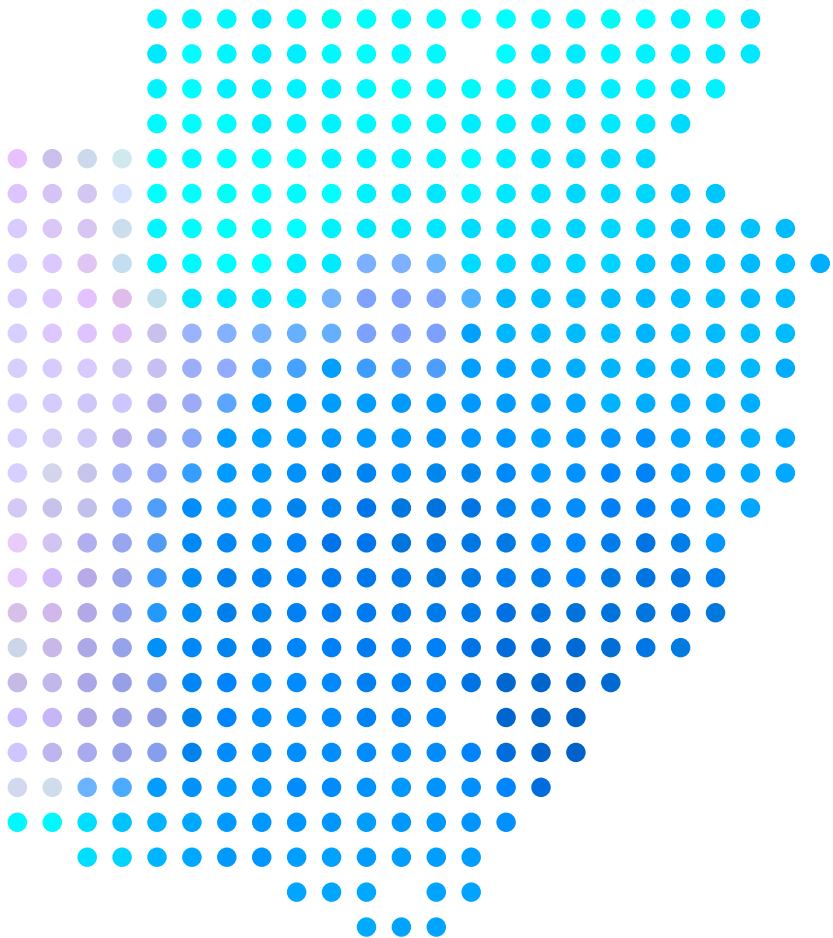

Supplement: Supplementary file 8 — Supplementary Data 5 [file 41467_2018_4724_MOESM8_ESM.zip › Supplementary Dataset 7/joint-field-dimensionality-reduction-tSNE-dots-split.pdf]

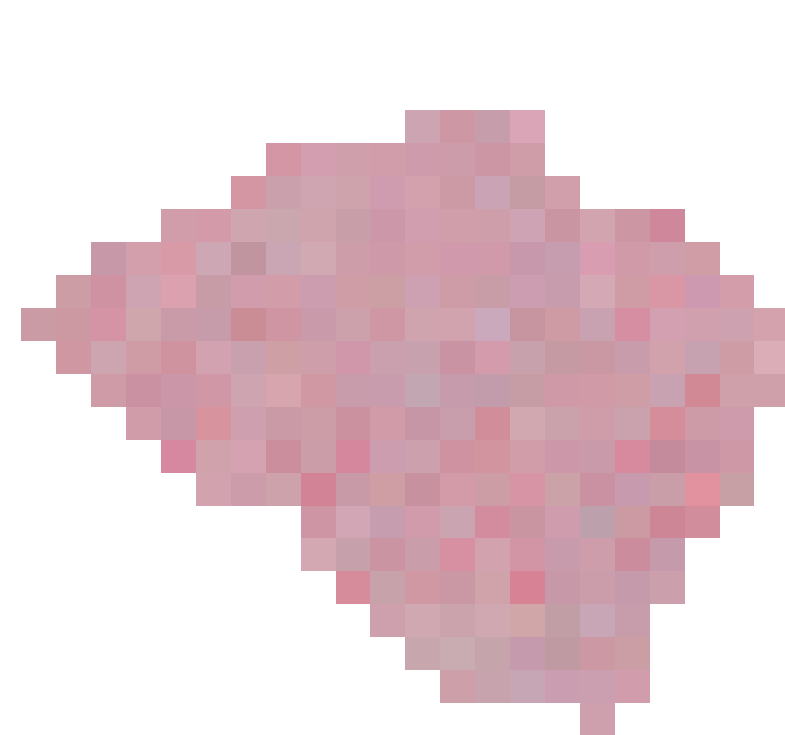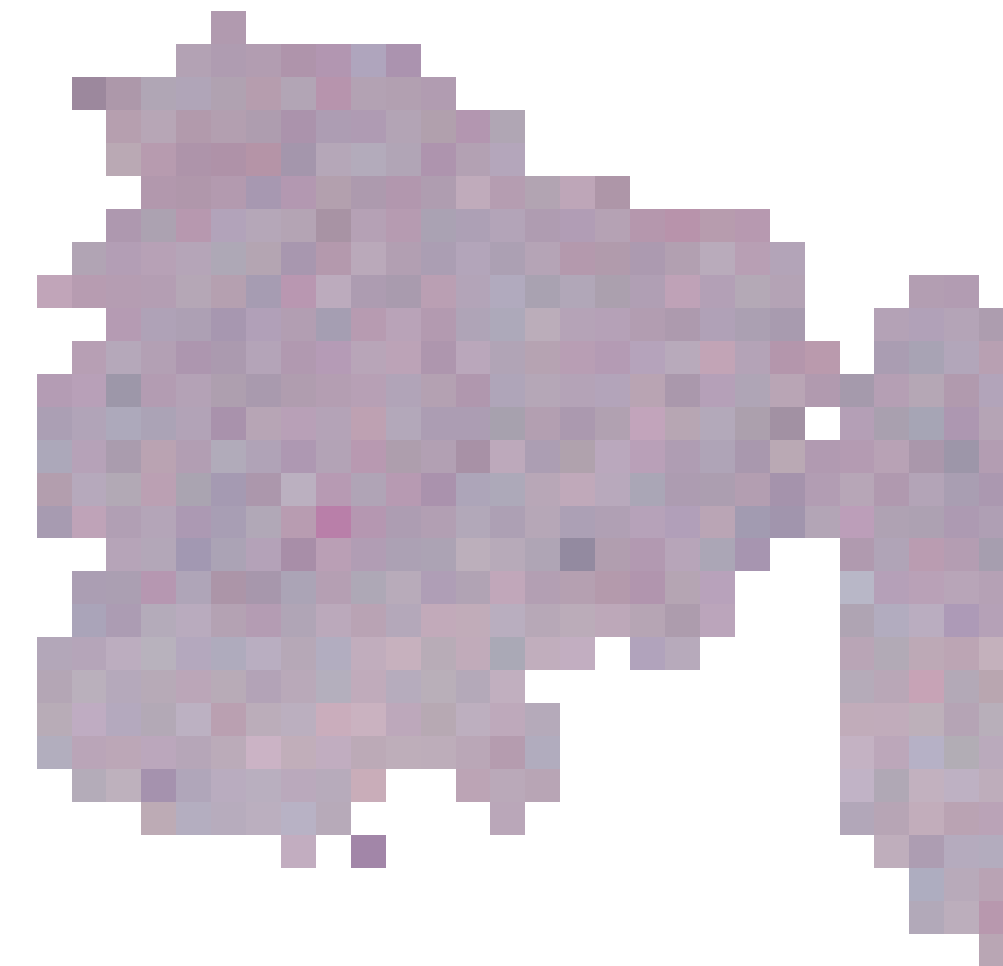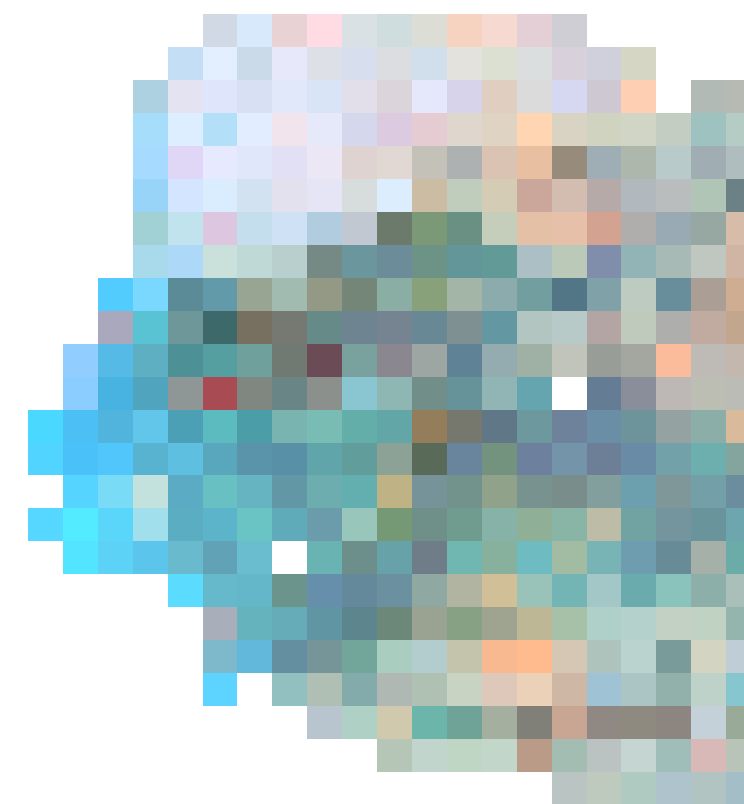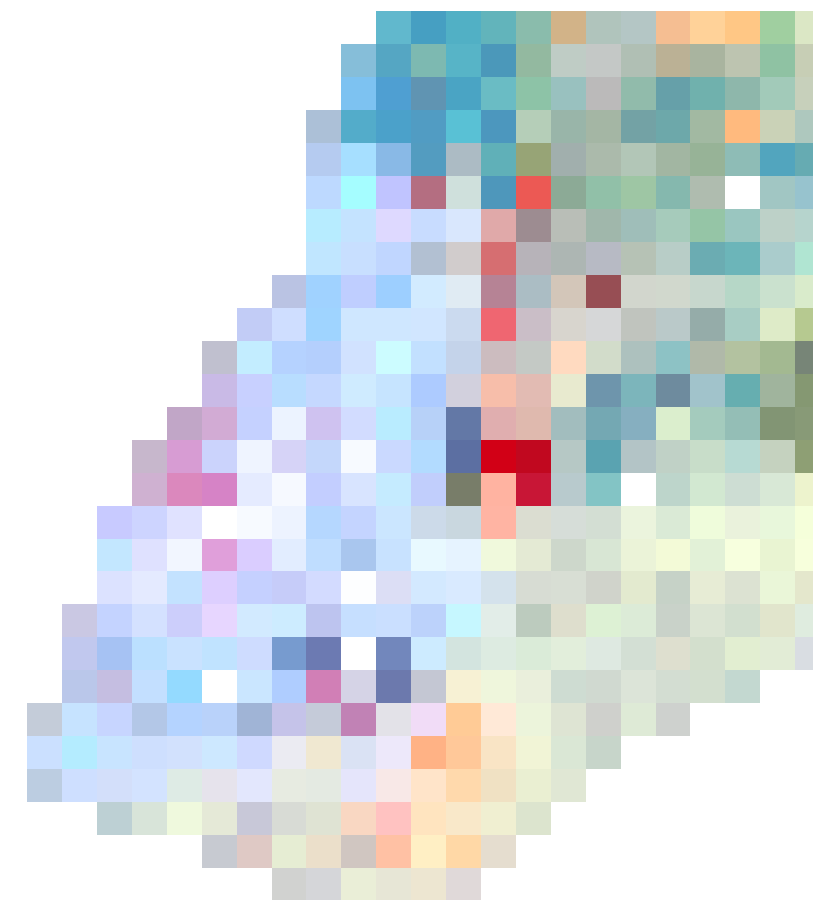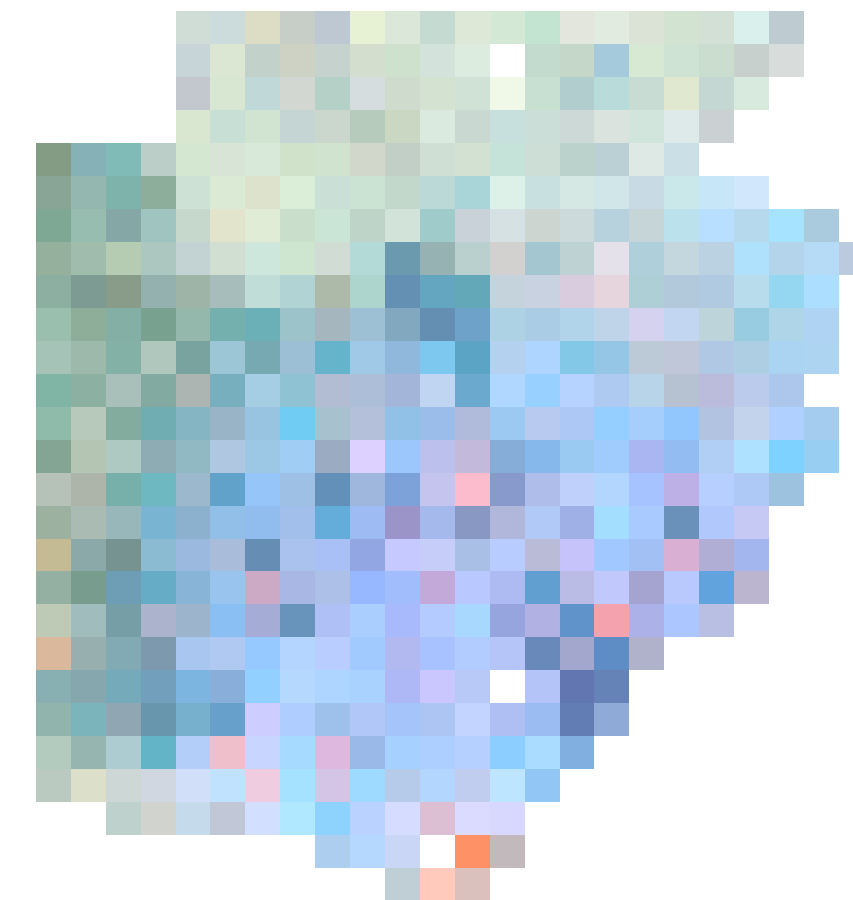

Supplement: Supplementary file 8 — Supplementary Data 5 [file 41467_2018_4724_MOESM8_ESM.zip › Supplementary Dataset 7/joint-mix-dimensionality-reduction-PCA-matrix.pdf]

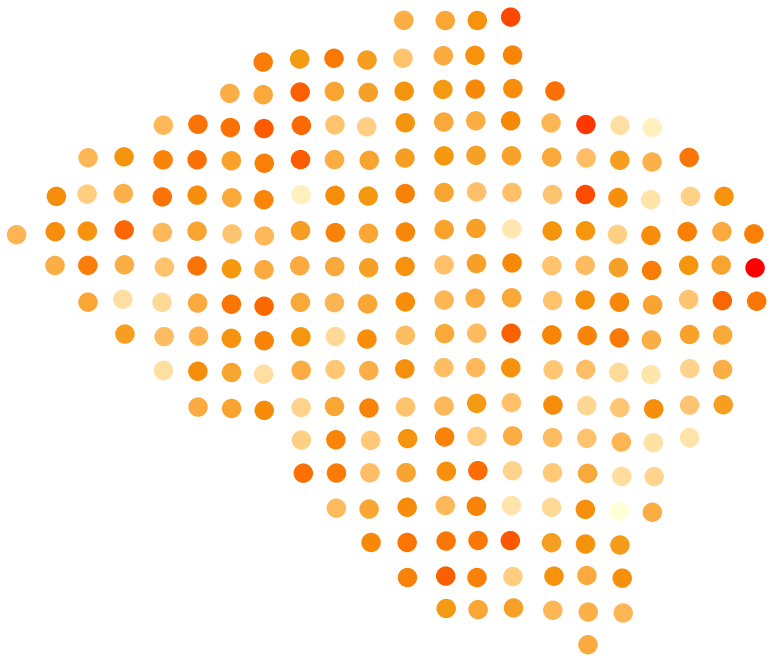

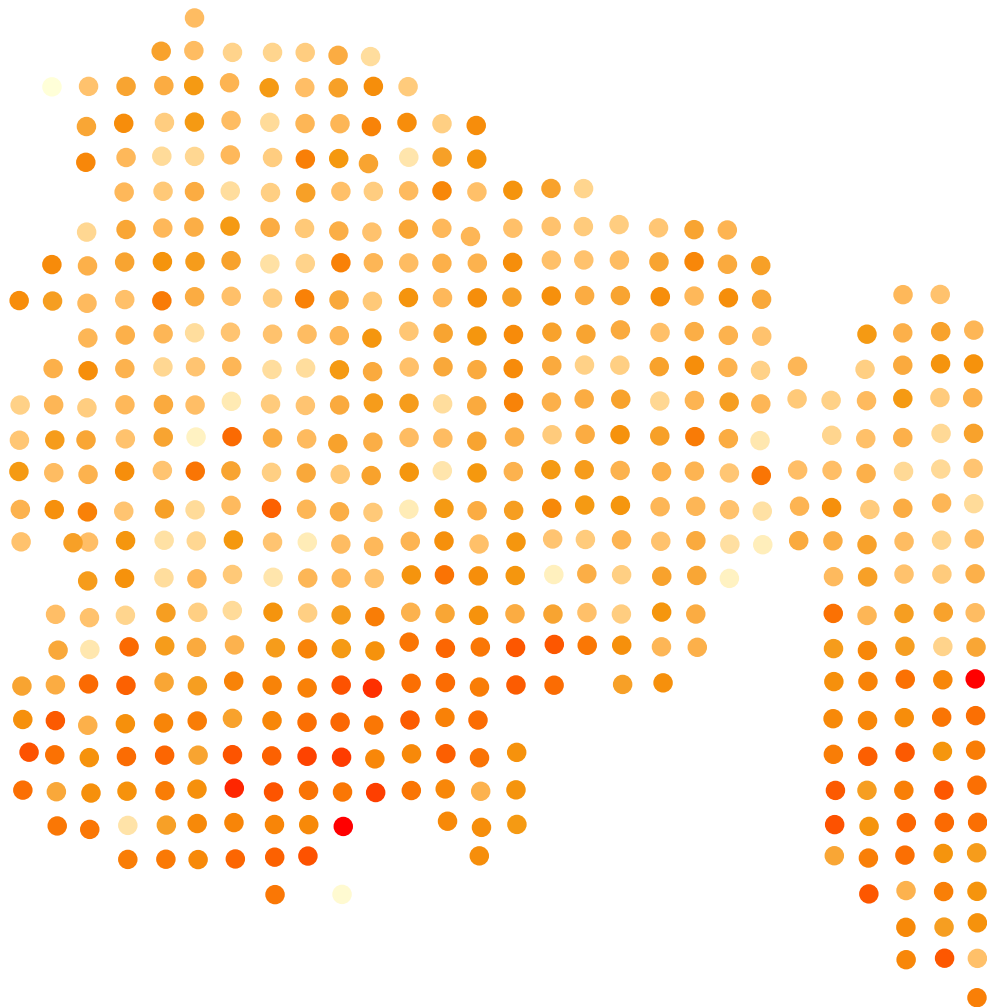

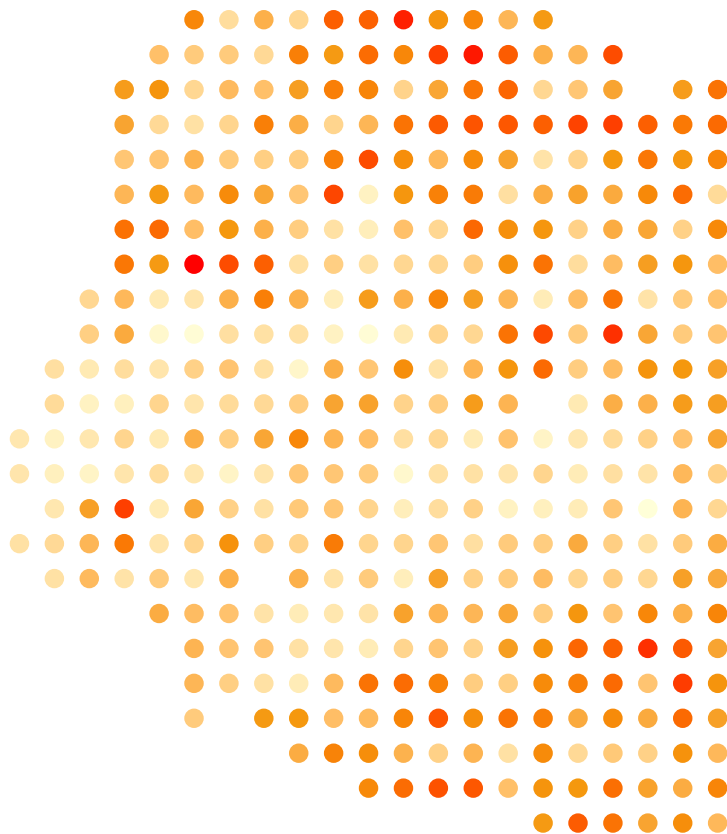

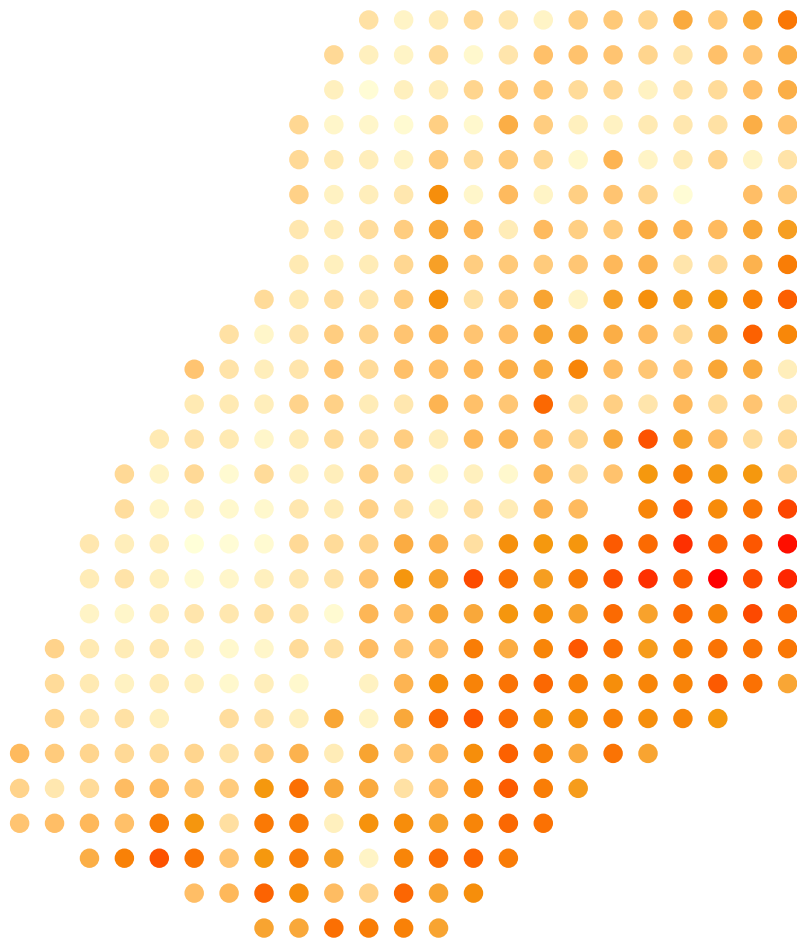

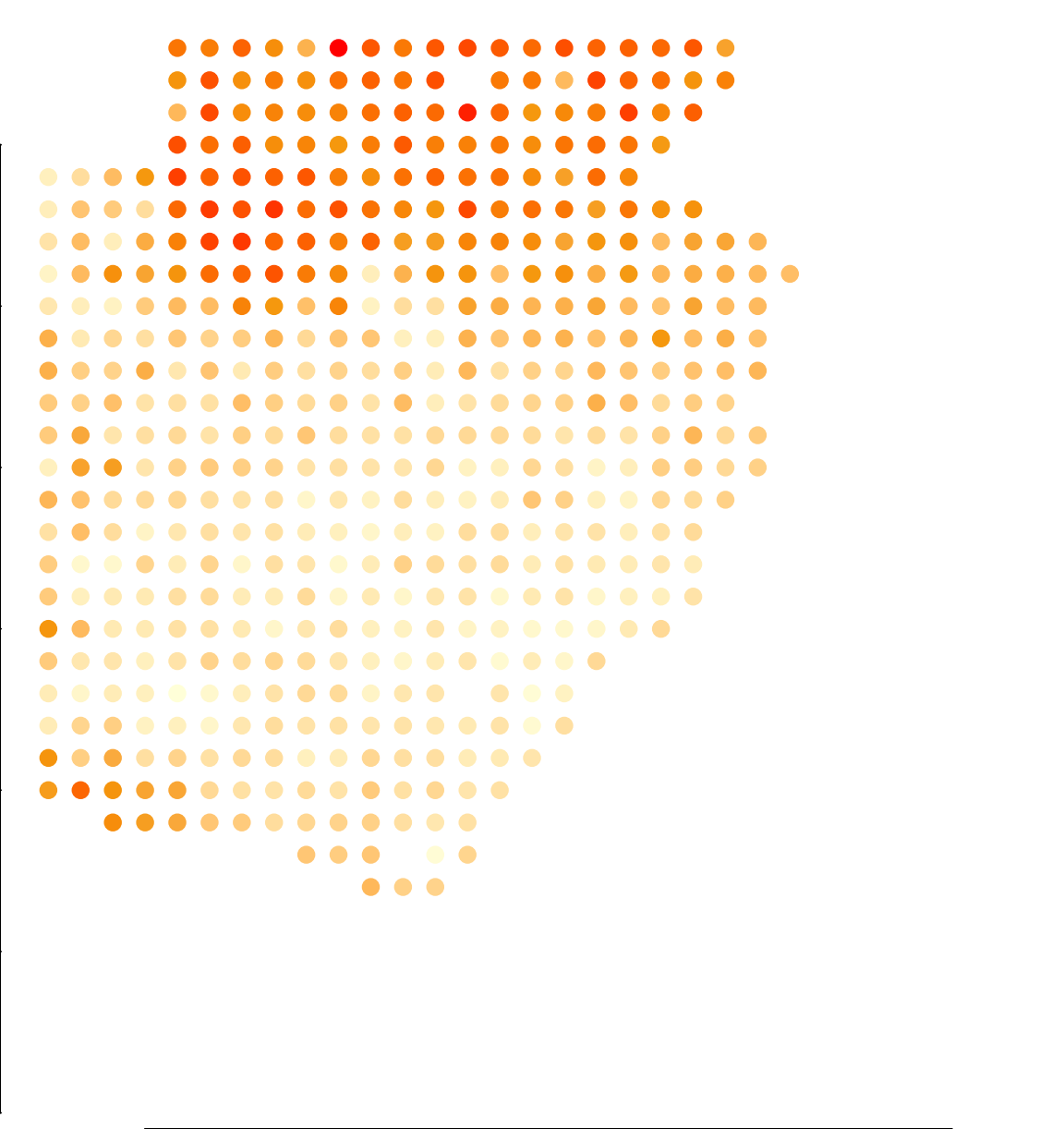

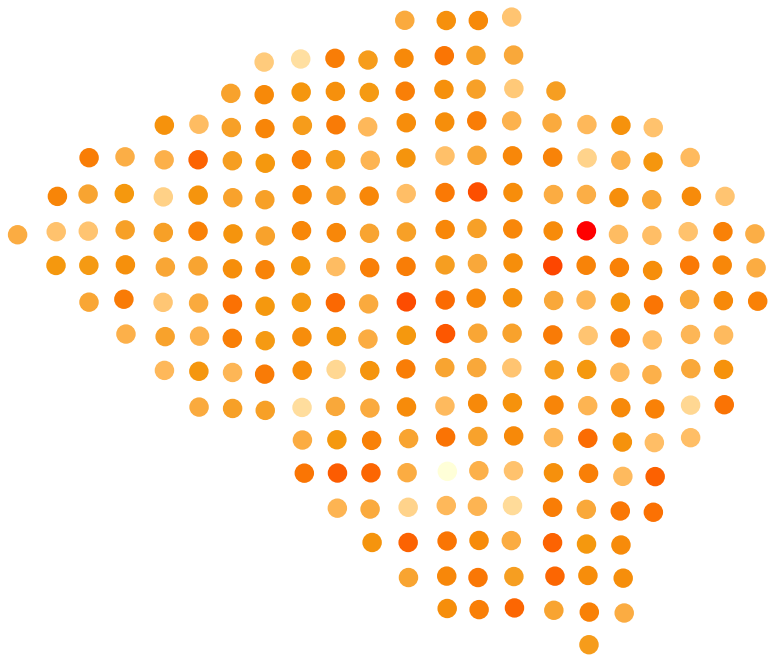

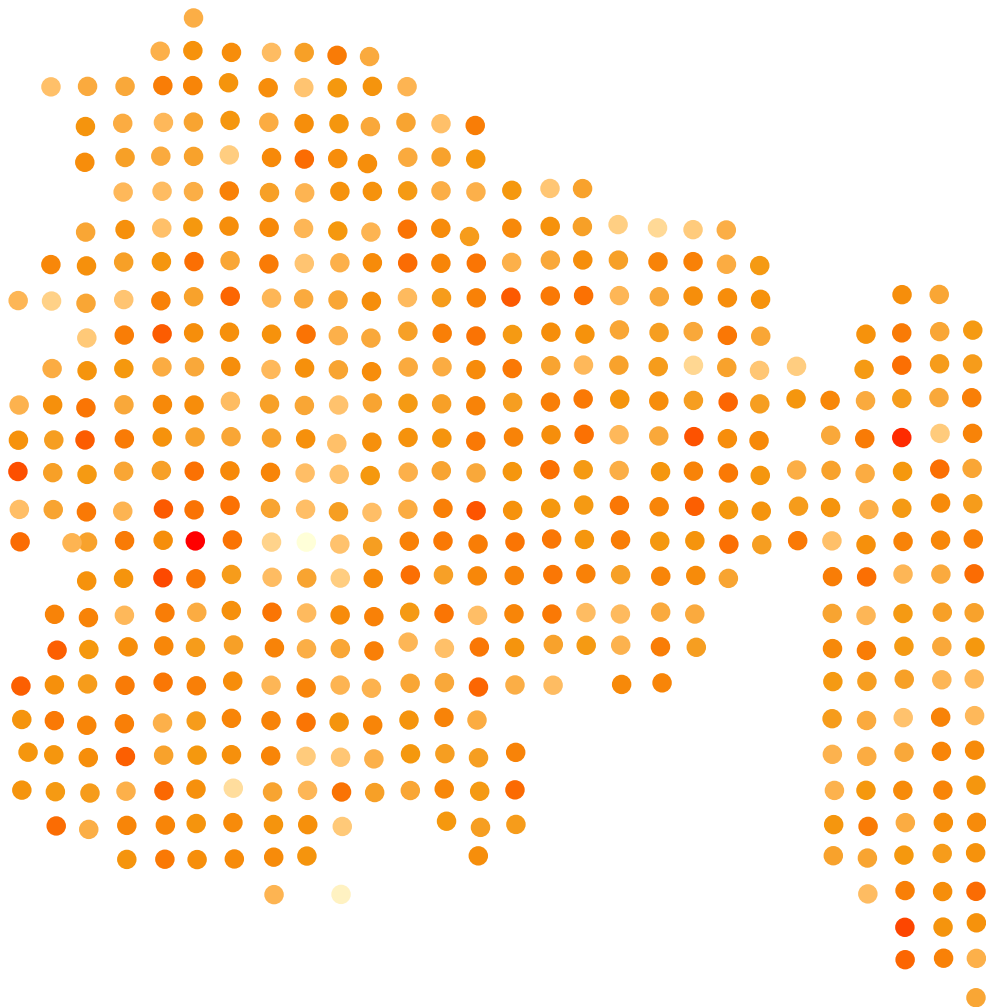

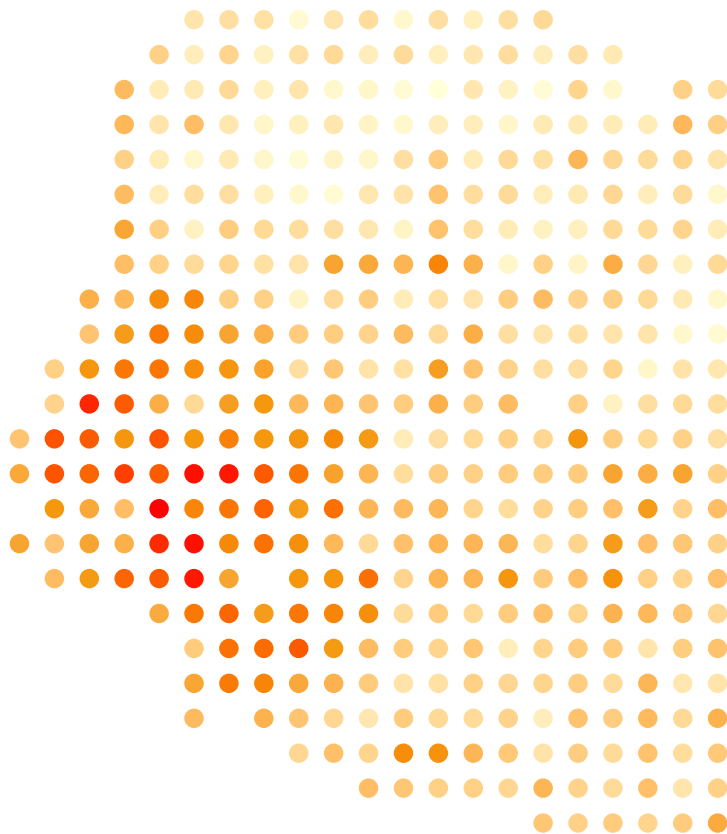

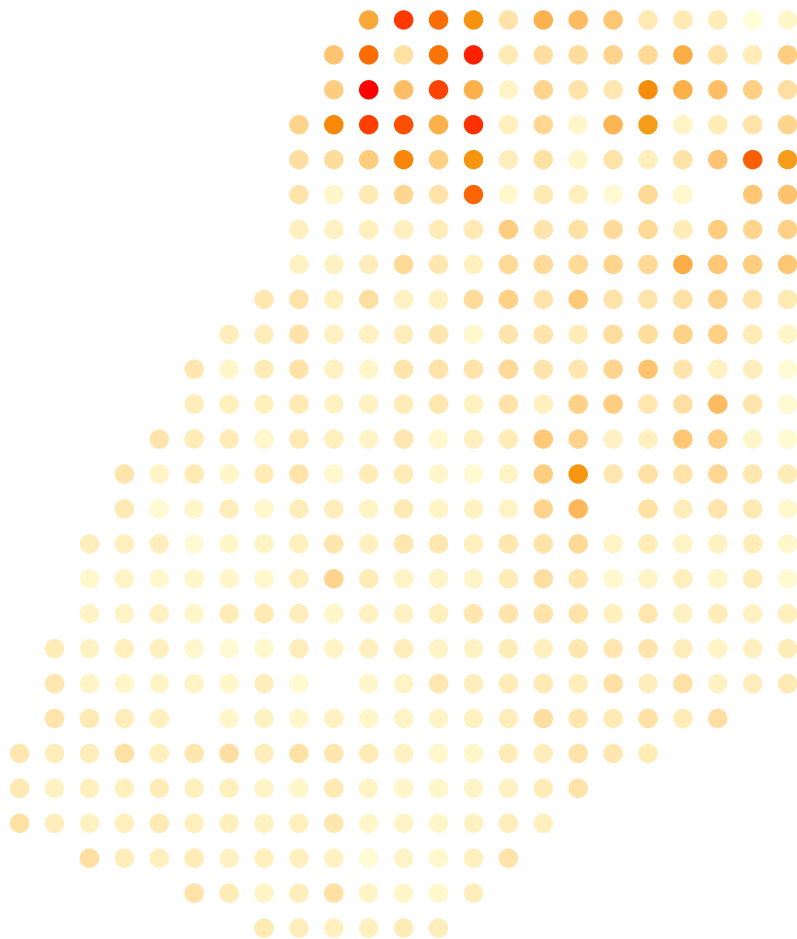

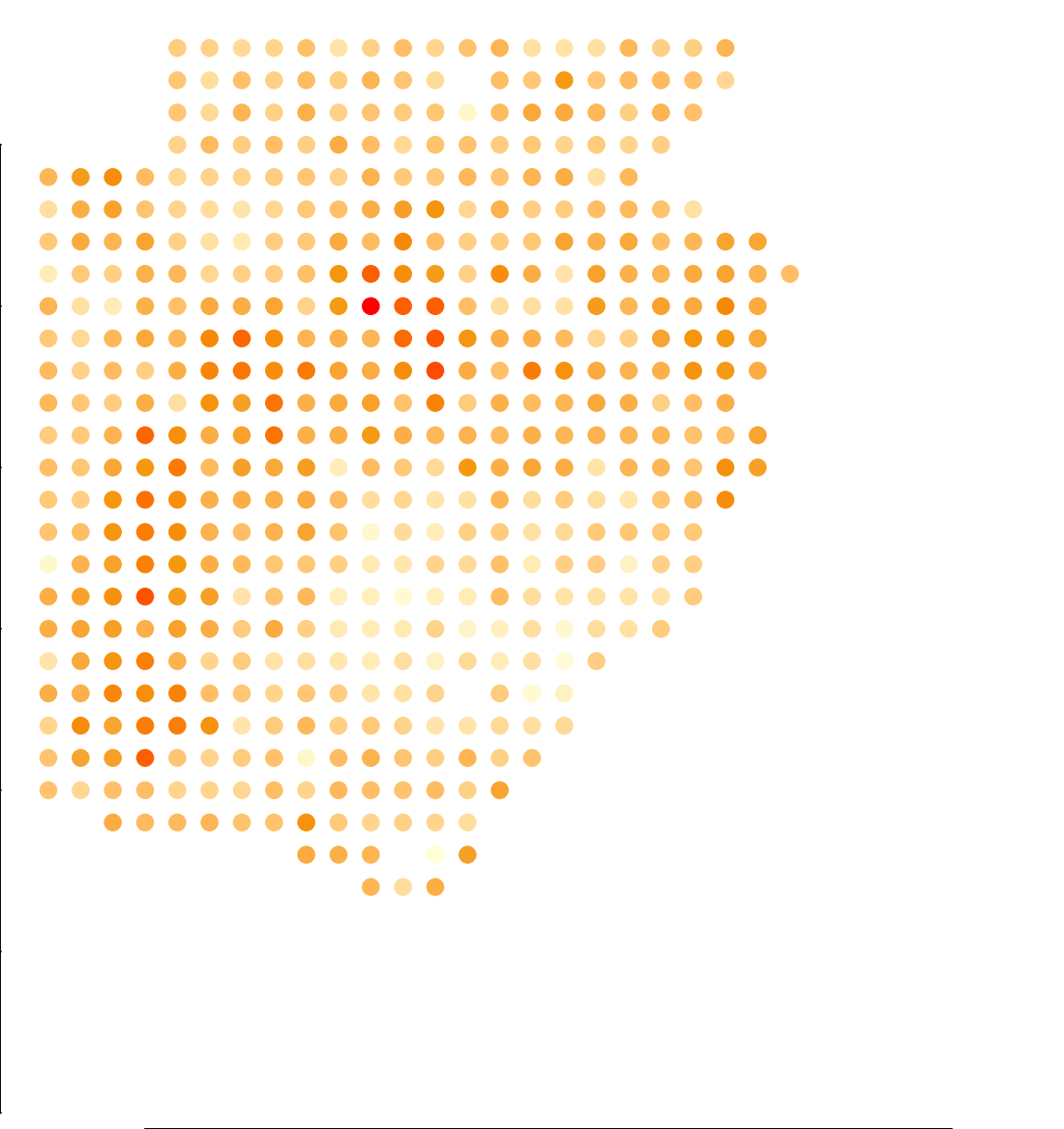

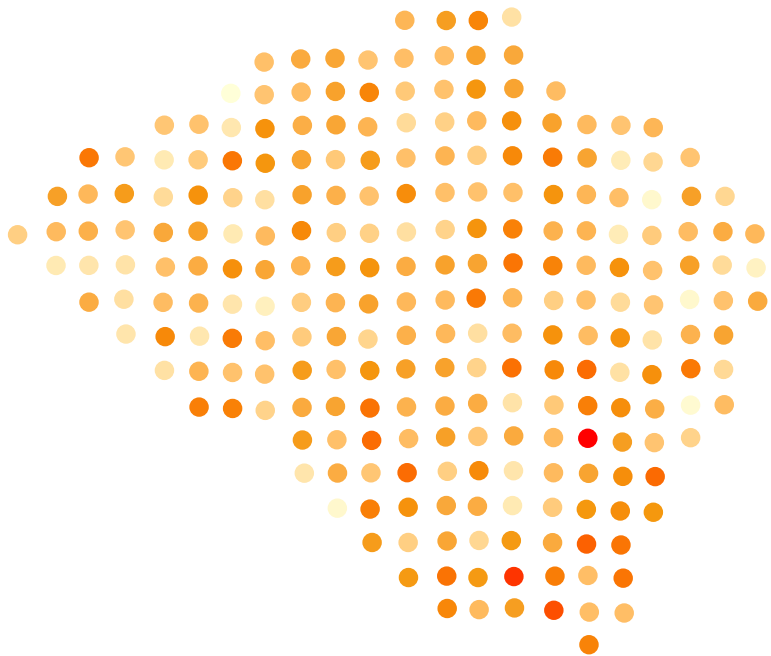

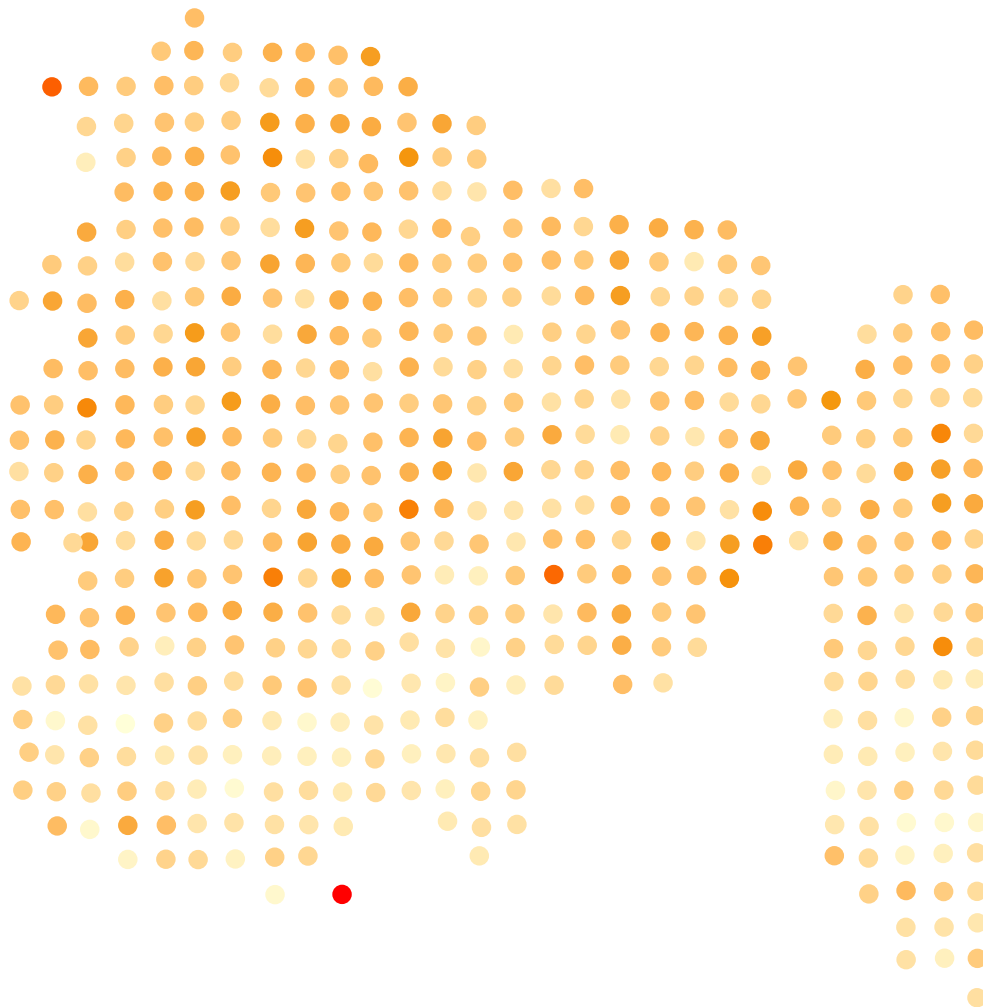

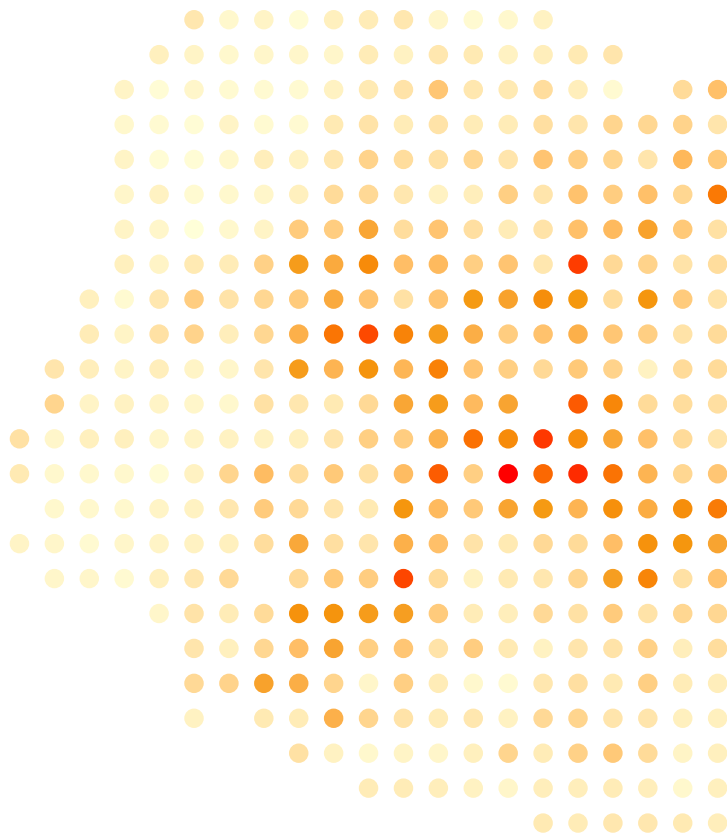

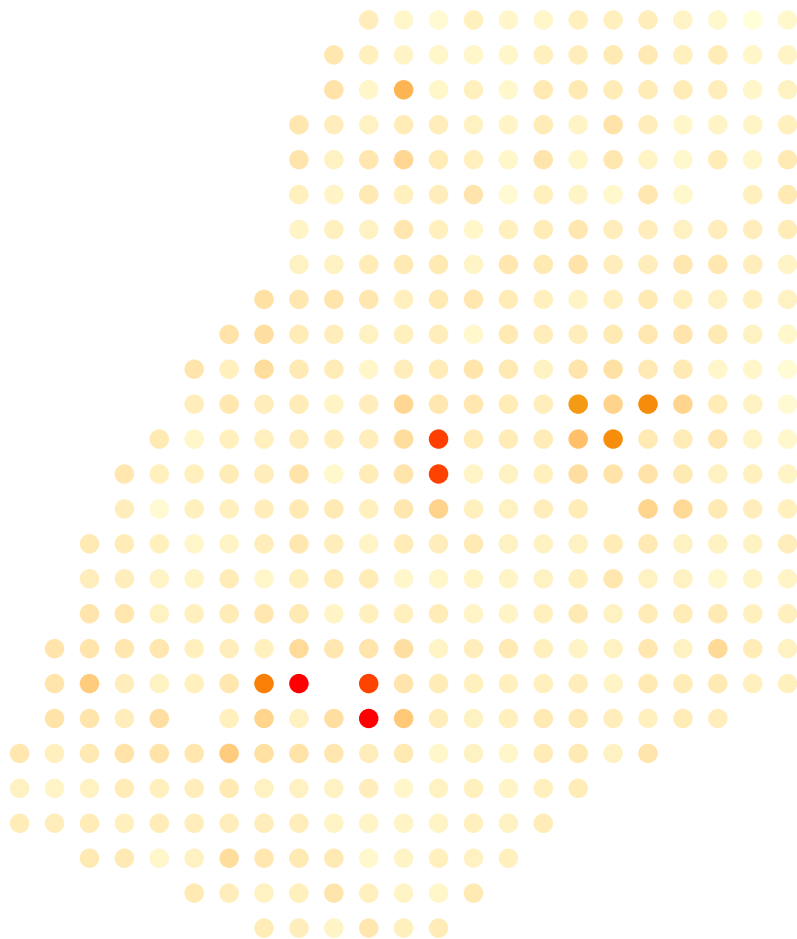

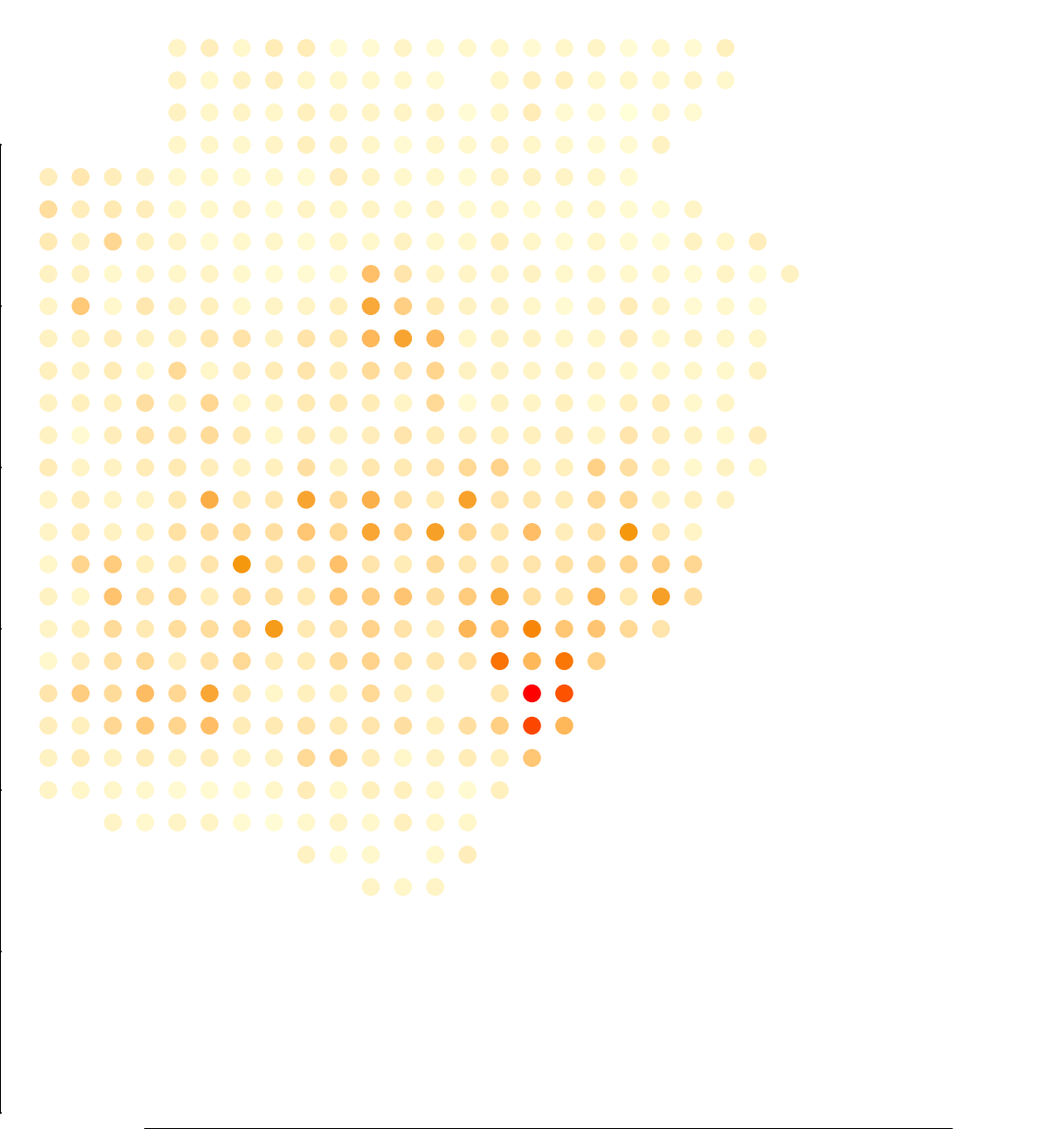

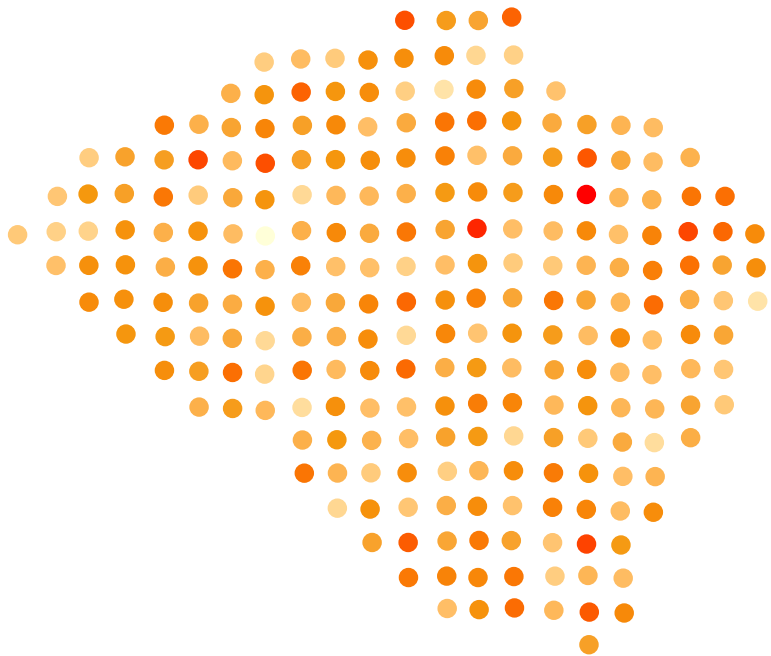

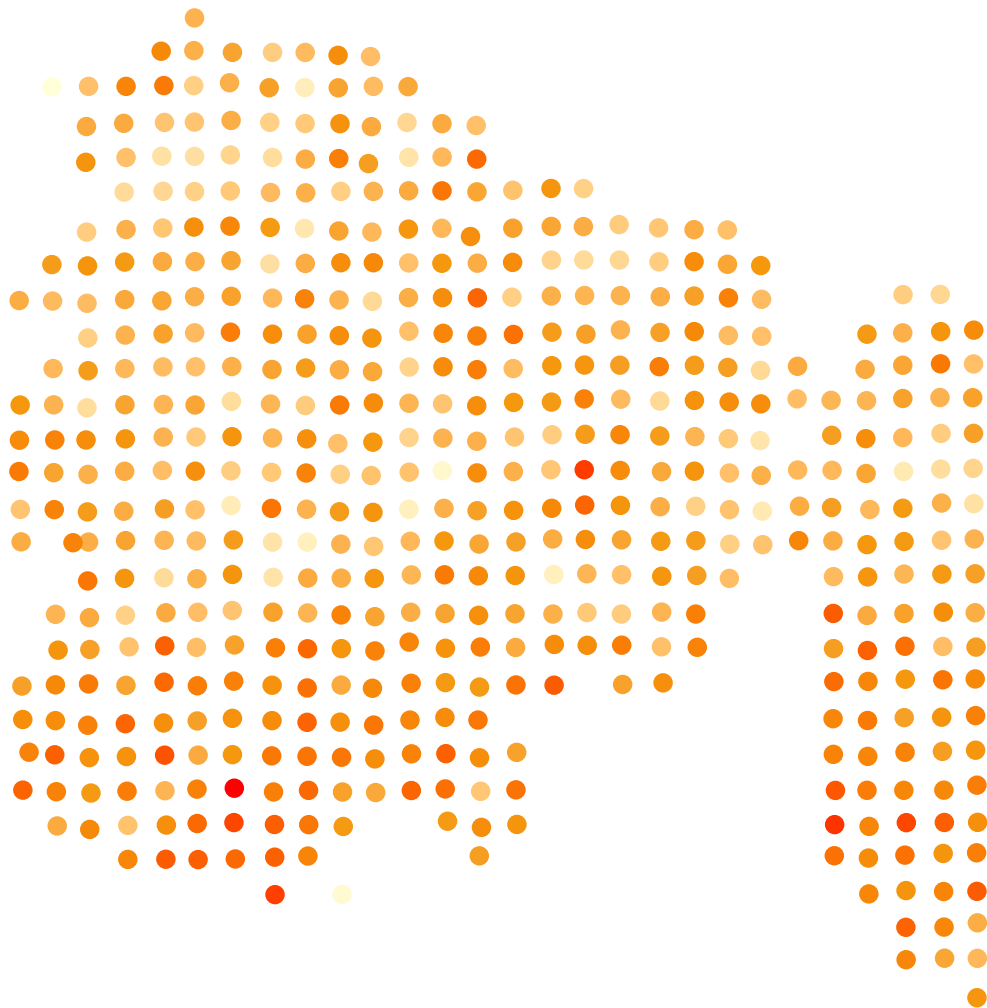

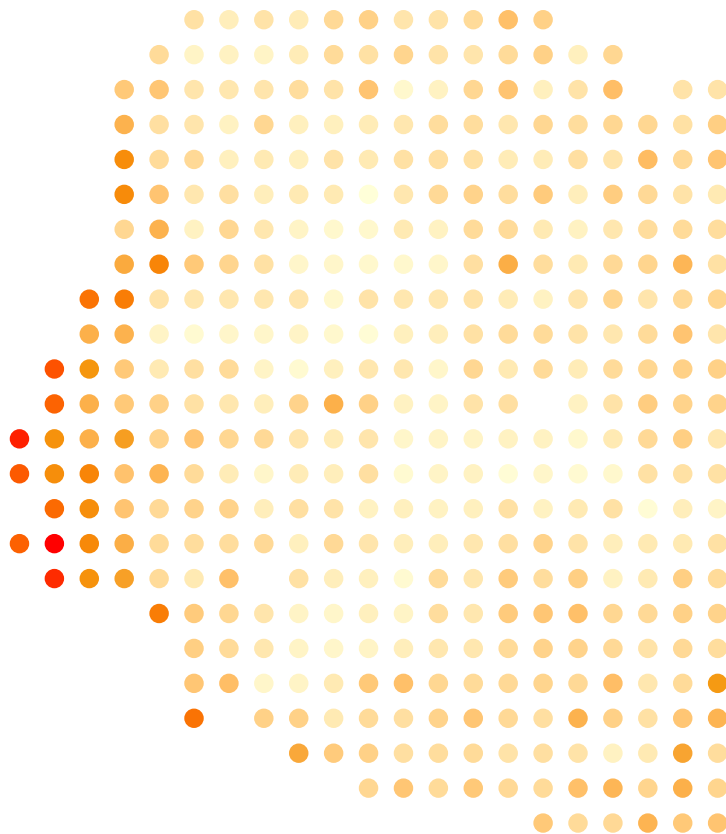

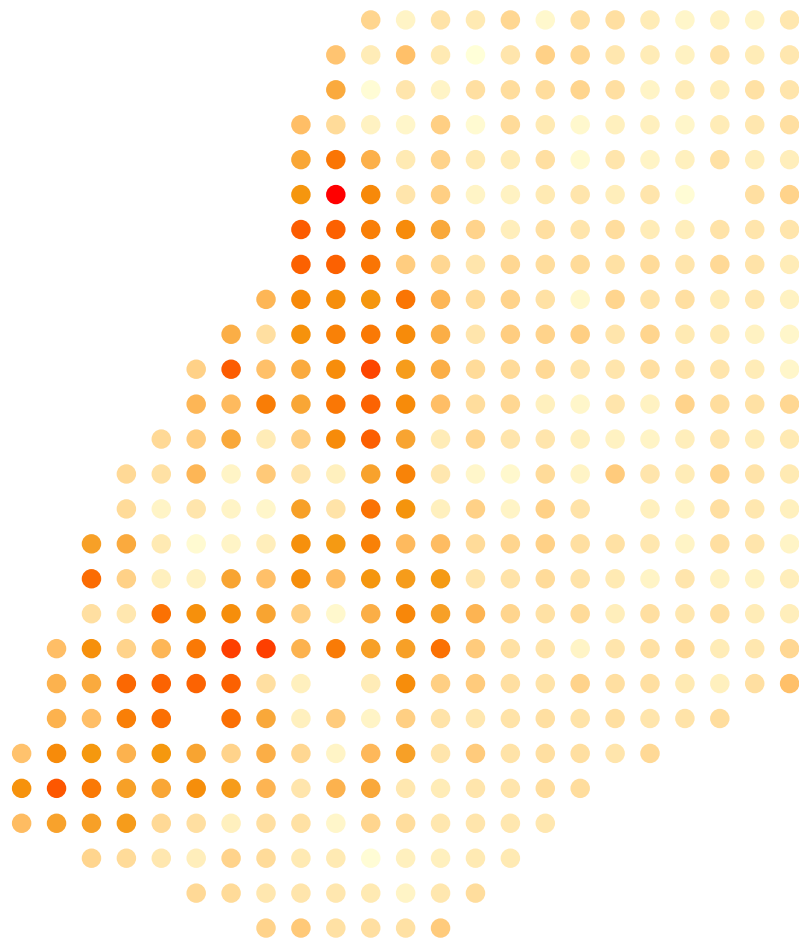

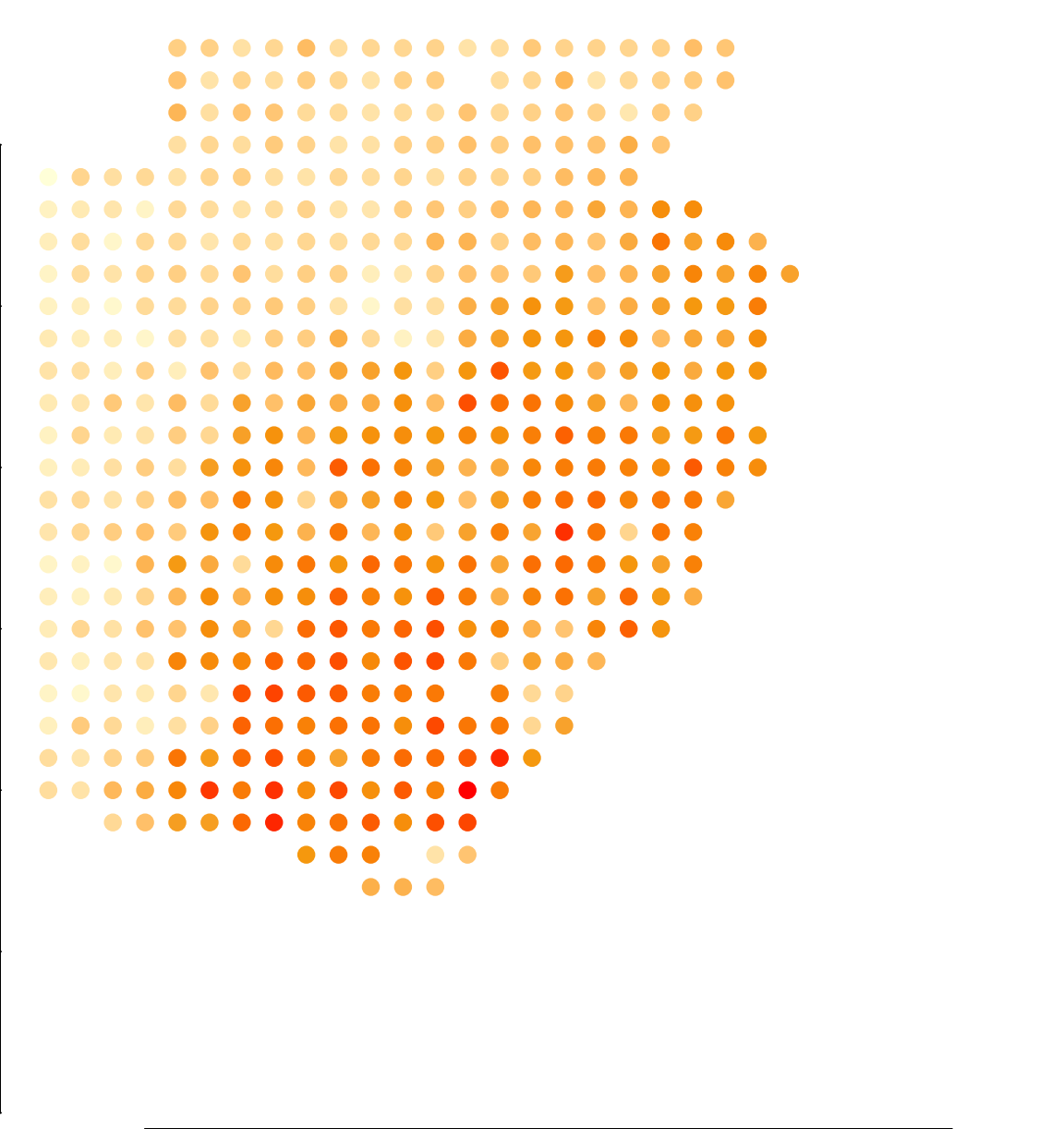

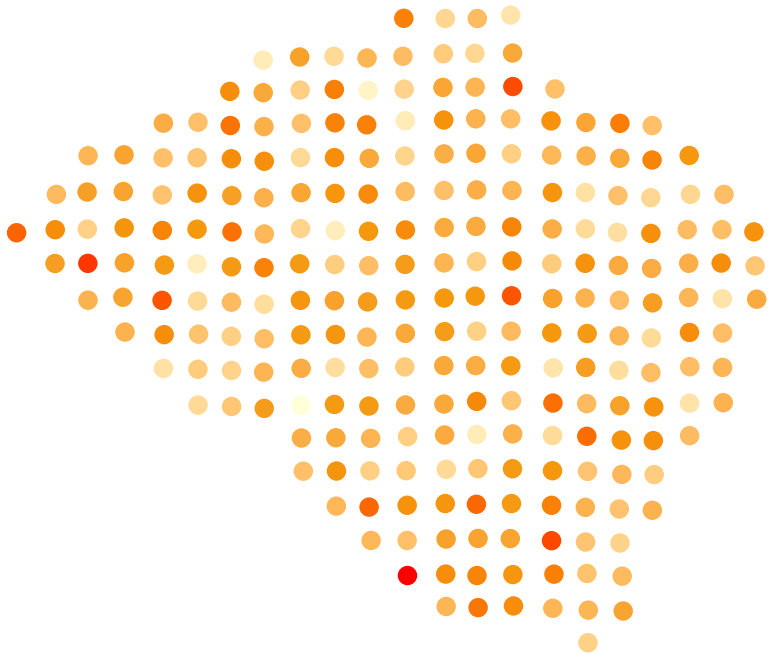

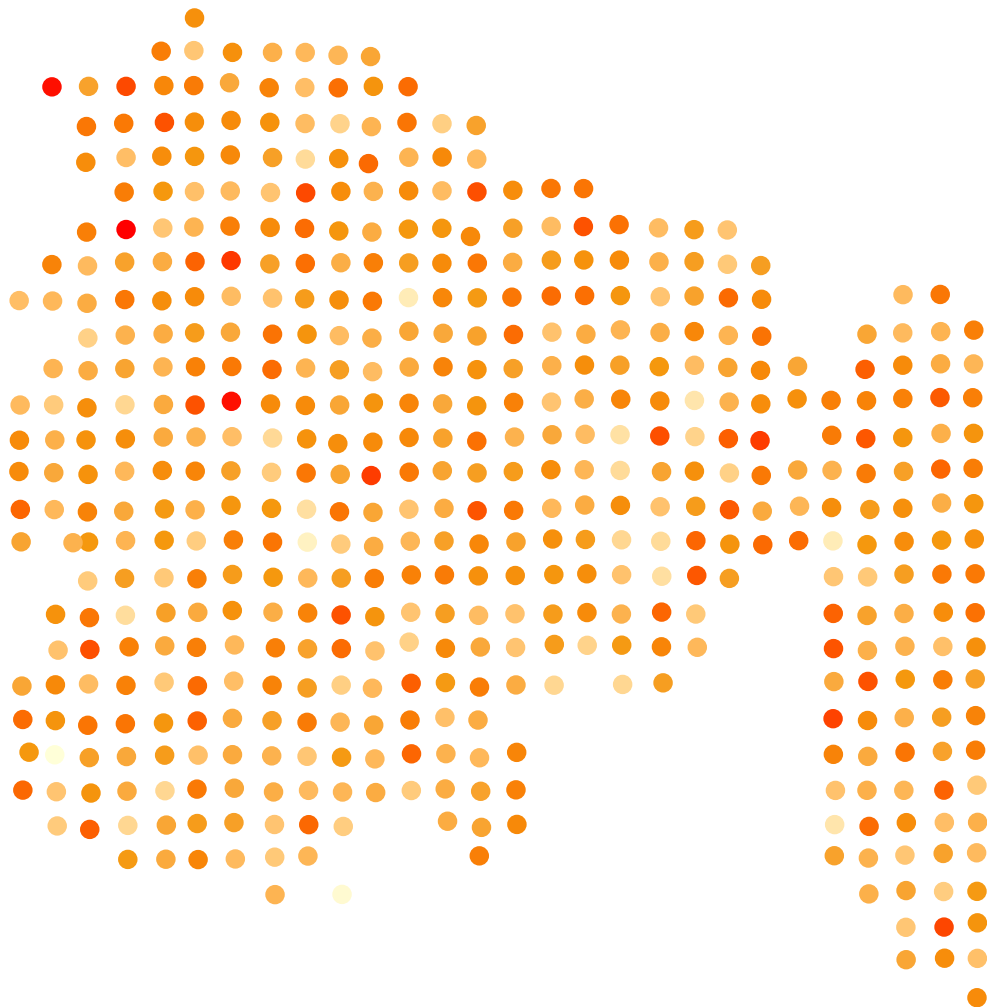

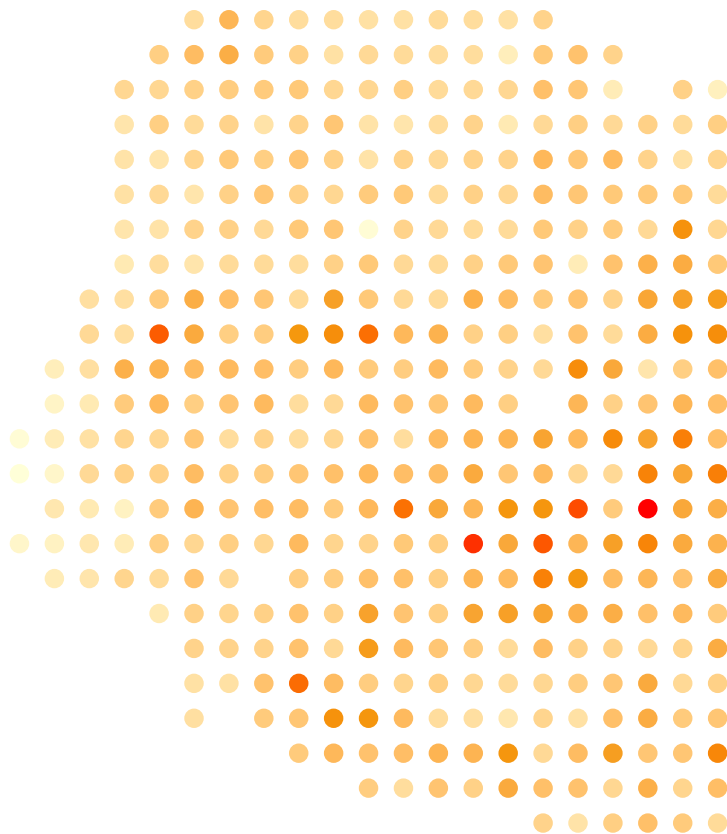

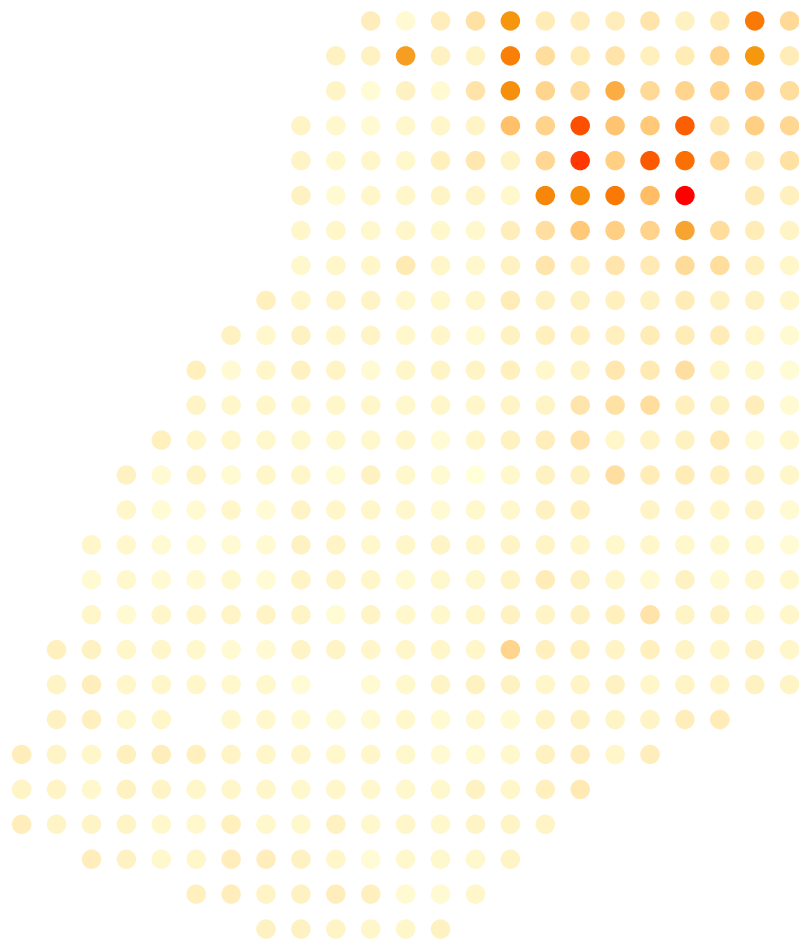

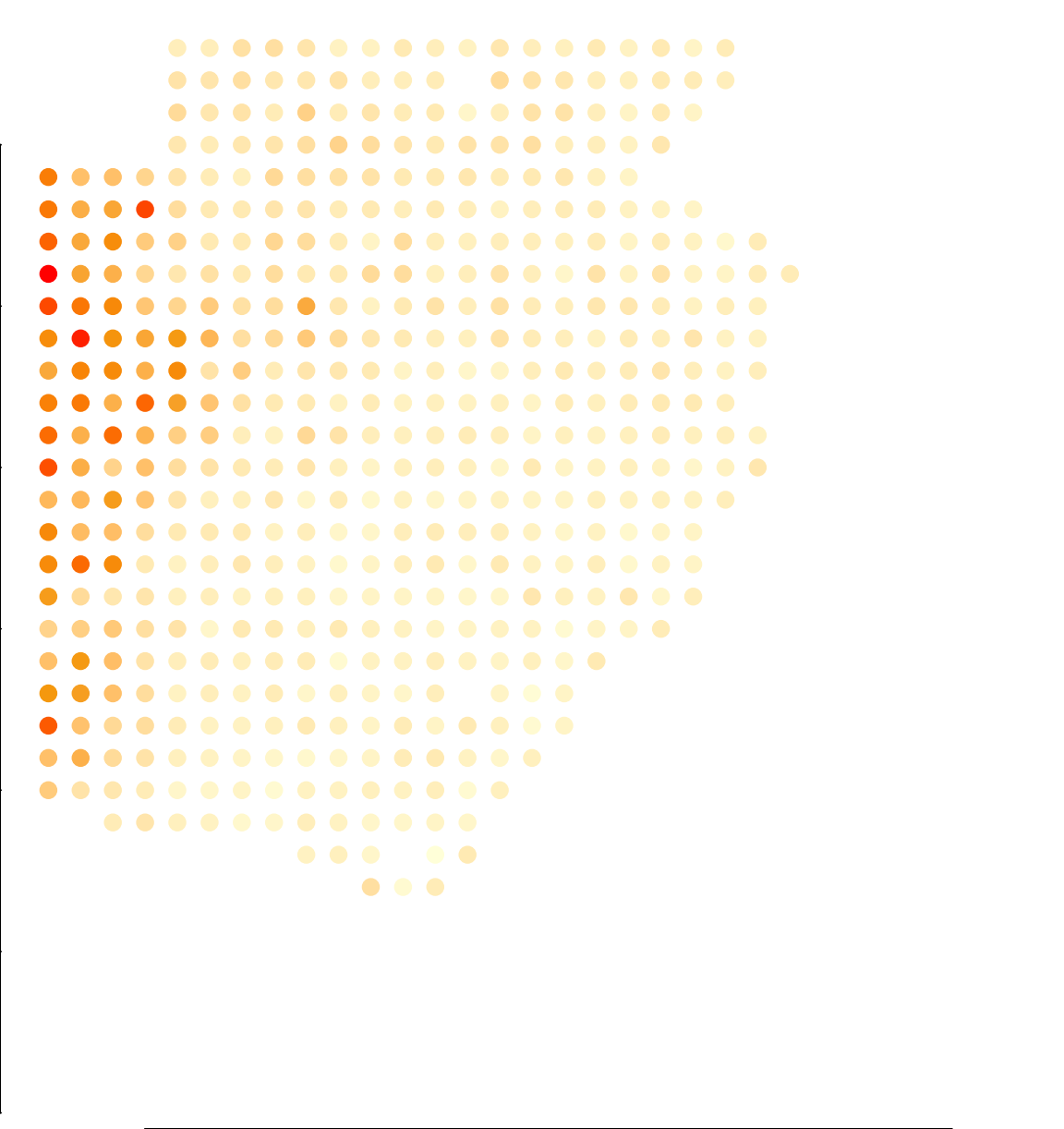

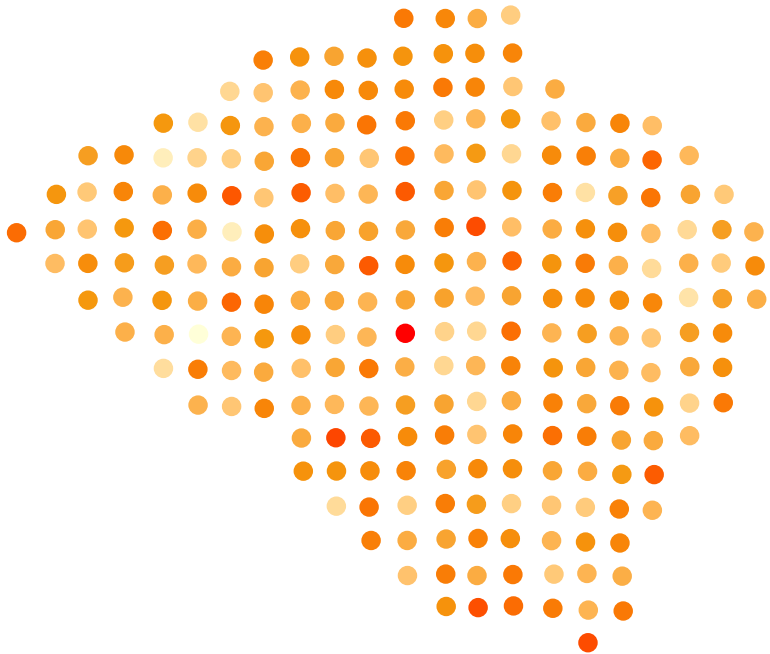

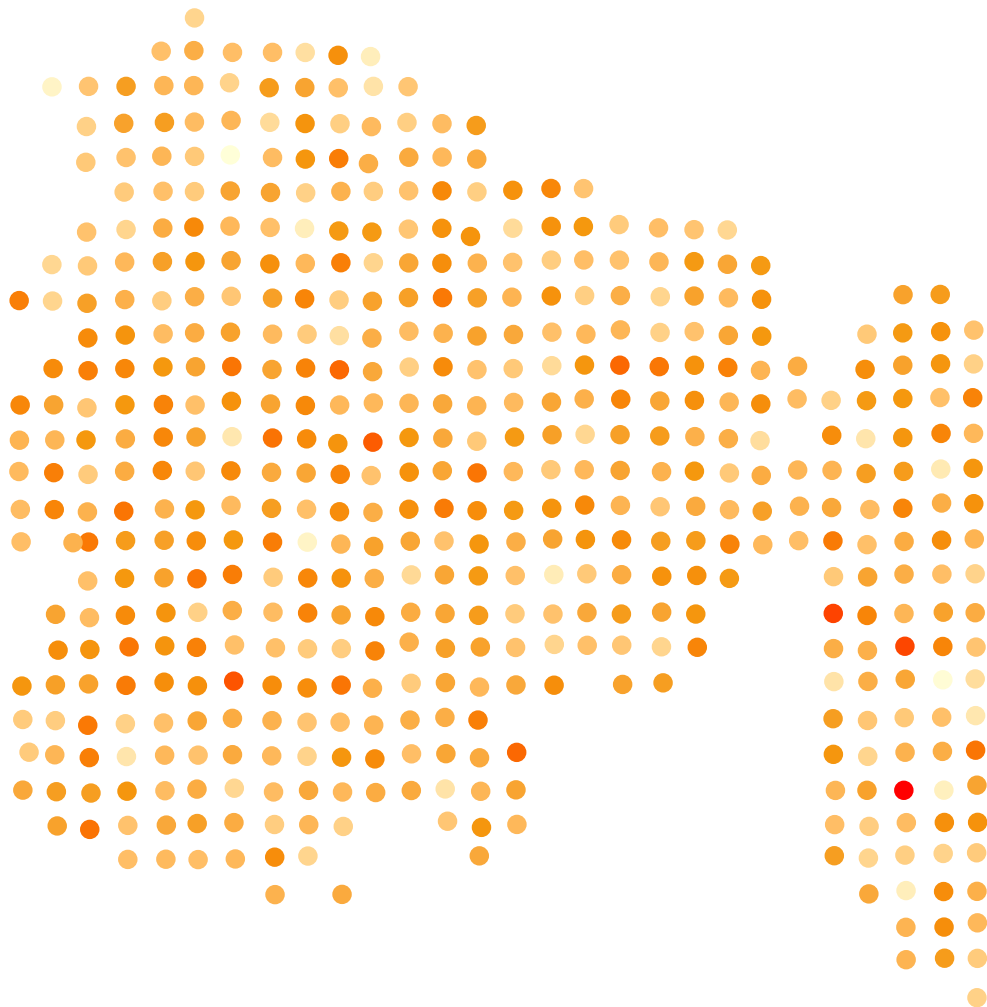

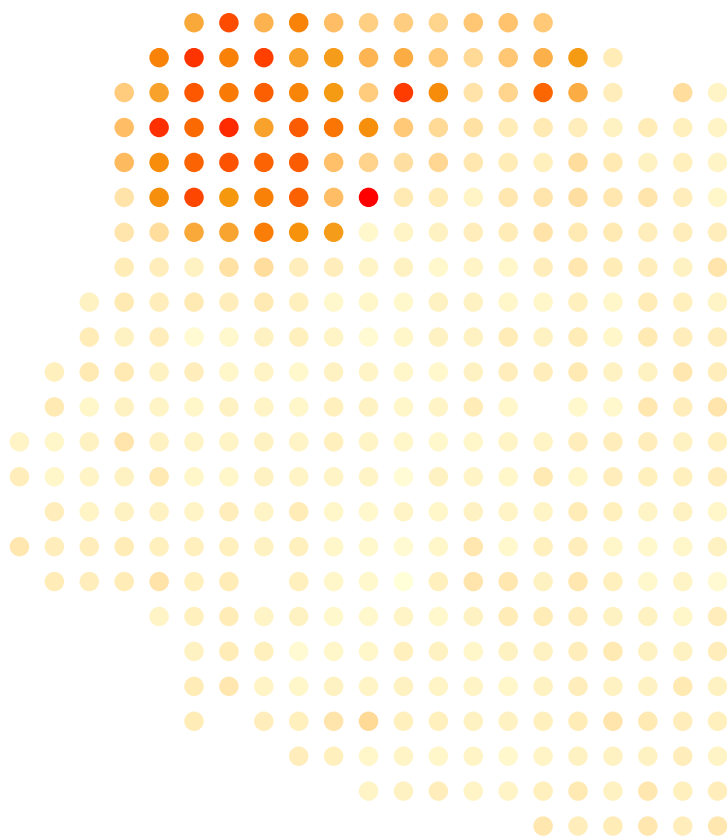

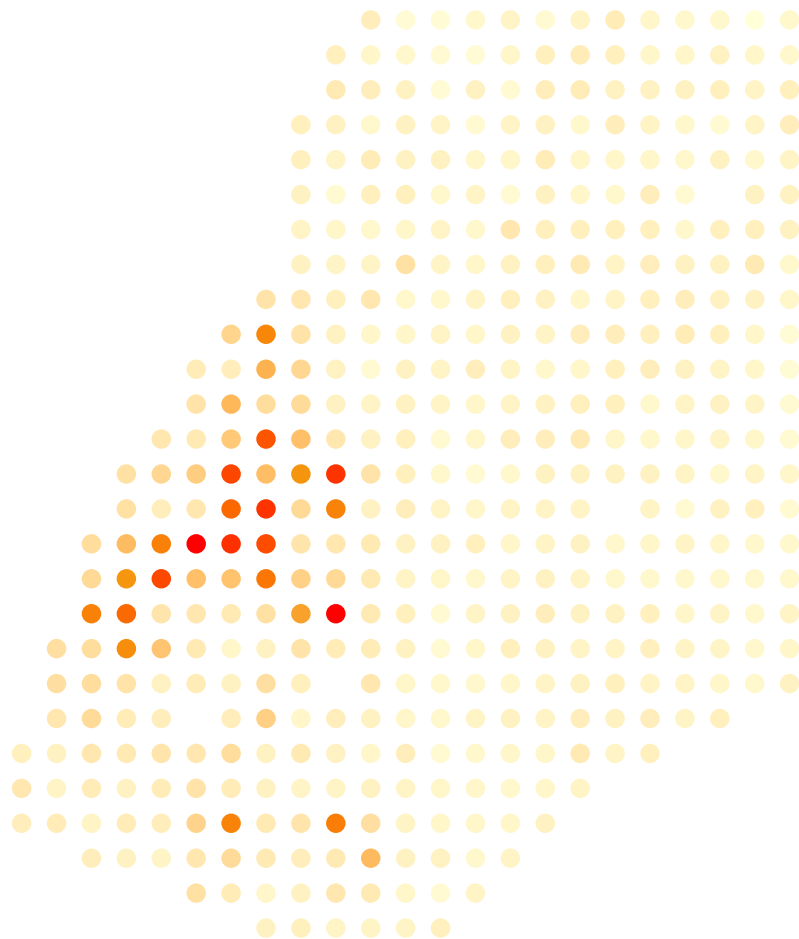

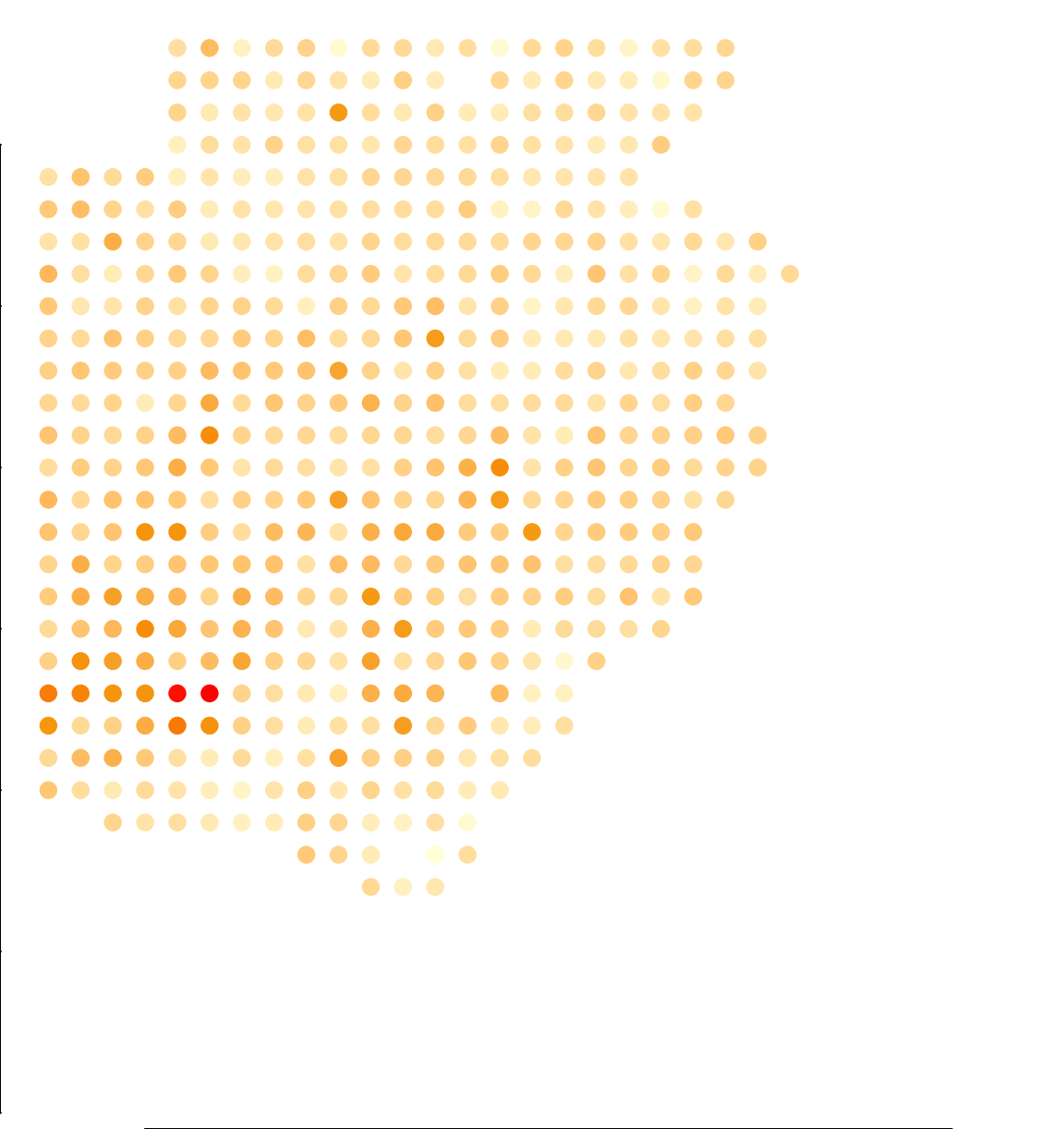

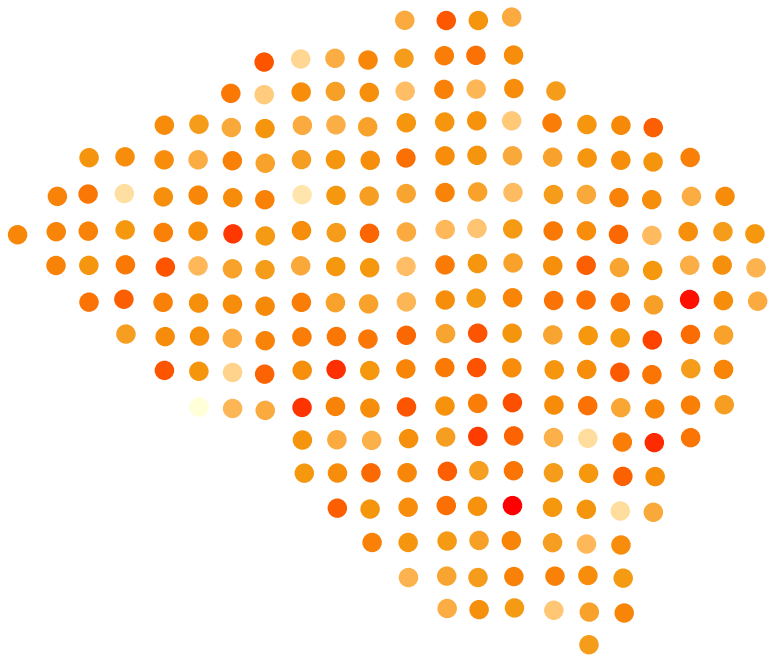

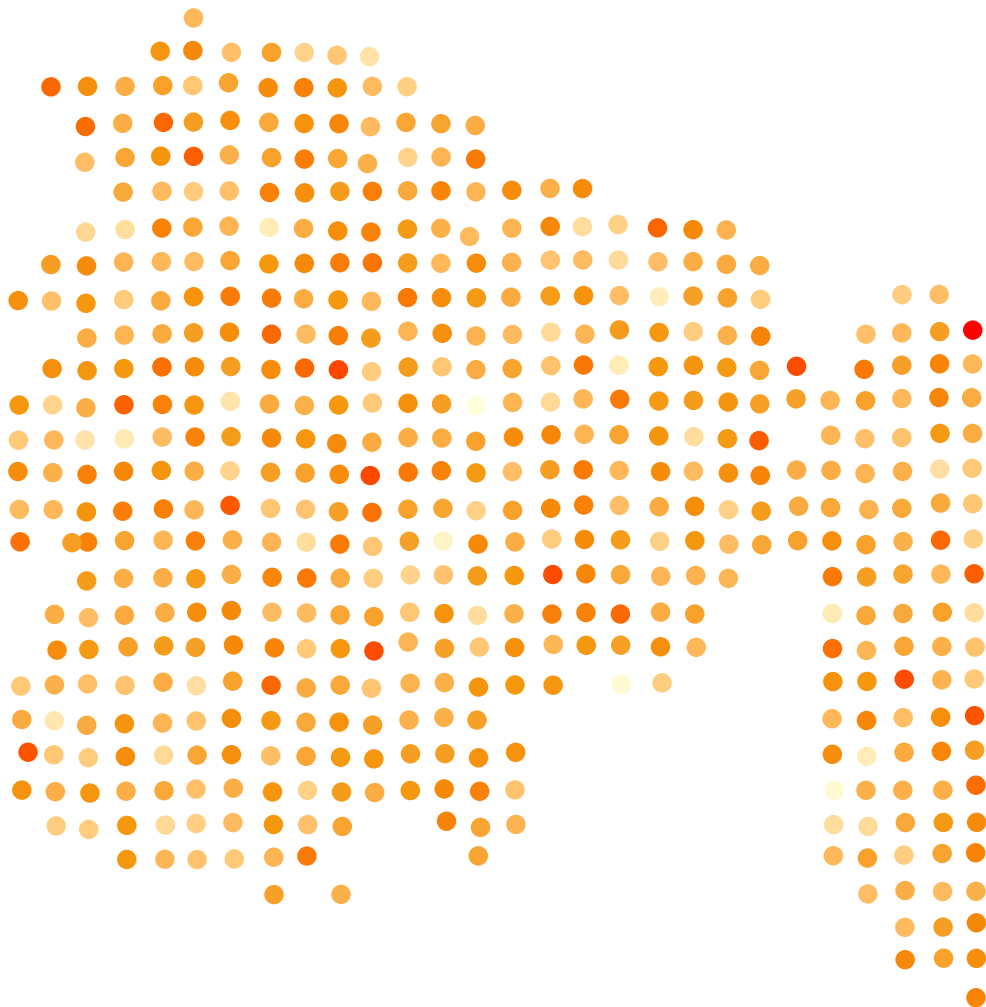

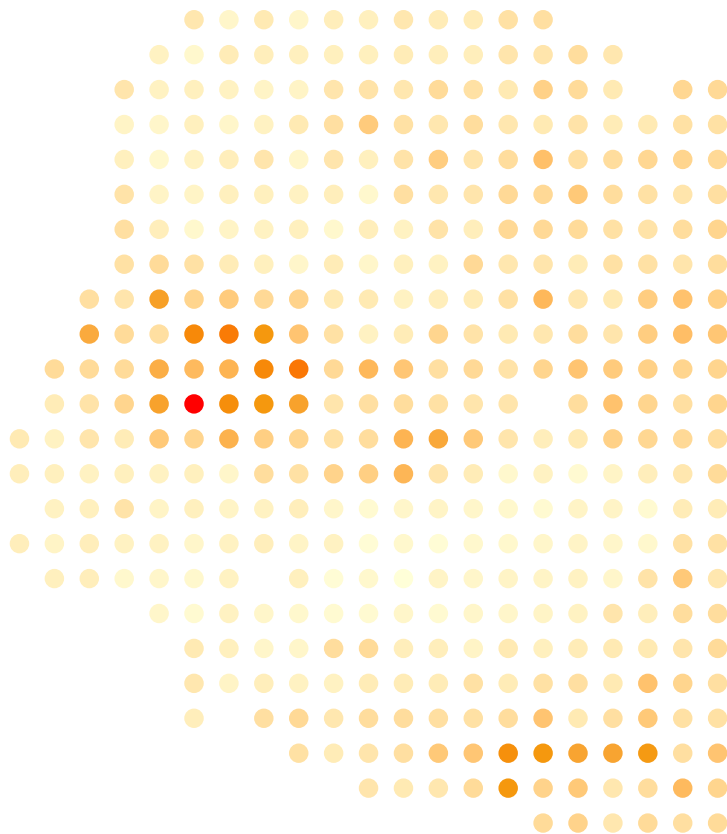

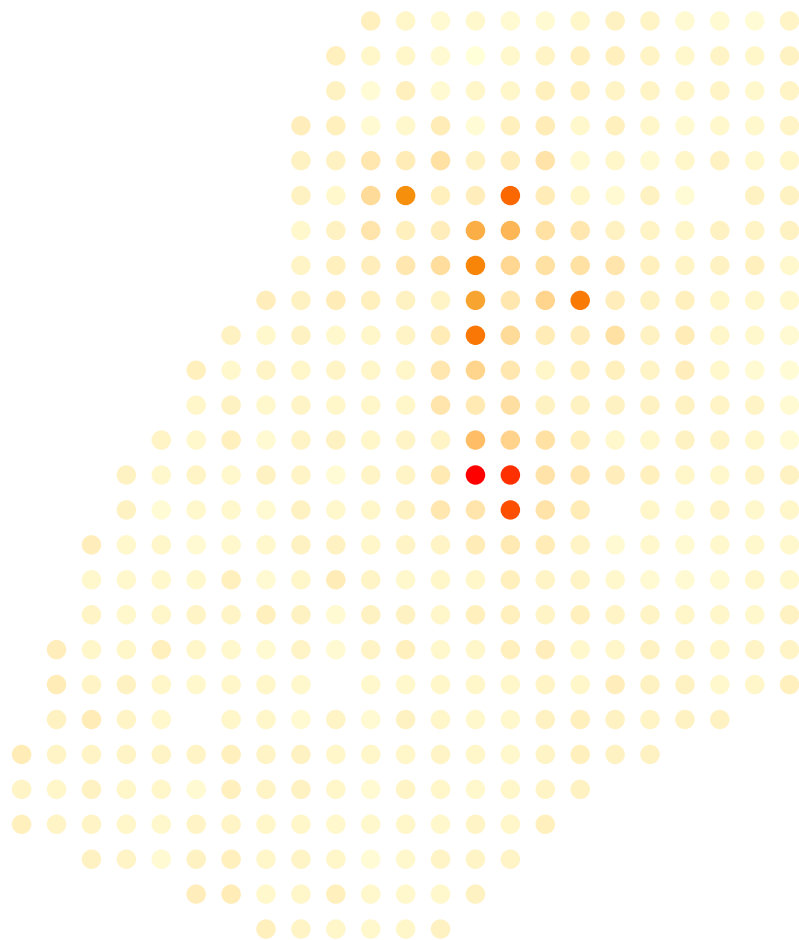

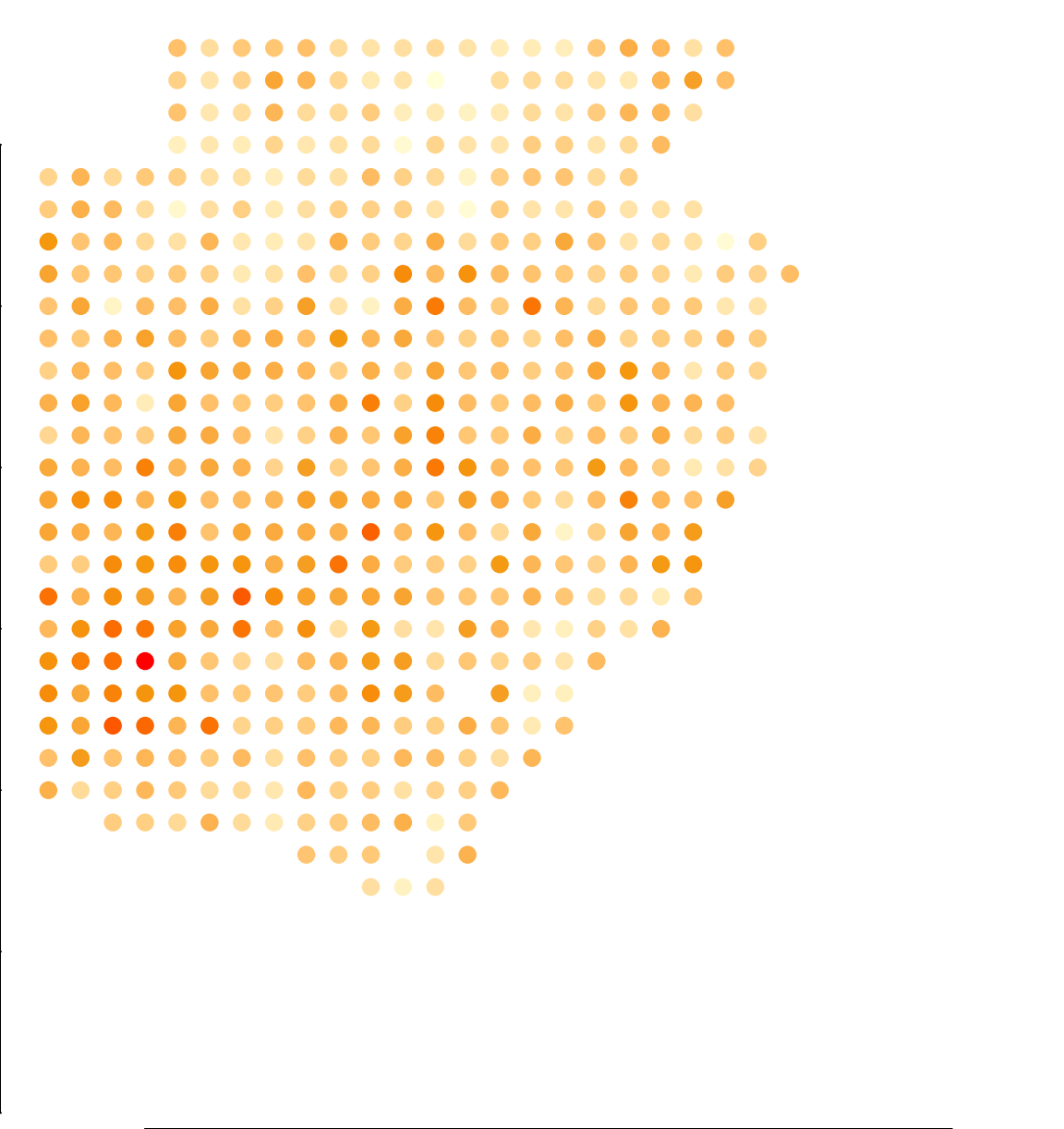

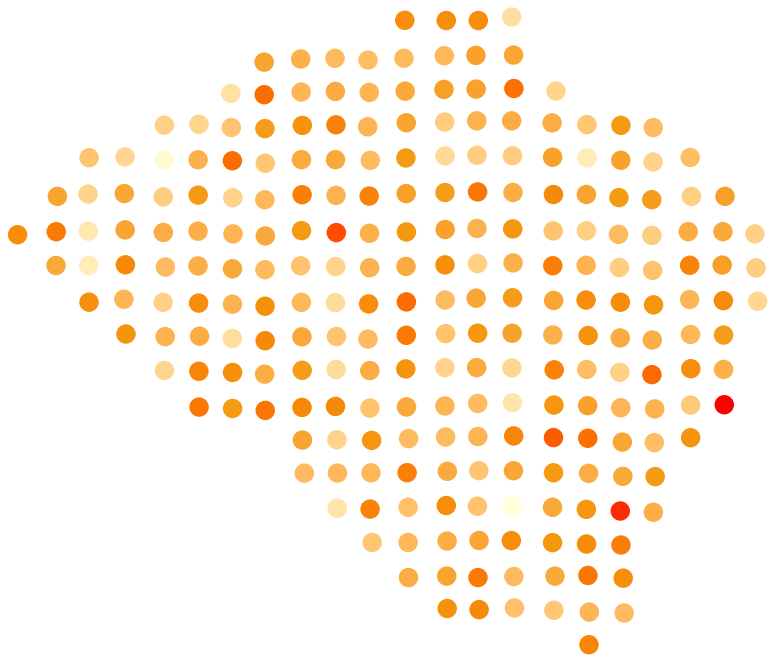

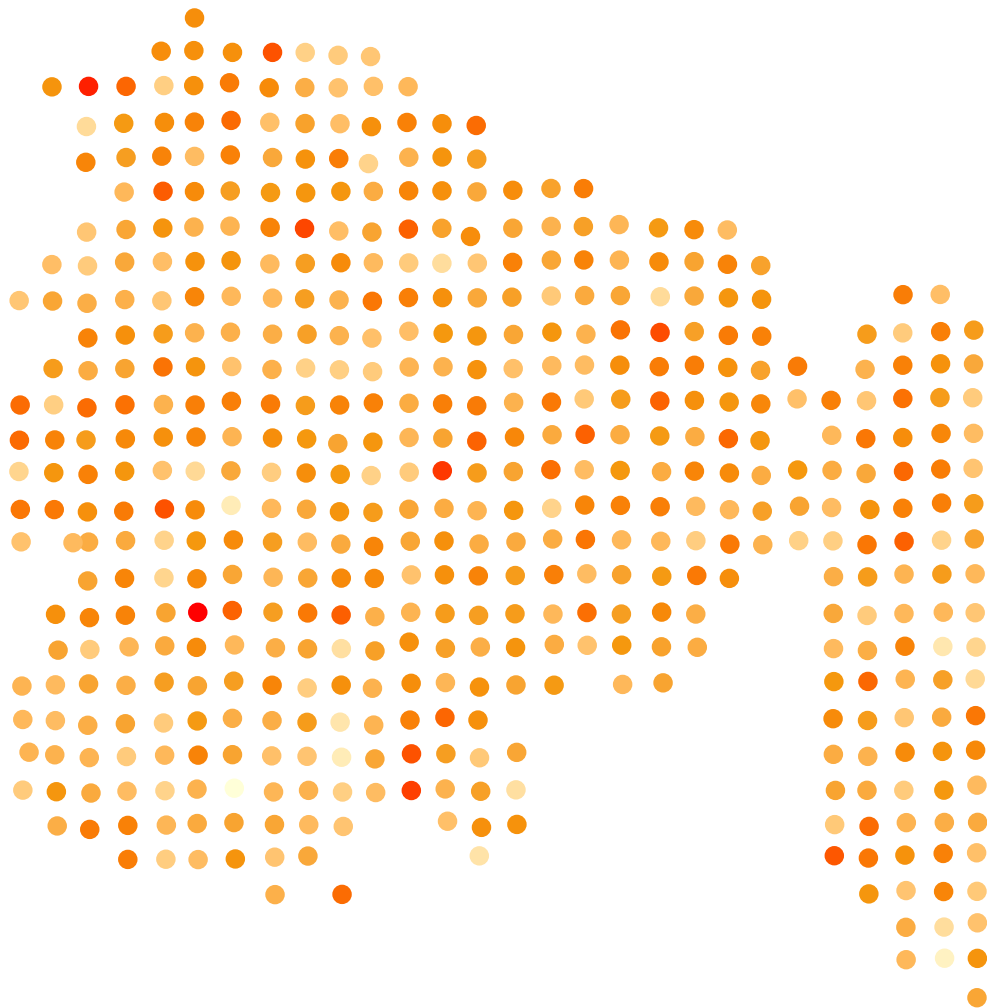

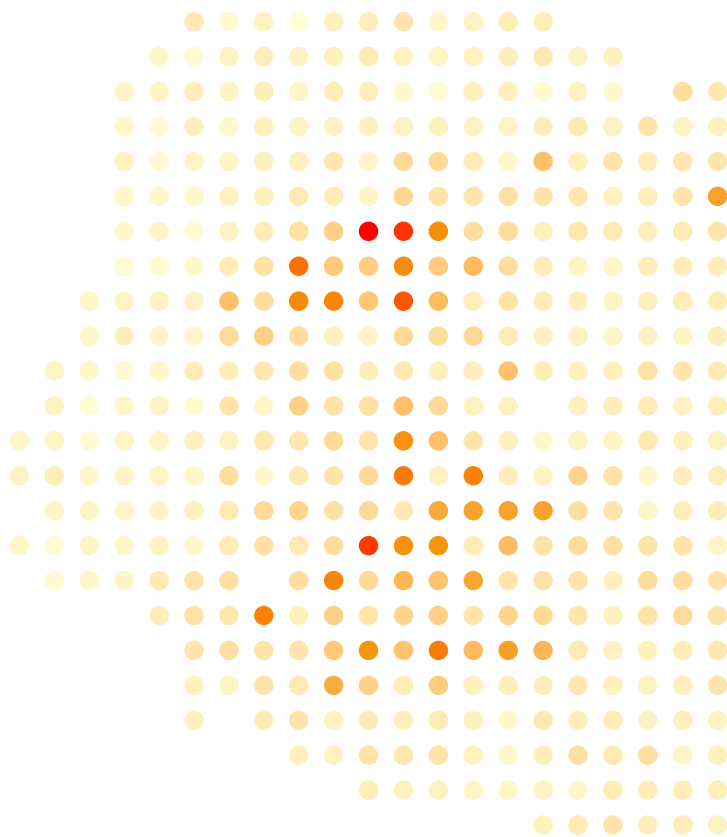

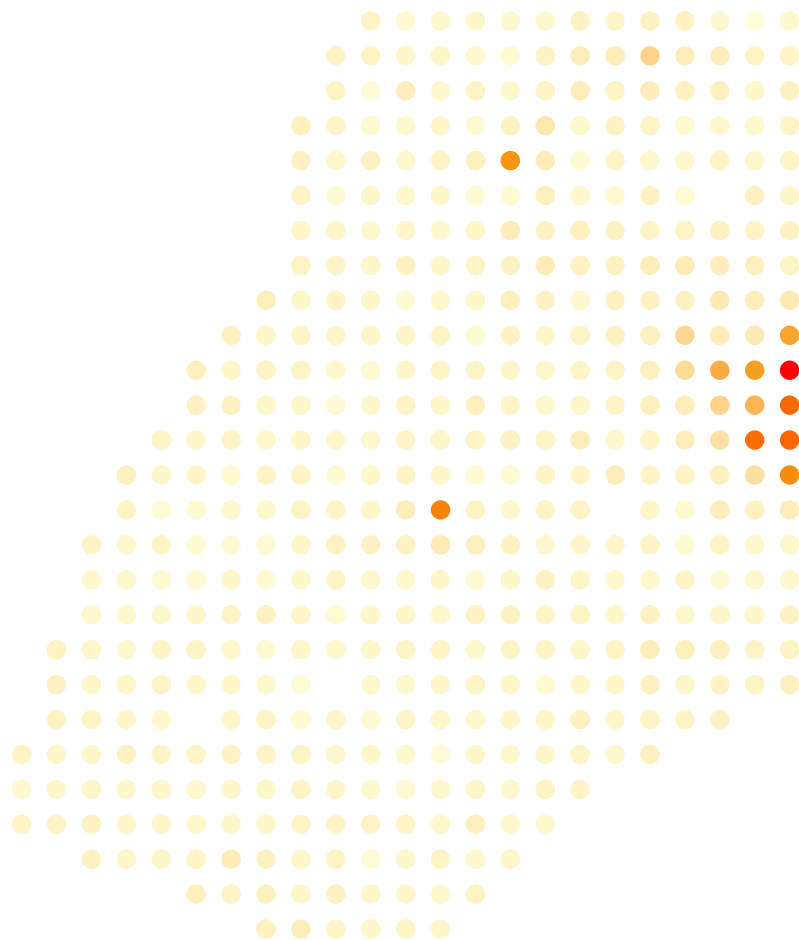

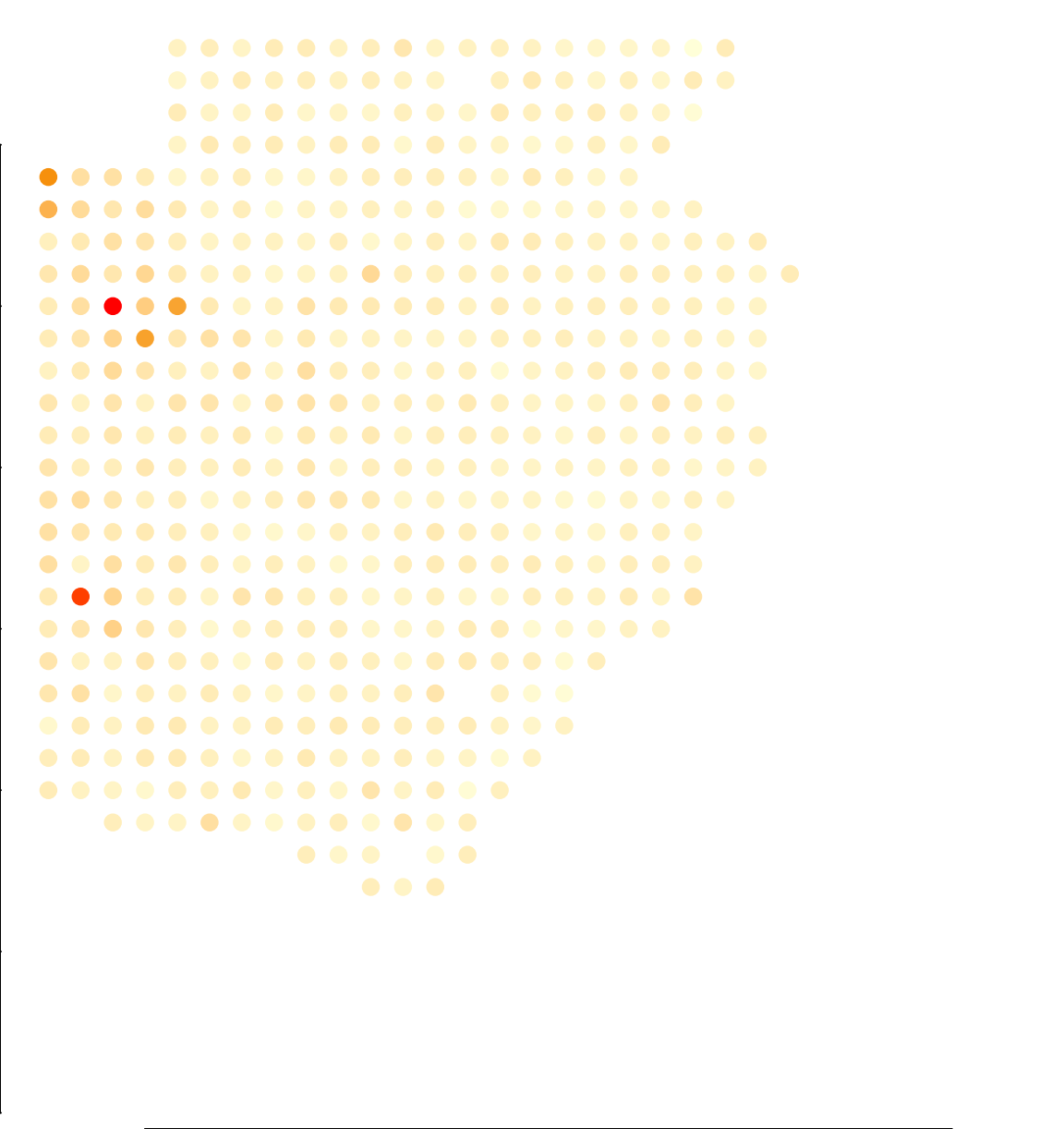

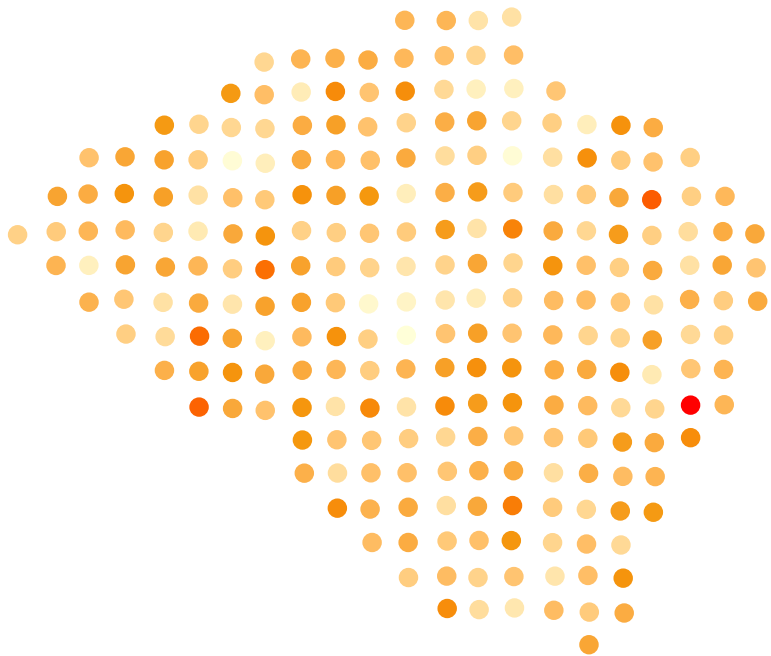

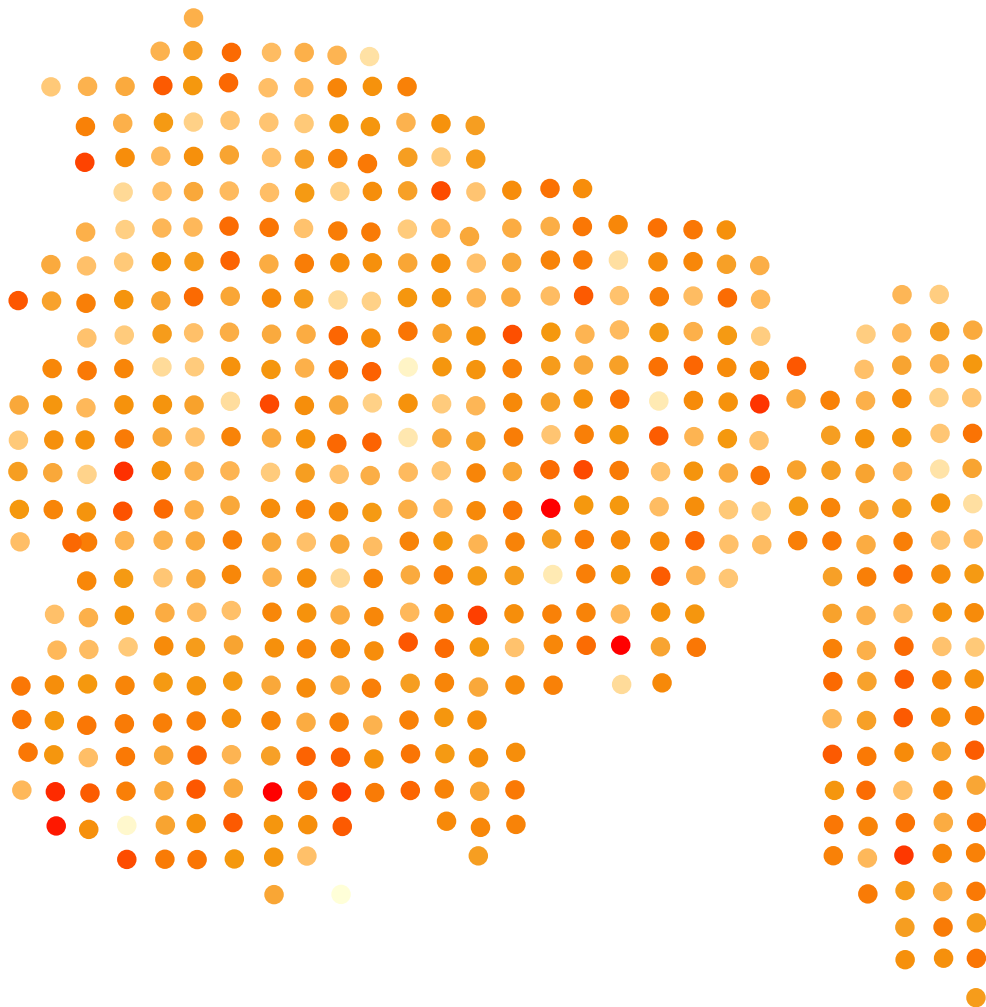

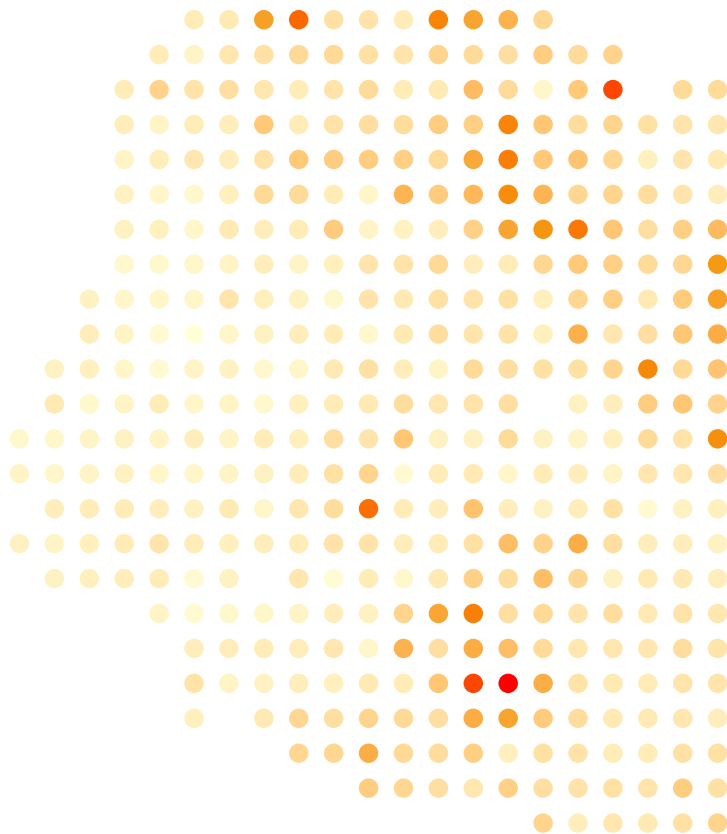

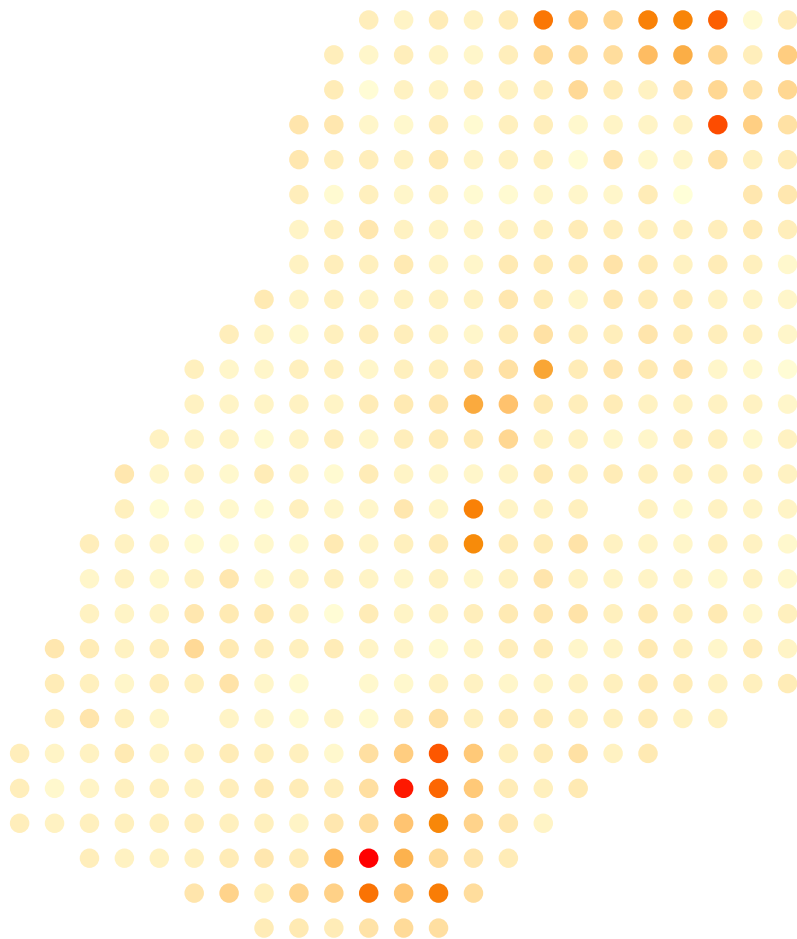

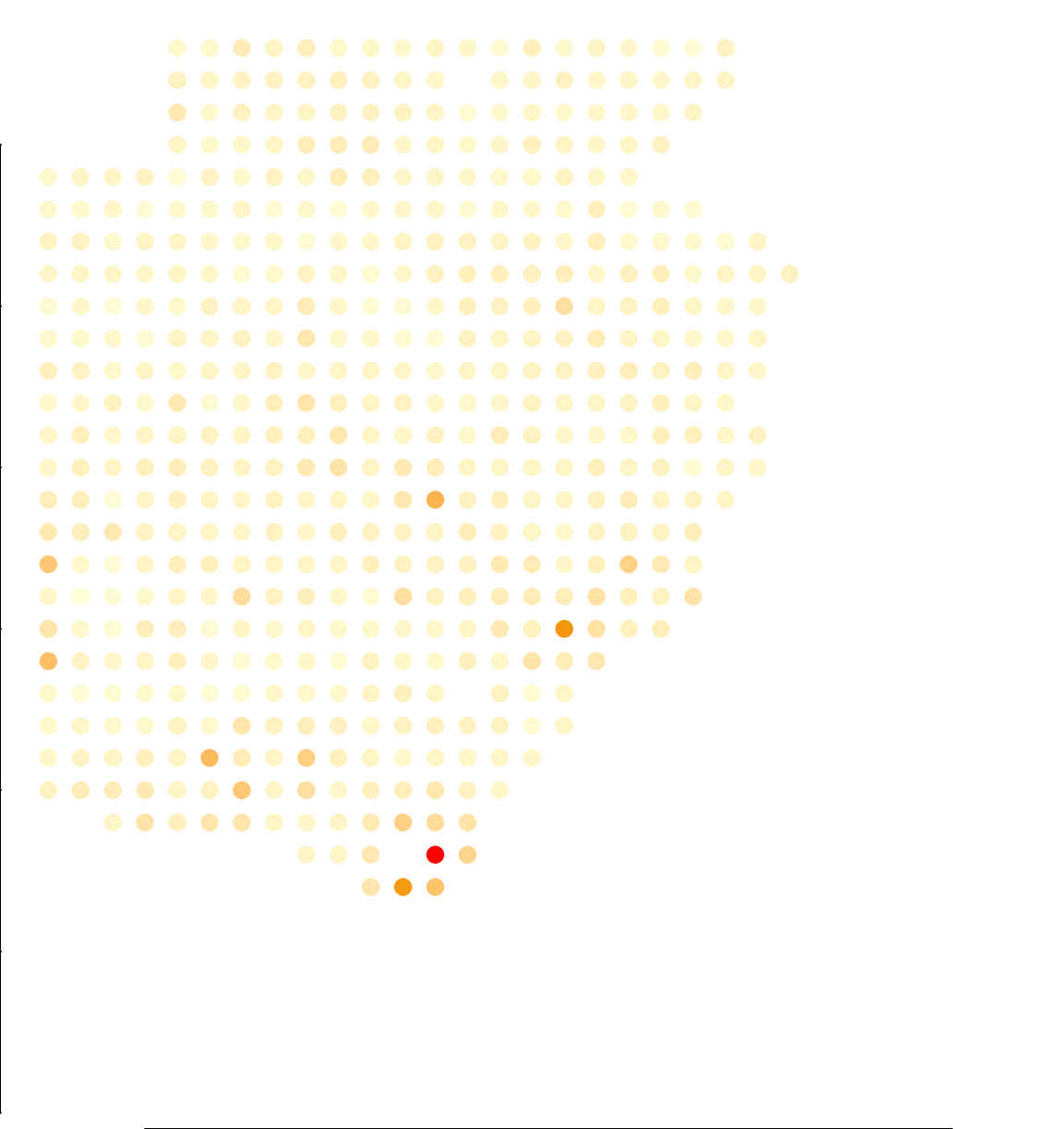

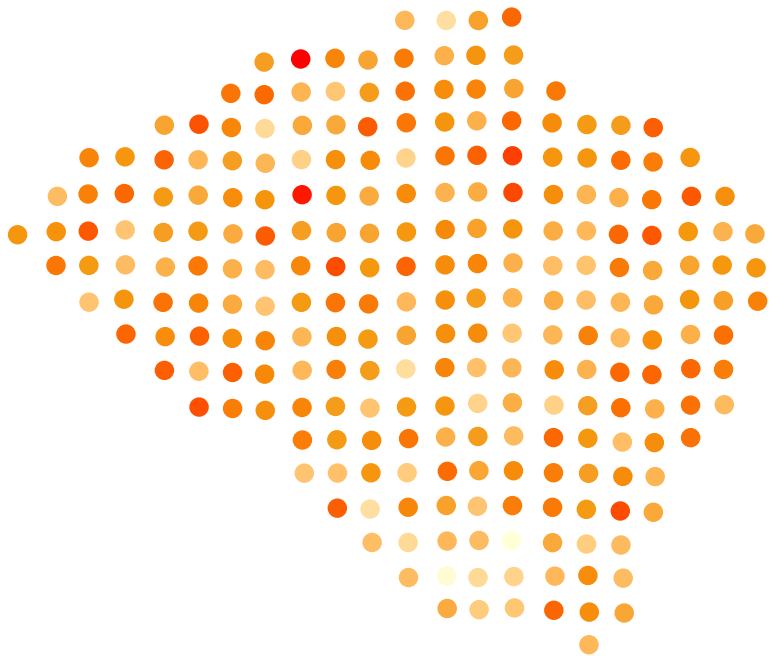

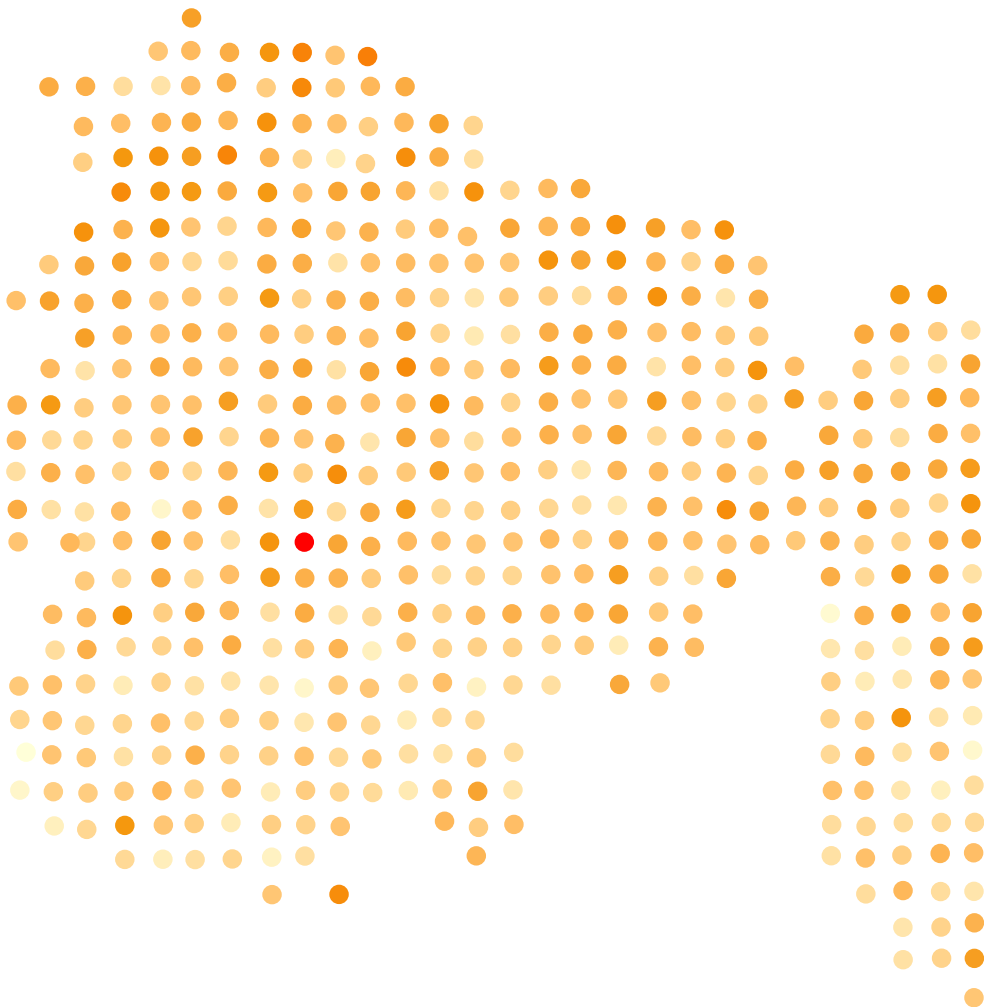

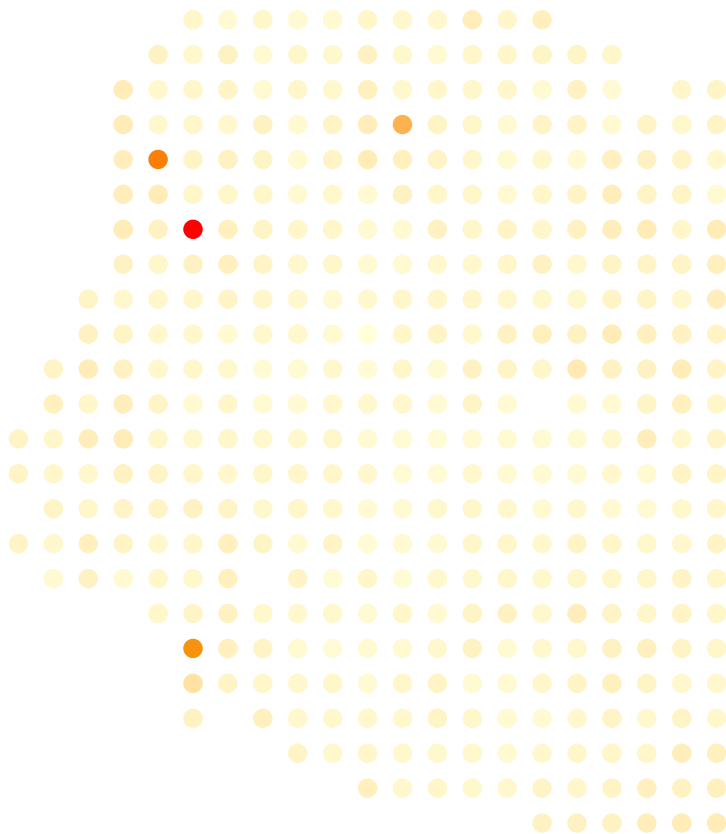

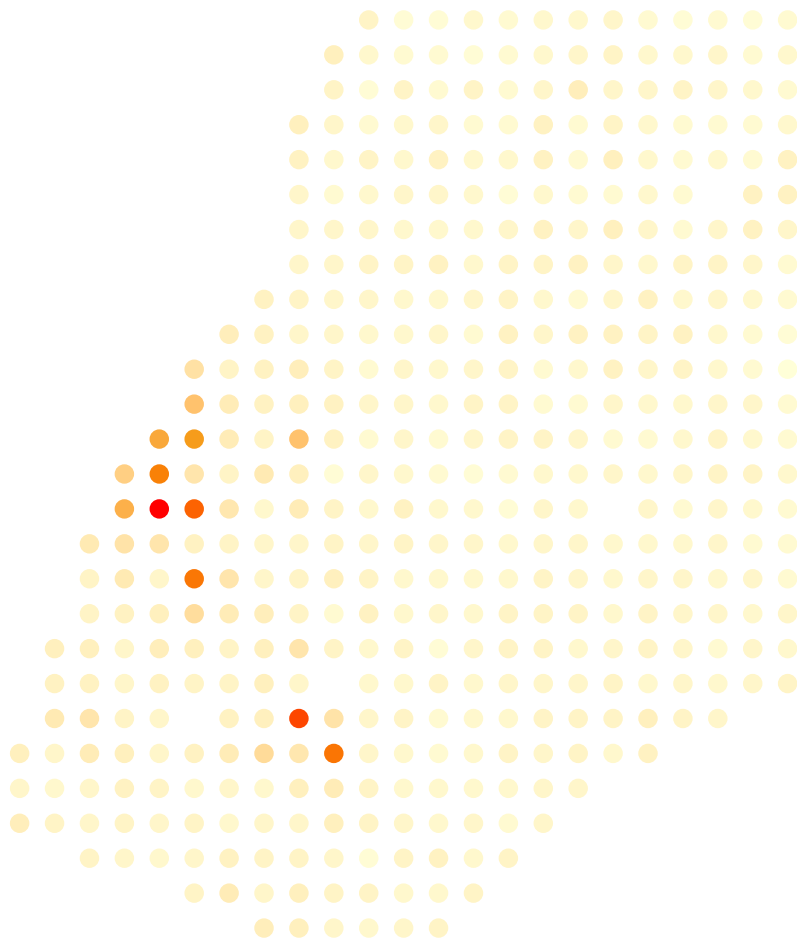

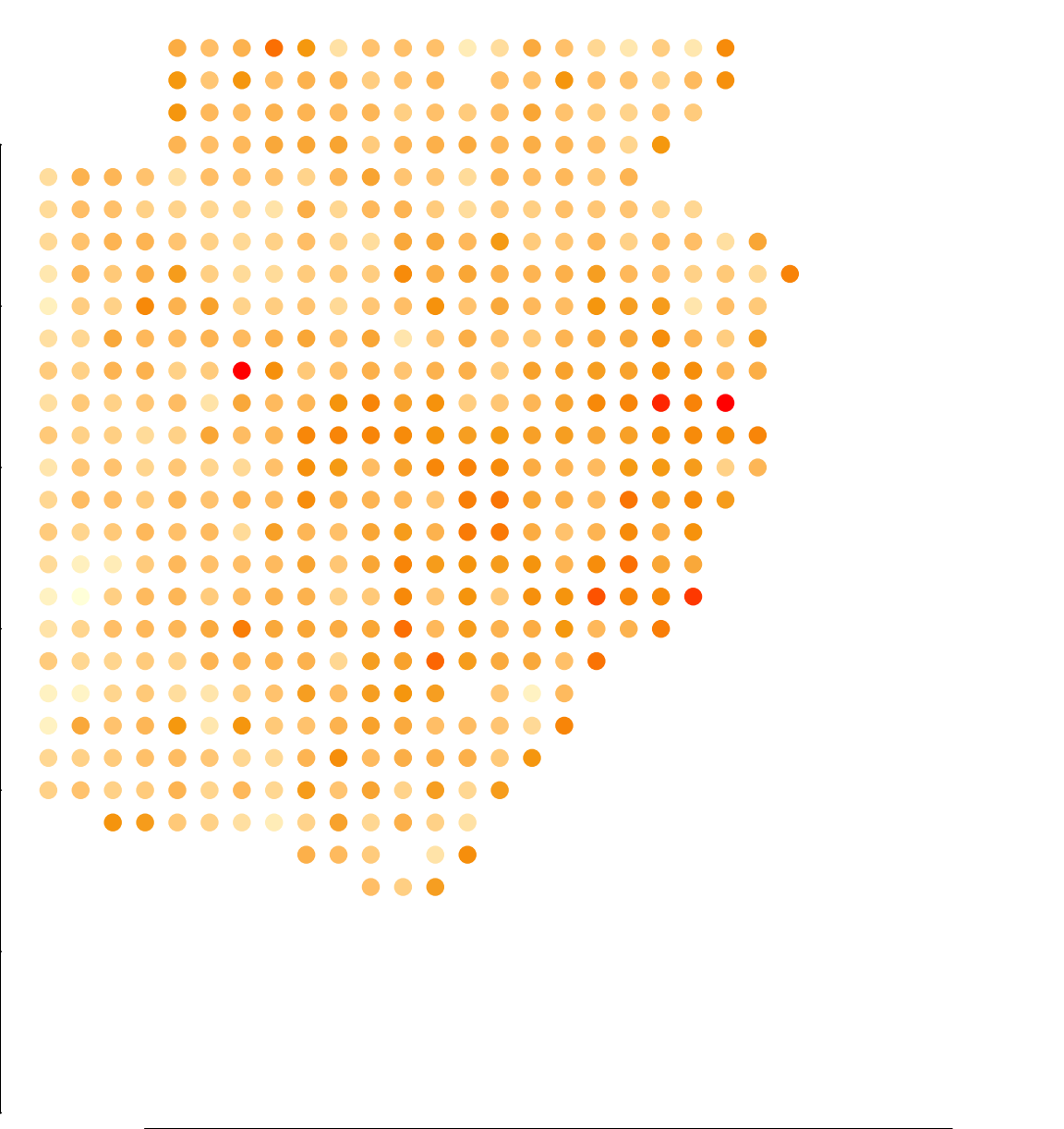

Supplement: Supplementary file 8 — Supplementary Data 5 [file 41467_2018_4724_MOESM8_ESM.zip › Supplementary Dataset 7/joint-mix-profiles-rel-individual-scale-dots-split.pdf]

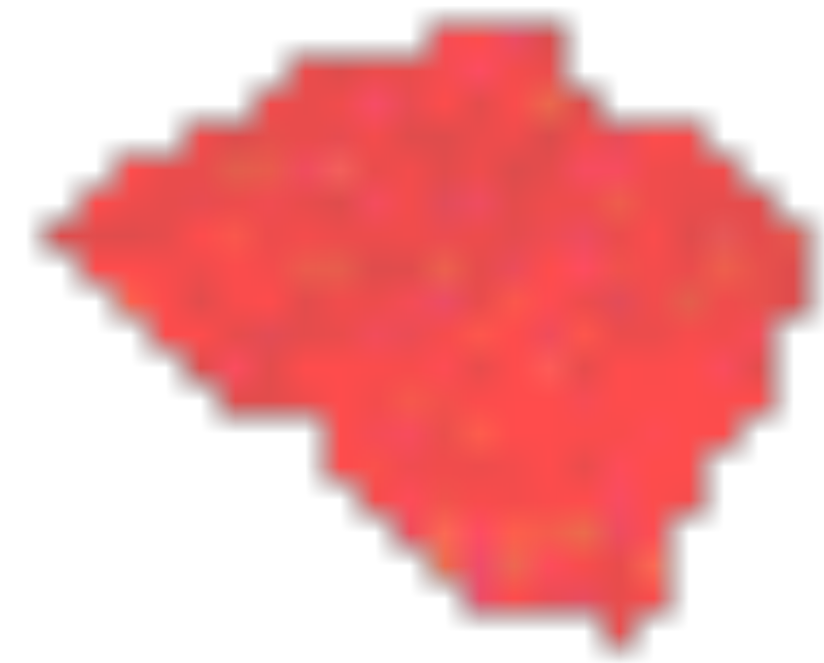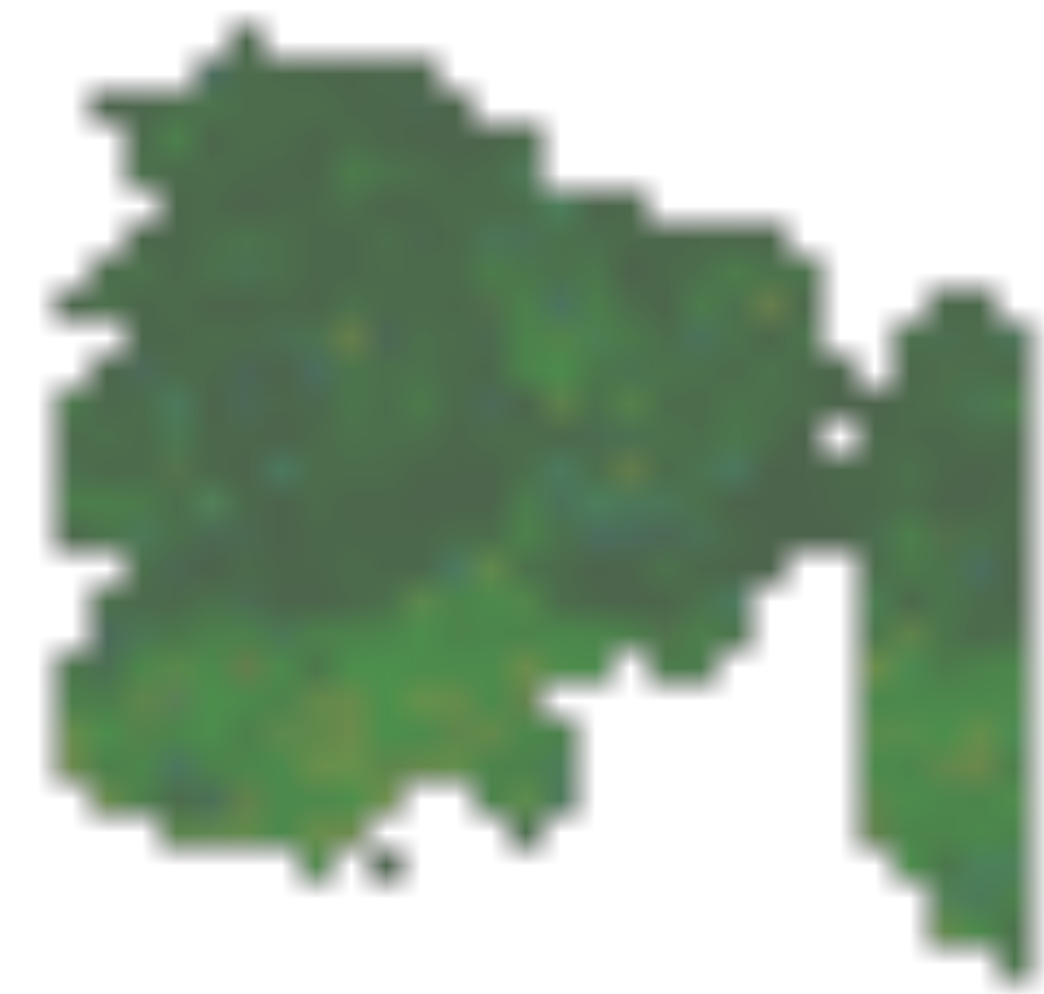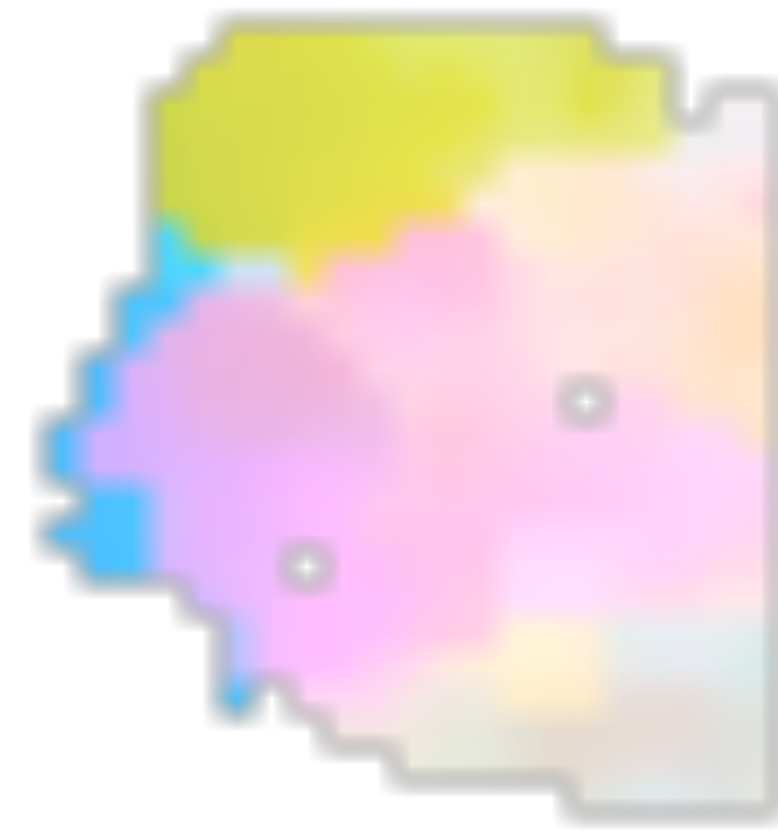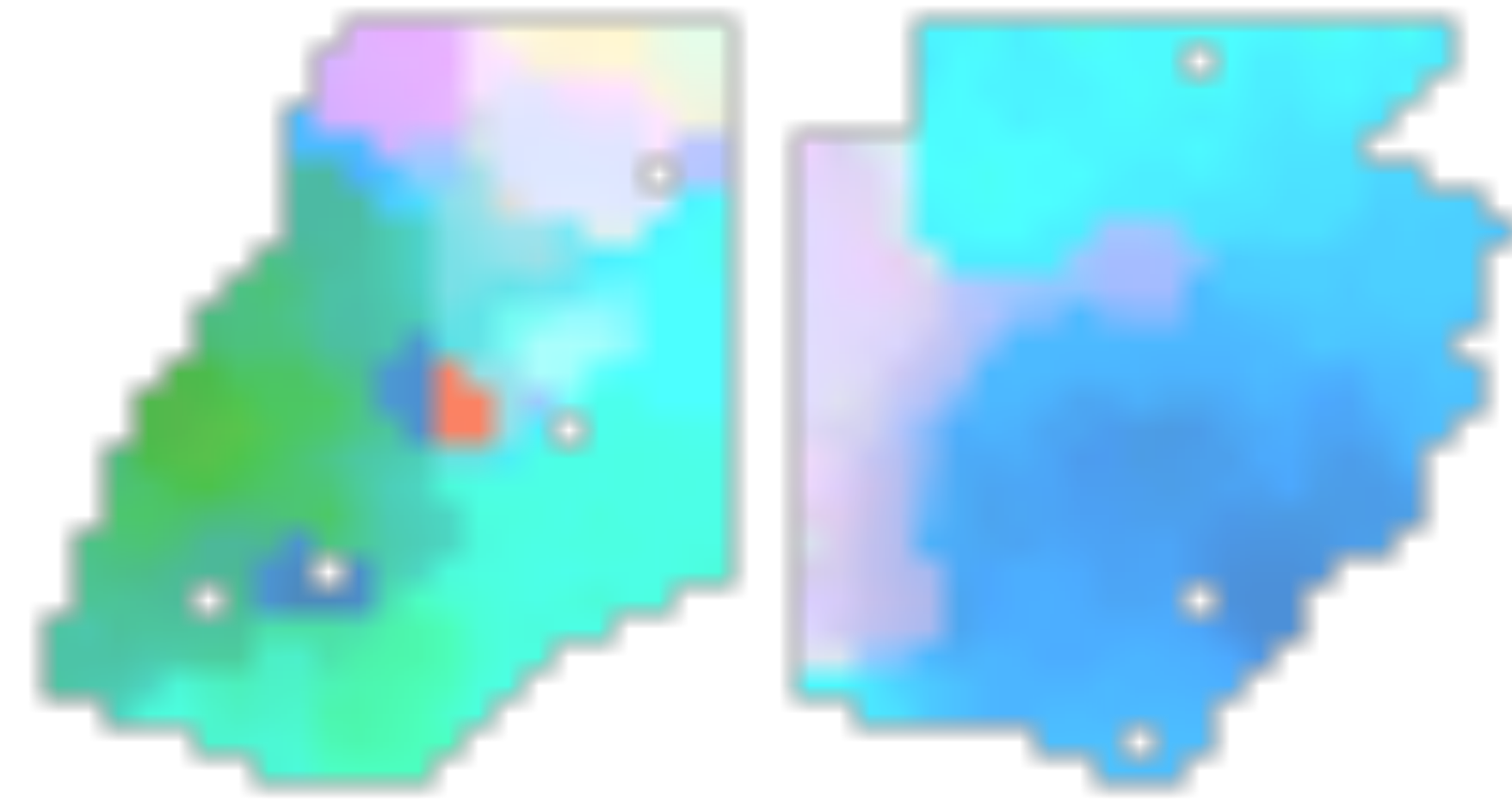

Supplement: Supplementary file 8 — Supplementary Data 5 [file 41467_2018_4724_MOESM8_ESM.zip › Supplementary Dataset 7/joint-field-dimensionality-reduction-tSNE-matrix.pdf.interpolated.pdf]

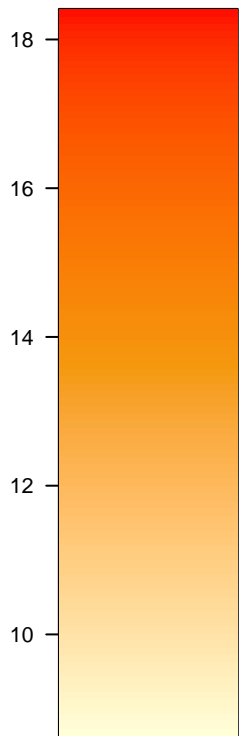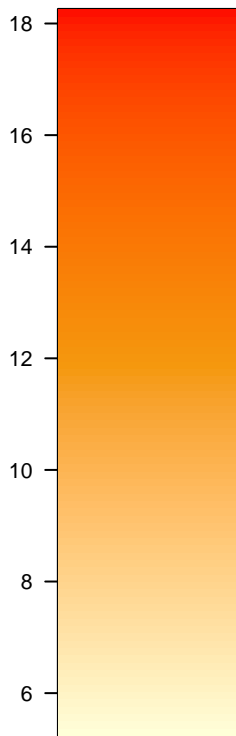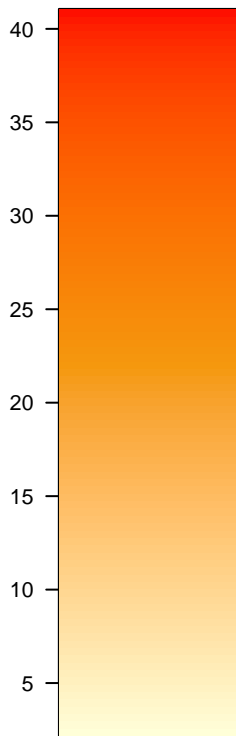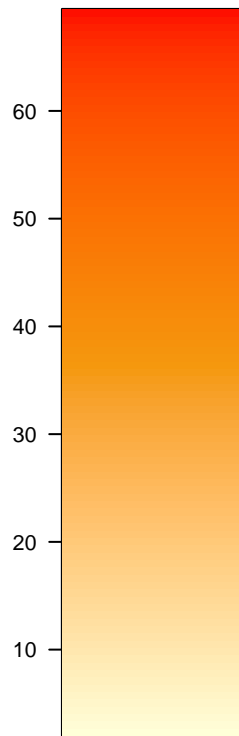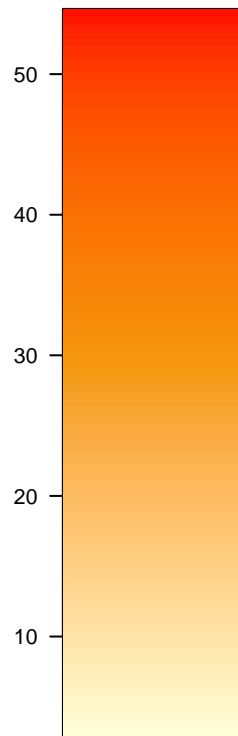

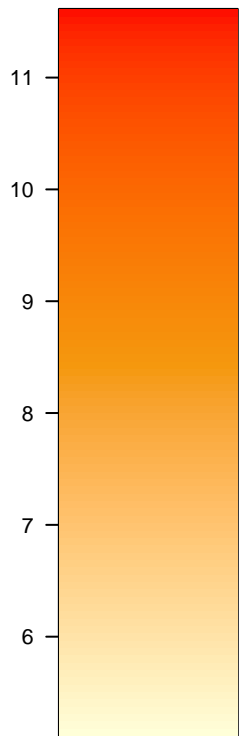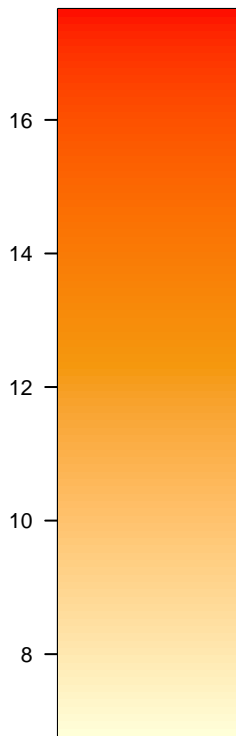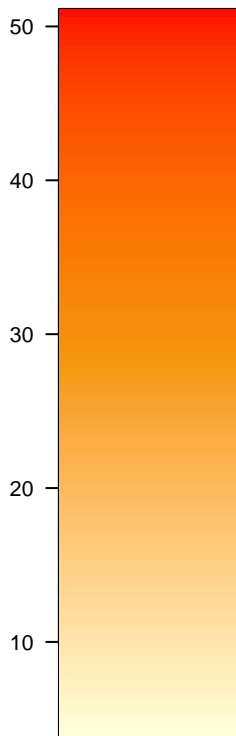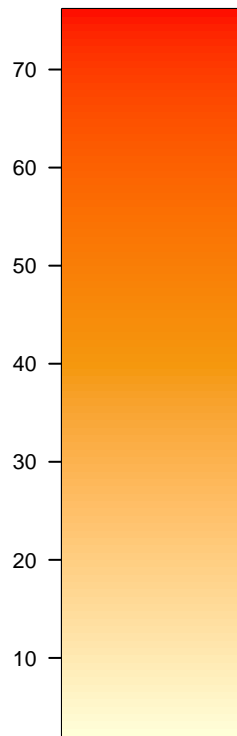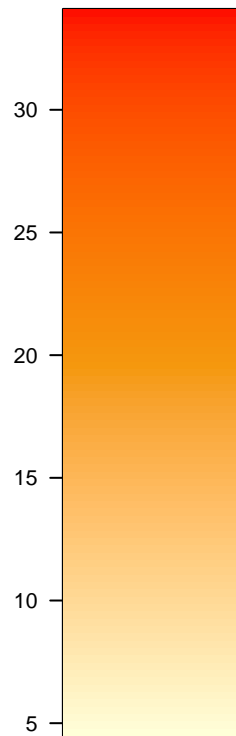

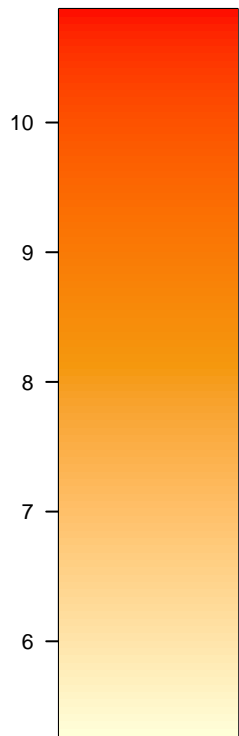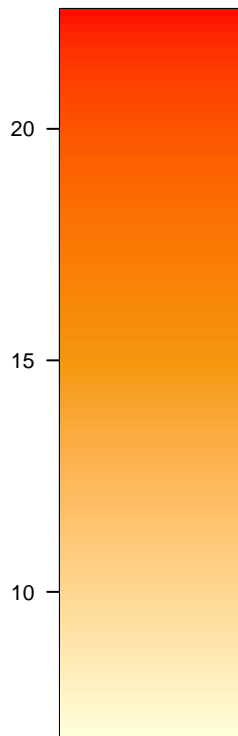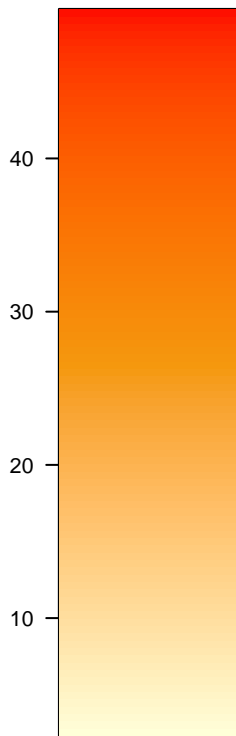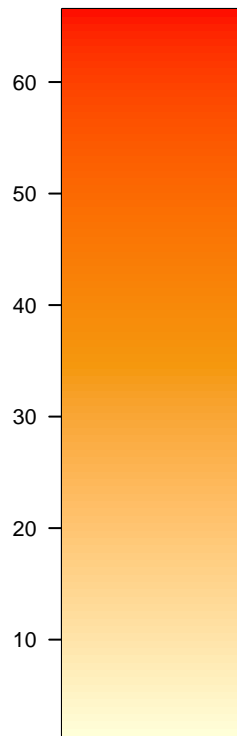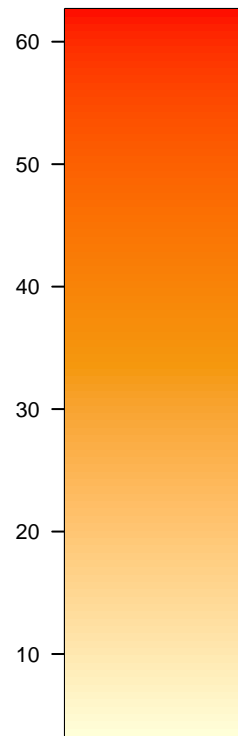

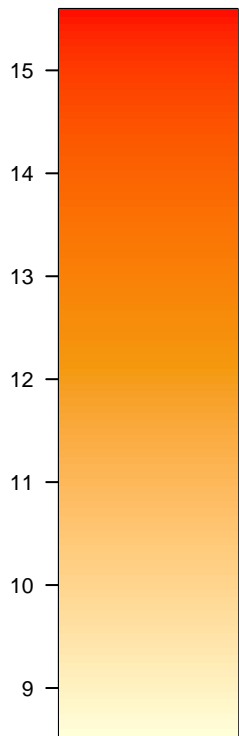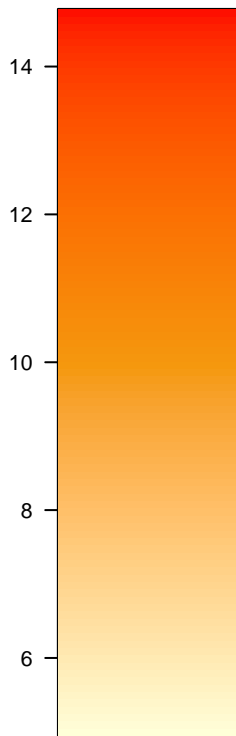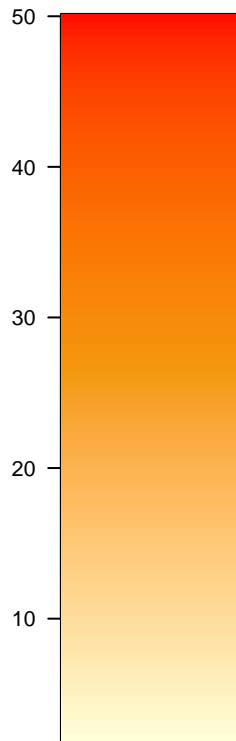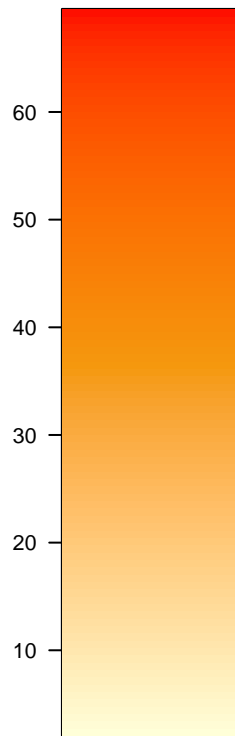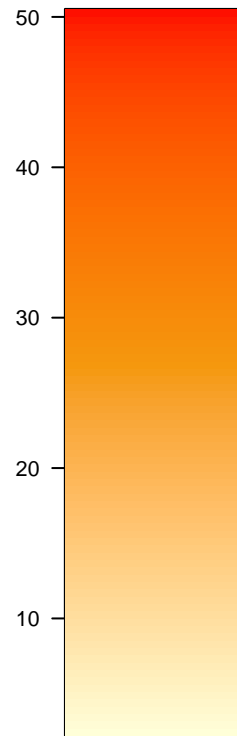

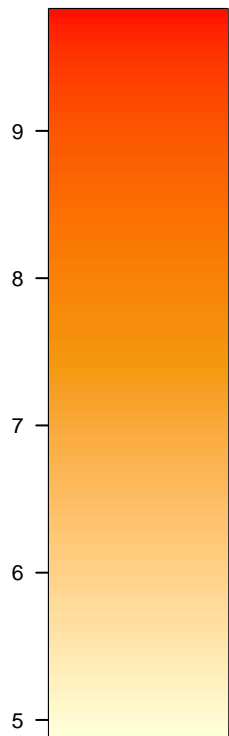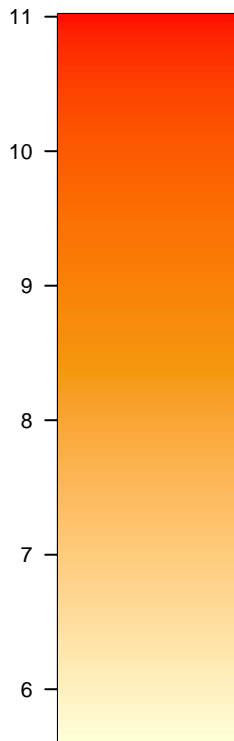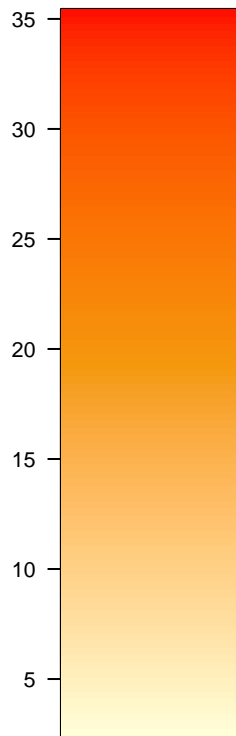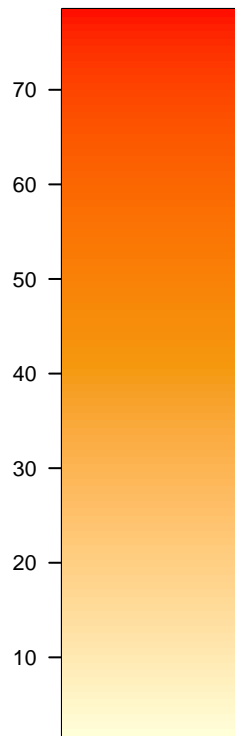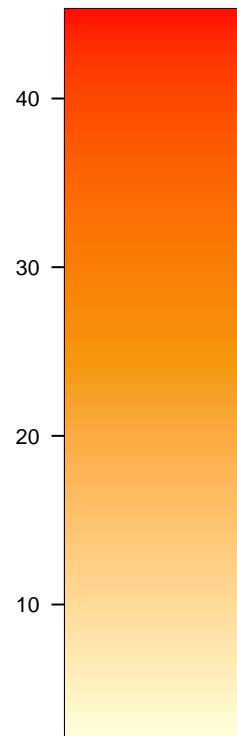

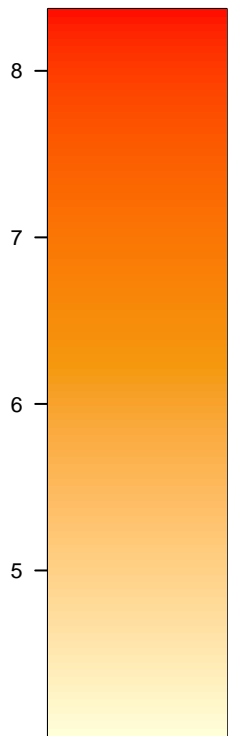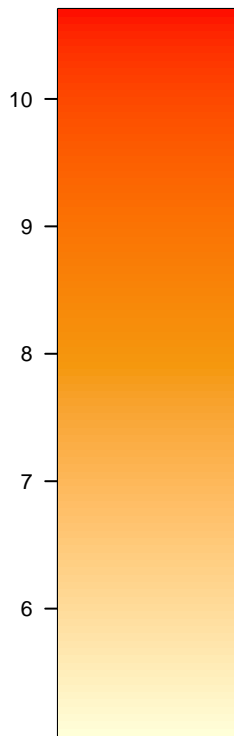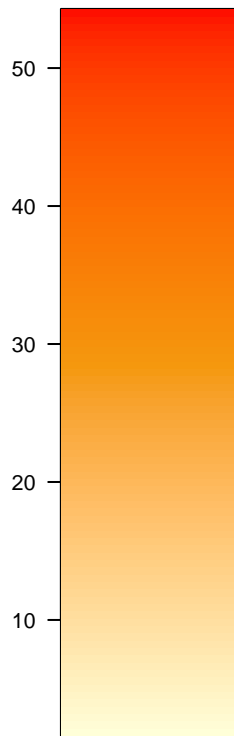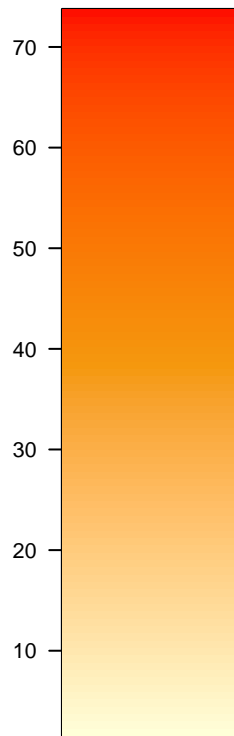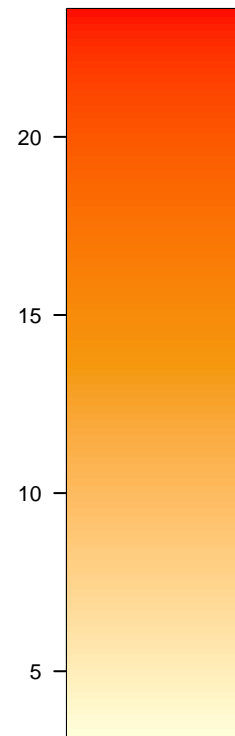

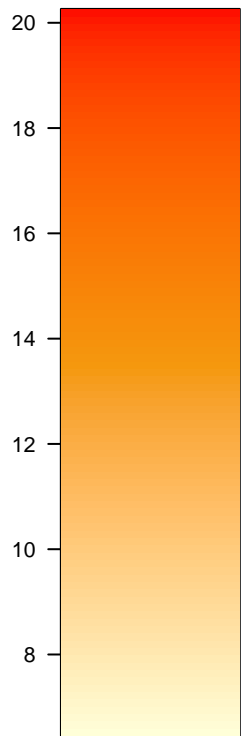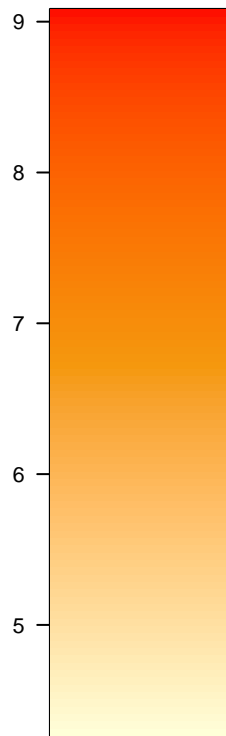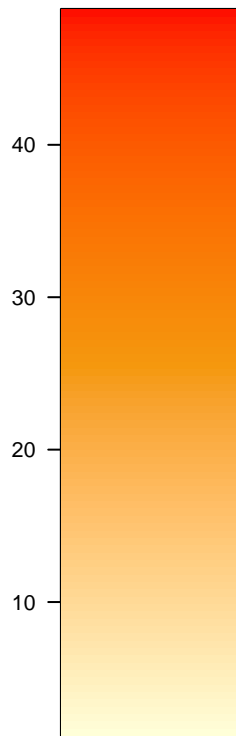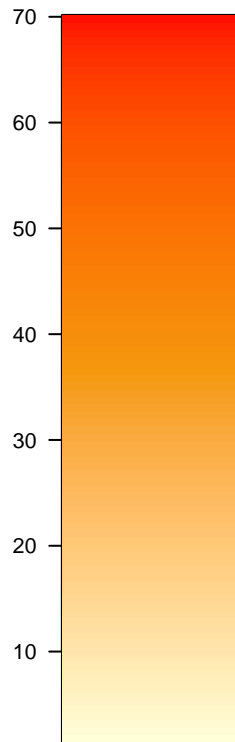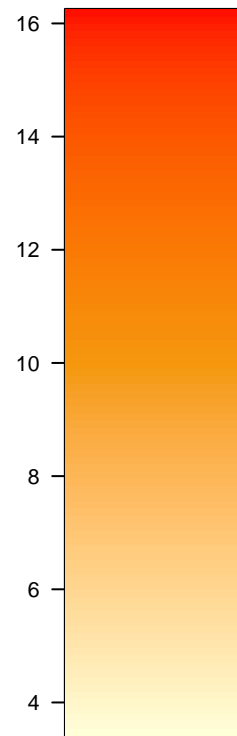

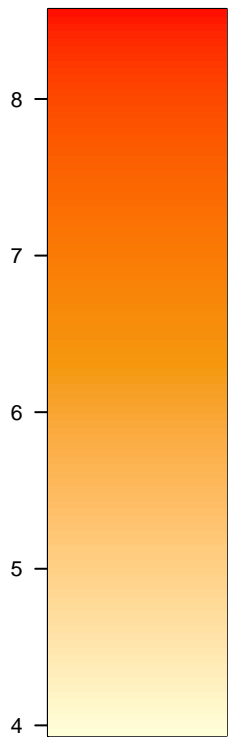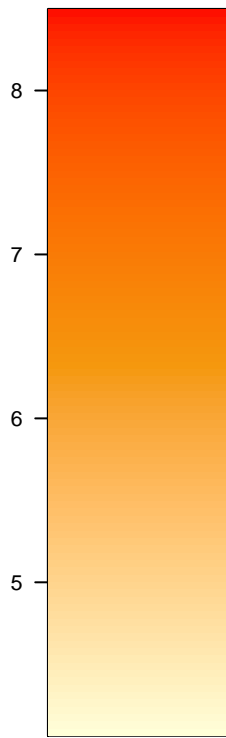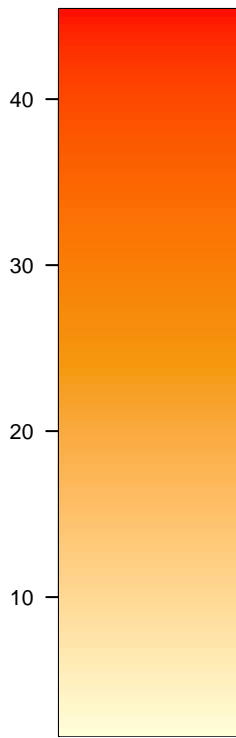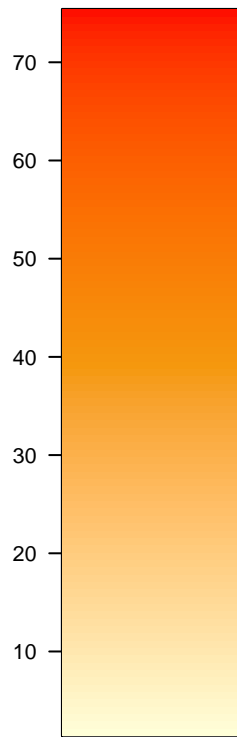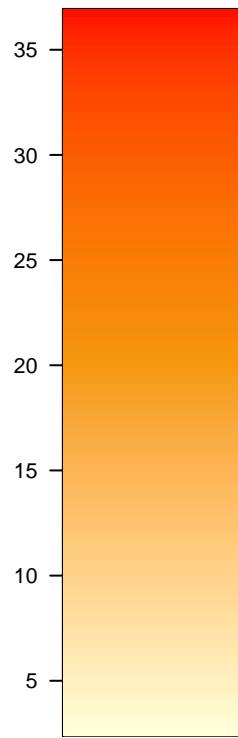

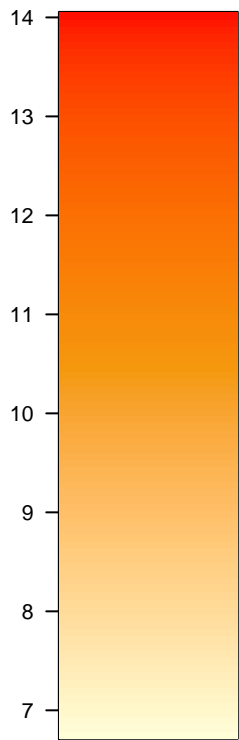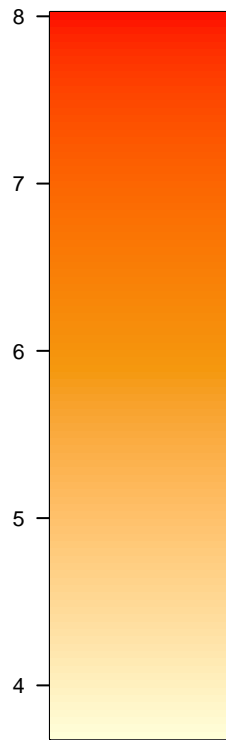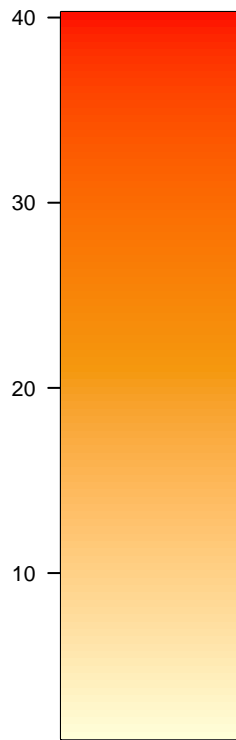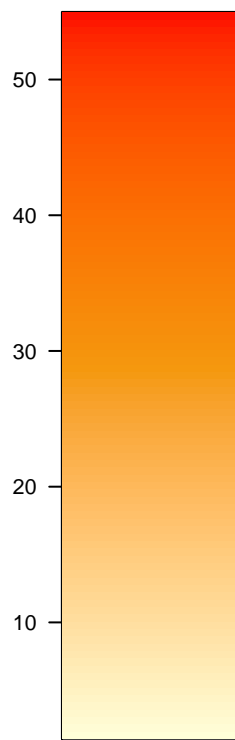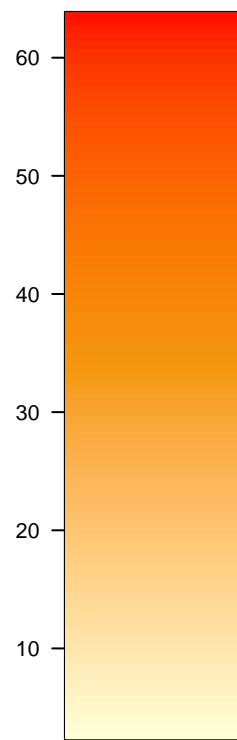

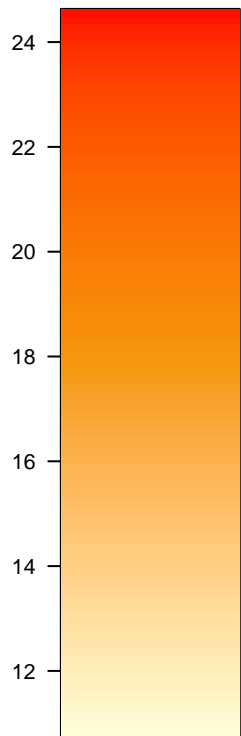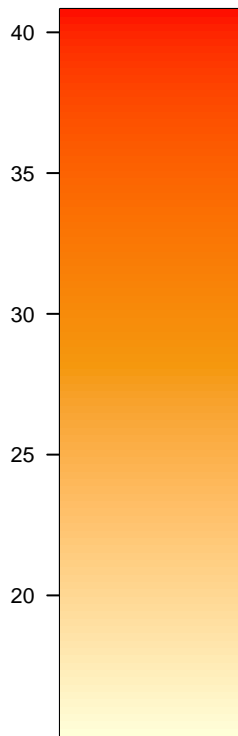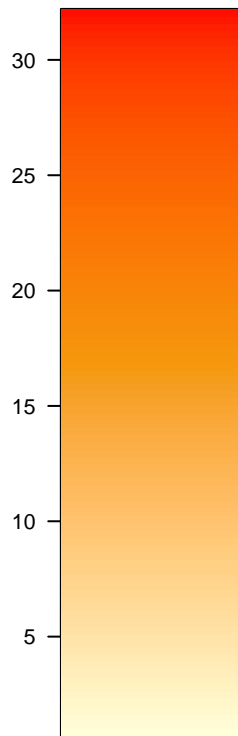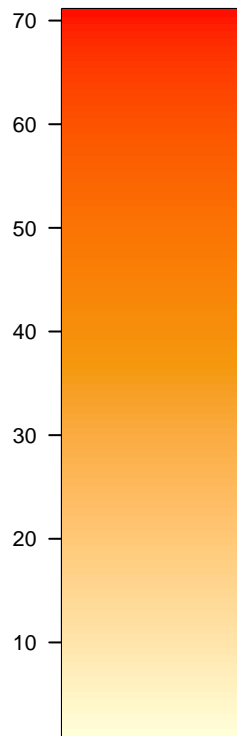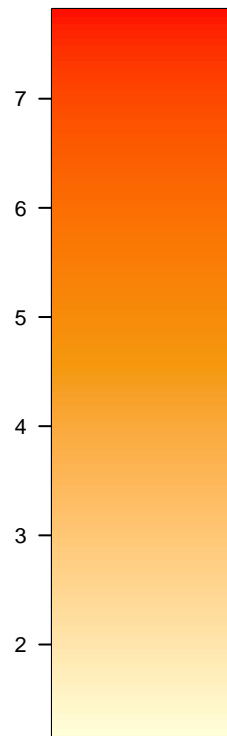

Supplement: Supplementary file 8 — Supplementary Data 5 [file 41467_2018_4724_MOESM8_ESM.zip › Supplementary Dataset 7/joint-mix-profiles-rel-individual-scale-dots-colorbar.pdf]

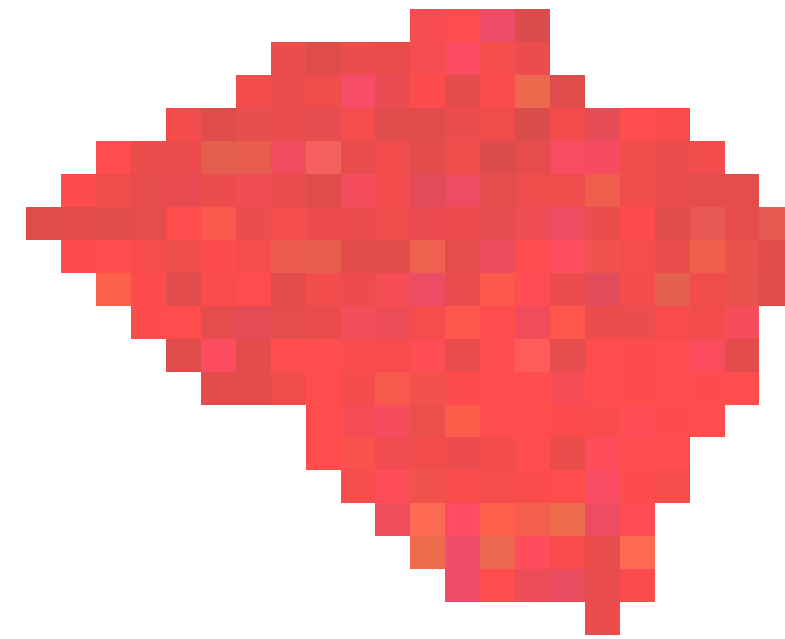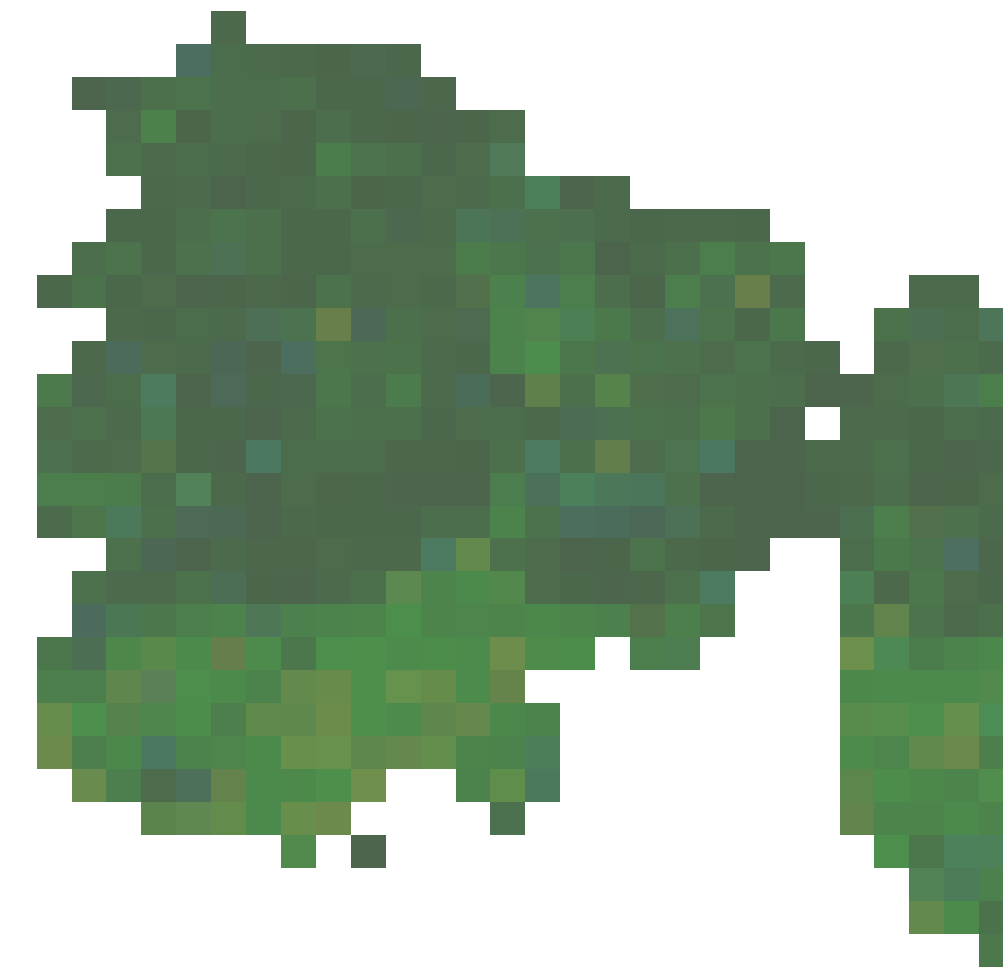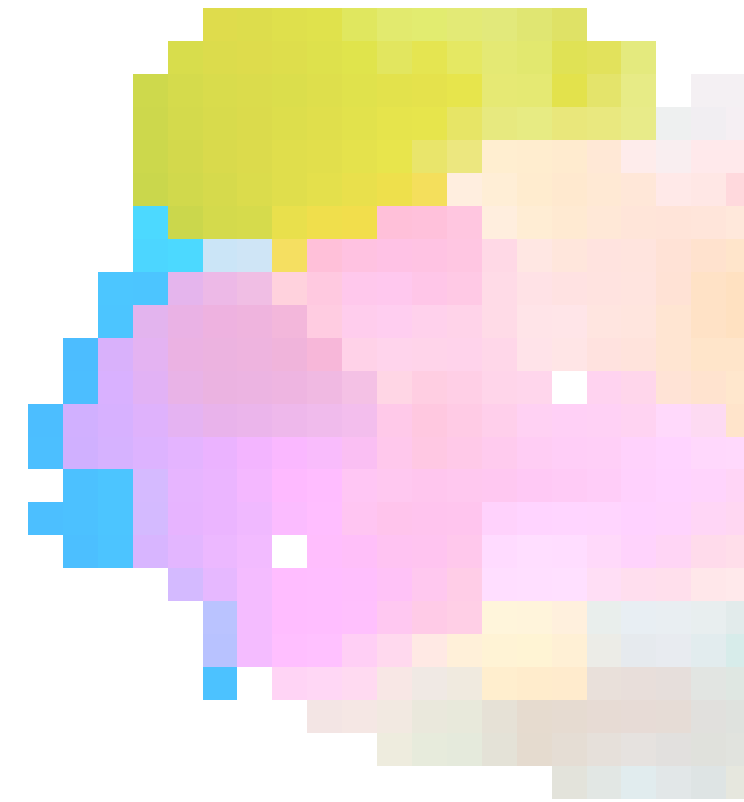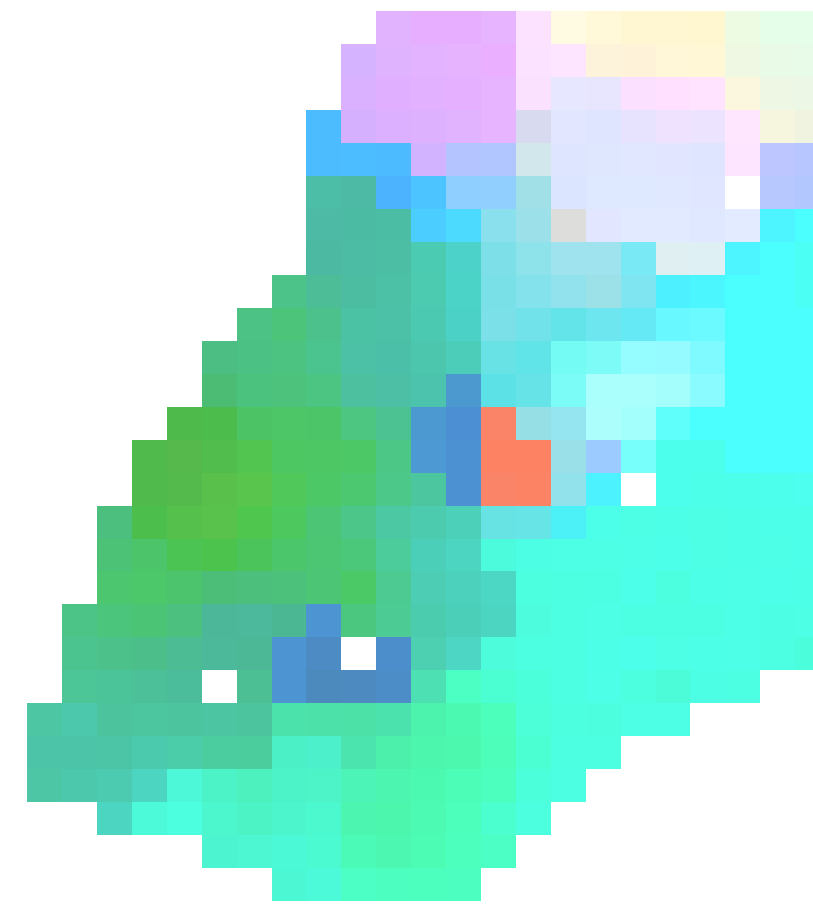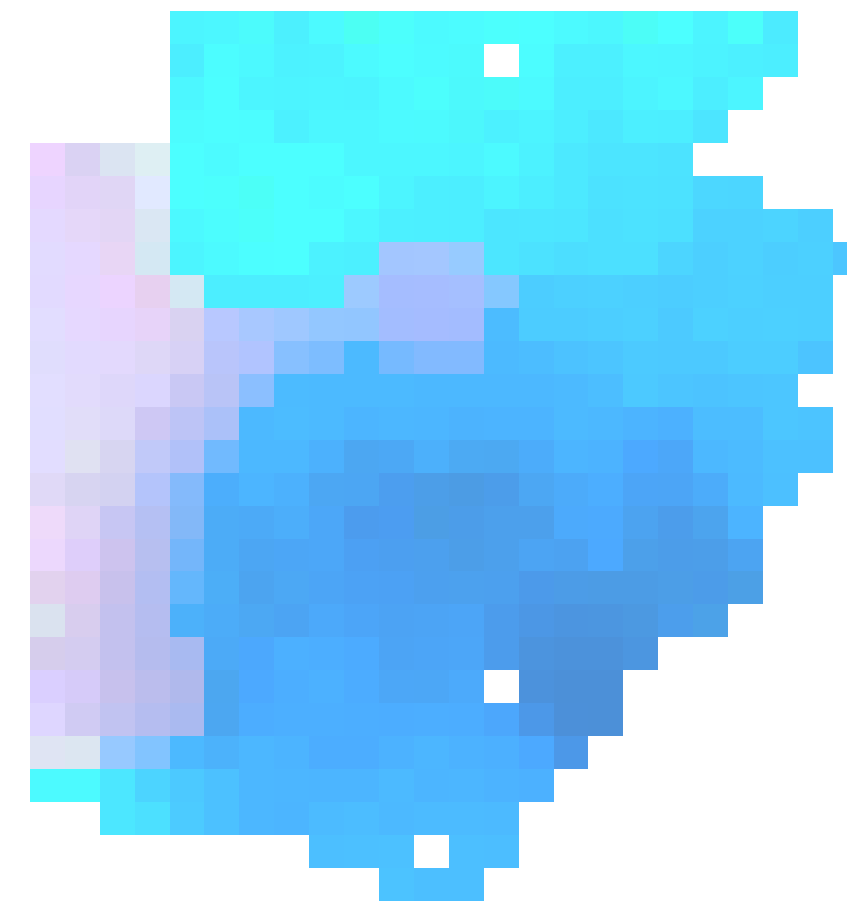

Supplement: Supplementary file 8 — Supplementary Data 5 [file 41467_2018_4724_MOESM8_ESM.zip › Supplementary Dataset 7/joint-field-dimensionality-reduction-tSNE-matrix.pdf]

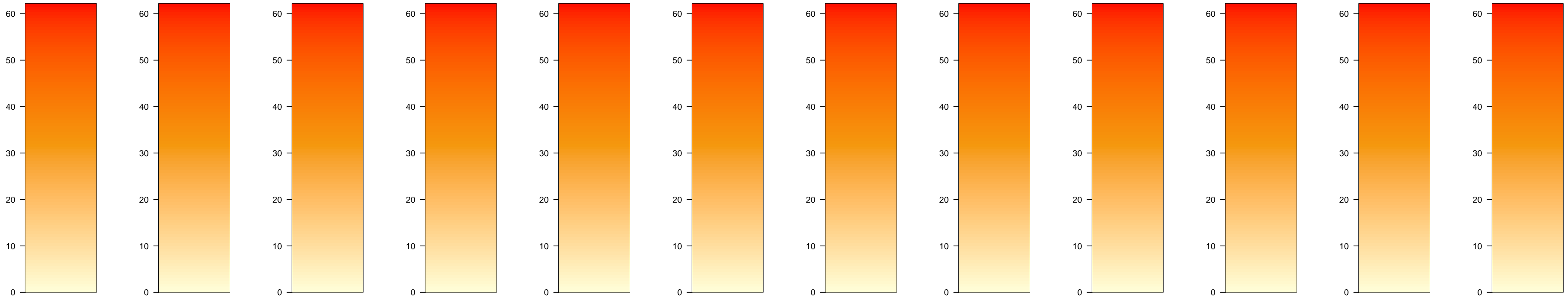

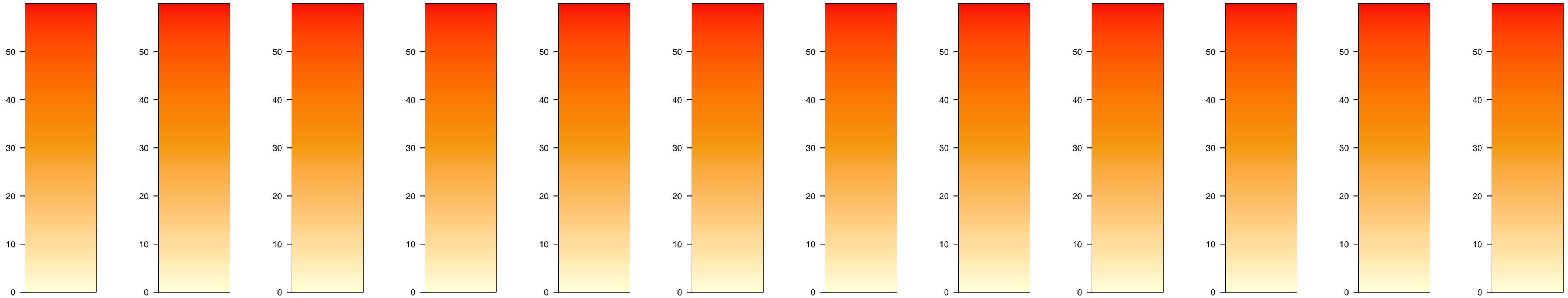

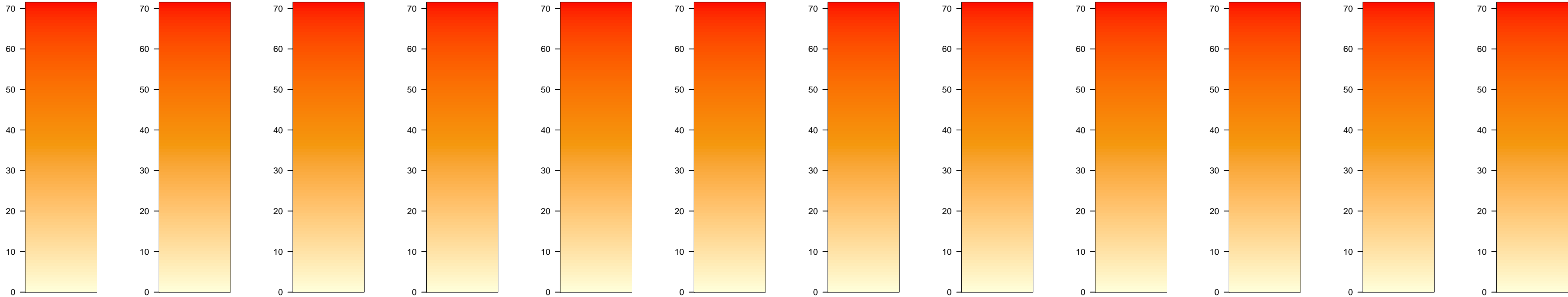

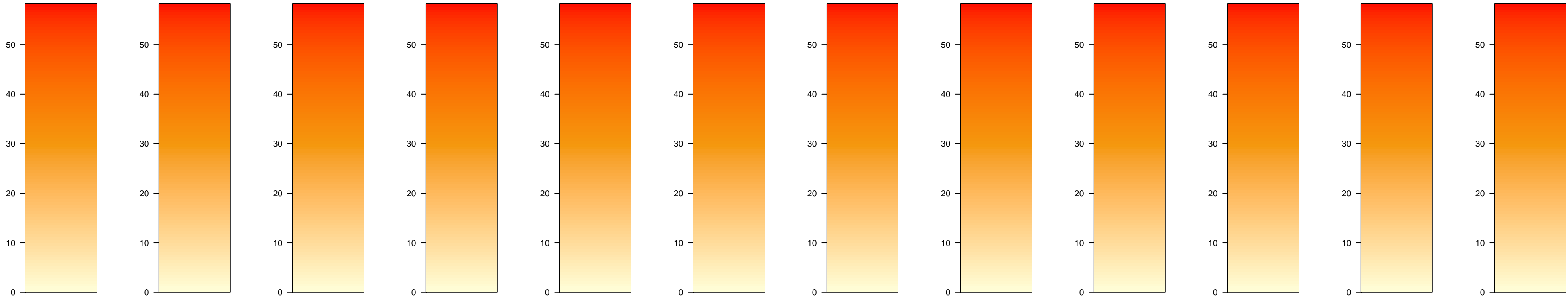

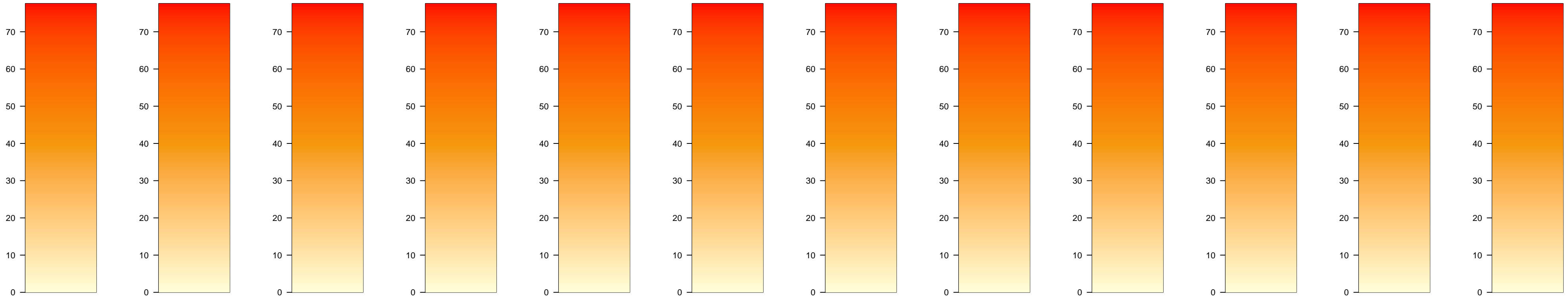

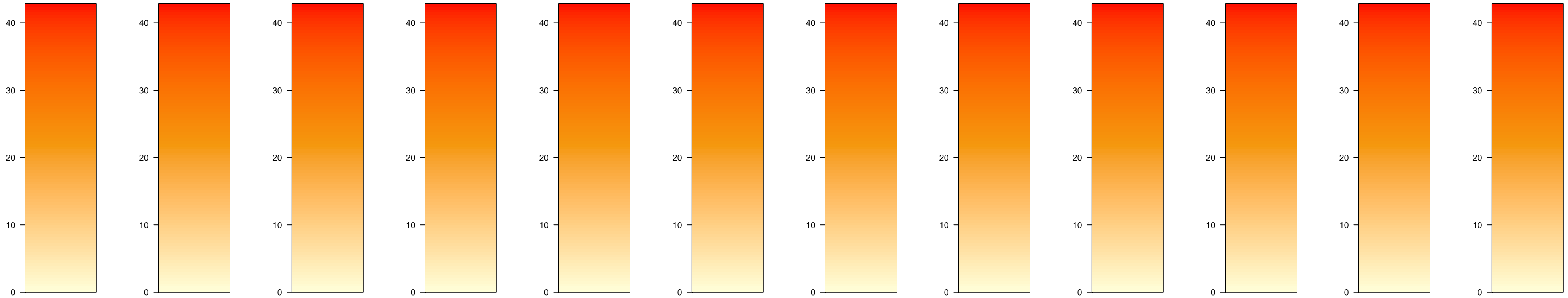

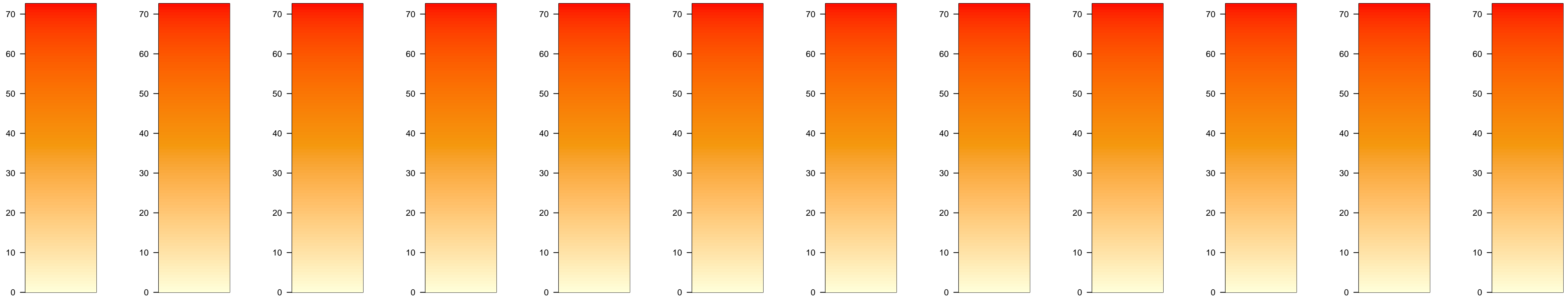

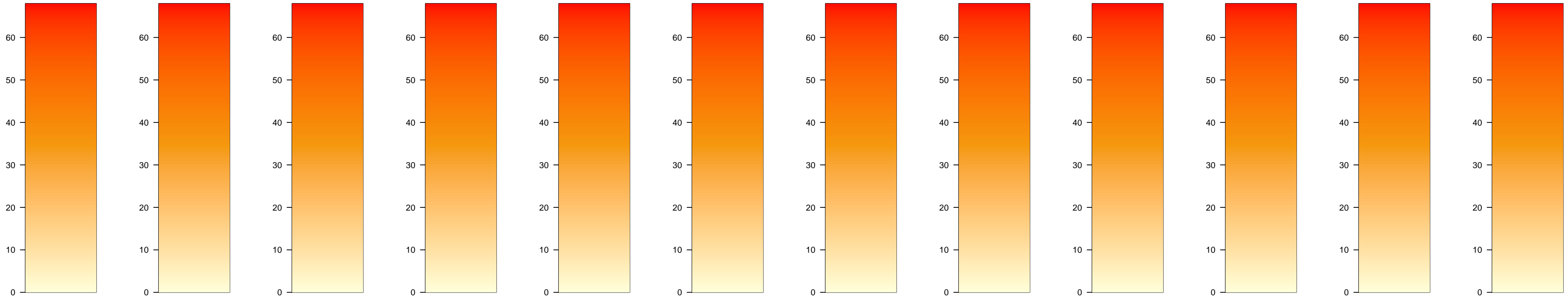

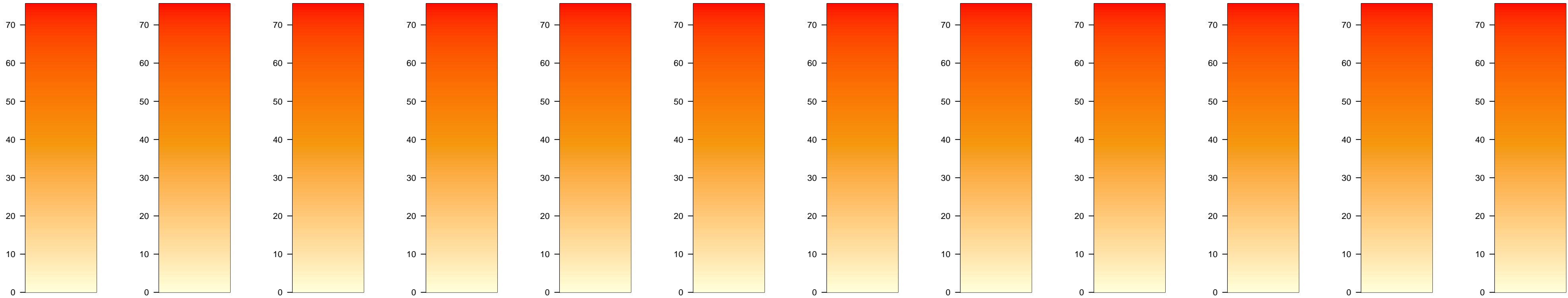

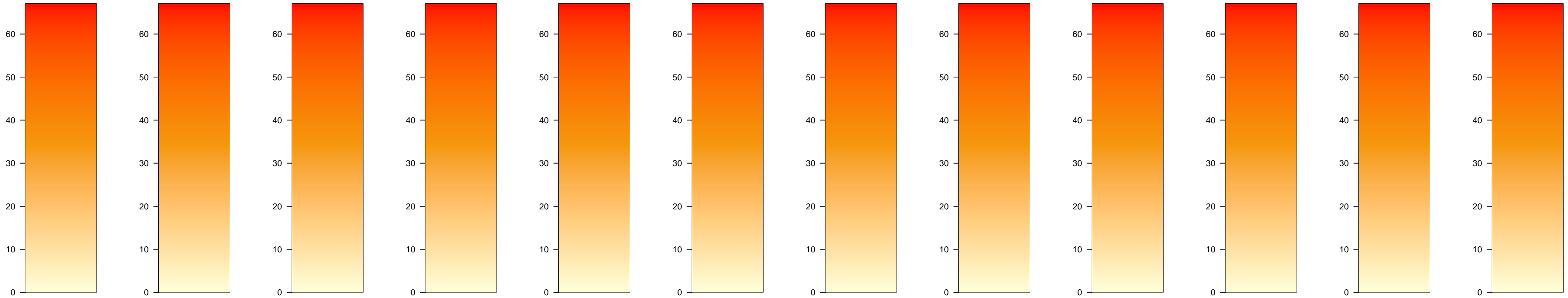

Supplement: Supplementary file 9 — Supplementary Data 6 [file 41467_2018_4724_MOESM9_ESM.zip › Supplementary Dataset 3/joint-mix-profiles-rel-common-scale-matrix-colorbar.pdf]

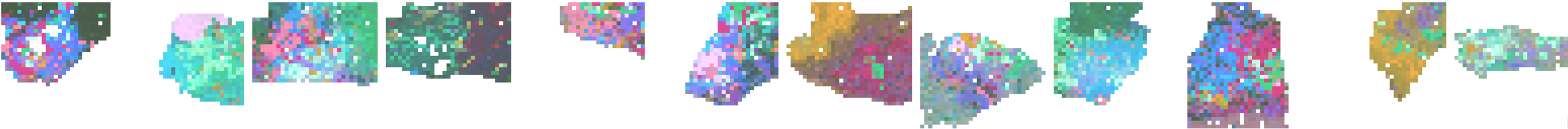

Supplement: Supplementary file 9 — Supplementary Data 6 [file 41467_2018_4724_MOESM9_ESM.zip › Supplementary Dataset 3/joint-mix-dimensionality-reduction-tSNE-matrix.pdf]

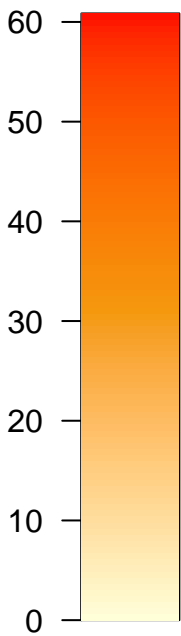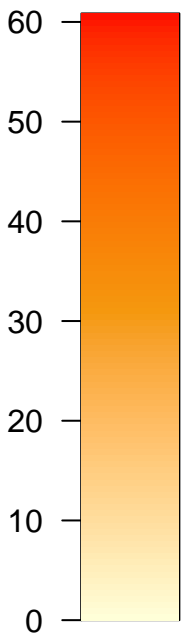

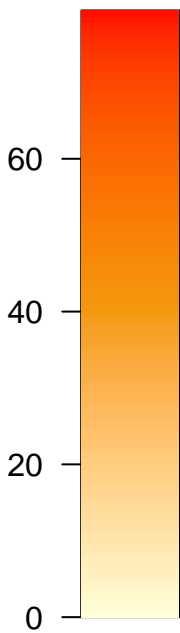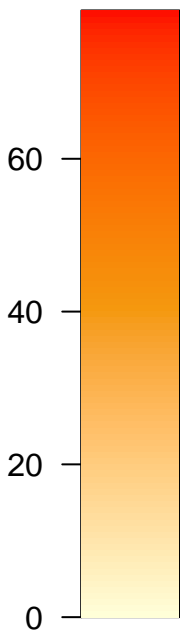

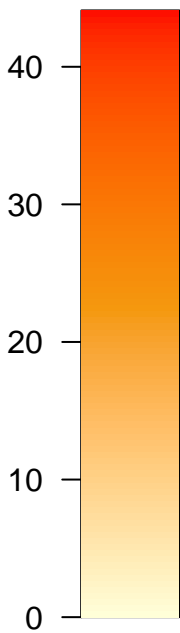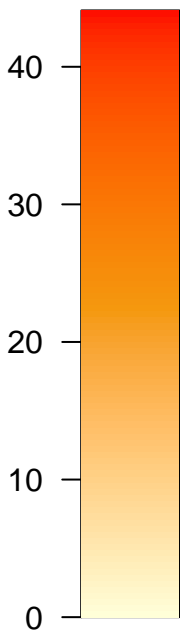

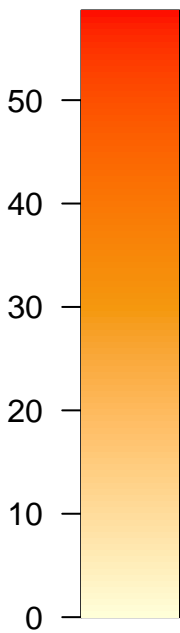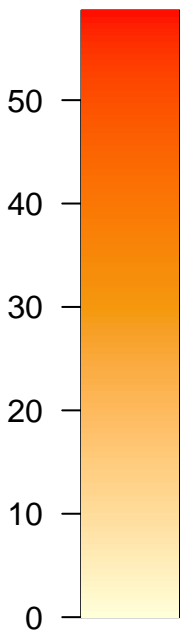

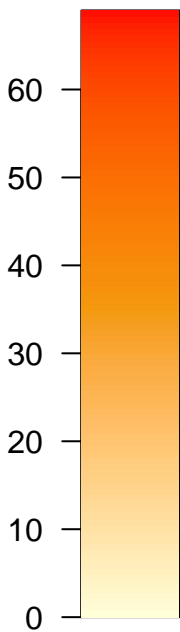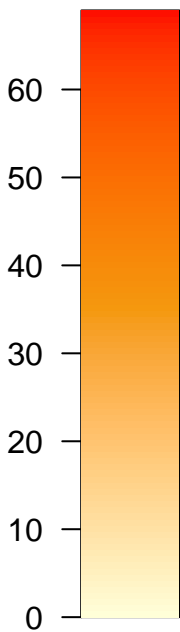

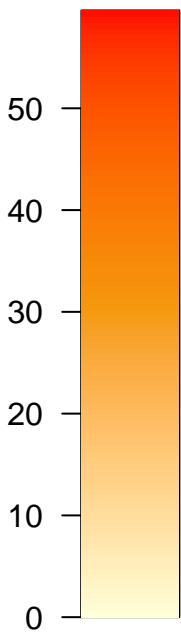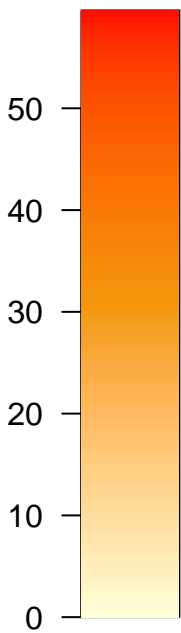

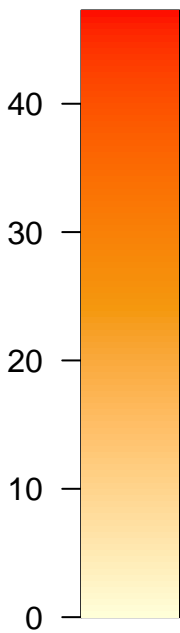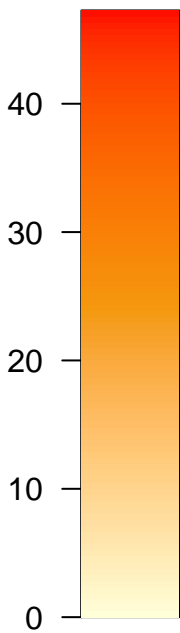

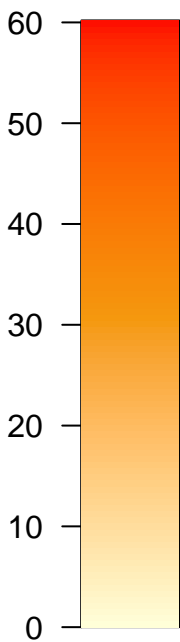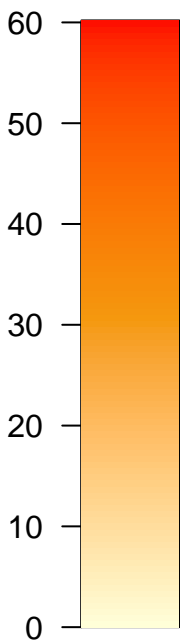

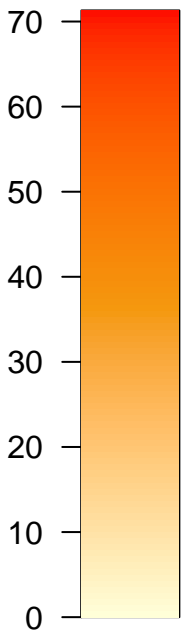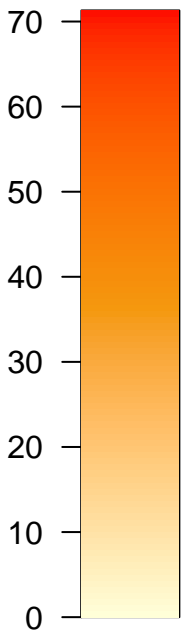

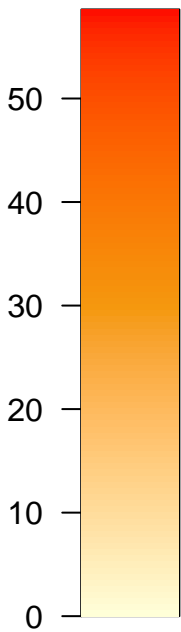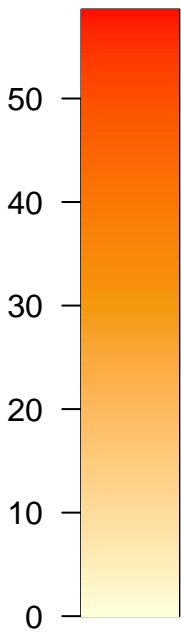

Supplement: Supplementary file 10 — Supplementary Data 7 [file 41467_2018_4724_MOESM10_ESM.zip › Supplementary Dataset 4/joint-mix-profiles-rel-common-scale-matrix-colorbar.pdf]

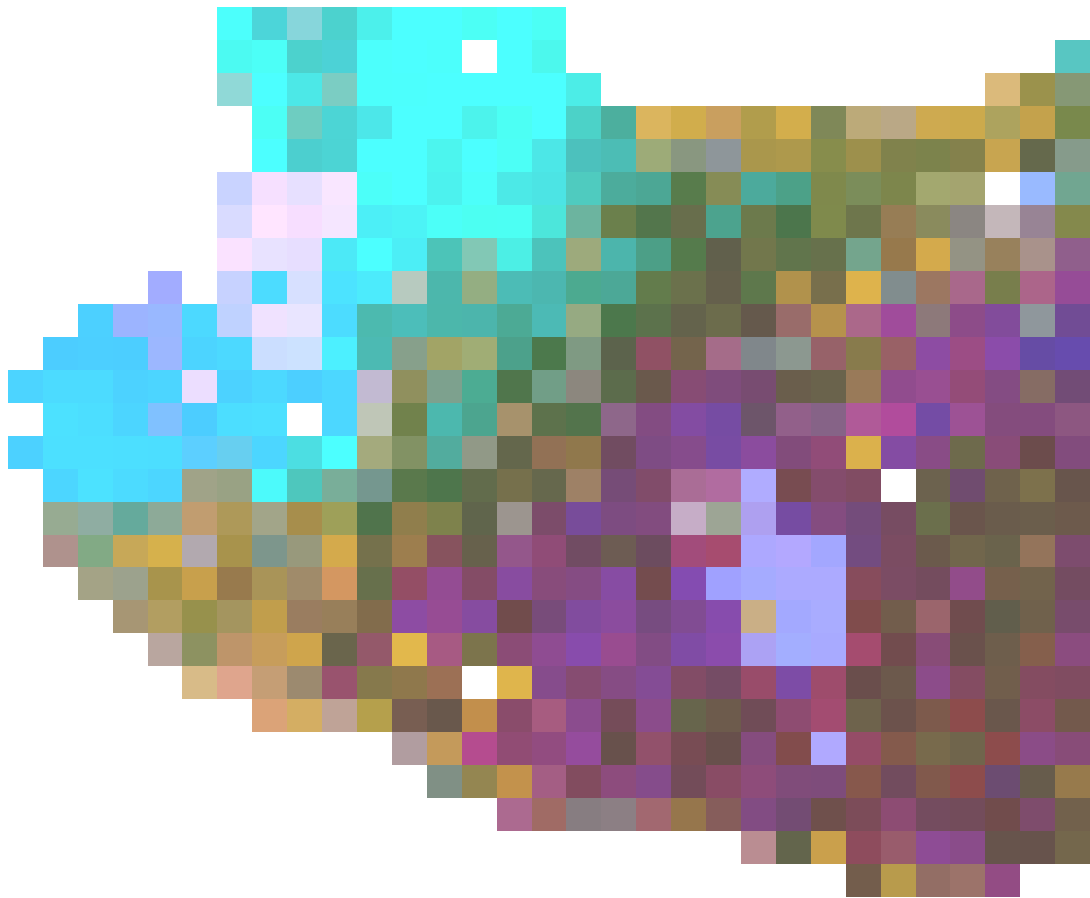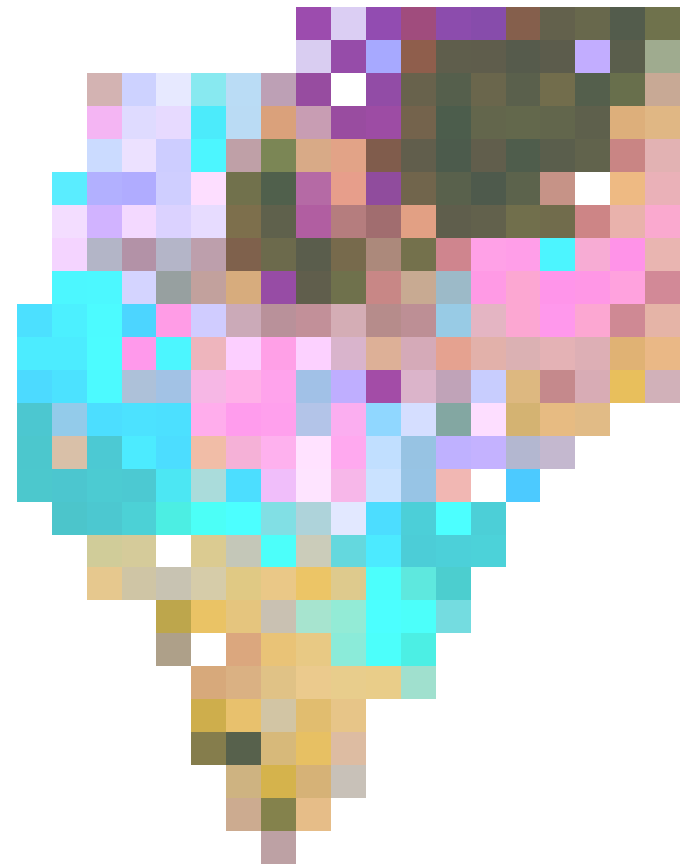

Supplement: Supplementary file 10 — Supplementary Data 7 [file 41467_2018_4724_MOESM10_ESM.zip › Supplementary Dataset 4/joint-mix-dimensionality-reduction-tSNE-matrix.pdf]

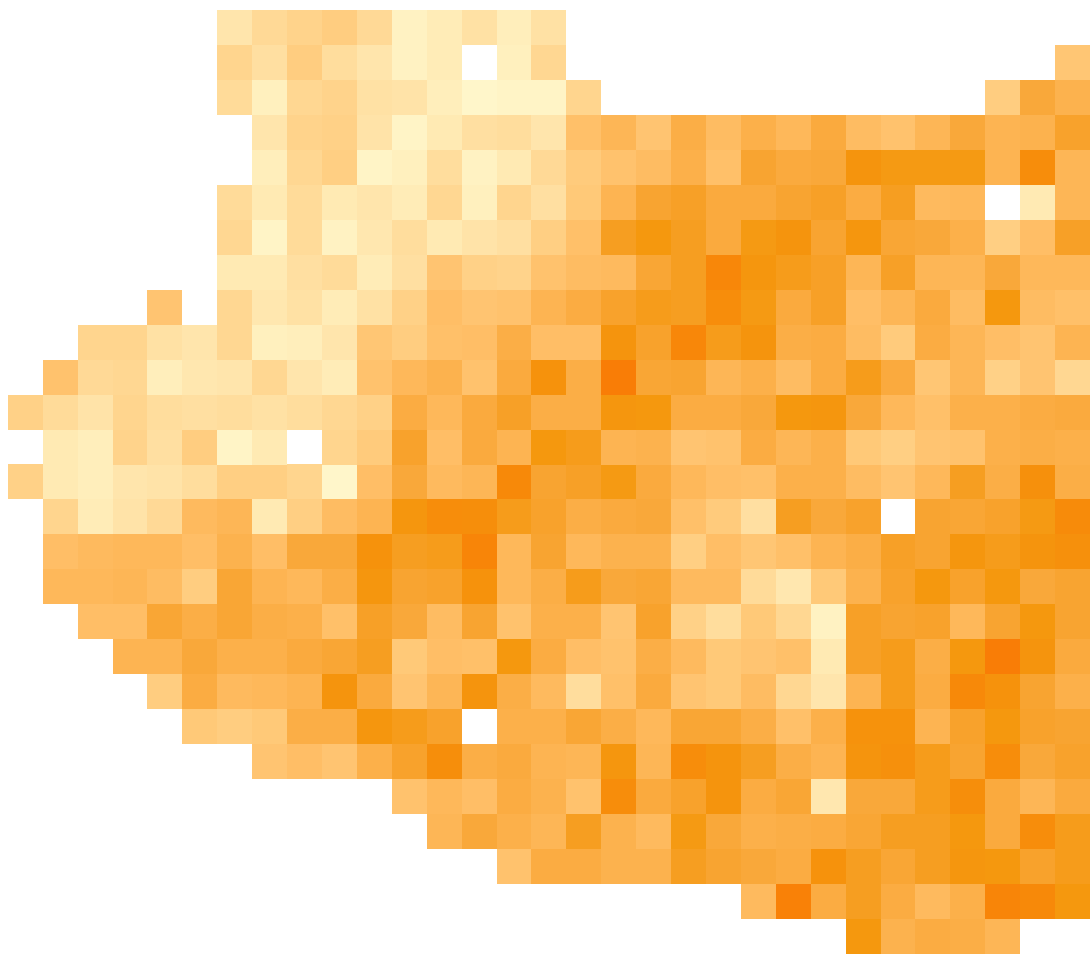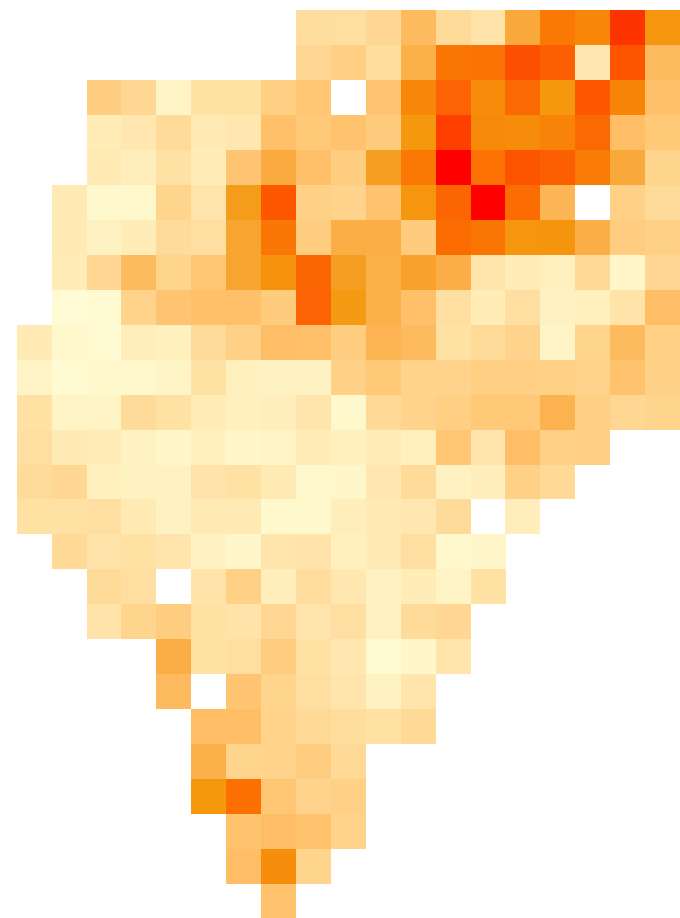

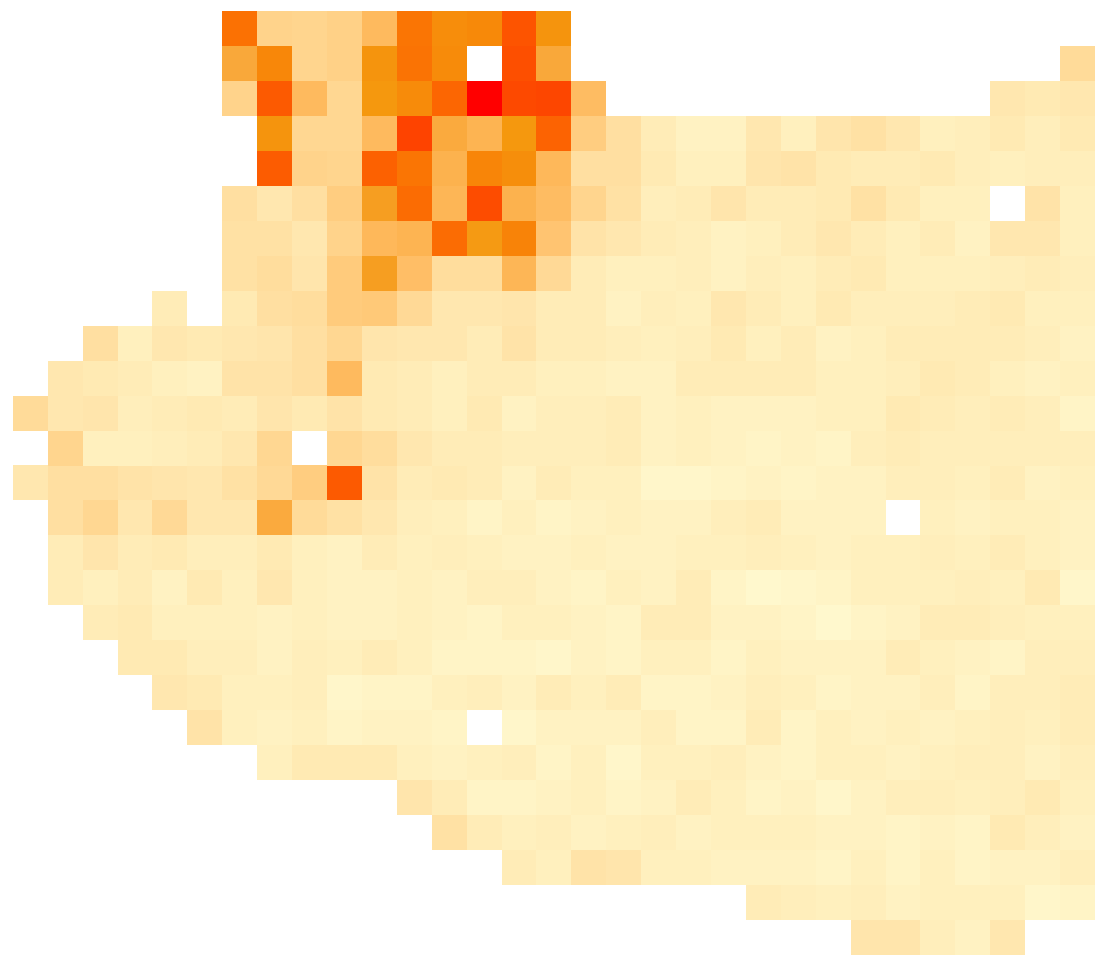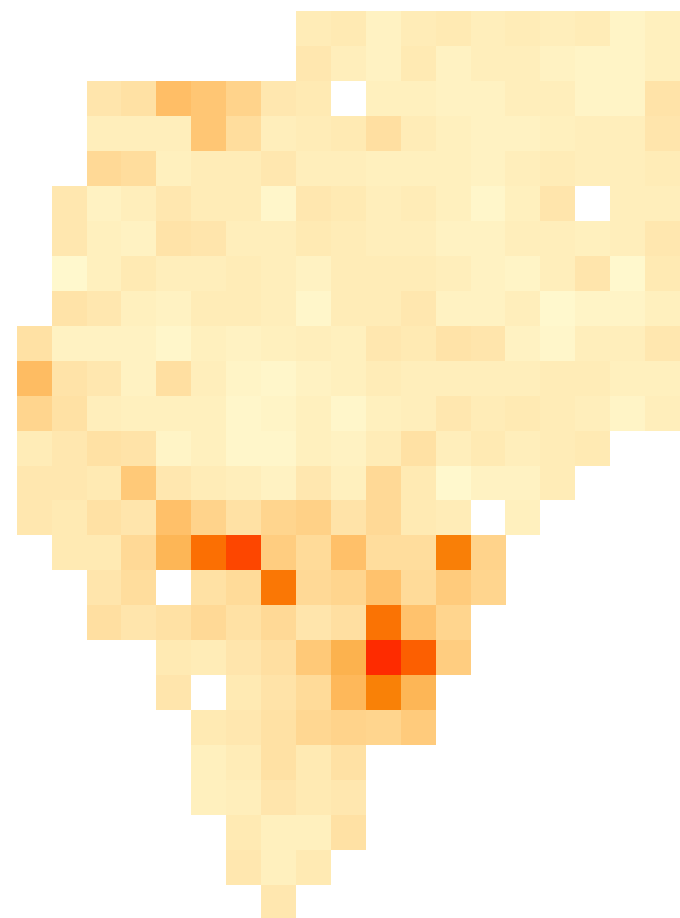

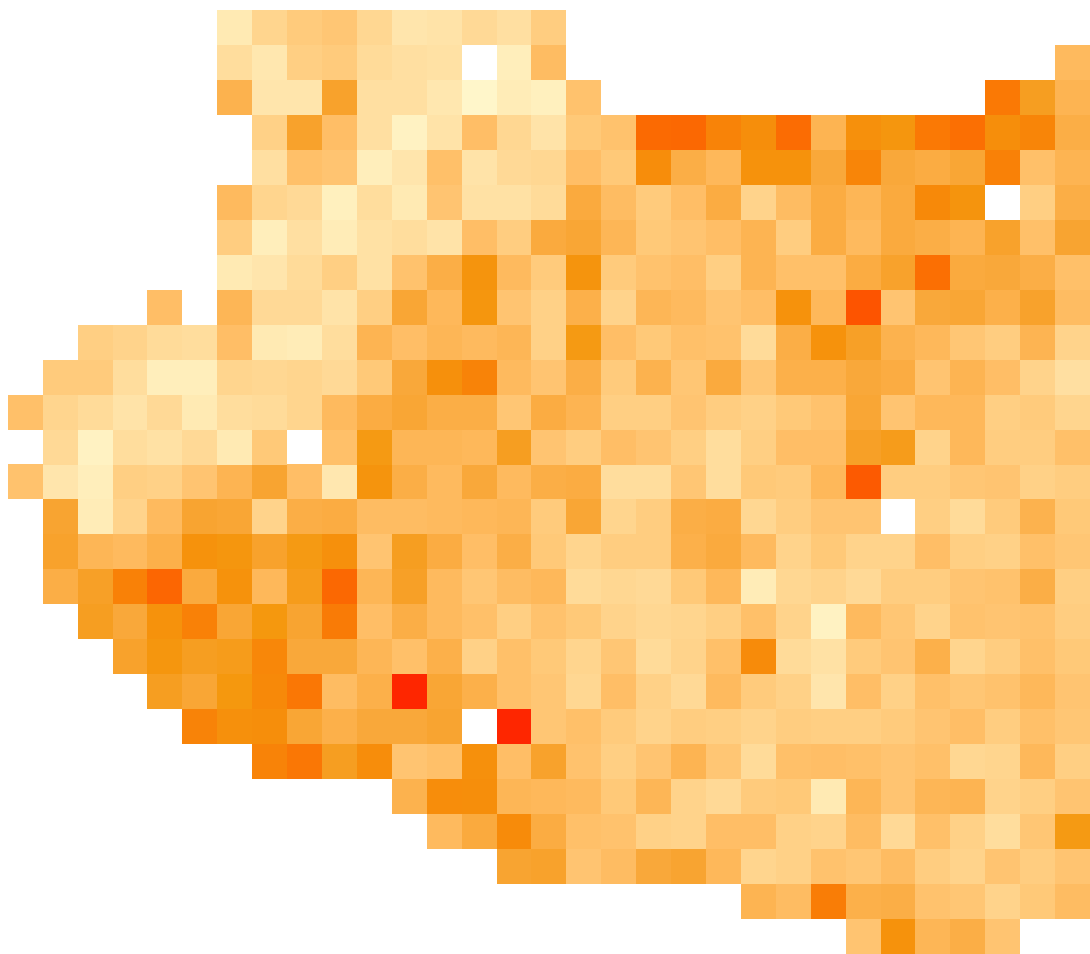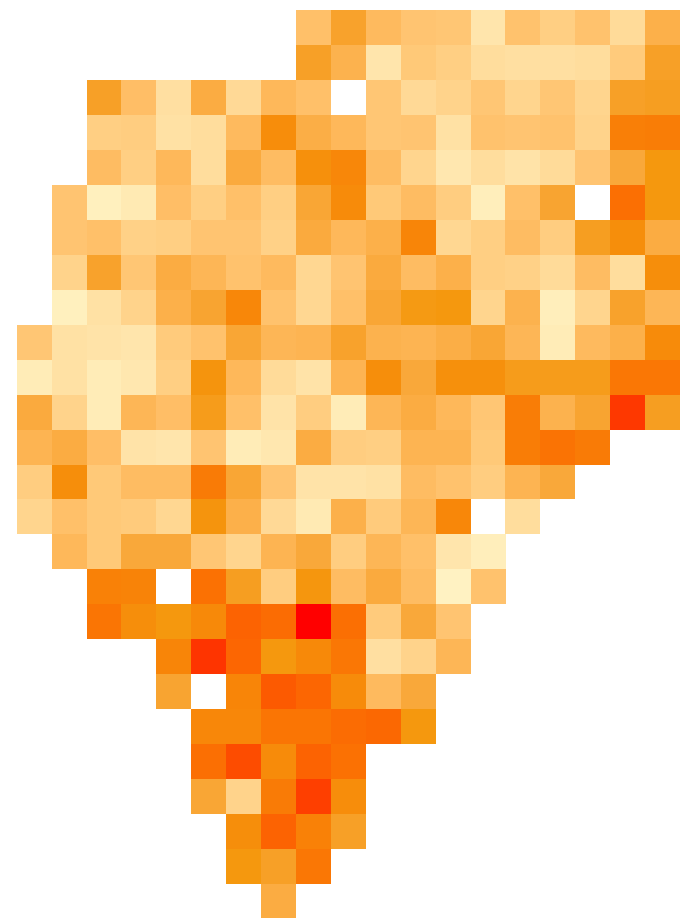

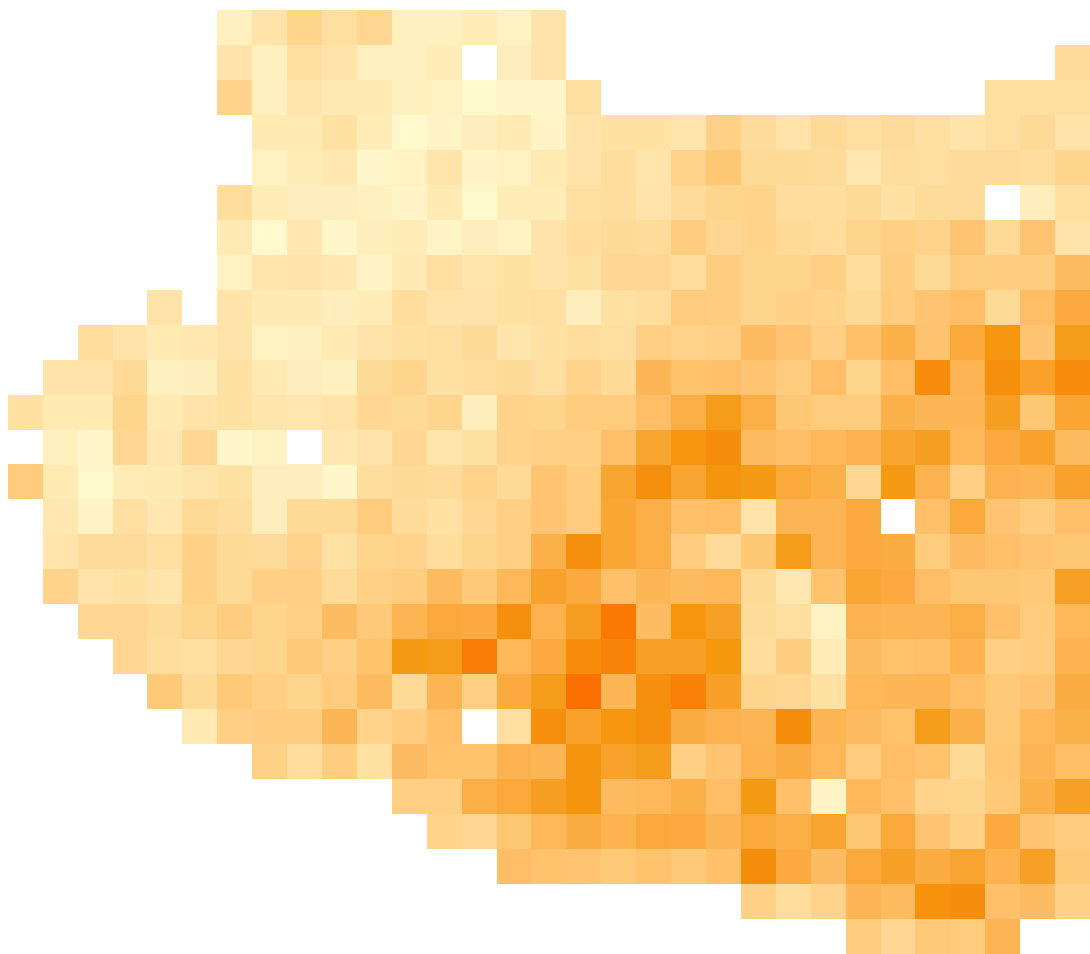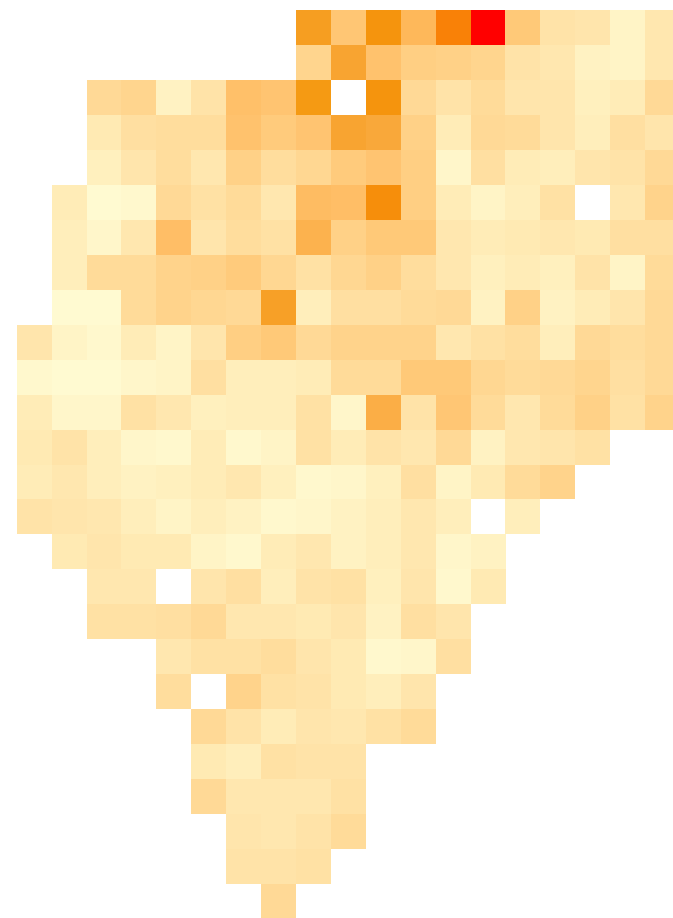

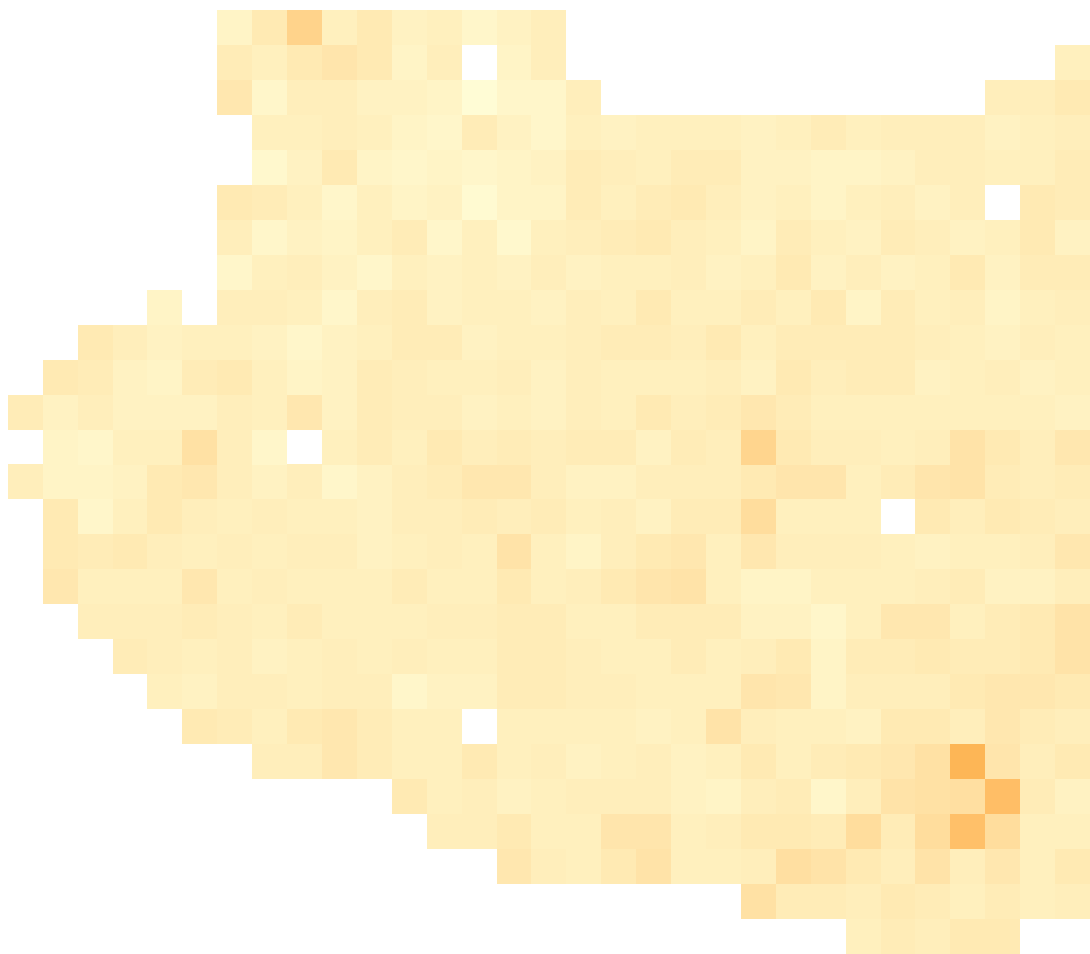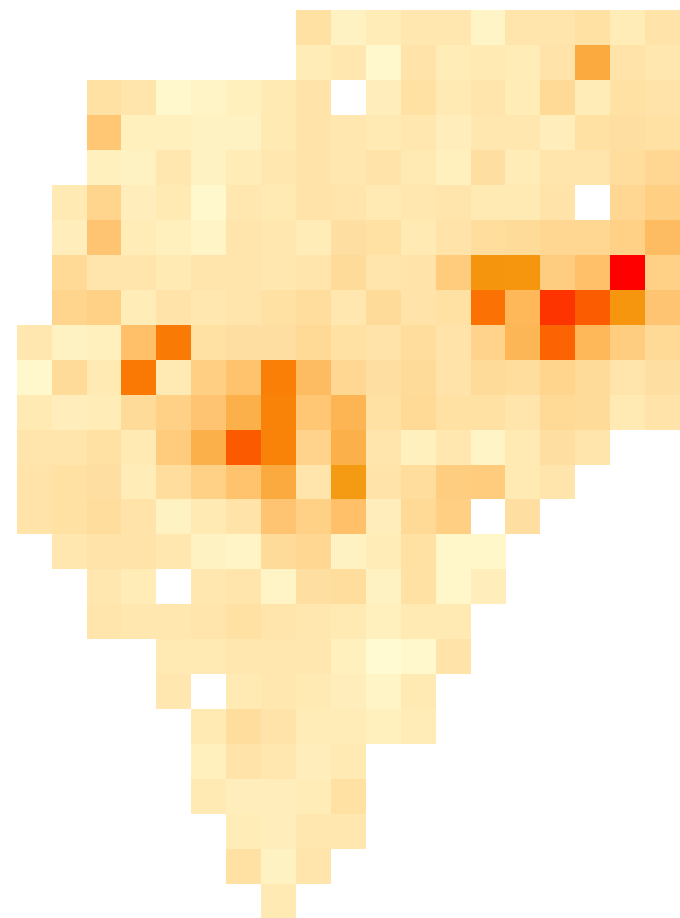

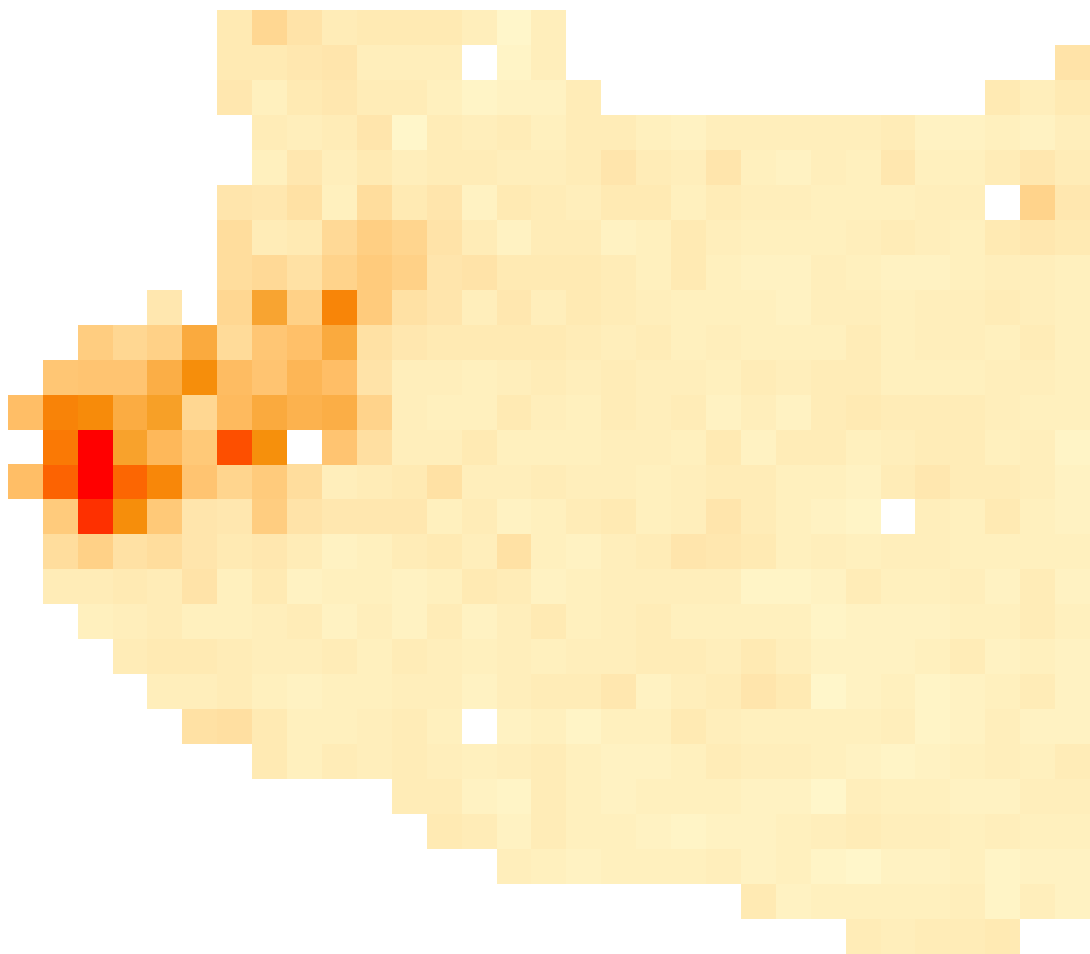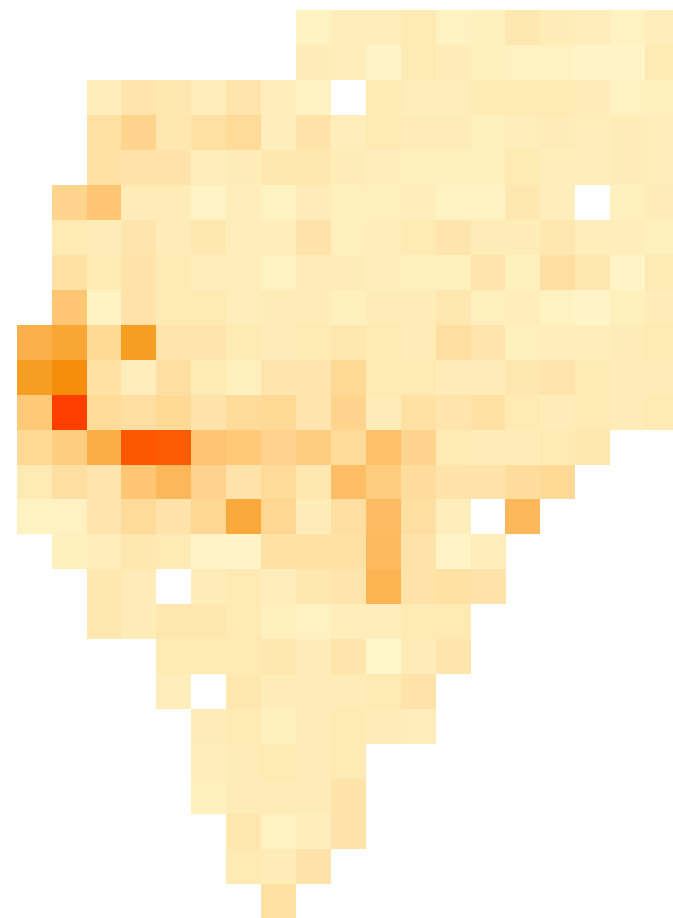

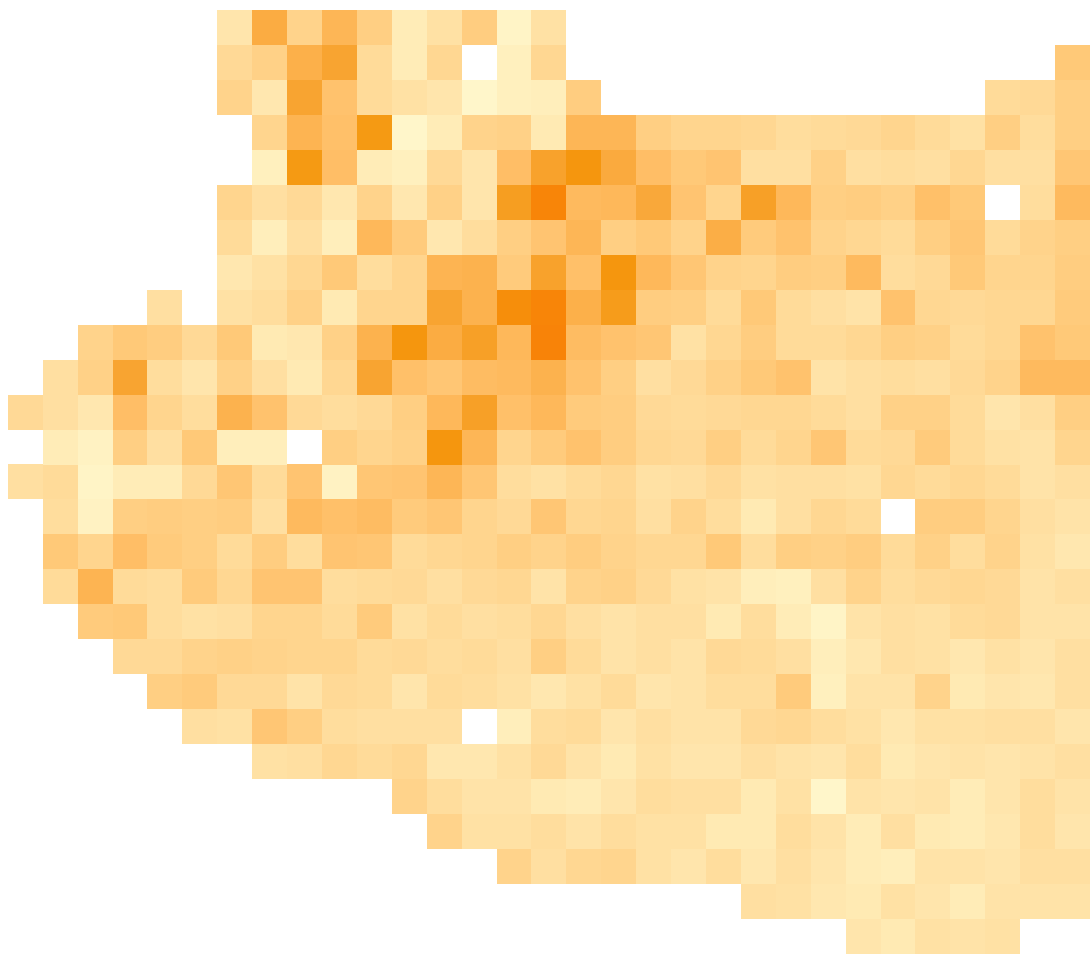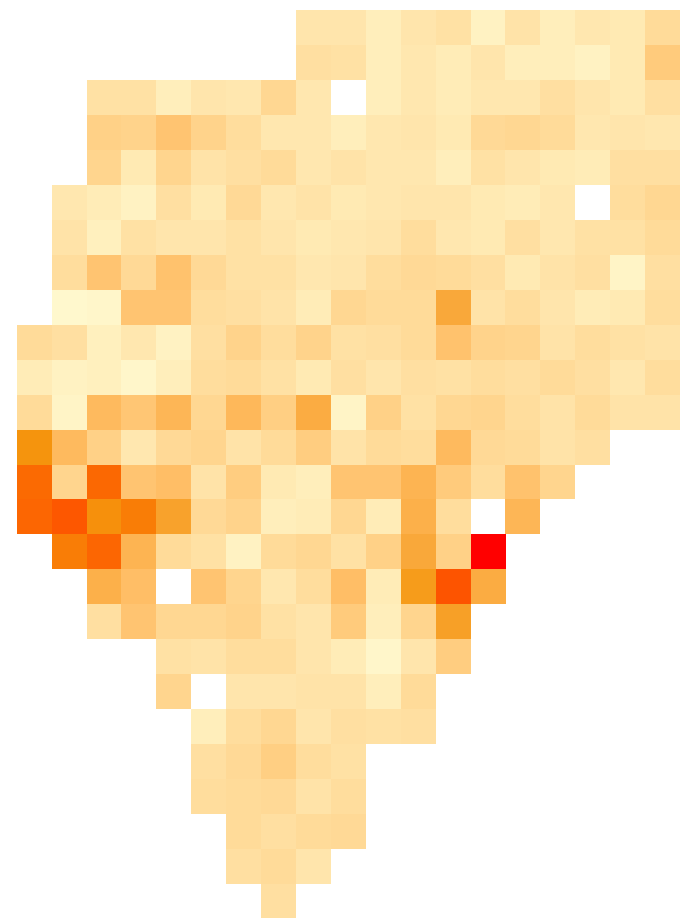

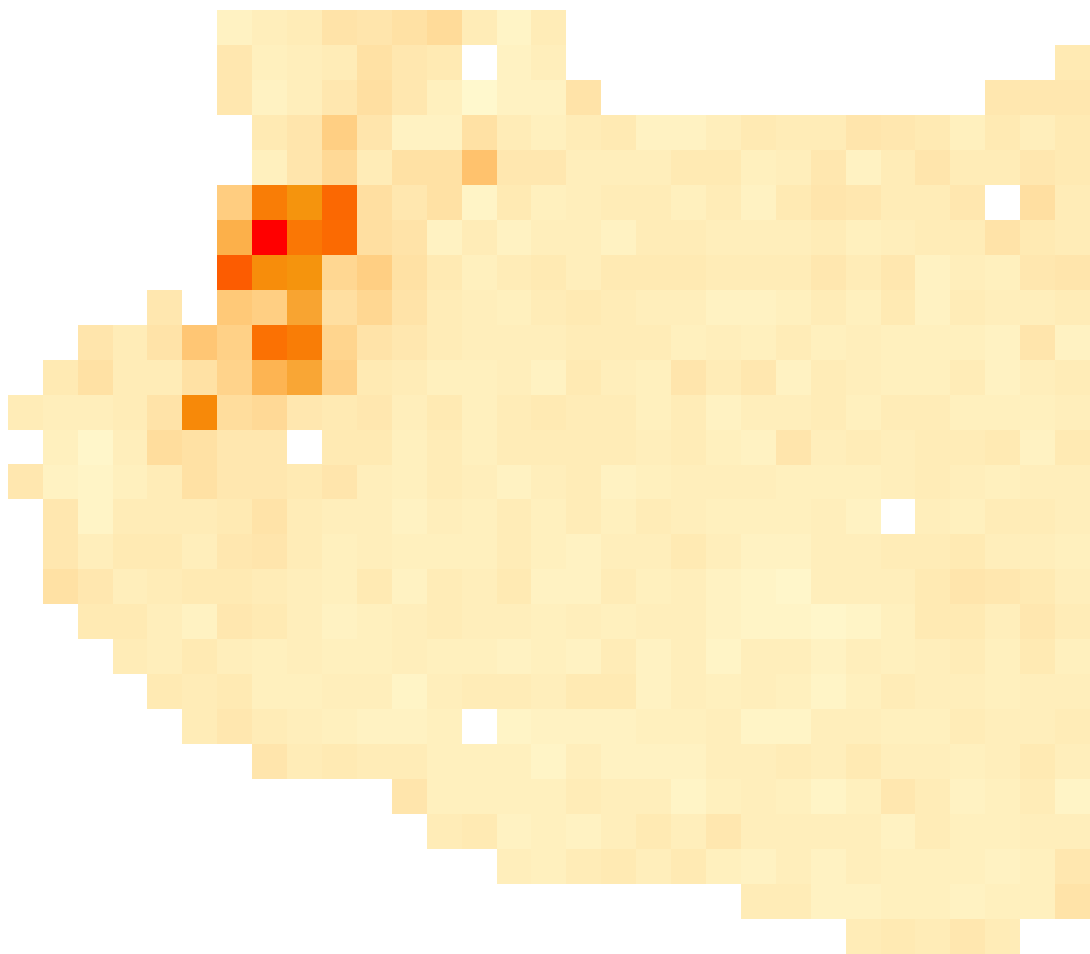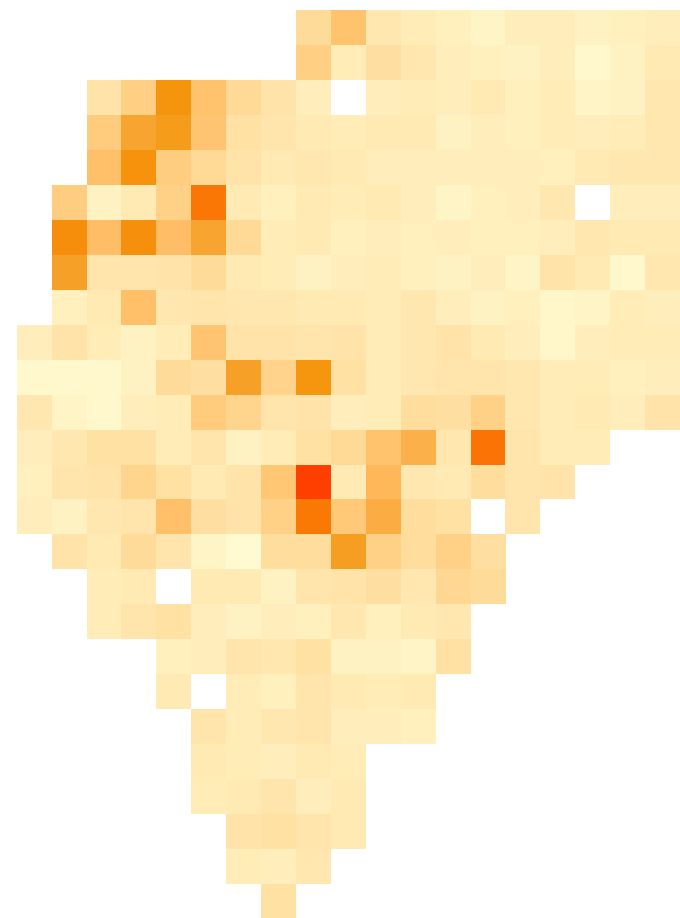

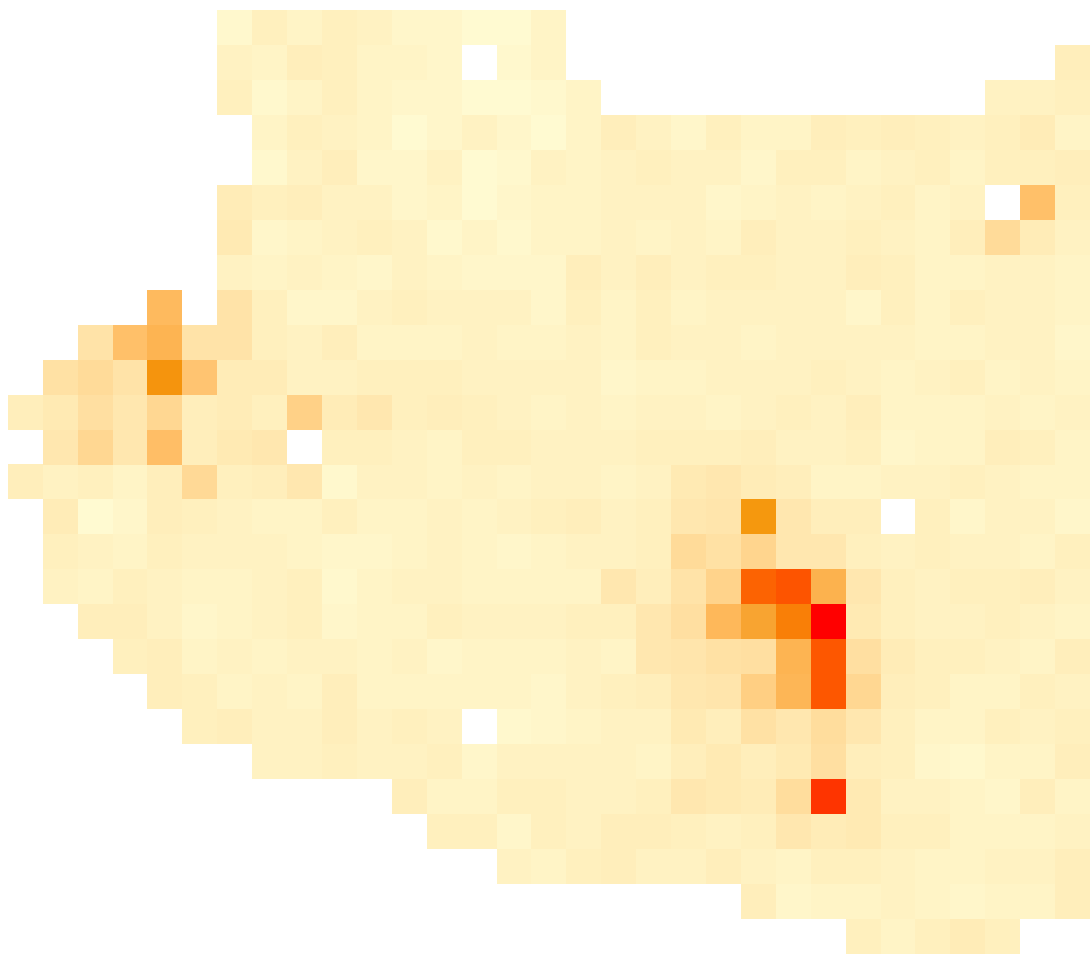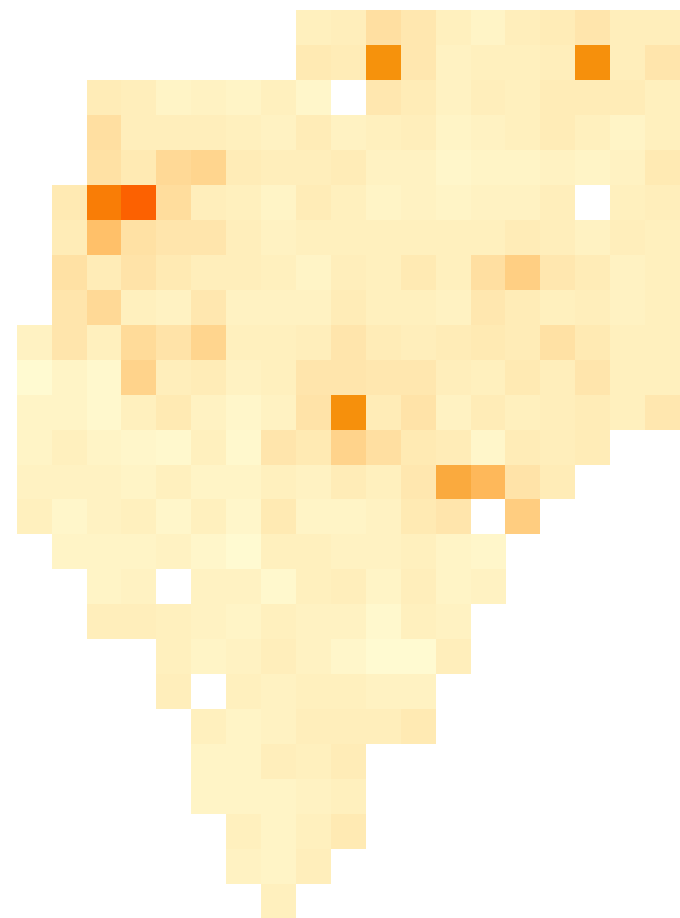

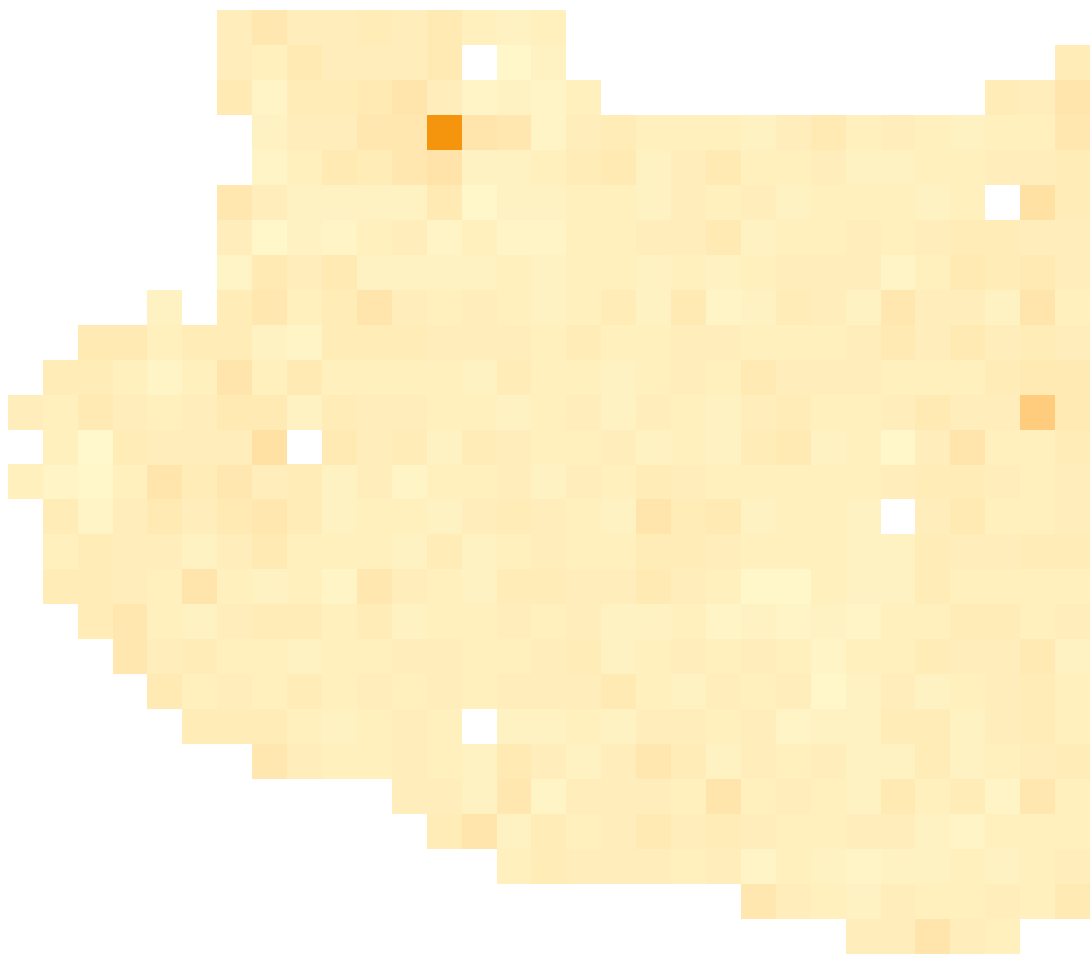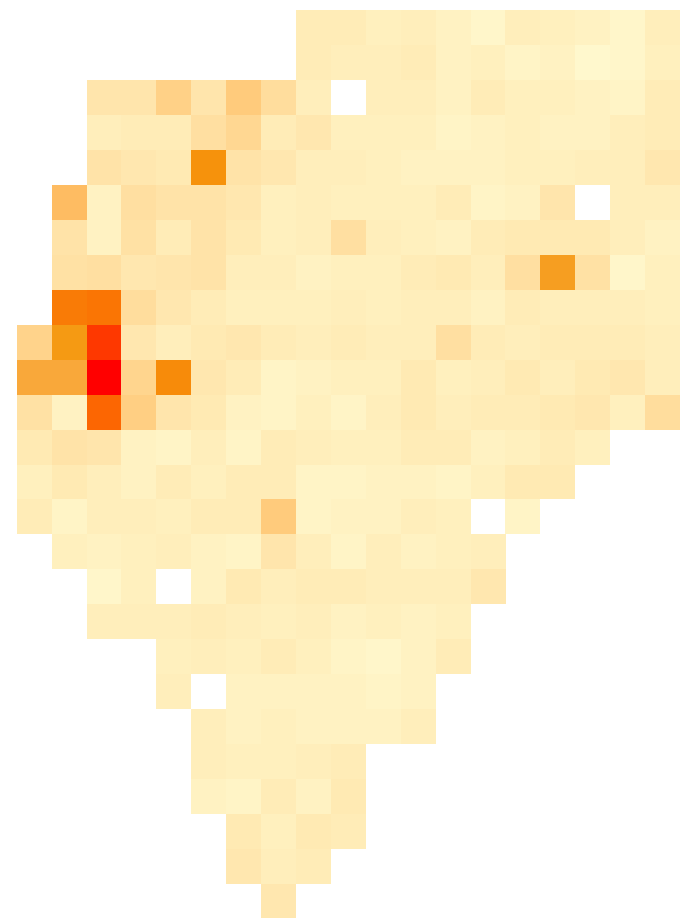

Supplement: Supplementary file 10 — Supplementary Data 7 [file 41467_2018_4724_MOESM10_ESM.zip › Supplementary Dataset 4/joint-mix-profiles-rel-common-scale-matrix.pdf]

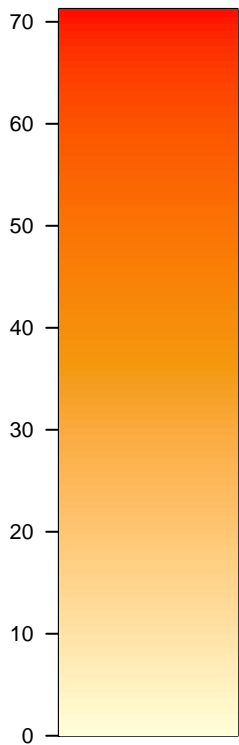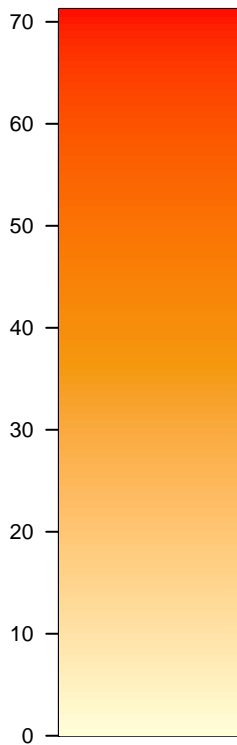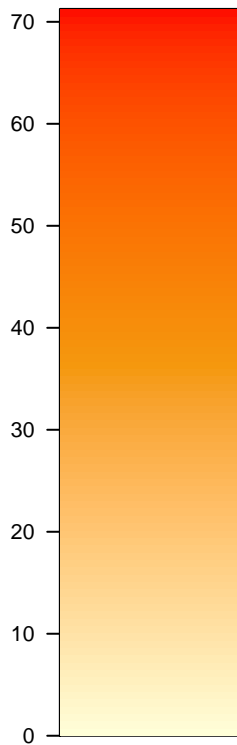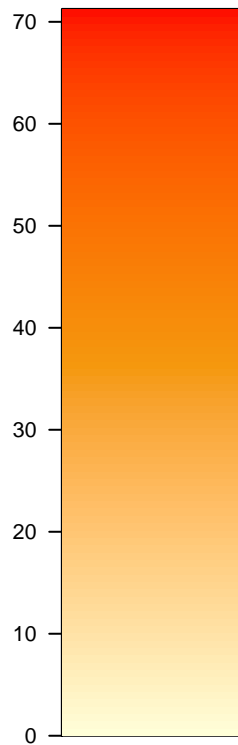

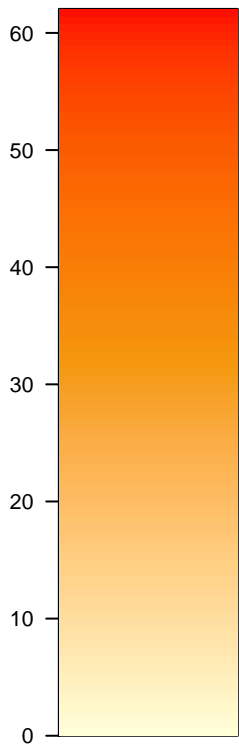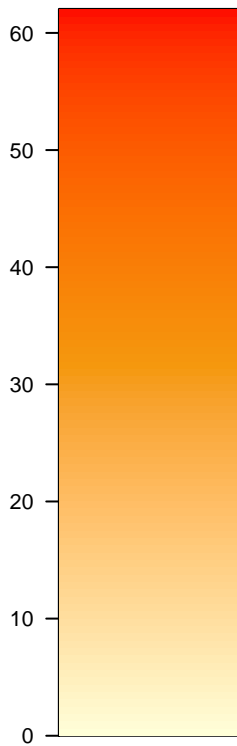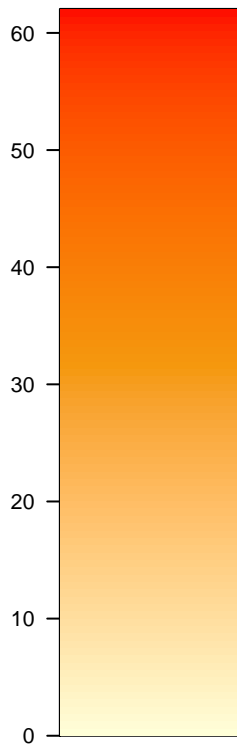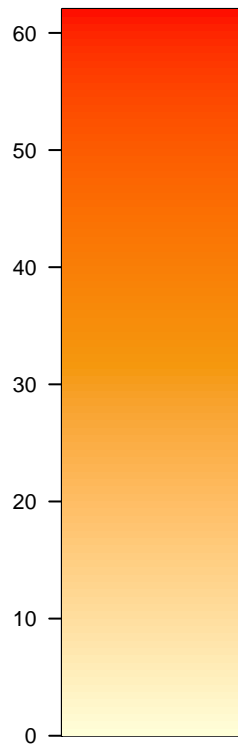

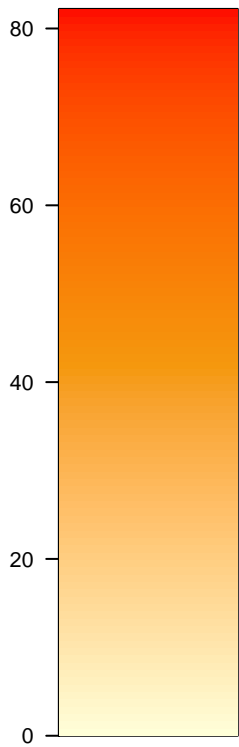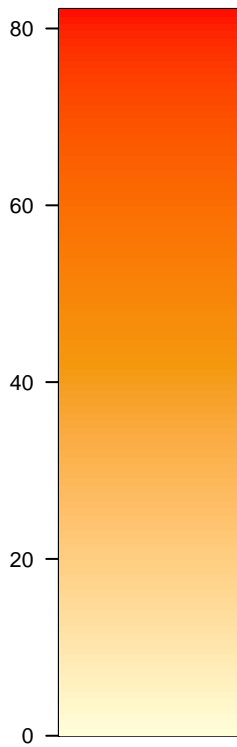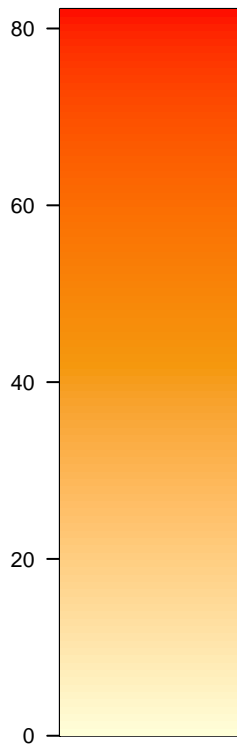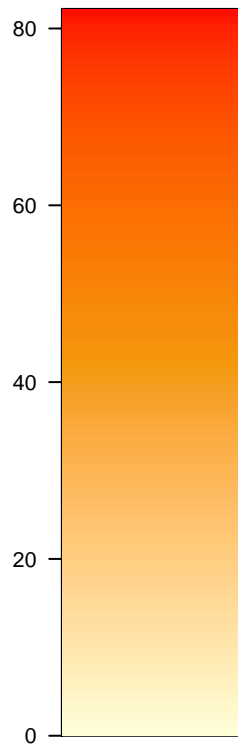

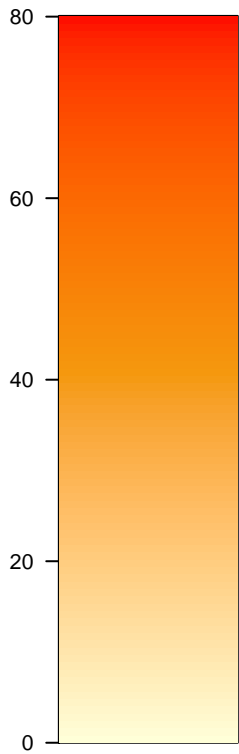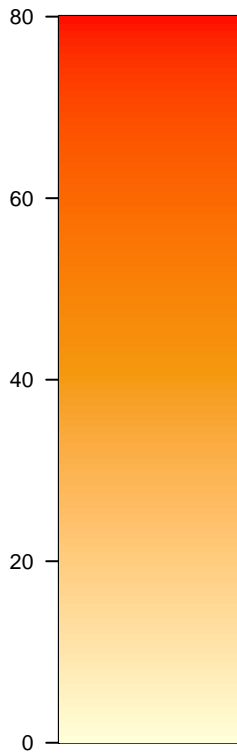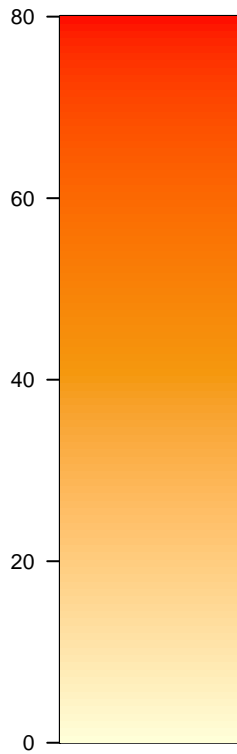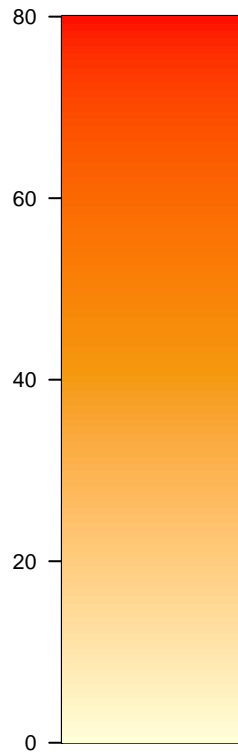

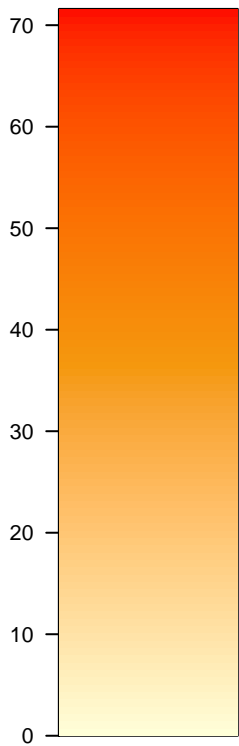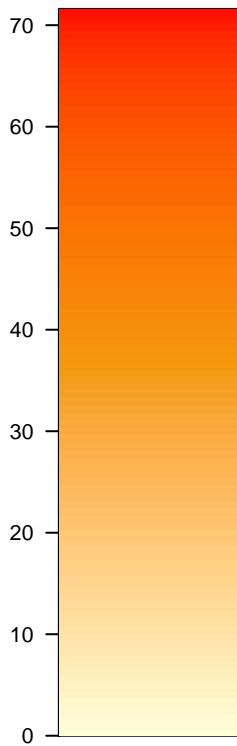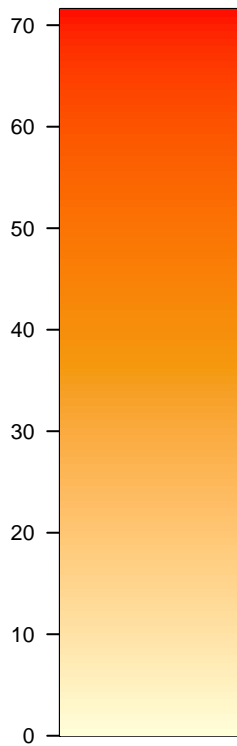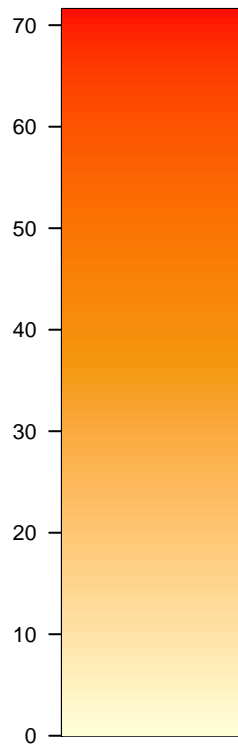

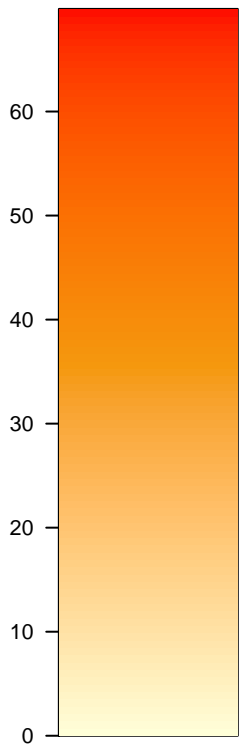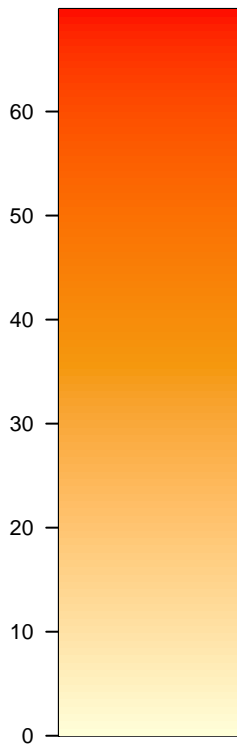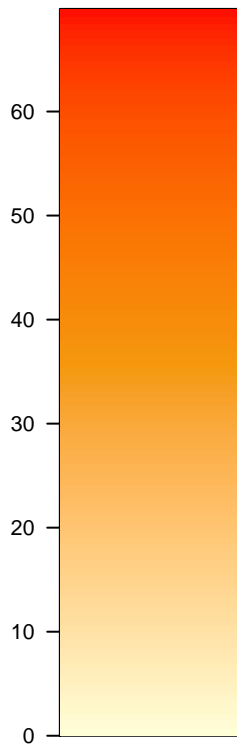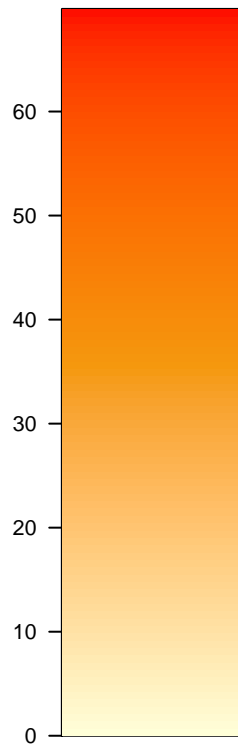

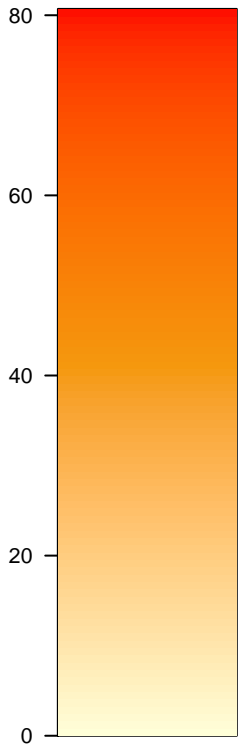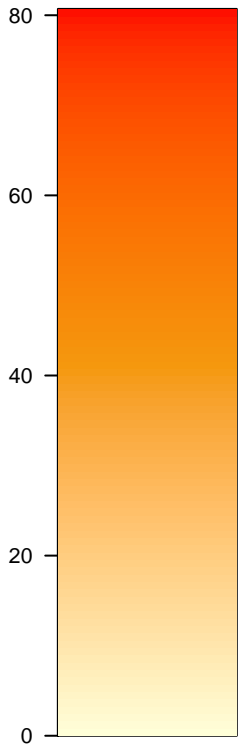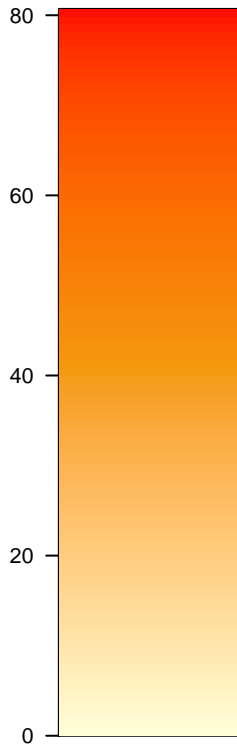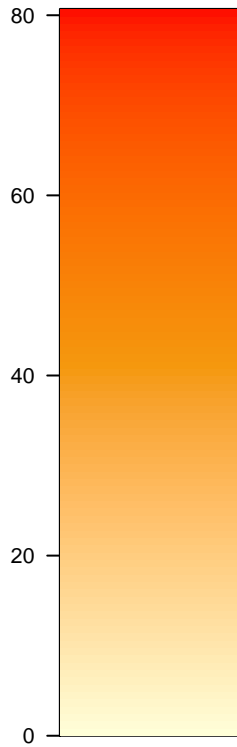

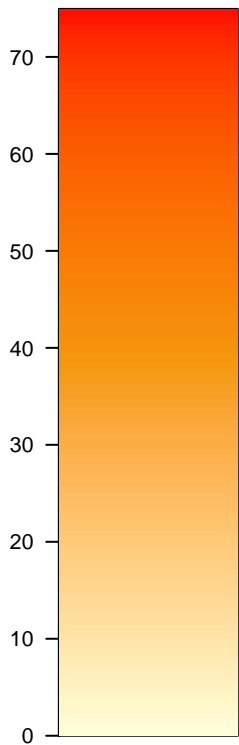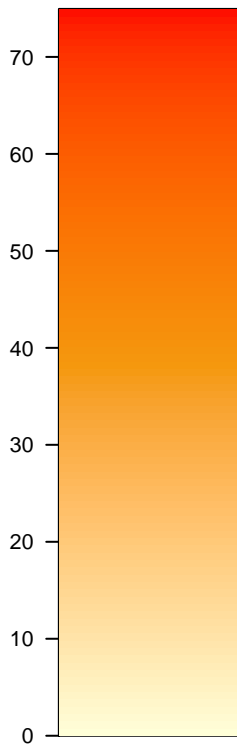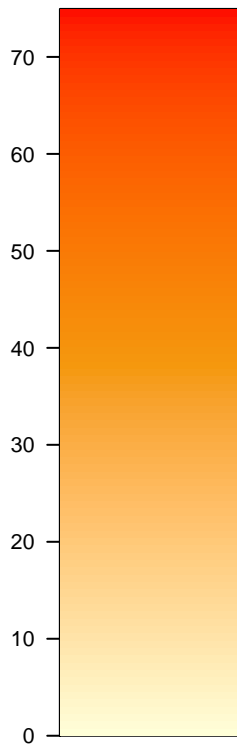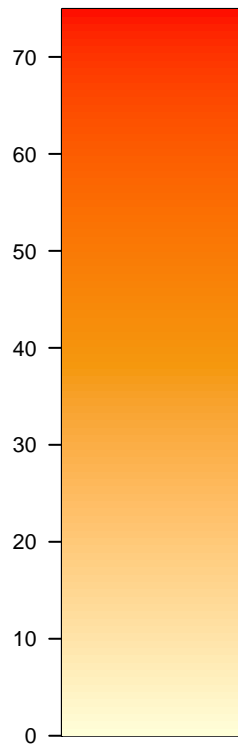

Supplement: Supplementary file 13 — Supplementary Data 10 [file 41467_2018_4724_MOESM13_ESM.zip › Supplementary Dataset 5/joint-mix-profiles-rel-common-scale-matrix-colorbar.pdf]

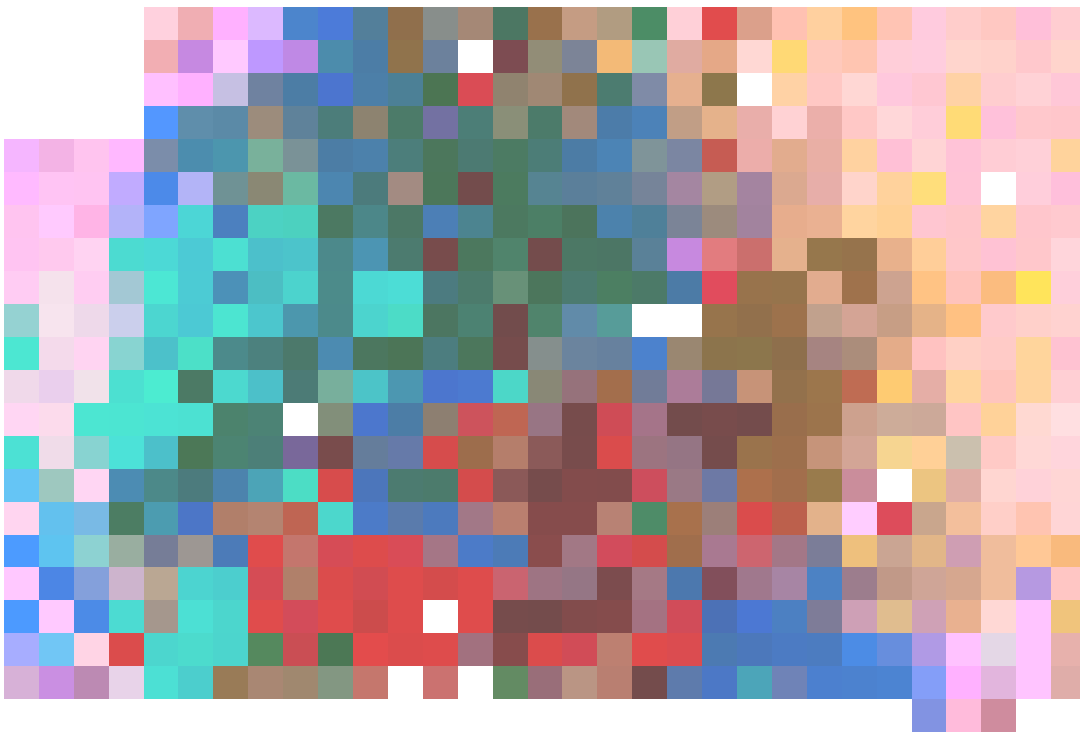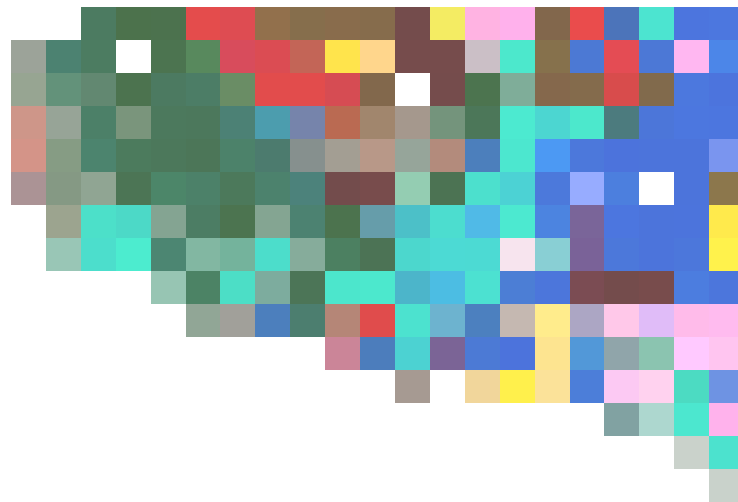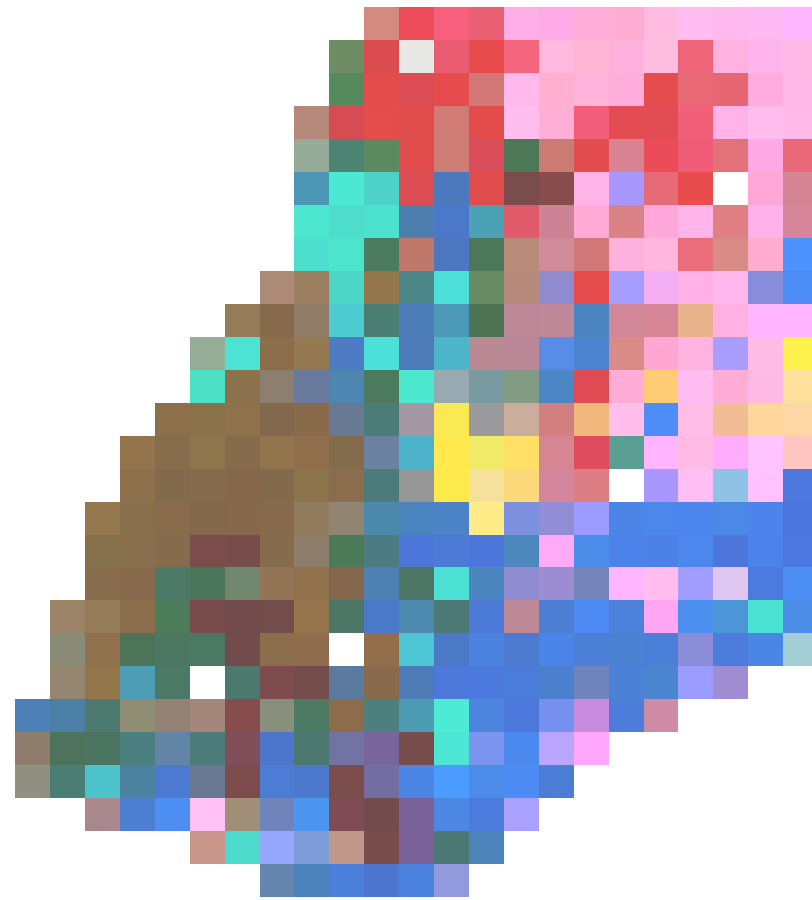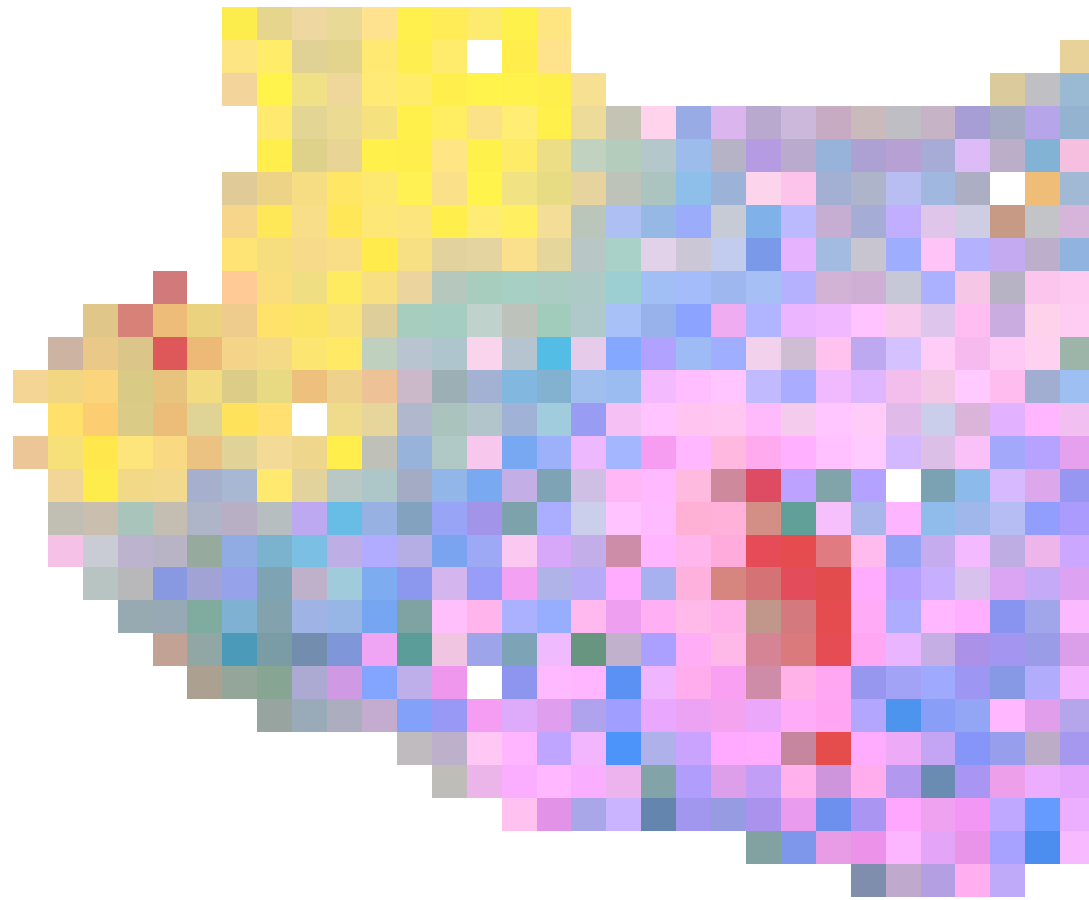

Supplement: Supplementary file 13 — Supplementary Data 10 [file 41467_2018_4724_MOESM13_ESM.zip › Supplementary Dataset 5/joint-mix-dimensionality-reduction-tSNE-matrix.pdf]

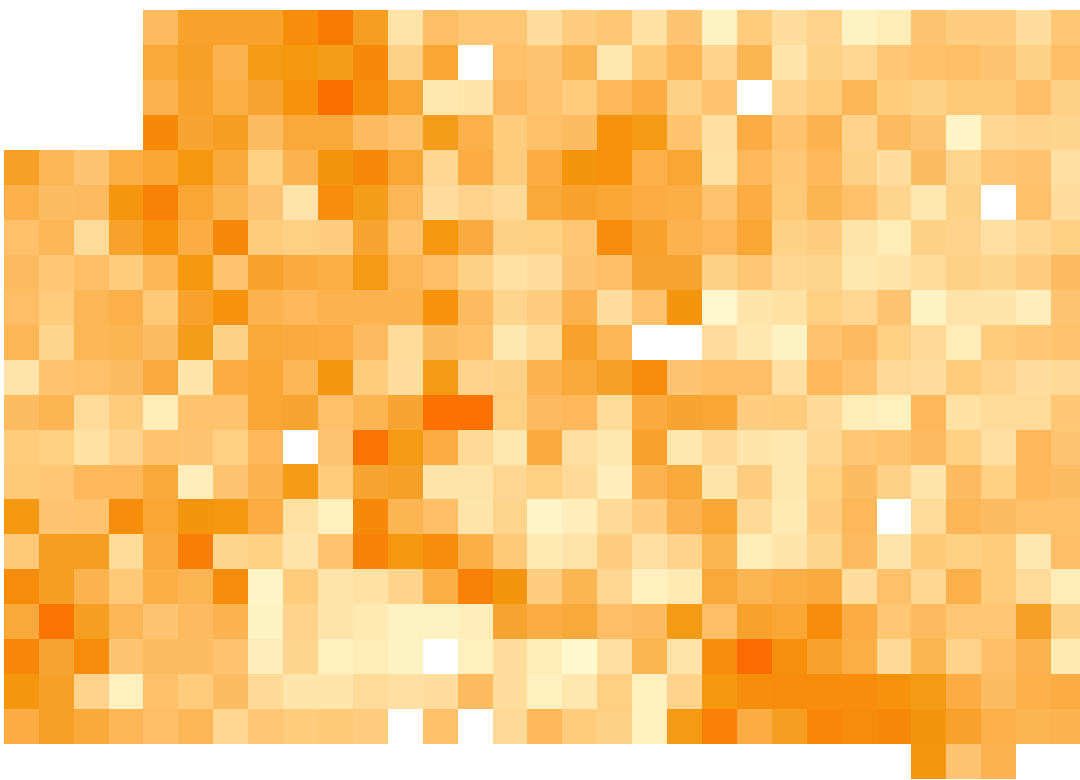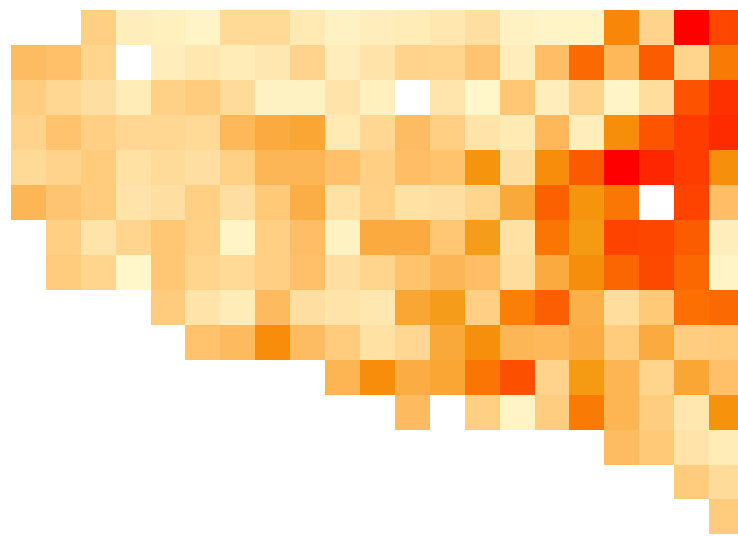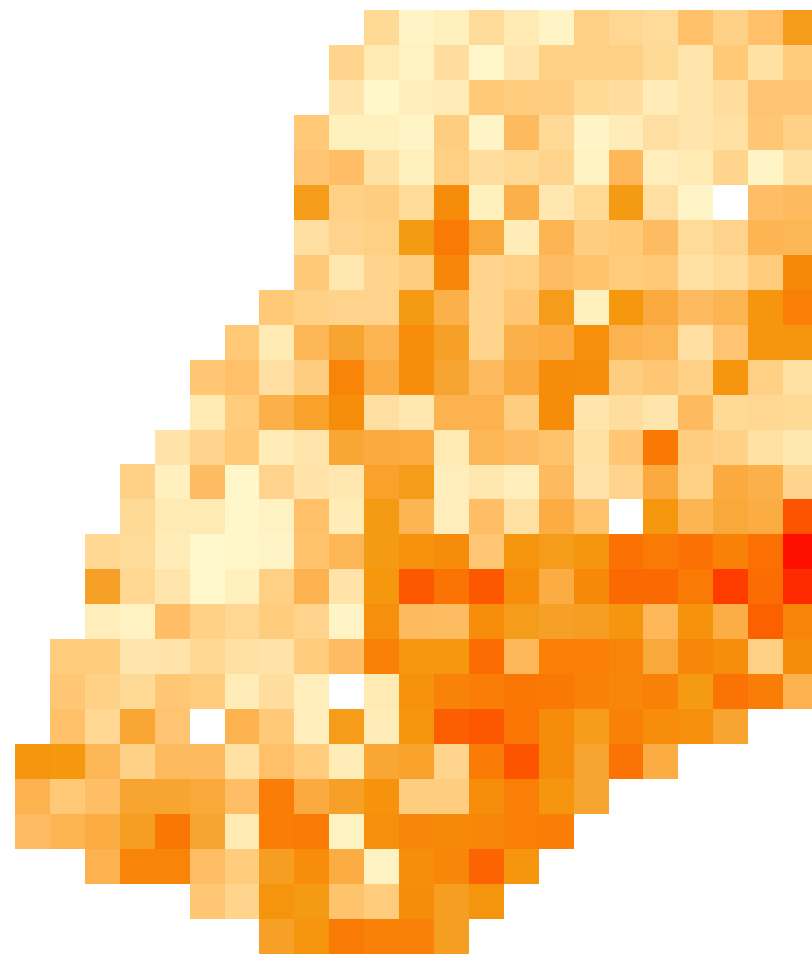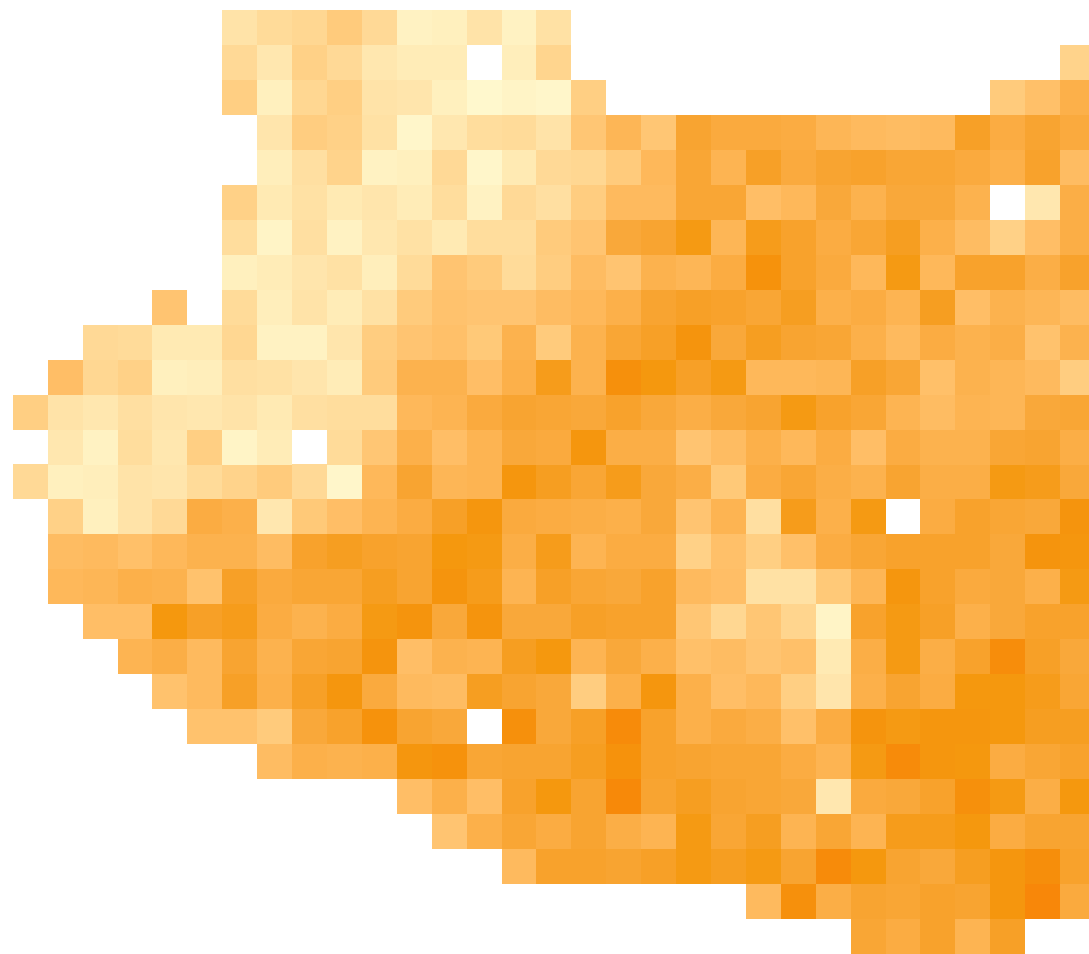

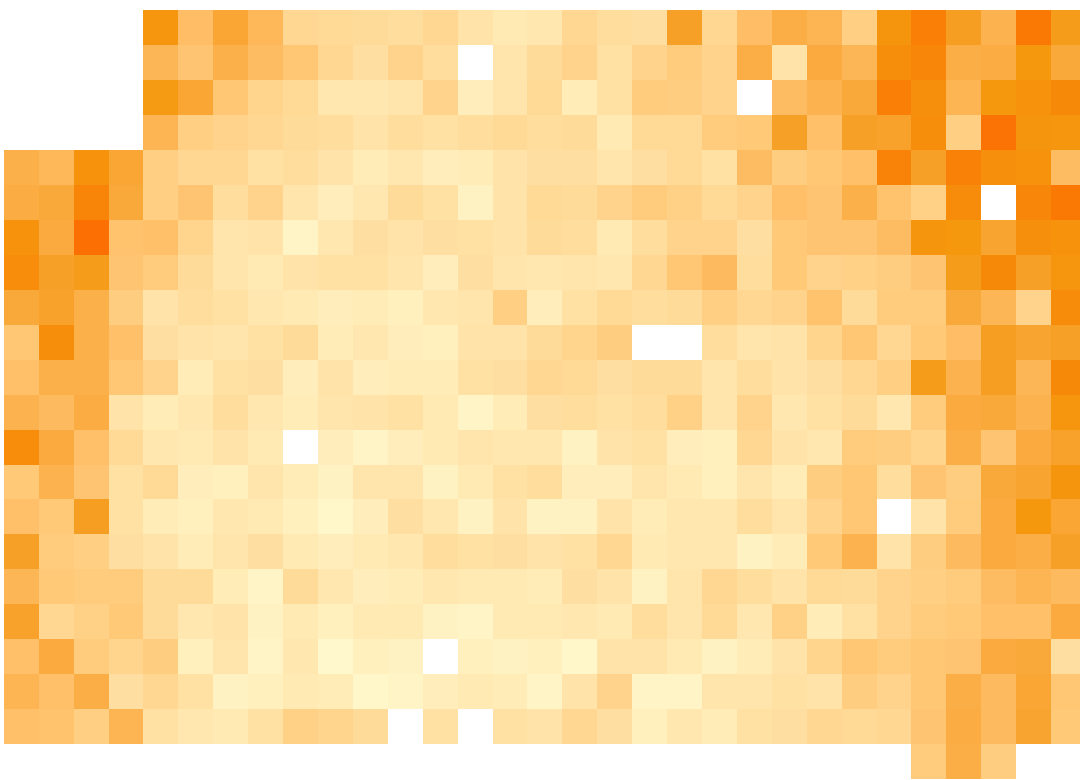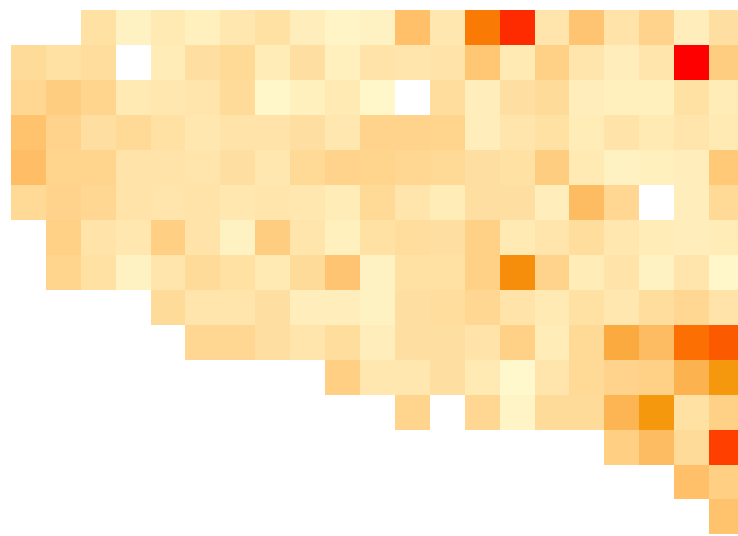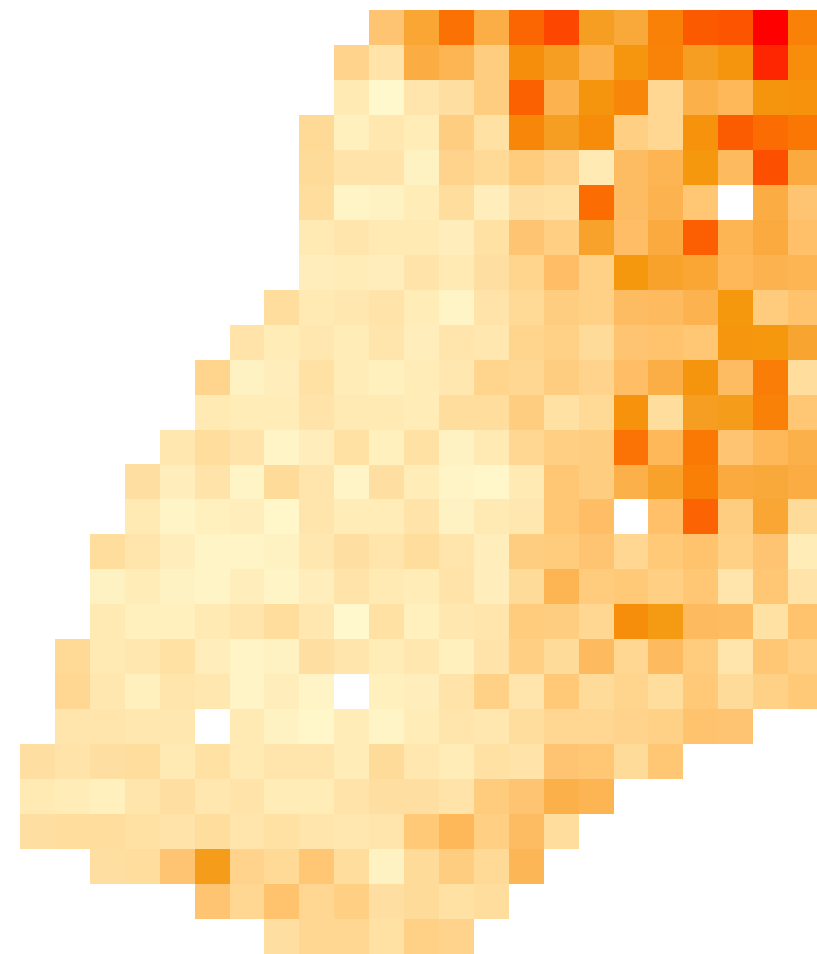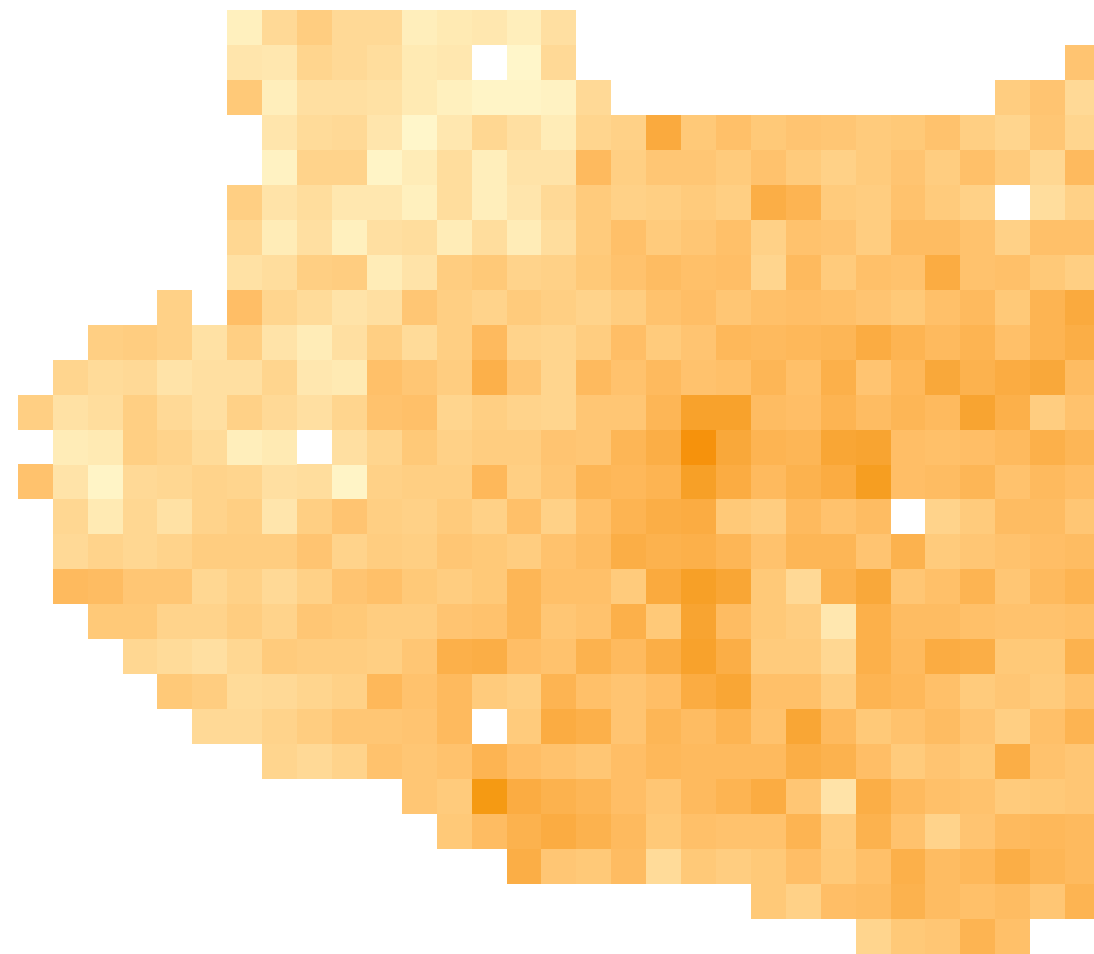

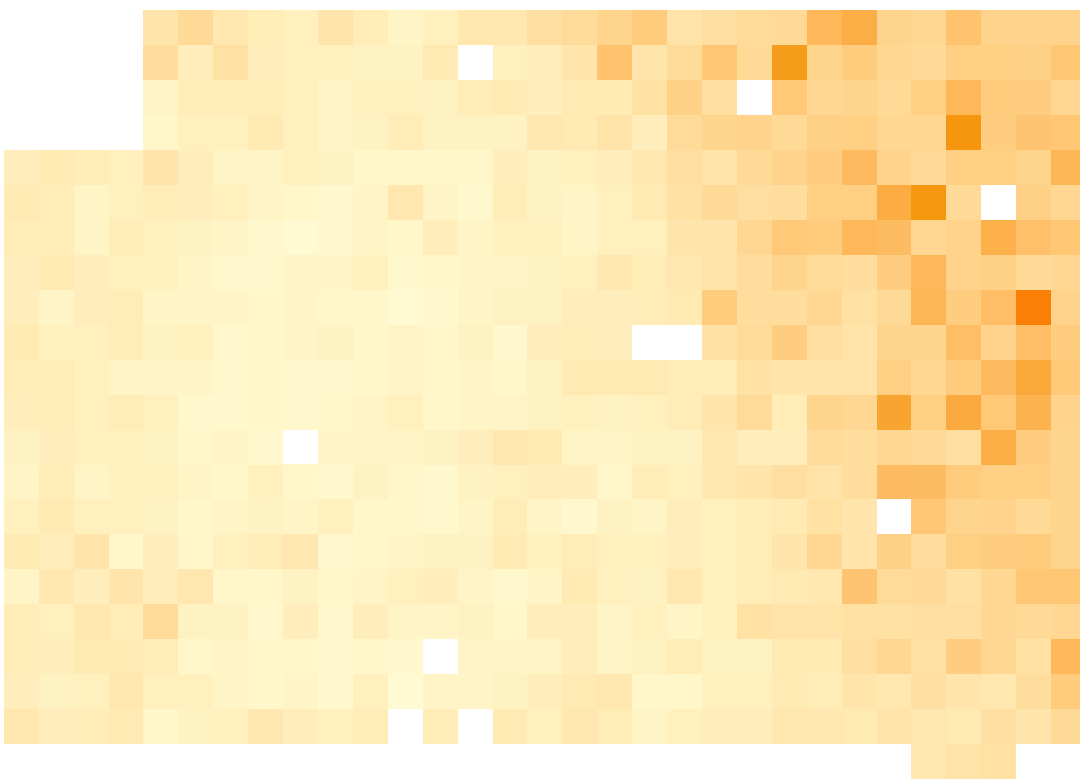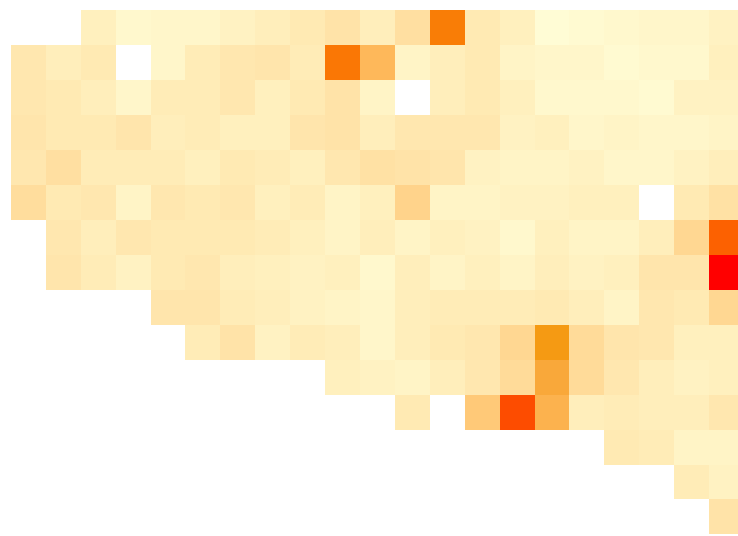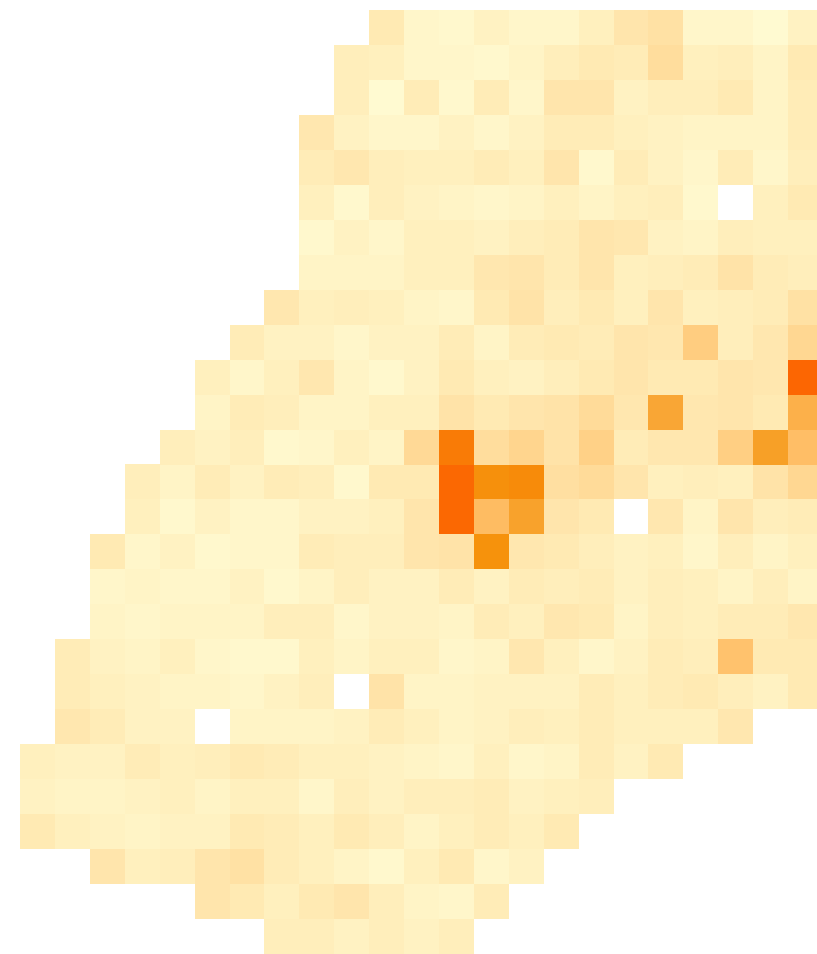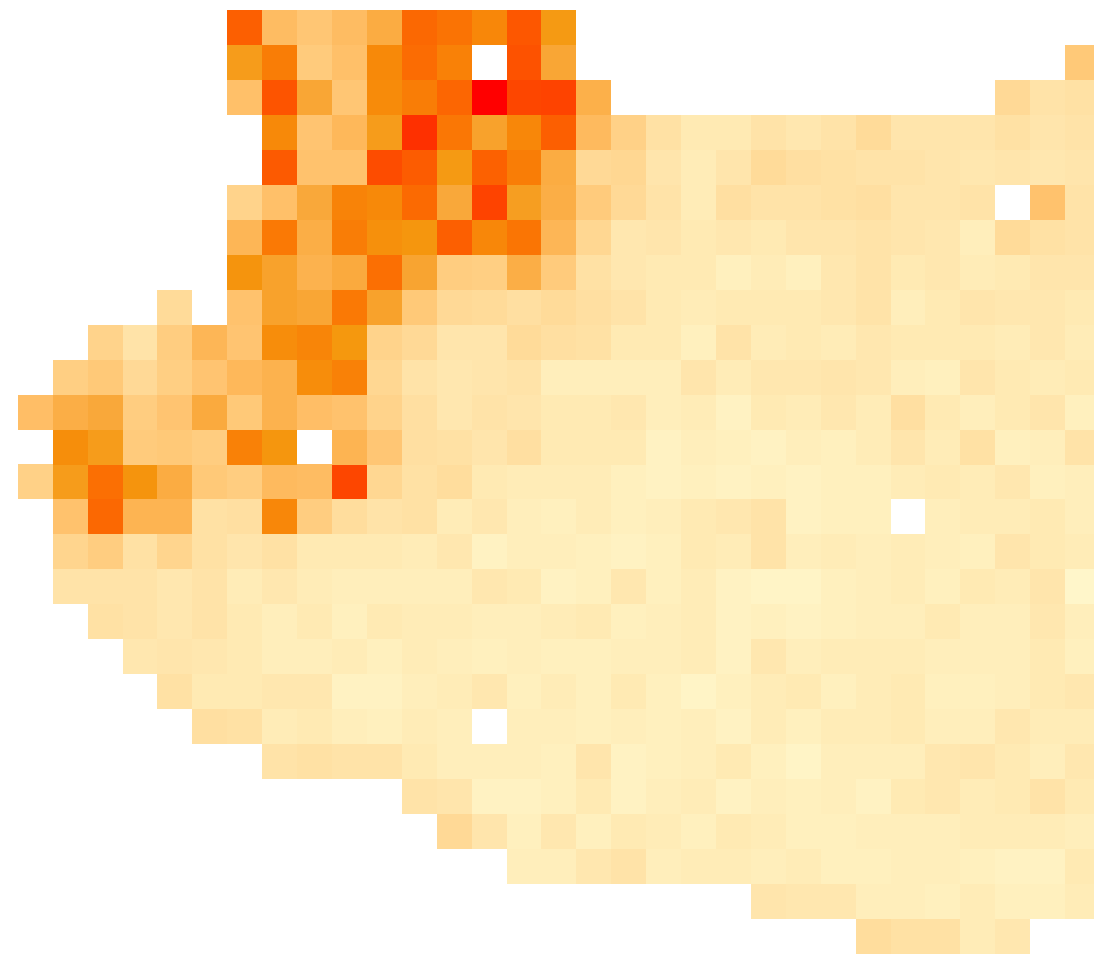

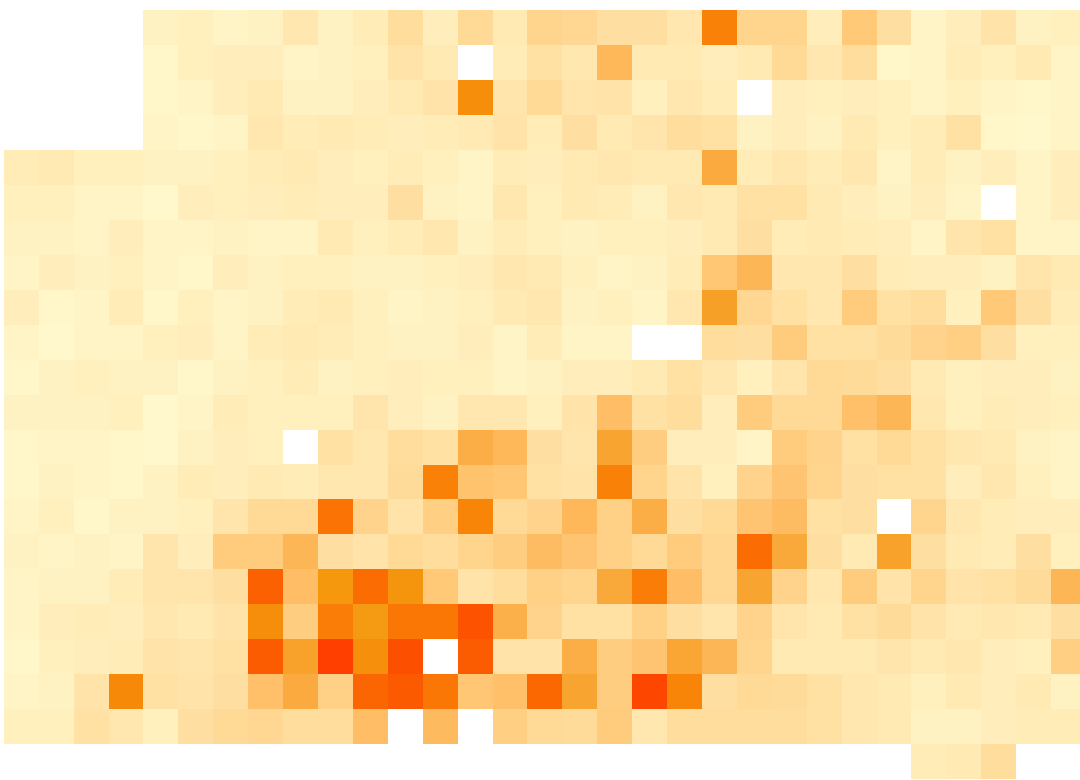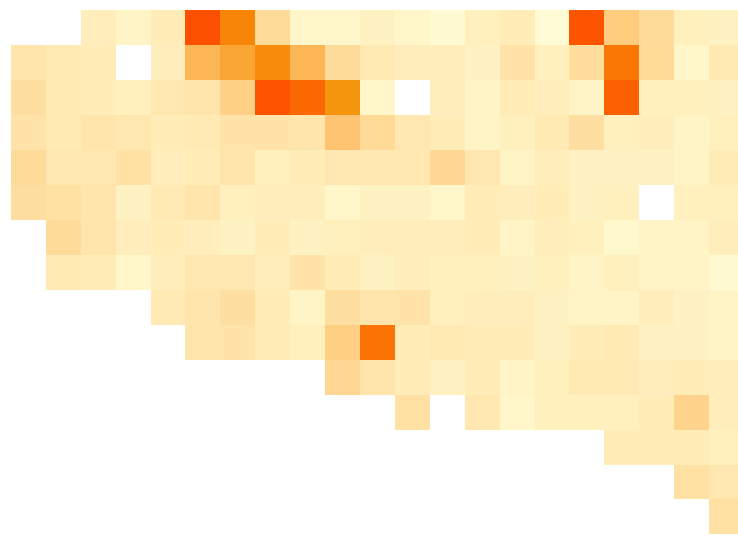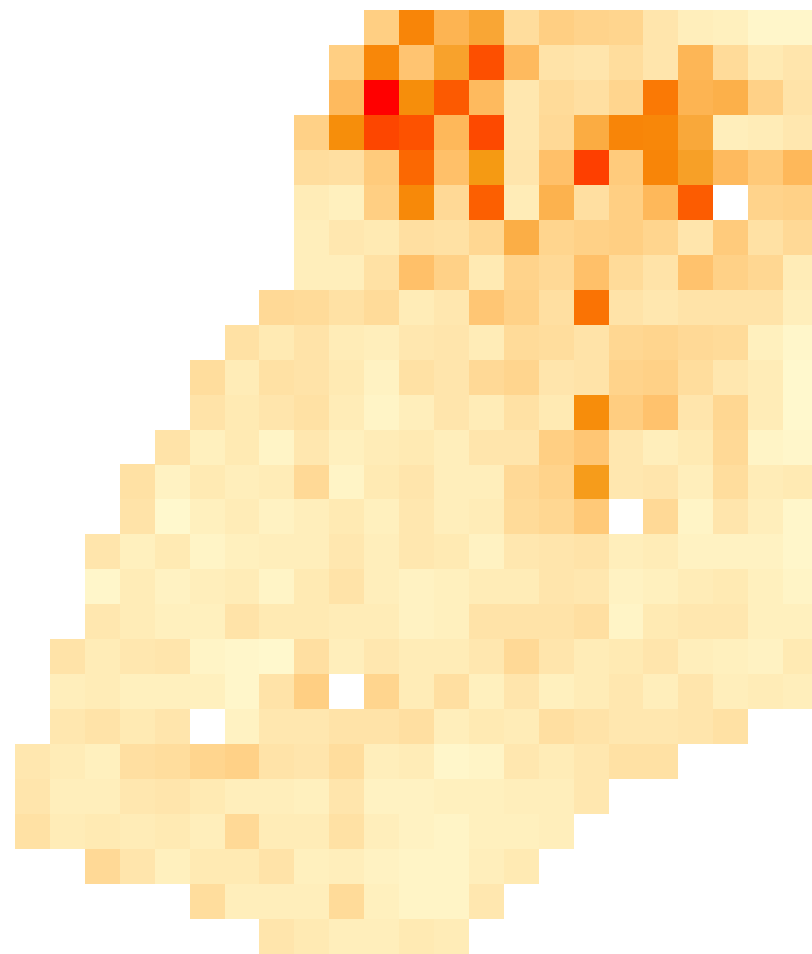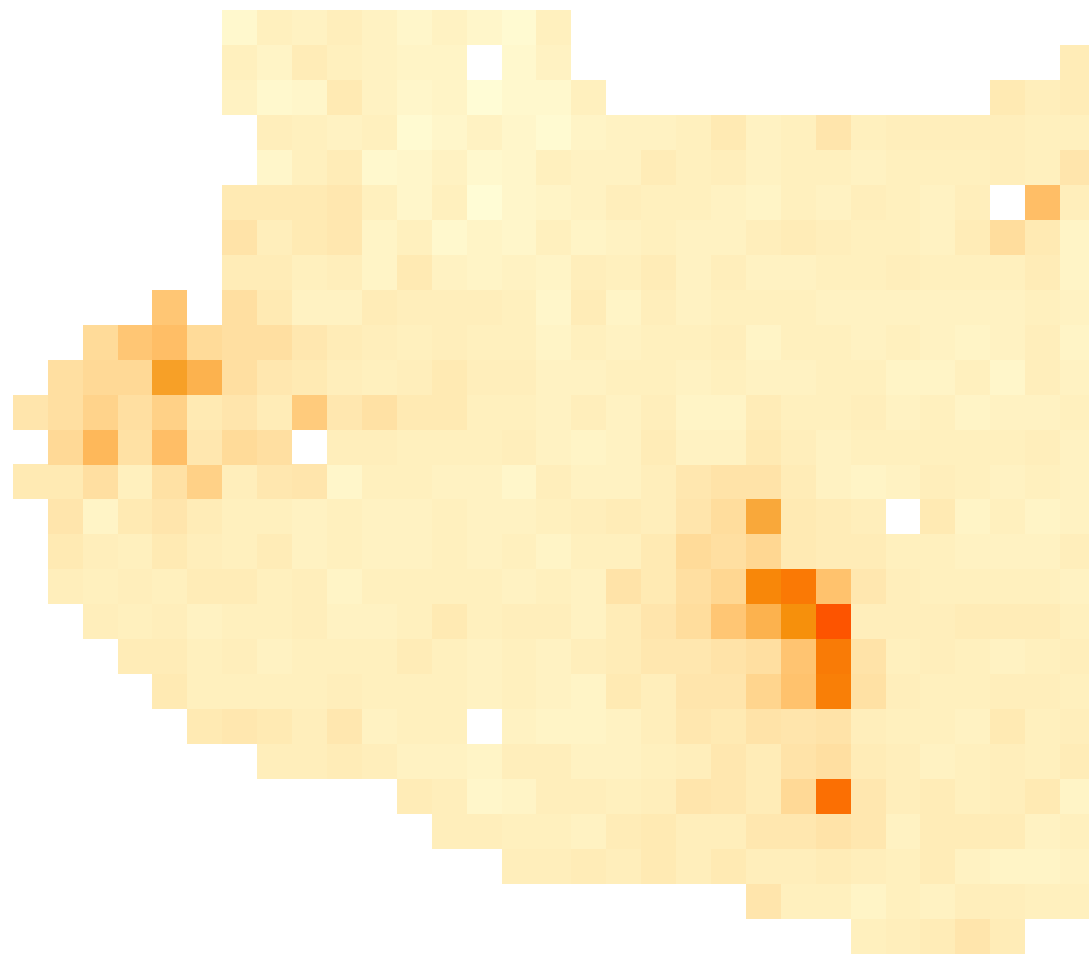

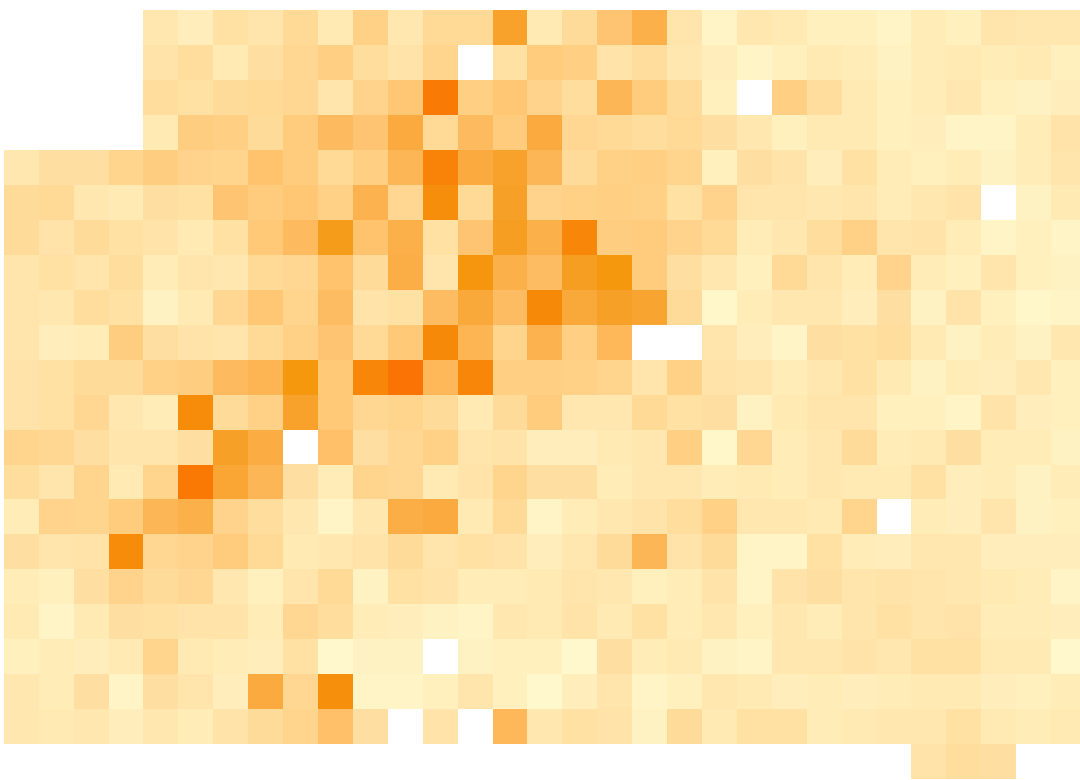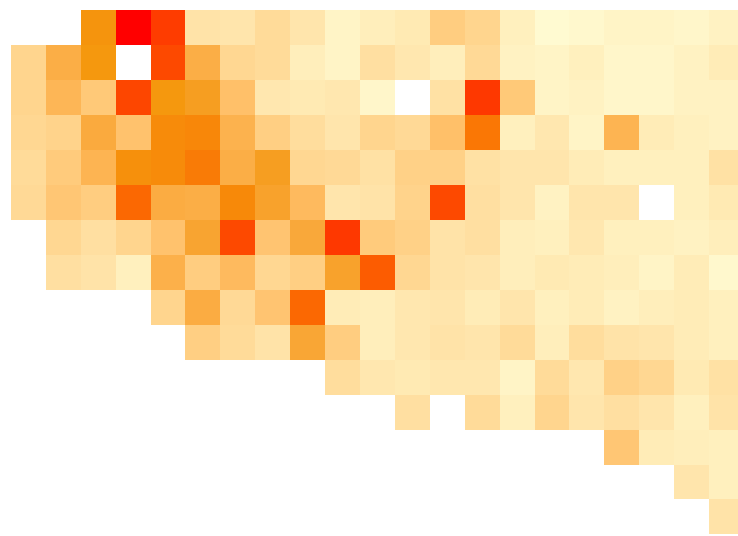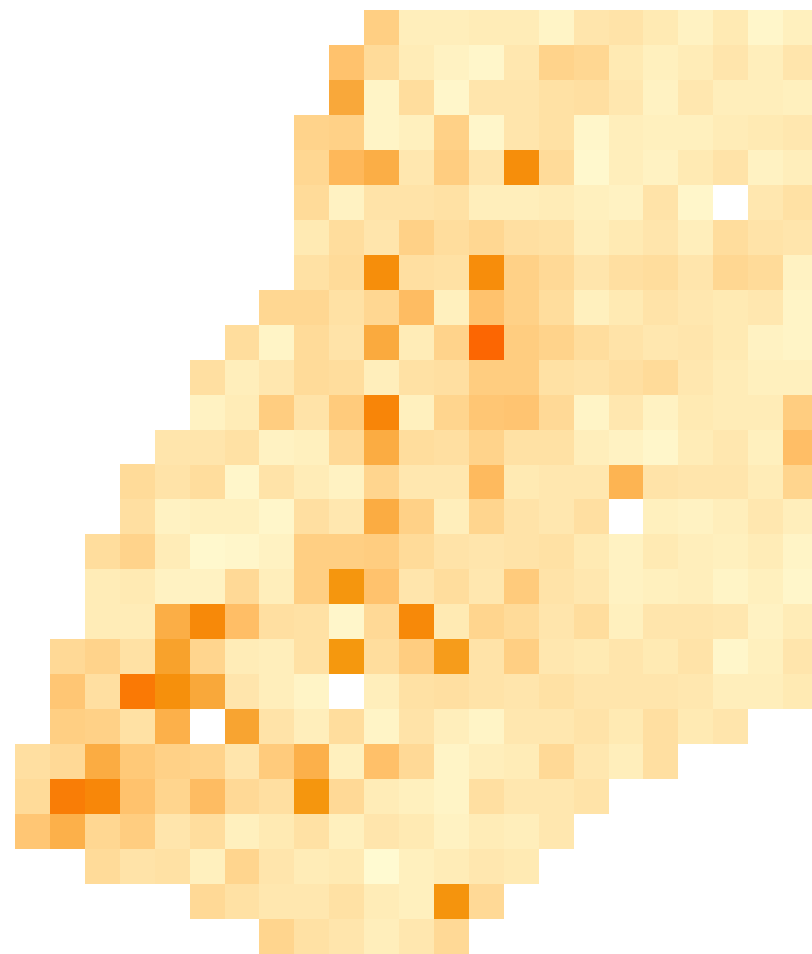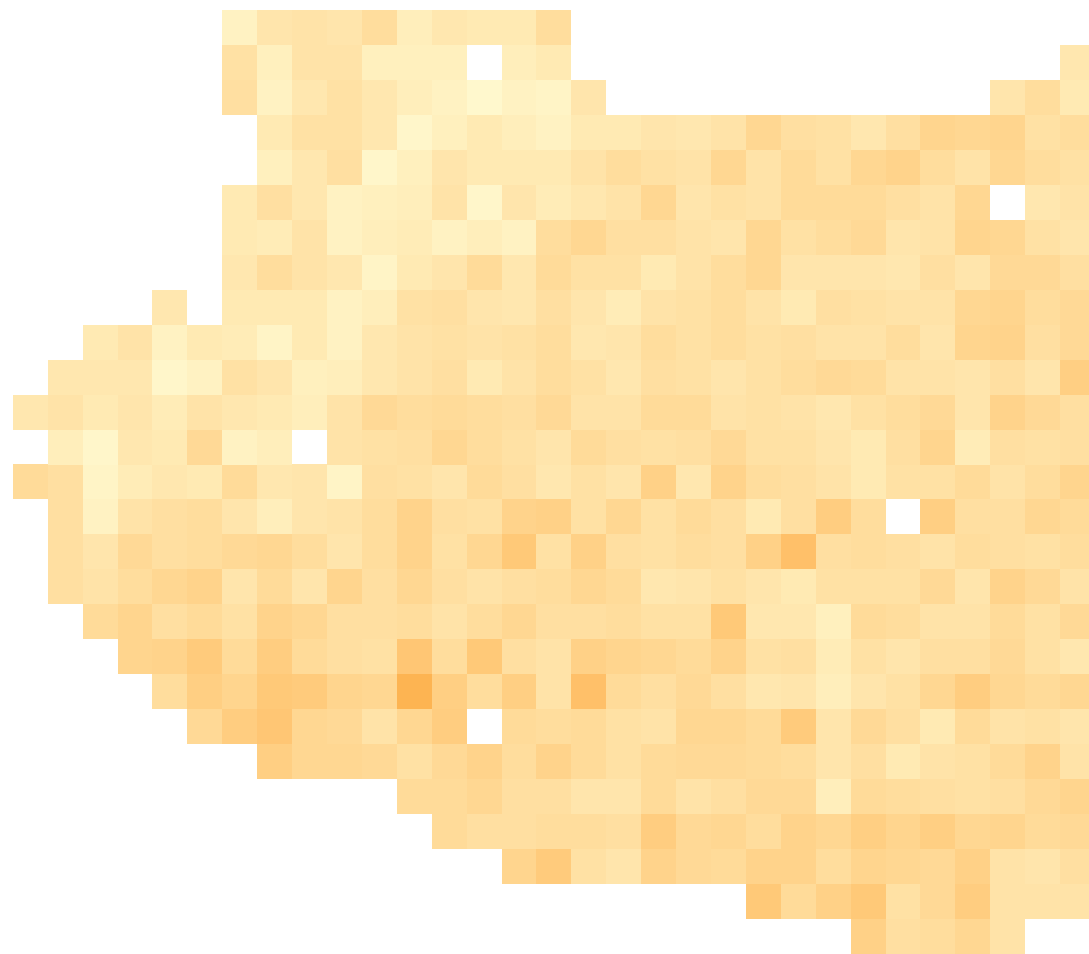

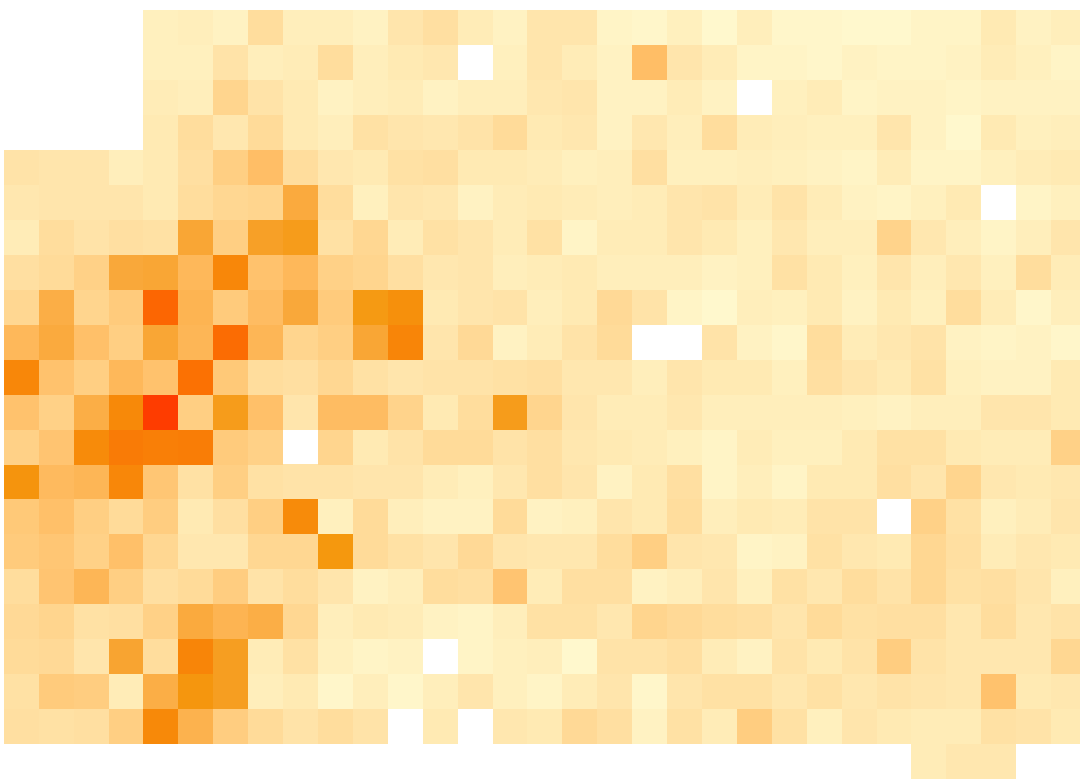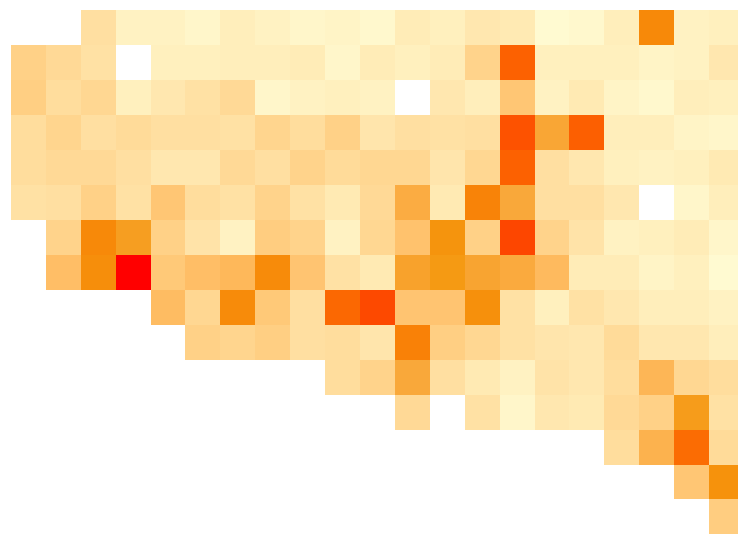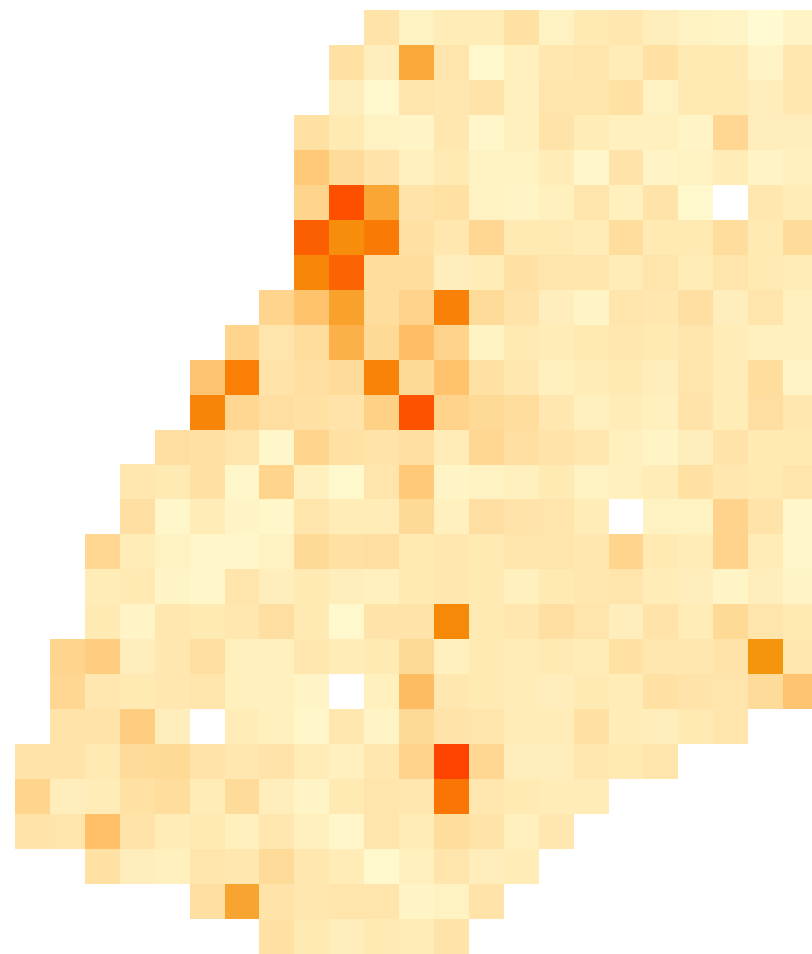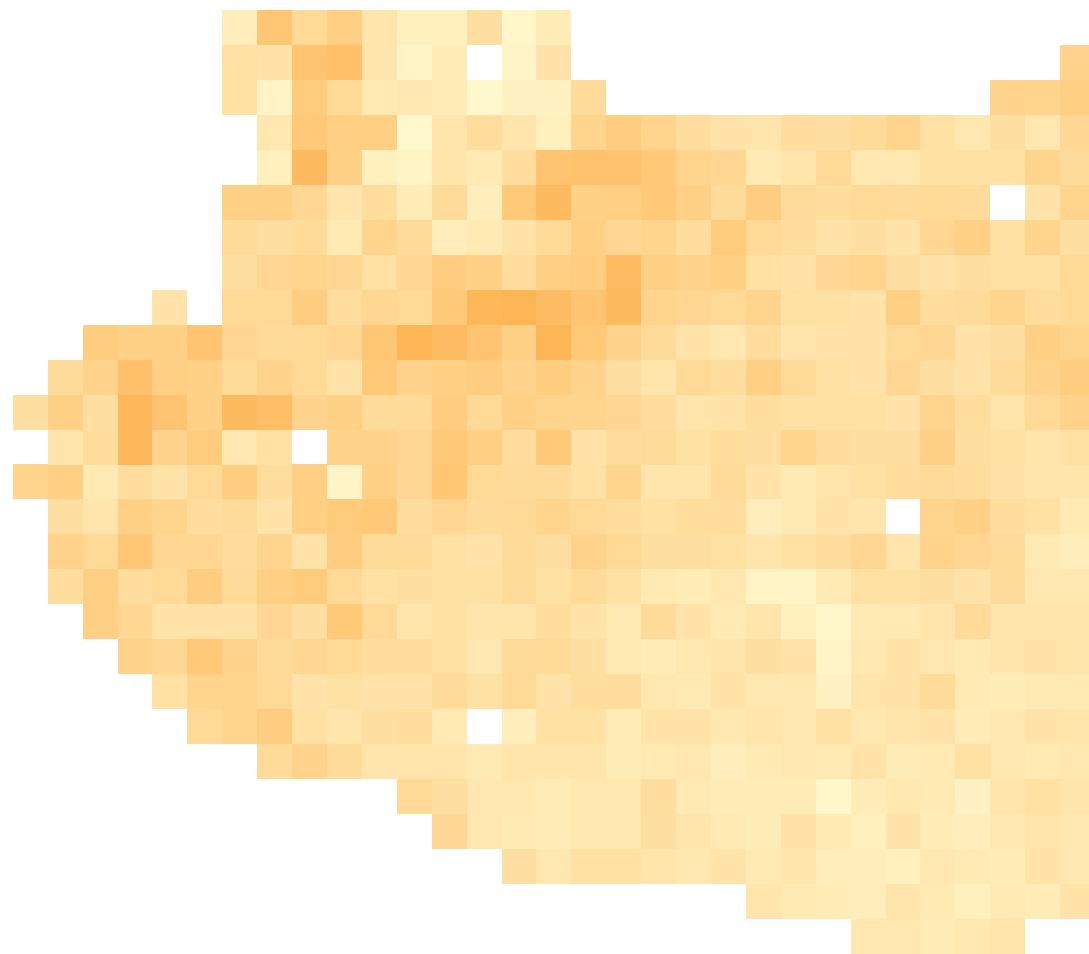

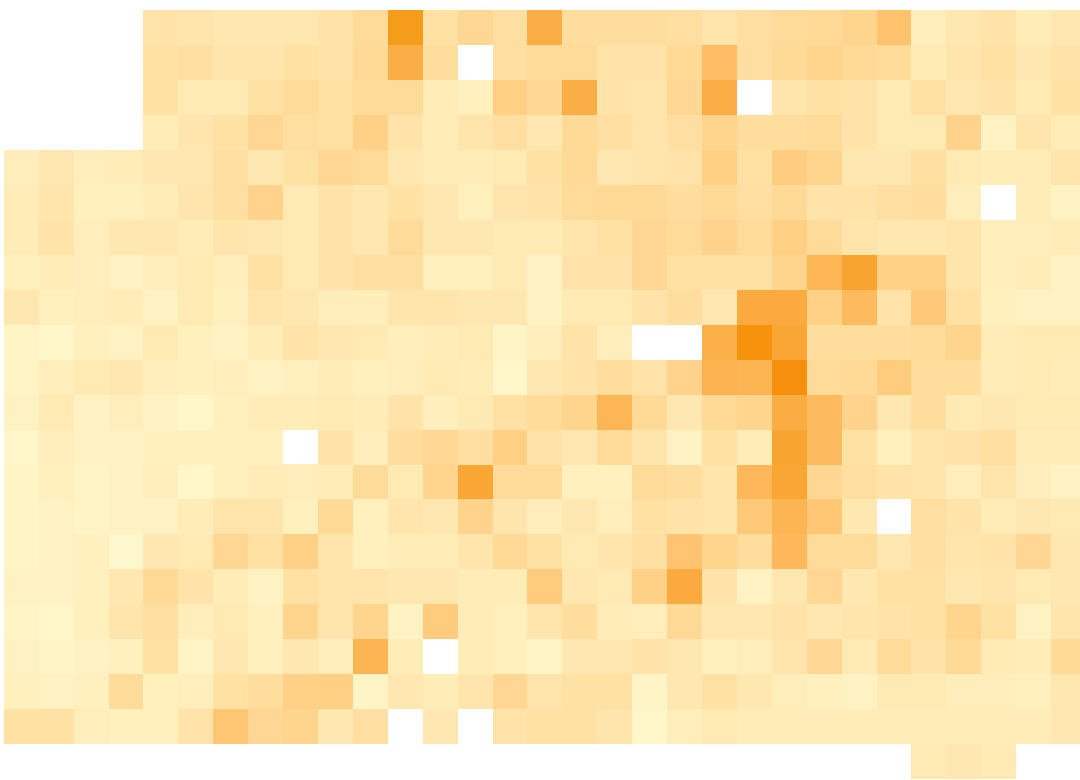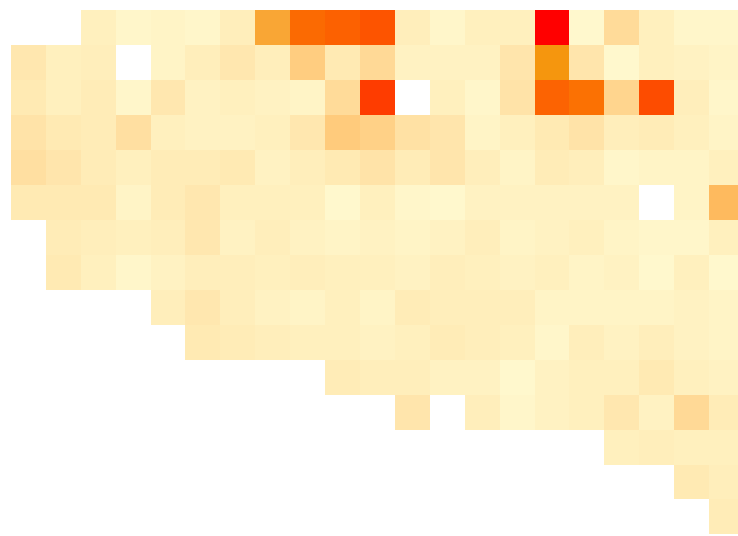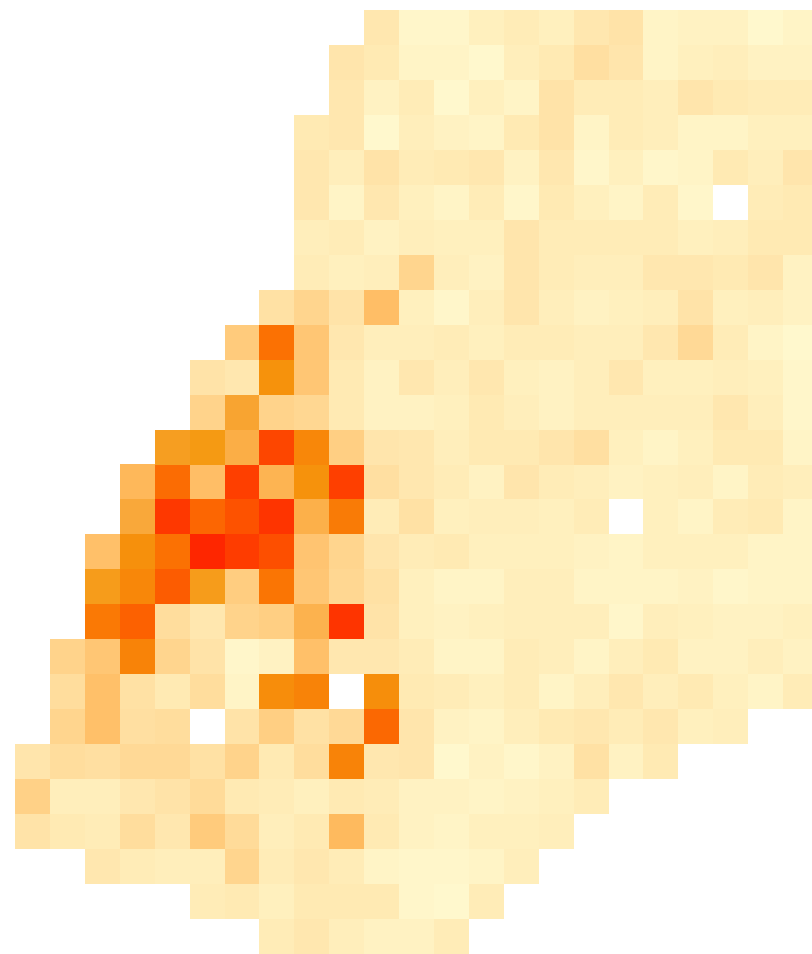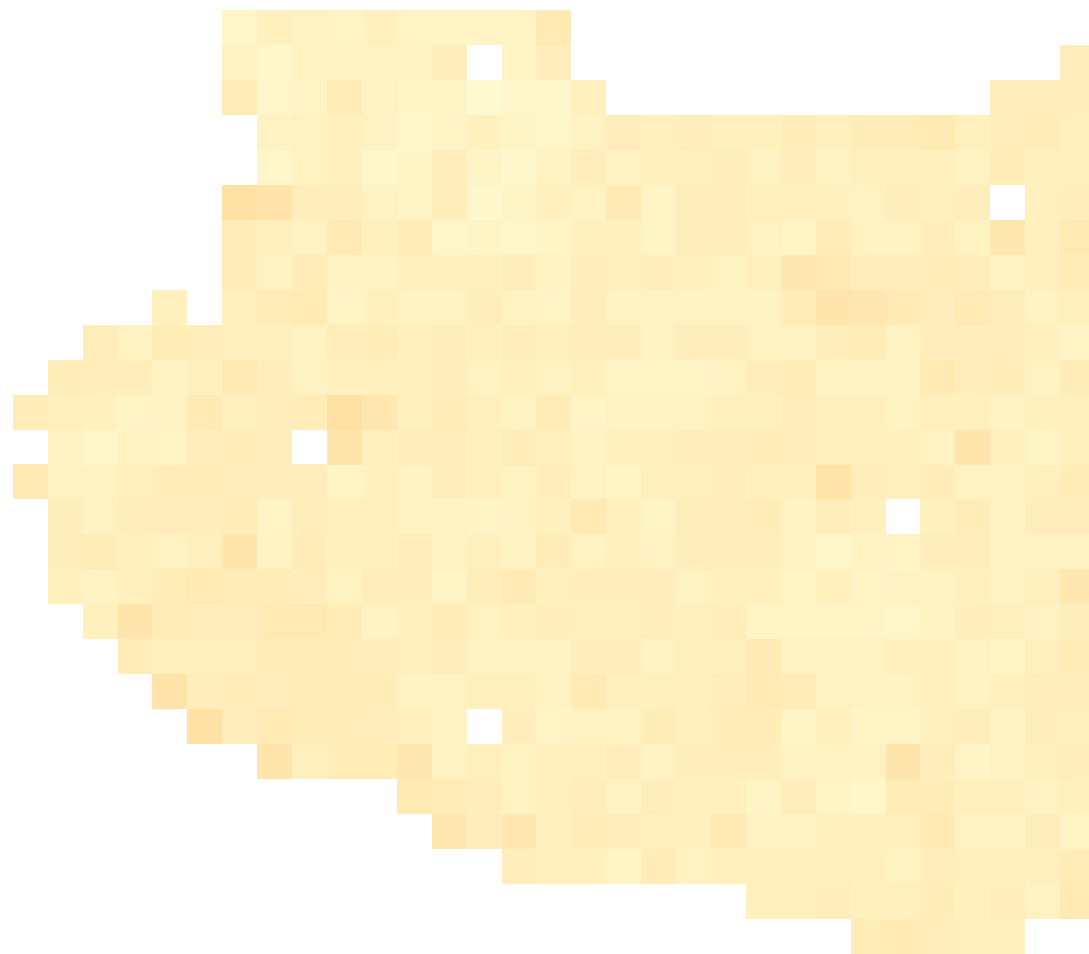

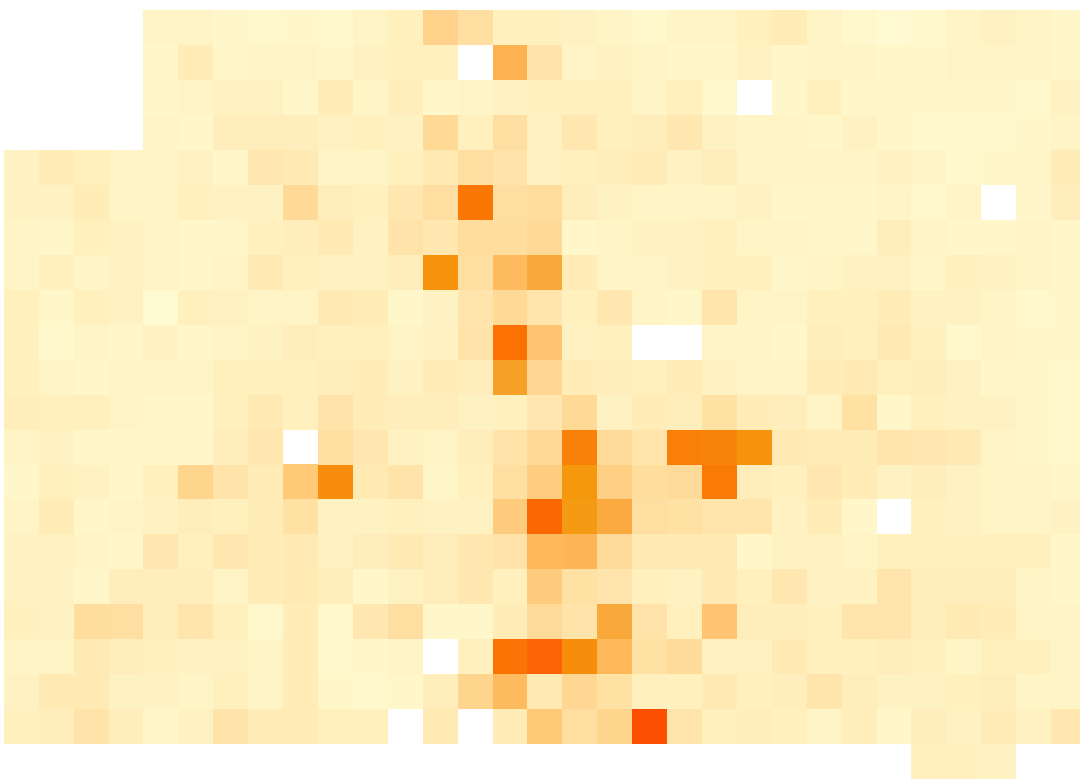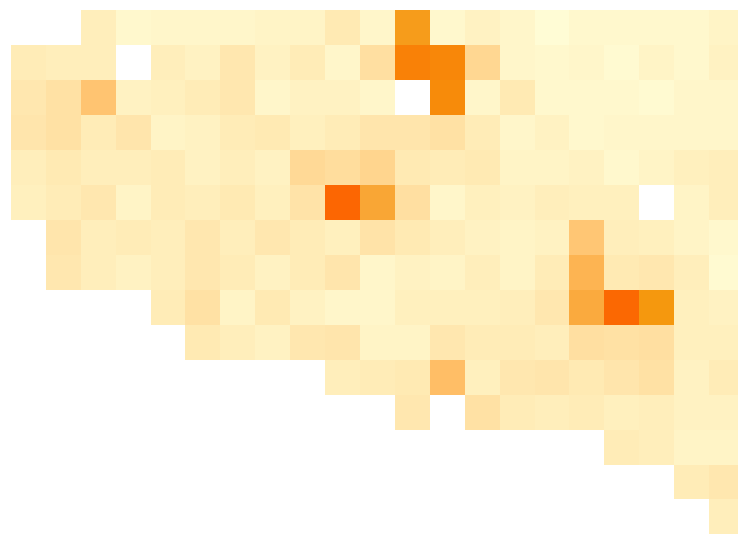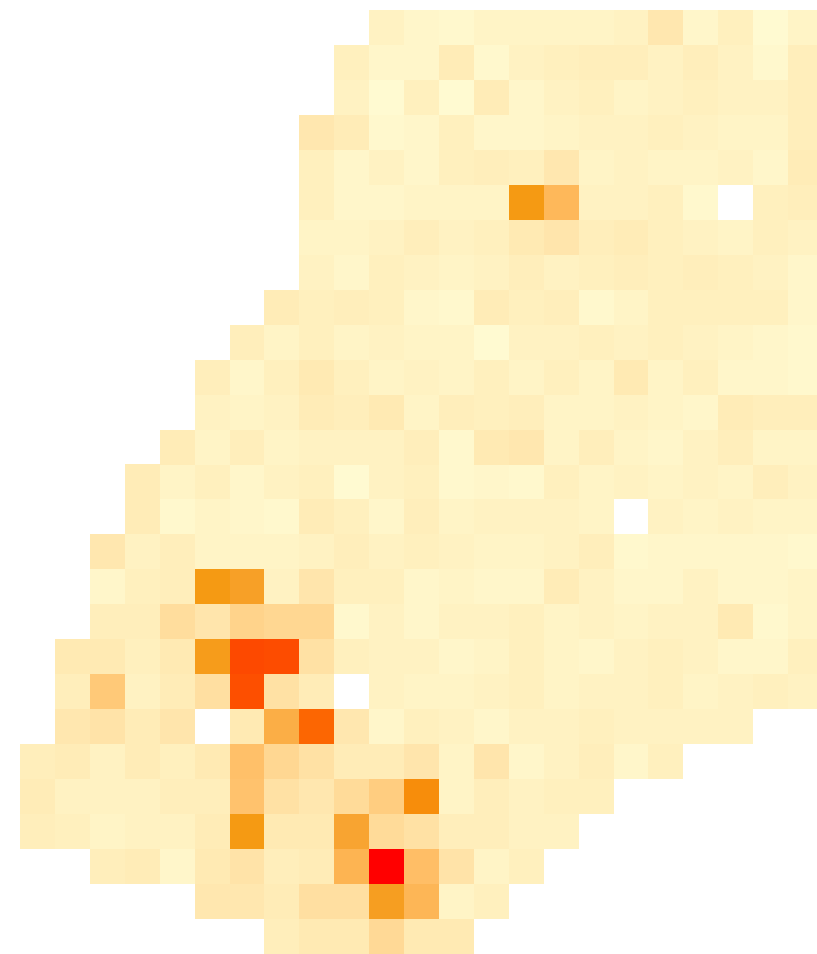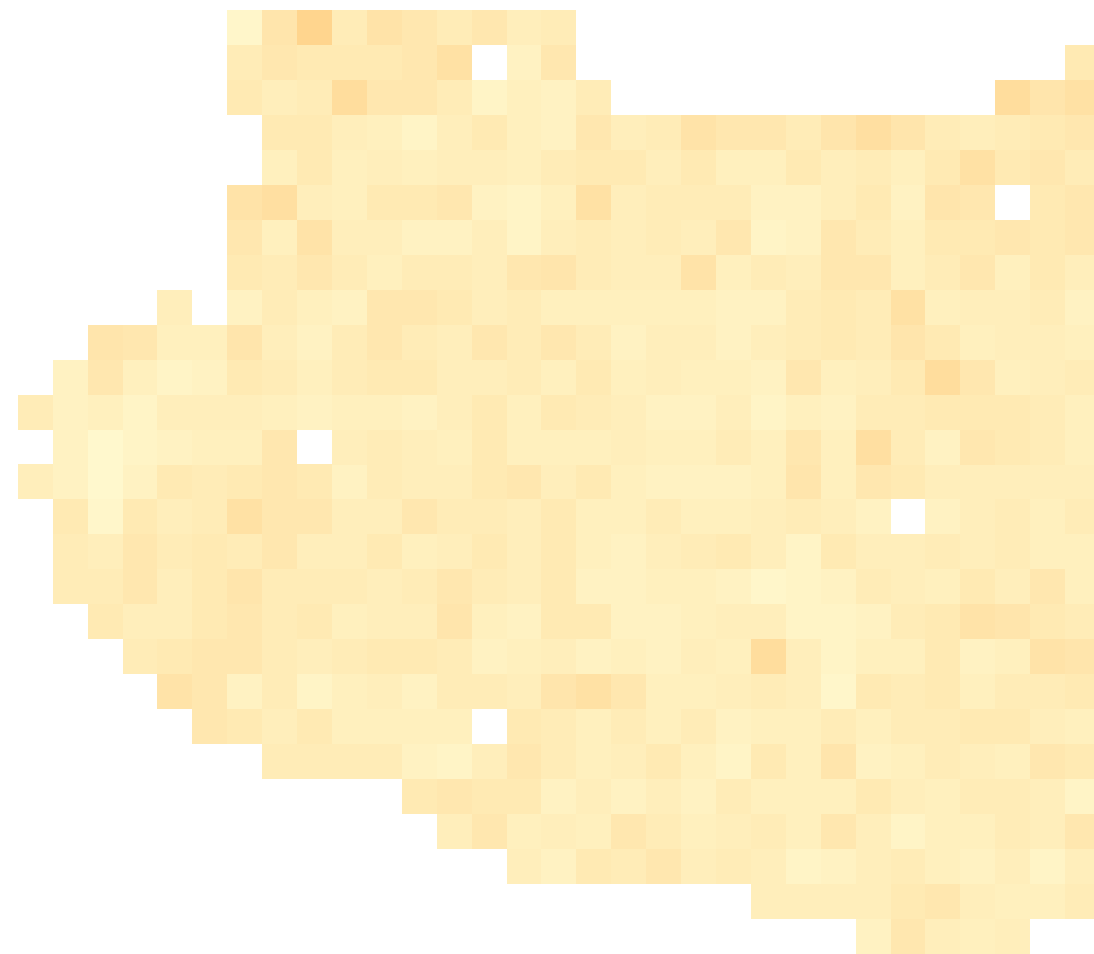

Supplement: Supplementary file 13 — Supplementary Data 10 [file 41467_2018_4724_MOESM13_ESM.zip › Supplementary Dataset 5/joint-mix-profiles-rel-common-scale-matrix.pdf]
