# Supplementary material for: Spatial maps of prostate cancer transcriptomes reveal an unexplored landscape of heterogeneity
Source: Nat Commun. 2018 Jun 20;9:2419. doi: 10.1038/s41467-018-04724-5 (PMC6010471; doi:10.1038/s41467-018-04724-5)
Supplement: Supplementary file 8 — Supplementary Data 5 [file 41467_2018_4724_MOESM8_ESM.zip › Supplementary Dataset 7/top-genes.pdf]

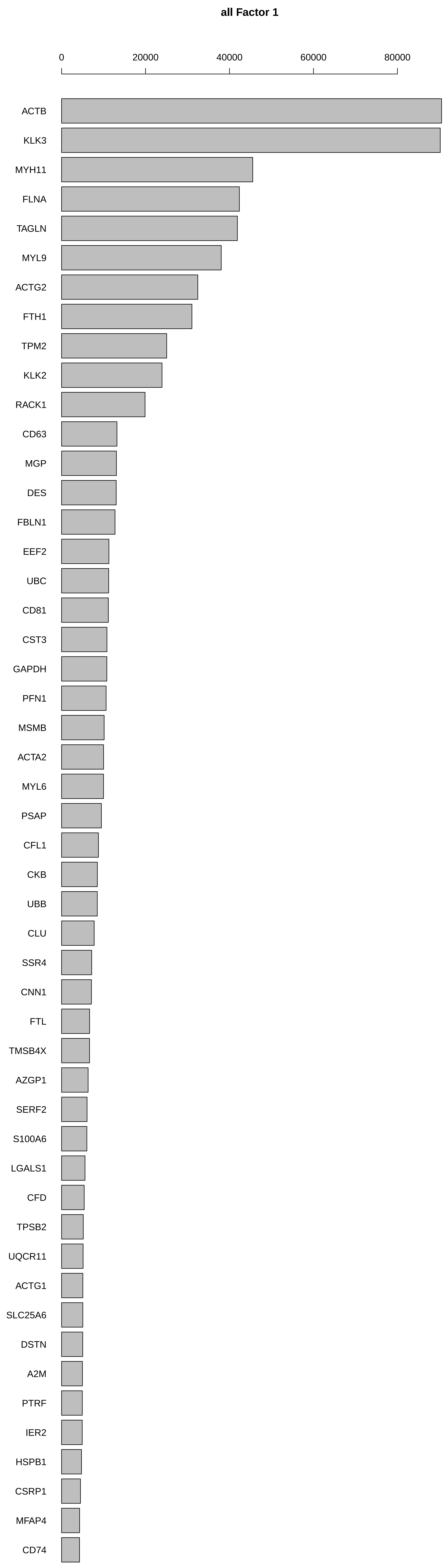
all Factor 1
0
20000
40000
60000
80000
ACTB
KLK3
MYH11
FLNA
TAGLN
MYL9
ACTG2
FTH1
TPM2
KLK2
RACK1
CD63
MGP
DES
FBLN1
EEF2
UBC
CD81
CST3
GAPDH
PFN1
MSMB
ACTA2
MYL6
PSAP
CFL1
CKB
UBB
CLU
SSR4
CNN1
FTL
TMSB4X
AZGP1
SERF2
S100A6
LGALS1
CFD
TPSB2
UQCR11
ACTG1
SLC25A6
DSTN
A2M
PTRF
IER2
HSPB1
CSRP1
MFAP4
CD74

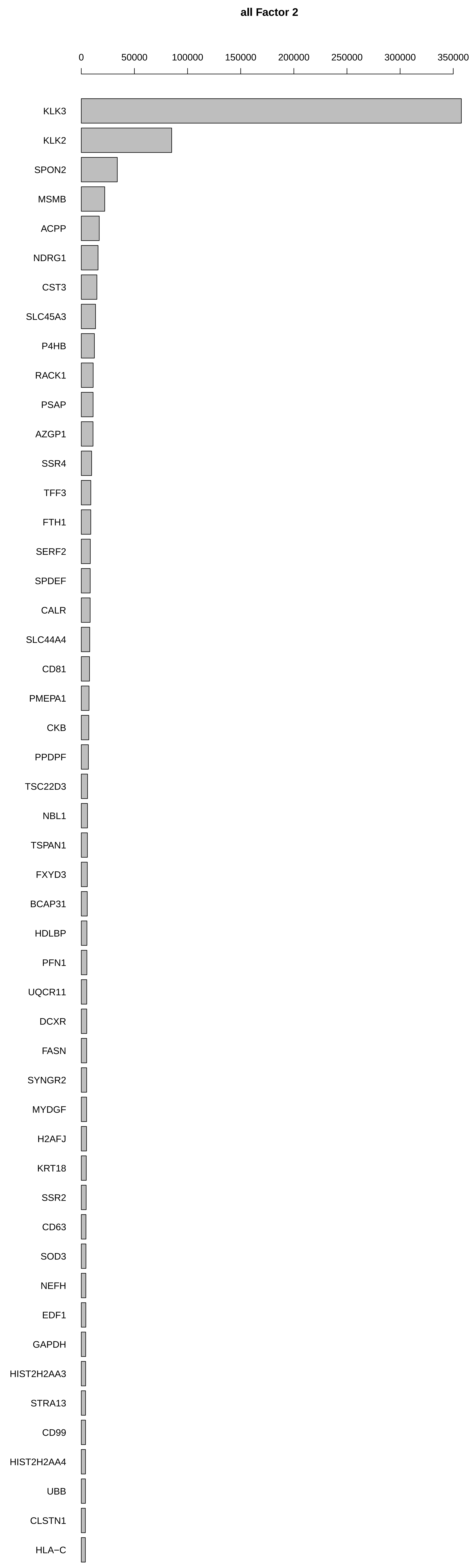
all Factor 2
0
50000
100000
150000
200000
250000
300000
350000
KLK3
KLK2
SPON2
MSMB
ACPP
NDRG1
CST3
SLC45A3
P4HB
RACK1
PSAP
AZGP1
SSR4
TFF3
FTH1
SERF2
SPDEF
CALR
SLC44A4
CD81
PMEPA1
CKB
PPDPF
TSC22D3
NBL1
TSPAN1
FXYD3
BCAP31
HDLBP
PFN1
UQCR11
DCXR
FASN
SYNGR2
MYDGF
H2AFJ
KRT18
SSR2
CD63
SOD3
NEFH
EDF1
GAPDH
HIST2H2AA3
STRA13
CD99
HIST2H2AA4
UBB
CLSTN1
HLA−C

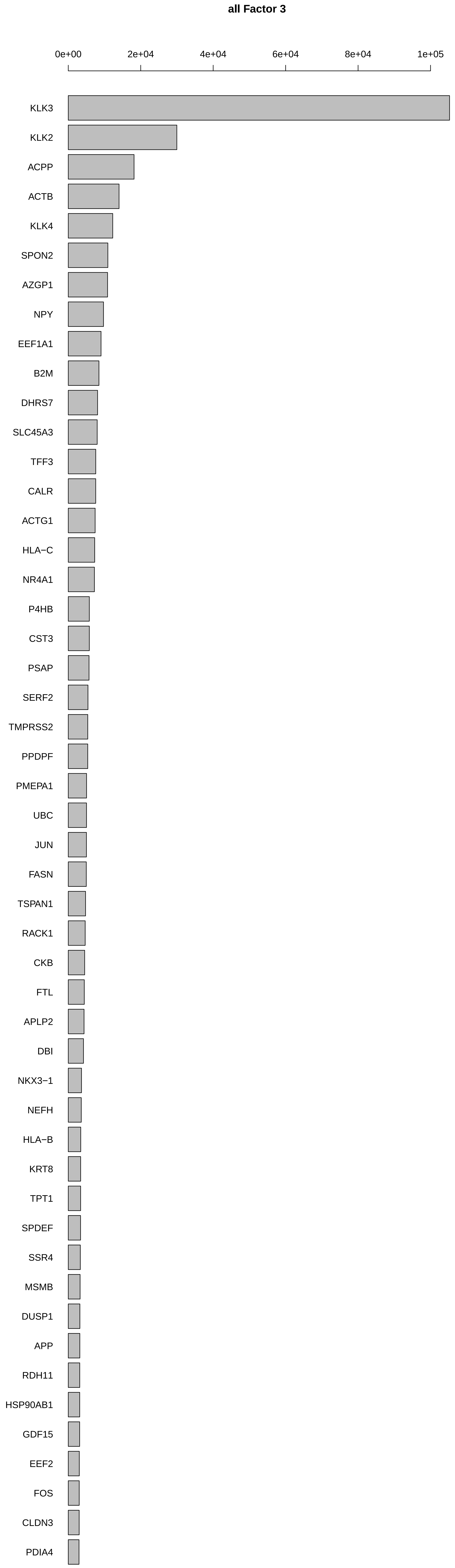

all Factor 3
0e+00
2e+04
4e+04
6e+04
8e+04
1e+05
KLK3
KLK2
ACPP
ACTB
KLK4
SPON2
AZGP1
NPY
EEF1A1
B2M
DHRS7
SLC45A3
TFF3
CALR
ACTG1
HLA−C
NR4A1
P4HB
CST3
PSAP
SERF2
TMPRSS2
PPDPF
PMEPA1
UBC
JUN
FASN
TSPAN1
RACK1
CKB
FTL
APLP2
DBI
NKX3−1
NEFH
HLA−B
KRT8
TPT1
SPDEF
SSR4
MSMB
DUSP1
APP
RDH11
HSP90AB1
GDF15
EEF2
FOS
CLDN3
PDIA4

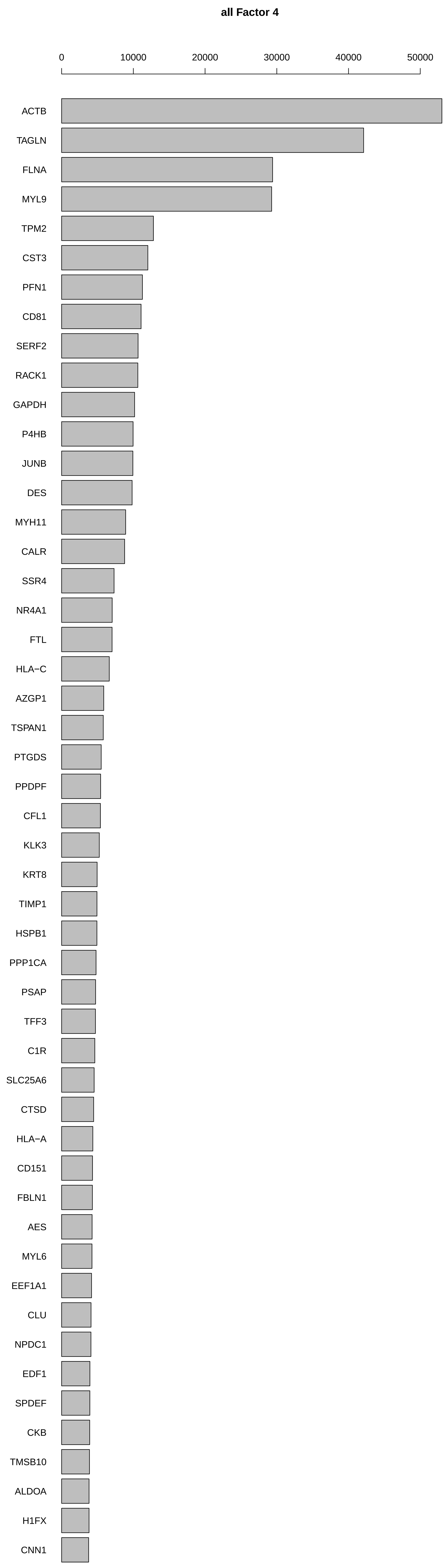
all Factor 4
0
10000
20000
30000
40000
50000
ACTB
TAGLN
FLNA
MYL9
TPM2
CST3
PFN1
CD81
SERF2
RACK1
GAPDH
P4HB
JUNB
DES
MYH11
CALR
SSR4
NR4A1
FTL
HLA−C
AZGP1
TSPAN1
PTGDS
PPDPF
CFL1
KLK3
KRT8
TIMP1
HSPB1
PPP1CA
PSAP
TFF3
C1R
SLC25A6
CTSD
HLA−A
CD151
FBLN1
AES
MYL6
EEF1A1
CLU
NPDC1
EDF1
SPDEF
CKB
TMSB10
ALDOA
H1FX
CNN1

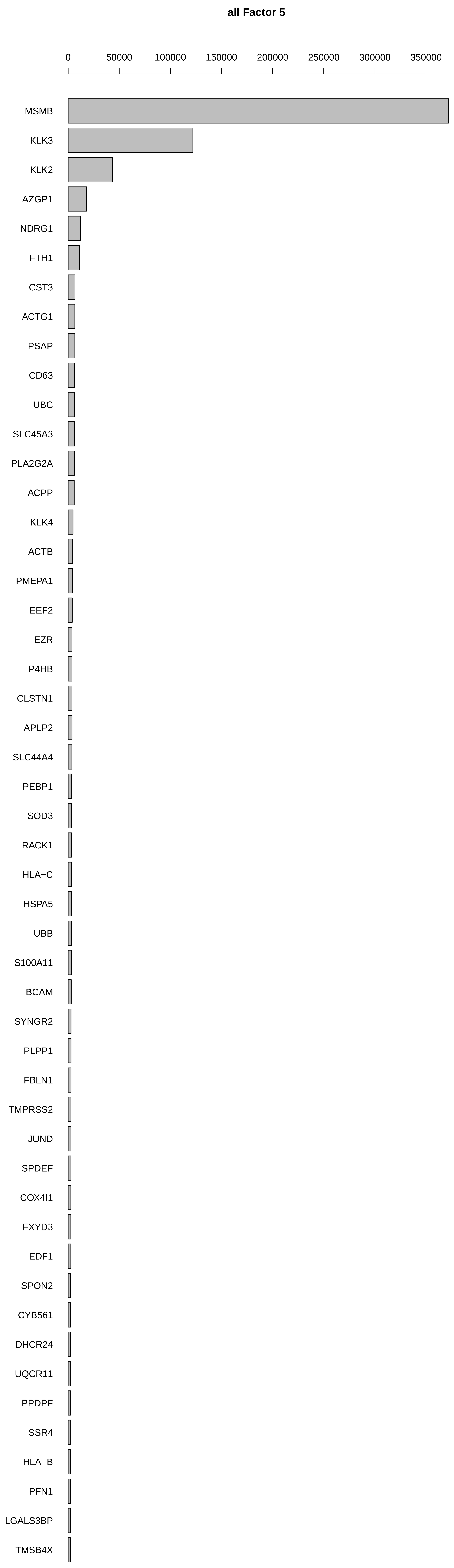
all Factor 5
0
50000
100000
150000
200000
250000
300000
350000
MSMB
KLK3
KLK2
AZGP1
NDRG1
FTH1
CST3
ACTG1
PSAP
CD63
UBC
SLC45A3
PLA2G2A
ACPP
KLK4
ACTB
PMEPA1
EEF2
EZR
P4HB
CLSTN1
APLP2
SLC44A4
PEBP1
SOD3
RACK1
HLA−C
HSPA5
UBB
S100A11
BCAM
SYNGR2
PLPP1
FBLN1
TMPRSS2
JUND
SPDEF
COX4I1
FXYD3
EDF1
SPON2
CYB561
DHCR24
UQCR11
PPDPF
SSR4
HLA−B
PFN1
LGALS3BP
TMSB4X

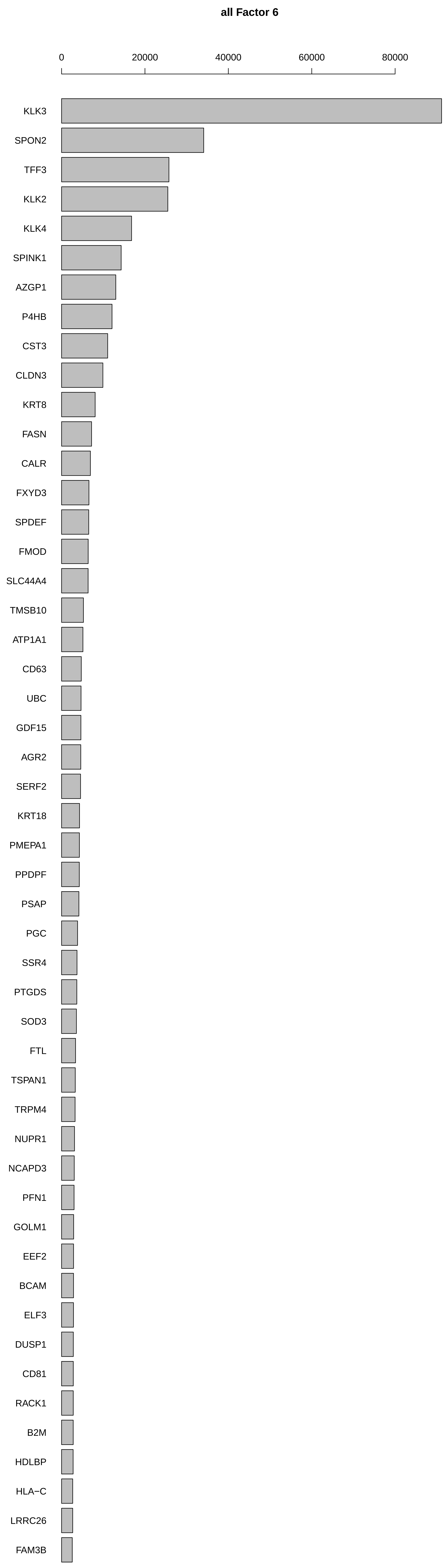
all Factor 6
0
20000
40000
60000
80000
KLK3
SPON2
TFF3
KLK2
KLK4
SPINK1
AZGP1
P4HB
CST3
CLDN3
KRT8
FASN
CALR
FXYD3
SPDEF
FMOD
SLC44A4
TMSB10
ATP1A1
CD63
UBC
GDF15
AGR2
SERF2
KRT18
PMEPA1
PPDPF
PSAP
PGC
SSR4
PTGDS
SOD3
FTL
TSPAN1
TRPM4
NUPR1
NCAPD3
PFN1
GOLM1
EEF2
BCAM
ELF3
DUSP1
CD81
RACK1
B2M
HDLBP
HLA−C
LRRC26
FAM3B

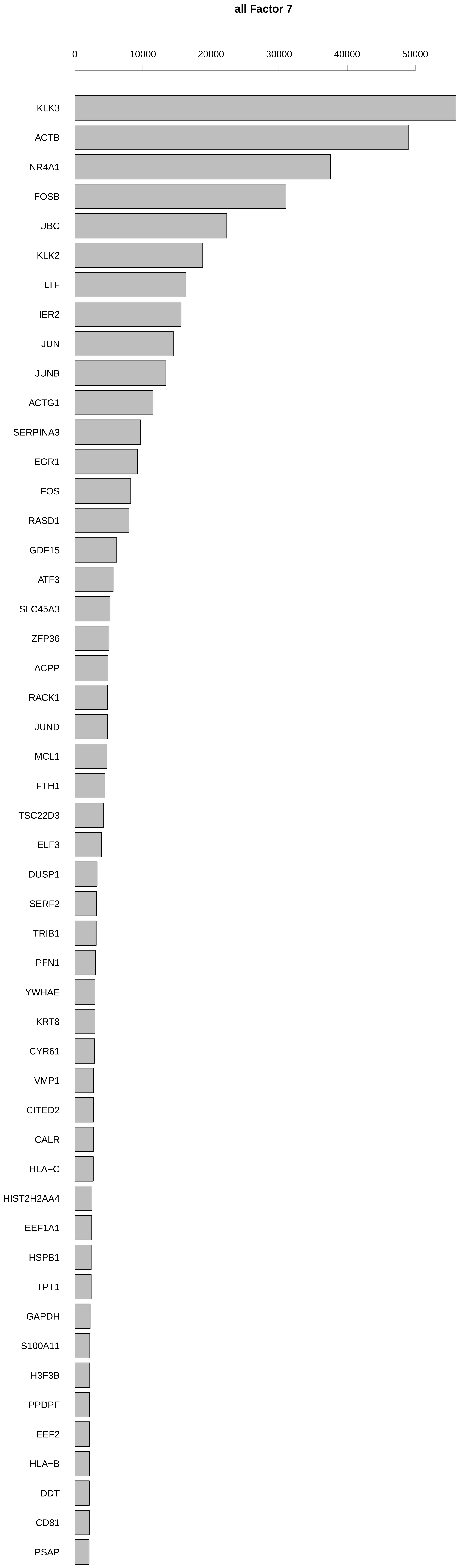

all Factor 7
0
10000
20000
30000
40000
50000
KLK3
ACTB
NR4A1
FOSB
UBC
KLK2
LTF
IER2
JUN
JUNB
ACTG1
SERPINA3
EGR1
FOS
RASD1
GDF15
ATF3
SLC45A3
ZFP36
ACPP
RACK1
JUND
MCL1
FTH1
TSC22D3
ELF3
DUSP1
SERF2
TRIB1
PFN1
YWHAE
KRT8
CYR61
VMP1
CITED2
CALR
HLA−C
HIST2H2AA4
EEF1A1
HSPB1
TPT1
GAPDH
S100A11
H3F3B
PPDPF
EEF2
HLA−B
DDT
CD81
PSAP

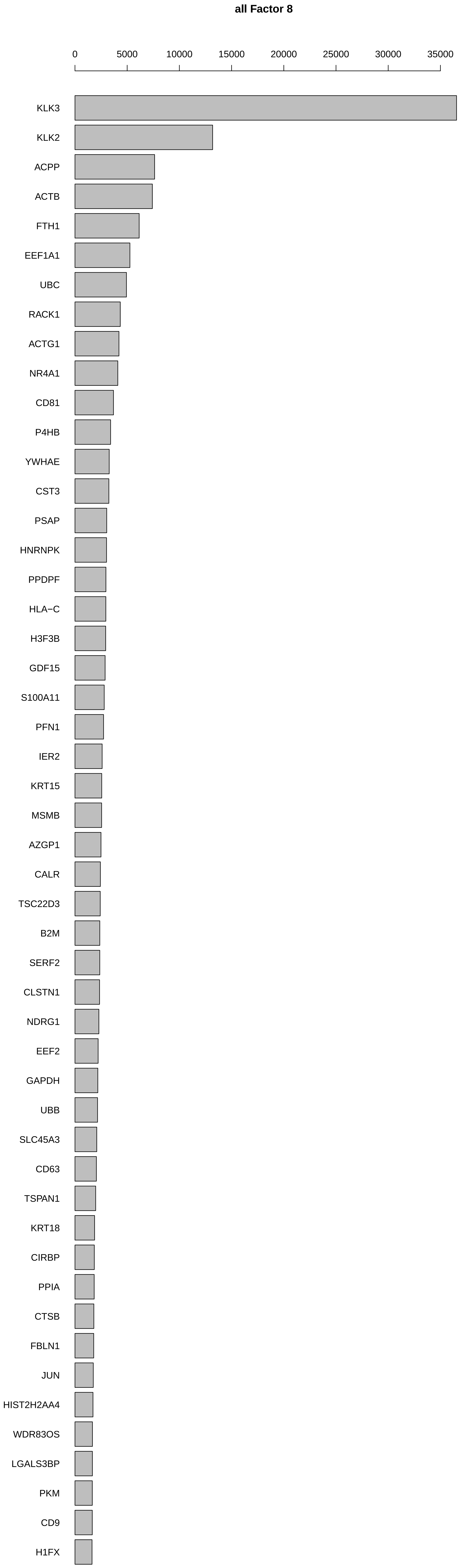
all Factor 8
0
5000
10000
15000
20000
25000
30000
35000
KLK3
KLK2
ACPP
ACTB
FTH1
EEF1A1
UBC
RACK1
ACTG1
NR4A1
CD81
P4HB
YWHAE
CST3
PSAP
HNRNPK
PPDPF
HLA−C
H3F3B
GDF15
S100A11
PFN1
IER2
KRT15
MSMB
AZGP1
CALR
TSC22D3
B2M
SERF2
CLSTN1
NDRG1
EEF2
GAPDH
UBB
SLC45A3
CD63
TSPAN1
KRT18
CIRBP
PPIA
CTSB
FBLN1
JUN
HIST2H2AA4
WDR83OS
LGALS3BP
PKM
CD9
H1FX

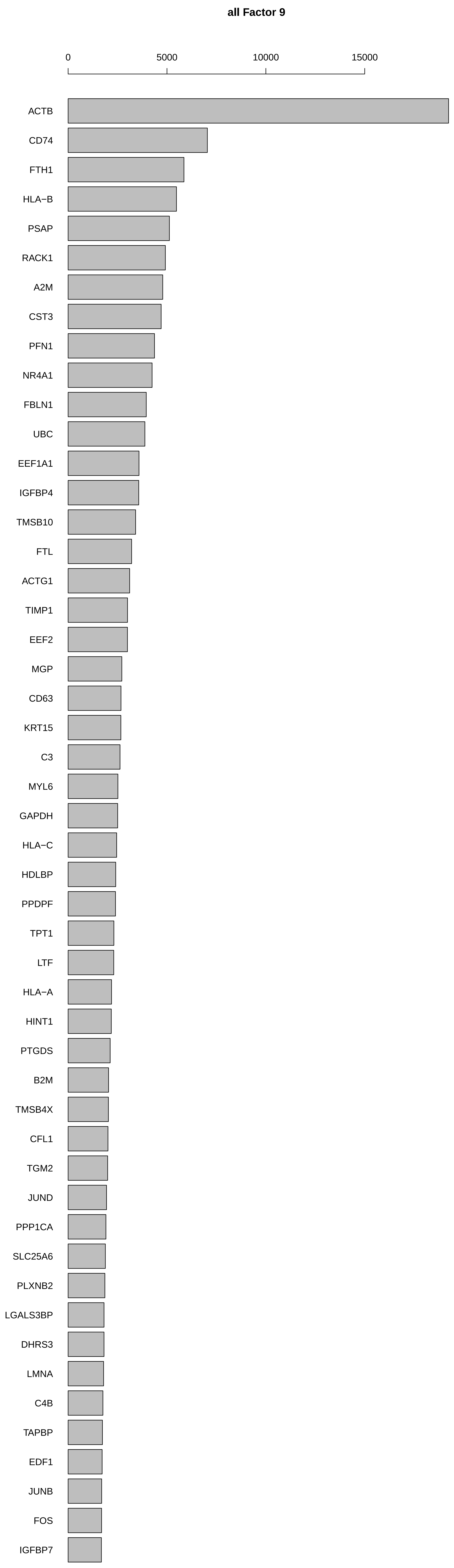
all Factor 9
0
5000
10000
15000
ACTB
CD74
FTH1
HLA−B
PSAP
RACK1
A2M
CST3
PFN1
NR4A1
FBLN1
UBC
EEF1A1
IGFBP4
TMSB10
FTL
ACTG1
TIMP1
EEF2
MGP
CD63
KRT15
C3
MYL6
GAPDH
HLA−C
HDLBP
PPDPF
TPT1
LTF
HLA−A
HINT1
PTGDS
B2M
TMSB4X
CFL1
TGM2
JUND
PPP1CA
SLC25A6
PLXNB2
LGALS3BP
DHRS3
LMNA
C4B
TAPBP
EDF1
JUNB
FOS
IGFBP7

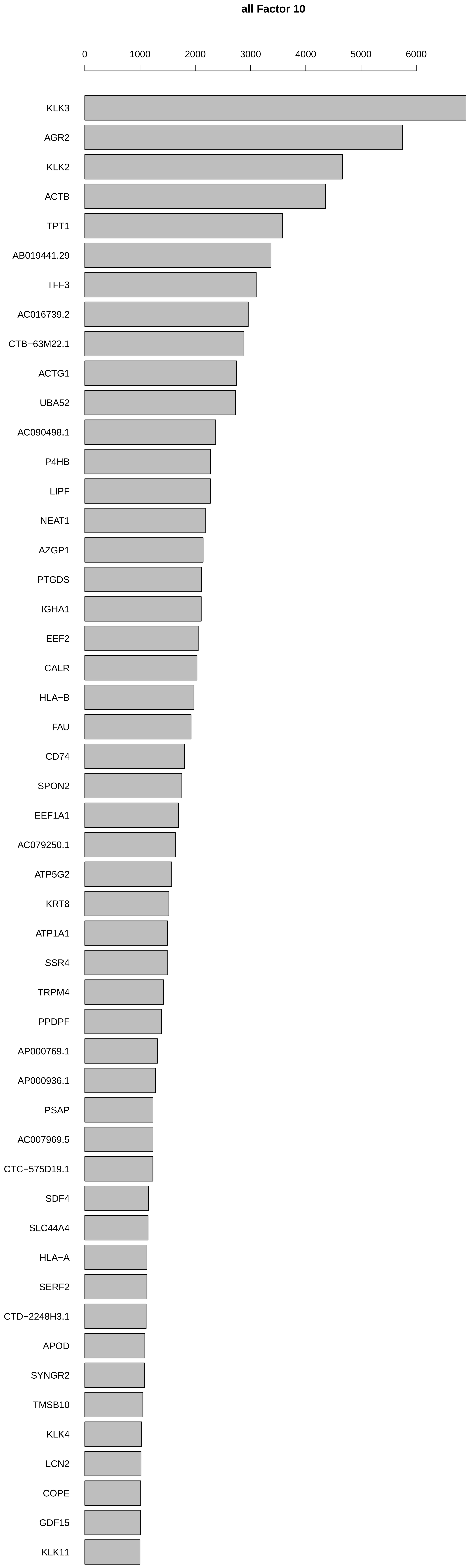
all Factor 10
0
1000
2000
3000
4000
5000
6000
KLK3
AGR2
KLK2
ACTB
TPT1
AB019441.29
TFF3
AC016739.2
CTB−63M22.1
ACTG1
UBA52
AC090498.1
P4HB
LIPF
NEAT1
AZGP1
PTGDS
IGHA1
EEF2
CALR
HLA−B
FAU
CD74
SPON2
EEF1A1
AC079250.1
ATP5G2
KRT8
ATP1A1
SSR4
TRPM4
PPDPF
AP000769.1
AP000936.1
PSAP
AC007969.5
CTC−575D19.1
SDF4
SLC44A4
HLA−A
SERF2
CTD−2248H3.1
APOD
SYNGR2
TMSB10
KLK4
LCN2
COPE
GDF15
KLK11

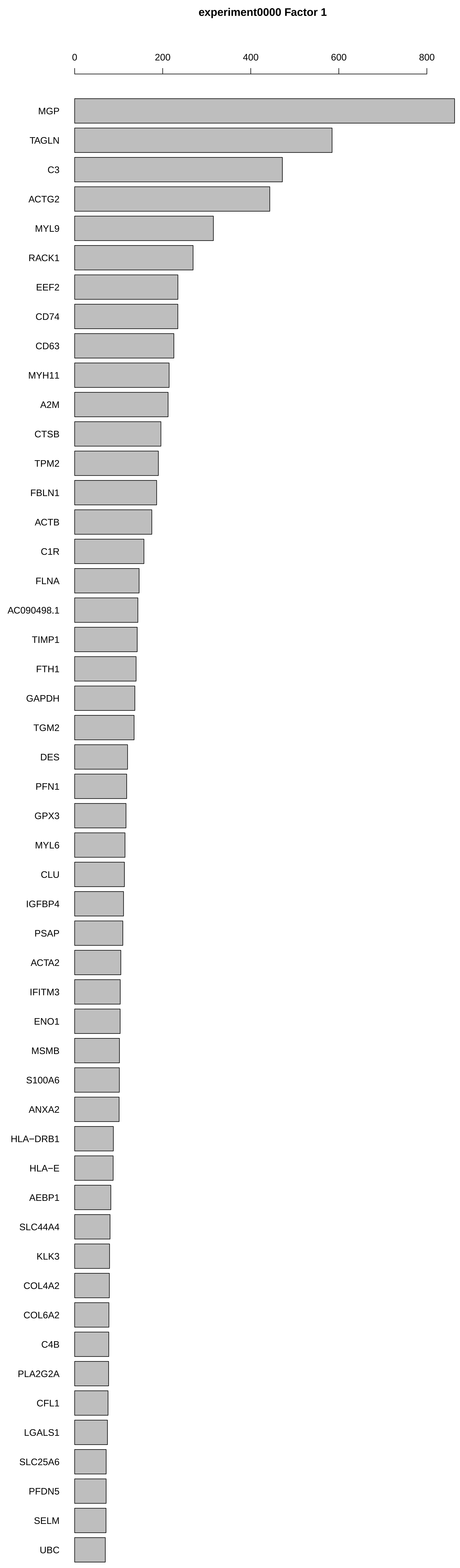
experiment0000 Factor 1
0
200
400
600
800
MGP
TAGLN
C3
ACTG2
MYL9
RACK1
EEF2
CD74
CD63
MYH11
A2M
CTSB
TPM2
FBLN1
ACTB
C1R
FLNA
AC090498.1
TIMP1
FTH1
GAPDH
TGM2
DES
PFN1
GPX3
MYL6
CLU
IGFBP4
PSAP
ACTA2
IFITM3
ENO1
MSMB
S100A6
ANXA2
HLA−DRB1
HLA−E
AEBP1
SLC44A4
KLK3
COL4A2
COL6A2
C4B
PLA2G2A
CFL1
LGALS1
SLC25A6
PFDN5
SELM
UBC

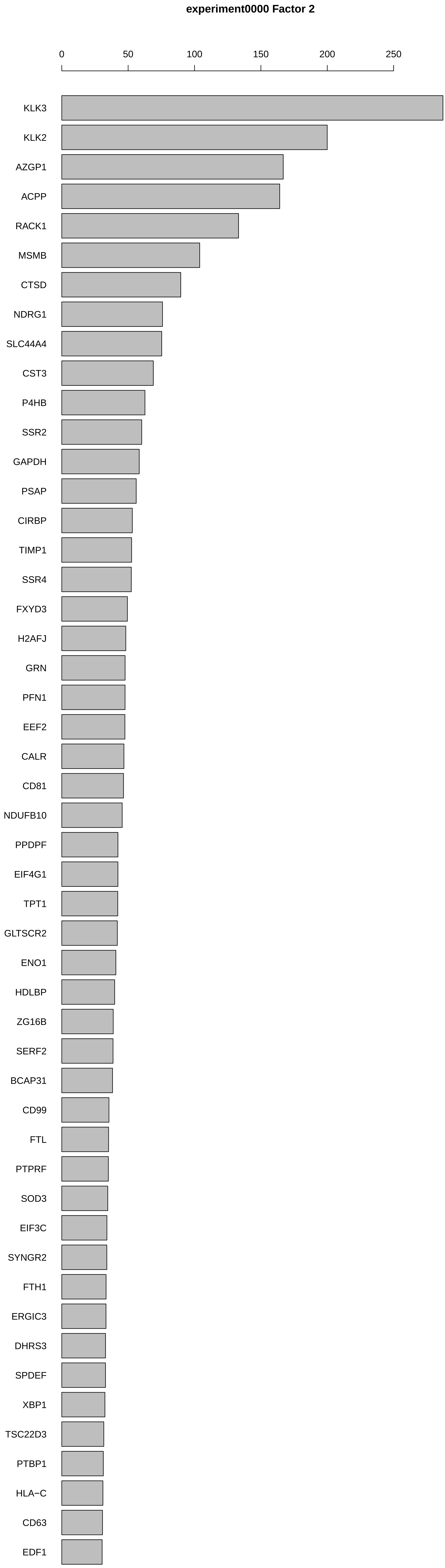
experiment0000 Factor 2
0
50
100
150
200
250
KLK3
KLK2
AZGP1
ACPP
RACK1
MSMB
CTSD
NDRG1
SLC44A4
CST3
P4HB
SSR2
GAPDH
PSAP
CIRBP
TIMP1
SSR4
FXYD3
H2AFJ
GRN
PFN1
EEF2
CALR
CD81
NDUFB10
PPDPF
EIF4G1
TPT1
GLTSCR2
ENO1
HDLBP
ZG16B
SERF2
BCAP31
CD99
FTL
PTPRF
SOD3
EIF3C
SYNGR2
FTH1
ERGIC3
DHRS3
SPDEF
XBP1
TSC22D3
PTBP1
HLA−C
CD63
EDF1

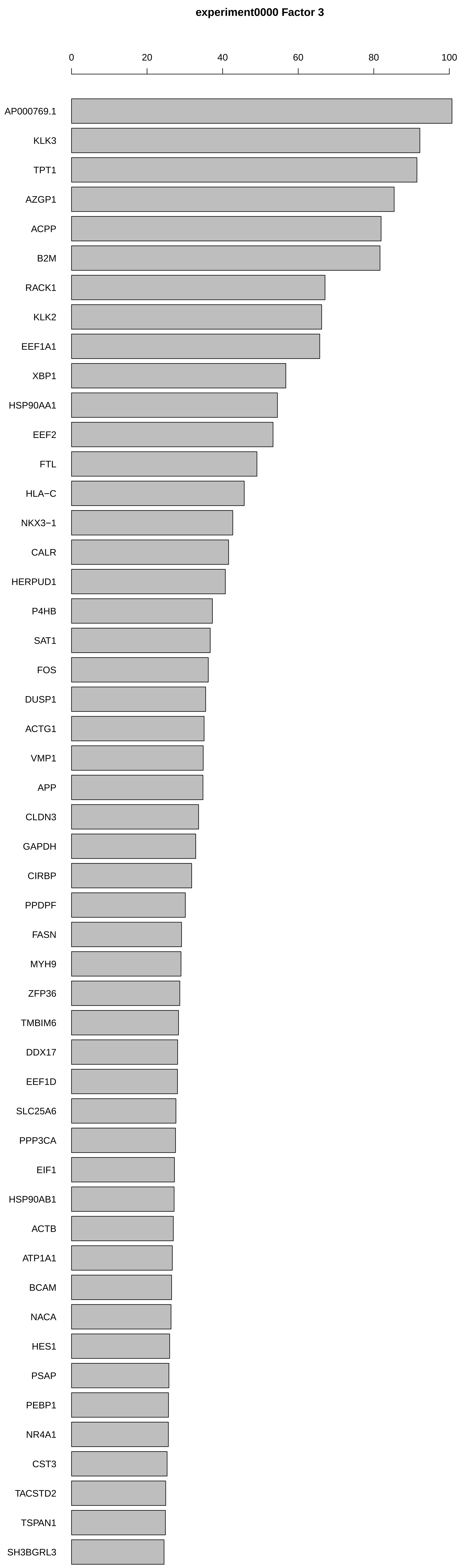

experiment0000 Factor 3
0
20
40
60
80
100
AP000769.1
KLK3
TPT1
AZGP1
ACPP
B2M
RACK1
KLK2
EEF1A1
XBP1
HSP90AA1
EEF2
FTL
HLA−C
NKX3−1
CALR
HERPUD1
P4HB
SAT1
FOS
DUSP1
ACTG1
VMP1
APP
CLDN3
GAPDH
CIRBP
PPDPF
FASN
MYH9
ZFP36
TMBIM6
DDX17
EEF1D
SLC25A6
PPP3CA
EIF1
HSP90AB1
ACTB
ATP1A1
BCAM
NACA
HES1
PSAP
PEBP1
NR4A1
CST3
TACSTD2
TSPAN1
SH3BGRL3

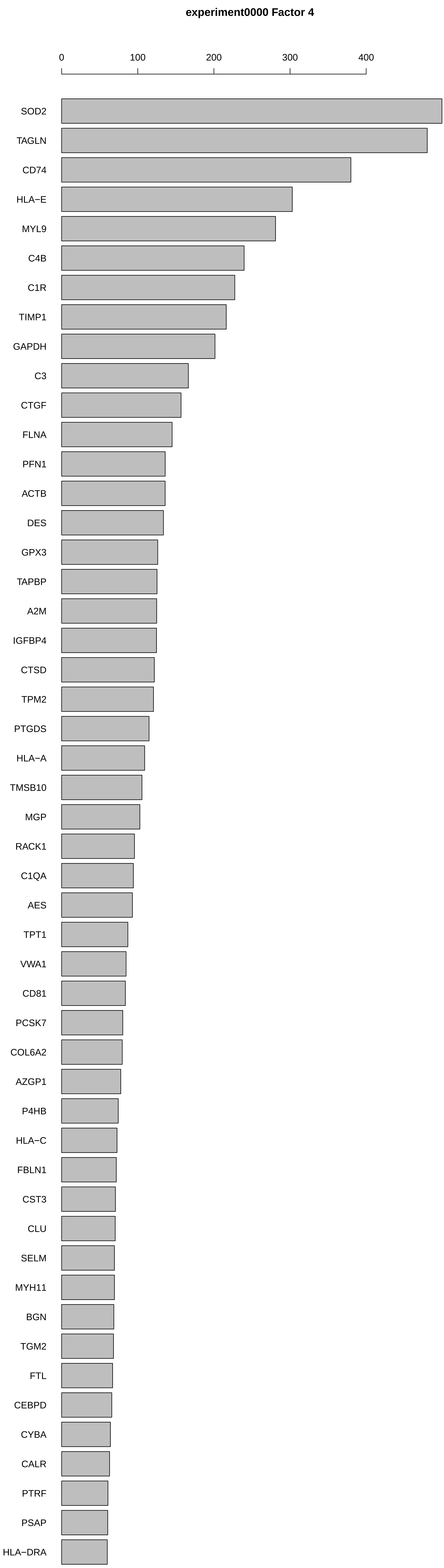

experiment0000 Factor 4
0
100
200
300
400
SOD2
TAGLN
CD74
HLA−E
MYL9
C4B
C1R
TIMP1
GAPDH
C3
CTGF
FLNA
PFN1
ACTB
DES
GPX3
TAPBP
A2M
IGFBP4
CTSD
TPM2
PTGDS
HLA−A
TMSB10
MGP
RACK1
C1QA
AES
TPT1
VWA1
CD81
PCSK7
COL6A2
AZGP1
P4HB
HLA−C
FBLN1
CST3
CLU
SELM
MYH11
BGN
TGM2
FTL
CEBPD
CYBA
CALR
PTRF
PSAP
HLA−DRA

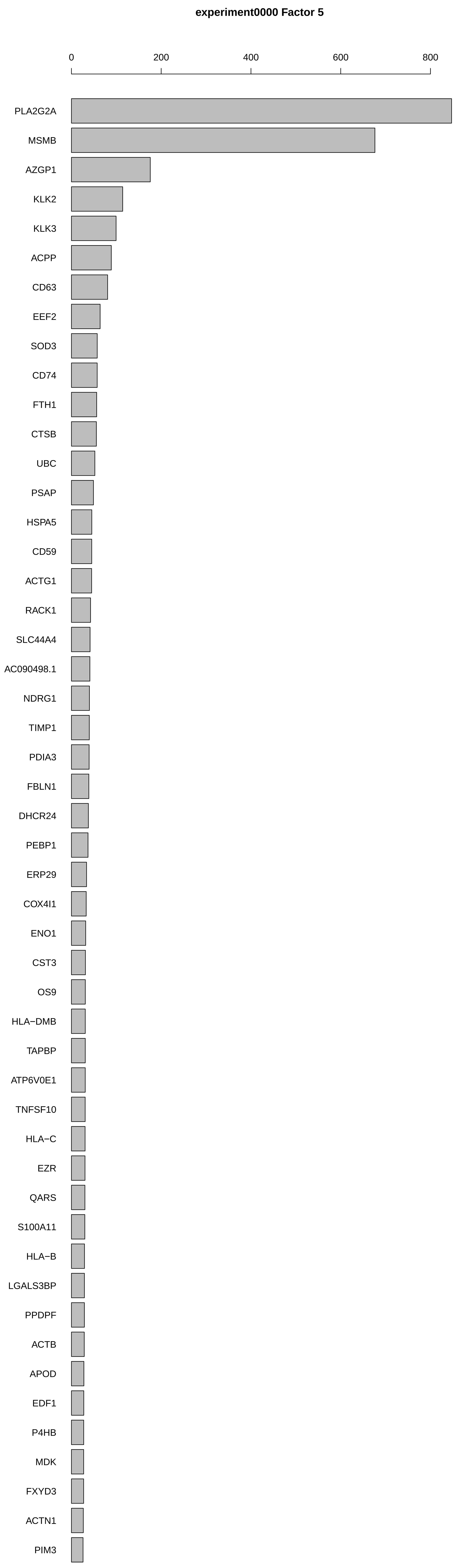

experiment0000 Factor 5
0
200
400
600
800
PLA2G2A
MSMB
AZGP1
KLK2
KLK3
ACPP
CD63
EEF2
SOD3
CD74
FTH1
CTSB
UBC
PSAP
HSPA5
CD59
ACTG1
RACK1
SLC44A4
AC090498.1
NDRG1
TIMP1
PDIA3
FBLN1
DHCR24
PEBP1
ERP29
COX4I1
ENO1
CST3
OS9
HLA−DMB
TAPBP
ATP6V0E1
TNFSF10
HLA−C
EZR
QARS
S100A11
HLA−B
LGALS3BP
PPDPF
ACTB
APOD
EDF1
P4HB
MDK
FXYD3
ACTN1
PIM3

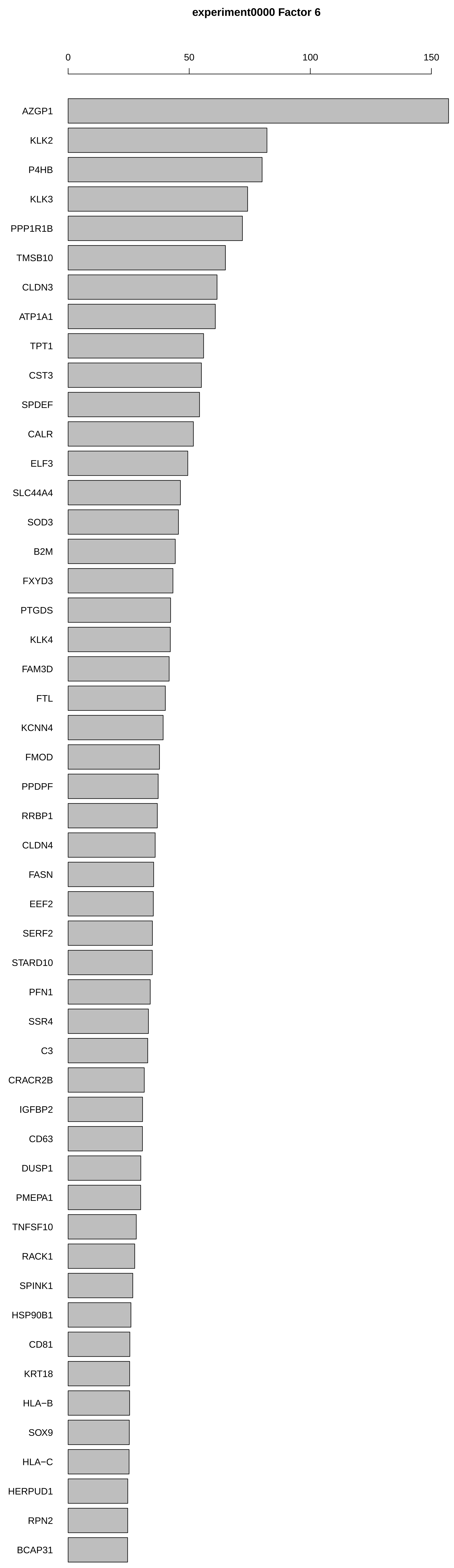
experiment0000 Factor 6
0
50
100
150
AZGP1
KLK2
P4HB
KLK3
PPP1R1B
TMSB10
CLDN3
ATP1A1
TPT1
CST3
SPDEF
CALR
ELF3
SLC44A4
SOD3
B2M
FXYD3
PTGDS
KLK4
FAM3D
FTL
KCNN4
FMOD
PPDPF
RRBP1
CLDN4
FASN
EEF2
SERF2
STARD10
PFN1
SSR4
C3
CRACR2B
IGFBP2
CD63
DUSP1
PMEPA1
TNFSF10
RACK1
SPINK1
HSP90B1
CD81
KRT18
HLA−B
SOX9
HLA−C
HERPUD1
RPN2
BCAP31

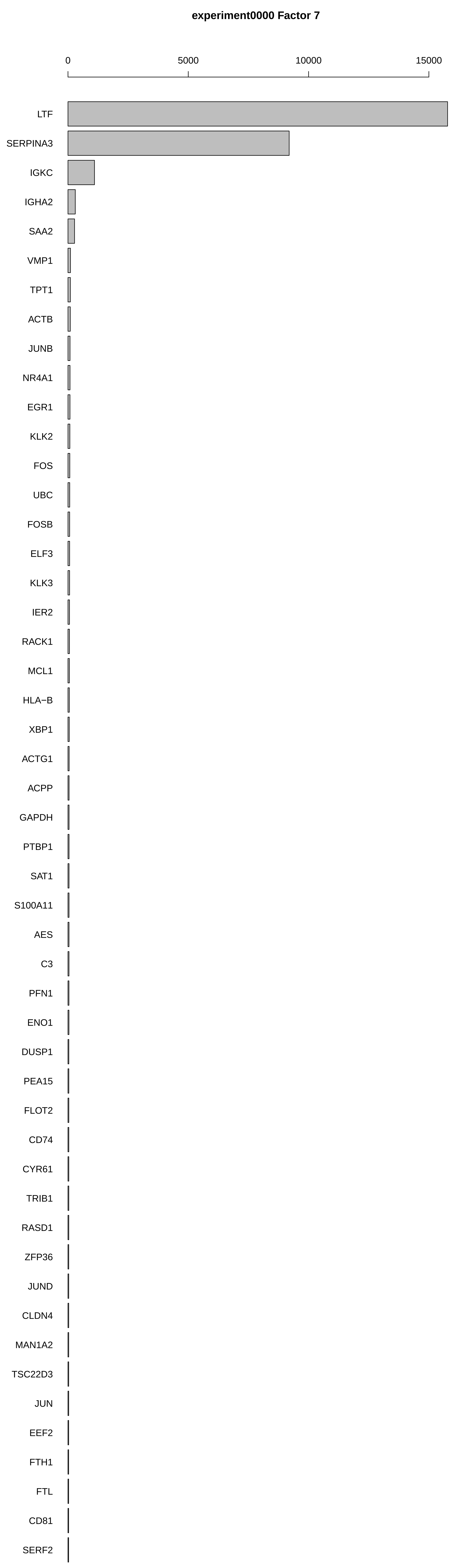
experiment0000 Factor 7
0
5000
10000
15000
LTF
SERPINA3
IGKC
IGHA2
SAA2
VMP1
TPT1
ACTB
JUNB
NR4A1
EGR1
KLK2
FOS
UBC
FOSB
ELF3
KLK3
IER2
RACK1
MCL1
HLA−B
XBP1
ACTG1
ACPP
GAPDH
PTBP1
SAT1
S100A11
AES
C3
PFN1
ENO1
DUSP1
PEA15
FLOT2
CD74
CYR61
TRIB1
RASD1
ZFP36
JUND
CLDN4
MAN1A2
TSC22D3
JUN
EEF2
FTH1
FTL
CD81
SERF2

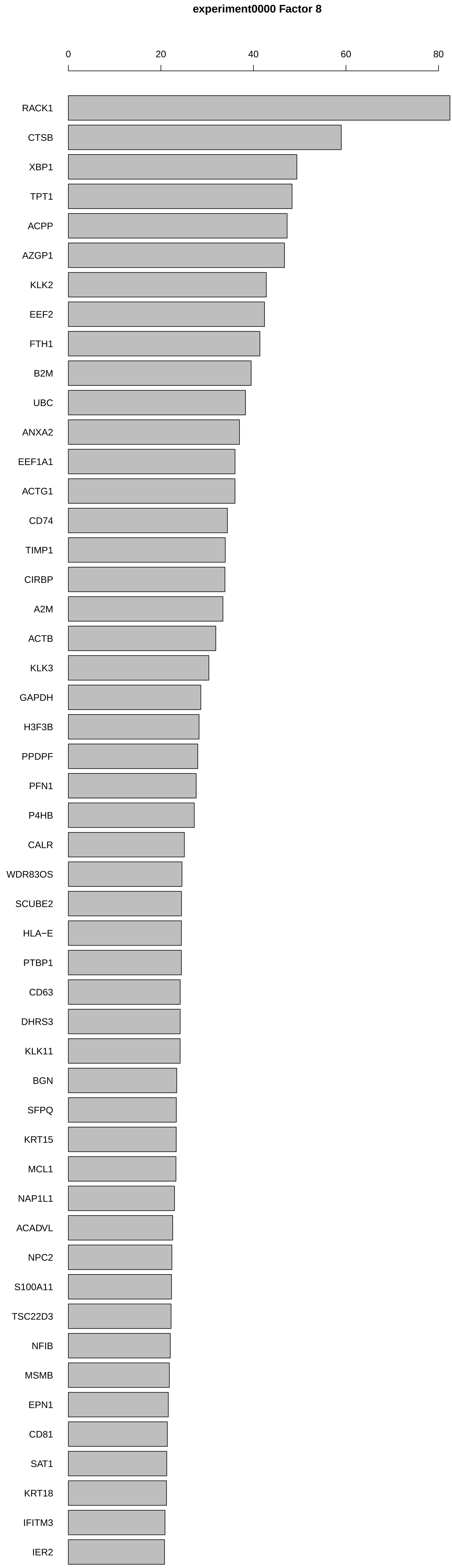
experiment0000 Factor 8
0
20
40
60
80
RACK1
CTSB
XBP1
TPT1
ACPP
AZGP1
KLK2
EEF2
FTH1
B2M
UBC
ANXA2
EEF1A1
ACTG1
CD74
TIMP1
CIRBP
A2M
ACTB
KLK3
GAPDH
H3F3B
PPDPF
PFN1
P4HB
CALR
WDR83OS
SCUBE2
HLA−E
PTBP1
CD63
DHRS3
KLK11
BGN
SFPQ
KRT15
MCL1
NAP1L1
ACADVL
NPC2
S100A11
TSC22D3
NFIB
MSMB
EPN1
CD81
SAT1
KRT18
IFITM3
IER2

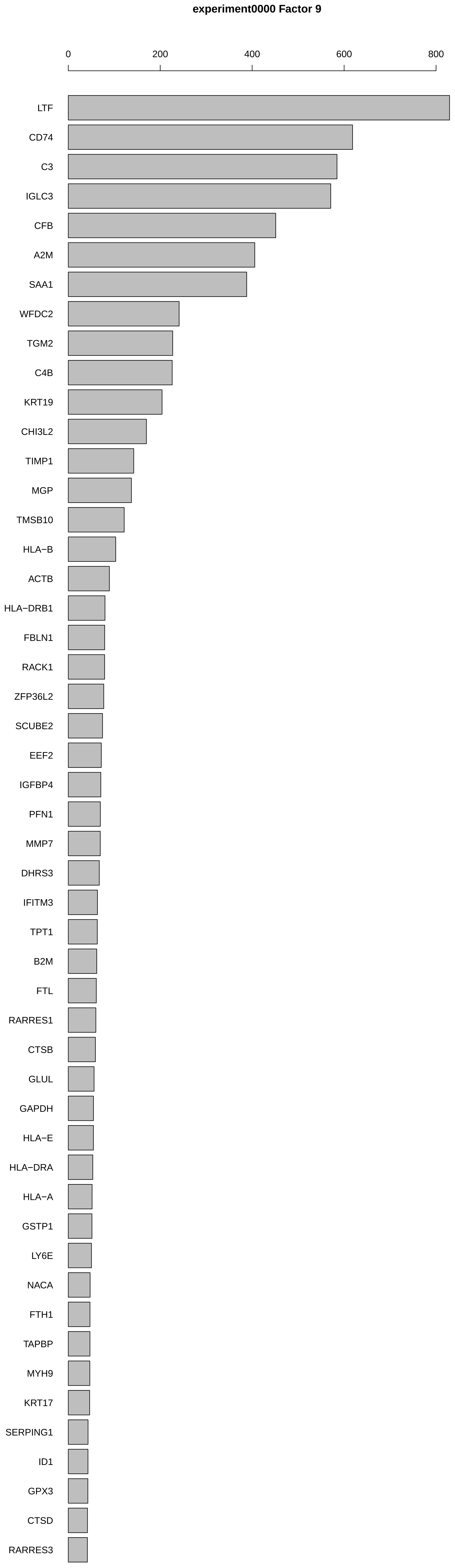

experiment0000 Factor 9
0
200
400
600
800
LTF
CD74
C3
IGLC3
CFB
A2M
SAA1
WFDC2
TGM2
C4B
KRT19
CHI3L2
TIMP1
MGP
TMSB10
HLA−B
ACTB
HLA−DRB1
FBLN1
RACK1
ZFP36L2
SCUBE2
EEF2
IGFBP4
PFN1
MMP7
DHRS3
IFITM3
TPT1
B2M
FTL
RARRES1
CTSB
GLUL
GAPDH
HLA−E
HLA−DRA
HLA−A
GSTP1
LY6E
NACA
FTH1
TAPBP
MYH9
KRT17
SERPING1
ID1
GPX3
CTSD
RARRES3

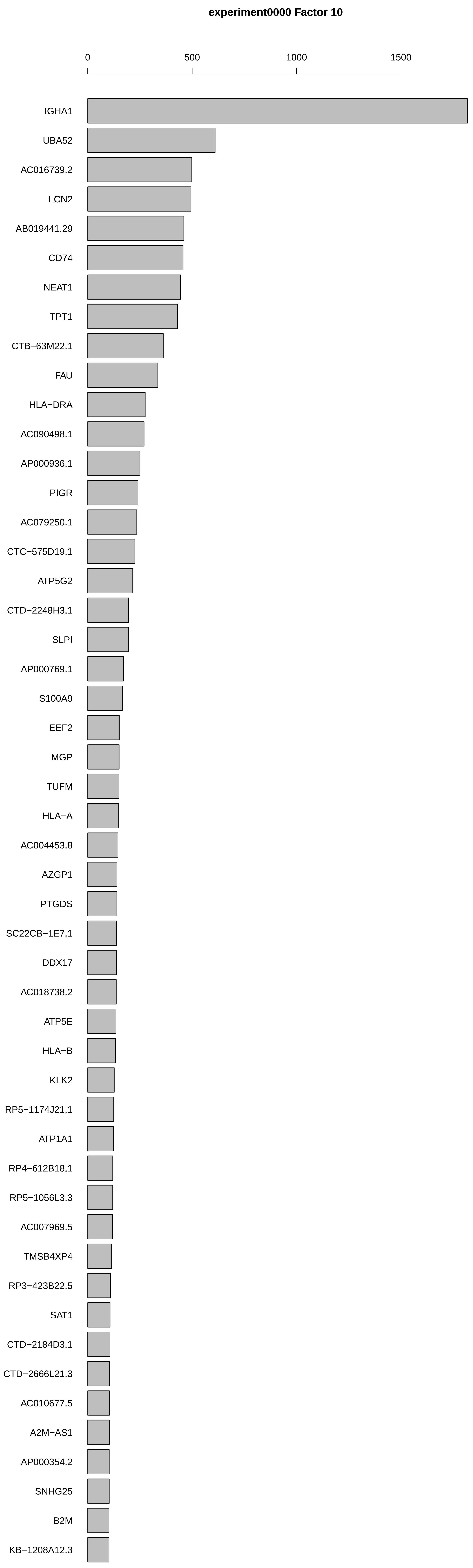
experiment0000 Factor 10
0
500
1000
1500
IGHA1
UBA52
AC016739.2
LCN2
AB019441.29
CD74
NEAT1
TPT1
CTB−63M22.1
FAU
HLA−DRA
AC090498.1
AP000936.1
PIGR
AC079250.1
CTC−575D19.1
ATP5G2
CTD−2248H3.1
SLPI
AP000769.1
S100A9
EEF2
MGP
TUFM
HLA−A
AC004453.8
AZGP1
PTGDS
SC22CB−1E7.1
DDX17
AC018738.2
ATP5E
HLA−B
KLK2
RP5−1174J21.1
ATP1A1
RP4−612B18.1
RP5−1056L3.3
AC007969.5
TMSB4XP4
RP3−423B22.5
SAT1
CTD−2184D3.1
CTD−2666L21.3
AC010677.5
A2M−AS1
AP000354.2
SNHG25
B2M
KB−1208A12.3

experiment0001 Factor 1

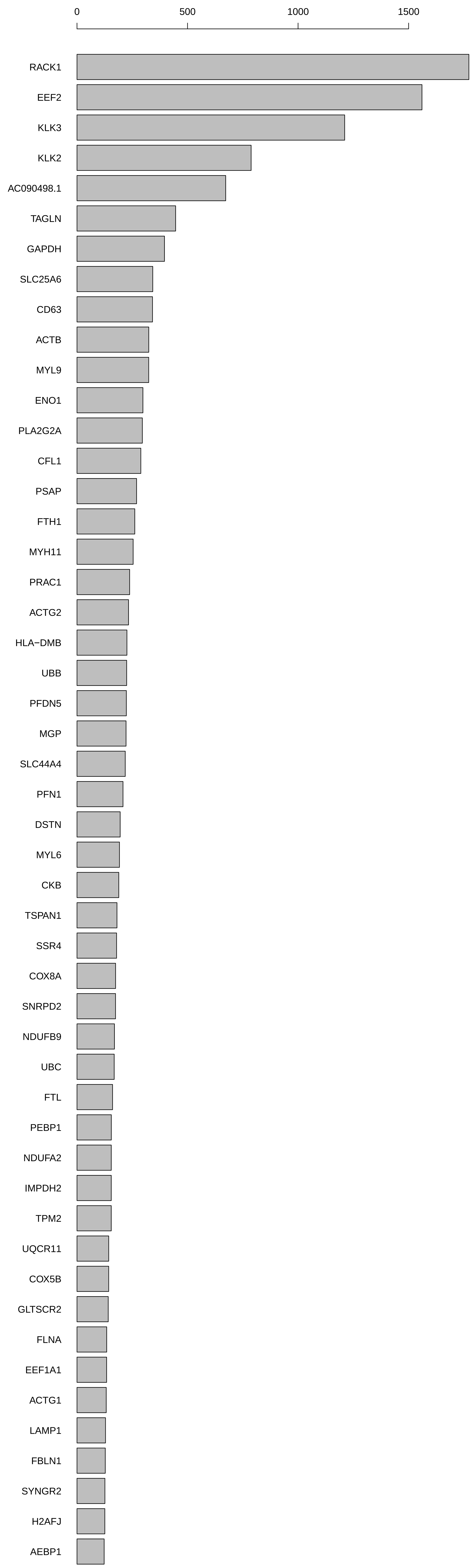

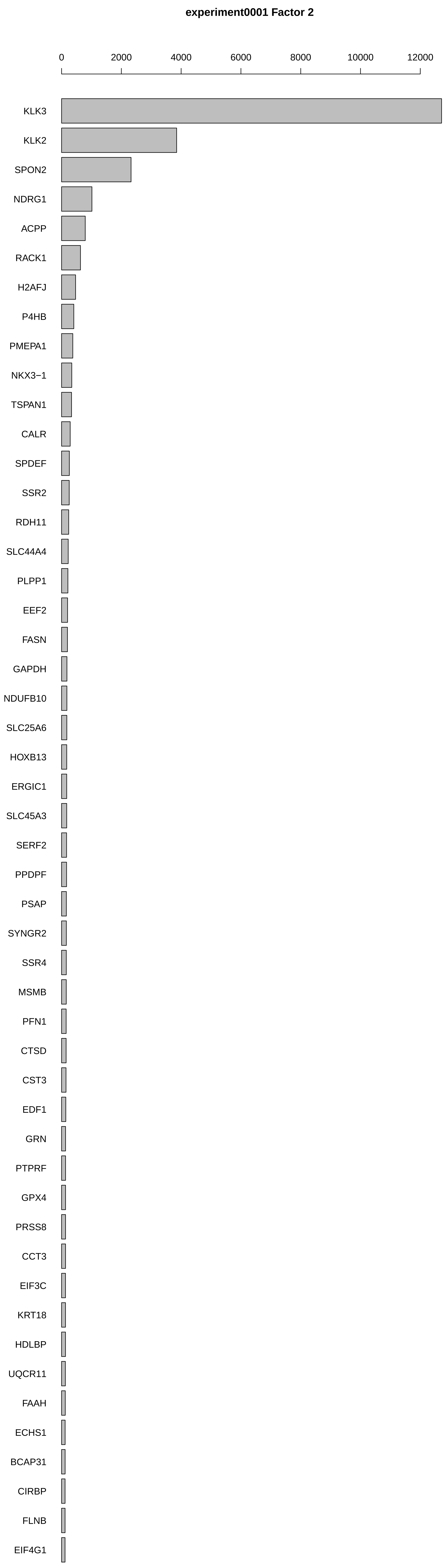
experiment0001 Factor 2
0
2000
4000
6000
8000
10000
12000
KLK3
KLK2
SPON2
NDRG1
ACPP
RACK1
H2AFJ
P4HB
PMEPA1
NKX3−1
TSPAN1
CALR
SPDEF
SSR2
RDH11
SLC44A4
PLPP1
EEF2
FASN
GAPDH
NDUFB10
SLC25A6
HOXB13
ERGIC1
SLC45A3
SERF2
PPDPF
PSAP
SYNGR2
SSR4
MSMB
PFN1
CTSD
CST3
EDF1
GRN
PTPRF
GPX4
PRSS8
CCT3
EIF3C
KRT18
HDLBP
UQCR11
FAAH
ECHS1
BCAP31
CIRBP
FLNB
EIF4G1

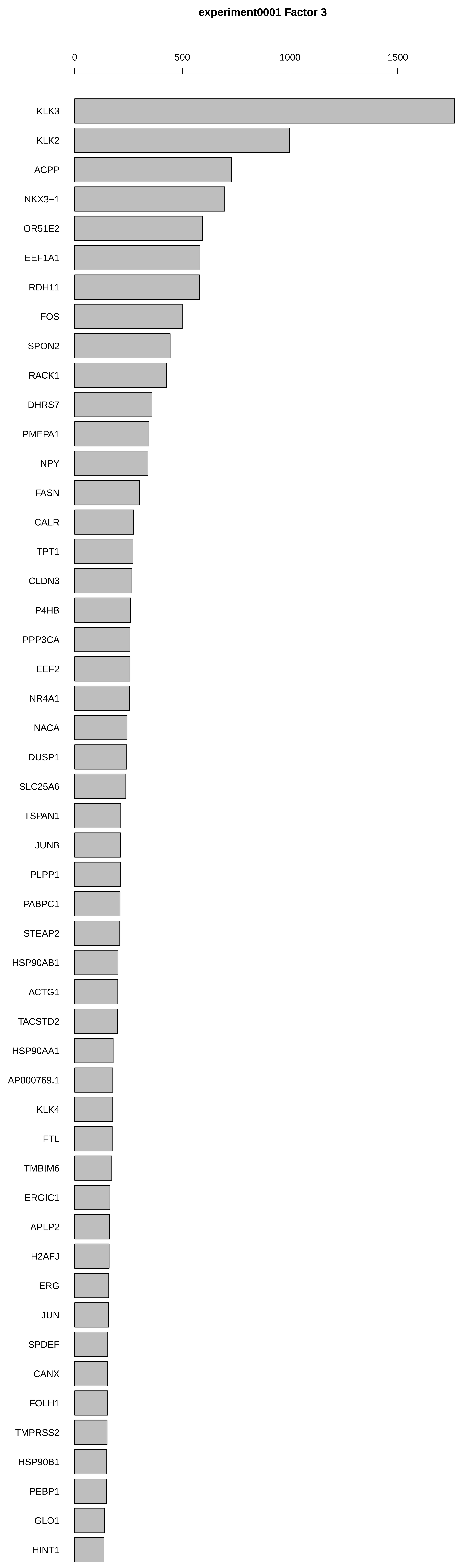
experiment0001 Factor 3
0
500
1000
1500
KLK3
KLK2
ACPP
NKX3−1
OR51E2
EEF1A1
RDH11
FOS
SPON2
RACK1
DHRS7
PMEPA1
NPY
FASN
CALR
TPT1
CLDN3
P4HB
PPP3CA
EEF2
NR4A1
NACA
DUSP1
SLC25A6
TSPAN1
JUNB
PLPP1
PABPC1
STEAP2
HSP90AB1
ACTG1
TACSTD2
HSP90AA1
AP000769.1
KLK4
FTL
TMBIM6
ERGIC1
APLP2
H2AFJ
ERG
JUN
SPDEF
CANX
FOLH1
TMPRSS2
HSP90B1
PEBP1
GLO1
HINT1

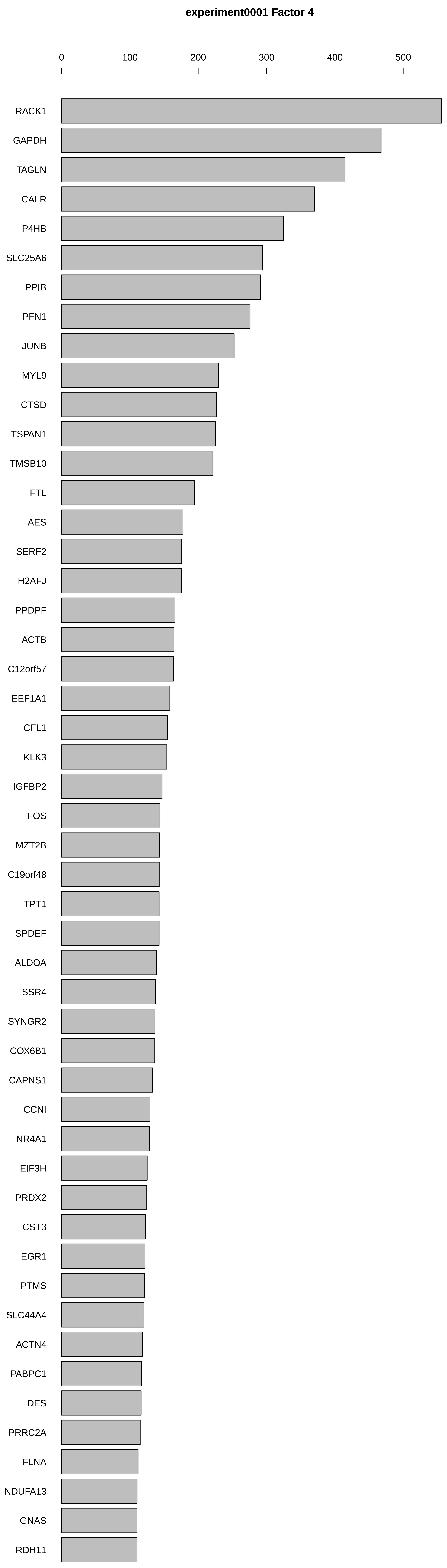
experiment0001 Factor 4
0
100
200
300
400
500
RACK1
GAPDH
TAGLN
CALR
P4HB
SLC25A6
PPIB
PFN1
JUNB
MYL9
CTSD
TSPAN1
TMSB10
FTL
AES
SERF2
H2AFJ
PPDPF
ACTB
C12orf57
EEF1A1
CFL1
KLK3
IGFBP2
FOS
MZT2B
C19orf48
TPT1
SPDEF
ALDOA
SSR4
SYNGR2
COX6B1
CAPNS1
CCNI
NR4A1
EIF3H
PRDX2
CST3
EGR1
PTMS
SLC44A4
ACTN4
PABPC1
DES
PRRC2A
FLNA
NDUFA13
GNAS
RDH11

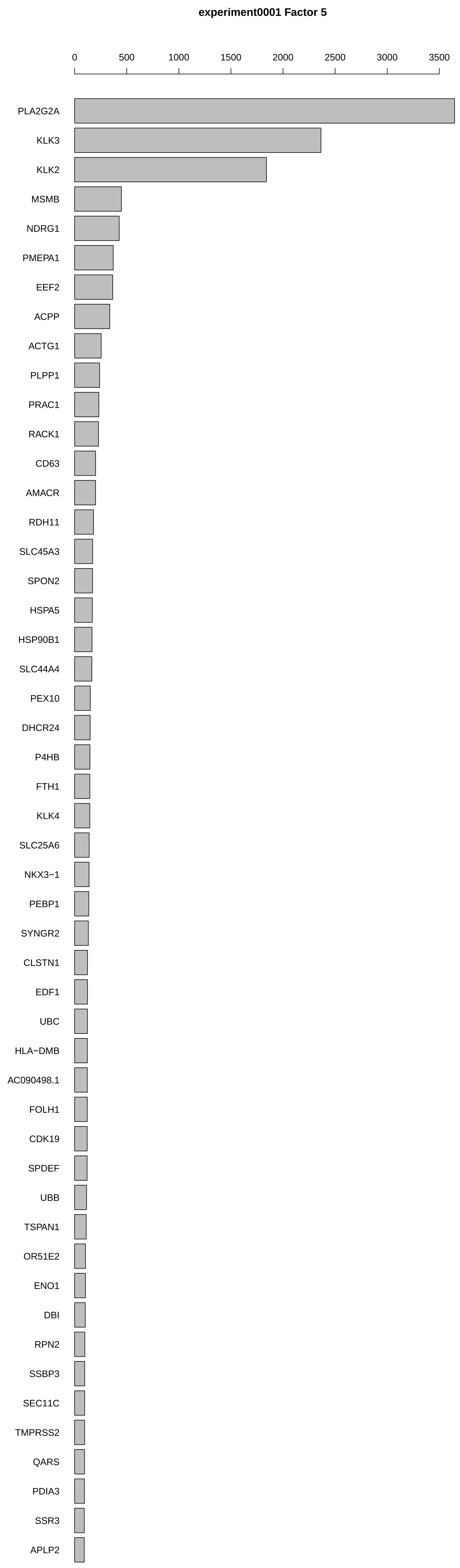
experiment0001 Factor 5
0
500
1000
1500
2000
2500
3000
3500
PLA2G2A
KLK3
KLK2
MSMB
NDRG1
PMEPA1
EEF2
ACPP
ACTG1
PLPP1
PRAC1
RACK1
CD63
AMACR
RDH11
SLC45A3
SPON2
HSPA5
HSP90B1
SLC44A4
PEX10
DHCR24
P4HB
FTH1
KLK4
SLC25A6
NKX3−1
PEBP1
SYNGR2
CLSTN1
EDF1
UBC
HLA−DMB
AC090498.1
FOLH1
CDK19
SPDEF
UBB
TSPAN1
OR51E2
ENO1
DBI
RPN2
SSBP3
SEC11C
TMPRSS2
QARS
PDIA3
SSR3
APLP2

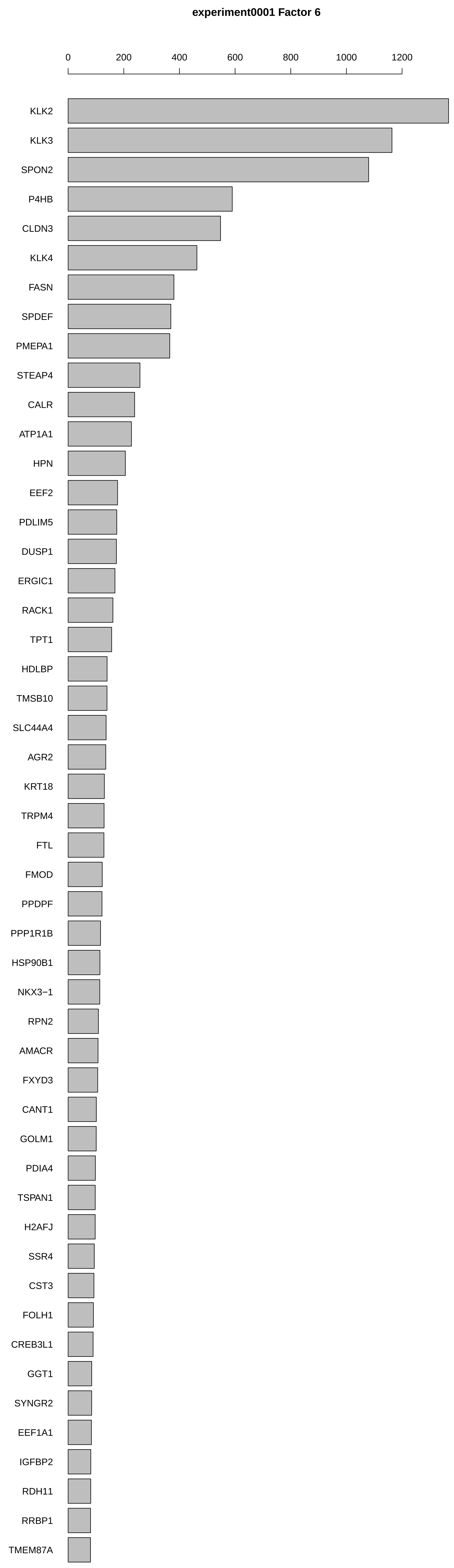
experiment0001 Factor 6
0
200
400
600
800
1000
1200
KLK2
KLK3
SPON2
P4HB
CLDN3
KLK4
FASN
SPDEF
PMEPA1
STEAP4
CALR
ATP1A1
HPN
EEF2
PDLIM5
DUSP1
ERGIC1
RACK1
TPT1
HDLBP
TMSB10
SLC44A4
AGR2
KRT18
TRPM4
FTL
FMOD
PPDPF
PPP1R1B
HSP90B1
NKX3−1
RPN2
AMACR
FXYD3
CANT1
GOLM1
PDIA4
TSPAN1
H2AFJ
SSR4
CST3
FOLH1
CREB3L1
GGT1
SYNGR2
EEF1A1
IGFBP2
RDH11
RRBP1
TMEM87A

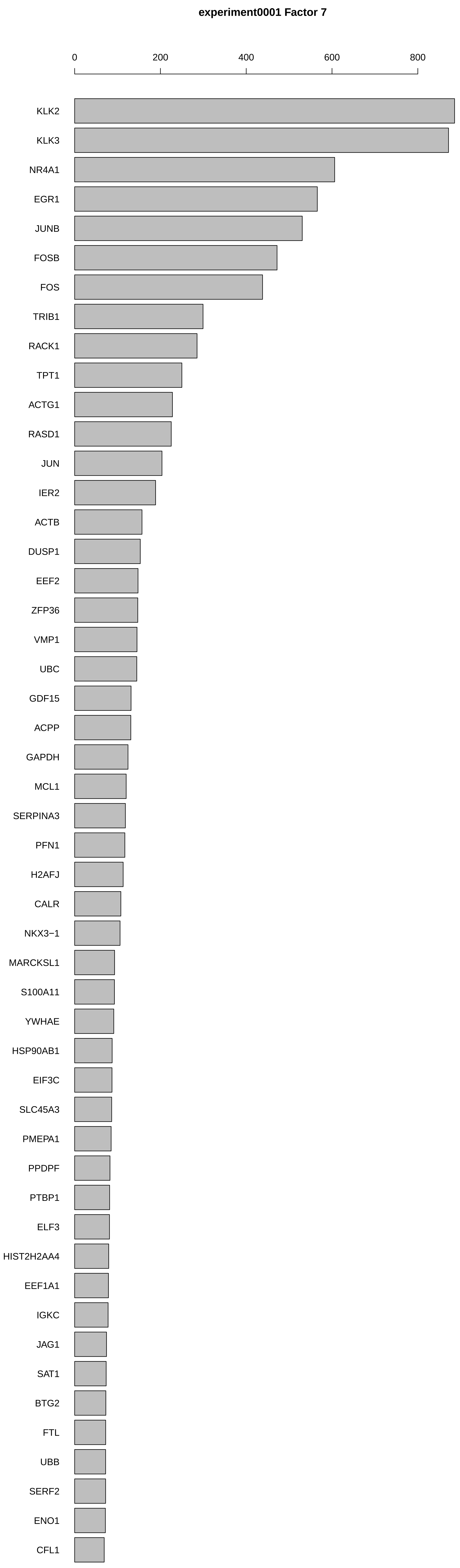
experiment0001 Factor 7
0
200
400
600
800
KLK2
KLK3
NR4A1
EGR1
JUNB
FOSB
FOS
TRIB1
RACK1
TPT1
ACTG1
RASD1
JUN
IER2
ACTB
DUSP1
EEF2
ZFP36
VMP1
UBC
GDF15
ACPP
GAPDH
MCL1
SERPINA3
PFN1
H2AFJ
CALR
NKX3−1
MARCKSL1
S100A11
YWHAE
HSP90AB1
EIF3C
SLC45A3
PMEPA1
PPDPF
PTBP1
ELF3
HIST2H2AA4
EEF1A1
IGKC
JAG1
SAT1
BTG2
FTL
UBB
SERF2
ENO1
CFL1

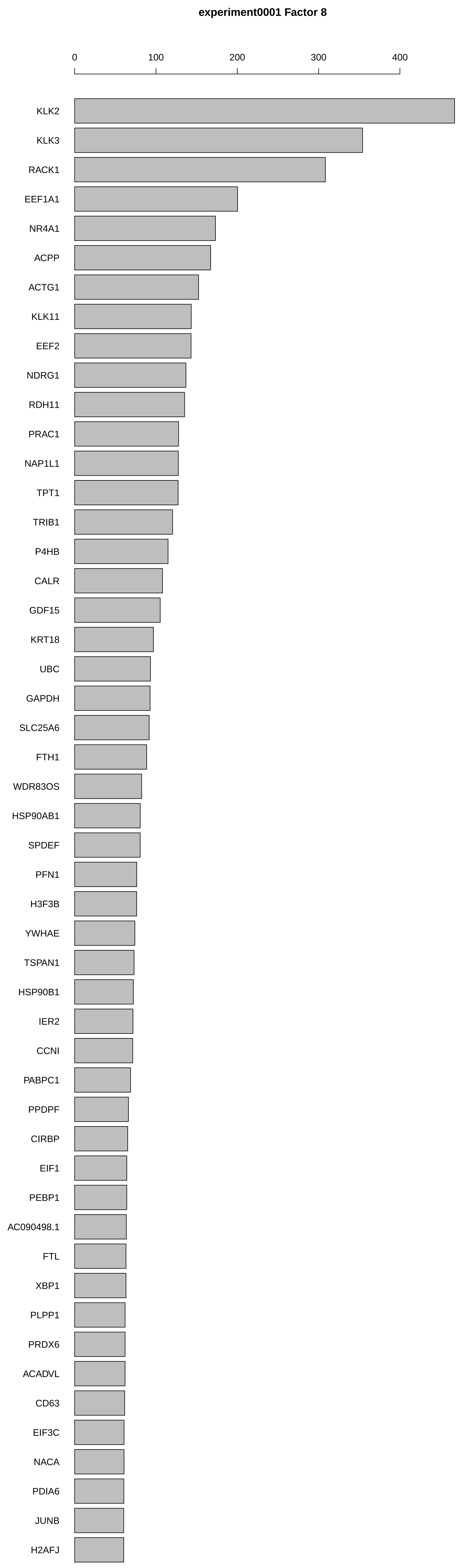
experiment0001 Factor 8
0
100
200
300
400
KLK2
KLK3
RACK1
EEF1A1
NR4A1
ACPP
ACTG1
KLK11
EEF2
NDRG1
RDH11
PRAC1
NAP1L1
TPT1
TRIB1
P4HB
CALR
GDF15
KRT18
UBC
GAPDH
SLC25A6
FTH1
WDR83OS
HSP90AB1
SPDEF
PFN1
H3F3B
YWHAE
TSPAN1
HSP90B1
IER2
CCNI
PABPC1
PPDPF
CIRBP
EIF1
PEBP1
AC090498.1
FTL
XBP1
PLPP1
PRDX6
ACADVL
CD63
EIF3C
NACA
PDIA6
JUNB
H2AFJ

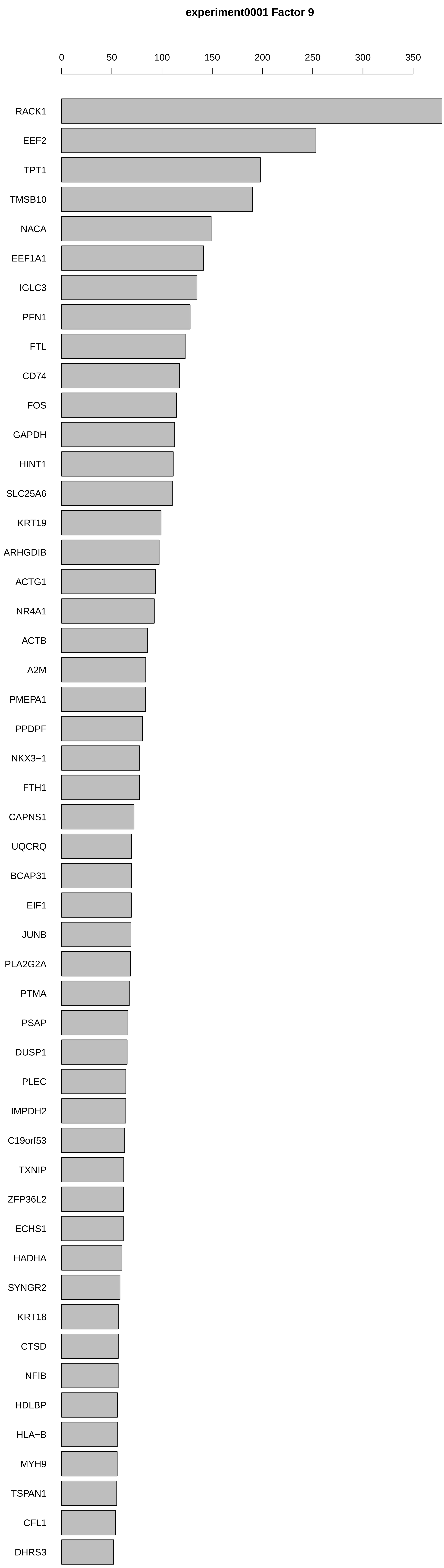
experiment0001 Factor 9
0
50
100
150
200
250
300
350
RACK1
EEF2
TPT1
TMSB10
NACA
EEF1A1
IGLC3
PFN1
FTL
CD74
FOS
GAPDH
HINT1
SLC25A6
KRT19
ARHGDIB
ACTG1
NR4A1
ACTB
A2M
PMEPA1
PPDPF
NKX3−1
FTH1
CAPNS1
UQCRQ
BCAP31
EIF1
JUNB
PLA2G2A
PTMA
PSAP
DUSP1
PLEC
IMPDH2
C19orf53
TXNIP
ZFP36L2
ECHS1
HADHA
SYNGR2
KRT18
CTSD
NFIB
HDLBP
HLA−B
MYH9
TSPAN1
CFL1
DHRS3

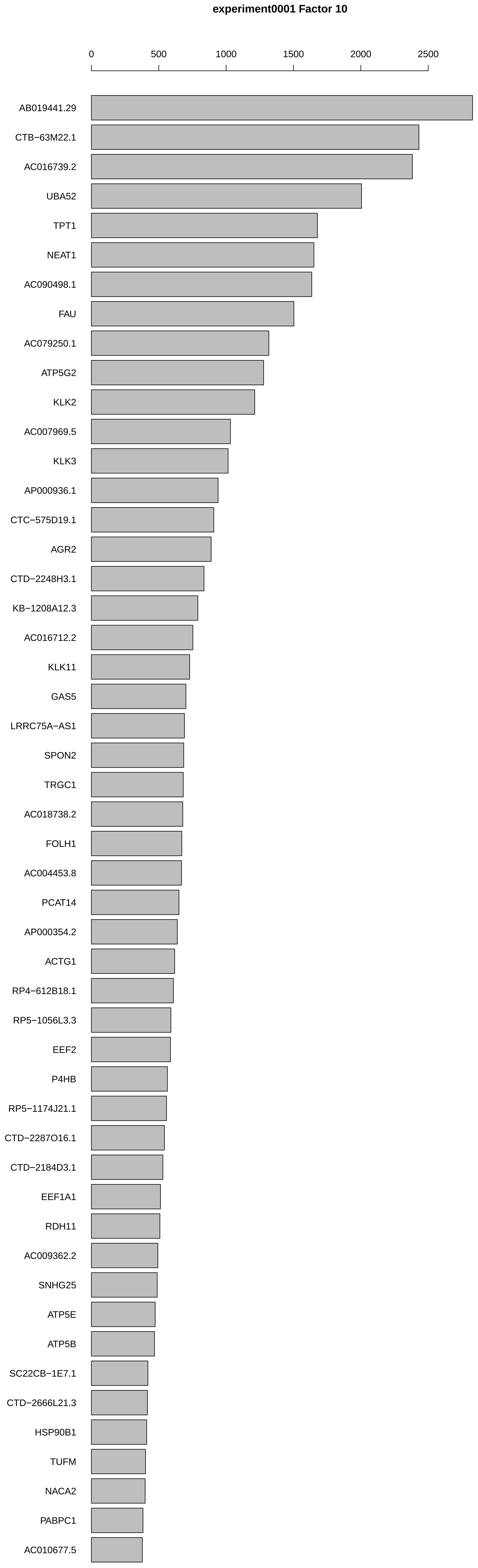
experiment0001 Factor 10
0
500
1000
1500
2000
2500
AB019441.29
CTB−63M22.1
AC016739.2
UBA52
TPT1
NEAT1
AC090498.1
FAU
AC079250.1
ATP5G2
KLK2
AC007969.5
KLK3
AP000936.1
CTC−575D19.1
AGR2
CTD−2248H3.1
KB−1208A12.3
AC016712.2
KLK11
GAS5
LRRC75A−AS1
SPON2
TRGC1
AC018738.2
FOLH1
AC004453.8
PCAT14
AP000354.2
ACTG1
RP4−612B18.1
RP5−1056L3.3
EEF2
P4HB
RP5−1174J21.1
CTD−2287O16.1
CTD−2184D3.1
EEF1A1
RDH11
AC009362.2
SNHG25
ATP5E
ATP5B
SC22CB−1E7.1
CTD−2666L21.3
HSP90B1
TUFM
NACA2
PABPC1
AC010677.5

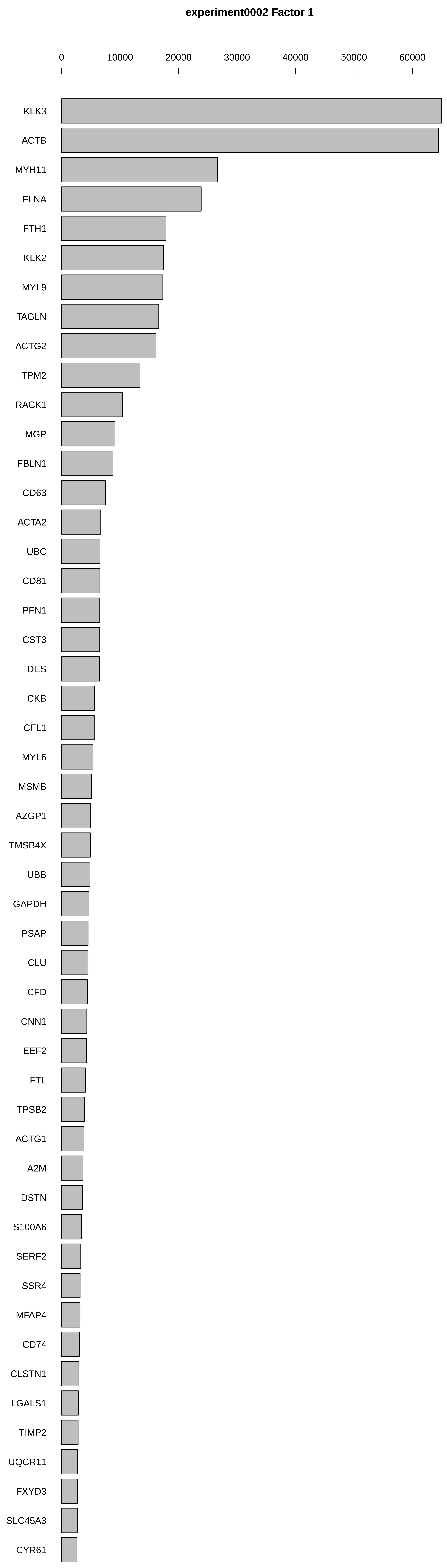
experiment0002 Factor 1
0
10000
20000
30000
40000
50000
60000
KLK3
ACTB
MYH11
FLNA
FTH1
KLK2
MYL9
TAGLN
ACTG2
TPM2
RACK1
MGP
FBLN1
CD63
ACTA2
UBC
CD81
PFN1
CST3
DES
CKB
CFL1
MYL6
MSMB
AZGP1
TMSB4X
UBB
GAPDH
PSAP
CLU
CFD
CNN1
EEF2
FTL
TPSB2
ACTG1
A2M
DSTN
S100A6
SERF2
SSR4
MFAP4
CD74
CLSTN1
LGALS1
TIMP2
UQCR11
FXYD3
SLC45A3
CYR61

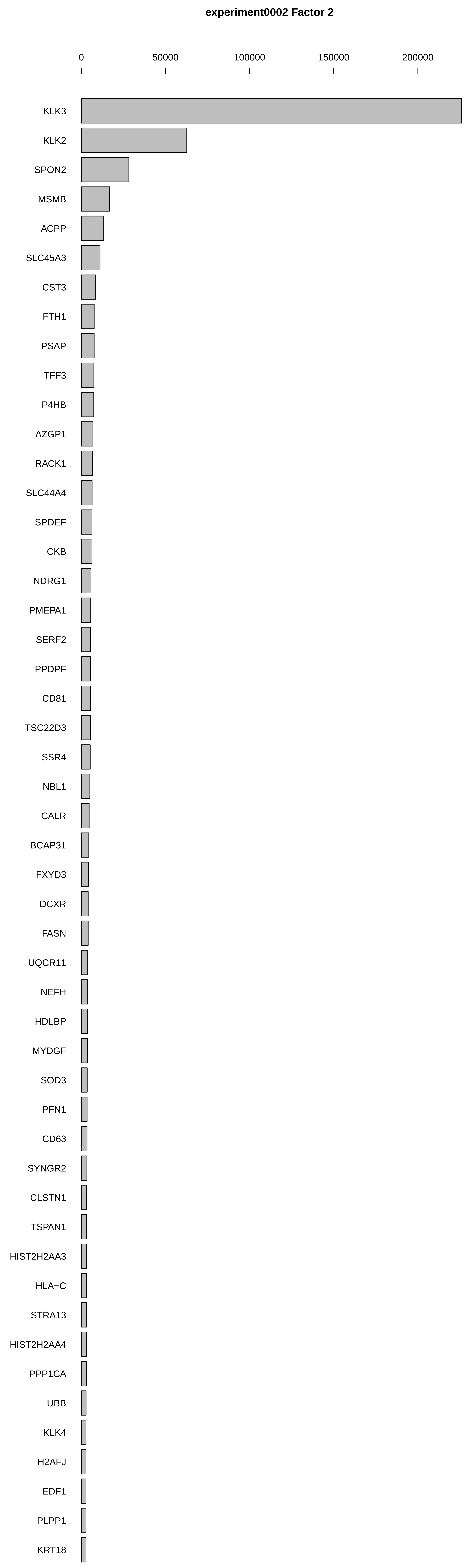

experiment0002 Factor 2
0
50000
100000
150000
200000
KLK3
KLK2
SPON2
MSMB
ACPP
SLC45A3
CST3
FTH1
PSAP
TFF3
P4HB
AZGP1
RACK1
SLC44A4
SPDEF
CKB
NDRG1
PMEPA1
SERF2
PPDPF
CD81
TSC22D3
SSR4
NBL1
CALR
BCAP31
FXYD3
DCXR
FASN
UQCR11
NEFH
HDLBP
MYDGF
SOD3
PFN1
CD63
SYNGR2
CLSTN1
TSPAN1
HIST2H2AA3
HLA−C
STRA13
HIST2H2AA4
PPP1CA
UBB
KLK4
H2AFJ
EDF1
PLPP1
KRT18

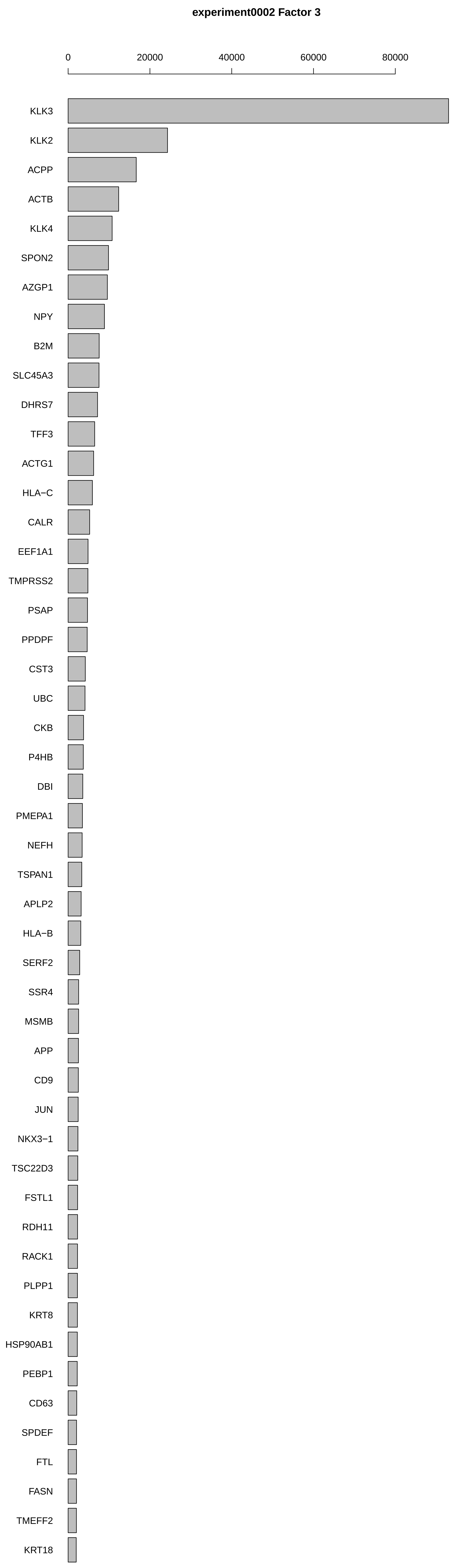

experiment0002 Factor 3
0
20000
40000
60000
80000
KLK3
KLK2
ACPP
ACTB
KLK4
SPON2
AZGP1
NPY
B2M
SLC45A3
DHRS7
TFF3
ACTG1
HLA−C
CALR
EEF1A1
TMPRSS2
PSAP
PPDPF
CST3
UBC
CKB
P4HB
DBI
PMEPA1
NEFH
TSPAN1
APLP2
HLA−B
SERF2
SSR4
MSMB
APP
CD9
JUN
NKX3−1
TSC22D3
FSTL1
RDH11
RACK1
PLPP1
KRT8
HSP90AB1
PEBP1
CD63
SPDEF
FTL
FASN
TMEFF2
KRT18

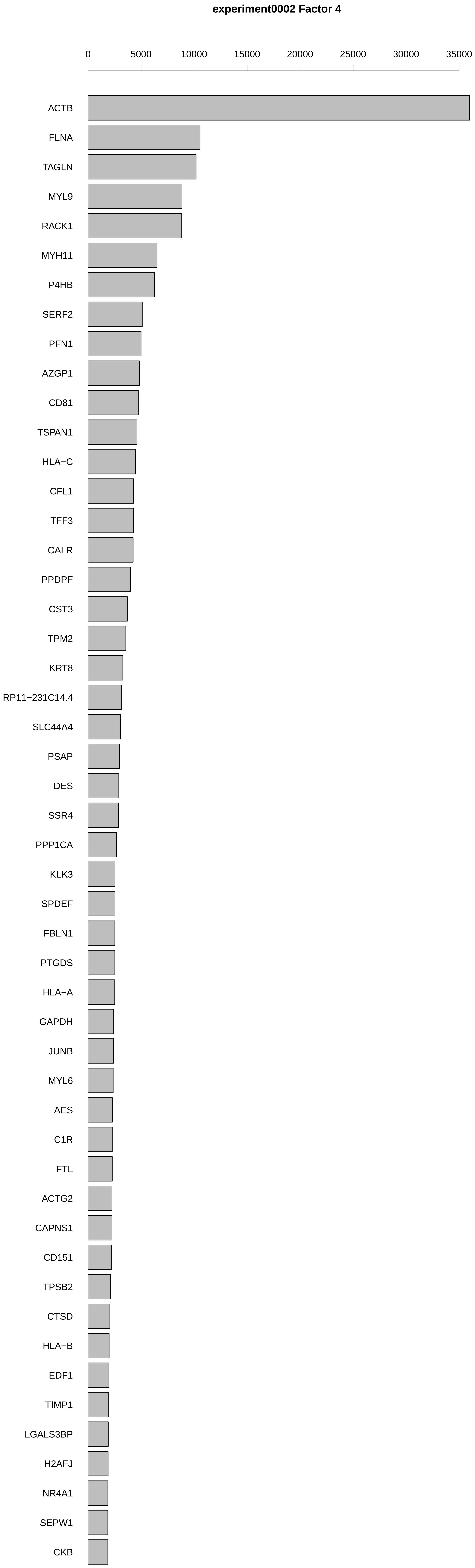
experiment0002 Factor 4
0
5000
10000
15000
20000
25000
30000
35000
ACTB
FLNA
TAGLN
MYL9
RACK1
MYH11
P4HB
SERF2
PFN1
AZGP1
CD81
TSPAN1
HLA−C
CFL1
TFF3
CALR
PPDPF
CST3
TPM2
KRT8
RP11−231C14.4
SLC44A4
PSAP
DES
SSR4
PPP1CA
KLK3
SPDEF
FBLN1
PTGDS
HLA−A
GAPDH
JUNB
MYL6
AES
C1R
FTL
ACTG2
CAPNS1
CD151
TPSB2
CTSD
HLA−B
EDF1
TIMP1
LGALS3BP
H2AFJ
NR4A1
SEPW1
CKB

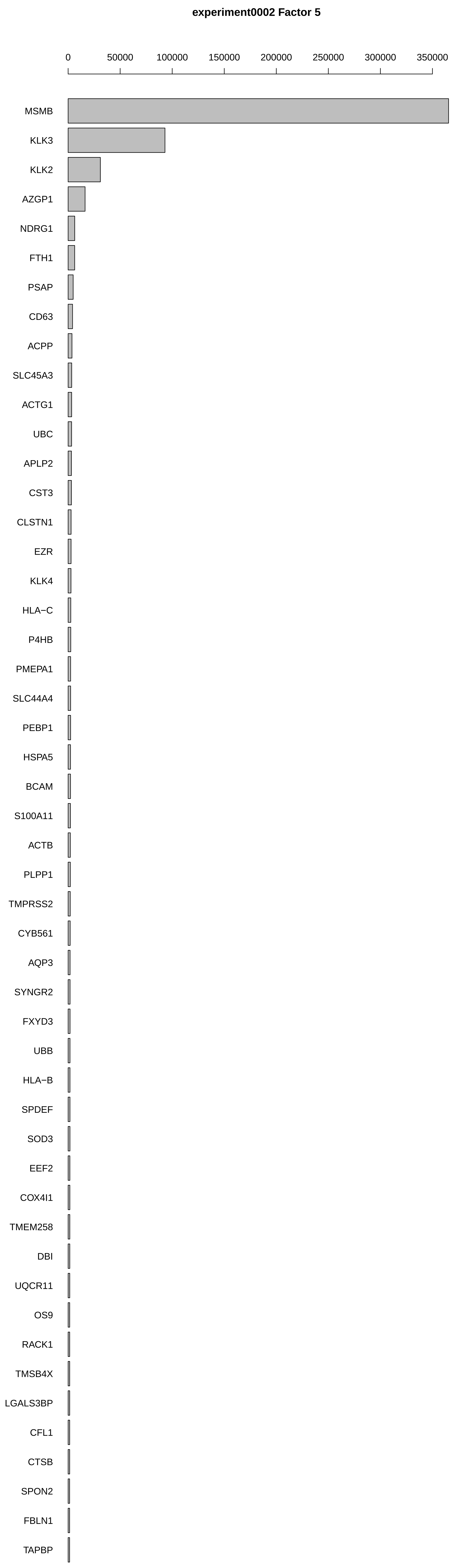
experiment0002 Factor 5
0
50000
100000
150000
200000
250000
300000
350000
MSMB
KLK3
KLK2
AZGP1
NDRG1
FTH1
PSAP
CD63
ACPP
SLC45A3
ACTG1
UBC
APLP2
CST3
CLSTN1
EZR
KLK4
HLA−C
P4HB
PMEPA1
SLC44A4
PEBP1
HSPA5
BCAM
S100A11
ACTB
PLPP1
TMPRSS2
CYB561
AQP3
SYNGR2
FXYD3
UBB
HLA−B
SPDEF
SOD3
EEF2
COX4I1
TMEM258
DBI
UQCR11
OS9
RACK1
TMSB4X
LGALS3BP
CFL1
CTSB
SPON2
FBLN1
TAPBP

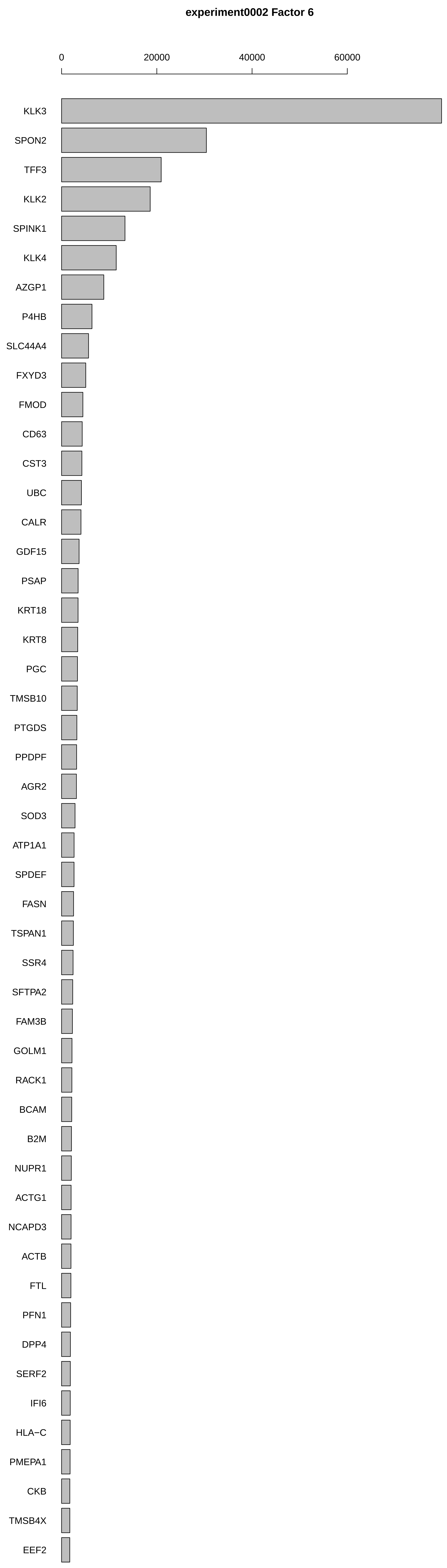

experiment0002 Factor 6
0
20000
40000
60000
KLK3
SPON2
TFF3
KLK2
SPINK1
KLK4
AZGP1
P4HB
SLC44A4
FXYD3
FMOD
CD63
CST3
UBC
CALR
GDF15
PSAP
KRT18
KRT8
PGC
TMSB10
PTGDS
PPDPF
AGR2
SOD3
ATP1A1
SPDEF
FASN
TSPAN1
SSR4
SFTPA2
FAM3B
GOLM1
RACK1
BCAM
B2M
NUPR1
ACTG1
NCAPD3
ACTB
FTL
PFN1
DPP4
SERF2
IFI6
HLA−C
PMEPA1
CKB
TMSB4X
EEF2

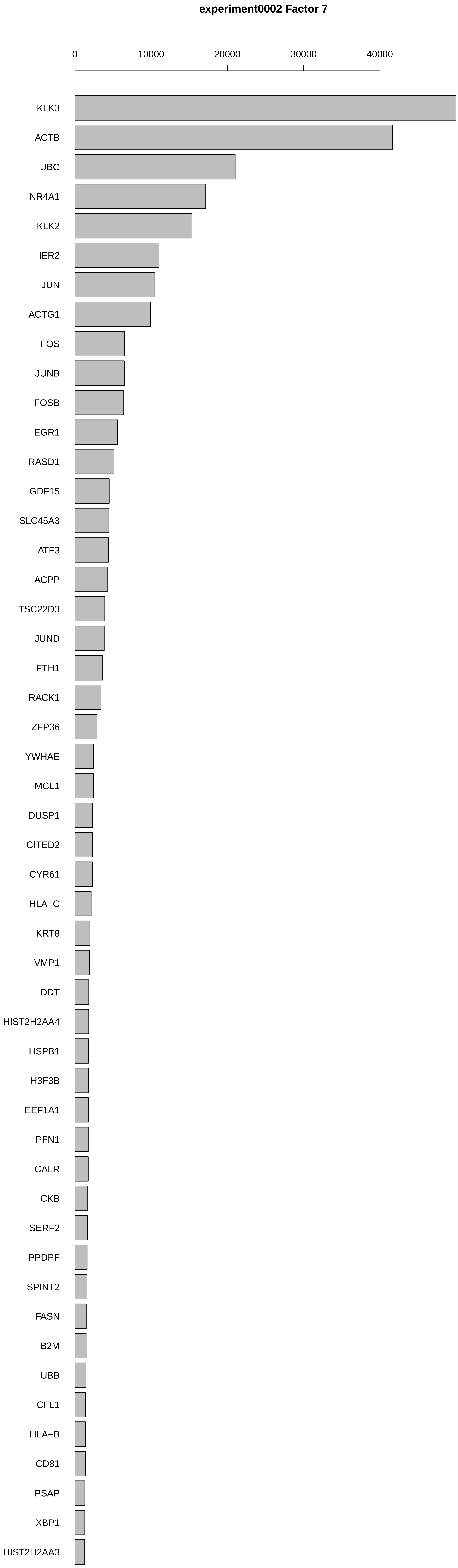

experiment0002 Factor 7
0
10000
20000
30000
40000
KLK3
ACTB
UBC
NR4A1
KLK2
IER2
JUN
ACTG1
FOS
JUNB
FOSB
EGR1
RASD1
GDF15
SLC45A3
ATF3
ACPP
TSC22D3
JUND
FTH1
RACK1
ZFP36
YWHAE
MCL1
DUSP1
CITED2
CYR61
HLA−C
KRT8
VMP1
DDT
HIST2H2AA4
HSPB1
H3F3B
EEF1A1
PFN1
CALR
CKB
SERF2
PPDPF
SPINT2
FASN
B2M
UBB
CFL1
HLA−B
CD81
PSAP
XBP1
HIST2H2AA3

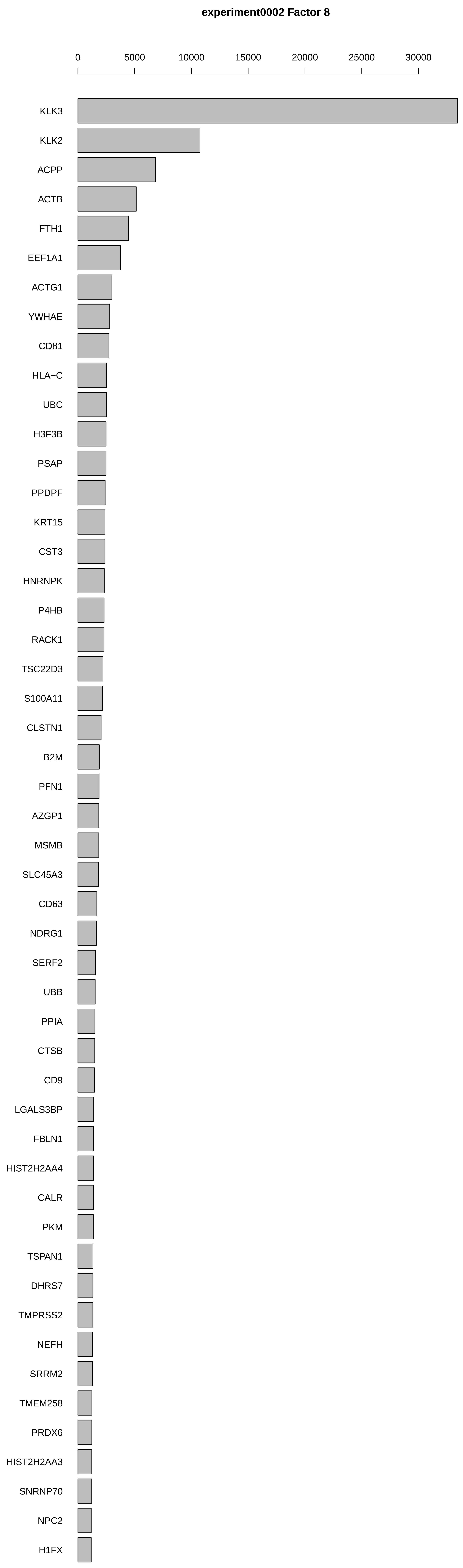
experiment0002 Factor 8
0
5000
10000
15000
20000
25000
30000
KLK3
KLK2
ACPP
ACTB
FTH1
EEF1A1
ACTG1
YWHAE
CD81
HLA−C
UBC
H3F3B
PSAP
PPDPF
KRT15
CST3
HNRNPK
P4HB
RACK1
TSC22D3
S100A11
CLSTN1
B2M
PFN1
AZGP1
MSMB
SLC45A3
CD63
NDRG1
SERF2
UBB
PPIA
CTSB
CD9
LGALS3BP
FBLN1
HIST2H2AA4
CALR
PKM
TSPAN1
DHRS7
TMPRSS2
NEFH
SRRM2
TMEM258
PRDX6
HIST2H2AA3
SNRNP70
NPC2
H1FX

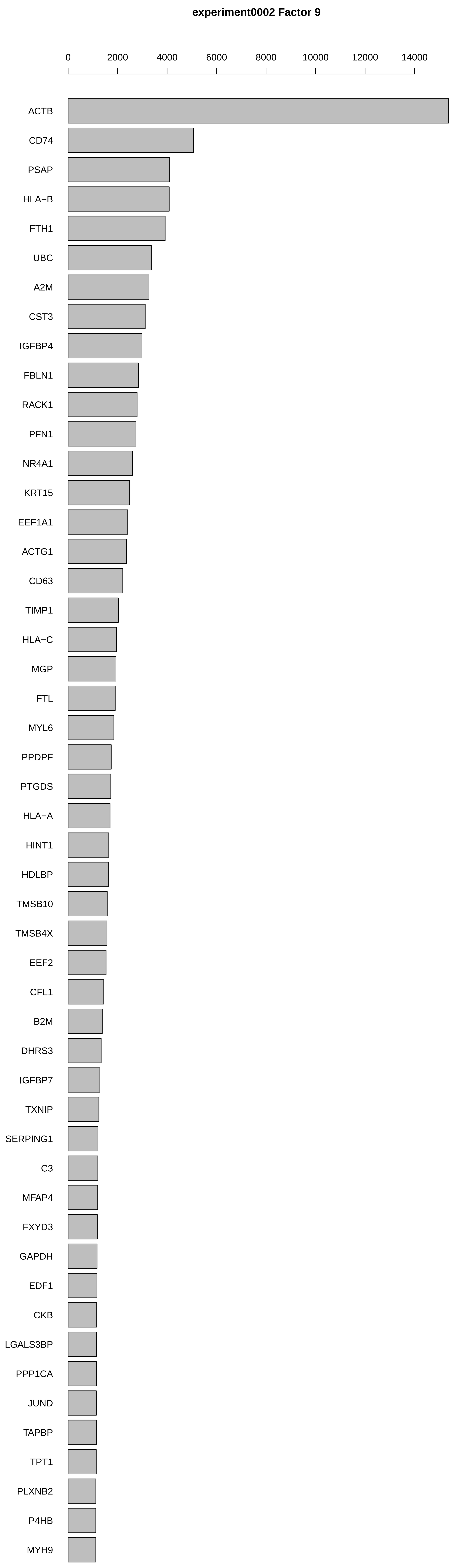
experiment0002 Factor 9
0
2000
4000
6000
8000
10000
12000
14000
ACTB
CD74
PSAP
HLA−B
FTH1
UBC
A2M
CST3
IGFBP4
FBLN1
RACK1
PFN1
NR4A1
KRT15
EEF1A1
ACTG1
CD63
TIMP1
HLA−C
MGP
FTL
MYL6
PPDPF
PTGDS
HLA−A
HINT1
HDLBP
TMSB10
TMSB4X
EEF2
CFL1
B2M
DHRS3
IGFBP7
TXNIP
SERPING1
C3
MFAP4
FXYD3
GAPDH
EDF1
CKB
LGALS3BP
PPP1CA
JUND
TAPBP
TPT1
PLXNB2
P4HB
MYH9

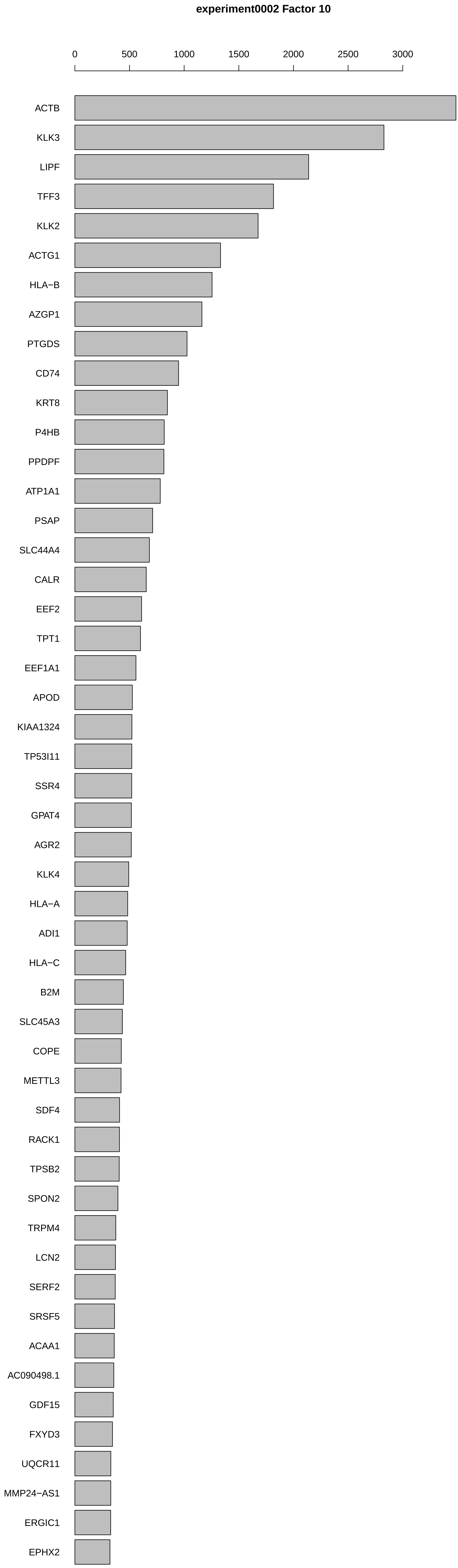
experiment0002 Factor 10
0
500
1000
1500
2000
2500
3000
ACTB
KLK3
LIPF
TFF3
KLK2
ACTG1
HLA−B
AZGP1
PTGDS
CD74
KRT8
P4HB
PPDPF
ATP1A1
PSAP
SLC44A4
CALR
EEF2
TPT1
EEF1A1
APOD
KIAA1324
TP53I11
SSR4
GPAT4
AGR2
KLK4
HLA−A
ADI1
HLA−C
B2M
SLC45A3
COPE
METTL3
SDF4
RACK1
TPSB2
SPON2
TRPM4
LCN2
SERF2
SRSF5
ACAA1
AC090498.1
GDF15
FXYD3
UQCR11
MMP24−AS1
ERGIC1
EPHX2

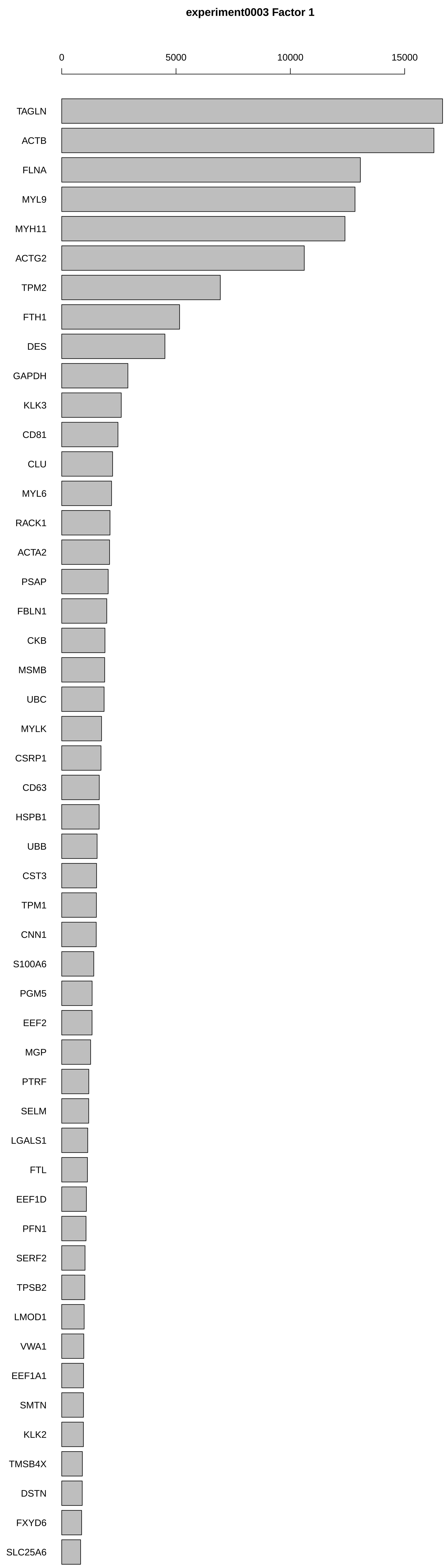
experiment0003 Factor 1
0
5000
10000
15000
TAGLN
ACTB
FLNA
MYL9
MYH11
ACTG2
TPM2
FTH1
DES
GAPDH
KLK3
CD81
CLU
MYL6
RACK1
ACTA2
PSAP
FBLN1
CKB
MSMB
UBC
MYLK
CSRP1
CD63
HSPB1
UBB
CST3
TPM1
CNN1
S100A6
PGM5
EEF2
MGP
PTRF
SELM
LGALS1
FTL
EEF1D
PFN1
SERF2
TPSB2
LMOD1
VWA1
EEF1A1
SMTN
KLK2
TMSB4X
DSTN
FXYD6
SLC25A6

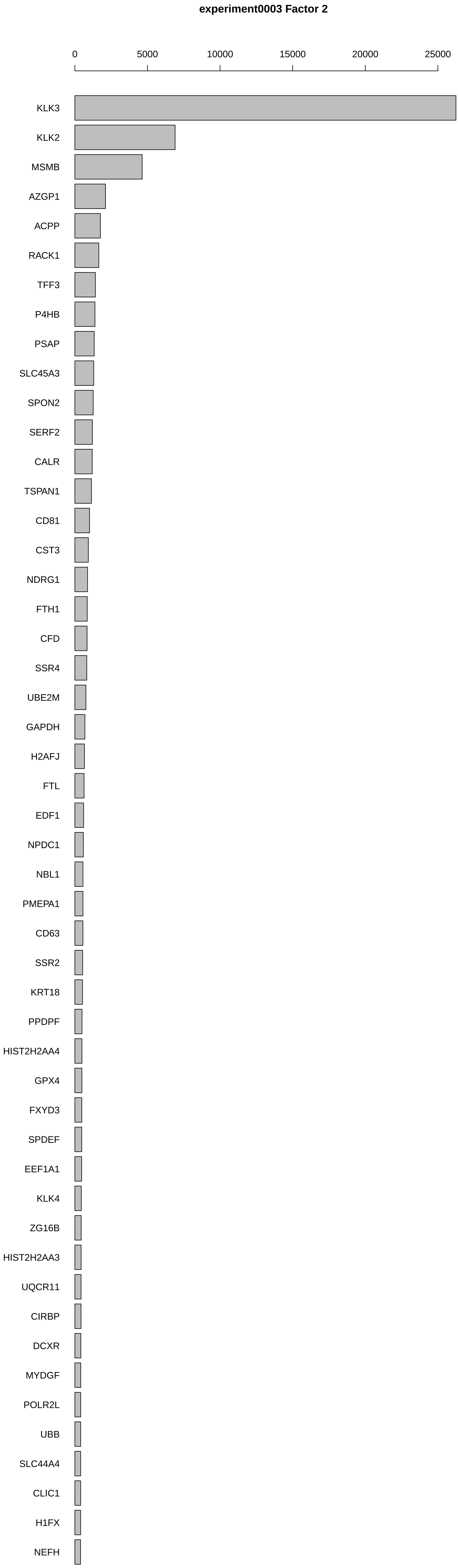
experiment0003 Factor 2
0
5000
10000
15000
20000
25000
KLK3
KLK2
MSMB
AZGP1
ACPP
RACK1
TFF3
P4HB
PSAP
SLC45A3
SPON2
SERF2
CALR
TSPAN1
CD81
CST3
NDRG1
FTH1
CFD
SSR4
UBE2M
GAPDH
H2AFJ
FTL
EDF1
NPDC1
NBL1
PMEPA1
CD63
SSR2
KRT18
PPDPF
HIST2H2AA4
GPX4
FXYD3
SPDEF
EEF1A1
KLK4
ZG16B
HIST2H2AA3
UQCR11
CIRBP
DCXR
MYDGF
POLR2L
UBB
SLC44A4
CLIC1
H1FX
NEFH

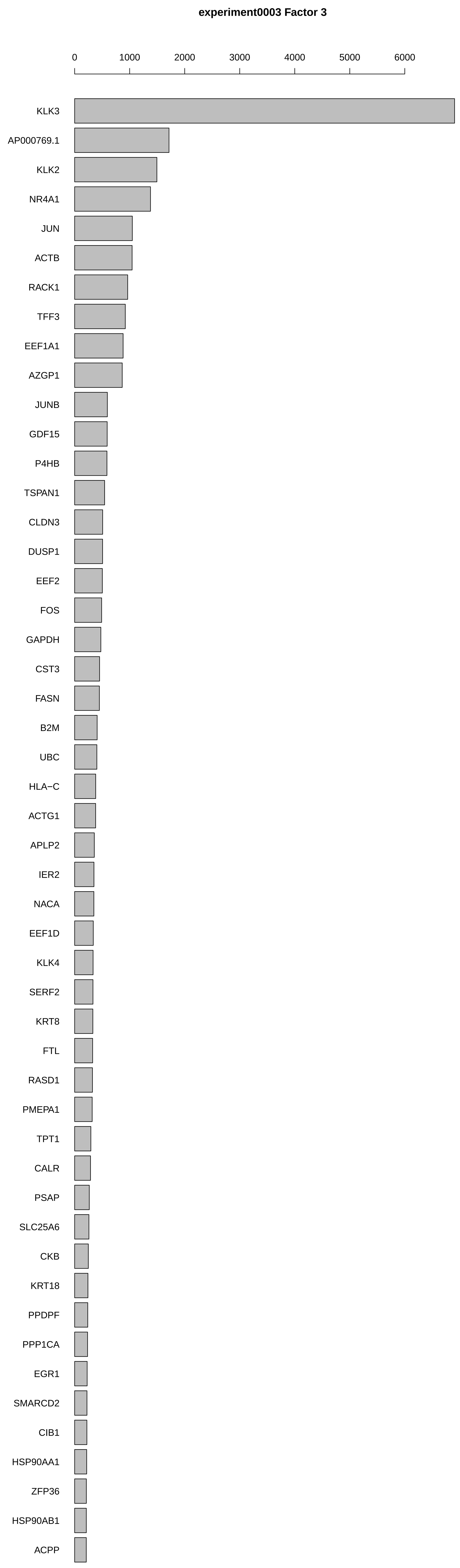
experiment0003 Factor 3
0
1000
2000
3000
4000
5000
6000
KLK3
AP000769.1
KLK2
NR4A1
JUN
ACTB
RACK1
TFF3
EEF1A1
AZGP1
JUNB
GDF15
P4HB
TSPAN1
CLDN3
DUSP1
EEF2
FOS
GAPDH
CST3
FASN
B2M
UBC
HLA−C
ACTG1
APLP2
IER2
NACA
EEF1D
KLK4
SERF2
KRT8
FTL
RASD1
PMEPA1
TPT1
CALR
PSAP
SLC25A6
CKB
KRT18
PPDPF
PPP1CA
EGR1
SMARCD2
CIB1
HSP90AA1
ZFP36
HSP90AB1
ACPP

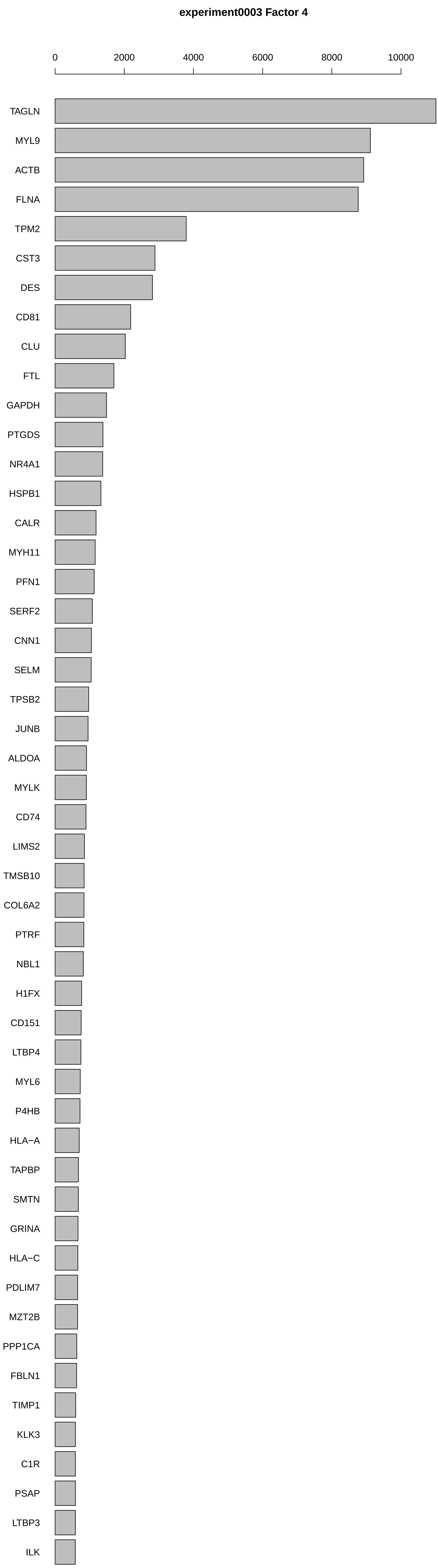
experiment0003 Factor 4
0
2000
4000
6000
8000
10000
TAGLN
MYL9
ACTB
FLNA
TPM2
CST3
DES
CD81
CLU
FTL
GAPDH
PTGDS
NR4A1
HSPB1
CALR
MYH11
PFN1
SERF2
CNN1
SELM
TPSB2
JUNB
ALDOA
MYLK
CD74
LIMS2
TMSB10
COL6A2
PTRF
NBL1
H1FX
CD151
LTBP4
MYL6
P4HB
HLA−A
TAPBP
SMTN
GRINA
HLA−C
PDLIM7
MZT2B
PPP1CA
FBLN1
TIMP1
KLK3
C1R
PSAP
LTBP3
ILK

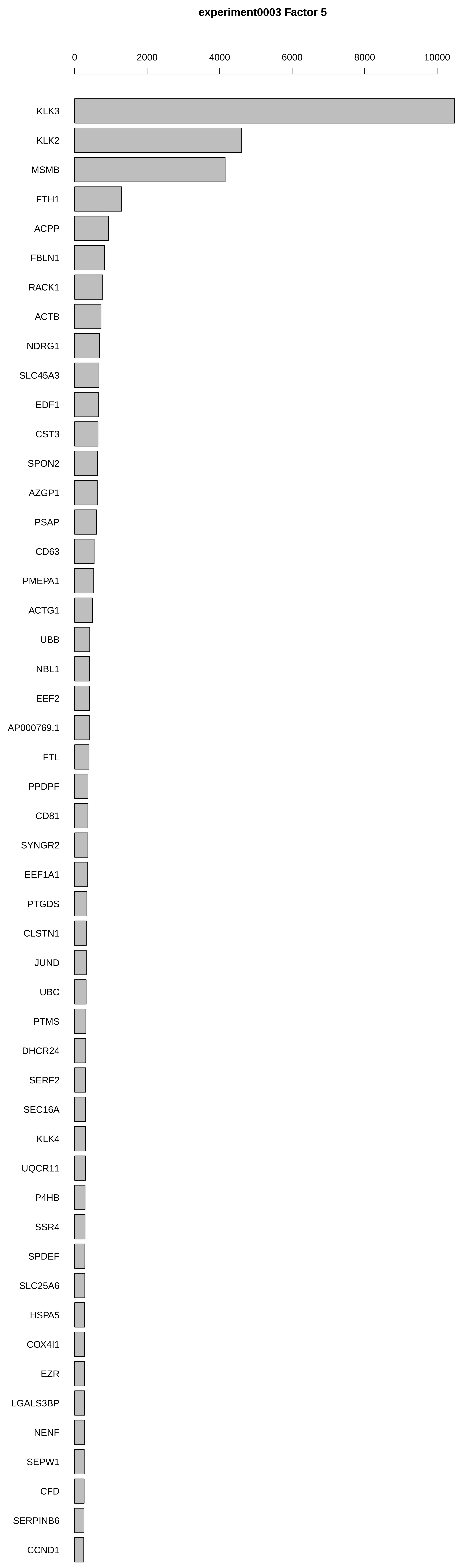
experiment0003 Factor 5
0
2000
4000
6000
8000
10000
KLK3
KLK2
MSMB
FTH1
ACPP
FBLN1
RACK1
ACTB
NDRG1
SLC45A3
EDF1
CST3
SPON2
AZGP1
PSAP
CD63
PMEPA1
ACTG1
UBB
NBL1
EEF2
AP000769.1
FTL
PPDPF
CD81
SYNGR2
EEF1A1
PTGDS
CLSTN1
JUND
UBC
PTMS
DHCR24
SERF2
SEC16A
KLK4
UQCR11
P4HB
SSR4
SPDEF
SLC25A6
HSPA5
COX4I1
EZR
LGALS3BP
NENF
SEPW1
CFD
SERPINB6
CCND1

experiment0003 Factor 6

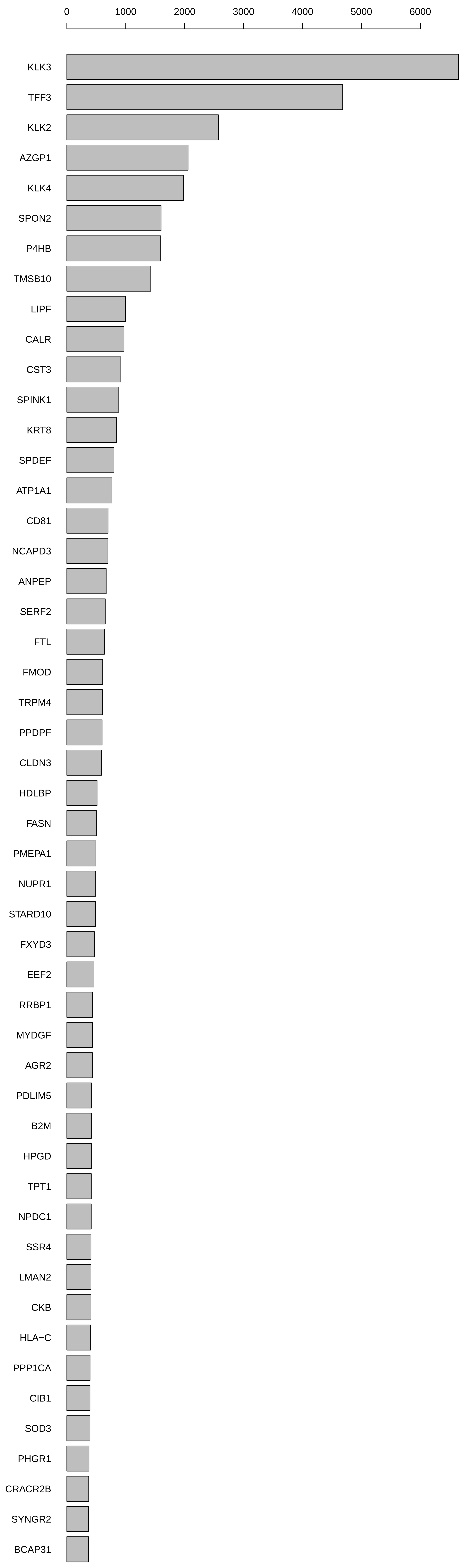

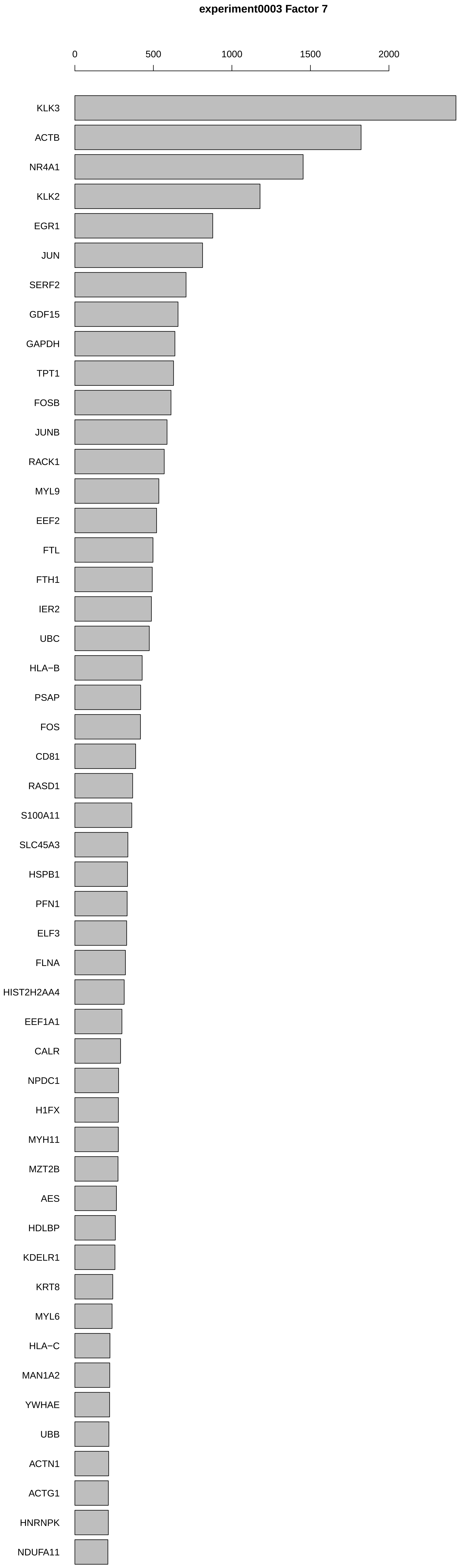
experiment0003 Factor 7
0
500
1000
1500
2000
KLK3
ACTB
NR4A1
KLK2
EGR1
JUN
SERF2
GDF15
GAPDH
TPT1
FOSB
JUNB
RACK1
MYL9
EEF2
FTL
FTH1
IER2
UBC
HLA−B
PSAP
FOS
CD81
RASD1
S100A11
SLC45A3
HSPB1
PFN1
ELF3
FLNA
HIST2H2AA4
EEF1A1
CALR
NPDC1
H1FX
MYH11
MZT2B
AES
HDLBP
KDELR1
KRT8
MYL6
HLA−C
MAN1A2
YWHAE
UBB
ACTN1
ACTG1
HNRNPK
NDUFA11

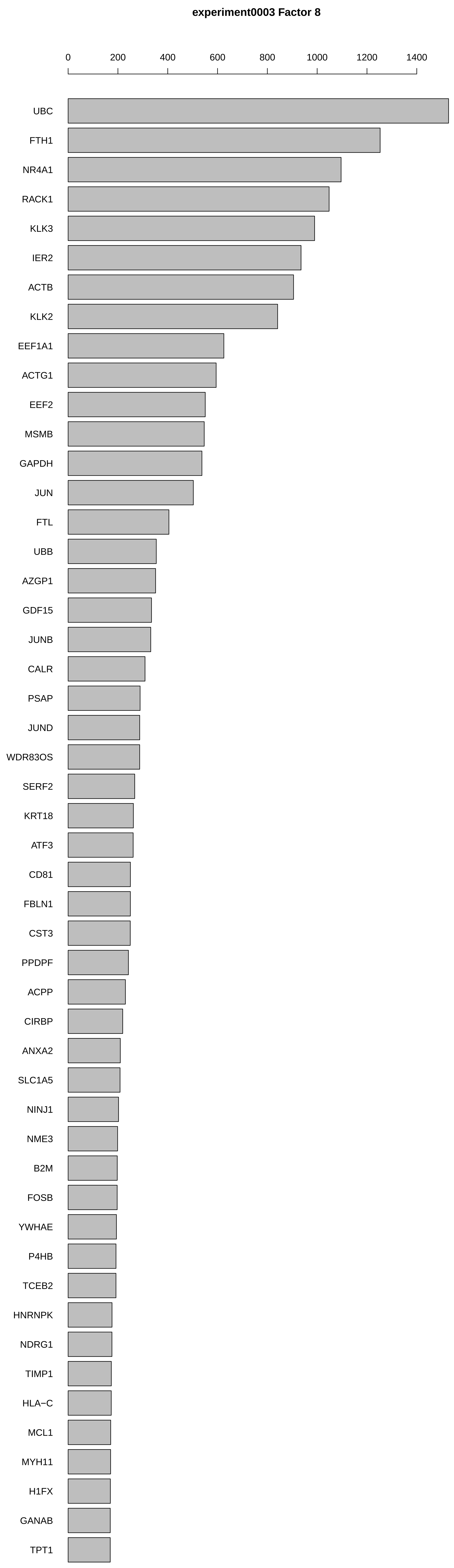
experiment0003 Factor 8
0
200
400
600
800
1000
1200
1400
UBC
FTH1
NR4A1
RACK1
KLK3
IER2
ACTB
KLK2
EEF1A1
ACTG1
EEF2
MSMB
GAPDH
JUN
FTL
UBB
AZGP1
GDF15
JUNB
CALR
PSAP
JUND
WDR83OS
SERF2
KRT18
ATF3
CD81
FBLN1
CST3
PPDPF
ACPP
CIRBP
ANXA2
SLC1A5
NINJ1
NME3
B2M
FOSB
YWHAE
P4HB
TCEB2
HNRNPK
NDRG1
TIMP1
HLA−C
MCL1
MYH11
H1FX
GANAB
TPT1

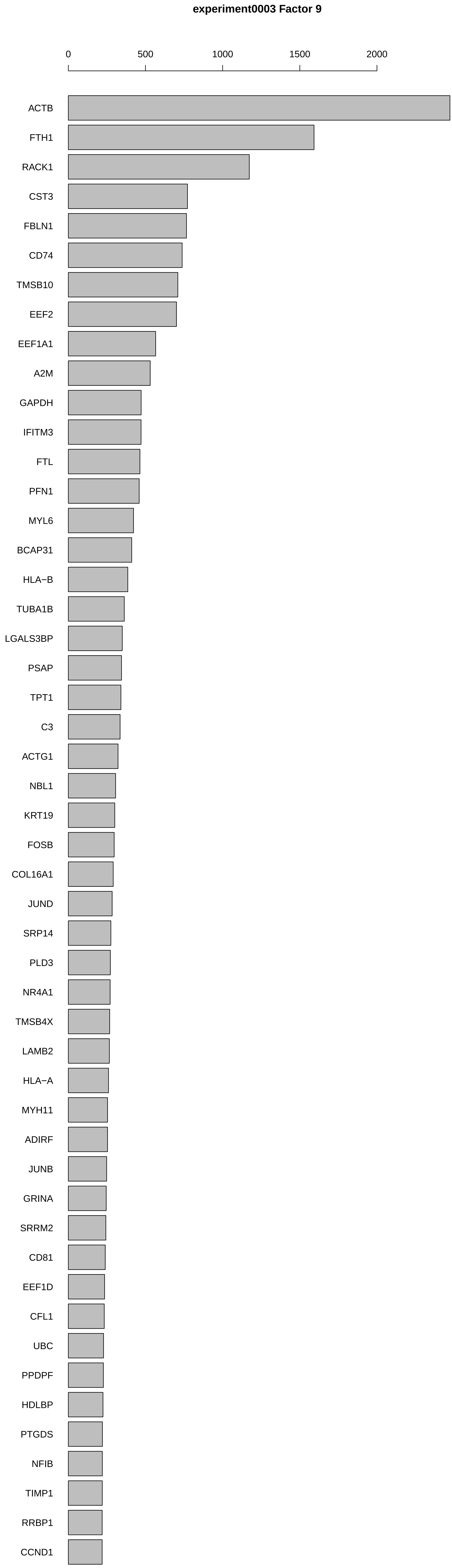
experiment0003 Factor 9
0
500
1000
1500
2000
ACTB
FTH1
RACK1
CST3
FBLN1
CD74
TMSB10
EEF2
EEF1A1
A2M
GAPDH
IFITM3
FTL
PFN1
MYL6
BCAP31
HLA−B
TUBA1B
LGALS3BP
PSAP
TPT1
C3
ACTG1
NBL1
KRT19
FOSB
COL16A1
JUND
SRP14
PLD3
NR4A1
TMSB4X
LAMB2
HLA−A
MYH11
ADIRF
JUNB
GRINA
SRRM2
CD81
EEF1D
CFL1
UBC
PPDPF
HDLBP
PTGDS
NFIB
TIMP1
RRBP1
CCND1

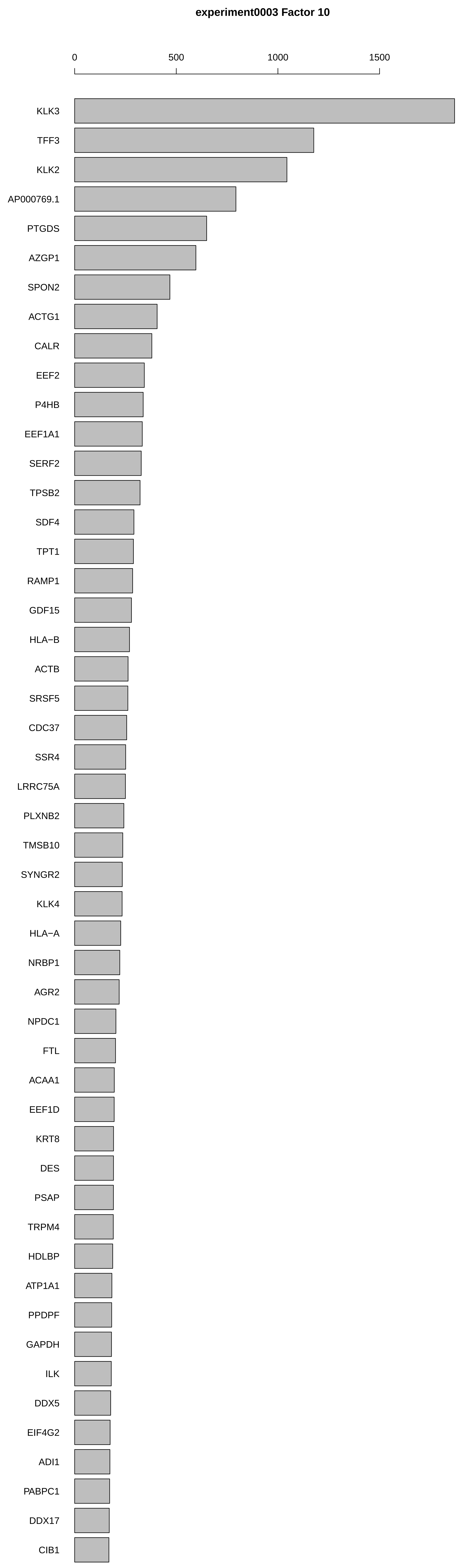
experiment0003 Factor 10
0
500
1000
1500
KLK3
TFF3
KLK2
AP000769.1
PTGDS
AZGP1
SPON2
ACTG1
CALR
EEF2
P4HB
EEF1A1
SERF2
TPSB2
SDF4
TPT1
RAMP1
GDF15
HLA−B
ACTB
SRSF5
CDC37
SSR4
LRRC75A
PLXNB2
TMSB10
SYNGR2
KLK4
HLA−A
NRBP1
AGR2
NPDC1
FTL
ACAA1
EEF1D
KRT8
DES
PSAP
TRPM4
HDLBP
ATP1A1
PPDPF
GAPDH
ILK
DDX5
EIF4G2
ADI1
PABPC1
DDX17
CIB1

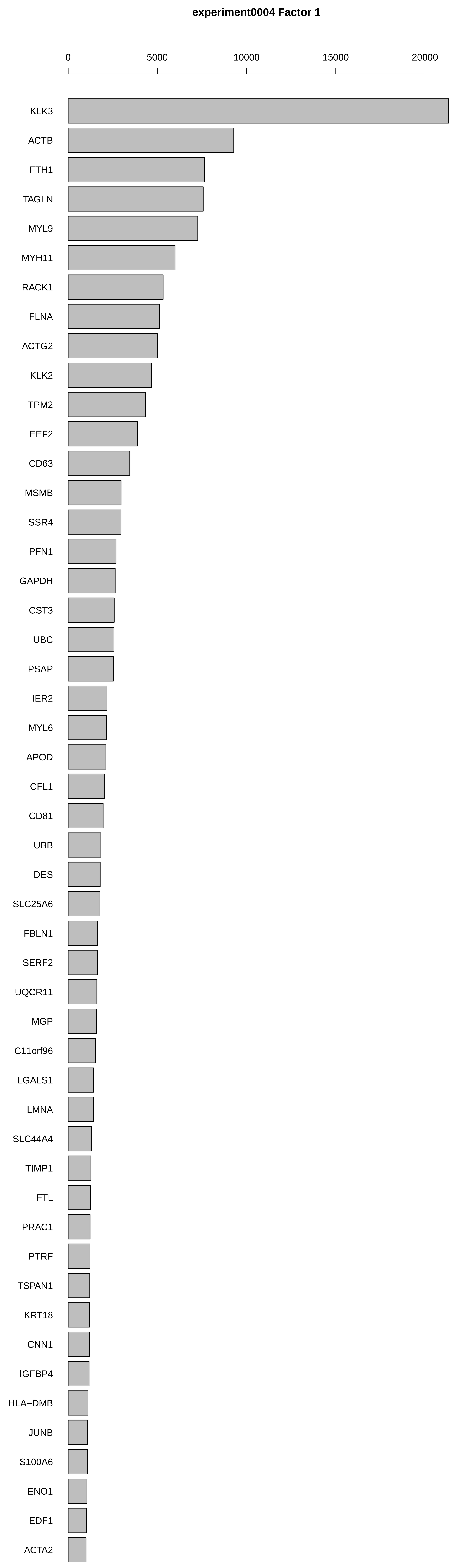
experiment0004 Factor 1
0
5000
10000
15000
20000
KLK3
ACTB
FTH1
TAGLN
MYL9
MYH11
RACK1
FLNA
ACTG2
KLK2
TPM2
EEF2
CD63
MSMB
SSR4
PFN1
GAPDH
CST3
UBC
PSAP
IER2
MYL6
APOD
CFL1
CD81
UBB
DES
SLC25A6
FBLN1
SERF2
UQCR11
MGP
C11orf96
LGALS1
LMNA
SLC44A4
TIMP1
FTL
PRAC1
PTRF
TSPAN1
KRT18
CNN1
IGFBP4
HLA−DMB
JUNB
S100A6
ENO1
EDF1
ACTA2

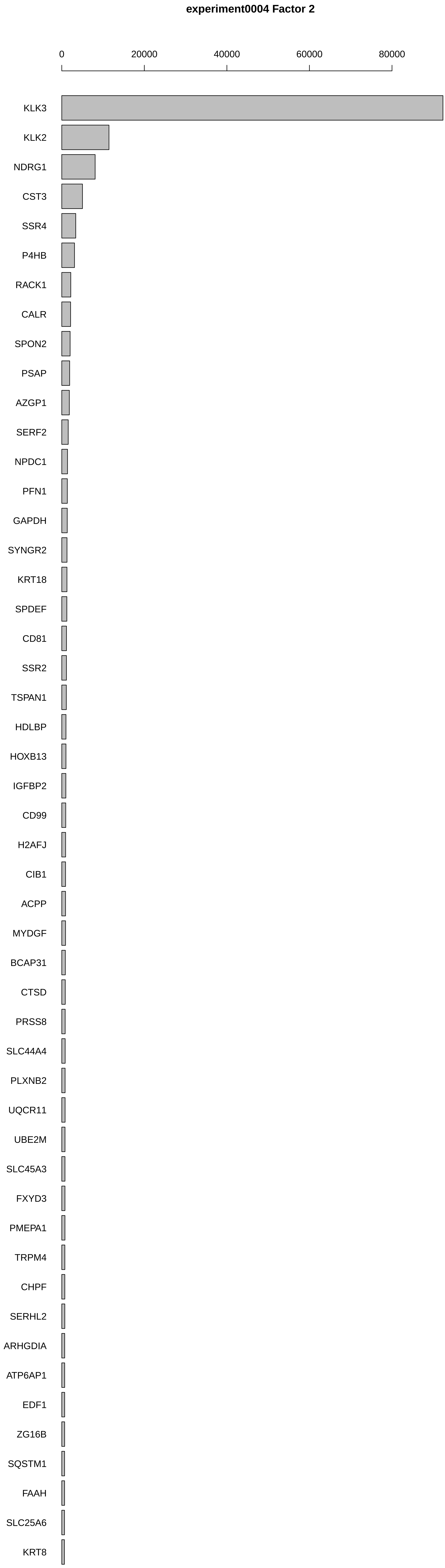
experiment0004 Factor 2
0
20000
40000
60000
80000
KLK3
KLK2
NDRG1
CST3
SSR4
P4HB
RACK1
CALR
SPON2
PSAP
AZGP1
SERF2
NPDC1
PFN1
GAPDH
SYNGR2
KRT18
SPDEF
CD81
SSR2
TSPAN1
HDLBP
HOXB13
IGFBP2
CD99
H2AFJ
CIB1
ACPP
MYDGF
BCAP31
CTSD
PRSS8
SLC44A4
PLXNB2
UQCR11
UBE2M
SLC45A3
FXYD3
PMEPA1
TRPM4
CHPF
SERHL2
ARHGDIA
ATP6AP1
EDF1
ZG16B
SQSTM1
FAAH
SLC25A6
KRT8

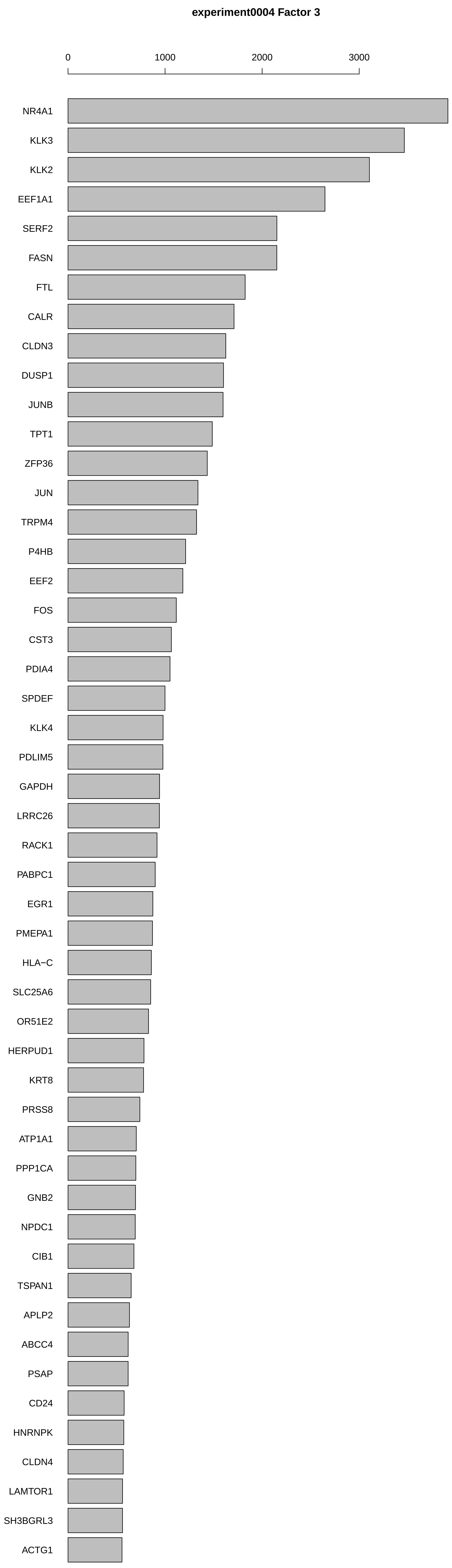
experiment0004 Factor 3
0
1000
2000
3000
NR4A1
KLK3
KLK2
EEF1A1
SERF2
FASN
FTL
CALR
CLDN3
DUSP1
JUNB
TPT1
ZFP36
JUN
TRPM4
P4HB
EEF2
FOS
CST3
PDIA4
SPDEF
KLK4
PDLIM5
GAPDH
LRRC26
RACK1
PABPC1
EGR1
PMEPA1
HLA−C
SLC25A6
OR51E2
HERPUD1
KRT8
PRSS8
ATP1A1
PPP1CA
GNB2
NPDC1
CIB1
TSPAN1
APLP2
ABCC4
PSAP
CD24
HNRNPK
CLDN4
LAMTOR1
SH3BGRL3
ACTG1

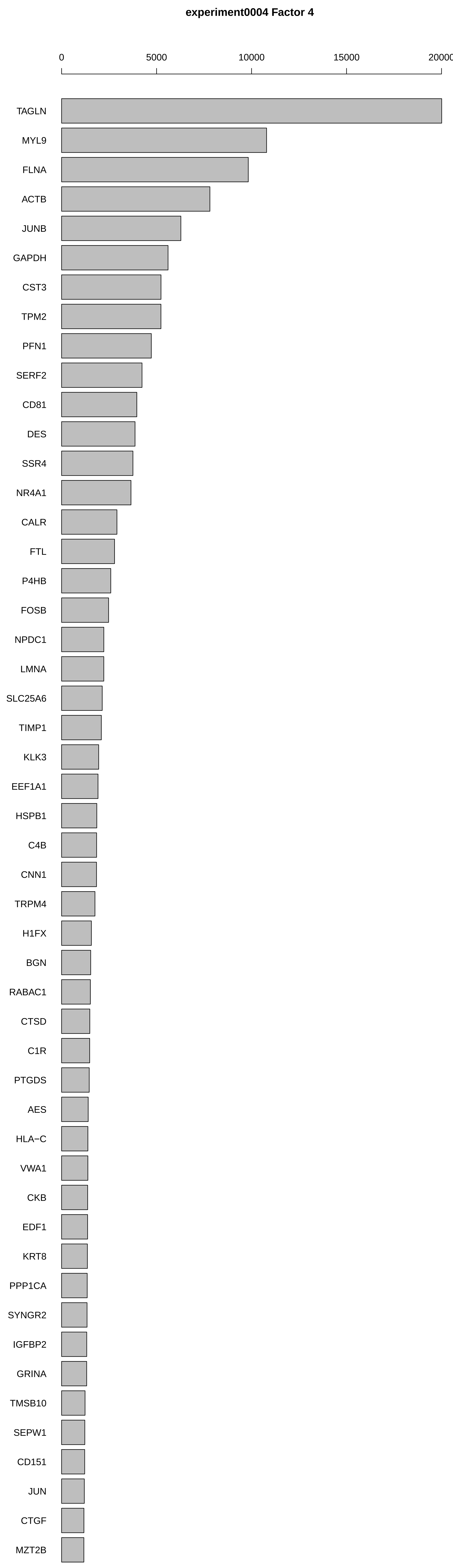
experiment0004 Factor 4
0
5000
10000
15000
20000
TAGLN
MYL9
FLNA
ACTB
JUNB
GAPDH
CST3
TPM2
PFN1
SERF2
CD81
DES
SSR4
NR4A1
CALR
FTL
P4HB
FOSB
NPDC1
LMNA
SLC25A6
TIMP1
KLK3
EEF1A1
HSPB1
C4B
CNN1
TRPM4
H1FX
BGN
RABAC1
CTSD
C1R
PTGDS
AES
HLA−C
VWA1
CKB
EDF1
KRT8
PPP1CA
SYNGR2
IGFBP2
GRINA
TMSB10
SEPW1
CD151
JUN
CTGF
MZT2B

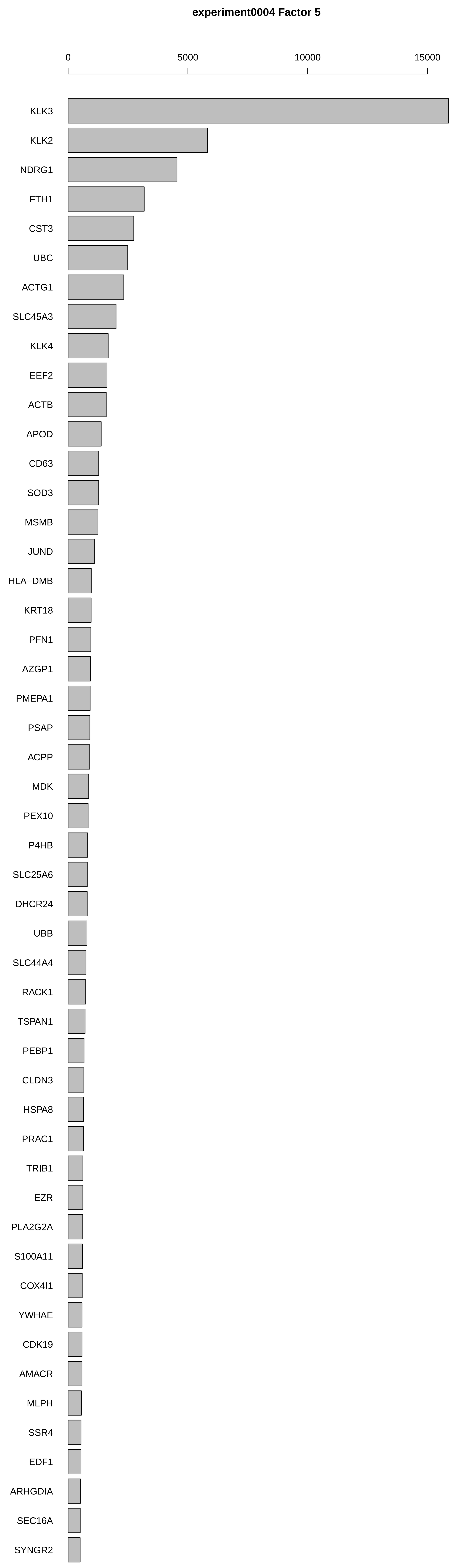
experiment0004 Factor 5
0
5000
10000
15000
KLK3
KLK2
NDRG1
FTH1
CST3
UBC
ACTG1
SLC45A3
KLK4
EEF2
ACTB
APOD
CD63
SOD3
MSMB
JUND
HLA−DMB
KRT18
PFN1
AZGP1
PMEPA1
PSAP
ACPP
MDK
PEX10
P4HB
SLC25A6
DHCR24
UBB
SLC44A4
RACK1
TSPAN1
PEBP1
CLDN3
HSPA8
PRAC1
TRIB1
EZR
PLA2G2A
S100A11
COX4I1
YWHAE
CDK19
AMACR
MLPH
SSR4
EDF1
ARHGDIA
SEC16A
SYNGR2

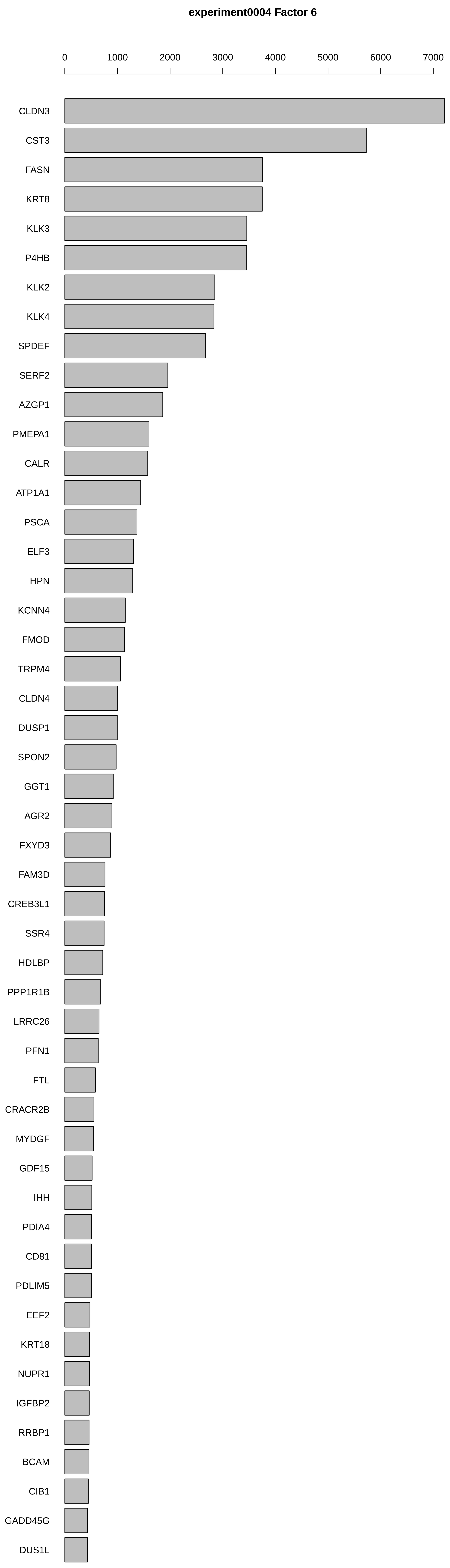

experiment0004 Factor 6
0
1000
2000
3000
4000
5000
6000
7000
CLDN3
CST3
FASN
KRT8
KLK3
P4HB
KLK2
KLK4
SPDEF
SERF2
AZGP1
PMEPA1
CALR
ATP1A1
PSCA
ELF3
HPN
KCNN4
FMOD
TRPM4
CLDN4
DUSP1
SPON2
GGT1
AGR2
FXYD3
FAM3D
CREB3L1
SSR4
HDLBP
PPP1R1B
LRRC26
PFN1
FTL
CRACR2B
MYDGF
GDF15
IHH
PDIA4
CD81
PDLIM5
EEF2
KRT18
NUPR1
IGFBP2
RRBP1
BCAM
CIB1
GADD45G
DUS1L

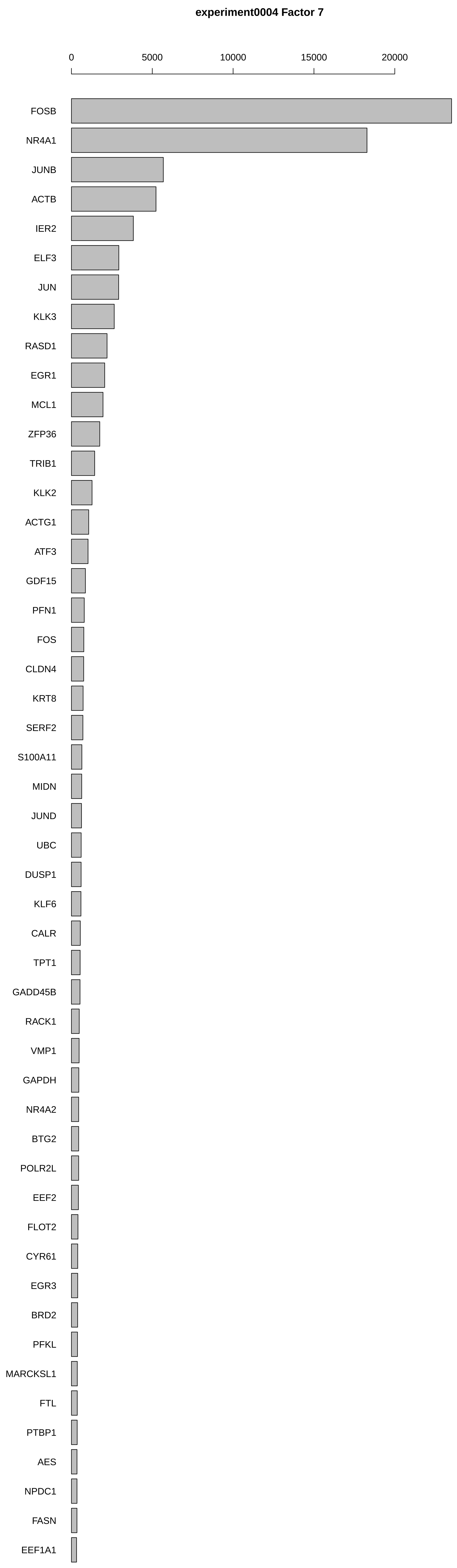
experiment0004 Factor 7
0
5000
10000
15000
20000
FOSB
NR4A1
JUNB
ACTB
IER2
ELF3
JUN
KLK3
RASD1
EGR1
MCL1
ZFP36
TRIB1
KLK2
ACTG1
ATF3
GDF15
PFN1
FOS
CLDN4
KRT8
SERF2
S100A11
MIDN
JUND
UBC
DUSP1
KLF6
CALR
TPT1
GADD45B
RACK1
VMP1
GAPDH
NR4A2
BTG2
POLR2L
EEF2
FLOT2
CYR61
EGR3
BRD2
PFKL
MARCKSL1
FTL
PTBP1
AES
NPDC1
FASN
EEF1A1

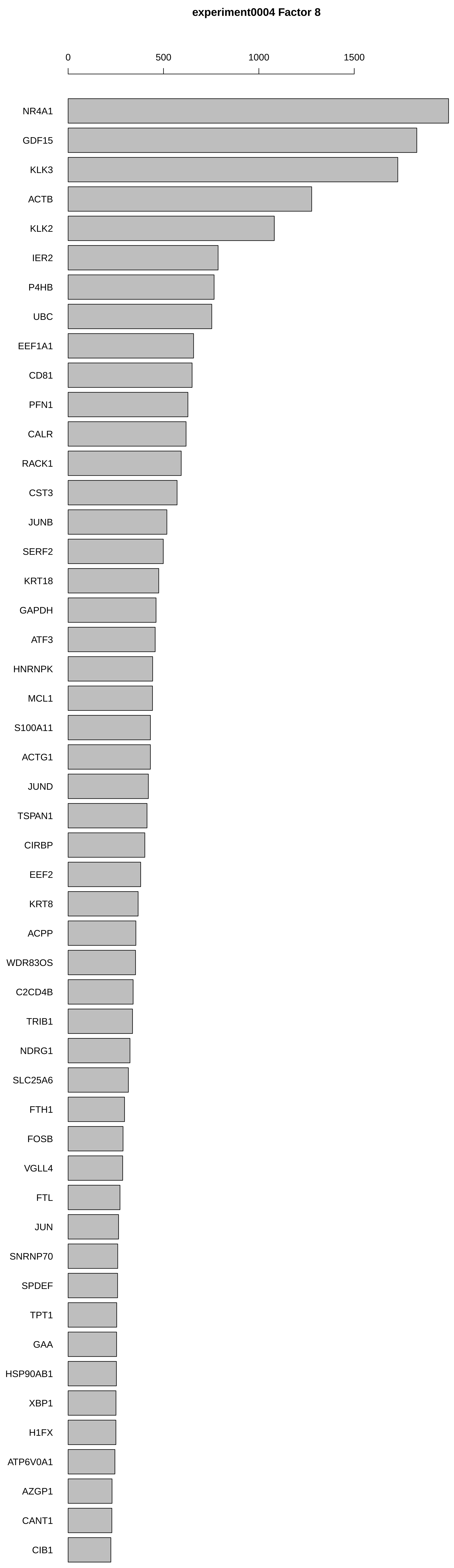
experiment0004 Factor 8
0
500
1000
1500
NR4A1
GDF15
KLK3
ACTB
KLK2
IER2
P4HB
UBC
EEF1A1
CD81
PFN1
CALR
RACK1
CST3
JUNB
SERF2
KRT18
GAPDH
ATF3
HNRNPK
MCL1
S100A11
ACTG1
JUND
TSPAN1
CIRBP
EEF2
KRT8
ACPP
WDR83OS
C2CD4B
TRIB1
NDRG1
SLC25A6
FTH1
FOSB
VGLL4
FTL
JUN
SNRNP70
SPDEF
TPT1
GAA
HSP90AB1
XBP1
H1FX
ATP6V0A1
AZGP1
CANT1
CIB1

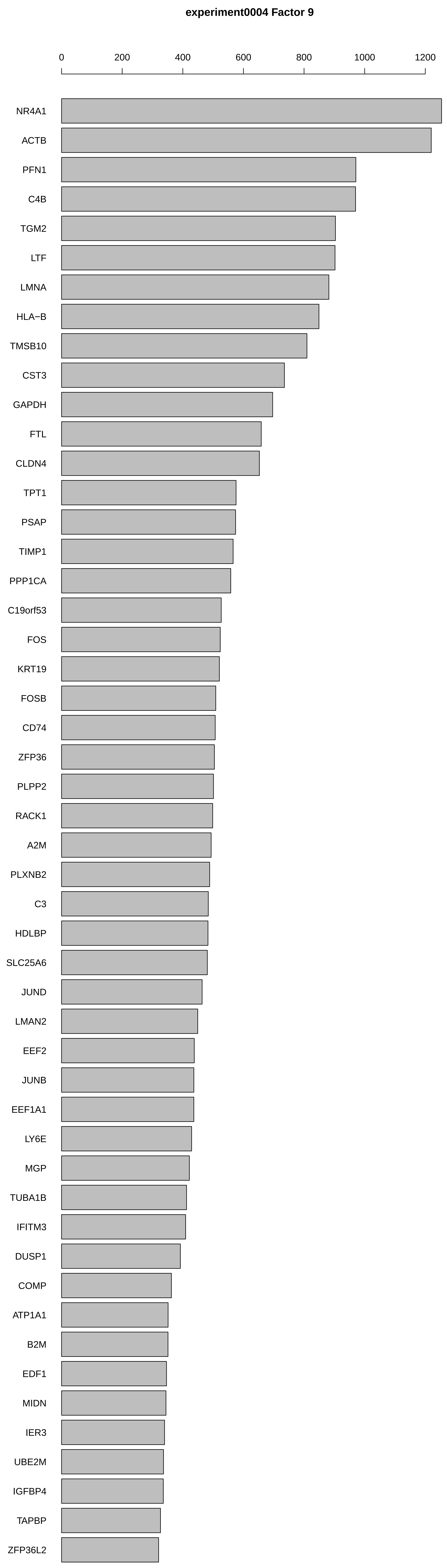

experiment0004 Factor 9
0
200
400
600
800
1000
1200
NR4A1
ACTB
PFN1
C4B
TGM2
LTF
LMNA
HLA−B
TMSB10
CST3
GAPDH
FTL
CLDN4
TPT1
PSAP
TIMP1
PPP1CA
C19orf53
FOS
KRT19
FOSB
CD74
ZFP36
PLPP2
RACK1
A2M
PLXNB2
C3
HDLBP
SLC25A6
JUND
LMAN2
EEF2
JUNB
EEF1A1
LY6E
MGP
TUBA1B
IFITM3
DUSP1
COMP
ATP1A1
B2M
EDF1
MIDN
IER3
UBE2M
IGFBP4
TAPBP
ZFP36L2

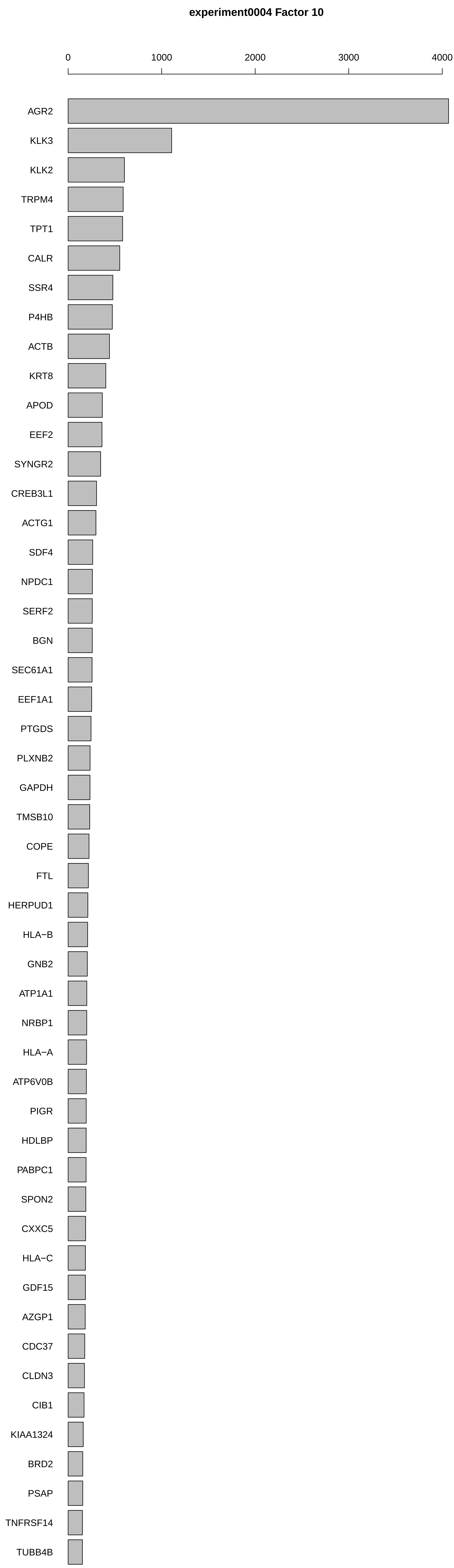
experiment0004 Factor 10
0
1000
2000
3000
4000
AGR2
KLK3
KLK2
TRPM4
TPT1
CALR
SSR4
P4HB
ACTB
KRT8
APOD
EEF2
SYNGR2
CREB3L1
ACTG1
SDF4
NPDC1
SERF2
BGN
SEC61A1
EEF1A1
PTGDS
PLXNB2
GAPDH
TMSB10
COPE
FTL
HERPUD1
HLA−B
GNB2
ATP1A1
NRBP1
HLA−A
ATP6V0B
PIGR
HDLBP
PABPC1
SPON2
CXXC5
HLA−C
GDF15
AZGP1
CDC37
CLDN3
CIB1
KIAA1324
BRD2
PSAP
TNFRSF14
TUBB4B
