# Supplementary material for: Spatial maps of prostate cancer transcriptomes reveal an unexplored landscape of heterogeneity
Source: Nat Commun. 2018 Jun 20;9:2419. doi: 10.1038/s41467-018-04724-5 (PMC6010471; doi:10.1038/s41467-018-04724-5)
Supplement: Supplementary file 8 — Supplementary Data 5 [file 41467_2018_4724_MOESM8_ESM.zip › Supplementary Dataset 7/joint-field-factor-strength-spotbarplot-abs-freq.pdf]

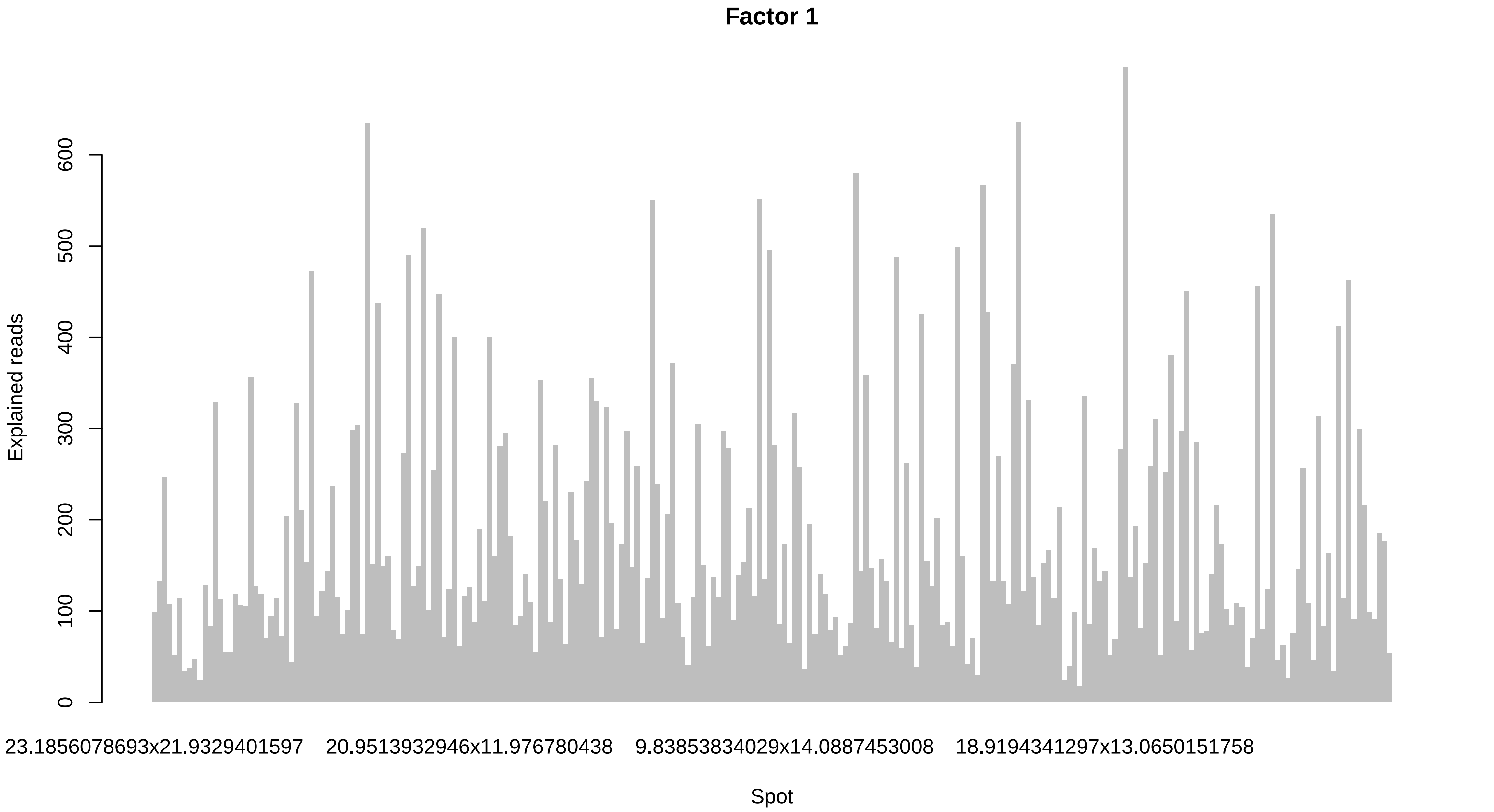

Factor 1
Explained reads
0
100
200
300
400
500
600
23.1856078693x21.9329401597
20.9513932946x11.976780438
9.83853834029x14.0887453008
18.9194341297x13.0650151758
Spot

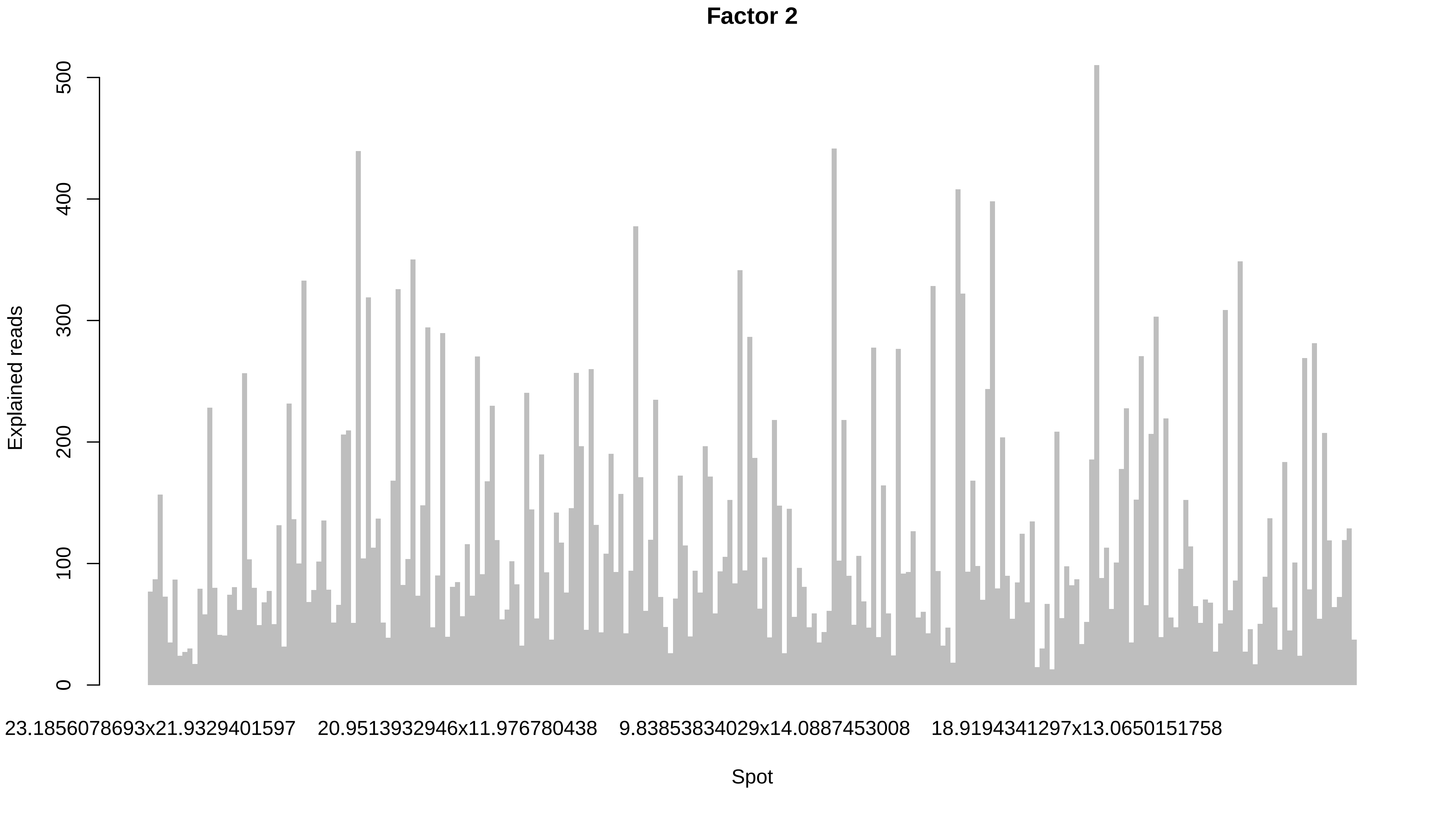
Factor 2
Explained reads
0
100
200
300
400
500
Spot
23.1856078693x21.9329401597
20.9513932946x11.976780438
9.83853834029x14.0887453008
18.9194341297x13.0650151758

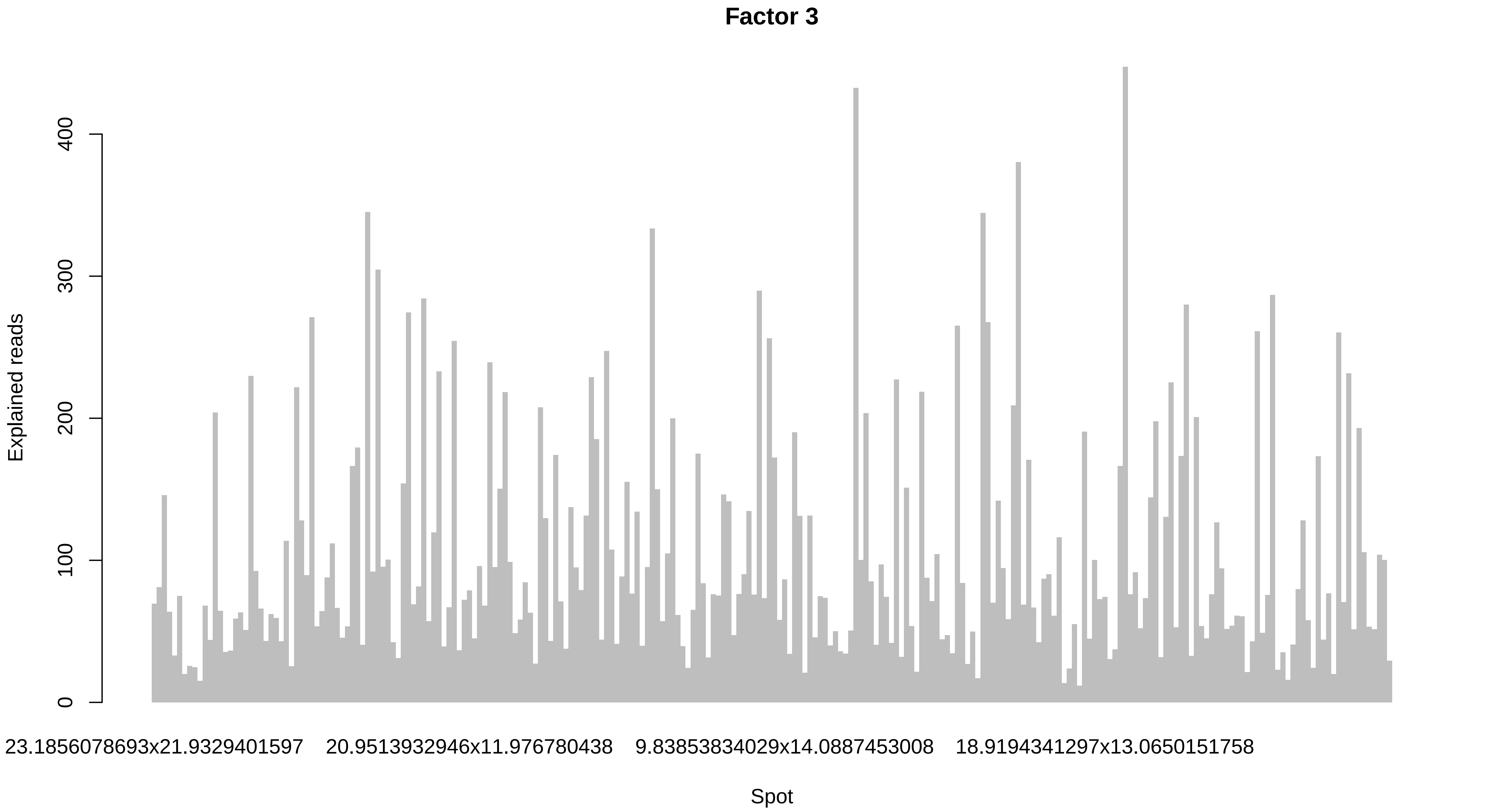

Factor 3
Explained reads
0
100
200
300
400
23.1856078693x21.9329401597
20.9513932946x11.976780438
9.83853834029x14.0887453008
18.9194341297x13.0650151758
Spot

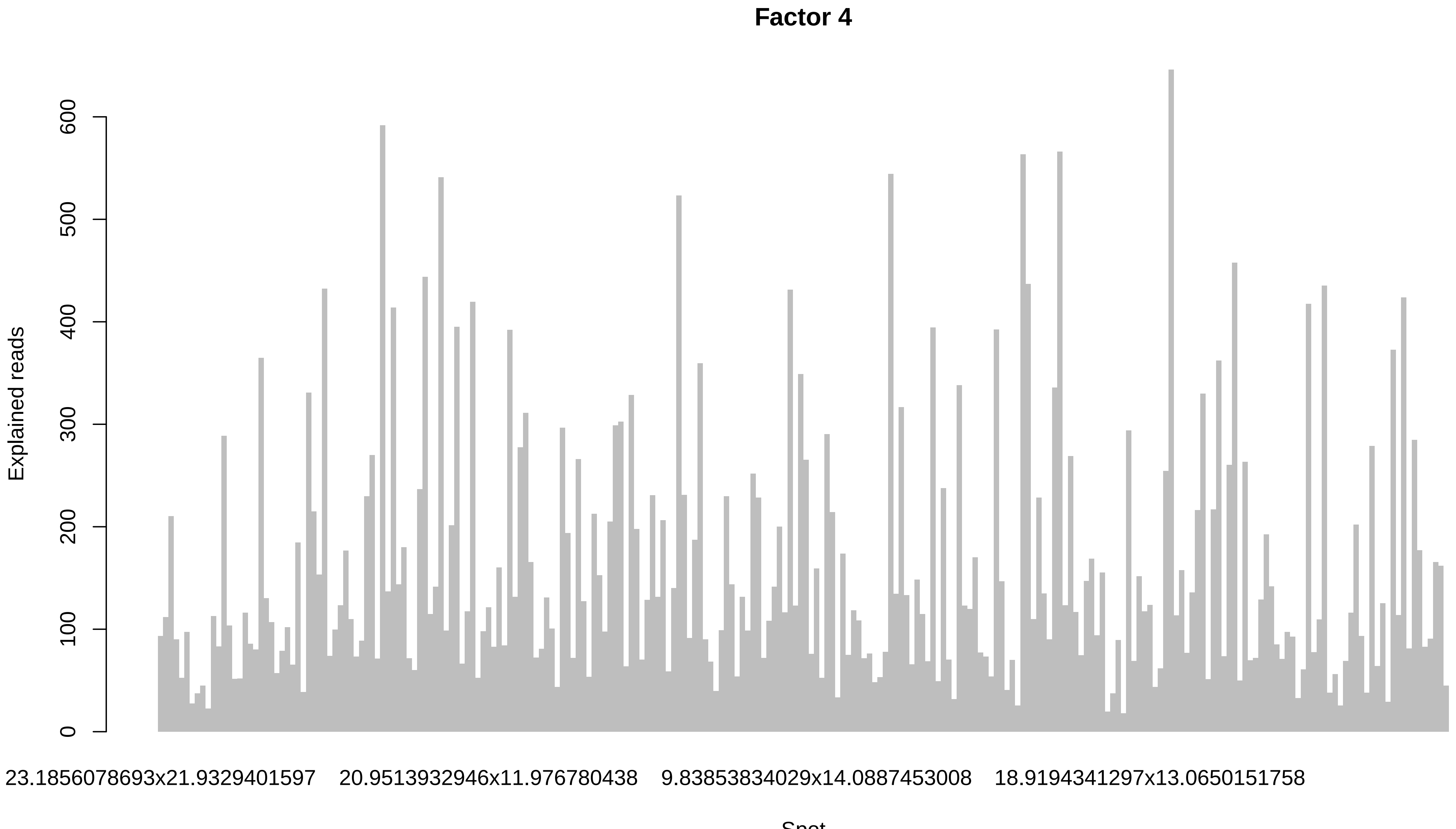
Factor 4
Explained reads
0
100
200
300
400
500
600
23.1856078693x21.9329401597
20.9513932946x11.976780438
9.83853834029x14.0887453008
18.9194341297x13.0650151758
Spot

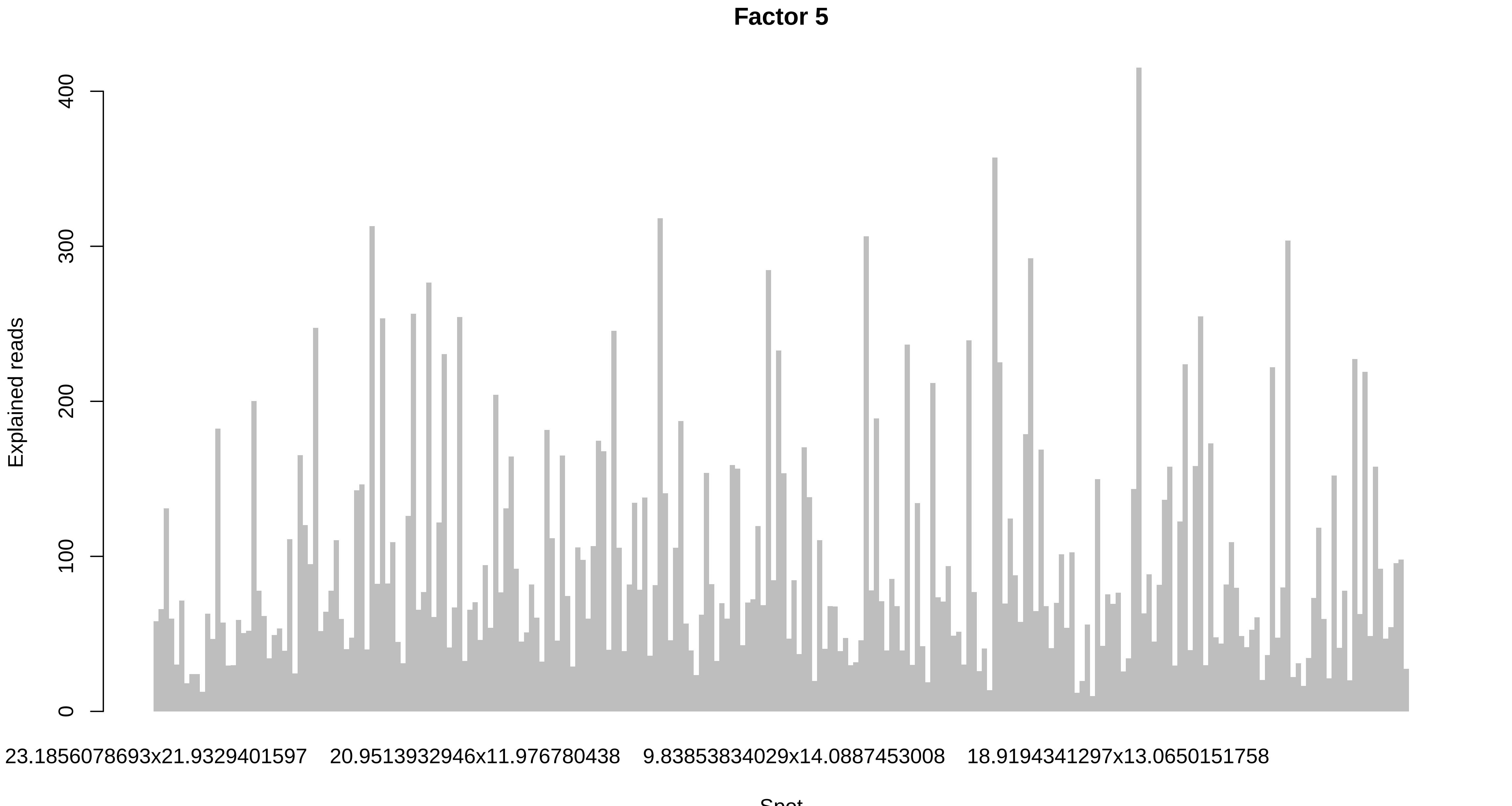

Factor 5
Explained reads
0
100
200
300
400
23.1856078693x21.9329401597
20.9513932946x11.976780438
9.83853834029x14.0887453008
18.9194341297x13.0650151758
Spot

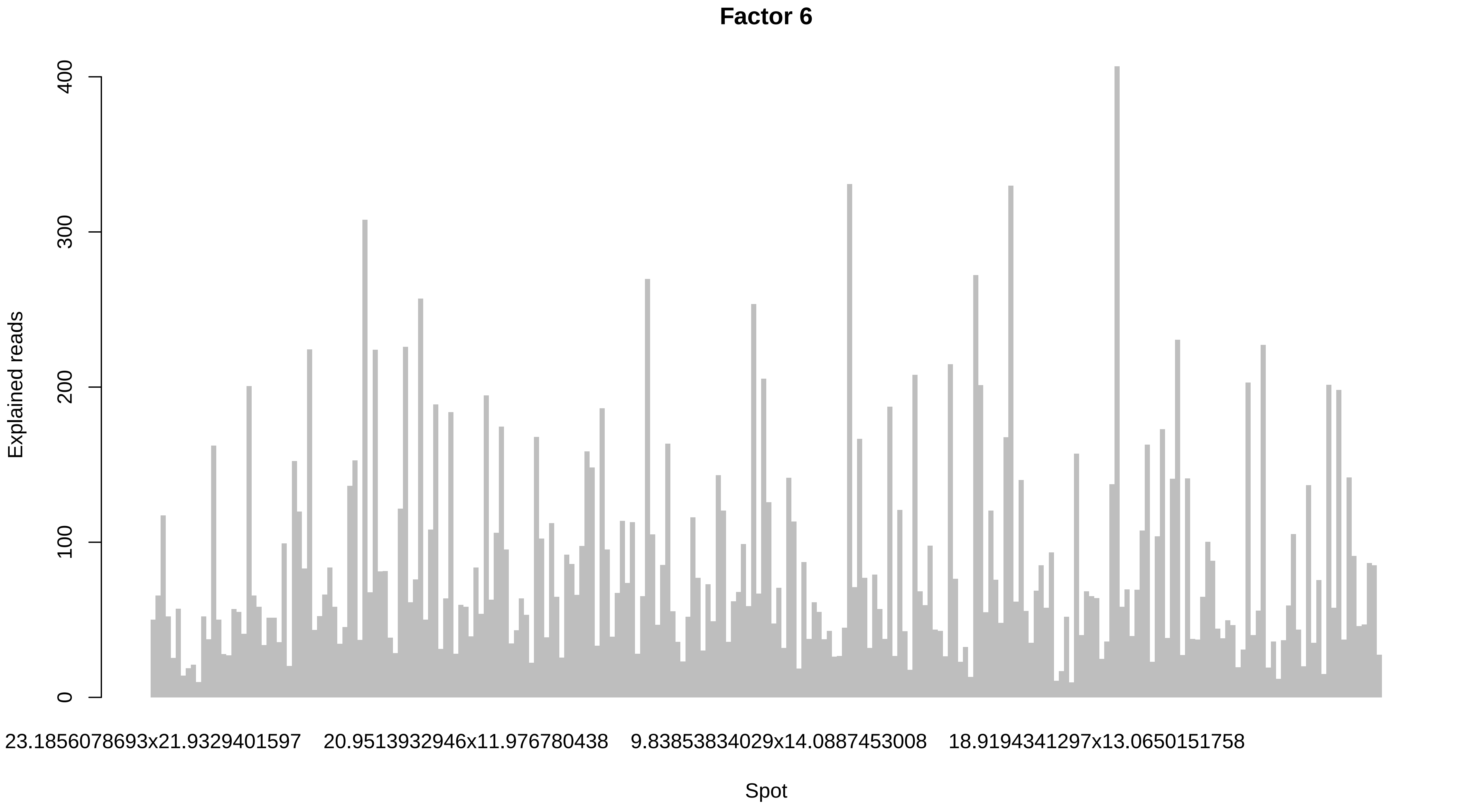
Factor 6
Explained reads
0
100
200
300
400
23.1856078693x21.9329401597
20.9513932946x11.976780438
9.83853834029x14.0887453008
18.9194341297x13.0650151758
Spot

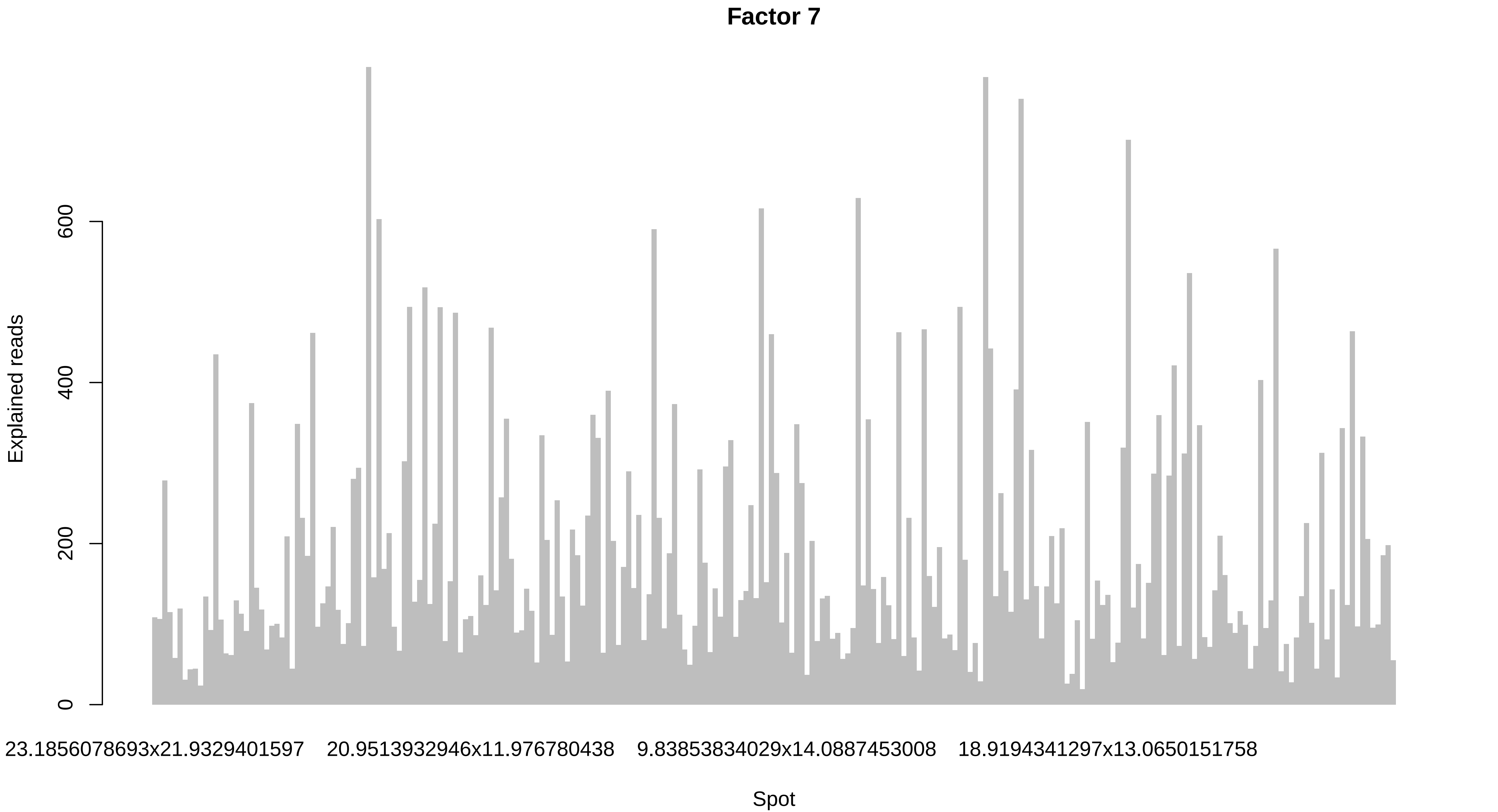
Factor 7
Explained reads
0
200
400
600
23.1856078693x21.9329401597
20.9513932946x11.976780438
9.83853834029x14.0887453008
18.9194341297x13.0650151758
Spot

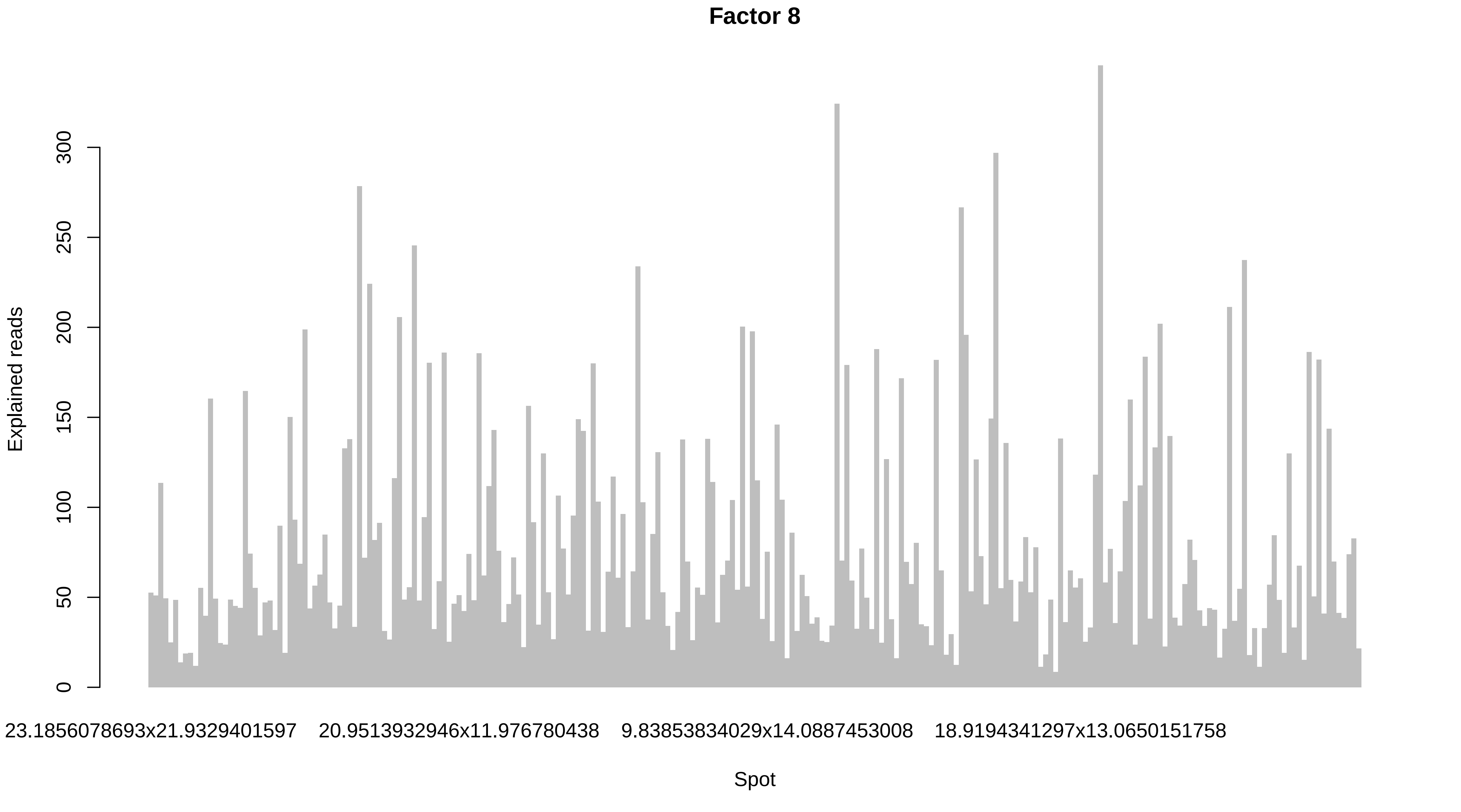
Factor 8
Explained reads
0
50
100
150
200
250
300
23.1856078693x21.9329401597
20.9513932946x11.976780438
9.83853834029x14.0887453008
18.9194341297x13.0650151758
Spot

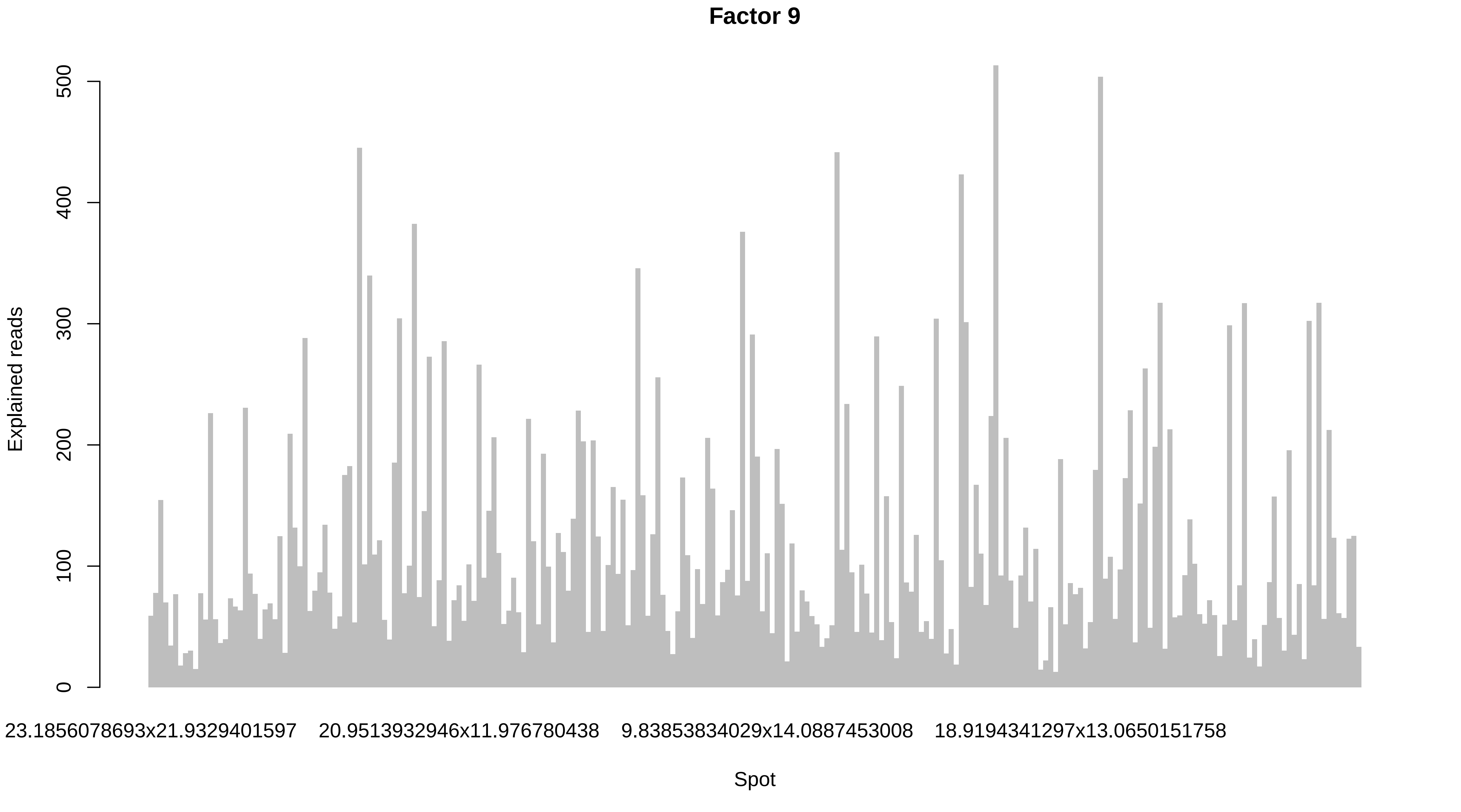
Factor 9
Explained reads
0
100
200
300
400
500
23.1856078693x21.9329401597
20.9513932946x11.976780438
9.83853834029x14.0887453008
18.9194341297x13.0650151758
Spot

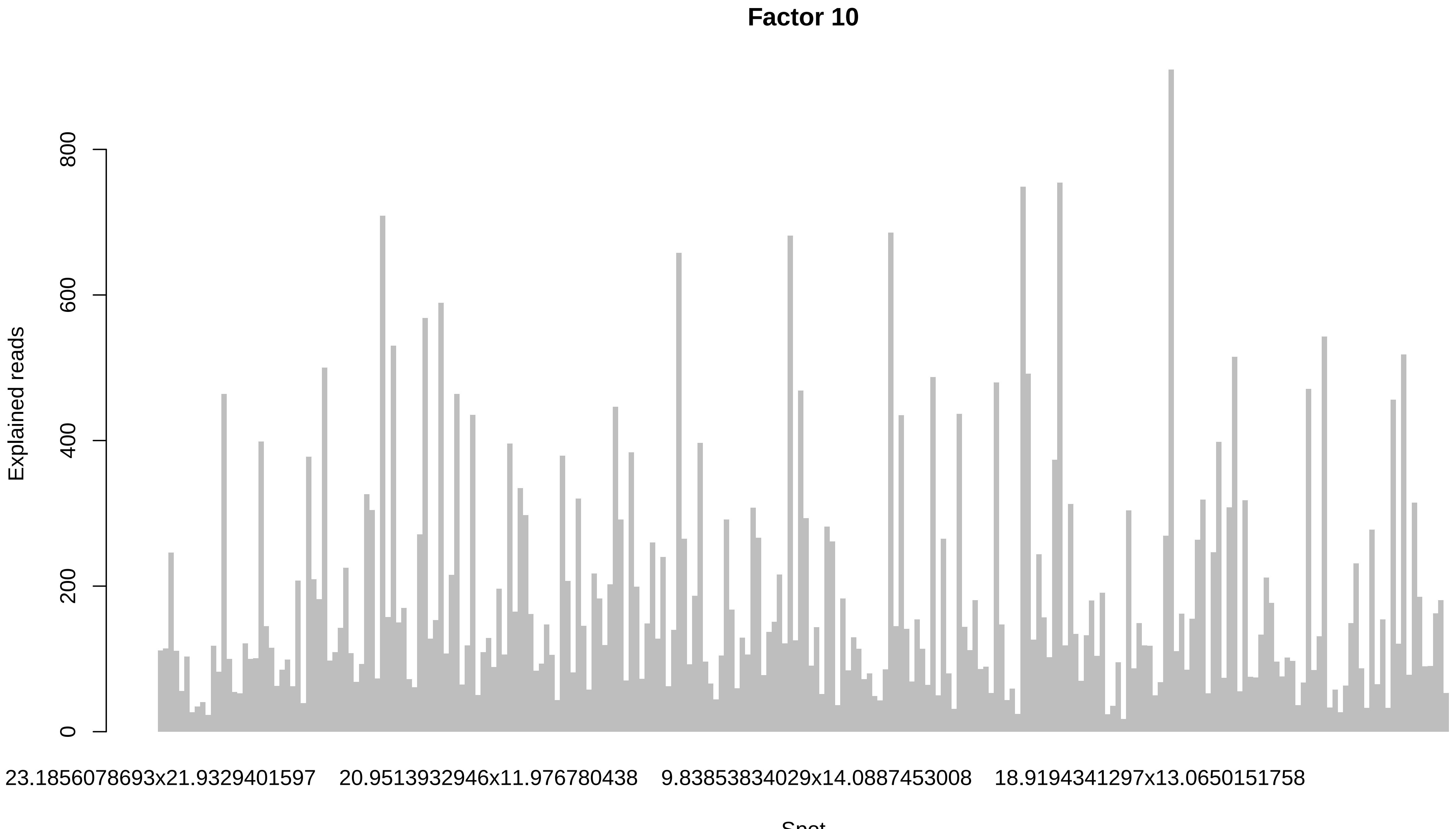

Factor 10
Explained reads
0
200
400
600
800
23.1856078693x21.9329401597
20.9513932946x11.976780438
9.83853834029x14.0887453008
18.9194341297x13.0650151758
Spot

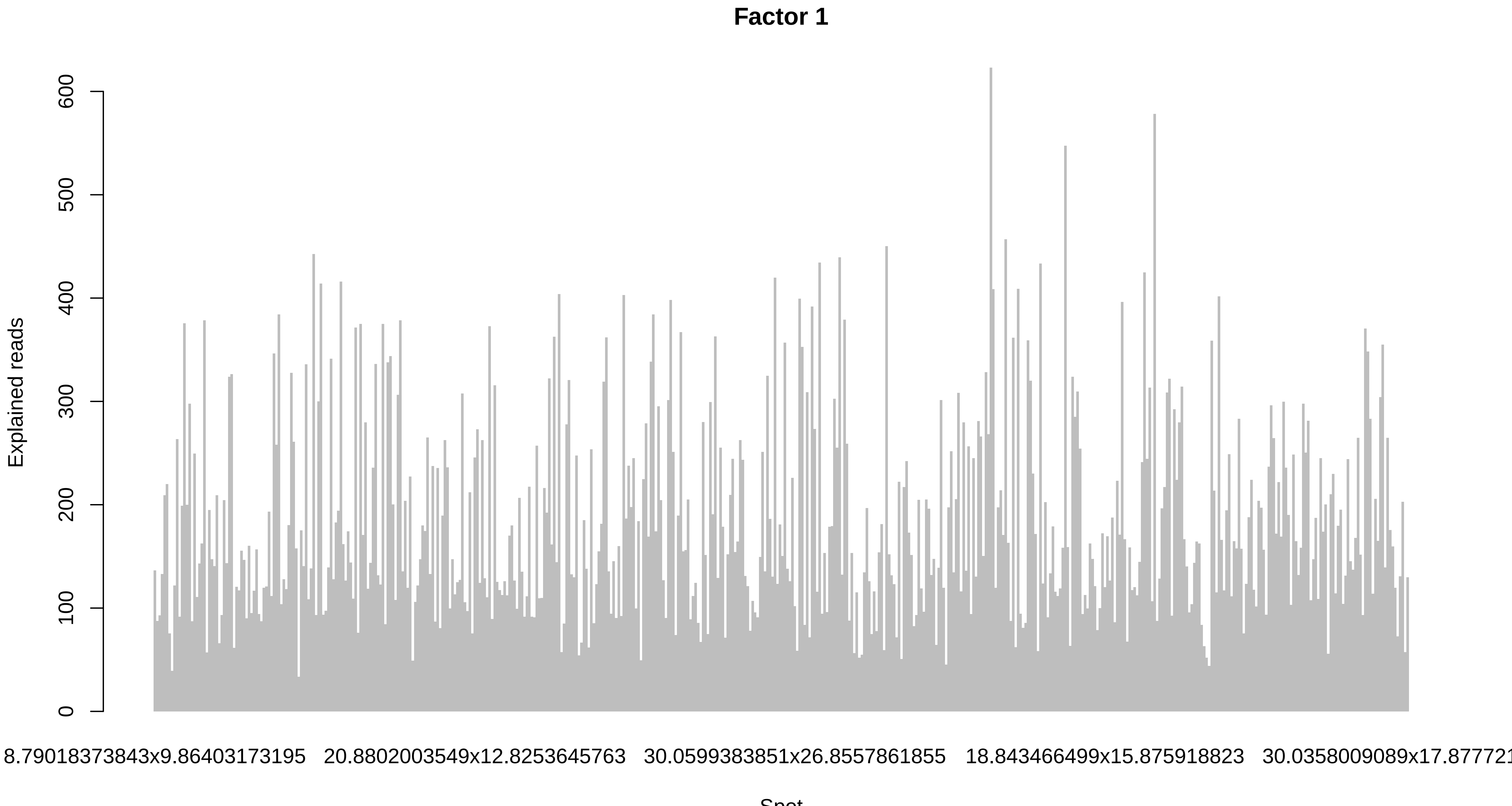
Factor 1
Explained reads
0
100
200
300
400
500
600
8.79018373843x9.86403173195
20.8802003549x12.8253645763
30.0599383851x26.8557861855
18.843466499x15.875918823
30.0358009089x17.877721
Spot

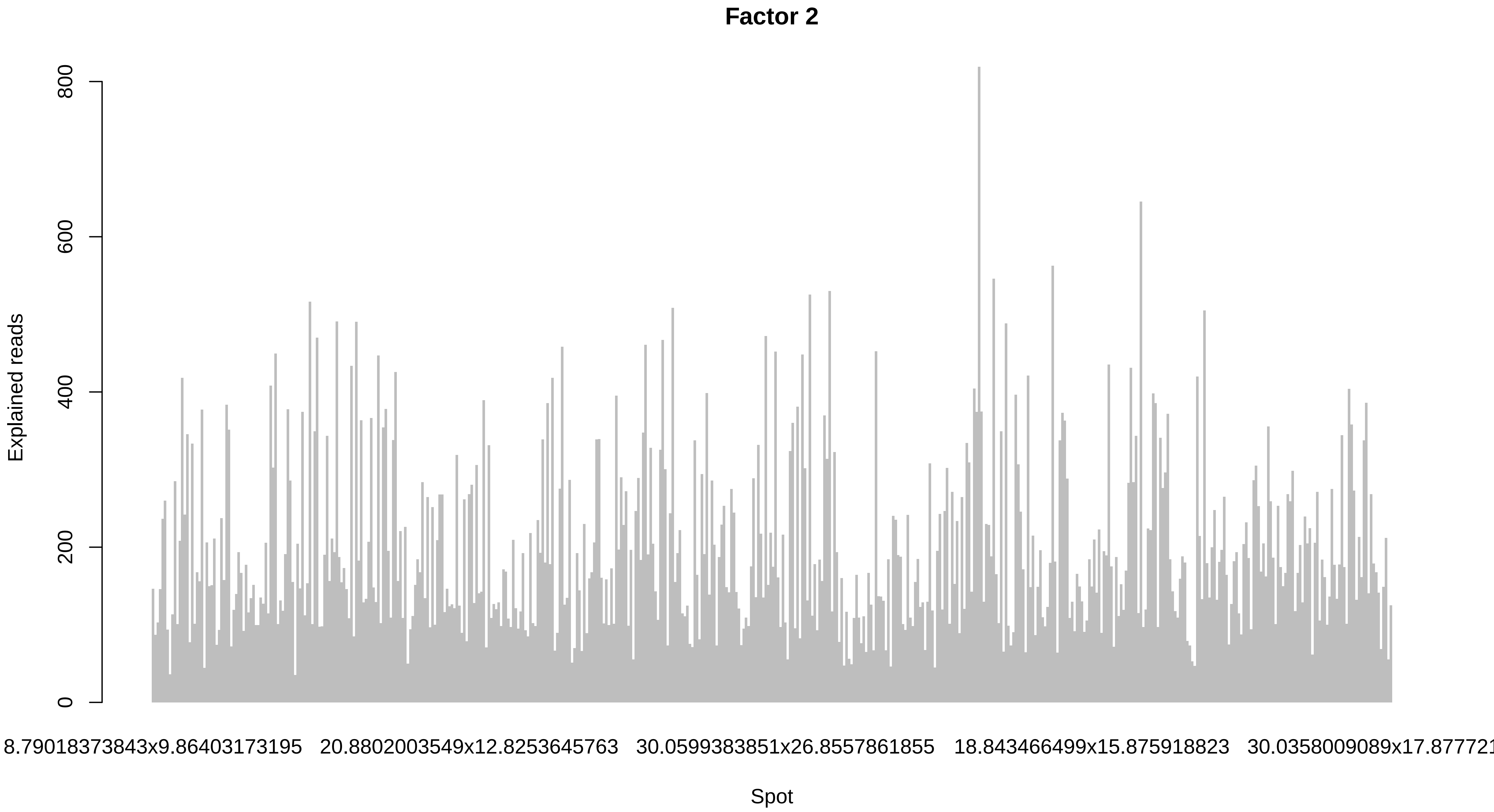
Factor 2
Explained reads
0
200
400
600
800
8.79018373843x9.86403173195
20.8802003549x12.8253645763
30.0599383851x26.8557861855
18.843466499x15.875918823
30.0358009089x17.87772
Spot

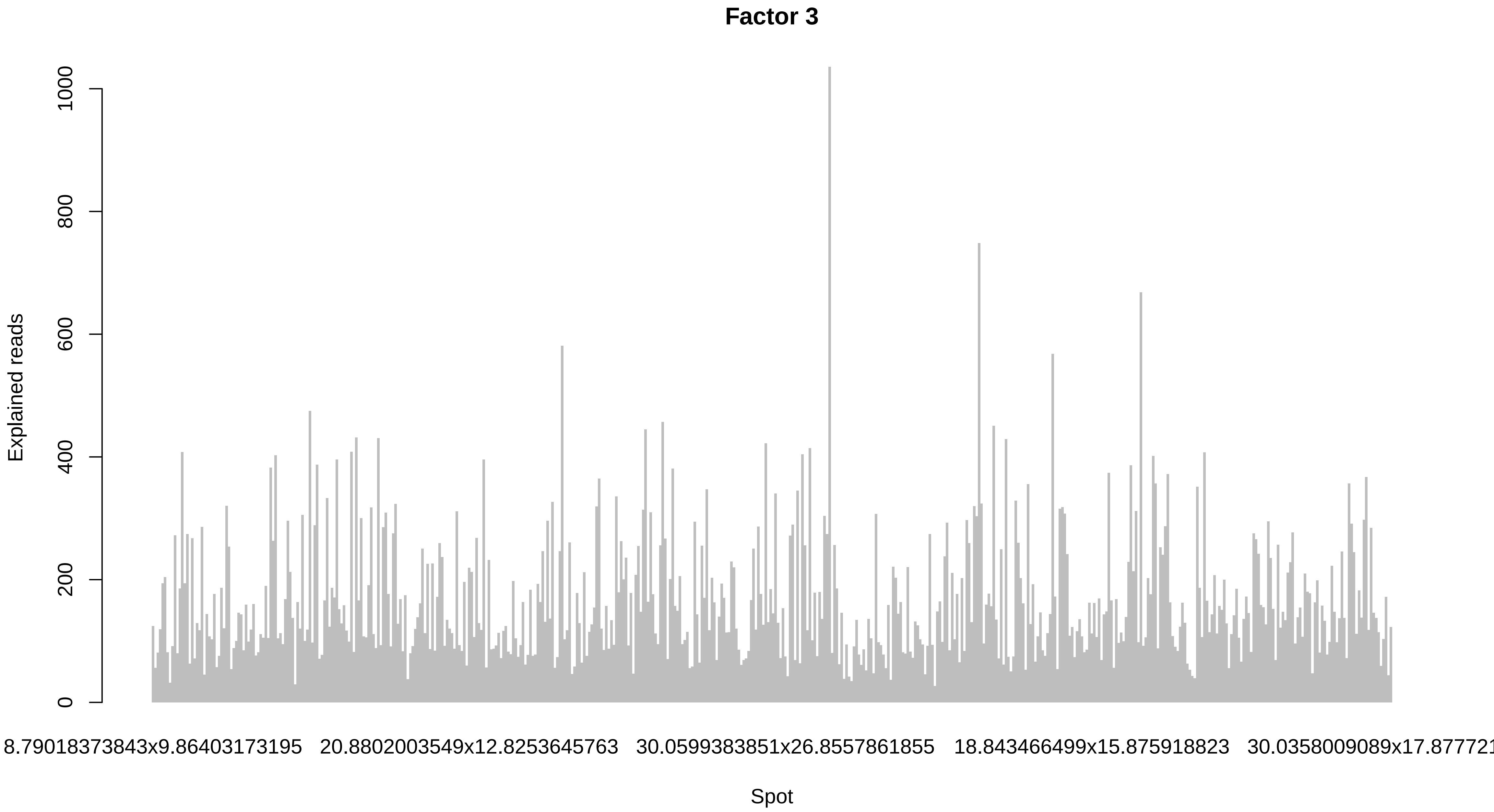
Factor 3
Explained reads
0
200
400
600
800
1000
8.79018373843x9.86403173195
20.8802003549x12.8253645763
30.0599383851x26.8557861855
18.843466499x15.875918823
30.0358009089x17.877721
Spot

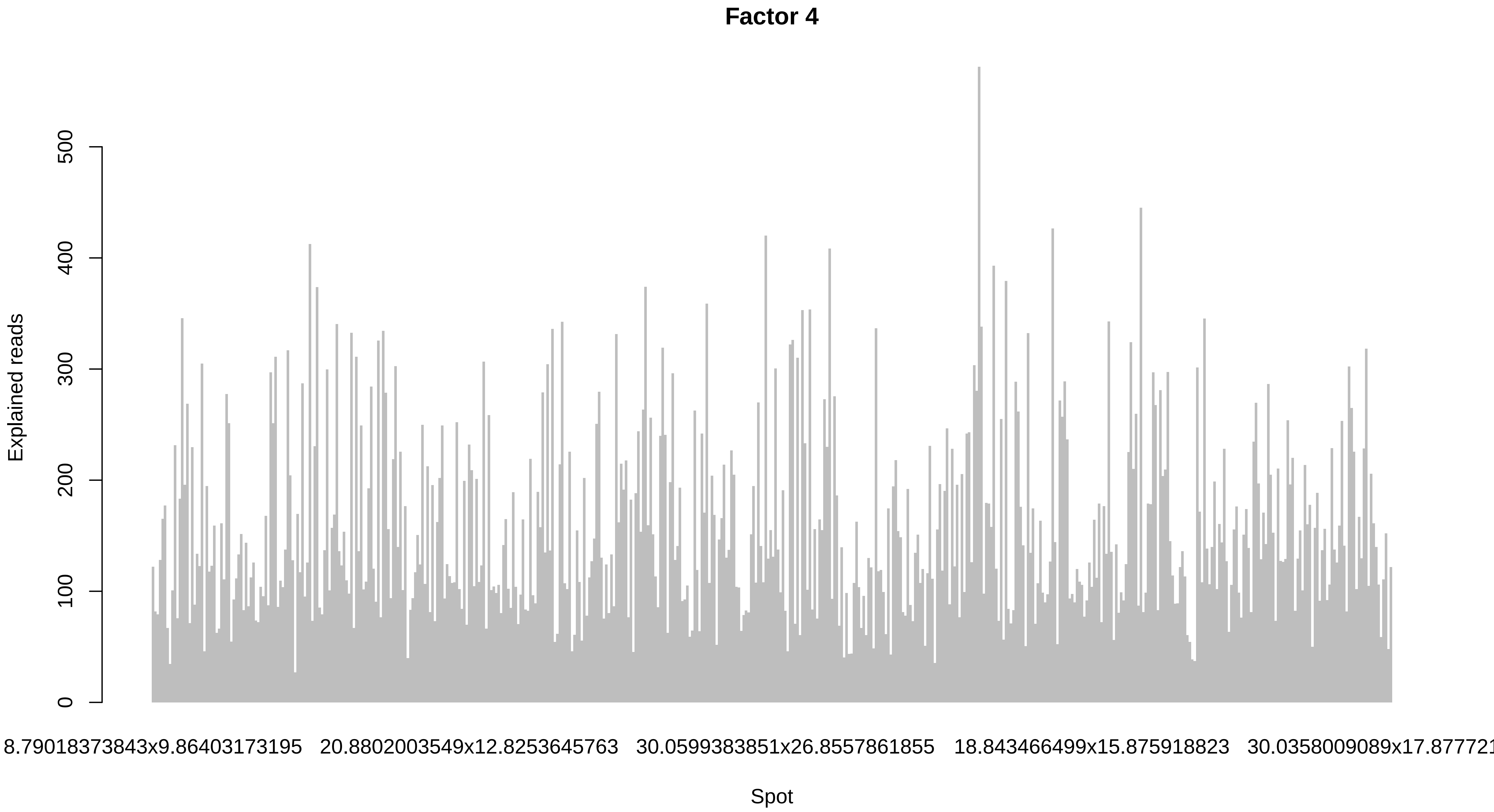

Factor 4
Explained reads
0
100
200
300
400
500
8.79018373843x9.86403173195
20.8802003549x12.8253645763
30.0599383851x26.8557861855
18.843466499x15.875918823
30.0358009089x17.87772
Spot

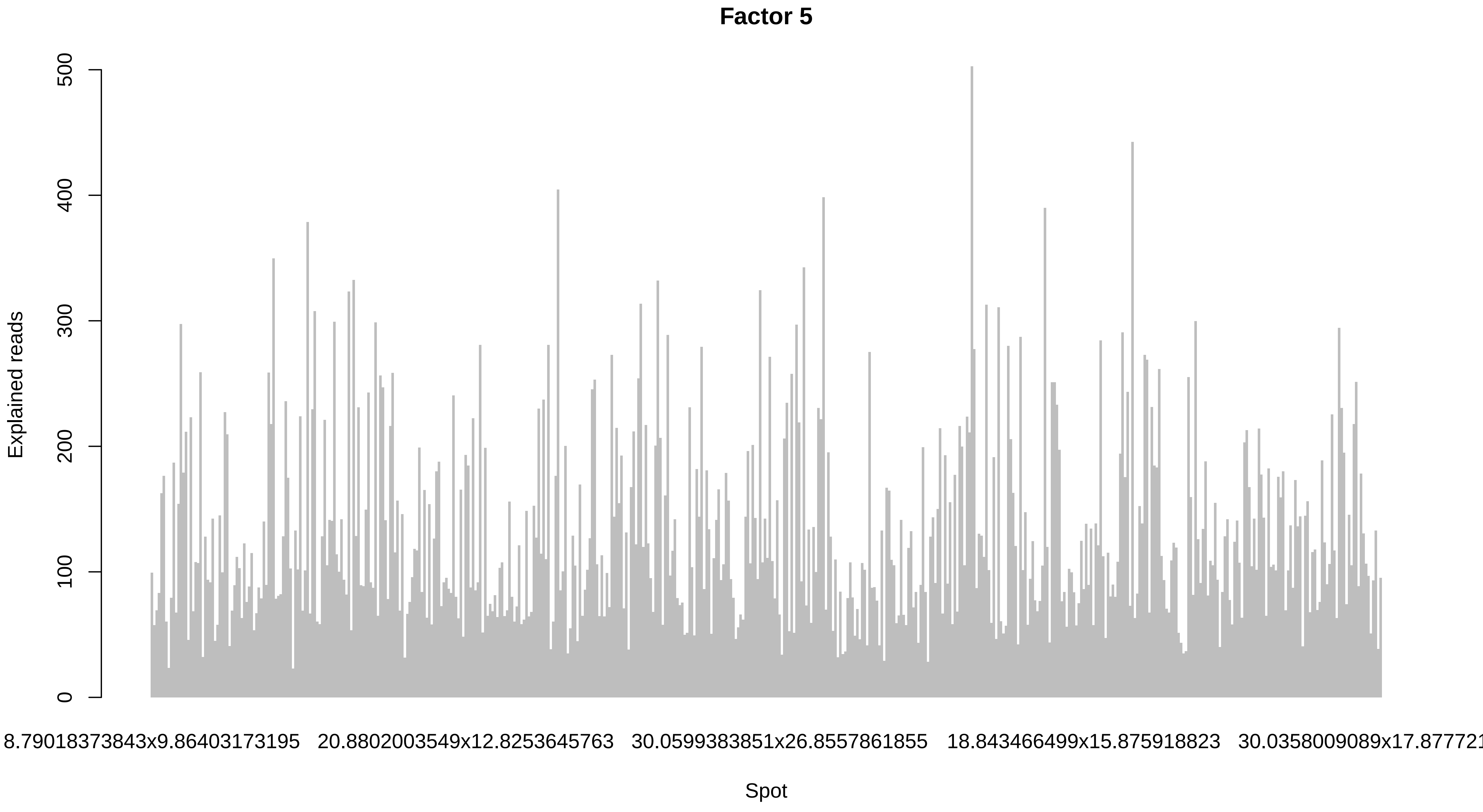

Factor 5
Explained reads
0
100
200
300
400
500
8.79018373843x9.86403173195
20.8802003549x12.8253645763
30.0599383851x26.8557861855
18.843466499x15.875918823
30.0358009089x17.877721
Spot

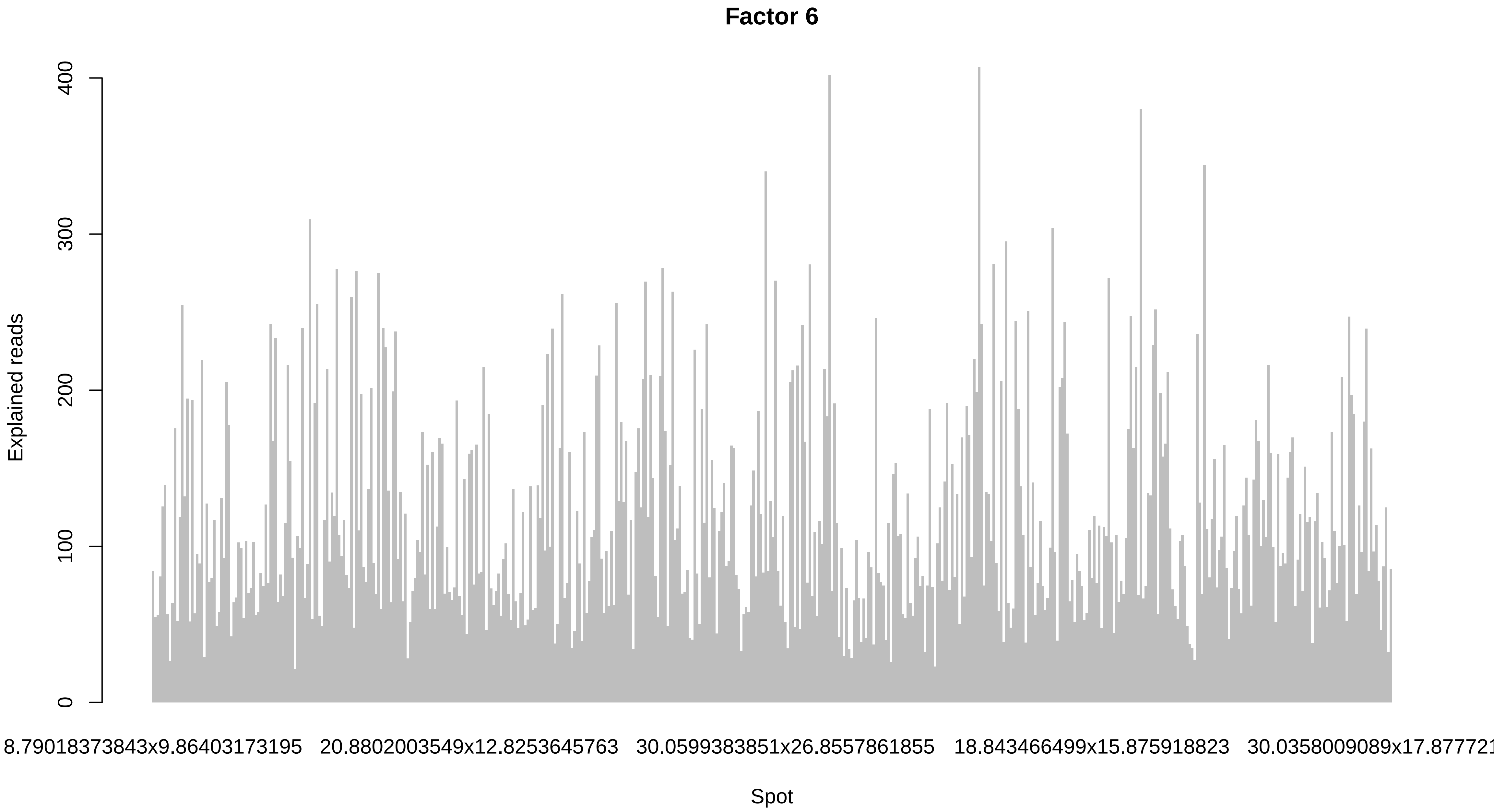

Factor 6
Explained reads
0
100
200
300
400
8.79018373843x9.86403173195
20.8802003549x12.8253645763
30.0599383851x26.8557861855
18.843466499x15.875918823
30.0358009089x17.877721
Spot

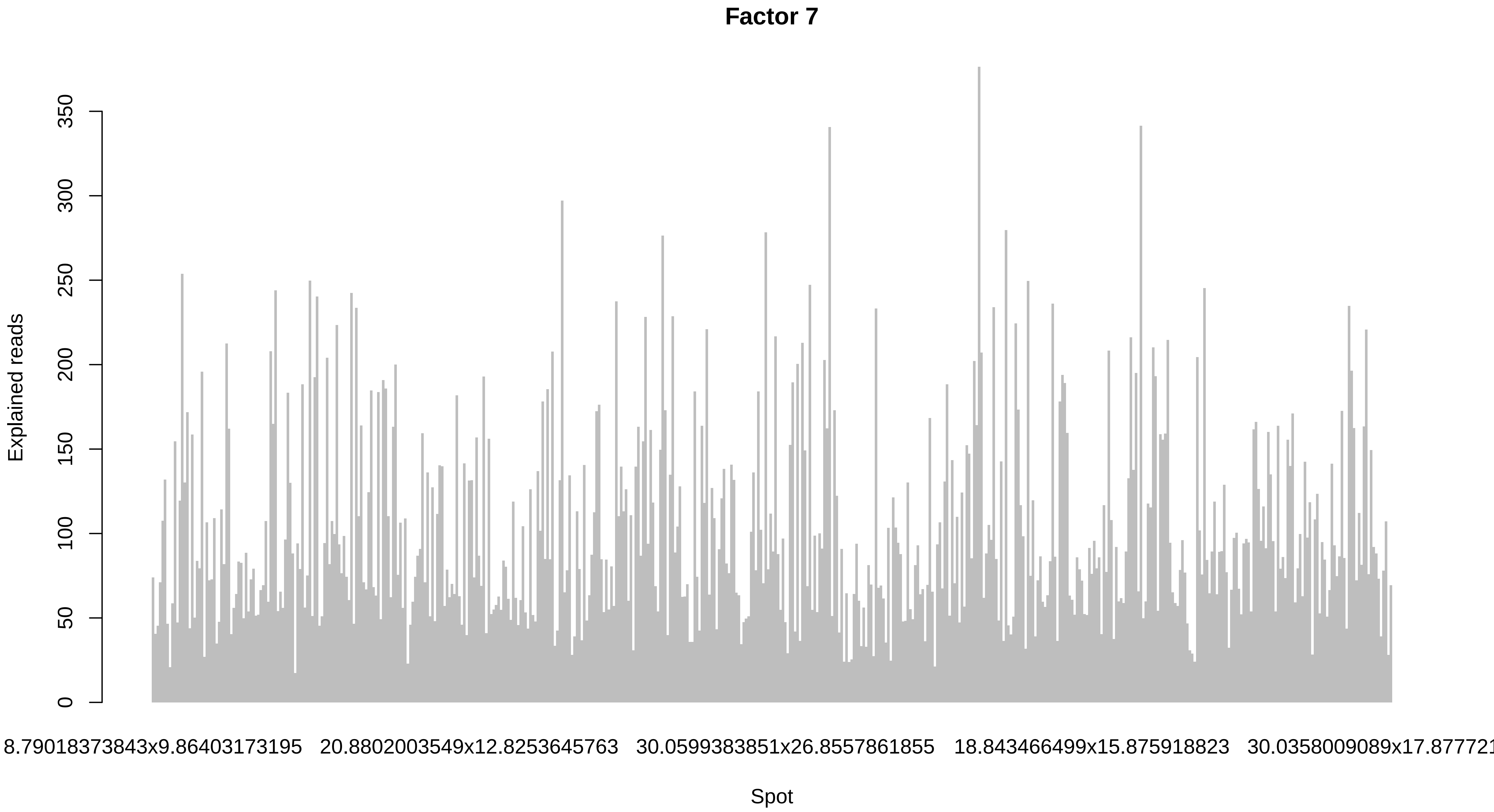

Factor 7
Explained reads
0
50
100
150
200
250
300
350
Spot
8.79018373843x9.86403173195
20.8802003549x12.8253645763
30.0599383851x26.8557861855
18.843466499x15.875918823
30.0358009089x17.877721

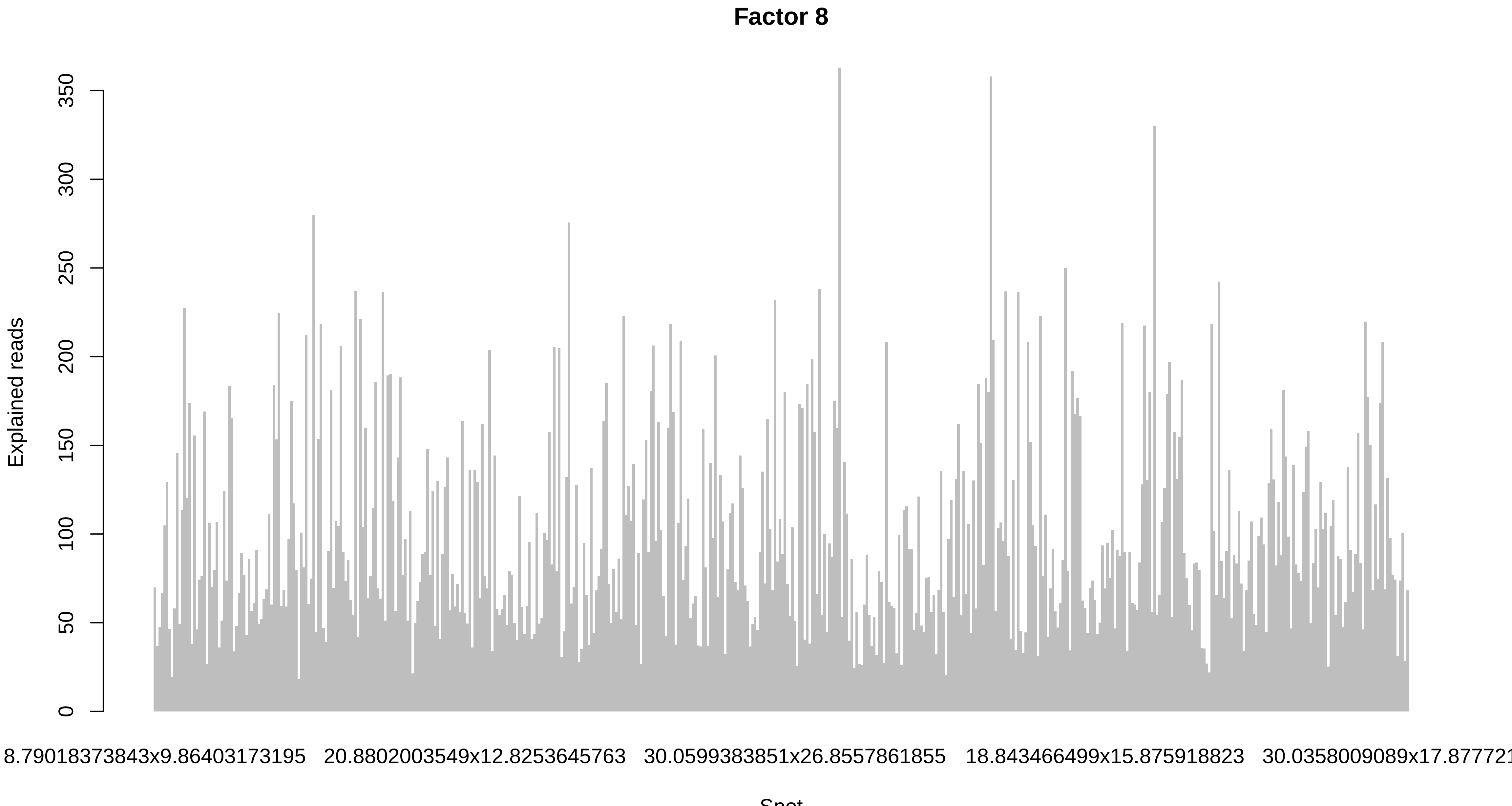
Factor 8
Explained reads
0
50
100
150
200
250
300
350
8.79018373843x9.86403173195
20.8802003549x12.8253645763
30.0599383851x26.8557861855
18.843466499x15.875918823
30.0358009089x17.87772
Spot

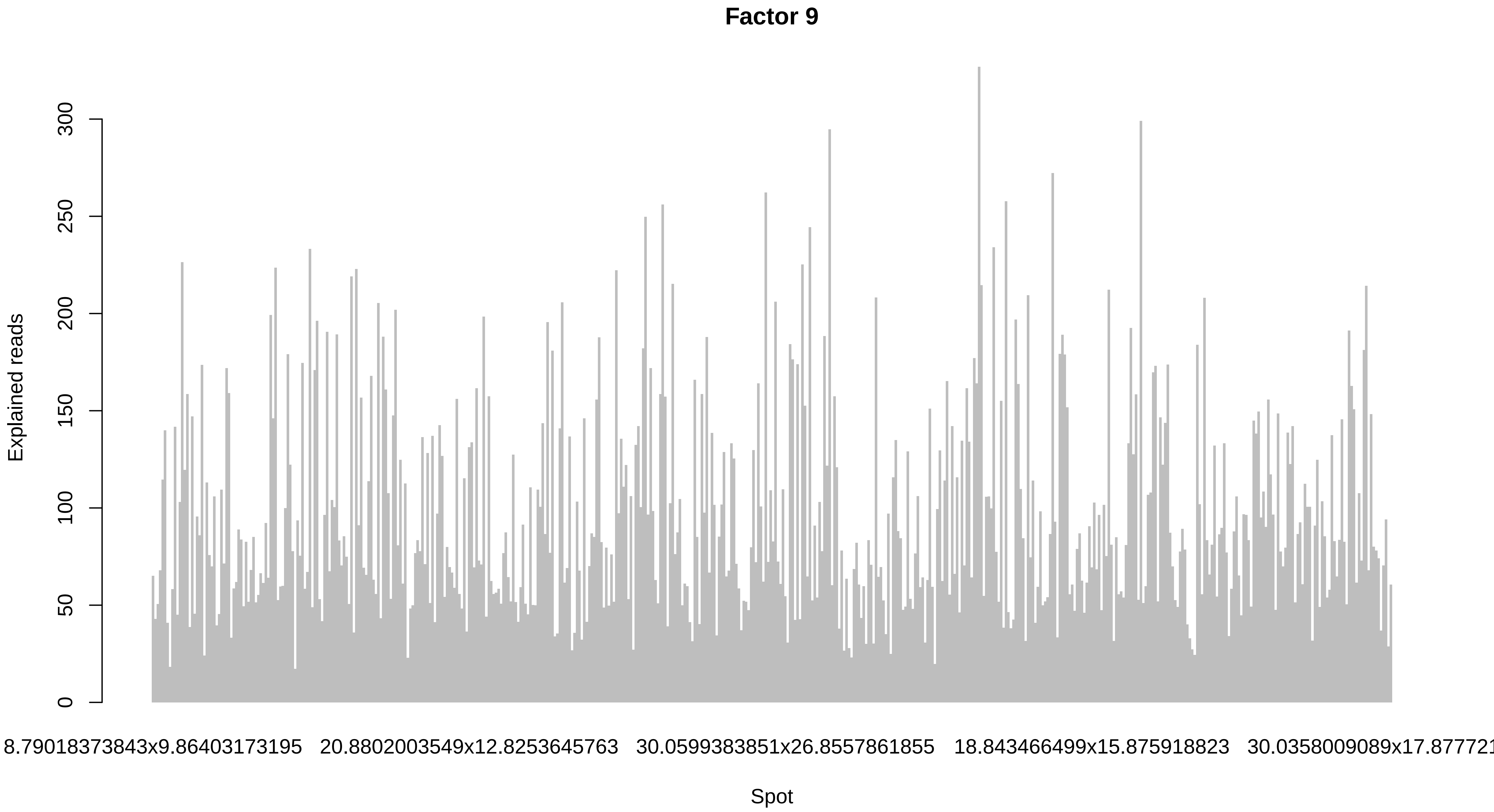
Factor 9
Explained reads
0
50
100
150
200
250
300
8.79018373843x9.86403173195
20.8802003549x12.8253645763
30.0599383851x26.8557861855
18.843466499x15.875918823
30.0358009089x17.87772
Spot

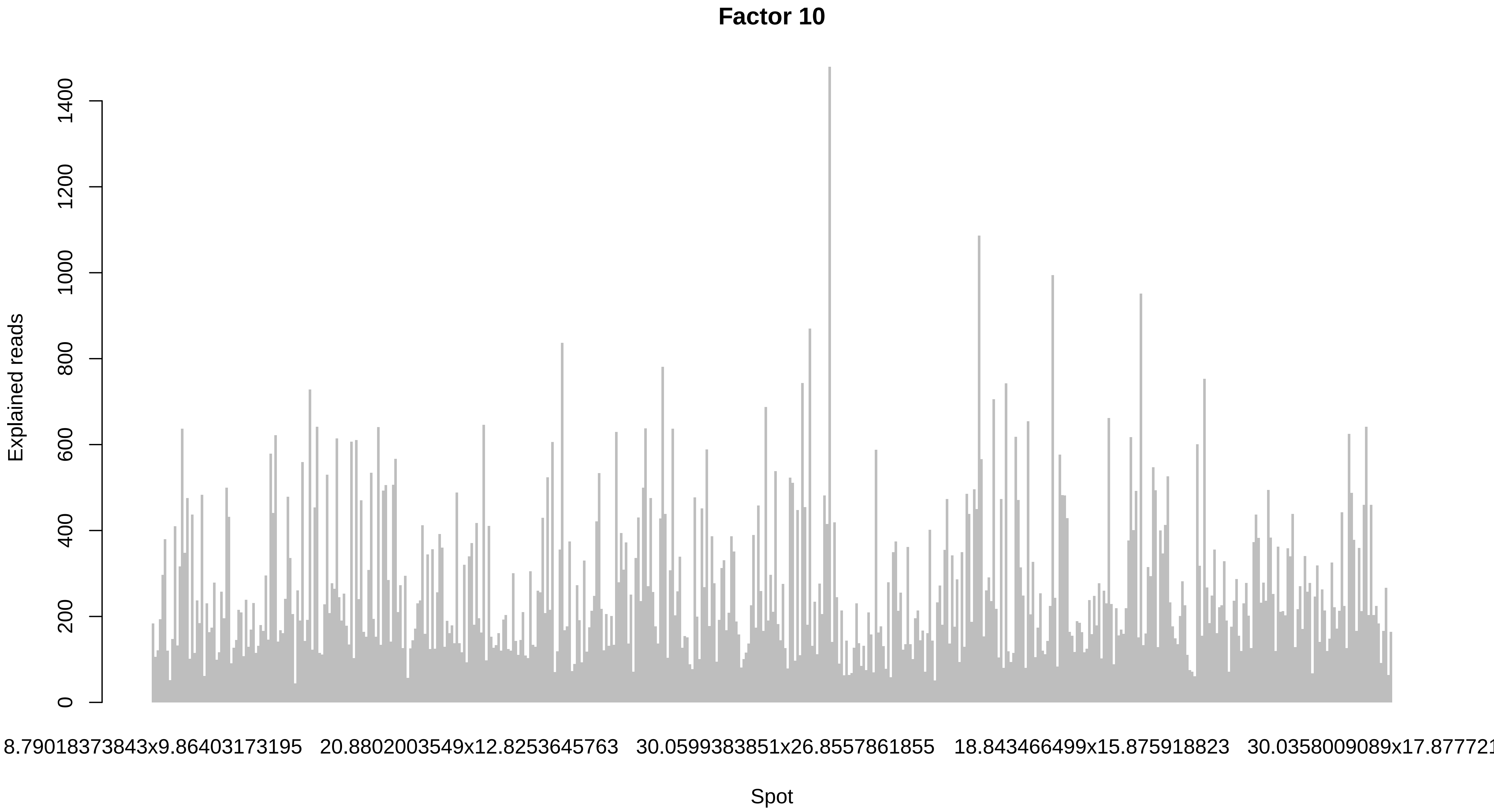

Factor 10
Explained reads
0
200
400
600
800
1000
1200
1400
8.79018373843x9.86403173195
20.8802003549x12.8253645763
30.0599383851x26.8557861855
18.843466499x15.875918823
30.0358009089x17.877721
Spot

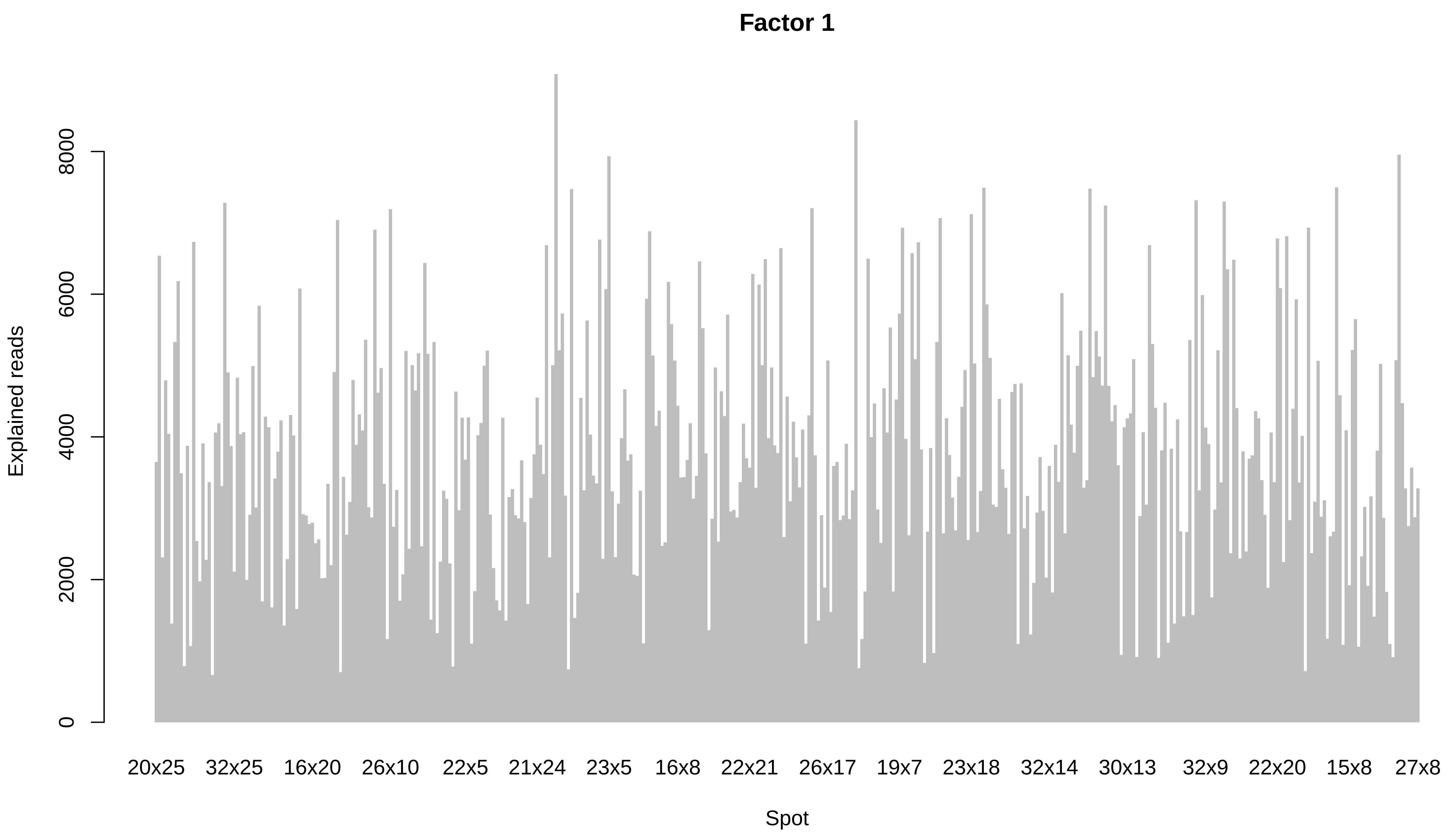

Factor 1
Explained reads
0
2000
4000
6000
8000
20x25
32x25
16x20
26x10
22x5
21x24
23x5
16x8
22x21
26x17
19x7
23x18
32x14
30x13
32x9
22x20
15x8
27x8
Spot

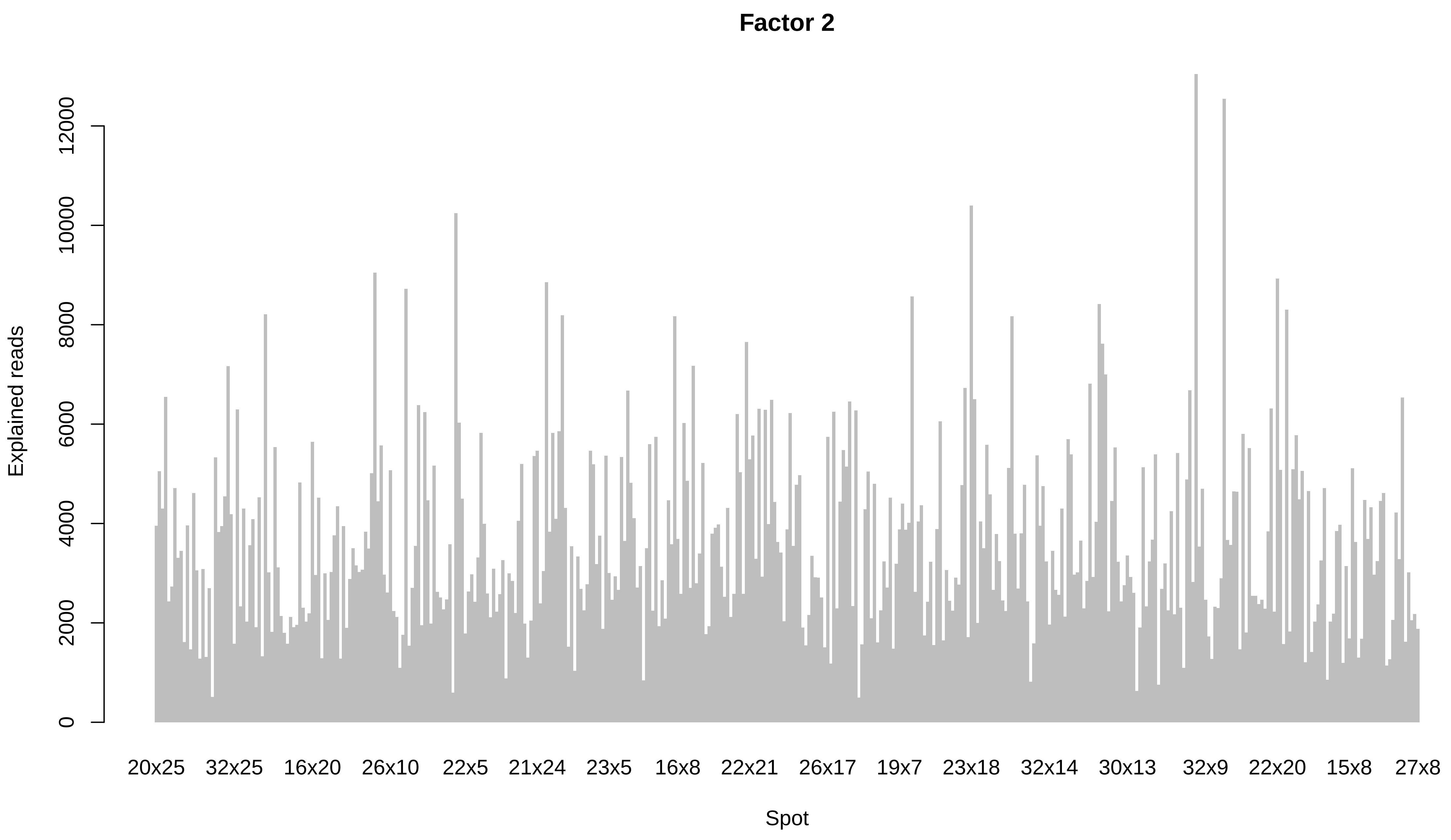
Factor 2
Explained reads
0
2000
4000
6000
8000
10000
12000
Spot
20x25
32x25
16x20
26x10
22x5
21x24
23x5
16x8
22x21
26x17
19x7
23x18
32x14
30x13
32x9
22x20
15x8
27x8

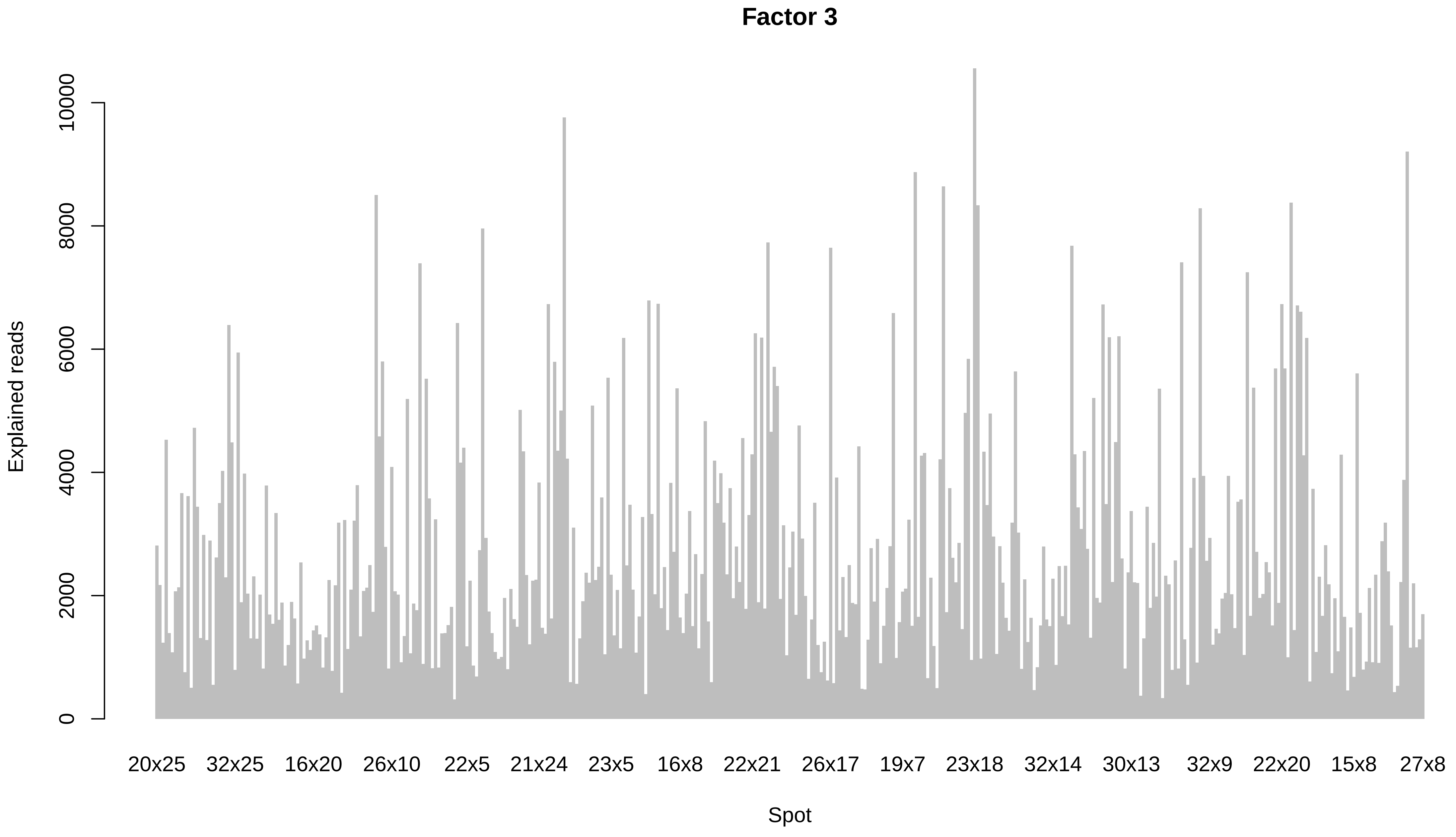

Factor 3
Explained reads
0
2000
4000
6000
8000
10000
Spot
20x25
32x25
16x20
26x10
22x5
21x24
23x5
16x8
22x21
26x17
19x7
23x18
32x14
30x13
32x9
22x20
15x8
27x8

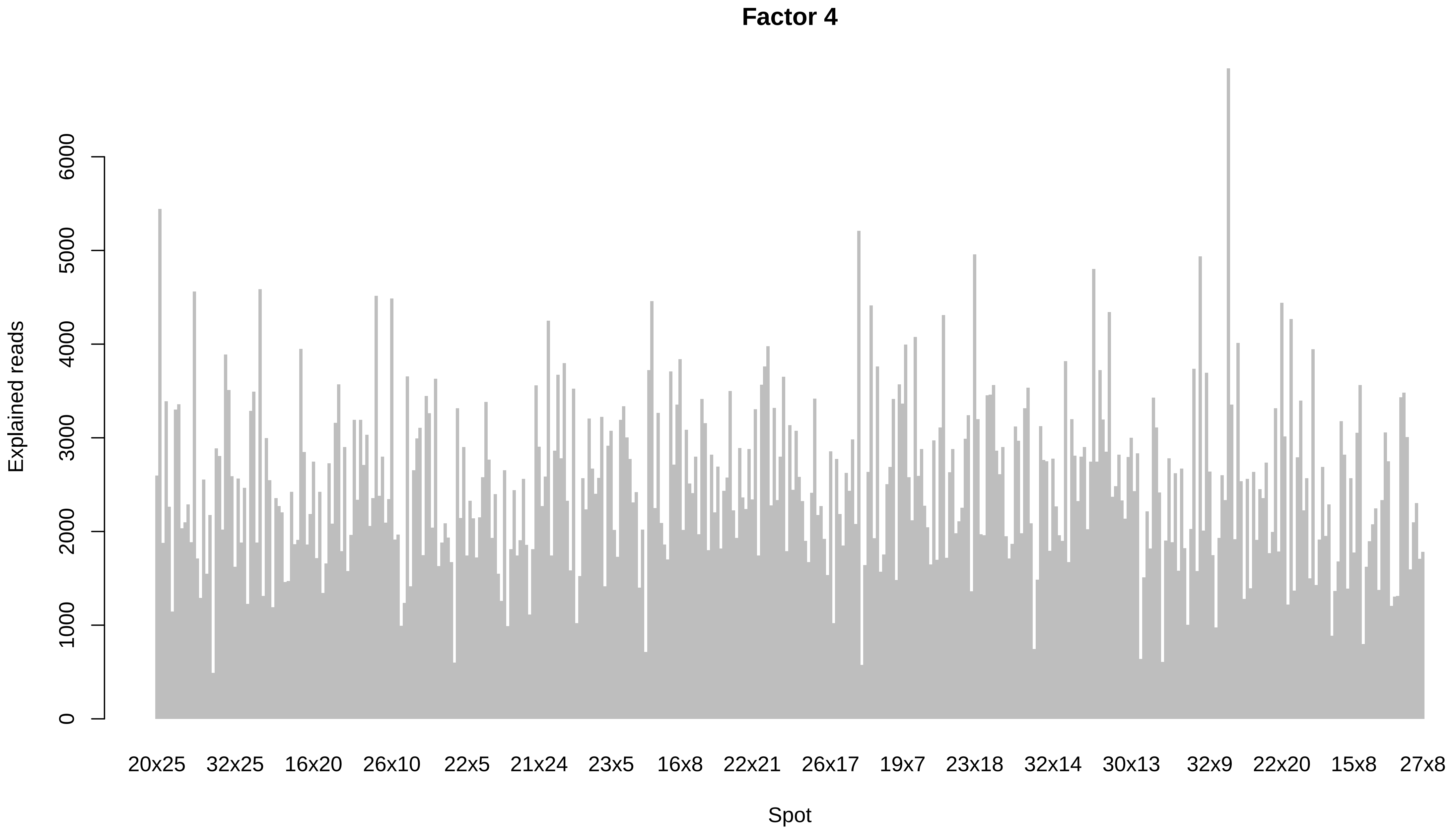

Factor 4
Explained reads
0
1000
2000
3000
4000
5000
6000
20x25
32x25
16x20
26x10
22x5
21x24
23x5
16x8
22x21
26x17
19x7
23x18
32x14
30x13
32x9
22x20
15x8
27x8
Spot

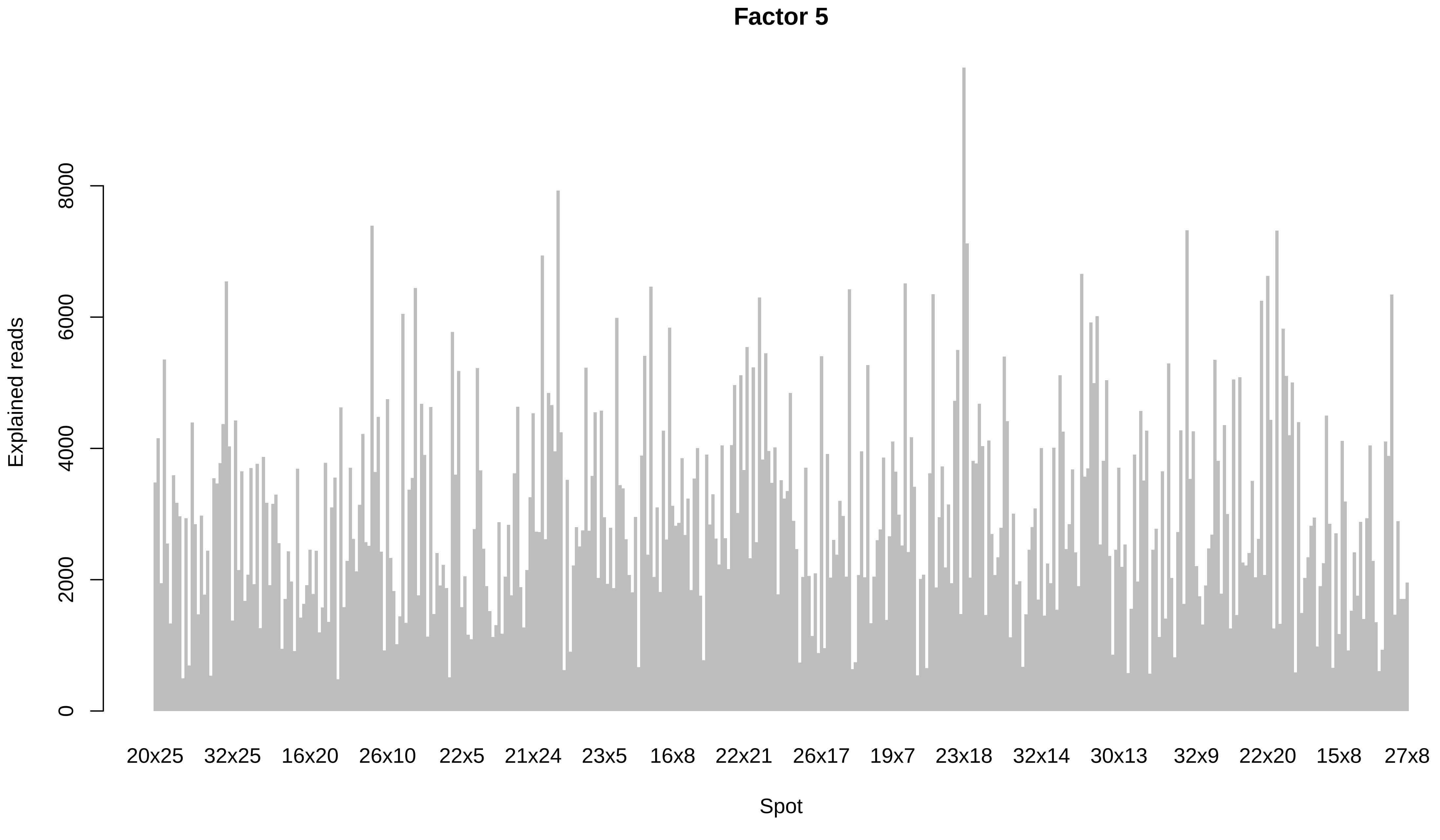

Factor 5
Explained reads
0
2000
4000
6000
8000
20x25
32x25
16x20
26x10
22x5
21x24
23x5
16x8
22x21
26x17
19x7
23x18
32x14
30x13
32x9
22x20
15x8
27x8
Spot

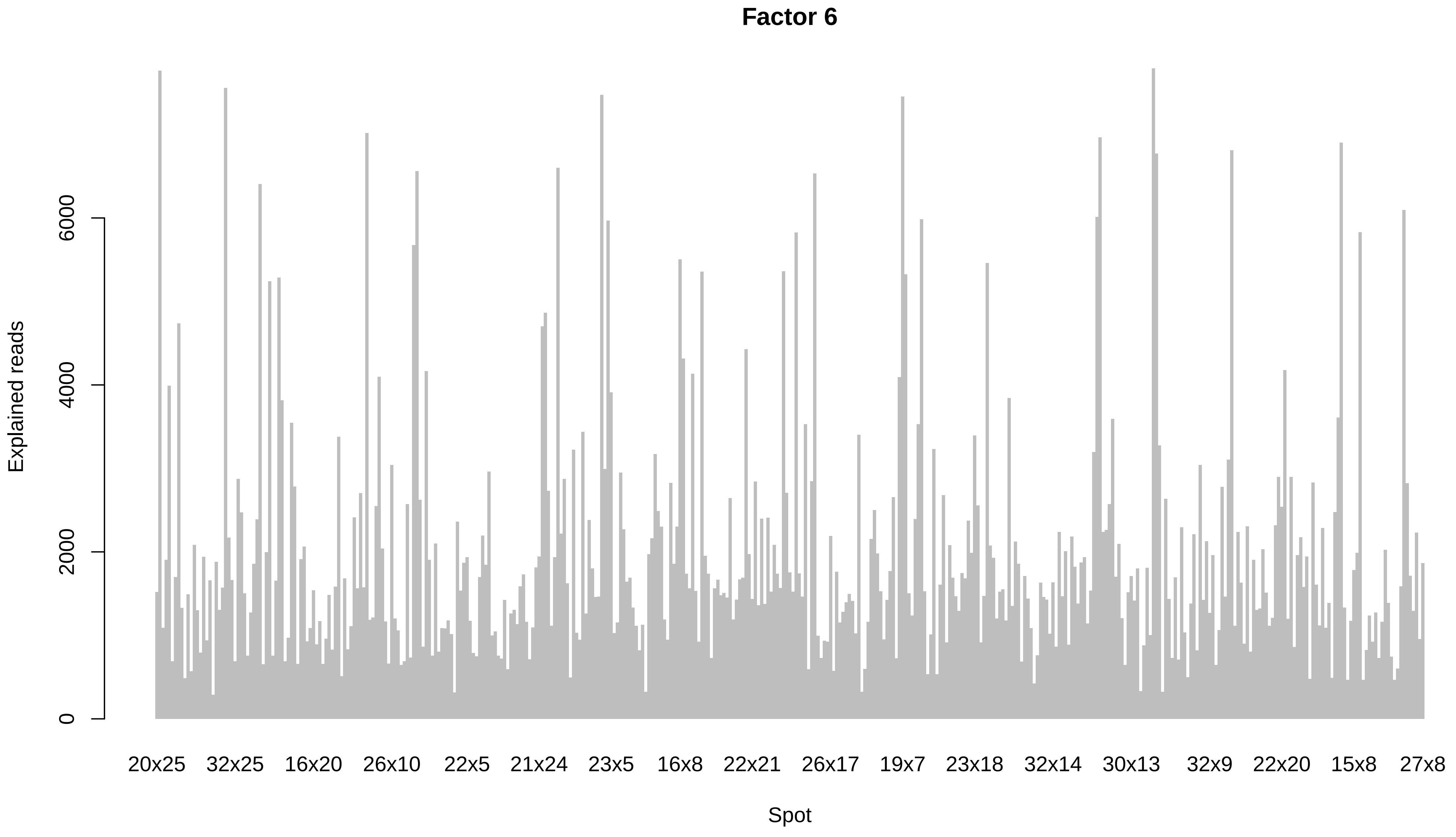

Factor 6
Explained reads
0
2000
4000
6000
20x25
32x25
16x20
26x10
22x5
21x24
23x5
16x8
22x21
26x17
19x7
23x18
32x14
30x13
32x9
22x20
15x8
27x8
Spot

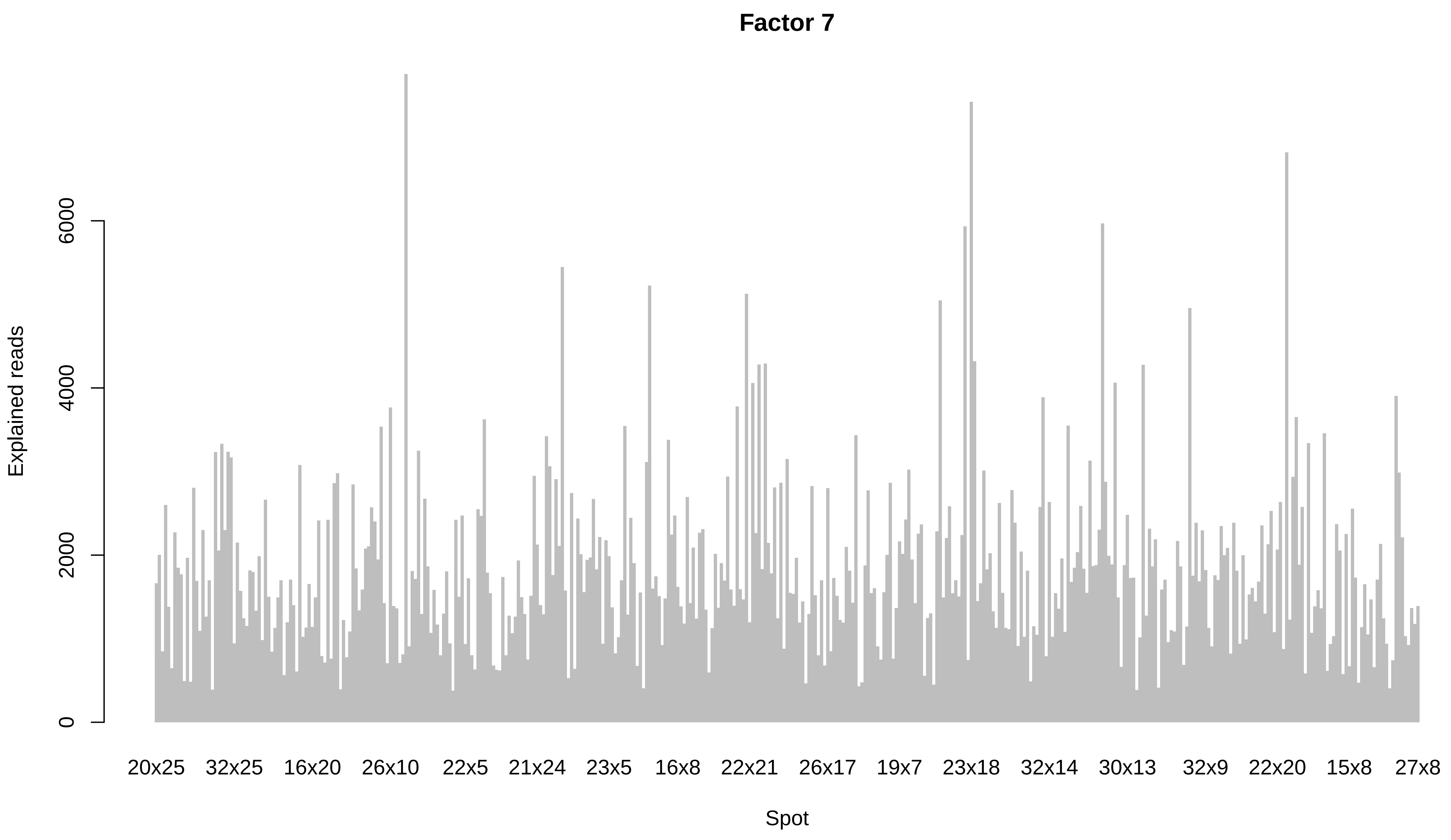
Factor 7
Explained reads
0
2000
4000
6000
20x25
32x25
16x20
26x10
22x5
21x24
23x5
16x8
22x21
26x17
19x7
23x18
32x14
30x13
32x9
22x20
15x8
27x8
Spot

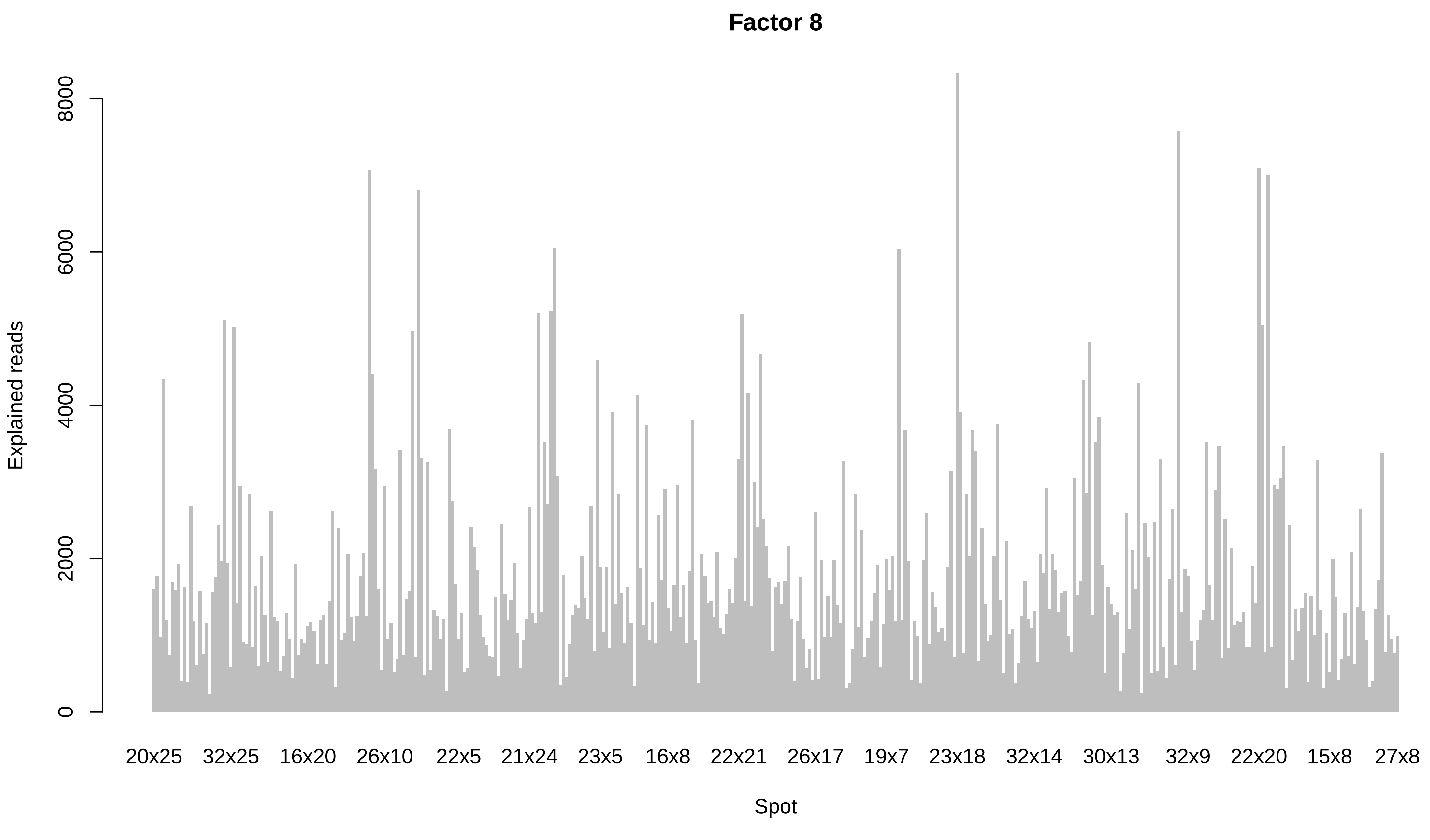

Factor 8
Explained reads
0
2000
4000
6000
8000
20x25
32x25
16x20
26x10
22x5
21x24
23x5
16x8
22x21
26x17
19x7
23x18
32x14
30x13
32x9
22x20
15x8
27x8
Spot

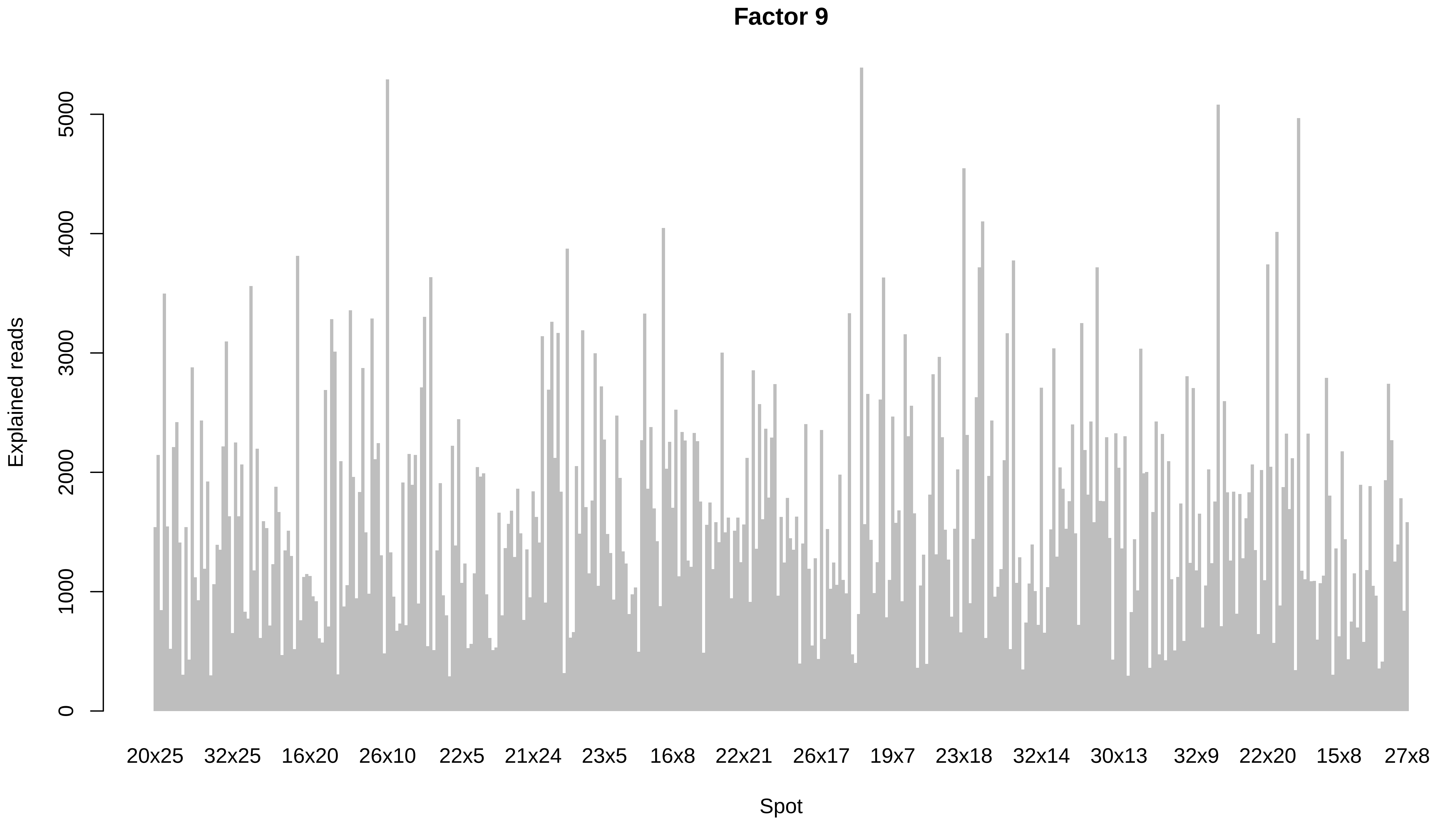
Factor 9
Explained reads
0
1000
2000
3000
4000
5000
20x25
32x25
16x20
26x10
22x5
21x24
23x5
16x8
22x21
26x17
19x7
23x18
32x14
30x13
32x9
22x20
15x8
27x8
Spot

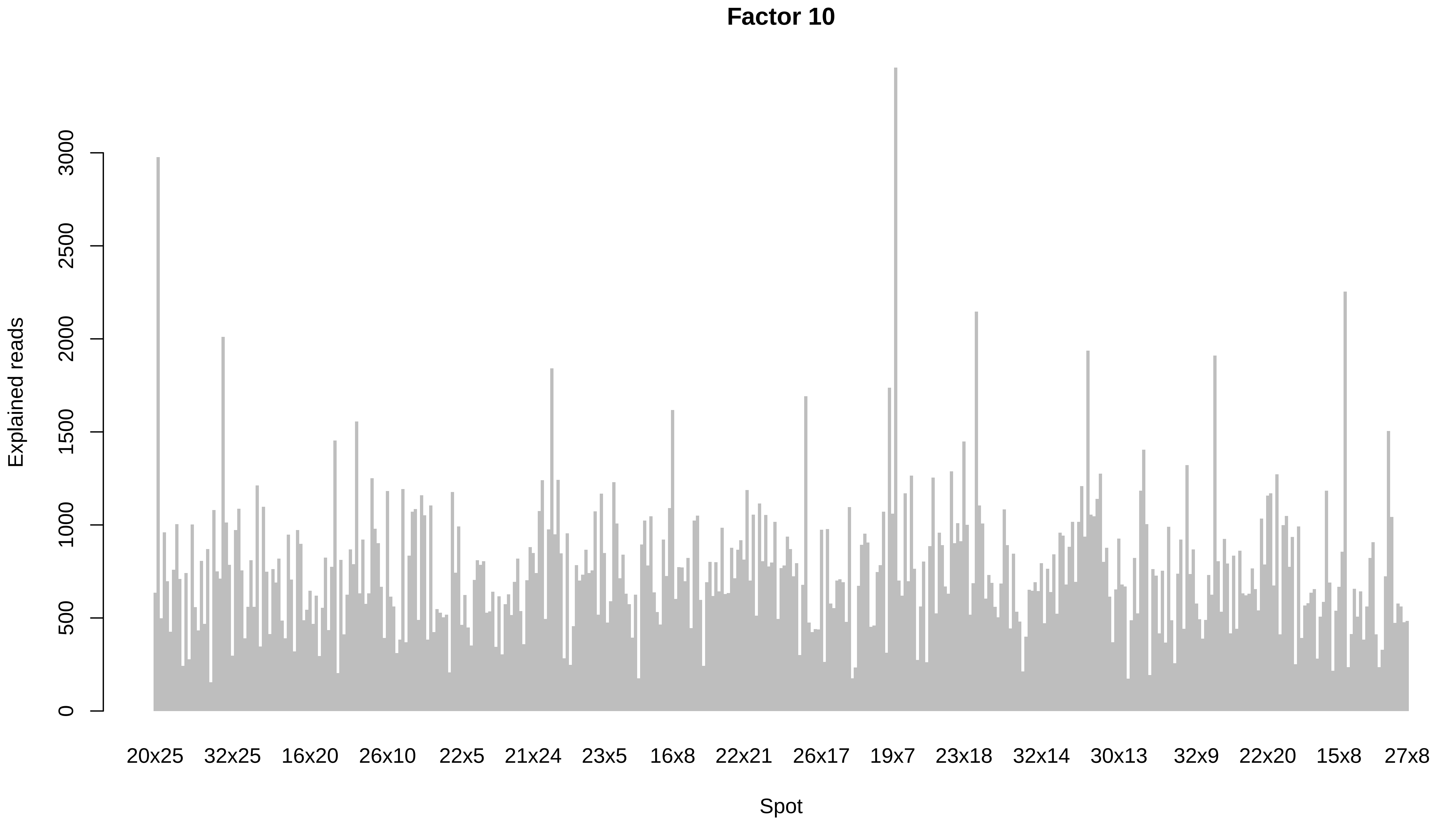
Factor 10
Explained reads
0
500
1000
1500
2000
2500
3000
Spot
20x25
32x25
16x20
26x10
22x5
21x24
23x5
16x8
22x21
26x17
19x7
23x18
32x14
30x13
32x9
22x20
15x8
27x8

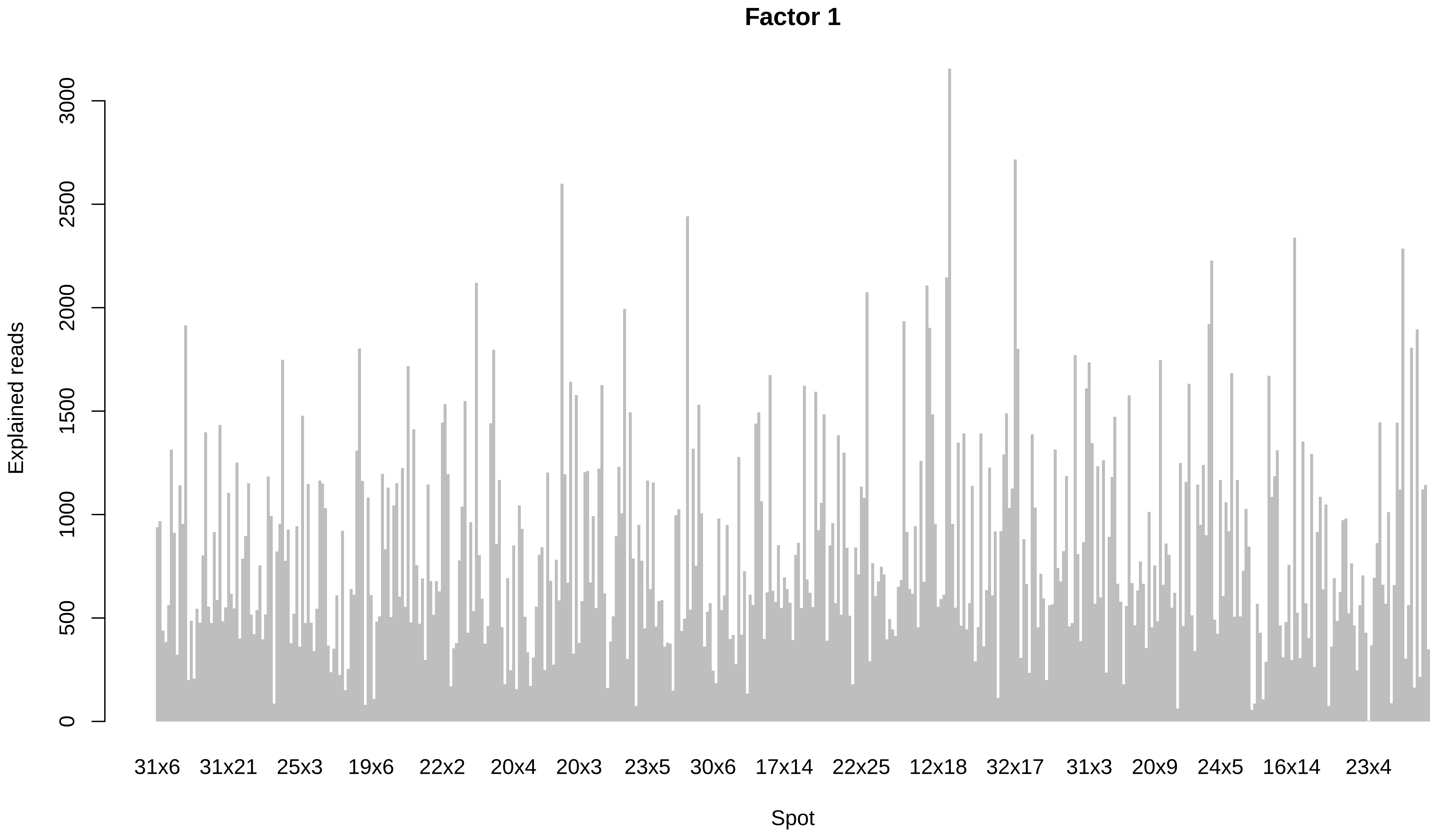
Factor 1
Explained reads
0
500
1000
1500
2000
2500
3000
Spot
31x6
31x21
25x3
19x6
22x2
20x4
20x3
23x5
30x6
17x14
22x25
12x18
32x17
31x3
20x9
24x5
16x14
23x4

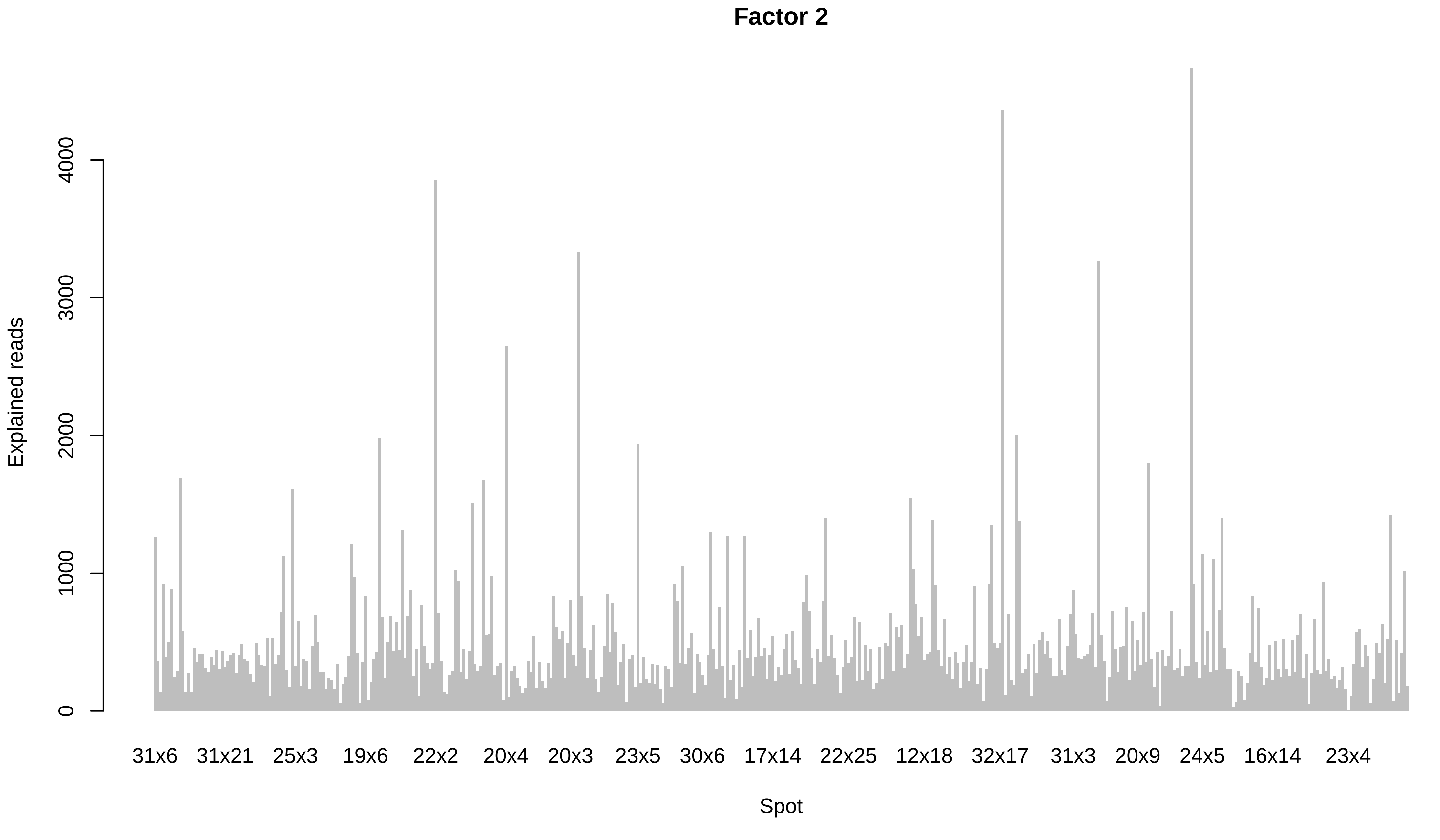

Factor 2
Explained reads
0
1000
2000
3000
4000
31x6
31x21
25x3
19x6
22x2
20x4
20x3
23x5
30x6
17x14
22x25
12x18
32x17
31x3
20x9
24x5
16x14
23x4
Spot

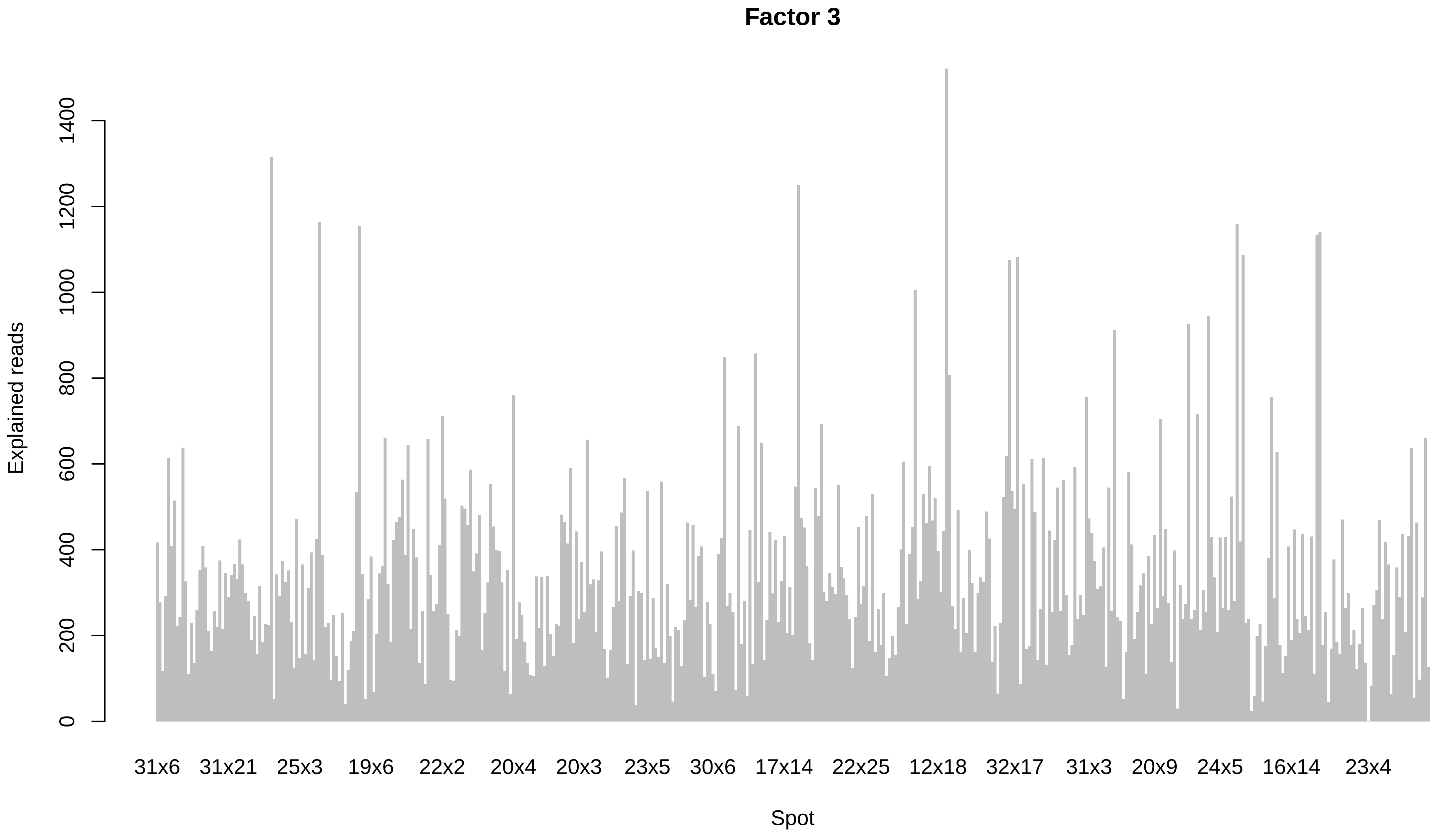
Factor 3
Explained reads
0
200
400
600
800
1000
1200
1400
31x6
31x21
25x3
19x6
22x2
20x4
20x3
23x5
30x6
17x14
22x25
12x18
32x17
31x3
20x9
24x5
16x14
23x4
Spot

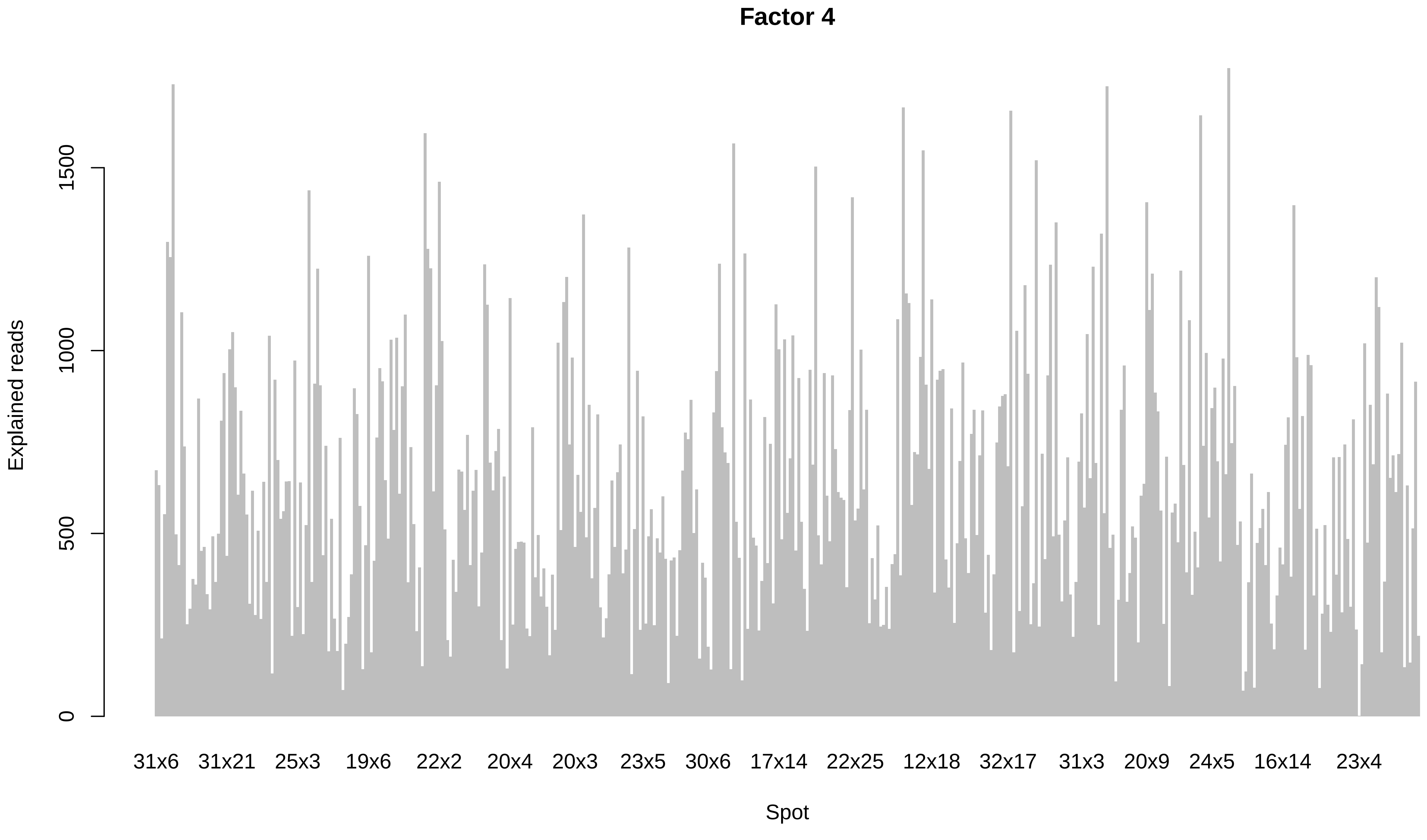
Factor 4
Explained reads
0
500
1000
1500
31x6
31x21
25x3
19x6
22x2
20x4
20x3
23x5
30x6
17x14
22x25
12x18
32x17
31x3
20x9
24x5
16x14
23x4
Spot

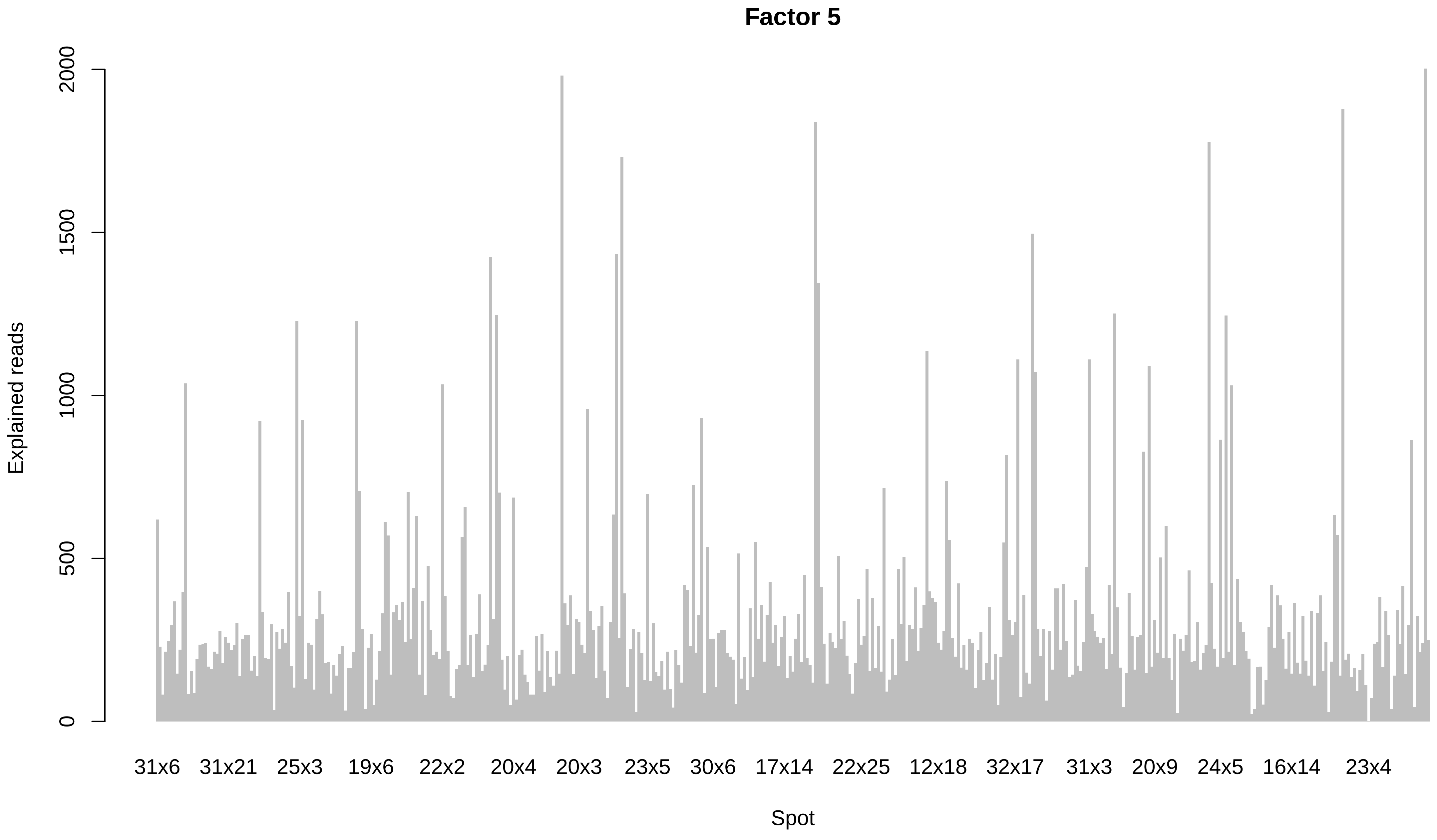

Factor 5
Explained reads
2000
1500
1000
500
0
Spot
31x6
31x21
25x3
19x6
22x2
20x4
20x3
23x5
30x6
17x14
22x25
12x18
32x17
31x3
20x9
24x5
16x14
23x4

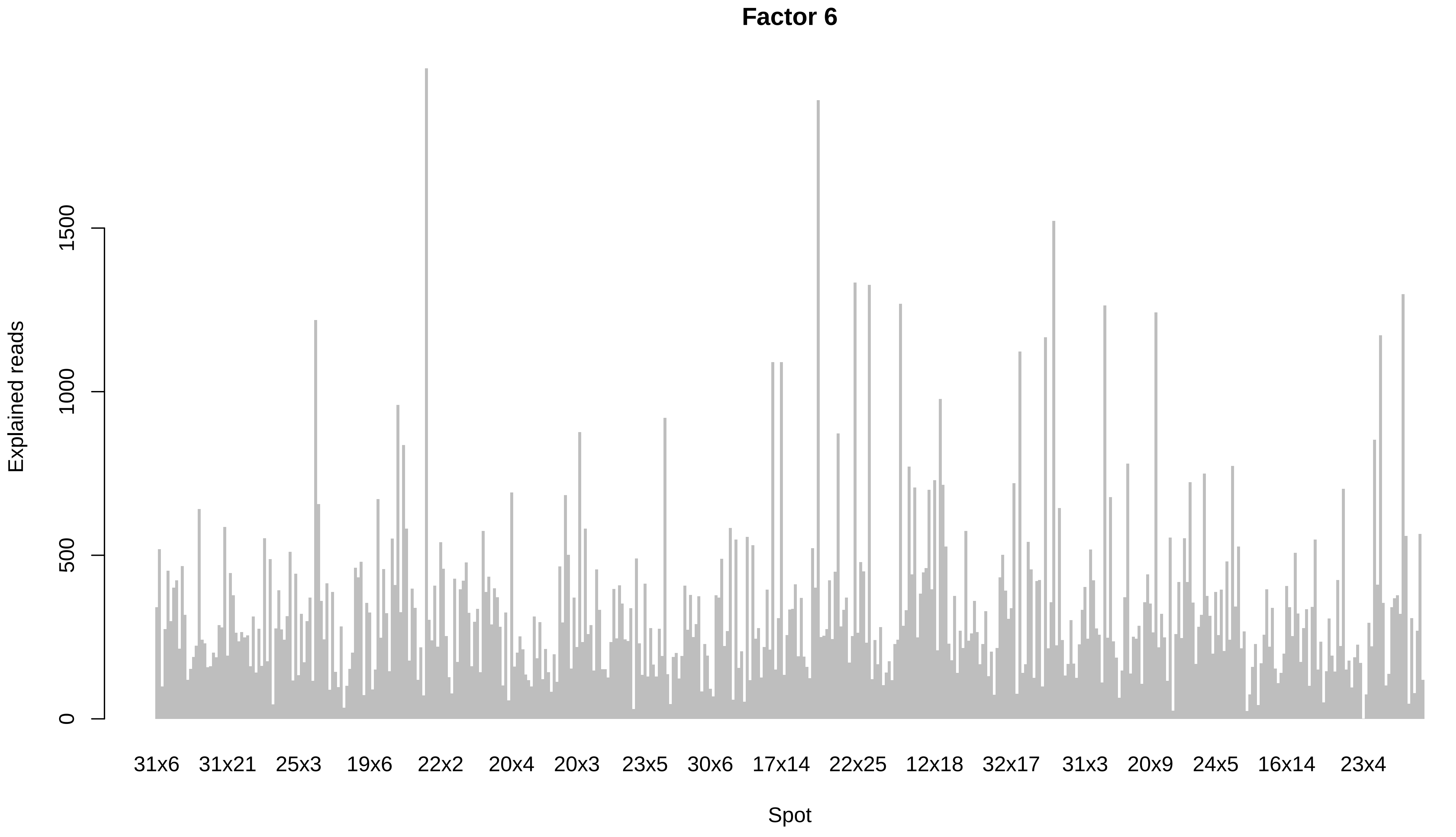
Factor 6
Explained reads
0
500
1000
1500
31x6
31x21
25x3
19x6
22x2
20x4
20x3
23x5
30x6
17x14
22x25
12x18
32x17
31x3
20x9
24x5
16x14
23x4
Spot

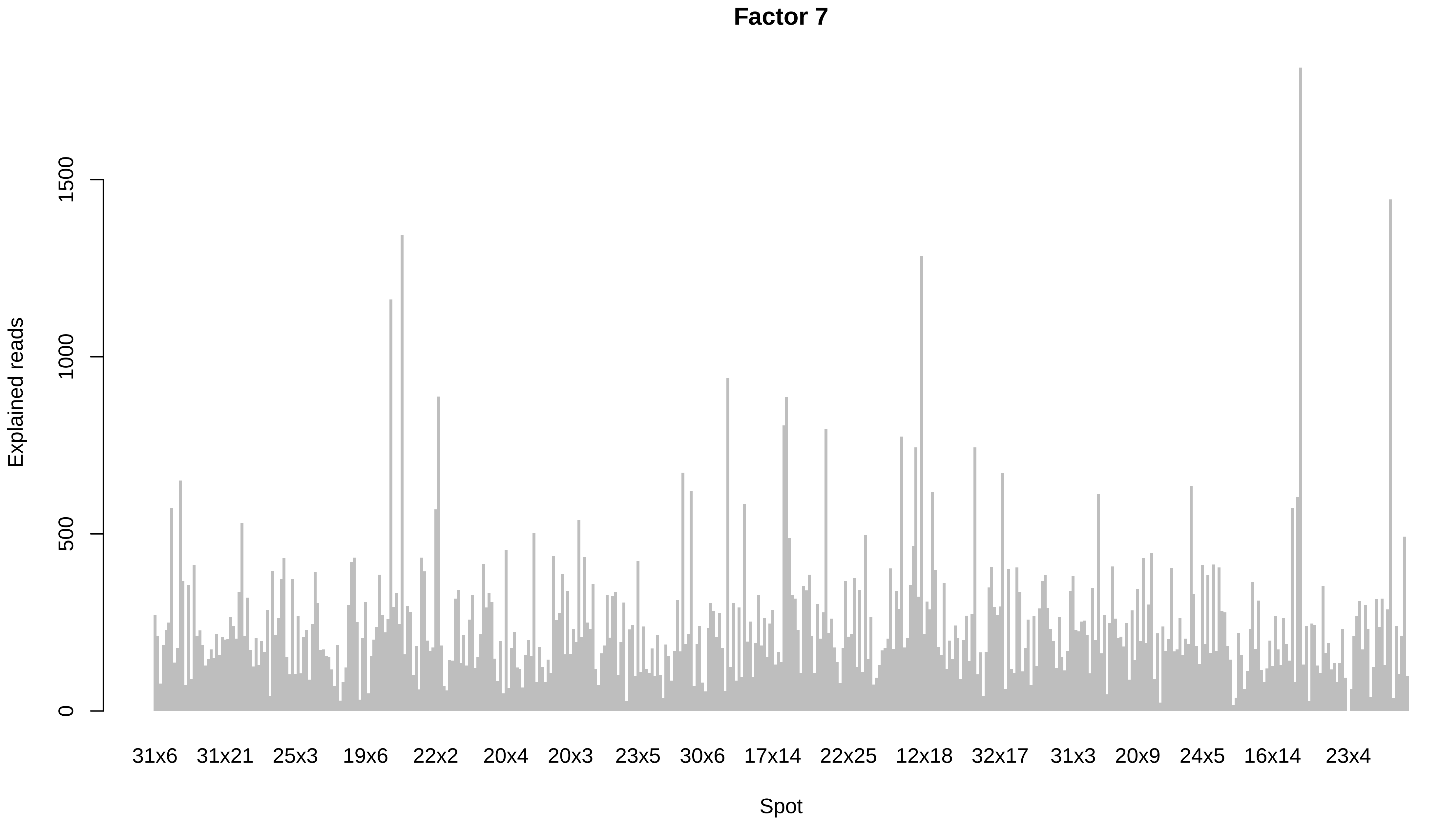
Factor 7
Explained reads
0
500
1000
1500
31x6
31x21
25x3
19x6
22x2
20x4
20x3
23x5
30x6
17x14
22x25
12x18
32x17
31x3
20x9
24x5
16x14
23x4
Spot

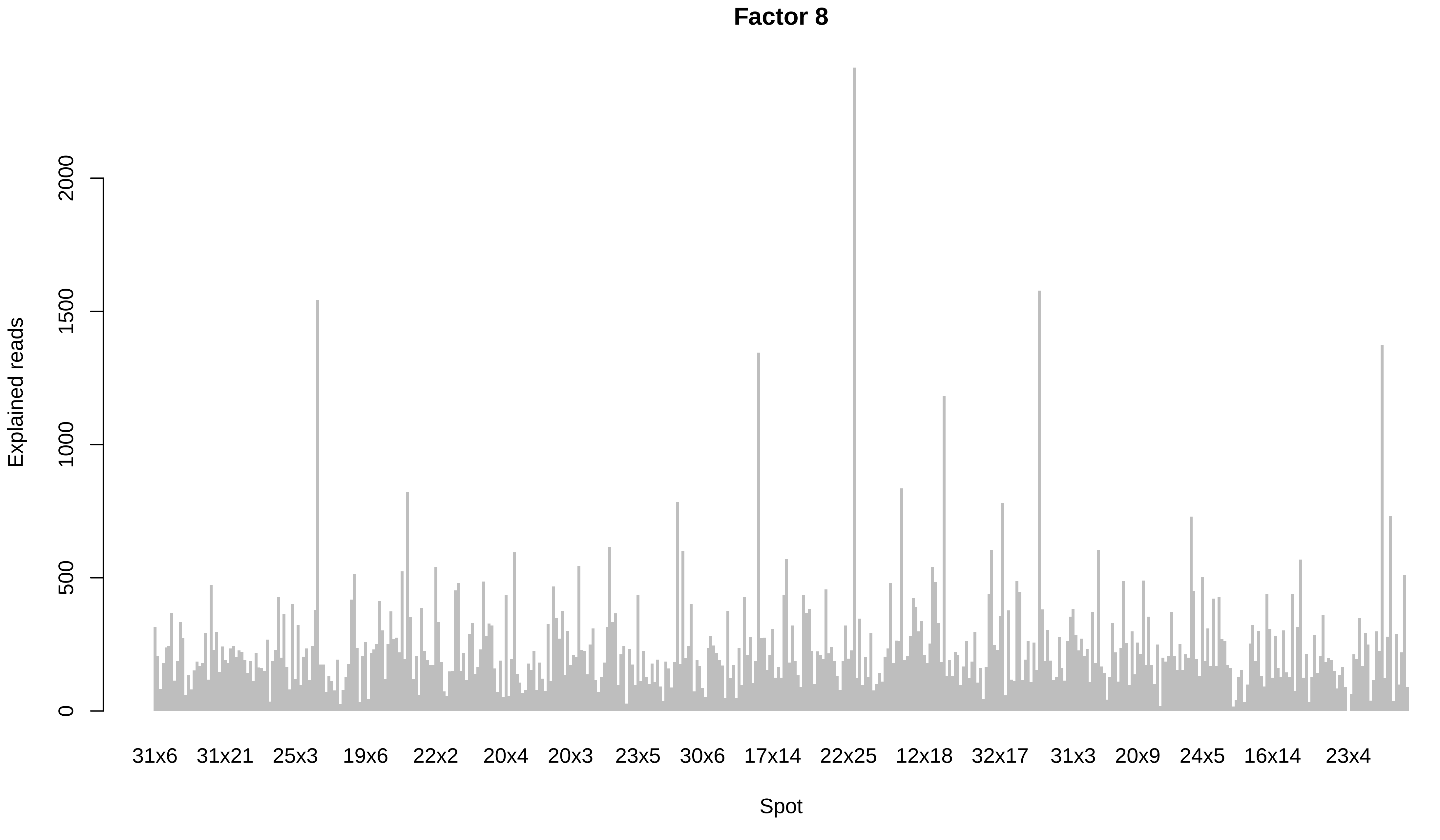

Factor 8
Explained reads
0
500
1000
1500
2000
31x6
31x21
25x3
19x6
22x2
20x4
20x3
23x5
30x6
17x14
22x25
12x18
32x17
31x3
20x9
24x5
16x14
23x4
Spot

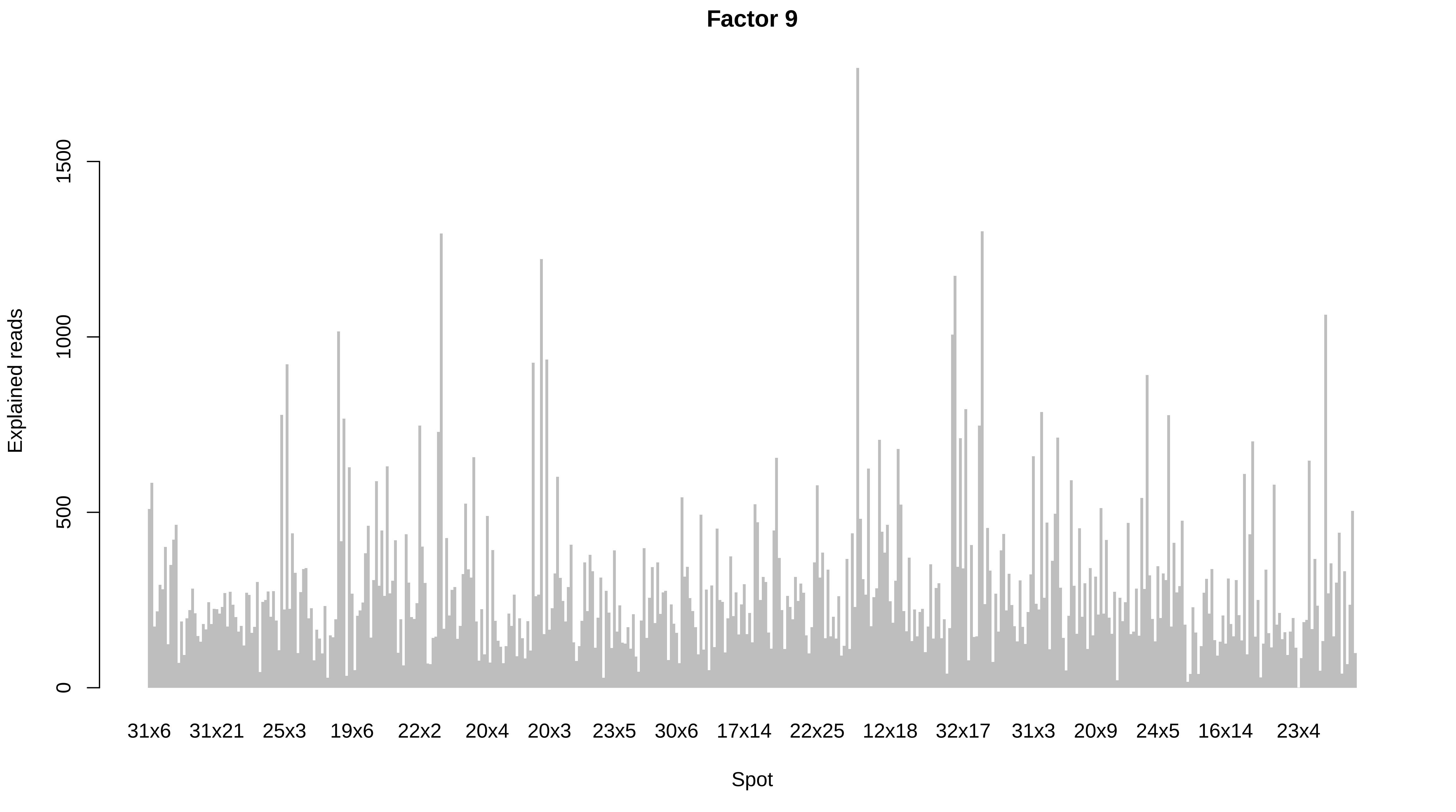
Factor 9
Explained reads
0
500
1000
1500
Spot
31x6
31x21
25x3
19x6
22x2
20x4
20x3
23x5
30x6
17x14
22x25
12x18
32x17
31x3
20x9
24x5
16x14
23x4

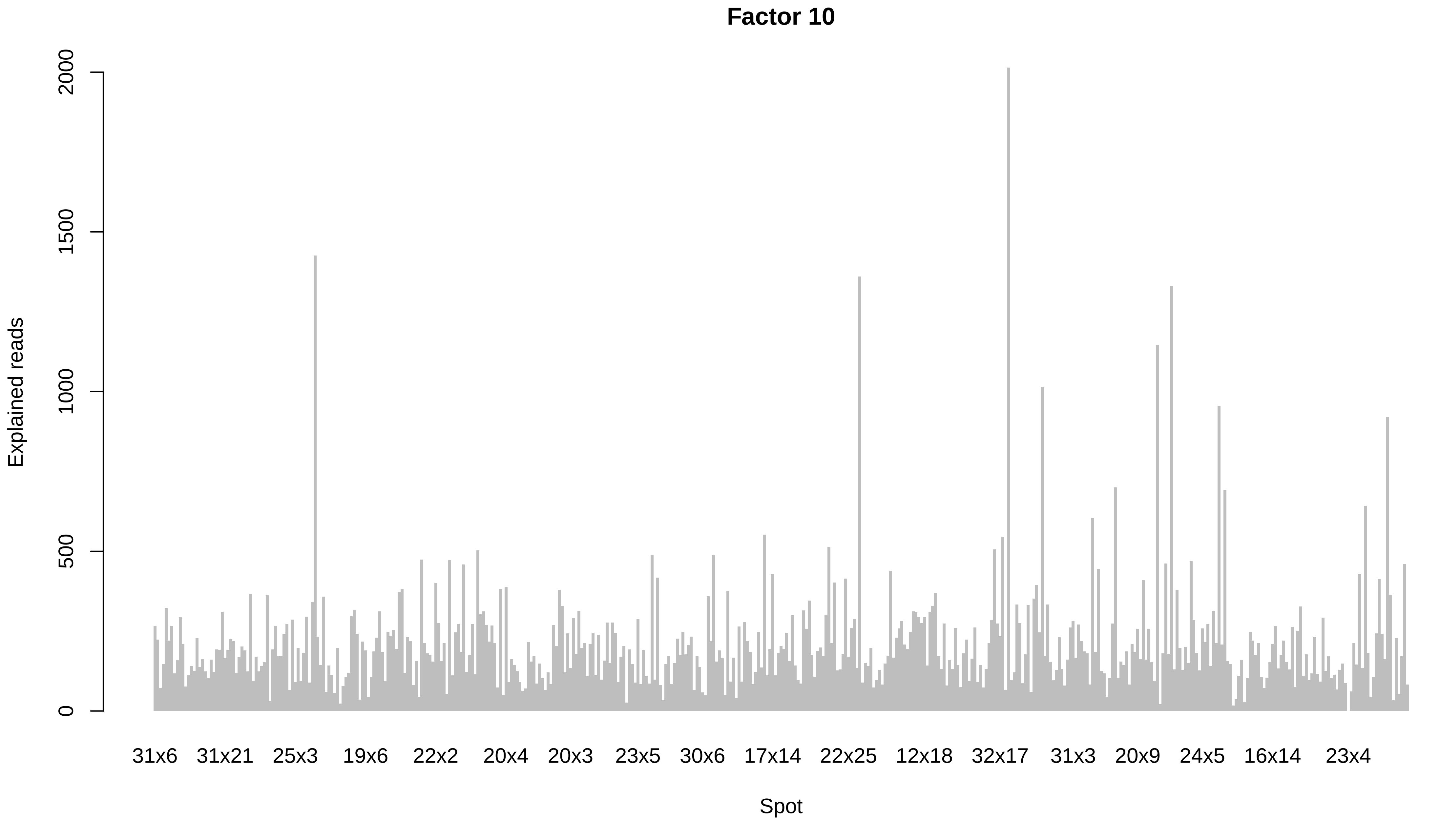
Factor 10
Explained reads
0
500
1000
1500
2000
31x6
31x21
25x3
19x6
22x2
20x4
20x3
23x5
30x6
17x14
22x25
12x18
32x17
31x3
20x9
24x5
16x14
23x4
Spot

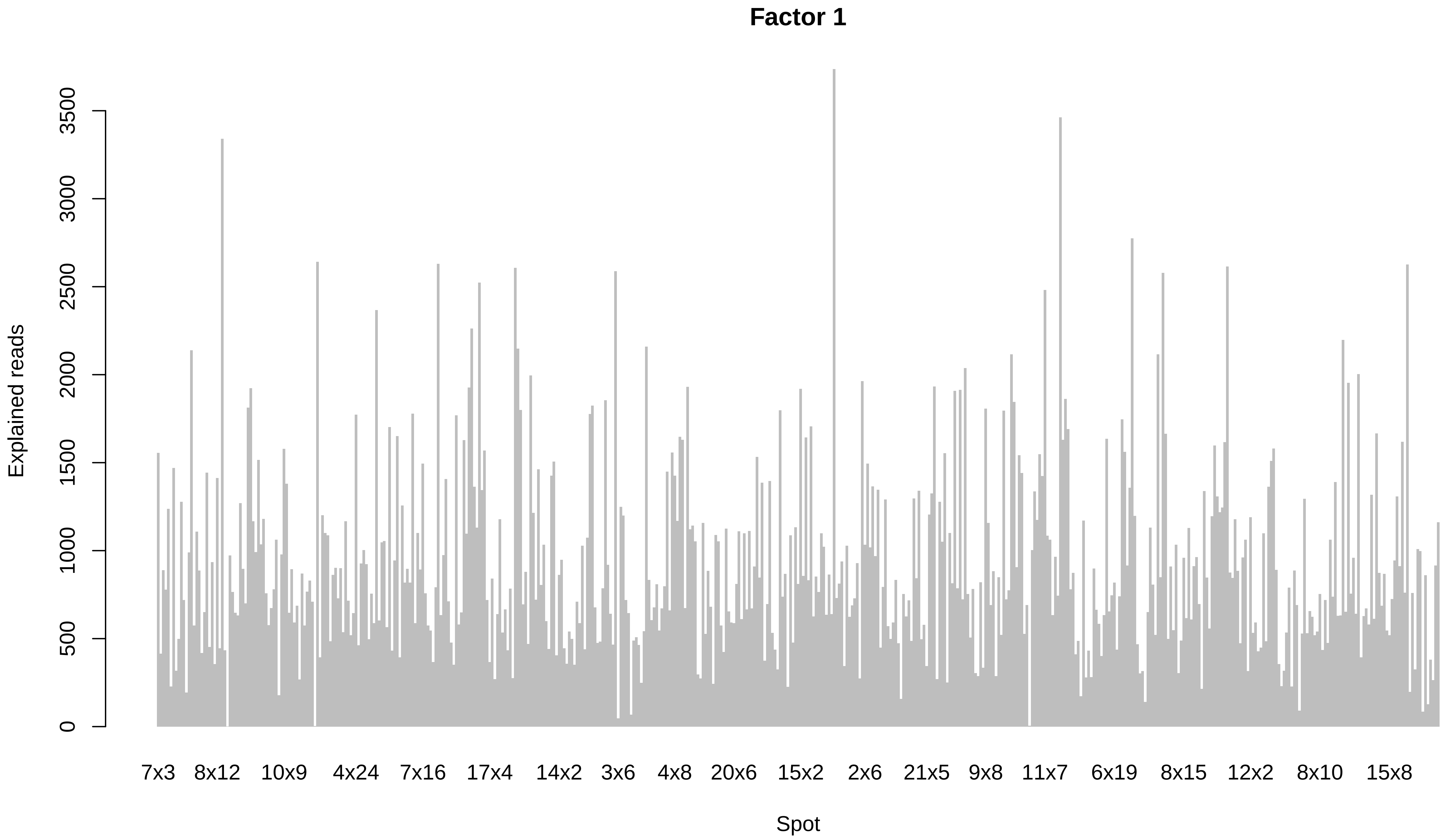
Factor 1
Explained reads
0
500
1000
1500
2000
2500
3000
3500
7x3
8x12
10x9
4x24
7x16
17x4
14x2
3x6
4x8
20x6
15x2
2x6
21x5
9x8
11x7
6x19
8x15
12x2
8x10
15x8
Spot

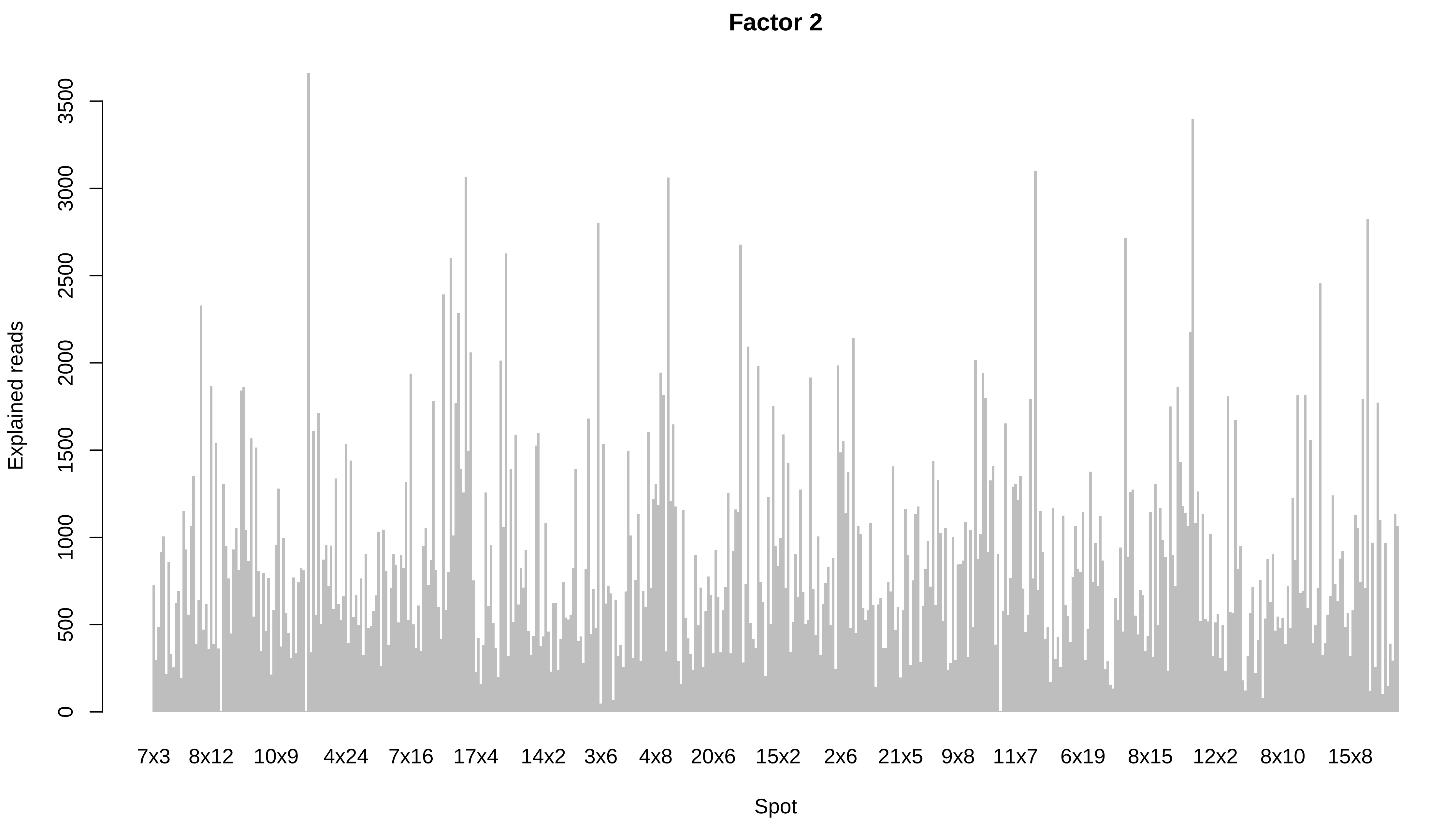

Factor 2
Explained reads
0
500
1000
1500
2000
2500
3000
3500
7x3
8x12
10x9
4x24
7x16
17x4
14x2
3x6
4x8
20x6
15x2
2x6
21x5
9x8
11x7
6x19
8x15
12x2
8x10
15x8
Spot

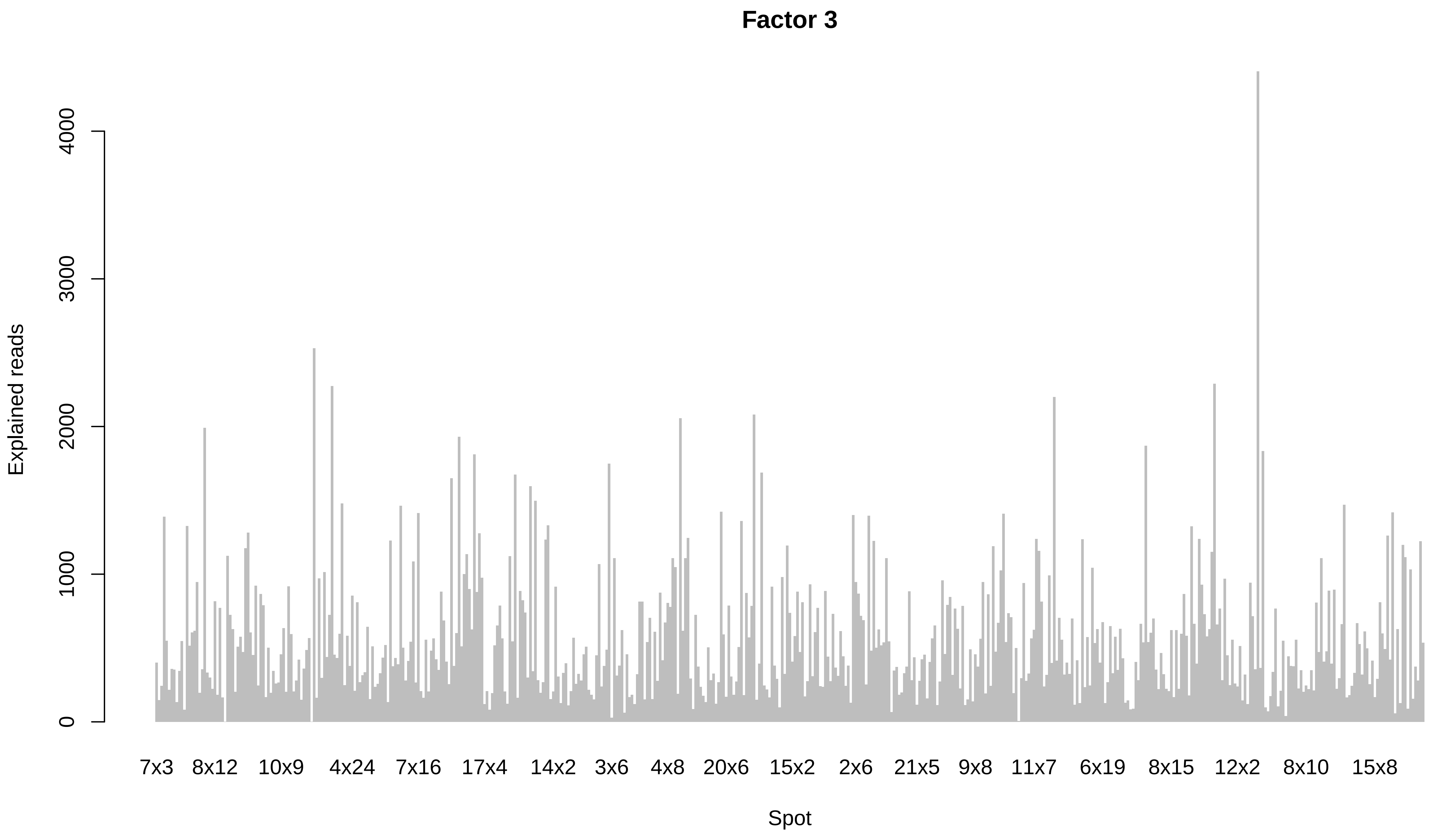
Factor 3
Explained reads
0
1000
2000
3000
4000
7x3
8x12
10x9
4x24
7x16
17x4
14x2
3x6
4x8
20x6
15x2
2x6
21x5
9x8
11x7
6x19
8x15
12x2
8x10
15x8
Spot

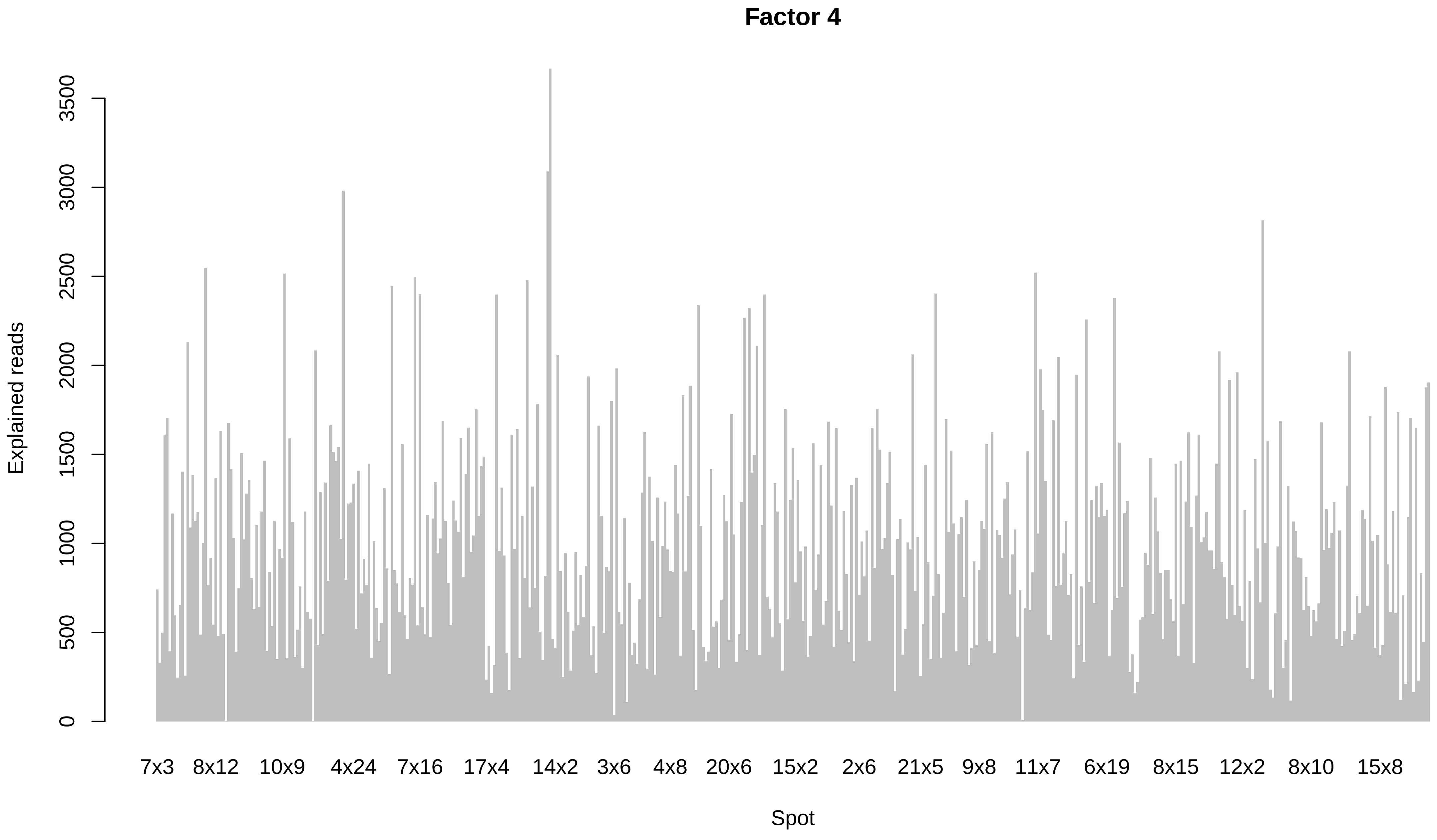

Factor 4
Explained reads
0
500
1000
1500
2000
2500
3000
3500
7x3
8x12
10x9
4x24
7x16
17x4
14x2
3x6
4x8
20x6
15x2
2x6
21x5
9x8
11x7
6x19
8x15
12x2
8x10
15x8
Spot

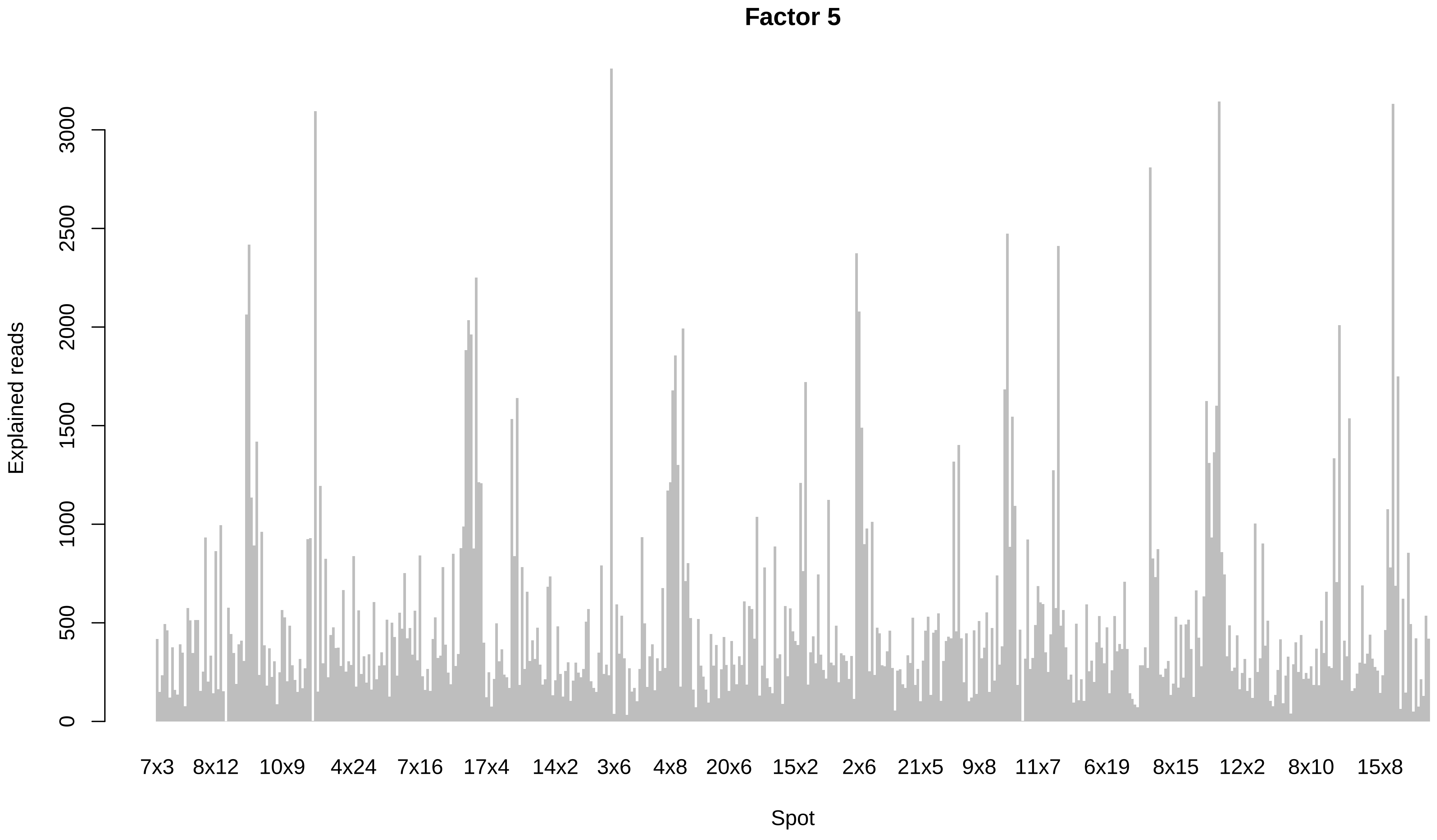
Factor 5
Explained reads
0
500
1000
1500
2000
2500
3000
Spot
7x3
8x12
10x9
4x24
7x16
17x4
14x2
3x6
4x8
20x6
15x2
2x6
21x5
9x8
11x7
6x19
8x15
12x2
8x10
15x8

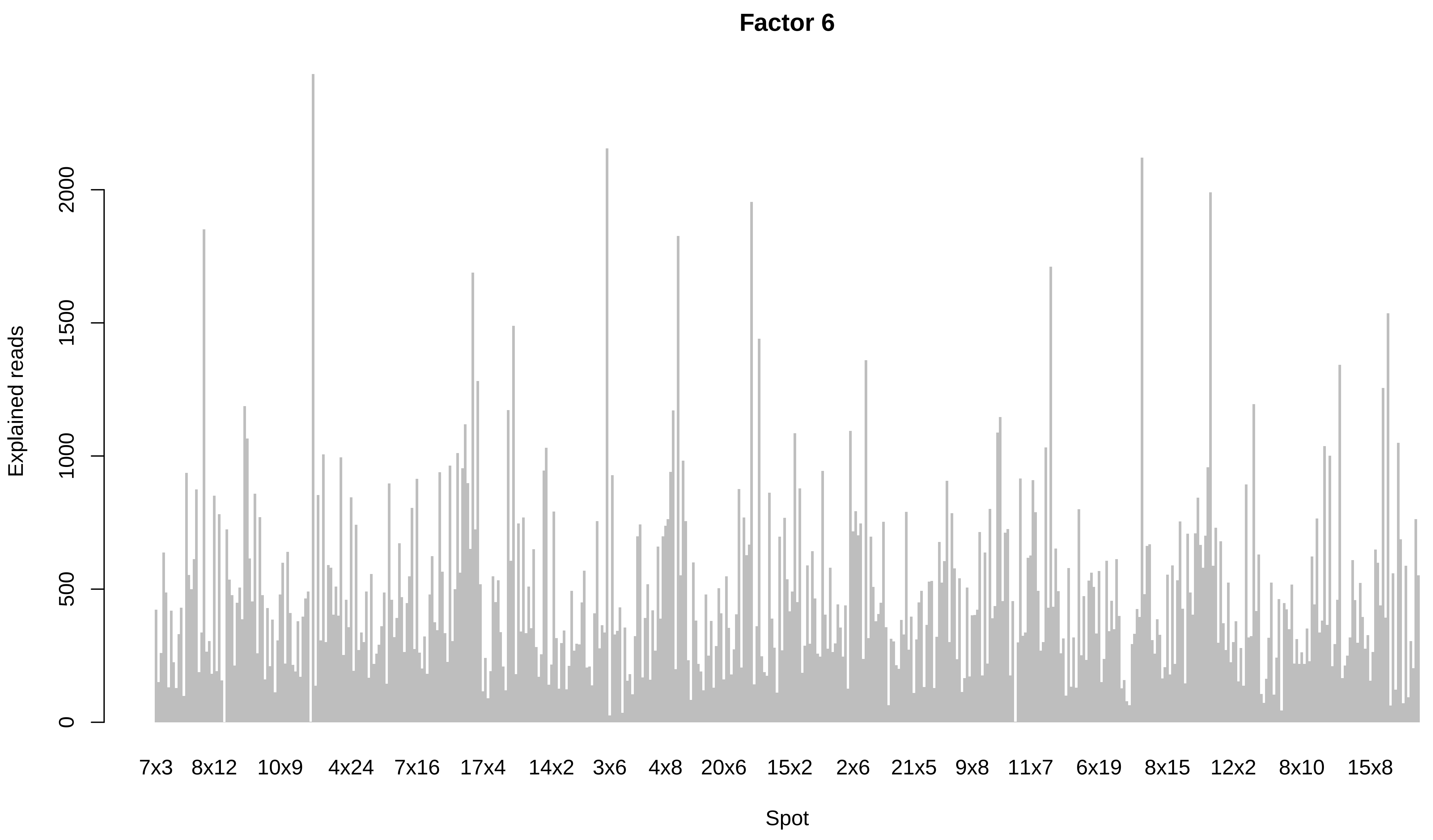
Factor 6
Explained reads
0
500
1000
1500
2000
Spot
7x3
8x12
10x9
4x24
7x16
17x4
14x2
3x6
4x8
20x6
15x2
2x6
21x5
9x8
11x7
6x19
8x15
12x2
8x10
15x8

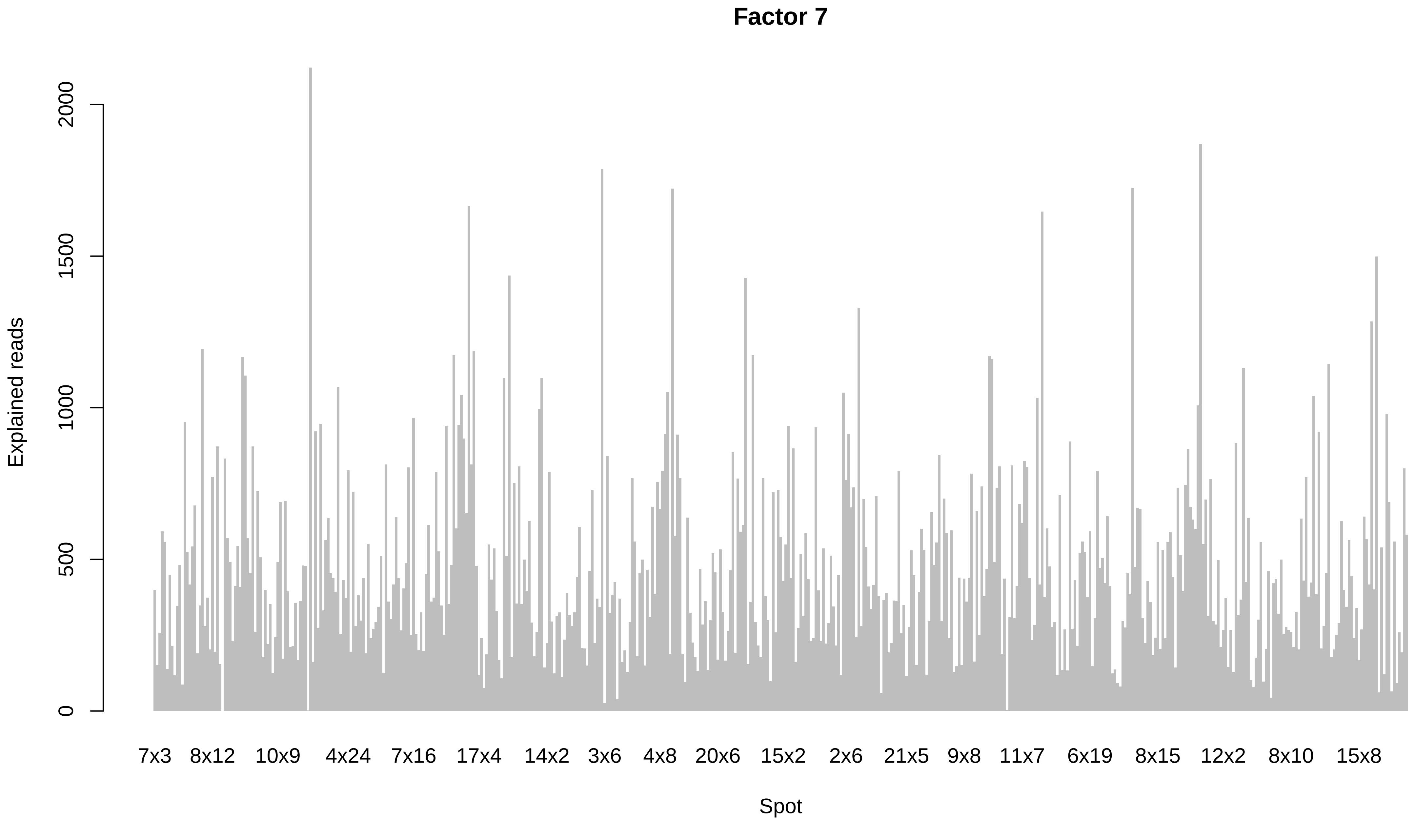

Factor 7
Explained reads
0
500
1000
1500
2000
7x3
8x12
10x9
4x24
7x16
17x4
14x2
3x6
4x8
20x6
15x2
2x6
21x5
9x8
11x7
6x19
8x15
12x2
8x10
15x8
Spot

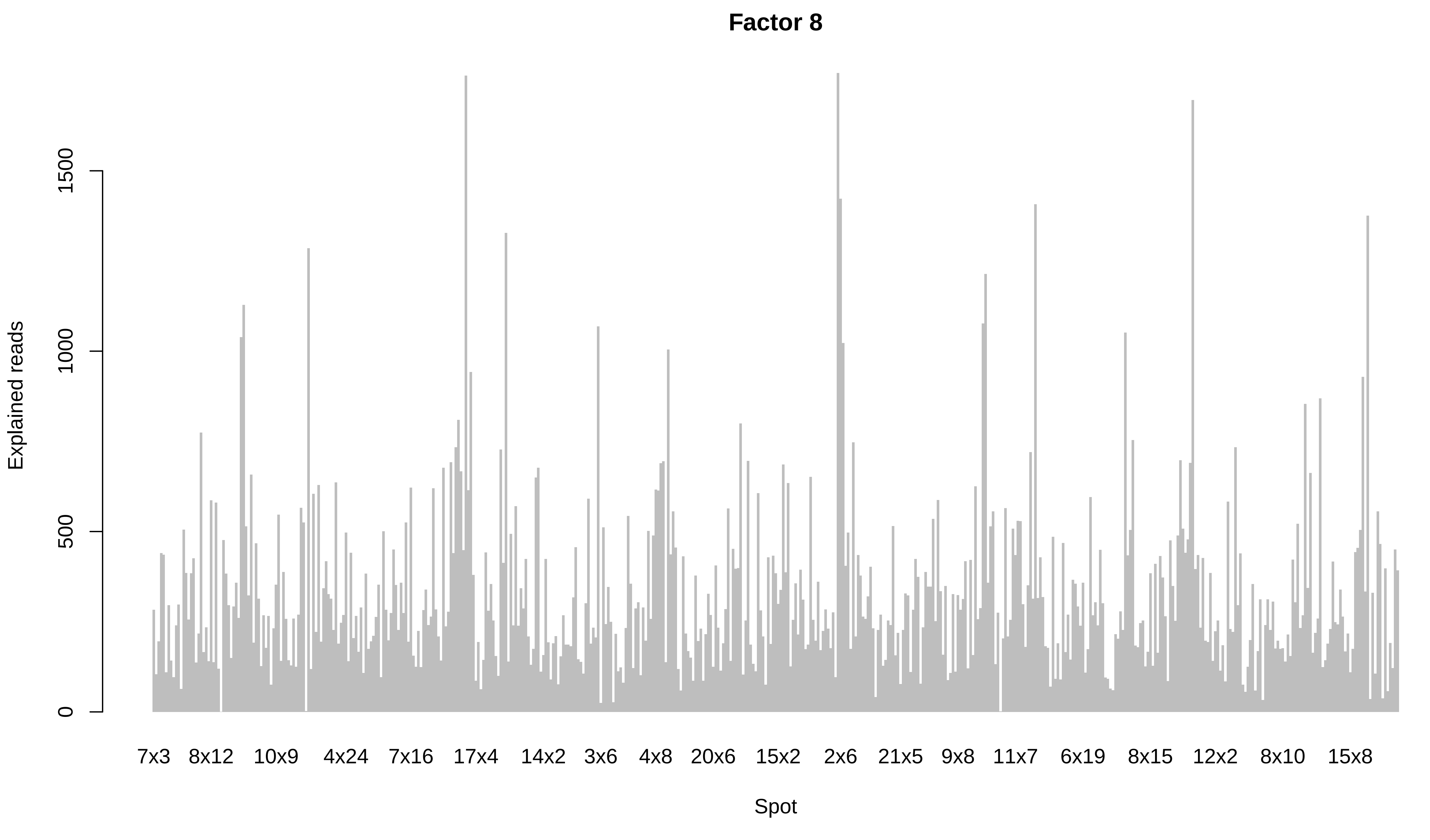
Factor 8
Explained reads
0
500
1000
1500
Spot
7x3
8x12
10x9
4x24
7x16
17x4
14x2
3x6
4x8
20x6
15x2
2x6
21x5
9x8
11x7
6x19
8x15
12x2
8x10
15x8

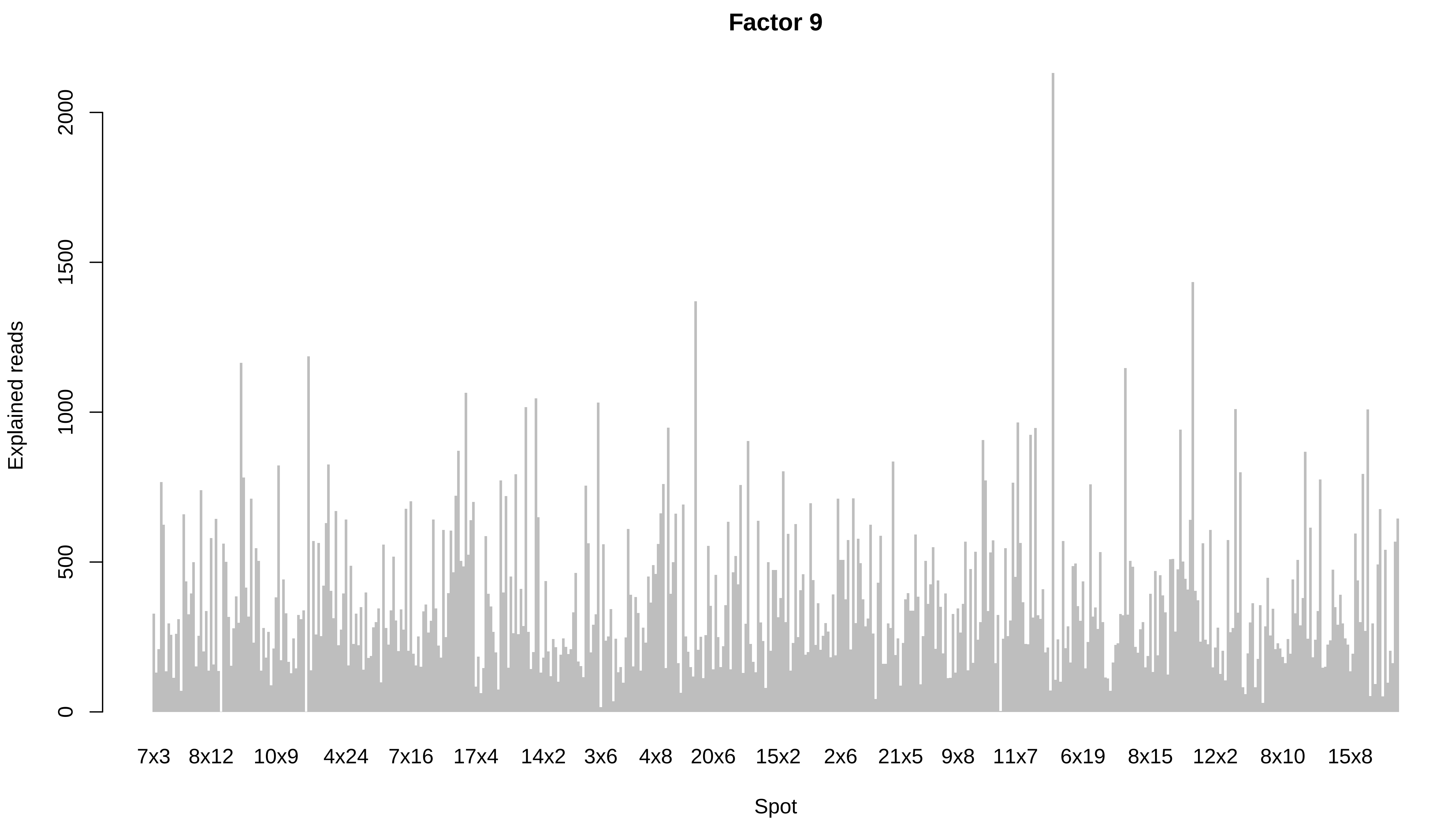
Factor 9
Explained reads
0
500
1000
1500
2000
7x3
8x12
10x9
4x24
7x16
17x4
14x2
3x6
4x8
20x6
15x2
2x6
21x5
9x8
11x7
6x19
8x15
12x2
8x10
15x8
Spot

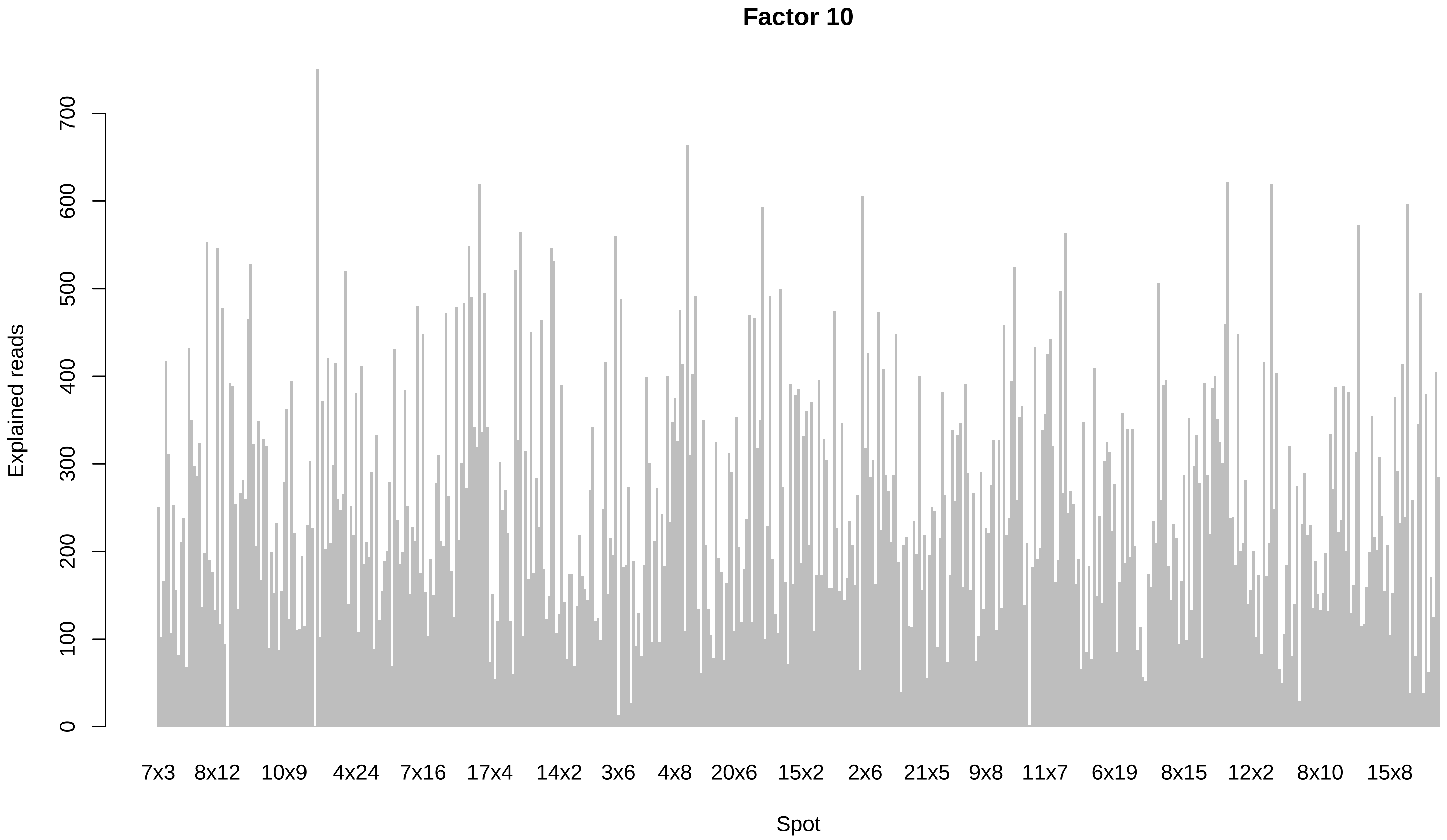
Factor 10
Explained reads
0
100
200
300
400
500
600
700
7x3
8x12
10x9
4x24
7x16
17x4
14x2
3x6
4x8
20x6
15x2
2x6
21x5
9x8
11x7
6x19
8x15
12x2
8x10
15x8
Spot
