# Supplementary material for: Spatial maps of prostate cancer transcriptomes reveal an unexplored landscape of heterogeneity
Source: Nat Commun. 2018 Jun 20;9:2419. doi: 10.1038/s41467-018-04724-5 (PMC6010471; doi:10.1038/s41467-018-04724-5)
Supplement: Supplementary file 8 — Supplementary Data 5 [file 41467_2018_4724_MOESM8_ESM.zip › Supplementary Dataset 7/joint-mix-factor-strength-barplot-expected-spots.pdf]

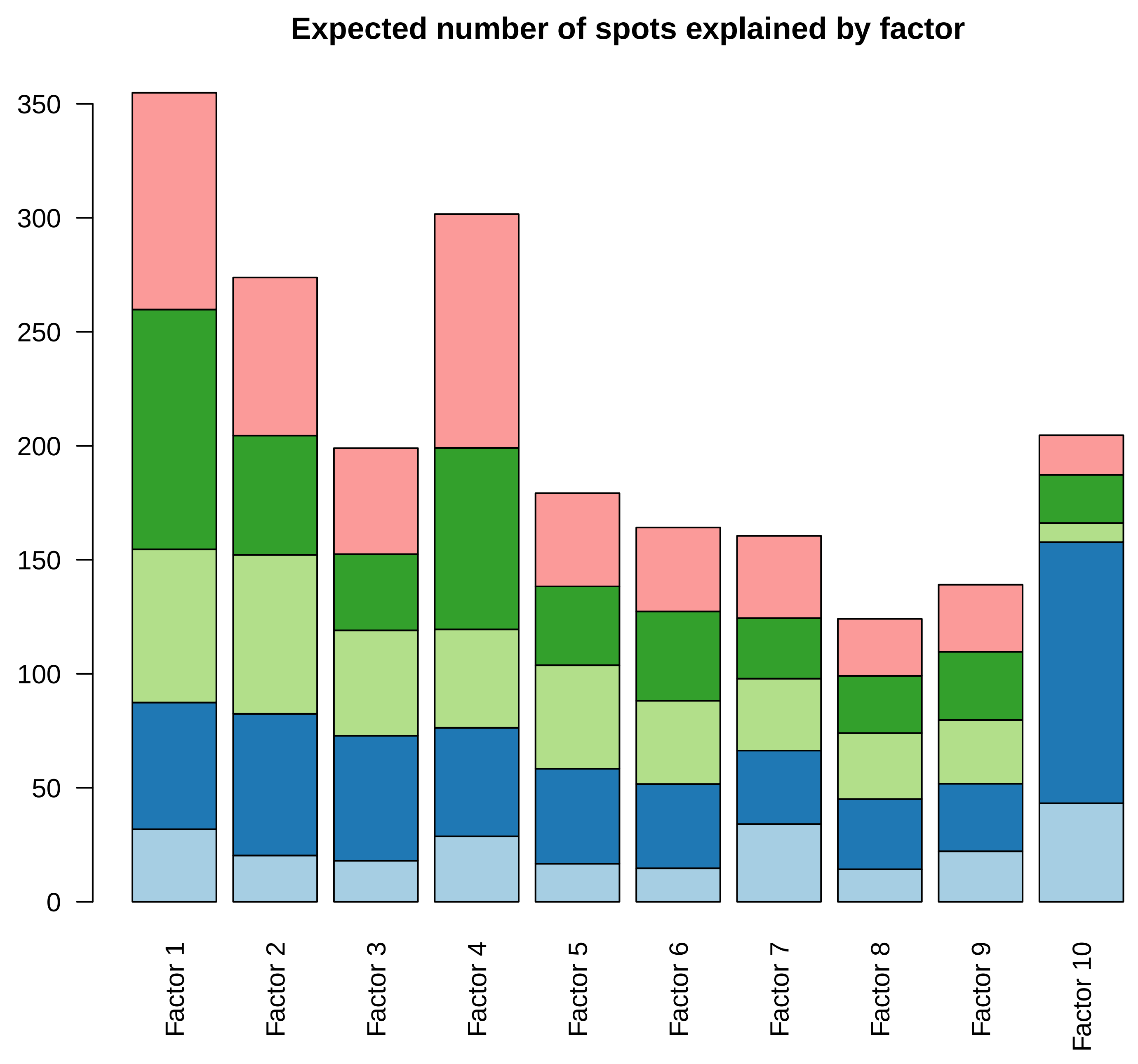
Expected number of spots explained by factor
350
300
250
200
150
100
50
0
Factor 1
Factor 2
Factor 3
Factor 4
Factor 5
Factor 6
Factor 7
Factor 8
Factor 9
Factor 10

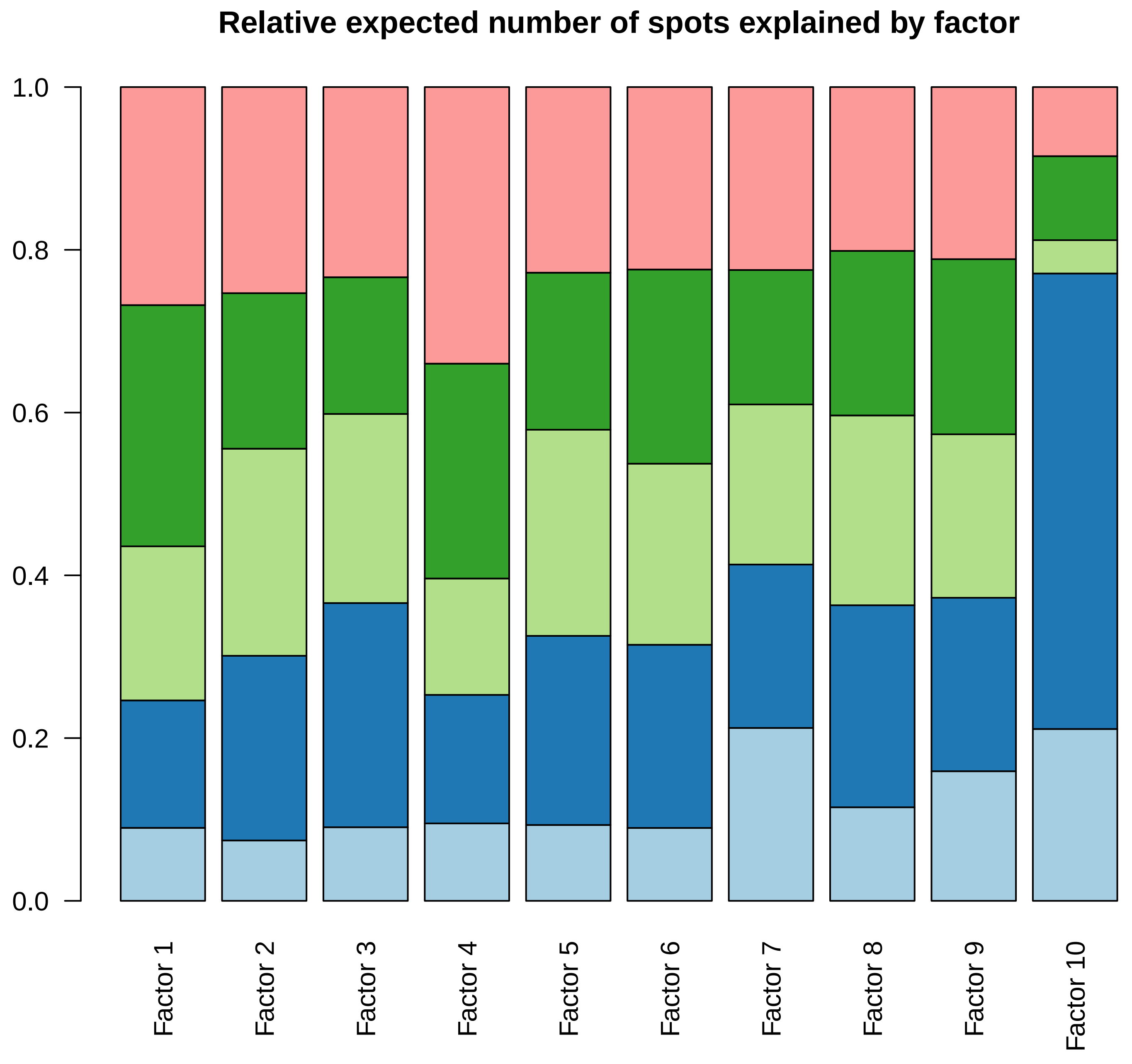
Relative expected number of spots explained by factor
0.0
0.2
0.4
0.6
0.8
1.0
Factor 1
Factor 2
Factor 3
Factor 4
Factor 5
Factor 6
Factor 7
Factor 8
Factor 9
Factor 10

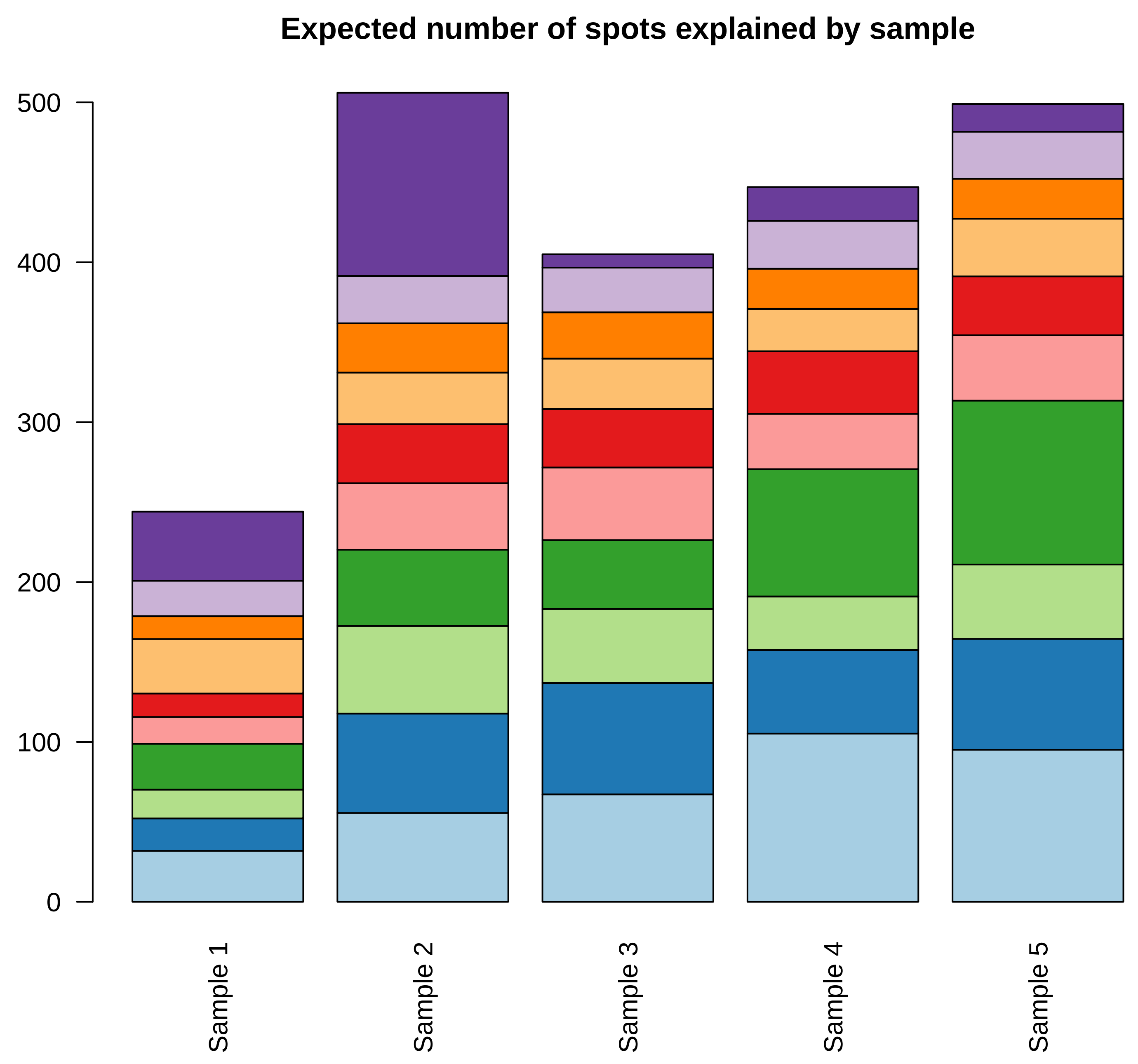
Expected number of spots explained by sample
0
100
200
300
400
500
Sample 1
Sample 2
Sample 3
Sample 4
Sample 5

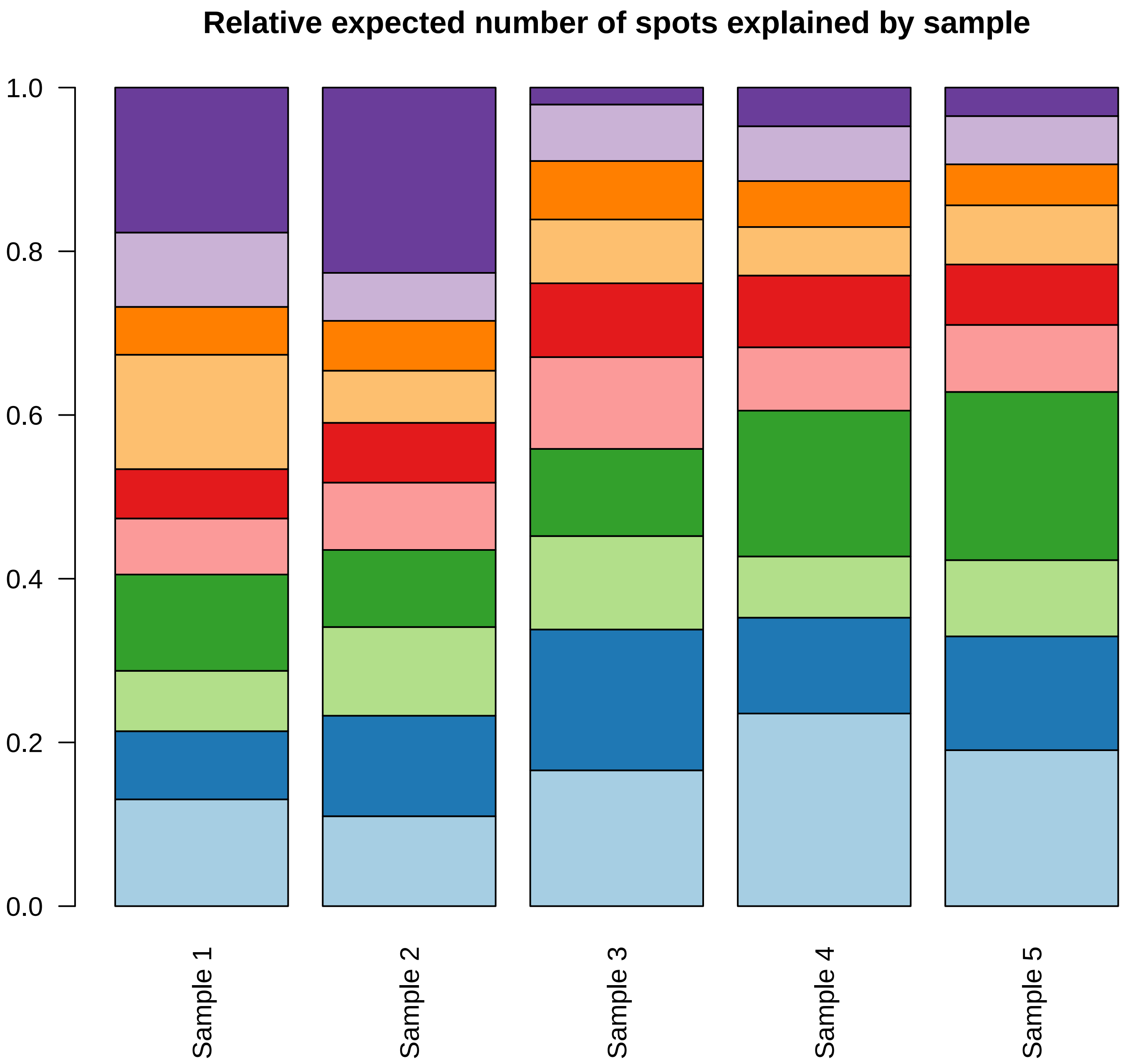
Relative expected number of spots explained by sample
1.0
0.8
0.6
0.4
0.2
0.0
Sample 1
Sample 2
Sample 3
Sample 4
Sample 5

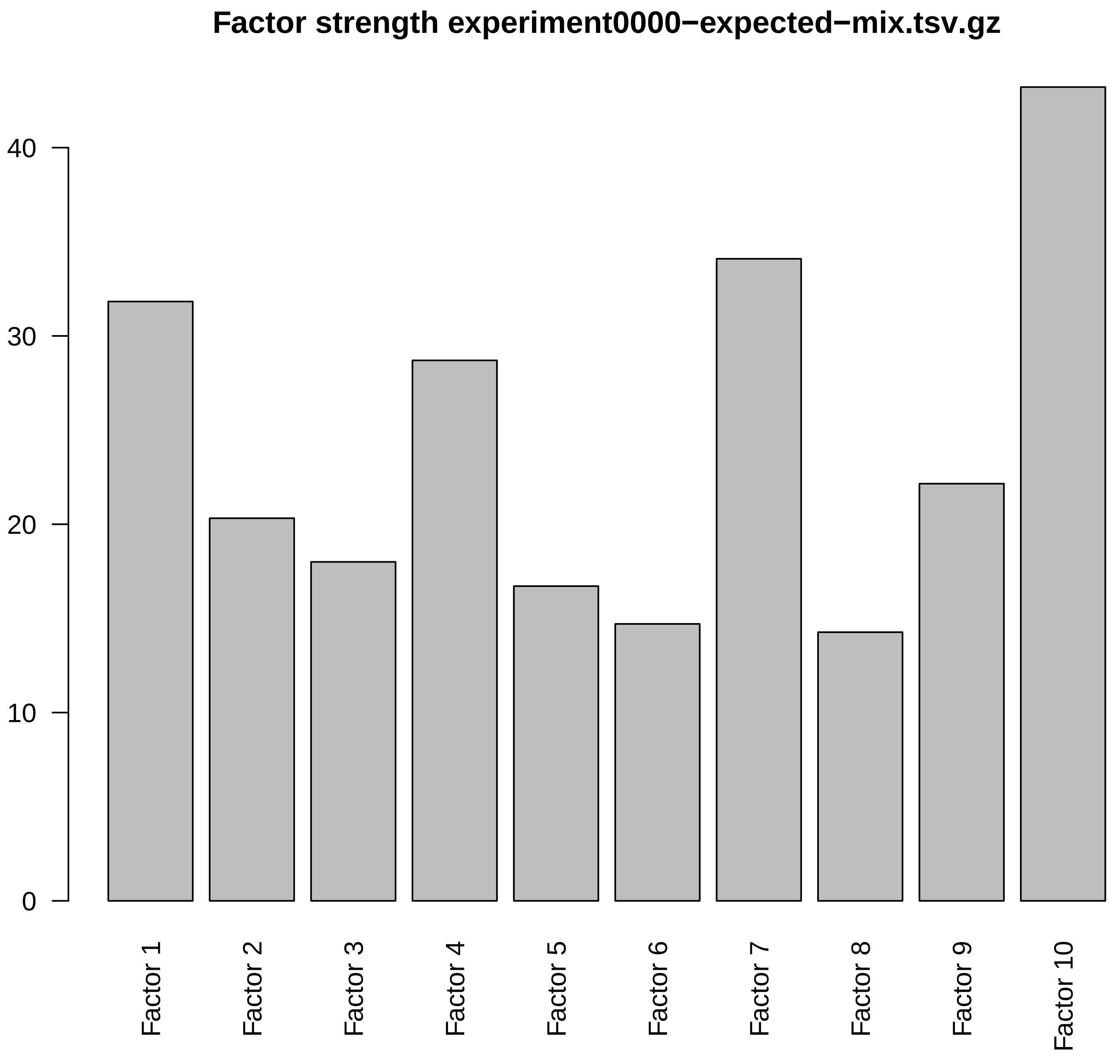
Factor strength experiment0000−expected−mix.tsv.gz
0
10
20
30
40
Factor 1
Factor 2
Factor 3
Factor 4
Factor 5
Factor 6
Factor 7
Factor 8
Factor 9
Factor 10

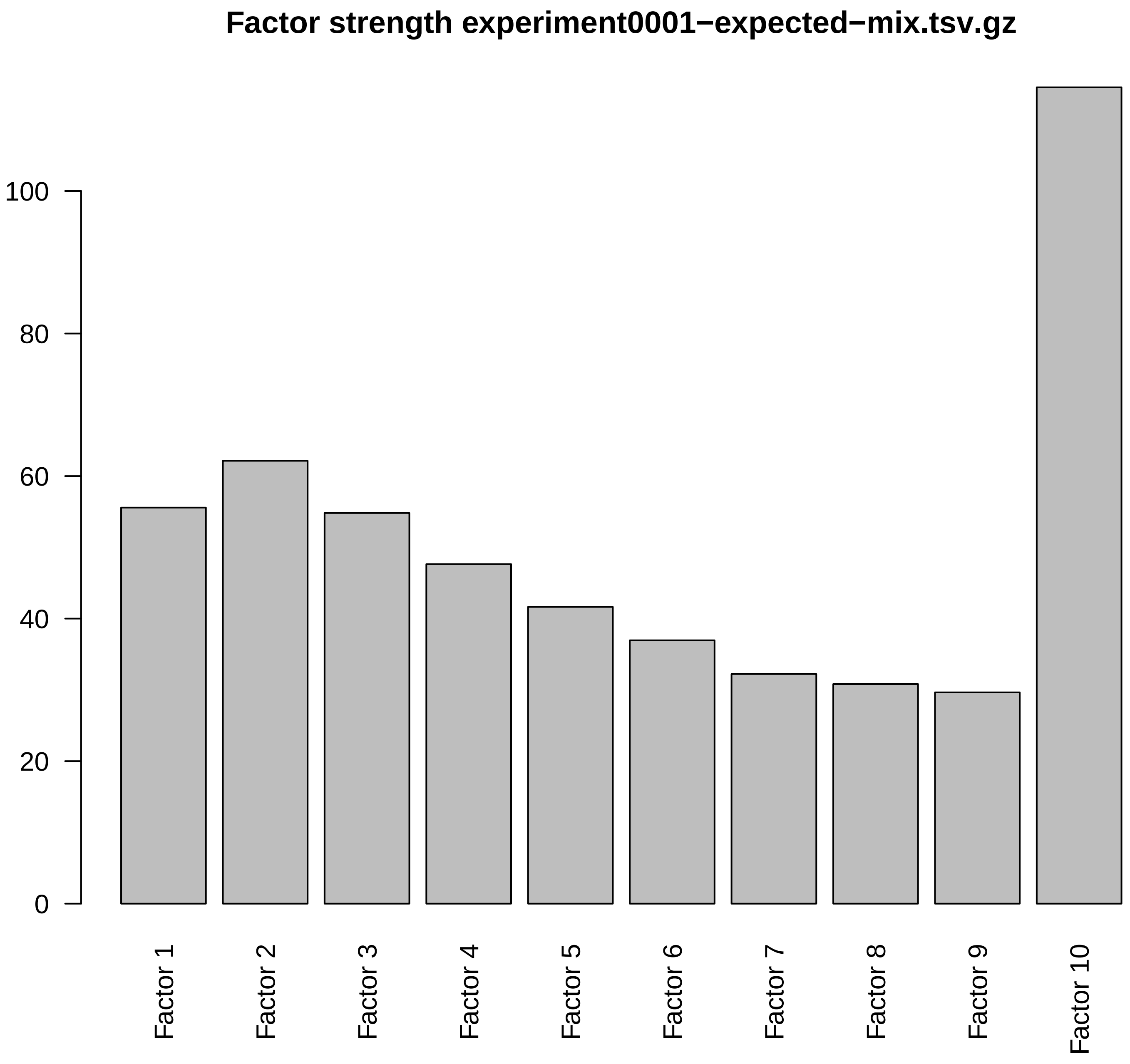
Factor strength experiment0001−expected−mix.tsv.gz
100
80
60
40
20
0
Factor 1
Factor 2
Factor 3
Factor 4
Factor 5
Factor 6
Factor 7
Factor 8
Factor 9
Factor 10

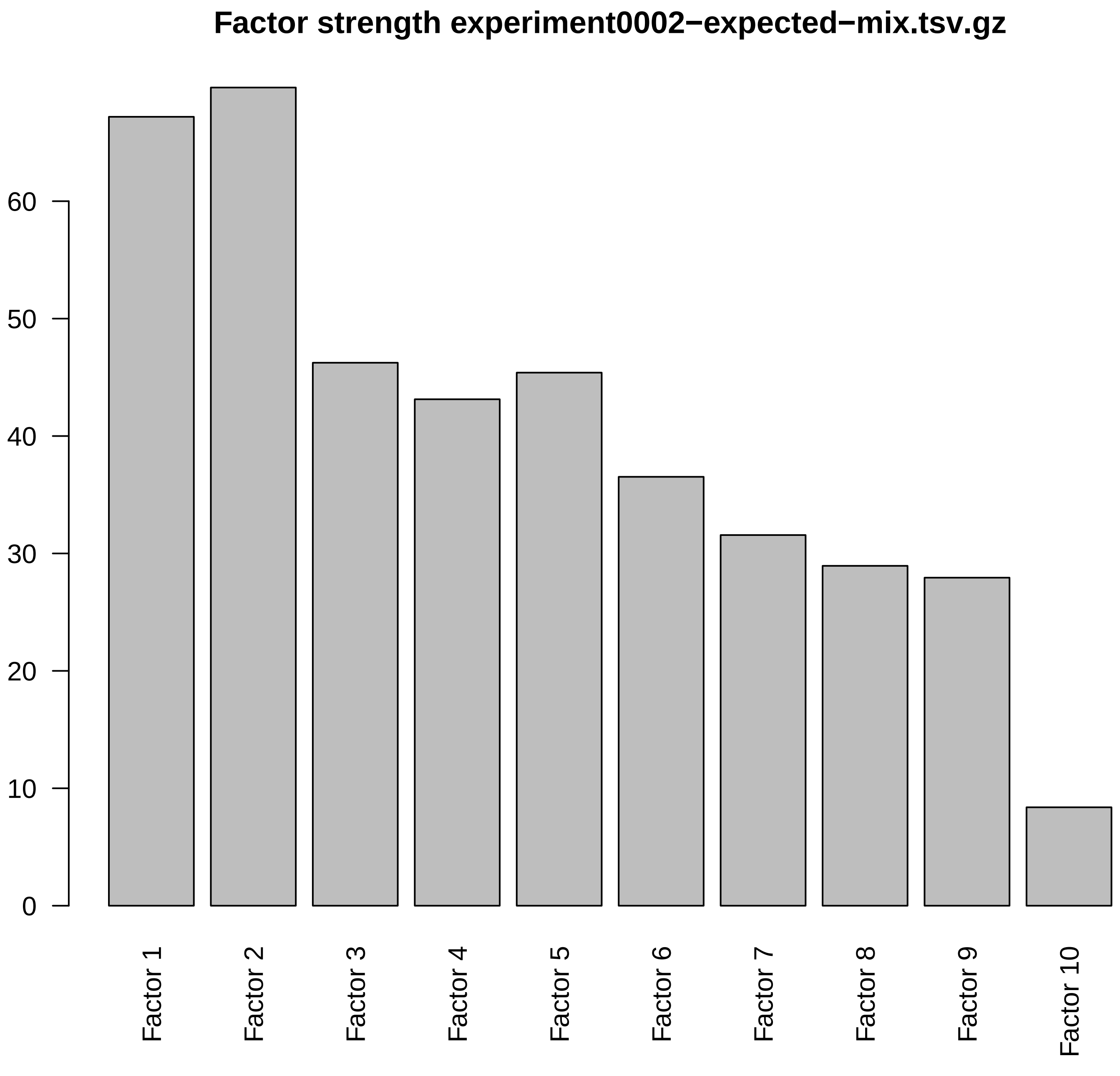
Factor strength experiment0002−expected−mix.tsv.gz
0
10
20
30
40
50
60
Factor 1
Factor 2
Factor 3
Factor 4
Factor 5
Factor 6
Factor 7
Factor 8
Factor 9
Factor 10

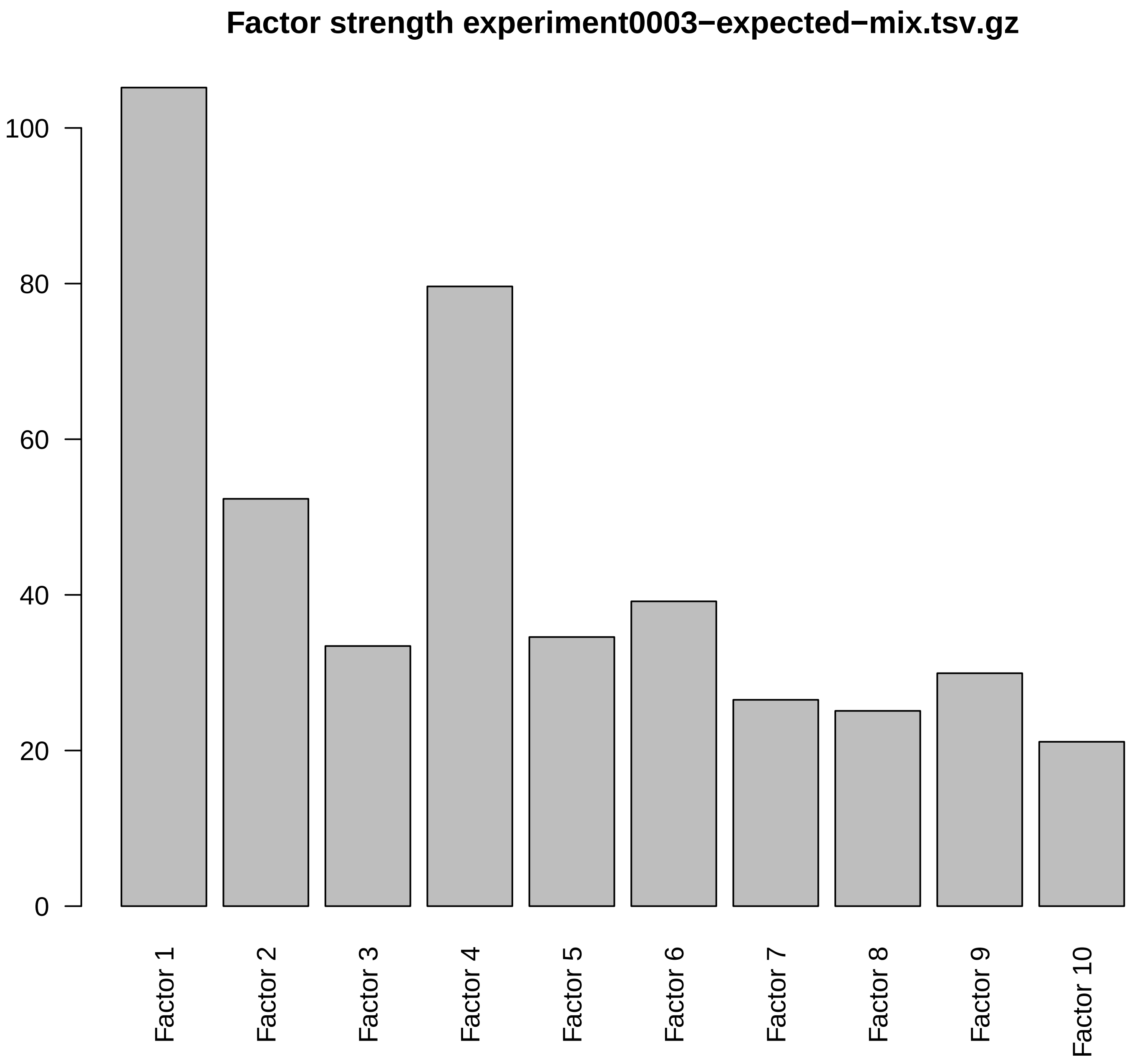

Factor strength experiment0003−expected−mix.tsv.gz
100
80
60
40
20
0
Factor 1
Factor 2
Factor 3
Factor 4
Factor 5
Factor 6
Factor 7
Factor 8
Factor 9
Factor 10

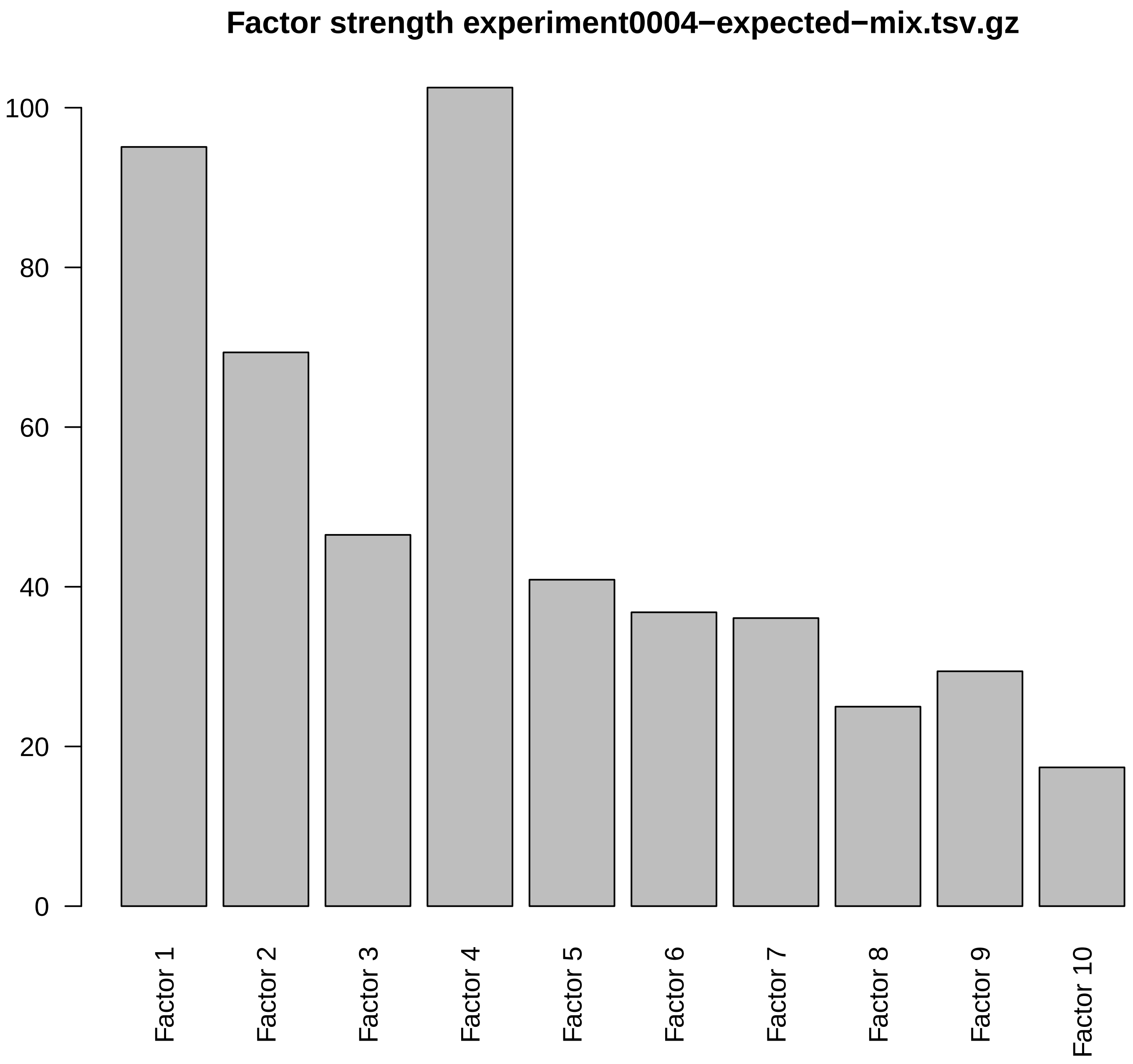
Factor strength experiment0004−expected−mix.tsv.gz
0
20
40
60
80
100
Factor 1
Factor 2
Factor 3
Factor 4
Factor 5
Factor 6
Factor 7
Factor 8
Factor 9
Factor 10
