# Supplementary material for: Spatial maps of prostate cancer transcriptomes reveal an unexplored landscape of heterogeneity
Source: Nat Commun. 2018 Jun 20;9:2419. doi: 10.1038/s41467-018-04724-5 (PMC6010471; doi:10.1038/s41467-018-04724-5)
Supplement: Supplementary file 8 — Supplementary Data 5 [file 41467_2018_4724_MOESM8_ESM.zip › Supplementary Dataset 7/joint-mix-factor-strength-barplot-expected-reads.pdf]

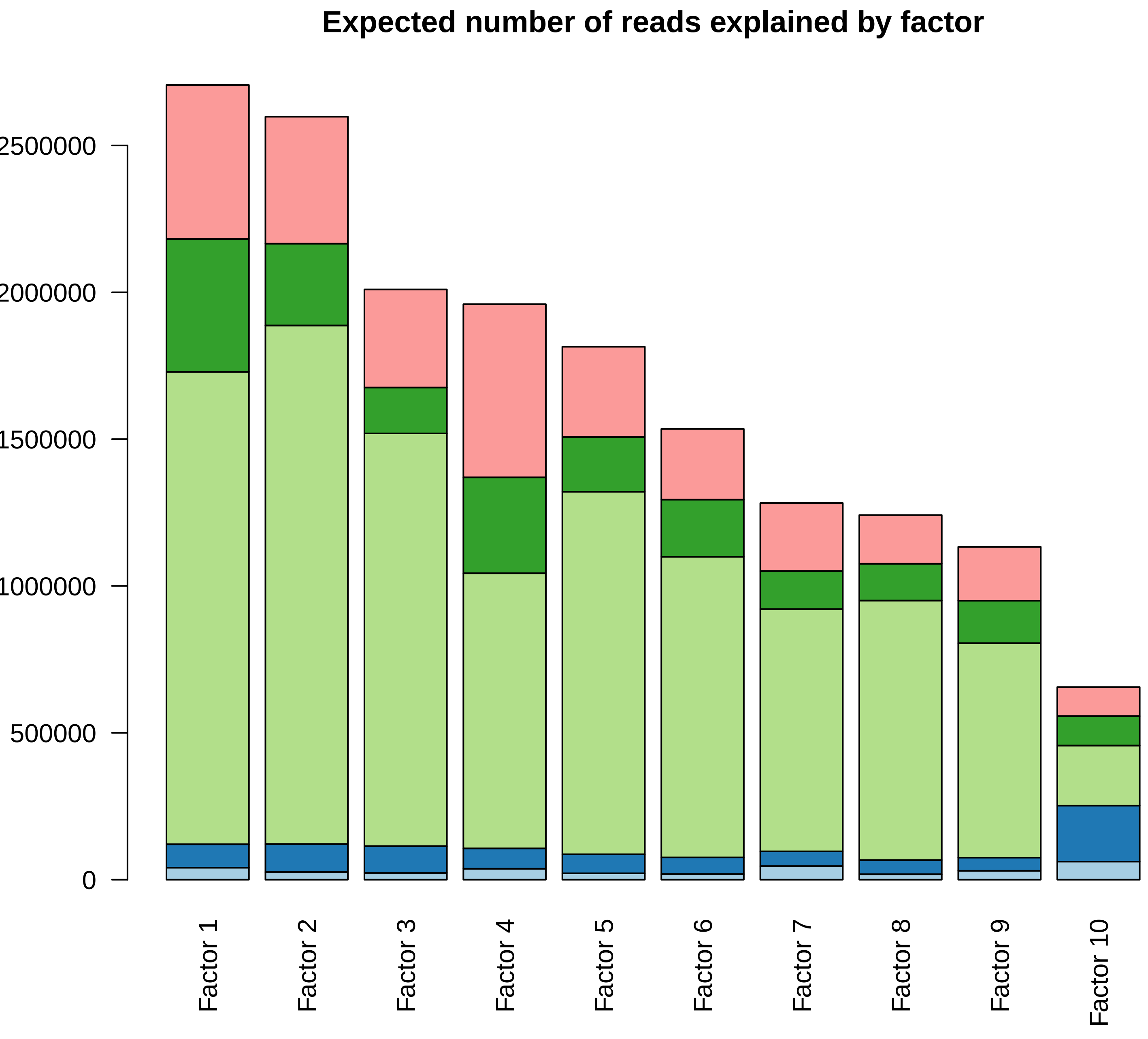
Expected number of reads explained by factor
2500000
2000000
1500000
1000000
500000
0
Factor 1
Factor 2
Factor 3
Factor 4
Factor 5
Factor 6
Factor 7
Factor 8
Factor 9
Factor 10

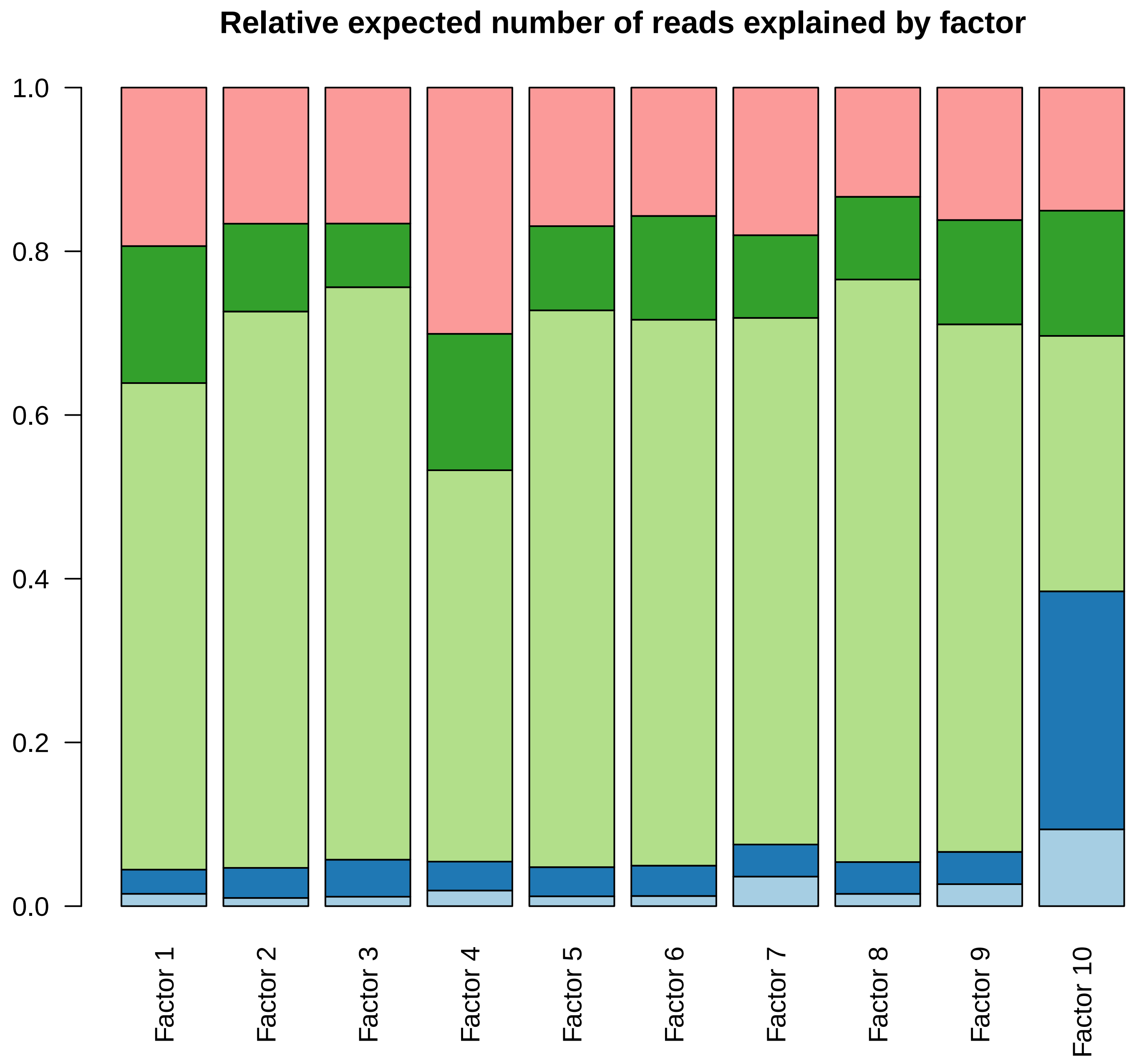
Relative expected number of reads explained by factor
1.0
0.8
0.6
0.4
0.2
0.0
Factor 1
Factor 2
Factor 3
Factor 4
Factor 5
Factor 6
Factor 7
Factor 8
Factor 9
Factor 10

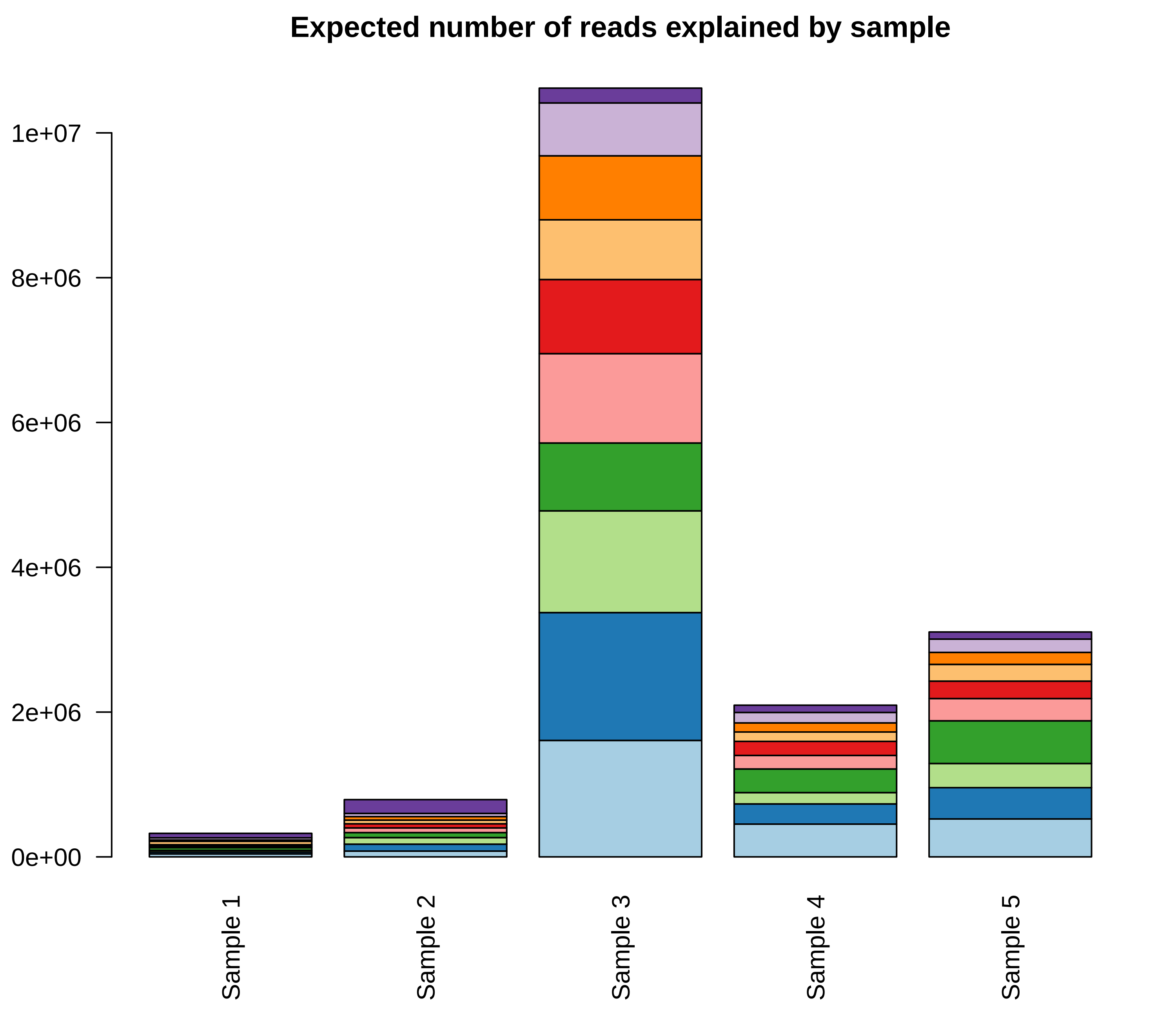
Expected number of reads explained by sample
0e+00
2e+06
4e+06
6e+06
8e+06
1e+07
Sample 1
Sample 2
Sample 3
Sample 4
Sample 5

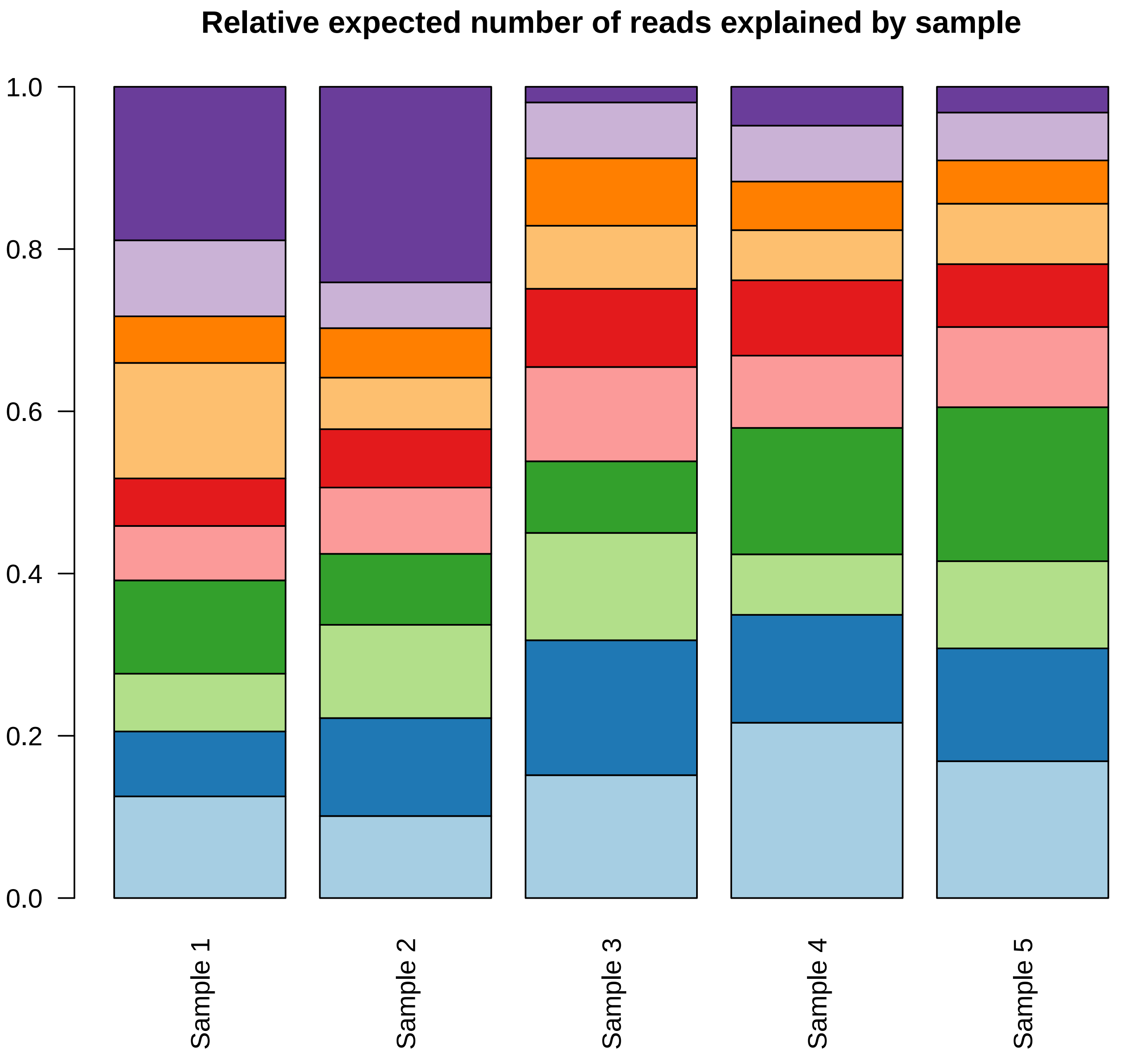

Relative expected number of reads explained by sample
1.0
0.8
0.6
0.4
0.2
0.0
Sample 1
Sample 2
Sample 3
Sample 4
Sample 5

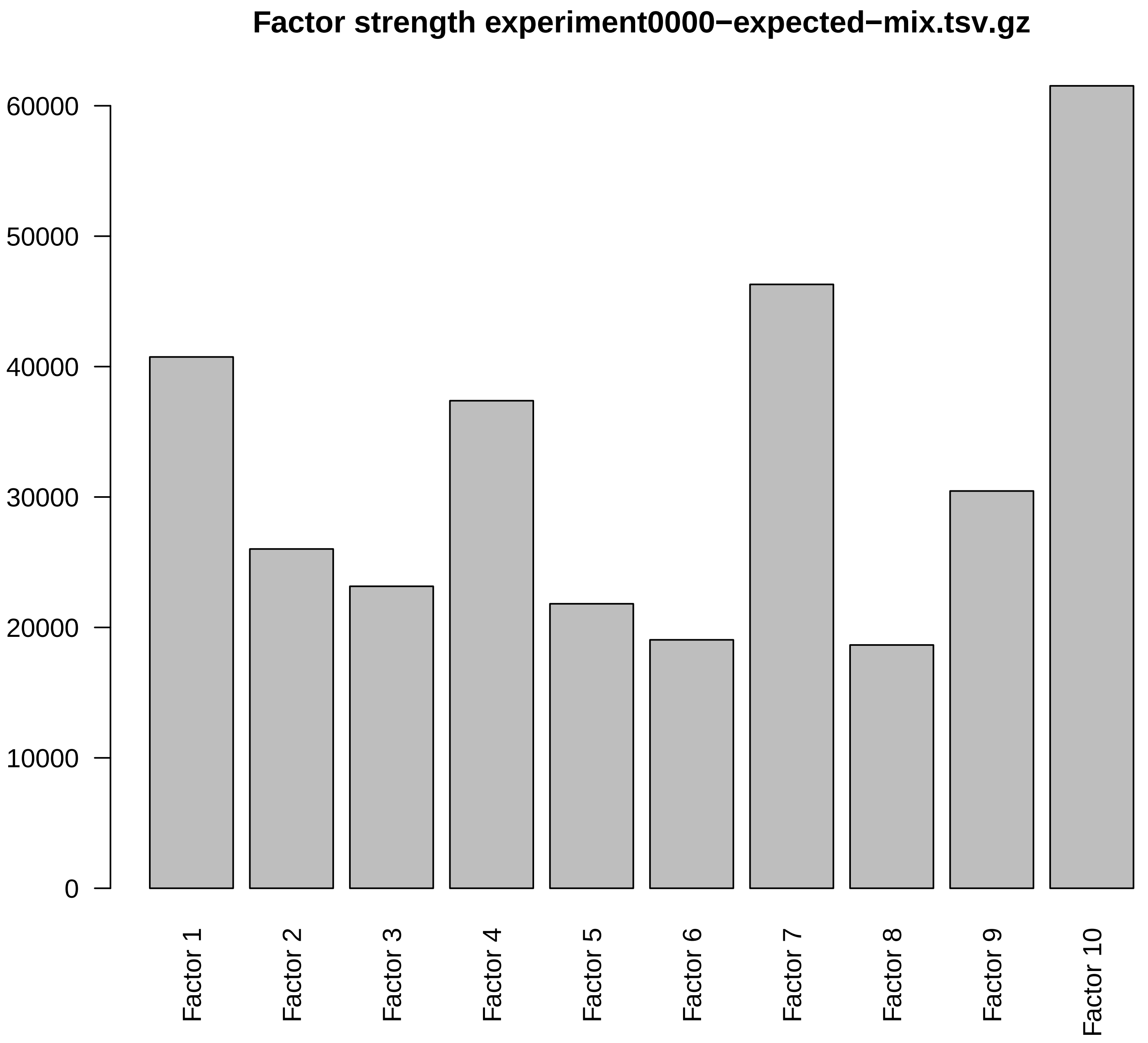

Factor strength experiment0000−expected−mix.tsv.gz
60000
50000
40000
30000
20000
10000
0
Factor 1
Factor 2
Factor 3
Factor 4
Factor 5
Factor 6
Factor 7
Factor 8
Factor 9
Factor 10

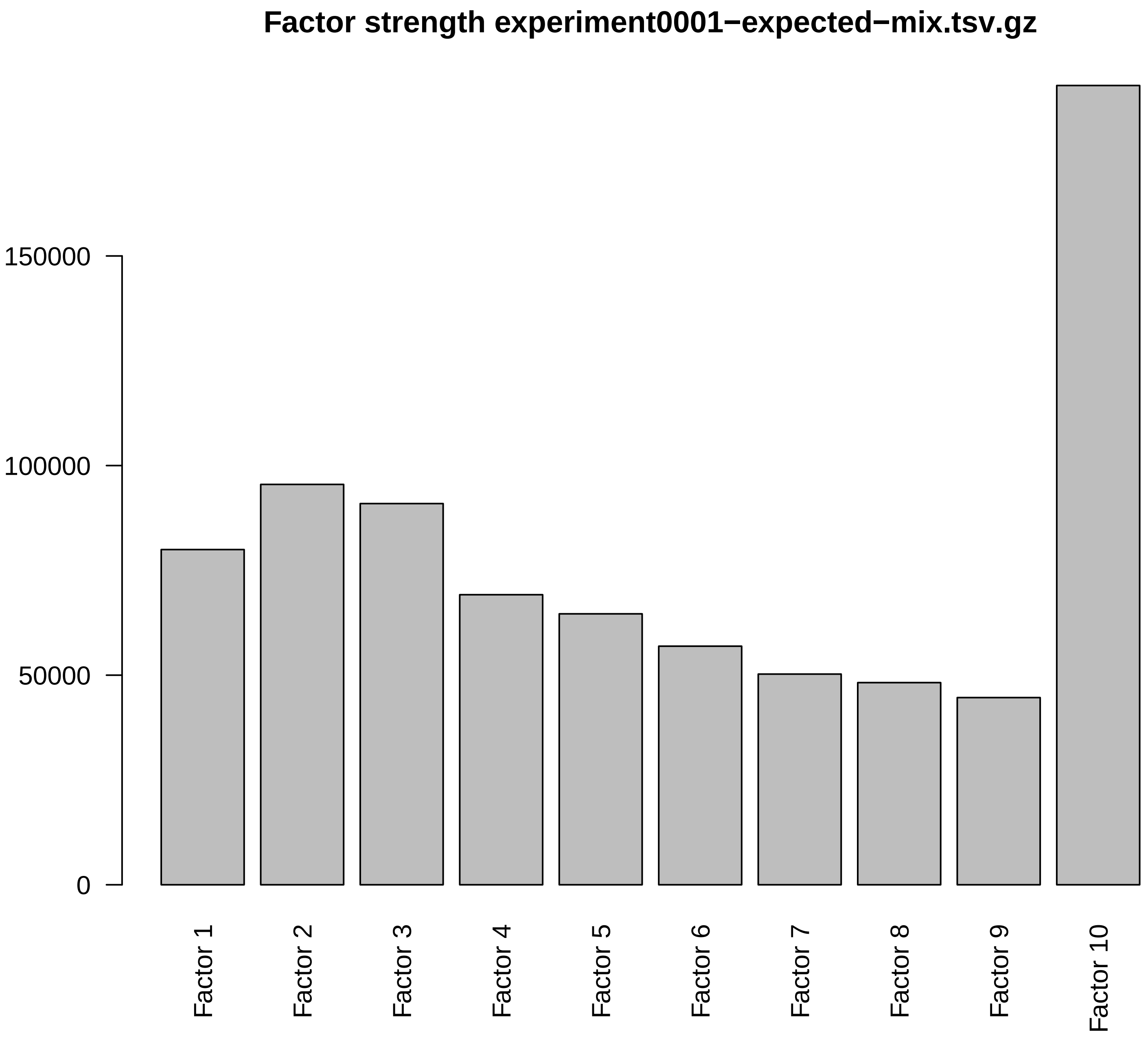
Factor strength experiment0001−expected−mix.tsv.gz
150000
100000
50000
0
Factor 1
Factor 2
Factor 3
Factor 4
Factor 5
Factor 6
Factor 7
Factor 8
Factor 9
Factor 10

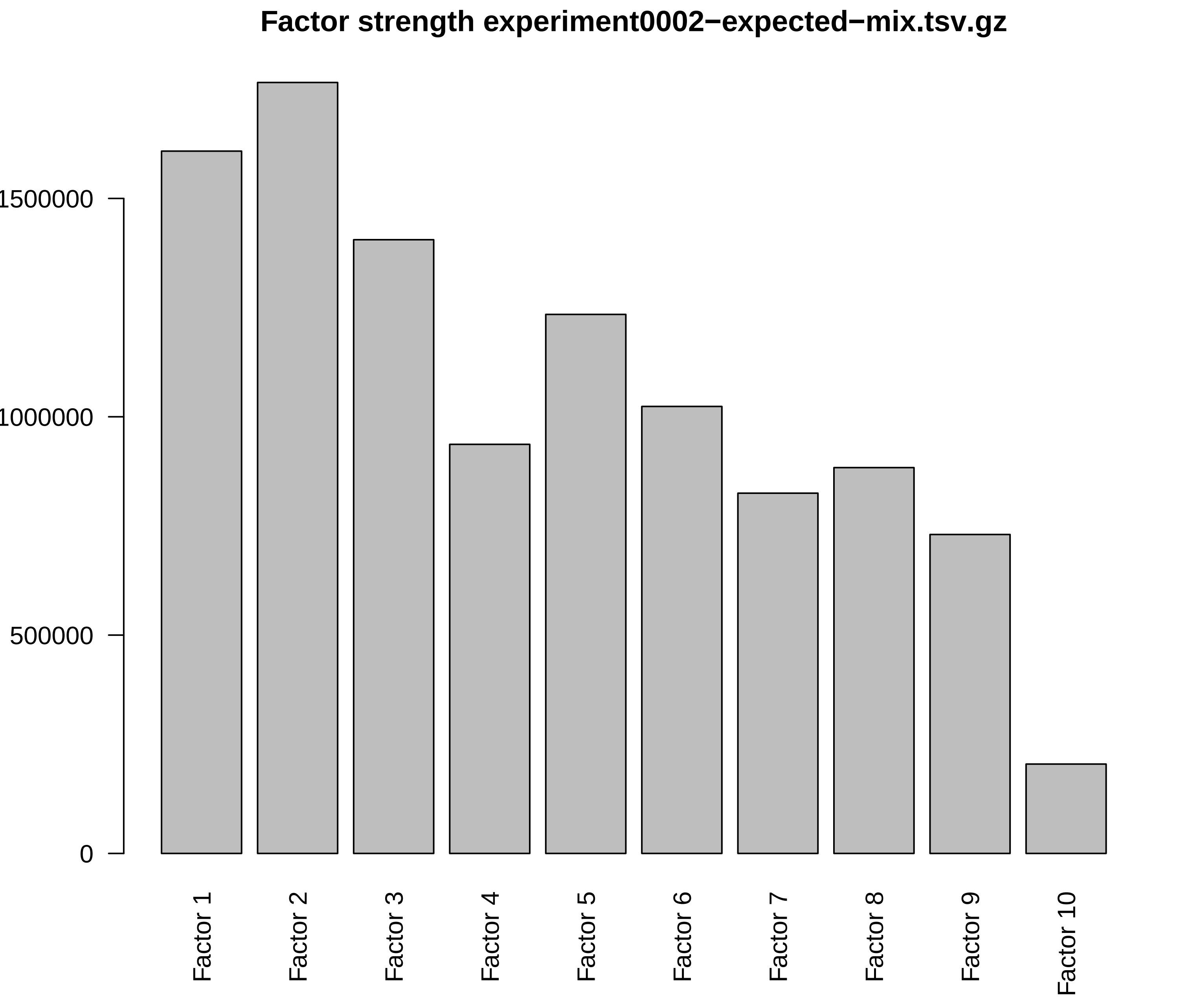
Factor strength experiment0002−expected−mix.tsv.gz
1500000
1000000
500000
0
Factor 1
Factor 2
Factor 3
Factor 4
Factor 5
Factor 6
Factor 7
Factor 8
Factor 9
Factor 10

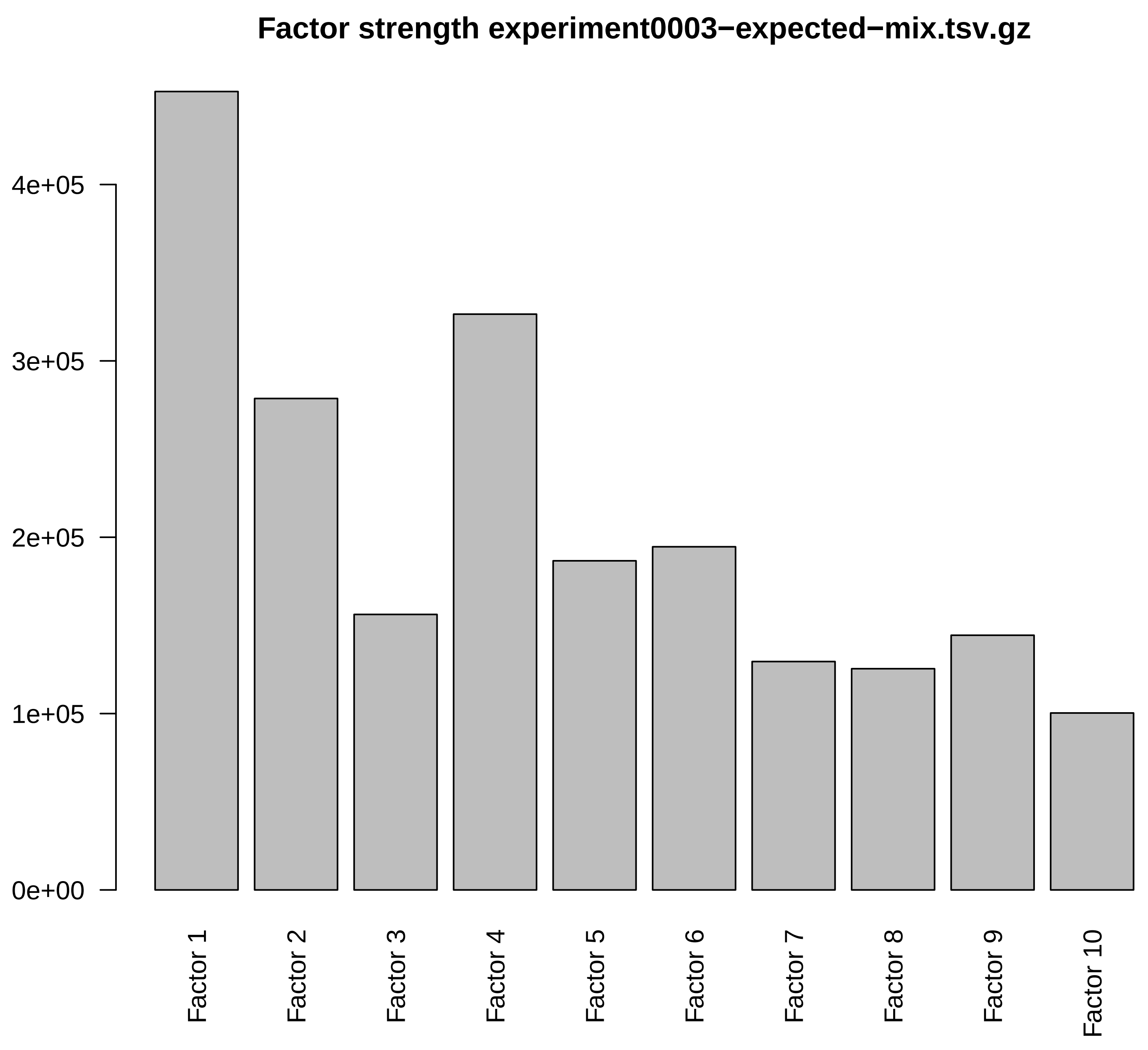
Factor strength experiment0003−expected−mix.tsv.gz
0e+00
1e+05
2e+05
3e+05
4e+05
Factor 1
Factor 2
Factor 3
Factor 4
Factor 5
Factor 6
Factor 7
Factor 8
Factor 9
Factor 10

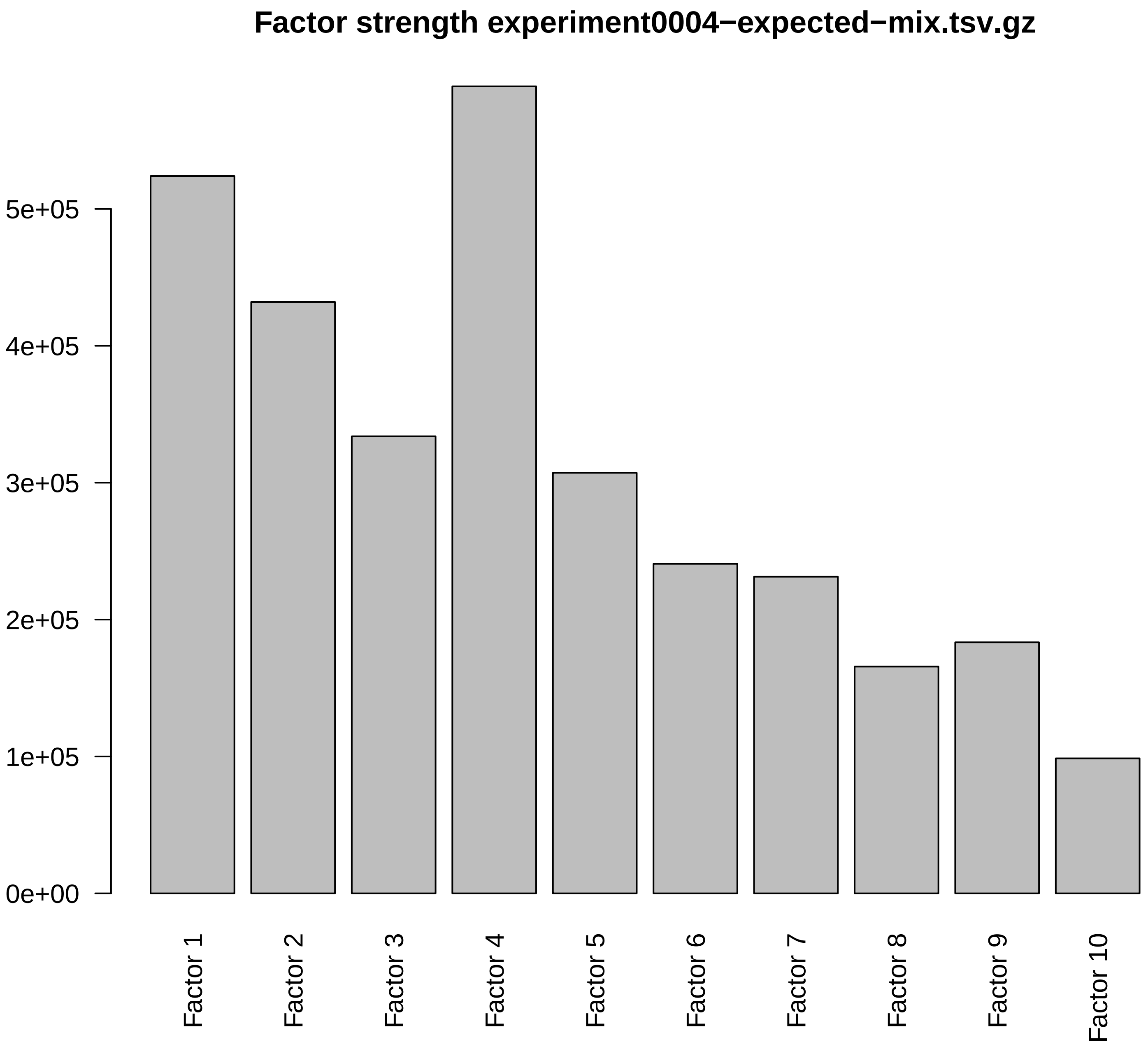
Factor strength experiment0004−expected−mix.tsv.gz
0e+00
1e+05
2e+05
3e+05
4e+05
5e+05
Factor 1
Factor 2
Factor 3
Factor 4
Factor 5
Factor 6
Factor 7
Factor 8
Factor 9
Factor 10
