# Supplementary material for: Spatial maps of prostate cancer transcriptomes reveal an unexplored landscape of heterogeneity
Source: Nat Commun. 2018 Jun 20;9:2419. doi: 10.1038/s41467-018-04724-5 (PMC6010471; doi:10.1038/s41467-018-04724-5)
Supplement: Supplementary file 8 — Supplementary Data 5 [file 41467_2018_4724_MOESM8_ESM.zip › Supplementary Dataset 7/joint-field-factor-strength-spotbarplot-split-rel-freq.pdf]

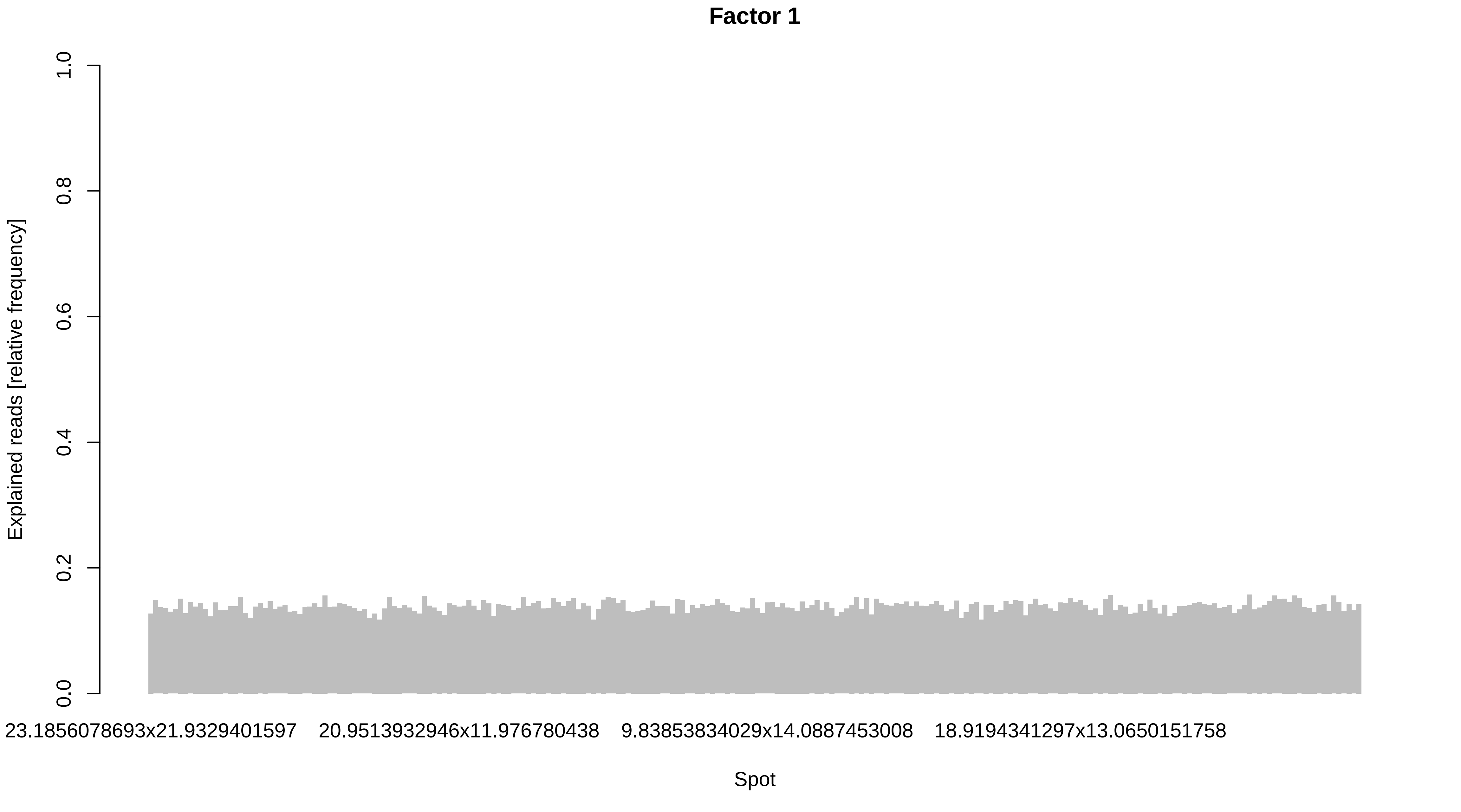
Factor 1
1.0
0.8
0.6
0.4
0.2
0.0
Explained reads [relative frequency]
23.1856078693x21.9329401597
20.9513932946x11.976780438
9.83853834029x14.0887453008
18.9194341297x13.0650151758
Spot

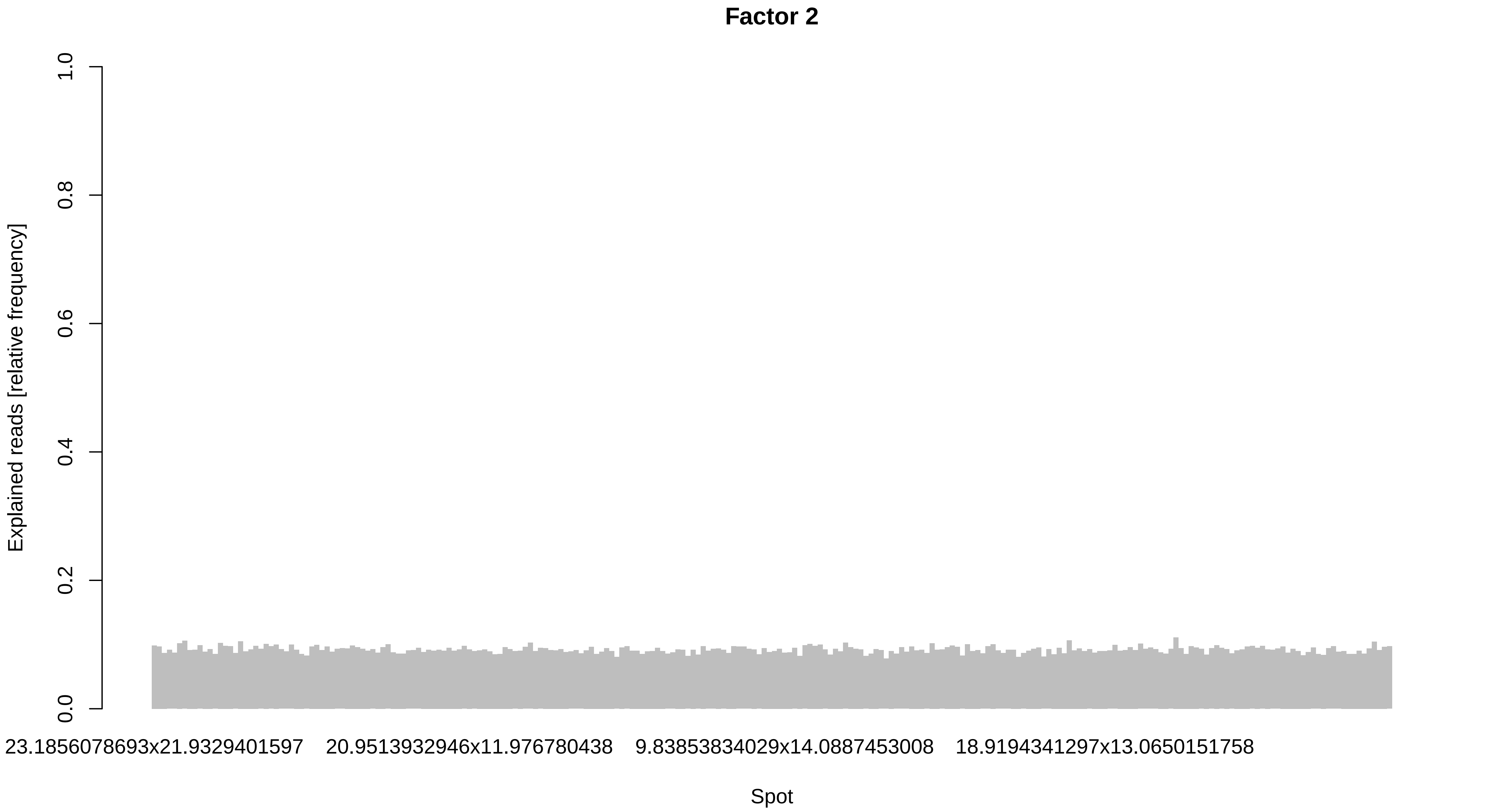

Factor 2
Explained reads [relative frequency]
1.0
0.8
0.6
0.4
0.2
0.0
23.1856078693x21.9329401597
20.9513932946x11.976780438
9.83853834029x14.0887453008
18.9194341297x13.0650151758
Spot

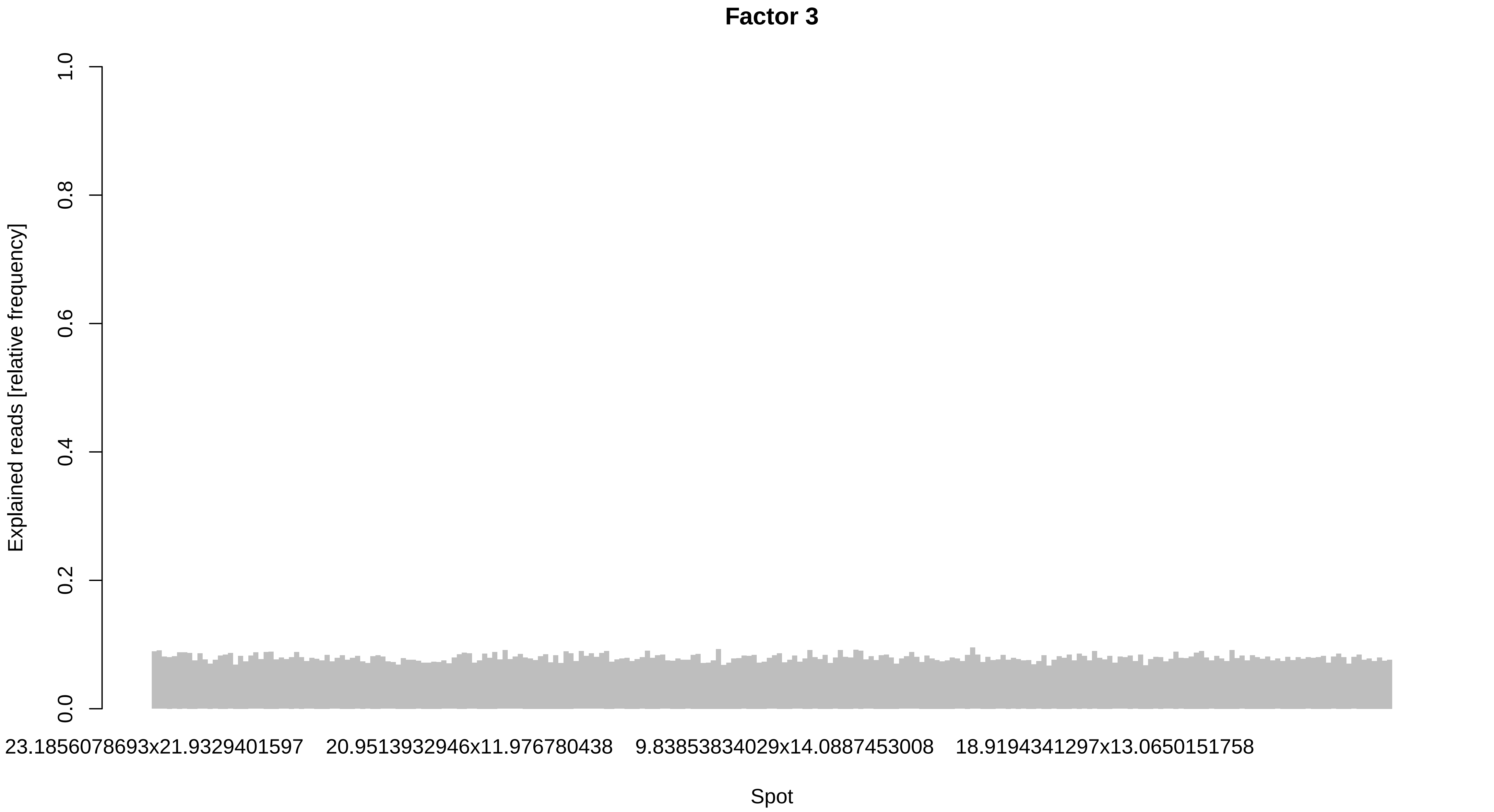

Factor 3
Explained reads [relative frequency]
1.0
0.8
0.6
0.4
0.2
0.0
23.1856078693x21.9329401597
20.9513932946x11.976780438
9.83853834029x14.0887453008
18.9194341297x13.0650151758
Spot

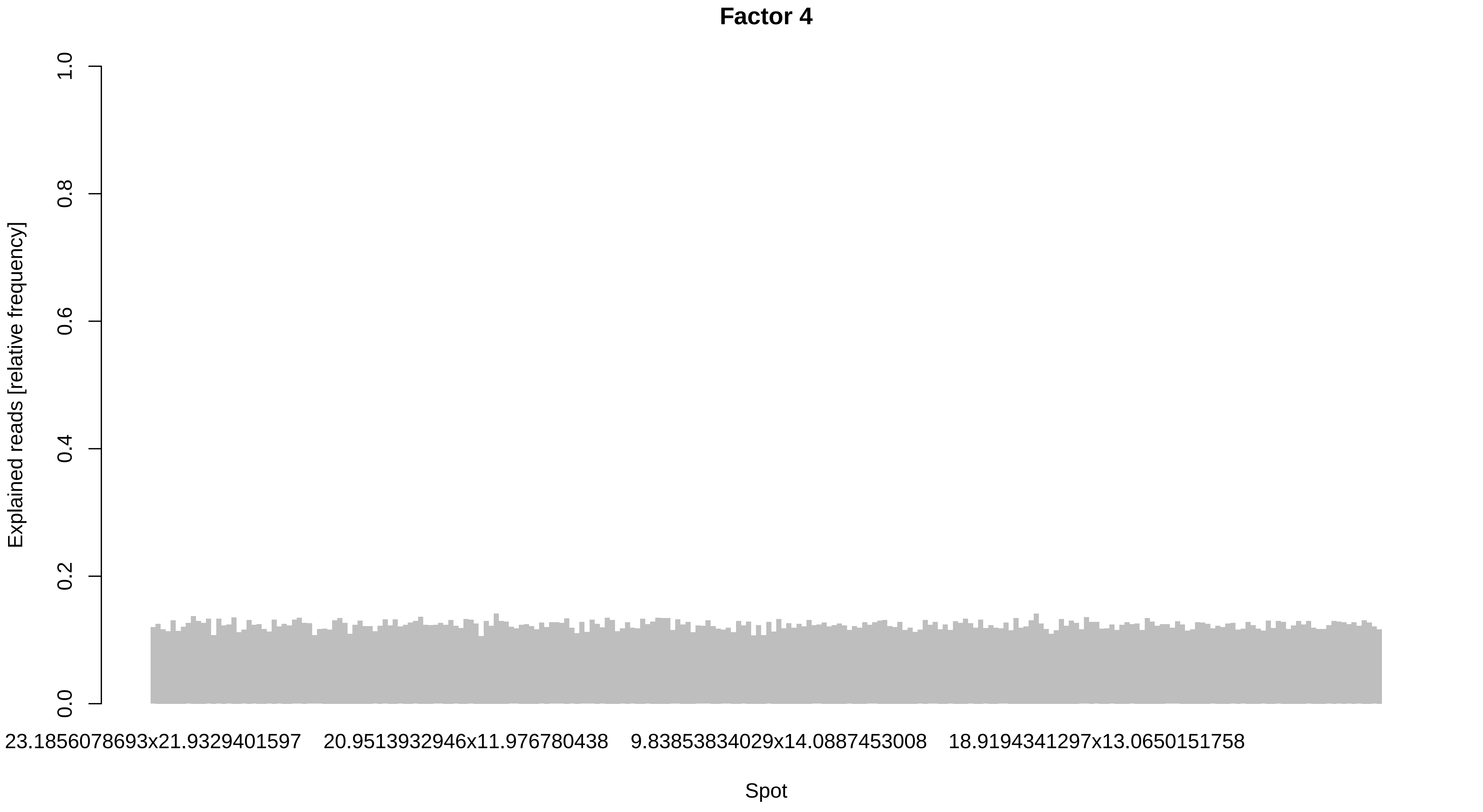

Factor 4
Explained reads [relative frequency]
1.0
0.8
0.6
0.4
0.2
0.0
23.1856078693x21.9329401597
20.9513932946x11.976780438
9.83853834029x14.0887453008
18.9194341297x13.0650151758
Spot

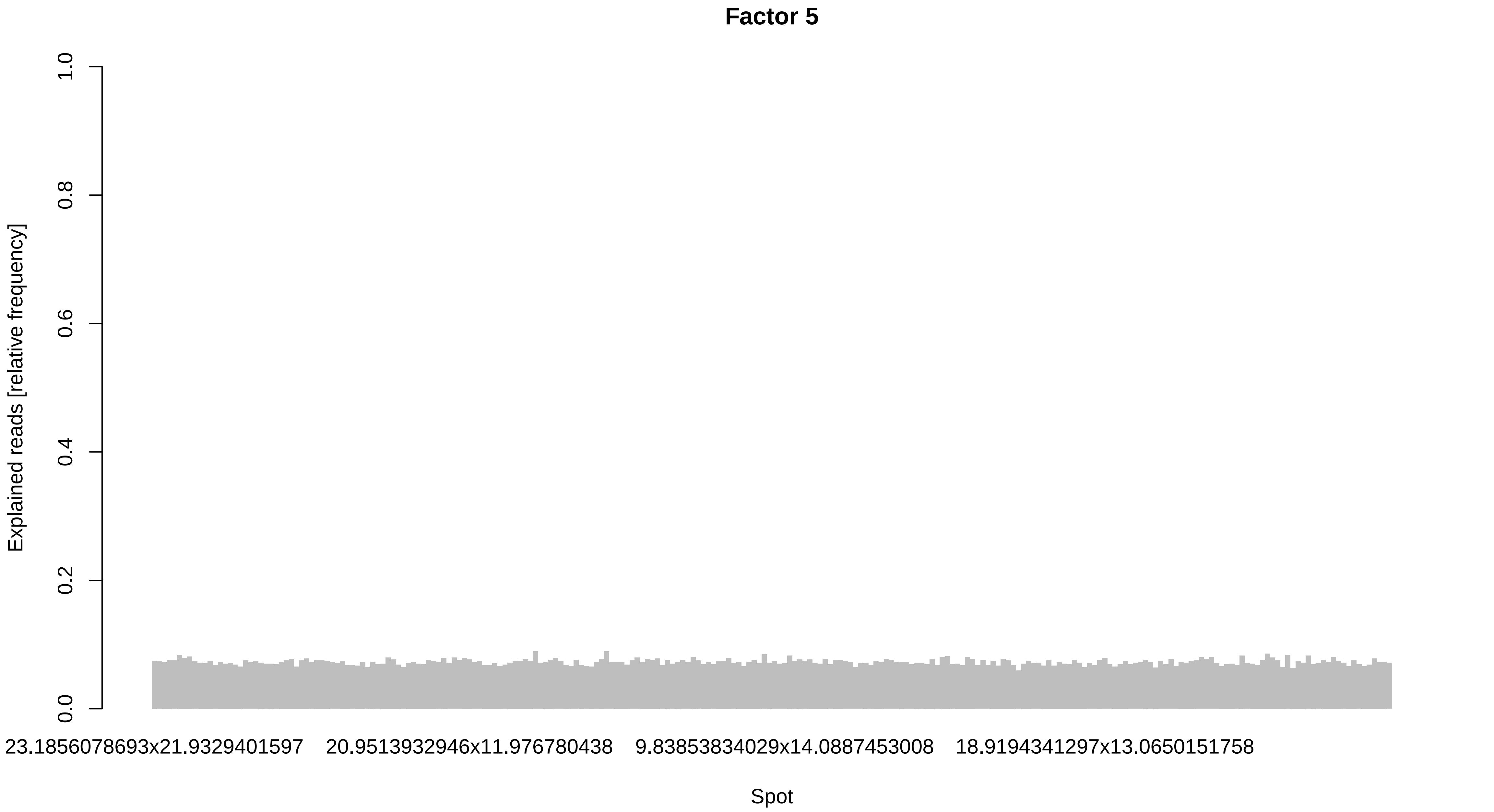

Factor 5
Explained reads [relative frequency]
1.0
0.8
0.6
0.4
0.2
0.0
23.1856078693x21.9329401597
20.9513932946x11.976780438
9.83853834029x14.0887453008
18.9194341297x13.0650151758
Spot

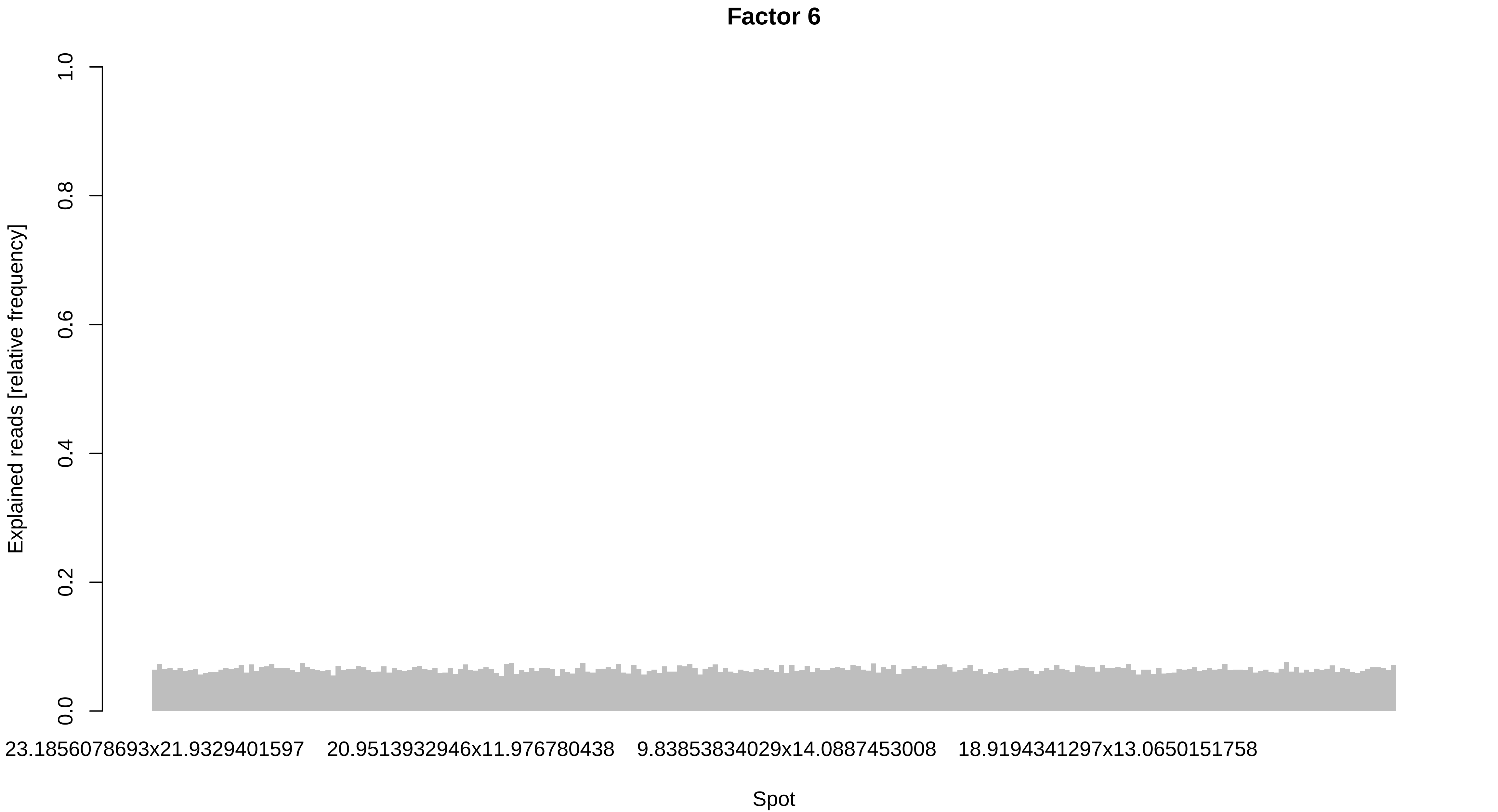

Factor 6
Explained reads [relative frequency]
1.0
0.8
0.6
0.4
0.2
0.0
23.1856078693x21.9329401597
20.9513932946x11.976780438
9.83853834029x14.0887453008
18.9194341297x13.0650151758
Spot

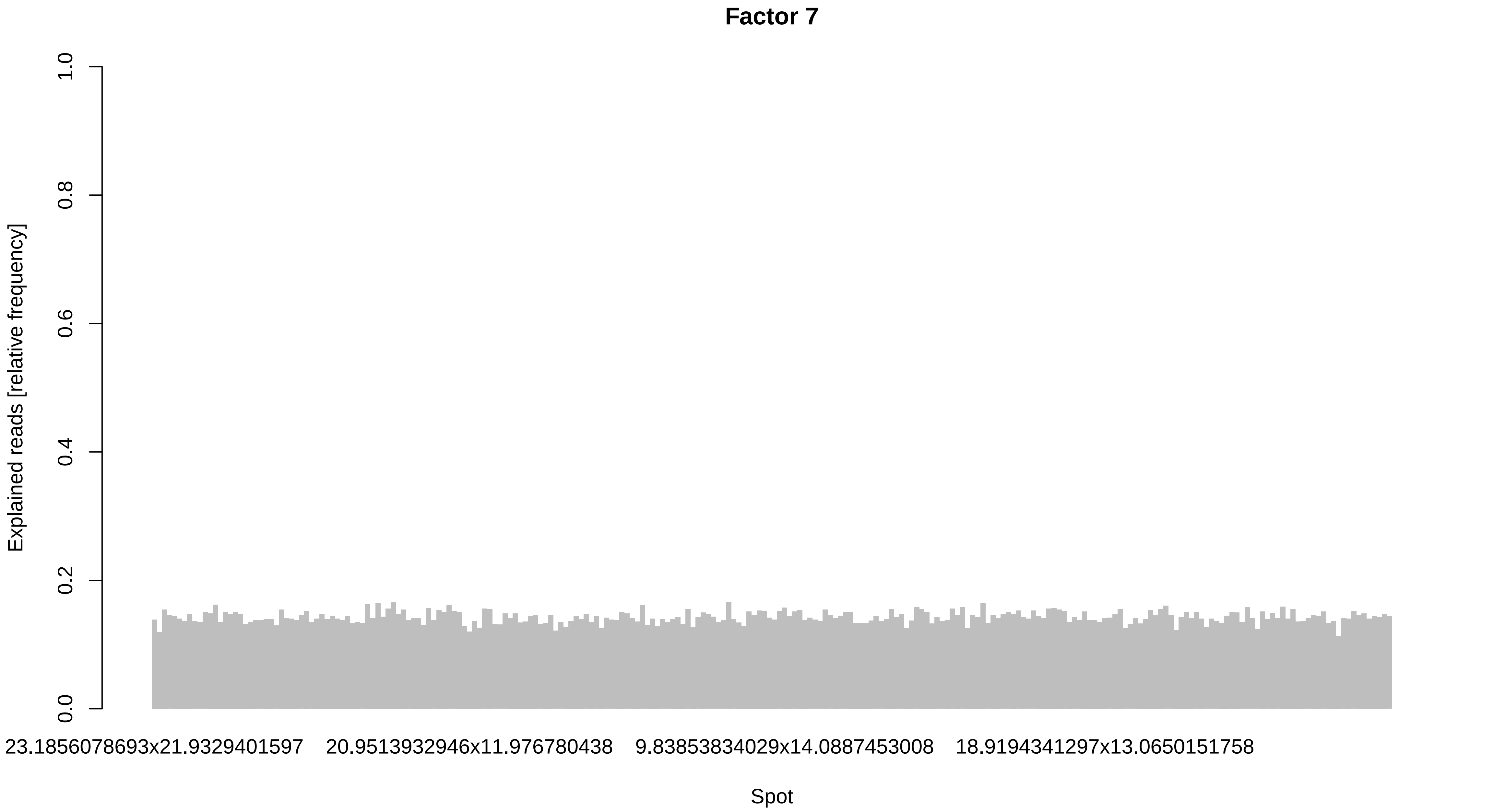
Factor 7
Explained reads [relative frequency]
1.0
0.8
0.6
0.4
0.2
0.0
23.1856078693x21.9329401597
20.9513932946x11.976780438
9.83853834029x14.0887453008
18.9194341297x13.0650151758
Spot

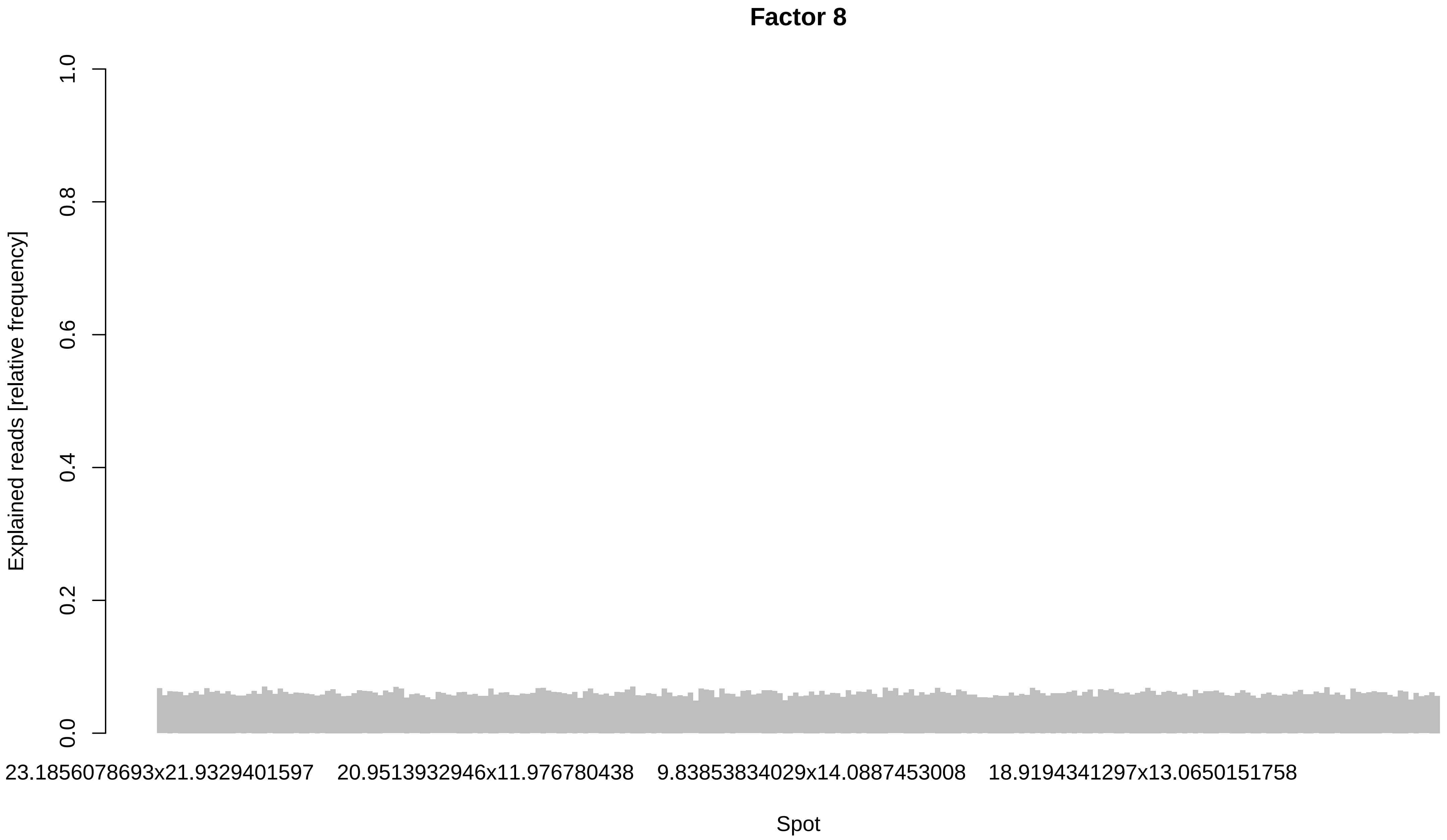

Factor 8
Explained reads [relative frequency]
1.0
0.8
0.6
0.4
0.2
0.0
23.1856078693x21.9329401597
20.9513932946x11.976780438
9.83853834029x14.0887453008
18.9194341297x13.0650151758
Spot

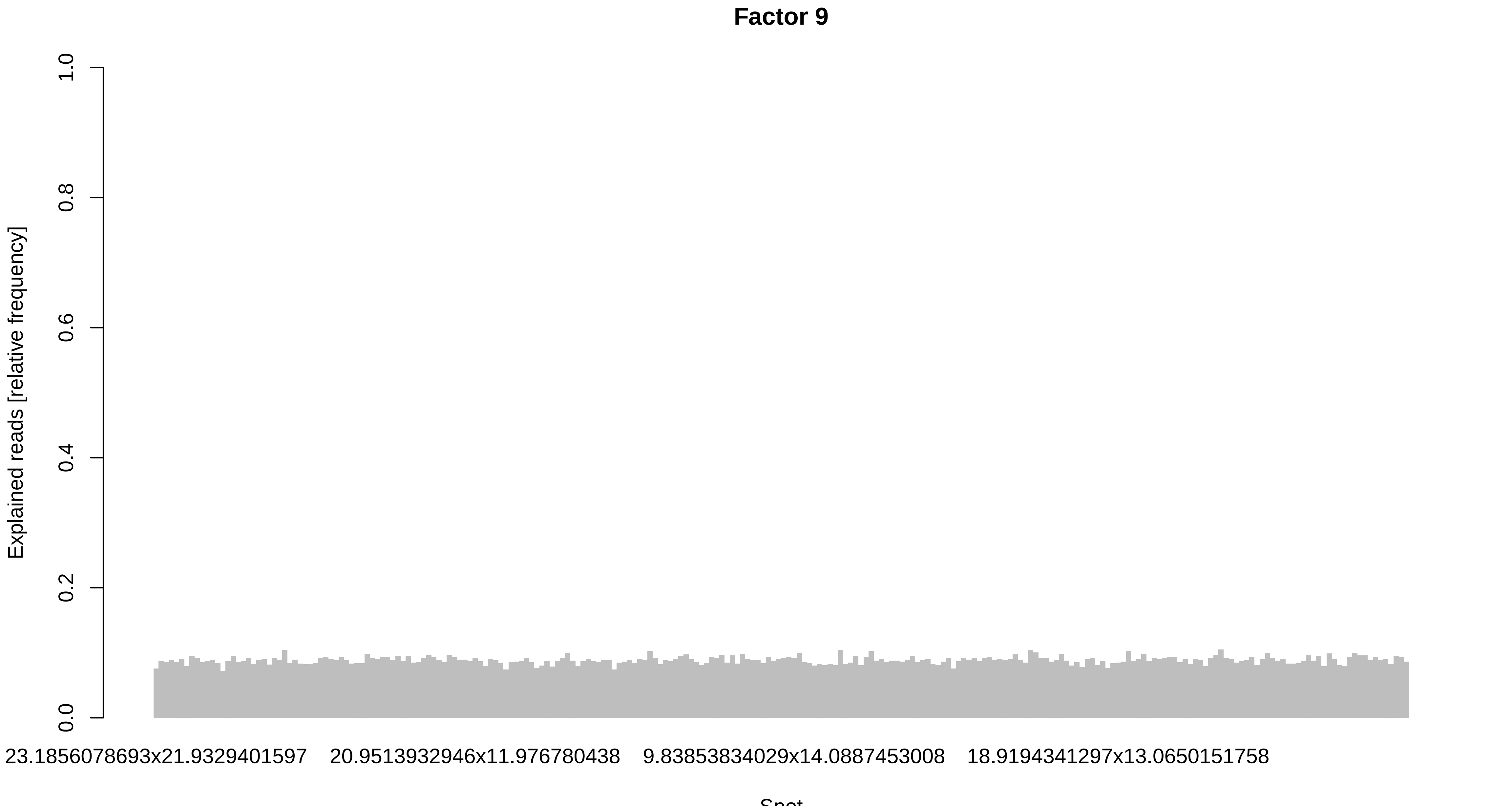
Factor 9
Explained reads [relative frequency]
1.0
0.8
0.6
0.4
0.2
0.0
23.1856078693x21.9329401597
20.9513932946x11.976780438
9.83853834029x14.0887453008
18.9194341297x13.0650151758
Spot

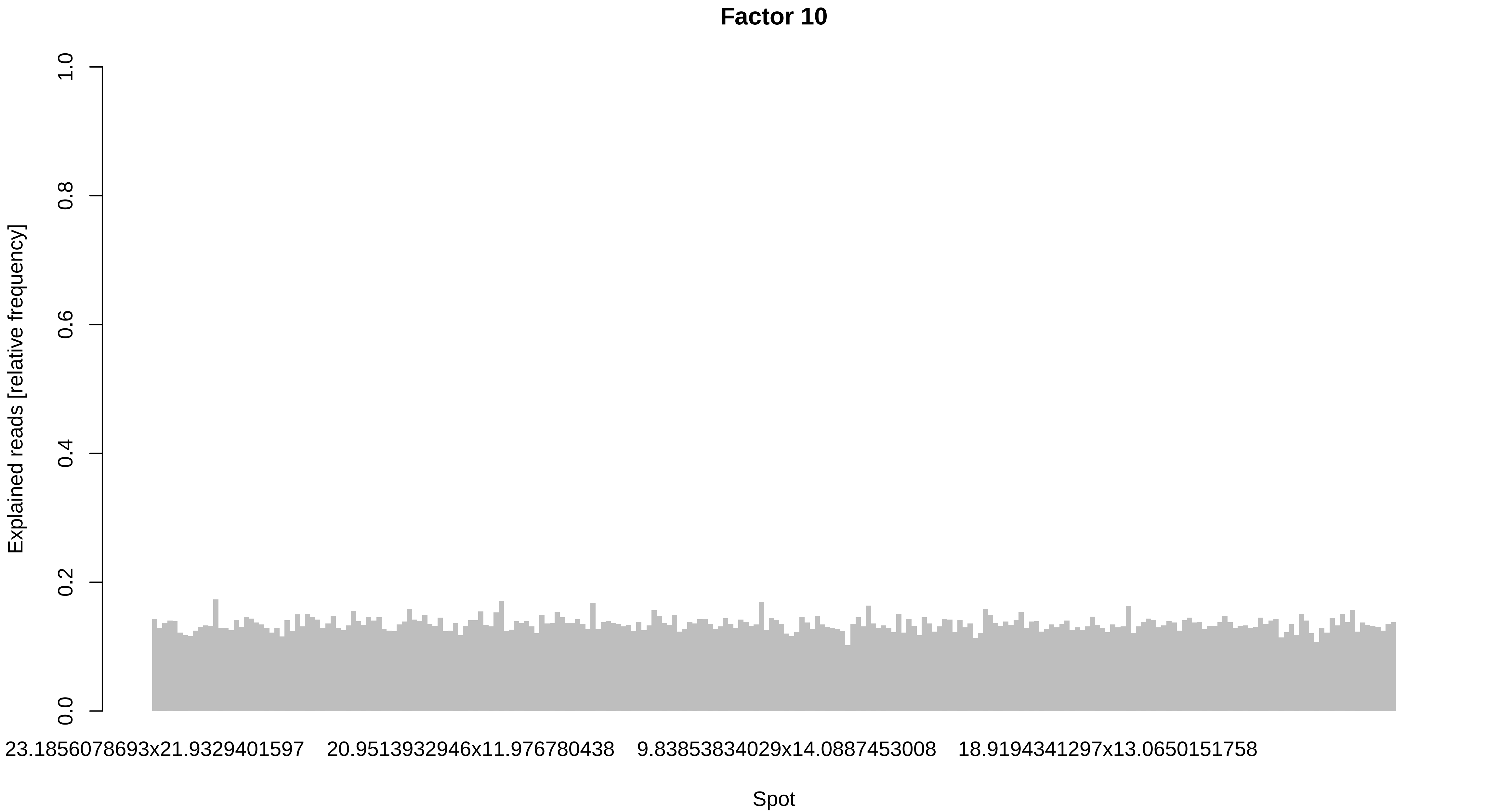

Factor 10
Explained reads [relative frequency]
0.0
0.2
0.4
0.6
0.8
1.0
23.1856078693x21.9329401597
20.9513932946x11.976780438
9.83853834029x14.0887453008
18.9194341297x13.0650151758
Spot

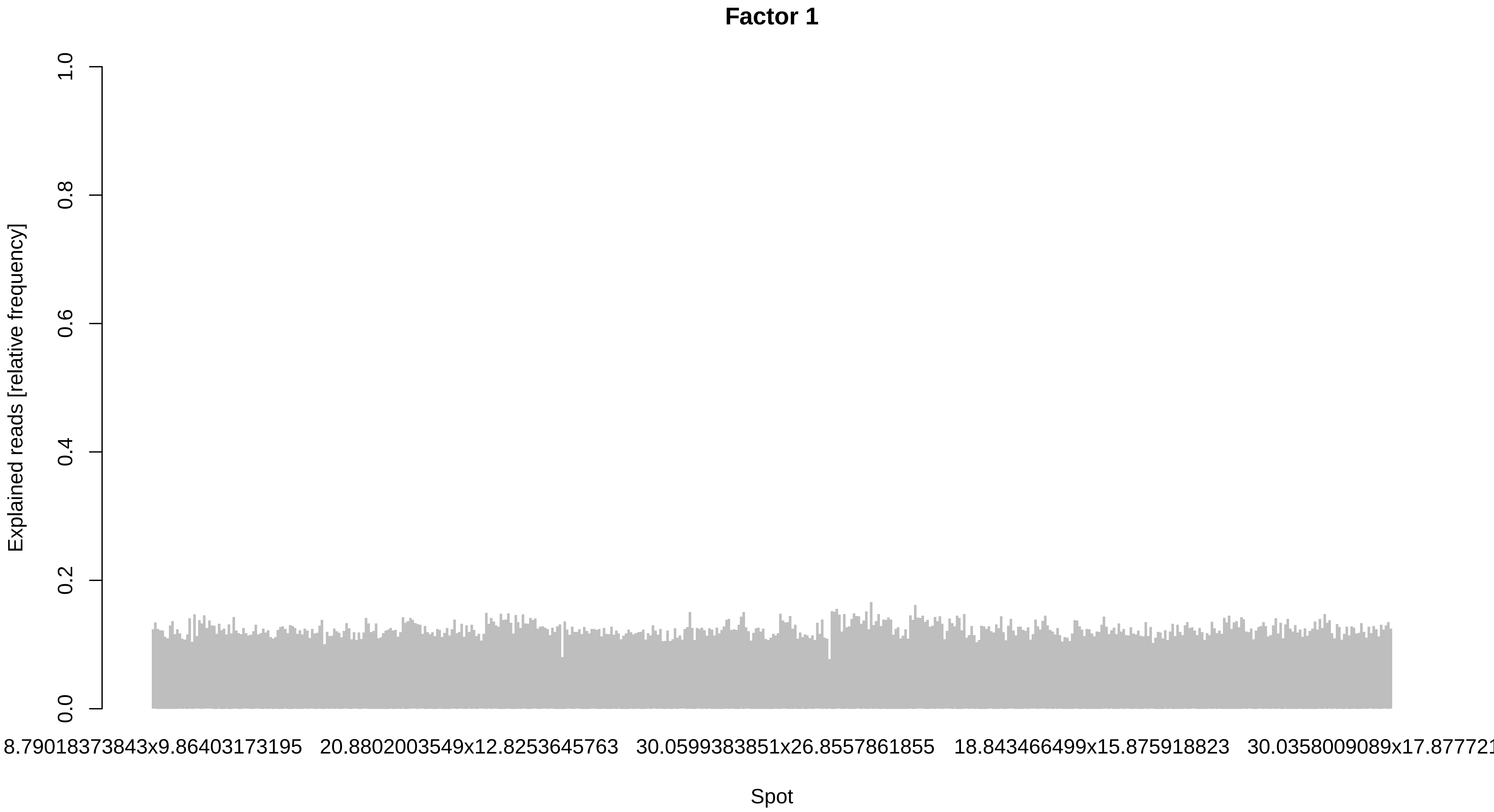
Factor 1
Explained reads [relative frequency]
1.0
0.8
0.6
0.4
0.2
0.0
8.79018373843x9.86403173195
20.8802003549x12.8253645763
30.0599383851x26.8557861855
18.843466499x15.875918823
30.0358009089x17.87772
Spot

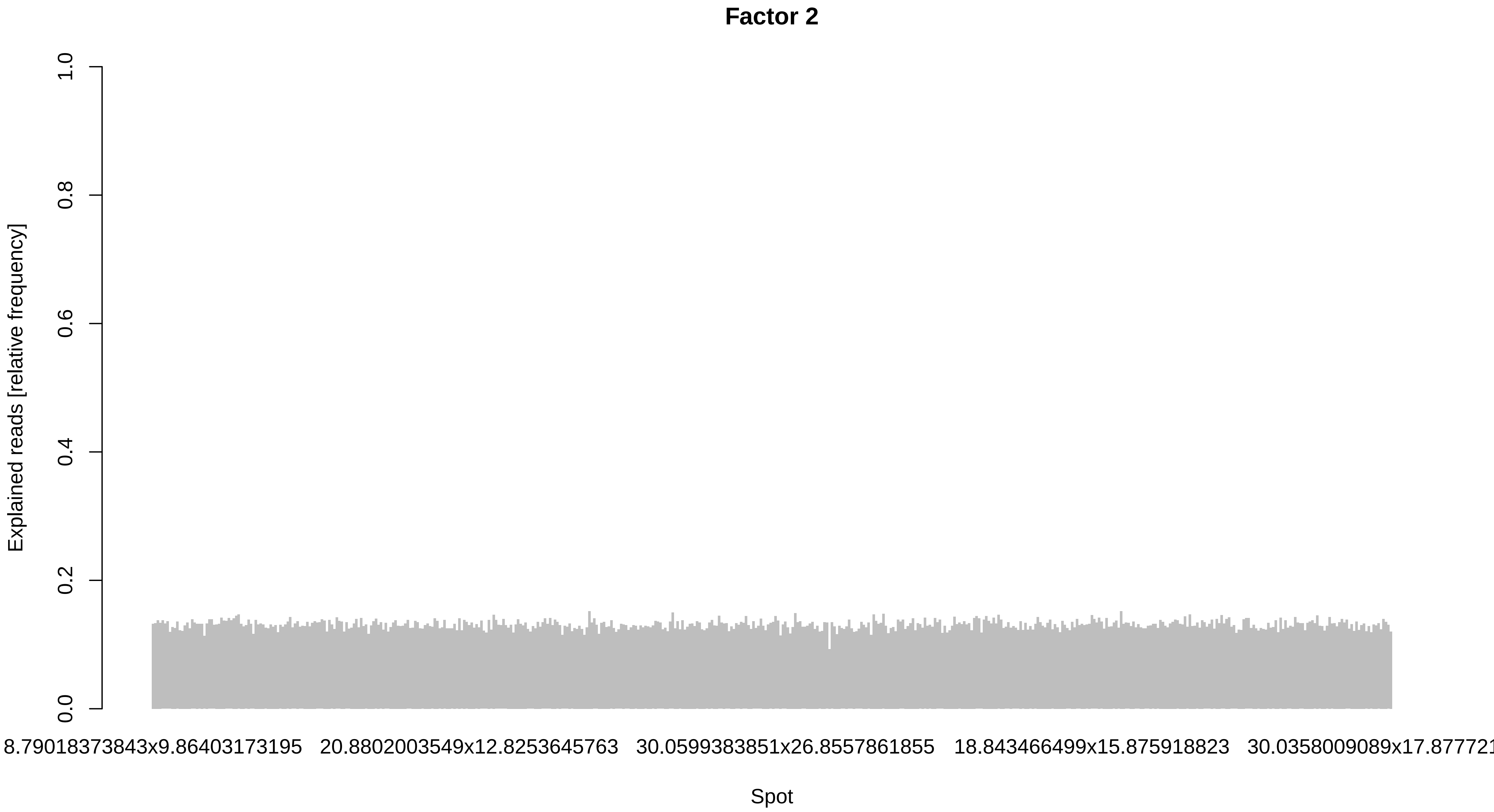

Factor 2
Explained reads [relative frequency]
0.0
0.2
0.4
0.6
0.8
1.0
8.79018373843x9.86403173195
20.8802003549x12.8253645763
30.0599383851x26.8557861855
18.843466499x15.875918823
30.0358009089x17.877721
Spot

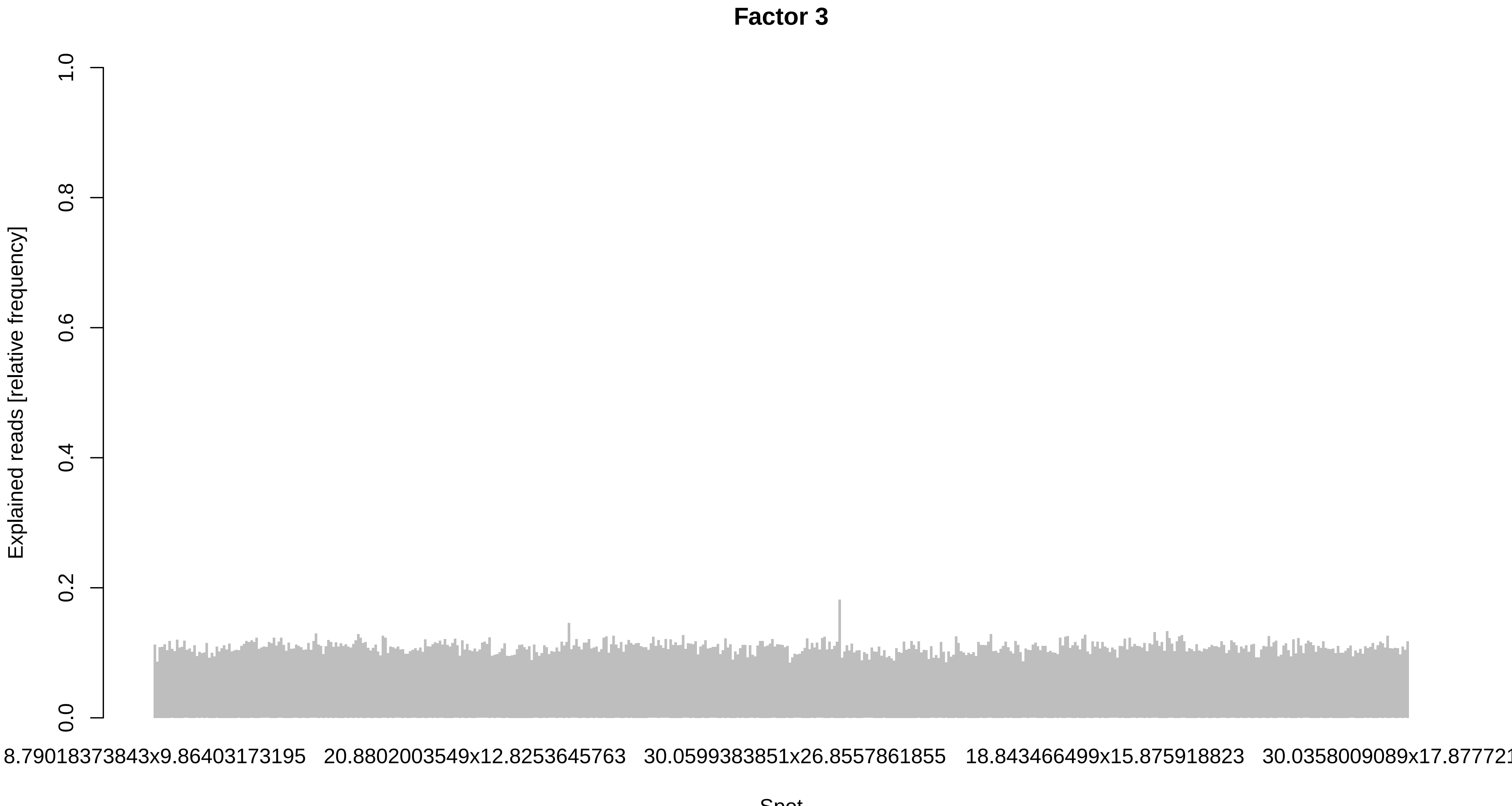
Factor 3
Explained reads [relative frequency]
0.0
0.2
0.4
0.6
0.8
1.0
8.79018373843x9.86403173195
20.8802003549x12.8253645763
30.0599383851x26.8557861855
18.843466499x15.875918823
30.0358009089x17.87772
Spot

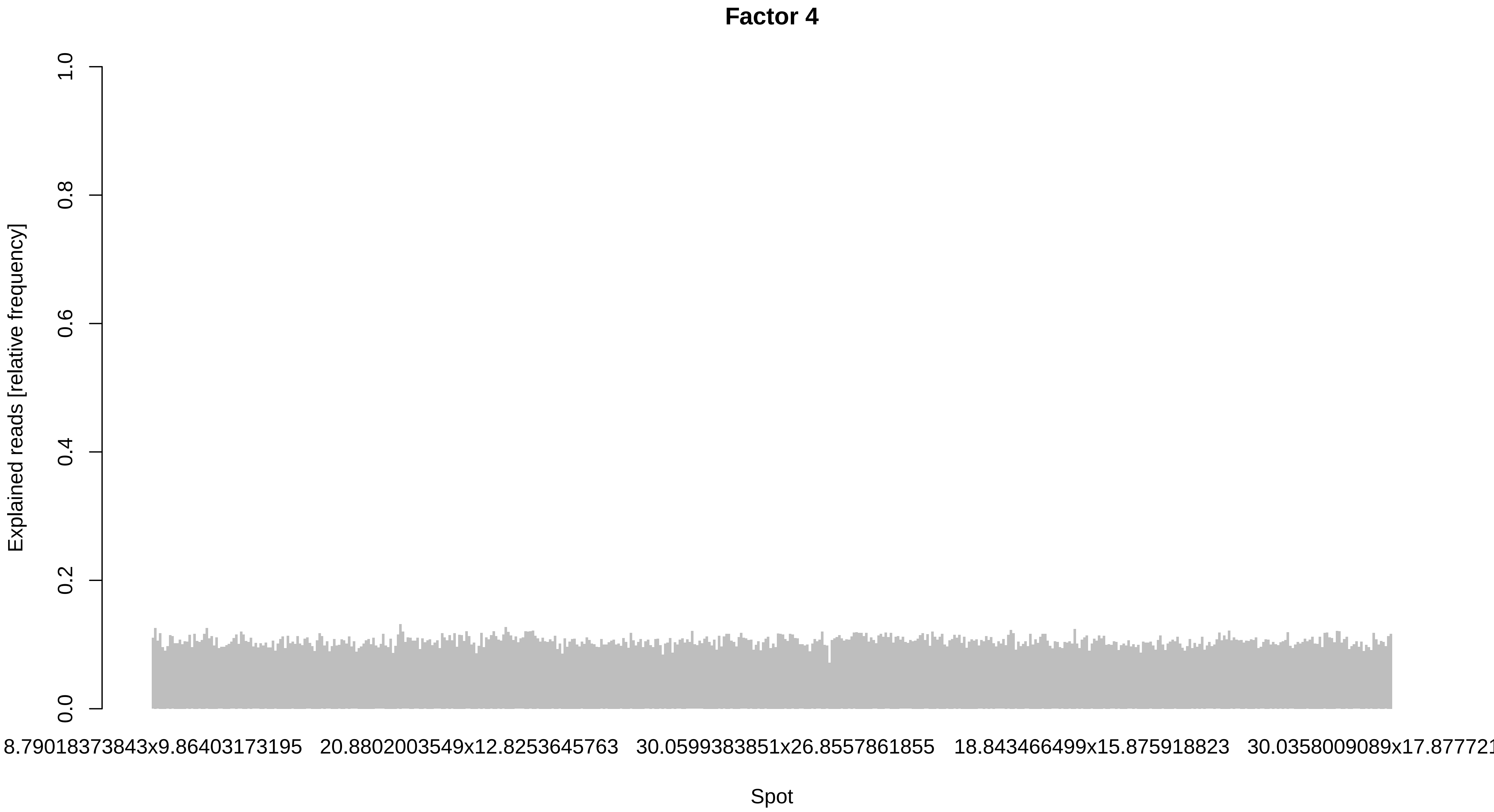

Factor 4
Explained reads [relative frequency]
0.0
0.2
0.4
0.6
0.8
1.0
8.79018373843x9.86403173195
20.8802003549x12.8253645763
30.0599383851x26.8557861855
18.843466499x15.875918823
30.0358009089x17.87772
Spot

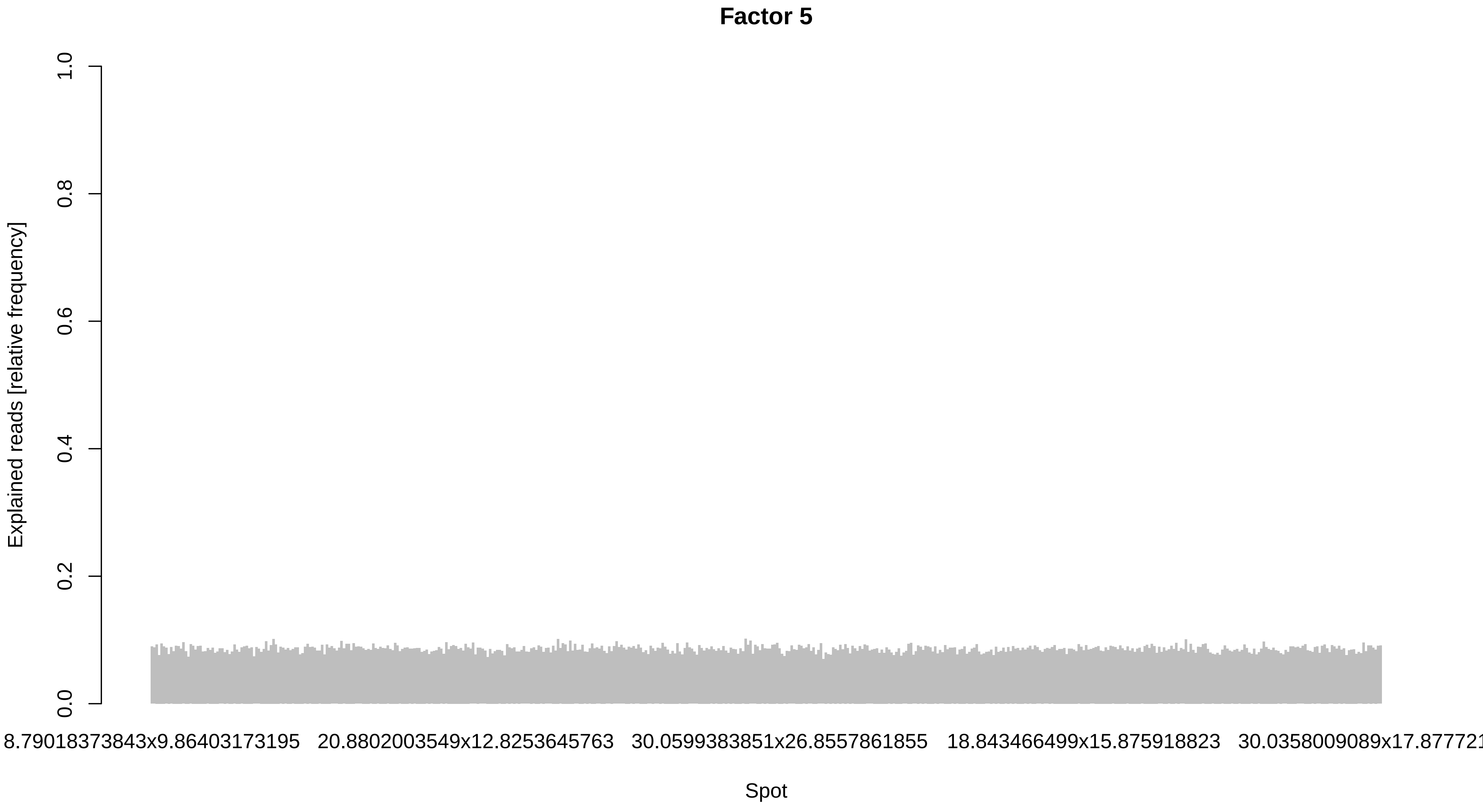
Factor 5
Explained reads [relative frequency]
0.0
0.2
0.4
0.6
0.8
1.0
8.79018373843x9.86403173195
20.8802003549x12.8253645763
30.0599383851x26.8557861855
18.843466499x15.875918823
30.0358009089x17.87772
Spot

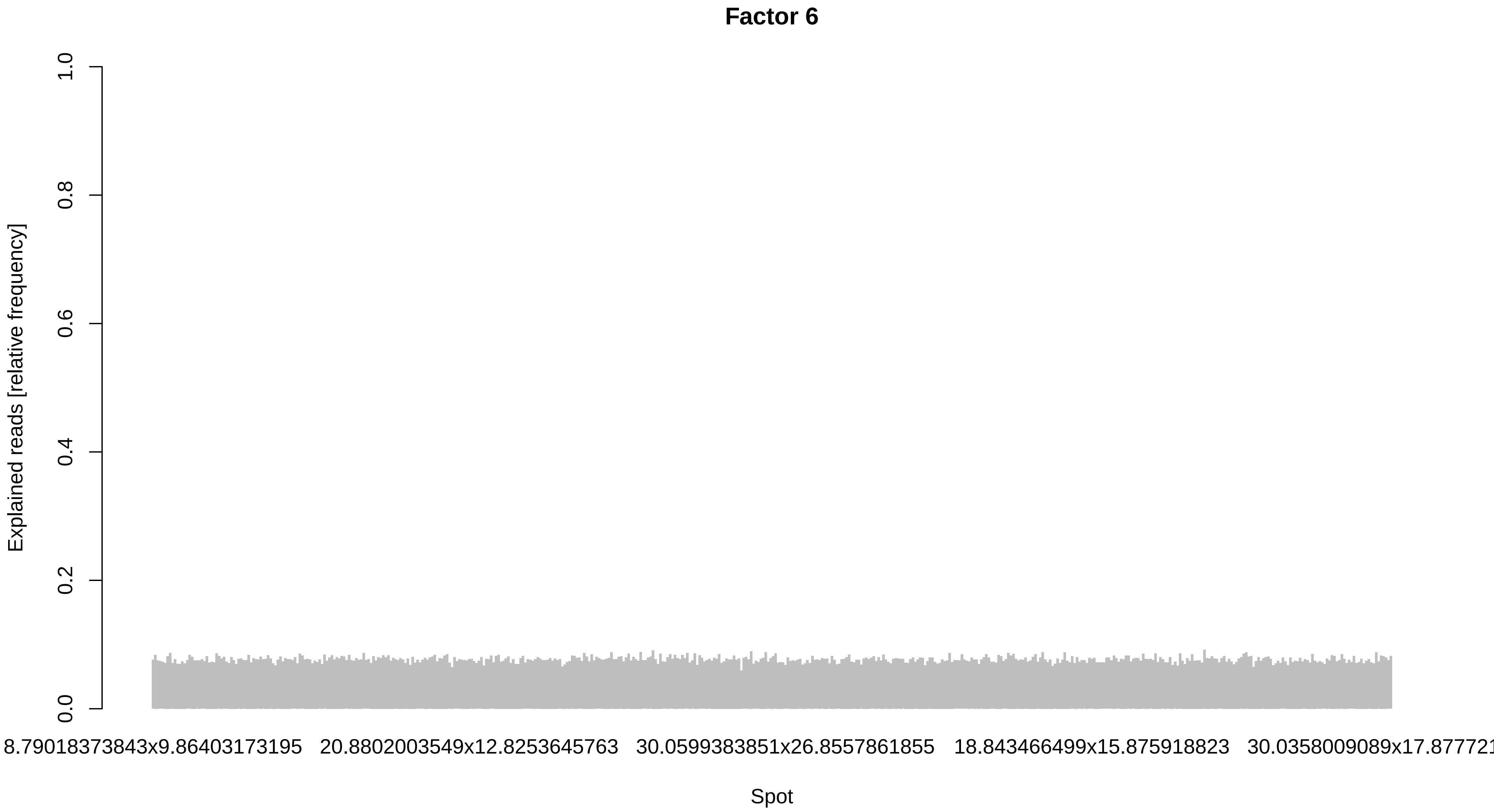
Factor 6
Explained reads [relative frequency]
0.0
0.2
0.4
0.6
0.8
1.0
8.79018373843x9.86403173195
20.8802003549x12.8253645763
30.0599383851x26.8557861855
18.843466499x15.875918823
30.0358009089x17.87772
Spot

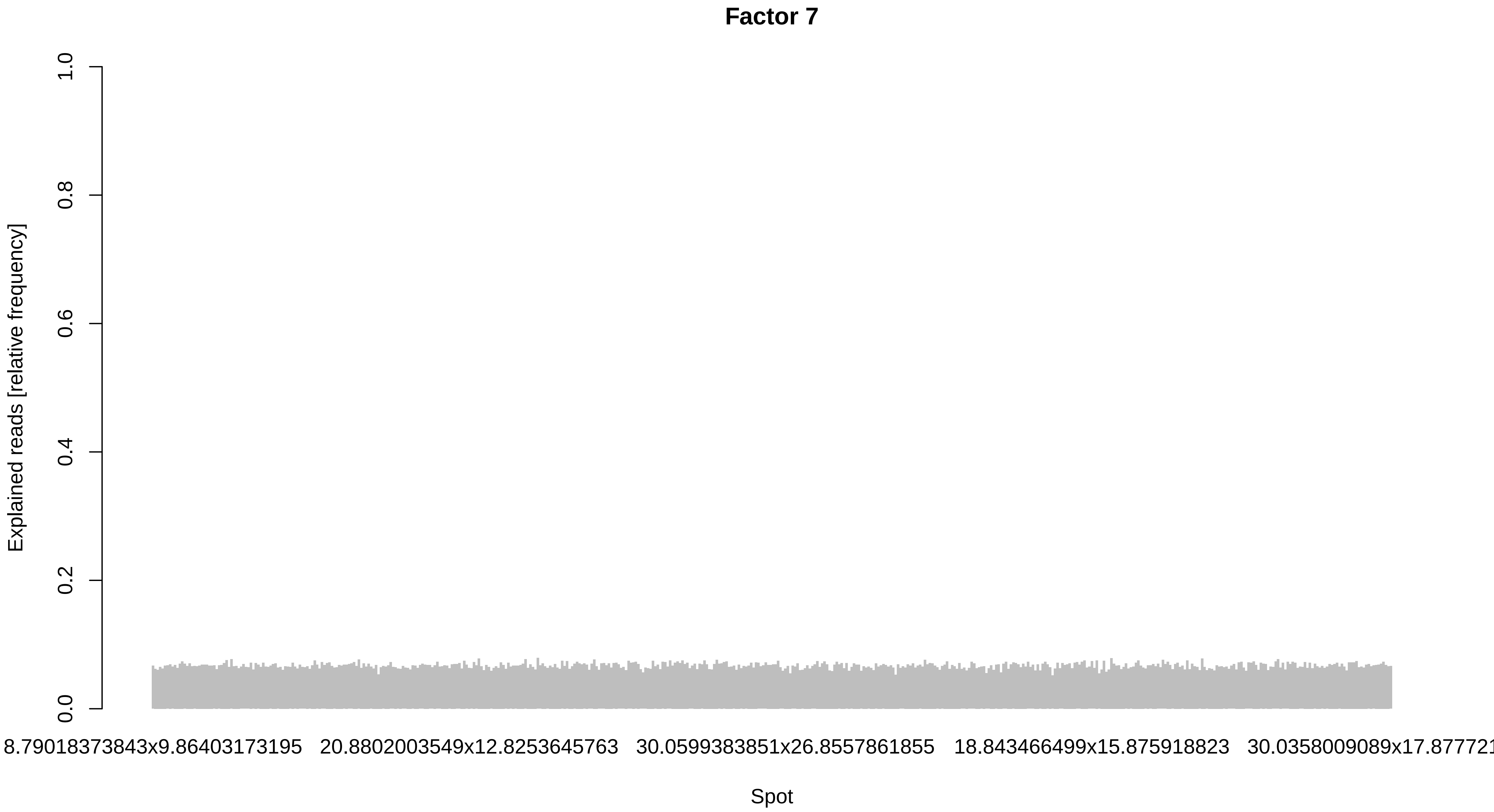
Factor 7
Explained reads [relative frequency]
0.0
0.2
0.4
0.6
0.8
1.0
8.79018373843x9.86403173195
20.8802003549x12.8253645763
30.0599383851x26.8557861855
18.843466499x15.875918823
30.0358009089x17.87772
Spot

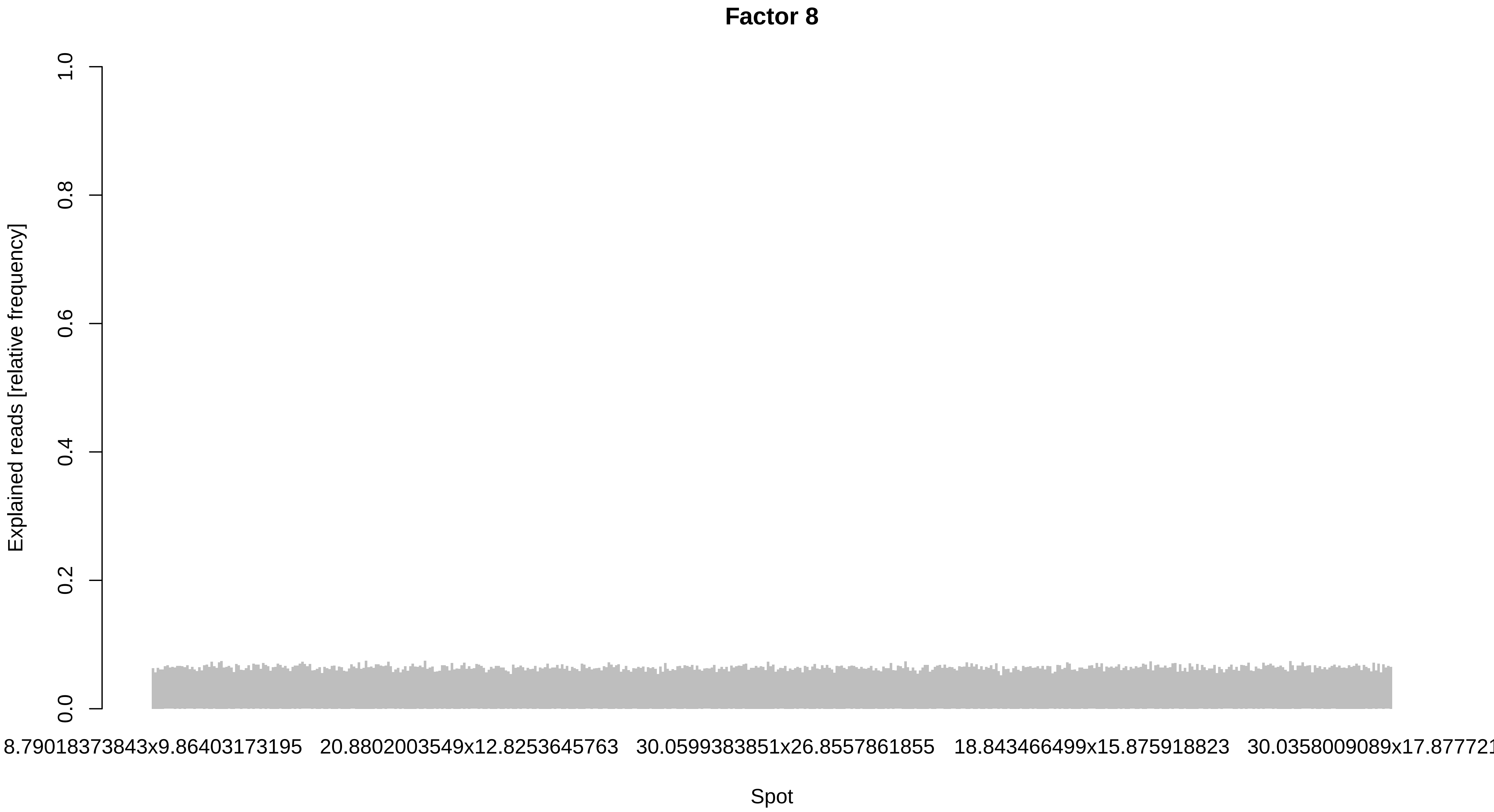

Factor 8
Explained reads [relative frequency]
1.0
0.8
0.6
0.4
0.2
0.0
8.79018373843x9.86403173195
20.8802003549x12.8253645763
30.0599383851x26.8557861855
18.843466499x15.875918823
30.0358009089x17.87772
Spot

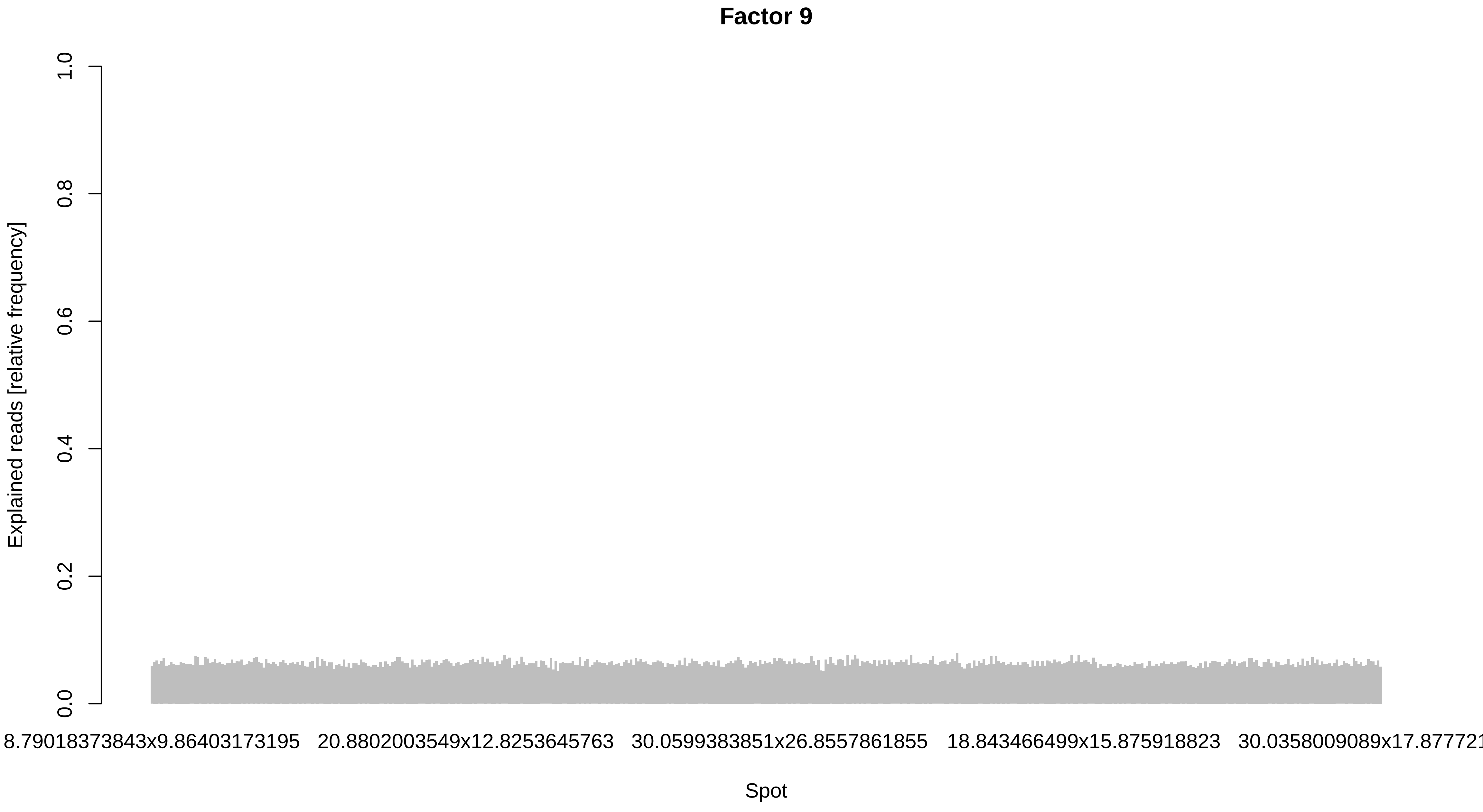

Factor 9
Explained reads [relative frequency]
0.0
0.2
0.4
0.6
0.8
1.0
8.79018373843x9.86403173195
20.8802003549x12.8253645763
30.0599383851x26.8557861855
18.843466499x15.875918823
30.0358009089x17.87772
Spot

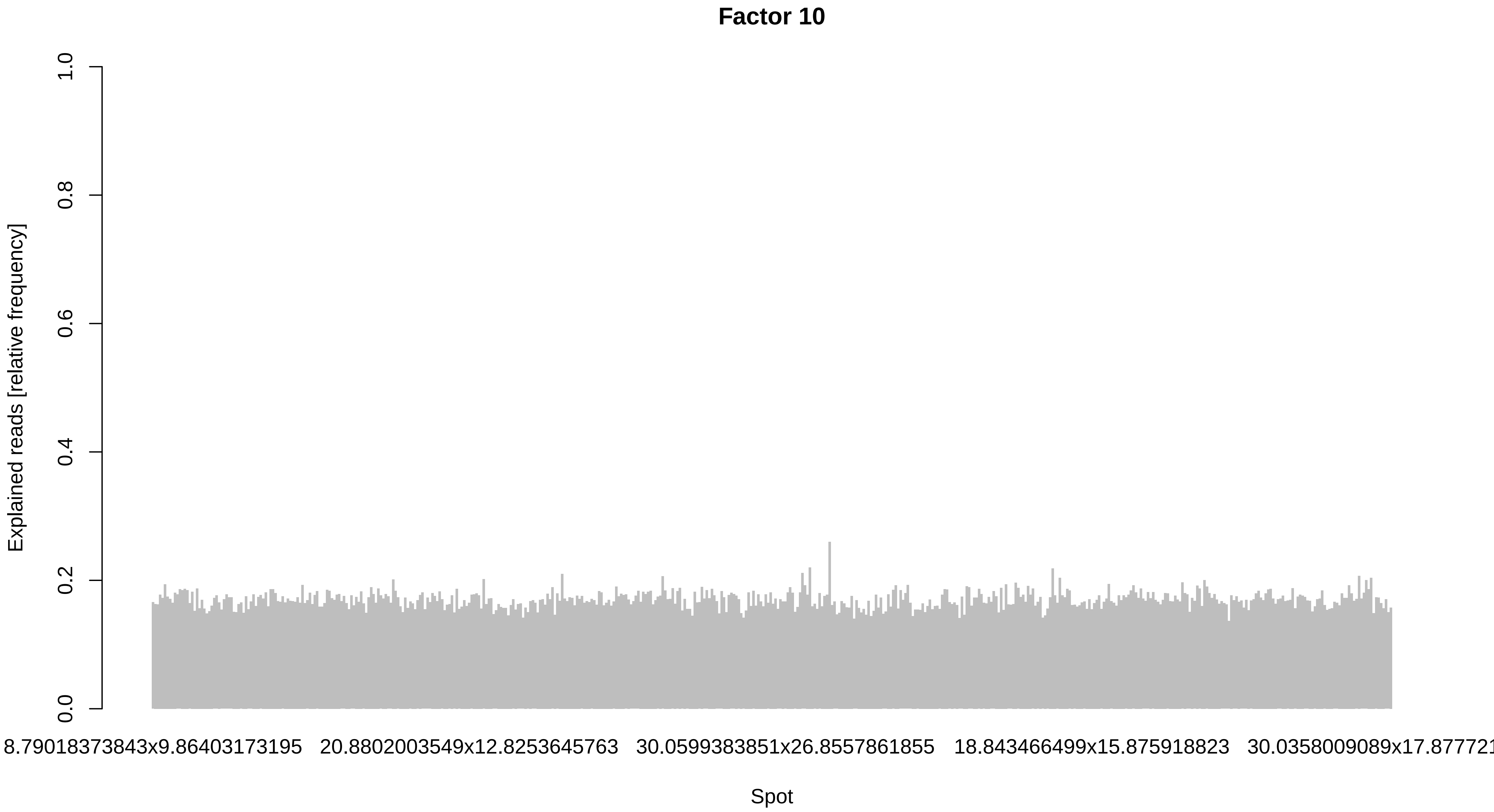

Factor 10
Explained reads [relative frequency]
0.0
0.2
0.4
0.6
0.8
1.0
8.79018373843x9.86403173195
20.8802003549x12.8253645763
30.0599383851x26.8557861855
18.843466499x15.875918823
30.0358009089x17.877721
Spot

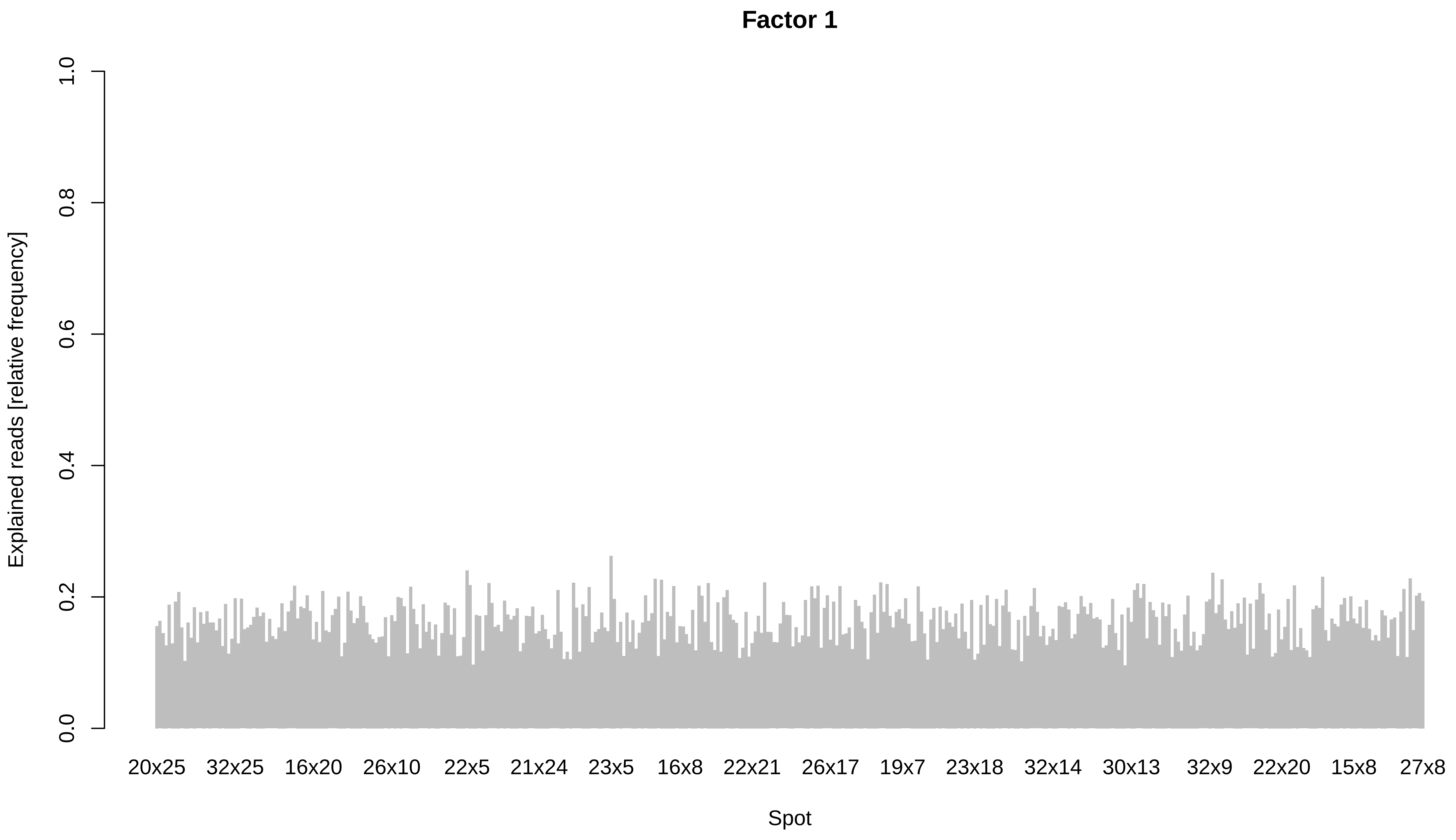

Factor 1
Explained reads [relative frequency]
1.0
0.8
0.6
0.4
0.2
0.0
20x25
32x25
16x20
26x10
22x5
21x24
23x5
16x8
22x21
26x17
19x7
23x18
32x14
30x13
32x9
22x20
15x8
27x8
Spot

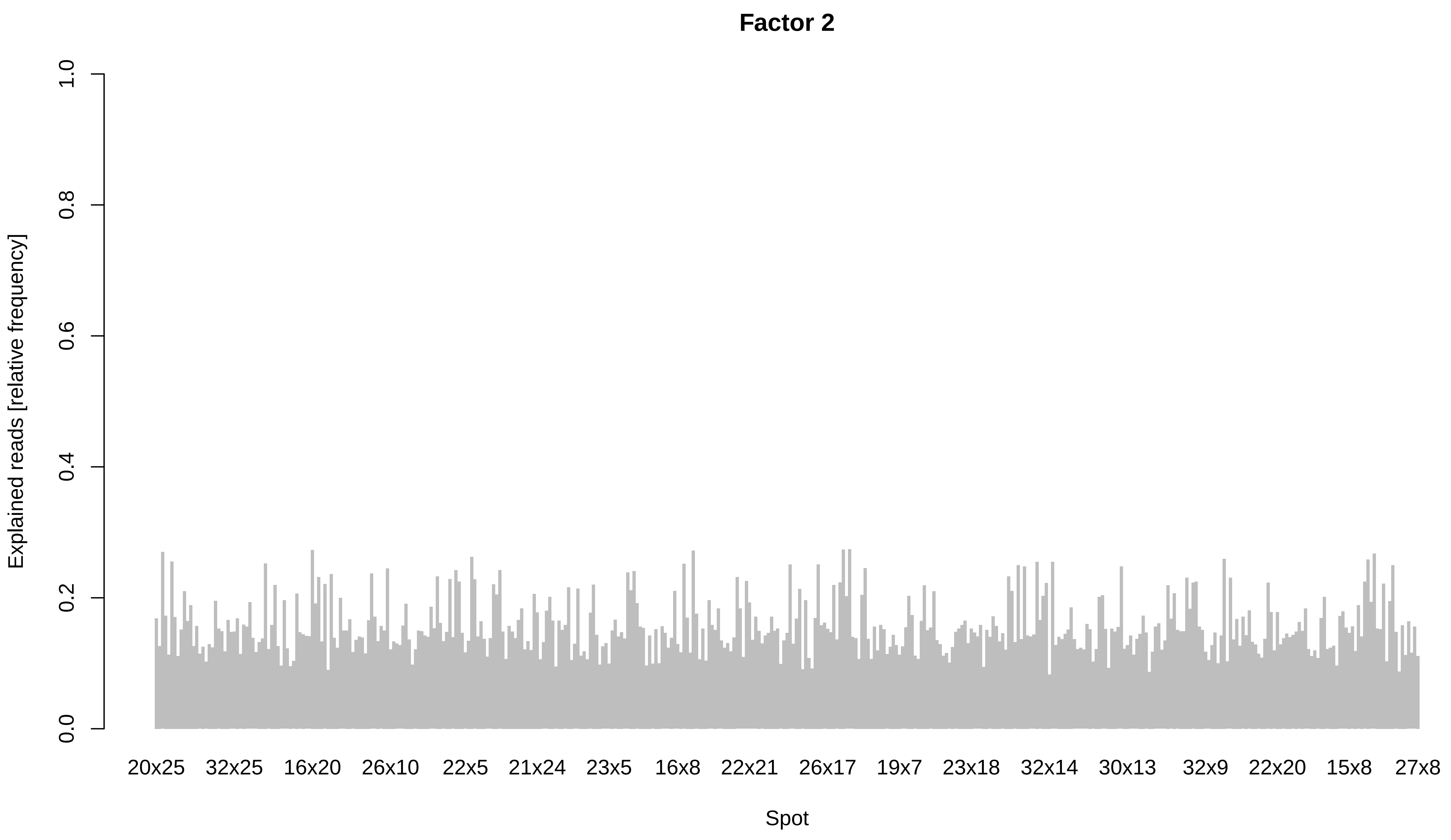

Factor 2
Explained reads [relative frequency]
0.0
0.2
0.4
0.6
0.8
1.0
20x25
32x25
16x20
26x10
22x5
21x24
23x5
16x8
22x21
26x17
19x7
23x18
32x14
30x13
32x9
22x20
15x8
27x8
Spot

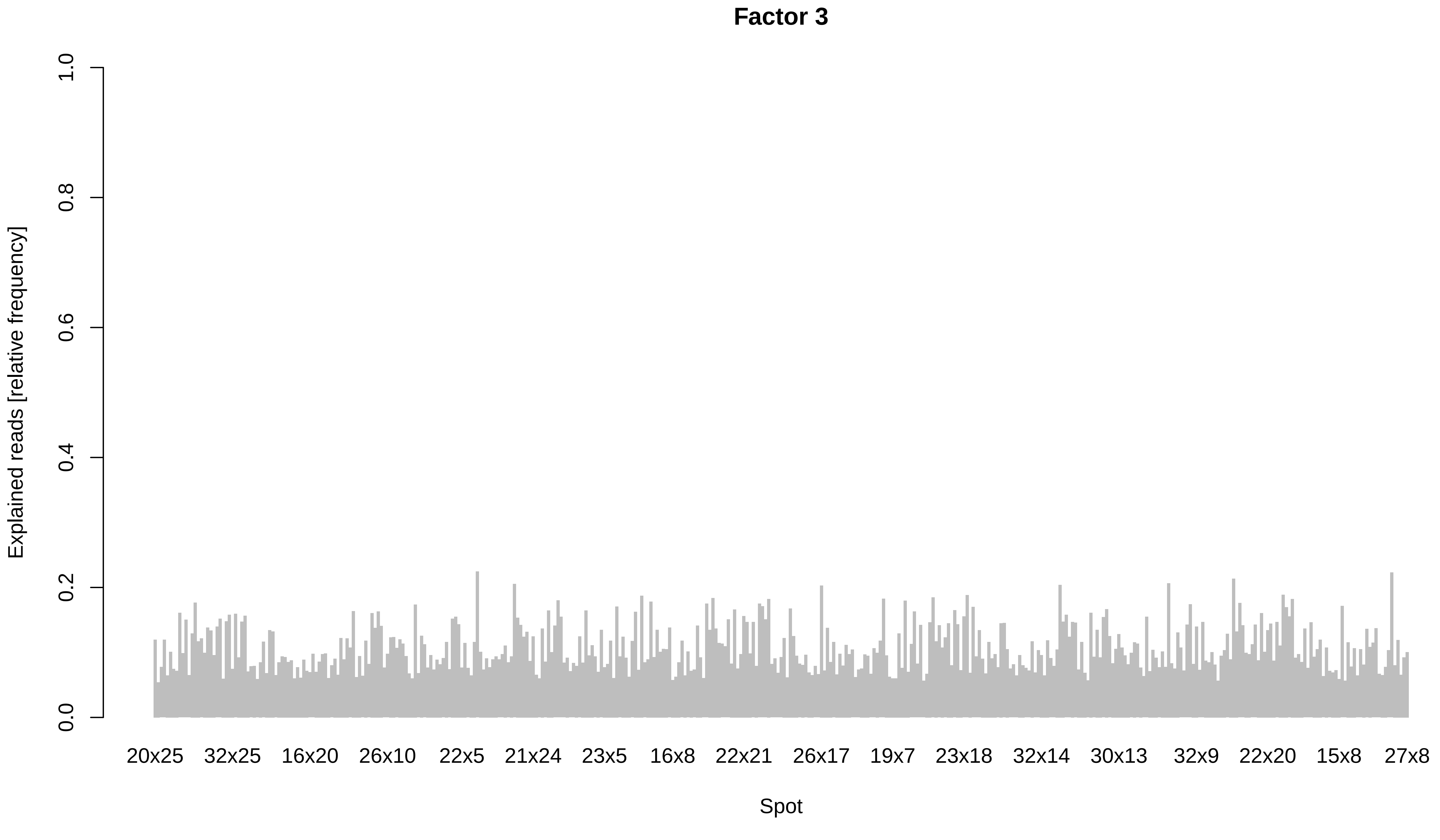

Factor 3
Explained reads [relative frequency]
0.0
0.2
0.4
0.6
0.8
1.0
20x25
32x25
16x20
26x10
22x5
21x24
23x5
16x8
22x21
26x17
19x7
23x18
32x14
30x13
32x9
22x20
15x8
27x8
Spot

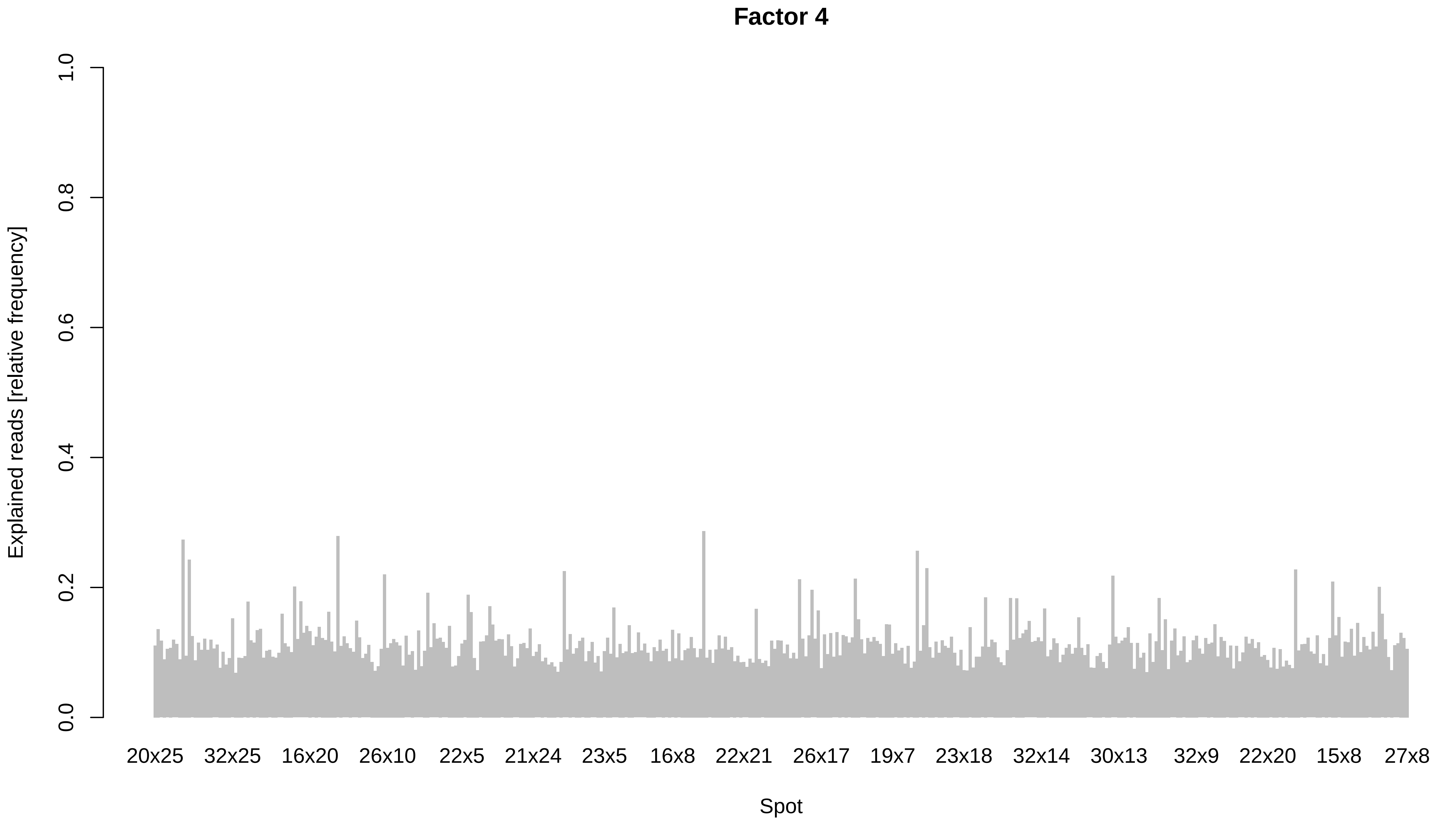

Factor 4
Explained reads [relative frequency]
0.0
0.2
0.4
0.6
0.8
1.0
20x25
32x25
16x20
26x10
22x5
21x24
23x5
16x8
22x21
26x17
19x7
23x18
32x14
30x13
32x9
22x20
15x8
27x8
Spot

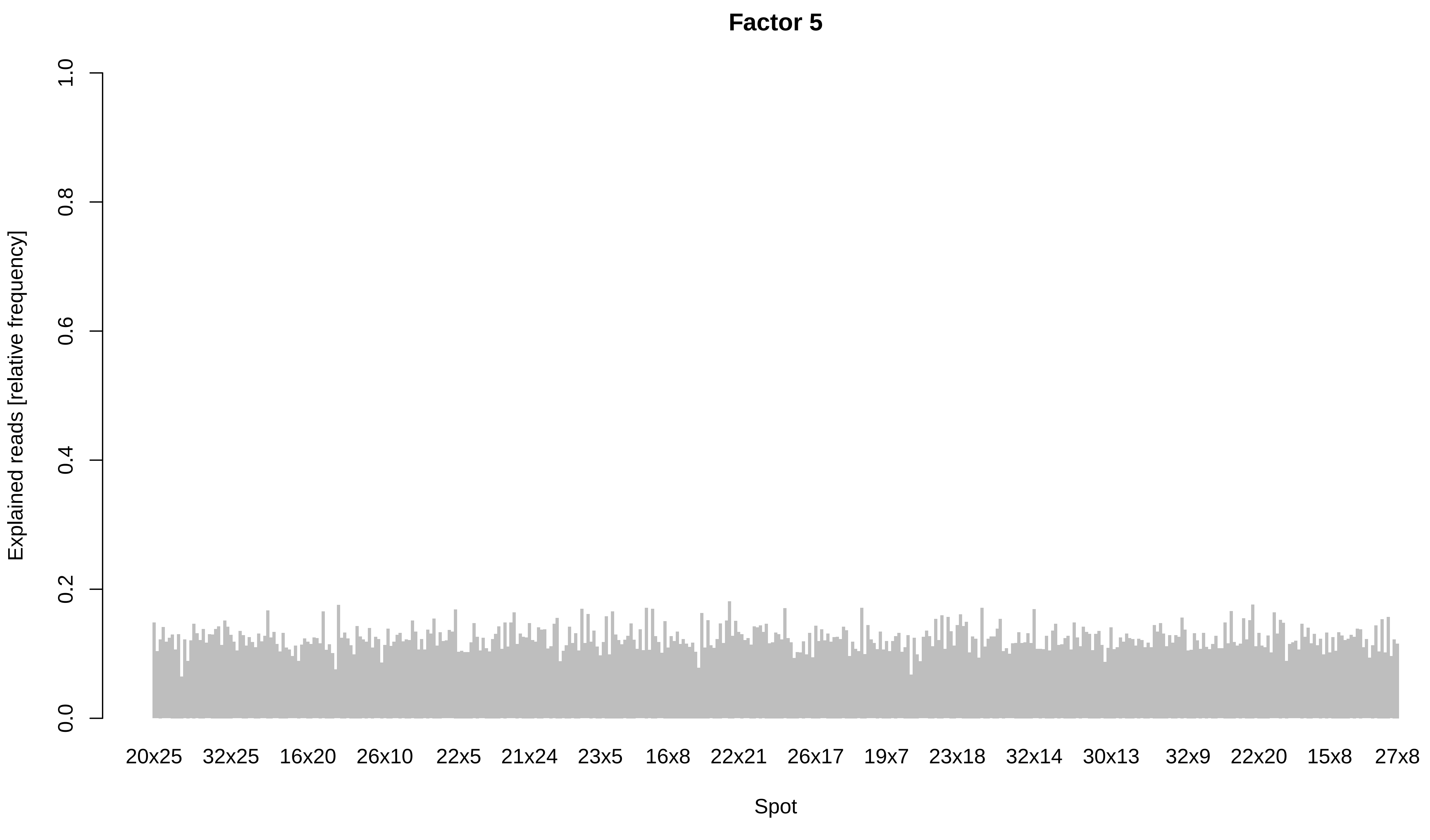

Factor 5
Explained reads [relative frequency]
1.0
0.8
0.6
0.4
0.2
0.0
20x25
32x25
16x20
26x10
22x5
21x24
23x5
16x8
22x21
26x17
19x7
23x18
32x14
30x13
32x9
22x20
15x8
27x8
Spot

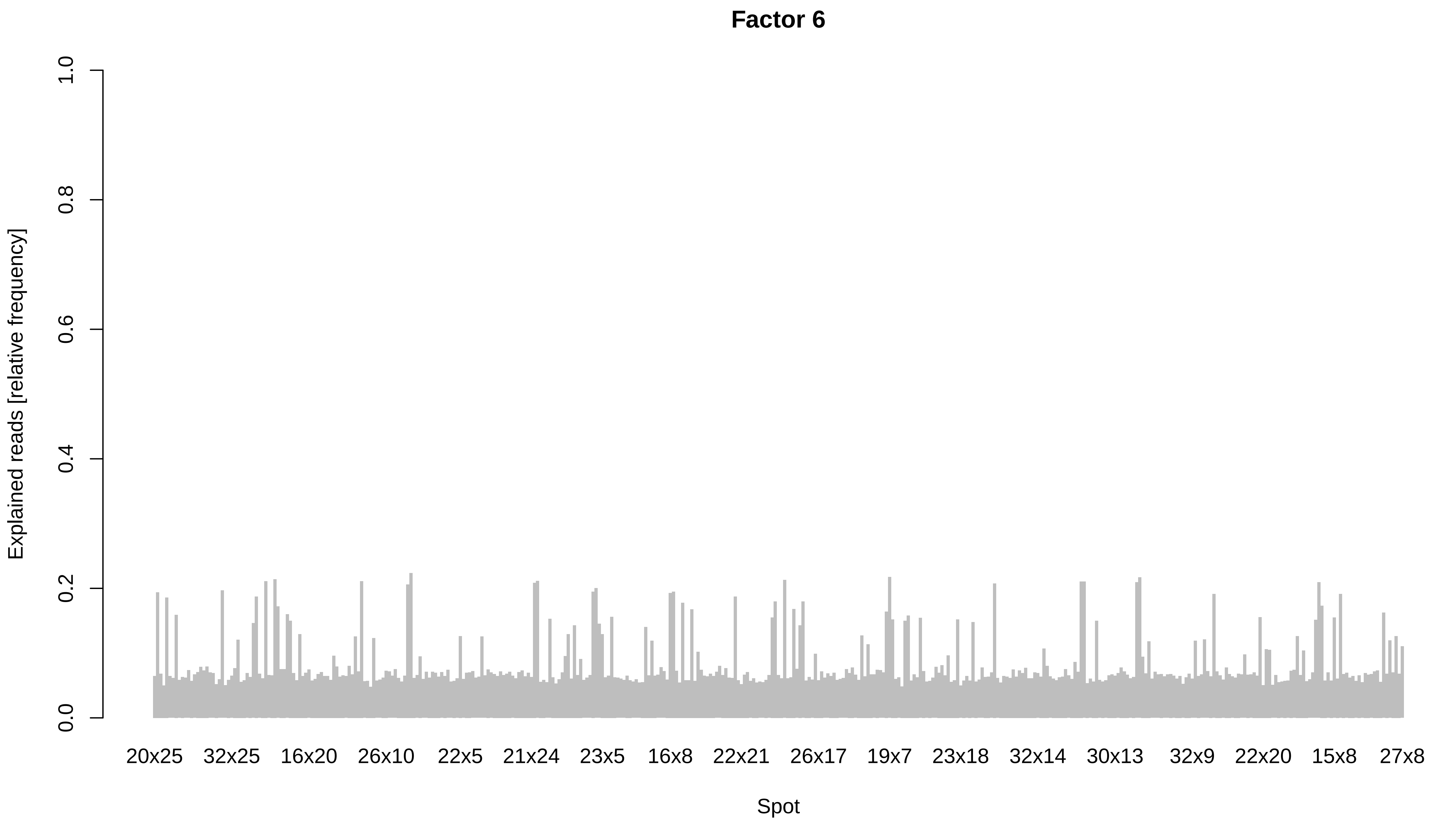

Factor 6
1.0
0.8
0.6
0.4
0.2
0.0
Explained reads [relative frequency]
20x25
32x25
16x20
26x10
22x5
21x24
23x5
16x8
22x21
26x17
19x7
23x18
32x14
30x13
32x9
22x20
15x8
27x8
Spot

Factor 7

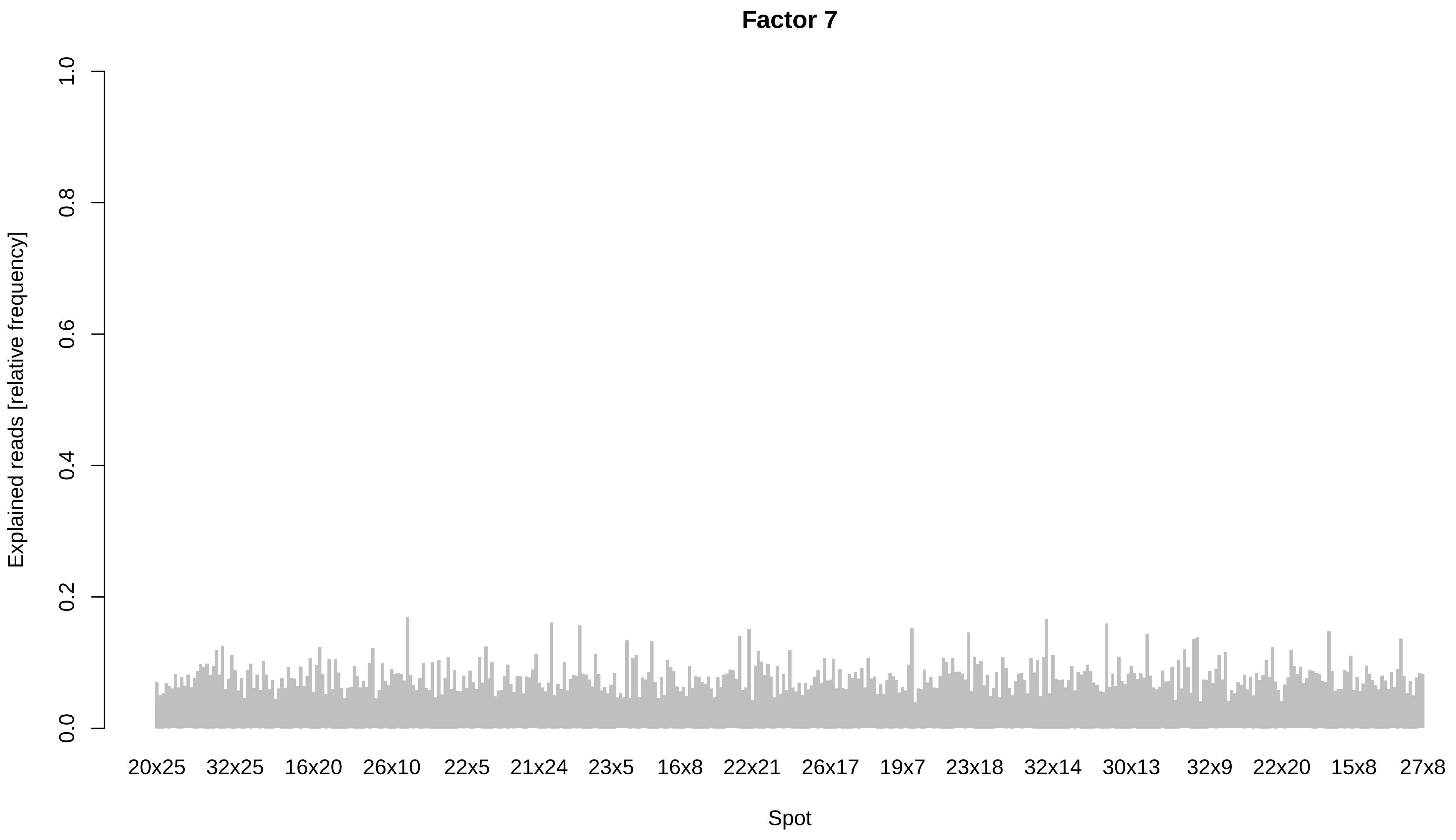

Factor 7
Explained reads [relative frequency]
0.0
0.2
0.4
0.6
0.8
1.0
20x25
32x25
16x20
26x10
22x5
21x24
23x5
16x8
22x21
26x17
19x7
23x18
32x14
30x13
32x9
22x20
15x8
27x8
Spot

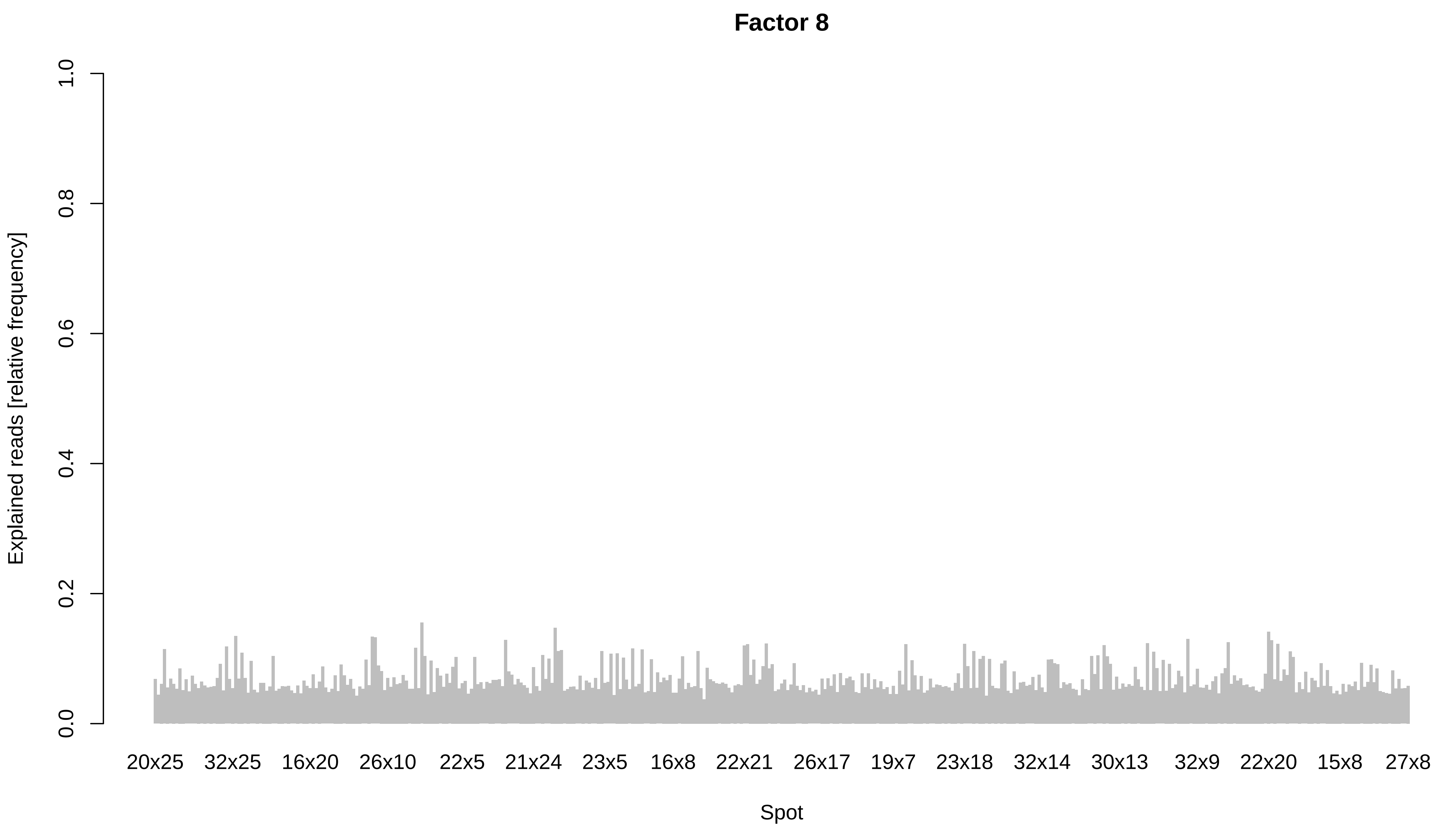

Factor 8
Explained reads [relative frequency]
0.0
0.2
0.4
0.6
0.8
1.0
20x25
32x25
16x20
26x10
22x5
21x24
23x5
16x8
22x21
26x17
19x7
23x18
32x14
30x13
32x9
22x20
15x8
27x8
Spot

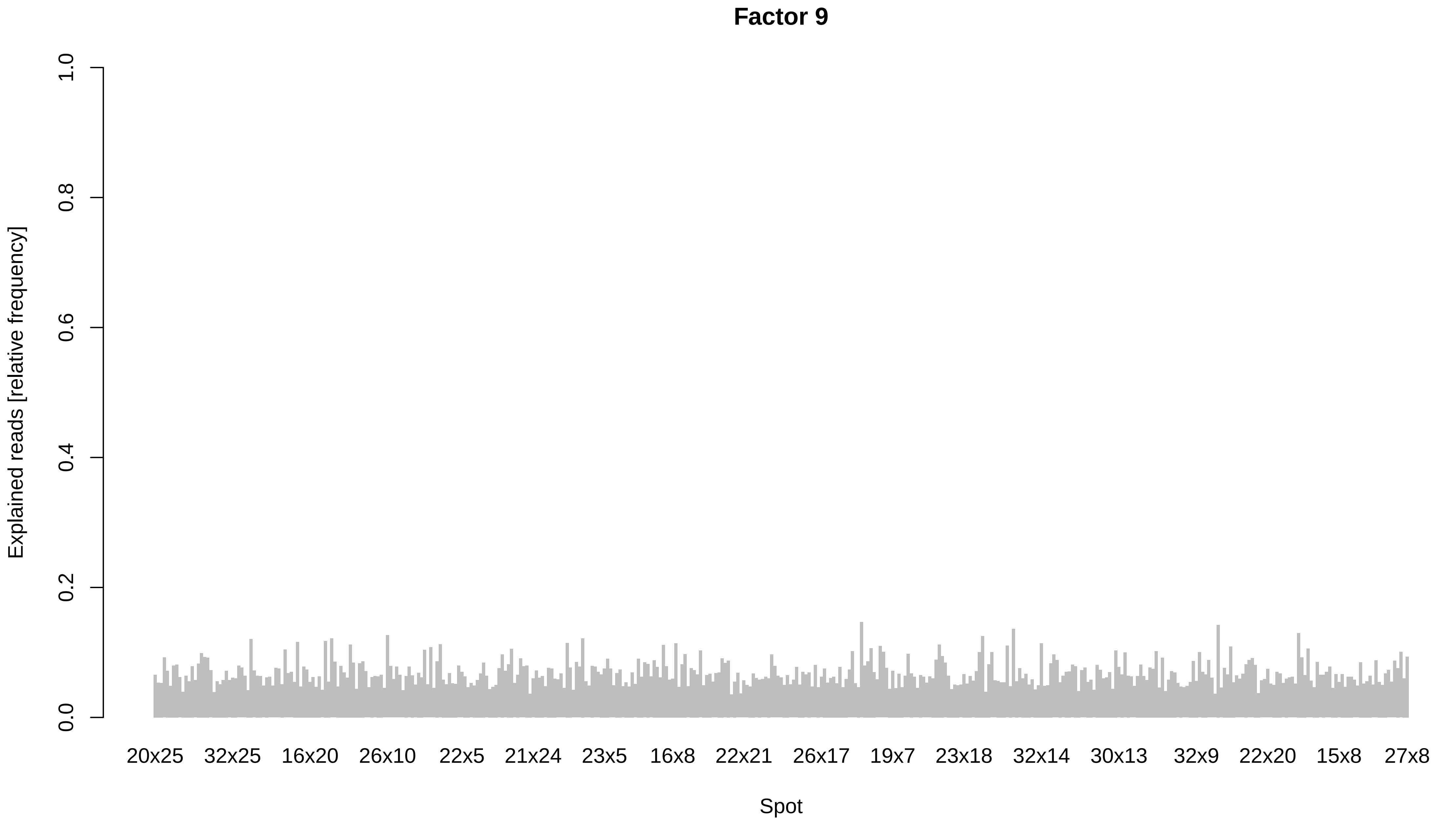

Factor 9
Explained reads [relative frequency]
0.0
0.2
0.4
0.6
0.8
1.0
20x25
32x25
16x20
26x10
22x5
21x24
23x5
16x8
22x21
26x17
19x7
23x18
32x14
30x13
32x9
22x20
15x8
27x8
Spot

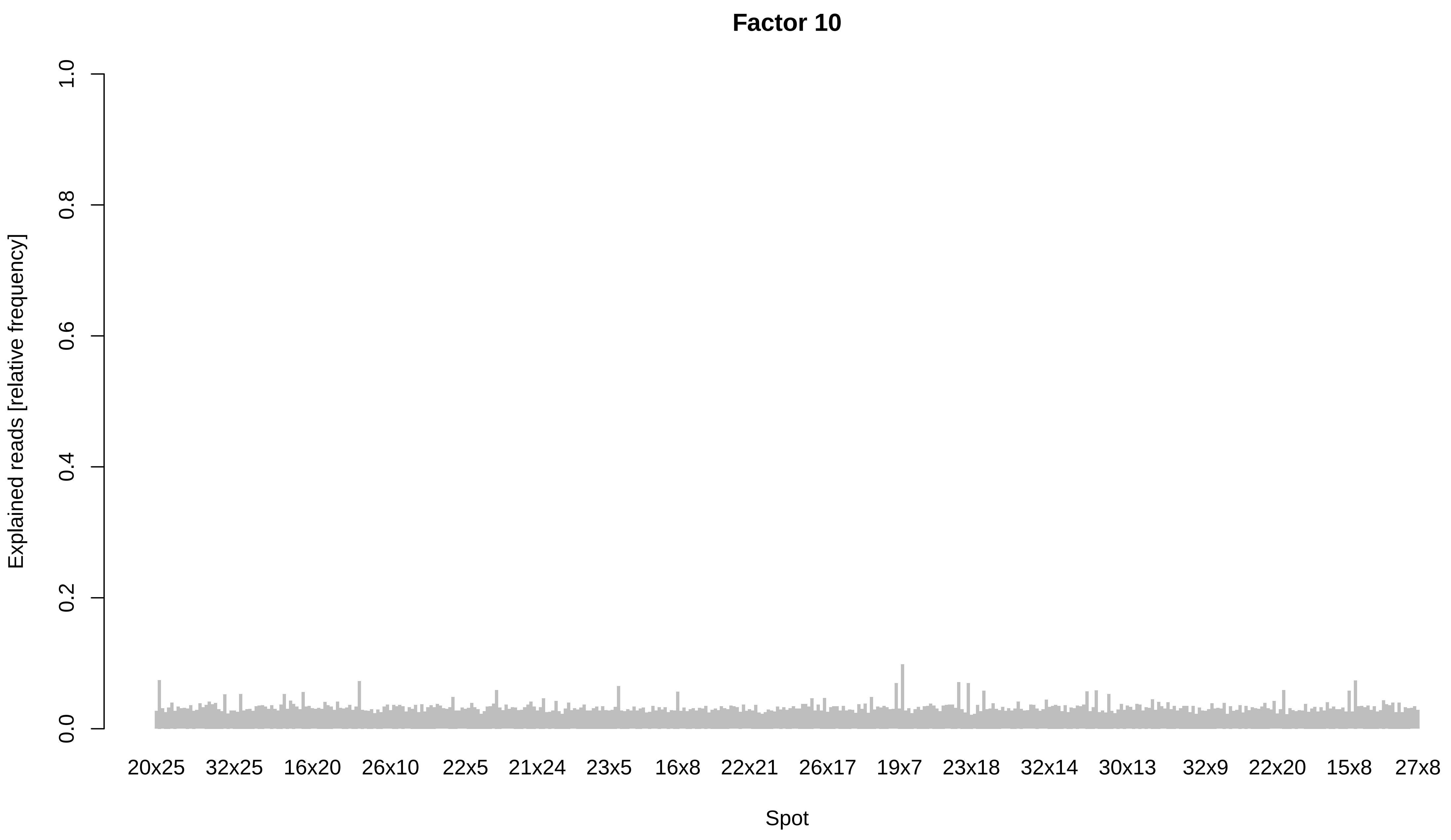
Factor 10
Explained reads [relative frequency]
0.0
0.2
0.4
0.6
0.8
1.0
20x25
32x25
16x20
26x10
22x5
21x24
23x5
16x8
22x21
26x17
19x7
23x18
32x14
30x13
32x9
22x20
15x8
27x8
Spot

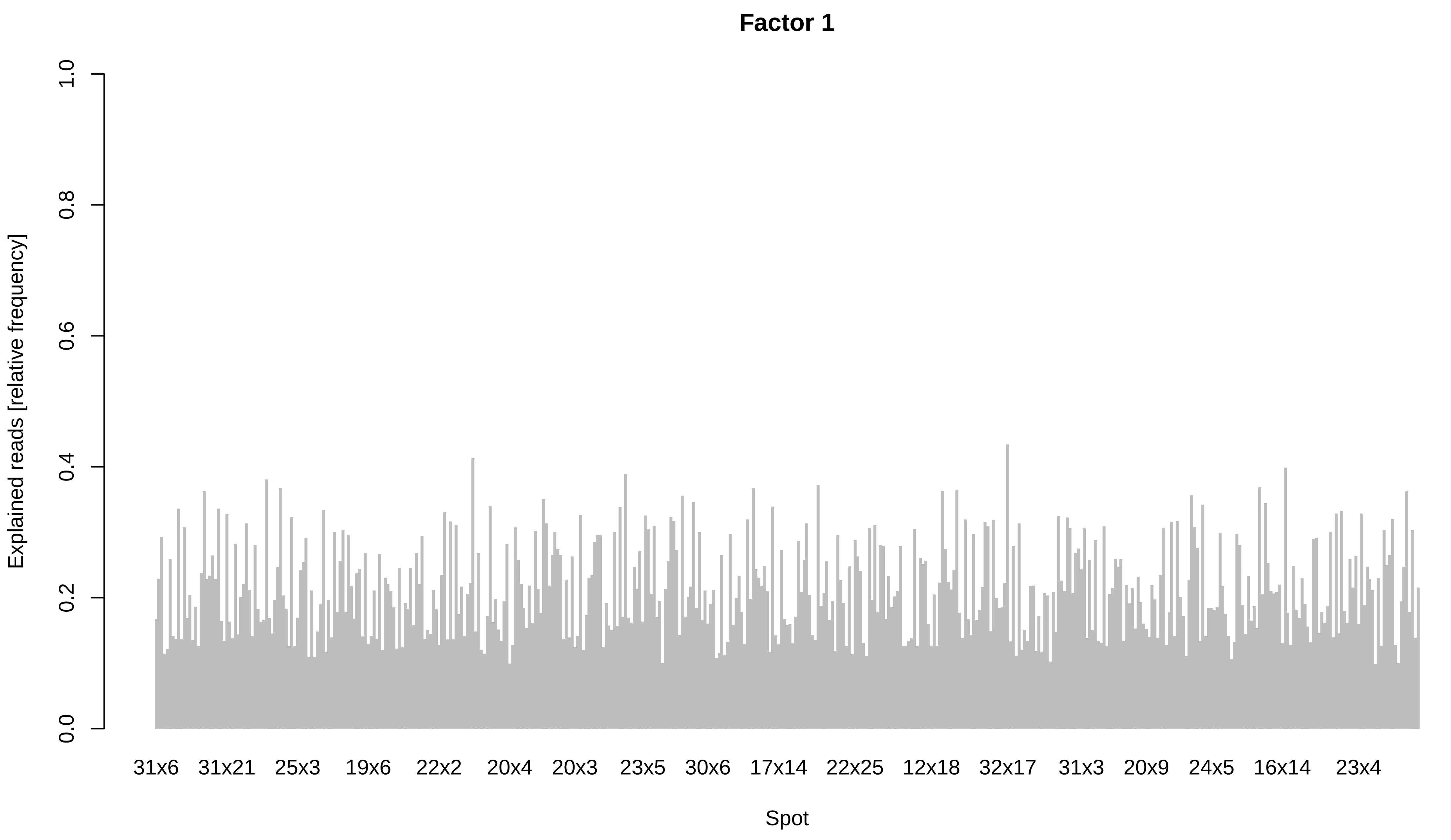

Factor 1
Explained reads [relative frequency]
0.0
0.2
0.4
0.6
0.8
1.0
31x6
31x21
25x3
19x6
22x2
20x4
20x3
23x5
30x6
17x14
22x25
12x18
32x17
31x3
20x9
24x5
16x14
23x4
Spot

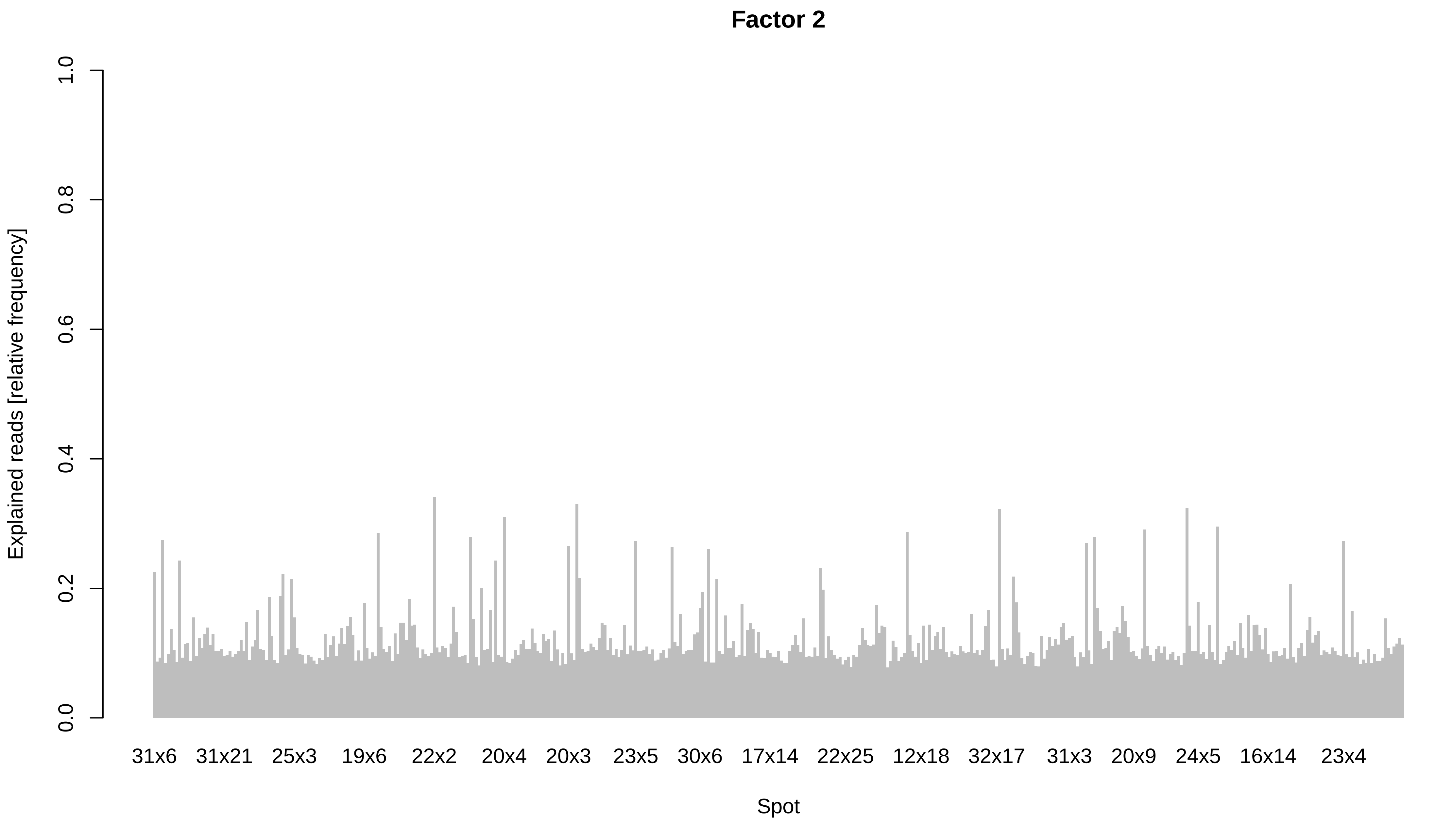
Factor 2
Explained reads [relative frequency]
0.0
0.2
0.4
0.6
0.8
1.0
31x6
31x21
25x3
19x6
22x2
20x4
20x3
23x5
30x6
17x14
22x25
12x18
32x17
31x3
20x9
24x5
16x14
23x4
Spot

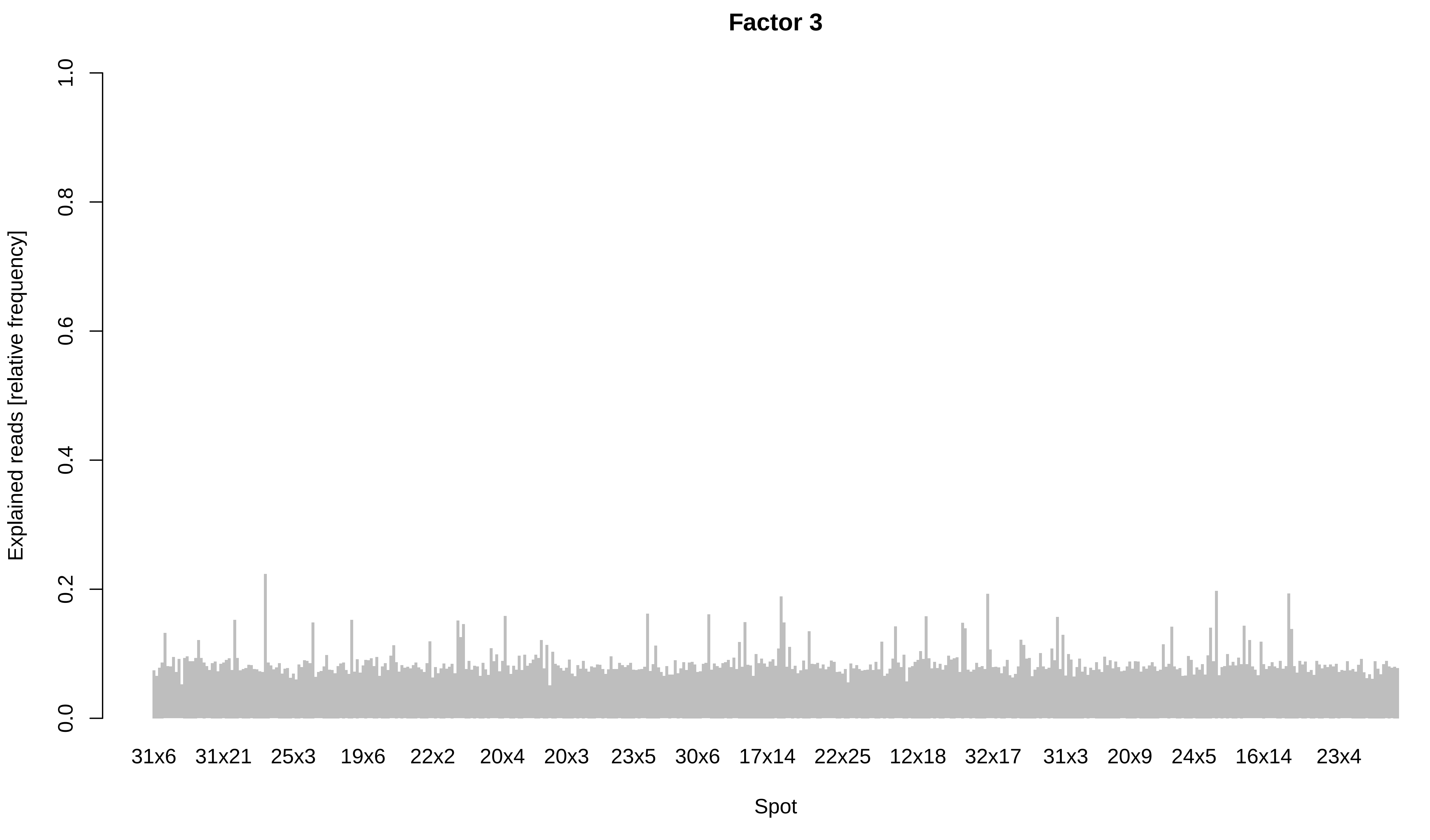

Factor 3
Explained reads [relative frequency]
0.0
0.2
0.4
0.6
0.8
1.0
31x6
31x21
25x3
19x6
22x2
20x4
20x3
23x5
30x6
17x14
22x25
12x18
32x17
31x3
20x9
24x5
16x14
23x4
Spot

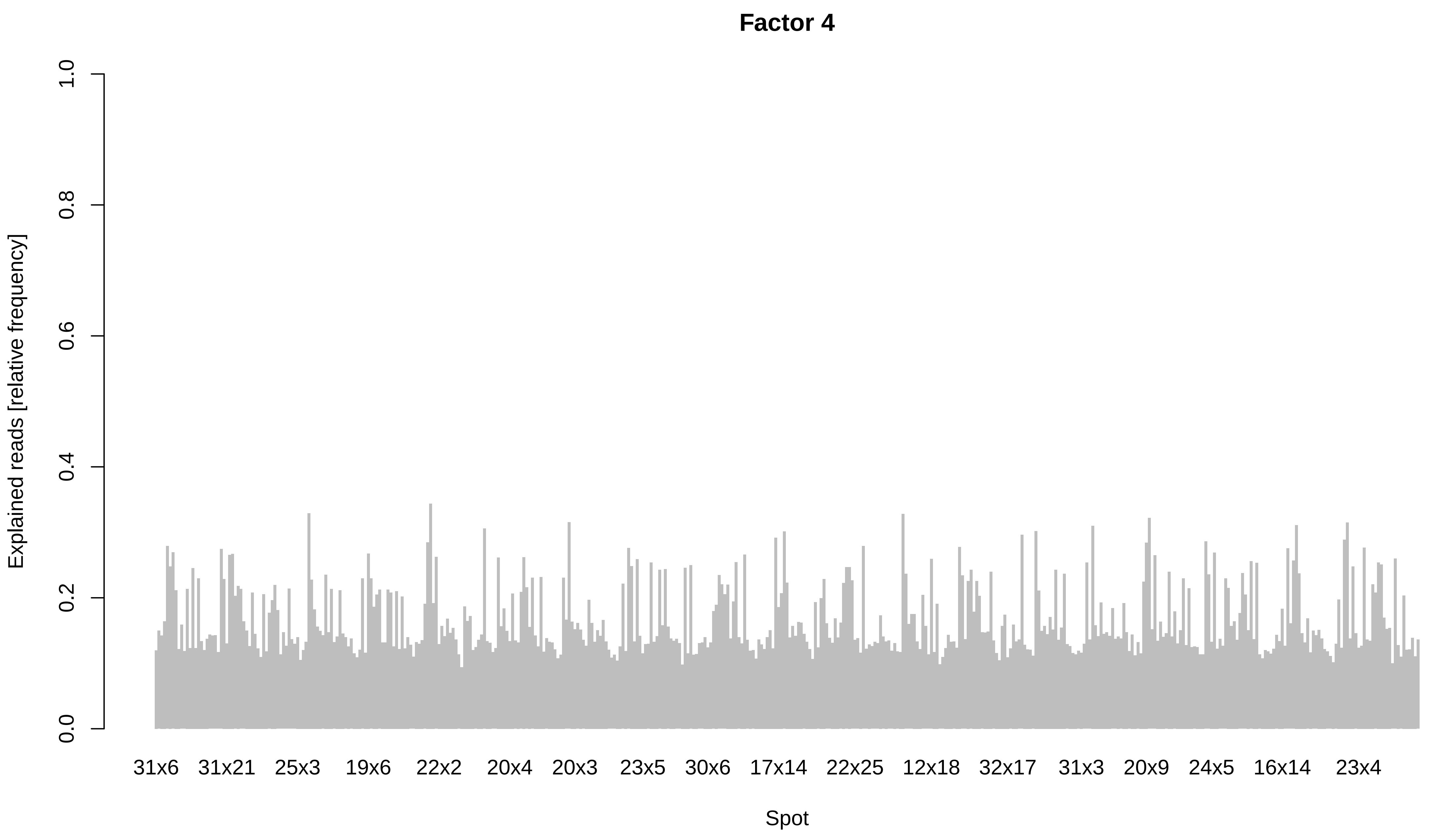

Factor 4
Explained reads [relative frequency]
1.0
0.8
0.6
0.4
0.2
0.0
31x6
31x21
25x3
19x6
22x2
20x4
20x3
23x5
30x6
17x14
22x25
12x18
32x17
31x3
20x9
24x5
16x14
23x4
Spot

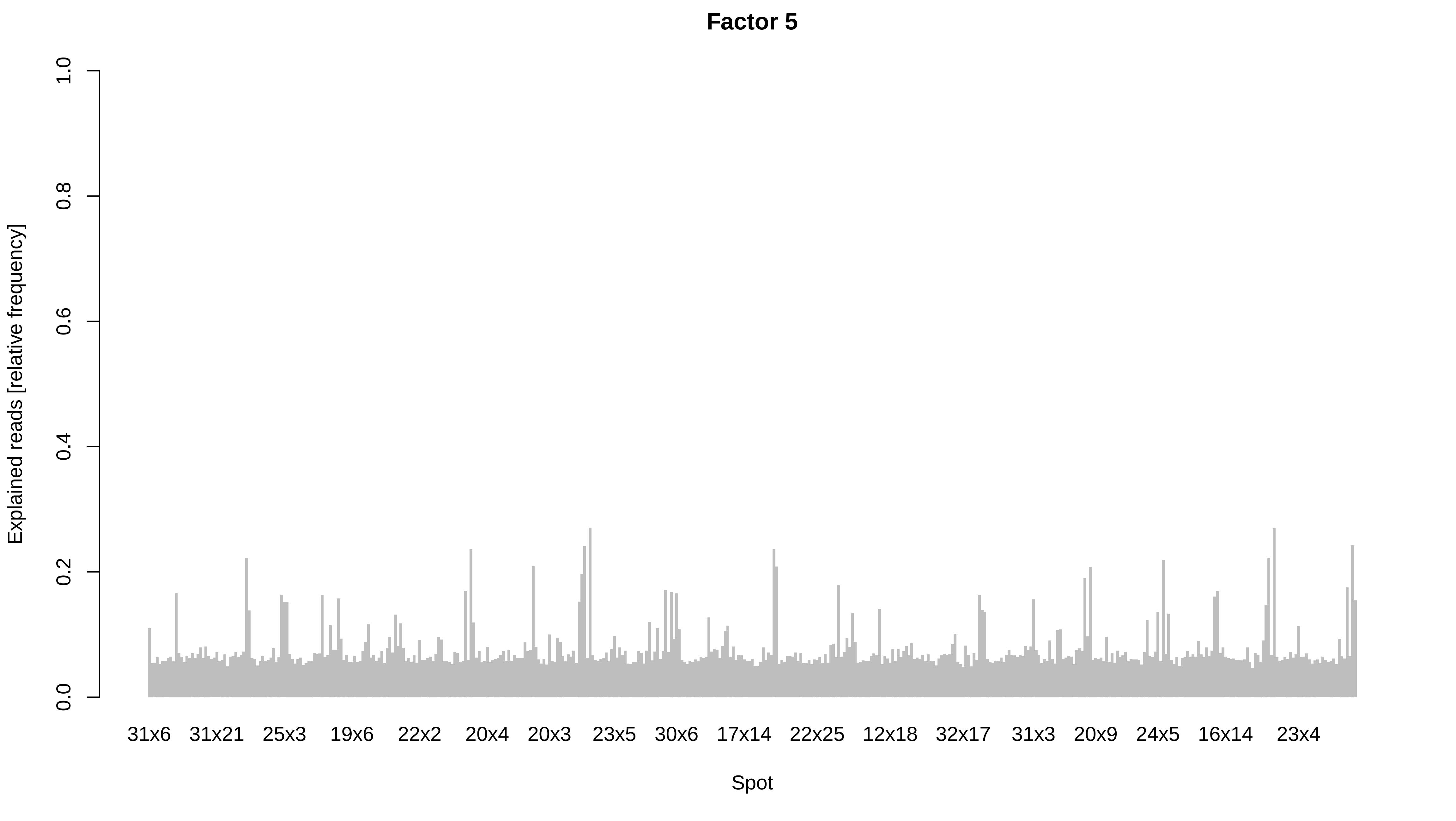

Factor 5
Explained reads [relative frequency]
0.0
0.2
0.4
0.6
0.8
1.0
31x6
31x21
25x3
19x6
22x2
20x4
20x3
23x5
30x6
17x14
22x25
12x18
32x17
31x3
20x9
24x5
16x14
23x4
Spot

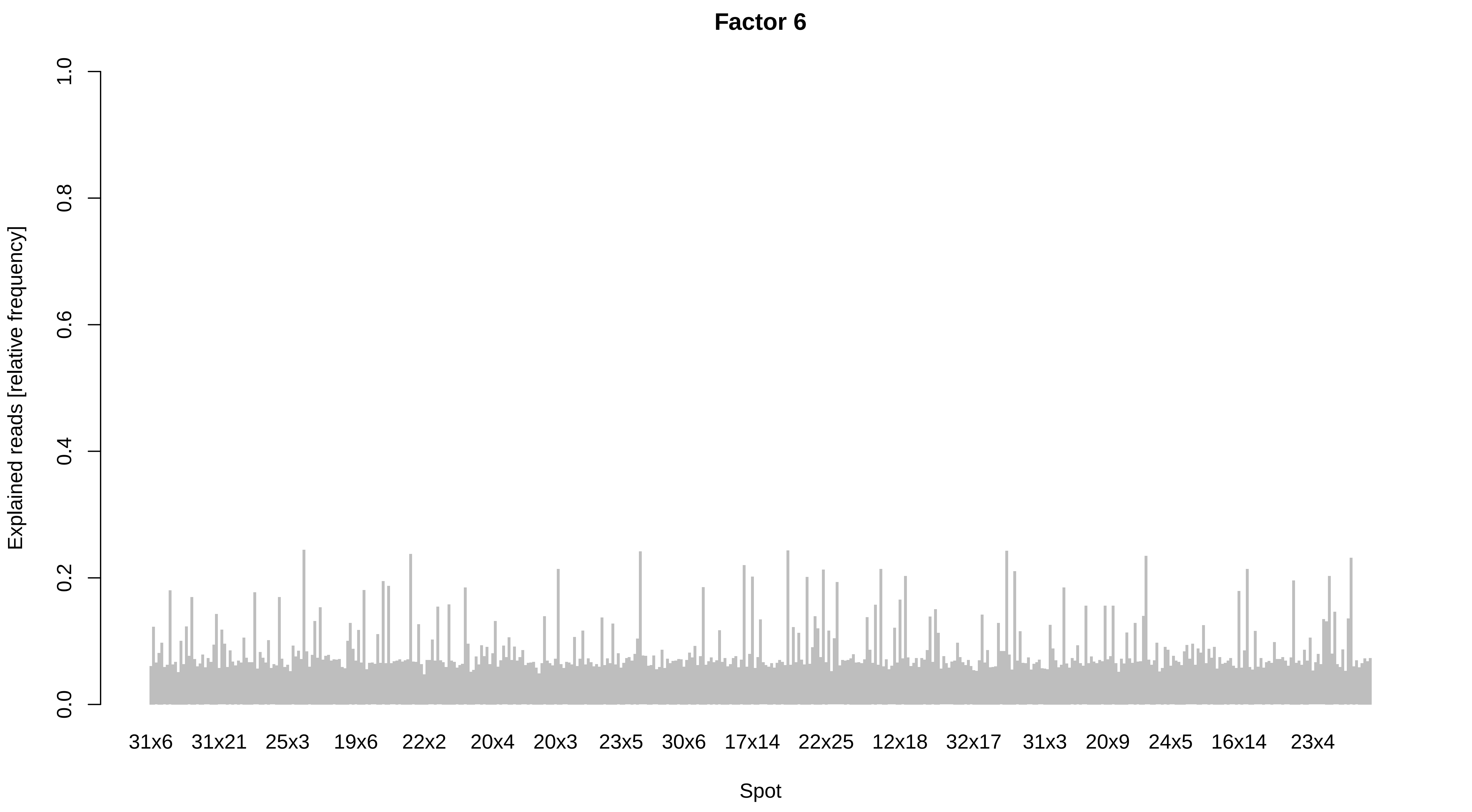
Factor 6
Explained reads [relative frequency]
0.0
0.2
0.4
0.6
0.8
1.0
31x6
31x21
25x3
19x6
22x2
20x4
20x3
23x5
30x6
17x14
22x25
12x18
32x17
31x3
20x9
24x5
16x14
23x4
Spot

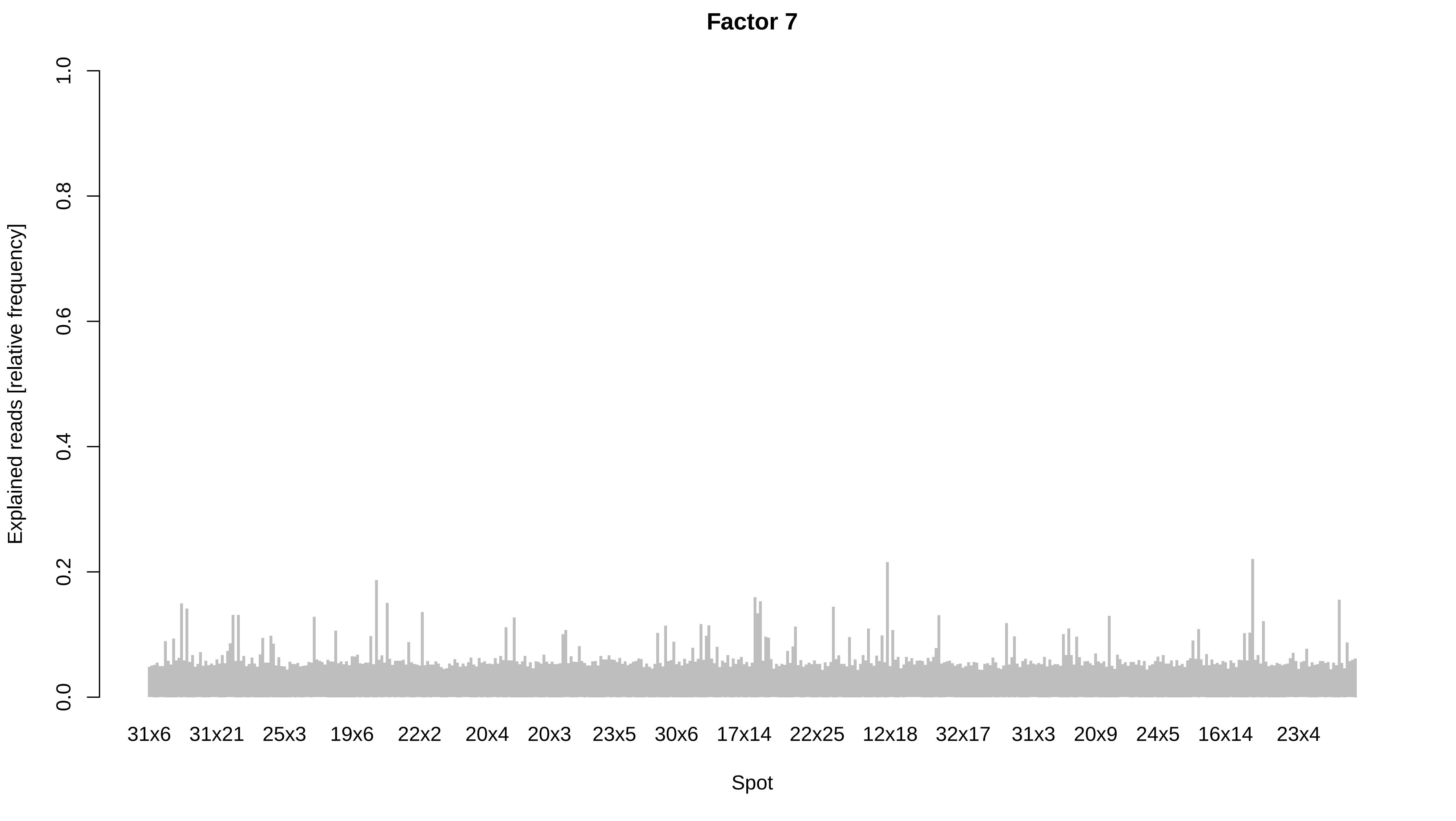
Factor 7
Explained reads [relative frequency]
0.0
0.2
0.4
0.6
0.8
1.0
31x6
31x21
25x3
19x6
22x2
20x4
20x3
23x5
30x6
17x14
22x25
12x18
32x17
31x3
20x9
24x5
16x14
23x4
Spot

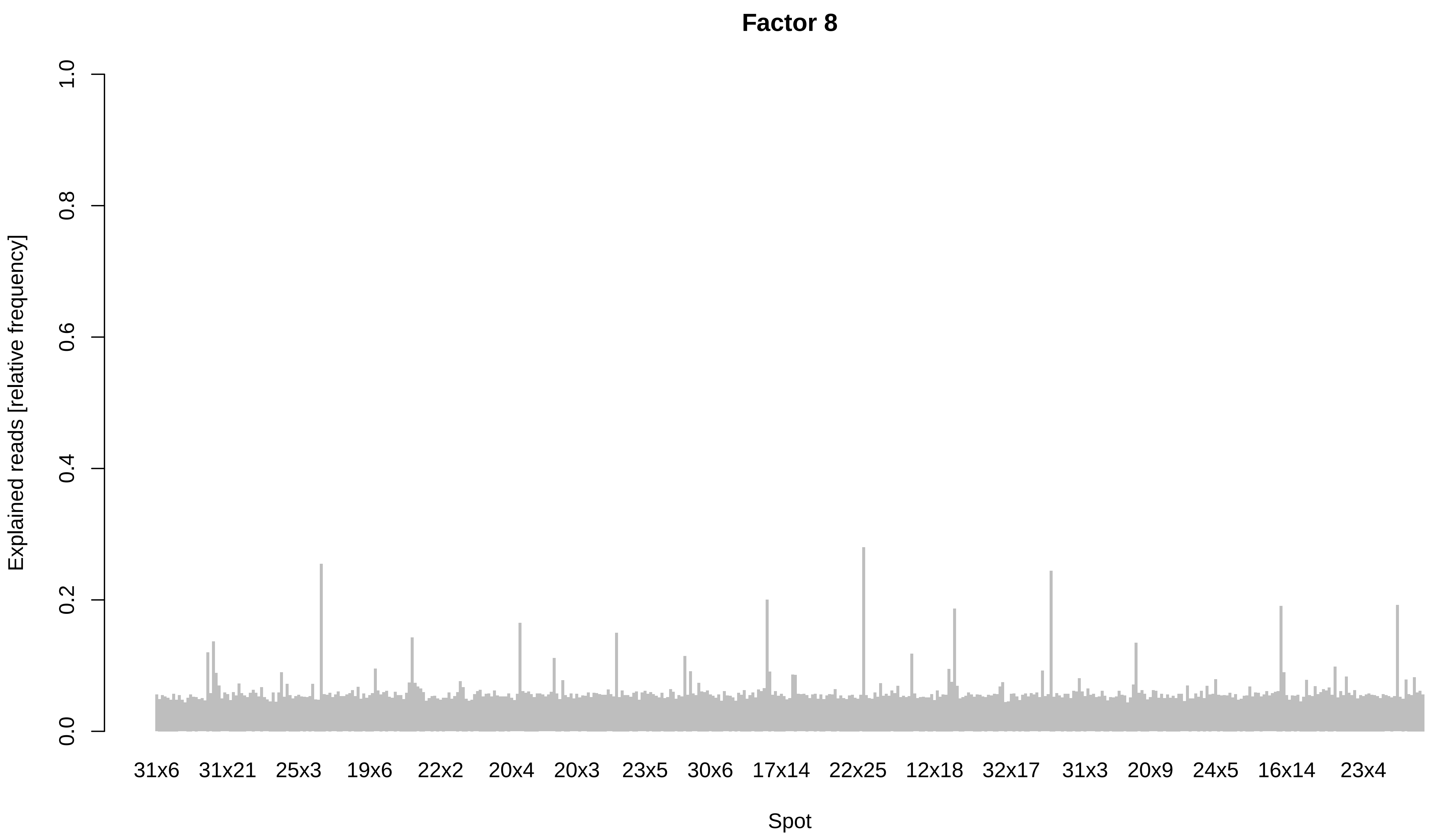
Factor 8
Explained reads [relative frequency]
0.0
0.2
0.4
0.6
0.8
1.0
31x6
31x21
25x3
19x6
22x2
20x4
20x3
23x5
30x6
17x14
22x25
12x18
32x17
31x3
20x9
24x5
16x14
23x4
Spot

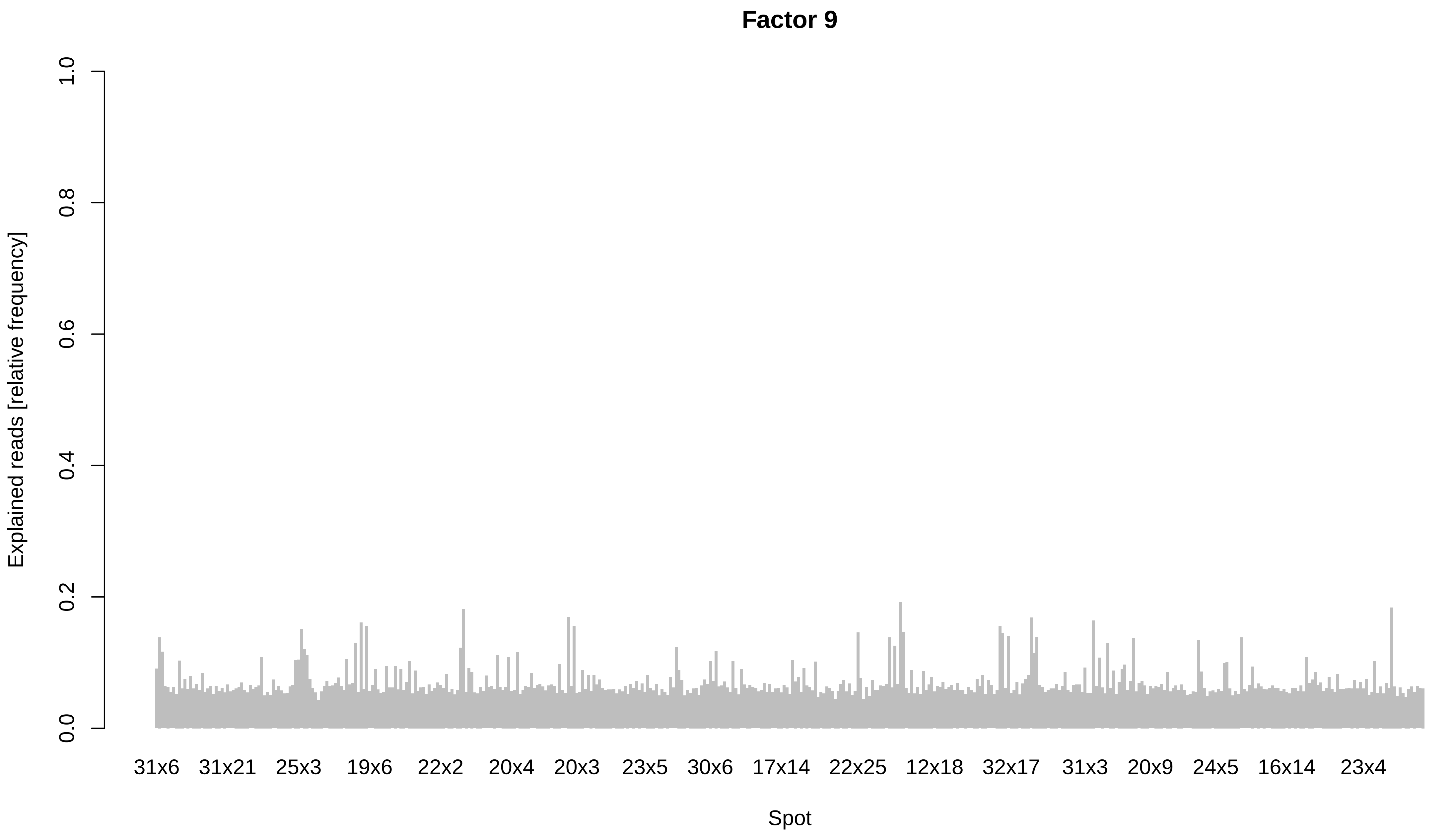
Factor 9
Explained reads [relative frequency]
0.0
0.2
0.4
0.6
0.8
1.0
31x6
31x21
25x3
19x6
22x2
20x4
20x3
23x5
30x6
17x14
22x25
12x18
32x17
31x3
20x9
24x5
16x14
23x4
Spot

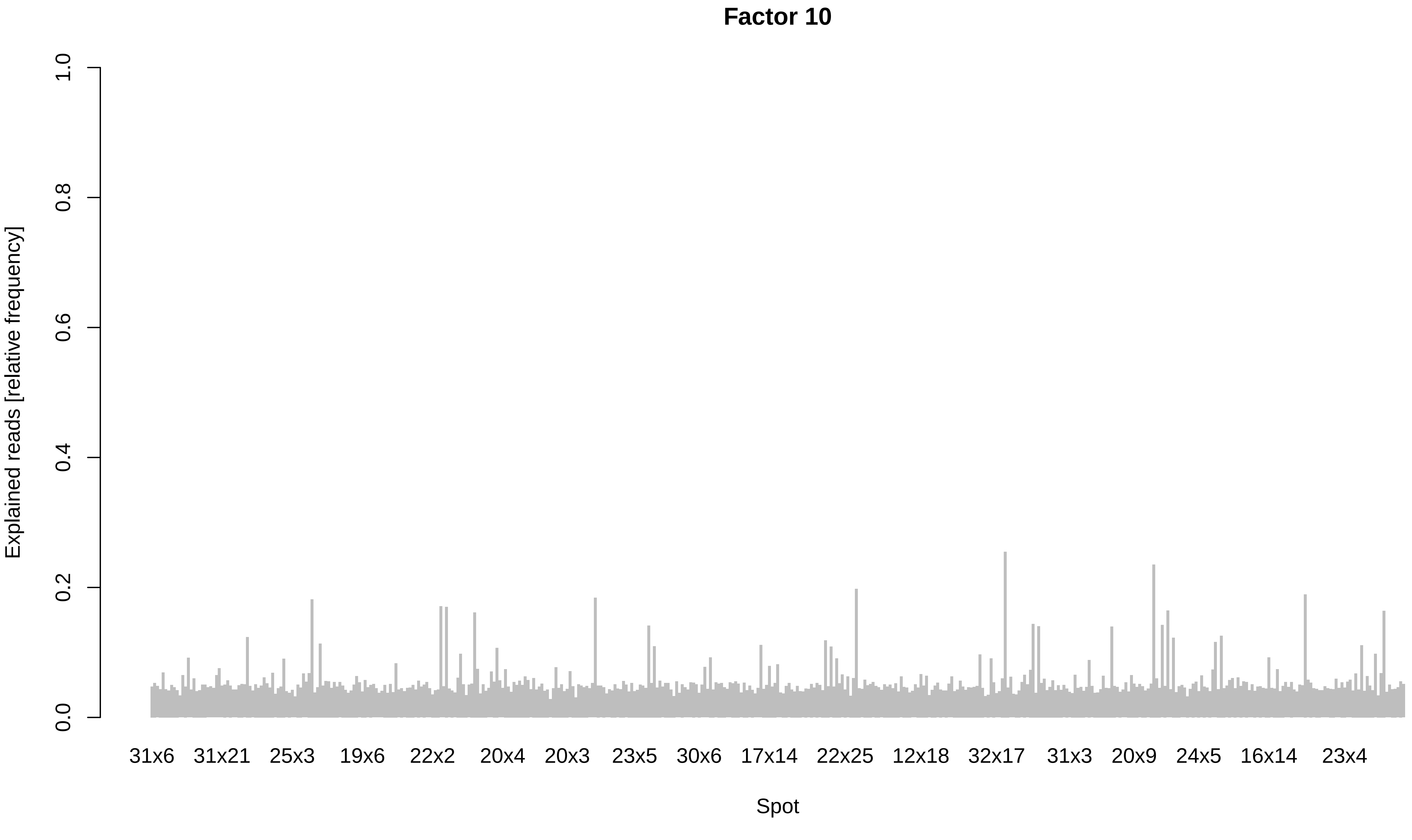
Factor 10
Explained reads [relative frequency]
0.0
0.2
0.4
0.6
0.8
1.0
31x6
31x21
25x3
19x6
22x2
20x4
20x3
23x5
30x6
17x14
22x25
12x18
32x17
31x3
20x9
24x5
16x14
23x4
Spot

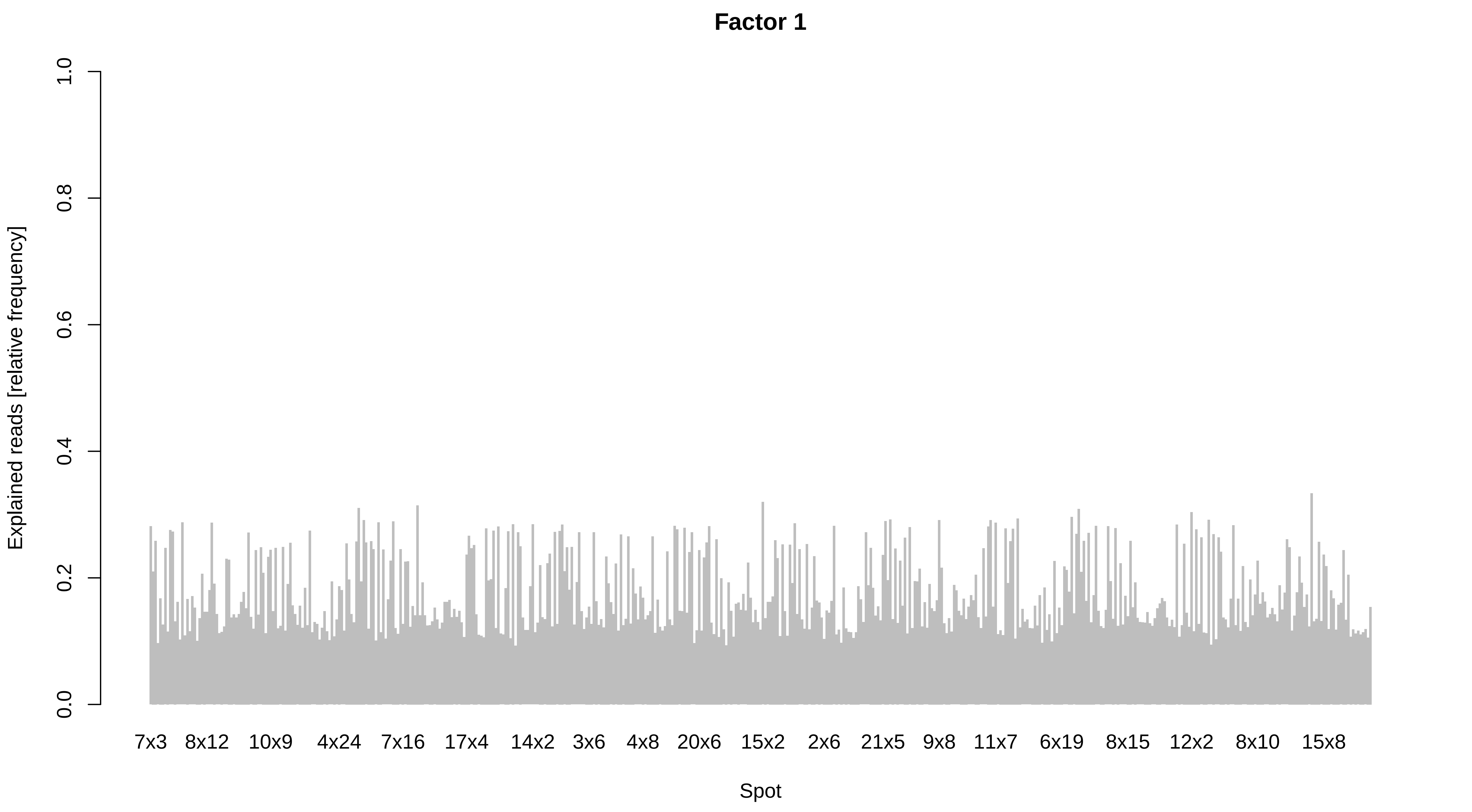

Factor 1
Explained reads [relative frequency]
0.0
0.2
0.4
0.6
0.8
1.0
7x3
8x12
10x9
4x24
7x16
17x4
14x2
3x6
4x8
20x6
15x2
2x6
21x5
9x8
11x7
6x19
8x15
12x2
8x10
15x8
Spot

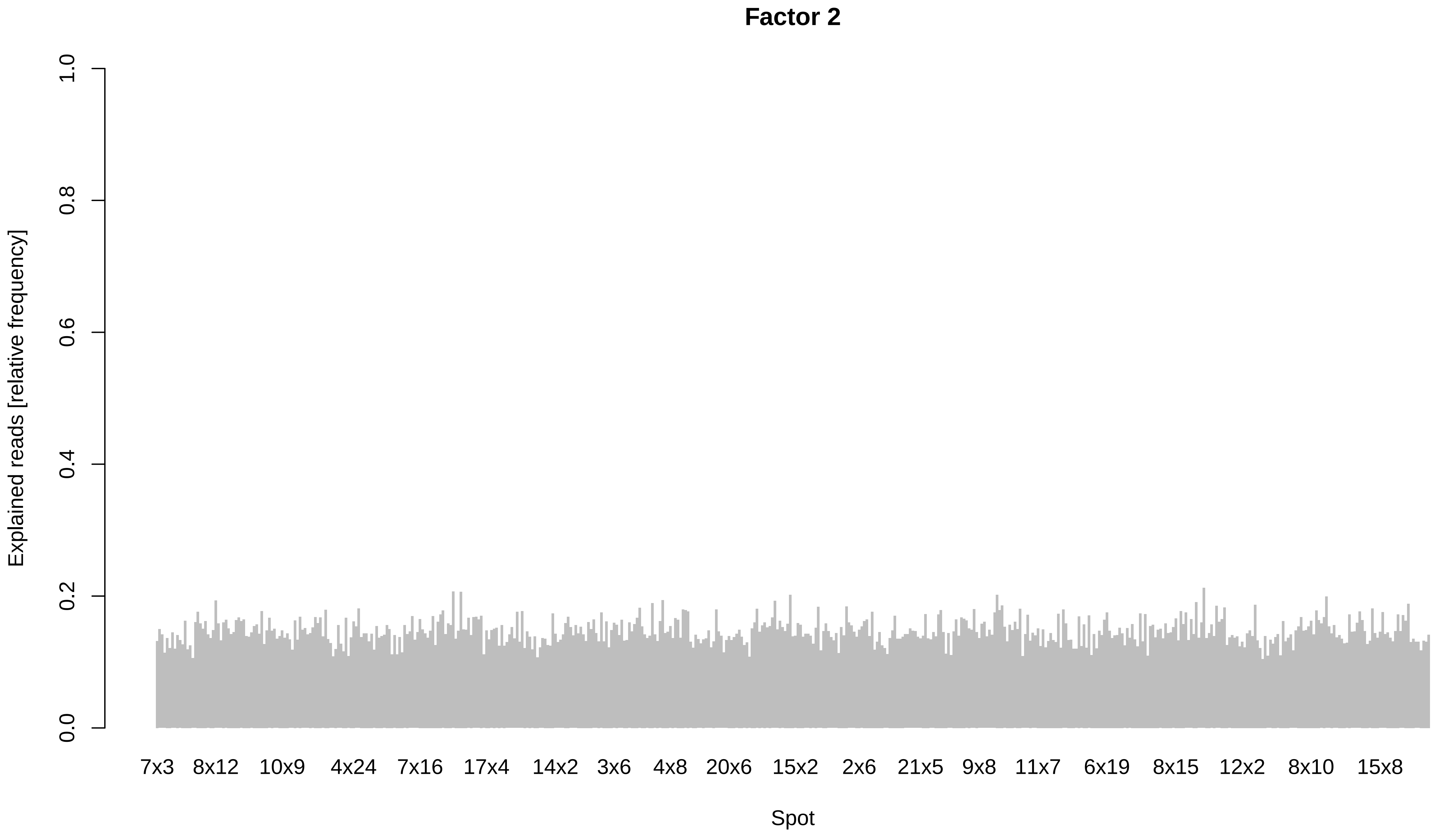

Factor 2
Explained reads [relative frequency]
1.0
0.8
0.6
0.4
0.2
0.0
7x3
8x12
10x9
4x24
7x16
17x4
14x2
3x6
4x8
20x6
15x2
2x6
21x5
9x8
11x7
6x19
8x15
12x2
8x10
15x8
Spot

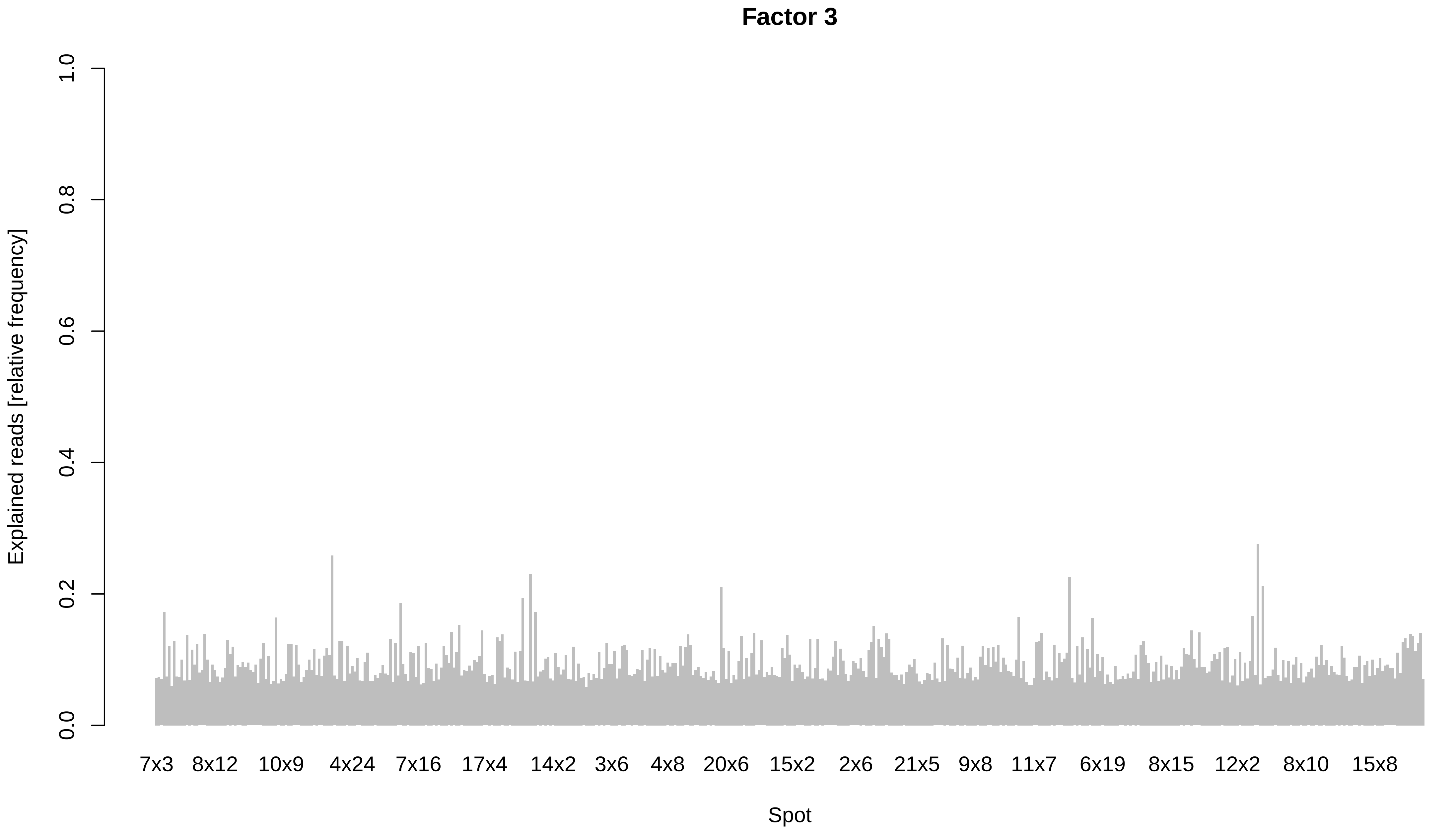

Factor 3
Explained reads [relative frequency]
0.0
0.2
0.4
0.6
0.8
1.0
7x3
8x12
10x9
4x24
7x16
17x4
14x2
3x6
4x8
20x6
15x2
2x6
21x5
9x8
11x7
6x19
8x15
12x2
8x10
15x8
Spot

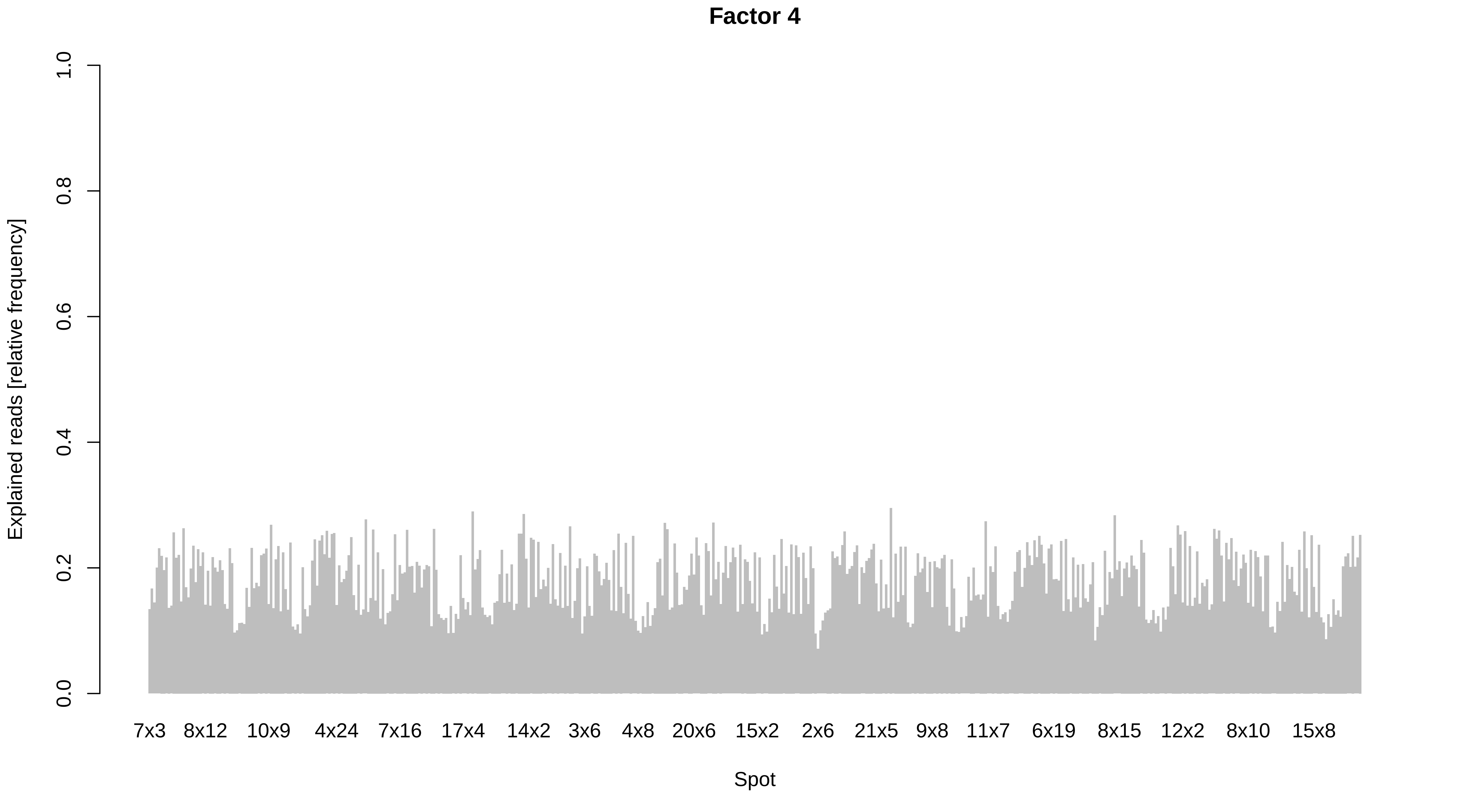
Factor 4
Explained reads [relative frequency]
0.0
0.2
0.4
0.6
0.8
1.0
7x3
8x12
10x9
4x24
7x16
17x4
14x2
3x6
4x8
20x6
15x2
2x6
21x5
9x8
11x7
6x19
8x15
12x2
8x10
15x8
Spot

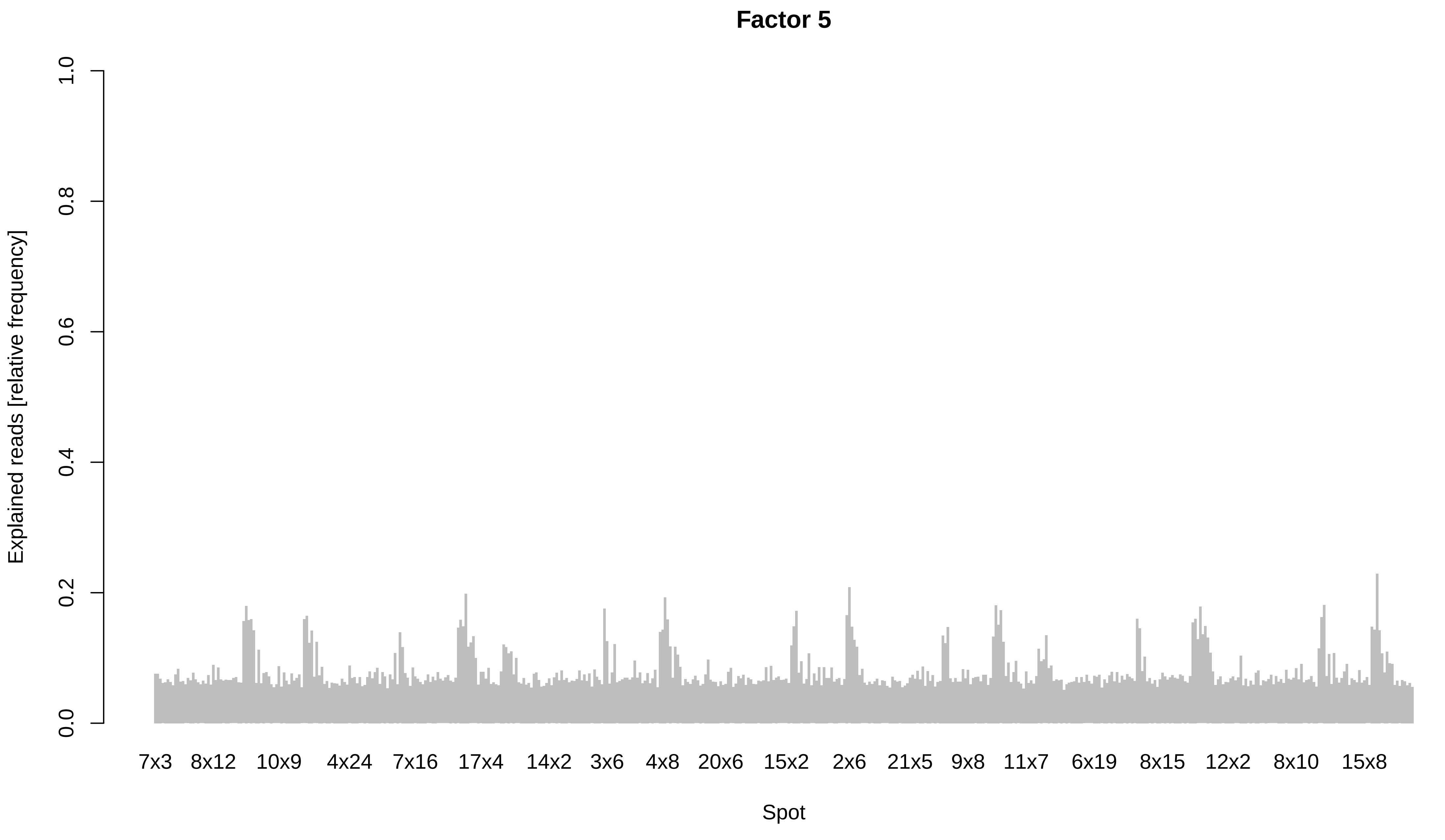
Factor 5
Explained reads [relative frequency]
0.0
0.2
0.4
0.6
0.8
1.0
7x3
8x12
10x9
4x24
7x16
17x4
14x2
3x6
4x8
20x6
15x2
2x6
21x5
9x8
11x7
6x19
8x15
12x2
8x10
15x8
Spot

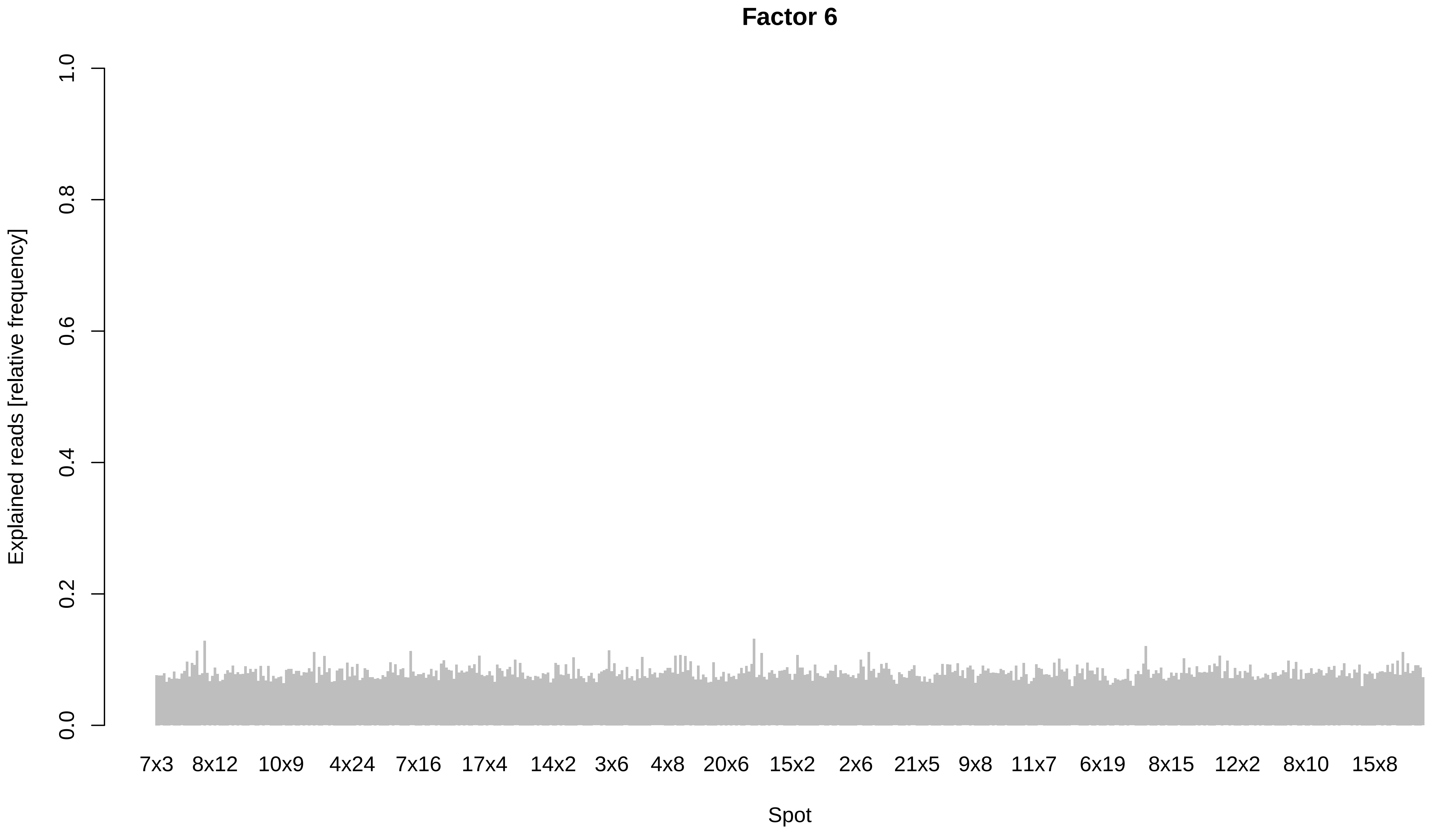
Factor 6
Explained reads [relative frequency]
1.0
0.8
0.6
0.4
0.2
0.0
7x3
8x12
10x9
4x24
7x16
17x4
14x2
3x6
4x8
20x6
15x2
2x6
21x5
9x8
11x7
6x19
8x15
12x2
8x10
15x8
Spot

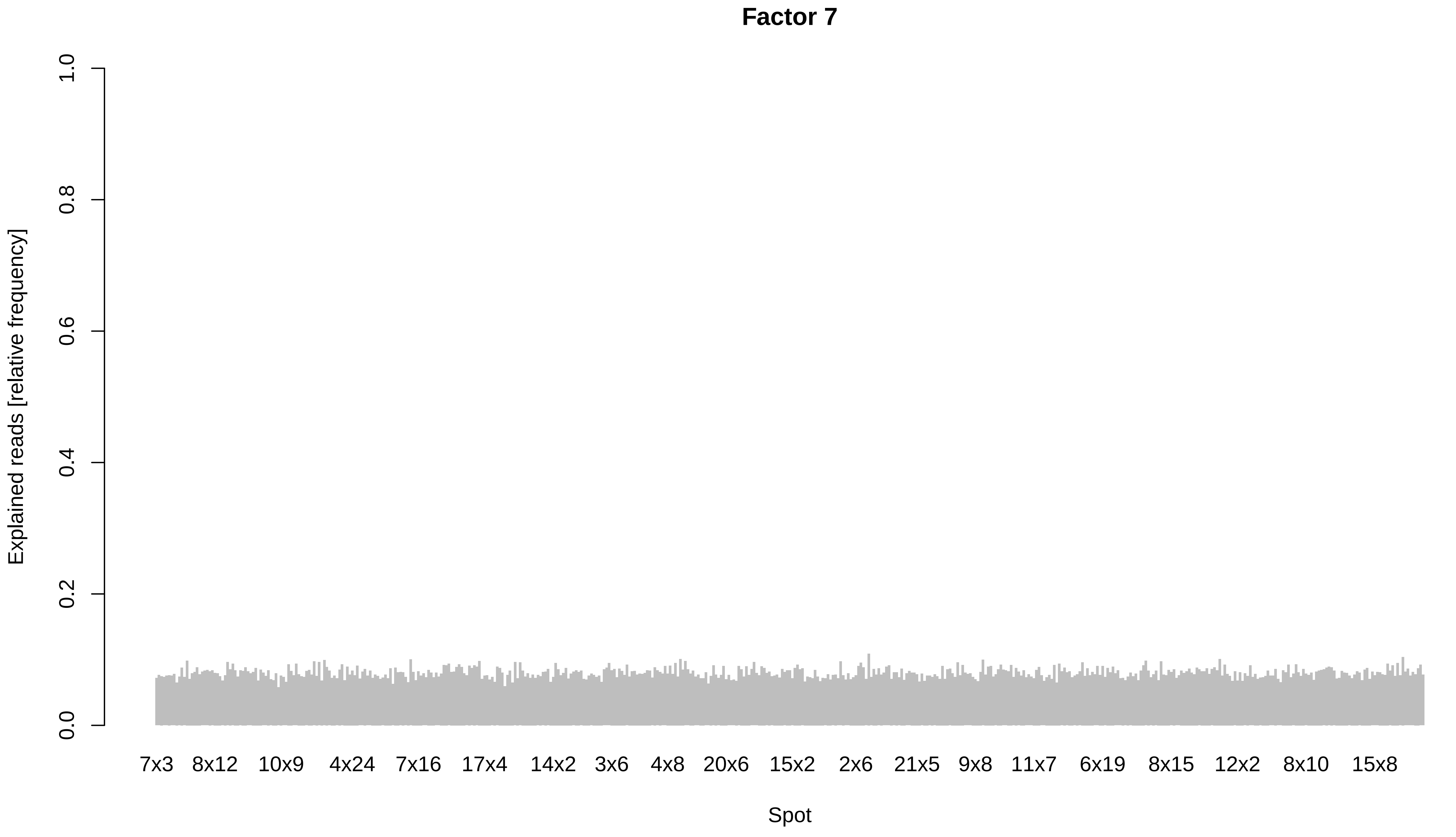

Factor 7
Explained reads [relative frequency]
0.0
0.2
0.4
0.6
0.8
1.0
7x3
8x12
10x9
4x24
7x16
17x4
14x2
3x6
4x8
20x6
15x2
2x6
21x5
9x8
11x7
6x19
8x15
12x2
8x10
15x8
Spot

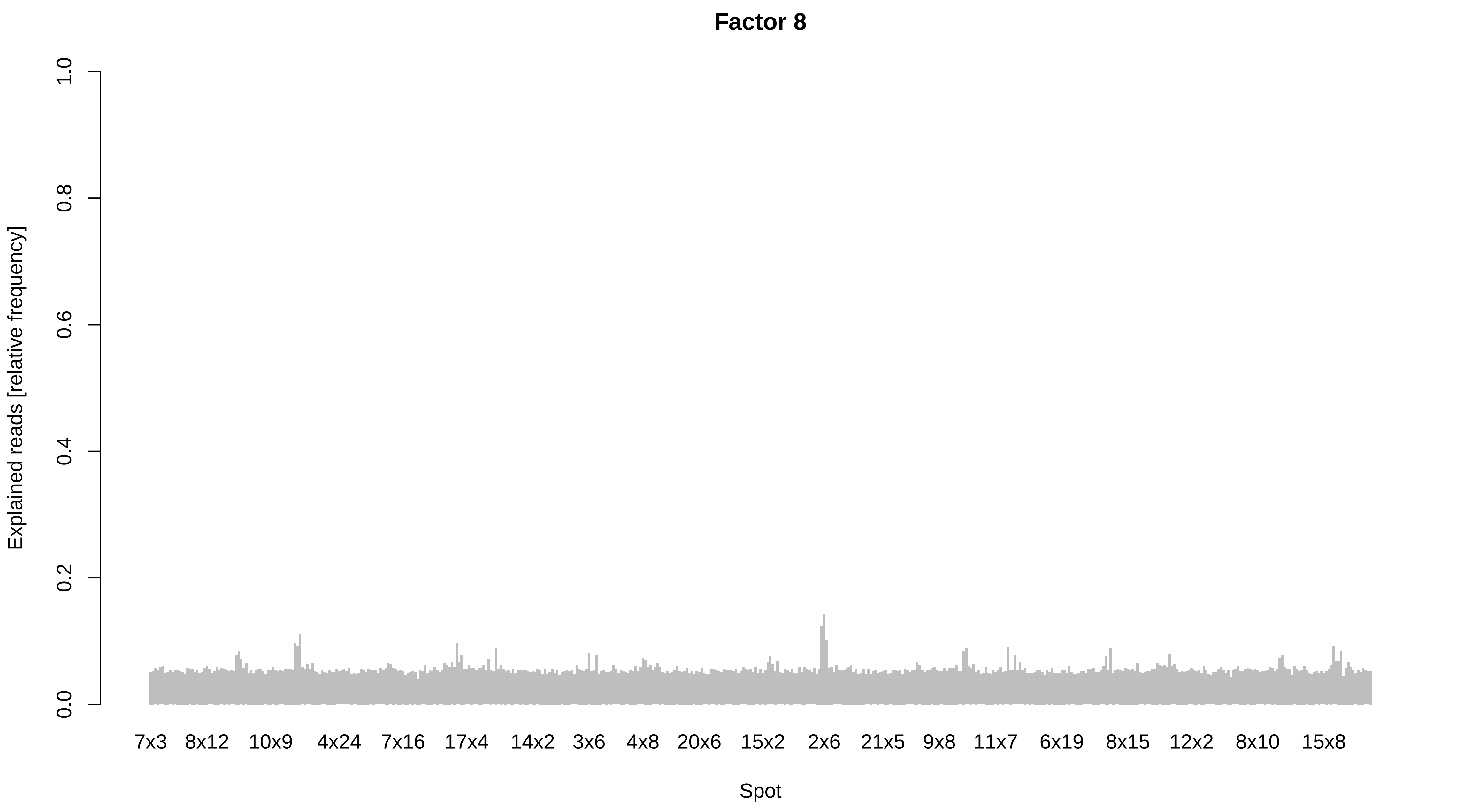
Factor 8
Explained reads [relative frequency]
0.0
0.2
0.4
0.6
0.8
1.0
7x3
8x12
10x9
4x24
7x16
17x4
14x2
3x6
4x8
20x6
15x2
2x6
21x5
9x8
11x7
6x19
8x15
12x2
8x10
15x8
Spot

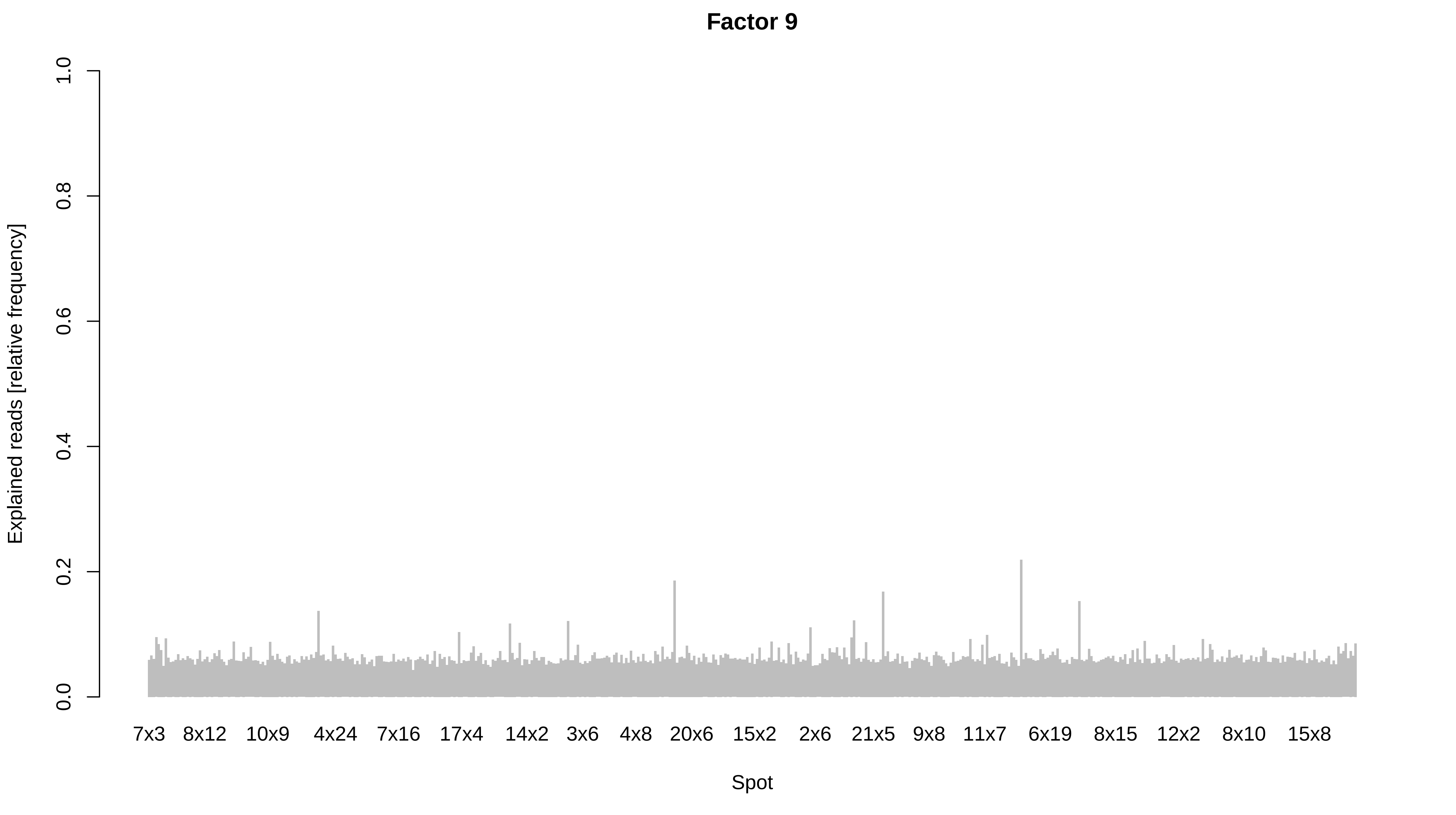
Factor 9
Explained reads [relative frequency]
0.0
0.2
0.4
0.6
0.8
1.0
7x3
8x12
10x9
4x24
7x16
17x4
14x2
3x6
4x8
20x6
15x2
2x6
21x5
9x8
11x7
6x19
8x15
12x2
8x10
15x8
Spot

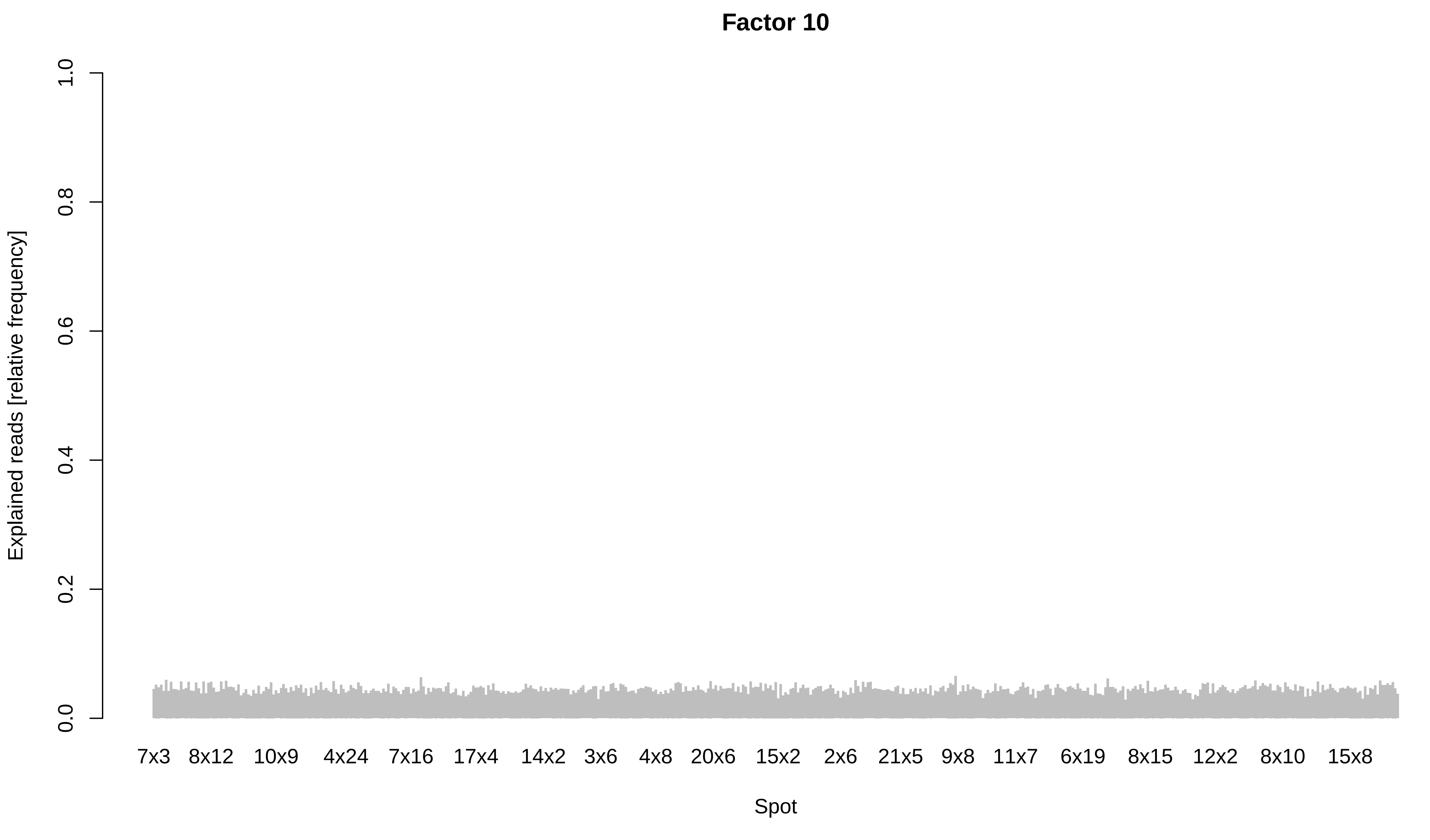

Factor 10
Explained reads [relative frequency]
0.0
0.2
0.4
0.6
0.8
1.0
7x3
8x12
10x9
4x24
7x16
17x4
14x2
3x6
4x8
20x6
15x2
2x6
21x5
9x8
11x7
6x19
8x15
12x2
8x10
15x8
Spot
