# Supplementary material for: Spatial maps of prostate cancer transcriptomes reveal an unexplored landscape of heterogeneity
Source: Nat Commun. 2018 Jun 20;9:2419. doi: 10.1038/s41467-018-04724-5 (PMC6010471; doi:10.1038/s41467-018-04724-5)
Supplement: Supplementary file 9 — Supplementary Data 6 [file 41467_2018_4724_MOESM9_ESM.zip › Supplementary Dataset 3/top-genes.pdf]

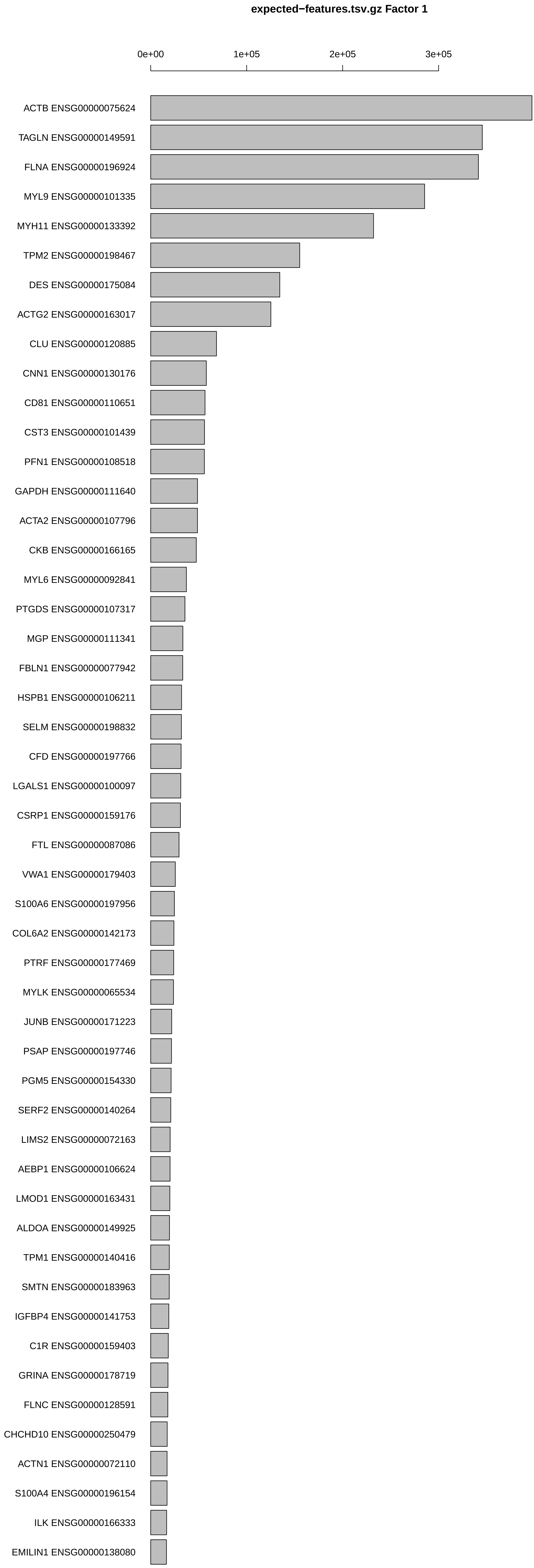
expected−features.tsv.gz Factor 1
0e+00
1e+05
2e+05
3e+05
ACTB ENSG00000075624
TAGLN ENSG00000149591
FLNA ENSG00000196924
MYL9 ENSG00000101335
MYH11 ENSG00000133392
TPM2 ENSG00000198467
DES ENSG00000175084
ACTG2 ENSG00000163017
CLU ENSG00000120885
CNN1 ENSG00000130176
CD81 ENSG00000110651
CST3 ENSG00000101439
PFN1 ENSG00000108518
GAPDH ENSG00000111640
ACTA2 ENSG00000107796
CKB ENSG00000166165
MYL6 ENSG00000092841
PTGDS ENSG00000107317
MGP ENSG00000111341
FBLN1 ENSG00000077942
HSPB1 ENSG00000106211
SELM ENSG00000198832
CFD ENSG00000197766
LGALS1 ENSG00000100097
CSRP1 ENSG00000159176
FTL ENSG00000087086
VWA1 ENSG00000179403
S100A6 ENSG00000197956
COL6A2 ENSG00000142173
PTRF ENSG00000177469
MYLK ENSG00000065534
JUNB ENSG00000171223
PSAP ENSG00000197746
PGM5 ENSG00000154330
SERF2 ENSG00000140264
LIMS2 ENSG00000072163
AEBP1 ENSG00000106624
LMOD1 ENSG00000163431
ALDOA ENSG00000149925
TPM1 ENSG00000140416
SMTN ENSG00000183963
IGFBP4 ENSG00000141753
C1R ENSG00000159403
GRINA ENSG00000178719
FLNC ENSG00000128591
CHCHD10 ENSG00000250479
ACTN1 ENSG00000072110
S100A4 ENSG00000196154
ILK ENSG00000166333
EMILIN1 ENSG00000138080

expected−features.tsv.gz Factor 2

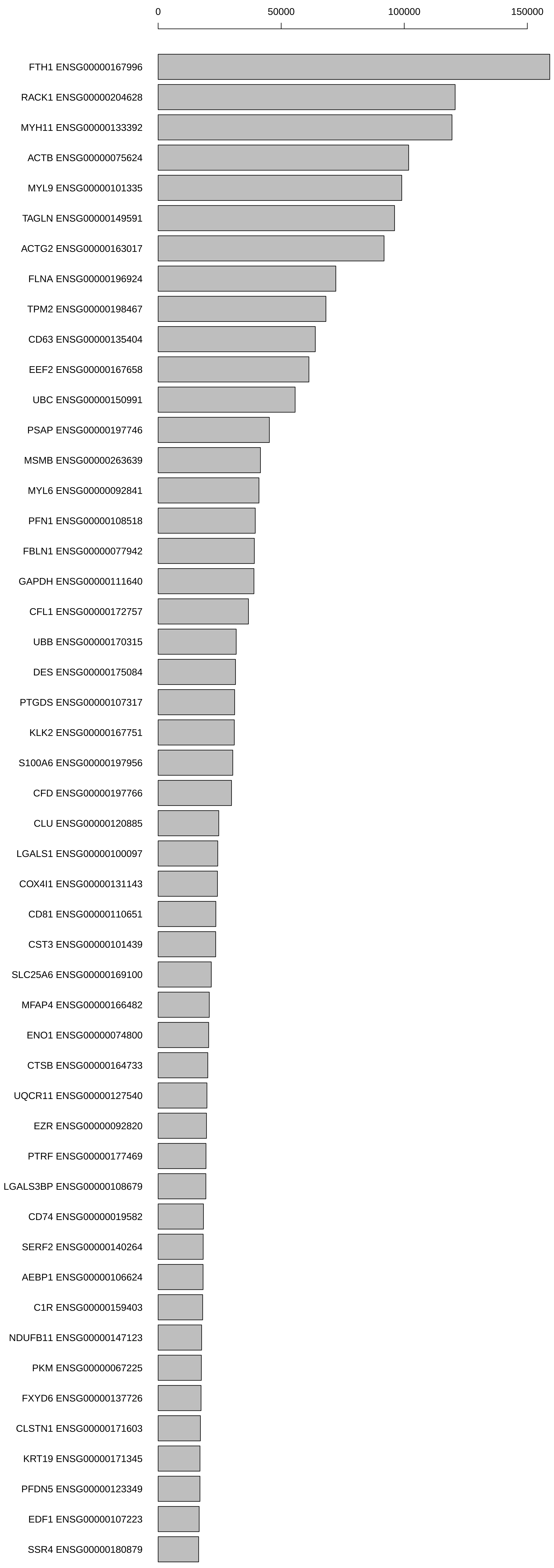

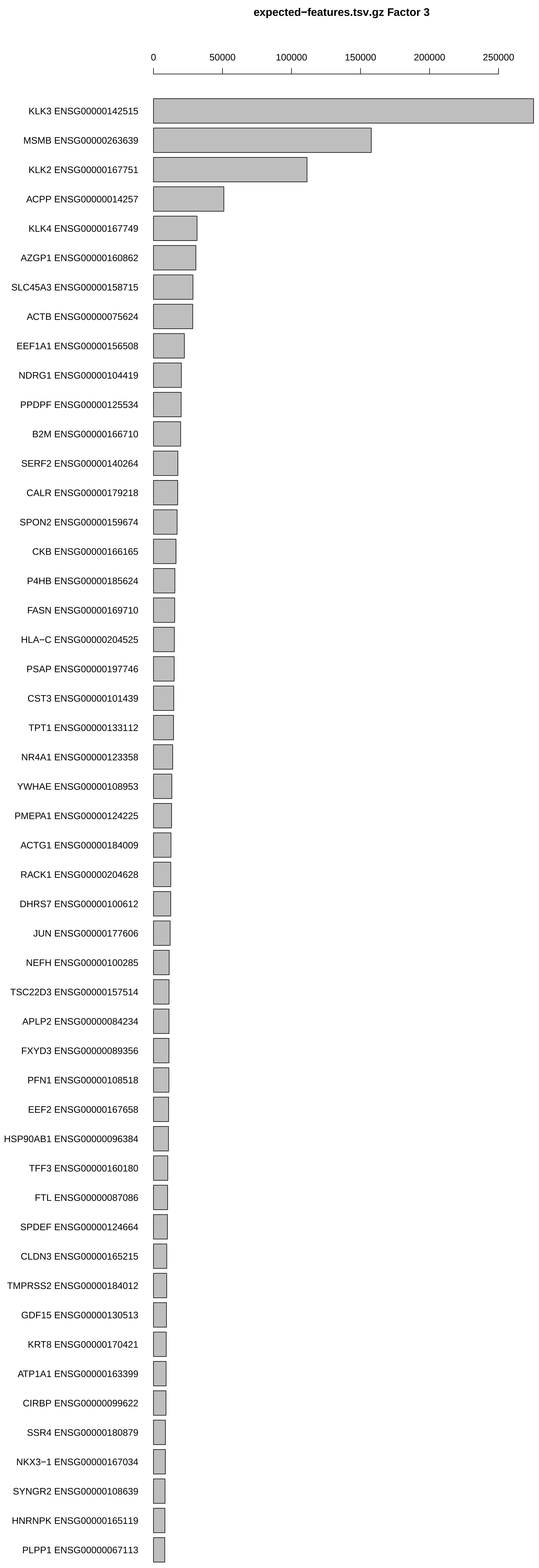
expected−features.tsv.gz Factor 3
0
50000
100000
150000
200000
250000
KLK3 ENSG00000142515
MSMB ENSG00000263639
KLK2 ENSG00000167751
ACPP ENSG00000014257
KLK4 ENSG00000167749
AZGP1 ENSG00000160862
SLC45A3 ENSG00000158715
ACTB ENSG00000075624
EEF1A1 ENSG00000156508
NDRG1 ENSG00000104419
PPDPF ENSG00000125534
B2M ENSG00000166710
SERF2 ENSG00000140264
CALR ENSG00000179218
SPON2 ENSG00000159674
CKB ENSG00000166165
P4HB ENSG00000185624
FASN ENSG00000169710
HLA−C ENSG00000204525
PSAP ENSG00000197746
CST3 ENSG00000101439
TPT1 ENSG00000133112
NR4A1 ENSG00000123358
YWHAE ENSG00000108953
PMEPA1 ENSG00000124225
ACTG1 ENSG00000184009
RACK1 ENSG00000204628
DHRS7 ENSG00000100612
JUN ENSG00000177606
NEFH ENSG00000100285
TSC22D3 ENSG00000157514
APLP2 ENSG00000084234
FXYD3 ENSG00000089356
PFN1 ENSG00000108518
EEF2 ENSG00000167658
HSP90AB1 ENSG00000096384
TFF3 ENSG00000160180
FTL ENSG00000087086
SPDEF ENSG00000124664
CLDN3 ENSG00000165215
TMPRSS2 ENSG00000184012
GDF15 ENSG00000130513
KRT8 ENSG00000170421
ATP1A1 ENSG00000163399
CIRBP ENSG00000099622
SSR4 ENSG00000180879
NKX3−1 ENSG00000167034
SYNGR2 ENSG00000108639
HNRNPK ENSG00000165119
PLPP1 ENSG00000067113

expected−features.tsv.gz Factor 4

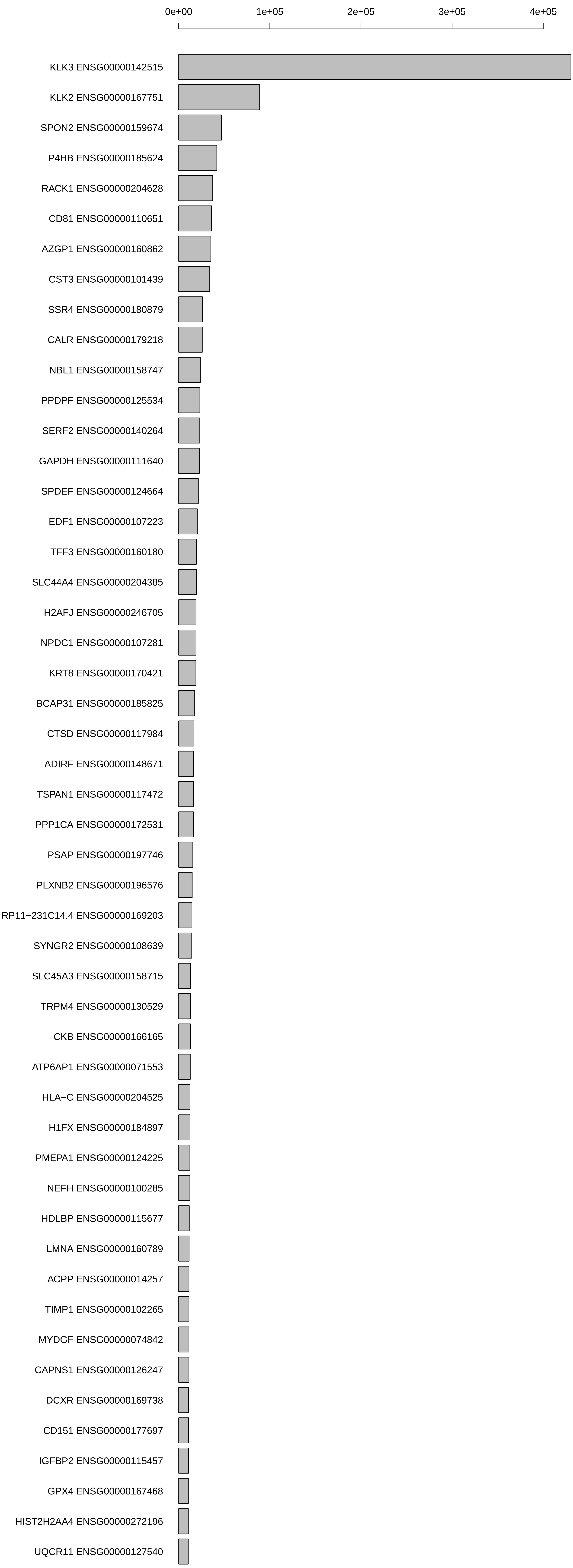

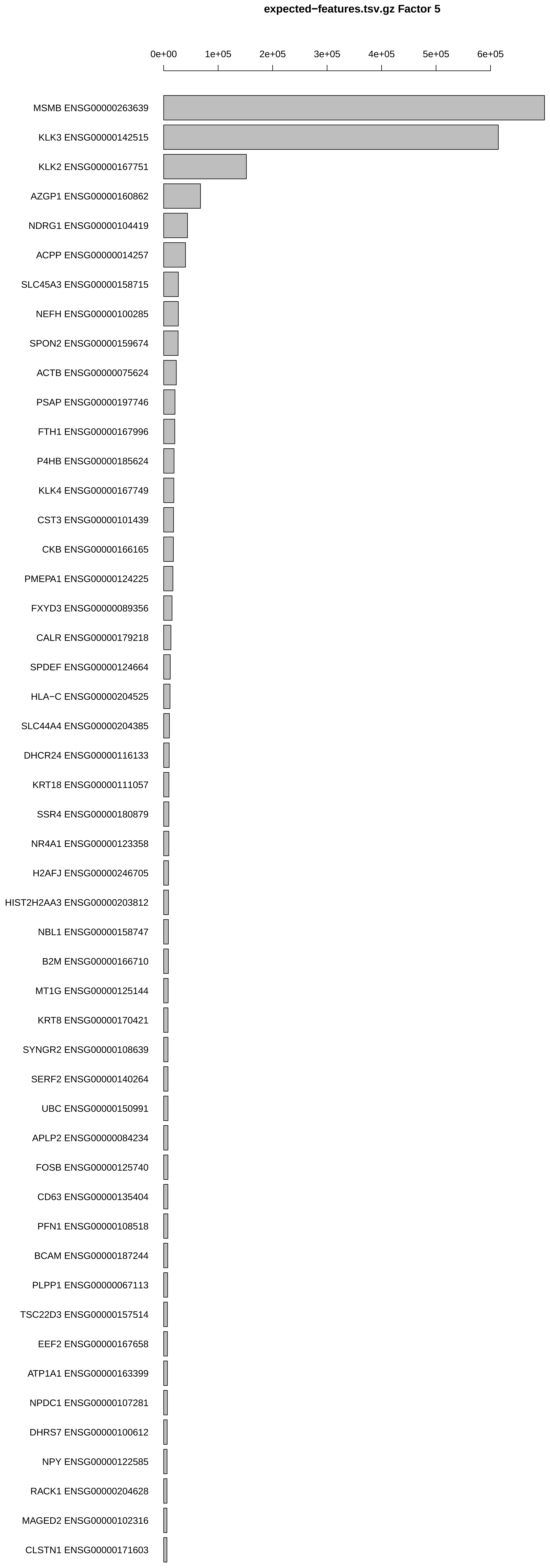
expected−features.tsv.gz Factor 5
0e+00
1e+05
2e+05
3e+05
4e+05
5e+05
6e+05
MSMB ENSG00000263639
KLK3 ENSG00000142515
KLK2 ENSG00000167751
AZGP1 ENSG00000160862
NDRG1 ENSG00000104419
ACPP ENSG00000014257
SLC45A3 ENSG00000158715
NEFH ENSG00000100285
SPON2 ENSG00000159674
ACTB ENSG00000075624
PSAP ENSG00000197746
FTH1 ENSG00000167996
P4HB ENSG00000185624
KLK4 ENSG00000167749
CST3 ENSG00000101439
CKB ENSG00000166165
PMEPA1 ENSG00000124225
FXYD3 ENSG00000089356
CALR ENSG00000179218
SPDEF ENSG00000124664
HLA−C ENSG00000204525
SLC44A4 ENSG00000204385
DHCR24 ENSG00000116133
KRT18 ENSG00000111057
SSR4 ENSG00000180879
NR4A1 ENSG00000123358
H2AFJ ENSG00000246705
HIST2H2AA3 ENSG00000203812
NBL1 ENSG00000158747
B2M ENSG00000166710
MT1G ENSG00000125144
KRT8 ENSG00000170421
SYNGR2 ENSG00000108639
SERF2 ENSG00000140264
UBC ENSG00000150991
APLP2 ENSG00000084234
FOSB ENSG00000125740
CD63 ENSG00000135404
PFN1 ENSG00000108518
BCAM ENSG00000187244
PLPP1 ENSG00000067113
TSC22D3 ENSG00000157514
EEF2 ENSG00000167658
ATP1A1 ENSG00000163399
NPDC1 ENSG00000107281
DHRS7 ENSG00000100612
NPY ENSG00000122585
RACK1 ENSG00000204628
MAGED2 ENSG00000102316
CLSTN1 ENSG00000171603

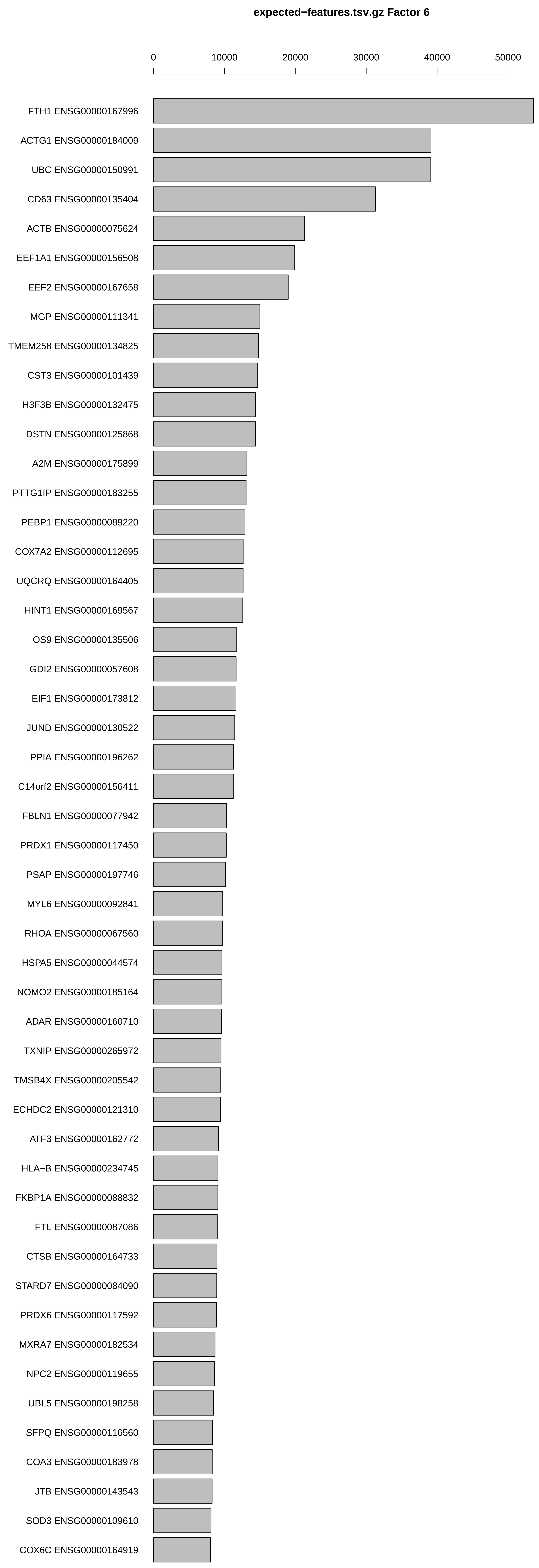

expected−features.tsv.gz Factor 6
0
10000
20000
30000
40000
50000
FTH1 ENSG00000167996
ACTG1 ENSG00000184009
UBC ENSG00000150991
CD63 ENSG00000135404
ACTB ENSG00000075624
EEF1A1 ENSG00000156508
EEF2 ENSG00000167658
MGP ENSG00000111341
TMEM258 ENSG00000134825
CST3 ENSG00000101439
H3F3B ENSG00000132475
DSTN ENSG00000125868
A2M ENSG00000175899
PTTG1IP ENSG00000183255
PEBP1 ENSG00000089220
COX7A2 ENSG00000112695
UQCRQ ENSG00000164405
HINT1 ENSG00000169567
OS9 ENSG00000135506
GDI2 ENSG00000057608
EIF1 ENSG00000173812
JUND ENSG00000130522
PPIA ENSG00000196262
C14orf2 ENSG00000156411
FBLN1 ENSG00000077942
PRDX1 ENSG00000117450
PSAP ENSG00000197746
MYL6 ENSG00000092841
RHOA ENSG00000067560
HSPA5 ENSG00000044574
NOMO2 ENSG00000185164
ADAR ENSG00000160710
TXNIP ENSG00000265972
TMSB4X ENSG00000205542
ECHDC2 ENSG00000121310
ATF3 ENSG00000162772
HLA−B ENSG00000234745
FKBP1A ENSG00000088832
FTL ENSG00000087086
CTSB ENSG00000164733
STARD7 ENSG00000084090
PRDX6 ENSG00000117592
MXRA7 ENSG00000182534
NPC2 ENSG00000119655
UBL5 ENSG00000198258
SFPQ ENSG00000116560
COA3 ENSG00000183978
JTB ENSG00000143543
SOD3 ENSG00000109610
COX6C ENSG00000164919

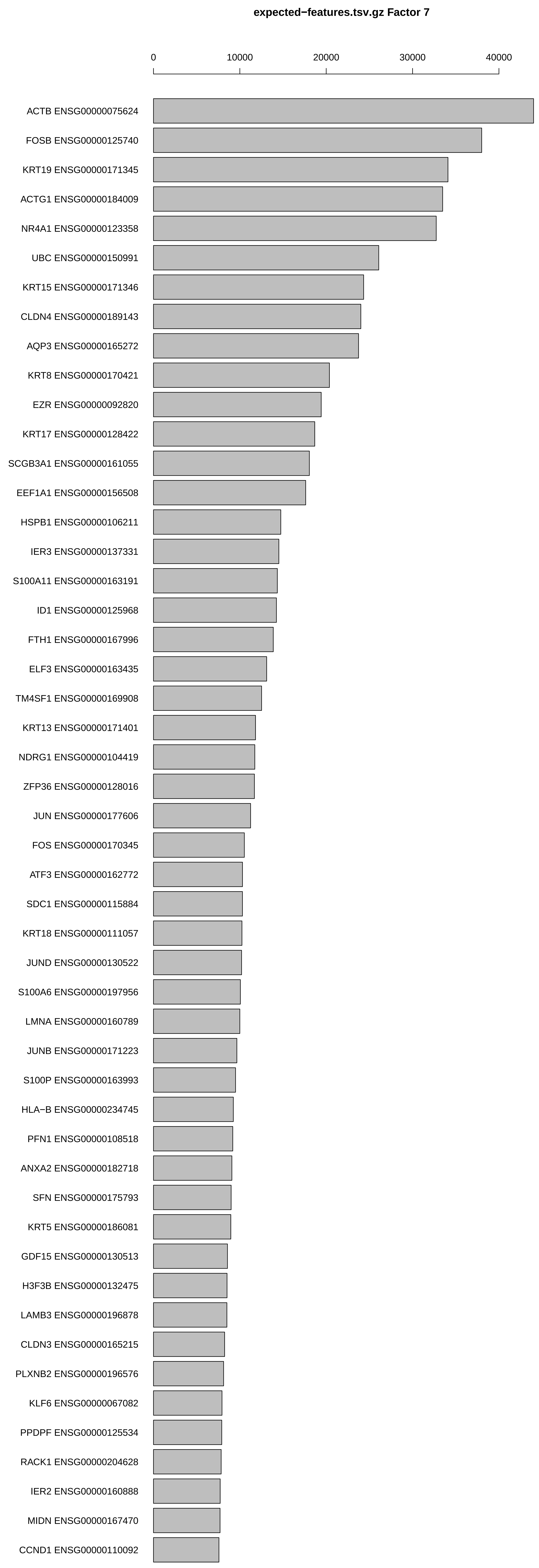
expected−features.tsv.gz Factor 7
0
10000
20000
30000
40000
ACTB ENSG00000075624
FOSB ENSG00000125740
KRT19 ENSG00000171345
ACTG1 ENSG00000184009
NR4A1 ENSG00000123358
UBC ENSG00000150991
KRT15 ENSG00000171346
CLDN4 ENSG00000189143
AQP3 ENSG00000165272
KRT8 ENSG00000170421
EZR ENSG00000092820
KRT17 ENSG00000128422
SCGB3A1 ENSG00000161055
EEF1A1 ENSG00000156508
HSPB1 ENSG00000106211
IER3 ENSG00000137331
S100A11 ENSG00000163191
ID1 ENSG00000125968
FTH1 ENSG00000167996
ELF3 ENSG00000163435
TM4SF1 ENSG00000169908
KRT13 ENSG00000171401
NDRG1 ENSG00000104419
ZFP36 ENSG00000128016
JUN ENSG00000177606
FOS ENSG00000170345
ATF3 ENSG00000162772
SDC1 ENSG00000115884
KRT18 ENSG00000111057
JUND ENSG00000130522
S100A6 ENSG00000197956
LMNA ENSG00000160789
JUNB ENSG00000171223
S100P ENSG00000163993
HLA−B ENSG00000234745
PFN1 ENSG00000108518
ANXA2 ENSG00000182718
SFN ENSG00000175793
KRT5 ENSG00000186081
GDF15 ENSG00000130513
H3F3B ENSG00000132475
LAMB3 ENSG00000196878
CLDN3 ENSG00000165215
PLXNB2 ENSG00000196576
KLF6 ENSG00000067082
PPDPF ENSG00000125534
RACK1 ENSG00000204628
IER2 ENSG00000160888
MIDN ENSG00000167470
CCND1 ENSG00000110092

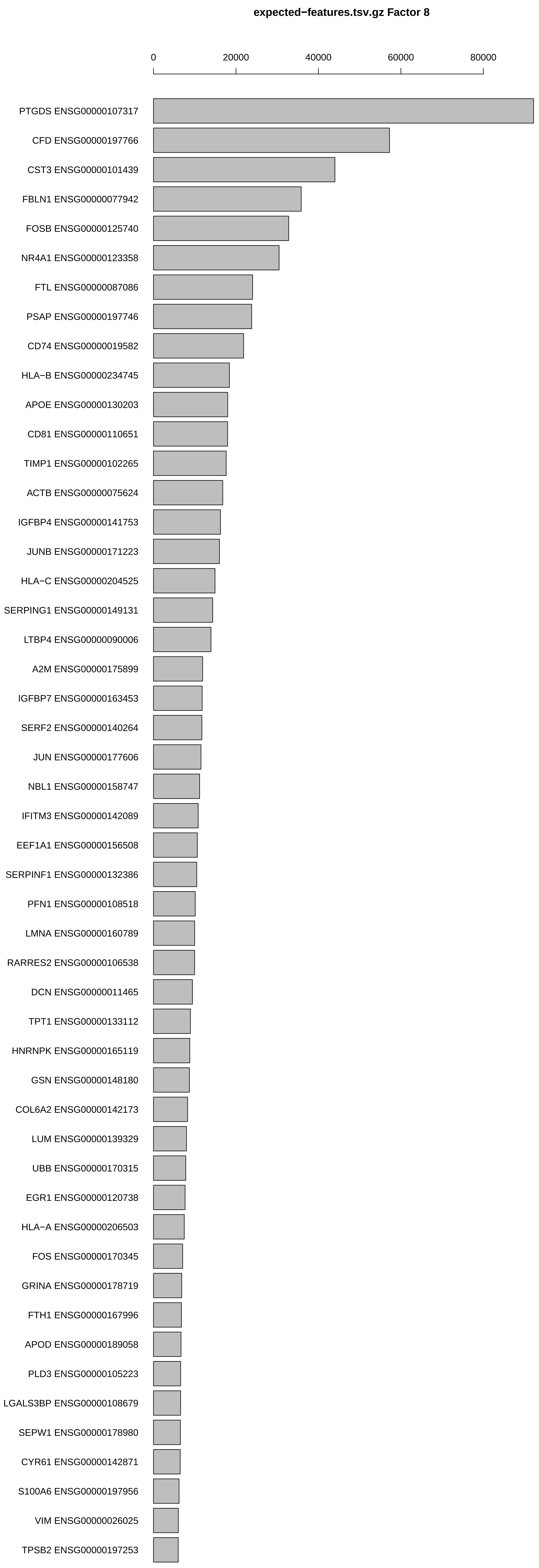

expected−features.tsv.gz Factor 8
0
20000
40000
60000
80000
PTGDS ENSG00000107317
CFD ENSG00000197766
CST3 ENSG00000101439
FBLN1 ENSG00000077942
FOSB ENSG00000125740
NR4A1 ENSG00000123358
FTL ENSG00000087086
PSAP ENSG00000197746
CD74 ENSG00000019582
HLA−B ENSG00000234745
APOE ENSG00000130203
CD81 ENSG00000110651
TIMP1 ENSG00000102265
ACTB ENSG00000075624
IGFBP4 ENSG00000141753
JUNB ENSG00000171223
HLA−C ENSG00000204525
SERPING1 ENSG00000149131
LTBP4 ENSG00000090006
A2M ENSG00000175899
IGFBP7 ENSG00000163453
SERF2 ENSG00000140264
JUN ENSG00000177606
NBL1 ENSG00000158747
IFITM3 ENSG00000142089
EEF1A1 ENSG00000156508
SERPINF1 ENSG00000132386
PFN1 ENSG00000108518
LMNA ENSG00000160789
RARRES2 ENSG00000106538
DCN ENSG00000011465
TPT1 ENSG00000133112
HNRNPK ENSG00000165119
GSN ENSG00000148180
COL6A2 ENSG00000142173
LUM ENSG00000139329
UBB ENSG00000170315
EGR1 ENSG00000120738
HLA−A ENSG00000206503
FOS ENSG00000170345
GRINA ENSG00000178719
FTH1 ENSG00000167996
APOD ENSG00000189058
PLD3 ENSG00000105223
LGALS3BP ENSG00000108679
SEPW1 ENSG00000178980
CYR61 ENSG00000142871
S100A6 ENSG00000197956
VIM ENSG00000026025
TPSB2 ENSG00000197253

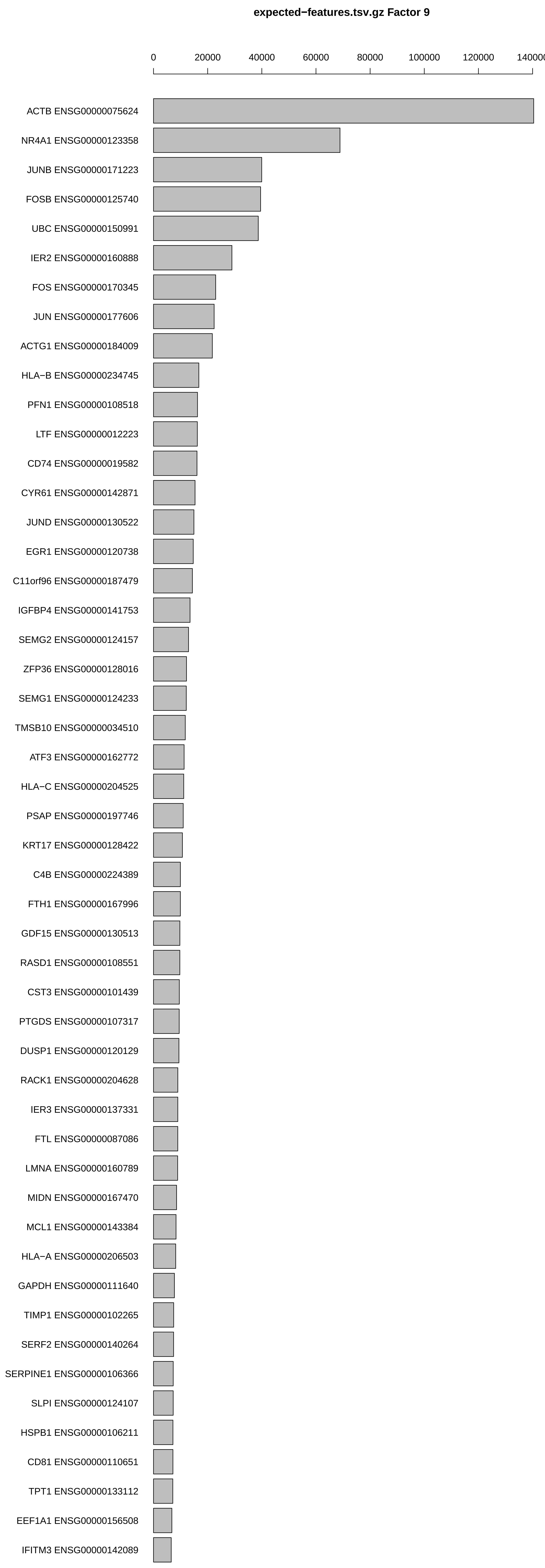

expected−features.tsv.gz Factor 9
0
20000
40000
60000
80000
100000
120000
140000
ACTB ENSG00000075624
NR4A1 ENSG00000123358
JUNB ENSG00000171223
FOSB ENSG00000125740
UBC ENSG00000150991
IER2 ENSG00000160888
FOS ENSG00000170345
JUN ENSG00000177606
ACTG1 ENSG00000184009
HLA−B ENSG00000234745
PFN1 ENSG00000108518
LTF ENSG00000012223
CD74 ENSG00000019582
CYR61 ENSG00000142871
JUND ENSG00000130522
EGR1 ENSG00000120738
C11orf96 ENSG00000187479
IGFBP4 ENSG00000141753
SEMG2 ENSG00000124157
ZFP36 ENSG00000128016
SEMG1 ENSG00000124233
TMSB10 ENSG00000034510
ATF3 ENSG00000162772
HLA−C ENSG00000204525
PSAP ENSG00000197746
KRT17 ENSG00000128422
C4B ENSG00000224389
FTH1 ENSG00000167996
GDF15 ENSG00000130513
RASD1 ENSG00000108551
CST3 ENSG00000101439
PTGDS ENSG00000107317
DUSP1 ENSG00000120129
RACK1 ENSG00000204628
IER3 ENSG00000137331
FTL ENSG00000087086
LMNA ENSG00000160789
MIDN ENSG00000167470
MCL1 ENSG00000143384
HLA−A ENSG00000206503
GAPDH ENSG00000111640
TIMP1 ENSG00000102265
SERF2 ENSG00000140264
SERPINE1 ENSG00000106366
SLPI ENSG00000124107
HSPB1 ENSG00000106211
CD81 ENSG00000110651
TPT1 ENSG00000133112
EEF1A1 ENSG00000156508
IFITM3 ENSG00000142089

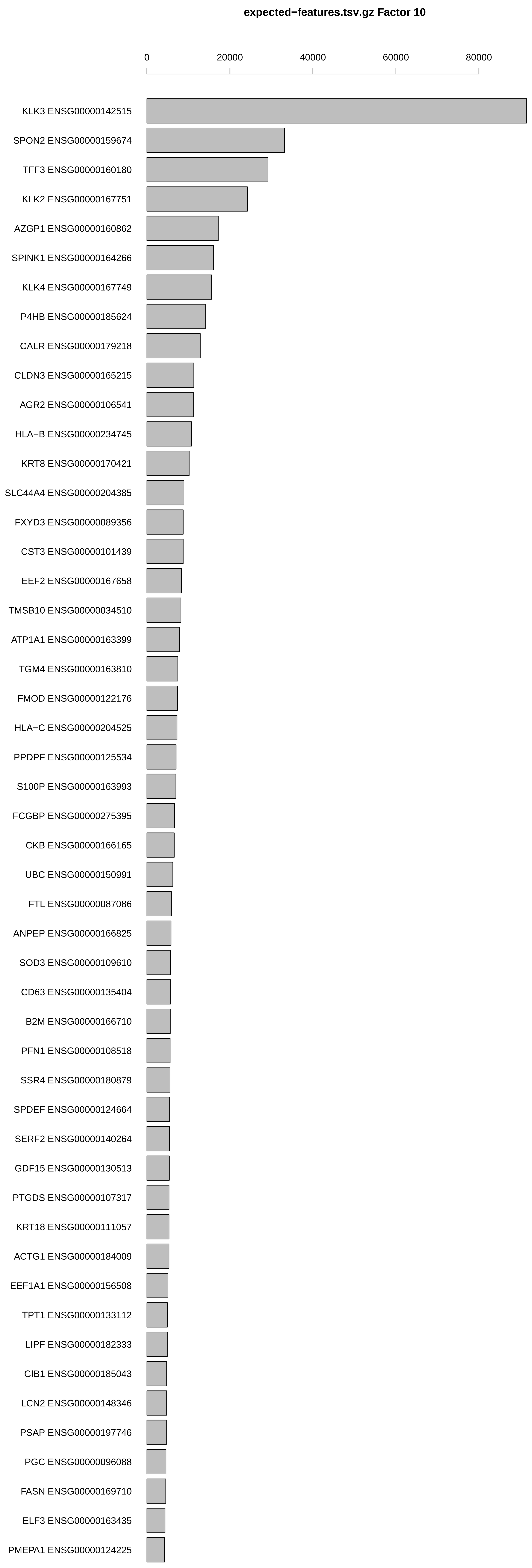
expected−features.tsv.gz Factor 10
0
20000
40000
60000
80000
KLK3 ENSG00000142515
SPON2 ENSG00000159674
TFF3 ENSG00000160180
KLK2 ENSG00000167751
AZGP1 ENSG00000160862
SPINK1 ENSG00000164266
KLK4 ENSG00000167749
P4HB ENSG00000185624
CALR ENSG00000179218
CLDN3 ENSG00000165215
AGR2 ENSG00000106541
HLA−B ENSG00000234745
KRT8 ENSG00000170421
SLC44A4 ENSG00000204385
FXYD3 ENSG00000089356
CST3 ENSG00000101439
EEF2 ENSG00000167658
TMSB10 ENSG00000034510
ATP1A1 ENSG00000163399
TGM4 ENSG00000163810
FMOD ENSG00000122176
HLA−C ENSG00000204525
PPDPF ENSG00000125534
S100P ENSG00000163993
FCGBP ENSG00000275395
CKB ENSG00000166165
UBC ENSG00000150991
FTL ENSG00000087086
ANPEP ENSG00000166825
SOD3 ENSG00000109610
CD63 ENSG00000135404
B2M ENSG00000166710
PFN1 ENSG00000108518
SSR4 ENSG00000180879
SPDEF ENSG00000124664
SERF2 ENSG00000140264
GDF15 ENSG00000130513
PTGDS ENSG00000107317
KRT18 ENSG00000111057
ACTG1 ENSG00000184009
EEF1A1 ENSG00000156508
TPT1 ENSG00000133112
LIPF ENSG00000182333
CIB1 ENSG00000185043
LCN2 ENSG00000148346
PSAP ENSG00000197746
PGC ENSG00000096088
FASN ENSG00000169710
ELF3 ENSG00000163435
PMEPA1 ENSG00000124225

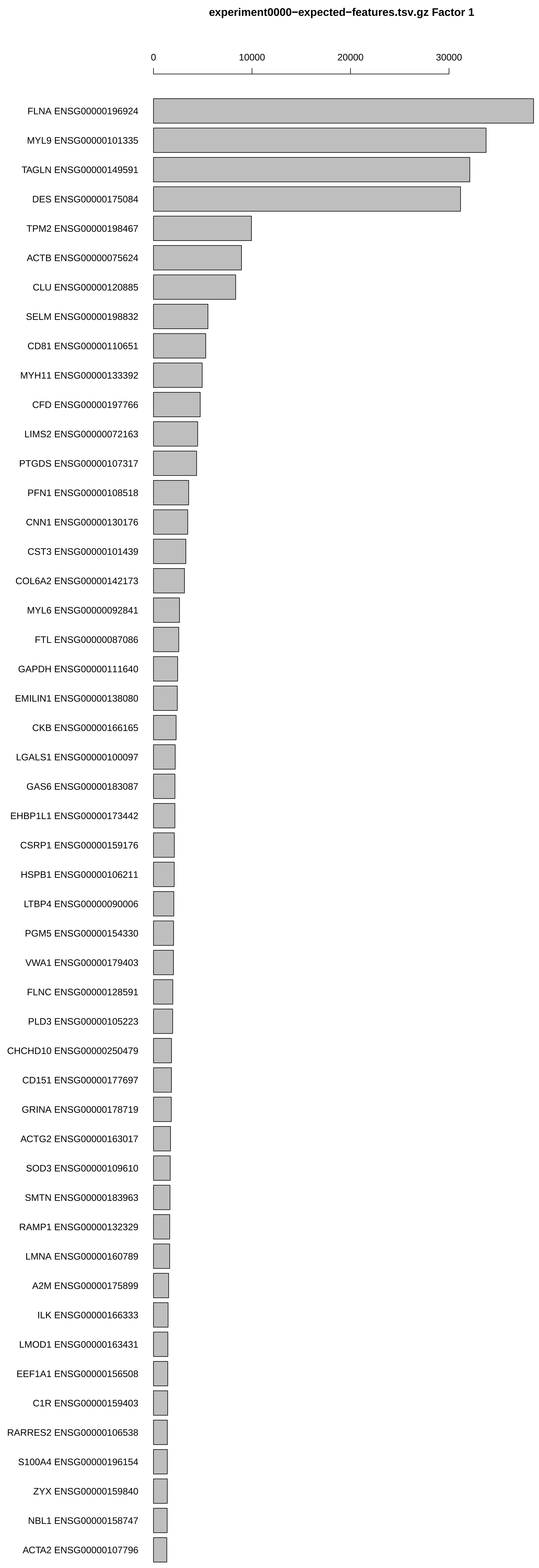
experiment0000−expected−features.tsv.gz Factor 1
0
10000
20000
30000
FLNA ENSG00000196924
MYL9 ENSG00000101335
TAGLN ENSG00000149591
DES ENSG00000175084
TPM2 ENSG00000198467
ACTB ENSG00000075624
CLU ENSG00000120885
SELM ENSG00000198832
CD81 ENSG00000110651
MYH11 ENSG00000133392
CFD ENSG00000197766
LIMS2 ENSG00000072163
PTGDS ENSG00000107317
PFN1 ENSG00000108518
CNN1 ENSG00000130176
CST3 ENSG00000101439
COL6A2 ENSG00000142173
MYL6 ENSG00000092841
FTL ENSG00000087086
GAPDH ENSG00000111640
EMILIN1 ENSG00000138080
CKB ENSG00000166165
LGALS1 ENSG00000100097
GAS6 ENSG00000183087
EHBP1L1 ENSG00000173442
CSRP1 ENSG00000159176
HSPB1 ENSG00000106211
LTBP4 ENSG00000090006
PGM5 ENSG00000154330
VWA1 ENSG00000179403
FLNC ENSG00000128591
PLD3 ENSG00000105223
CHCHD10 ENSG00000250479
CD151 ENSG00000177697
GRINA ENSG00000178719
ACTG2 ENSG00000163017
SOD3 ENSG00000109610
SMTN ENSG00000183963
RAMP1 ENSG00000132329
LMNA ENSG00000160789
A2M ENSG00000175899
ILK ENSG00000166333
LMOD1 ENSG00000163431
EEF1A1 ENSG00000156508
C1R ENSG00000159403
RARRES2 ENSG00000106538
S100A4 ENSG00000196154
ZYX ENSG00000159840
NBL1 ENSG00000158747
ACTA2 ENSG00000107796

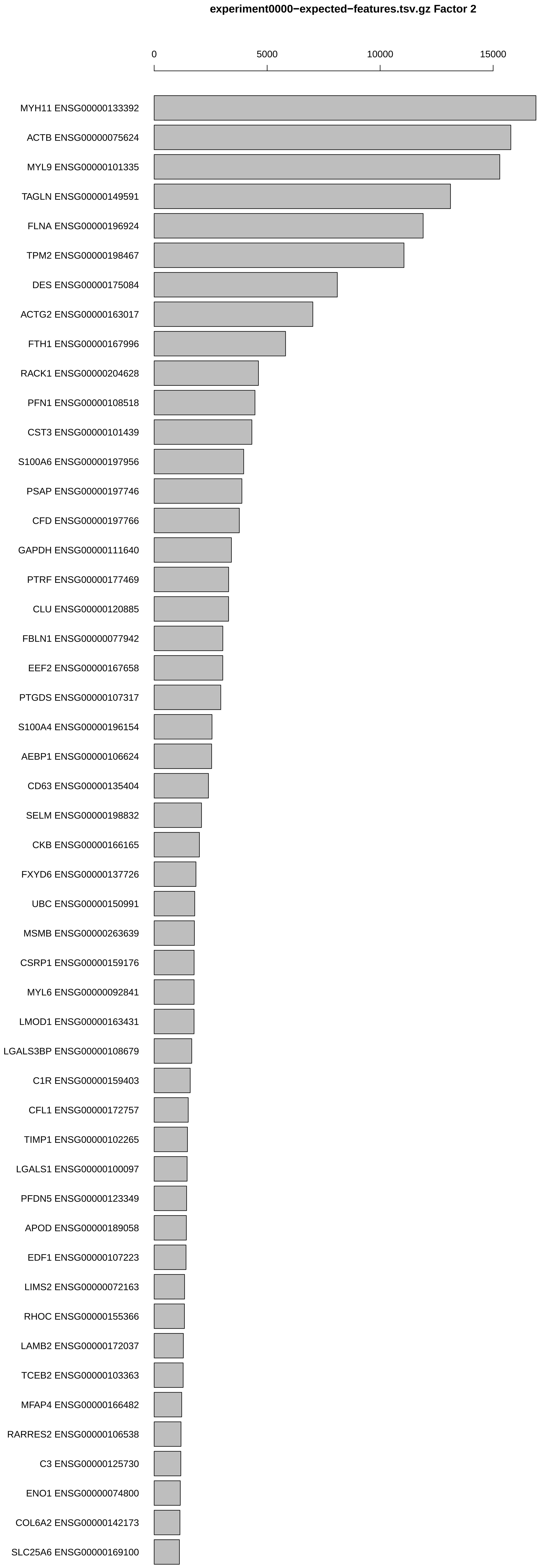
experiment0000−expected−features.tsv.gz Factor 2
0
5000
10000
15000
MYH11 ENSG00000133392
ACTB ENSG00000075624
MYL9 ENSG00000101335
TAGLN ENSG00000149591
FLNA ENSG00000196924
TPM2 ENSG00000198467
DES ENSG00000175084
ACTG2 ENSG00000163017
FTH1 ENSG00000167996
RACK1 ENSG00000204628
PFN1 ENSG00000108518
CST3 ENSG00000101439
S100A6 ENSG00000197956
PSAP ENSG00000197746
CFD ENSG00000197766
GAPDH ENSG00000111640
PTRF ENSG00000177469
CLU ENSG00000120885
FBLN1 ENSG00000077942
EEF2 ENSG00000167658
PTGDS ENSG00000107317
S100A4 ENSG00000196154
AEBP1 ENSG00000106624
CD63 ENSG00000135404
SELM ENSG00000198832
CKB ENSG00000166165
FXYD6 ENSG00000137726
UBC ENSG00000150991
MSMB ENSG00000263639
CSRP1 ENSG00000159176
MYL6 ENSG00000092841
LMOD1 ENSG00000163431
LGALS3BP ENSG00000108679
C1R ENSG00000159403
CFL1 ENSG00000172757
TIMP1 ENSG00000102265
LGALS1 ENSG00000100097
PFDN5 ENSG00000123349
APOD ENSG00000189058
EDF1 ENSG00000107223
LIMS2 ENSG00000072163
RHOC ENSG00000155366
LAMB2 ENSG00000172037
TCEB2 ENSG00000103363
MFAP4 ENSG00000166482
RARRES2 ENSG00000106538
C3 ENSG00000125730
ENO1 ENSG00000074800
COL6A2 ENSG00000142173
SLC25A6 ENSG00000169100

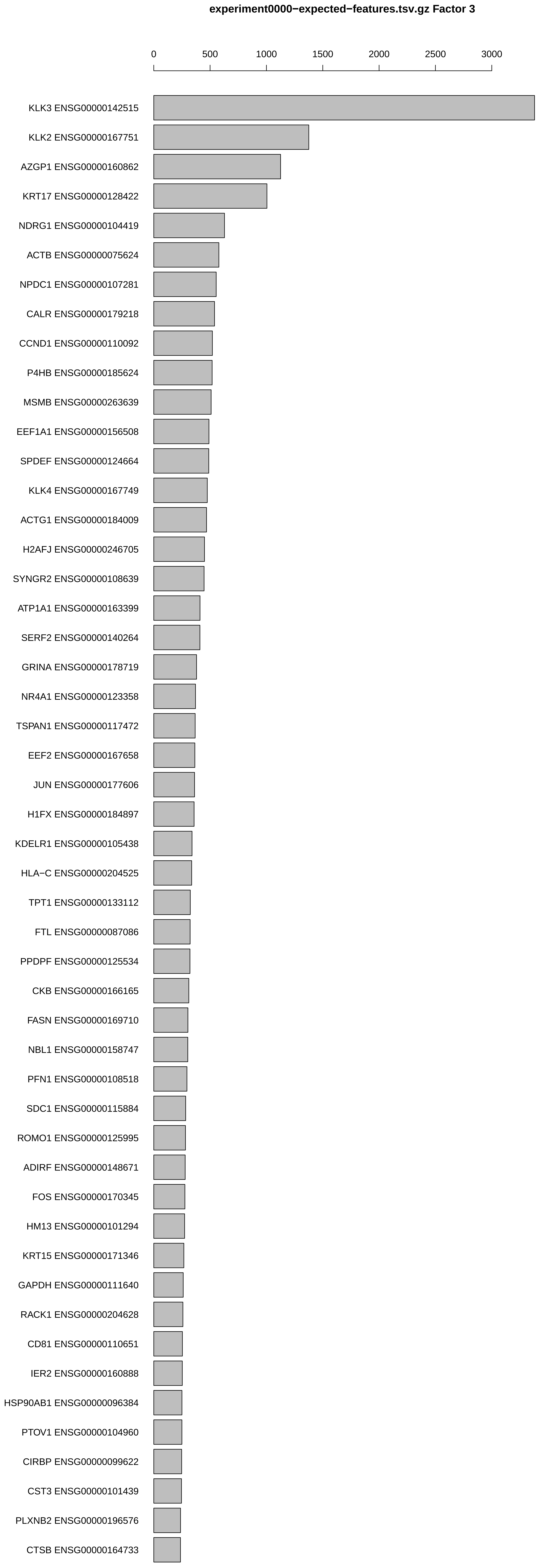
experiment0000−expected−features.tsv.gz Factor 3
0
500
1000
1500
2000
2500
3000
KLK3 ENSG00000142515
KLK2 ENSG00000167751
AZGP1 ENSG00000160862
KRT17 ENSG00000128422
NDRG1 ENSG00000104419
ACTB ENSG00000075624
NPDC1 ENSG00000107281
CALR ENSG00000179218
CCND1 ENSG00000110092
P4HB ENSG00000185624
MSMB ENSG00000263639
EEF1A1 ENSG00000156508
SPDEF ENSG00000124664
KLK4 ENSG00000167749
ACTG1 ENSG00000184009
H2AFJ ENSG00000246705
SYNGR2 ENSG00000108639
ATP1A1 ENSG00000163399
SERF2 ENSG00000140264
GRINA ENSG00000178719
NR4A1 ENSG00000123358
TSPAN1 ENSG00000117472
EEF2 ENSG00000167658
JUN ENSG00000177606
H1FX ENSG00000184897
KDELR1 ENSG00000105438
HLA−C ENSG00000204525
TPT1 ENSG00000133112
FTL ENSG00000087086
PPDPF ENSG00000125534
CKB ENSG00000166165
FASN ENSG00000169710
NBL1 ENSG00000158747
PFN1 ENSG00000108518
SDC1 ENSG00000115884
ROMO1 ENSG00000125995
ADIRF ENSG00000148671
FOS ENSG00000170345
HM13 ENSG00000101294
KRT15 ENSG00000171346
GAPDH ENSG00000111640
RACK1 ENSG00000204628
CD81 ENSG00000110651
IER2 ENSG00000160888
HSP90AB1 ENSG00000096384
PTOV1 ENSG00000104960
CIRBP ENSG00000099622
CST3 ENSG00000101439
PLXNB2 ENSG00000196576
CTSB ENSG00000164733

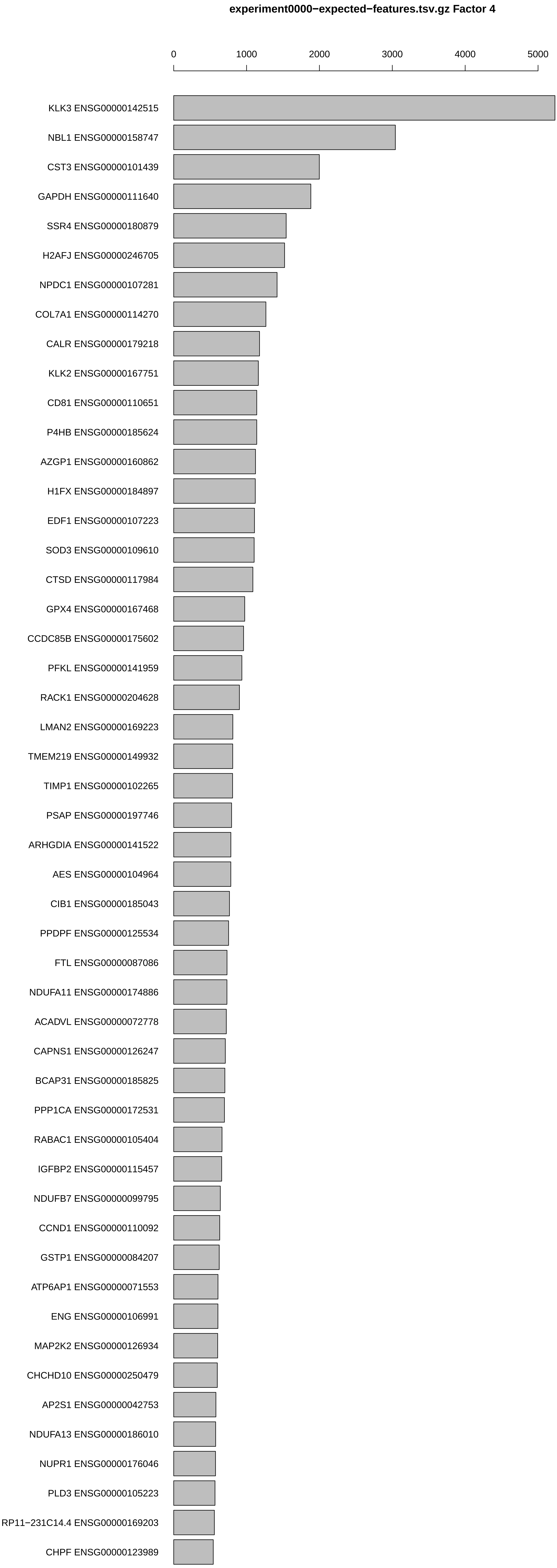
experiment0000−expected−features.tsv.gz Factor 4
0
1000
2000
3000
4000
5000
KLK3 ENSG00000142515
NBL1 ENSG00000158747
CST3 ENSG00000101439
GAPDH ENSG00000111640
SSR4 ENSG00000180879
H2AFJ ENSG00000246705
NPDC1 ENSG00000107281
COL7A1 ENSG00000114270
CALR ENSG00000179218
KLK2 ENSG00000167751
CD81 ENSG00000110651
P4HB ENSG00000185624
AZGP1 ENSG00000160862
H1FX ENSG00000184897
EDF1 ENSG00000107223
SOD3 ENSG00000109610
CTSD ENSG00000117984
GPX4 ENSG00000167468
CCDC85B ENSG00000175602
PFKL ENSG00000141959
RACK1 ENSG00000204628
LMAN2 ENSG00000169223
TMEM219 ENSG00000149932
TIMP1 ENSG00000102265
PSAP ENSG00000197746
ARHGDIA ENSG00000141522
AES ENSG00000104964
CIB1 ENSG00000185043
PPDPF ENSG00000125534
FTL ENSG00000087086
NDUFA11 ENSG00000174886
ACADVL ENSG00000072778
CAPNS1 ENSG00000126247
BCAP31 ENSG00000185825
PPP1CA ENSG00000172531
RABAC1 ENSG00000105404
IGFBP2 ENSG00000115457
NDUFB7 ENSG00000099795
CCND1 ENSG00000110092
GSTP1 ENSG00000084207
ATP6AP1 ENSG00000071553
ENG ENSG00000106991
MAP2K2 ENSG00000126934
CHCHD10 ENSG00000250479
AP2S1 ENSG00000042753
NDUFA13 ENSG00000186010
NUPR1 ENSG00000176046
PLD3 ENSG00000105223
RP11−231C14.4 ENSG00000169203
CHPF ENSG00000123989

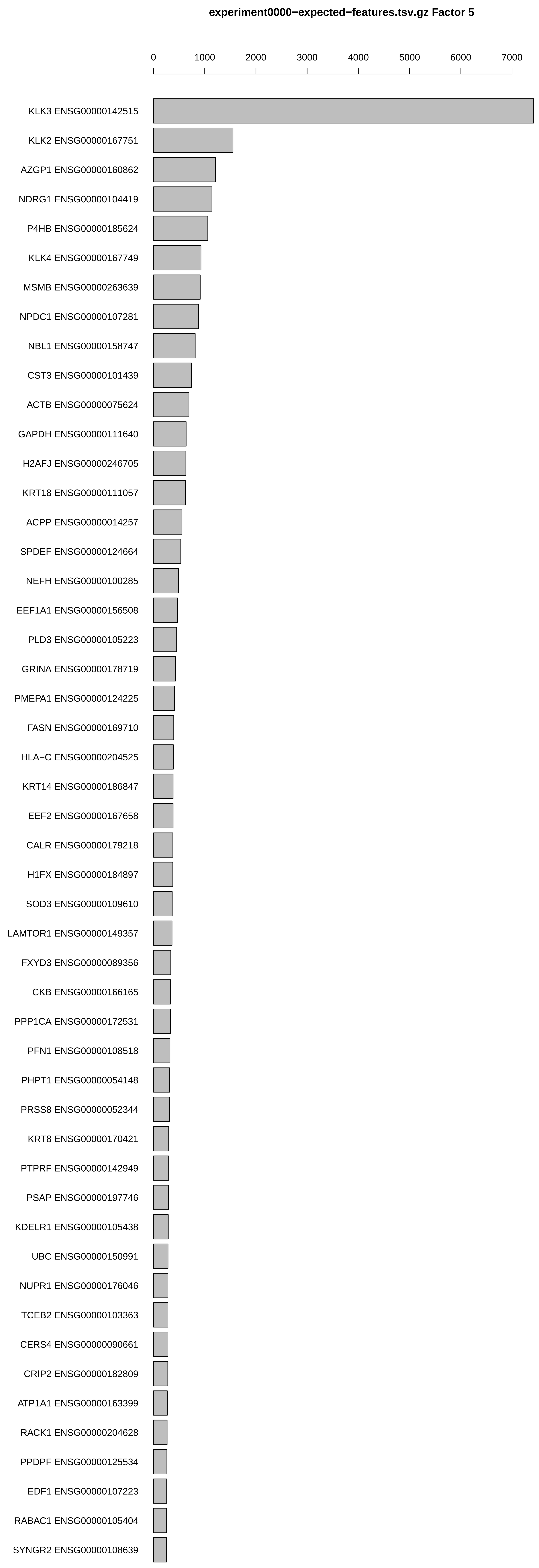
experiment0000−expected−features.tsv.gz Factor 5
0
1000
2000
3000
4000
5000
6000
7000
KLK3 ENSG00000142515
KLK2 ENSG00000167751
AZGP1 ENSG00000160862
NDRG1 ENSG00000104419
P4HB ENSG00000185624
KLK4 ENSG00000167749
MSMB ENSG00000263639
NPDC1 ENSG00000107281
NBL1 ENSG00000158747
CST3 ENSG00000101439
ACTB ENSG00000075624
GAPDH ENSG00000111640
H2AFJ ENSG00000246705
KRT18 ENSG00000111057
ACPP ENSG00000014257
SPDEF ENSG00000124664
NEFH ENSG00000100285
EEF1A1 ENSG00000156508
PLD3 ENSG00000105223
GRINA ENSG00000178719
PMEPA1 ENSG00000124225
FASN ENSG00000169710
HLA−C ENSG00000204525
KRT14 ENSG00000186847
EEF2 ENSG00000167658
CALR ENSG00000179218
H1FX ENSG00000184897
SOD3 ENSG00000109610
LAMTOR1 ENSG00000149357
FXYD3 ENSG00000089356
CKB ENSG00000166165
PPP1CA ENSG00000172531
PFN1 ENSG00000108518
PHPT1 ENSG00000054148
PRSS8 ENSG00000052344
KRT8 ENSG00000170421
PTPRF ENSG00000142949
PSAP ENSG00000197746
KDELR1 ENSG00000105438
UBC ENSG00000150991
NUPR1 ENSG00000176046
TCEB2 ENSG00000103363
CERS4 ENSG00000090661
CRIP2 ENSG00000182809
ATP1A1 ENSG00000163399
RACK1 ENSG00000204628
PPDPF ENSG00000125534
EDF1 ENSG00000107223
RABAC1 ENSG00000105404
SYNGR2 ENSG00000108639

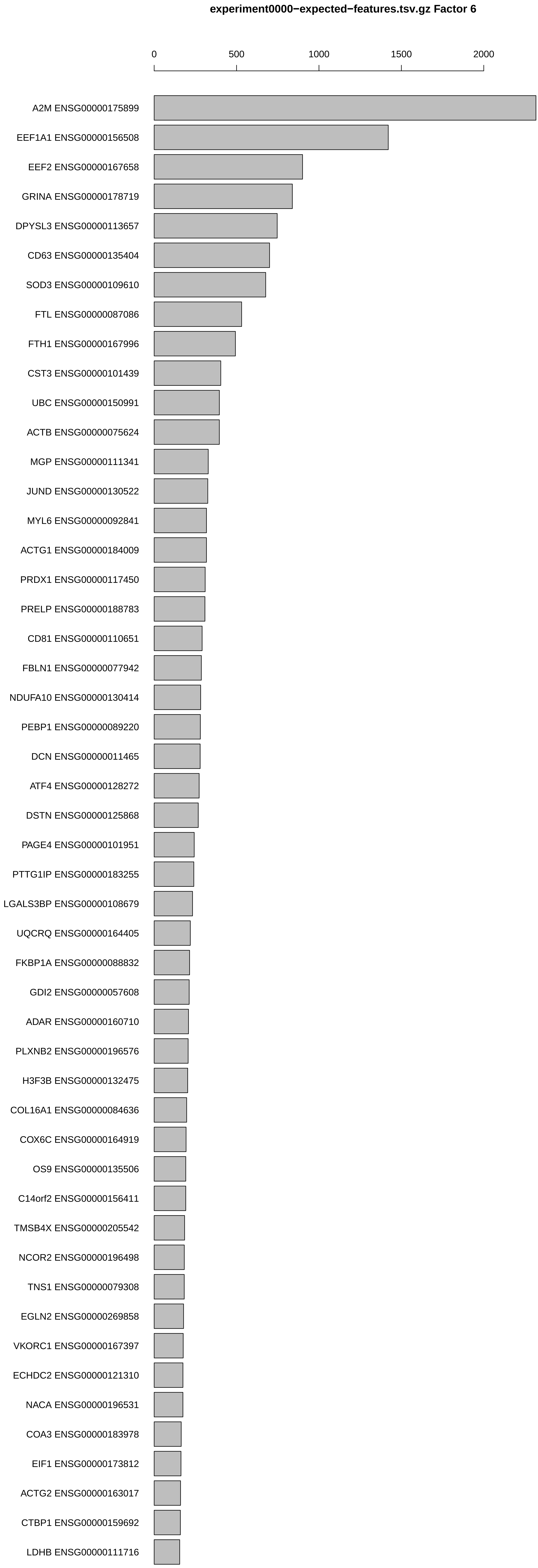
experiment0000−expected−features.tsv.gz Factor 6
0
500
1000
1500
2000
A2M ENSG00000175899
EEF1A1 ENSG00000156508
EEF2 ENSG00000167658
GRINA ENSG00000178719
DPYSL3 ENSG00000113657
CD63 ENSG00000135404
SOD3 ENSG00000109610
FTL ENSG00000087086
FTH1 ENSG00000167996
CST3 ENSG00000101439
UBC ENSG00000150991
ACTB ENSG00000075624
MGP ENSG00000111341
JUND ENSG00000130522
MYL6 ENSG00000092841
ACTG1 ENSG00000184009
PRDX1 ENSG00000117450
PRELP ENSG00000188783
CD81 ENSG00000110651
FBLN1 ENSG00000077942
NDUFA10 ENSG00000130414
PEBP1 ENSG00000089220
DCN ENSG00000011465
ATF4 ENSG00000128272
DSTN ENSG00000125868
PAGE4 ENSG00000101951
PTTG1IP ENSG00000183255
LGALS3BP ENSG00000108679
UQCRQ ENSG00000164405
FKBP1A ENSG00000088832
GDI2 ENSG00000057608
ADAR ENSG00000160710
PLXNB2 ENSG00000196576
H3F3B ENSG00000132475
COL16A1 ENSG00000084636
COX6C ENSG00000164919
OS9 ENSG00000135506
C14orf2 ENSG00000156411
TMSB4X ENSG00000205542
NCOR2 ENSG00000196498
TNS1 ENSG00000079308
EGLN2 ENSG00000269858
VKORC1 ENSG00000167397
ECHDC2 ENSG00000121310
NACA ENSG00000196531
COA3 ENSG00000183978
EIF1 ENSG00000173812
ACTG2 ENSG00000163017
CTBP1 ENSG00000159692
LDHB ENSG00000111716

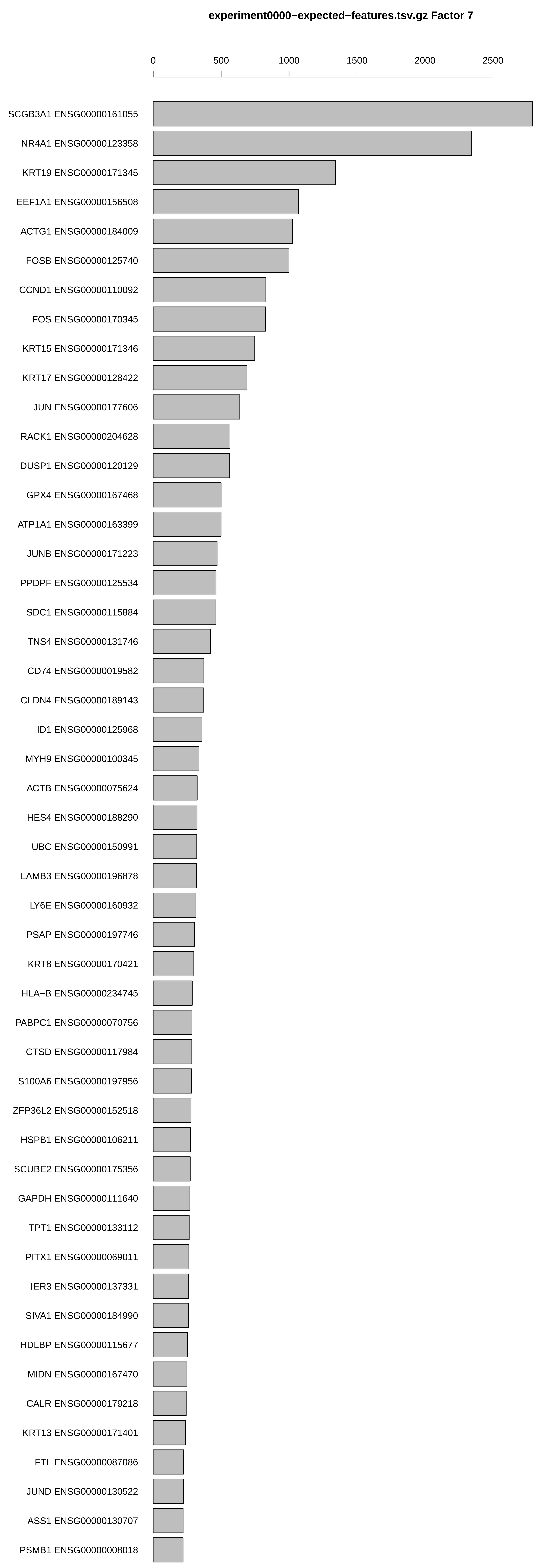
experiment0000−expected−features.tsv.gz Factor 7
0
500
1000
1500
2000
2500
SCGB3A1 ENSG00000161055
NR4A1 ENSG00000123358
KRT19 ENSG00000171345
EEF1A1 ENSG00000156508
ACTG1 ENSG00000184009
FOSB ENSG00000125740
CCND1 ENSG00000110092
FOS ENSG00000170345
KRT15 ENSG00000171346
KRT17 ENSG00000128422
JUN ENSG00000177606
RACK1 ENSG00000204628
DUSP1 ENSG00000120129
GPX4 ENSG00000167468
ATP1A1 ENSG00000163399
JUNB ENSG00000171223
PPDPF ENSG00000125534
SDC1 ENSG00000115884
TNS4 ENSG00000131746
CD74 ENSG00000019582
CLDN4 ENSG00000189143
ID1 ENSG00000125968
MYH9 ENSG00000100345
ACTB ENSG00000075624
HES4 ENSG00000188290
UBC ENSG00000150991
LAMB3 ENSG00000196878
LY6E ENSG00000160932
PSAP ENSG00000197746
KRT8 ENSG00000170421
HLA−B ENSG00000234745
PABPC1 ENSG00000070756
CTSD ENSG00000117984
S100A6 ENSG00000197956
ZFP36L2 ENSG00000152518
HSPB1 ENSG00000106211
SCUBE2 ENSG00000175356
GAPDH ENSG00000111640
TPT1 ENSG00000133112
PITX1 ENSG00000069011
IER3 ENSG00000137331
SIVA1 ENSG00000184990
HDLBP ENSG00000115677
MIDN ENSG00000167470
CALR ENSG00000179218
KRT13 ENSG00000171401
FTL ENSG00000087086
JUND ENSG00000130522
ASS1 ENSG00000130707
PSMB1 ENSG00000008018

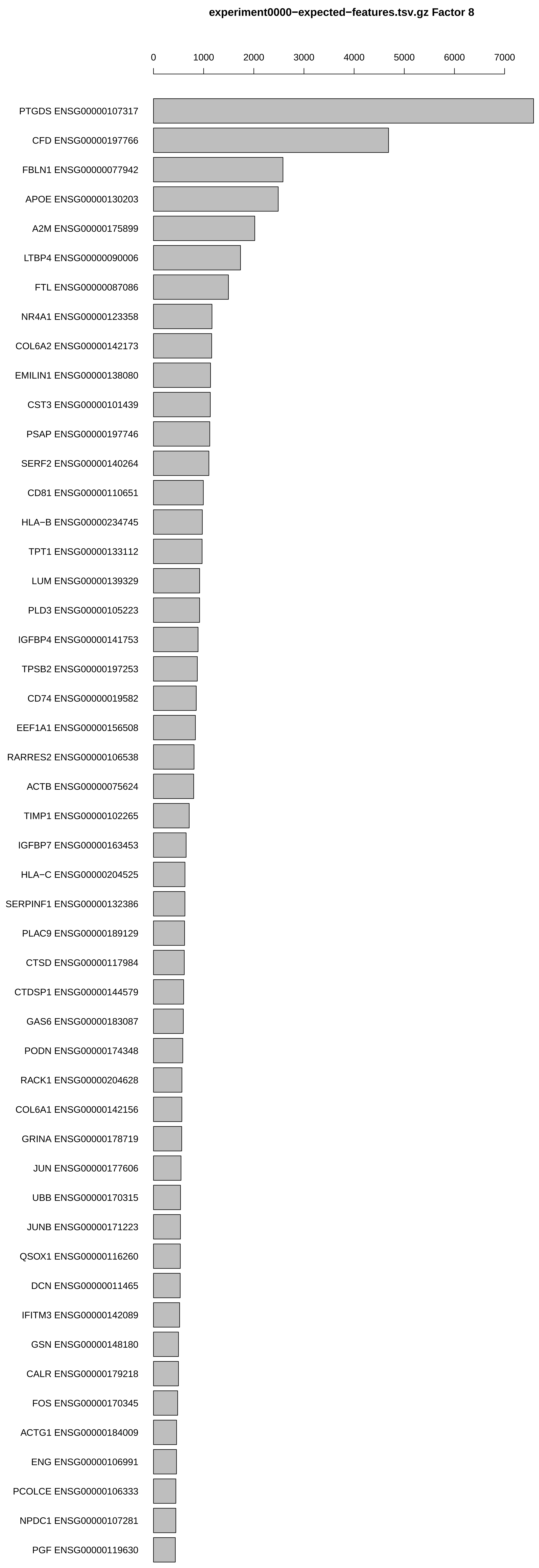
experiment0000−expected−features.tsv.gz Factor 8
0
1000
2000
3000
4000
5000
6000
7000
PTGDS ENSG00000107317
CFD ENSG00000197766
FBLN1 ENSG00000077942
APOE ENSG00000130203
A2M ENSG00000175899
LTBP4 ENSG00000090006
FTL ENSG00000087086
NR4A1 ENSG00000123358
COL6A2 ENSG00000142173
EMILIN1 ENSG00000138080
CST3 ENSG00000101439
PSAP ENSG00000197746
SERF2 ENSG00000140264
CD81 ENSG00000110651
HLA−B ENSG00000234745
TPT1 ENSG00000133112
LUM ENSG00000139329
PLD3 ENSG00000105223
IGFBP4 ENSG00000141753
TPSB2 ENSG00000197253
CD74 ENSG00000019582
EEF1A1 ENSG00000156508
RARRES2 ENSG00000106538
ACTB ENSG00000075624
TIMP1 ENSG00000102265
IGFBP7 ENSG00000163453
HLA−C ENSG00000204525
SERPINF1 ENSG00000132386
PLAC9 ENSG00000189129
CTSD ENSG00000117984
CTDSP1 ENSG00000144579
GAS6 ENSG00000183087
PODN ENSG00000174348
RACK1 ENSG00000204628
COL6A1 ENSG00000142156
GRINA ENSG00000178719
JUN ENSG00000177606
UBB ENSG00000170315
JUNB ENSG00000171223
QSOX1 ENSG00000116260
DCN ENSG00000011465
IFITM3 ENSG00000142089
GSN ENSG00000148180
CALR ENSG00000179218
FOS ENSG00000170345
ACTG1 ENSG00000184009
ENG ENSG00000106991
PCOLCE ENSG00000106333
NPDC1 ENSG00000107281
PGF ENSG00000119630

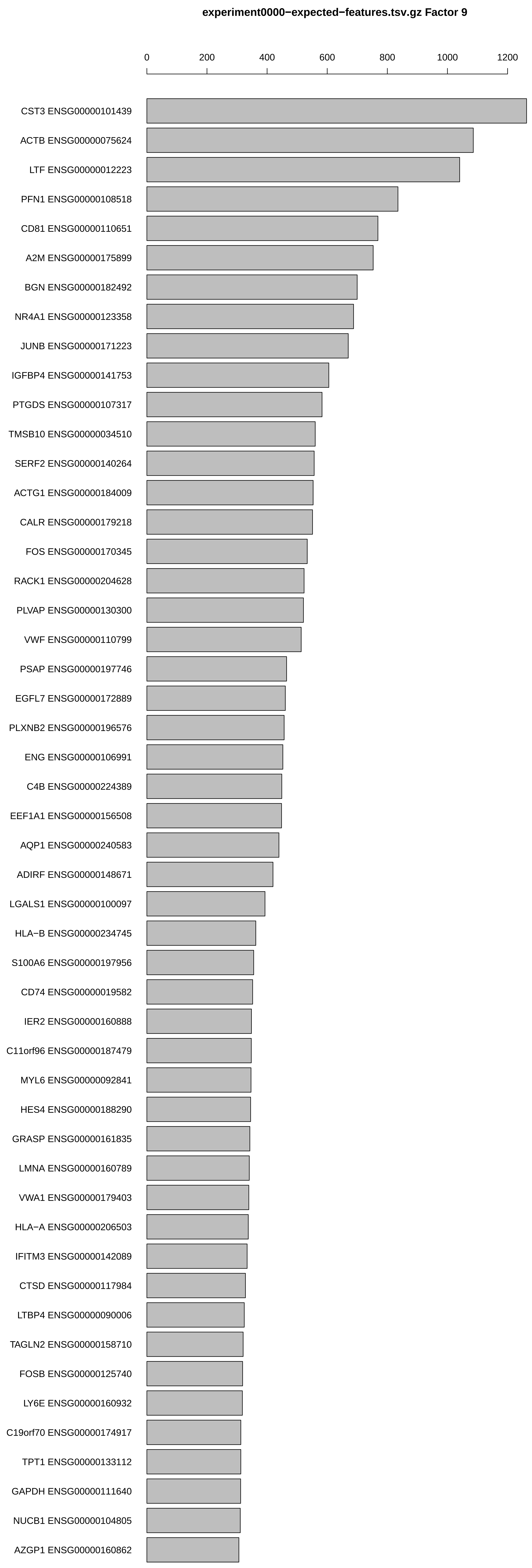
experiment0000−expected−features.tsv.gz Factor 9
0
200
400
600
800
1000
1200
CST3 ENSG00000101439
ACTB ENSG00000075624
LTF ENSG00000012223
PFN1 ENSG00000108518
CD81 ENSG00000110651
A2M ENSG00000175899
BGN ENSG00000182492
NR4A1 ENSG00000123358
JUNB ENSG00000171223
IGFBP4 ENSG00000141753
PTGDS ENSG00000107317
TMSB10 ENSG00000034510
SERF2 ENSG00000140264
ACTG1 ENSG00000184009
CALR ENSG00000179218
FOS ENSG00000170345
RACK1 ENSG00000204628
PLVAP ENSG00000130300
VWF ENSG00000110799
PSAP ENSG00000197746
EGFL7 ENSG00000172889
PLXNB2 ENSG00000196576
ENG ENSG00000106991
C4B ENSG00000224389
EEF1A1 ENSG00000156508
AQP1 ENSG00000240583
ADIRF ENSG00000148671
LGALS1 ENSG00000100097
HLA−B ENSG00000234745
S100A6 ENSG00000197956
CD74 ENSG00000019582
IER2 ENSG00000160888
C11orf96 ENSG00000187479
MYL6 ENSG00000092841
HES4 ENSG00000188290
GRASP ENSG00000161835
LMNA ENSG00000160789
VWA1 ENSG00000179403
HLA−A ENSG00000206503
IFITM3 ENSG00000142089
CTSD ENSG00000117984
LTBP4 ENSG00000090006
TAGLN2 ENSG00000158710
FOSB ENSG00000125740
LY6E ENSG00000160932
C19orf70 ENSG00000174917
TPT1 ENSG00000133112
GAPDH ENSG00000111640
NUCB1 ENSG00000104805
AZGP1 ENSG00000160862

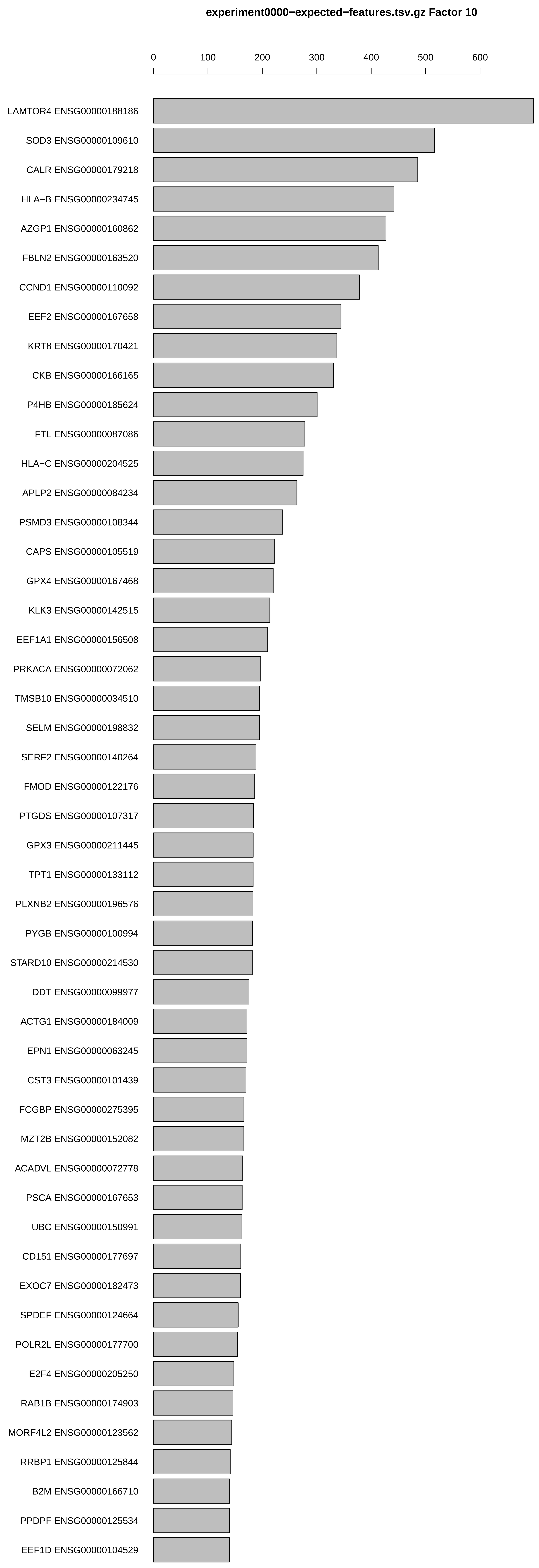
experiment0000−expected−features.tsv.gz Factor 10
0
100
200
300
400
500
600
LAMTOR4 ENSG00000188186
SOD3 ENSG00000109610
CALR ENSG00000179218
HLA−B ENSG00000234745
AZGP1 ENSG00000160862
FBLN2 ENSG00000163520
CCND1 ENSG00000110092
EEF2 ENSG00000167658
KRT8 ENSG00000170421
CKB ENSG00000166165
P4HB ENSG00000185624
FTL ENSG00000087086
HLA−C ENSG00000204525
APLP2 ENSG00000084234
PSMD3 ENSG00000108344
CAPS ENSG00000105519
GPX4 ENSG00000167468
KLK3 ENSG00000142515
EEF1A1 ENSG00000156508
PRKACA ENSG00000072062
TMSB10 ENSG00000034510
SELM ENSG00000198832
SERF2 ENSG00000140264
FMOD ENSG00000122176
PTGDS ENSG00000107317
GPX3 ENSG00000211445
TPT1 ENSG00000133112
PLXNB2 ENSG00000196576
PYGB ENSG00000100994
STARD10 ENSG00000214530
DDT ENSG00000099977
ACTG1 ENSG00000184009
EPN1 ENSG00000063245
CST3 ENSG00000101439
FCGBP ENSG00000275395
MZT2B ENSG00000152082
ACADVL ENSG00000072778
PSCA ENSG00000167653
UBC ENSG00000150991
CD151 ENSG00000177697
EXOC7 ENSG00000182473
SPDEF ENSG00000124664
POLR2L ENSG00000177700
E2F4 ENSG00000205250
RAB1B ENSG00000174903
MORF4L2 ENSG00000123562
RRBP1 ENSG00000125844
B2M ENSG00000166710
PPDPF ENSG00000125534
EEF1D ENSG00000104529

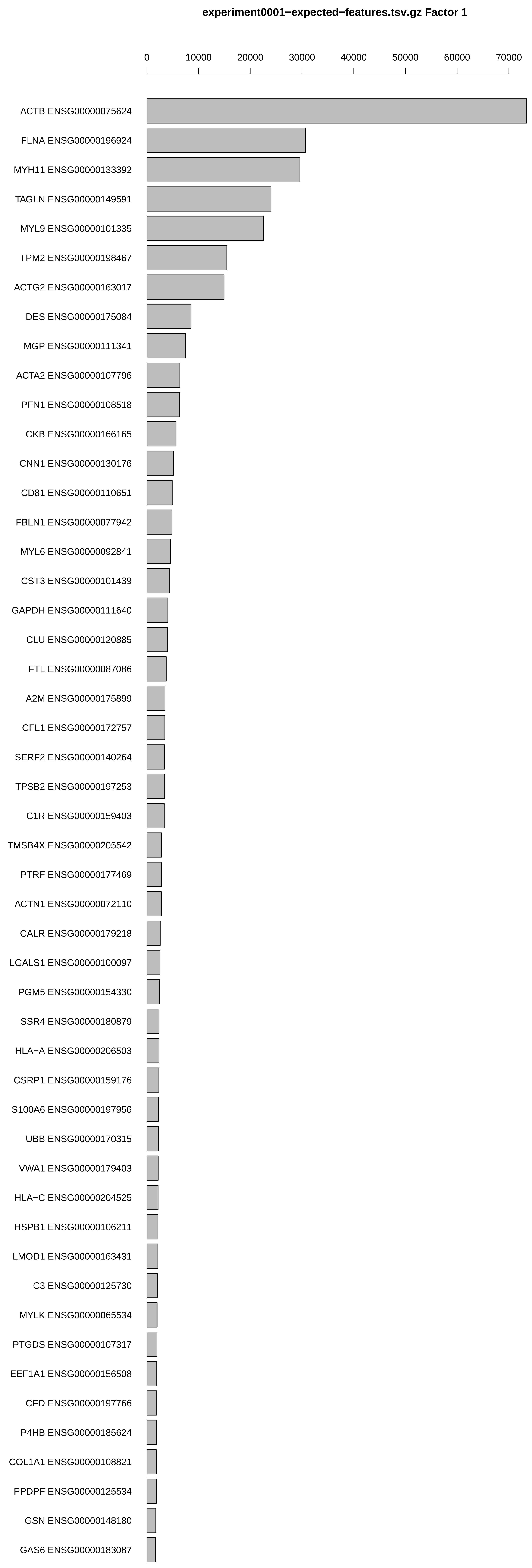

experiment0001−expected−features.tsv.gz Factor 1
0
10000
20000
30000
40000
50000
60000
70000
ACTB ENSG00000075624
FLNA ENSG00000196924
MYH11 ENSG00000133392
TAGLN ENSG00000149591
MYL9 ENSG00000101335
TPM2 ENSG00000198467
ACTG2 ENSG00000163017
DES ENSG00000175084
MGP ENSG00000111341
ACTA2 ENSG00000107796
PFN1 ENSG00000108518
CKB ENSG00000166165
CNN1 ENSG00000130176
CD81 ENSG00000110651
FBLN1 ENSG00000077942
MYL6 ENSG00000092841
CST3 ENSG00000101439
GAPDH ENSG00000111640
CLU ENSG00000120885
FTL ENSG00000087086
A2M ENSG00000175899
CFL1 ENSG00000172757
SERF2 ENSG00000140264
TPSB2 ENSG00000197253
C1R ENSG00000159403
TMSB4X ENSG00000205542
PTRF ENSG00000177469
ACTN1 ENSG00000072110
CALR ENSG00000179218
LGALS1 ENSG00000100097
PGM5 ENSG00000154330
SSR4 ENSG00000180879
HLA−A ENSG00000206503
CSRP1 ENSG00000159176
S100A6 ENSG00000197956
UBB ENSG00000170315
VWA1 ENSG00000179403
HLA−C ENSG00000204525
HSPB1 ENSG00000106211
LMOD1 ENSG00000163431
C3 ENSG00000125730
MYLK ENSG00000065534
PTGDS ENSG00000107317
EEF1A1 ENSG00000156508
CFD ENSG00000197766
P4HB ENSG00000185624
COL1A1 ENSG00000108821
PPDPF ENSG00000125534
GSN ENSG00000148180
GAS6 ENSG00000183087

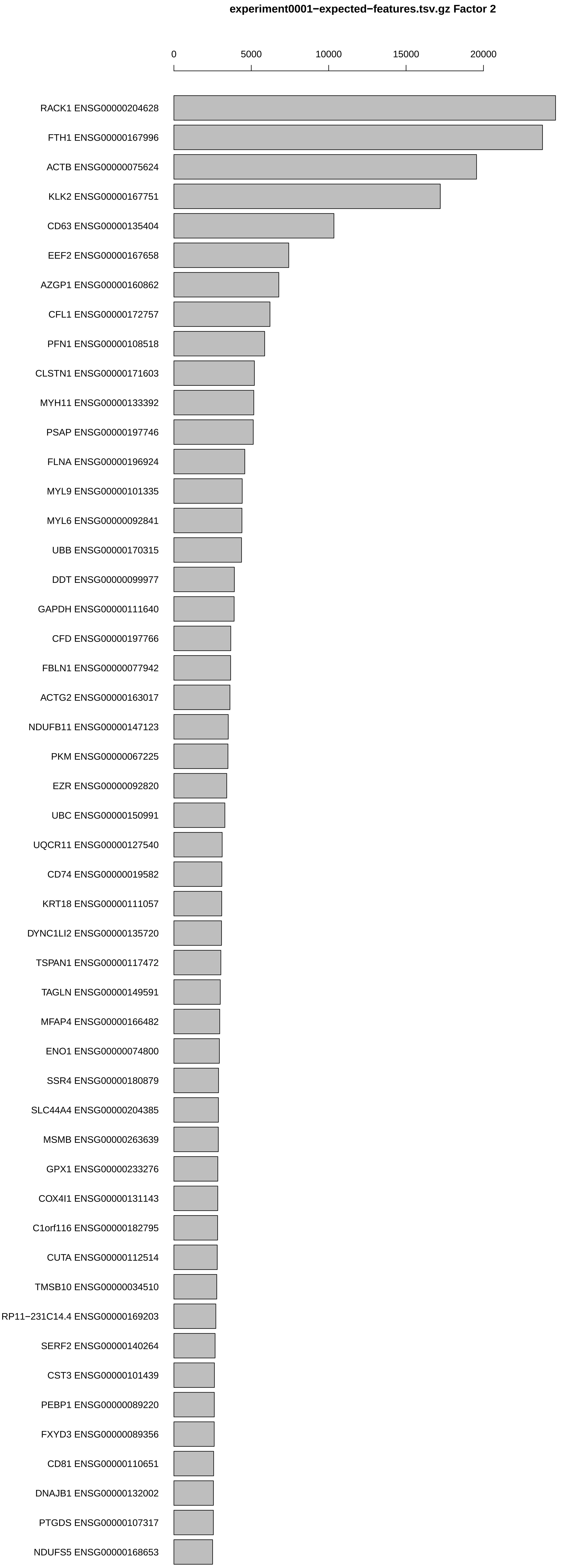
experiment0001−expected−features.tsv.gz Factor 2
0
5000
10000
15000
20000
RACK1 ENSG00000204628
FTH1 ENSG00000167996
ACTB ENSG00000075624
KLK2 ENSG00000167751
CD63 ENSG00000135404
EEF2 ENSG00000167658
AZGP1 ENSG00000160862
CFL1 ENSG00000172757
PFN1 ENSG00000108518
CLSTN1 ENSG00000171603
MYH11 ENSG00000133392
PSAP ENSG00000197746
FLNA ENSG00000196924
MYL9 ENSG00000101335
MYL6 ENSG00000092841
UBB ENSG00000170315
DDT ENSG00000099977
GAPDH ENSG00000111640
CFD ENSG00000197766
FBLN1 ENSG00000077942
ACTG2 ENSG00000163017
NDUFB11 ENSG00000147123
PKM ENSG00000067225
EZR ENSG00000092820
UBC ENSG00000150991
UQCR11 ENSG00000127540
CD74 ENSG00000019582
KRT18 ENSG00000111057
DYNC1LI2 ENSG00000135720
TSPAN1 ENSG00000117472
TAGLN ENSG00000149591
MFAP4 ENSG00000166482
ENO1 ENSG00000074800
SSR4 ENSG00000180879
SLC44A4 ENSG00000204385
MSMB ENSG00000263639
GPX1 ENSG00000233276
COX4I1 ENSG00000131143
C1orf116 ENSG00000182795
CUTA ENSG00000112514
TMSB10 ENSG00000034510
RP11−231C14.4 ENSG00000169203
SERF2 ENSG00000140264
CST3 ENSG00000101439
PEBP1 ENSG00000089220
FXYD3 ENSG00000089356
CD81 ENSG00000110651
DNAJB1 ENSG00000132002
PTGDS ENSG00000107317
NDUFS5 ENSG00000168653

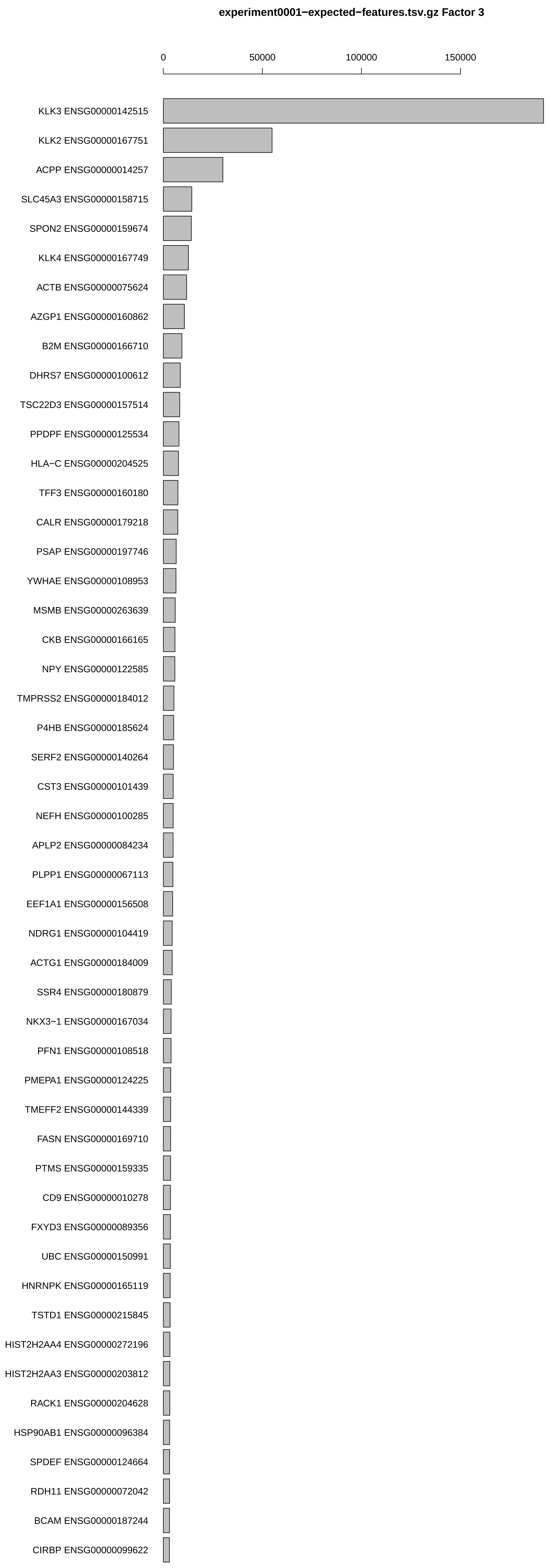

experiment0001−expected−features.tsv.gz Factor 3
0
50000
100000
150000
KLK3 ENSG00000142515
KLK2 ENSG00000167751
ACPP ENSG00000014257
SLC45A3 ENSG00000158715
SPON2 ENSG00000159674
KLK4 ENSG00000167749
ACTB ENSG00000075624
AZGP1 ENSG00000160862
B2M ENSG00000166710
DHRS7 ENSG00000100612
TSC22D3 ENSG00000157514
PPDPF ENSG00000125534
HLA−C ENSG00000204525
TFF3 ENSG00000160180
CALR ENSG00000179218
PSAP ENSG00000197746
YWHAE ENSG00000108953
MSMB ENSG00000263639
CKB ENSG00000166165
NPY ENSG00000122585
TMPRSS2 ENSG00000184012
P4HB ENSG00000185624
SERF2 ENSG00000140264
CST3 ENSG00000101439
NEFH ENSG00000100285
APLP2 ENSG00000084234
PLPP1 ENSG00000067113
EEF1A1 ENSG00000156508
NDRG1 ENSG00000104419
ACTG1 ENSG00000184009
SSR4 ENSG00000180879
NKX3−1 ENSG00000167034
PFN1 ENSG00000108518
PMEPA1 ENSG00000124225
TMEFF2 ENSG00000144339
FASN ENSG00000169710
PTMS ENSG00000159335
CD9 ENSG00000010278
FXYD3 ENSG00000089356
UBC ENSG00000150991
HNRNPK ENSG00000165119
TSTD1 ENSG00000215845
HIST2H2AA4 ENSG00000272196
HIST2H2AA3 ENSG00000203812
RACK1 ENSG00000204628
HSP90AB1 ENSG00000096384
SPDEF ENSG00000124664
RDH11 ENSG00000072042
BCAM ENSG00000187244
CIRBP ENSG00000099622

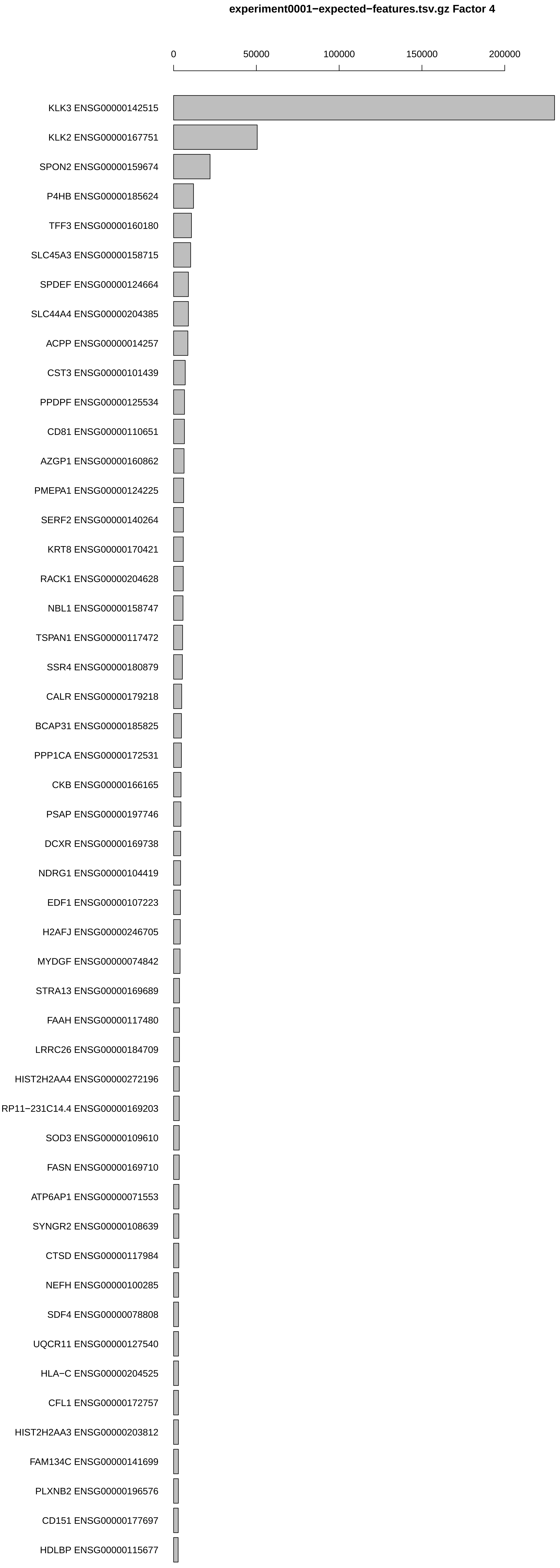
experiment0001−expected−features.tsv.gz Factor 4
0
50000
100000
150000
200000
KLK3 ENSG00000142515
KLK2 ENSG00000167751
SPON2 ENSG00000159674
P4HB ENSG00000185624
TFF3 ENSG00000160180
SLC45A3 ENSG00000158715
SPDEF ENSG00000124664
SLC44A4 ENSG00000204385
ACPP ENSG00000014257
CST3 ENSG00000101439
PPDPF ENSG00000125534
CD81 ENSG00000110651
AZGP1 ENSG00000160862
PMEPA1 ENSG00000124225
SERF2 ENSG00000140264
KRT8 ENSG00000170421
RACK1 ENSG00000204628
NBL1 ENSG00000158747
TSPAN1 ENSG00000117472
SSR4 ENSG00000180879
CALR ENSG00000179218
BCAP31 ENSG00000185825
PPP1CA ENSG00000172531
CKB ENSG00000166165
PSAP ENSG00000197746
DCXR ENSG00000169738
NDRG1 ENSG00000104419
EDF1 ENSG00000107223
H2AFJ ENSG00000246705
MYDGF ENSG00000074842
STRA13 ENSG00000169689
FAAH ENSG00000117480
LRRC26 ENSG00000184709
HIST2H2AA4 ENSG00000272196
RP11−231C14.4 ENSG00000169203
SOD3 ENSG00000109610
FASN ENSG00000169710
ATP6AP1 ENSG00000071553
SYNGR2 ENSG00000108639
CTSD ENSG00000117984
NEFH ENSG00000100285
SDF4 ENSG00000078808
UQCR11 ENSG00000127540
HLA−C ENSG00000204525
CFL1 ENSG00000172757
HIST2H2AA3 ENSG00000203812
FAM134C ENSG00000141699
PLXNB2 ENSG00000196576
CD151 ENSG00000177697
HDLBP ENSG00000115677

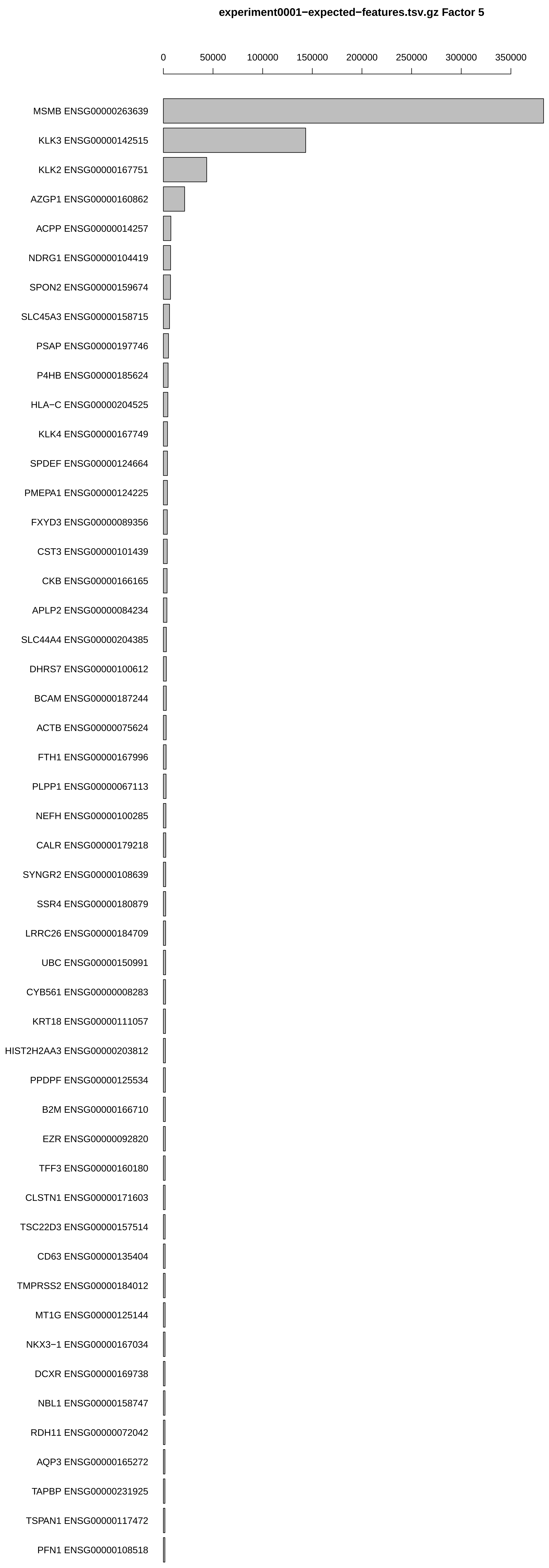
experiment0001−expected−features.tsv.gz Factor 5
0
50000
100000
150000
200000
250000
300000
350000
MSMB ENSG00000263639
KLK3 ENSG00000142515
KLK2 ENSG00000167751
AZGP1 ENSG00000160862
ACPP ENSG00000014257
NDRG1 ENSG00000104419
SPON2 ENSG00000159674
SLC45A3 ENSG00000158715
PSAP ENSG00000197746
P4HB ENSG00000185624
HLA−C ENSG00000204525
KLK4 ENSG00000167749
SPDEF ENSG00000124664
PMEPA1 ENSG00000124225
FXYD3 ENSG00000089356
CST3 ENSG00000101439
CKB ENSG00000166165
APLP2 ENSG00000084234
SLC44A4 ENSG00000204385
DHRS7 ENSG00000100612
BCAM ENSG00000187244
ACTB ENSG00000075624
FTH1 ENSG00000167996
PLPP1 ENSG00000067113
NEFH ENSG00000100285
CALR ENSG00000179218
SYNGR2 ENSG00000108639
SSR4 ENSG00000180879
LRRC26 ENSG00000184709
UBC ENSG00000150991
CYB561 ENSG00000008283
KRT18 ENSG00000111057
HIST2H2AA3 ENSG00000203812
PPDPF ENSG00000125534
B2M ENSG00000166710
EZR ENSG00000092820
TFF3 ENSG00000160180
CLSTN1 ENSG00000171603
TSC22D3 ENSG00000157514
CD63 ENSG00000135404
TMPRSS2 ENSG00000184012
MT1G ENSG00000125144
NKX3−1 ENSG00000167034
DCXR ENSG00000169738
NBL1 ENSG00000158747
RDH11 ENSG00000072042
AQP3 ENSG00000165272
TAPBP ENSG00000231925
TSPAN1 ENSG00000117472
PFN1 ENSG00000108518

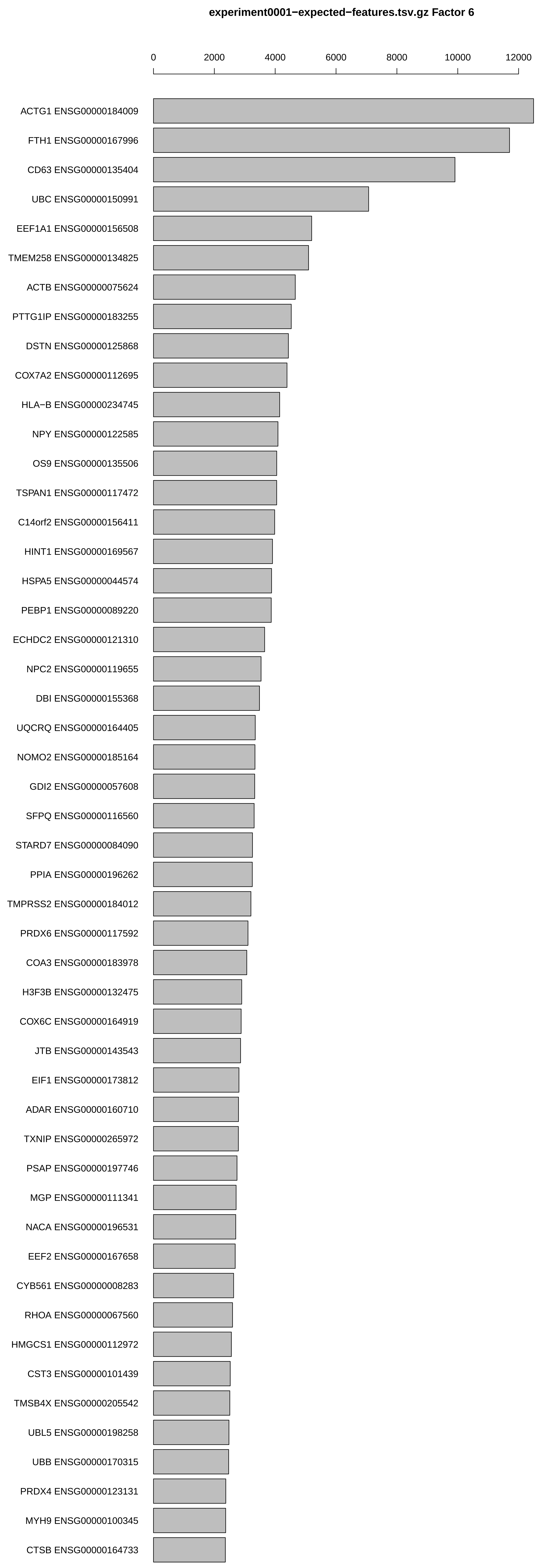
experiment0001−expected−features.tsv.gz Factor 6
0
2000
4000
6000
8000
10000
12000
ACTG1 ENSG00000184009
FTH1 ENSG00000167996
CD63 ENSG00000135404
UBC ENSG00000150991
EEF1A1 ENSG00000156508
TMEM258 ENSG00000134825
ACTB ENSG00000075624
PTTG1IP ENSG00000183255
DSTN ENSG00000125868
COX7A2 ENSG00000112695
HLA−B ENSG00000234745
NPY ENSG00000122585
OS9 ENSG00000135506
TSPAN1 ENSG00000117472
C14orf2 ENSG00000156411
HINT1 ENSG00000169567
HSPA5 ENSG00000044574
PEBP1 ENSG00000089220
ECHDC2 ENSG00000121310
NPC2 ENSG00000119655
DBI ENSG00000155368
UQCRQ ENSG00000164405
NOMO2 ENSG00000185164
GDI2 ENSG00000057608
SFPQ ENSG00000116560
STARD7 ENSG00000084090
PPIA ENSG00000196262
TMPRSS2 ENSG00000184012
PRDX6 ENSG00000117592
COA3 ENSG00000183978
H3F3B ENSG00000132475
COX6C ENSG00000164919
JTB ENSG00000143543
EIF1 ENSG00000173812
ADAR ENSG00000160710
TXNIP ENSG00000265972
PSAP ENSG00000197746
MGP ENSG00000111341
NACA ENSG00000196531
EEF2 ENSG00000167658
CYB561 ENSG00000008283
RHOA ENSG00000067560
HMGCS1 ENSG00000112972
CST3 ENSG00000101439
TMSB4X ENSG00000205542
UBL5 ENSG00000198258
UBB ENSG00000170315
PRDX4 ENSG00000123131
MYH9 ENSG00000100345
CTSB ENSG00000164733

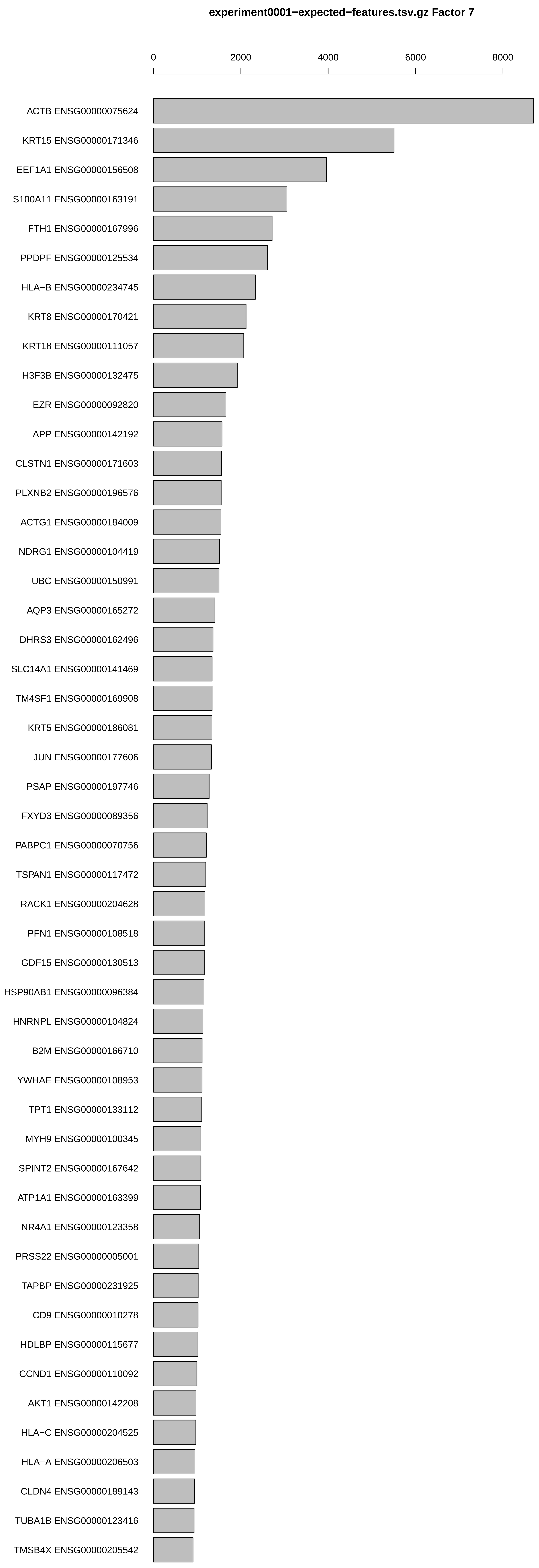

experiment0001−expected−features.tsv.gz Factor 7
0
2000
4000
6000
8000
ACTB ENSG00000075624
KRT15 ENSG00000171346
EEF1A1 ENSG00000156508
S100A11 ENSG00000163191
FTH1 ENSG00000167996
PPDPF ENSG00000125534
HLA−B ENSG00000234745
KRT8 ENSG00000170421
KRT18 ENSG00000111057
H3F3B ENSG00000132475
EZR ENSG00000092820
APP ENSG00000142192
CLSTN1 ENSG00000171603
PLXNB2 ENSG00000196576
ACTG1 ENSG00000184009
NDRG1 ENSG00000104419
UBC ENSG00000150991
AQP3 ENSG00000165272
DHRS3 ENSG00000162496
SLC14A1 ENSG00000141469
TM4SF1 ENSG00000169908
KRT5 ENSG00000186081
JUN ENSG00000177606
PSAP ENSG00000197746
FXYD3 ENSG00000089356
PABPC1 ENSG00000070756
TSPAN1 ENSG00000117472
RACK1 ENSG00000204628
PFN1 ENSG00000108518
GDF15 ENSG00000130513
HSP90AB1 ENSG00000096384
HNRNPL ENSG00000104824
B2M ENSG00000166710
YWHAE ENSG00000108953
TPT1 ENSG00000133112
MYH9 ENSG00000100345
SPINT2 ENSG00000167642
ATP1A1 ENSG00000163399
NR4A1 ENSG00000123358
PRSS22 ENSG00000005001
TAPBP ENSG00000231925
CD9 ENSG00000010278
HDLBP ENSG00000115677
CCND1 ENSG00000110092
AKT1 ENSG00000142208
HLA−C ENSG00000204525
HLA−A ENSG00000206503
CLDN4 ENSG00000189143
TUBA1B ENSG00000123416
TMSB4X ENSG00000205542

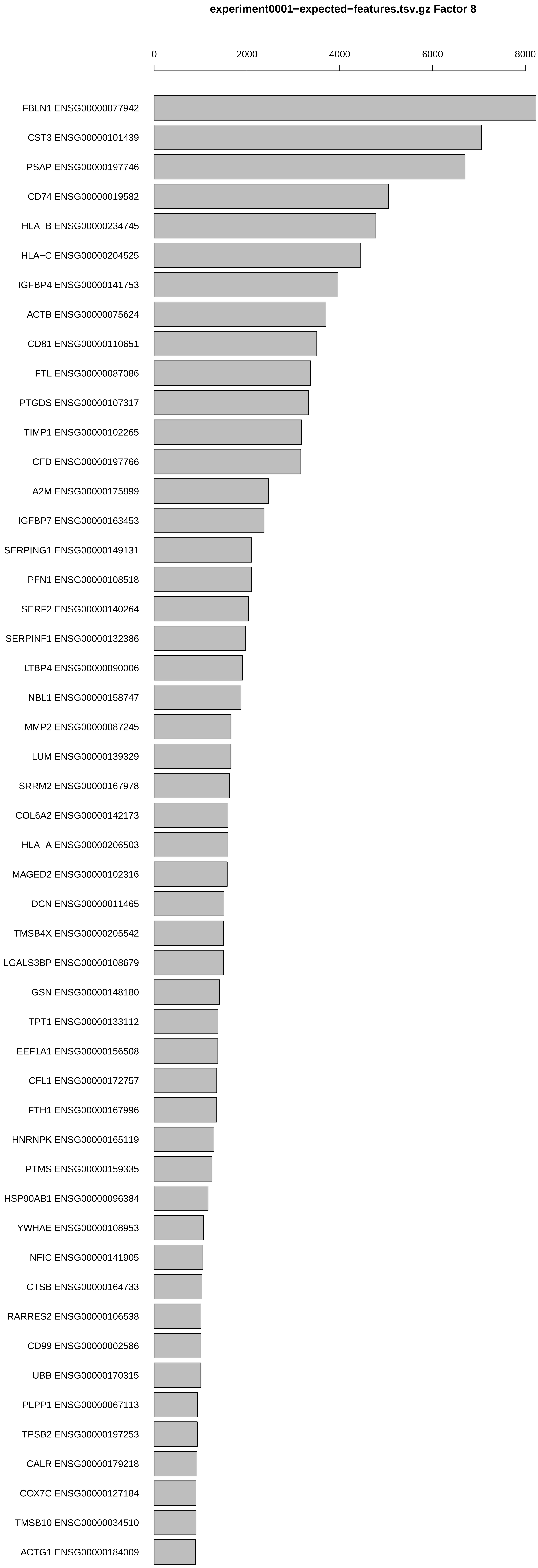
experiment0001−expected−features.tsv.gz Factor 8
0
2000
4000
6000
8000
FBLN1 ENSG00000077942
CST3 ENSG00000101439
PSAP ENSG00000197746
CD74 ENSG00000019582
HLA−B ENSG00000234745
HLA−C ENSG00000204525
IGFBP4 ENSG00000141753
ACTB ENSG00000075624
CD81 ENSG00000110651
FTL ENSG00000087086
PTGDS ENSG00000107317
TIMP1 ENSG00000102265
CFD ENSG00000197766
A2M ENSG00000175899
IGFBP7 ENSG00000163453
SERPING1 ENSG00000149131
PFN1 ENSG00000108518
SERF2 ENSG00000140264
SERPINF1 ENSG00000132386
LTBP4 ENSG00000090006
NBL1 ENSG00000158747
MMP2 ENSG00000087245
LUM ENSG00000139329
SRRM2 ENSG00000167978
COL6A2 ENSG00000142173
HLA−A ENSG00000206503
MAGED2 ENSG00000102316
DCN ENSG00000011465
TMSB4X ENSG00000205542
LGALS3BP ENSG00000108679
GSN ENSG00000148180
TPT1 ENSG00000133112
EEF1A1 ENSG00000156508
CFL1 ENSG00000172757
FTH1 ENSG00000167996
HNRNPK ENSG00000165119
PTMS ENSG00000159335
HSP90AB1 ENSG00000096384
YWHAE ENSG00000108953
NFIC ENSG00000141905
CTSB ENSG00000164733
RARRES2 ENSG00000106538
CD99 ENSG00000002586
UBB ENSG00000170315
PLPP1 ENSG00000067113
TPSB2 ENSG00000197253
CALR ENSG00000179218
COX7C ENSG00000127184
TMSB10 ENSG00000034510
ACTG1 ENSG00000184009

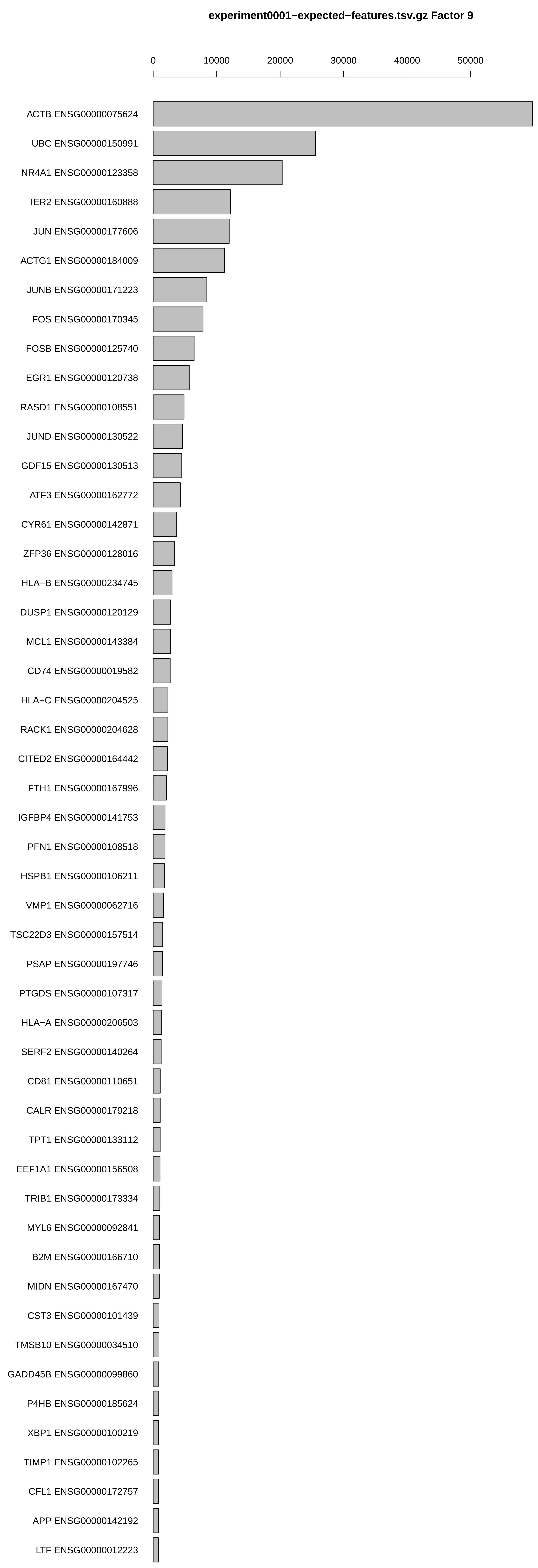
experiment0001−expected−features.tsv.gz Factor 9
0
10000
20000
30000
40000
50000
ACTB ENSG00000075624
UBC ENSG00000150991
NR4A1 ENSG00000123358
IER2 ENSG00000160888
JUN ENSG00000177606
ACTG1 ENSG00000184009
JUNB ENSG00000171223
FOS ENSG00000170345
FOSB ENSG00000125740
EGR1 ENSG00000120738
RASD1 ENSG00000108551
JUND ENSG00000130522
GDF15 ENSG00000130513
ATF3 ENSG00000162772
CYR61 ENSG00000142871
ZFP36 ENSG00000128016
HLA−B ENSG00000234745
DUSP1 ENSG00000120129
MCL1 ENSG00000143384
CD74 ENSG00000019582
HLA−C ENSG00000204525
RACK1 ENSG00000204628
CITED2 ENSG00000164442
FTH1 ENSG00000167996
IGFBP4 ENSG00000141753
PFN1 ENSG00000108518
HSPB1 ENSG00000106211
VMP1 ENSG00000062716
TSC22D3 ENSG00000157514
PSAP ENSG00000197746
PTGDS ENSG00000107317
HLA−A ENSG00000206503
SERF2 ENSG00000140264
CD81 ENSG00000110651
CALR ENSG00000179218
TPT1 ENSG00000133112
EEF1A1 ENSG00000156508
TRIB1 ENSG00000173334
MYL6 ENSG00000092841
B2M ENSG00000166710
MIDN ENSG00000167470
CST3 ENSG00000101439
TMSB10 ENSG00000034510
GADD45B ENSG00000099860
P4HB ENSG00000185624
XBP1 ENSG00000100219
TIMP1 ENSG00000102265
CFL1 ENSG00000172757
APP ENSG00000142192
LTF ENSG00000012223

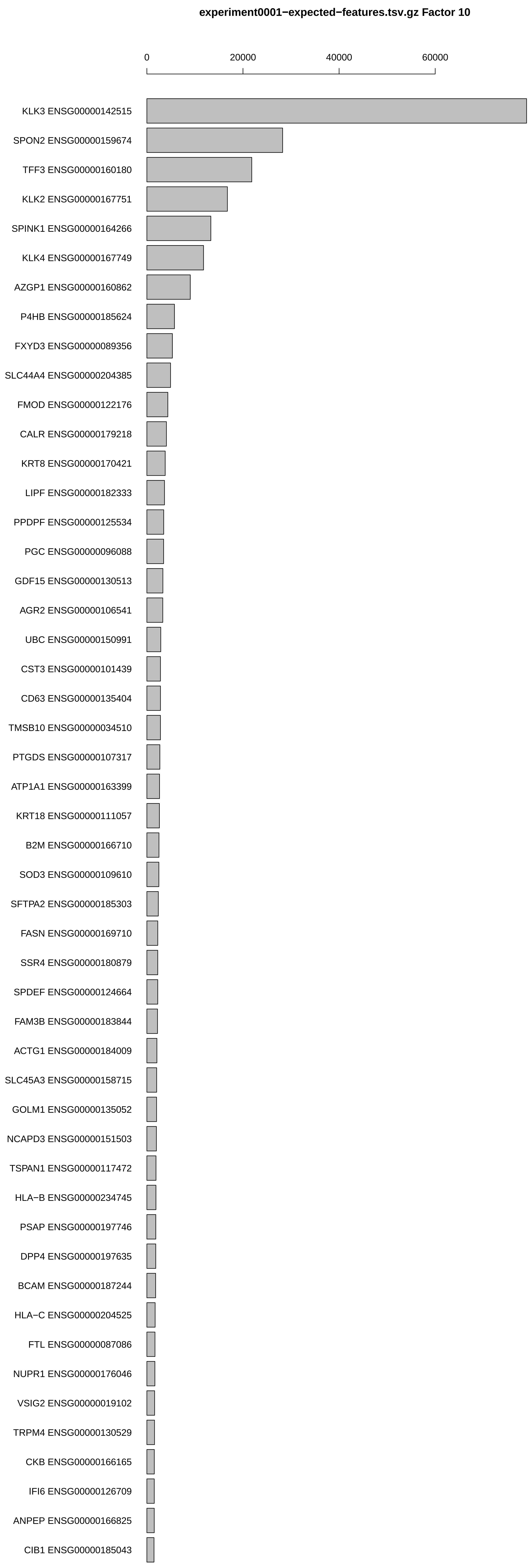
experiment0001−expected−features.tsv.gz Factor 10
0
20000
40000
60000
KLK3 ENSG00000142515
SPON2 ENSG00000159674
TFF3 ENSG00000160180
KLK2 ENSG00000167751
SPINK1 ENSG00000164266
KLK4 ENSG00000167749
AZGP1 ENSG00000160862
P4HB ENSG00000185624
FXYD3 ENSG00000089356
SLC44A4 ENSG00000204385
FMOD ENSG00000122176
CALR ENSG00000179218
KRT8 ENSG00000170421
LIPF ENSG00000182333
PPDPF ENSG00000125534
PGC ENSG00000096088
GDF15 ENSG00000130513
AGR2 ENSG00000106541
UBC ENSG00000150991
CST3 ENSG00000101439
CD63 ENSG00000135404
TMSB10 ENSG00000034510
PTGDS ENSG00000107317
ATP1A1 ENSG00000163399
KRT18 ENSG00000111057
B2M ENSG00000166710
SOD3 ENSG00000109610
SFTPA2 ENSG00000185303
FASN ENSG00000169710
SSR4 ENSG00000180879
SPDEF ENSG00000124664
FAM3B ENSG00000183844
ACTG1 ENSG00000184009
SLC45A3 ENSG00000158715
GOLM1 ENSG00000135052
NCAPD3 ENSG00000151503
TSPAN1 ENSG00000117472
HLA−B ENSG00000234745
PSAP ENSG00000197746
DPP4 ENSG00000197635
BCAM ENSG00000187244
HLA−C ENSG00000204525
FTL ENSG00000087086
NUPR1 ENSG00000176046
VSIG2 ENSG00000019102
TRPM4 ENSG00000130529
CKB ENSG00000166165
IFI6 ENSG00000126709
ANPEP ENSG00000166825
CIB1 ENSG00000185043

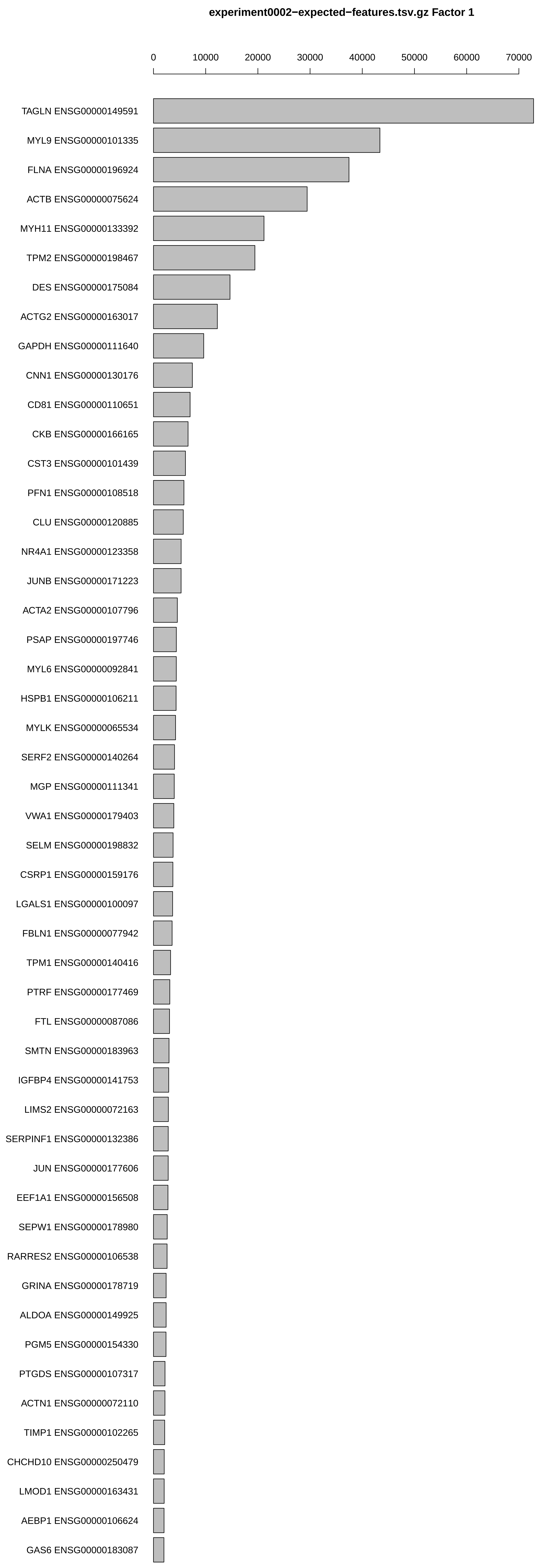
experiment0002−expected−features.tsv.gz Factor 1
0
10000
20000
30000
40000
50000
60000
70000
TAGLN ENSG00000149591
MYL9 ENSG00000101335
FLNA ENSG00000196924
ACTB ENSG00000075624
MYH11 ENSG00000133392
TPM2 ENSG00000198467
DES ENSG00000175084
ACTG2 ENSG00000163017
GAPDH ENSG00000111640
CNN1 ENSG00000130176
CD81 ENSG00000110651
CKB ENSG00000166165
CST3 ENSG00000101439
PFN1 ENSG00000108518
CLU ENSG00000120885
NR4A1 ENSG00000123358
JUNB ENSG00000171223
ACTA2 ENSG00000107796
PSAP ENSG00000197746
MYL6 ENSG00000092841
HSPB1 ENSG00000106211
MYLK ENSG00000065534
SERF2 ENSG00000140264
MGP ENSG00000111341
VWA1 ENSG00000179403
SELM ENSG00000198832
CSRP1 ENSG00000159176
LGALS1 ENSG00000100097
FBLN1 ENSG00000077942
TPM1 ENSG00000140416
PTRF ENSG00000177469
FTL ENSG00000087086
SMTN ENSG00000183963
IGFBP4 ENSG00000141753
LIMS2 ENSG00000072163
SERPINF1 ENSG00000132386
JUN ENSG00000177606
EEF1A1 ENSG00000156508
SEPW1 ENSG00000178980
RARRES2 ENSG00000106538
GRINA ENSG00000178719
ALDOA ENSG00000149925
PGM5 ENSG00000154330
PTGDS ENSG00000107317
ACTN1 ENSG00000072110
TIMP1 ENSG00000102265
CHCHD10 ENSG00000250479
LMOD1 ENSG00000163431
AEBP1 ENSG00000106624
GAS6 ENSG00000183087

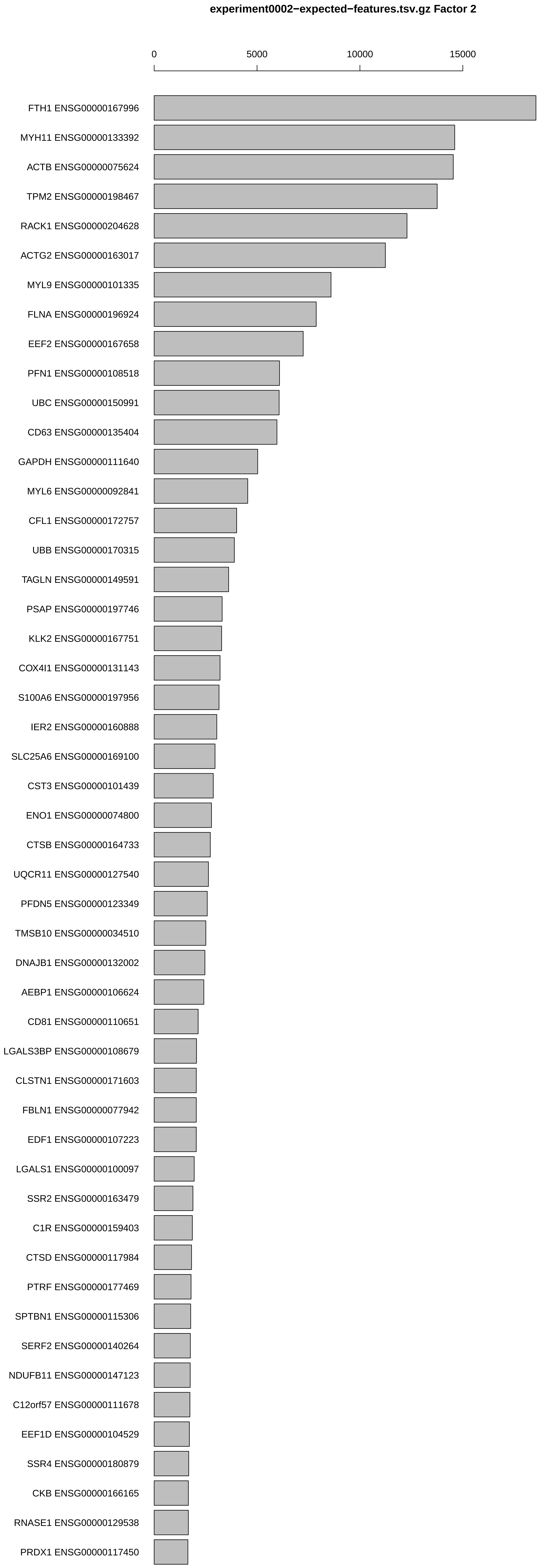

experiment0002−expected−features.tsv.gz Factor 2
0
5000
10000
15000
FTH1 ENSG00000167996
MYH11 ENSG00000133392
ACTB ENSG00000075624
TPM2 ENSG00000198467
RACK1 ENSG00000204628
ACTG2 ENSG00000163017
MYL9 ENSG00000101335
FLNA ENSG00000196924
EEF2 ENSG00000167658
PFN1 ENSG00000108518
UBC ENSG00000150991
CD63 ENSG00000135404
GAPDH ENSG00000111640
MYL6 ENSG00000092841
CFL1 ENSG00000172757
UBB ENSG00000170315
TAGLN ENSG00000149591
PSAP ENSG00000197746
KLK2 ENSG00000167751
COX4I1 ENSG00000131143
S100A6 ENSG00000197956
IER2 ENSG00000160888
SLC25A6 ENSG00000169100
CST3 ENSG00000101439
ENO1 ENSG00000074800
CTSB ENSG00000164733
UQCR11 ENSG00000127540
PFDN5 ENSG00000123349
TMSB10 ENSG00000034510
DNAJB1 ENSG00000132002
AEBP1 ENSG00000106624
CD81 ENSG00000110651
LGALS3BP ENSG00000108679
CLSTN1 ENSG00000171603
FBLN1 ENSG00000077942
EDF1 ENSG00000107223
LGALS1 ENSG00000100097
SSR2 ENSG00000163479
C1R ENSG00000159403
CTSD ENSG00000117984
PTRF ENSG00000177469
SPTBN1 ENSG00000115306
SERF2 ENSG00000140264
NDUFB11 ENSG00000147123
C12orf57 ENSG00000111678
EEF1D ENSG00000104529
SSR4 ENSG00000180879
CKB ENSG00000166165
RNASE1 ENSG00000129538
PRDX1 ENSG00000117450

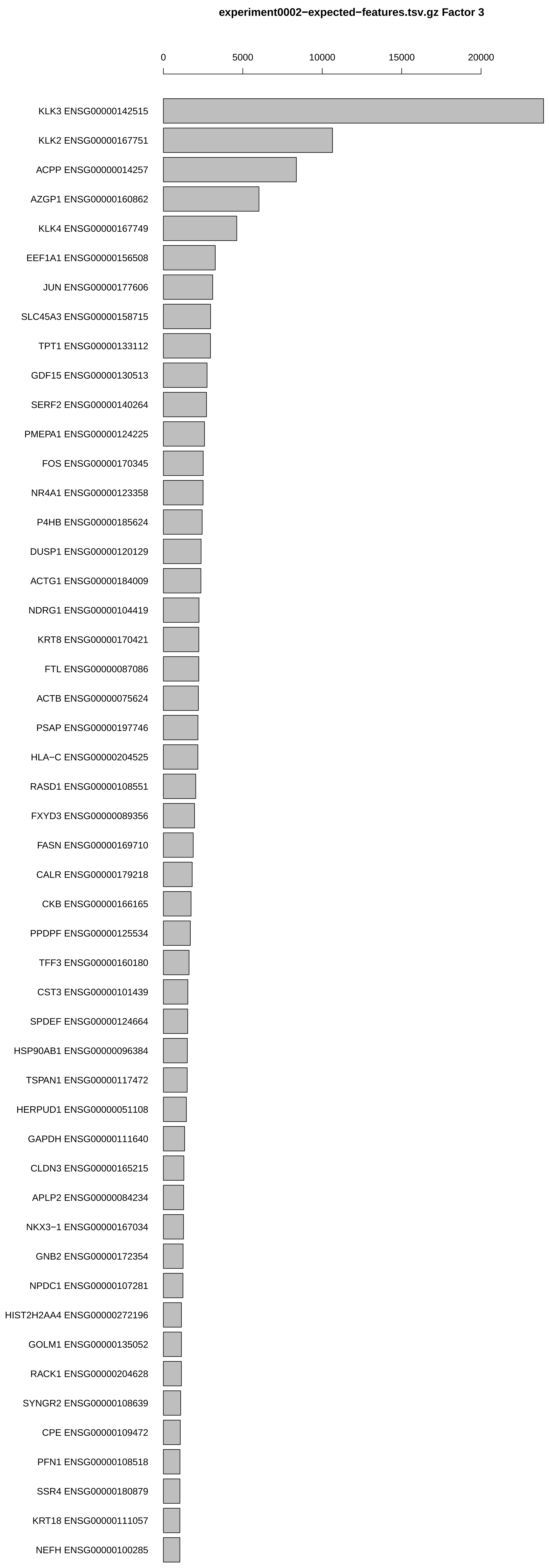

experiment0002−expected−features.tsv.gz Factor 3
0
5000
10000
15000
20000
KLK3 ENSG00000142515
KLK2 ENSG00000167751
ACPP ENSG00000014257
AZGP1 ENSG00000160862
KLK4 ENSG00000167749
EEF1A1 ENSG00000156508
JUN ENSG00000177606
SLC45A3 ENSG00000158715
TPT1 ENSG00000133112
GDF15 ENSG00000130513
SERF2 ENSG00000140264
PMEPA1 ENSG00000124225
FOS ENSG00000170345
NR4A1 ENSG00000123358
P4HB ENSG00000185624
DUSP1 ENSG00000120129
ACTG1 ENSG00000184009
NDRG1 ENSG00000104419
KRT8 ENSG00000170421
FTL ENSG00000087086
ACTB ENSG00000075624
PSAP ENSG00000197746
HLA−C ENSG00000204525
RASD1 ENSG00000108551
FXYD3 ENSG00000089356
FASN ENSG00000169710
CALR ENSG00000179218
CKB ENSG00000166165
PPDPF ENSG00000125534
TFF3 ENSG00000160180
CST3 ENSG00000101439
SPDEF ENSG00000124664
HSP90AB1 ENSG00000096384
TSPAN1 ENSG00000117472
HERPUD1 ENSG00000051108
GAPDH ENSG00000111640
CLDN3 ENSG00000165215
APLP2 ENSG00000084234
NKX3−1 ENSG00000167034
GNB2 ENSG00000172354
NPDC1 ENSG00000107281
HIST2H2AA4 ENSG00000272196
GOLM1 ENSG00000135052
RACK1 ENSG00000204628
SYNGR2 ENSG00000108639
CPE ENSG00000109472
PFN1 ENSG00000108518
SSR4 ENSG00000180879
KRT18 ENSG00000111057
NEFH ENSG00000100285

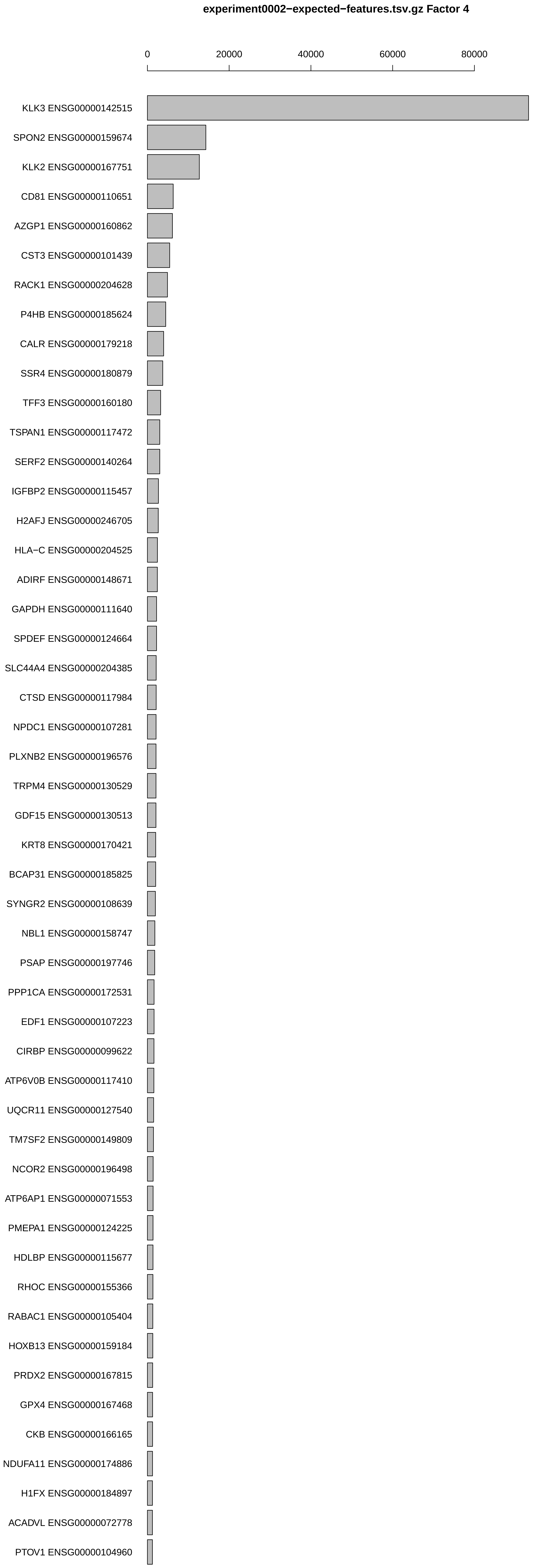
experiment0002−expected−features.tsv.gz Factor 4
0
20000
40000
60000
80000
KLK3 ENSG00000142515
SPON2 ENSG00000159674
KLK2 ENSG00000167751
CD81 ENSG00000110651
AZGP1 ENSG00000160862
CST3 ENSG00000101439
RACK1 ENSG00000204628
P4HB ENSG00000185624
CALR ENSG00000179218
SSR4 ENSG00000180879
TFF3 ENSG00000160180
TSPAN1 ENSG00000117472
SERF2 ENSG00000140264
IGFBP2 ENSG00000115457
H2AFJ ENSG00000246705
HLA−C ENSG00000204525
ADIRF ENSG00000148671
GAPDH ENSG00000111640
SPDEF ENSG00000124664
SLC44A4 ENSG00000204385
CTSD ENSG00000117984
NPDC1 ENSG00000107281
PLXNB2 ENSG00000196576
TRPM4 ENSG00000130529
GDF15 ENSG00000130513
KRT8 ENSG00000170421
BCAP31 ENSG00000185825
SYNGR2 ENSG00000108639
NBL1 ENSG00000158747
PSAP ENSG00000197746
PPP1CA ENSG00000172531
EDF1 ENSG00000107223
CIRBP ENSG00000099622
ATP6V0B ENSG00000117410
UQCR11 ENSG00000127540
TM7SF2 ENSG00000149809
NCOR2 ENSG00000196498
ATP6AP1 ENSG00000071553
PMEPA1 ENSG00000124225
HDLBP ENSG00000115677
RHOC ENSG00000155366
RABAC1 ENSG00000105404
HOXB13 ENSG00000159184
PRDX2 ENSG00000167815
GPX4 ENSG00000167468
CKB ENSG00000166165
NDUFA11 ENSG00000174886
H1FX ENSG00000184897
ACADVL ENSG00000072778
PTOV1 ENSG00000104960

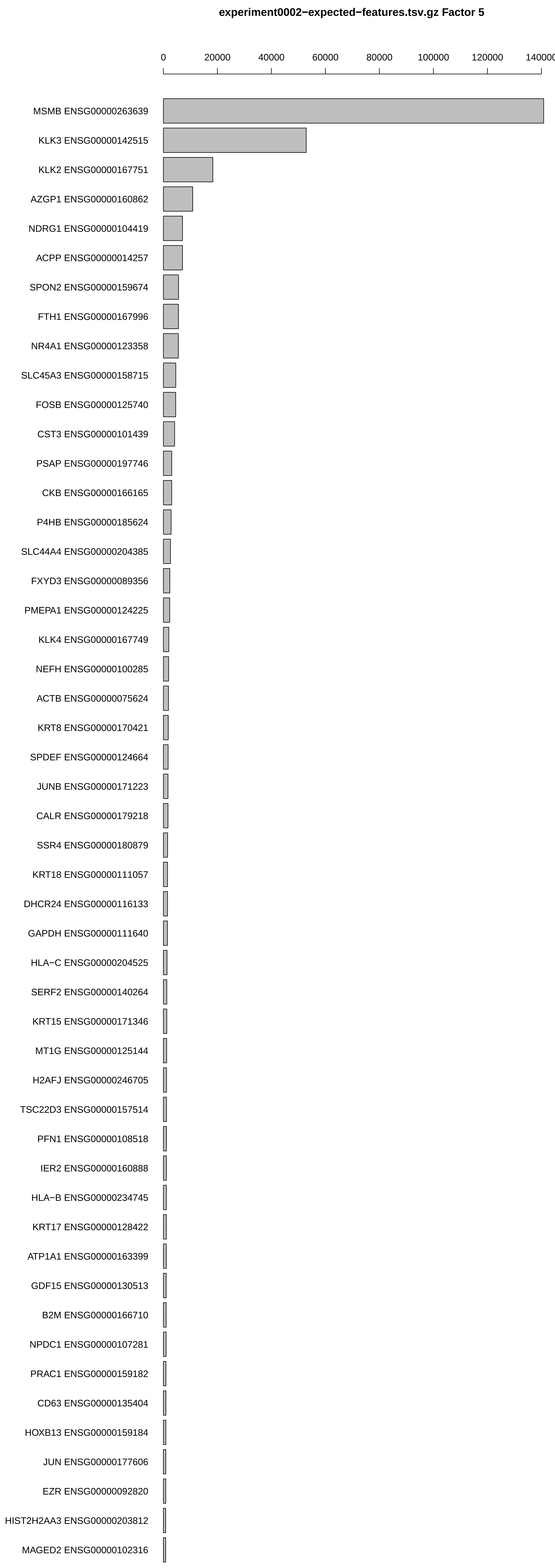

experiment0002−expected−features.tsv.gz Factor 5
0
20000
40000
60000
80000
100000
120000
140000
MSMB ENSG00000263639
KLK3 ENSG00000142515
KLK2 ENSG00000167751
AZGP1 ENSG00000160862
NDRG1 ENSG00000104419
ACPP ENSG00000014257
SPON2 ENSG00000159674
FTH1 ENSG00000167996
NR4A1 ENSG00000123358
SLC45A3 ENSG00000158715
FOSB ENSG00000125740
CST3 ENSG00000101439
PSAP ENSG00000197746
CKB ENSG00000166165
P4HB ENSG00000185624
SLC44A4 ENSG00000204385
FXYD3 ENSG00000089356
PMEPA1 ENSG00000124225
KLK4 ENSG00000167749
NEFH ENSG00000100285
ACTB ENSG00000075624
KRT8 ENSG00000170421
SPDEF ENSG00000124664
JUNB ENSG00000171223
CALR ENSG00000179218
SSR4 ENSG00000180879
KRT18 ENSG00000111057
DHCR24 ENSG00000116133
GAPDH ENSG00000111640
HLA−C ENSG00000204525
SERF2 ENSG00000140264
KRT15 ENSG00000171346
MT1G ENSG00000125144
H2AFJ ENSG00000246705
TSC22D3 ENSG00000157514
PFN1 ENSG00000108518
IER2 ENSG00000160888
HLA−B ENSG00000234745
KRT17 ENSG00000128422
ATP1A1 ENSG00000163399
GDF15 ENSG00000130513
B2M ENSG00000166710
NPDC1 ENSG00000107281
PRAC1 ENSG00000159182
CD63 ENSG00000135404
HOXB13 ENSG00000159184
JUN ENSG00000177606
EZR ENSG00000092820
HIST2H2AA3 ENSG00000203812
MAGED2 ENSG00000102316

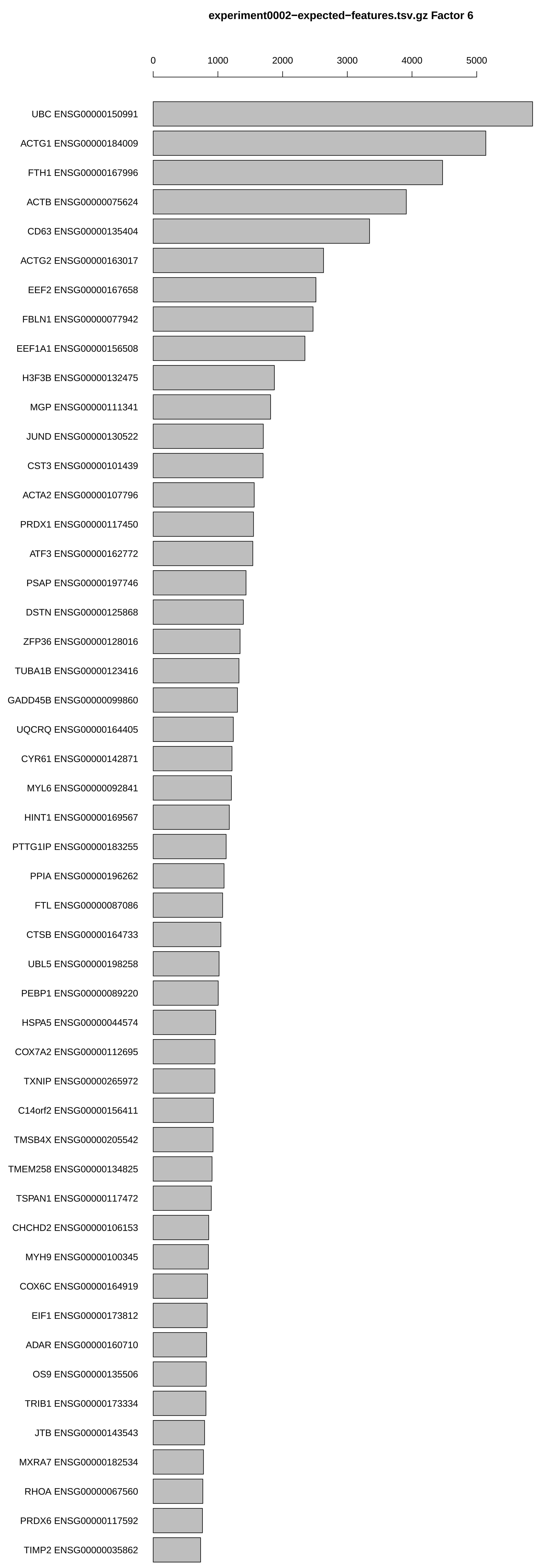
experiment0002−expected−features.tsv.gz Factor 6
0
1000
2000
3000
4000
5000
UBC ENSG00000150991
ACTG1 ENSG00000184009
FTH1 ENSG00000167996
ACTB ENSG00000075624
CD63 ENSG00000135404
ACTG2 ENSG00000163017
EEF2 ENSG00000167658
FBLN1 ENSG00000077942
EEF1A1 ENSG00000156508
H3F3B ENSG00000132475
MGP ENSG00000111341
JUND ENSG00000130522
CST3 ENSG00000101439
ACTA2 ENSG00000107796
PRDX1 ENSG00000117450
ATF3 ENSG00000162772
PSAP ENSG00000197746
DSTN ENSG00000125868
ZFP36 ENSG00000128016
TUBA1B ENSG00000123416
GADD45B ENSG00000099860
UQCRQ ENSG00000164405
CYR61 ENSG00000142871
MYL6 ENSG00000092841
HINT1 ENSG00000169567
PTTG1IP ENSG00000183255
PPIA ENSG00000196262
FTL ENSG00000087086
CTSB ENSG00000164733
UBL5 ENSG00000198258
PEBP1 ENSG00000089220
HSPA5 ENSG00000044574
COX7A2 ENSG00000112695
TXNIP ENSG00000265972
C14orf2 ENSG00000156411
TMSB4X ENSG00000205542
TMEM258 ENSG00000134825
TSPAN1 ENSG00000117472
CHCHD2 ENSG00000106153
MYH9 ENSG00000100345
COX6C ENSG00000164919
EIF1 ENSG00000173812
ADAR ENSG00000160710
OS9 ENSG00000135506
TRIB1 ENSG00000173334
JTB ENSG00000143543
MXRA7 ENSG00000182534
RHOA ENSG00000067560
PRDX6 ENSG00000117592
TIMP2 ENSG00000035862

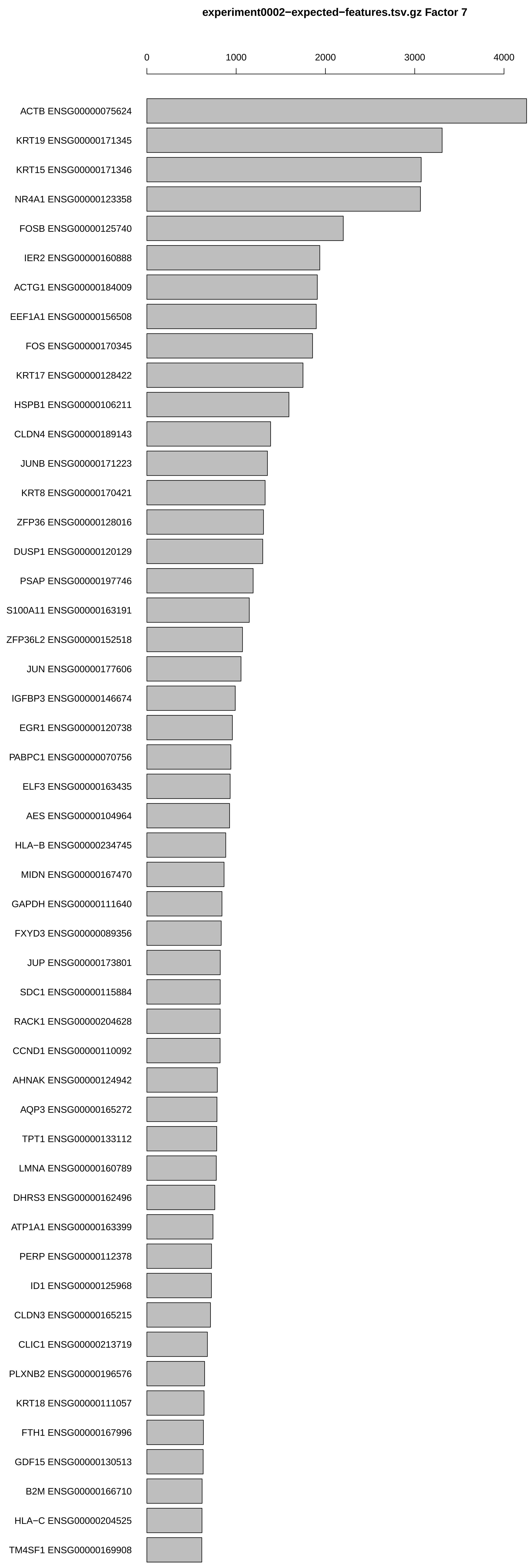

experiment0002−expected−features.tsv.gz Factor 7
0
1000
2000
3000
4000
ACTB ENSG00000075624
KRT19 ENSG00000171345
KRT15 ENSG00000171346
NR4A1 ENSG00000123358
FOSB ENSG00000125740
IER2 ENSG00000160888
ACTG1 ENSG00000184009
EEF1A1 ENSG00000156508
FOS ENSG00000170345
KRT17 ENSG00000128422
HSPB1 ENSG00000106211
CLDN4 ENSG00000189143
JUNB ENSG00000171223
KRT8 ENSG00000170421
ZFP36 ENSG00000128016
DUSP1 ENSG00000120129
PSAP ENSG00000197746
S100A11 ENSG00000163191
ZFP36L2 ENSG00000152518
JUN ENSG00000177606
IGFBP3 ENSG00000146674
EGR1 ENSG00000120738
PABPC1 ENSG00000070756
ELF3 ENSG00000163435
AES ENSG00000104964
HLA−B ENSG00000234745
MIDN ENSG00000167470
GAPDH ENSG00000111640
FXYD3 ENSG00000089356
JUP ENSG00000173801
SDC1 ENSG00000115884
RACK1 ENSG00000204628
CCND1 ENSG00000110092
AHNAK ENSG00000124942
AQP3 ENSG00000165272
TPT1 ENSG00000133112
LMNA ENSG00000160789
DHRS3 ENSG00000162496
ATP1A1 ENSG00000163399
PERP ENSG00000112378
ID1 ENSG00000125968
CLDN3 ENSG00000165215
CLIC1 ENSG00000213719
PLXNB2 ENSG00000196576
KRT18 ENSG00000111057
FTH1 ENSG00000167996
GDF15 ENSG00000130513
B2M ENSG00000166710
HLA−C ENSG00000204525
TM4SF1 ENSG00000169908

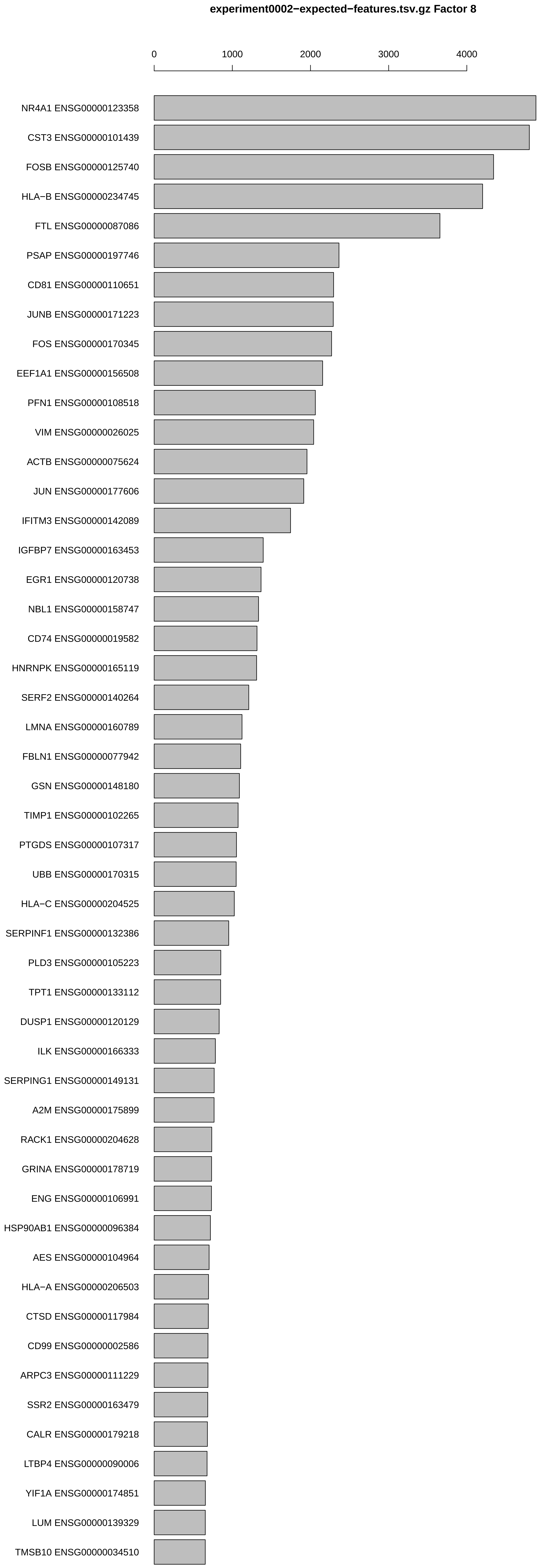
experiment0002−expected−features.tsv.gz Factor 8
0
1000
2000
3000
4000
NR4A1 ENSG00000123358
CST3 ENSG00000101439
FOSB ENSG00000125740
HLA−B ENSG00000234745
FTL ENSG00000087086
PSAP ENSG00000197746
CD81 ENSG00000110651
JUNB ENSG00000171223
FOS ENSG00000170345
EEF1A1 ENSG00000156508
PFN1 ENSG00000108518
VIM ENSG00000026025
ACTB ENSG00000075624
JUN ENSG00000177606
IFITM3 ENSG00000142089
IGFBP7 ENSG00000163453
EGR1 ENSG00000120738
NBL1 ENSG00000158747
CD74 ENSG00000019582
HNRNPK ENSG00000165119
SERF2 ENSG00000140264
LMNA ENSG00000160789
FBLN1 ENSG00000077942
GSN ENSG00000148180
TIMP1 ENSG00000102265
PTGDS ENSG00000107317
UBB ENSG00000170315
HLA−C ENSG00000204525
SERPINF1 ENSG00000132386
PLD3 ENSG00000105223
TPT1 ENSG00000133112
DUSP1 ENSG00000120129
ILK ENSG00000166333
SERPING1 ENSG00000149131
A2M ENSG00000175899
RACK1 ENSG00000204628
GRINA ENSG00000178719
ENG ENSG00000106991
HSP90AB1 ENSG00000096384
AES ENSG00000104964
HLA−A ENSG00000206503
CTSD ENSG00000117984
CD99 ENSG00000002586
ARPC3 ENSG00000111229
SSR2 ENSG00000163479
CALR ENSG00000179218
LTBP4 ENSG00000090006
YIF1A ENSG00000174851
LUM ENSG00000139329
TMSB10 ENSG00000034510

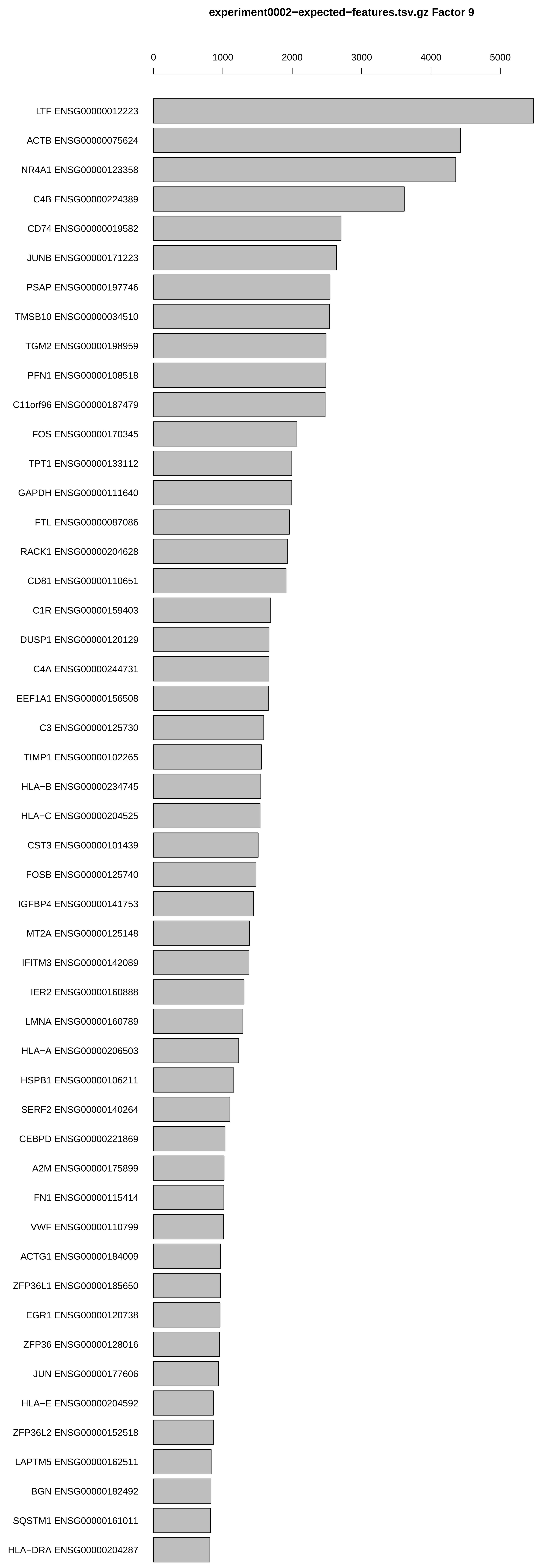
experiment0002−expected−features.tsv.gz Factor 9
0
1000
2000
3000
4000
5000
LTF ENSG00000012223
ACTB ENSG00000075624
NR4A1 ENSG00000123358
C4B ENSG00000224389
CD74 ENSG00000019582
JUNB ENSG00000171223
PSAP ENSG00000197746
TMSB10 ENSG00000034510
TGM2 ENSG00000198959
PFN1 ENSG00000108518
C11orf96 ENSG00000187479
FOS ENSG00000170345
TPT1 ENSG00000133112
GAPDH ENSG00000111640
FTL ENSG00000087086
RACK1 ENSG00000204628
CD81 ENSG00000110651
C1R ENSG00000159403
DUSP1 ENSG00000120129
C4A ENSG00000244731
EEF1A1 ENSG00000156508
C3 ENSG00000125730
TIMP1 ENSG00000102265
HLA−B ENSG00000234745
HLA−C ENSG00000204525
CST3 ENSG00000101439
FOSB ENSG00000125740
IGFBP4 ENSG00000141753
MT2A ENSG00000125148
IFITM3 ENSG00000142089
IER2 ENSG00000160888
LMNA ENSG00000160789
HLA−A ENSG00000206503
HSPB1 ENSG00000106211
SERF2 ENSG00000140264
CEBPD ENSG00000221869
A2M ENSG00000175899
FN1 ENSG00000115414
VWF ENSG00000110799
ACTG1 ENSG00000184009
ZFP36L1 ENSG00000185650
EGR1 ENSG00000120738
ZFP36 ENSG00000128016
JUN ENSG00000177606
HLA−E ENSG00000204592
ZFP36L2 ENSG00000152518
LAPTM5 ENSG00000162511
BGN ENSG00000182492
SQSTM1 ENSG00000161011
HLA−DRA ENSG00000204287

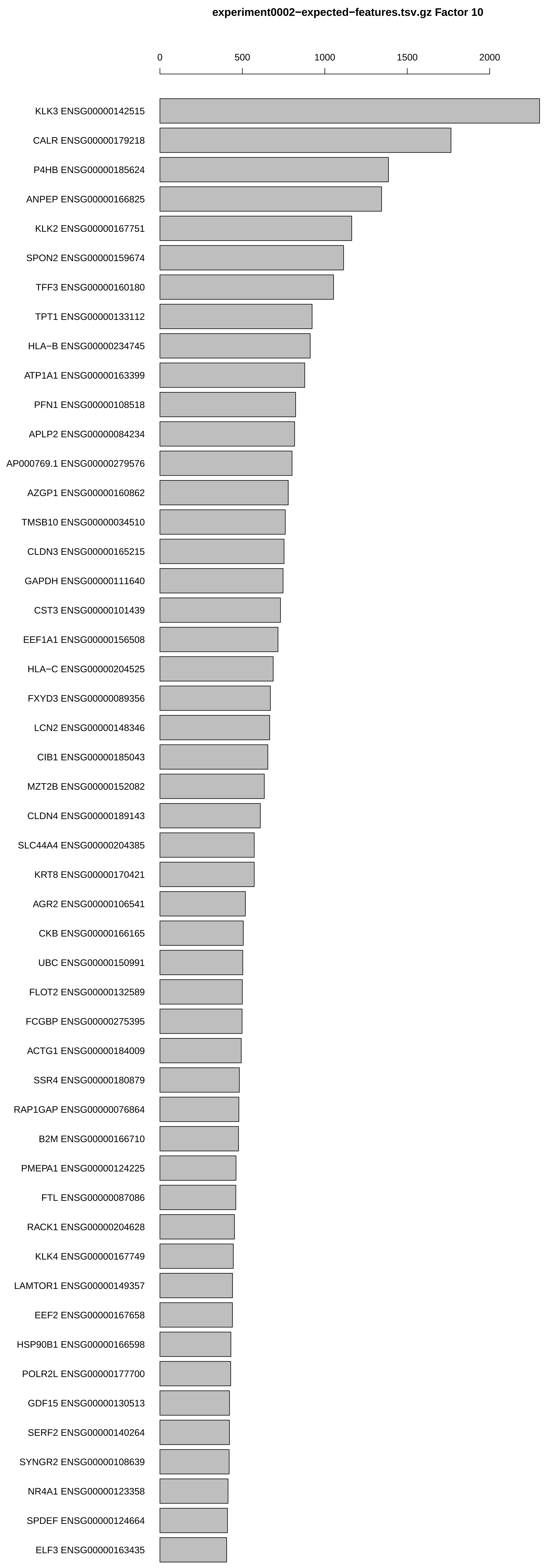
experiment0002−expected−features.tsv.gz Factor 10
0
500
1000
1500
2000
KLK3 ENSG00000142515
CALR ENSG00000179218
P4HB ENSG00000185624
ANPEP ENSG00000166825
KLK2 ENSG00000167751
SPON2 ENSG00000159674
TFF3 ENSG00000160180
TPT1 ENSG00000133112
HLA−B ENSG00000234745
ATP1A1 ENSG00000163399
PFN1 ENSG00000108518
APLP2 ENSG00000084234
AP000769.1 ENSG00000279576
AZGP1 ENSG00000160862
TMSB10 ENSG00000034510
CLDN3 ENSG00000165215
GAPDH ENSG00000111640
CST3 ENSG00000101439
EEF1A1 ENSG00000156508
HLA−C ENSG00000204525
FXYD3 ENSG00000089356
LCN2 ENSG00000148346
CIB1 ENSG00000185043
MZT2B ENSG00000152082
CLDN4 ENSG00000189143
SLC44A4 ENSG00000204385
KRT8 ENSG00000170421
AGR2 ENSG00000106541
CKB ENSG00000166165
UBC ENSG00000150991
FLOT2 ENSG00000132589
FCGBP ENSG00000275395
ACTG1 ENSG00000184009
SSR4 ENSG00000180879
RAP1GAP ENSG00000076864
B2M ENSG00000166710
PMEPA1 ENSG00000124225
FTL ENSG00000087086
RACK1 ENSG00000204628
KLK4 ENSG00000167749
LAMTOR1 ENSG00000149357
EEF2 ENSG00000167658
HSP90B1 ENSG00000166598
POLR2L ENSG00000177700
GDF15 ENSG00000130513
SERF2 ENSG00000140264
SYNGR2 ENSG00000108639
NR4A1 ENSG00000123358
SPDEF ENSG00000124664
ELF3 ENSG00000163435

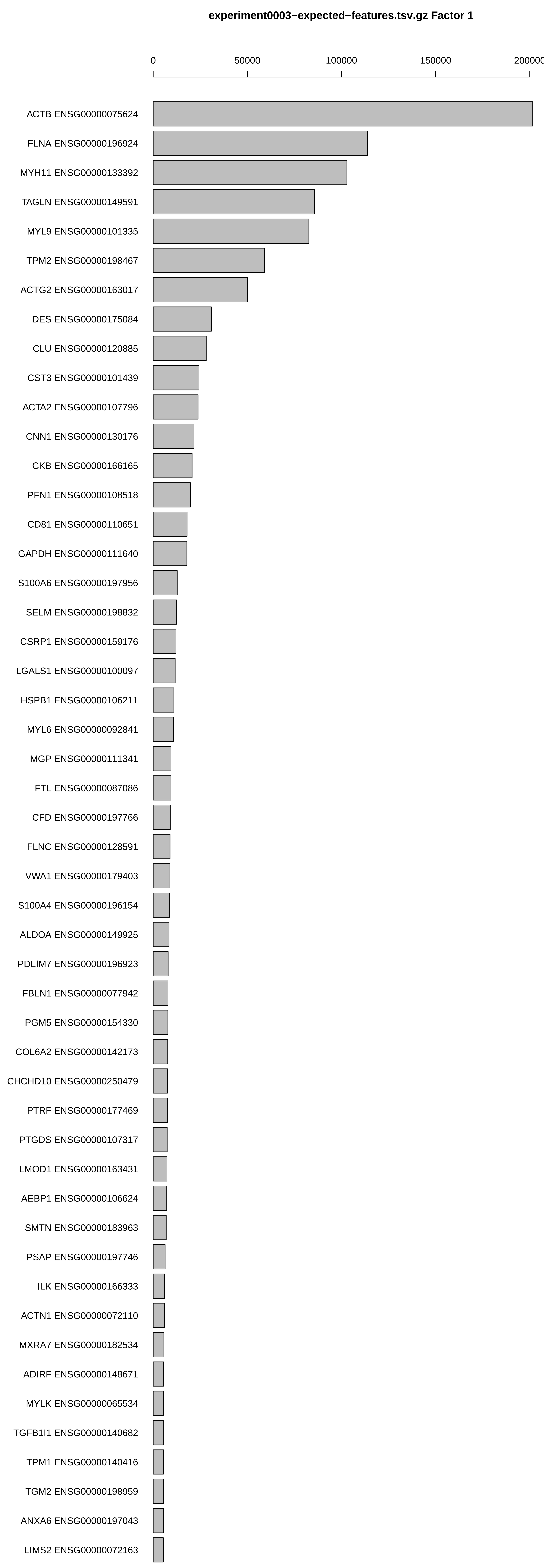
experiment0003−expected−features.tsv.gz Factor 1
0
50000
100000
150000
200000
ACTB ENSG00000075624
FLNA ENSG00000196924
MYH11 ENSG00000133392
TAGLN ENSG00000149591
MYL9 ENSG00000101335
TPM2 ENSG00000198467
ACTG2 ENSG00000163017
DES ENSG00000175084
CLU ENSG00000120885
CST3 ENSG00000101439
ACTA2 ENSG00000107796
CNN1 ENSG00000130176
CKB ENSG00000166165
PFN1 ENSG00000108518
CD81 ENSG00000110651
GAPDH ENSG00000111640
S100A6 ENSG00000197956
SELM ENSG00000198832
CSRP1 ENSG00000159176
LGALS1 ENSG00000100097
HSPB1 ENSG00000106211
MYL6 ENSG00000092841
MGP ENSG00000111341
FTL ENSG00000087086
CFD ENSG00000197766
FLNC ENSG00000128591
VWA1 ENSG00000179403
S100A4 ENSG00000196154
ALDOA ENSG00000149925
PDLIM7 ENSG00000196923
FBLN1 ENSG00000077942
PGM5 ENSG00000154330
COL6A2 ENSG00000142173
CHCHD10 ENSG00000250479
PTRF ENSG00000177469
PTGDS ENSG00000107317
LMOD1 ENSG00000163431
AEBP1 ENSG00000106624
SMTN ENSG00000183963
PSAP ENSG00000197746
ILK ENSG00000166333
ACTN1 ENSG00000072110
MXRA7 ENSG00000182534
ADIRF ENSG00000148671
MYLK ENSG00000065534
TGFB1I1 ENSG00000140682
TPM1 ENSG00000140416
TGM2 ENSG00000198959
ANXA6 ENSG00000197043
LIMS2 ENSG00000072163

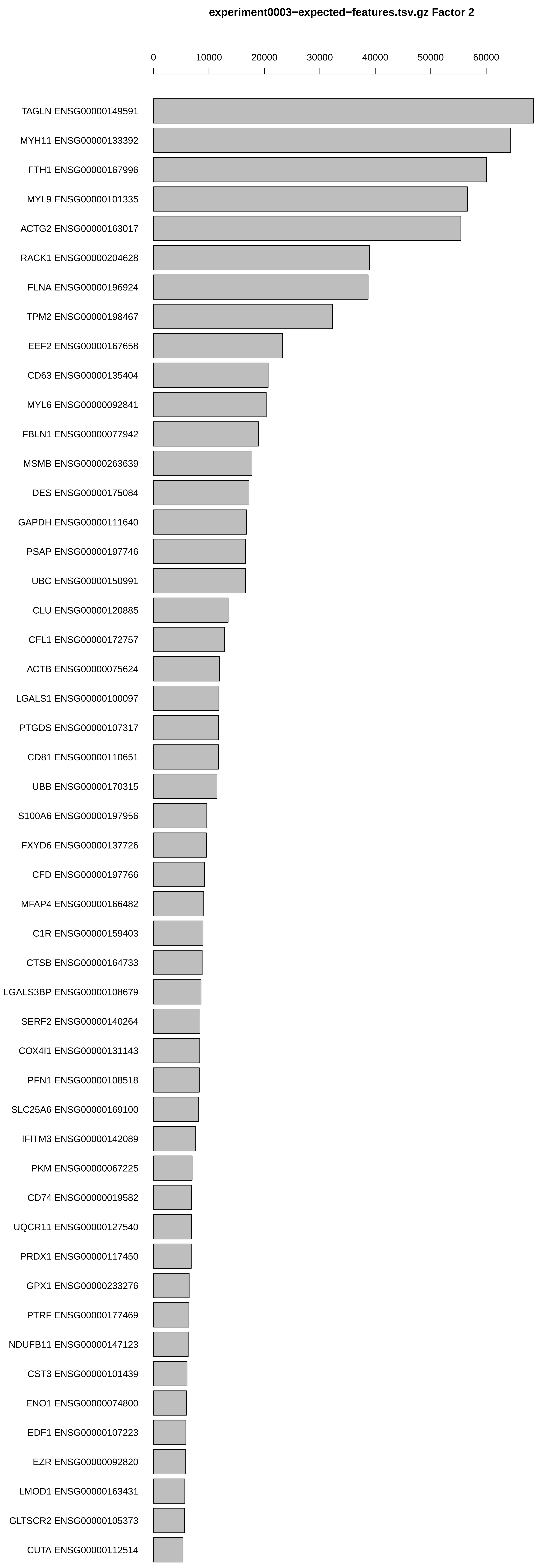
experiment0003−expected−features.tsv.gz Factor 2
0
10000
20000
30000
40000
50000
60000
TAGLN ENSG00000149591
MYH11 ENSG00000133392
FTH1 ENSG00000167996
MYL9 ENSG00000101335
ACTG2 ENSG00000163017
RACK1 ENSG00000204628
FLNA ENSG00000196924
TPM2 ENSG00000198467
EEF2 ENSG00000167658
CD63 ENSG00000135404
MYL6 ENSG00000092841
FBLN1 ENSG00000077942
MSMB ENSG00000263639
DES ENSG00000175084
GAPDH ENSG00000111640
PSAP ENSG00000197746
UBC ENSG00000150991
CLU ENSG00000120885
CFL1 ENSG00000172757
ACTB ENSG00000075624
LGALS1 ENSG00000100097
PTGDS ENSG00000107317
CD81 ENSG00000110651
UBB ENSG00000170315
S100A6 ENSG00000197956
FXYD6 ENSG00000137726
CFD ENSG00000197766
MFAP4 ENSG00000166482
C1R ENSG00000159403
CTSB ENSG00000164733
LGALS3BP ENSG00000108679
SERF2 ENSG00000140264
COX4I1 ENSG00000131143
PFN1 ENSG00000108518
SLC25A6 ENSG00000169100
IFITM3 ENSG00000142089
PKM ENSG00000067225
CD74 ENSG00000019582
UQCR11 ENSG00000127540
PRDX1 ENSG00000117450
GPX1 ENSG00000233276
PTRF ENSG00000177469
NDUFB11 ENSG00000147123
CST3 ENSG00000101439
ENO1 ENSG00000074800
EDF1 ENSG00000107223
EZR ENSG00000092820
LMOD1 ENSG00000163431
GLTSCR2 ENSG00000105373
CUTA ENSG00000112514

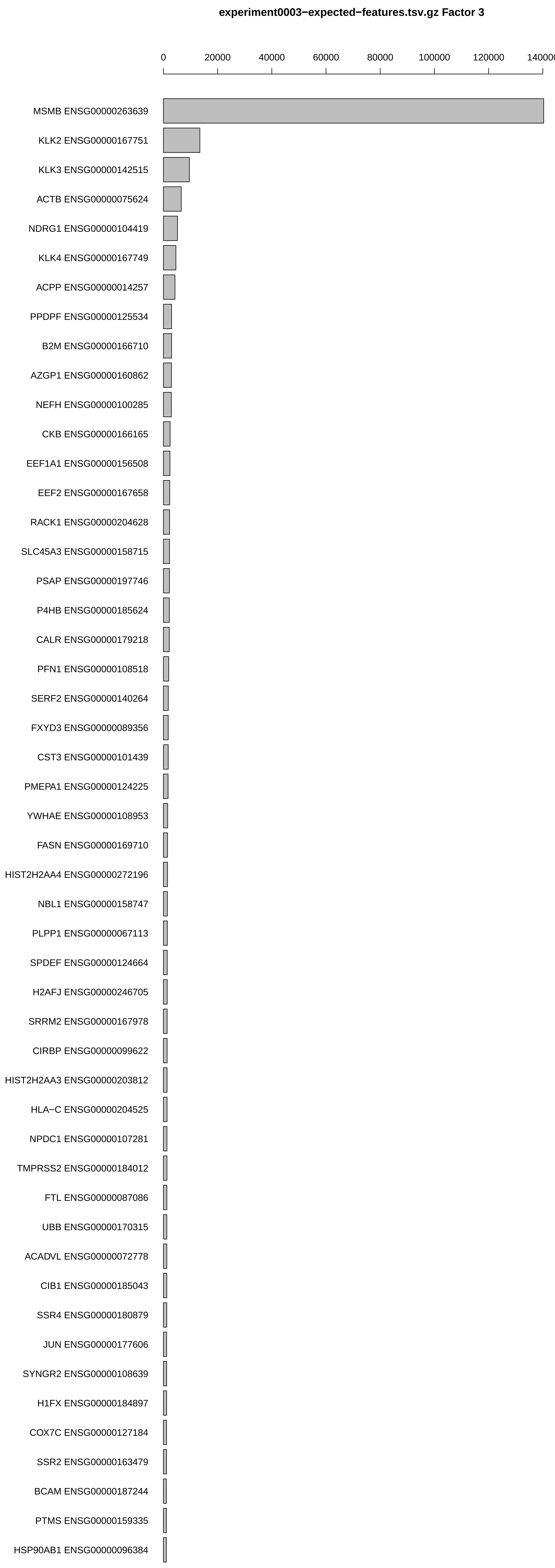

experiment0003−expected−features.tsv.gz Factor 3
0
20000
40000
60000
80000
100000
120000
140000
MSMB ENSG00000263639
KLK2 ENSG00000167751
KLK3 ENSG00000142515
ACTB ENSG00000075624
NDRG1 ENSG00000104419
KLK4 ENSG00000167749
ACPP ENSG00000014257
PPDPF ENSG00000125534
B2M ENSG00000166710
AZGP1 ENSG00000160862
NEFH ENSG00000100285
CKB ENSG00000166165
EEF1A1 ENSG00000156508
EEF2 ENSG00000167658
RACK1 ENSG00000204628
SLC45A3 ENSG00000158715
PSAP ENSG00000197746
P4HB ENSG00000185624
CALR ENSG00000179218
PFN1 ENSG00000108518
SERF2 ENSG00000140264
FXYD3 ENSG00000089356
CST3 ENSG00000101439
PMEPA1 ENSG00000124225
YWHAE ENSG00000108953
FASN ENSG00000169710
HIST2H2AA4 ENSG00000272196
NBL1 ENSG00000158747
PLPP1 ENSG00000067113
SPDEF ENSG00000124664
H2AFJ ENSG00000246705
SRRM2 ENSG00000167978
CIRBP ENSG00000099622
HIST2H2AA3 ENSG00000203812
HLA−C ENSG00000204525
NPDC1 ENSG00000107281
TMPRSS2 ENSG00000184012
FTL ENSG00000087086
UBB ENSG00000170315
ACADVL ENSG00000072778
CIB1 ENSG00000185043
SSR4 ENSG00000180879
JUN ENSG00000177606
SYNGR2 ENSG00000108639
H1FX ENSG00000184897
COX7C ENSG00000127184
SSR2 ENSG00000163479
BCAM ENSG00000187244
PTMS ENSG00000159335
HSP90AB1 ENSG00000096384

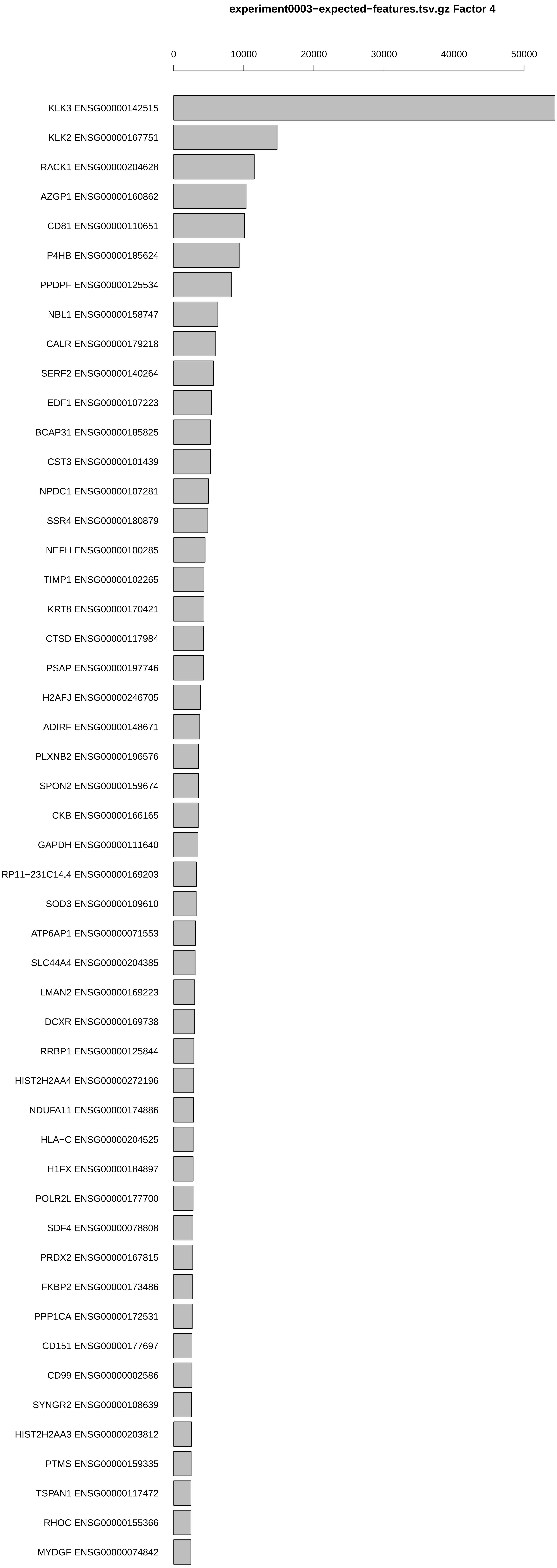
experiment0003−expected−features.tsv.gz Factor 4
0
10000
20000
30000
40000
50000
KLK3 ENSG00000142515
KLK2 ENSG00000167751
RACK1 ENSG00000204628
AZGP1 ENSG00000160862
CD81 ENSG00000110651
P4HB ENSG00000185624
PPDPF ENSG00000125534
NBL1 ENSG00000158747
CALR ENSG00000179218
SERF2 ENSG00000140264
EDF1 ENSG00000107223
BCAP31 ENSG00000185825
CST3 ENSG00000101439
NPDC1 ENSG00000107281
SSR4 ENSG00000180879
NEFH ENSG00000100285
TIMP1 ENSG00000102265
KRT8 ENSG00000170421
CTSD ENSG00000117984
PSAP ENSG00000197746
H2AFJ ENSG00000246705
ADIRF ENSG00000148671
PLXNB2 ENSG00000196576
SPON2 ENSG00000159674
CKB ENSG00000166165
GAPDH ENSG00000111640
RP11−231C14.4 ENSG00000169203
SOD3 ENSG00000109610
ATP6AP1 ENSG00000071553
SLC44A4 ENSG00000204385
LMAN2 ENSG00000169223
DCXR ENSG00000169738
RRBP1 ENSG00000125844
HIST2H2AA4 ENSG00000272196
NDUFA11 ENSG00000174886
HLA−C ENSG00000204525
H1FX ENSG00000184897
POLR2L ENSG00000177700
SDF4 ENSG00000078808
PRDX2 ENSG00000167815
FKBP2 ENSG00000173486
PPP1CA ENSG00000172531
CD151 ENSG00000177697
CD99 ENSG00000002586
SYNGR2 ENSG00000108639
HIST2H2AA3 ENSG00000203812
PTMS ENSG00000159335
TSPAN1 ENSG00000117472
RHOC ENSG00000155366
MYDGF ENSG00000074842

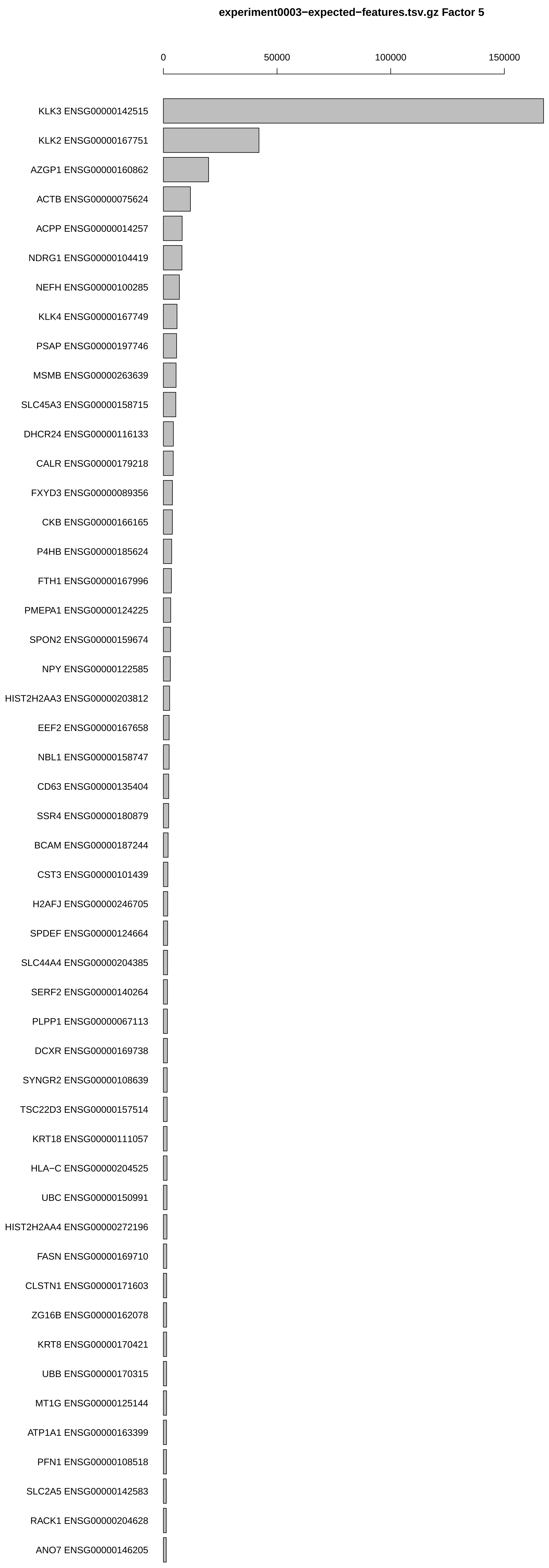
experiment0003−expected−features.tsv.gz Factor 5
0
50000
100000
150000
KLK3 ENSG00000142515
KLK2 ENSG00000167751
AZGP1 ENSG00000160862
ACTB ENSG00000075624
ACPP ENSG00000014257
NDRG1 ENSG00000104419
NEFH ENSG00000100285
KLK4 ENSG00000167749
PSAP ENSG00000197746
MSMB ENSG00000263639
SLC45A3 ENSG00000158715
DHCR24 ENSG00000116133
CALR ENSG00000179218
FXYD3 ENSG00000089356
CKB ENSG00000166165
P4HB ENSG00000185624
FTH1 ENSG00000167996
PMEPA1 ENSG00000124225
SPON2 ENSG00000159674
NPY ENSG00000122585
HIST2H2AA3 ENSG00000203812
EEF2 ENSG00000167658
NBL1 ENSG00000158747
CD63 ENSG00000135404
SSR4 ENSG00000180879
BCAM ENSG00000187244
CST3 ENSG00000101439
H2AFJ ENSG00000246705
SPDEF ENSG00000124664
SLC44A4 ENSG00000204385
SERF2 ENSG00000140264
PLPP1 ENSG00000067113
DCXR ENSG00000169738
SYNGR2 ENSG00000108639
TSC22D3 ENSG00000157514
KRT18 ENSG00000111057
HLA−C ENSG00000204525
UBC ENSG00000150991
HIST2H2AA4 ENSG00000272196
FASN ENSG00000169710
CLSTN1 ENSG00000171603
ZG16B ENSG00000162078
KRT8 ENSG00000170421
UBB ENSG00000170315
MT1G ENSG00000125144
ATP1A1 ENSG00000163399
PFN1 ENSG00000108518
SLC2A5 ENSG00000142583
RACK1 ENSG00000204628
ANO7 ENSG00000146205

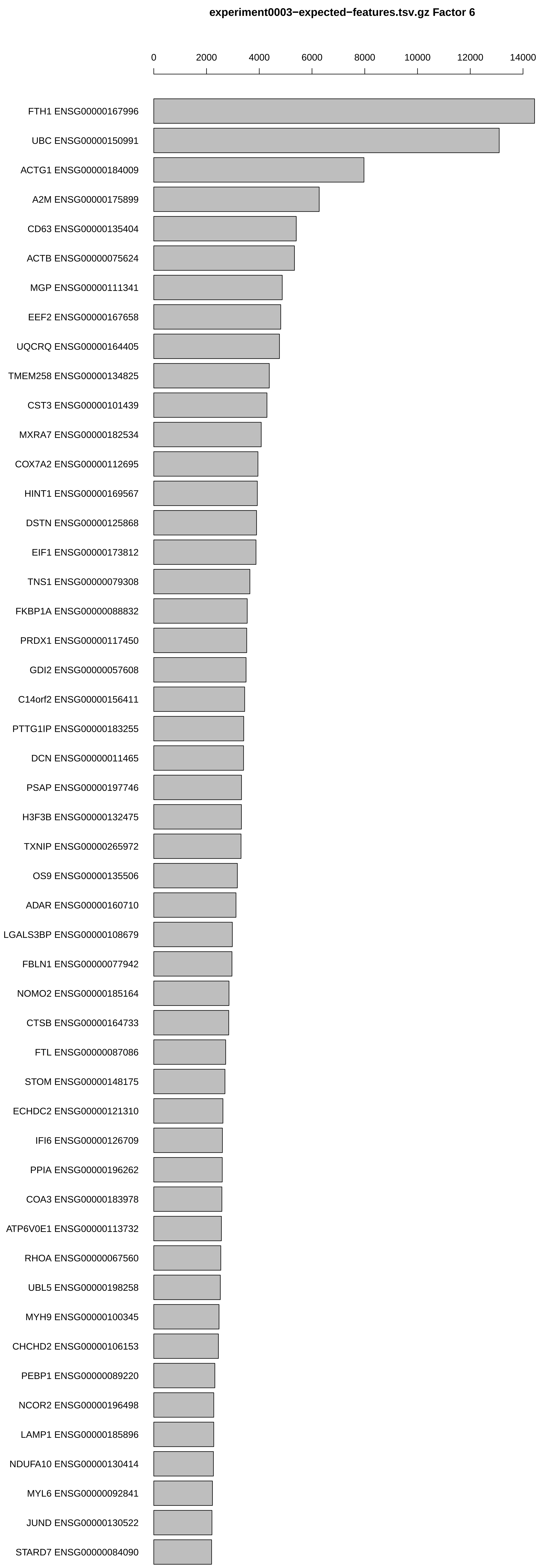
experiment0003−expected−features.tsv.gz Factor 6
0
2000
4000
6000
8000
10000
12000
14000
FTH1 ENSG00000167996
UBC ENSG00000150991
ACTG1 ENSG00000184009
A2M ENSG00000175899
CD63 ENSG00000135404
ACTB ENSG00000075624
MGP ENSG00000111341
EEF2 ENSG00000167658
UQCRQ ENSG00000164405
TMEM258 ENSG00000134825
CST3 ENSG00000101439
MXRA7 ENSG00000182534
COX7A2 ENSG00000112695
HINT1 ENSG00000169567
DSTN ENSG00000125868
EIF1 ENSG00000173812
TNS1 ENSG00000079308
FKBP1A ENSG00000088832
PRDX1 ENSG00000117450
GDI2 ENSG00000057608
C14orf2 ENSG00000156411
PTTG1IP ENSG00000183255
DCN ENSG00000011465
PSAP ENSG00000197746
H3F3B ENSG00000132475
TXNIP ENSG00000265972
OS9 ENSG00000135506
ADAR ENSG00000160710
LGALS3BP ENSG00000108679
FBLN1 ENSG00000077942
NOMO2 ENSG00000185164
CTSB ENSG00000164733
FTL ENSG00000087086
STOM ENSG00000148175
ECHDC2 ENSG00000121310
IFI6 ENSG00000126709
PPIA ENSG00000196262
COA3 ENSG00000183978
ATP6V0E1 ENSG00000113732
RHOA ENSG00000067560
UBL5 ENSG00000198258
MYH9 ENSG00000100345
CHCHD2 ENSG00000106153
PEBP1 ENSG00000089220
NCOR2 ENSG00000196498
LAMP1 ENSG00000185896
NDUFA10 ENSG00000130414
MYL6 ENSG00000092841
JUND ENSG00000130522
STARD7 ENSG00000084090

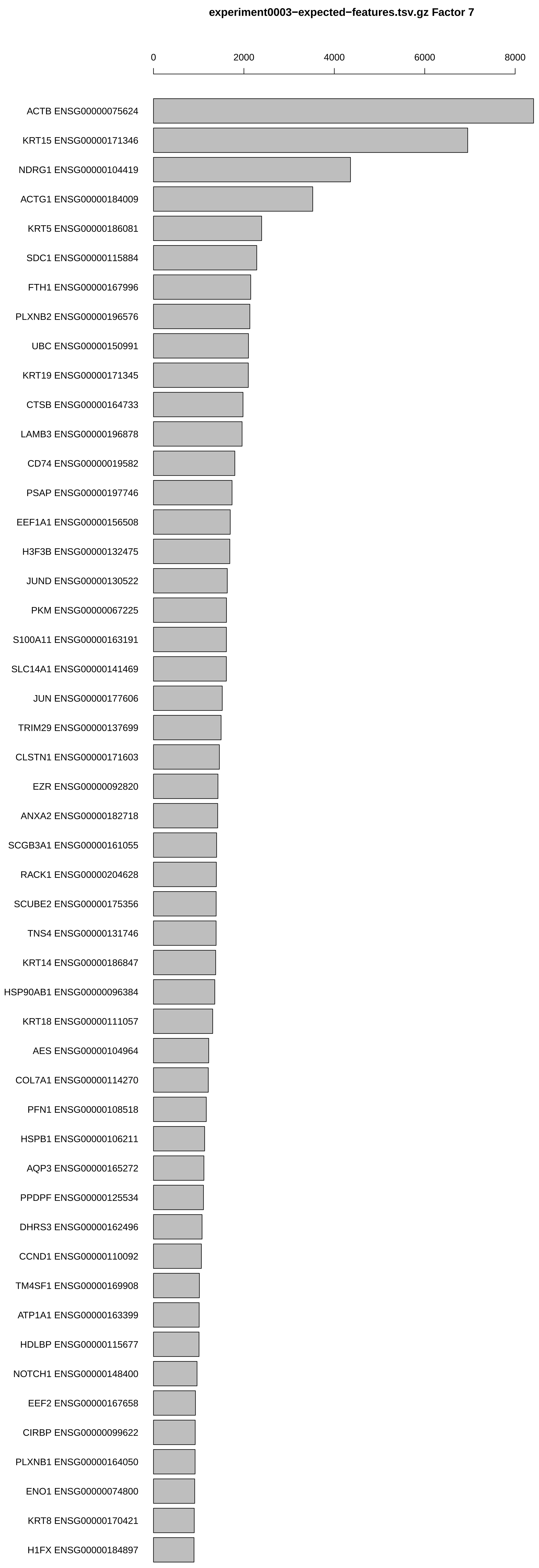
experiment0003−expected−features.tsv.gz Factor 7
0
2000
4000
6000
8000
ACTB ENSG00000075624
KRT15 ENSG00000171346
NDRG1 ENSG00000104419
ACTG1 ENSG00000184009
KRT5 ENSG00000186081
SDC1 ENSG00000115884
FTH1 ENSG00000167996
PLXNB2 ENSG00000196576
UBC ENSG00000150991
KRT19 ENSG00000171345
CTSB ENSG00000164733
LAMB3 ENSG00000196878
CD74 ENSG00000019582
PSAP ENSG00000197746
EEF1A1 ENSG00000156508
H3F3B ENSG00000132475
JUND ENSG00000130522
PKM ENSG00000067225
S100A11 ENSG00000163191
SLC14A1 ENSG00000141469
JUN ENSG00000177606
TRIM29 ENSG00000137699
CLSTN1 ENSG00000171603
EZR ENSG00000092820
ANXA2 ENSG00000182718
SCGB3A1 ENSG00000161055
RACK1 ENSG00000204628
SCUBE2 ENSG00000175356
TNS4 ENSG00000131746
KRT14 ENSG00000186847
HSP90AB1 ENSG00000096384
KRT18 ENSG00000111057
AES ENSG00000104964
COL7A1 ENSG00000114270
PFN1 ENSG00000108518
HSPB1 ENSG00000106211
AQP3 ENSG00000165272
PPDPF ENSG00000125534
DHRS3 ENSG00000162496
CCND1 ENSG00000110092
TM4SF1 ENSG00000169908
ATP1A1 ENSG00000163399
HDLBP ENSG00000115677
NOTCH1 ENSG00000148400
EEF2 ENSG00000167658
CIRBP ENSG00000099622
PLXNB1 ENSG00000164050
ENO1 ENSG00000074800
KRT8 ENSG00000170421
H1FX ENSG00000184897

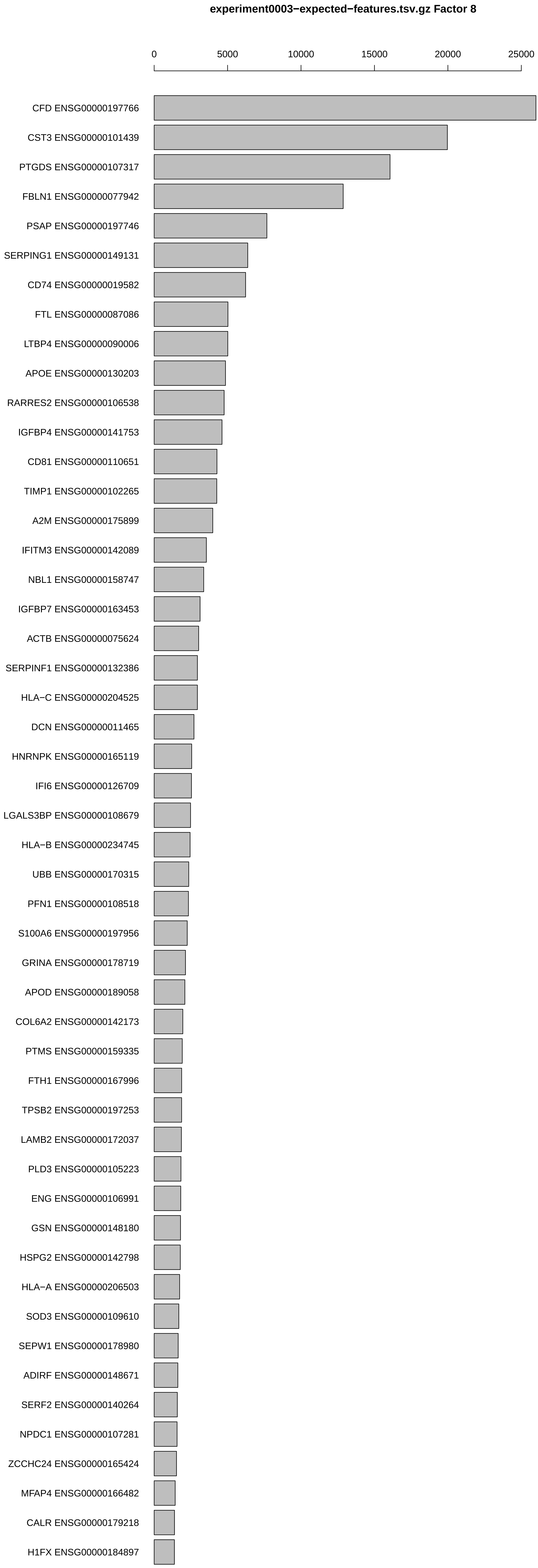
experiment0003−expected−features.tsv.gz Factor 8
0
5000
10000
15000
20000
25000
CFD ENSG00000197766
CST3 ENSG00000101439
PTGDS ENSG00000107317
FBLN1 ENSG00000077942
PSAP ENSG00000197746
SERPING1 ENSG00000149131
CD74 ENSG00000019582
FTL ENSG00000087086
LTBP4 ENSG00000090006
APOE ENSG00000130203
RARRES2 ENSG00000106538
IGFBP4 ENSG00000141753
CD81 ENSG00000110651
TIMP1 ENSG00000102265
A2M ENSG00000175899
IFITM3 ENSG00000142089
NBL1 ENSG00000158747
IGFBP7 ENSG00000163453
ACTB ENSG00000075624
SERPINF1 ENSG00000132386
HLA−C ENSG00000204525
DCN ENSG00000011465
HNRNPK ENSG00000165119
IFI6 ENSG00000126709
LGALS3BP ENSG00000108679
HLA−B ENSG00000234745
UBB ENSG00000170315
PFN1 ENSG00000108518
S100A6 ENSG00000197956
GRINA ENSG00000178719
APOD ENSG00000189058
COL6A2 ENSG00000142173
PTMS ENSG00000159335
FTH1 ENSG00000167996
TPSB2 ENSG00000197253
LAMB2 ENSG00000172037
PLD3 ENSG00000105223
ENG ENSG00000106991
GSN ENSG00000148180
HSPG2 ENSG00000142798
HLA−A ENSG00000206503
SOD3 ENSG00000109610
SEPW1 ENSG00000178980
ADIRF ENSG00000148671
SERF2 ENSG00000140264
NPDC1 ENSG00000107281
ZCCHC24 ENSG00000165424
MFAP4 ENSG00000166482
CALR ENSG00000179218
H1FX ENSG00000184897

experiment0003−expected−features.tsv.gz Factor 9

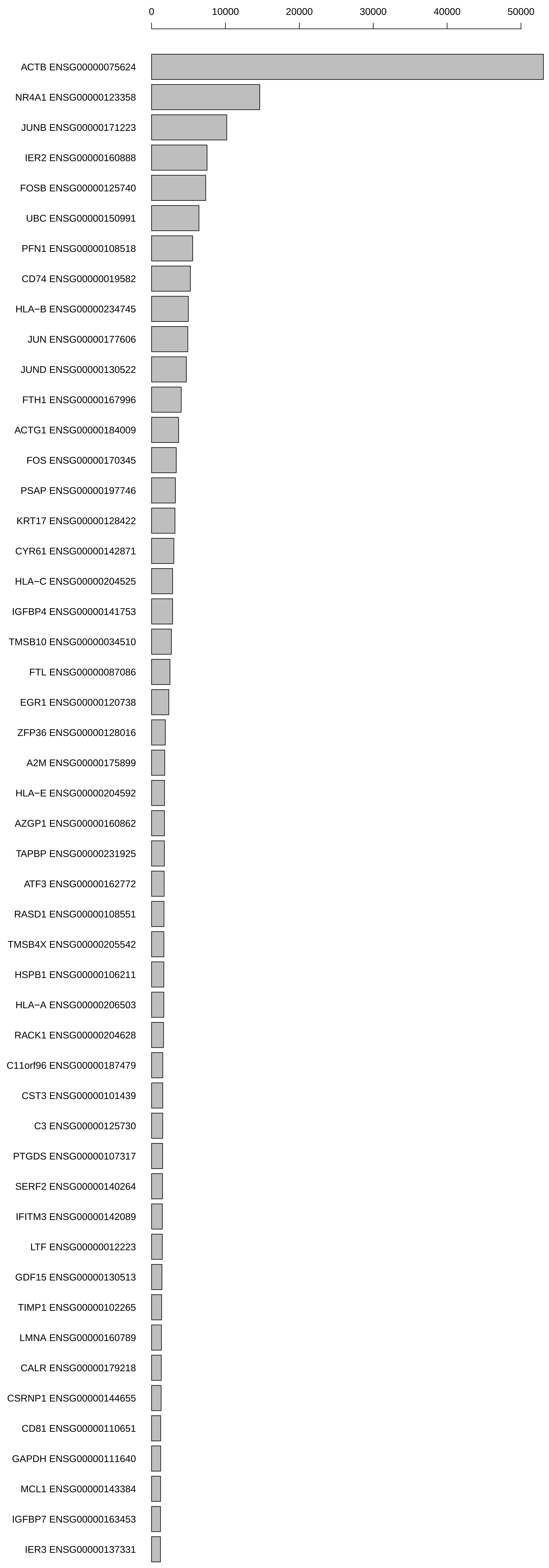

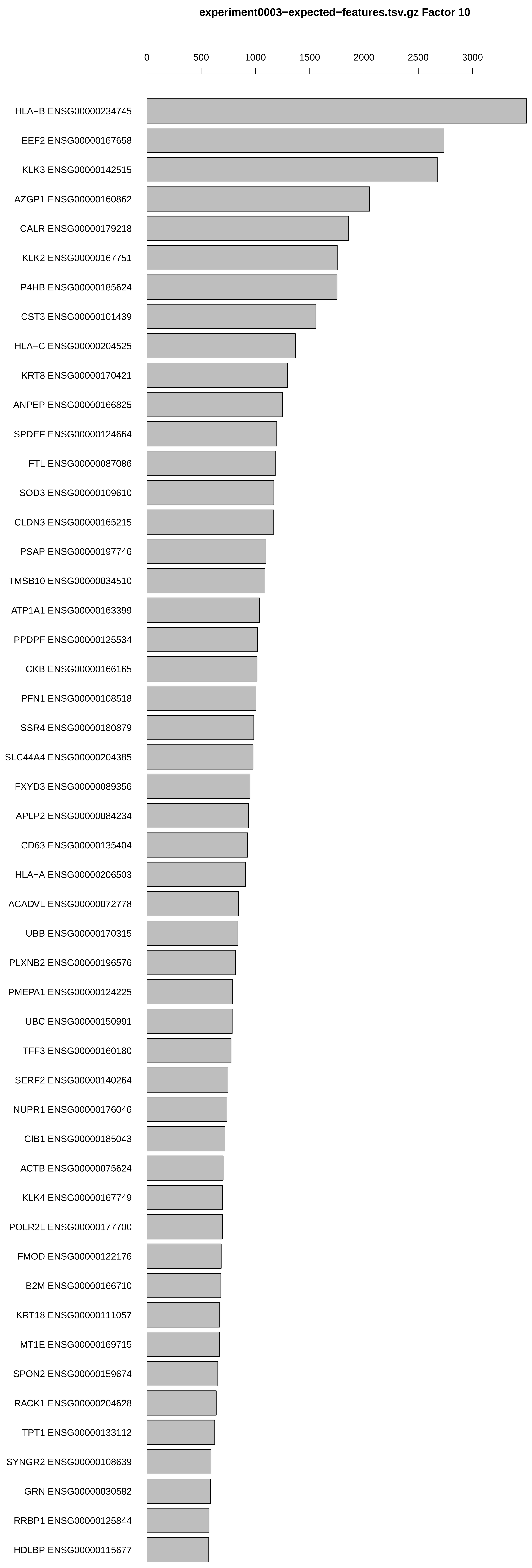

experiment0003−expected−features.tsv.gz Factor 10
0
500
1000
1500
2000
2500
3000
HLA−B ENSG00000234745
EEF2 ENSG00000167658
KLK3 ENSG00000142515
AZGP1 ENSG00000160862
CALR ENSG00000179218
KLK2 ENSG00000167751
P4HB ENSG00000185624
CST3 ENSG00000101439
HLA−C ENSG00000204525
KRT8 ENSG00000170421
ANPEP ENSG00000166825
SPDEF ENSG00000124664
FTL ENSG00000087086
SOD3 ENSG00000109610
CLDN3 ENSG00000165215
PSAP ENSG00000197746
TMSB10 ENSG00000034510
ATP1A1 ENSG00000163399
PPDPF ENSG00000125534
CKB ENSG00000166165
PFN1 ENSG00000108518
SSR4 ENSG00000180879
SLC44A4 ENSG00000204385
FXYD3 ENSG00000089356
APLP2 ENSG00000084234
CD63 ENSG00000135404
HLA−A ENSG00000206503
ACADVL ENSG00000072778
UBB ENSG00000170315
PLXNB2 ENSG00000196576
PMEPA1 ENSG00000124225
UBC ENSG00000150991
TFF3 ENSG00000160180
SERF2 ENSG00000140264
NUPR1 ENSG00000176046
CIB1 ENSG00000185043
ACTB ENSG00000075624
KLK4 ENSG00000167749
POLR2L ENSG00000177700
FMOD ENSG00000122176
B2M ENSG00000166710
KRT18 ENSG00000111057
MT1E ENSG00000169715
SPON2 ENSG00000159674
RACK1 ENSG00000204628
TPT1 ENSG00000133112
SYNGR2 ENSG00000108639
GRN ENSG00000030582
RRBP1 ENSG00000125844
HDLBP ENSG00000115677

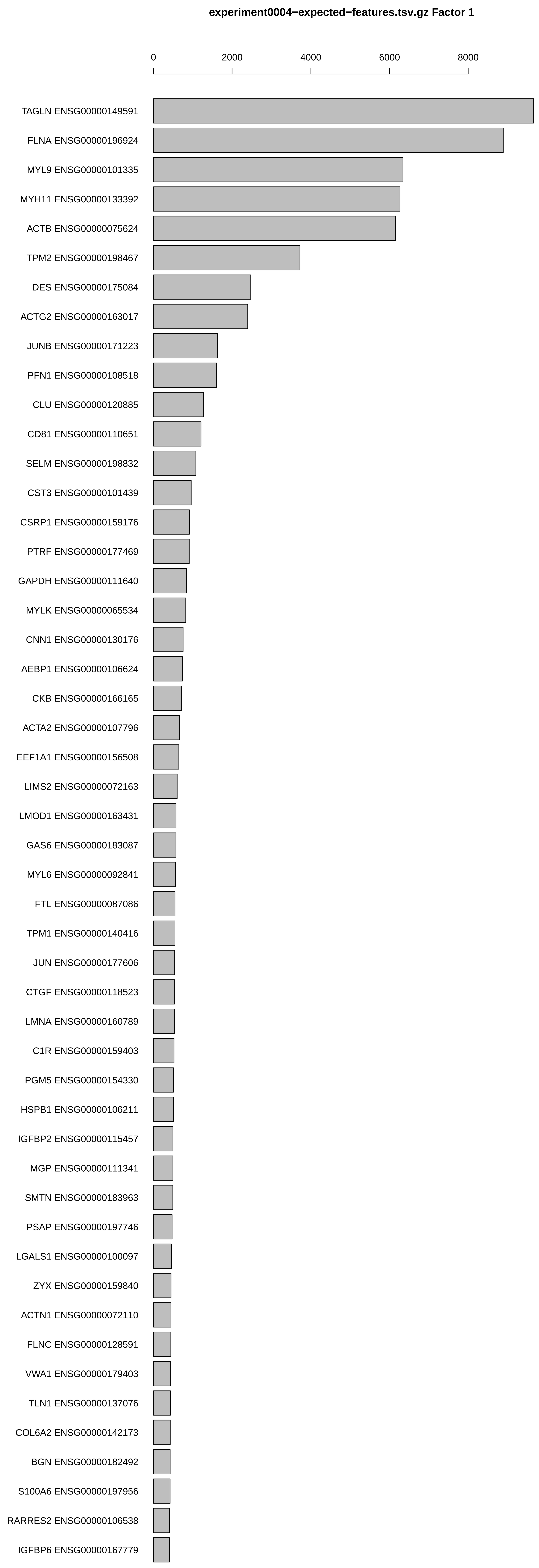
experiment0004−expected−features.tsv.gz Factor 1
0
2000
4000
6000
8000
TAGLN ENSG00000149591
FLNA ENSG00000196924
MYL9 ENSG00000101335
MYH11 ENSG00000133392
ACTB ENSG00000075624
TPM2 ENSG00000198467
DES ENSG00000175084
ACTG2 ENSG00000163017
JUNB ENSG00000171223
PFN1 ENSG00000108518
CLU ENSG00000120885
CD81 ENSG00000110651
SELM ENSG00000198832
CST3 ENSG00000101439
CSRP1 ENSG00000159176
PTRF ENSG00000177469
GAPDH ENSG00000111640
MYLK ENSG00000065534
CNN1 ENSG00000130176
AEBP1 ENSG00000106624
CKB ENSG00000166165
ACTA2 ENSG00000107796
EEF1A1 ENSG00000156508
LIMS2 ENSG00000072163
LMOD1 ENSG00000163431
GAS6 ENSG00000183087
MYL6 ENSG00000092841
FTL ENSG00000087086
TPM1 ENSG00000140416
JUN ENSG00000177606
CTGF ENSG00000118523
LMNA ENSG00000160789
C1R ENSG00000159403
PGM5 ENSG00000154330
HSPB1 ENSG00000106211
IGFBP2 ENSG00000115457
MGP ENSG00000111341
SMTN ENSG00000183963
PSAP ENSG00000197746
LGALS1 ENSG00000100097
ZYX ENSG00000159840
ACTN1 ENSG00000072110
FLNC ENSG00000128591
VWA1 ENSG00000179403
TLN1 ENSG00000137076
COL6A2 ENSG00000142173
BGN ENSG00000182492
S100A6 ENSG00000197956
RARRES2 ENSG00000106538
IGFBP6 ENSG00000167779

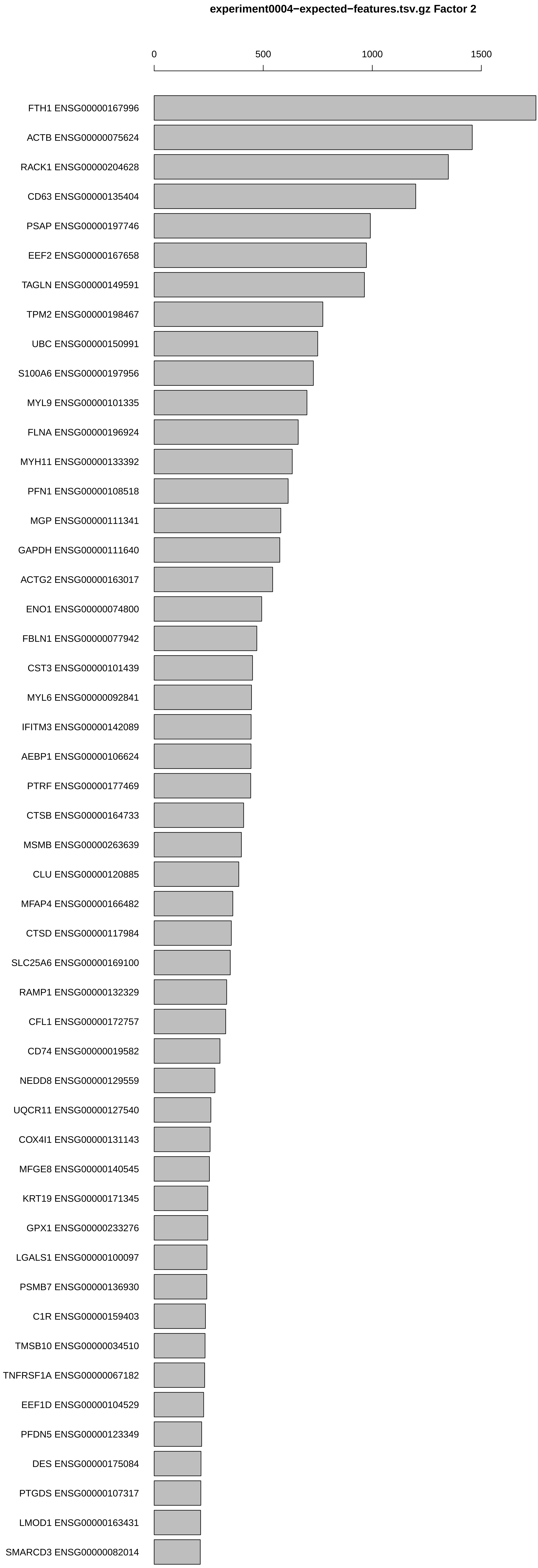
experiment0004−expected−features.tsv.gz Factor 2
0
500
1000
1500
FTH1 ENSG00000167996
ACTB ENSG00000075624
RACK1 ENSG00000204628
CD63 ENSG00000135404
PSAP ENSG00000197746
EEF2 ENSG00000167658
TAGLN ENSG00000149591
TPM2 ENSG00000198467
UBC ENSG00000150991
S100A6 ENSG00000197956
MYL9 ENSG00000101335
FLNA ENSG00000196924
MYH11 ENSG00000133392
PFN1 ENSG00000108518
MGP ENSG00000111341
GAPDH ENSG00000111640
ACTG2 ENSG00000163017
ENO1 ENSG00000074800
FBLN1 ENSG00000077942
CST3 ENSG00000101439
MYL6 ENSG00000092841
IFITM3 ENSG00000142089
AEBP1 ENSG00000106624
PTRF ENSG00000177469
CTSB ENSG00000164733
MSMB ENSG00000263639
CLU ENSG00000120885
MFAP4 ENSG00000166482
CTSD ENSG00000117984
SLC25A6 ENSG00000169100
RAMP1 ENSG00000132329
CFL1 ENSG00000172757
CD74 ENSG00000019582
NEDD8 ENSG00000129559
UQCR11 ENSG00000127540
COX4I1 ENSG00000131143
MFGE8 ENSG00000140545
KRT19 ENSG00000171345
GPX1 ENSG00000233276
LGALS1 ENSG00000100097
PSMB7 ENSG00000136930
C1R ENSG00000159403
TMSB10 ENSG00000034510
TNFRSF1A ENSG00000067182
EEF1D ENSG00000104529
PFDN5 ENSG00000123349
DES ENSG00000175084
PTGDS ENSG00000107317
LMOD1 ENSG00000163431
SMARCD3 ENSG00000082014

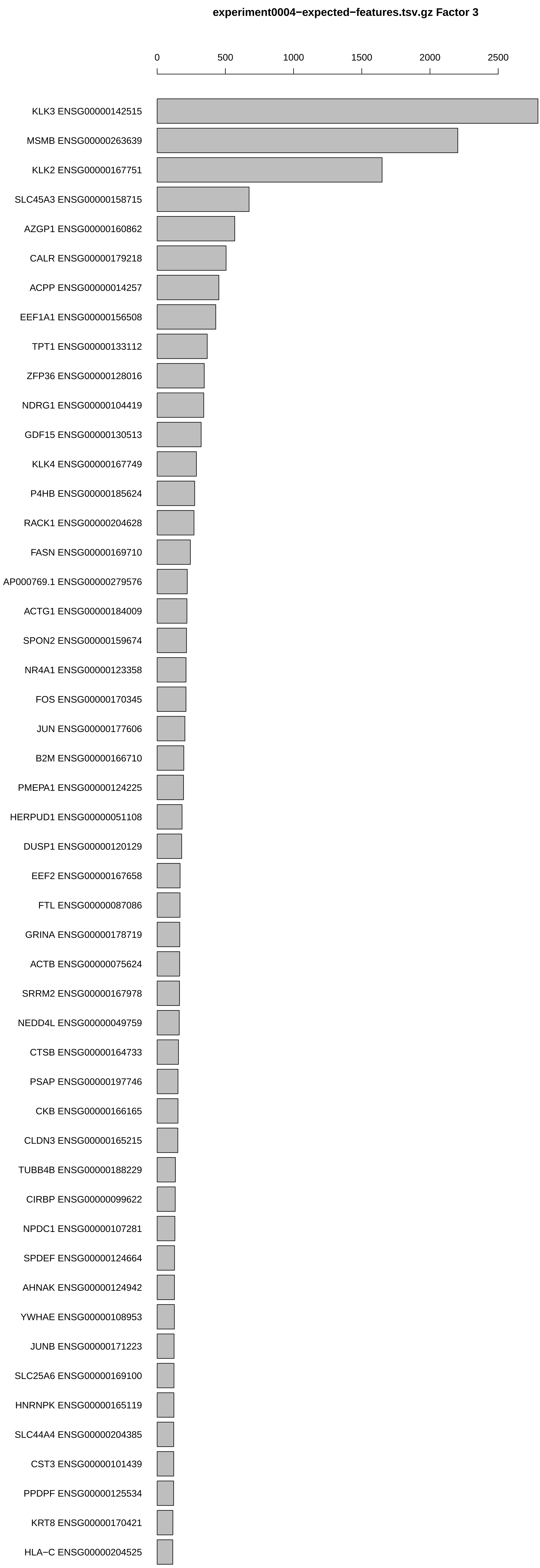

experiment0004−expected−features.tsv.gz Factor 3
0
500
1000
1500
2000
2500
KLK3 ENSG00000142515
MSMB ENSG00000263639
KLK2 ENSG00000167751
SLC45A3 ENSG00000158715
AZGP1 ENSG00000160862
CALR ENSG00000179218
ACPP ENSG00000014257
EEF1A1 ENSG00000156508
TPT1 ENSG00000133112
ZFP36 ENSG00000128016
NDRG1 ENSG00000104419
GDF15 ENSG00000130513
KLK4 ENSG00000167749
P4HB ENSG00000185624
RACK1 ENSG00000204628
FASN ENSG00000169710
AP000769.1 ENSG00000279576
ACTG1 ENSG00000184009
SPON2 ENSG00000159674
NR4A1 ENSG00000123358
FOS ENSG00000170345
JUN ENSG00000177606
B2M ENSG00000166710
PMEPA1 ENSG00000124225
HERPUD1 ENSG00000051108
DUSP1 ENSG00000120129
EEF2 ENSG00000167658
FTL ENSG00000087086
GRINA ENSG00000178719
ACTB ENSG00000075624
SRRM2 ENSG00000167978
NEDD4L ENSG00000049759
CTSB ENSG00000164733
PSAP ENSG00000197746
CKB ENSG00000166165
CLDN3 ENSG00000165215
TUBB4B ENSG00000188229
CIRBP ENSG00000099622
NPDC1 ENSG00000107281
SPDEF ENSG00000124664
AHNAK ENSG00000124942
YWHAE ENSG00000108953
JUNB ENSG00000171223
SLC25A6 ENSG00000169100
HNRNPK ENSG00000165119
SLC44A4 ENSG00000204385
CST3 ENSG00000101439
PPDPF ENSG00000125534
KRT8 ENSG00000170421
HLA−C ENSG00000204525

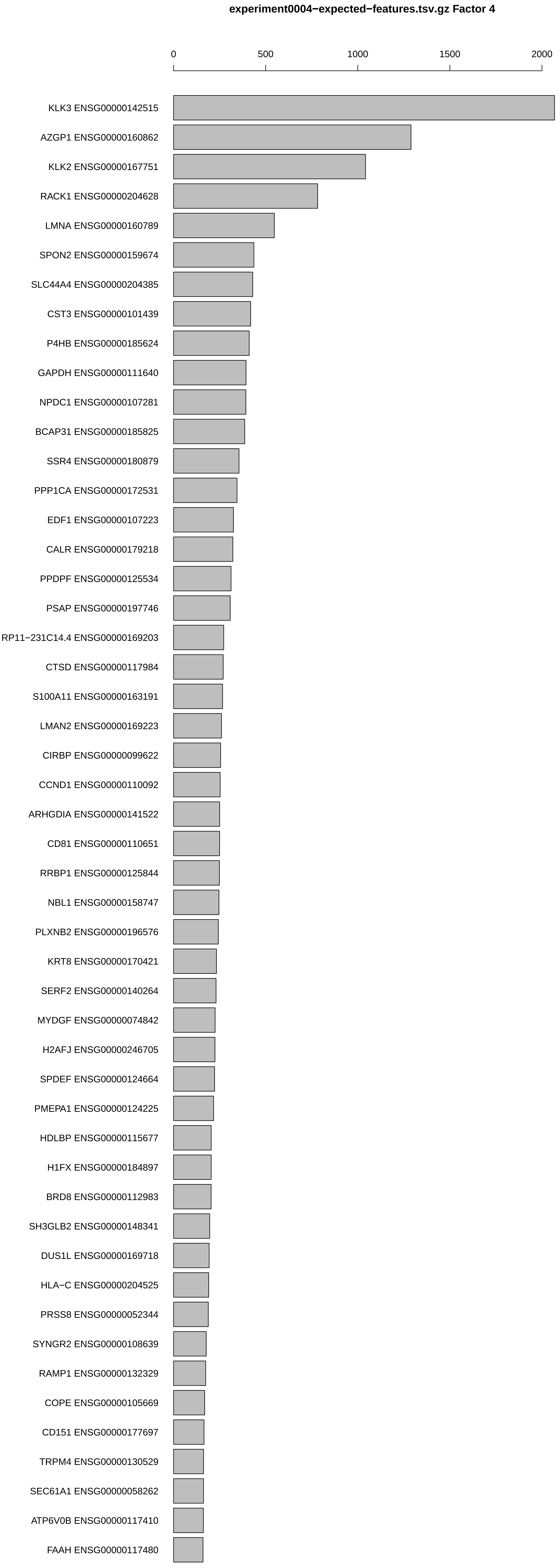
experiment0004−expected−features.tsv.gz Factor 4
0
500
1000
1500
2000
KLK3 ENSG00000142515
AZGP1 ENSG00000160862
KLK2 ENSG00000167751
RACK1 ENSG00000204628
LMNA ENSG00000160789
SPON2 ENSG00000159674
SLC44A4 ENSG00000204385
CST3 ENSG00000101439
P4HB ENSG00000185624
GAPDH ENSG00000111640
NPDC1 ENSG00000107281
BCAP31 ENSG00000185825
SSR4 ENSG00000180879
PPP1CA ENSG00000172531
EDF1 ENSG00000107223
CALR ENSG00000179218
PPDPF ENSG00000125534
PSAP ENSG00000197746
RP11−231C14.4 ENSG00000169203
CTSD ENSG00000117984
S100A11 ENSG00000163191
LMAN2 ENSG00000169223
CIRBP ENSG00000099622
CCND1 ENSG00000110092
ARHGDIA ENSG00000141522
CD81 ENSG00000110651
RRBP1 ENSG00000125844
NBL1 ENSG00000158747
PLXNB2 ENSG00000196576
KRT8 ENSG00000170421
SERF2 ENSG00000140264
MYDGF ENSG00000074842
H2AFJ ENSG00000246705
SPDEF ENSG00000124664
PMEPA1 ENSG00000124225
HDLBP ENSG00000115677
H1FX ENSG00000184897
BRD8 ENSG00000112983
SH3GLB2 ENSG00000148341
DUS1L ENSG00000169718
HLA−C ENSG00000204525
PRSS8 ENSG00000052344
SYNGR2 ENSG00000108639
RAMP1 ENSG00000132329
COPE ENSG00000105669
CD151 ENSG00000177697
TRPM4 ENSG00000130529
SEC61A1 ENSG00000058262
ATP6V0B ENSG00000117410
FAAH ENSG00000117480

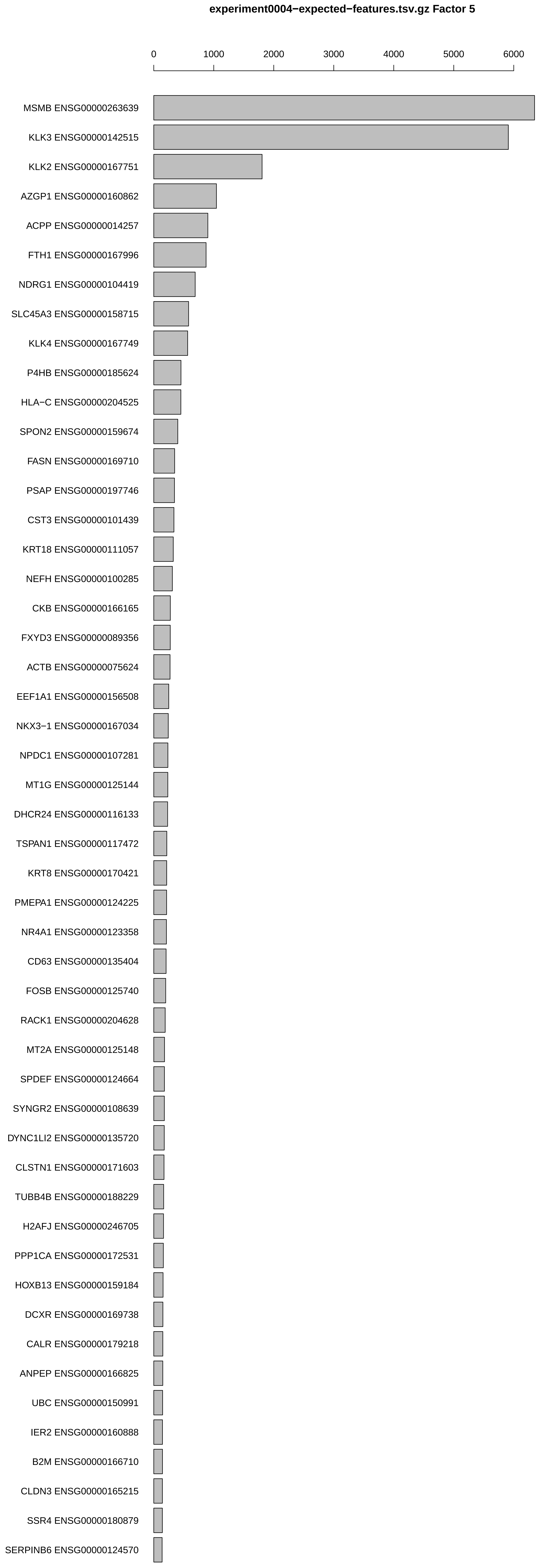
experiment0004−expected−features.tsv.gz Factor 5
0
1000
2000
3000
4000
5000
6000
MSMB ENSG00000263639
KLK3 ENSG00000142515
KLK2 ENSG00000167751
AZGP1 ENSG00000160862
ACPP ENSG00000014257
FTH1 ENSG00000167996
NDRG1 ENSG00000104419
SLC45A3 ENSG00000158715
KLK4 ENSG00000167749
P4HB ENSG00000185624
HLA−C ENSG00000204525
SPON2 ENSG00000159674
FASN ENSG00000169710
PSAP ENSG00000197746
CST3 ENSG00000101439
KRT18 ENSG00000111057
NEFH ENSG00000100285
CKB ENSG00000166165
FXYD3 ENSG00000089356
ACTB ENSG00000075624
EEF1A1 ENSG00000156508
NKX3−1 ENSG00000167034
NPDC1 ENSG00000107281
MT1G ENSG00000125144
DHCR24 ENSG00000116133
TSPAN1 ENSG00000117472
KRT8 ENSG00000170421
PMEPA1 ENSG00000124225
NR4A1 ENSG00000123358
CD63 ENSG00000135404
FOSB ENSG00000125740
RACK1 ENSG00000204628
MT2A ENSG00000125148
SPDEF ENSG00000124664
SYNGR2 ENSG00000108639
DYNC1LI2 ENSG00000135720
CLSTN1 ENSG00000171603
TUBB4B ENSG00000188229
H2AFJ ENSG00000246705
PPP1CA ENSG00000172531
HOXB13 ENSG00000159184
DCXR ENSG00000169738
CALR ENSG00000179218
ANPEP ENSG00000166825
UBC ENSG00000150991
IER2 ENSG00000160888
B2M ENSG00000166710
CLDN3 ENSG00000165215
SSR4 ENSG00000180879
SERPINB6 ENSG00000124570

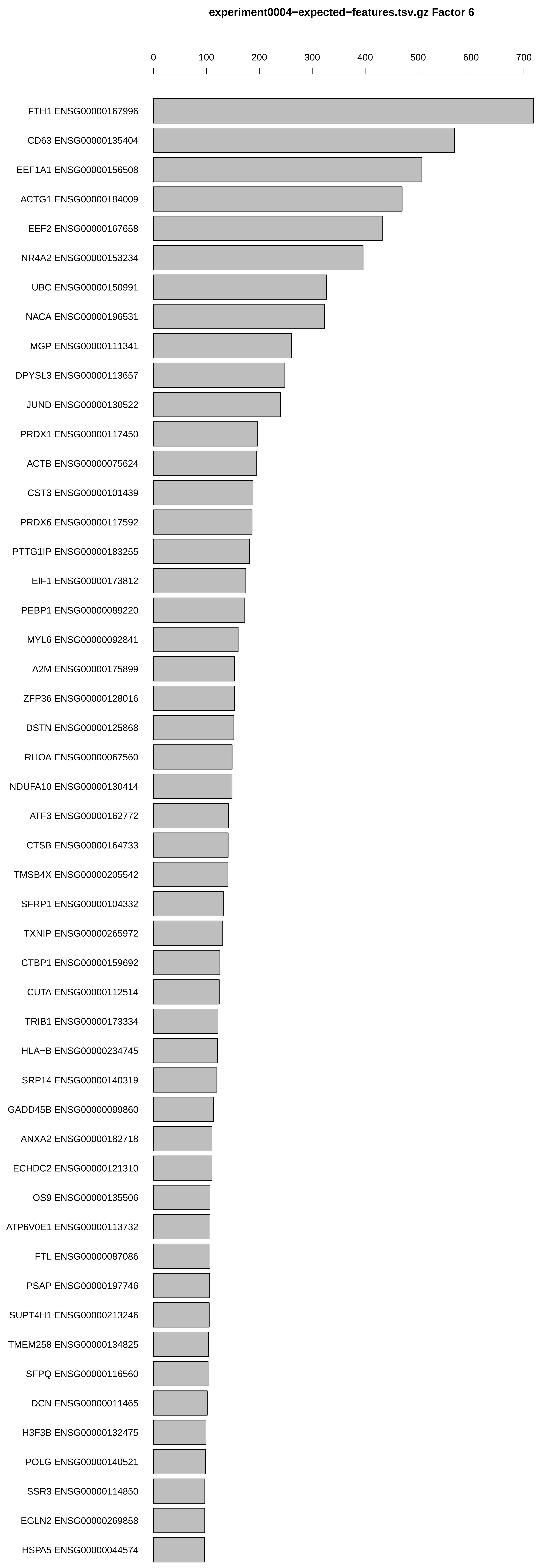
experiment0004−expected−features.tsv.gz Factor 6
0
100
200
300
400
500
600
700
FTH1 ENSG00000167996
CD63 ENSG00000135404
EEF1A1 ENSG00000156508
ACTG1 ENSG00000184009
EEF2 ENSG00000167658
NR4A2 ENSG00000153234
UBC ENSG00000150991
NACA ENSG00000196531
MGP ENSG00000111341
DPYSL3 ENSG00000113657
JUND ENSG00000130522
PRDX1 ENSG00000117450
ACTB ENSG00000075624
CST3 ENSG00000101439
PRDX6 ENSG00000117592
PTTG1IP ENSG00000183255
EIF1 ENSG00000173812
PEBP1 ENSG00000089220
MYL6 ENSG00000092841
A2M ENSG00000175899
ZFP36 ENSG00000128016
DSTN ENSG00000125868
RHOA ENSG00000067560
NDUFA10 ENSG00000130414
ATF3 ENSG00000162772
CTSB ENSG00000164733
TMSB4X ENSG00000205542
SFRP1 ENSG00000104332
TXNIP ENSG00000265972
CTBP1 ENSG00000159692
CUTA ENSG00000112514
TRIB1 ENSG00000173334
HLA−B ENSG00000234745
SRP14 ENSG00000140319
GADD45B ENSG00000099860
ANXA2 ENSG00000182718
ECHDC2 ENSG00000121310
OS9 ENSG00000135506
ATP6V0E1 ENSG00000113732
FTL ENSG00000087086
PSAP ENSG00000197746
SUPT4H1 ENSG00000213246
TMEM258 ENSG00000134825
SFPQ ENSG00000116560
DCN ENSG00000011465
H3F3B ENSG00000132475
POLG ENSG00000140521
SSR3 ENSG00000114850
EGLN2 ENSG00000269858
HSPA5 ENSG00000044574

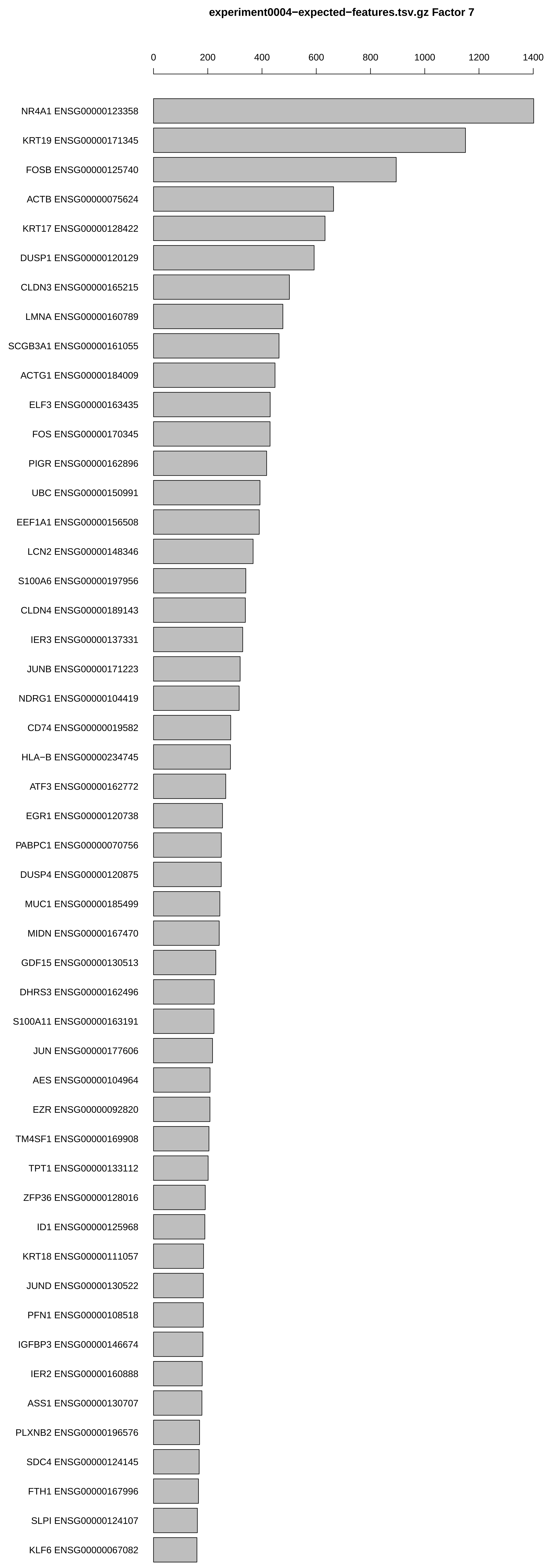
experiment0004−expected−features.tsv.gz Factor 7
0
200
400
600
800
1000
1200
1400
NR4A1 ENSG00000123358
KRT19 ENSG00000171345
FOSB ENSG00000125740
ACTB ENSG00000075624
KRT17 ENSG00000128422
DUSP1 ENSG00000120129
CLDN3 ENSG00000165215
LMNA ENSG00000160789
SCGB3A1 ENSG00000161055
ACTG1 ENSG00000184009
ELF3 ENSG00000163435
FOS ENSG00000170345
PIGR ENSG00000162896
UBC ENSG00000150991
EEF1A1 ENSG00000156508
LCN2 ENSG00000148346
S100A6 ENSG00000197956
CLDN4 ENSG00000189143
IER3 ENSG00000137331
JUNB ENSG00000171223
NDRG1 ENSG00000104419
CD74 ENSG00000019582
HLA−B ENSG00000234745
ATF3 ENSG00000162772
EGR1 ENSG00000120738
PABPC1 ENSG00000070756
DUSP4 ENSG00000120875
MUC1 ENSG00000185499
MIDN ENSG00000167470
GDF15 ENSG00000130513
DHRS3 ENSG00000162496
S100A11 ENSG00000163191
JUN ENSG00000177606
AES ENSG00000104964
EZR ENSG00000092820
TM4SF1 ENSG00000169908
TPT1 ENSG00000133112
ZFP36 ENSG00000128016
ID1 ENSG00000125968
KRT18 ENSG00000111057
JUND ENSG00000130522
PFN1 ENSG00000108518
IGFBP3 ENSG00000146674
IER2 ENSG00000160888
ASS1 ENSG00000130707
PLXNB2 ENSG00000196576
SDC4 ENSG00000124145
FTH1 ENSG00000167996
SLPI ENSG00000124107
KLF6 ENSG00000067082

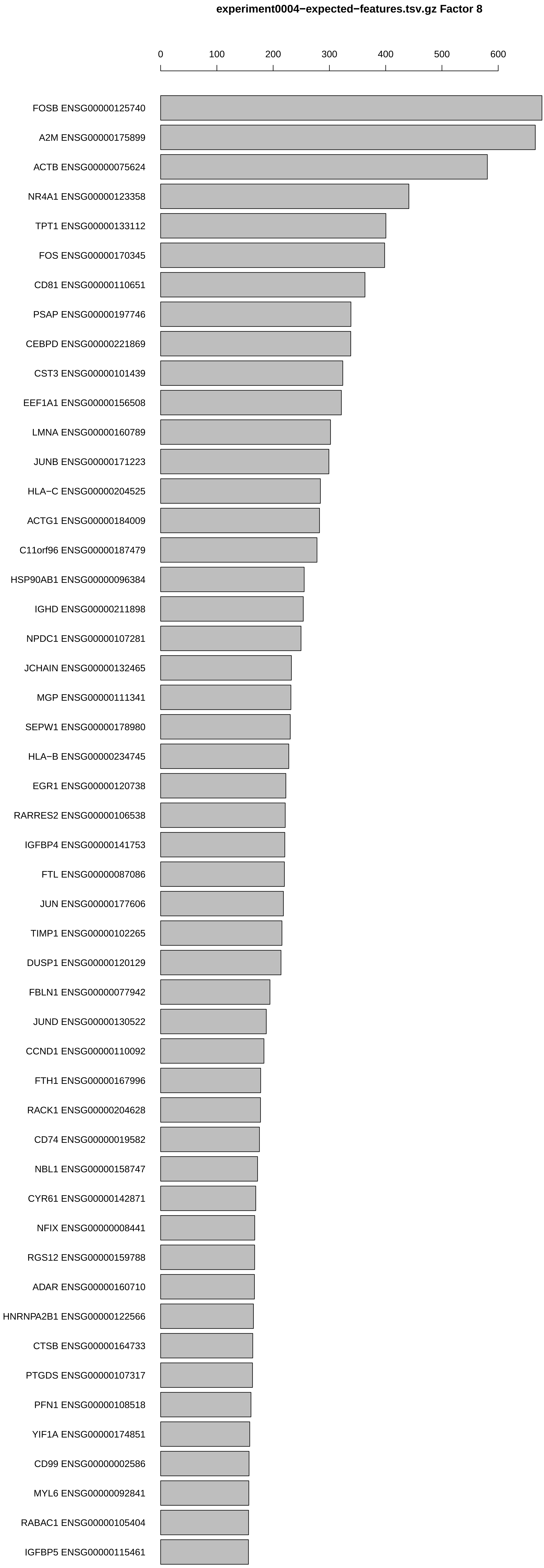
experiment0004−expected−features.tsv.gz Factor 8
0
100
200
300
400
500
600
FOSB ENSG00000125740
A2M ENSG00000175899
ACTB ENSG00000075624
NR4A1 ENSG00000123358
TPT1 ENSG00000133112
FOS ENSG00000170345
CD81 ENSG00000110651
PSAP ENSG00000197746
CEBPD ENSG00000221869
CST3 ENSG00000101439
EEF1A1 ENSG00000156508
LMNA ENSG00000160789
JUNB ENSG00000171223
HLA−C ENSG00000204525
ACTG1 ENSG00000184009
C11orf96 ENSG00000187479
HSP90AB1 ENSG00000096384
IGHD ENSG00000211898
NPDC1 ENSG00000107281
JCHAIN ENSG00000132465
MGP ENSG00000111341
SEPW1 ENSG00000178980
HLA−B ENSG00000234745
EGR1 ENSG00000120738
RARRES2 ENSG00000106538
IGFBP4 ENSG00000141753
FTL ENSG00000087086
JUN ENSG00000177606
TIMP1 ENSG00000102265
DUSP1 ENSG00000120129
FBLN1 ENSG00000077942
JUND ENSG00000130522
CCND1 ENSG00000110092
FTH1 ENSG00000167996
RACK1 ENSG00000204628
CD74 ENSG00000019582
NBL1 ENSG00000158747
CYR61 ENSG00000142871
NFIX ENSG00000008441
RGS12 ENSG00000159788
ADAR ENSG00000160710
HNRNPA2B1 ENSG00000122566
CTSB ENSG00000164733
PTGDS ENSG00000107317
PFN1 ENSG00000108518
YIF1A ENSG00000174851
CD99 ENSG00000002586
MYL6 ENSG00000092841
RABAC1 ENSG00000105404
IGFBP5 ENSG00000115461

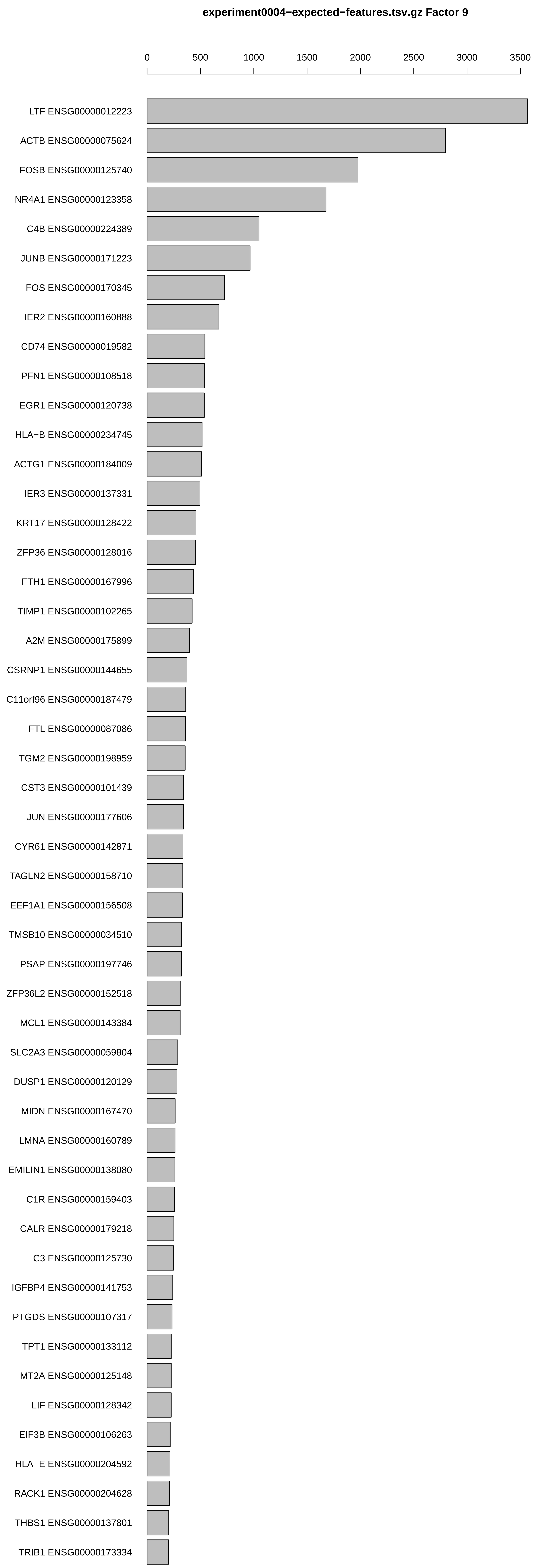

experiment0004−expected−features.tsv.gz Factor 9
0
500
1000
1500
2000
2500
3000
3500
LTF ENSG00000012223
ACTB ENSG00000075624
FOSB ENSG00000125740
NR4A1 ENSG00000123358
C4B ENSG00000224389
JUNB ENSG00000171223
FOS ENSG00000170345
IER2 ENSG00000160888
CD74 ENSG00000019582
PFN1 ENSG00000108518
EGR1 ENSG00000120738
HLA−B ENSG00000234745
ACTG1 ENSG00000184009
IER3 ENSG00000137331
KRT17 ENSG00000128422
ZFP36 ENSG00000128016
FTH1 ENSG00000167996
TIMP1 ENSG00000102265
A2M ENSG00000175899
CSRNP1 ENSG00000144655
C11orf96 ENSG00000187479
FTL ENSG00000087086
TGM2 ENSG00000198959
CST3 ENSG00000101439
JUN ENSG00000177606
CYR61 ENSG00000142871
TAGLN2 ENSG00000158710
EEF1A1 ENSG00000156508
TMSB10 ENSG00000034510
PSAP ENSG00000197746
ZFP36L2 ENSG00000152518
MCL1 ENSG00000143384
SLC2A3 ENSG00000059804
DUSP1 ENSG00000120129
MIDN ENSG00000167470
LMNA ENSG00000160789
EMILIN1 ENSG00000138080
C1R ENSG00000159403
CALR ENSG00000179218
C3 ENSG00000125730
IGFBP4 ENSG00000141753
PTGDS ENSG00000107317
TPT1 ENSG00000133112
MT2A ENSG00000125148
LIF ENSG00000128342
EIF3B ENSG00000106263
HLA−E ENSG00000204592
RACK1 ENSG00000204628
THBS1 ENSG00000137801
TRIB1 ENSG00000173334

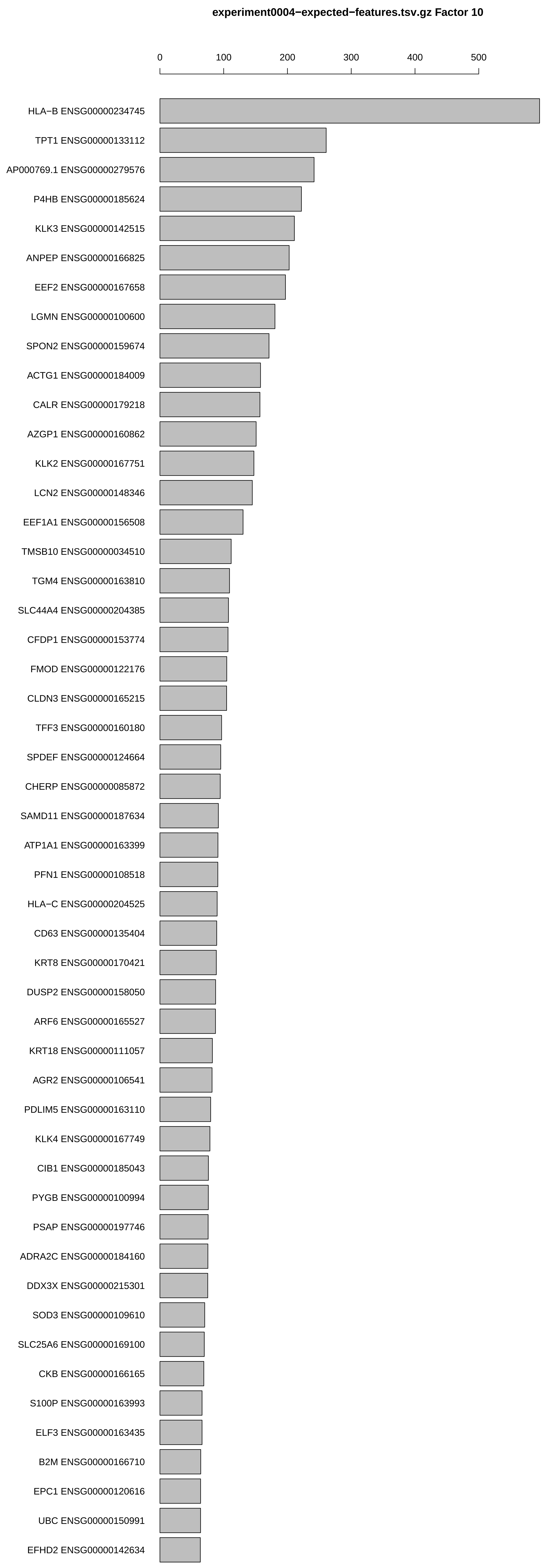

experiment0004−expected−features.tsv.gz Factor 10
0
100
200
300
400
500
HLA−B ENSG00000234745
TPT1 ENSG00000133112
AP000769.1 ENSG00000279576
P4HB ENSG00000185624
KLK3 ENSG00000142515
ANPEP ENSG00000166825
EEF2 ENSG00000167658
LGMN ENSG00000100600
SPON2 ENSG00000159674
ACTG1 ENSG00000184009
CALR ENSG00000179218
AZGP1 ENSG00000160862
KLK2 ENSG00000167751
LCN2 ENSG00000148346
EEF1A1 ENSG00000156508
TMSB10 ENSG00000034510
TGM4 ENSG00000163810
SLC44A4 ENSG00000204385
CFDP1 ENSG00000153774
FMOD ENSG00000122176
CLDN3 ENSG00000165215
TFF3 ENSG00000160180
SPDEF ENSG00000124664
CHERP ENSG00000085872
SAMD11 ENSG00000187634
ATP1A1 ENSG00000163399
PFN1 ENSG00000108518
HLA−C ENSG00000204525
CD63 ENSG00000135404
KRT8 ENSG00000170421
DUSP2 ENSG00000158050
ARF6 ENSG00000165527
KRT18 ENSG00000111057
AGR2 ENSG00000106541
PDLIM5 ENSG00000163110
KLK4 ENSG00000167749
CIB1 ENSG00000185043
PYGB ENSG00000100994
PSAP ENSG00000197746
ADRA2C ENSG00000184160
DDX3X ENSG00000215301
SOD3 ENSG00000109610
SLC25A6 ENSG00000169100
CKB ENSG00000166165
S100P ENSG00000163993
ELF3 ENSG00000163435
B2M ENSG00000166710
EPC1 ENSG00000120616
UBC ENSG00000150991
EFHD2 ENSG00000142634

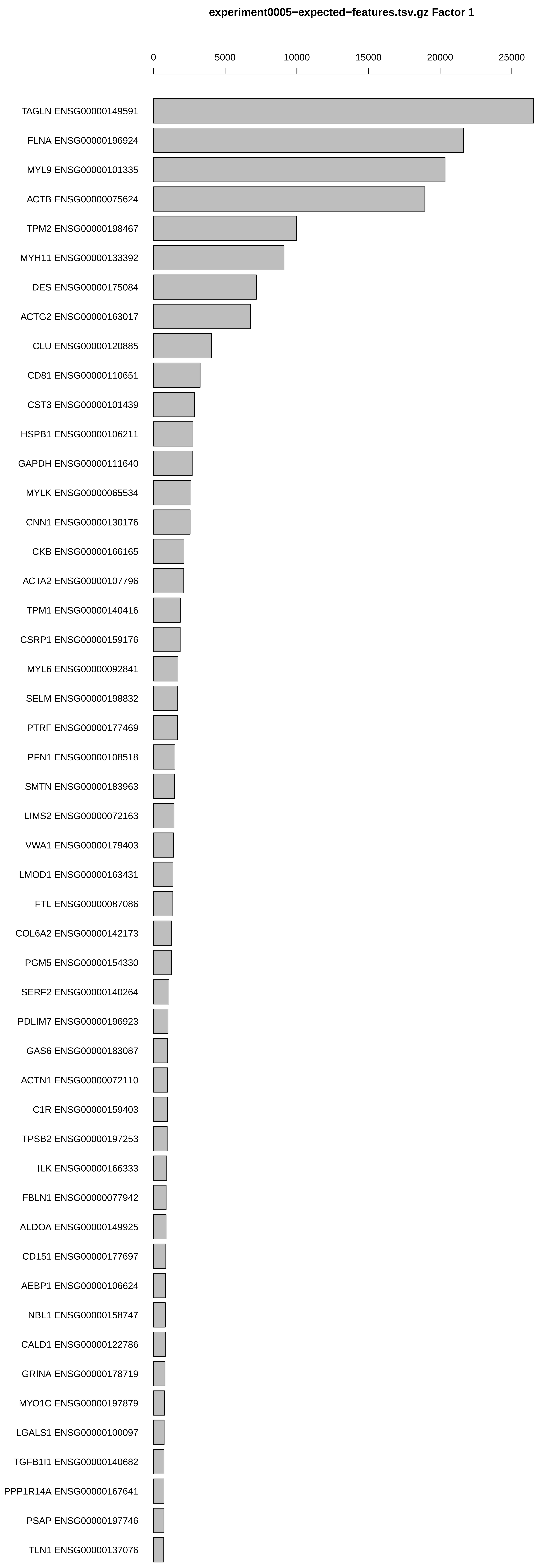
experiment0005−expected−features.tsv.gz Factor 1
0
5000
10000
15000
20000
25000
TAGLN ENSG00000149591
FLNA ENSG00000196924
MYL9 ENSG00000101335
ACTB ENSG00000075624
TPM2 ENSG00000198467
MYH11 ENSG00000133392
DES ENSG00000175084
ACTG2 ENSG00000163017
CLU ENSG00000120885
CD81 ENSG00000110651
CST3 ENSG00000101439
HSPB1 ENSG00000106211
GAPDH ENSG00000111640
MYLK ENSG00000065534
CNN1 ENSG00000130176
CKB ENSG00000166165
ACTA2 ENSG00000107796
TPM1 ENSG00000140416
CSRP1 ENSG00000159176
MYL6 ENSG00000092841
SELM ENSG00000198832
PTRF ENSG00000177469
PFN1 ENSG00000108518
SMTN ENSG00000183963
LIMS2 ENSG00000072163
VWA1 ENSG00000179403
LMOD1 ENSG00000163431
FTL ENSG00000087086
COL6A2 ENSG00000142173
PGM5 ENSG00000154330
SERF2 ENSG00000140264
PDLIM7 ENSG00000196923
GAS6 ENSG00000183087
ACTN1 ENSG00000072110
C1R ENSG00000159403
TPSB2 ENSG00000197253
ILK ENSG00000166333
FBLN1 ENSG00000077942
ALDOA ENSG00000149925
CD151 ENSG00000177697
AEBP1 ENSG00000106624
NBL1 ENSG00000158747
CALD1 ENSG00000122786
GRINA ENSG00000178719
MYO1C ENSG00000197879
LGALS1 ENSG00000100097
TGFB1I1 ENSG00000140682
PPP1R14A ENSG00000167641
PSAP ENSG00000197746
TLN1 ENSG00000137076

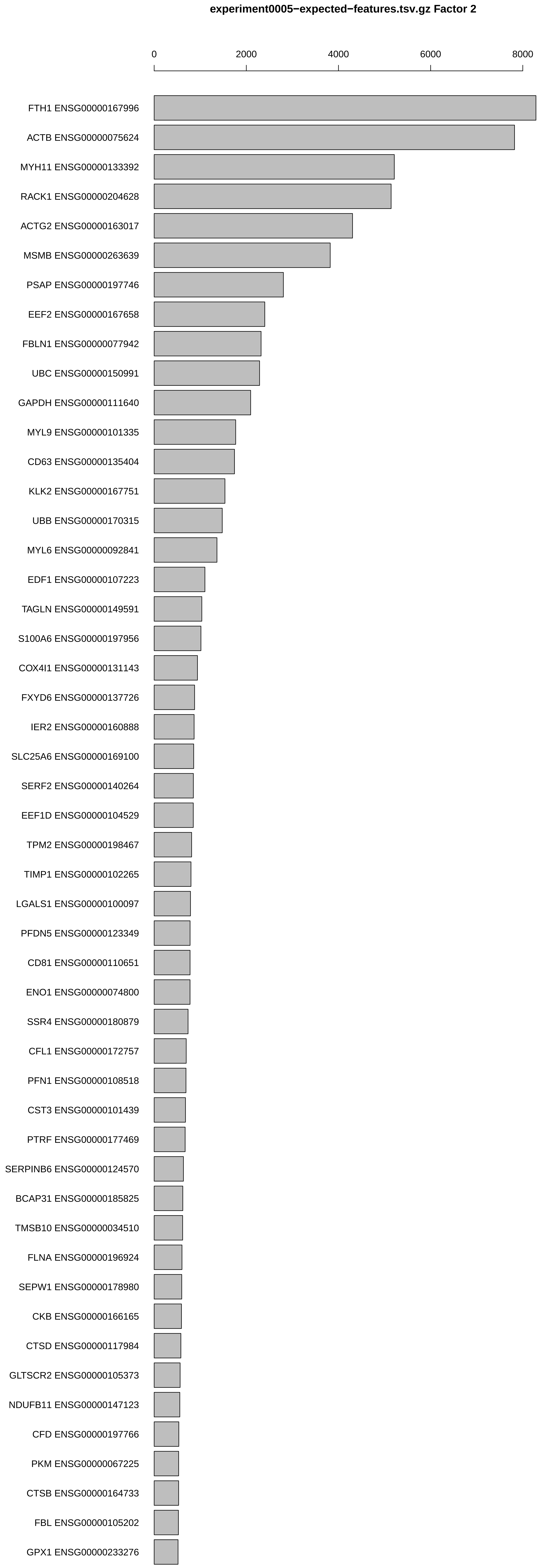
experiment0005−expected−features.tsv.gz Factor 2
0
2000
4000
6000
8000
FTH1 ENSG00000167996
ACTB ENSG00000075624
MYH11 ENSG00000133392
RACK1 ENSG00000204628
ACTG2 ENSG00000163017
MSMB ENSG00000263639
PSAP ENSG00000197746
EEF2 ENSG00000167658
FBLN1 ENSG00000077942
UBC ENSG00000150991
GAPDH ENSG00000111640
MYL9 ENSG00000101335
CD63 ENSG00000135404
KLK2 ENSG00000167751
UBB ENSG00000170315
MYL6 ENSG00000092841
EDF1 ENSG00000107223
TAGLN ENSG00000149591
S100A6 ENSG00000197956
COX4I1 ENSG00000131143
FXYD6 ENSG00000137726
IER2 ENSG00000160888
SLC25A6 ENSG00000169100
SERF2 ENSG00000140264
EEF1D ENSG00000104529
TPM2 ENSG00000198467
TIMP1 ENSG00000102265
LGALS1 ENSG00000100097
PFDN5 ENSG00000123349
CD81 ENSG00000110651
ENO1 ENSG00000074800
SSR4 ENSG00000180879
CFL1 ENSG00000172757
PFN1 ENSG00000108518
CST3 ENSG00000101439
PTRF ENSG00000177469
SERPINB6 ENSG00000124570
BCAP31 ENSG00000185825
TMSB10 ENSG00000034510
FLNA ENSG00000196924
SEPW1 ENSG00000178980
CKB ENSG00000166165
CTSD ENSG00000117984
GLTSCR2 ENSG00000105373
NDUFB11 ENSG00000147123
CFD ENSG00000197766
PKM ENSG00000067225
CTSB ENSG00000164733
FBL ENSG00000105202
GPX1 ENSG00000233276

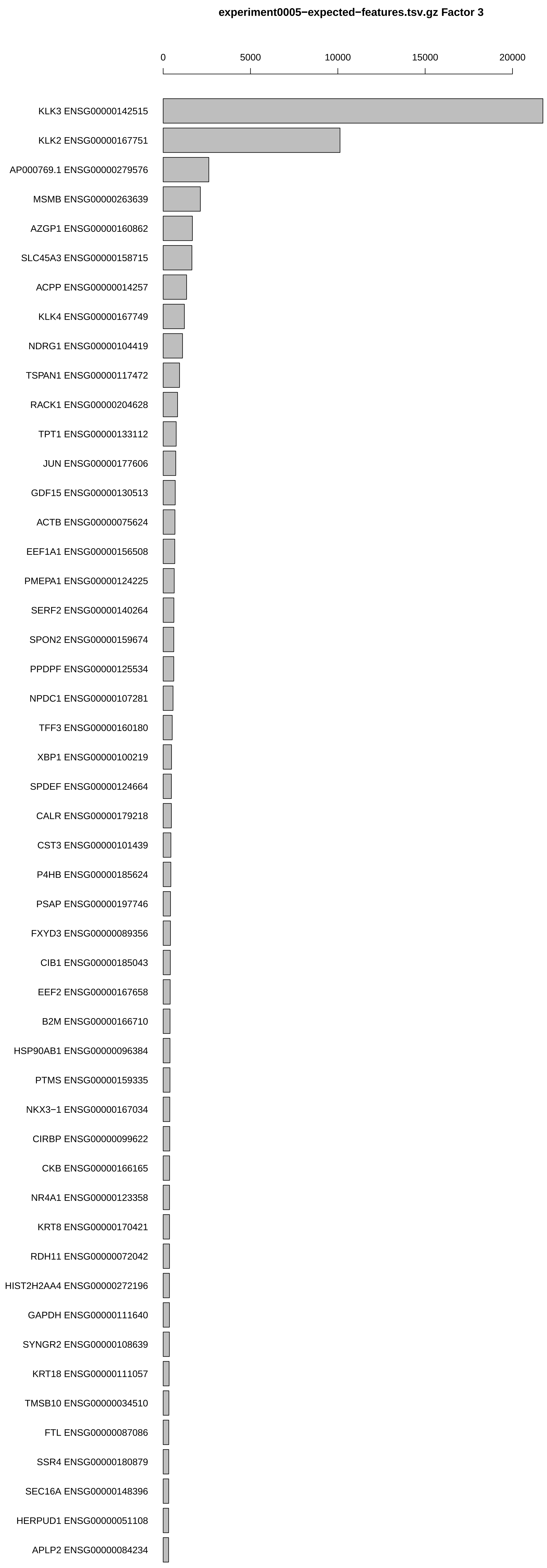
experiment0005−expected−features.tsv.gz Factor 3
0
5000
10000
15000
20000
KLK3 ENSG00000142515
KLK2 ENSG00000167751
AP000769.1 ENSG00000279576
MSMB ENSG00000263639
AZGP1 ENSG00000160862
SLC45A3 ENSG00000158715
ACPP ENSG00000014257
KLK4 ENSG00000167749
NDRG1 ENSG00000104419
TSPAN1 ENSG00000117472
RACK1 ENSG00000204628
TPT1 ENSG00000133112
JUN ENSG00000177606
GDF15 ENSG00000130513
ACTB ENSG00000075624
EEF1A1 ENSG00000156508
PMEPA1 ENSG00000124225
SERF2 ENSG00000140264
SPON2 ENSG00000159674
PPDPF ENSG00000125534
NPDC1 ENSG00000107281
TFF3 ENSG00000160180
XBP1 ENSG00000100219
SPDEF ENSG00000124664
CALR ENSG00000179218
CST3 ENSG00000101439
P4HB ENSG00000185624
PSAP ENSG00000197746
FXYD3 ENSG00000089356
CIB1 ENSG00000185043
EEF2 ENSG00000167658
B2M ENSG00000166710
HSP90AB1 ENSG00000096384
PTMS ENSG00000159335
NKX3−1 ENSG00000167034
CIRBP ENSG00000099622
CKB ENSG00000166165
NR4A1 ENSG00000123358
KRT8 ENSG00000170421
RDH11 ENSG00000072042
HIST2H2AA4 ENSG00000272196
GAPDH ENSG00000111640
SYNGR2 ENSG00000108639
KRT18 ENSG00000111057
TMSB10 ENSG00000034510
FTL ENSG00000087086
SSR4 ENSG00000180879
SEC16A ENSG00000148396
HERPUD1 ENSG00000051108
APLP2 ENSG00000084234

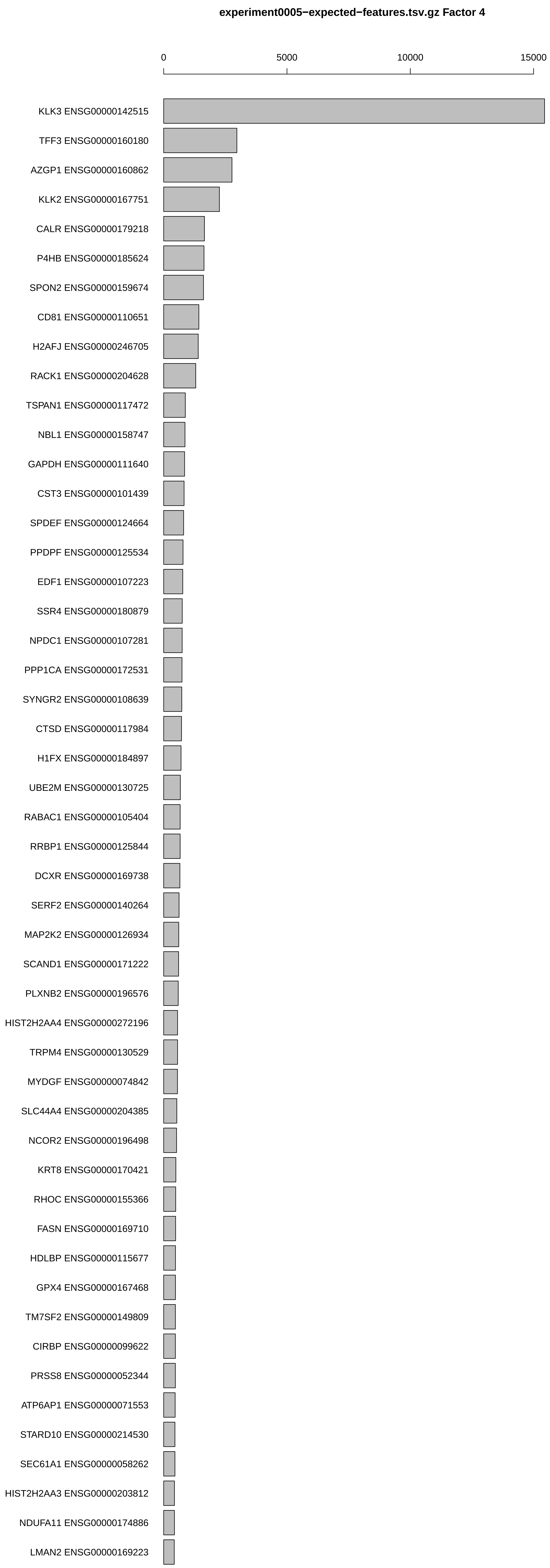
experiment0005−expected−features.tsv.gz Factor 4
0
5000
10000
15000
KLK3 ENSG00000142515
TFF3 ENSG00000160180
AZGP1 ENSG00000160862
KLK2 ENSG00000167751
CALR ENSG00000179218
P4HB ENSG00000185624
SPON2 ENSG00000159674
CD81 ENSG00000110651
H2AFJ ENSG00000246705
RACK1 ENSG00000204628
TSPAN1 ENSG00000117472
NBL1 ENSG00000158747
GAPDH ENSG00000111640
CST3 ENSG00000101439
SPDEF ENSG00000124664
PPDPF ENSG00000125534
EDF1 ENSG00000107223
SSR4 ENSG00000180879
NPDC1 ENSG00000107281
PPP1CA ENSG00000172531
SYNGR2 ENSG00000108639
CTSD ENSG00000117984
H1FX ENSG00000184897
UBE2M ENSG00000130725
RABAC1 ENSG00000105404
RRBP1 ENSG00000125844
DCXR ENSG00000169738
SERF2 ENSG00000140264
MAP2K2 ENSG00000126934
SCAND1 ENSG00000171222
PLXNB2 ENSG00000196576
HIST2H2AA4 ENSG00000272196
TRPM4 ENSG00000130529
MYDGF ENSG00000074842
SLC44A4 ENSG00000204385
NCOR2 ENSG00000196498
KRT8 ENSG00000170421
RHOC ENSG00000155366
FASN ENSG00000169710
HDLBP ENSG00000115677
GPX4 ENSG00000167468
TM7SF2 ENSG00000149809
CIRBP ENSG00000099622
PRSS8 ENSG00000052344
ATP6AP1 ENSG00000071553
STARD10 ENSG00000214530
SEC61A1 ENSG00000058262
HIST2H2AA3 ENSG00000203812
NDUFA11 ENSG00000174886
LMAN2 ENSG00000169223

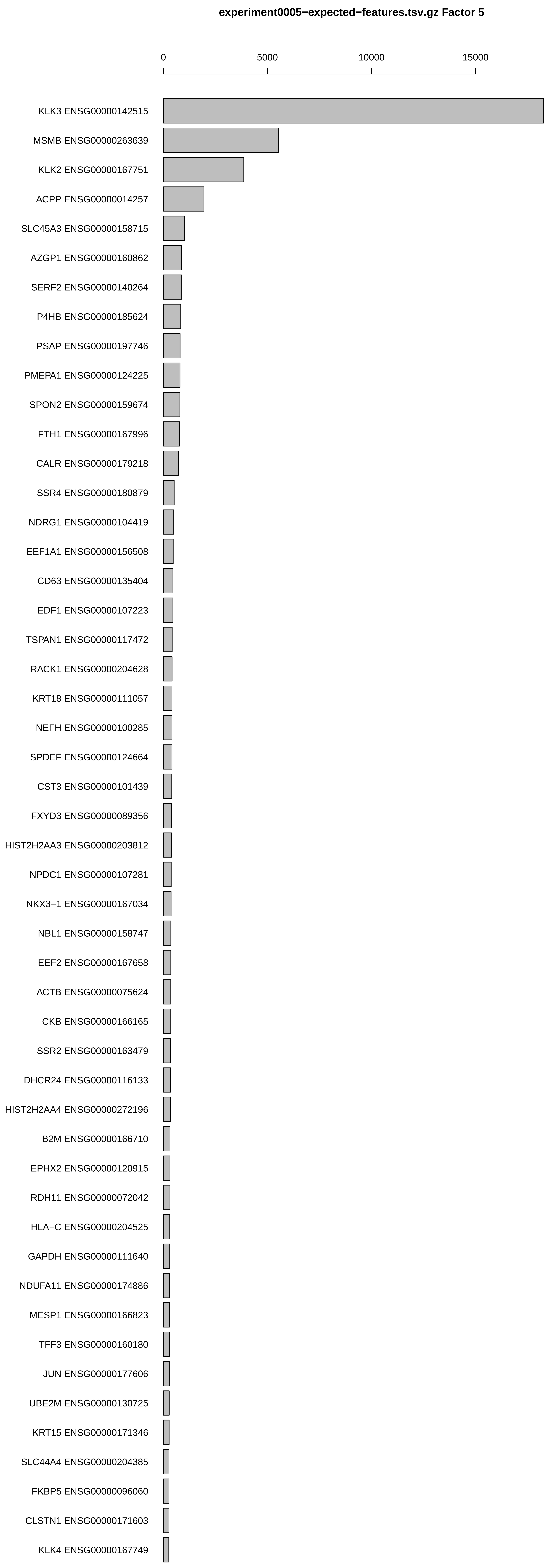
experiment0005−expected−features.tsv.gz Factor 5
0
5000
10000
15000
KLK3 ENSG00000142515
MSMB ENSG00000263639
KLK2 ENSG00000167751
ACPP ENSG00000014257
SLC45A3 ENSG00000158715
AZGP1 ENSG00000160862
SERF2 ENSG00000140264
P4HB ENSG00000185624
PSAP ENSG00000197746
PMEPA1 ENSG00000124225
SPON2 ENSG00000159674
FTH1 ENSG00000167996
CALR ENSG00000179218
SSR4 ENSG00000180879
NDRG1 ENSG00000104419
EEF1A1 ENSG00000156508
CD63 ENSG00000135404
EDF1 ENSG00000107223
TSPAN1 ENSG00000117472
RACK1 ENSG00000204628
KRT18 ENSG00000111057
NEFH ENSG00000100285
SPDEF ENSG00000124664
CST3 ENSG00000101439
FXYD3 ENSG00000089356
HIST2H2AA3 ENSG00000203812
NPDC1 ENSG00000107281
NKX3−1 ENSG00000167034
NBL1 ENSG00000158747
EEF2 ENSG00000167658
ACTB ENSG00000075624
CKB ENSG00000166165
SSR2 ENSG00000163479
DHCR24 ENSG00000116133
HIST2H2AA4 ENSG00000272196
B2M ENSG00000166710
EPHX2 ENSG00000120915
RDH11 ENSG00000072042
HLA−C ENSG00000204525
GAPDH ENSG00000111640
NDUFA11 ENSG00000174886
MESP1 ENSG00000166823
TFF3 ENSG00000160180
JUN ENSG00000177606
UBE2M ENSG00000130725
KRT15 ENSG00000171346
SLC44A4 ENSG00000204385
FKBP5 ENSG00000096060
CLSTN1 ENSG00000171603
KLK4 ENSG00000167749

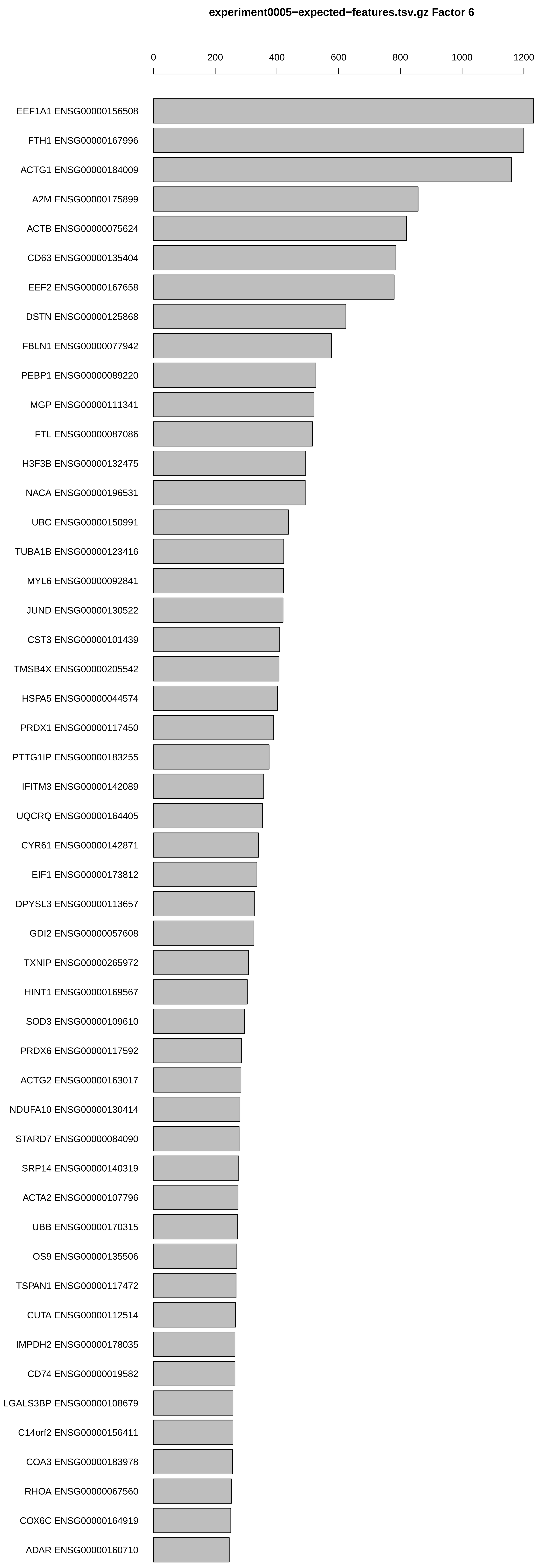

experiment0005−expected−features.tsv.gz Factor 6
0
200
400
600
800
1000
1200
EEF1A1 ENSG00000156508
FTH1 ENSG00000167996
ACTG1 ENSG00000184009
A2M ENSG00000175899
ACTB ENSG00000075624
CD63 ENSG00000135404
EEF2 ENSG00000167658
DSTN ENSG00000125868
FBLN1 ENSG00000077942
PEBP1 ENSG00000089220
MGP ENSG00000111341
FTL ENSG00000087086
H3F3B ENSG00000132475
NACA ENSG00000196531
UBC ENSG00000150991
TUBA1B ENSG00000123416
MYL6 ENSG00000092841
JUND ENSG00000130522
CST3 ENSG00000101439
TMSB4X ENSG00000205542
HSPA5 ENSG00000044574
PRDX1 ENSG00000117450
PTTG1IP ENSG00000183255
IFITM3 ENSG00000142089
UQCRQ ENSG00000164405
CYR61 ENSG00000142871
EIF1 ENSG00000173812
DPYSL3 ENSG00000113657
GDI2 ENSG00000057608
TXNIP ENSG00000265972
HINT1 ENSG00000169567
SOD3 ENSG00000109610
PRDX6 ENSG00000117592
ACTG2 ENSG00000163017
NDUFA10 ENSG00000130414
STARD7 ENSG00000084090
SRP14 ENSG00000140319
ACTA2 ENSG00000107796
UBB ENSG00000170315
OS9 ENSG00000135506
TSPAN1 ENSG00000117472
CUTA ENSG00000112514
IMPDH2 ENSG00000178035
CD74 ENSG00000019582
LGALS3BP ENSG00000108679
C14orf2 ENSG00000156411
COA3 ENSG00000183978
RHOA ENSG00000067560
COX6C ENSG00000164919
ADAR ENSG00000160710

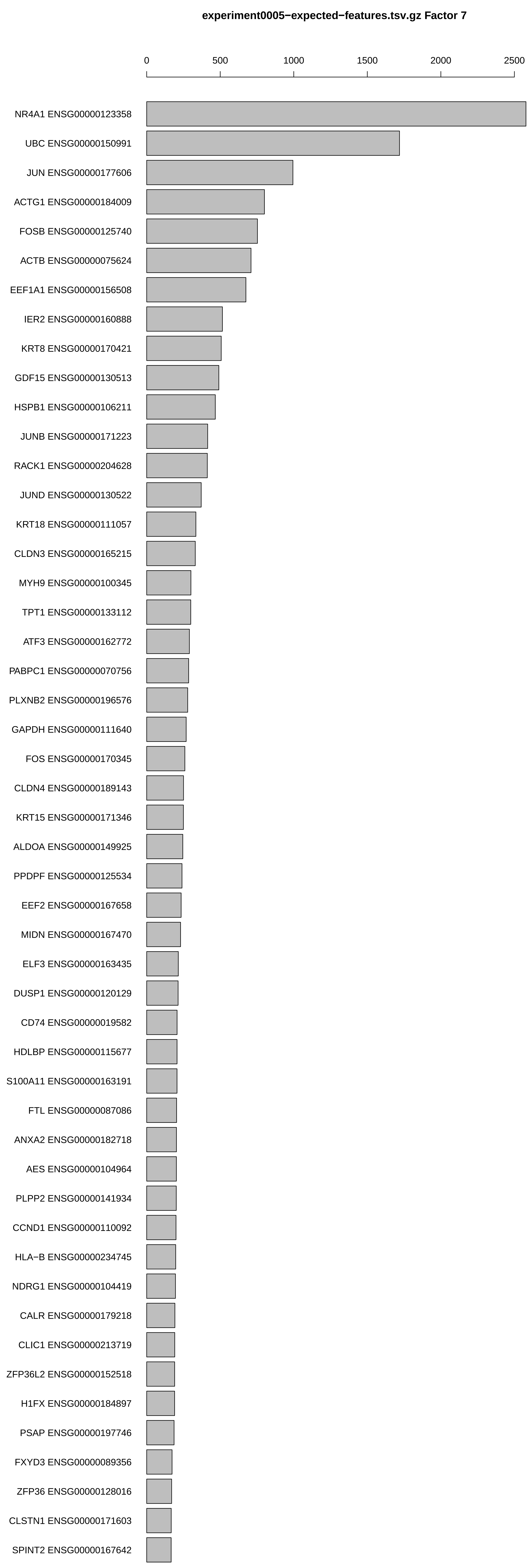
experiment0005−expected−features.tsv.gz Factor 7
0
500
1000
1500
2000
2500
NR4A1 ENSG00000123358
UBC ENSG00000150991
JUN ENSG00000177606
ACTG1 ENSG00000184009
FOSB ENSG00000125740
ACTB ENSG00000075624
EEF1A1 ENSG00000156508
IER2 ENSG00000160888
KRT8 ENSG00000170421
GDF15 ENSG00000130513
HSPB1 ENSG00000106211
JUNB ENSG00000171223
RACK1 ENSG00000204628
JUND ENSG00000130522
KRT18 ENSG00000111057
CLDN3 ENSG00000165215
MYH9 ENSG00000100345
TPT1 ENSG00000133112
ATF3 ENSG00000162772
PABPC1 ENSG00000070756
PLXNB2 ENSG00000196576
GAPDH ENSG00000111640
FOS ENSG00000170345
CLDN4 ENSG00000189143
KRT15 ENSG00000171346
ALDOA ENSG00000149925
PPDPF ENSG00000125534
EEF2 ENSG00000167658
MIDN ENSG00000167470
ELF3 ENSG00000163435
DUSP1 ENSG00000120129
CD74 ENSG00000019582
HDLBP ENSG00000115677
S100A11 ENSG00000163191
FTL ENSG00000087086
ANXA2 ENSG00000182718
AES ENSG00000104964
PLPP2 ENSG00000141934
CCND1 ENSG00000110092
HLA−B ENSG00000234745
NDRG1 ENSG00000104419
CALR ENSG00000179218
CLIC1 ENSG00000213719
ZFP36L2 ENSG00000152518
H1FX ENSG00000184897
PSAP ENSG00000197746
FXYD3 ENSG00000089356
ZFP36 ENSG00000128016
CLSTN1 ENSG00000171603
SPINT2 ENSG00000167642

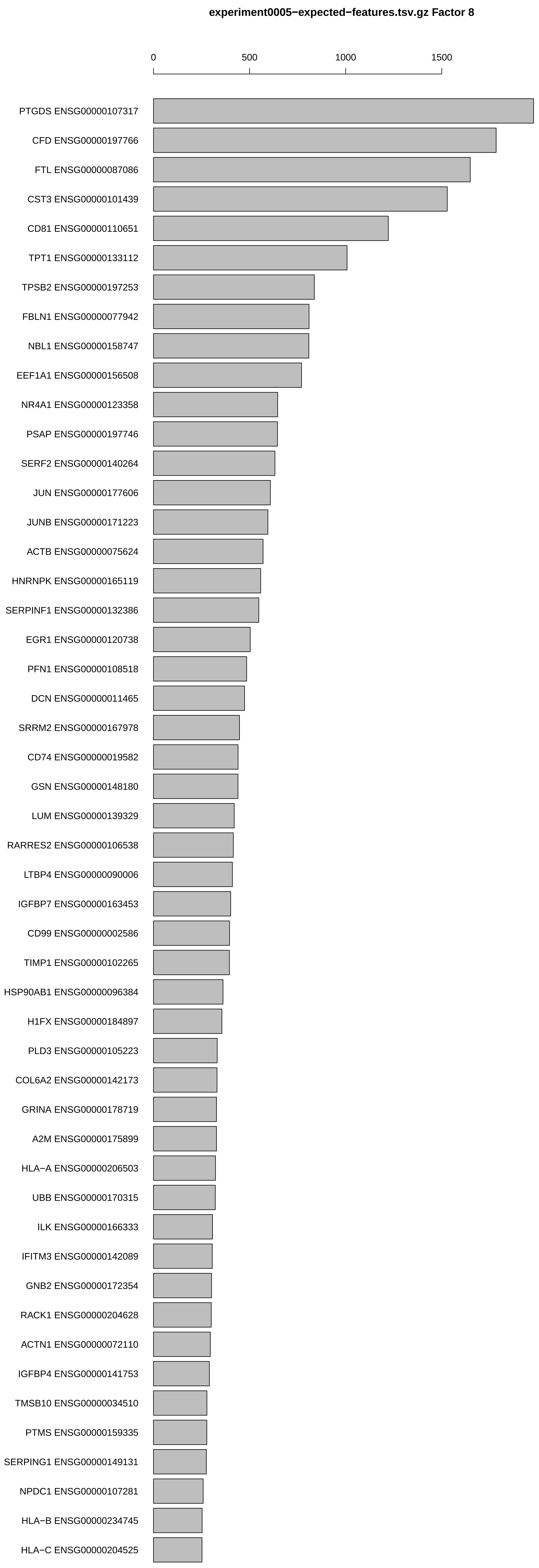
experiment0005−expected−features.tsv.gz Factor 8
0
500
1000
1500
PTGDS ENSG00000107317
CFD ENSG00000197766
FTL ENSG00000087086
CST3 ENSG00000101439
CD81 ENSG00000110651
TPT1 ENSG00000133112
TPSB2 ENSG00000197253
FBLN1 ENSG00000077942
NBL1 ENSG00000158747
EEF1A1 ENSG00000156508
NR4A1 ENSG00000123358
PSAP ENSG00000197746
SERF2 ENSG00000140264
JUN ENSG00000177606
JUNB ENSG00000171223
ACTB ENSG00000075624
HNRNPK ENSG00000165119
SERPINF1 ENSG00000132386
EGR1 ENSG00000120738
PFN1 ENSG00000108518
DCN ENSG00000011465
SRRM2 ENSG00000167978
CD74 ENSG00000019582
GSN ENSG00000148180
LUM ENSG00000139329
RARRES2 ENSG00000106538
LTBP4 ENSG00000090006
IGFBP7 ENSG00000163453
CD99 ENSG00000002586
TIMP1 ENSG00000102265
HSP90AB1 ENSG00000096384
H1FX ENSG00000184897
PLD3 ENSG00000105223
COL6A2 ENSG00000142173
GRINA ENSG00000178719
A2M ENSG00000175899
HLA−A ENSG00000206503
UBB ENSG00000170315
ILK ENSG00000166333
IFITM3 ENSG00000142089
GNB2 ENSG00000172354
RACK1 ENSG00000204628
ACTN1 ENSG00000072110
IGFBP4 ENSG00000141753
TMSB10 ENSG00000034510
PTMS ENSG00000159335
SERPING1 ENSG00000149131
NPDC1 ENSG00000107281
HLA−B ENSG00000234745
HLA−C ENSG00000204525

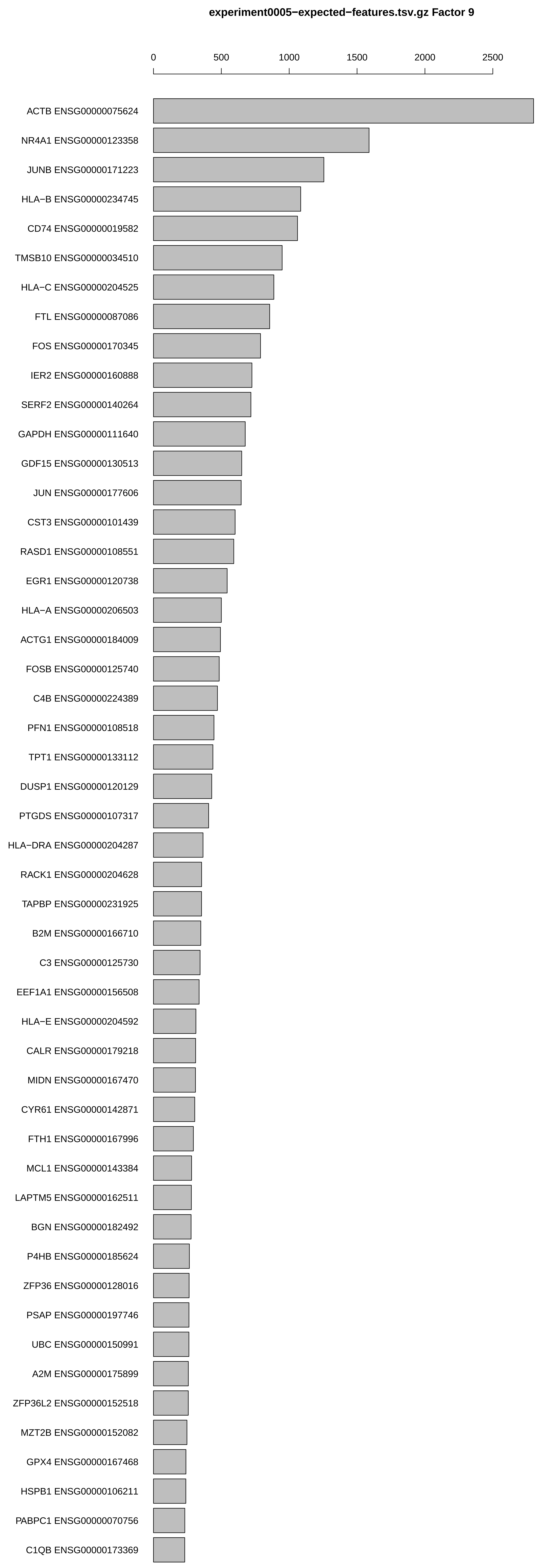
experiment0005−expected−features.tsv.gz Factor 9
0
500
1000
1500
2000
2500
ACTB ENSG00000075624
NR4A1 ENSG00000123358
JUNB ENSG00000171223
HLA−B ENSG00000234745
CD74 ENSG00000019582
TMSB10 ENSG00000034510
HLA−C ENSG00000204525
FTL ENSG00000087086
FOS ENSG00000170345
IER2 ENSG00000160888
SERF2 ENSG00000140264
GAPDH ENSG00000111640
GDF15 ENSG00000130513
JUN ENSG00000177606
CST3 ENSG00000101439
RASD1 ENSG00000108551
EGR1 ENSG00000120738
HLA−A ENSG00000206503
ACTG1 ENSG00000184009
FOSB ENSG00000125740
C4B ENSG00000224389
PFN1 ENSG00000108518
TPT1 ENSG00000133112
DUSP1 ENSG00000120129
PTGDS ENSG00000107317
HLA−DRA ENSG00000204287
RACK1 ENSG00000204628
TAPBP ENSG00000231925
B2M ENSG00000166710
C3 ENSG00000125730
EEF1A1 ENSG00000156508
HLA−E ENSG00000204592
CALR ENSG00000179218
MIDN ENSG00000167470
CYR61 ENSG00000142871
FTH1 ENSG00000167996
MCL1 ENSG00000143384
LAPTM5 ENSG00000162511
BGN ENSG00000182492
P4HB ENSG00000185624
ZFP36 ENSG00000128016
PSAP ENSG00000197746
UBC ENSG00000150991
A2M ENSG00000175899
ZFP36L2 ENSG00000152518
MZT2B ENSG00000152082
GPX4 ENSG00000167468
HSPB1 ENSG00000106211
PABPC1 ENSG00000070756
C1QB ENSG00000173369

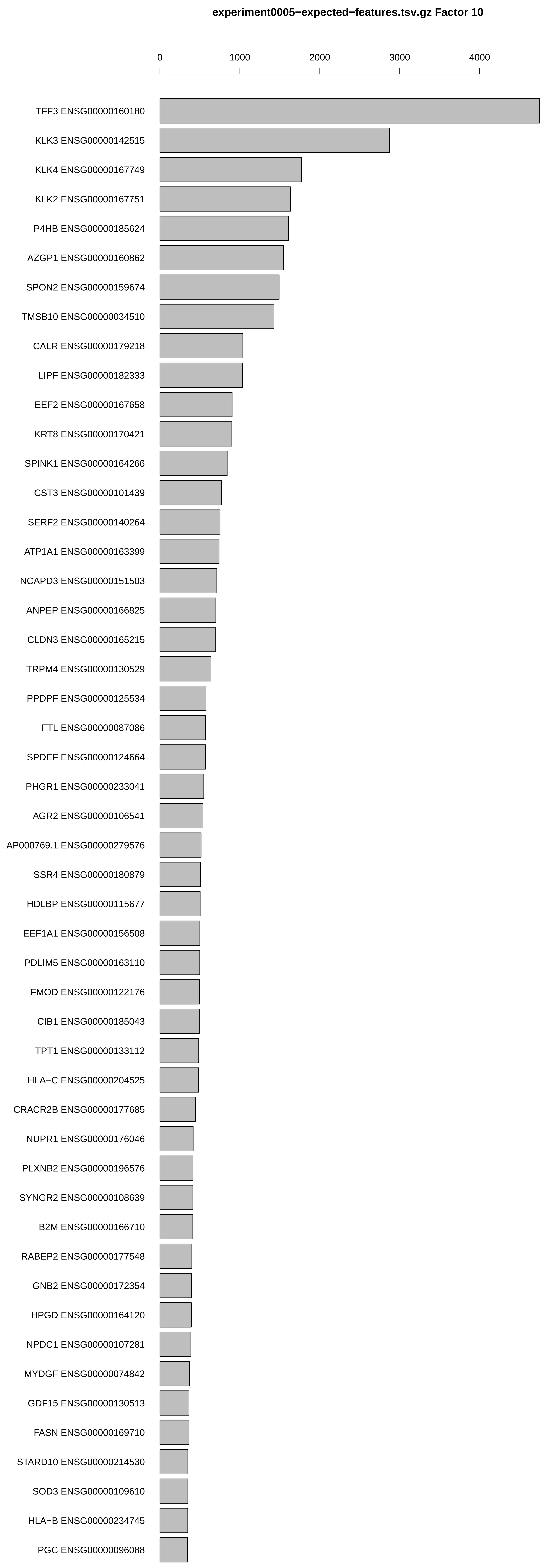

experiment0005−expected−features.tsv.gz Factor 10
0
1000
2000
3000
4000
TFF3 ENSG00000160180
KLK3 ENSG00000142515
KLK4 ENSG00000167749
KLK2 ENSG00000167751
P4HB ENSG00000185624
AZGP1 ENSG00000160862
SPON2 ENSG00000159674
TMSB10 ENSG00000034510
CALR ENSG00000179218
LIPF ENSG00000182333
EEF2 ENSG00000167658
KRT8 ENSG00000170421
SPINK1 ENSG00000164266
CST3 ENSG00000101439
SERF2 ENSG00000140264
ATP1A1 ENSG00000163399
NCAPD3 ENSG00000151503
ANPEP ENSG00000166825
CLDN3 ENSG00000165215
TRPM4 ENSG00000130529
PPDPF ENSG00000125534
FTL ENSG00000087086
SPDEF ENSG00000124664
PHGR1 ENSG00000233041
AGR2 ENSG00000106541
AP000769.1 ENSG00000279576
SSR4 ENSG00000180879
HDLBP ENSG00000115677
EEF1A1 ENSG00000156508
PDLIM5 ENSG00000163110
FMOD ENSG00000122176
CIB1 ENSG00000185043
TPT1 ENSG00000133112
HLA−C ENSG00000204525
CRACR2B ENSG00000177685
NUPR1 ENSG00000176046
PLXNB2 ENSG00000196576
SYNGR2 ENSG00000108639
B2M ENSG00000166710
RABEP2 ENSG00000177548
GNB2 ENSG00000172354
HPGD ENSG00000164120
NPDC1 ENSG00000107281
MYDGF ENSG00000074842
GDF15 ENSG00000130513
FASN ENSG00000169710
STARD10 ENSG00000214530
SOD3 ENSG00000109610
HLA−B ENSG00000234745
PGC ENSG00000096088

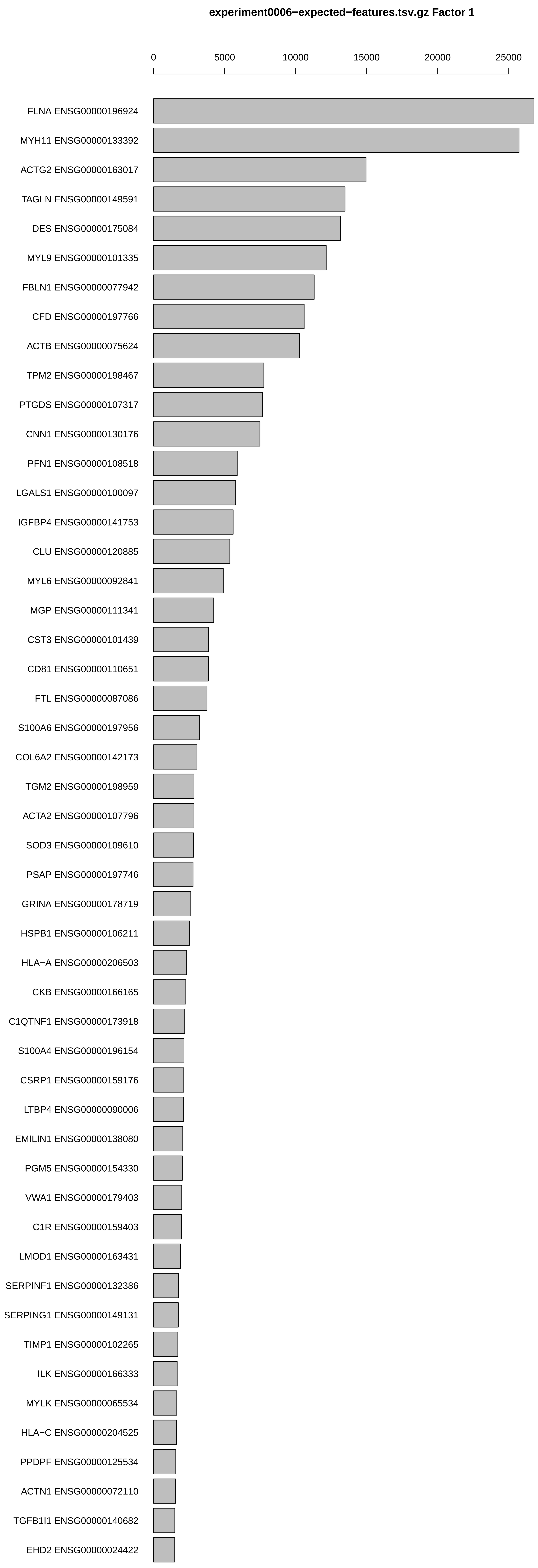
experiment0006−expected−features.tsv.gz Factor 1
0
5000
10000
15000
20000
25000
FLNA ENSG00000196924
MYH11 ENSG00000133392
ACTG2 ENSG00000163017
TAGLN ENSG00000149591
DES ENSG00000175084
MYL9 ENSG00000101335
FBLN1 ENSG00000077942
CFD ENSG00000197766
ACTB ENSG00000075624
TPM2 ENSG00000198467
PTGDS ENSG00000107317
CNN1 ENSG00000130176
PFN1 ENSG00000108518
LGALS1 ENSG00000100097
IGFBP4 ENSG00000141753
CLU ENSG00000120885
MYL6 ENSG00000092841
MGP ENSG00000111341
CST3 ENSG00000101439
CD81 ENSG00000110651
FTL ENSG00000087086
S100A6 ENSG00000197956
COL6A2 ENSG00000142173
TGM2 ENSG00000198959
ACTA2 ENSG00000107796
SOD3 ENSG00000109610
PSAP ENSG00000197746
GRINA ENSG00000178719
HSPB1 ENSG00000106211
HLA−A ENSG00000206503
CKB ENSG00000166165
C1QTNF1 ENSG00000173918
S100A4 ENSG00000196154
CSRP1 ENSG00000159176
LTBP4 ENSG00000090006
EMILIN1 ENSG00000138080
PGM5 ENSG00000154330
VWA1 ENSG00000179403
C1R ENSG00000159403
LMOD1 ENSG00000163431
SERPINF1 ENSG00000132386
SERPING1 ENSG00000149131
TIMP1 ENSG00000102265
ILK ENSG00000166333
MYLK ENSG00000065534
HLA−C ENSG00000204525
PPDPF ENSG00000125534
ACTN1 ENSG00000072110
TGFB1I1 ENSG00000140682
EHD2 ENSG00000024422

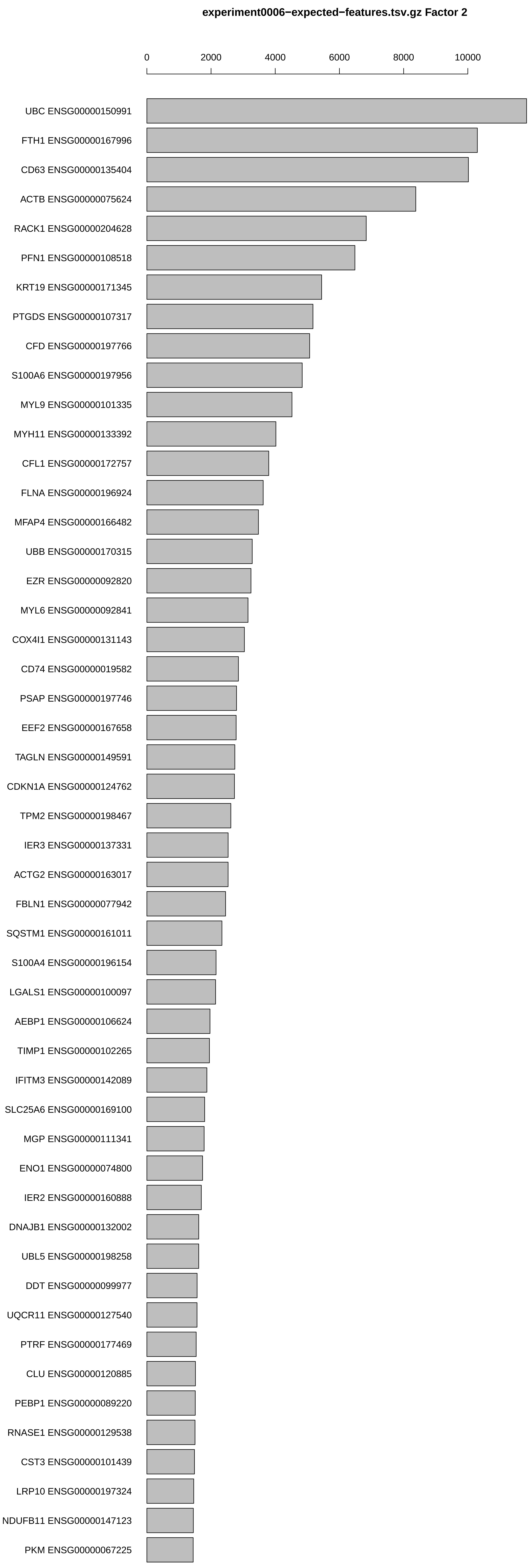
experiment0006−expected−features.tsv.gz Factor 2
0
2000
4000
6000
8000
10000
UBC ENSG00000150991
FTH1 ENSG00000167996
CD63 ENSG00000135404
ACTB ENSG00000075624
RACK1 ENSG00000204628
PFN1 ENSG00000108518
KRT19 ENSG00000171345
PTGDS ENSG00000107317
CFD ENSG00000197766
S100A6 ENSG00000197956
MYL9 ENSG00000101335
MYH11 ENSG00000133392
CFL1 ENSG00000172757
FLNA ENSG00000196924
MFAP4 ENSG00000166482
UBB ENSG00000170315
EZR ENSG00000092820
MYL6 ENSG00000092841
COX4I1 ENSG00000131143
CD74 ENSG00000019582
PSAP ENSG00000197746
EEF2 ENSG00000167658
TAGLN ENSG00000149591
CDKN1A ENSG00000124762
TPM2 ENSG00000198467
IER3 ENSG00000137331
ACTG2 ENSG00000163017
FBLN1 ENSG00000077942
SQSTM1 ENSG00000161011
S100A4 ENSG00000196154
LGALS1 ENSG00000100097
AEBP1 ENSG00000106624
TIMP1 ENSG00000102265
IFITM3 ENSG00000142089
SLC25A6 ENSG00000169100
MGP ENSG00000111341
ENO1 ENSG00000074800
IER2 ENSG00000160888
DNAJB1 ENSG00000132002
UBL5 ENSG00000198258
DDT ENSG00000099977
UQCR11 ENSG00000127540
PTRF ENSG00000177469
CLU ENSG00000120885
PEBP1 ENSG00000089220
RNASE1 ENSG00000129538
CST3 ENSG00000101439
LRP10 ENSG00000197324
NDUFB11 ENSG00000147123
PKM ENSG00000067225

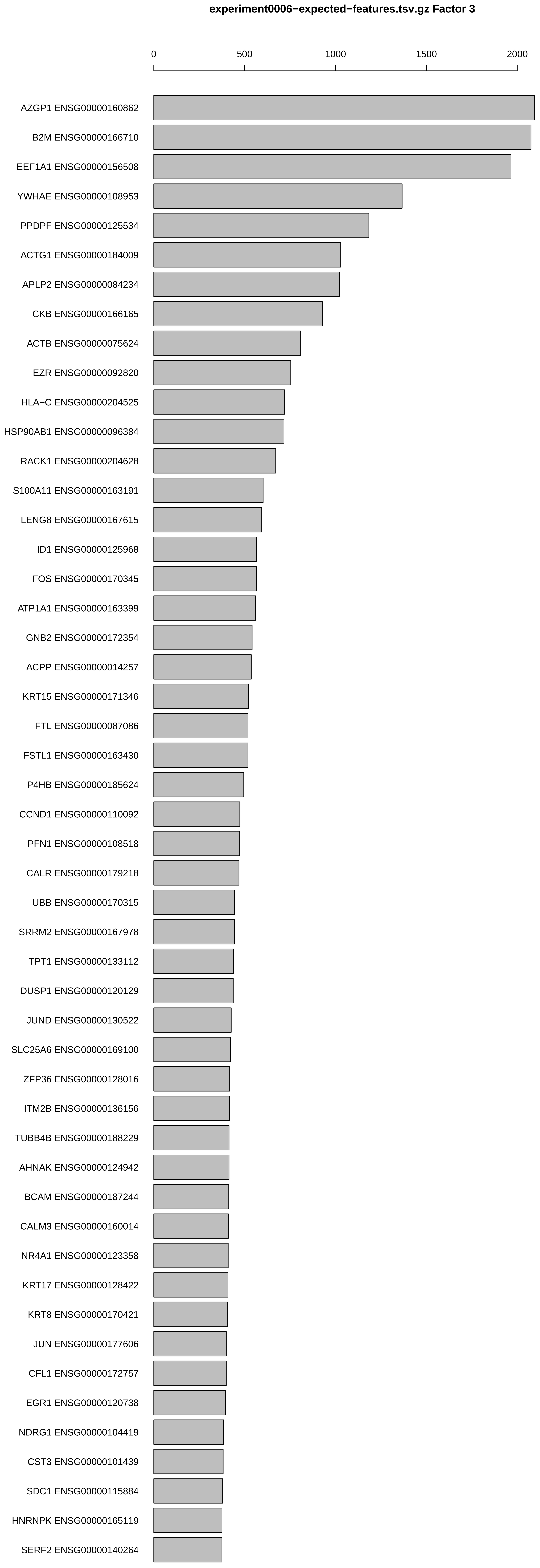
experiment0006−expected−features.tsv.gz Factor 3
0
500
1000
1500
2000
AZGP1 ENSG00000160862
B2M ENSG00000166710
EEF1A1 ENSG00000156508
YWHAE ENSG00000108953
PPDPF ENSG00000125534
ACTG1 ENSG00000184009
APLP2 ENSG00000084234
CKB ENSG00000166165
ACTB ENSG00000075624
EZR ENSG00000092820
HLA−C ENSG00000204525
HSP90AB1 ENSG00000096384
RACK1 ENSG00000204628
S100A11 ENSG00000163191
LENG8 ENSG00000167615
ID1 ENSG00000125968
FOS ENSG00000170345
ATP1A1 ENSG00000163399
GNB2 ENSG00000172354
ACPP ENSG00000014257
KRT15 ENSG00000171346
FTL ENSG00000087086
FSTL1 ENSG00000163430
P4HB ENSG00000185624
CCND1 ENSG00000110092
PFN1 ENSG00000108518
CALR ENSG00000179218
UBB ENSG00000170315
SRRM2 ENSG00000167978
TPT1 ENSG00000133112
DUSP1 ENSG00000120129
JUND ENSG00000130522
SLC25A6 ENSG00000169100
ZFP36 ENSG00000128016
ITM2B ENSG00000136156
TUBB4B ENSG00000188229
AHNAK ENSG00000124942
BCAM ENSG00000187244
CALM3 ENSG00000160014
NR4A1 ENSG00000123358
KRT17 ENSG00000128422
KRT8 ENSG00000170421
JUN ENSG00000177606
CFL1 ENSG00000172757
EGR1 ENSG00000120738
NDRG1 ENSG00000104419
CST3 ENSG00000101439
SDC1 ENSG00000115884
HNRNPK ENSG00000165119
SERF2 ENSG00000140264

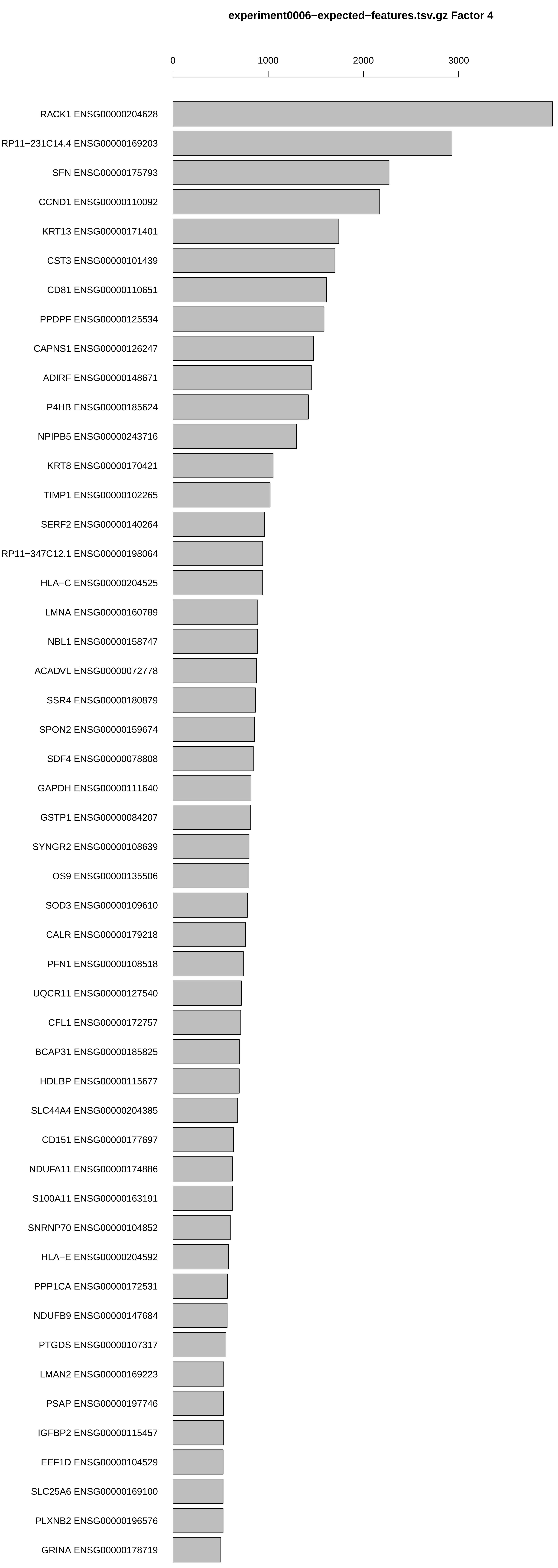
experiment0006−expected−features.tsv.gz Factor 4
0
1000
2000
3000
RACK1 ENSG00000204628
RP11−231C14.4 ENSG00000169203
SFN ENSG00000175793
CCND1 ENSG00000110092
KRT13 ENSG00000171401
CST3 ENSG00000101439
CD81 ENSG00000110651
PPDPF ENSG00000125534
CAPNS1 ENSG00000126247
ADIRF ENSG00000148671
P4HB ENSG00000185624
NPIPB5 ENSG00000243716
KRT8 ENSG00000170421
TIMP1 ENSG00000102265
SERF2 ENSG00000140264
RP11−347C12.1 ENSG00000198064
HLA−C ENSG00000204525
LMNA ENSG00000160789
NBL1 ENSG00000158747
ACADVL ENSG00000072778
SSR4 ENSG00000180879
SPON2 ENSG00000159674
SDF4 ENSG00000078808
GAPDH ENSG00000111640
GSTP1 ENSG00000084207
SYNGR2 ENSG00000108639
OS9 ENSG00000135506
SOD3 ENSG00000109610
CALR ENSG00000179218
PFN1 ENSG00000108518
UQCR11 ENSG00000127540
CFL1 ENSG00000172757
BCAP31 ENSG00000185825
HDLBP ENSG00000115677
SLC44A4 ENSG00000204385
CD151 ENSG00000177697
NDUFA11 ENSG00000174886
S100A11 ENSG00000163191
SNRNP70 ENSG00000104852
HLA−E ENSG00000204592
PPP1CA ENSG00000172531
NDUFB9 ENSG00000147684
PTGDS ENSG00000107317
LMAN2 ENSG00000169223
PSAP ENSG00000197746
IGFBP2 ENSG00000115457
EEF1D ENSG00000104529
SLC25A6 ENSG00000169100
PLXNB2 ENSG00000196576
GRINA ENSG00000178719

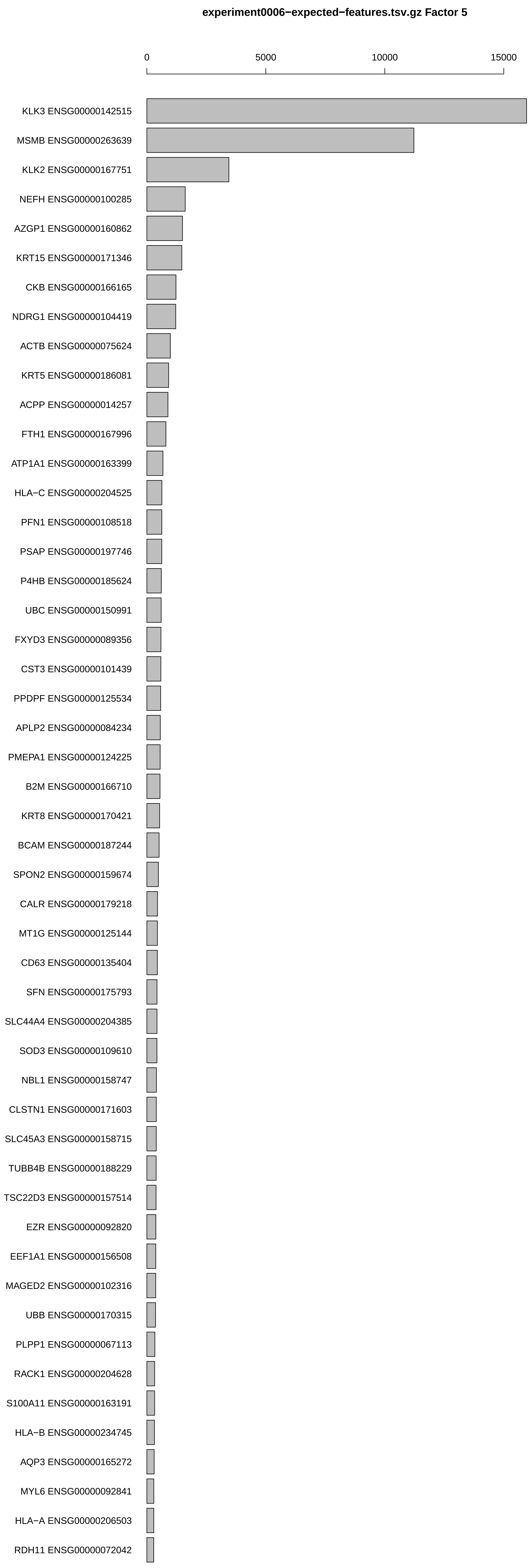
experiment0006−expected−features.tsv.gz Factor 5
0
5000
10000
15000
KLK3 ENSG00000142515
MSMB ENSG00000263639
KLK2 ENSG00000167751
NEFH ENSG00000100285
AZGP1 ENSG00000160862
KRT15 ENSG00000171346
CKB ENSG00000166165
NDRG1 ENSG00000104419
ACTB ENSG00000075624
KRT5 ENSG00000186081
ACPP ENSG00000014257
FTH1 ENSG00000167996
ATP1A1 ENSG00000163399
HLA−C ENSG00000204525
PFN1 ENSG00000108518
PSAP ENSG00000197746
P4HB ENSG00000185624
UBC ENSG00000150991
FXYD3 ENSG00000089356
CST3 ENSG00000101439
PPDPF ENSG00000125534
APLP2 ENSG00000084234
PMEPA1 ENSG00000124225
B2M ENSG00000166710
KRT8 ENSG00000170421
BCAM ENSG00000187244
SPON2 ENSG00000159674
CALR ENSG00000179218
MT1G ENSG00000125144
CD63 ENSG00000135404
SFN ENSG00000175793
SLC44A4 ENSG00000204385
SOD3 ENSG00000109610
NBL1 ENSG00000158747
CLSTN1 ENSG00000171603
SLC45A3 ENSG00000158715
TUBB4B ENSG00000188229
TSC22D3 ENSG00000157514
EZR ENSG00000092820
EEF1A1 ENSG00000156508
MAGED2 ENSG00000102316
UBB ENSG00000170315
PLPP1 ENSG00000067113
RACK1 ENSG00000204628
S100A11 ENSG00000163191
HLA−B ENSG00000234745
AQP3 ENSG00000165272
MYL6 ENSG00000092841
HLA−A ENSG00000206503
RDH11 ENSG00000072042

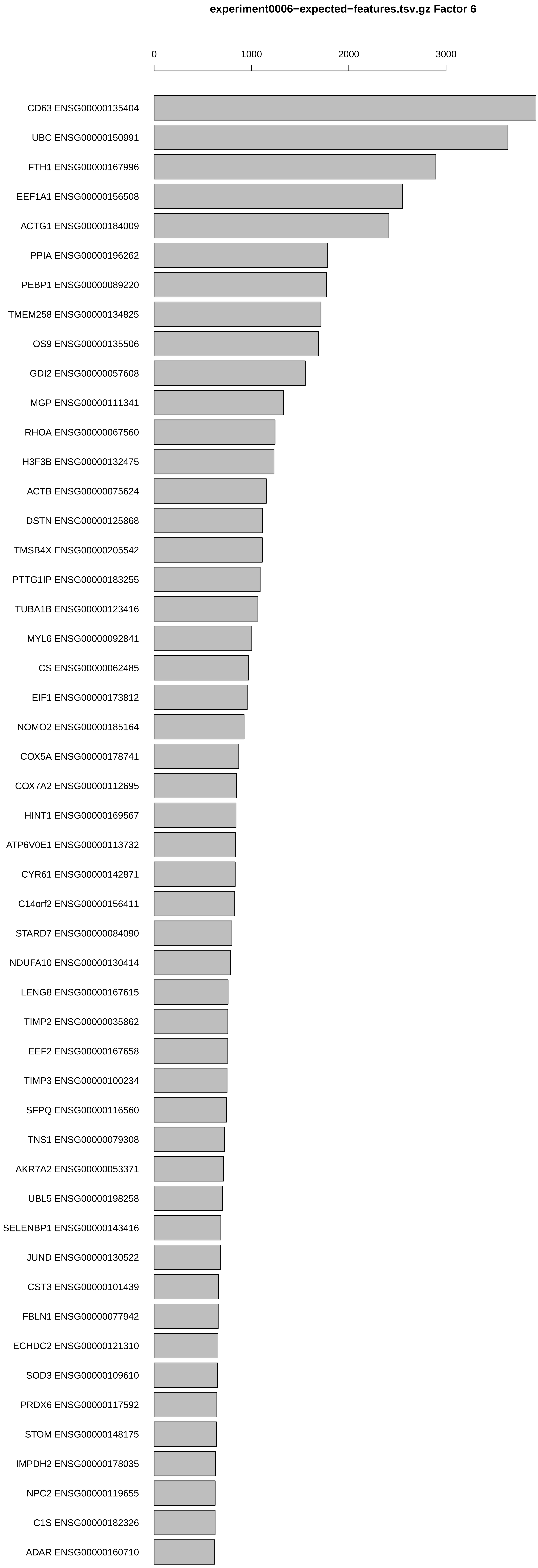
experiment0006−expected−features.tsv.gz Factor 6
0
1000
2000
3000
CD63 ENSG00000135404
UBC ENSG00000150991
FTH1 ENSG00000167996
EEF1A1 ENSG00000156508
ACTG1 ENSG00000184009
PPIA ENSG00000196262
PEBP1 ENSG00000089220
TMEM258 ENSG00000134825
OS9 ENSG00000135506
GDI2 ENSG00000057608
MGP ENSG00000111341
RHOA ENSG00000067560
H3F3B ENSG00000132475
ACTB ENSG00000075624
DSTN ENSG00000125868
TMSB4X ENSG00000205542
PTTG1IP ENSG00000183255
TUBA1B ENSG00000123416
MYL6 ENSG00000092841
CS ENSG00000062485
EIF1 ENSG00000173812
NOMO2 ENSG00000185164
COX5A ENSG00000178741
COX7A2 ENSG00000112695
HINT1 ENSG00000169567
ATP6V0E1 ENSG00000113732
CYR61 ENSG00000142871
C14orf2 ENSG00000156411
STARD7 ENSG00000084090
NDUFA10 ENSG00000130414
LENG8 ENSG00000167615
TIMP2 ENSG00000035862
EEF2 ENSG00000167658
TIMP3 ENSG00000100234
SFPQ ENSG00000116560
TNS1 ENSG00000079308
AKR7A2 ENSG00000053371
UBL5 ENSG00000198258
SELENBP1 ENSG00000143416
JUND ENSG00000130522
CST3 ENSG00000101439
FBLN1 ENSG00000077942
ECHDC2 ENSG00000121310
SOD3 ENSG00000109610
PRDX6 ENSG00000117592
STOM ENSG00000148175
IMPDH2 ENSG00000178035
NPC2 ENSG00000119655
C1S ENSG00000182326
ADAR ENSG00000160710

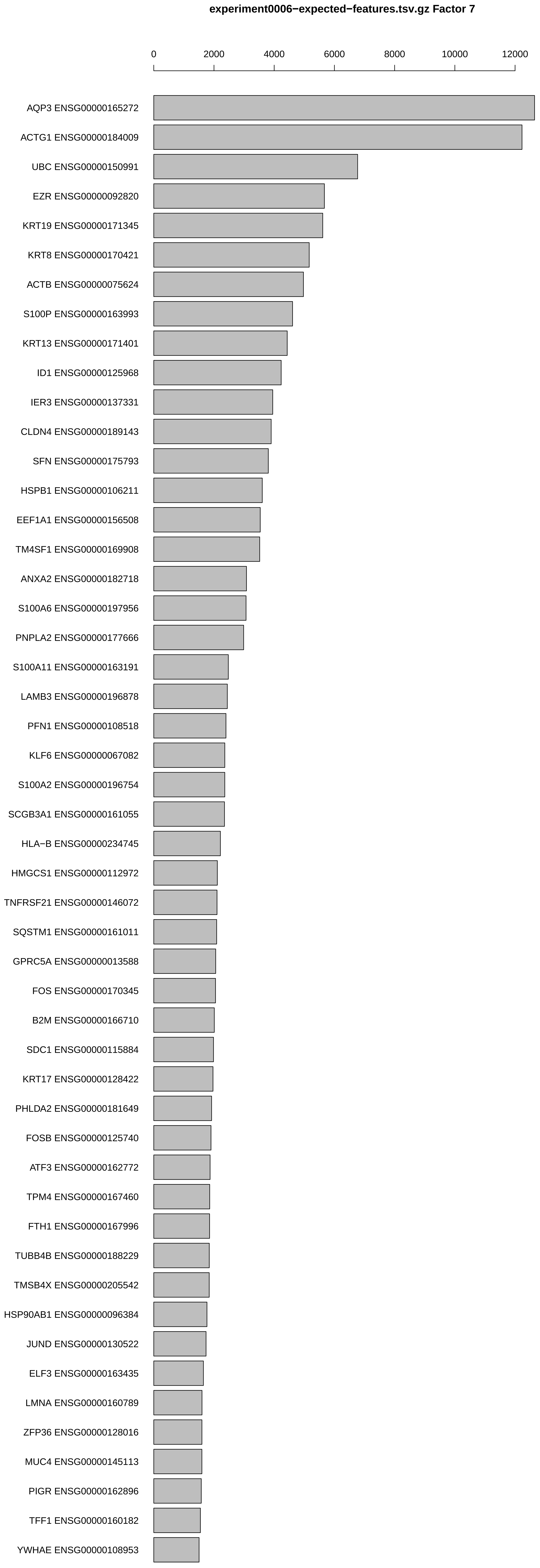
experiment0006−expected−features.tsv.gz Factor 7
0
2000
4000
6000
8000
10000
12000
AQP3 ENSG00000165272
ACTG1 ENSG00000184009
UBC ENSG00000150991
EZR ENSG00000092820
KRT19 ENSG00000171345
KRT8 ENSG00000170421
ACTB ENSG00000075624
S100P ENSG00000163993
KRT13 ENSG00000171401
ID1 ENSG00000125968
IER3 ENSG00000137331
CLDN4 ENSG00000189143
SFN ENSG00000175793
HSPB1 ENSG00000106211
EEF1A1 ENSG00000156508
TM4SF1 ENSG00000169908
ANXA2 ENSG00000182718
S100A6 ENSG00000197956
PNPLA2 ENSG00000177666
S100A11 ENSG00000163191
LAMB3 ENSG00000196878
PFN1 ENSG00000108518
KLF6 ENSG00000067082
S100A2 ENSG00000196754
SCGB3A1 ENSG00000161055
HLA−B ENSG00000234745
HMGCS1 ENSG00000112972
TNFRSF21 ENSG00000146072
SQSTM1 ENSG00000161011
GPRC5A ENSG00000013588
FOS ENSG00000170345
B2M ENSG00000166710
SDC1 ENSG00000115884
KRT17 ENSG00000128422
PHLDA2 ENSG00000181649
FOSB ENSG00000125740
ATF3 ENSG00000162772
TPM4 ENSG00000167460
FTH1 ENSG00000167996
TUBB4B ENSG00000188229
TMSB4X ENSG00000205542
HSP90AB1 ENSG00000096384
JUND ENSG00000130522
ELF3 ENSG00000163435
LMNA ENSG00000160789
ZFP36 ENSG00000128016
MUC4 ENSG00000145113
PIGR ENSG00000162896
TFF1 ENSG00000160182
YWHAE ENSG00000108953

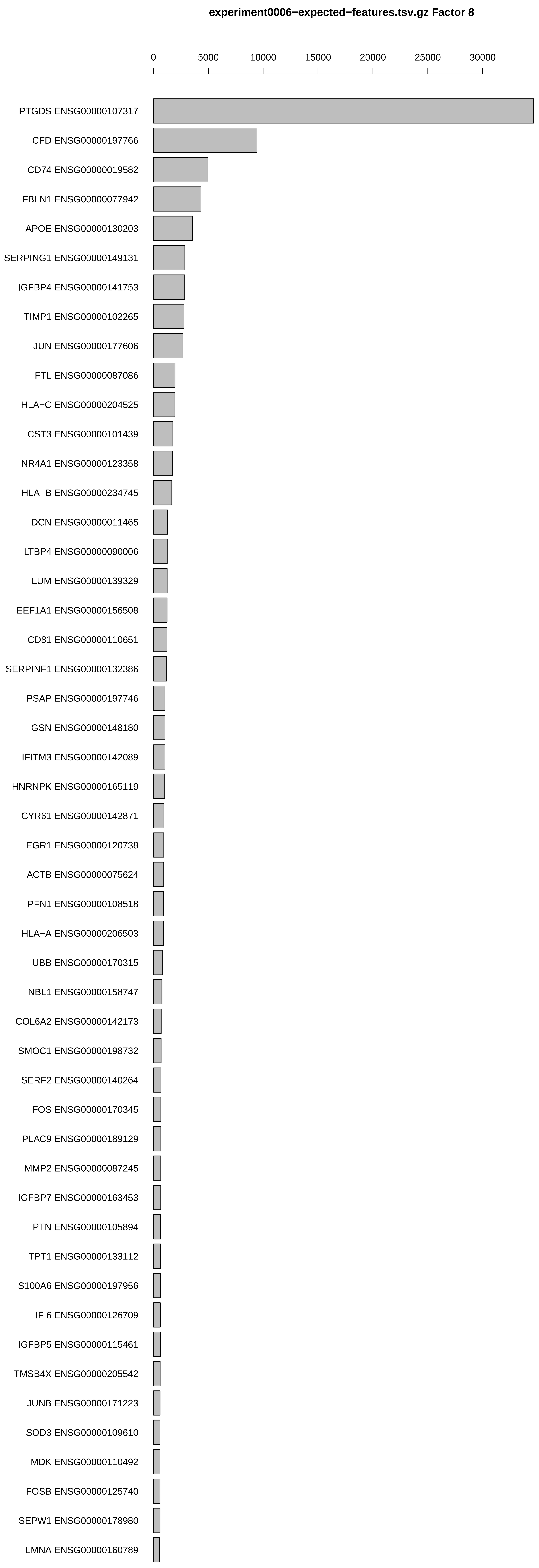

experiment0006−expected−features.tsv.gz Factor 8
0
5000
10000
15000
20000
25000
30000
PTGDS ENSG00000107317
CFD ENSG00000197766
CD74 ENSG00000019582
FBLN1 ENSG00000077942
APOE ENSG00000130203
SERPING1 ENSG00000149131
IGFBP4 ENSG00000141753
TIMP1 ENSG00000102265
JUN ENSG00000177606
FTL ENSG00000087086
HLA−C ENSG00000204525
CST3 ENSG00000101439
NR4A1 ENSG00000123358
HLA−B ENSG00000234745
DCN ENSG00000011465
LTBP4 ENSG00000090006
LUM ENSG00000139329
EEF1A1 ENSG00000156508
CD81 ENSG00000110651
SERPINF1 ENSG00000132386
PSAP ENSG00000197746
GSN ENSG00000148180
IFITM3 ENSG00000142089
HNRNPK ENSG00000165119
CYR61 ENSG00000142871
EGR1 ENSG00000120738
ACTB ENSG00000075624
PFN1 ENSG00000108518
HLA−A ENSG00000206503
UBB ENSG00000170315
NBL1 ENSG00000158747
COL6A2 ENSG00000142173
SMOC1 ENSG00000198732
SERF2 ENSG00000140264
FOS ENSG00000170345
PLAC9 ENSG00000189129
MMP2 ENSG00000087245
IGFBP7 ENSG00000163453
PTN ENSG00000105894
TPT1 ENSG00000133112
S100A6 ENSG00000197956
IFI6 ENSG00000126709
IGFBP5 ENSG00000115461
TMSB4X ENSG00000205542
JUNB ENSG00000171223
SOD3 ENSG00000109610
MDK ENSG00000110492
FOSB ENSG00000125740
SEPW1 ENSG00000178980
LMNA ENSG00000160789

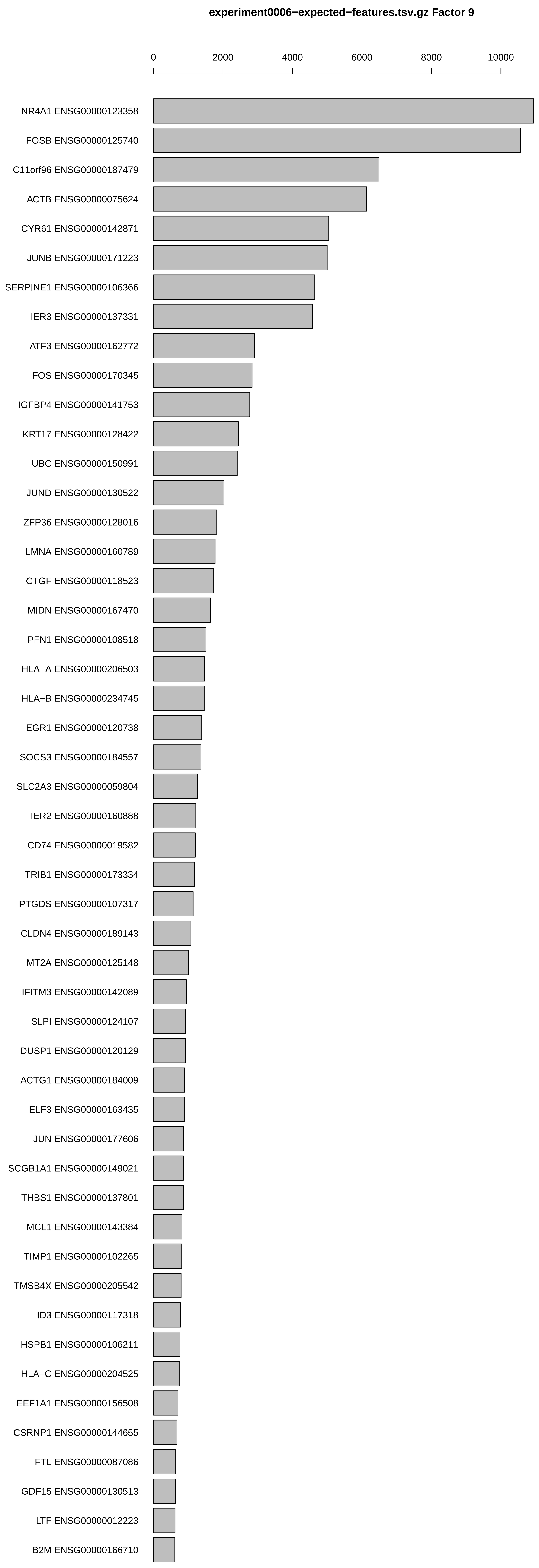
experiment0006−expected−features.tsv.gz Factor 9
0
2000
4000
6000
8000
10000
NR4A1 ENSG00000123358
FOSB ENSG00000125740
C11orf96 ENSG00000187479
ACTB ENSG00000075624
CYR61 ENSG00000142871
JUNB ENSG00000171223
SERPINE1 ENSG00000106366
IER3 ENSG00000137331
ATF3 ENSG00000162772
FOS ENSG00000170345
IGFBP4 ENSG00000141753
KRT17 ENSG00000128422
UBC ENSG00000150991
JUND ENSG00000130522
ZFP36 ENSG00000128016
LMNA ENSG00000160789
CTGF ENSG00000118523
MIDN ENSG00000167470
PFN1 ENSG00000108518
HLA−A ENSG00000206503
HLA−B ENSG00000234745
EGR1 ENSG00000120738
SOCS3 ENSG00000184557
SLC2A3 ENSG00000059804
IER2 ENSG00000160888
CD74 ENSG00000019582
TRIB1 ENSG00000173334
PTGDS ENSG00000107317
CLDN4 ENSG00000189143
MT2A ENSG00000125148
IFITM3 ENSG00000142089
SLPI ENSG00000124107
DUSP1 ENSG00000120129
ACTG1 ENSG00000184009
ELF3 ENSG00000163435
JUN ENSG00000177606
SCGB1A1 ENSG00000149021
THBS1 ENSG00000137801
MCL1 ENSG00000143384
TIMP1 ENSG00000102265
TMSB4X ENSG00000205542
ID3 ENSG00000117318
HSPB1 ENSG00000106211
HLA−C ENSG00000204525
EEF1A1 ENSG00000156508
CSRNP1 ENSG00000144655
FTL ENSG00000087086
GDF15 ENSG00000130513
LTF ENSG00000012223
B2M ENSG00000166710

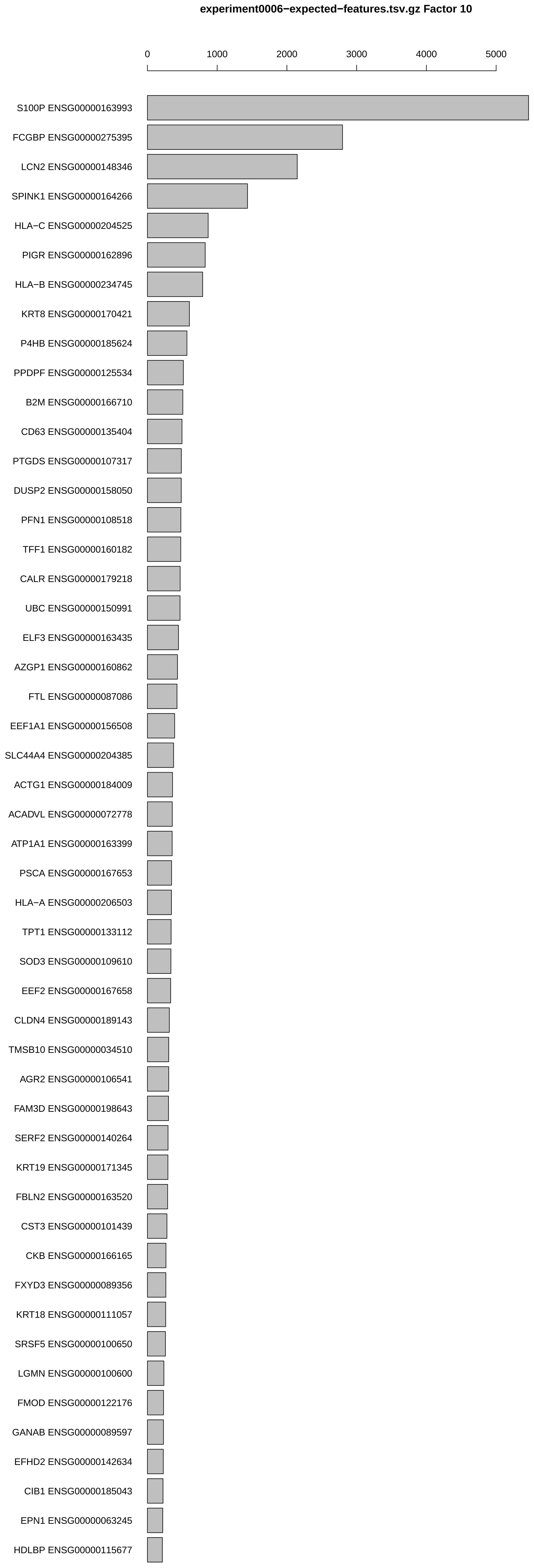
experiment0006−expected−features.tsv.gz Factor 10
0
1000
2000
3000
4000
5000
S100P ENSG00000163993
FCGBP ENSG00000275395
LCN2 ENSG00000148346
SPINK1 ENSG00000164266
HLA−C ENSG00000204525
PIGR ENSG00000162896
HLA−B ENSG00000234745
KRT8 ENSG00000170421
P4HB ENSG00000185624
PPDPF ENSG00000125534
B2M ENSG00000166710
CD63 ENSG00000135404
PTGDS ENSG00000107317
DUSP2 ENSG00000158050
PFN1 ENSG00000108518
TFF1 ENSG00000160182
CALR ENSG00000179218
UBC ENSG00000150991
ELF3 ENSG00000163435
AZGP1 ENSG00000160862
FTL ENSG00000087086
EEF1A1 ENSG00000156508
SLC44A4 ENSG00000204385
ACTG1 ENSG00000184009
ACADVL ENSG00000072778
ATP1A1 ENSG00000163399
PSCA ENSG00000167653
HLA−A ENSG00000206503
TPT1 ENSG00000133112
SOD3 ENSG00000109610
EEF2 ENSG00000167658
CLDN4 ENSG00000189143
TMSB10 ENSG00000034510
AGR2 ENSG00000106541
FAM3D ENSG00000198643
SERF2 ENSG00000140264
KRT19 ENSG00000171345
FBLN2 ENSG00000163520
CST3 ENSG00000101439
CKB ENSG00000166165
FXYD3 ENSG00000089356
KRT18 ENSG00000111057
SRSF5 ENSG00000100650
LGMN ENSG00000100600
FMOD ENSG00000122176
GANAB ENSG00000089597
EFHD2 ENSG00000142634
CIB1 ENSG00000185043
EPN1 ENSG00000063245
HDLBP ENSG00000115677

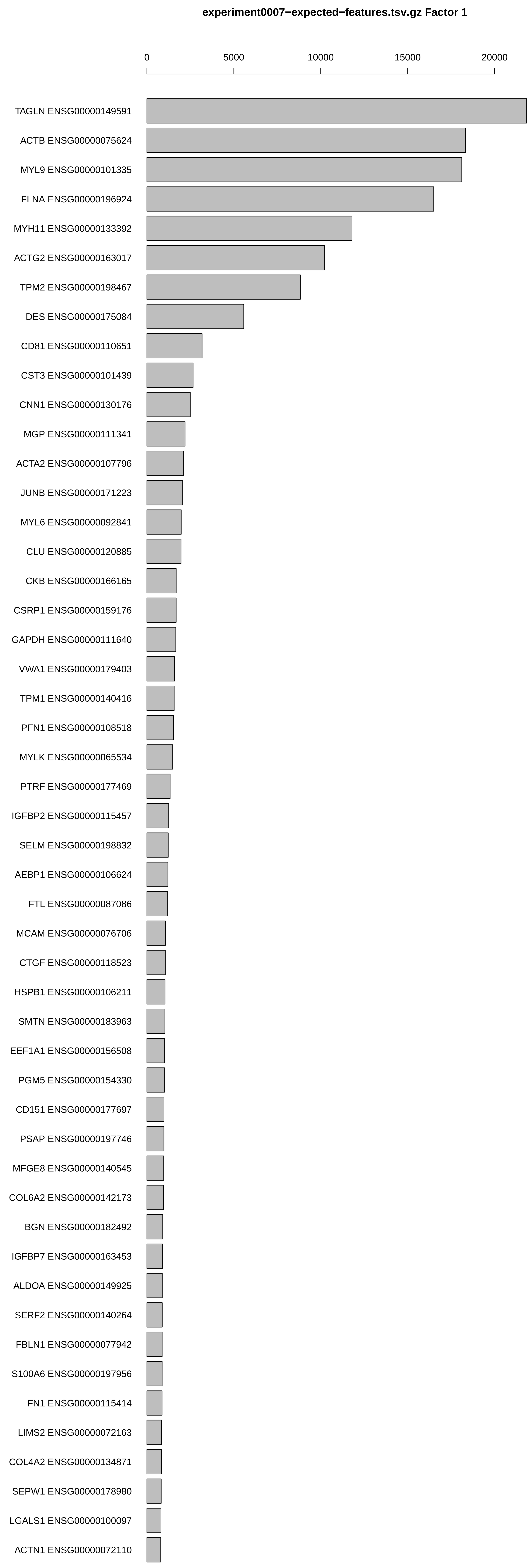

experiment0007−expected−features.tsv.gz Factor 1
0
5000
10000
15000
20000
TAGLN ENSG00000149591
ACTB ENSG00000075624
MYL9 ENSG00000101335
FLNA ENSG00000196924
MYH11 ENSG00000133392
ACTG2 ENSG00000163017
TPM2 ENSG00000198467
DES ENSG00000175084
CD81 ENSG00000110651
CST3 ENSG00000101439
CNN1 ENSG00000130176
MGP ENSG00000111341
ACTA2 ENSG00000107796
JUNB ENSG00000171223
MYL6 ENSG00000092841
CLU ENSG00000120885
CKB ENSG00000166165
CSRP1 ENSG00000159176
GAPDH ENSG00000111640
VWA1 ENSG00000179403
TPM1 ENSG00000140416
PFN1 ENSG00000108518
MYLK ENSG00000065534
PTRF ENSG00000177469
IGFBP2 ENSG00000115457
SELM ENSG00000198832
AEBP1 ENSG00000106624
FTL ENSG00000087086
MCAM ENSG00000076706
CTGF ENSG00000118523
HSPB1 ENSG00000106211
SMTN ENSG00000183963
EEF1A1 ENSG00000156508
PGM5 ENSG00000154330
CD151 ENSG00000177697
PSAP ENSG00000197746
MFGE8 ENSG00000140545
COL6A2 ENSG00000142173
BGN ENSG00000182492
IGFBP7 ENSG00000163453
ALDOA ENSG00000149925
SERF2 ENSG00000140264
FBLN1 ENSG00000077942
S100A6 ENSG00000197956
FN1 ENSG00000115414
LIMS2 ENSG00000072163
COL4A2 ENSG00000134871
SEPW1 ENSG00000178980
LGALS1 ENSG00000100097
ACTN1 ENSG00000072110

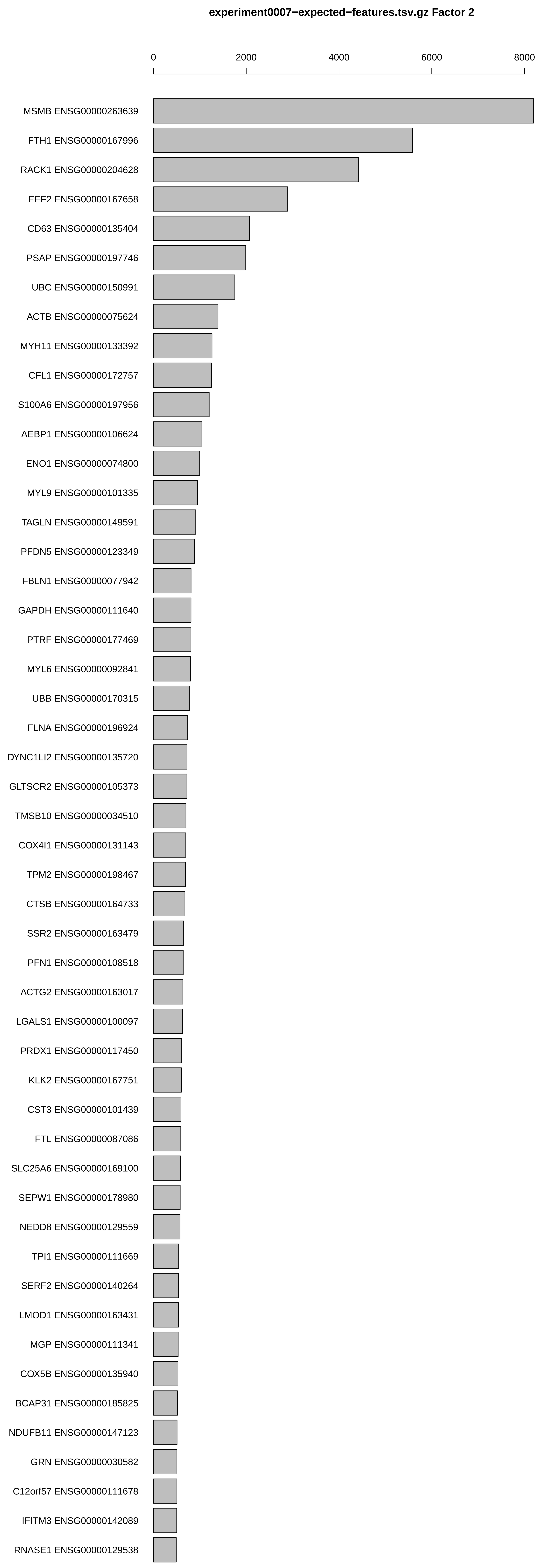
experiment0007−expected−features.tsv.gz Factor 2
0
2000
4000
6000
8000
MSMB ENSG00000263639
FTH1 ENSG00000167996
RACK1 ENSG00000204628
EEF2 ENSG00000167658
CD63 ENSG00000135404
PSAP ENSG00000197746
UBC ENSG00000150991
ACTB ENSG00000075624
MYH11 ENSG00000133392
CFL1 ENSG00000172757
S100A6 ENSG00000197956
AEBP1 ENSG00000106624
ENO1 ENSG00000074800
MYL9 ENSG00000101335
TAGLN ENSG00000149591
PFDN5 ENSG00000123349
FBLN1 ENSG00000077942
GAPDH ENSG00000111640
PTRF ENSG00000177469
MYL6 ENSG00000092841
UBB ENSG00000170315
FLNA ENSG00000196924
DYNC1LI2 ENSG00000135720
GLTSCR2 ENSG00000105373
TMSB10 ENSG00000034510
COX4I1 ENSG00000131143
TPM2 ENSG00000198467
CTSB ENSG00000164733
SSR2 ENSG00000163479
PFN1 ENSG00000108518
ACTG2 ENSG00000163017
LGALS1 ENSG00000100097
PRDX1 ENSG00000117450
KLK2 ENSG00000167751
CST3 ENSG00000101439
FTL ENSG00000087086
SLC25A6 ENSG00000169100
SEPW1 ENSG00000178980
NEDD8 ENSG00000129559
TPI1 ENSG00000111669
SERF2 ENSG00000140264
LMOD1 ENSG00000163431
MGP ENSG00000111341
COX5B ENSG00000135940
BCAP31 ENSG00000185825
NDUFB11 ENSG00000147123
GRN ENSG00000030582
C12orf57 ENSG00000111678
IFITM3 ENSG00000142089
RNASE1 ENSG00000129538

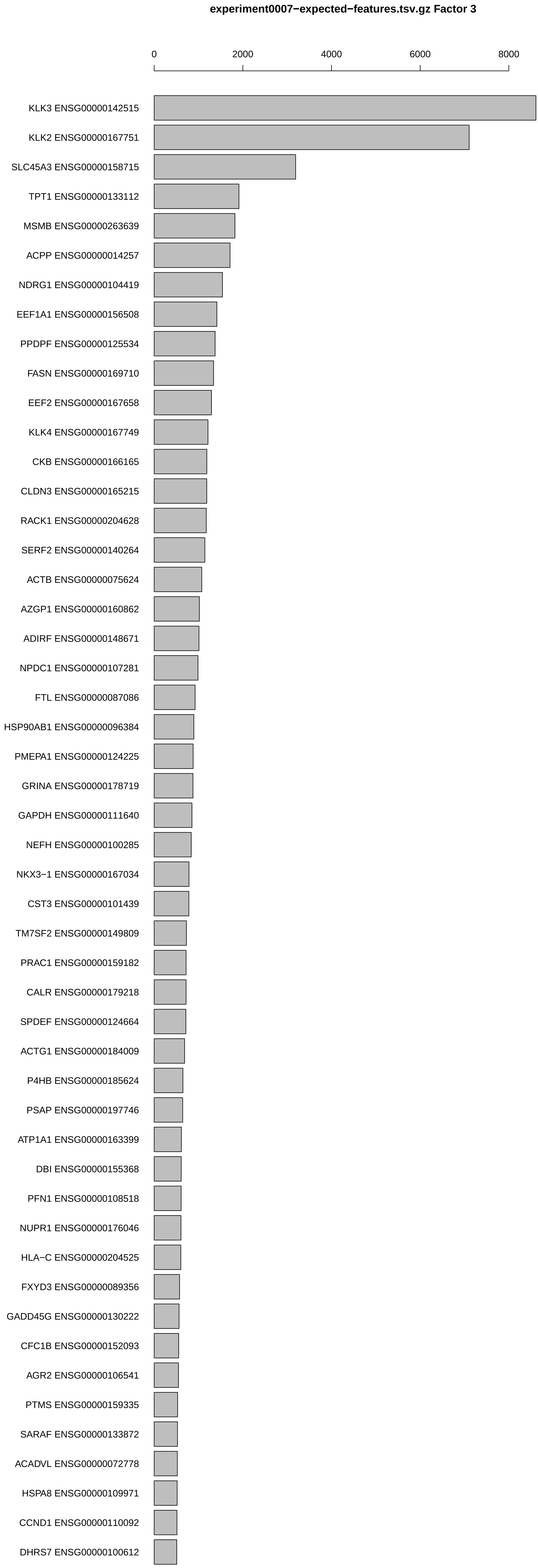

experiment0007−expected−features.tsv.gz Factor 3
0
2000
4000
6000
8000
KLK3 ENSG00000142515
KLK2 ENSG00000167751
SLC45A3 ENSG00000158715
TPT1 ENSG00000133112
MSMB ENSG00000263639
ACPP ENSG00000014257
NDRG1 ENSG00000104419
EEF1A1 ENSG00000156508
PPDPF ENSG00000125534
FASN ENSG00000169710
EEF2 ENSG00000167658
KLK4 ENSG00000167749
CKB ENSG00000166165
CLDN3 ENSG00000165215
RACK1 ENSG00000204628
SERF2 ENSG00000140264
ACTB ENSG00000075624
AZGP1 ENSG00000160862
ADIRF ENSG00000148671
NPDC1 ENSG00000107281
FTL ENSG00000087086
HSP90AB1 ENSG00000096384
PMEPA1 ENSG00000124225
GRINA ENSG00000178719
GAPDH ENSG00000111640
NEFH ENSG00000100285
NKX3−1 ENSG00000167034
CST3 ENSG00000101439
TM7SF2 ENSG00000149809
PRAC1 ENSG00000159182
CALR ENSG00000179218
SPDEF ENSG00000124664
ACTG1 ENSG00000184009
P4HB ENSG00000185624
PSAP ENSG00000197746
ATP1A1 ENSG00000163399
DBI ENSG00000155368
PFN1 ENSG00000108518
NUPR1 ENSG00000176046
HLA−C ENSG00000204525
FXYD3 ENSG00000089356
GADD45G ENSG00000130222
CFC1B ENSG00000152093
AGR2 ENSG00000106541
PTMS ENSG00000159335
SARAF ENSG00000133872
ACADVL ENSG00000072778
HSPA8 ENSG00000109971
CCND1 ENSG00000110092
DHRS7 ENSG00000100612

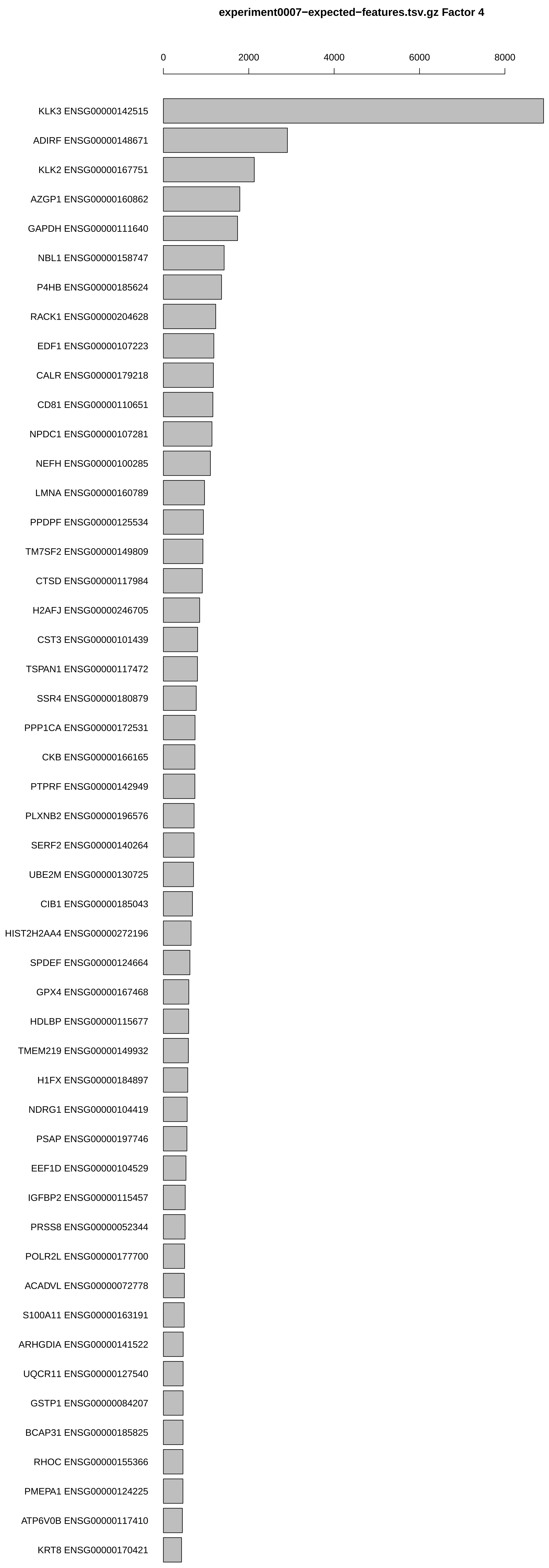
experiment0007−expected−features.tsv.gz Factor 4
0
2000
4000
6000
8000
KLK3 ENSG00000142515
ADIRF ENSG00000148671
KLK2 ENSG00000167751
AZGP1 ENSG00000160862
GAPDH ENSG00000111640
NBL1 ENSG00000158747
P4HB ENSG00000185624
RACK1 ENSG00000204628
EDF1 ENSG00000107223
CALR ENSG00000179218
CD81 ENSG00000110651
NPDC1 ENSG00000107281
NEFH ENSG00000100285
LMNA ENSG00000160789
PPDPF ENSG00000125534
TM7SF2 ENSG00000149809
CTSD ENSG00000117984
H2AFJ ENSG00000246705
CST3 ENSG00000101439
TSPAN1 ENSG00000117472
SSR4 ENSG00000180879
PPP1CA ENSG00000172531
CKB ENSG00000166165
PTPRF ENSG00000142949
PLXNB2 ENSG00000196576
SERF2 ENSG00000140264
UBE2M ENSG00000130725
CIB1 ENSG00000185043
HIST2H2AA4 ENSG00000272196
SPDEF ENSG00000124664
GPX4 ENSG00000167468
HDLBP ENSG00000115677
TMEM219 ENSG00000149932
H1FX ENSG00000184897
NDRG1 ENSG00000104419
PSAP ENSG00000197746
EEF1D ENSG00000104529
IGFBP2 ENSG00000115457
PRSS8 ENSG00000052344
POLR2L ENSG00000177700
ACADVL ENSG00000072778
S100A11 ENSG00000163191
ARHGDIA ENSG00000141522
UQCR11 ENSG00000127540
GSTP1 ENSG00000084207
BCAP31 ENSG00000185825
RHOC ENSG00000155366
PMEPA1 ENSG00000124225
ATP6V0B ENSG00000117410
KRT8 ENSG00000170421

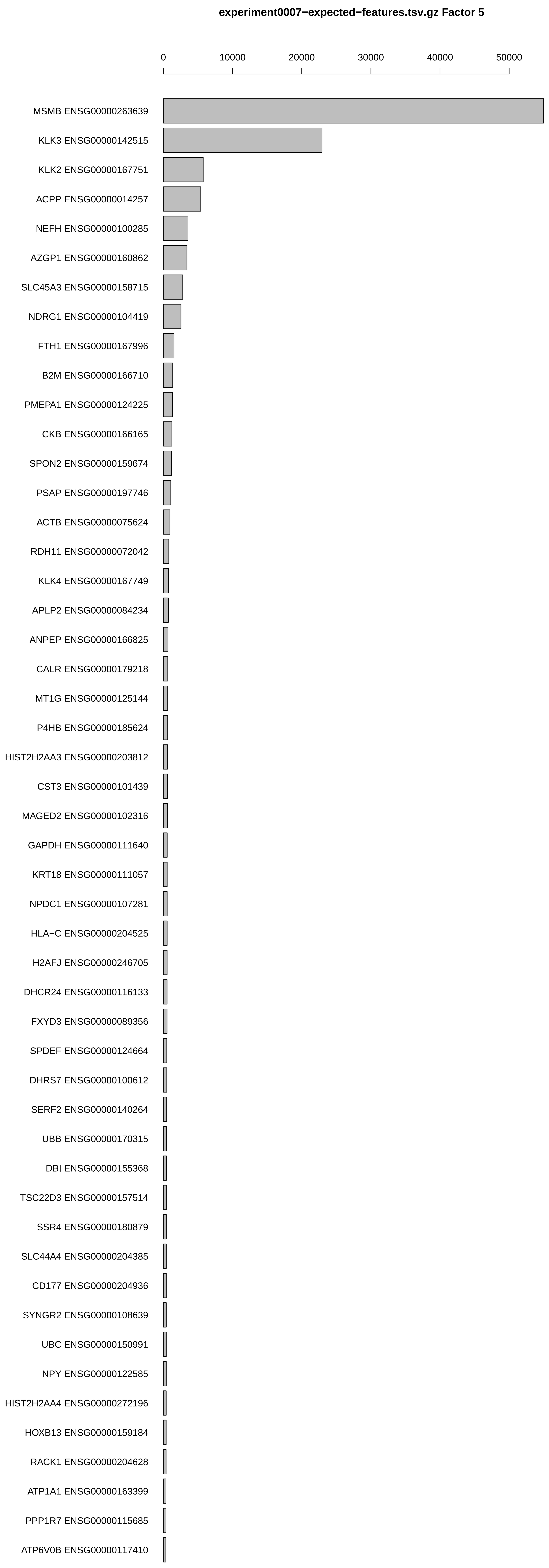
experiment0007−expected−features.tsv.gz Factor 5
0
10000
20000
30000
40000
50000
MSMB ENSG00000263639
KLK3 ENSG00000142515
KLK2 ENSG00000167751
ACPP ENSG00000014257
NEFH ENSG00000100285
AZGP1 ENSG00000160862
SLC45A3 ENSG00000158715
NDRG1 ENSG00000104419
FTH1 ENSG00000167996
B2M ENSG00000166710
PMEPA1 ENSG00000124225
CKB ENSG00000166165
SPON2 ENSG00000159674
PSAP ENSG00000197746
ACTB ENSG00000075624
RDH11 ENSG00000072042
KLK4 ENSG00000167749
APLP2 ENSG00000084234
ANPEP ENSG00000166825
CALR ENSG00000179218
MT1G ENSG00000125144
P4HB ENSG00000185624
HIST2H2AA3 ENSG00000203812
CST3 ENSG00000101439
MAGED2 ENSG00000102316
GAPDH ENSG00000111640
KRT18 ENSG00000111057
NPDC1 ENSG00000107281
HLA−C ENSG00000204525
H2AFJ ENSG00000246705
DHCR24 ENSG00000116133
FXYD3 ENSG00000089356
SPDEF ENSG00000124664
DHRS7 ENSG00000100612
SERF2 ENSG00000140264
UBB ENSG00000170315
DBI ENSG00000155368
TSC22D3 ENSG00000157514
SSR4 ENSG00000180879
SLC44A4 ENSG00000204385
CD177 ENSG00000204936
SYNGR2 ENSG00000108639
UBC ENSG00000150991
NPY ENSG00000122585
HIST2H2AA4 ENSG00000272196
HOXB13 ENSG00000159184
RACK1 ENSG00000204628
ATP1A1 ENSG00000163399
PPP1R7 ENSG00000115685
ATP6V0B ENSG00000117410

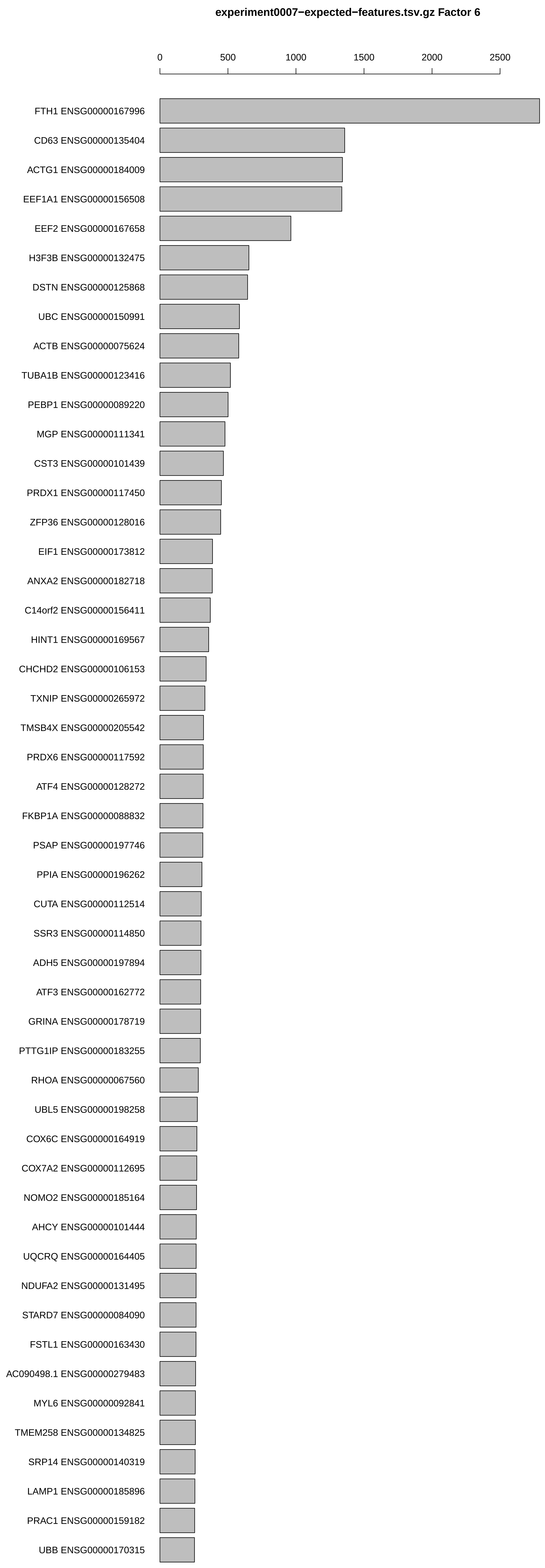
experiment0007−expected−features.tsv.gz Factor 6
0
500
1000
1500
2000
2500
FTH1 ENSG00000167996
CD63 ENSG00000135404
ACTG1 ENSG00000184009
EEF1A1 ENSG00000156508
EEF2 ENSG00000167658
H3F3B ENSG00000132475
DSTN ENSG00000125868
UBC ENSG00000150991
ACTB ENSG00000075624
TUBA1B ENSG00000123416
PEBP1 ENSG00000089220
MGP ENSG00000111341
CST3 ENSG00000101439
PRDX1 ENSG00000117450
ZFP36 ENSG00000128016
EIF1 ENSG00000173812
ANXA2 ENSG00000182718
C14orf2 ENSG00000156411
HINT1 ENSG00000169567
CHCHD2 ENSG00000106153
TXNIP ENSG00000265972
TMSB4X ENSG00000205542
PRDX6 ENSG00000117592
ATF4 ENSG00000128272
FKBP1A ENSG00000088832
PSAP ENSG00000197746
PPIA ENSG00000196262
CUTA ENSG00000112514
SSR3 ENSG00000114850
ADH5 ENSG00000197894
ATF3 ENSG00000162772
GRINA ENSG00000178719
PTTG1IP ENSG00000183255
RHOA ENSG00000067560
UBL5 ENSG00000198258
COX6C ENSG00000164919
COX7A2 ENSG00000112695
NOMO2 ENSG00000185164
AHCY ENSG00000101444
UQCRQ ENSG00000164405
NDUFA2 ENSG00000131495
STARD7 ENSG00000084090
FSTL1 ENSG00000163430
AC090498.1 ENSG00000279483
MYL6 ENSG00000092841
TMEM258 ENSG00000134825
SRP14 ENSG00000140319
LAMP1 ENSG00000185896
PRAC1 ENSG00000159182
UBB ENSG00000170315

experiment0007−expected−features.tsv.gz Factor 7

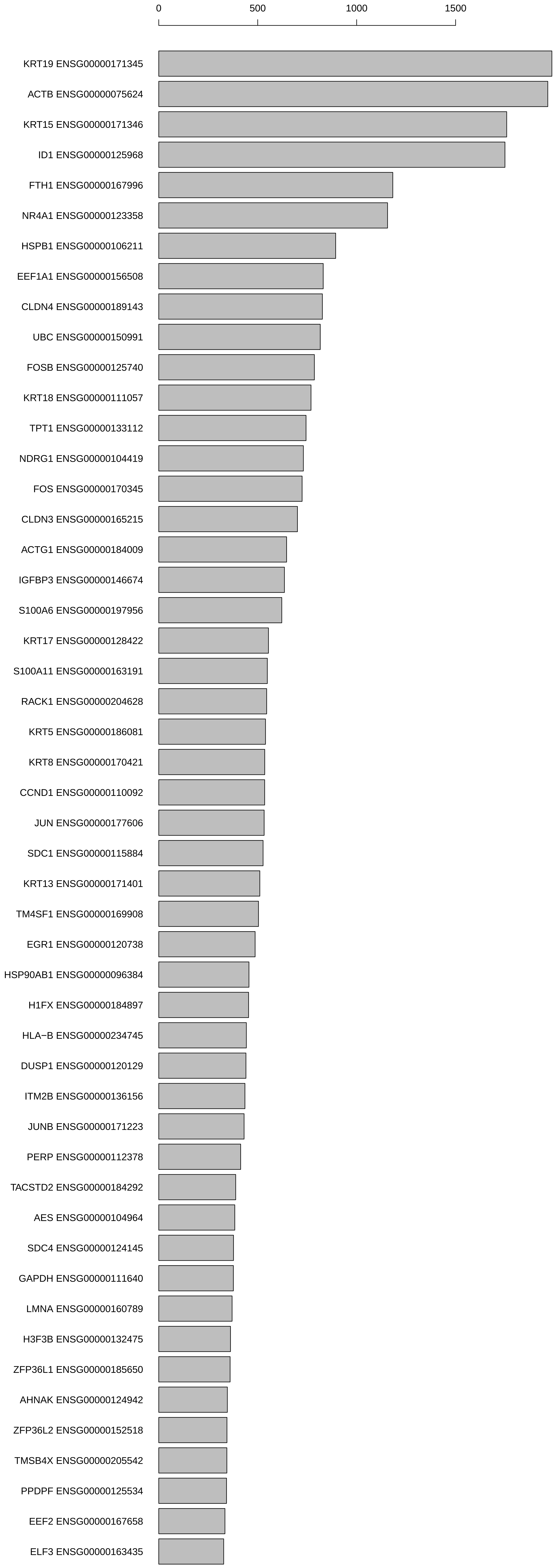

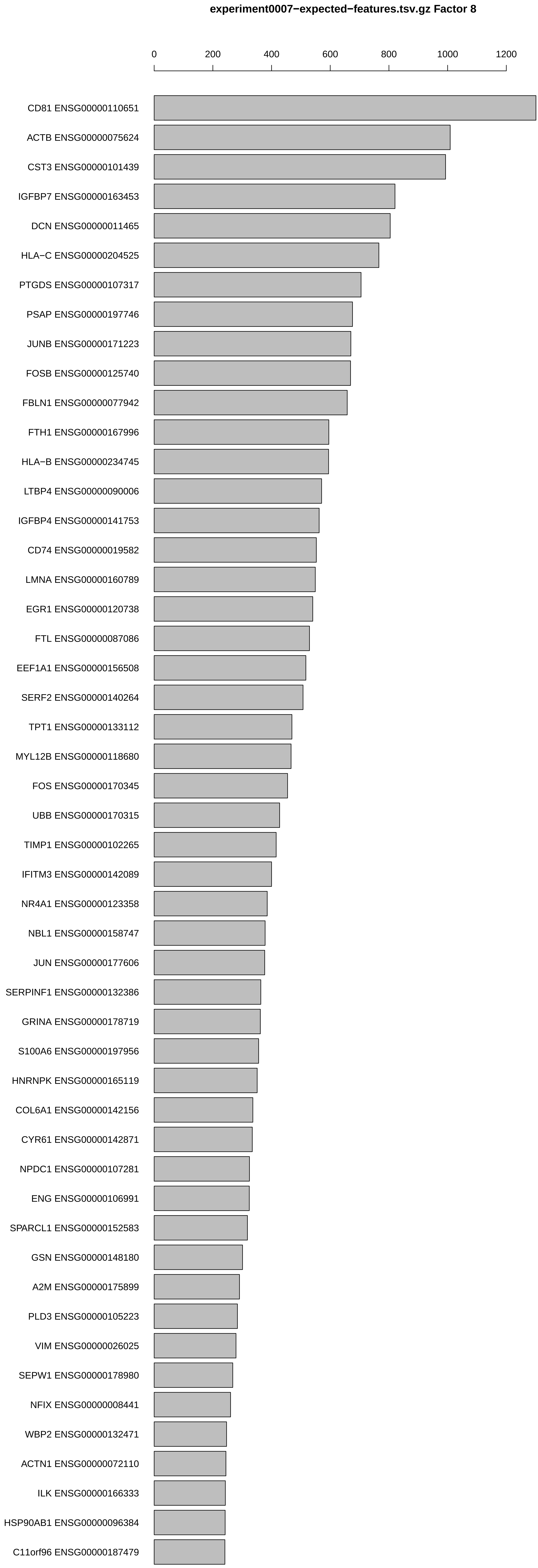
experiment0007−expected−features.tsv.gz Factor 8
0
200
400
600
800
1000
1200
CD81 ENSG00000110651
ACTB ENSG00000075624
CST3 ENSG00000101439
IGFBP7 ENSG00000163453
DCN ENSG00000011465
HLA−C ENSG00000204525
PTGDS ENSG00000107317
PSAP ENSG00000197746
JUNB ENSG00000171223
FOSB ENSG00000125740
FBLN1 ENSG00000077942
FTH1 ENSG00000167996
HLA−B ENSG00000234745
LTBP4 ENSG00000090006
IGFBP4 ENSG00000141753
CD74 ENSG00000019582
LMNA ENSG00000160789
EGR1 ENSG00000120738
FTL ENSG00000087086
EEF1A1 ENSG00000156508
SERF2 ENSG00000140264
TPT1 ENSG00000133112
MYL12B ENSG00000118680
FOS ENSG00000170345
UBB ENSG00000170315
TIMP1 ENSG00000102265
IFITM3 ENSG00000142089
NR4A1 ENSG00000123358
NBL1 ENSG00000158747
JUN ENSG00000177606
SERPINF1 ENSG00000132386
GRINA ENSG00000178719
S100A6 ENSG00000197956
HNRNPK ENSG00000165119
COL6A1 ENSG00000142156
CYR61 ENSG00000142871
NPDC1 ENSG00000107281
ENG ENSG00000106991
SPARCL1 ENSG00000152583
GSN ENSG00000148180
A2M ENSG00000175899
PLD3 ENSG00000105223
VIM ENSG00000026025
SEPW1 ENSG00000178980
NFIX ENSG00000008441
WBP2 ENSG00000132471
ACTN1 ENSG00000072110
ILK ENSG00000166333
HSP90AB1 ENSG00000096384
C11orf96 ENSG00000187479

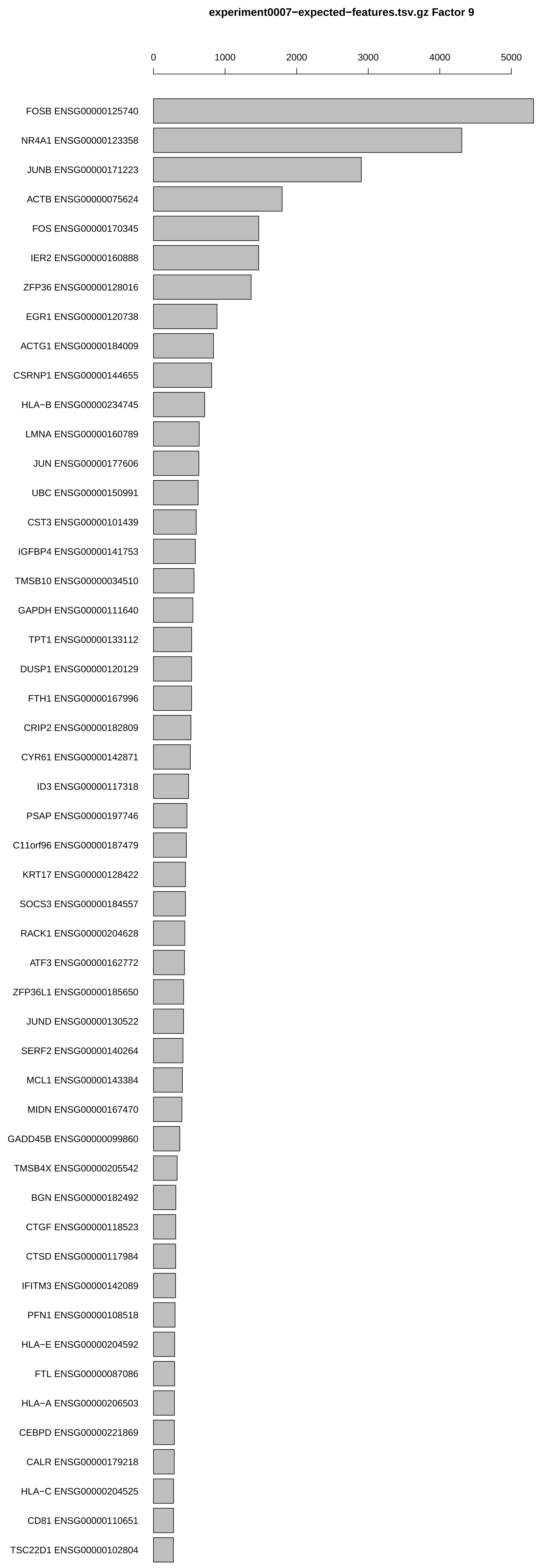
experiment0007−expected−features.tsv.gz Factor 9
0
1000
2000
3000
4000
5000
FOSB ENSG00000125740
NR4A1 ENSG00000123358
JUNB ENSG00000171223
ACTB ENSG00000075624
FOS ENSG00000170345
IER2 ENSG00000160888
ZFP36 ENSG00000128016
EGR1 ENSG00000120738
ACTG1 ENSG00000184009
CSRNP1 ENSG00000144655
HLA−B ENSG00000234745
LMNA ENSG00000160789
JUN ENSG00000177606
UBC ENSG00000150991
CST3 ENSG00000101439
IGFBP4 ENSG00000141753
TMSB10 ENSG00000034510
GAPDH ENSG00000111640
TPT1 ENSG00000133112
DUSP1 ENSG00000120129
FTH1 ENSG00000167996
CRIP2 ENSG00000182809
CYR61 ENSG00000142871
ID3 ENSG00000117318
PSAP ENSG00000197746
C11orf96 ENSG00000187479
KRT17 ENSG00000128422
SOCS3 ENSG00000184557
RACK1 ENSG00000204628
ATF3 ENSG00000162772
ZFP36L1 ENSG00000185650
JUND ENSG00000130522
SERF2 ENSG00000140264
MCL1 ENSG00000143384
MIDN ENSG00000167470
GADD45B ENSG00000099860
TMSB4X ENSG00000205542
BGN ENSG00000182492
CTGF ENSG00000118523
CTSD ENSG00000117984
IFITM3 ENSG00000142089
PFN1 ENSG00000108518
HLA−E ENSG00000204592
FTL ENSG00000087086
HLA−A ENSG00000206503
CEBPD ENSG00000221869
CALR ENSG00000179218
HLA−C ENSG00000204525
CD81 ENSG00000110651
TSC22D1 ENSG00000102804

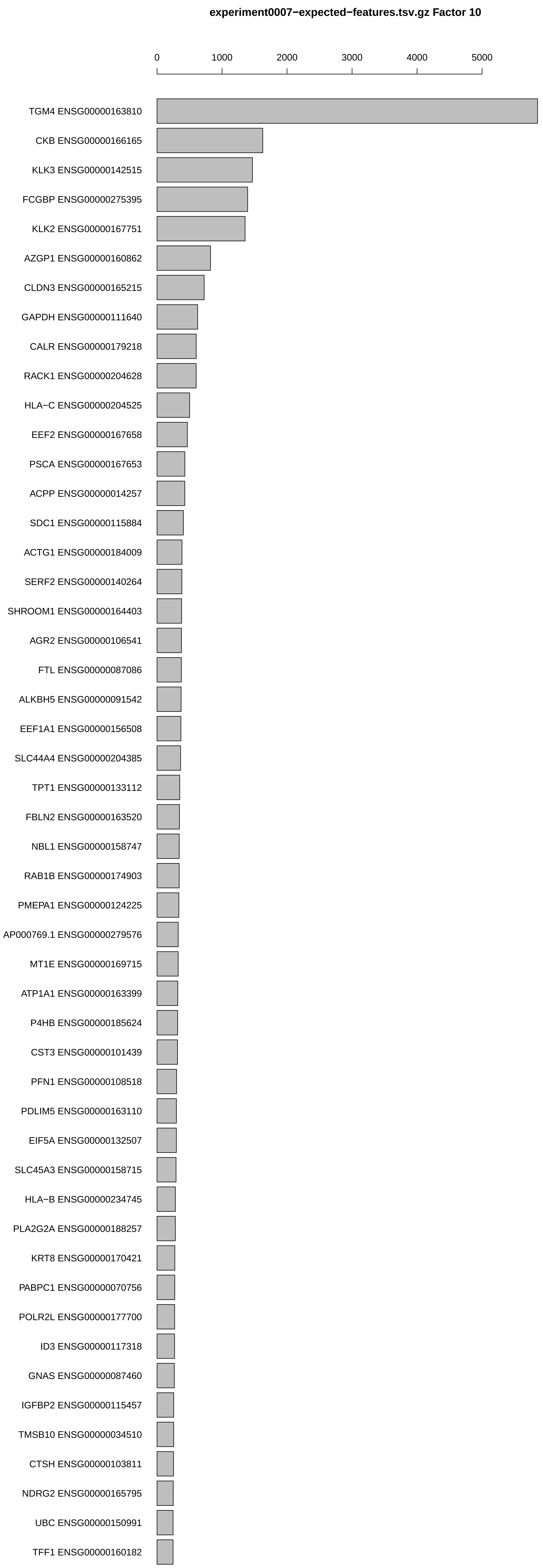
experiment0007−expected−features.tsv.gz Factor 10
0
1000
2000
3000
4000
5000
TGM4 ENSG00000163810
CKB ENSG00000166165
KLK3 ENSG00000142515
FCGBP ENSG00000275395
KLK2 ENSG00000167751
AZGP1 ENSG00000160862
CLDN3 ENSG00000165215
GAPDH ENSG00000111640
CALR ENSG00000179218
RACK1 ENSG00000204628
HLA−C ENSG00000204525
EEF2 ENSG00000167658
PSCA ENSG00000167653
ACPP ENSG00000014257
SDC1 ENSG00000115884
ACTG1 ENSG00000184009
SERF2 ENSG00000140264
SHROOM1 ENSG00000164403
AGR2 ENSG00000106541
FTL ENSG00000087086
ALKBH5 ENSG00000091542
EEF1A1 ENSG00000156508
SLC44A4 ENSG00000204385
TPT1 ENSG00000133112
FBLN2 ENSG00000163520
NBL1 ENSG00000158747
RAB1B ENSG00000174903
PMEPA1 ENSG00000124225
AP000769.1 ENSG00000279576
MT1E ENSG00000169715
ATP1A1 ENSG00000163399
P4HB ENSG00000185624
CST3 ENSG00000101439
PFN1 ENSG00000108518
PDLIM5 ENSG00000163110
EIF5A ENSG00000132507
SLC45A3 ENSG00000158715
HLA−B ENSG00000234745
PLA2G2A ENSG00000188257
KRT8 ENSG00000170421
PABPC1 ENSG00000070756
POLR2L ENSG00000177700
ID3 ENSG00000117318
GNAS ENSG00000087460
IGFBP2 ENSG00000115457
TMSB10 ENSG00000034510
CTSH ENSG00000103811
NDRG2 ENSG00000165795
UBC ENSG00000150991
TFF1 ENSG00000160182

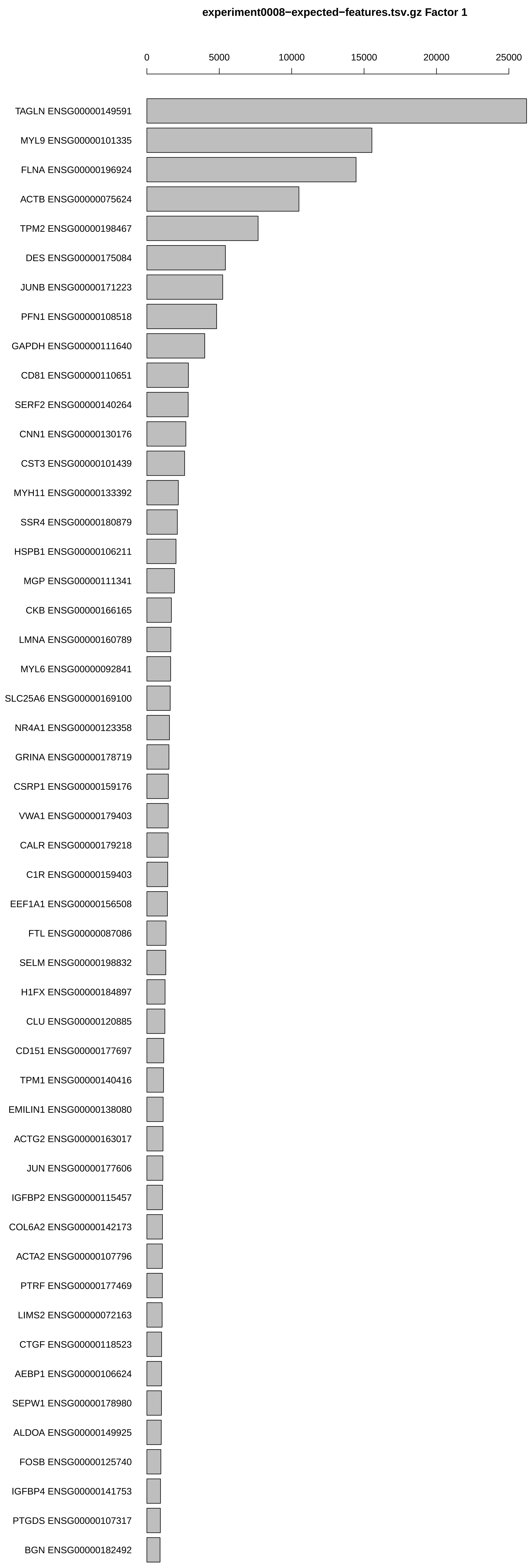
experiment0008−expected−features.tsv.gz Factor 1
0
5000
10000
15000
20000
25000
TAGLN ENSG00000149591
MYL9 ENSG00000101335
FLNA ENSG00000196924
ACTB ENSG00000075624
TPM2 ENSG00000198467
DES ENSG00000175084
JUNB ENSG00000171223
PFN1 ENSG00000108518
GAPDH ENSG00000111640
CD81 ENSG00000110651
SERF2 ENSG00000140264
CNN1 ENSG00000130176
CST3 ENSG00000101439
MYH11 ENSG00000133392
SSR4 ENSG00000180879
HSPB1 ENSG00000106211
MGP ENSG00000111341
CKB ENSG00000166165
LMNA ENSG00000160789
MYL6 ENSG00000092841
SLC25A6 ENSG00000169100
NR4A1 ENSG00000123358
GRINA ENSG00000178719
CSRP1 ENSG00000159176
VWA1 ENSG00000179403
CALR ENSG00000179218
C1R ENSG00000159403
EEF1A1 ENSG00000156508
FTL ENSG00000087086
SELM ENSG00000198832
H1FX ENSG00000184897
CLU ENSG00000120885
CD151 ENSG00000177697
TPM1 ENSG00000140416
EMILIN1 ENSG00000138080
ACTG2 ENSG00000163017
JUN ENSG00000177606
IGFBP2 ENSG00000115457
COL6A2 ENSG00000142173
ACTA2 ENSG00000107796
PTRF ENSG00000177469
LIMS2 ENSG00000072163
CTGF ENSG00000118523
AEBP1 ENSG00000106624
SEPW1 ENSG00000178980
ALDOA ENSG00000149925
FOSB ENSG00000125740
IGFBP4 ENSG00000141753
PTGDS ENSG00000107317
BGN ENSG00000182492

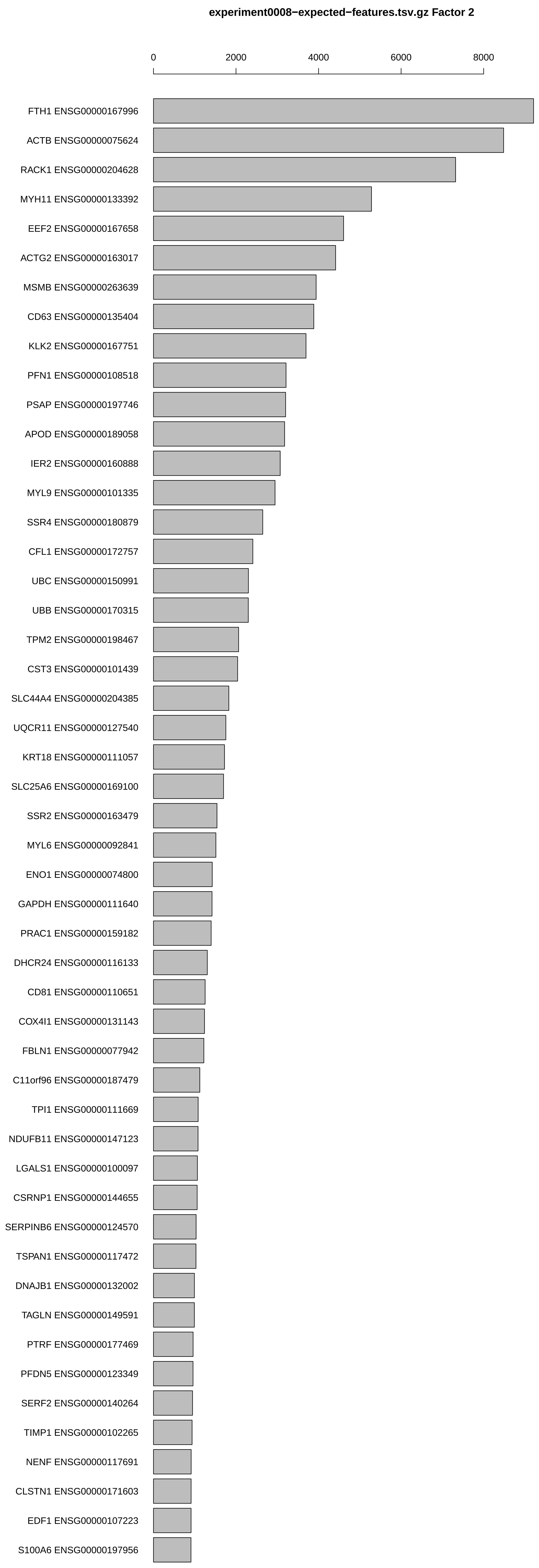
experiment0008−expected−features.tsv.gz Factor 2
0
2000
4000
6000
8000
FTH1 ENSG00000167996
ACTB ENSG00000075624
RACK1 ENSG00000204628
MYH11 ENSG00000133392
EEF2 ENSG00000167658
ACTG2 ENSG00000163017
MSMB ENSG00000263639
CD63 ENSG00000135404
KLK2 ENSG00000167751
PFN1 ENSG00000108518
PSAP ENSG00000197746
APOD ENSG00000189058
IER2 ENSG00000160888
MYL9 ENSG00000101335
SSR4 ENSG00000180879
CFL1 ENSG00000172757
UBC ENSG00000150991
UBB ENSG00000170315
TPM2 ENSG00000198467
CST3 ENSG00000101439
SLC44A4 ENSG00000204385
UQCR11 ENSG00000127540
KRT18 ENSG00000111057
SLC25A6 ENSG00000169100
SSR2 ENSG00000163479
MYL6 ENSG00000092841
ENO1 ENSG00000074800
GAPDH ENSG00000111640
PRAC1 ENSG00000159182
DHCR24 ENSG00000116133
CD81 ENSG00000110651
COX4I1 ENSG00000131143
FBLN1 ENSG00000077942
C11orf96 ENSG00000187479
TPI1 ENSG00000111669
NDUFB11 ENSG00000147123
LGALS1 ENSG00000100097
CSRNP1 ENSG00000144655
SERPINB6 ENSG00000124570
TSPAN1 ENSG00000117472
DNAJB1 ENSG00000132002
TAGLN ENSG00000149591
PTRF ENSG00000177469
PFDN5 ENSG00000123349
SERF2 ENSG00000140264
TIMP1 ENSG00000102265
NENF ENSG00000117691
CLSTN1 ENSG00000171603
EDF1 ENSG00000107223
S100A6 ENSG00000197956

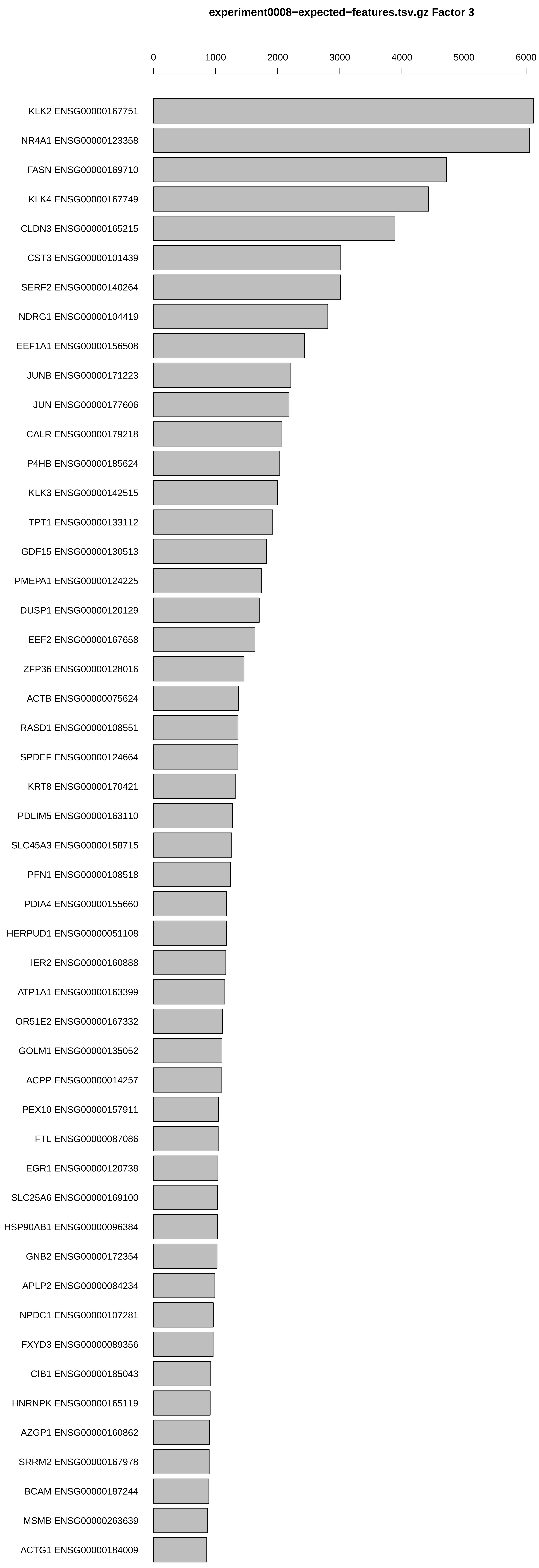
experiment0008−expected−features.tsv.gz Factor 3
0
1000
2000
3000
4000
5000
6000
KLK2 ENSG00000167751
NR4A1 ENSG00000123358
FASN ENSG00000169710
KLK4 ENSG00000167749
CLDN3 ENSG00000165215
CST3 ENSG00000101439
SERF2 ENSG00000140264
NDRG1 ENSG00000104419
EEF1A1 ENSG00000156508
JUNB ENSG00000171223
JUN ENSG00000177606
CALR ENSG00000179218
P4HB ENSG00000185624
KLK3 ENSG00000142515
TPT1 ENSG00000133112
GDF15 ENSG00000130513
PMEPA1 ENSG00000124225
DUSP1 ENSG00000120129
EEF2 ENSG00000167658
ZFP36 ENSG00000128016
ACTB ENSG00000075624
RASD1 ENSG00000108551
SPDEF ENSG00000124664
KRT8 ENSG00000170421
PDLIM5 ENSG00000163110
SLC45A3 ENSG00000158715
PFN1 ENSG00000108518
PDIA4 ENSG00000155660
HERPUD1 ENSG00000051108
IER2 ENSG00000160888
ATP1A1 ENSG00000163399
OR51E2 ENSG00000167332
GOLM1 ENSG00000135052
ACPP ENSG00000014257
PEX10 ENSG00000157911
FTL ENSG00000087086
EGR1 ENSG00000120738
SLC25A6 ENSG00000169100
HSP90AB1 ENSG00000096384
GNB2 ENSG00000172354
APLP2 ENSG00000084234
NPDC1 ENSG00000107281
FXYD3 ENSG00000089356
CIB1 ENSG00000185043
HNRNPK ENSG00000165119
AZGP1 ENSG00000160862
SRRM2 ENSG00000167978
BCAM ENSG00000187244
MSMB ENSG00000263639
ACTG1 ENSG00000184009

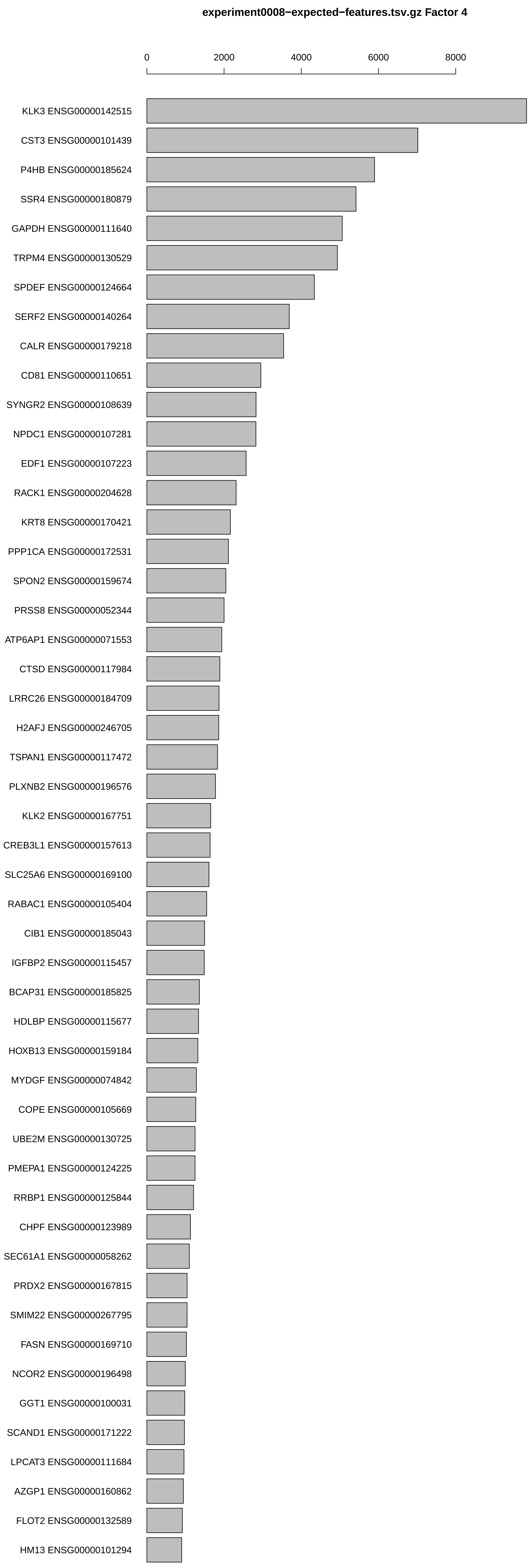

experiment0008−expected−features.tsv.gz Factor 4
0
2000
4000
6000
8000
KLK3 ENSG00000142515
CST3 ENSG00000101439
P4HB ENSG00000185624
SSR4 ENSG00000180879
GAPDH ENSG00000111640
TRPM4 ENSG00000130529
SPDEF ENSG00000124664
SERF2 ENSG00000140264
CALR ENSG00000179218
CD81 ENSG00000110651
SYNGR2 ENSG00000108639
NPDC1 ENSG00000107281
EDF1 ENSG00000107223
RACK1 ENSG00000204628
KRT8 ENSG00000170421
PPP1CA ENSG00000172531
SPON2 ENSG00000159674
PRSS8 ENSG00000052344
ATP6AP1 ENSG00000071553
CTSD ENSG00000117984
LRRC26 ENSG00000184709
H2AFJ ENSG00000246705
TSPAN1 ENSG00000117472
PLXNB2 ENSG00000196576
KLK2 ENSG00000167751
CREB3L1 ENSG00000157613
SLC25A6 ENSG00000169100
RABAC1 ENSG00000105404
CIB1 ENSG00000185043
IGFBP2 ENSG00000115457
BCAP31 ENSG00000185825
HDLBP ENSG00000115677
HOXB13 ENSG00000159184
MYDGF ENSG00000074842
COPE ENSG00000105669
UBE2M ENSG00000130725
PMEPA1 ENSG00000124225
RRBP1 ENSG00000125844
CHPF ENSG00000123989
SEC61A1 ENSG00000058262
PRDX2 ENSG00000167815
SMIM22 ENSG00000267795
FASN ENSG00000169710
NCOR2 ENSG00000196498
GGT1 ENSG00000100031
SCAND1 ENSG00000171222
LPCAT3 ENSG00000111684
AZGP1 ENSG00000160862
FLOT2 ENSG00000132589
HM13 ENSG00000101294

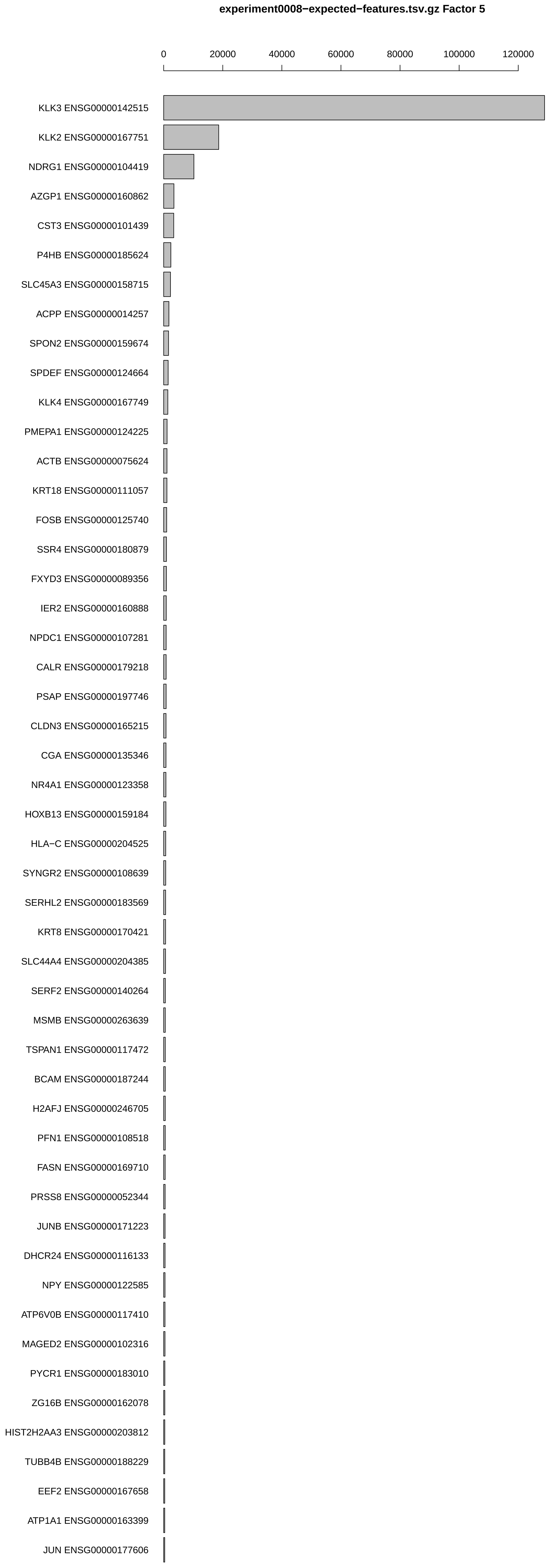
experiment0008−expected−features.tsv.gz Factor 5
0
20000
40000
60000
80000
100000
120000
KLK3 ENSG00000142515
KLK2 ENSG00000167751
NDRG1 ENSG00000104419
AZGP1 ENSG00000160862
CST3 ENSG00000101439
P4HB ENSG00000185624
SLC45A3 ENSG00000158715
ACPP ENSG00000014257
SPON2 ENSG00000159674
SPDEF ENSG00000124664
KLK4 ENSG00000167749
PMEPA1 ENSG00000124225
ACTB ENSG00000075624
KRT18 ENSG00000111057
FOSB ENSG00000125740
SSR4 ENSG00000180879
FXYD3 ENSG00000089356
IER2 ENSG00000160888
NPDC1 ENSG00000107281
CALR ENSG00000179218
PSAP ENSG00000197746
CLDN3 ENSG00000165215
CGA ENSG00000135346
NR4A1 ENSG00000123358
HOXB13 ENSG00000159184
HLA−C ENSG00000204525
SYNGR2 ENSG00000108639
SERHL2 ENSG00000183569
KRT8 ENSG00000170421
SLC44A4 ENSG00000204385
SERF2 ENSG00000140264
MSMB ENSG00000263639
TSPAN1 ENSG00000117472
BCAM ENSG00000187244
H2AFJ ENSG00000246705
PFN1 ENSG00000108518
FASN ENSG00000169710
PRSS8 ENSG00000052344
JUNB ENSG00000171223
DHCR24 ENSG00000116133
NPY ENSG00000122585
ATP6V0B ENSG00000117410
MAGED2 ENSG00000102316
PYCR1 ENSG00000183010
ZG16B ENSG00000162078
HIST2H2AA3 ENSG00000203812
TUBB4B ENSG00000188229
EEF2 ENSG00000167658
ATP1A1 ENSG00000163399
JUN ENSG00000177606

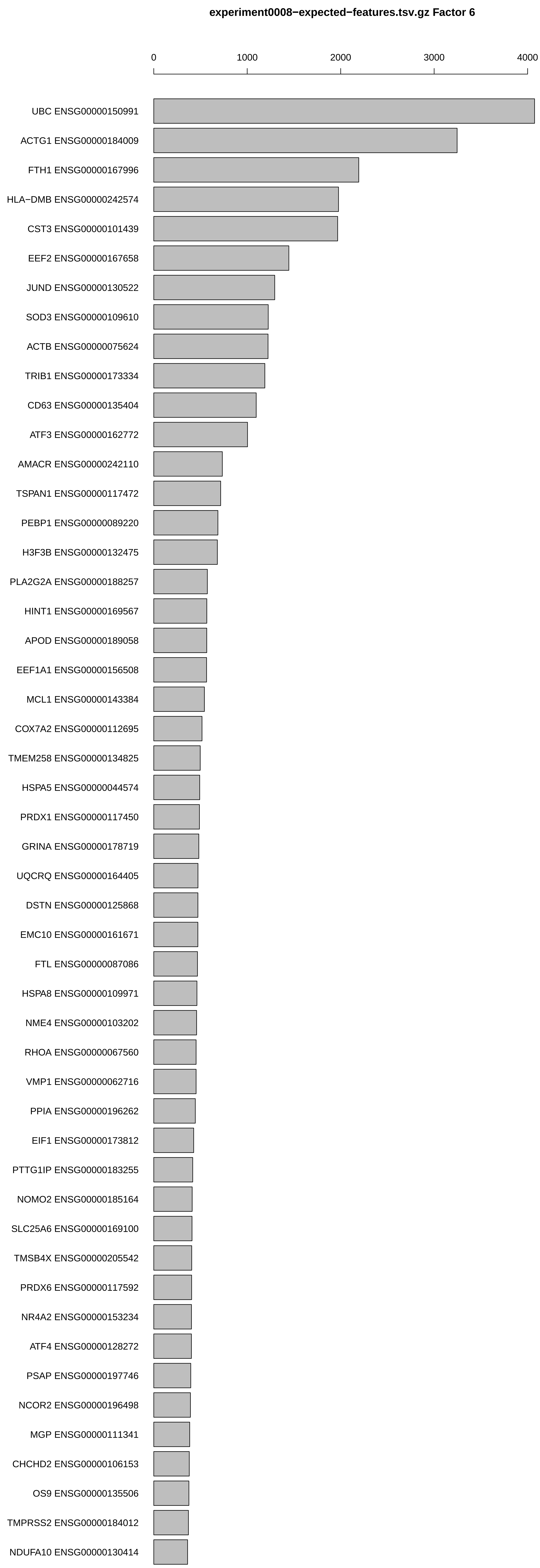
experiment0008−expected−features.tsv.gz Factor 6
0
1000
2000
3000
4000
UBC ENSG00000150991
ACTG1 ENSG00000184009
FTH1 ENSG00000167996
HLA−DMB ENSG00000242574
CST3 ENSG00000101439
EEF2 ENSG00000167658
JUND ENSG00000130522
SOD3 ENSG00000109610
ACTB ENSG00000075624
TRIB1 ENSG00000173334
CD63 ENSG00000135404
ATF3 ENSG00000162772
AMACR ENSG00000242110
TSPAN1 ENSG00000117472
PEBP1 ENSG00000089220
H3F3B ENSG00000132475
PLA2G2A ENSG00000188257
HINT1 ENSG00000169567
APOD ENSG00000189058
EEF1A1 ENSG00000156508
MCL1 ENSG00000143384
COX7A2 ENSG00000112695
TMEM258 ENSG00000134825
HSPA5 ENSG00000044574
PRDX1 ENSG00000117450
GRINA ENSG00000178719
UQCRQ ENSG00000164405
DSTN ENSG00000125868
EMC10 ENSG00000161671
FTL ENSG00000087086
HSPA8 ENSG00000109971
NME4 ENSG00000103202
RHOA ENSG00000067560
VMP1 ENSG00000062716
PPIA ENSG00000196262
EIF1 ENSG00000173812
PTTG1IP ENSG00000183255
NOMO2 ENSG00000185164
SLC25A6 ENSG00000169100
TMSB4X ENSG00000205542
PRDX6 ENSG00000117592
NR4A2 ENSG00000153234
ATF4 ENSG00000128272
PSAP ENSG00000197746
NCOR2 ENSG00000196498
MGP ENSG00000111341
CHCHD2 ENSG00000106153
OS9 ENSG00000135506
TMPRSS2 ENSG00000184012
NDUFA10 ENSG00000130414

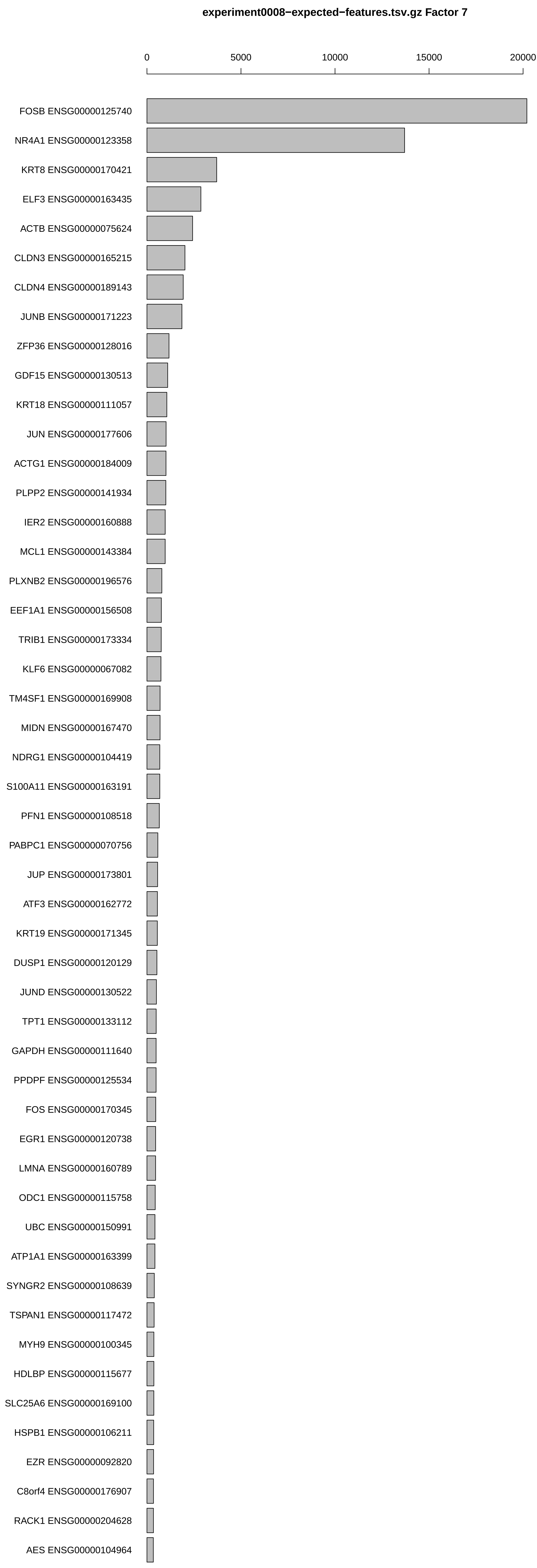
experiment0008−expected−features.tsv.gz Factor 7
0
5000
10000
15000
20000
FOSB ENSG00000125740
NR4A1 ENSG00000123358
KRT8 ENSG00000170421
ELF3 ENSG00000163435
ACTB ENSG00000075624
CLDN3 ENSG00000165215
CLDN4 ENSG00000189143
JUNB ENSG00000171223
ZFP36 ENSG00000128016
GDF15 ENSG00000130513
KRT18 ENSG00000111057
JUN ENSG00000177606
ACTG1 ENSG00000184009
PLPP2 ENSG00000141934
IER2 ENSG00000160888
MCL1 ENSG00000143384
PLXNB2 ENSG00000196576
EEF1A1 ENSG00000156508
TRIB1 ENSG00000173334
KLF6 ENSG00000067082
TM4SF1 ENSG00000169908
MIDN ENSG00000167470
NDRG1 ENSG00000104419
S100A11 ENSG00000163191
PFN1 ENSG00000108518
PABPC1 ENSG00000070756
JUP ENSG00000173801
ATF3 ENSG00000162772
KRT19 ENSG00000171345
DUSP1 ENSG00000120129
JUND ENSG00000130522
TPT1 ENSG00000133112
GAPDH ENSG00000111640
PPDPF ENSG00000125534
FOS ENSG00000170345
EGR1 ENSG00000120738
LMNA ENSG00000160789
ODC1 ENSG00000115758
UBC ENSG00000150991
ATP1A1 ENSG00000163399
SYNGR2 ENSG00000108639
TSPAN1 ENSG00000117472
MYH9 ENSG00000100345
HDLBP ENSG00000115677
SLC25A6 ENSG00000169100
HSPB1 ENSG00000106211
EZR ENSG00000092820
C8orf4 ENSG00000176907
RACK1 ENSG00000204628
AES ENSG00000104964

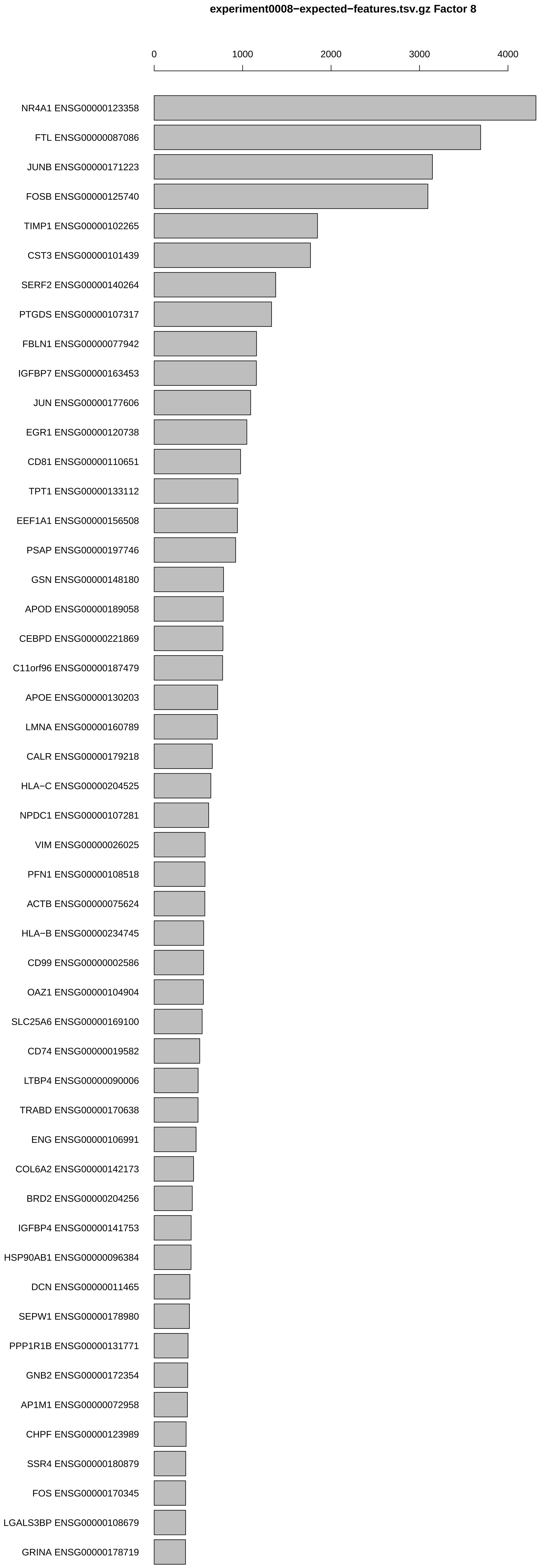
experiment0008−expected−features.tsv.gz Factor 8
0
1000
2000
3000
4000
NR4A1 ENSG00000123358
FTL ENSG00000087086
JUNB ENSG00000171223
FOSB ENSG00000125740
TIMP1 ENSG00000102265
CST3 ENSG00000101439
SERF2 ENSG00000140264
PTGDS ENSG00000107317
FBLN1 ENSG00000077942
IGFBP7 ENSG00000163453
JUN ENSG00000177606
EGR1 ENSG00000120738
CD81 ENSG00000110651
TPT1 ENSG00000133112
EEF1A1 ENSG00000156508
PSAP ENSG00000197746
GSN ENSG00000148180
APOD ENSG00000189058
CEBPD ENSG00000221869
C11orf96 ENSG00000187479
APOE ENSG00000130203
LMNA ENSG00000160789
CALR ENSG00000179218
HLA−C ENSG00000204525
NPDC1 ENSG00000107281
VIM ENSG00000026025
PFN1 ENSG00000108518
ACTB ENSG00000075624
HLA−B ENSG00000234745
CD99 ENSG00000002586
OAZ1 ENSG00000104904
SLC25A6 ENSG00000169100
CD74 ENSG00000019582
LTBP4 ENSG00000090006
TRABD ENSG00000170638
ENG ENSG00000106991
COL6A2 ENSG00000142173
BRD2 ENSG00000204256
IGFBP4 ENSG00000141753
HSP90AB1 ENSG00000096384
DCN ENSG00000011465
SEPW1 ENSG00000178980
PPP1R1B ENSG00000131771
GNB2 ENSG00000172354
AP1M1 ENSG00000072958
CHPF ENSG00000123989
SSR4 ENSG00000180879
FOS ENSG00000170345
LGALS3BP ENSG00000108679
GRINA ENSG00000178719

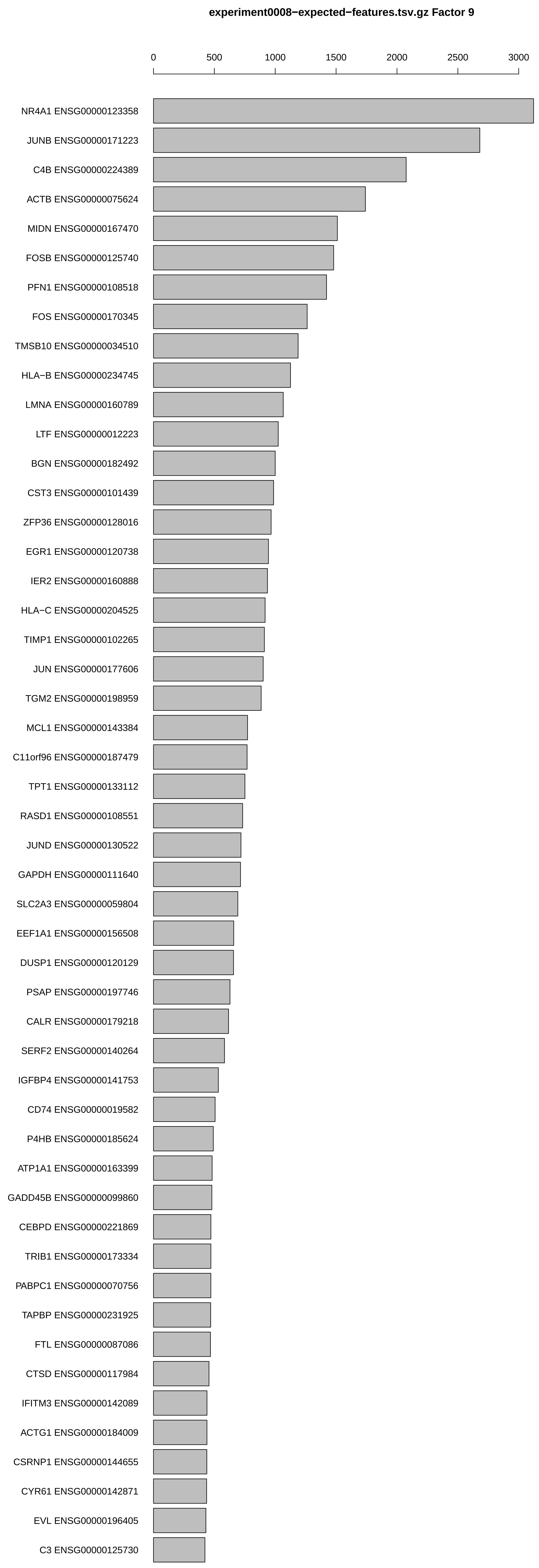

experiment0008−expected−features.tsv.gz Factor 9
0
500
1000
1500
2000
2500
3000
NR4A1 ENSG00000123358
JUNB ENSG00000171223
C4B ENSG00000224389
ACTB ENSG00000075624
MIDN ENSG00000167470
FOSB ENSG00000125740
PFN1 ENSG00000108518
FOS ENSG00000170345
TMSB10 ENSG00000034510
HLA−B ENSG00000234745
LMNA ENSG00000160789
LTF ENSG00000012223
BGN ENSG00000182492
CST3 ENSG00000101439
ZFP36 ENSG00000128016
EGR1 ENSG00000120738
IER2 ENSG00000160888
HLA−C ENSG00000204525
TIMP1 ENSG00000102265
JUN ENSG00000177606
TGM2 ENSG00000198959
MCL1 ENSG00000143384
C11orf96 ENSG00000187479
TPT1 ENSG00000133112
RASD1 ENSG00000108551
JUND ENSG00000130522
GAPDH ENSG00000111640
SLC2A3 ENSG00000059804
EEF1A1 ENSG00000156508
DUSP1 ENSG00000120129
PSAP ENSG00000197746
CALR ENSG00000179218
SERF2 ENSG00000140264
IGFBP4 ENSG00000141753
CD74 ENSG00000019582
P4HB ENSG00000185624
ATP1A1 ENSG00000163399
GADD45B ENSG00000099860
CEBPD ENSG00000221869
TRIB1 ENSG00000173334
PABPC1 ENSG00000070756
TAPBP ENSG00000231925
FTL ENSG00000087086
CTSD ENSG00000117984
IFITM3 ENSG00000142089
ACTG1 ENSG00000184009
CSRNP1 ENSG00000144655
CYR61 ENSG00000142871
EVL ENSG00000196405
C3 ENSG00000125730

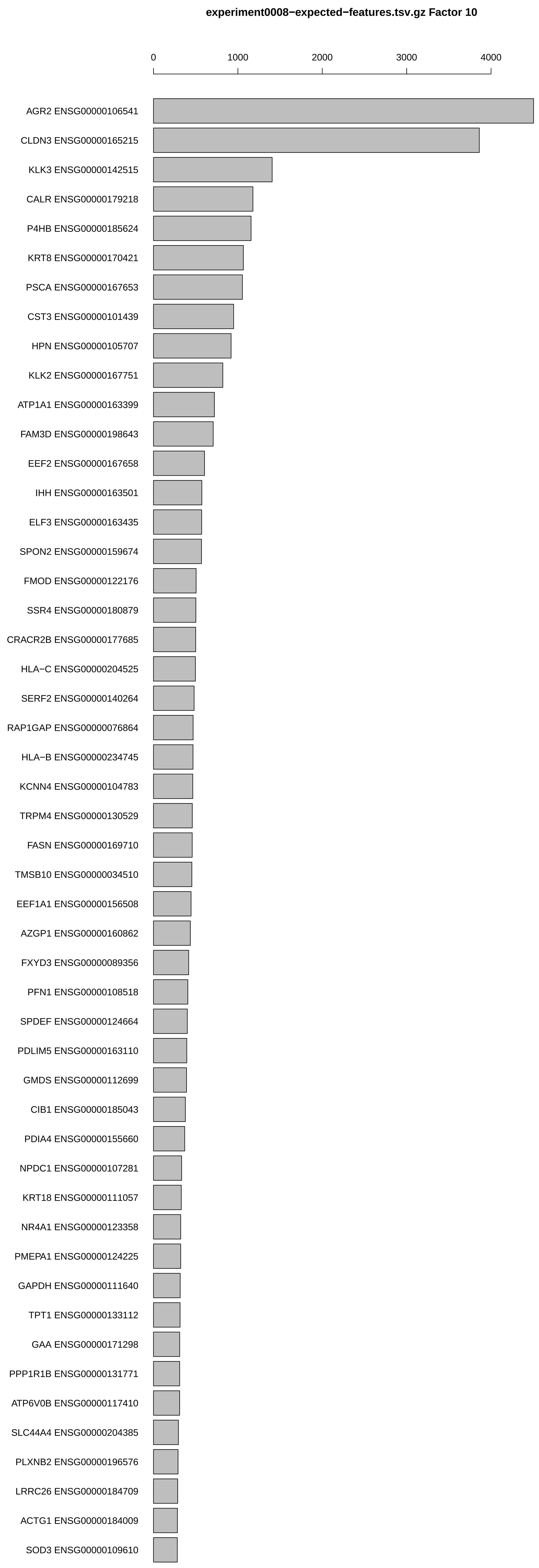
experiment0008−expected−features.tsv.gz Factor 10
0
1000
2000
3000
4000
AGR2 ENSG00000106541
CLDN3 ENSG00000165215
KLK3 ENSG00000142515
CALR ENSG00000179218
P4HB ENSG00000185624
KRT8 ENSG00000170421
PSCA ENSG00000167653
CST3 ENSG00000101439
HPN ENSG00000105707
KLK2 ENSG00000167751
ATP1A1 ENSG00000163399
FAM3D ENSG00000198643
EEF2 ENSG00000167658
IHH ENSG00000163501
ELF3 ENSG00000163435
SPON2 ENSG00000159674
FMOD ENSG00000122176
SSR4 ENSG00000180879
CRACR2B ENSG00000177685
HLA−C ENSG00000204525
SERF2 ENSG00000140264
RAP1GAP ENSG00000076864
HLA−B ENSG00000234745
KCNN4 ENSG00000104783
TRPM4 ENSG00000130529
FASN ENSG00000169710
TMSB10 ENSG00000034510
EEF1A1 ENSG00000156508
AZGP1 ENSG00000160862
FXYD3 ENSG00000089356
PFN1 ENSG00000108518
SPDEF ENSG00000124664
PDLIM5 ENSG00000163110
GMDS ENSG00000112699
CIB1 ENSG00000185043
PDIA4 ENSG00000155660
NPDC1 ENSG00000107281
KRT18 ENSG00000111057
NR4A1 ENSG00000123358
PMEPA1 ENSG00000124225
GAPDH ENSG00000111640
TPT1 ENSG00000133112
GAA ENSG00000171298
PPP1R1B ENSG00000131771
ATP6V0B ENSG00000117410
SLC44A4 ENSG00000204385
PLXNB2 ENSG00000196576
LRRC26 ENSG00000184709
ACTG1 ENSG00000184009
SOD3 ENSG00000109610

experiment0009−expected−features.tsv.gz Factor 1

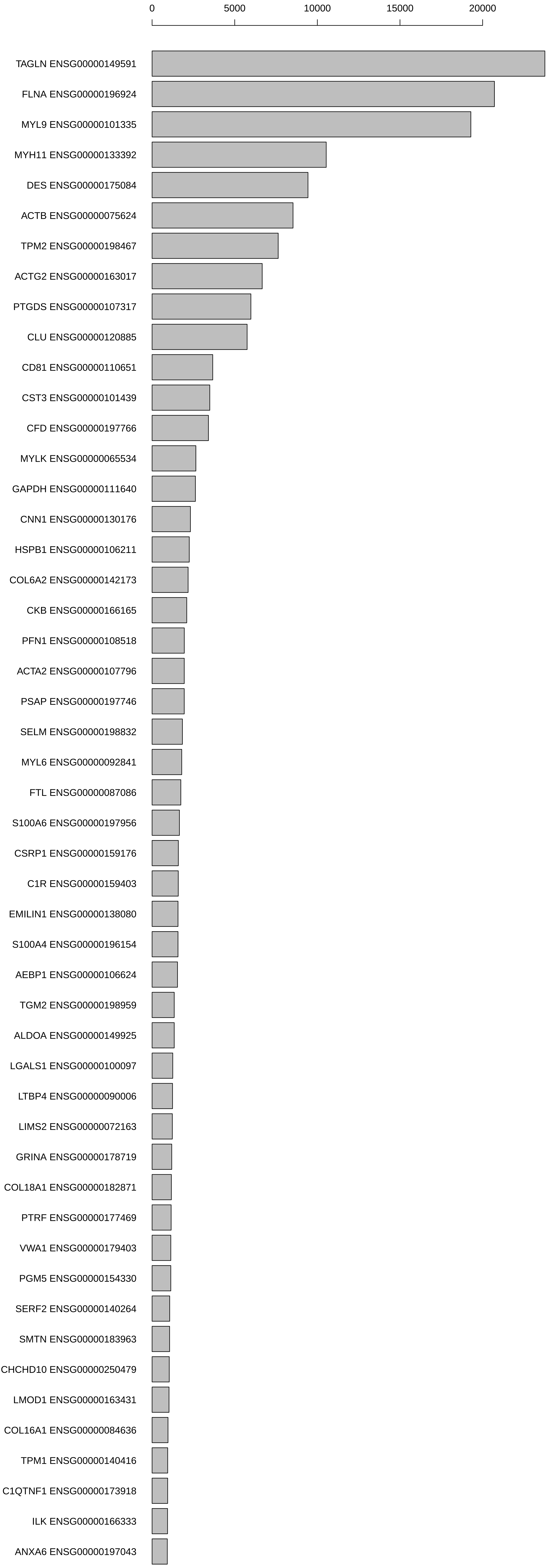

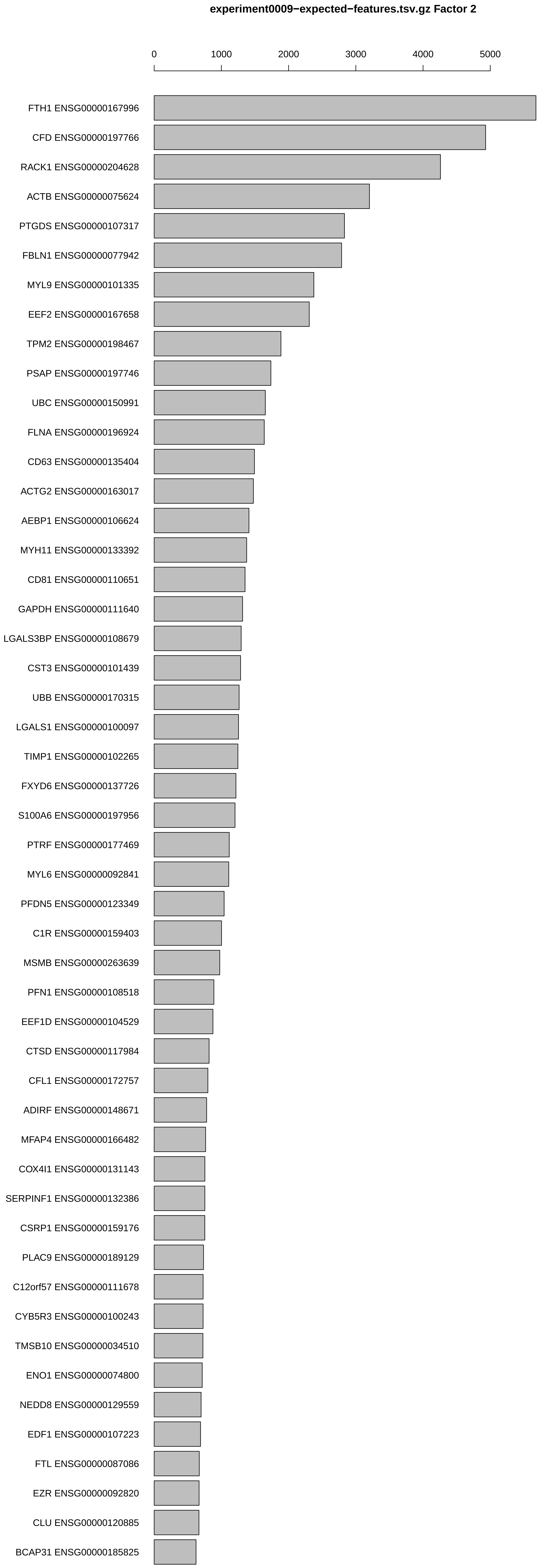

experiment0009−expected−features.tsv.gz Factor 2
0
1000
2000
3000
4000
5000
FTH1 ENSG00000167996
CFD ENSG00000197766
RACK1 ENSG00000204628
ACTB ENSG00000075624
PTGDS ENSG00000107317
FBLN1 ENSG00000077942
MYL9 ENSG00000101335
EEF2 ENSG00000167658
TPM2 ENSG00000198467
PSAP ENSG00000197746
UBC ENSG00000150991
FLNA ENSG00000196924
CD63 ENSG00000135404
ACTG2 ENSG00000163017
AEBP1 ENSG00000106624
MYH11 ENSG00000133392
CD81 ENSG00000110651
GAPDH ENSG00000111640
LGALS3BP ENSG00000108679
CST3 ENSG00000101439
UBB ENSG00000170315
LGALS1 ENSG00000100097
TIMP1 ENSG00000102265
FXYD6 ENSG00000137726
S100A6 ENSG00000197956
PTRF ENSG00000177469
MYL6 ENSG00000092841
PFDN5 ENSG00000123349
C1R ENSG00000159403
MSMB ENSG00000263639
PFN1 ENSG00000108518
EEF1D ENSG00000104529
CTSD ENSG00000117984
CFL1 ENSG00000172757
ADIRF ENSG00000148671
MFAP4 ENSG00000166482
COX4I1 ENSG00000131143
SERPINF1 ENSG00000132386
CSRP1 ENSG00000159176
PLAC9 ENSG00000189129
C12orf57 ENSG00000111678
CYB5R3 ENSG00000100243
TMSB10 ENSG00000034510
ENO1 ENSG00000074800
NEDD8 ENSG00000129559
EDF1 ENSG00000107223
FTL ENSG00000087086
EZR ENSG00000092820
CLU ENSG00000120885
BCAP31 ENSG00000185825

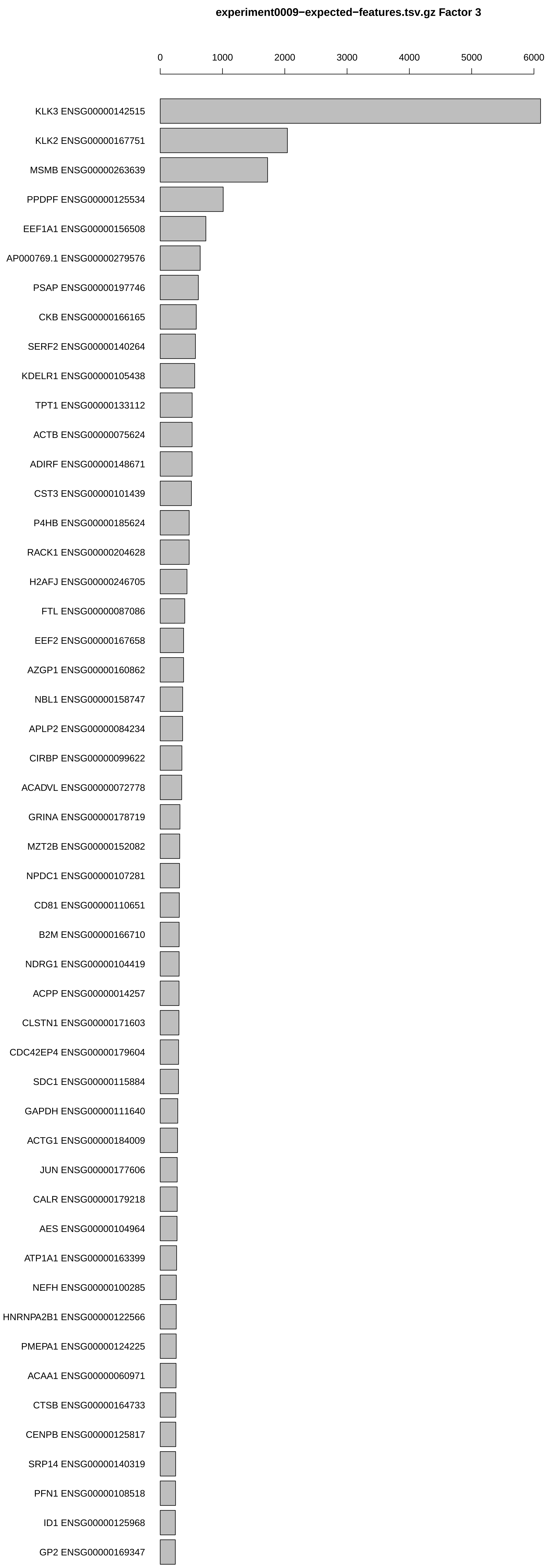
experiment0009−expected−features.tsv.gz Factor 3
0
1000
2000
3000
4000
5000
6000
KLK3 ENSG00000142515
KLK2 ENSG00000167751
MSMB ENSG00000263639
PPDPF ENSG00000125534
EEF1A1 ENSG00000156508
AP000769.1 ENSG00000279576
PSAP ENSG00000197746
CKB ENSG00000166165
SERF2 ENSG00000140264
KDELR1 ENSG00000105438
TPT1 ENSG00000133112
ACTB ENSG00000075624
ADIRF ENSG00000148671
CST3 ENSG00000101439
P4HB ENSG00000185624
RACK1 ENSG00000204628
H2AFJ ENSG00000246705
FTL ENSG00000087086
EEF2 ENSG00000167658
AZGP1 ENSG00000160862
NBL1 ENSG00000158747
APLP2 ENSG00000084234
CIRBP ENSG00000099622
ACADVL ENSG00000072778
GRINA ENSG00000178719
MZT2B ENSG00000152082
NPDC1 ENSG00000107281
CD81 ENSG00000110651
B2M ENSG00000166710
NDRG1 ENSG00000104419
ACPP ENSG00000014257
CLSTN1 ENSG00000171603
CDC42EP4 ENSG00000179604
SDC1 ENSG00000115884
GAPDH ENSG00000111640
ACTG1 ENSG00000184009
JUN ENSG00000177606
CALR ENSG00000179218
AES ENSG00000104964
ATP1A1 ENSG00000163399
NEFH ENSG00000100285
HNRNPA2B1 ENSG00000122566
PMEPA1 ENSG00000124225
ACAA1 ENSG00000060971
CTSB ENSG00000164733
CENPB ENSG00000125817
SRP14 ENSG00000140319
PFN1 ENSG00000108518
ID1 ENSG00000125968
GP2 ENSG00000169347

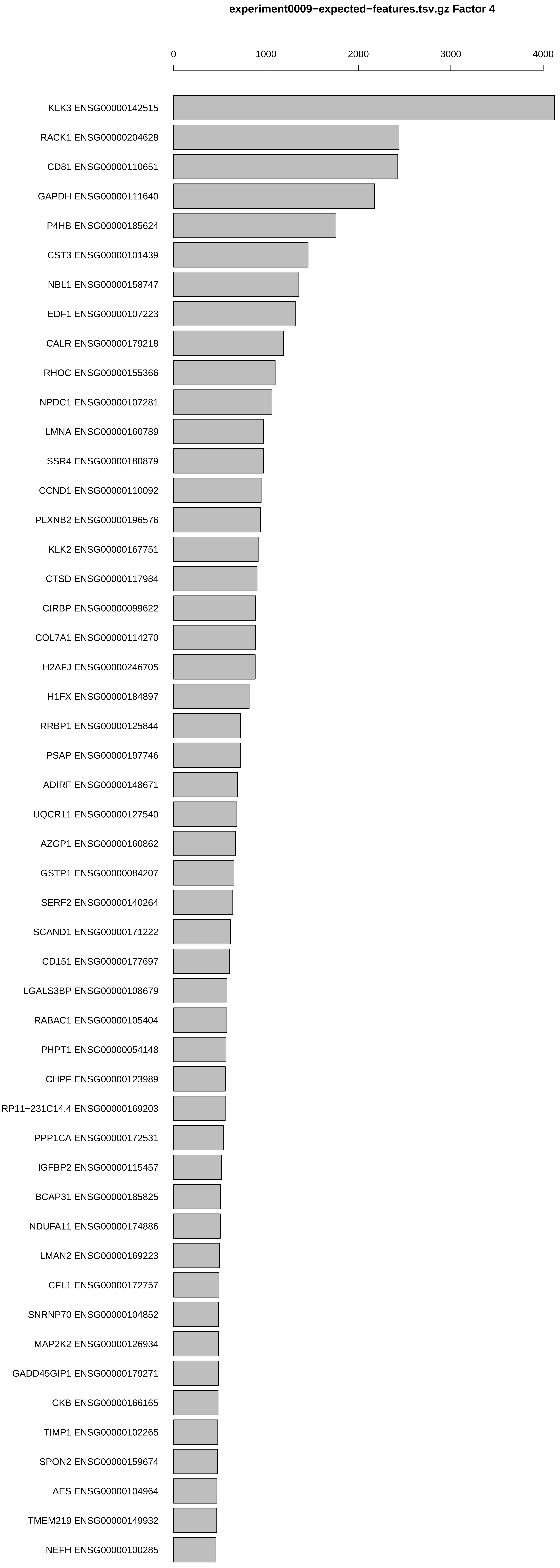
experiment0009−expected−features.tsv.gz Factor 4
0
1000
2000
3000
4000
KLK3 ENSG00000142515
RACK1 ENSG00000204628
CD81 ENSG00000110651
GAPDH ENSG00000111640
P4HB ENSG00000185624
CST3 ENSG00000101439
NBL1 ENSG00000158747
EDF1 ENSG00000107223
CALR ENSG00000179218
RHOC ENSG00000155366
NPDC1 ENSG00000107281
LMNA ENSG00000160789
SSR4 ENSG00000180879
CCND1 ENSG00000110092
PLXNB2 ENSG00000196576
KLK2 ENSG00000167751
CTSD ENSG00000117984
CIRBP ENSG00000099622
COL7A1 ENSG00000114270
H2AFJ ENSG00000246705
H1FX ENSG00000184897
RRBP1 ENSG00000125844
PSAP ENSG00000197746
ADIRF ENSG00000148671
UQCR11 ENSG00000127540
AZGP1 ENSG00000160862
GSTP1 ENSG00000084207
SERF2 ENSG00000140264
SCAND1 ENSG00000171222
CD151 ENSG00000177697
LGALS3BP ENSG00000108679
RABAC1 ENSG00000105404
PHPT1 ENSG00000054148
CHPF ENSG00000123989
RP11−231C14.4 ENSG00000169203
PPP1CA ENSG00000172531
IGFBP2 ENSG00000115457
BCAP31 ENSG00000185825
NDUFA11 ENSG00000174886
LMAN2 ENSG00000169223
CFL1 ENSG00000172757
SNRNP70 ENSG00000104852
MAP2K2 ENSG00000126934
GADD45GIP1 ENSG00000179271
CKB ENSG00000166165
TIMP1 ENSG00000102265
SPON2 ENSG00000159674
AES ENSG00000104964
TMEM219 ENSG00000149932
NEFH ENSG00000100285

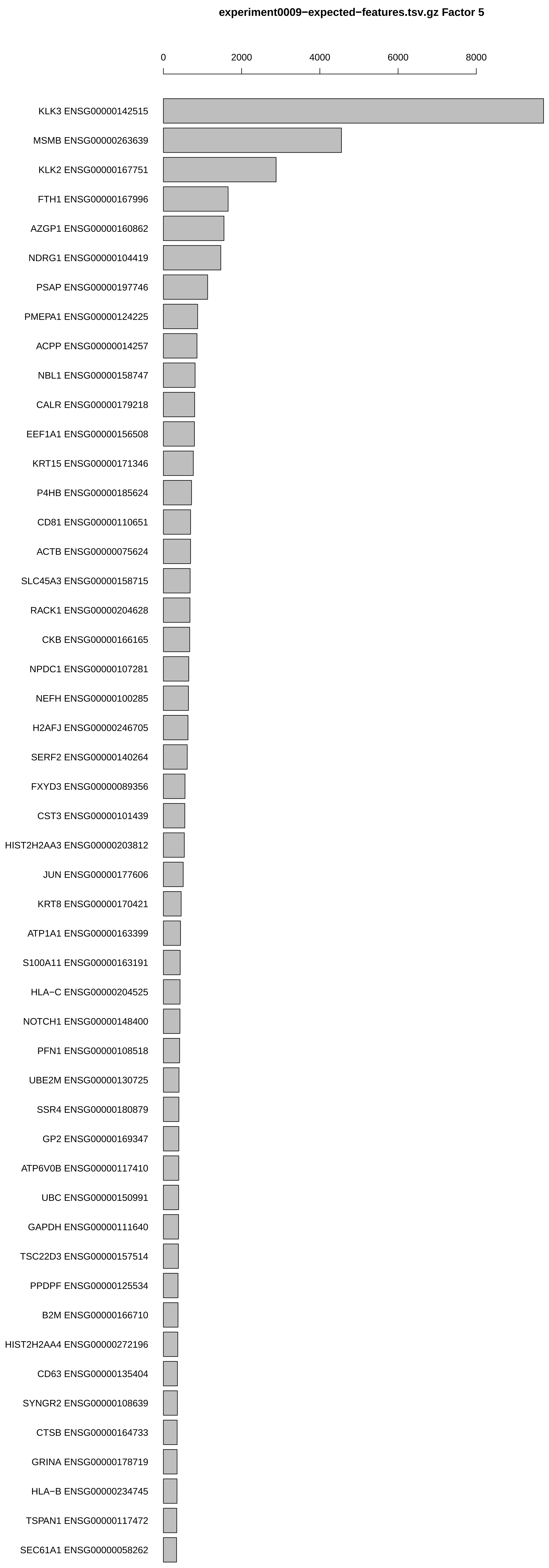
experiment0009−expected−features.tsv.gz Factor 5
0
2000
4000
6000
8000
KLK3 ENSG00000142515
MSMB ENSG00000263639
KLK2 ENSG00000167751
FTH1 ENSG00000167996
AZGP1 ENSG00000160862
NDRG1 ENSG00000104419
PSAP ENSG00000197746
PMEPA1 ENSG00000124225
ACPP ENSG00000014257
NBL1 ENSG00000158747
CALR ENSG00000179218
EEF1A1 ENSG00000156508
KRT15 ENSG00000171346
P4HB ENSG00000185624
CD81 ENSG00000110651
ACTB ENSG00000075624
SLC45A3 ENSG00000158715
RACK1 ENSG00000204628
CKB ENSG00000166165
NPDC1 ENSG00000107281
NEFH ENSG00000100285
H2AFJ ENSG00000246705
SERF2 ENSG00000140264
FXYD3 ENSG00000089356
CST3 ENSG00000101439
HIST2H2AA3 ENSG00000203812
JUN ENSG00000177606
KRT8 ENSG00000170421
ATP1A1 ENSG00000163399
S100A11 ENSG00000163191
HLA−C ENSG00000204525
NOTCH1 ENSG00000148400
PFN1 ENSG00000108518
UBE2M ENSG00000130725
SSR4 ENSG00000180879
GP2 ENSG00000169347
ATP6V0B ENSG00000117410
UBC ENSG00000150991
GAPDH ENSG00000111640
TSC22D3 ENSG00000157514
PPDPF ENSG00000125534
B2M ENSG00000166710
HIST2H2AA4 ENSG00000272196
CD63 ENSG00000135404
SYNGR2 ENSG00000108639
CTSB ENSG00000164733
GRINA ENSG00000178719
HLA−B ENSG00000234745
TSPAN1 ENSG00000117472
SEC61A1 ENSG00000058262

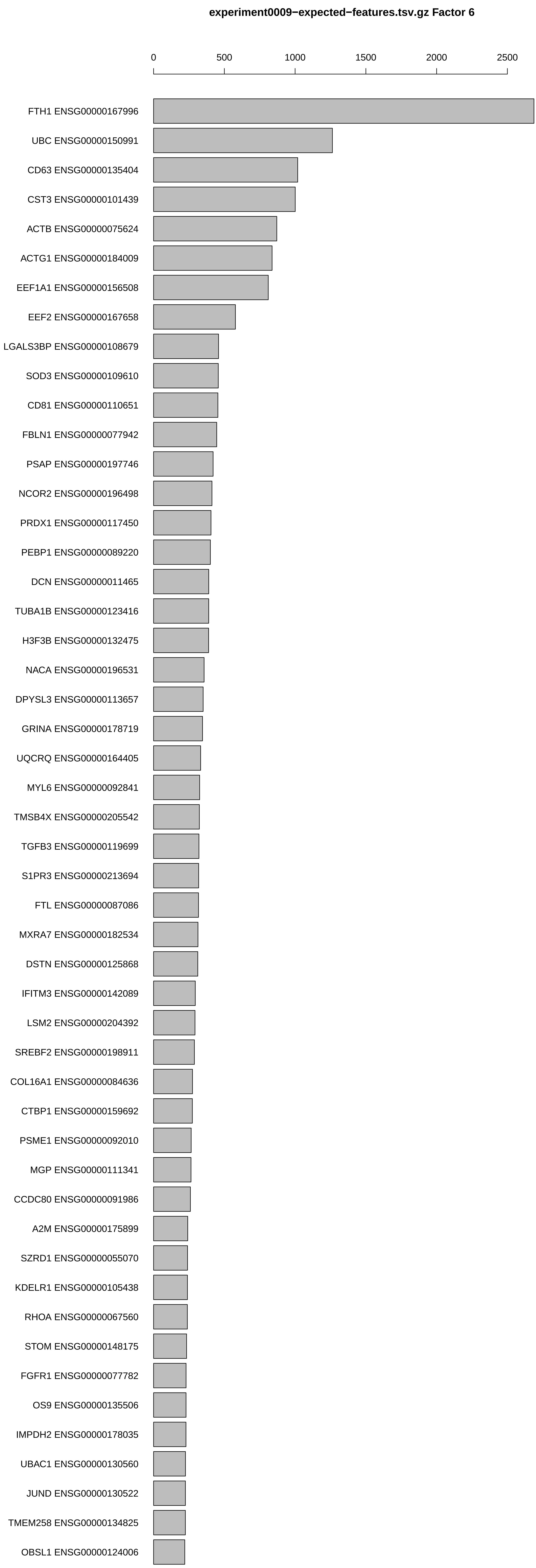
experiment0009−expected−features.tsv.gz Factor 6
0
500
1000
1500
2000
2500
FTH1 ENSG00000167996
UBC ENSG00000150991
CD63 ENSG00000135404
CST3 ENSG00000101439
ACTB ENSG00000075624
ACTG1 ENSG00000184009
EEF1A1 ENSG00000156508
EEF2 ENSG00000167658
LGALS3BP ENSG00000108679
SOD3 ENSG00000109610
CD81 ENSG00000110651
FBLN1 ENSG00000077942
PSAP ENSG00000197746
NCOR2 ENSG00000196498
PRDX1 ENSG00000117450
PEBP1 ENSG00000089220
DCN ENSG00000011465
TUBA1B ENSG00000123416
H3F3B ENSG00000132475
NACA ENSG00000196531
DPYSL3 ENSG00000113657
GRINA ENSG00000178719
UQCRQ ENSG00000164405
MYL6 ENSG00000092841
TMSB4X ENSG00000205542
TGFB3 ENSG00000119699
S1PR3 ENSG00000213694
FTL ENSG00000087086
MXRA7 ENSG00000182534
DSTN ENSG00000125868
IFITM3 ENSG00000142089
LSM2 ENSG00000204392
SREBF2 ENSG00000198911
COL16A1 ENSG00000084636
CTBP1 ENSG00000159692
PSME1 ENSG00000092010
MGP ENSG00000111341
CCDC80 ENSG00000091986
A2M ENSG00000175899
SZRD1 ENSG00000055070
KDELR1 ENSG00000105438
RHOA ENSG00000067560
STOM ENSG00000148175
FGFR1 ENSG00000077782
OS9 ENSG00000135506
IMPDH2 ENSG00000178035
UBAC1 ENSG00000130560
JUND ENSG00000130522
TMEM258 ENSG00000134825
OBSL1 ENSG00000124006

experiment0009−expected−features.tsv.gz Factor 7

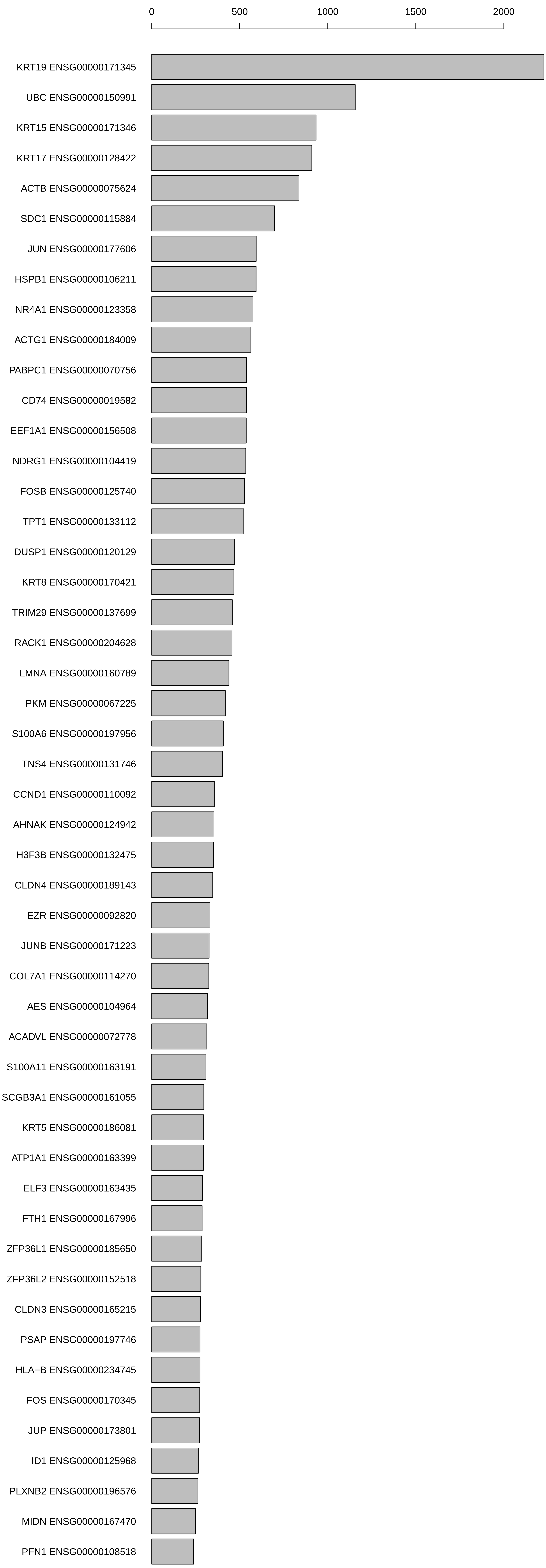

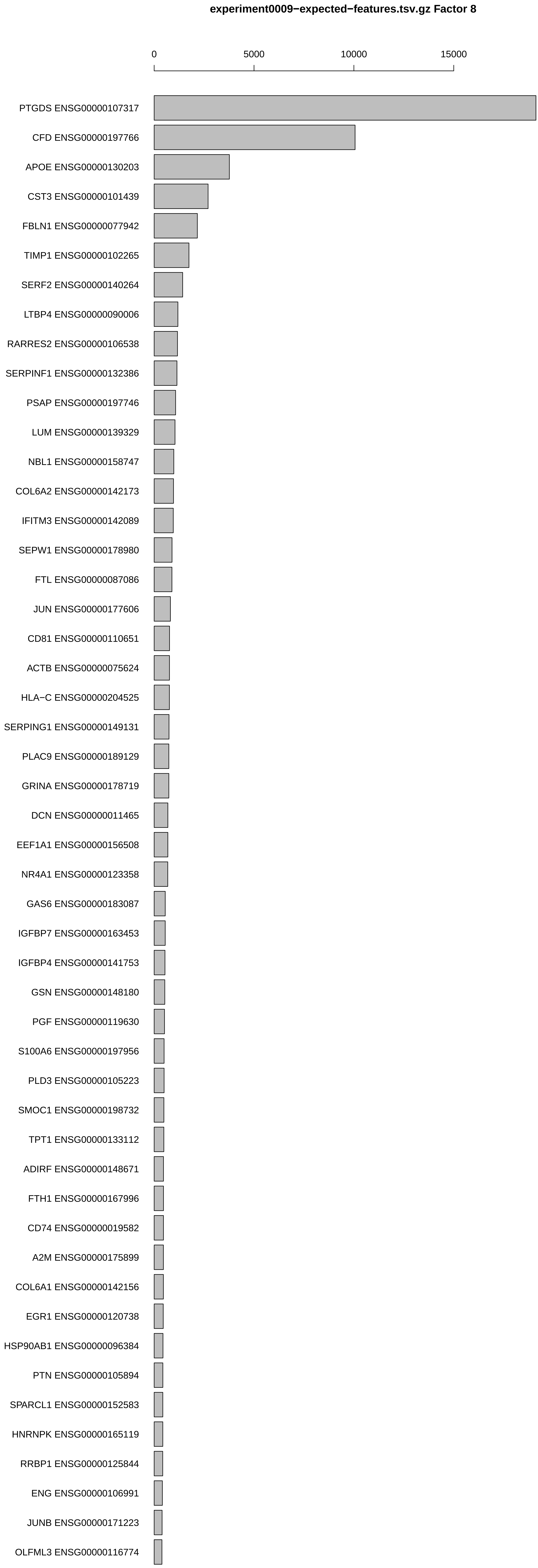
experiment0009−expected−features.tsv.gz Factor 8
0
5000
10000
15000
PTGDS ENSG00000107317
CFD ENSG00000197766
APOE ENSG00000130203
CST3 ENSG00000101439
FBLN1 ENSG00000077942
TIMP1 ENSG00000102265
SERF2 ENSG00000140264
LTBP4 ENSG00000090006
RARRES2 ENSG00000106538
SERPINF1 ENSG00000132386
PSAP ENSG00000197746
LUM ENSG00000139329
NBL1 ENSG00000158747
COL6A2 ENSG00000142173
IFITM3 ENSG00000142089
SEPW1 ENSG00000178980
FTL ENSG00000087086
JUN ENSG00000177606
CD81 ENSG00000110651
ACTB ENSG00000075624
HLA−C ENSG00000204525
SERPING1 ENSG00000149131
PLAC9 ENSG00000189129
GRINA ENSG00000178719
DCN ENSG00000011465
EEF1A1 ENSG00000156508
NR4A1 ENSG00000123358
GAS6 ENSG00000183087
IGFBP7 ENSG00000163453
IGFBP4 ENSG00000141753
GSN ENSG00000148180
PGF ENSG00000119630
S100A6 ENSG00000197956
PLD3 ENSG00000105223
SMOC1 ENSG00000198732
TPT1 ENSG00000133112
ADIRF ENSG00000148671
FTH1 ENSG00000167996
CD74 ENSG00000019582
A2M ENSG00000175899
COL6A1 ENSG00000142156
EGR1 ENSG00000120738
HSP90AB1 ENSG00000096384
PTN ENSG00000105894
SPARCL1 ENSG00000152583
HNRNPK ENSG00000165119
RRBP1 ENSG00000125844
ENG ENSG00000106991
JUNB ENSG00000171223
OLFML3 ENSG00000116774

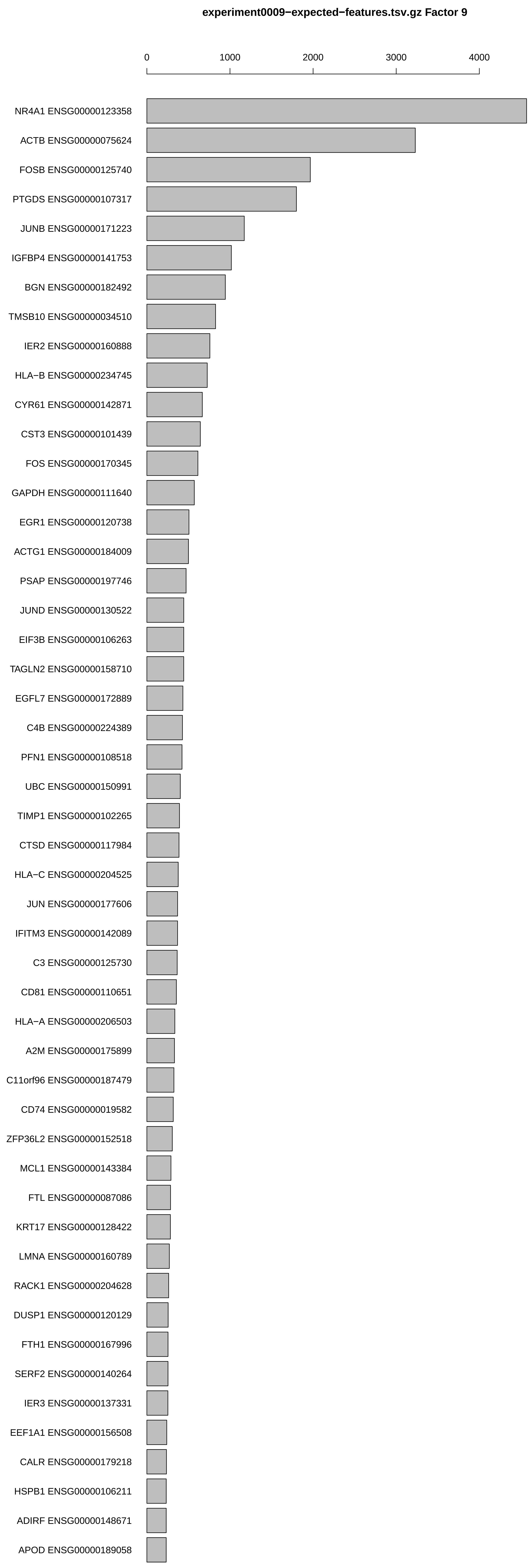
experiment0009−expected−features.tsv.gz Factor 9
0
1000
2000
3000
4000
NR4A1 ENSG00000123358
ACTB ENSG00000075624
FOSB ENSG00000125740
PTGDS ENSG00000107317
JUNB ENSG00000171223
IGFBP4 ENSG00000141753
BGN ENSG00000182492
TMSB10 ENSG00000034510
IER2 ENSG00000160888
HLA−B ENSG00000234745
CYR61 ENSG00000142871
CST3 ENSG00000101439
FOS ENSG00000170345
GAPDH ENSG00000111640
EGR1 ENSG00000120738
ACTG1 ENSG00000184009
PSAP ENSG00000197746
JUND ENSG00000130522
EIF3B ENSG00000106263
TAGLN2 ENSG00000158710
EGFL7 ENSG00000172889
C4B ENSG00000224389
PFN1 ENSG00000108518
UBC ENSG00000150991
TIMP1 ENSG00000102265
CTSD ENSG00000117984
HLA−C ENSG00000204525
JUN ENSG00000177606
IFITM3 ENSG00000142089
C3 ENSG00000125730
CD81 ENSG00000110651
HLA−A ENSG00000206503
A2M ENSG00000175899
C11orf96 ENSG00000187479
CD74 ENSG00000019582
ZFP36L2 ENSG00000152518
MCL1 ENSG00000143384
FTL ENSG00000087086
KRT17 ENSG00000128422
LMNA ENSG00000160789
RACK1 ENSG00000204628
DUSP1 ENSG00000120129
FTH1 ENSG00000167996
SERF2 ENSG00000140264
IER3 ENSG00000137331
EEF1A1 ENSG00000156508
CALR ENSG00000179218
HSPB1 ENSG00000106211
ADIRF ENSG00000148671
APOD ENSG00000189058

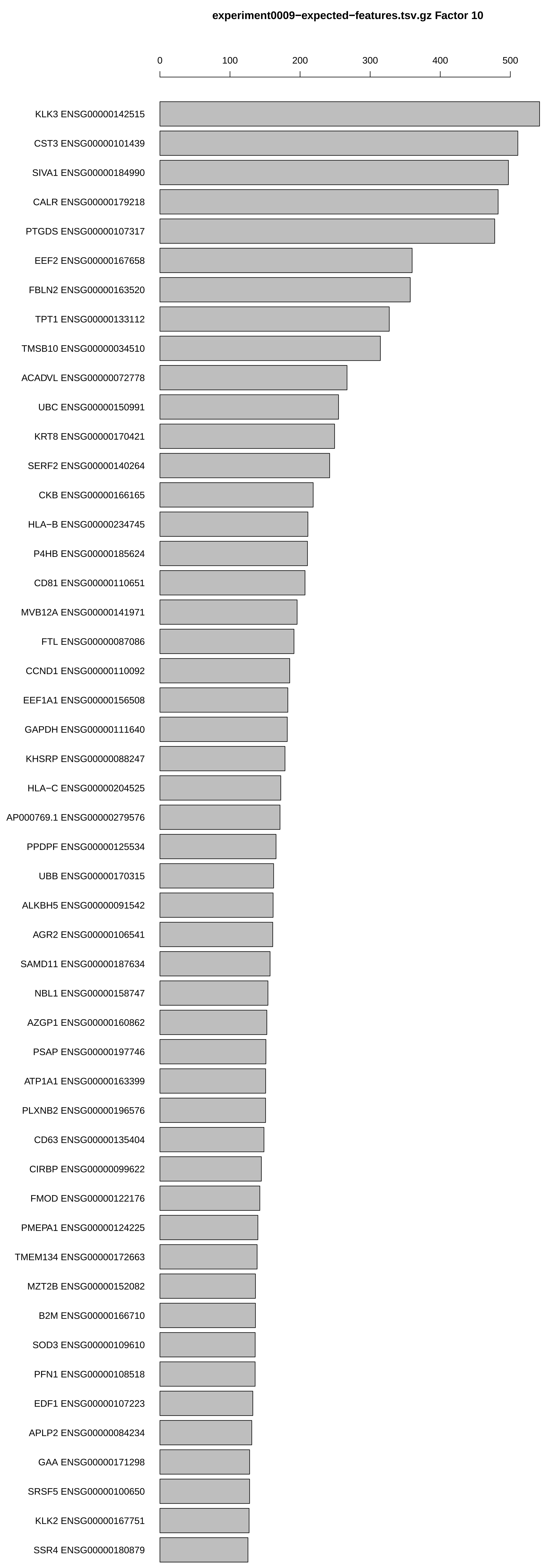
experiment0009−expected−features.tsv.gz Factor 10
0
100
200
300
400
500
KLK3 ENSG00000142515
CST3 ENSG00000101439
SIVA1 ENSG00000184990
CALR ENSG00000179218
PTGDS ENSG00000107317
EEF2 ENSG00000167658
FBLN2 ENSG00000163520
TPT1 ENSG00000133112
TMSB10 ENSG00000034510
ACADVL ENSG00000072778
UBC ENSG00000150991
KRT8 ENSG00000170421
SERF2 ENSG00000140264
CKB ENSG00000166165
HLA−B ENSG00000234745
P4HB ENSG00000185624
CD81 ENSG00000110651
MVB12A ENSG00000141971
FTL ENSG00000087086
CCND1 ENSG00000110092
EEF1A1 ENSG00000156508
GAPDH ENSG00000111640
KHSRP ENSG00000088247
HLA−C ENSG00000204525
AP000769.1 ENSG00000279576
PPDPF ENSG00000125534
UBB ENSG00000170315
ALKBH5 ENSG00000091542
AGR2 ENSG00000106541
SAMD11 ENSG00000187634
NBL1 ENSG00000158747
AZGP1 ENSG00000160862
PSAP ENSG00000197746
ATP1A1 ENSG00000163399
PLXNB2 ENSG00000196576
CD63 ENSG00000135404
CIRBP ENSG00000099622
FMOD ENSG00000122176
PMEPA1 ENSG00000124225
TMEM134 ENSG00000172663
MZT2B ENSG00000152082
B2M ENSG00000166710
SOD3 ENSG00000109610
PFN1 ENSG00000108518
EDF1 ENSG00000107223
APLP2 ENSG00000084234
GAA ENSG00000171298
SRSF5 ENSG00000100650
KLK2 ENSG00000167751
SSR4 ENSG00000180879

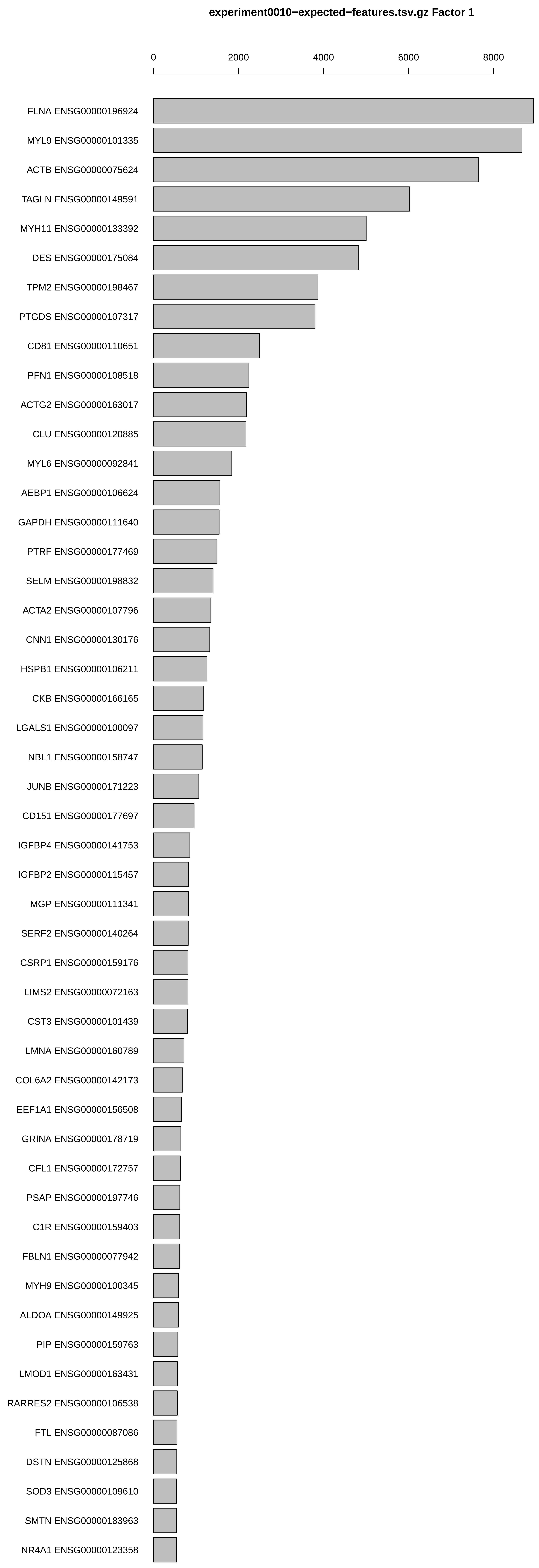
experiment0010−expected−features.tsv.gz Factor 1
0
2000
4000
6000
8000
FLNA ENSG00000196924
MYL9 ENSG00000101335
ACTB ENSG00000075624
TAGLN ENSG00000149591
MYH11 ENSG00000133392
DES ENSG00000175084
TPM2 ENSG00000198467
PTGDS ENSG00000107317
CD81 ENSG00000110651
PFN1 ENSG00000108518
ACTG2 ENSG00000163017
CLU ENSG00000120885
MYL6 ENSG00000092841
AEBP1 ENSG00000106624
GAPDH ENSG00000111640
PTRF ENSG00000177469
SELM ENSG00000198832
ACTA2 ENSG00000107796
CNN1 ENSG00000130176
HSPB1 ENSG00000106211
CKB ENSG00000166165
LGALS1 ENSG00000100097
NBL1 ENSG00000158747
JUNB ENSG00000171223
CD151 ENSG00000177697
IGFBP4 ENSG00000141753
IGFBP2 ENSG00000115457
MGP ENSG00000111341
SERF2 ENSG00000140264
CSRP1 ENSG00000159176
LIMS2 ENSG00000072163
CST3 ENSG00000101439
LMNA ENSG00000160789
COL6A2 ENSG00000142173
EEF1A1 ENSG00000156508
GRINA ENSG00000178719
CFL1 ENSG00000172757
PSAP ENSG00000197746
C1R ENSG00000159403
FBLN1 ENSG00000077942
MYH9 ENSG00000100345
ALDOA ENSG00000149925
PIP ENSG00000159763
LMOD1 ENSG00000163431
RARRES2 ENSG00000106538
FTL ENSG00000087086
DSTN ENSG00000125868
SOD3 ENSG00000109610
SMTN ENSG00000183963
NR4A1 ENSG00000123358

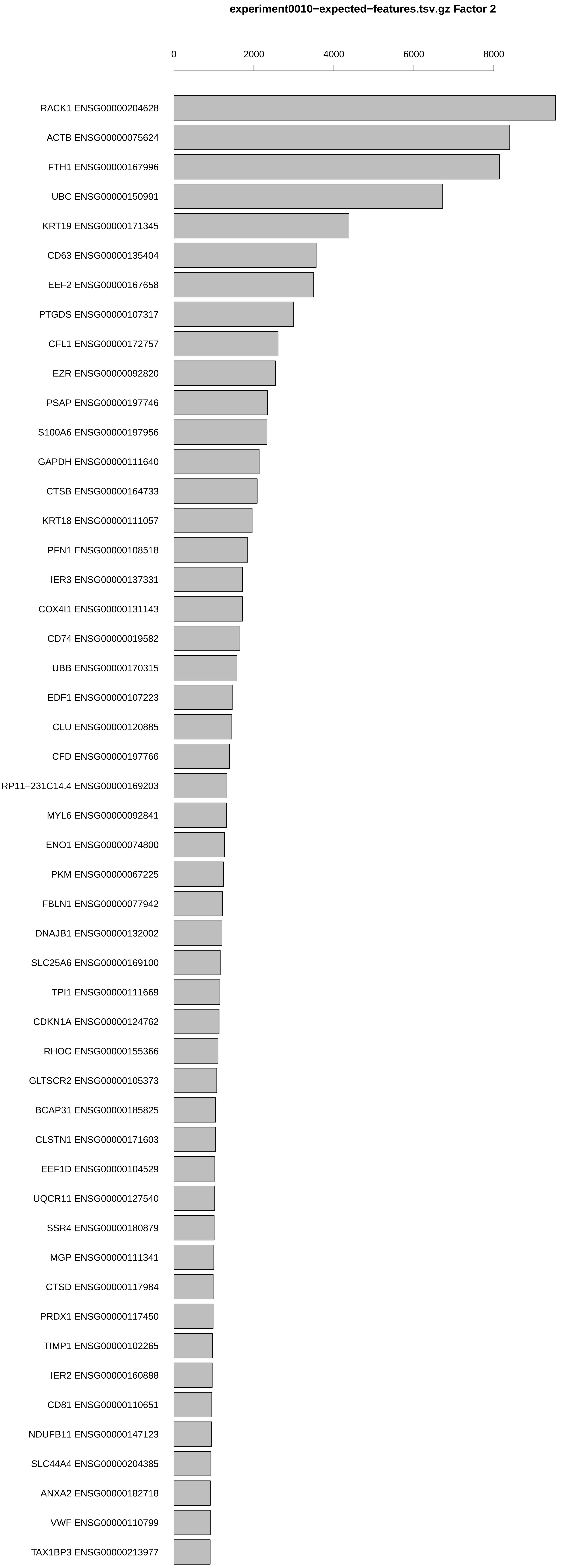
experiment0010−expected−features.tsv.gz Factor 2
0
2000
4000
6000
8000
RACK1 ENSG00000204628
ACTB ENSG00000075624
FTH1 ENSG00000167996
UBC ENSG00000150991
KRT19 ENSG00000171345
CD63 ENSG00000135404
EEF2 ENSG00000167658
PTGDS ENSG00000107317
CFL1 ENSG00000172757
EZR ENSG00000092820
PSAP ENSG00000197746
S100A6 ENSG00000197956
GAPDH ENSG00000111640
CTSB ENSG00000164733
KRT18 ENSG00000111057
PFN1 ENSG00000108518
IER3 ENSG00000137331
COX4I1 ENSG00000131143
CD74 ENSG00000019582
UBB ENSG00000170315
EDF1 ENSG00000107223
CLU ENSG00000120885
CFD ENSG00000197766
RP11−231C14.4 ENSG00000169203
MYL6 ENSG00000092841
ENO1 ENSG00000074800
PKM ENSG00000067225
FBLN1 ENSG00000077942
DNAJB1 ENSG00000132002
SLC25A6 ENSG00000169100
TPI1 ENSG00000111669
CDKN1A ENSG00000124762
RHOC ENSG00000155366
GLTSCR2 ENSG00000105373
BCAP31 ENSG00000185825
CLSTN1 ENSG00000171603
EEF1D ENSG00000104529
UQCR11 ENSG00000127540
SSR4 ENSG00000180879
MGP ENSG00000111341
CTSD ENSG00000117984
PRDX1 ENSG00000117450
TIMP1 ENSG00000102265
IER2 ENSG00000160888
CD81 ENSG00000110651
NDUFB11 ENSG00000147123
SLC44A4 ENSG00000204385
ANXA2 ENSG00000182718
VWF ENSG00000110799
TAX1BP3 ENSG00000213977

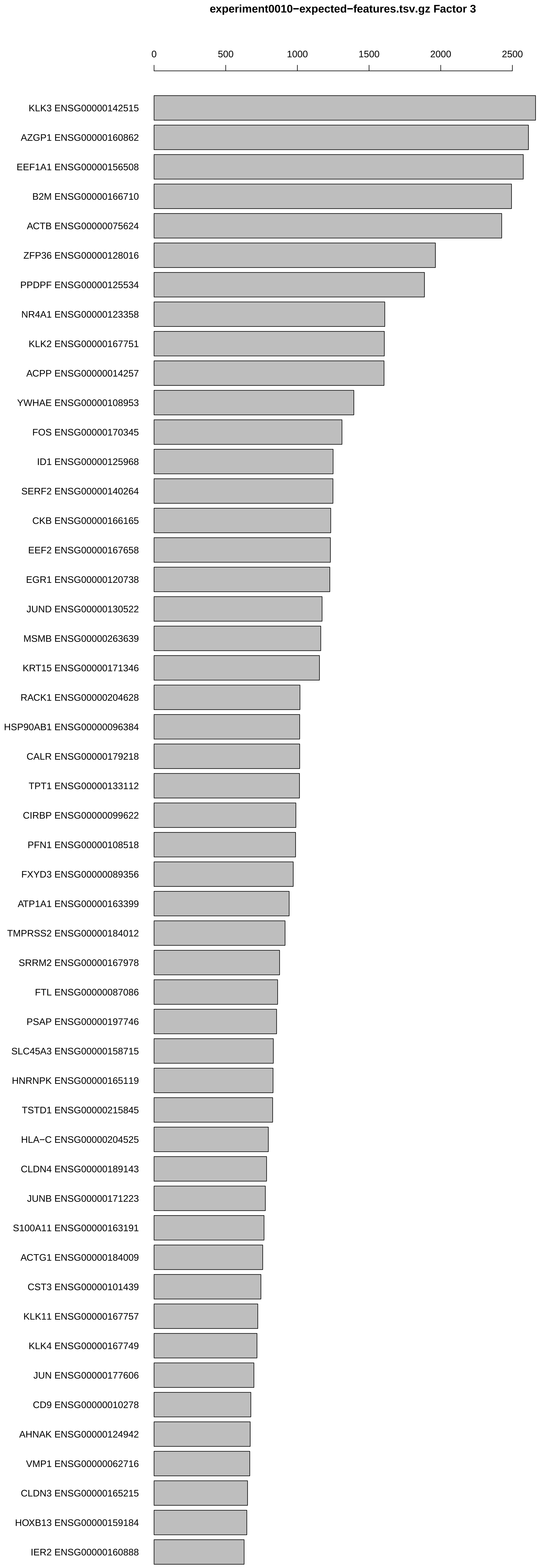
experiment0010−expected−features.tsv.gz Factor 3
0
500
1000
1500
2000
2500
KLK3 ENSG00000142515
AZGP1 ENSG00000160862
EEF1A1 ENSG00000156508
B2M ENSG00000166710
ACTB ENSG00000075624
ZFP36 ENSG00000128016
PPDPF ENSG00000125534
NR4A1 ENSG00000123358
KLK2 ENSG00000167751
ACPP ENSG00000014257
YWHAE ENSG00000108953
FOS ENSG00000170345
ID1 ENSG00000125968
SERF2 ENSG00000140264
CKB ENSG00000166165
EEF2 ENSG00000167658
EGR1 ENSG00000120738
JUND ENSG00000130522
MSMB ENSG00000263639
KRT15 ENSG00000171346
RACK1 ENSG00000204628
HSP90AB1 ENSG00000096384
CALR ENSG00000179218
TPT1 ENSG00000133112
CIRBP ENSG00000099622
PFN1 ENSG00000108518
FXYD3 ENSG00000089356
ATP1A1 ENSG00000163399
TMPRSS2 ENSG00000184012
SRRM2 ENSG00000167978
FTL ENSG00000087086
PSAP ENSG00000197746
SLC45A3 ENSG00000158715
HNRNPK ENSG00000165119
TSTD1 ENSG00000215845
HLA−C ENSG00000204525
CLDN4 ENSG00000189143
JUNB ENSG00000171223
S100A11 ENSG00000163191
ACTG1 ENSG00000184009
CST3 ENSG00000101439
KLK11 ENSG00000167757
KLK4 ENSG00000167749
JUN ENSG00000177606
CD9 ENSG00000010278
AHNAK ENSG00000124942
VMP1 ENSG00000062716
CLDN3 ENSG00000165215
HOXB13 ENSG00000159184
IER2 ENSG00000160888

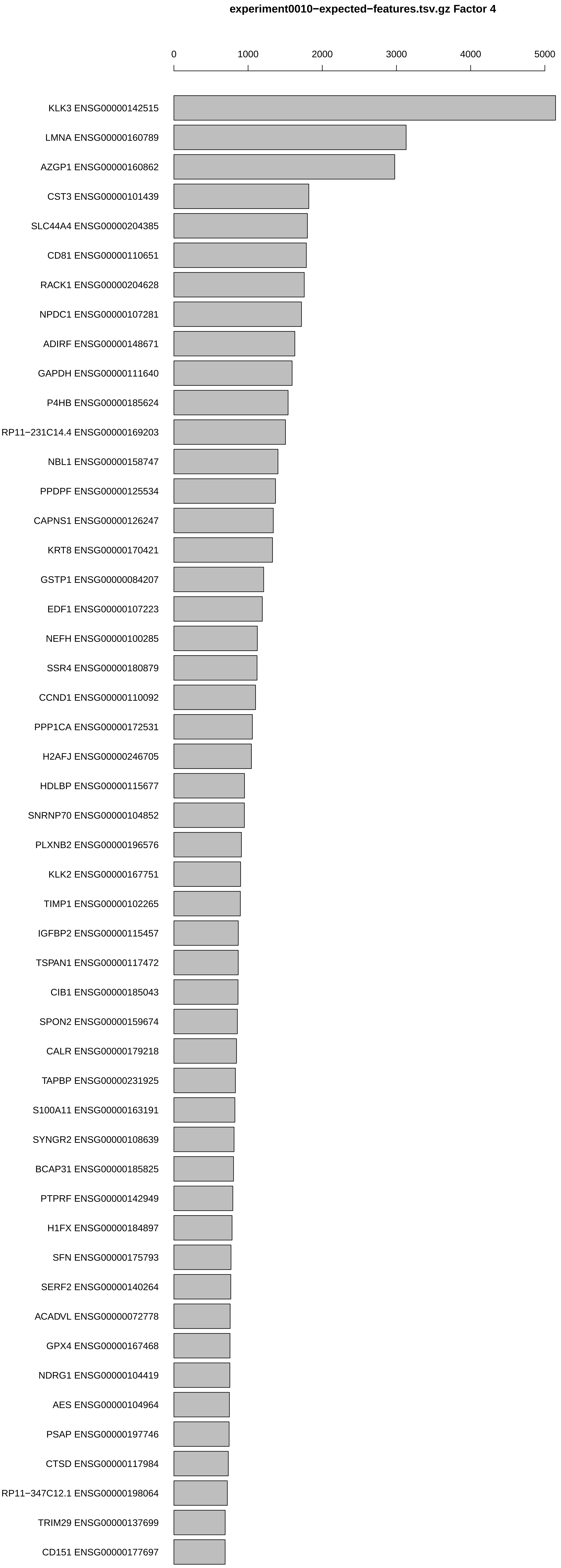
experiment0010−expected−features.tsv.gz Factor 4
0
1000
2000
3000
4000
5000
KLK3 ENSG00000142515
LMNA ENSG00000160789
AZGP1 ENSG00000160862
CST3 ENSG00000101439
SLC44A4 ENSG00000204385
CD81 ENSG00000110651
RACK1 ENSG00000204628
NPDC1 ENSG00000107281
ADIRF ENSG00000148671
GAPDH ENSG00000111640
P4HB ENSG00000185624
RP11−231C14.4 ENSG00000169203
NBL1 ENSG00000158747
PPDPF ENSG00000125534
CAPNS1 ENSG00000126247
KRT8 ENSG00000170421
GSTP1 ENSG00000084207
EDF1 ENSG00000107223
NEFH ENSG00000100285
SSR4 ENSG00000180879
CCND1 ENSG00000110092
PPP1CA ENSG00000172531
H2AFJ ENSG00000246705
HDLBP ENSG00000115677
SNRNP70 ENSG00000104852
PLXNB2 ENSG00000196576
KLK2 ENSG00000167751
TIMP1 ENSG00000102265
IGFBP2 ENSG00000115457
TSPAN1 ENSG00000117472
CIB1 ENSG00000185043
SPON2 ENSG00000159674
CALR ENSG00000179218
TAPBP ENSG00000231925
S100A11 ENSG00000163191
SYNGR2 ENSG00000108639
BCAP31 ENSG00000185825
PTPRF ENSG00000142949
H1FX ENSG00000184897
SFN ENSG00000175793
SERF2 ENSG00000140264
ACADVL ENSG00000072778
GPX4 ENSG00000167468
NDRG1 ENSG00000104419
AES ENSG00000104964
PSAP ENSG00000197746
CTSD ENSG00000117984
RP11−347C12.1 ENSG00000198064
TRIM29 ENSG00000137699
CD151 ENSG00000177697

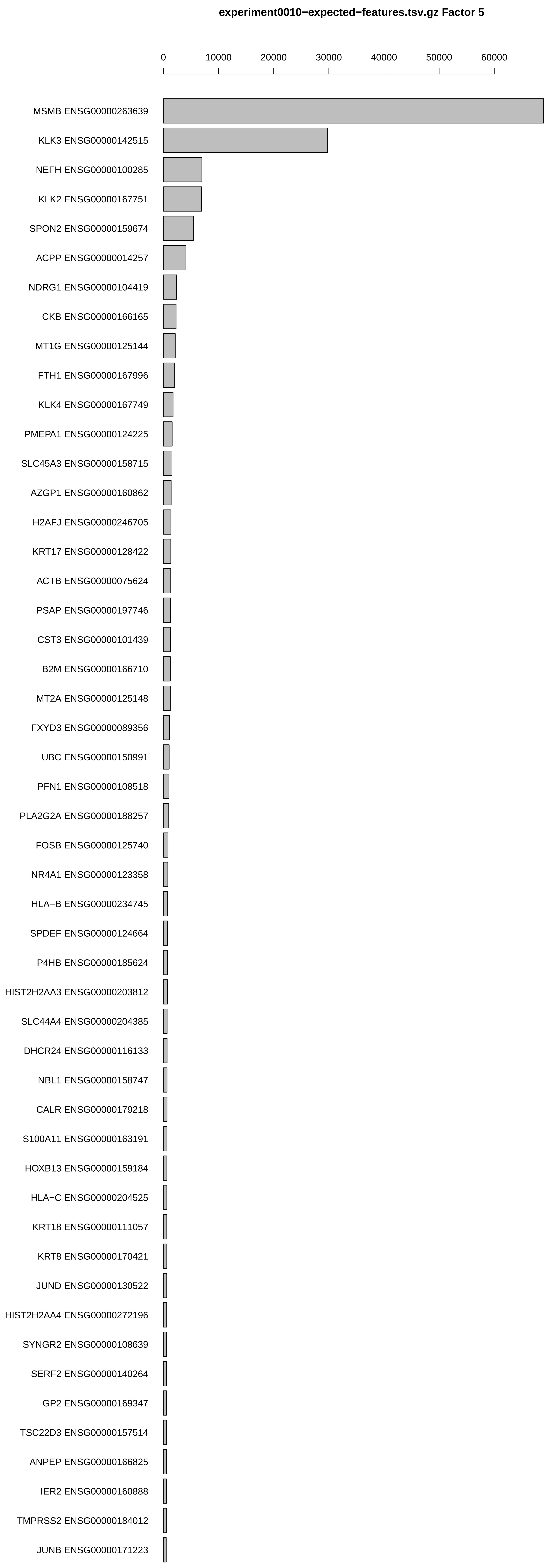
experiment0010−expected−features.tsv.gz Factor 5
0
10000
20000
30000
40000
50000
60000
MSMB ENSG00000263639
KLK3 ENSG00000142515
NEFH ENSG00000100285
KLK2 ENSG00000167751
SPON2 ENSG00000159674
ACPP ENSG00000014257
NDRG1 ENSG00000104419
CKB ENSG00000166165
MT1G ENSG00000125144
FTH1 ENSG00000167996
KLK4 ENSG00000167749
PMEPA1 ENSG00000124225
SLC45A3 ENSG00000158715
AZGP1 ENSG00000160862
H2AFJ ENSG00000246705
KRT17 ENSG00000128422
ACTB ENSG00000075624
PSAP ENSG00000197746
CST3 ENSG00000101439
B2M ENSG00000166710
MT2A ENSG00000125148
FXYD3 ENSG00000089356
UBC ENSG00000150991
PFN1 ENSG00000108518
PLA2G2A ENSG00000188257
FOSB ENSG00000125740
NR4A1 ENSG00000123358
HLA−B ENSG00000234745
SPDEF ENSG00000124664
P4HB ENSG00000185624
HIST2H2AA3 ENSG00000203812
SLC44A4 ENSG00000204385
DHCR24 ENSG00000116133
NBL1 ENSG00000158747
CALR ENSG00000179218
S100A11 ENSG00000163191
HOXB13 ENSG00000159184
HLA−C ENSG00000204525
KRT18 ENSG00000111057
KRT8 ENSG00000170421
JUND ENSG00000130522
HIST2H2AA4 ENSG00000272196
SYNGR2 ENSG00000108639
SERF2 ENSG00000140264
GP2 ENSG00000169347
TSC22D3 ENSG00000157514
ANPEP ENSG00000166825
IER2 ENSG00000160888
TMPRSS2 ENSG00000184012
JUNB ENSG00000171223

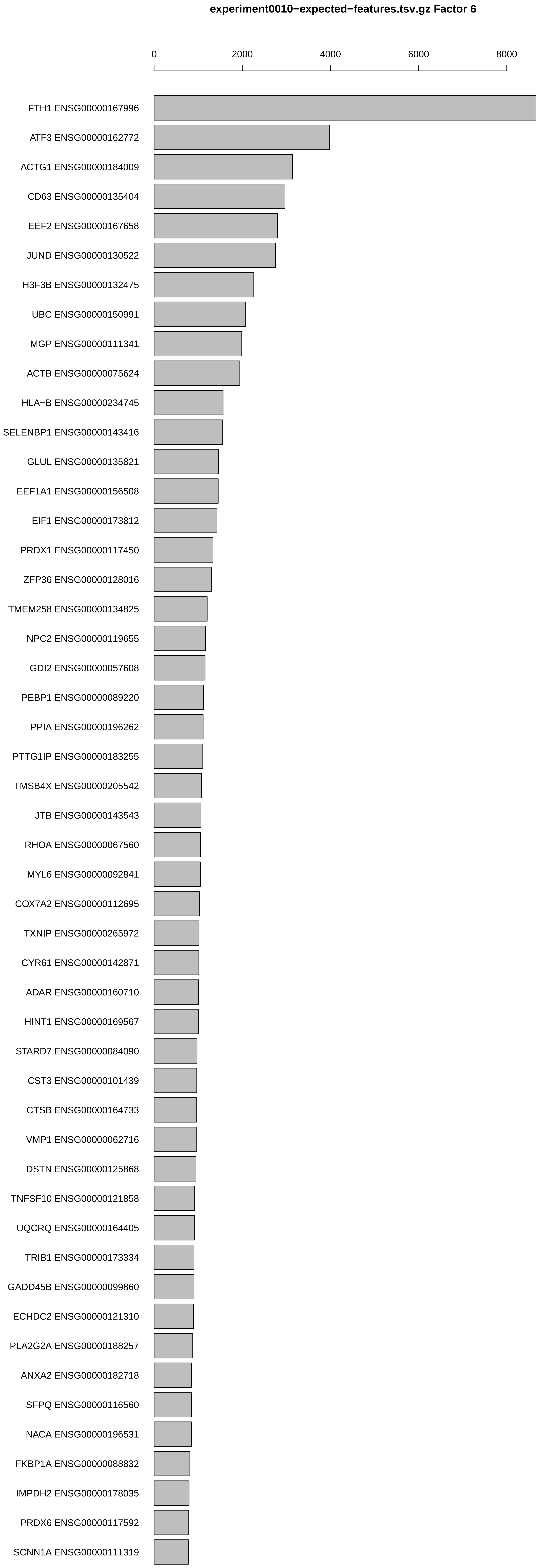
experiment0010−expected−features.tsv.gz Factor 6
0
2000
4000
6000
8000
FTH1 ENSG00000167996
ATF3 ENSG00000162772
ACTG1 ENSG00000184009
CD63 ENSG00000135404
EEF2 ENSG00000167658
JUND ENSG00000130522
H3F3B ENSG00000132475
UBC ENSG00000150991
MGP ENSG00000111341
ACTB ENSG00000075624
HLA−B ENSG00000234745
SELENBP1 ENSG00000143416
GLUL ENSG00000135821
EEF1A1 ENSG00000156508
EIF1 ENSG00000173812
PRDX1 ENSG00000117450
ZFP36 ENSG00000128016
TMEM258 ENSG00000134825
NPC2 ENSG00000119655
GDI2 ENSG00000057608
PEBP1 ENSG00000089220
PPIA ENSG00000196262
PTTG1IP ENSG00000183255
TMSB4X ENSG00000205542
JTB ENSG00000143543
RHOA ENSG00000067560
MYL6 ENSG00000092841
COX7A2 ENSG00000112695
TXNIP ENSG00000265972
CYR61 ENSG00000142871
ADAR ENSG00000160710
HINT1 ENSG00000169567
STARD7 ENSG00000084090
CST3 ENSG00000101439
CTSB ENSG00000164733
VMP1 ENSG00000062716
DSTN ENSG00000125868
TNFSF10 ENSG00000121858
UQCRQ ENSG00000164405
TRIB1 ENSG00000173334
GADD45B ENSG00000099860
ECHDC2 ENSG00000121310
PLA2G2A ENSG00000188257
ANXA2 ENSG00000182718
SFPQ ENSG00000116560
NACA ENSG00000196531
FKBP1A ENSG00000088832
IMPDH2 ENSG00000178035
PRDX6 ENSG00000117592
SCNN1A ENSG00000111319

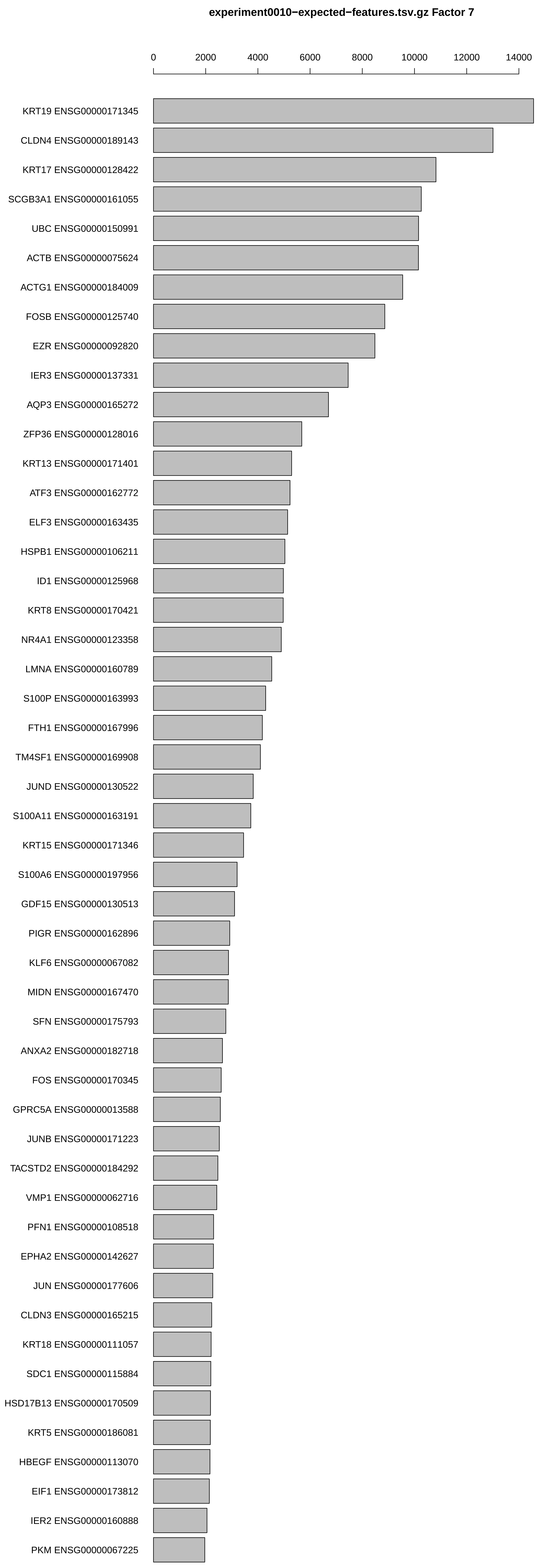
experiment0010−expected−features.tsv.gz Factor 7
0
2000
4000
6000
8000
10000
12000
14000
KRT19 ENSG00000171345
CLDN4 ENSG00000189143
KRT17 ENSG00000128422
SCGB3A1 ENSG00000161055
UBC ENSG00000150991
ACTB ENSG00000075624
ACTG1 ENSG00000184009
FOSB ENSG00000125740
EZR ENSG00000092820
IER3 ENSG00000137331
AQP3 ENSG00000165272
ZFP36 ENSG00000128016
KRT13 ENSG00000171401
ATF3 ENSG00000162772
ELF3 ENSG00000163435
HSPB1 ENSG00000106211
ID1 ENSG00000125968
KRT8 ENSG00000170421
NR4A1 ENSG00000123358
LMNA ENSG00000160789
S100P ENSG00000163993
FTH1 ENSG00000167996
TM4SF1 ENSG00000169908
JUND ENSG00000130522
S100A11 ENSG00000163191
KRT15 ENSG00000171346
S100A6 ENSG00000197956
GDF15 ENSG00000130513
PIGR ENSG00000162896
KLF6 ENSG00000067082
MIDN ENSG00000167470
SFN ENSG00000175793
ANXA2 ENSG00000182718
FOS ENSG00000170345
GPRC5A ENSG00000013588
JUNB ENSG00000171223
TACSTD2 ENSG00000184292
VMP1 ENSG00000062716
PFN1 ENSG00000108518
EPHA2 ENSG00000142627
JUN ENSG00000177606
CLDN3 ENSG00000165215
KRT18 ENSG00000111057
SDC1 ENSG00000115884
HSD17B13 ENSG00000170509
KRT5 ENSG00000186081
HBEGF ENSG00000113070
EIF1 ENSG00000173812
IER2 ENSG00000160888
PKM ENSG00000067225

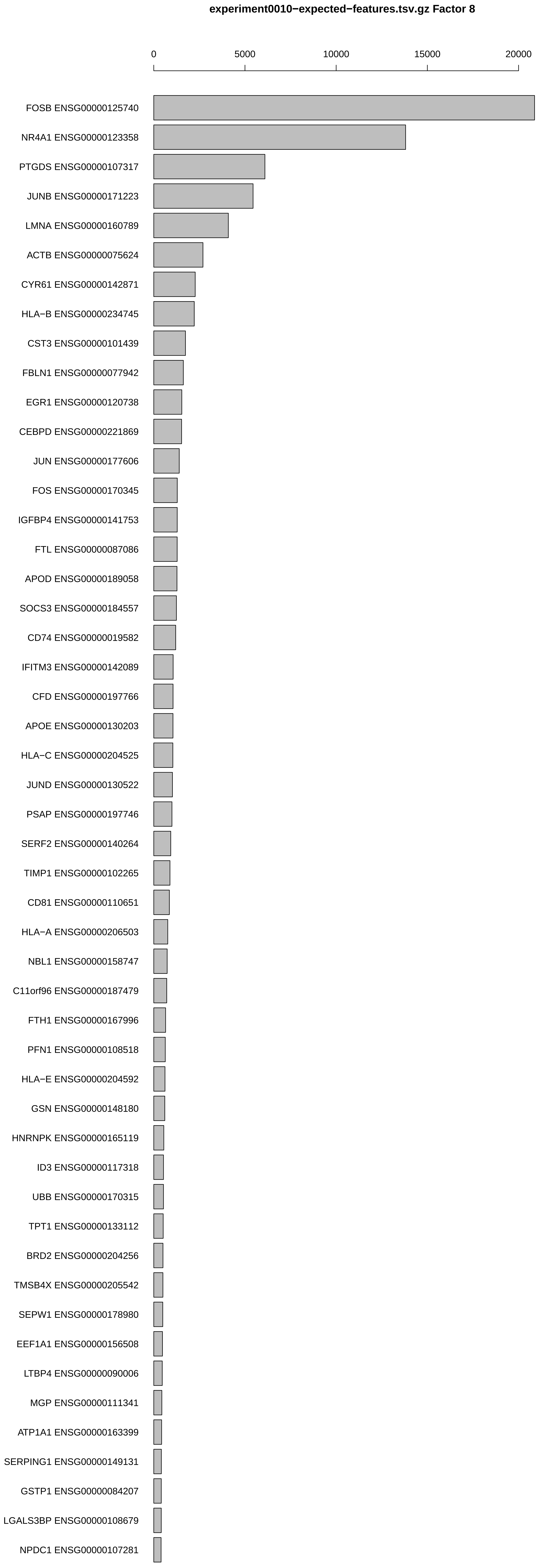
experiment0010−expected−features.tsv.gz Factor 8
0
5000
10000
15000
20000
FOSB ENSG00000125740
NR4A1 ENSG00000123358
PTGDS ENSG00000107317
JUNB ENSG00000171223
LMNA ENSG00000160789
ACTB ENSG00000075624
CYR61 ENSG00000142871
HLA−B ENSG00000234745
CST3 ENSG00000101439
FBLN1 ENSG00000077942
EGR1 ENSG00000120738
CEBPD ENSG00000221869
JUN ENSG00000177606
FOS ENSG00000170345
IGFBP4 ENSG00000141753
FTL ENSG00000087086
APOD ENSG00000189058
SOCS3 ENSG00000184557
CD74 ENSG00000019582
IFITM3 ENSG00000142089
CFD ENSG00000197766
APOE ENSG00000130203
HLA−C ENSG00000204525
JUND ENSG00000130522
PSAP ENSG00000197746
SERF2 ENSG00000140264
TIMP1 ENSG00000102265
CD81 ENSG00000110651
HLA−A ENSG00000206503
NBL1 ENSG00000158747
C11orf96 ENSG00000187479
FTH1 ENSG00000167996
PFN1 ENSG00000108518
HLA−E ENSG00000204592
GSN ENSG00000148180
HNRNPK ENSG00000165119
ID3 ENSG00000117318
UBB ENSG00000170315
TPT1 ENSG00000133112
BRD2 ENSG00000204256
TMSB4X ENSG00000205542
SEPW1 ENSG00000178980
EEF1A1 ENSG00000156508
LTBP4 ENSG00000090006
MGP ENSG00000111341
ATP1A1 ENSG00000163399
SERPING1 ENSG00000149131
GSTP1 ENSG00000084207
LGALS3BP ENSG00000108679
NPDC1 ENSG00000107281

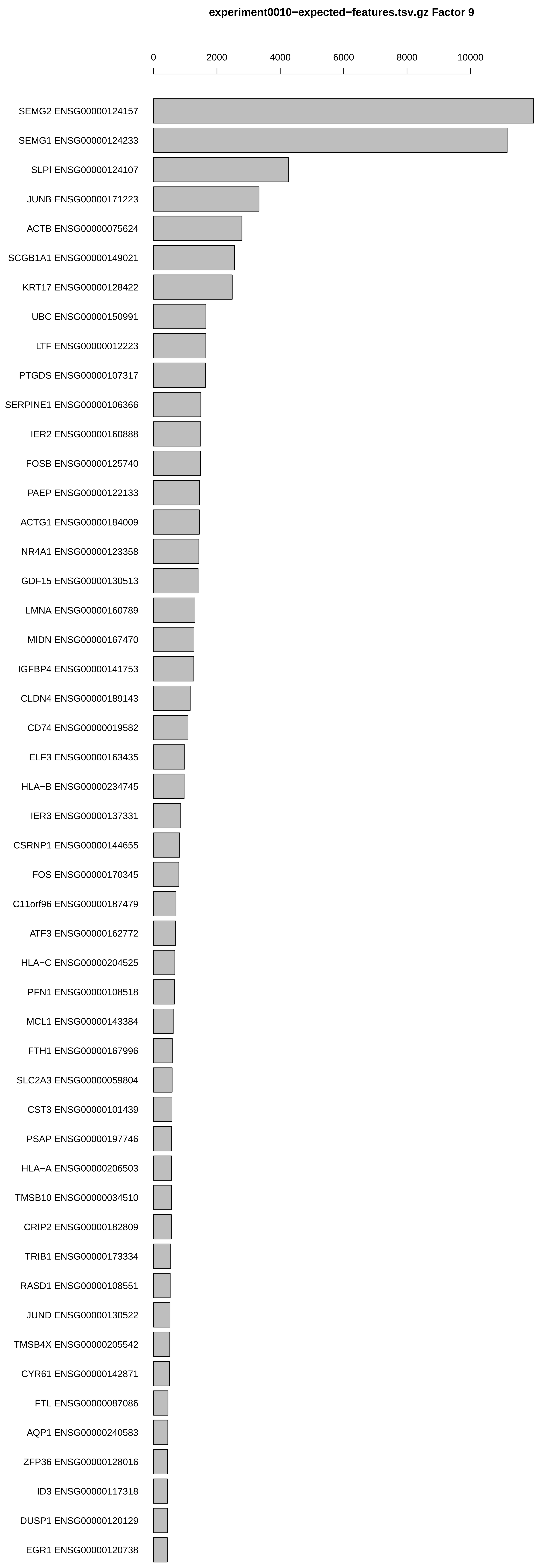

experiment0010−expected−features.tsv.gz Factor 9
0
2000
4000
6000
8000
10000
SEMG2 ENSG00000124157
SEMG1 ENSG00000124233
SLPI ENSG00000124107
JUNB ENSG00000171223
ACTB ENSG00000075624
SCGB1A1 ENSG00000149021
KRT17 ENSG00000128422
UBC ENSG00000150991
LTF ENSG00000012223
PTGDS ENSG00000107317
SERPINE1 ENSG00000106366
IER2 ENSG00000160888
FOSB ENSG00000125740
PAEP ENSG00000122133
ACTG1 ENSG00000184009
NR4A1 ENSG00000123358
GDF15 ENSG00000130513
LMNA ENSG00000160789
MIDN ENSG00000167470
IGFBP4 ENSG00000141753
CLDN4 ENSG00000189143
CD74 ENSG00000019582
ELF3 ENSG00000163435
HLA−B ENSG00000234745
IER3 ENSG00000137331
CSRNP1 ENSG00000144655
FOS ENSG00000170345
C11orf96 ENSG00000187479
ATF3 ENSG00000162772
HLA−C ENSG00000204525
PFN1 ENSG00000108518
MCL1 ENSG00000143384
FTH1 ENSG00000167996
SLC2A3 ENSG00000059804
CST3 ENSG00000101439
PSAP ENSG00000197746
HLA−A ENSG00000206503
TMSB10 ENSG00000034510
CRIP2 ENSG00000182809
TRIB1 ENSG00000173334
RASD1 ENSG00000108551
JUND ENSG00000130522
TMSB4X ENSG00000205542
CYR61 ENSG00000142871
FTL ENSG00000087086
AQP1 ENSG00000240583
ZFP36 ENSG00000128016
ID3 ENSG00000117318
DUSP1 ENSG00000120129
EGR1 ENSG00000120738

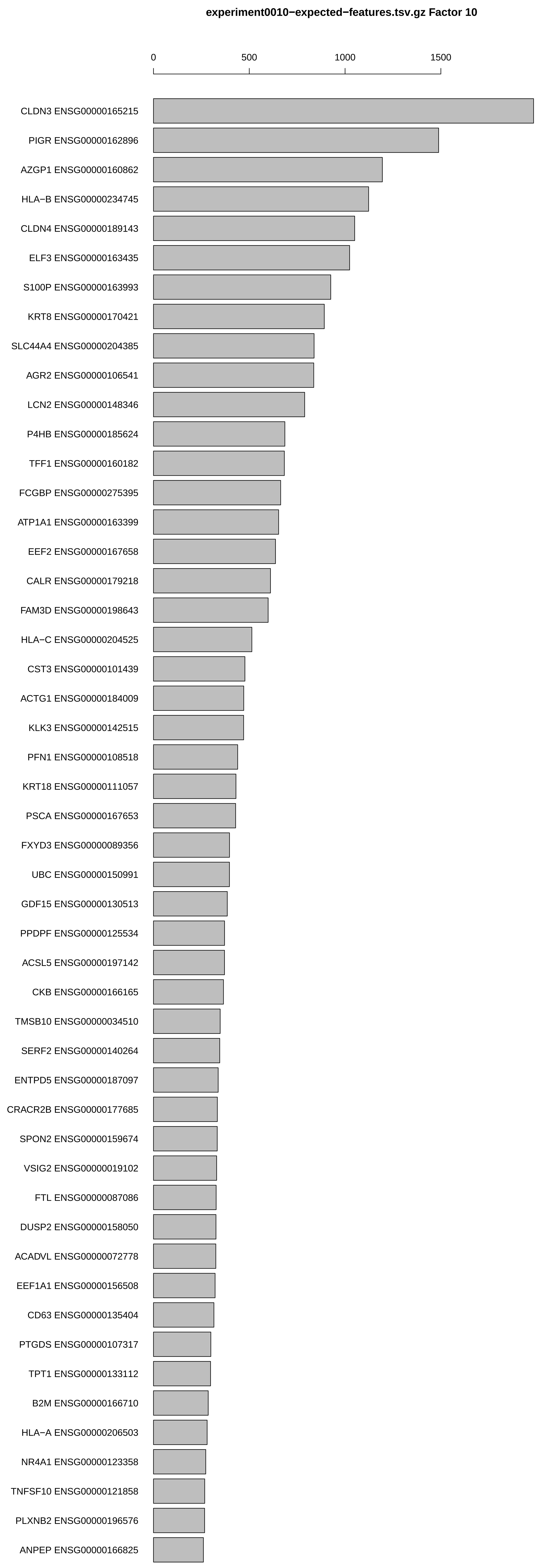

experiment0010−expected−features.tsv.gz Factor 10
0
500
1000
1500
CLDN3 ENSG00000165215
PIGR ENSG00000162896
AZGP1 ENSG00000160862
HLA−B ENSG00000234745
CLDN4 ENSG00000189143
ELF3 ENSG00000163435
S100P ENSG00000163993
KRT8 ENSG00000170421
SLC44A4 ENSG00000204385
AGR2 ENSG00000106541
LCN2 ENSG00000148346
P4HB ENSG00000185624
TFF1 ENSG00000160182
FCGBP ENSG00000275395
ATP1A1 ENSG00000163399
EEF2 ENSG00000167658
CALR ENSG00000179218
FAM3D ENSG00000198643
HLA−C ENSG00000204525
CST3 ENSG00000101439
ACTG1 ENSG00000184009
KLK3 ENSG00000142515
PFN1 ENSG00000108518
KRT18 ENSG00000111057
PSCA ENSG00000167653
FXYD3 ENSG00000089356
UBC ENSG00000150991
GDF15 ENSG00000130513
PPDPF ENSG00000125534
ACSL5 ENSG00000197142
CKB ENSG00000166165
TMSB10 ENSG00000034510
SERF2 ENSG00000140264
ENTPD5 ENSG00000187097
CRACR2B ENSG00000177685
SPON2 ENSG00000159674
VSIG2 ENSG00000019102
FTL ENSG00000087086
DUSP2 ENSG00000158050
ACADVL ENSG00000072778
EEF1A1 ENSG00000156508
CD63 ENSG00000135404
PTGDS ENSG00000107317
TPT1 ENSG00000133112
B2M ENSG00000166710
HLA−A ENSG00000206503
NR4A1 ENSG00000123358
TNFSF10 ENSG00000121858
PLXNB2 ENSG00000196576
ANPEP ENSG00000166825

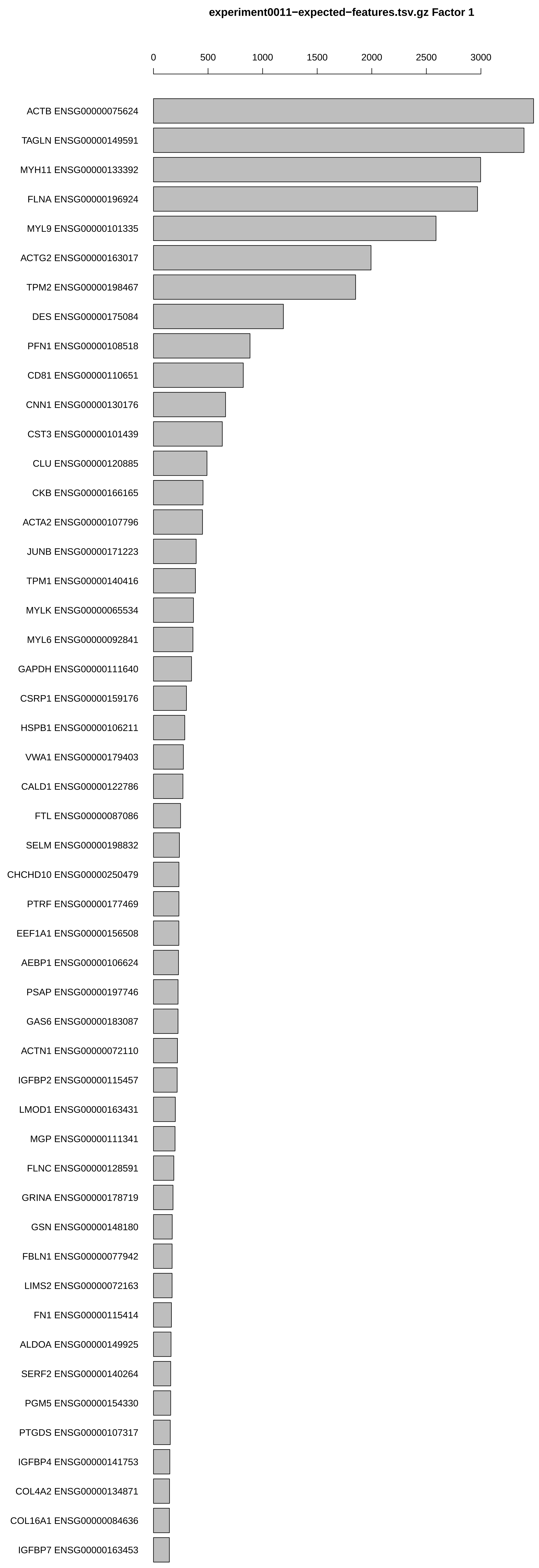
experiment0011−expected−features.tsv.gz Factor 1
0
500
1000
1500
2000
2500
3000
ACTB ENSG00000075624
TAGLN ENSG00000149591
MYH11 ENSG00000133392
FLNA ENSG00000196924
MYL9 ENSG00000101335
ACTG2 ENSG00000163017
TPM2 ENSG00000198467
DES ENSG00000175084
PFN1 ENSG00000108518
CD81 ENSG00000110651
CNN1 ENSG00000130176
CST3 ENSG00000101439
CLU ENSG00000120885
CKB ENSG00000166165
ACTA2 ENSG00000107796
JUNB ENSG00000171223
TPM1 ENSG00000140416
MYLK ENSG00000065534
MYL6 ENSG00000092841
GAPDH ENSG00000111640
CSRP1 ENSG00000159176
HSPB1 ENSG00000106211
VWA1 ENSG00000179403
CALD1 ENSG00000122786
FTL ENSG00000087086
SELM ENSG00000198832
CHCHD10 ENSG00000250479
PTRF ENSG00000177469
EEF1A1 ENSG00000156508
AEBP1 ENSG00000106624
PSAP ENSG00000197746
GAS6 ENSG00000183087
ACTN1 ENSG00000072110
IGFBP2 ENSG00000115457
LMOD1 ENSG00000163431
MGP ENSG00000111341
FLNC ENSG00000128591
GRINA ENSG00000178719
GSN ENSG00000148180
FBLN1 ENSG00000077942
LIMS2 ENSG00000072163
FN1 ENSG00000115414
ALDOA ENSG00000149925
SERF2 ENSG00000140264
PGM5 ENSG00000154330
PTGDS ENSG00000107317
IGFBP4 ENSG00000141753
COL4A2 ENSG00000134871
COL16A1 ENSG00000084636
IGFBP7 ENSG00000163453

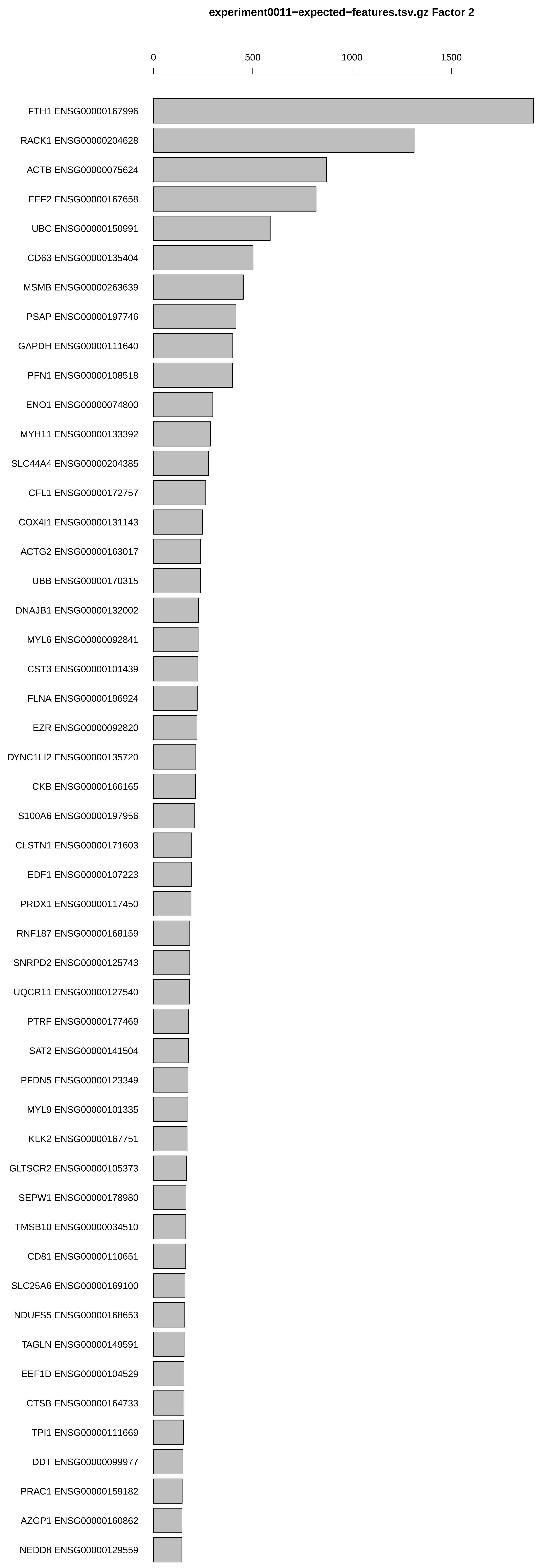
experiment0011−expected−features.tsv.gz Factor 2
0
500
1000
1500
FTH1 ENSG00000167996
RACK1 ENSG00000204628
ACTB ENSG00000075624
EEF2 ENSG00000167658
UBC ENSG00000150991
CD63 ENSG00000135404
MSMB ENSG00000263639
PSAP ENSG00000197746
GAPDH ENSG00000111640
PFN1 ENSG00000108518
ENO1 ENSG00000074800
MYH11 ENSG00000133392
SLC44A4 ENSG00000204385
CFL1 ENSG00000172757
COX4I1 ENSG00000131143
ACTG2 ENSG00000163017
UBB ENSG00000170315
DNAJB1 ENSG00000132002
MYL6 ENSG00000092841
CST3 ENSG00000101439
FLNA ENSG00000196924
EZR ENSG00000092820
DYNC1LI2 ENSG00000135720
CKB ENSG00000166165
S100A6 ENSG00000197956
CLSTN1 ENSG00000171603
EDF1 ENSG00000107223
PRDX1 ENSG00000117450
RNF187 ENSG00000168159
SNRPD2 ENSG00000125743
UQCR11 ENSG00000127540
PTRF ENSG00000177469
SAT2 ENSG00000141504
PFDN5 ENSG00000123349
MYL9 ENSG00000101335
KLK2 ENSG00000167751
GLTSCR2 ENSG00000105373
SEPW1 ENSG00000178980
TMSB10 ENSG00000034510
CD81 ENSG00000110651
SLC25A6 ENSG00000169100
NDUFS5 ENSG00000168653
TAGLN ENSG00000149591
EEF1D ENSG00000104529
CTSB ENSG00000164733
TPI1 ENSG00000111669
DDT ENSG00000099977
PRAC1 ENSG00000159182
AZGP1 ENSG00000160862
NEDD8 ENSG00000129559

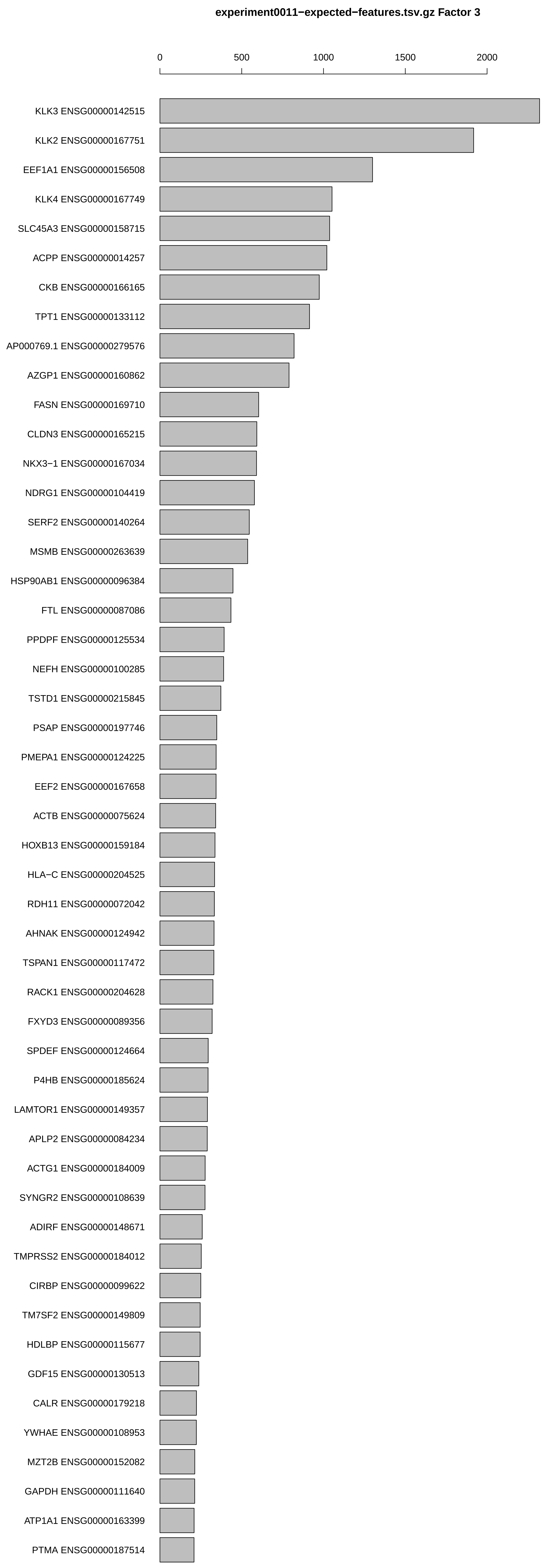
experiment0011−expected−features.tsv.gz Factor 3
0
500
1000
1500
2000
KLK3 ENSG00000142515
KLK2 ENSG00000167751
EEF1A1 ENSG00000156508
KLK4 ENSG00000167749
SLC45A3 ENSG00000158715
ACPP ENSG00000014257
CKB ENSG00000166165
TPT1 ENSG00000133112
AP000769.1 ENSG00000279576
AZGP1 ENSG00000160862
FASN ENSG00000169710
CLDN3 ENSG00000165215
NKX3−1 ENSG00000167034
NDRG1 ENSG00000104419
SERF2 ENSG00000140264
MSMB ENSG00000263639
HSP90AB1 ENSG00000096384
FTL ENSG00000087086
PPDPF ENSG00000125534
NEFH ENSG00000100285
TSTD1 ENSG00000215845
PSAP ENSG00000197746
PMEPA1 ENSG00000124225
EEF2 ENSG00000167658
ACTB ENSG00000075624
HOXB13 ENSG00000159184
HLA−C ENSG00000204525
RDH11 ENSG00000072042
AHNAK ENSG00000124942
TSPAN1 ENSG00000117472
RACK1 ENSG00000204628
FXYD3 ENSG00000089356
SPDEF ENSG00000124664
P4HB ENSG00000185624
LAMTOR1 ENSG00000149357
APLP2 ENSG00000084234
ACTG1 ENSG00000184009
SYNGR2 ENSG00000108639
ADIRF ENSG00000148671
TMPRSS2 ENSG00000184012
CIRBP ENSG00000099622
TM7SF2 ENSG00000149809
HDLBP ENSG00000115677
GDF15 ENSG00000130513
CALR ENSG00000179218
YWHAE ENSG00000108953
MZT2B ENSG00000152082
GAPDH ENSG00000111640
ATP1A1 ENSG00000163399
PTMA ENSG00000187514

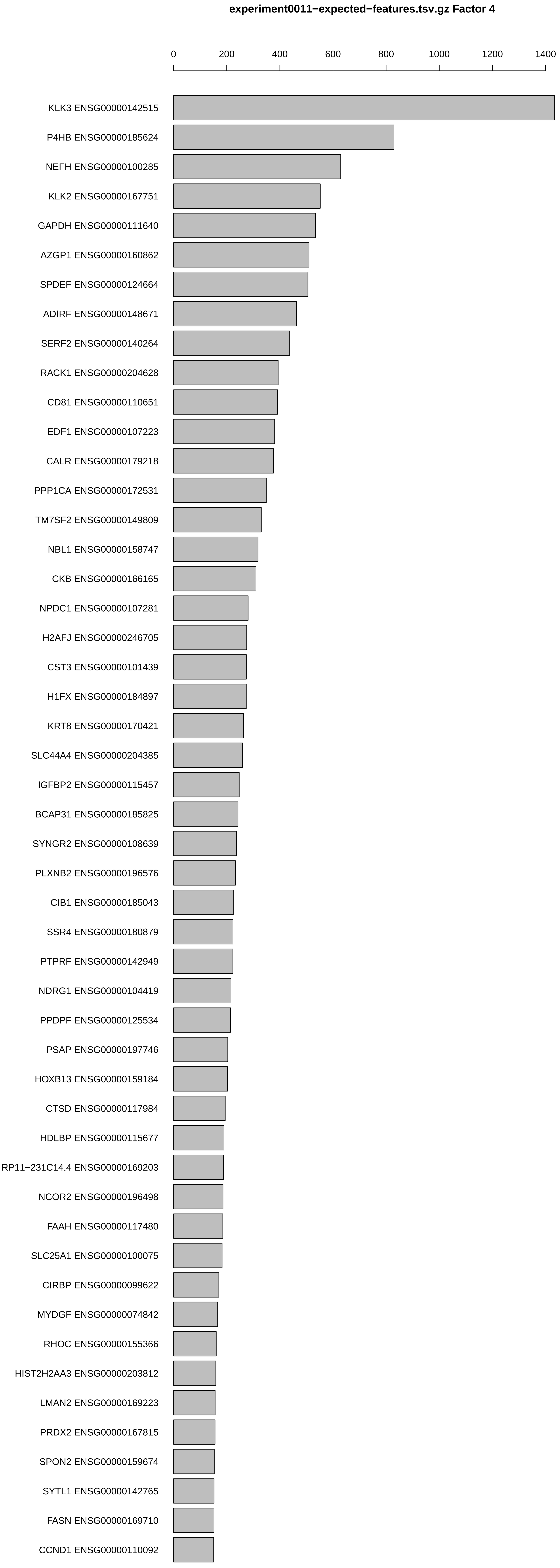
experiment0011−expected−features.tsv.gz Factor 4
0
200
400
600
800
1000
1200
1400
KLK3 ENSG00000142515
P4HB ENSG00000185624
NEFH ENSG00000100285
KLK2 ENSG00000167751
GAPDH ENSG00000111640
AZGP1 ENSG00000160862
SPDEF ENSG00000124664
ADIRF ENSG00000148671
SERF2 ENSG00000140264
RACK1 ENSG00000204628
CD81 ENSG00000110651
EDF1 ENSG00000107223
CALR ENSG00000179218
PPP1CA ENSG00000172531
TM7SF2 ENSG00000149809
NBL1 ENSG00000158747
CKB ENSG00000166165
NPDC1 ENSG00000107281
H2AFJ ENSG00000246705
CST3 ENSG00000101439
H1FX ENSG00000184897
KRT8 ENSG00000170421
SLC44A4 ENSG00000204385
IGFBP2 ENSG00000115457
BCAP31 ENSG00000185825
SYNGR2 ENSG00000108639
PLXNB2 ENSG00000196576
CIB1 ENSG00000185043
SSR4 ENSG00000180879
PTPRF ENSG00000142949
NDRG1 ENSG00000104419
PPDPF ENSG00000125534
PSAP ENSG00000197746
HOXB13 ENSG00000159184
CTSD ENSG00000117984
HDLBP ENSG00000115677
RP11−231C14.4 ENSG00000169203
NCOR2 ENSG00000196498
FAAH ENSG00000117480
SLC25A1 ENSG00000100075
CIRBP ENSG00000099622
MYDGF ENSG00000074842
RHOC ENSG00000155366
HIST2H2AA3 ENSG00000203812
LMAN2 ENSG00000169223
PRDX2 ENSG00000167815
SPON2 ENSG00000159674
SYTL1 ENSG00000142765
FASN ENSG00000169710
CCND1 ENSG00000110092

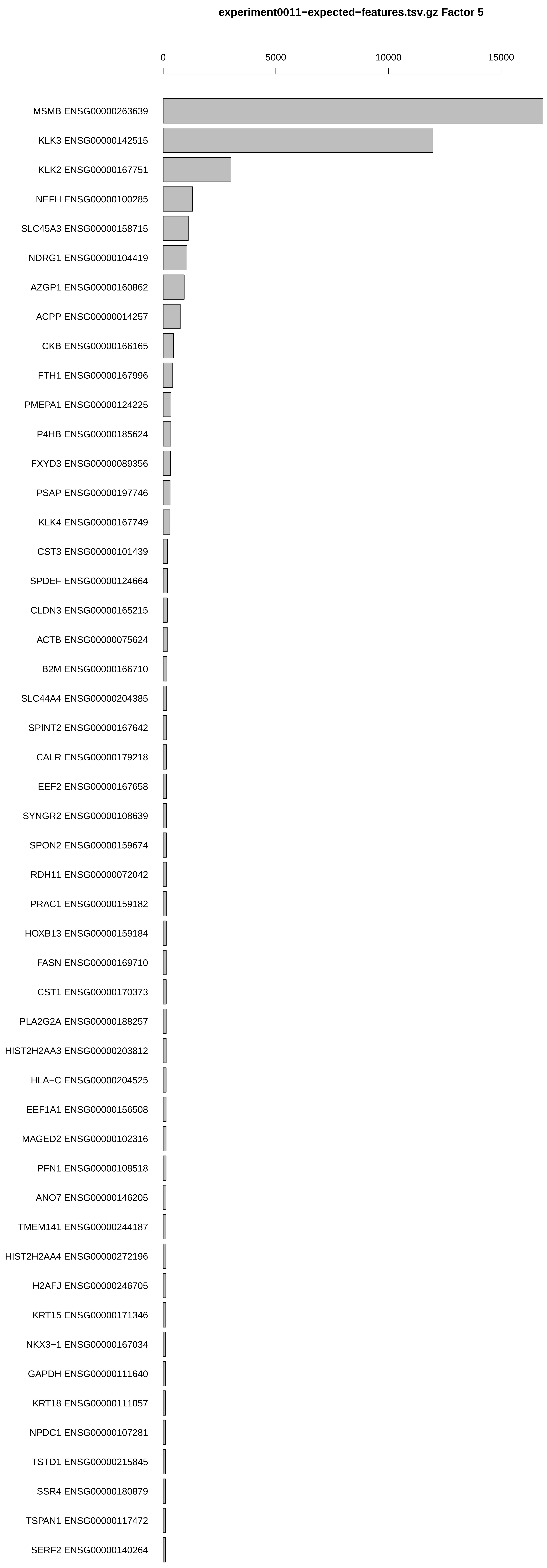

experiment0011−expected−features.tsv.gz Factor 5
0
5000
10000
15000
MSMB ENSG00000263639
KLK3 ENSG00000142515
KLK2 ENSG00000167751
NEFH ENSG00000100285
SLC45A3 ENSG00000158715
NDRG1 ENSG00000104419
AZGP1 ENSG00000160862
ACPP ENSG00000014257
CKB ENSG00000166165
FTH1 ENSG00000167996
PMEPA1 ENSG00000124225
P4HB ENSG00000185624
FXYD3 ENSG00000089356
PSAP ENSG00000197746
KLK4 ENSG00000167749
CST3 ENSG00000101439
SPDEF ENSG00000124664
CLDN3 ENSG00000165215
ACTB ENSG00000075624
B2M ENSG00000166710
SLC44A4 ENSG00000204385
SPINT2 ENSG00000167642
CALR ENSG00000179218
EEF2 ENSG00000167658
SYNGR2 ENSG00000108639
SPON2 ENSG00000159674
RDH11 ENSG00000072042
PRAC1 ENSG00000159182
HOXB13 ENSG00000159184
FASN ENSG00000169710
CST1 ENSG00000170373
PLA2G2A ENSG00000188257
HIST2H2AA3 ENSG00000203812
HLA−C ENSG00000204525
EEF1A1 ENSG00000156508
MAGED2 ENSG00000102316
PFN1 ENSG00000108518
ANO7 ENSG00000146205
TMEM141 ENSG00000244187
HIST2H2AA4 ENSG00000272196
H2AFJ ENSG00000246705
KRT15 ENSG00000171346
NKX3−1 ENSG00000167034
GAPDH ENSG00000111640
KRT18 ENSG00000111057
NPDC1 ENSG00000107281
TSTD1 ENSG00000215845
SSR4 ENSG00000180879
TSPAN1 ENSG00000117472
SERF2 ENSG00000140264

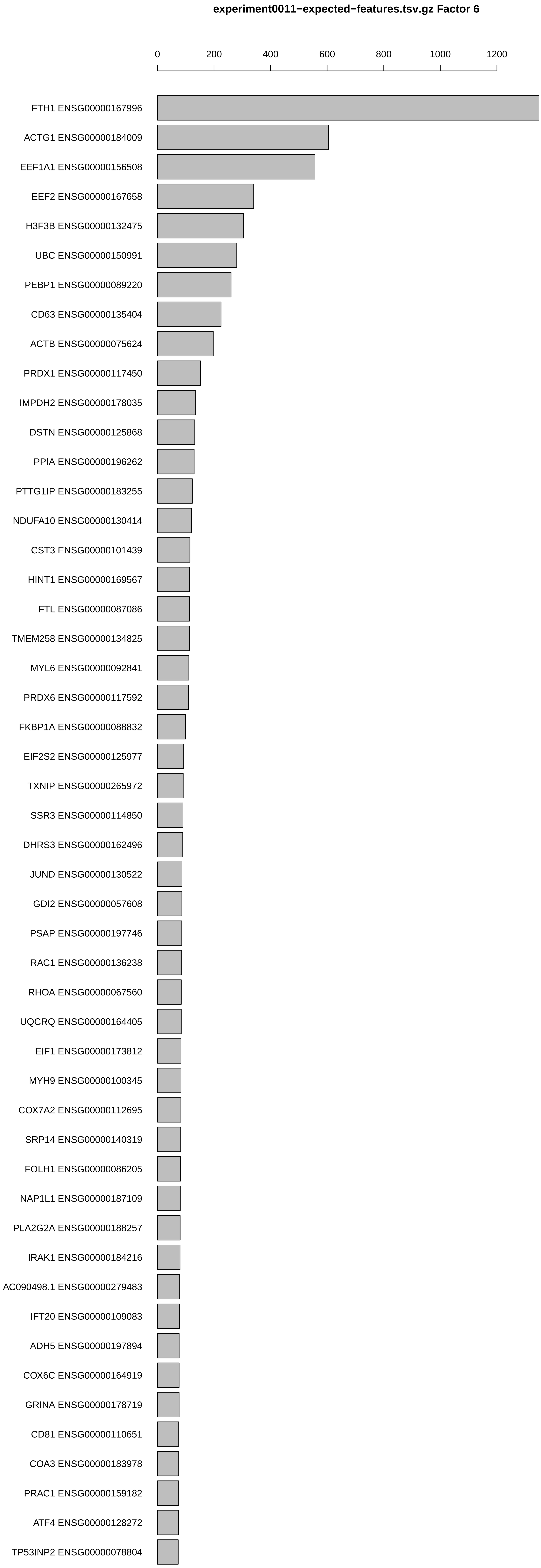
experiment0011−expected−features.tsv.gz Factor 6
0
200
400
600
800
1000
1200
FTH1 ENSG00000167996
ACTG1 ENSG00000184009
EEF1A1 ENSG00000156508
EEF2 ENSG00000167658
H3F3B ENSG00000132475
UBC ENSG00000150991
PEBP1 ENSG00000089220
CD63 ENSG00000135404
ACTB ENSG00000075624
PRDX1 ENSG00000117450
IMPDH2 ENSG00000178035
DSTN ENSG00000125868
PPIA ENSG00000196262
PTTG1IP ENSG00000183255
NDUFA10 ENSG00000130414
CST3 ENSG00000101439
HINT1 ENSG00000169567
FTL ENSG00000087086
TMEM258 ENSG00000134825
MYL6 ENSG00000092841
PRDX6 ENSG00000117592
FKBP1A ENSG00000088832
EIF2S2 ENSG00000125977
TXNIP ENSG00000265972
SSR3 ENSG00000114850
DHRS3 ENSG00000162496
JUND ENSG00000130522
GDI2 ENSG00000057608
PSAP ENSG00000197746
RAC1 ENSG00000136238
RHOA ENSG00000067560
UQCRQ ENSG00000164405
EIF1 ENSG00000173812
MYH9 ENSG00000100345
COX7A2 ENSG00000112695
SRP14 ENSG00000140319
FOLH1 ENSG00000086205
NAP1L1 ENSG00000187109
PLA2G2A ENSG00000188257
IRAK1 ENSG00000184216
AC090498.1 ENSG00000279483
IFT20 ENSG00000109083
ADH5 ENSG00000197894
COX6C ENSG00000164919
GRINA ENSG00000178719
CD81 ENSG00000110651
COA3 ENSG00000183978
PRAC1 ENSG00000159182
ATF4 ENSG00000128272
TP53INP2 ENSG00000078804

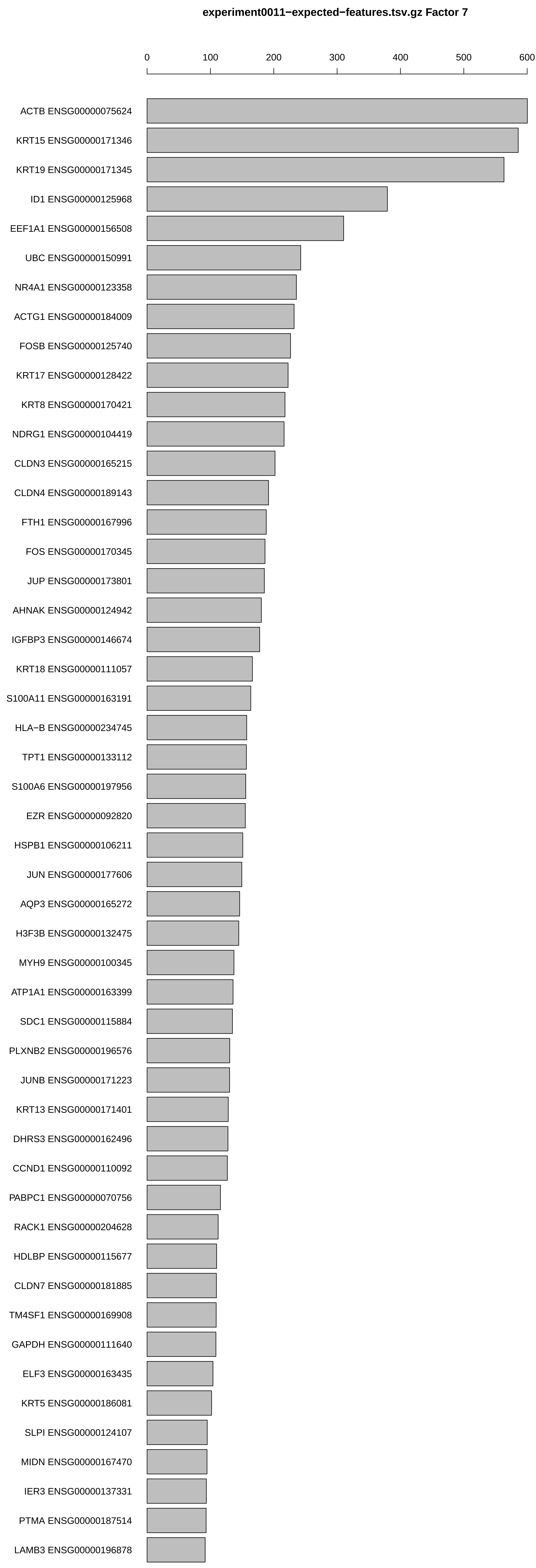
experiment0011−expected−features.tsv.gz Factor 7
0
100
200
300
400
500
600
ACTB ENSG00000075624
KRT15 ENSG00000171346
KRT19 ENSG00000171345
ID1 ENSG00000125968
EEF1A1 ENSG00000156508
UBC ENSG00000150991
NR4A1 ENSG00000123358
ACTG1 ENSG00000184009
FOSB ENSG00000125740
KRT17 ENSG00000128422
KRT8 ENSG00000170421
NDRG1 ENSG00000104419
CLDN3 ENSG00000165215
CLDN4 ENSG00000189143
FTH1 ENSG00000167996
FOS ENSG00000170345
JUP ENSG00000173801
AHNAK ENSG00000124942
IGFBP3 ENSG00000146674
KRT18 ENSG00000111057
S100A11 ENSG00000163191
HLA−B ENSG00000234745
TPT1 ENSG00000133112
S100A6 ENSG00000197956
EZR ENSG00000092820
HSPB1 ENSG00000106211
JUN ENSG00000177606
AQP3 ENSG00000165272
H3F3B ENSG00000132475
MYH9 ENSG00000100345
ATP1A1 ENSG00000163399
SDC1 ENSG00000115884
PLXNB2 ENSG00000196576
JUNB ENSG00000171223
KRT13 ENSG00000171401
DHRS3 ENSG00000162496
CCND1 ENSG00000110092
PABPC1 ENSG00000070756
RACK1 ENSG00000204628
HDLBP ENSG00000115677
CLDN7 ENSG00000181885
TM4SF1 ENSG00000169908
GAPDH ENSG00000111640
ELF3 ENSG00000163435
KRT5 ENSG00000186081
SLPI ENSG00000124107
MIDN ENSG00000167470
IER3 ENSG00000137331
PTMA ENSG00000187514
LAMB3 ENSG00000196878

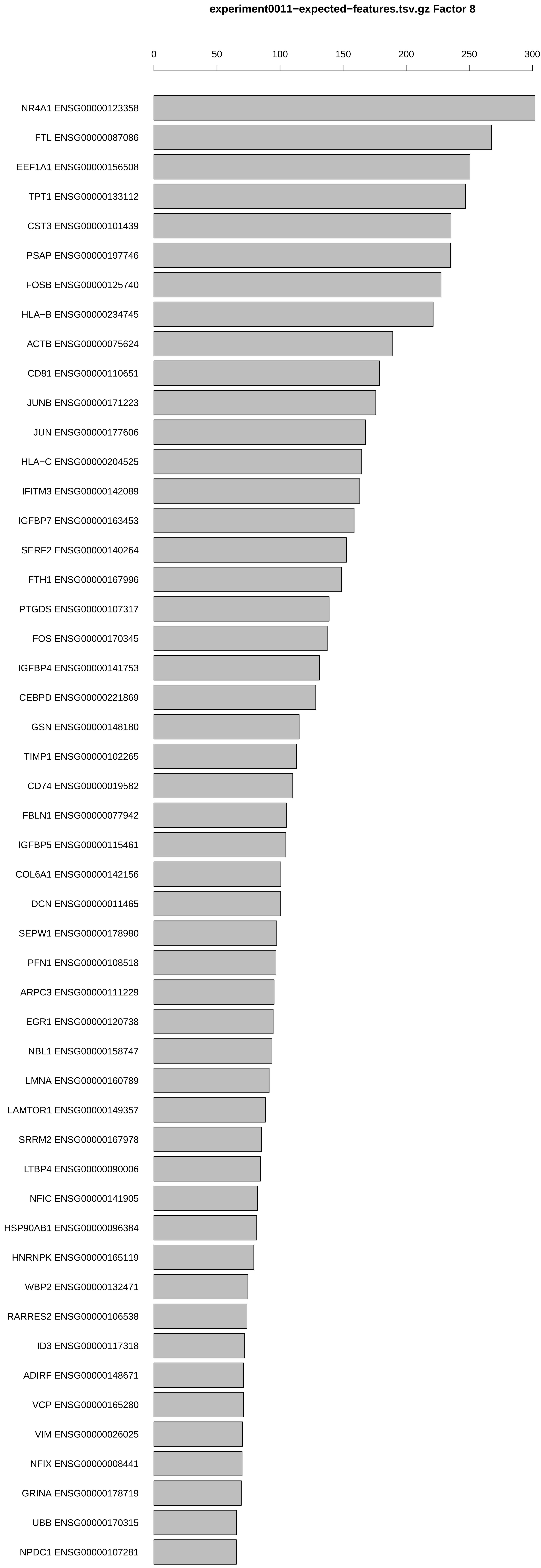

experiment0011−expected−features.tsv.gz Factor 8
0
50
100
150
200
250
300
NR4A1 ENSG00000123358
FTL ENSG00000087086
EEF1A1 ENSG00000156508
TPT1 ENSG00000133112
CST3 ENSG00000101439
PSAP ENSG00000197746
FOSB ENSG00000125740
HLA−B ENSG00000234745
ACTB ENSG00000075624
CD81 ENSG00000110651
JUNB ENSG00000171223
JUN ENSG00000177606
HLA−C ENSG00000204525
IFITM3 ENSG00000142089
IGFBP7 ENSG00000163453
SERF2 ENSG00000140264
FTH1 ENSG00000167996
PTGDS ENSG00000107317
FOS ENSG00000170345
IGFBP4 ENSG00000141753
CEBPD ENSG00000221869
GSN ENSG00000148180
TIMP1 ENSG00000102265
CD74 ENSG00000019582
FBLN1 ENSG00000077942
IGFBP5 ENSG00000115461
COL6A1 ENSG00000142156
DCN ENSG00000011465
SEPW1 ENSG00000178980
PFN1 ENSG00000108518
ARPC3 ENSG00000111229
EGR1 ENSG00000120738
NBL1 ENSG00000158747
LMNA ENSG00000160789
LAMTOR1 ENSG00000149357
SRRM2 ENSG00000167978
LTBP4 ENSG00000090006
NFIC ENSG00000141905
HSP90AB1 ENSG00000096384
HNRNPK ENSG00000165119
WBP2 ENSG00000132471
RARRES2 ENSG00000106538
ID3 ENSG00000117318
ADIRF ENSG00000148671
VCP ENSG00000165280
VIM ENSG00000026025
NFIX ENSG00000008441
GRINA ENSG00000178719
UBB ENSG00000170315
NPDC1 ENSG00000107281

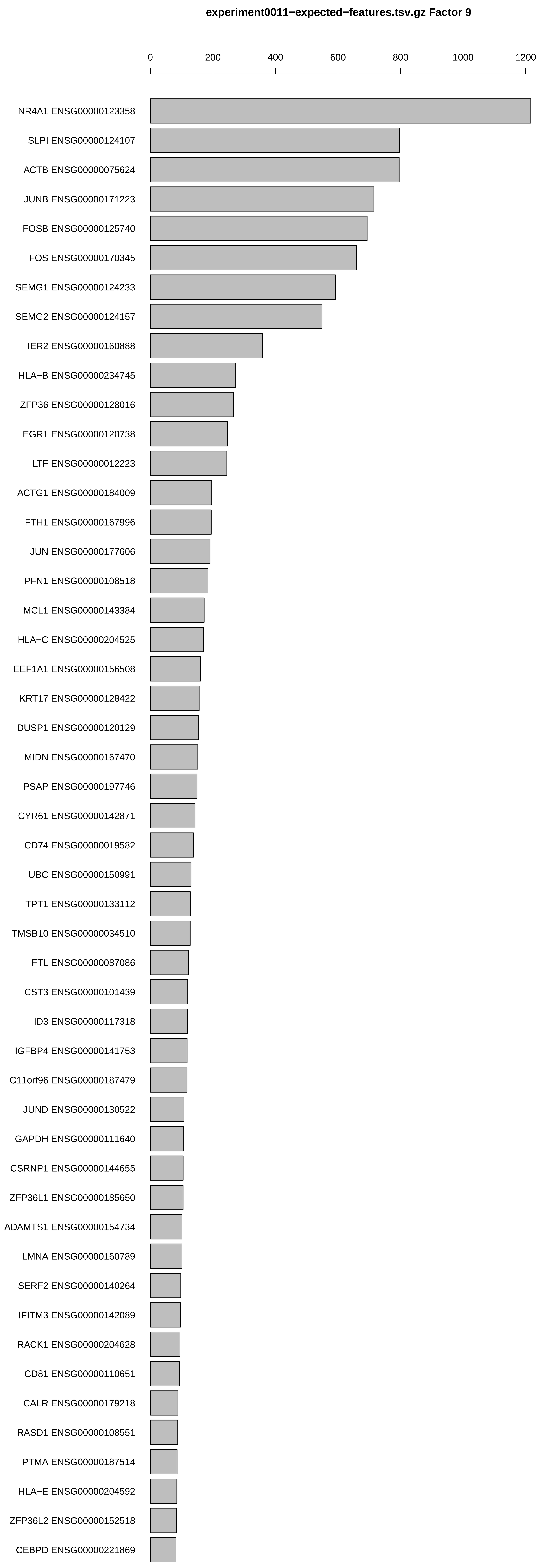
experiment0011−expected−features.tsv.gz Factor 9
0
200
400
600
800
1000
1200
NR4A1 ENSG00000123358
SLPI ENSG00000124107
ACTB ENSG00000075624
JUNB ENSG00000171223
FOSB ENSG00000125740
FOS ENSG00000170345
SEMG1 ENSG00000124233
SEMG2 ENSG00000124157
IER2 ENSG00000160888
HLA−B ENSG00000234745
ZFP36 ENSG00000128016
EGR1 ENSG00000120738
LTF ENSG00000012223
ACTG1 ENSG00000184009
FTH1 ENSG00000167996
JUN ENSG00000177606
PFN1 ENSG00000108518
MCL1 ENSG00000143384
HLA−C ENSG00000204525
EEF1A1 ENSG00000156508
KRT17 ENSG00000128422
DUSP1 ENSG00000120129
MIDN ENSG00000167470
PSAP ENSG00000197746
CYR61 ENSG00000142871
CD74 ENSG00000019582
UBC ENSG00000150991
TPT1 ENSG00000133112
TMSB10 ENSG00000034510
FTL ENSG00000087086
CST3 ENSG00000101439
ID3 ENSG00000117318
IGFBP4 ENSG00000141753
C11orf96 ENSG00000187479
JUND ENSG00000130522
GAPDH ENSG00000111640
CSRNP1 ENSG00000144655
ZFP36L1 ENSG00000185650
ADAMTS1 ENSG00000154734
LMNA ENSG00000160789
SERF2 ENSG00000140264
IFITM3 ENSG00000142089
RACK1 ENSG00000204628
CD81 ENSG00000110651
CALR ENSG00000179218
RASD1 ENSG00000108551
PTMA ENSG00000187514
HLA−E ENSG00000204592
ZFP36L2 ENSG00000152518
CEBPD ENSG00000221869

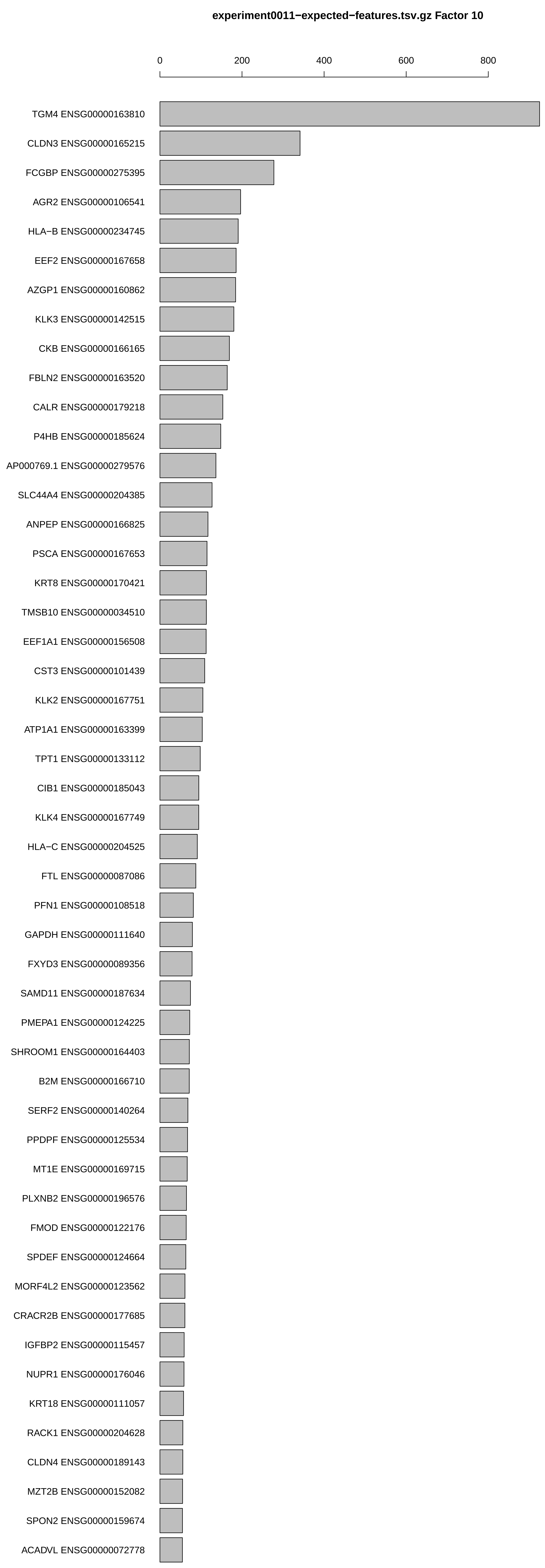
experiment0011−expected−features.tsv.gz Factor 10
0
200
400
600
800
TGM4 ENSG00000163810
CLDN3 ENSG00000165215
FCGBP ENSG00000275395
AGR2 ENSG00000106541
HLA−B ENSG00000234745
EEF2 ENSG00000167658
AZGP1 ENSG00000160862
KLK3 ENSG00000142515
CKB ENSG00000166165
FBLN2 ENSG00000163520
CALR ENSG00000179218
P4HB ENSG00000185624
AP000769.1 ENSG00000279576
SLC44A4 ENSG00000204385
ANPEP ENSG00000166825
PSCA ENSG00000167653
KRT8 ENSG00000170421
TMSB10 ENSG00000034510
EEF1A1 ENSG00000156508
CST3 ENSG00000101439
KLK2 ENSG00000167751
ATP1A1 ENSG00000163399
TPT1 ENSG00000133112
CIB1 ENSG00000185043
KLK4 ENSG00000167749
HLA−C ENSG00000204525
FTL ENSG00000087086
PFN1 ENSG00000108518
GAPDH ENSG00000111640
FXYD3 ENSG00000089356
SAMD11 ENSG00000187634
PMEPA1 ENSG00000124225
SHROOM1 ENSG00000164403
B2M ENSG00000166710
SERF2 ENSG00000140264
PPDPF ENSG00000125534
MT1E ENSG00000169715
PLXNB2 ENSG00000196576
FMOD ENSG00000122176
SPDEF ENSG00000124664
MORF4L2 ENSG00000123562
CRACR2B ENSG00000177685
IGFBP2 ENSG00000115457
NUPR1 ENSG00000176046
KRT18 ENSG00000111057
RACK1 ENSG00000204628
CLDN4 ENSG00000189143
MZT2B ENSG00000152082
SPON2 ENSG00000159674
ACADVL ENSG00000072778
