# Supplementary material for: Spatial maps of prostate cancer transcriptomes reveal an unexplored landscape of heterogeneity
Source: Nat Commun. 2018 Jun 20;9:2419. doi: 10.1038/s41467-018-04724-5 (PMC6010471; doi:10.1038/s41467-018-04724-5)
Supplement: Supplementary file 10 — Supplementary Data 7 [file 41467_2018_4724_MOESM10_ESM.zip › Supplementary Dataset 4/top-genes.pdf]

expected−features.tsv.gz Factor 1

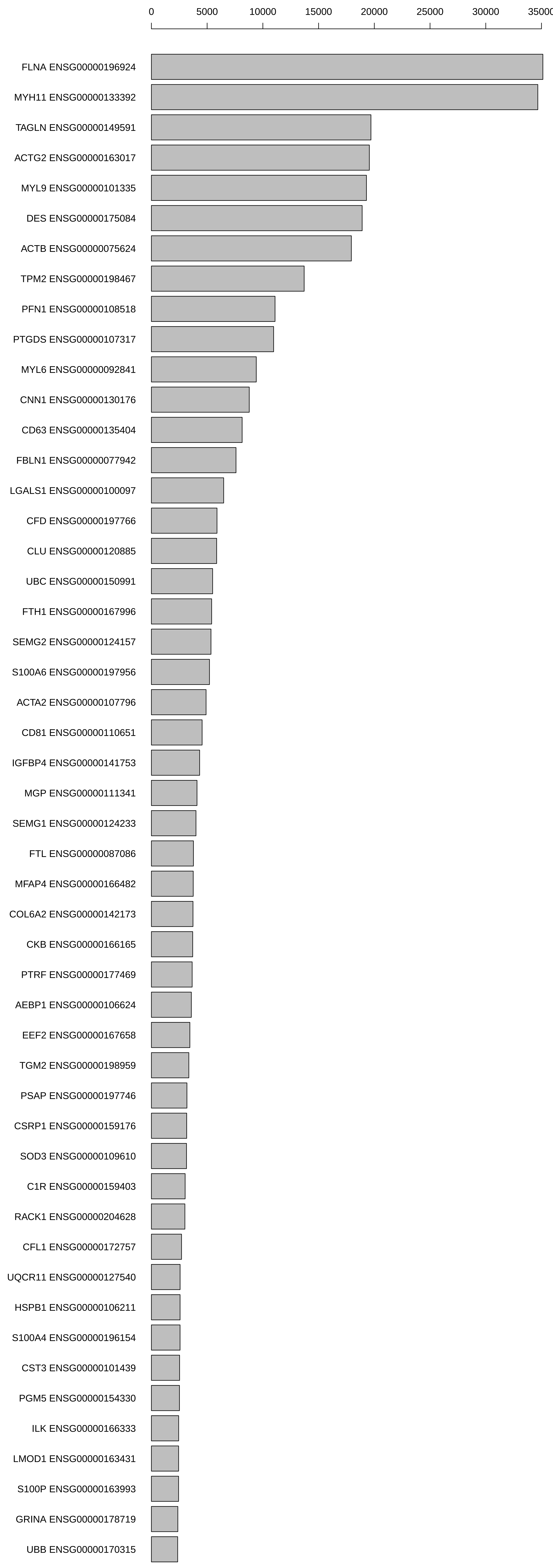

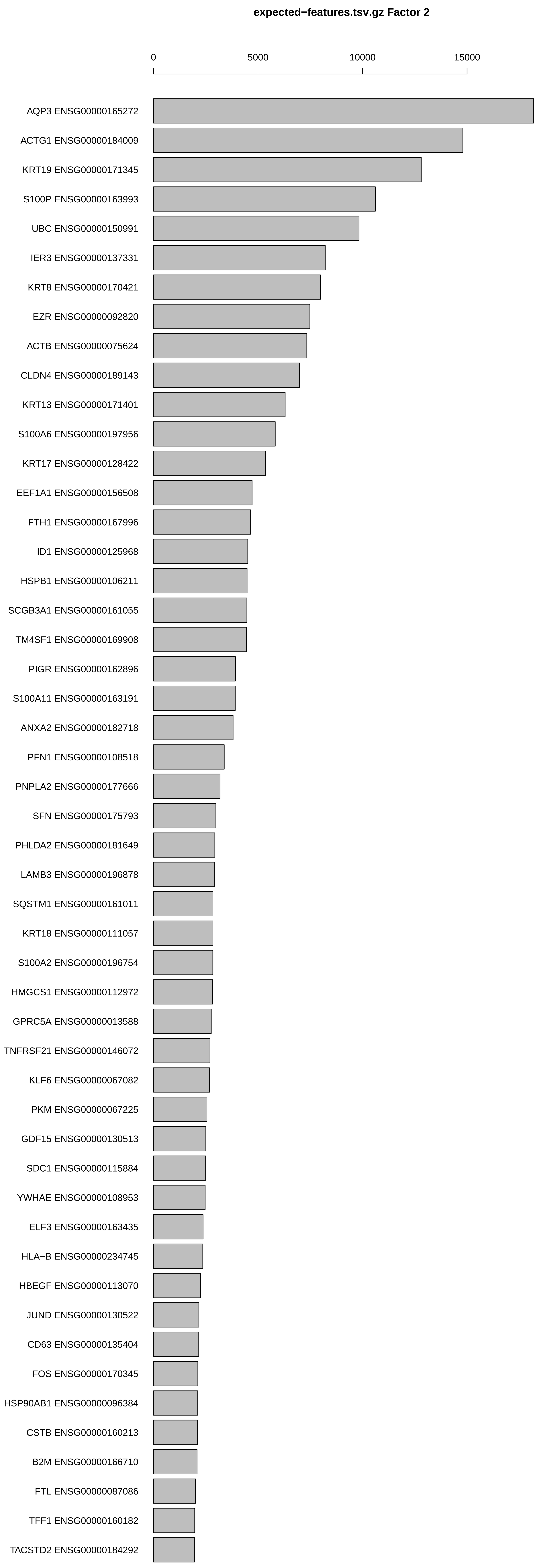

expected−features.tsv.gz Factor 2
0
5000
10000
15000
AQP3 ENSG00000165272
ACTG1 ENSG00000184009
KRT19 ENSG00000171345
S100P ENSG00000163993
UBC ENSG00000150991
IER3 ENSG00000137331
KRT8 ENSG00000170421
EZR ENSG00000092820
ACTB ENSG00000075624
CLDN4 ENSG00000189143
KRT13 ENSG00000171401
S100A6 ENSG00000197956
KRT17 ENSG00000128422
EEF1A1 ENSG00000156508
FTH1 ENSG00000167996
ID1 ENSG00000125968
HSPB1 ENSG00000106211
SCGB3A1 ENSG00000161055
TM4SF1 ENSG00000169908
PIGR ENSG00000162896
S100A11 ENSG00000163191
ANXA2 ENSG00000182718
PFN1 ENSG00000108518
PNPLA2 ENSG00000177666
SFN ENSG00000175793
PHLDA2 ENSG00000181649
LAMB3 ENSG00000196878
SQSTM1 ENSG00000161011
KRT18 ENSG00000111057
S100A2 ENSG00000196754
HMGCS1 ENSG00000112972
GPRC5A ENSG00000013588
TNFRSF21 ENSG00000146072
KLF6 ENSG00000067082
PKM ENSG00000067225
GDF15 ENSG00000130513
SDC1 ENSG00000115884
YWHAE ENSG00000108953
ELF3 ENSG00000163435
HLA−B ENSG00000234745
HBEGF ENSG00000113070
JUND ENSG00000130522
CD63 ENSG00000135404
FOS ENSG00000170345
HSP90AB1 ENSG00000096384
CSTB ENSG00000160213
B2M ENSG00000166710
FTL ENSG00000087086
TFF1 ENSG00000160182
TACSTD2 ENSG00000184292

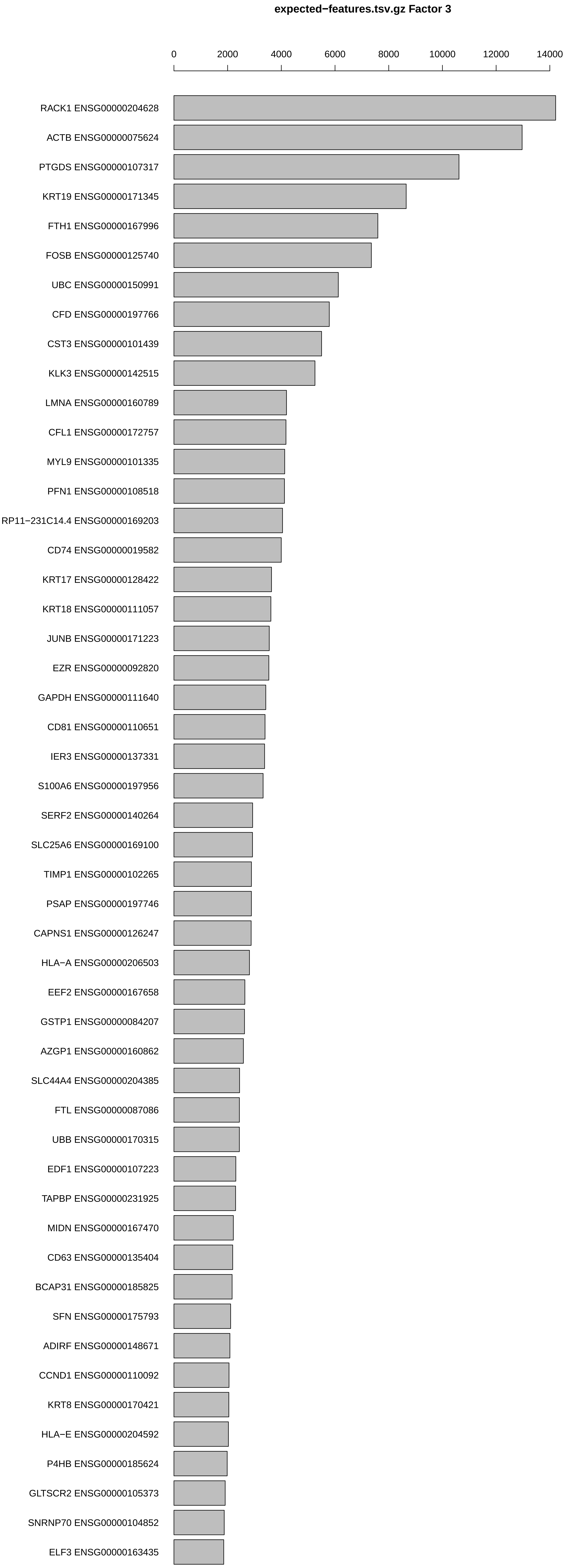
expected−features.tsv.gz Factor 3
0
2000
4000
6000
8000
10000
12000
14000
RACK1 ENSG00000204628
ACTB ENSG00000075624
PTGDS ENSG00000107317
KRT19 ENSG00000171345
FTH1 ENSG00000167996
FOSB ENSG00000125740
UBC ENSG00000150991
CFD ENSG00000197766
CST3 ENSG00000101439
KLK3 ENSG00000142515
LMNA ENSG00000160789
CFL1 ENSG00000172757
MYL9 ENSG00000101335
PFN1 ENSG00000108518
RP11−231C14.4 ENSG00000169203
CD74 ENSG00000019582
KRT17 ENSG00000128422
KRT18 ENSG00000111057
JUNB ENSG00000171223
EZR ENSG00000092820
GAPDH ENSG00000111640
CD81 ENSG00000110651
IER3 ENSG00000137331
S100A6 ENSG00000197956
SERF2 ENSG00000140264
SLC25A6 ENSG00000169100
TIMP1 ENSG00000102265
PSAP ENSG00000197746
CAPNS1 ENSG00000126247
HLA−A ENSG00000206503
EEF2 ENSG00000167658
GSTP1 ENSG00000084207
AZGP1 ENSG00000160862
SLC44A4 ENSG00000204385
FTL ENSG00000087086
UBB ENSG00000170315
EDF1 ENSG00000107223
TAPBP ENSG00000231925
MIDN ENSG00000167470
CD63 ENSG00000135404
BCAP31 ENSG00000185825
SFN ENSG00000175793
ADIRF ENSG00000148671
CCND1 ENSG00000110092
KRT8 ENSG00000170421
HLA−E ENSG00000204592
P4HB ENSG00000185624
GLTSCR2 ENSG00000105373
SNRNP70 ENSG00000104852
ELF3 ENSG00000163435

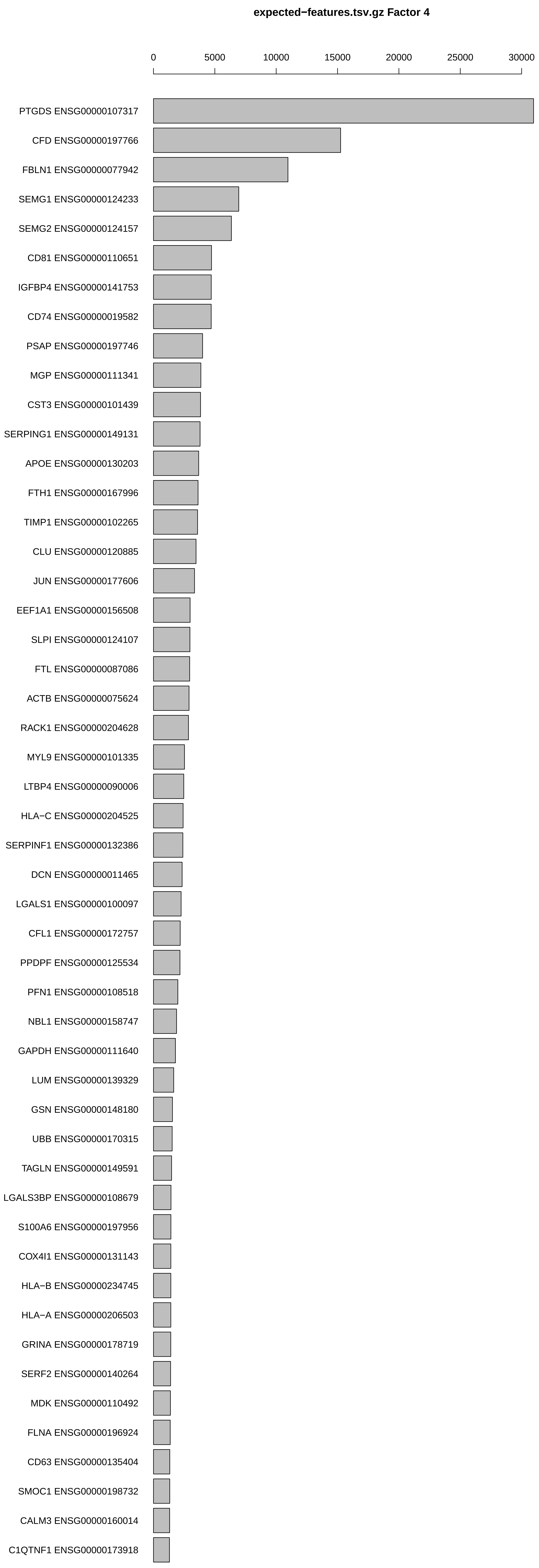
expected−features.tsv.gz Factor 4
0
5000
10000
15000
20000
25000
30000
PTGDS ENSG00000107317
CFD ENSG00000197766
FBLN1 ENSG00000077942
SEMG1 ENSG00000124233
SEMG2 ENSG00000124157
CD81 ENSG00000110651
IGFBP4 ENSG00000141753
CD74 ENSG00000019582
PSAP ENSG00000197746
MGP ENSG00000111341
CST3 ENSG00000101439
SERPING1 ENSG00000149131
APOE ENSG00000130203
FTH1 ENSG00000167996
TIMP1 ENSG00000102265
CLU ENSG00000120885
JUN ENSG00000177606
EEF1A1 ENSG00000156508
SLPI ENSG00000124107
FTL ENSG00000087086
ACTB ENSG00000075624
RACK1 ENSG00000204628
MYL9 ENSG00000101335
LTBP4 ENSG00000090006
HLA−C ENSG00000204525
SERPINF1 ENSG00000132386
DCN ENSG00000011465
LGALS1 ENSG00000100097
CFL1 ENSG00000172757
PPDPF ENSG00000125534
PFN1 ENSG00000108518
NBL1 ENSG00000158747
GAPDH ENSG00000111640
LUM ENSG00000139329
GSN ENSG00000148180
UBB ENSG00000170315
TAGLN ENSG00000149591
LGALS3BP ENSG00000108679
S100A6 ENSG00000197956
COX4I1 ENSG00000131143
HLA−B ENSG00000234745
HLA−A ENSG00000206503
GRINA ENSG00000178719
SERF2 ENSG00000140264
MDK ENSG00000110492
FLNA ENSG00000196924
CD63 ENSG00000135404
SMOC1 ENSG00000198732
CALM3 ENSG00000160014
C1QTNF1 ENSG00000173918

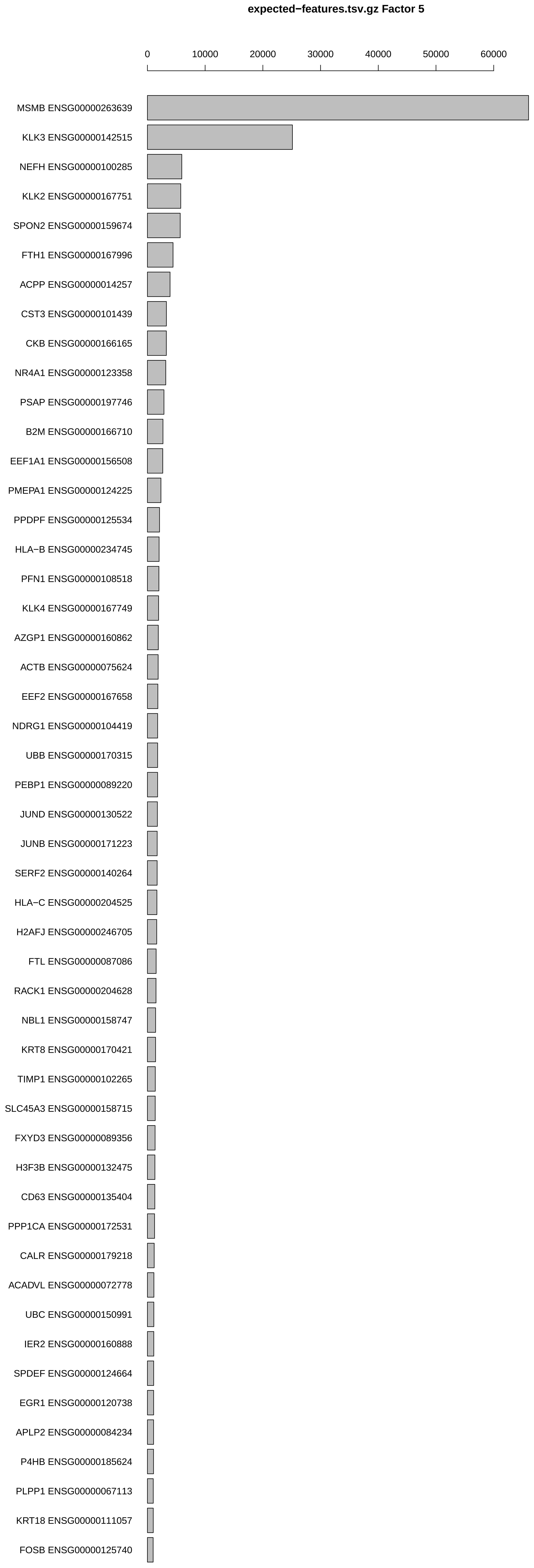

expected−features.tsv.gz Factor 5
0
10000
20000
30000
40000
50000
60000
MSMB ENSG00000263639
KLK3 ENSG00000142515
NEFH ENSG00000100285
KLK2 ENSG00000167751
SPON2 ENSG00000159674
FTH1 ENSG00000167996
ACPP ENSG00000014257
CST3 ENSG00000101439
CKB ENSG00000166165
NR4A1 ENSG00000123358
PSAP ENSG00000197746
B2M ENSG00000166710
EEF1A1 ENSG00000156508
PMEPA1 ENSG00000124225
PPDPF ENSG00000125534
HLA−B ENSG00000234745
PFN1 ENSG00000108518
KLK4 ENSG00000167749
AZGP1 ENSG00000160862
ACTB ENSG00000075624
EEF2 ENSG00000167658
NDRG1 ENSG00000104419
UBB ENSG00000170315
PEBP1 ENSG00000089220
JUND ENSG00000130522
JUNB ENSG00000171223
SERF2 ENSG00000140264
HLA−C ENSG00000204525
H2AFJ ENSG00000246705
FTL ENSG00000087086
RACK1 ENSG00000204628
NBL1 ENSG00000158747
KRT8 ENSG00000170421
TIMP1 ENSG00000102265
SLC45A3 ENSG00000158715
FXYD3 ENSG00000089356
H3F3B ENSG00000132475
CD63 ENSG00000135404
PPP1CA ENSG00000172531
CALR ENSG00000179218
ACADVL ENSG00000072778
UBC ENSG00000150991
IER2 ENSG00000160888
SPDEF ENSG00000124664
EGR1 ENSG00000120738
APLP2 ENSG00000084234
P4HB ENSG00000185624
PLPP1 ENSG00000067113
KRT18 ENSG00000111057
FOSB ENSG00000125740

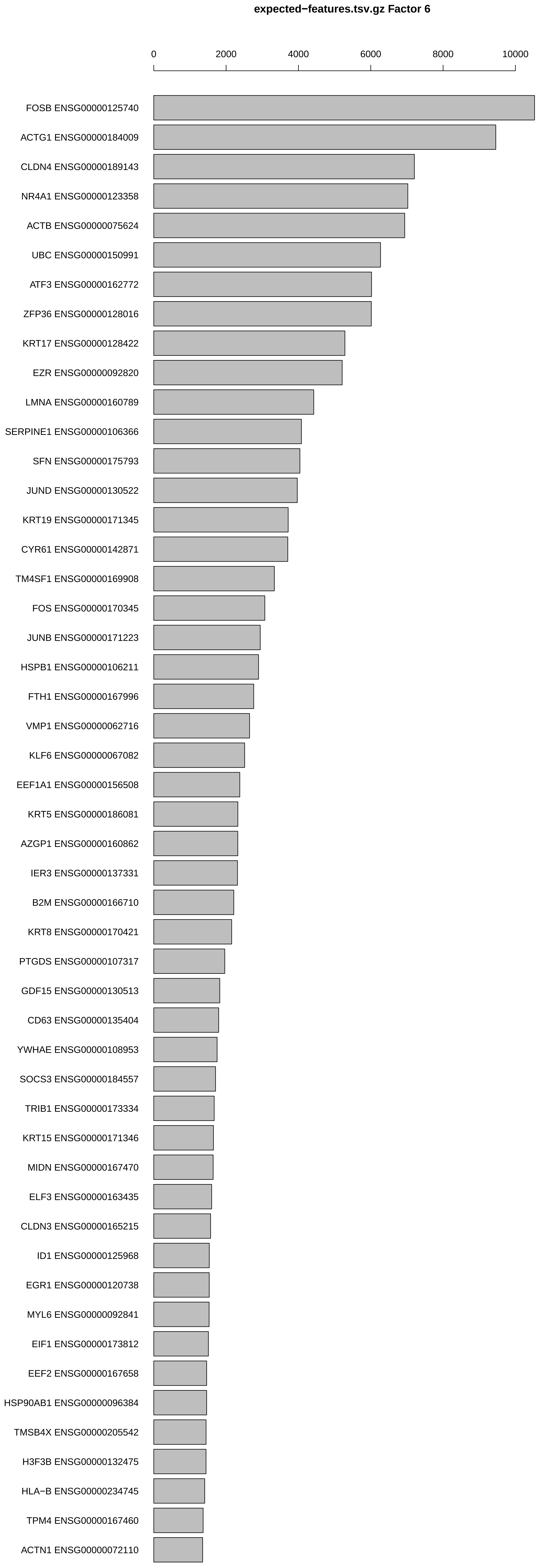
expected−features.tsv.gz Factor 6
0
2000
4000
6000
8000
10000
FOSB ENSG00000125740
ACTG1 ENSG00000184009
CLDN4 ENSG00000189143
NR4A1 ENSG00000123358
ACTB ENSG00000075624
UBC ENSG00000150991
ATF3 ENSG00000162772
ZFP36 ENSG00000128016
KRT17 ENSG00000128422
EZR ENSG00000092820
LMNA ENSG00000160789
SERPINE1 ENSG00000106366
SFN ENSG00000175793
JUND ENSG00000130522
KRT19 ENSG00000171345
CYR61 ENSG00000142871
TM4SF1 ENSG00000169908
FOS ENSG00000170345
JUNB ENSG00000171223
HSPB1 ENSG00000106211
FTH1 ENSG00000167996
VMP1 ENSG00000062716
KLF6 ENSG00000067082
EEF1A1 ENSG00000156508
KRT5 ENSG00000186081
AZGP1 ENSG00000160862
IER3 ENSG00000137331
B2M ENSG00000166710
KRT8 ENSG00000170421
PTGDS ENSG00000107317
GDF15 ENSG00000130513
CD63 ENSG00000135404
YWHAE ENSG00000108953
SOCS3 ENSG00000184557
TRIB1 ENSG00000173334
KRT15 ENSG00000171346
MIDN ENSG00000167470
ELF3 ENSG00000163435
CLDN3 ENSG00000165215
ID1 ENSG00000125968
EGR1 ENSG00000120738
MYL6 ENSG00000092841
EIF1 ENSG00000173812
EEF2 ENSG00000167658
HSP90AB1 ENSG00000096384
TMSB4X ENSG00000205542
H3F3B ENSG00000132475
HLA−B ENSG00000234745
TPM4 ENSG00000167460
ACTN1 ENSG00000072110

expected−features.tsv.gz Factor 7

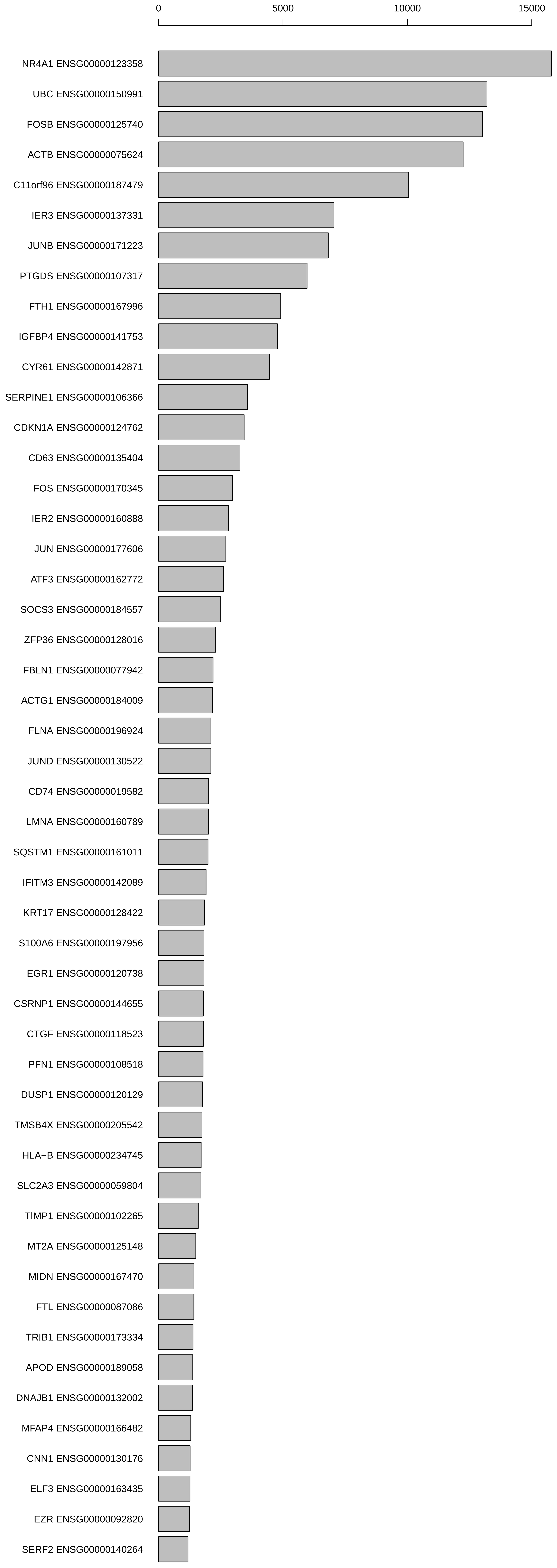

expected−features.tsv.gz Factor 8

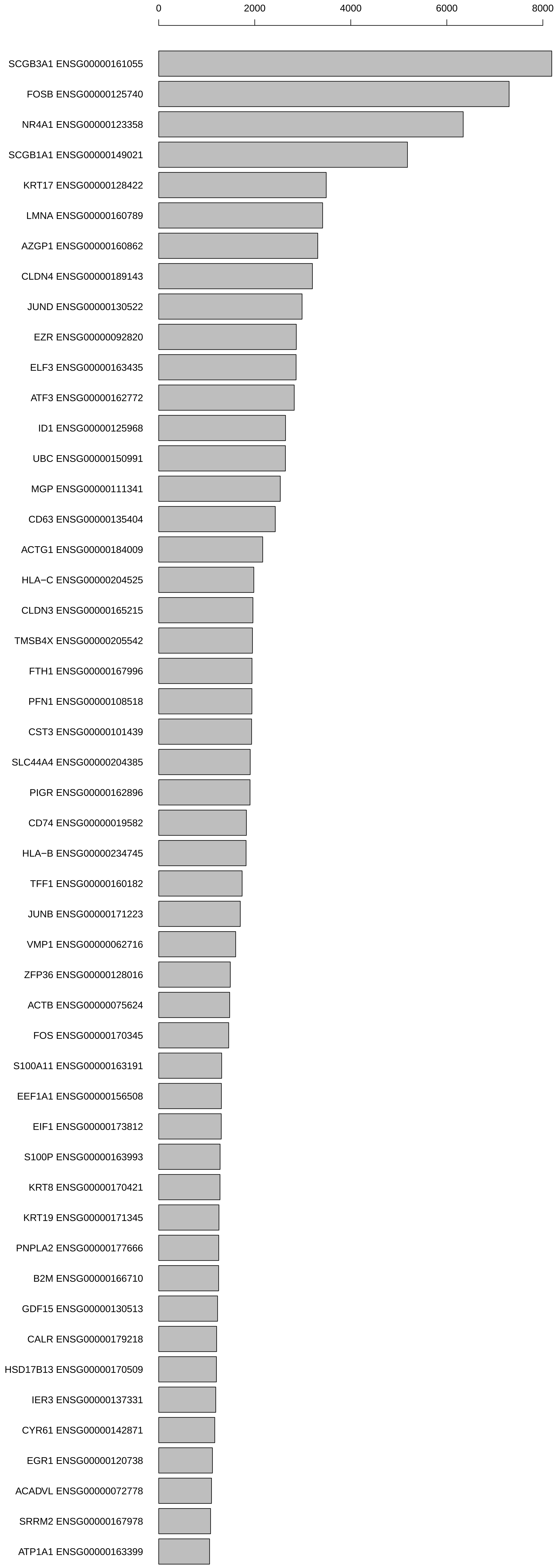

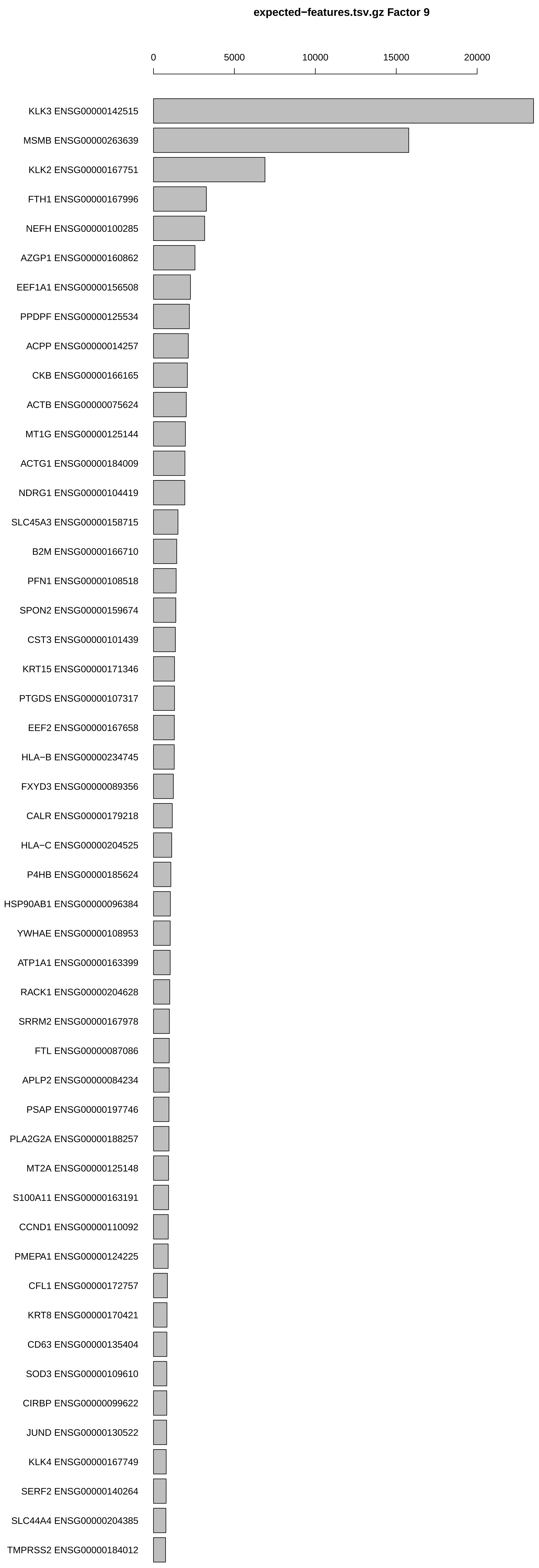
expected−features.tsv.gz Factor 9
0
5000
10000
15000
20000
KLK3 ENSG00000142515
MSMB ENSG00000263639
KLK2 ENSG00000167751
FTH1 ENSG00000167996
NEFH ENSG00000100285
AZGP1 ENSG00000160862
EEF1A1 ENSG00000156508
PPDPF ENSG00000125534
ACPP ENSG00000014257
CKB ENSG00000166165
ACTB ENSG00000075624
MT1G ENSG00000125144
ACTG1 ENSG00000184009
NDRG1 ENSG00000104419
SLC45A3 ENSG00000158715
B2M ENSG00000166710
PFN1 ENSG00000108518
SPON2 ENSG00000159674
CST3 ENSG00000101439
KRT15 ENSG00000171346
PTGDS ENSG00000107317
EEF2 ENSG00000167658
HLA−B ENSG00000234745
FXYD3 ENSG00000089356
CALR ENSG00000179218
HLA−C ENSG00000204525
P4HB ENSG00000185624
HSP90AB1 ENSG00000096384
YWHAE ENSG00000108953
ATP1A1 ENSG00000163399
RACK1 ENSG00000204628
SRRM2 ENSG00000167978
FTL ENSG00000087086
APLP2 ENSG00000084234
PSAP ENSG00000197746
PLA2G2A ENSG00000188257
MT2A ENSG00000125148
S100A11 ENSG00000163191
CCND1 ENSG00000110092
PMEPA1 ENSG00000124225
CFL1 ENSG00000172757
KRT8 ENSG00000170421
CD63 ENSG00000135404
SOD3 ENSG00000109610
CIRBP ENSG00000099622
JUND ENSG00000130522
KLK4 ENSG00000167749
SERF2 ENSG00000140264
SLC44A4 ENSG00000204385
TMPRSS2 ENSG00000184012

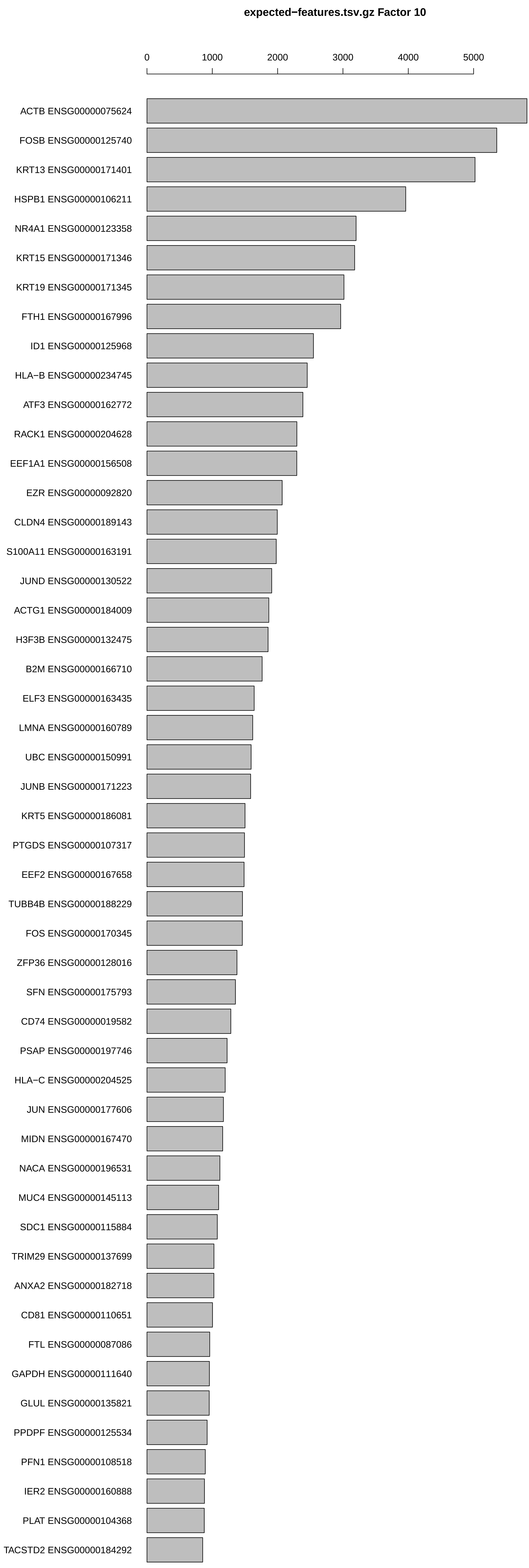

expected−features.tsv.gz Factor 10
0
1000
2000
3000
4000
5000
ACTB ENSG00000075624
FOSB ENSG00000125740
KRT13 ENSG00000171401
HSPB1 ENSG00000106211
NR4A1 ENSG00000123358
KRT15 ENSG00000171346
KRT19 ENSG00000171345
FTH1 ENSG00000167996
ID1 ENSG00000125968
HLA−B ENSG00000234745
ATF3 ENSG00000162772
RACK1 ENSG00000204628
EEF1A1 ENSG00000156508
EZR ENSG00000092820
CLDN4 ENSG00000189143
S100A11 ENSG00000163191
JUND ENSG00000130522
ACTG1 ENSG00000184009
H3F3B ENSG00000132475
B2M ENSG00000166710
ELF3 ENSG00000163435
LMNA ENSG00000160789
UBC ENSG00000150991
JUNB ENSG00000171223
KRT5 ENSG00000186081
PTGDS ENSG00000107317
EEF2 ENSG00000167658
TUBB4B ENSG00000188229
FOS ENSG00000170345
ZFP36 ENSG00000128016
SFN ENSG00000175793
CD74 ENSG00000019582
PSAP ENSG00000197746
HLA−C ENSG00000204525
JUN ENSG00000177606
MIDN ENSG00000167470
NACA ENSG00000196531
MUC4 ENSG00000145113
SDC1 ENSG00000115884
TRIM29 ENSG00000137699
ANXA2 ENSG00000182718
CD81 ENSG00000110651
FTL ENSG00000087086
GAPDH ENSG00000111640
GLUL ENSG00000135821
PPDPF ENSG00000125534
PFN1 ENSG00000108518
IER2 ENSG00000160888
PLAT ENSG00000104368
TACSTD2 ENSG00000184292

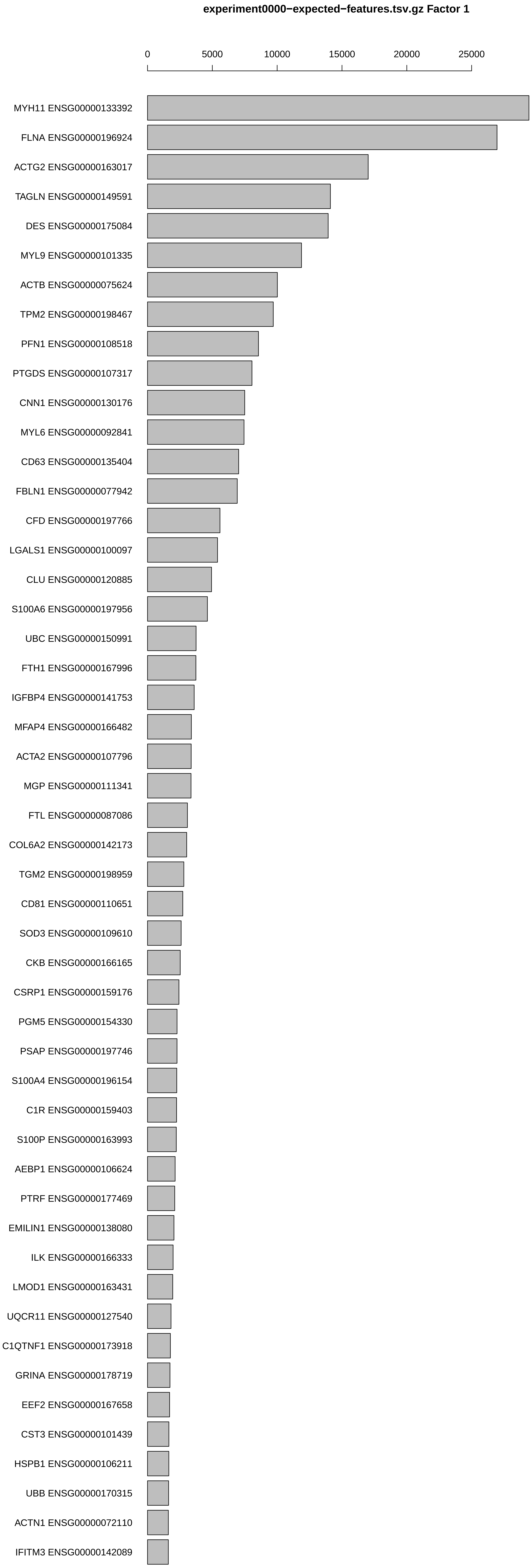
experiment0000−expected−features.tsv.gz Factor 1
0
5000
10000
15000
20000
25000
MYH11 ENSG00000133392
FLNA ENSG00000196924
ACTG2 ENSG00000163017
TAGLN ENSG00000149591
DES ENSG00000175084
MYL9 ENSG00000101335
ACTB ENSG00000075624
TPM2 ENSG00000198467
PFN1 ENSG00000108518
PTGDS ENSG00000107317
CNN1 ENSG00000130176
MYL6 ENSG00000092841
CD63 ENSG00000135404
FBLN1 ENSG00000077942
CFD ENSG00000197766
LGALS1 ENSG00000100097
CLU ENSG00000120885
S100A6 ENSG00000197956
UBC ENSG00000150991
FTH1 ENSG00000167996
IGFBP4 ENSG00000141753
MFAP4 ENSG00000166482
ACTA2 ENSG00000107796
MGP ENSG00000111341
FTL ENSG00000087086
COL6A2 ENSG00000142173
TGM2 ENSG00000198959
CD81 ENSG00000110651
SOD3 ENSG00000109610
CKB ENSG00000166165
CSRP1 ENSG00000159176
PGM5 ENSG00000154330
PSAP ENSG00000197746
S100A4 ENSG00000196154
C1R ENSG00000159403
S100P ENSG00000163993
AEBP1 ENSG00000106624
PTRF ENSG00000177469
EMILIN1 ENSG00000138080
ILK ENSG00000166333
LMOD1 ENSG00000163431
UQCR11 ENSG00000127540
C1QTNF1 ENSG00000173918
GRINA ENSG00000178719
EEF2 ENSG00000167658
CST3 ENSG00000101439
HSPB1 ENSG00000106211
UBB ENSG00000170315
ACTN1 ENSG00000072110
IFITM3 ENSG00000142089

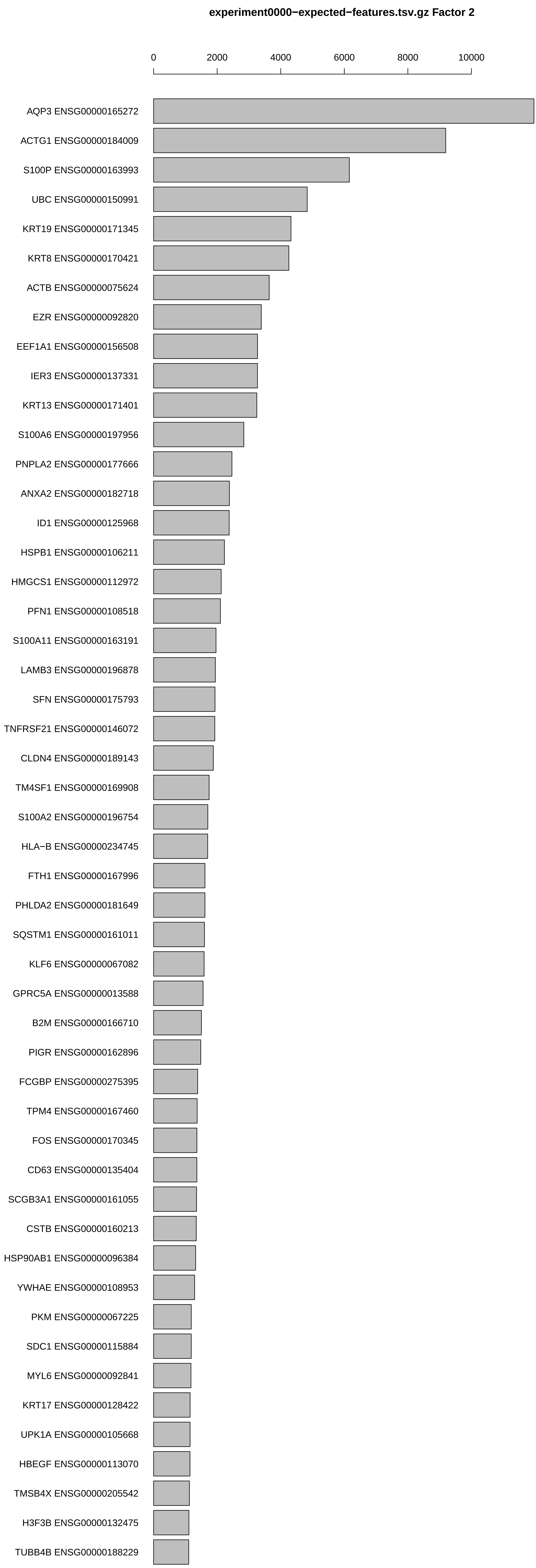
experiment0000−expected−features.tsv.gz Factor 2
0
2000
4000
6000
8000
10000
AQP3 ENSG00000165272
ACTG1 ENSG00000184009
S100P ENSG00000163993
UBC ENSG00000150991
KRT19 ENSG00000171345
KRT8 ENSG00000170421
ACTB ENSG00000075624
EZR ENSG00000092820
EEF1A1 ENSG00000156508
IER3 ENSG00000137331
KRT13 ENSG00000171401
S100A6 ENSG00000197956
PNPLA2 ENSG00000177666
ANXA2 ENSG00000182718
ID1 ENSG00000125968
HSPB1 ENSG00000106211
HMGCS1 ENSG00000112972
PFN1 ENSG00000108518
S100A11 ENSG00000163191
LAMB3 ENSG00000196878
SFN ENSG00000175793
TNFRSF21 ENSG00000146072
CLDN4 ENSG00000189143
TM4SF1 ENSG00000169908
S100A2 ENSG00000196754
HLA−B ENSG00000234745
FTH1 ENSG00000167996
PHLDA2 ENSG00000181649
SQSTM1 ENSG00000161011
KLF6 ENSG00000067082
GPRC5A ENSG00000013588
B2M ENSG00000166710
PIGR ENSG00000162896
FCGBP ENSG00000275395
TPM4 ENSG00000167460
FOS ENSG00000170345
CD63 ENSG00000135404
SCGB3A1 ENSG00000161055
CSTB ENSG00000160213
HSP90AB1 ENSG00000096384
YWHAE ENSG00000108953
PKM ENSG00000067225
SDC1 ENSG00000115884
MYL6 ENSG00000092841
KRT17 ENSG00000128422
UPK1A ENSG00000105668
HBEGF ENSG00000113070
TMSB4X ENSG00000205542
H3F3B ENSG00000132475
TUBB4B ENSG00000188229

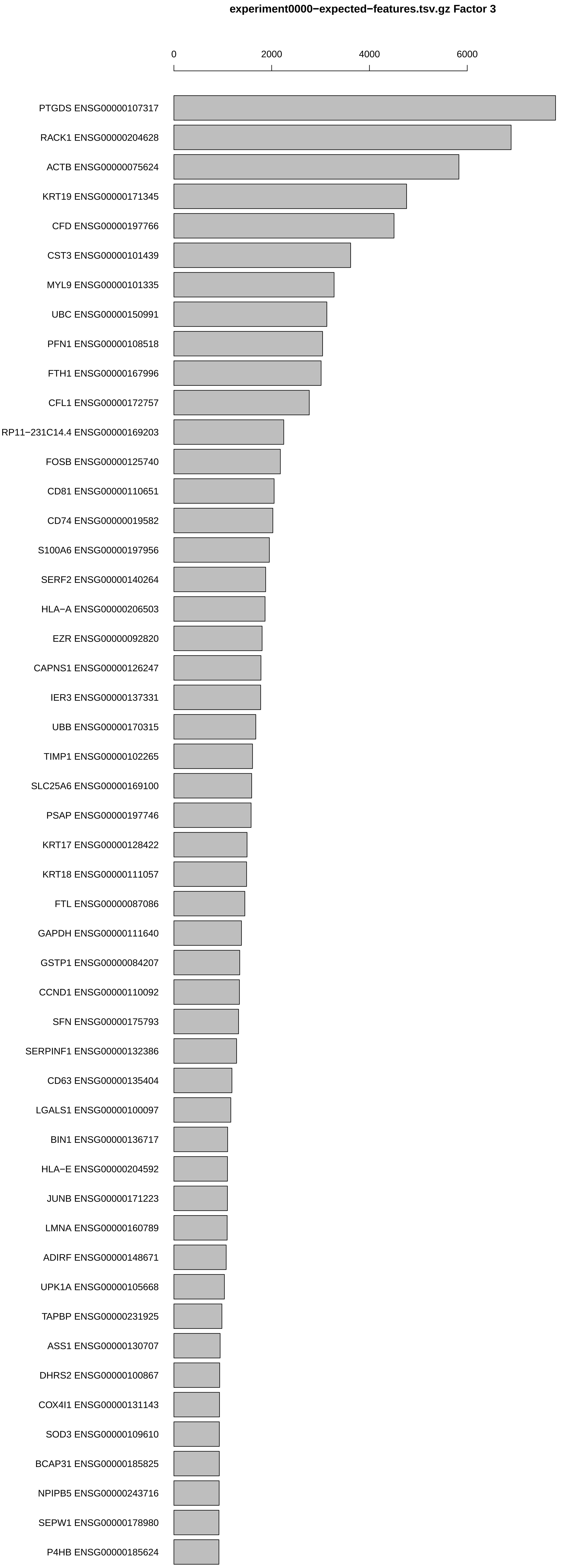
experiment0000−expected−features.tsv.gz Factor 3
0
2000
4000
6000
PTGDS ENSG00000107317
RACK1 ENSG00000204628
ACTB ENSG00000075624
KRT19 ENSG00000171345
CFD ENSG00000197766
CST3 ENSG00000101439
MYL9 ENSG00000101335
UBC ENSG00000150991
PFN1 ENSG00000108518
FTH1 ENSG00000167996
CFL1 ENSG00000172757
RP11−231C14.4 ENSG00000169203
FOSB ENSG00000125740
CD81 ENSG00000110651
CD74 ENSG00000019582
S100A6 ENSG00000197956
SERF2 ENSG00000140264
HLA−A ENSG00000206503
EZR ENSG00000092820
CAPNS1 ENSG00000126247
IER3 ENSG00000137331
UBB ENSG00000170315
TIMP1 ENSG00000102265
SLC25A6 ENSG00000169100
PSAP ENSG00000197746
KRT17 ENSG00000128422
KRT18 ENSG00000111057
FTL ENSG00000087086
GAPDH ENSG00000111640
GSTP1 ENSG00000084207
CCND1 ENSG00000110092
SFN ENSG00000175793
SERPINF1 ENSG00000132386
CD63 ENSG00000135404
LGALS1 ENSG00000100097
BIN1 ENSG00000136717
HLA−E ENSG00000204592
JUNB ENSG00000171223
LMNA ENSG00000160789
ADIRF ENSG00000148671
UPK1A ENSG00000105668
TAPBP ENSG00000231925
ASS1 ENSG00000130707
DHRS2 ENSG00000100867
COX4I1 ENSG00000131143
SOD3 ENSG00000109610
BCAP31 ENSG00000185825
NPIPB5 ENSG00000243716
SEPW1 ENSG00000178980
P4HB ENSG00000185624

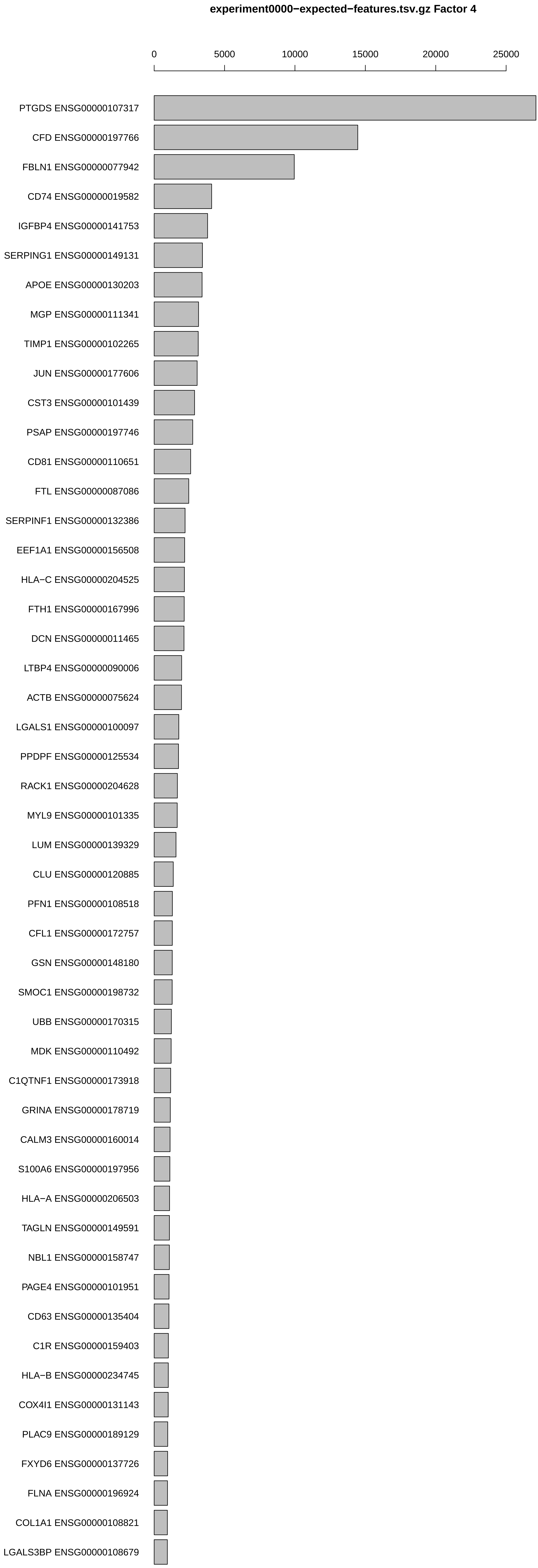
experiment0000−expected−features.tsv.gz Factor 4
0
5000
10000
15000
20000
25000
PTGDS ENSG00000107317
CFD ENSG00000197766
FBLN1 ENSG00000077942
CD74 ENSG00000019582
IGFBP4 ENSG00000141753
SERPING1 ENSG00000149131
APOE ENSG00000130203
MGP ENSG00000111341
TIMP1 ENSG00000102265
JUN ENSG00000177606
CST3 ENSG00000101439
PSAP ENSG00000197746
CD81 ENSG00000110651
FTL ENSG00000087086
SERPINF1 ENSG00000132386
EEF1A1 ENSG00000156508
HLA−C ENSG00000204525
FTH1 ENSG00000167996
DCN ENSG00000011465
LTBP4 ENSG00000090006
ACTB ENSG00000075624
LGALS1 ENSG00000100097
PPDPF ENSG00000125534
RACK1 ENSG00000204628
MYL9 ENSG00000101335
LUM ENSG00000139329
CLU ENSG00000120885
PFN1 ENSG00000108518
CFL1 ENSG00000172757
GSN ENSG00000148180
SMOC1 ENSG00000198732
UBB ENSG00000170315
MDK ENSG00000110492
C1QTNF1 ENSG00000173918
GRINA ENSG00000178719
CALM3 ENSG00000160014
S100A6 ENSG00000197956
HLA−A ENSG00000206503
TAGLN ENSG00000149591
NBL1 ENSG00000158747
PAGE4 ENSG00000101951
CD63 ENSG00000135404
C1R ENSG00000159403
HLA−B ENSG00000234745
COX4I1 ENSG00000131143
PLAC9 ENSG00000189129
FXYD6 ENSG00000137726
FLNA ENSG00000196924
COL1A1 ENSG00000108821
LGALS3BP ENSG00000108679

experiment0000−expected−features.tsv.gz Factor 5

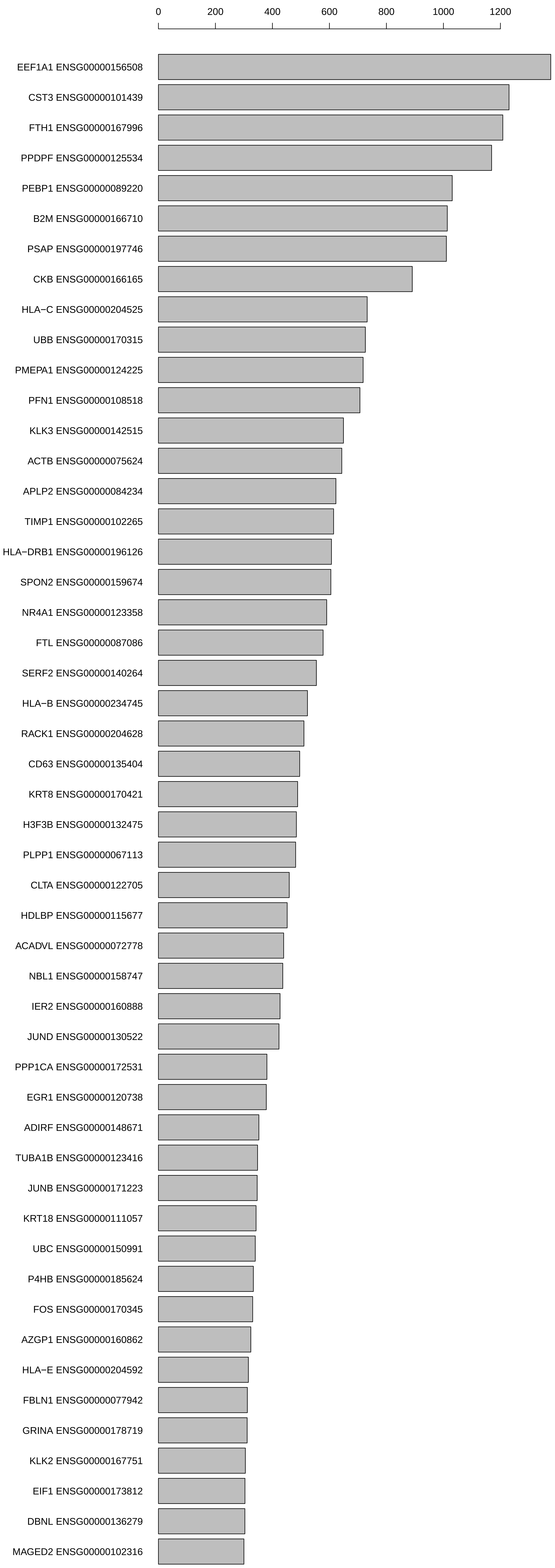

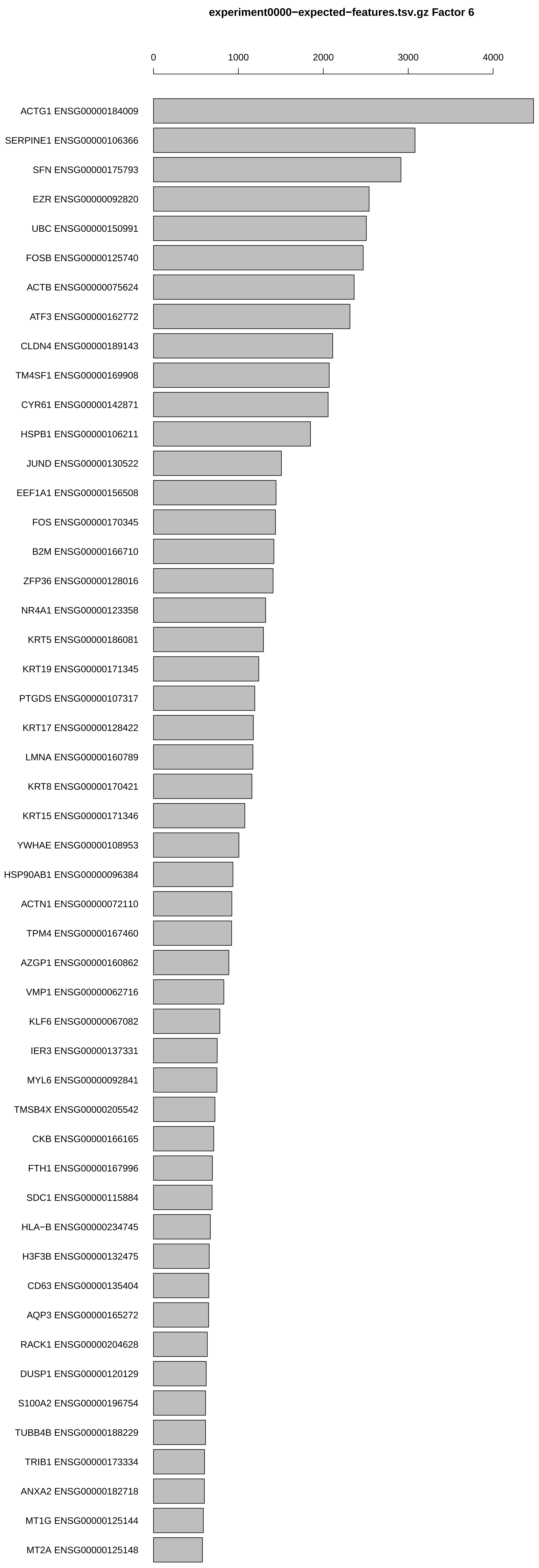
experiment0000−expected−features.tsv.gz Factor 6
0
1000
2000
3000
4000
ACTG1 ENSG00000184009
SERPINE1 ENSG00000106366
SFN ENSG00000175793
EZR ENSG00000092820
UBC ENSG00000150991
FOSB ENSG00000125740
ACTB ENSG00000075624
ATF3 ENSG00000162772
CLDN4 ENSG00000189143
TM4SF1 ENSG00000169908
CYR61 ENSG00000142871
HSPB1 ENSG00000106211
JUND ENSG00000130522
EEF1A1 ENSG00000156508
FOS ENSG00000170345
B2M ENSG00000166710
ZFP36 ENSG00000128016
NR4A1 ENSG00000123358
KRT5 ENSG00000186081
KRT19 ENSG00000171345
PTGDS ENSG00000107317
KRT17 ENSG00000128422
LMNA ENSG00000160789
KRT8 ENSG00000170421
KRT15 ENSG00000171346
YWHAE ENSG00000108953
HSP90AB1 ENSG00000096384
ACTN1 ENSG00000072110
TPM4 ENSG00000167460
AZGP1 ENSG00000160862
VMP1 ENSG00000062716
KLF6 ENSG00000067082
IER3 ENSG00000137331
MYL6 ENSG00000092841
TMSB4X ENSG00000205542
CKB ENSG00000166165
FTH1 ENSG00000167996
SDC1 ENSG00000115884
HLA−B ENSG00000234745
H3F3B ENSG00000132475
CD63 ENSG00000135404
AQP3 ENSG00000165272
RACK1 ENSG00000204628
DUSP1 ENSG00000120129
S100A2 ENSG00000196754
TUBB4B ENSG00000188229
TRIB1 ENSG00000173334
ANXA2 ENSG00000182718
MT1G ENSG00000125144
MT2A ENSG00000125148

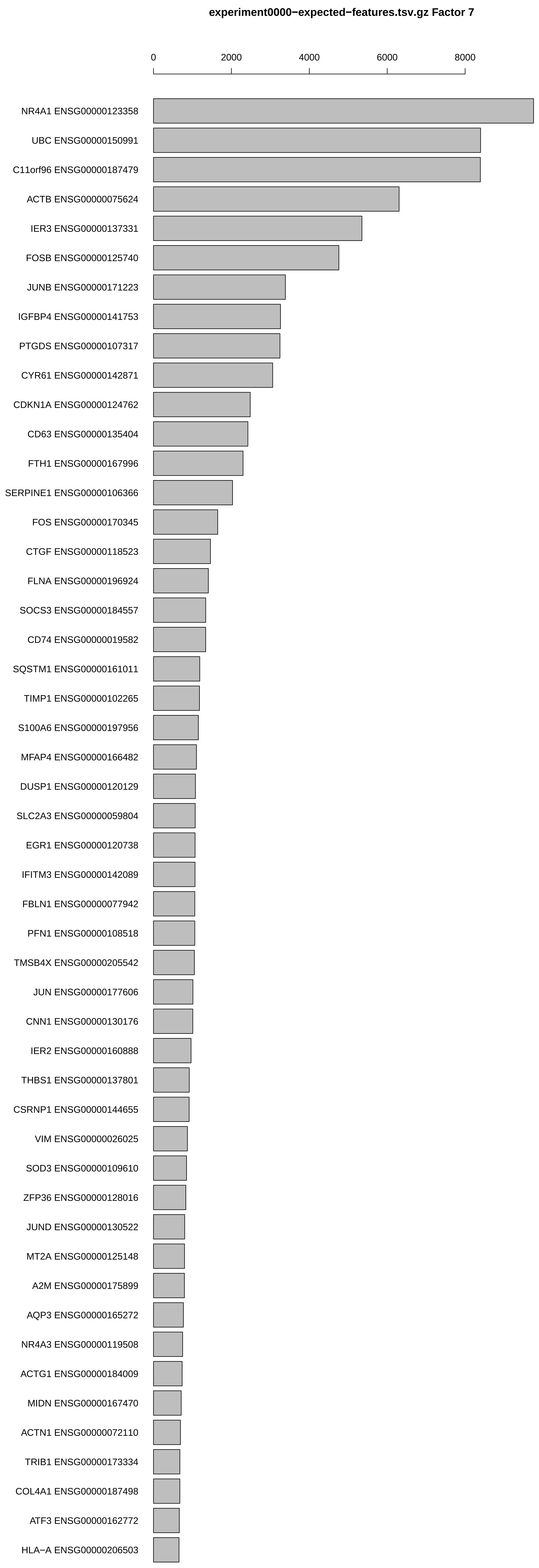
experiment0000−expected−features.tsv.gz Factor 7
0
2000
4000
6000
8000
NR4A1 ENSG00000123358
UBC ENSG00000150991
C11orf96 ENSG00000187479
ACTB ENSG00000075624
IER3 ENSG00000137331
FOSB ENSG00000125740
JUNB ENSG00000171223
IGFBP4 ENSG00000141753
PTGDS ENSG00000107317
CYR61 ENSG00000142871
CDKN1A ENSG00000124762
CD63 ENSG00000135404
FTH1 ENSG00000167996
SERPINE1 ENSG00000106366
FOS ENSG00000170345
CTGF ENSG00000118523
FLNA ENSG00000196924
SOCS3 ENSG00000184557
CD74 ENSG00000019582
SQSTM1 ENSG00000161011
TIMP1 ENSG00000102265
S100A6 ENSG00000197956
MFAP4 ENSG00000166482
DUSP1 ENSG00000120129
SLC2A3 ENSG00000059804
EGR1 ENSG00000120738
IFITM3 ENSG00000142089
FBLN1 ENSG00000077942
PFN1 ENSG00000108518
TMSB4X ENSG00000205542
JUN ENSG00000177606
CNN1 ENSG00000130176
IER2 ENSG00000160888
THBS1 ENSG00000137801
CSRNP1 ENSG00000144655
VIM ENSG00000026025
SOD3 ENSG00000109610
ZFP36 ENSG00000128016
JUND ENSG00000130522
MT2A ENSG00000125148
A2M ENSG00000175899
AQP3 ENSG00000165272
NR4A3 ENSG00000119508
ACTG1 ENSG00000184009
MIDN ENSG00000167470
ACTN1 ENSG00000072110
TRIB1 ENSG00000173334
COL4A1 ENSG00000187498
ATF3 ENSG00000162772
HLA−A ENSG00000206503

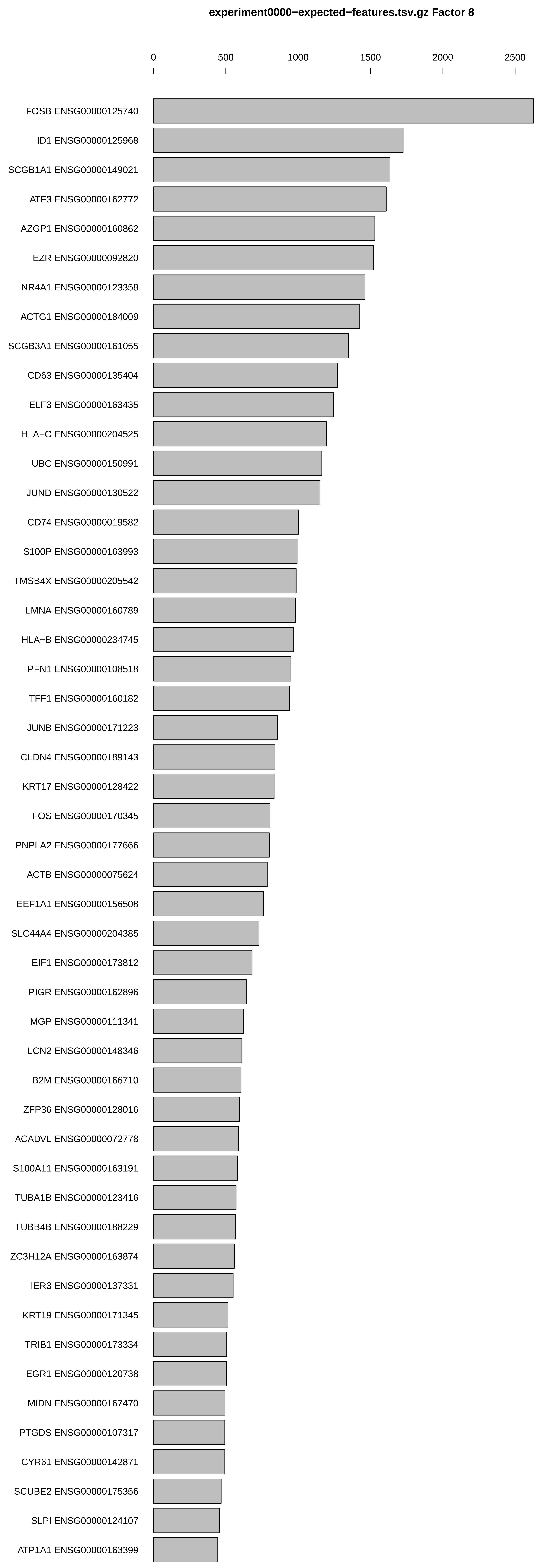
experiment0000−expected−features.tsv.gz Factor 8
0
500
1000
1500
2000
2500
FOSB ENSG00000125740
ID1 ENSG00000125968
SCGB1A1 ENSG00000149021
ATF3 ENSG00000162772
AZGP1 ENSG00000160862
EZR ENSG00000092820
NR4A1 ENSG00000123358
ACTG1 ENSG00000184009
SCGB3A1 ENSG00000161055
CD63 ENSG00000135404
ELF3 ENSG00000163435
HLA−C ENSG00000204525
UBC ENSG00000150991
JUND ENSG00000130522
CD74 ENSG00000019582
S100P ENSG00000163993
TMSB4X ENSG00000205542
LMNA ENSG00000160789
HLA−B ENSG00000234745
PFN1 ENSG00000108518
TFF1 ENSG00000160182
JUNB ENSG00000171223
CLDN4 ENSG00000189143
KRT17 ENSG00000128422
FOS ENSG00000170345
PNPLA2 ENSG00000177666
ACTB ENSG00000075624
EEF1A1 ENSG00000156508
SLC44A4 ENSG00000204385
EIF1 ENSG00000173812
PIGR ENSG00000162896
MGP ENSG00000111341
LCN2 ENSG00000148346
B2M ENSG00000166710
ZFP36 ENSG00000128016
ACADVL ENSG00000072778
S100A11 ENSG00000163191
TUBA1B ENSG00000123416
TUBB4B ENSG00000188229
ZC3H12A ENSG00000163874
IER3 ENSG00000137331
KRT19 ENSG00000171345
TRIB1 ENSG00000173334
EGR1 ENSG00000120738
MIDN ENSG00000167470
PTGDS ENSG00000107317
CYR61 ENSG00000142871
SCUBE2 ENSG00000175356
SLPI ENSG00000124107
ATP1A1 ENSG00000163399

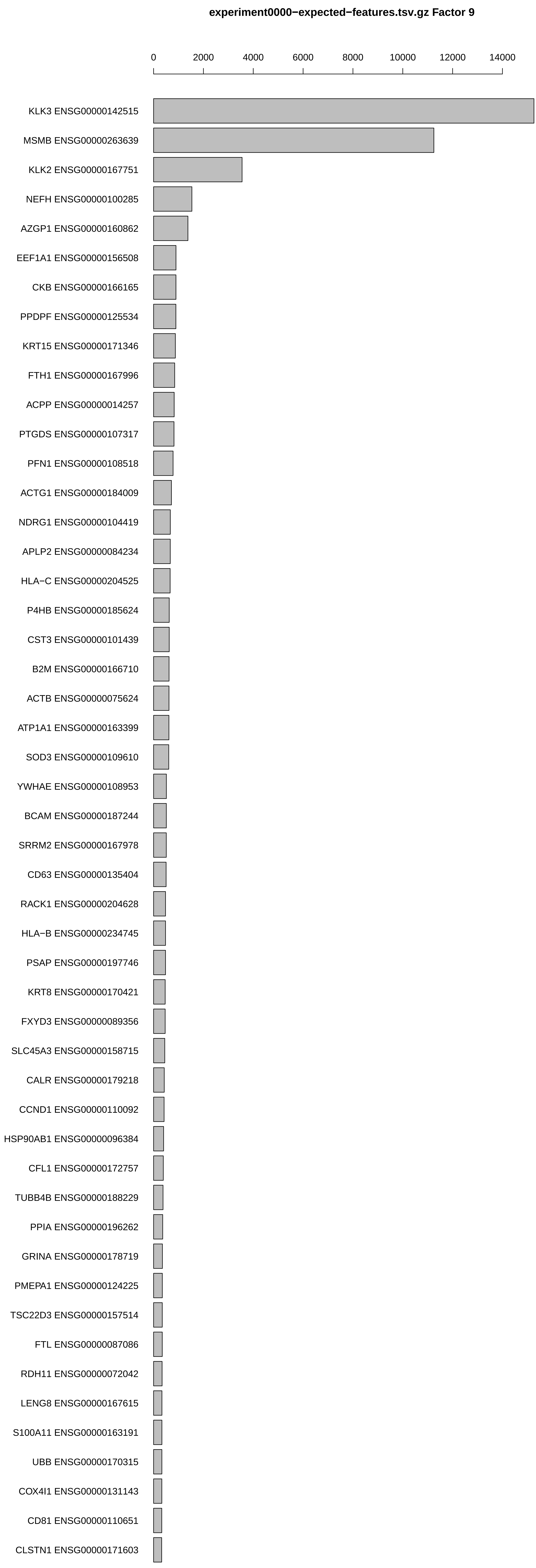
experiment0000−expected−features.tsv.gz Factor 9
0
2000
4000
6000
8000
10000
12000
14000
KLK3 ENSG00000142515
MSMB ENSG00000263639
KLK2 ENSG00000167751
NEFH ENSG00000100285
AZGP1 ENSG00000160862
EEF1A1 ENSG00000156508
CKB ENSG00000166165
PPDPF ENSG00000125534
KRT15 ENSG00000171346
FTH1 ENSG00000167996
ACPP ENSG00000014257
PTGDS ENSG00000107317
PFN1 ENSG00000108518
ACTG1 ENSG00000184009
NDRG1 ENSG00000104419
APLP2 ENSG00000084234
HLA−C ENSG00000204525
P4HB ENSG00000185624
CST3 ENSG00000101439
B2M ENSG00000166710
ACTB ENSG00000075624
ATP1A1 ENSG00000163399
SOD3 ENSG00000109610
YWHAE ENSG00000108953
BCAM ENSG00000187244
SRRM2 ENSG00000167978
CD63 ENSG00000135404
RACK1 ENSG00000204628
HLA−B ENSG00000234745
PSAP ENSG00000197746
KRT8 ENSG00000170421
FXYD3 ENSG00000089356
SLC45A3 ENSG00000158715
CALR ENSG00000179218
CCND1 ENSG00000110092
HSP90AB1 ENSG00000096384
CFL1 ENSG00000172757
TUBB4B ENSG00000188229
PPIA ENSG00000196262
GRINA ENSG00000178719
PMEPA1 ENSG00000124225
TSC22D3 ENSG00000157514
FTL ENSG00000087086
RDH11 ENSG00000072042
LENG8 ENSG00000167615
S100A11 ENSG00000163191
UBB ENSG00000170315
COX4I1 ENSG00000131143
CD81 ENSG00000110651
CLSTN1 ENSG00000171603

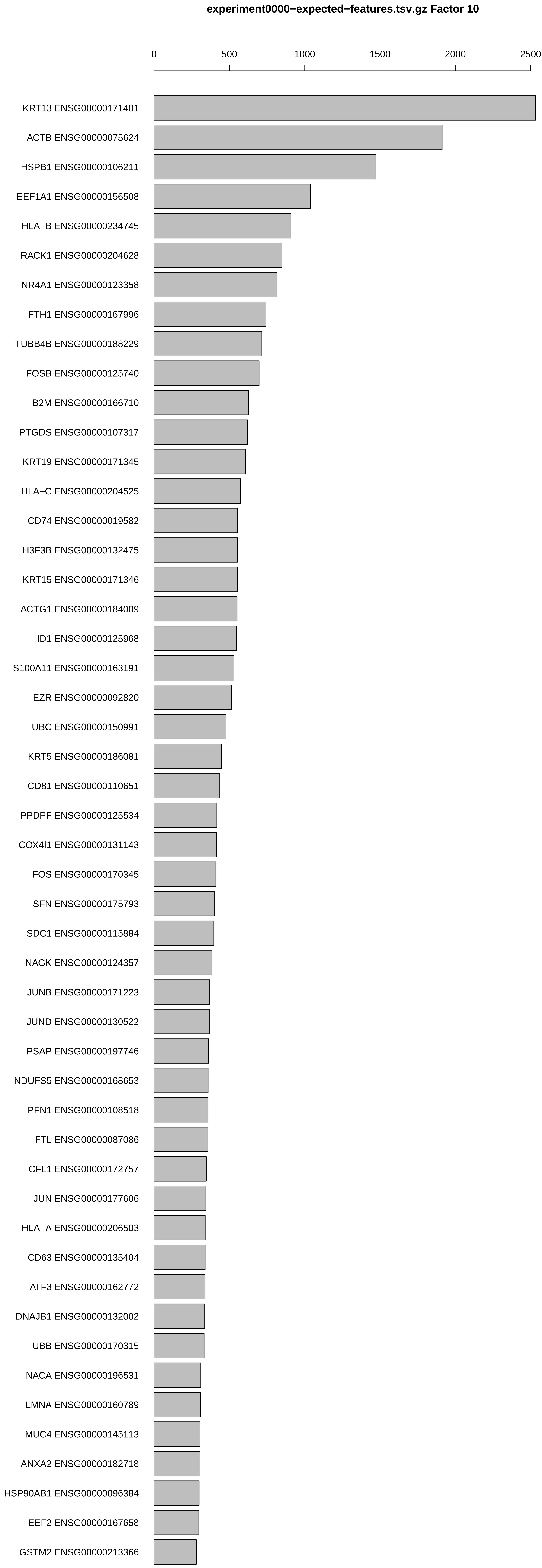
experiment0000−expected−features.tsv.gz Factor 10
0
500
1000
1500
2000
2500
KRT13 ENSG00000171401
ACTB ENSG00000075624
HSPB1 ENSG00000106211
EEF1A1 ENSG00000156508
HLA−B ENSG00000234745
RACK1 ENSG00000204628
NR4A1 ENSG00000123358
FTH1 ENSG00000167996
TUBB4B ENSG00000188229
FOSB ENSG00000125740
B2M ENSG00000166710
PTGDS ENSG00000107317
KRT19 ENSG00000171345
HLA−C ENSG00000204525
CD74 ENSG00000019582
H3F3B ENSG00000132475
KRT15 ENSG00000171346
ACTG1 ENSG00000184009
ID1 ENSG00000125968
S100A11 ENSG00000163191
EZR ENSG00000092820
UBC ENSG00000150991
KRT5 ENSG00000186081
CD81 ENSG00000110651
PPDPF ENSG00000125534
COX4I1 ENSG00000131143
FOS ENSG00000170345
SFN ENSG00000175793
SDC1 ENSG00000115884
NAGK ENSG00000124357
JUNB ENSG00000171223
JUND ENSG00000130522
PSAP ENSG00000197746
NDUFS5 ENSG00000168653
PFN1 ENSG00000108518
FTL ENSG00000087086
CFL1 ENSG00000172757
JUN ENSG00000177606
HLA−A ENSG00000206503
CD63 ENSG00000135404
ATF3 ENSG00000162772
DNAJB1 ENSG00000132002
UBB ENSG00000170315
NACA ENSG00000196531
LMNA ENSG00000160789
MUC4 ENSG00000145113
ANXA2 ENSG00000182718
HSP90AB1 ENSG00000096384
EEF2 ENSG00000167658
GSTM2 ENSG00000213366

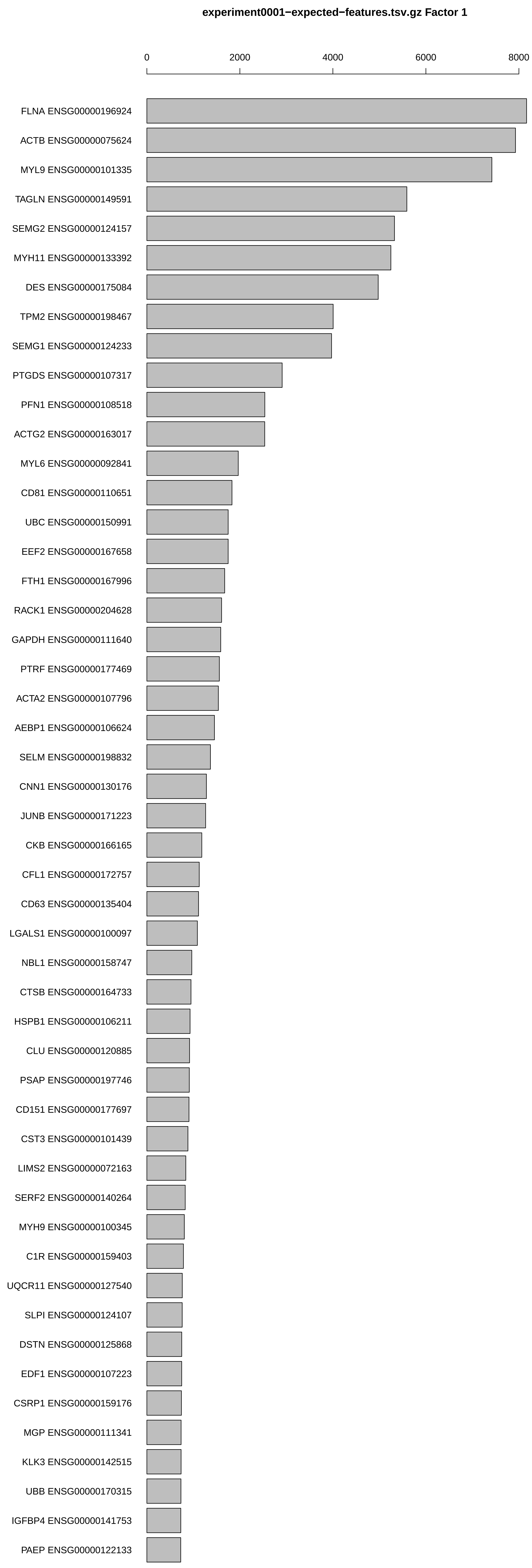
experiment0001−expected−features.tsv.gz Factor 1
0
2000
4000
6000
8000
FLNA ENSG00000196924
ACTB ENSG00000075624
MYL9 ENSG00000101335
TAGLN ENSG00000149591
SEMG2 ENSG00000124157
MYH11 ENSG00000133392
DES ENSG00000175084
TPM2 ENSG00000198467
SEMG1 ENSG00000124233
PTGDS ENSG00000107317
PFN1 ENSG00000108518
ACTG2 ENSG00000163017
MYL6 ENSG00000092841
CD81 ENSG00000110651
UBC ENSG00000150991
EEF2 ENSG00000167658
FTH1 ENSG00000167996
RACK1 ENSG00000204628
GAPDH ENSG00000111640
PTRF ENSG00000177469
ACTA2 ENSG00000107796
AEBP1 ENSG00000106624
SELM ENSG00000198832
CNN1 ENSG00000130176
JUNB ENSG00000171223
CKB ENSG00000166165
CFL1 ENSG00000172757
CD63 ENSG00000135404
LGALS1 ENSG00000100097
NBL1 ENSG00000158747
CTSB ENSG00000164733
HSPB1 ENSG00000106211
CLU ENSG00000120885
PSAP ENSG00000197746
CD151 ENSG00000177697
CST3 ENSG00000101439
LIMS2 ENSG00000072163
SERF2 ENSG00000140264
MYH9 ENSG00000100345
C1R ENSG00000159403
UQCR11 ENSG00000127540
SLPI ENSG00000124107
DSTN ENSG00000125868
EDF1 ENSG00000107223
CSRP1 ENSG00000159176
MGP ENSG00000111341
KLK3 ENSG00000142515
UBB ENSG00000170315
IGFBP4 ENSG00000141753
PAEP ENSG00000122133

experiment0001−expected−features.tsv.gz Factor 2

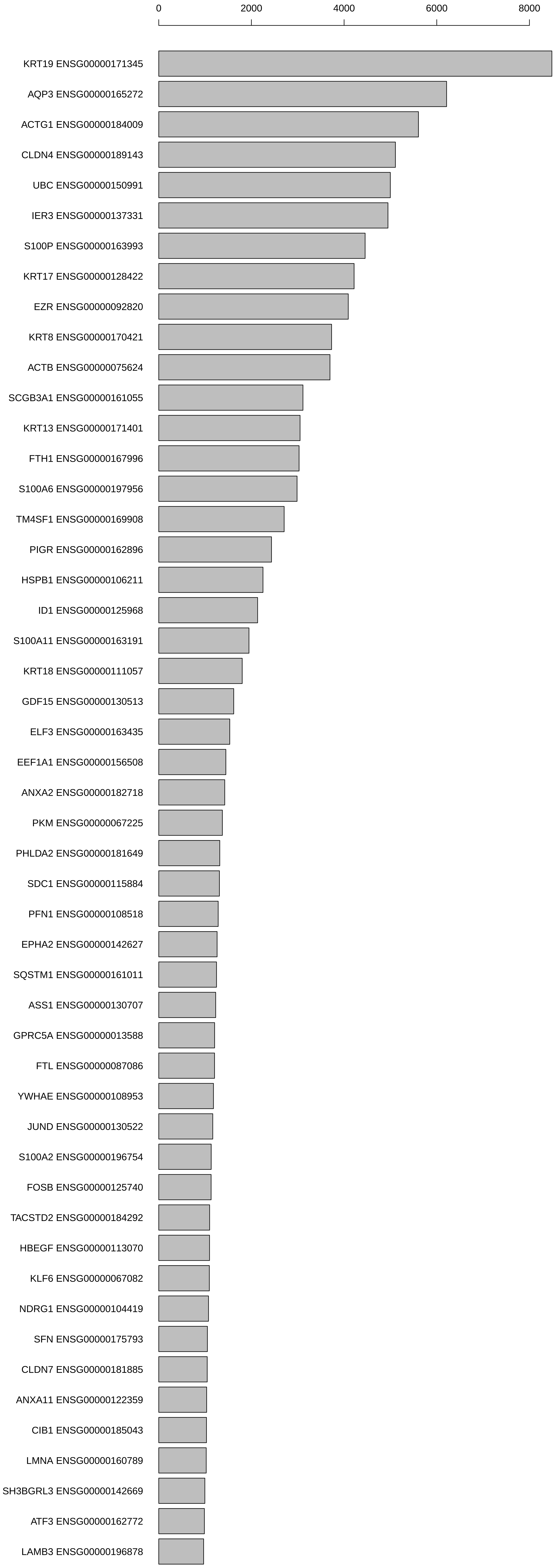

experiment0001−expected−features.tsv.gz Factor 3

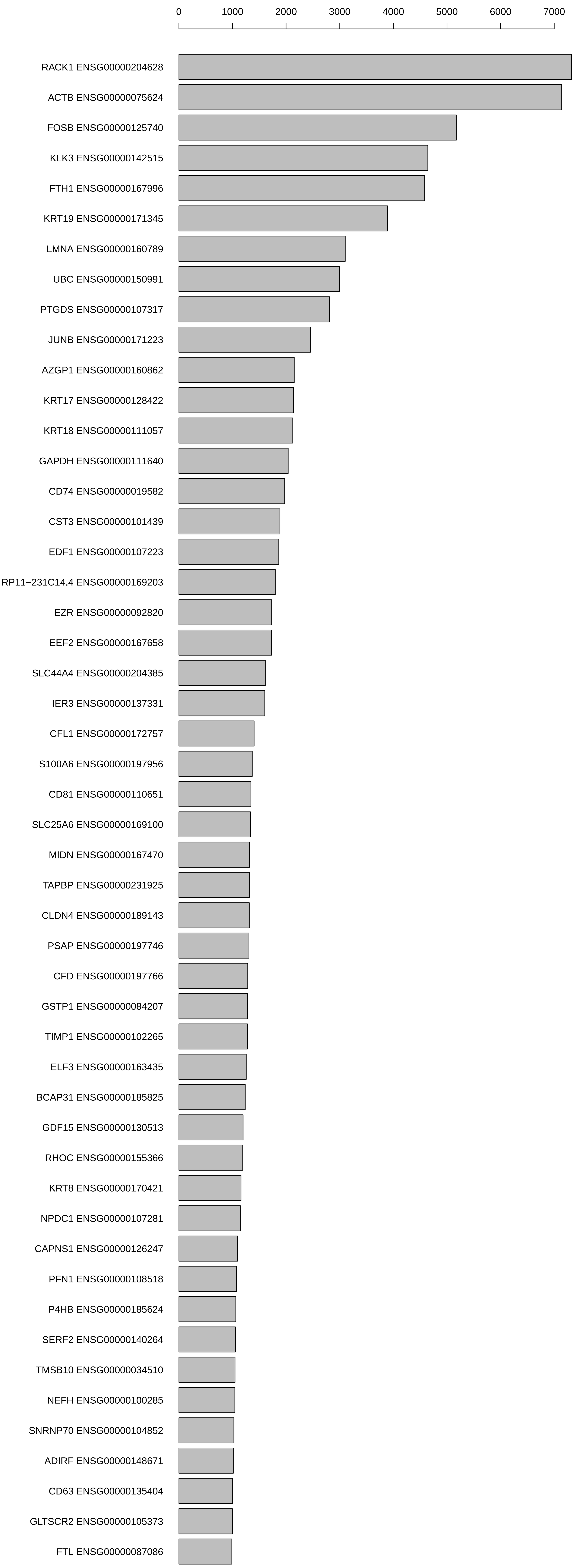

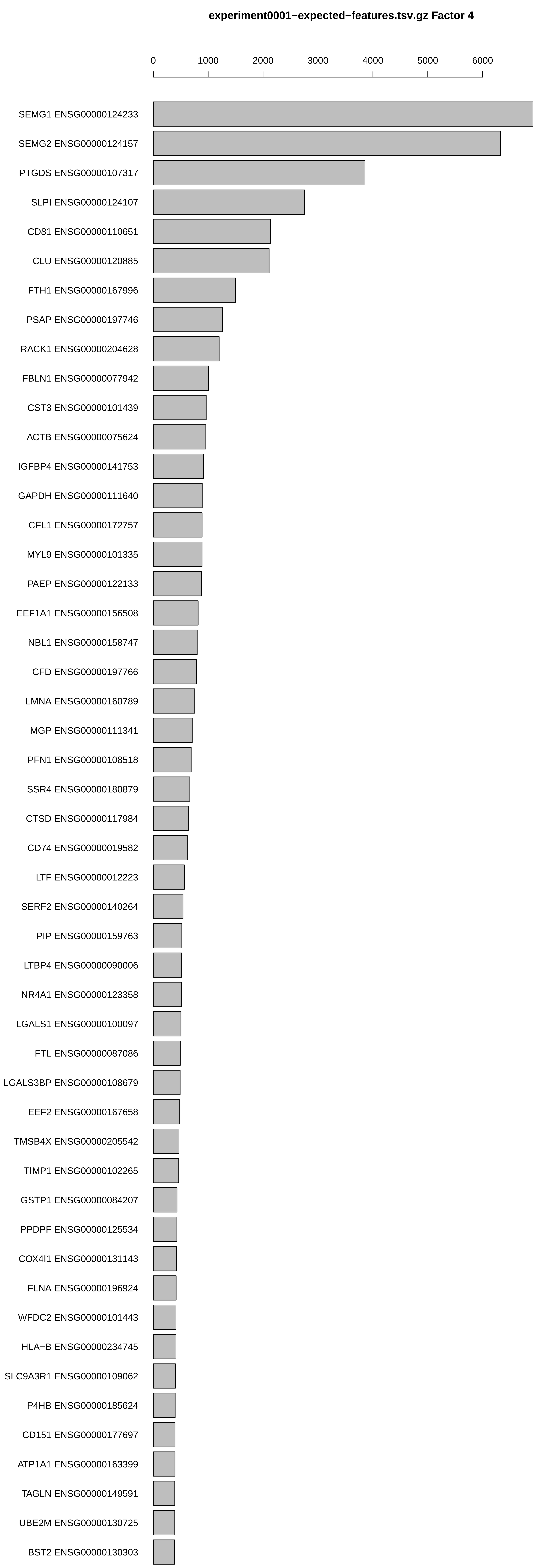
experiment0001−expected−features.tsv.gz Factor 4
0
1000
2000
3000
4000
5000
6000
SEMG1 ENSG00000124233
SEMG2 ENSG00000124157
PTGDS ENSG00000107317
SLPI ENSG00000124107
CD81 ENSG00000110651
CLU ENSG00000120885
FTH1 ENSG00000167996
PSAP ENSG00000197746
RACK1 ENSG00000204628
FBLN1 ENSG00000077942
CST3 ENSG00000101439
ACTB ENSG00000075624
IGFBP4 ENSG00000141753
GAPDH ENSG00000111640
CFL1 ENSG00000172757
MYL9 ENSG00000101335
PAEP ENSG00000122133
EEF1A1 ENSG00000156508
NBL1 ENSG00000158747
CFD ENSG00000197766
LMNA ENSG00000160789
MGP ENSG00000111341
PFN1 ENSG00000108518
SSR4 ENSG00000180879
CTSD ENSG00000117984
CD74 ENSG00000019582
LTF ENSG00000012223
SERF2 ENSG00000140264
PIP ENSG00000159763
LTBP4 ENSG00000090006
NR4A1 ENSG00000123358
LGALS1 ENSG00000100097
FTL ENSG00000087086
LGALS3BP ENSG00000108679
EEF2 ENSG00000167658
TMSB4X ENSG00000205542
TIMP1 ENSG00000102265
GSTP1 ENSG00000084207
PPDPF ENSG00000125534
COX4I1 ENSG00000131143
FLNA ENSG00000196924
WFDC2 ENSG00000101443
HLA−B ENSG00000234745
SLC9A3R1 ENSG00000109062
P4HB ENSG00000185624
CD151 ENSG00000177697
ATP1A1 ENSG00000163399
TAGLN ENSG00000149591
UBE2M ENSG00000130725
BST2 ENSG00000130303

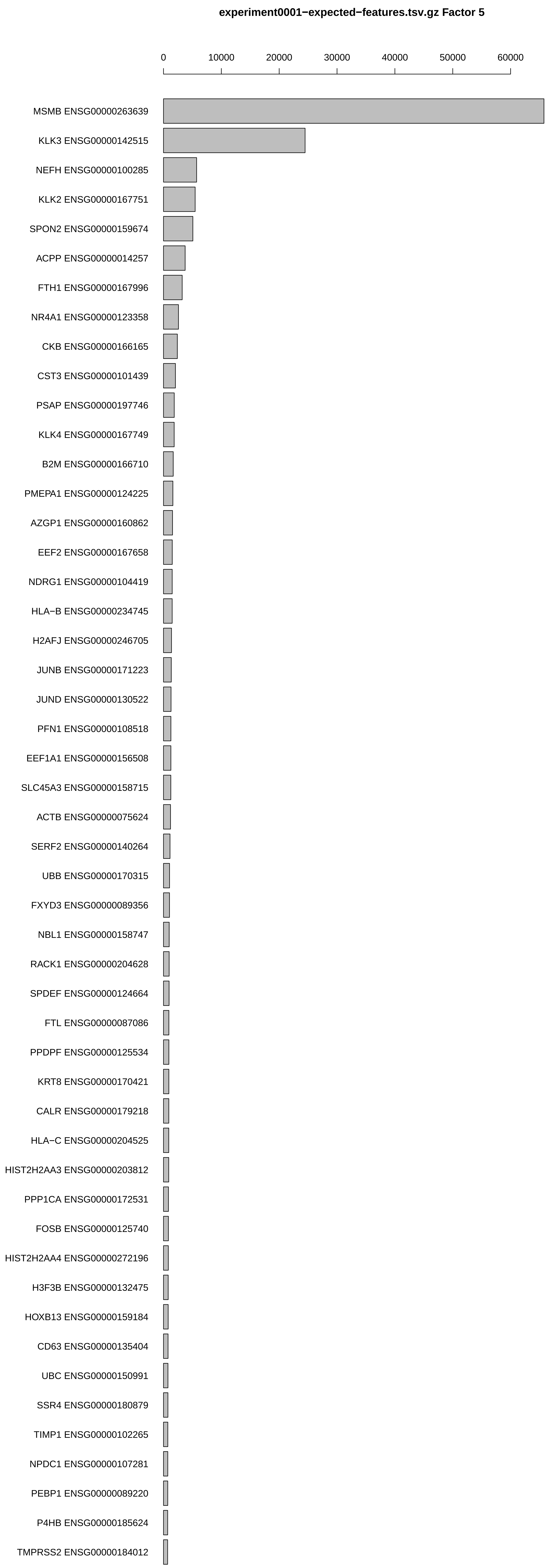
experiment0001−expected−features.tsv.gz Factor 5
0
10000
20000
30000
40000
50000
60000
MSMB ENSG00000263639
KLK3 ENSG00000142515
NEFH ENSG00000100285
KLK2 ENSG00000167751
SPON2 ENSG00000159674
ACPP ENSG00000014257
FTH1 ENSG00000167996
NR4A1 ENSG00000123358
CKB ENSG00000166165
CST3 ENSG00000101439
PSAP ENSG00000197746
KLK4 ENSG00000167749
B2M ENSG00000166710
PMEPA1 ENSG00000124225
AZGP1 ENSG00000160862
EEF2 ENSG00000167658
NDRG1 ENSG00000104419
HLA−B ENSG00000234745
H2AFJ ENSG00000246705
JUNB ENSG00000171223
JUND ENSG00000130522
PFN1 ENSG00000108518
EEF1A1 ENSG00000156508
SLC45A3 ENSG00000158715
ACTB ENSG00000075624
SERF2 ENSG00000140264
UBB ENSG00000170315
FXYD3 ENSG00000089356
NBL1 ENSG00000158747
RACK1 ENSG00000204628
SPDEF ENSG00000124664
FTL ENSG00000087086
PPDPF ENSG00000125534
KRT8 ENSG00000170421
CALR ENSG00000179218
HLA−C ENSG00000204525
HIST2H2AA3 ENSG00000203812
PPP1CA ENSG00000172531
FOSB ENSG00000125740
HIST2H2AA4 ENSG00000272196
H3F3B ENSG00000132475
HOXB13 ENSG00000159184
CD63 ENSG00000135404
UBC ENSG00000150991
SSR4 ENSG00000180879
TIMP1 ENSG00000102265
NPDC1 ENSG00000107281
PEBP1 ENSG00000089220
P4HB ENSG00000185624
TMPRSS2 ENSG00000184012

experiment0001−expected−features.tsv.gz Factor 6

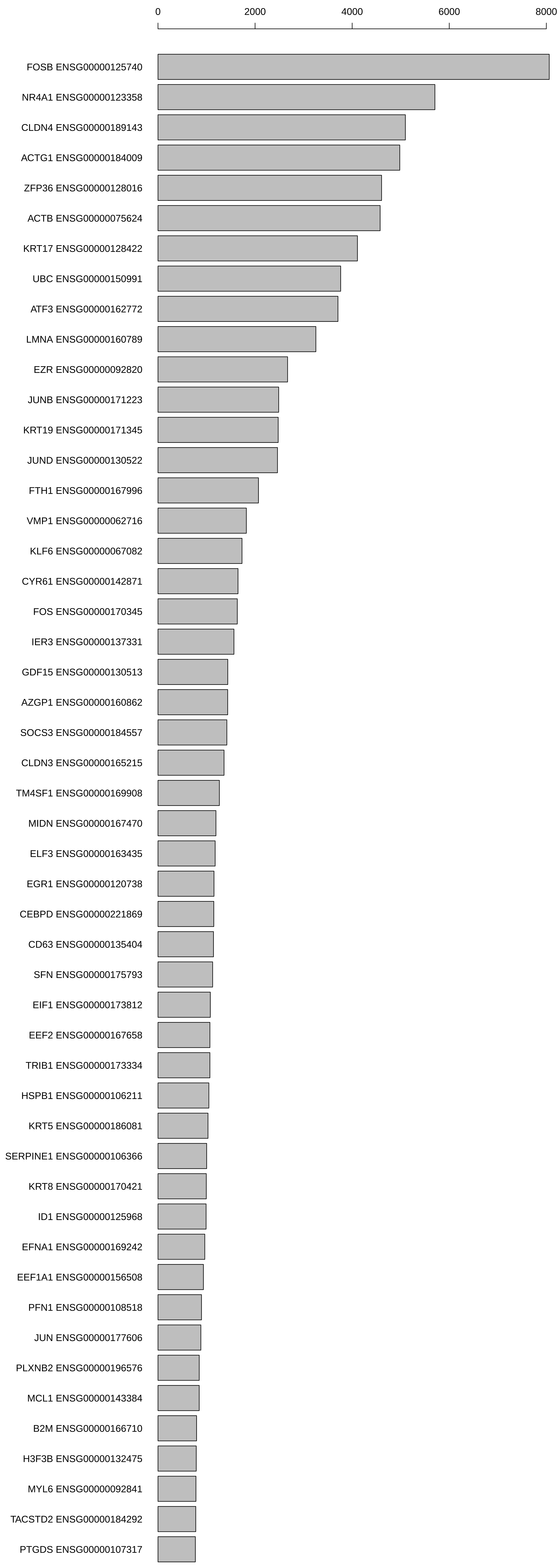

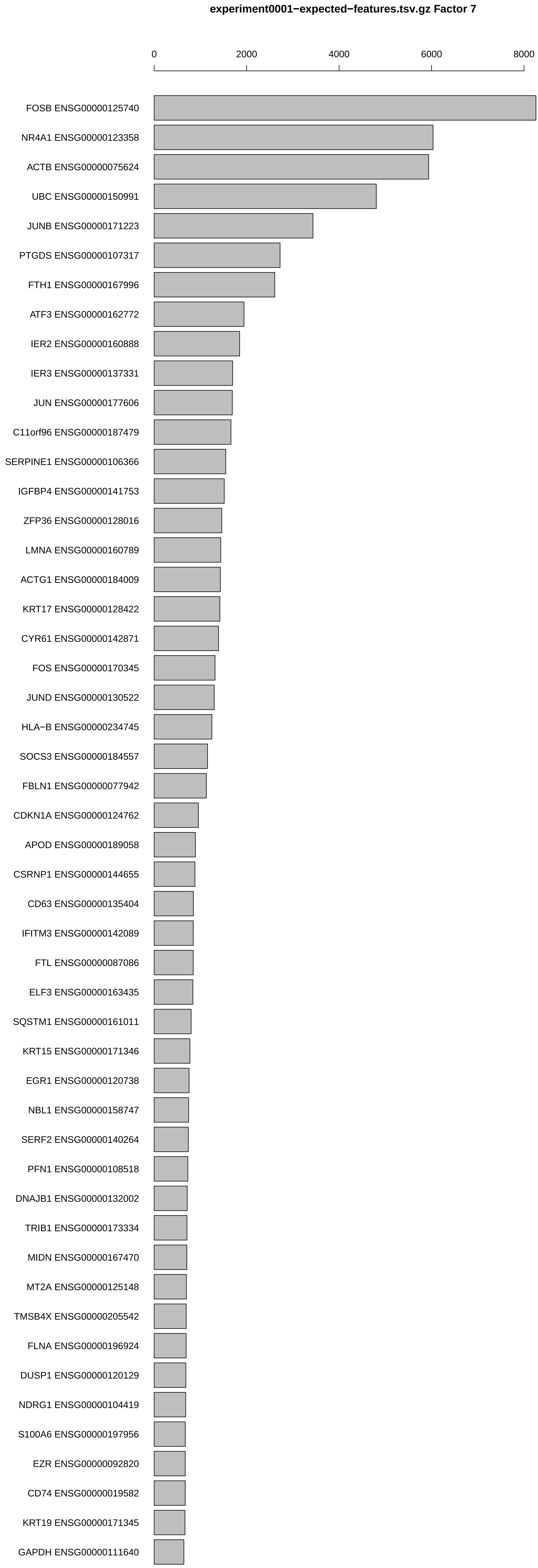
experiment0001−expected−features.tsv.gz Factor 7
0
2000
4000
6000
8000
FOSB ENSG00000125740
NR4A1 ENSG00000123358
ACTB ENSG00000075624
UBC ENSG00000150991
JUNB ENSG00000171223
PTGDS ENSG00000107317
FTH1 ENSG00000167996
ATF3 ENSG00000162772
IER2 ENSG00000160888
IER3 ENSG00000137331
JUN ENSG00000177606
C11orf96 ENSG00000187479
SERPINE1 ENSG00000106366
IGFBP4 ENSG00000141753
ZFP36 ENSG00000128016
LMNA ENSG00000160789
ACTG1 ENSG00000184009
KRT17 ENSG00000128422
CYR61 ENSG00000142871
FOS ENSG00000170345
JUND ENSG00000130522
HLA−B ENSG00000234745
SOCS3 ENSG00000184557
FBLN1 ENSG00000077942
CDKN1A ENSG00000124762
APOD ENSG00000189058
CSRNP1 ENSG00000144655
CD63 ENSG00000135404
IFITM3 ENSG00000142089
FTL ENSG00000087086
ELF3 ENSG00000163435
SQSTM1 ENSG00000161011
KRT15 ENSG00000171346
EGR1 ENSG00000120738
NBL1 ENSG00000158747
SERF2 ENSG00000140264
PFN1 ENSG00000108518
DNAJB1 ENSG00000132002
TRIB1 ENSG00000173334
MIDN ENSG00000167470
MT2A ENSG00000125148
TMSB4X ENSG00000205542
FLNA ENSG00000196924
DUSP1 ENSG00000120129
NDRG1 ENSG00000104419
S100A6 ENSG00000197956
EZR ENSG00000092820
CD74 ENSG00000019582
KRT19 ENSG00000171345
GAPDH ENSG00000111640

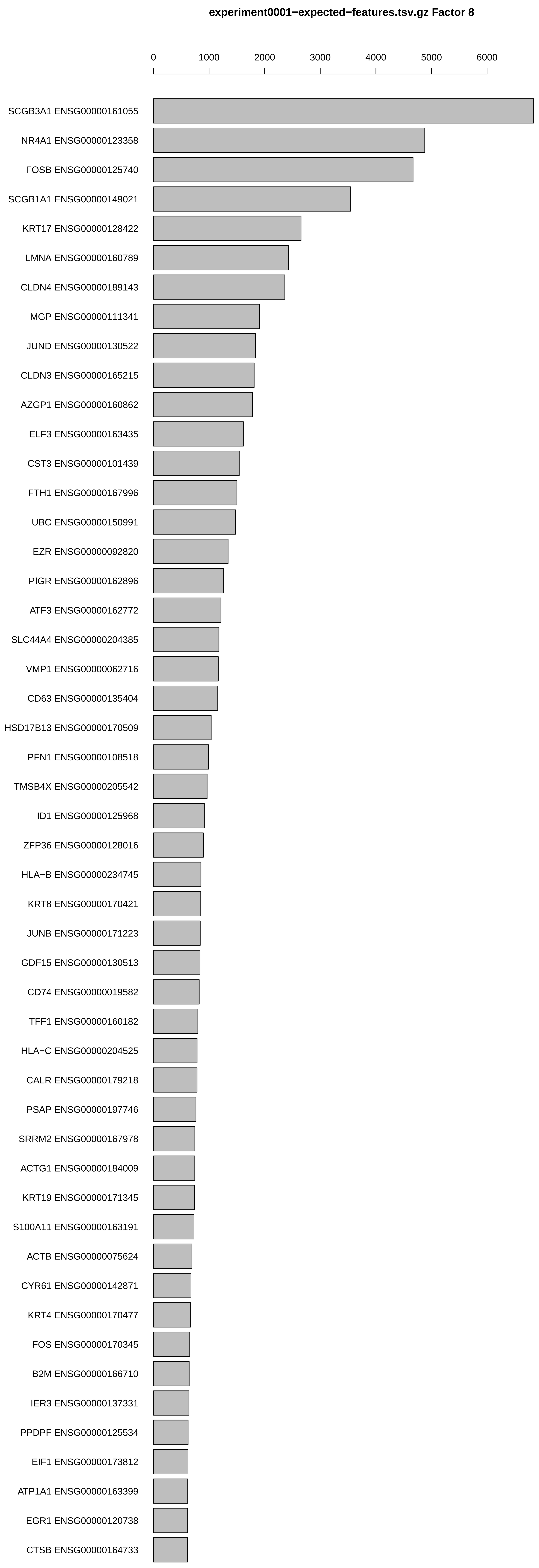
experiment0001−expected−features.tsv.gz Factor 8
0
1000
2000
3000
4000
5000
6000
SCGB3A1 ENSG00000161055
NR4A1 ENSG00000123358
FOSB ENSG00000125740
SCGB1A1 ENSG00000149021
KRT17 ENSG00000128422
LMNA ENSG00000160789
CLDN4 ENSG00000189143
MGP ENSG00000111341
JUND ENSG00000130522
CLDN3 ENSG00000165215
AZGP1 ENSG00000160862
ELF3 ENSG00000163435
CST3 ENSG00000101439
FTH1 ENSG00000167996
UBC ENSG00000150991
EZR ENSG00000092820
PIGR ENSG00000162896
ATF3 ENSG00000162772
SLC44A4 ENSG00000204385
VMP1 ENSG00000062716
CD63 ENSG00000135404
HSD17B13 ENSG00000170509
PFN1 ENSG00000108518
TMSB4X ENSG00000205542
ID1 ENSG00000125968
ZFP36 ENSG00000128016
HLA−B ENSG00000234745
KRT8 ENSG00000170421
JUNB ENSG00000171223
GDF15 ENSG00000130513
CD74 ENSG00000019582
TFF1 ENSG00000160182
HLA−C ENSG00000204525
CALR ENSG00000179218
PSAP ENSG00000197746
SRRM2 ENSG00000167978
ACTG1 ENSG00000184009
KRT19 ENSG00000171345
S100A11 ENSG00000163191
ACTB ENSG00000075624
CYR61 ENSG00000142871
KRT4 ENSG00000170477
FOS ENSG00000170345
B2M ENSG00000166710
IER3 ENSG00000137331
PPDPF ENSG00000125534
EIF1 ENSG00000173812
ATP1A1 ENSG00000163399
EGR1 ENSG00000120738
CTSB ENSG00000164733

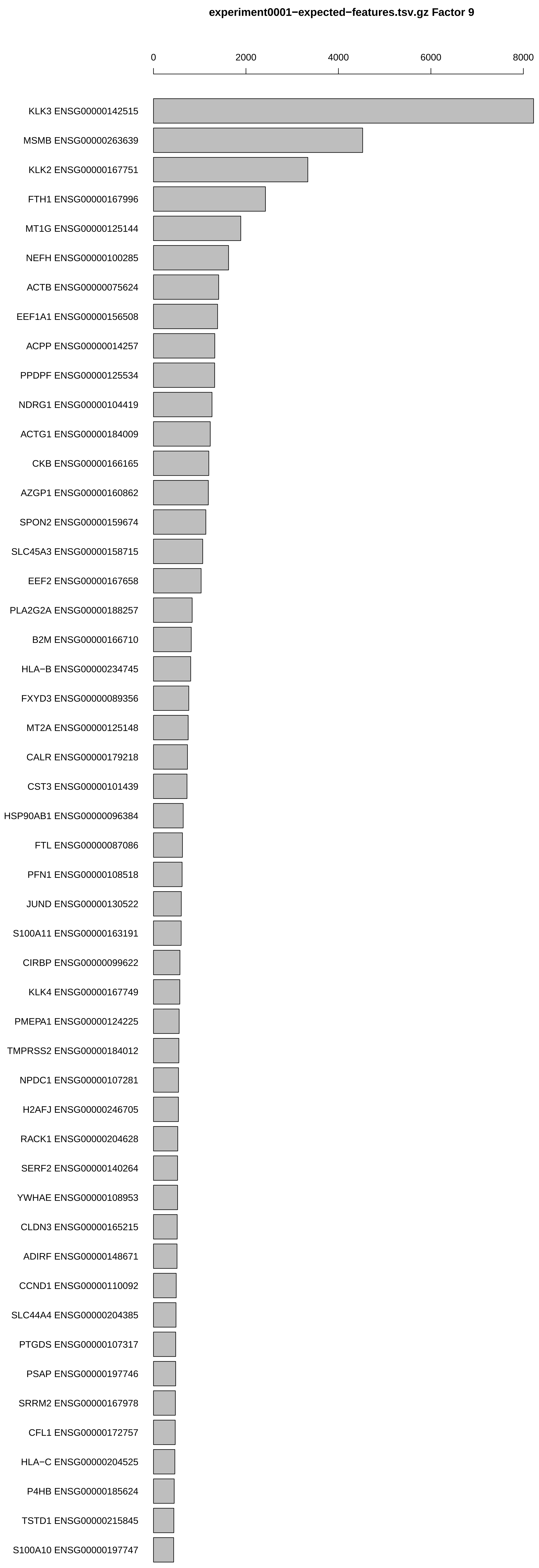
experiment0001−expected−features.tsv.gz Factor 9
0
2000
4000
6000
8000
KLK3 ENSG00000142515
MSMB ENSG00000263639
KLK2 ENSG00000167751
FTH1 ENSG00000167996
MT1G ENSG00000125144
NEFH ENSG00000100285
ACTB ENSG00000075624
EEF1A1 ENSG00000156508
ACPP ENSG00000014257
PPDPF ENSG00000125534
NDRG1 ENSG00000104419
ACTG1 ENSG00000184009
CKB ENSG00000166165
AZGP1 ENSG00000160862
SPON2 ENSG00000159674
SLC45A3 ENSG00000158715
EEF2 ENSG00000167658
PLA2G2A ENSG00000188257
B2M ENSG00000166710
HLA−B ENSG00000234745
FXYD3 ENSG00000089356
MT2A ENSG00000125148
CALR ENSG00000179218
CST3 ENSG00000101439
HSP90AB1 ENSG00000096384
FTL ENSG00000087086
PFN1 ENSG00000108518
JUND ENSG00000130522
S100A11 ENSG00000163191
CIRBP ENSG00000099622
KLK4 ENSG00000167749
PMEPA1 ENSG00000124225
TMPRSS2 ENSG00000184012
NPDC1 ENSG00000107281
H2AFJ ENSG00000246705
RACK1 ENSG00000204628
SERF2 ENSG00000140264
YWHAE ENSG00000108953
CLDN3 ENSG00000165215
ADIRF ENSG00000148671
CCND1 ENSG00000110092
SLC44A4 ENSG00000204385
PTGDS ENSG00000107317
PSAP ENSG00000197746
SRRM2 ENSG00000167978
CFL1 ENSG00000172757
HLA−C ENSG00000204525
P4HB ENSG00000185624
TSTD1 ENSG00000215845
S100A10 ENSG00000197747

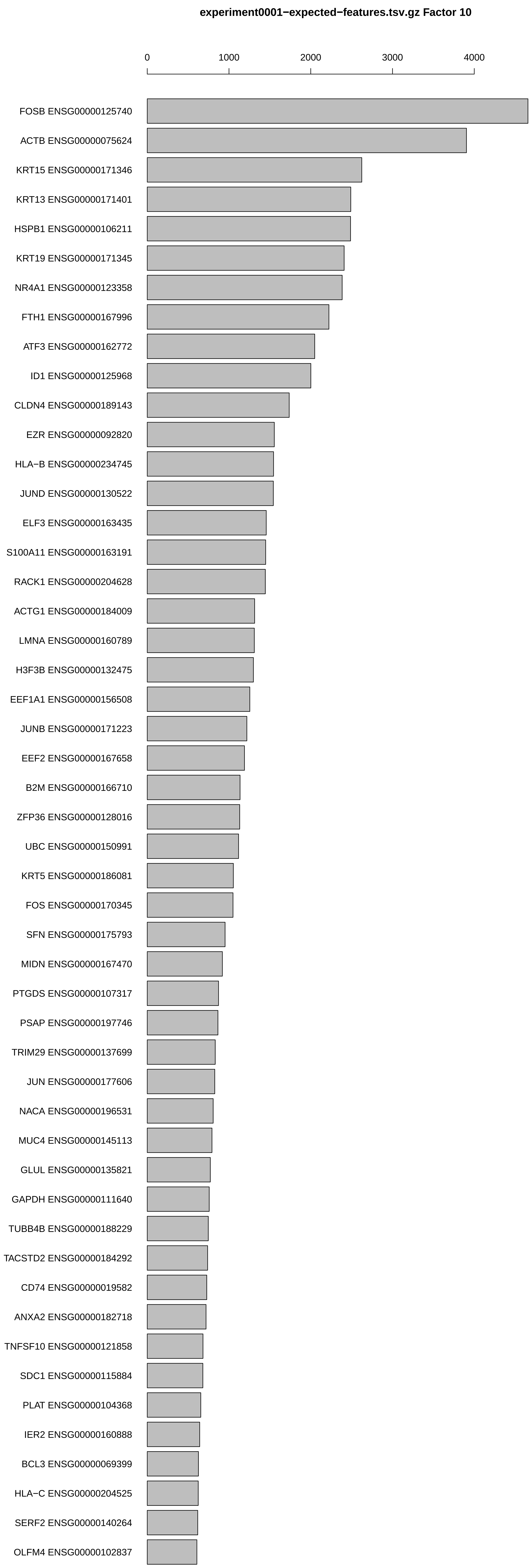
experiment0001−expected−features.tsv.gz Factor 10
0
1000
2000
3000
4000
FOSB ENSG00000125740
ACTB ENSG00000075624
KRT15 ENSG00000171346
KRT13 ENSG00000171401
HSPB1 ENSG00000106211
KRT19 ENSG00000171345
NR4A1 ENSG00000123358
FTH1 ENSG00000167996
ATF3 ENSG00000162772
ID1 ENSG00000125968
CLDN4 ENSG00000189143
EZR ENSG00000092820
HLA−B ENSG00000234745
JUND ENSG00000130522
ELF3 ENSG00000163435
S100A11 ENSG00000163191
RACK1 ENSG00000204628
ACTG1 ENSG00000184009
LMNA ENSG00000160789
H3F3B ENSG00000132475
EEF1A1 ENSG00000156508
JUNB ENSG00000171223
EEF2 ENSG00000167658
B2M ENSG00000166710
ZFP36 ENSG00000128016
UBC ENSG00000150991
KRT5 ENSG00000186081
FOS ENSG00000170345
SFN ENSG00000175793
MIDN ENSG00000167470
PTGDS ENSG00000107317
PSAP ENSG00000197746
TRIM29 ENSG00000137699
JUN ENSG00000177606
NACA ENSG00000196531
MUC4 ENSG00000145113
GLUL ENSG00000135821
GAPDH ENSG00000111640
TUBB4B ENSG00000188229
TACSTD2 ENSG00000184292
CD74 ENSG00000019582
ANXA2 ENSG00000182718
TNFSF10 ENSG00000121858
SDC1 ENSG00000115884
PLAT ENSG00000104368
IER2 ENSG00000160888
BCL3 ENSG00000069399
HLA−C ENSG00000204525
SERF2 ENSG00000140264
OLFM4 ENSG00000102837
