# Supplementary material for: Spatial maps of prostate cancer transcriptomes reveal an unexplored landscape of heterogeneity
Source: Nat Commun. 2018 Jun 20;9:2419. doi: 10.1038/s41467-018-04724-5 (PMC6010471; doi:10.1038/s41467-018-04724-5)
Supplement: Supplementary file 13 — Supplementary Data 10 [file 41467_2018_4724_MOESM13_ESM.zip › Supplementary Dataset 5/top-genes.pdf]

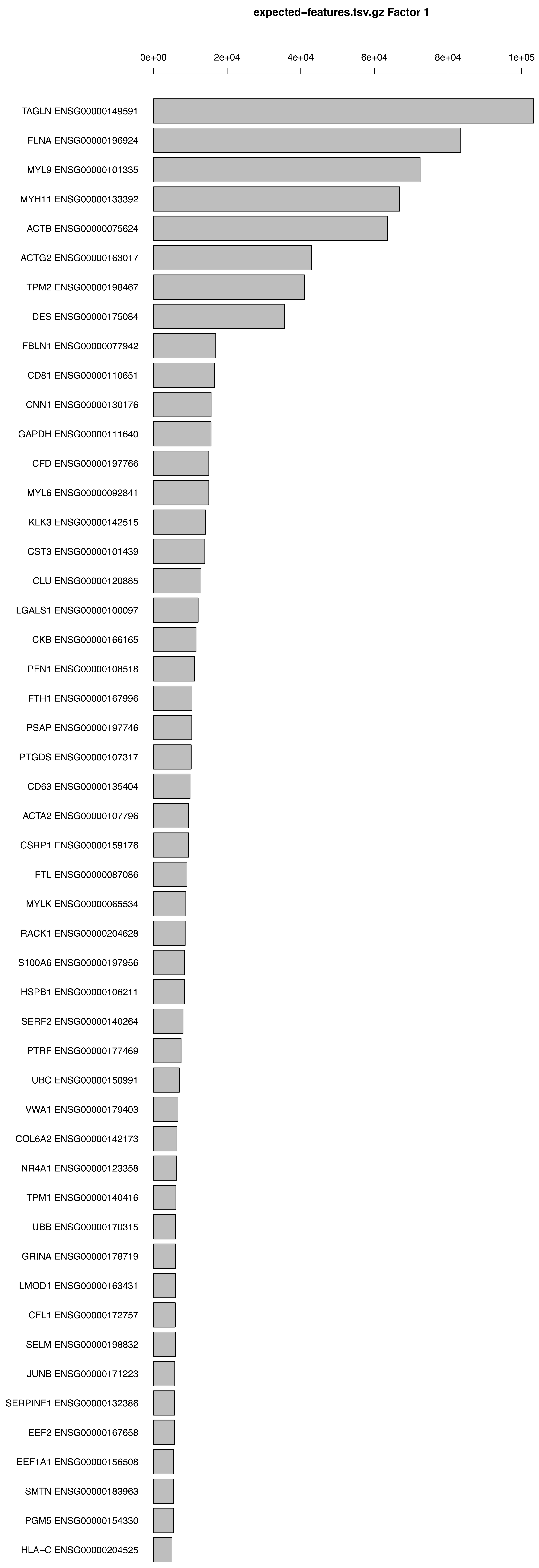
expected−features.tsv.gz Factor 1
0e+00
2e+04
4e+04
6e+04
8e+04
1e+05
TAGLN ENSG00000149591
FLNA ENSG00000196924
MYL9 ENSG00000101335
MYH11 ENSG00000133392
ACTB ENSG00000075624
ACTG2 ENSG00000163017
TPM2 ENSG00000198467
DES ENSG00000175084
FBLN1 ENSG00000077942
CD81 ENSG00000110651
CNN1 ENSG00000130176
GAPDH ENSG00000111640
CFD ENSG00000197766
MYL6 ENSG00000092841
KLK3 ENSG00000142515
CST3 ENSG00000101439
CLU ENSG00000120885
LGALS1 ENSG00000100097
CKB ENSG00000166165
PFN1 ENSG00000108518
FTH1 ENSG00000167996
PSAP ENSG00000197746
PTGDS ENSG00000107317
CD63 ENSG00000135404
ACTA2 ENSG00000107796
CSRP1 ENSG00000159176
FTL ENSG00000087086
MYLK ENSG00000065534
RACK1 ENSG00000204628
S100A6 ENSG00000197956
HSPB1 ENSG00000106211
SERF2 ENSG00000140264
PTRF ENSG00000177469
UBC ENSG00000150991
VWA1 ENSG00000179403
COL6A2 ENSG00000142173
NR4A1 ENSG00000123358
TPM1 ENSG00000140416
UBB ENSG00000170315
GRINA ENSG00000178719
LMOD1 ENSG00000163431
CFL1 ENSG00000172757
SELM ENSG00000198832
JUNB ENSG00000171223
SERPINF1 ENSG00000132386
EEF2 ENSG00000167658
EEF1A1 ENSG00000156508
SMTN ENSG00000183963
PGM5 ENSG00000154330
HLA−C ENSG00000204525

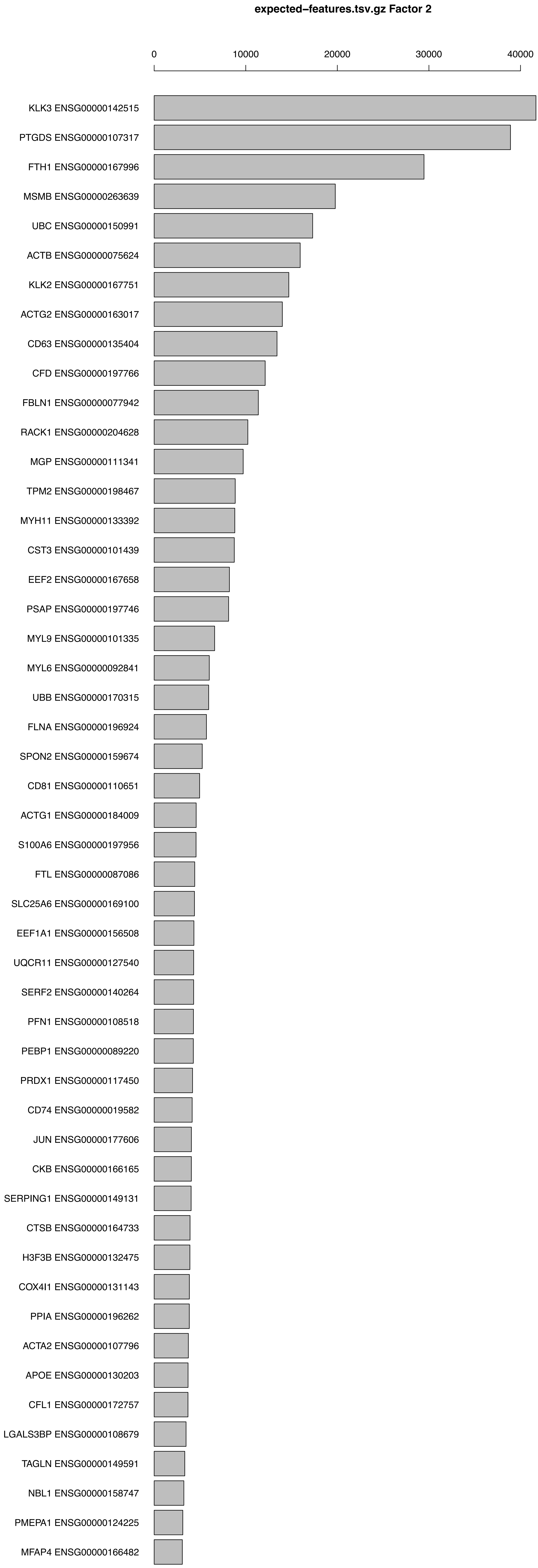
expected−features.tsv.gz Factor 2
0
10000
20000
30000
40000
KLK3 ENSG00000142515
PTGDS ENSG00000107317
FTH1 ENSG00000167996
MSMB ENSG00000263639
UBC ENSG00000150991
ACTB ENSG00000075624
KLK2 ENSG00000167751
ACTG2 ENSG00000163017
CD63 ENSG00000135404
CFD ENSG00000197766
FBLN1 ENSG00000077942
RACK1 ENSG00000204628
MGP ENSG00000111341
TPM2 ENSG00000198467
MYH11 ENSG00000133392
CST3 ENSG00000101439
EEF2 ENSG00000167658
PSAP ENSG00000197746
MYL9 ENSG00000101335
MYL6 ENSG00000092841
UBB ENSG00000170315
FLNA ENSG00000196924
SPON2 ENSG00000159674
CD81 ENSG00000110651
ACTG1 ENSG00000184009
S100A6 ENSG00000197956
FTL ENSG00000087086
SLC25A6 ENSG00000169100
EEF1A1 ENSG00000156508
UQCR11 ENSG00000127540
SERF2 ENSG00000140264
PFN1 ENSG00000108518
PEBP1 ENSG00000089220
PRDX1 ENSG00000117450
CD74 ENSG00000019582
JUN ENSG00000177606
CKB ENSG00000166165
SERPING1 ENSG00000149131
CTSB ENSG00000164733
H3F3B ENSG00000132475
COX4I1 ENSG00000131143
PPIA ENSG00000196262
ACTA2 ENSG00000107796
APOE ENSG00000130203
CFL1 ENSG00000172757
LGALS3BP ENSG00000108679
TAGLN ENSG00000149591
NBL1 ENSG00000158747
PMEPA1 ENSG00000124225
MFAP4 ENSG00000166482

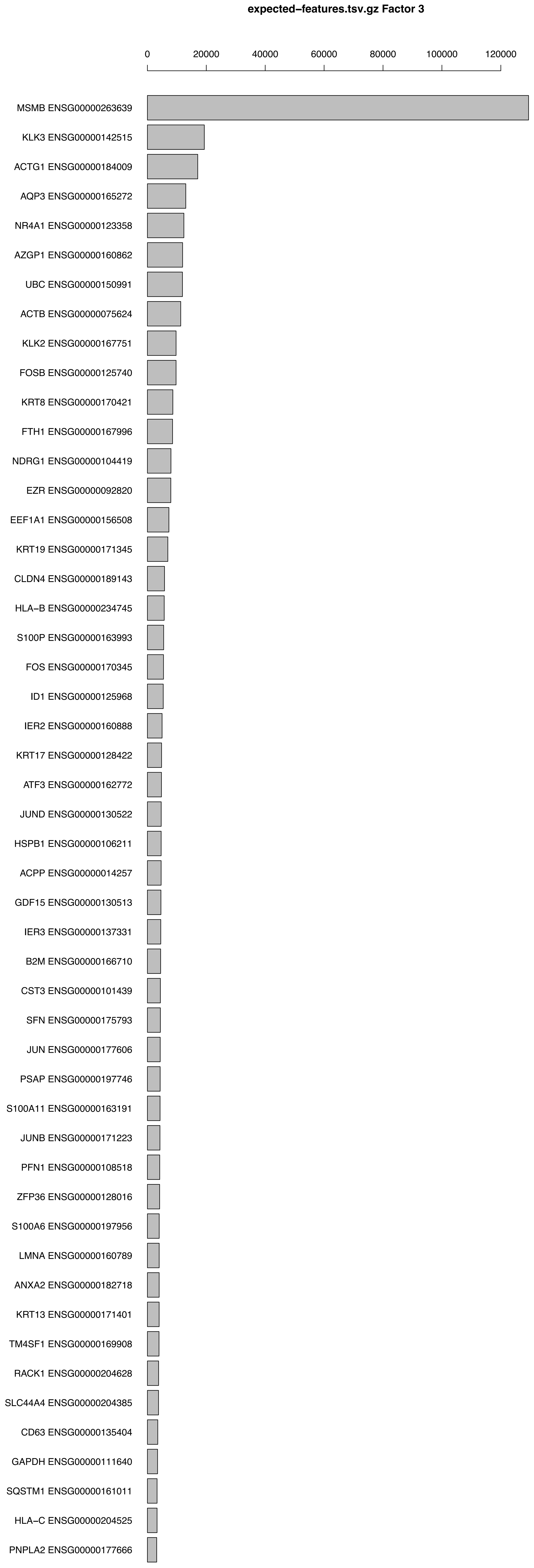
expected−features.tsv.gz Factor 3
0
20000
40000
60000
80000
100000
120000
MSMB ENSG00000263639
KLK3 ENSG00000142515
ACTG1 ENSG00000184009
AQP3 ENSG00000165272
NR4A1 ENSG00000123358
AZGP1 ENSG00000160862
UBC ENSG00000150991
ACTB ENSG00000075624
KLK2 ENSG00000167751
FOSB ENSG00000125740
KRT8 ENSG00000170421
FTH1 ENSG00000167996
NDRG1 ENSG00000104419
EZR ENSG00000092820
EEF1A1 ENSG00000156508
KRT19 ENSG00000171345
CLDN4 ENSG00000189143
HLA−B ENSG00000234745
S100P ENSG00000163993
FOS ENSG00000170345
ID1 ENSG00000125968
IER2 ENSG00000160888
KRT17 ENSG00000128422
ATF3 ENSG00000162772
JUND ENSG00000130522
HSPB1 ENSG00000106211
ACPP ENSG00000014257
GDF15 ENSG00000130513
IER3 ENSG00000137331
B2M ENSG00000166710
CST3 ENSG00000101439
SFN ENSG00000175793
JUN ENSG00000177606
PSAP ENSG00000197746
S100A11 ENSG00000163191
JUNB ENSG00000171223
PFN1 ENSG00000108518
ZFP36 ENSG00000128016
S100A6 ENSG00000197956
LMNA ENSG00000160789
ANXA2 ENSG00000182718
KRT13 ENSG00000171401
TM4SF1 ENSG00000169908
RACK1 ENSG00000204628
SLC44A4 ENSG00000204385
CD63 ENSG00000135404
GAPDH ENSG00000111640
SQSTM1 ENSG00000161011
HLA−C ENSG00000204525
PNPLA2 ENSG00000177666

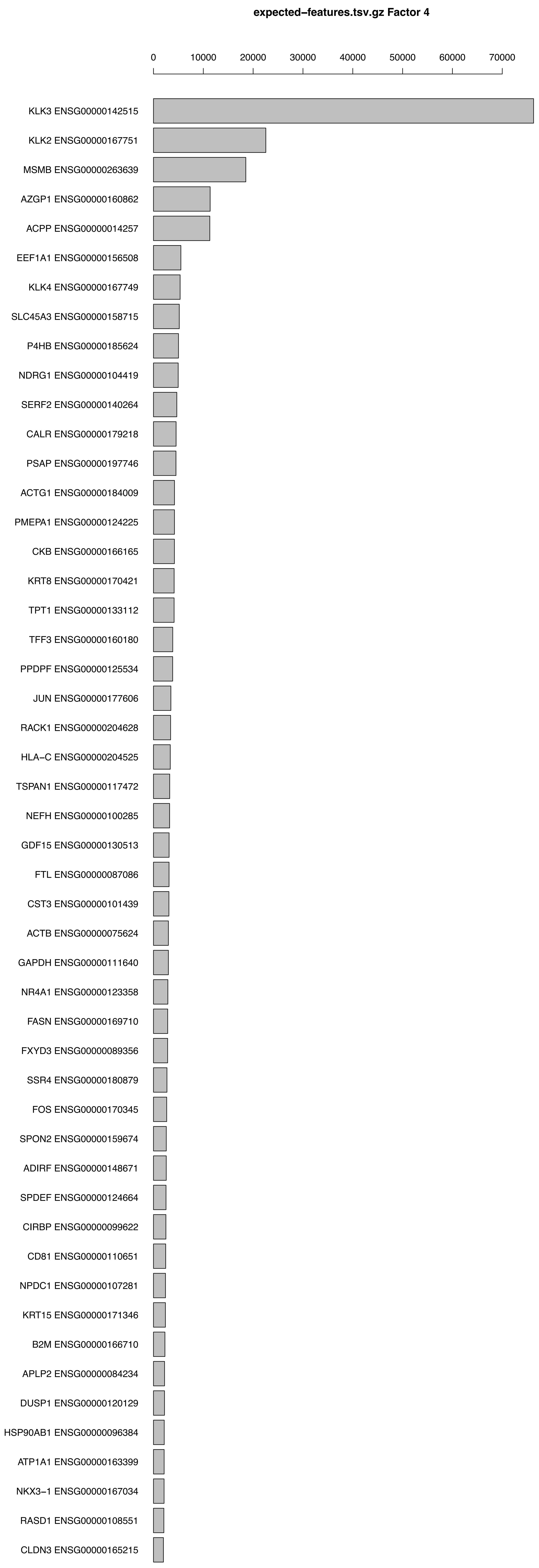
expected−features.tsv.gz Factor 4
0
10000
20000
30000
40000
50000
60000
70000
KLK3 ENSG00000142515
KLK2 ENSG00000167751
MSMB ENSG00000263639
AZGP1 ENSG00000160862
ACPP ENSG00000014257
EEF1A1 ENSG00000156508
KLK4 ENSG00000167749
SLC45A3 ENSG00000158715
P4HB ENSG00000185624
NDRG1 ENSG00000104419
SERF2 ENSG00000140264
CALR ENSG00000179218
PSAP ENSG00000197746
ACTG1 ENSG00000184009
PMEPA1 ENSG00000124225
CKB ENSG00000166165
KRT8 ENSG00000170421
TPT1 ENSG00000133112
TFF3 ENSG00000160180
PPDPF ENSG00000125534
JUN ENSG00000177606
RACK1 ENSG00000204628
HLA−C ENSG00000204525
TSPAN1 ENSG00000117472
NEFH ENSG00000100285
GDF15 ENSG00000130513
FTL ENSG00000087086
CST3 ENSG00000101439
ACTB ENSG00000075624
GAPDH ENSG00000111640
NR4A1 ENSG00000123358
FASN ENSG00000169710
FXYD3 ENSG00000089356
SSR4 ENSG00000180879
FOS ENSG00000170345
SPON2 ENSG00000159674
ADIRF ENSG00000148671
SPDEF ENSG00000124664
CIRBP ENSG00000099622
CD81 ENSG00000110651
NPDC1 ENSG00000107281
KRT15 ENSG00000171346
B2M ENSG00000166710
APLP2 ENSG00000084234
DUSP1 ENSG00000120129
HSP90AB1 ENSG00000096384
ATP1A1 ENSG00000163399
NKX3−1 ENSG00000167034
RASD1 ENSG00000108551
CLDN3 ENSG00000165215

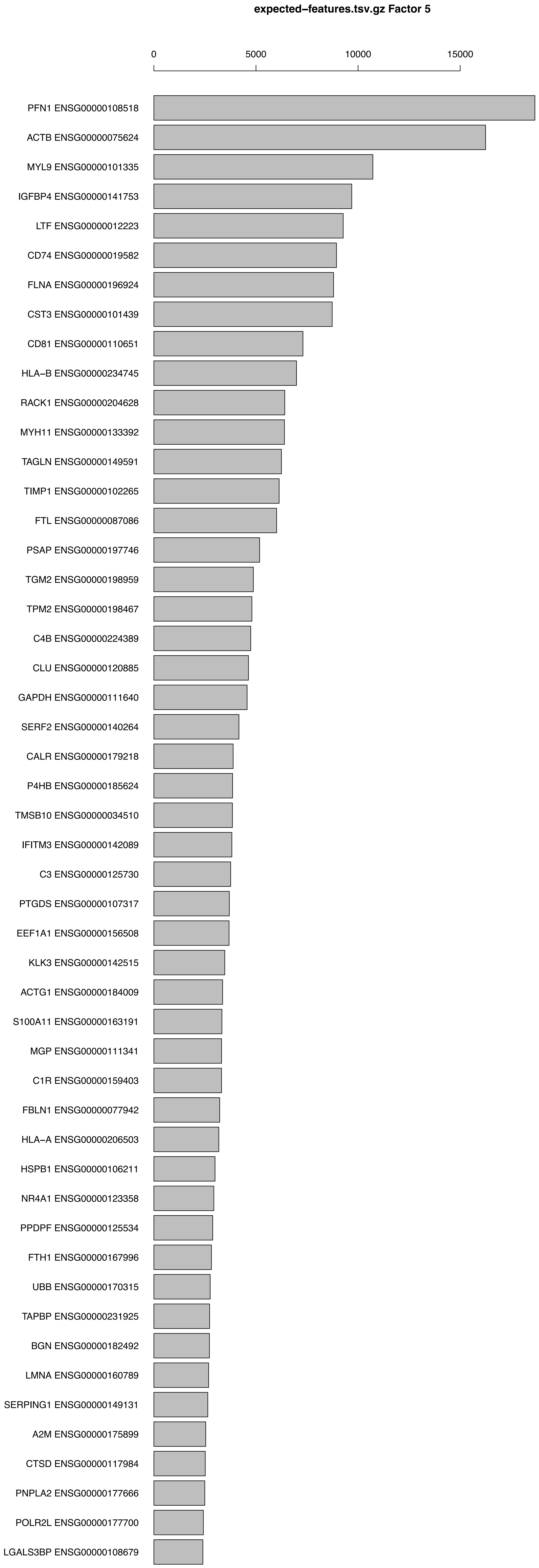
expected−features.tsv.gz Factor 5
0
5000
10000
15000
PFN1 ENSG00000108518
ACTB ENSG00000075624
MYL9 ENSG00000101335
IGFBP4 ENSG00000141753
LTF ENSG00000012223
CD74 ENSG00000019582
FLNA ENSG00000196924
CST3 ENSG00000101439
CD81 ENSG00000110651
HLA−B ENSG00000234745
RACK1 ENSG00000204628
MYH11 ENSG00000133392
TAGLN ENSG00000149591
TIMP1 ENSG00000102265
FTL ENSG00000087086
PSAP ENSG00000197746
TGM2 ENSG00000198959
TPM2 ENSG00000198467
C4B ENSG00000224389
CLU ENSG00000120885
GAPDH ENSG00000111640
SERF2 ENSG00000140264
CALR ENSG00000179218
P4HB ENSG00000185624
TMSB10 ENSG00000034510
IFITM3 ENSG00000142089
C3 ENSG00000125730
PTGDS ENSG00000107317
EEF1A1 ENSG00000156508
KLK3 ENSG00000142515
ACTG1 ENSG00000184009
S100A11 ENSG00000163191
MGP ENSG00000111341
C1R ENSG00000159403
FBLN1 ENSG00000077942
HLA−A ENSG00000206503
HSPB1 ENSG00000106211
NR4A1 ENSG00000123358
PPDPF ENSG00000125534
FTH1 ENSG00000167996
UBB ENSG00000170315
TAPBP ENSG00000231925
BGN ENSG00000182492
LMNA ENSG00000160789
SERPING1 ENSG00000149131
A2M ENSG00000175899
CTSD ENSG00000117984
PNPLA2 ENSG00000177666
POLR2L ENSG00000177700
LGALS3BP ENSG00000108679

expected−features.tsv.gz Factor 6

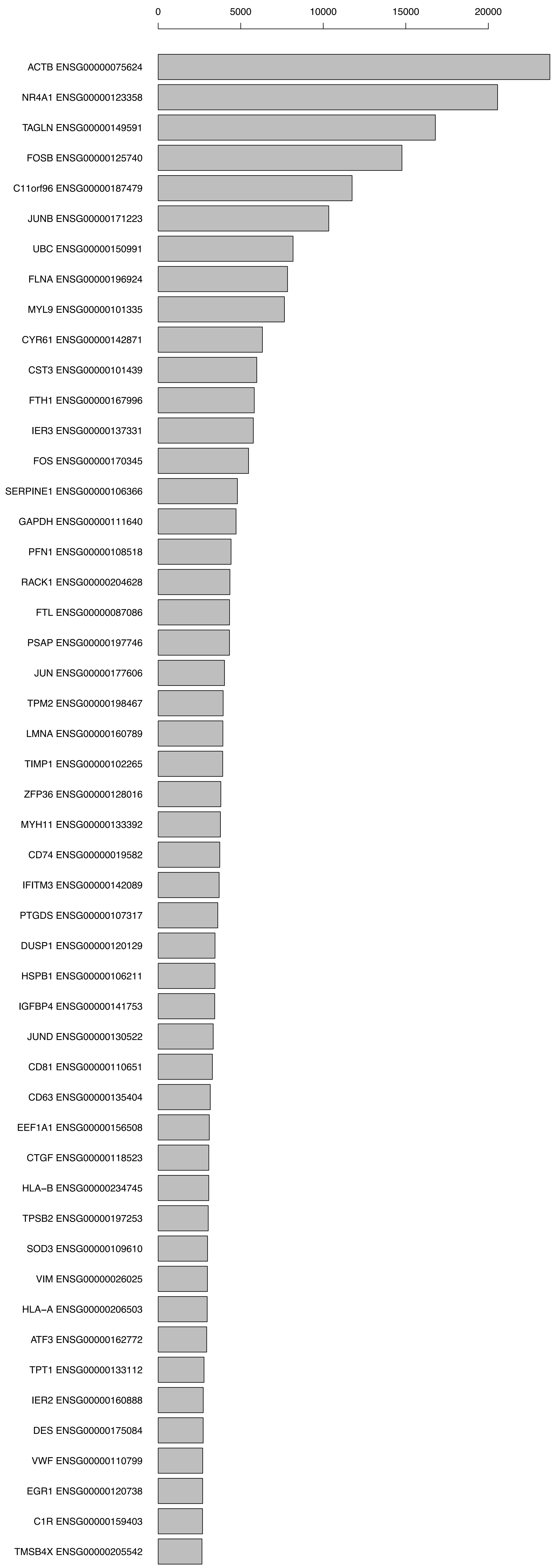

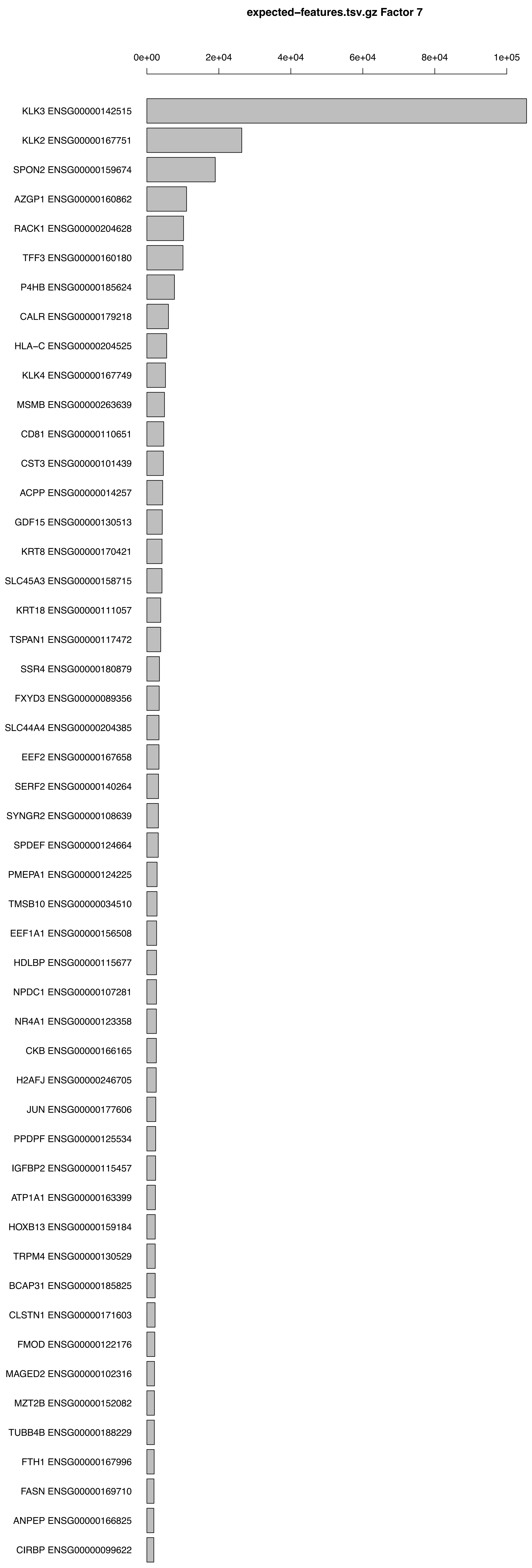
expected−features.tsv.gz Factor 7
0e+00
2e+04
4e+04
6e+04
8e+04
1e+05
KLK3 ENSG00000142515
KLK2 ENSG00000167751
SPON2 ENSG00000159674
AZGP1 ENSG00000160862
RACK1 ENSG00000204628
TFF3 ENSG00000160180
P4HB ENSG00000185624
CALR ENSG00000179218
HLA−C ENSG00000204525
KLK4 ENSG00000167749
MSMB ENSG00000263639
CD81 ENSG00000110651
CST3 ENSG00000101439
ACPP ENSG00000014257
GDF15 ENSG00000130513
KRT8 ENSG00000170421
SLC45A3 ENSG00000158715
KRT18 ENSG00000111057
TSPAN1 ENSG00000117472
SSR4 ENSG00000180879
FXYD3 ENSG00000089356
SLC44A4 ENSG00000204385
EEF2 ENSG00000167658
SERF2 ENSG00000140264
SYNGR2 ENSG00000108639
SPDEF ENSG00000124664
PMEPA1 ENSG00000124225
TMSB10 ENSG00000034510
EEF1A1 ENSG00000156508
HDLBP ENSG00000115677
NPDC1 ENSG00000107281
NR4A1 ENSG00000123358
CKB ENSG00000166165
H2AFJ ENSG00000246705
JUN ENSG00000177606
PPDPF ENSG00000125534
IGFBP2 ENSG00000115457
ATP1A1 ENSG00000163399
HOXB13 ENSG00000159184
TRPM4 ENSG00000130529
BCAP31 ENSG00000185825
CLSTN1 ENSG00000171603
FMOD ENSG00000122176
MAGED2 ENSG00000102316
MZT2B ENSG00000152082
TUBB4B ENSG00000188229
FTH1 ENSG00000167996
FASN ENSG00000169710
ANPEP ENSG00000166825
CIRBP ENSG00000099622

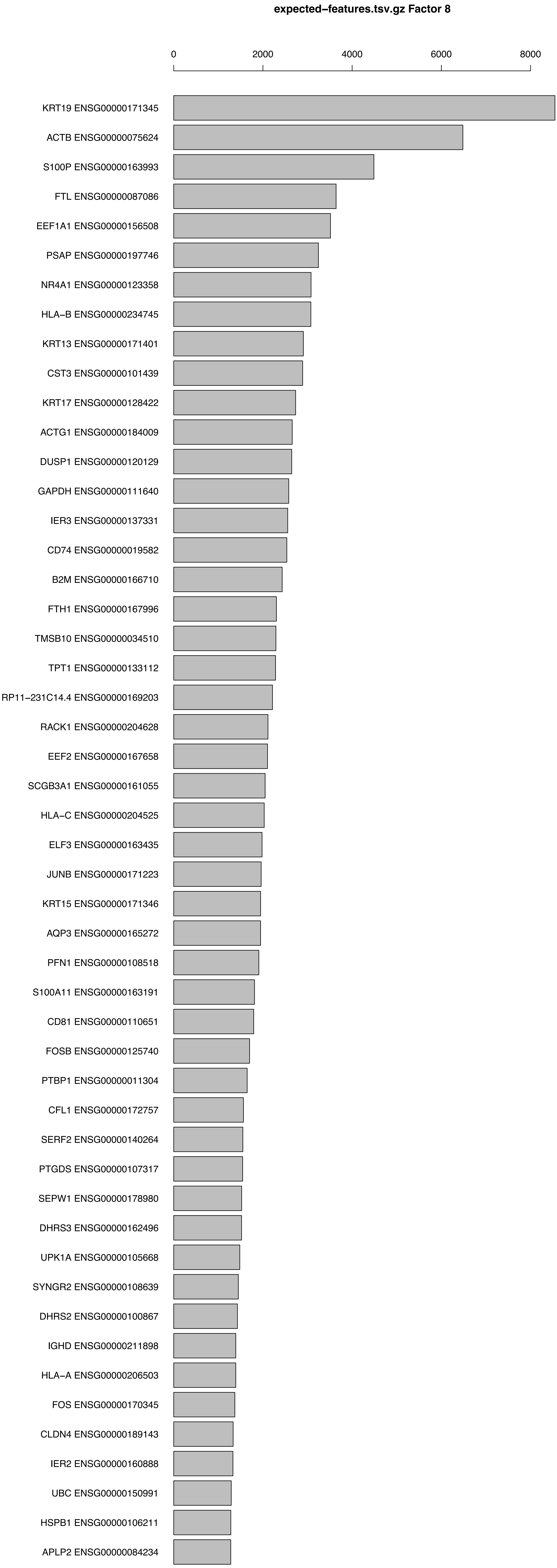
expected−features.tsv.gz Factor 8
0
2000
4000
6000
8000
KRT19 ENSG00000171345
ACTB ENSG00000075624
S100P ENSG00000163993
FTL ENSG00000087086
EEF1A1 ENSG00000156508
PSAP ENSG00000197746
NR4A1 ENSG00000123358
HLA−B ENSG00000234745
KRT13 ENSG00000171401
CST3 ENSG00000101439
KRT17 ENSG00000128422
ACTG1 ENSG00000184009
DUSP1 ENSG00000120129
GAPDH ENSG00000111640
IER3 ENSG00000137331
CD74 ENSG00000019582
B2M ENSG00000166710
FTH1 ENSG00000167996
TMSB10 ENSG00000034510
TPT1 ENSG00000133112
RP11−231C14.4 ENSG00000169203
RACK1 ENSG00000204628
EEF2 ENSG00000167658
SCGB3A1 ENSG00000161055
HLA−C ENSG00000204525
ELF3 ENSG00000163435
JUNB ENSG00000171223
KRT15 ENSG00000171346
AQP3 ENSG00000165272
PFN1 ENSG00000108518
S100A11 ENSG00000163191
CD81 ENSG00000110651
FOSB ENSG00000125740
PTBP1 ENSG00000011304
CFL1 ENSG00000172757
SERF2 ENSG00000140264
PTGDS ENSG00000107317
SEPW1 ENSG00000178980
DHRS3 ENSG00000162496
UPK1A ENSG00000105668
SYNGR2 ENSG00000108639
DHRS2 ENSG00000100867
IGHD ENSG00000211898
HLA−A ENSG00000206503
FOS ENSG00000170345
CLDN4 ENSG00000189143
IER2 ENSG00000160888
UBC ENSG00000150991
HSPB1 ENSG00000106211
APLP2 ENSG00000084234

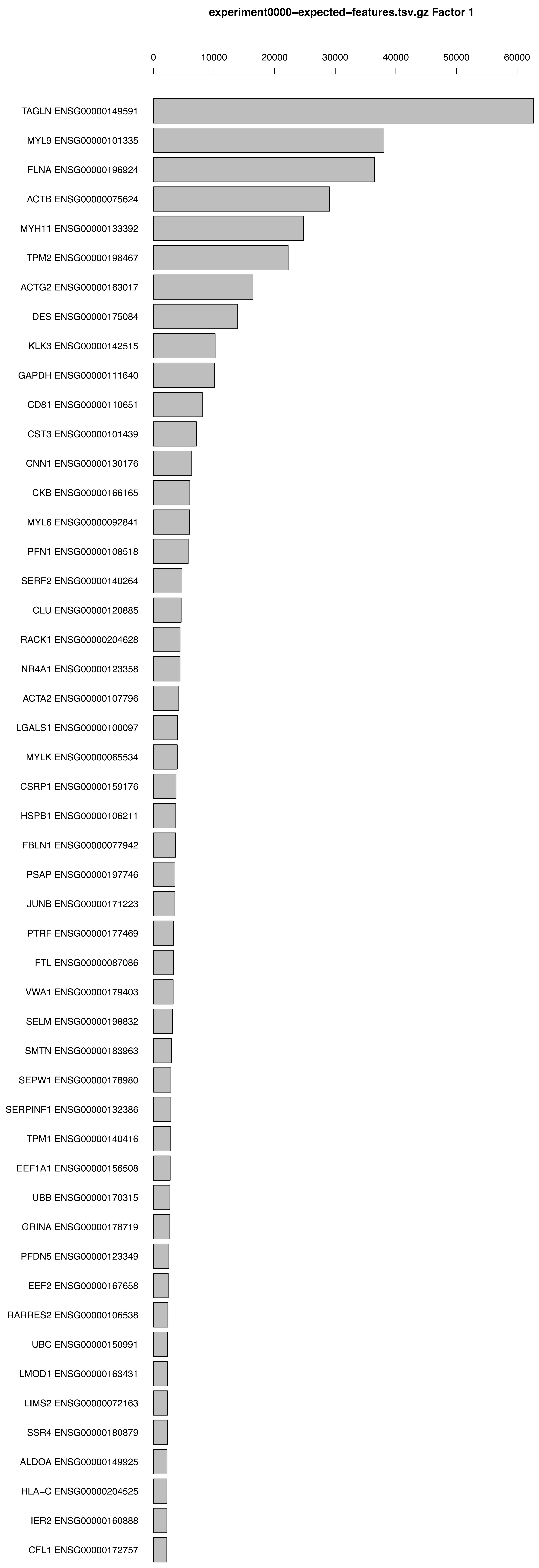
experiment0000−expected−features.tsv.gz Factor 1
0
10000
20000
30000
40000
50000
60000
TAGLN ENSG00000149591
MYL9 ENSG00000101335
FLNA ENSG00000196924
ACTB ENSG00000075624
MYH11 ENSG00000133392
TPM2 ENSG00000198467
ACTG2 ENSG00000163017
DES ENSG00000175084
KLK3 ENSG00000142515
GAPDH ENSG00000111640
CD81 ENSG00000110651
CST3 ENSG00000101439
CNN1 ENSG00000130176
CKB ENSG00000166165
MYL6 ENSG00000092841
PFN1 ENSG00000108518
SERF2 ENSG00000140264
CLU ENSG00000120885
RACK1 ENSG00000204628
NR4A1 ENSG00000123358
ACTA2 ENSG00000107796
LGALS1 ENSG00000100097
MYLK ENSG00000065534
CSRP1 ENSG00000159176
HSPB1 ENSG00000106211
FBLN1 ENSG00000077942
PSAP ENSG00000197746
JUNB ENSG00000171223
PTRF ENSG00000177469
FTL ENSG00000087086
VWA1 ENSG00000179403
SELM ENSG00000198832
SMTN ENSG00000183963
SEPW1 ENSG00000178980
SERPINF1 ENSG00000132386
TPM1 ENSG00000140416
EEF1A1 ENSG00000156508
UBB ENSG00000170315
GRINA ENSG00000178719
PFDN5 ENSG00000123349
EEF2 ENSG00000167658
RARRES2 ENSG00000106538
UBC ENSG00000150991
LMOD1 ENSG00000163431
LIMS2 ENSG00000072163
SSR4 ENSG00000180879
ALDOA ENSG00000149925
HLA−C ENSG00000204525
IER2 ENSG00000160888
CFL1 ENSG00000172757

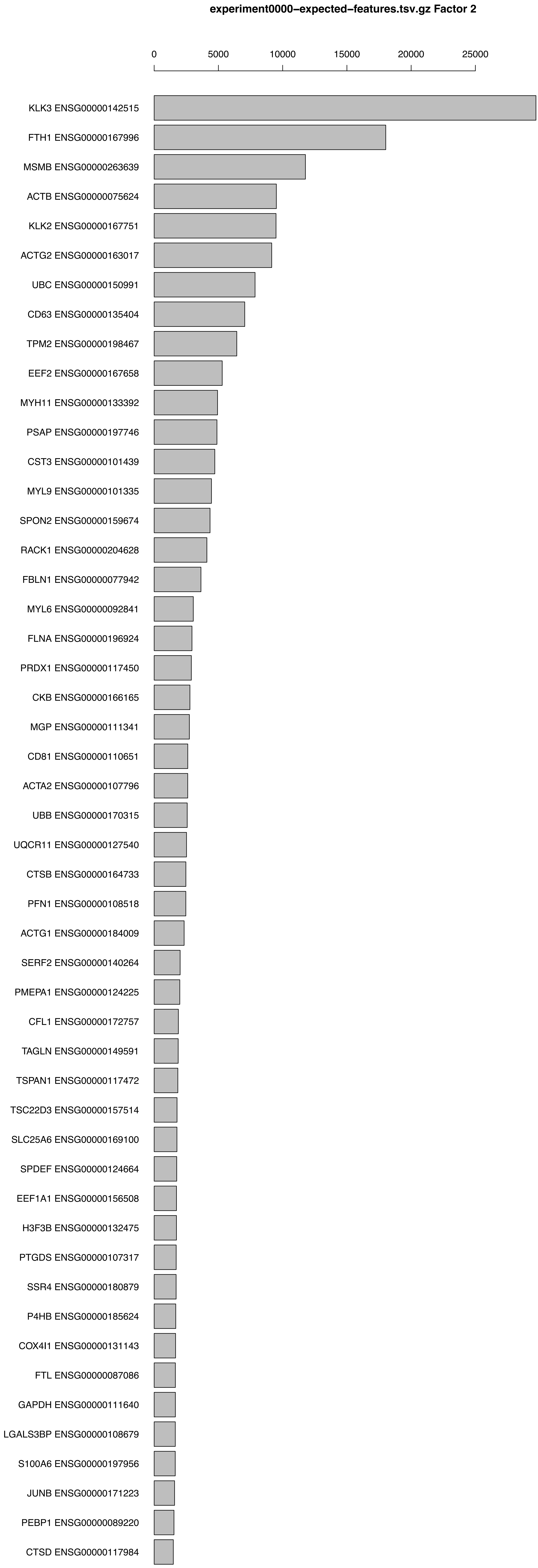
experiment0000−expected−features.tsv.gz Factor 2
0
5000
10000
15000
20000
25000
KLK3 ENSG00000142515
FTH1 ENSG00000167996
MSMB ENSG00000263639
ACTB ENSG00000075624
KLK2 ENSG00000167751
ACTG2 ENSG00000163017
UBC ENSG00000150991
CD63 ENSG00000135404
TPM2 ENSG00000198467
EEF2 ENSG00000167658
MYH11 ENSG00000133392
PSAP ENSG00000197746
CST3 ENSG00000101439
MYL9 ENSG00000101335
SPON2 ENSG00000159674
RACK1 ENSG00000204628
FBLN1 ENSG00000077942
MYL6 ENSG00000092841
FLNA ENSG00000196924
PRDX1 ENSG00000117450
CKB ENSG00000166165
MGP ENSG00000111341
CD81 ENSG00000110651
ACTA2 ENSG00000107796
UBB ENSG00000170315
UQCR11 ENSG00000127540
CTSB ENSG00000164733
PFN1 ENSG00000108518
ACTG1 ENSG00000184009
SERF2 ENSG00000140264
PMEPA1 ENSG00000124225
CFL1 ENSG00000172757
TAGLN ENSG00000149591
TSPAN1 ENSG00000117472
TSC22D3 ENSG00000157514
SLC25A6 ENSG00000169100
SPDEF ENSG00000124664
EEF1A1 ENSG00000156508
H3F3B ENSG00000132475
PTGDS ENSG00000107317
SSR4 ENSG00000180879
P4HB ENSG00000185624
COX4I1 ENSG00000131143
FTL ENSG00000087086
GAPDH ENSG00000111640
LGALS3BP ENSG00000108679
S100A6 ENSG00000197956
JUNB ENSG00000171223
PEBP1 ENSG00000089220
CTSD ENSG00000117984

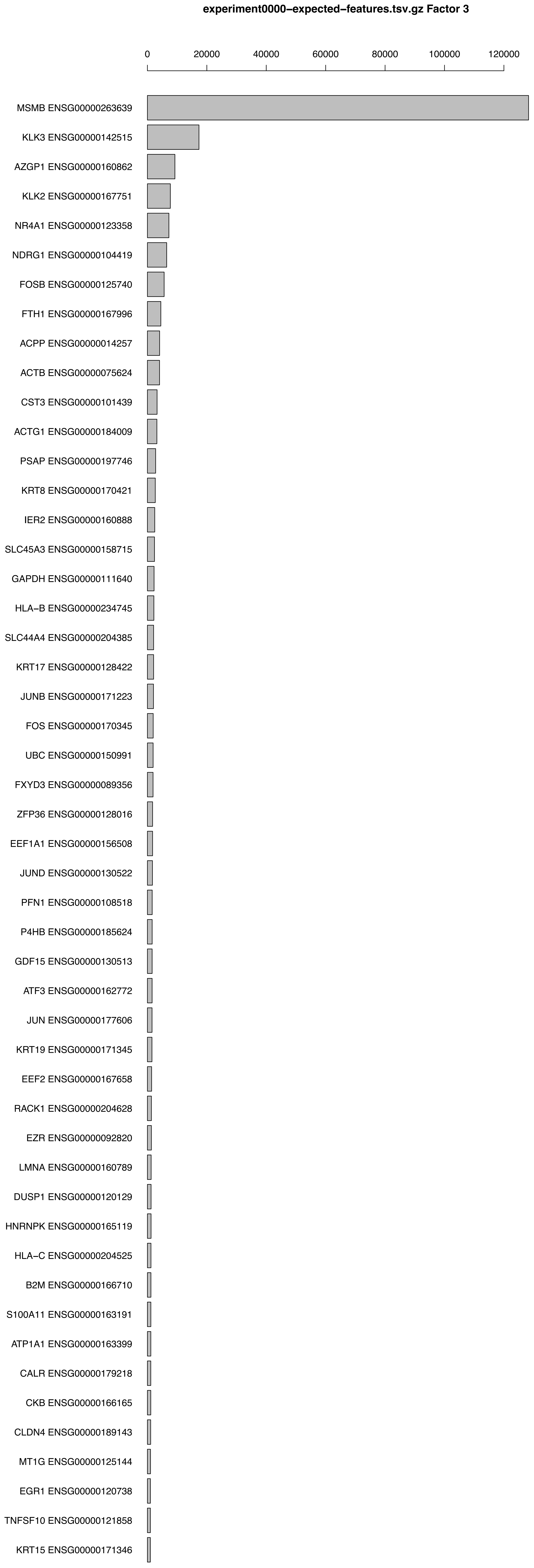
experiment0000−expected−features.tsv.gz Factor 3
0
20000
40000
60000
80000
100000
120000
MSMB ENSG00000263639
KLK3 ENSG00000142515
AZGP1 ENSG00000160862
KLK2 ENSG00000167751
NR4A1 ENSG00000123358
NDRG1 ENSG00000104419
FOSB ENSG00000125740
FTH1 ENSG00000167996
ACPP ENSG00000014257
ACTB ENSG00000075624
CST3 ENSG00000101439
ACTG1 ENSG00000184009
PSAP ENSG00000197746
KRT8 ENSG00000170421
IER2 ENSG00000160888
SLC45A3 ENSG00000158715
GAPDH ENSG00000111640
HLA−B ENSG00000234745
SLC44A4 ENSG00000204385
KRT17 ENSG00000128422
JUNB ENSG00000171223
FOS ENSG00000170345
UBC ENSG00000150991
FXYD3 ENSG00000089356
ZFP36 ENSG00000128016
EEF1A1 ENSG00000156508
JUND ENSG00000130522
PFN1 ENSG00000108518
P4HB ENSG00000185624
GDF15 ENSG00000130513
ATF3 ENSG00000162772
JUN ENSG00000177606
KRT19 ENSG00000171345
EEF2 ENSG00000167658
RACK1 ENSG00000204628
EZR ENSG00000092820
LMNA ENSG00000160789
DUSP1 ENSG00000120129
HNRNPK ENSG00000165119
HLA−C ENSG00000204525
B2M ENSG00000166710
S100A11 ENSG00000163191
ATP1A1 ENSG00000163399
CALR ENSG00000179218
CKB ENSG00000166165
CLDN4 ENSG00000189143
MT1G ENSG00000125144
EGR1 ENSG00000120738
TNFSF10 ENSG00000121858
KRT15 ENSG00000171346

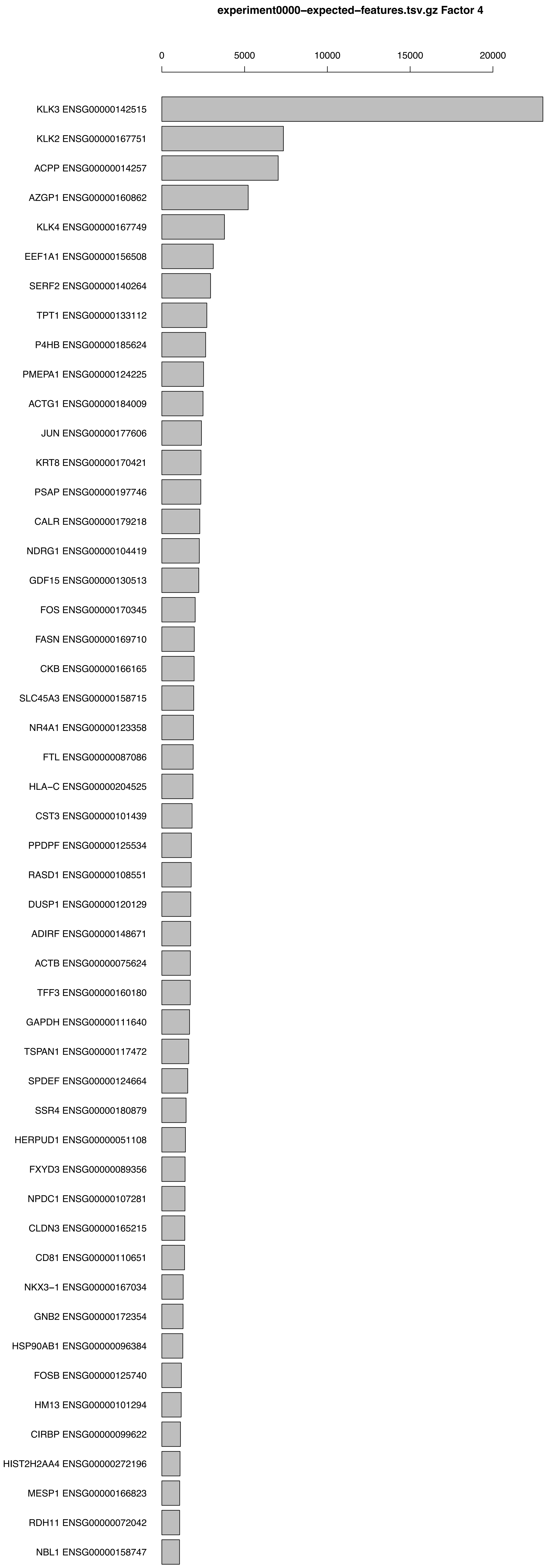
experiment0000−expected−features.tsv.gz Factor 4
0
5000
10000
15000
20000
KLK3 ENSG00000142515
KLK2 ENSG00000167751
ACPP ENSG00000014257
AZGP1 ENSG00000160862
KLK4 ENSG00000167749
EEF1A1 ENSG00000156508
SERF2 ENSG00000140264
TPT1 ENSG00000133112
P4HB ENSG00000185624
PMEPA1 ENSG00000124225
ACTG1 ENSG00000184009
JUN ENSG00000177606
KRT8 ENSG00000170421
PSAP ENSG00000197746
CALR ENSG00000179218
NDRG1 ENSG00000104419
GDF15 ENSG00000130513
FOS ENSG00000170345
FASN ENSG00000169710
CKB ENSG00000166165
SLC45A3 ENSG00000158715
NR4A1 ENSG00000123358
FTL ENSG00000087086
HLA−C ENSG00000204525
CST3 ENSG00000101439
PPDPF ENSG00000125534
RASD1 ENSG00000108551
DUSP1 ENSG00000120129
ADIRF ENSG00000148671
ACTB ENSG00000075624
TFF3 ENSG00000160180
GAPDH ENSG00000111640
TSPAN1 ENSG00000117472
SPDEF ENSG00000124664
SSR4 ENSG00000180879
HERPUD1 ENSG00000051108
FXYD3 ENSG00000089356
NPDC1 ENSG00000107281
CLDN3 ENSG00000165215
CD81 ENSG00000110651
NKX3−1 ENSG00000167034
GNB2 ENSG00000172354
HSP90AB1 ENSG00000096384
FOSB ENSG00000125740
HM13 ENSG00000101294
CIRBP ENSG00000099622
HIST2H2AA4 ENSG00000272196
MESP1 ENSG00000166823
RDH11 ENSG00000072042
NBL1 ENSG00000158747

experiment0000–expected–features.tsv.gz Factor 5

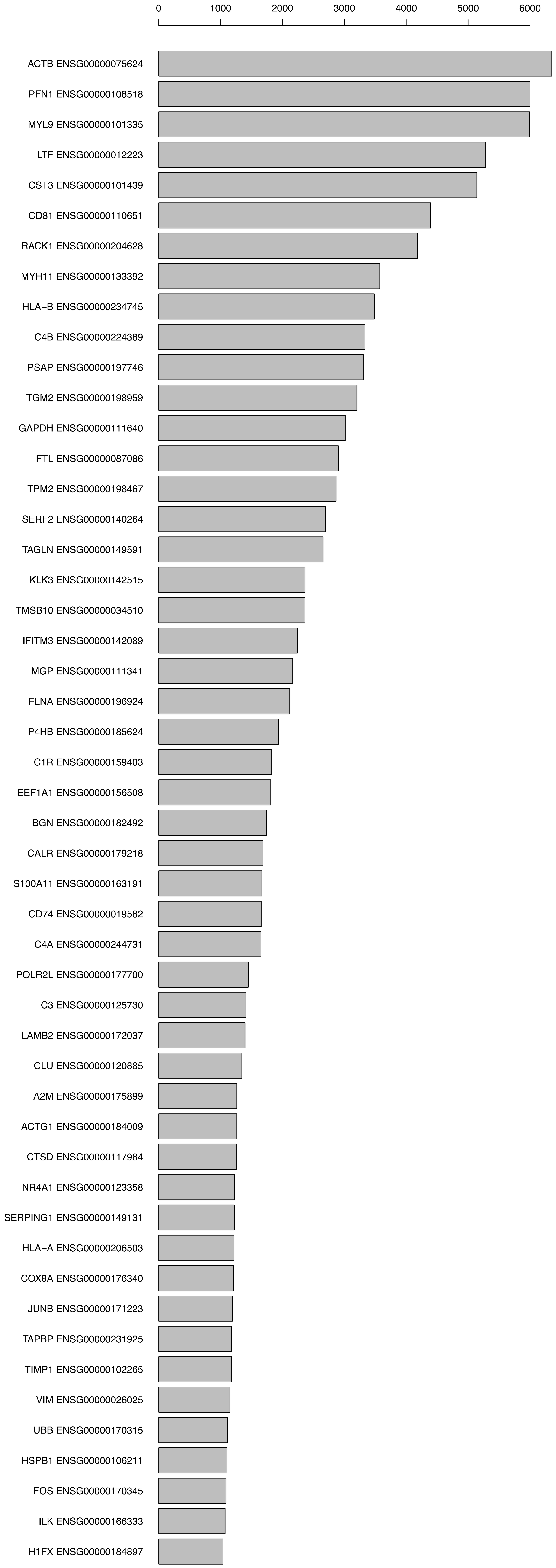

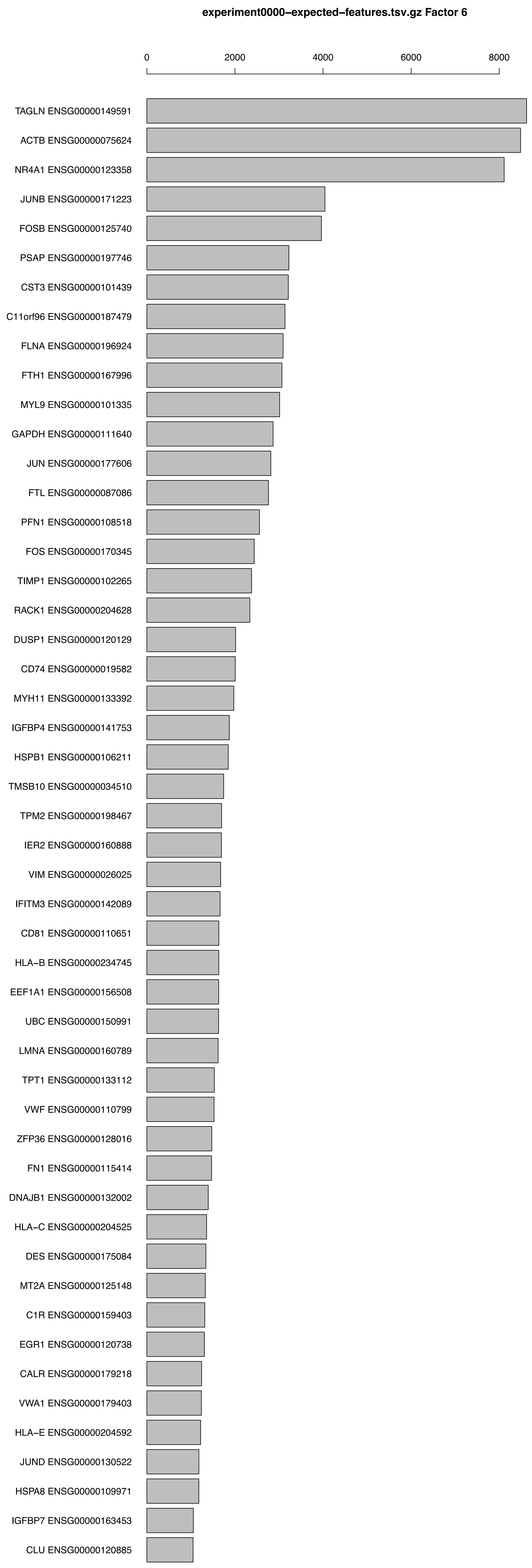
experiment0000−expected−features.tsv.gz Factor 6
0
2000
4000
6000
8000
TAGLN ENSG00000149591
ACTB ENSG00000075624
NR4A1 ENSG00000123358
JUNB ENSG00000171223
FOSB ENSG00000125740
PSAP ENSG00000197746
CST3 ENSG00000101439
C11orf96 ENSG00000187479
FLNA ENSG00000196924
FTH1 ENSG00000167996
MYL9 ENSG00000101335
GAPDH ENSG00000111640
JUN ENSG00000177606
FTL ENSG00000087086
PFN1 ENSG00000108518
FOS ENSG00000170345
TIMP1 ENSG00000102265
RACK1 ENSG00000204628
DUSP1 ENSG00000120129
CD74 ENSG00000019582
MYH11 ENSG00000133392
IGFBP4 ENSG00000141753
HSPB1 ENSG00000106211
TMSB10 ENSG00000034510
TPM2 ENSG00000198467
IER2 ENSG00000160888
VIM ENSG00000026025
IFITM3 ENSG00000142089
CD81 ENSG00000110651
HLA−B ENSG00000234745
EEF1A1 ENSG00000156508
UBC ENSG00000150991
LMNA ENSG00000160789
TPT1 ENSG00000133112
VWF ENSG00000110799
ZFP36 ENSG00000128016
FN1 ENSG00000115414
DNAJB1 ENSG00000132002
HLA−C ENSG00000204525
DES ENSG00000175084
MT2A ENSG00000125148
C1R ENSG00000159403
EGR1 ENSG00000120738
CALR ENSG00000179218
VWA1 ENSG00000179403
HLA−E ENSG00000204592
JUND ENSG00000130522
HSPA8 ENSG00000109971
IGFBP7 ENSG00000163453
CLU ENSG00000120885

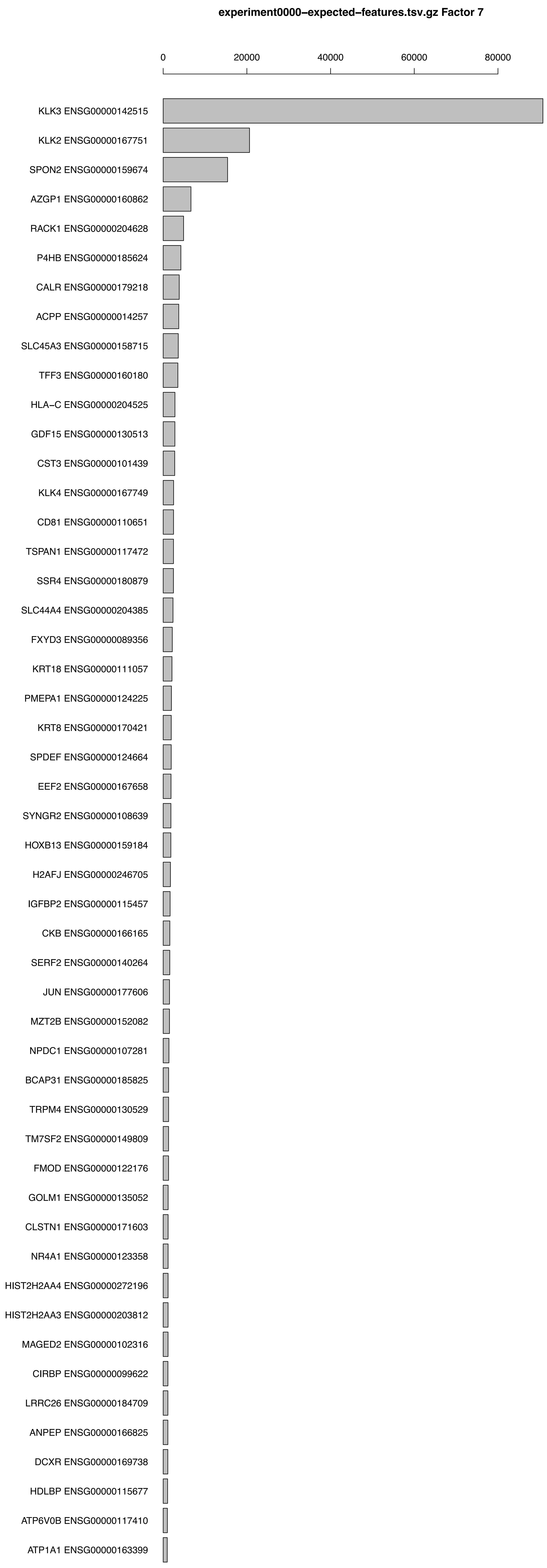
experiment0000−expected−features.tsv.gz Factor 7
0
20000
40000
60000
80000
KLK3 ENSG00000142515
KLK2 ENSG00000167751
SPON2 ENSG00000159674
AZGP1 ENSG00000160862
RACK1 ENSG00000204628
P4HB ENSG00000185624
CALR ENSG00000179218
ACPP ENSG00000014257
SLC45A3 ENSG00000158715
TFF3 ENSG00000160180
HLA−C ENSG00000204525
GDF15 ENSG00000130513
CST3 ENSG00000101439
KLK4 ENSG00000167749
CD81 ENSG00000110651
TSPAN1 ENSG00000117472
SSR4 ENSG00000180879
SLC44A4 ENSG00000204385
FXYD3 ENSG00000089356
KRT18 ENSG00000111057
PMEPA1 ENSG00000124225
KRT8 ENSG00000170421
SPDEF ENSG00000124664
EEF2 ENSG00000167658
SYNGR2 ENSG00000108639
HOXB13 ENSG00000159184
H2AFJ ENSG00000246705
IGFBP2 ENSG00000115457
CKB ENSG00000166165
SERF2 ENSG00000140264
JUN ENSG00000177606
MZT2B ENSG00000152082
NPDC1 ENSG00000107281
BCAP31 ENSG00000185825
TRPM4 ENSG00000130529
TM7SF2 ENSG00000149809
FMOD ENSG00000122176
GOLM1 ENSG00000135052
CLSTN1 ENSG00000171603
NR4A1 ENSG00000123358
HIST2H2AA4 ENSG00000272196
HIST2H2AA3 ENSG00000203812
MAGED2 ENSG00000102316
CIRBP ENSG00000099622
LRRC26 ENSG00000184709
ANPEP ENSG00000166825
DCXR ENSG00000169738
HDLBP ENSG00000115677
ATP6V0B ENSG00000117410
ATP1A1 ENSG00000163399

experiment0000−expected−features.tsv.gz Factor 8

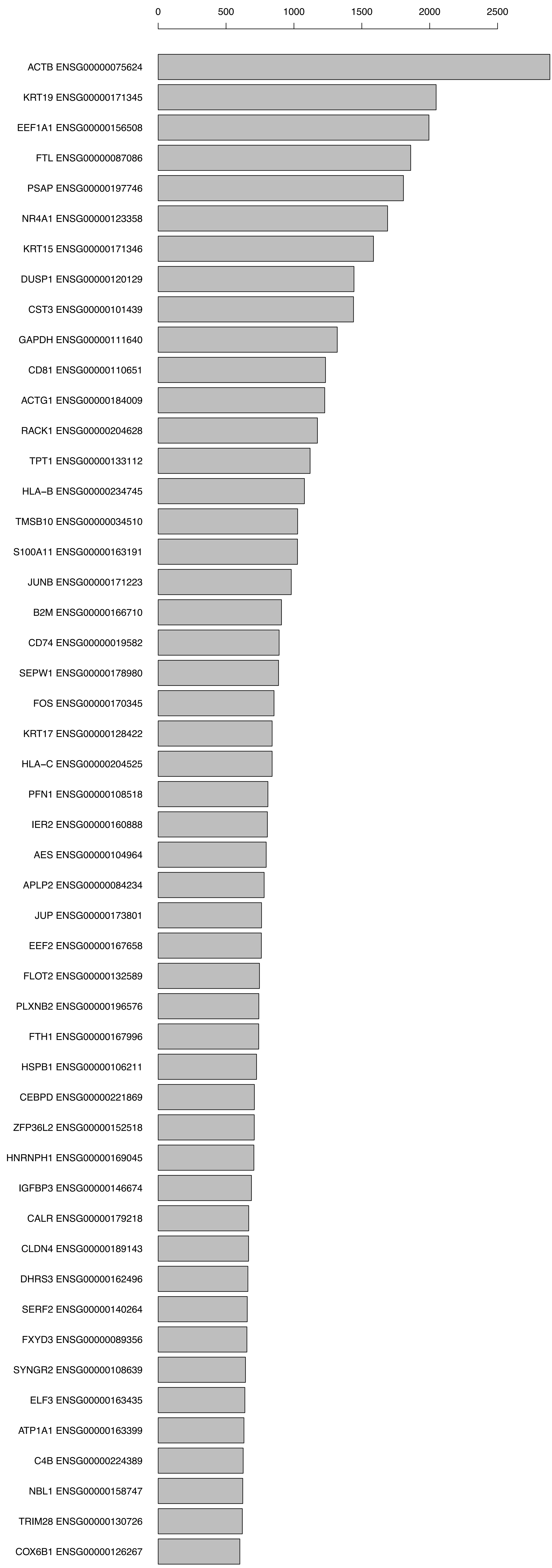

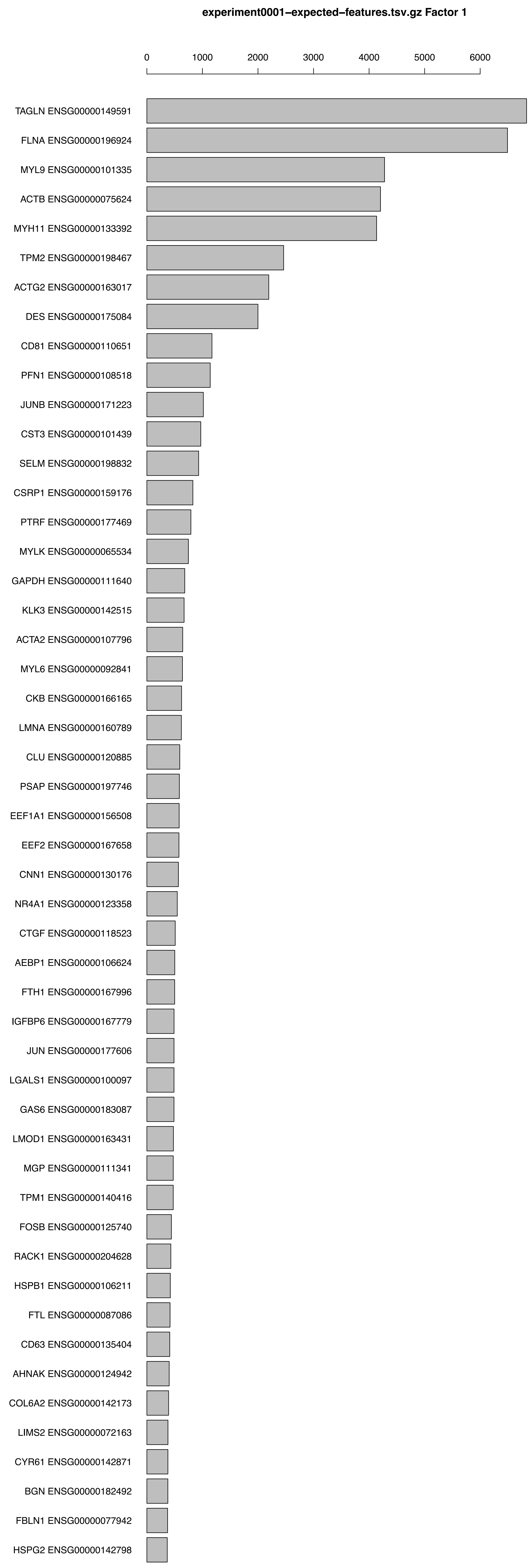
experiment0001−expected−features.tsv.gz Factor 1
0
1000
2000
3000
4000
5000
6000
TAGLN ENSG00000149591
FLNA ENSG00000196924
MYL9 ENSG00000101335
ACTB ENSG00000075624
MYH11 ENSG00000133392
TPM2 ENSG00000198467
ACTG2 ENSG00000163017
DES ENSG00000175084
CD81 ENSG00000110651
PFN1 ENSG00000108518
JUNB ENSG00000171223
CST3 ENSG00000101439
SELM ENSG00000198832
CSRP1 ENSG00000159176
PTRF ENSG00000177469
MYLK ENSG00000065534
GAPDH ENSG00000111640
KLK3 ENSG00000142515
ACTA2 ENSG00000107796
MYL6 ENSG00000092841
CKB ENSG00000166165
LMNA ENSG00000160789
CLU ENSG00000120885
PSAP ENSG00000197746
EEF1A1 ENSG00000156508
EEF2 ENSG00000167658
CNN1 ENSG00000130176
NR4A1 ENSG00000123358
CTGF ENSG00000118523
AEBP1 ENSG00000106624
FTH1 ENSG00000167996
IGFBP6 ENSG00000167779
JUN ENSG00000177606
LGALS1 ENSG00000100097
GAS6 ENSG00000183087
LMOD1 ENSG00000163431
MGP ENSG00000111341
TPM1 ENSG00000140416
FOSB ENSG00000125740
RACK1 ENSG00000204628
HSPB1 ENSG00000106211
FTL ENSG00000087086
CD63 ENSG00000135404
AHNAK ENSG00000124942
COL6A2 ENSG00000142173
LIMS2 ENSG00000072163
CYR61 ENSG00000142871
BGN ENSG00000182492
FBLN1 ENSG00000077942
HSPG2 ENSG00000142798

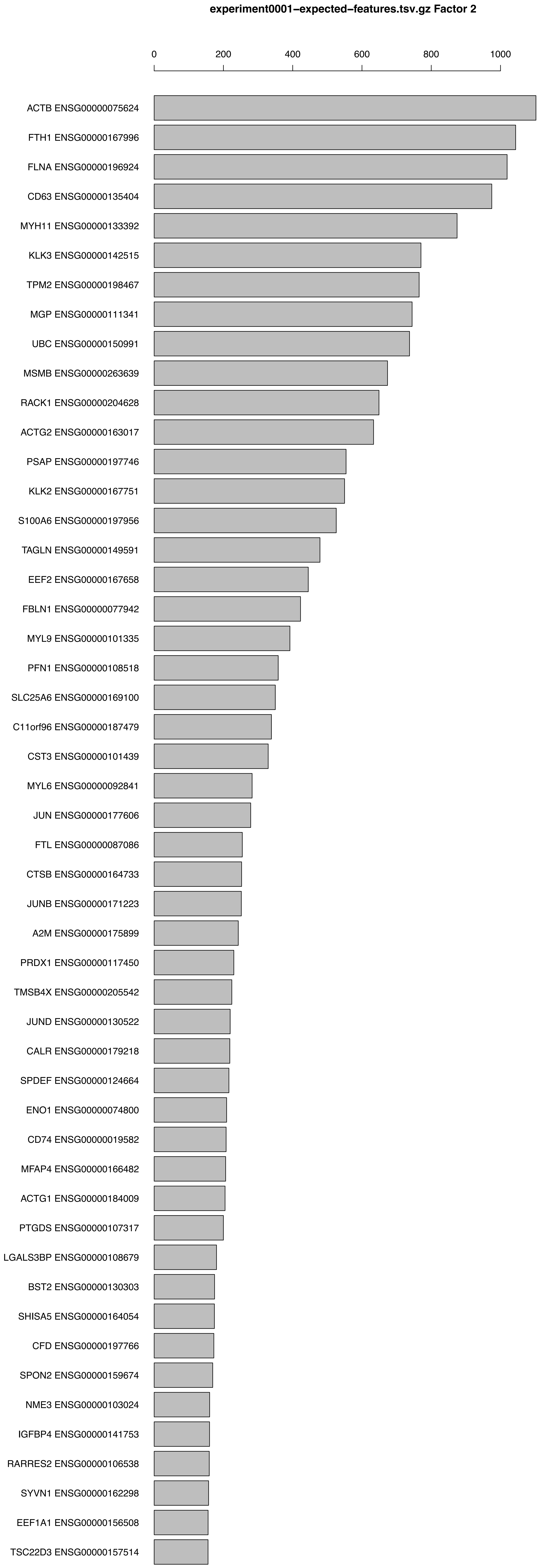
experiment0001−expected−features.tsv.gz Factor 2
0
200
400
600
800
1000
ACTB ENSG00000075624
FTH1 ENSG00000167996
FLNA ENSG00000196924
CD63 ENSG00000135404
MYH11 ENSG00000133392
KLK3 ENSG00000142515
TPM2 ENSG00000198467
MGP ENSG00000111341
UBC ENSG00000150991
MSMB ENSG00000263639
RACK1 ENSG00000204628
ACTG2 ENSG00000163017
PSAP ENSG00000197746
KLK2 ENSG00000167751
S100A6 ENSG00000197956
TAGLN ENSG00000149591
EEF2 ENSG00000167658
FBLN1 ENSG00000077942
MYL9 ENSG00000101335
PFN1 ENSG00000108518
SLC25A6 ENSG00000169100
C11orf96 ENSG00000187479
CST3 ENSG00000101439
MYL6 ENSG00000092841
JUN ENSG00000177606
FTL ENSG00000087086
CTSB ENSG00000164733
JUNB ENSG00000171223
A2M ENSG00000175899
PRDX1 ENSG00000117450
TMSB4X ENSG00000205542
JUND ENSG00000130522
CALR ENSG00000179218
SPDEF ENSG00000124664
ENO1 ENSG00000074800
CD74 ENSG00000019582
MFAP4 ENSG00000166482
ACTG1 ENSG00000184009
PTGDS ENSG00000107317
LGALS3BP ENSG00000108679
BST2 ENSG00000130303
SHISA5 ENSG00000164054
CFD ENSG00000197766
SPON2 ENSG00000159674
NME3 ENSG00000103024
IGFBP4 ENSG00000141753
RARRES2 ENSG00000106538
SYVN1 ENSG00000162298
EEF1A1 ENSG00000156508
TSC22D3 ENSG00000157514

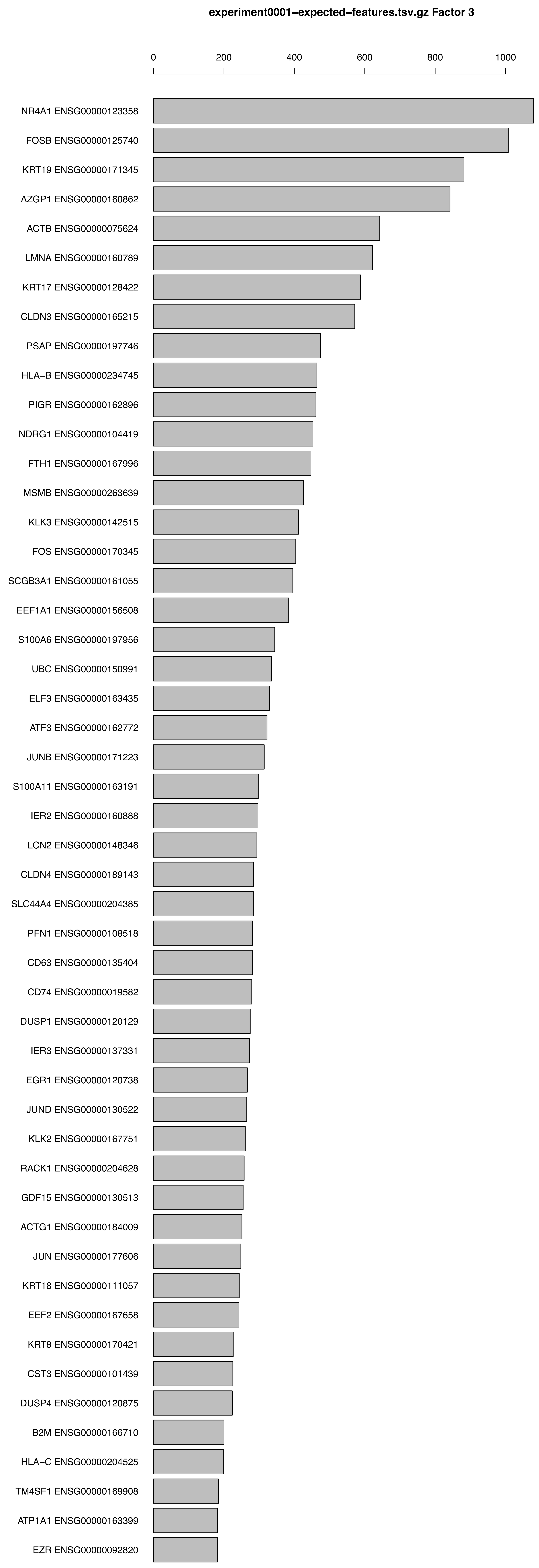
experiment0001−expected−features.tsv.gz Factor 3
0
200
400
600
800
1000
NR4A1 ENSG00000123358
FOSB ENSG00000125740
KRT19 ENSG00000171345
AZGP1 ENSG00000160862
ACTB ENSG00000075624
LMNA ENSG00000160789
KRT17 ENSG00000128422
CLDN3 ENSG00000165215
PSAP ENSG00000197746
HLA−B ENSG00000234745
PIGR ENSG00000162896
NDRG1 ENSG00000104419
FTH1 ENSG00000167996
MSMB ENSG00000263639
KLK3 ENSG00000142515
FOS ENSG00000170345
SCGB3A1 ENSG00000161055
EEF1A1 ENSG00000156508
S100A6 ENSG00000197956
UBC ENSG00000150991
ELF3 ENSG00000163435
ATF3 ENSG00000162772
JUNB ENSG00000171223
S100A11 ENSG00000163191
IER2 ENSG00000160888
LCN2 ENSG00000148346
CLDN4 ENSG00000189143
SLC44A4 ENSG00000204385
PFN1 ENSG00000108518
CD63 ENSG00000135404
CD74 ENSG00000019582
DUSP1 ENSG00000120129
IER3 ENSG00000137331
EGR1 ENSG00000120738
JUND ENSG00000130522
KLK2 ENSG00000167751
RACK1 ENSG00000204628
GDF15 ENSG00000130513
ACTG1 ENSG00000184009
JUN ENSG00000177606
KRT18 ENSG00000111057
EEF2 ENSG00000167658
KRT8 ENSG00000170421
CST3 ENSG00000101439
DUSP4 ENSG00000120875
B2M ENSG00000166710
HLA−C ENSG00000204525
TM4SF1 ENSG00000169908
ATP1A1 ENSG00000163399
EZR ENSG00000092820

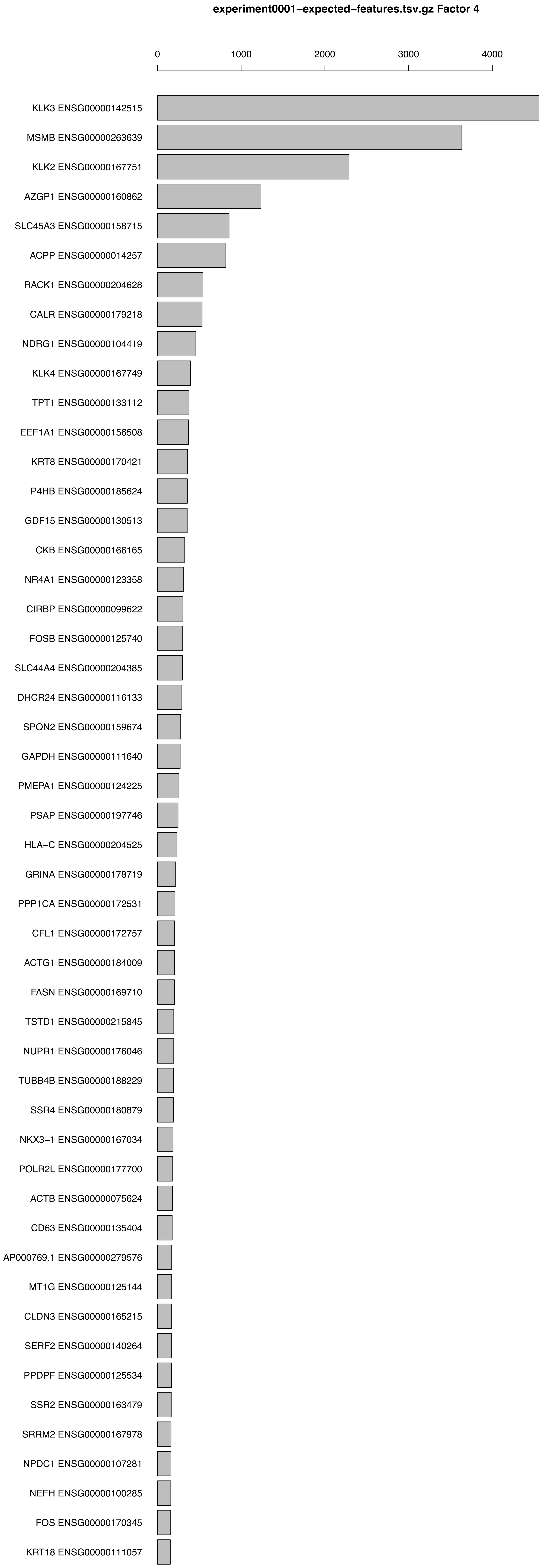
experiment0001−expected−features.tsv.gz Factor 4
0
1000
2000
3000
4000
KLK3 ENSG00000142515
MSMB ENSG00000263639
KLK2 ENSG00000167751
AZGP1 ENSG00000160862
SLC45A3 ENSG00000158715
ACPP ENSG00000014257
RACK1 ENSG00000204628
CALR ENSG00000179218
NDRG1 ENSG00000104419
KLK4 ENSG00000167749
TPT1 ENSG00000133112
EEF1A1 ENSG00000156508
KRT8 ENSG00000170421
P4HB ENSG00000185624
GDF15 ENSG00000130513
CKB ENSG00000166165
NR4A1 ENSG00000123358
CIRBP ENSG00000099622
FOSB ENSG00000125740
SLC44A4 ENSG00000204385
DHCR24 ENSG00000116133
SPON2 ENSG00000159674
GAPDH ENSG00000111640
PMEPA1 ENSG00000124225
PSAP ENSG00000197746
HLA−C ENSG00000204525
GRINA ENSG00000178719
PPP1CA ENSG00000172531
CFL1 ENSG00000172757
ACTG1 ENSG00000184009
FASN ENSG00000169710
TSTD1 ENSG00000215845
NUPR1 ENSG00000176046
TUBB4B ENSG00000188229
SSR4 ENSG00000180879
NKX3−1 ENSG00000167034
POLR2L ENSG00000177700
ACTB ENSG00000075624
CD63 ENSG00000135404
AP000769.1 ENSG00000279576
MT1G ENSG00000125144
CLDN3 ENSG00000165215
SERF2 ENSG00000140264
PPDPF ENSG00000125534
SSR2 ENSG00000163479
SRRM2 ENSG00000167978
NPDC1 ENSG00000107281
NEFH ENSG00000100285
FOS ENSG00000170345
KRT18 ENSG00000111057

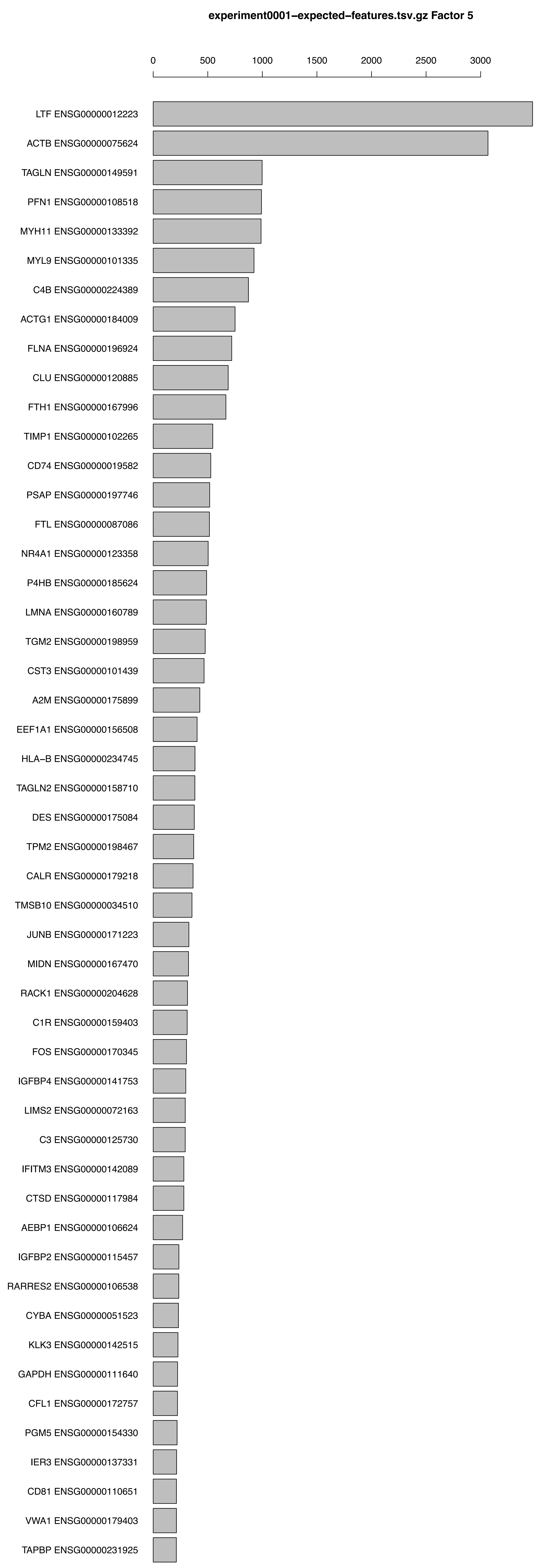
experiment0001−expected−features.tsv.gz Factor 5
0
500
1000
1500
2000
2500
3000
LTF ENSG00000012223
ACTB ENSG00000075624
TAGLN ENSG00000149591
PFN1 ENSG00000108518
MYH11 ENSG00000133392
MYL9 ENSG00000101335
C4B ENSG00000224389
ACTG1 ENSG00000184009
FLNA ENSG00000196924
CLU ENSG00000120885
FTH1 ENSG00000167996
TIMP1 ENSG00000102265
CD74 ENSG00000019582
PSAP ENSG00000197746
FTL ENSG00000087086
NR4A1 ENSG00000123358
P4HB ENSG00000185624
LMNA ENSG00000160789
TGM2 ENSG00000198959
CST3 ENSG00000101439
A2M ENSG00000175899
EEF1A1 ENSG00000156508
HLA−B ENSG00000234745
TAGLN2 ENSG00000158710
DES ENSG00000175084
TPM2 ENSG00000198467
CALR ENSG00000179218
TMSB10 ENSG00000034510
JUNB ENSG00000171223
MIDN ENSG00000167470
RACK1 ENSG00000204628
C1R ENSG00000159403
FOS ENSG00000170345
IGFBP4 ENSG00000141753
LIMS2 ENSG00000072163
C3 ENSG00000125730
IFITM3 ENSG00000142089
CTSD ENSG00000117984
AEBP1 ENSG00000106624
IGFBP2 ENSG00000115457
RARRES2 ENSG00000106538
CYBA ENSG00000051523
KLK3 ENSG00000142515
GAPDH ENSG00000111640
CFL1 ENSG00000172757
PGM5 ENSG00000154330
IER3 ENSG00000137331
CD81 ENSG00000110651
VWA1 ENSG00000179403
TAPBP ENSG00000231925

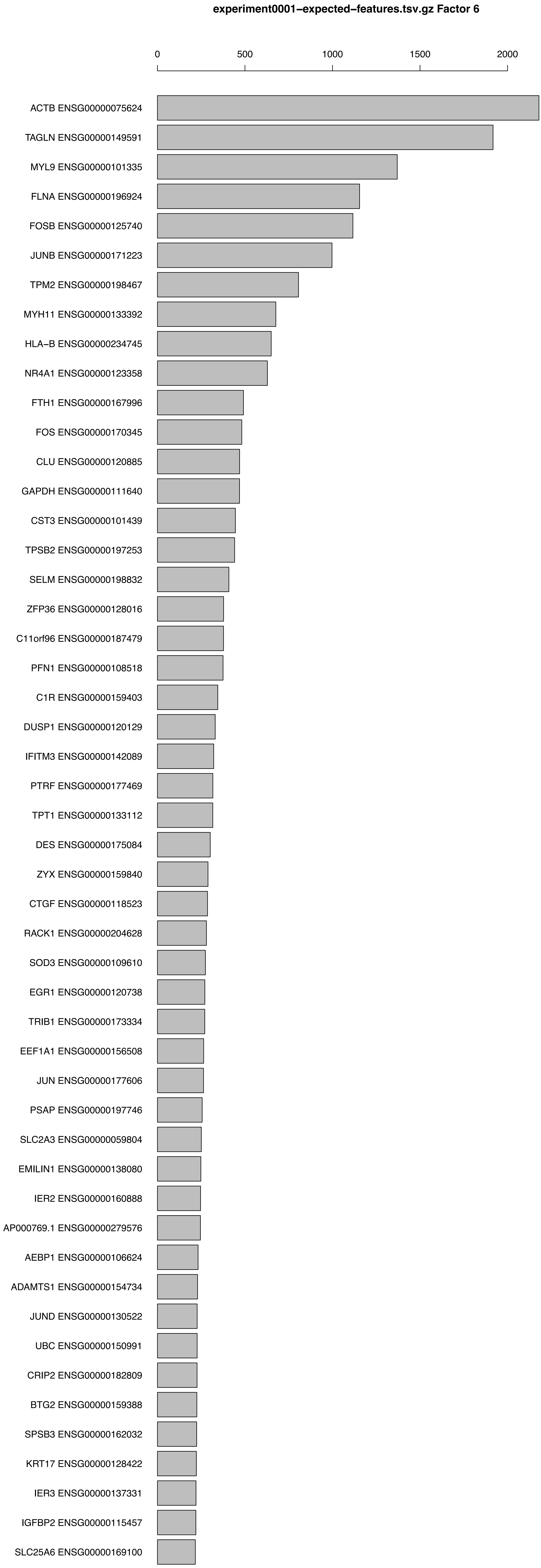
experiment0001−expected−features.tsv.gz Factor 6
0
500
1000
1500
2000
ACTB ENSG00000075624
TAGLN ENSG00000149591
MYL9 ENSG00000101335
FLNA ENSG00000196924
FOSB ENSG00000125740
JUNB ENSG00000171223
TPM2 ENSG00000198467
MYH11 ENSG00000133392
HLA−B ENSG00000234745
NR4A1 ENSG00000123358
FTH1 ENSG00000167996
FOS ENSG00000170345
CLU ENSG00000120885
GAPDH ENSG00000111640
CST3 ENSG00000101439
TPSB2 ENSG00000197253
SELM ENSG00000198832
ZFP36 ENSG00000128016
C11orf96 ENSG00000187479
PFN1 ENSG00000108518
C1R ENSG00000159403
DUSP1 ENSG00000120129
IFITM3 ENSG00000142089
PTRF ENSG00000177469
TPT1 ENSG00000133112
DES ENSG00000175084
ZYX ENSG00000159840
CTGF ENSG00000118523
RACK1 ENSG00000204628
SOD3 ENSG00000109610
EGR1 ENSG00000120738
TRIB1 ENSG00000173334
EEF1A1 ENSG00000156508
JUN ENSG00000177606
PSAP ENSG00000197746
SLC2A3 ENSG00000059804
EMILIN1 ENSG00000138080
IER2 ENSG00000160888
AP000769.1 ENSG00000279576
AEBP1 ENSG00000106624
ADAMTS1 ENSG00000154734
JUND ENSG00000130522
UBC ENSG00000150991
CRIP2 ENSG00000182809
BTG2 ENSG00000159388
SPSB3 ENSG00000162032
KRT17 ENSG00000128422
IER3 ENSG00000137331
IGFBP2 ENSG00000115457
SLC25A6 ENSG00000169100

experiment0001−expected−features.tsv.gz Factor 7

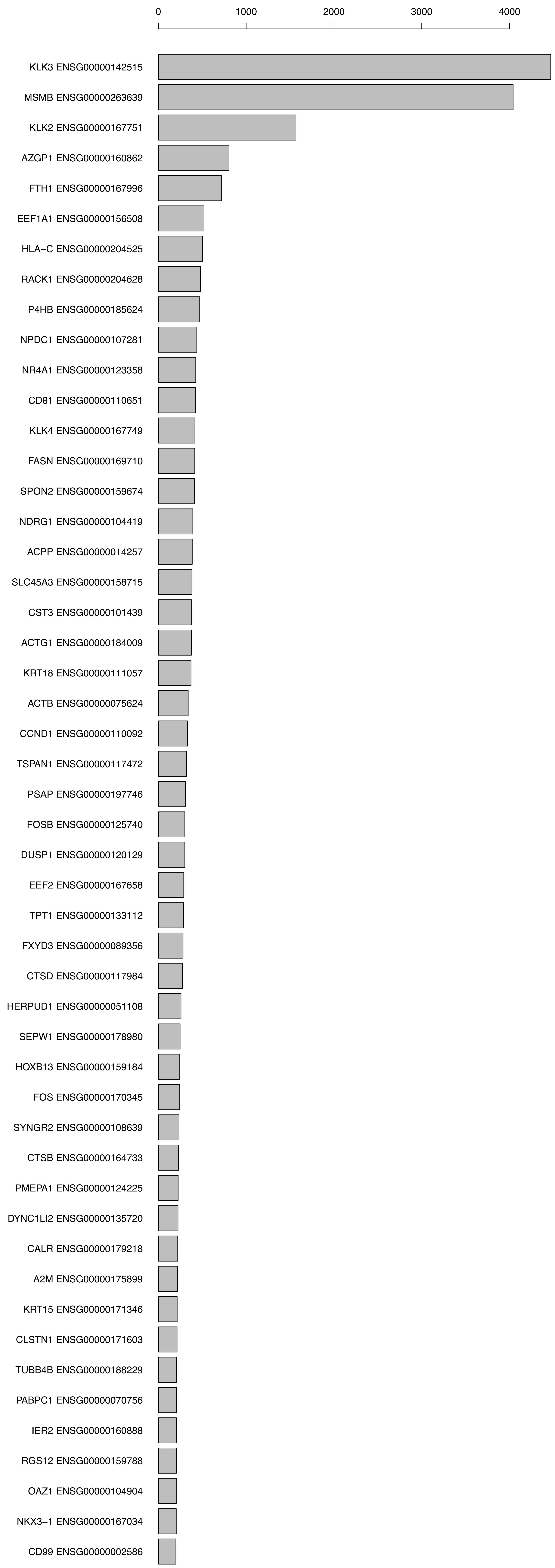

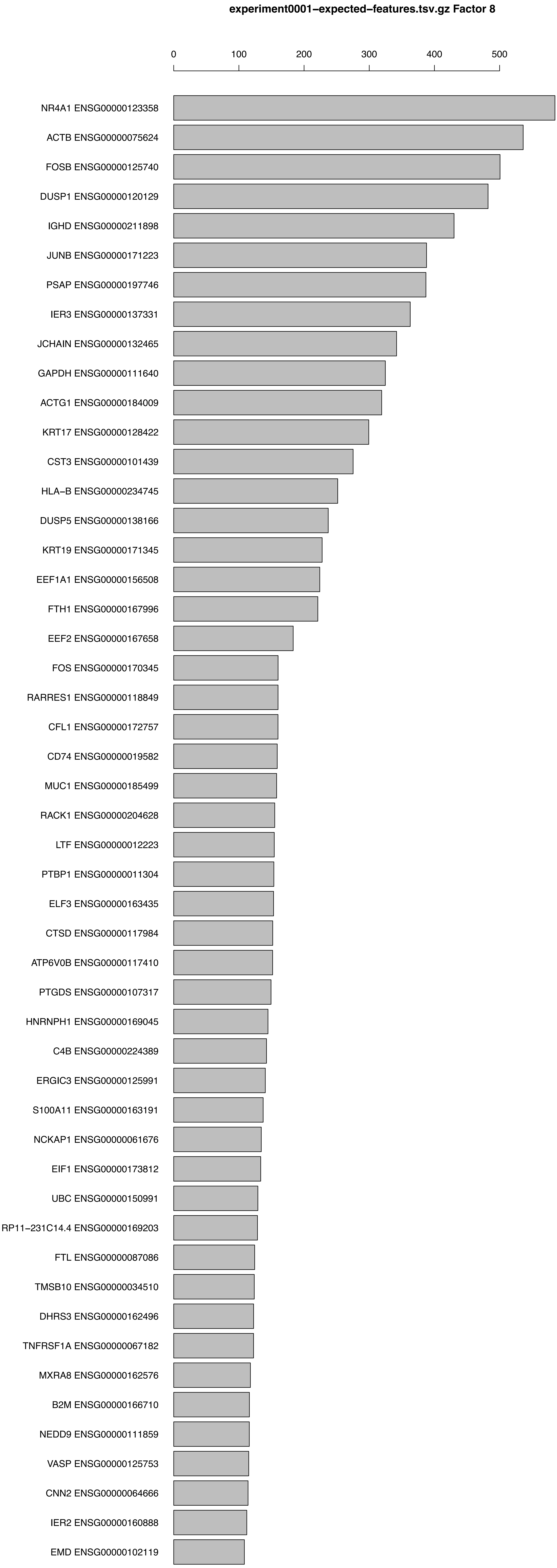
experiment0001−expected−features.tsv.gz Factor 8
0
100
200
300
400
500
NR4A1 ENSG00000123358
ACTB ENSG00000075624
FOSB ENSG00000125740
DUSP1 ENSG00000120129
IGHD ENSG00000211898
JUNB ENSG00000171223
PSAP ENSG00000197746
IER3 ENSG00000137331
JCHAIN ENSG00000132465
GAPDH ENSG00000111640
ACTG1 ENSG00000184009
KRT17 ENSG00000128422
CST3 ENSG00000101439
HLA−B ENSG00000234745
DUSP5 ENSG00000138166
KRT19 ENSG00000171345
EEF1A1 ENSG00000156508
FTH1 ENSG00000167996
EEF2 ENSG00000167658
FOS ENSG00000170345
RARRES1 ENSG00000118849
CFL1 ENSG00000172757
CD74 ENSG00000019582
MUC1 ENSG00000185499
RACK1 ENSG00000204628
LTF ENSG00000012223
PTBP1 ENSG00000011304
ELF3 ENSG00000163435
CTSD ENSG00000117984
ATP6V0B ENSG00000117410
PTGDS ENSG00000107317
HNRNPH1 ENSG00000169045
C4B ENSG00000224389
ERGIC3 ENSG00000125991
S100A11 ENSG00000163191
NCKAP1 ENSG00000061676
EIF1 ENSG00000173812
UBC ENSG00000150991
RP11−231C14.4 ENSG00000169203
FTL ENSG00000087086
TMSB10 ENSG00000034510
DHRS3 ENSG00000162496
TNFRSF1A ENSG00000067182
MXRA8 ENSG00000162576
B2M ENSG00000166710
NEDD9 ENSG00000111859
VASP ENSG00000125753
CNN2 ENSG00000064666
IER2 ENSG00000160888
EMD ENSG00000102119

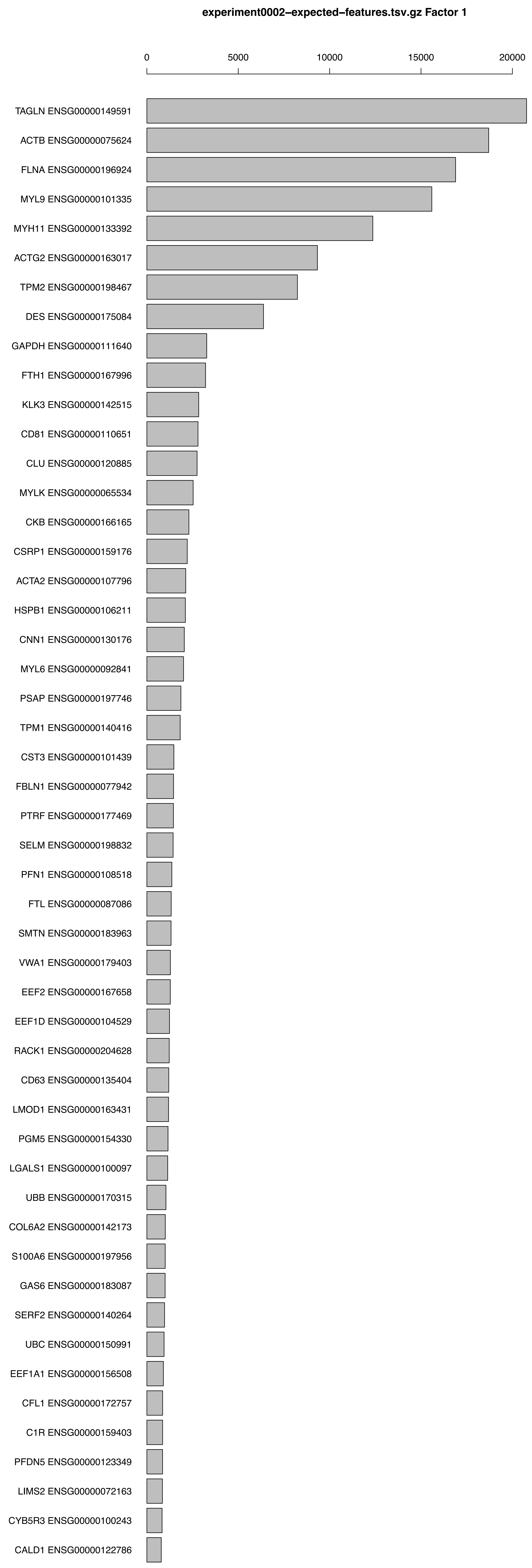
experiment0002−expected−features.tsv.gz Factor 1
0
5000
10000
15000
20000
TAGLN ENSG00000149591
ACTB ENSG00000075624
FLNA ENSG00000196924
MYL9 ENSG00000101335
MYH11 ENSG00000133392
ACTG2 ENSG00000163017
TPM2 ENSG00000198467
DES ENSG00000175084
GAPDH ENSG00000111640
FTH1 ENSG00000167996
KLK3 ENSG00000142515
CD81 ENSG00000110651
CLU ENSG00000120885
MYLK ENSG00000065534
CKB ENSG00000166165
CSRP1 ENSG00000159176
ACTA2 ENSG00000107796
HSPB1 ENSG00000106211
CNN1 ENSG00000130176
MYL6 ENSG00000092841
PSAP ENSG00000197746
TPM1 ENSG00000140416
CST3 ENSG00000101439
FBLN1 ENSG00000077942
PTRF ENSG00000177469
SELM ENSG00000198832
PFN1 ENSG00000108518
FTL ENSG00000087086
SMTN ENSG00000183963
VWA1 ENSG00000179403
EEF2 ENSG00000167658
EEF1D ENSG00000104529
RACK1 ENSG00000204628
CD63 ENSG00000135404
LMOD1 ENSG00000163431
PGM5 ENSG00000154330
LGALS1 ENSG00000100097
UBB ENSG00000170315
COL6A2 ENSG00000142173
S100A6 ENSG00000197956
GAS6 ENSG00000183087
SERF2 ENSG00000140264
UBC ENSG00000150991
EEF1A1 ENSG00000156508
CFL1 ENSG00000172757
C1R ENSG00000159403
PFDN5 ENSG00000123349
LIMS2 ENSG00000072163
CYB5R3 ENSG00000100243
CALD1 ENSG00000122786

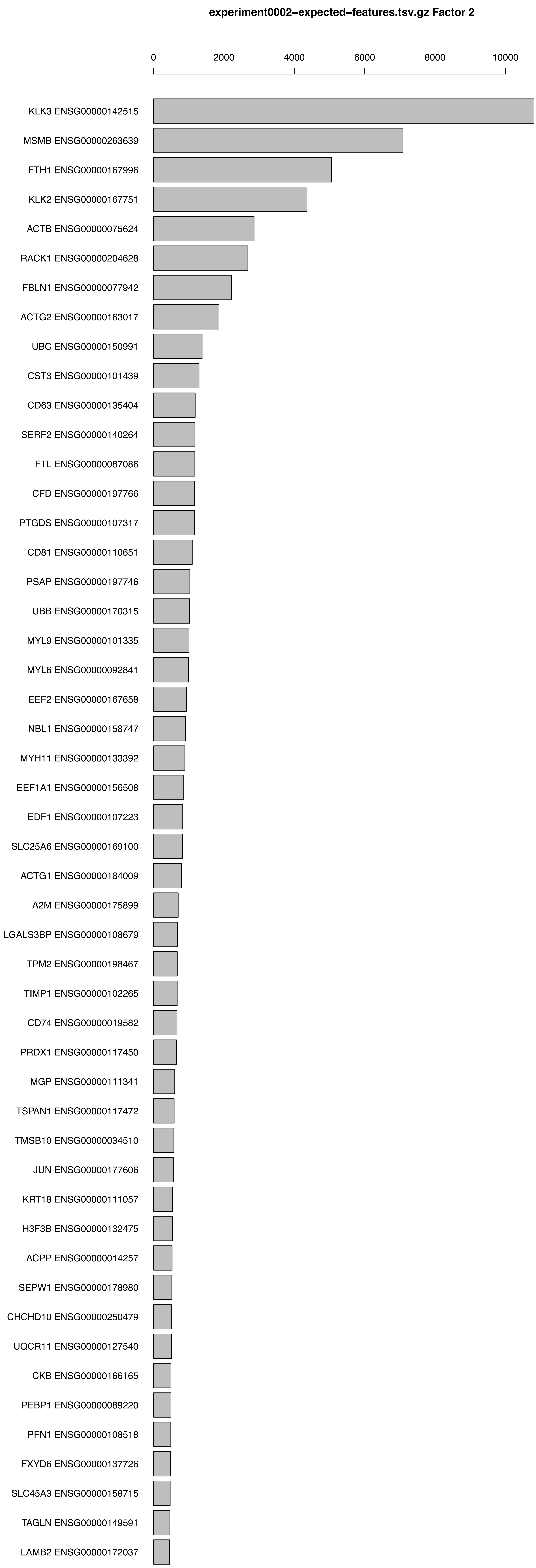
experiment0002−expected−features.tsv.gz Factor 2
0
2000
4000
6000
8000
10000
KLK3 ENSG00000142515
MSMB ENSG00000263639
FTH1 ENSG00000167996
KLK2 ENSG00000167751
ACTB ENSG00000075624
RACK1 ENSG00000204628
FBLN1 ENSG00000077942
ACTG2 ENSG00000163017
UBC ENSG00000150991
CST3 ENSG00000101439
CD63 ENSG00000135404
SERF2 ENSG00000140264
FTL ENSG00000087086
CFD ENSG00000197766
PTGDS ENSG00000107317
CD81 ENSG00000110651
PSAP ENSG00000197746
UBB ENSG00000170315
MYL9 ENSG00000101335
MYL6 ENSG00000092841
EEF2 ENSG00000167658
NBL1 ENSG00000158747
MYH11 ENSG00000133392
EEF1A1 ENSG00000156508
EDF1 ENSG00000107223
SLC25A6 ENSG00000169100
ACTG1 ENSG00000184009
A2M ENSG00000175899
LGALS3BP ENSG00000108679
TPM2 ENSG00000198467
TIMP1 ENSG00000102265
CD74 ENSG00000019582
PRDX1 ENSG00000117450
MGP ENSG00000111341
TSPAN1 ENSG00000117472
TMSB10 ENSG00000034510
JUN ENSG00000177606
KRT18 ENSG00000111057
H3F3B ENSG00000132475
ACPP ENSG00000014257
SEPW1 ENSG00000178980
CHCHD10 ENSG00000250479
UQCR11 ENSG00000127540
CKB ENSG00000166165
PEBP1 ENSG00000089220
PFN1 ENSG00000108518
FXYD6 ENSG00000137726
SLC45A3 ENSG00000158715
TAGLN ENSG00000149591
LAMB2 ENSG00000172037

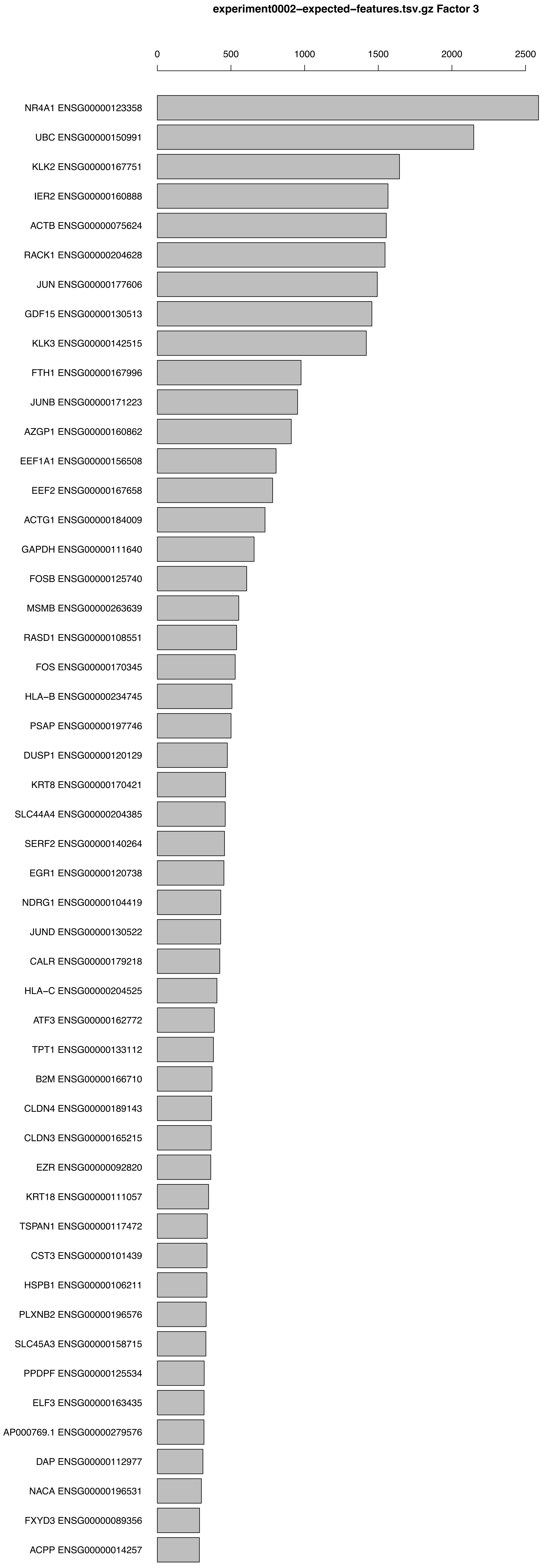
experiment0002−expected−features.tsv.gz Factor 3
0
500
1000
1500
2000
2500
NR4A1 ENSG00000123358
UBC ENSG00000150991
KLK2 ENSG00000167751
IER2 ENSG00000160888
ACTB ENSG00000075624
RACK1 ENSG00000204628
JUN ENSG00000177606
GDF15 ENSG00000130513
KLK3 ENSG00000142515
FTH1 ENSG00000167996
JUNB ENSG00000171223
AZGP1 ENSG00000160862
EEF1A1 ENSG00000156508
EEF2 ENSG00000167658
ACTG1 ENSG00000184009
GAPDH ENSG00000111640
FOSB ENSG00000125740
MSMB ENSG00000263639
RASD1 ENSG00000108551
FOS ENSG00000170345
HLA−B ENSG00000234745
PSAP ENSG00000197746
DUSP1 ENSG00000120129
KRT8 ENSG00000170421
SLC44A4 ENSG00000204385
SERF2 ENSG00000140264
EGR1 ENSG00000120738
NDRG1 ENSG00000104419
JUND ENSG00000130522
CALR ENSG00000179218
HLA−C ENSG00000204525
ATF3 ENSG00000162772
TPT1 ENSG00000133112
B2M ENSG00000166710
CLDN4 ENSG00000189143
CLDN3 ENSG00000165215
EZR ENSG00000092820
KRT18 ENSG00000111057
TSPAN1 ENSG00000117472
CST3 ENSG00000101439
HSPB1 ENSG00000106211
PLXNB2 ENSG00000196576
SLC45A3 ENSG00000158715
PPDPF ENSG00000125534
ELF3 ENSG00000163435
AP000769.1 ENSG00000279576
DAP ENSG00000112977
NACA ENSG00000196531
FXYD3 ENSG00000089356
ACPP ENSG00000014257

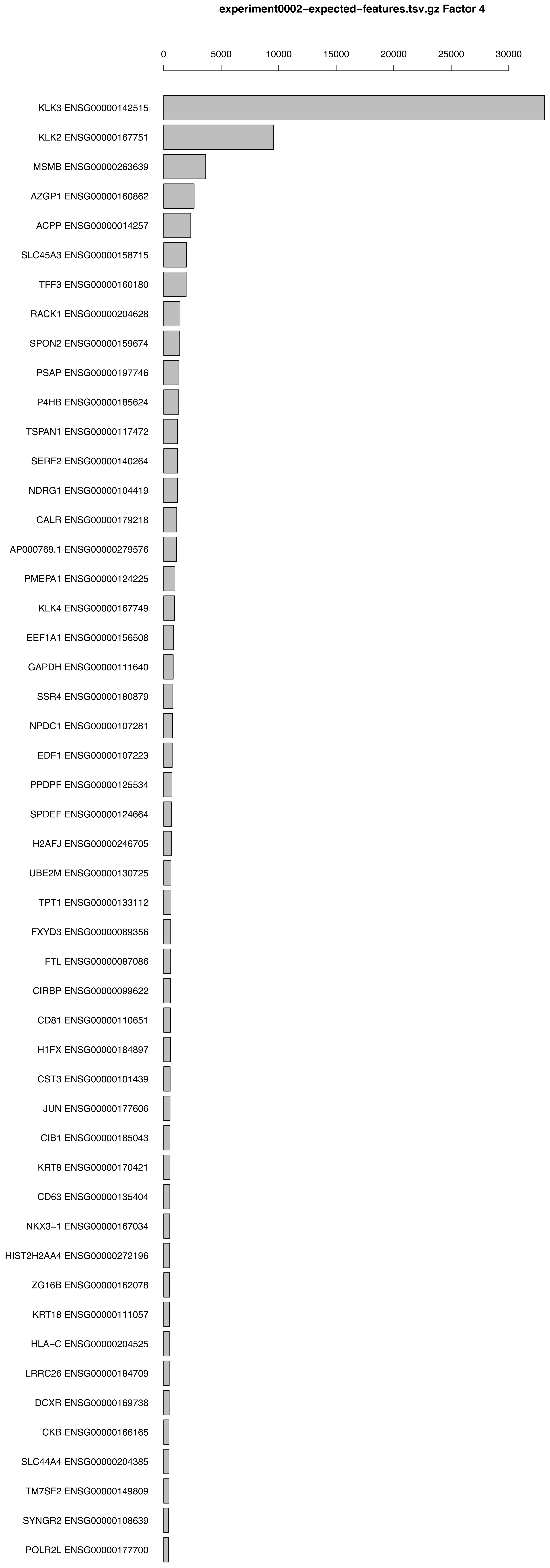
experiment0002−expected−features.tsv.gz Factor 4
0
5000
10000
15000
20000
25000
30000
KLK3 ENSG00000142515
KLK2 ENSG00000167751
MSMB ENSG00000263639
AZGP1 ENSG00000160862
ACPP ENSG00000014257
SLC45A3 ENSG00000158715
TFF3 ENSG00000160180
RACK1 ENSG00000204628
SPON2 ENSG00000159674
PSAP ENSG00000197746
P4HB ENSG00000185624
TSPAN1 ENSG00000117472
SERF2 ENSG00000140264
NDRG1 ENSG00000104419
CALR ENSG00000179218
AP000769.1 ENSG00000279576
PMEPA1 ENSG00000124225
KLK4 ENSG00000167749
EEF1A1 ENSG00000156508
GAPDH ENSG00000111640
SSR4 ENSG00000180879
NPDC1 ENSG00000107281
EDF1 ENSG00000107223
PPDPF ENSG00000125534
SPDEF ENSG00000124664
H2AFJ ENSG00000246705
UBE2M ENSG00000130725
TPT1 ENSG00000133112
FXYD3 ENSG00000089356
FTL ENSG00000087086
CIRBP ENSG00000099622
CD81 ENSG00000110651
H1FX ENSG00000184897
CST3 ENSG00000101439
JUN ENSG00000177606
CIB1 ENSG00000185043
KRT8 ENSG00000170421
CD63 ENSG00000135404
NKX3−1 ENSG00000167034
HIST2H2AA4 ENSG00000272196
ZG16B ENSG00000162078
KRT18 ENSG00000111057
HLA−C ENSG00000204525
LRRC26 ENSG00000184709
DCXR ENSG00000169738
CKB ENSG00000166165
SLC44A4 ENSG00000204385
TM7SF2 ENSG00000149809
SYNGR2 ENSG00000108639
POLR2L ENSG00000177700

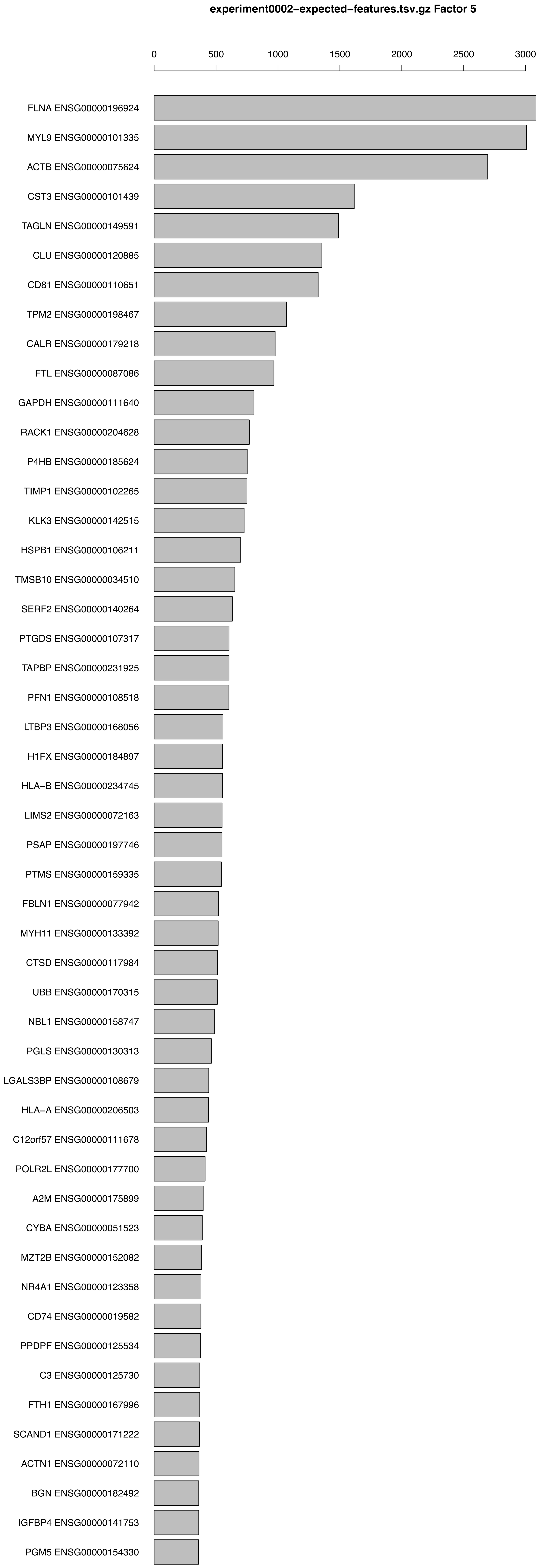
experiment0002−expected−features.tsv.gz Factor 5
0
500
1000
1500
2000
2500
3000
FLNA ENSG00000196924
MYL9 ENSG00000101335
ACTB ENSG00000075624
CST3 ENSG00000101439
TAGLN ENSG00000149591
CLU ENSG00000120885
CD81 ENSG00000110651
TPM2 ENSG00000198467
CALR ENSG00000179218
FTL ENSG00000087086
GAPDH ENSG00000111640
RACK1 ENSG00000204628
P4HB ENSG00000185624
TIMP1 ENSG00000102265
KLK3 ENSG00000142515
HSPB1 ENSG00000106211
TMSB10 ENSG00000034510
SERF2 ENSG00000140264
PTGDS ENSG00000107317
TAPBP ENSG00000231925
PFN1 ENSG00000108518
LTBP3 ENSG00000168056
H1FX ENSG00000184897
HLA−B ENSG00000234745
LIMS2 ENSG00000072163
PSAP ENSG00000197746
PTMS ENSG00000159335
FBLN1 ENSG00000077942
MYH11 ENSG00000133392
CTSD ENSG00000117984
UBB ENSG00000170315
NBL1 ENSG00000158747
PGLS ENSG00000130313
LGALS3BP ENSG00000108679
HLA−A ENSG00000206503
C12orf57 ENSG00000111678
POLR2L ENSG00000177700
A2M ENSG00000175899
CYBA ENSG00000051523
MZT2B ENSG00000152082
NR4A1 ENSG00000123358
CD74 ENSG00000019582
PPDPF ENSG00000125534
C3 ENSG00000125730
FTH1 ENSG00000167996
SCAND1 ENSG00000171222
ACTN1 ENSG00000072110
BGN ENSG00000182492
IGFBP4 ENSG00000141753
PGM5 ENSG00000154330

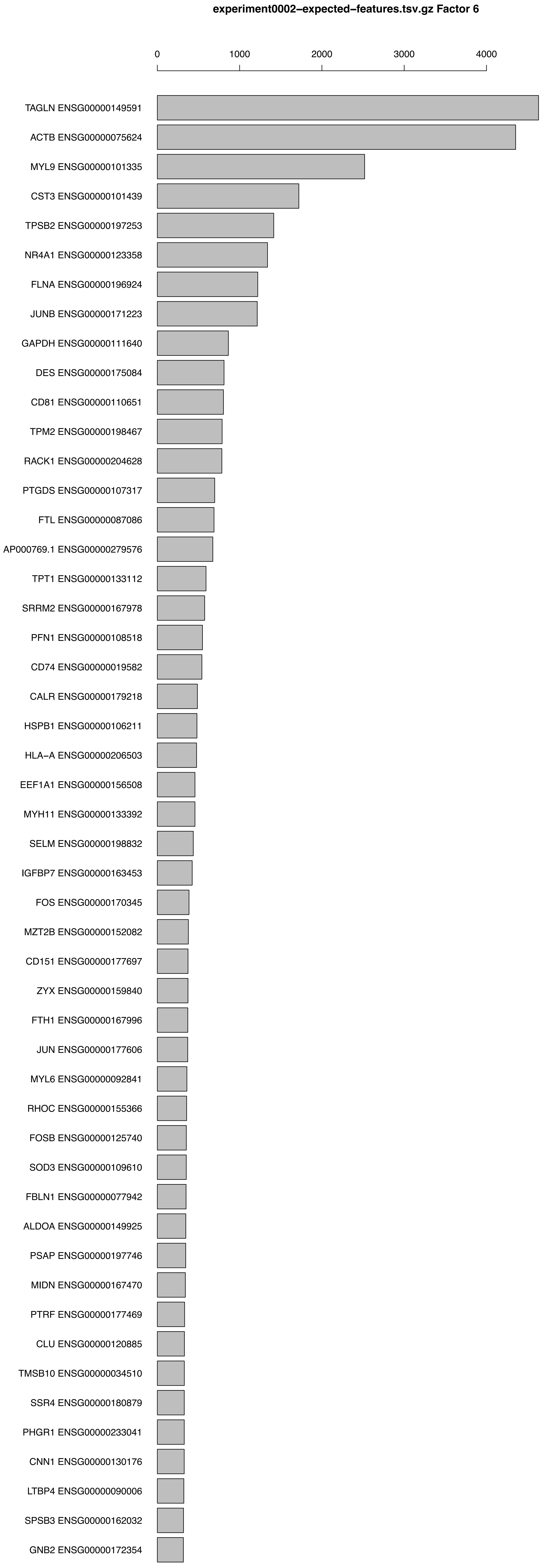
experiment0002−expected−features.tsv.gz Factor 6
0
1000
2000
3000
4000
TAGLN ENSG00000149591
ACTB ENSG00000075624
MYL9 ENSG00000101335
CST3 ENSG00000101439
TPSB2 ENSG00000197253
NR4A1 ENSG00000123358
FLNA ENSG00000196924
JUNB ENSG00000171223
GAPDH ENSG00000111640
DES ENSG00000175084
CD81 ENSG00000110651
TPM2 ENSG00000198467
RACK1 ENSG00000204628
PTGDS ENSG00000107317
FTL ENSG00000087086
AP000769.1 ENSG00000279576
TPT1 ENSG00000133112
SRRM2 ENSG00000167978
PFN1 ENSG00000108518
CD74 ENSG00000019582
CALR ENSG00000179218
HSPB1 ENSG00000106211
HLA−A ENSG00000206503
EEF1A1 ENSG00000156508
MYH11 ENSG00000133392
SELM ENSG00000198832
IGFBP7 ENSG00000163453
FOS ENSG00000170345
MZT2B ENSG00000152082
CD151 ENSG00000177697
ZYX ENSG00000159840
FTH1 ENSG00000167996
JUN ENSG00000177606
MYL6 ENSG00000092841
RHOC ENSG00000155366
FOSB ENSG00000125740
SOD3 ENSG00000109610
FBLN1 ENSG00000077942
ALDOA ENSG00000149925
PSAP ENSG00000197746
MIDN ENSG00000167470
PTRF ENSG00000177469
CLU ENSG00000120885
TMSB10 ENSG00000034510
SSR4 ENSG00000180879
PHGR1 ENSG00000233041
CNN1 ENSG00000130176
LTBP4 ENSG00000090006
SPSB3 ENSG00000162032
GNB2 ENSG00000172354

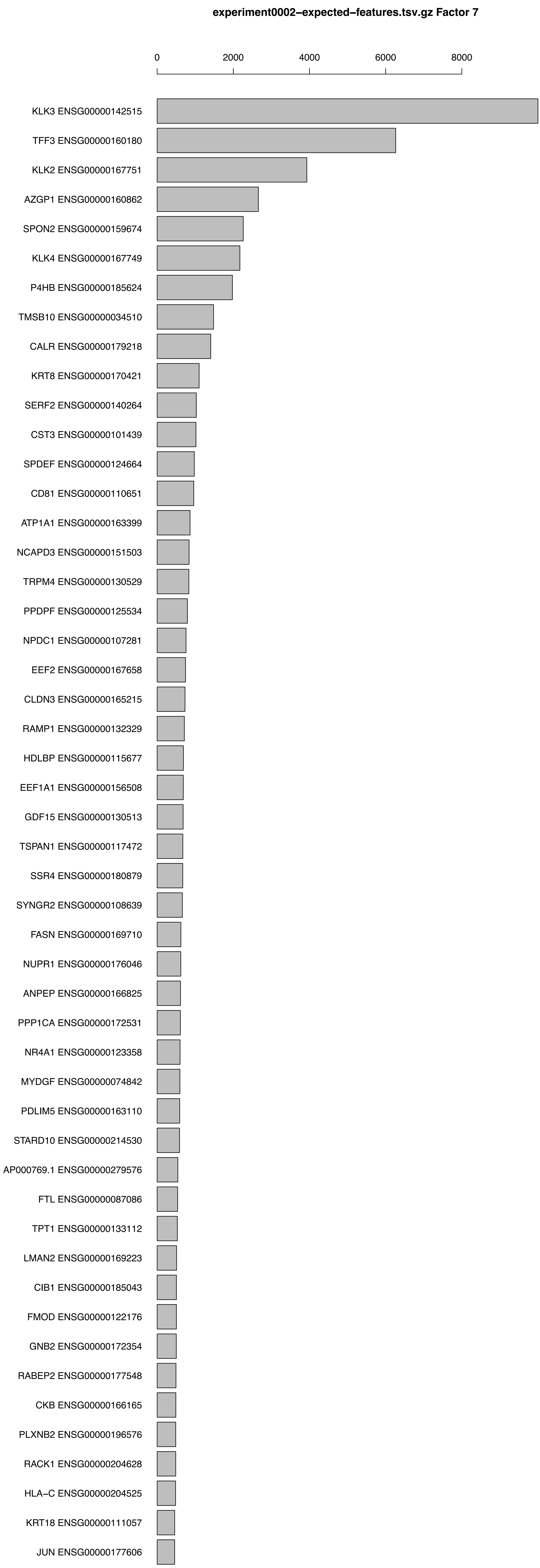
experiment0002−expected−features.tsv.gz Factor 7
0
2000
4000
6000
8000
KLK3 ENSG00000142515
TFF3 ENSG00000160180
KLK2 ENSG00000167751
AZGP1 ENSG00000160862
SPON2 ENSG00000159674
KLK4 ENSG00000167749
P4HB ENSG00000185624
TMSB10 ENSG00000034510
CALR ENSG00000179218
KRT8 ENSG00000170421
SERF2 ENSG00000140264
CST3 ENSG00000101439
SPDEF ENSG00000124664
CD81 ENSG00000110651
ATP1A1 ENSG00000163399
NCAPD3 ENSG00000151503
TRPM4 ENSG00000130529
PPDPF ENSG00000125534
NPDC1 ENSG00000107281
EEF2 ENSG00000167658
CLDN3 ENSG00000165215
RAMP1 ENSG00000132329
HDLBP ENSG00000115677
EEF1A1 ENSG00000156508
GDF15 ENSG00000130513
TSPAN1 ENSG00000117472
SSR4 ENSG00000180879
SYNGR2 ENSG00000108639
FASN ENSG00000169710
NUPR1 ENSG00000176046
ANPEP ENSG00000166825
PPP1CA ENSG00000172531
NR4A1 ENSG00000123358
MYDGF ENSG00000074842
PDLIM5 ENSG00000163110
STARD10 ENSG00000214530
AP000769.1 ENSG00000279576
FTL ENSG00000087086
TPT1 ENSG00000133112
LMAN2 ENSG00000169223
CIB1 ENSG00000185043
FMOD ENSG00000122176
GNB2 ENSG00000172354
RABEP2 ENSG00000177548
CKB ENSG00000166165
PLXNB2 ENSG00000196576
RACK1 ENSG00000204628
HLA−C ENSG00000204525
KRT18 ENSG00000111057
JUN ENSG00000177606

experiment0002−expected−features.tsv.gz Factor 8

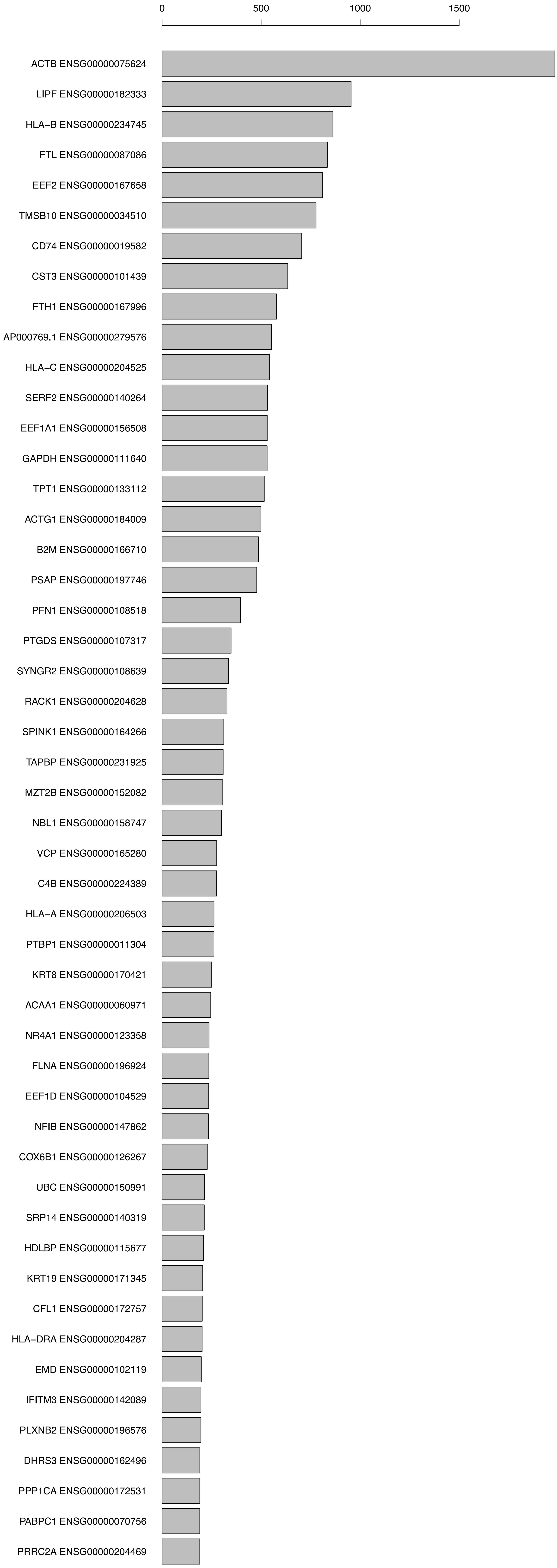

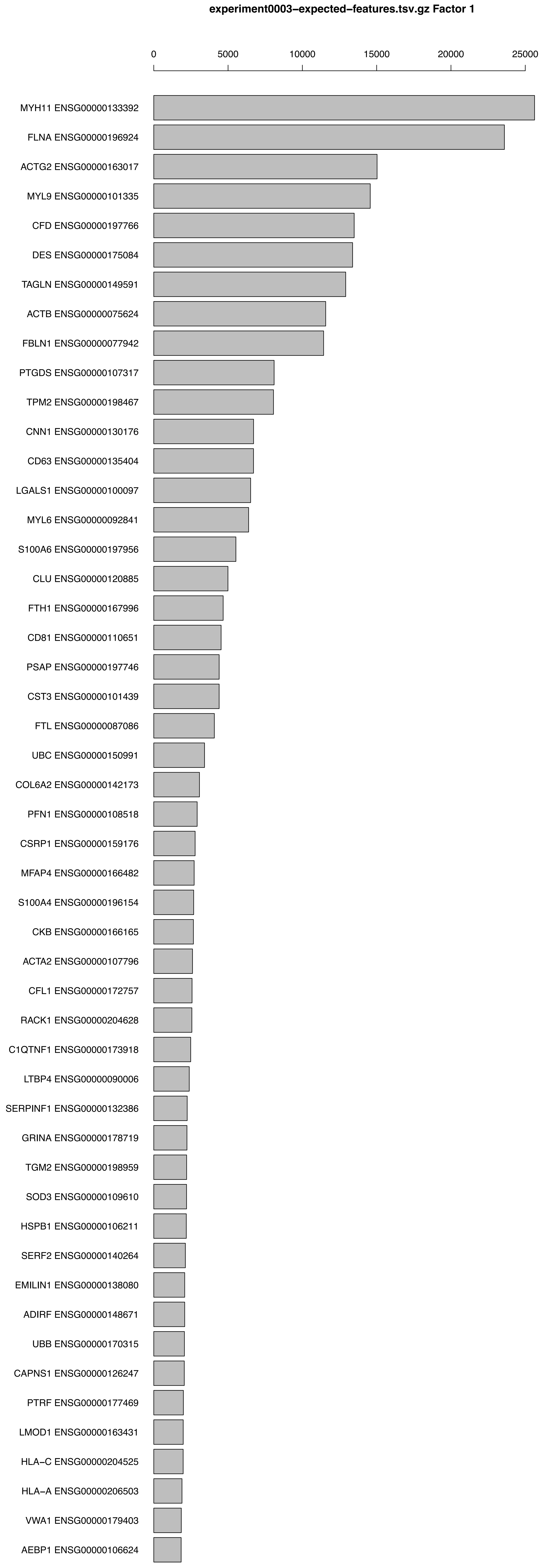
experiment0003−expected−features.tsv.gz Factor 1
0
5000
10000
15000
20000
25000
MYH11 ENSG00000133392
FLNA ENSG00000196924
ACTG2 ENSG00000163017
MYL9 ENSG00000101335
CFD ENSG00000197766
DES ENSG00000175084
TAGLN ENSG00000149591
ACTB ENSG00000075624
FBLN1 ENSG00000077942
PTGDS ENSG00000107317
TPM2 ENSG00000198467
CNN1 ENSG00000130176
CD63 ENSG00000135404
LGALS1 ENSG00000100097
MYL6 ENSG00000092841
S100A6 ENSG00000197956
CLU ENSG00000120885
FTH1 ENSG00000167996
CD81 ENSG00000110651
PSAP ENSG00000197746
CST3 ENSG00000101439
FTL ENSG00000087086
UBC ENSG00000150991
COL6A2 ENSG00000142173
PFN1 ENSG00000108518
CSRP1 ENSG00000159176
MFAP4 ENSG00000166482
S100A4 ENSG00000196154
CKB ENSG00000166165
ACTA2 ENSG00000107796
CFL1 ENSG00000172757
RACK1 ENSG00000204628
C1QTNF1 ENSG00000173918
LTBP4 ENSG00000090006
SERPINF1 ENSG00000132386
GRINA ENSG00000178719
TGM2 ENSG00000198959
SOD3 ENSG00000109610
HSPB1 ENSG00000106211
SERF2 ENSG00000140264
EMILIN1 ENSG00000138080
ADIRF ENSG00000148671
UBB ENSG00000170315
CAPNS1 ENSG00000126247
PTRF ENSG00000177469
LMOD1 ENSG00000163431
HLA−C ENSG00000204525
HLA−A ENSG00000206503
VWA1 ENSG00000179403
AEBP1 ENSG00000106624

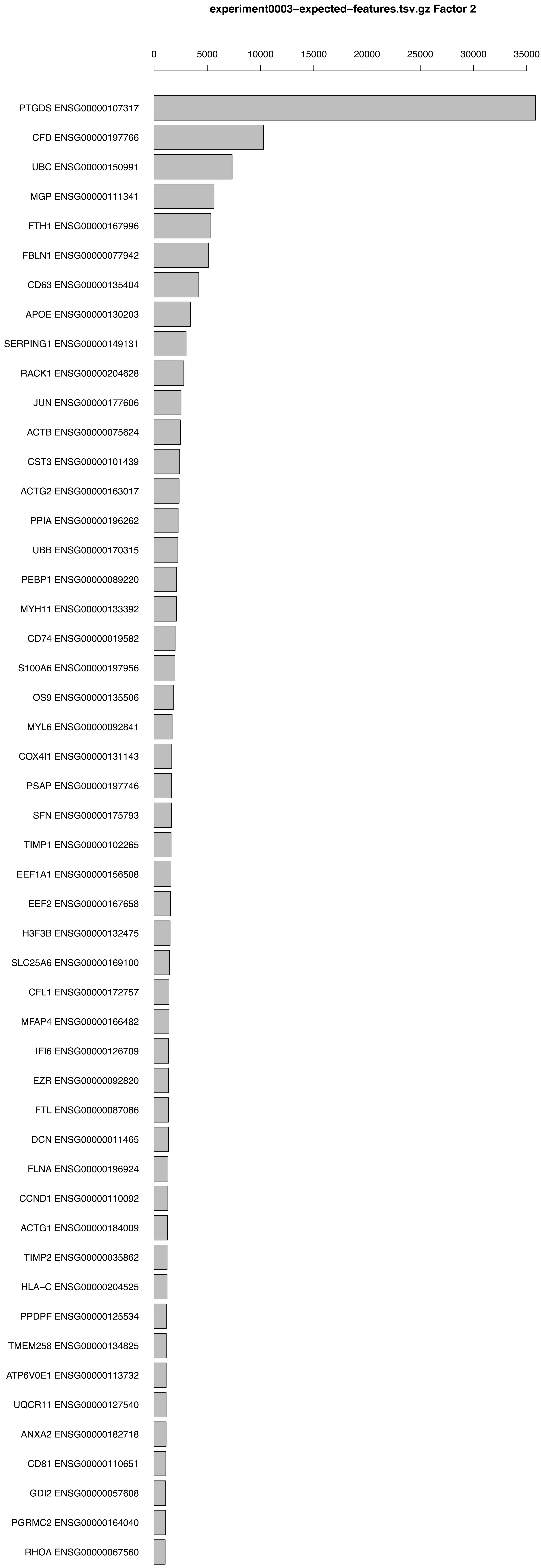
experiment0003−expected−features.tsv.gz Factor 2
0
5000
10000
15000
20000
25000
30000
35000
PTGDS ENSG00000107317
CFD ENSG00000197766
UBC ENSG00000150991
MGP ENSG00000111341
FTH1 ENSG00000167996
FBLN1 ENSG00000077942
CD63 ENSG00000135404
APOE ENSG00000130203
SERPING1 ENSG00000149131
RACK1 ENSG00000204628
JUN ENSG00000177606
ACTB ENSG00000075624
CST3 ENSG00000101439
ACTG2 ENSG00000163017
PPIA ENSG00000196262
UBB ENSG00000170315
PEBP1 ENSG00000089220
MYH11 ENSG00000133392
CD74 ENSG00000019582
S100A6 ENSG00000197956
OS9 ENSG00000135506
MYL6 ENSG00000092841
COX4I1 ENSG00000131143
PSAP ENSG00000197746
SFN ENSG00000175793
TIMP1 ENSG00000102265
EEF1A1 ENSG00000156508
EEF2 ENSG00000167658
H3F3B ENSG00000132475
SLC25A6 ENSG00000169100
CFL1 ENSG00000172757
MFAP4 ENSG00000166482
IFI6 ENSG00000126709
EZR ENSG00000092820
FTL ENSG00000087086
DCN ENSG00000011465
FLNA ENSG00000196924
CCND1 ENSG00000110092
ACTG1 ENSG00000184009
TIMP2 ENSG00000035862
HLA−C ENSG00000204525
PPDPF ENSG00000125534
TMEM258 ENSG00000134825
ATP6V0E1 ENSG00000113732
UQCR11 ENSG00000127540
ANXA2 ENSG00000182718
CD81 ENSG00000110651
GDI2 ENSG00000057608
PGRMC2 ENSG00000164040
RHOA ENSG00000067560

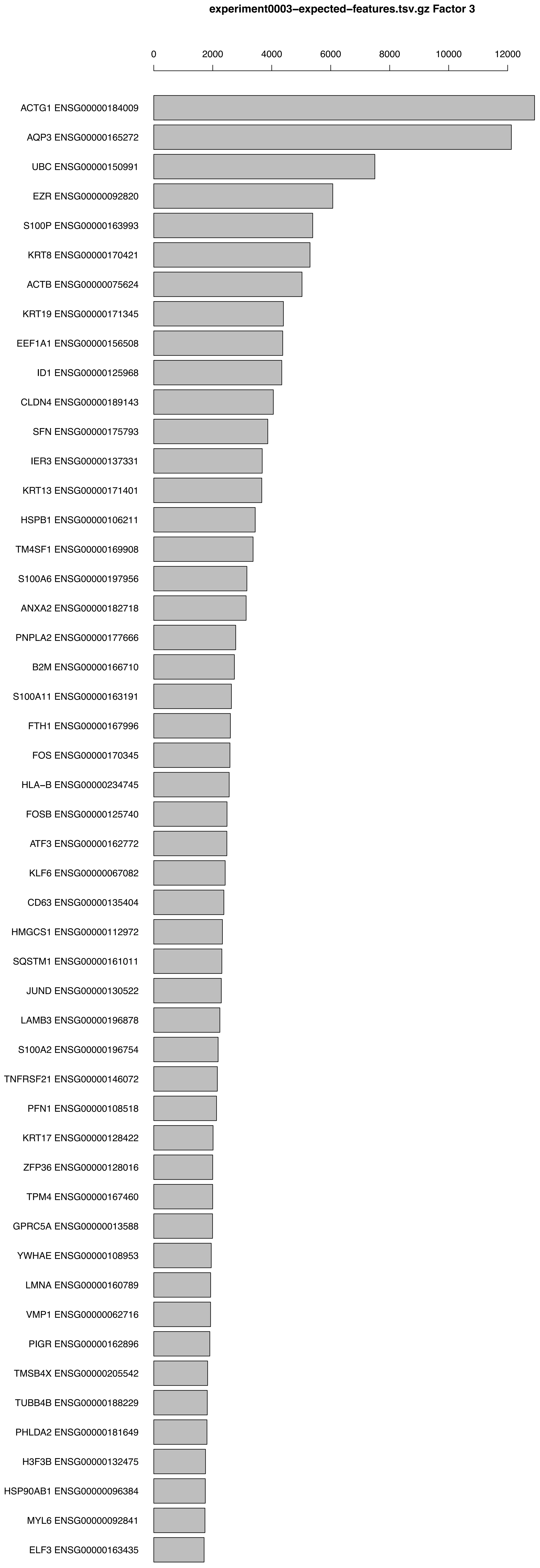
experiment0003−expected−features.tsv.gz Factor 3
0
2000
4000
6000
8000
10000
12000
ACTG1 ENSG00000184009
AQP3 ENSG00000165272
UBC ENSG00000150991
EZR ENSG00000092820
S100P ENSG00000163993
KRT8 ENSG00000170421
ACTB ENSG00000075624
KRT19 ENSG00000171345
EEF1A1 ENSG00000156508
ID1 ENSG00000125968
CLDN4 ENSG00000189143
SFN ENSG00000175793
IER3 ENSG00000137331
KRT13 ENSG00000171401
HSPB1 ENSG00000106211
TM4SF1 ENSG00000169908
S100A6 ENSG00000197956
ANXA2 ENSG00000182718
PNPLA2 ENSG00000177666
B2M ENSG00000166710
S100A11 ENSG00000163191
FTH1 ENSG00000167996
FOS ENSG00000170345
HLA−B ENSG00000234745
FOSB ENSG00000125740
ATF3 ENSG00000162772
KLF6 ENSG00000067082
CD63 ENSG00000135404
HMGCS1 ENSG00000112972
SQSTM1 ENSG00000161011
JUND ENSG00000130522
LAMB3 ENSG00000196878
S100A2 ENSG00000196754
TNFRSF21 ENSG00000146072
PFN1 ENSG00000108518
KRT17 ENSG00000128422
ZFP36 ENSG00000128016
TPM4 ENSG00000167460
GPRC5A ENSG00000013588
YWHAE ENSG00000108953
LMNA ENSG00000160789
VMP1 ENSG00000062716
PIGR ENSG00000162896
TMSB4X ENSG00000205542
TUBB4B ENSG00000188229
PHLDA2 ENSG00000181649
H3F3B ENSG00000132475
HSP90AB1 ENSG00000096384
MYL6 ENSG00000092841
ELF3 ENSG00000163435

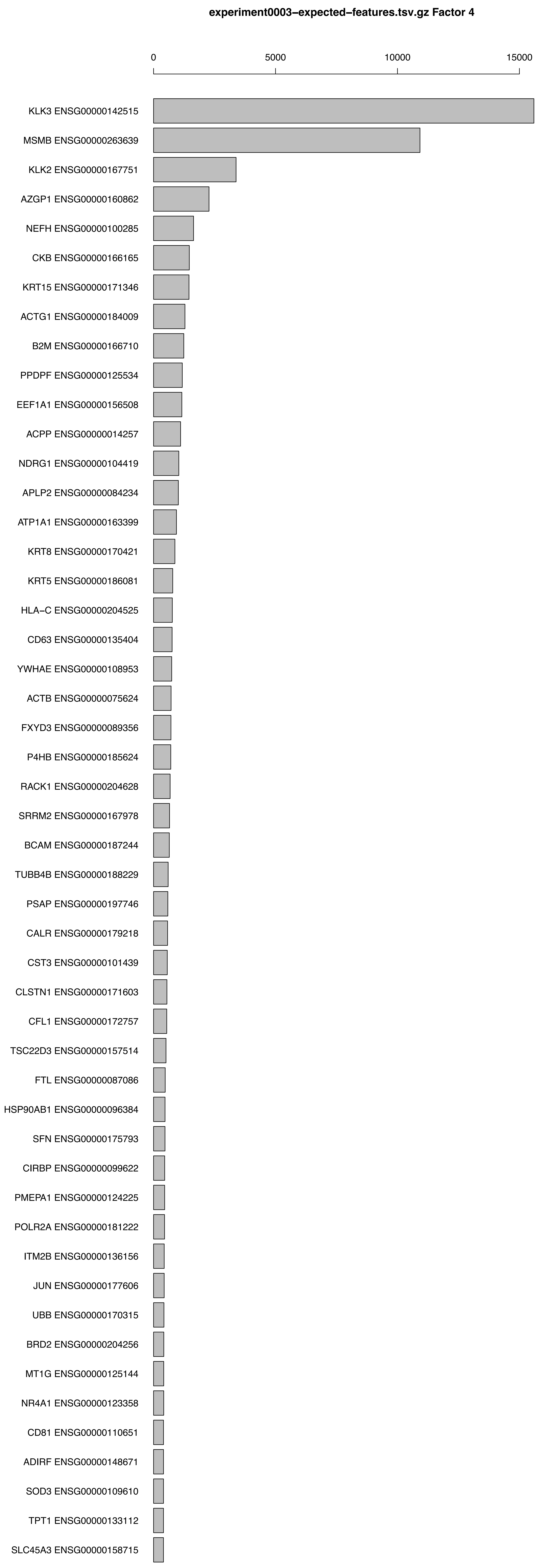

experiment0003−expected−features.tsv.gz Factor 4
0
5000
10000
15000
KLK3 ENSG00000142515
MSMB ENSG00000263639
KLK2 ENSG00000167751
AZGP1 ENSG00000160862
NEFH ENSG00000100285
CKB ENSG00000166165
KRT15 ENSG00000171346
ACTG1 ENSG00000184009
B2M ENSG00000166710
PPDPF ENSG00000125534
EEF1A1 ENSG00000156508
ACPP ENSG00000014257
NDRG1 ENSG00000104419
APLP2 ENSG00000084234
ATP1A1 ENSG00000163399
KRT8 ENSG00000170421
KRT5 ENSG00000186081
HLA−C ENSG00000204525
CD63 ENSG00000135404
YWHAE ENSG00000108953
ACTB ENSG00000075624
FXYD3 ENSG00000089356
P4HB ENSG00000185624
RACK1 ENSG00000204628
SRRM2 ENSG00000167978
BCAM ENSG00000187244
TUBB4B ENSG00000188229
PSAP ENSG00000197746
CALR ENSG00000179218
CST3 ENSG00000101439
CLSTN1 ENSG00000171603
CFL1 ENSG00000172757
TSC22D3 ENSG00000157514
FTL ENSG00000087086
HSP90AB1 ENSG00000096384
SFN ENSG00000175793
CIRBP ENSG00000099622
PMEPA1 ENSG00000124225
POLR2A ENSG00000181222
ITM2B ENSG00000136156
JUN ENSG00000177606
UBB ENSG00000170315
BRD2 ENSG00000204256
MT1G ENSG00000125144
NR4A1 ENSG00000123358
CD81 ENSG00000110651
ADIRF ENSG00000148671
SOD3 ENSG00000109610
TPT1 ENSG00000133112
SLC45A3 ENSG00000158715

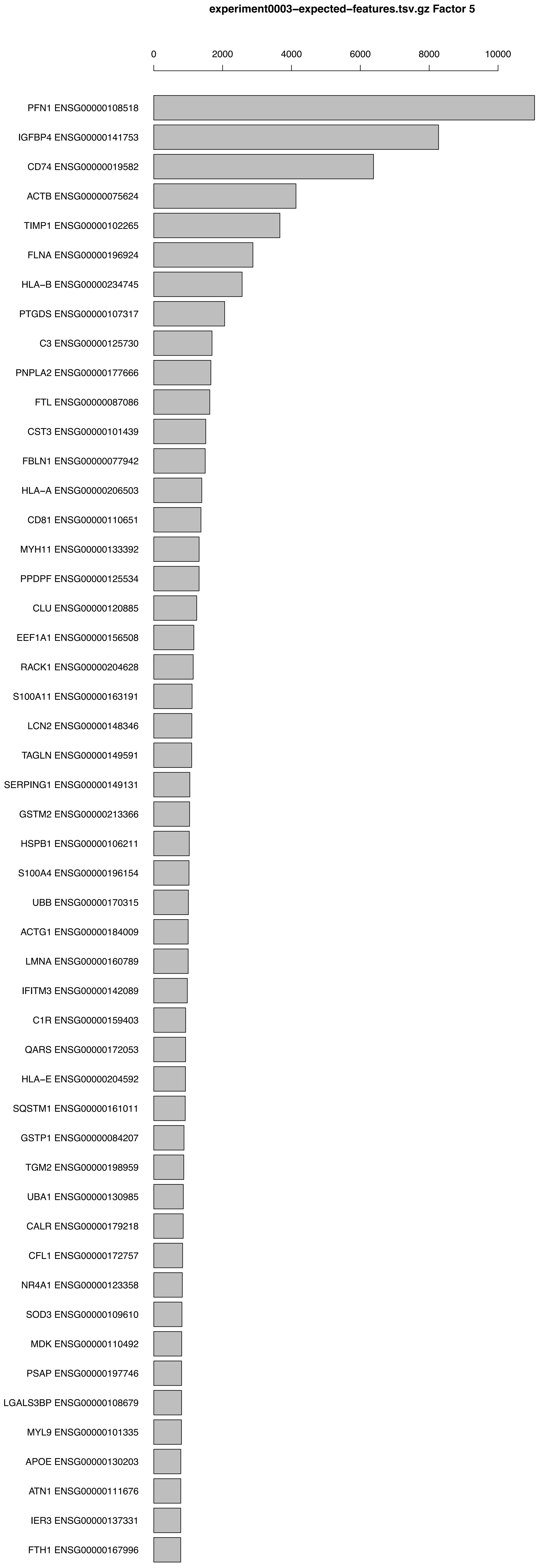

experiment0003−expected−features.tsv.gz Factor 5
0
2000
4000
6000
8000
10000
PFN1 ENSG00000108518
IGFBP4 ENSG00000141753
CD74 ENSG00000019582
ACTB ENSG00000075624
TIMP1 ENSG00000102265
FLNA ENSG00000196924
HLA−B ENSG00000234745
PTGDS ENSG00000107317
C3 ENSG00000125730
PNPLA2 ENSG00000177666
FTL ENSG00000087086
CST3 ENSG00000101439
FBLN1 ENSG00000077942
HLA−A ENSG00000206503
CD81 ENSG00000110651
MYH11 ENSG00000133392
PPDPF ENSG00000125534
CLU ENSG00000120885
EEF1A1 ENSG00000156508
RACK1 ENSG00000204628
S100A11 ENSG00000163191
LCN2 ENSG00000148346
TAGLN ENSG00000149591
SERPING1 ENSG00000149131
GSTM2 ENSG00000213366
HSPB1 ENSG00000106211
S100A4 ENSG00000196154
UBB ENSG00000170315
ACTG1 ENSG00000184009
LMNA ENSG00000160789
IFITM3 ENSG00000142089
C1R ENSG00000159403
QARS ENSG00000172053
HLA−E ENSG00000204592
SQSTM1 ENSG00000161011
GSTP1 ENSG00000084207
TGM2 ENSG00000198959
UBA1 ENSG00000130985
CALR ENSG00000179218
CFL1 ENSG00000172757
NR4A1 ENSG00000123358
SOD3 ENSG00000109610
MDK ENSG00000110492
PSAP ENSG00000197746
LGALS3BP ENSG00000108679
MYL9 ENSG00000101335
APOE ENSG00000130203
ATN1 ENSG00000111676
IER3 ENSG00000137331
FTH1 ENSG00000167996

experiment0003−expected−features.tsv.gz Factor 6

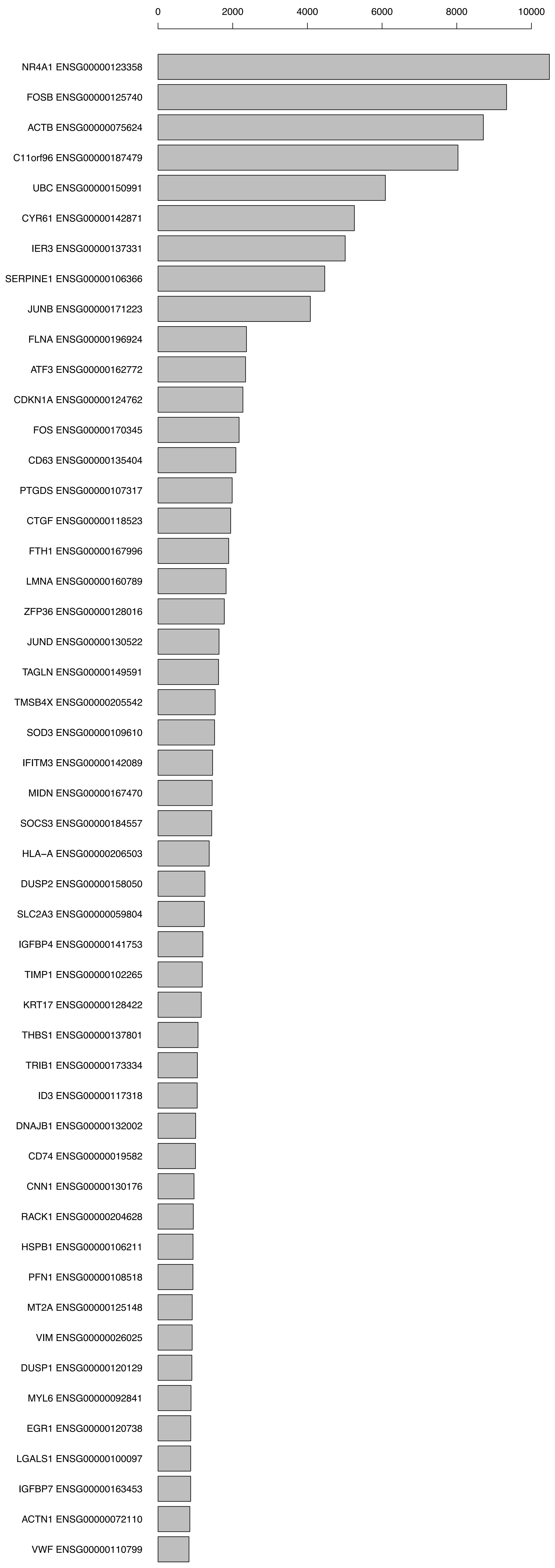

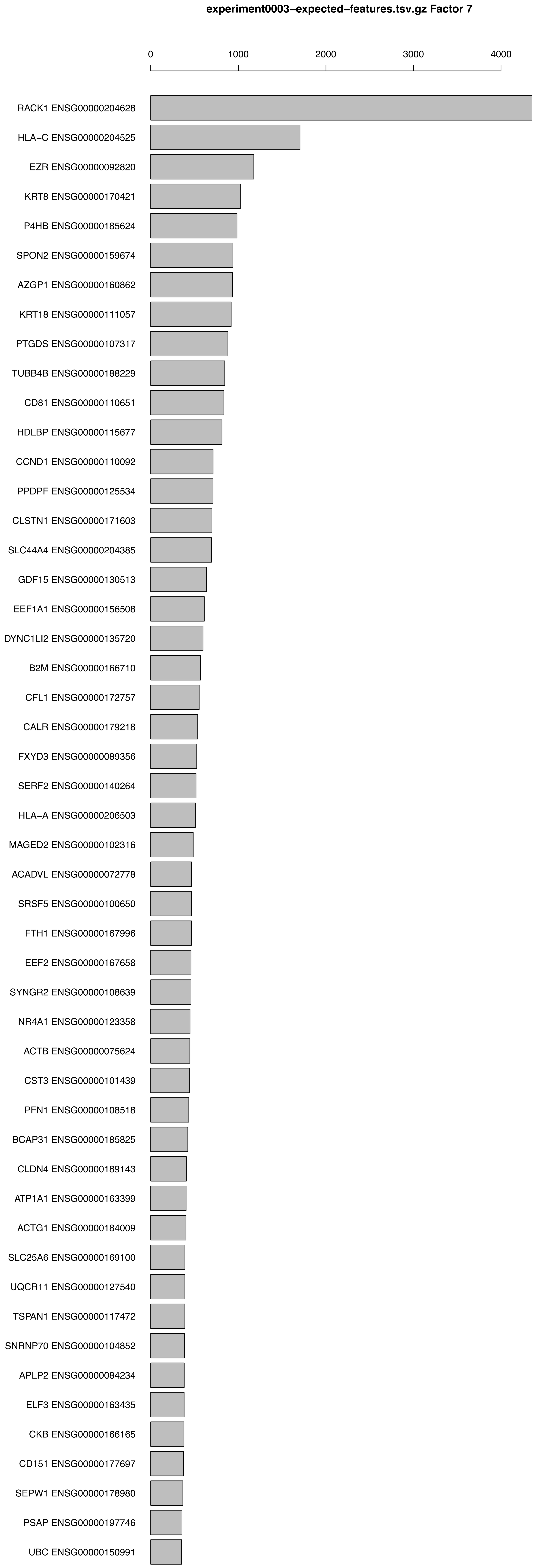
experiment0003−expected−features.tsv.gz Factor 7
0
1000
2000
3000
4000
RACK1 ENSG00000204628
HLA−C ENSG00000204525
EZR ENSG00000092820
KRT8 ENSG00000170421
P4HB ENSG00000185624
SPON2 ENSG00000159674
AZGP1 ENSG00000160862
KRT18 ENSG00000111057
PTGDS ENSG00000107317
TUBB4B ENSG00000188229
CD81 ENSG00000110651
HDLBP ENSG00000115677
CCND1 ENSG00000110092
PPDPF ENSG00000125534
CLSTN1 ENSG00000171603
SLC44A4 ENSG00000204385
GDF15 ENSG00000130513
EEF1A1 ENSG00000156508
DYNC1LI2 ENSG00000135720
B2M ENSG00000166710
CFL1 ENSG00000172757
CALR ENSG00000179218
FXYD3 ENSG00000089356
SERF2 ENSG00000140264
HLA−A ENSG00000206503
MAGED2 ENSG00000102316
ACADVL ENSG00000072778
SRSF5 ENSG00000100650
FTH1 ENSG00000167996
EEF2 ENSG00000167658
SYNGR2 ENSG00000108639
NR4A1 ENSG00000123358
ACTB ENSG00000075624
CST3 ENSG00000101439
PFN1 ENSG00000108518
BCAP31 ENSG00000185825
CLDN4 ENSG00000189143
ATP1A1 ENSG00000163399
ACTG1 ENSG00000184009
SLC25A6 ENSG00000169100
UQCR11 ENSG00000127540
TSPAN1 ENSG00000117472
SNRNP70 ENSG00000104852
APLP2 ENSG00000084234
ELF3 ENSG00000163435
CKB ENSG00000166165
CD151 ENSG00000177697
SEPW1 ENSG00000178980
PSAP ENSG00000197746
UBC ENSG00000150991

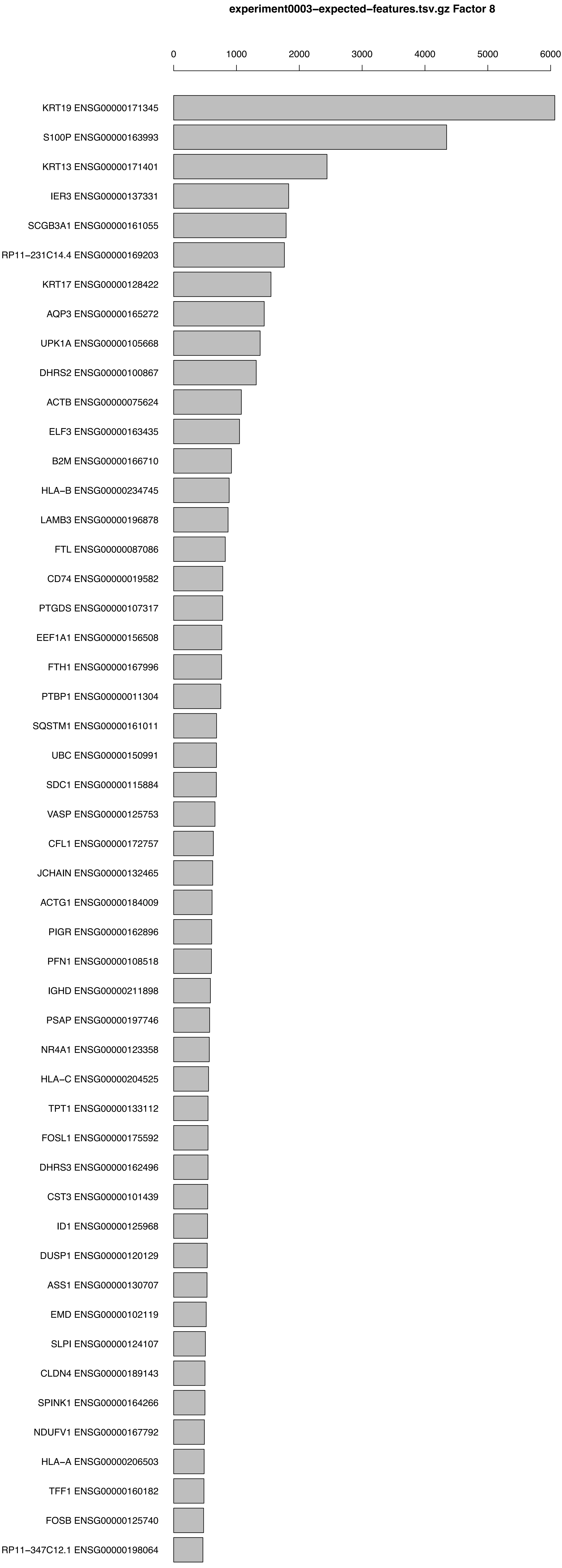
experiment0003−expected−features.tsv.gz Factor 8
0
1000
2000
3000
4000
5000
6000
KRT19 ENSG00000171345
S100P ENSG00000163993
KRT13 ENSG00000171401
IER3 ENSG00000137331
SCGB3A1 ENSG00000161055
RP11−231C14.4 ENSG00000169203
KRT17 ENSG00000128422
AQP3 ENSG00000165272
UPK1A ENSG00000105668
DHRS2 ENSG00000100867
ACTB ENSG00000075624
ELF3 ENSG00000163435
B2M ENSG00000166710
HLA−B ENSG00000234745
LAMB3 ENSG00000196878
FTL ENSG00000087086
CD74 ENSG00000019582
PTGDS ENSG00000107317
EEF1A1 ENSG00000156508
FTH1 ENSG00000167996
PTBP1 ENSG00000011304
SQSTM1 ENSG00000161011
UBC ENSG00000150991
SDC1 ENSG00000115884
VASP ENSG00000125753
CFL1 ENSG00000172757
JCHAIN ENSG00000132465
ACTG1 ENSG00000184009
PIGR ENSG00000162896
PFN1 ENSG00000108518
IGHD ENSG00000211898
PSAP ENSG00000197746
NR4A1 ENSG00000123358
HLA−C ENSG00000204525
TPT1 ENSG00000133112
FOSL1 ENSG00000175592
DHRS3 ENSG00000162496
CST3 ENSG00000101439
ID1 ENSG00000125968
DUSP1 ENSG00000120129
ASS1 ENSG00000130707
EMD ENSG00000102119
SLPI ENSG00000124107
CLDN4 ENSG00000189143
SPINK1 ENSG00000164266
NDUFV1 ENSG00000167792
HLA−A ENSG00000206503
TFF1 ENSG00000160182
FOSB ENSG00000125740
RP11−347C12.1 ENSG00000198064
