# Supplementary material for: Spatial maps of prostate cancer transcriptomes reveal an unexplored landscape of heterogeneity
Source: Nat Commun. 2018 Jun 20;9:2419. doi: 10.1038/s41467-018-04724-5 (PMC6010471; doi:10.1038/s41467-018-04724-5)
Supplement: Supplementary file 6 — Supplementary Data 3 [file top-genes.pdf › Supplementary Dataset 2/top-genes.pdf]

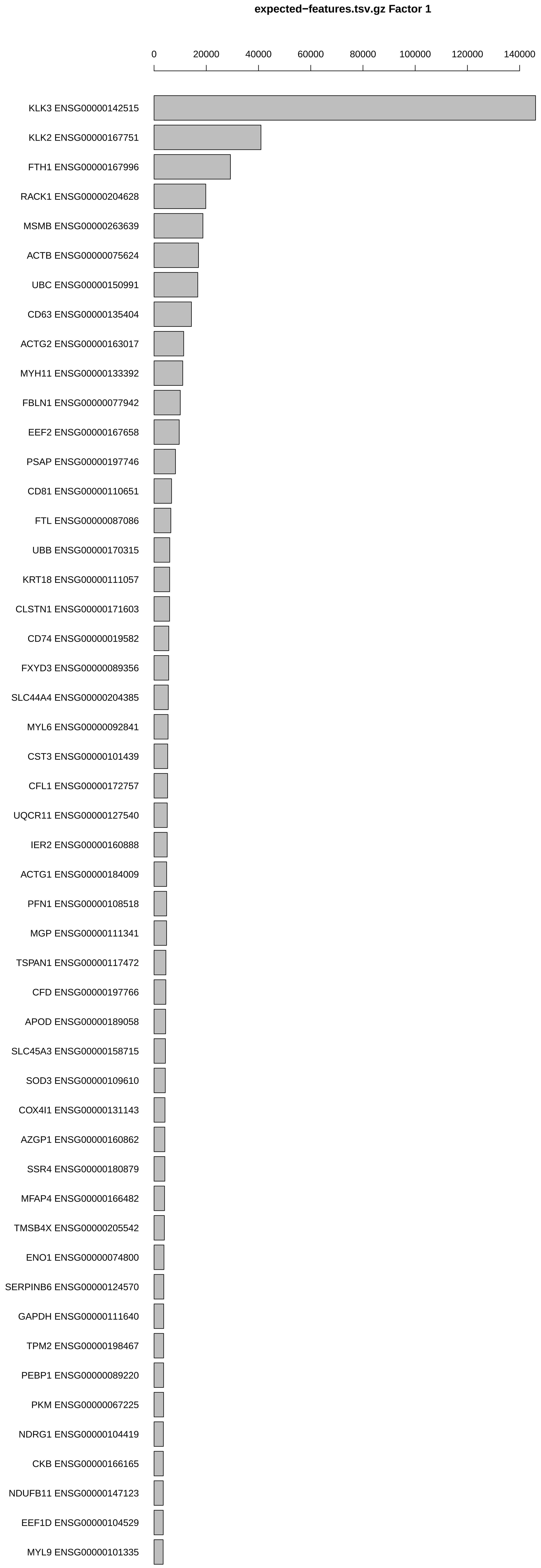
expected−features.tsv.gz Factor 1
0
20000
40000
60000
80000
100000
120000
140000
KLK3 ENSG00000142515
KLK2 ENSG00000167751
FTH1 ENSG00000167996
RACK1 ENSG00000204628
MSMB ENSG00000263639
ACTB ENSG00000075624
UBC ENSG00000150991
CD63 ENSG00000135404
ACTG2 ENSG00000163017
MYH11 ENSG00000133392
FBLN1 ENSG00000077942
EEF2 ENSG00000167658
PSAP ENSG00000197746
CD81 ENSG00000110651
FTL ENSG00000087086
UBB ENSG00000170315
KRT18 ENSG00000111057
CLSTN1 ENSG00000171603
CD74 ENSG00000019582
FXYD3 ENSG00000089356
SLC44A4 ENSG00000204385
MYL6 ENSG00000092841
CST3 ENSG00000101439
CFL1 ENSG00000172757
UQCR11 ENSG00000127540
IER2 ENSG00000160888
ACTG1 ENSG00000184009
PFN1 ENSG00000108518
MGP ENSG00000111341
TSPAN1 ENSG00000117472
CFD ENSG00000197766
APOD ENSG00000189058
SLC45A3 ENSG00000158715
SOD3 ENSG00000109610
COX4I1 ENSG00000131143
AZGP1 ENSG00000160862
SSR4 ENSG00000180879
MFAP4 ENSG00000166482
TMSB4X ENSG00000205542
ENO1 ENSG00000074800
SERPINB6 ENSG00000124570
GAPDH ENSG00000111640
TPM2 ENSG00000198467
PEBP1 ENSG00000089220
PKM ENSG00000067225
NDRG1 ENSG00000104419
CKB ENSG00000166165
NDUFB11 ENSG00000147123
EEF1D ENSG00000104529
MYL9 ENSG00000101335

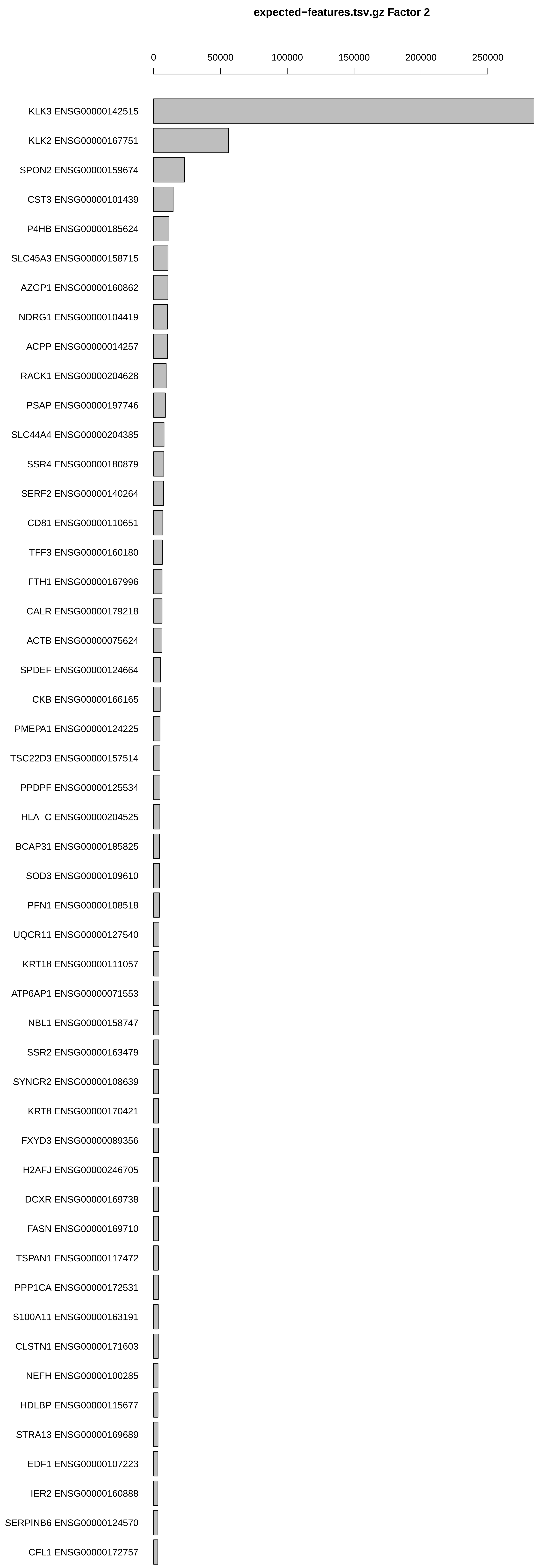
expected−features.tsv.gz Factor 2
0
50000
100000
150000
200000
250000
KLK3 ENSG00000142515
KLK2 ENSG00000167751
SPON2 ENSG00000159674
CST3 ENSG00000101439
P4HB ENSG00000185624
SLC45A3 ENSG00000158715
AZGP1 ENSG00000160862
NDRG1 ENSG00000104419
ACPP ENSG00000014257
RACK1 ENSG00000204628
PSAP ENSG00000197746
SLC44A4 ENSG00000204385
SSR4 ENSG00000180879
SERF2 ENSG00000140264
CD81 ENSG00000110651
TFF3 ENSG00000160180
FTH1 ENSG00000167996
CALR ENSG00000179218
ACTB ENSG00000075624
SPDEF ENSG00000124664
CKB ENSG00000166165
PMEPA1 ENSG00000124225
TSC22D3 ENSG00000157514
PPDPF ENSG00000125534
HLA−C ENSG00000204525
BCAP31 ENSG00000185825
SOD3 ENSG00000109610
PFN1 ENSG00000108518
UQCR11 ENSG00000127540
KRT18 ENSG00000111057
ATP6AP1 ENSG00000071553
NBL1 ENSG00000158747
SSR2 ENSG00000163479
SYNGR2 ENSG00000108639
KRT8 ENSG00000170421
FXYD3 ENSG00000089356
H2AFJ ENSG00000246705
DCXR ENSG00000169738
FASN ENSG00000169710
TSPAN1 ENSG00000117472
PPP1CA ENSG00000172531
S100A11 ENSG00000163191
CLSTN1 ENSG00000171603
NEFH ENSG00000100285
HDLBP ENSG00000115677
STRA13 ENSG00000169689
EDF1 ENSG00000107223
IER2 ENSG00000160888
SERPINB6 ENSG00000124570
CFL1 ENSG00000172757

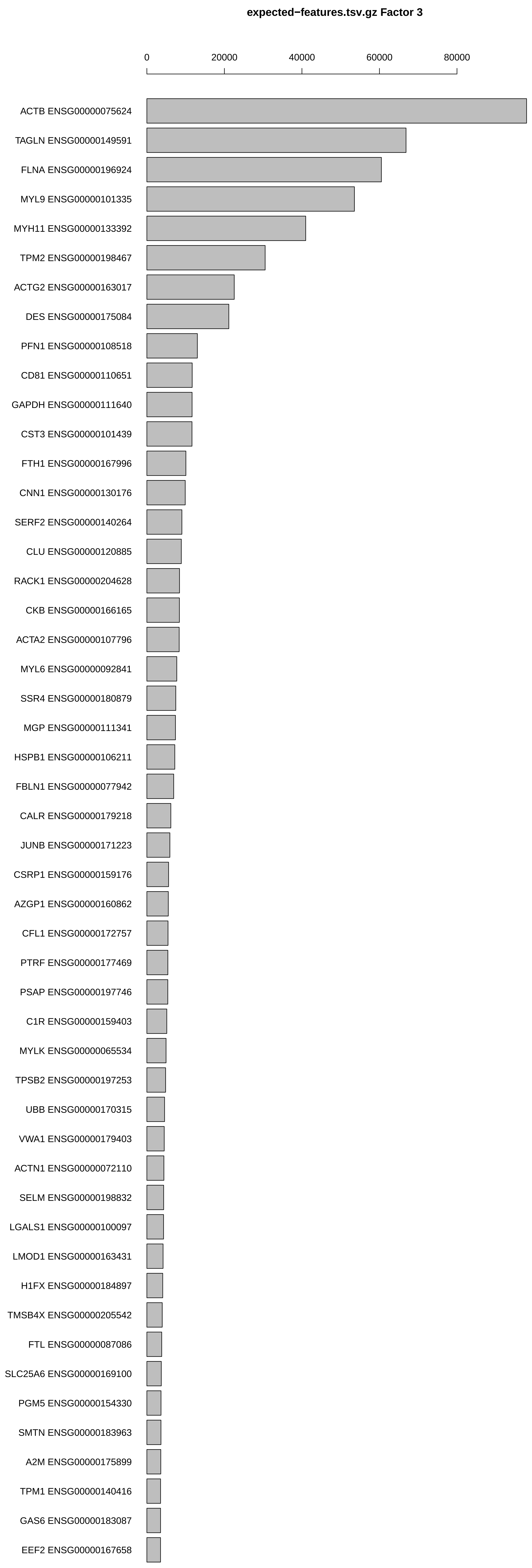
expected−features.tsv.gz Factor 3
0
20000
40000
60000
80000
ACTB ENSG00000075624
TAGLN ENSG00000149591
FLNA ENSG00000196924
MYL9 ENSG00000101335
MYH11 ENSG00000133392
TPM2 ENSG00000198467
ACTG2 ENSG00000163017
DES ENSG00000175084
PFN1 ENSG00000108518
CD81 ENSG00000110651
GAPDH ENSG00000111640
CST3 ENSG00000101439
FTH1 ENSG00000167996
CNN1 ENSG00000130176
SERF2 ENSG00000140264
CLU ENSG00000120885
RACK1 ENSG00000204628
CKB ENSG00000166165
ACTA2 ENSG00000107796
MYL6 ENSG00000092841
SSR4 ENSG00000180879
MGP ENSG00000111341
HSPB1 ENSG00000106211
FBLN1 ENSG00000077942
CALR ENSG00000179218
JUNB ENSG00000171223
CSRP1 ENSG00000159176
AZGP1 ENSG00000160862
CFL1 ENSG00000172757
PTRF ENSG00000177469
PSAP ENSG00000197746
C1R ENSG00000159403
MYLK ENSG00000065534
TPSB2 ENSG00000197253
UBB ENSG00000170315
VWA1 ENSG00000179403
ACTN1 ENSG00000072110
SELM ENSG00000198832
LGALS1 ENSG00000100097
LMOD1 ENSG00000163431
H1FX ENSG00000184897
TMSB4X ENSG00000205542
FTL ENSG00000087086
SLC25A6 ENSG00000169100
PGM5 ENSG00000154330
SMTN ENSG00000183963
A2M ENSG00000175899
TPM1 ENSG00000140416
GAS6 ENSG00000183087
EEF2 ENSG00000167658

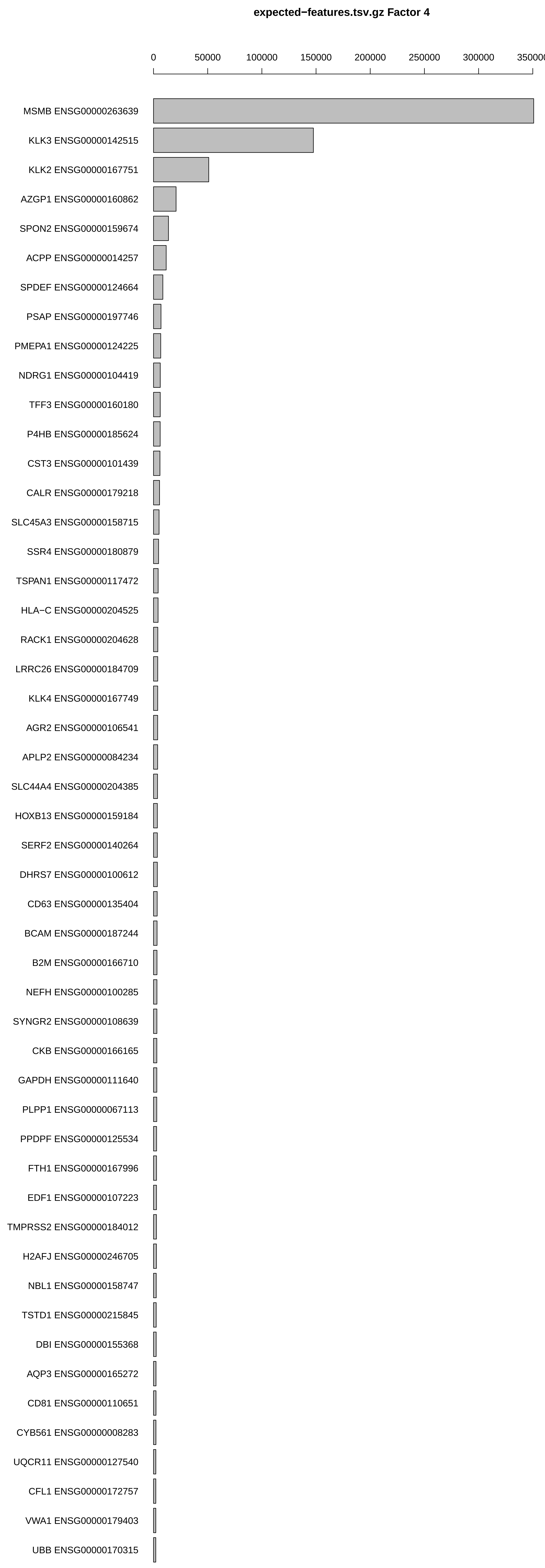
expected−features.tsv.gz Factor 4
0
50000
100000
150000
200000
250000
300000
350000
MSMB ENSG00000263639
KLK3 ENSG00000142515
KLK2 ENSG00000167751
AZGP1 ENSG00000160862
SPON2 ENSG00000159674
ACPP ENSG00000014257
SPDEF ENSG00000124664
PSAP ENSG00000197746
PMEPA1 ENSG00000124225
NDRG1 ENSG00000104419
TFF3 ENSG00000160180
P4HB ENSG00000185624
CST3 ENSG00000101439
CALR ENSG00000179218
SLC45A3 ENSG00000158715
SSR4 ENSG00000180879
TSPAN1 ENSG00000117472
HLA−C ENSG00000204525
RACK1 ENSG00000204628
LRRC26 ENSG00000184709
KLK4 ENSG00000167749
AGR2 ENSG00000106541
APLP2 ENSG00000084234
SLC44A4 ENSG00000204385
HOXB13 ENSG00000159184
SERF2 ENSG00000140264
DHRS7 ENSG00000100612
CD63 ENSG00000135404
BCAM ENSG00000187244
B2M ENSG00000166710
NEFH ENSG00000100285
SYNGR2 ENSG00000108639
CKB ENSG00000166165
GAPDH ENSG00000111640
PLPP1 ENSG00000067113
PPDPF ENSG00000125534
FTH1 ENSG00000167996
EDF1 ENSG00000107223
TMPRSS2 ENSG00000184012
H2AFJ ENSG00000246705
NBL1 ENSG00000158747
TSTD1 ENSG00000215845
DBI ENSG00000155368
AQP3 ENSG00000165272
CD81 ENSG00000110651
CYB561 ENSG00000008283
UQCR11 ENSG00000127540
CFL1 ENSG00000172757
VWA1 ENSG00000179403
UBB ENSG00000170315

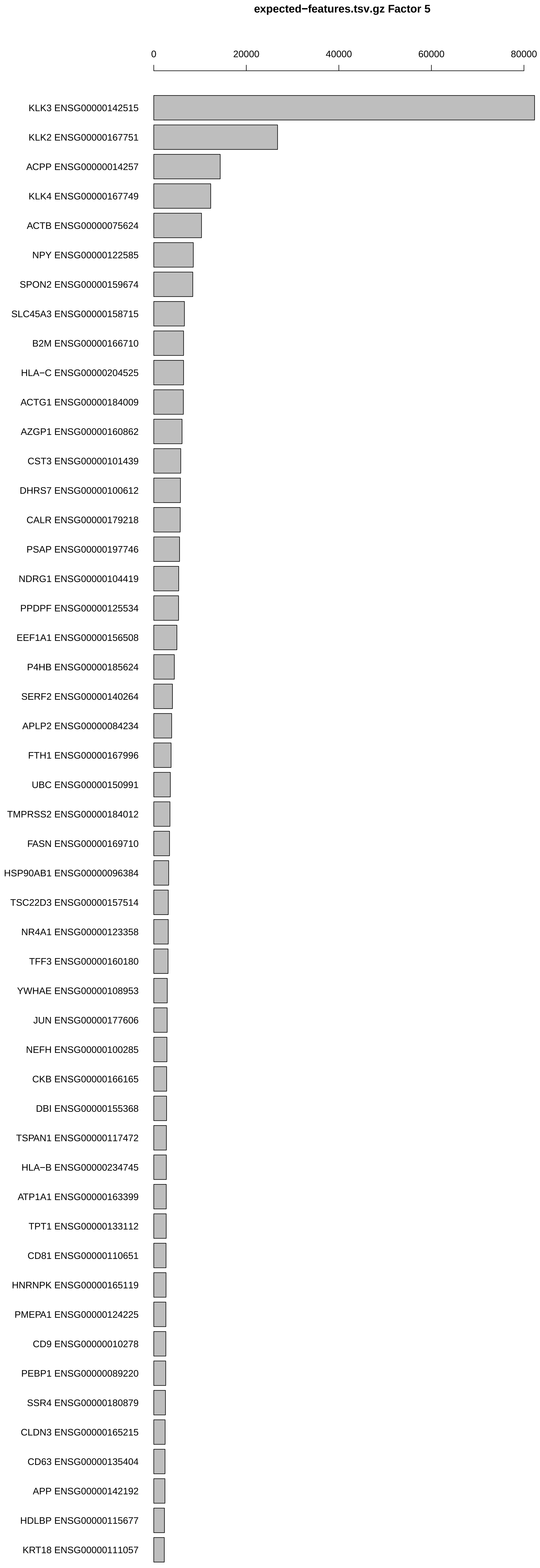
expected−features.tsv.gz Factor 5
0
20000
40000
60000
80000
KLK3 ENSG00000142515
KLK2 ENSG00000167751
ACPP ENSG00000014257
KLK4 ENSG00000167749
ACTB ENSG00000075624
NPY ENSG00000122585
SPON2 ENSG00000159674
SLC45A3 ENSG00000158715
B2M ENSG00000166710
HLA−C ENSG00000204525
ACTG1 ENSG00000184009
AZGP1 ENSG00000160862
CST3 ENSG00000101439
DHRS7 ENSG00000100612
CALR ENSG00000179218
PSAP ENSG00000197746
NDRG1 ENSG00000104419
PPDPF ENSG00000125534
EEF1A1 ENSG00000156508
P4HB ENSG00000185624
SERF2 ENSG00000140264
APLP2 ENSG00000084234
FTH1 ENSG00000167996
UBC ENSG00000150991
TMPRSS2 ENSG00000184012
FASN ENSG00000169710
HSP90AB1 ENSG00000096384
TSC22D3 ENSG00000157514
NR4A1 ENSG00000123358
TFF3 ENSG00000160180
YWHAE ENSG00000108953
JUN ENSG00000177606
NEFH ENSG00000100285
CKB ENSG00000166165
DBI ENSG00000155368
TSPAN1 ENSG00000117472
HLA−B ENSG00000234745
ATP1A1 ENSG00000163399
TPT1 ENSG00000133112
CD81 ENSG00000110651
HNRNPK ENSG00000165119
PMEPA1 ENSG00000124225
CD9 ENSG00000010278
PEBP1 ENSG00000089220
SSR4 ENSG00000180879
CLDN3 ENSG00000165215
CD63 ENSG00000135404
APP ENSG00000142192
HDLBP ENSG00000115677
KRT18 ENSG00000111057

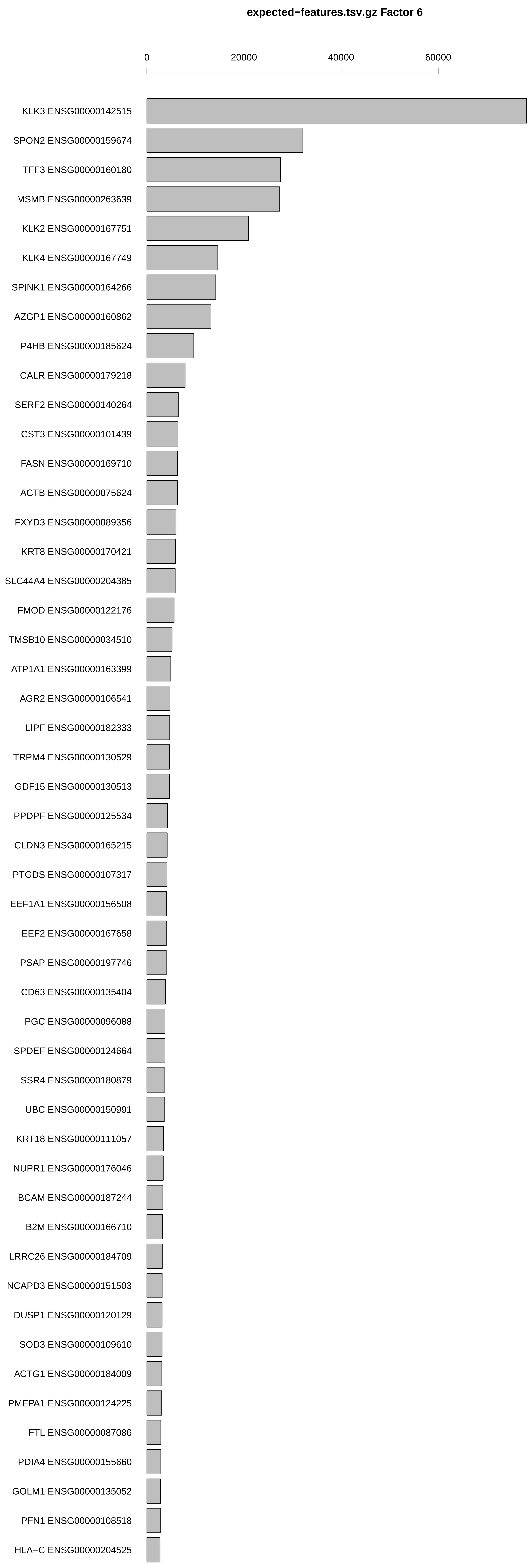
expected−features.tsv.gz Factor 6
0
20000
40000
60000
KLK3 ENSG00000142515
SPON2 ENSG00000159674
TFF3 ENSG00000160180
MSMB ENSG00000263639
KLK2 ENSG00000167751
KLK4 ENSG00000167749
SPINK1 ENSG00000164266
AZGP1 ENSG00000160862
P4HB ENSG00000185624
CALR ENSG00000179218
SERF2 ENSG00000140264
CST3 ENSG00000101439
FASN ENSG00000169710
ACTB ENSG00000075624
FXYD3 ENSG00000089356
KRT8 ENSG00000170421
SLC44A4 ENSG00000204385
FMOD ENSG00000122176
TMSB10 ENSG00000034510
ATP1A1 ENSG00000163399
AGR2 ENSG00000106541
LIPF ENSG00000182333
TRPM4 ENSG00000130529
GDF15 ENSG00000130513
PPDPF ENSG00000125534
CLDN3 ENSG00000165215
PTGDS ENSG00000107317
EEF1A1 ENSG00000156508
EEF2 ENSG00000167658
PSAP ENSG00000197746
CD63 ENSG00000135404
PGC ENSG00000096088
SPDEF ENSG00000124664
SSR4 ENSG00000180879
UBC ENSG00000150991
KRT18 ENSG00000111057
NUPR1 ENSG00000176046
BCAM ENSG00000187244
B2M ENSG00000166710
LRRC26 ENSG00000184709
NCAPD3 ENSG00000151503
DUSP1 ENSG00000120129
SOD3 ENSG00000109610
ACTG1 ENSG00000184009
PMEPA1 ENSG00000124225
FTL ENSG00000087086
PDIA4 ENSG00000155660
GOLM1 ENSG00000135052
PFN1 ENSG00000108518
HLA−C ENSG00000204525

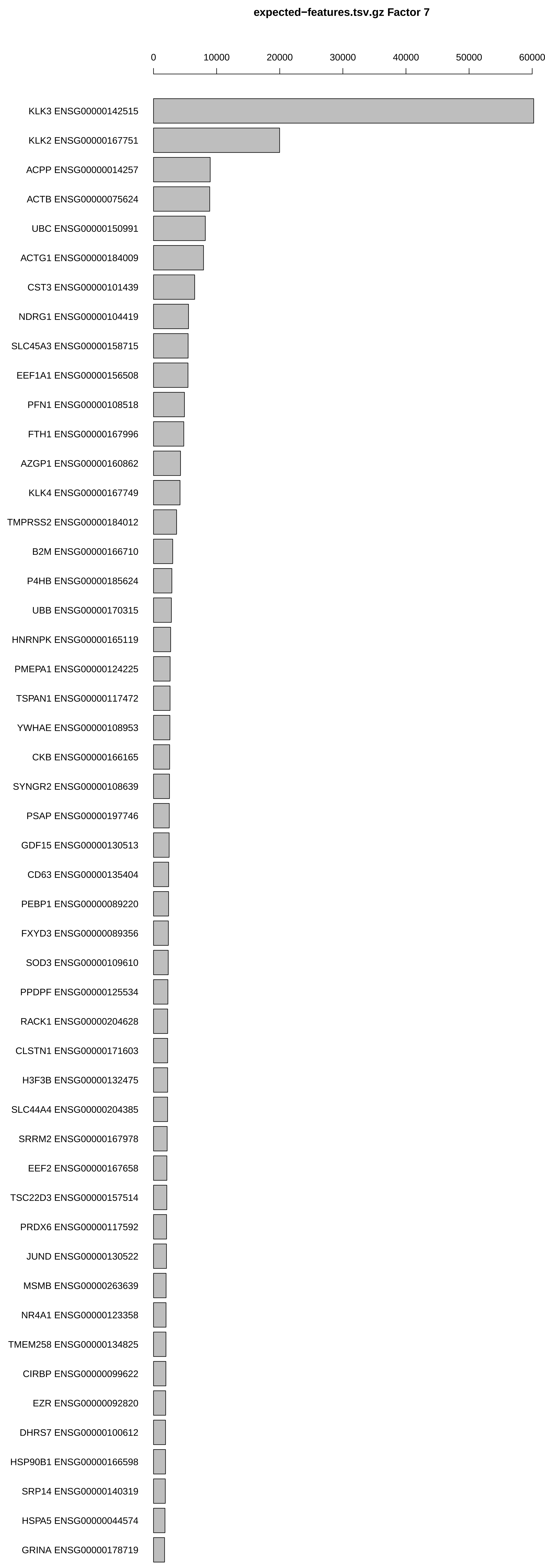
expected−features.tsv.gz Factor 7
0
10000
20000
30000
40000
50000
60000
KLK3 ENSG00000142515
KLK2 ENSG00000167751
ACPP ENSG00000014257
ACTB ENSG00000075624
UBC ENSG00000150991
ACTG1 ENSG00000184009
CST3 ENSG00000101439
NDRG1 ENSG00000104419
SLC45A3 ENSG00000158715
EEF1A1 ENSG00000156508
PFN1 ENSG00000108518
FTH1 ENSG00000167996
AZGP1 ENSG00000160862
KLK4 ENSG00000167749
TMPRSS2 ENSG00000184012
B2M ENSG00000166710
P4HB ENSG00000185624
UBB ENSG00000170315
HNRNPK ENSG00000165119
PMEPA1 ENSG00000124225
TSPAN1 ENSG00000117472
YWHAE ENSG00000108953
CKB ENSG00000166165
SYNGR2 ENSG00000108639
PSAP ENSG00000197746
GDF15 ENSG00000130513
CD63 ENSG00000135404
PEBP1 ENSG00000089220
FXYD3 ENSG00000089356
SOD3 ENSG00000109610
PPDPF ENSG00000125534
RACK1 ENSG00000204628
CLSTN1 ENSG00000171603
H3F3B ENSG00000132475
SLC44A4 ENSG00000204385
SRRM2 ENSG00000167978
EEF2 ENSG00000167658
TSC22D3 ENSG00000157514
PRDX6 ENSG00000117592
JUND ENSG00000130522
MSMB ENSG00000263639
NR4A1 ENSG00000123358
TMEM258 ENSG00000134825
CIRBP ENSG00000099622
EZR ENSG00000092820
DHRS7 ENSG00000100612
HSP90B1 ENSG00000166598
SRP14 ENSG00000140319
HSPA5 ENSG00000044574
GRINA ENSG00000178719

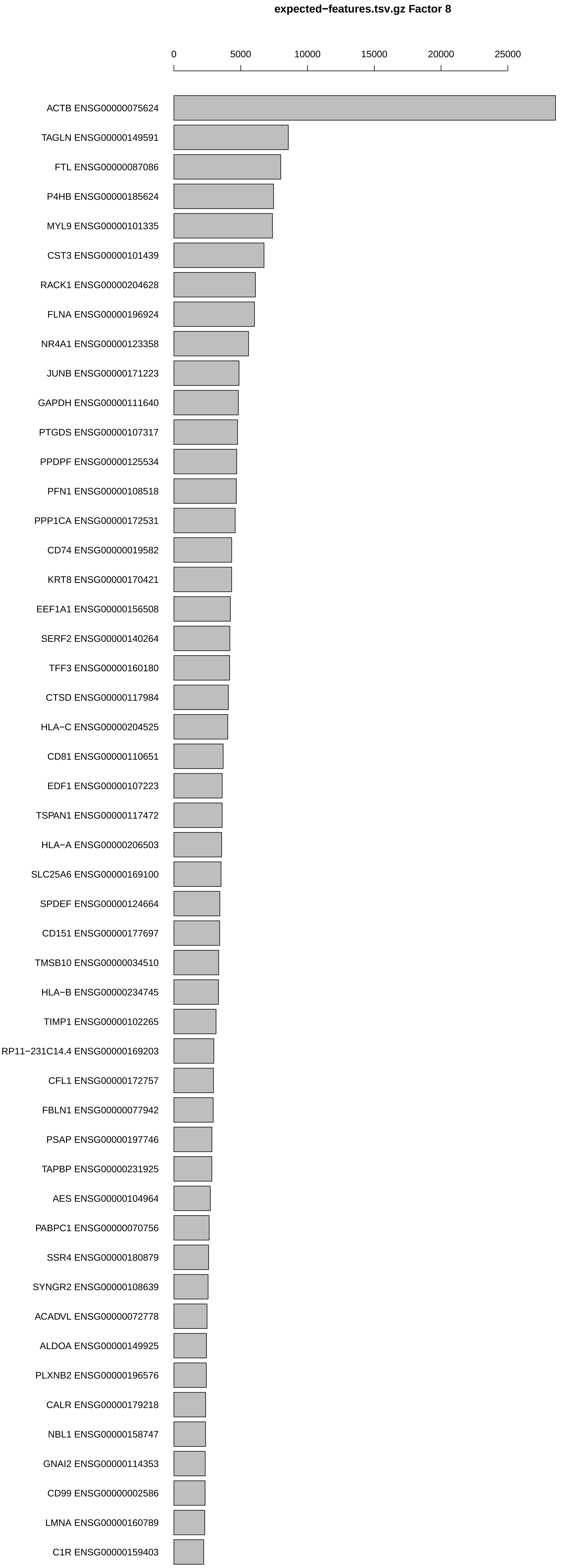
expected−features.tsv.gz Factor 8
0
5000
10000
15000
20000
25000
ACTB ENSG00000075624
TAGLN ENSG00000149591
FTL ENSG00000087086
P4HB ENSG00000185624
MYL9 ENSG00000101335
CST3 ENSG00000101439
RACK1 ENSG00000204628
FLNA ENSG00000196924
NR4A1 ENSG00000123358
JUNB ENSG00000171223
GAPDH ENSG00000111640
PTGDS ENSG00000107317
PPDPF ENSG00000125534
PFN1 ENSG00000108518
PPP1CA ENSG00000172531
CD74 ENSG00000019582
KRT8 ENSG00000170421
EEF1A1 ENSG00000156508
SERF2 ENSG00000140264
TFF3 ENSG00000160180
CTSD ENSG00000117984
HLA−C ENSG00000204525
CD81 ENSG00000110651
EDF1 ENSG00000107223
TSPAN1 ENSG00000117472
HLA−A ENSG00000206503
SLC25A6 ENSG00000169100
SPDEF ENSG00000124664
CD151 ENSG00000177697
TMSB10 ENSG00000034510
HLA−B ENSG00000234745
TIMP1 ENSG00000102265
RP11−231C14.4 ENSG00000169203
CFL1 ENSG00000172757
FBLN1 ENSG00000077942
PSAP ENSG00000197746
TAPBP ENSG00000231925
AES ENSG00000104964
PABPC1 ENSG00000070756
SSR4 ENSG00000180879
SYNGR2 ENSG00000108639
ACADVL ENSG00000072778
ALDOA ENSG00000149925
PLXNB2 ENSG00000196576
CALR ENSG00000179218
NBL1 ENSG00000158747
GNAI2 ENSG00000114353
CD99 ENSG00000002586
LMNA ENSG00000160789
C1R ENSG00000159403

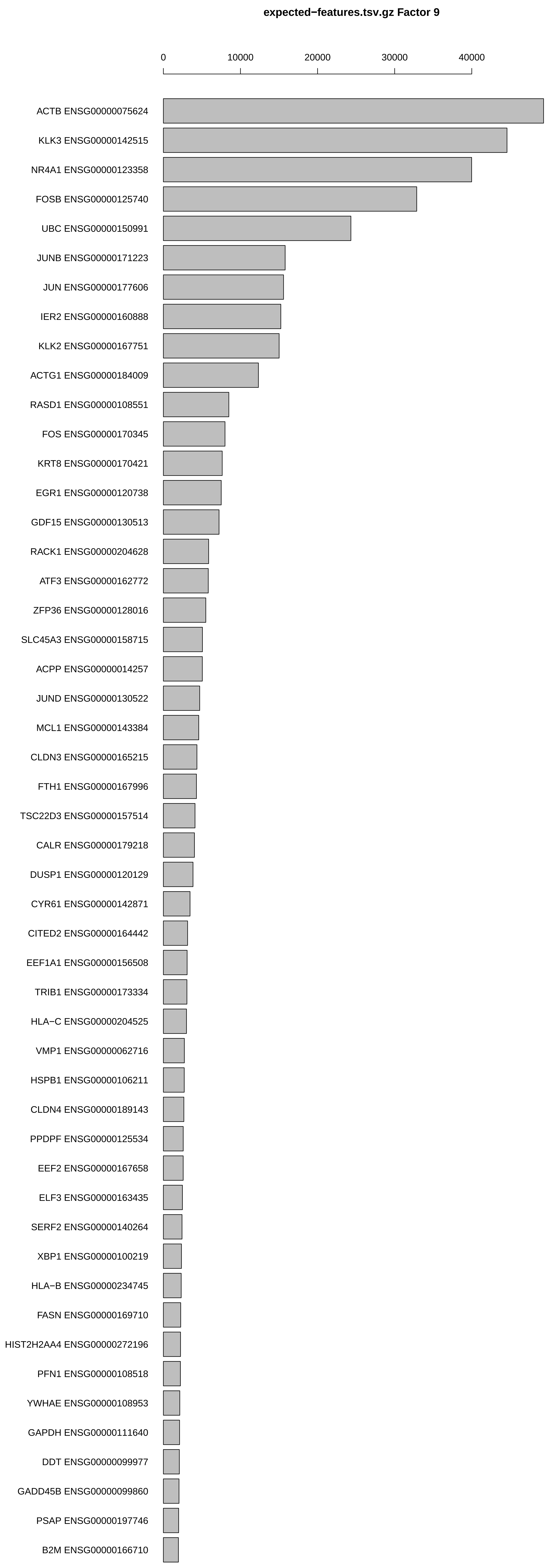
expected−features.tsv.gz Factor 9
0
10000
20000
30000
40000
ACTB ENSG00000075624
KLK3 ENSG00000142515
NR4A1 ENSG00000123358
FOSB ENSG00000125740
UBC ENSG00000150991
JUNB ENSG00000171223
JUN ENSG00000177606
IER2 ENSG00000160888
KLK2 ENSG00000167751
ACTG1 ENSG00000184009
RASD1 ENSG00000108551
FOS ENSG00000170345
KRT8 ENSG00000170421
EGR1 ENSG00000120738
GDF15 ENSG00000130513
RACK1 ENSG00000204628
ATF3 ENSG00000162772
ZFP36 ENSG00000128016
SLC45A3 ENSG00000158715
ACPP ENSG00000014257
JUND ENSG00000130522
MCL1 ENSG00000143384
CLDN3 ENSG00000165215
FTH1 ENSG00000167996
TSC22D3 ENSG00000157514
CALR ENSG00000179218
DUSP1 ENSG00000120129
CYR61 ENSG00000142871
CITED2 ENSG00000164442
EEF1A1 ENSG00000156508
TRIB1 ENSG00000173334
HLA−C ENSG00000204525
VMP1 ENSG00000062716
HSPB1 ENSG00000106211
CLDN4 ENSG00000189143
PPDPF ENSG00000125534
EEF2 ENSG00000167658
ELF3 ENSG00000163435
SERF2 ENSG00000140264
XBP1 ENSG00000100219
HLA−B ENSG00000234745
FASN ENSG00000169710
HIST2H2AA4 ENSG00000272196
PFN1 ENSG00000108518
YWHAE ENSG00000108953
GAPDH ENSG00000111640
DDT ENSG00000099977
GADD45B ENSG00000099860
PSAP ENSG00000197746
B2M ENSG00000166710

expected−features.tsv.gz Factor 10

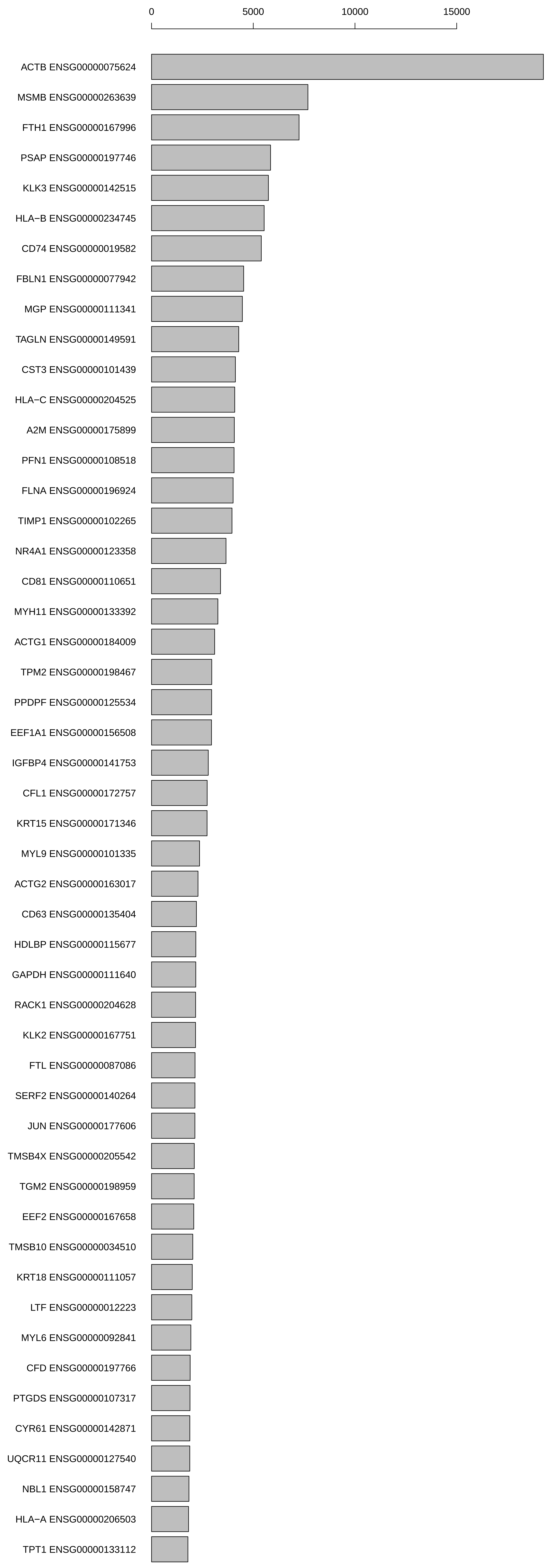

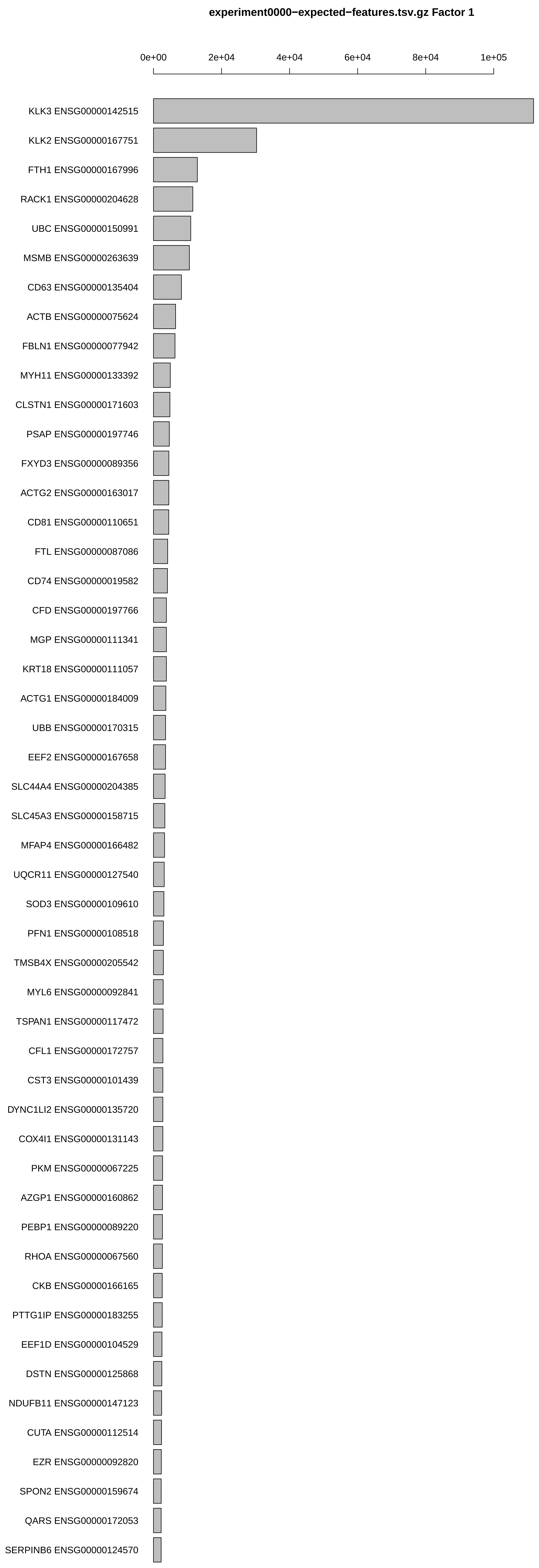
experiment0000−expected−features.tsv.gz Factor 1
0e+00
2e+04
4e+04
6e+04
8e+04
1e+05
KLK3 ENSG00000142515
KLK2 ENSG00000167751
FTH1 ENSG00000167996
RACK1 ENSG00000204628
UBC ENSG00000150991
MSMB ENSG00000263639
CD63 ENSG00000135404
ACTB ENSG00000075624
FBLN1 ENSG00000077942
MYH11 ENSG00000133392
CLSTN1 ENSG00000171603
PSAP ENSG00000197746
FXYD3 ENSG00000089356
ACTG2 ENSG00000163017
CD81 ENSG00000110651
FTL ENSG00000087086
CD74 ENSG00000019582
CFD ENSG00000197766
MGP ENSG00000111341
KRT18 ENSG00000111057
ACTG1 ENSG00000184009
UBB ENSG00000170315
EEF2 ENSG00000167658
SLC44A4 ENSG00000204385
SLC45A3 ENSG00000158715
MFAP4 ENSG00000166482
UQCR11 ENSG00000127540
SOD3 ENSG00000109610
PFN1 ENSG00000108518
TMSB4X ENSG00000205542
MYL6 ENSG00000092841
TSPAN1 ENSG00000117472
CFL1 ENSG00000172757
CST3 ENSG00000101439
DYNC1LI2 ENSG00000135720
COX4I1 ENSG00000131143
PKM ENSG00000067225
AZGP1 ENSG00000160862
PEBP1 ENSG00000089220
RHOA ENSG00000067560
CKB ENSG00000166165
PTTG1IP ENSG00000183255
EEF1D ENSG00000104529
DSTN ENSG00000125868
NDUFB11 ENSG00000147123
CUTA ENSG00000112514
EZR ENSG00000092820
SPON2 ENSG00000159674
QARS ENSG00000172053
SERPINB6 ENSG00000124570

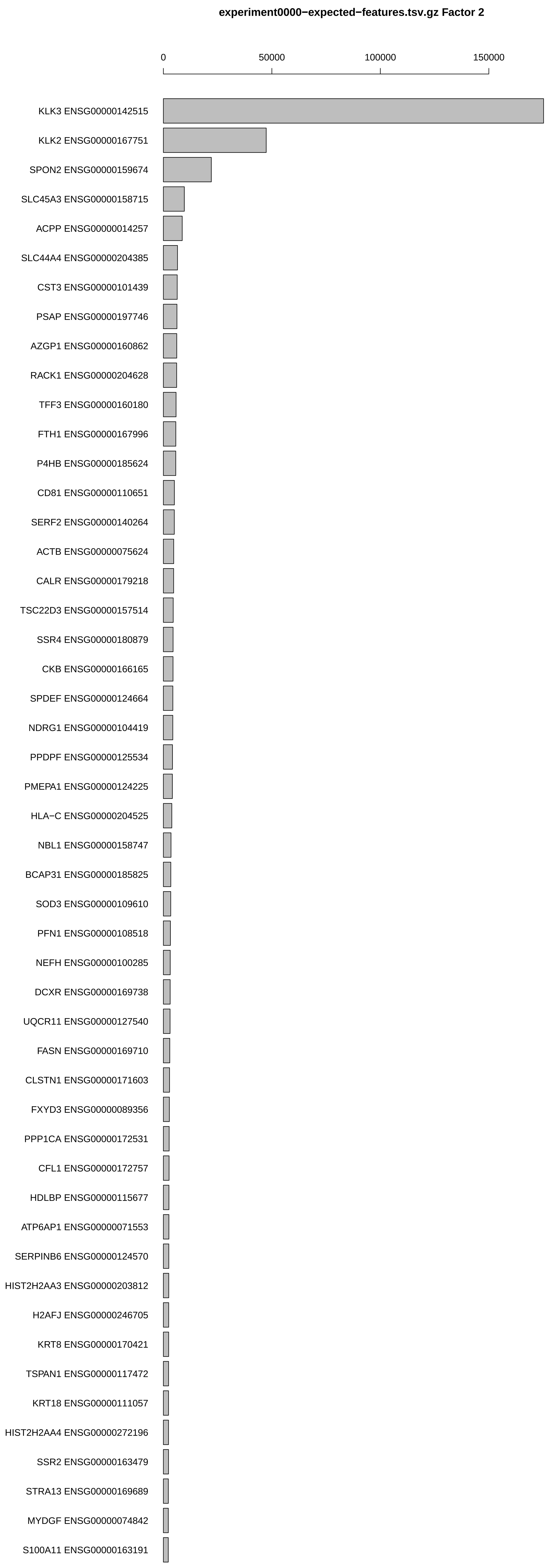
experiment0000−expected−features.tsv.gz Factor 2
0
50000
100000
150000
KLK3 ENSG00000142515
KLK2 ENSG00000167751
SPON2 ENSG00000159674
SLC45A3 ENSG00000158715
ACPP ENSG00000014257
SLC44A4 ENSG00000204385
CST3 ENSG00000101439
PSAP ENSG00000197746
AZGP1 ENSG00000160862
RACK1 ENSG00000204628
TFF3 ENSG00000160180
FTH1 ENSG00000167996
P4HB ENSG00000185624
CD81 ENSG00000110651
SERF2 ENSG00000140264
ACTB ENSG00000075624
CALR ENSG00000179218
TSC22D3 ENSG00000157514
SSR4 ENSG00000180879
CKB ENSG00000166165
SPDEF ENSG00000124664
NDRG1 ENSG00000104419
PPDPF ENSG00000125534
PMEPA1 ENSG00000124225
HLA−C ENSG00000204525
NBL1 ENSG00000158747
BCAP31 ENSG00000185825
SOD3 ENSG00000109610
PFN1 ENSG00000108518
NEFH ENSG00000100285
DCXR ENSG00000169738
UQCR11 ENSG00000127540
FASN ENSG00000169710
CLSTN1 ENSG00000171603
FXYD3 ENSG00000089356
PPP1CA ENSG00000172531
CFL1 ENSG00000172757
HDLBP ENSG00000115677
ATP6AP1 ENSG00000071553
SERPINB6 ENSG00000124570
HIST2H2AA3 ENSG00000203812
H2AFJ ENSG00000246705
KRT8 ENSG00000170421
TSPAN1 ENSG00000117472
KRT18 ENSG00000111057
HIST2H2AA4 ENSG00000272196
SSR2 ENSG00000163479
STRA13 ENSG00000169689
MYDGF ENSG00000074842
S100A11 ENSG00000163191

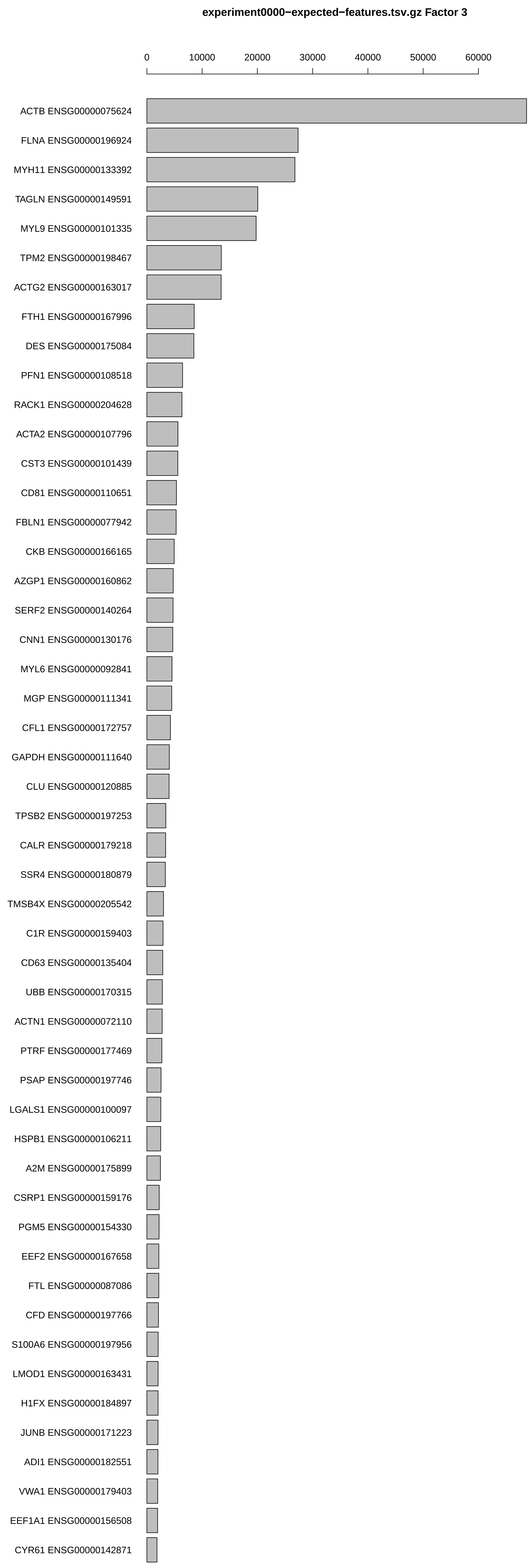
experiment0000−expected−features.tsv.gz Factor 3
0
10000
20000
30000
40000
50000
60000
ACTB ENSG00000075624
FLNA ENSG00000196924
MYH11 ENSG00000133392
TAGLN ENSG00000149591
MYL9 ENSG00000101335
TPM2 ENSG00000198467
ACTG2 ENSG00000163017
FTH1 ENSG00000167996
DES ENSG00000175084
PFN1 ENSG00000108518
RACK1 ENSG00000204628
ACTA2 ENSG00000107796
CST3 ENSG00000101439
CD81 ENSG00000110651
FBLN1 ENSG00000077942
CKB ENSG00000166165
AZGP1 ENSG00000160862
SERF2 ENSG00000140264
CNN1 ENSG00000130176
MYL6 ENSG00000092841
MGP ENSG00000111341
CFL1 ENSG00000172757
GAPDH ENSG00000111640
CLU ENSG00000120885
TPSB2 ENSG00000197253
CALR ENSG00000179218
SSR4 ENSG00000180879
TMSB4X ENSG00000205542
C1R ENSG00000159403
CD63 ENSG00000135404
UBB ENSG00000170315
ACTN1 ENSG00000072110
PTRF ENSG00000177469
PSAP ENSG00000197746
LGALS1 ENSG00000100097
HSPB1 ENSG00000106211
A2M ENSG00000175899
CSRP1 ENSG00000159176
PGM5 ENSG00000154330
EEF2 ENSG00000167658
FTL ENSG00000087086
CFD ENSG00000197766
S100A6 ENSG00000197956
LMOD1 ENSG00000163431
H1FX ENSG00000184897
JUNB ENSG00000171223
ADI1 ENSG00000182551
VWA1 ENSG00000179403
EEF1A1 ENSG00000156508
CYR61 ENSG00000142871

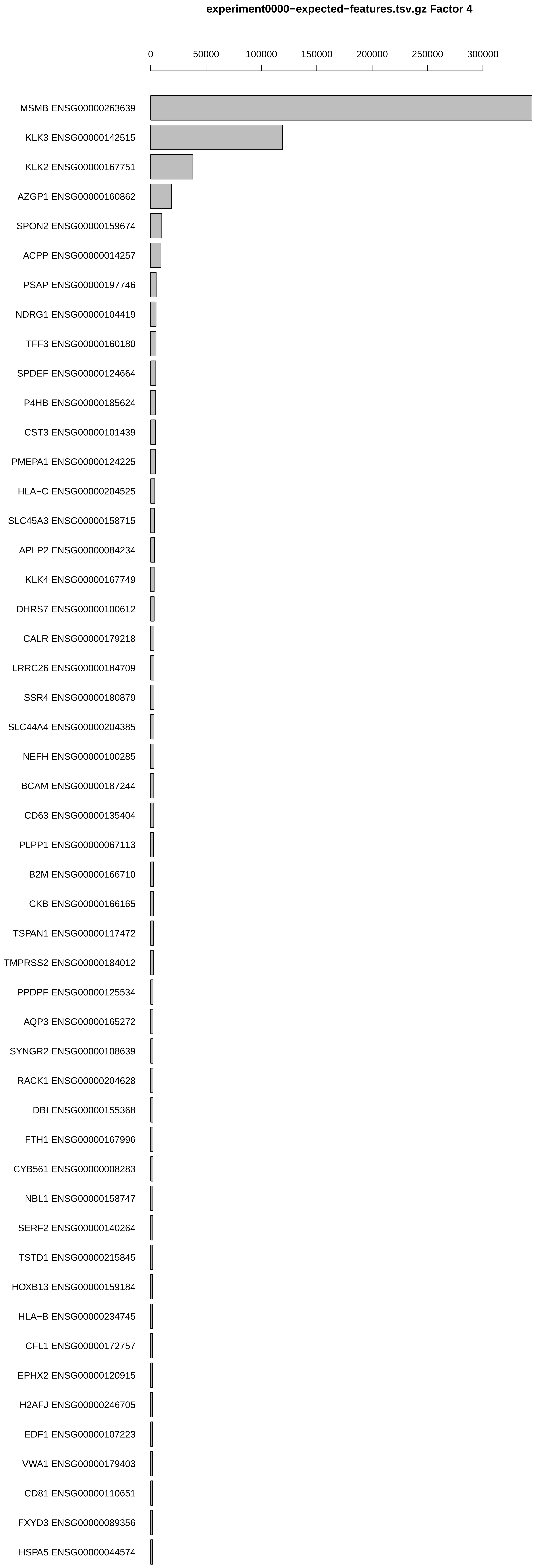
experiment0000−expected−features.tsv.gz Factor 4
0
50000
100000
150000
200000
250000
300000
MSMB ENSG00000263639
KLK3 ENSG00000142515
KLK2 ENSG00000167751
AZGP1 ENSG00000160862
SPON2 ENSG00000159674
ACPP ENSG00000014257
PSAP ENSG00000197746
NDRG1 ENSG00000104419
TFF3 ENSG00000160180
SPDEF ENSG00000124664
P4HB ENSG00000185624
CST3 ENSG00000101439
PMEPA1 ENSG00000124225
HLA−C ENSG00000204525
SLC45A3 ENSG00000158715
APLP2 ENSG00000084234
KLK4 ENSG00000167749
DHRS7 ENSG00000100612
CALR ENSG00000179218
LRRC26 ENSG00000184709
SSR4 ENSG00000180879
SLC44A4 ENSG00000204385
NEFH ENSG00000100285
BCAM ENSG00000187244
CD63 ENSG00000135404
PLPP1 ENSG00000067113
B2M ENSG00000166710
CKB ENSG00000166165
TSPAN1 ENSG00000117472
TMPRSS2 ENSG00000184012
PPDPF ENSG00000125534
AQP3 ENSG00000165272
SYNGR2 ENSG00000108639
RACK1 ENSG00000204628
DBI ENSG00000155368
FTH1 ENSG00000167996
CYB561 ENSG00000008283
NBL1 ENSG00000158747
SERF2 ENSG00000140264
TSTD1 ENSG00000215845
HOXB13 ENSG00000159184
HLA−B ENSG00000234745
CFL1 ENSG00000172757
EPHX2 ENSG00000120915
H2AFJ ENSG00000246705
EDF1 ENSG00000107223
VWA1 ENSG00000179403
CD81 ENSG00000110651
FXYD3 ENSG00000089356
HSPA5 ENSG00000044574

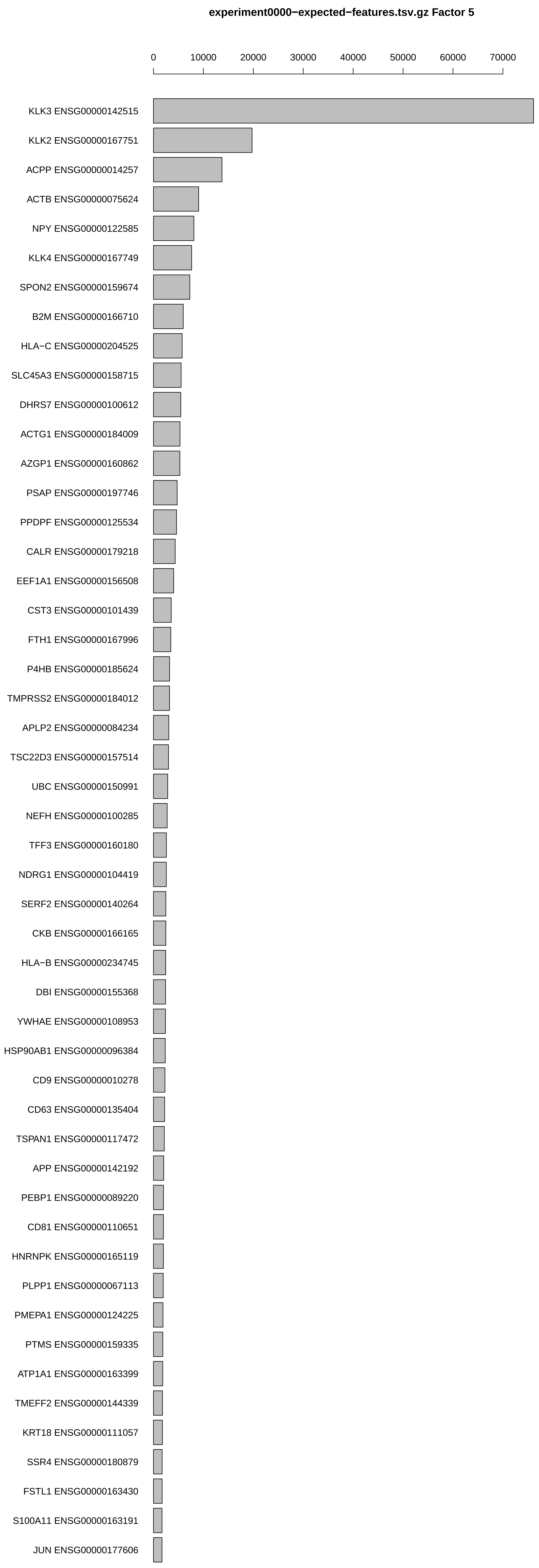

experiment0000−expected−features.tsv.gz Factor 5
0
10000
20000
30000
40000
50000
60000
70000
KLK3 ENSG00000142515
KLK2 ENSG00000167751
ACPP ENSG00000014257
ACTB ENSG00000075624
NPY ENSG00000122585
KLK4 ENSG00000167749
SPON2 ENSG00000159674
B2M ENSG00000166710
HLA−C ENSG00000204525
SLC45A3 ENSG00000158715
DHRS7 ENSG00000100612
ACTG1 ENSG00000184009
AZGP1 ENSG00000160862
PSAP ENSG00000197746
PPDPF ENSG00000125534
CALR ENSG00000179218
EEF1A1 ENSG00000156508
CST3 ENSG00000101439
FTH1 ENSG00000167996
P4HB ENSG00000185624
TMPRSS2 ENSG00000184012
APLP2 ENSG00000084234
TSC22D3 ENSG00000157514
UBC ENSG00000150991
NEFH ENSG00000100285
TFF3 ENSG00000160180
NDRG1 ENSG00000104419
SERF2 ENSG00000140264
CKB ENSG00000166165
HLA−B ENSG00000234745
DBI ENSG00000155368
YWHAE ENSG00000108953
HSP90AB1 ENSG00000096384
CD9 ENSG00000010278
CD63 ENSG00000135404
TSPAN1 ENSG00000117472
APP ENSG00000142192
PEBP1 ENSG00000089220
CD81 ENSG00000110651
HNRNPK ENSG00000165119
PLPP1 ENSG00000067113
PMEPA1 ENSG00000124225
PTMS ENSG00000159335
ATP1A1 ENSG00000163399
TMEFF2 ENSG00000144339
KRT18 ENSG00000111057
SSR4 ENSG00000180879
FSTL1 ENSG00000163430
S100A11 ENSG00000163191
JUN ENSG00000177606

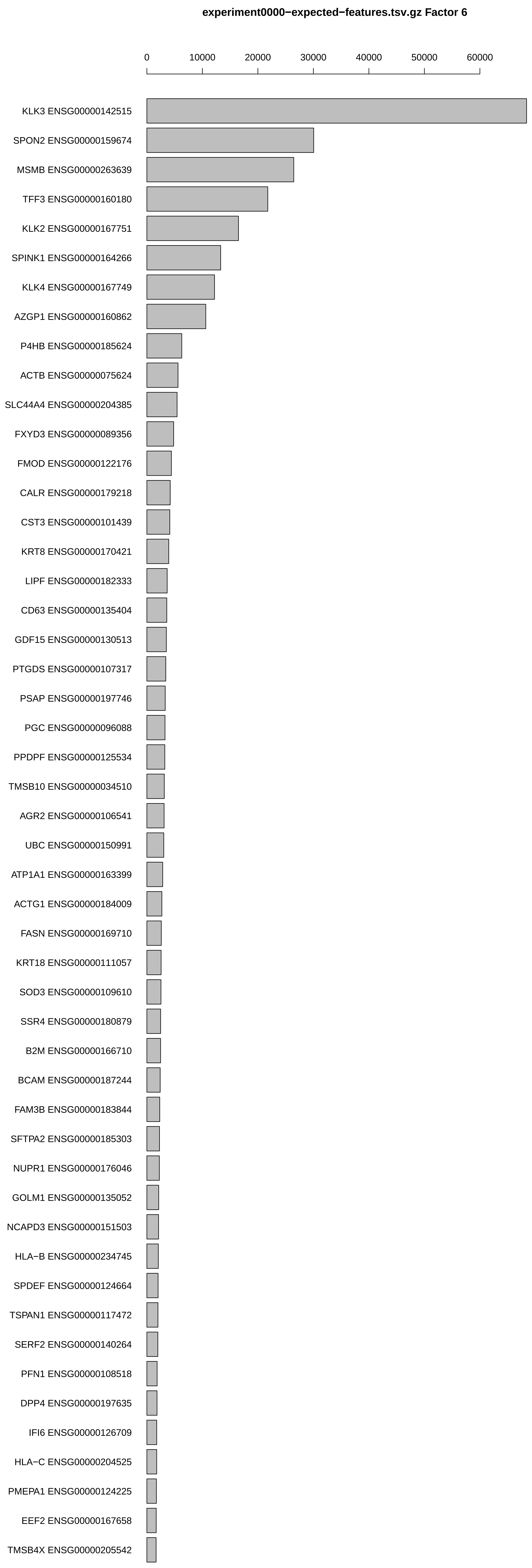

experiment0000−expected−features.tsv.gz Factor 6
0
10000
20000
30000
40000
50000
60000
KLK3 ENSG00000142515
SPON2 ENSG00000159674
MSMB ENSG00000263639
TFF3 ENSG00000160180
KLK2 ENSG00000167751
SPINK1 ENSG00000164266
KLK4 ENSG00000167749
AZGP1 ENSG00000160862
P4HB ENSG00000185624
ACTB ENSG00000075624
SLC44A4 ENSG00000204385
FXYD3 ENSG00000089356
FMOD ENSG00000122176
CALR ENSG00000179218
CST3 ENSG00000101439
KRT8 ENSG00000170421
LIPF ENSG00000182333
CD63 ENSG00000135404
GDF15 ENSG00000130513
PTGDS ENSG00000107317
PSAP ENSG00000197746
PGC ENSG00000096088
PPDPF ENSG00000125534
TMSB10 ENSG00000034510
AGR2 ENSG00000106541
UBC ENSG00000150991
ATP1A1 ENSG00000163399
ACTG1 ENSG00000184009
FASN ENSG00000169710
KRT18 ENSG00000111057
SOD3 ENSG00000109610
SSR4 ENSG00000180879
B2M ENSG00000166710
BCAM ENSG00000187244
FAM3B ENSG00000183844
SFTPA2 ENSG00000185303
NUPR1 ENSG00000176046
GOLM1 ENSG00000135052
NCAPD3 ENSG00000151503
HLA−B ENSG00000234745
SPDEF ENSG00000124664
TSPAN1 ENSG00000117472
SERF2 ENSG00000140264
PFN1 ENSG00000108518
DPP4 ENSG00000197635
IFI6 ENSG00000126709
HLA−C ENSG00000204525
PMEPA1 ENSG00000124225
EEF2 ENSG00000167658
TMSB4X ENSG00000205542

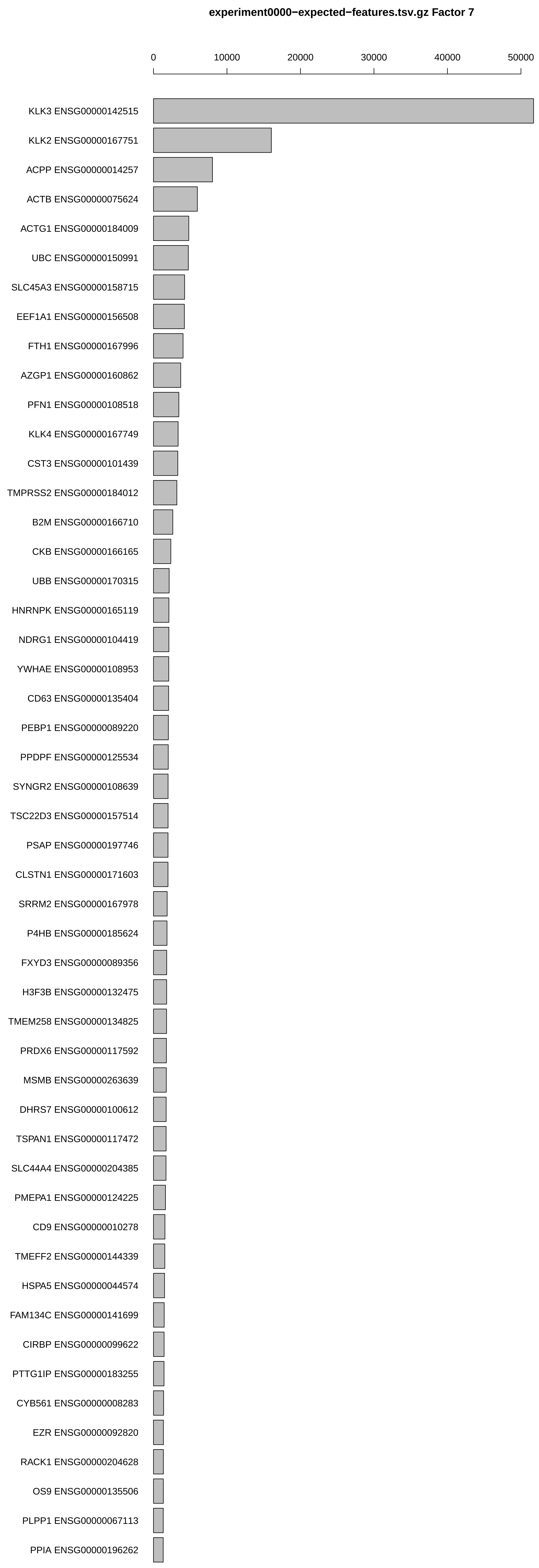
experiment0000−expected−features.tsv.gz Factor 7
0
10000
20000
30000
40000
50000
KLK3 ENSG00000142515
KLK2 ENSG00000167751
ACPP ENSG00000014257
ACTB ENSG00000075624
ACTG1 ENSG00000184009
UBC ENSG00000150991
SLC45A3 ENSG00000158715
EEF1A1 ENSG00000156508
FTH1 ENSG00000167996
AZGP1 ENSG00000160862
PFN1 ENSG00000108518
KLK4 ENSG00000167749
CST3 ENSG00000101439
TMPRSS2 ENSG00000184012
B2M ENSG00000166710
CKB ENSG00000166165
UBB ENSG00000170315
HNRNPK ENSG00000165119
NDRG1 ENSG00000104419
YWHAE ENSG00000108953
CD63 ENSG00000135404
PEBP1 ENSG00000089220
PPDPF ENSG00000125534
SYNGR2 ENSG00000108639
TSC22D3 ENSG00000157514
PSAP ENSG00000197746
CLSTN1 ENSG00000171603
SRRM2 ENSG00000167978
P4HB ENSG00000185624
FXYD3 ENSG00000089356
H3F3B ENSG00000132475
TMEM258 ENSG00000134825
PRDX6 ENSG00000117592
MSMB ENSG00000263639
DHRS7 ENSG00000100612
TSPAN1 ENSG00000117472
SLC44A4 ENSG00000204385
PMEPA1 ENSG00000124225
CD9 ENSG00000010278
TMEFF2 ENSG00000144339
HSPA5 ENSG00000044574
FAM134C ENSG00000141699
CIRBP ENSG00000099622
PTTG1IP ENSG00000183255
CYB561 ENSG00000008283
EZR ENSG00000092820
RACK1 ENSG00000204628
OS9 ENSG00000135506
PLPP1 ENSG00000067113
PPIA ENSG00000196262

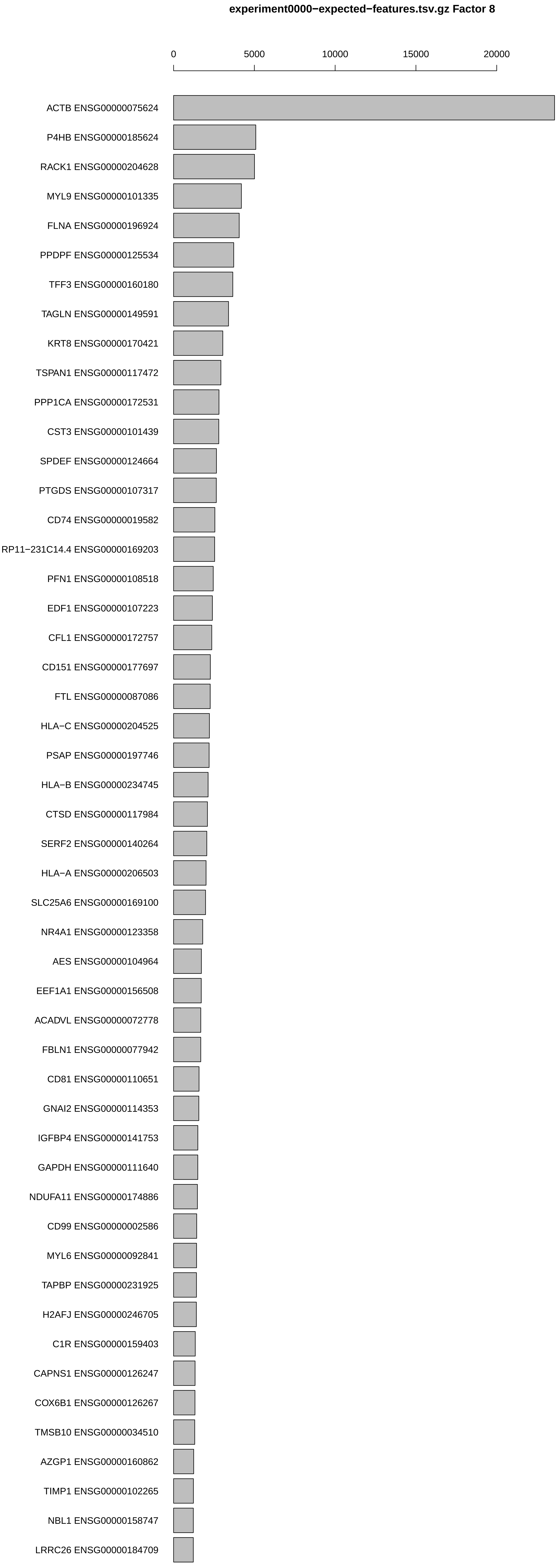

experiment0000−expected−features.tsv.gz Factor 8
0
5000
10000
15000
20000
ACTB ENSG00000075624
P4HB ENSG00000185624
RACK1 ENSG00000204628
MYL9 ENSG00000101335
FLNA ENSG00000196924
PPDPF ENSG00000125534
TFF3 ENSG00000160180
TAGLN ENSG00000149591
KRT8 ENSG00000170421
TSPAN1 ENSG00000117472
PPP1CA ENSG00000172531
CST3 ENSG00000101439
SPDEF ENSG00000124664
PTGDS ENSG00000107317
CD74 ENSG00000019582
RP11−231C14.4 ENSG00000169203
PFN1 ENSG00000108518
EDF1 ENSG00000107223
CFL1 ENSG00000172757
CD151 ENSG00000177697
FTL ENSG00000087086
HLA−C ENSG00000204525
PSAP ENSG00000197746
HLA−B ENSG00000234745
CTSD ENSG00000117984
SERF2 ENSG00000140264
HLA−A ENSG00000206503
SLC25A6 ENSG00000169100
NR4A1 ENSG00000123358
AES ENSG00000104964
EEF1A1 ENSG00000156508
ACADVL ENSG00000072778
FBLN1 ENSG00000077942
CD81 ENSG00000110651
GNAI2 ENSG00000114353
IGFBP4 ENSG00000141753
GAPDH ENSG00000111640
NDUFA11 ENSG00000174886
CD99 ENSG00000002586
MYL6 ENSG00000092841
TAPBP ENSG00000231925
H2AFJ ENSG00000246705
C1R ENSG00000159403
CAPNS1 ENSG00000126247
COX6B1 ENSG00000126267
TMSB10 ENSG00000034510
AZGP1 ENSG00000160862
TIMP1 ENSG00000102265
NBL1 ENSG00000158747
LRRC26 ENSG00000184709

experiment0000−expected−features.tsv.gz Factor 9

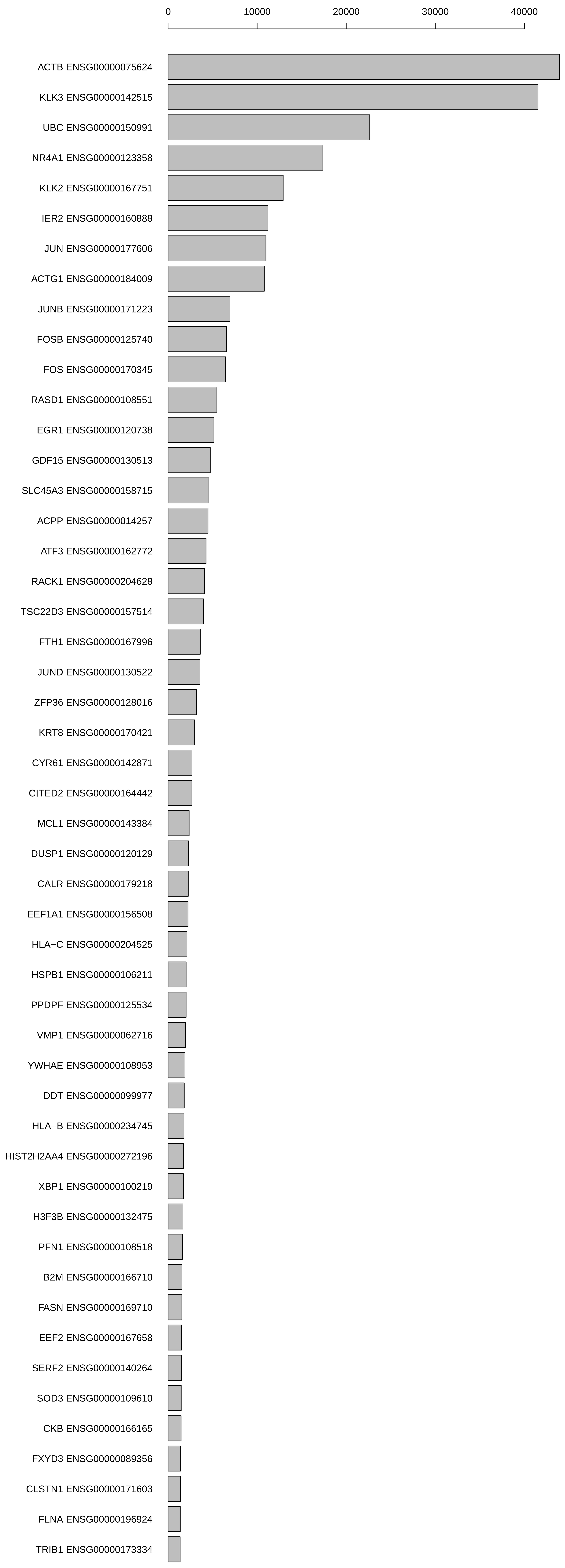

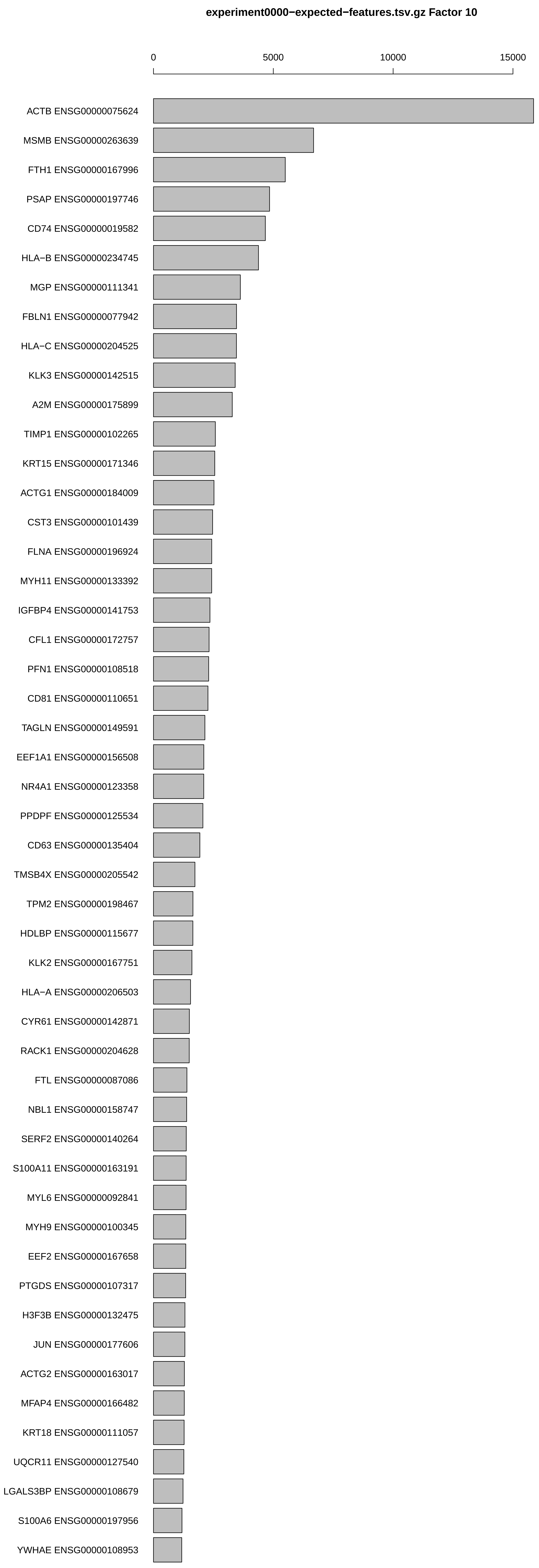
experiment0000−expected−features.tsv.gz Factor 10
0
5000
10000
15000
ACTB ENSG00000075624
MSMB ENSG00000263639
FTH1 ENSG00000167996
PSAP ENSG00000197746
CD74 ENSG00000019582
HLA−B ENSG00000234745
MGP ENSG00000111341
FBLN1 ENSG00000077942
HLA−C ENSG00000204525
KLK3 ENSG00000142515
A2M ENSG00000175899
TIMP1 ENSG00000102265
KRT15 ENSG00000171346
ACTG1 ENSG00000184009
CST3 ENSG00000101439
FLNA ENSG00000196924
MYH11 ENSG00000133392
IGFBP4 ENSG00000141753
CFL1 ENSG00000172757
PFN1 ENSG00000108518
CD81 ENSG00000110651
TAGLN ENSG00000149591
EEF1A1 ENSG00000156508
NR4A1 ENSG00000123358
PPDPF ENSG00000125534
CD63 ENSG00000135404
TMSB4X ENSG00000205542
TPM2 ENSG00000198467
HDLBP ENSG00000115677
KLK2 ENSG00000167751
HLA−A ENSG00000206503
CYR61 ENSG00000142871
RACK1 ENSG00000204628
FTL ENSG00000087086
NBL1 ENSG00000158747
SERF2 ENSG00000140264
S100A11 ENSG00000163191
MYL6 ENSG00000092841
MYH9 ENSG00000100345
EEF2 ENSG00000167658
PTGDS ENSG00000107317
H3F3B ENSG00000132475
JUN ENSG00000177606
ACTG2 ENSG00000163017
MFAP4 ENSG00000166482
KRT18 ENSG00000111057
UQCR11 ENSG00000127540
LGALS3BP ENSG00000108679
S100A6 ENSG00000197956
YWHAE ENSG00000108953

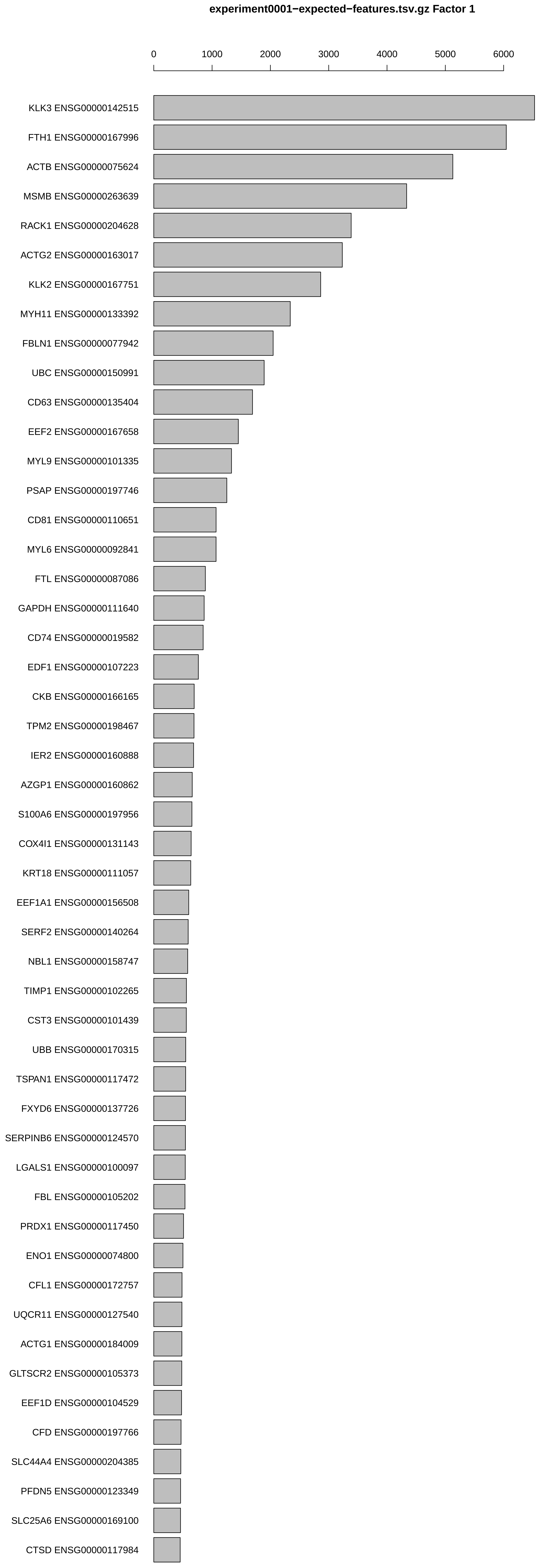
experiment0001−expected−features.tsv.gz Factor 1
0
1000
2000
3000
4000
5000
6000
KLK3 ENSG00000142515
FTH1 ENSG00000167996
ACTB ENSG00000075624
MSMB ENSG00000263639
RACK1 ENSG00000204628
ACTG2 ENSG00000163017
KLK2 ENSG00000167751
MYH11 ENSG00000133392
FBLN1 ENSG00000077942
UBC ENSG00000150991
CD63 ENSG00000135404
EEF2 ENSG00000167658
MYL9 ENSG00000101335
PSAP ENSG00000197746
CD81 ENSG00000110651
MYL6 ENSG00000092841
FTL ENSG00000087086
GAPDH ENSG00000111640
CD74 ENSG00000019582
EDF1 ENSG00000107223
CKB ENSG00000166165
TPM2 ENSG00000198467
IER2 ENSG00000160888
AZGP1 ENSG00000160862
S100A6 ENSG00000197956
COX4I1 ENSG00000131143
KRT18 ENSG00000111057
EEF1A1 ENSG00000156508
SERF2 ENSG00000140264
NBL1 ENSG00000158747
TIMP1 ENSG00000102265
CST3 ENSG00000101439
UBB ENSG00000170315
TSPAN1 ENSG00000117472
FXYD6 ENSG00000137726
SERPINB6 ENSG00000124570
LGALS1 ENSG00000100097
FBL ENSG00000105202
PRDX1 ENSG00000117450
ENO1 ENSG00000074800
CFL1 ENSG00000172757
UQCR11 ENSG00000127540
ACTG1 ENSG00000184009
GLTSCR2 ENSG00000105373
EEF1D ENSG00000104529
CFD ENSG00000197766
SLC44A4 ENSG00000204385
PFDN5 ENSG00000123349
SLC25A6 ENSG00000169100
CTSD ENSG00000117984

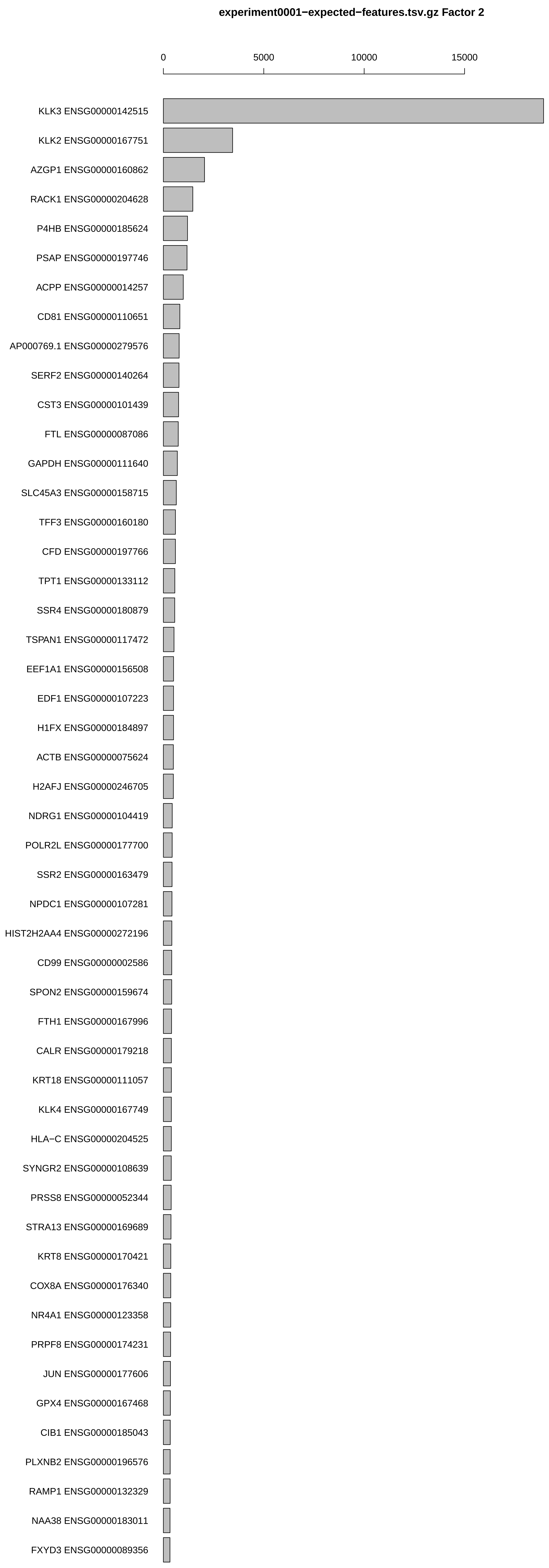
experiment0001−expected−features.tsv.gz Factor 2
0
5000
10000
15000
KLK3 ENSG00000142515
KLK2 ENSG00000167751
AZGP1 ENSG00000160862
RACK1 ENSG00000204628
P4HB ENSG00000185624
PSAP ENSG00000197746
ACPP ENSG00000014257
CD81 ENSG00000110651
AP000769.1 ENSG00000279576
SERF2 ENSG00000140264
CST3 ENSG00000101439
FTL ENSG00000087086
GAPDH ENSG00000111640
SLC45A3 ENSG00000158715
TFF3 ENSG00000160180
CFD ENSG00000197766
TPT1 ENSG00000133112
SSR4 ENSG00000180879
TSPAN1 ENSG00000117472
EEF1A1 ENSG00000156508
EDF1 ENSG00000107223
H1FX ENSG00000184897
ACTB ENSG00000075624
H2AFJ ENSG00000246705
NDRG1 ENSG00000104419
POLR2L ENSG00000177700
SSR2 ENSG00000163479
NPDC1 ENSG00000107281
HIST2H2AA4 ENSG00000272196
CD99 ENSG00000002586
SPON2 ENSG00000159674
FTH1 ENSG00000167996
CALR ENSG00000179218
KRT18 ENSG00000111057
KLK4 ENSG00000167749
HLA−C ENSG00000204525
SYNGR2 ENSG00000108639
PRSS8 ENSG00000052344
STRA13 ENSG00000169689
KRT8 ENSG00000170421
COX8A ENSG00000176340
NR4A1 ENSG00000123358
PRPF8 ENSG00000174231
JUN ENSG00000177606
GPX4 ENSG00000167468
CIB1 ENSG00000185043
PLXNB2 ENSG00000196576
RAMP1 ENSG00000132329
NAA38 ENSG00000183011
FXYD3 ENSG00000089356

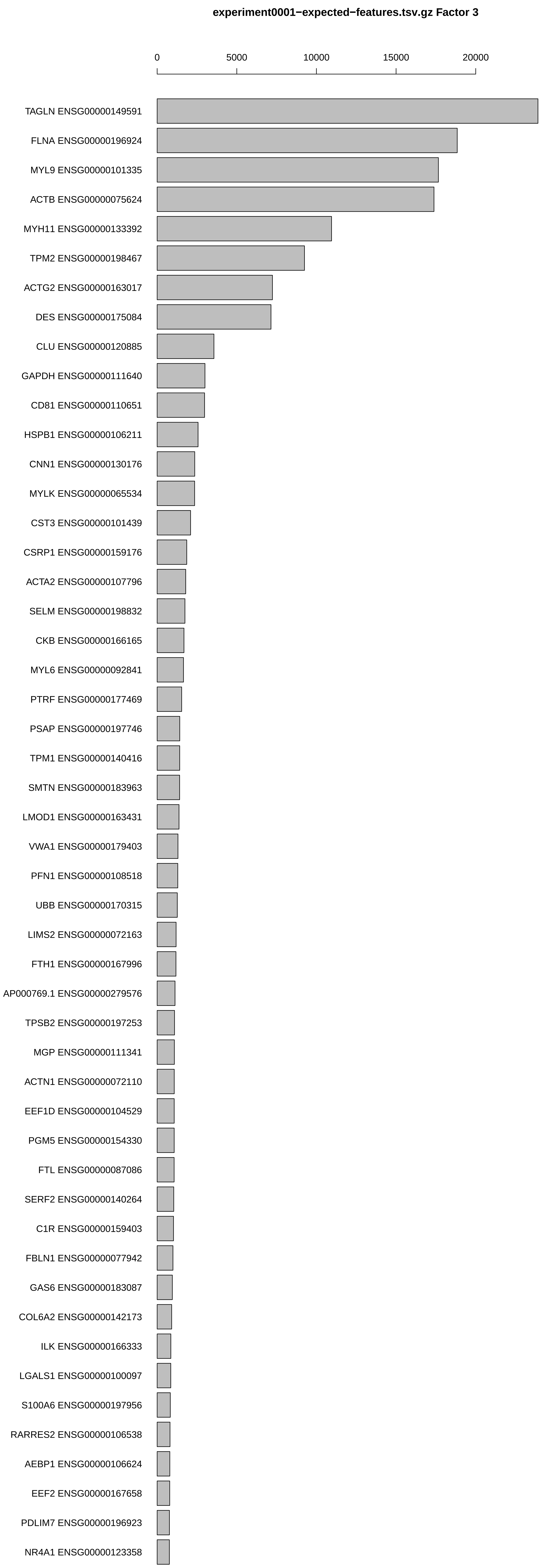
experiment0001−expected−features.tsv.gz Factor 3
0
5000
10000
15000
20000
TAGLN ENSG00000149591
FLNA ENSG00000196924
MYL9 ENSG00000101335
ACTB ENSG00000075624
MYH11 ENSG00000133392
TPM2 ENSG00000198467
ACTG2 ENSG00000163017
DES ENSG00000175084
CLU ENSG00000120885
GAPDH ENSG00000111640
CD81 ENSG00000110651
HSPB1 ENSG00000106211
CNN1 ENSG00000130176
MYLK ENSG00000065534
CST3 ENSG00000101439
CSRP1 ENSG00000159176
ACTA2 ENSG00000107796
SELM ENSG00000198832
CKB ENSG00000166165
MYL6 ENSG00000092841
PTRF ENSG00000177469
PSAP ENSG00000197746
TPM1 ENSG00000140416
SMTN ENSG00000183963
LMOD1 ENSG00000163431
VWA1 ENSG00000179403
PFN1 ENSG00000108518
UBB ENSG00000170315
LIMS2 ENSG00000072163
FTH1 ENSG00000167996
AP000769.1 ENSG00000279576
TPSB2 ENSG00000197253
MGP ENSG00000111341
ACTN1 ENSG00000072110
EEF1D ENSG00000104529
PGM5 ENSG00000154330
FTL ENSG00000087086
SERF2 ENSG00000140264
C1R ENSG00000159403
FBLN1 ENSG00000077942
GAS6 ENSG00000183087
COL6A2 ENSG00000142173
ILK ENSG00000166333
LGALS1 ENSG00000100097
S100A6 ENSG00000197956
RARRES2 ENSG00000106538
AEBP1 ENSG00000106624
EEF2 ENSG00000167658
PDLIM7 ENSG00000196923
NR4A1 ENSG00000123358

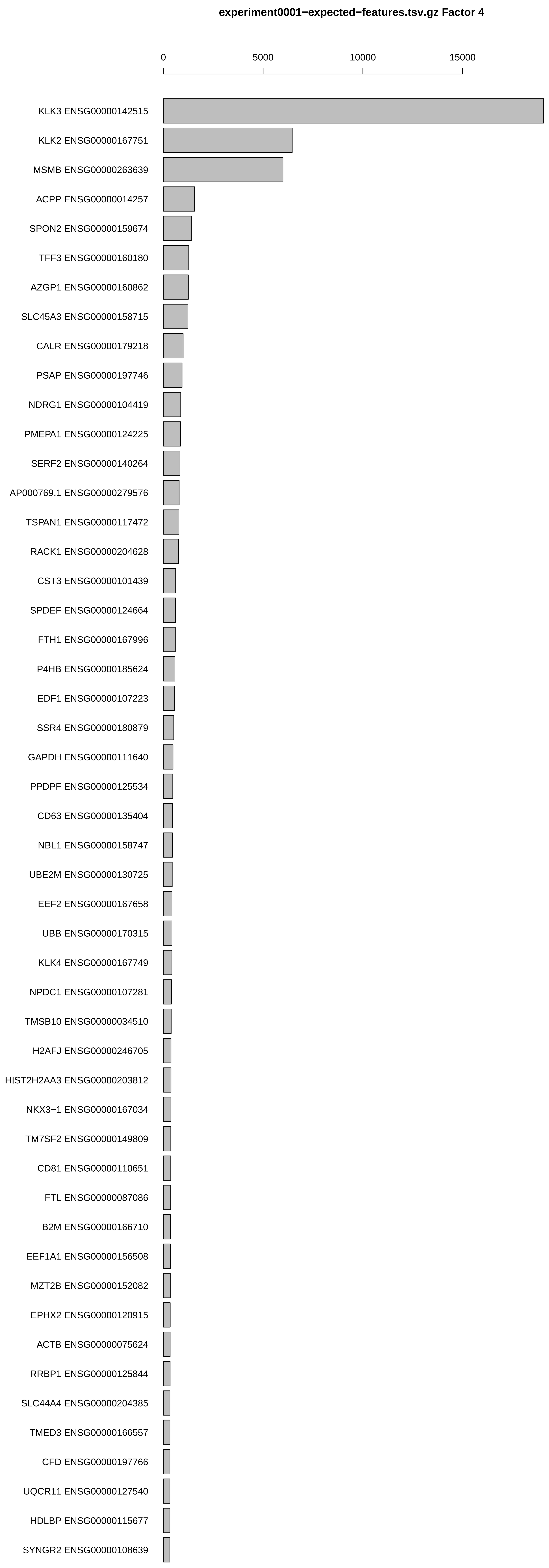
experiment0001−expected−features.tsv.gz Factor 4
0
5000
10000
15000
KLK3 ENSG00000142515
KLK2 ENSG00000167751
MSMB ENSG00000263639
ACPP ENSG00000014257
SPON2 ENSG00000159674
TFF3 ENSG00000160180
AZGP1 ENSG00000160862
SLC45A3 ENSG00000158715
CALR ENSG00000179218
PSAP ENSG00000197746
NDRG1 ENSG00000104419
PMEPA1 ENSG00000124225
SERF2 ENSG00000140264
AP000769.1 ENSG00000279576
TSPAN1 ENSG00000117472
RACK1 ENSG00000204628
CST3 ENSG00000101439
SPDEF ENSG00000124664
FTH1 ENSG00000167996
P4HB ENSG00000185624
EDF1 ENSG00000107223
SSR4 ENSG00000180879
GAPDH ENSG00000111640
PPDPF ENSG00000125534
CD63 ENSG00000135404
NBL1 ENSG00000158747
UBE2M ENSG00000130725
EEF2 ENSG00000167658
UBB ENSG00000170315
KLK4 ENSG00000167749
NPDC1 ENSG00000107281
TMSB10 ENSG00000034510
H2AFJ ENSG00000246705
HIST2H2AA3 ENSG00000203812
NKX3−1 ENSG00000167034
TM7SF2 ENSG00000149809
CD81 ENSG00000110651
FTL ENSG00000087086
B2M ENSG00000166710
EEF1A1 ENSG00000156508
MZT2B ENSG00000152082
EPHX2 ENSG00000120915
ACTB ENSG00000075624
RRBP1 ENSG00000125844
SLC44A4 ENSG00000204385
TMED3 ENSG00000166557
CFD ENSG00000197766
UQCR11 ENSG00000127540
HDLBP ENSG00000115677
SYNGR2 ENSG00000108639

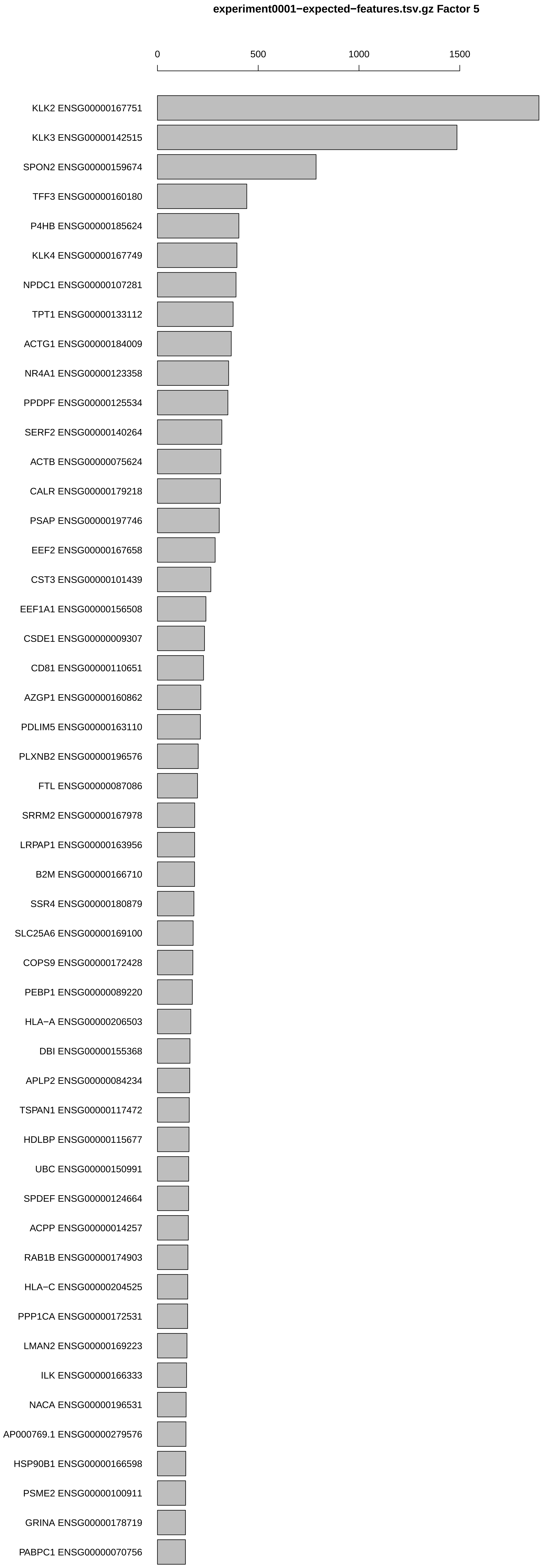
experiment0001−expected−features.tsv.gz Factor 5
0
500
1000
1500
KLK2 ENSG00000167751
KLK3 ENSG00000142515
SPON2 ENSG00000159674
TFF3 ENSG00000160180
P4HB ENSG00000185624
KLK4 ENSG00000167749
NPDC1 ENSG00000107281
TPT1 ENSG00000133112
ACTG1 ENSG00000184009
NR4A1 ENSG00000123358
PPDPF ENSG00000125534
SERF2 ENSG00000140264
ACTB ENSG00000075624
CALR ENSG00000179218
PSAP ENSG00000197746
EEF2 ENSG00000167658
CST3 ENSG00000101439
EEF1A1 ENSG00000156508
CSDE1 ENSG00000009307
CD81 ENSG00000110651
AZGP1 ENSG00000160862
PDLIM5 ENSG00000163110
PLXNB2 ENSG00000196576
FTL ENSG00000087086
SRRM2 ENSG00000167978
LRPAP1 ENSG00000163956
B2M ENSG00000166710
SSR4 ENSG00000180879
SLC25A6 ENSG00000169100
COPS9 ENSG00000172428
PEBP1 ENSG00000089220
HLA−A ENSG00000206503
DBI ENSG00000155368
APLP2 ENSG00000084234
TSPAN1 ENSG00000117472
HDLBP ENSG00000115677
UBC ENSG00000150991
SPDEF ENSG00000124664
ACPP ENSG00000014257
RAB1B ENSG00000174903
HLA−C ENSG00000204525
PPP1CA ENSG00000172531
LMAN2 ENSG00000169223
ILK ENSG00000166333
NACA ENSG00000196531
AP000769.1 ENSG00000279576
HSP90B1 ENSG00000166598
PSME2 ENSG00000100911
GRINA ENSG00000178719
PABPC1 ENSG00000070756

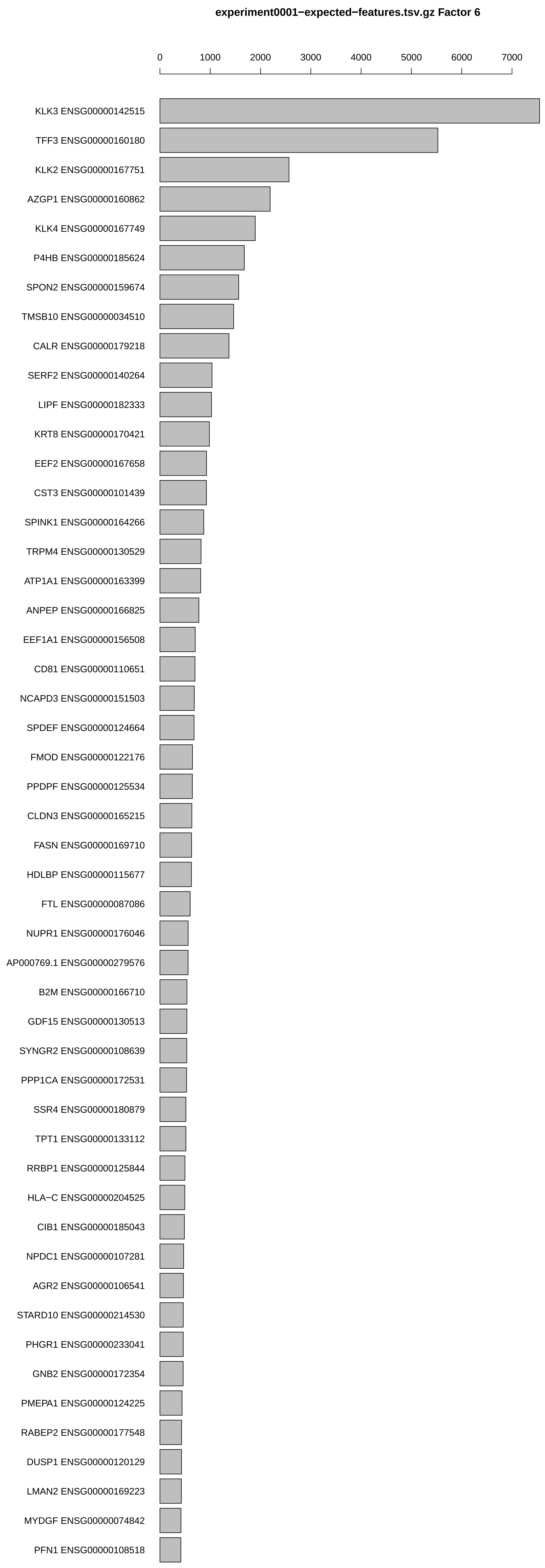
experiment0001−expected−features.tsv.gz Factor 6
0
1000
2000
3000
4000
5000
6000
7000
KLK3 ENSG00000142515
TFF3 ENSG00000160180
KLK2 ENSG00000167751
AZGP1 ENSG00000160862
KLK4 ENSG00000167749
P4HB ENSG00000185624
SPON2 ENSG00000159674
TMSB10 ENSG00000034510
CALR ENSG00000179218
SERF2 ENSG00000140264
LIPF ENSG00000182333
KRT8 ENSG00000170421
EEF2 ENSG00000167658
CST3 ENSG00000101439
SPINK1 ENSG00000164266
TRPM4 ENSG00000130529
ATP1A1 ENSG00000163399
ANPEP ENSG00000166825
EEF1A1 ENSG00000156508
CD81 ENSG00000110651
NCAPD3 ENSG00000151503
SPDEF ENSG00000124664
FMOD ENSG00000122176
PPDPF ENSG00000125534
CLDN3 ENSG00000165215
FASN ENSG00000169710
HDLBP ENSG00000115677
FTL ENSG00000087086
NUPR1 ENSG00000176046
AP000769.1 ENSG00000279576
B2M ENSG00000166710
GDF15 ENSG00000130513
SYNGR2 ENSG00000108639
PPP1CA ENSG00000172531
SSR4 ENSG00000180879
TPT1 ENSG00000133112
RRBP1 ENSG00000125844
HLA−C ENSG00000204525
CIB1 ENSG00000185043
NPDC1 ENSG00000107281
AGR2 ENSG00000106541
STARD10 ENSG00000214530
PHGR1 ENSG00000233041
GNB2 ENSG00000172354
PMEPA1 ENSG00000124225
RABEP2 ENSG00000177548
DUSP1 ENSG00000120129
LMAN2 ENSG00000169223
MYDGF ENSG00000074842
PFN1 ENSG00000108518

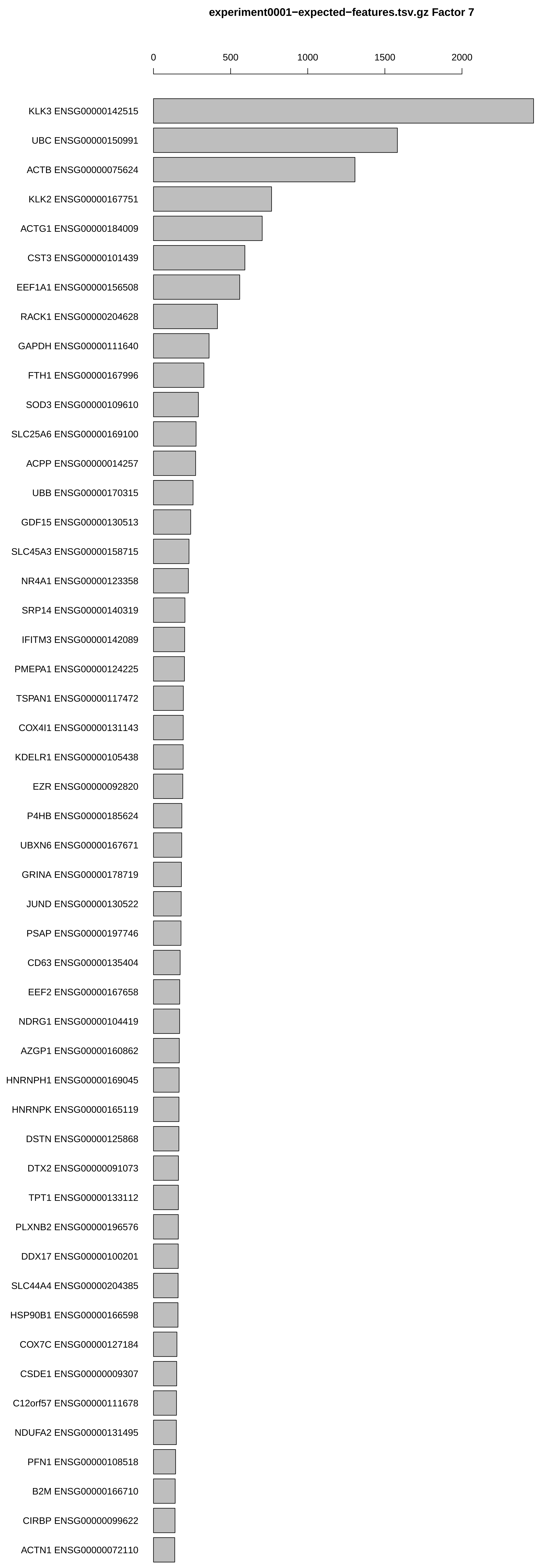
experiment0001−expected−features.tsv.gz Factor 7
0
500
1000
1500
2000
KLK3 ENSG00000142515
UBC ENSG00000150991
ACTB ENSG00000075624
KLK2 ENSG00000167751
ACTG1 ENSG00000184009
CST3 ENSG00000101439
EEF1A1 ENSG00000156508
RACK1 ENSG00000204628
GAPDH ENSG00000111640
FTH1 ENSG00000167996
SOD3 ENSG00000109610
SLC25A6 ENSG00000169100
ACPP ENSG00000014257
UBB ENSG00000170315
GDF15 ENSG00000130513
SLC45A3 ENSG00000158715
NR4A1 ENSG00000123358
SRP14 ENSG00000140319
IFITM3 ENSG00000142089
PMEPA1 ENSG00000124225
TSPAN1 ENSG00000117472
COX4I1 ENSG00000131143
KDELR1 ENSG00000105438
EZR ENSG00000092820
P4HB ENSG00000185624
UBXN6 ENSG00000167671
GRINA ENSG00000178719
JUND ENSG00000130522
PSAP ENSG00000197746
CD63 ENSG00000135404
EEF2 ENSG00000167658
NDRG1 ENSG00000104419
AZGP1 ENSG00000160862
HNRNPH1 ENSG00000169045
HNRNPK ENSG00000165119
DSTN ENSG00000125868
DTX2 ENSG00000091073
TPT1 ENSG00000133112
PLXNB2 ENSG00000196576
DDX17 ENSG00000100201
SLC44A4 ENSG00000204385
HSP90B1 ENSG00000166598
COX7C ENSG00000127184
CSDE1 ENSG00000009307
C12orf57 ENSG00000111678
NDUFA2 ENSG00000131495
PFN1 ENSG00000108518
B2M ENSG00000166710
CIRBP ENSG00000099622
ACTN1 ENSG00000072110

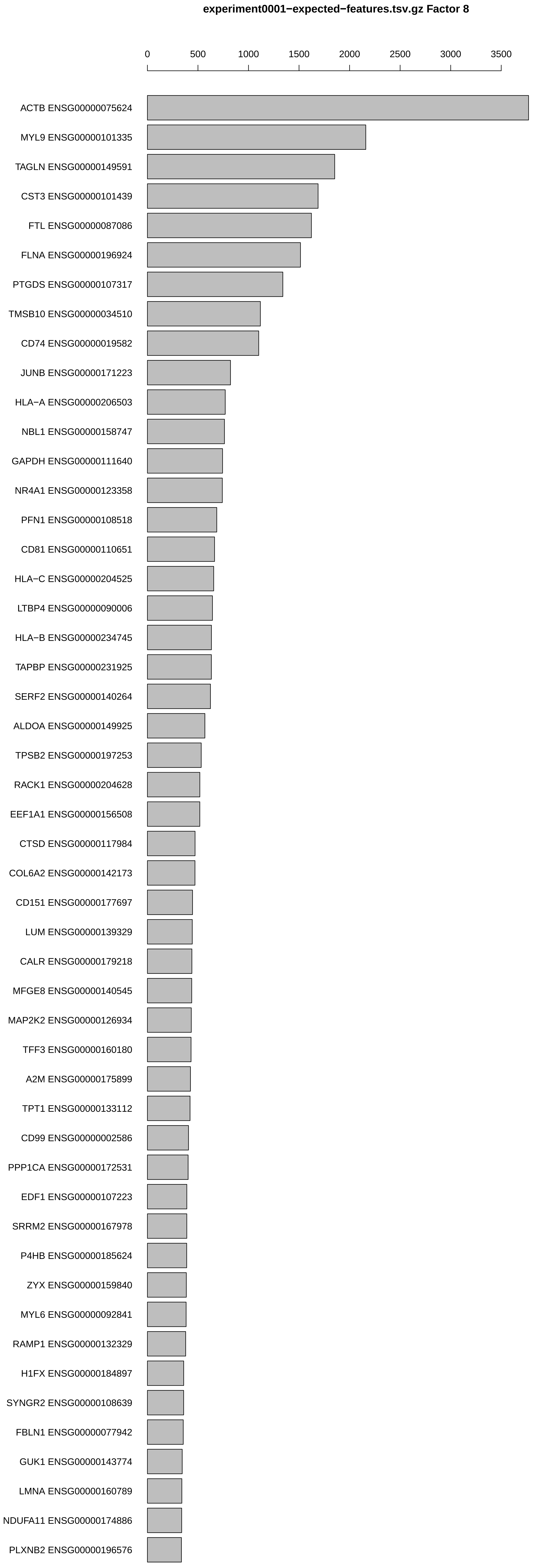
experiment0001−expected−features.tsv.gz Factor 8
0
500
1000
1500
2000
2500
3000
3500
ACTB ENSG00000075624
MYL9 ENSG00000101335
TAGLN ENSG00000149591
CST3 ENSG00000101439
FTL ENSG00000087086
FLNA ENSG00000196924
PTGDS ENSG00000107317
TMSB10 ENSG00000034510
CD74 ENSG00000019582
JUNB ENSG00000171223
HLA−A ENSG00000206503
NBL1 ENSG00000158747
GAPDH ENSG00000111640
NR4A1 ENSG00000123358
PFN1 ENSG00000108518
CD81 ENSG00000110651
HLA−C ENSG00000204525
LTBP4 ENSG00000090006
HLA−B ENSG00000234745
TAPBP ENSG00000231925
SERF2 ENSG00000140264
ALDOA ENSG00000149925
TPSB2 ENSG00000197253
RACK1 ENSG00000204628
EEF1A1 ENSG00000156508
CTSD ENSG00000117984
COL6A2 ENSG00000142173
CD151 ENSG00000177697
LUM ENSG00000139329
CALR ENSG00000179218
MFGE8 ENSG00000140545
MAP2K2 ENSG00000126934
TFF3 ENSG00000160180
A2M ENSG00000175899
TPT1 ENSG00000133112
CD99 ENSG00000002586
PPP1CA ENSG00000172531
EDF1 ENSG00000107223
SRRM2 ENSG00000167978
P4HB ENSG00000185624
ZYX ENSG00000159840
MYL6 ENSG00000092841
RAMP1 ENSG00000132329
H1FX ENSG00000184897
SYNGR2 ENSG00000108639
FBLN1 ENSG00000077942
GUK1 ENSG00000143774
LMNA ENSG00000160789
NDUFA11 ENSG00000174886
PLXNB2 ENSG00000196576

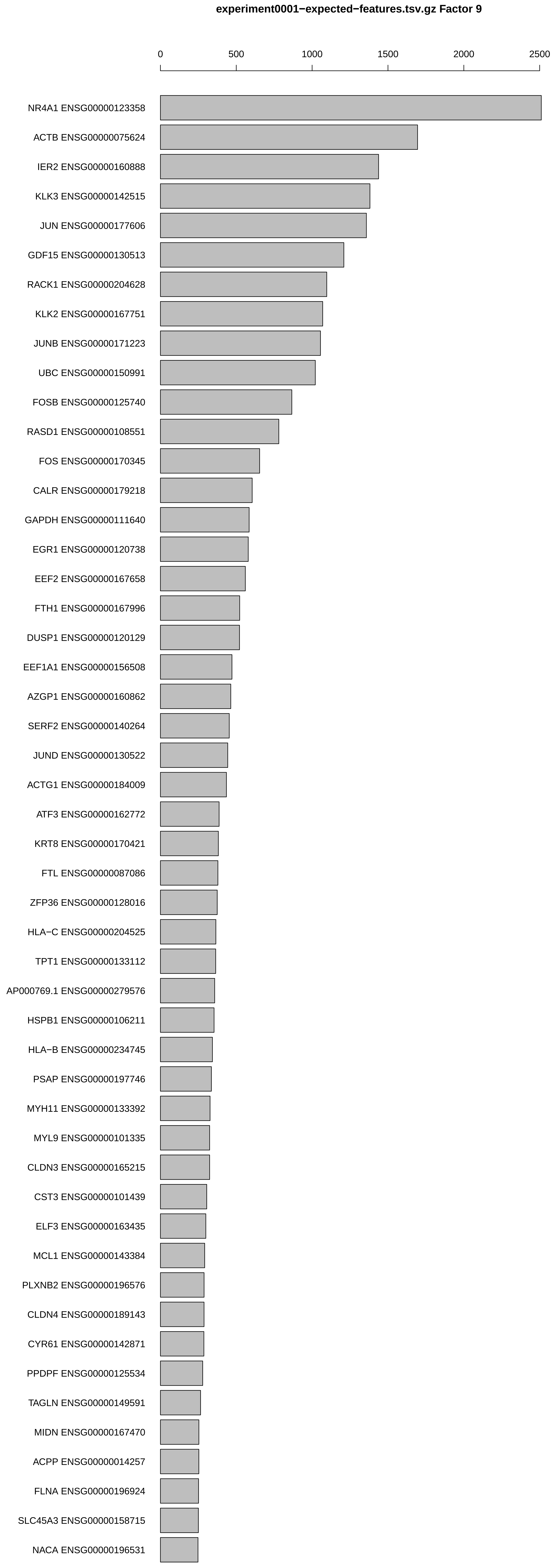
experiment0001−expected−features.tsv.gz Factor 9
0
500
1000
1500
2000
2500
NR4A1 ENSG00000123358
ACTB ENSG00000075624
IER2 ENSG00000160888
KLK3 ENSG00000142515
JUN ENSG00000177606
GDF15 ENSG00000130513
RACK1 ENSG00000204628
KLK2 ENSG00000167751
JUNB ENSG00000171223
UBC ENSG00000150991
FOSB ENSG00000125740
RASD1 ENSG00000108551
FOS ENSG00000170345
CALR ENSG00000179218
GAPDH ENSG00000111640
EGR1 ENSG00000120738
EEF2 ENSG00000167658
FTH1 ENSG00000167996
DUSP1 ENSG00000120129
EEF1A1 ENSG00000156508
AZGP1 ENSG00000160862
SERF2 ENSG00000140264
JUND ENSG00000130522
ACTG1 ENSG00000184009
ATF3 ENSG00000162772
KRT8 ENSG00000170421
FTL ENSG00000087086
ZFP36 ENSG00000128016
HLA−C ENSG00000204525
TPT1 ENSG00000133112
AP000769.1 ENSG00000279576
HSPB1 ENSG00000106211
HLA−B ENSG00000234745
PSAP ENSG00000197746
MYH11 ENSG00000133392
MYL9 ENSG00000101335
CLDN3 ENSG00000165215
CST3 ENSG00000101439
ELF3 ENSG00000163435
MCL1 ENSG00000143384
PLXNB2 ENSG00000196576
CLDN4 ENSG00000189143
CYR61 ENSG00000142871
PPDPF ENSG00000125534
TAGLN ENSG00000149591
MIDN ENSG00000167470
ACPP ENSG00000014257
FLNA ENSG00000196924
SLC45A3 ENSG00000158715
NACA ENSG00000196531

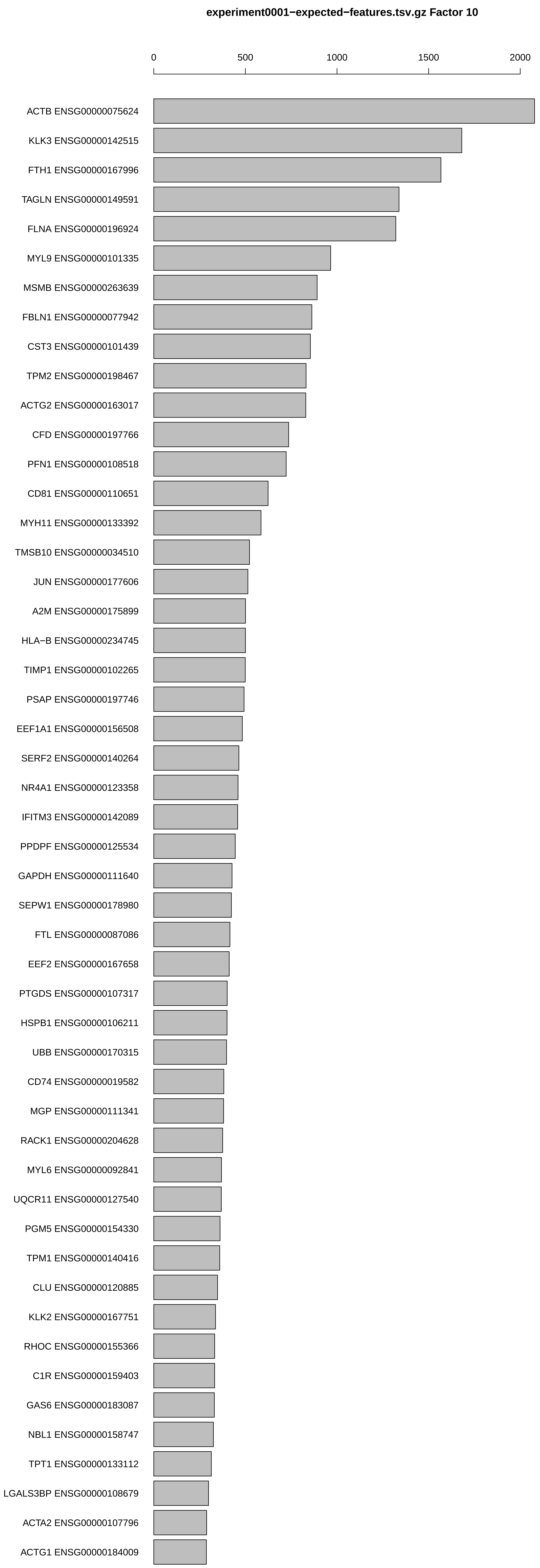
experiment0001−expected−features.tsv.gz Factor 10
0
500
1000
1500
2000
ACTB ENSG00000075624
KLK3 ENSG00000142515
FTH1 ENSG00000167996
TAGLN ENSG00000149591
FLNA ENSG00000196924
MYL9 ENSG00000101335
MSMB ENSG00000263639
FBLN1 ENSG00000077942
CST3 ENSG00000101439
TPM2 ENSG00000198467
ACTG2 ENSG00000163017
CFD ENSG00000197766
PFN1 ENSG00000108518
CD81 ENSG00000110651
MYH11 ENSG00000133392
TMSB10 ENSG00000034510
JUN ENSG00000177606
A2M ENSG00000175899
HLA−B ENSG00000234745
TIMP1 ENSG00000102265
PSAP ENSG00000197746
EEF1A1 ENSG00000156508
SERF2 ENSG00000140264
NR4A1 ENSG00000123358
IFITM3 ENSG00000142089
PPDPF ENSG00000125534
GAPDH ENSG00000111640
SEPW1 ENSG00000178980
FTL ENSG00000087086
EEF2 ENSG00000167658
PTGDS ENSG00000107317
HSPB1 ENSG00000106211
UBB ENSG00000170315
CD74 ENSG00000019582
MGP ENSG00000111341
RACK1 ENSG00000204628
MYL6 ENSG00000092841
UQCR11 ENSG00000127540
PGM5 ENSG00000154330
TPM1 ENSG00000140416
CLU ENSG00000120885
KLK2 ENSG00000167751
RHOC ENSG00000155366
C1R ENSG00000159403
GAS6 ENSG00000183087
NBL1 ENSG00000158747
TPT1 ENSG00000133112
LGALS3BP ENSG00000108679
ACTA2 ENSG00000107796
ACTG1 ENSG00000184009

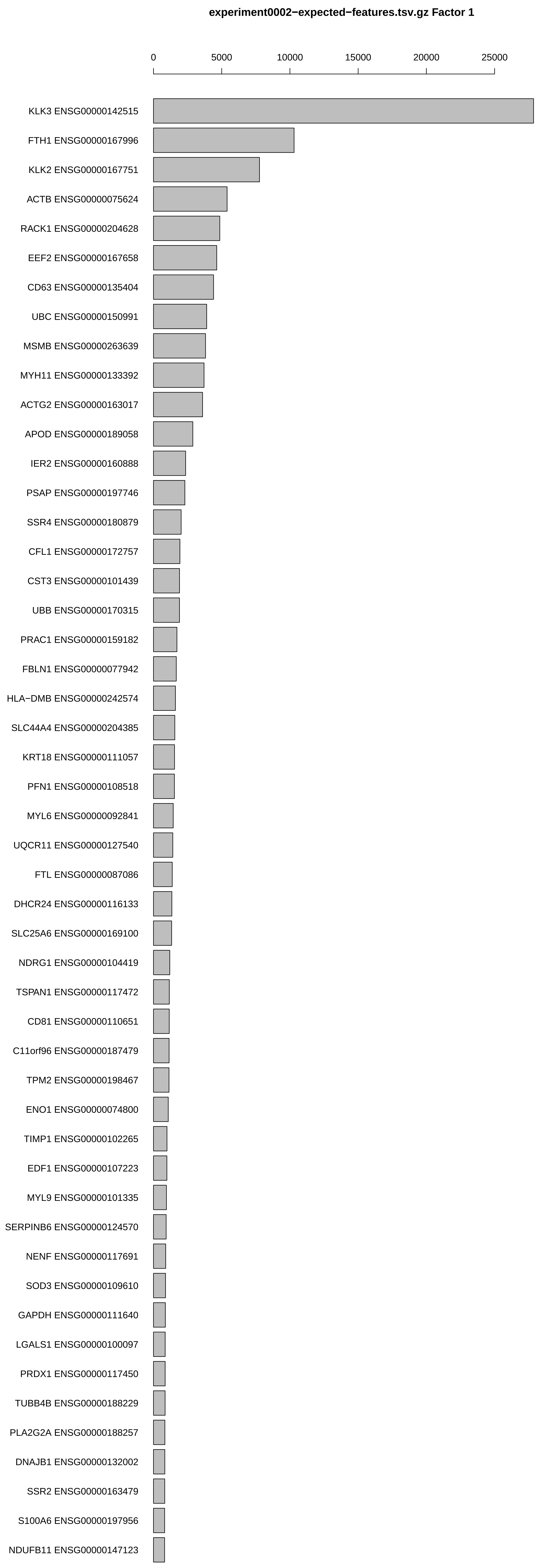
experiment0002−expected−features.tsv.gz Factor 1
0
5000
10000
15000
20000
25000
KLK3 ENSG00000142515
FTH1 ENSG00000167996
KLK2 ENSG00000167751
ACTB ENSG00000075624
RACK1 ENSG00000204628
EEF2 ENSG00000167658
CD63 ENSG00000135404
UBC ENSG00000150991
MSMB ENSG00000263639
MYH11 ENSG00000133392
ACTG2 ENSG00000163017
APOD ENSG00000189058
IER2 ENSG00000160888
PSAP ENSG00000197746
SSR4 ENSG00000180879
CFL1 ENSG00000172757
CST3 ENSG00000101439
UBB ENSG00000170315
PRAC1 ENSG00000159182
FBLN1 ENSG00000077942
HLA−DMB ENSG00000242574
SLC44A4 ENSG00000204385
KRT18 ENSG00000111057
PFN1 ENSG00000108518
MYL6 ENSG00000092841
UQCR11 ENSG00000127540
FTL ENSG00000087086
DHCR24 ENSG00000116133
SLC25A6 ENSG00000169100
NDRG1 ENSG00000104419
TSPAN1 ENSG00000117472
CD81 ENSG00000110651
C11orf96 ENSG00000187479
TPM2 ENSG00000198467
ENO1 ENSG00000074800
TIMP1 ENSG00000102265
EDF1 ENSG00000107223
MYL9 ENSG00000101335
SERPINB6 ENSG00000124570
NENF ENSG00000117691
SOD3 ENSG00000109610
GAPDH ENSG00000111640
LGALS1 ENSG00000100097
PRDX1 ENSG00000117450
TUBB4B ENSG00000188229
PLA2G2A ENSG00000188257
DNAJB1 ENSG00000132002
SSR2 ENSG00000163479
S100A6 ENSG00000197956
NDUFB11 ENSG00000147123

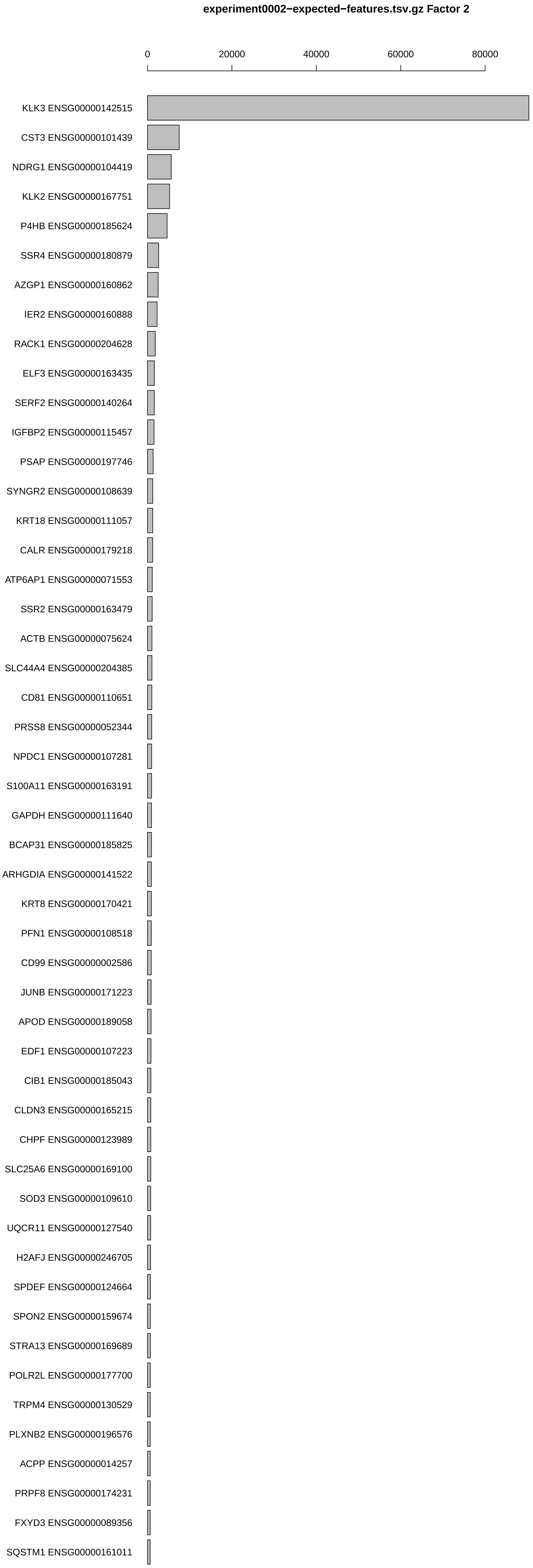
experiment0002−expected−features.tsv.gz Factor 2
0
20000
40000
60000
80000
KLK3 ENSG00000142515
CST3 ENSG00000101439
NDRG1 ENSG00000104419
KLK2 ENSG00000167751
P4HB ENSG00000185624
SSR4 ENSG00000180879
AZGP1 ENSG00000160862
IER2 ENSG00000160888
RACK1 ENSG00000204628
ELF3 ENSG00000163435
SERF2 ENSG00000140264
IGFBP2 ENSG00000115457
PSAP ENSG00000197746
SYNGR2 ENSG00000108639
KRT18 ENSG00000111057
CALR ENSG00000179218
ATP6AP1 ENSG00000071553
SSR2 ENSG00000163479
ACTB ENSG00000075624
SLC44A4 ENSG00000204385
CD81 ENSG00000110651
PRSS8 ENSG00000052344
NPDC1 ENSG00000107281
S100A11 ENSG00000163191
GAPDH ENSG00000111640
BCAP31 ENSG00000185825
ARHGDIA ENSG00000141522
KRT8 ENSG00000170421
PFN1 ENSG00000108518
CD99 ENSG00000002586
JUNB ENSG00000171223
APOD ENSG00000189058
EDF1 ENSG00000107223
CIB1 ENSG00000185043
CLDN3 ENSG00000165215
CHPF ENSG00000123989
SLC25A6 ENSG00000169100
SOD3 ENSG00000109610
UQCR11 ENSG00000127540
H2AFJ ENSG00000246705
SPDEF ENSG00000124664
SPON2 ENSG00000159674
STRA13 ENSG00000169689
POLR2L ENSG00000177700
TRPM4 ENSG00000130529
PLXNB2 ENSG00000196576
ACPP ENSG00000014257
PRPF8 ENSG00000174231
FXYD3 ENSG00000089356
SQSTM1 ENSG00000161011

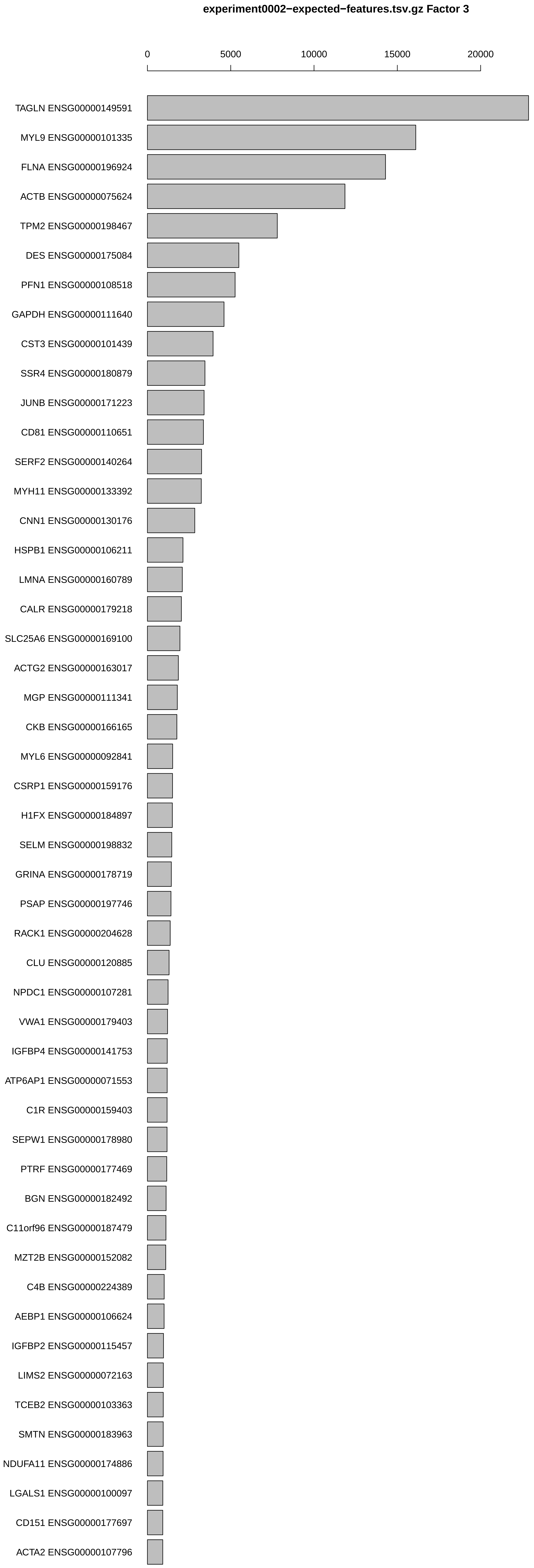
experiment0002−expected−features.tsv.gz Factor 3
0
5000
10000
15000
20000
TAGLN ENSG00000149591
MYL9 ENSG00000101335
FLNA ENSG00000196924
ACTB ENSG00000075624
TPM2 ENSG00000198467
DES ENSG00000175084
PFN1 ENSG00000108518
GAPDH ENSG00000111640
CST3 ENSG00000101439
SSR4 ENSG00000180879
JUNB ENSG00000171223
CD81 ENSG00000110651
SERF2 ENSG00000140264
MYH11 ENSG00000133392
CNN1 ENSG00000130176
HSPB1 ENSG00000106211
LMNA ENSG00000160789
CALR ENSG00000179218
SLC25A6 ENSG00000169100
ACTG2 ENSG00000163017
MGP ENSG00000111341
CKB ENSG00000166165
MYL6 ENSG00000092841
CSRP1 ENSG00000159176
H1FX ENSG00000184897
SELM ENSG00000198832
GRINA ENSG00000178719
PSAP ENSG00000197746
RACK1 ENSG00000204628
CLU ENSG00000120885
NPDC1 ENSG00000107281
VWA1 ENSG00000179403
IGFBP4 ENSG00000141753
ATP6AP1 ENSG00000071553
C1R ENSG00000159403
SEPW1 ENSG00000178980
PTRF ENSG00000177469
BGN ENSG00000182492
C11orf96 ENSG00000187479
MZT2B ENSG00000152082
C4B ENSG00000224389
AEBP1 ENSG00000106624
IGFBP2 ENSG00000115457
LIMS2 ENSG00000072163
TCEB2 ENSG00000103363
SMTN ENSG00000183963
NDUFA11 ENSG00000174886
LGALS1 ENSG00000100097
CD151 ENSG00000177697
ACTA2 ENSG00000107796

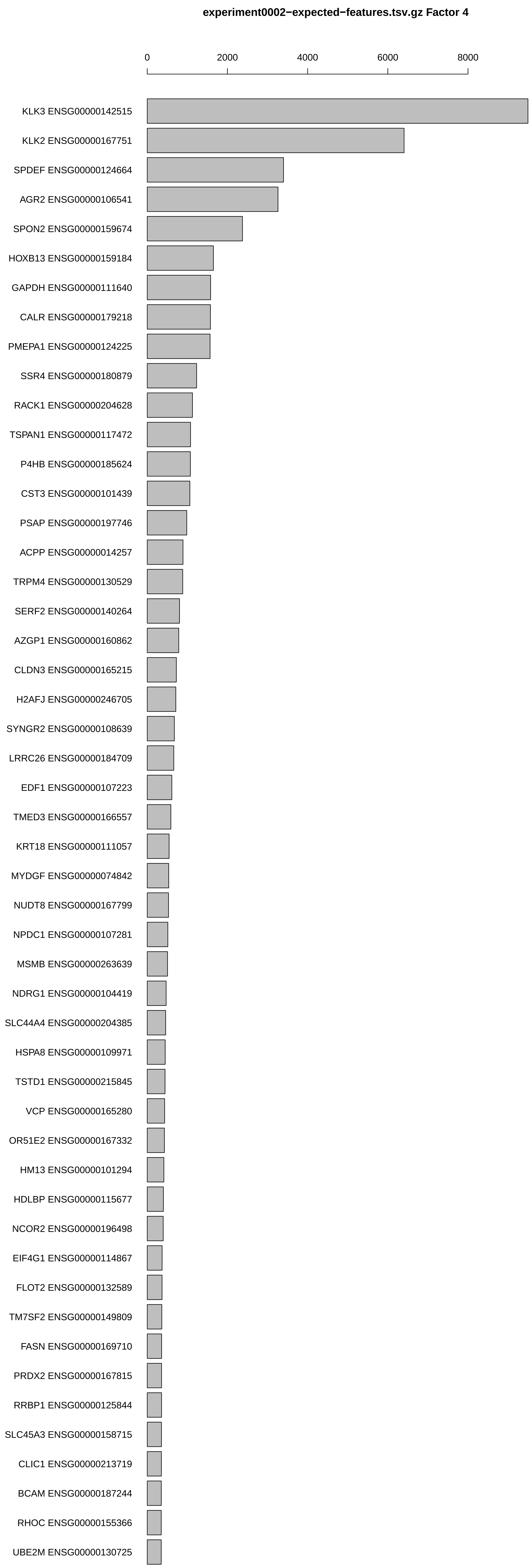
experiment0002−expected−features.tsv.gz Factor 4
0
2000
4000
6000
8000
KLK3 ENSG00000142515
KLK2 ENSG00000167751
SPDEF ENSG00000124664
AGR2 ENSG00000106541
SPON2 ENSG00000159674
HOXB13 ENSG00000159184
GAPDH ENSG00000111640
CALR ENSG00000179218
PMEPA1 ENSG00000124225
SSR4 ENSG00000180879
RACK1 ENSG00000204628
TSPAN1 ENSG00000117472
P4HB ENSG00000185624
CST3 ENSG00000101439
PSAP ENSG00000197746
ACPP ENSG00000014257
TRPM4 ENSG00000130529
SERF2 ENSG00000140264
AZGP1 ENSG00000160862
CLDN3 ENSG00000165215
H2AFJ ENSG00000246705
SYNGR2 ENSG00000108639
LRRC26 ENSG00000184709
EDF1 ENSG00000107223
TMED3 ENSG00000166557
KRT18 ENSG00000111057
MYDGF ENSG00000074842
NUDT8 ENSG00000167799
NPDC1 ENSG00000107281
MSMB ENSG00000263639
NDRG1 ENSG00000104419
SLC44A4 ENSG00000204385
HSPA8 ENSG00000109971
TSTD1 ENSG00000215845
VCP ENSG00000165280
OR51E2 ENSG00000167332
HM13 ENSG00000101294
HDLBP ENSG00000115677
NCOR2 ENSG00000196498
EIF4G1 ENSG00000114867
FLOT2 ENSG00000132589
TM7SF2 ENSG00000149809
FASN ENSG00000169710
PRDX2 ENSG00000167815
RRBP1 ENSG00000125844
SLC45A3 ENSG00000158715
CLIC1 ENSG00000213719
BCAM ENSG00000187244
RHOC ENSG00000155366
UBE2M ENSG00000130725

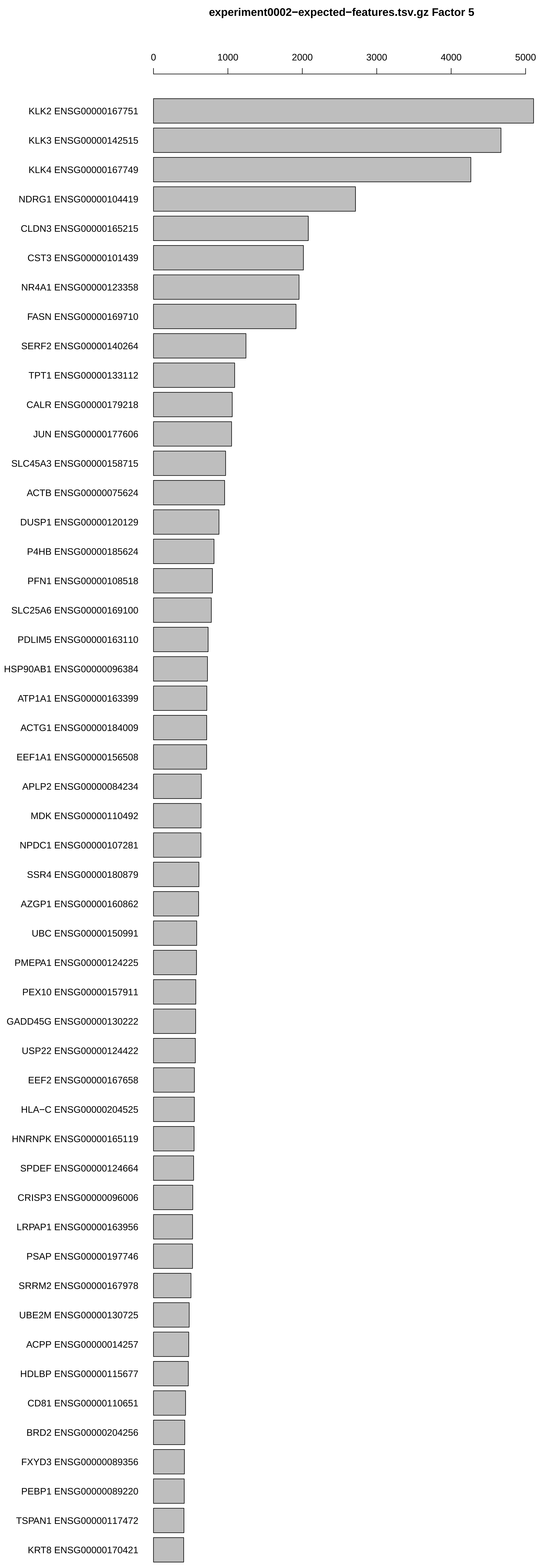
experiment0002−expected−features.tsv.gz Factor 5
0
1000
2000
3000
4000
5000
KLK2 ENSG00000167751
KLK3 ENSG00000142515
KLK4 ENSG00000167749
NDRG1 ENSG00000104419
CLDN3 ENSG00000165215
CST3 ENSG00000101439
NR4A1 ENSG00000123358
FASN ENSG00000169710
SERF2 ENSG00000140264
TPT1 ENSG00000133112
CALR ENSG00000179218
JUN ENSG00000177606
SLC45A3 ENSG00000158715
ACTB ENSG00000075624
DUSP1 ENSG00000120129
P4HB ENSG00000185624
PFN1 ENSG00000108518
SLC25A6 ENSG00000169100
PDLIM5 ENSG00000163110
HSP90AB1 ENSG00000096384
ATP1A1 ENSG00000163399
ACTG1 ENSG00000184009
EEF1A1 ENSG00000156508
APLP2 ENSG00000084234
MDK ENSG00000110492
NPDC1 ENSG00000107281
SSR4 ENSG00000180879
AZGP1 ENSG00000160862
UBC ENSG00000150991
PMEPA1 ENSG00000124225
PEX10 ENSG00000157911
GADD45G ENSG00000130222
USP22 ENSG00000124422
EEF2 ENSG00000167658
HLA−C ENSG00000204525
HNRNPK ENSG00000165119
SPDEF ENSG00000124664
CRISP3 ENSG00000096006
LRPAP1 ENSG00000163956
PSAP ENSG00000197746
SRRM2 ENSG00000167978
UBE2M ENSG00000130725
ACPP ENSG00000014257
HDLBP ENSG00000115677
CD81 ENSG00000110651
BRD2 ENSG00000204256
FXYD3 ENSG00000089356
PEBP1 ENSG00000089220
TSPAN1 ENSG00000117472
KRT8 ENSG00000170421

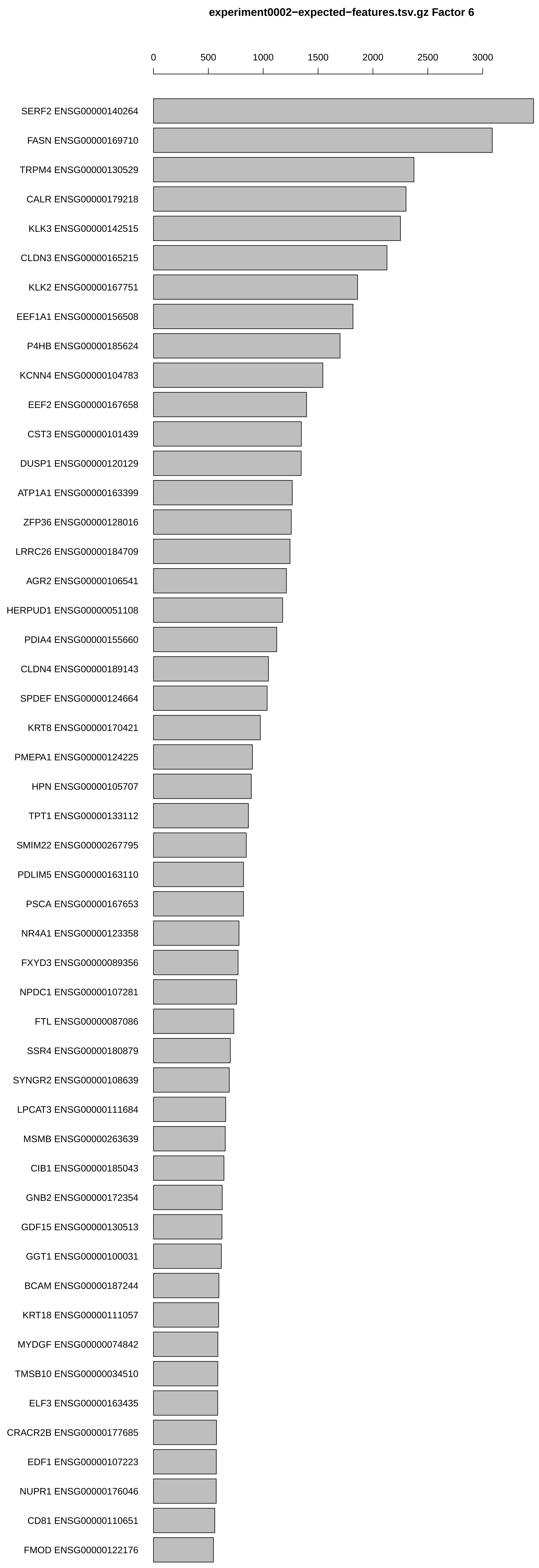
experiment0002−expected−features.tsv.gz Factor 6
0
500
1000
1500
2000
2500
3000
SERF2 ENSG00000140264
FASN ENSG00000169710
TRPM4 ENSG00000130529
CALR ENSG00000179218
KLK3 ENSG00000142515
CLDN3 ENSG00000165215
KLK2 ENSG00000167751
EEF1A1 ENSG00000156508
P4HB ENSG00000185624
KCNN4 ENSG00000104783
EEF2 ENSG00000167658
CST3 ENSG00000101439
DUSP1 ENSG00000120129
ATP1A1 ENSG00000163399
ZFP36 ENSG00000128016
LRRC26 ENSG00000184709
AGR2 ENSG00000106541
HERPUD1 ENSG00000051108
PDIA4 ENSG00000155660
CLDN4 ENSG00000189143
SPDEF ENSG00000124664
KRT8 ENSG00000170421
PMEPA1 ENSG00000124225
HPN ENSG00000105707
TPT1 ENSG00000133112
SMIM22 ENSG00000267795
PDLIM5 ENSG00000163110
PSCA ENSG00000167653
NR4A1 ENSG00000123358
FXYD3 ENSG00000089356
NPDC1 ENSG00000107281
FTL ENSG00000087086
SSR4 ENSG00000180879
SYNGR2 ENSG00000108639
LPCAT3 ENSG00000111684
MSMB ENSG00000263639
CIB1 ENSG00000185043
GNB2 ENSG00000172354
GDF15 ENSG00000130513
GGT1 ENSG00000100031
BCAM ENSG00000187244
KRT18 ENSG00000111057
MYDGF ENSG00000074842
TMSB10 ENSG00000034510
ELF3 ENSG00000163435
CRACR2B ENSG00000177685
EDF1 ENSG00000107223
NUPR1 ENSG00000176046
CD81 ENSG00000110651
FMOD ENSG00000122176

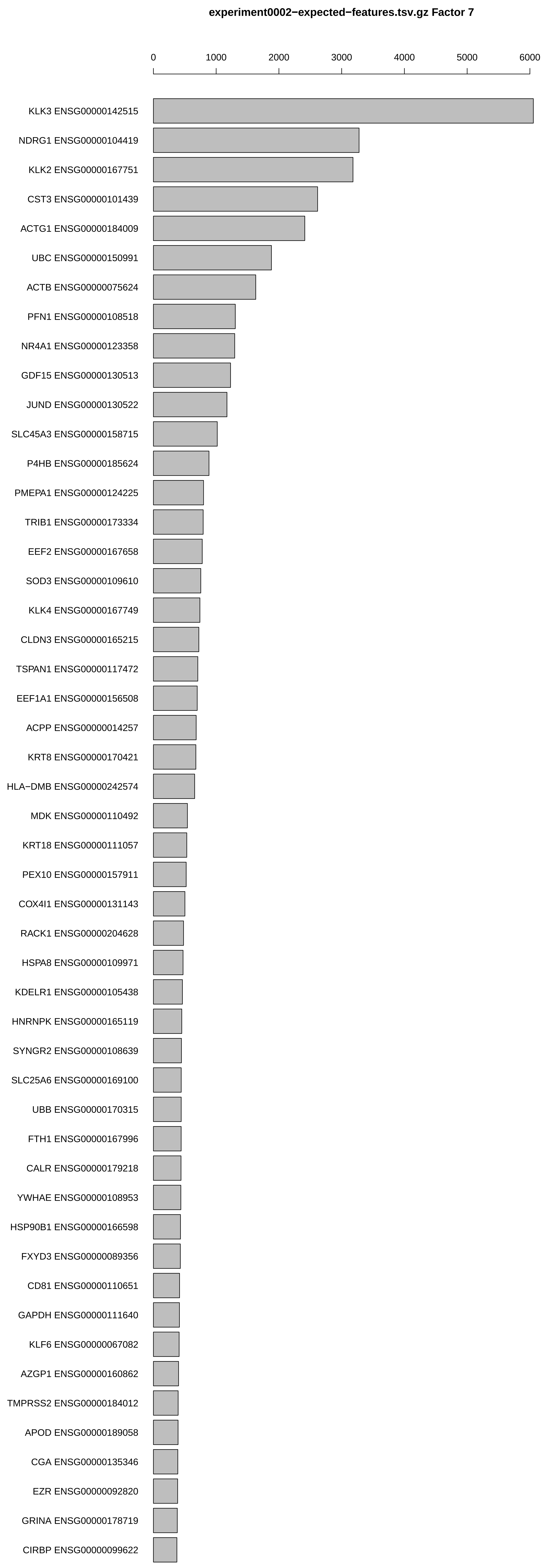
experiment0002−expected−features.tsv.gz Factor 7
0
1000
2000
3000
4000
5000
6000
KLK3 ENSG00000142515
NDRG1 ENSG00000104419
KLK2 ENSG00000167751
CST3 ENSG00000101439
ACTG1 ENSG00000184009
UBC ENSG00000150991
ACTB ENSG00000075624
PFN1 ENSG00000108518
NR4A1 ENSG00000123358
GDF15 ENSG00000130513
JUND ENSG00000130522
SLC45A3 ENSG00000158715
P4HB ENSG00000185624
PMEPA1 ENSG00000124225
TRIB1 ENSG00000173334
EEF2 ENSG00000167658
SOD3 ENSG00000109610
KLK4 ENSG00000167749
CLDN3 ENSG00000165215
TSPAN1 ENSG00000117472
EEF1A1 ENSG00000156508
ACPP ENSG00000014257
KRT8 ENSG00000170421
HLA−DMB ENSG00000242574
MDK ENSG00000110492
KRT18 ENSG00000111057
PEX10 ENSG00000157911
COX4I1 ENSG00000131143
RACK1 ENSG00000204628
HSPA8 ENSG00000109971
KDELR1 ENSG00000105438
HNRNPK ENSG00000165119
SYNGR2 ENSG00000108639
SLC25A6 ENSG00000169100
UBB ENSG00000170315
FTH1 ENSG00000167996
CALR ENSG00000179218
YWHAE ENSG00000108953
HSP90B1 ENSG00000166598
FXYD3 ENSG00000089356
CD81 ENSG00000110651
GAPDH ENSG00000111640
KLF6 ENSG00000067082
AZGP1 ENSG00000160862
TMPRSS2 ENSG00000184012
APOD ENSG00000189058
CGA ENSG00000135346
EZR ENSG00000092820
GRINA ENSG00000178719
CIRBP ENSG00000099622

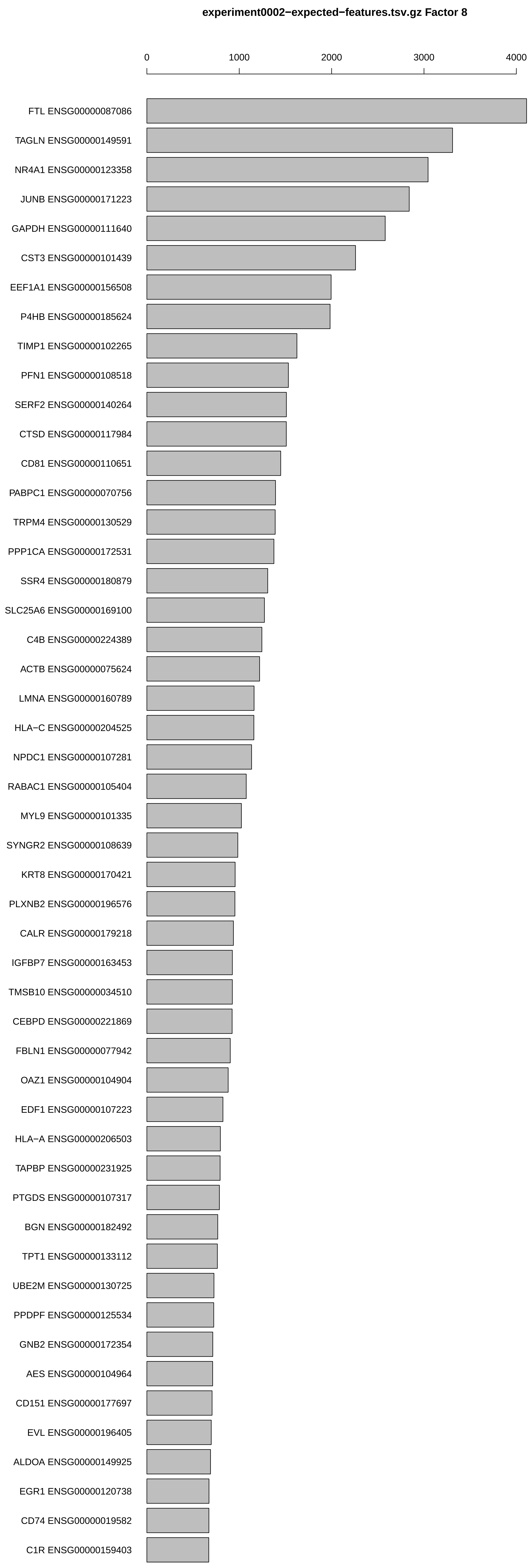
experiment0002−expected−features.tsv.gz Factor 8
0
1000
2000
3000
4000
FTL ENSG00000087086
TAGLN ENSG00000149591
NR4A1 ENSG00000123358
JUNB ENSG00000171223
GAPDH ENSG00000111640
CST3 ENSG00000101439
EEF1A1 ENSG00000156508
P4HB ENSG00000185624
TIMP1 ENSG00000102265
PFN1 ENSG00000108518
SERF2 ENSG00000140264
CTSD ENSG00000117984
CD81 ENSG00000110651
PABPC1 ENSG00000070756
TRPM4 ENSG00000130529
PPP1CA ENSG00000172531
SSR4 ENSG00000180879
SLC25A6 ENSG00000169100
C4B ENSG00000224389
ACTB ENSG00000075624
LMNA ENSG00000160789
HLA−C ENSG00000204525
NPDC1 ENSG00000107281
RABAC1 ENSG00000105404
MYL9 ENSG00000101335
SYNGR2 ENSG00000108639
KRT8 ENSG00000170421
PLXNB2 ENSG00000196576
CALR ENSG00000179218
IGFBP7 ENSG00000163453
TMSB10 ENSG00000034510
CEBPD ENSG00000221869
FBLN1 ENSG00000077942
OAZ1 ENSG00000104904
EDF1 ENSG00000107223
HLA−A ENSG00000206503
TAPBP ENSG00000231925
PTGDS ENSG00000107317
BGN ENSG00000182492
TPT1 ENSG00000133112
UBE2M ENSG00000130725
PPDPF ENSG00000125534
GNB2 ENSG00000172354
AES ENSG00000104964
CD151 ENSG00000177697
EVL ENSG00000196405
ALDOA ENSG00000149925
EGR1 ENSG00000120738
CD74 ENSG00000019582
C1R ENSG00000159403

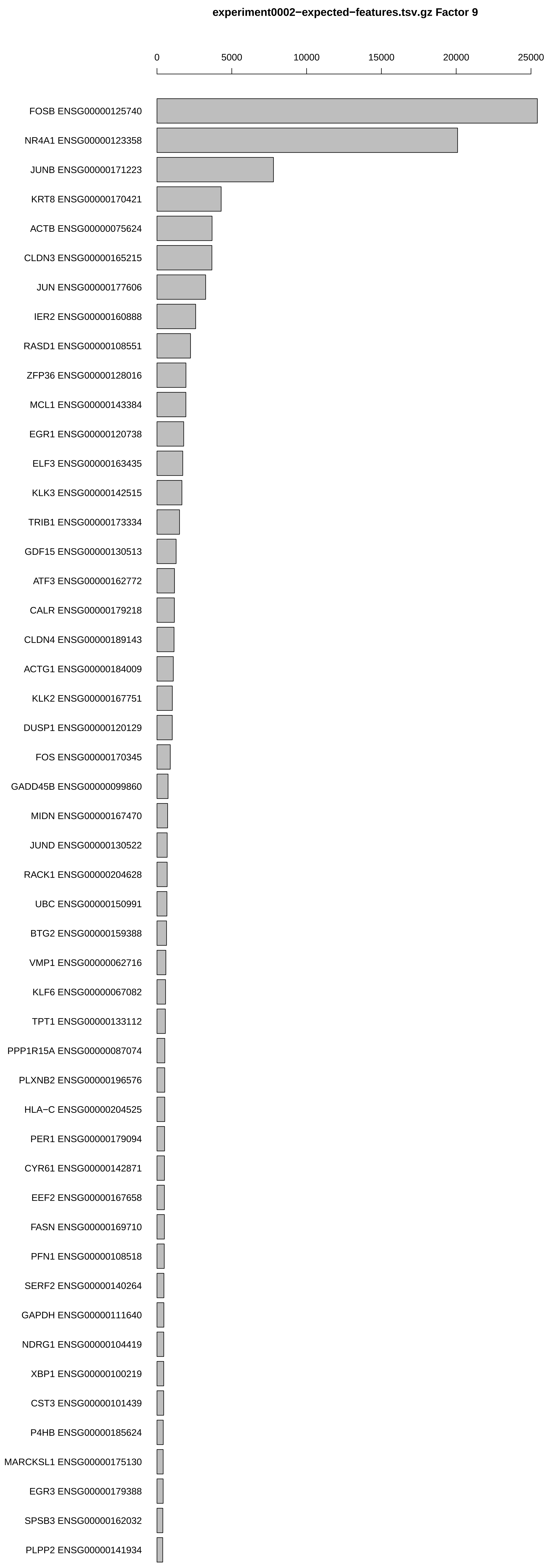
experiment0002−expected−features.tsv.gz Factor 9
0
5000
10000
15000
20000
25000
FOSB ENSG00000125740
NR4A1 ENSG00000123358
JUNB ENSG00000171223
KRT8 ENSG00000170421
ACTB ENSG00000075624
CLDN3 ENSG00000165215
JUN ENSG00000177606
IER2 ENSG00000160888
RASD1 ENSG00000108551
ZFP36 ENSG00000128016
MCL1 ENSG00000143384
EGR1 ENSG00000120738
ELF3 ENSG00000163435
KLK3 ENSG00000142515
TRIB1 ENSG00000173334
GDF15 ENSG00000130513
ATF3 ENSG00000162772
CALR ENSG00000179218
CLDN4 ENSG00000189143
ACTG1 ENSG00000184009
KLK2 ENSG00000167751
DUSP1 ENSG00000120129
FOS ENSG00000170345
GADD45B ENSG00000099860
MIDN ENSG00000167470
JUND ENSG00000130522
RACK1 ENSG00000204628
UBC ENSG00000150991
BTG2 ENSG00000159388
VMP1 ENSG00000062716
KLF6 ENSG00000067082
TPT1 ENSG00000133112
PPP1R15A ENSG00000087074
PLXNB2 ENSG00000196576
HLA−C ENSG00000204525
PER1 ENSG00000179094
CYR61 ENSG00000142871
EEF2 ENSG00000167658
FASN ENSG00000169710
PFN1 ENSG00000108518
SERF2 ENSG00000140264
GAPDH ENSG00000111640
NDRG1 ENSG00000104419
XBP1 ENSG00000100219
CST3 ENSG00000101439
P4HB ENSG00000185624
MARCKSL1 ENSG00000175130
EGR3 ENSG00000179388
SPSB3 ENSG00000162032
PLPP2 ENSG00000141934

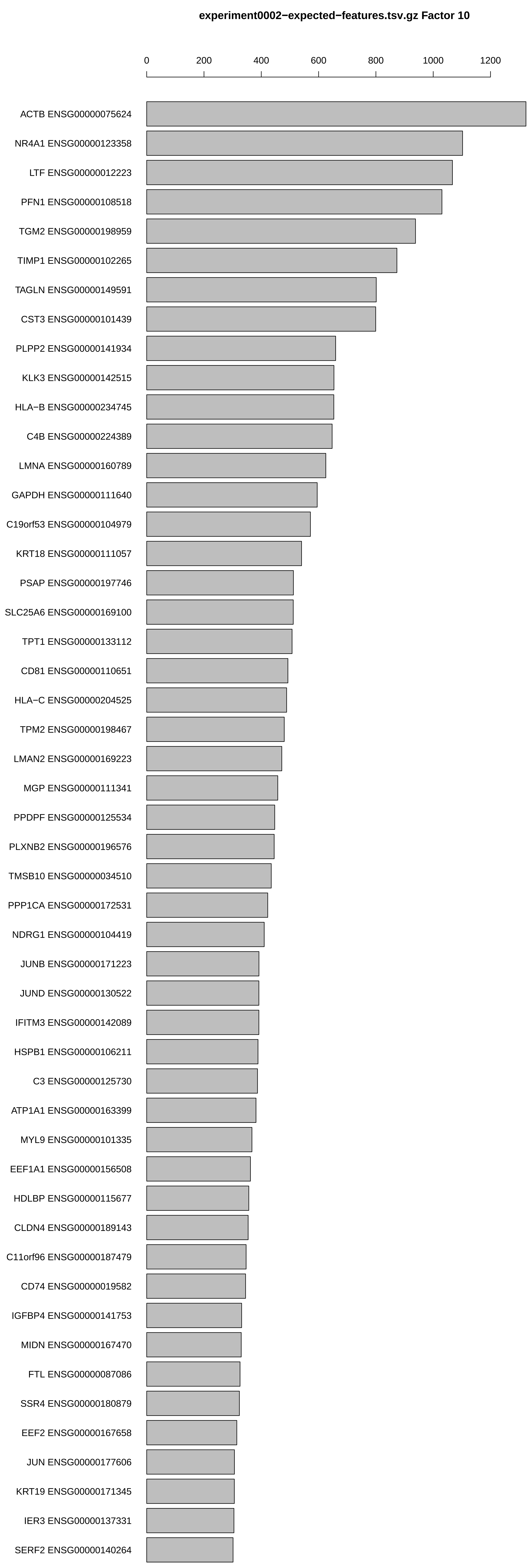
experiment0002−expected−features.tsv.gz Factor 10
0
200
400
600
800
1000
1200
ACTB ENSG00000075624
NR4A1 ENSG00000123358
LTF ENSG00000012223
PFN1 ENSG00000108518
TGM2 ENSG00000198959
TIMP1 ENSG00000102265
TAGLN ENSG00000149591
CST3 ENSG00000101439
PLPP2 ENSG00000141934
KLK3 ENSG00000142515
HLA−B ENSG00000234745
C4B ENSG00000224389
LMNA ENSG00000160789
GAPDH ENSG00000111640
C19orf53 ENSG00000104979
KRT18 ENSG00000111057
PSAP ENSG00000197746
SLC25A6 ENSG00000169100
TPT1 ENSG00000133112
CD81 ENSG00000110651
HLA−C ENSG00000204525
TPM2 ENSG00000198467
LMAN2 ENSG00000169223
MGP ENSG00000111341
PPDPF ENSG00000125534
PLXNB2 ENSG00000196576
TMSB10 ENSG00000034510
PPP1CA ENSG00000172531
NDRG1 ENSG00000104419
JUNB ENSG00000171223
JUND ENSG00000130522
IFITM3 ENSG00000142089
HSPB1 ENSG00000106211
C3 ENSG00000125730
ATP1A1 ENSG00000163399
MYL9 ENSG00000101335
EEF1A1 ENSG00000156508
HDLBP ENSG00000115677
CLDN4 ENSG00000189143
C11orf96 ENSG00000187479
CD74 ENSG00000019582
IGFBP4 ENSG00000141753
MIDN ENSG00000167470
FTL ENSG00000087086
SSR4 ENSG00000180879
EEF2 ENSG00000167658
JUN ENSG00000177606
KRT19 ENSG00000171345
IER3 ENSG00000137331
SERF2 ENSG00000140264
