# Supplementary material for: Spatial maps of prostate cancer transcriptomes reveal an unexplored landscape of heterogeneity
Source: Nat Commun. 2018 Jun 20;9:2419. doi: 10.1038/s41467-018-04724-5 (PMC6010471; doi:10.1038/s41467-018-04724-5)

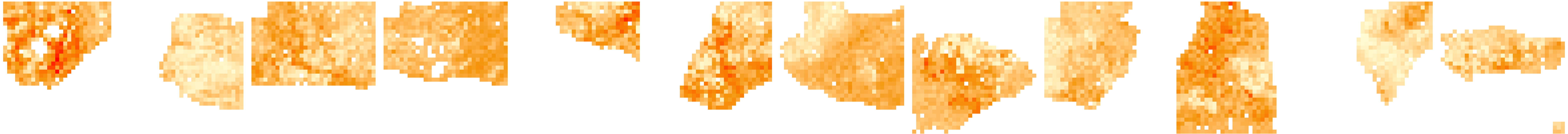

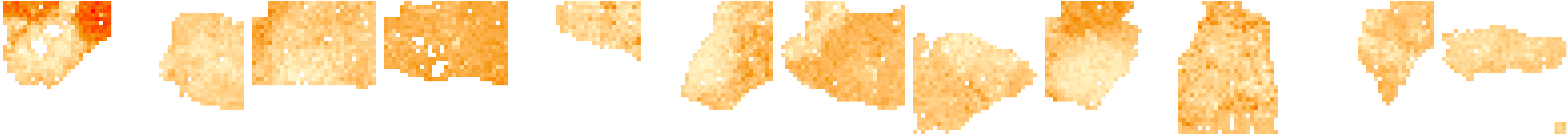

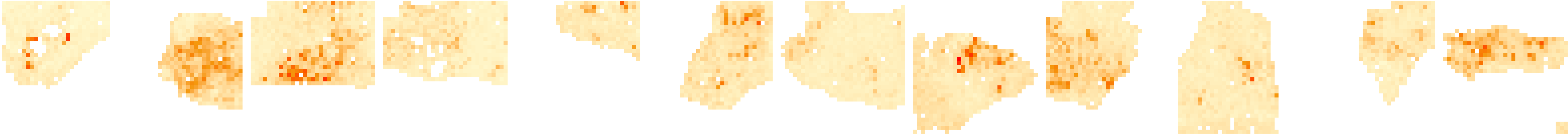

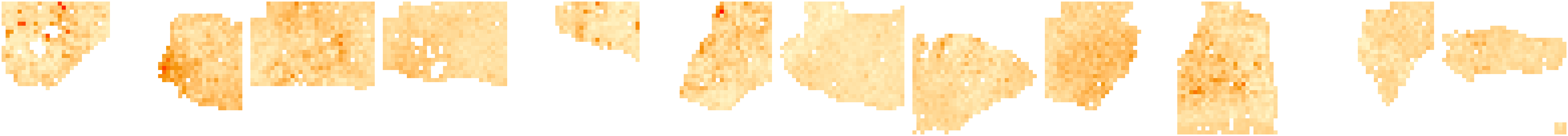

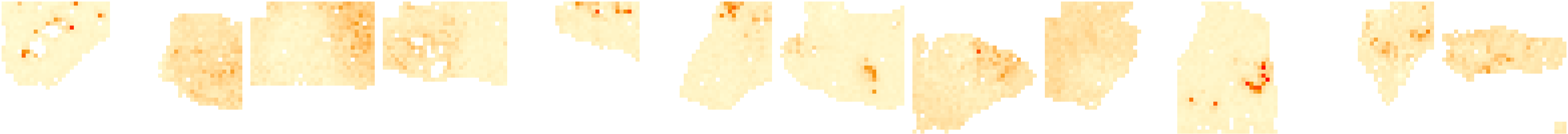

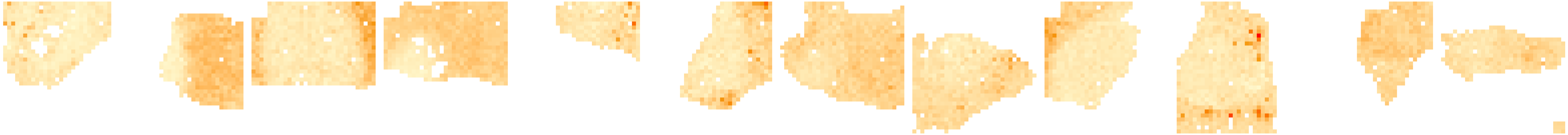

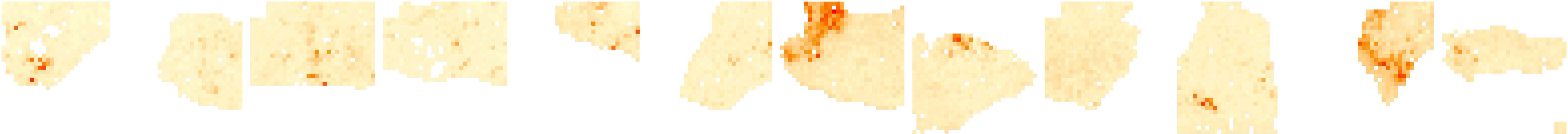

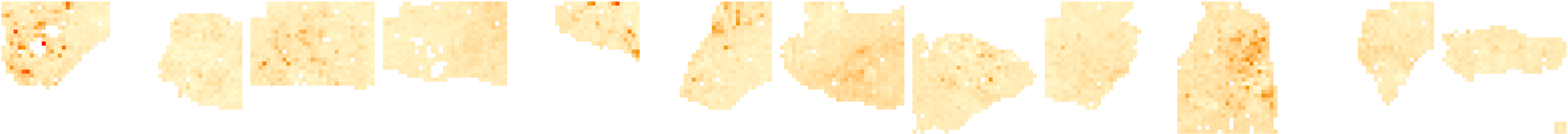

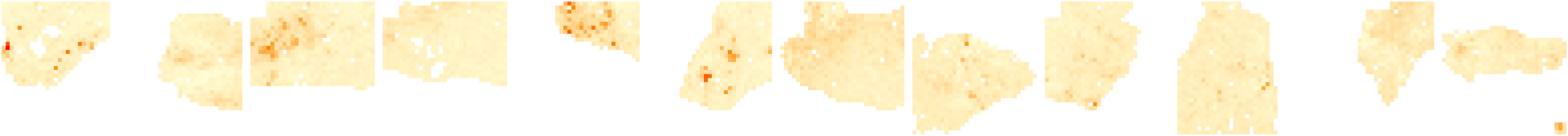

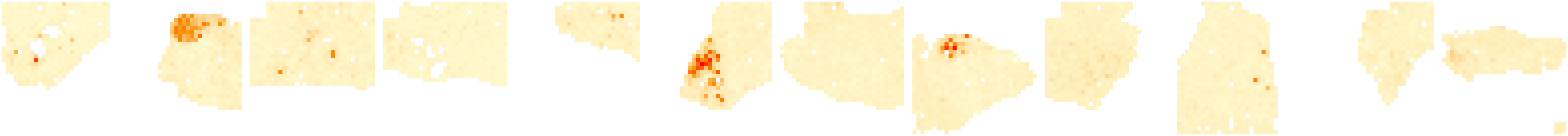

Supplement: Supplementary file 9 — Supplementary Data 6 [file 41467_2018_4724_MOESM9_ESM.zip › Supplementary Dataset 3/joint-mix-profiles-rel-common-scale-matrix.pdf]
